# Current status and future directions of nanovaccine for cancer: a bibliometric analysis during 2004-2023

**DOI:** 10.3389/fimmu.2024.1423212

**Published:** 2024-07-29

**Authors:** Yuhui Hou, Yue Li, Youao Zhang, Juan Zhang, Dinglan Wu

**Affiliations:** ^1^ The Second School of Clinical Medicine, Southern Medical University, Guangzhou, Guangdong, China; ^2^ The First School of Clinical Medicine, Southern Medical University, Guangzhou, Guangdong, China; ^3^ Shenzhen Key Laboratory of Oncology, The Clinical Innovation & Research Center (CIRC), Shenzhen Hospital, Southern Medical University, Shenzhen, Guangdong, China

**Keywords:** bibliometric analysis, nanovaccine, cancer, immunotherapy, neoantigen

## Abstract

**Background:**

Nanovaccine treatment is an exciting area of research in immunology and personalized medicine, holding great promise for enhancing immune responses and targeting specific diseases. Their small size allows efficient uptake by immune cells, leading to robust immune activation. They can incorporate immune-stimulating molecules to boost vaccine efficacy. Therefore, nanovaccine can be personalized to target tumor-specific antigens, activating the immune system against cancer cells. Currently, there have been ample evidence showing the effectiveness and potential of nanovaccine as a treatment for cancer. However, there was rare bibliometric analysis of nanovaccine for cancer. Here we performed a bibliometric and visual analysis of published studies related to nanovaccine treatment for cancer, providing the trend of future development of nanovaccine.

**Methods:**

We collected the literatures based on the Web of Science Core Collection SCI-Expanded database. The bibliometric analysis was performed via utilizing visualization analysis tools VOSviewer, Co-Occurrence (COOC), Citespace, Bibliometrix (R-Tool of R-Studio), and HitCite.

**Results:**

A total of 517 literatures were included in this study. China is the country with the most publications and the highest total local citation score (TLCS). The Chinese Academy of Sciences holds the largest research count in this field and the most prolific author is Deling Kong from Nankai University. The most prominent journal for publishing in this area is Biomaterials. The researches mainly focus on the therapeutic process of tumor nanovaccines, the particle composition and the application of nanovaccines, suggesting the potential hotspots and trends of nanovaccine.

**Conclusion:**

In this study, we summarized the characteristics and variation trends of publications involved in nanovaccine, and categorized the most influential countries, institutions, authors, journals, hotspots and trends regarding the nanovaccine for cancer. With the continuous development of nanomaterials and tumor immunotherapy, nanovaccine for cancer provides a research field of significant clinical value and potential application.

## 1 Introduction

Nanovaccines are vaccines based on nanotechnology, consisting of antigens, adjuvants, and nanocarriers. Nanoparticle-based delivery vehicles can facilitate shared delivery of antigens and adjuvants, significantly enhancing the effectiveness and safety of vaccines, achieving targeted delivery of nanovaccines, and stimulating the body’s immune response (1, 2). These delivery systems can be classified by their chemical composition into inorganic (gold, metal oxides, silica, etc.) and organic nanoparticles (such as hyaluronic acid, alginic acid, ferritin, polymer nanoparticles, liposomes, VLPs, viral particles, and micelles), which exert diverse effects on MHC class I and II immune responses and are used in different immunological microenvironments (3).

Immunotherapy for cancer involves activating antigen-specific T cells by antigen-presenting cells (APCs), mobilizing different cell types, breaking through inhibitory tumor microenvironments, and maintaining immune responses (4). However, malignant tumor cells can evade immune surveillance due to their complex growth environment, inhibitory tumor immune microenvironment, inherent heterogeneity, and low immunogenicity, making tumor immunotherapy challenging (5, 6). Additional tools for tumor therapy, such as immune checkpoint blockade (CPB) and adoptive cell transfer (ACT), have been widely used. Unfortunately, for solid tumors, Chimeric antigen receptor (CAR)-T cell therapy is limited due to the lack of common surface antigen targets (7, 8), and the toxicity of CPB cannot be ignored due to the blockade of CTLA-4 (9), not to mention its resistance and inability to maintain a lasting immune response (10).

Compared to traditional cancer vaccines, nanotechnology can prevent the degradation of tumor antigens, provide a stable delivery platform, prolong the effect of antigens in the tumor microenvironment, and enhance the lasting efficacy of the immune response (11). Moreover, due to their variable size and the ability to carry multiple antigens simultaneously, nanovaccines can achieve multiple antigen stimulation, enhance immunogenicity, and can be applied to various solid tumor environments, thereby improving the stability and activity of the vaccine (12, 13). In recent years, with the development of genomics, personalized and precise cancer therapy has become a trend. Vaccines based on nanoscale particles can be combined with tumor neoantigens (14, 15) to exhibit their good stability, targeted delivery ability, high drug delivery efficiency and ability to achieve multiple immune stimulation while enhancing anti-tumor CTL responses (1, 16). Nanovaccines are therefore gaining widespread attention. In order to adapt to different individuals and achieve effective cell-mediated immune responses, nanovaccines can be carefully designed and optimized in terms of carriers, antigens, and adjuvants to achieve synergistic immune therapeutic effects, thus showing great potential for application in the field of cancer.

Bibliometric analysis employs mathematical and statistical methods to qualitatively or quantitatively explore the distribution, structure, quantity, and evolution of bibliographic information. It holds significant value in delineating the current landscape across various research disciplines, revealing publishing trends, showcasing scientific achievements of researchers, institutions, and countries, and identifying potential future research areas, academic frontiers, and knowledge maps. These insights provide both researchers and clinicians with a comprehensive overview of the prevailing state of development within a specific research domain. Furthermore, bibliometrics has found extensive application in the fields of vaccine, nanomaterial and cancer (17–21). CiteSpace, developed by Prof. Meichao Chen, is an interactive analysis tool that allows for visual analysis through a combination of bibliometrics, visual analytics methods, and data mining algorithms (22). Bibliometrix is a commonly used open-source bibliometrics tool (23), VOSviewer is used for bibliometric network graph analysis (24), COOC13.2 and HistCite are also commonly used bibliometric softwares (25).

## 2 Materials and methods

### 2.1 Data retrieval, extraction, and cleaning

**Figure 1:**
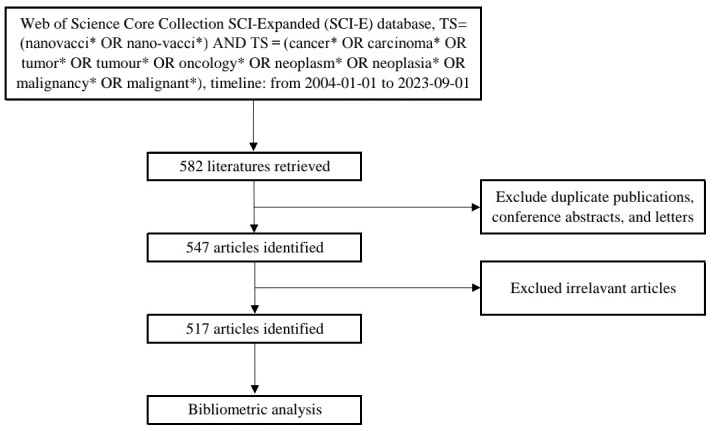
Screening flowchart for inclusion of studies.

The Web of Science Core Collection was selected as the data source due to its comprehensive and authoritative coverage, encompassing over 12,000 high-quality journals. We utilized the SCI-Expanded (SCI-E) database for our search. The search query (**Figure 1**) was set as the following formula: TS= (nanovacci* OR nano-vacci*) AND TS = (cancer* OR carcinoma* OR tumor* OR tumor* OR oncology* OR neoplasm* OR neoplasia* OR malignancy* OR malignant*). The timeline was set from 2004-01-01 to 2023-09-01. 582 literatures were retrieved. After excluding duplicate publications, conference abstracts, and letters, etc. there were 547 articles left (the plain text format is attached in the **Supplementary Material**). Meanwhile, we excluded the irrelevant literature by reading the title, abstract and keywords among the 547 literatures, and a total of 517 articles were left.

### 2.2 Scientometric analysis

The 517 literatures were exported in plain text format. For overall trend analysis, we utilized Excel 2019, visualization analysis tools VOSviewer, COOC13.2, Citespace, Bibliometrix (R-Tool of R-Studio), and HistCite. To explore research hotspots and frontier directions of nanovaccine treatment for cancer, we employed methods including synonym merging, frequency and total local citation score (TLCS) analysis of countries/regions, institutions, and authors, cluster analysis of co-occurrence matrices, citation analysis, two-mode matrix analysis, and burst keywords map.

## 3 Results

### 3.1 Annual analysis of publications

**Figure 2:**
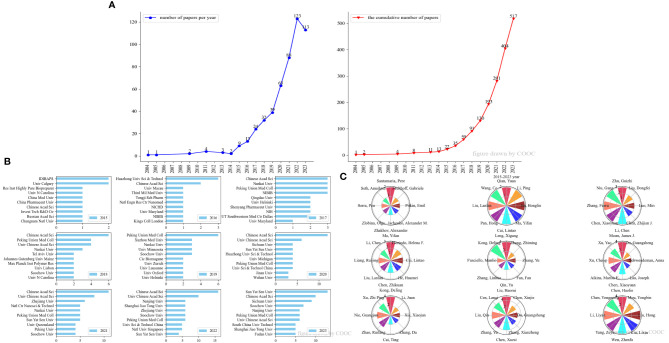
Annual analysis of the publications on nanovaccine for cancer. **(A)** Number of papers per year and cumulative number of papers per year from 2004 to 2023. **(B)** Top 10 institutions with the highest number of publications from 2015 to 2023. **(C)** Top 8 authors with the highest number of publications from 2015 to 2023.

From 2004 to 2014, research on nanovaccines and cancer was in its nascent stage, with only 13 publications and minimal attention. However, the research increased rapidly after 2014 (**Figure 2A**). By September 1, 2023, the number of publications had reached 517. Due to the limited number of publications from 2004 to 2014, statistical significance is relatively low. **Figures 2B, C** showed the institutions and authors with the highest publication counts each year, primarily focusing on the results after 2014.

In terms of institutions, the Chinese Academy of Sciences has consistently led in research on nanovaccine treatment for cancer since 2015. In 2023, Sun Yat-sen University and Sichuan University demonstrated significant growth in research output. Regarding authors, there is considerable diversity in individuals with the highest annual publication counts, reflecting broad interest from researchers across various fields.

### 3.2 Frequency analysis of countries/regions, institutions, authors, and journals

Frequency analysis of countries/regions (**Table 1**) reveals that China has the largest number of research publications on nanovaccine treatment for cancer. The Chinese Academy of Sciences has the largest research count, and Deling Kong from Nankai University is the most prolific author. The three leading journals are Biomaterials, Journal of Controlled Release, and ACS Nano, with the top ten journals primarily focusing on materials and immunology. All top ten journals are JCR Q1 journals, indicating high-impact recognition of research in this field.

**Table 1:** Top 10 countries/regions, institutions, authors and journals.

Rank	Country /Region	Count	Institution	Count	Author	Count	Journal	Count	2022 Impact Factor/JCR quartile
1	China	366	Chinese Acad Sci	62	Kong, Deling	14	Biomaterials	33	14.0/Q1
2	USA	98	Chinese Acad Med Sci & Peking Union Med Coll	37	Zhu, Guizhi	11	Journal of Controlled Release	28	10.8/Q1
3	Germany	25	Univ Chinese Acad Sci	34	Zhang, Yu	11	ACS Nano	27	17.1/Q1
4	Australia	23	Sun Yat Sen Univ	28	Chen, Xiaoyuan	10	Advanced Healthcare Materials	19	10.0/Q1
5	India	20	Soochow Univ	23	Chen, Xuesi	10	Nano Letters	18	10.8/Q1
6	South Korea	19	Nankai Univ	23	Zhang, Chuangnian	9	Advanced Materials	17	29.4/Q1
7	Iran	15	Sichuan Univ	22	Liu, Zhuang	9	Small	16	13.3/Q1
8	Singapore	15	Huazhong Univ Sci & Technol	17	Chen, Yongming	8	Advanced Science	15	15.1/Q1
9	Netherlands	14	Nanjing Univ	16	Nie, Guangjun	8	Frontiers in Immunology	15	7.3/Q1
10	England	13	Shanghai Jiao Tong Univ	16	Liu, Lixin	8	ACS Applied Materials & Interfaces	13	9.5/Q1

### 3.3 TLCS analysis of countries/regions, institutions, authors, and journals

Total Local Citation Score (TLCS) represents the number of citations in all relevant literatures. It provides a measurement to reflect the influence of a country/region, institution and authors on a particular topic. TLCS analysis using HistCite (**Table 2**) shows that China and the USA have the highest TLCS scores. The highly cited institutions are concentrated in China, with Soochow University ranking at the top. Among authors, Jun Xu from Soochow University has the highest TLCS, reflecting that his research on the topic is well recognized. Institutions and authors with high TLCS are mainly centralized in China, reflecting that research on this topic is highly regarded in this region, but it may be due to the high Local Citation Score (LCS) of individual articles. The three journals with the highest TLCS are ACS Nano, Nature Nanotechnology, and Biomaterials.

**Table 2:** Top 10 TLCS countries/regions, institutions, authors and journals.

Rank	Country /Region	TLCS	Institution	TLCS	Author	TLCS	Journal	TLCS	2022 Impact Factor/JCR quartile
1	China	1027	Soochow Univ (China)	205	Xu, Jun	184	ACS Nano	341	17.1/Q1
2	USA	500	Chinese Acad Sci (China)	198	Liu, Zhuang	188	Nature Nanotechnology	237	38.3/Q1
3	UK	55	Chinese Acad Med Sci/Peking Union Med Coll (China)	152	Peng, Rui	178	Biomaterials	206	14.0/Q1
4	Australia	48	Univ Texas Southwestern Med Ctr Dallas (USA)	134	Xu, Ligeng	157	Nano Letters	142	10.8/Q1
5	Canada	45	Nankai Univ (China)	121	Luo, Min	138	Journal of Controlled Release	109	10.8/Q1
6	Israel	42	Huazhong Univ Sci & Technol (China)	118	Gao, Jinming	134	ACS Applied Materials & Interfaces	40	9.5/Q1
7	Portugal	42	Univ Chinese Acad Sci (China)	114	Chen, Zhijian J.	126	Acta Biomaterialia	30	9.7/Q1
8	Spain	38	South China Univ Technol (China)	83	Wang, Zhaohui	126	Nature Materials	29	41.2/Q1
9	Italy	36	East China Normal Univ (China)	82	Kong, Deling	121	International Journal of Nanomedicine	28	8.0/Q1
10	Netherlands	33	Natl Ctr Nanosci & Technol (China)	81	Fu, Yangxin	114	Nanoscale	24	6.7/Q1

### 3.4 Cooperation analysis of countries/regions, institutions, and authors

**Figure 3:**
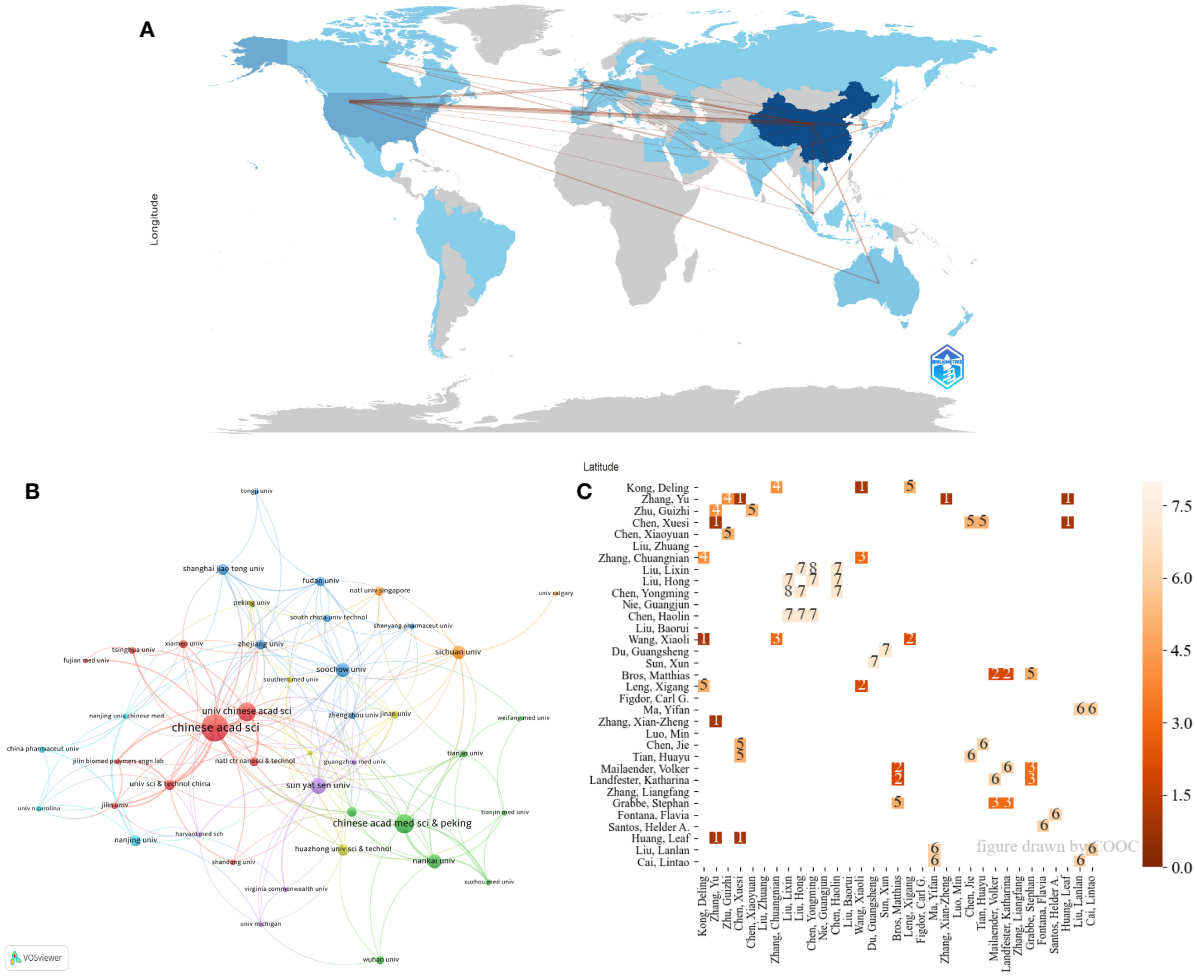
The collaboration among countries/regions, institutions, and authors. **(A)** Distribution and collaboration of publications among countries/regions. **(B)** Visualization map of institutional collaboration. **(C)** The collaboration between authors. The number on the square indicates times of cooperation.

**Figure 3A** illustrates that China has the most extensive cooperation with the USA. Institutional collaboration (**Figure 3B**) shows frequent interactions between the Chinese Academy of Sciences and the University of Chinese Academy of Sciences, as well as multiple collaborations among Nankai University, the Chinese Academy of Medical Sciences, and Peking Union Medical College. **Figure 3C** reveals frequent collaborations among professors Yongming Chen, Lixin Liu, Hong Liu, Haolin Chen, Guangsheng Du, and Xun Sun, indicating concentrated author cooperation.

### 3.5 Citation analysis

#### 3.5.1 Usage rate analysis of citations in 180 days

The 180-day usage count (**Table 3**) provided by Web of Science measures user attention to specific items on the platform. This count reflects how many times users have accessed information related to a particular article within the last 180 days. Specifically, this includes instances where users clicked on links leading to the full text from the publisher (via direct link or Open URL), or saved the paper for later use in bibliographic management tools (via direct export or saving in another format for later re-import). High citation counts can reflect authoritative discoveries in the field but may struggle to uncover newer directions. In contrast, high 180-day usage counts reveal new research hotspots, allowing researchers to capture trends by exploring the latest literature discovered through this metric (36). The 180 days here refer to the period ending on the day we retrieved the publications (i.e., September 1, 2023). The top 3 used article in the past 180 days were a 2023 review by Rui Liu and colleagues in Chinese Chemical Letters, Jun Xu et al. in 2020 in Nature Nanotechnology, and Fangmin Chen et al. (2023) in Advanced Materials.

**Table 3:** Ranking of the top 10 highest 180 days usage.

Rank	Year	Title	Journal	First Author	Usage count	References
1	2023	Advances of Nanoparticles as Drug Delivery Systems for Disease Diagnosis and Treatment	Chinese Chemical Letters	Liu, Rui	86	(26)
2	2020	A General Strategy Towards Personalized Nanovaccines Based on Fluoropolymers for Post-Surgical Cancer Immunotherapy	Nature Nanotechnology	Xu, Jun	80	(27)
3	2023	Acid-Ionizable Iron Nanoadjuvant Augments Sting Activation for Personalized Vaccination Immunotherapy of Cancer	Advanced Materials	Chen, Fangmin	79	(28)
4	2021	A DNA Nanodevice-Based Vaccine for Cancer Immunotherapy	Nature Materials	Liu, Shaoli	69	(29)
5	2023	A Tumor Cell Membrane-Coated Self-Amplified Nanosystem as a Nanovaccine to Boost the Therapeutic Effect of Anti-Pd-L1 Antibody	Bioactive Materials	Li, Zhilin	69	(30)
6	2023	Immune-Regulating Camouflaged Nanoplatforms: A Promising Strategy to Improve Cancer Nano-Immunotherapy	Bioactive Materials	Chen, Biaoqi	66	(31)
7	2022	A Nanovaccine for Antigen Self-Presentation and Immunosuppression Reversal as a Personalized Cancer Immunotherapy Strategy	Nature Nanotechnology	Liu, Chao	64	(32)
8	2023	Photothermal-Triggered Dendrimer Nanovaccines Boost Systemic Antitumor Immunity	Journal of Controlled Release	Shen, Siyan	63	(33)
9	2022	Polyacrylic Acid Nanoplatforms: Antimicrobial, Tissue Engineering, And Cancer Theranostic Applications	Polymers	Arkaban, Hassan	57	(34)
10	2022	A Minimalist Binary Vaccine Carrier for Personalized Postoperative Cancer Vaccine Therapy	Advanced Materials	Zhao, Jiayu	55	(35)

#### 3.5.2 Local citation score analysis of citations

LCS (**Table 4**) refers to the number of citations within a specific topic area. LCS provides a more focused evaluation within the specific area discussed, whereas the global citation score (GCS) includes values from various fields. Therefore, LCS offers a more accurate analytical perspective for our discussion. The articles with the top 3 highest LCS were an article published by Min Luo et al. in Nature Nanotechnology in 2017, an article by Guizhi Zhu et al. in 2017 in ACS Nano, and an article by Rong Yang et al. in 2018 in ACS Nano.

**Table 4:** Ranking of the top 10 LCS cited references.

Rank	Year	Title	Journal	First Author	LCS	References
1	2017	A STING-activating nanovaccine for cancer immunotherapy	Nature Nanotechnology	Luo, Min	106	(37)
2	2017	Efficient Nanovaccine Delivery in Cancer Immunotherapy	ACS Nano	Zhu, Guizhi	79	(1)
3	2018	Cancer Cell Membrane-Coated Adjuvant Nanoparticles with Mannose Modification for Effective Anticancer Vaccination	ACS Nano	Yang, Rong	74	(38)
4	2020	A general strategy towards personalized nanovaccines based on fluoropolymers for post-surgical cancer immunotherapy	Nature Nanotechnology	Xu, Jun	55	(27)
5	2015	Erythrocyte Membrane-Enveloped Polymeric Nanoparticles as Nanovaccine for Induction of Antitumor Immunity against Melanoma	ACS Nano	Guo, Yuanyuan	48	(39)
6	2020	Proton-driven transformable nanovaccine for cancer immunotherapy	Nature Nanotechnology	Gong, Ningqiang	35	(40)
7	2017	Enhanced antitumor immunity by targeting dendritic cells with tumor cell lysate-loaded chitosan nanoparticles vaccine	Biomaterials	Shi, Gaona	32	(41)
8	2015	Nanoparticle-Based Immunotherapy for Cancer	ACS Nano	Shao, Kun	30	(42)
9	2019	Immunization with mannosylated nanovaccines and inhibition of the immune-suppressing microenvironment sensitizes melanoma to immune checkpoint modulators	Nature Nanotechnology	Conniot, Joao	30	(43)
10	2021	A DNA Nanodevice-Based Vaccine for Cancer Immunotherapy	Nature Materials	Liu, Shaoli	29	(29)

#### 3.5.3 Citation map, citation burst and co-citation analysis

**Figure 4:**
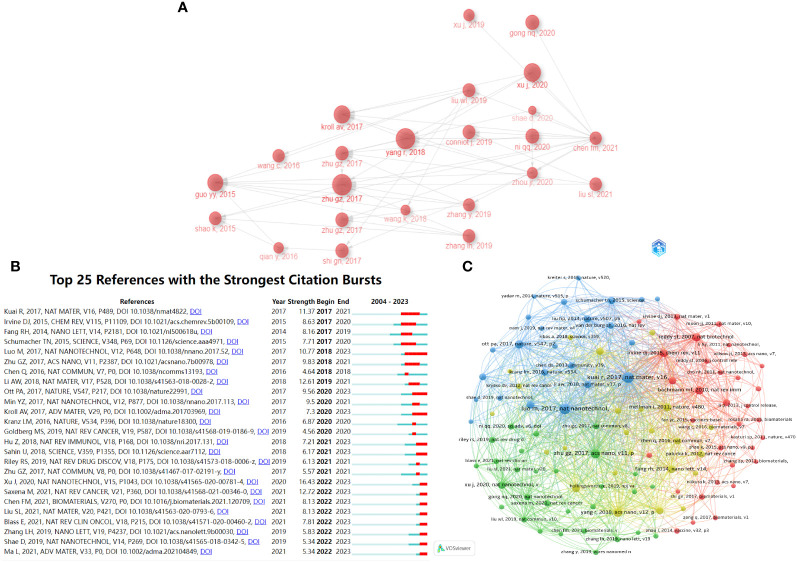
Citation analysis. **(A)** Map of inter-citation relationship between highly cited references. The arrow points to the cited literature. **(B)** Top 25 references with the strongest citation bursts. The light blue line segment represents the period from 2004 to 2023. The dark blue line segment represents the period from the publication year of the article to 2023. The red line segment represents the period of a citation burst. **(C)** Co-citation network analysis of most cited references.

Bibliometrix was used to draw the inter-citation relationships between highly cited articles (**Figure 4A**). This chart shows how these articles are interconnected and how they refer to one another. Through the citation map, we can identify the main centers of connections among the included articles, divided into citation centers and cited literature centers. The citation center refers to the most cited article among those with high LCS, representing authority and pioneering research in the field. The cited literature center refers to articles with high LCS that cite the most other articles, providing a comprehensive view of the field. **Figure 4A** shows that the citation centers include an article by Guizhi Zhu et al. in ACS Nano in 2017. Guizhi Zhu, as the first author, also published two significant papers in Nature Communications in 2017, attracting substantial attention. The cited literature center is a review by Fangmin Chen et al. in Biomaterials in 2021, which cites a large number of highly cited articles. This article may be a good choice for a comprehensive understanding of this field.

Additionally, CiteSpace was used to list the top 25 references with the strongest citation bursts in chronological order (**Figure 4B**). The light blue line segment indicates the period from 2004 to 2023, while the dark blue line segment represents the period from the publication year of the article to 2023. The red line segment denotes the timeframe during which the article experienced a citation burst. **Figure 4B** shows the publication by Jun Xu et al. in Nature Nanotechnology in 2020 and the review in Nature Reviews Cancer by Mansi Saxena et al. in 2021 have the strongest citation bursts based on strength scores.

References receiving multiple citations over time reflect current trending articles and future directions. **Figure 4C** presents a co-citation analysis using VOSviewer. Articles with higher co-citation levels are more closely connected and exhibit greater similarity between them. In **Figure 4C**, references can be categorized into four colors, representing different research directions: blue primarily focuses on the immunotherapeutic effects of nanovaccines, red on the transport and delivery of nanovaccines, green on the applications of nanovaccines, and yellow on the design of nanovaccines. Of course, there are some overlaps between these areas, as these four major directions are intricately connected in the development of tumor nanovaccines. Therefore, **Figure 4C** allows a better understanding of the subject’s development and the direction of further attention.

### 3.6 Keywords analysis

#### 3.6.1 Co-occurrence analysis of keywords frequency

**Figure 5:**
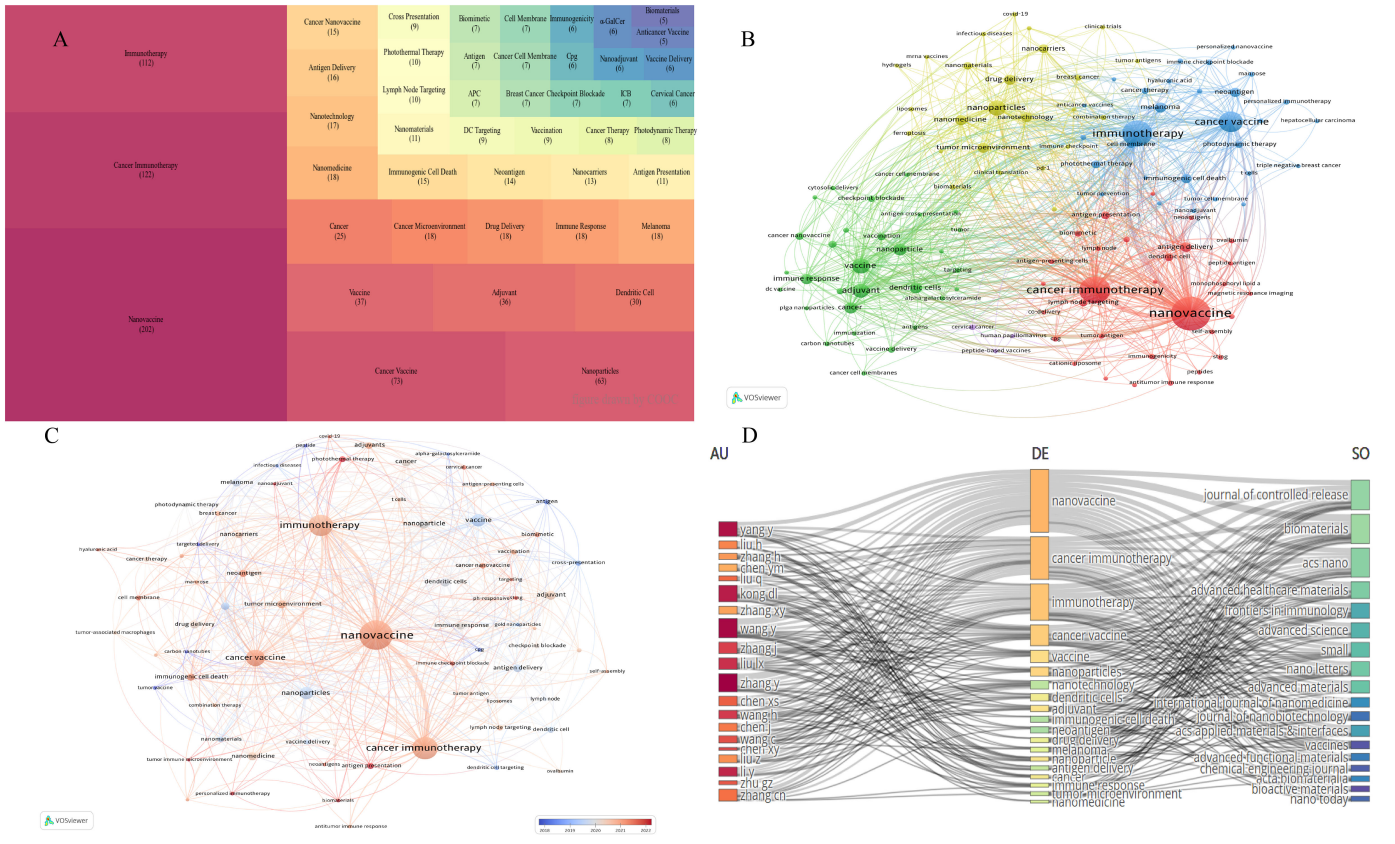
Keywords frequency and co-occurrence analysis. **(A)** Tree map of the 45 most frequent keywords. **(B)** Clustering of keywords through co-occurrence analysis. **(C)** Timeline visualization of collaboration among keywords. **(D)** Three-field plot of top 20 most productive authors (left), top 20 keywords (center) and top 18 journals (right).

COOC13.2 was used to extract and synonymously combine keywords, with the top 45 keyword frequencies displayed in **Figure 5A**. Keyword frequency is an important indicator that directly reflects a particular field’s research content, hotspots and frontier directions. There may be some correlations among the keywords mentioned in the papers, which can be represented by the co-occurrence frequency (**Figures 5B, C**). **Figure 5B** showed that the keywords are mainly divided into four categories: yellow for composition of tumor nanovaccines, green for adjuvants in tumor nanovaccines, red for the role of nanovaccines in tumor immunity, and blue for therapeutic applications of nanovaccines.

**Figure 5C** illustrates the timeline of keyword appearances from 2018 to 2022. The predominant red color indicates that the field is undergoing rapid development, with constant introductions of new keywords. Major red keywords, such as nanovaccine, cancer immunotherapy, immunotherapy, and cancer vaccine, reflect cutting-edge hotspots in this field.

It is generally believed that the more frequently two keywords appear together in the same literature, the stronger their relationship. **Figure 5D** is a three-field diagram, revealing the links between the top 20 most productive authors (left), the top 20 keywords (middle), and the top 18 journals (right). The size of the region reflects the linear density of the number of articles published and indicates the strength of the relationship. This figure provides insights into the research direction of each author and the focus of the journals.

#### 3.6.2 Cluster analysis of keywords

**Figure 6:**
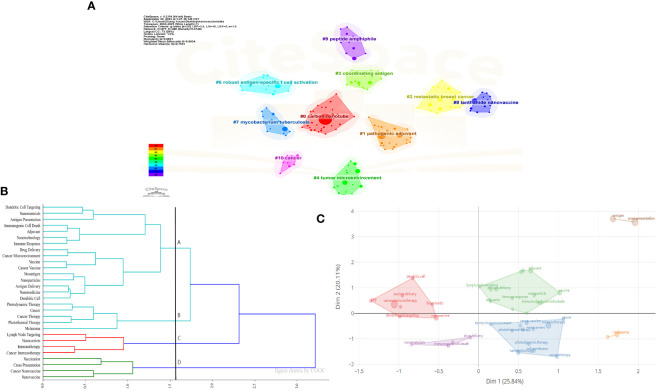
Cluster analysis of keywords. **(A)** Cluster diagram of references using title words in reference by CiteSpace. **(B)** Cosine matrix cluster analysis by COOC. **(C)** Cluster diagram of references using keywords by Bibliometrix.

Cluster analysis helps in gaining a more comprehensive understanding of a particular topic. Citespace, COOC 13.2 and Bibliometrix software were used for cluster analysis to classify keywords. **Figure 6** presents the results of this analysis. **Figure 6A** can be divided into #0 carbon nanotube, #1 pathogenic adjuvant, #2 metastatic breast cancer, #3 coordinating antigen, #4 tumor microenvironment, #6 robust antigen-specific t cell activation, #7 mycobacterium tuberculosis #8 lanthanide nanovaccine #9 peptide amphiphile, and #10 cancer. **Figure 6B** uses a cosine matrix to divide high-frequency keywords into four directions. In **Figure 6C**, six clusters were obtained using Bibliometrix.

#### 3.6.3 Time analysis of keywords

**Figure 7:**
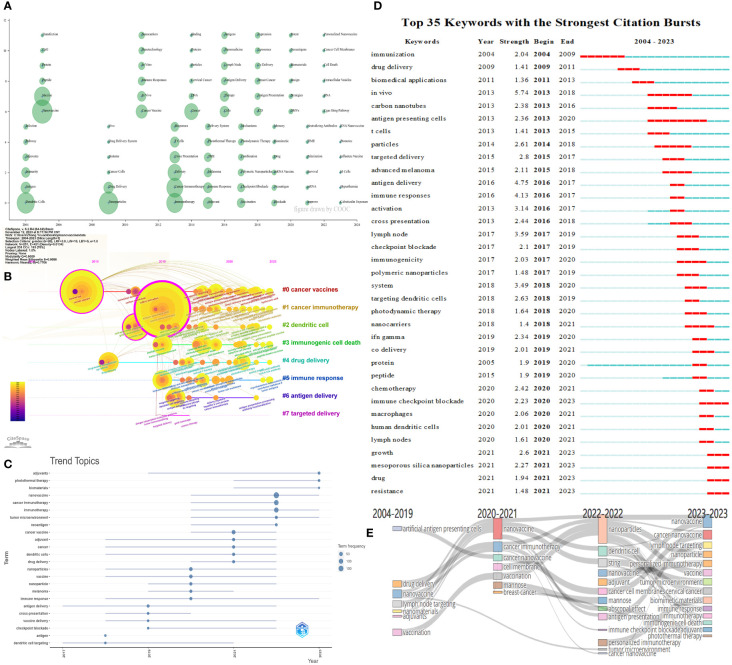
Time analysis of keywords. **(A)** Time zone diagram of research topics evolution. **(B)** Timeline view of keywords. **(C)** Topics trend analysis. The dots in the graph represent the peak of topic frequency; the dark blue lines refer to periods with active topic. **(D)** Top 35 keywords with the strongest citation bursts. The light blue represents periods without citation burst; dark blue represents periods of slight mutation; red represents periods with citation bursts. **(E)** Themes evolution chart.

Citespace and COOC13.2 software were used to generate **Figure 7**, which illustrates the evolving trends of research topics in the field over time. In **Figure 7A**, each circle represents a keyword, with larger circles indicating higher keyword frequency. Keywords were analyzed based on their initial appearance year in the dataset. Once a keyword appears, its reference year is fixed, even if it continues to appear in subsequent papers. Consequently, keywords are displayed in the figure only in the year they first emerged. If a keyword reappears in subsequent years, its frequency is incremented in the position corresponding to its initial appearance, resulting in a proportional increase in frequency. This approach accounts for keyword overlaps in the visual representation. COOC software was employed to construct **Figure 7A**, depicting the evolution of research topics over time. **Figure 7B** enables the observation of keyword changes within each cluster, complementing the cluster analysis. Based on the keywords, the clusters can be categorized as follows: #0 cancer vaccines, #1 cancer immunotherapy, #2 dendritic cell, #3 immunogenic cell death, #4 drug delivery, #5 immune response, #6 antigen delivery, and #7 targeted delivery. **Figures 7C, D** concentrate on annual keyword mutations, facilitating a better understanding of annual hot topics and serving as a reference for future industry research and development based on recent keyword shifts. As shown in 7D, light blue represents periods without keywords burst; dark blue represents the periods of “slight mutation” in its keywords, during which the growth of keywords occurred but did not exceed the threshold; red represents the periods that experience keyword bursts in the Citespace. **Figure 7E** offers insights into the directional evolution of these keywords at different time points, aiding in a deeper understanding of the research’s conceptual evolution and direction.

## 4 Discussion

### 4.1 General information

In this study, we traced 517 articles from 2004 to 2023 and conducted a hotspot analysis of the application of nanovaccines in cancer. We used VOSviewer, COOC13.2, Citespace, Bibliometrix (R-Tool of R-Studio), and HistCite for data analysis. Before 2014, the number of articles on nanovaccines in the field of cancer was very small. Overall, the field of nanovaccines and cancer has shown an explosive growth trend after 2015 (**Figure 2A**). This may be due to the development of genomics and the attention given to tumor neoantigens (44), which increases the accuracy of tumor treatment. Since 2015, the Chinese Academy of Sciences has led in research on nanovaccine for cancer. In 2023, the number of articles from Sun Yat-sen University and Sichuan University increased rapidly (**Figure 2B**). Every year, the authors who publish the most articles are different and scattered, reflecting that this field is being observed by researchers from different fields (**Figure 2C**).

The top three journals with the largest number of publications are Biomaterials, Journal of Controlled Release, and ACS Nano (**Table 1**). Furthermore, the top 10 journals are mainly categorized into materials and immunology. The top ten journals are all JCR Q1 journals. To some extent, it proves that research on nanovaccines and cancer has received attention from high-impact journals.

TLCS analysis (**Table 2**) shows that China and the USA have the highest TLCS scores. The highly cited research institutions are concentrated in China, among which Soochow University ranks on the top, reflecting that the region’s research on the topic is well recognized. Suzhou’s leading position in China’s nanomaterial industry chain is the reason for its rapid development. **Figure 3** shows the cooperation between countries/regions, institutions and authors. **Figure 3A** shows that China and the United States have close cooperative relationship, which created a conducive academic environment. Institutional collaboration (**Figure 3B**) shows frequent interactions between the Chinese Academy of Sciences and the University of Chinese Academy of Sciences, as well as multiple collaborations among Nankai University, the Chinese Academy of Medical Sciences, and Peking Union Medical College. The frequent cooperation of various institutions in China, combined with the close geographical location, provides a convenient, interactive and broad research platform for the research of tumor nanovaccines. Additionally, almost all the listed institutions have interdisciplinary research institutes in medicine and other fields such as materials chemistry or biomedical engineering. This combination provides a platform for the rapid development of nanovaccines in the field of oncology. The Chinese Academy of Sciences has the National Center for Nanoscience and Technology, which is a key factor in the strong development of nanovaccines. The close collaboration between Chinese Academy of Sciences and various major clinical institutions reflects the significant emphasis on clinical research for tumor nanovaccines. It is likely that in the near future, tumor nanovaccines will be widely applied in clinical research. Therefore, in the future, interdisciplinary collaborations should be fostered to integrate insights from nanotechnology, immunology, and oncology. Additionally, several national policies have been introduced to encourage the development of the nanomaterial industry, which is also a significant reason for the substantial development of nanomaterials in China. **Figure 3C** reveals frequent collaborations among professors Yongming Chen, Lixin Liu, Hong Liu, Haolin Chen, Guangsheng Du, and Xun Sun. The authors interact closely, creating a more focused research platform.

**Table 3** shows the articles of the top 10 highest 180 days usage. The most used article in the past 180 days was a 2023 review by Rui Liu and colleagues in Chinese Chemical Letters. The article summarized recent advances in nanoparticles, illustrating their components, strategies, and functions. Besides, Rui Liu and his colleagues provided critical illustrations about nanoparticles-based delivery of these specific therapeutic agents, including protein, nucleic acid, gas, and metals-involved artificial nanoenzyme (26). The second most accessed article was published by Jun Xu et al. in 2020 in Nature Nanotechnology, which demonstrates a general strategy to fabricate personalized nanovaccines by mixing the fluoropolymer with a model antigen ovalbumin. This nanovaccine inhibits established ovalbumin-expressing B16-OVA melanoma. Moreover, a mix of the fluoropolymer with cell membranes from resected autologous primary tumors can synergize with checkpoint blockade therapy to inhibit post-surgical tumor recurrence and metastases in two subcutaneous tumor models and an orthotopic breast tumor (27). The third article, by Fangmin Chen et al. in 2023 in Advanced Materials, discussed an acid-ionizable iron nanoadjuvant developed to enhance antigen cross-presentation in CD169+ APCs with robust STING cascade activation for personalized cancer vaccination immunotherapy (28). The remaining articles primarily focused on the activation of cancer cytotoxicity, and the composition, performance, and mechanisms of various immune-regulating nanoplatforms.

**Table 4** refers to the number of citations within the nanovaccine and cancer area. The article with the highest LCS published by Min Luo et al. in Nature Nanotechnology in 2017, discovered a synthetic nanoparticle named PC7A that enhances antigen delivery and cross-presentation, stimulating the STING pathway to boost antitumor immunity for cancer immunotherapy (37). The second highest LCS article by Guizhi Zhu et al. in 2017 in ACS Nano addressed the challenge of efficiently delivering nanovaccines in cancer immunotherapy. They also discussed peptide antigens in four parts, including passengers, vehicle, teaming up passengers, and the passport to cells. The article indicates that nanovaccines can efficiently co-deliver adjuvants and multiepitope antigens into lymphoid organs and APCs. The intracellular release of vaccine and antigens cross-presentation can be finely-tuned via nanovaccine engineering. Additionally, mRNA shows its unique potential (1). The third-ranked article by Rong Yang et al. in 2018 in ACS Nano observed that cancer cell membrane-coated adjuvant nanoparticles with mannose modification facilitate APC binding and uptake, enhancing antitumor immune responses (38). In the eighth article, Shao et al. reviewed the current status of nanoparticle-based cancer immunotherapy strategies, providing a comprehensive knowledge base for subsequent research (42). The other six articles studied different kinds of nanovaccines, including fluoropolymer-based nanovaccines (27), erythrocyte membrane-enveloped polymeric nanovaccines (39), proton-driven transformable nanovaccines (40), tumor cell lysate-loaded chitosan nanovaccines (41), mannosylated nanovaccines (43) and DNA nanodevice-based vaccines (29).

**Figure 4A** shows that the citation center is an article by Guizhi Zhu et al. in ACS Nano in 2017, representing authority and pioneering research in the field. The cited literature center is a review by Fangmin Chen et al. in Biomaterials in 2021, which cites a large number of highly cited articles. This article may be a good choice for a comprehensive understanding of this field. **Figure 4B** shows references with the strongest citation bursts according to the strength scores. The publication by Jun Xu et al. in Nature Nanotechnology in 2020 received the highest strength score and experienced the strongest citation burst in 2022-2023, indicating a period of significant interest. Jun Xu et al.’s study garnered considerable attention during this period for two primary reasons: it demonstrated the high efficacy of anti-tumor immune responses using tumor nanovaccines constructed from halothane-grafted cationic polymer, thereby providing a novel approach to vaccine design; furthermore, their research has potential applications in other vaccine types, including COVID-19 (26). In the review by Mansi Saxena et al., they provided a profound analysis of vaccine antigen pool and platform development, summarized past failures of tumor vaccines, and proposed relevant strategies. This comprehensive review, which highlights current obstacles and difficulties in vaccine design, has received significant attention (4). **Figure 4C** presents a co-citation analysis. References can be categorized into four different research directions: blue for the immunotherapeutic effects of nanovaccines, red for the transport and delivery of nanovaccines, green for the applications of nanovaccines, and yellow for the design of nanovaccines. Before 2014, research primarily focused on vaccine delivery and cancer immunotherapy (45, 46). Nanoparticle vaccines began to show promise in lymphatic transport and complement activation (47). Since then, research has increasingly focused on tumor neoantigens, personalized therapy, and STING pathway activation (37, 48, 49). The design of nanovaccines has also become a key area of interest (4, 50), including synthetic nanoparticle vaccines (51), membrane-coated nanovaccines (38, 52), and nanodiscs (49). Furthermore, triple-negative breast cancer and melanoma have emerged as focal points in cancer research within this field (4, 48, 53). Therefore, **Figure 4C** allows a better understanding of the subject’s development and the direction of further attention in terms of citations, journals and authors of interest.

**Figure 5** presents the co-occurrence analysis of keyword frequencies, reflecting the research content, hotspots, and frontier directions within a specific field, and helping us classify the keywords. Cluster analysis is helpful for a more comprehensive understanding of a particular topic. Time analysis illustrates the evolving trends of research topics in the field of time. **Figure 6** presents the cluster analysis of keywords. **Figure 7** illustrates the evolving trends of research topics in the field over time. For instance, **Figure 7D** shows that before 2014, “immunization”, “drug delivery”, “biomedical applications” had shown citation burst. Subsequently,”*in vivo*”, “antigen delivery”, “immune responses”, and “lymph node” received the highest strength score and experienced the strongest citation burst. **Figure 7E** shows that prior to 2020, the field had fewer keywords of interest, primarily focusing on the immune processes of nanoparticles. After 2020, there has been widespread attention on topics like “nanovaccines” and “nanoparticles”. Besides, the variety of keywords has increased, progressing from a focus on the immune processes of nanoparticles to areas such as “adjuvants”, “the STING pathway”, “personalized immunotherapy”, “biomimetic materials”, and “photothermal therapy”. This indicates that globally, research on tumor nanovaccines has gained extensive attention and has delved into more detailed and profound aspects.

### 4.2 Analysis of research hotspots and frontier

Keyword analysis is instrumental in understanding the frontier of the field. **Figure 5B** categorizes keywords related to the content, hotspots, and frontier directions in nanovaccine research for cancer. Cluster yellow mainly covers the composition of tumor vaccines, including “nanoparticles”, “nanocarriers”, “biomaterials”, “liposomes”, and “nanotechnology”. Cluster green focuses on tumor nanovaccine adjuvants, including “adjuvants”, “nanoparticles”, “dendritic cells”, and “immune response”. Cluster red primarily highlights the role of nanovaccines in tumor immunity, including “cancer immunotherapy”, “nanovaccine”, “antigen delivery”, and “antigen presentation”. Cluster blue mainly shows the therapeutic effects of nanovaccines in the tumor field, including “immunotherapy”, “cancer vaccine”, “personalized immunotherapy”, and “melanoma”. **Figures 6A, C**, through cluster analysis, roughly divide the keywords into four major directions: adjuvants, antigens, tumor microenvironment, and diseases in which nanovaccines are applied. Therefore, based on keyword clustering and our analysis of the articles, we divided the discussion section into four parts: carriers, adjuvants, antigens, and therapeutic applications and prospects of nanovaccines. This division aims to analyze the development and hotspots of nanovaccines based on bibliometric analysis, offering more comprehensive insights.

Generally, in the tumor immune process, nanovaccines load tumor-specific antigens (L) through a nano-delivery system, initiate the tumor antigen in lymph nodes (D), are internalized by dendritic cells (DCs) (I), stimulate DC maturation (M), and present peptide-MHC class I complexes to CD8+ T lymphocytes (P), a process referred to as the LDIMP cascade (54). Obstacles faced by nanovaccine treatment include immune system clearance of the nanovaccine, insufficient intensity of tumor-specific immune reactions, insufficient targeting of vaccine delivery, insufficient stability of nanovaccine formulations, and difficulties in large-scale production (31). Current research mainly focuses on designing and preparing nanovaccine schemes. This involves exploring suitable and effective antigens, using carriers that can target and deliver antigens, and loading adjuvants to enhance immune response intensity. Researchers also aim to improve the tumor treatment efficacy of nanovaccines by combining them with other immune therapies (55).

#### 4.2.1 Carriers

As shown in **Figure 5B**, research on nanovaccines in the yellow cluster primarily focuses on “nanoparticles” and “nanocarriers.” Currently, carriers for tumor nanovaccines include a range of materials such as polymeric nanomaterials, lipid-based nanoparticles, biomimetic nanomaterials, endogenous nanocarriers, and other special carriers. The primary research focus on nanovaccine carrier centers on the transportation and delivery of the nanovaccine.

##### 4.2.1.1 Polymeric nanomaterials

Polymers are often used as nanocarriers due to their excellent stability, high biocompatibility, and capability to provide targeting ability and stimulus responsiveness (31, 34). These are often combined with biomimetic technology. For instance, polymeric materials can be wrapped in tumor cell membranes to prepare nanovaccines (52, 55). An example is Fang et al.’s cancer cell membrane-coated nanoparticles (CCNPs), created by coating mouse melanoma cell membranes with poly lactic-co-glycolic acid (PLGA) nanoparticle cores. These CCNPs replicate the diversity of surface antigens from the source cells, promoting the uptake of membrane-bound tumor antigens and activating downstream immunity. This membrane-coating technology can be extended to other cell types (52). In a 2023 study, a self-amplified biomimetic nanosystem, mEHGZ, was constructed by encapsulation of epirubicin (EPI), glucose oxidase (Gox) and hemin in ZIF-8 nanoparticles and coating them with calreticulin (CRT) over-expressed tumor cell membrane. This triggers a cascade reaction for ROS generation to amplify the immunogenic cell death (ICD) effect and boost the sensitivity of tumor cells to the treatment with anti-PD-L1 antibody (30).

Dendrimers, other widely used polymeric materials in nanotechnology, offer a higher structural flexibility and a higher density of functional groups compared to linear molecules. Drug molecules can be flexibly bound to dendrimers either covalently or non-covalently on their exterior or core (56). Their controllable size and uniformly symmetrical structure allow for controlled biodistribution and targeting in cancer therapy applications, providing a significant advantage (57). Shen et al. reported an advanced nanosystem integrated with phenylboronic acid-functionalized poly(amidoamine) dendrimers of generation 5, copper sulfide nanoparticles, and cyclic GMP-AMP, an immune adjuvant to act as a photothermal triggered nanovaccine (33). This nanosystem can be adopted for photothermal therapy of primary melanoma tumors, absorbing the whole tumor cell antigens and creating photothermal-triggered dendrimeric nanovaccine *in situ* to induce antitumor immune responses, inhibiting the distal tumors.

Polymeric applications extend beyond this. Certain types of polymeric nanoparticles are responsive to stimuli, such as PC7A, which responds to specific pH levels to ensure the on-demand release of loaded vaccine components (37). Notably, a nanovaccine based on fluoropolymers, in combination with the mixture of membranes from excised autologous primary tumor cells and checkpoint blockade therapy, can effectively inhibit postoperative tumor recurrence and metastasis (27). Additionally, nanotransformers, formed by the conjugation of polymers and peptides, can adapt and deliver effectively in the slightly acidic environment of endosomes/lysosomes, demonstrating strong environmental adaptability and delivery capabilities (40). For polymer carriers such as chitosan nanoparticles and PLGA/polylactic acid (PLA), surface modification with mannose can help target tumor antigen-specific dendritic cells, enhancing their effective presentation (38, 41, 43).

##### 4.2.1.2 Lipid-based nanoparticles

Lipid nanoparticles have gained increasing attention as prospective nanocarriers for delivering various therapeutics, including small molecules, peptides and nucleic acids (26).Lipid-based nanoparticles provide a feasible method for improving the nanomaterial stability and large-scale production of cancer nanovaccines (58). Lena M. Kranz and colleagues utilized widely used cationic liposomes composed of DOTMA and DOPE lipids and RNA to prepare colloidally stable RNA-lipid complex (RNA-LPX) nanoparticles. These nanoparticles protect RNA from extracellular ribonucleases and mediate effective uptake and expression of encoded antigens by DCs and various lymphoid zone macrophages. By optimizing the overall particle charge, these nanoparticles can be precisely targeted to DCs *in vivo* (59). Researchers have also developed lipid-polymer hybrid nanoparticles (LPNPs), which are effective vesicles composed of single or multiple layers of lipid shells that encapsulate polymer core. LPNPs combine the advantages of lipid and polymer nanoparticles while alleviating their inherent limitations, exhibiting high stability and biocompatibility, making them an effective vaccine delivery platform (60).

##### 4.2.1.3 Biomimetic nanomaterials

Biomimetic high-density lipoprotein (sHDL) has been shown to be safe in clinical trials (61), with a maximum tolerated dose higher than that of most polymer or inorganic nanoparticles. Rui Kuai et al. synthesized sHDL nanodiscs composed of phospholipids and apolipoprotein A1-mimetic peptides, combined with adjuvants and antigen peptides. These synthesized nanodiscs are non-homologous with endogenous ApoA1, avoiding the potential triggering of self-immunity and enhancing their intracellular transport (49).

The red blood cell (RBC) membrane, due to its long circulation time, high membrane flexibility and stability, good biocompatibility, ability to retain basic cellular functions, and potential for functional group modification on the membrane surface (62), exhibits unique advantages as a biomimetic material carrier. Nanoparticles delivered by RBCs to the spleen can enhance antibody response to antigens, increase the central memory T cell responses, and reduce the regulatory T cell responses (63). Recently, Jing Zhao et al. utilized an immune strategy involving senescent RBCs (63) to temporarily saturate liver macrophages, achieving accumulation of the nanovaccine in the spleen and solid tumors (64), potentially regulating the tumor immune microenvironment. Although RBC membrane-coated nanovaccines have not yet been widely introduced into clinical research, their efficacy and safety have been verified in animal models (39, 63–66). The unique biological properties of these biomimetic materials demonstrate significant potential in tumor immunotherapy.

Due to their inherent immune properties, immune cells can also serve as biomimetic carriers for nanovaccines. The safety of these immune cell-based carriers has been validated in animal models, and their capabilities in drug delivery, biological detoxification, and immune regulation have been widely utilized (67). One of the obstacles that tumor nanovaccines need to overcome is effectively eliciting robust antigen cross-presentation and inducing a strong T-cell immune response (42). DCs, which can present specific antigen epitopes through MHC-I molecules and co-deliver anti-PD-1 and B7 co-stimulatory molecules, have become ideal nanovaccine carriers (32). Similarly, macrophages possess efficient lymphatic transport and the ability to deliver antigens in their natural form. When loaded with antigens, macrophages can co-deliver antigens and adjuvants, significantly enhancing the stimulation of immune cells (68). However, current cancer vaccines utilizing DCs and macrophages primarily use T-cell epitopes to mediate T-cell immune responses, providing limited stimulation to B-cell immunity. Therefore, their long-term anti-cancer efficacy may be suboptimal.

##### 4.2.1.4 Endogenous carriers

Endogenous carriers offer unique advantages in nanovaccine design, such as high biocompatibility, good safety, and ease of manufacture (69). The albumin/AlbiVax nanocomposite, which self-assembles *in vivo* from AlbiVax and endogenous albumin, can also be used for effective vaccine delivery, inducing the expansion of CD8+CTL and T cell memory. When used in combination with other immunotherapies, it can significantly suppress tumor progression in various tumor models, increasing the possibility of clinical translation (70).

##### 4.2.1.5 Special carriers

DNA-based nanodevices represent an innovative design, often prepared using bacteriophages. Shaoli Liu et al. used M13 bacteriophage for DNA sequence modification, leading to the development of a DNA-based nanodevice. This device addresses the issue of extracellular ribonuclease attacks on antigen payloads and enables effective lymph node drainage. The precise arrangement of antigens (peptides) and Toll-like receptor (TLR) agonists (double-stranded RNA [dsRNA] and cytosine-phosphate-guanosine oligonucleotide [CpG DNA]) on the DNA nanodevice results in an effective and robust CTL response (29).

Adeno-associated virus (AAV) is a highly promising gene therapy vector that can mediate gene expression, gene silencing, and gene editing, and has achieved significant success in both preclinical and clinical applications (71, 72). Initially, poor transduction of APCs by AAV produced functionally impaired CD8+ cells, limiting its use in vaccine platforms (73). However, the advent of COVID-19 has greatly promoted its development. Through bioengineering of AAV vectors and manipulation of their carrying antigens, Karina Krotova et al. developed a strategy to load tumor antigens onto AAV vaccines to drive protective immunity in a mouse model of melanoma (74). In addition, AAV-based anti-tumor drugs have produced good efficacy with the assistance of nanoparticles. For example, iron oxide nanoparticles coupled with the AAV platform have demonstrated significant growth inhibitory effects on non-small cell lung cancer (75). By chemically modifying AAV to attach to ions, effective delivery to *in situ* liver cancer can be achieved, leading to drug accumulation and localized tumor treatment (76). Although it has good efficacy as a cancer vaccine and cancer treatment drug, and it has high transduction efficiency, suitable for non-dividing cells, and integrated stability (77), the AAV platform has not been widely used in the production of nanovaccines. If the AAV platform is to be used in nanovaccines, restricted cargo capacity and the potential risk of integration into the host genome (77) should be taken into account. Additionally, the effects of this nano-engineered virus on the tumor microenvironment require further elucidation. Despite these limitations, its superiority as a vector has been well demonstrated.

In summary, we present five different types of nanovaccine carriers. The current design of these vectors focuses on generating a strong and effective immune response while ensuring safety. These different perspectives provide research directions for the future design of nanovaccine vectors.

#### 4.2.2 Adjuvants

As shown in **Figure 5B**, the green cluster focuses on tumor nanovaccine adjuvants, including “adjuvants”, “nanoparticles”, “dendritic cells”, and “immune response”, indicating the research trend on the adjuvant part. It is well known that individual antigens typically exhibit weak immunogenicity. Nanotechnology enables the co-encapsulation of multiple components in the same preparation, allowing nanovaccines to deliver both antigens and adjuvants to the corresponding APCs. The use of adjuvants with antigens can significantly induce powerful innate and adaptive immune responses, enhance antigen-specific immune reactions, and even reduce tolerance of APCs (78).

Common adjuvants in nanovaccines can be broadly classified into two categories: inorganic and organic. Numerous studies have shown that metal ions (Ca2+, Fe2+/3+, Zn2+, Mn2+) can play crucial roles in immunoregulation (79). Aluminum adjuvants have been extensively studied and commonly used in FDA-approved vaccines. Aluminum hydroxide induces immune activation by causing inflammatory cell death at the injection site and producing inflammatory cytokines in the draining lymph nodes (78, 80). Additionally, its low cost, high safety, ease of availability, and flexibility as a modification platform have led to its widespread use. However, its cellular immune response is limited, making it a relatively weak adjuvant in terms of immunogenicity. Therefore, numerous attempts have been made to enhance its efficacy. For instance, chemically conjugating alum with CpG can promote the activation of bone marrow-derived dendritic cells and the secretion of Th1 and Th2 cytokines (81). Furthermore, incorporating active compounds into nanoscale salt precipitates for modifying aluminum adjuvants has also emerged as a method. Building on the base of aluminum salts, the safety and antigen adsorption capacity can be further improved (82). Zinc ions (Zn2+) play a significant role in promoting antigen cross-presentation by DCs and inducing T-cell activation, enhancing T-cell responses through the induction of ICD (64, 83, 84). Therefore, zinc-aluminum composite adjuvants also show advantages. Although the complete mechanisms of action of aluminum adjuvants are not yet fully understood, their safety and strong operability suggest that their potential might be far greater than we currently realize. A 2023 study first discovered that iron oxide nanoparticles (IONPs) markedly augment the type-I interferon (IFN-I) production profile of the stimulator of interferon genes (STING) agonist MSA-2 and achieve a 16-fold dosage-sparing effect in the human STING haplotype (28). Acid-ionizable copolymers are coassembled with IONPs and MSA-2 into iron nanoadjuvants to concentrate STING activation in the draining lymph nodes.

Organic adjuvants include Monophosphoryl lipid A (MPLA), CpG, cyclic diguanylate monophosphate (cdGMP), and others. MPLA(TLR4a), imiquimod (R837, TLR7a), CpG (TLR9a), imiquimod (IMQ, TLR7/8a) can recognize and activate Toll-like receptors on immune cells, thereby triggering adaptive immune responses (49, 52, 55, 60, 70, 85). In recent years, STING pathway research has gained popularity, becoming a target for various immunotherapies. For instance, cdGMP can activate the STING pathway, leading to significant type I interferon (IFN- I) production (86). Consequently, many STING activators are used as immune adjuvants in designing nanovaccines for cancer immunotherapy.

Recent studies have found that nanocarriers themselves can also provide adjuvant effects. For example, the proton-driven nanotransformer-based vaccine mentioned in the polymer section also serves as an adjuvant while acting as a carrier. It enhances innate immunity by activating the NLRP3 inflammasome pathway (40). Also, a pH-sensitive polymer, PC7A, used as a vaccine carrier, can activate the IFN-I gene pathway through the STING pathway (37). Jiayu Zhao et al. found that pentazole-terminated PEI (PEI-M) can effectively stimulate DCs to secrete IFN-γ through the STING pathway (35). Alum, the only inorganic adjuvant approved by the US Food and Drug Administration, is widely used in various nanoparticles and nanovaccines as adjuvants, as well as carriers. Metal ions are often added to alum to enhance its cellular immune response for metal immunotherapy (64). Although effective in many vaccines, alum-induced anti-tumor immune responses are still suboptimal compared to other adjuvants in clinical studies (87), and its molecular structure is not conducive to modulation. Recently, Mayer et al. developed a novel porous hydrogel that can increase infiltration and reduce host foreign body response, showing a stronger immune response and longer duration (88). Self-adjuvanting nanovaccines can be developed without the need for additional adjuvants or with the minimal dosage of additional adjuvants to improve the efficiency of immune responses and simplify the preparation of nanovaccines (89).

In summary, loading adjuvants into tumor nanovaccines can help to break the immune-suppressive microenvironment and enhance the intensity of immune responses. Related research has important implications for designing more effective nanovaccines.

#### 4.2.3 Antigens

“Antigen” is the keyword showing as “antigens”, “tumor antigens”, “neantigens”, “peptide antigen”, “antigen presentation” and “antigen delivery” in different color parts in **Figure 5B**, and “coordinating antigen”, “antigen-specific” in **Figure 6A**. Common antigens include tumor cell lysate, dendritic cells, nucleic acid (such as mRNA) and neoantigens (90). With the advent of feasible second-generation sequencing, the previous selection of antigens has also been evolved. Neoantigens, which are completely absent from the human normal genome, can be simply referred to as tumor specific mutations. These neoantigens are widely applied in tumor immunotherapy for their specificity, making targeted treatments of tumors more precise (1). However, the complexity of tumor growth often distorts the body’s immune system and suppresses the specific immune response to neoantigens, presenting a major challenge.

Single-stranded RNA has inherent adjuvant function stimulated by TLR7 and TLR8, and its relative ease in encoding multiple vaccine epitopes on the same RNA molecule indicates its advantages as a cancer vaccine formulation (8). Moreover, RNA vaccine production is fast, cost-effective, and can encode almost all tumor antigens (59), which, combined with its low toxicity, makes it an attractive option for tumor vaccines. The combination of nanotechnology and mRNA vaccines offers unique advantages, such as preventing mRNA from being degraded by RNases and enhancing its uptake by APCs, thereby accelerating the transformation process of RNA vaccines within the human body (1). In recent years, an individualized neoantigen vaccine based on uridine mRNA–lipoplex nanoparticles underwent a phase I trial in the treatment of pancreatic ductal adenocarcinoma, showing substantial T cell activity that may delay its recurrence (14). The advent of the COVID-19 mRNA vaccine demonstrates the overcoming of many challenges, indicating that nanotechnology-based mRNA vaccines are advancing toward various diseases, including cancer.

#### 4.2.4 Application and future prospect of nanovaccines

In recent years, the most studied cancers in the field of nanovaccines are melanoma, hepatocellular carcinoma, and triple-negative breast cancer, as indicated by **Figures 5B** and **6A**. With the molecular mechanisms of melanoma and its profound interaction with the immune system being uncovered, there has been a growing focus on immunotherapy. At present, research on nanovaccines in melanoma still faces identification difficulties due to the reasons such as the immune suppression mediated by regulatory T cells, defects in antigen presentation, and immune suppression in the medium (91). In addition, nanovaccines combined with other immunotherapies, such as immunomodulatory CPB, have demonstrated significant control of tumor growth in mouse models (49).

Auxiliary nanovaccine technology has also become a hot topic. Cluster blue in **Figure 5B** collects terms like “photothermal therapy” and “photodynamic therapy,” highlighting their research trend, especially in the field of triple-negative breast cancer. Photothermal therapy uses photoactivatable agents at specific wavelengths of light to kill tumor cells. For example, combining TLR-7 agonists with photoactivatable agents such as indocyanine green to attack tumor cells in mice can significantly inhibit tumor cell migration in mice and provide strong immune memory to prevent cancer recurrence, applicable to various tumor cells (55). Additionally, photodynamic therapy, which produces reactive oxygen species and induces irreversible damage to tumor cells and microvasculature, is another form of phototherapy based on nanoparticles. This therapy kills tumors through direct killing and immunogenic cell death mechanisms (92).

In primary hepatocellular carcinoma, the concept of solid tumors is often emphasized due to high selectivity in permeability and retention of lipid particles and large molecules. This selectivity is related to the blood supply and physical properties of the solid tumor itself and the tumor microenvironment, including surrounding prostaglandins, nitric oxide, peptides, vascular endothelial growth factors, and other factors and their interactions (93, 94). In clinical applications, a large part of nanomedicine will be non-specifically absorbed by cells during the blood circulation and metabolized by organs such as the liver and kidney (92). Therefore, optimizing the physical properties of nanomaterials, such as size, shape, and pH value, and their interactions with the microenvironment is crucial to improving delivery effectiveness and treatment efficiency.

So far, many cancer vaccines have been applied in the clinic, such as the therapeutic cancer vaccine Sipuleucel-T (Provenge^®^) (95). Over a decade ago, nanotechnology-based cancer drugs entered clinical trials, such as the albumin-bound paclitaxel nanoparticle used to treat metastatic breast cancer, which showed better efficacy and safety than solvent-formulated paclitaxel (96). This confirms the good targeting effect of nanoparticles in tumor therapy and its good clinical translation ability. In recent years, nano-sized products have gradually entered the public’s vision, such as gadolinium-chelated polysiloxane nanoparticles (AGuIX^®^) for cancer radiotherapy. Nowadays, advances in genomics make it possible to understand individual cancer mutations, which can be used to identify multiple antigenic epitopes at a personalized level. The high mutation characteristics of tumors also make new antigen targets possible (1, 49). Therefore, nanotechnology-based tumor vaccines are gradually attracting attention. Since 2022, personalized treatment has become popular (**Figures 5C**, **7E**). However, the complex formation environment of tumors and their heterogeneity can be a double-edged sword. Designing a nanoparticle-based vaccine that combines the characteristics of nanoparticles and tumor vaccines to achieve personalized and precise treatment has become the current and future trend. To achieve personalization, predecessors have tried to improve the tumor immunogenicity, such as using polyethyleneimine (PEI) to enhance the immunogenicity of antigens in mesoporous silica rods (MSR) vaccines (97), or using neoantigens in combination with other immune interventions. In addition, the tumor immune microenvironment is dynamic and changes over the course of treatment, necessitating the development of personalized, stage-specific intervention techniques based on different immune treatment stages.

Many clinical trial data (**Table 5**) have shown that nanotechnology-based therapeutic cancer drugs or regimens have achieved some success. However, most nanotechnology-based drugs are still in clinical trials in combination with other therapies or drugs. Therefore, although the clinical translation of nanovaccines has become more accessible, challenges remain. These include optimizing their safety and stability, determining the most suitable clinical environment, shortening production turnaround time, scaling up production, and ensuring affordability and availability (98). In this case, future trend will be focusing on conducting longitudinal studies to evaluate the long-term outcomes and potential side effects of nanovaccines in clinical settings.

**Table 5:** Recent clinical trials of cancer vaccines or drugs with nanotechnology.

Cancer	ClinicalTrials.gov ID	Category	Intervention/Treatment	Target	Clinical phase	Start
Melanoma	NCT04079166	DNA plasmid	Biological: SCIB1 DNA vaccine	Patients with advanced melanoma	Phase II	2019-08
	NCT05264974	RNA	Biological: Autologous total tumor mRNA loaded DOTAP liposome vaccine	Patients with Early Melanoma Recurrence Following Adjuvant Anti-PD-1 Antibody Therapy	Phase I	2024-04
Breast Cancer	NCT06218303	peptide	Biological: MUC1 Peptide Vaccine Drug: Hiltonol^®^ Drug: Aromatase Inhibitor	Post-menopausal women with biopsy-proven DCIS	phase I	2024-02
	NCT06048367	Carbon Iron/Carbon Nanoparticle-Loaded Iron [CNSI-Fe(II)]	Drug: CNSI-Fe(II) 30 mg Drug: CNSI-Fe(II) 60 mg Drug: CNSI-Fe(II) 90 mg	Patients with Advanced Solid Tumor	Phase I	2022-10
Hepatocellular Carcinoma	NCT06309485	Antisense nucleic acid AKT-1 inhibitor	Drug: WGI-0301 at MTD/RP2D dose IV infusion, QW Drug: WGI-0301 at MTD/RP2D -1 dose IV infusion, QW Drug: Sorafenib 400 mg PO, BID continuously Drug: Sorafenib 400 mg PO, BID	Patients With advanced Hepatocellular Carcinoma	Phase II	2024-04
	NCT05497453	mRNA, lipid	Drug: OTX-2002 Drug: Tyrosine kinase inhibitor One Drug: Tyrosine kinase inhibitor Two Drug: Checkpoint Inhibitor, Immune	Patients With Hepatocellular Carcinoma and Other Solid Tumor Types Known for Association With the MYC Oncogene	phase I/II	2022-08
	NCT02716012	dsRNA, liposomal nanoparticle	Drug: MTL-CEBPA Drug: Sorafenib 200mg	Patients With Advanced Liver Cancer	phase I	2016-03
Brain Metastases	NCT03818386	AGuIX Gadolinium	Drug: AGuIX^®^ Radiation: Whole Brain Radiation Therapy	Patients With Multiple Brain Metastases	Phase II	2019-03
Lung cancer	NCT03970746	Peptide	Biological: PDC*lung01 Drug: Keytruda Injectable Product Drug: Alimta Injectable Product	Patients with Non-Small-Cell Lung Cancer	phase I/II	2019-09
Pancreatic cancer	NCT05013216	Peptide	Drug: KRAS peptide vaccine	Patients at High Risk of Developing Pancreatic Cancer	phase I	2022-04
	NCT04161755	mRNA/lipoplex	Drug: Atezolizumab Biological: RO7198457 Drug: mFOLFIRINOX	Patients With Surgically Resected Pancreatic Cancer	phase I	2019-12
	NCT03153410	GVAX/a GM-CSF gene-transduced tumor vaccine	Drug: Cyclophosphamide Drug: GVAX Drug: Pembrolizumab Drug: IMC-CS4	patients with borderline resectable pancreatic cancer	Early Phase I	2018-09
Prostate Cancer	NCT06343077	Viral mimic poly-ICLC/peptide	Drug: Poly-ICLC intramuscular (IM) injection Drug: Poly-ICLC, Intertumoral (IT) injection	Patients with Prostate Cancer	Phase II	2024-01
	NCT05544227	SV-102	Drug: SV-102	Patients with Advanced/​Metastatic Castration-Resistant Prostate Cancer	phase I	2023-02

### 4.3 Strength and limitation

In this study, we provide the first systematic, objective and accurate analysis of nanovaccine and cancer, helping readers to easily access the status and trends of research in this field.

Inevitably, there are some limitations. The literature included in our study may not be exhaustive as we only examined data from the Web of Science SCI-E database, and included only English papers. Besides, new papers published after the search date were not included in the study. Therefore, the articles included may not adequately reflect all the researches on nanovaccine and cancer.

## 5 Conclusion

Since 2014, there has been an explosive growth in research articles in the fields of nanovaccines and cancer. By mapping the current landscape of nanovaccine research, our study indicates that nanovaccine acts as a promising approach in cancer immunotherapy. Besides, it offers potential advantages in terms of targeted delivery, enhanced immunogenicity, and reduced side effects compared to traditional vaccines. With the continuous development and improvement of genomics, research in the field is moving towards individualization and personalized treatments, and immunotherapy for cancer will enter a new stage. More and more treatment methods are also being implemented clinically, and the role of nanovaccines in various aspects of tumor immunity will continue to be optimized. Interdisciplinary collaborative research efforts have played a crucial role in advancing nanovaccine technologies, as evidenced by the extensive co-authorship networks and joint publications. Future research may need to continue to focus on themes such as “personalized,” “robust antigen-specific T cell activation,” “tumor environment,” “dendritic cells,” “nanomaterials,” and “immunogenic cell death.”

## Author contributions

YH: Writing – original draft, Writing – review & editing. YL: Writing – original draft, Writing – review & editing. YZ: Writing – original draft, Writing – review & editing. JZ: Supervision, Writing – review & editing. DW: Supervision, Writing – review & editing.

## Supplementary material

Supplementary Data Sheet 1 Literature data source file (1).

Supplementary Data Sheet 2 Literature data source file (2).
