# Supplementary material for: Current status and future directions of nanovaccine for cancer: a bibliometric analysis during 2004-2023
Source: Front Immunol. 2024 Jul 29;15:1423212. doi: 10.3389/fimmu.2024.1423212 (PMC11317272; doi:10.3389/fimmu.2024.1423212)
Supplement: Supplementary Data Sheet 2 — Literature data source file (2). [file DataSheet_2.pdf]

FN Clarivate Analytics Web of Science
VR 1.0
PT J
AU Saini, S
Dagar, P
Gupta, S
Kumar, Y
AF Saini, Samvedna
Dagar, Pranjal
Gupta, Sakshi
Kumar, Yatender
TI A nanotherapeutic approach for fighting the odds against the malignant disorders
SO JOURNAL OF NANOPARTICLE RESEARCH
LA English
DT Review
DE Inorganic nanomaterials; Immunotherapy; Nanoparticles; Nano-carriers; T-cell expansion; Nanomedicine; Comparative study
ID NANOSTRUCTURED LIPID CARRIERS; IRON-OXIDE NANOPARTICLES; DRUG-DELIVERY SYSTEM; T-CELL IMMUNOTHERAPY; MAGNETIC NANOPARTICLES; CANCER-IMMUNOTHERAPY; SURFACE MODIFICATION; GOLD NANOPARTICLES; BASEMENT-MEMBRANE; QUANTUM DOTS
AB Cancer, a combination of haematological and neoplastic malignancies, is a dreadful disease accounting for major fatalities worldwide. The domain of innovative nanoparticle-based technology has revolutionized the field of cancer therapeutics and imaging. With an emphasis on various nano-immunotherapeutic approaches, this study highlights the most recent developments in nano-immune engineering for metastatic tumours. Nanotechnology-based cancer immunotherapy has powered the (i) activation of T-cells in the tumour microenvironment (TME), (ii) preparation of efficient nanovaccines via nano-carriers and (iii) generation of smart nanomaterials which change their size/shape (size range of 1 to 1000 nm) and functionality upon activation in TME. The tumour microenvironment has an important, albeit contentious, role in controlling nanoparticle (NP) dispersion and subsequent biological consequences. The current study promotes the harnessing of potential peripheral immune cells by avoiding the creation of a pre-metastatic niche and, thus, suppressing tumour recurrence. This review descriptively accounts for a wide array of nanomaterials based on their polymeric constituents. Moreover, the current article explores the obstacles of integrating nanoscale immunomodulators and presents a forward-looking view of the novel nanotechnology-based approaches that may eventually prove helpful in eliminating metastatic illnesses.
C1 [Saini, Samvedna; Dagar, Pranjal; Gupta, Sakshi; Kumar, Yatender] Netaji Subhas Univ Technol NSUT, Dept Biol Sci & Engn BSE, New Delhi 110078, India.
C3 Netaji Subhas University of Technology
RP Kumar, Y (通讯作者)，Netaji Subhas Univ Technol NSUT, Dept Biol Sci & Engn BSE, New Delhi 110078, India.
EM yatender.kumar@nsut.ac.in
OI Kumar, Yatender/0000-0002-9835-9892
CR Ahmad F, 2021, CHEMOSPHERE, V262, DOI 10.1016/j.chemosphere.2020.128058
Ahmad F, 2019, ADV FUNCT MATER, V29, DOI 10.1002/adfm.201904268
Ahuja R, 2020, ENVIRON CHEM LETT, V18, P2021, DOI 10.1007/s10311-020-01056-z
Aikins ME, 2020, ACCOUNTS CHEM RES, V53, P2094, DOI 10.1021/acs.accounts.0c00456
Akbarzadeh A, 2013, NANOSCALE RES LETT, V8, DOI 10.1186/1556-276X-8-102
Anju S, 2020, MATER TODAY CHEM, V16, DOI 10.1016/j.mtchem.2019.100236
[Anonymous], 2021, NAT NANOTECHNOL, V16, P1, DOI 10.1038/s41565-020-00842-8
[Anonymous], CANCER
Apostolico JD, 2016, J IMMUNOL RES, V2016, DOI 10.1155/2016/1459394
Armulik A, 2011, DEV CELL, V21, P193, DOI 10.1016/j.devcel.2011.07.001
Avramovic N, 2020, PHARMACEUTICS, V12, DOI 10.3390/pharmaceutics12040298
Balkwill FR, 2012, J CELL SCI, V125, P5591, DOI 10.1242/jcs.116392
Baluk P, 2003, AM J PATHOL, V163, P1801, DOI 10.1016/S0002-9440(10)63540-7
Banday AH, 2015, IMMUNOPHARM IMMUNOT, V37, P1, DOI 10.3109/08923973.2014.971963
Bayda S, 2018, CURR MED CHEM, V25, P4269, DOI 10.2174/0929867325666171229141156
Bernhard H, 2002, ENDOCR-RELAT CANCER, V9, P33, DOI 10.1677/erc.0.0090033

Bignold LP, 2020, PRINCIPLES OF TUMORS: A TRANSLATIONAL APPROACH TO FOUNDATIONS, 2ND EDITION, P419, DOI 10.1016/B978-0-12-816920-9.00016-X
Blanco E, 2015, NAT BIOTECHNOL, V33, P941, DOI 10.1038/nbt.3330
Bouchkouj N, 2019, CLIN CANCER RES, V25, P1702, DOI 10.1158/1078-0432.CCR-18-2743
Cao LL, 2010, J ENVIRON SCI, V22, P454, DOI 10.1016/S1001-0742(09)60129-7
Cevaal PM, 2021, ACS NANO, V15, P3736, DOI 10.1021/acsnano.0c09514
Chang HI, 2012, INT J NANOMED, V7, P49, DOI 10.2147/IJN.S26766
Chauhan I, 2020, ADV PHARM BULL, V10, P150, DOI 10.34172/apb.2020.021
Chen FG, 2018, CERAM INT, V44, pS34, DOI 10.1016/j.ceramint.2018.08.276
Chen L, 2020, MAT SCI ENG C-MATER, V112, DOI 10.1016/j.msec.2020.110924
Choi Y, 2020, ACS NANO, V14, P12195, DOI 10.1021/acsnano.0c05902
Dai Q, 2018, ADV HEALTHC MATER, V7, DOI 10.1002/adhm.201700575
Dang Yu, 2020, Smart Mater Med, V1, P10, DOI 10.1016/j.smaim.2020.04.001
Devi S, 2022, FRONT MATER, V8, DOI 10.3389/fmats.2021.798440
Dhiman N, 2021, FRONT CHEM, V9, DOI 10.3389/fchem.2021.580118
Ding Q, 2015, J CONTROL RELEASE, V207, P86, DOI 10.1016/j.jconrel.2015.03.035
Dubensky TW, 2010, SEMIN IMMUNOL, V22, P155, DOI 10.1016/j.smim.2010.04.007
Dulinska-Litewka J, 2019, MATERIALS, V12, DOI 10.3390/ma12040617
Dunn ZS, 2019, BIOMATERIALS, V217, DOI 10.1016/j.biomaterials.2019.119265
ELKAREH AW, 1995, INT J RADIAT ONCOL, V32, P1419, DOI 10.1016/0360-3016(95)00110-K
Eskandari P, 2021, ADV COLLOID INTERFAC, V294, DOI 10.1016/j.cis.2021.102471
Evans ER, 2018, MATER TODAY, V21, P673, DOI 10.1016/j.mattod.2017.11.022
Faghfuri E, 2021, EXPERT OPIN BIOL TH, V21, P201, DOI 10.1080/14712598.2020.1815704
Ferrara F, 2013, LANCET, V381, P484, DOI 10.1016/S0140-6736(12)61727-9
Fioretti D, 2010, J BIOMED BIOTECHNOL, DOI 10.1155/2010/174378
Gagliardi A, 2021, FRONT PHARMACOL, V12, DOI 10.3389/fphar.2021.601626
Gagliardi M, 2017, THER DELIV, V8, P289, DOI 10.4155/tde-2017-0013
Garcia KP, 2014, SMALL, V10, P2516, DOI 10.1002/smll.201303540
Garcia-Pinel B, 2019, NANOMATERIALS-BASEL, V9, DOI 10.3390/nano9040638
Gill P, 2013, SCI IRAN, V20, P1003, DOI 10.1016/j.scient.2013.05.012
Gong NQ, 2021, NAT NANOTECHNOL, V16, P25, DOI 10.1038/s41565-020-00822-y
Guedan S, 2019, ANNU REV IMMUNOL, V37, P145, DOI 10.1146/annurev-immunol-042718-041407
Cisneros CG, 2021, POLYMERS-BASEL, V13, DOI 10.3390/polym13050760
Han X, 2018, BIOCONJUGATE CHEM, V29, P852, DOI 10.1021/acs.bioconjchem.7b00758
Hao MQ, 2020, MATER CHEM FRONT, V4, P2571, DOI 10.1039/d0qm00323a
Hesp NCH, 2021, NAT COMMUN, V12, DOI 10.1038/s41467-021-21862-5
Hess KL, 2019, NANO TODAY, V27, P73, DOI 10.1016/j.nantod.2019.04.005
Hu HG, 2020, FRONT CHEM, V8, DOI 10.3389/fchem.2020.00601
Hua S, 2013, FRONT PHARMACOL, V4, DOI 10.3389/fphar.2013.00143
Hussein-Al-Ali SH, 2021, INT J NANOMED, V16, P6205, DOI 10.2147/IJN.S312752
INOUE S, 1989, INT REV CYTOL, V117, P57
Irvine DJ, 2015, CHEM REV, V115, P11109, DOI 10.1021/acs.chemrev.5b00109
JAIN RK, 1987, CANCER RES, V47, P3039
June CH, 2018, SCIENCE, V359, P1361, DOI 10.1126/science.aar6711
Kalos M, 2011, SCI TRANSL MED, V3, DOI 10.1126/scitranslmed.3002842
Kanapathipillai M, 2014, ADV DRUG DELIVER REV, V79-80, P107, DOI 10.1016/j.addr.2014.05.005
Kandil R, 2019, ADV THER-GERMANY, V2, DOI 10.1002/adtp.201900047
Karlsson J, 2018, ANNU REV CHEM BIOMOL, V9, P105, DOI 10.1146/annurev-chembioeng-060817-084055
Kawai S, 2019, HELIYON, V5, DOI 10.1016/j.heliyon.2019.e02165
Kenchegowda M, 2022, MOLECULES, V27, DOI 10.3390/molecules27010146
Kennedy LC, 2011, NANOSCALE RES LETT, V6, DOI 10.1186/1556-276X-6-283
Keskin DB, 2019, NATURE, V565, P234, DOI 10.1038/s41586-018-0792-9
Kim SS, 2010, MOL THER, V18, P370, DOI 10.1038/mt.2009.271
Kitaoka M, 2016, BIOTECHNOL J, V11, P1375, DOI 10.1002/biot.201600081
Kosmides AK, 2017, ACS NANO, V11, P5417, DOI 10.1021/acsnano.6b08152
Kulkarni JA, 2018, NUCLEIC ACID THER, V28, P146, DOI 10.1089/nat.2018.0721
Lee Christina K., 2022, Nanotheranostics, V6, P243, DOI 10.7150/ntno.65544
Lee J, 2014, CANCER LETT, V347, P46, DOI 10.1016/j.canlet.2014.02.006
Li BB, 2017, FRONT PHARMACOL, V8, DOI 10.3389/fphar.2017.00051
Li N, 2011, VACCINE, V29, P6179, DOI 10.1016/j.vaccine.2011.06.086

Lim J, 2013, NANOSCALE RES LETT, V8, DOI 10.1186/1556-276X-8-381
Lobo GCNB, 2021, PHARMACEUTICS, V13, DOI 10.3390/pharmaceutics13081167
Luk BT, 2014, ACS APPL MATER INTER, V6, P21859, DOI 10.1021/am5036225
Mahmoud K, 2022, J NANOBIOTECHNOL, V20, DOI 10.1186/s12951-022-01309-9
McMahon HT, 2011, NAT REV MOL CELL BIO, V12, P517, DOI 10.1038/nrm3151
Mehanna MM, 2021, ADV PHARM BULL, V11, P56, DOI 10.34172/apb.2021.006
Mellman I, 2011, NATURE, V480, P480, DOI 10.1038/nature10673
Miao Lei, 2015, Cancer Treat Res, V166, P193, DOI 10.1007/978-3-319-16555-4_9
Le MQ, 2018, INT J PHARMACEUT, V550, P316, DOI 10.1016/j.ijpharm.2018.08.054
Montaseri H, 2021, PHARMACEUTICS, V13, DOI 10.3390/pharmaceutics13030296
Montomoli E, 2014, CURRENT ADJUVANTS NE, V10, P1053, DOI [10.1586/ERV.11.48, DOI 10.1586/ERV.11.48]
Mukherjee S, 2020, PHARMACEUTICS, V12, DOI 10.3390/pharmaceutics12020147
Naseri N, 2015, ADV PHARM BULL, V5, P305, DOI 10.15171/apb.2015.043
Navya PN, 2019, NANO CONVERG, V6, DOI 10.1186/s40580-019-0193-2
Nel AE, 2009, NAT MATER, V8, P543, DOI [10.1038/nmat2442, 10.1038/NMAT2442]
Ni QQ, 2020, SCI ADV, V6, DOI 10.1126/sciadv.aaw6071
Nicoletta FP, 2023, NANOMATERIALS-BASEL, V13, DOI 10.3390/nano13010207
Niikura K, 2013, ACS NANO, V7, P3926, DOI 10.1021/nn3057005
Nikmaneshi MR, 2020, SCI REP-UK, V10, DOI 10.1038/s41598-020-59658-0
O'Leary MC, 2019, CLIN CANCER RES, V25, P1142, DOI 10.1158/1078-0432.CCR-18-2035
Ou W, 2018, J CONTROL RELEASE, V281, P84, DOI 10.1016/j.jconrel.2018.05.018
Paul W, 2020, WOODH PUBL SER BIOM, P333, DOI 10.1016/B978-0-08-102680-9.00013-5
Peng M, 2019, MOL CANCER, V18, DOI 10.1186/s12943-019-1055-6
Perez-Herrero E, 2015, EUR J PHARM BIOPHARM, V93, P52, DOI 10.1016/j.ejpb.2015.03.018
Persano S, 2021, CANCERS, V13, DOI 10.3390/cancers13112735
Pettitt ME, 2013, ENVIRON INT, V52, P41, DOI 10.1016/j.envint.2012.11.009
Puri A, 2009, CRIT REV THER DRUG, V26, P523, DOI 10.1615/CritRevTherDrugCarrierSyst.v26.i6.10
Quail DF, 2013, NAT MED, V19, P1423, DOI 10.1038/nm.3394
Rahman M., 2019, SYNTHETIC BIOPOLYMER, V9, P1, DOI DOI 10.1007/978-3-319-95990-0_1
Ramishetti S, 2015, ACS NANO, V9, P6706, DOI 10.1021/acsnano.5b02796
Rasmussen MK, 2020, NAT COMMUN, V11, DOI 10.1038/s41467-020-15889-3
Rivera-Rodriguez Angelie, 2021, Nanotheranostics, V5, P431, DOI 10.7150/ntno.55165
Rossi L, 2016, ADV DRUG DELIVER REV, V106, P73, DOI 10.1016/j.addr.2016.05.008
Saini S, 2021, TRANSLATIONAL BIOTEC, P233, DOI [10.1016/B978-0-12-821972-0.00014-9, DOI 10.1016/B978-0-12-821972-0.00014-9]
Salatin S, 2015, CELL BIOL INT, V39, P881, DOI 10.1002/cbin.10459
Salunkhe AB, 2014, CURR TOP MED CHEM, V14, P572, DOI 10.2174/1568026614666140118203550
Samir A, 2015, ONCOL REP, V34, P1097, DOI 10.3892/or.2015.4100
Santos H.A., 2015, TARGETED DRUG DELIVE, P571
Schmid D, 2017, NAT COMMUN, V8, DOI 10.1038/s41467-017-01830-8
Schuster Manfred, 2006, Biotechnology Journal, V1, P138, DOI 10.1002/biot.200500044
Selby LI, 2017, WIRES NANOMED NANOBI, V9, DOI 10.1002/wnan.1452
Sercombe L, 2015, FRONT PHARMACOL, V6, DOI 10.3389/fphar.2015.00286
Shams F, 2022, MOL BIOL REP, V49, P1389, DOI 10.1007/s11033-021-06876-y
Shariatzadeh S, 2022, FRONT BIOENG BIOTECH, V10, DOI 10.3389/fbioe.2022.847433
Sharma A, 2018, J DRUG TARGET, V26, P617, DOI 10.1080/1061186X.2017.1400553
Sharma H, 2020, INT J MOL SCI, V21, DOI 10.3390/ijms21176280
Shervedani RK, 2018, BIOSENS BIOELECTRON, V117, P794, DOI 10.1016/j.bios.2018.07.011
Shrestha H, 2014, J PHARMACEUTICS, V2014, DOI 10.1155/2014/801820
Singh A, 2014, DRUG DISCOV TODAY, V19, P474, DOI 10.1016/j.drudis.2013.10.005
Singh M, 2021, MED BIOL ENG COMPUT, V59, P301, DOI 10.1007/s11517-020-02308-4
Singh R, 2022, NANO CONVERG, V9, DOI 10.1186/s40580-022-00313-x
Sithole MN, 2017, PHARM DEV TECHNOL, V22, P283, DOI 10.1080/10837450.2016.1212882
Smith DM, 2013, NAT REV IMMUNOL, V13, P592, DOI 10.1038/nri3488
Smith TT, 2017, NAT NANOTECHNOL, V12, P813, DOI [10.1038/NNANO.2017.57, 10.1038/nnano.2017.57]
Soenen SJ, 2011, NANO TODAY, V6, P446, DOI 10.1016/j.nantod.2011.08.001
Song GD, 2019, DENT MATER J, V38, P127, DOI 10.4012/dmj.2017-313
Sperling RA, 2010, PHILOS T R SOC A, V368, P1333, DOI 10.1098/rsta.2009.0273
Su S, 2020, NANOMATERIALS-BASEL, V10, DOI 10.3390/nano10040656

Sun C, 2008, ADV DRUG DELIVER REV, V60, P1252, DOI 10.1016/j.addr.2008.03.018
Sushnitha M, 2020, FRONT BIOENG BIOTECH, V8, DOI 10.3389/fbioe.2020.00627
Swami A, 2014, P NATL ACAD SCI USA, V111, P10287, DOI 10.1073/pnas.1401337111
Tang J, 2015, EXPERT OPIN BIOL TH, V15, P1251, DOI 10.1517/14712598.2015.1049944
Tang L, 2022, CANCER COMMUN, V42, P141, DOI 10.1002/cac2.12255
Tang L, 2021, PHARMACEUTICS, V13, DOI 10.3390/pharmaceutics13081151
Temizoz B, 2016, INT IMMUNOL, V28, P329, DOI 10.1093/intimm/dxw015
Tope S., 2014, PHARM INNOVATION J, V2
Uner M, 2006, PHARMAZIE, V61, P375
Wakabayashi R, 2018, MOL PHARMACEUT, V15, P955, DOI 10.1021/acs.molpharmaceut.7b00894
Wang F, 2018, MAT SCI ENG C-MATER, V90, P46, DOI 10.1016/j.msec.2018.04.030
Wang LY, 2018, ADV HEALTHC MATER, V7, DOI 10.1002/adhm.201701156
Wang YH, 2013, J CONTROL RELEASE, V172, P179, DOI 10.1016/j.jconrel.2013.08.015
Wen L, 2014, BIOMATERIALS, V35, P2295, DOI 10.1016/j.biomaterials.2013.11.069
Westmeier D, 2018, NANOSCALE, V10, P1453, DOI 10.1039/c7nr06573f
Wu F, 2016, BIOSENS BIOELECTRON, V77, P464, DOI 10.1016/j.bios.2015.10.002
Xia Q, 2019, ACTA PHARM SIN B, V9, P675, DOI 10.1016/j.apsb.2019.01.011
Xie YR, 2016, J CONTROL RELEASE, V229, P120, DOI 10.1016/j.jconrel.2016.03.029
Yang MY, 2021, BIOACT MATER, V6, P1973, DOI 10.1016/j.bioactmat.2020.12.010
Yang YSS, 2019, BIOMATER SCI-UK, V7, P113, DOI 10.1039/c8bm01208c
Yang ZG, 2020, WIRES NANOMED NANOBI, V12, DOI 10.1002/wnan.1590
Yokoi K, 2014, CANCER RES, V74, P4239, DOI 10.1158/0008-5472.CAN-13-3494
YURCHENCO PD, 1987, J CELL BIOL, V105, P2559, DOI 10.1083/jcb.105.6.2559
Zanganeh S, 2016, NAT NANOTECHNOL, V11, P986, DOI [10.1038/nnano.2016.168, 10.1038/NNANO.2016.168]
Zhang D, 2020, FRONT CHEM, V8, DOI 10.3389/fchem.2020.00799
Zhang PS, 2021, INNOVATION-AMSTERDAM, V2, DOI 10.1016/j.xinn.2021.100174
Zhang Y, 2019, WIRES NANOMED NANOBI, V11, DOI 10.1002/wnan.1559
Zhang ZY, 2021, FRONT IMMUNOL, V12, DOI 10.3389/fimmu.2021.672356
Zheng Y, 2013, J CONTROL RELEASE, V172, P426, DOI 10.1016/j.jconrel.2013.05.037
Zheng YY, 2021, ADV HEALTHC MATER, V10, DOI 10.1002/adhm.202001743
Zhuang J, 2019, THERANOSTICS, V9, P7826, DOI 10.7150/thno.37216
NR 164
TC 0
Z9 0
U1 3
U2 3
PU SPRINGER
PI DORDRECHT
PA VAN GODEWIJCKSTRAAT 30, 3311 GZ DORDRECHT, NETHERLANDS
SN 1388-0764
EI 1572-896X
J9 J NANOPART RES
JI J. Nanopart. Res.
PD JUN
PY 2023
VL 25
IS 6
AR 116
DI 10.1007/s11051-023-05754-x
PG 20
WC Chemistry, Multidisciplinary; Nanoscience & Nanotechnology; Materials Science, Multidisciplinary
WE Science Citation Index Expanded (SCI-EXPANDED)
SC Chemistry; Science & Technology - Other Topics; Materials Science
GA H8UA4
UT WOS:000998634500003
DA 2023-09-14
ER

PT J

AU Sarkar, S
Mishra, A
Periasamy, S
Dyett, B
Dogra, P
Ball, AS
Yeo, LY
White, JF
Wang, ZH
Cristini, V
Jagannath, C
Khan, A
Soni, SK
Drummond, CJ
Conn, CE
AF Sarkar, Sampa
Mishra, Abhishek
Periasamy, Selvakannan
Dyett, Brendan
Dogra, Prashant
Ball, Andrew S. S.
Yeo, Leslie Y. Y.
White, Jacinta F. F.
Wang, Zhihui
Cristini, Vittorio
Jagannath, Chinnaswamy
Khan, Arshad
Soni, Sarvesh K. K.
Drummond, Calum J. J.
Conn, Charlotte E. E.
TI Prospective Subunit Nanovaccine against Mycobacterium tuberculosis Infection-Cubosome Lipid Nanocarriers of Cord Factor, Trehalose 6,6 ' Dimycolate
SO ACS APPLIED MATERIALS & INTERFACES
LA English
DT Article
DE Mycobacterium tuberculosis (H37Rv); cord factor trehalose 6,6 ' dimycolate (TDM); cubosomes; nanovaccine; immune response
ID INNATE IMMUNITY; CUBIC PHASES; 6,6'-DIMYCOLATE; BCG; PHARMACOKINETICS; FORMULATIONS; PATHOGENESIS; VACCINATION; GENERATION; GRANULOMAS
AB An improved vaccine is urgently needed to replace the now more than 100-year-old Bacillus Calmette-Guerin (BCG) vaccine against tuberculosis (TB) disease, which represents a significant burden on global public health. Mycolic acid, or cord factor trehalose 6,6 ' dimycolate (TDM), a lipid component abundant in the cell wall of the pathogen Mycobacterium tuberculosis (MTB), has been shown to have strong immunostimulatory activity but remains underexplored due to its high toxicity and poor solubility. Herein, we employed a novel strategy to encapsulate TDM within a cubosome lipid nanocarrier as a potential subunit nanovaccine candidate against TB. This strategy not only increased the solubility and reduced the toxicity of TDM but also elicited a protective immune response to control MTB growth in macrophages. Both pre-treatment and concurrent treatment of the TDM encapsulated in lipid monoolein (MO) cubosomes (MO-TDM) (1 mol %) induced a strong proinflammatory cytokine response in MTB-infected macrophages, due to epigenetic changes at the promoters of tumor necrosis factor alpha (TNF-a) and interleukin 6 (IL-6) in comparison to the untreated control. Furthermore, treatment with MO-TDM (1 mol %) cubosomes significantly improved antigen processing and presentation capabilities of MTB-infected macrophages to CD4 T cells. The ability of MO-TDM (1 mol %) cubosomes to induce a robust innate and adaptive response in vitro was further supported by a mathematical modeling study predicting the vaccine efficacy in vivo. Overall, these results indicate a strong immunostimulatory effect of TDM when delivered through the lipid nanocarrier, suggesting its potential as a novel TB vaccine.
C1 [Sarkar, Sampa; Periasamy, Selvakannan; Dyett, Brendan; Ball, Andrew S. S.; Yeo, Leslie Y. Y.; Soni, Sarvesh K. K.; Drummond, Calum J. J.; Conn, Charlotte E. E.] RMIT Univ, STEM Coll, Sch Sci, Melbourne, Vic 3001, Australia.
[Mishra, Abhishek; Jagannath, Chinnaswamy; Khan, Arshad] Houston Methodist Res Inst, Dept Pathol

& Genom Med, Houston, TX 77030 USA.
[White, Jacinta F. F.] Commonwealth Sci & Ind Res Org, Clayton, Vic 3169, Australia.
[Dogra, Prashant; Wang, Zhihui; Cristini, Vittorio] Houston Methodist Res Inst, Math Med Program, Houston, TX 77030 USA.
[Dogra, Prashant; Wang, Zhihui] Weill Cornell Med Coll, Dept Physiol & Biophys, New York, NY 10021 USA.
[Wang, Zhihui; Cristini, Vittorio] Houston Methodist Res Inst, Neal Canc Ctr, Houston, TX 77030 USA.
[Cristini, Vittorio] Univ Texas MD Anderson Canc Ctr, Dept Imaging Phys, Houston, TX 77030 USA.
[Cristini, Vittorio] Weill Cornell Med, Grad Sch Med Sci, Physiol Biophys & Syst Biol Program, New York, NY 10021 USA.
C3 Royal Melbourne Institute of Technology (RMIT); The Methodist Hospital System; The Methodist Hospital - Houston; Commonwealth Scientific & Industrial Research Organisation (CSIRO); The Methodist Hospital System; The Methodist Hospital - Houston; Cornell University; Weill Cornell Medicine; The Methodist Hospital System; The Methodist Hospital - Houston; University of Texas System; UTMD Anderson Cancer Center; Cornell University; Weill Cornell Medicine
RP Soni, SK; Drummond, CJ; Conn, CE (通讯作者)，RMIT Univ, STEM Coll, Sch Sci, Melbourne, Vic 3001, Australia.; Khan, A (通讯作者)，Houston Methodist Res Inst, Dept Pathol & Genom Med, Houston, TX 77030 USA.
EM akhan5@houstonmethodist.org; sarvesh.soni@rmit.edu.au; calum.drummond@rmit.edu.au; charlotte.conn@rmit.edu.au
RI Drummond, Calum/AAR-9854-2020; Dogra, Prashant/Y-1921-2019; Mishra, Abhishek/I-5362-2019; Wang, Zhihui/B-2464-2009; wang, zhihui/HSF-6639-2023
OI Dogra, Prashant/0000-0001-6722-7371; Mishra, Abhishek/0000-0003-2077-0937; Wang, Zhihui/0000-0001-6262-700X; Cristini, Vittorio/0000-0002-7909-4278
FU CSIRO Manufacturing Morphology and Structure Group within the Materials Characterisation and Modelling Program; Cockrell Foundation; ARC DECRA Fellowship [DE160101281]; RMIT
FX The authors acknowledge the RMIT Micro Nano Research Facility (MNRF) for providing access to equipment and resources as well as the use other facilities within the RMIT. We would like to acknowledge the CSIRO Manufacturing Morphology and Structure Group within the Materials Characterisation and Modelling Program for the TEM contribution to this project. Part of this research was undertaken on the SAXS/WAXS beamline at the Australian Synchrotron, part of ANSTO. The mathematical modeling was partially supported by internal funding from the Cockrell Foundation (Dogra, Cristini). Thanks to the Rebecca L Cooper Medical Research Foundation (S. Soni); C.E.C. acknowledges support from an ARC DECRA Fellowship (DE160101281) and RMIT for a Vice Chancellor's Senior Research Fellowship.
CR Alley SH, 2008, CHEM PHYS LIPIDS, V154, P64, DOI 10.1016/j.chemphyslip.2008.03.007
Angelova A, 2018, ACS OMEGA, V3, P3235, DOI 10.1021/acsomega.7b01935
Arts RJW, 2016, CELL REP, V17, P2562, DOI 10.1016/j.celrep.2016.11.011
Barriga HMG, 2019, ANGEW CHEM INT EDIT, V58, P2958, DOI 10.1002/anie.201804067
Behar SM, 2007, EXPERT REV VACCINES, V6, P441, DOI 10.1586/14760584.6.3.441
Bekkering S, 2018, CELL, V172, P135, DOI 10.1016/j.cell.2017.11.025
Bekkering S, 2016, CLIN VACCINE IMMUNOL, V23, P926, DOI 10.1128/CVI.00349-16
Bisset NB, 2015, INT J PHARMACEUT, V495, P241, DOI 10.1016/j.ijpharm.2015.08.072
Blok BA, 2019, EUR J CLIN MICROBIOL, V38, P449, DOI 10.1007/s10096-018-03449-z
Caminero JA, 2019, EUR RESPIR J, V54, DOI 10.1183/13993003.01272-2019
Canaday DH, 2003, J IMMUNOL METHODS, V281, P129, DOI 10.1016/j.jim.2003.07.004
Choi HS, 2007, NAT BIOTECHNOL, V25, P1165, DOI 10.1038/nbt1340
Choi JH, 2007, J IND ENG CHEM, V13, P380
Dienz O, 2009, CLIN IMMUNOL, V130, P27, DOI 10.1016/j.clim.2008.08.018
Dogra P, 2021, ACS PHARMACOL TRANSL, V4, P248, DOI 10.1021/acsptsci.0c00183
Dogra P, 2020, COMPUT STRUCT BIOTEC, V18, P518, DOI 10.1016/j.csbj.2020.02.014
Dogra P, 2018, NAT COMMUN, V9, DOI 10.1038/s41467-018-06730-z
Dorhoi A, 2014, SEMIN IMMUNOL, V26, P203, DOI 10.1016/j.smim.2014.04.003

Dyett BP, 2019, NAT COMMUN, V10, DOI 10.1038/s41467-019-12508-8
Falchi AM, 2015, TOXICOL RES-UK, V4, P1025, DOI 10.1039/c5tx00078e
FINE PEM, 1995, LANCET, V346, P1339, DOI 10.1016/S0140-6736(95)92348-9
Freire RVM, 2021, J COLLOID INTERF SCI, V596, P352, DOI 10.1016/j.jcis.2021.03.060
Geller AE, 2022, NAT COMMUN, V13, DOI 10.1038/s41467-022-28407-4
Goel S, 2019, SMALL, V15, DOI 10.1002/smll.201903747
GU ZT, 1994, J AM CHEM SOC, V116, P6368, DOI 10.1021/ja00093a042
Hoshyar N, 2016, NANOMEDICINE-UK, V11, P673, DOI 10.2217/nnm.16.5
Hou XC, 2021, NAT REV MATER, V6, P1078, DOI 10.1038/s41578-021-00358-0
Hunter RL, 2006, AM J PATHOL, V168, P1249, DOI 10.2353/ajpath.2006.050848
Hunter RL, 2006, ANN CLIN LAB SCI, V36, P371
Indrigo J, 2003, MICROBIOL-SGM, V149, P2049, DOI 10.1099/mic.0.26226-0
KATO M, 1973, INFECT IMMUN, V7, P14, DOI 10.1128/IAI.7.1.14-21.1973
KATO M, 1972, INFECT IMMUN, V5, P203, DOI 10.1128/IAI.5.2.203-212.1972
Kaveh DA, 2014, VACCINE, V32, P6911, DOI 10.1016/j.vaccine.2014.10.041
Kim HJ, 2015, ACS NANO, V9, P10214, DOI 10.1021/acsnano.5b03902
Kirby NM, 2013, J APPL CRYSTALLOGR, V46, P1670, DOI 10.1107/S002188981302774X
Larsson K, 2005, CURR OPIN COLLOID IN, V9, P365, DOI 10.1016/j.cocis.2004.12.002
Lerias JR, 2020, FRONT MICROBIOL, V10, DOI 10.3389/fmicb.2019.02924
Lima VMF, 2001, INFECT IMMUN, V69, P5305, DOI 10.1128/IAI.69.9.5305-5312.2001
Litjens NHR, 2008, J IMMUNOL, V181, P3665, DOI 10.4049/jimmunol.181.5.3665
Liu J, 1996, J BIOL CHEM, V271, P29545, DOI 10.1074/jbc.271.47.29545
Martinez AN, 2013, J INFECT DIS, V207, P1253, DOI 10.1093/infdis/jit037
McKinstry KK, 2014, NAT COMMUN, V5, DOI 10.1038/ncomms6377
Moorlag SJCFM, 2020, CELL REP, V31, DOI 10.1016/j.celrep.2020.107634
Mulet X, 2013, ACCOUNTS CHEM RES, V46, P1497, DOI 10.1021/ar300285u
Mustfa SA, 2021, ADV THER-GERMANY, V4, DOI 10.1002/adtp.202000160
Okada M, 2013, HUM VACC IMMUNOTHER, V9, P515, DOI 10.4161/hv.23229
Oliveira C, 2022, NANOMATERIALS-BASEL, V12, DOI 10.3390/nano12132224
Olsen A, 2016, MBIO, V7, DOI 10.1128/mBio.01023-15
Ottenhoff THM, 2012, PLOS PATHOG, V8, DOI 10.1371/journal.ppat.1002607
Rakotoarisoa M, 2021, ACS SUSTAIN CHEM ENG, V9, P14821, DOI 10.1021/acssuschemeng.1c04706
Saeed S, 2014, SCIENCE, V345, P1578, DOI 10.1126/science.1251086
Sarkar S, 2021, ACS APPL MATER INTER, V13, P2336, DOI 10.1021/acsami.0c20956
Sarkar S, 2019, ACS APPL BIO MATER, V2, P182, DOI 10.1021/acsabm.8b00539
Sasindran SJ, 2011, FRONT MICROBIOL, V2, DOI 10.3389/fmicb.2011.00002
Schoenmaker L, 2021, INT J PHARMACEUT, V601, DOI 10.1016/j.ijpharm.2021.120586
Sharma A, 2012, J HAZARD MATER, V221, P275, DOI 10.1016/j.jhazmat.2012.04.045
Staquicini DI, 2021, MED-CAMBRIDGE, V2, P321, DOI 10.1016/j.medj.2020.10.005
Steigler P, 2019, IMMUNOL CELL BIOL, V97, P647, DOI 10.1111/imcb.12278
Strachan JB, 2020, AUST J CHEM, V73, P1042, DOI 10.1071/CH19573
Torres M, 2006, INFECT IMMUN, V74, P1621, DOI 10.1128/IAI.74.3.1621-1630.2006
Trunz BB, 2006, LANCET, V367, P1173, DOI 10.1016/S0140-6736(06)68507-3
Vermeulen I, 2017, J LIPID RES, V58, P709, DOI 10.1194/jlr.M073171
Welsh KJ, 2013, TUBERCULOSIS, V93, pS3, DOI 10.1016/S1472-9792(13)70003-9
WHO, 2012, GLOBAL TUBERCULOSIS REPORT 2012, P1
World Health Organization, 2020, TOP 10 CAUS DEATH
Yamagami H, 2001, INFECT IMMUN, V69, P810, DOI 10.1128/IAI.69.2.810-815.2001
Zhai JL, 2019, ACS NANO, V13, P6178, DOI 10.1021/acsnano.8b07961
Zhai JL, 2018, ACS APPL MATER INTER, V10, P25174, DOI 10.1021/acsami.8b08125
NR 68
TC 0
Z9 0
U1 5
U2 5
PU AMER CHEMICAL SOC
PI WASHINGTON
PA 1155 16TH ST, NW, WASHINGTON, DC 20036 USA
SN 1944-8244
EI 1944-8252
J9 ACS APPL MATER INTER

JI ACS Appl. Mater. Interfaces
PD JUN 1
PY 2023
VL 15
IS 23
BP 27670
EP 27686
DI 10.1021/acsami.3c04063
PG 17
WC Nanoscience & Nanotechnology; Materials Science, Multidisciplinary
WE Science Citation Index Expanded (SCI-EXPANDED)
SC Science & Technology - Other Topics; Materials Science
GA J6ZB2
UT WOS:001011071200001
PM 37262346
DA 2023-09-14
ER

PT J
AU Sarkar, S
   Mishra, A
   Periasamy, S
   Dyett, B
   Dogra, P
   Ball, AS
   Yeo, LY
   White, JF
   Wang, ZH
   Cristini, V
   Jagannath, C
   Khan, A
   Soni, SK
   Drummond, CJ
   Conn, CE
AF Sarkar, Sampa
   Mishra, Abhishek
   Periasamy, Selvakannan
   Dyett, Brendan
   Dogra, Prashant
   Ball, Andrew S.
   Yeo, Leslie Y.
   White, Jacinta F.
   Wang, Zhihui
   Cristini, Vittorio
   Jagannath, Chinnaswamy
   Khan, Arshad
   Soni, Sarvesh K.
   Drummond, Calum J.
   Conn, Charlotte E.
TI Prospective Subunit Nanovaccine against Mycobacterium tuberculosis
   Infection-Cubosome Lipid Nanocarriers of Cord Factor, Trehalose 6,6'
   Dimycolate
SO ACS APPLIED MATERIALS & INTERFACES
LA English
DT Article; Early Access
DE Mycobacterium tuberculosis (H37Rv); cord factor trehalose 6,6'
   dimycolate (TDM); cubosomes; nanovaccine; imm un e response
ID INNATE IMMUNITY; CUBIC PHASES; 6,6'-DIMYCOLATE; BCG; PHARMACOKINETICS;
   FORMULATIONS; PATHOGENESIS; VACCINATION; GENERATION; GRANULOMAS
AB An improved vaccine is urgently needed to replace the now more than 100-year-old Bacillus Calmette-Gue ' rin (BCG) vaccine against tuberculosis (TB) disease, which represents a significant burden on global

public health. Mycolic acid, or cord factor trehalose 6,6' dimycolate (TDM), a lipid component abundant in the cell wall of the pathogen Mycobacterium tuberculosis (MTB), has been shown to have strong immunostimulatory activity but remains underexplored due to its high toxicity and poor solubility. Herein, we employed a novel strategy to encapsulate TDM within a cubosome lipid nanocarrier as a potential subunit nanovaccine candidate against TB. This strategy not only increased the solubility and reduced the toxicity of TDM but also elicited a protective immune response to control MTB growth in macrophages. Both pre-treatment and concurrent treatment of the TDM encapsulated in lipid monoolein (MO) cubosomes (MO-TDM) (1 mol %) induced a strong proinflammatory cytokine response in MTB-infected macrophages, due to epigenetic changes at the promoters of tumor necrosis factor alpha (TNF-alpha) and interleukin 6 (IL-6) in comparison to the untreated control. Furthermore, treatment with MO-TDM (1 mol %) cubosomes significantly improved antigen processing and presentation capabilities of MTB-infected macrophages to CD4 T cells. The ability of MO-TDM (1 mol %) cubosomes to induce a robust innate and adaptive response in vitro was further supported by a mathematical modeling study predicting the vaccine efficacy in vivo. Overall, these results indicate a strong immunostimulatory effect of TDM when delivered through the lipid nanocarrier, suggesting its potential as a novel TB vaccine.
C1 [Sarkar, Sampa; Periasamy, Selvakannan; Dyett, Brendan; Ball, Andrew S.; Yeo, Leslie Y.; Soni, Sarvesh K.; Drummond, Calum J.; Conn, Charlotte E.] RMIT Univ, STEM Coll, Sch Sci, Melbourne, Vic 3001, Australia.
[Mishra, Abhishek; Jagannath, Chinnaswamy; Khan, Arshad] Houston Methodist Res Inst, Dept Pathol & Genom Med, Houston, TX 77030 USA.
[Dogra, Prashant; Wang, Zhihui; Cristini, Vittorio] Houston Methodist Res Inst, Math Med Program, Houston, TX 77030 USA.
[Dogra, Prashant; Wang, Zhihui] Weill Cornell Med Coll, Dept Physiol & Biophys, New York, NY 10021 USA.
[White, Jacinta F.] Commonwealth Sci & Ind Res Org, Clayton, Vic 3169, Australia.
[Wang, Zhihui; Cristini, Vittorio] Houston Methodist Res Inst, Neal Canc Ctr, Houston, TX 77030 USA.
[Cristini, Vittorio] Univ Texas MD Anderson Canc Ctr, Dept Imaging Phys, Houston, TX 77030 USA.
[Cristini, Vittorio] Weill Cornell Med, Grad Sch Med Sci, Physiol Biophys & Syst Biol Program, New York, NY 10021 USA.
C3 Royal Melbourne Institute of Technology (RMIT); The Methodist Hospital System; The Methodist Hospital - Houston; The Methodist Hospital System; The Methodist Hospital - Houston; Cornell University; Weill Cornell Medicine; Commonwealth Scientific & Industrial Research Organisation (CSIRO); The Methodist Hospital System; The Methodist Hospital - Houston; University of Texas System; UTMD Anderson Cancer Center; Cornell University; Weill Cornell Medicine
RP Soni, SK; Drummond, CJ; Conn, CE (通讯作者)，RMIT Univ, STEM Coll, Sch Sci, Melbourne, Vic 3001, Australia.; Khan, A (通讯作者)，Houston Methodist Res Inst, Dept Pathol & Genom Med, Houston, TX 77030 USA.
EM akhan5@houstonmethodist.org; sarvesh.soni@rmit.edu.au; calum.drummond@rmit.edu.au; charlotte.conn@rmit.edu.au
RI Drummond, Calum/AAR-9854-2020; Dogra, Prashant/Y-1921-2019; Mishra, Abhishek/I-5362-2019; Wang, Zhihui/B-2464-2009; wang, zhihui/HSF-6639-2023
OI Dogra, Prashant/0000-0001-6722-7371; Mishra, Abhishek/0000-0003-2077-0937; Wang, Zhihui/0000-0001-6262-700X; Cristini, Vittorio/0000-0002-7909-4278
FU CSIRO Manufacturing Morphology and Structure Group within the Materials Characterisation and Modelling Program; Cockrell Foundation; Rebecca L Cooper Medical Research Foundation; ARC DECRA Fellowship [DE160101281]; RMIT
FX The authors acknowledge the RMIT Micro Nano Research Facility (MNRF) for providing access to equipment and resources as well as the use other facilities within the RMIT. We would like to acknowledge the CSIRO Manufacturing Morphology and Structure Group within the Materials Characterisation and Modelling Program for the TEM contribution to this project. Part of this research was undertaken on the SAXS/WAXS beamline at the Australian Synchrotron, part of ANSTO. The mathematical modeling was partially supported by internal funding from the Cockrell Foundation (Dogra, Cristini). Thanks to the Rebecca L Cooper Medical Research Foundation (S. Soni); C.E.C. acknowledges support from an ARC DECRA

Fellowship (DE160101281) and RMIT for a Vice Chancellor's Senior
Research Fellowship.
CR Alley SH, 2008, CHEM PHYS LIPIDS, V154, P64, DOI 10.1016/j.chemphyslip.2008.03.007
Angelova A, 2018, ACS OMEGA, V3, P3235, DOI 10.1021/acsomega.7b01935
Arts RJW, 2016, CELL REP, V17, P2562, DOI 10.1016/j.celrep.2016.11.011
Barriga HMG, 2019, ANGEW CHEM INT EDIT, V58, P2958, DOI 10.1002/anie.201804067
Behar SM, 2007, EXPERT REV VACCINES, V6, P441, DOI 10.1586/14760584.6.3.441
Bekkering S, 2018, CELL, V172, P135, DOI 10.1016/j.cell.2017.11.025
Bekkering S, 2016, CLIN VACCINE IMMUNOL, V23, P926, DOI 10.1128/CVI.00349-16
Bisset NB, 2015, INT J PHARMACEUT, V495, P241, DOI 10.1016/j.ijpharm.2015.08.072
Blok BA, 2019, EUR J CLIN MICROBIOL, V38, P449, DOI 10.1007/s10096-018-03449-z
Caminero JA, 2019, EUR RESPIR J, V54, DOI 10.1183/13993003.01272-2019
Canaday DH, 2003, J IMMUNOL METHODS, V281, P129, DOI 10.1016/j.jim.2003.07.004
Choi HS, 2007, NAT BIOTECHNOL, V25, P1165, DOI 10.1038/nbt1340
Choi JH, 2007, J IND ENG CHEM, V13, P380
Dienz O, 2009, CLIN IMMUNOL, V130, P27, DOI 10.1016/j.clim.2008.08.018
Dogra P, 2021, ACS PHARMACOL TRANSL, V4, P248, DOI 10.1021/acsptsci.0c00183
Dogra P, 2020, COMPUT STRUCT BIOTEC, V18, P518, DOI 10.1016/j.csbj.2020.02.014
Dogra P, 2018, NAT COMMUN, V9, DOI 10.1038/s41467-018-06730-z
Dorhoi A, 2014, SEMIN IMMUNOL, V26, P203, DOI 10.1016/j.smim.2014.04.003
Dyett BP, 2019, NAT COMMUN, V10, DOI 10.1038/s41467-019-12508-8
Falchi AM, 2015, TOXICOL RES-UK, V4, P1025, DOI 10.1039/c5tx00078e
FINE PEM, 1995, LANCET, V346, P1339, DOI 10.1016/S0140-6736(95)92348-9
Freire RVM, 2021, J COLLOID INTERF SCI, V596, P352, DOI 10.1016/j.jcis.2021.03.060
Geller AE, 2022, NAT COMMUN, V13, DOI 10.1038/s41467-022-28407-4
Goel S, 2019, SMALL, V15, DOI 10.1002/smll.201903747
GU ZT, 1994, J AM CHEM SOC, V116, P6368, DOI 10.1021/ja00093a042
Hoshyar N, 2016, NANOMEDICINE-UK, V11, P673, DOI 10.2217/nnm.16.5
Hou XC, 2021, NAT REV MATER, V6, P1078, DOI 10.1038/s41578-021-00358-0
Hunter RL, 2006, AM J PATHOL, V168, P1249, DOI 10.2353/ajpath.2006.050848
Hunter RL, 2006, ANN CLIN LAB SCI, V36, P371
Indrigo J, 2003, MICROBIOL-SGM, V149, P2049, DOI 10.1099/mic.0.26226-0
KATO M, 1973, INFECT IMMUN, V7, P14, DOI 10.1128/IAI.7.1.14-21.1973
KATO M, 1972, INFECT IMMUN, V5, P203, DOI 10.1128/IAI.5.2.203-212.1972
Kaveh DA, 2014, VACCINE, V32, P6911, DOI 10.1016/j.vaccine.2014.10.041
Kim HJ, 2015, ACS NANO, V9, P10214, DOI 10.1021/acsnano.5b03902
Kirby NM, 2013, J APPL CRYSTALLOGR, V46, P1670, DOI 10.1107/S002188981302774X
Larsson K, 2005, CURR OPIN COLLOID IN, V9, P365, DOI 10.1016/j.cocis.2004.12.002
Lerias JR, 2020, FRONT MICROBIOL, V10, DOI 10.3389/fmicb.2019.02924
Lima VMF, 2001, INFECT IMMUN, V69, P5305, DOI 10.1128/IAI.69.9.5305-5312.2001
Litjens NHR, 2008, J IMMUNOL, V181, P3665, DOI 10.4049/jimmunol.181.5.3665
Liu J, 1996, J BIOL CHEM, V271, P29545, DOI 10.1074/jbc.271.47.29545
Martinez AN, 2013, J INFECT DIS, V207, P1253, DOI 10.1093/infdis/jit037
McKinstry KK, 2014, NAT COMMUN, V5, DOI 10.1038/ncomms6377
Moorlag SJCFM, 2020, CELL REP, V31, DOI 10.1016/j.celrep.2020.107634
Mulet X, 2013, ACCOUNTS CHEM RES, V46, P1497, DOI 10.1021/ar300285u
Mustfa SA, 2021, ADV THER-GERMANY, V4, DOI 10.1002/adtp.202000160
Okada M, 2013, HUM VACC IMMUNOTHER, V9, P515, DOI 10.4161/hv.23229
Oliveira C, 2022, NANOMATERIALS-BASEL, V12, DOI 10.3390/nano12132224
Olsen A, 2016, MBIO, V7, DOI 10.1128/mBio.01023-15
Ottenhoff THM, 2012, PLOS PATHOG, V8, DOI 10.1371/journal.ppat.1002607
Rakotoarisoa M, 2021, ACS SUSTAIN CHEM ENG, V9, P14821, DOI 10.1021/acssuschemeng.1c04706
Saeed S, 2014, SCIENCE, V345, P1578, DOI 10.1126/science.1251086
Sarkar S, 2021, ACS APPL MATER INTER, V13, P2336, DOI 10.1021/acsami.0c20956
Sarkar S, 2019, ACS APPL BIO MATER, V2, P182, DOI 10.1021/acsabm.8b00539
Sasindran SJ, 2011, FRONT MICROBIOL, V2, DOI 10.3389/fmicb.2011.00002
Schoenmaker L, 2021, INT J PHARMACEUT, V601, DOI 10.1016/j.ijpharm.2021.120586
Sharma A, 2012, J HAZARD MATER, V221, P275, DOI 10.1016/j.jhazmat.2012.04.045
Staquicini DI, 2021, MED-CAMBRIDGE, V2, P321, DOI 10.1016/j.medj.2020.10.005
Steigler P, 2019, IMMUNOL CELL BIOL, V97, P647, DOI 10.1111/imcb.12278
Strachan JB, 2020, AUST J CHEM, V73, P1042, DOI 10.1071/CH19573

Torres M, 2006, INFECT IMMUN, V74, P1621, DOI 10.1128/IAI.74.3.1621-1630.2006
Trunz BB, 2006, LANCET, V367, P1173, DOI 10.1016/S0140-6736(06)68507-3
Vermeulen I, 2017, J LIPID RES, V58, P709, DOI 10.1194/jlr.M073171
Welsh KJ, 2013, TUBERCULOSIS, V93, pS3, DOI 10.1016/S1472-9792(13)70003-9
WHO, 2012, GLOBAL TUBERCULOSIS REPORT 2012, P1
World Health Organization, 2020, TOP 10 CAUS DEATH
Yamagami H, 2001, INFECT IMMUN, V69, P810, DOI 10.1128/IAI.69.2.810-815.2001
Zhai JL, 2019, ACS NANO, V13, P6178, DOI 10.1021/acsnano.8b07961
Zhai JL, 2018, ACS APPL MATER INTER, V10, P25174, DOI 10.1021/acsami.8b08125
NR 68
TC 0
Z9 0
U1 5
U2 5
PU AMER CHEMICAL SOC
PI WASHINGTON
PA 1155 16TH ST, NW, WASHINGTON, DC 20036 USA
SN 1944-8244
EI 1944-8252
J9 ACS APPL MATER INTER
JI ACS Appl. Mater. Interfaces
PD 2023 JUN 1
PY 2023
DI 10.1021/acsami.3c04063
EA JUN 2023
PG 17
WC Nanoscience & Nanotechnology; Materials Science, Multidisciplinary
WE Science Citation Index Expanded (SCI-EXPANDED)
SC Science & Technology - Other Topics; Materials Science
GA K2OW7
UT WOS:001014894700001
PM 37262346
DA 2023-09-14
ER

PT J
AU Ji, P
Deng, XC
Jin, XK
Zhang, SM
Wang, JW
Feng, J
Chen, WH
Zhang, XZ
AF Ji, Ping
Deng, Xin-Chen
Jin, Xiao-Kang
Zhang, Shi-Man
Wang, Jia-Wei
Feng, Jun
Chen, Wei-Hai
Zhang, Xian-Zheng
TI Fused Cytomembrane-Camouflaged Nanoparticles for Tumor-Specific
Immunotherapy
SO ADVANCED HEALTHCARE MATERIALS
LA English
DT Article; Early Access
DE adjuvants; fused cytomembranes; nanovaccines; tumor immunotherapy; whole
antigens
ID DENDRITIC CELLS; ANTITUMOR IMMUNITY; CANCER; NANOVACCINES; CD80;
NANOMATERIALS; MACROPHAGES; ACTIVATION; BLOCKADE; DELIVERY

AB Tumor immunotherapy is commonly hindered by inefficient delivery and presentation of tumor antigens as well as immunosuppressive tumor microenvironment. To overcome these barriers, a tumor-specific nanovaccine capable of delivering tumor antigens and adjuvants to antigen-presenting cells and modulating the immune microenvironment to elicit strong antitumor immunity is reported. This nanovaccine, named FCM@4RM, is designed by coating the nanocore (FCM) with a bioreconstituted cytomembrane (4RM). The 4RM, which is derived from fused cells of tumorous 4T1 cells and RAW264.7 macrophages, enables effective antigen presentation and stimulation of effector T cells. FCM is self-assembled from Fe(II), unmethylated cytosine-phosphate-guanine oligodeoxynucleotide (CpG), and metformin (MET). CpG, as the stimulator of toll-like receptor 9, induces the production of pro-inflammatory cytokine and the maturation of cytotoxic T lymphocytes (CTLs), thereby enhancing antitumor immunity. Meanwhile, MET functions as the programmed cell death ligand 1 inhibitor and can restore the immune responses of T cells against tumor cells. Therefore, FCM@4RM exhibits high targeting capabilities toward homologous tumors that develop from 4T1 cells. This work offers a paradigm for developing a nanovaccine that systematically regulates multiple immune-related processes to achieve optimal antitumor immunotherapy.
C1 [Ji, Ping; Deng, Xin-Chen; Jin, Xiao-Kang; Zhang, Shi-Man; Wang, Jia-Wei; Feng, Jun; Chen, Wei-Hai; Zhang, Xian-Zheng] Wuhan Univ, Key Lab Biomed Polymers, Minist Educ, Wuhan 430072, Peoples R China.
[Ji, Ping; Deng, Xin-Chen; Jin, Xiao-Kang; Zhang, Shi-Man; Wang, Jia-Wei; Feng, Jun; Chen, Wei-Hai; Zhang, Xian-Zheng] Wuhan Univ, Dept Chem, Wuhan 430072, Peoples R China.
[Chen, Wei-Hai; Zhang, Xian-Zheng] Wuhan Univ, Zhongnan Hosp, Canc Precis Diag & Treatment & Translat Med Hubei, Wuhan 430071, Peoples R China.
C3 Wuhan University; Wuhan University; Wuhan University
RP Chen, WH; Zhang, XZ (通讯作者)，Wuhan Univ, Key Lab Biomed Polymers, Minist Educ, Wuhan 430072, Peoples R China.; Chen, WH; Zhang, XZ (通讯作者)，Wuhan Univ, Dept Chem, Wuhan 430072, Peoples R China.; Chen, WH; Zhang, XZ (通讯作者)，Wuhan Univ, Zhongnan Hosp, Canc Precis Diag & Treatment & Translat Med Hubei, Wuhan 430071, Peoples R China.
EM chenweihai@whu.edu.cn; xz-zhang@whu.edu.cn
OI Chen, Wei-Hai/0000-0003-3100-2319
FU National Key Research and Development Program of China [2022YFB3804600]; National Natural Science Foundation of China [52273301, 52073218, 22135005, 51833007, 51988102]; Jiangsu Province Science Foundation for Youths [BK20200241]; Fundamental Research Funds for the Central Universities [2042023kfyq05]; Institutional Animal Care and Use Committee (IACUC) [WP20210503]
FX This work was supported by the National Key Research and Development Program of China (2022YFB3804600), the National Natural Science Foundation of China (52273301, 52073218, 22135005, 51833007, and 51988102), the Jiangsu Province Science Foundation for Youths (BK20200241), and the Fundamental Research Funds for the Central Universities (2042023kfyq05). All of the animal experiments were conducted under protocols (WP20210503) approved by the Institutional Animal Care and Use Committee (IACUC) of the Animal Experiment Center of Wuhan University (Wuhan, China).
CR Awate S, 2013, FRONT IMMUNOL, V4, DOI 10.3389/fimmu.2013.00114
Blass E, 2021, NAT REV CLIN ONCOL, V18, P215, DOI 10.1038/s41571-020-00460-2
Brentjens RJ, 2003, NAT MED, V9, P279, DOI 10.1038/nm827
Cha JH, 2018, MOL CELL, V71, P606, DOI 10.1016/j.molcel.2018.07.030
Chen J, 2022, BIOACT MATER, V7, P167, DOI 10.1016/j.bioactmat.2021.05.036
Chen WQ, 2021, INT J NANOMED, V16, P5281, DOI 10.2147/IJN.S317626
Chen Z, 2001, INT J CANCER, V93, P539, DOI 10.1002/ijc.1365
Cotzomi-Ortega I, 2021, CELL SIGNAL, V86, DOI 10.1016/j.cellsig.2021.110075
De Koker S, 2011, CHEM SOC REV, V40, P320, DOI 10.1039/b914943k
Deng XX, 2021, J ETHNOPHARMACOL, V274, DOI 10.1016/j.jep.2021.113978
Hess KL, 2019, NANO TODAY, V27, P73, DOI 10.1016/j.nantod.2019.04.005
Hiam-Galvez KJ, 2021, NAT REV CANCER, V21, P345, DOI 10.1038/s41568-021-00347-z
Hochweller K, 2010, P NATL ACAD SCI USA, V107, P5931, DOI 10.1073/pnas.0911877107
Hu C, 2021, ADV FUNCT MATER, V31, DOI 10.1002/adfm.202007149
Islam MA, 2021, BIOMATERIALS, V266, DOI 10.1016/j.biomaterials.2020.120431
Jiang Y, 2020, ADV MATER, V32, DOI 10.1002/adma.202001808
Landesman-Milo D, 2012, J CONTROL RELEASE, V161, P600, DOI 10.1016/j.jconrel.2011.12.034

Leifer C. A., 2019, ACS CENTRAL SCI, V5, P796
Li MY, 2019, ANGEW CHEM INT EDIT, V58, P1350, DOI 10.1002/anie.201810735
Liu JY, 2021, CANCER CELL INT, V21, DOI 10.1186/s12935-021-02089-2
Liu MG, 2019, NAT IMMUNOL, V20, P265, DOI 10.1038/s41590-018-0292-y
Liu WL, 2019, NAT COMMUN, V10, DOI 10.1038/s41467-019-11157-1
Lv S, 2022, ADV HEALTHC MATER, V11, DOI 10.1002/adhm.202101651
Obst R, 2007, P NATL ACAD SCI USA, V104, P15460, DOI 10.1073/pnas.0707331104
Orabona C, 2004, NAT IMMUNOL, V5, P1134, DOI 10.1038/ni1124
Park W., 2018, BIOMATER RES, V22, P29
Pulendran B, 2021, NAT REV DRUG DISCOV, V20, P454, DOI 10.1038/s41573-021-00163-y
Qian HQ, 2018, MATER TODAY CHEM, V7, P53, DOI 10.1016/j.mtchem.2018.01.001
Rescigno M, 1999, IMMUNOL TODAY, V20, P200, DOI 10.1016/S0167-5699(98)01427-3
Sommariva M, 2011, CANCER RES, V71, P6382, DOI 10.1158/0008-5472.CAN-11-1285
Sousa S, 2015, BREAST CANCER RES, V17, DOI 10.1186/s13058-015-0621-0
Thery C, 2002, NAT IMMUNOL, V3, P1156, DOI 10.1038/ni854
Wang J, 2019, NANO-MICRO LETT, V11, DOI 10.1007/s40820-019-0305-x
Wu JY, 2020, SCI ADV, V6, DOI 10.1126/sciadv.abc7828
Wu XR, 2017, CHINESE J POLYM SCI, V35, P1, DOI 10.1007/s10118-017-1871-3
Xu J, 2017, ACS NANO, V11, P4463, DOI 10.1021/acsnano.7b00715
Yang R, 2018, ACS NANO, V12, P5121, DOI 10.1021/acsnano.7b09041
Zeng ZL, 2020, ADV FUNCT MATER, V30, DOI 10.1002/adfm.202004397
Zheng Y, 2004, J IMMUNOL, V172, P2778, DOI 10.4049/jimmunol.172.5.2778
Zhu GZ, 2017, ACS NANO, V11, P2387, DOI 10.1021/acsnano.7b00978
Zhuang J, 2019, THERANOSTICS, V9, P7826, DOI 10.7150/thno.37216
NR 41
TC 0
Z9 0
U1 23
U2 23
PU WILEY
PI HOBOKEN
PA 111 RIVER ST, HOBOKEN 07030-5774, NJ USA
SN 2192-2640
EI 2192-2659
J9 ADV HEALTHC MATER
JI Adv. Healthc. Mater.
PD 2023 MAY 28
PY 2023
DI 10.1002/adhm.202300323
EA MAY 2023
PG 15
WC Engineering, Biomedical; Nanoscience & Nanotechnology; Materials
Science, Biomaterials
WE Science Citation Index Expanded (SCI-EXPANDED)
SC Engineering; Science & Technology - Other Topics; Materials Science
GA H4UY2
UT WOS:000995942900001
PM 37212324
DA 2023-09-14
ER

PT J
AU Ye, H
Wang, KY
Zhao, J
Lu, Q
Wang, ML
Sun, BJ
Shen, Y
Liu, HC
Pane, S

Chen, XZ
He, ZG
Sun, J
AF Ye, Hao
Wang, Kaiyuan
Zhao, Jian
Lu, Qi
Wang, Menglin
Sun, Bingjun
Shen, Yang
Liu, Hongchen
Pane, Salvador
Chen, Xiang-Zhong
He, Zhonggui
Sun, Jin
TI In Situ Sprayed Nanovaccine Suppressing Exosomal PD-L1 by Golgi Apparatus Disorganization for Postsurgical Melanoma Immunotherapy
SO ACS NANO
LA English
DT Article
DE biomimetic; immunotherapy; Golgiapparatusdisorganization; exosomal PD-L1; in situ sprayed hydrogel vaccine
ID ACQUIRED-RESISTANCE; CANCER; BLOCKADE; METASTASIS; SURVIVAL; THERAPY; DRIVEN; ROLES
AB Theanti-PD-L1 immunotherapy has shown promise in treatingcancer.However, certain patients with metastatic cancer have low responseand high relapse rates. A main reason is systemic immunosuppressioncaused by exosomal PD-L1, which can circulate in the body and inhibitT cell functions. Here, we show that Golgi apparatus-Pd-l1 (-/-) exosome hybrid membrane coated nanoparticles(GENPs) can significantly reduce the secretion of PD-L1. The GENPscan accumulate in tumors through homotypic targeting and effectivelydeliver retinoic acid, inducing disorganization of the Golgi apparatusand a sequence of intracellular events including alteration of endoplasmicreticulum (ER)-to-Golgi trafficking and subsequent ER stress, whichfinally disrupts the PD-L1 production and the release of exosomes.Furthermore, GENPs could mimic exosomes to access draining lymph nodes.The membrane antigen of PD-l1 (-/-) exosome on GENPs can activate T cells through a vaccine-like effect,strongly promoting systemic immune responses. By combining GENPs withanti-PD-L1 treatment in the sprayable in situ hydrogel,we have successfully realized a low recurrence rate and substantiallyextended survival periods in mice models with incomplete metastaticmelanoma resection.
C1 [Ye, Hao; Wang, Kaiyuan; Zhao, Jian; Lu, Qi; Sun, Bingjun; He, Zhonggui; Sun, Jin] Shenyang Pharmaceut Univ, Wuya Coll Innovat, Dept Pharmaceut, Shenyang 110016, Liaoning, Peoples R China.
[Ye, Hao; Pane, Salvador; Chen, Xiang-Zhong] Swiss Fed Inst Technol, IRIS, MSRL, CH-8092 Zurich, Switzerland.
[Wang, Menglin] Univ North Carolina Chapel Hill, Eshelman Sch Pharm, Div Pharmacoengn & Mol Pharmaceut, Chapel Hill, NC 27599 USA.
[Shen, Yang] Swiss Fed Inst Technol, Inst Food Nutr & Hlth, CH-8092 Zurich, Switzerland.
[Liu, Hongchen] South China Univ Technol, State Key Lab Pulp & Paper Engn, Guangzhou 510641, Peoples R China.
[Chen, Xiang-Zhong] Fudan Univ, Inst Optoelect, Songhu Rd 2005, Shanghai 200438, Peoples R China.
C3 Shenyang Pharmaceutical University; Swiss Federal Institutes of Technology Domain; ETH Zurich; University of North Carolina; University of North Carolina Chapel Hill; Swiss Federal Institutes of Technology Domain; ETH Zurich; South China University of Technology; Fudan University
RP He, ZG; Sun, J (通讯作者), Shenyang Pharmaceut Univ, Wuya Coll Innovat, Dept Pharmaceut, Shenyang 110016, Liaoning, Peoples R China.; Chen, XZ (通讯作者), Swiss Fed Inst Technol, IRIS, MSRL, CH-8092 Zurich, Switzerland.; Liu, HC (通讯作者), South China Univ Technol, State Key Lab Pulp & Paper Engn, Guangzhou 510641, Peoples R China.; Chen, XZ (通讯作者), Fudan Univ, Inst Optoelect, Songhu Rd 2005, Shanghai 200438, Peoples R China.
EM liuhch@scut.edu.cn; chenxian@ethz.ch; hezhonggui@vip.163.com; sunjin@syphu.edu.cn
RI ; shen, yang/X-2665-2018
OI Pane, Salvador/0000-0003-0147-8287; /0000-0002-1114-6481; Ye,

Hao/0000-0003-3526-9627; shen, yang/0000-0003-4278-1477
FU National Natural Science Foundation of China [82073777]; Liaoning Revitalization Talents Program [XLYC1808017]; Shenyang Youth Science and Technology Innovation Talents Program [RC190454]; Swiss National Science Foundation [206033]; National Natural Science Foundations of China [82161138029]; Sino-Swiss Science and Technology Cooperation project; Open Fund of the State Key Laboratory on Integrated Optoelectronics [IOSKL2020KF03]
FX This work was financially supported by the National Natural Science Foundation of China (No. 82073777), Liaoning Revitalization Talents Program (No. XLYC1808017), Shenyang Youth Science and Technology Innovation Talents Program (No. RC190454), Swiss National Science Foundation (No. 206033) and National Natural Science Foundations of China (Grant 82161138029) for a Sino-Swiss Science and Technology Cooperation project, and the Open Fund of the State Key Laboratory on Integrated Optoelectronics (IOSKL2020KF03). We thank Q. Chen (Soochow University) for providing the dual-cartridge sprayer device.
CR Adams CJ, 2019, FRONT MOL BIOSCI, V6, DOI 10.3389/fmolb.2019.00011
Anderson RL, 2019, NAT REV CLIN ONCOL, V16, P185, DOI 10.1038/s41571-018-0134-8
Chen G, 2018, NATURE, V560, P382, DOI 10.1038/s41586-018-0392-8
Chen LP, 2015, J CLIN INVEST, V125, P3384, DOI 10.1172/JCI80011
Chen Q, 2019, NAT NANOTECHNOL, V14, P89, DOI 10.1038/s41565-018-0319-4
Diskin B, 2020, NAT IMMUNOL, V21, P442, DOI 10.1038/s41590-020-0620-x
Fathi P, 2021, VIEW-CHINA, V2, DOI 10.1002/VIW.20200187
Fichter KM, 2013, ACS NANO, V7, P347, DOI 10.1021/nn304218q
Galen J., 2013, BIO-PROTOCOL, DOI [10.21769/BioProtoc.906, DOI 10.21769/BIOPROTOC.906]
Glick BS, 2009, ANNU REV CELL DEV BI, V25, P113, DOI 10.1146/annurev.cellbio.24.110707.175421
Ibrahim A, 2016, ANNU REV PHYSIOL, V78, P67, DOI 10.1146/annurev-physiol-021115-104929
Jiang Q, 2019, BIOMATERIALS, V192, P292, DOI 10.1016/j.biomaterials.2018.11.021
Kalluri R, 2020, SCIENCE, V367, P640, DOI 10.1126/science.aau6977
Kong HM, 2021, ADV SCI, V8, DOI 10.1002/advs.202102051
Kwon ED, 1999, P NATL ACAD SCI USA, V96, P15074, DOI 10.1073/pnas.96.26.15074
Li HH, 2019, ACS NANO, V13, P9386, DOI 10.1021/acsnano.9b04166
Liu C, 2019, NANO LETT, V19, P7836, DOI 10.1021/acs.nanolett.9b02841
Lu Y, 2017, NAT REV MATER, V2, DOI 10.1038/natrevmats.2016.75
Luo JW, 2019, ACS NANO, V13, P3910, DOI 10.1021/acsnano.8b06924
Ma DH, 2011, CELL, V145, P1102, DOI 10.1016/j.cell.2011.06.007
Malfitano AM, 2020, CANCERS, V12, DOI 10.3390/cancers12071987
Mallard F, 2002, J CELL BIOL, V156, P653, DOI 10.1083/jcb.200110081
Marban E, 2018, J AM COLL CARDIOL, V71, P193, DOI 10.1016/j.jacc.2017.11.013
Morrissey SM, 2020, TRENDS CANCER, V6, P550, DOI 10.1016/j.trecan.2020.03.002
Murray RZ, 2005, J BIOL CHEM, V280, P10478, DOI 10.1074/jbc.M414420200
Murray RZ, 2005, SCIENCE, V310, P1492, DOI 10.1126/science.1120225
Page DB, 2014, ANNU REV MED, V65, P185, DOI 10.1146/annurev-med-092012-112807
Pardoll DM, 2012, NAT REV CANCER, V12, P252, DOI 10.1038/nrc3239
Poggio M, 2019, CELL, V177, P414, DOI 10.1016/j.cell.2019.02.016
Qiu C, 2019, NAT COMMUN, V10, DOI 10.1038/s41467-019-10562-w
Ribas A, 2016, JAMA-J AM MED ASSOC, V315, P1600, DOI 10.1001/jama.2016.4059
Sano R, 2013, BBA-MOL CELL RES, V1833, P3460, DOI 10.1016/j.bbamcr.2013.06.028
Sharma P, 2017, CELL, V168, P707, DOI 10.1016/j.cell.2017.01.017
Shitara A, 2013, MOL CELL BIOCHEM, V380, P11, DOI 10.1007/s11010-013-1652-4
Stephan SB, 2015, NAT BIOTECHNOL, V33, P97, DOI 10.1038/nbt.3104
Tabas I, 2011, NAT CELL BIOL, V13, P184, DOI 10.1038/ncb0311-184
Topalian SL, 2016, NAT REV CANCER, V16, P275, DOI 10.1038/nrc.2016.36
Tsang KY, 2010, J CELL SCI, V123, P2145, DOI 10.1242/jcs.068833
Turajlic S, 2016, SCIENCE, V352, P169, DOI 10.1126/science.aaf2784
Villarroya-Beltri C, 2016, NAT COMMUN, V7, DOI 10.1038/ncomms13588
von Blume J, 2012, J CELL BIOL, V199, P1057, DOI 10.1083/jcb.201207180
Wang C, 2017, ADV MATER, V29, DOI 10.1002/adma.201606036
Wang C, 2017, NAT BIOMED ENG, V1, DOI 10.1038/s41551-016-0011
Wang DD, 2018, ACS NANO, V12, P5241, DOI 10.1021/acsnano.7b08355

Wang GH, 2021, NAT COMMUN, V12, DOI 10.1038/s41467-021-25990-w
Wang KY, 2020, BIOMATERIALS, V257, DOI 10.1016/j.biomaterials.2020.120224
Wang R, 2015, CELL PHYSIOL BIOCHEM, V35, P969, DOI 10.1159/000369753
Wang YZ, 2011, CSH PERSPECT BIOL, V3, DOI 10.1101/cshperspect.a005330
Wang YN, 2020, J BIOMED SCI, V27, DOI 10.1186/s12929-020-00670-x
Wang YT, 2018, FRONT PHARMACOL, V9, DOI 10.3389/fphar.2018.00536
Ye H, 2020, BIOMATERIALS, V242, DOI 10.1016/j.biomaterials.2020.119932
Ye H, 2019, BIOMATERIALS, V206, P1, DOI 10.1016/j.biomaterials.2019.03.024
Yong TY, 2019, NAT COMMUN, V10, DOI 10.1038/s41467-019-11718-4
Yu RY, 2018, BIOMATER SCI-UK, V6, P2144, DOI 10.1039/c8bm00381e
Zaretsky JM, 2016, NEW ENGL J MED, V375, P819, DOI 10.1056/NEJMoa1604958
Zhang XM, 2017, J CELL BIOL, V216, P2151, DOI 10.1083/jcb.201702099
Zhao MN, 2023, ACTA PHARM SIN B, V13, P916, DOI [10.1016/j.apsb.2022.10.019, 10.1016/j.apsb.2022.10.0192211-3835]
NR 57
TC 0
Z9 0
U1 26
U2 26
PU AMER CHEMICAL SOC
PI WASHINGTON
PA 1155 16TH ST, NW, WASHINGTON, DC 20036 USA
SN 1936-0851
EI 1936-086X
J9 ACS NANO
JI ACS Nano
PD MAY 22
PY 2023
VL 17
IS 11
BP 10637
EP 10650
DI 10.1021/acsnano.3c01733
EA MAY 2023
PG 14
WC Chemistry, Multidisciplinary; Chemistry, Physical; Nanoscience & Nanotechnology; Materials Science, Multidisciplinary
WE Science Citation Index Expanded (SCI-EXPANDED)
SC Chemistry; Science & Technology - Other Topics; Materials Science
GA J0MI9
UT WOS:001012204400001
PM 37213184
DA 2023-09-14
ER

PT J
AU Mao, LZ
Ma, PQ
Luo, X
Cheng, HW
Wang, ZX
Ye, EY
Loh, XJ
Wu, YL
Li, ZB
AF Mao, Liuzhou
Ma, Panqin
Luo, Xi
Cheng, Hongwei
Wang, Zhanxiang
Ye, Enyi

Loh, Xian Jun
Wu, Yun-Long
Li, Zibiao
TI Stimuli-Responsive Polymeric Nanovaccines Toward Next-Generation Immunotherapy
SO ACS NANO
LA English
DT Review
DE stimuli-responsive; polymer nanoparticle; nanovaccine; immunotherapy; cancer; antigen-presenting cells; adjuvants; antigens
ID DELIVERY-SYSTEM; CANCER-IMMUNOTHERAPY; ANTIGEN-DELIVERY; IN-VITRO; DENDRITIC CELLS; CO-DELIVERY; IMMUNE-RESPONSE; NANOPARTICLES; VACCINE; PH
AB The development of nanovaccines that employ polymericdeliverycarriers has garnered substantial interest in therapeutic treatmentof cancer and a variety of infectious diseases due to their superiorbiocompatibility, lower toxicity and reduced immunogenicity. Particularly,stimuli-responsive polymeric nanocarriers show great promise for deliveringantigens and adjuvants to targeted immune cells, preventing antigendegradation and clearance, and increasing the uptake of specific antigen-presentingcells, thereby sustaining adaptive immune responses and improvingimmunotherapy for certain diseases. In this review, the most recentadvances in the utilization of stimulus-responsive polymer-based nanovaccinesfor immunotherapeutic applications are presented. These sophisticatedpolymeric nanovaccines with diverse functions, aimed at therapeuticadministration for disease prevention and immunotherapy, are furtherclassified into several active domains, including pH, temperature,redox, light and ultrasound-sensitive intelligent nanodelivery systems.Finally, the potential strategies for the future design of multifunctionalnext-generation polymeric nanovaccines by integrating materials sciencewith biological interface are proposed.
C1 [Mao, Liuzhou; Ma, Panqin; Wu, Yun-Long] Xiamen Univ, Sch Pharmaceut Sci, Fujian Prov Key Lab Innovat Drug Target Res, Xiamen 361102, Peoples R China.
[Mao, Liuzhou; Ma, Panqin; Wu, Yun-Long] Xiamen Univ, Sch Pharmaceut Sci, State Key Lab Cellular Stress Biol, Xiamen 361102, Peoples R China.
[Luo, Xi; Wang, Zhanxiang] Xiamen Univ, Affiliated Hosp, BE Phase Clin Ctr 1, Sch Med, Xiamen 361000, Peoples R China.
[Cheng, Hongwei] Xiamen Univ, Sch Publ Hlth, State Key Lab Mol Vaccinol & Mol Diagnost, Xiamen 361102, Peoples R China.
[Cheng, Hongwei] Xiamen Univ, Ctr Mol Imaging & Translat Med, Sch Publ Hlth, Xiamen 361102, Peoples R China.
[Ye, Enyi; Loh, Xian Jun; Li, Zibiao] ASTAR, Inst Mat Res & Engn IMRE, Singapore 138634, Singapore.
[Ye, Enyi; Loh, Xian Jun; Li, Zibiao] ASTAR, Inst Sustainabil Chem Energy & Environm (ISCE2), Singapore 627833, Singapore.
C3 Xiamen University; Xiamen University; Xiamen University; Xiamen University; Xiamen University; Agency for Science Technology & Research (A*STAR); A*STAR - Institute of Materials Research & Engineering (IMRE); Agency for Science Technology & Research (A*STAR)
RP Wu, YL (通讯作者)，Xiamen Univ, Sch Pharmaceut Sci, Fujian Prov Key Lab Innovat Drug Target Res, Xiamen 361102, Peoples R China.; Wu, YL (通讯作者)，Xiamen Univ, Sch Pharmaceut Sci, State Key Lab Cellular Stress Biol, Xiamen 361102, Peoples R China.; Wang, ZX (通讯作者)，Xiamen Univ, Affiliated Hosp, BE Phase Clin Ctr 1, Sch Med, Xiamen 361000, Peoples R China.; Li, ZB (通讯作者)，ASTAR, Inst Mat Res & Engn IMRE, Singapore 138634, Singapore.; Li, ZB (通讯作者)，ASTAR, Inst Sustainabil Chem Energy & Environm (ISCE2), Singapore 627833, Singapore.
EM wangzx@xmu.edu.cn; wuyl@xmu.edu.cn; lizb@imre.a-star.edu.sg
OI Wu, Yun-Long/0000-0001-6426-6340; YE, Enyi/0000-0002-2398-0676
FU National Key R&D Program of China [2020YFA0908100]; Natural Science Foundation of China [81971724, 82173750]; Leading Innovative and Entrepreneur Team Introduction Program of Zhejiang [2020R01018, 2021B42001, 2022C02037]; A*STAR Science and Engineering Research Council (SERC) Central Research Fund
FX The authors would like to express gratitude to the National Key R&D Program of China (grant no. 2020YFA0908100), and the Natural Science Foundation of China (81971724 and 82173750), the Leading Innovative and Entrepreneur Team Introduction Program of Zhejiang (2020R01018, 2021B42001, 2022C02037), and the A*STAR Science and Engineering Research

Council (SERC) Central Research Fund (Use-inspired Basic Research) for
support of this project.
CR Allard B, 2016, CURR OPIN PHARMACOL, V29, P7, DOI 10.1016/j.coph.2016.04.001
Appel EA, 2015, NAT COMMUN, V6, DOI 10.1038/ncomms7295
Appel EA, 2012, CHEM SOC REV, V41, P6195, DOI 10.1039/c2cs35264h
Arpaia N, 2011, CURR OPIN VIROL, V1, P447, DOI 10.1016/j.coviro.2011.10.006
Bauhuber S, 2009, ADV MATER, V21, P3286, DOI 10.1002/adma.200802453
Bhardwaj P, 2020, ACTA BIOMATER, V108, P1, DOI 10.1016/j.actbio.2020.03.020
Blakney AK, 2021, J CONTROL RELEASE, V338, P201, DOI 10.1016/j.jconrel.2021.08.029
Blakney AK, 2020, ACS NANO, V14, P5711, DOI 10.1021/acsnano.0c00326
Blass E, 2021, NAT REV CLIN ONCOL, V18, P215, DOI 10.1038/s41571-020-00460-2
BREM H, 1992, J CONTROL RELEASE, V19, P325, DOI 10.1016/0168-3659(92)90087-8
Brito LA, 2014, J CONTROL RELEASE, V190, P563, DOI 10.1016/j.jconrel.2014.06.027
Brito LA, 2013, SEMIN IMMUNOL, V25, P130, DOI 10.1016/j.smim.2013.05.007
Cajot S, 2011, J CONTROL RELEASE, V152, P30, DOI 10.1016/j.jconrel.2011.03.026
Carson CS, 2022, J CONTROL RELEASE, V345, P354, DOI 10.1016/j.jconrel.2022.03.020
Chao Y, 2018, NAT BIOMED ENG, V2, P611, DOI 10.1038/s41551-018-0262-6
Chen DS, 2013, IMMUNITY, V39, P1, DOI 10.1016/j.immuni.2013.07.012
Chen DL, 2020, DRUG DELIV, V27, P1094, DOI 10.1080/10717544.2020.1797245
Chen G, 2019, CHEM SOC REV, V48, P1434, DOI 10.1039/c8cs00801a
Chen JA, 2011, INT J NANOMED, V6, P77, DOI 10.2147/IJN.S15457
Chen Q, 2019, NAT NANOTECHNOL, V14, P89, DOI 10.1038/s41565-018-0319-4
Chen W, 2014, J CONTROL RELEASE, V190, P398, DOI 10.1016/j.jconrel.2014.05.023
Couzin-Frankel J, 2013, SCIENCE, V342, P1432, DOI 10.1126/science.342.6165.1432
Da Silva CG, 2019, BIOMATERIALS, V220, DOI 10.1016/j.biomaterials.2019.119417
Danhier F, 2012, J CONTROL RELEASE, V161, P505, DOI 10.1016/j.jconrel.2012.01.043
de Figueiredo P, 2015, AM J PATHOL, V185, P1505, DOI 10.1016/j.ajpath.2015.03.003
Diwan M, 2002, J CONTROL RELEASE, V85, P247, DOI 10.1016/S0168-3659(02)00275-4
Dong YX, 2017, ADV FUNCT MATER, V27, DOI 10.1002/adfm.201606619
Du LL, 2018, BIOMATERIALS, V176, P84, DOI 10.1016/j.biomaterials.2018.05.046
Duan F, 2017, BIOMATERIALS, V122, P23, DOI 10.1016/j.biomaterials.2017.01.017
Fang RH, 2014, NANO LETT, V14, P2181, DOI 10.1021/nl500618u
Frankiw L, 2019, NAT REV IMMUNOL, V19, P675, DOI 10.1038/s41577-019-0195-7
Fu DJ, 2021, DRUG DELIV, V28, P195, DOI 10.1080/10717544.2020.1870583
Fu J, 2015, SCI TRANSL MED, V7, DOI 10.1126/scitranslmed.aaa4306
Giussani M, 2015, SEMIN CANCER BIOL, V35, P3, DOI 10.1016/j.semcancer.2015.09.012
Gong LL, 2018, NANO RES, V11, P4574, DOI [10.1007/s12274-018-2037-5, 10.1007/s12274-018-2117-6]
Gong NQ, 2020, NAT NANOTECHNOL, V15, P1053, DOI 10.1038/s41565-020-00782-3
Gou SS, 2021, NANO LETT, V21, P9939, DOI 10.1021/acs.nanolett.1c03243
Gou SS, 2021, THERANOSTICS, V11, P7308, DOI 10.7150/thno.56406
Grant EV, 2012, EUR J IMMUNOL, V42, P2937, DOI 10.1002/eji.201242410
Guan QQ, 2020, J CONTROL RELEASE, V320, P392, DOI 10.1016/j.jconrel.2020.01.048
Guan XW, 2018, BIOMATERIALS, V171, P198, DOI 10.1016/j.biomaterials.2018.04.039
Guo X, 2013, BIOMATERIALS, V34, P4544, DOI 10.1016/j.biomaterials.2013.02.071
Guo YY, 2015, ACS NANO, V9, P6918, DOI 10.1021/acsnano.5b01042
Hassan HAFM, 2016, BIOMATERIALS, V104, P310, DOI 10.1016/j.biomaterials.2016.07.005
Hassan HAFM, 2016, J CONTROL RELEASE, V225, P205, DOI 10.1016/j.jconrel.2016.01.030
He CB, 2016, NAT COMMUN, V7, DOI 10.1038/ncomms12499
He K, 2020, ADV MATER, V32, DOI 10.1002/adma.202001130
Hu YY, 2020, BIOMATERIALS, V252, DOI 10.1016/j.biomaterials.2020.120114
Kaneko M, 2021, ACS MACRO LETT, V10, P926, DOI 10.1021/acsmacrolett.1c00336
Kaneko M, 2017, BIOELECTROCHEMISTRY, V114, P8, DOI 10.1016/j.bioelechem.2016.11.001
Kasturi SP, 2011, NATURE, V470, P543, DOI 10.1038/nature09737
Ke LJ, 2020, ADV THER-GERMANY, V3, DOI 10.1002/adtp.201900213
Khan N, 2017, FRONT MICROBIOL, V8, DOI 10.3389/fmicb.2017.00742
Kim M, 2019, ADV SCI, V6, DOI 10.1002/advs.201900471
KREUTER J, 1976, EXP CELL BIOL, V44, P12
Krieg AM, 2008, ONCOGENE, V27, P161, DOI 10.1038/sj.onc.1210911
Krieg AM, 2006, NAT REV DRUG DISCOV, V5, P471, DOI 10.1038/nrd2059
Kultti Anne, 2012, Cancers (Basel), V4, P873, DOI 10.3390/cancers4030873

Kumar S, 2016, J MATER CHEM B, V4, P7950, DOI 10.1039/c6tb02181f
Lan GX, 2018, J AM CHEM SOC, V140, P5670, DOI 10.1021/jacs.8b01072
Lawrence DA, 1996, J LEUKOCYTE BIOL, V60, P611, DOI 10.1002/jlb.60.5.611
Le Quoc-Viet, 2018, Journal of Pharmaceutical Investigation, V48, P527, DOI 10.1007/s40005-018-0399-z
Li DD, 2020, ADV HEALTHC MATER, V9, DOI 10.1002/adhm.202000605
Li P, 2013, J CONTROL RELEASE, V168, P271, DOI 10.1016/j.jconrel.2013.03.025
Li T, 2022, NANO LETT, V22, P3095, DOI 10.1021/acs.nanolett.2c00500
Li X, 2022, ACS NANO, V16, P14982, DOI 10.1021/acsnano.2c06026
Li X, 2022, SMALL, V18, DOI 10.1002/smll.202203100
Li X, 2020, NANO LETT, V20, P4454, DOI 10.1021/acs.nanolett.0c01230
Liang RJ, 2017, BIOMATERIALS, V149, P41, DOI 10.1016/j.biomaterials.2017.09.029
Liang XY, 2018, NANOSCALE, V10, P9489, DOI 10.1039/c8nr00355f
Liang XY, 2018, MOL PHARMACEUT, V15, P508, DOI 10.1021/acs.molpharmaceut.7b00890
Lin LCW, 2019, ADV FUNCT MATER, V29, DOI 10.1002/adfm.201807616
Liu D, 2016, THERANOSTICS, V6, P1306, DOI 10.7150/thno.14858
Liu H, 2022, J CONTROL RELEASE, V345, P91, DOI 10.1016/j.jconrel.2022.03.006
Liu J, 2022, ADV MATER, V34, DOI 10.1002/adma.202106654
Liu J, 2020, NANO LETT, V20, P4882, DOI 10.1021/acs.nanolett.0c00893
Liu MT, 2023, CHEM ENG J, V453, DOI 10.1016/j.cej.2022.139879
Liu MT, 2021, SMALL METHODS, V5, DOI 10.1002/smtd.202100347
Liu QQ, 2019, ACS APPL MATER INTER, V11, P47798, DOI 10.1021/acsami.9b19446
Liu X, 2021, BIOMATERIALS, V275, DOI 10.1016/j.biomaterials.2021.120921
Liu X, 2020, BIOMATERIALS, V230, DOI 10.1016/j.biomaterials.2019.119649
Liu YR, 2014, BIOMACROMOLECULES, V15, P3836, DOI 10.1021/bm501166j
Liu YT, 2022, ADV HEALTHC MATER, V11, DOI 10.1002/adhm.202102781
Lizotte PH, 2016, NAT NANOTECHNOL, V11, P295, DOI [10.1038/NNANO.2015.292, 10.1038/nnano.2015.292]
Lou B, 2019, J CONTROL RELEASE, V293, P48, DOI 10.1016/j.jconrel.2018.11.006
Lu YX, 2020, MAT SCI ENG C-MATER, V106, DOI 10.1016/j.msec.2019.110294
Luo M, 2017, NAT NANOTECHNOL, V12, P648, DOI [10.1038/nnano.2017.52, 10.1038/NNANO.2017.52]
Luo YF, 2023, ACS NANO, V17, P5211, DOI 10.1021/acsnano.2c12606
Lybaert L, 2018, J CONTROL RELEASE, V289, P125, DOI 10.1016/j.jconrel.2018.09.009
Ma FH, 2017, ACS NANO, V11, P10549, DOI 10.1021/acsnano.7b05947
Matsiko A, 2018, NAT MATER, V17, P472, DOI 10.1038/s41563-018-0091-8
McCarley RL, 2012, ANNU REV ANAL CHEM, V5, P391, DOI 10.1146/annurev-anchem-062011-143157
Mellman I, 2011, NATURE, V480, P480, DOI 10.1038/nature10673
Meng FH, 2009, BIOMATERIALS, V30, P2180, DOI 10.1016/j.biomaterials.2009.01.026
Meng ZQ, 2021, NANO LETT, V21, P1228, DOI 10.1021/acs.nanolett.0c03646
Merkel OM, 2011, BIOMATERIALS, V32, P4936, DOI 10.1016/j.biomaterials.2011.03.035
Miao J, 2019, CARBOHYD POLYM, V212, P215, DOI 10.1016/j.carbpol.2019.02.047
Miao L, 2021, MOL CANCER, V20, DOI 10.1186/s12943-021-01335-5
Min YZ, 2017, NAT NANOTECHNOL, V12, P877, DOI [10.1038/nnano.2017.113, 10.1038/NNANO.2017.113]
Minn AJ, 2016, CELL, V165, P272, DOI 10.1016/j.cell.2016.03.031
Mishra B, 2010, NANOMED-NANOTECHNOL, V6, P9, DOI 10.1016/j.nano.2009.04.008
Molino NM, 2016, BIOMATERIALS, V86, P83, DOI 10.1016/j.biomaterials.2016.01.056
Nathan C, 2013, NAT REV IMMUNOL, V13, P349, DOI 10.1038/nri3423
Oelke M, 2003, NAT MED, V9, P619, DOI 10.1038/nm869
Olson B, 2018, CANCER DISCOV, V8, P1358, DOI 10.1158/2159-8290.CD-18-0044
Oroojalian F, 2021, SMALL, V17, DOI 10.1002/smll.202006484
Orr MT, 2013, J CONTROL RELEASE, V172, P190, DOI 10.1016/j.jconrel.2013.07.030
Palucka K, 2012, NAT REV CANCER, V12, P265, DOI 10.1038/nrc3258
Pan SW, 2020, ADV FUNCT MATER, V30, DOI 10.1002/adfm.201909540
Pang X, 2016, J CONTROL RELEASE, V222, P116, DOI 10.1016/j.jconrel.2015.12.024
Papa AL, 2017, BIOMATERIALS, V139, P187, DOI 10.1016/j.biomaterials.2017.06.003
Phan VHG, 2019, BIOMATERIALS, V195, P100, DOI 10.1016/j.biomaterials.2018.12.034
Plank C, 1996, HUM GENE THER, V7, P1437, DOI 10.1089/hum.1996.7.12-1437
Potineni A, 2003, J CONTROL RELEASE, V86, P223, DOI 10.1016/S0168-3659(02)00374-7
Rahim MA, 2019, ANGEW CHEM INT EDIT, V58, P1904, DOI 10.1002/anie.201807804

Ratput MKS, 2018, ACS APPL MATER INTER, V10, P27589, DOI 10.1021/acsami.8b02019
Reed SG, 2013, NAT MED, V19, P1597, DOI 10.1038/nm.3409
Rezvantalab S, 2018, FRONT PHARMACOL, V9, DOI 10.3389/fphar.2018.01260
Riley RS, 2019, NAT REV DRUG DISCOV, V18, P175, DOI 10.1038/s41573-018-0006-z
Roche PA, 2015, NAT REV IMMUNOL, V15, P203, DOI 10.1038/nri3818
Rodell CB, 2018, NAT BIOMED ENG, V2, P578, DOI 10.1038/s41551-018-0236-8
Rosenberg SA, 2004, NAT MED, V10, P909, DOI 10.1038/nm1100
Roth GA, 2020, ACS CENTRAL SCI, V6, P1800, DOI 10.1021/acscentsci.0c00732
Roy D, 2013, CHEM SOC REV, V42, P7214, DOI 10.1039/c3cs35499g
Ruterbusch M, 2020, ANNU REV IMMUNOL, V38, P705, DOI 10.1146/annurev-immunol-103019-085803
Vallecillo MFS, 2014, BIOMATERIALS, V35, P2529, DOI 10.1016/j.biomaterials.2013.12.002
Shae D, 2020, ACS NANO, V14, P9904, DOI 10.1021/acsnano.0c02765
Shae D, 2019, NAT NANOTECHNOL, V14, P269, DOI 10.1038/s41565-018-0342-5
Shao JW, 2020, ASIAN J PHARM SCI, V15, P685, DOI 10.1016/j.ajps.2020.03.001
Simon-Vazquez R, 2020, EXPERT OPIN DRUG DEL, V17, P1007, DOI 10.1080/17425247.2020.1776259
Singh A, 2021, NAT NANOTECHNOL, V16, P16, DOI 10.1038/s41565-020-00790-3
Singh A, 2014, ADV MATER, V26, P6530, DOI 10.1002/adma.201402105
Singh D, 2015, MOL IMMUNOL, V68, P272, DOI 10.1016/j.molimm.2015.09.011
Singh M, 2000, P NATL ACAD SCI USA, V97, P811, DOI 10.1073/pnas.97.2.811
Stickdorn J, 2022, ACS NANO, V16, P4426, DOI 10.1021/acsnano.1c10709
Su TQ, 2022, J CONTROL RELEASE, V343, P152, DOI 10.1016/j.jconrel.2021.12.036
Su T, 2022, ADV SCI, V9, DOI 10.1002/advs.202201895
Sui Y, 2022, ASIAN J PHARM SCI, V17, P583, DOI 10.1016/j.ajps.2022.05.004
Tang L, 2018, NAT BIOTECHNOL, V36, P707, DOI 10.1038/nbt.4181
Tang M, 2017, CELL MOL LIFE SCI, V74, P761, DOI 10.1007/s00018-016-2317-8
Thomas CE, 2003, NAT REV GENET, V4, P346, DOI 10.1038/nrg1066
Thomas C, 2011, MOL PHARMACEUT, V8, P405, DOI 10.1021/mp100255c
Wagner-Muniz DA, 2018, FRONT IMMUNOL, V9, DOI 10.3389/fimmu.2018.00325
Wang C, 2016, BIOMATERIALS, V79, P88, DOI 10.1016/j.biomaterials.2015.11.040
Wang DG, 2016, NANO LETT, V16, P5503, DOI 10.1021/acs.nanolett.6b01994
Wang HX, 2016, NANO TODAY, V11, P133, DOI 10.1016/j.nantod.2016.04.008
Wang JW, 2021, ACS NANO, V15, P18671, DOI 10.1021/acsnano.1c10313
Wang J, 2014, BIOMATERIALS, V35, P3080, DOI 10.1016/j.biomaterials.2013.12.025
Wang M, 2019, SMALL METHODS, V3, DOI 10.1002/smtd.201900025
Wang X, 2020, NAT COMMUN, V11, DOI 10.1038/s41467-020-19651-7
Wang X, 2022, J CONTROL RELEASE, V349, P18, DOI 10.1016/j.jconrel.2022.06.054
Wang Y, 2021, MOL CANCER, V20, DOI 10.1186/s12943-021-01311-z
Woodford N, 2011, FEMS MICROBIOL REV, V35, P736, DOI 10.1111/j.1574-6976.2011.00268.x
Xiong X, 2021, NANO LETT, V21, P8418, DOI 10.1021/acs.nanolett.1c03004
Xu J, 2020, NAT NANOTECHNOL, V15, P1043, DOI 10.1038/s41565-020-00781-4
Xu J, 2019, BIOMATERIALS, V207, P1, DOI 10.1016/j.biomaterials.2019.03.037
Xu SW, 2022, INT J BIOL MACROMOL, V222, P1936, DOI 10.1016/j.ijbiomac.2022.09.283
Xu SW, 2019, INT J BIOL MACROMOL, V125, P865, DOI 10.1016/j.ijbiomac.2018.12.156
Xu YD, 2022, BIOMATERIALS, V284, DOI 10.1016/j.biomaterials.2022.121489
Yamamoto TN, 2019, NAT MED, V25, P1488, DOI 10.1038/s41591-019-0596-y
Yan P, 2021, ADV HEALTHC MATER, V10, DOI 10.1002/adhm.202101222
Yang PX, 2018, NANO LETT, V18, P4377, DOI 10.1021/acs.nanolett.8b01406
Yang R, 2018, ACS NANO, V12, P5121, DOI 10.1021/acsnano.7b09041
Yang WJ, 2020, SCI ADV, V6, DOI 10.1126/sciadv.abd1631
Yin Y, 2021, NANO LETT, V21, P2224, DOI 10.1021/acs.nanolett.0c05039
Yu XY, 2019, J COLLOID INTERF SCI, V556, P258, DOI 10.1016/j.jcis.2019.08.027
Yuba E, 2013, BIOMATERIALS, V34, P5711, DOI 10.1016/j.biomaterials.2013.04.007
Zeng Q, 2017, BIOMATERIALS, V122, P105, DOI 10.1016/j.biomaterials.2017.01.010
Zhang BD, 2022, NANO RES, V15, P6328, DOI 10.1007/s12274-022-4282-x
Zhang CN, 2017, J CONTROL RELEASE, V256, P170, DOI 10.1016/j.jconrel.2017.04.020
Zhang LH, 2019, NANO LETT, V19, P4237, DOI 10.1021/acs.nanolett.9b00030
Zhang PJ, 2017, MICROB PATHOGENESIS, V111, P81, DOI 10.1016/j.micpath.2017.08.023
Zhang YX, 2023, MATER HORIZ, V10, P361, DOI 10.1039/d2mh01358d
Zhang Y, 2020, ADV THER-GERMANY, V3, DOI 10.1002/adtp.202000083
Zhang ZP, 2011, BIOMATERIALS, V32, P3666, DOI 10.1016/j.biomaterials.2011.01.067
Zhao H, 2019, ACS NANO, V13, P13127, DOI 10.1021/acsnano.9b05974

Zhou JR, 2020, ADV MATER, V32, DOI 10.1002/adma.201901255
Zhou L, 2020, NANO LETT, V20, P4393, DOI 10.1021/acs.nanolett.0c01140
Zhou QH, 2021, ADV THER-GERMANY, V4, DOI 10.1002/adtp.202100130
Zhou X, 2020, MOL PHARMACEUT, V17, P4603, DOI 10.1021/acs.molpharmaceut.0c00802
Zhou Y, 2019, ADV FUNCT MATER, V29, DOI 10.1002/adfm.201806220
Zhu HJ, 2017, ACS NANO, V11, P8998, DOI 10.1021/acsnano.7b03507
Zhuang WH, 2018, ACS APPL MATER INTER, V10, P18489, DOI 10.1021/acsami.8b02890
Zong JB, 2016, CANCER IMMUNOL IMMUN, V65, P821, DOI 10.1007/s00262-016-1820-y
NR 184
TC 0
Z9 0
U1 22
U2 22
PU AMER CHEMICAL SOC
PI WASHINGTON
PA 1155 16TH ST, NW, WASHINGTON, DC 20036 USA
SN 1936-0851
EI 1936-086X
J9 ACS NANO
JI ACS Nano
PD MAY 19
PY 2023
VL 17
IS 11
BP 9826
EP 9849
DI 10.1021/acsnano.3c02273
EA MAY 2023
PG 24
WC Chemistry, Multidisciplinary; Chemistry, Physical; Nanoscience &
Nanotechnology; Materials Science, Multidisciplinary
WE Science Citation Index Expanded (SCI-EXPANDED)
SC Chemistry; Science & Technology - Other Topics; Materials Science
GA J0HG6
UT WOS:001012173400001
PM 37207347
DA 2023-09-14
ER

PT J
AU Shin, H
Kim, Y
Jon, S
AF Shin, Hocheol
Kim, Yujin
Jon, Sangyong
TI Nanovaccine Displaying Immunodominant T Cell Epitopes of Fibroblast
Activation Protein Is Effective Against Desmoplastic Tumors
SO ACS NANO
LA English
DT Article
DE cancer-associated fibroblast; fibroblast activationprotein; cancer
vaccines; lipid nanoparticles; nanovaccines
ID STROMAL CELLS; CANCER; DEPLETION; IMMUNITY
AB Cancer-associated fibroblasts (CAFs), which are dominantcell typesin the tumor microenvironment (TME), support tumor growth by secretingcytokines and forming an extracellular matrix (ECM) that hampers thepenetration of chemical and biological therapeutics within the tumorand thereby limits their therapeutic efficacy. Here, we report a cancernanovaccine targeting fibroblast activation protein alpha (FAP)-expressingCAFs as a potential pan-tumor vaccine. We predicted immunodominantFAP-specific epitope peptides in silico and selectedtwo candidate peptides after in vitro and in vivo screening for immunogenicity and antitumor efficacy.Next, we developed a nanoparticle-based vaccine that displays

thetwo selected epitope peptides on the surface of lipid nanoparticlesencapsulating CpG adjuvant (FAP(PEP)-SLNPs). Immunizationwith one of two FAP(PEP)-SLNP nanovaccines led to considerablegrowth inhibition of various tumors, including desmoplastic tumors,by depleting FAP(+) CAFs and thereby reducing ECM productionin the TME while causing little appreciable adverse effects. Furthermore,when combined with a chemotherapeutic drug, the FAP(PEP)-SLNPnanovaccine increased drug accumulation and resulted in a synergisticantitumor efficacy far better than that of each corresponding monotherapy.These findings suggest that our FAP(PEP)-SLNP nanovaccinehas potential for use as an "off-the-shelf" pan-tumorvaccine applicable to a variety of tumors and may be a suitable platformfor use in various combination therapies.
C1 [Shin, Hocheol; Kim, Yujin; Jon, Sangyong] Korea Adv Inst Sci & Technol KAIST, KAIST Inst BioCentury, Dept Biol Sci, 291 Daehak Ro, Daejeon 34141, South Korea.
[Shin, Hocheol; Kim, Yujin; Jon, Sangyong] Korea Adv Inst Sci & Technol KAIST, Ctr Precis Bionanomed, 291 Daehak Ro, Daejeon 34141, South Korea.
C3 Korea Advanced Institute of Science & Technology (KAIST); Korea Advanced Institute of Science & Technology (KAIST)
RP Jon, S (通讯作者)，Korea Adv Inst Sci & Technol KAIST, KAIST Inst BioCentury, Dept Biol Sci, 291 Daehak Ro, Daejeon 34141, South Korea.; Jon, S (通讯作者)，Korea Adv Inst Sci & Technol KAIST, Ctr Precis Bionanomed, 291 Daehak Ro, Daejeon 34141, South Korea.
EM syjon@kaist.ac.kr
OI Shin, Hocheol/0000-0002-9774-0704; Kim, Yujin/0000-0002-7186-221X; Jon, Sangyong/0000-0002-6971-586X
FU Basic Science Research Program through the National Research Foundation of Korea (NRF) - Ministry of Science and ICT [NRF-2018R1A3B1052661]; Korea Health Technology R&D Project through the Korea Health Industry Development Institute (KHIDI) - Ministry of Health Welfare [HI22C0497]
FX The authors thank Prof. Suk-Jo Kang (KAIST, Daejeon, Republic of Korea) and Prof. Chan Hyuk Kim (KAIST) for kindly providing Panc02 and MC38 cancer cell lines. This research was supported by the Basic Science Research Program through the National Research Foundation of Korea (NRF) funded by the Ministry of Science and ICT (NRF-2018R1A3B1052661) and a grant of the Korea Health Technology R&D Project through the Korea Health Industry Development Institute (KHIDI) funded by the Ministry of Health & Welfare (HI22C0497).
CR Baghba R, 2020, CELL COMMUN SIGNAL, V18, DOI 10.1186/s12964-020-0530-4
Bareke H, 2021, INT J MOL SCI, V22, DOI 10.3390/ijms22158030
Bharadwaj AG, 2009, AM J PATHOL, V174, P1027, DOI 10.2353/ajpath.2009.080501
Blass E, 2021, NAT REV CLIN ONCOL, V18, P215, DOI 10.1038/s41571-020-00460-2
Borst J, 2018, NAT REV IMMUNOL, V18, P635, DOI 10.1038/s41577-018-0044-0
Busek P, 2018, FRONT BIOSCI-LANDMRK, V23, P1933, DOI 10.2741/4682
Calis JJA, 2013, PLOS COMPUT BIOL, V9, DOI 10.1371/journal.pcbi.1003266
Chauhan VP, 2019, P NATL ACAD SCI USA, V116, P10674, DOI 10.1073/pnas.1819889116
Chen Y, 2021, NAT REV CLIN ONCOL, V18, P792, DOI 10.1038/s41571-021-00546-5
Chiodetti AL, 2018, FRONT IMMUNOL, V9, DOI 10.3389/fimmu.2018.02319
Chowell D, 2015, P NATL ACAD SCI USA, V112, pE1754, DOI 10.1073/pnas.1500973112
Dauer P, 2018, CANCER RES, V78, P1321, DOI 10.1158/0008-5472.CAN-17-2320
Duperret EK, 2018, CLIN CANCER RES, V24, P1190, DOI 10.1158/1078-0432.CCR-17-2033
Fabre M, 2020, CLIN CANCER RES, V26, P3420, DOI 10.1158/1078-0432.CCR-19-2238
Fan YC, 2015, VACCINES-BASEL, V3, P662, DOI 10.3390/vaccines3030662
Fischer C, 2020, MABS-AUSTIN, V12, DOI 10.1080/19420862.2020.1834818
Gong NQ, 2021, NAT NANOTECHNOL, V16, P25, DOI 10.1038/s41565-020-00822-y
Gong NQ, 2020, NAT NANOTECHNOL, V15, P1053, DOI 10.1038/s41565-020-00782-3
Hu ZT, 2021, NAT MED, V27, P515, DOI 10.1038/s41591-020-01206-4
Huang JC, 2021, SIGNAL TRANSDUCT TAR, V6, DOI 10.1038/s41392-021-00544-0
Kalluri R, 2016, NAT REV CANCER, V16, P582, DOI 10.1038/nrc.2016.73
Kim Y, 2020, ANGEW CHEM INT EDIT, V59, P14628, DOI 10.1002/anie.202006117
Kong J, 2019, MOL CANCER, V18, DOI 10.1186/s12943-019-1101-4
Kraman M, 2010, SCIENCE, V330, P827, DOI 10.1126/science.1195300
Kreiter S, 2015, NATURE, V520, P692, DOI 10.1038/nature14426
Kroll AV, 2017, ADV MATER, V29, DOI 10.1002/adma.201703969
Kuai R, 2017, NAT MATER, V16, P489, DOI [10.1038/nmat4822, 10.1038/NMAT4822]
Lee J, 2005, CANCER RES, V65, P11156, DOI 10.1158/0008-5472.CAN-05-2805

Lee J, 2016, THERANOSTICS, V6, P192, DOI 10.7150/thno.13657
Liu HP, 2014, NATURE, V507, P519, DOI 10.1038/nature12978
Liu TY, 2019, J HEMATOL ONCOL, V12, DOI 10.1186/s13045-019-0770-1
Nam J, 2021, ADV THER-GERMANY, V4, DOI 10.1002/adtp.202100093
Ozdemir BC, 2014, CANCER CELL, V25, P719, DOI 10.1016/j.ccr.2014.04.005
Oh DY, 2021, IMMUNITY, V54, P2701, DOI 10.1016/j.immuni.2021.11.015
Ott PA, 2018, NATURE, V555, P402, DOI 10.1038/nature25145
Phan GQ, 2003, P NATL ACAD SCI USA, V100, P8372, DOI 10.1073/pnas.1533209100
Roberts EW, 2013, J EXP MED, V210, P1137, DOI 10.1084/jem.20122344
Roth GA, 2022, NAT REV MATER, V7, P174, DOI 10.1038/s41578-021-00372-2
Santos AM, 2009, J CLIN INVEST, V119, P3613, DOI 10.1172/JCI38988
Sewe MO, 2017, SCI REP-UK, V7, DOI 10.1038/s41598-017-02560-z
Son S, 2022, NAT BIOMED ENG, DOI 10.1038/s41551-022-00973-4
Stephens AJ, 2021, FRONT IMMUNOL, V12, DOI 10.3389/fimmu.2021.696791
Tigno-Aranjuez JT, 2009, J IMMUNOL, V183, P5654, DOI 10.4049/jimmunol.0900645
Torres MP, 2013, PLOS ONE, V8, DOI 10.1371/journal.pone.0080580
Tran E, 2013, J EXP MED, V210, P1125, DOI 10.1084/jem.20130110
Turley SJ, 2015, NAT REV IMMUNOL, V15, P669, DOI 10.1038/nri3902
Wang LCS, 2014, CANCER IMMUNOL RES, V2, P154, DOI 10.1158/2326-6066.CIR-13-0027
Yu M, 2020, NAT COMMUN, V11, DOI 10.1038/s41467-019-14060-x
Zeng H, 2022, THERANOSTICS, V12, P7351, DOI 10.7150/thno.74753
Zhang XQ, 2021, NAT COMMUN, V12, DOI 10.1038/s41467-021-22898-3
Zhao YX, 2019, P NATL ACAD SCI USA, V116, P2210, DOI 10.1073/pnas.1818357116
Zinger A, 2019, ACS NANO, V13, P11008, DOI 10.1021/acsnano.9b02395
NR 52
TC 0
Z9 0
U1 9
U2 9
PU AMER CHEMICAL SOC
PI WASHINGTON
PA 1155 16TH ST, NW, WASHINGTON, DC 20036 USA
SN 1936-0851
EI 1936-086X
J9 ACS NANO
JI ACS Nano
PD MAY 15
PY 2023
VL 17
IS 11
BP 10337
EP 10352
DI 10.1021/acsnano.3c00764
PG 16
WC Chemistry, Multidisciplinary; Chemistry, Physical; Nanoscience &
Nanotechnology; Materials Science, Multidisciplinary
WE Science Citation Index Expanded (SCI-EXPANDED)
SC Chemistry; Science & Technology - Other Topics; Materials Science
GA J0FS6
UT WOS:001006454500001
PM 37184372
DA 2023-09-14
ER

PT J
AU Bhattacharjee, B
Syeda, AF
Rynjah, D
Hussain, SM
Bora, SC
Pegu, P

Sahu, RK
Khan, J
AF Bhattacharjee, Bedanta
Syeda, Ayesha Farhana
Rynjah, Damanbhalang
Hussain, Shalam M.
Bora, Shekhar Chandra
Pegu, Padmanath
Sahu, Ram Kumar
Khan, Jiyauddin
TI Pharmacological impact of microRNAs in head and neck squamous cell carcinoma: Prevailing insights on molecular pathways, diagnosis, and nanomedicine treatment
SO FRONTIERS IN PHARMACOLOGY
LA English
DT Review
DE head and neck squamous cell carcinoma; microRNA; molecular signaling; nanomedicine; nanovaccines
ID GOLD NANOPARTICLES; IN-VITRO; MAGNETIC NANOPARTICLES; CIRCULATING MICRORNAS; EXPRESSION PROFILES; RADIATION-THERAPY; DOWN-REGULATION; DRUG-DELIVERY; STEM-CELLS; CANCER
AB Head and neck squamous cell carcinoma is a disease that most commonly produce tumours from the lining of the epithelial cells of the lips, larynx, nasopharynx, mouth, or oro-pharynx. It is one of the most deadly forms of cancer. About one to two percent of all neo-plasm-related deaths are attributed to head and neck squamous cell carcinoma, which is responsible for about six percent of all cancers. MicroRNAs play a critical role in cell proliferation, differentiation, tumorigenesis, stress response, triggering apoptosis, and other physiological process. MicroRNAs regulate gene expression and provide new diagnostic, prognostic, and therapeutic options for head and neck squamous cell carcinoma. In this work, the role of molecular signaling pathways related to head and neck squamous cell carcinoma is emphasized. We also provide an overview of MicroRNA downregulation and overexpression and its role as a diagnostic and prognostic marker in head and neck squamous cell carcinoma. In recent years, MicroRNA nano-based therapies for head and neck squamous cell carcinoma have been explored. In addition, nanotechnology-based alternatives have been discussed as a promising strategy in exploring therapeutic paradigms aimed at improving the efficacy of conventional cytotoxic chemotherapeutic agents against head and neck squamous cell carcinoma and attenuating their cytotoxicity. This article also provides information on ongoing and recently completed clinical trials for therapies based on nanotechnology.
C1 [Bhattacharjee, Bedanta; Rynjah, Damanbhalang; Bora, Shekhar Chandra; Pegu, Padmanath] Girijananda Chowdhury Inst Pharmaceut Sci, Tezpur, India.
[Syeda, Ayesha Farhana] Qassim Univ, Unaiza Coll Pharm, Dept Pharmaceut, Unaizah, Saudi Arabia.
[Hussain, Shalam M.] Al Rayyan Med Coll, Coll Nursing & Hlth Sci, Dept Clin Pharm, Madinah, Saudi Arabia.
[Sahu, Ram Kumar] Hemvati Nandan Bahuguna Garhwal Univ, Cent Univ, Dept Pharmaceut Sci, Chauras Campus, Tehri Garhwal, Uttarakhand, India.
[Khan, Jiyauddin] Management & Sci Univ, Sch Pharm, Shah Alam, Malaysia.
C3 Qassim University; Hemwati Nandan Bahuguna Garhwal University; Management Science University
RP Syeda, AF (通讯作者)，Qassim Univ, Unaiza Coll Pharm, Dept Pharmaceut, Unaizah, Saudi Arabia.; Sahu, RK (通讯作者)，Hemvati Nandan Bahuguna Garhwal Univ, Cent Univ, Dept Pharmaceut Sci, Chauras Campus, Tehri Garhwal, Uttarakhand, India.; Khan, J (通讯作者)，Management & Sci Univ, Sch Pharm, Shah Alam, Malaysia.
EM a.farhana@qu.edu.sa; ramkumar.sahu@hnbgu.ac.in; jiyauddin_khan@msu.edu
RI Sahu, Ram Kumar/F-9292-2011
OI Sahu, Ram Kumar/0000-0001-5671-6591; Mohamed Hussain, Shalam/0000-0002-2398-5485; Rynjah, Damanbhalang/0000-0002-9880-7632
FU Qassim University, Buraydah, Saudi Arabia
FX The authors would like to thank the Deanship of Scientific Research, Qassim University, Buraydah, Saudi Arabia, for funding the publication of this project.
CR Abu-Khalaf MM, 2015, CANCER-AM CANCER SOC, V121, P1817, DOI 10.1002/cncr.29254
Adkins D, 2021, ORAL ONCOL, V115, DOI 10.1016/j.oraloncology.2020.105173
Ahmad P, 2019, CANCER GENOM PROTEOM, V16, P139, DOI 10.21873/cgp.20119

Ahmed EA, 2022, INT J MOL SCI, V23, DOI 10.3390/ijms23115870
Alalaiwe A, 2021, NANOMEDICINE-UK, V16, P1791, DOI 10.2217/nnm-2021-0010
Aldaoud A, 2022, PHYSICA B, V639, DOI 10.1016/j.physb.2022.413993
Ali ES, 2022, CANCER CELL INT, V22, DOI 10.1186/s12935-022-02706-8
Ali Syeda Z, 2020, INT J MOL SCI, V21, DOI 10.3390/ijms21051723
Allen B, 2018, FRONT ONCOL, V8, DOI 10.3389/fonc.2018.00217
Alsahafi E, 2019, CELL DEATH DIS, V10, DOI 10.1038/s41419-019-1769-9
Andrade F, 2016, NANOMEDICINE-UK, V11, P2305, DOI 10.2217/nnm-2016-0045
Antra, 2022, CRIT REV ONCOL HEMAT, V178, DOI 10.1016/j.critrevonc.2022.103778
Basak SK, 2015, ONCOTARGET, V6, P18504, DOI 10.18632/oncotarget.4181
Bauml JM, 2019, ANN TRANSL MED, V7, DOI 10.21037/atm.2019.03.58
Bharadwaj R., 2020, MULTIDISCIPLINARY CA, V4, P36, DOI 10.30699/mci.4.2.36
Bhatia A, 2022, CANCER J, V28, P331, DOI 10.1097/PPO.0000000000000623
Bonvalot S, 2019, LANCET ONCOL, V20, P1148, DOI 10.1016/S1470-2045(19)30326-2
Boza F., 1999, PAPILLOMAVIRUS INFEC
Braga TV, 2022, BRAZ J PHARM SCI, V58, DOI 10.1590/s2175-97902022e19332
Bukovszky B, 2022, STRAHLENTHER ONKOL, V198, P820, DOI 10.1007/s00066-022-01917-2
Canning M, 2019, FRONT CELL DEV BIOL, V7, DOI 10.3389/fcell.2019.00052
Cao H, 2022, IN VITRO CELL DEV-AN, V58, P384, DOI 10.1007/s11626-022-00682-x
Caparica R, 2020, BIOMOLECULES, V10, DOI 10.3390/biom10020233
Capodanno Y, 2021, NEOPLASIA, V23, P1, DOI 10.1016/j.neo.2021.07.008
Carbajo-Pescador S, 2013, BRIT J CANCER, V109, P83, DOI 10.1038/bjc.2013.285
Cataldo A, 2016, ONCOTARGET, V7, P786, DOI 10.18632/oncotarget.6381
Caudell JJ, 2022, J NATL COMPR CANC NE, V20, P224, DOI 10.6004/jnccn.2022.0016
Centuori SM, 2022, CANCER J, V28, P346, DOI 10.1097/PPO.0000000000000619
Chan JYK, 2019, SEMIN CANCER BIOL, V55, P1, DOI 10.1016/j.semcancer.2018.07.008
Chandrakala V, 2022, EMERGENT MATER, V5, P1593, DOI 10.1007/s42247-021-00335-x
Chang WM, 2016, CANCER RES, V76, P7140, DOI 10.1158/0008-5472.CAN-16-1188
Chantre-Justino M, 2022, HUM PATHOL, V119, P94, DOI 10.1016/j.humpath.2021.11.008
Chen FA, 2003, HEAD NECK-J SCI SPEC, V25, P554, DOI 10.1002/hed.10241
Chen HM, 2022, BMC MED GENOMICS, V15, DOI 10.1186/s12920-022-01392-9
Chen Y, 2022, NAT COMMUN, V13, DOI 10.1038/s41467-022-28158-2
Chen Y, 2020, INT J NANOMED, V15, P9407, DOI 10.2147/IJN.S272902
Chen YP, 2017, ACCOUNTS CHEM RES, V50, P310, DOI 10.1021/acs.accounts.6b00506
Cheng YW, 2020, J IMMUNOTHER CANCER, V8, DOI 10.1136/jitc-2020-000940
Choi YH, 2022, BIOTECHNOL BIOPROC E, V27, P183, DOI 10.1007/s12257-021-0199-6
Chung CH, 2015, ANN ONCOL, V26, P1216, DOI 10.1093/annonc/mdv109
Chung CH, 2009, CLIN CANCER RES, V15, P6758, DOI 10.1158/1078-0432.CCR-09-0784
Cluff E, 2022, CANCER IMMUNOL IMMUN, V71, P1989, DOI 10.1007/s00262-021-03126-9
Codony VL, 2021, TRANSL ONCOL, V14, DOI 10.1016/j.tranon.2021.101017
Cortez MA, 2019, GENE CHROMOSOME CANC, V58, P244, DOI 10.1002/gcc.22725
Creemers JHA, 2021, BMJ OPEN, V11, DOI 10.1136/bmjopen-2021-050725
D'Assoro AB, 2022, INT J MOL SCI, V23, DOI 10.3390/ijms23116241
Das D, 2022, P NATL ACAD SCI USA, V119, DOI 10.1073/pnas.2113649119
Das P, 2019, TRENDS BIOTECHNOL, V37, P995, DOI 10.1016/j.tibtech.2019.02.005
Das V, 2019, J CELL PHYSIOL, V234, P14535, DOI 10.1002/jcp.28160
Davidi ES, 2018, HEAD NECK-J SCI SPEC, V40, P70, DOI 10.1002/hed.24935
de Bakker T, 2022, FRONT ONCOL, V11, DOI 10.3389/fonc.2021.799993
De Martino M, 2022, HEMATOL ONCOL, V40, P2, DOI 10.1002/hon.2934
De Palma FDE, 2022, BIOMEDICINES, V10, DOI 10.3390/biomedicines10030725
Dias AMM, 2022, PHARMACEUTICS, V14, DOI 10.3390/pharmaceutics14112388
Ding JH, 2019, BIOSCIENCE REP, V39, DOI 10.1042/BSR20182214
Dioguardi M, 2022, BIOLOGY-BASEL, V11, DOI 10.3390/biology11050651
Dong YB, 2018, CANCER CELL INT, V18, DOI 10.1186/s12935-018-0619-7
Elumalai P, 2022, EVID-BASED COMPL ALT, V2022, DOI 10.1155/2022/5901191
Endo K, 2013, CANCER SCI, V104, P369, DOI 10.1111/cas.12079
Ertin B, 2022, EUR J ONCOL NURS, V61, DOI 10.1016/j.ejon.2022.102239
Eusebio D, 2021, DRUG DISCOV TODAY, V26, P2575, DOI 10.1016/j.drudis.2021.06.008
Fabbrizi MR, 2020, CANCER DRUG RESIST, V3, P775, DOI 10.20517/cdr.2020.49
Faiella A, 2022, J ONCOL, V2022, DOI 10.1155/2022/5179182
Falzone L, 2019, CANCERS, V11, DOI 10.3390/cancers11050610

Farzanegan Z., 2022, MAGNETIC NANOPARTICL
Feng CJ, 2019, FRONT PHARMACOL, V10, DOI 10.3389/fphar.2019.00940
Feng J, 2022, CELLS-BASEL, V11, DOI 10.3390/cells11131981
Fitzmaurice C, 2017, JAMA ONCOL, V3, P524, DOI 10.1001/jamaoncol.2016.5688
Florido J, 2022, J PINEAL RES, V73, DOI 10.1111/jpi.12824
Fruman DA, 2017, CELL, V170, P605, DOI 10.1016/j.cell.2017.07.029
Fung SYH, 2016, CHIN CLIN ONCOL, V5, DOI 10.21037/cco.2016.03.07
Gao SY, 2016, ONCOTARGET, V7, P57367, DOI 10.18632/oncotarget.10350
Gayol A, 2023, INT J MOL SCI, V24, DOI 10.3390/ijms24010514
Ghafouri-Fard S, 2020, HELIYON, V6, DOI 10.1016/j.heliyon.2020.e05436
Gharat SA, 2016, CRIT REV THER DRUG, V33, P363, DOI 10.1615/CritRevTherDrugCarrierSyst.2016016272
Gislon LC, 2022, CANCER EPIDEMIOL, V78, DOI 10.1016/j.canep.2022.102143
Goldberg MS, 2019, NAT REV CANCER, V19, P587, DOI 10.1038/s41568-019-0186-9
Gougis P, 2019, JNCI CANCER SPECT, V3, DOI 10.1093/jncics/pkz055
Goyal N, 2023, ORAL DIS, V29, P1565, DOI 10.1111/odi.14196
Greither T, 2017, ONCOL REP, V38, P1268, DOI 10.3892/or.2017.5764
Grippin AJ, 2017, ONCOIMMUNOLOGY, V6, DOI 10.1080/2162402X.2017.1290036
Gurin D, 2020, J ORAL PATHOL MED, V49, P886, DOI 10.1111/jop.13055
Gursel Urun Y., 2022, MOL MECH CANC, V2022, DOI [10.5772/INTECHOPEN.103134, DOI 10.5772/INTECHOPEN.103134]
Hagege A, 2022, CELL REP MED, V3, DOI 10.1016/j.xcrm.2022.100659
Hainfeld JF, 2010, PHYS MED BIOL, V55, P3045, DOI 10.1088/0031-9155/55/11/004
Han YY, 2022, FRONT GENET, V12, DOI 10.3389/fgene.2021.721199
Hashim D, 2019, ANN ONCOL, V30, P744, DOI 10.1093/annonc/mdz084
Hauser B, 2015, PLOS ONE, V10, DOI 10.1371/journal.pone.0116321
Heo SK, 2022, PLOS ONE, V17, DOI 10.1371/journal.pone.0265958
Hinge N, 2020, WOODH PUBL SER BIOM, P219, DOI 10.1016/B978-0-12-818471-4.00008-X
Hou B, 2015, CANCER BIOL THER, V16, P1042, DOI 10.1080/15384047.2015.1045692
Hsing EW, 2019, PLOS ONE, V14, DOI 10.1371/journal.pone.0213463
Hu Y, 2021, INT J RADIAT ONCOL, V111, P647, DOI 10.1016/j.ijrobp.2021.06.041
Huang HY, 2017, ADV FUNCT MATER, V27, DOI 10.1002/adfm.201603524
Huang J, 2022, AM J TRANSL RES, V14, P2162
Huang SH, 2015, J CLIN ONCOL, V33, P836, DOI 10.1200/JCO.2014.58.6412
Hudcova K, 2016, TUMOR BIOL, V37, P12627, DOI 10.1007/s13277-016-5147-2
Hutchinson MKND, 2020, ONCOGENE, V39, P3638, DOI 10.1038/s41388-020-1250-3
Irshad M., 2018, HEPATOMA RES, V4, P23, DOI [10.20517/2394-5079.2018.25, DOI 10.20517/2394-5079.2018.25]
Jayaseelan VP, 2022, GLOB MED GENET, V09, P277, DOI 10.1055/s-0042-1758204
Jiang XX, 2019, LEUKEMIA LYMPHOMA, V60, P1709, DOI 10.1080/10428194.2018.1543875
Jiang ZX, 2019, BIOMATER SCI-UK, V7, P461, DOI 10.1039/c8bm01340c
Jinesh GG, 2022, SIGNAL TRANSDUCT TAR, V7, DOI 10.1038/s41392-022-01132-6
Jurkovicova D, 2022, INT J MOL SCI, V23, DOI 10.3390/ijms232314672
Kabzinski J, 2021, BIOMOLECULES, V11, DOI 10.3390/biom11060844
Kalfert D, 2015, ANTICANCER RES, V35, P2455
Kamalabadi MA, 2022, MED ONCOL, V39, DOI 10.1007/s12032-022-01780-2
Kang JJ, 2023, BBA-REV CANCER, V1878, DOI 10.1016/j.bbcan.2022.188827
Kannaiyan R, 2018, EXPERT REV ANTICANC, V18, P1249, DOI 10.1080/14737140.2018.1527688
Kaurav M, 2023, FRONT PHARMACOL, V14, DOI 10.3389/fphar.2023.1159131
Khademi S, 2019, INT J BIOCHEM CELL B, V114, DOI 10.1016/j.biocel.2019.06.002
Khan I, 2022, CANCERS, V14, DOI 10.3390/cancers14102523
Khan MI, 2022, ACS APPL BIO MATER, V5, P971, DOI 10.1021/acsabm.2c00002
Khatoon E, 2022, ARCH PHARM RES, V45, P507, DOI 10.1007/s12272-022-01398-y
Knights O, 2020, NANOMATERIALS-BASEL, V10, DOI 10.3390/nano10071307
Kwapisz D, 2017, ANN TRANSL MED, V5, DOI 10.21037/atm.2017.01.32
Kwizera EA, 2022, J HEAT TRANS-T ASME, V144, DOI 10.1115/1.4053007
Lajer CB, 2012, BRIT J CANCER, V106, P1526, DOI 10.1038/bjc.2012.109
Lang LW, 2018, J HEMATOL ONCOL, V11, DOI 10.1186/s13045-018-0623-3
Lee JS, 2022, CELL REP, V41, DOI 10.1016/j.celrep.2022.111759
Lee SY, 2022, J DERMATOL SCI, V106, P70, DOI 10.1016/j.jdermsci.2022.03.007
Li F, 2022, SEMIN CANCER BIOL, V83, P335, DOI 10.1016/j.semcancer.2020.11.018

Li HQ, 2022, ORAL DIS, V28, P142, DOI 10.1111/odi.13751
Li HQ, 2016, COLLOID SURFACE B, V147, P65, DOI 10.1016/j.colsurfb.2016.07.053
Li L, 2018, J CLIN ONCOL, V36, DOI 10.1200/JCO.2018.36.15_suppl.e17508
Li QF, 2023, SIGNAL TRANSDUCT TAR, V8, DOI 10.1038/s41392-022-01297-0
Li R, 2020, SCI REP-UK, V10, DOI 10.1038/s41598-020-68203-y
Li W, 2019, CANCER BIOMARK, V25, P79, DOI 10.3233/CBM-182315
Li YX, 2022, FRONT GENET, V13, DOI 10.3389/fgene.2022.850038
Lin LT, 2016, ONCOTARGET, V7, P65782, DOI 10.18632/oncotarget.11666
Lin SC, 2016, ONCOTARGET, V7, P61355, DOI 10.18632/oncotarget.11349
Liu C, 2019, EBIOMEDICINE, V50, P135, DOI 10.1016/j.ebiom.2019.11.016
Liu J, 2023, BIOMOLECULES, V13, DOI 10.3390/biom13010119
Liu YF, 2016, SCI REP-UK, V6, DOI 10.1038/srep24014
Liu Y, 2020, FRONT GENET, V11, DOI 10.3389/fgene.2020.00853
Lo WL, 2011, J PATHOL, V223, P482, DOI 10.1002/path.2826
Lo YL, 2020, THERANOSTICS, V10, P6695, DOI 10.7150/thno.45164
Lu JA, 2011, CANCER RES, V71, P225, DOI 10.1158/0008-5472.CAN-10-1850
Lu M, 2018, DNA CELL BIOL, V37, P381, DOI 10.1089/dna.2017.4095
Lukianova-Hleb EY, 2015, HEAD NECK-J SCI SPEC, V37, P1547, DOI 10.1002/hed.24018
Ma GF, 2022, J CANCER, V13, P1830, DOI 10.7150/jca.69064
Ma MW, 2022, FRONT ONCOL, V12, DOI 10.3389/fonc.2022.858892
Ma YX, 2021, CELLS-BASEL, V10, DOI 10.3390/cells10092415
Maia D, 2015, J TRANSL MED, V13, DOI 10.1186/s12967-015-0621-y
Mamot C, 2012, LANCET ONCOL, V13, P1234, DOI 10.1016/S1470-2045(12)70476-X
Marquard FE, 2020, BIOCHEM PHARMACOL, V172, DOI 10.1016/j.bcp.2019.113729
Martinez BV, 2015, ONCOTARGET, V6, P19246, DOI 10.18632/oncotarget.4266
Matthews S, 2022, PSYCHO-ONCOLOGY, V31, P2009, DOI 10.1002/pon.5984
Mattox AK, 2019, SCI TRANSL MED, V11, DOI 10.1126/scitranslmed.aay1984
Mazumder S, 2019, CANCER EPIDEMIOL, V58, P137, DOI 10.1016/j.canep.2018.12.008
Miller B, 2022, FRONT CARDIOVASC MED, V8, DOI 10.3389/fcvm.2021.804934
Mirestean CC, 2022, CURR ONCOL, V29, P816, DOI 10.3390/curroncol29020069
Mohan CD, 2022, SEMIN CANCER BIOL, V80, P157, DOI 10.1016/j.semcancer.2020.03.016
Mondal P, 2022, SEMIN CANCER BIOL, V83, P503, DOI 10.1016/j.semcancer.2020.12.006
Moulder DE, 2018, CANCERS, V10, DOI 10.3390/cancers10060189
Mourad M, 2017, J ORAL MAXIL SURG, V75, P2562, DOI 10.1016/j.joms.2017.05.008
Mudra SE, 2021, CANCERS, V13, DOI 10.3390/cancers13205098
Muhanna N, 2015, THERANOSTICS, V5, P1428, DOI 10.7150/thno.13451
Najjar YG, 2017, CLIN CANCER RES, V23, P2346, DOI 10.1158/1078-0432.CCR-15-1823
Nathan CA, 2022, MOL CARCINOGEN, V61, P385, DOI 10.1002/mc.23385
Nguyen KA, 2022, MOL CARCINOGEN, V61, P664, DOI 10.1002/mc.23409
Nowicka Z, 2019, INT J MOL SCI, V20, DOI 10.3390/ijms20194799
Oppelt P, 2021, MED ONCOL, V38, DOI 10.1007/s12032-021-01479-w
Osude C, 2022, CELLS-BASEL, V11, DOI 10.3390/cells11101694
Pavuluri S, 2022, CANCER GENE THER, V29, P1847, DOI 10.1038/s41417-022-00529-3
Pellionisz PA, 2018, OTOLARYNG HEAD NECK, V158, P110, DOI 10.1177/0194599817730304
Perrotti V, 2022, INT J MOL SCI, V23, DOI 10.3390/ijms231810238
Pesce S, 2020, FRONT CELL DEV BIOL, V8, DOI 10.3389/fcell.2020.00119
Philips RL, 2022, CELL, V185, P3857, DOI 10.1016/j.cell.2022.09.023
Piao LZ, 2012, MOL THER, V20, P1261, DOI 10.1038/mt.2012.67
Pinto I, 2020, ONCOGENE, V39, P975, DOI 10.1038/s41388-019-1042-9
Popovtzer A, 2016, NANOSCALE, V8, P2678, DOI 10.1039/c5nr07496g
Provenzano M, 2020, FRONT ONCOL, V10, DOI 10.3389/fonc.2020.00778
Puigdevall L, 2022, J CELL MOL MED, V26, P1865, DOI 10.1111/jcmm.17168
Qureshy Z, 2022, CLIN CANCER RES, V28, P4737, DOI 10.1158/1078-0432.CCR-22-0744
Raj S., 2022, MOL CANCER, V21, P1
Rajabi-Moghaddam M, 2022, REP PRACT ONCOL RADI, V27, P1045, DOI 10.5603/RPOR.a2022.0109
Ramdas L, 2009, HEAD NECK-J SCI SPEC, V31, P642, DOI 10.1002/hed.21017
Cajal SRY, 2020, J MOL MED, V98, P161, DOI 10.1007/s00109-020-01874-2
Rao L, 2018, ANGEW CHEM INT EDIT, V57, P986, DOI 10.1002/anie.201709457
Rapado-Gonzalez O, 2019, ORAL ONCOL, V99, DOI 10.1016/j.oraloncology.2019.104465
Rastogi B, 2017, CANCER INVEST, V35, P152, DOI 10.1080/07357907.2017.1286669
Riestra-Ayora J, 2021, DRUG DELIV, V28, P1376, DOI 10.1080/10717544.2021.1923863

Rose PG, 2020, ANTI-CANCER DRUG, V31, P747, DOI 10.1097/CAD.0000000000000950
Sahu RK, 2021, ANTI-CANCER AGENT ME, V21, P1403, DOI 10.2174/1871520620999201110191741
Salazar-Ruales C, 2018, BIOMED RES INT, V2018, DOI 10.1155/2018/9792730
Saleh AD, 2019, CLIN CANCER RES, V25, P2860, DOI 10.1158/1078-0432.CCR-18-0716
Sannigrahi MK, 2017, CLIN CANCER RES, V23, P3884, DOI 10.1158/1078-0432.CCR-16-2936
Sayed N, 2022, LIFE SCI, V294, DOI 10.1016/j.lfs.2022.120375
Sayyed AA, 2022, CURR PHARM DESIGN, V28, P471, DOI 10.2174/1381612827666211109113305
Schneider A, 2018, SCI REP-UK, V8, DOI 10.1038/s41598-017-18945-z
Schubert AD, 2018, CURR CANCER RES, P229, DOI 10.1007/978-3-319-78762-6_8
Seiwert TY, 2015, CLIN CANCER RES, V21, P632, DOI 10.1158/1078-0432.CCR-13-3310
Setia A, 2022, CURR DRUG METAB, V23, P757, DOI 10.2174/1389200223666220627110049
Shen JJ, 2020, ACTA PHARMACOL SIN, V41, P110, DOI 10.1038/s41401-019-0290-0
Shi JB, 2019, THERANOSTICS, V9, P920, DOI 10.7150/thno.30339
Shukuya T, 2020, J THORAC ONCOL, V15, P1773, DOI 10.1016/j.jtho.2020.05.022
Simonet S, 2020, J BIOMED NANOTECHNOL, V16, P111, DOI 10.1166/jbn.2020.2871
Solomon MC, 2020, JPN DENT SCI REV, V56, P85, DOI 10.1016/j.jdsr.2020.04.001
Song HJ, 2022, NANOSCALE, V14, P11429, DOI 10.1039/d2nr02620a
Squarize CH, 2013, NEOPLASIA, V15, P461, DOI 10.1593/neo.121024
Stanciu S, 2022, INT J MOL SCI, V23, DOI 10.3390/ijms231710132
Su Z, 2019, INT J NANOMED, V14, P7549, DOI 10.2147/IJN.S215087
Subash A, 2022, ORAL ONCOL, V125, DOI 10.1016/j.oraloncology.2021.105677
Subbiah V, 2018, CLIN CANCER RES, V24, P43, DOI 10.1158/1078-0432.CCR-17-1114
Subha ST, 2022, MOL BIOL REP, V49, P1501, DOI 10.1007/s11033-021-06954-1
Summerer I, 2015, BRIT J CANCER, V113, P76, DOI 10.1038/bjc.2015.111
Sun X, 2021, J INT MED RES, V49, DOI 10.1177/0300060520939031
Sun Z, 2022, INT J PEPT RES THER, V28, DOI 10.1007/s10989-021-10334-5
Sung H, 2021, CA-CANCER J CLIN, V71, P209, DOI 10.3322/caac.21660
Swain S, 2016, CURR DRUG DELIV, V13, P1290, DOI 10.2174/1567201813666160713121122
Tawk B, 2022, FRONT PHARMACOL, V12, DOI 10.3389/fphar.2021.753387
Tchekneva EE, 2019, J IMMUNOTHER CANCER, V7, DOI 10.1186/s40425-019-0566-4
Thomaidou AC, 2022, INT J MOL SCI, V23, DOI 10.3390/ijms23158257
Thompson A, 2017, INT J RADIAT ONCOL, V99, pE621, DOI 10.1016/j.ijrobp.2017.06.2098
Tong FJ, 2020, CANCER LETT, V478, P34, DOI 10.1016/j.canlet.2020.02.037
Torres BPG, 2023, AM J OTOLARYNG, V44, DOI 10.1016/j.amjoto.2022.103771
Trinidad AJ, 2014, LASER SURG MED, V46, P310, DOI 10.1002/lsm.22235
Tseng HH, 2017, ANTICANCER RES, V37, P1059, DOI 10.21873/anticanres.11417
Turai PI, 2022, CANCERS, V14, DOI 10.3390/cancers14040895
Ulusan M, 2022, PATHOL RES PRACT, V239, DOI 10.1016/j.prp.2022.154147
Usman S, 2021, FRONT ORAL HEALTH, V1, DOI 10.3389/froh.2020.603160
Uzuner Erez, 2022, Methods Mol Biol, V2257, P375, DOI 10.1007/978-1-0716-1170-8_18
van Poelgeest MIE, 2016, CLIN CANCER RES, V22, P2342, DOI 10.1158/1078-0432.CCR-15-2594
Venugopal I, 2016, MATER RES EXPRESS, V3, DOI 10.1088/2053-1591/3/9/095010
von der Grun J, 2019, CANCERS, V11, DOI 10.3390/cancers11040472
Voskuil FJ, 2020, NAT COMMUN, V11, DOI 10.1038/s41467-020-16814-4
Wang BK, 2014, BIOMATERIALS, V35, P1954, DOI 10.1016/j.biomaterials.2013.11.066
Wang Y, 2018, INT J ONCOL, V52, P1149, DOI 10.3892/ijo.2018.4293
Wang Y, 2017, MOL CANCER THER, V16, P578, DOI 10.1158/1535-7163.MCT-16-0606
Wefers C, 2018, FRONT IMMUNOL, V9, DOI 10.3389/fimmu.2018.01412
Weizman B, 2021, HEAD NECK-J SCI SPEC, V43, P3001, DOI 10.1002/hed.26788
Windon MJ, 2018, CANCER-AM CANCER SOC, V124, P2993, DOI 10.1002/cncr.31385
Wojtynek NE, 2020, WIRES NANOMED NANOBI, V12, DOI 10.1002/wnan.1624
Wong GL, 2022, SEMIN CANCER BIOL, V86, P84, DOI 10.1016/j.semcancer.2022.08.003
Wong XY, 2020, ACS NANO, V14, P2585, DOI 10.1021/acsnano.9b08133
Xie R, 2022, DNA REPAIR, V114, DOI 10.1016/j.dnarep.2022.103330
Yamashita F, 2022, FEBS J, V289, P3422, DOI 10.1111/febs.16348
Yang CX, 2019, CANCERS, V11, DOI 10.3390/cancers11030395
Yang JY, 2017, DRUG DELIV, V24, P792, DOI 10.1080/10717544.2016.1236849
Yang ML, 2022, LIPIDS HEALTH DIS, V21, DOI 10.1186/s12944-022-01693-z
Yang Zejia, 2022, Oral Oncol, V131, P105939, DOI 10.1016/j.oraloncology.2022.105939
Yeh LY, 2015, ONCOTARGET, V6, P6062, DOI 10.18632/oncotarget.3340
Yu D, 2019, CELL COMMUN SIGNAL, V17, DOI 10.1186/s12964-019-0490-8

Yuan GY, 2019, ONCOL LETT, V17, P2063, DOI 10.3892/ol.2018.9820
Yuan HX, 2017, ONCOL LETT, V14, P3371, DOI 10.3892/ol.2017.6605
Zarrintaj P, 2019, CURR STEM CELL RES T, V14, P137, DOI 10.2174/1574888X13666181002152247
Zeshan B, 2016, J NANOSCI NANOTECHNO, V16, P6622, DOI 10.1166/jnn.2016.11360
Zhang P, 2020, INT J NANOMED, V15, P3843, DOI 10.2147/IJN.S250490
Zhang YZ, 2018, MOL CANCER, V17, DOI 10.1186/s12943-018-0796-y
Zhang ZQ, 2020, AM J CANCER RES, V10, P350
Zhao LY, 2013, CHINESE PHYS B, V22, DOI 10.1088/1674-1056/22/10/108104
Zhao Q, 2012, THERANOSTICS, V2, P113, DOI 10.7150/thno.3854
Zhao YY, 2017, WIRES NANOMED NANOBI, V9, DOI 10.1002/wnan.1469
Zheng XQ, 2015, ORAL ONCOL, V51, P998, DOI 10.1016/j.oraloncology.2015.08.002
Zhou JZ, 2022, CANCERS, V14, DOI 10.3390/cancers14010033
Zhu DJ, 2021, J NANOBIOTECHNOL, V19, DOI 10.1186/s12951-021-01190-y
Zhu Jia-Yu, 2012, Rev Obstet Gynecol, V5, pe137
Zhu L., 2022, IDENTIFICATION BIOMA
NR 259
TC 0
Z9 0
U1 3
U2 3
PU FRONTIERS MEDIA SA
PI LAUSANNE
PA AVENUE DU TRIBUNAL FEDERAL 34, LAUSANNE, CH-1015, SWITZERLAND
EI 1663-9812
J9 FRONT PHARMACOL
JI Front. Pharmacol.
PD MAY 3
PY 2023
VL 14
AR 1174330
DI 10.3389/fphar.2023.1174330
PG 40
WC Pharmacology & Pharmacy
WE Science Citation Index Expanded (SCI-EXPANDED)
SC Pharmacology & Pharmacy
GA G5CZ0
UT WOS:000989345500001
PM 37205904
OA gold, Green Published
DA 2023-09-14
ER

PT J
AU Wang, QT
Liu, YX
Wang, J
Wang, H
AF Wang, Qian-Ting
Liu, Yi-Xuan
Wang, Jie
Wang, Hao
TI Advances in Cancer Nanovaccines: Harnessing Nanotechnology for
Broadening Cancer Immune Response
SO CHEMMEDCHEM
LA English
DT Review
DE cancer nanovaccines; immune microenvironment; immune response;
nanocarriers; tumor antigens
ID METASTATIC MELANOMA; CROSS-PRESENTATION; CTLA-4 BLOCKADE; VACCINE;
IMMUNOTHERAPY; COMBINATION; DELIVERY; CELLS; INTERLEUKIN-2; STRATEGIES
AB Many advances have been made recently in the field of cancer immunotherapy, particularly with the

development of treatments such as immune checkpoint inhibitors and adoptive cellular immunotherapy. The efficacy of immunotherapy is limited, however, owing to high levels of tumor heterogeneity and the immunosuppressive environments of advanced malignant tumors. Therefore, therapeutic anticancer vaccines have gradually become powerful tools for inducing valid antitumor immune responses and regulating the immune microenvironment. Tumor vaccines loaded in nanocarriers have become an indispensable delivery platform for tumor treatment because of their enhanced stability, targeting capability, and high level of safety. Through a unique design, cancer nanovaccines activate innate immunity and tumor-specific immunity simultaneously. For example, the design of cancer vaccines can incorporate strategies such as enhancing the stability and targeting of tumor antigens, combining effective adjuvants, cytokines, and immune microenvironment regulators, and promoting the maturation and cross-presentation of antigen-presenting cells (APCs). In this review, we discuss the design and preparation of nanovaccines for remodeling tumor antigen immunogenicity and regulating the immunosuppressive microenvironment.
C1 [Wang, Qian-Ting; Liu, Yi-Xuan; Wang, Jie; Wang, Hao] Natl Ctr Nanosci & Technol NCNST, CAS Ctr Excellence Nanosci, CAS Key Lab Biomed Effects Nanomat & Nano Safety, Beijing 100190, Peoples R China.
[Liu, Yi-Xuan] Univ Chinese Acad Sci UCAS, Beijing 100049, Peoples R China.
C3 Chinese Academy of Sciences; National Center for Nanoscience & Technology - China; Chinese Academy of Sciences; University of Chinese Academy of Sciences, CAS
RP Wang, H (通讯作者)，Natl Ctr Nanosci & Technol NCNST, CAS Ctr Excellence Nanosci, CAS Key Lab Biomed Effects Nanomat & Nano Safety, Beijing 100190, Peoples R China.
EM wanghao@nanoctr.cn
OI WANG, QIANTING/0000-0002-5366-9141
FU Strategic Priority Research Program of the Chinese Academy of Sciences [XDA16021200]
FX Acknowledgments The authors thank the Strategic Priority Research Program of the Chinese Academy of Sciences (XDA16021200).
CR Abramson JS, 2020, TRANSFUS MED REV, V34, P29, DOI 10.1016/j.tmrv.2019.08.003
Akinc A, 2019, NAT NANOTECHNOL, V14, P1084, DOI 10.1038/s41565-019-0591-y
Akinc A, 2010, MOL THER, V18, P1357, DOI 10.1038/mt.2010.85
Ali S, 2020, ESMO OPEN, V5, DOI 10.1136/esmoopen-2020-000798
Anderson RP, 2013, CURR OPIN IMMUNOL, V25, P410, DOI 10.1016/j.coi.2013.02.004
Anwar MA, 2019, MED RES REV, V39, P1053, DOI 10.1002/med.21553
Atkins MB, 1999, J CLIN ONCOL, V17, P2105, DOI 10.1200/JCO.1999.17.7.2105
Bachmann MF, 2010, NAT REV IMMUNOL, V10, P787, DOI 10.1038/nri2868
Baharom F, 2021, NAT IMMUNOL, V22, P41, DOI 10.1038/s41590-020-00810-3
Basith S, 2012, ARCH PHARM RES, V35, P1297, DOI 10.1007/s12272-012-0802-7
Berti C, 2022, MACROMOL BIOSCI, V22, DOI 10.1002/mabi.202100356
Bowen WS, 2018, EXPERT REV VACCINES, V17, P207, DOI 10.1080/14760584.2018.1434000
Burch PA, 2000, CLIN CANCER RES, V6, P2175
Chen DY, 2019, NANOSCALE, V11, P18806, DOI 10.1039/c9nr05788a
Chen L, 2021, SCI TRANSL MED, V13, DOI 10.1126/scitranslmed.abc2816
Conlon KC, 2019, J INTERF CYTOK RES, V39, P6, DOI 10.1089/jir.2018.0019
Dane EL, 2022, NAT MATER, V21, P710, DOI 10.1038/s41563-022-01251-z
DeMaria PJ, 2019, HEMATOL ONCOL CLIN N, V33, P199, DOI 10.1016/j.hoc.2018.12.001
Domingos-Pereira S, 2021, ONCOIMMUNOLOGY, V10, DOI 10.1080/2162402X.2021.1912473
Eisenberg G, 2010, CELL IMMUNOL, V266, P98, DOI 10.1016/j.cellimm.2010.09.003
Embgenbroich M, 2018, FRONT IMMUNOL, V9, DOI 10.3389/fimmu.2018.01643
Fehres CM, 2014, FRONT IMMUNOL, V5, DOI 10.3389/fimmu.2014.00149
Feng YJ, 2022, BIOMATER SCI-UK, V10, P6230, DOI 10.1039/d2bm01211a
Finn OJ, 2018, CSH PERSPECT BIOL, V10, DOI 10.1101/cshperspect.a028829
Gao YN, 2021, BIOMATERIALS, V273, DOI 10.1016/j.biomaterials.2021.120792
Gautam A, 2021, INT J MOL SCI, V22, DOI 10.3390/ijms22189733
Gong NQ, 2020, NAT NANOTECHNOL, V15, P1053, DOI 10.1038/s41565-020-00782-3
Gravitz L, 2011, NATURE, V478, P163, DOI 10.1038/478163a
Gupta J, 2021, SEMIN CANCER BIOL, V69, P307, DOI 10.1016/j.semcancer.2020.03.015
Herlyn D, 1999, ANN MED, V31, P66, DOI 10.3109/07853899909019264
Hewitt SL, 2019, SCI TRANSL MED, V11, DOI 10.1126/scitranslmed.aat9143
Hilf N, 2019, NATURE, V565, P240, DOI 10.1038/s41586-018-0810-y
HUANG KW, 2020, INT EL DEVICES MEET, V6

Hunder NN, 2008, NEW ENGL J MED, V358, P2698, DOI 10.1056/NEJMoa0800251
Irvine DJ, 2015, CHEM REV, V115, P11109, DOI 10.1021/acs.chemrev.5b00109
Islam MA, 2021, BIOMATERIALS, V266, DOI 10.1016/j.biomaterials.2020.120431
Jemal A, 2009, CA-CANCER J CLIN, V59, P225, DOI [10.3322/caac.20006, 10.3322/caac.21601, 10.3322/caac.21254, 10.3322/caac.21387, 10.3322/caac.20073]
Jiang DD, 2018, ACS APPL MATER INTER, V10, P37797, DOI 10.1021/acsami.8b09946
Joffre OP, 2012, NAT REV IMMUNOL, V12, P557, DOI 10.1038/nri3254
Johnson DT, 2022, LEUKEMIA, V36, P994, DOI 10.1038/s41375-021-01432-w
Keskin DB, 2019, NATURE, V565, P234, DOI 10.1038/s41586-018-0792-9
Kreiter S, 2008, J IMMUNOL, V180, P309, DOI 10.4049/jimmunol.180.1.309
Kwok G, 2016, HUM VACC IMMUNOTHER, V12, P2777, DOI 10.1080/21645515.2016.1199310
Lee JY, 2020, ACS APPL MATER INTER, V12, P34658, DOI 10.1021/acsami.0c09484
Li Q, 2021, DRUG DES DEV THER, V15, P3953, DOI 10.2147/DDDT.S325790
Li X, 2022, SMALL, V18, DOI 10.1002/smll.202203100
Li Y, 2022, ADV MATER, V34, DOI 10.1002/adma.202109984
Liau LM, 2018, J TRANSL MED, V16, DOI 10.1186/s12967-018-1507-6
Liu LN, 2018, MOL THER, V26, P45, DOI 10.1016/j.ymthe.2017.10.020
Liu QJ, 2020, PROTEIN CELL, V11, P549, DOI 10.1007/s13238-020-00707-9
Liu SL, 2021, NAT MATER, V20, P421, DOI 10.1038/s41563-020-0793-6
Lu Ligong, 2022, Curr Res Immunol, V3, P118, DOI 10.1016/j.crimmu.2022.05.003
Lu LG, 2021, HEPATOLOGY, V73, P414, DOI 10.1002/hep.31279
Lynn GM, 2020, NAT BIOTECHNOL, V38, P320, DOI 10.1038/s41587-019-0390-x
Ma L, 2021, ADV MATER, V33, DOI 10.1002/adma.202104849
Mai JH, 2021, ADV SCI, V8, DOI 10.1002/advs.202100166
Marchand M, 1999, INT J CANCER, V80, P219, DOI 10.1002/(SICI)1097-0215(19990118)80:2<219::AID-IJC10>3.3.CO;2-J
Maerkl F, 2022, TRENDS CANCER, V8, P670, DOI 10.1016/j.trecan.2022.04.001
McCarthy Edward F, 2006, Iowa Orthop J, V26, P154
Miao L, 2021, MOL CANCER, V20, DOI 10.1186/s12943-021-01335-5
Morse MA, 2021, TARGET ONCOL, V16, P121, DOI 10.1007/s11523-020-00788-w
Ni J, 2020, BIOMED PHARMACOTHER, V126, DOI 10.1016/j.biopha.2020.110046
Ni QQ, 2020, SCI ADV, V6, DOI 10.1126/sciadv.aaw6071
Novellino L, 2005, CANCER IMMUNOL IMMUN, V54, P187, DOI 10.1007/s00262-004-0560-6
O'Neill RE, 2019, ADV CANCER RES, V143, P145, DOI 10.1016/bs.acr.2019.03.003
Oberli MA, 2017, NANO LETT, V17, P1326, DOI 10.1021/acs.nanolett.6b03329
Parmiani G, 2002, JNCI-J NATL CANCER I, V94, P805, DOI 10.1093/jnci/94.11.805
Patel M, 2021, J IMMUNOTHER CANCER, V9, pA569, DOI 10.1136/jitc-2021-SITC2021.539
QUESADA JR, 1986, BLOOD, V68, P493, DOI 10.1182/blood.V68.2.493.493
Reuven EM, 2019, ACS NANO, V13, P2936, DOI 10.1021/acsnano.8b07241
Sahin U, 2020, NATURE, V585, P107, DOI 10.1038/s41586-020-2537-9
Sahin U, 2017, NATURE, V547, P222, DOI 10.1038/nature23003
Saxena M, 2021, NAT REV CANCER, V21, P360, DOI 10.1038/s41568-021-00346-0
Shah NN, 2019, NAT REV CLIN ONCOL, V16, P372, DOI 10.1038/s41571-019-0184-6
Sharma P, 2015, CELL, V161, P205, DOI 10.1016/j.cell.2015.03.030
Small EJ, 2000, J CLIN ONCOL, V18, P3894, DOI 10.1200/JCO.2000.18.23.3894
Sosman JA, 2008, J CLIN ONCOL, V26, P2292, DOI 10.1200/JCO.2007.13.3165
Sun B, 2021, J MATER CHEM B, V9, P7435, DOI 10.1039/d1tb01408k
Sun XQ, 2021, NAT NANOTECHNOL, V16, P1260, DOI 10.1038/s41565-021-00962-9
Takaba H, 2017, TRENDS IMMUNOL, V38, P805, DOI 10.1016/j.it.2017.07.010
Nguyen TL, 2020, BIOMATERIALS, V239, DOI 10.1016/j.biomaterials.2020.119859
Tse SW, 2021, MOL THER, V29, P2227, DOI 10.1016/j.ymthe.2021.03.002
Tsoras AN, 2018, BIOCONJUGATE CHEM, V29, P776, DOI 10.1021/acs.bioconjchem.8b00079
Tuo Z, 2022, CHEM ENG J, V433, DOI 10.1016/j.cej.2021.134437
Van Allen EM, 2015, SCIENCE, V350, P207, DOI 10.1126/science.aad0095
Van Lysebetten D, 2021, ACS APPL MATER INTER, V13, P6011, DOI 10.1021/acsami.0c20607
Vermaelen K, 2019, FRONT IMMUNOL, V10, DOI 10.3389/fimmu.2019.00008
Vormehr M, 2019, ANNU REV MED, V70, P395, DOI 10.1146/annurev-med-042617-101816
Wang HW, 2022, NAT BIOMED ENG, V6, DOI 10.1038/s41551-021-00815-9
Wang J, 2020, ACS BIOMATER SCI ENG, V6, P6036, DOI 10.1021/acsbiomaterials.0c01201
Wang QT, 2020, CANCER IMMUNOL IMMUN, V69, P1375, DOI 10.1007/s00262-020-02496-w
Wang S, 2021, SCI TRANSL MED, V13, DOI 10.1126/scitranslmed.abb6981

Wang WW, 2022, ADV MATER, V34, DOI 10.1002/adma.202109354
Wang WJ, 2019, NANOMED-NANOTECHNOL, V16, P69, DOI 10.1016/j.nano.2018.11.009
Wang YL, 2021, NANO TODAY, V38, DOI 10.1016/j.nantod.2021.101139
Xu J, 2020, NAT NANOTECHNOL, V15, P1043, DOI 10.1038/s41565-020-00781-4
Yang R, 2018, ACS NANO, V12, P5121, DOI 10.1021/acsnano.7b09041
Yang XY, 2021, NANOMED-NANOTECHNOL, V37, DOI 10.1016/j.nano.2021.102443
Yang YP, 2015, J CLIN INVEST, V125, P3335, DOI 10.1172/JCI83871
Ye XY, 2019, ACS NANO, V13, P2956, DOI 10.1021/acsnano.8b07371
Yu M, 2019, ADV SCI, V6, DOI 10.1002/advs.201900037
Zhao Y, 2018, MOL PHARMACEUT, V15, P1791, DOI 10.1021/acs.molpharmaceut.7b01103
Zhou SL, 2020, BIOMATERIALS, V235, DOI 10.1016/j.biomaterials.2020.119795
Zou MZ, 2021, NANO LETT, V21, P8609, DOI 10.1021/acs.nanolett.1c02482
NR 104
TC 0
Z9 0
U1 12
U2 12
PU WILEY-V C H VERLAG GMBH
PI WEINHEIM
PA POSTFACH 101161, 69451 WEINHEIM, GERMANY
SN 1860-7179
EI 1860-7187
J9 CHEMMEDCHEM
JI ChemMedChem
PD JUL 3
PY 2023
VL 18
IS 13
DI 10.1002/cmdc.202200673
EA MAY 2023
PG 15
WC Chemistry, Medicinal; Pharmacology & Pharmacy
WE Science Citation Index Expanded (SCI-EXPANDED)
SC Pharmacology & Pharmacy
GA M1OU3
UT WOS:000979131700001
PM 37088719
DA 2023-09-14
ER

PT J
AU Zhang, BB
Zhang, J
Li, YQ
Li, N
Wang, YZ
Jang, R
Xu, XX
Li, RF
Chen, ZZ
Duan, SB
Wang, YC
Zhang, LZ
AF Zhang, Beibei
Zhang, Juan
Li, Yaqiong
Li, Na
Wang, Yuzhou
Jang, Ru
Xu, Xiaoxia
Li, Ruifang

Chen, Zhenzhen
Duan, Shaobo
Wang, Yongchao
Zhang, Lianzhong
TI In Situ STING-Activating Nanovaccination with TIGIT Blockade for Enhanced Immunotherapy of Anti-PD-1-Resistant Tumors
SO ADVANCED MATERIALS
LA English
DT Article
DE anti-PD-1-resistant tumors; biomimetic materials; immunotherapy; nanovaccination; STING
ID ANTITUMOR; RESISTANCE; PATHWAY
AB Immunotherapies comprising programmed cell death protein 1/PD ligand 1 (PD-1/PD-L1) immune checkpoint inhibitors are effective cancer treatments. However, the low response rate and immunoresistance resulting from alternative immune checkpoint upregulation and inefficient immune stimulation by T cells are problematic. The present report describes a biomimetic nanoplatform that simultaneously blocks the alternative T-cell immunoglobulin and immunoreceptor tyrosine-based inhibitory motif domain (TIGIT) checkpoint and activates the stimulator of interferon genes (STING) signaling pathway in situ for enhanced antitumor immunity. The nanoplatform is engineered by fusing a red blood cell membrane with glutathione-responsive liposome-encapsulated cascade-activating chemoagents (beta-lapachone and tirapazamine), and anchoring them with a detachable TIGIT block peptide (named as RTLT). In the tumor environment, the peptide is spatiotemporally released to reverse T-cell exhaustion and restore antitumor immunity. The cascade activation of chemotherapeutic agents causes DNA damage and inhibits the repair of double-stranded DNA, which induces robust in situ STING activation for an efficient immune response. The RTLT inhibits anti-PD-1-resistant tumor growth, and prevents tumor metastasis and recurrence in vivo by inducing antigen-specific immune memory. This biomimetic nanoplatform thus provides a promising strategy for in situ cancer vaccination.
C1 [Zhang, Beibei; Zhang, Juan; Li, Yaqiong; Li, Na; Wang, Yuzhou; Jang, Ru; Xu, Xiaoxia; Duan, Shaobo; Zhang, Lianzhong] Henan Univ, Zhengzhou Univ, Peoples Hosp, Henan Prov Peoples Hosp,Dept Ultrasound, Zhengzhou 450003, Peoples R China.
[Chen, Zhenzhen; Wang, Yongchao] Zhengzhou Univ, Sch Life Sci, Zhengzhou 450001, Peoples R China.
[Zhang, Beibei; Li, Ruifang] Henan Univ Technol, Sch Biol Engn, Zhengzhou 450001, Peoples R China.
C3 Henan University; Zhengzhou University; Zhengzhou University; Henan University of Technology
RP Duan, SB; Zhang, LZ (通讯作者)，Henan Univ, Zhengzhou Univ, Peoples Hosp, Henan Prov Peoples Hosp,Dept Ultrasound, Zhengzhou 450003, Peoples R China.; Wang, YC (通讯作者)，Zhengzhou Univ, Sch Life Sci, Zhengzhou 450001, Peoples R China.
EM dustin2662@zzu.edu.cn; wangyongchao@zzu.edu.cn; zlz8777@zzu.edu.cn
OI Wang, Yongchao/0000-0001-8152-2350
FU Science and Technology Department of Henan Province [212102310322]; Postdoctoral Science Foundation of China [2022M711068, 2022M712913, 2022TQ0309]; Zhengzhou University [32213161]; Key Research and Development Projects in Henan Province [221111310400]; National Natural Science Foundation of China [32201156]; Medical Science and Technology Breakthrough Plan Project of Henan Province [LHGJ20210031, LHGJ20210020]
FX This work was supported by grants from the Science and Technology Department of Henan Province (Grant No. 212102310322), the Postdoctoral Science Foundation of China (Grant Nos. 2022M711068, 2022M712913, 2022TQ0309), the Start-up Grant from Zhengzhou University (Grant No. 32213161), the Key Research and Development Projects in Henan Province (Grant No. 221111310400), the National Natural Science Foundation of China (Grant No. 32201156), the Medical Science and Technology Breakthrough Plan Project of Henan Province (Grant Nos. LHGJ20210031, LHGJ20210020). The authors also thank for the technical support of the Modern Analysis and Computer Center of Zhengzhou University.
CR Banta KL, 2022, IMMUNITY, V55, P512, DOI 10.1016/j.immuni.2022.02.005
Chen J, 2021, SCI ADV, V7, DOI 10.1126/sciadv.abc5267
Chen L, 2022, CLIN IMMUNOL, V237, DOI 10.1016/j.clim.2022.108962
Chin EN, 2020, SCIENCE, V369, P993, DOI 10.1126/science.abb4255
Cho BC, 2022, LANCET ONCOL, V23, P781, DOI 10.1016/S1470-2045(22)00226-1

Florou V, 2022, CURR ONCOL REP, V24, P1107, DOI 10.1007/s11912-022-01281-5
Galluzzi L, 2018, CELL, V173, DOI 10.1016/j.cell.2018.03.015
Han JC, 2020, CANCER CELL, V38, P844, DOI 10.1016/j.ccell.2020.10.009
Han X, 2019, SCI ADV, V5, DOI 10.1126/sciadv.aaw6870
Johnston RJ, 2014, CANCER CELL, V26, P923, DOI 10.1016/j.ccell.2014.10.018
Kalbasi Anusha, 2020, Nat Rev Immunol, V20, P25, DOI 10.1038/s41577-019-0218-4
Korman AJ, 2022, NAT REV DRUG DISCOV, V21, P509, DOI 10.1038/s41573-021-00345-8
Koyama S, 2016, NAT COMMUN, V7, DOI 10.1038/ncomms10501
Kraehenbuehl L, 2022, NAT REV CLIN ONCOL, V19, P37, DOI 10.1038/s41571-021-00552-7
Kwon J, 2020, CANCER DISCOV, V10, P26, DOI 10.1158/2159-8290.CD-19-0761
Liang JJ, 2020, SCI ADV, V6, DOI 10.1126/sciadv.abc3646
Lioux T, 2016, J MED CHEM, V59, P10253, DOI 10.1021/acs.jmedchem.6b01300
Liu GN, 2019, ADV MATER, V31, DOI 10.1002/adma.201900795
Manieri NA, 2017, TRENDS IMMUNOL, V38, P20, DOI 10.1016/j.it.2016.10.002
Morad G, 2021, CELL, V184, P5309, DOI 10.1016/j.cell.2021.09.020
Motwani M, 2019, NAT REV GENET, V20, P657, DOI 10.1038/s41576-019-0151-1
Ramanjulu JM, 2018, NATURE, V564, P439, DOI 10.1038/s41586-018-0705-y
Ribas A, 2018, SCIENCE, V359, P1350, DOI 10.1126/science.aar4060
Rudin C. M., 2022, J CLIN ONCOL, V1, P2
Samson N, 2022, NAT CANCER, DOI 10.1038/s43018-022-00468-w
Sharma P, 2017, CELL, V168, P707, DOI 10.1016/j.cell.2017.01.017
Tigano M, 2021, NATURE, V591, P477, DOI 10.1038/s41586-021-03269-w
Waldman AD, 2020, NAT REV IMMUNOL, V20, P651, DOI 10.1038/s41577-020-0306-5
Wein L, 2018, BRIT J CANCER, V119, P4, DOI 10.1038/s41416-018-0126-6
Xue G, 2021, NAT BIOMED ENG, V5, DOI 10.1038/s41551-021-00799-6
Yang YQ, 2021, SCI ADV, V7, DOI 10.1126/sciadv.abf6290
Yu JX, 2020, NAT REV DRUG DISCOV, V19, P163, DOI 10.1038/d41573-019-00182-w
Yu X, 2009, NAT IMMUNOL, V10, P48, DOI 10.1038/ni.1674
Zhang Q, 2018, NAT IMMUNOL, V19, P723, DOI 10.1038/s41590-018-0132-0
Zhou C, 2021, CANCER LETT, V521, P119, DOI 10.1016/j.canlet.2021.07.023
Zhou XM, 2020, ANGEW CHEM INT EDIT, V59, P15114, DOI 10.1002/anie.202002783
NR 36
TC 0
Z9 0
U1 43
U2 43
PU WILEY-V C H VERLAG GMBH
PI WEINHEIM
PA POSTFACH 101161, 69451 WEINHEIM, GERMANY
SN 0935-9648
EI 1521-4095
J9 ADV MATER
JI Adv. Mater.
PD JUN
PY 2023
VL 35
IS 24
DI 10.1002/adma.202300171
EA APR 2023
PG 15
WC Chemistry, Multidisciplinary; Chemistry, Physical; Nanoscience &
Nanotechnology; Materials Science, Multidisciplinary; Physics, Applied;
Physics, Condensed Matter
WE Science Citation Index Expanded (SCI-EXPANDED)
SC Chemistry; Science & Technology - Other Topics; Materials Science;
Physics
GA J2PJ2
UT WOS:000978001600001
PM 37053496
DA 2023-09-14
ER

PT J
AU Sun, WQ
Ji, PP
Zhou, T
Li, ZL
Xing, CY
Zhang, L
Wei, MY
Yang, GD
Yuan, LJ
AF Sun, Wenqi
Ji, Panpan
Zhou, Tian
Li, Zhelong
Xing, Changyang
Zhang, Liang
Wei, Mengying
Yang, Guodong
Yuan, Lijun
TI Ultrasound Responsive Nanovaccine Armed with Engineered Cancer Cell Membrane and RNA to Prevent Foreseeable Metastasis
SO ADVANCED SCIENCE
LA English
DT Article
DE alternative splicing; antigen presentation; cancer vaccine; sonosensitizer
ID ANTIGEN PRESENTATION; DENDRITIC CELLS; VACCINES; BREAST; NANOPARTICLES; GENERATION
AB Cancer vaccine has been considered as a promising immunotherapy by inducing specific anti-tumor immune response. Rational vaccination at suitable time to efficiently present tumor associated antigen will boost tumor immunity and is badly needed. Here, a poly (lactic-co-glycolic acid) (PLGA)-based cancer vaccine of nanoscale is designed, in which engineered tumor cell membrane proteins, mRNAs, and sonosensitizer chlorin e6 (Ce6) are encapsulated at high efficiency. The nanosized vaccine can be efficiently delivered into antigen presentation cells (APCs) in lymph nodes after subcutaneous injection. In the APCs, the encapsulated cell membrane and RNA from engineered cells, which have disturbed splicing resembling the metastatic cells, provide neoantigens of metastatic cancer in advance. Moreover, the sonosensitizer Ce6 together with ultrasound irradiation promotes mRNA escape from endosome, and augments antigen presentation. Through 4T1 syngeneic mouse model, it has been proved that the proposed nanovaccine is efficient to elicit antitumor immunity and thus prevent cancer metastasis.
C1 [Sun, Wenqi; Zhou, Tian; Li, Zhelong; Xing, Changyang; Zhang, Liang; Yuan, Lijun] Fourth Mil Med Univ, Tangdu Hosp, Dept Ultrasound Diagnost, Xian 710038, Shaanxi, Peoples R China.
[Sun, Wenqi; Li, Zhelong; Wei, Mengying; Yang, Guodong] Fourth Mil Med Univ, Dept Biochem & Mol Biol, State Lab Canc Biol, Xian 710032, Shaanxi, Peoples R China.
[Ji, Panpan] Fourth Mil Med Univ, Xijing Hosp, Dept Digest Surg, Xian 710032, Shaanxi, Peoples R China.
C3 Air Force Military Medical University; Air Force Military Medical University; Air Force Military Medical University
RP Yuan, LJ (通讯作者)，Fourth Mil Med Univ, Tangdu Hosp, Dept Ultrasound Diagnost, Xian 710038, Shaanxi, Peoples R China.; Yang, GD (通讯作者)，Fourth Mil Med Univ, Dept Biochem & Mol Biol, State Lab Canc Biol, Xian 710032, Shaanxi, Peoples R China.
EM yanggd@fmmu.edu.cn; yuanlj@fmmu.edu.cn
OI Sun, Wenqi/0000-0002-3837-5647; Ji, Panpan/0000-0002-7786-6844
FU National Natural Science Foundation of China (NSFC) [82272010, 32271199, 82202195]
FX Acknowledgements W.S. and P.J. contributed equally to this work. The authors gratefully acknowledge Jing Zhang and Zhenzhen Hao for technical assistance. This work was supported by the National Natural Science Foundation of China (NSFC) (82272010, 32271199, 82202195).
CR Allen BM, 2020, NAT MED, V26, P1125, DOI 10.1038/s41591-020-0892-6
Alsafadi S, 2016, NAT COMMUN, V7, DOI 10.1038/ncomms10615

Anczukow O, 2012, NAT STRUCT MOL BIOL, V19, P220, DOI 10.1038/nsmb.2207
Banday AH, 2015, IMMUNOPHARM IMMUNOT, V37, P1, DOI 10.3109/08923973.2014.971963
Bigot J, 2021, CANCER DISCOV, V11, P1938, DOI 10.1158/2159-8290.CD-20-0555
Blander JM, 2018, ANNU REV IMMUNOL, V36, P717, DOI 10.1146/annurev-immunol-041015-055523
Casbon AJ, 2015, P NATL ACAD SCI USA, V112, pE566, DOI 10.1073/pnas.1424927112
Danhier F, 2012, J CONTROL RELEASE, V161, P505, DOI 10.1016/j.jconrel.2012.01.043
Desterro J, 2020, NAT REV DRUG DISCOV, V19, P112, DOI 10.1038/s41573-019-0042-3
Du JX, 2021, J EXP CLIN CANC RES, V40, DOI 10.1186/s13046-021-01978-8
Enokida T, 2021, J CLIN INVEST, V131, DOI 10.1172/JCI146956
Fang RH, 2014, NANO LETT, V14, P2181, DOI 10.1021/nl500618u
Finn OJ, 2003, NAT REV IMMUNOL, V3, P630, DOI 10.1038/nri1150
Fish L, 2021, SCIENCE, V372, P702, DOI 10.1126/science.abc7531
Gonzales J, 2016, J BIOMED OPT, V21, DOI 10.1117/1.JBO.21.7.078002
Guermonprez P, 2002, ANNU REV IMMUNOL, V20, P621, DOI
10.1146/annurev.immunol.20.100301.064828
Islam MA, 2021, BIOMATERIALS, V266, DOI 10.1016/j.biomaterials.2020.120431
Kettern N, 2011, PLOS ONE, V6, DOI 10.1371/journal.pone.0016398
Le DT, 2013, J NATL COMPR CANC NE, V11, P766, DOI 10.6004/jnccn.2013.0099
Lee WJ, 2022, FRONT IMMUNOL, V13, DOI 10.3389/fimmu.2022.940047
LI B, 2022, ACS EST WATER, V46
Li GG, 2023, ULTRASON SONOCHEM, V92, DOI 10.1016/j.ultsonch.2022.106262
Li P, 2005, IMMUNOL REV, V207, P206, DOI 10.1111/j.0105-2896.2005.00297.x
Li WH, 2020, CHEM REV, V120, P11420, DOI 10.1021/acs.chemrev.9b00833
Li Y, 2022, ADV MATER, V34, DOI 10.1002/adma.202109984
Lin MJ, 2022, NAT CANCER, V3, P911, DOI 10.1038/s43018-022-00418-6
Liu C, 2022, NAT NANOTECHNOL, V17, P531, DOI 10.1038/s41565-022-01098-0
Liu J, 2022, J HEMATOL ONCOL, V15, DOI 10.1186/s13045-022-01247-x
Lu SX, 2021, CELL, V184, P4032, DOI 10.1016/j.cell.2021.05.038
Ma YT, 2011, CURR OPIN IMMUNOL, V23, P146, DOI 10.1016/j.coi.2010.09.008
Matsue H, 2003, J IMMUNOL, V171, P3010, DOI 10.4049/jimmunol.171.6.3010
MATSUO K, 1969, BIOCHIM BIOPHYS ACTA, V179, P39, DOI 10.1016/0005-2787(69)90119-1
Maugeri M, 2019, NAT COMMUN, V10, DOI 10.1038/s41467-019-12275-6
McAllister SS, 2014, NAT CELL BIOL, V16, P717, DOI 10.1038/ncb3015
Meng FQ, 2022, J EXTRACELL VESICLES, V11, DOI 10.1002/jev2.12289
Meng JL, 2022, ADV MATER, V34, DOI 10.1002/adma.202202168
Meyer MA, 2018, NAT COMMUN, V9, DOI 10.1038/s41467-018-03600-6
Morse MA, 2021, TARGET ONCOL, V16, P121, DOI 10.1007/s11523-020-00788-w
Nasirmoghadas P, 2021, BIOTECHNOL PROGR, V37, DOI 10.1002/btpr.3070
Nguyen Le J., 2020, CLIN ONCOL RES, DOI [10.31487/j.COR.2020.05.10, DOI
10.31487/J.COR.2020.05.10]
Qin H, 2021, ADV MATER, V33, DOI 10.1002/adma.202006007
Sahin U, 2018, SCIENCE, V359, P1355, DOI 10.1126/science.aar7112
Saxena M, 2021, NAT REV CANCER, V21, P360, DOI 10.1038/s41568-021-00346-0
Schumacher TN, 2015, SCIENCE, V348, P69, DOI 10.1126/science.aaa4971
Shemesh CS, 2021, MOL THER, V29, P555, DOI 10.1016/j.ymthe.2020.09.038
Smith CC, 2019, NAT REV CANCER, V19, P465, DOI 10.1038/s41568-019-0162-4
Taha EA, 2019, INT J MOL SCI, V20, DOI 10.3390/ijms20184588
Wang C, 2016, BIOMATERIALS, V79, P88, DOI 10.1016/j.biomaterials.2015.11.040
Wang E, 2020, TRENDS CANCER, V6, P631, DOI 10.1016/j.trecan.2020.04.011
Wang HJ, 2020, BIOMATERIALS, V244, DOI 10.1016/j.biomaterials.2020.119964
WCULEK SK, 2020, NAT REV IMMUNOL, V20, P7, DOI DOI 10.1038/S41577-019-0210-Z
Xiong X, 2021, NANO LETT, V21, P8418, DOI 10.1021/acs.nanolett.1c03004
Zhang S, 2016, PLOS ONE, V11, DOI 10.1371/journal.pone.0164514
Zhang YJ, 2021, SIGNAL TRANSDUCT TAR, V6, DOI 10.1038/s41392-021-00486-7
Zhou HZ, 2022, HEPATOLOGY, V75, P847, DOI 10.1002/hep.32195
NR 55
TC 0
Z9 0
U1 22
U2 22
PU WILEY

PI HOBOKEN
PA 111 RIVER ST, HOBOKEN 07030-5774, NJ USA
EI 2198-3844
J9 ADV SCI
JI Adv. Sci.
PD JUL
PY 2023
VL 10
IS 19
DI 10.1002/advs.202301107
EA APR 2023
PG 15
WC Chemistry, Multidisciplinary; Nanoscience & Nanotechnology; Materials
Science, Multidisciplinary
WE Science Citation Index Expanded (SCI-EXPANDED)
SC Chemistry; Science & Technology - Other Topics; Materials Science
GA L4IG2
UT WOS:000974070600001
PM 37097746
OA gold, Green Published
DA 2023-09-14
ER

PT J
AU Xu, JB
Liu, H
Wang, T
Wen, ZF
Chen, HL
Yang, ZY
Li, LY
Yu, S
Gao, SY
Yang, L
Li, K
Li, JY
Li, X
Liu, LX
Liao, GQ
Chen, YM
Liang, YJ
AF Xu, Jiabin
Liu, Hong
Wang, Tao
Wen, Zhenfu
Chen, Haolin
Yang, Zeyu
Li, Liyan
Yu, Shan
Gao, Siyong
Yang, Le
Li, Kan
Li, Jingyuan
Li, Xiang
Liu, Lixin
Liao, Guiqing
Chen, Yongming
Liang, Yujie
TI CCR7 Mediated Mimetic Dendritic Cell Vaccine Homing in Lymph Node for
Head and Neck Squamous Cell Carcinoma Therapy
SO ADVANCED SCIENCE

LA English
DT Article
DE CCR7; head and neck squamous cell carcinoma; lymph node targeting;
nanovaccines; tumor-derived exosomes
ID TUMOR-DERIVED EXOSOMES; NANOPARTICLES; ACTIVATION; TRANSPORT; ANTIGENS
AB Immunotherapy has been recognized as one of the most promising treatment strategies for head and neck squamous cell carcinoma (HNSCC). As a pioneering trend of immunotherapy, dendritic cell (DC) vaccines have displayed the ability to prime an immune response, while the insufficient immunogenicity and low lymph node (LN) targeting efficiency, resulted in an unsubstantiated therapeutic efficacy in clinical trials. Herein, a hybrid nanovaccine (Hy-M-Exo) is developed via fusing tumor-derived exosome (TEX) and dendritic cell membrane vesicle (DCMV). The hybrid nanovaccine inherited the key protein for lymphatic homing, CCR7, from DCMV and demonstrated an enhanced efficiency of LN targeting. Meanwhile, the reserved tumor antigens and endogenous danger signals in the hybrid nanovaccine activated antigen presenting cells (APCs) elicited a robust T-cell response. Moreover, the nanovaccine Hy-M-Exo displayed good therapeutic efficacy in a mouse model of HNSCC. These results indicated that Hy-M-Exo is of high clinical value to serve as a feasible strategy for antitumor immunotherapy.
C1 [Xu, Jiabin; Wang, Tao; Yu, Shan; Gao, Siyong; Yang, Le; Li, Kan; Li, Jingyuan; Li, Xiang; Liao, Guiqing; Liang, Yujie] Sun Yat sen Univ, Hosp Stomatol, Guangzhou 510030, Peoples R China.
[Liu, Hong; Wen, Zhenfu; Chen, Haolin; Yang, Zeyu; Li, Liyan; Liu, Lixin; Chen, Yongming] Sun Yat sen Univ, Sch Mat Sci & Engn, Key Lab Polymer Composite, Funct Mat Minist Educ, Guangzhou 510275, Peoples R China.
[Xu, Jiabin; Wang, Tao; Yu, Shan; Gao, Siyong; Yang, Le; Li, Kan; Li, Jingyuan; Li, Xiang; Liao, Guiqing; Liang, Yujie] Guangdong Prov Key Lab Stomatol, Guangzhou 510030, Peoples R China.
[Xu, Jiabin; Wang, Tao; Yu, Shan; Gao, Siyong; Yang, Le; Li, Kan; Li, Jingyuan; Li, Xiang; Liao, Guiqing; Liang, Yujie] Sun Yat sen Univ, Inst Stomatol, Guangzhou 510030, Peoples R China.
[Xu, Jiabin] Xuzhou Med Univ, Sch Stomatol, Xuzhou 221004, Peoples R China.
[Xu, Jiabin] Xuzhou Med Univ, Affiliated Stomatol Hosp, Xuzhou 221004, Peoples R China.
C3 Sun Yat Sen University; Sun Yat Sen University; Sun Yat Sen University;
Xuzhou Medical University; Xuzhou Medical University
RP Liao, GQ; Liang, YJ (通讯作者)，Sun Yat sen Univ, Hosp Stomatol, Guangzhou 510030, Peoples R China.; Liu, H; Chen, YM (通讯作者)，Sun Yat sen Univ, Sch Mat Sci & Engn, Key Lab Polymer Composite, Funct Mat Minist Educ, Guangzhou 510275, Peoples R China.; Liao, GQ; Liang, YJ (通讯作者)，Guangdong Prov Key Lab Stomatol, Guangzhou 510030, Peoples R China.; Liao, GQ; Liang, YJ (通讯作者)，Sun Yat sen Univ, Inst Stomatol, Guangzhou 510030, Peoples R China.
EM liuh258@mail2.sysu.edu.cn; liaogq@mail.sysu.edu.cn;
chenym35@mail.sysu.edu.cn; liangyj35@mail.sysu.edu.cn
FU National Natural Science Foundation of China [81972544, 82072995];
Program of Xuzhou Science and Technology [KC22040]; Scientific Research
Start-up Fund for Talent in Xuzhou Medical University [D2022023]
FX Acknowledgements Financial support from the National Natural Science
Foundation of China (81972544 and 82072995), the Program of Xuzhou
Science and Technology (KC22040), and the Scientific Research Start-up
Fund for Talent in Xuzhou Medical University (D2022023) was gratefully
acknowledged.
CR Chaput N, 2004, CANCER IMMUNOL IMMUN, V53, P234, DOI 10.1007/s00262-003-0472-x
Chen FM, 2021, BIOMATERIALS, V270, DOI 10.1016/j.biomaterials.2021.120709
Cheng SH, 2020, ADV SCI, V7, DOI 10.1002/advs.201903301
Chow LQM, 2020, NEW ENGL J MED, V382, P60, DOI 10.1056/NEJMra1715715
Ciesielska A, 2021, CELL MOL LIFE SCI, V78, P1233, DOI 10.1007/s00018-020-03656-y
Dong H, 2018, CANCER MANAG RES, V10, P493, DOI 10.2147/CMAR.S155914
Forster R, 2008, NAT REV IMMUNOL, V8, P362, DOI 10.1038/nri2297
Gu XY, 2015, INT J CANCER, V136, pE74, DOI 10.1002/ijc.29100
Hashimoto M, 2018, ANNU REV MED, V69, P301, DOI 10.1146/annurev-med-012017-043208
Hosseini R, 2022, MOL CANCER, V21, DOI 10.1186/s12943-021-01492-7
Howard GP, 2019, NANO RES, V12, P837, DOI 10.1007/s12274-019-2301-3
Kalluri R, 2020, SCIENCE, V367, P640, DOI 10.1126/science.aau6977
Liu H, 2022, J CONTROL RELEASE, V352, P497, DOI 10.1016/j.jconrel.2022.10.053
Liu H, 2020, BIOMATERIALS, V255, DOI 10.1016/j.biomaterials.2020.120158
Lu L, 2015, ONCOIMMUNOLOGY, V4, DOI 10.4161/2162402X.2014.990767
Lu TW, 2022, J EXTRACELL VESICLES, V11, DOI 10.1002/jev2.12218
Ludwig N, 2020, CANCERS, V12, DOI 10.3390/cancers12061602

Lybaert L, 2017, ADV SCI, V4, DOI 10.1002/advs.201700050
Mellman I, 2013, CANCER IMMUNOL RES, V1, P145, DOI 10.1158/2326-6066.CIR-13-0102
Mody MD, 2021, LANCET, V398, P2289, DOI 10.1016/S0140-6736(21)01550-6
Monypenny J, 2018, CELL REP, V24, P630, DOI 10.1016/j.celrep.2018.06.066
Moyer TJ, 2016, J CLIN INVEST, V126, P799, DOI 10.1172/JCI81083
Mulcahy LA, 2014, J EXTRACELL VESICLES, V3, DOI 10.3402/jev.v3.24641
Nam GH, 2020, ADV MATER, V32, DOI 10.1002/adma.202002440
Parodi A, 2013, NAT NANOTECHNOL, V8, P61, DOI 10.1038/nnano.2012.212
Rana J, 2020, CELL IMMUNOL, V356, DOI 10.1016/j.cellimm.2020.104193
Rao Q, 2016, HEPATOLOGY, V64, P456, DOI 10.1002/hep.28549
Reddy ST, 2007, NAT BIOTECHNOL, V25, P1159, DOI [10.1038/nbt1332, 10.1038/nbtl332]
Schineis P, 2021, J CONTROL RELEASE, V332, P96, DOI 10.1016/j.jconrel.2021.02.014
Singh AK, 2015, ORAL DIS, V21, pE105, DOI 10.1111/odi.12238
Steinhagen F, 2011, VACCINE, V29, P3341, DOI 10.1016/j.vaccine.2010.08.002
Tanaka A, 2017, CELL RES, V27, P109, DOI 10.1038/cr.2016.151
Tanyi JL, 2018, SCI TRANSL MED, V10, DOI 10.1126/scitranslmed.aao5931
Thery C, 2002, NAT REV IMMUNOL, V2, P569, DOI 10.1038/nri855
Tian T, 2014, J BIOL CHEM, V289, P22258, DOI 10.1074/jbc.M114.588046
Vincent-Schneider H, 2002, INT IMMUNOL, V14, P713, DOI 10.1093/intimm/dxf048
Wang YB, 2020, ACCOUNTS CHEM RES, V53, P1046, DOI 10.1021/acs.accounts.9b00631
Wolfers J, 2001, NAT MED, V7, P297, DOI 10.1038/85438
Xie F, 2019, ADV SCI, V6, DOI 10.1002/advs.201901779
Xu JB, 2022, PHARMACEUTICALS-BASE, V15, DOI 10.3390/ph15070876
Yoon HY, 2018, BIOMATERIALS, V178, P597, DOI 10.1016/j.biomaterials.2018.03.036
Zeng Q, 2017, BIOMATERIALS, V122, P105, DOI 10.1016/j.biomaterials.2017.01.010
Zhang M, 2021, TRANSL LUNG CANCER R, V10, P3079, DOI 10.21037/tlcr-21-1
Zheng R., 2022, J NATL CANC CTR, V2, P1, DOI [10.1016/j.jncc.2022.02.002, DOI 10.1016/J.JNCC.2022.02.002]
Zheng YJ, 2019, INT J ONCOL, V54, P1061, DOI 10.3892/ijo.2019.4685
NR 45
TC 0
Z9 0
U1 22
U2 22
PU WILEY
PI HOBOKEN
PA 111 RIVER ST, HOBOKEN 07030-5774, NJ USA
EI 2198-3844
J9 ADV SCI
JI Adv. Sci.
PD JUN
PY 2023
VL 10
IS 17
DI 10.1002/advs.202207017
EA APR 2023
PG 11
WC Chemistry, Multidisciplinary; Nanoscience & Nanotechnology; Materials
Science, Multidisciplinary
WE Science Citation Index Expanded (SCI-EXPANDED)
SC Chemistry; Science & Technology - Other Topics; Materials Science
GA I9OD0
UT WOS:000972967000001
PM 37092579
OA Green Published, gold
DA 2023-09-14
ER

PT J
AU Zhang, HL
Zhang, YW

Hu, HL
Yang, WQ
Xia, X
Lei, L
Lin, RY
Li, JM
Li, Y
Gao, HL
AF Zhang, Huilin
Zhang, Yiwei
Hu, Haili
Yang, Wenqin
Xia, Xue
Lei, Lei
Lin, Ruyi
Li, Jiamei
Li, Yuan
Gao, Huile
TI In Situ Tumor Vaccine for Lymph Nodes Delivery and Cancer Therapy Based on Small Size Nanoadjuvant
SO SMALL
LA English
DT Article; Early Access
DE antigen delivery; CTLA4; in situ vaccines; losartan; lymph node targets; tumor vaccines
ID CHECKPOINT BLOCKADE; BREAST-CANCER; NANOPARTICLES; MICROENVIRONMENT; HYDROGEL; IMMUNOTHERAPY; COMBINATION; NANOVACCINE; EFFICACY; RECEPTOR
AB Tumor vaccine is a promising cancer treatment modality, however, the convenient antigens loading in vivo and efficient delivery of vaccines to lymph nodes (LNs) still remain a formidable challenge. Herein, an in situ nanovaccine strategy targeting LNs to induce powerful antitumor immune responses by converting the primary tumor into whole-cell antigens and then delivering these antigens and nanoadjuvants simultaneously to LNs is proposed. The in situ nanovaccine is based on a hydrogel system, which loaded with doxorubicin (DOX) and nanoadjuvant CpG-P-ss-M. The gel system exhibits ROS-responsive release of DOX and CpG-P-ss-M, generating abundant in situ storage of whole-cell tumor antigens. CpG-P-ss-M adsorbs tumor antigens through the positive surface charge and achieves charge reversal, forming small-sized and negatively charged tumor vaccines in situ, which are then primed to LNs. Eventually, the tumor vaccine promotes antigens uptake by dendritic cells (DCs), maturation of DCs, and proliferation of T cells. Moreover, the vaccine combined with anti-CTLA4 antibody and losartan inhibits tumor growth by 50%, significantly increasing the percentage of splenic cytotoxic T cells (CTLs), and generating tumor-specific immune responses. Overall, the treatment effectively inhibits primary tumor growth and induces tumor-specific immune response. This study provides a scalable strategy for in situ tumor vaccination.
C1 [Zhang, Huilin; Li, Yuan] Peking Univ Third Hosp, Dept Obstet & Gynecol, Beijing 100191, Peoples R China.
[Zhang, Huilin; Zhang, Yiwei; Hu, Haili; Yang, Wenqin; Xia, Xue; Lei, Lei; Lin, Ruyi; Li, Jiamei; Gao, Huile] Sichuan Univ, West China Sch Pharm, Key Lab Drug Targeting & Drug Delivery Syst, Chengdu 610041, Peoples R China.
C3 Peking University; Sichuan University
RP Li, Y (通讯作者)，Peking Univ Third Hosp, Dept Obstet & Gynecol, Beijing 100191, Peoples R China.; Gao, HL (通讯作者)，Sichuan Univ, West China Sch Pharm, Key Lab Drug Targeting & Drug Delivery Syst, Chengdu 610041, Peoples R China.
EM yuanli@bjmu.edu.cn; gaohuile@scu.edu.cn
FU Key Research and Development Program of Science and Technology Department of Sichuan Province [2022JDJQ0050, 2022YFS0334]; 111 Project [B18035]; Fundamental of Research Funds for the Central Universities; Beijing Natural Science Foundation [L212054]
FX Acknowledgements The work was supported by the Key Research and Development Program of Science and Technology Department of Sichuan Province (2022JDJQ0050 and 2022YFS0334), the 111 Project (B18035), the Fundamental of Research Funds for the Central Universities and Beijing Natural Science Foundation No. L212054. The authors acknowledge the

assistance provided by Dr. Guangjun Yu for support in the synthesis of
TSPBA and Jing Wang for support in the animal experiments.
CR Baharom F, 2021, NAT IMMUNOL, V22, P41, DOI 10.1038/s41590-020-00810-3
Chen JJ, 2021, SCI ADV, V7, DOI 10.1126/sciadv.abf1244
Chen WQ, 2021, INT J NANOMED, V16, P5281, DOI 10.2147/IJN.S317626
Chu YC, 2022, CHINESE CHEM LETT, V33, P4157, DOI 10.1016/j.cclet.2022.02.051
Cun XL, 2016, ACTA BIOMATER, V31, P186, DOI 10.1016/j.actbio.2015.12.002
Diop-Frimpong B, 2011, P NATL ACAD SCI USA, V108, P2909, DOI 10.1073/pnas.1018892108
Dovedi SJ, 2021, CANCER DISCOV, V11, P1100, DOI 10.1158/2159-8290.CD-20-1445
Duan XP, 2019, ANGEW CHEM INT EDIT, V58, P670, DOI 10.1002/anie.201804882
East L, 2002, BBA-GEN SUBJECTS, V1572, P364, DOI 10.1016/S0304-4165(02)00319-7
Engering AJ, 1997, EUR J IMMUNOL, V27, P2417, DOI 10.1002/eji.1830270941
Fucikova J, 2020, CELL DEATH DIS, V11, DOI 10.1038/s41419-020-03221-2
Gide TN, 2019, CANCER CELL, V35, P238, DOI 10.1016/j.ccell.2019.01.003
Griffiths KL, 2016, NAT COMMUN, V7, DOI 10.1038/ncomms13894
Grohmann U, 2002, NAT IMMUNOL, V3, P1097, DOI 10.1038/ni846
Hammerich L, 2019, NAT MED, V25, P814, DOI 10.1038/s41591-019-0410-x
Han X, 2020, ACCOUNTS CHEM RES, V53, P2521, DOI 10.1021/acs.accounts.0c00339
Hollingsworth RE, 2019, NPJ VACCINES, V4, DOI 10.1038/s41541-019-0103-y
Hong XY, 2020, SCI ADV, V6, DOI 10.1126/sciadv.aaz4462
Hu C, 2021, ADV FUNCT MATER, V31, DOI 10.1002/adfm.202007149
Irvine DJ, 2020, NAT REV IMMUNOL, V20, P321, DOI 10.1038/s41577-019-0269-6
Jagodinsky JC, 2022, J IMMUNOTHER CANCER, V10, DOI 10.1136/jitc-2022-005103
Ji XY, 2021, ADV MATER, V33, DOI 10.1002/adma.202100949
Jia YP, 2020, ADV FUNCT MATER, V30, DOI 10.1002/adfm.202001059
Jiang H, 2017, J CONTROL RELEASE, V267, P47, DOI 10.1016/j.jconrel.2017.08.009
Jin LJ, 2022, ACS NANO, DOI 10.1021/acsnano.2c06560
Jou J, 2021, CLIN CANCER RES, V27, P689, DOI 10.1158/1078-0432.CCR-20-0245
Kvakova K, 2022, ADV FUNCT MATER, V32, DOI 10.1002/adfm.202109960
Lavrador P, 2021, ADV FUNCT MATER, V31, DOI 10.1002/adfm.202005941
Lee HJ, 2020, SMALL, V16, DOI 10.1002/smll.201903045
Li JM, 2022, J CONTROL RELEASE, V352, P313, DOI 10.1016/j.jconrel.2022.10.025
Liang S, 2021, ADV FUNCT MATER, V31, DOI 10.1002/adfm.202106123
Lin MJ, 2022, NAT CANCER, V3, P911, DOI 10.1038/s43018-022-00418-6
Liu R, 2023, CHINESE CHEM LETT, V34, DOI [10.1016/j.cclet.2022.05.032,
10.1016/j.cclet.2022.05.007]
Luo M, 2017, NAT NANOTECHNOL, V12, P648, DOI [10.1038/nnano.2017.52,
10.1038/NNANO.2017.52]
Martinez-Pomares L, 2001, IMMUNOBIOLOGY, V204, P527, DOI 10.1078/0171-2985-00089
Mei L, 2018, J CONTROL RELEASE, V292, P67, DOI 10.1016/j.jconrel.2018.04.053
Morse MA, 2021, TARGET ONCOL, V16, P121, DOI 10.1007/s11523-020-00788-w
Nam J, 2021, ADV SCI, V8, DOI 10.1002/advs.202002577
Nam J, 2018, NAT COMMUN, V9, DOI 10.1038/s41467-018-03473-9
Pan Y, 2021, ADV FUNCT MATER, V31, DOI 10.1002/adfm.202105742
Qi YQ, 2018, ACS APPL MATER INTER, V10, P6972, DOI 10.1021/acsami.7b19258
Qiao DD, 2022, ACS APPL MATER INTER, V14, P50592, DOI 10.1021/acsami.2c15644
Qin L, 2021, SMALL, V17, DOI 10.1002/smll.202006000
Qin L, 2020, SCI ADV, V6, DOI 10.1126/sciadv.abb3116
Sahin U, 2018, SCIENCE, V359, P1355, DOI 10.1126/science.aar7112
Sasso MS, 2021, SCI ADV, V7, DOI 10.1126/sciadv.abe4362
Saxena M, 2021, NAT REV CANCER, V21, P360, DOI 10.1038/s41568-021-00346-0
Scheetz L, 2019, NAT BIOMED ENG, V3, P768, DOI 10.1038/s41551-019-0436-x
Shetty K, 2021, ANNU REV CANC BIOL, V5, P259, DOI 10.1146/annurev-cancerbio-060820-111701
Shi W, 2023, ADV THER-GERMANY, V6, DOI 10.1002/adtp.202200239
Shi YY, 2021, ADV HEALTHC MATER, V10, DOI 10.1002/adhm.202001934
Sultan H, 2020, J IMMUNOTHER CANCER, V8, DOI 10.1136/jitc-2020-001224
Sun Y., 2020, CLIN CANCER RES, V26, P13
Tardon MC, 2019, CURR OPIN PHARMACOL, V47, P20, DOI 10.1016/j.coph.2019.01.007
Thomas SN, 2014, BIOMATERIALS, V35, P814, DOI 10.1016/j.biomaterials.2013.10.003
Vedove ED, 2018, ADV HEALTHC MATER, V7, DOI 10.1002/adhm.201701398
Wada H, 2018, CHEM ENG J, V340, P51, DOI 10.1016/j.cej.2018.01.008

Wang C, 2017, NAT BIOMED ENG, V1, DOI 10.1038/s41551-016-0011
Wang JH, 2020, ADV SCI, V7, DOI 10.1002/advs.202001108
Wang J, 2022, NATL SCI REV, V9, DOI 10.1093/nsr/nwab159
Wang JQ, 2018, ACS NANO, V12, P2466, DOI 10.1021/acsnano.7b08152
Wang S, 2018, SMALL, V14, DOI 10.1002/smll.201704272
Wang X, 2022, J CONTROL RELEASE, V349, P18, DOI 10.1016/j.jconrel.2022.06.054
Wang YF, 2022, NAT COMMUN, V13, DOI 10.1038/s41467-022-29120-y
Wawrzyniecka PA, 2022, LEUKEMIA, V36, P577, DOI 10.1038/s41375-021-01385-0
Xiong X, 2021, NANO LETT, V21, P8418, DOI 10.1021/acs.nanolett.1c03004
Xu JL, 2022, NAT COMMUN, V13, DOI 10.1038/s41467-021-27750-2
Xu J, 2020, NAT NANOTECHNOL, V15, P1043, DOI 10.1038/s41565-020-00781-4
Xu J, 2017, ACS NANO, V11, P4463, DOI 10.1021/acsnano.7b00715
Xu YY, 2022, ACTA PHARM SIN B, V12, P4327, DOI 10.1016/j.apsb.2022.11.001
Yang Y, 2022, INT J NANOMED, V17, P4791, DOI 10.2147/IJN.S372048
Yu WQ, 2019, BIOMATERIALS, V217, DOI 10.1016/j.biomaterials.2019.119309
Yu X, 2020, NAT COMMUN, V11, DOI 10.1038/s41467-020-14906-9
Zhang W, 2020, ACTA PHARMACOL SIN B, V10, P2037, DOI 10.1016/j.apsb.2020.07.013
Zhang YY, 2022, NAT COMMUN, V13, DOI 10.1038/s41467-022-32160-z
Zhang Y, 2021, ADV MATER, V33, DOI 10.1002/adma.202007293
Zhao Q, 2022, FRONT IMMUNOL, V13, DOI 10.3389/fimmu.2022.938439
NR 77
TC 0
Z9 0
U1 43
U2 43
PU WILEY-V C H VERLAG GMBH
PI WEINHEIM
PA POSTFACH 101161, 69451 WEINHEIM, GERMANY
SN 1613-6810
EI 1613-6829
J9 SMALL
JI Small
PD 2023 APR 20
PY 2023
DI 10.1002/smll.202301041
EA APR 2023
PG 14
WC Chemistry, Multidisciplinary; Chemistry, Physical; Nanoscience &
Nanotechnology; Materials Science, Multidisciplinary; Physics, Applied;
Physics, Condensed Matter
WE Science Citation Index Expanded (SCI-EXPANDED)
SC Chemistry; Science & Technology - Other Topics; Materials Science;
Physics
GA D8NK2
UT WOS:000971237400001
PM 37078903
DA 2023-09-14
ER

PT J
AU Wen, ZF
Liu, H
Qiao, DD
Chen, HL
Li, LY
Yang, ZY
Zhu, CX
Zeng, ZP
Chen, YM
Liu, LX
AF Wen, Zhenfu

Liu, Hong
Qiao, Dongdong
Chen, Haolin
Li, Liyan
Yang, Zeyu
Zhu, Chenxu
Zeng, Zhipeng
Chen, Yongming
Liu, Lixin
TI Nanovaccines Fostering Tertiary Lymphoid Structure to Attack Mimicry Nasopharyngeal Carcinoma
SO ACS NANO
LA English
DT Article
DE Nanovaccine; Nasopharyngeal carcinoma; STING; Tertiary lymphoid structures; TLR9 agonist
ID IMMUNE-RESPONSES; DENDRITIC CELLS; IMMUNOTHERAPY; INDUCTION; SECONDARY; CANCER; TUMORS
AB Tertiary lymphoid structures (TLSs) are formed in inflamed tissues, and recent studies demonstrated that the appearance of TLSs in tumor sites is associated with a good prognosis for tumor patients. However, the process of natural TLSs' formation was slow and uncontrollable. Herein, we developed a nanovaccine consisting of Epstein-Barr virus nuclear antigen 1 (EBNA1) and a bi-adjuvant of Mn2+ and cytosine-phosphate-guanine (CpG) formulated with tannic acid that significantly inhibited the development of mimicry nasopharyngeal carcinoma by fostering TLS formation. The nanovaccine activated LT-alpha and LT-beta pathways, subsequently enhancing the expression of downstream chemokines, CCL19/ CCL21, CXCL10 and CXCL13, in the tumor microenvironment. In turn, normalized blood and lymph vessels were detected in the tumor tissues of the nanovaccine group, correlated with increased infiltration of lymphocytes. Especially, the proportion of the B220+ CD8+ T, which was produced via trogocytosis between T and B cells during activation of T cells, was increased in tumors of the nanovaccine group. Furthermore, the intratumoral effector memory T cells (Tem), CD45+, CD3+, CD8+, CD44+, and CD62L-, did not decrease after blocking the egress of T cells from tumor-draining lymph nodes by FTY-720. These results demonstrated that the nanovaccine can foster TLS formation, which thus enhances local immune responses significantly, delays tumor outgrowth, and prolongs the median survival time of murine models of mimicry nasopharyngeal carcinoma, demonstrating a promising strategy for nanovaccine development.
C1 [Wen, Zhenfu; Liu, Hong; Qiao, Dongdong; Chen, Haolin; Li, Liyan; Yang, Zeyu; Zhu, Chenxu; Zeng, Zhipeng; Chen, Yongming; Liu, Lixin] Sun Yat Sen Univ, Sch Mat Sci & Engn, Key Lab Polymer Composite & Funct Mat, Minist Educ, Guangzhou 510275, Peoples R China.
[Chen, Yongming; Liu, Lixin] Sun Yat Sen Univ, State Key Lab Oncol Southern China, Canc Ctr, Guangzhou 510060, Peoples R China.
[Chen, Yongming] Sun Yat Sen Univ, Affiliated Hosp 3, Ctr Nanomed, Lab Biomat & Translat Med, Guangzhou 510630, Peoples R China.
C3 Sun Yat Sen University; Sun Yat Sen University; State Key Lab Oncology South China; Sun Yat Sen University
RP Chen, YM; Liu, LX (通讯作者)，Sun Yat Sen Univ, Sch Mat Sci & Engn, Key Lab Polymer Composite & Funct Mat, Minist Educ, Guangzhou 510275, Peoples R China.; Chen, YM; Liu, LX (通讯作者)，Sun Yat Sen Univ, State Key Lab Oncol Southern China, Canc Ctr, Guangzhou 510060, Peoples R China.; Chen, YM (通讯作者)，Sun Yat Sen Univ, Affiliated Hosp 3, Ctr Nanomed, Lab Biomat & Translat Med, Guangzhou 510630, Peoples R China.
EM chenym35@mail.sysu.edu.cn; liulixin@mail.sysu.edu.cn
OI Liu, Lixin/0000-0001-6133-4977
FU National Natural Science Foundation of China [51820105004, 22075324]; Key Project of the Ministry of Science and Technology [2022YFC2304201]; Key Areas Research and Development Program of Guangzhou [202007020006]
FX We appreciate the financial support from the National Natural Science Foundation of China (51820105004, 22075324), the Key Project of the Ministry of Science and Technology (2022YFC2304201) and the Key Areas Research and Development Program of Guangzhou (202007020006).
CR Aloisi F, 2006, NAT REV IMMUNOL, V6, P205, DOI 10.1038/nri1786
Boivin G, 2018, FRONT ONCOL, V8, DOI 10.3389/fonc.2018.00256
Cabrita R, 2020, NATURE, V577, P561, DOI 10.1038/s41586-019-1914-8

Chelvanambi M, 2021, J IMMUNOTHER CANCER, V9, DOI 10.1136/jitc-2020-001906
Chen JQ, 2019, ACS NANO, V13, P11653, DOI 10.1021/acsnano.9b05521
Chen YP, 2019, LANCET, V394, P64, DOI 10.1016/S0140-6736(19)30956-0
Chu X, 2016, J PHARMACOL SCI, V130, P15, DOI 10.1016/j.jphs.2015.12.002
Coppola D, 2011, AM J PATHOL, V179, P37, DOI 10.1016/j.ajpath.2011.03.007
Cupedo T, 2004, IMMUNITY, V21, P655, DOI 10.1016/j.immuni.2004.09.006
Dieu-Nosjean MC, 2014, TRENDS IMMUNOL, V35, P571, DOI 10.1016/j.it.2014.09.006
Dijk NV, 2021, FRONT IMMUNOL, V12, DOI 10.3389/fimmu.2021.793964
Fridman WH, 2022, NAT REV CLIN ONCOL, V19, P441, DOI 10.1038/s41571-022-00619-z
Fukurnura D, 2018, NAT REV CLIN ONCOL, V15, P325, DOI 10.1038/nrclinonc.2018.29
Germain C, 2015, FRONT IMMUNOL, V6, DOI 10.3389/fimmu.2015.00067
Girard JP, 2012, NAT REV IMMUNOL, V12, P762, DOI 10.1038/nri3298
Goc J, 2013, ONCOIMMUNOLOGY, V2, DOI 10.4161/onci.26836
Gong LQ, 2021, NAT COMMUN, V12, DOI 10.1038/s41467-021-21795-z
He Z., 2019, J CONTROL RELEASE, V301
He ZY, 2020, J CONTROL RELEASE, V318, P86, DOI 10.1016/j.jconrel.2019.12.014
Johansson-Percival A, 2017, NAT IMMUNOL, V18, P1207, DOI 10.1038/ni.3836
Joshi NS, 2015, IMMUNITY, V43, P579, DOI 10.1016/j.immuni.2015.08.006
Kroeger DR, 2016, CLIN CANCER RES, V22, P3005, DOI 10.1158/1078-0432.CCR-15-2762
Kumar V, 2015, IMMUNITY, V42, P719, DOI 10.1016/j.immuni.2015.03.015
Le ZC, 2018, ACS APPL MATER INTER, V10, P42186, DOI 10.1021/acsami.8b18979
Li JP, 2021, J IMMUNOTHER CANCER, V9, DOI 10.1136/jitc-2020-002101
Li R, 2021, FRONT IMMUNOL, V12, DOI 10.3389/fimmu.2021.694079
Liu H, 2020, BIOMATERIALS, V255, DOI 10.1016/j.biomaterials.2020.120158
Liu Y, 2011, VACCINE, V29, P5778, DOI 10.1016/j.vaccine.2011.05.087
Liu ZJ, 2019, NANOSCALE, V11, P9410, DOI 10.1039/c9nr02185j
Lv M.Z., CELL RES
Martinet L, 2013, J IMMUNOL, V191, P2001, DOI 10.4049/jimmunol.1300872
Moussion C, 2011, NATURE, V479, P542, DOI 10.1038/nature10540
Moyron-Quiroz JE, 2006, IMMUNITY, V25, P643, DOI 10.1016/j.immuni.2006.08.022
Munoz-Erazo L, 2020, CELL MOL IMMUNOL, V17, P570, DOI 10.1038/s41423-020-0457-0
Ochs J, 2022, SCI TRANSL MED, V14, DOI 10.1126/scitranslmed.abi4632
Peres TV, 2016, BMC PHARMACOL TOXICO, V17, DOI 10.1186/s40360-016-0099-0
Pitzalis C, 2014, NAT REV IMMUNOL, V14, P447, DOI 10.1038/nri3700
Pulendran B, 2021, NAT REV DRUG DISCOV, V20, P454, DOI 10.1038/s41573-021-00163-y
Qiao DD, 2018, NANO LETT, V18, P3007, DOI 10.1021/acs.nanolett.8b00478
Ruffin AT, 2021, NAT COMMUN, V12, DOI 10.1038/s41467-021-23355-x
Ruuskanen M, 2018, ACTA ONCOL, V57, P251, DOI 10.1080/0284186X.2017.1346378
Sautes-Fridman C, 2020, SEMIN IMMUNOL, V48, DOI 10.1016/j.smim.2020.101406
Sautes-Fridman C, 2019, NAT REV CANCER, V19, P307, DOI 10.1038/s41568-019-0144-6
Schumacher TN, 2022, SCIENCE, V375, P39, DOI 10.1126/science.abf9419
Stowman AM, 2018, MELANOMA RES, V28, P237, DOI 10.1097/CMR.0000000000000439
Stylianopoulos T, 2018, TRENDS CANCER, V4, P292, DOI 10.1016/j.trecan.2018.02.005
Suematsu S, 2004, NAT BIOTECHNOL, V22, P1539, DOI 10.1038/nbt1039
Sun YZ, 2021, NANO RES, V14, P1260, DOI 10.1007/s12274-020-3243-5
Zhang CX, 2019, CELL DEATH DIFFER, V26, P2314, DOI 10.1038/s41418-019-0302-0
Zhu GY, 2018, FRONT IMMUNOL, V9, DOI 10.3389/fimmu.2018.01609
NR 50
TC 0
Z9 0
U1 16
U2 16
PU AMER CHEMICAL SOC
PI WASHINGTON
PA 1155 16TH ST, NW, WASHINGTON, DC 20036 USA
SN 1936-0851
EI 1936-086X
J9 ACS NANO
JI ACS Nano
PD APR 25
PY 2023

VL 17
IS 8
BP 7194
EP 7206
DI 10.1021/acsnano.2c09619
EA APR 2023
PG 13
WC Chemistry, Multidisciplinary; Chemistry, Physical; Nanoscience &
Nanotechnology; Materials Science, Multidisciplinary
WE Science Citation Index Expanded (SCI-EXPANDED)
SC Chemistry; Science & Technology - Other Topics; Materials Science
GA E5WB8
UT WOS:000974329000001
PM 37057967
DA 2023-09-14
ER

PT J
AU Martinez-Perez, A
Diego-Gonzalez, L
Vilanova, M
Correia, A
Simon-Vazquez, R
Gonzalez-Fernandez, A
AF Martinez-Perez, Amparo
Diego-Gonzalez, Lara
Vilanova, Manuel
Correia, Alexandra
Simon-Vazquez, Rosana
Gonzalez-Fernandez, Africa
TI Immunization with nanovaccines containing mutated K-Ras peptides and
imiquimod aggravates heterotopic pancreatic cancer induced in mice
SO FRONTIERS IN IMMUNOLOGY
LA English
DT Article
DE KRAS; nanovaccine; pancreatic cancer; imiquimod (IMQ); chitosan;
polyarginine
ID LOADED CHITOSAN NANOPARTICLES; TLR7 AGONIST; VACCINE; CELLS;
IMMUNOSUPPRESSION; NANOCAPSULES; MECHANISM; CARCINOMA; PROTEINS;
DELIVERY
AB PurposeThe growing incidence and lethality of pancreatic cancer urges the development of new therapeutic approaches. Anti-tumoral vaccines can potentiate the immune response against the tumor, targeting specific antigens expressed only on tumor cells. In this work, we designed new vaccines for pancreatic cancer, composed by chitosan nanocapsules (CS NCs) containing imiquimod (IMQ) as adjuvant, and targeting the K-Ras mutation G12V. Experimental designWe tested the immunogenicity of our vaccines in mice, carrying different combinations of K-Ras mutated peptides. Then, we analyzed their prophylactic and therapeutic efficacy in mice bearing heterotopic pancreatic cancer. ResultsUnexpectedly, although good results were observed at short time points, the different combinations of our CS NCs vaccines seemed to potentiate tumor growth and reduce survival rate. We propose that this effect could be due to an inadequate immune response, partially because of the induction of a regulatory tolerogenic response. ConclusionOur results call for caution in the use of some NCs containing IMQ in the immunotherapy against pancreatic cancer.
C1 [Martinez-Perez, Amparo; Diego-Gonzalez, Lara; Simon-Vazquez, Rosana; Gonzalez-Fernandez, Africa] Univ Vigo, CINBIO, Inmunol Grp, Vigo, Spain.
[Martinez-Perez, Amparo; Diego-Gonzalez, Lara; Simon-Vazquez, Rosana; Gonzalez-Fernandez, Africa] SERGAS UVIGO, Inst Invest Sanitaria Galicia IIS Galicia Sur, Vigo, Spain.
[Vilanova, Manuel; Correia, Alexandra] Univ Porto, I3S Inst Invest & Inovacao Saude, Porto, Portugal.
[Vilanova, Manuel; Correia, Alexandra] Univ Porto, IBMC Inst Biol Mol & Celular, Porto, Portugal.
[Vilanova, Manuel; Correia, Alexandra] Univ Porto, ICBAS Inst Ciencias Biomed Abel Salazar, Porto, Portugal.
C3 Universidade de Vigo; CINBIO; Universidade do Porto; i3S - Instituto de

Investigacao e Inovacao em Saude, Universidade do Porto; Universidade do Porto; Universidade do Porto
RP Martinez-Perez, A (通讯作者)，Univ Vigo, CINBIO, Inmunol Grp, Vigo, Spain.; Martinez-Perez, A (通讯作者)，SERGAS UVIGO, Inst Invest Sanitaria Galicia IIS Galicia Sur, Vigo, Spain.
EM ammartinez@uvigo.es
RI González-Fernández, África/E-2641-2012; Correia, Alexandra/J-7926-2013
OI González-Fernández, África/0000-0002-9226-4825; Correia, Alexandra/0000-0003-0408-6262
CR Abrams SI, 1997, CELL IMMUNOL, V182, P137, DOI 10.1006/cimm.1997.1224
Adams S, 2008, J IMMUNOL, V181, P776, DOI 10.4049/jimmunol.181.1.776
Aggarwal P, 2009, ADV DRUG DELIVER REV, V61, P428, DOI 10.1016/j.addr.2009.03.009
Almand B, 2001, J IMMUNOL, V166, P678, DOI 10.4049/jimmunol.166.1.678
Beutner KR, 1999, J AM ACAD DERMATOL, V41, P1002, DOI 10.1016/S0190-9622(99)70261-6
Briukhovetska D, 2021, NAT REV CANCER, V21, P481, DOI 10.1038/s41568-021-00363-z
Chang PH, 2017, SCI REP-UK, V7, DOI 10.1038/srep45751
Crecente-Campo J, 2019, NANOMEDICINE-UK, V14, P3013, DOI 10.2217/nnm-2019-0206
Dang YS, 2012, CLIN CANCER RES, V18, P3122, DOI 10.1158/1078-0432.CCR-12-0113
David KI, 2015, COLLOID SURFACE B, V135, P689, DOI 10.1016/j.colsurfb.2015.08.038
Di Sciullo P, 2019, CLIN IMMUNOL, V203, P154, DOI 10.1016/j.clim.2019.04.013
Diego-Gonzalez L, 2020, PHARMACEUTICS, V12, DOI 10.3390/pharmaceutics12060489
Ding YY, 2021, ADV DRUG DELIVER REV, V179, DOI 10.1016/j.addr.2021.113914
Dobrovolskaia MA, 2013, J CONTROL RELEASE, V172, P456, DOI 10.1016/j.jconrel.2013.05.025
Frank LA, 2019, EUR J PHARM BIOPHARM, V136, P9, DOI 10.1016/j.ejpb.2019.01.001
Fu SZ, 2016, J BIOMED NANOTECHNOL, V12, P1585, DOI 10.1166/jbn.2016.2228
Gjertsen MK, 1998, VOX SANG, V74, P489, DOI 10.1111/j.1423-0410.1998.tb05462.x
Guerra C, 2011, CANCER CELL, V19, P728, DOI 10.1016/j.ccr.2011.05.011
Hatzioannou A, 2021, FRONT IMMUNOL, V12, DOI 10.3389/fimmu.2021.731947
Hu Y, 2020, PEDIATR INVEST, V4, P133, DOI 10.1002/ped4.12205
Jesus S, 2020, FRONT BIOENG BIOTECH, V8, DOI 10.3389/fbioe.2020.00100
Jiang YB, 2019, ARTIF CELL NANOMED B, V47, P747, DOI 10.1080/21691401.2019.1577876
Kazimierczak P, 2021, INT J MOL SCI, V22, DOI 10.3390/ijms22031109
Khan MA, 2016, INT J BIOL MACROMOL, V93, P242, DOI 10.1016/j.ijbiomac.2016.08.050
Kim JH, 2008, J CONTROL RELEASE, V127, P41, DOI 10.1016/j.jconrel.2007.12.014
Lebre F, 2012, METHOD ENZYMOL, V509, P127, DOI 10.1016/B978-0-12-391858-1.00007-1
Lippi G, 2020, ARCH MED SCI, V16, P820, DOI 10.5114/aoms.2020.94845
Malumbres M, 2003, NAT REV CANCER, V3, P459, DOI 10.1038/nrc1097
Markowitz LE, 2012, VACCINE, V30, pF139, DOI 10.1016/j.vaccine.2012.05.039
Ohue Y, 2019, CANCER SCI, V110, P2080, DOI 10.1111/cas.14069
Pati R, 2018, FRONT IMMUNOL, V9, DOI 10.3389/fimmu.2018.02224
Potdar PD., 2016, J CANCER METAS TREAT, V2, P259, DOI [10.20517/2394-4722.2016.25, DOI 10.20517/2394-4722.2016.25]
PREHN RT, 1994, CANCER RES, V54, P908
PREHN RT, 1972, SCIENCE, V176, P170, DOI 10.1126/science.176.4031.170
Prior IA, 2012, CANCER RES, V72, P2457, DOI 10.1158/0008-5472.CAN-11-2612
Rahma OE, 2014, J TRANSL MED, V12, DOI 10.1186/1479-5876-12-55
Salasche SJ, 2002, J AM ACAD DERMATOL, V47, P571, DOI 10.1067/mjd.2002.126257
Siegel CT, 2000, J EXP MED, V191, P1945, DOI 10.1084/jem.191.11.1945
Siegel RL, 2019, CA-CANCER J CLIN, V69, P7, DOI 10.3322/caac.21551
Singhi AD, 2019, GASTROENTEROLOGY, V156, P2024, DOI 10.1053/j.gastro.2019.01.259
Smyth EC, 2011, J CLIN ONCOL, V29, pE809, DOI 10.1200/JCO.2011.36.8829
Stanley MA, 2002, CLIN EXP DERMATOL, V27, P571, DOI 10.1046/j.1365-2230.2002.01151.x
Takiguchi H, 2021, SCI REP-UK, V11, DOI 10.1038/s41598-021-87720-y
Togashi Y, 2019, NAT REV CLIN ONCOL, V16, P356, DOI 10.1038/s41571-019-0175-7
Vasconcelos DP, 2013, BIOMATERIALS, V34, P9952, DOI 10.1016/j.biomaterials.2013.09.012
Vicente S, 2013, J CONTROL RELEASE, V172, P773, DOI 10.1016/j.jconrel.2013.09.012
von Boxberg Y, 2022, J BIOMED MATER RES A, V110, P773, DOI 10.1002/jbm.a.37326
Wedemeyer H, 2009, VACCINE, V27, P5142, DOI 10.1016/j.vaccine.2009.06.027
Zhang RH, 2019, CANCER CELL INT, V19, DOI 10.1186/s12935-019-1027-3
Zhang Y, 2020, EXPERT REV VACCINES, V19, P163, DOI 10.1080/14760584.2020.1733420
NR 50
TC 0

Z9 0
U1 1
U2 1
PU FRONTIERS MEDIA SA
PI LAUSANNE
PA AVENUE DU TRIBUNAL FEDERAL 34, LAUSANNE, CH-1015, SWITZERLAND
SN 1664-3224
J9 FRONT IMMUNOL
JI Front. Immunol.
PD APR 12
PY 2023
VL 14
AR 1153724
DI 10.3389/fimmu.2023.1153724
PG 14
WC Immunology
WE Science Citation Index Expanded (SCI-EXPANDED)
SC Immunology
GA E2RM1
UT WOS:000974070000001
PM 37122717
OA Green Accepted, gold
DA 2023-09-14
ER

PT J
AU Su, RP
Gu, JJ
Sun, JJ
Zang, J
Zhao, YG
Zhang, TT
Chen, YN
Chong, GW
Yin, WM
Zheng, X
Liu, BB
Huang, L
Ruan, SR
Dong, HQ
Li, Y
Li, YY
AF Su, Runping
Gu, Jingjing
Sun, Juanjuan
Zang, Jie
Zhao, Yuge
Zhang, Tingting
Chen, Yingna
Chong, Gaowei
Yin, Weimin
Zheng, Xiao
Liu, Bingbing
Huang, Li
Ruan, Shuangrong
Dong, Haiqing
Li, Yan
Li, Yongyong
TI $CaCO_3$ powder-mediated biomineralization of antigen nanosponges synergize with PD-1 blockade to potentiate anti-tumor immunity
SO JOURNAL OF NANOBIOTECHNOLOGY

LA English
DT Article
DE Nanovaccine; CaCO3 powder; Biomineralization; Antigen-presenting cells;
Antigen-specific CD8(+) T cell responses
ID CALCIUM-CARBONATE; STABILIZATION; INFECTION; OVALBUMIN; CATALYST;
VATERITE
AB Antigen self-assembly nanovaccines advance the minimalist design of therapeutic cancer vaccines, but the issue of inefficient cross-presentation has not yet been fully addressed. Herein, we report a unique approach by combining the concepts of "antigen multi-copy display" and "calcium carbonate (CaCO3) biomineralization" to increase cross-presentation. Based on this strategy, we successfully construct sub-100 nm biomineralized antigen nanosponges (BANSs) with high CaCO3 loading (38.13 wt%) and antigen density (61.87%). BANSs can be effectively uptaken by immature antigen-presenting cells (APCs) in the lymph node upon subcutaneous injection. Achieving efficient spatiotemporal coordination of antigen cross-presentation and immune effects, BANSs induce the production of CD4(+) T helper cells and cytotoxic T lymphocytes, resulting in effective tumor growth inhibition. BANSs combined with anti-PD-1 antibodies synergistically enhance anti-tumor immunity and reverse the tumor immunosuppressive microenvironment. Overall, this CaCO3 powder-mediated biomineralization of antigen nanosponges offer a robust and safe strategy for cancer immunotherapy.
C1 [Su, Runping; Gu, Jingjing; Sun, Juanjuan; Zang, Jie; Zhao, Yuge; Zhang, Tingting; Chong, Gaowei; Yin, Weimin; Zheng, Xiao; Liu, Bingbing; Huang, Li; Ruan, Shuangrong; Dong, Haiqing; Li, Yan; Li, Yongyong] Tongji Univ, Shanghai Skin Dis Hosp, Inst Biomed Engn & Nano Sci, Sch Med, Shanghai 200092, Peoples R China.
[Su, Runping] Fudan Univ, Sch Basic Med Sci, Key Lab Med Mol Virol MOE NHC CAMS, Shanghai 200032, Peoples R China.
[Chen, Yingna] Tongji Univ, Inst Acoust, Sch Phys Sci & Engn, Shanghai 200092, Peoples R China.
C3 Tongji University; Fudan University; Tongji University
RP Li, YY (通讯作者)，Tongji Univ, Shanghai Skin Dis Hosp, Inst Biomed Engn & Nano Sci, Sch Med, Shanghai 200092, Peoples R China.
EM yongyong_li@tongji.edu.cn
RI 陈, 鹰娜/HDO-7357-2022
FU National Natural Science Foundation of China [32271387]; Talent Program
of Shanghai Municipal Health Commission [2022XD052]; Shanghai Natural
Science Foundation [23ZR1465200]; Sci-Tech Innovation 2030 - Major
Project of Brain science and brain-inspired intelligence technology
[2021ZD0202003]; Fundamental Research Funds for the Central Universities
[2022-4-YB-09]
FX This work was financially supported through grants from the National
Natural Science Foundation of China (32271387), Talent Program of
Shanghai Municipal Health Commission(2022XD052), Shanghai Natural
Science Foundation(23ZR1465200), Sci-Tech Innovation 2030 - Major
Project of Brain science and brain-inspired intelligence technology
(2021ZD0202003), Fundamental Research Funds for the Central Universities
(2022-4-YB-09).
CR An J, 2020, ACS NANO
Artzy-Randrup Y, 2012, ELIFE, V1, DOI 10.7554/eLife.00093
Azzi J, 2016, CELL REP, V15, P1202, DOI 10.1016/j.celrep.2016.04.007
Bachmann MF, 2010, NAT REV IMMUNOL, V10, P787, DOI 10.1038/nri2868
Bahmani B, 2018, J CLIN INVEST, V128, P4770, DOI 10.1172/JCI120923
Castiello L, 2011, CANCER IMMUNOL IMMUN, V60, P457, DOI 10.1007/s00262-010-0954-6
Chen Y, 2019, MINERALS-BASEL, V9
Chen YY, 2019, MINERALS-BASEL, V9, DOI 10.3390/min9020068
Chiang CS, 2018, NAT NANOTECHNOL, V13, P746, DOI 10.1038/s41565-018-0146-7
Chiuppesi F, 2020, NAT COMMUN, V11, DOI 10.1038/s41467-020-19819-1
Duce C, 2012, ANAL BIOANAL CHEM, V402, P2183, DOI 10.1007/s00216-011-5684-x
Ghosh S, 2020, CELL, V183, P1520, DOI 10.1016/j.cell.2020.10.039
Gopi S, 2013, MATER RES BULL, V48, P1906, DOI 10.1016/j.materresbull.2013.01.048
Green AM, 2013, J IMMUNOL, V190, P270, DOI 10.4049/jimmunol.1200061
Judge SJ, 2020, J IMMUNOTHER CANCER, V8, DOI 10.1136/jitc-2020-001355
Keselman A, 2016, INFECT IMMUN, V84, P2853, DOI 10.1128/IAI.00348-16
Matyjaszewski K, 2006, P NATL ACAD SCI USA, V103, P15309, DOI 10.1073/pnas.0602675103
Misinzo G, 2008, J VIROL, V82, P1128, DOI 10.1128/JVI.01229-07

Pulendran B, 2021, NAT REV DRUG DISCOV, V20, P454, DOI 10.1038/s41573-021-00163-y
Qiu NS, 2021, BIOMATERIALS, V269, DOI 10.1016/j.biomaterials.2020.120604
Saboury AA, 2005, INT J BIOL MACROMOL, V36, P305, DOI 10.1016/j.ijbiomac.2005.07.003
Saxena M, 2021, NAT REV CANCER, V21, P360, DOI 10.1038/s41568-021-00346-0
Schudel A, 2019, NAT REV MATER, V4, P415, DOI 10.1038/s41578-019-0110-7
Song SM, 2011, KOREAN J CHEM ENG, V28, P1749, DOI 10.1007/s11814-011-0022-8
Su RP, 2021, BIOMATERIALS, V277, DOI 10.1016/j.biomaterials.2021.121089
Tang HD, 2006, J AM CHEM SOC, V128, P16277, DOI 10.1021/ja0653369
Terry TD, 2005, VACCINE, V23, P4521, DOI 10.1016/j.vaccine.2005.04.028
Wagner CS, 2012, FRONT IMMUNOL, V3, DOI 10.3389/fimmu.2012.00138
Wang CG, 2020, ADV IMMUNOL, V145, P187, DOI 10.1016/bs.ai.2019.11.007
Wang K, 2018, ACS NANO, V12, P6398, DOI 10.1021/acsnano.8b00558
Wang S, 2018, SMALL, V14, DOI 10.1002/smll.201704272
Wang XQ, 2009, J PHYS CHEM B, V113, P8975, DOI 10.1021/jp810281f
Wolf SE, 2011, J AM CHEM SOC, V133, P12642, DOI 10.1021/ja202622g
Yao SS, 2017, ADV MATER, V29, DOI 10.1002/adma.201605903
NR 34
TC 0
Z9 0
U1 11
U2 11
PU BMC
PI LONDON
PA CAMPUS, 4 CRINAN ST, LONDON N1 9XW, ENGLAND
EI 1477-3155
J9 J NANOBIOTECHNOL
JI J. Nanobiotechnol.
PD APR 7
PY 2023
VL 21
IS 1
AR 120
DI 10.1186/s12951-023-01870-x
PG 15
WC Biotechnology & Applied Microbiology; Nanoscience & Nanotechnology
WE Science Citation Index Expanded (SCI-EXPANDED)
SC Biotechnology & Applied Microbiology; Science & Technology - Other
Topics
GA C7UH5
UT WOS:000963920000001
PM 37024939
OA gold, Green Published
DA 2023-09-14
ER

PT J
AU Zheng, LY
Wu, H
Wen, NC
Zhang, Y
Wang, ZM
Peng, XY
Tan, Y
Qiu, LP
Qu, FL
Tan, WH
AF Zheng, Liyan
Wu, Hui
Wen, Nachuan
Zhang, Yue
Wang, Zhimin

Peng, Xueyu
Tan, Yan
Qiu, Liping
Qu, Fengli
Tan, Weihong
TI Aptamer-Functionalized Nanovaccines: Targeting In Vivo DC Subsets for Enhanced Antitumor Immunity
SO ACS APPLIED MATERIALS & INTERFACES
LA English
DT Article
DE aptamer; nanovaccine; DC subset-targeting; T cell activation; cancer immunoprevention
ID EPIGALLOCATECHIN GALLATE EGCG; PLASMACYTOID DENDRITIC CELLS; ADJUVANTS; POLYPHENOL; SELECTION; ANTIGENS; SELEX
AB Cancer vaccines, which directly pulsed in vivo dendritic cells (DCs) with specific antigens and immunostimulatory adjuvants, showed great potential for cancer immunoprevention. However, most of them were limited by suboptimal outcomes, mainly owing to overlooking the complex biology of DC phenotypes. Herein, based on adjuvant-induced antigen assembly, we developed aptamer-functionalized nanovaccines for in vivo DC subset-targeted codelivery of tumor-related antigens and immunostimulatory adjuvants. We chose two aptamers, iDC and CD209, and tested their performance on DC targeting. Our results verified that these aptamer-functionalized nanovaccines could specifically recognize circulating classical DCs (cDCs), a subset of DCs capable of priming nai"ve T cells, noting that iDC outperformed CD209 in this regard. With excellent cDC-targeting capability, the iDC-functionalized nanovaccine induced potent antitumor immunity, leading to effective inhibition of tumor occurrence and metastasis, thus providing a promising platform for cancer immunoprevention.
C1 [Zheng, Liyan; Wu, Hui; Wen, Nachuan; Zhang, Yue; Wang, Zhimin; Peng, Xueyu; Tan, Yan; Qiu, Liping; Tan, Weihong] Hunan Univ, Coll Chem & Chem Engn, Coll Biol, State Key Lab Chemo BioSensing & Chemometr,Aptamer, Changsha 410082, Hunan, Peoples R China.
[Zheng, Liyan; Wu, Hui; Wen, Nachuan; Zhang, Yue; Peng, Xueyu; Tan, Yan; Qiu, Liping; Qu, Fengli; Tan, Weihong] Chinese Acad Sci, Zhejiang Canc Hosp, Hangzhou Inst Med HIM, Key Lab Zhejiang Prov Aptamers & Theranost, Hangzhou 310022, Zhejiang, Peoples R China.
[Tan, Weihong] Shanghai Jiao Tong Univ, Renji Hosp, Inst Mol Med IMM, Sch Med, Shanghai 200240, Peoples R China.
[Tan, Weihong] Shanghai Jiao Tong Univ, Coll Chem & Chem Engn, Shanghai 200240, Peoples R China.
C3 Hunan University; Zhejiang Cancer Hospital; Chinese Academy of Sciences; Shanghai Jiao Tong University; Shanghai Jiao Tong University
RP Qiu, LP; Tan, WH (通讯作者)，Hunan Univ, Coll Chem & Chem Engn, Coll Biol, State Key Lab Chemo BioSensing & Chemometr,Aptamer, Changsha 410082, Hunan, Peoples R China.; Qiu, LP; Tan, WH (通讯作者)，Chinese Acad Sci, Zhejiang Canc Hosp, Hangzhou Inst Med HIM, Key Lab Zhejiang Prov Aptamers & Theranost, Hangzhou 310022, Zhejiang, Peoples R China.; Tan, WH (通讯作者)，Shanghai Jiao Tong Univ, Renji Hosp, Inst Mol Med IMM, Sch Med, Shanghai 200240, Peoples R China.; Tan, WH (通讯作者)，Shanghai Jiao Tong Univ, Coll Chem & Chem Engn, Shanghai 200240, Peoples R China.
EM qiuliping@hnu.edu.cn; tan@hnu.edu.cn
RI Tan, Weihong/AAA-4536-2020
FU National Key Research Program [2021YFA0910100, 2019YFA0905800]; National Natural Science Foundation of China [NSFC 21922404, 22174039, 21827811]; Science and Technology Project of Hunan Province [2021RC4022, 2019SK2201, 2017XK2103]
FX Funding This work is supported by the National Key Research Program (2021YFA0910100 and 2019YFA0905800) , the National Natural Science Foundation of China (NSFC 21922404, 22174039 and 21827811) , and the Science and Technology Project of Hunan Province (2021RC4022, 2019SK2201 and 2017XK2103) .
CR Banchereau J, 1998, NATURE, V392, P245, DOI 10.1038/32588
Barratt-Boyes SM, 2005, AM J TRANSPLANT, V5, P2807, DOI 10.1111/j.1600-6143.2005.01116.x
Bonifaz LC, 2004, J EXP MED, V199, P815, DOI 10.1084/jem.20032220
Burzio LA, 2000, BIOCHEMISTRY-US, V39, P11147, DOI 10.1021/bi0002434
Caminschi I, 2012, FRONT IMMUNOL, V3, DOI 10.3389/fimmu.2012.00013
Chang DH, 2005, J EXP MED, V201, P1503, DOI 10.1084/jem.20042592
Coffman RL, 2010, IMMUNITY, V33, P492, DOI 10.1016/j.immuni.2010.10.002
Collin M, 2013, IMMUNOLOGY, V140, P22, DOI 10.1111/imm.12117

Colonna M, 2004, NAT IMMUNOL, V5, P1219, DOI 10.1038/ni1141
Du GJ, 2012, NUTRIENTS, V4, P1679, DOI 10.3390/nu4111679
Fang XH, 2010, ACCOUNTS CHEM RES, V43, P48, DOI 10.1021/ar900101s
Laus R, 2000, NAT BIOTECHNOL, V18, P1269, DOI 10.1038/82377
Li J, 2016, NANOSCALE, V8, P8600, DOI 10.1039/c6nr00600k
Lu YC, 2008, CYTOKINE, V42, P145, DOI 10.1016/j.cyto.2008.01.006
Luo M, 2017, NAT NANOTECHNOL, V12, P648, DOI [10.1038/nnano.2017.52, 10.1038/NNANO.2017.52]
Melssen MM, 2019, J IMMUNOTHER CANCER, V7, DOI 10.1186/s40425-019-0625-x
Merad M, 2013, ANNU REV IMMUNOL, V31, P563, DOI 10.1146/annurev-immunol-020711-074950
Moghadam M, 2016, INT IMMUNOPHARMACOL, V36, P324, DOI 10.1016/j.intimp.2016.04.042
Pardoll DM, 1998, NAT MED, V4, P525, DOI 10.1038/nm0598supp-525
Park HY, 2017, NPJ VACCINES, V2, DOI 10.1038/s41541-017-0033-5
Qiu LP, 2018, SMALL, V14, DOI 10.1002/smll.201703539
Sousa CR, 2006, NAT REV IMMUNOL, V6, P476, DOI 10.1038/nri1845
Reizis B, 2011, ANNU REV IMMUNOL, V29, P163, DOI 10.1146/annurev-immunol-031210-101345
Scheiermann J, 2014, VACCINE, V32, P6377, DOI 10.1016/j.vaccine.2014.06.065
Schlitzer A, 2015, NAT IMMUNOL, V16, P718, DOI 10.1038/ni.3200
Smith JJ, 2010, GASTROENTEROLOGY, V138, P958, DOI 10.1053/j.gastro.2009.11.005
Tacken PJ, 2007, NAT REV IMMUNOL, V7, P790, DOI 10.1038/nri2173
Weiner GJ, 1997, P NATL ACAD SCI USA, V94, P10833, DOI 10.1073/pnas.94.20.10833
Xie JP, 2009, J AM CHEM SOC, V131, P888, DOI 10.1021/ja806804u
Yan H, 2007, MOL CELL BIOCHEM, V306, P71, DOI 10.1007/s11010-007-9555-x
Zhong Y, 2012, FOOD CHEM, V131, P22, DOI 10.1016/j.foodchem.2011.07.089
NR 31
TC 0
Z9 0
U1 20
U2 20
PU AMER CHEMICAL SOC
PI WASHINGTON
PA 1155 16TH ST, NW, WASHINGTON, DC 20036 USA
SN 1944-8244
EI 1944-8252
J9 ACS APPL MATER INTER
JI ACS Appl. Mater. Interfaces
PD APR 19
PY 2023
VL 15
IS 15
BP 18590
EP 18597
DI 10.1021/acsami.2c20846
EA APR 2023
PG 8
WC Nanoscience & Nanotechnology; Materials Science, Multidisciplinary
WE Science Citation Index Expanded (SCI-EXPANDED)
SC Science & Technology - Other Topics; Materials Science
GA D8NE4
UT WOS:000967552100001
PM 37017594
DA 2023-09-14
ER

PT J
AU Yan, H
Lin, GB
Liu, ZY
Gu, F
Zhang, Y
AF Yan, Huan

Lin, Guibin
Liu, Zhanyan
Gu, Fei
Zhang, Yuan
TI Nano-adjuvants and immune agonists promote antitumor immunity of peptide amphiphiles
SO ACTA BIOMATERIALIA
LA English
DT Article
DE PolyIC; Anti-CD40 antibody; Nanovaccine; Peptide amphiphile; Antitumor immunity; Anti-4-1BB antibody
ID NF-KAPPA-B; TUMOR-ANTIGEN; INNATE; RESPONSES; ACTIVATION; INDUCTION; CELLS; EXPRESSION; RESISTANCE; RECEPTORS
AB Immunostimulatory cues play an important role in priming antitumor immunity and promoting the efficacy of subunit cancer vaccines. However, the clinical use of many immunostimulatory agents is often hampered by their inefficient in vivo delivery which may decrease immune response to the vaccination. To promote vaccine efficacy, we develop vaccine formulations which integrate three key elements: (1) a nano-adjuvant formulated by conjugating an agonistic anti-CD40 monoclonal antibody (alpha CD40) to the surface of a polyIC-loaded lipid nanoparticle, (2) a peptide amphiphile containing an optimized CD8 + T-cell epitope that derived from a melanoma antigen gp100, (3) an agonistic anti-4-1BB monoclonal antibody (alpha 4-1BB) that boosts the efficacy of vaccinations. In a syngeneic mouse model of melanoma, the vaccine formulations enhanced innate immunity and activated multiple innate immune signaling pathways within draining lymph nodes, as well as promoted antigen-specific immune responses and reduced immunosuppression in the tumor microenvironment, leading to profound tumor growth inhibition and prolonged survival. Thus, our vaccine formulations represent an attractive strategy to stimulate antitumor immunity and control tumor progression.
C1 [Yan, Huan; Lin, Guibin; Liu, Zhanyan; Gu, Fei; Zhang, Yuan] South China Univ Technol, Sch Biomed Sci & Engn, Guangzhou Int Campus, Guangzhou 511442, Peoples R China.
[Yan, Huan; Lin, Guibin; Liu, Zhanyan; Gu, Fei; Zhang, Yuan] South China Univ Technol, Natl Engn Res Ctr Tissue Restorat & Reconstruct, Guangzhou 510006, Peoples R China.
[Yan, Huan; Lin, Guibin; Liu, Zhanyan; Gu, Fei; Zhang, Yuan] South China Univ Technol, Guangdong Prov Key Lab Biomed Engn, Guangzhou 510006, Peoples R China.
[Yan, Huan; Lin, Guibin; Liu, Zhanyan; Gu, Fei; Zhang, Yuan] South China Univ Technol, Key Lab Biomed Mat & Engn, Minist Educ, Guangzhou 510006, Peoples R China.
C3 South China University of Technology; South China University of Technology; South China University of Technology; South China University of Technology
RP Zhang, Y (通讯作者)，South China Univ Technol, Sch Biomed Sci & Engn, Guangzhou Int Campus, Guangzhou 511442, Peoples R China.; Zhang, Y (通讯作者)，South China Univ Technol, Natl Engn Res Ctr Tissue Restorat & Reconstruct, Guangzhou 510006, Peoples R China.; Zhang, Y (通讯作者)，South China Univ Technol, Guangdong Prov Key Lab Biomed Engn, Guangzhou 510006, Peoples R China.; Zhang, Y (通讯作者)，South China Univ Technol, Key Lab Biomed Mat & Engn, Minist Educ, Guangzhou 510006, Peoples R China.
EM mczhang@scut.edu.cn
FU National Natural Science Foundation of China [82172080]; Basic and Applied Basic Research Foundation of Guangdong Province [2020A1515111204]; Fundamental Research Funds for the Central Universities [2020ZYGXZR099]; Recruitment Program of Global Experts
FX This work was supported by National Natural Science Founda- tion of China (82172080) , Basic and Applied Basic Research Foun- dation of Guangdong Province (2020A1515111204) , the Fundamen- tal Research Funds for the Central Universities (2020ZYGXZR099) and the Recruitment Program of Global Experts. We would like to thank the Research Core Facilities at the South China University of Technology.
CR Adams S, 2009, IMMUNOTHERAPY-UK, V1, P949, DOI [10.2217/imt.09.70, 10.2217/IMT.09.70]
Bartkowiak T, 2015, FRONT ONCOL, V5, DOI 10.3389/fonc.2015.00117
Bennett SRM, 1998, NATURE, V393, P478, DOI 10.1038/30996
Bocanegra Gondan A.I., BIOMATERIALS
Byrne KT, 2016, CELL REP, V15, P2719, DOI 10.1016/j.celrep.2016.05.058
Careaga M, 2018, J NEUROIMMUNOL, V323, P87, DOI 10.1016/j.jneuroim.2018.06.014
Chen Q, 2016, NAT IMMUNOL, V17, P1142, DOI 10.1038/ni.3558

Chono S, 2008, J CONTROL RELEASE, V131, P64, DOI 10.1016/j.jconrel.2008.07.006
Civril F, 2013, NATURE, V498, P332, DOI 10.1038/nature12305
D'Arcangelo E, 2020, BRIT J CANCER, V122, P931, DOI 10.1038/s41416-019-0705-1
de Silva S, 2020, CANCER IMMUNOL RES, V8, P230, DOI 10.1158/2326-6066.CIR-19-0493
de Titta A, 2013, P NATL ACAD SCI USA, V110, P19902, DOI 10.1073/pnas.1313152110
Di SM, 2019, FRONT ONCOL, V9, DOI 10.3389/fonc.2019.00241
Forghani P, 2015, BREAST CANCER RES TR, V153, P21, DOI 10.1007/s10549-015-3508-y
Freen-van Heeren JJ, 2021, SCAND J IMMUNOL, V93, DOI 10.1111/sji.13019
Hatzifoti C, 2007, IMMUNOLOGY, V122, P98, DOI 10.1111/j.1365-2567.2007.02617.x
Inoue C, 2019, CANCERS, V11, DOI 10.3390/cancers11091257
Jalali SA, 2012, NANOMED-NANOTECHNOL, V8, P692, DOI 10.1016/j.nano.2011.09.010
Jang JS, 2018, CELL DEATH DIS, V9, DOI 10.1038/s41419-018-0889-y
Jiang ZF, 2004, P NATL ACAD SCI USA, V101, P3533, DOI 10.1073/pnas.0308496101
Kawai T, 2011, IMMUNITY, V34, P637, DOI 10.1016/j.immuni.2011.05.006
Kwong B, 2011, BIOMATERIALS, V32, P5134, DOI 10.1016/j.biomaterials.2011.03.067
Lee S.C., INFECT IMMUN
Li YZ, 2020, NAT CANCER, V1, P882, DOI 10.1038/s43018-020-0095-6
Liu HP, 2014, NATURE, V507, P519, DOI 10.1038/nature12978
Liu SQ, 2015, SCIENCE, V347, P1217, DOI 10.1126/science.aaa2630
Llopiz D, 2008, CANCER IMMUNOL IMMUN, V57, P19, DOI 10.1007/s00262-007-0346-8
Long KB, 2016, CANCER DISCOV, V6, P400, DOI 10.1158/2159-8290.CD-15-1032
Lousberg EL, 2010, J VIROL, V84, P6549, DOI 10.1128/JVI.02618-09
Marraco SAF, 2011, PLOS ONE, V6, DOI 10.1371/journal.pone.0020189
Mazurkiewicz J, 2022, CELL COMMUN SIGNAL, V20, DOI 10.1186/s12964-022-00871-x
Meylan E, 2006, NATURE, V442, P39, DOI 10.1038/nature04946
Mogensen TH, 2019, FRONT IMMUNOL, V9, DOI 10.3389/fimmu.2018.03047
Morrison AH, 2020, P NATL ACAD SCI USA, V117, P8022, DOI 10.1073/pnas.1918971117
Moschonas A, 2012, J IMMUNOL, V188, P5521, DOI 10.4049/jimmunol.1200133
Nakata M., NUCL INSTRUM METH A, V35, P2021
Qi CJ, 2011, BLOOD, V117, P6825, DOI 10.1182/blood-2011-02-339812
Rotzschke O., 2012, PLOS ONE, V7
Schmidt W, 1997, P NATL ACAD SCI USA, V94, P3262, DOI 10.1073/pnas.94.7.3262
Shae D, 2020, ACS NANO, V14, P9904, DOI 10.1021/acsnano.0c02765
Sultan H, 2020, J IMMUNOTHER CANCER, V8, DOI 10.1136/jitc-2020-001224
Takeda Y, 2017, CELL REP, V19, P1874, DOI 10.1016/j.celrep.2017.05.015
van Stipdonk MJB, 2009, CANCER RES, V69, P7784, DOI 10.1158/0008-5472.CAN-09-1724
Verma P, 2021, MOL PHARMACEUT, V18, P3832, DOI 10.1021/acs.molpharmaceut.1c00451
Vonderheide RH, 2020, ANNU REV MED, V71, P47, DOI 10.1146/annurev-med-062518-045435
Wattenberg MM, 2021, JCI INSIGHT, V6, DOI 10.1172/jci.insight.145389
Wenthe J, 2021, CANCER IMMUNOL IMMUN, V70, P2851, DOI 10.1007/s00262-021-02895-7
Yalamanoglu A, 2021, FRONT IMMUNOL, V12, DOI 10.3389/fimmu.2021.627944
Yamashita M, 2021, MOL THER-ONCOLYTICS, V20, P422, DOI 10.1016/j.omto.2021.01.016
Yin W., EBIOMEDICINE
Zhang Y, 2018, NAT COMMUN, V9, DOI [10.1038/s41467-017-02251-3, 10.1038/s41467-018-06126-z]
Zhang Y, 2012, MOL THER, V20, P1298, DOI 10.1038/mt.2012.79
Zhu Q, 2010, J CLIN INVEST, V120, P607, DOI 10.1172/JCI39293
Zippelius A, 2015, CANCER IMMUNOL RES, V3, P236, DOI 10.1158/2326-6066.CIR-14-0226
NR 54
TC 0
Z9 0
U1 4
U2 5
PU ELSEVIER SCI LTD
PI OXFORD
PA THE BOULEVARD, LANGFORD LANE, KIDLINGTON, OXFORD OX5 1GB, OXON, ENGLAND
SN 1742-7061
EI 1878-7568
J9 ACTA BIOMATER
JI Acta Biomater.
PD APR 15
PY 2023

VL 161
BP 213
EP 225
DI 10.1016/j.actbio.2023.02.034
EA MAR 2023
PG 13
WC Engineering, Biomedical; Materials Science, Biomaterials
WE Science Citation Index Expanded (SCI-EXPANDED)
SC Engineering; Materials Science
GA D5JW5
UT WOS:000969104900001
PM 36858163
DA 2023-09-14
ER

PT J
AU Liu, HX
Xie, ZG
Zheng, M
AF Liu, Hongxin
Xie, Zhigang
Zheng, Min
TI Carbon Dots and Tumor Antigen Conjugates as Nanovaccines for Elevated Cancer Immunotherapy
SO SMALL
LA English
DT Article
DE cancer immunotherapy; carbon dots; nanovaccines; tumor antigens
ID BLOCKADE
AB Cancer immunotherapy has become one of the current research hotspots. However, the deficiencies including restricted immunogenicity, insufficient antigen presentation, and low responsive rate limited their therapeutic applications. Own to the small size and excellent biocompatibility, carbon dots (CDs) can serve as nanovectors to improve the efficacy of cancer immunotherapy. Herein, a tumor antigen-based nanovaccines (GMal+B16F10-Ag and GMal+CT26-Ag) by the conjugation of CDs with the tumor cell-derived antigens (B16F10-Ag and CT26-Ag) is constructed. These nanovaccines can be effectively taken up by dendritic cells (DC2.4), promote DC cell maturation, cross-present the antigen to T cells, specifically target B16F10 melanoma or CT26 colon cancers, and inhibit tumor growth distinctly. This work illustrates the promise of CDs acting as versatile carriers for antigen delivery to achieve the optimal immunotherapeutic outcomes.
C1 [Liu, Hongxin; Zheng, Min] Changchun Univ Technol, Adv Inst Mat Sci, Sch Chem & Life Sci, 2055 Yanan St, Changchun 130012, Jilin, Peoples R China.
[Xie, Zhigang] Chinese Acad Sci, Changchun Inst Appl Chem, State Key Lab Polymer Chem & Phys, 5625 Renmin St, Changchun 130022, Jilin, Peoples R China.
C3 Changchun University of Technology; Chinese Academy of Sciences; Changchun Institute of Applied Chemistry, CAS
RP Zheng, M (通讯作者)，Changchun Univ Technol, Adv Inst Mat Sci, Sch Chem & Life Sci, 2055 Yanan St, Changchun 130012, Jilin, Peoples R China.; Xie, ZG (通讯作者)，Chinese Acad Sci, Changchun Inst Appl Chem, State Key Lab Polymer Chem & Phys, 5625 Renmin St, Changchun 130022, Jilin, Peoples R China.
EM xiez@ciac.ac.cn; zhengm@ciac.ac.cn
RI Zheng, Min/B-6267-2013; Xie, Zhigang/E-6501-2010
OI Zheng, Min/0000-0002-4985-2680; Xie, Zhigang/0000-0003-2974-1825
FU National Natural Science Foundation of China [51873023]; Science and Technology Development Plan of Changchun City [21ZY38]
FX Acknowledgements The financial support from the National Natural Science Foundation of China (No. 51873023), and the Science and Technology Development Plan of Changchun City (No. 21ZY38).
CR Chen AH, 2022, SMALL, V18, DOI 10.1002/smll.202200993
Chen S, 2020, ADV FUNCT MATER, V30, DOI 10.1002/adfm.202004680
Chong GW, 2022, CHEM ENG J, V435, DOI 10.1016/j.cej.2022.134993
Chong GW, 2021, NANO RES, V14, P1244, DOI 10.1007/s12274-020-3179-9

Conniot J, 2019, NAT NANOTECHNOL, V14, P891, DOI 10.1038/s41565-019-0512-0
Das K, 2021, NAT COMMUN, V12, DOI 10.1038/s41467-021-25506-6
Dong X, 2022, ACS APPL MATER INTER, V14, P4808, DOI 10.1021/acsami.1c25198
Feng Q, 2022, SENSOR ACTUAT B-CHEM, V351, DOI 10.1016/j.snb.2021.130976
Feng XR, 2021, SCI BULL, V66, P362, DOI 10.1016/j.scib.2020.07.013
Gao PL, 2021, ACS APPL MATER INTER, V13, P56456, DOI 10.1021/acsami.1c16091
Gao PL, 2020, BIOCONJUGATE CHEM, V31, P646, DOI 10.1021/acs.bioconjchem.9b00801
Guan XW, 2018, BIOMATERIALS, V171, P198, DOI 10.1016/j.biomaterials.2018.04.039
Hua XW, 2019, ACS APPL MATER INTER, V11, P32647, DOI 10.1021/acsami.9b09590
Jiang H, 2018, ADV SCI, V5, DOI 10.1002/advs.201700426
Jiang MX, 2022, J ADV RES, V35, P49, DOI 10.1016/j.jare.2021.08.011
Kim Y, 2020, ANGEW CHEM INT EDIT, V59, P14628, DOI 10.1002/anie.202006117
Li HJ, 2022, THERANOSTICS, V12, P6422, DOI 10.7150/thno.77350
Li T, 2022, NANO LETT, V22, P3095, DOI 10.1021/acs.nanolett.2c00500
Li X, 2022, SMALL, V18, DOI 10.1002/smll.202203100
Liang XL, 2022, SCI BULL, V67, P1611, DOI 10.1016/j.scib.2022.07.018
Liu GN, 2021, ADV DRUG DELIVER REV, V176, DOI 10.1016/j.addr.2021.113889
Liu HX, 2022, ACS APPL MATER INTER, V14, P39858, DOI 10.1021/acsami.2c11596
Liu J, 2021, NAT COMMUN, V12, DOI 10.1038/s41467-021-22407-6
Liu JJ, 2020, ADV MATER, V32, DOI 10.1002/adma.201906641
Lu SY, 2017, ADV MATER, V29, DOI 10.1002/adma.201603443
Reda M, 2022, NAT COMMUN, V13, DOI 10.1038/s41467-022-31926-9
Saeed M, 2021, ADV MATER, V33, DOI 10.1002/adma.202008094
Song HJ, 2022, ACTA BIOMATER, V141, P398, DOI 10.1016/j.actbio.2022.01.004
Song YQ, 2021, ACS APPL MATER INTER, V13, P49737, DOI 10.1021/acsami.1c15361
Su T, 2022, ADV SCI, V9, DOI 10.1002/advs.202201895
Taghikhani A, 2020, FRONT IMMUNOL, V11, DOI 10.3389/fimmu.2020.00221
Wu QH, 2022, ADV SCI, V9, DOI 10.1002/advs.202200999
Wu Y., 2022, CHEM ENG J, P436
Xiao YL, 2022, NAT COMMUN, V13, DOI 10.1038/s41467-022-28279-8
Xie X., 2022, CHEM ENG J, V437
Xie XX, 2022, BIOACT MATER, V16, P107, DOI 10.1016/j.bioactmat.2022.03.008
Xiong X, 2021, NANO LETT, V21, P8418, DOI 10.1021/acs.nanolett.1c03004
Zhang D, 2021, ADV SCI, V8, DOI 10.1002/advs.202003504
Zhang LSW, 2011, NANOMEDICINE-UK, V6, P777, DOI [10.2217/nnm.11.73, 10.2217/NNM.11.73]
Zheng C., 2022, CHEM ENG J, P435
NR 40
TC 0
Z9 0
U1 38
U2 38
PU WILEY-V C H VERLAG GMBH
PI WEINHEIM
PA POSTFACH 101161, 69451 WEINHEIM, GERMANY
SN 1613-6810
EI 1613-6829
J9 SMALL
JI Small
PD AUG
PY 2023
VL 19
IS 31
SI SI
DI 10.1002/smll.202206683
EA MAR 2023
PG 11
WC Chemistry, Multidisciplinary; Chemistry, Physical; Nanoscience & Nanotechnology; Materials Science, Multidisciplinary; Physics, Applied; Physics, Condensed Matter
WE Science Citation Index Expanded (SCI-EXPANDED)
SC Chemistry; Science & Technology - Other Topics; Materials Science;

Physics
GA O1WV4
UT WOS:000956121600001
PM 36978241
DA 2023-09-14
ER

PT J
AU Lv, XJ
Huang, J
Min, J
Wang, HR
Xu, YY
Zhang, ZM
Zhou, XX
Wang, J
Liu, Z
Zhao, H
AF Lv, Xinjing
Huang, Jie
Min, Jie
Wang, Hairong
Xu, Yunyun
Zhang, Zimu
Zhou, Xiuxia
Wang, Jian
Liu, Zhuang
Zhao, He
TI Multi-signaling pathway activation by pH responsive manganese particles for enhanced vaccination
SO JOURNAL OF CONTROLLED RELEASE
LA English
DT Article
DE cancer nanovaccine; Immunotherapy; Checkpoint blockade; Metalloimmunotherapy
ID DNA VACCINES
AB As metal ions play important roles in the process of immunomodulation, immunotherapy based on metal ions has attracted tremendous interests in recent years. Here, we screened common metal ions and found that Mn2+ could enhance the immune function in vitro. A new type of nanovaccine is thus fabricated by a biomimetic approach using nanoscale coordination polymer formed by Mn2+ and 2-methylimidazole (2-MI) to encapsulate ovalbumin (OVA) protein, a model antigen, obtaining OVA@MM nanoparticles. Compared to free OVA, OVA@MM nano -particles could more effectively induce the maturation of bone marrow-derived dendritic cells (BMDCs) and their subsequent antigen cross-presentation. The particles made of Mn2+ and 2-MI could activate immune-regulated signal pathways to enhance the immune functions of BMDCs. Such OVA@MM nanovaccine could not only provide prophylactic effect to inhibit the growth of B16-OVA tumor on immunized mice, but also significantly inhibit tumor growth in the mice with B16-OVA tumor combined with anti-programmed cell death protein 1 (anti-PD-1) antibody. Therefore, this nanovaccine platform based on Mn2+, 2-MI and antigen may provide a simple, effective and broadly applicable strategy to enhance adaptive immunity against cancer and other diseases.
C1 [Lv, Xinjing; Huang, Jie; Min, Jie; Wang, Hairong; Xu, Yunyun; Zhang, Zimu; Zhou, Xiuxia; Wang, Jian; Zhao, He] Soochow Univ, Childrens Hosp, Pediat Res Inst, Suzhou 215123, Jiangsu, Peoples R China.
[Liu, Zhuang] Soochow Univ, Inst Funct Nano & Soft Mat FUNSOM, Jiangsu Key Lab Carbon Based Funct Mat & Devices, Suzhou 215123, Jiangsu, Peoples R China.
C3 Soochow University - China; Soochow University - China
RP Wang, J; Zhao, H (通讯作者)，Soochow Univ, Childrens Hosp, Pediat Res Inst, Suzhou 215123, Jiangsu, Peoples R China.; Liu, Z (通讯作者)，Soochow Univ, Inst Funct Nano & Soft Mat FUNSOM, Jiangsu Key Lab Carbon Based Funct Mat & Devices, Suzhou 215123, Jiangsu, Peoples R China.
EM wj196312@vip.163.com; zliu@suda.edu.cn; zh2021@suda.edu.cn
RI Wang, Jian/ITW-0790-2023

FU National Key Research and Development Program of China [2021YFF0701800]; National Natural Science Foundation of China [52032008, 21927803]; Jiangsu Social Development Project [BE2019658, BE2020659]; Suzhou science and technology project [SKJY2021107]; Jiangsu health commission project [M2022120, Szlcyxzx202104]; Natural Science Foundation of the Jiangsu Higher Education Institutions of China [21KJB320010]; Collaborative Innovation Center of Suzhou Nano Science and Technology; Ministry of Education of China
FX This work was supported by the National Key Research and Development Program of China (2021YFF0701800), the National Natural Science Foundation of China (52032008, 21927803), the Jiangsu Social Development Project (BE2019658, BE2020659), Suzhou science and technology project (SKJY2021107), the Jiangsu health commission project (M2022120, Szlcyxzx202104), the Natural Science Foundation of the Jiangsu Higher Education Institutions of China (21KJB320010), the Collaborative Innovation Center of Suzhou Nano Science and Technology and the '111' program from the Ministry of Education of China. The scheme was created by BioRender.
CR Chandrangsu P, 2017, NAT REV MICROBIOL, V15, P338, DOI 10.1038/nrmicro.2017.15
Chen CY, 2021, SMALL, V17, DOI 10.1002/smll.202006970
Chen X, 2022, NANO TODAY, V46, DOI 10.1016/j.nantod.2022.101583
Dudev T, 2003, CHEM REV, V103, P773, DOI 10.1021/cr020467n
Englinger B, 2019, CHEM REV, V119, P1519, DOI 10.1021/acs.chemrev.8b00396
Fang RH, 2014, NANO LETT, V14, P2181, DOI 10.1021/nl500618u
Gao M, 2021, ADV THER-GERMANY, V4, DOI 10.1002/adtp.202100065
Gao S, 2021, ACS NANO, V15, P12567, DOI 10.1021/acsnano.1c02103
Geng ZM, 2021, BIOMATERIALS, V275, DOI 10.1016/j.biomaterials.2021.120897
Gong F, 2020, ADV FUNCT MATER, V30, DOI 10.1002/adfm.202002753
Gong XC, 2022, VACCINES-BASEL, V10, DOI 10.3390/vaccines10111906
Gurunathan S, 2000, CURR OPIN IMMUNOL, V12, P442, DOI 10.1016/S0952-7915(00)00118-7
Haase H, 2018, IMMUNITY, V48, P616, DOI 10.1016/j.immuni.2018.03.031
Hayat SMG, 2019, J CELL PHYSIOL, V234, P12530, DOI 10.1002/jcp.28120
Hu QL, 2015, NANO LETT, V15, P2732, DOI 10.1021/acs.nanolett.5b00570
Hu XM, 2019, ADV HEALTHC MATER, V8, DOI 10.1002/adhm.201800837
Huang LH, 2022, MATER TODAY BIO, V17, DOI 10.1016/j.mtbio.2022.100497
Kramps T, 2013, WIRES RNA, V4, P737, DOI 10.1002/wrna.1189
Krishnan N, 2022, ADV DRUG DELIVER REV, V185, DOI 10.1016/j.addr.2022.114294
Kroll AV, 2017, ADV MATER, V29, DOI 10.1002/adma.201703969
Li JX, 2022, COORDIN CHEM REV, V454, DOI 10.1016/j.ccr.2021.214345
Li SL, 2021, ADV FUNCT MATER, V31, DOI 10.1002/adfm.202100243
Li X., 2022, MOLECULES
Lv MZ, 2020, CELL RES, V30, P966, DOI 10.1038/s41422-020-00395-4
Meng X, 2019, NANO LETT, V19, P7866, DOI 10.1021/acs.nanolett.9b02904
Mohapatra A, 2021, PHARMACEUTICS, V13, DOI 10.3390/pharmaceutics13111867
O'Neal Stefanie L, 2015, Curr Environ Health Rep, V2, P315, DOI 10.1007/s40572-015-0056-x
Pan JB, 2018, ADV MATER, V30, DOI 10.1002/adma.201704408
Racette BA, 2005, NEUROLOGY, V64, P230, DOI 10.1212/01.WNL.0000149511.19487.44
Racette BA, 2012, NEUROTOXICOLOGY, V33, P881, DOI 10.1016/j.neuro.2011.12.010
Sang W, 2019, CHEM SOC REV, V48, P3771, DOI 10.1039/c8cs00896e
Shi YH, 2023, CHEM ENG J, V453, DOI 10.1016/j.cej.2022.139885
Song RX, 2018, BIOMATERIALS, V175, P123, DOI 10.1016/j.biomaterials.2018.05.018
Stelling MP, 2019, FEBS J, V286, P2950, DOI 10.1111/febs.14986
Sun K, 2021, SMALL, V17, DOI 10.1002/smll.202101897
Sun XQ, 2021, NAT NANOTECHNOL, V16, P1260, DOI 10.1038/s41565-021-00962-9
Tuschl K, 2013, INT REV NEUROBIOL, V110, P277, DOI 10.1016/B978-0-12-410502-7.00013-2
Waldron KJ, 2009, NATURE, V460, P823, DOI 10.1038/nature08300
Wang CG, 2020, ADV IMMUNOL, V145, P187, DOI 10.1016/bs.ai.2019.11.007
Wang CG, 2018, IMMUNITY, V48, P675, DOI 10.1016/j.immuni.2018.03.017
Wang DD, 2022, COORDIN CHEM REV, V472, DOI 10.1016/j.ccr.2022.214788
Wang P, 2019, ACS APPL MATER INTER, V11, P41140, DOI 10.1021/acsami.9b16617
Wei XL, 2019, NANO LETT, V19, P4760, DOI 10.1021/acs.nanolett.9b01844

Wen D, 2019, BIOCONJUGATE CHEM, V30, P515, DOI 10.1021/acs.bioconjchem.9b00061
Xu JT, 2018, ADV FUNCT MATER, V28, DOI 10.1002/adfm.201803804
Xu J, 2020, NAT NANOTECHNOL, V15, P1043, DOI 10.1038/s41565-020-00781-4
Yang X, 2021, NANO TODAY, V38, DOI 10.1016/j.nantod.2021.101109
Yang Y, 2022, NANO LETT, V22, P2826, DOI 10.1021/acs.nanolett.1c04888
Yuan PY, 2018, ADV HEALTHC MATER, V7, DOI 10.1002/adhm.201701392
Zhang L., 2022, ADV MATER, DOI [10.1002/adma.202206915,2206915, DOI 10.1002/ADMA.202206915,2206915]
Zhang R, 2021, CELL MOL IMMUNOL, V18, P1222, DOI 10.1038/s41423-021-00669-w
Zhang XD, 2022, ACS APPL NANO MATER, V5, P13959, DOI 10.1021/acsanm.2c01380
Zhao ZL, 2014, J AM CHEM SOC, V136, P11220, DOI 10.1021/ja5029364
Zhong XF, 2020, ACTA PHARMACOL SIN, V41, P928, DOI 10.1038/s41401-020-0414-6
Zhu GZ, 2017, ACS NANO, V11, P2387, DOI 10.1021/acsnano.7b00978
NR 55
TC 0
Z9 0
U1 13
U2 13
PU ELSEVIER
PI AMSTERDAM
PA RADARWEG 29, 1043 NX AMSTERDAM, NETHERLANDS
SN 0168-3659
EI 1873-4995
J9 J CONTROL RELEASE
JI J. Control. Release
PD MAY
PY 2023
VL 357
BP 109
EP 119
DI 10.1016/j.jconrel.2023.01.078
EA MAR 2023
PG 11
WC Chemistry, Multidisciplinary; Pharmacology & Pharmacy
WE Science Citation Index Expanded (SCI-EXPANDED)
SC Chemistry; Pharmacology & Pharmacy
GA D2JB2
UT WOS:000967025200001
PM 36738971
DA 2023-09-14
ER

PT J
AU Shin, H
Kang, S
Chae, SY
Won, C
Min, DH
AF Shin, Hojeong
Kang, Seounghun
Chae, Se-Youl
Won, Cheolhee
Min, Dal-Hee
TI Development of a Cancer Nanovaccine to Induce Antigen-specific Immune Responses Based on Large-Sized Porous Silica Nanoparticles
SO ACS APPLIED MATERIALS & INTERFACES
LA English
DT Article
DE anticancer immunity; antigen depot; cancer immunotherapy; nanovaccine; silica nanoparticles
ID VACCINE; IMMUNOTHERAPY; ACTIVATION; CELLS

AB Cancer vaccine is one of the immunotherapeutic strategies aiming to effectively deliver cancer antigens to professional antigen-presenting cells such as dendritic cells (DCs), macrophages, and B cells to elicit a cancer-specific immune response. Despite the advantages of the cancer vaccine that can be applied to various cancer types, the clinical approach is limited due to the non-specific or adverse immune responses, stability, and safety issues. In this study, we report an injectable nanovaccine platform based on large-sized (similar to 350 nm) porous silica nanoparticles (PSNs). We found that large-sized PSNs, called PS3, facilitated the formation of an antigen supply depot at the site of injection so that a single injection of PSN-based nanovaccine elicited sufficient tumor-specific cell-mediated and humoral immune response. As a result, antigen-loaded PS3 induced successful tumor regression in prophylactic and therapeutic vaccination.
C1 [Shin, Hojeong; Kang, Seounghun; Chae, Se-Youl; Min, Dal-Hee] Seoul Natl Univ, Dept Chem, Seoul 08826, South Korea.
[Won, Cheolhee; Min, Dal-Hee] Lemonex Inc, Inst Biotherapeut Convergence Technol, Seoul 06683, South Korea.
[Min, Dal-Hee] Seoul Natl Univ, Dept Biol Sci, Seoul 08826, South Korea.
C3 Seoul National University (SNU); Seoul National University (SNU)
RP Min, DH (通讯作者)，Seoul Natl Univ, Dept Chem, Seoul 08826, South Korea.; Min, DH (通讯作者)，Lemonex Inc, Inst Biotherapeut Convergence Technol, Seoul 06683, South Korea.; Min, DH (通讯作者)，Seoul Natl Univ, Dept Biol Sci, Seoul 08826, South Korea.
EM dalheemin@snu.ac.kr
OI Chae, Se-Youl/0000-0002-7754-3914
FU Lemonex Inc. [LEMONEX-SN201709-3-12, LEMONEX-SN201901-3-30, LEMON-EX-SN202003-3-60]
FX Funding This work was supported by Lemonex Inc. (LEMONEX-SN201709-3-12, LEMONEX-SN201901-3-30, and LEMON-EX-SN202003-3-60) .
CR An M, 2017, ACS APPL MATER INTER, V9, P23466, DOI 10.1021/acsami.7b06024
Arima Y, 2009, ACS APPL MATER INTER, V1, P2400, DOI 10.1021/am9005463
Benezra M, 2011, J CLIN INVEST, V121, P2768, DOI 10.1172/JCI45600
Chae SY, 2022, J CONTROL RELEASE, V345, P108, DOI 10.1016/j.jconrel.2022.02.035
Croissant JG, 2017, ADV MATER, V29, DOI 10.1002/adma.201604634
Eisenbarth SC, 2019, NAT REV IMMUNOL, V19, P89, DOI 10.1038/s41577-018-0088-1
Goldman B, 2009, NAT BIOTECHNOL, V27, P129, DOI 10.1038/nbt0209-129
Gomez DM, 2017, INNATE IMMUN-LONDON, V23, P697, DOI 10.1177/1753425917738331
Han DH, 2014, NAT COMMUN, V5, DOI 10.1038/ncomms6633
Hong XY, 2020, SCI ADV, V6, DOI 10.1126/sciadv.aaz4462
Jeannot V, 2016, NANOMED-NANOTECHNOL, V12, P921, DOI 10.1016/j.nano.2015.11.018
Jia JB, 2018, FRONT ONCOL, V8, DOI 10.3389/fonc.2018.00404
Joffre OP, 2012, NAT REV IMMUNOL, V12, P557, DOI 10.1038/nri3254
Kang S, 2020, NANOSCALE, V12, P6385, DOI 10.1039/c9nr10305h
Ke XY, 2019, ADV DRUG DELIVER REV, V151, P72, DOI 10.1016/j.addr.2019.09.005
Keskin DB, 2019, NATURE, V565, P234, DOI 10.1038/s41586-018-0792-9
Kim J, 2015, NAT BIOTECHNOL, V33, P64, DOI 10.1038/nbt.3071
Kim J, 2022, BIOMATERIALS, V280, DOI 10.1016/j.biomaterials.2021.121257
Lin ZB, 2021, ACTA PHARM SIN B, V11, P1047, DOI 10.1016/j.apsb.2020.11.008
Manzano M, 2020, ADV FUNCT MATER, V30, DOI 10.1002/adfm.201902634
Martin JD, 2020, NAT REV CLIN ONCOL, V17, P251, DOI 10.1038/s41571-019-0308-z
McHugh KJ, 2015, J CONTROL RELEASE, V219, P596, DOI 10.1016/j.jconrel.2015.07.029
Mellman I, 2011, NATURE, V480, P480, DOI 10.1038/nature10673
Na HK, 2012, SMALL, V8, P1752, DOI 10.1002/smll.201200028
Ott PA, 2017, NATURE, V547, P217, DOI 10.1038/nature22991
Ow H, 2005, NANO LETT, V5, P113, DOI 10.1012/nl0482478
Palucka K, 2012, NAT REV CANCER, V12, P265, DOI 10.1038/nrc3258
Peng M, 2019, MOL CANCER, V18, DOI 10.1186/s12943-019-1055-6
Reddy ST, 2007, NAT BIOTECHNOL, V25, P1159, DOI [10.1038/nbt1332, 10.1038/nbtl332]
Riley RS, 2019, NAT REV DRUG DISCOV, V18, P175, DOI 10.1038/s41573-018-0006-z
ROBBINS A, 1988, SCI AM, V259, P126, DOI 10.1038/scientificamerican1188-126
Romero P, 2016, SCI TRANSL MED, V8, DOI 10.1126/scitranslmed.aaf0685
Rosenberg SA, 2004, NAT MED, V10, P909, DOI 10.1038/nm1100
Sagiv-Barfi I, 2022, SCI IMMUNOL, V7, DOI 10.1126/sciimmunol.abn5859
Sandberg WJ, 2012, PART FIBRE TOXICOL, V9, DOI 10.1186/1743-8977-9-32
Schudel A, 2019, NAT REV MATER, V4, P415, DOI 10.1038/s41578-019-0110-7

Schumacher TN, 2015, SCIENCE, V348, P69, DOI 10.1126/science.aaa4971
Shortman K, 2002, NAT REV IMMUNOL, V2, P151, DOI 10.1038/nri746
Nguyen TL, 2019, ADV MATER, V31, DOI 10.1002/adma.201803953
Yu A, 2022, BIOSENSORS-BASEL, V12, DOI 10.3390/bios12020109
NR 40
TC 0
Z9 0
U1 6
U2 6
PU AMER CHEMICAL SOC
PI WASHINGTON
PA 1155 16TH ST, NW, WASHINGTON, DC 20036 USA
SN 1944-8244
EI 1944-8252
J9 ACS APPL MATER INTER
JI ACS Appl. Mater. Interfaces
PD MAR 22
PY 2023
VL 15
IS 11
BP 13860
EP 13868
DI 10.1021/acsami.2c19526
EA MAR 2023
PG 9
WC Nanoscience & Nanotechnology; Materials Science, Multidisciplinary
WE Science Citation Index Expanded (SCI-EXPANDED)
SC Science & Technology - Other Topics; Materials Science
GA A2DZ9
UT WOS:000950558800001
PM 36913611
DA 2023-09-14
ER

PT J
AU Zhao, XY
Zhang, JT
Chen, BB
Ding, XK
Zhao, NN
Xu, FJ
AF Zhao, Xiaoyi
Zhang, Jiatong
Chen, Beibei
Ding, Xiaokang
Zhao, Nana
Xu, Fu-Jian
TI Rough Nanovaccines Boost Antitumor Immunity Through the Enhancement of
Vaccination Cascade and Immunogenic Cell Death Induction
SO SMALL METHODS
LA English
DT Article; Early Access
DE cascade; immunogenic cell death; intrinsic properties; MnOx
nanoparticles; nanovaccines
ID SILICA NANOPARTICLES; CANCER VACCINES; DELIVERY; IMMUNOTHERAPY
AB Nanovaccines have attracted intense interests for efficient antigen delivery and tumor-specific immunity. It is challenging to develop a more efficient and personalized nanovaccine to maximize all steps of the vaccination cascade by exploiting the intrinsic properties of nanoparticles. Here, biodegradable nanohybrids (MP) composed of manganese oxide nanoparticles and cationic polymers are synthesized to load a model antigen ovalbumin to form MPO nanovaccines. More interestingly, MPO could serve as autologous nanovaccines for personalized tumor treatment taking advantage of in situ released

tumor-associated antigens induced by immunogenic cell death (ICD). The intrinsic properties of MP nanohybrids including morphology, size, surface charge, chemical, and immunoregulatory functions are fully exploited to enhance of all steps of the cascade and induce ICD. MP nanohybrids are designed to efficiently encapsulate antigens by cationic polymers, drain to lymph nodes by appropriate size, be internalized by dendritic cells (DCs) by rough morphology, induce DC maturation through cGAS-STING pathway, and enhance lysosomal escape and antigen cross-presentation through the "proton sponge effect". The MPO nanovaccines are found to efficiently accumulate in lymph nodes and elicit robust specific T-cell immune responses to inhibit the occurrence of ovalbumin-expressing B16-OVA melanoma. Furthermore, MPO demonstrate great potential to serve as personalized cancer vaccines through the generation of autologous antigen depot through ICD induction, activation of potent antitumor immunity, and reversal of immunosuppression. This work provides a facile strategy for the construction of personalized nanovaccines by exploiting the intrinsic properties of nanohybrids.
C1 [Zhao, Xiaoyi; Zhang, Jiatong; Chen, Beibei; Ding, Xiaokang; Zhao, Nana; Xu, Fu-Jian] Beijing Univ Chem Technol, State Key Lab Chem Resource Engn, Key Lab Biomed Mat Nat Macromol, Beijing Lab Biomed Mat,Coll Mat Sci Engn, Beijing 100029, Peoples R China.
C3 Beijing University of Chemical Technology
RP Zhao, NN; Xu, FJ (通讯作者)，Beijing Univ Chem Technol, State Key Lab Chem Resource Engn, Key Lab Biomed Mat Nat Macromol, Beijing Lab Biomed Mat,Coll Mat Sci Engn, Beijing 100029, Peoples R China.
EM zhaonn@mail.buct.edu.cn; xufj@mail.buct.edu.cn
RI Zhang, Jiatong/HJA-0177-2022
FU National Key R&D Program of China [2021YFC2400500]; National Natural Science Foundation of China [52173271, 52221006, 51922022]; Beijing Outstanding Young Scientist Program [BJJWZYJH01201910010024]; Fundamental Research Funds for the Central Universities [BHYC1705A, XK1802-2]; Postdoctoral Science Foundation of Beijing University of Chemical Technology [CMSE202203]
FX X.Z. and J.Z. contributed equally to this work. This work was supported by the National Key R&D Program of China (Grant No. 2021YFC2400500), National Natural Science Foundation of China (Nos. 52173271, 52221006, and 51922022), Beijing Outstanding Young Scientist Program (No. BJJWZYJH01201910010024), the Fundamental Research Funds for the Central Universities (Nos. BHYC1705A and XK1802-2), and Postdoctoral Science Foundation of Beijing University of Chemical Technology (No. CMSE202203). All animal experiments were approved by the Ethical Committee of Chinese Academy of Medical Sciences and Peking Union Medical College.
CR Abbaraju PL, 2017, J AM CHEM SOC, V139, P6321, DOI 10.1021/jacs.6b12622
Bachmann MF, 2010, NAT REV IMMUNOL, V10, P787, DOI 10.1038/nri2868
Banchereau J, 2005, NAT REV IMMUNOL, V5, P296, DOI 10.1038/nri1592
Chen FM, 2021, BIOMATERIALS, V270, DOI 10.1016/j.biomaterials.2021.120709
Chen J, 2022, BIOACT MATER, V7, P167, DOI 10.1016/j.bioactmat.2021.05.036
Chen XY, 2018, ACS NANO, V12, P5646, DOI 10.1021/acsnano.8b01440
Deng CF, 2019, ADV SCI, V6, DOI 10.1002/advs.201801868
Ding BB, 2020, ANGEW CHEM INT EDIT, V59, P16381, DOI 10.1002/anie.202005111
Fang L, 2020, SCI ADV, V6, DOI 10.1126/sciadv.aba4024
Gong NQ, 2020, NAT NANOTECHNOL, V15, P1053, DOI 10.1038/s41565-020-00782-3
Guo YX, 2022, NAT COMMUN, V13, DOI 10.1038/s41467-022-33831-7
He LY, 2022, CANCER IMMUNOL IMMUN, V71, P2007, DOI 10.1007/s00262-021-03136-7
Hong XY, 2020, SCI ADV, V6, DOI 10.1126/sciadv.aaz4462
Hou L, 2020, ACS NANO, V14, P3927, DOI 10.1021/acsnano.9b06111
Hu YY, 2020, BIOMATERIALS, V252, DOI 10.1016/j.biomaterials.2020.120114
Irvine DJ, 2015, CHEM REV, V115, P11109, DOI 10.1021/acs.chemrev.5b00109
Krysko DV, 2012, NAT REV CANCER, V12, P860, DOI 10.1038/nrc3380
Li JC, 2022, ADV MATER, V34, DOI 10.1002/adma.202108012
Li Q., 2022, NAT COMMUN, V18
Li X, 2020, NANO LETT, V20, P4454, DOI 10.1021/acs.nanolett.0c01230
Lin LS, 2018, ANGEW CHEM INT EDIT, V57, P4902, DOI 10.1002/anie.201712027
Lin MJ, 2022, NAT CANCER, V3, P911, DOI 10.1038/s43018-022-00418-6
Lin XH, 2020, ANGEW CHEM INT EDIT, V59, P1682, DOI 10.1002/anie.201912768
Liu B, 2021, NANO LETT, V21, P9410, DOI 10.1021/acs.nanolett.1c02582

Liu C, 2022, NAT NANOTECHNOL, V17, P531, DOI 10.1038/s41565-022-01098-0
Liu SL, 2021, NAT MATER, V20, P421, DOI 10.1038/s41563-020-0793-6
Meng ZQ, 2021, NANO LETT, V21, P1228, DOI 10.1021/acs.nanolett.0c03646
Qin L, 2021, SMALL, V17, DOI 10.1002/smll.202006000
Su T, 2022, ADV SCI, V9, DOI 10.1002/advs.202201895
Sun XQ, 2021, NAT NANOTECHNOL, V16, P1260, DOI 10.1038/s41565-021-00962-9
Tang W, 2019, ADV MATER, V31, DOI 10.1002/adma.201900401
Teng ZG, 2018, J AM CHEM SOC, V140, P1385, DOI 10.1021/jacs.7b10694
Wang X, 2022, NAT COMMUN, V13, DOI 10.1038/s41467-022-33301-0
Xiao B, 2021, BIOMATERIALS, V274, DOI 10.1016/j.biomaterials.2021.120893
Xu C, 2020, ACS NANO, V14, P13268, DOI 10.1021/acsnano.0c05062
Xu J, 2020, NAT NANOTECHNOL, V15, P1043, DOI 10.1038/s41565-020-00781-4
Xu YD, 2022, BIOMATERIALS, V284, DOI 10.1016/j.biomaterials.2022.121489
Xue J, 2022, ACTA PHARM SIN B, V12, P2934, DOI 10.1016/j.apsb.2021.11.020
Yang GB, 2017, NAT COMMUN, V8, DOI 10.1038/s41467-017-01050-0
Yang X, 2021, NANO TODAY, V38, DOI 10.1016/j.nantod.2021.101109
Yang YN, 2021, ADV SCI, V8, DOI 10.1002/advs.202002667
Ye XY, 2019, ACS NANO, V13, P2956, DOI 10.1021/acsnano.8b07371
Zhang LM, 2022, BIOMATERIALS, V282, DOI 10.1016/j.biomaterials.2022.121411
Zhang Q, 2017, ADV FUNCT MATER, V27, DOI 10.1002/adfm.201606229
Zhang SJ, 2022, BIOMATERIALS, V289, DOI 10.1016/j.biomaterials.2022.121794
Zhang YN, 2019, NANO LETT, V19, P7226, DOI 10.1021/acs.nanolett.9b02834
Zhao NN, 2021, NANO TODAY, V38, DOI 10.1016/j.nantod.2021.101118
Zhao NN, 2015, SMALL, V11, P6467, DOI 10.1002/smll.201502760
Zhao XY, 2022, ADV MATER, V34, DOI 10.1002/adma.202108263
Zhou LH, 2018, SMALL, V14, DOI 10.1002/smll.201801008
Zhu GZ, 2017, ACS NANO, V11, P2387, DOI 10.1021/acsnano.7b00978
NR 51
TC 0
Z9 0
U1 45
U2 49
PU WILEY-V C H VERLAG GMBH
PI WEINHEIM
PA POSTFACH 101161, 69451 WEINHEIM, GERMANY
SN 2366-9608
J9 SMALL METHODS
JI Small Methods
PD 2023 MAR 3
PY 2023
DI 10.1002/smtd.202201595
EA MAR 2023
PG 12
WC Chemistry, Physical; Nanoscience & Nanotechnology; Materials Science,
Multidisciplinary
WE Science Citation Index Expanded (SCI-EXPANDED)
SC Chemistry; Science & Technology - Other Topics; Materials Science
GA 9N5GW
UT WOS:000942942800001
PM 36869418
DA 2023-09-14
ER

PT J
AU Hong, JHY
Jung, MY
Kim, C
Kang, MKY
Go, S
Sohn, H
Moon, S

Kwon, S
Song, SY
Kim, BS
AF Hong, Jihye
Jung, Mungyo
Kim, Cheesue
Kang, Mikyung
Go, Seokhyeong
Sohn, Heesu
Moon, Sangjun
Kwon, Sungpil
Song, Seuk Young
Kim, Byung-Soo
TI Senescent cancer cell-derived nanovesicle as a personalized therapeutic cancer vaccine
SO EXPERIMENTAL AND MOLECULAR MEDICINE
LA English
DT Article
ID IMMUNOTHERAPY; EXOSOMES; 1ST
AB Cancer: Turning tumors into vaccinesTherapeutic vaccines derived from tumor cells could provide a personalized strategy for training the immune system to find and destroy cancer. Past efforts to develop such vaccines have been hampered by the challenge of identifying tumor-specific proteins that elicit a strong immune response. To overcome this, Byung-Soo Kim and colleagues at Seoul National University pursued a strategy in which they subjected tumor cells to chemical treatments that halted proliferation and stimulated production of immunity-activating signaling molecules. The cells were then broken up into membrane-encased fragments filled with tumor-derived biomolecules. Mice dosed with such vaccines, which carry a diverse mix of antigens, showed a potent anti-cancer response, resulting in tumor cell death and significantly improved survival. By treating cells from patient biopsies in this fashion, clinicians could potentially derive potent, individually tailored tumor vaccines.

The development of therapeutic cancer vaccines (TCVs) that provide clinical benefits is challenging mainly due to difficulties in identifying immunogenic tumor antigens and effectively inducing antitumor immunity. Furthermore, there is an urgent need for personalized TCVs because only a limited number of tumor antigens are shared among cancer patients. Several autologous nanovaccines that do not require the identification of immunogenic tumor antigens have been proposed as personalized TCVs. However, these nanovaccines generally require exogenous adjuvants (e.g., Toll-like receptor agonists) to improve vaccine immunogenicity, which raises safety concerns. Here, we present senescent cancer cell-derived nanovesicle (SCCNV) as a personalized TCV that provides patient-specific tumor antigens and improved vaccine immunogenicity without the use of exogenous adjuvants. SCCNVs are prepared by inducing senescence in cancer cells ex vivo and subsequently extruding the senescent cancer cells through nanoporous membranes. In the clinical setting, SCCNVs can be prepared from autologous cancer cells from the blood of liquid tumor patients or from tumors surgically removed from solid cancer patients. SCCNVs also contain interferon-gamma and tumor necrosis factor-alpha, which are expressed during senescence. These endogenous cytokines act as adjuvants and enhance vaccine immunogenicity, avoiding the need for exogenous adjuvants. Intradermally injected SCCNVs effectively activate dendritic cells and tumor-specific T cells and inhibit primary and metastatic tumor growth and tumor recurrence. SCCNV therapy showed an efficacy similar to that of immune checkpoint blockade (ICB) therapy and synergized with ICB. SCCNVs, which can be prepared using a simple and facile procedure, show potential as personalized TCVs.
C1 [Hong, Jihye; Kang, Mikyung; Go, Seokhyeong; Kim, Byung-Soo] Seoul Natl Univ, Interdisciplinary Program Bioengn, Seoul 08826, South Korea.
[Jung, Mungyo; Kim, Cheesue; Sohn, Heesu; Moon, Sangjun; Kwon, Sungpil; Song, Seuk Young; Kim, Byung-Soo] Seoul Natl Univ, Sch Chem & Biol Engn, Seoul 08826, South Korea.
[Kim, Byung-Soo] Seoul Natl Univ, Inst Chem Proc, Inst Engn Res, BioMAX, Seoul 08826, South Korea.
C3 Seoul National University (SNU); Seoul National University (SNU); Seoul National University (SNU)
RP Kim, BS (通讯作者)，Seoul Natl Univ, Interdisciplinary Program Bioengn, Seoul 08826, South Korea.; Kim, BS (通讯作者)，Seoul Natl Univ, Sch Chem & Biol Engn, Seoul 08826, South Korea.; Kim, BS (通讯作者)，Seoul Natl Univ, Inst Chem Proc, Inst Engn Res, BioMAX, Seoul 08826, South Korea.
EM byungskim@snu.ac.kr
RI Kang, Mikyung/ITU-4433-2023
OI Kang, Mikyung/0000-0002-0592-1648; Jung, Mungyo/0000-0001-9366-9026

FU National Research Foundation of Korea [SNU-200630-2-4,
2019M3A9H1103651]; National Research Foundation of Korea
[2019M3A9H1103651] Funding Source: Korea Institute of Science &
Technology Information (KISTI), National Science & Technology
Information Service (NTIS)
FX AcknowledgementsAll animal experiments were approved by the
Institutional Animal Care and Use Committee (IACUC) of Seoul National
University (SNU-200630-2-4) and performed in compliance with the
guidelines of the IACUC. This study was supported by a grant
(2019M3A9H1103651) from the National Research Foundation of Korea.
CR Accolla RS, 2020, FRONT IMMUNOL, V10, DOI 10.3389/fimmu.2019.03117
Admyre C, 2006, EUR J IMMUNOL, V36, P1772, DOI 10.1002/eji.200535615
Banstola A, 2020, ACTA BIOMATER, V114, P16, DOI 10.1016/j.actbio.2020.07.063
Bodey B, 2000, ANTICANCER RES, V20, P2665
Bowen WS, 2018, EXPERT REV VACCINES, V17, P207, DOI 10.1080/14760584.2018.1434000
Buonaguro L, 2020, VACCINES-BASEL, V8, DOI 10.3390/vaccines8040615
Cheever MA, 2011, CLIN CANCER RES, V17, P3520, DOI 10.1158/1078-0432.CCR-10-3126
Choo YW, 2018, ACS NANO, V12, P8977, DOI 10.1021/acsnano.8b02446
Crescenzi E, 2011, ONCOGENE, V30, P2707, DOI 10.1038/onc.2011.1
Cuzzubbo S, 2021, FRONT IMMUNOL, V11, DOI 10.3389/fimmu.2020.615240
Dudek AZ, 2007, CLIN CANCER RES, V13, P7119, DOI 10.1158/1078-0432.CCR-07-1443
Fang RH, 2014, NANO LETT, V14, P2181, DOI 10.1021/nl500618u
Fry TJ, 2018, NAT MED, V24, P20, DOI 10.1038/nm.4441
Gardner JK, 2018, PLOS ONE, V13, DOI 10.1371/journal.pone.0195313
Ghorashian S, 2019, NAT MED, V25, P1408, DOI 10.1038/s41591-019-0549-5
Gu XY, 2015, INT J CANCER, V136, pE74, DOI 10.1002/ijc.29100
Hong J, 2021, ADV MATER, V33, DOI 10.1002/adma.202101110
Hsu CH, 2019, CELL, V178, P361, DOI 10.1016/j.cell.2019.05.041
Hsu DH, 2003, J IMMUNOTHER, V26, P440, DOI 10.1097/00002371-200309000-00007
Jang SC, 2013, ACS NANO, V7, P7698, DOI 10.1021/nn402232g
Jimbu L, 2021, PHARMACEUTICALS-BASE, V14, DOI 10.3390/ph14040288
Jung M, 2022, ADV MATER, V34, DOI 10.1002/adma.202106516
Kaiser J, 2017, SCIENCE, V356, P122, DOI 10.1126/science.356.6334.122
Kang M, 2021, ADV MATER, V33, DOI 10.1002/adma.202103258
Kang M, 2020, ADV MATER, V32, DOI 10.1002/adma.202003368
Karpanen T, 2017, FRONT IMMUNOL, V8, DOI 10.3389/fimmu.2017.01718
Kersten K, 2017, EMBO MOL MED, V9, P137, DOI 10.15252/emmm.201606857
Kim HY, 2019, NANO LETT, V19, P5185, DOI 10.1021/acs.nanolett.9b01571
Kim HY, 2018, NANO LETT, V18, P4965, DOI 10.1021/acs.nanolett.8b01816
Kroll AV, 2017, ADV MATER, V29, DOI 10.1002/adma.201703969
Link BK, 2006, J IMMUNOTHER, V29, P558, DOI 10.1097/01.cji.0000211304.60126.8f
Liu C, 2022, NAT NANOTECHNOL, V17, P531, DOI 10.1038/s41565-022-01098-0
Liu HL, 2020, STEM CELL RES THER, V11, DOI 10.1186/s13287-020-01798-1
Mardiana S, 2020, FRONT ONCOL, V10, DOI 10.3389/fonc.2020.00697
Morse MA, 2021, TARGET ONCOL, V16, P121, DOI 10.1007/s11523-020-00788-w
Peng M, 2019, MOL CANCER, V18, DOI 10.1186/s12943-019-1055-6
Reddy ST, 2007, NAT BIOTECHNOL, V25, P1159, DOI [10.1038/nbt1332, 10.1038/nbtl332]
Rosenberg SA, 2015, SCIENCE, V348, P62, DOI 10.1126/science.aaa4967
Schlickeiser S, 2011, J IMMUNOL, V186, P5201, DOI 10.4049/jimmunol.1003410
Schudel A, 2019, NAT REV MATER, V4, P415, DOI 10.1038/s41578-019-0110-7
Schumacher TN, 2015, SCIENCE, V348, P69, DOI 10.1126/science.aaa4971
Shin S, 2022, TISSUE ENG REGEN MED, V19, P205, DOI 10.1007/s13770-021-00403-7
Swartz MA, 2001, ADV DRUG DELIVER REV, V50, P3, DOI 10.1016/S0169-409X(01)00150-8
van Deursen JM, 2014, NATURE, V509, P439, DOI 10.1038/nature13193
Waldman AD, 2020, NAT REV IMMUNOL, V20, P651, DOI 10.1038/s41577-020-0306-5
Whiteside TL, 2016, ADV CLIN CHEM, V74, P103, DOI 10.1016/bs.acc.2015.12.005
Ye ZL, 2018, J CANCER, V9, P263, DOI 10.7150/jca.20059
NR 47
TC 0
Z9 0
U1 8

U2 9
PU SPRINGERNATURE
PI LONDON
PA CAMPUS, 4 CRINAN ST, LONDON, N1 9XW, ENGLAND
SN 1226-3613
EI 2092-6413
J9 EXP MOL MED
JI Exp. Mol. Med.
PD MAR
PY 2023
VL 55
IS 3
BP 541
EP 554
DI 10.1038/s12276-023-00951-z
EA MAR 2023
PG 14
WC Biochemistry & Molecular Biology; Medicine, Research & Experimental
WE Science Citation Index Expanded (SCI-EXPANDED)
SC Biochemistry & Molecular Biology; Research & Experimental Medicine
GA C2UN2
UT WOS:000940763400003
PM 36854774
OA gold, Green Published
DA 2023-09-14
ER

PT J
AU Chen, YC
AF Chen, Yunching
TI Nanotechnology for next-generation cancer immunotherapy: State of the
art and future perspectives
SO JOURNAL OF CONTROLLED RELEASE
LA English
DT Article
DE Immunotherapy; Nanotechnology; Cancer vaccine; Cancer therapy;
Immunosuppressive tumor microenvironment
ID NANOPARTICLES; DELIVERY; CELLS; PEPTIDE; SYSTEM; CPG
AB Over the past decade, immunotherapy aiming to activate an effective antitumor immune response has ushered in a new era of cancer treatment. However, the efficacy of cancer immunotherapy is limited by low response rates and high systemic toxicity. Nanotechnology is an encouraging platform for the development of next-generation cancer immunotherapy to effectively treat advanced cancer. Nanotechnology-enabled immunotherapy has remarkable advantages, ranging from the increased bioavailability and stability of immunotherapeutic agents to the enhanced activation of immune cells and favorable safety profiles. Nanotechnology-enabled immunotherapy can target solid tumors through reprogramming or stimulating immune cells (i.e., nanovaccines); modulating the immunosuppressive tumor microenvironment; or targeting tumor cells and altering their responses to immune cells to generate effective antitumor immunity. In this Oration, I introduce the advanced strategies currently being pursued by our laboratory and other groups to improve the therapeutic efficacy of cancer immunotherapy and discuss the potential challenges and future directions.
C1 [Chen, Yunching] Natl Tsing Hua Univ, Inst Biomed Engn, Hsinchu 30013, Taiwan.
[Chen, Yunching] Natl Tsing Hua Univ, Frontier Res Ctr Fundamental & Appl Sci Matters, Hsinchu 30013, Taiwan.
[Chen, Yunching] Natl Tsing Hua Univ, Dept Chem, Hsinchu 30013, Taiwan.
C3 National Tsing Hua University; National Tsing Hua University; National
Tsing Hua University
RP Chen, YC (通讯作者)，Natl Tsing Hua Univ, Inst Biomed Engn, Hsinchu 30013, Taiwan.
EM yunching@mx.nthu.edu.tw
RI chen, yu-cheng/IQT-1648-2023
OI Chen, Yunching/0000-0001-6228-5169
FU Ministry of Science and Technology (MOST) [108-2221-E-007-104-MY5,

111-2628-E-007-015, 111QR001I5]; National Health Research Institutes
[111- 2634-F-007-023]; Frontier Research Center on Fundamental and
"Applied Sciences of Matters" of the Featured Areas Research Center
Program [NHRI-EX112-11015BI]
FX This study was supported by the Ministry of Science and Technology (MOST
108-2221-E-007-104-MY5 and 111-2628-E-007-015), the National Health
Research Institutes (NHRI-EX112-11015BI) and "Frontier Research Center
on Fundamental and Applied Sciences of Matters" of the Featured Areas
Research Center Program within the framework of the Higher Education
Sprout Project by the Ministry of Education (MOE 111QR001I5) and the
Ministry of Science and Technology (MOST 111- 2634-F-007-023).
CR Bai ST, 2022, J CONTROL RELEASE, V344, P134, DOI 10.1016/j.jconrel.2022.02.027
Beck JD, 2021, MOL CANCER, V20, DOI 10.1186/s12943-021-01348-0
Berraondo P, 2019, BRIT J CANCER, V120, P6, DOI 10.1038/s41416-018-0328-y
Berti C, 2022, MACROMOL BIOSCI, V22, DOI 10.1002/mabi.202100356
Blanco E, 2015, NAT BIOTECHNOL, V33, P941, DOI 10.1038/nbt.3330
Bulik-Sullivan B, 2019, NAT BIOTECHNOL, V37, P55, DOI 10.1038/nbt.4313
Cafri G, 2020, J CLIN INVEST, V130, P5976, DOI 10.1172/JCI134915
Chauhan V.P., 2019, P NATL ACAD SCI USA, V116
Chen WHC, 2012, PLOS ONE, V7, DOI 10.1371/journal.pone.0039039
Choi Y, 2022, J CONTROL RELEASE, V342, P321, DOI 10.1016/j.jconrel.2022.01.004
Darvin P, 2018, EXP MOL MED, V50, DOI 10.1038/s12276-018-0191-1
DeMaria PJ, 2019, HEMATOL ONCOL CLIN N, V33, P199, DOI 10.1016/j.hoc.2018.12.001
Deng YD, 2021, J CONTROL RELEASE, V340, P87, DOI 10.1016/j.jconrel.2021.10.016
Depil S, 2020, NAT REV DRUG DISCOV, V19, P185, DOI 10.1038/s41573-019-0051-2
Doherty GJ, 2018, FUTURE ONCOL, V14, P13, DOI 10.2217/fon-2017-0338
Doshi AS, 2022, J CONTROL RELEASE, V345, P721, DOI 10.1016/j.jconrel.2022.03.054
Goel S, 2012, CSH PERSPECT MED, V2, DOI 10.1101/cshperspect.a006486
Guasch J, 2017, NANO LETT, V17, P6110, DOI 10.1021/acs.nanolett.7b02636
Han B, 2022, J CONTROL RELEASE, V343, P379, DOI 10.1016/j.jconrel.2022.01.049
Hollingsworth RE, 2019, NPJ VACCINES, V4, DOI 10.1038/s41541-019-0103-y
Hope A, 2022, J CONTROL RELEASE, V341, P399, DOI 10.1016/j.jconrel.2021.11.043
Hsieh HT, 2022, J CONTROL RELEASE, V352, P920, DOI 10.1016/j.jconrel.2022.10.047
Huang HC, 2022, GUT, V71, P1843, DOI 10.1136/gutjnl-2021-325180
Huang KW, 2020, SCI ADV, V6, DOI 10.1126/sciadv.aax5032
Ishikawa T, 2021, CANCER IMMUNOL IMMUN, V70, P3081, DOI 10.1007/s00262-021-02892-w
Japir AMM, 2021, J CONTROL RELEASE, V339, P418, DOI 10.1016/j.jconrel.2021.10.015
Jiang CT, 2021, NAT COMMUN, V12, DOI 10.1038/s41467-021-21497-6
Kang M., ADV MATER
Kang Y, 2022, J CONTROL RELEASE, V343, P78, DOI 10.1016/j.jconrel.2022.01.021
Kelly G, 2022, J CONTROL RELEASE, V343, P267, DOI 10.1016/j.jconrel.2022.01.024
Lai CH, 2018, THERANOSTICS, V8, P1723, DOI 10.7150/thno.22056
Lan XM, 2020, NANOSCALE, V12, P18885, DOI 10.1039/d0nr04213g
Le Naour J, 2020, ONCOIMMUNOLOGY, V9, DOI 10.1080/2162402X.2020.1777624
Lee SH, 2019, J AM CHEM SOC, V141, P13829, DOI 10.1021/jacs.9b04621
Li MM, 2021, J CONTROL RELEASE, V340, P35, DOI 10.1016/j.jconrel.2021.10.026
Li Q, 2022, J CONTROL RELEASE, V347, P183, DOI 10.1016/j.jconrel.2022.05.003
Li SX, 2021, NAT BIOMED ENG, V5, P455, DOI 10.1038/s41551-020-00675-9
Li T, 2022, NANO LETT, V22, P3095, DOI 10.1021/acs.nanolett.2c00500
Li WQ, 2021, NAT COMMUN, V12, DOI 10.1038/s41467-021-27434-x
Liao ZY, 2022, J CONTROL RELEASE, V344, P272, DOI 10.1016/j.jconrel.2022.02.023
Liu JQ, 2022, J CONTROL RELEASE, V345, P306, DOI 10.1016/j.jconrel.2022.03.021
Liu SL, 2021, NAT MATER, V20, P421, DOI 10.1038/s41563-020-0793-6
Liu YQ, 2022, BIOMATERIALS, V284, DOI 10.1016/j.biomaterials.2022.121516
Lu X., SCI TRANSL MED
Meng FF, 2022, J CONTROL RELEASE, V345, P586, DOI 10.1016/j.jconrel.2022.03.045
Mi Y, 2018, ADV MATER, V30, DOI 10.1002/adma.201706098
Miao L, 2021, MOL CANCER, V20, DOI 10.1186/s12943-021-01335-5
Mpekris F, 2022, J CONTROL RELEASE, V345, P190, DOI 10.1016/j.jconrel.2022.03.008
Muraoka D, 2022, J CONTROL RELEASE, V347, P175, DOI 10.1016/j.jconrel.2022.05.004
Nakamura T, 2022, J CONTROL RELEASE, V345, P200, DOI 10.1016/j.jconrel.2022.03.026

Nam J, 2021, ADV SCI, V8, DOI 10.1002/advs.202002577
Neek M, 2020, ADV THER-GERMANY, V3, DOI 10.1002/adtp.202000122
Ni QK, 2022, J CONTROL RELEASE, V342, P210, DOI 10.1016/j.jconrel.2022.01.003
Ou W, 2018, J CONTROL RELEASE, V281, P84, DOI 10.1016/j.jconrel.2018.05.018
Petrovic M, 2021, J CONTROL RELEASE, V339, P235, DOI 10.1016/j.jconrel.2021.09.033
Pulendran B, 2021, NAT REV DRUG DISCOV, V20, P454, DOI 10.1038/s41573-021-00163-y
Qian Y, 2016, BIOMATERIALS, V98, P171, DOI 10.1016/j.biomaterials.2016.05.008
Reda M, 2022, NAT COMMUN, V13, DOI 10.1038/s41467-022-31926-9
Rios-Doria J, 2015, NEOPLASIA, V17, P661, DOI 10.1016/j.neo.2015.08.004
Roth GA, 2022, NAT REV MATER, V7, P174, DOI 10.1038/s41578-021-00372-2
Ruan SB, 2019, NANO LETT, V19, P8318, DOI 10.1021/acs.nanolett.9b03968
Sahin U, 2020, NATURE, V585, P107, DOI 10.1038/s41586-020-2537-9
Schudel A, 2019, NAT REV MATER, V4, P415, DOI 10.1038/s41578-019-0110-7
Shields CW, 2020, SCI ADV, V6, DOI 10.1126/sciadv.aaz6579
Su T, 2022, ADV SCI, V9, DOI 10.1002/advs.202201895
Sun JY, 2020, BIOMARK RES, V8, DOI 10.1186/s40364-020-00212-5
Sung YC, 2019, NAT NANOTECHNOL, V14, P1160, DOI 10.1038/s41565-019-0570-3
Tang L, 2018, NAT BIOTECHNOL, V36, P707, DOI 10.1038/nbt.4181
Tureci O, 2018, NAT BIOMED ENG, V2, P566, DOI 10.1038/s41551-018-0266-2
Van Cutsem E, 2020, J CLIN ONCOL, V38, P3185, DOI 10.1200/JCO.20.00590
Waldman AD, 2020, NAT REV IMMUNOL, V20, P651, DOI 10.1038/s41577-020-0306-5
Wei JJ, 2022, J CONTROL RELEASE, V347, P68, DOI 10.1016/j.jconrel.2022.04.048
Xu J, 2019, BIOMATERIALS, V207, P1, DOI 10.1016/j.biomaterials.2019.03.037
Yang R, 2018, ACS NANO, V12, P5121, DOI 10.1021/acsnano.7b09041
Ye T, 2021, ADV DRUG DELIVER REV, V177, DOI 10.1016/j.addr.2021.113927
Yoo YJ, 2022, J CONTROL RELEASE, V343, P564, DOI 10.1016/j.jconrel.2022.01.047
Zang XL, 2022, J MATER CHEM B, V10, P2019, DOI 10.1039/d2tb00197g
Zhang H., 2021, P NATL ACAD SCI USA, V118
Zhang P., P NATL ACAD SCI
Zhang YR, 2020, SMALL, V16, DOI 10.1002/smll.202004240
Zhang YR, 2020, CHEM SCI, V11, P5323, DOI 10.1039/d0sc01146k
Zhang ZZ, 2019, ADV MATER, V31, DOI 10.1002/adma.201905751
Zhao CY, 2022, J CONTROL RELEASE, V341, P272, DOI 10.1016/j.jconrel.2021.11.028
Zhou H, 2016, NANO LETT, V16, P3268, DOI 10.1021/acs.nanolett.6b00820
Zhou S., ADV FUNCT MATER, V31
Zinger A, 2019, ACS NANO, V13, P11008, DOI 10.1021/acsnano.9b02395
NR 86
TC 0
Z9 0
U1 11
U2 11
PU ELSEVIER
PI AMSTERDAM
PA RADARWEG 29, 1043 NX AMSTERDAM, NETHERLANDS
SN 0168-3659
EI 1873-4995
J9 J CONTROL RELEASE
JI J. Control. Release
PD APR
PY 2023
VL 356
BP 14
EP 25
DI 10.1016/j.jconrel.2023.02.016
EA FEB 2023
PG 12
WC Chemistry, Multidisciplinary; Pharmacology & Pharmacy
WE Science Citation Index Expanded (SCI-EXPANDED)
SC Chemistry; Pharmacology & Pharmacy
GA A1CP8
UT WOS:000952581900001

PM 36805873
DA 2023-09-14
ER

PT J
AU Manna, S
Maiti, S
Shen, JJ
Weiss, A
Mulder, E
Du, WJ
Esser-Kahn, AP
AF Manna, Saikat
Maiti, Sampa
Shen, Jingjing
Weiss, Adam
Mulder, Elizabeth
Du, Wenjun
Esser-Kahn, Aaron P.
TI Nanovaccine that activates the NLRP3 inflammasome enhances tumor
specific activation of anti-cancer immunity
SO BIOMATERIALS
LA English
DT Article
DE Nanovaccine; Biomaterials; Adjuvant; Inflammasome; Neoantigen therapy
ID DENDRITIC CELLS; VACCINE
AB Neoantigen cancer vaccines that target tumor specific mutations are emerging as a promising modality for cancer immunotherapy. To date, various approaches have been adopted to enhance efficacy of these therapies, but the low immunogenicity of neoantigens has hindered clinical application. To address this challenge, we developed a polymeric nanovaccine platform that activates the NLRP3 inflammasome, a key immunological signaling pathway in pathogen recognition and clearance. The nanovaccine is comprised of a poly (orthoester) scaffold engrafted with a small-molecule TLR7/8 agonist and an endosomal escape peptide that facilitates lysosomal rupture and NLRP3 inflammasome activation. Upon solvent transfer, the polymer self-assembles with neo-antigens to form similar to 50 nm nanoparticles that facilitate co-delivery to antigen-presenting cells. This polymeric activator of the inflammasome (PAI) was found to induce potent antigen-specific CD8(+) T cell responses char-acterized by IFN-gamma and GranzymeB secretion. Moreover, in combination with immune checkpoint blockade therapy, the nanovaccine stimulated robust anti-tumor immune responses against established tumors in EG.7-OVA, B16.F10, and CT-26 models. Results from our studies indicate that NLRP3 inflammasome activating nanovaccines demonstrate promise for development as a robust platform to enhance immunogenicity of neo-antigen therapies.
C1 [Manna, Saikat; Maiti, Sampa; Shen, Jingjing; Weiss, Adam; Mulder, Elizabeth; Esser-Kahn, Aaron P.] Univ Chicago, Pritzker Sch Mol Engn, 5640 S Ellis Ave, Chicago, IL 60637 USA.
[Maiti, Sampa; Du, Wenjun] Cent Michigan Univ, Dept Chem & Biochem, Sci Adv Mat, Mt Pleasant, MI 48858 USA.
[Weiss, Adam] Univ Chicago, Dept Chem, 5735 S Ellis Ave, Chicago, IL 60637 USA.
[Manna, Saikat; Maiti, Sampa] Sanofi US, Bridgewater, NJ USA.
C3 University of Chicago; Central Michigan University; University of
Chicago; Sanofi-Aventis
RP Esser-Kahn, AP (通讯作者)，Univ Chicago, Pritzker Sch Mol Engn, 5640 S Ellis Ave, Chicago, IL 60637 USA.
EM aesserkahn@uchicago.edu
RI Esser-Kahn, Aaron/P-2591-2016
OI Esser-Kahn, Aaron/0000-0003-1273-0951; Weiss, Adam/0000-0002-4972-1402
FU NIH [T32 GM008720, U01AI124286]; DTRA [1-18-1-0052]; NSF [CHE-1413033]
FX Sai.M., J.S., A.M.W., E.J.M., and A.P.E.-K. acknowledge the support of
NIH (U01AI124286) and DTRA (1-18-1-0052) . Sam. M and W.D. acknowledge
the support of NSF (CHE-1413033) . A.M.W. acknowledges partial support
of NIH T32 GM008720. All authors acknowledge University of Chicago's
Soft Matter Characterization, NMR, Integrated Light Microscopy, and
Human Tissue Resource facilities. The authors thank Yimei Chen for

assistance with TEM imaging, Dr. Vytas Bindokas for assistance with
confocal microscopy, Anthony Fernald for assistance with Hemavet, Tyler
Lieberthal for graphical design services, and the veterinary technicians
at University of Chicago for exceptional animal care.
CR Bae JY, 2017, ONCOTARGET, V8, P48972, DOI 10.18632/oncotarget.16903
Black M, 2012, ADV MATER, V24, P3845, DOI 10.1002/adma.201200209
Bowen WS, 2018, EXPERT REV VACCINES, V17, P207, DOI 10.1080/14760584.2018.1434000
Deets KA, 2021, NAT IMMUNOL, V22, P412, DOI 10.1038/s41590-021-00869-6
Dupaul-Chicoine J, 2015, IMMUNITY, V43, P751, DOI 10.1016/j.immuni.2015.08.013
Engel Abbi L, 2011, Expert Rev Clin Pharmacol, V4, P275, DOI 10.1586/ecp.11.5
Giunta EF, 2020, SCI REP-UK, V10, DOI 10.1038/s41598-020-72711-2
Gong NQ, 2020, NAT NANOTECHNOL, V15, P1053, DOI 10.1038/s41565-020-00782-3
Han CAH, 2021, SCI IMMUNOL, V6, DOI 10.1126/sciimmunol.abc6998
Hatscher L, 2021, SCI SIGNAL, V14, DOI 10.1126/scisignal.abe1757
Ishihara J, 2019, SCI TRANSL MED, V11, DOI 10.1126/scitranslmed.aau3259
Kang M, 2021, J EXTRACELL VESICLES, V10, DOI 10.1002/jev2.12085
Kuai R, 2017, NAT MATER, V16, P489, DOI [10.1038/nmat4822, 10.1038/NMAT4822]
Li AW, 2018, NAT MATER, V17, P528, DOI 10.1038/s41563-018-0028-2
Lonn P, 2016, SCI REP-UK, V6, DOI 10.1038/srep32301
Lynn GM, 2020, NAT BIOTECHNOL, V38, P320, DOI 10.1038/s41587-019-0390-x
Ma ZF, 2016, CLIN CANCER RES, V22, P2969, DOI 10.1158/1078-0432.CCR-15-1655
Maiti S, 2019, J AM CHEM SOC, V141, P4510, DOI 10.1021/jacs.8b12205
Manna S, 2020, ACS CENTRAL SCI, V6, P2071, DOI 10.1021/acscentsci.0c01001
Manna S, 2018, ACS CENTRAL SCI, V4, P982, DOI 10.1021/acscentsci.8b00218
Moser BA, 2020, SCI ADV, V6, DOI 10.1126/sciadv.aaz8700
Ni QQ, 2020, SCI ADV, V6, DOI 10.1126/sciadv.aaw6071
Nuhn L, 2018, ADV MATER, V30, DOI 10.1002/adma.201803397
Ott PA, 2017, NATURE, V547, P217, DOI 10.1038/nature22991
Paston SJ, 2021, FRONT IMMUNOL, V12, DOI 10.3389/fimmu.2021.627932
Reddy ST, 2006, J CONTROL RELEASE, V112, P26, DOI 10.1016/j.jconrel.2006.01.006
Robertson MJ, 2006, CLIN CANCER RES, V12, P4265, DOI 10.1158/1078-0432.CCR-06-0121
Schumacher TN, 2015, SCIENCE, V348, P69, DOI 10.1126/science.aaa4971
Seydoux E, 2018, J IMMUNOL, V201, P98, DOI 10.4049/jimmunol.1701604
Shae D, 2020, J LEUKOCYTE BIOL, V108, P1435, DOI 10.1002/JLB.5BT0119-016R
Sharp FA, 2009, P NATL ACAD SCI USA, V106, P870, DOI 10.1073/pnas.0804897106
Shen H, 2006, IMMUNOLOGY, V117, P78, DOI 10.1111/j.1365-2567.2005.02268.x
Swanson KV, 2019, NAT REV IMMUNOL, V19, P477, DOI 10.1038/s41577-019-0165-0
Tahtinen S, 2022, NAT IMMUNOL, V23, P532, DOI 10.1038/s41590-022-01160-y
van der Burg SH, 2016, NAT REV CANCER, V16, P219, DOI 10.1038/nrc.2016.16
Van Herck S, 2020, ACTA PHARMACOL SIN, V41, P881, DOI 10.1038/s41401-020-0425-3
Wang J, 2018, NAT NANOTECHNOL, V13, P1078, DOI 10.1038/s41565-018-0274-0
Wang JL, 1998, J IMMUNOL, V161, P5516
Wei SS, 2021, FRONT CELL DEV BIOL, V9, DOI 10.3389/fcell.2021.634607
Weiss AM, 2022, MACROMOLECULES, V55, P6913, DOI 10.1021/acs.macromol.2c00854
Zhao BG, 2014, J IMMUNOTHER CANCER, V2, DOI 10.1186/2051-1426-2-12
Zhou T, 2020, NATURE, V583, P609, DOI 10.1038/s41586-020-2422-6
NR 42
TC 0
Z9 0
U1 11
U2 12
PU ELSEVIER SCI LTD
PI OXFORD
PA THE BOULEVARD, LANGFORD LANE, KIDLINGTON, OXFORD OX5 1GB, OXON, ENGLAND
SN 0142-9612
EI 1878-5905
J9 BIOMATERIALS
JI Biomaterials
PD MAY
PY 2023
VL 296

AR 122062
DI 10.1016/j.biomaterials.2023.122062
EA FEB 2023
PG 11
WC Engineering, Biomedical; Materials Science, Biomaterials
WE Science Citation Index Expanded (SCI-EXPANDED)
SC Engineering; Materials Science
GA 9X3FU
UT WOS:000949658300001
PM 36863071
DA 2023-09-14
ER

PT J
AU Zhuang, XD
Li, B
Liu, XY
Fan, TT
Lan, XY
Wang, XY
Yu, M
AF Zhuang, Xiaoduan
Li, Bang
Liu, Xiaoyan
Fan, Tingting
Lan, Xinyue
Wang, Xinying
Yu, Meng
TI The colorectal cancer-specific microbiome regulation and immune response
activation via an artificial biomimetic nanovaccine
SO CHEMICAL ENGINEERING JOURNAL
LA English
DT Article
DE Microbiome regulation; Biomimetic drug carrier; Drug -resistance; Tumor
microenvironment; Orthotopic colorectal cancer
ID FUSOBACTERIUM-NUCLEATUM; TUMOR; PROLIFERATION; CELLS; ROS
AB The tumor microbiome has been reported to be composed of tumor-type-specific intracellular bacteria and is closely related to tumor progression and the response to anticancer therapies. Although antitumor treatments focusing on the abnormal tumor microenvironment (TME), such as anti-bacterial or TME regulatory approaches, have been shown to significantly improve antitumor efficacy, strategies that can interfere with the microbial environment to inhibit tumor growth and activate immune response are rare. Here, we have constructed an artificially modified biomimetic nanovaccine PMO for the first time using an inactivated anaerobic bacterium P. anaerobius, a colorectal cancer (CRC)-specific bacterium carrying therapeutical agents to regulate the microbial environment. The PMO could sneak into tumors due to its natural affinity to CRC, which has a particularly rich and diverse microbiome that results in therapy resistance. The PMO could not only achieve significant tumor growth suppression by precisely delivering therapeutical MnO2 and OXA to CRC tissues but also effectively prevent tumor recurrence by the cascade reaction of activating the tumor immune responses after reshaping the microbial environment. This nanovaccine has provided the tremendous clinical potential for designing new treatment strategies for drug-resistant tumors from the perspective of microbial environmental regulation and improving the prognosis of patients.
C1 [Zhuang, Xiaoduan; Li, Bang; Liu, Xiaoyan; Fan, Tingting; Wang, Xinying; Yu, Meng] Southern Med Univ, Zhujiang Hosp, Dept Gastroenterol, Guangzhou 510282, Peoples R China.
[Lan, Xinyue; Yu, Meng] Southern Med Univ, NMPA Key Lab Res & Evaluat Drug Metab, Guangzhou 510515, Peoples R China.
[Liu, Xiaoyan; Yu, Meng] Southern Med Univ, Sch Pharmaceut Sci, Guangdong Prov Key Lab New Drug Screening, Guangzhou 510515, Peoples R China.
[Lan, Xinyue] Southern Med Univ, Nanfang Hosp, Breast Ctr, Dept Gen Surg, Guangzhou 510515, Peoples R China.
C3 Southern Medical University - China; Southern Medical University -
China; Southern Medical University - China; Southern Medical University

file:///D/Joyce/计量学/frontiers/修/Supplementary materials/savedrecs (95).txt[2024/6/27 16:50:09]

- China
RP Wang, XY; Yu, M (通讯作者)，Southern Med Univ, Zhujiang Hosp, Dept Gastroenterol, Guangzhou 510282, Peoples R China.
EM sunwingwxy@163.com; yumeng999@smu.edu.cn
FU National Natural Science Foundation of China [82170532]; Natural Science Foundation of Guangdong Province [2023A1515030291, 2019A1515011498]; Science and Technology Program of Guangzhou [202201011130280065]
FX Acknowledgments This work was financially supported by the National Natural Science Foundation of China (82170532) , the Natural Science Foundation of Guangdong Province (2023A1515030291 and 2019A1515011498) and the Science and Technology Program of Guangzhou (202201011130280065) .
CR Brennan CA, 2016, ANNU REV MICROBIOL, V70, P395, DOI 10.1146/annurev-micro-102215-095513
Bullman S, 2017, SCIENCE, V358, P1443, DOI 10.1126/science.aal5240
Chen Y, 2008, CELL DEATH DIFFER, V15, P171, DOI 10.1038/sj.cdd.4402233
de Martel C, 2012, LANCET ONCOL, V13, DOI 10.1016/S1470-2045(12)70137-7
Dharmaraja AT, 2017, J MED CHEM, V60, P3221, DOI 10.1021/acs.jmedchem.6b01243
Garajova I, 2021, EXPERT REV ANTICANC, V21, P165, DOI 10.1080/14737140.2021.1844007
Garrett WS, 2015, SCIENCE, V348, P80, DOI 10.1126/science.aaa4972
Ge ZS, 2013, CHEM SOC REV, V42, P7289, DOI 10.1039/c3cs60048c
Gopalakrishnan V, 2018, CANCER CELL, V33, P570, DOI 10.1016/j.ccell.2018.03.015
Goradel NH, 2019, J CELL PHYSIOL, V234, P2337, DOI 10.1002/jcp.27250
Gur C, 2019, ONCOIMMUNOLOGY, V8, DOI 10.1080/2162402X.2019.1581531
Helmink BA, 2019, NAT MED, V25, P377, DOI 10.1038/s41591-019-0377-7
Li R, 2019, CELL DEATH DIFFER, V26, P2447, DOI 10.1038/s41418-019-0312-y
Liu ZP, 2020, CANCER LETT, V469, P173, DOI 10.1016/j.canlet.2019.10.020
Long XH, 2019, NAT MICROBIOL, V4, P2319, DOI 10.1038/s41564-019-0541-3
Lopes A, 2020, INT J CANCER, V146, P3147, DOI 10.1002/ijc.32920
Malekghasemi S, 2020, ADV PHARM BULL, V10, P556, DOI 10.34172/apb.2020.066
Prorok-Hamon M, 2014, GUT, V63, P761, DOI 10.1136/gutjnl-2013-304739
Rabinovich GA, 2007, ANNU REV IMMUNOL, V25, P267, DOI 10.1146/annurev.immunol.25.022106.141609
Rehman S, 2019, REV MED VIROL, V29, DOI 10.1002/rmv.2041
Sena LA, 2013, IMMUNITY, V38, P225, DOI 10.1016/j.immuni.2012.10.020
Sun W, 2017, FREE RADICAL BIO MED, V108, P433, DOI 10.1016/j.freeradbiomed.2017.04.010
Tsay JCJ, 2018, AM J RESP CRIT CARE, V198, P1188, DOI 10.1164/rccm.201710-2118OC
Tsoi H, 2017, GASTROENTEROLOGY, V152, P1419, DOI 10.1053/j.gastro.2017.01.009
Wang HR, 2019, NANO LETT, V19, P2935, DOI 10.1021/acs.nanolett.9b00021
Wang L., 1988, GUT, P69
Wilson M. R., 2019, SCIENCE, P363
Yamamura K, 2016, CLIN CANCER RES, V22, P5574, DOI 10.1158/1078-0432.CCR-16-1786
Yang YZ, 2017, GASTROENTEROLOGY, V152, P851, DOI 10.1053/j.gastro.2016.11.018
Yu TC, 2017, CELL, V170, P548, DOI 10.1016/j.cell.2017.07.008
Zhang QF, 2021, CANCER DISCOV, V11, P1248, DOI 10.1158/2159-8290.CD-20-0304
Zhao PF, 2020, BIOMATERIALS, V254, DOI 10.1016/j.biomaterials.2020.120142
Zheng DW, 2019, NAT BIOMED ENG, V3, P717, DOI 10.1038/s41551-019-0423-2
NR 33
TC 0
Z9 0
U1 17
U2 17
PU ELSEVIER SCIENCE SA
PI LAUSANNE
PA PO BOX 564, 1001 LAUSANNE, SWITZERLAND
SN 1385-8947
EI 1873-3212
J9 CHEM ENG J
JI Chem. Eng. J.
PD APR 1
PY 2023
VL 461

AR 141982
DI 10.1016/j.cej.2023.141982
EA FEB 2023
PG 12
WC Engineering, Environmental; Engineering, Chemical
WE Science Citation Index Expanded (SCI-EXPANDED)
SC Engineering
GA 9U5OM
UT WOS:000947759900001
DA 2023-09-14
ER

PT J
AU Goradel, NH
Nemati, M
Bakhshandeh, A
Arashkia, A
Negahdari, B
AF Goradel, Nasser Hashemi
Nemati, Mahnaz
Bakhshandeh, Azam
Arashkia, Arash
Negahdari, Babak
TI Nanovaccines for cancer immunotherapy: Focusing on complex formation between adjuvant and antigen
SO INTERNATIONAL IMMUNOPHARMACOLOGY
LA English
DT Review
DE Immunotherapy; Cancer vaccine; Nanovaccine; Nanocomplex; Immune response
ID CO-DELIVERY; ONCOLYTIC ADENOVIRUS; CELLULAR-IMMUNITY; DENDRITIC CELLS; NANOPARTICLES; VACCINE; ANTITUMOR; RECEPTOR; DNA; PROTEIN
AB As an interesting cancer immunotherapy approach, cancer vaccines have been developed to deliver tumor antigens and adjuvants to antigen-presenting cells (APCs). Although the safety and easy production shifted the vaccine designing platforms toward the subunit vaccines, their efficacy is limited due to inefficient vaccine delivery. Nanotechnology-based vaccines, called nanovaccines, address the delivery limitations through codelivery of antigens and adjuvants into lymphoid organs and APCs and their intracellular release, leading to cross-presentation of antigens and induction of potent anti-tumor immune responses. Although the nanovaccines, either as encapsulating agents or biomimetic nanoparticles, exert the desired anti-tumor activities, there is evidence that the mixing formulation to form nanocomplexes between antigens and adjuvants based on the electrostatic interactions provokes high levels of immune responses owing to Ags' availability and faster release. Here, we summarized the various platforms for developing cancer vaccines and the advantages of using delivery systems. The cancer nanovaccines, including nanoparticle-based and biomimetic-based nanovaccines, are discussed in detail. Finally, we focused on the nanocomplexes formation between antigens and adjuvants as promising cancer nanovaccine platforms.
C1 [Goradel, Nasser Hashemi] Maragheh Univ Med Sci, Dept Med Biotechnol, Maragheh, Iran.
[Nemati, Mahnaz] Shiraz Univ Med Sci, Amir Oncol Hosp, Shiraz, Iran.
[Bakhshandeh, Azam] Amirkabir Univ Technol, Dept Ind Engn & Management Syst, Tehran, Iran.
[Arashkia, Arash] Pasteur Inst Iran, Dept Mol Virol, Tehran, Iran.
[Negahdari, Babak] Univ Tehran Med Sci, Sch Adv Technol Med, Dept Med Biotechnol, Tehran, Iran.
[Negahdari, Babak] Univ Tehran Med Sci, Sch Adv Technol Med, Dept Med Biotechnol, 87 Third Floor,Bldg 2,Italia st,Keshavarz Blv, Tehran, Iran.
C3 Shiraz University of Medical Science; Amirkabir University of Technology; Le Reseau International des Instituts Pasteur (RIIP); Pasteur Institute of Iran; Tehran University of Medical Sciences; Tehran University of Medical Sciences
RP Goradel, NH (通讯作者)，Maragheh Univ Med Sci, Dept Med Biotechnol, Maragheh, Iran.; Negahdari, B (通讯作者)，Univ Tehran Med Sci, Sch Adv Technol Med, Dept Med Biotechnol, 87 Third Floor,Bldg 2,Italia st,Keshavarz Blv, Tehran, Iran.
EM Nassergoradel@yahoo.com; b-negahdari@sina.tums.ac.ir
CR Ajay F, 2021, J COMPL MED RES, V12, P7, DOI 10.5455/jcmr.2021.12.04.02

Alexis F, 2008, MOL PHARMACEUT, V5, P505, DOI 10.1021/mp800051m
Ammi R, 2015, PHARMACOL THERAPEUT, V146, P120, DOI 10.1016/j.pharmthera.2014.09.010
Anselmo AC, 2015, ACS NANO, V9, P3169, DOI 10.1021/acsnano.5b00147
Awate S, 2013, FRONT IMMUNOL, V4, DOI 10.3389/fimmu.2013.00114
Bapsy PP, 2014, CYTOTHERAPY, V16, P234, DOI 10.1016/j.jcyt.2013.11.013
Beck Z, 2018, J CONTROL RELEASE, V275, P12, DOI 10.1016/j.jconrel.2018.02.006
Beg S, 2021, DRUG DISCOV TODAY, V26, P1891, DOI 10.1016/j.drudis.2021.02.013
Blass E, 2021, NAT REV CLIN ONCOL, V18, P215, DOI 10.1038/s41571-020-00460-2
Bonilla WV, 2021, CELL REP MED, V2, DOI 10.1016/j.xcrm.2021.100209
Capasso C, 2016, ONCOIMMUNOLOGY, V5, DOI 10.1080/2162402X.2015.1105429
Cecco S, 2011, CURR CANCER DRUG TAR, V11, P85, DOI 10.2174/156800911793743664
Cha BG, 2018, ACS CENTRAL SCI, V4, P484, DOI 10.1021/acscentsci.8b00035
Champion CR, 2021, ANN PHARMACOTHER, V55, P783, DOI 10.1177/1060028020962050
Chen FJ, 2022, SMALL, V18, DOI [10.1002/smll.202203066, 10.1002/smll.202104402]
Cheng KM, 2021, NAT COMMUN, V12, DOI 10.1038/s41467-021-22308-8
Cheng SH, 2020, ADV SCI, V7, DOI 10.1002/advs.201903301
Coughlan L, 2020, FRONT IMMUNOL, V11, DOI 10.3389/fimmu.2020.00909
Danaei M, 2018, PHARMACEUTICS, V10, DOI 10.3390/pharmaceutics10020057
Demento SL, 2012, BIOMATERIALS, V33, P4957, DOI 10.1016/j.biomaterials.2012.03.041
Deng GJ, 2018, ACS NANO, V12, P12096, DOI 10.1021/acsnano.8b05292
Dhakal S, 2019, VET RES, V50, DOI 10.1186/s13567-019-0712-5
Dong H, 2018, INT J NANOMED, V13, P3353, DOI 10.2147/IJN.S164097
Dorostkar F., INFECT AGENTS CANCER, V16
Faghfuri E, 2021, EXPERT OPIN BIOL TH, V21, P201, DOI 10.1080/14712598.2020.1815704
Fang RH, 2014, NANO LETT, V14, P2181, DOI 10.1021/nl500618u
Francica JR, 2015, NAT COMMUN, V6, DOI 10.1038/ncomms7565
Gan JY, 2020, J CONTROL RELEASE, V326, P297, DOI 10.1016/j.jconrel.2020.07.008
Garofalo M, 2016, MOL CANCER THER, V15, P651, DOI 10.1158/1535-7163.MCT-15-0559
Ghanaat M, 2021, ACTA PHARMACOL SIN, V42, P1981, DOI 10.1038/s41401-021-00616-5
Gong NQ, 2020, NAT NANOTECHNOL, V15, P1053, DOI 10.1038/s41565-020-00782-3
Goradel NH, 2021, FUTURE ONCOL, V18, P245, DOI 10.2217/fon-2021-0802
Goradel NH, 2021, CURR PROB CANCER, V45, DOI 10.1016/j.currproblcancer.2020.100639
Goradel NH, 2020, PHARMACOL THERAPEUT, V213, DOI 10.1016/j.pharmthera.2020.107586
Goradel NH, 2019, J CELL PHYSIOL, V234, P8636, DOI 10.1002/jcp.27850
Grandi A, 2017, FRONT ONCOL, V7, DOI 10.3389/fonc.2017.00253
Gulley JL, 2019, J CLIN ONCOL, V37, P1051, DOI 10.1200/JCO.18.02031
Hailemichael Y, 2013, NAT MED, V19, P465, DOI 10.1038/nm.3105
Hamdy S, 2008, VACCINE, V26, P5046, DOI 10.1016/j.vaccine.2008.07.035
Hammerstrom AE, 2011, PHARMACOTHERAPY, V31, P813, DOI 10.1592/phco.31.8.813
Han YT, 2019, ADV SCI, V6, DOI 10.1002/advs.201900251
Hanagata N, 2017, INT J NANOMED, V12, P515, DOI 10.2147/IJN.S114477
He CB, 2010, BIOMATERIALS, V31, P3657, DOI 10.1016/j.biomaterials.2010.01.065
He HL, 2018, NANO LETT, V18, P6164, DOI 10.1021/acs.nanolett.8b01892
Hobernik D, 2018, INT J MOL SCI, V19, DOI 10.3390/ijms19113605
HogenEsch H, 2018, NPJ VACCINES, V3, DOI 10.1038/s41541-018-0089-x
Hollingsworth RE, 2019, NPJ VACCINES, V4, DOI 10.1038/s41541-019-0103-y
Hong XY, 2020, SCI ADV, V6, DOI 10.1126/sciadv.aaz4462
Huang SM, 2020, ACS APPL NANO MATER, V3, P9449, DOI 10.1021/acsanm.0c02062
Hubbell JA, 2009, NATURE, V462, P449, DOI 10.1038/nature08604
Iacobelli-Martinez M, 2007, J VIROL, V81, P1305, DOI 10.1128/JVI.01926-06
Irvine DJ, 2015, CHEM REV, V115, P11109, DOI 10.1021/acs.chemrev.5b00109
Jalali SA, 2012, NANOMED-NANOTECHNOL, V8, P692, DOI 10.1016/j.nano.2011.09.010
Ji ZH, 2021, DRUG DEV IND PHARM, V47, P1744, DOI 10.1080/03639045.2022.2045306
Jia JB, 2018, FRONT ONCOL, V8, DOI 10.3389/fonc.2018.00404
Kang T, 2018, BIOMATERIALS, V164, P80, DOI 10.1016/j.biomaterials.2018.02.033
Kantoff PW, 2017, J CLIN ONCOL, V35, P124, DOI 10.1200/JCO.2016.69.7748
Kantoff PW, 2010, NEW ENGL J MED, V363, P411, DOI 10.1056/NEJMoa1001294
Kapadia CH, 2015, J CONTROL RELEASE, V219, P167, DOI 10.1016/j.jconrel.2015.09.062
Kariko K, 2008, MOL THER, V16, P1833, DOI 10.1038/mt.2008.200
Kariko K, 2007, CURR OPIN DRUG DISC, V10, P523
Karimi H, 2020, SCI REP-UK, V10, DOI 10.1038/s41598-020-62448-3

Khong H, 2016, J IMMUNOTHER CANCER, V4, DOI 10.1186/s40425-016-0160-y
Khurana S, 2010, SCI TRANSL MED, V2, DOI 10.1126/scitranslmed.3000624
Kim SY, 2015, ANGEW CHEM INT EDIT, V54, P8139, DOI 10.1002/anie.201501380
Kool M, 2012, J MED MICROBIOL, V61, P927, DOI 10.1099/jmm.0.038943-0
Koster B.D., CANCER IMMUNOL IMMUN
Krishnamachari Y, 2009, ADV DRUG DELIVER REV, V61, P205, DOI 10.1016/j.addr.2008.12.013
Kubler H, 2015, J IMMUNOTHER CANCER, V3, DOI 10.1186/s40425-015-0068-y
Kumar C, 2017, IMMUNOTHERAPY-UK, V9, P889, DOI 10.2217/imt-2017-0064
Lee GH, 2021, EXPERT REV VACCINES, V20, P487, DOI 10.1080/14760584.2021.1908133
Leroux-Roels G, 2016, CLIN IMMUNOL, V169, P16, DOI 10.1016/j.clim.2016.05.007
Lesterhuis WJ, 2011, NAT REV DRUG DISCOV, V10, P591, DOI 10.1038/nrd3500
Li J, 2011, ACS NANO, V5, P8783, DOI 10.1021/nn202774x
Li S, 1997, GENE THER, V4, P891, DOI 10.1038/sj.gt.3300482
Li XR, 2014, J CONTROL RELEASE, V173, P148, DOI 10.1016/j.jconrel.2013.10.032
Liang J, 2022, FUND RES-CHINA, V2, P23, DOI 10.1016/j.fmre.2021.11.032
Liang ZY, 2021, INT J PHARMACEUT, V608, DOI 10.1016/j.ijpharm.2021.121091
Liu B, 2021, NANO LETT, V21, P9410, DOI 10.1021/acs.nanolett.1c02582
Liu JL, 2018, ACS APPL MATER INTER, V10, P30983, DOI 10.1021/acsami.8b09348
Lu Y, 2023, HUM RESOUR MANAGE-US, V62, P331, DOI 10.1002/hrm.22153
Ma WJ, 2020, THERANOSTICS, V10, P1281, DOI 10.7150/thno.40291
Molinier-Frenkel V, 2002, J VIROL, V76, P127, DOI 10.1128/JVI.76.1.127-135.2002
Moyer TJ, 2020, NAT MED, V26, P430, DOI 10.1038/s41591-020-0753-3
Mueller SN, 2015, MOL PHARMACEUT, V12, P1356, DOI 10.1021/mp500589c
Nam J, 2021, ADV SCI, V8, DOI 10.1002/advs.202002577
Nazarizadeh A, 2022, INT J MOL SCI, V23, DOI 10.3390/ijms23094707
Ochyl LJ, 2018, BIOMATERIALS, V182, P157, DOI 10.1016/j.biomaterials.2018.08.016
Oroojalian F, 2021, SMALL, V17, DOI 10.1002/smll.202006484
Palucka K, 2012, NAT REV CANCER, V12, P265, DOI 10.1038/nrc3258
Park KS, 2021, J EXTRACELL VESICLES, V10, DOI 10.1002/jev2.12120
Sanchez LP, 2015, HUM VACC IMMUNOTHER, V11, P2030, DOI 10.1080/21645515.2015.1029213
Rahimian S, 2015, J CONTROL RELEASE, V203, P16, DOI 10.1016/j.jconrel.2015.02.006
Rajendrakumar SK, 2018, POLYMERS-BASEL, V10, DOI 10.3390/polym10101063
Raponi A, 2021, SEMIN IMMUNOL, V56, DOI 10.1016/j.smim.2021.101544
Rosalia RA, 2013, EUR J IMMUNOL, V43, P2554, DOI 10.1002/eji.201343324
Sarin H, 2009, J TRANSL MED, V7, DOI 10.1186/1479-5876-7-51
Saxena M, 2021, NAT REV CANCER, V21, P360, DOI 10.1038/s41568-021-00346-0
Schoggins JW, 2005, J VIROL, V79, P11627, DOI 10.1128/JVI.79.18.11627-11637.2005
Shi XG, 2020, CHEM ENG J, V399, DOI 10.1016/j.cej.2020.125854
Shirota H, 2014, EXPERT REV VACCINES, V13, P299, DOI 10.1586/14760584.2014.863715
Silva JM, 2014, NANOMEDICINE-UK, V9, P2639, DOI [10.2217/NNM.14.135, 10.2217/nnm.14.135]
Song WT, 2017, BIOMATERIALS, V148, P16, DOI 10.1016/j.biomaterials.2017.09.017
Tamanini A, 2006, J VIROL, V80, P11241, DOI 10.1128/JVI.00721-06
Trimble CL, 2015, LANCET, V386, P2078, DOI 10.1016/S0140-6736(15)00239-1
Tsai HJ, 2015, TAIWAN J OBSTET GYNE, V54, P112, DOI 10.1016/j.tjog.2013.11.009
Tuo Z, 2022, CHEM ENG J, V433, DOI 10.1016/j.cej.2021.134437
Vijayan V, 2019, PHARMACEUTICS, V11, DOI 10.3390/pharmaceutics11100534
Vijayan V, 2018, POLYMERS-BASEL, V10, DOI 10.3390/polym10090983
Vo MC, 2021, CELL MOL IMMUNOL, V18, P1599, DOI 10.1038/s41423-021-00666-z
Wang JM, 2018, DRUG DELIV, V25, P1319, DOI 10.1080/10717544.2018.1477857
Wang SJ, 2017, INT J NANOMED, V12, P6813, DOI 10.2147/IJN.S143264
Wang XL, 2019, ACTA BIOMATER, V83, P390, DOI 10.1016/j.actbio.2018.11.023
Wang ZB, 2020, VACCINES-BASEL, V8, DOI 10.3390/vaccines8010128
Wani S. D., 2021, INT J PHARM RES TECH, V11, P1, DOI DOI 10.31838/IJPRT/11.01.01
Whitmore MM, 2001, CANCER IMMUNOL IMMUN, V50, P503, DOI 10.1007/s002620100227
Xiang SD, 2006, METHODS, V40, P1, DOI 10.1016/j.ymeth.2006.05.016
Xu JJ, 2017, SMALL, V13, DOI 10.1002/smll.201700666
Yang R, 2018, ACS NANO, V12, P5121, DOI 10.1021/acsnano.7b09041
Zhai YH, 2017, THERANOSTICS, V7, P2575, DOI 10.7150/thno.20118
Zhang QS, 2020, ACS APPL MATER INTER, V12, P54399, DOI 10.1021/acsami.0c15522
Zhang WF, 2014, BIOMATERIALS, V35, P6086, DOI 10.1016/j.biomaterials.2014.04.022
Zhang YX, 2019, CANCERS, V11, DOI 10.3390/cancers11091314

Zhong XF, 2019, J CONTROL RELEASE, V300, P81, DOI 10.1016/j.jconrel.2019.02.035
Zhou SL, 2020, BIOMATERIALS, V235, DOI 10.1016/j.biomaterials.2020.119795
Zom GGP, 2012, ADV IMMUNOL, V114, P177, DOI 10.1016/B978-0-12-396548-6.00007-X
NR 126
TC 0
Z9 0
U1 10
U2 12
PU ELSEVIER
PI AMSTERDAM
PA RADARWEG 29, 1043 NX AMSTERDAM, NETHERLANDS
SN 1567-5769
EI 1878-1705
J9 INT IMMUNOPHARMACOL
JI Int. Immunopharmacol.
PD APR
PY 2023
VL 117
AR 109887
DI 10.1016/j.intimp.2023.109887
EA FEB 2023
PG 11
WC Immunology; Pharmacology & Pharmacy
WE Science Citation Index Expanded (SCI-EXPANDED)
SC Immunology; Pharmacology & Pharmacy
GA C8YA2
UT WOS:000964698200001
PM 36841155
DA 2023-09-14
ER

PT J
AU Chen, HL
Li, YH
Li, LY
Yang, ZY
Wen, ZF
Liu, LX
Liu, H
Chen, YM
AF Chen, Haolin
Li, Yuhui
Li, Liyan
Yang, Zeyu
Wen, Zhenfu
Liu, Lixin
Liu, Hong
Chen, Yongming
TI Carrier-free subunit nanovaccine amplifies immune responses against tumors and viral infections
SO ACTA BIOMATERIALIA
LA English
DT Article
DE Adjuvant; Diprovocim; Immune therapy; Nanovaccine; Subunit antigens
ID DELIVERY SYSTEMS; NANOPARTICLES; CANCER; ADJUVANTS; VACCINE
AB Codelivering subunit antigens and Toll-like receptor (TLR) molecular adjuvants via nanocarriers can stim-ulate potent innate and specific immune responses. Simple and effective nanovaccines fabrication is cru-cial for application. However, most nanovaccines were fabricated by introducing additional delivery mate-rials, increasing safety risk, cost and processing complexity. Herein, a carrier-free nanovaccine was facilely prepared using a TLR1/TLR2 adjuvant, Diprovocim, rich in benzene rings that could interact with aromatic residues in subunit antigens through pi-pi stacking without additional materials. The carrier-free

nanovac-cines with a narrow size distribution could target lymph nodes (LNs) after intravenous injection to mice. The carrier-free nanovaccines based on ovalbumin (OVA) can stimulate strong antibody titers and CD4+ and CD8+ T cell immune responses in mice, and it synergized with anti-PD1 showing a potent tumor suppression in B16F10-OVA tumor model of mice. Furthermore, the carrier-free nanovaccine with glyco-protein E (gE), a glycoprotein of the varicella-zoster virus (VZV), also showed potent humoral and cellular immune responses. Therefore, using subunit proteins to support Diprovocim by pi-pi stacking provides a new approach for the preparation and application of novel vaccines for tumor therapy and prevention of infectious diseases.Statement of significance Codelivering subunit antigens and adjuvants via nanocarriers stimulate potent innate and specific im-mune responses. However, existing delivery materials for fabricating nanovaccines will inevitably increase the cost of preparation, controllability, process complexity and safety assessment. Therefore, this study easily prepared carrier-free nanovaccines using the benzene ring-rich TLR1/TLR2 adjuvant Diprovocim, which can interact with aromatic residues in subunit antigens via pi-pi stacking without additional mate-rials. The carrier-free nanovaccines of OVA demonstrated a potent tumor inhibition in treating melanoma in combination with anti-PD1. And the nanovaccines of gE stimulated a strong antibody titer and cellular immune response for herpes zoster. Thus, the present study provides a new approach for the preparation of subunit vaccines to combat various cancers and virus infections.(c) 2022 Acta Materialia Inc. Published by Elsevier Ltd. All rights reserved.
C1 [Chen, Haolin; Li, Liyan; Yang, Zeyu; Wen, Zhenfu; Liu, Lixin; Liu, Hong; Chen, Yongming] Sun Yat sen Univ, Sch Mat Sci & Engn, Key Lab Polymer Composite & Funct Mat, Minist Educ, Guangzhou 510275, Peoples R China.
[Li, Yuhui] Sun Yat sen Univ, Sun Yat sen Mem Hosp, Dept Pathol, Guangdong Prov Key Lab Malignant Tumor Epigenet &, Guangzhou 510275, Peoples R China.
[Liu, Lixin; Chen, Yongming] Sun Yat sen Univ, Canc Ctr, State Key Lab Oncol Southern China, Guangzhou 510060, Peoples R China.
[Chen, Yongming] Sun Yat sen Univ, Affiliated Hosp 3, Ctr Nanomed, Lab Biomat & Translat Med, Guangzhou 510630, Peoples R China.
C3 Sun Yat Sen University; Sun Yat Sen University; Sun Yat Sen University;
State Key Lab Oncology South China; Sun Yat Sen University
RP Liu, LX; Liu, H; Chen, YM (通讯作者)，Sun Yat sen Univ, Sch Mat Sci & Engn, Key Lab Polymer Composite & Funct Mat, Minist Educ, Guangzhou 510275, Peoples R China.; Liu, LX; Chen, YM (通讯作者)，Sun Yat sen Univ, Canc Ctr, State Key Lab Oncol Southern China, Guangzhou 510060, Peoples R China.; Chen, YM (通讯作者)，Sun Yat sen Univ, Affiliated Hosp 3, Ctr Nanomed, Lab Biomat & Translat Med, Guangzhou 510630, Peoples R China.
EM liulixin@mail.sysu.edu.cn; liuhong@jnu.edu.cn; chenym35@mail.sysu.edu.cn
OI Liu, Hong/0000-0003-0350-7475
FU Key Project of Ministry of Science and Technology [2022YFC2304201];
National Natural Science Foundation of China [51820105004, 22075324];
Key Areas Research and Development Program of Guangzhou [2020 07020 0 06]
FX We appreciate the financial support from Key Project of Ministry of Science and Technology (2022YFC2304201) and the National Natural Science Foundation of China (51820105004 , 22075324) , and the Key Areas Research and Development Program of Guangzhou (2020 07020 0 06) is gratefully acknowledged. Also, we thank BioRender.com for copyright agreements allowing the use of the image.
CR Ahmad MZ, 2020, EXPERT REV VACCINES, V19, P1053, DOI 10.1080/14760584.2020.1858058
Al Mughram MH, 2021, J CHEM INF MODEL, V61, P2937, DOI 10.1021/acs.jcim.1c00235
An MG, 2018, NANOSCALE, V10, P9311, DOI 10.1039/c8nr01376d
Bal SM, 2011, VACCINE, V29, P1045, DOI 10.1016/j.vaccine.2010.11.061
Bhardwaj P, 2020, ACTA BIOMATER, V108, P1, DOI 10.1016/j.actbio.2020.03.020
Chen HL, 2022, SCI CHINA TECHNOL SC, V65, P989, DOI 10.1007/s11431-021-2011-7
Duthie MS, 2011, IMMUNOL REV, V239, P178, DOI 10.1111/j.1600-065X.2010.00978.x
Fan YC, 2015, VACCINES-BASEL, V3, P662, DOI 10.3390/vaccines3030662
Grupping K, 2017, J INFECT DIS, V216, P1343, DOI 10.1093/infdis/jix482
Huang PS, 2019, ACTA BIOMATER, V85, P1, DOI 10.1016/j.actbio.2018.12.028
Jiang H, 2017, J CONTROL RELEASE, V267, P47, DOI 10.1016/j.jconrel.2017.08.009
Lau J, 2017, NAT COMMUN, V8, DOI 10.1038/ncomms14572
Liu D, 2021, NANO LETT, V21, P3965, DOI 10.1021/acs.nanolett.1c00648
Liu H, 2022, J CONTROL RELEASE, V352, P497, DOI 10.1016/j.jconrel.2022.10.053
Liu H, 2022, J CONTROL RELEASE, V345, P91, DOI 10.1016/j.jconrel.2022.03.006

Liu H, 2020, BIOMATERIALS, V255, DOI 10.1016/j.biomaterials.2020.120158
Liu LH, 2022, PROG MATER SCI, V125, DOI 10.1016/j.pmatsci.2021.100919
Lu LT, 2021, VACCINES-BASEL, V9, DOI 10.3390/vaccines9060563
Luo M, 2017, J CONTROL RELEASE, V263, P200, DOI 10.1016/j.jconrel.2017.03.033
Medzhitov R, 2001, NAT REV IMMUNOL, V1, P135, DOI 10.1038/35100529
Morin MD, 2018, J AM CHEM SOC, V140, P14440, DOI 10.1021/jacs.8b09223
Moyer TJ, 2016, J CLIN INVEST, V126, P799, DOI 10.1172/JCI81083
Pati R, 2018, FRONT IMMUNOL, V9, DOI 10.3389/fimmu.2018.02224
Qiao DD, 2021, BIOMATERIALS, V269, DOI 10.1016/j.biomaterials.2021.120674
Qiao DD, 2018, NANO LETT, V18, P3007, DOI 10.1021/acs.nanolett.8b00478
Qin SY, 2017, BIOMATERIALS, V112, P234, DOI 10.1016/j.biomaterials.2016.10.016
Riwar LJ, 2017, ANGEW CHEM INT EDIT, V56, P11252, DOI 10.1002/anie.201703744
Salmaninejad A, 2019, J CELL PHYSIOL, V234, P16824, DOI 10.1002/jcp.28358
Schmidt ST, 2016, PHARMACEUTICS, V8, DOI 10.3390/pharmaceutics8010007
Shi ST, 2019, VACCINE, V37, P3167, DOI 10.1016/j.vaccine.2019.04.055
Silva JM, 2013, J CONTROL RELEASE, V168, P179, DOI 10.1016/j.jconrel.2013.03.010
Singha S, 2018, ACS NANO, V12, P10621, DOI 10.1021/acsnano.8b05950
Song HJ, 2022, ACTA BIOMATER, V141, P398, DOI 10.1016/j.actbio.2022.01.004
Steinhagen F, 2011, VACCINE, V29, P3341, DOI 10.1016/j.vaccine.2010.08.002
Le TT, 2020, NAT REV DRUG DISCOV, V19, P305, DOI 10.1038/d41573-020-00073-5
Toy R, 2016, BIOENG TRANSL MED, V1, P47, DOI 10.1002/btm2.10005
Trevaskis NL, 2015, NAT REV DRUG DISCOV, V14, P781, DOI 10.1038/nrd4608
Wang Y, 2018, P NATL ACAD SCI USA, V115, pE8698, DOI 10.1073/pnas.1809232115
Wang ZB, 2020, VACCINES-BASEL, V8, DOI 10.3390/vaccines8010128
Wu XJ, 2019, J MATER CHEM B, V7, P4854, DOI 10.1039/c9tb00448c
Yang DJ, 2018, NANOMEDICINE-UK, V13, P3159, DOI 10.2217/nnm-2018-0288
Ye T, 2021, ADV DRUG DELIVER REV, V177, DOI 10.1016/j.addr.2021.113927
Zazo H, 2016, J CONTROL RELEASE, V224, P86, DOI 10.1016/j.jconrel.2016.01.008
Zhang XQ, 2012, ADV DRUG DELIVER REV, V64, P1363, DOI 10.1016/j.addr.2012.08.005
Zhou JR, 2021, NANO TODAY, V36, DOI 10.1016/j.nantod.2020.101031
Zhou ZZ, 2020, NANO RES, V13, P1509, DOI 10.1007/s12274-020-2737-5
Zhu MZ, 2021, ADV DRUG DELIVER REV, V178, DOI 10.1016/j.addr.2021.113966
NR 47
TC 0
Z9 0
U1 16
U2 22
PU ELSEVIER SCI LTD
PI OXFORD
PA THE BOULEVARD, LANGFORD LANE, KIDLINGTON, OXFORD OX5 1GB, OXON, ENGLAND
SN 1742-7061
EI 1878-7568
J9 ACTA BIOMATER
JI Acta Biomater.
PD MAR 1
PY 2023
VL 158
BP 525
EP 534
DI 10.1016/j.actbio.2022.12.042
EA FEB 2023
PG 10
WC Engineering, Biomedical; Materials Science, Biomaterials
WE Science Citation Index Expanded (SCI-EXPANDED)
SC Engineering; Materials Science
GA 9N4AY
UT WOS:000942858100001
PM 36572250
DA 2023-09-14
ER

PT J
AU Su, QH
Liu, ZW
Du, RL
Chen, XL
Chen, LL
Fu, ZM
Luo, XY
Yang, Y
Shi, XQ
AF Su, Qianhong
Liu, Zuwei
Du, Ruolin
Chen, Xiaolu
Chen, Longlong
Fu, Zhaoming
Luo, Xingyu
Yang, Yong
Shi, Xuequn
TI Facile preparation of a metal-phenolic network-based lymph node targeting nanovaccine for antitumor immunotherapy
SO ACTA BIOMATERIALIA
LA English
DT Article
DE Metal -phenolic networks; Cancer vaccine; Lymph node targeting; Cellular immune response; Chlorogenic acid; Regulatory T cells
ID CHECKPOINT; POLARIZATION; RESISTANCE; CELLS
AB Cancer vaccines are being explored for enhanced cancer immunotherapy and prophylaxis. Some of their prevailing weaknesses, however, such as complicated preparation, poor biocompatibility, and failure to elicit strong cellular immune responses, have limited their further clinical applications. Here, we reported a multifunctional nanovaccine that was prepared in a quick and simple way. During the self-assembly of metal-phenolic networks (MPNs), the antigen ovalbumin (OVA) and immunoreactive chlorogenic acid (CHA) were simultaneously loaded. Owing to its dual pH and reduction sensitivities, the nanovaccine could deliver antigens into the cytoplasm of dendritic cells (DCs) and facilitate the cross-presentation of antigens. Moreover, the results of in vivo immunization assays demonstrated that the nanovaccine signifi-cantly excited the antigen presentation of DCs and provoked a robust cellular immune response with the restrained activation of regulatory T cells (Tregs), by targeting lymph nodes and executing the function of CHA. In vivo antitumor assays indicated that the nanovaccine with good biocompatibility afforded con-spicuous cancer treatment and prevention effects. Overall, the nanovaccine presented in this study shows a promise for potentiating cancer immunotherapy by the lymph node-targeted delivery.Statement of significanceCancer nanovaccines can be used for cancer immunotherapy. However, some existing shortcomings, such as cumbersome preparation, poor biocompatibility, and failure to elicit strong immune responses, limit the clinical application of cancer nanovaccines. This study developed a multifunctional nanovaccine that was readily prepared through the self-assembly of metal-phenolic networks. The nanovaccine with dual pH and reduction sensitivities could efficiently promote the antigen lysosome escape and cross -presentation. In vivo , it efficiently delivered antigen into lymph nodes and provoked strong cellular im-mune responses, and thus it showed significant cancer immunotherapy and prevention effect.(c) 2022 Acta Materialia Inc. Published by Elsevier Ltd. All rights reserved.
C1 [Su, Qianhong; Liu, Zuwei; Du, Ruolin; Chen, Longlong; Fu, Zhaoming; Luo, Xingyu; Yang, Yong; Shi, Xuequn] Hainan Univ, Coll Food Sci & Engn, Key Lab Food Nutr & Funct Food Hainan Prov, Haikou 570228, Peoples R China.
[Chen, Xiaolu] Chinese Acad Trop Agr Sci, Trop Crops Genet Resources Inst, Haikou 570100, Peoples R China.
C3 Hainan University; Chinese Academy of Tropical Agricultural Sciences
RP Yang, Y (通讯作者)，Hainan Univ, Coll Food Sci & Engn, Key Lab Food Nutr & Funct Food Hainan Prov, Haikou 570228, Peoples R China.
EM yangyong@hainanu.edu.cn
RI yang, yong/ADH-0488-2022; Su, Qianhong/HTN-4068-2023
FU Hainan Provincial Natural Science Foundation of China [2019RC008, 520MS016]; National Natural Science Foundation of China [32060225];

Central Public-interest Scientific Institution Basal Research Fund for Chinese Academy of Tropical Agricultural Sciences [1630032022022]; State Key Laboratory of Advanced Technology for Materials Synthesis and Processing (Wuhan University of Technology) [2021-KF-21]
FX This study was financially supported by the Hainan Provincial Natural Science Foundation of China (2019RC008 , 520MS016) , the National Natural Science Foundation of China (32060225) , and the Central Public-interest Scientific Institution Basal Research Fund for Chinese Academy of Tropical Agricultural Sciences (No. 1630032022022) . The project was supported by State Key Laboratory of Advanced Technology for Materials Synthesis and Processing (Wuhan University of Technology) (2021-KF-21) .
CR Ahmad S, 2017, CANCER RES, V77, P1892, DOI 10.1158/0008-5472.CAN-16-1839
Cabrera G, 2019, VACCINE, V37, P3628, DOI 10.1016/j.vaccine.2019.05.015
Cai T, 2021, J NANOBIOTECHNOL, V19, DOI 10.1186/s12951-021-01146-2
Chen FM, 2021, BIOMATERIALS, V270, DOI 10.1016/j.biomaterials.2021.120709
Chen JQ, 2019, ACS NANO, V13, P11653, DOI 10.1021/acsnano.9b05521
Chen Q, 2016, NAT COMMUN, V7, DOI 10.1038/ncomms13193
Dai Y.L., ADV MATER
Dees S, 2021, EUR J IMMUNOL, V51, P280, DOI 10.1002/eji.202048992
Ejima H, 2017, NANO TODAY, V12, P136, DOI 10.1016/j.nantod.2016.12.012
Eroglu Z, 2018, NATURE, V553, P347, DOI 10.1038/nature25187
de Carvalho JTG, 2020, J NUTR BIOCHEM, V85, DOI 10.1016/j.jnutbio.2020.108428
Gu TT, 2020, POULTRY SCI, V99, P5461, DOI 10.1016/j.psj.2020.08.069
Guan XW, 2018, BIOMATERIALS, V171, P198, DOI 10.1016/j.biomaterials.2018.04.039
Guo YX, 2019, CHEM MATER, V31, P10071, DOI 10.1021/acs.chemmater.9b03042
Hu HZ, 2021, ADV SCI, V8, DOI 10.1002/advs.202002020
Jiang H, 2018, ADV SCI, V5, DOI 10.1002/advs.201700426
Jiang H, 2017, J CONTROL RELEASE, V267, P47, DOI 10.1016/j.jconrel.2017.08.009
Joffre OP, 2012, NAT REV IMMUNOL, V12, P557, DOI 10.1038/nri3254
Kalbasi A, 2020, NAT REV IMMUNOL, V20, P25, DOI 10.1038/s41577-019-0218-4
Li K, 2019, ADV SCI, V6, DOI 10.1002/advs.201801688
Li Q, 2022, SMALL, V18, DOI 10.1002/smll.202201108
Li WB, 2018, J CLIN ONCOL, V36, DOI 10.1200/JCO.2018.36.15_suppl.e14081
Liu H, 2020, BIOMATERIALS, V255, DOI 10.1016/j.biomaterials.2020.120158
O'Donnell JS, 2017, CANCER TREAT REV, V52, P71, DOI 10.1016/j.ctrv.2016.11.007
Orsolic N, 2016, CHEM-BIOL INTERACT, V256, P111, DOI 10.1016/j.cbi.2016.06.027
Pan C, 2020, ADV MATER, V32, DOI 10.1002/adma.202002940
Pezeshki PS, 2021, EXPERT REV ANTICANC, V21, P1003, DOI 10.1080/14737140.2021.1918548
Qiao DD, 2018, NANO LETT, V18, P3007, DOI 10.1021/acs.nanolett.8b00478
Ribas A, 2018, SCIENCE, V359, P1350, DOI 10.1126/science.aar4060
Sarode A, 2020, BIOMATERIALS, V242, DOI 10.1016/j.biomaterials.2020.119929
Tanaka A, 2017, CELL RES, V27, P109, DOI 10.1038/cr.2016.151
Wen Y, 2021, CHEM MATER, V33, P7089, DOI 10.1021/acs.chemmater.1c02267
Xiang Z, 2019, FRONT MED-PRC, V13, P24, DOI 10.1007/s11684-019-0679-7
Xie WS, 2021, THERANOSTICS, V11, P6407, DOI 10.7150/thno.58711
Ye J, 2020, BIOACT MATER, V5, P694, DOI 10.1016/j.bioactmat.2020.05.001
Zhang Y, 2021, SCI CHINA LIFE SCI, V64, P1097, DOI 10.1007/s11427-020-1739-6
Zhang Z, 2021, SMALL, V17, DOI 10.1002/smll.202100314
Zhang Z, 2021, ANGEW CHEM INT EDIT, V60, P1967, DOI 10.1002/anie.202013406
Zheng DW, 2017, NANO LETT, V17, P284, DOI 10.1021/acs.nanolett.6b04060
Zhou BH, 2021, FRONT IMMUNOL, V12, DOI 10.3389/fimmu.2021.693609
Zhou LL, 2021, SMALL, V17, DOI 10.1002/smll.202103919
Zhou X, 2020, MOL PHARMACEUT, V17, P4603, DOI 10.1021/acs.molpharmaceut.0c00802
Zou JY, 2018, J CELL BIOCHEM, V119, P1420, DOI 10.1002/jcb.26302
NR 43
TC 0
Z9 0
U1 22
U2 32
PU ELSEVIER SCI LTD

PI OXFORD
PA THE BOULEVARD, LANGFORD LANE, KIDLINGTON, OXFORD OX5 1GB, OXON, ENGLAND
SN 1742-7061
EI 1878-7568
J9 ACTA BIOMATER
JI Acta Biomater.
PD MAR 1
PY 2023
VL 158
BP 510
EP 524
DI 10.1016/j.actbio.2022.12.066
EA FEB 2023
PG 15
WC Engineering, Biomedical; Materials Science, Biomaterials
WE Science Citation Index Expanded (SCI-EXPANDED)
SC Engineering; Materials Science
GA 9N6RV
UT WOS:000943041000001
PM 36603733
DA 2023-09-14
ER

PT J
AU Jyoti, K
Malik, G
Chaudhary, M
Madan, J
Kamboj, A
AF Jyoti, Kiran
Malik, Garima
Chaudhary, Monika
Madan, Jitender
Kamboj, Anjoo
TI Hyaluronate decorated polyethylene glycol linked poly
(lactide-co-glycolide) nanoparticles encapsulating MUC-1 peptide
augmented mucosal immune response in Balb/c mice through inhalation
route
SO BIOCHIMICA ET BIOPHYSICA ACTA-GENERAL SUBJECTS
LA English
DT Article
DE Non small cell lung cancer; MUC-1 peptide; Hyaluronic acid; Cellular
uptake; Cytokine production; Poly (D; L-lactide-co-glycolide)
ID THERAPEUTIC CANCER VACCINES; CELL; PROTEINS; MICROPARTICLES;
NANOCARRIERS; PEGYLATION; SURVIVAL; L-BLP25; GROWTH; FLUID
AB Background and objectives: NSCLC (Non-Small Cell Lung Cancer) clutches highest mortality rate in man and women globally. The present study was conducted to target MUC-1 peptide (M-1) into antigen presenting cells by cargo the peptide into hyaluronic acid decorated polyethylene glycol linked poly (D, L-lactide-co-glycolide) nanoparticles (M-1-PL-co-GA-PEG-sHA-NPs) for generating mucosal immunity through inhalation (i.h.) route. Methodology and results: The mean particle size and surface charge of M-1-PL-co-GA-PEG-sHA-NPs was measured to be 136.2 +/- 18.38-nm and -28.34 +/- 6.77-mV, respectively, prepared by non-aggregated emulsion-diffusion evaporation method. The 28.42% percentage release of M-1 peptide from M-1-PL-co-GA-PEG-NPs was observed to be at 2 h and 95.29% at 8 h while the percentage release of M-1 peptide from M-1-PL-co-GA-PEG-sHA-NPs was observed to be 26.02% at 4 h and 97.95% at 24 h that proved the prolonged release of antigen. M-1-PL-co-GA-PEG-sHA-NPs demonstrated higher (P < 0.05) cellular uptake of 86.2% in RAW 264.7 cells in comparison to 27.6% of M-1-PL-co-GA-PEG-NPs. In addition, M-1-PL-co-GA-PEG-sHA-NPs induced remarkably (P < 0.05) elevated release of 80.6-pg/ml of TNF-alpha in comparison to 5-pg/ml by culture medium and 57.9-pg/ml of TNF-alpha by M-1-PL-co-GA-PEG-NPs. Similarly, M-1-PL-co-GA-PEG-sHA-NPs persuade remarkably (P < 0.05) elevated release of 225-pg/ml of IL-1 beta in comparison to 47-pg/ml by culture medium and 161.9-pg/ml of IL-1 beta by M-1-PL-co-GA-PEG-NPs. M-1-PL-co-GA-PEG-sHA-NPs might have been endocytosed

through receptor mediated pathway owing to presence of sHA. Mice immunized through i.h. route with M-1-PL-co-GA-PEG-sHA-NPs induced strong (P < 0.05) IgA antibody titre as compared to M-1-PL-co-GA-PEG-NPs and M-1 peptide in dose-dosage regimen. Conclusion: M-1-PL-co-GA-PEG-sHA-NPs nanovaccine warrants further analysis in xenograft model of NSCLC to showcase its antitumor capability.
C1 [Jyoti, Kiran] IKG Punjab Tech Univ, Jalandhar, Punjab, India.
[Jyoti, Kiran; Kamboj, Anjoo] Chandigarh Coll Pharm, Dept Pharmaceut, Mohali, Punjab, India.
[Malik, Garima] Maharishi Markandeshwar Univ, MM Coll Pharm, Ambala, Haryana, India.
[Chaudhary, Monika] Amity Univ, Amity Inst Pharm, Panchgaon, Haryana, India.
[Madan, Jitender] Natl Inst Pharmaceut Educ & Res, Dept Pharmaceut, Hyderabad, Telangana, India.
[Jyoti, Kiran] Chandigarh Coll Pharm, Mohali, Punjab, India.
C3 I. K. Gujral Punjab Technical University; Maharishi Markandeshwar University; National Institute of Pharmaceutical Education & Research, Hyderabad; National Institute of Pharmaceutical Education & Research (NIPER)
RP Jyoti, K (通讯作者)，Chandigarh Coll Pharm, Mohali, Punjab, India.
EM jyotikiran2411@gmail.com
FU Indian Council of Medical Research, New Delhi, India [5/13/40/NCD-III]
FX The authors are highly grateful to Indian Council of Medical Research, New Delhi, India for offering financial support to Chandigarh College of Pharmacy) , Mohali Punjab, India as extramural adhoc project (5/13/40/NCD-III) .
CR American Cancer Society, 2019, CANC FACTS FIG
Arpicco S, 2013, J DRUG DELIV, V2013, DOI 10.1155/2013/860780
Bajwa N., 2021, NANOMATERIALS EVOLUT, V13, P246
Blank F, 2011, EXPERT OPIN DRUG DEL, V8, P547, DOI 10.1517/17425247.2011.565326
Brandsma AM, 2015, CANCER IMMUNOL RES, V3, P1316, DOI 10.1158/2326-6066.CIR-15-0099-T
Buono C, 2009, J CLIN INVEST, V119, P1373, DOI 10.1172/JCI35548
Butts C, 2014, LANCET ONCOL, V15, P59, DOI 10.1016/S1470-2045(13)70510-2
Chen C, 2018, J HEMATOL ONCOL, V11, DOI 10.1186/s13045-018-0605-5
Choi KY, 2012, COLLOID SURFACE B, V99, P82, DOI 10.1016/j.colsurfb.2011.10.029
Choi KY, 2011, BIOMATERIALS, V32, P1880, DOI 10.1016/j.biomaterials.2010.11.010
Dalvi H, 2021, INT J PEPT RES THER, V27, P2965, DOI 10.1007/s10989-021-10303-y
Daubeuf Francois, 2012, Curr Protoc Mouse Biol, V2, P167, DOI 10.1002/9780470942390.mo110201
Day AJ, 2002, J BIOL CHEM, V277, P4585, DOI 10.1074/jbc.R100036200
Dev A, 2012, ARTIF CELL BLOOD SUB, V40, P400, DOI 10.3109/10731199.2012.696063
Dondulkar A, 2022, CURR PHARM DESIGN, V28, P395, DOI 10.2174/1381612827666211104155604
Guan HH, 1998, BIOCONJUGATE CHEM, V9, P451, DOI 10.1021/bc970183n
Guo CQ, 2013, ADV CANCER RES, V119, P421, DOI 10.1016/B978-0-12-407190-2.00007-1
Harde H, 2014, NANOMEDICINE-UK, V9, P2511, DOI [10.2217/nnm.13.225, 10.2217/NNM.13.225]
Hardy CL, 2013, J IMMUNOL, V191, P5278, DOI 10.4049/jimmunol.1203131
Harris DT, 1999, SEMIN ONCOL, V26, P439
Hart DNJ, 1997, BLOOD, V90, P3245, DOI 10.1182/blood.V90.9.3245
Hussain Z, 2019, DRUG DELIV TRANSL RE, V9, P721, DOI 10.1007/s13346-019-00631-4
Johansen P, 2000, EUR J PHARM BIOPHARM, V50, P129, DOI 10.1016/S0939-6411(00)00079-5
Jyoti K, 2020, J MICROENCAPSUL, V37, P566, DOI 10.1080/02652048.2020.1823500
Jyoti K, 2020, J MICROENCAPSUL, V37, P14, DOI 10.1080/02652048.2019.1692943
Katoh S, 1999, ALLERGY, V54, P1286, DOI 10.1034/j.1398-9995.1999.00277.x
Kaur A, 2016, J MICROENCAPSUL, V33, P263, DOI 10.3109/02652048.2016.1169324
Kelly RJ, 2011, CANCER J, V17, P302, DOI 10.1097/PPO.0b013e318233e6b4
Kelly SM, 2005, BBA-PROTEINS PROTEOM, V1751, P119, DOI 10.1016/j.bbapap.2005.06.005
Kim T.H., BIOL PHARMACOL
Limacher JM, 2013, EXPERT REV VACCINES, V12, P263, DOI [10.1586/erv.13.14, 10.1586/ERV.13.14]
Metcalf D, 2008, BLOOD, V111, P485, DOI 10.1182/blood-2007-03-079681
Mizrahy S, 2011, J CONTROL RELEASE, V156, P231, DOI 10.1016/j.jconrel.2011.06.031
MOSMANN T, 1983, J IMMUNOL METHODS, V65, P55, DOI 10.1016/0022-1759(83)90303-4
Obermann HL, 2022, FRONT IMMUNOL, V12, DOI 10.3389/fimmu.2021.658895
Palmer M, 2001, Clin Lung Cancer, V3, P49, DOI 10.3816/CLC.2001.n.018
Park JH, 2014, INT J PHARMACEUT, V473, P426, DOI 10.1016/j.ijpharm.2014.07.038
Pawar D, 2010, AAPS J, V12, P130, DOI 10.1208/s12248-009-9169-1
Powell Erin, 2008, Expert Rev Respir Med, V2, P37, DOI 10.1586/17476348.2.1.37
Qhattal HSS, 2011, MOL PHARMACEUT, V8, P1233, DOI 10.1021/mp2000428

Rakhra K, 2021, SCI IMMUNOL, V6, DOI 10.1126/sciimmunol.abd8003
Remy-Ziller C, 2018, HUM VACC IMMUNOTHER, V14, P140, DOI 10.1080/21645515.2017.1373921
Roswell Park Cancer Institute, 2023, VACC CIMAVAX EGF PRE
Sangha R, 2007, CLIN CANCER RES, V13, p4652S, DOI 10.1158/1078-0432.CCR-07-0213
Sharma S, 2011, EXPERT OPIN BIOL TH, V11, P987, DOI 10.1517/14712598.2011.598146
Shojaee Samira, 2017, F1000Res, V6, P2110, DOI 10.12688/f1000research.11471.1
Shortman K, 1997, STEM CELLS, V15, P409, DOI 10.1002/stem.150409
Siefert AL, 2016, BIOMATERIALS, V97, P85, DOI 10.1016/j.biomaterials.2016.03.039
Siegel RL, 2016, CA-CANCER J CLIN, V66, P7, DOI DOI 10.3322/CAAC.21442
Stano A, 2011, VACCINE, V29, P804, DOI 10.1016/j.vaccine.2010.11.010
Tagliamento M, 2018, EXPERT OPIN PHARMACO, V19, P2055, DOI 10.1080/14656566.2018.1540591
Tang W, 2022, NANOSCALE, V14, P263, DOI 10.1039/d1nr06512b
Turecek PL, 2016, J PHARM SCI-US, V105, P460, DOI 10.1016/j.xphs.2015.11.015
Wickens JM, 2017, DRUG DISCOV TODAY, V22, P665, DOI 10.1016/j.drudis.2016.12.009
Youn YS, 2008, DIABETES OBES METAB, V10, P343, DOI 10.1111/j.1463-1326.2007.00823.x
ZALIPSKY S, 1983, EUR POLYM J, V19, P1177, DOI 10.1016/0014-3057(83)90016-2
NR 56
TC 0
Z9 0
U1 3
U2 3
PU ELSEVIER
PI AMSTERDAM
PA RADARWEG 29, 1043 NX AMSTERDAM, NETHERLANDS
SN 0304-4165
EI 1872-8006
J9 BBA-GEN SUBJECTS
JI Biochim. Biophys. Acta-Gen. Subj.
PD APR
PY 2023
VL 1867
IS 4
AR 130317
DI 10.1016/j.bbagen.2023.130317
EA FEB 2023
PG 10
WC Biochemistry & Molecular Biology; Biophysics
WE Science Citation Index Expanded (SCI-EXPANDED)
SC Biochemistry & Molecular Biology; Biophysics
GA H6TM5
UT WOS:000997263900001
PM 36731729
DA 2023-09-14
ER

PT J
AU Donkor, M
Choe, J
Reid, DM
Quinn, B
Pulse, M
Ranjan, A
Chaudhary, P
Jones, HP
AF Donkor, Michael
Choe, Jamie
Reid, Danielle Marie
Quinn, Byron
Pulse, Mark
Ranjan, Amalendu
Chaudhary, Pankaj

Jones, Harlan P.
TI Nasal Tumor Vaccination Protects against Lung Tumor Development by
Induction of Resident Effector and Memory Anti-Tumor Immune Responses
SO PHARMACEUTICS
LA English
DT Article
DE breast cancer; lung metastasis; vaccination; immune response;
nanoparticles
ID NEGATIVE BREAST-CANCER; T-CELL ANERGY; INFILTRATING LYMPHOCYTES;
DENDRITIC CELLS; SURVIVAL; METASTASIS; HYPERTHERMIA; EXPRESSION;
DELIVERY; DORMANCY
AB Lung metastasis is a leading cause of cancer-related deaths. Here, we show that intranasal delivery of our engineered CpG-coated tumor antigen (Tag)-encapsulated nanoparticles (NPs)-nasal nano-vaccine-significantly reduced lung colonization by intravenous challenge of an extra-pulmonary tumor. Protection against tumor-cell lung colonization was linked to the induction of localized mucosal-associated effector and resident memory T cells as well as increased bronchiolar alveolar lavage-fluid IgA and serum IgG antibody responses. The nasal nano-vaccine-induced T-cell-mediated antitumor mucosal immune response was shown to increase tumor-specific production of IFN-gamma and granzyme B by lung-derived CD8(+) T cells. These findings demonstrate that our engineered nasal nano-vaccine has the potential to be used as a prophylactic approach prior to the seeding of tumors in the lungs, and thereby prevent overt lung metastases from existing extra pulmonary tumors.
C1 [Donkor, Michael; Choe, Jamie; Reid, Danielle Marie; Ranjan, Amalendu; Chaudhary, Pankaj; Jones, Harlan P.] Univ North Texas Hlth Sci Ctr, Dept Microbiol Immunol & Genet, Ft Worth, TX 76107 USA.
[Quinn, Byron] Langston Univ, Dept Biol, Langston, OK 73050 USA.
[Pulse, Mark] Univ North Texas Hlth Sci Ctr, Dept Pharmaceut Sci, Ft Worth, TX 76107 USA.
C3 Langston University
RP Jones, HP (通讯作者)，Univ North Texas Hlth Sci Ctr, Dept Microbiol Immunol & Genet, Ft Worth, TX 76107 USA.
EM harlan.jones@unthsc.edu
OI Reid, Danielle/0000-0002-3316-9115; Donkor, Michael/0000-0002-5488-0328;
Choe, Jamie/0000-0002-7803-5962
FU National Cancer Institute of the National Institutes of Health [1 P20
CA233355-01]; National Institute on Drug Abuse [5 R25 DA043225-04];
National Institutes on Minority Health, Health Disparities
[5S21MD012472-05]; Cancer Prevention and Research Institute of Texas
[RP210046]
FX Research reported in this work was supported by the National Cancer
Institute of the National Institutes of Health under Award Number 1 P20
CA233355-01, the National Institute on Drug Abuse under award number 5
R25 DA043225-04 and the National Institutes on Minority Health, Health
Disparities under award number 5S21MD012472-05 and the Cancer Prevention
and Research Institute of Texas (Award #: RP210046). The content is
solely the responsibility of the authors and does not necessarily
represent the official views of the National Institutes of Health.
CR Andtbacka RHI, 2015, J CLIN ONCOL, V33, P2780, DOI 10.1200/JCO.2014.58.3377
Baumjohann D, 2021, J IMMUNOTHER CANCER, V9, DOI 10.1136/jitc-2021-002588
Blanc C, 2018, FRONT IMMUNOL, V9, DOI 10.3389/fimmu.2018.01722
Cameron MD, 2000, CANCER RES, V60, P2541
Chambers AF, 2002, NAT REV CANCER, V2, P563, DOI 10.1038/nrc865
Chen AH, 2022, SMALL, V18, DOI 10.1002/smll.202200993
Chin AR, 2016, CLIN CANCER RES, V22, P3725, DOI 10.1158/1078-0432.CCR-16-0028
Couch FJ, 2015, J CLIN ONCOL, V33, P304, DOI 10.1200/JCO.2014.57.1414
Cuenca A, 2003, CANCER RES, V63, P9007
Cuzzubbo S, 2021, FRONT IMMUNOL, V11, DOI 10.3389/fimmu.2020.615240
Dent R, 2007, CLIN CANCER RES, V13, P4429, DOI 10.1158/1078-0432.CCR-06-3045
Djenidi F, 2015, J IMMUNOL, V194, P3475, DOI 10.4049/jimmunol.1402711
Donkor M, 2021, FRONT NANOTECHNOL, V3, DOI 10.3389/fnano.2021.635194
Dubensky TW, 2010, SEMIN IMMUNOL, V22, P155, DOI 10.1016/j.smim.2010.04.007
Egelston CA, 2019, JCI INSIGHT, V4, DOI 10.1172/jci.insight.130000
Gutierro I, 2002, VACCINE, V21, P67, DOI 10.1016/S0264-410X(02)00435-8
Hammerich L, 2019, NAT MED, V25, P814, DOI 10.1038/s41591-019-0410-x

Hiam-Galvez KJ, 2021, NAT REV CANCER, V21, P345, DOI 10.1038/s41568-021-00347-z
Jamil A, 2022, STATPEARLS
Kaplan RN, 2005, NATURE, V438, P820, DOI 10.1038/nature04186
Kitamura T, 2015, NAT REV IMMUNOL, V15, P73, DOI 10.1038/nri3789
Kokate RA, 2016, NANOMEDICINE-UK, V11, P479, DOI 10.2217/nnm.15.213
Kokate RA, 2015, NANOMEDICINE-UK, V10, P915, DOI [10.2217/NNM.14.144, 10.2217/nnm.14.144]
Kreutz M, 2013, BLOOD, V121, P2836, DOI 10.1182/blood-2012-09-452078
Kurts C, 2010, NAT REV IMMUNOL, V10, P403, DOI 10.1038/nri2780
Lambert AW, 2017, CELL, V168, P670, DOI 10.1016/j.cell.2016.11.037
Lee YJ, 2022, SCI IMMUNOL, V7, DOI 10.1126/sciimmunol.abn8390
Lelekakis M, 1999, CLIN EXP METASTAS, V17, P163, DOI 10.1023/A:1006689719505
Liu Y, 2016, CANCER CELL, V30, P668, DOI 10.1016/j.ccell.2016.09.011
Luzzi KJ, 1998, AM J PATHOL, V153, P865, DOI 10.1016/S0002-9440(10)65628-3
Malik BT, 2017, SCI IMMUNOL, V2, DOI 10.1126/sciimmunol.aam6346
Massague J, 2016, NATURE, V529, P298, DOI 10.1038/nature17038
Nagrath S, 2007, NATURE, V450, P1235, DOI 10.1038/nature06385
Ngamcherdtrakul W, 2021, ADV MATER, V33, DOI 10.1002/adma.202100628
Nizard M, 2017, NAT COMMUN, V8, DOI 10.1038/ncomms15221
Olkhanud PB, 2009, CANCER RES, V69, P5996, DOI 10.1158/0008-5472.CAN-08-4619
Palucka K, 2013, IMMUNITY, V39, P38, DOI 10.1016/j.immuni.2013.07.004
Peinado H, 2017, NAT REV CANCER, V17, P302, DOI 10.1038/nrc.2017.6
Perry JL, 2020, ACS NANO, V14, P7200, DOI 10.1021/acsnano.0c02207
Sandoval F, 2013, SCI TRANSL MED, V5, DOI 10.1126/scitranslmed.3004888
Sasson SC, 2020, CELL MOL IMMUNOL, V17, P113, DOI 10.1038/s41423-019-0359-1
Sato T, 2015, MOL CANCER THER, V14, P2198, DOI 10.1158/1535-7163.MCT-15-0401
Savas P, 2018, NAT MED, V24, P986, DOI 10.1038/s41591-018-0078-7
Schenkel JM, 2014, IMMUNITY, V41, P886, DOI 10.1016/j.immuni.2014.12.007
Shi HZ, 2006, J IMMUNOL, V176, P2134, DOI 10.4049/jimmunol.176.4.2134
Staveley-O'Carroll K, 1998, P NATL ACAD SCI USA, V95, P1178, DOI 10.1073/pnas.95.3.1178
Takahashi T, 1995, ANTICANCER RES, V15, P2601
Tohme S, 2017, CANCER RES, V77, P1548, DOI 10.1158/0008-5472.CAN-16-1536
Vadala M, 2017, EPMA J, V8, P295, DOI 10.1007/s13167-017-0101-y
Wculek SK, 2020, NAT REV IMMUNOL, V20, P7, DOI 10.1038/s41577-019-0210-z
Webb JR, 2015, CANCER IMMUNOL RES, V3, P926, DOI 10.1158/2326-6066.CIR-14-0239
Webb JR, 2014, CLIN CANCER RES, V20, P434, DOI 10.1158/1078-0432.CCR-13-1877
Wu T, 2014, J LEUKOCYTE BIOL, V95, P215, DOI 10.1189/jlb.0313180
Zheng MZM, 2022, MUCOSAL IMMUNOL, V15, P379, DOI 10.1038/s41385-021-00461-z
NR 54
TC 0
Z9 0
U1 4
U2 4
PU MDPI
PI BASEL
PA ST ALBAN-ANLAGE 66, CH-4052 BASEL, SWITZERLAND
EI 1999-4923
J9 PHARMACEUTICS
JI Pharmaceutics
PD FEB
PY 2023
VL 15
IS 2
AR 445
DI 10.3390/pharmaceutics15020445
PG 16
WC Pharmacology & Pharmacy
WE Science Citation Index Expanded (SCI-EXPANDED)
SC Pharmacology & Pharmacy
GA 9L6FN
UT WOS:000941644200001
PM 36839766

OA Green Published, gold
DA 2023-09-14
ER

PT J
AU Xie, YJ
Liu, WQ
Li, D
Hou, JC
Coghi, PS
Fan, XX
AF Xie, Ya-Jia
Liu, Wen-Qian
Li, Dan
Hou, Jin-Cai
Coghi, Paolo Saul
Fan, Xing-Xing
TI Overcoming Suppressive Tumor Microenvironment by Vaccines in Solid Tumor
SO VACCINES
LA English
DT Review
DE cancer vaccines; immunosuppressive TME; solid tumor; immunotherapy;
nanovaccines
ID ANTITUMOR IMMUNE-RESPONSES; MESSENGER-RNA VACCINES; LAHERPAREPVEC T-VEC;
HUMAN-PAPILLOMAVIRUS; DENDRITIC CELLS; CERVICAL-CANCER; GLOBAL BURDEN;
DNA; IMMUNOGENICITY; IMMUNOTHERAPY
AB Conventional vaccines are widely used to boost human natural ability to defend against foreign invaders, such as bacteria and viruses. Recently, therapeutic cancer vaccines attracted the most attention for anti-cancer therapy. According to the main components, it can be divided into five types: cell, DNA, RNA, peptide, and virus-based vaccines. They mainly perform through two rationales: (1) it trains the host immune system to protect itself and effectively eradicate cancer cells; (2) these vaccines expose the immune system to molecules associated with cancer that enable the immune system to recognize and destroy cancer cells. In this review, we thoroughly summarized the potential strategies and technologies for developing cancer vaccines, which may provide critical achievements for overcoming the suppressive tumor microenvironment through vaccines in solid tumors.
C1 [Xie, Ya-Jia; Liu, Wen-Qian; Coghi, Paolo Saul; Fan, Xing-Xing] Macau Univ Sci & Technol, State Key Lab Qual Res Chinese Med, Macau 999078, Peoples R China.
[Li, Dan; Hou, Jin-Cai] Beijing Wanteer Biol Pharmaceut Co Ltd, 32 yard,East 2nd Rd, Beijing 101400, Peoples R China.
C3 Macau University of Science & Technology
RP Coghi, PS; Fan, XX (通讯作者)，Macau Univ Sci & Technol, State Key Lab Qual Res Chinese Med, Macau 999078, Peoples R China.
EM coghips@must.edu.mo; xxfan@must.edu.mo
RI cogh, pao/AAE-9122-2019
OI cogh, pao/0000-0002-0015-1453
CR Abd-Aziz N, 2022, J ONCOL, V2022, DOI 10.1155/2022/9749363
Achmad H, 2022, INT IMMUNOPHARMACOL, V113, DOI 10.1016/j.intimp.2022.109434
Ammi R, 2015, PHARMACOL THERAPEUT, V146, P120, DOI 10.1016/j.pharmthera.2014.09.010
Andtbacka RHI, 2015, J CLIN ONCOL, V33, P2780, DOI 10.1200/JCO.2014.58.3377
[Anonymous], MRNA 4157 CANC VACCI
Baldin AV, 2020, CANCERS, V12, DOI 10.3390/cancers12030590
Barbier AJ, 2022, NAT BIOTECHNOL, V40, P840, DOI 10.1038/s41587-022-01294-2
Bidram M, 2021, VACCINES-BASEL, V9, DOI 10.3390/vaccines9101060
Bijker MS, 2007, J IMMUNOL, V179, P5033, DOI 10.4049/jimmunol.179.8.5033
Bode C, 2011, EXPERT REV VACCINES, V10, P499, DOI [10.1586/ERV.10.174, 10.1586/erv.10.174]
Bol KF, 2015, ONCOIMMUNOLOGY, V4, DOI 10.1080/2162402X.2015.1019197
Bommareddy PK, 2017, AM J CLIN DERMATOL, V18, P1, DOI 10.1007/s40257-016-0238-9
Chakraborty C, 2021, FRONT IMMUNOL, V12, DOI 10.3389/fimmu.2021.679344
Chen J, 2022, BIOACT MATER, V7, P167, DOI 10.1016/j.bioactmat.2021.05.036
Chen LJ, 2021, THERANOSTICS, V11, P6668, DOI 10.7150/thno.56494
Chen YP, 2021, TAIWAN J OBSTET GYNE, V60, P700, DOI 10.1016/j.tjog.2021.05.020

Ciesielska U, 2012, ADV CLIN EXP MED, V21, P235
Constantino J, 2017, IMMUNOL RES, V65, P798, DOI 10.1007/s12026-017-8931-1
Crosby EJ, 2019, CLIN CANCER RES, V25, P2725, DOI 10.1158/1078-0432.CCR-18-3102
Cui ZR, 2005, ADV GENET, V54, P257, DOI 10.1016/S0065-2660(05)54011-2
de Martel C, 2020, LANCET GLOB HEALTH, V8, pE180, DOI 10.1016/S2214-109X(19)30488-7
Draghiciu O, 2015, ONCOIMMUNOLOGY, V4, DOI 10.1080/2162402X.2015.1029699
Eusebio D, 2021, DRUG DISCOV TODAY, V26, P2575, DOI 10.1016/j.drudis.2021.06.008
Ferrucci PF, 2021, CANCERS, V13, DOI 10.3390/cancers13061383
Fu CM, 2018, FRONT IMMUNOL, V9, DOI 10.3389/fimmu.2018.03059
Gibney GT, 2015, CLIN CANCER RES, V21, P712, DOI 10.1158/1078-0432.CCR-14-2468
Glebe D, 2021, ANTIVIR RES, V186, DOI 10.1016/j.antiviral.2020.104973
Guo ZS, 2019, J IMMUNOTHER CANCER, V7, DOI 10.1186/s40425-018-0495-7
Gupta M, 2022, VACCINES-BASEL, V10, DOI 10.3390/vaccines10122011
Handy CE, 2018, FUTURE ONCOL, V14, P907, DOI 10.2217/fon-2017-0531
Hu SC, 2021, ACTA BIOMATER, V135, P567, DOI 10.1016/j.actbio.2021.09.003
Hu YY, 2020, BIOMATERIALS, V252, DOI 10.1016/j.biomaterials.2020.120114
Huang DQ, 2022, BIOMATERIALS, V289, DOI 10.1016/j.biomaterials.2022.121808
Jhaveri R, 2021, CLIN THER, V43, P549, DOI 10.1016/j.clinthera.2021.01.014
Johnson DB, 2015, IMMUNOTHERAPY-UK, V7, P611, DOI [10.2217/IMT.15.35, 10.2217/imt.15.35]
Jorritsma SHT, 2016, VACCINE, V34, P5488, DOI 10.1016/j.vaccine.2016.09.062
Joshi S, 2019, J ONCOL, V2019, DOI 10.1155/2019/5245034
Ju F, 2022, J IMMUNOTHER CANCER, V10, DOI 10.1136/jitc-2022-004762
Kano Y, 2016, CANCER SCI, V107, P398, DOI 10.1111/cas.12861
Kantoff PW, 2010, NEW ENGL J MED, V363, P411, DOI 10.1056/NEJMoa1001294
Kaur A, 2022, CURR OPIN CHEM BIOL, V70, DOI 10.1016/j.cbpa.2022.102172
Kim CG, 2021, INT J MOL SCI, V22, DOI 10.3390/ijms22158035
Kowalski PS, 2019, MOL THER, V27, P710, DOI 10.1016/j.ymthe.2019.02.012
Kumar V, 2016, TRENDS IMMUNOL, V37, P208, DOI 10.1016/j.it.2016.01.004
Kyte JA, 2016, ONCOIMMUNOLOGY, V5, DOI 10.1080/2162402X.2016.1232237
Larocca C, 2011, CANCER J, V17, P359, DOI 10.1097/PPO.0b013e3182325e63
Laureano RS, 2022, ONCOIMMUNOLOGY, V11, DOI 10.1080/2162402X.2022.2096363
Li CX, 2022, PHARMACEUTICALS-BASE, V15, DOI 10.3390/ph15111411
Li JC, 2022, ADV MATER, V34, DOI 10.1002/adma.202108012
Li T, 2022, NANO LETT, V22, P3095, DOI 10.1021/acs.nanolett.2c00500
Li ZL, 2023, BIOACT MATER, V21, P299, DOI 10.1016/j.bioactmat.2022.08.028
Li ZL, 2022, ADV SCI, V9, DOI 10.1002/advs.202201734
Liang ZY, 2021, INT J PHARMACEUT, V608, DOI 10.1016/j.ijpharm.2021.121091
Liu SY, 2023, BIOACT MATER, V22, P211, DOI 10.1016/j.bioactmat.2022.09.017
Liu WS, 2021, CELL PROLIFERAT, V54, DOI 10.1111/cpr.13025
Lopes A, 2019, J EXP CLIN CANC RES, V38, DOI 10.1186/s13046-019-1154-7
Lorentzen CL, 2022, LANCET ONCOL, V23, pE450, DOI 10.1016/S1470-2045(22)00372-2
Ma Y, 2013, J CANCER, V4, P36, DOI 10.7150/jca.5046
Maisonneuve C, 2014, P NATL ACAD SCI USA, V111, P12294, DOI 10.1073/pnas.1400478111
Mhaidly R, 2020, SEMIN IMMUNOL, V48, DOI 10.1016/j.smim.2020.101417
Miao L, 2021, MOL CANCER, V20, DOI 10.1186/s12943-021-01335-5
Mildner A, 2014, IMMUNITY, V40, P642, DOI 10.1016/j.immuni.2014.04.016
Nakao S, 2020, SCI TRANSL MED, V12, DOI 10.1126/scitranslmed.aax7992
Ohshio Y, 2015, CANCER SCI, V106, P134, DOI 10.1111/cas.12584
Ohue Y, 2019, CANCER SCI, V110, P2080, DOI 10.1111/cas.14069
Pattyn J, 2021, J INFECT DIS, V224, pS343, DOI 10.1093/infdis/jiaa668
Pellom ST, 2021, JCI INSIGHT, V6, DOI 10.1172/jci.insight.141912
Plummer M, 2016, LANCET GLOB HEALTH, V4, pE609, DOI 10.1016/S2214-109X(16)30143-7
Ramachandran S, 2022, PHARM MED, V36, P11, DOI 10.1007/s40290-021-00417-5
Randolph GJ, 2021, J EXP MED, V218, DOI 10.1084/jem.20202077
Sousa CR, 2006, NAT REV IMMUNOL, V6, P476, DOI 10.1038/nri1845
Sabado RL, 2010, IMMUNOTHERAPY-UK, V2, P37, DOI 10.2217/IMT.09.43
Semmrich M, 2022, J IMMUNOTHER CANCER, V10, DOI 10.1136/jitc-2021-003488
Shi W, 2021, CANCER LETT, V522, P184, DOI 10.1016/j.canlet.2021.09.028
Shrestha AC, 2019, VACCINES-BASEL, V7, DOI 10.3390/vaccines7020038
Skwarczynski M, 2016, CHEM SCI, V7, P842, DOI 10.1039/c5sc03892h
Sprooten J, 2019, ONCOIMMUNOLOGY, V8, DOI 10.1080/2162402X.2019.1638212

Tanaka Y, 2020, SCI REP-UK, V10, DOI 10.1038/s41598-020-74187-6
Tashiro H, 2017, CELL RES, V27, P59, DOI 10.1038/cr.2016.153
Tie Y, 2022, J HEMATOL ONCOL, V15, DOI 10.1186/s13045-022-01282-8
Tiptiri-Kourpeti A, 2016, PHARMACOL THERAPEUT, V165, P32, DOI 10.1016/j.pharmthera.2016.05.004
Vellios N, 2020, DATA BRIEF, V32, DOI [10.1016/j.dib.2020.106260, 10.1016/j.dib.020.106260]
Wang C, 2020, INT J BIOL SCI, V16, P633, DOI 10.7150/ijbs.38414
Wang RJ, 2020, CANCER LETT, V471, P88, DOI 10.1016/j.canlet.2019.11.039
Wu ZM, 2021, PHARM RES-DORDR, V38, P473, DOI 10.1007/s11095-021-03015-x
Xia JY, 2022, BIOMED PHARMACOTHER, V152, DOI 10.1016/j.biopha.2022.113250
Xie XX, 2022, BIOACT MATER, V16, P107, DOI 10.1016/j.bioactmat.2022.03.008
Xu SQ, 2020, INT J MOL SCI, V21, DOI 10.3390/ijms21186582
Yang B, 2014, HUM VACC IMMUNOTHER, V10, P3153, DOI 10.4161/21645515.2014.980686
Yang J, 2019, PATHOL RES PRACT, V215, DOI 10.1016/j.prp.2019.152691
Yang J, 2015, INT J MOL MED, V35, P17, DOI 10.3892/ijmm.2014.2000
Ye JF, 2017, CELL IMMUNOL, V318, P35, DOI 10.1016/j.cellimm.2017.06.002
Zhang JH, 2022, INT J NANOMED, V17, P869, DOI 10.2147/IJN.S269986
Zhang YL, 2021, CANCER CELL INT, V21, DOI 10.1186/s12935-021-01972-2
Zhou J, 2023, CANCER MED-US, V12, P7207, DOI 10.1002/cam4.5511
NR 95
TC 0
Z9 0
U1 18
U2 21
PU MDPI
PI BASEL
PA ST ALBAN-ANLAGE 66, CH-4052 BASEL, SWITZERLAND
EI 2076-393X
J9 VACCINES-BASEL
JI Vaccines
PD FEB
PY 2023
VL 11
IS 2
AR 394
DI 10.3390/vaccines11020394
PG 19
WC Immunology; Medicine, Research & Experimental
WE Science Citation Index Expanded (SCI-EXPANDED)
SC Immunology; Research & Experimental Medicine
GA 9L0QK
UT WOS:000941262300001
PM 36851271
OA Green Published, gold
DA 2023-09-14
ER

PT J
AU Huang, FL
Zhang, Q
Xiao, J
Zhang, X
Han, XZ
Shi, X
Hu, J
Li, L
Qian, XP
AF Huang, Fengli
Zhang, Qun
Xiao, Jie
Zhang, Xin
Han, Xingzhi

Shi, Xiao
Hu, Jing
Li, Li
Qian, Xiaoping
TI Cancer Cell Membrane-Coated Gambogic Acid Nanoparticles for Effective
Anticancer Vaccination by Activating Dendritic Cells
SO INTERNATIONAL JOURNAL OF NANOMEDICINE
LA English
DT Article
DE cancer immunotherapy; gambogic acid; nano-vaccine; tumor immune
microenvironment; colorectal cancer
AB Purpose: Recent studies have shown that traditional Chinese medicine (TCM), such as gambogic acid (GA), is involved in the regulation of tumor immune microenvironment and can be combined with other anti-tumor treatment strategies. Here, we used GA as an adjuvant to construct a nano-vaccine to improve the anti-tumor immune response of colorectal cancer (CRC). Materials and Methods: We used a previously reported two-step emulsification method to obtain poly (lactic-co-glycolic acid) /GA nanoparticles (PLGA/GA NPs), and then CT26 colon cancer cell membrane (CCM) was used to obtain CCM-PLGA/GA NPs. This novel nano-vaccine, CCM-PLGA/GA NPs, was co-synthesized with GA as an adjuvant and neoantigen provided by CT26 CCM. We further confirmed the stability, tumor targeting, and cytotoxicity of CCM-PLGA/GA NPs. The regulatory effect on the tumor immune microenvironment, the anti-tumor efficacy, and the combined anti-tumor efficacy with anti-PD-1 monoclonal Antibodies (mAbs) of this novel nano-vaccine was also detected in vivo. Results: We successfully constructed the CCM-PLGA/GA NPs. In vitro and in vivo tests showed low biological toxicity, as well as the high tumor-targeting ability of the CCM-PLGA/GA NPs. Besides, we revealed a remarkable effect of CCM-PLGA/GA NPs to activate the maturation of dendritic cells (DCs) and the formation of a positive anti-tumor immune microenvironment. Conclusion: This novel nano-vaccine constructed with GA as the adjuvant and CCM providing the tumor antigen can not only directly kill tumors by enhancing the ability of GA to target tumors, but also indirectly kill tumors by regulating tumor immune microenvironment, providing a new strategy for immunotherapy of CRC.
C1 [Huang, Fengli; Zhang, Xin; Qian, Xiaoping] Nanjing Univ Chinese Med, Dept Oncol, Nanjing Drum Tower Hosp, Clin Coll, 321 Zhongshan Rd, Nanjing 210008, Jiangsu, Peoples R China.
[Zhang, Qun; Hu, Jing; Li, Li; Qian, Xiaoping] Nanjing Drum Tower Hosp, Dept Oncol, Nanjing, Peoples R China.
[Xiao, Jie; Han, Xingzhi; Shi, Xiao] Nanjing Univ, Nanjing Drum Tower Hosp, Affiliated Hosp Med Sch, Dept Oncol, Nanjing, Peoples R China.
C3 Nanjing University of Chinese Medicine; Nanjing University; Nanjing
University; Nanjing University
RP Qian, XP (通讯作者)，Nanjing Univ Chinese Med, Dept Oncol, Nanjing Drum Tower Hosp, Clin Coll, 321 Zhongshan Rd, Nanjing 210008, Jiangsu, Peoples R China.
EM xiaopingqian@nju.edu.cn
FU Nanjing health science and technology development key program
[ZKX21028]; Provincial Natural Science Foundation of Jiangsu
[BK20211007]; Jiangsu scientific and technological development of
traditional Chinese medicine Key projects [ZD202227]
FX This study was supported by grants from Nanjing health science and
technology development key program (no ZKX21028) , Provincial Natural
Science Foundation of Jiangsu (no BK20211007) , Jiangsu scientific and
technological development of traditional Chinese medicine Key projects
(no ZD202227) .
CR Bray F, 2018, CA-CANCER J CLIN, V68, P394, DOI 10.3322/caac.21492
Cao M, 2019, J IMMUNOTHER CANCER, V7, DOI 10.1186/s40425-019-0817-4
Chang WT, 2015, PLOS ONE, V10, DOI 10.1371/journal.pone.0122374
Chen XM, 2022, J CONTROL RELEASE, V351, P381, DOI 10.1016/j.jconrel.2022.09.010
Deng GL, 2022, J IMMUNOTHER CANCER, V10, DOI 10.1136/jitc-2022-004874
Fang RH, 2014, NANO LETT, V14, P2181, DOI 10.1021/nl500618u
Feng SN, 2021, INT J NANOMED, V16, P2123, DOI 10.2147/IJN.S266948
Gu PF, 2019, INT J PHARMACEUT, V554, P72, DOI 10.1016/j.ijpharm.2018.11.008
Hegde PS, 2018, SEMIN CANCER BIOL, V52, P117, DOI 10.1016/j.semcancer.2017.12.002
Horn L, 2018, NEW ENGL J MED, V379, P2220, DOI 10.1056/NEJMoa1809064
Huang YH, 2018, NAT REV IMMUNOL, V18, P195, DOI 10.1038/nri.2017.145
Jiang XJ, 2019, MOL CANCER, V18, DOI 10.1186/s12943-018-0928-4

Jiang XY, 2022, J IMMUNOTHER CANCER, V10, DOI 10.1136/jitc-2021-003960
Lee WS, 2020, EXP MOL MED, V52, P1475, DOI 10.1038/s12276-020-00500-y
Liu HY, 2023, CHINA ANIMAL HUSBAND, V50, P390, DOI [10.16431/j.cnki.1671-7236.2023.01.039, DOI 10.16431/J.CNKI.1671-7236.2023.01.039]
Luk BT, 2015, J CONTROL RELEASE, V220, P600, DOI 10.1016/j.jconrel.2015.07.019
Mo FZ, 2021, SIGNAL TRANSDUCT TAR, V6, DOI 10.1038/s41392-021-00462-1
Nie D, 2020, NANO LETT, V20, P936, DOI 10.1021/acs.nanolett.9b03817
Ren SJ, 2021, FRONT IMMUNOL, V12, DOI 10.3389/fimmu.2021.689132
Ren XL, 2021, J COLLOID INTERF SCI, V591, P229, DOI 10.1016/j.jcis.2021.02.006
Wan L, 2019, PHYTOTHER RES, V33, P1579, DOI 10.1002/ptr.6350
Wen CY, 2015, INT J ONCOL, V47, P1663, DOI 10.3892/ijo.2015.3166
Xie YF, 2021, ADV SCI, V8, DOI 10.1002/advs.202101672
Yang DD, 2015, JILIN J CHINE MED, V35, P753, DOI [10.13463/j.cnki.jlzyy.2015.07.032, DOI 10.13463/J.CNKI.JLZYY.2015.07.032]
Yang ML, 2016, TUMORI, V102, P135, DOI 10.5301/tj.5000411
Yi CH, 2021, HEPATOLOGY, V74, P2544, DOI 10.1002/hep.31921
Yi M, 2022, MOL CANCER, V21, DOI 10.1186/s12943-021-01489-2
Zhang L, 2022, MATER TODAY BIO, V15, DOI 10.1016/j.mtbio.2022.100294
Zhang Z, 2017, INT J NANOMED, V12, P1593, DOI 10.2147/IJN.S127256
NR 29
TC 0
Z9 0
U1 14
U2 14
PU DOVE MEDICAL PRESS LTD
PI ALBANY
PA PO BOX 300-008, ALBANY, AUCKLAND 0752, NEW ZEALAND
SN 1178-2013
J9 INT J NANOMED
JI Int. J. Nanomed.
PY 2023
VL 18
BP 2261
EP 2273
DI 10.2147/IJN.S408521
PG 13
WC Nanoscience & Nanotechnology; Pharmacology & Pharmacy
WE Science Citation Index Expanded (SCI-EXPANDED)
SC Science & Technology - Other Topics; Pharmacology & Pharmacy
GA F8ZZ1
UT WOS:000985187300001
PM 37159807
OA Green Published, gold
DA 2023-09-14
ER

PT J
AU Khojini, JY
Babaei, B
Shakarami, M
Mofidi, M
Tahershamsi, Z
Arjmand, TF
Tajbakhsh, A
Gheibihayat, SM
AF Khojini, Javad Yaghmoorian
Babaei, Benjamin
Shakarami, Maryam
Mofidi, Mahdis
Tahershamsi, Zahra
Arjmand, Tahura Fayeghi

Tajbakhsh, Amir
Gheibihayat, Seyed Mohammad
TI Biomimetic Nanovaccines: A Novel Approach in Immunization
SO CURRENT PHARMACEUTICAL DESIGN
LA English
DT Review
DE Biomimetic nanoparticles; nanovaccines; immunization; conventional vaccines; vaccine delivery; nano-based drug delivery system; adjuvants; cancer cell membranes
ID OUTER-MEMBRANE VESICLES; VIRUS-LIKE PARTICLES; POLYMERIC NANOPARTICLES; TARGETED THERAPY; DRUG-DELIVERY; MESSENGER-RNA; GOLD NANOPARTICLES; SUBUNIT VACCINES; IMMUNE-RESPONSE; CHOLERA-TOXIN
AB As the World Health Organization (WHO) declared, vaccines prevent an average of 2-3 million deaths yearly from diseases. However, effective prophylactic and therapeutic vaccines have yet to be developed for eradicating the deadliest diseases, viz., types of cancer, malaria, human immunodeficiency virus (HIV), and most serious microbial infections. Furthermore, scores of the existing vaccines have disadvantages, such as failure to completely stimulate the immune system, in vivo instability, high toxicity, need for the cold chain, and multiple administrations. Thus, good vaccine candidates need to be designed to elicit adaptive immune responses. In this line, the integration of sciences along with the use of various technologies has led to the emergence of a new field in vaccine production called biomimetic nanovaccines (BNVs). Given that, nanotechnology can significantly contribute to the design of such vaccines, providing them with enhanced specificity and potency. Nanoparticles (NPs) and biomimetic NPs (BNPs) are now exploited as the main carriers for drug delivery systems, especially BNPs, whose biological mimicry makes them escape the immune system and transport drugs to the desired target. The drug accordingly seeks to camouflage itself with the help of NPs and the membranes taken from cells in the human body, including red blood cells (RBCs), white blood cells (WBCs), platelets, and cancer cells, for more effective and ideal delivery. As BNPs have recently become the center of attention in vaccine design, this review deliberates on the advances in BNVs.
C1 [Khojini, Javad Yaghmoorian] Shahid Sadoughi Univ Med Sci, Sch Med, Dept Med Biotechnol, Yazd, Iran.
[Babaei, Benjamin] North Khorasan Univ Med Sci, Sch Med, Dept Adv Technol, Bojnurd, Iran.
[Shakarami, Maryam] Islamic Azad Univ, Fac Sci, Dept Biol, East Tehran Branch, Tehran, Iran.
[Mofidi, Mahdis] Shahid Beheshti Univ, Fac Life Sci & Biotechnol, Dept Microbiol & Microbial Biotechnol, Tehran, Iran.
[Tahershamsi, Zahra] Tarbiat Modares Univ, Fac Biol Sci, Dept Biophys, Tehran, Iran.
[Arjmand, Tahura Fayeghi] Tabriz Univ Med Sci, Fac Adv Med Sci, Dept Med Biotechnol, Tabriz, Iran.
[Tajbakhsh, Amir] Shiraz Univ Med Sci, Pharmaceut Sci Res Ctr, Shiraz, Iran.
[Tajbakhsh, Amir] Shiraz Univ Med Sci, Sch Adv Med Sci & Technol, Dept Mol Med, Shiraz, Iran.
[Gheibihayat, Seyed Mohammad] Shahid Sadoughi Univ Med Sci, Sch Med, Dept Med Biotechnol, Yazd, Iran.
[Gheibihayat, Seyed Mohammad] Universal Sci Educ & Res Network USERN, Network Immun Infect Malignancy & Autoimmun NIIMA, Munich, Germany.
C3 North Khorasan University of Medical Sciences; Islamic Azad University; Shahid Beheshti University; Tarbiat Modares University; Tabriz University of Medical Science; Tehran University of Medical Sciences; Shiraz University of Medical Science; Shiraz University of Medical Science; Universal Scientific Education & Research Network (USERN)
RP Gheibihayat, SM (通讯作者)，Shahid Sadoughi Univ Med Sci, Sch Med, Dept Med Biotechnol, Yazd, Iran.; Gheibihayat, SM (通讯作者)，Universal Sci Educ & Res Network USERN, Network Immun Infect Malignancy & Autoimmun NIIMA, Munich, Germany.
EM gheibi65@yahoo.com
RI Yaghmoorian Khojini, Javad/AEN-4898-2022; Gheibihayat, Seyed Mohammad ./T-7187-2017; Tajbakhsh, Amir/HMV-4969-2023
OI Yaghmoorian Khojini, Javad/0000-0003-3445-7984; Gheibihayat, Seyed Mohammad ./0000-0002-1378-118X; Fayeghi, Tahura/0000-0001-6207-6674; Tahershamsi, Zahra/0000-0002-4407-0209; Tajbakhsh, Amir/0000-0002-2311-6554; Babaei, Benjamin/0000-0001-7455-1460
CR ABRAHAM E, 1992, VACCINE, V10, P461, DOI 10.1016/0264-410X(92)90395-Z
Al-Dulaimi KAK., 2018, MICROSCOPY SCI LAST
Al-Halifa S, 2019, FRONT IMMUNOL, V10, DOI 10.3389/fimmu.2019.00022
ALVING CR, 1986, VACCINE, V4, P166, DOI 10.1016/0264-410X(86)90005-8

Amina SJ, 2020, INT J NANOMED, V15, P9823, DOI 10.2147/IJN.S279094
Antonio M, 2018, NANOMATERIALS-BASEL, V8, DOI 10.3390/nano8040200
Arora S, 2012, TOXICOL APPL PHARM, V258, P151, DOI 10.1016/j.taap.2011.11.010
Badgett MR, 2002, J VIROL, V76, P10524, DOI 10.1128/JVI.76.20.10524-10529.2002
Bai SF, 2009, INT J BIOL MACROMOL, V45, P80, DOI 10.1016/j.ijbiomac.2009.04.008
Ball JM, 1999, GASTROENTEROLOGY, V117, P40, DOI 10.1016/S0016-5085(99)70548-2
BANGHAM AD, 1965, J MOL BIOL, V13, P253, DOI 10.1016/S0022-2836(65)80094-8
Baxter D, 2007, OCCUP MED-OXFORD, V57, P552, DOI 10.1093/occmed/kqm110
Beh CY, 2021, MOLECULES, V26, DOI 10.3390/molecules26165052
Behzadi M, 2021, EUR J PHARM SCI, V159, DOI 10.1016/j.ejps.2021.105718
Ben-Akiva E, 2020, SCI ADV, V6, DOI 10.1126/sciadv.aay9035
Benko A, 2021, CANCER DRUG RESIST, V4, P264, DOI 10.20517/cdr.2020.81
Bielinska AU, 2007, INFECT IMMUN, V75, P4020, DOI 10.1128/IAI.00070-07
Bishop AL, 2012, J INFECT DIS, V205, P412, DOI 10.1093/infdis/jir756
Bishop CJ, 2015, J CONTROL RELEASE, V219, P488, DOI 10.1016/j.jconrel.2015.09.046
Bisso S, 2020, INT J PHARMACEUT, V578, DOI 10.1016/j.ijpharm.2020.119098
Cai JX, 2022, INT J NANOMED, V17, P837, DOI 10.2147/IJN.S346685
Caillet C, 2010, VACCINE, V28, P3076, DOI 10.1016/j.vaccine.2010.02.050
Candas-Green D, 2020, NAT COMMUN, V11, DOI 10.1038/s41467-020-18245-7
Chatzikleanthous D, 2021, MOL PHARMACEUT, V18, P2867, DOI 10.1021/acs.molpharmaceut.1c00447
Chen H, 2018, INT J NANOMED, V13, P5347, DOI 10.2147/IJN.S165109
Chen I, 2005, NAT METHODS, V2, P99, DOI 10.1038/NMETH735
Chen PF, 2016, HUM VACC IMMUNOTHER, V12, P612, DOI 10.1080/21645515.2015.1105415
Chen Q, 2011, CANCER CELL, V20, P538, DOI 10.1016/j.ccr.2011.08.025
Chen XN, 2022, SIGNAL TRANSDUCT TAR, V7, DOI 10.1038/s41392-022-00942-y
Chen Z, 2016, ACS NANO, V10, P10049, DOI 10.1021/acsnano.6b04695
Cheuk DKL, 2011, COCHRANE DB SYST REV, DOI 10.1002/14651858.CD006505.pub2
Cloutier N, 2018, P NATL ACAD SCI USA, V115, pE1550, DOI 10.1073/pnas.1720553115
Dahlberg CIM, 2015, FRONT IMMUNOL, V6, DOI 10.3389/fimmu.2015.00605
Dai J, 2022, MATER TODAY BIO, V15, DOI 10.1016/j.mtbio.2022.100279
Dai LP, 2020, CELL, V182, P722, DOI 10.1016/j.cell.2020.06.035
Dai X, 2019, VACCINES HIST FUTURE, DOI [10.5772/intechopen.84626, DOI 10.5772/INTECHOPEN.84626]
Das I, 2017, NANOTECHNOLOGY, V28, DOI 10.1088/1361-6528/aa60fd
De Pasquale D, 2020, MATER DESIGN, V192, DOI 10.1016/j.matdes.2020.108742
Delany I, 2014, EMBO MOL MED, V6, P708, DOI 10.1002/emmm.201403876
Diaz-Arevalo D, 2020, NANOPHARMACEUTICALS, VOL. 1, P135, DOI 10.1016/B978-0-12-817778-5.00007-5
Dumpa N, 2019, AAPS PHARMSCITECH, V20, DOI 10.1208/s12249-018-1254-2
Ebermeyer T, 2021, INT J MOL SCI, V22, DOI 10.3390/ijms22157894
Ebert D, 1998, SCIENCE, V282, P1432, DOI 10.1126/science.282.5393.1432
Ernst LM, 2021, NANOMATERIALS-BASEL, V11, DOI 10.3390/nano11112991
Fang RH, 2018, ADV MATER, V30, DOI 10.1002/adma.201706759
Fang RNH, 2013, NANOSCALE, V5, P8884, DOI 10.1039/c3nr03064d
Feng C, 2022, ACTA PHARM SIN B, V12, P2206, DOI [10.1016/j.apsb.2021.12.0212211-3835, 10.1016/j.apsb.2021.12.021]
Feng GZ, 2013, PLOS ONE, V8, DOI 10.1371/journal.pone.0061135
Foged C, 2011, THER DELIV, V2, P1057, DOI 10.4155/TDE.11.68
Fotin-Mleczek M, 2012, J GENE MED, V14, P428, DOI 10.1002/jgm.2605
Fotin-Mleczek M, 2011, J IMMUNOTHER, V34, P1, DOI 10.1097/CJI.0b013e3181f7dbe8
Geldmacher A, 2005, VACCINE, V23, P3973, DOI 10.1016/j.vaccine.2005.02.025
Gianazza E, 2020, INT J MOL SCI, V21, DOI 10.3390/ijms21124541
Glenny AT, 1926, J PATHOL BACTERIOL, V29, P31, DOI 10.1002/path.1700290106
Goodman JL, 2020, JAMA-J AM MED ASSOC, V324, P2027, DOI 10.1001/jama.2020.20590
Guerrini G, 2022, NAT NANOTECHNOL, V17, P570, DOI 10.1038/s41565-022-01129-w
Guido C, 2020, BIOENGINEERING-BASEL, V7, DOI 10.3390/bioengineering7030111
Guo YY, 2015, ACS NANO, V9, P6918, DOI 10.1021/acsnano.5b01042
Gupta R, 2023, FRONT IMMUNOL, V14, DOI 10.3389/fimmu.2023.1123805
Halwani AA, 2022, PHARMACEUTICS, V14, DOI 10.3390/pharmaceutics14010106
Han HJ, 2022, EUR J PHARM BIOPHARM, V172, P1, DOI 10.1016/j.ejpb.2022.01.004
Han X, 2019, SCI ADV, V5, DOI 10.1126/sciadv.aaw6870

Han YT, 2019, ADV SCI, V6, DOI 10.1002/advs.201900251
Hanley Kathryn A, 2011, Evolution (N Y), V4, P635
Harish V, 2022, NANOMATERIALS-BASEL, V12, DOI 10.3390/nano12030457
Hayat SMG, 2020, CELL ONCOL, V43, P19, DOI 10.1007/s13402-019-00469-5
Hayat SMG, 2019, J CELL PHYSIOL, V234, P12530, DOI 10.1002/jcp.28120
Heaton PM, 2020, FRONT IMMUNOL, V11, DOI 10.3389/fimmu.2020.517290
HogenEsch H, 2002, VACCINE, V20, pS34, DOI 10.1016/S0264-410X(02)00169-X
Holstein SA, 2020, CLIN PHARMACOL THER, V107, P112, DOI 10.1002/cpt.1674
Hottz ED, 2022, PLATELETS, V33, P200, DOI 10.1080/09537104.2021.1952179
Hu CMJ, 2011, P NATL ACAD SCI USA, V108, P10980, DOI 10.1073/pnas.1106634108
Hu QY, 2015, ADV MATER, V27, P7043, DOI 10.1002/adma.201503323
Hu XP, 2020, FRONT BIOENG BIOTECH, V8, DOI 10.3389/fbioe.2020.00990
Hwang J, 2015, INT J NANOMED, V10, P5701, DOI 10.2147/IJN.S83642
Ibaraki H, 2022, SENSOR MATER, V34, P987, DOI 10.18494/SAM3673
Idris S.A., 2022, INDONESIAN J MED LAB, V4, P71, DOI [10.33086/ijmlst.v4i1.2105, DOI 10.33086/IJMLST.V4I1.2105]
Jiang ZW, 2017, FRONT IMMUNOL, V7, DOI 10.3389/fimmu.2016.00690
Jiao XX, 2022, CURR PHARM BIOTECHNO, V23, P835, DOI 10.2174/1389201022666210719164538
Jin JF, 2020, FRONT ONCOL, V9, DOI 10.3389/fonc.2019.01560
Jing LJ, 2018, THERANOSTICS, V8, P2683, DOI 10.7150/thno.23654
Kamath AT, 2009, PLOS ONE, V4, DOI 10.1371/journal.pone.0005771
Kariko K, 2004, J BIOL CHEM, V279, P12542, DOI 10.1074/jbc.M310175200
Karlsson J, 2018, ANNU REV CHEM BIOMOL, V9, P105, DOI 10.1146/annurev-chembioeng-060817-084055
Kaushik S, 2019, HDB POLYM CERAMIC NA, P1
Khan I, 2019, ARAB J CHEM, V12, P908, DOI 10.1016/j.arabjc.2017.05.011
Khan Kishwar Hayat, 2013, Germs, V3, P26, DOI 10.11599/germs.2013.1034
Kheirollahpour Mehdi, 2020, Pharmaceutical Nanotechnology, V8, P6, DOI 10.2174/2211738507666191024162042
Kim MG, 2014, ASIAN J PHARM SCI, V9, P227, DOI 10.1016/j.ajps.2014.06.002
KREUTER J, 1988, J MICROENCAPSUL, V5, P115, DOI 10.3109/02652048809056475
Krishnamurthy S, 2016, NANOSCALE, V8, P6981, DOI 10.1039/c5nr07588b
Langer HF, 2007, ARTERIOSCL THROM VAS, V27, P1463, DOI 10.1161/ATVBAHA.107.141515
Lappann M, 2013, J BACTERIOL, V195, P4425, DOI 10.1128/JB.00625-13
Li LL, 2021, ARCH ORAL BIOL, V124, DOI 10.1016/j.archoralbio.2021.105064
Li S, 1998, GENE THER, V5, P930, DOI 10.1038/sj.gt.3300683
Li XD, 2013, ONCOTARGETS THER, V6, P629, DOI 10.2147/OTT.S41902
Liang ZH, 2020, FRONT IMMUNOL, V11, DOI 10.3389/fimmu.2020.589833
Lindblad EB, 2004, VACCINE, V22, P3658, DOI 10.1016/j.vaccine.2004.03.032
Liu Y., 2022, EUR J PHARM BIOPHARM
Liu YC, 2017, SEMIN IMMUNOL, V34, P25, DOI 10.1016/j.smim.2017.09.011
Lobo GCNB, 2021, PHARMACEUTICS, V13, DOI 10.3390/pharmaceutics13081167
Lombardo D, 2022, PHARMACEUTICS, V14, DOI 10.3390/pharmaceutics14030543
Longmire M, 2008, NANOMEDICINE-UK, V3, P703, DOI 10.2217/17435889.3.5.703
Luk BT, 2014, NANOSCALE, V6, P2730, DOI 10.1039/c3nr06371b
Ma WJ, 2020, THERANOSTICS, V10, P1281, DOI 10.7150/thno.40291
Mahalingam M, 2015, ADV PHARM BULL, V5, P57, DOI 10.5681/apb.2015.008
Mahmoud K, 2022, J NANOBIOTECHNOL, V20, DOI 10.1186/s12951-022-01309-9
Makela P Helena, 2002, Expert Rev Vaccines, V1, P399, DOI 10.1586/14760584.1.3.399
Makidon PE, 2010, MED MICROBIOL IMMUN, V199, P81, DOI 10.1007/s00430-009-0137-2
Mancini F, 2021, FRONT IMMUNOL, V12, DOI 10.3389/fimmu.2021.715393
Micoli F, 2020, VACCINES-BASEL, V8, DOI 10.3390/vaccines8030540
Micoli F, 2018, P NATL ACAD SCI USA, V115, P10428, DOI 10.1073/pnas.1807655115
Milan A, 2022, INT J MOL SCI, V23, DOI 10.3390/ijms23031140
Mills JA, 2022, BIOMATER SCI-UK, V10, P3029, DOI 10.1039/d2bm00181k
Minor PD, 2015, VIROLOGY, V479, P379, DOI 10.1016/j.virol.2015.03.032
Mitchell MJ, 2021, NAT REV DRUG DISCOV, V20, P101, DOI 10.1038/s41573-020-0090-8
Mohanraj V. J., 2006, Tropical Journal of Pharmaceutical Research, V5, P561
Mohsen MO, 2017, SEMIN IMMUNOL, V34, P123, DOI 10.1016/j.smim.2017.08.014
Moon JJ, 2012, P NATL ACAD SCI USA, V109, P1080, DOI 10.1073/pnas.1112648109
Morrell CN, 2007, AM J TRANSPLANT, V7, P2447, DOI 10.1111/j.1600-6143.2007.01958.x

Moyle PM, 2013, CHEMMEDCHEM, V8, P360, DOI 10.1002/cmdc.201200487
Murata Y, 2014, J BIOCHEM, V155, P335, DOI 10.1093/jb/mvu017
Nascimento IP, 2012, BRAZ J MED BIOL RES, V45, P1102, DOI 10.1590/S0100-879X2012007500142
Nathanael AJ, 2020, POLYMERS-BASEL, V12, DOI 10.3390/polym12123061
Nienhaus K, 2020, MATER TODAY ADV, V5, DOI 10.1016/j.mtadv.2019.100036
Niza E, 2021, CANCERS, V13, DOI 10.3390/cancers13143387
Nunes D, 2022, POLYMERS-BASEL, V14, DOI 10.3390/polym14051010
Oliu-Barton M, 2022, LANCET GLOB HEALTH, V10, pE142, DOI 10.1016/S2214-109X(21)00494-0
Ong GH, 2021, FRONT CELL INFECT MI, V11, DOI 10.3389/fcimb.2021.745016
Opie EL, 1937, J EXP MED, V66, P761, DOI 10.1084/jem.66.6.761
Oroojalian F, 2021, SMALL, V17, DOI 10.1002/smll.202006484
Orr MT, 2019, NPJ VACCINES, V4, DOI 10.1038/s41541-018-0094-0
PARHAM P, 2014, IMMUNE SYSTEM
Parodi A, 2013, NAT NANOTECHNOL, V8, P61, DOI 10.1038/nnano.2012.212
Pati R, 2018, FRONT IMMUNOL, V9, DOI 10.3389/fimmu.2018.02224
Pereira-Silva M, 2021, EXPERT OPIN DRUG DEL, V18, P1395, DOI 10.1080/17425247.2021.1922387
Pierscionek BK, 2010, NANOTECHNOLOGY, V21, DOI 10.1088/0957-4484/21/3/035102
Pitchaimani A, 2018, BIOMATERIALS, V160, P124, DOI 10.1016/j.biomaterials.2018.01.018
Plotkin S.A., 2012, VACCINES E BOOK
Pollard AJ, 2021, NAT REV IMMUNOL, V21, P83, DOI 10.1038/s41577-020-00479-7
Popp MW, 2007, NAT CHEM BIOL, V3, P707, DOI 10.1038/nchembio.2007.31
Prego C, 2010, VACCINE, V28, P2607, DOI 10.1016/j.vaccine.2010.01.011
Probst Jochen, 2006, Genet Vaccines Ther, V4, P4, DOI 10.1186/1479-0556-4-4
Rao M, 2013, TISSUE ENG REGEN MED, V10, P223, DOI 10.1007/s13770-013-1081-1
Raso MM, 2020, VACCINES-BASEL, V8, DOI 10.3390/vaccines8020160
Reddy ST, 2007, NAT BIOTECHNOL, V25, P1159, DOI [10.1038/nbt1332, 10.1038/nbtl332]
Richardson CD, 2022, LANCET, V399, P3, DOI 10.1016/S0140-6736(21)02757-4
Robinson HL, 2000, ADV VIRUS RES, V55, P1, DOI 10.1016/S0065-3527(00)55001-5
Rosenthal JA, 2014, CURR OPIN BIOTECH, V28, P51, DOI 10.1016/j.copbio.2013.11.005
Sackstein R, 2008, NAT MED, V14, P181, DOI 10.1038/nm1703
Sadek SH, 2021, MATERIALS, V14, DOI 10.3390/ma14092451
Sahu T, 2021, J DRUG DELIV SCI TEC, V63, DOI 10.1016/j.jddst.2021.102487
Sanders B., 2014, VACCINE ANAL STRATEG, P45, DOI [10.1007/978-3-662-45024-6_2, DOI 10.1007/978-3-662-45024-6_2]
Savina A, 2007, IMMUNOL REV, V219, P143, DOI 10.1111/j.1600-065X.2007.00552.x
Saxena, 2015, APPL MICROBIOL, P173, DOI [10.1007/978-81-322-2259-0_11, DOI 10.1007/978-81-322-2259-0_11]
Scheel B, 2005, EUR J IMMUNOL, V35, P1557, DOI 10.1002/eji.200425656
Schwechheimer C, 2015, NAT REV MICROBIOL, V13, P605, DOI 10.1038/nrmicro3525
Seth L, 2017, VACCINE, V35, P5448, DOI 10.1016/j.vaccine.2017.02.040
Sharpe SW, 2011, INFECT IMMUN, V79, P4361, DOI 10.1128/IAI.05332-11
Shen YB, 2022, CELL PROLIFERAT, V55, DOI 10.1111/cpr.13192
Shimizu H, 2004, J VIROL, V78, P13512, DOI 10.1128/JVI.78.24.13512-13521.2004
Slifka MK, 2014, VACCINE, V32, P2948, DOI 10.1016/j.vaccine.2014.03.078
Stauffer Fausto, 2006, Recent Pat Antiinfect Drug Discov, V1, P291, DOI 10.2174/157489106778777673
Steinke JW, 2004, IMMUNOL ALLERGY CLIN, V24, P599, DOI 10.1016/j.iac.2004.06.008
Sun HP, 2016, ADV MATER, V28, P9581, DOI 10.1002/adma.201602173
Tao WQ, 2015, VACCINE, V33, P2307, DOI 10.1016/j.vaccine.2015.03.063
Tariq H, 2022, FRONT MICROBIOL, V12, DOI 10.3389/fmicb.2021.790121
Tian H, 2017, ADV FUNCT MATER, V27, DOI 10.1002/adfm.201703197
Tian W, 2019, POLYM ADVAN TECHNOL, V30, P1051, DOI 10.1002/pat.4538
Tretyakova I, 2013, VACCINE, V31, P1019, DOI 10.1016/j.vaccine.2012.12.050
Tseng CT, 2012, PLOS ONE, V7, DOI 10.1371/journal.pone.0035421
Tyler M, 2014, BIOTECHNOL BIOENG, V111, P2398, DOI 10.1002/bit.25311
Ulmer JB, 2012, VACCINE, V30, P4414, DOI 10.1016/j.vaccine.2012.04.060
Vartak A, 2016, VACCINES-BASEL, V4, DOI 10.3390/vaccines4020012
Vetter V, 2018, ANN MED, V50, P110, DOI 10.1080/07853890.2017.1407035
Vijayan V, 2019, PHARMACEUTICS, V11, DOI 10.3390/pharmaceutics11100534
Vincy A, 2022, FRONT CHEM, V10, DOI 10.3389/fchem.2022.905256
von Hoegen P, 2001, ADV DRUG DELIVER REV, V51, P113, DOI 10.1016/S0169-409X(01)00175-2

Wang F, 2016, ADV FUNCT MATER, V26, P1628, DOI 10.1002/adfm.201505231
Wang JJ, 2011, INT J NANOMED, V6, P765, DOI 10.2147/IJN.S17296
Wang LS, 2015, NAT COMMUN, V6, DOI 10.1038/ncomms8712
Wang Q, 2015, ADV DRUG DELIVER REV, V91, P125, DOI 10.1016/j.addr.2014.12.003
Wilkins AL, 2017, FRONT IMMUNOL, V8, DOI 10.3389/fimmu.2017.01760
Xie XP, 2017, SCI REP-UK, V7, DOI 10.1038/s41598-017-04229-z
Xu JC, 2020, ADV MATER, V32, DOI 10.1002/adma.201905145
Xu LT, 2022, ADV NANOBIOMED RES, V2, DOI 10.1002/anbr.202100109
Xu LG, 2012, NANO LETT, V12, P2003, DOI 10.1021/nl300027p
Yadav D.K., 2020, ANIM BIOTECHNOL, P523, DOI DOI 10.1016/B978-0-12-811710-1.00024-0
Yadav HKS, 2018, J DRUG DELIV SCI TEC, V44, P380, DOI 10.1016/j.jddst.2018.01.015
Yang GY, 2019, FRONT PHARMACOL, V10, DOI 10.3389/fphar.2019.00751
Yang R, 2018, ACS NANO, V12, P5121, DOI 10.1021/acsnano.7b09041
Zhang L, 2018, ACS NANO, V12, P10201, DOI 10.1021/acsnano.8b05200
Zhang ML, 2020, DRUG DES DEV THER, V14, P5495, DOI 10.2147/DDDT.S282368
Zhang MH, 2021, CLIN TRANSL MED, V11, DOI 10.1002/ctm2.292
Zhang NR, 2022, VIRUSES-BASEL, V14, DOI 10.3390/v14020387
Zhang WT, 2020, FRONT IMMUNOL, V11, DOI 10.3389/fimmu.2020.00018
Zhang Z, 2018, INT J NANOMED, V13, P4961, DOI 10.2147/IJN.S170148
Zhang Z, 2017, INT J NANOMED, V12, P1593, DOI 10.2147/IJN.S127256
Zhao CY, 2022, APPL MATER TODAY, V27, DOI 10.1016/j.apmt.2022.101412
Zhao K, 2012, PLOS ONE, V7, DOI 10.1371/journal.pone.0053314
Zhao L, 2014, VACCINE, V32, P327, DOI 10.1016/j.vaccine.2013.11.069
Zhao WF, 2007, VACCINE, V25, P7664, DOI 10.1016/j.vaccine.2007.08.034
Zhao XF, 2022, NANOSCALE RES LETT, V17, DOI 10.1186/s11671-022-03673-9
Zheng B, 2021, CHEM ENG J, V418, DOI 10.1016/j.cej.2021.129392
Zhou H, 2016, THERANOSTICS, V6, P1012, DOI 10.7150/thno.15095
Zhou SL, 2020, BIOMATERIALS, V235, DOI 10.1016/j.biomaterials.2020.119795
Zou J, 2018, EBIOMEDICINE, V36, P92, DOI 10.1016/j.ebiom.2018.08.056
NR 207
TC 0
Z9 0
U1 0
U2 0
PU BENTHAM SCIENCE PUBL LTD
PI SHARJAH
PA EXECUTIVE STE Y-2, PO BOX 7917, SAIF ZONE, 1200 BR SHARJAH, U ARAB
EMIRATES
SN 1381-6128
EI 1873-4286
J9 CURR PHARM DESIGN
JI Curr. Pharm. Design
PY 2023
VL 29
IS 18
BP 1391
EP 1408
DI 10.2174/1381612829666230529094128
PG 18
WC Pharmacology & Pharmacy
WE Science Citation Index Expanded (SCI-EXPANDED)
SC Pharmacology & Pharmacy
GA N5LN1
UT WOS:001037425900001
PM 37254540
DA 2023-09-14
ER

PT J
AU Makandar, AI
Jain, M

Yuba, E
Sethi, G
Gupta, RK
AF Makandar, Amina I.
Jain, Mannat
Yuba, Eiji
Sethi, Gautam
Gupta, Rajesh Kumar
TI Canvassing Prospects of Glyco-Nanovaccines for Developing
Cross-Presentation Mediated Anti-Tumor Immunotherapy
SO VACCINES
LA English
DT Review
DE lectin; galectin; vaccine; antigen cross-presentation; dendritic cells;
C-type lectin receptors; glycan; CD8(+) T lymphocytes; cancer
immunotherapy
ID C-TYPE LECTIN; T-CELL RESPONSES; MHC-CLASS-I; TOLL-LIKE RECEPTORS;
PROLONGED ANTIGEN PRESENTATION; TARGETING DC-SIGN; DENDRITIC CELLS;
STEADY-STATE; MANNOSE RECEPTOR; NANOPARTICLE VACCINES
AB In view of the severe downsides of conventional cancer therapies, the quest of developing alternative strategies still remains of critical importance. In this regard, antigen cross-presentation, usually employed by dendritic cells (DCs), has been recognized as a potential solution to overcome the present impasse in anti-cancer therapeutic strategies. It has been established that an elevated cytotoxic T lymphocyte (CTL) response against cancer cells can be achieved by targeting receptors expressed on DCs with specific ligands. Glycans are known to serve as ligands for C-type lectin receptors (CLRs) expressed on DCs, and are also known to act as a tumor-associated antigen (TAA), and, thus, can be harnessed as a potential immunotherapeutic target. In this scenario, integrating the knowledge of cross-presentation and glycan-conjugated nanovaccines can help us to develop so called 'glyco-nanovaccines' (GNVs) for targeting DCs. Here, we briefly review and analyze the potential of GNVs as the next-generation anti-tumor immunotherapy. We have compared different antigen-presenting cells (APCs) for their ability to cross-present antigens and described the potential nanocarriers for tumor antigen cross-presentation. Further, we discuss the role of glycans in targeting of DCs, the immune response due to pathogens, and imitative approaches, along with parameters, strategies, and challenges involved in cross-presentation-based GNVs for cancer immunotherapy. It is known that the effectiveness of GNVs in eradicating tumors by inducing strong CTL response in the tumor microenvironment (TME) has been largely hindered by tumor glycosylation and the expression of different lectin receptors (such as galectins) by cancer cells. Tumor glycan signatures can be sensed by a variety of lectins expressed on immune cells and mediate the immune suppression which, in turn, facilitates immune evasion. Therefore, a sound understanding of the glycan language of cancer cells, and glycan-lectin interaction between the cancer cells and immune cells, would help in strategically designing the next-generation GNVs for anti-tumor immunotherapy.
C1 [Makandar, Amina I.; Jain, Mannat; Gupta, Rajesh Kumar] Dr DY Patil Vidyapeeth, Dr DY Patil Biotechnol & Bioinformat Inst, Prot Biochem Res Ctr, Pune 411033, Maharashtra, India.
[Yuba, Eiji] Osaka Metropolitan Univ, Grad Sch Engn, Dept Appl Chem, 1-1 Gakuen cho,Naka ku, Osaka 5998531, Japan.
[Sethi, Gautam] Natl Univ Singapore, Yong Loo Lin Sch Med, Dept Pharmacol, Singapore 117600, Singapore.
C3 Dr DY Patil Vidyapeeth Pune; Dr D Y Patil Biotechnology & Bioinformatics
Institute; National University of Singapore
RP Gupta, RK (通讯作者)，Dr DY Patil Vidyapeeth, Dr DY Patil Biotechnol & Bioinformat Inst, Prot Biochem Res Ctr, Pune 411033, Maharashtra, India.; Yuba, E (通讯作者)，Osaka Metropolitan Univ, Grad Sch Engn, Dept Appl Chem, 1-1 Gakuen cho,Naka ku, Osaka 5998531, Japan.; Sethi, G (通讯作者)，Natl Univ Singapore, Yong Loo Lin Sch Med, Dept Pharmacol, Singapore 117600, Singapore.
EM yuba@omu.ac.jp; phcgs@nus.edu.sg; rajeshkumar.gupta@dpu.edu.in
OI Eiji, Yuba/0000-0003-4984-2113
FU DST [DST/INT/JSPS/P-307/2020]; JSPS [DST/INT/JSPS/P-307/2020];
Department of Science and Technology, Science and Engineering Research
Board (DST-SERB), Government of India, under the ECRA scheme
[ECR/2016/001187]; Patil Vidyapeeth, Pune [DPU/17/2016]
FX The authors wish to express their gratitude to DST and JSPS for funding
under the India-Japan collaborative project (DST/INT/JSPS/P-307/2020),
Department of Science and Technology, Science and Engineering Research

Board (DST-SERB), Government of India, under the ECRA scheme (grant
number ECR/2016/001187), and by D.Y. Patil Vidyapeeth, Pune (grant
number DPU/17/2016) to Rajesh Kumar Gupta.
CR Abensee JEB, 2008, SEMIN IMMUNOL, V20, P101, DOI 10.1016/j.smim.2007.10.013
Affandi AJ, 2020, P NATL ACAD SCI USA, V117, P27528, DOI 10.1073/pnas.2006186117
Aguilar LK, 2011, J CELL BIOCHEM, V112, P1969, DOI 10.1002/jcb.23126
Ahmadi M, 2015, PHARM RES-DORDR, V32, P1383, DOI 10.1007/s11095-014-1541-x
Ahsan FL, 2002, J CONTROL RELEASE, V79, P29, DOI 10.1016/S0168-3659(01)00549-1
Ajay Vidyasagar KS, 2013, J PHYS CHEM BIOPHYS, V3, P2161
Akira S, 2006, CELL, V124, P783, DOI 10.1016/j.cell.2006.02.015
Akira S, 2001, NAT IMMUNOL, V2, P675, DOI 10.1038/90609
Alloatti A, 2016, IMMUNOL REV, V272, P97, DOI 10.1111/imr.12432
Anderluh M, 2021, FEBS J, V288, P4746, DOI 10.1111/febs.15830
Apostolopoulos V, 2003, P NATL ACAD SCI USA, V100, P15029, DOI 10.1073/pnas.2432220100
Apostolopoulos V, 2000, VACCINE, V18, P3174, DOI 10.1016/S0264-410X(00)00090-6
Apostolopoulos V, 2000, EUR J IMMUNOL, V30, P1714, DOI 10.1002/1521-
4141(200006)30:6<1714::AID-IMMU1714>3.0.CO;2-C
Arina A, 2002, EXP HEMATOL, V30, P1355, DOI 10.1016/S0301-472X(02)00956-6
Ashrafizadeh M, 2022, SEMIN IMMUNOL, V59, DOI 10.1016/j.smim.2022.101606
Ashrafizadeh M, 2019, CELLS-BASEL, V8, DOI 10.3390/cells8101158
Avci FY, 2011, NAT MED, V17, P1602, DOI 10.1038/nm.2535
Baar J, 1999, Oncologist, V4, P140
Bachem A, 2012, FRONT IMMUNOL, V3, DOI 10.3389/fimmu.2012.00214
Backer R, 2010, P NATL ACAD SCI USA, V107, P216, DOI 10.1073/pnas.0909541107
Baek SH, 2016, PHYTOMEDICINE, V23, P566, DOI 10.1016/j.phymed.2016.02.011
Baker GJ, 2014, CANCER RES, V74, P5079, DOI 10.1158/0008-5472.CAN-14-1203
Banchereau J, 1998, NATURE, V392, P245, DOI 10.1038/32588
Belizaire R, 2009, P NATL ACAD SCI USA, V106, P17463, DOI 10.1073/pnas.0908583106
Bevan MJ, 2005, J IMMUNOL, V175, P7069
Biricova V, 2009, BIOORG CHEM, V37, P185, DOI 10.1016/j.bioorg.2009.07.006
Bloem K, 2014, IMMUNOL LETT, V158, P33, DOI 10.1016/j.imlet.2013.11.007
Boks MA, 2015, J INVEST DERMATOL, V135, P2697, DOI 10.1038/jid.2015.226
Boks MA, 2015, J CONTROL RELEASE, V216, P37, DOI 10.1016/j.jconrel.2015.06.033
Boltjes A, 2014, FRONT IMMUNOL, V5, DOI 10.3389/fimmu.2014.00131
Bonifaz L, 2002, J EXP MED, V196, P1627, DOI 10.1084/jem.20021598
Bouteau A, 2019, FRONT IMMUNOL, V10, DOI 10.3389/fimmu.2019.01134
Brahmer JR, 2012, NEW ENGL J MED, V366, P2455, DOI 10.1056/NEJMoa1200694
Brown GD, 2006, NAT REV IMMUNOL, V6, P33, DOI 10.1038/nri1745
Brown GD, 2001, NATURE, V413, P36, DOI 10.1038/35092620
Burgdorf S, 2008, NAT IMMUNOL, V9, P558, DOI 10.1038/ni.1601
Burgdorf S, 2007, SCIENCE, V316, P612, DOI 10.1126/science.1137971
Caparros E, 2006, BLOOD, V107, P3950, DOI 10.1182/blood-2005-03-1252
Carter RW, 2006, J IMMUNOL, V177, P2276, DOI 10.4049/jimmunol.177.4.2276
Cella M, 1999, NAT MED, V5, P919, DOI 10.1038/11360
Chaitanya TS., 2022, NANOTHERAPEUTICS CAN, P349, DOI [10.1016/B978-0-12-823686-4.00015-X,
DOI 10.1016/B978-0-12-823686-4.00015-X]
Chattopadhyay Saborni, 2017, Nanotheranostics, V1, P244, DOI 10.7150/ntno.19796
Chen PF, 2016, HUM VACC IMMUNOTHER, V12, P612, DOI 10.1080/21645515.2015.1105415
Chi GY, 2022, INT J NANOMED, V17, P1567, DOI 10.2147/IJN.S337082
CHIBA M, 1990, J PHARM SCI, V79, P281, DOI 10.1002/jps.2600790402
Cho JH, 2001, J IMMUNOL, V167, P5549, DOI 10.4049/jimmunol.167.10.5549
Clayton K, 2017, FRONT IMMUNOL, V8, DOI 10.3389/fimmu.2017.01676
Collin M, 2018, IMMUNOLOGY, V154, P3, DOI 10.1111/imm.12888
Cruz FM, 2017, ANNU REV IMMUNOL, V35, P149, DOI 10.1146/annurev-immunol-041015-055254
Cruz LJ, 2017, NANOMEDICINE-UK, V12, P491, DOI 10.2217/nnm-2016-0295
Cullen SP, 2010, CELL DEATH DIFFER, V17, P616, DOI 10.1038/cdd.2009.206
Martinez JD, 2020, PHARMACEUTICALS-BASE, V13, DOI 10.3390/ph13080179
Dardalhon V, 2010, J IMMUNOL, V185, P1383, DOI 10.4049/jimmunol.0903275
Darvin P, 2018, EXP MOL MED, V50, DOI 10.1038/s12276-018-0191-1
de Jong JMH, 2006, MOL IMMUNOL, V43, P2045, DOI 10.1016/j.molimm.2006.01.002
de Jong MAWP, 2010, MOL IMMUNOL, V47, P1216, DOI 10.1016/j.molimm.2009.12.016

Dehshahri A, 2020, PHARMACOL RES, V151, DOI 10.1016/j.phrs.2019.104551
Delamarre L, 2005, SCIENCE, V307, P1630, DOI 10.1126/science.1108003
den Haan JMM, 2002, J EXP MED, V196, P817, DOI 10.1084/jem.20020295
Denda-Nagai K, 2010, J BIOL CHEM, V285, P19193, DOI 10.1074/jbc.M110.113613
Dhodapkar MV, 2008, CELL DEATH DIFFER, V15, P39, DOI 10.1038/sj.cdd.4402247
Drickamer K, 2015, CURR OPIN STRUC BIOL, V34, P26, DOI 10.1016/j.sbi.2015.06.003
Drummond RA, 2016, MUCOSAL IMMUNOL, V9, P492, DOI 10.1038/mi.2015.79
Duinkerken S, 2019, THERANOSTICS, V9, P5797, DOI 10.7150/thno.35059
Duncan R, 2006, NAT REV CANCER, V6, P688, DOI 10.1038/nrc1958
Engering A, 2002, J IMMUNOL, V168, P2118, DOI 10.4049/jimmunol.168.5.2118
Engering AJ, 1997, ADV EXP MED BIOL, V417, P183
FALO LD, 1995, NAT MED, V1, P649, DOI 10.1038/nm0795-649
Fasting C, 2012, ANGEW CHEM INT EDIT, V51, P10472, DOI 10.1002/anie.201201114
Fehres CM, 2017, CELL MOL IMMUNOL, V14, P360, DOI 10.1038/cmi.2015.87
Fehres CM, 2015, J CONTROL RELEASE, V203, P67, DOI 10.1016/j.jconrel.2015.01.040
Fehres CM, 2014, FRONT IMMUNOL, V5, DOI 10.3389/fimmu.2014.00149
Feinberg H, 2021, J BIOL CHEM, V296, DOI 10.1016/j.jbc.2021.100368
Feng SS, 2003, CHEM ENG SCI, V58, P4087, DOI 10.1016/S0009-2509(03)00234-3
Figdor CG, 2002, NAT REV IMMUNOL, V2, P77, DOI 10.1038/nri723
Flacher V, 2010, J INVEST DERMATOL, V130, P755, DOI 10.1038/jid.2009.343
Fritz JH, 2005, EUR J IMMUNOL, V35, P2459, DOI 10.1002/eji.200526286
Fuss M, 2008, LANGMUIR, V24, P5124, DOI 10.1021/la703716g
Gao YA, 2021, ADV FUNCT MATER, V31, DOI 10.1002/adfm.202105059
Garcia I, 2010, NANOMEDICINE-UK, V5, P777, DOI 10.2217/NNM.10.48
Garcia-Vallejo JJ, 2013, ONCOIMMUNOLOGY, V2, DOI 10.4161/onci.23040
Garcia-Vallejo JJ, 2013, MOL IMMUNOL, V53, P387, DOI 10.1016/j.molimm.2012.09.012
Gardner A, 2020, FRONT IMMUNOL, V11, DOI 10.3389/fimmu.2020.00924
Geijtenbeek TBH, 2004, ANNU REV IMMUNOL, V22, P33, DOI 10.1146/annurev.immunol.22.012703.104558
Glaffig M, 2018, CHEMMEDCHEM, V13, P25, DOI 10.1002/cmdc.201700646
Goedhart M, 2022, CELLS-BASEL, V11, DOI 10.3390/cells11010055
Goldberg AL, 2002, MOL IMMUNOL, V39, P147, DOI 10.1016/S0161-5890(02)00098-6
Gotwals P, 2017, NAT REV CANCER, V17, P286, DOI 10.1038/nrc.2017.17
Gringhuis SI, 2007, IMMUNITY, V26, P605, DOI 10.1016/j.immuni.2007.03.012
Guo Y, 2004, NAT STRUCT MOL BIOL, V11, P591, DOI 10.1038/nsmb784
Guo YH, 2020, CELL HOST MICROBE, V28, P104, DOI 10.1016/j.chom.2020.05.002
Gupta B, 2022, SEMIN CANCER BIOL, V80, P87, DOI 10.1016/j.semcancer.2020.02.005
Harshyne LA, 2001, J IMMUNOL, V166, P3717, DOI 10.4049/jimmunol.166.6.3717
Hawiger D, 2001, J EXP MED, V194, P769, DOI 10.1084/jem.194.6.769
Heath WR, 2004, IMMUNOL REV, V199, P9, DOI 10.1111/j.0105-2896.2004.00142.x
Heath WR, 2001, NAT REV IMMUNOL, V1, P126, DOI 10.1038/35100512
Heipertz EL, 2014, J IMMUNOL, V193, P4169, DOI 10.4049/jimmunol.1302565
Heit A, 2004, J IMMUNOL, V172, P1501, DOI 10.4049/jimmunol.172.3.1501
Henry S, 1998, J PHARM SCI, V87, P922, DOI 10.1021/js980042+
Hirosue S, 2014, J IMMUNOL, V192, P5002, DOI 10.4049/jimmunol.1302492
Holla A, 2011, PROTEIN ENG DES SEL, V24, P659, DOI 10.1093/protein/gzr016
Hon H, 2005, J IMMUNOL, V174, P5233, DOI 10.4049/jimmunol.174.9.5233
Hossain MK, 2019, CANCERS, V11, DOI 10.3390/cancers11030418
Iborra S, 2015, IMMUNOBIOLOGY, V220, P175, DOI 10.1016/j.imbio.2014.09.013
Ignacio BJ, 2018, BIOCONJUGATE CHEM, V29, P587, DOI 10.1021/acs.bioconjchem.7b00808
Ilarregui JM, 2009, NAT IMMUNOL, V10, P981, DOI 10.1038/ni.1772
Ito T, 2008, IMMUNITY, V28, P870, DOI 10.1016/j.immuni.2008.03.018
Iwama S, 2014, SCI TRANSL MED, V6, DOI 10.1126/scitranslmed.3008002
Jandus C, 2014, J CLIN INVEST, V124, P1810, DOI 10.1172/JCI65899
Jegouzo SAF, 2015, J BIOL CHEM, V290, P16759, DOI 10.1074/jbc.M115.660613
Joffre OP, 2012, NAT REV IMMUNOL, V12, P557, DOI 10.1038/nri3254
Johnson JL, 2013, TRENDS IMMUNOL, V34, P290, DOI 10.1016/j.it.2013.01.006
Joshi MD, 2012, J CONTROL RELEASE, V161, P25, DOI 10.1016/j.jconrel.2012.05.010
Joshi MD, 2011, INT J PHARMACEUT, V416, P426, DOI 10.1016/j.ijpharm.2011.02.055
Kamphorst AO, 2010, J IMMUNOL, V185, P3426, DOI 10.4049/jimmunol.1001205
Keir ME, 2008, ANNU REV IMMUNOL, V26, P677, DOI 10.1146/annurev.immunol.26.021607.090331

Khan S, 2007, J BIOL CHEM, V282, P21145, DOI 10.1074/jbc.M701705200
Khatoon E, 2022, LIFE SCI, V306, DOI 10.1016/j.lfs.2022.120827
Kim H, 2019, J PHARMACOL EXP THER, V370, P715, DOI 10.1124/jpet.118.254953
Kim R, 2006, CLIN EXP IMMUNOL, V146, P189, DOI 10.1111/j.1365-2249.2006.03215.x
Kirtonia A, 2021, SEMIN CANCER BIOL, V68, P258, DOI 10.1016/j.semcancer.2020.04.006
Klechevsky E, 2010, BLOOD, V116, P1685, DOI 10.1182/blood-2010-01-264960
Kou PM, 2011, J BIOMED MATER RES A, V96A, P239, DOI 10.1002/jbm.a.32971
KOVACSOVICSBANKOWSKI M, 1993, P NATL ACAD SCI USA, V90, P4942, DOI 10.1073/pnas.90.11.4942
Kurts C, 2000, J MOL MED, V78, P326, DOI 10.1007/s001090000108
Lehmann CHK, 2016, VACCINES-BASEL, V4, DOI 10.3390/vaccines4020008
Leon B, 2014, J EXP MED, V211, P1637, DOI 10.1084/jem.20131692
Lepenies B, 2013, ADV DRUG DELIVER REV, V65, P1271, DOI 10.1016/j.addr.2013.05.007
Li HY, 2011, NAT NANOTECHNOL, V6, P645, DOI [10.1038/nnano.2011.153, 10.1038/NNANO.2011.153]
Li K, 2020, CURR TOP MICROBIOL, V429, P1, DOI 10.1007/82_2020_198
Li M, 2001, J IMMUNOL, V166, P6099, DOI 10.4049/jimmunol.166.10.6099
Li RJE, 2018, CURR OPIN BIOTECH, V51, P24, DOI 10.1016/j.copbio.2017.11.003
Liu CB, 2018, INT J PHARMACEUT, V553, P327, DOI 10.1016/j.ijpharm.2018.10.054
Liu FT, 2005, NAT REV CANCER, V5, P29, DOI 10.1038/nrc1527
Liu LX, 2016, ACS APPL MATER INTER, V8, P11969, DOI 10.1021/acsami.6b01135
Liu Q, 2016, SMALL, V12, P1744, DOI 10.1002/smll.201503662
Ma ZW, 2022, SEMIN CANCER BIOL, V80, P379, DOI 10.1016/j.semcancer.2020.09.011
MACATONIA SE, 1995, J IMMUNOL, V154, P5071
Mahnke K, 2000, J CELL BIOL, V151, P673, DOI 10.1083/jcb.151.3.673
Maji M, 2016, SCI REP-UK, V6, DOI 10.1038/srep27206
Malaker SA, 2017, CANCER IMMUNOL RES, V5, P376, DOI 10.1158/2326-6066.CIR-16-0280
Manu KA, 2014, J MOL MED, V92, P267, DOI 10.1007/s00109-013-1095-0
Martin-Gayo E, 2010, BLOOD, V115, P5366, DOI 10.1182/blood-2009-10-248260
Martinez-Lostao L, 2015, CLIN CANCER RES, V21, P5047, DOI 10.1158/1078-0432.CCR-15-0685
Martinez-Pomares L, 2012, J LEUKOCYTE BIOL, V92, P1177, DOI 10.1189/jlb.0512231
Matheoud D, 2010, BLOOD, V115, P4412, DOI 10.1182/blood-2009-11-255935
Mayer CT, 2012, FRONT IMMUNOL, V3, DOI 10.3389/fimmu.2012.00183
McGreal EP, 2005, CURR OPIN IMMUNOL, V17, P18, DOI 10.1016/j.coi.2004.12.001
McKenzie EJ, 2007, J IMMUNOL, V178, P4975, DOI 10.4049/jimmunol.178.8.4975
Medzhitov R, 2001, NAT REV IMMUNOL, V1, P135, DOI 10.1038/35100529
Mendez-Huergo SP, 2017, CURR OPIN IMMUNOL, V45, P8, DOI 10.1016/j.coi.2016.12.003
Menon S, 2009, P NATL ACAD SCI USA, V106, P11524, DOI 10.1073/pnas.0901783106
Mestas J, 2004, J IMMUNOL, V172, P2731, DOI 10.4049/jimmunol.172.5.2731
Met O, 2003, CELL IMMUNOL, V222, P126, DOI 10.1016/S0008-8749(03)00128-X
Miller JC, 2012, NAT IMMUNOL, V13, P888, DOI 10.1038/ni.2370
Mirzaei S, 2021, CELLS-BASEL, V10, DOI 10.3390/cells10123348
Miyazaki M, 2019, ACS BIOMATER SCI ENG, V5, P5790, DOI 10.1021/acsbiomaterials.9b01278
Molino NM, 2013, ACS NANO, V7, P9743, DOI 10.1021/nn403085w
Mollaei H, 2019, J CELL PHYSIOL, V234, P12369, DOI 10.1002/jcp.28058
Moon JJ, 2012, P NATL ACAD SCI USA, V109, P1080, DOI 10.1073/pnas.1112648109
Moore C, 2022, JCI INSIGHT, V7, DOI 10.1172/jci.insight.159479
Mukhopadhaya A, 2008, P NATL ACAD SCI USA, V105, P6374, DOI 10.1073/pnas.0802644105
Muntjewerff EM, 2020, FRONT IMMUNOL, V11, DOI 10.3389/fimmu.2020.01276
Murakami R, 2013, PLOS ONE, V8, DOI 10.1371/journal.pone.0073270
Nandi S., 2021, LECTINS INNATE IMMUN, P215, DOI [10.1007/978-981-16-7462-4_11, DOI 10.1007/978-981-16-7462-4_11]
Nasery MM, 2020, MOLECULES, V25, DOI 10.3390/molecules25030689
NESTLE FO, 1993, J IMMUNOL, V151, P6535
Ni L, 2010, J IMMUNOL, V185, P3504, DOI 10.4049/jimmunol.1000999
Twilhaar MNK, 2022, FRONT IMMUNOL, V13, DOI 10.3389/fimmu.2022.842241
Nonaka M, 2011, J BIOL CHEM, V286, P22403, DOI 10.1074/jbc.M110.215301
Oberkampf M, 2018, NAT COMMUN, V9, DOI 10.1038/s41467-018-04686-8
Okubo M, 2019, BIOCONJUGATE CHEM, V30, P1518, DOI 10.1021/acs.bioconjchem.9b00221
Orth M, 2014, RADIAT ENVIRON BIOPH, V53, P1, DOI 10.1007/s00411-013-0497-2
Overwijk WW, 2013, J IMMUNOTHER CANCER, V1, DOI 10.1186/2051-1426-1-11

Palmer DH, 2009, HEPATOLOGY, V49, P124, DOI 10.1002/hep.22626
Panda AK, 2011, EXPERT REV VACCINES, V10, P155, DOI [10.1586/erv.10.164, 10.1586/ERV.10.164]
Papaioannou NE, 2016, ANN TRANSL MED, V4, DOI 10.21037/atm.2016.04.01
Park R, 2019, BRIEF FUNCT GENOMICS, V18, P133, DOI 10.1093/bfgp/ely027
Parmiani G, 2007, J IMMUNOL, V178, P1975, DOI 10.4049/jimmunol.178.4.1975
Paulis LE, 2013, CURR OPIN IMMUNOL, V25, P389, DOI 10.1016/j.coi.2013.03.001
Peer D, 2007, NAT NANOTECHNOL, V2, P751, DOI 10.1038/nnano.2007.387
Perdicchio M, 2016, ONCOTARGET, V7, P8771, DOI 10.18632/oncotarget.6822
Pereira CF, 2007, J IMMUNOTHER, V30, P705, DOI 10.1097/CJI.0b013e31812e6256
Perrin P, 2019, CURR OPIN IMMUNOL, V58, P1, DOI 10.1016/j.coi.2018.12.004
Pinho SS, 2015, NAT REV CANCER, V15, P540, DOI 10.1038/nrc3982
Pozzi LAM, 2005, J IMMUNOL, V175, P2071, DOI 10.4049/jimmunol.175.4.2071
Prausnitz MR, 2004, ADV DRUG DELIVER REV, V56, P581, DOI 10.1016/j.addr.2003.10.023
Pyz E, 2006, ANN MED, V38, P242, DOI 10.1080/07853890600608985
Quetard C, 1998, BIOCONJUGATE CHEM, V9, P268, DOI 10.1021/bc970122p
Qureshi OS, 2011, SCIENCE, V332, P600, DOI 10.1126/science.1202947
Rajendran P, 2011, CLIN CANCER RES, V17, P1425, DOI 10.1158/1078-0432.CCR-10-1123
Rajesh A, 2020, IMMUNOLOGY, V160, P366, DOI 10.1111/imm.13202
Ramasubramanian MK, 2008, BIOINSPIR BIOMIM, V3, DOI 10.1088/1748-3182/3/4/046001
Rapoport N, 2002, J PHARM SCI, V91, P157, DOI 10.1002/jps.10006
Rauen J, 2014, PLOS ONE, V9, DOI 10.1371/journal.pone.0103755
Ren A, 2021, J INFLAMM RES, V14, P63, DOI 10.2147/JIR.S287453
Reuven EM, 2019, ACS NANO, V13, P2936, DOI 10.1021/acsnano.8b07241
Rissoan MC, 1999, SCIENCE, V283, P1183, DOI 10.1126/science.283.5405.1183
Roche PA, 2015, NAT REV IMMUNOL, V15, P203, DOI 10.1038/nri3818
Rock KL, 2016, TRENDS IMMUNOL, V37, P724, DOI 10.1016/j.it.2016.08.010
ROCK KL, 1990, SCIENCE, V249, P918, DOI 10.1126/science.2392683
Rodriguez A, 1999, NAT CELL BIOL, V1, P362, DOI 10.1038/14058
Rodriguez E, 2018, NAT REV IMMUNOL, V18, P204, DOI 10.1038/nri.2018.3
Roy NK, 2019, INT J MOL SCI, V20, DOI 10.3390/ijms20174101
Rubinstein N, 2004, CANCER CELL, V5, P241, DOI 10.1016/S1535-6108(04)00024-8
Sakuishi K, 2011, TRENDS IMMUNOL, V32, P345, DOI 10.1016/j.it.2011.05.003
Sartorius R, 2011, EUR J IMMUNOL, V41, P2573, DOI 10.1002/eji.201141526
SATO Y, 1993, NEW ENGL J MED, V328, P1802, DOI 10.1056/NEJM199306243282502
Scatena R, 2008, EXPERT OPIN INV DRUG, V17, P1533, DOI 10.1517/13543784.17.10.1533
Schuurhuis DH, 2002, J IMMUNOL, V168, P2240, DOI 10.4049/jimmunol.168.5.2240
Segura E, 2015, ADV IMMUNOL, V127, P1, DOI 10.1016/bs.ai.2015.03.002
Segura E, 2009, P NATL ACAD SCI USA, V106, P20377, DOI 10.1073/pnas.0910295106
Sever R, 2015, CSH PERSPECT MED, V5, DOI 10.1101/cshperspect.a006098
Shanmugam MK, 2018, FRONT PHARMACOL, V9, DOI 10.3389/fphar.2018.01294
Shanmugam Muthu K, 2018, Oncotarget, V9, P11414, DOI 10.18632/oncotarget.23356
Shen H, 2006, IMMUNOLOGY, V117, P78, DOI 10.1111/j.1365-2567.2005.02268.x
Shi JJ, 2010, NANO LETT, V10, P3223, DOI 10.1021/nl102184c
Shin KS, 2019, FRONT IMMUNOL, V10, DOI 10.3389/fimmu.2019.01887
Shortman K, 2010, IMMUNOL REV, V234, P18, DOI 10.1111/j.0105-2896.2009.00870.x
Shrimpton RE, 2009, MOL IMMUNOL, V46, P1229, DOI 10.1016/j.molimm.2008.11.016
Singh SK, 2009, MOL IMMUNOL, V47, P164, DOI 10.1016/j.molimm.2009.09.026
Singh SK, 2009, MOL IMMUNOL, V46, P1240, DOI 10.1016/j.molimm.2008.11.021
Singh SS, 2015, WORLD J GASTROENTERO, V21, P12261, DOI 10.3748/wjg.v21.i43.12261
Song C, 2016, INT J NANOMED, V11, P3753, DOI 10.2147/IJN.S110796
Srinivas O, 2007, BIOCONJUGATE CHEM, V18, P1547, DOI 10.1021/bc070026g
Stambach NS, 2003, GLYCOBIOLOGY, V13, P401, DOI 10.1093/glycob/cwg045
Steinhagen F, 2011, VACCINE, V29, P3341, DOI 10.1016/j.vaccine.2010.08.002
Steinman RM, 2003, ANN NY ACAD SCI, V987, P15, DOI 10.1111/j.1749-6632.2003.tb06029.x
Stoitzner P, 2022, EUR J IMMUNOL, V52, P1909, DOI 10.1002/eji.202149515
Stolk DA, 2020, FRONT IMMUNOL, V11, DOI 10.3389/fimmu.2020.00990
Streng-Ouwehand I, 2016, ELIFE, V5, DOI 10.7554/eLife.11765
Stylianou E, 2011, VACCINE, V29, P2279, DOI 10.1016/j.vaccine.2011.01.030
Sun XM, 2008, NANO RES, V1, P203, DOI 10.1007/s12274-008-8021-8
Tacken PJ, 2011, BLOOD, V118, P4111, DOI 10.1182/blood-2011-04-346957
Teran-Navarro H, 2022, CANCERS, V14, DOI 10.3390/cancers14102413

Tiburcio R, 2021, FRONT IMMUNOL, V12, DOI 10.3389/fimmu.2021.750648
Topalian SL, 2012, NEW ENGL J MED, V366, P2443, DOI 10.1056/NEJMoa1200690
Toscano MA, 2007, NAT IMMUNOL, V8, P825, DOI 10.1038/ni1482
Tsuboi S, 2011, EMBO J, V30, P3173, DOI 10.1038/emboj.2011.215
Tsuji T, 2011, J IMMUNOL, V186, P1218, DOI 10.4049/jimmunol.1000808
Ujita M, 2009, BIOSCI BIOTECH BIOCH, V73, P237, DOI 10.1271/bbb.80503
Unger WWJ, 2015, ONCOIMMUNOLOGY, V4, DOI 10.4161/21624011.2014.970462
Unger WWJ, 2012, J CONTROL RELEASE, V160, P88, DOI 10.1016/j.jconrel.2012.02.007
Unger WWJ, 2011, CURR OPIN IMMUNOL, V23, P131, DOI 10.1016/j.coi.2010.11.011
Valladeau J, 1999, EUR J IMMUNOL, V29, P2695, DOI 10.1002/(SICI)1521-4141(199909)29:09<2695::AID-IMMU2695>3.0.CO;2-Q
van Broekhoven CL, 2004, CANCER RES, V64, P4357, DOI 10.1158/0008-5472.CAN-04-0138
van Hateren A, 2021, CURR OPIN IMMUNOL, V70, P138, DOI 10.1016/j.coi.2021.06.016
van Kooyk Y, 2008, NAT IMMUNOL, V9, P593, DOI 10.1038/ni.f.203
van Kooyk Y, 2013, MOL IMMUNOL, V55, P143, DOI 10.1016/j.molimm.2012.10.031
van Liempt E, 2006, FEBS LETT, V580, P6123, DOI 10.1016/j.febslet.2006.10.009
van Vliet SJ, 2008, TRENDS IMMUNOL, V29, P83, DOI 10.1016/j.it.2007.10.010
van Vliet SJ, 2005, INT IMMUNOL, V17, P661, DOI 10.1093/intimm/dxh246
Vassilaros S, 2013, IMMUNOTHERAPY-UK, V5, P1177, DOI [10.2217/IMT.13.126, 10.2217/imt.13.126]
Veglia F, 2017, CURR OPIN IMMUNOL, V45, P43, DOI 10.1016/j.coi.2017.01.002
Visintin A, 2001, J IMMUNOL, V166, P249, DOI 10.4049/jimmunol.166.1.249
Vlad AM, 2002, J EXP MED, V196, P1435, DOI 10.1084/jem.20020493
Wang B, 2012, CHINESE SCI BULL, V57, P3985, DOI 10.1007/s11434-012-5419-1
Wang J, 2019, NAT MED, V25, P656, DOI 10.1038/s41591-019-0374-x
Wang QT, 2020, CANCER IMMUNOL IMMUN, V69, P1375, DOI 10.1007/s00262-020-02496-w
Wang XW, 2021, CHEM SOC REV, V50, P8669, DOI 10.1039/d0cs00461h
Wang XW, 2021, COORDIN CHEM REV, V430, DOI 10.1016/j.ccr.2020.213662
Wang YY, 2020, J HEMATOL ONCOL, V13, DOI 10.1186/s13045-020-00939-6
Warrier S, 2021, BBA-REV CANCER, V1875, DOI 10.1016/j.bbcan.2020.188475
Warrier VU, 2019, BIOSCIENCE REP, V39, DOI 10.1042/BSR20193220
Weis WI, 1996, ANNU REV BIOCHEM, V65, P441, DOI 10.1146/annurev.bi.65.070196.002301
Wieczorek M, 2017, FRONT IMMUNOL, V8, DOI 10.3389/fimmu.2017.00292
Wilson JT, 2013, ACS NANO, V7, P3912, DOI 10.1021/nn305466z
Wu A, 2010, NEURO-ONCOLOGY, V12, P1113, DOI 10.1093/neuonc/noq082
Wu QH, 2022, J HEMATOL ONCOL, V15, DOI 10.1186/s13045-022-01292-6
Xu YD, 2022, BIOMATERIALS, V284, DOI 10.1016/j.biomaterials.2022.121489
Yang R, 2018, ACS NANO, V12, P5121, DOI 10.1021/acsnano.7b09041
Yang ZJ, 2021, NAT COMMUN, V12, DOI 10.1038/s41467-021-24604-9
Yewdell JW, 2001, TRENDS CELL BIOL, V11, P294, DOI 10.1016/S0962-8924(01)02030-X
Yuba Eiji, 2022, J Control Release, DOI 10.1016/j.jconrel.2022.10.016
Yuba E, 2020, PHARMACEUTICS, V12, DOI 10.3390/pharmaceutics12080754
Yuba E, 2020, J MATER CHEM B, V8, P1093, DOI 10.1039/c9tb02470k
Yuba E, 2018, MOL IMMUNOL, V98, P8, DOI 10.1016/j.molimm.2017.11.001
Yuba E, 2017, MEMBRANES-BASEL, V7, DOI 10.3390/membranes7030041
Yuba E, 2017, BIOMATERIALS, V120, P32, DOI 10.1016/j.biomaterials.2016.12.021
Zehner M, 2013, MOL IMMUNOL, V55, P146, DOI 10.1016/j.molimm.2012.10.010
Zhang H, 2022, BIOMATERIALS, V287, DOI 10.1016/j.biomaterials.2022.121673
Zhang L, 2008, CLIN PHARMACOL THER, V83, P761, DOI 10.1038/sj.clpt.6100400
Zhou F, 2002, BIOSCIENCE REP, V22, P355, DOI 10.1023/A:1020103109483
ZHOU LJ, 1995, BLOOD, V86, P3295, DOI 10.1182/blood.V86.9.3295.bloodjournal8693295
Zhou Z, 2021, SMALL, V17, DOI 10.1002/smll.202007486
Zhu YZ, 2022, CLIN TRANSL MED, V12, DOI 10.1002/ctm2.887
NR 286
TC 0
Z9 0
U1 3
U2 18
PU MDPI
PI BASEL
PA ST ALBAN-ANLAGE 66, CH-4052 BASEL, SWITZERLAND
EI 2076-393X

J9 VACCINES-BASEL
JI Vaccines
PD DEC
PY 2022
VL 10
IS 12
AR 2049
DI 10.3390/vaccines10122049
PG 39
WC Immunology; Medicine, Research & Experimental
WE Science Citation Index Expanded (SCI-EXPANDED)
SC Immunology; Research & Experimental Medicine
GA 7I8FT
UT WOS:000904124800001
PM 36560459
OA Green Published, gold
DA 2023-09-14
ER

PT J
AU Liu, C
Yan, B
Wen, NN
Li, WQ
Wang, J
Li, HG
Wang, SY
Shi, KJ
Liu, XL
AF Liu, Chen
Yan, Bin
Wen, Nana
Li, Wenqi
Wang, Jing
Li, Hugang
Wang, Siyao
Shi, Kejian
Liu, Xiaoli
TI Engineering Energy-Responsive Magnetic Nanomaterials to Improve the
Efficacy of Dendritic Cell-Based Immunotherapy
SO ADVANCED THERAPEUTICS
LA English
DT Review
DE antigen presentation; dendritic cell; immunogenic death; magnetic
nanoparticles; tumor immunotherapy
ID SUPERPARAMAGNETIC IRON-OXIDE; CANCER-THERAPY; SIRNA DELIVERY;
CHEMOTHERAPEUTIC-AGENTS; ANTIGEN PRESENTATION; IMMUNE-RESPONSE;
BREAST-CANCER; NANOPARTICLES; HYPERTHERMIA; NANOVACCINE
AB Effective diagnosis and treatment of cancer is still a great challenge to modern medicine. The advent of immunotherapy is revolutionizing the field of cancer research while drug toxicity and low responsive rates have become the most serious concerns in current treatment regimes. Dendritic cells (DCs) are the strongest antigen-presenting immune cells and play an important role in the initiation and regulation of the immune response. They can engulf a variety of foreign substances, including cell lysates and nucleic acids, and present antigens to activate the immune system. Some nanomaterials in vitro activation systems for DCs provide new ideas for immune-related therapies for tumors. Among these, magnetic nanoparticles (MNPs) have gradually become a major focus of basic scientific research and clinical treatment due to their advantages of good biocompatibility, low toxic side effects, and high ability to respond to external stimulus. The emergence of MNPs provides a novel, safe, and effective way to enhance the anti-tumor immune response. In this review, the application of MNPs as platform materials combined with DC immunotherapy is discussed, with the intention of providing a reference for future disease immunotherapy.

C1 [Liu, Chen; Yan, Bin; Wen, Nana; Li, Wenqi; Li, Hugang; Wang, Siyao; Liu, Xiaoli] Northwest Univ, Minist Educ, Prov Key Lab Biotechnol Shaanxi Prov, Xian 710069, Shaanxi, Peoples R China.
[Wang, Jing] Natl Med Prod Adm, Ctr Drug Evaluat, Beijing 100022, Peoples R China.
[Shi, Kejian] Capital Med Univ, Xuanwu Hosp, Dept Thorac Surg, Beijing 100053, Peoples R China.
[Liu, Xiaoli] Xi An Jiao Tong Univ, Affiliated Hosp 1, Med X Inst, Inst Regenerat & Reconstruct Med, Xian 710049, Shaanxi, Peoples R China.
[Liu, Xiaoli] Xi An Jiao Tong Univ, Affiliated Hosp 1, Shaanxi Prov Ctr Regenerat Med & Surg Engn, Natl Local Joint Engn Res Ctr Precis Surg & Regen, Xian 710061, Shaanxi, Peoples R China.
C3 Northwest University Xi'an; Capital Medical University; Xi'an Jiaotong University; Xi'an Jiaotong University
RP Liu, XL (通讯作者), Northwest Univ, Minist Educ, Prov Key Lab Biotechnol Shaanxi Prov, Xian 710069, Shaanxi, Peoples R China.; Shi, KJ (通讯作者), Capital Med Univ, Xuanwu Hosp, Dept Thorac Surg, Beijing 100053, Peoples R China.; Liu, XL (通讯作者), Xi An Jiao Tong Univ, Affiliated Hosp 1, Med X Inst, Inst Regenerat & Reconstruct Med, Xian 710049, Shaanxi, Peoples R China.; Liu, XL (通讯作者), Xi An Jiao Tong Univ, Affiliated Hosp 1, Shaanxi Prov Ctr Regenerat Med & Surg Engn, Natl Local Joint Engn Res Ctr Precis Surg & Regen, Xian 710061, Shaanxi, Peoples R China.
EM xiaotian.tian2008@163.com; liuxiaoli@nwu.edu.cn
OI LIU, XIAOLI/0000-0002-2585-7559
FU National Natural Science Foundation of China (NSFC) projects [82072063, 31901003]; Shaanxi Province Funds for Distinguished Young Scholars [2019JC-27]; Shaanxi Province Youth Science and Technology New Star [2022KJXX-09]; Natural Science Foundation of Shaanxi Province [2020JQ610]
FX C.L., B.Y., and J.W. contributed equally to this work. This work was supported by the National Natural Science Foundation of China (NSFC) projects (grant numbers 82072063 and 31901003), the Shaanxi Province Funds for Distinguished Young Scholars (grant number 2019JC-27), the Shaanxi Province Youth Science and Technology New Star (grant number 2022KJXX-09), and the Natural Science Foundation of Shaanxi Province (grant numbers 2020JQ610).
CR Albakova Z, 2020, CELLS-BASEL, V9, DOI 10.3390/cells9030587
Aloysius MM, 2006, SURG-J R COLL SURG E, V4, P195, DOI 10.1016/S1479-666X(06)80061-2
Amstad E, 2011, NANO LETT, V11, P1664, DOI 10.1021/nl2001499
Anguille S, 2014, LANCET ONCOL, V15, pE257, DOI 10.1016/S1470-2045(13)70585-0
[Anonymous], 2018, IRON OXIDE NANOPART, DOI 10.1016/b978-0-08-101925-2.00007-3
Beik J, 2016, J CONTROL RELEASE, V235, P205, DOI 10.1016/j.jconrel.2016.05.062
Blanco-Andujar C, 2016, NANOMEDICINE-UK, V11, P1889, DOI 10.2217/nnm-2016-5001
Gondan AIB, 2018, BIOMATERIALS, V170, P95, DOI 10.1016/j.biomaterials.2018.04.003
Cabeza-Cabrerizo M, 2021, ANNU REV IMMUNOL, V39, P131, DOI 10.1146/annurev-immunol-061020-053707
Cabral H, 2015, ACS NANO, V9, P4957, DOI 10.1021/nn5070259
Carreno BM, 2015, SCIENCE, V348, P803, DOI 10.1126/science.aaa3828
Castanotto D, 2009, NATURE, V457, P426, DOI 10.1038/nature07758
Chahal JS, 2016, P NATL ACAD SCI USA, V113, pE4133, DOI 10.1073/pnas.1600299113
Chao Y, 2019, NANO LETT, V19, P4287, DOI 10.1021/acs.nanolett.9b00579
Cheever MAM, 2008, IMMUNOL REV, V222, P357, DOI 10.1111/j.1600-065X.2008.00604.x
Chen Q, 2015, ADV MATER, V27, P903, DOI 10.1002/adma.201404308
Chen ZW, 2012, ACS NANO, V6, P4001, DOI 10.1021/nn300291r
Chinen T, 2011, NAT COMMUN, V2, DOI 10.1038/ncomms1181
Collin M, 2013, IMMUNOLOGY, V140, P22, DOI 10.1111/imm.12117
Cruz FM, 2017, ANNU REV IMMUNOL, V35, P149, DOI 10.1146/annurev-immunol-041015-055254
Cui Xiao, 2022, Exploration (Beijing), V2, P20210264, DOI 10.1002/EXP.20210264
Dalmina M, 2019, MAT SCI ENG C-MATER, V99, P1182, DOI 10.1016/j.msec.2019.02.026
Demaria O, 2019, NATURE, V574, P45, DOI 10.1038/s41586-019-1593-5
Ding F, 2020, BIOMATERIALS, V245, DOI 10.1016/j.biomaterials.2020.119976
Dong YZ, 2019, ADV DRUG DELIVER REV, V144, P133, DOI 10.1016/j.addr.2019.05.004
Gabrilovich DI, 2012, NAT REV IMMUNOL, V12, P253, DOI 10.1038/nri3175
Galluzzi L, 2012, EMBO J, V31, P1055, DOI 10.1038/emboj.2012.2
Gandhi NS, 2014, J CONTROL RELEASE, V194, P238, DOI 10.1016/j.jconrel.2014.09.001
Gardner A, 2016, TRENDS IMMUNOL, V37, P855, DOI 10.1016/j.it.2016.09.006
Garg AD, 2015, INT J DEV BIOL, V59, P131, DOI 10.1387/ijdb.150061pa

Garg AD, 2010, BBA-REV CANCER, V1805, P53, DOI 10.1016/j.bbcan.2009.08.003
Ge R, 2018, ACS APPL MATER INTER, V10, P20342, DOI 10.1021/acsami.8b05876
GILCHRIST RK, 1957, ANN SURG, V146, P596, DOI 10.1097/00000658-195710000-00007
Gostner JM, 2013, REDOX REP, V18, P88, DOI 10.1179/1351000213Y.0000000044
Grippin AJ, 2019, ACS NANO, V13, P13884, DOI 10.1021/acsnano.9b05037
Guevara ML, 2021, SEMIN CANCER BIOL, V69, P238, DOI 10.1016/j.semcancer.2019.11.010
Gyparaki MT, 2014, TRENDS MOL MED, V20, P239, DOI 10.1016/j.molmed.2014.01.009
Hashimoto M, 2009, CANCER SCI, V100, P730, DOI 10.1111/j.1349-7006.2009.01098.x
Hergt R, 2006, J PHYS-CONDENS MAT, V18, pS2919, DOI 10.1088/0953-8984/18/38/S26
Hong BX, 2009, CANCER RES, V69, P8076, DOI 10.1158/0008-5472.CAN-09-1507
Huang CR, 2021, NATL SCI REV, V8, DOI 10.1093/nsr/nwaa232
Huang XY, 2021, BIOMATER SCI-UK, V9, P6282, DOI 10.1039/d1bm00474c
Huang ZS, 2021, ACS NANO, V15, P8450, DOI 10.1021/acsnano.0c10764
Jaque D, 2014, NANOSCALE, V6, P9494, DOI 10.1039/c4nr00708e
Joshi MD, 2012, J CONTROL RELEASE, V161, P25, DOI 10.1016/j.jconrel.2012.05.010
Juliano R, 2009, MOL PHARMACEUT, V6, P686, DOI 10.1021/mp900093r
Kanasty R, 2013, NAT MATER, V12, P967, DOI [10.1038/NMAT3765, 10.1038/nmat3765]
Ke HT, 2014, THERANOSTICS, V4, P12, DOI 10.7150/thno.7275
Keskin DB, 2019, NATURE, V565, P234, DOI 10.1038/s41586-018-0792-9
Kobayashi T, 2005, TRENDS IMMUNOL, V26, P177, DOI 10.1016/j.it.2005.02.004
Kotsias F, 2013, ANTIOXID REDOX SIGN, V18, P714, DOI 10.1089/ars.2012.4557
Kubiatowicz, 2022, EXPLORATION, DOI [10.1002/EXP.20210217, DOI 10.1002/EXP.20210217]
Kumar CSSR, 2011, ADV DRUG DELIVER REV, V63, P789, DOI 10.1016/j.addr.2011.03.008
Li F, 2019, ACS CENTRAL SCI, V5, P796, DOI 10.1021/acscentsci.9b00060
Li JC, 2021, ANGEW CHEM INT EDIT, V60, P12682, DOI 10.1002/anie.202008386
Li W, 2019, NAT COMMUN, V10, DOI 10.1038/s41467-019-11269-8
Liu Yanjie, 2022, Exploration (Beijing), V2, P20210274, DOI 10.1002/EXP.20210274
Liu YJ, 2019, CHEM SOC REV, V48, P2053, DOI 10.1039/c8cs00618k
Luo LJ, 2019, BIOMATERIALS, V223, DOI 10.1016/j.biomaterials.2019.119464
Luo M, 2017, NAT NANOTECHNOL, V12, P648, DOI [10.1038/nnano.2017.52, 10.1038/NNANO.2017.52]
Ma Y, 2013, J CANCER, V4, P36, DOI 10.7150/jca.5046
Maier B, 2020, NATURE, V580, P257, DOI 10.1038/s41586-020-2134-y
Makela SM, 2009, J LEUKOCYTE BIOL, V85, P664, DOI 10.1189/jlb.0808503
Matsue H, 2003, J IMMUNOL, V171, P3010, DOI 10.4049/jimmunol.171.6.3010
Michaud M, 2011, SCIENCE, V334, P1573, DOI 10.1126/science.1208347
Miller JB, 2018, NANO RES, V11, P5310, DOI 10.1007/s12274-018-2099-4
Moroz P, 2002, INT J HYPERTHER, V18, P267, DOI 10.1080/02656730110108785
Mou YB, 2011, INT J NANOMED, V6, P1779, DOI 10.2147/IJN.S23240
Mura S, 2013, NAT MATER, V12, P991, DOI [10.1038/NMAT3776, 10.1038/nmat3776]
Nam J, 2019, NAT REV MATER, V4, P398, DOI 10.1038/s41578-019-0108-1
Napolitani G, 2005, NAT IMMUNOL, V6, P769, DOI 10.1038/ni1223
Nathan C, 2013, NAT REV IMMUNOL, V13, P349, DOI 10.1038/nri3423
Ni L, 2022, FRONT IMMUNOL, V13, DOI 10.3389/fimmu.2022.887189
Norum OJ, 2009, J PHOTOCH PHOTOBIO B, V96, P83, DOI 10.1016/j.jphotobiol.2009.04.012
Oberkampf M, 2018, NAT COMMUN, V9, DOI 10.1038/s41467-018-04686-8
Ott PA, 2017, NATURE, V547, P217, DOI 10.1038/nature22991
Pan J, 2020, ACS NANO, V14, P1033, DOI 10.1021/acsnano.9b08550
Pardoll DM, 2012, NAT REV CANCER, V12, P252, DOI 10.1038/nrc3239
Park W, 2020, INT MATER REV, V65, P445, DOI 10.1080/09506608.2020.1735117
Peng XC, 2012, MATER LETT, V81, P102, DOI 10.1016/j.matlet.2012.04.127
Perez CR, 2019, NAT COMMUN, V10, DOI 10.1038/s41467-019-13368-y
Perigo EA, 2015, APPL PHYS REV, V2, DOI 10.1063/1.4935688
Pradhan P, 2010, J CONTROL RELEASE, V142, P108, DOI 10.1016/j.jconrel.2009.10.002
Radogna F, 2018, BIOCHEM PHARMACOL, V153, P12, DOI 10.1016/j.bcp.2018.02.006
Reddy ST, 2006, TRENDS IMMUNOL, V27, P573, DOI 10.1016/j.it.2006.10.005
Riley RS, 2019, NAT REV DRUG DISCOV, V18, P175, DOI 10.1038/s41573-018-0006-z
Rius M, 2012, ONCOGENE, V31, P4257, DOI 10.1038/onc.2011.601
Rodriguez A, 1999, NAT CELL BIOL, V1, P362, DOI 10.1038/14058
Rodriguez-Luccioni HL, 2011, INT J NANOMED, V6, P373, DOI 10.2147/IJN.S14613
Roussakow S., 2013, C PAP MED, V2013

Sabado RL, 2017, CELL RES, V27, P74, DOI 10.1038/cr.2016.157
Sahay G, 2013, NAT BIOTECHNOL, V31, P653, DOI 10.1038/nbt.2614
Sahle FF, 2018, DRUG DISCOV TODAY, V23, P992, DOI 10.1016/j.drudis.2018.04.003
Sanchez-Paulete AR, 2017, ANN ONCOL, V28, P44, DOI 10.1093/annonc/mdx237
Sato M, 2009, J INVEST DERMATOL, V129, P2233, DOI 10.1038/jid.2009.39
Shevtsov MA, 2015, J CONTROL RELEASE, V220, P329, DOI 10.1016/j.jconrel.2015.10.051
Shi Y, 2019, ACCOUNTS CHEM RES, V52, P1543, DOI 10.1021/acs.accounts.9b00148
STEINMAN RM, 1973, J EXP MED, V137, P1142, DOI 10.1084/jem.137.5.1142
Stupp R, 2005, NEW ENGL J MED, V352, P987, DOI 10.1056/NEJMoa043330
Teoh CY, 2004, ARCH BIOCHEM BIOPHYS, V423, P88, DOI 10.1016/j.abb.2003.12.001
Tong HX, 2016, CHEM COMMUN, V52, P3966, DOI 10.1039/c6cc00450d
Toraya-Brown S, 2014, NANOMED-NANOTECHNOL, V10, P1273, DOI 10.1016/j.nano.2014.01.011
Wang C, 2016, BIOMATERIALS, V79, P88, DOI 10.1016/j.biomaterials.2015.11.040
Wang H, 2018, NAT MATER, V17, P761, DOI 10.1038/s41563-018-0147-9
Wang L, 2019, J MATER CHEM B, V7, P7406, DOI 10.1039/c9tb00630c
Wang N, 2019, ACS BIOMATER SCI ENG, V5, P2330, DOI 10.1021/acsbiomaterials.9b00359
Wang YY, 2020, J HEMATOL ONCOL, V13, DOI 10.1186/s13045-020-00939-6
Wang YH, 2022, ADV SCI, V9, DOI 10.1002/advs.202105631
Wang Z, 2019, ADV SCI, V6, DOI 10.1002/advs.201901690
Warger T, 2006, BLOOD, V108, P544, DOI 10.1182/blood-2005-10-4015
Warnatsch A, 2013, MOL IMMUNOL, V55, P106, DOI 10.1016/j.molimm.2012.10.007
Wculek SK, 2019, J IMMUNOTHER CANCER, V7, DOI 10.1186/s40425-019-0565-5
Whitehead KA, 2009, NAT REV DRUG DISCOV, V8, P129, DOI 10.1038/nrd2742
Woo SR, 2015, ANNU REV IMMUNOL, V33, P445, DOI 10.1146/annurev-immunol-032414-112043
Wu CE, 2020, FRONT ONCOL, V10, DOI 10.3389/fonc.2020.00905
Wu HA, 2019, ACS NANO, V13, P14013, DOI 10.1021/acsnano.9b06134
Wu J, 2014, J MATER CHEM B, V2, P7756, DOI 10.1039/c4tb01264j
Xie LS, 2021, BIOMATERIALS, V269, DOI 10.1016/j.biomaterials.2020.120638
Xie W, 2019, ACS NANO, V13, P2849, DOI 10.1021/acsnano.8b03788
Xu C, 2021, ADV MATER, V33, DOI 10.1002/adma.202008061
Xu J, 2020, NAT NANOTECHNOL, V15, P1043, DOI 10.1038/s41565-020-00781-4
Yanase M, 1998, JPN J CANCER RES, V89, P775, DOI 10.1111/j.1349-7006.1998.tb03283.x
Yang MY, 2021, BIOACT MATER, V6, P1973, DOI 10.1016/j.bioactmat.2020.12.010
Yu MK, 2008, ANGEW CHEM INT EDIT, V47, P5362, DOI 10.1002/anie.200800857
Yusuf N, 2014, FRONT IMMUNOL, V5, DOI 10.3389/fimmu.2014.00224
Zeng BJ, 2018, J CLIN INVEST, V128, P1971, DOI 10.1172/JCI96791
Zeng YY, 2020, J IMMUNOTHER CANCER, V8, DOI 10.1136/jitc-2019-000217
Zhang CN, 2017, J CONTROL RELEASE, V256, P170, DOI 10.1016/j.jconrel.2017.04.020
Zhang F, 2020, J CONTROL RELEASE, V326, P131, DOI 10.1016/j.jconrel.2020.06.015
Zhang HM, 2018, J CANCER, V9, P1773, DOI 10.7150/jca.24577
Zhao Y, 2018, MOL PHARMACEUT, V15, P1791, DOI 10.1021/acs.molpharmaceut.7b01103
Zhou Q, 2017, NANOTECHNOL REV, V6, P473, DOI 10.1515/ntrev-2016-0102
Zhou YF, 2020, ADV MATER, V32, DOI 10.1002/adma.202003708
Zhu GZ, 2017, ACS NANO, V11, P2387, DOI 10.1021/acsnano.7b00978
Zhu RR, 2014, MAT SCI ENG C-MATER, V39, P305, DOI 10.1016/j.msec.2014.03.005
Zou LL, 2016, THERANOSTICS, V6, P762, DOI 10.7150/thno.14988
NR 136
TC 0
Z9 0
U1 12
U2 37
PU WILEY
PI HOBOKEN
PA 111 RIVER ST, HOBOKEN 07030-5774, NJ USA
EI 2366-3987
J9 ADV THER-GERMANY
JI Adv. Therap.
PD JAN
PY 2023
VL 6
IS 1

DI 10.1002/adtp.202200234
EA NOV 2022
PG 15
WC Pharmacology & Pharmacy
WE Science Citation Index Expanded (SCI-EXPANDED)
SC Pharmacology & Pharmacy
GA 8B7YT
UT WOS:000883301800001
DA 2023-09-14
ER

PT J
AU Gao, XY
   Feng, QQ
   Wang, J
   Zhao, X
AF Gao, Xiaoyu
   Feng, Qingqing
   Wang, Jing
   Zhao, Xiao
TI Bacterial outer membrane vesicle-based cancer nanovaccines
SO CANCER BIOLOGY & MEDICINE
LA English
DT Review
DE Cancer; cancer vaccines; outer membrane vesicles; nanocarriers; tumor
   antigen
ID VIRULENCE FACTORS; VACCINE; PLATFORM; NANOPARTICLES; ASSOCIATION;
   MECHANISM; GROWTH
AB Tumor vaccines, a type of personalized tumor immunotherapy, have developed rapidly in recent decades. These vaccines evoke tumor antigen-specific T cells to achieve immune recognition and killing of tumor cells. Because the immunogenicity of tumor antigens alone is insufficient, immune adjuvants and nanocarriers are often required to enhance anti-tumor immune responses. At present, vaccine carrier development often integrates nanocarriers and immune adjuvants. Among them, outer membrane vesicles (OMVs) are receiving increasing attention as a delivery platform for tumor vaccines. OMVs are natural nanovesicles derived from Gram-negative bacteria, which have adjuvant function because they contain pathogen associated molecular patterns. Importantly, OMVs can be functionally modified by genetic engineering of bacteria, thus laying a foundation for applications as a delivery platform for tumor nanovaccines. This review summarizes 5 aspects of recent progress in, and future development of, OMV-based tumor nanovaccines: strain selection, heterogeneity, tumor antigen loading, immunogenicity and safety, and mass production of OMVs.
C1 [Gao, Xiaoyu; Feng, Qingqing; Zhao, Xiao] Natl Ctr Nanosci & Technol China, CAS Key Lab Biomed Effects Nanomat & Nanosafety, Beijing 100190, Peoples R China.
   [Gao, Xiaoyu; Feng, Qingqing; Zhao, Xiao] Natl Ctr Nanosci & Technol China, CAS Ctr Excellence Nanosci, Beijing 100190, Peoples R China.
   [Gao, Xiaoyu; Zhao, Xiao] Univ Chinese Acad Sci, Beijing 100049, Peoples R China.
   [Wang, Jing] Natl Med Prod Adm, Ctr Drug Evaluat, Beijing 100022, Peoples R China.
   [Zhao, Xiao] Chinese Acad Sci, IGDB NCNST Joint Res Ctr, Inst Genet & Dev Biol, Beijing 100101, Peoples R China.
C3 Chinese Academy of Sciences; National Center for Nanoscience &
   Technology - China; Chinese Academy of Sciences; National Center for
   Nanoscience & Technology - China; Chinese Academy of Sciences;
   University of Chinese Academy of Sciences, CAS; Chinese Academy of
   Sciences; Institute of Genetics & Developmental Biology, CAS
RP Zhao, X (通讯作者)，Natl Ctr Nanosci & Technol China, CAS Key Lab Biomed Effects Nanomat & Nanosafety, Beijing 100190, Peoples R China.; Zhao, X (通讯作者)，Natl Ctr Nanosci & Technol China, CAS Ctr Excellence Nanosci, Beijing 100190, Peoples R China.; Zhao, X (通讯作者)，Univ Chinese Acad Sci, Beijing 100049, Peoples R China.; Wang, J (通讯作者)，Natl Med Prod Adm, Ctr Drug Evaluat, Beijing 100022, Peoples R China.; Zhao, X (通讯作者)，Chinese Acad Sci, IGDB NCNST Joint Res Ctr, Inst Genet & Dev Biol, Beijing 100101, Peoples R China.
EM wangjing01@cde.org.cn; zhaox@nanoctr.cn
RI Feng, Qingqing/HTO-5554-2023

OI Feng, Qingqing/0000-0001-9640-7187
FU National Key R&D Program of China [2021YFA0909900]; CAS Project for
Young Scientists in Basic Research [YSBR-010]; Beijing Natural Science
Foundation [Z200020]; Beijing Nova Program [Z201100006820031]; National
Natural Science Foundation of China [32171384]
FX This work was supported by grants from the National Key R&D Program of
China (Grant No. 2021YFA0909900, X. Z.), the CAS Project for Young
Scientists in Basic Research (Grant No. YSBR-010, X. Z.), the Beijing
Natural Science Foundation (Grant No. Z200020, X. Z.), the Beijing Nova
Program (Grant No. Z201100006820031, X. Z.), and the National Natural
Science Foundation of China (Grant No. 32171384, X. Z.).
CR Arenas J, 2010, CLIN VACCINE IMMUNOL, V17, P487, DOI 10.1128/CVI.00423-09
Arnold R, 2011, VACCINE, V29, P7100, DOI 10.1016/j.vaccine.2011.06.120
Bos MP, 2004, P NATL ACAD SCI USA, V101, P9417, DOI 10.1073/pnas.0402340101
Bowen WS, 2018, EXPERT REV VACCINES, V17, P207, DOI 10.1080/14760584.2018.1434000
CHATTERJ.SN, 1967, J GEN MICROBIOL, V49, P1
Cheng KM, 2021, NAT COMMUN, V12, DOI 10.1038/s41467-021-22308-8
Cheng KM, 2020, WIRES NANOMED NANOBI, V12, DOI 10.1002/wnan.1646
Chowdhury C, 2013, BBA-PROTEINS PROTEOM, V1834, P231, DOI 10.1016/j.bbapap.2012.09.015
Couzin-Frankel J, 2013, SCIENCE, V342, P1432, DOI 10.1126/science.342.6165.1432
Gerritzen MJH, 2019, APPL MICROBIOL BIOT, V103, P9401, DOI 10.1007/s00253-019-10163-z
Gerritzen MJH, 2018, MICROB CELL FACT, V17, DOI 10.1186/s12934-018-1007-7
Gerritzen MJH, 2017, BIOTECHNOL ADV, V35, P565, DOI 10.1016/j.biotechadv.2017.05.003
Grandi A, 2017, FRONT ONCOL, V7, DOI 10.3389/fonc.2017.00253
Grizot S, 2004, MOL MICROBIOL, V51, P1027, DOI 10.1111/j.1365-2958.2003.03903.x
Haen SP, 2020, NAT REV CLIN ONCOL, V17, P595, DOI 10.1038/s41571-020-0387-x
Handy CE, 2018, FUTURE ONCOL, V14, P907, DOI 10.2217/fon-2017-0531
Hellmann MD, 2019, NEW ENGL J MED, V381, P2020, DOI 10.1056/NEJMoa1910231
HOEKSTRA D, 1976, BIOCHIM BIOPHYS ACTA, V455, P889, DOI 10.1016/0005-2736(76)90058-4
Hong JW, 2019, J EXTRACELL VESICLES, V8, DOI 10.1080/20013078.2019.1632099
Hu Z, 2018, NAT REV IMMUNOL, V18, P168, DOI 10.1038/nri.2017.131
Huang C, 2013, ACS NANO, V7, P5791, DOI 10.1021/nn400683s
KADURUGAMUWA JL, 1995, J BACTERIOL, V177, P3998, DOI 10.1128/jb.177.14.3998-4008.1995
Kaparakis-Liaskos M, 2015, NAT REV IMMUNOL, V15, P375, DOI 10.1038/nri3837
Kennedy LB, 2020, CA-CANCER J CLIN, V70, P86, DOI 10.3322/caac.21596
Kranz LM, 2016, NATURE, V534, P396, DOI 10.1038/nature18300
Kuerban K, 2020, ACTA PHARM SIN B, V10, P1534, DOI 10.1016/j.apsb.2020.02.002
Kushnir N, 2012, VACCINE, V31, P58, DOI 10.1016/j.vaccine.2012.10.083
Lee EY, 2007, PROTEOMICS, V7, P3143, DOI [10.1002/pmic.200700196, 10.1002/pmic.200790083]
Li M, 2020, J CONTROL RELEASE, V323, P253, DOI 10.1016/j.jconrel.2020.04.031
Li SP, 2018, NAT BIOTECHNOL, V36, P258, DOI 10.1038/nbt.4071
Li Y, 2022, ADV MATER, V34, DOI 10.1002/adma.202109984
Li Y, 2022, SMALL, V18, DOI 10.1002/smll.202107461
Liang J, 2022, FUND RES-CHINA, V2, P23, DOI 10.1016/j.fmre.2021.11.032
Liang J, 2021, CANCER BIOL MED, V18, P352, DOI 10.20892/j.issn.2095-3941.2021.0004
Liu GN, 2021, ADV DRUG DELIVER REV, V176, DOI 10.1016/j.addr.2021.113889
Luo M, 2017, J CONTROL RELEASE, V263, P200, DOI 10.1016/j.jconrel.2017.03.033
Luo M, 2017, NAT NANOTECHNOL, V12, P648, DOI [10.1038/nnano.2017.52,
10.1038/NNANO.2017.52]
Miao L, 2019, NAT BIOTECHNOL, V37, P1174, DOI 10.1038/s41587-019-0247-3
Morse MA, 2005, NAT CLIN PRACT ONCOL, V2, P108, DOI 10.1038/ncponc0098
Mun EJ, 2018, CLIN CANCER RES, V24, P266, DOI 10.1158/1078-0432.CCR-17-1117
O'Ryan M, 2014, DRUGS, V74, P15, DOI 10.1007/s40265-013-0155-7
Olofsson A, 2010, MOL MICROBIOL, V77, P1539, DOI 10.1111/j.1365-2958.2010.07307.x
Ott PA, 2017, NATURE, V547, P217, DOI 10.1038/nature22991
Pan JM, 2022, ADV MATER, V34, DOI 10.1002/adma.202106307
Poltorak A, 1998, SCIENCE, V282, P2085, DOI 10.1126/science.282.5396.2085
Qing S, 2020, ADV MATER, V32, DOI 10.1002/adma.202002085
Rini BI, 2019, NEW ENGL J MED, V380, P1116, DOI 10.1056/NEJMoa1816714
Rumbo C, 2011, ANTIMICROB AGENTS CH, V55, P3084, DOI 10.1128/AAC.00929-10
Sahin U, 2018, SCIENCE, V359, P1355, DOI 10.1126/science.aar7112

Sahin U, 2017, NATURE, V547, P222, DOI 10.1038/nature23003
Sanders H, 2011, EXPERT REV VACCINES, V10, P323, DOI [10.1586/erv.11.10, 10.1586/ERV.11.10]
Sanmamed MF, 2018, CELL, V175, P313, DOI 10.1016/j.cell.2018.09.035
Schumacher TN, 2015, SCIENCE, V348, P69, DOI 10.1126/science.aaa4971
Schwechheimer C, 2015, NAT REV MICROBIOL, V13, P605, DOI 10.1038/nrmicro3525
Schwechheimer C, 2014, BMC MICROBIOL, V14, DOI 10.1186/s12866-014-0324-1
Siegel RL, 2022, CA-CANCER J CLIN, V72, P7, DOI 10.3322/caac.21708
SIERRA G V G, 1991, NIPH (National Institute of Public Health) Annals (Oslo), V14, P195
Tashiro Y, 2010, MICROBES ENVIRON, V25, P120, DOI 10.1264/jsme2.ME09182
Toyofuku M, 2019, NAT REV MICROBIOL, V17, P13, DOI 10.1038/s41579-018-0112-2
Toyofuku M, 2017, NAT COMMUN, V8, DOI 10.1038/s41467-017-00492-w
van de Waterbeemd B, 2013, PLOS ONE, V8, DOI 10.1371/journal.pone.0065157
van de Waterbeemd B, 2010, VACCINE, V28, P4810, DOI 10.1016/j.vaccine.2010.04.082
Van der Jeught K, 2018, ACS NANO, V12, P9815, DOI 10.1021/acsnano.8b00966
van der Pol L, 2015, BIOTECHNOL J, V10, P1689, DOI 10.1002/biot.201400395
Vaughan TE, 2006, VACCINE, V24, P5277, DOI 10.1016/j.vaccine.2006.03.013
Wang SH, 2022, NANO TODAY, V45, DOI 10.1016/j.nantod.2022.101512
Weber J, 2017, NEW ENGL J MED, V377, P1824, DOI 10.1056/NEJMoa1709030
Xie JH, 2022, TRENDS BIOTECHNOL, V40, P1173, DOI 10.1016/j.tibtech.2022.03.005
Yamamoto TN, 2019, NAT MED, V25, P1488, DOI 10.1038/s41591-019-0596-y
Yue YL, 2022, NAT BIOMED ENG, V6, DOI 10.1038/s41551-022-00886-2
Zariri A, 2016, INFECT IMMUN, V84, P3024, DOI 10.1128/IAI.00635-16
Zavan L, 2019, PROTEOMICS, V19, DOI 10.1002/pmic.201800209
Zhai YH, 2017, THERANOSTICS, V7, P2575, DOI 10.7150/thno.20118
Zhang JY, 2022, INT J NANOMED, V17, P1971, DOI 10.2147/IJN.S353330
Zhao X, 2022, NAT PROTOC, V17, P2240, DOI 10.1038/s41596-022-00713-7
Zheng BB, 2019, ADV FUNCT MATER, V29, DOI 10.1002/adfm.201901437
Zhuang Q, 2021, BIOMATERIALS, V268, DOI 10.1016/j.biomaterials.2020.120550
Zingl FG, 2020, CELL HOST MICROBE, V27, P225, DOI 10.1016/j.chom.2019.12.002
NR 78
TC 0
Z9 0
U1 8
U2 25
PU CHINA ANTI-CANCER ASSOC
PI TIANJIN
PA TIANJIN MEDICAL UNIV, CANCER INST & HOSPITAL TI-YUAN-BEI, HUANHU XI LU,
HEXIQU, TIANJIN, 300060, PEOPLES R CHINA
SN 2095-3941
J9 CANCER BIOL MED
JI Cancer Biol. Med.
PD SEP 15
PY 2022
VL 19
IS 9
BP 1290
EP 1300
DI 10.20892/j.issn.2095-3941.2022.0452
PG 11
WC Oncology; Medicine, Research & Experimental
WE Science Citation Index Expanded (SCI-EXPANDED)
SC Oncology; Research & Experimental Medicine
GA 8F1SP
UT WOS:000919448400002
PM 36172794
OA gold, Green Published
DA 2023-09-14
ER

PT J
AU Bruckner, M

Fichter, M
Marques, RD
Landfester, K
Mailander, V
AF Bruckner, Maximilian
Fichter, Michael
da Costa Marques, Richard
Landfester, Katharina
Mailaender, Volker
TI PEG Spacer Length Substantially Affects Antibody-Based Nanocarrier
Targeting of Dendritic Cell Subsets
SO PHARMACEUTICS
LA English
DT Article
DE antibody functionalization; nanoparticles; nanovaccine; dendritic cell
targeting; PEG
ID PROTEIN ADSORPTION; LINKER LENGTH; NANOPARTICLES; CORONA;
PHARMACOKINETICS; OPSONIZATION; GENERATION; DENSITY; KEY
AB Successful cell targeting depends on the controlled positioning of cell-type-specific antibodies on the nanocarrier's (NC) surface. Uncontrolled antibody immobilization results in unintended cell uptake due to Fc-mediated cell interaction. Consequently, precise immobilization of the Fc region towards the nanocarrier surface is needed with the Fab regions staying freely accessible for antigen binding. Moreover, the antibody needs to be a certain distance from the nanocarrier surface, influencing the targeting performance after formation of the biomolecular corona. This can be achieved by using PEG linker molecules. Here we demonstrate cell type-specific targeting for dendritic cells (DC) as cellular key regulators of immune responses. However, to date, dendritic cell targeting experiments using different linker lengths still need to be conducted. Consequently, we focused on the surface modification of nanocarriers with different molecular weight PEG linkers (0.65, 2, and 5 kDa), and their ability to reduce undesired cell uptake, while achieving efficient DC targeting via covalently immobilized antibodies (stealth targeting). Our findings demonstrate that the PEG linker length significantly affects active dendritic cell targeting from cell lines (DC2.4) to primary cells (BMDCs, splenocytic conventional DCs type 1 (cDC1)). While antibody-functionalized nanocarriers with a shorter PEG length (0.65 kDa) showed the best targeting in DC2.4, a longer PEG length (5 kDa) was required to specifically accumulate in BMDCs and splenocytic cDC1. Our study highlights that these crucial aspects must be considered when targeting dendritic cell subsets, which are of great importance in the fields of cancer immunotherapy and vaccine development.
C1 [Bruckner, Maximilian; Fichter, Michael; da Costa Marques, Richard; Mailaender, Volker] Johannes Gutenberg Univ Mainz, Univ Med Ctr, Dept Dermatol, Langenbeckstr 1, D-55131 Mainz, Germany.
[Bruckner, Maximilian; Fichter, Michael; da Costa Marques, Richard; Landfester, Katharina; Mailaender, Volker] Max Planck Inst Polymer Res, Ackermannweg 10, D-55128 Mainz, Germany.
C3 Johannes Gutenberg University of Mainz; Max Planck Society
RP Mailander, V (通讯作者)，Johannes Gutenberg Univ Mainz, Univ Med Ctr, Dept Dermatol, Langenbeckstr 1, D-55131 Mainz, Germany.; Mailander, V (通讯作者)，Max Planck Inst Polymer Res, Ackermannweg 10, D-55128 Mainz, Germany.
EM brueckner@mpip-mainz.mpg.de; fichter@uni-mainz.de;
dacostamarques@mpip-mainz.mpg.de; landfester@mpip-mainz.mpg.de;
volker.mailaender@unimedizin-mainz.de
RI Landfester, Katharina/F-9449-2011
OI Landfester, Katharina/0000-0001-9591-4638
FU German Research Foundation (DFG) [Sonderforschungsbereich 1066 (SFB
1066)]
FX This research was funded by the German Research Foundation (DFG) within
the framework of the Sonderforschungsbereich 1066 (SFB 1066).
CR Ammon C, 2000, IMMUNOLOGY, V100, P364, DOI 10.1046/j.1365-2567.2000.00056.x
Anselmo AC, 2019, BIOENG TRANSL MED, V4, DOI 10.1002/btm2.10143
Banchereau J, 2000, ANNU REV IMMUNOL, V18, P767, DOI 10.1146/annurev.immunol.18.1.767
Berry JD, 2003, HYBRIDOMA HYBRIDOM, V22, P23, DOI 10.1089/153685903321538053
Bradshaw RA, 2006, MOL CELL PROTEOMICS, V5, P787, DOI 10.1074/mcp.E600005-MCP200
Bros M, 2016, INT IMMUNOPHARMACOL, V35, P174, DOI 10.1016/j.intimp.2016.03.025
Bruckner M, 2021, NANOSCALE, V13, P9816, DOI 10.1039/d0nr08191d
Calmeiro J, 2020, PHARMACEUTICS, V12, DOI 10.3390/pharmaceutics12020158

Caracciolo G, 2015, LANGMUIR, V31, P10764, DOI 10.1021/acs.langmuir.5b02158
Cifuentes-Rius A, 2013, ACS NANO, V7, P10066, DOI 10.1021/nn404166q
Collin M, 2018, IMMUNOLOGY, V154, P3, DOI 10.1111/imm.12888
Cruz LJ, 2014, J CONTROL RELEASE, V192, P209, DOI 10.1016/j.jconrel.2014.07.040
Cruz LJ, 2011, BIOMATERIALS, V32, P6791, DOI 10.1016/j.biomaterials.2011.04.082
Dolcetti R, 2020, FRONT IMMUNOL, V11, DOI 10.3389/fimmu.2020.00507
Fam SY, 2020, NANOMATERIALS-BASEL, V10, DOI 10.3390/nano10040787
Friedman AD, 2013, CURR PHARM DESIGN, V19, P6315
He HL, 2019, ACCOUNTS CHEM RES, V52, P2445, DOI 10.1021/acs.accounts.9b00228
Horejs C, 2021, NAT REV MATER, V6, P1075, DOI 10.1038/s41578-021-00379-9
Ilinskaya AN, 2016, TOXICOL APPL PHARM, V299, P70, DOI 10.1016/j.taap.2016.01.005
Jeong HS, 2014, J BIOMED MATER RES A, V102, P4545, DOI 10.1002/jbm.a.35112
Johnston Michael C, 2018, Drug Discov Today Technol, V30, P63, DOI 10.1016/j.ddtec.2018.10.003
Juan AL, 2020, INT J MOL SCI, V21, DOI 10.3390/ijms21176018
Kapadia CH, 2019, ACS OMEGA, V4, P5547, DOI 10.1021/acsomega.8b03391
Kokkinopoulou M, 2017, NANOSCALE, V9, P8858, DOI 10.1039/c7nr02977b
Kozma GT, 2020, ADV DRUG DELIVER REV, V154, P163, DOI 10.1016/j.addr.2020.07.024
Ledford H, 2016, NATURE, V533, P304, DOI 10.1038/533304a
Li MY, 2021, NANO LETT, V21, P1591, DOI 10.1021/acs.nanolett.0c03756
Li SD, 2010, J CONTROL RELEASE, V145, P178, DOI 10.1016/j.jconrel.2010.03.016
Mahmoudi M, 2018, TRENDS BIOTECHNOL, V36, P755, DOI 10.1016/j.tibtech.2018.02.014
Miao ZH, 2019, CHEM SCI, V10, P5435, DOI 10.1039/c9sc00729f
Moghimi SM, 2003, PROG LIPID RES, V42, P463, DOI 10.1016/S0163-7827(03)00033-X
Muller LK, 2018, BIOMACROMOLECULES, V19, P374, DOI 10.1021/acs.biomac.7b01472
Owens DE, 2006, INT J PHARMACEUT, V307, P93, DOI 10.1016/j.ijpharm.2005.10.010
Passlick D, 2018, J CONTROL RELEASE, V289, P23, DOI 10.1016/j.jconrel.2018.09.008
Ricklin D, 2010, NAT IMMUNOL, V11, P785, DOI 10.1038/ni.1923
Rosenblum D, 2018, NAT COMMUN, V9, DOI 10.1038/s41467-018-03705-y
Salmaso S, 2013, J DRUG DELIV, V2013, DOI 10.1155/2013/374252
Salvati A, 2013, NAT NANOTECHNOL, V8, P137, DOI [10.1038/nnano.2012.237, 10.1038/NNANO.2012.237]
Schottler S, 2016, NAT NANOTECHNOL, V11, P372, DOI [10.1038/NNANO.2015.330, 10.1038/nnano.2015.330]
Silva JC, 2006, MOL CELL PROTEOMICS, V5, P144, DOI 10.1074/mcp.M500230-MCP200
Simon J, 2022, NANO TODAY, V43, DOI 10.1016/j.nantod.2022.101375
Stefanick JF, 2013, ACS NANO, V7, P2935, DOI 10.1021/nn305663e
Suk JS, 2016, ADV DRUG DELIVER REV, V99, P28, DOI 10.1016/j.addr.2015.09.012
Tenzer S, 2011, ACS NANO, V5, P7155, DOI 10.1021/nn201950e
Vonarbourg A, 2006, BIOMATERIALS, V27, P4356, DOI 10.1016/j.biomaterials.2006.03.039
Walkey CD, 2012, J AM CHEM SOC, V134, P2139, DOI 10.1021/ja2084338
Wang XW, 2021, CHEM SOC REV, V50, P8669, DOI 10.1039/d0cs00461h
Wilhelm S, 2016, NAT REV MATER, V1, DOI 10.1038/natrevmats.2016.14
NR 48
TC 0
Z9 0
U1 1
U2 6
PU MDPI
PI BASEL
PA ST ALBAN-ANLAGE 66, CH-4052 BASEL, SWITZERLAND
EI 1999-4923
J9 PHARMACEUTICS
JI Pharmaceutics
PD AUG
PY 2022
VL 14
IS 8
AR 1614
DI 10.3390/pharmaceutics14081614
PG 19
WC Pharmacology & Pharmacy

WE Science Citation Index Expanded (SCI-EXPANDED)
SC Pharmacology & Pharmacy
GA 4C8SO
UT WOS:000846715900001
PM 36015239
OA Green Published, gold
DA 2023-09-14
ER

PT J
AU Operti, MC
Bernhardt, A
Pots, J
Sincari, V
Jager, E
Grimm, S
Engel, A
Benedikt, A
Hruby, M
De Vries, IJM
Figdor, CG
Tagit, O
AF Operti, Maria Camilla
Bernhardt, Alexander
Pots, Jeanette
Sincari, Vladimir
Jager, Eliezer
Grimm, Silko
Engel, Andrea
Benedikt, Anne
Hruby, Martin
De Vries, Ingrid Jolanda M.
Figdor, Carl G.
Tagit, Oya
TI Translating the Manufacture of Immunotherapeutic PLGA Nanoparticles from Lab to Industrial Scale: Process Transfer and In Vitro Testing
SO PHARMACEUTICS
LA English
DT Article
DE drug delivery; PLGA; nanoparticles; nanomedicine; scale-up manufacturing; clinical translation
AB Poly(lactic-co-glycolic acid) (PLGA) nanoparticle-based drug delivery systems are known to offer a plethora of potential therapeutic benefits. However, challenges related to large-scale manufacturing, such as the difficulty of reproducing complex formulations and high manufacturing costs, hinder their clinical and commercial development. In this context, a reliable manufacturing technique suitable for the scale-up production of nanoformulations without altering efficacy and safety profiles is highly needed. In this paper, we develop an inline sonication process and adapt it to the industrial scale production of immunomodulating PLGA nanovaccines developed using a batch sonication method at the laboratory scale. The investigated formulations contain three distinct synthetic peptides derived from the carcinogenic antigen New York Esophageal Squamous Cell Carcinoma-1 (NY-ESO-1) together with an invariant natural killer T-cell (iNKT) activator, threitolceramide-6 (IMM60). Process parameters were optimized to obtain polymeric nanovaccine formulations with a mean diameter of 150 +/- 50 nm and a polydispersity index <0.2. Formulation characteristics, including encapsulation efficiencies, release profiles and in vitro functional and toxicological profiles, are assessed and statistically compared for each formulation. Overall, scale-up formulations obtained by inline sonication method could replicate the colloidal and functional properties of the nanovaccines developed using batch sonication at the laboratory scale. Both types of formulations induced specific T-cell and iNKT cell responses in vitro without any toxicity, highlighting the suitability of the inline sonication method for the continuous scale-up of nanomedicine formulations in terms of efficacy and safety.
C1 [Operti, Maria Camilla; Pots, Jeanette; De Vries, Ingrid Jolanda M.; Figdor, Carl G.; Tagit, Oya] Radboud Univ Nijmegen, Radboud Inst Mol Life Sci, Dept Tumor Immunol, Med Ctr, NL-6500 HB

Nijmegen, Netherlands.
[Operti, Maria Camilla; Bernhardt, Alexander; Grimm, Silko; Benedikt, Anne] Evonik Operat GmbH, Res Dev & Innovat, D-64293 Darmstadt, Germany.
[Hruby, Martin] CAS, Inst Macromol Chem, Heyrovsky Sq 2, Prague 16206, Czech Republic.
[Engel, Andrea] Evonik Corp, Birmingham Labs, Birmingham, AL 35211 USA.
C3 Radboud University Nijmegen; Czech Academy of Sciences; Institute of Macromolecular Chemistry of the Czech Academy of Sciences
RP Tagit, O (通讯作者)，Radboud Univ Nijmegen, Radboud Inst Mol Life Sci, Dept Tumor Immunol, Med Ctr, NL-6500 HB Nijmegen, Netherlands.
EM oya.tagit@fhnw.ch
RI Jäger, Eliézer/D-8924-2016; Sincari, Vladimir/P-2977-2017; Hruby, Martin/H-6479-2014; de Vries, I. Jolanda M./F-5563-2010; Tagit, Oya/A-2194-2016
OI Jäger, Eliézer/0000-0001-9939-2355; Sincari, Vladimir/0000-0002-7379-066X; Hruby, Martin/0000-0002-5075-261X; de Vries, I. Jolanda M./0000-0002-8653-4040; Operti, Maria Camilla/0000-0002-2217-6096; Tagit, Oya/0000-0002-5773-6647
FU NWO Spinoza grant; ERC Advanced Grant Pathfinder [269019]; Dutch cancer society award [2009-4402]
FX The authors acknowledge Ian Walters for kindly providing the IMM60 and Yusuf Dolen for developing the in vitro antigen presentation assay used in this work. CF received the NWO Spinoza grant, ERC Advanced Grant Pathfinder (269019) and Dutch cancer society award 2009-4402.
CR Bairwa M, 2014, HUM VACC IMMUNOTHER, V10, P708, DOI 10.4161/hv.27520
Bosshart H, 2016, ANN TRANSL MED, V4, DOI 10.21037/atm.2016.08.53
Burn Olivia K, 2021, Oxf Open Immunol, V2, piqab013, DOI 10.1093/oxfimm/iqab013
Castro F, 2018, FRONT IMMUNOL, V9, DOI 10.3389/fimmu.2018.00847
Chackerian B, 2010, HUM VACCINES, V6, P926, DOI [10.4161/hv.6.11.12655, 10.4161/hv.7.1.12655]
Chauhan G, 2020, ACS NANO, V14, P7760, DOI 10.1021/acsnano.0c04006
Choo SY, 2007, YONSEI MED J, V48, P11, DOI 10.3349/ymj.2007.48.1.11
clinicaltrials, DOSE ESCALATION STUD
Dolen Y, 2021, FRONT IMMUNOL, V12, DOI 10.3389/fimmu.2021.641703
Dolen Y, 2020, ONCOIMMUNOLOGY, V9, DOI 10.1080/2162402X.2020.1738813
Dolen Y, 2016, ONCOIMMUNOLOGY, V5, DOI 10.1080/2162402X.2015.1068493
Dordevic S, 2022, DRUG DELIV TRANSL RE, V12, P500, DOI 10.1007/s13346-021-01024-2
Dutta D, 2016, J BIOMED MATER RES A, V104, P688, DOI 10.1002/jbm.a.35608
expasy, PROTPARAM TOOL EXPAS
Gasteiger E., 2005, PROTEOMICS PROTOCOLS, P571
Hu Y, 2014, HUM VACC IMMUNOTHER, V10, P64, DOI 10.4161/hv.26635
Hua S, 2018, FRONT PHARMACOL, V9, DOI 10.3389/fphar.2018.00790
Jensen KK, 2019, SCI REP-UK, V9, DOI 10.1038/s41598-019-50932-4
KYTE J, 1982, J MOL BIOL, V157, P105, DOI 10.1016/0022-2836(82)90515-0
Maleki M, 2019, ARTIF CELL NANOMED B, V47, P4248, DOI 10.1080/21691401.2019.1687490
Oliver RC, 2013, PLOS ONE, V8, DOI 10.1371/journal.pone.0062488
Operti MC, 2022, PHARMACEUTICS, V14, DOI 10.3390/pharmaceutics14020276
Operti MC, 2021, INT J PHARMACEUT, V605, DOI 10.1016/j.ijpharm.2021.120807
Operti MC, 2019, PHARMACEUTICS, V11, DOI 10.3390/pharmaceutics11110590
Operti MC, 2018, INT J PHARMACEUT, V550, P140, DOI 10.1016/j.ijpharm.2018.08.044
PubChem Threitolceramide, US
Sahu R, 2020, NANOMED-NANOTECHNOL, V29, DOI 10.1016/j.nano.2020.102257
Thomas R, 2018, FRONT IMMUNOL, V9, DOI 10.3389/fimmu.2018.00947
Thukral A, 2020, NPJ VACCINES, V5, DOI 10.1038/s41541-020-0164-y
Wieczorek M, 2017, FRONT IMMUNOL, V8, DOI 10.3389/fimmu.2017.00292
Zhao L, 2014, VACCINE, V32, P327, DOI 10.1016/j.vaccine.2013.11.069
NR 31
TC 0
Z9 0
U1 3
U2 5
PU MDPI
PI BASEL

PA ST ALBAN-ANLAGE 66, CH-4052 BASEL, SWITZERLAND
EI 1999-4923
J9 PHARMACEUTICS
JI Pharmaceutics
PD AUG
PY 2022
VL 14
IS 8
AR 1690
DI 10.3390/pharmaceutics14081690
PG 16
WC Pharmacology & Pharmacy
WE Science Citation Index Expanded (SCI-EXPANDED)
SC Pharmacology & Pharmacy
GA 4C6AP
UT WOS:000846534000001
PM 36015316
OA Green Published, gold
DA 2023-09-14
ER

PT J
AU Wang, QQ
Muhammad, TA
Muhammad, WH
Muhammad, AM
Muhammad, H
Yan, RF
Xu, LX
Song, XK
Li, XR
AF Wang, QiangQiang
Muhammad, Tahir Aleem
Muhammad, Waqqas Hasan
Muhammad, Ali Memon
Muhammad, Haseeb
Yan, RuoFeng
Xu, LiXin
Song, XiaoKai
Li, XiangRui
TI Hepatocellular carcinoma-associated antigen 59 and ADP-ribosylation factor 1 with poly (lactic-co-glycolic acid): A promising candidate as nanovaccine against haemonchosis
SO MICROBIAL PATHOGENESIS
LA English
DT Article
DE PLGA nanoparticles; Haemonchus contortus; Hc-HCA59; Hc-ARF1; Nanovaccines; Goats
ID HAEMONCHUS-CONTORTUS; EXCRETORY/SECRETORY PRODUCTS; IMMUNE-RESPONSE; ANTHELMINTIC RESISTANCE; INFECTION; NANOPARTICLES; GTPASES; DESIGN; ARF1
AB Haemonchus contortus (H. contortus) ADP-ribosylation factor 1 (Hc-ARF1) and Hepatocellular carcinomaassociated antigen 59 (Hc-HCA59) are recognized to largely regulate the immune responses of host cells. However, studies about the protective efficacy of the two molecules are poorly unknown. In this research, combinations of recombinant Hc-HCA59 (rHc-HCA59) and Hc-ARF1 (rHc-ARF1) proteins were amalgamated with poly (lactic-co-glycolic acid) (PLGA) nanoparticles adjuvant in order to investigate their protection potential against H. contortus in goats. The results demonstrated that the levels of IgG, IgA, IgE, and IL-4 were noticeably enhanced in the rHc-HCA59 and rHc-ARF1 (rHc-HCA59+rHc-ARF1) group before H. contortus thirdstage larvae (L3) challenge. After the L3 challenge, the levels of IL-17, IL-9, and TGF-beta were considerably upregulated in the rHc-HCA59+rHc-ARF1 group. In the meantime, the abomasal worm burdens and the fecal eggs were reduced by 63.2% and 69.4% respectively in the rHc-HCA59+rHc-ARF1 group. According to the studies, PLGA nanoparticles immobilized with rHc-HCA59 and

rHc-ARF1 proteins conferred partial protection and were expected to be a potential candidate for developing nano vaccines to combat goat haemonchosis.
C1 [Wang, QiangQiang; Muhammad, Tahir Aleem; Muhammad, Waqqas Hasan; Muhammad, Ali Memon; Muhammad, Haseeb; Yan, RuoFeng; Xu, LiXin; Song, XiaoKai; Li, XiangRui] Nanjing Agr Univ, Coll Vet Med, MOE Joint Int Res Lab Anim Hlth & Food Safety, Nanjing, Jiangsu, Peoples R China.
C3 Nanjing Agricultural University
RP Li, XR (通讯作者)，Nanjing Agr Univ, Coll Vet Med, MOE Joint Int Res Lab Anim Hlth & Food Safety, Nanjing, Jiangsu, Peoples R China.
EM lixiangrui@njau.edu.cn
OI hasan, Muhammad waqqas/0000-0001-8560-7714
FU Policy Guidance Project of Jiangsu Province for International Scientific and Technological Coop-eration [BZ2019013]; National Key Research and Devel-opment Program of China [2017YFD0501200]; National Key Basic Research Program (973 Program) of P.R. China [2015CB150300]
FX Acknowledgments This work was funded by grants from the Policy Guidance Project of Jiangsu Province for International Scientific and Technological Coop-eration (Grant No. BZ2019013) , the National Key Research and Devel-opment Program of China (Grant No.2017YFD0501200) and the National Key Basic Research Program (973 Program) of P.R. China (Grant No.2015CB150300) .
CR Abbas RZ, 2020, PAK VET J, V40, P455, DOI 10.29261/pakvetj/2020.083
Amarante AFT, 2005, VET PARASITOL, V128, P99, DOI 10.1016/j.vetpar.2004.11.021
Aryal S, 2021, ARTIF CELL NANOMED B, V49, P390, DOI 10.1080/21691401.2021.1909605
Bambou JC, 2008, VET PARASITOL, V158, P311, DOI 10.1016/j.vetpar.2008.09.020
Bressani FA, 2014, GENET MOL RES, V13, P8530, DOI 10.4238/2014.October.20.29
Casalou C, 2020, FRONT CELL DEV BIOL, V8, DOI 10.3389/fcell.2020.00217
Ceriac S, 2019, SCI REP-UK, V9, DOI 10.1038/s41598-018-37800-3
Cetin G, 2021, J VET PHARMACOL THER, V44, P961, DOI 10.1111/jvp.13009
Chen JL, 2019, PARASITOL RES, V118, P1239, DOI 10.1007/s00436-019-06230-z
Cherfils J, 2014, CURR OPIN STRUC BIOL, V29, P67, DOI 10.1016/j.sbi.2014.09.007
Chuang SC, 2017, PARASITE, V24, DOI 10.1051/parasite/2017004
De Vries E, 2009, VET RES, V40, DOI 10.1051/vetres/2009025
Diliani N, 2017, PARASITE IMMUNOL, V39, DOI 10.1111/pim.12459
Estrada-Reyes Z, 2017, PARASITE IMMUNOL, V39, DOI 10.1111/pim.12427
Gadahi JA, 2017, ONCOTARGET, V8, P112211, DOI 10.18632/oncotarget.22662
Gadahi JA, 2016, PLOS ONE, V11, DOI 10.1371/journal.pone.0159796
Gadahi JA, 2016, ONCOTARGET, V7, P35670, DOI 10.18632/oncotarget.9589
Hasan MW, 2021, RES VET SCI, V136, P247, DOI 10.1016/j.rvsc.2021.03.007
Hasan MW, 2020, VACCINES-BASEL, V8, DOI 10.3390/vaccines8040726
Hua YB, 2021, DRUG DELIV, V28, P1342, DOI 10.1080/10717544.2021.1943056
Huber A, 2005, PARASITOL RES, V96, P290, DOI 10.1007/s00436-005-1321-y
Isailovic N, 2015, J AUTOIMMUN, V60, P1, DOI 10.1016/j.jaut.2015.04.006
Jacobs JR, 2016, PARASITE IMMUNOL, V38, P333, DOI 10.1111/pim.12321
Jacobs JR, 2015, VET PARASITOL, V211, P102, DOI 10.1016/j.vetpar.2015.04.024
Johnston CJC, 2017, NAT COMMUN, V8, DOI 10.1038/s41467-017-01886-6
Kalyanasundaram A, 2015, EXP PARASITOL, V154, P98, DOI 10.1016/j.exppara.2015.04.016
Kim ES, 2015, PLOS ONE, V10, DOI 10.1371/journal.pone.0119380
Kong F, 1997, LIVESTOCK PARASITOLO, Vthird, P365
Kotze AC, 2016, ADV PARASIT, V93, P397, DOI 10.1016/bs.apar.2016.02.012
Lamb J, 2017, VET PARASITOL, V241, P48, DOI 10.1016/j.vetpar.2017.05.008
Lee JE, 2020, PATHOL ONCOL RES, V26, P2017, DOI 10.1007/s12253-019-00665-6
Li B, 2005, CLIN EXP IMMUNOL, V140, P310, DOI 10.1111/j.1365-2249.2005.02786.x
Licona-Limon P, 2017, SEMIN IMMUNOPATHOL, V39, P29, DOI 10.1007/s00281-016-0606-9
Merkelbach P, 2002, VET PARASITOL, V104, P217, DOI 10.1016/S0304-4017(01)00635-5
Mir M, 2017, COLLOID SURFACE B, V159, P217, DOI 10.1016/j.colsurfb.2017.07.038
Naeem M, 2021, TROP ANIM HEALTH PRO, V53, DOI 10.1007/s11250-020-02439-8
Nazish A, 2021, ANIMALS-BASEL, V11, DOI 10.3390/ani11061843
Nehra AK, 2019, VET PARASITOL, V267, P47, DOI 10.1016/j.vetpar.2019.01.005
Ngongeh Lucas Atehmengo, 2017, J Parasit Dis, V41, P997, DOI 10.1007/s12639-017-0924-4
Noormehr H, 2018, INT IMMUNOPHARMACOL, V59, P97, DOI 10.1016/j.intimp.2018.03.012
Peres C, 2017, ACTA BIOMATER, V48, P41, DOI 10.1016/j.actbio.2016.11.012

Brandan CP, 2017, BMC INFECT DIS, V17, DOI 10.1186/s12879-017-2834-6
Przemeck S, 2005, PARASITOL RES, V95, P213, DOI 10.1007/s00436-004-1280-8
Rehman T, 2021, VET ARHIV, V91, P307, DOI 10.24099/vet.arhiv.1024
Rehman ZU, 2016, VET PARASITOL, V221, P104, DOI 10.1016/j.vetpar.2016.03.017
Robinson N, 2011, INT J PARASITOL, V41, P487, DOI 10.1016/j.ijpara.2010.11.006
Sarkar I, 2019, EXPERT REV VACCINES, V18, P505, DOI 10.1080/14760584.2019.1604231
Schijns V, 2020, IMMUNOL REV, V296, P169, DOI 10.1111/imr.12889
Serbzhinskiy DA, 2015, ACTA CRYSTALLOGR F, V71, P594, DOI 10.1107/S2053230X15004677
Tian XW, 2019, VET RES, V50, DOI 10.1186/s13567-019-0661-z
Tuo WB, 2021, VET PARASITOL, V296, DOI 10.1016/j.vetpar.2021.109510
Vedamurthy GV, 2019, GENE, V703, P102, DOI 10.1016/j.gene.2019.03.056
Wang QQ, 2021, INT J NANOMED, V16, P3125, DOI 10.2147/IJN.S301851
Wang QQ, 2021, VET PARASITOL, V292, DOI 10.1016/j.vetpar.2021.109398
Wang QQ, 2019, PARASITE VECTOR, V12, DOI 10.1186/s13071-019-3375-1
Widiarso BP, 2018, VET WORLD, V11, P921, DOI 10.14202/vetworld.2018.921-925
Wusiman A, 2019, CARBOHYD POLYM, V211, P217, DOI 10.1016/j.carbpol.2019.01.102
Yan RF, 2013, RES VET SCI, V95, P189, DOI 10.1016/j.rvsc.2013.02.020
Yan XF, 2021, PLOS ONE, V16, DOI 10.1371/journal.pone.0251307
Yavuz S, 2017, MOL BIOL CELL, V28, P1782, DOI [10.1091/mbc.E17-03-0151, 10.1091/mbc.e17-03-0151]
Zhang NZ, 2016, BMC INFECT DIS, V16, DOI 10.1186/s12879-016-1496-0
Zhao GW, 2012, VET J, V191, P94, DOI 10.1016/j.tvjl.2010.12.023
Zhao K, 2012, PLOS ONE, V7, DOI 10.1371/journal.pone.0053314
Zupancic E, 2017, J CONTROL RELEASE, V258, P182, DOI 10.1016/j.jconrel.2017.05.014
NR 64
TC 0
Z9 0
U1 0
U2 4
PU ACADEMIC PRESS LTD- ELSEVIER SCIENCE LTD
PI LONDON
PA 24-28 OVAL RD, LONDON NW1 7DX, ENGLAND
SN 0882-4010
EI 1096-1208
J9 MICROB PATHOGENESIS
JI Microb. Pathog.
PD JUL
PY 2022
VL 168
AR 105614
DI 10.1016/j.micpath.2022.105614
EA JUN 2022
PG 8
WC Immunology; Microbiology
WE Science Citation Index Expanded (SCI-EXPANDED)
SC Immunology; Microbiology
GA 3L8VK
UT WOS:000835040000006
PM 35662672
DA 2023-09-14
ER

PT J
AU Song, SL
Xu, HB
Yang, Y
Wan, QK
He, B
Cai, F
Yin, HM
Zhou, YC

Jin, XX
He, ZL
AF Song, Shilong
Xu, Hongbo
Yang, Yan
Wan, Qiangkun
He, Bin
Cai, Feng
Yin, Hongmei
Zhou, Yongchun
Jin, Xiaoxiao
He, Zelai
TI Assessing the Efficacy of a Tumor Nanovaccine and Artificial Antigen Presenting Cell-Based System as a Combination Therapy in a Mouse Model of Melanoma
SO JOURNAL OF BIOMEDICAL NANOTECHNOLOGY
LA English
DT Article
DE Melanoma; Tumor Vaccine; aAPC; Immunotherapy
ID CD4(+) T-CELLS; LYSATE VACCINE; NANOPARTICLES; RESPONSES; POLYMERS
AB Tumor cell lysate (TCL)-based vaccines contain a large number of tumor-specific and related antigens, albeit at low levels, that require active transfer and presentation by antigen-presenting cells (APCs) in vivo, which stimulate a weak immune response. The artificial APC (aAPC) system presented herein is a cell-based therapeutic system that can significantly enhance the immune response compared to TCL-based vaccines. This study combines these two treatment strategies to assess their in vitro and in vivo effects. We successfully prepared TCL-poly(lactic-co-glycolic acid)-PEI (TPP) and demonstrated that it was phagocytosedIP:by203.8.109.20theAPCsO:andThu, enhanced22 Septhe2022maturation09:47:02ofDCsin vitro. The use of TPP in combination with the aAPCs resulted in better antitumor effects compared to tindividual therapies. The combination Copyright: American Sc entific Publishe rs therapy induced a higher proportion of CD4+ T, C8+ T, and TRP2180-188-specific CD8+ T cells in comparison with the Delive ed by Inge a individual therapies. Additionally, the combination therapy enhanced the in vitro proliferation activity; greater inhibited regulatory T cells; and promoted inflammatory cytokine secretion, while reduced the production of inhibitory cytokines. In conclusion, the combination therapy consisting of the TPP tumor nanovaccine and the aAPC system enabled a broader immune response and achieve better antitumor effects compared to treatment with the individual therapies.
C1 [Song, Shilong; Xu, Hongbo; Yang, Yan; Wan, Qiangkun; He, Bin; Cai, Feng; Yin, Hongmei; Zhou, Yongchun; He, Zelai] Bengbu Med Coll, Dept Radiat Oncol, Affiliated Hosp 1, Bengbu 233000, Anhui, Peoples R China.
[Song, Shilong; He, Zelai] Bengbu Med Coll, Anhui Prov Key Lab Translat Canc Res, Bengbu 233000, Anhui, Peoples R China.
[Jin, Xiaoxiao] Southeast Univ, Med Sch, Dept Microbiol & Immunol, Nanjing 210009, Jiangsu, Peoples R China.
C3 Bengbu Medical College; Bengbu Medical College; Southeast University - China
RP He, ZL (通讯作者)，Bengbu Med Coll, Dept Radiat Oncol, Affiliated Hosp 1, Bengbu 233000, Anhui, Peoples R China.; He, ZL (通讯作者)，Bengbu Med Coll, Anhui Prov Key Lab Translat Canc Res, Bengbu 233000, Anhui, Peoples R China.
EM hezelai@alumni.situ.edu.cn
FU Natural Science Research Project of Universities of Anhui Province [KJ2021A0781]; Scientific Research Project of Anhui Province of Health Commission [AHWJ2021b075]; National Natural Science Foundation of China [81602727]; First Affiliated Hospital of Bengbu Medical College Science Fund for Distinguished Young Scholars [2019BYY-FYJQ04]; Bengbu Medical College Science Fund for "Excellent Young Teachers in 512 Talent Development Programme" [BY51201314]; Translation Medicine Key Project of Bengbu Medical College [BYTM2019013]; Bengbu-Bengbu Medical College Joint Research Project [BYLK201810]; Bengbu Municipality: Scientific and technological innovation guidance program [20200341]
FX These studies were supported by the Natural Science Research Project of Universities of Anhui Province (No. KJ2021A0781) , the Scientific Research Project of Anhui Province of Health Commission (No.

AHWJ2021b075) , the National Natural Science Foundation of China (No. 81602727) , the First Affiliated Hospital of Bengbu Medical College Science Fund for Distinguished Young Scholars (No. 2019BYY- FYJQ04) , the Bengbu Medical College Science Fund for "Excellent Young Teachers in 512 Talent Development Programme" (No. BY51201314) , the Translation Medicine Key Project of Bengbu Medical College (No. BYTM2019013) , the Bengbu-Bengbu Medical College Joint Research Project (No. BYLK201810) , the Bengbu Municipality: Scientific and technological innovation guidance program (No. 20200341) .
CR Chen HA, 2010, BIOMATERIALS, V31, P8172, DOI 10.1016/j.biomaterials.2010.07.056
Dobrovolskaia MA, 2007, NAT NANOTECHNOL, V2, P469, DOI 10.1038/nnano.2007.223
Dong X, 2019, ACS APPL MATER INTER, V11, P4876, DOI 10.1021/acsami.8b20364
Grego EA, 2021, CURR TOP MICROBIOL, V433, P29, DOI 10.1007/82_2020_226
Gross BP, 2014, AAPS J, V16, P1194, DOI 10.1208/s12248-014-9662-z
Gutkin DW, 2014, CANCER IMMUNOL IMMUN, V63, P45, DOI 10.1007/s00262-013-1480-0
Ilinskaya AN, 2016, TOXICOL APPL PHARM, V299, P70, DOI 10.1016/j.taap.2016.01.005
Iranpour S, 2016, J EXP CLIN CANC RES, V35, DOI 10.1186/s13046-016-0444-6
Janssen EM, 2003, NATURE, V421, P852, DOI 10.1038/nature01441
Kawahara M, 2015, CANCER BIOL THER, V16, P1616, DOI 10.1080/15384047.2015.1078027
Lojk J, 2017, TOXICOL LETT, V270, P108, DOI 10.1016/j.toxlet.2017.02.010
Ma L, 2021, ADV MATER, V33, DOI 10.1002/adma.202104849
MacKerracher A, 2022, EUR J PHARM SCI, V175, DOI 10.1016/j.ejps.2022.106209
Oh DY, 2020, CELL, V181, P1612, DOI 10.1016/j.cell.2020.05.017
Ohue Y, 2019, CANCER SCI, V110, P2080, DOI 10.1111/cas.14069
Rabinovich GA, 2007, ANNU REV IMMUNOL, V25, P267, DOI
10.1146/annurev.immunol.25.022106.141609
Najafabadi SAS, 2022, IMMUNOTHERAPY-UK, V14, P639, DOI 10.2217/imt-2022-0036
Shedlock DJ, 2003, SCIENCE, V300, P337, DOI 10.1126/science.1082305
Shen C, 2017, INT J NANOMED, V12, P5443, DOI 10.2147/IJN.S137980
Silva AL, 2016, HUM VACC IMMUNOTHER, V12, P1056, DOI 10.1080/21645515.2015.1117714
Sondak VK, 2003, SEMIN CANCER BIOL, V13, P409, DOI 10.1016/j.semcancer.2003.09.004
Song C, 2016, INT J NANOMED, V11, P3753, DOI 10.2147/IJN.S110796
Song SL, 2019, INT J NANOMED, V14, P2465, DOI 10.2147/IJN.S195828
Tang XD, 2022, CANCER IMMUNOL IMMUN, V71, P2969, DOI 10.1007/s00262-022-03209-1
Xiang SD, 2006, METHODS, V40, P1, DOI 10.1016/j.ymeth.2006.05.016
Yuba E, 2013, BIOMATERIALS, V34, P3042, DOI 10.1016/j.biomaterials.2012.12.031
Zhang L, 2019, CANCER IMMUNOL RES, V7, P1188, DOI 10.1158/2326-6066.CIR-18-0881
Zhang Y, 2020, INT J NANOMED, V15, P5527, DOI 10.2147/IJN.S252515
Zhao LJ, 2018, ONCOL LETT, V16, P1180, DOI 10.3892/ol.2018.8785
Zou WP, 2005, NAT REV CANCER, V5, P263, DOI 10.1038/nrc1586
NR 30
TC 0
Z9 0
U1 1
U2 7
PU AMER SCIENTIFIC PUBLISHERS
PI VALENCIA
PA 26650 THE OLD RD, STE 208, VALENCIA, CA 91381-0751 USA
SN 1550-7033
EI 1550-7041
J9 J BIOMED NANOTECHNOL
JI J. Biomed. Nanotechnol.
PD MAY
PY 2022
VL 18
IS 5
BP 1362
EP 1375
DI 10.1166/jbn.2022.3347
PG 14
WC Nanoscience & Nanotechnology; Materials Science, Biomaterials

WE Science Citation Index Expanded (SCI-EXPANDED)
SC Science & Technology - Other Topics; Materials Science
GA 4Z9GA
UT WOS:000862506400002
DA 2023-09-14
ER

PT J
AU Shao, J
Liu, Q
Shen, J
Qian, XP
Yan, J
Zhu, YH
Qiu, X
Lu, CC
Cen, LQ
Tian, MM
Du, J
Liu, BR
AF Shao, Jie
Liu, Qin
Shen, Jie
Qian, Xiaoping
Yan, Jing
Zhu, Yahui
Qiu, Xin
Lu, Changchang
Cen, Lanqi
Tian, Manman
Du, Juan
Liu, Baorui
TI Advanced Pancreatic Cancer Patient Benefit From Personalized Neoantigen Nanovaccine Based Immunotherapy: A Case Report
SO FRONTIERS IN IMMUNOLOGY
LA English
DT Article
DE pancreatic cancer; neoantigen nanovaccine; immunotherapy; T-cell responses; benefit
ID SECRETING TUMOR VACCINE; TRIAL; NIVOLUMAB; SAFETY; ADENOCARCINOMA; ACTIVATION; SURVIVAL
AB Personal neoantigen vaccines are considered to be effective methods for inducing, amplifying and diversifying antitumor T cell responses. We recently conducted a clinical study that combined neoantigen nanovaccine with anti-PD-1 antibody. Here, we reported a case with a clear beneficial outcome from this treatment. We established a process that includes comprehensive identification of individual mutations, computational prediction of new epitopes, and design and manufacture of unique nanovaccines for this patient. Nanovaccine started after a relapse in third-line treatment. We assessed the patient's clinical outcome and circulating immune response. In this advanced pancreatic cancer patient, the OS associated with the vaccine treatment was 10.5 months. A peptide-specific T-cell response against 9 of the 12 vaccine peptides could be detected sequentially. Robust neoantigen-specific T cell responses were also detected by IFN-gamma ELISPOT and intracellular cytokine staining. In conclusion, sustained functional neoantigen-specific T cell therapy combined with immune checkpoint targeting may be well suited to help control progressive metastatic pancreatic cancer.
C1 [Shao, Jie; Liu, Qin; Shen, Jie; Qian, Xiaoping; Yan, Jing; Zhu, Yahui; Qiu, Xin; Lu, Changchang; Cen, Lanqi; Tian, Manman; Du, Juan; Liu, Baorui] Nanjing Univ, Sch Med, Drum Tower Hosp, Ctr Comprehens Canc, Nanjing, Peoples R China.
[Shao, Jie; Liu, Qin; Shen, Jie; Qian, Xiaoping; Yan, Jing; Zhu, Yahui; Qiu, Xin; Lu, Changchang; Cen, Lanqi; Tian, Manman; Du, Juan; Liu, Baorui] Nanjing Univ, Clin Canc Inst, Nanjing, Peoples R China.
C3 Nanjing University; Nanjing University
RP Du, J; Liu, BR (通讯作者)，Nanjing Univ, Sch Med, Drum Tower Hosp, Ctr Comprehens Canc, Nanjing, Peoples R China.; Du, J; Liu, BR (通讯作者)，Nanjing Univ, Clin Canc Inst, Nanjing, Peoples R China.

EM dujuanglyy@163.com; baoruiliu@nju.edu.cn
RI Lu, Changchang/HCI-0267-2022
FU National Natural Science Foundation of China [81902914]
FX Funding This work was supported by grants from the National Natural
Science Foundation of China (NO. 81902914).
CR Borghaei H, 2015, NEW ENGL J MED, V373, P1627, DOI 10.1056/NEJMoa1507643
Brahmer JR, 2012, NEW ENGL J MED, V366, P2455, DOI 10.1056/NEJMoa1200694
Cappellano G, 2021, VACCINES-BASEL, V9, DOI 10.3390/vaccines9060606
Chen FJ, 2019, J CLIN INVEST, V129, P2056, DOI 10.1172/JCI99538
Chu YH, 2018, THERANOSTICS, V8, P4238, DOI 10.7150/thno.24387
Conroy T, 2011, NEW ENGL J MED, V364, P1817, DOI 10.1056/NEJMoa1011923
Haanen JBAG, 2017, ANN ONCOL, V28, P119, DOI 10.1093/annonc/mdx225
Hilf N, 2019, NATURE, V565, P240, DOI 10.1038/s41586-018-0810-y
Hu ZT, 2021, NAT MED, V27, P515, DOI 10.1038/s41591-020-01206-4
Hu ZSI, 2018, CLIN CANCER RES, V24, P1326, DOI 10.1158/1078-0432.CCR-17-3099
Jaffee EM, 2001, J CLIN ONCOL, V19, P145, DOI 10.1200/JCO.2001.19.1.145
Lau MK, 2010, PANCREAS, V39, P458, DOI 10.1097/MPA.0b013e3181bd6489
Lin QJ, 2015, WORLD J GASTROENTERO, V21, P7988, DOI 10.3748/wjg.v21.i26.7988
Liu LN, 2018, MOL THER, V26, P45, DOI 10.1016/j.ymthe.2017.10.020
Lutz E, 2011, ANN SURG, V253, P328, DOI 10.1097/SLA.0b013e3181fd271c
Motzer RJ, 2015, NEW ENGL J MED, V373, P1803, DOI 10.1056/NEJMoa1510665
Ott PA, 2020, CELL, V183, P347, DOI 10.1016/j.cell.2020.08.053
Ott PA, 2017, NATURE, V547, P217, DOI 10.1038/nature22991
Rahib L, 2014, CANCER RES, V74, P2913, DOI 10.1158/0008-5472.CAN-14-0155
Royal RE, 2010, J IMMUNOTHER, V33, P828, DOI 10.1097/CJI.0b013e3181eec14c
Ryan DP, 2014, NEW ENGL J MED, V371, P1039, DOI 10.1056/NEJMra1404198
Santos PM, 2020, J EXP MED, V217, DOI 10.1084/jem.20191369
Sharma P, 2016, LANCET ONCOL, V17, P1590, DOI 10.1016/S1470-2045(16)30496-X
Siegel Rebecca L, 2017, CA Cancer J Clin, V67, P7, DOI 10.3322/caac.21387
Sun BB, 2013, SMALL, V9, P1595, DOI 10.1002/smll.201201962
Tanida T, 2015, INT J ONCOL, V46, P78, DOI 10.3892/ijo.2014.2717
Tao YC, 2020, INT IMMUNOPHARMACOL, V78, DOI 10.1016/j.intimp.2019.106023
Torphy RJ, 2018, ANN GASTROENT SURG, V2, P274, DOI 10.1002/ags3.12176
Von Hoff DD, 2013, NEW ENGL J MED, V369, P1691, DOI 10.1056/NEJMoa1304369
Wang GC, 2017, INT J BIOL MACROMOL, V101, P398, DOI 10.1016/j.ijbiomac.2017.03.060
NR 30
TC 0
Z9 0
U1 2
U2 9
PU FRONTIERS MEDIA SA
PI LAUSANNE
PA AVENUE DU TRIBUNAL FEDERAL 34, LAUSANNE, CH-1015, SWITZERLAND
SN 1664-3224
J9 FRONT IMMUNOL
JI Front. Immunol.
PD FEB 22
PY 2022
VL 13
AR 799026
DI 10.3389/fimmu.2022.799026
PG 7
WC Immunology
WE Science Citation Index Expanded (SCI-EXPANDED)
SC Immunology
GA ZU4AX
UT WOS:000769785500001
PM 35273594
OA Green Published, gold
DA 2023-09-14
ER

PT J
AU Mao, JR
Qian, Y
Shi, GP
Wang, JJ
Chen, YG
AF Mao Jia-Rong
Qian Ying
Shi Guo-Ping
Wang Jing-Jing
Chen Yu-Gen
TI Research Progress of Nanovaccine in Tumor Immunotherapy
SO PROGRESS IN BIOCHEMISTRY AND BIOPHYSICS
LA Chinese
DT Review
DE nano; vaccine; tumor immunotherapy; adjuvant; targeting
AB In recent years, tumor immunotherapy has become a hot spot in the field of cancer treatment. Among them, nanovaccines combining tumor vaccines and nanotechnology provide new ideas for tumor immunotherapy. Nanovaccine can realize the co-loading of vaccine and adjuvant. The intelligent nanocarriers further realize the effective targeted delivery of antigen, promote the uptake and presentation of antigen, specifically activate antigen-specific immune response and effectively kill tumor cells. This article reviews the principles, advantages, types of nano materials, and clinical efficacy of nanovaccine to provide a more reliable reference for the design of nano-vaccine in the later stage.
C1 [Mao Jia-Rong; Shi Guo-Ping; Chen Yu-Gen] Nanjing Univ Chinese Med, Affiliated Hosp, Nanjing 210029, Peoples R China.
[Qian Ying; Wang Jing-Jing] Nanjing Univ Chinese Med, Coll Pharm, Nanjing 210044, Peoples R China.
C3 Nanjing University of Chinese Medicine; Nanjing University of Chinese Medicine
RP Chen, YG (通讯作者)，Nanjing Univ Chinese Med, Affiliated Hosp, Nanjing 210029, Peoples R China.; Wang, JJ (通讯作者)，Nanjing Univ Chinese Med, Coll Pharm, Nanjing 210044, Peoples R China.
EM jingjingwang123456@163.com; yugen.chen@njucm.edu.cn
RI wang, jian/HRB-9588-2023; Wang, Jin/GYA-2019-2022; wang, jing/HJA-5384-2022; WANG, JINGYI/GSJ-1241-2022; wang, jing/GVT-8700-2022; wang, jie/HTQ-4920-2023; wang, jing/GRS-7509-2022; wang, jiahui/IXD-1197-2023; wang, juan/IUO-6218-2023; Wang, Jing/IQW-3496-2023; wang, xu/IAN-4886-2023; ZHOU, YUE/IZE-6277-2023
OI Wang, Jing/0000-0002-8296-2961;
FU National Natural Science Foundation of China [32000997]; Natural Science Foundation of Nanjing University of Chinese Medicine [NZY32000997]; Natural Science Fund for Colleges and Universities in Jiangsu Province [20KJB350004]; Advantage Discipline Project of Traditional Chinese Medicine [ZYX03KF038]; Innovation and Entrepreneurship Training Program for College Students in Jiangsu Province [202010315035Z]
FX This work was supported by grants from The National Natural Science Foundation of China(32000997), Natural Science Foundation of Nanjing University of Chinese Medicine (NZY32000997), Natural Science Fund for Colleges and Universities in Jiangsu Province (20KJB350004), Advantage Discipline Project of Traditional Chinese Medicine (ZYX03KF038), Innovation and Entrepreneurship Training Program for College Students in Jiangsu Province(202010315035Z).
NR 0
TC 0
Z9 0
U1 9
U2 69
PU CHINESE ACAD SCIENCES, INST BIOPHYSICS
PI BEIJING
PA 15 DATUN RD, CHAOYAND DISTRICT, BEIJING, 100101, PEOPLES R CHINA
SN 1000-3282
J9 PROG BIOCHEM BIOPHYS

JI Prog. Biochem. Biophys.
PD OCT
PY 2021
VL 48
IS 10
BP 1130
EP 1136
DI 10.16476/j.pibb.2021.0007
PG 7
WC Biochemistry & Molecular Biology; Biophysics
WE Science Citation Index Expanded (SCI-EXPANDED)
SC Biochemistry & Molecular Biology; Biophysics
GA WL7NZ
UT WOS:000710589300002
DA 2023-09-14
ER

PT S
AU Fontana, F
Bartolo, R
Santos, HA
AF Fontana, Flavia
Bartolo, Raquel
Santos, Helder A.
BE Fontana, F
Santos, HA
TI Biohybrid Nanosystems for Cancer Treatment: Merging the Best of Two Worlds
SO BIO-NANOMEDICINE FOR CANCER THERAPY
SE Advances in Experimental Medicine and Biology
LA English
DT Article; Book Chapter
DE Biohybrid; Nanoparticle; Cancer; Immunotherapy; Photothermal therapy
ID MESENCHYMAL STEM-CELLS; MEMBRANE-CAMOUFLAGED NANOPARTICLES; IN-VIVO BIODISTRIBUTION; RED-BLOOD-CELLS; BIOMIMETIC NANOPARTICLES; POLYMERIC NANOPARTICLES; PHOTOTHERMAL THERAPY; MACROPHAGE-MEMBRANE; TUMOR MICROENVIRONMENT; INTRACELLULAR DELIVERY
AB During the last 20+ years, research into the biomedical application of nanotechnology has helped in reshaping cancer treatment. The clinical use of several passively targeted nanosystems resulted in improved quality of care for patients. However, the therapeutic efficacy of these systems is not superior to the original drugs. Moreover, despite extensive investigations into actively targeted nanocarriers, numerous barriers still remain before their successful clinical translation, including sufficient blood-stream circulation time and efficient tumor targeting. The combination of synthetic nanomaterials with biological elements (e.g., cells, cell membranes, and macromolecules) is presently the cutting-edge research in cancer nanotechnology. The features provided by the biological moieties render the particles with prolonged bloodstream circulation time and homotopic targeting to the tumor site. Moreover, cancer cell membranes serve as sources of neoantigens, useful in the formulation of nanovaccines. In this chapter, we will discuss the advantages of biohybrid nanosystems in cancer chemotherapy, immunotherapy, and combined therapy, as well as highlight their preparation methods and clinical translatability.
C1 [Fontana, Flavia; Bartolo, Raquel; Santos, Helder A.] Univ Helsinki, Fac Pharm, Div Pharmaceut Chem & Technol, Drug Res Program, Helsinki, Finland.
C3 University of Helsinki
RP Santos, HA (通讯作者)，Univ Helsinki, Fac Pharm, Div Pharmaceut Chem & Technol, Drug Res Program, Helsinki, Finland.
EM helder.santos@helsinki.fi
RI Fontana, Flavia/AAB-4898-2020; Santos, Hélder A./AAC-6347-2022
OI Fontana, Flavia/0000-0001-9112-6917; Santos, Hélder A./0000-0001-7850-6309
FU HiLIFE Research Funds; Sigrid Juselius Foundation; European Research Council Proof-of-Concept Research Grant [825020]; European Research Council (ERC) [825020] Funding Source: European Research Council (ERC)

FX Prof. H. A. Santos acknowledges financial support from the HiLIFE
Research Funds, the Sigrid Juselius Foundation, and the European
Research Council Proof-of-Concept Research Grant (grant no. 825020).
CR Aldayel AM, 2018, J CONTROL RELEASE, V283, P280, DOI 10.1016/j.jconrel.2018.05.035
Altschuler SJ, 2010, CELL, V141, P559, DOI 10.1016/j.cell.2010.04.033
Anselmo AC, 2016, BIOENG TRANSL MED, V1, P10, DOI 10.1002/btm2.10003
Arnon TI, 2006, SEMIN CANCER BIOL, V16, P348, DOI 10.1016/j.semcancer.2006.07.005
Aryal S, 2013, NANOMEDICINE-UK, V8, P1271, DOI [10.2217/NNM.12.153, 10.2217/nnm.12.153]
Attia MF, 2019, J PHARM PHARMACOL, V71, P1185, DOI 10.1111/jphp.13098
Ayer M, 2017, J CONTROL RELEASE, V259, P92, DOI 10.1016/j.jconrel.2017.01.048
Balasubramanian V, 2017, ADV MATER, V29, DOI 10.1002/adma.201605375
Banskota S, 2017, MACROMOL BIOSCI, V17, DOI 10.1002/mabi.201600361
Banz A, 2010, VACCINE, V28, P2965, DOI 10.1016/j.vaccine.2010.02.013
BEZWODA WR, 1987, S AFR MED J, V72, P661
Boada C, 2020, CIRC RES, V126, P25, DOI 10.1161/CIRCRESAHA.119.315185
Bobo D, 2016, PHARM RES-DORDR, V33, P2373, DOI 10.1007/s11095-016-1958-5
Bommareddy PK, 2018, NAT REV IMMUNOL, V18, P498, DOI 10.1038/s41577-018-0014-6
Bose RJC, 2018, BIOMATERIALS, V185, P360, DOI 10.1016/j.biomaterials.2018.08.018
Bregoli L, 2016, NANOMED-NANOTECHNOL, V12, P81, DOI 10.1016/j.nano.2015.08.006
Brenner JS, 2018, NAT COMMUN, V9, DOI 10.1038/s41467-018-05079-7
Bronte V, 2016, NAT COMMUN, V7, DOI 10.1038/ncomms12150
Bu LL, 2019, ADV FUNCT MATER, V29, DOI 10.1002/adfm.201807733
Cao HQ, 2016, ACS NANO, V10, P7738, DOI 10.1021/acsnano.6b03148
Cao X, 2019, ACTA PHARM SIN B, V9, P575, DOI 10.1016/j.apsb.2018.12.009
Cheever MA, 2011, CLIN CANCER RES, V17, P3520, DOI 10.1158/1078-0432.CCR-10-3126
Chen MS, 2019, ARTIF CELL NANOMED B, V47, P1635, DOI 10.1080/21691401.2019.1608219
Chen WS, 2016, NANOSCALE, V8, P10364, DOI 10.1039/c6nr00535g
Chen Z, 2016, ACS NANO, V10, P10049, DOI 10.1021/acsnano.6b04695
Cheung AS, 2016, SMALL, V12, P2321, DOI 10.1002/smll.201600061
Cole C, 2005, NAT MED, V11, P1073, DOI 10.1038/nm1297
D'Avanzo N, 2020, ADV THER-GERMANY, V3, DOI 10.1002/adtp.201900170
Danhier F, 2016, J CONTROL RELEASE, V244, P108, DOI 10.1016/j.jconrel.2016.11.015
Dehaini D, 2017, ADV MATER, V29, DOI 10.1002/adma.201606209
Deng GJ, 2018, ACS NANO, V12, P12096, DOI 10.1021/acsnano.8b05292
Deshpande P, 2018, DRUG DELIV, V25, P517, DOI 10.1080/10717544.2018.1435747
Duchi S, 2013, J CONTROL RELEASE, V168, P225, DOI 10.1016/j.jconrel.2013.03.012
Dunn GP, 2002, NAT IMMUNOL, V3, P991, DOI 10.1038/ni1102-991
Fang RH, 2018, ADV MATER, V30, DOI 10.1002/adma.201706759
Fang RH, 2014, NANO LETT, V14, P2181, DOI 10.1021/nl500618u
Felismino CD, 2018, J CONTROL RELEASE, V281, P11, DOI 10.1016/j.jconrel.2018.05.003
Filley AC, 2017, FRONT ONCOL, V7, DOI 10.3389/fonc.2017.00106
Fontana F, 2019, ACS NANO, V13, P6477, DOI 10.1021/acsnano.8b09613
Fontana F, 2018, ADV FUNCT MATER, V28, DOI 10.1002/adfm.201801355
Fontana F, 2017, ADV MATER, V29, DOI 10.1002/adma.201603239
Fouad YA, 2017, AM J CANCER RES, V7, P1016
Furman NET, 2013, NANO LETT, V13, P3248, DOI 10.1021/nl401376w
Fusciello M, 2019, NAT COMMUN, V10, DOI 10.1038/s41467-019-13744-8
Gao CY, 2016, SMALL, V12, P4056, DOI 10.1002/smll.201600624
Gao J., 2014, ENCY POLYM NANOMATER
Gao JQ, 2008, PHARM RES-DORDR, V25, P752, DOI 10.1007/s11095-007-9443-9
Gao YL, 2017, RSC ADV, V7, P31588, DOI 10.1039/c7ra04349j
Guo YY, 2015, ACS NANO, V9, P6918, DOI 10.1021/acsnano.5b01042
Hadrys A, 2020, EUR J PHARMACOL, V874, DOI 10.1016/j.ejphar.2020.172991
Han X, 2019, SCI ADV, V5, DOI 10.1126/sciadv.aaw6870
Han X, 2018, BIOCONJUGATE CHEM, V29, P852, DOI 10.1021/acs.bioconjchem.7b00758
Hanahan D, 2011, CELL, V144, P646, DOI 10.1016/j.cell.2011.02.013
Hayat SMG, 2019, INT J PHARMACEUT, V569, DOI 10.1016/j.ijpharm.2019.118628
He HL, 2019, ACCOUNTS CHEM RES, V52, P2445, DOI 10.1021/acs.accounts.9b00228
He HL, 2018, NANO LETT, V18, P6164, DOI 10.1021/acs.nanolett.8b01892
He WP, 2016, LANGMUIR, V32, P3637, DOI 10.1021/acs.langmuir.5b04762
Herranz-Blanco B, 2015, ADV FUNCT MATER, V25, P1488, DOI 10.1002/adfm.201404122

Hirn S, 2011, EUR J PHARM BIOPHARM, V77, P407, DOI 10.1016/j.ejpb.2010.12.029
Hosseinidoust Z, 2016, ADV DRUG DELIVER REV, V106, P27, DOI 10.1016/j.addr.2016.09.007
Hu CMJ, 2015, NATURE, V526, P118, DOI 10.1038/nature15373
Hu CMJ, 2013, NAT NANOTECHNOL, V8, P336, DOI [10.1038/nnano.2013.54, 10.1038/NNANO.2013.54]
Hu CMJ, 2013, NANOSCALE, V5, P2664, DOI 10.1039/c3nr00015j
Hu CMJ, 2011, P NATL ACAD SCI USA, V108, P10980, DOI 10.1073/pnas.1106634108
Hu XM, 2019, ADV HEALTHC MATER, V8, DOI 10.1002/adhm.201800837
Huang GY, 2017, CHEM REV, V117, P12764, DOI 10.1021/acs.chemrev.7b00094
Jamal M, 2013, ADV HEALTHC MATER, V2, P1142, DOI 10.1002/adhm.201200458
Jiang Q, 2019, BIOMATERIALS, V192, P292, DOI 10.1016/j.biomaterials.2018.11.021
Jiang Q, 2017, BIOMATERIALS, V143, P29, DOI 10.1016/j.biomaterials.2017.07.027
Jin JF, 2019, ACS APPL MATER INTER, V11, P7850, DOI 10.1021/acsami.8b22309
Kamalabadi-Farahani M, 2018, ARTIF CELL NANOMED B, V46, pS1011, DOI 10.1080/21691401.2018.1527345
Kaneti L, 2016, NANO LETT, V16, P1574, DOI 10.1021/acs.nanolett.5b04237
Kang S, 2017, CHEM MATER, V29, P3461, DOI 10.1021/acs.chemmater.6b05164
Kang S, 2015, ACS NANO, V9, P9678, DOI 10.1021/acsnano.5b02207
Kang T, 2017, ACS NANO, V11, P1397, DOI 10.1021/acsnano.6b06477
Khait NL, 2019, J CONTROL RELEASE, V293, P215, DOI 10.1016/j.jconrel.2018.12.005
Khutoryanskiy VV, 2018, ADV DRUG DELIVER REV, V124, P140, DOI 10.1016/j.addr.2017.07.015
Kim R, 2007, IMMUNOLOGY, V121, P1, DOI 10.1111/j.1365-2567.2007.02587.x
Kim YK, 2016, J MATER CHEM B, V4, P1600, DOI 10.1039/c5tb01605c
Kleponis J, 2015, CANCER BIOL MED, V12, P201, DOI 10.7497/j.issn.2095-3941.2015.0046
Kong F, 2016, CLIN TRANSL ONCOL, V18, P1051, DOI 10.1007/s12094-016-1489-x
Koyama S, 2016, NAT COMMUN, V7, DOI 10.1038/ncomms10501
Krishnamurthy S, 2016, NANOSCALE, V8, P6981, DOI 10.1039/c5nr07588b
Kroll AV, 2017, ADV MATER, V29, DOI 10.1002/adma.201703969
Lawler SE, 2017, JAMA ONCOL, V3, P841, DOI 10.1001/jamaoncol.2016.2064
Layek B, 2018, MOL CANCER THER, V17, P1196, DOI 10.1158/1535-7163.MCT-17-0682
Leclerc M, 2019, FRONT IMMUNOL, V10, DOI 10.3389/fimmu.2019.01505
Li PY, 2018, BIOCONJUGATE CHEM, V29, P624, DOI 10.1021/acs.bioconjchem.7b00669
Li SY, 2017, ACS NANO, V11, P7006, DOI 10.1021/acsnano.7b02533
Liu DF, 2017, ADV FUNCT MATER, V27, DOI 10.1002/adfm.201604508
Liu WL, 2019, ADV MATER, V31, DOI 10.1002/adma.201900499
Liu Y, 2018, J MATER CHEM B, V6, P7033, DOI 10.1039/c8tb02143k
Lopes M, 2017, EUR J PHARM BIOPHARM, V113, P88, DOI 10.1016/j.ejpb.2016.11.037
Luk BT, 2014, NANOSCALE, V6, P2730, DOI 10.1039/c3nr06371b
Lundy DJ, 2016, SCI REP-UK, V6, DOI 10.1038/srep25613
Luo ZQ, 2018, NANO RES, V11, P3009, DOI 10.1007/s12274-018-2004-1
Lupu-Haber Y, 2019, ADV HEALTHC MATER, V8, DOI 10.1002/adhm.201801589
Lybaert L, 2018, J CONTROL RELEASE, V289, P125, DOI 10.1016/j.jconrel.2018.09.009
Ma L, 2016, ACS NANO, V10, P6952, DOI 10.1021/acsnano.6b02878
Ma WJ, 2020, THERANOSTICS, V10, P1281, DOI 10.7150/thno.40291
Marvel D, 2015, J CLIN INVEST, V125, P3356, DOI 10.1172/JCI80005
Mazzarella L, 2019, EUR J CANCER, V117, P14, DOI 10.1016/j.ejca.2019.04.035
Meng QF, 2018, NANOTECHNOLOGY, V29, DOI 10.1088/1361-6528/aaa7c7
Mittal D, 2014, CURR OPIN IMMUNOL, V27, P16, DOI 10.1016/j.coi.2014.01.004
Mohammadi MR, 2019, SMALL, V15, DOI 10.1002/smll.201902333
Mohr A, 2018, CANCER LETT, V414, P239, DOI 10.1016/j.canlet.2017.11.025
Molinaro R, 2016, NAT MATER, V15, P1037, DOI [10.1038/nmat4644, 10.1038/NMAT4644]
Morvan MG, 2016, NAT REV CANCER, V16, P7, DOI 10.1038/nrc.2015.5
Mu XP, 2018, ACS BIOMATER SCI ENG, V4, P3895, DOI 10.1021/acsbiomaterials.8b00858
Muller FJ, 2006, NAT REV NEUROSCI, V7, P75, DOI 10.1038/nrn1829
Nakamizo A, 2005, CANCER RES, V65, P3307, DOI 10.1158/0008-5472.CAN-04-1874
Nam J, 2019, NAT REV MATER, V4, P398, DOI 10.1038/s41578-019-0108-1
Oieni J, 2020, METHODS, V177, P126, DOI 10.1016/j.ymeth.2019.11.013
Ott PA, 2017, NATURE, V547, P217, DOI 10.1038/nature22991
Palomba R, 2016, SCI REP-UK, V6, DOI 10.1038/srep34422
Pan D, 2016, PLOS ONE, V11, DOI 10.1371/journal.pone.0152074
Pan DC, 2018, SCI REP-UK, V8, DOI 10.1038/s41598-018-19897-8

Paris JL, 2016, ACTA BIOMATER, V33, P275, DOI 10.1016/j.actbio.2016.01.017
Park J, 2019, THERANOSTICS, V9, P8073, DOI 10.7150/thno.37198
Parodi A, 2013, NAT NANOTECHNOL, V8, P61, DOI 10.1038/nnano.2012.212
Pei Q, 2018, ACS NANO, V12, P1630, DOI 10.1021/acsnano.7b08219
Pinheiro WO, 2019, INT J NANOMED, V14, P3375, DOI 10.2147/IJN.S197888
Pitchaimani A, 2019, ADV FUNCT MATER, V29, DOI 10.1002/adfm.201806817
Pitchaimani A, 2018, BIOMATERIALS, V160, P124, DOI 10.1016/j.biomaterials.2018.01.018
Prabha S., 2017, ABSTRACT 3103 NANOEN, P3103
Prabha S., 2019, ABSTRACT 3623 MESENC, P3623
Qie YQ, 2016, SCI REP-UK, V6, DOI 10.1038/srep26269
Raman R, 2020, ADV MATER, V32, DOI 10.1002/adma.201901969
Rao L, 2019, ADV FUNCT MATER, V29, DOI 10.1002/adfm.201905671
Rao L, 2018, ADV FUNCT MATER, V28, DOI 10.1002/adfm.201803531
Rao L, 2017, J BIOMED MATER RES A, V105, P521, DOI 10.1002/jbm.a.35927
Rao L, 2015, SMALL, V11, P6225, DOI 10.1002/smll.201502388
Ren XQ, 2016, BIOMATERIALS, V92, P13, DOI 10.1016/j.biomaterials.2016.03.026
Ricotti L, 2017, SCI ROBOT, V2, DOI 10.1126/scirobotics.aaq0495
Riley RS, 2019, NAT REV DRUG DISCOV, V18, P175, DOI 10.1038/s41573-018-0006-z
Rosenberg SA, 2014, J IMMUNOL, V192, P5451, DOI 10.4049/jimmunol.1490019
Ruoslahti E, 2017, ADV DRUG DELIVER REV, V110, P3, DOI 10.1016/j.addr.2016.03.008
Saha RN, 2010, MOL MEMBR BIOL, V27, P215, DOI 10.3109/09687688.2010.510804
Saldrnoto KK, 2017, ACCOUNTS CHEM RES, V50, P476, DOI 10.1021/acs.accounts.6b00483
Santos AG, 2019, SCI REP-UK, V9, DOI 10.1038/s41598-018-37186-2
Schmitt MW, 2016, NAT REV CLIN ONCOL, V13, P335, DOI 10.1038/nrclinonc.2015.175
Schreiber RD, 2011, SCIENCE, V331, P1565, DOI 10.1126/science.1203486
Schumacher TN, 2015, SCIENCE, V348, P69, DOI 10.1126/science.aaa4971
Shahzad KA, 2018, DRUG DELIV, V25, P703, DOI 10.1080/10717544.2018.1447049
Shi JJ, 2017, NAT REV CANCER, V17, P20, DOI 10.1038/nrc.2016.108
Simon J, 2018, NANOSCALE, V10, P10731, DOI 10.1039/c8nr03331e
Song SL, 2019, INT J NANOMED, V14, P2465, DOI 10.2147/IJN.S195828
Su JH, 2016, ADV FUNCT MATER, V26, P7495, DOI 10.1002/adfm.201603381
Su ZX, 2017, SCI REP-UK, V7, P1, DOI 10.1038/srep43026
Suk JS, 2016, ADV DRUG DELIVER REV, V99, P28, DOI 10.1016/j.addr.2015.09.012
Sun HP, 2017, ADV FUNCT MATER, V27, DOI 10.1002/adfm.201604300
Sun HP, 2016, ADV MATER, V28, P9581, DOI 10.1002/adma.201602173
Timaner M, 2018, CANCER RES, V78, P1253, DOI 10.1158/0008-5472.CAN-17-1547
Torchilin VP, 2008, ADV DRUG DELIVER REV, V60, P548, DOI 10.1016/j.addr.2007.10.008
Van Hove AH, 2015, J CONTROL RELEASE, V217, P191, DOI 10.1016/j.jconrel.2015.09.005
Ventola C Lee, 2017, P T, V42, P742
Villa CH, 2016, ADV DRUG DELIVER REV, V106, P88, DOI 10.1016/j.addr.2016.02.007
Wagner HJ, 2016, ADV DRUG DELIVER REV, V105, P77, DOI 10.1016/j.addr.2016.05.004
Wais U, 2016, NANOSCALE, V8, P1746, DOI 10.1039/c5nr07161e
Wang C, 2014, ADV MATER, V26, P4794, DOI 10.1002/adma.201400158
Wang DD, 2018, ACS NANO, V12, P5241, DOI 10.1021/acsnano.7b08355
Wang J, 2017, NANOSCALE RES LETT, V12, DOI 10.1186/s11671-017-1836-z
Wang XL, 2018, INT J NANOMED, V13, P5231, DOI 10.2147/IJN.S167142
Wehner R, 2011, J INNATE IMMUN, V3, P258, DOI 10.1159/000323923
Wilhelm S, 2016, NAT REV MATER, V1, DOI 10.1038/natrevmats.2016.14
Willmon C, 2009, MOL THER, V17, P1667, DOI 10.1038/mt.2009.194
Xia Q, 2019, ACTA PHARM SIN B, V9, P675, DOI 10.1016/j.apsb.2019.01.011
Xie W, 2019, ACS NANO, V13, P2849, DOI 10.1021/acsnano.8b03788
Xuan MJ, 2016, ACS APPL MATER INTER, V8, P9610, DOI 10.1021/acsami.6b00853
Xuan MJ, 2015, ADV HEALTHC MATER, V4, P1645, DOI 10.1002/adhm.201500129
Yan XH, 2017, SCI ROBOT, V2, DOI 10.1126/scirobotics.aaq1155
Yang PX, 2018, NANO LETT, V18, P4377, DOI 10.1021/acs.nanolett.8b01406
Yang Q, 2016, ANAL CHEM, V88, P11804, DOI 10.1021/acs.analchem.6b03437
Yong TY, 2019, NAT COMMUN, V10, DOI 10.1038/s41467-019-11718-4
Yoo J, 2019, CANCERS, V11, DOI 10.3390/cancers11050640
Yu GT, 2018, ADV FUNCT MATER, V28, DOI 10.1002/adfm.201801389
Zelepukin IV, 2019, NANOSCALE, V11, P1636, DOI 10.1039/c8nr07730d
Zhang K, 2019, J BONE ONCOL, V16, DOI 10.1016/j.jbo.2019.100238

Zhang LR, 2017, INT J NANOMED, V12, P2129, DOI 10.2147/IJN.S126016
Zhang N, 2018, BIOMATERIALS, V159, P25, DOI 10.1016/j.biomaterials.2018.01.007
Zhang QZ, 2018, NAT NANOTECHNOL, V13, P1182, DOI 10.1038/s41565-018-0254-4
Zhang WZ, 2018, ADV MATER, V30, DOI 10.1002/adma.201805557
Zhang Y, 2018, NANO LETT, V18, P1908, DOI 10.1021/acs.nanolett.7b05263
Zhang ZP, 2011, BIOMATERIALS, V32, P3666, DOI 10.1016/j.biomaterials.2011.01.067
Zhao ZM, 2019, SCI ADV, V5, DOI 10.1126/sciadv.aax9250
Zhou H, 2016, THERANOSTICS, V6, P1012, DOI 10.7150/thno.15095
Zhu JY, 2016, NANO LETT, V16, P5895, DOI 10.1021/acs.nanolett.6b02786
Zhu Y, 2018, ACTA BIOMATER, V73, P112, DOI 10.1016/j.actbio.2018.04.003
NR 188
TC 0
Z9 0
U1 1
U2 17
PU SPRINGER INTERNATIONAL PUBLISHING AG
PI CHAM
PA GEWERBESTRASSE 11, CHAM, CH-6330, SWITZERLAND
SN 0065-2598
EI 2214-8019
BN 978-3-030-58174-9; 978-3-030-58173-2
J9 ADV EXP MED BIOL
JI Adv.Exp.Med.Biol.
PY 2021
VL 1295
BP 135
EP 162
DI 10.1007/978-3-030-58174-9_7
D2 10.1007/978-3-030-58174-9
PG 28
WC Oncology; Nanoscience & Nanotechnology; Medicine, Research &
Experimental
WE Book Citation Index– Science (BKCI-S); Science Citation Index Expanded (SCI-EXPANDED)
SC Oncology; Science & Technology - Other Topics; Research & Experimental
Medicine
GA BQ9FI
UT WOS:000624289800008
PM 33543459
DA 2023-09-14
ER

EF
