# Supplementary material for: Current status and future directions of nanovaccine for cancer: a bibliometric analysis during 2004-2023
Source: Front Immunol. 2024 Jul 29;15:1423212. doi: 10.3389/fimmu.2024.1423212 (PMC11317272; doi:10.3389/fimmu.2024.1423212)
Supplement: Supplementary Data Sheet 1 — Literature data source file (1). [file DataSheet_1.pdf]

FN Clarivate Analytics Web of Science
VR 1.0
PT J
AU Acharya, S
Sahoo, SK
AF Acharya, Sarbari
Sahoo, Sanjeeb K.
TI PLGA nanoparticles containing various anticancer agents and tumour delivery by EPR effect
SO ADVANCED DRUG DELIVERY REVIEWS
LA English
DT Review
DE Chemotherapy; Nanotechnology; Drug Delivery; Drug-loaded nanoparticles; EPR Effects
AB As mortality due to cancer continues to rise, advances in nanotechnology have significantly become an effective approach for achieving efficient drug targeting to tumour tissues by circumventing all the shortcomings of conventional chemotherapy. During the past decade, the importance of polymeric drug-delivery systems in oncology has grown exponentially. In this context, poly(lactic-co-glycolic acid) (PLGA) is a widely used polymer for fabricating 'nanoparticles' because of biocompatibility, long-standing track record in biomedical applications and well-documented utility for sustained drug release, and hence has been the centre of focus for developing drug-loaded nanoparticles for cancer therapy. Such PLGA nanoparticles have also been used to develop proteins and peptides for nanomedicine, and nanovaccines, as well as a nanoparticle-based drug- and gene-delivery system for cancer therapy, and nanoantigens and growth factors. These drug-loaded nanoparticles extravasate through the tumour vasculature, delivering their payload into the cells by the enhanced permeability and retention (EPR) effect, thereby increasing their therapeutic effect. Ongoing research about drug-loaded nanoparticles and their delivery by the EPR effect to the tumour tissues has been elucidated in this review with clarity. (C) 2010 Elsevier B.V. All rights reserved.
C1 [Sahoo, Sanjeeb K.] Inst Life Sci, Lab Nanomed, Bhubaneswar 751023, Orissa, India.
C3 Department of Biotechnology (DBT) India; Institute of Life Sciences India (ILS)
RP Sahoo, SK (通讯作者)，Inst Life Sci, Lab Nanomed, Bhubaneswar 751023, Orissa, India.
EM sanjeebsahoo2005@gmail.com
RI Sanjeeb, Sahoo/ABF-3419-2020; Acharya, Sarbari/AAW-4877-2021
OI Sahoo, Sanjeeb/0000-0002-9096-6954
CR Wang XR, 2008, PHYS REV LETT, V100, DOI 10.1103/PhysRevLett.100.206803
NR 1
TC 822
Z9 856
U1 6
U2 506
PU ELSEVIER SCIENCE BV
PI AMSTERDAM
PA PO BOX 211, 1000 AE AMSTERDAM, NETHERLANDS
SN 0169-409X
J9 ADV DRUG DELIVER REV
JI Adv. Drug Deliv. Rev.
PD MAR 18
PY 2011
VL 63
IS 3
BP 170
EP 183
DI 10.1016/j.addr.2010.10.008
PG 14
WC Pharmacology & Pharmacy
WE Science Citation Index Expanded (SCI-EXPANDED)
SC Pharmacology & Pharmacy
GA 755KK
UT WOS:000289929200006

PM 20965219
DA 2023-09-14
ER

PT J
AU Luo, M
Wang, H
Wang, ZH
Cai, HC
Lu, ZG
Li, Y
Du, MJ
Huang, G
Wang, CS
Chen, X
Porembka, MR
Lea, J
Frankel, AE
Fu, YX
Chen, ZJJ
Gao, JM
AF Luo, Min
Wang, Hua
Wang, Zhaohui
Cai, Haocheng
Lu, Zhigang
Li, Yang
Du, Mingjian
Huang, Gang
Wang, Chensu
Chen, Xiang
Porembka, Matthew R.
Lea, Jayanthi
Frankel, Arthur E.
Fu, Yang-Xin
Chen, Zhijian J.
Gao, Jinming
TI A STING-activating nanovaccine for cancer immunotherapy
SO NATURE NANOTECHNOLOGY
LA English
DT Article
ID CYCLIC GMP-AMP; CYTOSOLIC DNA; NANOPARTICLE VACCINES; IMMUNE-RESPONSES;
I INTERFERONS; SENSOR; INDUCTION; CELLS; RECOGNITION; RESISTANCE
AB The generation of tumour-specific T cells is critically important for cancer immunotherapy(1,2). A major challenge in achieving a robust T-cell response is the spatiotemporal orchestration of antigen cross-presentation in antigen-presenting cells with innate stimulation. Here, we report a minimalist nanovaccine, comprising a simple physical mixture of an antigen and a synthetic polymeric nanoparticle, PC7A NP, which generates a strong cytotoxic T-cell response with low systemic cytokine expression. Mechanistically, the PC7A NP achieves efficient cytosolic delivery of tumour antigens to antigen-presenting cells in draining lymph nodes, leading to increased surface presentation while simultaneously activating type I interferon-stimulated genes. This effect is dependent on stimulator of interferon genes (STING), but not the Toll-like receptor or the mitochondrial antiviral-signalling protein (MAVS) pathway. The nanovaccine led to potent tumour growth inhibition in melanoma, colon cancer and human papilloma virusE6/E7 tumour models. The combination of the PC7A nanovaccine and an anti-PD-1 antibody showed great synergy, with 100% survival over 60 days in a TC-1 tumour model. Rechallenging of these tumour-free animals with TC-1 cells led to complete inhibition of tumour growth, suggesting the generation of long-term antitumour memory. The STING-activating nanovaccine offers a simple, safe and robust strategy in boosting anti-tumour immunity for cancer immunotherapy.
C1 [Luo, Min; Wang, Zhaohui; Li, Yang; Huang, Gang; Wang, Chensu; Gao, Jinming] Univ Texas Southwestern Med Ctr Dallas, Simmons Comprehens Canc Ctr, Dept Pharmacol, 5323 Harry Hines Blvd, Dallas, TX 75390 USA.

[Wang, Hua; Cai, Haocheng; Du, Mingjian; Chen, Xiang; Chen, Zhijian J.] Univ Texas Southwestern Med Ctr Dallas, Dept Mol Biol, 5323 Harry Hines Blvd, Dallas, TX 75390 USA.
[Lu, Zhigang] Univ Texas Southwestern Med Ctr Dallas, Dept Dev Biol, 5323 Harry Hines Blvd, Dallas, TX 75390 USA.
[Porembka, Matthew R.] Univ Texas Southwestern Med Ctr Dallas, Dept Surg, 5323 Harry Hines Blvd, Dallas, TX 75390 USA.
[Lea, Jayanthi] Univ Texas Southwestern Med Ctr Dallas, Dept Obstet & Gynecol, 5323 Harry Hines Blvd, Dallas, TX 75390 USA.
[Frankel, Arthur E.] Univ Texas Southwestern Med Ctr Dallas, Dept Internal Med, 5323 Harry Hines Blvd, Dallas, TX 75390 USA.
[Fu, Yang-Xin] Univ Texas Southwestern Med Ctr Dallas, Dept Pathol, 5323 Harry Hines Blvd, Dallas, TX 75390 USA.
[Chen, Zhijian J.] Univ Texas Southwestern Med Ctr Dallas, Howard Hughes Med Inst, 5323 Harry Hines Blvd, Dallas, TX 75390 USA.
C3 University of Texas System; University of Texas Southwestern Medical Center Dallas; University of Texas System; University of Texas Southwestern Medical Center Dallas; University of Texas System; University of Texas Southwestern Medical Center Dallas; University of Texas System; University of Texas Southwestern Medical Center Dallas; University of Texas System; University of Texas Southwestern Medical Center Dallas; University of Texas System; University of Texas Southwestern Medical Center Dallas; University of Texas System; University of Texas Southwestern Medical Center Dallas; Howard Hughes Medical Institute; University of Texas System; University of Texas Southwestern Medical Center Dallas
RP Gao, JM (通讯作者)，Univ Texas Southwestern Med Ctr Dallas, Simmons Comprehens Canc Ctr, Dept Pharmacol, 5323 Harry Hines Blvd, Dallas, TX 75390 USA.; Chen, ZJJ (通讯作者)，Univ Texas Southwestern Med Ctr Dallas, Dept Mol Biol, 5323 Harry Hines Blvd, Dallas, TX 75390 USA.; Chen, ZJJ (通讯作者)，Univ Texas Southwestern Med Ctr Dallas, Howard Hughes Med Inst, 5323 Harry Hines Blvd, Dallas, TX 75390 USA.
EM zhijian.chen@utsouthwestern.edu; jinming.gao@utsouthwestern.edu
RI Chen, Zhijian/C-6039-2012; Wang, Zhaohui/C-9795-2016; Cai, Haocheng/K-6114-2019; Li, Yang/W-5310-2018
OI Chen, Zhijian/0000-0002-8475-8251; Wang, Zhaohui/0000-0003-4650-9008; Cai, Haocheng/0000-0003-3827-8246; Li, Yang/0000-0001-8186-2435; Porembka, Matthew/0000-0003-1596-0058; Lu, Zhigang/0000-0002-2880-7848; Lea, Jayanthi/0000-0002-8657-4309; Du, Mingjian/0000-0002-9464-4098; Wang, Zhaohui/0000-0001-7994-7464
FU National Institutes of Health [R01AI093967, R01EB013149, R01CA192221]; Cancer Prevention and Research Institute of Texas [RP120718-P3, RP150498]; UT Southwestern Small Animal Imaging Resource Grant [U24 CA126608]; Simmons Cancer Center Support Grant [P30 CA142543]
FX This work was supported by grants from the National Institutes of Health (R01AI093967 to Z.J.C., R01EB013149 and R01CA192221 to J.G.) and the Cancer Prevention and Research Institute of Texas (RP120718-P3 and RP150498 to Z.J.C.). Z.J.C. is an Investigator of the Howard Hughes Medical Institute. M.R.P. is a Dedman Scholar in Clinical Care. Animal imaging work is supported by a UT Southwestern Small Animal Imaging Resource Grant (U24 CA126608) and a Simmons Cancer Center Support Grant (P30 CA142543). The authors thank Z. Zeng for cell culture, Q. Wei for polymer synthesis and T. Zhao for animal imaging. The authors also thank N. Yan for discussions on STING activation, T.C. Wu for providing the TC-1 tumour cells, P. Hwu for the B16-OVA cancer cells, Y. Peng for vaccine safety analysis, S. Tso for ITC experiments and the molecular pathology core of UT Southwestern for tissue toxicity analysis.
CR Alexopoulou L, 2001, NATURE, V413, P732, DOI 10.1038/35099560
Baccala R, 2007, NAT MED, V13, P543, DOI 10.1038/nm1590
BOUSSIF O, 1995, P NATL ACAD SCI USA, V92, P7297, DOI 10.1073/pnas.92.16.7297
Carroll EC, 2016, IMMUNITY, V44, P597, DOI 10.1016/j.immuni.2016.02.004
Collins AC, 2015, CELL HOST MICROBE, V17, P820, DOI 10.1016/j.chom.2015.05.005
Fuertes MB, 2013, TRENDS IMMUNOL, V34, P67, DOI 10.1016/j.it.2012.10.004

Heath WR, 2004, IMMUNOL REV, V199, P9, DOI 10.1111/j.0105-2896.2004.00142.x
Hemmi H, 2000, NATURE, V408, P740, DOI 10.1038/35047123
Hildner K, 2008, SCIENCE, V322, P1097, DOI 10.1126/science.1164206
Holmgaard RB, 2013, J EXP MED, V210, P1389, DOI 10.1084/jem.20130066
Hoshi M, 2010, J IMMUNOL, V185, P3305, DOI 10.4049/jimmunol.0901150
Huang L, 2012, J IMMUNOL, V188, P4913, DOI 10.4049/jimmunol.1103668
Kreiter S, 2015, NATURE, V520, P692, DOI 10.1038/nature14426
Kuai R, 2017, NAT MATER, V16, P489, DOI [10.1038/nmat4822, 10.1038/NMAT4822]
Lemos H, 2014, EUR J IMMUNOL, V44, P2847, DOI 10.1002/eji.201344407
Li XD, 2013, SCIENCE, V341, P1390, DOI 10.1126/science.1244040
Liechtenstein Therese, 2012, Immunology Endocrine & Metabolic Agents in Medicinal Chemistry, V12, P224
Liu HP, 2014, NATURE, V507, P519, DOI 10.1038/nature12978
Liu ZD, 2016, ONCOIMMUNOLOGY, V5, DOI 10.1080/2162402X.2016.1147641
Ma XP, 2014, J AM CHEM SOC, V136, P11085, DOI 10.1021/ja5053158
Maldonado RA, 2015, P NATL ACAD SCI USA, V112, pE156, DOI 10.1073/pnas.1408686111
Poltorak A, 1998, SCIENCE, V282, P2085, DOI 10.1126/science.282.5396.2085
Reddy ST, 2007, NAT BIOTECHNOL, V25, P1159, DOI [10.1038/nbt1332, 10.1038/nbtl332]
Rice AE, 2015, CANCER GENE THER, V22, P454, DOI 10.1038/cgt.2015.40
Rosenberg SA, 2015, SCIENCE, V348, P62, DOI 10.1126/science.aaa4967
Schumacher TN, 2015, SCIENCE, V348, P69, DOI 10.1126/science.aaa4971
Sharma P, 2015, CELL, V161, P205, DOI 10.1016/j.cell.2015.03.030
Sun LJ, 2013, SCIENCE, V339, P786, DOI 10.1126/science.1232458
Sun YY, 2016, CLIN CANCER RES, V22, P657, DOI 10.1158/1078-0432.CCR-15-0234
Trinchieri G, 2010, J EXP MED, V207, P2053, DOI 10.1084/jem.20101664
Tsarevsky NV, 2007, CHEM REV, V107, P2270, DOI 10.1021/cr050947p
Tumeh PC, 2014, NATURE, V515, P568, DOI 10.1038/nature13954
Wang CS, 2015, NAT COMMUN, V6, DOI 10.1038/ncomms9524
Wang ZH, 2017, ANGEW CHEM INT EDIT, V56, P1319, DOI 10.1002/anie.201610302
Wilson JT, 2013, ACS NANO, V7, P3912, DOI 10.1021/nn305466z
Woo SR, 2014, IMMUNITY, V41, P830, DOI 10.1016/j.immuni.2014.10.017
Yadav M, 2014, NATURE, V515, P572, DOI 10.1038/nature14001
Zhang X, 2013, MOL CELL, V51, P226, DOI 10.1016/j.molcel.2013.05.022
Zhou KJ, 2012, J AM CHEM SOC, V134, P7803, DOI 10.1021/ja300176w
Zhou KJ, 2011, ANGEW CHEM INT EDIT, V50, P6109, DOI 10.1002/anie.201100884
Zitvogel L, 2015, NAT REV IMMUNOL, V15, P405, DOI 10.1038/nri3845
NR 41
TC 522
Z9 539
U1 53
U2 995
PU NATURE PUBLISHING GROUP
PI LONDON
PA MACMILLAN BUILDING, 4 CRINAN ST, LONDON N1 9XW, ENGLAND
SN 1748-3387
EI 1748-3395
J9 NAT NANOTECHNOL
JI Nat. Nanotechnol.
PD JUL
PY 2017
VL 12
IS 7
BP 648
EP +
DI 10.1038/NNANO.2017.52
PG 10
WC Nanoscience & Nanotechnology; Materials Science, Multidisciplinary
WE Science Citation Index Expanded (SCI-EXPANDED)
SC Science & Technology - Other Topics; Materials Science
GA EZ7BG
UT WOS:000404874000014

PM 28436963
OA Green Accepted
HC Y
HP N
DA 2023-09-14
ER

PT J
AU Fifis, T
Gamvrellis, A
Crimeen-Irwin, B
Pietersz, GA
Li, J
Mottram, PL
McKenzie, IFC
Plebanski, M
AF Fifis, T
Gamvrellis, A
Crimeen-Irwin, B
Pietersz, GA
Li, J
Mottram, PL
McKenzie, IFC
Plebanski, M
TI Size-dependent immunogenicity: Therapeutic and protective properties of
nano-vaccines against tumors
SO JOURNAL OF IMMUNOLOGY
LA English
DT Article
ID SURFACE-CONNECTED COMPARTMENTS; CYTOTOXIC T-LYMPHOCYTES; DENDRITIC
CELLS; FUSION PROTEIN; ANTIGEN; IMMUNITY; PATHWAY; IMMUNIZATION;
ADJUVANTS; INFECTION
AB Infection can protect against subsequent disease by induction of both humoral and cellular immunity, but inert protein-based vaccines are not as effective. In this study, we present a new vaccine design, with Ag covalently conjugated to solid core nano-beads of narrowly defined size (0.04-0.05 mum) that localize to dendritic cells (DEC205(+) CD40(+), CD86(+)) in draining lymph nodes, inducing high levels of IFN-gamma production (CD8 T cells: precursor frequencies 115000 to 1/1000) and high Ab titers in mice. Conjugation of Ag to these nano-beads induced responses that were significantly higher (2- to 10-fold) than those elicited by other bead sizes, and higher than a range of currently used adjuvants (alum, QuilA, monophosphoryl lipid A). Responses were comparable to CFA/IFA immunization for Abs and ex vivo peptide-pulsed dendritic cell immunization for CD8 T cells. A single dose of Ag-conjugated beads protected mice from tumors in two different model challenges and caused rapid clearance of established tumors in mice. Thus, a range of Ags conjugated to nano-beads was effective as immunogens in both therapeutic and prophylactic scenarios.
C1 Austin Hosp, Austin Res Inst, Heidelberg, Vic 3084, Australia.
C3 Austin Research Institute; Florey Institute of Neuroscience & Mental
Health
RP Plebanski, M (通讯作者)，Austin & Repatriat Med Ctr, Austin Res Inst, Vaccine Dev & Infect Dis Unit, Heidelberg, Vic 3084, Australia.
EM mplebans@ari.unimelb.edu.au
RI Plebanski, Magdalena/AAU-3144-2021
OI Plebanski, Magdalena/0000-0001-6889-3667
CR Ada G, 2001, NEW ENGL J MED, V345, P1042, DOI 10.1056/NEJMra011223
Allsopp CEM, 1996, EUR J IMMUNOL, V26, P1951, DOI 10.1002/eji.1830260841
Berinstein NL, 2002, J CLIN ONCOL, V20, P2197, DOI 10.1200/JCO.2002.08.017
Brown GV, 1999, PARASSITOLOGIA, VOL 41, NOS 1-3, SEPTEMBER 1999, P429
Daemen T, 2003, VACCINE, V21, P1082, DOI 10.1016/S0264-410X(02)00558-3
FALO LD, 1995, NAT MED, V1, P649, DOI 10.1038/nm0795-649
Fernando GJP, 1998, J IMMUNOL, V161, P2421
FIFIS T, IN PRESS VACCINE
Gallucci S, 1999, NAT MED, V5, P1249, DOI 10.1038/15200

Guermonprez P, 2002, ANNU REV IMMUNOL, V20, P621, DOI 10.1146/annurev.immunol.20.100301.064828
Harty JT, 2000, ANNU REV IMMUNOL, V18, P275, DOI 10.1146/annurev.immunol.18.1.275
Houde M, 2003, NATURE, V425, P402, DOI 10.1038/nature01912
Jeannin P, 2000, NAT IMMUNOL, V1, P502, DOI 10.1038/82751
Kruth HS, 1999, J BIOL CHEM, V274, P7495, DOI 10.1074/jbc.274.11.7495
Kruth HS, 1999, EUR J CELL BIOL, V78, P91, DOI 10.1016/S0171-9335(99)80010-7
MOORE MW, 1988, CELL, V54, P777, DOI 10.1016/S0092-8674(88)91043-4
O'Hagan DT, 2001, BIOMOL ENG, V18, P69, DOI 10.1016/S1389-0344(01)00101-0
Pietersz GA, 1998, CANCER IMMUNOL IMMUN, V45, P321, DOI 10.1007/s002620050449
Plebanski M, 1998, EUR J IMMUNOL, V28, P4345, DOI 10.1002/(SICI)1521-4141(199812)28:12<4345::AID-IMMU4345>3.0.CO;2-P
Raychaudhuri S, 1998, NAT BIOTECHNOL, V16, P1025, DOI 10.1038/3469
Reimann J, 1997, CURR OPIN IMMUNOL, V9, P462, DOI 10.1016/S0952-7915(97)80096-9
SCHIRMBECK R, 1995, VACCINE, V13, P857, DOI 10.1016/0264-410X(94)00038-O
Schoenberger SP, 1998, NATURE, V393, P480, DOI 10.1038/31002
Shortman K, 2002, NAT REV IMMUNOL, V2, P151, DOI 10.1038/nri746
Sjolander A, 2001, VACCINE, V19, P2661, DOI 10.1016/S0264-410X(00)00497-7
Yeh KY, 1998, J IMMUNOL, V160, P5773
NR 26
TC 512
Z9 566
U1 2
U2 102
PU AMER ASSOC IMMUNOLOGISTS
PI BETHESDA
PA 9650 ROCKVILLE PIKE, BETHESDA, MD 20814 USA
SN 0022-1767
EI 1550-6606
J9 J IMMUNOL
JI J. Immunol.
PD SEP 1
PY 2004
VL 173
IS 5
BP 3148
EP 3154
DI 10.4049/jimmunol.173.5.3148
PG 7
WC Immunology
WE Science Citation Index Expanded (SCI-EXPANDED)
SC Immunology
GA 849HR
UT WOS:000223529800037
PM 15322175
OA Bronze
DA 2023-09-14
ER

PT J
AU Yang, R
Xu, J
Xu, LG
Sun, XQ
Chen, Q
Zhao, YH
Peng, R
Liu, Z
AF Yang, Rong
Xu, Jun
Xu, Ligeng

Sun, Xiaoqi
Chen, Qian
Zhao, Yuhuan
Peng, Rui
Liu, Zhuang
TI Cancer Cell Membrane-Coated Adjuvant Nanoparticles with Mannose Modification for Effective Anticancer Vaccination
SO ACS NANO
LA English
DT Article
DE vaccination; cancer cell membrane; cancer immunotherapy; checkpoint blockade; mannose modification
ID DENDRITIC CELLS; CHECKPOINT BLOCKADE; POLYMERIC NANOPARTICLES; IMMUNE-RESPONSE; IMMUNOTHERAPY; ANTITUMOR; DELIVERY; VACCINES; SYSTEM; IDENTIFICATION
AB Tumor vaccines for cancer prevention and treatment have attracted tremendous interests in the area of cancer immunotherapy in recent years. In this work, we present a strategy to construct cancer vaccines by encapsulating immune-adjuvant nanoparticles with cancer cell membranes modified by mannose. Poly(D,L-lactide-co-glycolide) nanoparticles are first loaded with toll-like receptor 7 agonist, imiquimod (R837). Those adjuvant nanoparticles (NP-R) are then coated with cancer cell membranes (NP-R@M), whose surface proteins could act as tumor-specific antigens. With further modification with mannose moiety (NP-R@M-M), the obtained nanovaccine shows enhanced uptake by antigen presenting cells such as dendritic cells, which would then be stimulated to the maturation status to trigger antitumor immune responses. With great efficacy to delay tumor development as a prevention vaccine, vaccination with such NP-R@M-M in combination with checkpoint blockade therapy further demonstrates outstanding therapeutic efficacy to treat established tumors. Therefore, our work presents an innovative way to fabricate cancer nanovaccines, which in principle may be applied for a wide range of tumor types.
C1 [Yang, Rong; Xu, Jun; Xu, Ligeng; Sun, Xiaoqi; Chen, Qian; Zhao, Yuhuan; Peng, Rui; Liu, Zhuang] Soochow Univ, Inst Funct Nano & Soft Mat FUNSOM, Jiangsu Key Lab Carbon Based Funct Mat & Devices, Suzhou 215123, Jiangsu, Peoples R China.
C3 Soochow University - China
RP Xu, LG; Peng, R; Liu, Z (通讯作者)，Soochow Univ, Inst Funct Nano & Soft Mat FUNSOM, Jiangsu Key Lab Carbon Based Funct Mat & Devices, Suzhou 215123, Jiangsu, Peoples R China.
EM lgxu@suda.edu.cn; rpeng@suda.edu.cn; zliu@suda.edu.cn
RI chen, qian/R-1324-2019; Xu, Ligeng/F-9844-2013; Chen, Qian/R-3275-2019; Liu, Zhuang/H-4352-2011; Sun, Xiaoqi/D-9875-2019
OI chen, qian/0000-0002-1487-5479; Xu, Ligeng/0000-0003-4946-2567; Liu, Zhuang/0000-0002-1629-1039; Sun, Xiaoqi/0000-0003-0752-1928
FU National Research Programs from Ministry of Science and Technology (MOST) of China [2016YFA0201200]; National Natural Science Foundation of China [51525203, 51761145041]; Collaborative Innovation Center of Suzhou Nano Science and Technology, the "111" program of the Ministry of Education (MOE) of China; Priority Academic Program Development (PAPD) of Jiangsu Higher Education Institutions
FX This work was supported by the National Research Programs from Ministry of Science and Technology (MOST) of China (2016YFA0201200), the National Natural Science Foundation of China (51525203, 51761145041), Collaborative Innovation Center of Suzhou Nano Science and Technology, the "111" program of the Ministry of Education (MOE) of China, and a Project Funded by the Priority Academic Program Development (PAPD) of Jiangsu Higher Education Institutions.
CR Betts MR, 2003, J IMMUNOL METHODS, V281, P65, DOI 10.1016/S0022-1759(03)00265-5
Blonder J, 2006, NAT PROTOC, V1, P2784, DOI 10.1038/nprot.2006.359
Branca MA, 2016, NAT BIOTECHNOL, V34, P1019, DOI 10.1038/nbt.3690
BRUNDA MJ, 1993, J EXP MED, V178, P1223, DOI 10.1084/jem.178.4.1223
Carreno BM, 2013, J CLIN INVEST, V123, P3383, DOI 10.1172/JCI68395
Carrillo-Conde B, 2011, MOL PHARMACEUT, V8, P1877, DOI 10.1021/mp200213r
Chen Q, 2016, NAT COMMUN, V7, DOI 10.1038/ncomms13193
Christensen D, 2012, J CONTROL RELEASE, V160, P468, DOI 10.1016/j.jconrel.2012.03.016
Fan ZY, 2014, J MATER CHEM B, V2, P8231, DOI 10.1039/c4tb00980k
Fang RH, 2014, NANO LETT, V14, P2181, DOI 10.1021/nl500618u

Finn OJ, 2003, NAT REV IMMUNOL, V3, P630, DOI 10.1038/nri1150
Frelinger J, 2010, CANCER IMMUNOL IMMUN, V59, P1435, DOI 10.1007/s00262-010-0883-4
Furman NET, 2013, NANO LETT, V13, P3248, DOI 10.1021/nl401376w
Gattinoni L, 2005, J CLIN INVEST, V115, P1616, DOI 10.1172/JCI24480
Gong H, 2015, NANOSCALE, V7, P19282, DOI 10.1039/c5nr06081h
Gubin MM, 2014, NATURE, V515, P577, DOI 10.1038/nature13988
Guo YY, 2015, ACS NANO, V9, P6918, DOI 10.1021/acsnano.5b01042
Hu CMJ, 2011, P NATL ACAD SCI USA, V108, P10980, DOI 10.1073/pnas.1106634108
Iclozan C, 2013, CANCER IMMUNOL IMMUN, V62, P909, DOI 10.1007/s00262-013-1396-8
Kasturi SP, 2011, NATURE, V470, P543, DOI 10.1038/nature09737
Kirkwood JM, 2012, CA-CANCER J CLIN, V62, P309, DOI 10.3322/caac.20132
Kroll AV, 2017, ADV MATER, V29, DOI 10.1002/adma.201703969
Kuai R, 2017, NAT MATER, V16, P489, DOI [10.1038/nmat4822, 10.1038/NMAT4822]
Le Mercier I, 2013, CANCER RES, V73, P4629, DOI 10.1158/0008-5472.CAN-12-3058
Li P, 2013, J CONTROL RELEASE, V168, P271, DOI 10.1016/j.jconrel.2013.03.025
Liu YJ, 2001, CELL, V106, P259, DOI 10.1016/S0092-8674(01)00456-1
Lodge PA, 2000, CANCER RES, V60, P829
Lutz MB, 1999, J IMMUNOL METHODS, V223, P77, DOI 10.1016/S0022-1759(98)00204-X
Mahoney KM, 2015, NAT REV DRUG DISCOV, V14, P561, DOI 10.1038/nrd4591
Manolova V, 2008, EUR J IMMUNOL, V38, P1404, DOI 10.1002/eji.200737984
Marrache S, 2013, ACS NANO, V7, P7392, DOI 10.1021/nn403158n
McCarthy SA, 2012, NAT PROTOC, V7, P1677, DOI 10.1038/nprot.2012.082
Palucka K, 2012, NAT REV CANCER, V12, P265, DOI 10.1038/nrc3258
Pardoll DM, 2012, NAT REV CANCER, V12, P252, DOI 10.1038/nrc3239
Parodi A, 2013, NAT NANOTECHNOL, V8, P61, DOI 10.1038/nnano.2012.212
Popovtzer R, 2008, NANO LETT, V8, P4593, DOI 10.1021/nl8029114
Randolph GJ, 2005, NAT REV IMMUNOL, V5, P617, DOI 10.1038/nri1670
Schirrmacher V, 1999, GENE THER, V6, P63, DOI 10.1038/sj.gt.3300787
SHEPHARD RJ, 1995, CAN J APPL PHYSIOL, V20, P1, DOI 10.1139/h95-001
Shevtsov MA, 2015, J CONTROL RELEASE, V220, P329, DOI 10.1016/j.jconrel.2015.10.051
Steinman RM, 2007, NAT MED, V13, P1155, DOI 10.1038/nm1643
TILLACK TW, 1970, J CELL BIOL, V45, P649, DOI 10.1083/jcb.45.3.649
Topalian SL, 2012, NEW ENGL J MED, V366, P2443, DOI 10.1056/NEJMoa1200690
Wang C, 2017, ADV MATER, V29, DOI 10.1002/adma.201606036
Wang C, 2017, NAT BIOMED ENG, V1, DOI 10.1038/s41551-016-0011
Wei SC, 2017, CELL, V170, P1120, DOI 10.1016/j.cell.2017.07.024
Woelders H, 1997, CRYOBIOLOGY, V35, P93, DOI 10.1006/cryo.1997.2028
Xiang J, 2015, ACS NANO, V9, P6401, DOI 10.1021/acsnano.5b02014
Xiang SD, 2008, EXPERT REV VACCINES, V7, P1103, DOI 10.1586/14760584.7.7.1103
Xu LG, 2013, ADV MATER, V25, P5928, DOI 10.1002/adma.201300583
Yang YP, 2015, J CLIN INVEST, V125, P3335, DOI 10.1172/JCI83871
Ye YQ, 2017, SCI IMMUNOL, V2, DOI 10.1126/sciimmunol.aan5692
Zhang ZP, 2011, BIOMATERIALS, V32, P3666, DOI 10.1016/j.biomaterials.2011.01.067
Zhao F, 2011, SMALL, V7, P1322, DOI 10.1002/smll.201100001
Zhou H, 2016, THERANOSTICS, V6, P1012, DOI 10.7150/thno.15095
NR 55
TC 413
Z9 427
U1 37
U2 795
PU AMER CHEMICAL SOC
PI WASHINGTON
PA 1155 16TH ST, NW, WASHINGTON, DC 20036 USA
SN 1936-0851
EI 1936-086X
J9 ACS NANO
JI ACS Nano
PD JUN
PY 2018
VL 12
IS 6

BP 5121
EP 5129
DI 10.1021/acsnano.7b09041
PG 9
WC Chemistry, Multidisciplinary; Chemistry, Physical; Nanoscience &
Nanotechnology; Materials Science, Multidisciplinary
WE Science Citation Index Expanded (SCI-EXPANDED)
SC Chemistry; Science & Technology - Other Topics; Materials Science
GA GL1YM
UT WOS:000436910200006
PM 29771487
HC Y
HP N
DA 2023-09-14
ER

PT J
AU Shao, K
Singha, S
Clemente-Casares, X
Tsai, S
Yang, Y
Santamaria, P
AF Shao, Kun
Singha, Santiswarup
Clemente-Casares, Xavier
Tsai, Sue
Yang, Yang
Santamaria, Pere
TI Nanoparticle-Based Immunotherapy for Cancer
SO ACS NANO
LA English
DT Review
DE cancer immunotherapy; tumor immune surveillance; tumor immune evasion;
tumor immunoediting; tumor-associated antigens; danger-associated
molecular patterns; T-lymphocytes; lymphocyte co-stimulators; vaccines;
nanoparticles; targeted delivery; environment-responsive nanoparticles
ID ANTIGEN-PRESENTING CELLS; CD8-ALPHA(+) DENDRITIC CELLS; DRAINING
LYMPH-NODE; T-CELLS; TARGETED DELIVERY; DRUG-DELIVERY; METASTATIC
MELANOMA; POLYMERIC MICELLES; IMMUNE CELLS; SOLID TUMORS
AB The design of nanovaccines capable of triggering effective antitumor immunity requires an understanding of how the immune system senses and responds to threats, including pathogens and tumors. Equally important is an understanding of the mechanisms employed by tumor cells to evade immunity and an appreciation of the deleterious effects that antitumor immune responses can have on tumor growth, such as by skewing tumor cell composition toward immunologically silent tumor cell variants. The immune system and tumors engage in a tug-of-war driven by competition where promoting antitumor immunity or tumor cell death alone may be therapeutically insufficient. Nanotechnology affords a unique opportunity to develop therapeutic compounds than can simultaneously tackle both aspects, favoring tumor eradication. Here, we review the current status of nanoparticle-based immunotherapeutic strategies for the treatment of cancer, ranging from antigen/adjuvant delivery vehicles (to professional antigen-presenting cell types of the immune system) to direct tumor antigen-specific T-lymphocyte-targeting compounds and their combinations thereof.
C1 [Shao, Kun; Singha, Santiswarup; Clemente-Casares, Xavier; Tsai, Sue; Yang, Yang; Santamaria, Pere] Univ Calgary, Cummings Sch Med, JMDRC, Calgary, AB T2N 4N1, Canada.
[Shao, Kun; Singha, Santiswarup; Clemente-Casares, Xavier; Tsai, Sue; Yang, Yang; Santamaria, Pere] Univ Calgary, Cummings Sch Med, Snyder Inst Chron Dis, Dept Microbiol Immunol & Infect Dis, Calgary, AB T2N 4N1, Canada.
[Shao, Kun; Singha, Santiswarup; Clemente-Casares, Xavier; Tsai, Sue; Yang, Yang; Santamaria, Pere] Univ Calgary, Cummings Sch Med, Hotchkiss Brain Inst, Calgary, AB T2N 4N1, Canada.
[Yang, Yang] Univ Calgary, Cummings Sch Med, Dept Biochem & Mol Biol, Calgary, AB T2N 4N1, Canada.

[Santamaria, Pere] Inst Invest Biomed August Pi & Sunyer, Barcelona 08036, Spain.
C3 University of Calgary; University of Barcelona; Hospital Clinic de Barcelona; IDIBAPS
RP Santamaria, P (通讯作者)，Univ Calgary, Cummings Sch Med, JMDRC, Calgary, AB T2N 4N1, Canada.
EM yyang@ucalgary.ca; psantama@ucalgary.ca
RI Santamaria, Pere/ABF-6823-2021
OI Clemente-Casares, Xavier/0000-0002-8627-4971; Tsai, Sue/0000-0002-6261-5233; Shao, Kun/0000-0001-6876-4697
FU Canadian Institutes of Health Research (CIHR); Natural Sciences and Engineering Research Council of Canada (NSERC); Diabetes Research Foundation (DRF); Juvenile Diabetes Research Foundation (JDRF); Canadian Diabetes Association (CDA); Multiple Sclerosis Society of Canada (MSSC); Brawn Family Foundation; Sarda Farriol Research Programme; European Community's Seventh Framework Programme (EC FP7) [229673]; Eyes' High/Alberta Innovates-Technology Futures postdoctoral fellowship; AXA Research Fund; endMS network; Alberta Heritage Foundation of Medical Research (AHFMR); Diabetes Association (Foothills); CDA
FX The authors' work relevant to this review was funded by the Canadian Institutes of Health Research (CIHR), the Natural Sciences and Engineering Research Council of Canada (NSERC), the Diabetes Research Foundation (DRF), the Juvenile Diabetes Research Foundation (JDRF), the Canadian Diabetes Association (CDA), the Multiple Sclerosis Society of Canada (MSSC), the Brawn Family Foundation, the Sarda Farriol Research Programme, and the European Community's Seventh Framework Programme (EC FP7/2007-2013; #229673). K.S. was funded by an Eyes' High/Alberta Innovates-Technology Futures postdoctoral fellowship. X.C.C. was supported by studentships from the AXA Research Fund and the endMS network. S.T. was supported by a studentship from the Alberta Heritage Foundation of Medical Research (AHFMR). P.S. is a Scientist of the Alberta Innovates-Health Solutions and a scholar of the Instituto de Investigaciones Sanitarias Carlos III. The JMDRC is supported by the Diabetes Association (Foothills) and the CDA.
CR Ahrens S, 2012, IMMUNITY, V36, P635, DOI 10.1016/j.immuni.2012.03.008
Ando K, 2011, EXPERT OPIN PHARMACO, V12, P285, DOI 10.1517/14656566.2011.543129
Ashkenazi A, 2002, NAT REV CANCER, V2, P420, DOI 10.1038/nrc821
Bae YH, 2011, J CONTROL RELEASE, V153, P198, DOI 10.1016/j.jconrel.2011.06.001
Bal SM, 2011, VACCINE, V29, P1045, DOI 10.1016/j.vaccine.2010.11.061
Bellocq NC, 2003, BIOCONJUGATE CHEM, V14, P1122, DOI 10.1021/bc034125f
Bertrand N, 2012, J CONTROL RELEASE, V161, P152, DOI 10.1016/j.jconrel.2011.09.098
Bianchi ME, 2014, CELL DEATH DIFFER, V21, P1, DOI 10.1038/cdd.2013.147
Bladergroen BA, 2002, BLOOD, V99, P232, DOI 10.1182/blood.V99.1.232
Boissonnas A, 2007, J EXP MED, V204, P345, DOI 10.1084/jem.20061890
Bombelli FB, 2014, LANCET ONCOL, V15, pE22, DOI 10.1016/S1470-2045(13)70333-4
Boon T, 2006, ANNU REV IMMUNOL, V24, P175, DOI 10.1146/annurev.immunol.24.021605.090733
Bourquin C, 2008, J IMMUNOL, V181, P2990, DOI 10.4049/jimmunol.181.5.2990
Byrne KT, 2011, J CLIN INVEST, V121, P1797, DOI 10.1172/JCI44849
Caserta S, 2008, CANCER RES, V68, P3010, DOI 10.1158/0008-5472.CAN-07-5796
Cho KJ, 2008, CLIN CANCER RES, V14, P1310, DOI 10.1158/1078-0432.CCR-07-1441
Cho NH, 2011, NAT NANOTECHNOL, V6, P675, DOI [10.1038/NNANO.2011.149, 10.1038/nnano.2011.149]
Choi HS, 2007, NAT BIOTECHNOL, V25, P1165, DOI 10.1038/nbt1340
Clemente-Casares X, 2011, J MOL MED, V89, P733, DOI 10.1007/s00109-011-0757-z
Cochran AJ, 2001, MODERN PATHOL, V14, P604, DOI 10.1038/modpathol.3880358
Curiel TJ, 2003, NAT MED, V9, P562, DOI 10.1038/nm863
D Basu G, 2006, J IMMUNOL, V177, P2391, DOI 10.4049/jimmunol.177.4.2391
Davis ME, 2008, NAT REV DRUG DISCOV, V7, P771, DOI 10.1038/nrd2614
Decuzzi P, 2009, PHARM RES-DORD, V26, P235, DOI 10.1007/s11095-008-9697-x
Delyon J, 2013, ANN ONCOL, V24, P1697, DOI 10.1093/annonc/mdt027
Di Costanzo F, 2009, ONCOTARGETS THER, V2, P179
Dixit V, 2006, BIOCONJUGATE CHEM, V17, P603, DOI 10.1021/bc050335b
Dougan M, 2009, ANNU REV IMMUNOL, V27, P83, DOI 10.1146/annurev.immunol.021908.132544

Dudley ME, 2002, SCIENCE, V298, P850, DOI 10.1126/science.1076514
Dwivedi PD, 2011, J BIOMED NANOTECHNOL, V7, P193, DOI 10.1166/jbn.2011.1264
Eggermonf AMM, 2009, EUR J CANCER, V45, P2087, DOI 10.1016/j.ejca.2009.05.004
Ernsting MJ, 2012, J CONTROL RELEASE, V162, P575, DOI 10.1016/j.jconrel.2012.07.043
FALO LD, 1995, NAT MED, V1, P649, DOI 10.1038/nm0795-649
Fang RH, 2014, NANO LETT, V14, P2181, DOI 10.1021/nl500618u
Farokhzad OC, 2006, P NATL ACAD SCI USA, V103, P6315, DOI 10.1073/pnas.0601755103
Fifis T, 2004, J IMMUNOL, V173, P3148, DOI 10.4049/jimmunol.173.5.3148
Flattery M P, 1998, J Transpl Coord, V8, P105
GABIZON A, 1994, CANCER RES, V54, P987
Gabrilovich D, 2004, NAT REV IMMUNOL, V4, P941, DOI 10.1038/nri1498
Gadiot J, 2011, CANCER-AM CANCER SOC, V117, P2192, DOI 10.1002/cncr.25747
Gajewski TF, 2013, NAT IMMUNOL, V14, P1014, DOI 10.1038/ni.2703
Galon J, 2006, SCIENCE, V313, P1960, DOI 10.1126/science.1129139
Gao HL, 2013, SCI REP-UK, V3, DOI 10.1038/srep02534
Gao WW, 2014, WIRES NANOMED NANOBI, V6, P532, DOI 10.1002/wnan.1282
Gettinger S, 2014, CANCER J, V20, P281, DOI 10.1097/PPO.0000000000000063
Harisinghani MG, 2003, NEW ENGL J MED, V348, P2491, DOI 10.1056/NEJMoa022749
Harrington KJ, 2001, CLIN CANCER RES, V7, P243
Hashizume H, 2000, AM J PATHOL, V156, P1363, DOI 10.1016/S0002-9440(10)65006-7
Heo MB, 2014, ACTA BIOMATER, V10, P2169, DOI 10.1016/j.actbio.2013.12.050
Heo MB, 2014, BIOMATERIALS, V35, P590, DOI 10.1016/j.biomaterials.2013.10.009
Hodi FS, 2010, NEW ENGL J MED, V363, P711, DOI 10.1056/NEJMoa1003466
Hong MH, 2009, J CONTROL RELEASE, V133, P96, DOI 10.1016/j.jconrel.2008.09.005
Idoyaga J, 2009, P NATL ACAD SCI USA, V106, P1524, DOI 10.1073/pnas.0812247106
Jeanbart L, 2014, CANCER IMMUNOL RES, V2, P436, DOI 10.1158/2326-6066.CIR-14-0019-T
Jhaveri AM, 2014, FRONT PHARMACOL, V5, DOI 10.3389/fphar.2014.00077
Jiang W, 2008, NAT NANOTECHNOL, V3, P145, DOI 10.1038/nnano.2008.30
Kantoff PW, 2010, J CLIN ONCOL, V28, P1099, DOI 10.1200/JCO.2009.25.0597
Kasturi SP, 2011, NATURE, V470, P543, DOI 10.1038/nature09737
Kim C, 2010, ACS NANO, V4, P4559, DOI 10.1021/nn100736c
Kim J, 2011, NANO TODAY, V6, P466, DOI 10.1016/j.nantod.2011.08.005
Kuang YY, 2013, INT J PHARMACEUT, V454, P11, DOI 10.1016/j.ijpharm.2013.07.019
Kwong B, 2011, BIOMATERIALS, V32, P5134, DOI 10.1016/j.biomaterials.2011.03.067
Lam AR, 2014, CANCER RES, V74, P2193, DOI 10.1158/0008-5472.CAN-13-1703
Lee H, 2010, MOL PHARMACEUT, V7, P1195, DOI 10.1021/mp100038h
Lee SY, 2009, J IMMUNOTHER, V32, P22, DOI 10.1097/CJI.0b013e31818ac2f7
Leleux J, 2013, ADV HEALTHC MATER, V2, P72, DOI 10.1002/adhm.201200268
Lu J, 2011, MACROMOLECULES, V44, P6002, DOI 10.1021/ma200675w
Lu W, 2009, CLIN CANCER RES, V15, P876, DOI 10.1158/1078-0432.CCR-08-1480
Macho-Fernandez E, 2014, J IMMUNOL, V193, P961, DOI 10.4049/jimmunol.1303029
Manolova V, 2008, EUR J IMMUNOL, V38, P1404, DOI 10.1002/eji.200737984
Maus MV, 2003, CLIN IMMUNOL, V106, P16, DOI 10.1016/S1521-6616(02)00017-7
McMahan RH, 2006, J CLIN INVEST, V116, P2543, DOI 10.1172/JCI26936
Mellman I, 2011, NATURE, V480, P480, DOI 10.1038/nature10673
Meng FH, 2014, NANOMEDICINE-UK, V9, P487, DOI [10.2217/NNM.13.212, 10.2217/nnm.13.212]
Milicic A, 2012, PLOS ONE, V7, DOI 10.1371/journal.pone.0034255
Mocellin S, 2004, LANCET ONCOL, V5, P681, DOI 10.1016/S1470-2045(04)01610-9
Mottram PL, 2007, MOL PHARMACEUT, V4, P73, DOI 10.1021/mp060096p
Munn DH, 2006, IMMUNOL REV, V213, P146, DOI 10.1111/j.1600-065X.2006.00444.x
Munn DH, 2004, J CLIN INVEST, V114, P280, DOI 10.1172/JCI200421583
Novellino L, 2005, CANCER IMMUNOL IMMUN, V54, P187, DOI 10.1007/s00262-004-0560-6
Obermajer N, 2011, BLOOD, V118, P5498, DOI 10.1182/blood-2011-07-365825
Oelke M, 2003, NAT MED, V9, P619, DOI 10.1038/nm869
Cobucci RNO, 2012, CANCER EPIDEMIOL, V36, pE69, DOI 10.1016/j.canep.2011.12.002
PAPAHADJOPOULOS D, 1991, P NATL ACAD SCI USA, V88, P11460, DOI 10.1073/pnas.88.24.11460
Pasare C, 2003, SCIENCE, V299, P1033, DOI 10.1126/science.1078231
Peggs KS, 2006, CURR OPIN IMMUNOL, V18, P206, DOI 10.1016/j.coi.2006.01.011
Perica K, 2014, NANOMED-NANOTECHNOL, V10, P119, DOI 10.1016/j.nano.2013.06.015
Perrault SD, 2009, NANO LETT, V9, P1909, DOI 10.1021/nl900031y
Pinzon-Charry A, 2005, IMMUNOL CELL BIOL, V83, P451, DOI 10.1111/j.1440-1711.2005.01371.x

Prabhakar U, 2013, CANCER RES, V73, P2412, DOI 10.1158/0008-5472.CAN-12-4561
Pradhan P, 2014, BIOMATERIALS, V35, P5491, DOI 10.1016/j.biomaterials.2014.03.039
Ray M, 2012, PROSTATE, V72, P846, DOI 10.1002/pros.21486
Reddy ST, 2007, NAT BIOTECHNOL, V25, P1159, DOI [10.1038/nbt1332, 10.1038/nbtl332]
Romero P, 1998, J EXP MED, V188, P1641, DOI 10.1084/jem.188.9.1641
Rosenberg SA, 2004, NAT MED, V10, P909, DOI 10.1038/nm1100
Rosenberg SA, 2008, NAT REV CANCER, V8, P299, DOI 10.1038/nrc2355
Schreiber RD, 2011, SCIENCE, V331, P1565, DOI 10.1126/science.1203486
Schwartzentruber DJ, 2011, NEW ENGL J MED, V364, P2119, DOI 10.1056/NEJMoa1012863
Seremet T, 2011, CANCER J, V17, P325, DOI 10.1097/PPO.0b013e3182326004
Shao K, 2014, ACS NANO, V8, P1191, DOI 10.1021/nn406285x
Sharpe M, 2002, DRUGS, V62, P2089, DOI 10.2165/00003495-200262140-00012
Shen CL, 2013, IMMUNOL LETT, V150, P1, DOI 10.1016/j.imlet.2013.01.003
Shimizu K, 2013, J IMMUNOL, V190, P5609, DOI 10.4049/jimmunol.1300033
Sica A, 2006, EUR J CANCER, V42, P717, DOI 10.1016/j.ejca.2006.01.003
Simpson TR, 2013, J EXP MED, V210, P1695, DOI 10.1084/jem.20130579
Steenblock ER, 2008, MOL THER, V16, P765, DOI 10.1038/mt.2008.11
Steenblock ER, 2011, J BIOL CHEM, V286, P34883, DOI 10.1074/jbc.M111.276329
Taurin S, 2012, J CONTROL RELEASE, V164, P265, DOI 10.1016/j.jconrel.2012.07.013
Thomas SN, 2014, BIOMATERIALS, V35, P814, DOI 10.1016/j.biomaterials.2013.10.003
Topfer K, 2011, J BIOMED BIOTECHNOL, DOI 10.1155/2011/918471
Tsai SE, 2010, IMMUNITY, V32, P568, DOI 10.1016/j.immuni.2010.03.015
Ugel S, 2009, CANCER RES, V69, P9376, DOI 10.1158/0008-5472.CAN-09-0400
Uto T, 2007, J IMMUNOL, V178, P2979, DOI 10.4049/jimmunol.178.5.2979
van Mierlo GJD, 2004, J IMMUNOL, V173, P6753, DOI 10.4049/jimmunol.173.11.6753
Vesely MD, 2011, ANNU REV IMMUNOL, V29, P235, DOI 10.1146/annurev-immunol-031210-101324
Vicari AP, 2002, J EXP MED, V196, P541, DOI 10.1084/jem.20020732
Vigneron Nathalie, 2013, Cancer Immun, V13, P15
Wang YH, 2013, MOL THER, V21, P1919, DOI 10.1038/mt.2013.135
Wilson JT, 2013, ACS NANO, V7, P3912, DOI 10.1021/nn305466z
Xu ZH, 2014, ACS NANO, V8, P3636, DOI 10.1021/nn500216y
Xu ZH, 2013, J CONTROL RELEASE, V172, P259, DOI 10.1016/j.jconrel.2013.08.021
Zaks K, 2006, J IMMUNOL, V176, P7335, DOI 10.4049/jimmunol.176.12.7335
Zamboni WC, 2012, CLIN CANCER RES, V18, P3229, DOI 10.1158/1078-0432.CCR-11-2938
Zhang L, 2003, NEW ENGL J MED, V348, P203, DOI 10.1056/NEJMoa020177
Zhang PS, 2007, CANCER RES, V67, P6468, DOI 10.1158/0008-5472.CAN-07-1264
Zolnik BS, 2010, ENDOCRINOLOGY, V151, P458, DOI 10.1210/en.2009-1082
Zwiorek K, 2008, PHARM RES-DORDR, V25, P551, DOI 10.1007/s11095-007-9410-5
NR 127
TC 339
Z9 358
U1 20
U2 524
PU AMER CHEMICAL SOC
PI WASHINGTON
PA 1155 16TH ST, NW, WASHINGTON, DC 20036 USA
SN 1936-0851
EI 1936-086X
J9 ACS NANO
JI ACS Nano
PD JAN
PY 2015
VL 9
IS 1
BP 16
EP 30
DI 10.1021/nn5062029
PG 15
WC Chemistry, Multidisciplinary; Chemistry, Physical; Nanoscience & Nanotechnology; Materials Science, Multidisciplinary
WE Science Citation Index Expanded (SCI-EXPANDED)

SC Chemistry; Science & Technology - Other Topics; Materials Science
GA CA0PJ
UT WOS:000348619000004
PM 25469470
OA hybrid
HC Y
HP N
DA 2023-09-14
ER

PT J
AU Kroll, AV
Fang, RH
Jiang, Y
Zhou, JR
Wei, XL
Yu, CL
Gao, J
Luk, BT
Dehaini, D
Gao, WW
Zhang, LF
AF Kroll, Ashley V.
Fang, Ronnie H.
Jiang, Yao
Zhou, Jiarong
Wei, Xiaoli
Yu, Chun Lai
Gao, Jie
Luk, Brian T.
Dehaini, Diana
Gao, Weiwei
Zhang, Liangfang
TI Nanoparticulate Delivery of Cancer Cell Membrane Elicits Multiantigenic Antitumor Immunity
SO ADVANCED MATERIALS
LA English
DT Article
DE anticancer vaccines; biomimetic nanoparticles; immunotherapies; nanomedicines; personalized medicines
ID T-CELLS; CHECKPOINT BLOCKADE; TUMOR-ANTIGENS; VACCINES; MELANOMA; IMMUNOTHERAPY; COMBINATION; THERAPY; IMMUNOSUPPRESSION; VACCINATION
AB Anticancer vaccines train the body's own immune system to recognize and eliminate malignant cells based on differential antigen expression. While conceptually attractive, clinical efficacy is lacking given several key challenges stemming from the similarities between cancerous and healthy tissue. Ideally, an effective vaccine formulation would deliver multiple tumor antigens in a fashion that potently stimulates endogenous immune responses against those antigens. Here, it is reported on the fabrication of a biomimetic, nanoparticulate anticancer vaccine that is capable of delivering autologously derived tumor antigen material together with a highly immunostimulatory adjuvant. The two major components, tumor antigens and adjuvant, are presented concurrently in a fashion that maximizes their ability to promote effective antigen presentation and activation of downstream immune processes. Ultimately, it is demonstrated that the formulation can elicit potent antitumor immune responses in vivo. When combined with additional immunotherapies such as checkpoint blockades, the nanovaccine demonstrates substantial therapeutic effect. Overall, the work represents the rational application of nanotechnology for immunoengineering and can provide a blueprint for the future development of personalized, autologous anticancer vaccines with broad applicability.
C1 [Kroll, Ashley V.; Fang, Ronnie H.; Jiang, Yao; Zhou, Jiarong; Wei, Xiaoli; Yu, Chun Lai; Gao, Jie; Luk, Brian T.; Dehaini, Diana; Gao, Weiwei; Zhang, Liangfang] Univ Calif San Diego, Dept NanoEngn, La Jolla, CA 92093 USA.
[Kroll, Ashley V.; Fang, Ronnie H.; Jiang, Yao; Zhou, Jiarong; Wei, Xiaoli; Yu, Chun Lai; Gao, Jie; Luk, Brian T.; Dehaini, Diana; Gao, Weiwei; Zhang, Liangfang] Univ Calif San Diego, Moores Canc Ctr, La Jolla,

CA 92093 USA.
C3 University of California System; University of California San Diego;
University of California System; University of California San Diego
RP Zhang, LF (通讯作者)，Univ Calif San Diego, Dept NanoEngn, La Jolla, CA 92093 USA.; Zhang, LF (通讯作者)，Univ Calif San Diego, Moores Canc Ctr, La Jolla, CA 92093 USA.
EM zhang@ucsd.edu
RI wei, xiao/ISB-6027-2023; jiang, lei/IWE-1124-2023; Fang, Ronnie H./HCH-5747-2022
OI Zhang, Liangfang/0000-0003-0637-0654
FU National Institutes of Health [R01CA200574]; National Science Foundation [DMR-1505699]; Direct For Mathematical & Physical Scien; Division Of Materials Research [1505699] Funding Source: National Science Foundation
FX A.V.K. and R.H.F. contributed equally to this work. This work was supported by the National Institutes of Health under Award No. R01CA200574 and the National Science Foundation Grant No. DMR-1505699.
CR Bode C, 2011, EXPERT REV VACCINES, V10, P499, DOI [10.1586/ERV.10.174, 10.1586/erv.10.174]
Buonaguro L, 2011, CLIN VACCINE IMMUNOL, V18, P23, DOI 10.1128/CVI.00286-10
Carreno BM, 2015, SCIENCE, V348, P803, DOI 10.1126/science.aaa3828
Castle JC, 2012, CANCER RES, V72, P1081, DOI 10.1158/0008-5472.CAN-11-3722
Chaturvedi A, 2009, TRAFFIC, V10, P621, DOI 10.1111/j.1600-0854.2009.00899.x
Curran MA, 2010, P NATL ACAD SCI USA, V107, P4275, DOI 10.1073/pnas.0915174107
de Gruijl TD, 2008, CANCER IMMUNOL IMMUN, V57, P1569, DOI 10.1007/s00262-008-0536-z
Fang RH, 2014, NANO LETT, V14, P2181, DOI 10.1021/nl500618u
Finn OJ, 2003, NAT REV IMMUNOL, V3, P630, DOI 10.1038/nri1150
Ghirelli C, 2013, J CLIN INVEST, V123, P2355, DOI 10.1172/JCI69999
Gibney GT, 2015, CLIN CANCER RES, V21, P712, DOI 10.1158/1078-0432.CCR-14-2468
Gottesman MM, 2002, ANNU REV MED, V53, P615, DOI 10.1146/annurev.med.53.082901.103929
Hu CMJ, 2015, NATURE, V526, P118, DOI 10.1038/nature15373
Hu CMJ, 2011, P NATL ACAD SCI USA, V108, P10980, DOI 10.1073/pnas.1106634108
Keenan BP, 2012, SEMIN ONCOL, V39, P276, DOI 10.1053/j.seminoncol.2012.02.007
Khalil DN, 2016, NAT REV CLIN ONCOL, V13, P394, DOI 10.1038/nrclinonc.2016.65
Kim R, 2007, IMMUNOLOGY, V121, P1, DOI 10.1111/j.1365-2567.2007.02587.x
Kleponis J, 2015, CANCER BIOL MED, V12, P201, DOI 10.7497/j.issn.2095-3941.2015.0046
Lokhov PG, 2010, J CANCER, V1, P230
Melief CJM, 2015, J CLIN INVEST, V125, P3401, DOI 10.1172/JCI80009
Mellman I, 2011, NATURE, V480, P480, DOI 10.1038/nature10673
Mittal D, 2014, CURR OPIN IMMUNOL, V27, P16, DOI 10.1016/j.coi.2014.01.004
Morse MA, 2015, VACCINE, V33, P7377, DOI 10.1016/j.vaccine.2015.10.057
Nathanson DA, 2014, SCIENCE, V343, P72, DOI 10.1126/science.1241328
Novellino L, 2005, CANCER IMMUNOL IMMUN, V54, P187, DOI 10.1007/s00262-004-0560-6
Oh N, 2014, INT J NANOMED, V9, P51, DOI 10.2147/IJN.S26592
Palucka K, 2012, NAT REV CANCER, V12, P265, DOI 10.1038/nrc3258
Parmiani G, 2002, JNCI-J NATL CANCER I, V94, P805, DOI 10.1093/jnci/94.11.805
Rabinovich GA, 2007, ANNU REV IMMUNOL, V25, P267, DOI 10.1146/annurev.immunol.25.022106.141609
Rizvi NA, 2015, SCIENCE, V348, P124, DOI 10.1126/science.aaa1348
Schlosser HA, 2014, IMMUNOTHERAPY-UK, V6, P973, DOI 10.2217/imt.14.58
Seliger B, 2001, CANCER RES, V61, P1095
Shang L, 2014, J NANOBIOTECHNOL, V12, DOI 10.1186/1477-3155-12-5
Slingluff CL, 2011, CANCER J, V17, P343, DOI 10.1097/PPO.0b013e318233e5b2
Snyder A, 2014, NEW ENGL J MED, V371, P2189, DOI 10.1056/NEJMoa1406498
Tabi Z, 2006, ADV DRUG DELIVER REV, V58, P902, DOI 10.1016/j.addr.2006.05.004
Twyman-Saint Victor C, 2015, NATURE, V520, P373, DOI 10.1038/nature14292
Valsecchi ME, 2015, NEW ENGL J MED, V373, P1270, DOI [10.1056/NEJMoa1504030, 10.1056/NEJMc1509660]
Vandenberk L, 2016, FRONT IMMUNOL, V6, DOI 10.3389/fimmu.2015.00663
vansder Burg S. H., 2016, NAT REV CANCER, V16, P219
Vinay DS, 2015, SEMIN CANCER BIOL, V35, pS185, DOI 10.1016/j.semcancer.2015.03.004
Vollmer J, 2004, EUR J IMMUNOL, V34, P251, DOI 10.1002/eji.200324032
Xu DP, 2004, INT J CANCER, V109, P499, DOI 10.1002/ijc.11696
Yang YP, 2015, J CLIN INVEST, V125, P3335, DOI 10.1172/JCI83871

Zou WP, 2005, NAT REV CANCER, V5, P263, DOI 10.1038/nrc1586
NR 45
TC 318
Z9 323
U1 23
U2 468
PU WILEY-V C H VERLAG GMBH
PI WEINHEIM
PA POSTFACH 101161, 69451 WEINHEIM, GERMANY
SN 0935-9648
EI 1521-4095
J9 ADV MATER
JI Adv. Mater.
PD DEC 20
PY 2017
VL 29
IS 47
AR 1703969
DI 10.1002/adma.201703969
PG 9
WC Chemistry, Multidisciplinary; Chemistry, Physical; Nanoscience &
Nanotechnology; Materials Science, Multidisciplinary; Physics, Applied;
Physics, Condensed Matter
WE Science Citation Index Expanded (SCI-EXPANDED)
SC Chemistry; Science & Technology - Other Topics; Materials Science;
Physics
GA FQ0TD
UT WOS:000418068700016
PM 29239517
OA Green Accepted, Bronze
HC Y
HP N
DA 2023-09-14
ER

PT J
AU Guo, YY
Wang, D
Song, QL
Wu, TT
Zhuang, XT
Bao, YL
Kong, M
Qj, Y
Tan, SW
Zhang, ZP
AF Guo, Yuanyuan
Wang, Dong
Song, Qingle
Wu, Tingting
Zhuang, Xiangting
Bao, Yuling
Kong, Miao
Qj, Yan
Tan, Songwei
Zhang, Zhiping
TI Erythrocyte Membrane-Enveloped Polymeric Nanoparticles as Nanovaccine
for Induction of Antitumor Immunity against Melanoma
SO ACS NANO
LA English
DT Article

DE cancer vaccine; red blood cell; nanoparticles; dendritic cells;
immunotherapy
ID RED-BLOOD-CELLS; DENDRITIC CELLS; IN-VIVO; DELIVERY-SYSTEM;
CANCER-IMMUNOTHERAPY; EXOGENOUS ANTIGENS; CROSS-PRESENTATION;
INTERFERON-GAMMA; MANNOSE RECEPTOR; DRUG-DELIVERY
AB Cancer immunotherapy is mainly focused on manipulating patient's own immune system to recognize and destroy cancer cells. Vaccine formulations based on nanotechnology have been developed to target delivery antigens to antigen presenting cells (APCs), especially dendritic cells (DCs) for efficiently induction of antigen specific T cells response. To enhance DC targeting and antigen presenting efficiency, we developed erythrocyte membrane-enveloped poly(D,L-lactide-co-glycolide) (PLGA) nanoparticles for antigenic peptide (hgp100(25-33)) and toll-like receptor 4 agonist, monophosphoryl lipid (MPLA). A Mannose-inserted membrane structure was constructed to actively target APCs in the lymphatic organ, and redox-sensitive peptide-conjugated PLGA nanoparticles were fabricated which prone to cleave in the intracellular milieu. The nanovaccine demonstrated the retained protein content in erythrocyte and enhanced in vitro cell uptake. An antigen-depot effect was observed in the administration site with promoted retention in draining lymph nodes. Compared with other formulations after intradermal injection, the nanovaccine prolonged tumor-occurring time, inhibited tumor growth, and suppressed tumor metastasis in prophylactic, therapeutic, and metastatic melanoma models, respectively. Additionally, we revealed that nanovaccine effectively enhanced IFN-gamma secretion and CD8(+) T cell response. Taken together, these results demonstrated the great potential in applying an erythrocyte membrane-enveloped polymeric nanoplatform for an antigen delivery system in cancer immunotherapy.
C1 [Guo, Yuanyuan; Wang, Dong; Song, Qingle; Wu, Tingting; Zhuang, Xiangting; Bao, Yuling; Kong, Miao; Qj, Yan; Tan, Songwei; Zhang, Zhiping] Huazhong Univ Sci & Technol, Tiongji Sch Pharm, Wuhan 430030, Peoples R China.
[Zhang, Zhiping] Huazhong Univ Sci & Technol, Hubei Engn Res Ctr Novel Drug Delivery Syst, Wuhan 430030, Peoples R China.
[Zhang, Zhiping] Huazhong Univ Sci & Technol, Natl Engn Res Ctr Nanomed, Wuhan 430030, Peoples R China.
C3 Huazhong University of Science & Technology; Huazhong University of
Science & Technology; Huazhong University of Science & Technology
RP Zhang, ZP (通讯作者)，Huazhong Univ Sci & Technol, Tiongji Sch Pharm, Wuhan 430030, Peoples R China.
EM zhipingzhang@mail.hust.edu.cn
RI Guo, Yuan/HKV-9606-2023; TAN, Songwei/K-2222-2019; zhang,
zhiping/H-1786-2011
OI TAN, Songwei/0000-0002-2986-8968; zhang, zhiping/0000-0001-9975-4862
FU National Basic Research Program of China (973 Program) [2012CB932501];
National Natural Science Foundation of China [81373360, 21474086];
Ministry of Education of China [20120142120093]; Fundamental Research
Funds for the Central Universities [2014TS091]; Chutian Scholar Award;
HUST
FX This work was supported by National Basic Research Program of China (973
Program, 2012CB932501), the National Natural Science Foundation of China
(81373360 and 21474086), the Doctoral Fund of Ministry of Education of
China (20120142120093), the Fundamental Research Funds for the Central
Universities (2014TS091), Chutian Scholar Award, and 2013 Youth Scholar
Award of HUST. We thank the Analytical and Testing Center of Huazhong
University of Science for performing the TEM.
CR Ali OA, 2009, NAT MATER, V8, P151, DOI [10.1038/NMAT2357, 10.1038/nmat2357]
Aryal S, 2013, NANOMEDICINE-UK, V8, P1271, DOI [10.2217/NNM.12.153, 10.2217/nnm.12.153]
Banchereau J, 1998, NATURE, V392, P245, DOI 10.1038/32588
Banz A, 2010, VACCINE, V28, P2965, DOI 10.1016/j.vaccine.2010.02.013
Cella M, 1997, CURR OPIN IMMUNOL, V9, P10, DOI 10.1016/S0952-7915(97)80153-7
Challa-Malladi M, 2011, CANCER CELL, V20, P728, DOI 10.1016/j.ccr.2011.11.006
Chambers AF, 2002, NAT REV CANCER, V2, P563, DOI 10.1038/nrc865
Chiarantini L, 1997, VACCINE, V15, P276, DOI 10.1016/S0264-410X(96)00181-8
Choi MR, 2007, NANO LETT, V7, P3759, DOI 10.1021/nl072209h
Christensen D, 2012, J CONTROL RELEASE, V160, P468, DOI 10.1016/j.jconrel.2012.03.016
Copp JA, 2014, P NATL ACAD SCI USA, V111, P13481, DOI 10.1073/pnas.1412420111
Corinti S, 2002, J LEUKOCYTE BIOL, V71, P652
Cremel M, 2013, INT J PHARMACEUT, V443, P39, DOI 10.1016/j.ijpharm.2012.12.044

Culp WD, 2006, J PROTEOME RES, V5, P1332, DOI 10.1021/pr060059q
Dewitte H, 2014, NANO TODAY, V9, P743, DOI 10.1016/j.nantod.2014.10.001
Dominici S, 2003, VACCINE, V21, P2073, DOI 10.1016/S0264-410X(02)00746-6
Dunn GP, 2006, NAT REV IMMUNOL, V6, P836, DOI 10.1038/nri1961
Egeblad M, 2002, NAT REV CANCER, V2, P161, DOI 10.1038/nrc745
Fang RH, 2014, NANO LETT, V14, P2181, DOI 10.1021/nl500618u
Fang RNH, 2013, NANOSCALE, V5, P8884, DOI 10.1039/c3nr03064d
Fogg DK, 2006, SCIENCE, V311, P83, DOI 10.1126/science.1117729
Godfrin Y, 2012, IMMUNOTHERAPY-UK, V4, P871, DOI [10.2217/IMT.12.88, 10.2217/imt.12.88]
Govender T, 1999, J CONTROL RELEASE, V57, P171, DOI 10.1016/S0168-3659(98)00116-3
Hamdy S, 2011, PHARM RES-DORDR, V28, P2288, DOI 10.1007/s11095-011-0459-9
Hanes J, 1997, ADV DRUG DELIVER REV, V28, P97, DOI 10.1016/S0169-409X(97)00053-7
Hirosue S, 2010, VACCINE, V28, P7897, DOI 10.1016/j.vaccine.2010.09.077
HOCHMUTH RM, 1983, SCIENCE, V220, P101, DOI 10.1126/science.6828875
Hu CMJ, 2013, NAT NANOTECHNOL, V8, P933, DOI [10.1038/nnano.2013.254, 10.1038/NNANO.2013.254]
Hu CMJ, 2013, NANOSCALE, V5, P2664, DOI 10.1039/c3nr00015j
Hu CMJ, 2012, ADV HEALTHC MATER, V1, P537, DOI 10.1002/adhm.201200138
Hu CMJ, 2011, P NATL ACAD SCI USA, V108, P10980, DOI 10.1073/pnas.1106634108
Jiang LY, 2013, BIOMATERIALS, V34, P7418, DOI 10.1016/j.biomaterials.2013.05.078
Kantoff PW, 2010, NEW ENGL J MED, V363, P411, DOI 10.1056/NEJMoa1001294
Kimberley FC, 2007, MOL IMMUNOL, V44, P73, DOI 10.1016/j.molimm.2006.06.019
Kirkwood JM, 2012, CA-CANCER J CLIN, V62, P309, DOI 10.3322/caac.20132
Kleindienst P, 2003, J IMMUNOL, V170, P2817, DOI 10.4049/jimmunol.170.6.2817
Krishnamachari Y, 2011, PHARM RES-DORDR, V28, P215, DOI 10.1007/s11095-010-0241-4
Lee IH, 2012, ANGEW CHEM INT EDIT, V51, P8800, DOI 10.1002/anie.201203193
Li P, 2013, J CONTROL RELEASE, V168, P271, DOI 10.1016/j.jconrel.2013.03.025
Manolova V, 2008, EUR J IMMUNOL, V38, P1404, DOI 10.1002/eji.200737984
Marrache S, 2013, ACS NANO, V7, P7392, DOI 10.1021/nn403158n
Martinez-Pomares L, 2012, J LEUKOCYTE BIOL, V92, P1177, DOI 10.1189/jlb.0512231
Meng FH, 2009, BIOMATERIALS, V30, P2180, DOI 10.1016/j.biomaterials.2009.01.026
Millan CG, 2004, J CONTROL RELEASE, V95, P27, DOI 10.1016/j.jconrel.2003.11.018
Mqadmi A, 2005, BLOOD, V105, P3746, DOI 10.1182/blood-2004-12-4692
Pan XY, 2012, INT J BIOCHEM CELL B, V44, P998, DOI 10.1016/j.biocel.2012.03.008
Papetti M, 2002, AM J PHYSIOL-CELL PH, V282, pC947, DOI 10.1152/ajpcell.00389.2001
Pasare C, 2003, SCIENCE, V299, P1033, DOI 10.1126/science.1078231
Popielarski SR, 2005, BIOCONJUGATE CHEM, V16, P1071, DOI 10.1021/bc0501146
Qiu H, 2009, CANCER BIOTHER RADIO, V24, P597, DOI 10.1089/cbr.2009.0642
Roger M, 2010, BIOMATERIALS, V31, P8393, DOI 10.1016/j.biomaterials.2010.07.048
ROTH C, 1994, ADV IMMUNOL, V57, P281, DOI 10.1016/S0065-2776(08)60675-6
Scarlett UK, 2012, J EXP MED, V209, P495, DOI 10.1084/jem.20111413
Shen H, 2006, IMMUNOLOGY, V117, P78, DOI 10.1111/j.1365-2567.2005.02268.x
Shen ZH, 1997, J IMMUNOL, V158, P2723
Sheng KC, 2008, EUR J IMMUNOL, V38, P424, DOI 10.1002/eji.200737578
Singh M, 2007, EXPERT REV VACCINES, V6, P797, DOI 10.1586/14760584.6.5.797
Stahl PD, 1998, CURR OPIN IMMUNOL, V10, P50, DOI 10.1016/S0952-7915(98)80031-9
Takami A, 1999, BRIT J HAEMATOL, V107, P791, DOI 10.1046/j.1365-2141.1999.01790.x
Tan SW, 2015, THERANOSTICS, V5, P863, DOI 10.7150/thno.11852
Tan SW, 2014, NANOMEDICINE-UK, V9, P635, DOI [10.2217/NNM.13.67, 10.2217/nnm.13.67]
Tang K, 2012, NAT COMMUN, V3, DOI 10.1038/ncomms2282
Tian X, 2012, BIOMATERIALS, V33, P6147, DOI 10.1016/j.biomaterials.2012.05.034
Wang YQ, 2014, ADV HEALTHC MATER, V3, P670, DOI 10.1002/adhm.201300335
Xiang SD, 2008, EXPERT REV VACCINES, V7, P1103, DOI 10.1586/14760584.7.7.1103
Zhang ZP, 2011, BIOMATERIALS, V32, P3666, DOI 10.1016/j.biomaterials.2011.01.067
Zhao YZ, 2014, J TOXICOL SCI, V39, P701, DOI 10.2131/jts.39.701
NR 67
TC 296
Z9 309
U1 8
U2 420
PU AMER CHEMICAL SOC

PI WASHINGTON
PA 1155 16TH ST, NW, WASHINGTON, DC 20036 USA
SN 1936-0851
EI 1936-086X
J9 ACS NANO
JI ACS Nano
PD JUL
PY 2015
VL 9
IS 7
BP 6918
EP 6933
DI 10.1021/acsnano.5b01042
PG 16
WC Chemistry, Multidisciplinary; Chemistry, Physical; Nanoscience &
   Nanotechnology; Materials Science, Multidisciplinary
WE Science Citation Index Expanded (SCI-EXPANDED)
SC Chemistry; Science & Technology - Other Topics; Materials Science
GA CO0EP
UT WOS:000358823200029
PM 26153897
DA 2023-09-14
ER

PT J
AU Xu, J
   Lv, J
   Zhuang, Q
   Yang, ZJ
   Cao, ZQ
   Xu, LG
   Pei, P
   Wang, CY
   Wu, HF
   Dong, ZL
   Chao, Y
   Wang, C
   Yang, K
   Peng, R
   Cheng, YY
   Liu, Z
AF Xu, Jun
   Lv, Jia
   Zhuang, Qi
   Yang, Zongjin
   Cao, Zhiqin
   Xu, Ligeng
   Pei, Pei
   Wang, Chenya
   Wu, Hanfei
   Dong, Ziliang
   Chao, Yu
   Wang, Chao
   Yang, Kai
   Peng, Rui
   Cheng, Yiyun
   Liu, Zhuang
TI A general strategy towards personalized nanovaccines based on
   fluoropolymers for post-surgical cancer immunotherapy
SO NATURE NANOTECHNOLOGY
LA English

DT Article
ID CHECKPOINT BLOCKADE; NANOPARTICLES; VIRUS; ANTIGENS; DELIVERY; IMMUNITY; THERAPY
AB A fluoropolymer-based cancer nanovaccine that delivers antigens directly to the cytosol of dendritic cells and elicits strong antitumour immune responses inhibiting tumour growth in animal models can be used to produce personalized treatment for post-surgical immunotherapy.
Cancer metastases and recurrence after surgical resection remain an important cause of treatment failure. Here we demonstrate a general strategy to fabricate personalized nanovaccines based on a cationic fluoropolymer for post-surgical cancer immunotherapy. Nanoparticles formed by mixing the fluoropolymer with a model antigen ovalbumin, induce dendritic cell maturation via the Toll-like receptor 4 (TLR4)-mediated signalling pathway, and promote antigen transportation into the cytosol of dendritic cells, which leads to an effective antigen cross-presentation. Such a nanovaccine inhibits established ovalbumin-expressing B16-OVA melanoma. More importantly, a mix of the fluoropolymer with cell membranes from resected autologous primary tumours synergizes with checkpoint blockade therapy to inhibit post-surgical tumour recurrence and metastases in two subcutaneous tumour models and an orthotopic breast cancer tumour. Furthermore, in the orthotopic tumour model, we observed a strong immune memory against tumour rechallenge. Our work offers a simple and general strategy for the preparation of personalized cancer vaccines to prevent post-operative cancer recurrence and metastasis.
C1 [Xu, Jun; Zhuang, Qi; Yang, Zongjin; Cao, Zhiqin; Xu, Ligeng; Wang, Chenya; Wu, Hanfei; Dong, Ziliang; Chao, Yu; Wang, Chao; Peng, Rui; Liu, Zhuang] Soochow Univ, Inst Funct Nano & Soft Mat FUNSOM, Jiangsu Key Lab Carbon Based Funct Mat & Devices, Suzhou, Peoples R China.
[Lv, Jia; Cheng, Yiyun] South China Univ Technol, South China Adv Inst Soft Matter Sci & Technol, Guangzhou, Peoples R China.
[Pei, Pei; Yang, Kai] Soochow Univ, State Key Lab Radiat Med & Protect, Sch Radiat Med & Protect, Suzhou, Peoples R China.
[Pei, Pei; Yang, Kai] Soochow Univ, Sch Radiol & Interdisciplinary Sci RAD X, Collaborat Innovat Ctr Radiat Med Jiangsu Higher, Suzhou, Peoples R China.
[Cheng, Yiyun] East China Normal Univ, Sch Life Sci, Shanghai Key Lab Regulatory Biol, Shanghai, Peoples R China.
C3 Soochow University - China; South China University of Technology; Soochow University - China; Soochow University - China; East China Normal University
RP Peng, R; Liu, Z (通讯作者)，Soochow Univ, Inst Funct Nano & Soft Mat FUNSOM, Jiangsu Key Lab Carbon Based Funct Mat & Devices, Suzhou, Peoples R China.; Cheng, YY (通讯作者)，South China Univ Technol, South China Adv Inst Soft Matter Sci & Technol, Guangzhou, Peoples R China.; Cheng, YY (通讯作者)，East China Normal Univ, Sch Life Sci, Shanghai Key Lab Regulatory Biol, Shanghai, Peoples R China.
EM rpeng@suda.edu.cn; yycheng@mail.ustc.edu.cn; zliu@suda.edu.cn
RI Wang, Chao/G-5951-2012; Liu, Zhuang/H-4352-2011; Xu, Ligeng/F-9844-2013; Dong, Ziliang/AEH-7615-2022
OI Wang, Chao/0000-0002-8054-3472; Liu, Zhuang/0000-0002-1629-1039; Xu, Ligeng/0000-0003-4946-2567; pei, pei/0000-0002-5261-793X; pei, pei/0000-0001-5676-9087
FU National Key Research and Development (R&D) Program of China [2016YFA0201200, 2017YFE0131700, 2019YFA0904500]; National Natural Science Foundation of China [51525203, 21725402, 51761145041]; 111 Project; Jiangsu Social Development Project [BE2019658]; Natural Science Foundation of Jiangsu Higher Education Institutions of China [19KJA310008]; Collaborative Innovation Center of Suzhou Nano Science and Technology (Nano-CIC)
FX This work was partially supported by the National Key Research and Development (R&D) Program of China (2016YFA0201200, 2017YFE0131700 and 2019YFA0904500), the National Natural Science Foundation of China (51525203, 21725402 and 51761145041), the 111 Project, the Jiangsu Social Development Project (BE2019658), the Natural Science Foundation of Jiangsu Higher Education Institutions of China (19KJA310008) and the Collaborative Innovation Center of Suzhou Nano Science and Technology (Nano-CIC). The authors thank H. Liu for B16-OVA tumour cells and X. Wang for the OT-I mice. Our gratitude also goes to Y. Huang for valuable comments and suggestions.
CR Bevan MJ, 2004, NAT REV IMMUNOL, V4, P595, DOI 10.1038/nri1413

Brahmer JR, 2010, J CLIN ONCOL, V28, P3167, DOI 10.1200/JCO.2009.26.7609
Cametti M, 2012, CHEM SOC REV, V41, P31, DOI 10.1039/c1cs15084g
Chao Y, 2018, NAT BIOMED ENG, V2, P611, DOI 10.1038/s41551-018-0262-6
Chen Q, 2019, NAT NANOTECHNOL, V14, P89, DOI 10.1038/s41565-018-0319-4
Cruz FM, 2017, ANNU REV IMMUNOL, V35, P149, DOI 10.1146/annurev-immunol-041015-055254
D'Alise AM, 2019, NAT COMMUN, V10, DOI 10.1038/s41467-019-10594-2
Dienz O, 2009, CLIN IMMUNOL, V130, P27, DOI 10.1016/j.clim.2008.08.018
FOLKS TM, 1989, P NATL ACAD SCI USA, V86, P2365, DOI 10.1073/pnas.86.7.2365
Grosso Joseph F, 2013, Cancer Immun, V13, P5
Guo YG, 2018, FRONT IMMUNOL, V9, DOI 10.3389/fimmu.2018.01499
He W, 2016, SCI REP-UK, V6, DOI 10.1038/srep24506
Hornung V, 2008, NAT IMMUNOL, V9, P847, DOI 10.1038/ni.1631
Issadore D, 2011, LAB CHIP, V11, P147, DOI [10.1039/c0lc00149j, 10.1039/c01c00149j]
JANEWAY CA, 1994, CELL, V76, P275, DOI 10.1016/0092-8674(94)90335-2
Klevorn LE, 2016, TRENDS IMMUNOL, V37, P354, DOI 10.1016/j.it.2016.03.010
Kroll AV, 2017, ADV MATER, V29, DOI 10.1002/adma.201703969
Kuai R, 2017, NAT MATER, V16, P489, DOI [10.1038/nmat4822, 10.1038/NMAT4822]
Lanzavecchia A, 2001, CELL, V106, P263, DOI 10.1016/S0092-8674(01)00455-X
Li AW, 2018, NAT MATER, V17, P528, DOI 10.1038/s41563-018-0028-2
Li F, 2017, ADV MATER, V29, DOI 10.1002/adma.201602432
Littman DR, 2015, CELL, V162, P1186, DOI 10.1016/j.cell.2015.08.038
Lu JQ, 2017, NAT COMMUN, V8, DOI 10.1038/s41467-017-01651-9
Luo M, 2017, NAT NANOTECHNOL, V12, P648, DOI [10.1038/nnano.2017.52,
10.1038/NNANO.2017.52]
Lv SJ, 2014, HEPATOLOGY, V59, P385, DOI 10.1002/hep.26654
Ochyl LJ, 2018, BIOMATERIALS, V182, P157, DOI 10.1016/j.biomaterials.2018.08.016
Rodriguez A, 1999, NAT CELL BIOL, V1, P362, DOI 10.1038/14058
Rosenberg SA, 2004, NAT MED, V10, P909, DOI 10.1038/nm1100
Stewart MP, 2018, CHEM REV, V118, P7409, DOI 10.1021/acs.chemrev.7b00678
Wang C, 2017, NAT BIOMED ENG, V1, DOI 10.1038/s41551-016-0011
Wang MM, 2014, NAT COMMUN, V5, DOI 10.1038/ncomms4053
Weichselbaum RR, 2017, NAT REV CLIN ONCOL, V14, P365, DOI 10.1038/nrclinonc.2016.211
Xu J, 2017, ACS NANO, V11, P4463, DOI 10.1021/acsnano.7b00715
Xu LG, 2013, ADV MATER, V25, P5928, DOI 10.1002/adma.201300583
Yamamoto A, 1998, CELL STRUCT FUNCT, V23, P33, DOI 10.1247/csf.23.33
Yang JP, 2015, CHEM REV, V115, P5274, DOI 10.1021/cr500542t
Yang R, 2018, ACS NANO, V12, P5121, DOI 10.1021/acsnano.7b09041
Yarchoan M, 2017, NAT REV CANCER, V17, DOI 10.1038/nrc.2017.74
Zhang ZJ, 2018, NAT COMMUN, V9, DOI 10.1038/s41467-018-03779-8
Zhu GZ, 2017, ACS NANO, V11, P2387, DOI 10.1021/acsnano.7b00978
NR 40
TC 245
Z9 248
U1 80
U2 513
PU NATURE RESEARCH
PI BERLIN
PA HEIDELBERGER PLATZ 3, BERLIN, 14197, GERMANY
SN 1748-3387
EI 1748-3395
J9 NAT NANOTECHNOL
JI Nat. Nanotechnol.
PD DEC
PY 2020
VL 15
IS 12
BP 1043
EP U92
DI 10.1038/s41565-020-00781-4
EA NOV 2020
PG 17

WC Nanoscience & Nanotechnology; Materials Science, Multidisciplinary
WE Science Citation Index Expanded (SCI-EXPANDED)
SC Science & Technology - Other Topics; Materials Science
GA PA6UC
UT WOS:000585909900003
PM 33139933
OA Bronze
HC Y
HP N
DA 2023-09-14
ER

PT J
AU Chen, ZK
Liu, LL
Liang, RJ
Luo, ZY
He, HM
Wu, ZH
Tian, H
Zheng, MB
Ma, YF
Cai, LT
AF Chen, Zhikuan
Liu, Lanlan
Liang, Ruijing
Luo, Zhenyu
He, Huamei
Wu, Zhihao
Tian, Hao
Zheng, Mingbin
Ma, Yifan
Cai, Lintao
TI Bioinspired Hybrid Protein Oxygen Nanocarrier Amplified Photodynamic Therapy for Eliciting Anti-tumor Immunity and Abscopal Effect
SO ACS NANO
LA English
DT Article
DE oxygen nanocarrier; photodynamic therapy; tumor hypoxia; immunogenic cell death; metastasis
ID IMMUNOGENIC CELL-DEATH; CHECKPOINT BLOCKADE; TUMOR MICROENVIRONMENT; CANCER-IMMUNOTHERAPY; NANOPARTICLES; CHEMOTHERAPY; NANOVACCINE; CARRIER
AB An ideal cancer therapeutic strategy is expected to possess potent ability to not only ablate primary tumors but also prevent distance metastasis and relapse. In this study, human serum albumin was hybridized with hemoglobin by intermolecular disulfide bonds to develop a hybrid protein oxygen nanocarrier with chlorine e6 encapsulated (C@HPOC) for oxygen self-sufficient photodynamic therapy (PDT). C@HPOC realized the tumor-targeted co-delivery of photosensitizer and oxygen, which remarkably relieved tumor hypoxia. C@HPOC was favorable for more efficient PDT and enhanced infiltration of CD8(+) T cells in tumors. Moreover, oxygen-boosted PDT of C@HPOC induced immunogenic cell death, with the release of danger-associated molecular patterns to activate dendritic cells, T lymphocytes, and natural killer cells in vivo. Notably, C@HPOC-mediated immunogenic PDT could destroy primary tumors and effectively suppress distant and lung metastasis in a metastatic triple-negative breast cancer model by evoking systemic anti-tumor immunity. This study provides a paradigm of oxygen augmented immunogenic PDT for metastatic cancer treatment.
C1 [Chen, Zhikuan; Liu, Lanlan; Liang, Ruijing; Luo, Zhenyu; He, Huamei; Wu, Zhihao; Zheng, Mingbin; Ma, Yifan; Cai, Lintao] Chinese Acad Sci, Guangdong Key Lab Nanomed, Shenzhen Engn Lab Nanomed & Nanoformulat, CAS Key Lab Hlth Informat,Shenzhen Inst Adv Techn, Shenzhen 518055, Peoples R China.
[Tian, Hao; Zheng, Mingbin] Guangdong Med Univ, Key Lab Nanomed, Dongguan Key Lab Drug Design & Formulat Technol, Dongguan 523808, Peoples R China.
[Chen, Zhikuan; Liu, Lanlan; Luo, Zhenyu] Univ Chinese Acad Sci, Beijing 100049, Peoples R China.
C3 Chinese Academy of Sciences; Shenzhen Institute of Advanced Technology,

CAS; Guangdong Medical University; Chinese Academy of Sciences;
University of Chinese Academy of Sciences, CAS
RP Zheng, MB; Cai, LT (通讯作者)，Chinese Acad Sci, Guangdong Key Lab Nanomed, Shenzhen Engn Lab Nanomed & Nanoformulat, CAS Key Lab Hlth Informat,Shenzhen Inst Adv Techn, Shenzhen 518055, Peoples R China.; Zheng, MB (通讯作者)，Guangdong Med Univ, Key Lab Nanomed, Dongguan Key Lab Drug Design & Formulat Technol, Dongguan 523808, Peoples R China.
EM mb.zheng@siat.ac.cn; lt.cai@siat.ac.cn
RI Tian, Hao/JAC-6906-2023; WU, ZHIHAO/GVU-5080-2022; Liang, Ruijing/AED-9686-2022
OI WU, ZHIHAO/0000-0003-4682-0031; Cai, Lintao/0000-0002-2461-6390; Liang, Ruijing/0000-0003-3248-2780
FU Shenzhen Science and Technology Program [JCYJ20170818162259843, JSGG2016-0331185422390, JCYJ20160429191503002]; Key International S&T Cooperation Project [2015DFH50230]; National Natural Science Foundation of China [81701816, 81501591, 81671758, 31571013, 51502333, 81501580]; Guangdong Natural Science Foundation of Research Team [2016A-030312006]; Natural Science Foundation of Guangdong Province [2018A030313013, 2017A030313079, 2017A-030313726]; Dongguan project on Social Science and Technology Development [2015108101019]; SIAT Innovation Program for Excellent Young Researchers [Y7G005]
FX This work was supported by Shenzhen Science and Technology Program (JCYJ20170818162259843, JSGG2016-0331185422390, and JCYJ20160429191503002), Key International S&T Cooperation Project (2015DFH50230), National Natural Science Foundation of China (81701816, 81501591, 81671758, 31571013, 51502333, and 81501580), Guangdong Natural Science Foundation of Research Team (2016A-030312006), Natural Science Foundation of Guangdong Province (2018A030313013, 2017A030313079, and 2017A-030313726), Dongguan project on Social Science and Technology Development (2015108101019), and SIAT Innovation Program for Excellent Young Researchers (Y7G005).
CR Adkins I, 2014, ONCOIMMUNOLOGY, V3, DOI 10.4161/21624011.2014.968434
Agostinis P, 2011, CA-CANCER J CLIN, V61, P250, DOI 10.3322/caac.20114
Castano AP, 2006, NAT REV CANCER, V6, P535, DOI 10.1038/nrc1894
Chen Q, 2016, ADV MATER, V28, P10557, DOI 10.1002/adma.201600038
Cheng H, 2016, ADV FUNCT MATER, V26, P7847, DOI 10.1002/adfm.201603212
Cheng YH, 2015, NAT COMMUN, V6, DOI 10.1038/ncomms9785
Chouaib S, 2017, ONCOGENE, V36, P439, DOI 10.1038/onc.2016.225
Duan XP, 2016, J AM CHEM SOC, V138, P16686, DOI 10.1021/jacs.6b09538
Fan WP, 2016, CHEM SOC REV, V45, P6488, DOI 10.1039/c6cs00616g
Fan WP, 2015, ADV MATER, V27, P4155, DOI 10.1002/adma.201405141
Galluzzi L, 2017, NAT REV IMMUNOL, V17, P97, DOI 10.1038/nri.2016.107
Gardai SJ, 2005, CELL, V123, P321, DOI 10.1016/j.cell.2005.08.032
Garg AD, 2014, CELL DEATH DIFFER, V21, P26, DOI 10.1038/cdd.2013.48
Grimaldi AM, 2017, NANOMEDICINE-UK, V12, P2349, DOI 10.2217/nnm-2017-0208
Hatfield SM, 2015, SCI TRANSL MED, V7, DOI 10.1126/scitranslmed.aaa1260
Hu DH, 2016, BIOMATERIALS, V93, P10, DOI 10.1016/j.biomaterials.2016.03.037
Jia Y, 2016, ADV MATER, V28, P1312, DOI 10.1002/adma.201502581
Kakwere H, 2017, BIOCONJUGATE CHEM, V28, P2756, DOI 10.1021/acs.bioconjchem.7b00502
Kim J, 2017, J AM CHEM SOC, V139, P10992, DOI 10.1021/jacs.7b05559
Kroemer G, 2013, ANNU REV IMMUNOL, V31, P51, DOI 10.1146/annurev-immunol-032712-100008
Krysko DV, 2012, NAT REV CANCER, V12, P860, DOI 10.1038/nrc3380
Ledford H, 2013, NATURE, V497, P544, DOI 10.1038/497544a
Leone RD, 2015, CANCER CELL, V27, P435, DOI 10.1016/j.ccell.2015.03.014
Li F, 2013, J MATER CHEM B, V1, P1678, DOI 10.1039/c3tb00506b
Lin TT, 2016, ACS NANO, V10, P9999, DOI 10.1021/acsnano.6b04268
Ling D, 2014, J AM CHEM SOC, V136, P5647, DOI 10.1021/ja4108287
Ling D, 2012, BIOMATERIALS, V33, P5478, DOI 10.1016/j.biomaterials.2012.04.023
Liu LL, 2016, J IMMUNOL, V197, P1231, DOI 10.4049/jimmunol.1600182
Liu LH, 2017, SMALL, V13, DOI 10.1002/smll.201701621
Lizotte PH, 2016, NAT NANOTECHNOL, V11, P295, DOI [10.1038/NNANO.2015.292, 10.1038/nnano.2015.292]

Lu KD, 2016, J AM CHEM SOC, V138, P12502, DOI 10.1021/jacs.6b06663
Lucky SS, 2015, CHEM REV, V115, P1990, DOI 10.1021/cr5004198
Luo M, 2017, NAT NANOTECHNOL, V12, P648, DOI [10.1038/nnano.2017.52, 10.1038/NNANO.2017.52]
Melief CJM, 2008, NAT REV CANCER, V8, P351, DOI 10.1038/nrc2373
Min YZ, 2017, NAT NANOTECHNOL, V12, P877, DOI [10.1038/nnano.2017.113, 10.1038/NNANO.2017.113]
O'Shaughnessy J, 2011, NEW ENGL J MED, V364, P205, DOI 10.1056/NEJMoa1011418
Rankin EB, 2016, SCIENCE, V352, P175, DOI 10.1126/science.aaf4405
Riener CK, 2002, ANAL BIOANAL CHEM, V373, P266, DOI 10.1007/s00216-002-1347-2
Rong PF, 2015, NANOSCALE, V7, P16330, DOI 10.1039/c5nr04428f
Rosenberg SA, 2004, NAT MED, V10, P909, DOI 10.1038/nm1100
Schroeder A, 2012, NAT REV CANCER, V12, P39, DOI 10.1038/nrc3180
Song QL, 2017, NANO LETT, V17, P6366, DOI 10.1021/acs.nanolett.7b03186
Steeg PS, 2006, NAT MED, V12, P895, DOI 10.1038/nm1469
Tian H, 2017, ADV FUNCT MATER, V27, DOI 10.1002/adfm.201703197
Vanneman M, 2012, NAT REV CANCER, V12, P237, DOI 10.1038/nrc3237
Wang SS, 2015, BIOMACROMOLECULES, V16, P2693, DOI 10.1021/acs.biomac.5b00571
Wang Y, 2015, P NATL ACAD SCI USA, V112, P208, DOI 10.1073/pnas.1414821112
Xu J, 2017, ACS NANO, V11, P4463, DOI 10.1021/acsnano.7b00715
Yang GB, 2017, NAT COMMUN, V8, DOI 10.1038/s41467-017-01050-0
Yang YM, 2016, OXID MED CELL LONGEV, V2016, DOI 10.1155/2016/5274084
Zhang Y, 2018, ACS NANO, V12, P651, DOI 10.1021/acsnano.7b07746
Zhao PF, 2016, ADV HEALTHC MATER, V5, P2161, DOI 10.1002/adhm.201600121
Zhao YD, 2018, J CONTROL RELEASE, V269, P322, DOI 10.1016/j.jconrel.2017.11.037
Zhou ZJ, 2016, CHEM SOC REV, V45, P6597, DOI 10.1039/c6cs00271d
Zitvogel L, 2013, IMMUNITY, V39, P74, DOI 10.1016/j.immuni.2013.06.014
NR 55
TC 238
Z9 241
U1 28
U2 449
PU AMER CHEMICAL SOC
PI WASHINGTON
PA 1155 16TH ST, NW, WASHINGTON, DC 20036 USA
SN 1936-0851
EI 1936-086X
J9 ACS NANO
JI ACS Nano
PD AUG
PY 2018
VL 12
IS 8
BP 8633
EP 8645
DI 10.1021/acsnano.8b04371
PG 13
WC Chemistry, Multidisciplinary; Chemistry, Physical; Nanoscience & Nanotechnology; Materials Science, Multidisciplinary
WE Science Citation Index Expanded (SCI-EXPANDED)
SC Chemistry; Science & Technology - Other Topics; Materials Science
GA GS3NM
UT WOS:000443525600117
PM 30005164
HC Y
HP N
DA 2023-09-14
ER

PT J
AU Zhu, GZ

Zhang, FW
Ni, QQ
Niu, G
Chen, XY
AF Zhu, Guizhi
Zhang, Fuwu
Ni, Qianqian
Niu, Gang
Chen, Xiaoyuan
TI Efficient Nanovaccine Delivery in Cancer Immunotherapy
SO ACS NANO
LA English
DT Article
ID DENDRITIC CELLS; CROSS-PRESENTATION; LYMPH-NODE; CTLA-4 BLOCKADE;
ANTIGEN; NANOPARTICLES; VACCINATION; ACTIVATION; VACCINES; MELANOMA
AB Vaccines hold tremendous potential for cancer immunotherapy by treating the immune system. Subunit vaccines, including molecular adjuvants and cancer-associated antigens or cancer-specific neoantigens, can elicit potent antitumor immunity. However, subunit vaccines have shown limited clinical benefit in cancer patients, which is in part attributed to inefficient vaccine delivery. In this Perspective, we discuss vaccine delivery by synthetic nanoparticles or naturally derived nanoparticles for cancer immunotherapy. Nano vaccines can efficiently codeliver adjuvants and multiepitope antigens into lymphoid organs and into antigen-presenting cells, and the intracellular release of vaccine and cross presentation of antigens can be fine-tuned via nanovaccine engineering. Aside from peptide antigens, antigen-encoding mRNA for cancer immunotherapy delivered by nanovaccine will also be discussed.
C1 [Zhu, Guizhi; Zhang, Fuwu; Ni, Qianqian; Niu, Gang; Chen, Xiaoyuan] NIBIB, Lab Mol Imaging & Nanomed, NIH, Bethesda, MD 20892 USA.
C3 National Institutes of Health (NIH) - USA; NIH National Institute of
Biomedical Imaging & Bioengineering (NIBIB)
RP Chen, XY (通讯作者)，NIBIB, Lab Mol Imaging & Nanomed, NIH, Bethesda, MD 20892 USA.
EM shawn.chen@nih.gov
RI Zhu, Guizhi/AAN-2888-2020; Chen, Xiao/GQA-8928-2022; Zhang,
Fuwu/B-6747-2014; Zhang, Fuwu/T-4066-2019; Chen, Xiaoyuan/D-1860-2014
OI Chen, Xiao/0000-0002-9797-8384; Zhang, Fuwu/0000-0002-1928-3472; Zhang,
Fuwu/0000-0002-1928-3472; Zhu, Guizhi/0000-0002-2945-7982; Chen,
Xiaoyuan/0000-0002-9622-0870
FU National Institute of Biomedical Imaging and Bioengineering (NIBIB),
National Institutes of Health (NIH)
FX This work was supported by intramural research program of the National
Institute of Biomedical Imaging and Bioengineering (NIBIB), National
Institutes of Health (NIH).
CR Alexandrov LB, 2013, NATURE, V500, P415, DOI 10.1038/nature12477
Barber GN, 2015, NAT REV IMMUNOL, V15, P760, DOI 10.1038/nri3921
Benteyn D, 2015, EXPERT REV VACCINES, V14, P161, DOI 10.1586/14760584.2014.957684
Burgdorf S, 2008, NAT IMMUNOL, V9, P558, DOI 10.1038/ni.1601
Cheung AS, 2016, SMALL, V12, P2321, DOI 10.1002/smll.201600061
Fang RH, 2014, NANO LETT, V14, P2181, DOI 10.1021/nl500618u
Fu J, 2015, SCI TRANSL MED, V7, DOI 10.1126/scitranslmed.aaa4306
Gill S, 2015, IMMUNOL REV, V263, P68, DOI 10.1111/imr.12243
Gubin MM, 2014, NATURE, V515, P577, DOI 10.1038/nature13988
Guermonprez P, 2003, NATURE, V425, P397, DOI 10.1038/nature01911
Guo YY, 2015, ACS NANO, V9, P6918, DOI 10.1021/acsnano.5b01042
Hailemichael Y, 2013, NAT MED, V19, P465, DOI 10.1038/nm.3105
Hanson MC, 2015, J CLIN INVEST, V125, P2532, DOI 10.1172/JCI79915
Hirosue S, 2010, VACCINE, V28, P7897, DOI 10.1016/j.vaccine.2010.09.077
Hubbell JA, 2009, NATURE, V462, P449, DOI 10.1038/nature08604
Irvine DJ, 2015, CHEM REV, V115, P11109, DOI 10.1021/acs.chemrev.5b00109
Jeanbart L, 2015, P NATL ACAD SCI USA, V112, P14467, DOI 10.1073/pnas.1508516112
Klinman DM, 2004, NAT REV IMMUNOL, V4, P248, DOI 10.1038/nri1329
Koshy ST, 2017, ADV BIOSYST, V1, DOI 10.1002/adbi.201600013
Kranz LM, 2016, NATURE, V534, P396, DOI 10.1038/nature18300
Kreiter S, 2015, NATURE, V520, P692, DOI 10.1038/nature14426

Kreiter S, 2010, CANCER RES, V70, P9031, DOI 10.1158/0008-5472.CAN-10-0699
Krieg AM, 2006, NAT REV DRUG DISCOV, V5, P471, DOI 10.1038/nrd2059
Kuai R, 2017, NAT MATER, V16, P489, DOI [10.1038/nmat4822, 10.1038/NMAT4822]
Leifer CA, 2004, J IMMUNOL, V173, P1179, DOI 10.4049/jimmunol.173.2.1179
Li JH, 2017, ACS NANO, V11, P2531, DOI 10.1021/acsnano.6b08447
Liu HP, 2014, NATURE, V507, P519, DOI 10.1038/nature12978
Lynn GM, 2015, NAT BIOTECHNOL, V33, P1201, DOI 10.1038/nbt.3371
Mellman I, 2001, CELL, V106, P255, DOI 10.1016/S0092-8674(01)00449-4
Moon JJ, 2012, ADV MATER, V24, P3724, DOI 10.1002/adma.201200446
Nguyen DN, 2012, P NATL ACAD SCI USA, V109, pE797, DOI 10.1073/pnas.1121423109
Radovic-Moreno AF, 2015, P NATL ACAD SCI USA, V112, P3892, DOI 10.1073/pnas.1502850112
Reddy ST, 2007, NAT BIOTECHNOL, V25, P1159, DOI [10.1038/nbt1332, 10.1038/nbtl332]
Rosenberg SA, 2014, J IMMUNOL, V192, P5451, DOI 10.4049/jimmunol.1490019
Sahin U, 2014, NAT REV DRUG DISCOV, V13, P759, DOI 10.1038/nrd4278
Sand KMK, 2015, FRONT IMMUNOL, V5, P1, DOI 10.3389/fimmu.2014.00682
Schlake T, 2012, RNA BIOL, V9, P1319, DOI 10.4161/rna.22269
Schumacher TN, 2015, SCIENCE, V348, P69, DOI 10.1126/science.aaa4971
Scott EA, 2012, BIOMATERIALS, V33, P6211, DOI 10.1016/j.biomaterials.2012.04.060
Shao K, 2015, ACS NANO, V9, P16, DOI 10.1021/nn5062029
Sharma P, 2015, SCIENCE, V348, P56, DOI 10.1126/science.aaa8172
Snyder A, 2014, NEW ENGL J MED, V371, P2189, DOI 10.1056/NEJMoa1406498
Thomas SN, 2014, BIOMATERIALS, V35, P814, DOI 10.1016/j.biomaterials.2013.10.003
Van Allen EM, 2015, SCIENCE, V350, P207, DOI 10.1126/science.aad0095
Yao S, 2013, NAT REV DRUG DISCOV, V12, P130, DOI 10.1038/nrd3877
Zhang LQ, 2015, ACS APPL MATER INTER, V7, P24069, DOI 10.1021/acsami.5b06987
Zhang ZP, 2011, BIOMATERIALS, V32, P3666, DOI 10.1016/j.biomaterials.2011.01.067
Zhu GZ, 2016, NANOSCALE, V8, P6684, DOI 10.1039/c5nr08821f
NR 48
TC 235
Z9 240
U1 18
U2 405
PU AMER CHEMICAL SOC
PI WASHINGTON
PA 1155 16TH ST, NW, WASHINGTON, DC 20036 USA
SN 1936-0851
EI 1936-086X
J9 ACS NANO
JI ACS Nano
PD MAR
PY 2017
VL 11
IS 3
BP 2387
EP 2392
DI 10.1021/acsnano.7b00978
PG 6
WC Chemistry, Multidisciplinary; Chemistry, Physical; Nanoscience &
Nanotechnology; Materials Science, Multidisciplinary
WE Science Citation Index Expanded (SCI-EXPANDED)
SC Chemistry; Science & Technology - Other Topics; Materials Science
GA EQ4AK
UT WOS:000398014900004
PM 28277646
DA 2023-09-14
ER

PT J
AU Liu, SL
Jiang, Q
Zhao, X

Zhao, RF
Wang, YN
Wang, YM
Liu, JB
Shang, YX
Zhao, S
Wu, TT
Zhang, YL
Nie, GJ
Ding, BQ
AF Liu, Shaoli
Jiang, Qiao
Zhao, Xiao
Zhao, Ruifang
Wang, Yuanning
Wang, Yiming
Liu, Jianbing
Shang, Yingxu
Zhao, Shuai
Wu, Tiantian
Zhang, Yinlong
Nie, Guangjun
Ding, Baoquan
TI A DNA nanodevice-based vaccine for cancer immunotherapy
SO NATURE MATERIALS
LA English
DT Article
ID DENDRITIC CELLS; NANOVACCINE; NANOROBOT; DELIVERY; ANTIGEN
AB A DNA nanodevice vaccine has been developed and utilized to stimulate a tumour-specific cytotoxic T lymphocyte response in vivo, leading to the inhibition of tumour growth as well as prevention of metastasis.
A major challenge in cancer vaccine therapy is the efficient delivery of antigens and adjuvants to stimulate a controlled yet robust tumour-specific T-cell response. Here, we describe a structurally well defined DNA nanodevice vaccine generated by precisely assembling two types of molecular adjuvants and an antigen peptide within the inner cavity of a tubular DNA nanostructure that can be activated in the subcellular environment to trigger T-cell activation and cancer cytotoxicity. The integration of low pH-responsive DNA 'locking strands' outside the nanostructures enables the opening of the vaccine in lysosomes in antigen-presenting cells, exposing adjuvants and antigens to activate a strong immune response. The DNA nanodevice vaccine elicited a potent antigen-specific T-cell response, with subsequent tumour regression in mouse cancer models. Nanodevice vaccination generated long-term T-cell responses that potently protected the mice against tumour rechallenge.
C1 [Liu, Shaoli; Jiang, Qiao; Zhao, Xiao; Zhao, Ruifang; Wang, Yuanning; Wang, Yiming; Liu, Jianbing; Shang, Yingxu; Zhao, Shuai; Wu, Tiantian; Zhang, Yinlong; Nie, Guangjun; Ding, Baoquan] Natl Ctr Nanosci & Technol, CAS Ctr Excellence Nanosci, CAS Key Lab Nanosyst & Hierarch Fabricat, Beijing, Peoples R China.
[Liu, Shaoli; Wang, Yiming; Shang, Yingxu; Zhao, Shuai; Wu, Tiantian; Nie, Guangjun; Ding, Baoquan] Univ Chinese Acad Sci, Beijing, Peoples R China.
[Ding, Baoquan] Zhengzhou Univ, Henan Inst Adv Technol, Sch Mat Sci & Engn, Zhengzhou, Peoples R China.
C3 Chinese Academy of Sciences; National Center for Nanoscience &
Technology - China; Chinese Academy of Sciences; University of Chinese
Academy of Sciences, CAS; Zhengzhou University
RP Ding, BQ (通讯作者)，Natl Ctr Nanosci & Technol, CAS Ctr Excellence Nanosci, CAS Key Lab Nanosyst & Hierarch Fabricat, Beijing, Peoples R China.; Ding, BQ (通讯作者)，Univ Chinese Acad Sci, Beijing, Peoples R China.; Ding, BQ (通讯作者)，Zhengzhou Univ, Henan Inst Adv Technol, Sch Mat Sci & Engn, Zhengzhou, Peoples R China.
EM dingbq@nanoctr.cn
FU Beijing Municipal Science & Technology Commission [Z191100004819008];
National Basic Research Program of China [2016YFA0201601,
2018YFA0208900]; National Natural Science Foundation of China [21573051,
31700871, 21708004, 51761145044]; Science Fund for Creative Research

Groups of the National Natural Science Foundation of China [21721002];
Key Research Program of Frontier Sciences, CAS [QYZDBSSW-SLH029]; CAS
Interdisciplinary Innovation Team; K. C. Wong Education Foundation
[GJTD-2018-03]; Strategic Priority Research Program of Chinese Academy
of Sciences [XDB36000000]
FX This work was supported by the Beijing Municipal Science & Technology
Commission (Z191100004819008), the National Basic Research Program of
China (2016YFA0201601, 2018YFA0208900), the National Natural Science
Foundation of China (21573051, 31700871, 21708004 and 51761145044), the
Science Fund for Creative Research Groups of the National Natural
Science Foundation of China (21721002), the Key Research Program of
Frontier Sciences, CAS, Grant QYZDBSSW-SLH029, the CAS Interdisciplinary
Innovation Team and K. C. Wong Education Foundation (GJTD-2018-03) and
the Strategic Priority Research Program of Chinese Academy of Sciences
(XDB36000000).
CR Banchereau J, 2005, NAT REV IMMUNOL, V5, P296, DOI 10.1038/nri1592
Barber DL, 2003, J IMMUNOL, V171, P27, DOI 10.4049/jimmunol.171.1.27
Conniot J, 2019, NAT NANOTECHNOL, V14, P891, DOI 10.1038/s41565-019-0512-0
Douglas SM, 2007, P NATL ACAD SCI USA, V104, P6644, DOI 10.1073/pnas.0700930104
Douglas SM, 2012, SCIENCE, V335, P831, DOI 10.1126/science.1214081
Green LN, 2017, NANO LETT, V17, P7283, DOI 10.1021/acs.nanolett.7b02787
Hailemichael Y, 2013, NAT MED, V19, P465, DOI 10.1038/nm.3105
Hamanishi J, 2007, P NATL ACAD SCI USA, V104, P3360, DOI 10.1073/pnas.0611533104
Jiang Q, 2019, ADV MATER, V31, DOI 10.1002/adma.201804785
Kaczanowska S, 2013, J LEUKOCYTE BIOL, V93, P847, DOI 10.1189/jlb.1012501
Kranz LM, 2016, NATURE, V534, P396, DOI 10.1038/nature18300
Kreiter S, 2015, NATURE, V520, P692, DOI 10.1038/nature14426
Kuai R, 2017, NAT MATER, V16, P489, DOI [10.1038/nmat4822, 10.1038/NMAT4822]
Latz E, 2004, NAT IMMUNOL, V5, P190, DOI 10.1038/ni1028
Li AW, 2018, NAT MATER, V17, P528, DOI 10.1038/s41563-018-0028-2
Li SP, 2018, NAT BIOTECHNOL, V36, P258, DOI 10.1038/nbt.4071
Liu HP, 2014, NATURE, V507, P519, DOI 10.1038/nature12978
Liu L, 2008, SCIENCE, V320, P379, DOI 10.1126/science.1155406
Liu YF, 2016, CANCER CELL, V30, P243, DOI 10.1016/j.ccell.2016.06.021
Luo M, 2017, NAT NANOTECHNOL, V12, P648, DOI [10.1038/nnano.2017.52,
10.1038/NNANO.2017.52]
Mellman I, 2011, NATURE, V480, P480, DOI 10.1038/nature10673
Min YZ, 2017, NAT NANOTECHNOL, V12, P877, DOI [10.1038/nnano.2017.113,
10.1038/NNANO.2017.113]
Ott PA, 2017, NATURE, V547, P217, DOI 10.1038/nature22991
Praetorius F, 2017, NATURE, V552, P84, DOI 10.1038/nature24650
Reed SG, 2009, TRENDS IMMUNOL, V30, P23, DOI 10.1016/j.it.2008.09.006
Rothemund PWK, 2006, NATURE, V440, P297, DOI 10.1038/nature04586
Sahin U, 2017, NATURE, V547, P222, DOI 10.1038/nature23003
Verdegaal EME, 2016, NATURE, V536, P91, DOI 10.1038/nature18945
Zhu GZ, 2017, NAT COMMUN, V8, DOI 10.1038/s41467-017-01386-7
Zhu GZ, 2017, ACS NANO, V11, P2387, DOI 10.1021/acsnano.7b00978
NR 30
TC 222
Z9 223
U1 69
U2 562
PU NATURE PORTFOLIO
PI BERLIN
PA HEIDELBERGER PLATZ 3, BERLIN, 14197, GERMANY
SN 1476-1122
EI 1476-4660
J9 NAT MATER
JI Nat. Mater.
PD MAR
PY 2021

VL 20
IS 3
BP 421
EP +
DI 10.1038/s41563-020-0793-6
EA SEP 2020
PG 17
WC Chemistry, Physical; Materials Science, Multidisciplinary; Physics,
Applied; Physics, Condensed Matter
WE Science Citation Index Expanded (SCI-EXPANDED)
SC Chemistry; Materials Science; Physics
GA QM7IZ
UT WOS:000566892000002
PM 32895504
HC Y
HP N
DA 2023-09-14
ER

PT J
AU Ding, BB
Shao, S
Yu, C
Teng, B
Wang, MF
Cheng, ZY
Wong, KL
Ma, PA
Lin, J
AF Ding, Binbin
Shao, Shuai
Yu, Chang
Teng, Bo
Wang, Meifang
Cheng, Ziyong
Wong, Ka-Leung
Ma, Ping'an
Lin, Jun
TI Large-Pore Mesoporous-Silica-Coated Upconversion Nanoparticles as
Multifunctional Immunoadjuvants with Ultrahigh Photosensitizer and
Antigen Loading Efficiency for Improved Cancer Photodynamic
Immunotherapy
SO ADVANCED MATERIALS
LA English
DT Article
DE adjuvants; immunotherapy; large-pore mesoporous silica; photodynamic
therapy; upconversion
ID CHECKPOINT BLOCKADE; IN-VIVO; THERAPY; NANOCRYSTALS; METASTASIS;
MECHANISM; IMMUNITY
AB Reported immunoadjuvants still have many limitations, such as inferior cellular uptake capacity and biocompatibility, overly large particle sizes, single function, and unsatisfactory therapeutic efficacy. Here, large-pore mesoporous-silica-coated upconversion nanoparticles (UCMSs) with a size of less than 100 nm are successfully prepared by a typical silica sol-gel reaction using mesitylene as a pore-swelling agent and are applied as a novel immunoadjuvant. The obtained UCMSs not only show significantly higher loadings for the photosensitizers merocyanine 540 (MC540), model proteins (chicken ovalbumin (OVA)), and tumor antigens (tumor cell fragment (TF)), but also are successfully employed for highly efficient in vivo vaccine delivery. The prepared UCMSs-MC540-OVA under 980 nm near-infrared irradiation shows the best synergistic immunopotentiation action, verified by the strongest Th1 and Th2 immune responses and the highest frequency of CD4(+), CD8(+), and effector-memory T cells. Additionally, nanovaccines UCMSs-MC540-TF can more effectively inhibit tumor growth and increase the survival of colon cancer (CT26)-tumor-bearing BALB/c mice compared with either photodynamic therapy or immunological therapy alone,

suggesting the enhanced immunotherapy efficacy and clinical potential of UCMSs as immunoadjuvants for cancer immunotherapy.
C1 [Ding, Binbin; Shao, Shuai; Yu, Chang; Wang, Meifang; Cheng, Ziyong; Ma, Ping'an; Lin, Jun] Chinese Acad Sci, Changchun Inst Appl Chem, State Key Lab Rare Earth Resource Utilizat, Changchun 130022, Jilin, Peoples R China.
[Ding, Binbin; Wang, Meifang] Univ Sci & Technol China, Hefei 230026, Anhui, Peoples R China.
[Shao, Shuai] Changchun Univ Sci & Technol, Changchun 130022, Jilin, Peoples R China.
[Teng, Bo] Jilin Univ, Hosp 2, Dept Otolaryngol Head & Neck Surg, Changchun 130041, Jilin, Peoples R China.
[Wong, Ka-Leung] Hong Kong Baptist Univ, Dept Chem, Kowloon, Hong Kong 999077, Peoples R China.
C3 Chinese Academy of Sciences; Changchun Institute of Applied Chemistry, CAS; Chinese Academy of Sciences; University of Science & Technology of China, CAS; Changchun University of Science & Technology; Jilin University; Hong Kong Baptist University
RP Ma, PA; Lin, J (通讯作者)，Chinese Acad Sci, Changchun Inst Appl Chem, State Key Lab Rare Earth Resource Utilizat, Changchun 130022, Jilin, Peoples R China.
EM mapa675@ciac.ac.cn; jlin@ciac.ac.cn
RI Lin, Jun/D-3750-2012
OI Ding, Binbin/0000-0002-3717-857X
FU National Natural Science Foundation of China; NSFC [51720105015, 51672269, 51332008, 51772124, 21521092, 51872282]; National Basic Research Program of China [2014CB643803]; Science and Technology Development Planning Project of Jilin Province [20170101188JC, 20180520163JH]; Youth Innovation Promotion Association of CAS [2017273]; Overseas, Hong Kong & Macao Scholars Collaborated Researching Fund [21728101]; CAS-Croucher Funding Scheme for Joint Laboratories [CAS18204]
FX This project is financially supported by the National Natural Science Foundation of China (Grant Nos. NSFC 51720105015, 51672269, 51332008, 51772124, 21521092, and 51872282), the National Basic Research Program of China (Grant No. 2014CB643803), Science and Technology Development Planning Project of Jilin Province (Grant Nos. 20170101188JC and 20180520163JH), Youth Innovation Promotion Association of CAS (Grant No. 2017273), Overseas, Hong Kong & Macao Scholars Collaborated Researching Fund (Grant No. 21728101), and the CAS-Croucher Funding Scheme for Joint Laboratories (Grant No. CAS18204). All animals in this study were handled according to a protocol approved by the Institutional Animal Care and Use Committee of Jilin University.
CR Abbas M, 2017, ADV MATER, V29, DOI 10.1002/adma.201605021
[Anonymous], 2016, ANGEW CHEM-GER EDIT
Brites CDS, 2016, NAT NANOTECHNOL, V11, P851, DOI [10.1038/NNANO.2016.111, 10.1038/nnano.2016.111]
Castano AP, 2006, NAT REV CANCER, V6, P535, DOI 10.1038/nrc1894
Chan MH, 2017, SMALL, V13, DOI 10.1002/smll.201700038
Chatterjee DK, 2008, ADV DRUG DELIVER REV, V60, P1627, DOI 10.1016/j.addr.2008.08.003
Chen S, 2018, SCIENCE, V359, P679, DOI 10.1126/science.aaq1144
Chen WS, 2017, ADV MATER, V29, DOI 10.1002/adma.201603864
Croissant JG, 2017, ADV MATER, V29, DOI 10.1002/adma.201604634
Dai LL, 2018, ADV FUNCT MATER, V28, DOI 10.1002/adfm.201707249
Ding BB, 2016, ADV MATER INTERFACES, V3, DOI 10.1002/admi.201500649
Dolmans DEJGJ, 2003, NAT REV CANCER, V3, P380, DOI 10.1038/nrc1071
Fan L, 2016, BIOMATERIALS, V76, P399, DOI 10.1016/j.biomaterials.2015.10.069
Fang RH, 2014, NANO LETT, V14, P2181, DOI 10.1021/nl500618u
Funt SA, 2017, NAT REV CLIN ONCOL, V14, P221, DOI 10.1038/nrclinonc.2016.188
Gai SL, 2014, CHEM REV, V114, P2343, DOI 10.1021/cr4001594
Garg AD, 2012, CANCER IMMUNOL IMMUN, V61, P215, DOI 10.1007/s00262-011-1184-2
HAMAOKA T, 1973, J EXP MED, V138, P306, DOI 10.1084/jem.138.1.306
He CB, 2016, NAT COMMUN, V7, DOI 10.1038/ncomms12499
HOWARD JA, 1975, CAN J CHEM, V53, P2199, DOI 10.1139/v75-307
Idris NM, 2012, NAT MED, V18, P1580, DOI 10.1038/nm.2933
Ishihara J, 2017, SCI TRANSL MED, V9, DOI 10.1126/scitranslmed.aan0401

Karimi M, 2016, CHEM SOC REV, V45, P1457, DOI 10.1039/c5cs00798d
Kim MH, 2011, ACS NANO, V5, P3568, DOI 10.1021/nn103130q
LEBEL CP, 1992, CHEM RES TOXICOL, V5, P227, DOI 10.1021/tx00026a012
Lee JE, 2011, ACCOUNTS CHEM RES, V44, P893, DOI 10.1021/ar2000259
Li X, 2013, ADV HEALTHC MATER, V2, P863, DOI 10.1002/adhm.201200149
Li XS, 2017, ADV FUNCT MATER, V27, DOI 10.1002/adfm.201604053
Liu B, 2017, ADV MATER, V29, DOI 10.1002/adma.201605434
Liu JN, 2014, J AM CHEM SOC, V136, P9701, DOI 10.1021/ja5042989
Liu YS, 2013, CHEM SOC REV, V42, P6924, DOI 10.1039/c3cs60060b
Lu F, 2016, ADV FUNCT MATER, V26, P4778, DOI 10.1002/adfm.201600464
Lu KD, 2016, J AM CHEM SOC, V138, P12502, DOI 10.1021/jacs.6b06663
Lucky SS, 2015, CHEM REV, V115, P1990, DOI 10.1021/cr5004198
Ottaviani MF, 2004, J PHYS CHEM B, V108, P12123, DOI 10.1021/jp049032p
Piette J, 2015, PHOTOCH PHOTOBIO SCI, V14, P1510, DOI 10.1039/c4pp00465e
Sakai-Kato K, 2014, BIOMATERIALS, V35, P1347, DOI 10.1016/j.biomaterials.2013.11.027
Shao K, 2015, ACS NANO, V9, P16, DOI 10.1021/nn5062029
Song W, 2018, ACS NANO, V12, P1978, DOI 10.1021/acsnano.7b09112
Tang FQ, 2012, ADV MATER, V24, P1504, DOI 10.1002/adma.201104763
Trewyn BG, 2007, ACCOUNTS CHEM RES, V40, P846, DOI 10.1021/ar600032u
Vallhov H, 2007, NANO LETT, V7, P3576, DOI 10.1021/nl0714785
Wang WN, 2018, APPL CATAL B-ENVIRON, V224, P854, DOI 10.1016/j.apcatb.2017.11.037
Wang XP, 2016, SMALL, V12, P3510, DOI 10.1002/smll.201600677
Wang XP, 2016, ADV HEALTHC MATER, V5, P1169, DOI 10.1002/adhm.201501013
Wang YZ, 2017, ACS NANO, V11, P2227, DOI 10.1021/acsnano.6b08731
Wu SH, 2013, CHEM SOC REV, V42, P3862, DOI 10.1039/c3cs35405a
Xu JT, 2017, ACS NANO, V11, P4133, DOI 10.1021/acsnano.7b00944
Xu J, 2017, ACS NANO, V11, P4463, DOI 10.1021/acsnano.7b00715
Yang GB, 2017, NAT COMMUN, V8, DOI 10.1038/s41467-017-01050-0
Yang PP, 2012, CHEM SOC REV, V41, P3679, DOI 10.1039/c2cs15308d
Yang YN, 2017, ANGEW CHEM INT EDIT, V56, P8446, DOI 10.1002/anie.201701550
Yu XH, 2017, ACS NANO, V11, P10147, DOI 10.1021/acsnano.7b04736
Zhou B, 2015, NAT NANOTECHNOL, V10, P924, DOI [10.1038/NNANO.2015.251, 10.1038/nnano.2015.251]
Zhou J, 2015, CHEM REV, V115, P395, DOI 10.1021/cr400478f
NR 55
TC 221
Z9 228
U1 17
U2 668
PU WILEY-V C H VERLAG GMBH
PI WEINHEIM
PA POSTFACH 101161, 69451 WEINHEIM, GERMANY
SN 0935-9648
EI 1521-4095
J9 ADV MATER
JI Adv. Mater.
PD DEC 27
PY 2018
VL 30
IS 52
AR 1802479
DI 10.1002/adma.201802479
PG 10
WC Chemistry, Multidisciplinary; Chemistry, Physical; Nanoscience & Nanotechnology; Materials Science, Multidisciplinary; Physics, Applied; Physics, Condensed Matter
WE Science Citation Index Expanded (SCI-EXPANDED)
SC Chemistry; Science & Technology - Other Topics; Materials Science; Physics
GA HF3GZ
UT WOS:000454124800016

PM 30387197
DA 2023-09-14
ER

PT J
AU Nooraei, S
Bahrulolum, H
Hoseini, ZS
Katalani, C
Hajizade, A
Easton, AJ
Ahmadian, G
AF Nooraei, Saghi
Bahrulolum, Howra
Hoseini, Zakieh Sadat
Katalani, Camellia
Hajizade, Abbas
Easton, Andrew J.
Ahmadian, Gholamreza
TI Virus-like particles: preparation, immunogenicity and their roles as
nanovaccines and drug nanocarriers
SO JOURNAL OF NANOBIOTECHNOLOGY
LA English
DT Review
DE Virus-like particles (VLPs); Subunit vaccine; Expression and
purification platforms; Infectious disease vaccine; Cancer vaccine;
Immune response
ID HUMAN-PAPILLOMAVIRUS TYPE-16; PROTECTIVE IMMUNE-RESPONSES; FIELD-FLOW
FRACTIONATION; TOBACCO-MOSAIC-VIRUS; HEPATITIS-B-VACCINE;
ESCHERICHIA-COLI; DENDRITIC CELLS; NEUTRALIZING ANTIBODIES;
CROSS-PRESENTATION; DISEASE VIRUS
AB Virus-like particles (VLPs) are virus-derived structures made up of one or more different molecules with the ability to self-assemble, mimicking the form and size of a virus particle but lacking the genetic material so they are not capable of infecting the host cell. Expression and self-assembly of the viral structural proteins can take place in various living or cell-free expression systems after which the viral structures can be assembled and reconstructed. VLPs are gaining in popularity in the field of preventive medicine and to date, a wide range of VLP-based candidate vaccines have been developed for immunization against various infectious agents, the latest of which is the vaccine against SARS-CoV-2, the efficacy of which is being evaluated. VLPs are highly immunogenic and are able to elicit both the antibody- and cell-mediated immune responses by pathways different from those elicited by conventional inactivated viral vaccines. However, there are still many challenges to this surface display system that need to be addressed in the future. VLPs that are classified as subunit vaccines are subdivided into enveloped and non- enveloped subtypes both of which are discussed in this review article. VLPs have also recently received attention for their successful applications in targeted drug delivery and for use in gene therapy. The development of more effective and targeted forms of VLP by modification of the surface of the particles in such a way that they can be introduced into specific cells or tissues or increase their half-life in the host is likely to expand their use in the future. Recent advances in the production and fabrication of VLPs including the exploration of different types of expression systems for their development, as well as their applications as vaccines in the prevention of infectious diseases and cancers resulting from their interaction with, and mechanism of activation of, the humoral and cellular immune systems are discussed in this review.
C1 [Nooraei, Saghi; Bahrulolum, Howra; Hoseini, Zakieh Sadat; Ahmadian, Gholamreza] Natl Inst Genet Engn & Biotechnol NIGEB, Dept Ind & Environm Biotechnol, POB 14155-6343, Tehran 1497716316, Iran.
[Easton, Andrew J.] Univ Warwick, Sch Life Sci, Gibbet Hill Campus, Coventry, W Midlands, England.
[Katalani, Camellia] Sari Agr Sci & Nat Resource Univ SANRU, Genet & Agr Biotechnol Inst Tabarestan GABIT, Sari, Iran.
[Hajizade, Abbas] Baqiyatallah Univ Med Sci, Syst Biol & Poisonings Inst, Appl Microbiol Res Ctr, Tehran, Iran.
C3 University of Warwick; Baqiyatallah University of Medical Sciences
(BMSU)
RP Ahmadian, G (通讯作者)，Natl Inst Genet Engn & Biotechnol NIGEB, Dept Ind & Environm Biotechnol,

POB 14155-6343, Tehran 1497716316, Iran.; Easton, AJ (通讯作者)，Univ Warwick, Sch Life Sci, Gibbet Hill Campus, Coventry, W Midlands, England.
EM a.j.easton@warwick.ac.uk; ahmadian@nigeb.ac.ir
RI katalani, Camellia/ABE-5358-2021
OI Bahrulolum, Howra/0000-0001-6061-1581
FU National Institute of Genetic Engineering and Biotechnology, Tehran, Iran
FX This research was supported by the National Institute of Genetic Engineering and Biotechnology, Tehran, Iran.
CR Abbing A, 2004, J BIOL CHEM, V279, P27410, DOI 10.1074/jbc.M313612200
Aires KA, 2006, APPL ENVIRON MICROB, V72, P745, DOI 10.1128/AEM.72.1.745-752.2006
Al-Barwani F, 2014, PLOS ONE, V9, DOI 10.1371/journal.pone.0104523
Aljabali AAA, 2013, MOL PHARMACEUT, V10, P3, DOI 10.1021/mp3002057
Ashley CE, 2011, ACS NANO, V5, P5729, DOI 10.1021/nn201397z
Atmar RL, 2011, NEW ENGL J MED, V365, P2178, DOI 10.1056/NEJMoa1101245
Bai BK, 2008, PLOS ONE, V3, DOI 10.1371/journal.pone.0002685
Balke I, 2020, VIRUSES-BASEL, V12, DOI 10.3390/v12030270
Balke I, 2019, ADV DRUG DELIVER REV, V145, P119, DOI 10.1016/j.addr.2018.08.007
Banchereau J, 1998, NATURE, V392, P245, DOI 10.1038/32588
Baumert TF, 1998, J VIROL, V72, P3827, DOI 10.1128/JVI.72.5.3827-3836.1998
BAYER ME, 1968, NATURE, V218, P1057, DOI 10.1038/2181057a0
Beran J, 2008, EXPERT OPIN BIOL TH, V8, P235, DOI [10.1517/14712598.8.2.235, 10.1517/14712598.8.2.235 ]
Bessa J, 2012, P NATL ACAD SCI USA, V109, P20566, DOI 10.1073/pnas.1206970109
Bianchini G, 2014, LANCET ONCOL, V15, pE58, DOI 10.1016/S1470-2045(13)70477-7
Block SL, 2006, PEDIATRICS, V118, P2135, DOI 10.1542/peds.2006-0461
Bolli E, 2018, ONCOIMMUNOLOGY, V7, DOI 10.1080/2162402X.2017.1408746
Branco LM., 2010, VIROL J, V7, P1, DOI [10.1186/1743-422X-7-1, DOI 10.1186/1743-422X-7-1]
BRAUTIGAM S, 1993, VIROLOGY, V192, P512, DOI 10.1006/viro.1993.1067
Bryant JE, 2007, VIROLOGY, V366, P415, DOI 10.1016/j.virol.2007.05.007
Bundy BC, 2008, BIOTECHNOL BIOENG, V100, P28, DOI 10.1002/bit.21716
Bundy BC, 2011, J BIOTECHNOL, V154, P230, DOI 10.1016/j.jbiotec.2011.04.011
Buonaguro L, 2006, J VIROL, V80, P9134, DOI 10.1128/JVI.00050-06
Cai WH, 2016, SCI REP-UK, V6, DOI 10.1038/srep26890
Chang GD, 2003, J BIOTECHNOL, V102, P61, DOI 10.1016/S0168-1656(02)00364-4
Chang GJJ, 2003, VIROLOGY, V306, P170, DOI 10.1016/S0042-6822(02)00028-4
Chen XJS, 2001, J MOL BIOL, V307, P173, DOI 10.1006/jmbi.2000.4464
Chichester JA, 2018, VACCINE, V36, P5865, DOI 10.1016/j.vaccine.2018.08.033
Choi KM, 2013, MOL PHARMACEUT, V10, P18, DOI 10.1021/mp300211a
Chuan YP, 2008, BIOTECHNOL BIOENG, V99, P1425, DOI 10.1002/bit.21710
Chung YC, 2008, VACCINE, V26, P1855, DOI 10.1016/j.vaccine.2008.01.058
Chung YH, 2020, ADV DRUG DELIVER REV, V156, P214, DOI 10.1016/j.addr.2020.06.024
Cimica V, 2017, CLIN IMMUNOL, V183, P99, DOI 10.1016/j.clim.2017.08.004
Conti L, 2020, CANCER IMMUNOL RES, V8, P1039, DOI 10.1158/2326-6066.CIR-20-0082
Crooke SN, 2019, MOL PHARMACEUT, V16, P2947, DOI 10.1021/acs.molpharmaceut.9b00180
Cubas R, 2011, J IMMUNOTHER, V34, P251, DOI 10.1097/CJI.0b013e318209ee72
D'Aoust MA, 2010, PLANT BIOTECHNOL J, V8, P607, DOI 10.1111/j.1467-7652.2009.00496.x
De Filette M, 2006, VACCINE, V24, P6597, DOI 10.1016/j.vaccine.2006.05.082
Dennis SJ, 2018, PLANT BIOTECHNOL J, V16, P442, DOI 10.1111/pbi.12783
Deschuyteneer M, 2010, HUM VACCINES, V6, P407, DOI 10.4161/hv.6.5.11023
Doan LX, 2005, REV MED VIROL, V15, P75, DOI 10.1002/rmv.449
Doring M, 2019, BLOOD ADV, V3, P839, DOI 10.1182/bloodadvances.2018027268
Donaldson B, 2018, EXPERT REV VACCINES, V17, P833, DOI 10.1080/14760584.2018.1516552
Donaldson B, 2015, ADV DEL SCI TECHNOL, P159, DOI 10.1007/978-1-4939-1417-3_9
Duffy PE, 2020, NPJ VACCINES, V5, DOI 10.1038/s41541-020-0196-3
Fasehee H, 2018, AMB EXPRESS, V8, DOI 10.1186/s13568-018-0638-8
Fasehee H, 2011, PROTEOMICS, V11, P3905, DOI 10.1002/pmic.201100174
Fernandes F, 2013, EXPERT REV VACCINES, V12, P225, DOI [10.1586/ERV.12.153, 10.1586/erv.12.153]
Fiebiger E, 2001, J EXP MED, V193, P881, DOI 10.1084/jem.193.8.881
Fong D, 2008, BRIT J CANCER, V99, P1290, DOI 10.1038/sj.bjc.6604677

Fontes-Garfias CR, 2017, CELL REP, V21, P1180, DOI 10.1016/j.celrep.2017.10.016
FRENCH TJ, 1990, J VIROL, V64, P1530, DOI 10.1128/JVI.64.4.1530-1536.1990
Fuenmayor J, 2017, NEW BIOTECHNOL, V39, P174, DOI 10.1016/j.nbt.2017.07.010
Galarza JM, 2005, VIRAL IMMUNOL, V18, P244, DOI 10.1089/vim.2005.18.244
Galaway FA, 2013, MOL PHARMACEUT, V10, P59, DOI 10.1021/mp3003368
Garg A, 2014, CANCER CHEMOTH PHARM, V74, P819, DOI 10.1007/s00280-014-2560-3
Glass PJ, 2000, J VIROL, V74, P6581, DOI 10.1128/JVI.74.14.6581-6591.2000
Gleba Y, 2005, VACCINE, V23, P2042, DOI 10.1016/j.vaccine.2005.01.006
GOELET P, 1982, P NATL ACAD SCI-BIOL, V79, P5818, DOI 10.1073/pnas.79.19.5818
Gu Y, 2017, VACCINE, V35, P4637, DOI 10.1016/j.vaccine.2017.06.084
Guo JJ, 2019, FRONT IMMUNOL, V10, DOI 10.3389/fimmu.2019.00592
Gutierrez-Granados S, 2016, APPL MICROBIOL BIOT, V100, P3935, DOI 10.1007/s00253-015-7213-x
Harrison BD, 1999, PHILOS T ROY SOC B, V354, P521, DOI 10.1098/rstb.1999.0403
Havlikova S, 2013, ACTA VIROL, V57, P123, DOI 10.4149/av_2013_02_123
Heath WR, 2001, NAT REV IMMUNOL, V1, P126, DOI 10.1038/35100512
Herbst-Kralovetz M, 2010, EXPERT REV VACCINES, V9, P299, DOI 10.1586/ERV.09.163
Hillebrandt N, 2020, FRONT BIOENG BIOTECH, V8, DOI 10.3389/fbioe.2020.00489
Hu YM, 2014, HUM VACC IMMUNOTHER, V10, P469, DOI 10.4161/hv.26846
Huang XF, 2017, NPJ VACCINES, V2, DOI 10.1038/s41541-017-0006-8
Huang Z, 2009, BIOTECHNOL BIOENG, V103, P706, DOI 10.1002/bit.22299
Hutchins B, 2000, MOL THER, V2, P532, DOI 10.1006/mthe.2000.0217
Janitzek CM, 2019, SCI REP-UK, V9, DOI 10.1038/s41598-019-41522-5
Jegerlehner A, 2002, VACCINE, V20, P3104, DOI 10.1016/S0264-410X(02)00266-9
JEMAL A, 2011, CA-CANCER J CLIN, V61, P69, DOI DOI 10.3322/CAAC.20107
Jeong H, 2017, J MICROBIOL, V55, P220, DOI 10.1007/s12275-017-7058-3
Joe CCD, 2020, VACCINE, V38, P3892, DOI 10.1016/j.vaccine.2020.03.007
Joung YH, 2016, INT J MOL SCI, V17, DOI 10.3390/ijms17101715
Kato T, 2015, PHARM RES-DORDR, V32, P3699, DOI 10.1007/s11095-015-1730-2
Keating GM, 2003, DRUGS, V63, P1021, DOI 10.2165/00003495-200363100-00006
Keech C, 2020, NEW ENGL J MED, V383, P2320, DOI 10.1056/NEJMoa2026920
KEENE JA, 1982, J EXP MED, V155, P768, DOI 10.1084/jem.155.3.768
Kibenge FSB, 1999, CAN J VET RES, V63, P49
KIRNBAUER R, 1993, J VIROL, V67, P6929, DOI 10.1128/JVI.67.12.6929-6936.1993
Kondo Kazunari, 2009, Nihon Rinsho, V67, P62
Kong DN, 2012, VIROLOGY, V432, P327, DOI 10.1016/j.virol.2012.06.001
Lacson Eduardo, 2005, Hemodial Int, V9, P367, DOI 10.1111/j.1492-7535.2005.01155.x
Lai CC, 2019, J BIOL ENG, V13, DOI 10.1186/s13036-019-0206-z
Langenkamp A, 2000, NAT IMMUNOL, V1, P311, DOI 10.1038/79758
Langeveld JPM, 2001, VACCINE, V19, P3661, DOI 10.1016/S0264-410X(01)00083-4
Latham T, 2001, J VIROL, V75, P6154, DOI 10.1128/JVI.75.13.6154-6165.2001
Le DT, 2019, SCI REP-UK, V9, DOI 10.1038/s41598-019-54928-y
Lee YT, 2018, PLOS ONE, V13, DOI 10.1371/journal.pone.0190868
Lenz P, 2005, EUR J IMMUNOL, V35, P1548, DOI 10.1002/eji.200425547
Leyland-Jones B, 2001, ONCOLOGY-BASEL, V61, P31, DOI 10.1159/000055399
Li CA, 2010, VACCINE, V28, P4294, DOI 10.1016/j.vaccine.2010.04.025
Li HY, 2013, VACCINE, V31, P3281, DOI 10.1016/j.vaccine.2013.05.019
Li LM, 2012, VIROL J, V9, DOI 10.1186/1743-422X-9-203
Li SW, 2005, VACCINE, V23, P2893, DOI 10.1016/j.vaccine.2004.11.064
Li TC, 2003, VIROLOGY, V311, P115, DOI 10.1016/S0042-6822(03)00141-7
Lin YL, 2012, VACCINE, V30, P1305, DOI 10.1016/j.vaccine.2011.12.081
Link A, 2012, J IMMUNOL, V188, P3724, DOI 10.4049/jimmunol.1103312
Liu WQ, 2010, VIRUS GENES, V40, P53, DOI 10.1007/s11262-009-0418-2
Lizotte PH, 2016, NAT NANOTECHNOL, V11, P295, DOI [10.1038/NNANO.2015.292, 10.1038/nnano.2015.292]
Lopez OJ, 2007, CLIN VACCINE IMMUNOL, V14, P269, DOI 10.1128/CVI.00304-06
Lopez-Macias Constantino, 2012, Hum Vaccin Immunother, V8, P411, DOI 10.4161/hv.18757
Lua LHL, 2014, BIOTECHNOL BIOENG, V111, P425, DOI 10.1002/bit.25159
Lunsdorf H, 2011, MICROB CELL FACT, V10, DOI 10.1186/1475-2859-10-48
Manolova V, 2008, EUR J IMMUNOL, V38, P1404, DOI 10.1002/eji.200737984
Marsian J, 2017, NAT COMMUN, V8, DOI 10.1038/s41467-017-00090-w
Marsian J, 2016, CURR OPIN BIOTECH, V37, P201, DOI 10.1016/j.copbio.2015.12.007

Martins KAO, 2016, EBIOMEDICINE, V3, P67, DOI 10.1016/j.ebiom.2015.11.041
McGinnes LW, 2011, J VIROL, V85, P366, DOI 10.1128/JVI.01861-10
MELL R, 2010, VIROLOGY, V397, P187, DOI DOI 10.1016/J.VIROL.2009.11.001
Mohsen MO., 2019, J IMMUNOTHER CANCER, V7, P1, DOI [10.1186/s40425-018-0484-x, DOI 10.1186/S40425-018-0484-X]
Mohsen MO, 2019, FRONT IMMUNOL, V10, DOI 10.3389/fimmu.2019.01015
Mohsen MO, 2018, VACCINES-BASEL, V6, DOI 10.3390/vaccines6030037
Moron VG, 2003, J IMMUNOL, V171, P2242, DOI 10.4049/jimmunol.171.5.2242
Mortola E, 2004, FEBS LETT, V576, P174, DOI 10.1016/j.febslet.2004.09.009
Munoz N, 2004, INT J CANCER, V111, P278, DOI 10.1002/ijc.20244
NAMBA K, 1986, SCIENCE, V231, P1401, DOI 10.1126/science.3952490
Nardin EH, 2004, INFECT IMMUN, V72, P6519, DOI 10.1128/IAI.72.11.6519-6527.2004
Naskalska A, 2015, POL J MICROBIOL, V64, P3
O'Donnell K, 2020, EXPERT REV VACCINES, V19, P267, DOI 10.1080/14760584.2020.1738225
Oldstone MBA, 2002, CURR TOP MICROBIOL, V262, pV
Oropesa R, 2013, ADV NAT SCI-NANOSCI, V4, DOI 10.1088/2043-6262/4/2/025007
Palladini A, 2018, ONCOIMMUNOLOGY, V7, DOI 10.1080/2162402X.2017.1408749
Pang HH, 2019, THERANOSTICS, V9, P1752, DOI 10.7150/thno.30977
PARKER DC, 1993, ANNU REV IMMUNOL, V11, P331, DOI 10.1146/annurev.iy.11.040193.001555
Pease LF, 2009, BIOTECHNOL BIOENG, V102, P845, DOI 10.1002/bit.22085
Peixoto C, 2007, J BIOTECHNOL, V127, P452, DOI 10.1016/j.jbiotec.2006.08.002
Phelps JP, 2007, J BIOTECHNOL, V128, P290, DOI 10.1016/j.jbiotec.2006.10.005
Pniewski T, 2011, J APPL GENET, V52, P125, DOI 10.1007/s13353-010-0001-5
Proffitt A., 2012, 1 HEV VACCINE APPROV, DOI [10.1038/nbt0412-300a, DOI 10.1038/NBT0412-300A]
Pulcini C, 2013, VACCINE, V31, P3943, DOI 10.1016/j.vaccine.2013.06.039
Pushko P, 2013, INTERVIROLOGY, V56, P141, DOI 10.1159/000346773
Qafari SM, 2017, RSC ADV, V7, P56006, DOI 10.1039/c7ra12128h
Qian CY, 2020, VACCINES-BASEL, V8, DOI 10.3390/vaccines8010139
Quan FS, 2013, VIRAL IMMUNOL, V26, P385, DOI 10.1089/vim.2013.0013
Ren J, 1997, J SURG RES, V71, P13, DOI 10.1006/jsre.1997.5097
Rockman S, 2020, MICROORGANISMS, V8, DOI 10.3390/microorganisms8111745
Rodriguez-Limas WA, 2011, MICROB CELL FACT, V10, DOI 10.1186/1475-2859-10-33
Rolih V, 2020, CANCERS, V12, DOI 10.3390/cancers12061492
Rosales-Mendoza S, 2020, EXPERT OPIN BIOL TH, V20, P545, DOI 10.1080/14712598.2020.1752177
Ross TM, 2009, PLOS ONE, V4, DOI 10.1371/journal.pone.0006032
Rudd PM, 1999, J MOL BIOL, V293, P351, DOI 10.1006/jmbi.1999.3104
Ruedl C, 2002, EUR J IMMUNOL, V32, P818, DOI 10.1002/1521-4141(200203)32:3<818::AID-IMMU818>3.0.CO;2-U
Rybicki EP, 2020, WIRES NANOMED NANOBI, V12, DOI 10.1002/wnan.1587
Sailaja G, 2007, VIROLOGY, V362, P331, DOI 10.1016/j.virol.2006.12.014
Sallusto F, 2000, IMMUNOL REV, V177, P134, DOI 10.1034/j.1600-065X.2000.17717.x
SALUNKE DM, 1986, CELL, V46, P895, DOI 10.1016/0092-8674(86)90071-1
Santi L, 2008, VACCINE, V26, P1846, DOI 10.1016/j.vaccine.2008.01.053
Saraswat S, 2016, PLOS NEGLECT TROP D, V10, DOI 10.1371/journal.pntd.0004782
Scotti N, 2013, EXPERT REV VACCINES, V12, P211, DOI [10.1586/ERV.12.147, 10.1586/erv.12.147]
Serradell MC, 2019, NAT COMMUN, V10, DOI 10.1038/s41467-018-08265-9
Shan LL, 2012, BIOMATERIALS, V33, P146, DOI 10.1016/j.biomaterials.2011.09.025
Sharma VK, 2020, MASS SPECTROM REV, V39, P83, DOI 10.1002/mas.21571
Shirbaghaee Z, 2016, BIOPOLYMERS, V105, P113, DOI 10.1002/bip.22759
Shiri F, 2020, ANAL BIOANAL CHEM, V412, P1563, DOI 10.1007/s00216-019-02383-z
Shytuhina A, 2014, J CHROMATOGR A, V1364, P192, DOI 10.1016/j.chroma.2014.05.087
SLAMON DJ, 1987, SCIENCE, V235, P177, DOI 10.1126/science.3798106
Smith JS, 2007, INT J CANCER, V121, P621, DOI 10.1002/ijc.22527
Song JM, 2011, PLOS ONE, V6, DOI 10.1371/journal.pone.0014538
Splawn LM, 2018, DRUG TODAY, V54, P399, DOI 10.1358/dot.2018.54.7.2833984
Steinmetz NF, 2010, NANOMED-NANOTECHNOL, V6, P634, DOI 10.1016/j.nano.2010.04.005
Syomin B V, 2019, Mol Biol (Mosk), V53, P367, DOI 10.1134/S0026898419030157
Temizoz B, 2016, INT IMMUNOL, V28, P329, DOI 10.1093/intimm/dxw015
Thiam F, 2015, MICROB PATHOGENESIS, V89, P27, DOI 10.1016/j.micpath.2015.08.013
Tregoning JS, 2020, LANCET, V396, P1464, DOI 10.1016/S0140-6736(20)32010-9
van Oers MM, 2011, J INVERTEBR PATHOL, V107, pS3, DOI 10.1016/j.jip.2011.05.001

Vicente T, 2011, J INVERTEBR PATHOL, V107, pS42, DOI 10.1016/j.jip.2011.05.004
Vieira HLA, 2005, J BIOTECHNOL, V120, P72, DOI 10.1016/j.jbiotec.2005.03.026
Walpita P, 2011, PLOS ONE, V6, DOI 10.1371/journal.pone.0018437
Wang C, 2017, ANTIVIR RES, V140, P55, DOI 10.1016/j.antiviral.2016.12.019
Warfield KL, 2003, P NATL ACAD SCI USA, V100, P15889, DOI 10.1073/pnas.2237038100
WEBER J, 1995, VACCINE, V13, P831, DOI 10.1016/0264-410X(94)00061-Q
Wei MX, 2018, EMERG MICROBES INFEC, V7, DOI 10.1038/s41426-018-0158-2
Welsch S, 2007, FEBS LETT, V581, P2089, DOI 10.1016/j.febslet.2007.03.060
Win SJ, 2011, IMMUNOL CELL BIOL, V89, P681, DOI 10.1038/icb.2010.161
Wu CY, 2010, PLOS ONE, V5, DOI 10.1371/journal.pone.0009784
Wu T, 2012, HUM VACC IMMUNOTHER, V8, P823, DOI 10.4161/hv.20042
Yacoby I, 2006, ANTIMICROB AGENTS CH, V50, P2087, DOI 10.1128/AAC.00169-06
Yadav R, 2020, VIRUSES-BASEL, V12, DOI 10.3390/v12010018
Yang CD, 2007, BMC BIOTECHNOL, V7, DOI 10.1186/1472-6750-7-62
Yang YL, 2020, ENG LIFE SCI, V20, P451, DOI 10.1002/elsc.202000033
Yang YL, 2015, VACCINE, V33, P1143, DOI 10.1016/j.vaccine.2015.01.031
Yao QZ, 2004, J IMMUNOL, V173, P1951, DOI 10.4049/jimmunol.173.3.1951
Yim Eun-Kyoung, 2005, Cancer Res Treat, V37, P319, DOI 10.4143/crt.2005.37.6.319
Yuen CYS, 2016, VACCINE, V34, P33, DOI 10.1016/j.vaccine.2015.11.032
Zabel F, 2013, CURR OPIN VIROL, V3, P357, DOI 10.1016/j.coviro.2013.05.004
Zdanowicz M, 2016, ACTA BIOCHIM POL, V63, P469, DOI 10.18388/abp.2016_1275
Zeltins A, 2013, MOL BIOTECHNOL, V53, P92, DOI 10.1007/s12033-012-9598-4
Zeng QB, 2013, BIOMATERIALS, V34, P4632, DOI 10.1016/j.biomaterials.2013.03.017
Zhai LK, 2019, ANTIVIR RES, V166, P56, DOI 10.1016/j.antiviral.2019.03.012
Zhai LK, 2017, ANTIVIR RES, V147, P116, DOI 10.1016/j.antiviral.2017.09.012
Zhang S., 2011, VIROL J, V8, P1, DOI [10.1186/1743-422X-8-1, DOI 10.1186/1743-422X-8-1]
Zhang S, 2013, PLOS ONE, V8, DOI 10.1371/journal.pone.0068303
Zhang T, 2006, PLOS BIOL, V4, P108, DOI 10.1371/journal.pbio.0040003
Zhang T, 2019, VACCINE, V37, P4022, DOI 10.1016/j.vaccine.2019.06.002
Zhang XO, 2014, VACCINE, V32, P4039, DOI 10.1016/j.vaccine.2014.05.064
Zhao QH, 2011, BIOCONJUGATE CHEM, V22, P346, DOI 10.1021/bc1002532
Zhao QJ, 2012, NANOMED-NANOTECHNOL, V8, P1182, DOI 10.1016/j.nano.2012.01.007
Zhou Zhao-rui, 2011, Virologica Sinica, V26, P338, DOI 10.1007/s12250-011-3209-6
Zhu FC, 2010, LANCET, V376, P895, DOI 10.1016/S0140-6736(10)61030-6
Zlotnick A, 2011, TRENDS MICROBIOL, V19, P14, DOI 10.1016/j.tim.2010.11.003
Zochowska M, 2015, NANOMED-NANOTECHNOL, V11, P67, DOI 10.1016/j.nano.2014.07.009
Zochowska M, 2009, PLOS ONE, V4, DOI 10.1371/journal.pone.0005569
NR 206
TC 218
Z9 225
U1 43
U2 206
PU BMC
PI LONDON
PA CAMPUS, 4 CRINAN ST, LONDON N1 9XW, ENGLAND
EI 1477-3155
J9 J NANOBIOTECHNOL
JI J. Nanobiotechnol.
PD FEB 25
PY 2021
VL 19
IS 1
AR 59
DI 10.1186/s12951-021-00806-7
PG 27
WC Biotechnology & Applied Microbiology; Nanoscience & Nanotechnology
WE Science Citation Index Expanded (SCI-EXPANDED)
SC Biotechnology & Applied Microbiology; Science & Technology - Other
Topics
GA QQ3WG
UT WOS:000624453900005

PM 33632278
OA Green Published, gold
HC Y
HP N
DA 2023-09-14
ER

PT J
AU Shi, GN
   Zhang, CN
   Xu, R
   Niu, JF
   Song, HJ
   Zhang, XY
   Wang, WW
   Wang, YM
   Li, C
   Wei, XQ
   Kong, DL
AF Shi, Gao-Na
   Zhang, Chuang-Nian
   Xu, Rong
   Niu, Jin-Feng
   Song, Hui-Juan
   Zhang, Xiu-Yuan
   Wang, Wei -Wei
   Wang, Yan-Ming
   Li, Chen
   Wei, Xiao-Qing
   Kong, De-Ling
TI Enhanced antitumor immunity by targeting dendritic cells with tumor cell
   lysate-loaded chitosan nanoparticles vaccine
SO BIOMATERIALS
LA English
DT Article
DE Dendritic cell targeting; Immunotherapy; Tumor cell lysates;
   Nano-vaccine; Chitosan
ID MOUSE BONE-MARROW; IN-VIVO; CANCER-IMMUNOTHERAPY; MANNOSE RECEPTOR;
   THERAPEUTIC VACCINES; CLINICAL-TRIALS; LYMPH-NODE; ANTIGEN; RESPONSES;
   INDUCTION
AB Whole tumor cell lysates (TCL) have been implemented as tumor antigens for cancer vaccine development, although clinical outcomes of TCL-based antitumor immunotherapy remain unsatisfactory. In order to improve the efficacy of TCL-based vaccines, biomaterials have been employed to enhance antigen delivery and presentation. Here, we have developed chitosan nanoparticles (CTS NPs) with surface mannose (Man) moieties for specific dendritic cells (DCs) targeting (Man-CTS NPs). The Man-CTS NPs were then loaded with TCL generated from B16 melanoma cells (Man-CTS-TCL NPs) for in vitro and in vivo assessment. Potency of the Man-CTS-TCL NPs as cancer vaccine was also assessed in vivo by immunization of mice with Man-CTS-TCL NPs followed by re-challenge with B16 melanoma cell inoculation. We have shown here that Man-CTS-TCL NPs promote bone marrow-derived dendritic cells (BMDCs) maturation and antigen presentation in vitro. In vivo evaluation further demonstrated that the Man-CTS-TCL NPs were readily taken up by endogenous DCs within the draining lymph node (DLN) following subcutaneous administration accompanied by increasing in serum IFN-gamma and IL-4 levels. Tumor growth was also significantly delayed in mice primed with Man-CTS-TCL NPs vaccine, attributable at least in part to cytotoxic T lymphocytes response. Moreover, Man-CTS-TCL NPs vaccine also exhibited therapeutic effects in mice with melanoma. Thus, we report here the Man-CTS-TCL NPs as effective anti-tumor vaccine for cancer immunotherapy. (C) 2016 Elsevier Ltd. All rights reserved.
C1 [Shi, Gao-Na; Zhang, Chuang-Nian; Song, Hui-Juan; Zhang, Xiu-Yuan; Wang, Wei -Wei; Li, Chen; Kong, De-Ling] Chinese Acad Med Sci, Inst Biomed Engn, Tianjin Key Lab Biomat Res, Tianjin 300192, Peoples R China.
   [Shi, Gao-Na; Zhang, Chuang-Nian; Song, Hui-Juan; Zhang, Xiu-Yuan; Wang, Wei -Wei; Li, Chen; Kong, De-Ling] Peking Union Med Coll, Tianjin 300192, Peoples R China.

[Xu, Rong] Univ Liverpool, Inst Infect & Global Hlth, Dept Clin Infect Microbiol & Immunol, Liverpool L69 7BE, Merseyside, England.
[Niu, Jin-Feng; Wang, Yan-Ming] Nankai Univ, State Key Lab Med Chem Biol, Coll Pharm, Tianjin, Peoples R China.
[Niu, Jin-Feng; Wang, Yan-Ming] Nankai Univ, Tianjin Key Lab Mol Drug Res, Tianjin, Peoples R China.
[Wei, Xiao-Qing] Cardiff Univ, Coll Biomed & Life Sci, Sch Dent, Cardiff Inst Tissue Engn & Repair, Cardiff, S Glam, Wales.
[Kong, De-Ling] Nankai Univ, Key Lab Bioact Mat, Minist Educ, Tianjin 300071, Peoples R China.
C3 Chinese Academy of Medical Sciences - Peking Union Medical College;
Institute of BioMedical Engineering - CAMS; Chinese Academy of Medical
Sciences - Peking Union Medical College; Peking Union Medical College;
University of Liverpool; Nankai University; Nankai University; Cardiff
University; Nankai University
RP Li, C (通讯作者)，Chinese Acad Med Sci, Inst Biomed Engn, Tianjin Key Lab Biomat Res, Tianjin 300192, Peoples R China.; Li, C (通讯作者)，Peking Union Med Coll, Tianjin 300192, Peoples R China.; Wei, XQ (通讯作者)，Cardiff Univ, Coll Biomed & Life Sci, Sch Dent, Cardiff Inst Tissue Engn & Repair, Cardiff, S Glam, Wales.; Kong, DL (通讯作者)，Nankai Univ, Key Lab Bioact Mat, Minist Educ, Tianjin 300071, Peoples R China.
EM cli0616826@126.com; weiX1@cardiff.ac.uk; kongdeling@nankai.edu.cn
RI Wang, Weiwei/GRF-3625-2022
FU National Natural Science Foundation of China [81301309, 51373199];
Peking Union Medical College Innovation Research Team Fund; Global
Innovation Initiative Consortium Fund; National Fund for Young
Investigator [31400833]; Peking Union Medical College Innovation Fund
FX This work was supported by the National Natural Science Foundation of
China (81301309 and 51373199), Peking Union Medical College Innovation
Research Team Fund, Global Innovation Initiative Consortium Fund,
National Fund for Young Investigator (31400833), and Peking Union
Medical College Innovation Fund.
CR Alaniz L, 2014, METHODS MOL BIOL, V1139, P27, DOI 10.1007/978-1-4939-0345-0_3
Bachmann MF, 2010, NAT REV IMMUNOL, V10, P787, DOI 10.1038/nri2868
Banchereau J, 1998, NATURE, V392, P245, DOI 10.1038/32588
Banchereau J, 2005, NAT REV IMMUNOL, V5, P296, DOI 10.1038/nri1592
Carroll EC, 2016, IMMUNITY, V44, P597, DOI 10.1016/j.immuni.2016.02.004
Celluzzi CM, 1996, J EXP MED, V183, P283, DOI 10.1084/jem.183.1.283
Cheung AS, 2015, NANO TODAY, V10, P511, DOI 10.1016/j.nantod.2015.06.007
Chiang CLL, 2010, SEMIN IMMUNOL, V22, P132, DOI 10.1016/j.smim.2010.02.004
Coffelt SB, 2015, TRENDS IMMUNOL, V36, P198, DOI 10.1016/j.it.2015.02.006
Coulie PG, 2014, NAT REV CANCER, V14, P135, DOI 10.1038/nrc3670
Engering AJ, 1997, EUR J IMMUNOL, V27, P2417, DOI 10.1002/eji.1830270941
Fridman WH, 2012, NAT REV CANCER, V12, P298, DOI 10.1038/nrc3245
Garcia-Fuentes M, 2012, J CONTROL RELEASE, V161, P496, DOI 10.1016/j.jconrel.2012.03.017
Garu A, 2016, MOL THER, V24, P385, DOI 10.1038/mt.2015.215
Guo FF, 2013, BIOMED RES INT, V2013, DOI 10.1155/2013/212315
INABA K, 1992, J EXP MED, V176, P1693, DOI 10.1084/jem.176.6.1693
Jager E, 1996, INT J CANCER, V66, P470, DOI 10.1002/(SICI)1097-0215(19960516)66:4<470::AID-IJC10>3.0.CO;2-C
Kambayashi T, 2014, NAT REV IMMUNOL, V14, P719, DOI 10.1038/nri3754
Kandalaft LE, 2013, ONCOIMMUNOLOGY, V2, DOI 10.4161/onci.22664
Kosaka N, 2011, CONTRAST MEDIA MOL I, V6, P55, DOI 10.1002/cmmi.395
Kwon YJ, 2005, P NATL ACAD SCI USA, V102, P18264, DOI 10.1073/pnas.0509541102
Livingston P, 2001, CLIN CANCER RES, V7, P1837
Lutz MB, 1999, J IMMUNOL METHODS, V223, P77, DOI 10.1016/S0022-1759(98)00204-X
Maharjan S, 2016, BIOMATERIALS, V84, P286, DOI 10.1016/j.biomaterials.2016.01.043
Manolova V, 2008, EUR J IMMUNOL, V38, P1404, DOI 10.1002/eji.200737984
Melero I, 2014, NAT REV CLIN ONCOL, V11, P509, DOI 10.1038/nrclinonc.2014.111
Milani A, 2013, ANN ONCOL, V24, P1740, DOI 10.1093/annonc/mdt133
Moon JJ, 2012, P NATL ACAD SCI USA, V109, P1080, DOI 10.1073/pnas.1112648109
Nestle FO, 2001, NAT MED, V7, P761, DOI 10.1038/89863
Palucka K, 2013, IMMUNITY, V39, P38, DOI 10.1016/j.immuni.2013.07.004
Qian Y, 2016, BIOMATERIALS, V98, P171, DOI 10.1016/j.biomaterials.2016.05.008

Rahimian S, 2015, BIOMATERIALS, V37, P469, DOI 10.1016/j.biomaterials.2014.10.043
Rao Q., 2016, HEPATOLOGY, V15, P1
Reddy ST, 2007, NAT BIOTECHNOL, V25, P1159, DOI [10.1038/nbt1332, 10.1038/nbtl332]
Reddy ST, 2006, TRENDS IMMUNOL, V27, P573, DOI 10.1016/j.it.2006.10.005
Rosalia RA, 2015, BIOMATERIALS, V40, P88, DOI 10.1016/j.biomaterials.2014.10.053
SALLUSTO F, 1995, J EXP MED, V182, P389, DOI 10.1084/jem.182.2.389
Segura E, 2009, CURR OPIN IMMUNOL, V21, P105, DOI 10.1016/j.coi.2009.03.011
Sixt M, 2005, IMMUNITY, V22, P19, DOI 10.1016/j.immuni.2004.11.013
Solbrig CM, 2007, MOL PHARMACEUT, V4, P47, DOI 10.1021/mp060107e
Sondak VK, 2003, SEMIN CANCER BIOL, V13, P409, DOI 10.1016/j.semcancer.2003.09.004
Thomann JS, 2011, BIOMATERIALS, V32, P4574, DOI 10.1016/j.biomaterials.2011.03.015
van Broekhoven CL, 2004, CANCER RES, V64, P4357, DOI 10.1158/0008-5472.CAN-04-0138
Xia XJ, 2015, CELL REP, V11, P957, DOI 10.1016/j.celrep.2015.04.009
Zhang HF, 2015, CANCER IMMUNOL RES, V3, P196, DOI 10.1158/2326-6066.CIR-14-0177
Zhang YF, 2007, BREAST CANCER RES TR, V105, P277, DOI 10.1007/s10549-006-9457-8
NR 46
TC 199
Z9 208
U1 11
U2 319
PU ELSEVIER SCI LTD
PI OXFORD
PA THE BOULEVARD, LANGFORD LANE, KIDLINGTON, OXFORD OX5 1GB, OXON, ENGLAND
SN 0142-9612
EI 1878-5905
J9 BIOMATERIALS
JI Biomaterials
PD JAN
PY 2017
VL 113
BP 191
EP 202
DI 10.1016/j.biomaterials.2016.10.047
PG 12
WC Engineering, Biomedical; Materials Science, Biomaterials
WE Science Citation Index Expanded (SCI-EXPANDED)
SC Engineering; Materials Science
GA EE2EK
UT WOS:000389396300016
PM 27816821
OA Green Accepted
DA 2023-09-14
ER

PT J
AU Liu, LN
Wang, YH
Miao, L
Liu, Q
Musetti, S
Li, J
Huang, L
AF Liu, Lina
Wang, Yuhua
Miao, Lei
Liu, Qi
Musetti, Sara
Li, Jun
Huang, Leaf
TI Combination Immunotherapy of MUC1 mRNA Nano-vaccine and CTLA-4 Blockade
Effectively Inhibits Growth of Triple Negative Breast Cancer

SO MOLECULAR THERAPY
LA English
DT Article
ID NEURAL-NETWORKS; DELIVERY; NANOPARTICLES; CELLS; EXPRESSION; GENE; PD-1;
MELANOMA; RECEPTOR; THERAPY
AB Triple negative breast cancer (TNBC), which constitutes 10%-20% of all breast cancers, is associated with aggressive progression, a high rate of metastasis, and poor prognosis. The treatment of patients with TNBC remains a great clinical challenge. Preclinical reports support the combination immunotherapy of cancer vaccines and immune checkpoint blockades in non-immunogenic tumors. In this study, we constructed nanoparticles (NPs) to deliver an mRNA vaccine encoding tumor antigen MUC1 to dendritic cells (DCs) in lymph nodes to activate and expand tumor-specific T cells. An anti-CTLA-4 (cytotoxic T-lymphocyte-associated protein 4) monoclonal antibody was combined with the mRNA vaccine to enhance the anti-tumor benefits. In vivo studies demonstrated that the NP-based mRNA vaccine, targeted to mannose receptors on DCs, could successfully express tumor antigen in the DCs of the lymph node; that the NP vaccine could induce a strong, antigen-specific, in vivo cytotoxic T lymphocyte response against TNBC 4T1 cells; and that combination immunotherapy of the vaccine and anti-CTLA-4 monoclonal antibody could significantly enhance anti-tumor immune response compared to the vaccine or monoclonal antibody alone. These data support both the NP as a carrier for delivery of mRNA vaccine and a potential combination immunotherapy of the NP-based mRNA vaccine and the CTLA-4 inhibitor for TNBC.
C1 [Liu, Lina; Wang, Yuhua; Miao, Lei; Liu, Qi; Musetti, Sara; Li, Jun; Huang, Leaf] Univ N Carolina, Eshelman Sch Pharm, Div Pharmacoengn & Mol Pharmaceut, Chapel Hill, NC 27599 USA.
[Liu, Lina; Wang, Yuhua; Miao, Lei; Liu, Qi; Musetti, Sara; Li, Jun; Huang, Leaf] Univ N Carolina, Ctr Nanotechnol Drug Delivery, Eshelman Sch Pharm, Chapel Hill, NC 27599 USA.
[Liu, Qi; Huang, Leaf] Univ N Carolina, UNC & NCSU Joint Dept Biomed Engn, Chapel Hill, NC 27599 USA.
[Liu, Lina] Guizhou Med Univ, Key Lab Biol & Med Engn, Guiyang 550025, Guizhou, Peoples R China.
[Liu, Lina] Guizhou Med Univ, Engn Res Ctr Med Biotechnol, Guiyang 550025, Guizhou, Peoples R China.
[Liu, Lina] Guizhou Med Univ, Guizhou Prov Engn Res Ctr Immune Cells & Antibody, Sch Biol & Engn, Guiyang 550025, Guizhou, Peoples R China.
C3 University of North Carolina; University of North Carolina Chapel Hill;
University of North Carolina; University of North Carolina Chapel Hill;
University of North Carolina; University of North Carolina Chapel Hill;
North Carolina State University; Guizhou Medical University; Guizhou
Medical University; Guizhou Medical University
RP Huang, L (通讯作者)，Univ N Carolina, Eshelman Sch Pharm, Div Pharmacoengn & Mol Pharmaceut, Chapel Hill, NC 27599 USA.
EM leafh@unc.edu
RI Miao, Lei/H-2700-2017; liu, li/HGC-0900-2022; Huang, Li/IUQ-0909-2023;
huang, lei/GQP-8739-2022; HUANG, LING/HTR-1819-2023
OI Miao, Lei/0000-0003-4235-2680; Musetti Jenkins, PhD,
Sara/0000-0002-5682-1225; Huang, Lan/0000-0001-9594-4722
FU NIH [CA149363, CA151652, CA149387]; Science and Technology Foundation of
Guizhou Province [LH-2015-7318, J-2016-1118]; State Scholarship Fund of
China Scholarship Council(CSC) [201308525104]
FX The work was supported by NIH grants CA149363, CA151652; and CA149387.
L.L. was supported by the State Scholarship Fund of China Scholarship
Council(CSC) (201308525104) and the Science and Technology Foundation of
Guizhou Province (LH-2015-7318, J-2016-1118). L.H. was a Senior Visiting
Scholar at the State Key Laboratory of Molecular Engineering of
Polymers, Fudan University, China.
CR Acres B, 2017, CURR TOP MICROBIOL, V405, P79, DOI 10.1007/82_2015_429
Andreatta M, 2016, BIOINFORMATICS, V32, P511, DOI 10.1093/bioinformatics/btv639
[Anonymous], 2015, CANCER DISCOV, V5, P570, DOI 10.1158/2159-8290.CD-NB2015-059
Apostolopoulos V, 2006, BREAST CANCER RES, V8, DOI 10.1186/bcr1505
Bauer KR, 2007, CANCER, V109, P1721, DOI 10.1002/cncr.22618
Bianchini G, 2016, NAT REV CLIN ONCOL, V13, P674, DOI 10.1038/nrclinonc.2016.66
Bringmann A, 2010, J BIOMED BIOTECHNOL, DOI 10.1155/2010/623687
Byers AM, 2003, J IMMUNOL, V171, P17, DOI 10.4049/jimmunol.171.1.17
Castro NP, 2015, ONCOTARGET, V6, P11910, DOI 10.18632/oncotarget.4182
Cheever MA, 2009, CLIN CANCER RES, V15, P5323, DOI 10.1158/1078-0432.CCR-09-0737

Curran MA, 2010, P NATL ACAD SCI USA, V107, P4275, DOI 10.1073/pnas.0915174107
Finn OJ, 2003, NAT REV IMMUNOL, V3, P630, DOI 10.1038/nri1150
Geall AJ, 2013, SEMIN IMMUNOL, V25, P152, DOI 10.1016/j.smim.2013.05.001
Hoerr I, 2000, EUR J IMMUNOL, V30, P1
Ismail-Khan R, 2010, CANCER CONTROL, V17, P173, DOI 10.1177/107327481001700305
Jonckheere N, 2010, BIOCHIMIE, V92, P1, DOI 10.1016/j.biochi.2009.09.018
Karaki S, 2016, VACCINES-BASEL, V4, DOI 10.3390/vaccines4040037
Kim IS, 2005, INT J PHARMACEUT, V298, P255, DOI 10.1016/j.ijpharm.2005.04.017
Kim SH, 2009, CANCER RES, V69, P5860, DOI 10.1158/0008-5472.CAN-08-4855
Koneru M, 2006, J IMMUNOL, V176, P6103, DOI 10.4049/jimmunol.176.10.6103
Kormann MSD, 2011, NAT BIOTECHNOL, V29, P154, DOI 10.1038/nbt.1733
Kranz LM, 2016, NATURE, V534, P396, DOI 10.1038/nature18300
Lakshminarayanan V, 2012, P NATL ACAD SCI USA, V109, P261, DOI 10.1073/pnas.1115166109
Le DT, 2013, J IMMUNOTHER, V36, P382, DOI 10.1097/CJI.0b013e31829fb7a2
Li J, 2012, J CONTROL RELEASE, V158, P108, DOI 10.1016/j.jconrel.2011.10.020
Li J, 2010, J CONTROL RELEASE, V142, P416, DOI 10.1016/j.jconrel.2009.11.008
Livingston PO, 1997, CANCER IMMUNOL IMMUN, V45, P10, DOI 10.1007/s002620050395
Machluf N, 2012, WORLD J VACCINES, V2, P109, DOI DOI 10.4236/WJV.2012.23015
McNamara MA, 2015, J IMMUNOL RES, V2015, DOI 10.1155/2015/794528
Melero I, 2014, NAT REV CLIN ONCOL, V11, P509, DOI 10.1038/nrclinonc.2014.111
Mukherjee P, 2007, VACCINE, V25, P1607, DOI 10.1016/j.vaccine.2006.11.007
Nielsen M, 2003, PROTEIN SCI, V12, P1007, DOI 10.1110/ps.0239403
Okazaki T, 2007, INT IMMUNOL, V19, P813, DOI 10.1093/intimm/dxm057
Palama IE, 2015, BIOMATER SCI-UK, V3, P144, DOI 10.1039/c4bm00242c
Parry RV, 2005, MOL CELL BIOL, V25, P9543, DOI 10.1128/MCB.25.21.9543-9553.2005
PEAT N, 1992, CANCER RES, V52, P1954
Peggs KS, 2008, IMMUNOL REV, V224, P141, DOI 10.1111/j.1600-065X.2008.00649.x
Philips GK, 2015, INT IMMUNOL, V27, P39, DOI 10.1093/intimm/dxu095
Phua KKL, 2013, J CONTROL RELEASE, V166, P227, DOI 10.1016/j.jconrel.2012.12.029
Picco G, 2014, EUR J IMMUNOL, V44, P1947, DOI 10.1002/eji.201344076
Powell Erin, 2008, Expert Rev Respir Med, V2, P37, DOI 10.1586/17476348.2.1.37
Roulois D, 2013, BIOMED RES INT, V2013, DOI 10.1155/2013/871936
SALLUSTO F, 1995, J EXP MED, V182, P389, DOI 10.1084/jem.182.2.389
Sharma P, 2015, SCIENCE, V348, P56, DOI 10.1126/science.aaa8172
Tacken PJ, 2007, NAT REV IMMUNOL, V7, P790, DOI 10.1038/nri2173
Wang XY, 2011, EXPERT OPIN PHARMACO, V12, P2695, DOI 10.1517/14656566.2011.629187
Warren L, 2012, SCI REP-UK, V2, DOI 10.1038/srep00657
Weide B, 2008, J IMMUNOTHER, V31, P180, DOI 10.1097/CJI.0b013e31815ce501
Woodrow KA, 2009, NAT MATER, V8, P526, DOI [10.1038/NMAT2444, 10.1038/nmat2444]
Xu ZH, 2013, J CONTROL RELEASE, V172, P259, DOI 10.1016/j.jconrel.2013.08.021
Zhao Y, 2015, BIOMATERIALS, V68, P54, DOI 10.1016/j.biomaterials.2015.07.053
NR 51
TC 196
Z9 209
U1 14
U2 283
PU CELL PRESS
PI CAMBRIDGE
PA 50 HAMPSHIRE ST, FLOOR 5, CAMBRIDGE, MA 02139 USA
SN 1525-0016
EI 1525-0024
J9 MOL THER
JI Mol. Ther.
PD JAN 3
PY 2018
VL 26
IS 1
BP 45
EP 55
DI 10.1016/j.ymthe.2017.10.020
PG 11

WC Biotechnology & Applied Microbiology; Genetics & Heredity; Medicine,
   Research & Experimental
WE Science Citation Index Expanded (SCI-EXPANDED)
SC Biotechnology & Applied Microbiology; Genetics & Heredity; Research &
   Experimental Medicine
GA FT2ZB
UT WOS:000423013500009
PM 29258739
OA hybrid, Green Published
HC Y
HP N
DA 2023-09-14
ER

PT J
AU Zhu, GZ
   Lynn, GM
   Jacobson, O
   Chen, K
   Liu, Y
   Zhang, HM
   Ma, Y
   Zhang, FW
   Tian, R
   Ni, QQ
   Cheng, SY
   Wang, ZT
   Lu, N
   Yung, BC
   Wang, Z
   Lang, LX
   Fu, X
   Jin, A
   Weiss, ID
   Vishwasrao, H
   Niu, G
   Shroff, H
   Klinman, DM
   Seder, RA
   Chen, XY
AF Zhu, Guizhi
   Lynn, Geoffrey M.
   Jacobson, Orit
   Chen, Kai
   Liu, Yi
   Zhang, Huimin
   Ma, Ying
   Zhang, Fuwu
   Tian, Rui
   Ni, Qianqian
   Cheng, Siyuan
   Wang, Zhantong
   Lu, Nan
   Yung, Bryant C.
   Wang, Zhe
   Lang, Lixin
   Fu, Xiao
   Jin, Albert
   Weiss, Ido D.
   Vishwasrao, Harshad
   Niu, Gang

Shroff, Hari
Klinman, Dennis M.
Seder, Robert A.
Chen, Xiaoyuan
TI Albumin/vaccine nanocomplexes that assemble in vivo for combination cancer immunotherapy
SO NATURE COMMUNICATIONS
LA English
DT Article
ID SERUM-ALBUMIN; CHECKPOINT BLOCKADE; IMMUNE-RESPONSES; SWISS-PDBVIEWER; LYMPH-NODE; T-CELLS; TUMOR; BINDING; DELIVERY; VACCINES
AB Subunit vaccines have been investigated in over 1000 clinical trials of cancer immunotherapy, but have shown limited efficacy. Nanovaccines may improve efficacy but have rarely been clinically translated. By conjugating molecular vaccines with Evans blue (EB) into albuminbinding vaccines (AlbiVax), here we develop clinically promising albumin/AlbiVax nanocomplexes that self-assemble in vivo from AlbiVax and endogenous albumin for efficient vaccine delivery and potent cancer immunotherapy. PET pharmacoimaging, super-resolution microscopies, and flow cytometry reveal almost 100-fold more efficient co-delivery of CpG and antigens (Ags) to lymph nodes (LNs) by albumin/AlbiVax than benchmark incomplete Freund's adjuvant (IFA). Albumin/AlbiVax elicits similar to 10 times more frequent peripheral antigenspecific CD8(+) cytotoxic T lymphocytes with immune memory than IFA-emulsifying vaccines. Albumin/AlbiVax specifically inhibits progression of established primary or metastatic EG7. OVA, B16F10, and MC38 tumors; combination with anti-PD-1 and/or Abraxane further potentiates immunotherapy and eradicates most MC38 tumors. Albumin/AlbiVax nanocomplexes are thus a robust platform for combination cancer immunotherapy.
C1 [Zhu, Guizhi; Jacobson, Orit; Liu, Yi; Zhang, Huimin; Ma, Ying; Zhang, Fuwu; Tian, Rui; Ni, Qianqian; Cheng, Siyuan; Wang, Zhantong; Lu, Nan; Yung, Bryant C.; Wang, Zhe; Lang, Lixin; Niu, Gang; Chen, Xiaoyuan] NIBIB, Lab Mol Imaging & Nanomed, NIH, Bethesda, MD 20892 USA.
[Lynn, Geoffrey M.; Seder, Robert A.] NIAID, Vaccine Res Ctr, NIH, Bethesda, MD 20892 USA.
[Chen, Kai] Univ Southern Calif, Keck Sch Med, Dept Radiol, Mol Imaging Ctr, Los Angeles, CA 90033 USA.
[Liu, Yi] China Pharmaceut Univ, Sch Engn, Nanjing 210009, Jiangsu, Peoples R China.
[Wang, Zhantong] Xiamen Univ, Sch Publ Hlth, Ctr Mol Imaging & Translat Med, State Key Lab Mol Vaccinol & Mol Diagnost, Xiamen 361102, Peoples R China.
[Fu, Xiao; Jin, Albert] NIBIB, Lab Cellular Imaging & Macromol Biophys, NIH, Bethesda, MD 20892 USA.
[Weiss, Ido D.] NIAID, Lab Mol Immunol, NIH, Bethesda, MD 20892 USA.
[Vishwasrao, Harshad; Shroff, Hari] NIH, Adv Imaging & Microscopy Resource, Bethesda, MD 20892 USA.
[Shroff, Hari] NIBIB, Sect High Resolut Opt Imaging, NIH, Bethesda, MD 20892 USA.
[Klinman, Dennis M.] NCI, Canc & Inflammat Program, Frederick, MD 21702 USA.
C3 National Institutes of Health (NIH) - USA; NIH National Institute of Biomedical Imaging & Bioengineering (NIBIB); National Institutes of Health (NIH) - USA; NIH National Institute of Allergy & Infectious Diseases (NIAID); University of Southern California; China Pharmaceutical University; Xiamen University; National Institutes of Health (NIH) - USA; NIH National Institute of Biomedical Imaging & Bioengineering (NIBIB); National Institutes of Health (NIH) - USA; NIH National Institute of Allergy & Infectious Diseases (NIAID); National Institutes of Health (NIH) - USA; National Institutes of Health (NIH) - USA; NIH National Institute of Biomedical Imaging & Bioengineering (NIBIB); National Institutes of Health (NIH) - USA; NIH National Cancer Institute (NCI)
RP Chen, XY (通讯作者)，NIBIB, Lab Mol Imaging & Nanomed, NIH, Bethesda, MD 20892 USA.
EM shawn.chen@nih.gov
RI wang, jing/GRS-7509-2022; Wang, ZT/GXN-3762-2022; Chen, Kai/ABE-7509-2020; chen, xia/GXM-5435-2022; Ma, Ying/HKN-2215-2023; chen, xi/GXH-3653-2022; chen, xin/IQW-3432-2023; chen, xia/GYR-3948-2022; Chen, Kai/C-7881-2016; Zhu, Guizhi/AAN-2888-2020; Jin, Albert/HKV-3135-2023; Zhang, Fuwu/T-4066-2019; Zhang, Fuwu/B-6747-2014
OI Chen, Kai/0000-0002-8647-1182; Jin, Albert/0000-0003-3826-1081; Zhang, Fuwu/0000-0002-1928-3472; Zhang, Fuwu/0000-0002-1928-3472; Zhu,

Guizhi/0000-0002-2945-7982
FU Intramural Research Programs of National Institutes of Health; National Science Foundation of China [81771900, 81601531, BK20160755]
FX We thank Dr Robert Langer (MIT) and Dr Henry Eden (NIBIB) for valuable suggestions and insightful comments on our manuscript. We thank Dr Xin Xu (NIAID) for research discussion and reagent contribution; Dr Meghan O. Altman (NIAID) for reagent contribution; Ms Lynne Holtzclaw and Dr Vincent Schram (NICHD) for their valuable expertise in tissue clearing and fluorescence microscopy; Dr Courtney Akitake (Carl Zeiss Microscopy LLC) and Mr Geoffrey Daniels (Leica Microsystems Inc.) for help with fluorescence microscopy; Mr Sam Darko (VRC of NIAID) for data analysis of RNA sequencing; Drs Jennie Garcia-Olivares (NIMH), Francisco J. Palma-Cerda (NINDS), and Joseph R Francica (NIAID) for help with BLI; and NIH Tetramer Core Facility for providing tetramers. This work was supported by Intramural Research Programs of National Institutes of Health, and National Science Foundation of China to Dr Yi Liu (81771900, 81601531, and BK20160755).
CR Alexandrov LB, 2013, NATURE, V500, P415, DOI 10.1038/nature12477
BALBONI PG, 1976, NATURE, V264, P181, DOI 10.1038/264181a0
Carreno BM, 2015, SCIENCE, V348, P803, DOI 10.1126/science.aaa3828
Chen HJ, 2017, J NUCL MED, V58, P590, DOI 10.2967/jnumed.116.182097
Chen HM, 2017, NAT REV MATER, V2, DOI 10.1038/natrevmats.2017.24
Chen K, 2007, J MED CHEM, V50, P663, DOI 10.1021/jm060944k
Cohen CJ, 2015, J CLIN INVEST, V125, P3981, DOI 10.1172/JCI82416
Cullis J, 2017, CANCER IMMUNOL RES, V5, P182, DOI 10.1158/2326-6066.CIR-16-0125
Ghuman J, 2005, J MOL BIOL, V353, P38, DOI 10.1016/j.jmb.2005.07.075
Gros A, 2016, NAT MED, V22, P433, DOI 10.1038/nm.4051
Gros A, 2014, J CLIN INVEST, V124, P2246, DOI [10.1172/JC173639, 10.1172/JCI73639]
Gubin MM, 2014, NATURE, V515, P577, DOI 10.1038/nature13988
Guex N, 1997, ELECTROPHORESIS, V18, P2714, DOI 10.1002/elps.1150181505
Guex N, 2009, ELECTROPHORESIS, V30, pS162, DOI 10.1002/elps.200900140
Hailemichael Y, 2013, NAT MED, V19, P465, DOI 10.1038/nm.3105
Harrell MI, 2008, J IMMUNOL METHODS, V332, P170, DOI 10.1016/j.jim.2007.11.012
Heikenwalder M, 2004, NAT MED, V10, P187, DOI 10.1038/nm987
Hubbell JA, 2009, NATURE, V462, P449, DOI 10.1038/nature08604
Irvine DJ, 2015, CHEM REV, V115, P11109, DOI 10.1021/acs.chemrev.5b00109
Kim J, 2015, NAT BIOTECHNOL, V33, P64, DOI 10.1038/nbt.3071
Klinman DM, 2004, NAT REV IMMUNOL, V4, P248, DOI 10.1038/nri1329
Kratz F, 2008, J CONTROL RELEASE, V132, P171, DOI 10.1016/j.jconrel.2008.05.010
Kreiter S, 2015, NATURE, V520, P692, DOI 10.1038/nature14426
Krieg AM, 2006, NAT REV DRUG DISCOV, V5, P471, DOI 10.1038/nrd2059
Kuai R, 2017, NAT MATER, V16, P489, DOI [10.1038/nmat4822, 10.1038/NMAT4822]
Larsen Maja Thim, 2016, Mol Cell Ther, V4, P3, DOI 10.1186/s40591-016-0048-8
LASKOWSKI RA, 1993, J APPL CRYSTALLOGR, V26, P283, DOI 10.1107/S0021889892009944
Leifer CA, 2004, J IMMUNOL, V173, P1179, DOI 10.4049/jimmunol.173.2.1179
Lincoff AM, 2016, LANCET, V387, P349, DOI 10.1016/S0140-6736(15)00515-2
Liu HP, 2014, NATURE, V507, P519, DOI 10.1038/nature12978
Liu HP, 2011, ANGEW CHEM INT EDIT, V50, P7052, DOI 10.1002/anie.201101266
Liu Y, 2016, BIOCONJUGATE CHEM, V27, P54, DOI 10.1021/acs.bioconjchem.5b00625
Lynn GM, 2015, NAT BIOTECHNOL, V33, P1201, DOI 10.1038/nbt.3371
MALAOWALLA A M, 1962, Oral Surg Oral Med Oral Pathol, V15, P1259, DOI 10.1016/0030-4220(62)90162-7
McGranahan N, 2016, SCIENCE, V351, P1463, DOI 10.1126/science.aaf1490
Mitragotri S, 2014, NAT REV DRUG DISCOV, V13, P655, DOI 10.1038/nrd4363
Morris GM, 2009, J COMPUT CHEM, V30, P2785, DOI 10.1002/jcc.21256
Moyer TJ, 2016, J CLIN INVEST, V126, P799, DOI 10.1172/JCI81083
Moynihan KD, 2016, NAT MED, V22, P1402, DOI 10.1038/nm.4200
Niu G, 2014, J NUCL MED, V55, P1150, DOI 10.2967/jnumed.114.139642
Perilla JR, 2015, CURR OPIN STRUC BIOL, V31, P64, DOI 10.1016/j.sbi.2015.03.007
Putta MR, 2010, BIOCONJUGATE CHEM, V21, P39, DOI 10.1021/bc900425s
Rizvi NA, 2015, SCIENCE, V348, P124, DOI 10.1126/science.aaa1348

Rosenberg SA, 2012, SCI TRANSL MED, V4, DOI 10.1126/scitranslmed.3003634
Saini SK, 2013, P NATL ACAD SCI USA, V110, P15383, DOI 10.1073/pnas.1308672110
Sand KMK, 2015, FRONT IMMUNOL, V5, P1, DOI 10.3389/fimmu.2014.00682
Schumacher TN, 2015, SCIENCE, V348, P69, DOI 10.1126/science.aaa4971
Sharma P, 2015, SCIENCE, V348, P56, DOI 10.1126/science.aaa8172
Sjolander A, 1997, J IMMUNOL METHODS, V201, P115, DOI 10.1016/S0022-1759(96)00217-7
Staak JO, 2004, BLOOD, V104, p242B, DOI 10.1182/blood.V104.11.4633.4633
Tanei T., 2016, CANCER RES, V76, P2
Tran E, 2017, NAT IMMUNOL, V18, P255, DOI 10.1038/ni.3682
Trevaskis NL, 2015, NAT REV DRUG DISCOV, V14, P781, DOI 10.1038/nrd4608
Tsopelas C, 2002, J NUCL MED, V43, P1377
Vanommeslaeghe K, 2010, J COMPUT CHEM, V31, P671, DOI 10.1002/jcc.21367
Wang Y, 2015, P NATL ACAD SCI USA, V112, P208, DOI 10.1073/pnas.1414821112
Yadav M, 2014, NATURE, V515, P572, DOI 10.1038/nature14001
Yang B, 2014, CELL, V158, P945, DOI 10.1016/j.cell.2014.07.017
Yardley DA, 2013, J CONTROL RELEASE, V170, P365, DOI 10.1016/j.jconrel.2013.05.041
York AG, 2013, NAT METHODS, V10, P1122, DOI [10.1038/NMETH.2687, 10.1038/nmeth.2687]
Zhang JJ, 2015, J NUCL MED, V56, P1609, DOI 10.2967/jnumed.115.159640
Zhang W, 2016, CLIN NUCL MED, V41, P302, DOI 10.1097/RLU.0000000000001171
Zhu EF, 2015, CANCER CELL, V27, P489, DOI 10.1016/j.ccell.2015.03.004
Zhu GZ, 2017, ACS NANO, V11, P2387, DOI 10.1021/acsnano.7b00978
Zhu GZ, 2016, NANOSCALE, V8, P6684, DOI 10.1039/c5nr08821f
Zorzi A, 2017, NAT COMMUN, V8, DOI 10.1038/ncomms16092
NR 66
TC 193
Z9 197
U1 15
U2 212
PU NATURE PUBLISHING GROUP
PI LONDON
PA MACMILLAN BUILDING, 4 CRINAN ST, LONDON N1 9XW, ENGLAND
SN 2041-1723
J9 NAT COMMUN
JI Nat. Commun.
PD DEC 5
PY 2017
VL 8
AR 1954
DI 10.1038/s41467-017-02191-y
PG 15
WC Multidisciplinary Sciences
WE Science Citation Index Expanded (SCI-EXPANDED)
SC Science & Technology - Other Topics
GA FO7LJ
UT WOS:000417055600003
PM 29203865
OA Green Published, gold
HC Y
HP N
DA 2023-09-14
ER

PT J
AU Zhu, GZ
Mei, L
Vishwasrao, HD
Jacobson, O
Wang, ZT
Liu, YJ
Yung, BC
Fu, X

Jin, A
Niu, G
Wang, Q
Zhang, FW
Shroff, H
Chen, XY
AF Zhu, Guizhi
Mei, Lei
Vishwasrao, Harshad D.
Jacobson, Orit
Wang, Zhantong
Liu, Yijing
Yung, Bryant C.
Fu, Xiao
Jin, Albert
Niu, Gang
Wang, Qin
Zhang, Fuwu
Shroff, Hari
Chen, Xiaoyuan
TI Intertwining DNA-RNA nanocapsules loaded with tumor neoantigens as synergistic nanovaccines for cancer immunotherapy
SO NATURE COMMUNICATIONS
LA English
DT Article
ID CHECKPOINT BLOCKADE; DELIVERY; NANOPARTICLES; ACTIVATION; IMMUNITY; GROWTH; SIGNAL; CELLS; SIRNA
AB Nanomedicines that co-deliver DNA, RNA, and peptide therapeutics are highly desirable yet remain underdeveloped for cancer theranostics. Herein, we report self-assembled intertwining DNA-RNA nanocapsules (iDR-NCs) that efficiently delivered synergistic DNA CpG and short hairpin RNA (shRNA) adjuvants, as well as tumor-specific peptide neoantigens into antigen presenting cells (APCs) in lymph nodes for cancer immunotherapy. These nanovaccines were prepared by (1) producing tandem CpG and shRNA via concurrent rolling circle replication and rolling circle transcription, (2) self-assembling CpG and shRNA into DNA-RNA microflowers, (3) shrinking microflowers into iDR-NCs using PEG-grafted cationic polypeptides, and (4) physically loading neoantigen into iDR-NCs. CpG and shRNA in iDR-NCs synergistically activate APCs for sustained antigen presentation. Remarkably, iDR-NC/neoantigen nanovaccines elicit 8-fold more frequent neoantigen-specific peripheral CD8(+) T cells than CpG, induce T cell memory, and significantly inhibit the progression of neoantigen-specific colorectal tumors. Collectively, iDR-NCs represent potential DNA/RNA/peptide triple-co-delivery nanocarriers and synergistic tumor immunotherapeutic nanovaccines.
C1 [Zhu, Guizhi; Jacobson, Orit; Wang, Zhantong; Liu, Yijing; Yung, Bryant C.; Niu, Gang; Zhang, Fuwu; Chen, Xiaoyuan] NIBIB, Lab Mol Imaging & Nanomed, NIH, Bethesda, MD 20892 USA.
[Mei, Lei; Wang, Qin] Univ Maryland, Dept Nutr & Food Sci, Coll Agr & Nat Resources, College Pk, MD 20742 USA.
[Vishwasrao, Harshad D.; Shroff, Hari] NIH, Adv Imaging & Microscopy Resource, Bethesda, MD 20892 USA.
[Fu, Xiao; Jin, Albert] NIBIB, Lab Cellular Imaging & Macromol Biophys, NIH, Bethesda, MD 20892 USA.
[Shroff, Hari] NIBIB, Sect High Resolut Opt Imaging, NIH, Bethesda, MD 20892 USA.
C3 National Institutes of Health (NIH) - USA; NIH National Institute of Biomedical Imaging & Bioengineering (NIBIB); University System of Maryland; University of Maryland College Park; National Institutes of Health (NIH) - USA; National Institutes of Health (NIH) - USA; NIH National Institute of Biomedical Imaging & Bioengineering (NIBIB); National Institutes of Health (NIH) - USA; NIH National Institute of Biomedical Imaging & Bioengineering (NIBIB)
RP Zhang, FW; Chen, XY (通讯作者)，NIBIB, Lab Mol Imaging & Nanomed, NIH, Bethesda, MD 20892 USA.
EM fuwu.zhang@nih.gov; shawn.chen@nih.gov
RI Chen, Xiao/GQA-8928-2022; Jin, Albert/HKV-3135-2023; Liu, Yijing/D-6666-2016; Zhang, Fuwu/T-4066-2019; Wang, ZT/GXN-3762-2022; Zhang, Fuwu/B-6747-2014; Zhu, Guizhi/AAN-2888-2020; Chen,

Xiaoyuan/D-1860-2014
OI Chen, Xiao/0000-0002-9797-8384; Jin, Albert/0000-0003-3826-1081; Zhang, Fuwu/0000-0002-1928-3472; Zhang, Fuwu/0000-0002-1928-3472; Liu, Yijing/0000-0002-7503-3910; Zhu, Guizhi/0000-0002-2945-7982; Chen, Xiaoyuan/0000-0002-9622-0870
FU National Institute of Biomedical Imaging and Bioengineering
FX We appreciate access to the Flow Cytometry Core Facility at the Vaccine Research Center (VRC) of National Institute of Allergy and Infectious Diseases (NIAID), and the provision of MC38 cells from the Dr Robert Seder Laboratory at VRC and DC2.4 cells from Dr Jonathan W. Yewdell Laboratory at NIAID. We thank Ms. Lynne Holtzclaw and Dr Vincent Schram at the Microscopy & Imaging Core (MIC) of National Institute of Child Health and Human Development (NICHD) for help with confocal microscopy. We thank Mr. Geoffrey Daniels (Leica Microsystems Inc.) for technical assistance with confocal microscopy. This work was supported by Intramural Research Program of the National Institute of Biomedical Imaging and Bioengineering.
CR Alexandrov LB, 2013, NATURE, V500, P415, DOI 10.1038/nature12477
Alshamsan A, 2010, BIOMATERIALS, V31, P1420, DOI 10.1016/j.biomaterials.2009.11.003
Carreno BM, 2015, SCIENCE, V348, P803, DOI 10.1126/science.aaa3828
Chen Q, 2016, NAT COMMUN, V7, DOI 10.1038/ncomms13193
Cheng FD, 2003, IMMUNITY, V19, P425, DOI 10.1016/S1074-7613(03)00232-2
Cohen CJ, 2015, J CLIN INVEST, V125, P3981, DOI 10.1172/JCI82416
Cutler JI, 2012, J AM CHEM SOC, V134, P1376, DOI 10.1021/ja209351u
Dan L, 2010, ADV DRUG DELIV REV, V62, P591
Dykxhoorn DM, 2003, NAT REV MOL CELL BIO, V4, P457, DOI 10.1038/nrm1129
ELLINGTON AD, 1990, NATURE, V346, P818, DOI 10.1038/346818a0
Engler AC, 2009, ANGEW CHEM INT EDIT, V48, P9334, DOI 10.1002/anie.200904070
Fan JW, 2014, POLYM CHEM-UK, V5, P3977, DOI 10.1039/c4py00628c
Gros A, 2016, NAT MED, V22, P433, DOI 10.1038/nm.4051
Gubin MM, 2014, NATURE, V515, P577, DOI 10.1038/nature13988
Hartmann G, 1999, P NATL ACAD SCI USA, V96, P9305, DOI 10.1073/pnas.96.16.9305
Hubbell JA, 2009, NATURE, V462, P449, DOI 10.1038/nature08604
Irvine DJ, 2015, CHEM REV, V115, P11109, DOI 10.1021/acs.chemrev.5b00109
Jang M, 2015, NAT COMMUN, V6, DOI 10.1038/ncomms8930
Kawai T, 2010, NAT IMMUNOL, V11, P373, DOI 10.1038/ni.1863
Keefe AD, 2010, NAT REV DRUG DISCOV, V9, P537, DOI 10.1038/nrd3141
Kim JH, 2012, ANGEW CHEM INT EDIT, V51, P9670, DOI 10.1002/anie.201204989
Klinman DM, 2004, NAT REV IMMUNOL, V4, P248, DOI 10.1038/nri1329
Kortylewski M, 2009, NAT BIOTECHNOL, V27, P925, DOI 10.1038/nbt.1564
Kortylewski M, 2009, CANCER RES, V69, P2497, DOI 10.1158/0008-5472.CAN-08-3031
Kuai R, 2017, NAT MATER, V16, P489, DOI [10.1038/nmat4822, 10.1038/NMAT4822]
Lee JB, 2012, NAT MATER, V11, P316, DOI [10.1038/NMAT3253, 10.1038/nmat3253]
Luo M., 2017, NAT NANOTECHNOL
McGranahan N, 2016, SCIENCE, V351, P1463, DOI 10.1126/science.aaf1490
Palucka AK, 2016, CELL, V164, P1233, DOI 10.1016/j.cell.2016.01.049
Pashenkov M, 2006, J CLIN ONCOL, V24, P5716, DOI 10.1200/JCO.2006.07.9129
Roh YH, 2016, ANGEW CHEM INT EDIT, V55, P3347, DOI 10.1002/anie.201508978
Rosenberg SA, 2012, SCI TRANSL MED, V4, DOI 10.1126/scitranslmed.3003634
Saini SK, 2013, P NATL ACAD SCI USA, V110, P15383, DOI 10.1073/pnas.1308672110
Salzano G, 2015, CURR PHARM DESIGN, V21, P4566, DOI 10.2174/1381612821131151013190410
Schumacher TN, 2015, SCIENCE, V348, P69, DOI 10.1126/science.aaa4971
Seeman NC, 2010, ANNU REV BIOCHEM, V79, P65, DOI 10.1146/annurev-biochem-060308-102244
Shu D, 2011, NAT NANOTECHNOL, V6, P658, DOI [10.1038/nnano.2011.105, 10.1038/NNANO.2011.105]
Tran E, 2017, NAT IMMUNOL, V18, P255, DOI 10.1038/ni.3682
TUERK C, 1990, SCIENCE, V249, P505, DOI 10.1126/science.2200121
Yadav M, 2014, NATURE, V515, P572, DOI 10.1038/nature14001
York AG, 2013, NAT METHODS, V10, P1122, DOI [10.1038/NMETH.2687, 10.1038/nmeth.2687]
Yu H, 2014, NAT REV CANCER, V14, P736, DOI 10.1038/nrc3818
Yu H, 2009, NAT REV CANCER, V9, P798, DOI 10.1038/nrc2734

Zhang LY, 2009, GLIA, V57, P1458, DOI 10.1002/glia.20863
Zhu GZ, 2017, ACS NANO, V11, P2387, DOI 10.1021/acsnano.7b00978
Zhu GZ, 2016, NANOSCALE, V8, P6684, DOI 10.1039/c5nr08821f
Zhu GZ, 2013, J AM CHEM SOC, V135, P16438, DOI 10.1021/ja406115e
Zhu GZ, 2013, P NATL ACAD SCI USA, V110, P7998, DOI 10.1073/pnas.1220817110
NR 48
TC 187
Z9 192
U1 10
U2 181
PU NATURE PUBLISHING GROUP
PI LONDON
PA MACMILLAN BUILDING, 4 CRINAN ST, LONDON N1 9XW, ENGLAND
SN 2041-1723
J9 NAT COMMUN
JI Nat. Commun.
PD NOV 14
PY 2017
VL 8
AR 1482
DI 10.1038/s41467-017-01386-7
PG 13
WC Multidisciplinary Sciences
WE Science Citation Index Expanded (SCI-EXPANDED)
SC Science & Technology - Other Topics
GA FM5ZU
UT WOS:000415124000009
PM 29133898
OA gold, Green Published
DA 2023-09-14
ER

PT J
AU Ding, BB
Zheng, P
Jiang, F
Zhao, YJ
Wang, MF
Chang, MY
Ma, PA
Lin, J
AF Ding, Binbin
Zheng, Pan
Jiang, Fan
Zhao, Yajie
Wang, Meifang
Chang, Mengyu
Ma, Ping'an
Lin, Jun
TI MnO(x)Nanospikes as Nanoadjuvants and Immunogenic Cell Death Drugs with Enhanced Antitumor Immunity and Antimetastatic Effect
SO ANGEWANDTE CHEMIE-INTERNATIONAL EDITION
LA English
DT Article
DE ferroptosis; immunogenic cell death; immunotherapy; manganese oxide; nanovaccines
ID IN-VIVO; CANCER; ARCHITECTURE
AB Despite the widespread applications of manganese oxide nanomaterials (MONs) in biomedicine, the intrinsic immunogenicity of MONs is still unclear. MnO(x)nanospikes (NSs) as tumor microenvironment (TME)-responsive nanoadjuvants and immunogenic cell death (ICD) drugs are proposed for cancer nanovaccine-based immunotherapy. MnO(x)NSs with large mesoporous structures show ultrahigh loading

efficiencies for ovalbumin and tumor cell fragment. The combination of ICD via chemodynamic therapy and ferroptosis inductions, as well as antigen stimulations, presents a better synergistic immunopotentiation action. Furthermore, the obtained nanovaccines achieve TME-responsive magnetic resonance/photoacoustic dual-mode imaging contrasts, while effectively inhibiting primary/distal tumor growth and tumor metastasis.
C1 [Ding, Binbin; Jiang, Fan; Zhao, Yajie; Wang, Meifang; Chang, Mengyu; Ma, Ping'an; Lin, Jun] Chinese Acad Sci, Changchun Inst Appl Chem, State Key Lab Rare Earth Resource Utilizat, Changchun 130022, Peoples R China.
[Ding, Binbin; Jiang, Fan; Zhao, Yajie; Wang, Meifang; Chang, Mengyu; Ma, Ping'an; Lin, Jun] Univ Sci & Technol China, Hefei 230026, Peoples R China.
[Zheng, Pan] Chinese Acad Sci, Changchun Inst Appl Chem, Key Lab Polymer Ecomat, Changchun 130022, Peoples R China.
C3 Chinese Academy of Sciences; Changchun Institute of Applied Chemistry, CAS; Chinese Academy of Sciences; University of Science & Technology of China, CAS; Chinese Academy of Sciences; Changchun Institute of Applied Chemistry, CAS
RP Ma, PA; Lin, J (通讯作者)，Chinese Acad Sci, Changchun Inst Appl Chem, State Key Lab Rare Earth Resource Utilizat, Changchun 130022, Peoples R China.; Ma, PA; Lin, J (通讯作者)，Univ Sci & Technol China, Hefei 230026, Peoples R China.
EM mapa675@ciac.ac.cn; jlin@ciac.ac.cn
RI Chang, Mengyu/HLG-3876-2023
OI Chang, Mengyu/0000-0001-6112-4451; Ding, Binbin/0000-0002-3717-857X
FU National Natural Science Foundation of China; NSFC [51720105015, 51672269, 51929201, 51922097, 51772124, 51872282]; Science and Technology Cooperation Project between Chinese and Australian Governments [2017YFE0132300]; Key Research Program of Frontier Sciences, CAS [YZDY-SSW-JSC018]; Youth Innovation Promotion Association of CAS [2017273]
FX This work is financially supported by the National Natural Science Foundation of China (Grant No. NSFC 51720105015, 51672269, 51929201, 51922097, 51772124, and 51872282), the Science and Technology Cooperation Project between Chinese and Australian Governments (2017YFE0132300), Key Research Program of Frontier Sciences, CAS (YZDY-SSW-JSC018), and Youth Innovation Promotion Association of CAS (Grant No. 2017273). All animals in this study were handled according to a protocol approved by the Institutional Animal Care and Use Committee of Jilin University.
CR [Anonymous], 2016, ANGEW CHEM-GER EDIT
[Anonymous], 2018, ANGEW CHEM
Askhatova D, 2018, ANGEW CHEM, V130, P1526, DOI DOI 10.1002/ANGE.201710144
Chen J, 2019, NANOSCALE HORIZ, V4, P321, DOI 10.1039/c8nh00274f
Chen QJ, 2019, ADV SCI, V6, DOI 10.1002/advs.201802134
Cho NH, 2011, NAT NANOTECHNOL, V6, P675, DOI [10.1038/NNANO.2011.149, 10.1038/nnano.2011.149]
Ding BB, 2020, ADV MATER, V32, DOI 10.1002/adma.201905823
Ding BB, 2019, CHEM MATER, V31, P2651, DOI 10.1021/acs.chemmater.9b00893
Ding BB, 2018, ADV MATER, V30, DOI 10.1002/adma.201802479
Dixon SJ, 2012, CELL, V149, P1060, DOI 10.1016/j.cell.2012.03.042
Duan X., 2019, ANGEW CHEM, V131, P680, DOI DOI 10.1002/ANGE.201804882
Duan XP, 2019, ANGEW CHEM INT EDIT, V58, P670, DOI 10.1002/anie.201804882
Feng B, 2018, ADV MATER, V30, DOI 10.1002/adma.201803001
He DG, 2017, ADV FUNCT MATER, V27, DOI 10.1002/adfm.201704089
He DG, 2015, CHEM COMMUN, V51, P776, DOI 10.1039/c4cc08172b
Ji XY, 2019, ADV SCI, V6, DOI 10.1002/advs.201901211
Kagan VE, 2017, NAT CHEM BIOL, V13, P81, DOI [10.1038/NCHEMBIO.2238, 10.1038/nchembio.2238]
Kim J, 2015, NAT BIOTECHNOL, V33, P64, DOI 10.1038/nbt.3071
Li HY, 2011, NAT NANOTECHNOL, V6, P645, DOI [10.1038/nnano.2011.153, 10.1038/NNANO.2011.153]
Li W, 2019, NAT COMMUN, V10, DOI 10.1038/s41467-019-11269-8
Li X, 2018, CHEM SOC REV, V47, P4954, DOI 10.1039/c8cs00028j
Lin LS, 2019, J AM CHEM SOC, V141, P9937, DOI 10.1021/jacs.9b03457

   Lin LS, 2018, ANGEW CHEM INT EDIT, V57, P4902, DOI 10.1002/anie.201712027
   Lin LS., 2018, ANGEW CHEM INT EDIT, V130, P4996, DOI [DOI 10.1002/ANGE, 10.1002/ange.201712027]
   Liu YL, 2018, ANGEW CHEM INT EDIT, V57, P1510, DOI 10.1002/anie.201710144
   Meng LT, 2018, ACS NANO, V12, P8308, DOI 10.1021/acsnano.8b03590
   Obeid M, 2007, NAT MED, V13, P54, DOI 10.1038/nm1523
   Pan JB, 2018, ADV MATER, V30, DOI 10.1002/adma.201704408
   Tang W, 2019, ADV MATER, V31, DOI 10.1002/adma.201900401
   Tao W, 2018, ADV MATER, V30, DOI 10.1002/adma.201802061
   Tao W, 2017, ADV MATER, V29, DOI 10.1002/adma.201603276
   Wang SF, 2018, ACS NANO, V12, P12380, DOI 10.1021/acsnano.8b06399
   Wang TT, 2018, NAT COMMUN, V9, DOI 10.1038/s41467-018-03915-4
   Wang XP, 2016, ANGEW CHEM INT EDIT, V55, P1899, DOI 10.1002/anie.201506179
   Wen M., 2019, ANGEW CHEM, V131, P17586, DOI DOI 10.1002/ANGE.201909729
   Wen M, 2019, ANGEW CHEM INT EDIT, V58, P17425, DOI 10.1002/anie.201909729
   Wong DYQ, 2015, ANGEW CHEM INT EDIT, V54, P6483, DOI 10.1002/anie.201500934
   Wong DYQ, 2015, ANGEW CHEM, V127, P6583
   Xia L, 2016, CELL CHEM BIOL, V23, P1515, DOI 10.1016/j.chembiol.2016.10.012
   Yang, 2017, ANGEW CHEM, V129, P8566, DOI DOI 10.1002/ange.201701550
   Yang WS, 2014, CELL, V156, P317, DOI 10.1016/j.cell.2013.12.010
   Yang YN, 2018, ANGEW CHEM INT EDIT, V57, P11764, DOI 10.1002/anie.201807595
   Yang YN, 2017, ANGEW CHEM INT EDIT, V56, P8446, DOI 10.1002/anie.201701550
   Zhang F, 2019, ACS NANO, V13, P5662, DOI 10.1021/acsnano.9b00892
NR 44
TC 179
Z9 180
U1 45
U2 472
PU WILEY-V C H VERLAG GMBH
PI WEINHEIM
PA POSTFACH 101161, 69451 WEINHEIM, GERMANY
SN 1433-7851
EI 1521-3773
J9 ANGEW CHEM INT EDIT
JI Angew. Chem.-Int. Edit.
PD SEP 14
PY 2020
VL 59
IS 38
BP 16381
EP 16384
DI 10.1002/anie.202005111
EA JUL 2020
PG 4
WC Chemistry, Multidisciplinary
WE Science Citation Index Expanded (SCI-EXPANDED)
SC Chemistry
GA NK7IG
UT WOS:000548131100001
PM 32484598
HC Y
HP N
DA 2023-09-14
ER

PT J
AU Zhou, JR
   Kroll, AV
   Holay, M
   Fang, RH
   Zhang, LF

AF Zhou, Jiarong
Kroll, Ashley, V
Holay, Maya
Fang, Ronnie H.
Zhang, Liangfang
TI Biomimetic Nanotechnology toward Personalized Vaccines
SO ADVANCED MATERIALS
LA English
DT Article
DE anticancer vaccinations; bacteria vaccinations; biomimetic
nanoparticles; nanomedicine; personalized medicine
ID ANTIGEN-PRESENTING CELLS; OUTER-MEMBRANE VESICLES; IN-SITU VACCINATION;
VIRUS-LIKE PARTICLES; DENDRITIC CELLS; POLYMER NANOPARTICLES;
CANCER-IMMUNOTHERAPY; DRUG-DELIVERY; ANTITUMOR IMMUNITY;
CROSS-PRESENTATION
AB While traditional approaches for disease management in the era of modern medicine have saved countless lives and enhanced patient well-being, it is clear that there is significant room to improve upon the current status quo. For infectious diseases, the steady rise of antibiotic resistance has resulted in super pathogens that do not respond to most approved drugs. In the field of cancer treatment, the idea of a cure-all silver bullet has long been abandoned. As a result of the challenges facing current treatment and prevention paradigms in the clinic, there is an increasing push for personalized therapeutics, where plans for medical care are established on a patient-by-patient basis. Along these lines, vaccines, both against bacteria and tumors, are a clinical modality that could benefit significantly from personalization. Effective vaccination strategies could help to address many challenging disease conditions, but current vaccines are limited by factors such as a lack of potency and antigenic breadth. Recently, researchers have turned toward the use of biomimetic nanotechnology as a means of addressing these hurdles. Recent progress in the development of biomimetic nanovaccines for antibacterial and anticancer applications is discussed, with an emphasis on their potential for personalized medicine.
C1 [Zhou, Jiarong; Kroll, Ashley, V; Holay, Maya; Fang, Ronnie H.; Zhang, Liangfang] Univ Calif San Diego, Dept NanoEngn, Chem Engn Program, La Jolla, CA 92093 USA.
[Zhou, Jiarong; Kroll, Ashley, V; Holay, Maya; Fang, Ronnie H.; Zhang, Liangfang] Univ Calif San Diego, Moores Canc Ctr, La Jolla, CA 92093 USA.
C3 University of California System; University of California San Diego;
University of California System; University of California San Diego
RP Fang, RH; Zhang, LF (通讯作者)，Univ Calif San Diego, Dept NanoEngn, Chem Engn Program, La Jolla, CA 92093 USA.; Fang, RH; Zhang, LF (通讯作者)，Univ Calif San Diego, Moores Canc Ctr, La Jolla, CA 92093 USA.
EM rhfang@ucsd.edu; zhang@ucsd.edu
RI Fang, Ronnie H./HCH-5747-2022
OI Zhang, Liangfang/0000-0003-0637-0654
FU Defense Threat Reduction Agency [HDTRA1-18-1-0014] Funding Source:
Medline; Defense Threat Reduction Agency Joint Science and Technology
Office for Chemical and Biological Defense [HDTRA1-18-1-0014] Funding
Source: Medline; NCI NIH HHS [T32 CA153915] Funding Source: Medline; NIH
HHS [5T32CA153915] Funding Source: Medline
CR Alanis AJ, 2005, ARCH MED RES, V36, P697, DOI 10.1016/j.arcmed.2005.06.009
Angsantikul P, 2018, ADV THER-GERMANY, V1, DOI 10.1002/adtp.201800016
Angsantikul P, 2018, BIOCONJUGATE CHEM, V29, P604, DOI 10.1021/acs.bioconjchem.7b00692
Bachmann MF, 2010, NAT REV IMMUNOL, V10, P787, DOI 10.1038/nri2868
Bale SS, 2010, ACS NANO, V4, P1493, DOI 10.1021/nn901586e
Banchereau J, 2005, NAT REV IMMUNOL, V5, P296, DOI 10.1038/nri1592
Bar-Cohen Y, 2006, BIOINSPIR BIOMIM, V1, pP1, DOI 10.1088/1748-3182/1/1/P01
Barbosa TM, 2000, DRUG RESIST UPDATE, V3, P303, DOI 10.1054/drup.2000.0167
Batista FD, 2009, NAT REV IMMUNOL, V9, P15, DOI 10.1038/nri2454
Bennadi Darshana, 2013, J Basic Clin Pharm, V5, P19, DOI 10.4103/0976-0105.128253
Bousso P, 2003, NAT IMMUNOL, V4, P579, DOI 10.1038/ni928
Camacho AI, 2013, VACCINE, V31, P3288, DOI 10.1016/j.vaccine.2013.05.020
Carreno BM, 2015, SCIENCE, V348, P803, DOI 10.1126/science.aaa3828
Cegelski L, 2008, NAT REV MICROBIOL, V6, P17, DOI 10.1038/nrmicro1818
Chackerian B, 2007, EXPERT REV VACCINES, V6, P381, DOI 10.1586/14760584.6.3.381
Chai ZL, 2017, J CONTROL RELEASE, V264, P102, DOI 10.1016/j.jconrel.2017.08.027

Chen DJ, 2010, P NATL ACAD SCI USA, V107, P3099, DOI 10.1073/pnas.0805532107
Chen L, 2016, P NATL ACAD SCI USA, V113, pE3609, DOI 10.1073/pnas.1518311113
Chen WS, 2016, NANOSCALE, V8, P10364, DOI 10.1039/c6nr00535g
Chen YJ, 2018, ADV HEALTHC MATER, V7, DOI 10.1002/adhm.201701366
Chen Z, 2016, ACS NANO, V10, P10049, DOI 10.1021/acsnano.6b04695
Cheng H, 2016, ADV FUNCT MATER, V26, P7847, DOI 10.1002/adfm.201603212
Cheung AS, 2016, SMALL, V12, P2321, DOI 10.1002/smll.201600061
Cho NH, 2011, NAT NANOTECHNOL, V6, P675, DOI [10.1038/NNANO.2011.149, 10.1038/nnano.2011.149]
Copp JA, 2014, P NATL ACAD SCI USA, V111, P13481, DOI 10.1073/pnas.1412420111
Czapar AE, 2018, ADV SCI, V5, DOI 10.1002/advs.201700991
Dagogo-Jack I, 2018, NAT REV CLIN ONCOL, V15, P81, DOI 10.1038/nrclinonc.2017.166
Dakterzada F, 2016, VACCINE, V34, P1472, DOI 10.1016/j.vaccine.2016.01.041
Danhier F, 2009, J CONTROL RELEASE, V140, P166, DOI 10.1016/j.jconrel.2009.08.011
DAVIES J, 1994, SCIENCE, V264, P375, DOI 10.1126/science.8153624
de la Fuente-Nunez C, 2017, CURR OPIN MICROBIOL, V37, P95, DOI 10.1016/j.mib.2017.05.014
Dehaini D, 2017, ADV MATER, V29, DOI 10.1002/adma.201606209
Dehaini D, 2016, BIOENG TRANSL MED, V1, P30, DOI 10.1002/btm2.10004
Demento SL, 2012, BIOMATERIALS, V33, P4957, DOI 10.1016/j.biomaterials.2012.03.041
Deng GJ, 2018, ACS NANO, V12, P12096, DOI 10.1021/acsnano.8b05292
DeWeerdt S, 2017, NATURE, V552, P576, DOI 10.1038/d41586-017-08706-3
Dickey SW, 2017, NAT REV DRUG DISCOV, V16, P457, DOI 10.1038/nrd.2017.23
Douglas T, 2006, SCIENCE, V312, P873, DOI 10.1126/science.1123223
Dranoff G, 2004, NAT REV CANCER, V4, P11, DOI 10.1038/nrc1252
Dziubla TD, 2005, J CONTROL RELEASE, V102, P427, DOI 10.1016/j.jconrel.2004.10.017
Eggermont LJ, 2014, TRENDS BIOTECHNOL, V32, P456, DOI 10.1016/j.tibtech.2014.06.007
Embgenbroich M, 2018, FRONT IMMUNOL, V9, DOI 10.3389/fimmu.2018.01643
Fan YC, 2017, NANO LETT, V17, P7387, DOI 10.1021/acs.nanolett.7b03218
Fang RH, 2018, ADV MATER, V30, DOI 10.1002/adma.201706759
Fang RH, 2017, BIOMATERIALS, V128, P69, DOI 10.1016/j.biomaterials.2017.02.041
Fang RNH, 2016, ANNU REV CHEM BIOMOL, V7, P305, DOI 10.1146/annurev-chembioeng-080615-034446
Fang RH, 2015, SMALL, V11, P5483, DOI 10.1002/smll.201501284
Fang RH, 2015, ADV DRUG DELIVER REV, V90, P69, DOI 10.1016/j.addr.2015.04.001
Fang RH, 2014, NANO LETT, V14, P2181, DOI 10.1021/nl500618u
Fang RNH, 2013, NANOSCALE, V5, P8884, DOI 10.1039/c3nr03064d
Fang RH, 2012, EXPERT OPIN BIOL TH, V12, P385, DOI 10.1517/14712598.2012.661710
Fernandez A, 2011, J IMMUNOL, V186, P264, DOI 10.4049/jimmunol.1001465
Fesnak AD, 2016, NAT REV CANCER, V16, P566, DOI 10.1038/nrc.2016.97
Fidler IJ, 2003, NAT REV CANCER, V3, P453, DOI 10.1038/nrc1098
Fischer NO, 2013, J AM CHEM SOC, V135, P2044, DOI 10.1021/ja3063293
Fitzgerald JR, 2006, NAT REV MICROBIOL, V4, P445, DOI 10.1038/nrmicro1425
Fontana F, 2017, ADV MATER, V29, DOI 10.1002/adma.201603239
Galluzzi L, 2017, NAT REV IMMUNOL, V17, P97, DOI 10.1038/nri.2016.107
Gao GP, 2002, P NATL ACAD SCI USA, V99, P11854, DOI 10.1073/pnas.182412299
Gao WW, 2015, NANO LETT, V15, P1403, DOI 10.1021/nl504798g
Gao WW, 2014, WIRES NANOMED NANOBI, V6, P532, DOI 10.1002/wnan.1282
Geddes K, 2009, NAT REV DRUG DISCOV, V8, P465, DOI 10.1038/nrd2783
Gerritzen MJH, 2017, BIOTECHNOL ADV, V35, P565, DOI 10.1016/j.biotechadv.2017.05.003
Gil-Torregrosa BC, 2004, EUR J IMMUNOL, V34, P398, DOI 10.1002/eji.200324508
Ginsburg GS, 2001, TRENDS BIOTECHNOL, V19, P491, DOI 10.1016/S0167-7799(01)01814-5
Glinsky VV, 2003, CANCER RES, V63, P3805
Grimm SK, 2013, CURR OPIN BIOTECH, V24, P1078, DOI 10.1016/j.copbio.2013.02.015
Guo YY, 2015, ACS NANO, V9, P6918, DOI 10.1021/acsnano.5b01042
Hamburg MA, 2010, NEW ENGL J MED, V363, P301, DOI 10.1056/NEJMp1006304
Hanlon DJ, 2011, AM J REPROD IMMUNOL, V65, P597, DOI 10.1111/j.1600-0897.2010.00968.x
He Q, 2000, CLIN DIAGN LAB IMMUN, V7, P899, DOI 10.1128/CDLI.7.6.899-903.2000
Hickey JW, 2017, NANO LETT, V17, P7045, DOI 10.1021/acs.nanolett.7b03734
Hoopes PJ, 2018, MOL PHARMACEUT, V15, P3717, DOI 10.1021/acs.molpharmaceut.8b00126
Hoshino Y, 2010, J AM CHEM SOC, V132, P6644, DOI 10.1021/ja102148f
Hoshino Y, 2009, SMALL, V5, P1562, DOI 10.1002/smll.200900186

Hoshino Y, 2008, J AM CHEM SOC, V130, P15242, DOI 10.1021/ja8062875
Hotchkiss RS, 2013, NAT REV IMMUNOL, V13, P862, DOI 10.1038/nri3552
Hu CMJ, 2015, NATURE, V526, P118, DOI 10.1038/nature15373
Hu CMJ, 2013, NAT NANOTECHNOL, V8, P933, DOI [10.1038/nnano.2013.254, 10.1038/NNANO.2013.254]
Hu CMJ, 2013, NAT NANOTECHNOL, V8, P336, DOI [10.1038/nnano.2013.54, 10.1038/NNANO.2013.54]
Hu CMJ, 2012, ADV HEALTHC MATER, V1, P537, DOI 10.1002/adhm.201200138
Hu CMJ, 2011, P NATL ACAD SCI USA, V108, P10980, DOI 10.1073/pnas.1106634108
Hu QY, 2018, NAT BIOMED ENG, V2, P831, DOI 10.1038/s41551-018-0310-2
Iwasaki A, 2010, SCIENCE, V327, P291, DOI 10.1126/science.1183021
Jackson HJ, 2016, NAT REV CLIN ONCOL, V13, P370, DOI 10.1038/nrclinonc.2016.36
Jiang Y, 2019, CURR OPIN BIOTECH, V58, P1, DOI 10.1016/j.copbio.2018.10.005
Jobsri J, 2015, PLOS ONE, V10, DOI 10.1371/journal.pone.0118096
Joffre OP, 2012, NAT REV IMMUNOL, V12, P557, DOI 10.1038/nri3254
June CH, 2018, NEW ENGL J MED, V379, P64, DOI 10.1056/NEJMra1706169
June CH, 2018, SCIENCE, V359, P1361, DOI 10.1126/science.aar6711
Kambayashi T, 2014, NAT REV IMMUNOL, V14, P719, DOI 10.1038/nri3754
Kang T, 2018, BIOMATERIALS, V164, P80, DOI 10.1016/j.biomaterials.2018.02.033
Kaufmann SHE, 2018, NAT REV DRUG DISCOV, V17, P35, DOI 10.1038/nrd.2017.162
Kawai T, 2010, NAT IMMUNOL, V11, P373, DOI 10.1038/ni.1863
Keenan BP, 2012, SEMIN ONCOL, V39, P276, DOI 10.1053/j.seminoncol.2012.02.007
Kenyon C, 2005, CELL, V120, P449, DOI 10.1016/j.cell.2005.02.002
Kim OY, 2017, NAT COMMUN, V8, DOI 10.1038/s41467-017-00729-8
Kosmides AK, 2018, NANO LETT, V18, P1916, DOI 10.1021/acs.nanolett.7b05284
Kotterman MA, 2014, NAT REV GENET, V15, P445, DOI 10.1038/nrg3742
Kroll AV, 2019, ADV BIOSYST, V3, DOI 10.1002/adbi.201800219
Kroll AV, 2017, ADV MATER, V29, DOI 10.1002/adma.201703969
Kroll AV, 2017, BIOCONJUGATE CHEM, V28, P23, DOI 10.1021/acs.bioconjchem.6b00569
Krysko DV, 2012, NAT REV CANCER, V12, P860, DOI 10.1038/nrc3380
Kuai R, 2018, SCI ADV, V4, DOI 10.1126/sciadv.aao1736
Kuai R, 2017, NAT MATER, V16, P489, DOI [10.1038/nmat4822, 10.1038/NMAT4822]
Kumar H, 2009, BIOCHEM J, V420, P1, DOI 10.1042/BJ20090272
Laidlaw BJ, 2016, NAT REV IMMUNOL, V16, P102, DOI 10.1038/nri.2015.10
Lapek JD, 2017, ACS NANO, V11, P11831, DOI 10.1021/acsnano.7b02650
Lebel ME, 2017, J IMMUNOL, V198, P292, DOI 10.4049/jimmunol.1601271
Lebel ME, 2016, NANO LETT, V16, P1826, DOI 10.1021/acs.nanolett.5b04877
Lee KL, 2017, NANO LETT, V17, P4019, DOI [10.1021/acs.nanolett.7b00107, 10.1021/acs.nanolett.7600107]
Leelatian N, 2017, CYTOM PART B-CLIN CY, V92, P68, DOI 10.1002/cyto.b.21481
Levy SB, 2004, NAT MED, V10, pS122, DOI 10.1038/nm1145
Lewis K, 2013, NAT REV DRUG DISCOV, V12, P371, DOI 10.1038/nrd3975
Li AV, 2013, SCI TRANSL MED, V5, DOI 10.1126/scitranslmed.3006516
Li HY, 2011, NAT NANOTECHNOL, V6, P645, DOI [10.1038/nnano.2011.153, 10.1038/NNANO.2011.153]
Li SY, 2018, BIOMATERIALS, V151, P1, DOI 10.1016/j.biomaterials.2017.10.021
Lichtenstein P, 2000, NEW ENGL J MED, V343, P78, DOI 10.1056/NEJM200007133430201
Lichty BD, 2014, NAT REV CANCER, V14, P559, DOI 10.1038/nrc3770
Lin JC, 2019, NAT COMMUN, V10, DOI 10.1038/s41467-019-09049-5
Lin LCW, 2018, ADV HEALTHC MATER, V7, DOI 10.1002/adhm.201701395
Liu SY, 2013, BIOMATERIALS, V34, P8291, DOI 10.1016/j.biomaterials.2013.07.020
Lizotte PH, 2016, NAT NANOTECHNOL, V11, P295, DOI [10.1038/NNANO.2015.292, 10.1038/nnano.2015.292]
Lokhov PG, 2010, J CANCER, V1, P230
Lovly CM, 2011, NAT REV CLIN ONCOL, V8, P68, DOI 10.1038/nrclinonc.2010.224
Luk BT, 2016, THERANOSTICS, V6, P1004, DOI 10.7150/thno.14471
Luo M, 2017, NAT NANOTECHNOL, V12, P648, DOI [10.1038/nnano.2017.52, 10.1038/NNANO.2017.52]
Manolova V, 2008, EUR J IMMUNOL, V38, P1404, DOI 10.1002/eji.200737984
Mastrobattista E, 2006, NAT REV DRUG DISCOV, V5, P115, DOI 10.1038/nrd1960
Meacham CE, 2013, NATURE, V501, P328, DOI 10.1038/nature12624

Mellbye B, 2011, MBIO, V2, DOI 10.1128/mBio.00131-11
Menendez JA, 2007, NAT REV CANCER, V7, P763, DOI 10.1038/nrc2222
Meric-Bernstam F, 2012, NAT REV CLIN ONCOL, V9, P542, DOI 10.1038/nrclinonc.2012.127
Meyer RA, 2015, SMALL, V11, P1519, DOI 10.1002/smll.201402369
Moon JJ, 2011, NAT MATER, V10, P243, DOI 10.1038/NMAT2960
Mosmann TR, 1996, IMMUNOL TODAY, V17, P138, DOI 10.1016/0167-5699(96)80606-2
MOSMANN TR, 1989, ANNU REV IMMUNOL, V7, P145, DOI 10.1146/annurev.immunol.7.1.145
Murray AA, 2018, MOL PHARMACEUT, V15, P3700, DOI 10.1021/acs.molpharmaceut.8b00316
Nieswandt B, 2003, BLOOD, V102, P449, DOI 10.1182/blood-2002-12-3882
Nirthanan S, 2002, J BIOL CHEM, V277, P17811, DOI 10.1074/jbc.M111152200
Nishino M, 2017, NAT REV CLIN ONCOL, V14, P655, DOI 10.1038/nrclinonc.2017.88
NOELLE RJ, 1992, IMMUNOL TODAY, V13, P431, DOI 10.1016/0167-5699(92)90068-I
Ochman H, 2000, NATURE, V405, P299, DOI 10.1038/35012500
Ochyl LJ, 2018, BIOMATERIALS, V182, P157, DOI 10.1016/j.biomaterials.2018.08.016
Ogino S, 2011, NAT REV CLIN ONCOL, V8, P711, DOI 10.1038/nrclinonc.2011.122
Ogretmen B, 2004, NAT REV CANCER, V4, P604, DOI 10.1038/nrc1411
Ott PA, 2017, NATURE, V547, P217, DOI 10.1038/nature22991
Pardoll DM, 2012, NAT REV CANCER, V12, P252, DOI 10.1038/nrc3239
Park J, 2012, CANCER GENE THER, V19, P741, DOI 10.1038/cgt.2012.64
Parodi A, 2013, NAT NANOTECHNOL, V8, P61, DOI 10.1038/nnano.2012.212
Patel R, 2018, ACS OMEGA, V3, P3702, DOI 10.1021/acsomega.8b00227
Perica K, 2014, NANOMED-NANOTECHNOL, V10, P119, DOI 10.1016/j.nano.2013.06.015
Peruzzi PP, 2016, NAT NANOTECHNOL, V11, P214, DOI 10.1038/nnano.2015.306
Petousis-Harris H, 2017, LANCET, V390, P1603, DOI 10.1016/S0140-6736(17)31449-6
Petrovsky N, 2004, IMMUNOL CELL BIOL, V82, P488, DOI 10.1111/j.0818-9641.2004.01272.x
PIERSCHBACHER MD, 1984, NATURE, V309, P30, DOI 10.1038/309030a0
Prasad S, 2011, NANOMED-NANOTECHNOL, V7, P1, DOI 10.1016/j.nano.2010.07.002
Rajewsky K, 1996, NATURE, V381, P751, DOI 10.1038/381751a0
Rao L, 2016, ADV MATER, V28, P3460, DOI 10.1002/adma.201506086
Rasko DA, 2010, NAT REV DRUG DISCOV, V9, P117, DOI 10.1038/nrd3013
Reddy ST, 2007, NAT BIOTECHNOL, V25, P1159, DOI [10.1038/nbt1332, 10.1038/nbtl332]
Sousa CR, 2006, NAT REV IMMUNOL, V6, P476, DOI 10.1038/nri1845
Ribas A, 2018, SCIENCE, V359, P1350, DOI 10.1126/science.aar4060
Roldao A, 2010, EXPERT REV VACCINES, V9, P1149, DOI [10.1586/erv.10.115, 10.1586/ERV.10.115]
Romero D, 2017, NAT REV CLIN ONCOL, V14, DOI 10.1038/nrclinonc.2017.16
Rosenberg SA, 2004, NAT MED, V10, P909, DOI 10.1038/nm1100
Rosenberg SA, 2008, NAT REV CANCER, V8, P299, DOI 10.1038/nrc2355
Rosenberg SA, 2015, SCIENCE, V348, P62, DOI 10.1126/science.aaa4967
Russell SJ, 2012, NAT BIOTECHNOL, V30, P658, DOI 10.1038/nbt.2287
Sahin U, 2017, NATURE, V547, P222, DOI 10.1038/nature23003
Schumacher TN, 2015, SCIENCE, V348, P69, DOI 10.1126/science.aaa4971
Schwechheimer C, 2015, NAT REV MICROBIOL, V13, P605, DOI 10.1038/nrmicro3525
Shae D, 2019, NAT NANOTECHNOL, V14, P269, DOI 10.1038/s41565-018-0342-5
Shi GN, 2017, BIOMATERIALS, V113, P191, DOI 10.1016/j.biomaterials.2016.10.047
Shukla S, 2017, BIOMATERIALS, V121, P15, DOI 10.1016/j.biomaterials.2016.12.030
Silva JM, 2015, J CONTROL RELEASE, V198, P91, DOI 10.1016/j.jconrel.2014.11.033
Solbrig CM, 2007, MOL PHARMACEUT, V4, P47, DOI 10.1021/mp060107e
Song QL, 2017, NANO LETT, V17, P6366, DOI 10.1021/acs.nanolett.7b03186
Sorlie T, 2001, P NATL ACAD SCI USA, V98, P10869, DOI 10.1073/pnas.191367098
Stahl PD, 1998, CURR OPIN IMMUNOL, V10, P50, DOI 10.1016/S0952-7915(98)80031-9
Stary G, 2015, SCIENCE, V348, DOI 10.1126/science.aaa8205
Stevenson TC, 2018, P NATL ACAD SCI USA, V115, pE3106, DOI 10.1073/pnas.1718341115
Stojdl DF, 2000, NAT MED, V6, P821, DOI 10.1038/77558
Tam HH, 2016, P NATL ACAD SCI USA, V113, pE6639, DOI 10.1073/pnas.1606050113
Tan K, 2018, FRONT MICROBIOL, V9, DOI 10.3389/fmicb.2018.00783
Thamphiwatana S, 2017, P NATL ACAD SCI USA, V114, P11488, DOI 10.1073/pnas.1714267114
Tumeh PC, 2014, NATURE, V515, P568, DOI 10.1038/nature13954
Umamaheshwari RB, 2003, J DRUG TARGET, V11, P415, DOI 10.1080/10611860310001647771
UNANUE ER, 1984, ANNU REV IMMUNOL, V2, P395, DOI 10.1146/annurev.iy.02.040184.002143
van der Burg SH, 2016, NAT REV CANCER, V16, P219, DOI 10.1038/nrc.2016.16
Vargo-Gogola T, 2007, NAT REV CANCER, V7, P659, DOI 10.1038/nrc2193

Waehler R, 2007, NAT REV GENET, V8, P573, DOI 10.1038/nrg2141
Walport MJ, 2001, NEW ENGL J MED, V344, P1058, DOI 10.1056/NEJM200104053441406
Wang C, 2017, NAT BIOMED ENG, V1, DOI 10.1038/s41551-016-0011
Wang F, 2016, ADV FUNCT MATER, V26, P1628, DOI 10.1002/adfm.201505231
Wang XG, 2018, NAT COMMUN, V9, DOI 10.1038/s41467-018-03847-z
Webster R, 2014, NAT REV DRUG DISCOV, V13, P883, DOI 10.1038/nrd4476
Wei XL, 2018, ACS NANO, V12, P109, DOI 10.1021/acsnano.7b07720
Wei XL, 2017, ADV MATER, V29, DOI 10.1002/adma.201701644
Wei XL, 2016, BIOMATERIALS, V111, P116, DOI 10.1016/j.biomaterials.2016.10.003
Wei XL, 2015, J CONTROL RELEASE, V218, P13, DOI 10.1016/j.jconrel.2015.09.059
Wei XL, 2015, ANGEW CHEM INT EDIT, V54, P3023, DOI 10.1002/anie.201411226
Wilson DR, 2018, NANOMED-NANOTECHNOL, V14, P237, DOI 10.1016/j.nano.2017.10.013
Yang R, 2018, ACS NANO, V12, P5121, DOI 10.1021/acsnano.7b09041
Yeaman MR, 2010, CELL MOL LIFE SCI, V67, P525, DOI 10.1007/s00018-009-0210-4
Ying M, 2018, ADV FUNCT MATER, V28, DOI 10.1002/adfm.201801032
Yoo JW, 2011, NAT REV DRUG DISCOV, V10, P521, DOI 10.1038/nrd3499
Yu GT, 2018, ADV FUNCT MATER, V28, DOI 10.1002/adfm.201801389
Zhan CY, 2011, ANGEW CHEM INT EDIT, V50, P5482, DOI 10.1002/anie.201100875
Zhang QB, 2017, ACS NANO, V11, P10724, DOI 10.1021/acsnano.7b04955
Zhang XL, 2018, VACCINE, V36, P1047, DOI 10.1016/j.vaccine.2018.01.034
Zhang XD, 2018, NANO LETT, V18, P5716, DOI 10.1021/acs.nanolett.8b02321
Zhang XD, 2018, ADV MATER, V30, DOI 10.1002/adma.201707112
Zhou H, 2016, THERANOSTICS, V6, P1012, DOI 10.7150/thno.15095
Zhuang J, 2018, ADV MATER, V30, DOI 10.1002/adma.201804693
NR 215
TC 166
Z9 170
U1 38
U2 505
PU WILEY-V C H VERLAG GMBH
PI WEINHEIM
PA POSTFACH 101161, 69451 WEINHEIM, GERMANY
SN 0935-9648
EI 1521-4095
J9 ADV MATER
JI Adv. Mater.
PD APR
PY 2020
VL 32
IS 13
SI SI
AR 1901255
DI 10.1002/adma.201901255
PG 22
WC Chemistry, Multidisciplinary; Chemistry, Physical; Nanoscience &
Nanotechnology; Materials Science, Multidisciplinary; Physics, Applied;
Physics, Condensed Matter
WE Science Citation Index Expanded (SCI-EXPANDED)
SC Chemistry; Science & Technology - Other Topics; Materials Science;
Physics
GA LB2XE
UT WOS:000524500400010
PM 31206841
OA Green Accepted
HC Y
HP N
DA 2023-09-14
ER

PT J
AU Musetti, S

Huang, L
AF Musetti, Sara
Huang, Leaf
TI Nanoparticle-Mediated Remodeling of the Tumor Microenvironment to
Enhance Immunotherapy
SO ACS NANO
LA English
DT Article
DE nanoparticles; cancer; immunotherapy; tumor microenvironment;
nanovaccine; cytokines; checkpoint inhibitors; immunogenic cell death;
cytotoxic T cells
ID IMMUNOGENIC CELL-DEATH; CANCER-ASSOCIATED FIBROBLASTS; REGULATORY
T-CELLS; INFILTRATING LYMPHOCYTES; PHOTODYNAMIC THERAPY; FAVORABLE
PROGNOSIS; ANTITUMOR IMMUNITY; DENDRITIC CELLS; DRUG-DELIVERY;
BREAST-CANCER
AB Nanoscience has long been lauded as a method through which tumor-associated barriers could be overcome. As successful as cancer immunotherapy has been, limitations associated with the tumor microenvironment or side effects of systemic treatment have become more apparent. In this Review, we seek to lay out the therapeutic challenges associated with the tumor microenvironment and the ways in which nanoscience is being applied to remodel the tumor microenvironment and increase the susceptibility of many cancer types to immunotherapy. We detail the nanomedicines on the cutting edge of cancer immunotherapy and how their interactions with the tumor microenvironment make them more effective than systemically administered immunotherapies.
C1 [Musetti, Sara; Huang, Leaf] Univ N Carolina, Eshelman Sch Pharm, Div Pharmacoengn & Mol Pharmaceut, Chapel Hill, NC 27599 USA.
C3 University of North Carolina; University of North Carolina Chapel Hill
RP Huang, L (通讯作者)，Univ N Carolina, Eshelman Sch Pharm, Div Pharmacoengn & Mol Pharmaceut, Chapel Hill, NC 27599 USA.
EM leafh@email.unc.edu
OI Musetti Jenkins, PhD, Sara/0000-0002-5682-1225
FU Carolina Center for Cancer Nanotechnology Excellence (NIH) [CA198999]
FX We thank and acknowledge the support of Dr. W. Song in the preparation
of this manuscript as well as the Carolina Center for Cancer
Nanotechnology Excellence (NIH grant no. CA198999) for their support in
funding our research. Graphics were created using BioRender.
CR AALTOMAA S, 1992, EUR J CANCER, V28A, P859, DOI 10.1016/0959-8049(92)90134-N
Alexe G, 2007, CANCER RES, V67, P10669, DOI 10.1158/0008-5472.CAN-07-0539
[Anonymous], NAT COMMUN
Arrowsmith J, 2013, NAT REV DRUG DISCOV, V12, P568, DOI 10.1038/nrd4090
Arvizo RR, 2011, NANOMED-NANOTECHNOL, V7, P580, DOI 10.1016/j.nano.2011.01.011
Azimi F, 2012, J CLIN ONCOL, V30, P2678, DOI 10.1200/JCO.2011.37.8539
Banerjee K, 2017, CANCER RES, V77, DOI 10.1158/1538-7445.AM2017-3678
Baras AS, 2016, ONCOIMMUNOLOGY, V5, DOI 10.1080/2162402X.2015.1134412
de Faria PCB, 2014, NANO LETT, V14, P5458, DOI 10.1021/nl502911a
Bezu L, 2015, FRONT IMMUNOL, V6, DOI 10.3389/fimmu.2015.00187
Bonertz A, 2009, J CLIN INVEST, V119, P3311, DOI 10.1172/JCI39608
Cao FQ, 2018, ADV HEALTHC MATER, V7, DOI 10.1002/adhm.201701439
Cekic C, 2014, CANCER RES, V74, P7250, DOI 10.1158/0008-5472.CAN-13-3583
Cha BG, 2018, ACS CENTRAL SCI, V4, P484, DOI 10.1021/acscentsci.8b00035
Chen BL, 2016, J CONTROL RELEASE, V241, P68, DOI 10.1016/j.jconrel.2016.09.014
Chen PG, 2017, PURE APPL CHEM, V89, P931, DOI 10.1515/pac-2016-0913
Chen Q, 2018, ADV HEALTHC MATER, V7, DOI 10.1002/adhm.201800424
Cheng T, 2018, ACS BIOMATER SCI ENG, V4, P1013, DOI 10.1021/acsbiomaterials.8b00049
Cieslewicz M, 2013, P NATL ACAD SCI USA, V110, P15919, DOI 10.1073/pnas.1312197110
Conde J, 2015, ADV FUNCT MATER, V25, P4183, DOI 10.1002/adfm.201501283
Corthay A, 2009, SCAND J IMMUNOL, V70, P326, DOI 10.1111/j.1365-3083.2009.02308.x
David JM, 2017, ONCOIMMUNOLOGY, V6, DOI 10.1080/2162402X.2017.1349589
Denkert C, 2010, J CLIN ONCOL, V28, P105, DOI 10.1200/JCO.2009.23.7370
Diamond MS, 2011, J EXP MED, V208, P1989, DOI 10.1084/jem.20101158
Duan XP, 2016, J AM CHEM SOC, V138, P16686, DOI 10.1021/jacs.6b09538
Dudek AM, 2013, CYTOKINE GROWTH F R, V24, P319, DOI 10.1016/j.cytogfr.2013.01.005

Erez N, 2010, CANCER CELL, V17, P135, DOI 10.1016/j.ccr.2009.12.041
Ernsting MJ, 2015, J CONTROL RELEASE, V206, P122, DOI 10.1016/j.jconrel.2015.03.023
Flies DB, 2016, ONCOIMMUNOLOGY, V5, DOI 10.1080/2162402X.2016.1185583
Fontana F, 2017, ADV MATER, V29, DOI 10.1002/adma.201603239
Frese KK, 2012, CANCER DISCOV, V2, P260, DOI 10.1158/2159-8290.CD-11-0242
Fuertes MB, 2011, J EXP MED, V208, P2005, DOI 10.1084/jem.20101159
Gammon JM, 2017, J CONTROL RELEASE, V263, P151, DOI 10.1016/j.jconrel.2017.02.034
Garcia-Rendon A, 2018, LIPID NANOCARRIERS FOR DRUG TARGETING, P231, DOI 10.1016/B978-0-12-813687-4.00006-2
Garg AD, 2015, INT J DEV BIOL, V59, P131, DOI 10.1387/ijdb.150061pa
Garg AD, 2012, EMBO J, V31, P1062, DOI 10.1038/emboj.2011.497
Gettinger SN, 2015, J CLIN ONCOL, V33, P2004, DOI 10.1200/JCO.2014.58.3708
Glentis A, 2017, NAT COMMUN, V8, DOI 10.1038/s41467-017-00985-8
Goodwin TJ, 2017, BIOMATERIALS, V141, P260, DOI 10.1016/j.biomaterials.2017.07.007
Goodwin TJ, 2017, VACCINE, V35, P2550, DOI 10.1016/j.vaccine.2017.03.067
Goodwin TJ, 2016, SCI TRANSL MED, V8, DOI 10.1126/scitranslmed.aag2306
Guan XW, 2018, BIOMATERIALS, V171, P198, DOI 10.1016/j.biomaterials.2018.04.039
Guo LR, 2014, ACS NANO, V8, P5670, DOI 10.1021/nn5002112
Guo ST, 2014, ACS NANO, V8, P4996, DOI 10.1021/nn5010815
Hendry S, 2017, ADV ANAT PATHOL, V24, P311, DOI 10.1097/PAP.0000000000000161
Hodge JW, 2013, INT J CANCER, V133, P624, DOI 10.1002/ijc.28070
Hodi FS, 2010, NEW ENGL J MED, V363, P711, DOI 10.1056/NEJMoa1003466
Holtzhausen A, 2015, CANCER IMMUNOL RES, V3, P1082, DOI 10.1158/2326-6066.CIR-14-0167
Hsu FJ, 1996, NAT MED, V2, P52, DOI 10.1038/nm0196-52
Hu CH, 2017, BIOMATERIALS, V144, P60, DOI 10.1016/j.biomaterials.2017.08.009
Hu YB, 2015, PLOS ONE, V10, DOI 10.1371/journal.pone.0125625
Huang FY, 2018, ONCOIMMUNOLOGY, V7, DOI 10.1080/2162402X.2018.1446720
Ji T. J., 2016, ANGEW CHEM, V128, P1062
Jinushi M, 2011, P NATL ACAD SCI USA, V108, P12425, DOI 10.1073/pnas.1106645108
Kang T, 2018, BIOMATERIALS, V164, P80, DOI 10.1016/j.biomaterials.2018.02.033
Kapadia CH, 2016, MOL PHARMACEUT, V13, P3381, DOI 10.1021/acs.molpharmaceut.6b00288
Karin M, 2006, CELL, V124, P823, DOI 10.1016/j.cell.2006.02.016
Kepp O, 2014, ONCOTARGET, V5, P5190, DOI 10.18632/oncotarget.2266
Klinman DM, 2004, NAT REV IMMUNOL, V4, P248, DOI 10.1038/nri1329
Kortylewski M, 2009, CANCER CELL, V15, P114, DOI 10.1016/j.ccr.2008.12.018
Koshy ST, 2017, ADV BIOSYST, V1, DOI 10.1002/adbi.201600013
Kroemer G, 2013, ANNU REV IMMUNOL, V31, P51, DOI 10.1146/annurev-immunol-032712-100008
Krysko DV, 2012, NAT REV CANCER, V12, P860, DOI 10.1038/nrc3380
Lan Y, 2018, SCI TRANSL MED, V10, DOI 10.1126/scitranslmed.aan5488
Latz E, 2007, NAT IMMUNOL, V8, P772, DOI 10.1038/ni1479
Lee H, 2017, CLIN CANCER RES, V23, P4190, DOI 10.1158/1078-0432.CCR-16-3193
Lei CH, 2010, J AM CHEM SOC, V132, P6906, DOI 10.1021/ja102414t
Li HJ, 2016, P NATL ACAD SCI USA, V113, P4164, DOI 10.1073/pnas.1522080113
Lin AY, 2013, PLOS ONE, V8, DOI 10.1371/journal.pone.0063550
Liu LN, 2018, MOL THER, V26, P45, DOI 10.1016/j.ymthe.2017.10.020
Liu Q, 2018, ACS NANO, V12, P1250, DOI [10.1021/acsnano.7b07384, 10.1021/acsnano.7607384]
Loeffler M, 2006, J CLIN INVEST, V116, P1955, DOI 10.1172/JCI26532
Loi S, 2014, ANN ONCOL, V25, P1544, DOI 10.1093/annonc/mdu112
Loi S, 2013, J CLIN ONCOL, V31, P860, DOI 10.1200/JCO.2011.41.0902
Lu J., 2017, NAT COMMUN, V8, P1
Lu KD, 2016, J AM CHEM SOC, V138, P12502, DOI 10.1021/jacs.6b06663
Lu-Emerson C, 2013, NEURO-ONCOLOGY, V15, P1079, DOI 10.1093/neuonc/not082
Luo M, 2017, NAT NANOTECHNOL, V12, P648, DOI [10.1038/nnano.2017.52, 10.1038/NNANO.2017.52]
Luo ZC, 2015, BIOMATERIALS, V38, P50, DOI 10.1016/j.biomaterials.2014.10.050
Maiti R, 2014, J PHARMACOL PHARMACO, V5, P186, DOI 10.4103/0976-500X.136098
Martinet L, 2011, CANCER RES, V71, P5678, DOI 10.1158/0008-5472.CAN-11-0431
Mastria EM, 2018, J CONTROL RELEASE, V269, P364, DOI 10.1016/j.jconrel.2017.11.021
McGranahan N, 2017, CELL, V168, P613, DOI 10.1016/j.cell.2017.01.018
Melamed JR, 2016, ACS NANO, V10, P10631, DOI 10.1021/acsnano.6b07673
Mi Y, 2018, ADV MATER, V30, DOI 10.1002/adma.201706098

Miao L, 2017, ACS NANO, V11, P8690, DOI 10.1021/acsnano.7b01786
Miao L, 2017, CANCER RES, V77, P719, DOI 10.1158/0008-5472.CAN-16-0866
Miao L, 2016, ACS NANO, V10, P9243, DOI 10.1021/acsnano.6b02776
Miao L, 2015, J CONTROL RELEASE, V217, P27, DOI 10.1016/j.jconrel.2015.08.027
Min YZ, 2017, NAT NANOTECHNOL, V12, P877, DOI [10.1038/nnano.2017.113, 10.1038/NNANO.2017.113]
Mitchem JB, 2013, CANCER RES, V73, P1128, DOI 10.1158/0008-5472.CAN-12-2731
Mukherjee P, 2005, CLIN CANCER RES, V11, P3530, DOI 10.1158/1078-0432.CCR-04-2482
Mulens-Arias V, 2015, BIOMATERIALS, V52, P494, DOI 10.1016/j.biomaterials.2015.02.068
Nakamura T, 2015, J CONTROL RELEASE, V216, P149, DOI 10.1016/j.jconrel.2015.08.026
Neubert NJ, 2018, SCI TRANSL MED, V10, DOI 10.1126/scitranslmed.aan3311
Neumann Tobias, 2018, Oncotarget, V9, P16775, DOI 10.18632/oncotarget.24697
Nielsen JS, 2012, ONCOIMMUNOLOGY, V1, P1623, DOI 10.4161/onci.21650
Nielsen JS, 2012, CLIN CANCER RES, V18, P3281, DOI 10.1158/1078-0432.CCR-12-0234
Nielsen SR, 2017, MEDIAT INFLAMM, V2017, DOI 10.1155/2017/9624760
Niu MM, 2016, MOL PHARMACEUT, V13, P1833, DOI 10.1021/acs.molpharmaceut.5b00987
Obeid M, 2007, CELL DEATH DIFFER, V14, P1848, DOI 10.1038/sj.cdd.4402201
Pal R, 2016, INT IMMUNOPHARMACOL, V38, P332, DOI 10.1016/j.intimp.2016.06.006
Pan JB, 2018, ADV MATER, V30, DOI 10.1002/adma.201704408
Parker KH, 2015, ADV CANCER RES, V128, P95, DOI 10.1016/bs.acr.2015.04.002
Piersma B, 2015, FRONT MED, V2, P1
Ping Y, 2013, BIOMATERIALS, V34, P6482, DOI 10.1016/j.biomaterials.2013.03.071
POLVERINI PJ, 1977, NATURE, V269, P804, DOI 10.1038/269804a0
Pruitt SK, 2011, EUR J IMMUNOL, V41, P3553, DOI 10.1002/eji.201141383
Qiu LP, 2018, SMALL, V14, DOI 10.1002/smll.201703539
Ramanathan RK, 2017, CLIN CANCER RES, V23, P3638, DOI 10.1158/1078-0432.CCR-16-1990
Richards KE, 2017, ONCOGENE, V36, P1770, DOI 10.1038/onc.2016.353
Roswall P, 2018, NAT MED, V24, P463, DOI 10.1038/nm.4494
Saha S, 2016, ACS NANO, V10, P10636, DOI 10.1021/acsnano.6b02231
Sanmamed MF, 2016, ANN ONCOL, V27, P1190, DOI 10.1093/annonc/mdw041
Sanovic R, 2011, PHOTODIAGN PHOTODYN, V8, P291, DOI 10.1016/j.pdpdt.2011.04.003
Santi A, 2015, BBA-MOL CELL RES, V1853, P3211, DOI 10.1016/j.bbamcr.2015.09.013
Schiffelers RM, 2004, NUCLEIC ACIDS RES, V32, DOI 10.1093/nar/gnh140
Schuller VJ, 2011, ACS NANO, V5, P9696, DOI 10.1021/nn203161y
Schuler T, 2003, J EXP MED, V198, P1487, DOI 10.1084/jem.20030849
Sellner S, 2017, BIOMATERIALS, V134, P78, DOI 10.1016/j.biomaterials.2017.04.031
Senovilla L, 2012, ONCOIMMUNOLOGY, V1, P1323, DOI 10.4161/onci.22009
Shang B, 2015, SCI REP-UK, V5, DOI 10.1038/srep15179
Shen S, 2017, NANO LETT, V17, P3822, DOI 10.1021/acs.nanolett.7b01193
Shi JY, 2013, CLIN CANCER RES, V19, P5994, DOI 10.1158/1078-0432.CCR-12-3497
Shojima K., 2015, SCI REP, V5, P1
SINGH S, 1992, J EXP MED, V175, P139, DOI 10.1084/jem.175.1.139
Son K.-J., 2013, CYTOKINE GROWTH F R, V24, P319
Song WT, 2017, BIOMATERIALS, V148, P16, DOI 10.1016/j.biomaterials.2017.09.017
Song W, 2018, ACS NANO, V12, P1978, DOI 10.1021/acsnano.7b09112
Song X, 2016, BIOMATERIALS, V108, P44, DOI 10.1016/j.biomaterials.2016.09.001
Spaeth EL, 2009, PLOS ONE, V4, DOI 10.1371/journal.pone.0004992
Spranger S, 2013, SCI TRANSL MED, V5, DOI 10.1126/scitranslmed.3006504
Squadrito ML, 2011, MOL ASPECTS MED, V32, P123, DOI 10.1016/j.mam.2011.04.005
Strop P, 2013, CHEM BIOL, V20, P161, DOI 10.1016/j.chembiol.2013.01.010
Sugahara KN, 2010, SCIENCE, V328, P1031, DOI 10.1126/science.1183057
Sun YL, 2017, ONCOIMMUNOLOGY, V6, DOI 10.1080/2162402X.2017.1309487
Tao Y, 2014, BIOMATERIALS, V35, P9963, DOI 10.1016/j.biomaterials.2014.08.036
Tao Y, 2014, BIOMATERIALS, V35, P6646, DOI 10.1016/j.biomaterials.2014.04.073
Teo PY, 2015, ADV HEALTHC MATER, V4, P1180, DOI 10.1002/adhm.201500089
Valsecchi ME, 2015, NEW ENGL J MED, V373, P1270, DOI [10.1056/NEJMoa1504030, 10.1056/NEJMc1509660]
Verginis P, 2005, J IMMUNOL, V174, P7433, DOI 10.4049/jimmunol.174.11.7433
Walsh TJ, 1999, NEW ENGL J MED, V340, P764, DOI 10.1056/NEJM199903113401004
Wang C, 2016, BIOMATERIALS, V79, P88, DOI 10.1016/j.biomaterials.2015.11.040
Wang C, 2018, NANOMEDICINE-UK, V13, P157, DOI 10.2217/nnm-2017-0266

Wang C, 2017, ADV MATER, V29, DOI 10.1002/adma.201606036
Wang C, 2016, ADV MATER, V28, P8912, DOI 10.1002/adma.201506312
Wang HY, 2007, CURR OPIN IMMUNOL, V19, P217, DOI 10.1016/j.coi.2007.02.004
Wang Z., 2016, SCI REPORTS, V6, P1
Xu ZH, 2014, ACS NANO, V8, P3636, DOI 10.1021/nn500216y
Yang G, 2017, NAT COMMUN, V8, DOI 10.1038/ncomms14941
Zanganeh S, 2016, NAT NANOTECHNOL, V11, P986, DOI [10.1038/nnano.2016.168, 10.1038/NNANO.2016.168]
Zhan XD, 2014, BIOMATERIALS, V35, P10046, DOI 10.1016/j.biomaterials.2014.09.007
Zhang CR, 2016, BIOMATERIALS, V84, P1, DOI 10.1016/j.biomaterials.2016.01.027
Zhang HX, 2018, IMMUNOTHERAPY-UK, V10, P631, DOI 10.2217/imt-2018-0052
Zhang R, 2014, P NATL ACAD SCI USA, V111, P12181, DOI 10.1073/pnas.1406233111
Zhang XD, 2018, ADV MATER, V30, DOI 10.1002/adma.201707112
Zhao DC, 2011, CLIN CANCER RES, V17, P771, DOI 10.1158/1078-0432.CCR-10-2444
Zhao XD, 2017, ONCOTARGET, V8, P54775, DOI 10.18632/oncotarget.18423
Zhao X, 2016, BIOMATERIALS, V102, P187, DOI 10.1016/j.biomaterials.2016.06.032
Zhen ZP, 2017, NANO LETT, V17, P862, DOI 10.1021/acs.nanolett.6b04150
Zhou S, 2014, INT J PHARMACEUT, V471, P214, DOI 10.1016/j.ijpharm.2014.05.037
Zhu G., 2017, NAT COMMUN, V8, P1
Zhu GZ, 2017, ACS NANO, V11, P2387, DOI 10.1021/acsnano.7b00978
Zhu GZ, 2016, NANOSCALE, V8, P6684, DOI 10.1039/c5nr08821f
NR 164
TC 152
Z9 155
U1 19
U2 358
PU AMER CHEMICAL SOC
PI WASHINGTON
PA 1155 16TH ST, NW, WASHINGTON, DC 20036 USA
SN 1936-0851
EI 1936-086X
J9 ACS NANO
JI ACS Nano
PD DEC
PY 2018
VL 12
IS 12
BP 11740
EP 11755
DI 10.1021/acsnano.8b05893
PG 16
WC Chemistry, Multidisciplinary; Chemistry, Physical; Nanoscience &
Nanotechnology; Materials Science, Multidisciplinary
WE Science Citation Index Expanded (SCI-EXPANDED)
SC Chemistry; Science & Technology - Other Topics; Materials Science
GA HF9NA
UT WOS:000454567500006
PM 30508378
DA 2023-09-14
ER

PT J
AU Liu, WL
Zou, MZ
Liu, T
Zeng, JY
Li, X
Yu, WY
Li, CX
Ye, JJ
Song, W

Feng, J
Zhang, XZ
AF Liu, Wen-Long
Zou, Mei-Zhen
Liu, Tao
Zeng, Jin-Yue
Li, Xue
Yu, Wu-Yang
Li, Chu-Xin
Ye, Jing-Jie
Song, Wen
Feng, Jun
Zhang, Xian-Zheng
TI Cytomembrane nanovaccines show therapeutic effects by mimicking tumor cells and antigen presenting cells
SO NATURE COMMUNICATIONS
LA English
DT Article
ID MEMBRANE-COATED NANOPARTICLES; DENDRITIC-CELL; ANTITUMOR IMMUNITY; PHOTODYNAMIC THERAPY; T-CELLS; IMMUNOTHERAPY; RESPONSES; STIMULATION; VACCINATION; INDUCTION
AB Most cancer vaccines are unsuccessful in eliciting clinically relevant effects. Without using exogenous antigens and adoptive cells, we show a concept of utilizing biologically reprogrammed cytomembranes of the fused cells (FCs) derived from dendritic cells (DCs) and cancer cells as tumor vaccines. The fusion of immunologically interrelated two types of cells results in strong expression of the whole tumor antigen complexes and the immunological co-stimulatory molecules on cytomembranes (FMs), allowing the nanoparticle-supported FM (NP@FM) to function like antigen presenting cells (APCs) for T cell immunoactivation. Moreover, tumor-antigen bearing NP@FM can be bio-recognized by DCs to induce DC-mediated T cell immunoactivation. The combination of these two immunoactivation pathways offers powerful antitumor immunoresponse. Through mimicking both APCs and cancer cells, this cytomembrane vaccine strategy can develop various vaccines toward multiple tumor types and provide chances for accommodating diverse functions originating from the supporters.
C1 [Liu, Wen-Long; Liu, Tao; Zeng, Jin-Yue; Li, Xue; Yu, Wu-Yang; Li, Chu-Xin; Ye, Jing-Jie; Song, Wen; Feng, Jun; Zhang, Xian-Zheng] Wuhan Univ, Dept Chem, Minist Educ, Key Lab Biomed Polymers, Wuhan 430072, Hubei, Peoples R China.
[Zou, Mei-Zhen; Zhang, Xian-Zheng] Wuhan Univ, Inst Adv Studies, Wuhan 430072, Hubei, Peoples R China.
C3 Wuhan University; Wuhan University
RP Feng, J; Zhang, XZ (通讯作者)，Wuhan Univ, Dept Chem, Minist Educ, Key Lab Biomed Polymers, Wuhan 430072, Hubei, Peoples R China.; Zhang, XZ (通讯作者)，Wuhan Univ, Inst Adv Studies, Wuhan 430072, Hubei, Peoples R China.
EM fengjun@whu.edu.cn; xz-zhang@whu.edu.cn
RI zhang, xian/GYA-0290-2022; Song, Wen/IAR-3557-2023; Zhang, XZ/HJA-4189-2022; Liu, Tao/ISA-9651-2023
OI Liu, Tao/0000-0002-6179-2051; Liu, Wen-Long/0000-0002-3993-4850
FU National Key Research and Development Program of China [2016YFC1100703]; National Natural Science foundation of China [51690152, 51533006, 51833007, 21374085]
FX This work was financially supported by the National Key Research and Development Program of China (2016YFC1100703) and the National Natural Science foundation of China (51690152, 51533006, 51833007, 21374085).
CR Anderson DM, 1997, NATURE, V390, P175, DOI 10.1038/36593
Anguille S, 2015, PHARMACOL REV, V67, P731, DOI 10.1124/pr.114.009456
Brunner C, 2000, J IMMUNOL, V165, P6278, DOI 10.4049/jimmunol.165.11.6278
Caruana I, 2015, NAT MED, V21, P524, DOI 10.1038/nm.3833
Chao M, 2016, ELIFE, V5, DOI [10.7754/eLife.15691, 10.7554/eLife.15691]
Chen WS, 2017, ADV FUNCT MATER, V27, DOI 10.1002/adfm.201605795
Chomarat P, 2000, NAT IMMUNOL, V1, P510, DOI 10.1038/82763
Choo YW, 2018, ACS NANO, V12, P8977, DOI 10.1021/acsnano.8b02446
Dehaini D, 2017, ADV MATER, V29, DOI 10.1002/adma.201606209
Deng H, 2015, ADV MATER, V27, P3645, DOI 10.1002/adma.201501420

Ding BB, 2018, ADV MATER, V30, DOI 10.1002/adma.201802479
Donin NM, 2017, J UROLOGY, V197, P14, DOI 10.1016/j.juro.2016.02.3005
Fang RH, 2014, NANO LETT, V14, P2181, DOI 10.1021/nl500618u
Fraietta JA, 2018, NAT MED, V24, P563, DOI 10.1038/s41591-018-0010-1
Garg AD, 2017, TRENDS IMMUNOL, V38, P577, DOI 10.1016/j.it.2017.05.006
GOLD P, 1965, J EXP MED, V121, P439, DOI 10.1084/jem.121.3.439
Gong JL, 1997, NAT MED, V3, P558, DOI 10.1038/nm0597-558
Gong JL, 1998, P NATL ACAD SCI USA, V95, P6279, DOI 10.1073/pnas.95.11.6279
Guillerey C, 2016, NAT IMMUNOL, V17, P1025, DOI 10.1038/ni.3518
Guo YY, 2015, ACS NANO, V9, P6918, DOI 10.1021/acsnano.5b01042
Han L, 2016, BIOMATERIALS, V105, P185, DOI 10.1016/j.biomaterials.2016.08.007
Jin HL, 2018, ACS NANO, V12, P3295, DOI 10.1021/acsnano.7b08148
Kalergis AM, 2001, NAT IMMUNOL, V2, P229, DOI 10.1038/85286
Kasturi SP, 2011, NATURE, V470, P543, DOI 10.1038/nature09737
Kim B, 2018, NAT COMMUN, V9, DOI 10.1038/s41467-018-04390-7
Koido S, 2005, CLIN CANCER RES, V11, P7891, DOI 10.1158/1078-0432.CCR-05-1330
Koido S, 2013, ONCOIMMUNOLOGY, V2, DOI 10.4161/onci.24437
Koido S, 2013, PLOS ONE, V8, DOI 10.1371/journal.pone.0063498
Kovar M, 2006, P NATL ACAD SCI USA, V103, P11671, DOI 10.1073/pnas.0603466103
Kroll AV, 2017, ADV MATER, V29, DOI 10.1002/adma.201703969
Li AW, 2018, NAT MATER, V17, P528, DOI 10.1038/s41563-018-0028-2
Li CX, 2019, ADV MATER, V31, DOI 10.1002/adma.201807211
Li JJ, 2017, ADV FUNCT MATER, V27, DOI 10.1002/adfm.201702108
Li SY, 2017, ACS NANO, V11, P7006, DOI 10.1021/acsnano.7b02533
Li SY, 2017, ADV FUNCT MATER, V27, DOI 10.1002/adfm.201604916
Liu T, 2018, ACS NANO, V12, P12181, DOI 10.1021/acsnano.8b05860
Liu T, 2018, ACS NANO, V12, P3917, DOI 10.1021/acsnano.8b01456
Liu WL, 2018, ADV MATER, V30, DOI 10.1002/adma.201802006
Luo M, 2017, NAT NANOTECHNOL, V12, P648, DOI [10.1038/nnano.2017.52, 10.1038/NNANO.2017.52]
Lutz MB, 1999, J IMMUNOL METHODS, V223, P77, DOI 10.1016/S0022-1759(98)00204-X
Lv YL, 2018, ACS NANO, V12, P1350, DOI 10.1021/acsnano.7b07716
Min YZ, 2017, NAT NANOTECHNOL, V12, P877, DOI [10.1038/nnano.2017.113, 10.1038/NNANO.2017.113]
Munn DH, 2016, TRENDS IMMUNOL, V37, P193, DOI 10.1016/j.it.2016.01.002
Nestle FO, 1998, NAT MED, V4, P328, DOI 10.1038/nm0398-328
O'Shea JJ, 2008, IMMUNITY, V28, P477, DOI 10.1016/j.immuni.2008.03.002
Pan JB, 2018, ADV MATER, V30, DOI 10.1002/adma.201704408
Perret R, 2013, CANCER RES, V73, P6597, DOI 10.1158/0008-5472.CAN-13-0875
Ridge JP, 1998, NATURE, V393, P474, DOI 10.1038/30989
Sabado RL, 2017, CELL RES, V27, P74, DOI 10.1038/cr.2016.157
Sagiv-Barfi I, 2018, SCI TRANSL MED, V10, DOI 10.1126/scitranslmed.aan4488
Shi GN, 2017, BIOMATERIALS, V113, P191, DOI 10.1016/j.biomaterials.2016.10.047
Song W, 2018, ACS NANO, V12, P1978, DOI 10.1021/acsnano.7b09112
Squadrito ML, 2018, NAT METHODS, V15, P183, DOI [10.1038/NMETH.4579, 10.1038/nmeth.4579]
Tacken PJ, 2007, NAT REV IMMUNOL, V7, P790, DOI 10.1038/nri2173
Tanaka A, 2017, CELL RES, V27, P109, DOI 10.1038/cr.2016.151
Thomas SN, 2014, BIOMATERIALS, V35, P814, DOI 10.1016/j.biomaterials.2013.10.003
Vermaelen KY, 2003, J IMMUNOL, V171, P1016, DOI 10.4049/jimmunol.171.2.1016
Wang C, 2016, BIOMATERIALS, V79, P88, DOI 10.1016/j.biomaterials.2015.11.040
Wang F, 2016, ADV FUNCT MATER, V26, P1628, DOI 10.1002/adfm.201505231
Wang K, 2018, ACS NANO, V12, P6398, DOI 10.1021/acsnano.8b00558
Wang XP, 2016, ANGEW CHEM INT EDIT, V55, P1899, DOI 10.1002/anie.201506179
Wu L, 2018, P NATL ACAD SCI USA, V115, pE4453, DOI 10.1073/pnas.1800550115
Xu J, 2017, ACS NANO, V11, P4463, DOI 10.1021/acsnano.7b00715
Zhai YH, 2018, ADV MATER, V30, DOI 10.1002/adma.201802378
Zhang XX, 2019, NUCLEIC ACIDS RES, V47, pD721, DOI 10.1093/nar/gky900
Zhang XD, 2018, NANO LETT, V18, P5716, DOI 10.1021/acs.nanolett.8b02321
Zhang Y, 2018, NANO LETT, V18, P1908, DOI 10.1021/acs.nanolett.7b05263
Zhu JY, 2016, NANO LETT, V16, P5895, DOI 10.1021/acs.nanolett.6b02786
NR 68

TC 148
Z9 154
U1 29
U2 293
PU NATURE PUBLISHING GROUP
PI LONDON
PA MACMILLAN BUILDING, 4 CRINAN ST, LONDON N1 9XW, ENGLAND
SN 2041-1723
J9 NAT COMMUN
JI Nat. Commun.
PD JUL 19
PY 2019
VL 10
AR 3199
DI 10.1038/s41467-019-11157-1
PG 12
WC Multidisciplinary Sciences
WE Science Citation Index Expanded (SCI-EXPANDED)
SC Science & Technology - Other Topics
GA IK3ED
UT WOS:000476471300008
PM 31324770
OA Green Published, gold
DA 2023-09-14
ER

PT J
AU Gong, NQ
   Zhang, YX
   Teng, XC
   Wang, YC
   Huo, SD
   Qing, GC
   Ni, QK
   Li, XL
   Wang, JJ
   Ye, XX
   Zhang, TB
   Chen, SZ
   Wang, YJ
   Yu, J
   Wang, PC
   Gan, YL
   Zhang, JC
   Mitchell, MJ
   Li, JH
   Liang, XJ
AF Gong, Ningqiang
   Zhang, Yuxuan
   Teng, Xucong
   Wang, Yongchao
   Huo, Shuaidong
   Qing, Guangchao
   Ni, Qiankun
   Li, Xianlei
   Wang, Jinjin
   Ye, Xiaoxia
   Zhang, Tingbin
   Chen, Shizhu
   Wang, Yongji
   Yu, Jie

Wang, Paul C.
Gan, Yaling
Zhang, Jinchao
Mitchell, Michael J.
Li, Jinghong
Liang, Xing-Jie
TI Proton-driven transformable nanovaccine for cancer immunotherapy
SO NATURE NANOTECHNOLOGY
LA English
DT Article
ID INTRACELLULAR DELIVERY; IN-VITRO; NEOANTIGEN VACCINE; NANOPARTICLES; RESPONSES; IMMUNITY; MICELLES; PLATFORM; NLRP3
AB Cancer vaccines hold great promise for improved cancer treatment. However, endosomal trapping and low immunogenicity of tumour antigens usually limit the efficiency of vaccination strategies. Here, we present a proton-driven nanotransformer-based vaccine, comprising a polymer-peptide conjugate-based nanotransformer and loaded antigenic peptide. The nanotransformer-based vaccine induces a strong immune response without substantial systemic toxicity. In the acidic endosomal environment, the nanotransformer-based vaccine undergoes a dramatic morphological change from nanospheres (about 100 nanometres in diameter) into nanosheets (several micrometres in length or width), which mechanically disrupts the endosomal membrane and directly delivers the antigenic peptide into the cytoplasm. The re-assembled nanosheets also boost tumour immunity via activation of specific inflammation pathways. The nanotransformer-based vaccine effectively inhibits tumour growth in the B16F10-OVA and human papilloma virus-E6/E7 tumour models in mice. Moreover, combining the nanotransformer-based vaccine with anti-PD-L1 antibodies results in over 83 days of survival and in about half of the mice produces complete tumour regression in the B16F10 model. This proton-driven transformable nanovaccine offers a robust and safe strategy for cancer immunotherapy.

A shape-changing pH-responsive cancer vaccine delivers antigenic peptides directly to the cytoplasm and boosts the cellular immune response upon activation of the inflammasome pathway, efficiently inhibiting tumour growth and extending survival in animal tumour models.
C1 [Gong, Ningqiang; Teng, Xucong; Ni, Qiankun; Wang, Yongji; Li, Jinghong] Tsinghua Univ, Dept Chem, Key Lab Bioorgan Phosphorus Chem & Chem Biol, Beijing, Peoples R China.

[Gong, Ningqiang; Zhang, Yuxuan; Wang, Yongchao; Huo, Shuaidong; Qing, Guangchao; Ni, Qiankun; Li, Xianlei; Wang, Jinjin; Ye, Xiaoxia; Zhang, Tingbin; Chen, Shizhu; Wang, Paul C.; Gan, Yaling; Liang, Xing-Jie] Chinese Acad Sci, Ctr Excellence Nanosci, Lab Controllable Nanopharmaceut, Beijing, Peoples R China.

[Gong, Ningqiang; Zhang, Yuxuan; Wang, Yongchao; Huo, Shuaidong; Qing, Guangchao; Ni, Qiankun; Li, Xianlei; Wang, Jinjin; Ye, Xiaoxia; Zhang, Tingbin; Chen, Shizhu; Wang, Paul C.; Gan, Yaling; Liang, Xing-Jie] Natl Ctr Nanosci & Technol, CAS Key Lab Biomed Effects Nanomat & Nanosafety, Beijing, Peoples R China.

[Gong, Ningqiang; Mitchell, Michael J.] Univ Penn, Dept Bioengn, Philadelphia, PA 19104 USA.

[Gong, Ningqiang; Zhang, Yuxuan; Ni, Qiankun; Li, Xianlei; Wang, Jinjin; Liang, Xing-Jie] Univ Chinese Acad Sci, Beijing, Peoples R China.

[Huo, Shuaidong] Xiamen Univ, Sch Pharmaceut Sci, Fujian Prov Key Lab Innovat Drug Target Res, Xiamen, Peoples R China.

[Chen, Shizhu; Zhang, Jinchao; Liang, Xing-Jie] Hebei Univ, Coll Chem & Environm Sci, Key Lab Med Chem & Mol Diag, Minist Educ, Baoding, Peoples R China.

[Yu, Jie] Chinese Peoples Liberat Army Gen Hosp, Dept Intervent Ultrasound, Beijing, Peoples R China.

[Wang, Paul C.] Howard Univ, Dept Radiol, Lab Mol Imaging, Washington, DC 20059 USA.

[Wang, Paul C.] Fu Jen Catholic Univ, Dept Elect Engn, Taipei, Taiwan.

C3 Tsinghua University; Chinese Academy of Sciences; Chinese Academy of Sciences; National Center for Nanoscience & Technology - China; University of Pennsylvania; Chinese Academy of Sciences; University of Chinese Academy of Sciences, CAS; Xiamen University; Hebei University; Chinese People's Liberation Army General Hospital; Howard University; Fu Jen Catholic University

RP Li, JH (通讯作者)，Tsinghua Univ, Dept Chem, Key Lab Bioorgan Phosphorus Chem & Chem Biol, Beijing, Peoples R China.; Liang, XJ (通讯作者)，Chinese Acad Sci, Ctr Excellence Nanosci, Lab Controllable Nanopharmaceut, Beijing, Peoples R China.; Liang, XJ (通讯作者)，Natl Ctr Nanosci & Technol, CAS Key Lab Biomed Effects Nanomat & Nanosafety, Beijing, Peoples R China.; Liang, XJ (通讯作者)，Univ Chinese Acad Sci, Beijing, Peoples R China.; Liang, XJ (通讯作者)，Hebei Univ, Coll Chem & Environm Sci, Key Lab Med Chem & Mol Diag, Minist Educ, Baoding, Peoples R China.

EM jhli@mail.tsinghua.edu.cn; liangxj@nanoctr.cn
RI Huo, Shuaidong/T-9026-2018; Liang, Richard/ABF-2708-2021; Wang, Jin/GYA-2019-2022; Mitchell, Michael/K-8420-2014
OI Huo, Shuaidong/0000-0003-3590-044X; Mitchell, Michael/0000-0002-3628-2244; Gong, Ningqiang/0000-0002-9444-8505; Wang, Paul/0000-0001-8219-7051
FU National Natural Science Foundation of China [31630027, 31430031, 21327806, 21621003, 21235004]; NSFC-German Research Foundation (DFG) project [31761133013]; Strategic Priority Research Program of the Chinese Academy of Sciences [XDA09030301, XDB36000000]; National Key Research and Development Program of China [2018YFE0117800, 2016YFA0203101]; NIH/NIMHHD [U54MD007597]
FX This work was financially supported by the National Natural Science Foundation of China (grant numbers 31630027, 31430031, 21327806, 21621003 and 21235004) and NSFC-German Research Foundation (DFG) project (grant number 31761133013). We are grateful for support from the Strategic Priority Research Program of the Chinese Academy of Sciences (grant numbers XDA09030301 and XDB36000000), the National Key Research and Development Program of China (grant numbers 2018YFE0117800 and 2016YFA0203101). We also acknowledge support from the NIH/NIMHHD (grant number U54MD007597). We thank Z. Ao for help with the AFM measurements and X. Hu and H. Guo for help with the TEM analysis.
CR Aits S, 2015, AUTOPHAGY, V11, P1408, DOI 10.1080/15548627.2015.1063871
Akishiba M, 2017, NAT CHEM, V9, P751, DOI [10.1038/NCHEM.2779, 10.1038/nchem.2779]
Alam MM, 2017, J CONTROL RELEASE, V252, P62, DOI 10.1016/j.jconrel.2017.03.012
Blanco E, 2015, NAT BIOTECHNOL, V33, P941, DOI 10.1038/nbt.3330
Brewer JM, 1999, J IMMUNOL, V163, P6448
Cheng CJ, 2015, NATURE, V518, P107, DOI 10.1038/nature13905
CHENG Y, 2016, ANGEW CHEM, V128, P12192, DOI DOI 10.1002/ANGE.201605958
Chiefari J, 1998, MACROMOLECULES, V31, P5559, DOI 10.1021/ma9804951
Chou LYT, 2011, CHEM SOC REV, V40, P233, DOI 10.1039/c0cs00003e
Chu YH, 2018, THERANOSTICS, V8, P4238, DOI 10.7150/thno.24387
Coll RC, 2015, NAT MED, V21, P248, DOI 10.1038/nm.3806
D'Astolfo DS, 2015, CELL, V161, P674, DOI 10.1016/j.cell.2015.03.028
Douat C, 2015, ANGEW CHEM INT EDIT, V54, P11133, DOI 10.1002/anie.201504884
Duan F, 2017, BIOMATERIALS, V122, P23, DOI 10.1016/j.biomaterials.2017.01.017
ELLENS H, 1984, BIOCHEMISTRY-US, V23, P1532, DOI 10.1021/bi00302a029
Fan YC, 2017, NANO LETT, V17, P7387, DOI 10.1021/acs.nanolett.7b03218
Garaude J, 2012, SCI TRANSL MED, V4, DOI 10.1126/scitranslmed.3002868
Ghiringhelli F, 2009, NAT MED, V15, P1170, DOI 10.1038/nm.2028
Goel S, 2017, NATURE, V548, P471, DOI 10.1038/nature23465
Goldberg MS, 2015, CELL, V161, P201, DOI 10.1016/j.cell.2015.03.037
Gong NQ, 2019, NAT NANOTECHNOL, V14, P379, DOI 10.1038/s41565-019-0373-6
Guo YG, 2018, FRONT IMMUNOL, V9, DOI 10.3389/fimmu.2018.01499
Heneka MT, 2013, NATURE, V493, P674, DOI 10.1038/nature11729
Irvine DJ, 2015, CHEM REV, V115, P11109, DOI 10.1021/acs.chemrev.5b00109
Kanasty R, 2013, NAT MATER, V12, P967, DOI [10.1038/NMAT3765, 10.1038/nmat3765]
Keller S, 2014, J CONTROL RELEASE, V191, P24, DOI 10.1016/j.jconrel.2014.03.041
Kuai R, 2017, NAT MATER, V16, P489, DOI [10.1038/nmat4822, 10.1038/NMAT4822]
Li AV, 2013, SCI TRANSL MED, V5, DOI 10.1126/scitranslmed.3006516
Li AW, 2018, NAT MATER, V17, P528, DOI 10.1038/s41563-018-0028-2
Luo M, 2017, NAT NANOTECHNOL, V12, P648, DOI [10.1038/nnano.2017.52, 10.1038/NNANO.2017.52]
Martens TF, 2014, NANO TODAY, V9, P344, DOI 10.1016/j.nantod.2014.04.011
Martinon F, 2006, NATURE, V440, P237, DOI 10.1038/nature04516
Morishita M, 2016, BIOMATERIALS, V111, P55, DOI 10.1016/j.biomaterials.2016.09.031
Niikura K, 2013, ACS NANO, V7, P3926, DOI 10.1021/nn3057005
Ott PA, 2017, NATURE, V547, P217, DOI 10.1038/nature22991
Petersen NH., 2014, BIO-PROTOCOL, V4, pe1162, DOI [10.21769/BioProtoc.1162, DOI 10.21769/BIOPROTOC.1162]
Qiu LP, 2014, ACTA BIOMATER, V10, P2024, DOI 10.1016/j.actbio.2013.12.025

Riley RS, 2019, NAT REV DRUG DISCOV, V18, P175, DOI 10.1038/s41573-018-0006-z
Rosenthal AK, 2016, NEW ENGL J MED, V374, P2575, DOI 10.1056/NEJMra1511117
Sahin U, 2017, NATURE, V547, P222, DOI 10.1038/nature23003
Shae D, 2019, NAT NANOTECHNOL, V14, P269, DOI 10.1038/s41565-018-0342-5
Stewart MP, 2016, NATURE, V538, P183, DOI 10.1038/nature19764
Tanyi JL, 2018, SCI TRANSL MED, V10, DOI 10.1126/scitranslmed.aao5931
Wilhelm C, 2003, EUR BIOPHYS J BIOPHY, V32, P655, DOI 10.1007/s00249-003-0312-0
Wilson DS, 2019, NAT MATER, V18, P175, DOI 10.1038/s41563-018-0256-5
Wilson JT, 2015, AAPS J, V17, P358, DOI 10.1208/s12248-014-9697-1
Xia XJ, 2015, CELL REP, V11, P957, DOI 10.1016/j.celrep.2015.04.009
Yang YY, 2001, BIOMATERIALS, V22, P231, DOI 10.1016/S0142-9612(00)00178-2
Yin H, 2014, NAT REV GENET, V15, P541, DOI 10.1038/nrg3763
Zambaux MF, 1998, J CONTROL RELEASE, V50, P31, DOI 10.1016/S0168-3659(97)00106-5
Zhang ZJ, 2012, ADV MATER, V24, P1418, DOI 10.1002/adma.201104714
Zhong YN, 2016, BIOMATERIALS, V84, P250, DOI 10.1016/j.biomaterials.2016.01.049
Zhou J, 2016, J AM CHEM SOC, V138, P3813, DOI 10.1021/jacs.5b13541
NR 53
TC 145
Z9 147
U1 50
U2 413
PU NATURE RESEARCH
PI BERLIN
PA HEIDELBERGER PLATZ 3, BERLIN, 14197, GERMANY
SN 1748-3387
EI 1748-3395
J9 NAT NANOTECHNOL
JI Nat. Nanotechnol.
PD DEC
PY 2020
VL 15
IS 12
BP 1053
EP U111
DI 10.1038/s41565-020-00782-3
EA OCT 2020
PG 19
WC Nanoscience & Nanotechnology; Materials Science, Multidisciplinary
WE Science Citation Index Expanded (SCI-EXPANDED)
SC Science & Technology - Other Topics; Materials Science
GA PA6UC
UT WOS:000584087000001
PM 33106640
OA Green Accepted
HC Y
HP N
DA 2023-09-14
ER

PT J
AU Conniot, J
Scomparin, A
Peres, C
Yeini, E
Pozzi, S
Matos, AI
Kleiner, R
Moura, LIF
Zupancic, E
Viana, AS
Doron, H

Gois, PMP
Erez, N
Jung, S
Satchi-Fainaro, R
Florindo, HF
AF Conniot, Joao
Scomparin, Anna
Peres, Carina
Yeini, Eilam
Pozzi, Sabina
Matos, Ana, I
Kleiner, Ron
Moura, Liane I. F.
Zupancic, Eva
Viana, Ana S.
Doron, Hila
Gois, Pedro M. P.
Erez, Neta
Jung, Steffen
Satchi-Fainaro, Ronit
Florindo, Helena F.
TI Immunization with mannosylated nanovaccines and inhibition of the immune-suppressing microenvironment sensitizes melanoma to immune checkpoint modulators
SO NATURE NANOTECHNOLOGY
LA English
DT Article
ID METASTATIC MELANOMA; CO-STIMULATION; T-CELLS; BLOCKADE; CANCER; NANOPARTICLES; PEPTIDES; OX40; IMMUNOTHERAPY; VACCINATION
AB A low response rate, acquired resistance and severe side effects have limited the clinical outcomes of immune checkpoint therapy. Here, we show that combining cancer nanovaccines with an anti-PD-1 antibody (alpha PD-1) for immunosuppression blockade and an anti-OX40 antibody (alpha OX40) for effector T-cell stimulation, expansion and survival can potentiate the efficacy of melanoma therapy. Prophylactic and therapeutic combination regimens of dendritic cell-targeted mannosylated nanovaccines with alpha PD-1/alpha OX40 demonstrate a synergism that stimulates T-cell infiltration into tumours at early treatment stages. However, this treatment at the therapeutic regimen does not result in an enhanced inhibition of tumour growth compared to alpha PD-1/alpha OX40 alone and is accompanied by an increased infiltration of myeloid-derived suppressor cells in tumours. Combining the double therapy with ibrutinib, a myeloid-derived suppressor cell inhibitor, leads to a remarkable tumour remission and prolonged survival in melanoma-bearing mice. The synergy between the mannosylated nanovaccines, ibrutinib and alpha PD-1/alpha OX40 provides essential insights to devise alternative regimens to improve the efficacy of immune checkpoint modulators in solid tumours by regulating the endogenous immune response.
C1 [Conniot, Joao; Scomparin, Anna; Yeini, Eilam; Pozzi, Sabina; Kleiner, Ron; Satchi-Fainaro, Ronit] Tel Aviv Univ, Sackler Fac Med, Dept Physiol & Pharmacol, Tel Aviv, Israel.
[Conniot, Joao; Peres, Carina; Matos, Ana, I; Moura, Liane I. F.; Zupancic, Eva; Gois, Pedro M. P.; Florindo, Helena F.] Univ Lisbon, Fac Pharm, Res Inst Med iMed ULisboa, Lisbon, Portugal.
[Scomparin, Anna] Univ Turin, Dept Drug Sci & Technol, Turin, Italy.
[Zupancic, Eva; Jung, Steffen] Weizmann Inst Sci, Dept Immunol, Rehovot, Israel.
[Viana, Ana S.] Univ Lisbon, Fac Sci, Ctr Chem & Biochem, Lisbon, Portugal.
[Doron, Hila; Erez, Neta] Tel Aviv Univ, Sackler Fac Med, Dept Pathol, Tel Aviv, Israel.
C3 Tel Aviv University; Sackler Faculty of Medicine; Universidade de Lisboa; University of Turin; Weizmann Institute of Science; Universidade de Lisboa; Tel Aviv University; Sackler Faculty of Medicine
RP Satchi-Fainaro, R (通讯作者)，Tel Aviv Univ, Sackler Fac Med, Dept Physiol & Pharmacol, Tel Aviv, Israel.; Florindo, HF (通讯作者)，Univ Lisbon, Fac Pharm, Res Inst Med iMed ULisboa, Lisbon, Portugal.
EM ronitsf@tauex.tau.ac.il; hflorindo@ff.ulisboa.pt
RI Peres, Carina/AAD-3718-2021; Pozzi, Sabina/JAC-8743-2023; Jung, Steffen/K-1409-2012; Satchi-Fainaro, Ronit/F-9137-2011; Viana, Ana S/D-7048-2012; Matos, Ana I/HMV-9020-2023; gois, pedro M P/A-9251-2014; Erez, Neta/G-8584-2012; Florindo, Helena F./AAB-3963-2019; Scomparin,

Anna/AAG-8832-2019; Moura, Liane/F-6534-2015
OI Peres, Carina/0000-0001-9399-9156; Jung, Steffen/0000-0003-4290-5716; Satchi-Fainaro, Ronit/0000-0002-7360-7837; Viana, Ana S/0000-0001-8405-9672; gois, pedro M P/0000-0002-7698-630X; Florindo, Helena F./0000-0002-4006-5840; Scomparin, Anna/0000-0003-4751-620X; Moura, Liane/0000-0003-4264-6865; Doron, Hila/0000-0001-9704-7240; Neves de Matos, Ana Isabel/0000-0002-7325-6328; Pozzi, Sabina/0000-0002-6299-9273; Conniot, Joao/0000-0001-8784-6110; Erez, Neta/0000-0001-6506-9074
FU Israeli Ministry of Health; Fundacao para a Ciencia e Tecnologia-Ministerio da Ciencia, Tecnologia e Ensino Superior (FCT-MCTES) [ENMed/0051/2016]; European Research Council (ERC) [617445]; ERC [835227]; Israel Science Foundation [918/14, 1969/18]; Melanoma Research Alliance (the Saban Family Foundation-MRA); Israel Cancer Research Fund (ICRF); FCT-MCTES [SFRH/BD/87150/2012, PD/BD/113959/2015, SFRH/BD/87591/2012, SFRH/BD/78480/2011, SFRH/BPD/94111/2013]; European Structural & Investment Funds through the COMPETE Programme; FCT [SAICTPAC/0019/2015]; Fundação para a Ciência e a Tecnologia [SFRH/BD/87591/2012, SFRH/BD/87150/2012, PD/BD/113959/2015, ENMed/0051/2016, SFRH/BPD/94111/2013, SFRH/BD/78480/2011] Funding Source: FCT; European Research Council (ERC) [835227] Funding Source: European Research Council (ERC)
FX The MultiNano@MBM project was supported by The Israeli Ministry of Health and The Fundacao para a Ciencia e Tecnologia-Ministerio da Ciencia, Tecnologia e Ensino Superior (FCT-MCTES) under the framework of EuroNanoMed-II (ENMed/0051/2016 to H.F.F., R.S.-F. and S.J.). R.S.-F. thanks the European Research Council (ERC) Consolidator Grant Agreement no. [617445]-PolyDorm and ERC Advanced Grant Agreement no. [835227] - 3DBrainStrom, The Israel Science Foundation (Grant nos. 918/14 and 1969/18), The Melanoma Research Alliance (the Saban Family Foundation-MRA Team Science Award to R.S.-F. and N.E., and Established Investigator Award to R.S.-F.) and the Israel Cancer Research Fund (ICRF). J.C., A.I.M., C.P., E.Z. and L.I.F.M. are supported by the FCT-MCTES (Fellowships SFRH/BD/87150/2012, PD/BD/113959/2015, SFRH/BD/87591/2012, SFRH/BD/78480/2011 and SFRH/BPD/94111/2013, respectively). This project has received funding from European Structural & Investment Funds through the COMPETE Programme and from National Funds through FCT under the Programme grant SAICTPAC/0019/2015 (H.F.F.). We thank E. Haimov and B. Redko from the Blavatnik Center for Drug Discovery at the Tel Aviv University for their professional and technical assistance with the peptide synthesis.
CR Alonso-Sande M, 2013, BIOMACROMOLECULES, V14, P4046, DOI 10.1021/bm401141u
[Anonymous], 2017, JAMA-J AM MED ASSOC, DOI DOI 10.1001/JAMA.2016.20324
Arch RH, 1998, MOL CELL BIOL, V18, P558, DOI 10.1128/MCB.18.1.558
Aspeslagh S, 2016, EUR J CANCER, V52, P50, DOI 10.1016/j.ejca.2015.08.021
Azzi J, 2016, CELL REP, V15, P1202, DOI 10.1016/j.celrep.2016.04.007
Beyersdorf N, 2015, IMMUNOTARGETS THER, V4, P111, DOI 10.2147/ITT.S61647
Burkholder B, 2014, BBA-REV CANCER, V1845, P182, DOI 10.1016/j.bbcan.2014.01.004
Chen LP, 2013, NAT REV IMMUNOL, V13, P227, DOI 10.1038/nri3405
De Koker S, 2016, ANGEW CHEM INT EDIT, V55, P1334, DOI 10.1002/anie.201508626
DRANOFF G, 1993, P NATL ACAD SCI USA, V90, P3539, DOI 10.1073/pnas.90.8.3539
Fang HL, 2014, CELL MOL IMMUNOL, V11, P150, DOI 10.1038/cmi.2013.59
Fridlender ZG, 2010, CANCER RES, V70, P109, DOI 10.1158/0008-5472.CAN-09-2326
Gabrilovich DI, 2009, NAT REV IMMUNOL, V9, P162, DOI 10.1038/nri2506
Gajewski TF, 2015, SEMIN ONCOL, V42, P663, DOI 10.1053/j.seminoncol.2015.05.011
Gramaglia I, 2000, J IMMUNOL, V165, P3043, DOI 10.4049/jimmunol.165.6.3043
Guo ZH, 2014, PLOS ONE, V9, DOI 10.1371/journal.pone.0087029
Hanahan D, 2012, CANCER CELL, V21, P309, DOI 10.1016/j.ccr.2012.02.022
Hodi FS, 2010, NEW ENGL J MED, V363, P711, DOI 10.1056/NEJMoa1003466
Kaiser AD, 2015, CANCER GENE THER, V22, P72, DOI 10.1038/cgt.2014.78
Kodumudi KN, 2012, J IMMUNOL, V189, P5147, DOI 10.4049/jimmunol.1200274

Kowalczyk DW, 2003, CANCER GENE THER, V10, P870, DOI 10.1038/sj.cgt.7700653
Maher Elizabeth A, 2009, Cancer Res, V69, P6015, DOI 10.1158/0008-5472.CAN-08-4347
Meyer C, 2014, CANCER IMMUNOL IMMUN, V63, P247, DOI 10.1007/s00262-013-1508-5
Minn AJ, 2016, CELL, V165, P272, DOI 10.1016/j.cell.2016.03.031
Nagaraj S, 2010, J IMMUNOL, V184, P3106, DOI 10.4049/jimmunol.0902661
Natarajan G., 2016, ONCOIMMUNOLOGY, V5
Pasare C, 2003, CURR OPIN IMMUNOL, V15, P677, DOI 10.1016/j.coi.2003.09.002
Phan GQ, 2003, J IMMUNOTHER, V26, P349, DOI 10.1097/00002371-200307000-00007
Sagiv-Barfi I, 2018, SCI TRANSL MED, V10, DOI 10.1126/scitranslmed.aan4488
Sagiv-Barfi I, 2015, P NATL ACAD SCI USA, V112, pE966, DOI 10.1073/pnas.1500712112
Santarelli Justin G, 2007, Neurosurg Focus, V22, pE1
Sarff M, 2008, AM J SURG, V195, P621, DOI 10.1016/j.amjsurg.2007.12.036
Scheller J, 2011, BBA-MOL CELL RES, V1813, P878, DOI 10.1016/j.bbamcr.2011.01.034
Schwartz H, 2016, CANCER RES, V76, P4359, DOI 10.1158/0008-5472.CAN-16-0485
Seliger B, 2008, ADV CANCER RES, V101, P249, DOI 10.1016/S0065-230X(08)00407-7
Shedlock DJ, 2003, SCIENCE, V300, P337, DOI 10.1126/science.1082305
Silva JM, 2015, J CONTROL RELEASE, V198, P91, DOI 10.1016/j.jconrel.2014.11.033
Slingluff CL, 2013, CLIN CANCER RES, V19, P4228, DOI 10.1158/1078-0432.CCR-13-0002
Spranger S, 2014, J IMMUNOTHER CANCER, V2, DOI 10.1186/2051-1426-2-3
Stiff A, 2016, CANCER RES, V76, P2125, DOI 10.1158/0008-5472.CAN-15-1490
Swart M, 2016, FRONT ONCOL, V6, DOI 10.3389/fonc.2016.00233
Topalian SL, 2012, NEW ENGL J MED, V366, P2443, DOI 10.1056/NEJMoa1200690
Tsui Ping, 2007, Human Antibodies, V16, P117
Tumeh PC, 2014, NATURE, V515, P568, DOI 10.1038/nature13954
van den Eertwegh AJM, 2012, LANCET ONCOL, V13, P509, DOI 10.1016/S1470-2045(12)70007-4
van Kooyk Y, 2008, BIOCHEM SOC T, V36, P1478, DOI 10.1042/BST0361478
Vetto JT, 1997, AM J SURG, V174, P258, DOI 10.1016/S0002-9610(97)00139-6
Wang X, 2011, ANALYST, V136, P4174, DOI 10.1039/c1an15469a
Wilson DS, 2019, NAT MATER, V18, P175, DOI 10.1038/s41563-018-0256-5
Woods DM, 2016, CANCER IMMUNOL RES, V4, DOI 10.1158/2326-6066.IMM2016-A067
Zhu Q, 2010, J CLIN INVEST, V120, P607, DOI 10.1172/JCI39293
NR 51
TC 136
Z9 137
U1 6
U2 157
PU NATURE PUBLISHING GROUP
PI LONDON
PA MACMILLAN BUILDING, 4 CRINAN ST, LONDON N1 9XW, ENGLAND
SN 1748-3387
EI 1748-3395
J9 NAT NANOTECHNOL
JI Nat. Nanotechnol.
PD SEP
PY 2019
VL 14
IS 9
BP 891
EP +
DI 10.1038/s41565-019-0512-0
PG 13
WC Nanoscience & Nanotechnology; Materials Science, Multidisciplinary
WE Science Citation Index Expanded (SCI-EXPANDED)
SC Science & Technology - Other Topics; Materials Science
GA IV9RL
UT WOS:000484601200015
PM 31384037
OA Green Submitted
DA 2023-09-14
ER

PT J
AU Fontana, F
Shahbazi, MA
Liu, DF
Zhang, HB
Makila, E
Salonen, J
Hirvonen, JT
Santos, HA
AF Fontana, Flavia
Shahbazi, Mohammad-Ali
Liu, Dongfei
Zhang, Hongbo
Makila, Ermei
Salonen, Jarno
Hirvonen, Jouni T.
Santos, Helder A.
TI Multistaged Nanovaccines Based on Porous Silicon@Acetalated
Dextran@Cancer Cell Membrane for Cancer Immunotherapy
SO ADVANCED MATERIALS
LA English
DT Article
ID SYNTHETIC NANOPARTICLES; CROSS-PRESENTATION; SURFACE-CHEMISTRY; DELIVERY
PLATFORM; DENDRITIC CELLS; DRUG-DELIVERY; MICROPARTICLES; FABRICATION;
VESICLES; IMMUNITY
C1 [Fontana, Flavia; Shahbazi, Mohammad-Ali; Liu, Dongfei; Zhang, Hongbo; Hirvonen, Jouni T.; Santos, Helder A.] Univ Helsinki, Fac Pharm, Div Pharmaceut Chem & Technol, FI-00014 Helsinki, Finland.
[Liu, Dongfei; Zhang, Hongbo] Harvard Univ, Harvard John A Paulson Sch Engn & Appl Sci, Cambridge, MA 02138 USA.
[Makila, Ermei; Salonen, Jarno] Univ Turku, Lab Ind Phys, FI-20014 Turku, Finland.
[Shahbazi, Mohammad-Ali] Tech Univ Denmark, BioLabChip, Dept Micro & Nanotechnol, DK-2800 Lyngby, Denmark.
[Zhang, Hongbo] Abo Akad Univ, Dept Pharmaceut Sci, FI-20520 Turku, Finland.
C3 University of Helsinki; Harvard University; University of Turku;
Technical University of Denmark; Abo Akademi University
RP Fontana, F; Santos, HA (通讯作者)，Univ Helsinki, Fac Pharm, Div Pharmaceut Chem & Technol, FI-00014 Helsinki, Finland.
EM flavia.fontana@helsinki.fi; helder.santos@helsinki.fi
RI Liu, Dongfei/B-1087-2011; shahbazi, Mohammad-Ali/M-5838-2013; Santos,
Hélder A./B-5253-2009; Fontana, Flavia/AAB-4898-2020; Santos, Hélder
A./AAC-6347-2022; Zhang, Hongbo/O-8330-2019
OI Liu, Dongfei/0000-0002-2426-134X; shahbazi,
Mohammad-Ali/0000-0002-4860-3017; Santos, Hélder A./0000-0001-7850-6309;
Fontana, Flavia/0000-0001-9112-6917; Santos, Hélder
A./0000-0001-7850-6309; Zhang, Hongbo/0000-0002-1071-4416; Makila,
Ermei/0000-0002-8300-6533; Hirvonen, Jouni Tapio/0000-0002-5029-1657
FU Jane and Aatos Erkko Foundation [4704010]; Academy of Finland [277423,
252215, 281300]; University of Helsinki Research Funds; Biocentrum
Helsinki; Sigrid Juselius Foundation [4704580]; European Research
Council under the European Union's Seventh Framework Programme (FP)
[310892]; Academy of Finland (AKA) [277423, 277423] Funding Source:
Academy of Finland (AKA)
FX H.Z. acknowledges the Jane and Aatos Erkko Foundation (Grant No.
4704010) for financial support. J.S. also acknowledges financial support
from the Academy of Finland (decision no. 277423). H.A.S. acknowledges
financial support from the Academy of Finland (decision nos. 252215 and
281300), the University of Helsinki Research Funds, the Biocentrum
Helsinki, Sigrid Juselius Foundation (decision no. 4704580), and the
European Research Council under the European Union's Seventh Framework
Programme (FP/2007-2013, Grant No. 310892). The authors thank the
Electron Microscopy Unit and the Flow Cytometry Unit of the Institute of

Biotechnology, University of Helsinki, for providing the necessary
laboratory facilities and assistance. The SEM analysis was carried out
in Harvard CNS and the authors thank Prof. David A. Weitz at Harvard
University for helpful discussions.
CR Bachelder EM, 2010, MOL PHARMACEUT, V7, P826, DOI 10.1021/mp900311x
Bachmann MF, 2010, NAT REV IMMUNOL, V10, P787, DOI 10.1038/nri2868
Bimbo LM, 2011, BIOMATERIALS, V32, P2625, DOI 10.1016/j.biomaterials.2010.12.011
Broaders KE, 2009, P NATL ACAD SCI USA, V106, P5497, DOI 10.1073/pnas.0901592106
Chen LP, 2013, NAT REV IMMUNOL, V13, P227, DOI 10.1038/nri3405
Chen WS, 2016, NANOSCALE, V8, P10364, DOI 10.1039/c6nr00535g
Cheung AS, 2016, SMALL, V12, P2321, DOI 10.1002/smll.201600061
Choi SYC, 2014, ADV DRUG DELIVER REV, V79-80, P222, DOI 10.1016/j.addr.2014.09.009
Cohen JL, 2011, BIOCONJUGATE CHEM, V22, P1056, DOI 10.1021/bc100542r
Corkum CP, 2015, BMC IMMUNOL, V16, DOI 10.1186/s12865-015-0113-0
Cui LN, 2011, BIOCONJUGATE CHEM, V22, P949, DOI 10.1021/bc100596w
Evangelopoulos M, 2016, BIOMATERIALS, V82, P168, DOI 10.1016/j.biomaterials.2015.11.054
Fang RH, 2015, SMALL, V11, P5483, DOI 10.1002/smll.201501284
Fang RH, 2014, NANO LETT, V14, P2181, DOI 10.1021/nl500618u
Flacher V, 2015, ACS CHEM BIOL, V10, P2697, DOI 10.1021/acschembio.5b00552
Garg AD, 2014, CELL DEATH DIFFER, V21, P26, DOI 10.1038/cdd.2013.48
Godin Biana, 2008, Controll Release Newsl, V25, P9
Godowski PJ, 2005, NAT IMMUNOL, V6, P544, DOI 10.1038/ni0605-544
Gonzalez FE, 2014, HUM VACC IMMUNOTHER, V10, P3261, DOI 10.4161/21645515.2014.982996
Gu L, 2016, NAT REV CANCER, V16, P56, DOI 10.1038/nrc.2015.3
Hu CMJ, 2015, NATURE, V526, P118, DOI 10.1038/nature15373
Hu CMJ, 2011, P NATL ACAD SCI USA, V108, P10980, DOI 10.1073/pnas.1106634108
Irvine DJ, 2015, CHEM REV, V115, P11109, DOI 10.1021/acs.chemrev.5b00109
Kelly PM, 2015, NAT NANOTECHNOL, V10, P472, DOI [10.1038/nnano.2015.47,
10.1038/NNANO.2015.47]
Kodali V, 2013, ACS NANO, V7, P6997, DOI 10.1021/nn402145t
Kong F, 2015, ADV FUNCT MATER, V25, P3330, DOI 10.1002/adfm.201500594
Krysko DV, 2012, NAT REV CANCER, V12, P860, DOI 10.1038/nrc3380
Kwong B, 2013, CANCER RES, V73, P1547, DOI 10.1158/0008-5472.CAN-12-3343
Leleux J, 2013, ADV HEALTHC MATER, V2, P72, DOI 10.1002/adhm.201200268
Liu DF, 2015, ADV MATER, V27, P2298, DOI 10.1002/adma.201405408
Liu DF, 2015, BIOMATERIALS, V39, P249, DOI 10.1016/j.biomaterials.2014.10.079
Mailliard RB, 2004, CANCER RES, V64, P5934, DOI 10.1158/0008-5472.CAN-04-1261
Mempel TR, 2004, NATURE, V427, P154, DOI 10.1038/nature02238
Meraz IM, 2014, PLOS ONE, V9, DOI 10.1371/journal.pone.0094703
Miller SI, 2005, NAT REV MICROBIOL, V3, P36, DOI 10.1038/nrmicro1068
Mitra A, 2013, TRENDS BIOTECHNOL, V31, P347, DOI 10.1016/j.tibtech.2013.03.006
Parodi A, 2013, NAT NANOTECHNOL, V8, P61, DOI 10.1038/nnano.2012.212
Paulis LE, 2013, CURR OPIN IMMUNOL, V25, P389, DOI 10.1016/j.coi.2013.03.001
Peine KJ, 2013, MOL PHARMACEUT, V10, P2849, DOI 10.1021/mp300643d
Rosenberg SA, 2015, SCIENCE, V348, P62, DOI 10.1126/science.aaa4967
Santos HA, 2010, ACTA BIOMATER, V6, P2721, DOI 10.1016/j.actbio.2009.12.043
Savage DJ, 2013, CURR OPIN PHARMACOL, V13, P834, DOI 10.1016/j.coph.2013.06.006
Shahbazi MA, 2014, BIOMATERIALS, V35, P9224, DOI 10.1016/j.biomaterials.2014.07.050
Shahbazi Mohammad-Ali, 2012, Biomatter, V2, P296, DOI 10.4161/biom.22347
Shen H, 2006, IMMUNOLOGY, V117, P78, DOI 10.1111/j.1365-2567.2005.02268.x
Smith DM, 2013, NAT REV IMMUNOL, V13, P592, DOI 10.1038/nri3488
Storni T, 2005, ADV DRUG DELIVER REV, V57, P333, DOI 10.1016/j.addr.2004.09.008
Tasciotti E, 2008, NAT NANOTECHNOL, V3, P151, DOI 10.1038/nnano.2008.34
Thery C, 2009, NAT REV IMMUNOL, V9, P581, DOI 10.1038/nri2567
van der Burg SH, 2016, NAT REV CANCER, V16, P219, DOI 10.1038/nrc.2016.16
Vegas AJ, 2016, NAT BIOTECHNOL, V34, P345, DOI 10.1038/nbt.3462
Veiseh O, 2015, NAT MATER, V14, P643, DOI [10.1038/NMAT4290, 10.1038/nmat4290]
Wong KK, 2016, ADV IMMUNOL, V130, P191, DOI 10.1016/bs.ai.2015.12.001
Woo SR, 2015, ANNU REV IMMUNOL, V33, P445, DOI 10.1146/annurev-immunol-032414-112043
Xia XJ, 2015, CELL REP, V11, P957, DOI 10.1016/j.celrep.2015.04.009
Yang YP, 2004, NAT IMMUNOL, V5, P508, DOI 10.1038/ni1059

Zhang HB, 2014, ADV MATER, V26, P4497, DOI 10.1002/adma.201400953
NR 57
TC 127
Z9 131
U1 4
U2 181
PU WILEY-V C H VERLAG GMBH
PI WEINHEIM
PA POSTFACH 101161, 69451 WEINHEIM, GERMANY
SN 0935-9648
EI 1521-4095
J9 ADV MATER
JI Adv. Mater.
PD FEB 17
PY 2017
VL 29
IS 7
AR 1603239
DI 10.1002/adma.201603239
PG 9
WC Chemistry, Multidisciplinary; Chemistry, Physical; Nanoscience &
Nanotechnology; Materials Science, Multidisciplinary; Physics, Applied;
Physics, Condensed Matter
WE Science Citation Index Expanded (SCI-EXPANDED)
SC Chemistry; Science & Technology - Other Topics; Materials Science;
Physics
GA EN6WR
UT WOS:000396144600001
PM 28009461
DA 2023-09-14
ER

PT J
AU Wang, C
Li, P
Liu, LL
Pan, H
Li, HC
Cai, LT
Ma, YF
AF Wang, Ce
Li, Ping
Liu, Lanlan
Pan, Hong
Li, Hongchang
Cai, Lintao
Ma, Yifan
TI Self-adjuvanted nanovaccine for cancer immunotherapy: Role of lysosomal
rupture-induced ROS in MHC class I antigen presentation
SO BIOMATERIALS
LA English
DT Article
DE pH-sensitive; Lysosomal rupture; ROS; Proteasome activity; MHC I antigen
presentation
ID TRANS-RETINOIC ACID; CROSS-PRESENTATION; DENDRITIC CELLS; CATIONIC
LIPOSOMES; OXIDATIVE STRESS; VACCINE ADJUVANT; DELIVERY-SYSTEM;
ACTIVATION; NANOTECHNOLOGY; NANOPARTICLES
AB MHC class I (MHC I) antigen presentation of exogenous antigens (so called "cross presentation") is a central mechanism of CD8(+) cytotoxic T lymphocyte (CTL) responses essential for successful vaccine based cancer immunotherapy. The present study constructed amphiphilic pH-sensitive galactosyl dextran-retinal (GDR) nanogels for cancer vaccine delivery, in which dextran was conjugated with all trans retinal

(a metabolite of vitamin A) through a pH-sensitive hydrazone bond, followed by galactosylation to acquire dendritic cell (DC)-targeting ability. Our results showed that pH-sensitive GDR nanogel was a self-adjuvanted vaccine carrier that not only promoted DC maturation through activating retinoic acid receptor (RAR) signaling, but also facilitated antigen uptake and cytosolic antigen release in DCs. Furthermore, pH-sensitive GDR nanogel effectively augmented MHC I antigen presentation and evoked potent anti-cancer immune responses in vivo. More importantly, we first reported that nanoparticle-triggered lysosome rupture could directly induce ROS production in DCs, which was found to be essential for augmenting proteasome activity and downstream MHC I antigen presentation. Hence, DC-targeted pH-sensitive GDR nanogels could be a potent delivery system for cancer vaccine development. Triggering lyososomal rupture in DCs with pH-sensitive nanoparticles might be a plausible strategy to elevate intracellular ROS production for promoting antigen cross presentation, thereby improving cancer vaccine efficacy. (C) 2015 Elsevier Ltd. All rights reserved.
C1 [Wang, Ce; Li, Ping; Liu, Lanlan; Pan, Hong; Li, Hongchang; Cai, Lintao; Ma, Yifan] Chinese Acad Sci, Shenzhen Inst Adv Technol, Key Lab Hlth Informat, Guangdong Key Lab Nanomed, Shenzhen 518055, Peoples R China.
C3 Chinese Academy of Sciences; Shenzhen Institute of Advanced Technology, CAS
RP Ma, YF (通讯作者)，Chinese Acad Sci, Shenzhen Inst Adv Technol, 1068 Xueyuan Ave, Shenzhen 518055, Peoples R China.
EM yf.ma@siat.ac.cn
RI Li, Hongchang/GQO-9662-2022; Pan, Hong/AAC-2326-2019
OI Cai, Lintao/0000-0002-2461-6390
FU National Natural Science Foundation of China [81171446, 81371679]; Shenzhen Overseas Outstanding Professional Talent [KQCX20140520154115 0]; Shenzhen Science and Technology Program [GJHS20140610151856702, CXZZ20130506140505859]; Guangdong leading talents program (Antibody/Protein Drugs for Major Diseases); Shenzhen Peacock Next-generation Monoclonal Antibody Drug research and development program [KQTD201210]
FX This work was supported by the National Natural Science Foundation of China (Grant No. 81171446, 81371679), Shenzhen Overseas Outstanding Professional Talent (KQCX20140520154115 0), Shenzhen Science and Technology Program (GJHS20140610151856702, CXZZ20130506140505859), Guangdong leading talents program (Antibody/Protein Drugs for Major Diseases), Shenzhen Peacock Next-generation Monoclonal Antibody Drug research and development program (KQTD201210).
CR Aiken CT, 2011, MOL CELL PROTEOMICS, V10, DOI 10.1074/mcp.M110.006924
Allie SR, 2013, J IMMUNOL, V190, P2178, DOI 10.4049/jimmunol.1201945
Andersen BM, 2012, CANCER LETT, V325, P155, DOI 10.1016/j.canlet.2012.07.012
Basta S, 2007, SCAND J IMMUNOL, V65, P311, DOI 10.1111/j.1365-3083.2007.01909.x
Bi Y, 2013, ARCH MED SCI, V9, P314, DOI 10.5114/aoms.2012.31410
CastroObregon S, 1996, FEBS LETT, V381, P93, DOI 10.1016/0014-5793(96)00088-9
Cheong TC, 2015, ACS CHEM BIOL, V10, P757, DOI 10.1021/cb5009124
den Haan JMM, 2014, IMMUNOL LETT, V162, P103, DOI 10.1016/j.imlet.2014.10.011
Denda-Nagai K, 2010, J BIOL CHEM, V285, P19193, DOI 10.1074/jbc.M110.113613
Fehres CM, 2014, FRONT IMMUNOL, V5, DOI 10.3389/fimmu.2014.00149
Geissmann F, 2003, J EXP MED, V198, P623, DOI 10.1084/jem.20030390
Ghosh M, 2011, FEBS LETT, V585, P623, DOI 10.1016/j.febslet.2010.12.043
Goldberg MS, 2015, CELL, V161, P201, DOI 10.1016/j.cell.2015.03.037
Gostner JM, 2013, REDOX REP, V18, P88, DOI 10.1179/1351000213Y.0000000044
Hirosue S, 2010, VACCINE, V28, P7897, DOI 10.1016/j.vaccine.2010.09.077
Jhaveri A, 2014, J CONTROL RELEASE, V190, P352, DOI 10.1016/j.jconrel.2014.05.002
Li P, 2013, J CONTROL RELEASE, V168, P271, DOI 10.1016/j.jconrel.2013.03.025
Lin YZ, 2010, INVEST OPHTH VIS SCI, V51, P6483, DOI 10.1167/iovs.10-5410
Liu Q, 2015, ACS NANO, V9, P4925, DOI 10.1021/nn5066793
Luo ZC, 2013, J CONTROL RELEASE, V170, P259, DOI 10.1016/j.jconrel.2013.05.027
Ma YF, 2011, NANOSCALE, V3, P2307, DOI 10.1039/c1nr10166h
Melero I, 2014, NAT REV CLIN ONCOL, V11, P509, DOI 10.1038/nrclinonc.2014.111
Melief CJM, 2002, IMMUNOL REV, V188, P177, DOI 10.1034/j.1600-065X.2002.18816.x
Moon JJ, 2011, NAT MATER, V10, P243, DOI 10.1038/NMAT2960
Nathan C, 2013, NAT REV IMMUNOL, V13, P349, DOI 10.1038/nri3423

Pino-Lagos K, 2011, J EXP MED, V208, P1767, DOI 10.1084/jem.20102358
Pino-Lagos K, 2010, BIOFACTORS, V36, P430, DOI 10.1002/biof.117
Poudel B, 2015, J BIOSCIENCES, V40, P71, DOI 10.1007/s12038-015-9507-7
Rock KL, 2005, IMMUNOL REV, V207, P166, DOI 10.1111/j.0105-2896.2005.00301.x
Ross AC, 2011, VITAM HORM, V86, P103, DOI 10.1016/B978-0-12-386960-9.00005-8
Saha B, 2010, CYTOKINE, V50, P1, DOI 10.1016/j.cyto.2009.11.021
Scott EA, 2012, BIOMATERIALS, V33, P6211, DOI 10.1016/j.biomaterials.2012.04.060
Seifert U, 2010, CELL, V142, P613, DOI 10.1016/j.cell.2010.07.036
Smith DM, 2013, NAT REV IMMUNOL, V13, P592, DOI 10.1038/nri3488
Son S, 2012, ACCOUNTS CHEM RES, V45, P1100, DOI 10.1021/ar200248u
Sun BB, 2013, ACS NANO, V7, P10834, DOI 10.1021/nn404211j
Tan XF, 2011, J VIROL, V85, P8316, DOI 10.1128/JVI.00781-11
Teoh CY, 2004, ARCH BIOCHEM BIOPHYS, V423, P88, DOI 10.1016/j.abb.2003.12.001
Trinchieri G, 1998, ADV IMMUNOL, V70, P83, DOI 10.1016/S0065-2776(08)60387-9
Wang C, 2014, VACCINE, V32, P5475, DOI 10.1016/j.vaccine.2014.07.081
Wang X, 2014, BIOCHIMIE, V106, P121, DOI 10.1016/j.biochi.2014.08.009
Warnatsch A, 2013, MOL IMMUNOL, V55, P106, DOI 10.1016/j.molimm.2012.10.007
Watts C, 1997, ANNU REV IMMUNOL, V15, P821, DOI 10.1146/annurev.immunol.15.1.821
Wick DA, 2011, VACCINE, V29, P984, DOI 10.1016/j.vaccine.2010.11.036
Yuan YY, 2015, ANGEW CHEM INT EDIT, V54, P11419, DOI 10.1002/anie.201503640
Zhuang Y, 2012, J CONTROL RELEASE, V159, P135, DOI 10.1016/j.jconrel.2011.12.017
NR 46
TC 125
Z9 127
U1 8
U2 256
PU ELSEVIER SCI LTD
PI OXFORD
PA THE BOULEVARD, LANGFORD LANE, KIDLINGTON, OXFORD OX5 1GB, OXON, ENGLAND
SN 0142-9612
EI 1878-5905
J9 BIOMATERIALS
JI Biomaterials
PD FEB
PY 2016
VL 79
BP 88
EP 100
DI 10.1016/j.biomaterials.2015.11.040
PG 13
WC Engineering, Biomedical; Materials Science, Biomaterials
WE Science Citation Index Expanded (SCI-EXPANDED)
SC Engineering; Materials Science
GA DB9WM
UT WOS:000368868400009
PM 26702587
DA 2023-09-14
ER

PT J
AU Ni, QQ
Zhang, FW
Liu, YJ
Wang, ZT
Yu, GC
Liang, B
Niu, G
Su, T
Zhu, GZ
Lu, GM
Zhang, LJ

Chen, XY
AF Ni, Qianqian
Zhang, Fuwu
Liu, Yijing
Wang, Zhantong
Yu, Guocan
Liang, Brian
Niu, Gang
Su, Ting
Zhu, Guizhi
Lu, Guangming
Zhang, Longjiang
Chen, Xiaoyuan
TI A bi-adjuvant nanovaccine that potentiates immunogenicity of neoantigen for combination immunotherapy of colorectal cancer
SO SCIENCE ADVANCES
LA English
DT Article
ID TLR AGONISTS; VACCINES; NANOPARTICLES; ACTIVATION; EFFICACY; IMMUNITY; ANTIGEN
AB Neoantigen vaccines have been enthusiastically pursued for personalized cancer immunotherapy while vast majority of neoantigens have no or low immunogenicity. Here, a bi-adjuvant neoantigen nanovaccine (banNV) that codelivered a peptide neoantigen (Adpgk) with two adjuvants [Toll-like receptor (TLR) 7/8 agonist R848 and TLR9 agonist CpG] was developed for potent cancer immunotherapy. Specifically, banNVs were prepared by a nanotemplated synthesis of concatemer CpG, nanocondensation with cationic polypeptides, and then physical loading with hydrophobic R848 and Adpgk. The immunogenicity of the neoantigen was profoundly potentiated by efficient codelivery of neoantigen and dual synergistic adjuvants, which is accompanied by reduced acute systemic toxicity. BanNVs sensitized immune checkpoint programmed death receptor 1 (PD-1) on T cells, therefore, a combination of banNVs with aPD-1 conspicuously induced the therapy response and led to complete regression of 70% neoantigen-specific tumors without recurrence. We conclude that banNVs are promising to optimize personalized therapeutic neoantigen vaccines for cancer immunotherapy.
C1 [Ni, Qianqian; Lu, Guangming; Zhang, Longjiang] Nanjing Univ, Jinling Hosp, Dept Med Imaging, Med Sch, Nanjing 210002, Jiangsu, Peoples R China.
[Ni, Qianqian; Zhang, Fuwu; Liu, Yijing; Wang, Zhantong; Yu, Guocan; Liang, Brian; Niu, Gang; Chen, Xiaoyuan] Natl Inst Biomed Imaging & Bioengn, Lab Mol Imaging & Nanomed, NIH, Bethesda, MD 20892 USA.
[Su, Ting; Zhu, Guizhi] Ctr Pharmaceut Engn & Sci, Inst Struct Biol Drug Discovery & Dev, Sch Pharm, Dept Pharmaceut, Richmond, VA 23219 USA.
[Su, Ting; Zhu, Guizhi] Massey Canc Ctr, Richmond, VA 23219 USA.
[Su, Ting; Zhu, Guizhi] Virginia Commonwealth Univ, Richmond, VA 23219 USA.
C3 Nanjing University; National Institutes of Health (NIH) - USA; NIH National Institute of Biomedical Imaging & Bioengineering (NIBIB); Virginia Commonwealth University; Virginia Commonwealth University
RP Lu, GM; Zhang, LJ (通讯作者)，Nanjing Univ, Jinling Hosp, Dept Med Imaging, Med Sch, Nanjing 210002, Jiangsu, Peoples R China.; Chen, XY (通讯作者)，Natl Inst Biomed Imaging & Bioengn, Lab Mol Imaging & Nanomed, NIH, Bethesda, MD 20892 USA.; Zhu, GZ (通讯作者)，Ctr Pharmaceut Engn & Sci, Inst Struct Biol Drug Discovery & Dev, Sch Pharm, Dept Pharmaceut, Richmond, VA 23219 USA.; Zhu, GZ (通讯作者)，Massey Canc Ctr, Richmond, VA 23219 USA.; Zhu, GZ (通讯作者)，Virginia Commonwealth Univ, Richmond, VA 23219 USA.
EM gzhu2@vcu.edu; cjr.luguangming@vip.163.com; kevinzhlj@163.com; shawn.chen@nih.gov
RI Zhu, Guizhi/AAN-2888-2020; Liu, Yijing/D-6666-2016; chen, xia/GXM-5435-2022; chen, xin/IQW-3432-2023; Wang, ZT/GXN-3762-2022; Zhang, Fuwu/T-4066-2019; chen, xi/GXH-3653-2022; chen, xia/GYR-3948-2022; Yu, Guocan/D-2963-2014
OI Zhang, Fuwu/0000-0002-1928-3472; Yu, Guocan/0000-0003-1157-4184; Liu, Yijing/0000-0002-7503-3910
FU Intramural Research Program (IRP) of the NIBIB, NIH [1ZIAEB000073-09]; National Key Basic Research Program of the PRC [2014CB744501, 2014CB744504]; Key projects of the National Natural Science Foundation

of China [81230032]; Major International (Regional) Joint Research
Program of China [81120108013]; VCU School of Pharmacy [KL2TR002648,
UL1TR002649]; NCATS Clinical and Translational Science Award (CTSA)
[KL2TR002648, UL1TR002649]; VCU Massey Cancer Center [P30 CA106059]
FX This research was supported partially by the Intramural Research Program
(IRP) of the NIBIB, NIH (1ZIAEB000073-09); the National Key Basic
Research Program of the PRC (2014CB744501 and 2014CB744504), Key
projects of the National Natural Science Foundation of China (81230032),
and the Major International (Regional) Joint Research Program of China
(81120108013); start-up funds from the VCU School of Pharmacy and NCATS
Clinical and Translational Science Award (CTSA) (no. KL2TR002648 under
UL1TR002649); and a pilot grant from VCU Massey Cancer Center (P30
CA106059). The contents of this publication are solely the
responsibility of the authors and do not necessarily represent the
official views of the National Institutes of Health.
CR Alizadeh AA, 2015, NAT MED, V21, P846, DOI 10.1038/nm.3915
Cho NH, 2011, NAT NANOTECHNOL, V6, P675, DOI [10.1038/NNANO.2011.149,
10.1038/nnano.2011.149]
Chu YH, 2018, THERANOSTICS, V8, P4238, DOI 10.7150/thno.24387
Coffman RL, 2010, IMMUNITY, V33, P492, DOI 10.1016/j.immuni.2010.10.002
Decker T, 2000, BLOOD, V95, P999, DOI 10.1182/blood.V95.3.999.003k10_999_1006
Fan YC, 2017, NANO LETT, V17, P7387, DOI 10.1021/acs.nanolett.7b03218
Gao S, 2019, THERANOSTICS, V9, P126, DOI 10.7150/thno.29431
Gubin MM, 2014, NATURE, V515, P577, DOI 10.1038/nature13988
Guy B, 2007, NAT REV MICROBIOL, V5, P505, DOI 10.1038/nrmicro1681
Ilyinskii PO, 2014, VACCINE, V32, P2882, DOI 10.1016/j.vaccine.2014.02.027
Irvine DJ, 2015, CHEM REV, V115, P11109, DOI 10.1021/acs.chemrev.5b00109
Kaczanowska S, 2013, J LEUKOCYTE BIOL, V93, P847, DOI 10.1189/jlb.1012501
Kasturi SP, 2011, NATURE, V470, P543, DOI 10.1038/nature09737
Kuai R, 2018, SCI ADV, V4, DOI 10.1126/sciadv.aao1736
Kuai R, 2017, NAT MATER, V16, P489, DOI [10.1038/nmat4822, 10.1038/NMAT4822]
Lee IH, 2012, ANGEW CHEM INT EDIT, V51, P8800, DOI 10.1002/anie.201203193
Li J, 2018, IMMUNITY, V48, P185, DOI 10.1016/j.immuni.2018.02.004
Liu HP, 2014, NATURE, V507, P519, DOI 10.1038/nature12978
Lu KD, 2016, J AM CHEM SOC, V138, P12502, DOI 10.1021/jacs.6b06663
Luo M, 2017, NAT NANOTECHNOL, V12, P648, DOI [10.1038/nnano.2017.52,
10.1038/NNANO.2017.52]
Lynn GM, 2015, NAT BIOTECHNOL, V33, P1201, DOI 10.1038/nbt.3371
Melero I, 2014, NAT REV CLIN ONCOL, V11, P509, DOI 10.1038/nrclinonc.2014.111
Mellman I, 2011, NATURE, V480, P480, DOI 10.1038/nature10673
Moon JJ, 2012, ADV MATER, V24, P3724, DOI 10.1002/adma.201200446
Neelapu SS, 2017, NEW ENGL J MED, V377, P2531, DOI 10.1056/NEJMoa1707447
O'Neill LAJ, 2013, NAT REV IMMUNOL, V13, P453, DOI 10.1038/nri3446
Oh N, 2014, INT J NANOMED, V9, P51, DOI 10.2147/IJN.S26592
Ott PA, 2017, NATURE, V547, P217, DOI 10.1038/nature22991
Pockros PJ, 2007, J HEPATOL, V47, P174, DOI 10.1016/j.jhep.2007.02.025
Rosenberg SA, 2015, SCIENCE, V348, P62, DOI 10.1126/science.aaa4967
Sahin U, 2017, NATURE, V547, P222, DOI 10.1038/nature23003
Saini SK, 2013, P NATL ACAD SCI USA, V110, P15383, DOI 10.1073/pnas.1308672110
Seidel JA, 2018, FRONT ONCOL, V8, DOI 10.3389/fonc.2018.00086
Shopsowitz KE, 2014, SMALL, V10, P1623, DOI 10.1002/smll.201302676
Skakuj K, 2018, J AM CHEM SOC, V140, P1227, DOI 10.1021/jacs.7b12579
Stephan SB, 2015, NAT BIOTECHNOL, V33, P97, DOI 10.1038/nbt.3104
Stronen E, 2016, SCIENCE, V352, P1337, DOI 10.1126/science.aaf2288
Suma T, 2017, J AM CHEM SOC, V139, P4009, DOI 10.1021/jacs.6b11302
Swartz MA, 2012, SCI TRANSL MED, V4, DOI 10.1126/scitranslmed.3003763
Tran E, 2017, NAT IMMUNOL, V18, P255, DOI 10.1038/ni.3682
Vacchelli E, 2012, ONCOIMMUNOLOGY, V1, P894, DOI 10.4161/onci.20931
Verma V, 2019, NAT IMMUNOL, V20, P1231, DOI 10.1038/s41590-019-0441-y
Yadav M, 2014, NATURE, V515, P572, DOI 10.1038/nature14001
Zhu GZ, 2017, NAT COMMUN, V8, DOI 10.1038/s41467-017-01386-7

Zitvogel L, 2015, NAT REV IMMUNOL, V15, P405, DOI 10.1038/nri3845
NR 45
TC 124
Z9 126
U1 16
U2 144
PU AMER ASSOC ADVANCEMENT SCIENCE
PI WASHINGTON
PA 1200 NEW YORK AVE, NW, WASHINGTON, DC 20005 USA
SN 2375-2548
J9 SCI ADV
JI Sci. Adv.
PD MAR
PY 2020
VL 6
IS 12
AR eaaw6071
DI 10.1126/sciadv.aaw6071
PG 12
WC Multidisciplinary Sciences
WE Science Citation Index Expanded (SCI-EXPANDED)
SC Science & Technology - Other Topics
GA KX5SB
UT WOS:000521937000002
PM 32206706
OA gold, Green Published
DA 2023-09-14
ER

PT J
AU Liang, RJ
Xie, J
Li, J
Wang, K
Liu, LP
Gao, YJ
Hussain, M
Shen, GX
Zhu, JT
Tao, J
AF Liang, Ruijing
Xie, Jun
Li, Jun
Wang, Ke
Liu, Liping
Gao, Yujie
Hussain, Mubashir
Shen, Guanxin
Zhu, Jintao
Tao, Juan
TI Liposomes-coated gold nanocages with antigens and adjuvants targeted
delivery to dendritic cells for enhancing antitumor immune response
SO BIOMATERIALS
LA English
DT Article
DE Gold nanocage; Peptide; Adjuvants; Targeted delivery; Bioimaging
ID CANCER-IMMUNOTHERAPY; FACILE SYNTHESIS; AU NANOCAGES; AG NANOCUBES;
IN-VITRO; VACCINES; NANOPARTICLES; TRACKING; THERAPY; MELANOMA
AB For nanovaccine-based cancer immunotherapy, dendritic cells (DCs) are one of the most powerful antigen presenting cells (APCs) that initiate and promote the maturation of antigen-specific cytotoxic T lymphocytes (e.g., CD8(+) T cells) to induce the local and systemic antitumor immunity and further

suppress the tumor metastasis and produce long-term protection against tumor. Thus, the activation and maturation of DCs is the prerequisite for efficient CD8(+) T cell-based antitumor immune responses, which is considered as a primary and promising task for nanovaccine engineering. Herein, we introduce a versatile nanovaccine of liposomes-coated gold nanocages (Lipos-AuNC5) Modified with DCs specific antibody aCD11c for targeted delivery of adjuvant MPLA and melanoma antigen peptide TRP2 to promote the activation and maturation of DCs, and enhance tumor specific T lymphocytes responses. Moreover, AuNCs accumulation and AuNCs-engulfed DCs migration to regional lymph nodes (RLNs) became realtime visualization through in vivo fluorescence and photoacoustic (PA) imaging to monitor the immunity process. In vivo experimental results demonstrated that the targeted antigen/adjuvants-loaded AuNCs exhibited enhanced antitumor immune response to inhibit tumor growth and metastasis in both B16-F10 prophylactic and lung metastasis models, which may act as a promising nanoplatform for antitumor immunotherapy and in vivo tracking. (C) 2017 Elsevier Ltd. All rights reserved.
C1 [Liang, Ruijing; Xie, Jun; Wang, Ke; Liu, Liping; Gao, Yujie; Hussain, Mubashir; Zhu, Jintao] Huazhong Univ Sci & Technol, Sch Chem & Chem Engn, Natl Engn Ctr Nanomed, Wuhan 430074, Hubei, Peoples R China.
[Xie, Jun; Li, Jun; Tao, Juan] HUST, Tongji Med Coll, Union Hosp, Dept Dermatol, Wuhan 430022, Hubei, Peoples R China.
[Shen, Guanxin] HUST, Tongji Med Coll, Dept Immunol, Wuhan 430030, Hubei, Peoples R China.
[Liang, Ruijing] Chinese Acad Sci, Inst Adv Technol, Inst Biomed & Biotechnol, Shenzhen 518055, Peoples R China.
C3 Huazhong University of Science & Technology; Huazhong University of Science & Technology; Huazhong University of Science & Technology; Chinese Academy of Sciences; Shenzhen Institute of Advanced Technology, CAS
RP Zhu, JT (通讯作者)，Huazhong Univ Sci & Technol, Sch Chem & Chem Engn, Natl Engn Ctr Nanomed, Wuhan 430074, Hubei, Peoples R China.; Tao, J (通讯作者)，HUST, Tongji Med Coll, Union Hosp, Dept Dermatol, Wuhan 430022, Hubei, Peoples R China.
EM jtzhu@mail.hust.edu.cn; tjhappy@126.com
RI liu, li/HGC-0900-2022; Liang, Ruijing/AED-9686-2022; Wang, Ke/T-6153-2017
OI Wang, Ke/0000-0003-1156-5296; Gao, Yujie/0000-0002-3168-6637; Liang, Ruijing/0000-0003-3248-2780
FU National Natural Science Foundation of China [51473059, 81241103, 81373360, 81501888]; Natural Science Foundation of Hubei Scientific Committee [2015BHE001]; Shenzhen Science and Technology Project [JCYJ20150630155150194]
FX We gratefully acknowledge funding for this work provided by National Natural Science Foundation of China (51473059, 81241103, 81373360 and 81501888) and Natural Science Foundation of Hubei Scientific Committee (2015BHE001) and Shenzhen Science and Technology Project (JCYJ20150630155150194). We also thank the HUST Analytical and Testing Center for allowing us to use its facilities.
CR Alter G, 2004, J IMMUNOL METHODS, V294, P15, DOI 10.1016/j.jim.2004.08.008
Alving CR, 2012, EXPERT REV VACCINES, V11, P733, DOI [10.1586/ERV.12.35, 10.1586/erv.12.35]
Au L, 2010, ACS NANO, V4, P35, DOI 10.1021/nn901392m
Black KCL, 2014, ACS NANO, V8, P4385, DOI 10.1021/nn406258m
Chen JY, 2007, NANO LETT, V7, P1318, DOI 10.1021/nl070345g
Chen JY, 2005, ADV MATER, V17, P2255, DOI 10.1002/adma.200500833
Couzin-Frankel J, 2013, SCIENCE, V342, P1432, DOI 10.1126/science.342.6165.1432
Cruz LJ, 2014, J CONTROL RELEASE, V192, P209, DOI 10.1016/j.jconrel.2014.07.040
de Vries IJM, 2005, NAT BIOTECHNOL, V23, P1407, DOI 10.1038/nbt1154
des Grottes JM, 2001, MELANOMA RES, V11, P477, DOI 10.1097/00008390-200110000-00006
Fan YC, 2015, VACCINES-BASEL, V3, P662, DOI 10.3390/vaccines3030662
Gao L, 2012, ACS NANO, V6, P8030, DOI 10.1021/nn302634m
Goldberg MS, 2015, CELL, V161, P201, DOI 10.1016/j.cell.2015.03.037
Herrera OB, 2002, VACCINE, V21, P231, DOI 10.1016/S0264-410X(02)00448-6
Iden DL, 2001, BBA-BIOMEMBRANES, V1513, P207, DOI 10.1016/S0005-2736(01)00357-1
Irvine DJ, 2015, CHEM REV, V115, P11109, DOI 10.1021/acs.chemrev.5b00109
Iwamoto H, 2014, INT J CANCER, V134, P332, DOI 10.1002/ijc.28367
Karde Supriya Ramesh, 2016, BMJ Case Rep, V2016, DOI 10.1136/bcr-2016-217503
Kwon YJ, 2005, P NATL ACAD SCI USA, V102, P18264, DOI 10.1073/pnas.0509541102

MULYE NV, 1995, DRUG DEV IND PHARM, V21, P943, DOI 10.3109/03639049509026658
Pedersen SR, 2013, J IMMUNOL, V191, P3955, DOI 10.4049/jimmunol.1300555
Prince HM, 2008, J IMMUNOTHER, V31, P166, DOI 10.1097/CJI.0b013e31815c5153
Reddy ST, 2006, TRENDS IMMUNOL, V27, P573, DOI 10.1016/j.it.2006.10.005
RITGER P L, 1987, Journal of Controlled Release, V5, P23, DOI 10.1016/0168-3659(87)90034-4
Shao K, 2015, ACS NANO, V9, P16, DOI 10.1021/nn5062029
Skrabalak SE, 2008, ACCOUNTS CHEM RES, V41, P1587, DOI 10.1021/ar800018v
Skrabalak SE, 2007, NAT PROTOC, V2, P2182, DOI 10.1038/nprot.2007.326
Subauste CS, 1998, J IMMUNOL, V160, P1831
van Broekhoven CL, 2004, CANCER RES, V64, P4357, DOI 10.1158/0008-5472.CAN-04-0138
Wang C, 2004, NAT MATER, V3, P190, DOI 10.1038/nmat1075
Xiang J, 2015, ACS NANO, V9, P6401, DOI 10.1021/acsnano.5b02014
Yavuz MS, 2009, NAT MATER, V8, P935, DOI [10.1038/NMAT2564, 10.1038/nmat2564]
Yi DH, 2013, SCAND J IMMUNOL, V78, P167, DOI 10.1111/sji.12060
Zhang QA, 2010, CHEM-EUR J, V16, P10234, DOI 10.1002/chem.201000341
Zhou QQ, 2016, ACS NANO, V10, P2678, DOI 10.1021/acsnano.5b07716
Zhu GZ, 2017, ACS NANO, V11, P2387, DOI 10.1021/acsnano.7b00978
NR 36
TC 117
Z9 118
U1 11
U2 344
PU ELSEVIER SCI LTD
PI OXFORD
PA THE BOULEVARD, LANGFORD LANE, KIDLINGTON, OXFORD OX5 1GB, OXON, ENGLAND
SN 0142-9612
EI 1878-5905
J9 BIOMATERIALS
JI Biomaterials
PD DEC
PY 2017
VL 149
BP 41
EP 50
DI 10.1016/j.biomaterials.2017.09.029
PG 10
WC Engineering, Biomedical; Materials Science, Biomaterials
WE Science Citation Index Expanded (SCI-EXPANDED)
SC Engineering; Materials Science
GA FM2LG
UT WOS:000414815700004
PM 28992509
DA 2023-09-14
ER

PT J
AU Pan, JB
Wang, YQ
Zhang, C
Wang, XY
Wang, HY
Wang, JJ
Yuan, YZ
Wang, X
Zhang, XJ
Yu, CS
Sun, SK
Yan, XP
AF Pan, Jinbin
Wang, Yaqiong
Zhang, Cai

Wang, Xiaoyi
Wang, Haoyu
Wang, Jiaojiao
Yuan, Yizhong
Wang, Xu
Zhang, Xuejun
Yu, Chunshui
Sun, Shao-Kai
Yan, Xiu-Ping
TI Antigen-Directed Fabrication of a Multifunctional Nanovaccine with Ultrahigh Antigen Loading Efficiency for Tumor Photothermal-Immunotherapy
SO ADVANCED MATERIALS
LA English
DT Article
DE antigen; dendritic cells; multifunctional nanovaccines; photothermal-immunotherapy; tumor prevention
ID CANCER-IMMUNOTHERAPY; NANOPARTICLES; THERAPY; VACCINES; COMBINATION; NANOPROBE; DELIVERY; AGENTS; GREEN; DYE
AB Current antigen-encapsulated multifunctional nanovaccines for oncotherapy suffer from limited antigen loading efficiency, low yield, tedious manufacture, and systemic toxicity. Here, an antigen-directed strategy for the fabrication of multifunctional nanovaccine with ultrahigh antigen loading efficiency in a facile way for tumor photothermal-immunotherapy is shown. As a proof of concept, a model antigen ovalbumin (OVA) is used as a natural carrier to load a representative theranostic agent indocyanine green (ICG). Mixing OVA and ICG in aqueous solution gives the simplest multifunctional nanovaccine so far. The nanovaccine owns antigen loading efficiency of 80.8%, high yield of >90%, intense near-infrared absorption and fluorescence, excellent reproducibility, good aqueous solubility and stability, and favorable biocompatibility. These merits not only guarantee sensitive labeling/tracking and efficient stimulation of dendritic cells, but also reliable imaging-guided photothermal-immunotherapy of tumors and tumor prevention. The proposed strategy provides a facile and robust method for large-scale and reproducible fabrication of multifunctional nanovaccines with ultrahigh antigen loading efficiency for tumor therapy.
C1 [Pan, Jinbin; Wang, Yaqiong; Wang, Xiaoyi; Yu, Chunshui] Tianjin Med Univ, Tianjin Key Lab Funct Imaging, Dept Radiol, Gen Hosp, Tianjin 300052, Peoples R China.
[Zhang, Cai] Nankai Univ, Res Ctr Analyt Sci, Coll Chem, State Key Lab Med Chem Biol, Tianjin 300071, Peoples R China.
[Zhang, Cai] Nankai Univ, Tianjin Key Lab Mol Recognit & Biosensing, Tianjin 300071, Peoples R China.
[Wang, Xiaoyi] Tianjin Med Univ, Hosp 2, Dept Ultrasound, Tianjin 300211, Peoples R China.
[Wang, Jiaojiao; Yuan, Yizhong; Zhang, Xuejun; Sun, Shao-Kai] Tianjin Med Univ, Sch Med Imaging, Tianjin 300203, Peoples R China.
[Wang, Xu] Tianjin Med Univ, Sch Pharm, Tianjin Key Lab Technol Enabling Dev Clin Therape, Tianjin 300070, Peoples R China.
[Yan, Xiu-Ping] Jiangnan Univ, Sch Food Sci & Technol, Inst Analyt Foodsafetiol, State Key Lab Food Sci & Technol, Wuxi 214122, Peoples R China.
[Yan, Xiu-Ping] Collaborat Innovat Ctr Chem Sci & Engn Tianjin, Tianjin 300071, Peoples R China.
C3 Tianjin Medical University; Nankai University; Nankai University; Tianjin Medical University; Tianjin Medical University; Tianjin Medical University; Jiangnan University
RP Sun, SK (通讯作者)，Tianjin Med Univ, Sch Med Imaging, Tianjin 300203, Peoples R China.
EM shaokaisun@163.com
RI Zhang, Cai/GXW-2590-2022; Pan, Jinbin/AAG-9617-2021; Sun, Shao-Kai/AAE-4714-2022; Yan, Xiu-Ping/A-8358-2011; 王, 雅琼/GRO-3202-2022
OI Pan, Jinbin/0000-0002-8398-760X; Yan, Xiu-Ping/0000-0001-9072-5125; 王, 雅琼/0000-0003-3336-2254; Yu, Chunshui/0000-0001-5648-5199; Zhang, Cai/0000-0001-9004-2543
FU National Natural Science Foundation of China [21435001, 21405112, 81401471]; Tianjin City High School Science & Technology Fund Planning Project [20140120]; China Postdoctoral Science Foundation [2015M581306]
FX J.P., Y.W., and C.Z. contributed equally to this work. This work was supported by the National Natural Science Foundation of China (Grants 21435001, 21405112, 81401471), the Tianjin City High School Science &

Technology Fund Planning Project (Grant 20140120), and the China
Postdoctoral Science Foundation (Grant 2015M581306).
CR [Anonymous], IMMUNITY
Chen Q, 2016, ADV MATER, V28, P10557, DOI 10.1002/adma.201600038
Chen Q, 2016, NAT COMMUN, V7, DOI 10.1038/ncomms13193
Chen Q, 2015, ADV MATER, V27, P903, DOI 10.1002/adma.201404308
Chen QW, 2016, BIOMATERIALS, V106, P144, DOI 10.1016/j.biomaterials.2016.08.022
Coulie PG, 2014, NAT REV CANCER, V14, P135, DOI 10.1038/nrc3670
Goldberg MS, 2015, CELL, V161, P201, DOI 10.1016/j.cell.2015.03.037
Guo LR, 2014, ACS NANO, V8, P5670, DOI 10.1021/nn5002112
Hayashi K, 2016, ADV FUNCT MATER, V26, P1708, DOI 10.1002/adfm.201504215
Huang P, 2014, ADV MATER, V26, P6401, DOI 10.1002/adma.201400914
Irvine DJ, 2015, CHEM REV, V115, P11109, DOI 10.1021/acs.chemrev.5b00109
Jaque D, 2014, NANOSCALE, V6, P9494, DOI 10.1039/c4nr00708e
Jemal A, 2009, CA-CANCER J CLIN, V59, P225, DOI [10.3322/caac.20006, 10.3322/caac.21601, 10.3322/caac.21254, 10.3322/caac.21387, 10.3322/caac.20073]
Jiang W, 2017, NAT BIOMED ENG, V1, DOI 10.1038/s41551-017-0029
Kim J, 2015, NAT BIOTECHNOL, V33, P64, DOI 10.1038/nbt.3071
Li XR, 2017, J FLUORESC, V27, P213, DOI 10.1007/s10895-016-1948-3
Lollini PL, 2006, NAT REV CANCER, V6, P204, DOI 10.1038/nrc1815
Luo M, 2017, J CONTROL RELEASE, V263, P200, DOI 10.1016/j.jconrel.2017.03.033
Luo M, 2017, NAT NANOTECHNOL, V12, P648, DOI [10.1038/nnano.2017.52, 10.1038/NNANO.2017.52]
Ma N, 2010, J AM CHEM SOC, V132, P6884, DOI 10.1021/ja101378g
Mao FX, 2016, ACS NANO, V10, P11145, DOI 10.1021/acsnano.6b06067
Meacham CE, 2013, NATURE, V501, P328, DOI 10.1038/nature12624
Mellman I, 2011, NATURE, V480, P480, DOI 10.1038/nature10673
Ni DZ, 2017, ADV SCI, V4, DOI 10.1002/advs.201700083
Palucka K, 2012, NAT REV CANCER, V12, P265, DOI 10.1038/nrc3258
Pan JB, 2017, ADV FUNCT MATER, V27, DOI 10.1002/adfm.201603440
Restifo NP, 2012, NAT REV IMMUNOL, V12, P269, DOI 10.1038/nri3191
Schreiber RD, 2011, SCIENCE, V331, P1565, DOI 10.1126/science.1203486
Schreiber TH, 2010, SEMIN IMMUNOL, V22, P105, DOI 10.1016/j.smim.2010.02.001
Shao K, 2015, ACS NANO, V9, P16, DOI 10.1021/nn5062029
Sheng ZH, 2014, ACS NANO, V8, P12310, DOI 10.1021/nn5062386
Stephan SB, 2015, NAT BIOTECHNOL, V33, P97, DOI 10.1038/nbt.3104
Sun SK, 2013, ANAL CHEM, V85, P8436, DOI 10.1021/ac401879y
Tao Y, 2015, NANOSCALE, V7, P12419, DOI 10.1039/c5nr02240a
Tsao CT, 2014, BIOMACROMOLECULES, V15, P2656, DOI 10.1021/bm500502n
Vanneman M, 2012, NAT REV CANCER, V12, P237, DOI 10.1038/nrc3237
Wang C, 2017, NAT BIOMED ENG, V1, DOI 10.1038/s41551-016-0011
Wang C, 2014, ADV MATER, V26, P8154, DOI 10.1002/adma.201402996
Wang SG, 2015, ADV MATER, V27, P7117, DOI 10.1002/adma.201503869
Wang Y, 2016, ADV FUNCT MATER, V26, P5335, DOI 10.1002/adfm.201601341
Wang Y, 2015, ADV MATER, V27, P3874, DOI 10.1002/adma.201500229
Xie JP, 2009, J AM CHEM SOC, V131, P888, DOI 10.1021/ja806804u
Yang T, 2016, ADV MATER, V28, P5923, DOI 10.1002/adma.201506119
Yang WT, 2017, J MATER CHEM B, V5, P401, DOI 10.1039/c6tb02308h
Yang WT, 2016, ACS NANO, V10, P10245, DOI 10.1021/acsnano.6b05760
Yuba E, 2014, BIOMATERIALS, V35, P3091, DOI 10.1016/j.biomaterials.2013.12.024
Zhang BB, 2012, J MATER CHEM, V22, P14494, DOI 10.1039/c2jm30629h
Zhang C, 2016, NANOSCALE, V8, P16204, DOI 10.1039/c6nr01845a
Zhang C, 2015, DALTON T, V44, P13112, DOI 10.1039/c5dt01467k
Zhang XF, 2015, LUMINESCENCE, V30, P1176, DOI 10.1002/bio.2873
Zhao WG, 2017, ACS APPL MATER INTER, V9, P23528, DOI 10.1021/acsami.7b06814
Zhou FF, 2015, CANCER LETT, V359, P169, DOI 10.1016/j.canlet.2015.01.029
Zhu GZ, 2017, ACS NANO, V11, P2387, DOI 10.1021/acsnano.7b00978
NR 53
TC 116
Z9 116
U1 12

U2 440
PU WILEY-V C H VERLAG GMBH
PI WEINHEIM
PA POSTFACH 101161, 69451 WEINHEIM, GERMANY
SN 0935-9648
EI 1521-4095
J9 ADV MATER
JI Adv. Mater.
PD FEB 22
PY 2018
VL 30
IS 8
AR 1704408
DI 10.1002/adma.201704408
PG 8
WC Chemistry, Multidisciplinary; Chemistry, Physical; Nanoscience &
Nanotechnology; Materials Science, Multidisciplinary; Physics, Applied;
Physics, Condensed Matter
WE Science Citation Index Expanded (SCI-EXPANDED)
SC Chemistry; Science & Technology - Other Topics; Materials Science;
Physics
GA FW6ST
UT WOS:000425449300006
PM 29318677
DA 2023-09-14
ER

PT J
AU Luo, ZC
Wang, C
Yi, HQ
Li, P
Pan, H
Liu, LL
Cai, LT
Ma, YF
AF Luo, Zichao
Wang, Ce
Yi, Huqiang
Li, Ping
Pan, Hong
Liu, Lanlan
Cai, Lintao
Ma, Yifan
TI Nanovaccine loaded with poly I:C and STAT3 siRNA robustly elicits
anti-tumor immune responses through modulating tumor-associated
dendritic cells in vivo
SO BIOMATERIALS
LA English
DT Article
DE Nanovaccine; Polypeptide micelles; TLR3 agonist; STAT3 siRNA;
Tumor-associated dendritic cells; Tumor immunosuppression
ID DRAINING LYMPH-NODES; CANCER; MICROENVIRONMENT; MICELLES; DELIVERY;
IMMUNOTHERAPY; NANOPARTICLES; INFLAMMATION; SUPPRESSION; PROGRESSION
AB Although cancer vaccine-based immunotherapy holds great potential for cancer treatment, tumorinduced dendritic cell (DC) dysfunction remains to be the major obstacle for developing effective vaccines. Compared with normal DCs, tumor-associated DCs (TADCs) are less matured with poor responsiveness to Toll-like receptor (TLR) stimulation, which has been related with STAT3 hyperactivity. In the present study, Poly I:C (PIC, a TLR3 agonist), STAT3 siRNA and OVA antigen were co-encapsulated by poly (ethylene glycol)-b-poly (L-lysine)-b-poly (L-leucine) (PEG-PLL-PLLeu) polypeptide micelles to generate PMP/OVA/siRNA nanovaccine, which was aimed to effectively overcome DC

dysfunction in vivo by deleting STAT3 gene in situ. The results showed that PMP/OVA/siRNA simultaneously facilitated the cellular uptake of OVA antigen and siRNA about 3-200 folds, and decreased STAT3 expression in TADCs over 50% both in vitro and in vivo. PMP/OVA/siRNA also elevated CD86 and CD40 expression as well as IL-12 production by TADCs more effectively than PMP/OVA did, indicating its strong potency of inducing TADC maturation and activation. Moreover, the immunization of PMP/OVA/siRNA rather than PMP/OVA effectively abrogated immunosuppression in the tumor microenvironment by increasing mature DCs and decreasing immunosuppressive cells in tumor-draining lymph nodes, which thereby led to potent antitumor immune responses and dramatic tumor regression with prolonged survival. Hence, in vivo codelivery of immunopotentiator (PIC) and immunosuppressive gene silencer (STAT3 siRNA) by nanovaccines are expected to be a promising strategy to improve the therapeutic efficacy of cancer vaccines by modulating TADCs and overcoming tumor immunosupression.
(C) 2014 Elsevier Ltd. All rights reserved.
C1 [Luo, Zichao; Wang, Ce; Yi, Huqiang; Li, Ping; Pan, Hong; Liu, Lanlan; Cai, Lintao; Ma, Yifan] Chinese Acad Sci, Shenzhen Inst Adv Technol, Guangdong Key Lab Nanomed, Key Lab Hlth Informat, Shenzhen 518055, Peoples R China.
C3 Chinese Academy of Sciences; Shenzhen Institute of Advanced Technology, CAS
RP Cai, LT (通讯作者)，Chinese Acad Sci, Shenzhen Inst Adv Technol, 1068 Xueyuan Ave,Shenzhen Univ Town, Shenzhen 518055, Peoples R China.
EM yf.ma@siat.ac.cn
RI Pan, Hong/AAC-2326-2019
FU National Natural Science Foundation of China [81171446, 81071249, 81371679]; Guangdong Innovation Research Team Fund for Low-cost Healthcare Technologies; Key Project of Science and Technology of Guangdong [20128090400043, 20128090600036]; Shenzhen Science and Technology Program [JC201005260247A, CXZZ20130506140505859]; Nanshan Innovative Technology Program [KC2013JSJS0012A]
FX This work was supported by the National Natural Science Foundation of China (Grant No. 81171446, 81071249, 81371679), Guangdong Innovation Research Team Fund for Low-cost Healthcare Technologies (GIRTF-LCHT), Key Project of Science and Technology of Guangdong (20128090400043, 20128090600036), Shenzhen Science and Technology Program (Grant No. JC201005260247A, CXZZ20130506140505859), Nanshan Innovative Technology Program (Grant No. KC2013JSJS0012A).
CR Alshamsan A, 2010, MOL PHARMACEUT, V7, P1643, DOI 10.1021/mp100067u
Alshamsan A, 2010, J PHARM PHARM SCI, V13, P21, DOI 10.18433/J37598
Aranda F, 2013, ONCOIMMUNOLOGY, V2, DOI 10.4161/onci.26621
Baxevanis CN, 2013, IMMUNOTHERAPY-UK, V5, P497, DOI [10.2217/IMT.13.24, 10.2217/imt.13.24]
Cochran AJ, 2006, NAT REV IMMUNOL, V6, P659, DOI 10.1038/nri1919
Demento SL, 2012, BIOMATERIALS, V33, P4957, DOI 10.1016/j.biomaterials.2012.03.041
Deng JZ, 2012, BIOMACROMOLECULES, V13, P3795, DOI 10.1021/bm3012538
Deng LF, 2010, CLIN CANCER RES, V16, P4105, DOI 10.1158/1078-0432.CCR-10-1073
Fransen MF, 2013, INT J CANCER, V132, P1971, DOI 10.1002/ijc.27755
Gabrilovich D, 2004, NAT REV IMMUNOL, V4, P941, DOI 10.1038/nri1498
Gao J, 2011, BIOMATERIALS, V32, P3459, DOI 10.1016/j.biomaterials.2011.01.034
Hargadon KM, 2013, FRONT IMMUNOL, V4, DOI 10.3389/fimmu.2013.00192
Heo MB, 2014, BIOMATERIALS, V35, P590, DOI 10.1016/j.biomaterials.2013.10.009
Hsu YL, 2011, J IMMUNOL, V187, P1733, DOI 10.4049/jimmunol.1100996
Hugues S, 2004, NAT IMMUNOL, V5, P1235, DOI 10.1038/ni1134
Imai K, 2012, J EXP CLIN CANC RES, V31, DOI 10.1186/1756-9966-31-3
Iwata-Kajihara T, 2011, J IMMUNOL, V187, P27, DOI 10.4049/jimmunol.1002067
Kortylewski M, 2009, CANCER CELL, V15, P114, DOI 10.1016/j.ccr.2008.12.018
Kuang DM, 2008, J IMMUNOL, V181, P3089, DOI 10.4049/jimmunol.181.5.3089
Liu QY, 2009, J IMMUNOL, V182, P6207, DOI 10.4049/jimmunol.0803926
Luo ZC, 2013, J CONTROL RELEASE, V170, P259, DOI 10.1016/j.jconrel.2013.05.027
Ma YF, 2005, P NATL ACAD SCI USA, V102, P13556, DOI 10.1073/pnas.0506438102
Musacchio T, 2010, BIOCONJUGATE CHEM, V21, P1530, DOI 10.1021/bc100199c
Norian LA, 2009, CANCER RES, V69, P3086, DOI 10.1158/0008-5472.CAN-08-2826
Rebe Cedric, 2013, JAKSTAT, V2, pe23010, DOI 10.4161/jkst.23010
Restifo NP, 2012, NAT REV IMMUNOL, V12, P269, DOI 10.1038/nri3191
Robinson-Smith TM, 2007, CANCER RES, V67, P5708, DOI 10.1158/0008-5472.CAN-06-4375

Shurin GV, 2013, CANCER MICROENVIRON, V6, P159, DOI 10.1007/s12307-013-0133-3
Wang C, 2012, VACCINE, V30, P4790, DOI 10.1016/j.vaccine.2012.05.027
Wang TH, 2004, NAT MED, V10, P48, DOI 10.1038/nm976
Watkins SK, 2011, J CLIN INVEST, V121, P1361, DOI 10.1172/JCI44325
Xu YH, 1996, BIOCHEMISTRY-US, V35, P5616, DOI 10.1021/bi9602019
Yu H, 2007, NAT REV IMMUNOL, V7, P41, DOI 10.1038/nri1995
Yu H, 2009, NAT REV CANCER, V9, P798, DOI 10.1038/nrc2734
Zeng B, 2008, CANCER RES, V68, P5397, DOI 10.1158/0008-5472.CAN-07-6792
Zheng CF, 2013, BIOMATERIALS, V34, P3431, DOI 10.1016/j.biomaterials.2013.01.053
NR 36
TC 115
Z9 117
U1 5
U2 162
PU ELSEVIER SCI LTD
PI OXFORD
PA THE BOULEVARD, LANGFORD LANE, KIDLINGTON, OXFORD OX5 1GB, OXON, ENGLAND
SN 0142-9612
EI 1878-5905
J9 BIOMATERIALS
JI Biomaterials
PD JAN
PY 2015
VL 38
BP 50
EP 60
DI 10.1016/j.biomaterials.2014.10.050
PG 11
WC Engineering, Biomedical; Materials Science, Biomaterials
WE Science Citation Index Expanded (SCI-EXPANDED)
SC Engineering; Materials Science
GA AY0GU
UT WOS:000347276300006
PM 25457983
DA 2023-09-14
ER

PT J
AU Zhang, LH
Wu, SJ
Qin, Y
Fan, F
Zhang, ZM
Huang, CL
Ji, WH
Lu, L
Wang, C
Sung, HF
Leng, XG
Kong, DL
Zhu, DW
AF Zhang, Linhua
Wu, Shengjie
Qin, Yu
Fan, Fan
Zhang, Zhiming
Huang, Chenlu
Ji, Weihang
Lu, Lu
Wang, Chun
Sung, Hongfan

Leng, Xigang
Kong, Deling
Zhu, Dunwan
TI Targeted Codelivery of an Antigen and Dual Agonists by Hybrid Nanoparticles for Enhanced Cancer Immunotherapy
SO NANO LETTERS
LA English
DT Article
DE Hybrid nanoparticles; antigen; TLR agonists; vaccine delivery; cancer immunotherapy
ID YELLOW-FEVER VACCINE; IN-VIVO TRACKING; DENDRITIC CELLS; DRUG-DELIVERY; CATIONIC LIPOSOMES; CROSS-PRESENTATION; POLYMERIC NANOPARTICLES; ADJUVANT; RECEPTOR; PH
AB Among approaches of current cancer immunotherapy, a dendritic cell (DC)-targeted vaccine based on nanotechnology could be a promising way to efficiently induce potent immune responses. To enhance DC targeting and vaccine efficiency, we included imiquimod (IMQ), a toll-like receptor 7/8 (TLR 7/8) agonist, and monophosphoryl lipid A (MPLA), a TLR4 agonist, to synthesize lipid-polymer hybrid nanoparticles using PCLPEG-PCL and DOTAP (IMNPs) as well as DSPE-PEG-mannose (MAN-IMNPS). The spatiotemporal delivery of MPLA (within the outer lipid layer) to extracellular TLR4 and IMQ (in the hydrophobic core of NPs) to intracellular TLR7/8 can activate DCs synergistically to improve vaccine efficacy. Ovalbumin (OVA) as a model antigen was readily absorbed by positively charged DOTAP and showed a quick release in vitro. Our results demonstrated that this novel nanovaccine enhanced cellular uptake, cytokine production, and maturation of DCs. Compared with the quick metabolism of free OVA-agonists, the depot effect of OVA-IMNPs was observed, whereas MAN-OVA-IMNPs promoted trafficking to secondary lymphoid organs. After immunization with a subcutaneous injection, the nanovaccine, especially MAN-OVA-IMNPs, induced more antigen-specific CD8(+) T cells, greater lymphocyte activation, stronger cross-presentation, and more generation of memory T cells, antibody, IFN-gamma, and granzyme B. Prophylactic vaccination of MAN-OVA-IMNPs significantly delayed tumor development and prolonged the survival in mice. The therapeutic tumor challenge indicated that MAN-OVA-IMNPs prohibited tumor progression more efficiently than other formulations, and the combination with an immune checkpoint blockade further enhanced antitumor effects. Hence, the DC-targeted vaccine codelivery with IMQ and MPLA adjuvants by hybrid cationic nanoparticles in a spatiotemporal manner is a promising multifunctional antigen delivery system in cancer immunotherapy.
C1 [Zhang, Linhua; Wu, Shengjie; Qin, Yu; Fan, Fan; Zhang, Zhiming; Huang, Chenlu; Sung, Hongfan; Leng, Xigang; Zhu, Dunwan] Chinese Acad Med Sci, Inst Biomed Engn, Tianjin Key Lab Biomed Mat, Tianjin 300192, Peoples R China.
[Zhang, Linhua; Wu, Shengjie; Qin, Yu; Fan, Fan; Zhang, Zhiming; Huang, Chenlu; Sung, Hongfan; Leng, Xigang; Zhu, Dunwan] Peking Union Med Coll, Tianjin 300192, Peoples R China.
[Ji, Weihang; Wang, Chun] Univ Minnesota, Dept Biomed Engn, 7-116 HasseImo Hall,312 Church St SE, Minneapolis, MN 55455 USA.
[Lu, Lu] Chinese Acad Med Sci, Inst Radiat Med, Tianjin 300192, Peoples R China.
[Lu, Lu] Peking Union Med Coll, Tianjin Key Lab Radiat Med & Mol Nucl Med, Tianjin 300192, Peoples R China.
[Kong, Deling] Nankai Univ, Key Lab Bioact Mat, Minist Educ, Coll Life Sci, Tianjin 300071, Peoples R China.
[Kong, Deling] Xuzhou Med Univ, Canc Inst, Jiangsu Ctr Collaborat & Innovat Canc Biotherapy, Xuzhou 221004, Jiangsu, Peoples R China.
C3 Chinese Academy of Medical Sciences - Peking Union Medical College; Institute of BioMedical Engineering - CAMS; Chinese Academy of Medical Sciences - Peking Union Medical College; Peking Union Medical College; University of Minnesota System; University of Minnesota Twin Cities; Chinese Academy of Medical Sciences - Peking Union Medical College; Institute of Radiation Medicine - CAMS; Chinese Academy of Medical Sciences - Peking Union Medical College; Peking Union Medical College; Nankai University; Xuzhou Medical University
RP Zhu, DW (通讯作者)，Chinese Acad Med Sci, Inst Biomed Engn, Tianjin Key Lab Biomed Mat, Tianjin 300192, Peoples R China.; Zhu, DW (通讯作者)，Peking Union Med Coll, Tianjin 300192, Peoples R China.
EM zhudunwan@bme.pumc.edu.cn
RI Ji, Weihang/I-7265-2012
OI Ji, Weihang/0000-0002-1054-6280; Qin, Yu/0000-0003-1130-7115

FU National Natural Science Foundation of China [81671806, 81571793, 81100100, 51103180]; CAMS Initiative for Innovative Medicine [2017-I2M-4-001, 2017-I2M-3-020]; Tianjin Municipal Natural Science Foundation [15JCZDJC38300]
FX This work was supported by the National Natural Science Foundation of China (81671806, 81571793, 81100100, and 51103180), CAMS Initiative for Innovative Medicine (2017-I2M-4-001 and 2017-I2M-3-020), and Tianjin Municipal Natural Science Foundation (15JCZDJC38300).
CR Arpaia N, 2011, CURR OPIN VIROL, V1, P447, DOI 10.1016/j.coviro.2011.10.006
Banchereau J, 2000, ANNU REV IMMUNOL, V18, P767, DOI 10.1146/annurev.immunol.18.1.767
Banchereau J, 1998, NATURE, V392, P245, DOI 10.1038/32588
Bayyurt B, 2017, J CONTROL RELEASE, V247, P134, DOI 10.1016/j.jconrel.2017.01.004
Black M, 2010, EXPERT REV VACCINES, V9, P157, DOI [10.1586/erv.09.160, 10.1586/ERV.09.160]
Cai S, 2011, ADV DRUG DELIVER REV, V63, P901, DOI 10.1016/j.addr.2011.05.017
Chen Q, 2016, NAT COMMUN, V7, DOI 10.1038/ncomms13193
Cheng CM, 2011, VACCINE, V29, P2437, DOI 10.1016/j.vaccine.2011.01.031
Christensen D, 2011, EXPERT REV VACCINES, V10, P513, DOI [10.1586/erv.11.17, 10.1586/ERV.11.17]
COLEY WB, 1991, CLIN ORTHOP RELAT R, P3
Dewitte H, 2014, NANO TODAY, V9, P743, DOI 10.1016/j.nantod.2014.10.001
Fan YC, 2017, NANO LETT, V17, P7387, DOI 10.1021/acs.nanolett.7b03218
Fan YC, 2015, J CONTROL RELEASE, V208, P121, DOI 10.1016/j.jconrel.2015.04.010
Fang RH, 2014, NANO LETT, V14, P2181, DOI 10.1021/nl500618u
Gao J, 2017, INT J NANOMED, V12, P1251, DOI 10.2147/IJN.S125866
Guo YY, 2015, ACS NANO, V9, P6918, DOI 10.1021/acsnano.5b01042
Hadinoto K, 2013, EUR J PHARM BIOPHARM, V85, P427, DOI 10.1016/j.ejpb.2013.07.002
Hirosue S, 2010, VACCINE, V28, P7897, DOI 10.1016/j.vaccine.2010.09.077
Irache JM, 2008, EXPERT OPIN DRUG DEL, V5, P703, DOI [10.1517/17425247.5.6.703, 10.1517/17425247.5.6.703 ]
Jiang PL, 2015, ACTA BIOMATER, V11, P356, DOI 10.1016/j.actbio.2014.09.019
Jin JW, 2018, ACTA BIOMATER, V78, P211, DOI 10.1016/j.actbio.2018.08.002
Jones CH, 2015, BIOMATERIALS, V37, P333, DOI 10.1016/j.biomaterials.2014.10.037
Kanamala M, 2016, BIOMATERIALS, V85, P152, DOI 10.1016/j.biomaterials.2016.01.061
Kasturi SP, 2011, NATURE, V470, P543, DOI 10.1038/nature09737
Kim SY, 2017, BIOMATERIALS, V130, P56, DOI 10.1016/j.biomaterials.2017.03.034
Kuai R, 2018, J CONTROL RELEASE, V282, P131, DOI 10.1016/j.jconrel.2018.04.041
Lin WJ, 2017, ACTA BIOMATER, V48, P378, DOI 10.1016/j.actbio.2016.10.038
Liu Q, 2015, ACS NANO, V9, P4925, DOI 10.1021/nn5066793
Lutz MB, 2002, TRENDS IMMUNOL, V23, P445, DOI 10.1016/S1471-4906(02)02281-0
Mandal B, 2013, NANOMED-NANOTECHNOL, V9, P474, DOI 10.1016/j.nano.2012.11.010
MOSMANN TR, 1989, ANNU REV IMMUNOL, V7, P145, DOI 10.1146/annurev.immunol.7.1.145
Napolitani G, 2005, NAT IMMUNOL, V6, P769, DOI 10.1038/ni1223
Ni DZ, 2017, ADV SCI, V4, DOI 10.1002/advs.201700083
Noh YW, 2011, BIOMATERIALS, V32, P6254, DOI 10.1016/j.biomaterials.2011.05.013
Peer D, 2012, ADV DRUG DELIVER REV, V64, P1738, DOI 10.1016/j.addr.2012.06.013
Querec T, 2006, J EXP MED, V203, P413, DOI 10.1084/jem.20051720
Querec TD, 2009, NAT IMMUNOL, V10, P116, DOI 10.1038/ni.1688
Rahimian S, 2015, BIOMATERIALS, V37, P469, DOI 10.1016/j.biomaterials.2014.10.043
Rose F, 2015, J CONTROL RELEASE, V210, P48, DOI 10.1016/j.jconrel.2015.05.004
Shao K, 2015, ACS NANO, V9, P16, DOI 10.1021/nn5062029
Shen KY, 2016, J CONTROL RELEASE, V233, P57, DOI 10.1016/j.jconrel.2016.05.008
Siefert AL, 2016, BIOMATERIALS, V97, P85, DOI 10.1016/j.biomaterials.2016.03.039
STAHL P, 1980, CELL, V19, P207, DOI 10.1016/0092-8674(80)90402-X
Taylor PR, 2005, TRENDS IMMUNOL, V26, P104, DOI 10.1016/j.it.2004.12.001
Trombetta ES, 2005, ANNU REV IMMUNOL, V23, P975, DOI 10.1146/annurev.immunol.22.012703.104538
Vyas JM, 2008, NAT REV IMMUNOL, V8, P607, DOI 10.1038/nri2368
Wang C, 2014, VACCINE, V32, P5475, DOI 10.1016/j.vaccine.2014.07.081
Wei C, 2019, J BIOMED NANOTECHNOL, V15, P100, DOI 10.1166/jbn.2019.2669
Wilson JT, 2013, ACS NANO, V7, P3912, DOI 10.1021/nn305466z
Xia QM, 2018, J BIOMED NANOTECHNOL, V14, P1613, DOI 10.1166/jbn.2018.2605
Yang PX, 2018, NANO LETT, V18, P4377, DOI 10.1021/acs.nanolett.8b01406

Zehner M, 2013, MOL IMMUNOL, V55, P146, DOI 10.1016/j.molimm.2012.10.010
Zhang LF, 2008, ACS NANO, V2, P1696, DOI 10.1021/nn800275r
Zhang LH, 2015, INT J NANOMED, V10, P2101, DOI 10.2147/IJN.S77667
Zhang WF, 2014, BIOMATERIALS, V35, P6086, DOI 10.1016/j.biomaterials.2014.04.022
Zhu GZ, 2017, NAT COMMUN, V8, DOI 10.1038/s41467-017-02191-y
Zhu GZ, 2017, ACS NANO, V11, P2387, DOI 10.1021/acsnano.7b00978
NR 57
TC 111
Z9 117
U1 15
U2 319
PU AMER CHEMICAL SOC
PI WASHINGTON
PA 1155 16TH ST, NW, WASHINGTON, DC 20036 USA
SN 1530-6984
EI 1530-6992
J9 NANO LETT
JI Nano Lett.
PD JUL
PY 2019
VL 19
IS 7
BP 4237
EP 4249
DI 10.1021/acs.nanolett.9b00030
PG 13
WC Chemistry, Multidisciplinary; Chemistry, Physical; Nanoscience & Nanotechnology; Materials Science, Multidisciplinary; Physics, Applied; Physics, Condensed Matter
WE Science Citation Index Expanded (SCI-EXPANDED)
SC Chemistry; Science & Technology - Other Topics; Materials Science; Physics
GA II9QA
UT WOS:000475533900004
PM 30868883
DA 2023-09-14
ER

PT J
AU Zhang, CN
Shi, GN
Zhang, J
Song, HJ
Niu, JF
Shi, SB
Huang, PS
Wang, YM
Wang, WW
Li, C
Kong, DL
AF Zhang, Chuangnian
Shi, Gaona
Zhang, Ju
Song, Huijuan
Niu, Jinfeng
Shi, Shengbin
Huang, Pingsheng
Wang, Yanming
Wang, Weiwei
Li, Chen
Kong, Deling

TI Targeted antigen delivery to dendritic cell via functionalized alginate
nanoparticles for cancer immunotherapy
SO JOURNAL OF CONTROLLED RELEASE
LA English
DT Article
DE Alginate; Ovalbumin; DC targeting; Antigen delivery; Cancer
immunotherapy
ID MOUSE BONE-MARROW; POLYMERIC NANOPARTICLES; GOLD NANOPARTICLE;
IMMUNE-RESPONSE; CHITOSAN NANOPARTICLES; RELEASE BEHAVIOR; VACCINE;
NANOGELS; DEXTRAN; CARRIERS
AB The purpose of the present study was to identify an "easy-to-adopt" strategy to enhance immune responses using functionalized alginate (ALG) nanoparticles (MAN-ALG/ALG = OVA NPs), which were prepared by CaCl2 cross-linking of two different types of ALG. The mannose (MAN) modified ALG (MAN-ALG) was used for dendritic cell targeting. The other component, composed of ovalbumin (OVA), a model antigen, is conjugated to ALG (ALG = OVA) via pH sensitive Schiff base bond. Grafting of alginate was demonstrated by FT-IR and H-1 NMR, while the morphological structure, particle size, Zeta potential of MAN-ALG/ALG = OVA NPs were measured using TEM and DLS. The OVA releasing behavior of MAN-ALG/ALG = OVA NPs was determined as a function of pH. Antigen uptake was examined by flow cytometry and confocal laser scanning microscopy in vitro using mouse bone marrow dendritic cells (BMDCs). The results showed that MAN-ALG/ALG = OVA NPs facilitated antigen uptake of BMDCs and cytosolic release of the antigen. Significant up-regulation of cytokine secretion and expression levels of the surface co-stimulatory molecules were also observed in MAN-ALG/ALG = OVA NPs-treated BMDCs, compared to free OVA. In vivo bio-distribution study using Cy7 (a near-infrared fluorescence dye) labeled MAN-ALG/ALG = OVA NPs showed efficient in vivo trafficking of the nanoparticles from the injection site to the draining lymph nodes. Moreover, MAN-ALG/ALG = OVA NPs were found to enhance cross-presentation of OVA to B3Z T cell hybridoma in vitro. Subcutaneous administration of MAN-ALG/ALG = OVA NPs also induced major cytotoxic T lymphocytes (CTL) response and inhibition of E.G7 tumor growth in C57BL/6 mice. In summary, we report here that the MAN-ALG/ALG = OVA NPs have the potential as a potent nanovaccine for cancer immunotherapy.
C1 [Zhang, Chuangnian; Shi, Gaona; Song, Huijuan; Shi, Shengbin; Huang, Pingsheng; Wang, Weiwei; Li, Chen; Kong, Deling] Chinese Acad Med Sci, Inst Biomed Engn, Tianjin Key Lab Biomat Res, Tianjin 300192, Peoples R China.
[Zhang, Chuangnian; Shi, Gaona; Song, Huijuan; Shi, Shengbin; Huang, Pingsheng; Wang, Weiwei; Li, Chen; Kong, Deling] Peking Union Med Coll, Tianjin 300192, Peoples R China.
[Kong, Deling] Nankai Univ, Minist Educ, Key Lab Bioact Mat, Tianjin 300071, Peoples R China.
[Zhang, Ju] Qingdao Univ, Sch Nursing, Basic Nursing T&R Sect, Qingdao 26000, Shandong, Peoples R China.
[Niu, Jinfeng; Wang, Yanming] Nankai Univ, Coll Pharm, State Key Lab Med Chem Biol, Tianjin 300353, Peoples R China.
[Niu, Jinfeng; Wang, Yanming] Nankai Univ, Tianjin Key Lab Mol Drug Res, Tianjin 300353, Peoples R China.
[Zhang, Chuangnian] Nankai Univ, Minist Educ, Key Lab Funct Polymer Mat, Tianjin 300071, Peoples R China.
C3 Chinese Academy of Medical Sciences - Peking Union Medical College;
Institute of BioMedical Engineering - CAMS; Chinese Academy of Medical
Sciences - Peking Union Medical College; Peking Union Medical College;
Nankai University; Qingdao University; Nankai University; Nankai
University; Nankai University
RP Li, C; Kong, DL (通讯作者)，Chinese Acad Med Sci, Inst Biomed Engn, Tianjin Key Lab Biomat Res, Tianjin 300192, Peoples R China.; Li, C; Kong, DL (通讯作者)，Peking Union Med Coll, Tianjin 300192, Peoples R China.
EM cli0616826@126.com; kongdeling@nankai.edu.cn
RI Wang, Weiwei/GRF-3625-2022
FU National Natural Science Foundation of China [21604095, 31300732,
31670977, 51473080]; China Postdoctoral Science Foundation
[2015M580066]; CAMS Initiative for Innovative Medicine [2016-I2M-3-022];
Key Laboratory of Functional Polymer Materials, Ministry of Education,
Nankai University [201606]
FX This work was financially supported by the National Natural Science
Foundation of China (21604095, 31300732, 31670977, 51473080), China
Postdoctoral Science Foundation (2015M580066), Program for Innovative

Research Team in Peking Union Medical College, CAMS Initiative for
Innovative Medicine (2016-I2M-3-022) and the Open Fund of Key Laboratory
of Functional Polymer Materials, Ministry of Education, Nankai
University (201606).
CR Akagi T, 2012, ADV POLYM SCI, V247, P31, DOI 10.1007/12_2011_150
Almeida JPM, 2015, SMALL, V11, P1453, DOI 10.1002/smll.201402179
Apostolopoulos V, 2014, CLIN TRANSL IMMUNOL, V3, DOI 10.1038/cti.2014.14
Bolhassani A, 2014, HUM VACC IMMUNOTHER, V10, P321, DOI 10.4161/hv.26796
Chang D, 2012, CARBOHYD POLYM, V88, P663, DOI 10.1016/j.carbpol.2012.01.020
Chen HY, 2016, INT J BIOL MACROMOL, V85, P596, DOI 10.1016/j.ijbiomac.2016.01.038
Craparo EF, 2012, CURR OPIN ALLERGY CL, V12, P658, DOI 10.1097/ACI.0b013e3283588c57
De Coen R, 2016, BIOMACROMOLECULES, V17, P2479, DOI 10.1021/acs.biomac.6b00685
Farjah A, 2015, APMIS, V123, P175, DOI 10.1111/apm.12337
Guo H, 2013, INT J PHARMACEUT, V451, P1, DOI 10.1016/j.ijpharm.2013.04.025
INABA K, 1992, J EXP MED, V176, P1693, DOI 10.1084/jem.176.6.1693
Jain S, 2015, BIOMATERIALS, V61, P162, DOI 10.1016/j.biomaterials.2015.05.028
Joffre OP, 2012, NAT REV IMMUNOL, V12, P557, DOI 10.1038/nri3254
Joshi MD, 2012, J CONTROL RELEASE, V161, P25, DOI 10.1016/j.jconrel.2012.05.010
Keller S, 2014, J CONTROL RELEASE, V191, P24, DOI 10.1016/j.jconrel.2014.03.041
Li DD, 2015, ADV FUNCT MATER, V25, P2993, DOI 10.1002/adfm.201500894
Li D, 2016, J MATER CHEM B, V4, P5167, DOI 10.1039/c6tb00991c
Li P, 2013, J CONTROL RELEASE, V168, P271, DOI 10.1016/j.jconrel.2013.03.025
Li ZY, 2015, RSC ADV, V5, P94248, DOI 10.1039/c5ra16912g
Liu Q, 2015, ACS NANO, V9, P4925, DOI 10.1021/nn5066793
Liu SG, 2016, LANGMUIR, V32, P1881, DOI 10.1021/acs.langmuir.6b00201
Lutz MB, 1999, J IMMUNOL METHODS, V223, P77, DOI 10.1016/S0022-1759(98)00204-X
Ma HL, 2007, INT J PHARMACEUT, V333, P177, DOI 10.1016/j.ijpharm.2006.10.006
Mellman I, 2011, NATURE, V480, P480, DOI 10.1038/nature10673
Mody N, 2015, EXPERT REV CLIN IMMU, V11, P213, DOI 10.1586/1744666X.2015.987663
Mueller SN, 2015, MOL PHARMACEUT, V12, P1356, DOI 10.1021/mp500589c
Oh IH, 2013, BIOMATERIALS, V34, P6454, DOI 10.1016/j.biomaterials.2013.05.017
Palucka K, 2012, NAT REV CANCER, V12, P265, DOI 10.1038/nrc3258
Pizzurro GA, 2015, FRONT IMMUNOL, V6, DOI 10.3389/fimmu.2015.00091
Rahimian S, 2015, BIOMATERIALS, V37, P469, DOI 10.1016/j.biomaterials.2014.10.043
Sau S, 2014, NANOSCALE, V6, P6745, DOI 10.1039/c4nr00974f
Shi GN, 2017, BIOMATERIALS, V113, P191, DOI 10.1016/j.biomaterials.2016.10.047
Silva JM, 2013, J CONTROL RELEASE, V168, P179, DOI 10.1016/j.jconrel.2013.03.010
Su HY, 2016, CARBOHYD POLYM, V152, P156, DOI 10.1016/j.carbpol.2016.06.091
Subbotin VM, 2014, DRUG DISCOV TODAY, V19, P834, DOI 10.1016/j.drudis.2014.02.008
Venkatesan J, 2015, INT J BIOL MACROMOL, V72, P269, DOI 10.1016/j.ijbiomac.2014.07.008
Wayteck L, 2016, BIOMATERIALS, V77, P243, DOI 10.1016/j.biomaterials.2015.11.016
Xu PP, 2016, J CONTROL RELEASE, V225, P252, DOI 10.1016/j.jconrel.2016.01.043
Xu ZH, 2014, ACS NANO, V8, P3636, DOI 10.1021/nn500216y
Yang JS, 2011, CARBOHYD POLYM, V84, P33, DOI 10.1016/j.carbpol.2010.11.048
Zhang WF, 2014, BIOMATERIALS, V35, P6086, DOI 10.1016/j.biomaterials.2014.04.022
ZHAO H, 1991, PHARMACEUT RES, V8, P400, DOI 10.1023/A:1015866104055
Zhou QQ, 2016, ACS NANO, V10, P2678, DOI 10.1021/acsnano.5b07716
NR 43
TC 109
Z9 111
U1 8
U2 218
PU ELSEVIER SCIENCE BV
PI AMSTERDAM
PA PO BOX 211, 1000 AE AMSTERDAM, NETHERLANDS
SN 0168-3659
EI 1873-4995
J9 J CONTROL RELEASE
JI J. Control. Release
PD JUN 28
PY 2017

VL 256
BP 170
EP 181
DI 10.1016/j.jconrel.2017.04.020
PG 12
WC Chemistry, Multidisciplinary; Pharmacology & Pharmacy
WE Science Citation Index Expanded (SCI-EXPANDED)
SC Chemistry; Pharmacology & Pharmacy
GA EX6AR
UT WOS:000403324800015
PM 28414151
DA 2023-09-14
ER

PT J
AU Zhang, W
Wang, F
Hu, C
Zhou, Y
Gao, HL
Hu, J
AF Zhang, Wei
Wang, Fei
Hu, Chuan
Zhou, Yang
Gao, Huile
Hu, Jiang
TI The progress and perspective of nanoparticle-enabled tumor metastasis treatment
SO ACTA PHARMACEUTICA SINICA B
LA English
DT Review
DE Nanoparticles; Tumor metastasis; Tumor targeting drug delivery; Immunotherapy; Photodynamic therapy; Ferroptosis; Nanovaccines; Stimulating immune response
ID DRUG-DELIVERY; MICROENVIRONMENTAL REGULATION; PHOTOTHERMAL THERAPY; CHECKPOINT BLOCKADE; CANCER METASTASIS; CELLS; SIZE; STRATEGIES; PROTEIN; NANOTHERAPEUTICS
AB As one of the most serious threats to human being, cancer is hard to be treated when metastasis happens. What's worse, there are few identified targets of metastasis for drug development. Therefore, it is important to develop strategies to prevent metastasis or treat existed metastasis. This review focuses on the procedure of metastasis, and first summarizes the targeting delivery strategies, including primary tumor targeting drug delivery, tumor metastasis targeting drug delivery and hijacking circulation cells. Then, as a promising treatment, the application of immunotherapy in tumor metastasis treatment is introduced, and strategies that stimulating immune response are reviewed, including chemotherapy, photothermal therapy, photodynamic therapy, ferroptosis, sonodynamic therapy, and nanovaccines. Finally, the challenges and perspective about nanoparticle-enabled tumor metastasis treatment are discussed. (C) 2020 Chinese Pharmaceutical Association and Institute of Materia Medica, Chinese Academy of Medical Sciences. Production and hosting by Elsevier B.V.
C1 [Zhang, Wei; Wang, Fei; Hu, Jiang] Sichuan Prov Peoples Hosp, Dept Orthoped, Chengdu 610072, Peoples R China.
[Zhang, Wei; Wang, Fei; Hu, Jiang] Sichuan Acad Med Sci, Chengdu 610072, Peoples R China.
[Zhang, Wei; Wang, Fei; Hu, Jiang] Univ Elect Sci & Technol, Affiliated Hosp, Chengdu 610072, Peoples R China.
[Hu, Chuan; Zhou, Yang; Gao, Huile] Sichuan Univ, Sichuan Engn Lab Plant Sourced Drug, Key Lab Drug Targeting & Drug Delivery Syst, Educ Minist,West China Sch Pharm, Chengdu 610041, Peoples R China.
[Hu, Chuan; Zhou, Yang; Gao, Huile] Sichuan Univ, West China Sch Pharm, Sichuan Res Ctr Drug Precis Ind Technol, Chengdu 610041, Peoples R China.
C3 University of Electronic Science & Technology of China; Sichuan University; Sichuan University

RP Hu, J (通讯作者)，Sichuan Prov Peoples Hosp, Dept Orthoped, Chengdu 610072, Peoples R China.; Hu, J (通讯作者)，Sichuan Acad Med Sci, Chengdu 610072, Peoples R China.; Hu, J (通讯作者)，Univ Elect Sci & Technol, Affiliated Hosp, Chengdu 610072, Peoples R China.; Gao, HL (通讯作者)，Sichuan Univ, Sichuan Engn Lab Plant Sourced Drug, Key Lab Drug Targeting & Drug Delivery Syst, Educ Minist,West China Sch Pharm, Chengdu 610041, Peoples R China.; Gao, HL (通讯作者)，Sichuan Univ, West China Sch Pharm, Sichuan Res Ctr Drug Precis Ind Technol, Chengdu 610041, Peoples R China.
EM gaohuile@scu.edu.cn; hujiang8711@163.com
RI gao, huile/G-8290-2012; Gao, Huile/H-9206-2019
OI gao, huile/0000-0002-5355-7238; Gao, Huile/0000-0002-5355-7238
FU National Natural Science Foundation of China [81961138009]; 111 Project
[B18035]
FX The work was supported by National Natural Science Foundation of China
(81961138009), and 111 Project (B18035, China).
CR Bai SB, 2020, NANOMEDICINE-UK, V15, P833, DOI 10.2217/nnm-2020-0024
Chaudhari KR, 2012, J CONTROL RELEASE, V158, P470, DOI 10.1016/j.jconrel.2011.11.020
Chen Q, 2016, NAT COMMUN, V7, DOI 10.1038/ncomms13193
Chen SH, 2020, J MATER CHEM B, V8, P3789, DOI 10.1039/d0tb00046a
Chen ZK, 2018, ACS NANO, V12, P8633, DOI 10.1021/acsnano.8b04371
Chu DF, 2016, ADV HEALTHC MATER, V5, P1088, DOI 10.1002/adhm.201500998
Cristofanilli M, 2004, NEW ENGL J MED, V351, P781, DOI 10.1056/NEJMoa040766
Del Bufalo D, 2006, CANCER RES, V66, P5549, DOI 10.1158/0008-5472.CAN-05-2825
Ding F, 2020, NANOSCALE HORIZ, V5, P999, DOI 10.1039/d0nh00148a
Dixon SJ, 2012, CELL, V149, P1060, DOI 10.1016/j.cell.2012.03.042
Dong XY, 2017, THERANOSTICS, V7, P751, DOI 10.7150/thno.18069
Eckhardt BL, 2012, NAT REV DRUG DISCOV, V11, P479, DOI 10.1038/nrd2372
Fein MR, 2013, DIS MODEL MECH, V6, P580, DOI 10.1242/dmm.009282
Fernandez-Trillo F, 2017, ANGEW CHEM INT EDIT, V56, P3142, DOI 10.1002/anie.201607825
Fidler IJ, 2015, CANCER METAST REV, V34, P635, DOI 10.1007/s10555-015-9586-9
Gao FL, 2020, BIOMATERIALS, V230, DOI 10.1016/j.biomaterials.2019.119635
Gao HL, 2016, CURR DRUG METAB, V17, P731, DOI 10.2174/1389200217666160630203600
Gao HL, 2017, J NEUROIMMUNE PHARM, V12, P6, DOI 10.1007/s11481-016-9687-4
Gao HL, 2016, ACTA PHARM SIN B, V6, P268, DOI 10.1016/j.apsb.2016.05.013
Gao HL, 2014, EXPERT OPIN DRUG DEL, V11, P409, DOI 10.1517/17425247.2014.877442
Gay LJ, 2011, NAT REV CANCER, V11, P123, DOI 10.1038/nrc3004
Goldberg MS, 2019, NAT REV CANCER, V19, P587, DOI 10.1038/s41568-019-0186-9
Gong NQ, 2019, ADV FUNCT MATER, V29, DOI 10.1002/adfm.201806087
Guan XM, 2015, ACTA PHARM SIN B, V5, P402, DOI 10.1016/j.apsb.2015.07.005
Guo JF, 2020, ACS NANO, V14, P5075, DOI 10.1021/acsnano.0c01676
Gupta GP, 2006, CELL, V127, P679, DOI 10.1016/j.cell.2006.11.001
Haemmerle M, 2018, CANCER CELL, V33, P965, DOI 10.1016/j.ccell.2018.03.002
Hernandez L, 2011, BREAST CANCER RES, V13, DOI 10.1186/bcr3074
Hoshino A, 2015, NATURE, V527, P329, DOI 10.1038/nature15756
Hu C, 2020, BIOMATERIALS, V255, DOI 10.1016/j.biomaterials.2020.120159
Hu C, 2018, ACS APPL MATER INTER, V10, P22571, DOI 10.1021/acsami.8b04847
Hu C, 2018, BIOMATERIALS, V168, P64, DOI 10.1016/j.biomaterials.2018.03.046
Huang YK, 2018, ACTA PHARM SIN B, V8, P4, DOI 10.1016/j.apsb.2017.12.001
Huo D, 2019, NAT COMMUN, V10, DOI 10.1038/s41467-019-11082-3
Jiang Q, 2020, SMALL, V16, DOI 10.1002/smll.202001704
Joyce JA, 2009, NAT REV CANCER, V9, P239, DOI 10.1038/nrc2618
Kalbasi A, 2020, NAT REV IMMUNOL, V20, P25, DOI 10.1038/s41577-019-0218-4
Kawatani M, 2009, CANCER SCI, V100, P1999, DOI 10.1111/j.1349-7006.2009.01294.x
Kuai R, 2018, SCI ADV, V4, DOI 10.1126/sciadv.aao1736
Labelle M, 2011, CANCER CELL, V20, P576, DOI 10.1016/j.ccr.2011.09.009
Lang JY, 2019, ACS NANO, V13, P12357, DOI 10.1021/acsnano.9b04857
Li C, 2019, ACTA PHARM SIN B, V9, P1145, DOI 10.1016/j.apsb.2019.08.003
Li RX, 2018, ACTA PHARM SIN B, V8, P14, DOI 10.1016/j.apsb.2017.11.009
Li X, 2010, CANCER BIOL THER, V10, P1081, DOI 10.4161/cbt.10.11.13434
Li YF, 2019, BIOMATERIALS, V188, P160, DOI 10.1016/j.biomaterials.2018.10.019
LIOTTA LA, 1986, CANCER RES, V46, P1
Liu DD, 2020, THERANOSTICS, V10, P1181, DOI 10.7150/thno.38989
Liu DC, 2019, NANO LETT, V19, P6964, DOI 10.1021/acs.nanolett.9b02306

Liu J, 2019, ADV SCI, V6, DOI 10.1002/advs.201801987
Liu J, 2019, ACS NANO, V13, P8648, DOI 10.1021/acsnano.9b03472
Liu MR, 2018, J CONTROL RELEASE, V290, P150, DOI 10.1016/j.jconrel.2018.10.007
Liu R, 2020, J CONTROL RELEASE, V321, P589, DOI 10.1016/j.jconrel.2020.02.043
Liu R, 2019, ADV FUNCT MATER, V29, DOI 10.1002/adfm.201808462
Liu R, 2019, ACTA PHARM SIN B, V9, P410, DOI 10.1016/j.apsb.2018.09.001
Liu R, 2018, J CONTROL RELEASE, V278, P127, DOI 10.1016/j.jconrel.2018.04.005
Liu X, 2020, BIOMATERIALS, V230, DOI 10.1016/j.biomaterials.2019.119649
Lo SS, 2011, NAT REV CLIN ONCOL, V8, P405, DOI 10.1038/nrclinonc.2011.75
Lu JQ, 2018, ACS NANO, V12, P11041, DOI 10.1021/acsnano.8b05189
Lu QL, 2019, J MATER CHEM B, V7, P2499, DOI 10.1039/c9tb00089e
Luo ZJ, 2019, ACTA PHARM SIN B, V9, P1099, DOI 10.1016/j.apsb.2019.06.004
Lynn GM, 2020, NAT BIOTECHNOL, V38, P320, DOI 10.1038/s41587-019-0390-x
Min YZ, 2017, NAT NANOTECHNOL, V12, P877, DOI [10.1038/nnano.2017.113, 10.1038/NNANO.2017.113]
Mishra SK, 2012, CANCER METAST REV, V31, P163, DOI 10.1007/s10555-011-9338-4
Mukherjee D, 2013, AM J CANCER RES, V3, P46
Nagy JA, 2012, CLIN EXP METASTAS, V29, P657, DOI 10.1007/s10585-012-9500-6
Nusse R, 2017, CELL, V169, P985, DOI 10.1016/j.cell.2017.05.016
Pan XT, 2018, SCI CHINA LIFE SCI, V61, P415, DOI 10.1007/s11427-017-9262-x
Peinado H, 2017, NAT REV CANCER, V17, P302, DOI 10.1038/nrc.2017.6
Peinado H, 2012, NAT MED, V18, P883, DOI 10.1038/nm.2753
Pohl SG, 2017, ONCOGENESIS, V6, DOI 10.1038/oncsis.2017.14
Qin L, 2020, SCI ADV, V6, DOI 10.1126/sciadv.abb3116
Qin L, 2019, ASIAN J PHARM SCI, V14, P380, DOI 10.1016/j.ajps.2018.10.005
Quail DF, 2013, NAT MED, V19, P1423, DOI 10.1038/nm.3394
Rachidi S, 2017, SCI IMMUNOL, V2, DOI 10.1126/sciimmunol.aai7911
Ribas A, 2018, SCIENCE, V359, P1350, DOI 10.1126/science.aar4060
Shi JJ, 2017, NAT REV CANCER, V17, P20, DOI 10.1038/nrc.2016.108
Steeg PS, 2016, NAT REV CANCER, V16, P201, DOI 10.1038/nrc.2016.25
Steeg PS, 2011, NAT REV CANCER, V11, P352, DOI 10.1038/nrc3053
Sun W, 2019, BIOMATERIALS, V217, DOI 10.1016/j.biomaterials.2019.119264
Sun WT, 2019, ACS NANO, V13, P7556, DOI 10.1021/acsnano.9b00097
van der Meel R, 2019, NAT NANOTECHNOL, V14, P1007, DOI 10.1038/s41565-019-0567-y
Vanderburgh J, 2020, ACS NANO, V14, P311, DOI 10.1021/acsnano.9b04571
Wang JB, 2014, ONCOTARGET, V5, P8478, DOI 10.18632/oncotarget.2328
Wang SF, 2019, CHINESE CHEM LETT, V30, P847, DOI 10.1016/j.cclet.2019.03.025
Wang WJ, 2015, INT J ONCOL, V47, P1912, DOI 10.3892/ijo.2015.3156
Xia Q, 2019, ACTA PHARM SIN B, V9, P675, DOI 10.1016/j.apsb.2019.01.011
Xiao W, 2018, INT J PHARMACEUT, V552, P328, DOI 10.1016/j.ijpharm.2018.10.011
Xiao W, 2018, INT J PHARMACEUT, V538, P105, DOI 10.1016/j.ijpharm.2018.01.011
Yang FJ, 2020, TRENDS CANCER, V6, P645, DOI 10.1016/j.trecan.2020.05.001
Yang LQ, 2020, SIGNAL TRANSDUCT TAR, V5, DOI 10.1038/s41392-020-0110-5
Yang S, 2017, PHARMACOL RES, V126, P97, DOI 10.1016/j.phrs.2017.05.004
Yang XT, 2019, ADV FUNCT MATER, V29, DOI 10.1002/adfm.201901896
Ye H, 2019, BIOMATERIALS, V206, P1, DOI 10.1016/j.biomaterials.2019.03.024
Yin HR, 2019, MOL THER, V27, P1252, DOI 10.1016/j.ymthe.2019.04.018
Yu HY, 2020, BIOMATER SCI-UK, V8, P325, DOI 10.1039/c9bm01398a
Yu WQ, 2020, ACS CENTRAL SCI, V6, P100, DOI 10.1021/acscentsci.9b01139
Yu WQ, 2019, BIOMATERIALS, V217, DOI 10.1016/j.biomaterials.2019.119309
Yue WW, 2019, NAT COMMUN, V10, DOI 10.1038/s41467-019-09760-3
Zhang C, 2018, ADV FUNCT MATER, V28
Zhang D, 2019, CHINESE CHEM LETT, V30, P7, DOI 10.1016/j.cclet.2018.06.011
Zhang DS, 2019, NANOSCALE, V11, P19912, DOI 10.1039/c9nr05684j
Zhang Y, 2019, ADV FUNCT MATER, V29
Zhao HJ, 2020, ADV HEALTHC MATER, V9, DOI 10.1002/adhm.201901335
Zhao LW, 2020, J CONTROL RELEASE, V318, P1, DOI 10.1016/j.jconrel.2019.12.005
Zhou Y, 2020, J MATER CHEM B, V8, P6765, DOI 10.1039/d0tb00649a
Zhou Y, 2020, ADV SCI, V7, DOI 10.1002/advs.201901866
Zou WP, 2005, NAT REV CANCER, V5, P263, DOI 10.1038/nrc1586
NR 107

TC 108
Z9 112
U1 24
U2 155
PU INST MATERIA MEDICA, CHINESE ACAD MEDICAL SCIENCES
PI BEIJING
PA C/O EDITORIAL BOARD OF ACTA PHARMACEUTICA SINICA, 1 XIANNONGTAN ST,
   BEIJING, 100050, PEOPLES R CHINA
SN 2211-3835
EI 2211-3843
J9 ACTA PHARMACOL SIN B
JI Acta Pharm. Sin. B
PD NOV
PY 2020
VL 10
IS 11
BP 2037
EP 2053
DI 10.1016/j.apsb.2020.07.013
PG 17
WC Pharmacology & Pharmacy
WE Science Citation Index Expanded (SCI-EXPANDED)
SC Pharmacology & Pharmacy
GA PA2YP
UT WOS:000595499500003
PM 33304778
OA gold, Green Published
HC Y
HP N
DA 2023-09-14
ER

PT J
AU Paulis, LE
   Mandal, S
   Kreutz, M
   Figdor, CG
AF Paulis, Leonie E.
   Mandal, Subhra
   Kreutz, Martin
   Figdor, Carl G.
TI Dendritic cell-based nanovaccines for cancer immunotherapy
SO CURRENT OPINION IN IMMUNOLOGY
LA English
DT Review
ID ANTIGEN-PRESENTING CELLS; T-CELLS; ANTITUMOR IMMUNITY;
   CROSS-PRESENTATION; NANOPARTICLE VACCINES; MEDIATED DELIVERY; IN-VIVO;
   PEPTIDE; INDUCTION; RESPONSES
AB Cancer immunotherapy critically relies on the efficient presentation of tumor antigens to T-cells to elicit a potent antitumor immune response aimed at life-long protection against cancer recurrence. Recent advances in the nanovaccine field have now resulted in formulations that trigger strong anti-tumor responses. Nanovaccines are assemblies that are able to present tumor antigens and appropriate immune-stimulatory signals either directly to T-cells or indirectly via antigen-presenting dendritic cells. This review focuses on important aspects of nanovaccine design for dendritic cells, including the synergistic and cytosolic delivery of immunogenic compounds, as well as their passive and active targeting to dendritic cells. In addition, nanoparticles for direct T-cell activation are discussed, addressing features necessary to effectively mimic dendritic cell/T-cell interactions.
C1 [Paulis, Leonie E.; Mandal, Subhra; Kreutz, Martin; Figdor, Carl G.] Radboud Univ Nijmegen, Dept Tumor Immunol, Nijmegen Ctr Mol Life Sci, Med Ctr, NL-6525 ED Nijmegen, Netherlands.
C3 Radboud University Nijmegen
RP Figdor, CG (通讯作者)，Radboud Univ Nijmegen, Dept Tumor Immunol, Nijmegen Ctr Mol Life Sci,

Med Ctr, NL-6525 ED Nijmegen, Netherlands.
EM C.Figdor@ncmls.ru.nl
RI Mandal, Subhra/A-9090-2013; Figdor, Carl G/A-4232-2010
OI Mandal, Subhra/0000-0003-0047-4305; Figdor, Carl G/0000-0002-2366-9212
FU EU (ERC advanced PATHFINDER) [269019]; Dutch Cancer Society [KUN2009-4402]; Dutch government to the Netherlands Institute for Regenerative Medicine (NIRM) [FES0908]; NWO Spinoza award
FX This work was supported by grants from the EU (ERC advanced PATHFINDER 269019), the Dutch Cancer Society (KUN2009-4402) and a grant from the Dutch government to the Netherlands Institute for Regenerative Medicine (NIRM, grant No. FES0908). Carl Figdor received the NWO Spinoza award.
CR Arranz A, 2013, J CONTROL RELEASE, V165, P163, DOI 10.1016/j.jconrel.2012.11.008
Blander JM, 2006, NATURE, V440, P808, DOI 10.1038/nature04596
Brenner MK, 2010, CURR OPIN IMMUNOL, V22, P251, DOI 10.1016/j.coi.2010.01.020
Bungener L, 2005, VACCINE, V23, P1232, DOI 10.1016/j.vaccine.2004.09.002
Butler MO, 2007, CLIN CANCER RES, V13, P1857, DOI 10.1158/1078-0432.CCR-06-1905
Butler MO, 2010, INT IMMUNOL, V22, P863, DOI 10.1093/intimm/dxq440
Chekmasova AA, 2010, DISCOV MED, V9, P62
Cho HJ, 2000, NAT BIOTECHNOL, V18, P509, DOI 10.1038/75365
Chu CS, 2012, CANCER IMMUNOL IMMUN, V61, P629, DOI 10.1007/s00262-011-1081-8
Cruz LJ, 2011, BIOMATERIALS, V32, P6791, DOI 10.1016/j.biomaterials.2011.04.082
Cubas R, 2009, J IMMUNOTHER, V32, P118, DOI 10.1097/CJI.0b013e31818f13c4
Daftarian P, 2011, CANCER RES, V71, P7452, DOI 10.1158/0008-5472.CAN-11-1766
Diken Mustafa, 2013, Methods Mol Biol, V969, P235, DOI 10.1007/978-1-62703-260-5_15
Ding Q, 2013, PHARM RES-DORDR, V30, P60, DOI 10.1007/s11095-012-0849-7
Dudziak D, 2007, SCIENCE, V315, P107, DOI 10.1126/science.1136080
Duijvesz D, 2011, EUR UROL, V59, P823, DOI 10.1016/j.eururo.2010.12.031
El Ansary M, 2013, J CANCER RES CLIN, V139, P39, DOI 10.1007/s00432-012-1298-8
Figdor CG, 2004, NAT MED, V10, P475, DOI 10.1038/nm1039
Han H, 2011, BIOCHEM BIOPH RES CO, V411, P530, DOI 10.1016/j.bbrc.2011.06.164
Hangalapura BN, 2011, CANCER RES, V71, P5827, DOI 10.1158/0008-5472.CAN-11-0804
Hasan AN, 2009, J IMMUNOL, V183, P2837, DOI 10.4049/jimmunol.0804178
Heath WR, 2004, IMMUNOL REV, V199, P9, DOI 10.1111/j.0105-2896.2004.00142.x
Idoyaga J, 2011, P NATL ACAD SCI USA, V108, P2384, DOI 10.1073/pnas.1019547108
Jerome V, 2006, J IMMUNOTHER, V29, P294
Jewell CM, 2011, P NATL ACAD SCI USA, V108, P15745, DOI 10.1073/pnas.1105200108
Kantoff PW, 2010, NEW ENGL J MED, V363, P411, DOI 10.1056/NEJMoa1001294
Kasturi SP, 2011, NATURE, V470, P543, DOI 10.1038/nature09737
Keller SA, 2010, J IMMUNOL, V184, P26, DOI 10.4049/jimmunol.0901540
Kreutz M, 2013, BLOOD, V121, P2836, DOI 10.1182/blood-2012-09-452078
Kreutz M, 2012, PLOS ONE, V7, DOI 10.1371/journal.pone.0040208
Lee EY, 2012, PLOS ONE, V7, DOI 10.1371/journal.pone.0033330
Li HY, 2011, NAT NANOTECHNOL, V6, P645, DOI [10.1038/nnano.2011.153, 10.1038/NNANO.2011.153]
Manolova V, 2008, EUR J IMMUNOL, V38, P1404, DOI 10.1002/eji.200737984
Marraco SAF, 2012, J IMMUNOL METHODS, V385, P90, DOI 10.1016/j.jim.2012.07.017
McKee SJ, 2012, J CONTROL RELEASE, V159, P338, DOI 10.1016/j.jconrel.2012.02.015
Moon JJ, 2012, ADV MATER, V24, P3724, DOI 10.1002/adma.201200446
Moon JJ, 2012, P NATL ACAD SCI USA, V109, P1080, DOI 10.1073/pnas.1112648109
Moon JJ, 2011, NAT MATER, V10, P243, DOI 10.1038/NMAT2960
Morse MA, 2011, CLIN CANCER RES, V17, P4844, DOI 10.1158/1078-0432.CCR-11-0891
Mukai Y, 2011, J IMMUNOL, V187, P6249, DOI 10.4049/jimmunol.1001093
Nakamura T, 2008, MOL THER, V16, P1507, DOI 10.1038/mt.2008.122
Oelke M, 2010, IMMUNOL RES, V47, P248, DOI 10.1007/s12026-009-8156-z
Oussoren C, 1997, BBA-BIOMEMBRANES, V1328, P261, DOI 10.1016/S0005-2736(97)00122-3
Palucka K, 2010, IMMUNITY, V33, P464, DOI 10.1016/j.immuni.2010.10.007
Perche F, 2011, NANOMED-NANOTECHNOL, V7, P445, DOI 10.1016/j.nano.2010.12.010
Randolph GJ, 1999, IMMUNITY, V11, P753, DOI 10.1016/S1074-7613(00)80149-1
Reddy ST, 2007, NAT BIOTECHNOL, V25, P1159, DOI [10.1038/nbt1332, 10.1038/nbtl332]
Reddy ST, 2006, J CONTROL RELEASE, V112, P26, DOI 10.1016/j.jconrel.2006.01.006
Rettig L, 2010, BLOOD, V115, P4533, DOI 10.1182/blood-2009-11-247817

Rosenberg SA, 2009, CURR OPIN IMMUNOL, V21, P233, DOI 10.1016/j.coi.2009.03.002
Schutz C, 2010, IMMUNOTHERAPY-UK, V2, P539, DOI [10.2217/imt.10.26, 10.2217/IMT.10.26]
Singh H, 2011, CANCER RES, V71, P3516, DOI 10.1158/0008-5472.CAN-10-3843
Speiser DE, 2010, J IMMUNOTHER, V33, P848, DOI 10.1097/CJI.0b013e3181f1d614
Steinman RM, 2012, ANNU REV IMMUNOL, V30, P1, DOI 10.1146/annurev-immunol-100311-102839
Stiff PJ, 2013, EXPERT REV ANTICANC, V13, P43, DOI [10.1586/ERA.12.153, 10.1586/era.12.153]
Tacken PJ, 2011, BLOOD, V118, P6836, DOI 10.1182/blood-2011-07-367615
Tacken PJ, 2011, SEMIN IMMUNOL, V23, P12, DOI 10.1016/j.smim.2011.01.001
Taieb J, 2005, CRIT REV IMMUNOL, V25, P215, DOI 10.1615/CritRevImmunol.v25.i3.30
Tanaka M, 2011, CLIN CANCER RES, V17, P5392, DOI 10.1158/1078-0432.CCR-10-3083
Tarasov SG, 2011, P NATL ACAD SCI USA, V108, P9798, DOI 10.1073/pnas.1014598108
Thomann JS, 2011, BIOMATERIALS, V32, P4574, DOI 10.1016/j.biomaterials.2011.03.015
Trumpfheller C, 2012, J INTERN MED, V271, P183, DOI 10.1111/j.1365-2796.2011.02496.x
Turtle CJ, 2010, CANCER J, V16, P374, DOI 10.1097/PPO.0b013e3181eb33a6
Ugel S, 2009, CANCER RES, V69, P9376, DOI 10.1158/0008-5472.CAN-09-0400
van Broekhoven CL, 2004, CANCER RES, V64, P4357, DOI 10.1158/0008-5472.CAN-04-0138
Villadangos JA, 2010, J EXP MED, V207, P1131, DOI 10.1084/jem.20100985
Wang C, 2012, VACCINE, V30, P4790, DOI 10.1016/j.vaccine.2012.05.027
Yuba E, 2010, BIOMATERIALS, V31, P943, DOI 10.1016/j.biomaterials.2009.10.006
Zhang ZP, 2011, BIOMATERIALS, V32, P3666, DOI 10.1016/j.biomaterials.2011.01.067
Zitvogel L, 1998, NAT MED, V4, P594, DOI 10.1038/nm0598-594
NR 70
TC 104
Z9 109
U1 4
U2 129
PU CURRENT BIOLOGY LTD
PI LONDON
PA 84 THEOBALDS RD, LONDON WC1X 8RR, ENGLAND
SN 0952-7915
EI 1879-0372
J9 CURR OPIN IMMUNOL
JI Curr. Opin. Immunol.
PD JUN
PY 2013
VL 25
IS 3
BP 389
EP 395
DI 10.1016/j.coi.2013.03.001
PG 7
WC Immunology
WE Science Citation Index Expanded (SCI-EXPANDED)
SC Immunology
GA 183ID
UT WOS:000321807100015
PM 23571027
DA 2023-09-14
ER

PT J
AU Liu, R
Luo, C
Pang, ZQ
Zhang, JM
Ruan, SB
Wu, MY
Wang, L
Sun, T
Li, N
Han, L

Shi, JJ
Huang, YY
Guo, WS
Peng, SJ
Zhou, WH
Gao, HL
AF Liu, Rui
Luo, Cong
Pang, Zhiqing
Zhang, Jinming
Ruan, Shaobo
Wu, Meiying
Wang, Lei
Sun, Tao
Li, Nan
Han, Liang
Shi, Jinjin
Huang, Yuanyu
Guo, Weisheng
Peng, Shaojun
Zhou, Wenhu
Gao, Huile
TI Advances of nanoparticles as drug delivery systems for disease diagnosis
and treatment
SO CHINESE CHEMICAL LETTERS
LA English
DT Article
DE Nanoparticles; Drug delivery; Nanomaterials; Delivery strategies;
Specific delivery; Disease diagnosis; Disease treatment
ID EFFICIENT HEPATIC DELIVERY; DEEP TUMOR PENETRATION; LIPID NANOPARTICLES;
MESSENGER-RNA; PHYSICOCHEMICAL PROPERTIES; PEPTIDE NANOPARTICLES;
PHOTOTHERMAL THERAPY; ORAL ABSORPTION; CELLULAR UPTAKE; SIZE
AB Decades have passed since the first nanoparticles-base medicine was approved for human cancer treat-ment, and the research and development of nanoparticles for drug delivery are always undergoing. Nowadays, the significant advances complicate nanoparticles' branches, including liposomes, solid lipid nanoparticles, inorganic nanoparticles, micelles, nanovaccines and nano-antibodies, etc. These nanoparti-cles show numerous capabilities in treatment and diagnosis of stubborn diseases like cancer and neurode-generative diseases, emerging as novel drug carriers or therapeutic agents in future. In this review, the complicated branches of nanoparticles are classified and summarized, with their property and functions concluded. Besides, there are also some delivery strategies that make nanoparticles smarter and more efficient in drug delivery, and frontiers in these strategies are also summarized in this review. Except these excellent works in newly-produced drug delivery nanoparticles, some points of view and future expectations are made in the end. (c) 2022 Published by Elsevier B.V. on behalf of Chinese Chemical Society and Institute of Materia Medica, Chinese Academy of Medical Sciences.
C1 [Liu, Rui; Gao, Huile] Sichuan Univ, Key Lab Drug Targeting & Drug Delivery Syst Educ M, Chengdu 610041, Peoples R China.
[Liu, Rui; Gao, Huile] Sichuan Univ, Sichuan Engn Lab Plant Sourced Drug, Chengdu 610041, Peoples R China.
[Liu, Rui; Gao, Huile] Sichuan Univ, Sichuan Res Ctr Drug Precis Ind Technol, West China Sch Pharm, Chengdu 610041, Peoples R China.
[Luo, Cong] Shenyang Pharmaceut Univ, Wuya Coll Innovat, Dept Pharmaceut, Shenyang 110016, Peoples R China.
[Pang, Zhiqing; Sun, Tao] Fudan Univ, Sch Pharm, Dept Pharmaceut, Key Lab Smart Drug Delivery,Minist Educ, Shanghai 200433, Peoples R China.
[Zhang, Jinming] Chengdu Univ Tradit Chinese Med, Pharm Sch, State Key Lab Southwestern Chinese Med Resources, Chengdu 611130, Peoples R China.
[Ruan, Shaobo] Beijing Inst Technol, Adv Res Inst Multidisciplinary Sci, Beijing 100081, Peoples R China.
[Wu, Meiying] Sun Yat sen Univ, Sch Pharmaceut Sci Shenzhen, Shenzhen 518107, Peoples R China.
[Wang, Lei; Shi, Jinjin] Zhengzhou Univ, Sch Pharmaceut Sci, Key Lab Targeting Therapy & Diag Crit Dis, Zhengzhou 450001, Peoples R China.

[Li, Nan] Guangzhou Med Univ, Sch Pharmaceut Sci & Technol, Tianjin Key Lab Drug Delivery & High Efficiency, Tianjin 300072, Peoples R China.
[Han, Liang] Soochow Univ, Sch Pharmaceut Sci, Clin Pharmacol, Guangzhou 510260, Peoples R China.
[Han, Liang] Guangzhou Med Univ, Affiliated Hosp 2, Guangzhou 510260, Peoples R China.
[Huang, Yuanyu] Jinan Univ, Zhuhai Hosp, Zhuhai Peoples Hosp, Zhuhai Inst Translat Med, Zhuhai 519000, Peoples R China.
[Zhou, Wenhu] Cent South Univ, Xiangya Sch Pharmaceut Sci, Changsha 410013, Peoples R China.
C3 Sichuan University; Sichuan University; Sichuan University; Shenyang Pharmaceutical University; Fudan University; Chengdu University of Traditional Chinese Medicine; Beijing Institute of Technology; Sun Yat Sen University; Zhengzhou University; Guangzhou Medical University; Soochow University - China; Guangzhou Medical University; Jinan University; Central South University
RP Gao, HL (通讯作者)，Sichuan Univ, Key Lab Drug Targeting & Drug Delivery Syst Educ M, Chengdu 610041, Peoples R China.; Gao, HL (通讯作者)，Sichuan Univ, Sichuan Engn Lab Plant Sourced Drug, Chengdu 610041, Peoples R China.; Gao, HL (通讯作者)，Sichuan Univ, Sichuan Res Ctr Drug Precis Ind Technol, West China Sch Pharm, Chengdu 610041, Peoples R China.
EM gaohuile@scu.edu.cn
RI Yao, Jingjing/GQG-9815-2022; Wu, Meiying/AAV-4636-2020; Gao, Huile/H-9206-2019; HAN, MINGMING/AAR-5381-2021
OI Yao, Jingjing/0000-0002-2608-0745; Wu, Meiying/0000-0003-2664-0498; Gao, Huile/0000-0002-5355-7238; Zhou, Jiang/0000-0003-0858-4533; Ruan, Shaobo/0000-0002-6300-4979
FU National Natural Science Founda-tion of China; 111 Project; Key Research and Development Program of Science and Technology Department of Sichuan Province; [81961138009]; [B18035]; [2020YFS0570]
FX Acknowledgments This work was supported by National Natural Science Founda-tion of China (No. 81961138009) , 111 Project (No. B18035) , and the Key Research and Development Program of Science and Technology Department of Sichuan Province (No. 2020YFS0570) .
CR Abu Abed OS, 2021, INT J PHARMACEUT, V592, DOI 10.1016/j.ijpharm.2020.120094
Acharya S, 2011, ADV DRUG DELIVER REV, V63, P170, DOI 10.1016/j.addr.2010.10.008
Akinc A, 2019, NAT NANOTECHNOL, V14, P1084, DOI 10.1038/s41565-019-0591-y
Ayer M, 2021, ADV HEALTHC MATER, V10, DOI 10.1002/adhm.202001375
Barclay TG, 2019, CARBOHYD POLYM, V221, P94, DOI 10.1016/j.carbpol.2019.05.067
Barenholz Y, 2012, J CONTROL RELEASE, V160, P117, DOI 10.1016/j.jconrel.2012.03.020
Beck A, 2017, NAT REV DRUG DISCOV, V16, P315, DOI 10.1038/nrd.2016.268
Burn GL, 2021, IMMUNITY, V54, P1377, DOI 10.1016/j.immuni.2021.06.006
Cai XQ, 2021, NANO TODAY, V36, DOI 10.1016/j.nantod.2020.101032
Cao ZP, 2019, NAT COMMUN, V10, DOI 10.1038/s41467-019-13727-9
Chang J, 2019, ACCOUNTS CHEM RES, V52, P665, DOI 10.1021/acs.accounts.8b00493
Chau CH, 2019, LANCET, V394, P793, DOI 10.1016/S0140-6736(19)31774-X
Che JY, 2020, ADV MATER, V32, DOI 10.1002/adma.202003598
Chen BL, 2017, THERANOSTICS, V7, P538, DOI 10.7150/thno.16684
Chen GY, 2014, CHEM REV, V114, P5161, DOI 10.1021/cr400425h
Chen LC, 2019, ACS NANO, V13, P10887, DOI 10.1021/acsnano.9b04954
Chen QW, 2021, CHEM SOC REV, V50, P12576, DOI 10.1039/d0cs01571g
Chen WH, 2021, ANGEW CHEM INT EDIT, V60, P5626, DOI 10.1002/anie.201914511
Cheng QK, 2021, ADV HEALTHC MATER, V10, DOI 10.1002/adhm.202001953
Choi KY, 2010, BIOMATERIALS, V31, P106, DOI 10.1016/j.biomaterials.2009.09.030
Chu DF, 2017, ADV MATER, V29, DOI 10.1002/adma.201701021
Chu LRC, 2021, VACCINE, V39, P2791, DOI 10.1016/j.vaccine.2021.02.007
Cong ZQ, 2020, ACS NANO, V14, P1958, DOI 10.1021/acsnano.9b08434
Dabholkar N, 2021, INT J BIOL MACROMOL, V170, P602, DOI 10.1016/j.ijbiomac.2020.12.177
Dai J, 2020, SIGNAL TRANSDUCT TAR, V5, DOI 10.1038/s41392-020-00261-0
Danhier F, 2016, J CONTROL RELEASE, V244, P108, DOI 10.1016/j.jconrel.2016.11.015
Dawidczyk CM, 2014, J CONTROL RELEASE, V187, P133, DOI 10.1016/j.jconrel.2014.05.036
Dimatteo R, 2018, ADV DRUG DELIVER REV, V127, P167, DOI 10.1016/j.addr.2018.03.007
DLUGI AM, 1990, FERTIL STERIL, V54, P419
Elsadek NE, 2020, POLYMER-PROTEIN CONJUGATES: FROM PEGYLATION AND BEYOND, P103, DOI 10.1016/B978-0-444-64081-9.00005-X

Elzoghby AO, 2020, BIOMATERIALS, V263, DOI 10.1016/j.biomaterials.2020.120355
Fu X, 2018, CHEM-US, V4, P2084, DOI 10.1016/j.chempr.2018.07.002
Gao CY, 2021, ADV MATER, V33, DOI 10.1002/adma.202000512
Gao C, 2021, ADV FUNCT MATER, V31, DOI 10.1002/adfm.202102440
Gao XH, 2017, ADV MATER, V29, DOI 10.1002/adma.201603917
Geng JL, 2012, SMALL, V8, P3655, DOI 10.1002/smll.201200814
Gillmore JD, 2021, NEW ENGL J MED, V385, P493, DOI 10.1056/NEJMoa2107454
Green MR, 2006, ANN ONCOL, V17, P1263, DOI 10.1093/annonc/mdl104
Guo Q, 2019, J CONTROL RELEASE, V303, P117, DOI 10.1016/j.jconrel.2019.04.031
Guo Shuai, 2021, Exploration (Beijing), V1, P35, DOI 10.1002/EXP.20210008
Guo S, 2021, CHINESE CHEM LETT, V32, P102, DOI 10.1016/j.cclet.2020.11.024
Han L, 2021, ACTA PHARM SIN B, V11, P2306, DOI 10.1016/j.apsb.2020.11.023
Haney MJ, 2019, ADV HEALTHC MATER, V8, DOI 10.1002/adhm.201801271
He HL, 2019, ACCOUNTS CHEM RES, V52, P2445, DOI 10.1021/acs.accounts.9b00228
He YW, 2019, ACS NANO, V13, P4148, DOI 10.1021/acsnano.8b08964
Hettiaratchi MH, 2020, SCI ADV, V6, DOI 10.1126/sciadv.aay1240
Honda Y, 2020, BIOMACROMOLECULES, V21, P3826, DOI 10.1021/acs.biomac.0c00903
Hou XC, 2021, NAT REV MATER, V6, P1078, DOI 10.1038/s41578-021-00358-0
Hu B, 2022, SCI ADV, V8, DOI 10.1126/sciadv.abm1418
Hu B, 2019, J GENE MED, V21, DOI 10.1002/jgm.3097
Hu CMJ, 2011, P NATL ACAD SCI USA, V108, P10980, DOI 10.1073/pnas.1106634108
Hu C, 2021, ADV FUNCT MATER, V31, DOI 10.1002/adfm.202007149
Hu C, 2018, BIOMATERIALS, V168, P64, DOI 10.1016/j.biomaterials.2018.03.046
Hu J, 2021, CHINESE CHEM LETT, V32, P1341, DOI 10.1016/j.cclet.2020.11.006
Hu XX, 2017, ACS NANO, V11, P4086, DOI 10.1021/acsnano.7b00781
Huang HY, 2021, THERANOSTICS, V11, P906, DOI 10.7150/thno.47446
Huang YY, 2017, MOL THER-NUCL ACIDS, V6, P116, DOI 10.1016/j.omtn.2016.12.003
Ji QJ, 2021, ADV MATER, V33, DOI 10.1002/adma.202007798
Jia WF, 2021, ADV FUNCT MATER, V31, DOI 10.1002/adfm.202009765
Jiang Q, 2019, BIOMATERIALS, V192, P292, DOI 10.1016/j.biomaterials.2018.11.021
Jiang YK, 2022, ADV DRUG DELIVER REV, V180, DOI 10.1016/j.addr.2021.114034
Jin LG, 2020, ADV MATER, V32, DOI 10.1002/adma.201906050
Jo H, 2020, J IND ENG CHEM, V82, P234, DOI 10.1016/j.jiec.2019.10.018
Jokerst JV, 2011, SMALL, V7, P625, DOI 10.1002/smll.201002291
Joseph A, 2017, SCI ADV, V3, DOI 10.1126/sciadv.1700362
Ju XF, 2021, MOL PHARMACEUT, V18, P2694, DOI 10.1021/acs.molpharmaceut.1c00224
Ju XF, 2021, ADV HEALTHC MATER, V10, DOI 10.1002/adhm.202001997
Kaczmarek JC, 2021, BIOMATERIALS, V275, DOI 10.1016/j.biomaterials.2021.120966
Kaczmarek JC, 2016, ANGEW CHEM INT EDIT, V55, P13808, DOI 10.1002/anie.201608450
Karimi M, 2016, CHEM SOC REV, V45, P1457, DOI 10.1039/c5cs00798d
Khan NEEL, 2021, ACTA PHARM SIN B, V11, P1341, DOI 10.1016/j.apsb.2020.10.015
Kim E, 2020, EBIOMEDICINE, V55, DOI 10.1016/j.ebiom.2020.102743
Kim H, 2018, ADV MATER, V30, DOI 10.1002/adma.201701460
Kim J, 2019, ACS NANO, V13, P3206, DOI 10.1021/acsnano.8b08785
Koli AR, 2021, INT J PHARMACEUT, V596, DOI 10.1016/j.ijpharm.2021.120202
Kuhn J, 2020, BIOCONJUGATE CHEM, V31, P729, DOI 10.1021/acs.bioconjchem.9b00853
Kulkarni JA, 2021, NAT NANOTECHNOL, V16, P630, DOI 10.1038/s41565-021-00898-0
Lau JL, 2018, BIOORGAN MED CHEM, V26, P2700, DOI 10.1016/j.bmc.2017.06.052
Lee JE, 2011, J MATER CHEM, V21, P16869, DOI 10.1039/c1jm11869b
Lee SH, 2008, J CONTROL RELEASE, V125, P25, DOI 10.1016/j.jconrel.2007.09.011
Li CW, 2019, ADV SCI, V6, DOI 10.1002/advs.201900610
Li CH, 2022, BIOACT MATER, V9, P590, DOI 10.1016/j.bioactmat.2021.05.051
Li CH, 2021, NANO LETT, V21, P3680, DOI 10.1021/acs.nanolett.0c04468
Li J, 2022, ADV HEALTHC MATER, V11, DOI 10.1002/adhm.202102118
Li K, 2021, CHINESE CHEM LETT, V32, P1010, DOI 10.1016/j.cclet.2020.09.010
Li LC, 2017, ADV EXP MED BIOL, V983, P1, DOI 10.1007/978-981-10-4310-9_1
Li MJ, 2021, ACS NANO, V15, P9808, DOI 10.1021/acsnano.1c00680
Li M, 2020, NAT COMMUN, V11, DOI 10.1038/s41467-020-14963-0
Li RX, 2019, NANO LETT, V19, P124, DOI 10.1021/acs.nanolett.8b03439
Li S., 2021, ADV MATER, V33
Li SY, 2021, DRUG DELIV, V28, P1120, DOI 10.1080/10717544.2021.1934189

Lin CC, 2020, ACTA PHARM SIN B, V10, P2348, DOI 10.1016/j.apsb.2020.10.009
Lin RY, 2021, ADV HEALTHC MATER, V10, DOI 10.1002/adhm.202001212
Liu BW, 2017, NANO RES, V10, P1125, DOI 10.1007/s12274-017-1426-5
Liu GQ, 2020, INT J MOL SCI, V21, DOI 10.3390/ijms21176380
Liu H., 2021, ADV SCI, V8
Liu HL, 2020, ADV FUNCT MATER, V30, DOI 10.1002/adfm.202006515
Liu J, 2021, ANGEW CHEM INT EDIT, V60, P26740, DOI 10.1002/anie.202111213
Liu JQ, 2021, CHEM ENG J, V426, DOI 10.1016/j.cej.2021.130163
Liu R, 2020, J CONTROL RELEASE, V321, P589, DOI 10.1016/j.jconrel.2020.02.043
Liu R, 2019, ADV FUNCT MATER, V29, DOI 10.1002/adfm.201808462
Liu R, 2019, ACTA PHARM SIN B, V9, P410, DOI 10.1016/j.apsb.2018.09.001
Liu R, 2018, J CONTROL RELEASE, V278, P127, DOI 10.1016/j.jconrel.2018.04.005
Liu S, 2021, J AM CHEM SOC, V143, P21321, DOI 10.1021/jacs.1c09822
Liu S, 2021, NAT MATER, V20, P701, DOI 10.1038/s41563-020-00886-0
Lostale-Seijo I, 2018, NAT REV CHEM, V2, P258, DOI 10.1038/s41570-018-0039-1
Loynachan CN, 2019, NAT NANOTECHNOL, V14, P883, DOI 10.1038/s41565-019-0527-6
Lundquist P, 2016, ADV DRUG DELIVER REV, V106, P256, DOI 10.1016/j.addr.2016.07.007
Lundqvist M, 2011, ACS NANO, V5, P7503, DOI 10.1021/nn202458g
Luo C, 2019, J CONTROL RELEASE, V302, P79, DOI 10.1016/j.jconrel.2019.04.001
Luo C, 2016, NANO LETT, V16, P5401, DOI 10.1021/acs.nanolett.6b01632
Luo YY, 2020, ACTA PHARMACOL SIN B, V10, P2227, DOI 10.1016/j.apsb.2020.05.011
Luo ZJ, 2019, ACTA PHARM SIN B, V9, P1099, DOI 10.1016/j.apsb.2019.06.004
Lv QJ, 2020, ADV SCI, V7, DOI 10.1002/advs.202000515
Ma J, 2020, ACS APPL NANO MATER, V3, P4925, DOI 10.1021/acsanm.0c00396
Ma TT, 2014, INT J PHARMACEUT, V473, P296, DOI 10.1016/j.ijpharm.2014.06.052
Maier MA, 2013, MOL THER, V21, P1570, DOI 10.1038/mt.2013.124
Mei H, 2022, BIOACT MATER, V8, P220, DOI 10.1016/j.bioactmat.2021.06.035
Meng J, 2020, SCI ADV, V6, DOI 10.1126/sciadv.aba1362
Meng XQ, 2021, ACS NANO, V15, P5735, DOI 10.1021/acsnano.1c01248
Meng YC, 2021, MATER HORIZ, V8, P972, DOI 10.1039/d0mh01372b
Merino M, 2021, J NANOBIOTECHNOL, V19, DOI 10.1186/s12951-021-00846-z
Miao L, 2019, NAT BIOTECHNOL, V37, P1174, DOI 10.1038/s41587-019-0247-3
Miao TT, 2019, ADV FUNCT MATER, V29, DOI 10.1002/adfm.201900259
Miao Y.B., 2021, ADV MATER, V33
Miao ZH, 2020, NANO LETT, V20, P3079, DOI 10.1021/acs.nanolett.9b05035
Mu YZ, 2019, CARBOHYD POLYM, V203, P10, DOI 10.1016/j.carbpol.2018.09.020
Ni J, 2021, J CONTROL RELEASE, V329, P934, DOI 10.1016/j.jconrel.2020.10.023
Ortiz N, 2020, NANOMEDICINE-UK, V15, P2771, DOI 10.2217/nnm-2020-0305
Ouyang B, 2020, NAT MATER, V19, P1362, DOI 10.1038/s41563-020-0755-z
Ouyang J, 2021, SMALL, V17, DOI 10.1002/smll.202102598
Pan H, 2021, ADV MATER, V33, DOI 10.1002/adma.202100241
Park H, 2022, J CONTROL RELEASE, V342, P53, DOI 10.1016/j.jconrel.2021.12.030
Patel AK, 2019, ADV MATER, V31, DOI 10.1002/adma.201805116
Peng SJ, 2021, NANO TODAY, V40, DOI 10.1016/j.nantod.2021.101240
Peng Z, 2020, J CLIN ONCOL, V38
Pfutzner Andreas, 2002, Diabetes Technol Ther, V4, P589, DOI 10.1089/152091502320798204
Piotrowski-Daspit AS, 2020, ADV DRUG DELIVER REV, V156, P119, DOI 10.1016/j.addr.2020.06.014
Popovic Z, 2010, ANGEW CHEM INT EDIT, V49, P8649, DOI 10.1002/anie.201003142
Povsic TJ, 2016, J ALLERGY CLIN IMMUN, V138, P1712, DOI 10.1016/j.jaci.2016.04.058
Qin L, 2019, ASIAN J PHARM SCI, V14, P380, DOI 10.1016/j.ajps.2018.10.005
Qin X, 2021, NAT COMMUN, V12, DOI 10.1038/s41467-021-25561-z
Qin Y, 2021, ADV FUNCT MATER, V31, DOI 10.1002/adfm.202104645
Rahim MA, 2021, CANCERS, V13, DOI 10.3390/cancers13040670
Raza A, 2019, MOLECULES, V24, DOI 10.3390/molecules24061117
Rohaizad N, 2021, CHEM SOC REV, V50, P619, DOI 10.1039/d0cs00150c
Roopenian DC, 2007, NAT REV IMMUNOL, V7, P715, DOI 10.1038/nri2155
Ruan SB, 2018, ADV FUNCT MATER, V28, DOI 10.1002/adfm.201802227
Ruan SB, 2016, ACS NANO, V10, P10086, DOI 10.1021/acsnano.6b05070
Ruan SB, 2015, BIOMATERIALS, V60, P100, DOI 10.1016/j.biomaterials.2015.05.006
Scott S, 2020, ACS CATAL, V10, P14315, DOI 10.1021/acscatal.0c05047
Shan XZ, 2021, J NANOBIOTECHNOL, V19, DOI 10.1186/s12951-021-01037-6

Shen J, 2021, ADV MATER, V33, DOI 10.1002/adma.202101993
Shen ZY, 2017, MOL PHARMACEUT, V14, P1352, DOI 10.1021/acs.molpharmaceut.6b00839
Sindhwani S, 2020, NAT MATER, V19, P566, DOI 10.1038/s41563-019-0566-2
Sonawane ND, 2003, J BIOL CHEM, V278, P44826, DOI 10.1074/jbc.M308643200
Song QL, 2019, ADV MATER, V31, DOI 10.1002/adma.201903793
Song YN, 2021, CHEM ENG J, V408, DOI 10.1016/j.cej.2020.127296
Stevens D A, 1994, J Infect, V28 Suppl 1, P45, DOI 10.1016/S0163-4453(94)95971-4
Subbiah R, 2020, ACTA BIOMATER, V114, P63, DOI 10.1016/j.actbio.2020.07.026
Sun T, 2021, ADV SCI, V8, DOI 10.1002/advs.202102256
Sun T, 2018, BIOMATERIALS, V183, P268, DOI 10.1016/j.biomaterials.2018.04.016
Taghipour-Sabzevar V, 2019, THER DELIV, V10, P527, DOI 10.4155/tde-2019-0044
Tan H., 2021, ADV SCI, V8
Tan T, 2019, NAT COMMUN, V10, DOI 10.1038/s41467-019-11235-4
Tang L, 2014, P NATL ACAD SCI USA, V111, P15344, DOI 10.1073/pnas.1411499111
Taranejoo S, 2015, J APPL POLYM SCI, V132, DOI 10.1002/app.42096
Tenchov R, 2021, ACS NANO, V15, P16982, DOI 10.1021/acsnano.1c04996
Tredan O, 2007, J NATL CANCER I, V99, P1441, DOI 10.1093/jnci/djm135
Varanko A, 2020, ADV DRUG DELIVER REV, V156, P133, DOI 10.1016/j.addr.2020.08.008
Veronese FM, 2005, DRUG DISCOV TODAY, V10, P1451, DOI 10.1016/S1359-6446(05)03575-0
Vinarov Z, 2021, ADV DRUG DELIVER REV, V171, P289, DOI 10.1016/j.addr.2021.02.001
Wan MM, 2020, SCI ADV, V6, DOI 10.1126/sciadv.aaz9014
Wang HR, 2019, ADV FUNCT MATER, V29, DOI 10.1002/adfm.201902440
Wang HM, 2015, SCI REP-UK, V5, DOI 10.1038/srep16680
Wang X, 2021, J CONTROL RELEASE, V331, P390, DOI 10.1016/j.jconrel.2021.01.032
Wang YG, 2020, NANOSCALE, V12, P1886, DOI 10.1039/c9nr09235h
Wang YS, 2020, NATL SCI REV, V7, P1485, DOI 10.1093/nsr/nwaa034
Wang Y, 2021, NATL SCI REV, V8, DOI 10.1093/nsr/nwaa268
Wang YP, 2021, ADV MATER, V33, DOI 10.1002/adma.202103497
Wang ZJ, 2020, P NATL ACAD SCI USA, V117, P29512, DOI 10.1073/pnas.2011099117
Wei H, 2021, NANO TODAY, V40, DOI 10.1016/j.nantod.2021.101269
Wei M, 2021, ACTA BIOMATER, V126, P15, DOI 10.1016/j.actbio.2021.02.036
Weng YH, 2021, ASIAN J PHARM SCI, V16, P263, DOI 10.1016/j.ajps.2021.02.005
Weng YH, 2020, BIOTECHNOL ADV, V40, DOI 10.1016/j.biotechadv.2020.107534
Weng YH, 2019, BIOTECHNOL ADV, V37, P801, DOI 10.1016/j.biotechadv.2019.04.012
Wilbie D, 2019, ACCOUNTS CHEM RES, V52, P1555, DOI 10.1021/acs.accounts.9b00106
Wilson AJ, 2020, CHEM SOC REV, V49, P6087, DOI 10.1039/d0cs00338g
Wu Y, 2021, ANGEW CHEM INT EDIT, V60, P12868, DOI 10.1002/anie.202100002
Xiao W, 2018, INT J PHARMACEUT, V552, P328, DOI 10.1016/j.ijpharm.2018.10.011
Xie A, 2020, EBIOMEDICINE, V56, DOI 10.1016/j.ebiom.2020.102821
Xie R, 2021, BIOMATERIALS, V275, DOI 10.1016/j.biomaterials.2021.120891
Xie ZW, 2017, SMALL, V13, DOI 10.1002/smll.201603121
Xu CF, 2019, ADV FUNCT MATER, V29, DOI 10.1002/adfm.201905213
Xu CF, 2021, ADV DRUG DELIVER REV, V168, P3, DOI 10.1016/j.addr.2019.11.005
Xu JK, 2021, ADV MATER, V33, DOI 10.1002/adma.202104355
Xu M, 2020, BIOMATERIALS, V250, DOI 10.1016/j.biomaterials.2020.120076
Xu XL, 2021, ADV MATER, V33, DOI 10.1002/adma.202102322
Yan Y, 2021, ADV SCI, V8, DOI 10.1002/advs.202002253
Yang BW, 2019, ADV MATER, V31, DOI 10.1002/adma.201901778
Yang DZ, 2020, COLLOID SURFACE B, V195, DOI 10.1016/j.colsurfb.2020.111252
Yang FJ, 2020, TRENDS CANCER, V6, P645, DOI 10.1016/j.trecan.2020.05.001
Yang G, 2021, SIGNAL TRANSDUCT TAR, V6, DOI 10.1038/s41392-021-00649-6
Yang G, 2021, CHINESE CHEM LETT, V32, P218, DOI 10.1016/j.cclet.2020.10.018
Yang HK, 2021, ACS APPL MATER INTER, V13, P54715, DOI 10.1021/acsami.1c15858
Yang PP, 2017, ADV MATER, V29, DOI 10.1002/adma.201605869
Yang Q, 2020, ADV MATER, V32, DOI 10.1002/adma.202001854
Yang S, 2017, PHARMACOL RES, V126, P97, DOI 10.1016/j.phrs.2017.05.004
Yang T, 2020, DRUG DELIV, V27, P575, DOI 10.1080/10717544.2020.1748762
Yang TR, 2021, NANO TODAY, V36, DOI 10.1016/j.nantod.2020.101017
Yang TR, 2020, BIOACT MATER, V5, P1053, DOI 10.1016/j.bioactmat.2020.07.003
Yang WP, 2021, COORDIN CHEM REV, V448, DOI 10.1016/j.ccr.2021.214170
Yang ZB, 2021, ADV FUNCT MATER, V31, DOI 10.1002/adfm.202007991

Yang Z, 2013, J AM CHEM SOC, V135, P11657, DOI 10.1021/ja405372k
Yao XX, 2019, BIOMATERIALS, V197, P268, DOI 10.1016/j.biomaterials.2019.01.026
Yin DX, 2021, CHINESE CHEM LETT, V32, P1731, DOI 10.1016/j.cclet.2020.12.005
Yong TY, 2019, NAT COMMUN, V10, DOI 10.1038/s41467-019-11718-4
Yu JC, 2020, NAT BIOMED ENG, V4, P499, DOI 10.1038/s41551-019-0508-y
Yu JC, 2015, P NATL ACAD SCI USA, V112, P8260, DOI 10.1073/pnas.1505405112
Yu J, 2019, ACS CATAL, V9, P9973, DOI 10.1021/acscatal.9b02457
Yu L, 2020, NANO LETT, V20, P8903, DOI 10.1021/acs.nanolett.0c03982
Yu LD, 2018, ADV MATER, V30, DOI 10.1002/adma.201801964
Yu WQ, 2021, ACTA PHARM SIN B, V11, P2924, DOI 10.1016/j.apsb.2021.04.006
Yu WQ, 2020, CHINESE CHEM LETT, V31, P1366, DOI 10.1016/j.cclet.2020.02.036
Yu WQ, 2020, ACS CENTRAL SCI, V6, P100, DOI 10.1021/acscentsci.9b01139
Zeng L, 2021, ANGEW CHEM INT EDIT, V60, P23569, DOI 10.1002/anie.202108076
Zhang DY, 2020, ACS APPL MATER INTER, V12, P56830, DOI 10.1021/acsami.0c17579
Zhang LL, 2022, ACTA PHARM SIN B, V12, P3427, DOI 10.1016/j.apsb.2022.01.005
Zhang L, 2020, NAT NANOTECHNOL, V15, P145, DOI 10.1038/s41565-019-0626-4
Zhang MJ, 2020, ACS APPL MATER INTER, V12, P32289, DOI 10.1021/acsami.0c06614
Zhang SQ, 2020, ADV FUNCT MATER, V30, DOI 10.1002/adfm.201910651
Zhang SW, 2021, ACTA PHARM SIN B, V11, P3636, DOI 10.1016/j.apsb.2021.04.005
Zhang SW, 2021, THERANOSTICS, V11, P6019, DOI 10.7150/thno.59065
Zhang X, 2019, ADV SCI, V6, DOI 10.1002/advs.201801122
Zhang XB, 2021, BIOACT MATER, V6, P2291, DOI 10.1016/j.bioactmat.2021.01.004
Zhang YB, 2021, CHEM REV, V121, P12181, DOI 10.1021/acs.chemrev.1c00244
Zhao DY, 2020, NANOSCALE, V12, P10939, DOI 10.1039/d0nr00301h
Zhao PH, 2018, NAT COMMUN, V9, DOI 10.1038/s41467-018-06630-2
Zhao YJ, 2021, PHARMACEUTICS, V13, DOI 10.3390/pharmaceutics13111917
Zhao Z., 2021, ADV SCI, V9
Zheng LY, 2020, J AM CHEM SOC, V142, P382, DOI 10.1021/jacs.9b11046
Zheng X, 2021, CHINESE CHEM LETT, V32, P243, DOI 10.1016/j.cclet.2020.11.029
Zhou Y, 2020, J MATER CHEM B, V8, P6765, DOI 10.1039/d0tb00649a
Zhou Y, 2020, BIOMATERIALS, V255, DOI 10.1016/j.biomaterials.2020.120193
Zhu MT, 2013, ACCOUNTS CHEM RES, V46, P622, DOI 10.1021/ar300031y
Zhuang J, 2020, SCI ADV, V6, DOI 10.1126/sciadv.aaz6108
Zielinska A, 2020, MOLECULES, V25, DOI 10.3390/molecules25163731
Zong Y, 2019, PROTEIN EXPRES PURIF, V153, P53, DOI 10.1016/j.pep.2018.08.012
Zou SJ, 2020, NANOMEDICINE-UK, V15, P625, DOI 10.2217/nnm-2019-0388
Zu MH, 2021, ADV DRUG DELIVER REV, V176, DOI 10.1016/j.addr.2021.113887
NR 250
TC 103
Z9 104
U1 86
U2 135
PU ELSEVIER SCIENCE INC
PI NEW YORK
PA STE 800, 230 PARK AVE, NEW YORK, NY 10169 USA
SN 1001-8417
EI 1878-5964
J9 CHINESE CHEM LETT
JI Chin. Chem. Lett.
PD FEB
PY 2023
VL 34
IS 2
AR 107518
DI 10.1016/j.cclet.2022.05.032
PG 24
WC Chemistry, Multidisciplinary
WE Science Citation Index Expanded (SCI-EXPANDED)
SC Chemistry
GA 7L5NH
UT WOS:000906011800001

HC Y
HP Y
DA 2023-09-14
ER

PT J
AU Qian, Y
Jin, HL
Qiao, S
Dai, YF
Huang, C
Lu, LS
Luo, QM
Zhang, ZH
AF Qian, Yuan
Jin, Honglin
Qiao, Sha
Dai, Yanfeng
Huang, Chuan
Lu, Lisen
Luo, Qingming
Zhang, Zhihong
TI Targeting dendritic cells in lymph node with an antigen peptide-based nanovaccine for cancer immunotherapy
SO BIOMATERIALS
LA English
DT Article
DE Cancer immunotherapy; Nanovaccine; Antigen delivery; Lymph node; Dendritic cell targeting
ID DELIVERY; VACCINES; NANOPARTICLE; RECEPTOR; MATURE; NANOTECHNOLOGY; IMMUNITY; CAPTURE
AB The design of peptide-based subunit vaccine formulations for the direct delivery of tumor antigen peptides (Aps) to dendritic cells (DCs) localized within draining lymph nodes (DLNs) is challenging. Mature DCs (mDCs) are abundantly distributed within DLNs but have dramatically reduced endocytic uptake and antigen-processing abilities, so their role as potential vaccine targets has been largely overlooked. Here we report an ultra-small biocompatible nanovaccine (alpha-Ap-FNP) functionalized by avidly targeting delivery of Ap via the scavenger receptor class B1 (SR-B1) pathway to mDCs. The self-assembly, small size (similar to 30 nm), SR-B1-targeting and optical properties of alpha-Ap-FNP resulted in its efficient Ap loading, substantial LN accumulation, targeting of mDCs and enhanced Ap presentation, and fluorescence trafficking, respectively. We also demonstrate that the alpha-Ap-FNP can be either used alone or encapsulated with CpG oligodeoxynucleotide as a prophylactic and therapeutic vaccine. Thus, the excellent properties of alpha-Ap-FNP provide it potential for clinical applications as a potent nanovaccine for cancer immunotherapy. (C) 2016 Elsevier Ltd. All rights reserved.
C1 [Qian, Yuan; Jin, Honglin; Qiao, Sha; Dai, Yanfeng; Huang, Chuan; Lu, Lisen; Luo, Qingming; Zhang, Zhihong] Huazhong Univ Sci & Technol, Britton Chance Ctr Biomed Photon, Wuhan Natl Lab Optoelect, Room G304, Wuhan 430074, Peoples R China.
[Qian, Yuan; Jin, Honglin; Qiao, Sha; Dai, Yanfeng; Huang, Chuan; Lu, Lisen; Luo, Qingming; Zhang, Zhihong] Huazhong Univ Sci & Technol, Dept Biomed Engn, MoE Key Lab Biomed Photon, Wuhan 430074, Peoples R China.
[Jin, Honglin] Huazhong Univ Sci & Technol, Union Hosp, Tongji Med Coll, Ctr Canc, 1277 JieFang Ave, Wuhan 430022, Peoples R China.
C3 Huazhong University of Science & Technology; Huazhong University of Science & Technology; Huazhong University of Science & Technology
RP Luo, QM; Zhang, ZH (通讯作者)，Huazhong Univ Sci & Technol, Britton Chance Ctr Biomed Photon, Wuhan Natl Lab Optoelect, Room G304, Wuhan 430074, Peoples R China.
EM qluo@mail.hust.edu.cn; czyzzh@mail.hust.edu.cn
RI Qian, Yuan/I-6922-2017
OI Qian, Yuan/0000-0002-3967-7727; Zhang, Zhihong/0000-0001-5227-8926; Luo, Qingming/0000-0002-6725-9311; Qiao, Sha/0000-0002-1400-9802; Jin, Honglin/0000-0002-4398-9539; , Chen/0000-0003-2093-4639
FU Major Research plan of the National Natural Science Foundation of China

[91442201]; National Natural Science Foundation of China [81172153]; Science Fund for Creative Research Groups of the National Natural Science Foundation of China [61421064]; Fundamental Research Funds for the Central Universities [HUST: 2015ZDTD014]; Open Research Fund of State Key Laboratory of Bioelectronics of Southeast University; Director Fund of WNLO
FX This study was supported by the Major Research plan of the National Natural Science Foundation of China (Grant No. 91442201), National Natural Science Foundation of China (Grant No. 81172153), Science Fund for Creative Research Groups of the National Natural Science Foundation of China (Grant No. 61421064), the Fundamental Research Funds for the Central Universities (HUST: 2015ZDTD014), the Open Research Fund of State Key Laboratory of Bioelectronics of Southeast University, and the Director Fund of WNLO. We thank Dr. Gang Zheng (University of Toronto, Toronto, ON, Canada) for paper discussion, and Dr. Monty Krieger of MIT for providing IdlA7 and IdlA(mSR-B1) cell lines. We also thank the Optical Bioimaging Core Facility of WNLO-HUST for the support in data acquisition, the Center for Nanoscale Characterization & Devices (CNCD, Tecnai G<SUP>2</SUP> 20 U-Twin) in WNLO-HUST for the facility support, and the Analytical and Testing Center of HUST for spectral measurements.
CR Allan RS, 2006, IMMUNITY, V25, P153, DOI 10.1016/j.immuni.2006.04.017
Apostolopoulos V, 2013, J DRUG DELIVERY, V2013, P1
Asano K, 2011, IMMUNITY, V34, P85, DOI 10.1016/j.immuni.2010.12.011
Bachmann MF, 2010, NAT REV IMMUNOL, V10, P787, DOI 10.1038/nri2868
Banchereau J, 1998, NATURE, V392, P245, DOI 10.1038/32588
Bonifaz L, 2002, J EXP MED, V196, P1627, DOI 10.1084/jem.20021598
Bonifaz LC, 2004, J EXP MED, V199, P815, DOI 10.1084/jem.20032220
Chen DS, 2013, IMMUNITY, V39, P1, DOI 10.1016/j.immuni.2013.07.012
Cho NH, 2011, NAT NANOTECHNOL, V6, P675, DOI [10.1038/NNANO.2011.149, 10.1038/nnano.2011.149]
De Temmerman ML, 2011, DRUG DISCOV TODAY, V16, P569, DOI 10.1016/j.drudis.2011.04.006
Drutman SB, 2010, J IMMUNOL, V185, P2140, DOI 10.4049/jimmunol.1000642
Forster R, 2012, TRENDS IMMUNOL, V33, P271, DOI 10.1016/j.it.2012.02.007
Goldberg MS, 2015, CELL, V161, P201, DOI 10.1016/j.cell.2015.03.037
Gray EE, 2012, J INNATE IMMUN, V4, P424, DOI 10.1159/000337007
Guo YY, 2015, ACS NANO, V9, P6918, DOI 10.1021/acsnano.5b01042
Henri S, 2001, J IMMUNOL, V167, P741, DOI 10.4049/jimmunol.167.2.741
Iyoda T, 2002, J EXP MED, V195, P1289, DOI 10.1084/jem.20020161
Jeanbart L, 2014, CANCER IMMUNOL RES, V2, P436, DOI 10.1158/2326-6066.CIR-14-0019-T
Johansen P, 2010, J CONTROL RELEASE, V148, P56, DOI 10.1016/j.jconrel.2010.05.019
Kreutz M, 2013, BLOOD, V121, P2836, DOI 10.1182/blood-2012-09-452078
Lee IH, 2012, ANGEW CHEM INT EDIT, V51, P8800, DOI 10.1002/anie.201203193
Lin Q, 2013, PHARM RES, V31, P1438
Lin QY, 2014, SMALL, V10, P3072, DOI 10.1002/smll.201303842
Lin QY, 2012, NANOMEDICINE-UK, V7, P1813, DOI [10.2217/NNM.12.73, 10.2217/nnm.12.73]
Liu HP, 2014, NATURE, V507, P519, DOI 10.1038/nature12978
Lutz MB, 2002, TRENDS IMMUNOL, V23, P445, DOI 10.1016/S1471-4906(02)02281-0
Mamo T, 2010, NANOMEDICINE-UK, V5, P269, DOI [10.2217/nnm.10.1, 10.2217/NNM.10.1]
Melero I, 2014, NAT REV CLIN ONCOL, V11, P509, DOI 10.1038/nrclinonc.2014.111
Mellman I, 2011, NATURE, V480, P480, DOI 10.1038/nature10673
Miller MJ, 2002, SCIENCE, V296, P1869, DOI 10.1126/science.1070051
Moon JJ, 2011, NAT MATER, V10, P243, DOI 10.1038/NMAT2960
Neefjes J, 2011, NAT REV IMMUNOL, V11, P823, DOI 10.1038/nri3084
Noh YW, 2012, ACS NANO, V6, P7820, DOI 10.1021/nn301949y
Noh YW, 2011, BIOMATERIALS, V32, P6254, DOI 10.1016/j.biomaterials.2011.05.013
Palucka K, 2013, IMMUNITY, V39, P38, DOI 10.1016/j.immuni.2013.07.004
Platt CD, 2010, P NATL ACAD SCI USA, V107, P4287, DOI 10.1073/pnas.0910609107
Proudfoot O, 2007, MOL PHARMACEUT, V4, P58, DOI 10.1021/mp0601087
Purcell AW, 2007, NAT REV DRUG DISCOV, V6, P404, DOI 10.1038/nrd2224
Rappuoli R, 2011, NAT REV IMMUNOL, V11, P865, DOI 10.1038/nri3085
Reddy ST, 2007, NAT BIOTECHNOL, V25, P1159, DOI [10.1038/nbt1332, 10.1038/nbtl332]

Sousa CR, 2006, NAT REV IMMUNOL, V6, P476, DOI 10.1038/nri1845
SALLUSTO F, 1995, J EXP MED, V182, P389, DOI 10.1084/jem.182.2.389
Schreiber TH, 2010, SEMIN IMMUNOL, V22, P105, DOI 10.1016/j.smim.2010.02.001
Shao K, 2015, ACS NANO, V9, P16, DOI 10.1021/nn5062029
Shao S, 2015, NAT CHEM, V7, P438, DOI [10.1038/nchem.2236, 10.1038/NCHEM.2236]
Skwarczynski M, 2011, CURR DRUG DELIV, V8, P282, DOI 10.2174/156720111795256192
Susaki EA, 2014, CELL, V157, P726, DOI 10.1016/j.cell.2014.03.042
van der Vlies AJ, 2010, BIOCONJUGATE CHEM, V21, P653, DOI 10.1021/bc9004443
Yang XQ, 2012, COMPUT MED IMAG GRAP, V36, P259, DOI 10.1016/j.compmedimag.2011.09.002
Zhang PF, 2015, P NATL ACAD SCI USA, V112, pE6129, DOI 10.1073/pnas.1505799112
Zhang ZH, 2010, SMALL, V6, P430, DOI 10.1002/smll.200901515
Zhang ZH, 2009, ANGEW CHEM INT EDIT, V48, P9171, DOI 10.1002/anie.200903112
Zheng Y., 2012, J INNOV OPT HEAL SCI, V5
Zheng Y., 2012, J INNOV OPT HEAL SCI, V5
Zheng Y, 2013, THERANOSTICS, V3, P477, DOI 10.7150/thno.6617
ZHOU F, 1992, CANCER RES, V52, P6287
Zhou FF, 2016, J INNOV OPT HEAL SCI, V9, DOI 10.1142/S1793545816300019
NR 57
TC 103
Z9 106
U1 5
U2 231
PU ELSEVIER SCI LTD
PI OXFORD
PA THE BOULEVARD, LANGFORD LANE, KIDLINGTON, OXFORD OX5 1GB, OXON, ENGLAND
SN 0142-9612
EI 1878-5905
J9 BIOMATERIALS
JI Biomaterials
PD AUG
PY 2016
VL 98
BP 171
EP 183
DI 10.1016/j.biomaterials.2016.05.008
PG 13
WC Engineering, Biomedical; Materials Science, Biomaterials
WE Science Citation Index Expanded (SCI-EXPANDED)
SC Engineering; Materials Science
GA DQ5XK
UT WOS:000379278400016
PM 27192420
DA 2023-09-14
ER

PT J
AU Yu, X
Dai, YF
Zhao, YF
Qi, SH
Liu, L
Lu, LS
Luo, QM
Zhang, ZH
AF Yu, Xiang
Dai, Yanfeng
Zhao, Yifan
Qi, Shuhong
Liu, Lei
Lu, Lisen
Luo, Qingming

Zhang, Zhihong
TI Melittin-lipid nanoparticles target to lymph nodes and elicit a systemic anti-tumor immune response
SO NATURE COMMUNICATIONS
LA English
DT Article
ID TUMOR-CELLS; PEPTIDE; VACCINES; DISCOVERY; DEFENSE
AB Targeted delivery of a nanovaccine loaded with a tumor antigen and adjuvant to the lymph nodes (LNs) is an attractive approach for improving cancer immunotherapy outcomes. However, the application of this technique is restricted by the paucity of suitable tumor-associated antigens (TAAs) and the sophisticated technology required to identify tumor neoantigens. Here, we demonstrate that a self-assembling melittin-lipid nanoparticle (alpha-melittin-NP) that is not loaded with extra tumor antigens promotes whole tumor antigen release in situ and results in the activation of antigen-presenting cells (APCs) in LNs. Compared with free melittin, alpha-melittin-NPs markedly enhance LN accumulation and activation of APCs, leading to a 3.6-fold increase in antigen-specific CD8(+) T cell responses. Furthermore, in a bilateral flank B16F10 tumor model, primary and distant tumor growth are significantly inhibited by alpha-melittin-NPs, with an inhibition rate of 95% and 92%, respectively. Thus, alpha-melittin-NPs induce a systemic anti-tumor response serving as an effective LN-targeted whole-cell nanovaccine. The bee venom melittin has anti-tumor properties but is unsuitable for therapeutic use on its own. Here, the authors generate melittin-nanoparticles and demonstrate that the nanoparticles reduce tumor growth and generate an anti-tumor immune response.
C1 [Yu, Xiang; Dai, Yanfeng; Zhao, Yifan; Qi, Shuhong; Liu, Lei; Lu, Lisen; Luo, Qingming; Zhang, Zhihong] Huazhong Univ Sci & Technol, Britton Chance Ctr Biomed Photon, Wuhan Natl Lab Optoelect, Wuhan 430074, Hubei, Peoples R China.
[Yu, Xiang; Dai, Yanfeng; Zhao, Yifan; Qi, Shuhong; Liu, Lei; Lu, Lisen; Luo, Qingming; Zhang, Zhihong] Huazhong Univ Sci & Technol, MoE Key Lab Biomed Photon, Collaborat Innovat Ctr Biomed Engn, Sch Engn Sci, Wuhan 430074, Hubei, Peoples R China.
C3 Huazhong University of Science & Technology; Huazhong University of Science & Technology
RP Zhang, ZH (通讯作者), Huazhong Univ Sci & Technol, Britton Chance Ctr Biomed Photon, Wuhan Natl Lab Optoelect, Wuhan 430074, Hubei, Peoples R China.; Zhang, ZH (通讯作者), Huazhong Univ Sci & Technol, MoE Key Lab Biomed Photon, Collaborat Innovat Ctr Biomed Engn, Sch Engn Sci, Wuhan 430074, Hubei, Peoples R China.
EM czyzzh@mail.hust.edu.cn
RI Liu, Lei/H-5754-2018; 赵, 一帆/GSM-8566-2022
OI Liu, Lei/0000-0003-3606-122X; Luo, Qingming/0000-0002-6725-9311; yanfeng, dai/0000-0003-3648-9429; Yu, Xiang/0000-0001-6541-1562
FU National Science Fund for Distinguished Young Scholars [81625012]; Science Fund for Creative Research Groups of the National Natural Science Foundation of China [61721092]; Fundamental Research Funds for the Central Universities [2019kfyXMBZ022]; Program for HUST Academic Frontier Youth Team; Innovation Fund of WNLO
FX We thank the Optical Bioimaging Core Facility of WNLO-HUST and the Research Core Facilities for Life Science (HUST) for the support in data acquisition, and the Analytical and Testing Center of HUST for the facility support. This work was supported by the National Science Fund for Distinguished Young Scholars (81625012), the Science Fund for Creative Research Groups of the National Natural Science Foundation of China (61721092), the Fundamental Research Funds for the Central Universities (2019kfyXMBZ022), Program for HUST Academic Frontier Youth Team (Zhang, Z.H.), and the Innovation Fund of WNLO.
CR [Anonymous], 2012, INT J ADV RES TECHNO
Gajski G, 2013, ENVIRON TOXICOL PHAR, V36, P697, DOI 10.1016/j.etap.2013.06.009
Hammerich L, 2019, NAT MED, V25, P814, DOI 10.1038/s41591-019-0410-x
Henninot A, 2018, J MED CHEM, V61, P1382, DOI 10.1021/acs.jmedchem.7b00318
Huang C, 2013, ACS NANO, V7, P5791, DOI 10.1021/nn400683s
Hubbell JA, 2009, NATURE, V462, P449, DOI 10.1038/nature08604
Irvine DJ, 2013, NAT MATER, V12, P978, DOI [10.1038/NMAT3775, 10.1038/nmat3775]
Junttila MR, 2013, NATURE, V501, P346, DOI 10.1038/nature12626
Kaufman HL, 2010, ANN SURG ONCOL, V17, P718, DOI 10.1245/s10434-009-0809-6
Keenan BP, 2012, SEMIN ONCOL, V39, P276, DOI 10.1053/j.seminoncol.2012.02.007

Keskin DB, 2019, NATURE, V565, P234, DOI 10.1038/s41586-018-0792-9
Kuai R, 2017, NAT MATER, V16, P489, DOI [10.1038/nmat4822, 10.1038/NMAT4822]
Kuka M, 2014, ANN NY ACAD SCI, V1319, P38, DOI 10.1111/nyas.12387
Lewis JD, 2003, INT REV IMMUNOL, V22, P81, DOI 10.1080/08830180305221
Liu MJ, 2016, J TRANSL MED, V14, DOI 10.1186/s12967-016-0910-0
Liu ZQ, 2018, NAT COMMUN, V9, DOI 10.1038/s41467-018-06954-z
Liu ZQ, 2017, NAT COMMUN, V8, DOI 10.1038/ncomms14754
Lynn GM, 2015, NAT BIOTECHNOL, V33, P1201, DOI 10.1038/nbt.3371
Melo MN, 2009, NAT REV MICROBIOL, V7, P245, DOI 10.1038/nrmicro2095
Nam S, 2005, INT IMMUNOPHARMACOL, V5, P1406, DOI 10.1016/j.intimp.2005.03.011
Orsolic N, 2012, CANCER METAST REV, V31, P173, DOI 10.1007/s10555-011-9339-3
Ott PA, 2017, NATURE, V547, P217, DOI 10.1038/nature22991
Palm NW, 2013, IMMUNITY, V39, P976, DOI 10.1016/j.immuni.2013.10.006
Palm NW, 2013, P NATL ACAD SCI USA, V110, P1809, DOI 10.1073/pnas.1221476110
Papo N, 2005, CELL MOL LIFE SCI, V62, P784, DOI 10.1007/s00018-005-4560-2
Pulido J, 2012, NAT BIOTECHNOL, V30, P336, DOI 10.1038/nbt.2157
Raghuraman H, 2005, CHEM PHYS LIPIDS, V134, P183, DOI 10.1016/j.chemphyslip.2004.12.011
Reddy ST, 2007, NAT BIOTECHNOL, V25, P1159, DOI [10.1038/nbt1332, 10.1038/nbtl332]
Ribas A, 2018, CELL, V174, P1031, DOI 10.1016/j.cell.2018.07.035
Sagiv-Barfi I, 2018, SCI TRANSL MED, V10, DOI 10.1126/scitranslmed.aan4488
Sahin U, 2018, SCIENCE, V359, P1355, DOI 10.1126/science.aar7112
Schumacher TN, 2015, SCIENCE, V348, P69, DOI 10.1126/science.aaa4971
Shi GN, 2017, BIOMATERIALS, V113, P191, DOI 10.1016/j.biomaterials.2016.10.047
Singh M, 2017, NAT COMMUN, V8, DOI 10.1038/s41467-017-01572-7
Soman NR, 2009, J CLIN INVEST, V119, P2830, DOI 10.1172/JCI38842
SUPERSAXO A, 1990, PHARMACEUT RES, V7, P167, DOI 10.1023/A:1015880819328
Trevaskis NL, 2015, NAT REV DRUG DISCOV, V14, P781, DOI 10.1038/nrd4608
UTSUGI T, 1991, CANCER RES, V51, P3062
von Andrian UH, 2003, NAT REV IMMUNOL, V3, P867, DOI 10.1038/nri1222
Wang H, 2018, NAT MATER, V17, P761, DOI 10.1038/s41563-018-0147-9
Wang S, 2016, P NATL ACAD SCI USA, V113, pE7240, DOI 10.1073/pnas.1608555113
Yang F, 2016, THERANOSTICS, V6, P1453, DOI 10.7150/thno.14145
Yu X, 2019, NAT COMMUN, V10, DOI 10.1038/s41467-019-08538-x
Zamarin D, 2014, SCI TRANSL MED, V6, DOI 10.1126/scitranslmed.3008095
Zhang YY, 2018, CELL RES, V28, P9, DOI 10.1038/cr.2017.133
Zhang ZH, 2009, ANGEW CHEM INT EDIT, V48, P9171, DOI 10.1002/anie.200903112
Zhu GZ, 2017, NAT COMMUN, V8, DOI 10.1038/s41467-017-01386-7
NR 47
TC 101
Z9 103
U1 25
U2 172
PU NATURE PUBLISHING GROUP
PI LONDON
PA MACMILLAN BUILDING, 4 CRINAN ST, LONDON N1 9XW, ENGLAND
SN 2041-1723
J9 NAT COMMUN
JI Nat. Commun.
PD FEB 28
PY 2020
VL 11
IS 1
AR 1110
DI 10.1038/s41467-020-14906-9
PG 14
WC Multidisciplinary Sciences
WE Science Citation Index Expanded (SCI-EXPANDED)
SC Science & Technology - Other Topics
GA KS9KD
UT WOS:000518626300003
PM 32111828

OA Green Published, gold
DA 2023-09-14
ER

PT J
AU Hassan, HAFM
Smyth, L
Wang, JTW
Costa, PM
Ratnasothy, K
Diebold, SS
Lombardi, G
Al-Jamal, KT
AF Hassan, Hatem A. F. M.
Smyth, Lesley
Wang, Julie T. -W.
Costa, Pedro M.
Ratnasothy, Kulachelvy
Diebold, Sandra S.
Lombardi, Giovanna
Al-Jamal, Khuloud T.
TI Dual stimulation of antigen presenting cells using carbon nanotube-based vaccine delivery system for cancer immunotherapy
SO BIOMATERIALS
LA English
DT Article
DE Carbon nanotubes; Dendritic cells; Nanomedicine; Vaccine delivery; Cancer vaccines
ID HUMAN DENDRITIC CELLS; CROSS-PRESENTATION; CPG OLIGODEOXYNUCLEOTIDES; THERMAL-STABILITY; EPITHELIAL-CELLS; PROTEIN ENHANCE; IN-VITRO; ACTIVATION; RESPONSES; CD40
AB Although anti cancer immuno based combinatorial therapeutic approaches have shown promising results, efficient tumour eradication demands further intensification of anti tumour immune response. With the emerging field of nanovaccinology, multi walled carbon nanotubes (MWNTs) have manifested prominent potentials as tumour antigen nanocarriers. Nevertheless, the utilization of MWNTs in co-delivering antigen along with different types of immunoadjuvants to antigen presenting cells (APCs) has not been investigated yet. We hypothesized that harnessing MWNT for concurrent delivery of cytosine phosphate guanine oligodeoxynucleotide (CpG) and anti-CD40 Ig (alpha CD40), as immunoadjuvants, along with the model antigen ovalbumin (OVA) could potentiate immune response induced against OVA expressing tumour cells. We initially investigated the effective method to co-deliver OVA and CpG using MWNT to the APC. Covalent conjugation of OVA and CpG prior to loading onto MWNTs markedly augmented the CpG mediated adjuvanticity, as demonstrated by the significantly increased OVA specific T cell responses in vitro and in C57BL/6 mice. alpha CD40 was then included as a second immunoadjuvant to further intensify the immune response. Immune response elicited in vitro and in vivo by OVA, CpG and alpha CD40 was significantly potentiated by their co-incorporation onto the MWNTs. Furthermore, MWNT remarkably improved the ability of co-loaded OVA, CpG and alpha CD40 in inhibiting the growth of OVA expressing B16F10 melanoma cells in subcutaneous or lung pseudo metastatic tumour models. Therefore, this study suggests that the utilization of MWNTs for the co-delivery of tumour-derived antigen, CpG and alpha CD40 could be a competent approach for efficient tumours eradication. (C) 2016 The Author(s). Published by Elsevier Ltd.
C1 [Hassan, Hatem A. F. M.; Wang, Julie T. -W.; Costa, Pedro M.; Al-Jamal, Khuloud T.] Kings Coll London, Fac Life Sci & Med, Inst Pharmaceut Sci, Franklin Wilkins Bldg, London SE1 9NH, England.
[Smyth, Lesley; Ratnasothy, Kulachelvy; Lombardi, Giovanna] Kings Coll London, Guys Hosp, MRC Ctr Transplantat, Immunoregulat Lab, London SE1 9RT, England.
[Diebold, Sandra S.] Kings Coll London, Guys Hosp, Div Immunol Infect & Inflammatory Dis, London SE1 9RT, England.
[Smyth, Lesley] Univ East London, Sch Hlth Sport & Biosci, Stratford Campus,Water Lane, London E15 4LZ, England.
[Diebold, Sandra S.] Natl Inst Biol Stand & Controls, Immunotoxicol Team, Biotherapeut Div, Blanche Lane, Potters Bar EN6 3QG, Herts, England.
C3 University of London; King's College London; Guy's & St Thomas' NHS

Foundation Trust; University of London; King's College London; Guy's &
St Thomas' NHS Foundation Trust; University of London; King's College
London; University of East London; National Institute for Biological
Standards & Control
RP Al-Jamal, KT (通讯作者)，Kings Coll London, Fac Life Sci & Med, Inst Pharmaceut Sci, Franklin Wilkins Bldg, London SE1 9NH, England.; Lombardi, G (通讯作者)，Kings Coll London, Guys Hosp, MRC Ctr Transplantat, Immunoregulat Lab, London SE1 9RT, England.
EM giovanna.lombardi@kcl.ac.uk; khuloud.al-jamal@kcl.ac.uk
RI Ahmed, Hassan Elzain Hassan/AGA-7917-2022; Wang, Julie
Tzu-Wen/O-4884-2016; Al-Jamal, Khuloud/ABD-5668-2021
OI AL-JAMAL, KHULOUD/0000-0001-5165-2699; Hassan,
Hatem/0009-0009-7493-8564; Coutinho da Costa, Pedro
Miguel/0000-0003-0196-8649; Wang, Tzu-Wen/0000-0001-5956-0123
FU Worldwide Cancer Research [12-1054]; Wellcome Trust [WT103913]; BHF
programme grant [RG13/12/30395]; YCCF; MRC [MR/L022699/1] Funding
Source: UKRI; Medical Research Council [MR/L022699/1] Funding Source:
researchfish; Worldwide Cancer Research [12-1054] Funding Source:
researchfish
FX We acknowledge funding from Worldwide Cancer Research (12-1054),
Wellcome Trust (WT103913) and BHF programme grant (RG13/12/30395).
H.A.F.M.H. acknowledges funding from YCCF. PMC is a Sir Henry Wellcome
Postdoctoral fellow.
CR Bachmann MF, 2010, NAT REV IMMUNOL, V10, P787, DOI 10.1038/nri2868
de Faria PCB, 2014, NANO LETT, V14, P5458, DOI 10.1021/nl502911a
Beaudette TT, 2009, MOL PHARMACEUT, V6, P1160, DOI 10.1021/mp900038e
Bennett SRM, 1998, NATURE, V393, P478, DOI 10.1038/30996
Bennett SRM, 1997, J EXP MED, V186, P65, DOI 10.1084/jem.186.1.65
Bianco A, 2005, J AM CHEM SOC, V127, P58, DOI 10.1021/ja044293y
Bianco A, 2005, CHEM COMMUN, P571, DOI 10.1039/b410943k
Blander JM, 2006, NATURE, V440, P808, DOI 10.1038/nature04596
Broos S, 2012, BIOMATERIALS, V33, P6230, DOI 10.1016/j.biomaterials.2012.05.011
Burgdorf S, 2007, SCIENCE, V316, P612, DOI 10.1126/science.1137971
Calvaresi M, 2013, ACCOUNTS CHEM RES, V46, P2454, DOI 10.1021/ar300347d
Cartiera MS, 2009, BIOMATERIALS, V30, P2790, DOI 10.1016/j.biomaterials.2009.01.057
Cataldo F, 2002, FULLER NANOTUB CAR N, V10, P293, DOI 10.1081/FST-120016451
Cella M, 1996, J EXP MED, V184, P747, DOI 10.1084/jem.184.2.747
Chatterjee B, 2012, BLOOD, V120, P2011, DOI 10.1182/blood-2012-01-402370
Cho HJ, 2000, NAT BIOTECHNOL, V18, P509, DOI 10.1038/75365
Cohn L, 2013, J EXP MED, V210, P1049, DOI 10.1084/jem.20121251
Fadel TR, 2014, NAT NANOTECHNOL, V9, P639, DOI [10.1038/NNANO.2014.154, 10.1038/nnano.2014.154]
Fan HT, 2012, CLIN CANCER RES, V18, P5628, DOI 10.1158/1078-0432.CCR-12-1911
Gu L, 2012, ADV MATER, V24, P3981, DOI 10.1002/adma.201200776
Hassan HAFM, 2016, J CONTROL RELEASE, V225, P205, DOI 10.1016/j.jconrel.2016.01.030
Haswell LE, 2001, EUR J IMMUNOL, V31, P3094, DOI 10.1002/1521-4141(2001010)31:10<3094::AID-IMMU3094>3.0.CO;2-F
Hatzifoti C, 2008, PLOS ONE, V3, DOI 10.1371/journal.pone.0002368
He CB, 2010, BIOMATERIALS, V31, P3657, DOI 10.1016/j.biomaterials.2010.01.065
Heit A, 2007, EUR J IMMUNOL, V37, P2063, DOI 10.1002/eji.200737169
Hennessy EJ, 2010, NAT REV DRUG DISCOV, V9, P293, DOI 10.1038/nrd3203
Kam NWS, 2006, ANGEW CHEM INT EDIT, V45, P577, DOI 10.1002/anie.200503389
Kastenmuller W, 2014, NAT REV IMMUNOL, V14, P705, DOI 10.1038/nri3727
Khanna R, 1997, J IMMUNOL, V159, P5782
Kostarelos K, 2007, NAT NANOTECHNOL, V2, P108, DOI 10.1038/nnano.2006.209
Kreutz M, 2012, PLOS ONE, V7, DOI 10.1371/journal.pone.0040208
Krieg AM, 2008, ONCOGENE, V27, P161, DOI 10.1038/sj.onc.1210911
Lehman JH, 2011, CARBON, V49, P2581, DOI 10.1016/j.carbon.2011.03.028
Li WM, 2003, VACCINE, V21, P3319, DOI 10.1016/S0264-410X(03)00172-5
Mahajan A, 2013, MATER LETT, V90, P165, DOI 10.1016/j.matlet.2012.08.120
Mao S, 2005, PHARM RES-DORDR, V22, P2058, DOI 10.1007/s11095-005-8175-y
Meng J, 2008, SMALL, V4, P1364, DOI 10.1002/smll.200701059

Palucka K, 2012, NAT REV CANCER, V12, P265, DOI 10.1038/nrc3258
Quail DF, 2013, NAT MED, V19, P1423, DOI 10.1038/nm.3394
Quezada SA, 2004, ANNU REV IMMUNOL, V22, P307, DOI
10.1146/annurev.immunol.22.012703.104533
Rosalia RA, 2015, BIOMATERIALS, V40, P88, DOI 10.1016/j.biomaterials.2014.10.053
Schlosser E, 2008, VACCINE, V26, P1626, DOI 10.1016/j.vaccine.2008.01.030
Schmidt V, 2005, POLYM DEGRAD STABIL, V87, P25, DOI 10.1016/j.polymdegradstab.2004.07.003
Schoenberger SP, 1998, NATURE, V393, P480, DOI 10.1038/31002
Smyth MJ, 2016, NAT REV CLIN ONCOL, V13, P143, DOI 10.1038/nrclinonc.2015.209
Sun Z, 2010, CELL RES, V20, P1170, DOI 10.1038/cr.2010.133
Tao Y, 2015, NANOSCALE, V7, P12419, DOI 10.1039/c5nr02240a
Van der Pereira A., 2014, J BIOL METHODS, V1
van Kooten C, 2000, J LEUKOCYTE BIOL, V67, P2, DOI 10.1002/jlb.67.1.2
Wilson NS, 2006, NAT IMMUNOL, V7, P165, DOI 10.1038/ni1300
Yarovinsky F, 2006, IMMUNITY, V25, P655, DOI 10.1016/j.immuni.2006.07.015
Zhang XQ, 2007, J IMMUNOTHER, V30, P469, DOI 10.1097/CJI.0b013e31802fd8c6
Zhao DC, 2011, CLIN CANCER RES, V17, P771, DOI 10.1158/1078-0432.CCR-10-2444
Zheng M, 2003, NAT MATER, V2, P338, DOI 10.1038/nmat877
NR 54
TC 97
Z9 97
U1 2
U2 113
PU ELSEVIER SCI LTD
PI OXFORD
PA THE BOULEVARD, LANGFORD LANE, KIDLINGTON, OXFORD OX5 1GB, OXON, ENGLAND
SN 0142-9612
EI 1878-5905
J9 BIOMATERIALS
JI Biomaterials
PD OCT
PY 2016
VL 104
BP 310
EP 322
DI 10.1016/j.biomaterials.2016.07.005
PG 13
WC Engineering, Biomedical; Materials Science, Biomaterials
WE Science Citation Index Expanded (SCI-EXPANDED)
SC Engineering; Materials Science
GA DU6SN
UT WOS:000382345000027
PM 27475727
OA Green Submitted, Green Accepted, Green Published, hybrid
DA 2023-09-14
ER

PT J
AU Xu, J
Wang, H
Xu, LG
Chao, Y
Wang, CY
Han, X
Dong, ZL
Chang, H
Peng, R
Cheng, YY
Liu, Z
AF Xu, Jun
Wang, Hui

Xu, Ligeng
Chao, Yu
Wang, Chenya
Han, Xiao
Dong, Ziliang
Chang, Hong
Peng, Rui
Cheng, Yiyun
Liu, Zhuang
TI Nanovaccine based on a protein-delivering dendrimer for effective antigen cross-presentation and cancer immunotherapy
SO BIOMATERIALS
LA English
DT Article
DE Dendrimer; Antigen delivery; Cross-presentation; Vaccine; Checkpoint blockade; Immunotherapy
ID IMMUNE CHECKPOINT BLOCKADE; IFN-GAMMA; T-CELLS; NANOPARTICLES; VACCINES; ALPHA; POLYMER; INNATE; SIRNA; IL-12
AB Cancer vaccines for prevention and treatment of tumors have attracted tremendous interests as a type of cancer immunotherapy strategy. A major challenge in achieving robust T-cell responses to destruct tumor cells after vaccination is the abilities of antigen cross-presentation for antigen-presenting cells (APCs) such as dendritic cells (DCs). Herein, we demonstrate that a polyarnidoamine dendrimer modified with guanidinobenzoic acid (DGBA) could serve as an effective protein carrier to enable delivery of protein antigen, thereby leading to effective antigen cross-presentation by DCs. With ovalbumin (OVA) as the model antigen and unmethylated cytosine-guanine dinucleotides (CpG) as the adjuvant, a unique type of tumor vaccine is formulated. Importantly, such DGBA-OVA-CpG nanovaccine can induce robust antigen-specific cellular immunities and further demonstrates outstanding prophylactic efficacy against B16-OVA melanoma. More significantly, the nanovaccine shows excellent therapeutic effect to treat established 816-OVA melanoma when used in combination with the programmed cell death protein 1 (PD-1) checkpoint-blockade immunotherapy. This study presents the great promises of employing rationally engineered cytosolic protein carriers for the development of tumor vaccines to achieve effective cancer immunotherapy.
C1 [Xu, Jun; Xu, Ligeng; Chao, Yu; Wang, Chenya; Han, Xiao; Dong, Ziliang; Peng, Rui; Liu, Zhuang] Soochow Univ, Collaborat Innovat Ctr Suzhou Nano Sci & Technol, Inst Inst Funct Nano & Soft Mat FUNSOM, Suzhou 215123, Jiangsu, Peoples R China.
[Wang, Hui; Cheng, Yiyun] South China Univ Technol, South China Adv Inst Soft Matter Sci & Technol, Guangzhou 510640, Guangdong, Peoples R China.
[Chang, Hong] East China Normal Univ, Sch Life Sci, Shanghai Key Lab Regulatory Biol, Shanghai 200241, Peoples R China.
C3 Soochow University - China; South China University of Technology; East China Normal University
RP Peng, R; Liu, Z (通讯作者)，Soochow Univ, Collaborat Innovat Ctr Suzhou Nano Sci & Technol, Inst Inst Funct Nano & Soft Mat FUNSOM, Suzhou 215123, Jiangsu, Peoples R China.; Cheng, YY (通讯作者)，South China Univ Technol, South China Adv Inst Soft Matter Sci & Technol, Guangzhou 510640, Guangdong, Peoples R China.
EM rpeng@suda.edu.cn; yycheng@mail.ustc.edu.cn; zliu@suda.edu.cn
RI Liu, Zhuang/H-4352-2011; Xu, Ligeng/F-9844-2013; Dong, Ziliang/AEH-7615-2022
OI Liu, Zhuang/0000-0002-1629-1039; Xu, Ligeng/0000-0003-4946-2567;
FU National Research Programs from Ministry of Science and Technology (MOST) of China [2016YFA0201200]; National Natural Science Foundation of China [51525203, 81602181, 31300824]; Key Laboratory of Biomedical Effects of Nanomaterials and Nanosafety, Chinese Academy of Sciences [NSKF201612]; Collaborative Innovation Center of Suzhou Nano Science and Technology; Priority Academic Program Development of Jiangsu Higher Education Institutions (PAPD) of Jiangsu Higher Education Institutions
FX This article was partially supported by the National Research Programs from Ministry of Science and Technology (MOST) of China (2016YFA0201200), the National Natural Science Foundation of China (51525203, 81602181, 31300824), the Key Laboratory of Biomedical Effects of Nanomaterials and Nanosafety, Chinese Academy of Sciences

(NSKF201612), the Collaborative Innovation Center of Suzhou Nano Science and Technology, and a project funded by the Priority Academic Program Development of Jiangsu Higher Education Institutions (PAPD) of Jiangsu Higher Education Institutions.
CR Ali OA, 2009, NAT MATER, V8, P151, DOI [10.1038/NMAT2357, 10.1038/nmat2357]
Alsaab HO, 2017, FRONT PHARMACOL, V8, DOI 10.3389/fphar.2017.00561
Alter G, 2004, J IMMUNOL METHODS, V294, P15, DOI 10.1016/j.jim.2004.08.008
Austin LM, 1999, J INVEST DERMATOL, V113, P752, DOI 10.1046/j.1523-1747.1999.00749.x
Banchereau J, 2000, ANNU REV IMMUNOL, V18, P767, DOI 10.1146/annurev.immunol.18.1.767
Banchereau J, 1998, NATURE, V392, P245, DOI 10.1038/32588
Banchereau J, 2018, NAT REV CLIN ONCOL, V15, P9, DOI 10.1038/nrclinonc.2017.149
Chang H, 2017, BIOMACROMOLECULES, V18, P2371, DOI 10.1021/acs.biomac.7b00567
Chang H, 2017, NANO LETT, V17, P1678, DOI 10.1021/acs.nanolett.6b04955
Chou LYT, 2011, CHEM SOC REV, V40, P233, DOI 10.1039/c0cs00003e
Couzin-Frankel J, 2013, SCIENCE, V342, P1432, DOI 10.1126/science.342.6165.1432
Drexhage RC, 2010, EXPERT REV NEUROTHER, V10, P59, DOI 10.1586/ERN.09.144
Farkona S, 2016, BMC MED, V14, DOI 10.1186/s12916-016-0623-5
Gajewski TF, 2013, NAT IMMUNOL, V14, P1014, DOI 10.1038/ni.2703
Gary DJ, 2007, J CONTROL RELEASE, V121, P64, DOI 10.1016/j.jconrel.2007.05.021
Gazzinelli RT, 1996, J IMMUNOL, V157, P798
Guermonprez P, 2003, NATURE, V425, P397, DOI 10.1038/nature01911
Guimaraes PPG, 2018, ACS NANO, V12, P912, DOI 10.1021/acsnano.7b05876
Kaech SM, 2002, NAT REV IMMUNOL, V2, P251, DOI 10.1038/nri778
Kang J, 2016, J IMMUNOTHER CANCER, V4, DOI 10.1186/s40425-016-0156-7
Kanzler H, 2007, NAT MED, V13, P552, DOI 10.1038/nm1589
Kesharwani P, 2014, PROG POLYM SCI, V39, P268, DOI 10.1016/j.progpolymsci.2013.07.005
Krieg AM, 2006, NAT REV DRUG DISCOV, V5, P471, DOI 10.1038/nrd2059
Kuai R, 2017, NAT MATER, V16, P489, DOI [10.1038/nmat4822, 10.1038/NMAT4822]
Li F, 2017, ADV MATER, V29, DOI 10.1002/adma.201602432
Li HY, 2011, NAT NANOTECHNOL, V6, P645, DOI [10.1038/nnano.2011.153, 10.1038/NNANO.2011.153]
Liu DF, 2009, IMMUNITY, V31, P99, DOI 10.1016/j.immuni.2009.05.009
Louis E, 1998, CLIN EXP IMMUNOL, V113, P401
Luo M, 2017, NAT NANOTECHNOL, V12, P648, DOI [10.1038/nnano.2017.52, 10.1038/NNANO.2017.52]
Migueles SA, 2002, NAT IMMUNOL, V3, P1061, DOI 10.1038/ni845
Molino NM, 2013, ACS NANO, V7, P9743, DOI 10.1021/nn403085w
Moon JJ, 2011, NAT MATER, V10, P243, DOI 10.1038/NMAT2960
NASTALA CL, 1994, J IMMUNOL, V153, P1697
Pardoll DM, 2012, NAT REV CANCER, V12, P252, DOI 10.1038/nrc3239
Parkin J, 2001, LANCET, V357, P1777, DOI 10.1016/S0140-6736(00)04904-7
Postow MA, 2015, J CLIN ONCOL, V33, P1974, DOI 10.1200/JCO.2014.59.4358
Randolph GJ, 2005, NAT REV IMMUNOL, V5, P617, DOI 10.1038/nri1670
Rizvi NA, 2015, LANCET ONCOL, V16, P257, DOI 10.1016/S1470-2045(15)70054-9
Rosenberg SA, 2015, SCIENCE, V348, P62, DOI 10.1126/science.aaa4967
Setaro F, 2015, NANO LETT, V15, P1245, DOI 10.1021/nl5044055
Tanaka Y, 2010, PLOS ONE, V5, DOI [10.1371/journal.pone.0015225, 10.1371/journal.pone.0015091]
Topalian SL, 2016, NAT REV CANCER, V16, P275, DOI 10.1038/nrc.2016.36
Topalian SL, 2015, CANCER CELL, V27, P450, DOI 10.1016/j.ccell.2015.03.001
van der Burg SH, 2016, NAT REV CANCER, V16, P219, DOI 10.1038/nrc.2016.16
Wang F, 2014, BIOMATERIALS, V35, P9187, DOI 10.1016/j.biomaterials.2014.07.027
Wang H, 2015, ANGEW CHEM INT EDIT, V54, P11647, DOI 10.1002/anie.201501461
Wang MM, 2014, NAT COMMUN, V5, DOI 10.1038/ncomms4053
Xie YQ, 2018, WIRES NANOMED NANOBI, V10, DOI 10.1002/wnan.1506
Xu LG, 2013, ADV MATER, V25, P5928, DOI 10.1002/adma.201300583
Yu T., 2012, ANGEW CHEM, V124, P8606, DOI 10.1002/ange.201203920
2012, NAT REV IMMUNOL, V12, P557, DOI DOI 10.1038/NRI3254
NR 51
TC 96
Z9 97
U1 19

U2 457
PU ELSEVIER SCI LTD
PI OXFORD
PA THE BOULEVARD, LANGFORD LANE, KIDLINGTON, OXFORD OX5 1GB, OXON, ENGLAND
SN 0142-9612
EI 1878-5905
J9 BIOMATERIALS
JI Biomaterials
PD JUL
PY 2019
VL 207
BP 1
EP 9
DI 10.1016/j.biomaterials.2019.03.037
PG 9
WC Engineering, Biomedical; Materials Science, Biomaterials
WE Science Citation Index Expanded (SCI-EXPANDED)
SC Engineering; Materials Science
GA HX8MS
UT WOS:000467660600001
PM 30947117
DA 2023-09-14
ER

PT J
AU Liu, LL
Yi, HQ
He, HM
Pan, H
Cai, LT
Ma, YF
AF Liu, Lanlan
Yi, Huqiang
He, Huamei
Pan, Hong
Cai, Lintao
Ma, Yifan
TI Tumor associated macrophage-targeted microRNA delivery with
dual-responsive polypeptide nanovectors for anti-cancer therapy
SO BIOMATERIALS
LA English
DT Article
DE Tumor-associated macrophages; Nanoparticles; Targeted delivery; miR155;
Cancer therapy
ID CELLS IN-VIVO; DENDRITIC CELLS; CYTOKINE PRODUCTION; IMMUNE-RESPONSES;
CANCER; NANOPARTICLES; MICROENVIRONMENT; IMMUNOTOXICITY; NANOVACCINE;
METASTASIS
AB Repolarizing Tumor-associated macrophages (TAMs) to anti-tumor M1 macrophages with microRNA (miR) is a plausible approach for cancer treatment. However, how to achieve TAM-targeted miR delivery remains a challenge. The present study generated redox/pH dual-responsive hybrid polypeptide nanovectors, which consisted of self-crosslinked redox-responsive nanoparticles based on galactosefunctionalized n-butylamine-poly(L-lysine)-b-poly(L-cysteine) polypeptides (GLC) coated with DCAgrafted sheddable PEG-PLL (sPEG) copolymers. The ex vivo study showed that sPEG shielded cationic GLC core at physiological pH but quickly shed off to re-expose GLC due to it charge reversible property. Encapsulation with sPEG/GLC nanovectors effectively facilitated macrophage-targeted miR delivery at the acidic condition but diminished miR uptake at neutral pH. Administration of miR155-loaded sPEG/GLC (sPEG/GLC/155) nanocomplexes increased miR155 expression in TAMs about 100-400 folds both in vitro and in vivo. sPEG/GLC/155 also effectively repolarized immunosuppressive TAMs to anti-tumor M1 macrophages through elevating M1 macrophage markers (IL-12, iNOS, MHC II) and suppressing M2 macrophage markers (Msr2 and Arg1) in TAMs. Moreover, the treatment of sPEG/GLC/155 significantly increased activated T lymphocytes and NK cells in tumors, which consequently led to robust tumor

regression. Hence, TAM-targeted delivery of miR with redox/pH dual-responsive sPEG/GLC nanovectors could be a promising approach to re-polarize TAMs to M1 macrophages in situ and induce tumor regression. (C) 2017 Published by Elsevier Ltd.
C1 [Liu, Lanlan; Yi, Huqiang; He, Huamei; Pan, Hong; Cai, Lintao; Ma, Yifan] Chinese Acad Sci, Shenzhen Inst Adv Technol, Key Lab Hlth Informat, Guangdong Key Lab Nanomed, Shenzhen 518055, Peoples R China.
[Yi, Huqiang] Southern Univ Sci & Technol, Dept Mat Sci & Engn, Shenzhen 518055, Peoples R China.
C3 Chinese Academy of Sciences; Shenzhen Institute of Advanced Technology, CAS; Southern University of Science & Technology
RP Cai, LT; Ma, YF (通讯作者)，Chinese Acad Sci, Shenzhen Inst Adv Technol, 1068 Xueyuan Ave, Shenzhen 518055, Peoples R China.
EM yf.ma@siat.ac.cn
RI Pan, Hong/AAC-2326-2019
FU National Natural Science Foundation of China [81371679, 31571013, 21375141, 81401509, 81501580]; Key International S&T Cooperation Project [2015DFH50230]; Instrument Developing Project of CAS [YZ201439]; Guangdong Natural Science Foundation of Research Team [2016A030312006]; Shenzhen Science and Technology Program [KQCX20140521115045447, JCYJ20150401145529015, JCYJ20130402092657771, JSGG20160331185422390, JCYJ20150 521094519473, JCYJ20160429191503002]; SIAT Innovation Program for Excellent Young Researchers [201506]
FX We gratefully acknowledge funding support from the National Natural Science Foundation of China (Grant No. 81371679, 31571013, 21375141, 81401509, 81501580), Key International S&T Cooperation Project (2015DFH50230), Instrument Developing Project of CAS (YZ201439), Guangdong Natural Science Foundation of Research Team (2016A030312006), Shenzhen Science and Technology Program (Grant No. KQCX20140521115045447, JCYJ20150401145529015, JCYJ20130402092657771, JSGG20160331185422390, JCYJ20150 521094519473, JCYJ20160429191503002) and SIAT Innovation Program for Excellent Young Researchers (201506).
CR Aagaard L, 2007, ADV DRUG DELIVER REV, V59, P75, DOI 10.1016/j.addr.2007.03.005
Allavena P, 2005, CANCER RES, V65, P2964, DOI 10.1158/0008-5472.CAN-04-4037
Cai X, 2012, J MOL CELL BIOL, V4, P341, DOI 10.1093/jmcb/mjs044
Chanmee T, 2014, CANCERS, V6, P1670, DOI 10.3390/cancers6031670
Chevre R, 2011, NUCLEIC ACIDS RES, V39, P1610, DOI 10.1093/nar/gkq922
Coffelt SB, 2009, BBA-REV CANCER, V1796, P11, DOI 10.1016/j.bbcan.2009.02.004
Condeelis J, 2006, CELL, V124, P263, DOI 10.1016/j.cell.2006.01.007
Cubillos-Ruiz JR, 2012, CANCER RES, V72, P1683, DOI 10.1158/0008-5472.CAN-11-3160
DALY WH, 1988, TETRAHEDRON LETT, V29, P5859, DOI 10.1016/S0040-4039(00)82209-1
Di Gioacchino M, 2011, INT J IMMUNOPATH PH, V24, P65
Elsabahy M, 2013, CHEM SOC REV, V42, P5552, DOI 10.1039/c3cs60064e
Eubank TD, 2009, CANCER RES, V69, P2133, DOI 10.1158/0008-5472.CAN-08-1405
Fransen MF, 2013, INT J CANCER, V132, P1971, DOI 10.1002/ijc.27755
Gandhi NS, 2014, J CONTROL RELEASE, V194, P238, DOI 10.1016/j.jconrel.2014.09.001
Gately MK, 1998, ANNU REV IMMUNOL, V16, P495, DOI 10.1146/annurev.immunol.16.1.495
Gordon S, 2005, NAT REV IMMUNOL, V5, P953, DOI 10.1038/nri1733
He CB, 2010, BIOMATERIALS, V31, P3657, DOI 10.1016/j.biomaterials.2010.01.065
He M, 2009, CELL MOL IMMUNOL, V6, P343, DOI 10.1038/cmi.2009.45
He YY, 2013, BIOMATERIALS, V34, P1235, DOI 10.1016/j.biomaterials.2012.09.049
Heyes J, 2005, J CONTROL RELEASE, V107, P276, DOI 10.1016/j.jconrel.2005.06.014
Higgs Gadareth, 2013, J Clin Bioinforma, V3, P17, DOI 10.1186/2043-9113-3-17
Huang Z, 2012, J CONTROL RELEASE, V158, P286, DOI 10.1016/j.jconrel.2011.11.013
Kedmi R, 2010, BIOMATERIALS, V31, P6867, DOI 10.1016/j.biomaterials.2010.05.027
Lind EF, 2015, J IMMUNOL, V195, P4632, DOI 10.4049/jimmunol.1302941
Liu LL, 2016, J IMMUNOL, V197, P1231, DOI 10.4049/jimmunol.1600182
Luo ZC, 2015, BIOMATERIALS, V38, P50, DOI 10.1016/j.biomaterials.2014.10.050
Mancino A, 2010, CLIN CANCER RES, V16, P784, DOI 10.1158/1078-0432.CCR-09-1015
Manthey CL, 2009, MOL CANCER THER, V8, P3151, DOI 10.1158/1535-7163.MCT-09-0255
Nagai T, 2009, CANCER IMMUNOL IMMUN, V58, P1577, DOI 10.1007/s00262-009-0667-x
Napoletano C, 2012, EUR J IMMUNOL, V42, P936, DOI 10.1002/eji.201142086
Price C, 2014, GENES DIS, V1, P53, DOI 10.1016/j.gendis.2014.06.004

Qian BZ, 2010, CELL, V141, P39, DOI 10.1016/j.cell.2010.03.014
Rogers TL, 2011, J TRANSL MED, V9, DOI 10.1186/1479-5876-9-177
Saeland E, 2007, CANCER IMMUNOL IMMUN, V56, P1225, DOI 10.1007/s00262-006-0274-z
Salomon B, 2001, ANNU REV IMMUNOL, V19, P225, DOI 10.1146/annurev.immunol.19.1.225
Shiri Sadaf, 2015, Asian Pac J Cancer Prev, V16, P3917
Soualhine H, 2007, J IMMUNOL, V179, P5137, DOI 10.4049/jimmunol.179.8.5137
Tang XQ, 2013, IMMUNOLOGY, V138, P93, DOI 10.1111/imm.12023
Watkins SK, 2007, J IMMUNOL, V178, P1357, DOI 10.4049/jimmunol.178.3.1357
Yi HQ, 2016, BIOMACROMOLECULES, V17, P1737, DOI 10.1021/acs.biomac.6b00141
Yi HQ, 2016, NANOSCALE, V8, P5985, DOI 10.1039/c5nr07348k
Yin H, 2014, NAT REV GENET, V15, P541, DOI 10.1038/nrg3763
Zheng CF, 2013, BIOMATERIALS, V34, P3431, DOI 10.1016/j.biomaterials.2013.01.053
Zhu SJ, 2013, MOL PHARMACEUT, V10, P3525, DOI 10.1021/mp400216r
NR 44
TC 95
Z9 98
U1 5
U2 217
PU ELSEVIER SCI LTD
PI OXFORD
PA THE BOULEVARD, LANGFORD LANE, KIDLINGTON, OXFORD OX5 1GB, OXON, ENGLAND
SN 0142-9612
EI 1878-5905
J9 BIOMATERIALS
JI Biomaterials
PD JUL
PY 2017
VL 134
BP 166
EP 179
DI 10.1016/j.biomaterials.2017.04.043
PG 14
WC Engineering, Biomedical; Materials Science, Biomaterials
WE Science Citation Index Expanded (SCI-EXPANDED)
SC Engineering; Materials Science
GA EV4GS
UT WOS:000401717500014
PM 28463694
DA 2023-09-14
ER

PT J
AU Skwarczynski, M
Toth, I
AF Skwarczynski, Mariusz
Toth, Istvan
TI Peptide-Based Subunit Nanovaccines
SO CURRENT DRUG DELIVERY
LA English
DT Review
DE Subunit vaccine; peptide epitope; nanotechnology; nanoparticles; infectious diseases
ID DRUG-DELIVERY SYSTEMS; FUNCTIONALIZED CARBON NANOTUBES; CROSS-LINKED NANOPARTICLES; GROUP-A STREPTOCOCCI; DENDRITIC CELLS; VACCINE DELIVERY; POLY(GAMMA-GLUTAMIC ACID); IMMUNE-RESPONSES; BIODEGRADABLE NANOPARTICLES; BIOMEDICAL APPLICATIONS
AB Classical vaccines incorporating live or attenuated microorganisms possess several disadvantages and cannot be applied against cancer and some pathogens. Modern vaccines utilizing immunogenic subunits derived from a particular pathogen are able to overcome these obstacles but need a specific delivery system for their efficacy. Nanotechnology has opened a new window into these delivery methodologies. A nano-sized formulation of subunit vaccines has been proven to be very effective in inducing cellular and

humoral immune responses. Here, we review a number of peptide vaccine delivery strategies based on nanoparticles composed of polymers, peptides, lipids, and inorganic materials.
C1 [Skwarczynski, Mariusz; Toth, Istvan] Univ Queensland, Sch Chem & Mol Biosci, St Lucia, Qld 4072, Australia.
C3 University of Queensland
RP Toth, I (通讯作者)，Univ Queensland, Sch Chem & Mol Biosci, St Lucia, Qld 4072, Australia.
EM i.toth@uq.edu.au
RI Skwarczynski, Mariusz/A-6707-2009; Toth, Istvan/C-1965-2013
OI Skwarczynski, Mariusz/0000-0001-7257-807X; Toth, Istvan/0000-0002-4572-397X
CR Adessi C, 2002, CURR MED CHEM, V9, P963, DOI 10.2174/0929867024606731
Akagi T, 2005, J CONTROL RELEASE, V108, P226, DOI 10.1016/j.jconrel.2005.08.003
Akagi T, 2007, POLYMER, V48, P6729, DOI 10.1016/j.polymer.2007.08.038
Batzloff MR, 2006, J INFECT DIS, V194, P325, DOI 10.1086/505146
Batzloff MR, 2004, CURR DRUG TARGETS, V5, P57, DOI 10.2174/1389450043490721
Becerra JC, 2003, SURGERY, V133, P404, DOI 10.1067/msy.2003.112
Beck A, 2007, J PEPT SCI, V13, P588, DOI 10.1002/psc.852
Becker ML, 2004, BIOCONJUGATE CHEM, V15, P699, DOI 10.1021/bc049946e
Becker ML, 2004, BIOCONJUGATE CHEM, V15, P710, DOI 10.1021/bc049945m
Bhattacharya R, 2008, ADV DRUG DELIVER REV, V60, P1289, DOI 10.1016/j.addr.2008.03.013
Boato F, 2007, ANGEW CHEM INT EDIT, V46, P9015, DOI 10.1002/anie.200702805
Boturyn D, 2008, J PEPT SCI, V14, P224, DOI 10.1002/psc.964
Chadwick S, 2010, ADV DRUG DELIVER REV, V62, P394, DOI 10.1016/j.addr.2009.11.012
Chua BY, 2007, VACCINE, V25, P92, DOI 10.1016/j.vaccine.2006.07.012
Collier JH, 2003, BIOCONJUGATE CHEM, V14, P748, DOI 10.1021/bc034017t
Combadiere B, 2008, COMP IMMUNOL MICROB, V31, P293, DOI 10.1016/j.cimid.2007.07.015
Cox JC, 1997, VACCINE, V15, P248, DOI 10.1016/S0264-410X(96)00183-1
Csaba N, 2009, ADV DRUG DELIVER REV, V61, P140, DOI 10.1016/j.addr.2008.09.005
Cui ZG, 2004, PHARM RES-DORDR, V21, P1018, DOI 10.1023/B:PHAM.0000029292.66792.4f
Cui ZR, 2005, MOL PHARMACEUT, V2, P22, DOI 10.1021/mp049907k
des Rieux A, 2006, J CONTROL RELEASE, V116, P1, DOI 10.1016/j.jconrel.2006.08.013
Elamanchili P, 2004, VACCINE, V22, P2406, DOI 10.1016/j.vaccine.2003.12.032
Elamanchili P, 2007, J IMMUNOTHER, V30, P378, DOI 10.1097/CJI.0b013e31802cf3e3
Ertl HCJ, 1996, J IMMUNOL, V156, P3579
Fifis T, 2004, VACCINE, V23, P258, DOI 10.1016/j.vaccine.2004.05.022
Fifis T, 2004, J IMMUNOL, V173, P3148, DOI 10.4049/jimmunol.173.5.3148
Foldvari M, 2008, NANOMED-NANOTECHNOL, V4, P173, DOI 10.1016/j.nano.2008.04.002
Foldvari M, 2008, NANOMED-NANOTECHNOL, V4, P183, DOI 10.1016/j.nano.2008.04.003
Frey A, 1997, BIOCONJUGATE CHEM, V8, P424, DOI 10.1021/bc970036p
Frey A, 1999, VACCINE, V17, P3007, DOI 10.1016/S0264-410X(99)00163-2
Garnett MC, 2006, OCCUP MED-OXFORD, V56, P307, DOI 10.1093/occmed/kql052
Gentilucci L, 2006, CURR MED CHEM, V13, P2449, DOI 10.2174/092986706777935041
Greenwood DLV, 2008, VACCINE, V26, P2706, DOI 10.1016/j.vaccine.2008.03.025
Hilgers LAT, 2000, VACCINE, V18, P3319, DOI 10.1016/S0264-410X(00)00114-6
HogenEsch H, 2002, VACCINE, V20, pS34, DOI 10.1016/S0264-410X(02)00169-X
Huang HY, 1997, J AM CHEM SOC, V119, P11653, DOI 10.1021/ja9717469
Huang HY, 1998, CHEM COMMUN, P1415, DOI 10.1039/a708686e
HUNTER R, 1981, J IMMUNOL, V127, P1244
Illum L, 2007, J PHARM SCI-US, V96, P473, DOI 10.1002/jps.20718
Irache JM, 2008, EXPERT OPIN DRUG DEL, V5, P703, DOI [10.1517/17425247.5.6.703, 10.1517/17425247.5.6.703 ]
Jiang WL, 2005, ADV DRUG DELIVER REV, V57, P391, DOI 10.1016/j.addr.2004.09.003
Jung JP, 2009, BIOMATERIALS, V30, P2400, DOI 10.1016/j.biomaterials.2009.01.033
Kaba SA, 2009, J IMMUNOL, V183, P7268, DOI 10.4049/jimmunol.0901957
Kabanov AV, 2006, ADV DRUG DELIVER REV, V58, P1597, DOI 10.1016/j.addr.2006.09.019
Kagan VE, 2010, NAT NANOTECHNOL, V5, P354, DOI [10.1038/NNANO.2010.44, 10.1038/nnano.2010.44]
Kakwere H, 2010, CHEM COMMUN, V46, P2188, DOI 10.1039/b924112d
KREUTER J, 1991, J CONTROL RELEASE, V16, P169, DOI 10.1016/0168-3659(91)90040-K
Lawson LB, 2007, CLIN PHARMACOL THER, V82, P641, DOI 10.1038/sj.clpt.6100425
Li C, 2002, ADV DRUG DELIVER REV, V54, P695, DOI 10.1016/S0169-409X(02)00045-5

Lutsiak MEC, 2006, J PHARM PHARMACOL, V58, P739, DOI 10.1211/jpp.58.6.0004
Lutsiak MEC, 2002, PHARM RES-DORDR, V19, P1480, DOI 10.1023/A:1020452531828
Malashkevich VN, 1996, SCIENCE, V274, P761, DOI 10.1126/science.274.5288.761
Malik Dhirendra Kumar, 2007, Current Drug Delivery, V4, P141
Matsuo K, 2007, BIOCHEM BIOPH RES CO, V362, P1069, DOI 10.1016/j.bbrc.2007.08.112
Medina C, 2007, BRIT J PHARMACOL, V150, P552, DOI 10.1038/sj.bjp.0707130
Morein B, 2004, ADV DRUG DELIVER REV, V56, P1367, DOI 10.1016/j.addr.2004.02.004
Mottram PL, 2007, MOL PHARMACEUT, V4, P73, DOI 10.1021/mp060096p
Moyle PM, 2008, CURR MED CHEM, V15, P506, DOI 10.2174/092986708783503249
Mundargi RC, 2008, J CONTROL RELEASE, V125, P193, DOI 10.1016/j.jconrel.2007.09.013
Murakami T, 2008, MINI-REV MED CHEM, V8, P175
Nair LS, 2007, J BIOMED NANOTECHNOL, V3, P301, DOI 10.1166/jbn.2007.041
Nandedkar TD, 2009, J BIOSCIENCES, V34, P995, DOI 10.1007/s12038-009-0114-3
Nishioka Y, 2001, ADV DRUG DELIVER REV, V47, P55, DOI 10.1016/S0169-409X(00)00121-6
O'Hagan Derek T, 2003, Expert Rev Vaccines, V2, P269, DOI 10.1586/14760584.2.2.269
OPPENHEIM RC, 1981, INT J PHARM, V8, P217, DOI 10.1016/0378-5173(81)90100-9
Oussoren C, 2001, ADV DRUG DELIVER REV, V50, P143, DOI 10.1016/S0169-409X(01)00154-5
Pagona G, 2006, CURR MED CHEM, V13, P1789, DOI 10.2174/092986706777452524
Pantarotto D, 2003, CHEM BIOL, V10, P961, DOI 10.1016/j.chembiol.2003.09.011
Pantarotto D, 2003, J AM CHEM SOC, V125, P6160, DOI 10.1021/ja034342r
Patarroyo ME, 2008, ACCOUNTS CHEM RES, V41, P377, DOI 10.1021/ar700120t
Peek LJ, 2008, ADV DRUG DELIVER REV, V60, P915, DOI 10.1016/j.addr.2007.05.017
Phillipps KSM, 2009, IMMUNOLOGY, V128, pe582, DOI 10.1111/j.1365-2567.2008.03038.x
Pimentel TAPF, 2009, CHEM BIOL DRUG DES, V73, P53, DOI 10.1111/j.1747-0285.2008.00746.x
Prato M, 2008, ACCOUNTS CHEM RES, V41, P60, DOI 10.1021/ar700089b
Prausnitz MR, 2008, NAT BIOTECHNOL, V26, P1261, DOI 10.1038/nbt.1504
PRUKSAKORN S, 1994, INT IMMUNOL, V6, P1235, DOI 10.1093/intimm/6.8.1235
PRUKSAKORN S, 1994, LANCET, V344, P639, DOI 10.1016/S0140-6736(94)92083-4
Purcell AW, 2007, NAT REV DRUG DISCOV, V6, P404, DOI 10.1038/nrd2224
Purcell AW, 2003, J PEPT SCI, V9, P255, DOI 10.1002/psc.456
Raman S, 2006, NANOMED-NANOTECHNOL, V2, P95, DOI 10.1016/j.nano.2006.04.007
Reddy ST, 2007, NAT BIOTECHNOL, V25, P1159, DOI [10.1038/nbt1332, 10.1038/nbtl332]
Reddy ST, 2006, TRENDS IMMUNOL, V27, P573, DOI 10.1016/j.it.2006.10.005
Reddy ST, 2006, J APPL PHYSIOL, V101, P1162, DOI 10.1152/japplphysiol.00389.2006
Reddy ST, 2006, J CONTROL RELEASE, V112, P26, DOI 10.1016/j.jconrel.2006.01.006
Reis CP, 2006, NANOMED-NANOTECHNOL, V2, P53, DOI 10.1016/j.nano.2006.04.009
Rudra JS, 2010, P NATL ACAD SCI USA, V107, P622, DOI 10.1073/pnas.0912124107
Sahoo SK, 2007, NANOMED-NANOTECHNOL, V3, P20, DOI 10.1016/j.nano.2006.11.008
Samuel J, 2003, FASEB J, V17, pC332
Sandhiya S, 2009, FUND CLIN PHARMACOL, V23, P263, DOI 10.1111/j.1472-8206.2009.00692.x
Scheerlinck JPY, 2008, DRUG DISCOV TODAY, V13, P882, DOI 10.1016/j.drudis.2008.06.016
Schroeder U, 2009, J MOL BIOL, V386, P1368, DOI 10.1016/j.jmb.2008.11.023
Seong SY, 2004, NAT REV IMMUNOL, V4, P469, DOI 10.1038/nri1372
Sharma S, 2009, J PHARM SCI-US, V98, P812, DOI 10.1002/jps.21493
Shih IL, 2004, MINI-REV MED CHEM, V4, P179, DOI 10.2174/1389557043487420
SIMERSKA P, 2010, MED RES REV IN PRESS
Singh R, 2009, EXP MOL PATHOL, V86, P215, DOI 10.1016/j.yexmp.2008.12.004
Skwarczynski M, 2006, J MED CHEM, V49, P7253, DOI 10.1021/jm0602155
Skwarczynski M, 2010, ANGEW CHEM INT EDIT, V49, P5742, DOI 10.1002/anie.201002221
Son SJ, 2007, DRUG DISCOV TODAY, V12, P650, DOI 10.1016/j.drudis.2007.06.002
Standley SM, 2004, BIOCONJUGATE CHEM, V15, P1281, DOI 10.1021/bc049956f
Sundaram R, 2002, BIOPOLYMERS, V66, P200, DOI 10.1002/bip.10258
Tsuji M, 2001, MOL IMMUNOL, V38, P433, DOI 10.1016/S0161-5890(01)00079-7
Uemura A, 2005, J VET MED SCI, V67, P1197, DOI 10.1292/jvms.67.1197
Uto T, 2007, J IMMUNOL, V178, P2979, DOI 10.4049/jimmunol.178.5.2979
Uto T, 2009, IMMUNOL LETT, V125, P46, DOI 10.1016/j.imlet.2009.05.008
Watford WT, 2010, J IMMUNOL, V184, P105, DOI 10.4049/jimmunol.0803730
Xiang SD, 2006, METHODS, V40, P1, DOI 10.1016/j.ymeth.2006.05.016
Xu ZP, 2006, CHEM ENG SCI, V61, P1027, DOI 10.1016/j.ces.2005.06.019
Yamaguchi S, 2010, CANCER IMMUNOL IMMUN, V59, P759, DOI 10.1007/s00262-009-0796-2
Yan W, 2007, POLYM REV, V47, P329, DOI 10.1080/15583720701455020

Yandara N, 2008, VACCINE, V26, P5864, DOI 10.1016/j.vaccine.2008.08.014
Zaman M, 2010, VACCINE, V28, P2243, DOI 10.1016/j.vaccine.2009.12.046
Zhong Wei, 2009, Current Drug Delivery, V6, P520
Zhong W, 2009, AUST J CHEM, V62, P956, DOI 10.1071/CH09149
Zhong W, 2009, AUST J CHEM, V62, P993, DOI 10.1071/CH09065
Zhong W, 2009, TETRAHEDRON, V65, P3459, DOI 10.1016/j.tet.2009.02.060
NR 116
TC 93
Z9 94
U1 2
U2 56
PU BENTHAM SCIENCE PUBL LTD
PI SHARJAH
PA EXECUTIVE STE Y-2, PO BOX 7917, SAIF ZONE, 1200 BR SHARJAH, U ARAB
EMIRATES
SN 1567-2018
EI 1875-5704
J9 CURR DRUG DELIV
JI Curr. Drug Deliv.
PD MAY
PY 2011
VL 8
IS 3
BP 282
EP 289
DI 10.2174/156720111795256192
PG 8
WC Pharmacology & Pharmacy
WE Science Citation Index Expanded (SCI-EXPANDED)
SC Pharmacology & Pharmacy
GA 768AW
UT WOS:000290904500008
PM 21291373
DA 2023-09-14
ER

PT J
AU Zhang, HX
You, XR
Wang, XJ
Cui, L
Wang, ZN
Xu, FF
Li, MY
Yang, ZG
Liu, JY
Huang, P
Kang, Y
Wu, J
Xia, XJ
AF Zhang, Hongxia
You, Xinru
Wang, Xiaojuan
Cui, Lei
Wang, Zining
Xu, Feifei
Li, Mengyun
Yang, Zhenggang
Liu, Jinyun
Huang, Peng
Kang, Yang

Wu, Jun
Xia, Xiaojun
TI Delivery of mRNA vaccine with a lipid-like material potentiates antitumor efficacy through Toll-like receptor 4 signaling
SO PROCEEDINGS OF THE NATIONAL ACADEMY OF SCIENCES OF THE UNITED STATES OF AMERICA
LA English
DT Article
DE mRNA nanovaccine; TLR4; self-adjuvant; cancer immunotherapy; lipid-like material
ID DENDRITIC CELLS; CANCER; ANTIGENS; IMMUNITY
AB Intracellular delivery of messenger RNA (mRNA)-based cancer vaccine has shown great potential to elicit antitumor immunity. To achieve robust antitumor efficacy, mRNA encoding tumor antigens needs to be efficiently delivered and translated in dendritic cells with concurrent innate immune stimulation to promote antigen presentation. Here, by screening a group of cationic lipid-like materials, we developed a minimalist nanovaccine with C1 lipid nanoparticle (LNP) that could efficiently deliver mRNA in antigen presenting cells with simultaneous Toll-like receptor 4 (TLR4) activation and induced robust T cell activation. The C1 nanovaccine entered cells via phagocytosis and showed efficient mRNA-encoded antigen expression and presentation. Furthermore, the C1 lipid nanoparticle itself induced the expression of inflammatory cytokines such as IL-12 via stimulating TLR4 signal pathway in dendritic cells. Importantly, the C1 mRNA nanovaccine exhibited significant antitumor efficacy in both tumor prevention and therapeutic vaccine settings. Overall, our work presents a C1 LNP-based mRNA cancer nanovaccine with efficient antigen expression as well as self-adjuvant property, which may provide a platform for developing cancer immunotherapy for a wide range of tumor types.
C1 [Zhang, Hongxia; Wang, Xiaojuan; Cui, Lei; Wang, Zining; Xu, Feifei; Li, Mengyun; Liu, Jinyun; Huang, Peng; Wu, Jun; Xia, Xiaojun] Sun Yat Sen Univ, Collaborat Innovat Ctr Canc Med, State Key Lab Oncol South China, Canc Ctr, Guangzhou 510060, Peoples R China.
[You, Xinru; Kang, Yang; Wu, Jun] Sun Yat Sen Univ, Sch Biomed Engn, Key Lab Sensing Technol & Biomed Instrument Guang, Guangzhou 510006, Peoples R China.
[Yang, Zhenggang] Zhejiang Univ, Affiliated Hosp 1, Coll Med,State Key Lab Diagnost & Treatment Infec, Collaborat Innovat Ctr Diag & Treatment Infect Di, Hangzhou 310003, Zhejiang, Peoples R China.
C3 State Key Lab Oncology South China; Sun Yat Sen University; Sun Yat Sen University; Zhejiang University; Collaborative Innovation Center for Diagnosis & Treatment of Infectious Diseases
RP Wu, J; Xia, XJ (通讯作者)，Sun Yat Sen Univ, Collaborat Innovat Ctr Canc Med, State Key Lab Oncol South China, Canc Ctr, Guangzhou 510060, Peoples R China.; Wu, J (通讯作者)，Sun Yat Sen Univ, Sch Biomed Engn, Key Lab Sensing Technol & Biomed Instrument Guang, Guangzhou 510006, Peoples R China.
EM wujun29@mail.sysu.edu.cn; xiaxj@sysucc.org.cn
RI Wu, Jun/IYJ-2133-2023
OI Xia, Xiaojun/0000-0003-4444-7472; Liu, Jinyun/0000-0002-4763-2684; Wang, Xiaojuan/0000-0002-9825-5567
FU National Key R&D Program of China [2017YFC0908500/2017YFC0908503]; National Natural Science Foundation of China [81773051, 81803005, 51973243]; Guangdong Innovative and Entrepreneurial Research Team Program [2016ZT06S638]; National Science and Technology Major Project of the Ministry of Science and Technology of China [2018ZX10301402]
FX We thank Dr. Seeruttun Sharvesh Raj for language editing. This work was supported by grants from the National Key R&D Program of China (2017YFC0908500/2017YFC0908503), the National Natural Science Foundation of China (Grants 81773051, 81803005, and 51973243), the Guangdong Innovative and Entrepreneurial Research Team Program (2016ZT06S638), and National Science and Technology Major Project of the Ministry of Science and Technology of China (2018ZX10301402).
CR Akinc A, 2008, NAT BIOTECHNOL, V26, P561, DOI 10.1038/nbt1402
Bachmann MF, 2010, NAT REV IMMUNOL, V10, P787, DOI 10.1038/nri2868
Blander JM, 2006, NATURE, V440, P808, DOI 10.1038/nature04596
Boushehri MAS, 2018, MOL PHARMACEUT, V15, P4777, DOI 10.1021/acs.molpharmaceut.8b00691
Coffman RL, 2010, IMMUNITY, V33, P492, DOI 10.1016/j.immuni.2010.10.002
Goldberg MS, 2019, NAT REV CANCER, V19, P587, DOI 10.1038/s41568-019-0186-9
Gubin MM, 2015, J CLIN INVEST, V125, P3413, DOI 10.1172/JCI80008

Holtkamp S, 2006, BLOOD, V108, P4009, DOI 10.1182/blood-2006-04-015024
Islam MA, 2015, BIOMATER SCI-UK, V3, P1519, DOI 10.1039/c5bm00198f
Kallen KJ, 2013, HUM VACC IMMUNOTHER, V9, P2263, DOI 10.4161/hv.25181
Kantoff PW, 2010, NEW ENGL J MED, V363, P411, DOI 10.1056/NEJMoa1001294
Kranz LM, 2016, NATURE, V534, P396, DOI 10.1038/nature18300
Kreiter S, 2008, J IMMUNOL, V180, P309, DOI 10.4049/jimmunol.180.1.309
Kroll AV, 2019, ADV BIOSYST, V3, DOI 10.1002/adbi.201800219
Kuai R, 2017, NAT MATER, V16, P489, DOI [10.1038/nmat4822, 10.1038/NMAT4822]
Liu HP, 2014, NATURE, V507, P519, DOI 10.1038/nature12978
Love KT, 2010, P NATL ACAD SCI USA, V107, P1864, DOI 10.1073/pnas.0910603106
Luo M, 2017, NAT NANOTECHNOL, V12, P648, DOI [10.1038/nnano.2017.52, 10.1038/NNANO.2017.52]
Mehta NK, 2015, CANCER IMMUNOL RES, V3, P836, DOI 10.1158/2326-6066.CIR-15-0112
Melero I, 2014, NAT REV CLIN ONCOL, V11, P509, DOI 10.1038/nrclinonc.2014.111
Miao L, 2019, NAT BIOTECHNOL, V37, P1174, DOI 10.1038/s41587-019-0247-3
Miller JFAP, 2015, CANCER CELL, V27, P439, DOI 10.1016/j.ccell.2015.03.007
Nguyen DN, 2012, P NATL ACAD SCI USA, V109, pE797, DOI 10.1073/pnas.1121423109
Oberli MA, 2017, NANO LETT, V17, P1326, DOI 10.1021/acs.nanolett.6b03329
Pardi N, 2018, NAT REV DRUG DISCOV, V17, P261, DOI 10.1038/nrd.2017.243
Sahin U, 2014, NAT REV DRUG DISCOV, V13, P759, DOI 10.1038/nrd4278
Schwartzentruber DJ, 2011, NEW ENGL J MED, V364, P2119, DOI 10.1056/NEJMoa1012863
Selmi A, 2016, CANCER IMMUNOL IMMUN, V65, P1075, DOI 10.1007/s00262-016-1869-7
Taniguchi K, 2018, NAT REV IMMUNOL, V18, P309, DOI 10.1038/nri.2017.142
Wang CC, 2017, ADV MATER, V29, DOI 10.1002/adma.201702212
Xu XY, 2013, P NATL ACAD SCI USA, V110, P18638, DOI 10.1073/pnas.1303958110
Yang R, 2018, ACS NANO, V12, P5121, DOI 10.1021/acsnano.7b09041
NR 32
TC 90
Z9 90
U1 17
U2 196
PU NATL ACAD SCIENCES
PI WASHINGTON
PA 2101 CONSTITUTION AVE NW, WASHINGTON, DC 20418 USA
SN 0027-8424
J9 P NATL ACAD SCI USA
JI Proc. Natl. Acad. Sci. U. S. A.
PD FEB 9
PY 2021
VL 118
IS 6
AR e2005191118
DI 10.1073/pnas.2005191118
PG 12
WC Multidisciplinary Sciences
WE Science Citation Index Expanded (SCI-EXPANDED)
SC Science & Technology - Other Topics
GA QG1MR
UT WOS:000617355300005
PM 33547233
OA Green Published
DA 2023-09-14
ER

PT J
AU Guan, XW
Chen, J
Hu, YY
Lin, L
Sun, PJ
Tian, H

Chen, XS
AF Guan, Xiuwen
Chen, Jie
Hu, Yingying
Lin, Lin
Sun, Pingjie
Tian, Huayu
Chen, Xuesi
TI Highly enhanced cancer immunotherapy by combining nanovaccine with hyaluronidase
SO BIOMATERIALS
LA English
DT Article
DE Immunotherapy; Nanovaccine; OVA; CpG; Hyaluronidase; PEI
ID T-CELL-ACTIVATION; DENDRITIC CELLS; DELIVERY-SYSTEMS; SUBUNIT VACCINES; TUMOR REJECTION; IN-VITRO; NANOPARTICLES; ANTIGEN; POLYETHYLENEIMINE; ADJUVANT
AB Tumor vaccine has been one of the research hotspots for cancer immunotherapy in recent years. By introducing tumor antigens into the body, the patient's own immune system will be specifically activated to induce effective immune responses for controlling or eliminating the malignant tumor cells. In this study, a simple nanovaccine was developed to induce antigen-specific anti-tumor immune responses. Polycationic polyethylenimine (PEI) was utilized to co-deliver the antigen ovalbumin (OVA) and the adjuvant unmethylated cytosine-phosphate-guanine (CpG) by electrostatic binding. The positively charged PEI could be beneficial to augment the PEI/CpG/OVA nanovaccine uptake in dendritic cells (DCs) and facilitate the endosomal escape of the nanovaccine for antigen delivering into the cytoplasm. The nanovaccine showed significant stimulation on DCs' maturation in vitro, and it was further applied for in vivo anti-tumor immunotherapy. To enhance the tumor infiltration of the nanovaccine-generated tumor-specific T cells, hyaluronidase (HAase) was employed to increase the permeability of the tumor tissues by breaking down the hyaluronan (HA) in the extracellular matrix (ECM) of tumors. Highly enhanced in vivo anti-tumor therapeutic efficiency was achieved by combining the PEI/CpG/OVA nanovaccine with HAase, which was attributed to the increased quantity of OVA-specific T cells in tumor tissues. The combination of nanovaccine with HAase has offered a simple and efficient strategy for inducing powerful anti-tumor effect in cancer immunotherapy. (C) 2018 Elsevier Ltd. All rights reserved.
C1 [Guan, Xiuwen; Chen, Jie; Hu, Yingying; Lin, Lin; Sun, Pingjie; Tian, Huayu; Chen, Xuesi] Chinese Acad Sci, Changchun Inst Appl Chem, Key Lab Polymer Ecomat, Changchun 130022, Jilin, Peoples R China.
[Guan, Xiuwen; Hu, Yingying] Univ Chinese Acad Sci, Beijing 100049, Peoples R China.
C3 Chinese Academy of Sciences; Changchun Institute of Applied Chemistry, CAS; Chinese Academy of Sciences; University of Chinese Academy of Sciences, CAS
RP Tian, H (通讯作者)，Chinese Acad Sci, Changchun Inst Appl Chem, Key Lab Polymer Ecomat, Changchun 130022, Jilin, Peoples R China.
EM thy@ciac.ac.cn
RI Chen, Xuesi/ABB-5039-2020; Chen, Jie/R-6737-2016; Hu, Ying/GQZ-8100-2022; hu, ying/IVV-6516-2023
OI Guan, Xiuwen/0000-0003-1796-8993
FU National Natural Science Foundation of China [21474104, 51233004, 51303173, 51390484, 51520105004]; National program for support of Top-notch young professionals; Jilin province science and technology development program [20160204032GX]
FX The authors are thankful to the National Natural Science Foundation of China (21474104, 51233004, 51303173, 51390484 and 51520105004), National program for support of Top-notch young professionals, Jilin province science and technology development program (20160204032GX) for financial support to this work.
CR Appay V, 2008, NAT MED, V14, P623, DOI 10.1038/nm.f.1774
Blank CU, 2016, SCIENCE, V352, P658, DOI 10.1126/science.aaf2834
Brunner C, 2000, J IMMUNOL, V165, P6278, DOI 10.4049/jimmunol.165.11.6278
Cappello P, 2002, J IMMUNOTHER, V25, P1, DOI 10.1097/00002371-200201000-00001
Chen JA, 2011, INT J NANOMED, V6, P77, DOI 10.2147/IJN.S15457
Choi B, 2016, ACS NANO, V10, P7339, DOI 10.1021/acsnano.5b08084

Dranoff G, 2004, NAT REV CANCER, V4, P11, DOI 10.1038/nrc1252
Fan YC, 2015, VACCINES-BASEL, V3, P662, DOI 10.3390/vaccines3030662
Foged C, 2005, INT J PHARMACEUT, V298, P315, DOI 10.1016/j.ijpharm.2005.03.035
Foged C, 2004, VACCINE, V22, P1903, DOI 10.1016/j.vaccine.2003.11.008
Foged C, 2011, THER DELIV, V2, P1057, DOI 10.4155/TDE.11.68
Fromen CA, 2015, P NATL ACAD SCI USA, V112, P488, DOI 10.1073/pnas.1422923112
Galluzzi L, 2014, ONCOTARGET, V5, P12472, DOI 10.18632/oncotarget.2998
Gause KT, 2017, ACS NANO, V11, P54, DOI 10.1021/acsnano.6b07343
Gong H, 2016, NANO LETT, V16, P2512, DOI 10.1021/acs.nanolett.6b00068
Guo YY, 2015, ACS NANO, V9, P6918, DOI 10.1021/acsnano.5b01042
Jacobetz MA, 2013, GUT, V62, P112, DOI 10.1136/gutjnl-2012-302529
Joyce JA, 2015, SCIENCE, V348, P74, DOI 10.1126/science.aaa6204
Krieg AM, 2008, ONCOGENE, V27, P161, DOI 10.1038/sj.onc.1210911
Kultti Anne, 2012, Cancers (Basel), V4, P873, DOI 10.3390/cancers4030873
Kwon YJ, 2005, J CONTROL RELEASE, V105, P199, DOI 10.1016/j.jconrel.2005.02.027
Lee RK, 1996, J IMMUNOL, V157, P1919
Liu S, 2016, ADV HEALTHC MATER, V5, P2872, DOI 10.1002/adhm.201600528
Liu SY, 2013, BIOMATERIALS, V34, P8291, DOI 10.1016/j.biomaterials.2013.07.020
Luo Z., 2017, ADV MAT, V29
Melief CJM, 2015, J CLIN INVEST, V125, P3401, DOI 10.1172/JCI80009
Mellman I, 2011, NATURE, V480, P480, DOI 10.1038/nature10673
Morishita M, 2016, BIOMATERIALS, V111, P55, DOI 10.1016/j.biomaterials.2016.09.031
Moyle PM, 2013, CHEMMEDCHEM, V8, P360, DOI 10.1002/cmdc.201200487
NAGATA S, 1995, SCIENCE, V267, P1449, DOI 10.1126/science.7533326
Noguchi M, 2013, CANCER IMMUNOL IMMUN, V62, P919, DOI 10.1007/s00262-012-1379-1
Palucka K, 2012, NAT REV CANCER, V12, P265, DOI 10.1038/nrc3258
PARDOLL DM, 1993, CURR OPIN IMMUNOL, V5, P719, DOI 10.1016/0952-7915(93)90127-E
Parish CR, 2003, IMMUNOL CELL BIOL, V81, P106, DOI 10.1046/j.0818-9641.2003.01151.x
Purcell AW, 2007, NAT REV DRUG DISCOV, V6, P404, DOI 10.1038/nrd2224
Qin T, 2015, CLIN VACCINE IMMUNOL, V22, P421, DOI 10.1128/CVI.00778-14
Qin ZH, 2003, CANCER RES, V63, P4095
Rosenberg SA, 2004, NAT MED, V10, P909, DOI 10.1038/nm1100
Sathiyajith C., 2017, INT J VACCINES VACCI, V4
Schumacher TN, 2015, SCIENCE, V348, P69, DOI 10.1126/science.aaa4971
Senovilla L, 2013, ONCOIMMUNOLOGY, V2, DOI 10.4161/onci.23803
Sharma P, 2011, NAT REV CANCER, V11, P805, DOI 10.1038/nrc3153
Skwarczynski M, 2014, NANOMEDICINE-UK, V9, P2657, DOI [10.2217/nnm.14.187, 10.2217/NNM.14.187]
Smith DM, 2013, NAT REV IMMUNOL, V13, P592, DOI 10.1038/nri3488
Sparwasser T, 1998, EUR J IMMUNOL, V28, P2045, DOI 10.1002/(SICI)1521-4141(199806)28:06<2045::AID-IMMU2045>3.0.CO;2-8
Thomas C, 2009, INT J PHARMACEUT, V379, P41, DOI 10.1016/j.ijpharm.2009.06.006
Toole BP, 2002, AM J PATHOL, V161, P745, DOI 10.1016/S0002-9440(10)64232-0
Uto T, 2007, J IMMUNOL, V178, P2979, DOI 10.4049/jimmunol.178.5.2979
Wang C, 2016, ADV MATER, V28, P8912, DOI 10.1002/adma.201506312
Wegmann F, 2012, NAT BIOTECHNOL, V30, P883, DOI 10.1038/nbt.2344
Zhang Y, 2017, ACTA POLYM SIN, P1150, DOI 10.11777/j.issn1000-3304.2017.17024
Zhang Y, 2016, ADV MATER, V28, P2699, DOI 10.1002/adma.201506232
Zhou H, 2016, NANO LETT, V16, P3268, DOI 10.1021/acs.nanolett.6b00820
Zhu GZ, 2017, ACS NANO, V11, P2387, DOI 10.1021/acsnano.7b00978
NR 54
TC 87
Z9 89
U1 2
U2 245
PU ELSEVIER SCI LTD
PI OXFORD
PA THE BOULEVARD, LANGFORD LANE, KIDLINGTON, OXFORD OX5 1GB, OXON, ENGLAND
SN 0142-9612
EI 1878-5905
J9 BIOMATERIALS

JI Biomaterials
PD JUL
PY 2018
VL 171
BP 198
EP 206
DI 10.1016/j.biomaterials.2018.04.039
PG 9
WC Engineering, Biomedical; Materials Science, Biomaterials
WE Science Citation Index Expanded (SCI-EXPANDED)
SC Engineering; Materials Science
GA GH7OJ
UT WOS:000433643000017
PM 29698869
DA 2023-09-14
ER

PT J
AU Liu, X
Feng, ZJ
Wang, CR
Su, Q
Song, HJ
Zhang, CN
Huang, PS
Liang, XJ
Dong, AJ
Kong, DL
Wang, WW
AF Liu, Xiang
Feng, Zujian
Wang, Changrong
Su, Qi
Song, Huijuan
Zhang, Chuangnian
Huang, Pingsheng
Liang, Xing-Jie
Dong, Anjie
Kong, Deling
Wang, Weiwei
TI Co-localized delivery of nanomedicine and nanovaccine augments the postoperative cancer immunotherapy by amplifying T-cell responses
SO BIOMATERIALS
LA English
DT Article
DE Cancer immunotherapy; Nanomedicine; Cancer vaccine; Immunogenic cell death; Drug delivery
ID INJECTABLE POLYPEPTIDE HYDROGEL; IN-VIVO; THERMOSENSITIVE HYDROGEL; DENDRITIC CELLS; CURCUMIN NANOPARTICLES; VACCINE; EFFICIENCY; MICELLES; KINETICS; PATHWAY
AB Immunotherapy in solid tumors is limited by the poor immunogenicity of tumors and limited T-cell immune response, resulting in low patient response rate. To increase the efficiency of cancer immunotherapy, a unique synergistic combination cancer immunotherapy by co-localized delivery of cancer nanomedicine for enhancing the tumor immunogenicity and nanovaccine for augmenting the antitumor T-cell immunity was developed for post-surgical tumor treatment. The thermo-responsive, curcumin-loaded polymer nanoparticles (nanomedicine)assembled hydrogel enabled the complete coverage of the surgical bed of primary tumor and the spatio-temporal delivery of cognate nanomedicines and encapsulated nanovaccines. Importantly, the nanomedicine efficiently induced the immunogenic cell death (ICD) of residual cancer cells, and consequently enhanced the tumor immunogenicity and sensitized the tumor to antitumor T-cell immunity. The cancer nanovaccine composed of antigenic peptide, CpG-ODN and cationic polymer nanoparticle significantly triggered the maturation of dendritic

cells (DCs) and elicited potent vaccine-specific T-cell immune responses. Using highly malignant postoperative breast carcinoma 4T1 models, we found that the combination immunotherapy strategy strikingly amplified the level of systemic host T-cell immunity, promoted the infiltration of CD8(+) T lymphocytes in tumor, and thus efficaciously attenuated the local tumor recurrence and pulmonary metastasis. Collectively, this work provided an advanced synergistic combination approach for post-surgical tumor immunotherapy. The self-assembled hydrogel should enable a broader combination of immunomodulating nanomedicines and vaccines for cancer immunotherapy.
C1 [Liu, Xiang; Feng, Zujian; Wang, Changrong; Su, Qi; Dong, Anjie] Tianjin Univ, Sch Chem Engn & Technol, Dept Polymer Sci & Engn, Key Lab Syst Bioengn,Minist Educ, Tianjin 300072, Peoples R China.
[Liu, Xiang; Feng, Zujian; Wang, Changrong; Su, Qi; Dong, Anjie] Collaborat Innovat Ctr Chem Sci & Engn Tianjin, Tianjin 300072, Peoples R China.
[Song, Huijuan; Zhang, Chuangnian; Huang, Pingsheng; Wang, Weiwei] Chinese Acad Med Sci & Peking Union Med Coll, Tianjin Key Lab Biomat Res, Inst Biomed Engn, Tianjin 300192, Peoples R China.
[Liang, Xing-Jie] Chinese Acad Sci, Natl Ctr Nanosci & Technol, Key Lab Biol Effects Nanomat & Nanosafety, Beijing 100190, Peoples R China.
[Kong, Deling] Nankai Univ, Coll Life Sci, State Key Lab Med Chem Biol, Tianjin 300071, Peoples R China.
C3 Tianjin University; Chinese Academy of Medical Sciences - Peking Union Medical College; Peking Union Medical College; Institute of BioMedical Engineering - CAMS; Chinese Academy of Sciences; National Center for Nanoscience & Technology - China; Nankai University
RP Dong, AJ (通讯作者)，Tianjin Univ, Sch Chem Engn & Technol, Dept Polymer Sci & Engn, Key Lab Syst Bioengn,Minist Educ, Tianjin 300072, Peoples R China.; Wang, WW (通讯作者)，Chinese Acad Med Sci & Peking Union Med Coll, Tianjin Key Lab Biomat Res, Inst Biomed Engn, Tianjin 300192, Peoples R China.; Liang, XJ (通讯作者)，Chinese Acad Sci, Natl Ctr Nanosci & Technol, Key Lab Biol Effects Nanomat & Nanosafety, Beijing 100190, Peoples R China.
EM liangxj@nanoctr.cn; ajdong@tju.edu.cn; wwwangtj@163.com
RI Liang, Richard/ABF-2708-2021; Wang, Weiwei/GRF-3625-2022
FU National Natural Science Foundation of China [31971306, 31671021, 31670977, 31870950, 51373199, 21604095]; National Natural Science Foundation key project [31630027, 31430031]; NSFC-DFG project [31761133013]; Key Projects of Advanced Manufacturing Technology for High Quality Veterinary Drugs [17ZXGSNC00080]; Non-profit Central Research Institute Fund of Chinese Academy of Medical Sciences [2018RC350017]; Young Elite Scientists Sponsorship Program by Tianjin [TJSQNTJ-2018-01]
FX We acknowledge the financial support from the National Natural Science Foundation of China (No. 31971306, 31671021, 31670977, 31870950, 51373199, and 21604095), National Natural Science Foundation key project (31630027 and 31430031), NSFC-DFG project (31761133013), Key Projects of Advanced Manufacturing Technology for High Quality Veterinary Drugs (No. 17ZXGSNC00080), Non-profit Central Research Institute Fund of Chinese Academy of Medical Sciences (No. 2018RC350017), and Young Elite Scientists Sponsorship Program by Tianjin (TJSQNTJ-2018-01).
CR Anand P, 2007, MOL PHARMACEUT, V4, P807, DOI 10.1021/mp700113r
Bachmann MF, 2010, NAT REV IMMUNOL, V10, P787, DOI 10.1038/nri2868
Banchereau J, 1998, NATURE, V392, P245, DOI 10.1038/32588
Begg AC, 2011, NAT REV CANCER, V11, P239, DOI 10.1038/nrc3007
Chen Q, 2019, ADV MATER, V31, DOI 10.1002/adma.201802228
Chopra D, 2016, BIOMATERIALS, V84, P25, DOI 10.1016/j.biomaterials.2016.01.018
Coolen AL, 2019, BIOMATERIALS, V195, P23, DOI 10.1016/j.biomaterials.2018.12.019
Du LL, 2018, BIOMATERIALS, V176, P84, DOI 10.1016/j.biomaterials.2018.05.046
Duan XP, 2019, ANGEW CHEM INT EDIT, V58, P670, DOI 10.1002/anie.201804882
Grivennikov S, 2009, CANCER CELL, V15, P103, DOI 10.1016/j.ccr.2009.01.001
Holohan C, 2013, NAT REV CANCER, V13, P714, DOI 10.1038/nrc3599
Hu Z, 2018, NAT REV IMMUNOL, V18, P168, DOI 10.1038/nri.2017.131
Huang PS, 2017, BIOMATERIALS, V145, P81, DOI 10.1016/j.biomaterials.2017.07.012
Huang PS, 2016, ACS APPL MATER INTER, V8, P29323, DOI 10.1021/acsami.6b10416
June CH, 2018, SCIENCE, V359, P1361, DOI 10.1126/science.aar6711
Keklikoglou I, 2019, NAT CELL BIOL, V21, P190, DOI 10.1038/s41556-018-0256-3
Kim J, 2015, NAT BIOTECHNOL, V33, P64, DOI 10.1038/nbt.3071

Krall JA, 2018, SCI TRANSL MED, V10, DOI 10.1126/scitranslmed.aan3464
Kulkarni A, 2018, NAT BIOMED ENG, V2, P589, DOI 10.1038/s41551-018-0254-6
Labanieh L, 2018, NAT BIOMED ENG, V2, P377, DOI 10.1038/s41551-018-0235-9
Li ZL, 2018, FOOD RES INT, V108, P246, DOI 10.1016/j.foodres.2018.03.048
Lu JQ, 2018, ACS NANO, V12, P11041, DOI 10.1021/acsnano.8b05189
Lu JQ, 2017, NAT COMMUN, V8, DOI 10.1038/s41467-017-01651-9
Lu Y, 2016, MOL THER, V24, P364, DOI 10.1038/mt.2015.165
Lynn GM, 2015, NAT BIOTECHNOL, V33, P1201, DOI 10.1038/nbt.3371
Melief CJM, 2008, NAT REV CANCER, V8, P351, DOI 10.1038/nrc2373
Pasare C, 2003, SCIENCE, V299, P1033, DOI 10.1126/science.1078231
Phuengkham H, 2018, ADV MATER, V30, DOI 10.1002/adma.201706719
Poon IKH, 2014, NAT REV IMMUNOL, V14, P166, DOI 10.1038/nri3607
Sahin U, 2017, NATURE, V547, P222, DOI 10.1038/nature23003
Schumacher TN, 2015, SCIENCE, V348, P69, DOI 10.1126/science.aaa4971
Schwartzentruber DJ, 2011, NEW ENGL J MED, V364, P2119, DOI 10.1056/NEJMoa1012863
Shae D, 2019, NAT NANOTECHNOL, V14, P269, DOI 10.1038/s41565-018-0342-5
Song HJ, 2019, THERANOSTICS, V9, P2299, DOI 10.7150/thno.30577
Song HJ, 2018, BIOMATERIALS, V159, P119, DOI 10.1016/j.biomaterials.2018.01.004
Turajlic S, 2016, SCIENCE, V352, P169, DOI 10.1126/science.aaf2784
Valsecchi ME, 2015, NEW ENGL J MED, V373, P1270, DOI [10.1056/NEJMoa1504030, 10.1056/NEJMc1509660]
van Horssen R, 2006, ONCOLOGIST, V11, P397, DOI 10.1634/theoncologist.11-4-397
Verbeke R, 2019, ACS NANO, V13, P1655, DOI 10.1021/acsnano.8b07660
Wang C, 2018, SCI TRANSL MED, V10, DOI 10.1126/scitranslmed.aan3682
Wang H, 2018, NAT MATER, V17, P761, DOI 10.1038/s41563-018-0147-9
Wang WW, 2013, J MATER CHEM B, V1, P552, DOI 10.1039/c2tb00068g
Wang WW, 2012, ACTA BIOMATER, V8, P3963, DOI 10.1016/j.actbio.2012.07.021
Wang WW, 2012, SOFT MATTER, V8, P1575, DOI 10.1039/c1sm06693e
Wang Z, 2015, EUR J PHARM BIOPHARM, V90, P53, DOI 10.1016/j.ejpb.2014.11.002
Weiden J, 2018, NAT REV IMMUNOL, V18, P212, DOI 10.1038/nri.2017.89
Yallapu MM, 2012, DRUG DISCOV TODAY, V17, P71, DOI 10.1016/j.drudis.2011.09.009
Yang PX, 2019, ADV THER-GERMANY, V2, DOI 10.1002/adtp.201900010
Yang PX, 2018, NANO LETT, V18, P4377, DOI 10.1021/acs.nanolett.8b01406
Yang WJ, 2019, ACS NANO, V13, P3083, DOI 10.1021/acsnano.8b08346
Yoshimoto Y, 2014, PLOS ONE, V9, DOI 10.1371/journal.pone.0092572
Zhang JF, 2015, NANOSCALE, V7, P13503, DOI 10.1039/c5nr03259h
Zhao ZG, 2015, CANCER CELL, V28, P415, DOI 10.1016/j.ccell.2015.09.004
Zhou FY, 2019, ADV MATER, V31, DOI 10.1002/adma.201805888
NR 54
TC 86
Z9 86
U1 18
U2 536
PU ELSEVIER SCI LTD
PI OXFORD
PA THE BOULEVARD, LANGFORD LANE, KIDLINGTON, OXFORD OX5 1GB, OXON, ENGLAND
SN 0142-9612
EI 1878-5905
J9 BIOMATERIALS
JI Biomaterials
PD FEB
PY 2020
VL 230
AR 119649
DI 10.1016/j.biomaterials.2019.119649
PG 13
WC Engineering, Biomedical; Materials Science, Biomaterials
WE Science Citation Index Expanded (SCI-EXPANDED)
SC Engineering; Materials Science
GA KE7QQ
UT WOS:000508746600049

PM 31791843
DA 2023-09-14
ER

PT J
AU Aikins, ME
Xu, C
Moon, JJ
AF Aikins, Marisa E.
Xu, Cheng
Moon, James J.
TI Engineered Nanoparticles for Cancer Vaccination and Immunotherapy
SO ACCOUNTS OF CHEMICAL RESEARCH
LA English
DT Review
ID THERAPY; COMBINATION; NANOMEDICINES; NEOANTIGENS
AB The immune system has evolved over time to protect the host from foreign microorganisms. Activation of the immune system is predicated on a distinction between self and nonseli Unfortunately, cancer is characterized by genetic alterations in the host's cells, leading to uncontrolled cellular proliferation and evasion of immune surveillance. Cancer immunotherapy aims to educate the host's immune system to not only recognize but also attack and kill mutated cancer cells. While immune checkpoint blockers have been proven to be effective against multiple types of advanced cancer, the overall patient response rate still remains below 30%. Therefore, there is an urgent need to improve current cancer immunotherapies. In this Account, we present an overview of our recent progress on nanoparticle-based strategies for improving cancer vaccines and immunotherapies. We also present other complementary strategies to give a well-rounded snapshot of the field of combination cancer immunotherapy. The versatility and tunability of nanopartides make them promising platforms for addressing individual challenges posed by various cancers. For example, nanoparticles can deliver cargo materials to specific cells, such as vaccines delivered to antigen-presenting cells for strong immune activation. Nanopartides also allow for stimuli-responsive delivery of various therapeutics to cancer cells, thus forming the basis for combination cancer immunotherapy. Here, we focus on nanoparticle platforms engineered to deliver tumor antigens, whole tumor cells, and chemotherapeutic or phototherapeutic agents in a manner to effectively and safely trigger the host's immune system against tumor cells. For each work, we discuss the nanoparticle platform developed, synthesis chemistry, and in vivo applications. Nanovaccines offer a unique platform for codelivery of personalized tumor neoantigens and adjuvants and elicitation of robust immune responses against aggressive tumors. Nanovaccines either delivering whole tumor cell lysate or formed from tumor cell lysate may increase the repertoire of tumor antigens as immune targets while exploiting immunogenic cell death to prime antitumor immune responses. We also discuss how antigen- and whole tumor cell-based approaches may open the door for personalized cancer vaccination and immunotherapy. On the other hand, chemotherapy, phototherapy, and radiotherapy are more standardized cancer therapies, and nanopartide-based approaches may promote their ability to initiate T cell activation against tumor cells and improve antitumor efficacy with minimal toxicity. Finally, building on the recent progress in nanoparticle-based cancer immunotherapy, the field should set the ultimate goal to be clinical translation and clinical efficacy. We will discuss regulatory, analytical, and manufacturing hurdles that should be addressed to expedite the clinical translation of nanomedicine-based cancer immunotherapy.
C1 [Aikins, Marisa E.; Xu, Cheng; Moon, James J.] Univ Michigan, Dept Pharmaceut Sci, Ann Arbor, MI 48109 USA.
[Aikins, Marisa E.; Xu, Cheng; Moon, James J.] Univ Michigan, Biointofaces Inst, Ann Arbor, MI 48109 USA.
[Moon, James J.] Univ Michigan, Dept Biomed Engn, Ann Arbor, MI 48109 USA.
C3 University of Michigan System; University of Michigan; University of Michigan System; University of Michigan; University of Michigan System; University of Michigan
RP Moon, JJ (通讯作者)，Univ Michigan, Dept Pharmaceut Sci, Ann Arbor, MI 48109 USA.; Moon, JJ (通讯作者)，Univ Michigan, Biointofaces Inst, Ann Arbor, MI 48109 USA.; Moon, JJ (通讯作者)，Univ Michigan, Dept Biomed Engn, Ann Arbor, MI 48109 USA.
EM moonjj@umich.edu
RI Moon, James J/ABE-4429-2021; Xu, Cheng/ACX-9795-2022
OI Moon, James J/0000-0003-2238-2372; Xu, Cheng/0000-0003-1000-733X
FU NIH [R01AI127070, R01CA210273, R01DK125087, U01CA210152]; NSF CAREER

Award [1553831]; Directorate For Engineering [1553831] Funding Source: National Science Foundation; Div Of Chem, Bioeng, Env, & Transp Sys [1553831] Funding Source: National Science Foundation
FX This work was supported in part by NIH (R01AI127070, R01CA210273, R01DK125087, U01CA210152). J.J. M. is supported by NSF CAREER Award (1553831).
CR Agrahari V, 2018, DRUG DISCOV TODAY, V23, P974, DOI 10.1016/j.drudis.2018.01.047
Chang MY, 2019, ADV MATER, V31, DOI 10.1002/adma.201905271
Chao Y, 2020, SCI ADV, V6, DOI 10.1126/sciadv.aaz4204
Chen Q, 2020, ADV MATER, V32, DOI 10.1002/adma.201908185
Chen Q, 2019, CHEM SOC REV, V48, P5506, DOI 10.1039/c9cs00271e
Chen Q, 2019, ADV MATER, V31, DOI 10.1002/adma.201802228
Chen Q, 2016, NAT COMMUN, V7, DOI 10.1038/ncomms13193
Chesson CB, 2017, NANOMEDICINE-UK, V12, P2693, DOI 10.2217/nnm-2017-0254
Ding BB, 2018, ADV MATER, V30, DOI 10.1002/adma.201802479
Dolmans DEJGJ, 2003, NAT REV CANCER, V3, P380, DOI 10.1038/nrc1071
Emens LA, 2015, CANCER IMMUNOL RES, V3, P436, DOI 10.1158/2326-6066.CIR-15-0064
Fan YC, 2017, NANO LETT, V17, P7387, DOI 10.1021/acs.nanolett.7b03218
Filley AC, 2017, ONCOTARGET, V8, P91779, DOI 10.18632/oncotarget.21586
Flickinger JC, 2018, VACCINES-BASEL, V6, DOI 10.3390/vaccines6030048
Gao D, 2020, MATER TODAY BIO, V5, DOI 10.1016/j.mtbio.2019.100035
Gaspar R, 2007, NANOMEDICINE-UK, V2, P143, DOI 10.2217/17435889.2.2.143
Gong F, 2020, NANO TODAY, V32, DOI 10.1016/j.nantod.2020.100851
Hanif Farina, 2017, Asian Pac J Cancer Prev, V18, P3
Hare JI, 2017, ADV DRUG DELIVER REV, V108, P25, DOI 10.1016/j.addr.2016.04.025
He CB, 2016, NAT COMMUN, V7, DOI 10.1038/ncomms12499
Hou XY, 2018, INT J CANCER, V143, P3050, DOI 10.1002/ijc.31717
Hua S, 2018, FRONT PHARMACOL, V9, DOI 10.3389/fphar.2018.00790
Irvine DJ, 2020, ADV DRUG DELIVER REV, V158, P91, DOI 10.1016/j.addr.2020.06.019
Jin JF, 2019, ACS APPL MATER INTER, V11, P7850, DOI 10.1021/acsami.8b22309
Johanns TM, 2016, CANCER IMMUNOL RES, V4, P1007, DOI 10.1158/2326-6066.CIR-16-0156
Jordan C. T., 2006, NEW ENGL J MED, V355, P61
Joshi VB, 2014, EXPERT REV VACCINES, V13, P9, DOI 10.1586/14760584.2014.851606
Kadiyala P, 2019, ACS NANO, V13, P1365, DOI 10.1021/acsnano.8b06842
Ko EC, 2019, INT J RADIAT BIOL, V95, P936, DOI 10.1080/09553002.2019.1623429
Kroemer G, 2013, ANNU REV IMMUNOL, V31, P51, DOI 10.1146/annurev-immunol-032712-100008
Kuai R, 2020, ADV THER-GERMANY, V3, DOI 10.1002/adtp.202000094
Kuai R, 2018, J CONTROL RELEASE, V282, P131, DOI 10.1016/j.jconrel.2018.04.041
Kuai R, 2018, SCI ADV, V4, DOI 10.1126/sciadv.aao1736
Kuai R, 2018, BIOCONJUGATE CHEM, V29, P771, DOI 10.1021/acs.bioconjchem.7b00761
Kuai R, 2017, NAT MATER, V16, P489, DOI [10.1038/nmat4822, 10.1038/NMAT4822]
Kuai R, 2016, ACS NANO, V10, P3015, DOI 10.1021/acsnano.5b07522
Li L, 2017, ANN ONCOL, V28, P11, DOI 10.1093/annonc/mdx681
Liu YJ, 2019, CHEM SOC REV, V48, P2053, DOI 10.1039/c8cs00618k
Lu KD, 2018, NAT BIOMED ENG, V2, P600, DOI 10.1038/s41551-018-0203-4
Lynn GM, 2020, NAT BIOTECHNOL, V38, P320, DOI 10.1038/s41587-019-0390-x
Madan-Lala R, 2017, SCI REP-UK, V7, DOI 10.1038/s41598-017-02804-y
Medina E, 2001, VACCINE, V19, P1573, DOI 10.1016/S0264-410X(00)00354-6
Melero I, 2014, NAT REV CLIN ONCOL, V11, P509, DOI 10.1038/nrclinonc.2014.111
Min YZ, 2017, NAT NANOTECHNOL, V12, P877, DOI [10.1038/nnano.2017.113, 10.1038/NNANO.2017.113]
Montico B, 2018, INT J MOL SCI, V19, DOI 10.3390/ijms19020594
Najafabadi AH, 2020, NANO LETT, V20, P7783, DOI 10.1021/acs.nanolett.0c03414
Nam J, 2019, NAT REV MATER, V4, P398, DOI 10.1038/s41578-019-0108-1
Nam J, 2018, NAT COMMUN, V9, DOI 10.1038/s41467-018-03473-9
Ochyl LJ, 2018, BIOMATERIALS, V182, P157, DOI 10.1016/j.biomaterials.2018.08.016
Sainz V, 2015, BIOCHEM BIOPH RES CO, V468, P504, DOI 10.1016/j.bbrc.2015.08.023
Sandoval F, 2013, SCI TRANSL MED, V5, DOI 10.1126/scitranslmed.3004888
Scheetz L, 2020, CLIN CANCER RES, V26, P4369, DOI 10.1158/1078-0432.CCR-20-0341
Scheetz L, 2019, NAT BIOMED ENG, V3, P768, DOI 10.1038/s41551-019-0436-x
Schumacher TN, 2015, SCIENCE, V348, P69, DOI 10.1126/science.aaa4971

Shao YL, 2020, J AM CHEM SOC, V142, P3939, DOI 10.1021/jacs.9b12788
Sondak VK, 2003, SEMIN CANCER BIOL, V13, P409, DOI 10.1016/j.semcancer.2003.09.004
Su H, 2018, J APPL TOXICOL, V38, P3, DOI 10.1002/jat.3476
Sun X, 2020, BIOMATERIALS, V240, DOI 10.1016/j.biomaterials.2020.119845
Teli MK, 2010, CURR PHARM DESIGN, V16, P1882
Tian QW, 2013, J AM CHEM SOC, V135, P8571, DOI 10.1021/ja4013497
Tinkle S, 2014, ANN NY ACAD SCI, V1313, P35, DOI 10.1111/nyas.12403
Vogelstein B, 2013, SCIENCE, V339, P1546, DOI 10.1126/science.1235122
Wu JJ, 2018, CANCER LETT, V419, P210, DOI 10.1016/j.canlet.2018.01.050
Xu C, 2020, ACS NANO, V14, P13268, DOI 10.1021/acsnano.0c05062
Xu C, 2019, ACS NANO, V13, P12148, DOI 10.1021/acsnano.9b06691
Xu C, 2013, ACS APPL MATER INTER, V5, P12911, DOI 10.1021/am404714w
Yong TY, 2019, NAT COMMUN, V10, DOI 10.1038/s41467-019-11718-4
Zhang D, 2019, NANO LETT, V19, P6635, DOI 10.1021/acs.nanolett.9b02903
Zhu XJ, 2016, NAT COMMUN, V7, DOI 10.1038/ncomms10437
NR 69
TC 83
Z9 84
U1 9
U2 90
PU AMER CHEMICAL SOC
PI WASHINGTON
PA 1155 16TH ST, NW, WASHINGTON, DC 20036 USA
SN 0001-4842
EI 1520-4898
J9 ACCOUNTS CHEM RES
JI Accounts Chem. Res.
PD OCT 20
PY 2020
VL 53
IS 10
BP 2094
EP 2105
DI 10.1021/acs.accounts.0c00456
PG 12
WC Chemistry, Multidisciplinary
WE Science Citation Index Expanded (SCI-EXPANDED)
SC Chemistry
GA OK1MM
UT WOS:000584415900007
PM 33017150
OA Green Accepted
DA 2023-09-14
ER

PT J
AU Luo, M
Samandi, LZ
Wang, ZH
Chen, ZJJ
Gao, JM
AF Luo, Min
Samandi, Layla Z.
Wang, Zhaohui
Chen, Zhijian J.
Gao, Jinming
TI Synthetic nanovaccines for immunotherapy
SO JOURNAL OF CONTROLLED RELEASE
LA English
DT Article; Proceedings Paper
CT 14th International Nanomedicine and Drug Delivery Symposium (NanoDDS)

CY SEP 16-18, 2016
CL Johns Hopkins Univ, Baltimore, MD
HO Johns Hopkins Univ
DE Synthetic nanoparticle; Nanovaccine; T cell response; Immunotherapy
ID T-CELL RESPONSES; TOLL-LIKE RECEPTORS; DENDRITIC CELLS; IMMUNE-RESPONSES; ANTIGEN-DELIVERY; ANTITUMOR IMMUNITY; VACCINE ADJUVANTS; DNA SENSOR; LYMPH-NODE; PHYSICOCHEMICAL PROPERTIES
AB Although vaccination is historically one of the most successful strategies for the prevention of infectious diseases, development of vaccines for cancer and many chronic infections, such as HIV, malaria, and tuberculosis, has remained a challenge. Strong and long-lasting antigen-specific Tcell responses are critical for therapy of these diseases. A major challenge in achieving a robust CD8+ T cell response is the requirement of spatio-temporal orchestration of antigen cross-presentation in antigen-presenting cells with innate stimulation. Here, we discuss the development of nanoparticle vaccine (nanovaccine) that modulates the innate immune system and enhances adaptive immunity with reduced toxicity. We address how nanovaccines can integrate multiple functions, such as lymph node targeting, antigen presentation, and stimulation of innate immunity, to achieve a robust T cell response for immunotherapy.
C1 [Luo, Min; Samandi, Layla Z.; Wang, Zhaohui; Gao, Jinming] Simmons Comprehens Canc Ctr, Dept Pharmacol, 5323 Harry Hines Blvd, Dallas, TX 75390 USA.
[Chen, Zhijian J.] Univ Texas Southwestern Med Ctr Dallas, Dept Mol Biol, 5323 Harry Hines Blvd, Dallas, TX 75390 USA.
[Chen, Zhijian J.] Howard Hughes Med Inst, 5323 Harry Hines Blvd, Dallas, TX 75390 USA.
C3 University of Texas System; University of Texas Southwestern Medical Center Dallas; University of Texas System; University of Texas Southwestern Medical Center Dallas; Howard Hughes Medical Institute; University of Texas System; University of Texas Southwestern Medical Center Dallas
RP Gao, JM (通讯作者)，Simmons Comprehens Canc Ctr, Dept Pharmacol, 5323 Harry Hines Blvd, Dallas, TX 75390 USA.
EM jinming.gao@utsouthwestern.edu
RI Wang, Zhaohui/C-9795-2016; Chen, Zhijian/C-6039-2012
OI Wang, Zhaohui/0000-0003-4650-9008; Chen, Zhijian/0000-0002-8475-8251; Samandi, Layla/0000-0001-8142-5841; Wang, Zhaohui/0000-0001-7994-7464
FU NCI NIH HHS [R01 CA192221] Funding Source: Medline; NIBIB NIH HHS [R01 EB013149] Funding Source: Medline
CR Abbas A. K., 2014, CELLULAR MOL IMMUNOL
Ablasser A, 2013, NATURE, V498, P380, DOI 10.1038/nature12306
Agrawal S, 2003, J IMMUNOL, V171, P4984, DOI 10.4049/jimmunol.171.10.4984
Ahn S, 2014, ADV HEALTHC MATER, V3, P1194, DOI 10.1002/adhm.201300597
Ahsan FL, 2002, J CONTROL RELEASE, V79, P29, DOI 10.1016/S0168-3659(01)00549-1
Akbarzadeh A, 2013, NANOSCALE RES LETT, V8, DOI 10.1186/1556-276X-8-102
Albanese A, 2012, ANNU REV BIOMED ENG, V14, P1, DOI [10.1146/annurev-bioeng-071811-150124, 10.1146/annurev.bioeng-071811-150124]
Alexopoulou L, 2001, NATURE, V413, P732, DOI 10.1038/35099560
Ali OA, 2011, PHARM RES-DORDR, V28, P1074, DOI 10.1007/s11095-010-0361-x
Ali OA, 2009, SCI TRANSL MED, V1, DOI 10.1126/scitranslmed.3000359
Ali OA, 2009, NAT MATER, V8, P151, DOI [10.1038/NMAT2357, 10.1038/nmat2357]
Ballester M, 2011, VACCINE, V29, P6959, DOI 10.1016/j.vaccine.2011.07.039
Basith S, 2011, EXPERT OPIN THER PAT, V21, P927, DOI 10.1517/13543776.2011.569494
BAZILE D, 1995, J PHARM SCI, V84, P493, DOI 10.1002/jps.2600840420
Benne N, 2016, J CONTROL RELEASE, V234, P124, DOI 10.1016/j.jconrel.2016.05.033
Blander JM, 2006, NATURE, V440, P808, DOI 10.1038/nature04596
BLUME G, 1990, BIOCHIM BIOPHYS ACTA, V1029, P91, DOI 10.1016/0005-2736(90)90440-Y
Bourquin C, 2008, J IMMUNOL, V181, P2990, DOI 10.4049/jimmunol.181.5.2990
Budhu S, 2014, CURR OPIN GENET DEV, V24, P46, DOI 10.1016/j.gde.2013.11.008
Bueter CL, 2011, J BIOL CHEM, V286, P35447, DOI 10.1074/jbc.M111.274936
Carroll EC, 2016, IMMUNITY, V44, P597, DOI 10.1016/j.immuni.2016.02.004
Coffman RL, 2010, IMMUNITY, V33, P492, DOI 10.1016/j.immuni.2010.10.002
Corrales L, 2015, CELL REP, V11, P1018, DOI 10.1016/j.celrep.2015.04.031
de Jong S, 2007, CANCER IMMUNOL IMMUN, V56, P1251, DOI 10.1007/s00262-006-0276-x
de Titta A, 2013, P NATL ACAD SCI USA, V110, P19902, DOI 10.1073/pnas.1313152110

Demento SL, 2010, J IMMUNOL, V185, P2989, DOI 10.4049/jimmunol.1000768
Demento SL, 2009, VACCINE, V27, P3013, DOI 10.1016/j.vaccine.2009.03.034
Deng LF, 2014, IMMUNITY, V41, P843, DOI 10.1016/j.immuni.2014.10.019
Diebold SS, 2004, SCIENCE, V303, P1529, DOI 10.1126/science.1093616
Diner EJ, 2013, CELL REP, V3, P1355, DOI 10.1016/j.celrep.2013.05.009
Drake CG, 2012, ANN ONCOL, V23, P41, DOI 10.1093/annonc/mds262
Dykman LA, 2011, ACTA NATURAE, V3, P34, DOI 10.32607/20758251-2011-3-2-34-56
FIDLER IJ, 1980, CANCER RES, V40, P4460
Flach TL, 2011, NAT MED, V17, P479, DOI 10.1038/nm.2306
Frohlich E, 2012, INT J NANOMED, V7, P5577, DOI 10.2147/IJN.S36111
Fu J, 2015, SCI TRANSL MED, V7, DOI 10.1126/scitranslmed.aaa4306
Gao P, 2013, CELL, V153, P1094, DOI 10.1016/j.cell.2013.04.046
Germain RN, 2010, IMMUNITY, V33, P441, DOI 10.1016/j.immuni.2010.09.014
Goldinger SM, 2012, EUR J IMMUNOL, V42, P3049, DOI 10.1002/eji.201142361
Gottardi R, 2013, J NANOBIOTECHNOL, V11, DOI 10.1186/1477-3155-11-30
Ha SJ, 2006, INFECT IMMUN, V74, P4954, DOI 10.1128/IAI.01781-05
Hafner AM, 2013, ADV DRUG DELIVER REV, V65, P1386, DOI 10.1016/j.addr.2013.05.013
Hanson MC, 2015, J CLIN INVEST, V125, P2532, DOI 10.1172/JCI79915
Heil F, 2004, SCIENCE, V303, P1526, DOI 10.1126/science.1093620
Hemmi H, 2000, NATURE, V408, P740, DOI 10.1038/35047123
Hildner K, 2008, SCIENCE, V322, P1097, DOI 10.1126/science.1164206
HORA MS, 1990, BIO-TECHNOL, V8, P755, DOI 10.1038/nbt0890-755
Hu Y, 2007, NANO LETT, V7, P3056, DOI 10.1021/nl071542i
Ignatius R, 2000, BLOOD, V96, P3505
Ilyinskii PO, 2014, VACCINE, V32, P2882, DOI 10.1016/j.vaccine.2014.02.027
Ioannou XP, 2003, ANTISENSE NUCLEIC A, V13, P157, DOI 10.1089/108729003768247628
Irvine DJ, 2015, CHEM REV, V115, P11109, DOI 10.1021/acs.chemrev.5b00109
Irvine DJ, 2013, NAT MATER, V12, P978, DOI [10.1038/NMAT3775, 10.1038/nmat3775]
Iwasaki A, 2004, NAT IMMUNOL, V5, P987, DOI 10.1038/ni1112
Jeanbart L, 2014, CANCER IMMUNOL RES, V2, P436, DOI 10.1158/2326-6066.CIR-14-0019-T
Johnson DA, 2008, CURR TOP MED CHEM, V8, P64, DOI 10.2174/156802608783378882
Johnston D, 2006, CANCER IMMUNOL IMMUN, V55, P412, DOI 10.1007/s00262-005-0013-x
Kamaly N, 2012, CHEM SOC REV, V41, P2971, DOI 10.1039/c2cs15344k
Kan P, 2011, J DRUG DELIV, V2011, DOI 10.1155/2011/629234
Kasturi SP, 2011, NATURE, V470, P543, DOI 10.1038/nature09737
Kawai T, 2011, IMMUNITY, V34, P637, DOI 10.1016/j.immuni.2011.05.006
Keller S, 2014, J CONTROL RELEASE, V191, P24, DOI 10.1016/j.jconrel.2014.03.041
KILLION JJ, 1994, CYTOKINE, V6, P443, DOI 10.1016/1043-4666(94)90069-8
KLEMPNER MS, 1990, NEW ENGL J MED, V322, P959, DOI 10.1056/NEJM199004053221404
KOFF WC, 1984, SCIENCE, V224, P1007, DOI 10.1126/science.6426057
Kreiter S, 2015, NATURE, V520, P692, DOI 10.1038/nature14426
Kuai R., 2016, NAT MAT
Le DT, 2015, J CLIN ONCOL, V33, P1325, DOI 10.1200/JCO.2014.57.4244
Leonard JP, 1997, BLOOD, V90, P2541
Li P, 2013, J CONTROL RELEASE, V168, P271, DOI 10.1016/j.jconrel.2013.03.025
Liu HP, 2014, NATURE, V507, P519, DOI 10.1038/nature12978
Lizotte PH, 2016, NAT NANOTECHNOL, V11, P295, DOI [10.1038/NNANO.2015.292, 10.1038/nnano.2015.292]
Luo M., 2017, NAT NANOTECHNOL
Luo X, 2012, J MATER SCI-MATER M, V23, P1685, DOI 10.1007/s10856-012-4632-4
Lynn GM, 2015, NAT BIOTECHNOL, V33, P1201, DOI 10.1038/nbt.3371
Malyala P, 2008, J PHARM SCI-US, V97, P1155, DOI 10.1002/jps.21065
Manolova V, 2008, EUR J IMMUNOL, V38, P1404, DOI 10.1002/eji.200737984
Marichal T, 2011, NAT MED, V17, P996, DOI 10.1038/nm.2403
Marshak-Rothstein A, 2007, ANNU REV IMMUNOL, V25, P419, DOI 10.1146/annurev.immunol.22.012703.104514
Medzhitov R, 2009, IMMUNITY, V30, P766, DOI 10.1016/j.immuni.2009.06.004
MELISSEN PMB, 1993, BIOTHERAPY, V6, P113, DOI 10.1007/BF01877424
Meng J, 2008, SMALL, V4, P1364, DOI 10.1002/smll.200701059
Merad M, 2013, ANNU REV IMMUNOL, V31, P563, DOI 10.1146/annurev-immunol-020711-074950
Miyabe H, 2014, J CONTROL RELEASE, V184, P20, DOI 10.1016/j.jconrel.2014.04.004

Moon JJ, 2012, P NATL ACAD SCI USA, V109, P1080, DOI 10.1073/pnas.1112648109
Moon JJ, 2011, NAT MATER, V10, P243, DOI 10.1038/NMAT2960
Moynihan KD, 2016, NAT MED, V22, P1402, DOI 10.1038/nm.4200
Nakamura T, 2015, J CONTROL RELEASE, V216, P149, DOI 10.1016/j.jconrel.2015.08.026
Neelapu SS, 2007, BLOOD, V109, P5160, DOI 10.1182/blood-2006-12-063594
Neelapu SS, 2004, CLIN CANCER RES, V10, P8309, DOI 10.1158/1078-0432.CCR-04-1071
Nembrini C, 2011, P NATL ACAD SCI USA, V108, pE989, DOI 10.1073/pnas.1104264108
Ng G, 2008, IMMUNITY, V29, P807, DOI 10.1016/j.immuni.2008.09.013
Nicholaou T, 2011, CANCER IMMUNOL IMMUN, V60, P1625, DOI 10.1007/s00262-011-1041-3
Orr MT, 2014, PLOS ONE, V9, DOI 10.1371/journal.pone.0083884
Palucka K, 2016, NAT MED, V22, P1390, DOI 10.1038/nm.4249
Petersen LK, 2011, BIOMATERIALS, V32, P6815, DOI 10.1016/j.biomaterials.2011.05.063
Poltorak A, 1998, SCIENCE, V282, P2085, DOI 10.1126/science.282.5396.2085
Popescu MC, 2007, BLOOD, V109, P5407, DOI 10.1182/blood-2006-08-039446
Pulendran B, 2011, NAT IMMUNOL, V12, P509, DOI 10.1038/ni.2039
Quandt D, 2015, SCI REP-UK, V5, DOI 10.1038/srep16719
Radovic-Moreno AF, 2015, P NATL ACAD SCI USA, V112, P3892, DOI 10.1073/pnas.1502850112
Raghuvanshi RS, 2002, INT J PHARMACEUT, V245, P109, DOI 10.1016/S0378-5173(02)00342-3
Rao DA, 2010, J PHARM SCI-US, V99, P2018, DOI 10.1002/jps.21970
Rappuoli R, 2011, NATURE, V473, P463, DOI 10.1038/nature10124
Reddy ST, 2007, NAT BIOTECHNOL, V25, P1159, DOI [10.1038/nbt1332, 10.1038/nbtl332]
Reddy ST, 2006, TRENDS IMMUNOL, V27, P573, DOI 10.1016/j.it.2006.10.005
Reddy ST, 2006, J CONTROL RELEASE, V112, P26, DOI 10.1016/j.jconrel.2006.01.006
Rietscher R, 2016, EUR J PHARM BIOPHARM, V102, P20, DOI 10.1016/j.ejpb.2016.02.014
Rosenberg SA, 2015, SCIENCE, V348, P62, DOI 10.1126/science.aaa4967
Rozenfeld JHK, 2012, J CONTROL RELEASE, V160, P367, DOI 10.1016/j.jconrel.2011.10.017
Ruizendaal L, 2009, NANOTOXICOLOGY, V3, P339, DOI 10.3109/17435390903288896
Sah H, 2013, INT J NANOMED, V8, P747, DOI 10.2147/IJN.S40579
Sahdev P, 2014, PHARM RES-DORDR, V31, P2563, DOI 10.1007/s11095-014-1419-y
Sauer JD, 2011, INFECT IMMUN, V79, P688, DOI 10.1128/IAI.00999-10
Schenten D, 2011, ADV IMMUNOL, V109, P87, DOI 10.1016/B978-0-12-387664-5.00003-0
Schnurr M, 2009, J IMMUNOL, V182, P1253, DOI 10.4049/jimmunol.182.3.1253
Schumacher TN, 2015, SCIENCE, V348, P69, DOI 10.1126/science.aaa4971
Seong SY, 2004, NAT REV IMMUNOL, V4, P469, DOI 10.1038/nri1372
Sharma P, 2015, SCIENCE, V348, P56, DOI 10.1126/science.aaa8172
Sharp FA, 2009, P NATL ACAD SCI USA, V106, P870, DOI 10.1073/pnas.0804897106
Smith DM, 2013, NAT REV IMMUNOL, V13, P592, DOI 10.1038/nri3488
Sporri R, 2005, NAT IMMUNOL, V6, P163, DOI 10.1038/ni1162
Standley SM, 2007, BIOCONJUGATE CHEM, V18, P77, DOI 10.1021/bc060165i
Stano A, 2011, VACCINE, V29, P804, DOI 10.1016/j.vaccine.2010.11.010
Steers NJ, 2009, VACCINE, V27, P6939, DOI 10.1016/j.vaccine.2009.08.105
Sun LJ, 2013, SCIENCE, V339, P786, DOI 10.1126/science.1232458
Tacken PJ, 2011, BLOOD, V118, P6836, DOI 10.1182/blood-2011-07-367615
Taneichi M, 2006, J IMMUNOL, V177, P2324, DOI 10.4049/jimmunol.177.4.2324
van der Burg SH, 2016, NAT REV CANCER, V16, P219, DOI 10.1038/nrc.2016.16
Varkouhi AK, 2011, J CONTROL RELEASE, V151, P220, DOI 10.1016/j.jconrel.2010.11.004
Vasdekis AE, 2012, ACS NANO, V6, P7850, DOI 10.1021/nn302122h
Vyas SP, 2004, INT J PHARM, V269, P37, DOI 10.1016/j.ijpharm.2003.08.017
Wang H, 2017, P NATL ACAD SCI USA, V114, P1637, DOI 10.1073/pnas.1621363114
Wang J, 2011, SMALL, V7, P1919, DOI 10.1002/smll.201100442
Watson DS, 2012, VACCINE, V30, P2256, DOI 10.1016/j.vaccine.2012.01.070
Wille-Reece U, 2006, J EXP MED, V203, P1249, DOI 10.1084/jem.20052433
Yan SY, 2013, J COLLOID INTERF SCI, V395, P1, DOI 10.1016/j.jcis.2012.11.061
Yuba E, 2013, BIOMATERIALS, V34, P3042, DOI 10.1016/j.biomaterials.2012.12.031
Zanganeh S, 2016, NAT NANOTECHNOL, V11, P986, DOI [10.1038/nnano.2016.168, 10.1038/NNANO.2016.168]
Zhang AH, 2016, CELL IMMUNOL, V301, P74, DOI 10.1016/j.cellimm.2015.11.004
NR 140
TC 77
Z9 77
U1 3

U2 113
PU ELSEVIER
PI AMSTERDAM
PA RADARWEG 29, 1043 NX AMSTERDAM, NETHERLANDS
SN 0168-3659
EI 1873-4995
J9 J CONTROL RELEASE
JI J. Control. Release
PD OCT 10
PY 2017
VL 263
SI SI
BP 200
EP 210
DI 10.1016/j.jconrel.2017.03.033
PG 11
WC Chemistry, Multidisciplinary; Pharmacology & Pharmacy
WE Science Citation Index Expanded (SCI-EXPANDED); Conference Proceedings Citation Index - Science (CPCI-S)
SC Chemistry; Pharmacology & Pharmacy
GA FH5JQ
UT WOS:000411202400021
PM 28336379
OA Green Accepted
DA 2023-09-14
ER

PT J
AU Zhou, L
Hou, B
Wang, DG
Sun, F
Song, RD
Shao, Q
Wang, H
Yu, HJ
Li, YP
AF Zhou, Lei
Hou, Bo
Wang, Dangge
Sun, Fang
Song, Rundi
Shao, Qian
Wang, Hao
Yu, Haijun
Li, Yaping
TI Engineering Polymeric Prodrug Nanoplatform for Vaccination Immunotherapy
of Cancer
SO NANO LETTERS
LA English
DT Article
DE Polymeric prodrug; Neoantigen; Adaptive immune response; Personalized
immunotherapy; Nanoparticles
ID INNATE IMMUNE RECOGNITION; DENDRITIC CELLS; POTENT; DELIVERY;
MICROENVIRONMENT; NANOPARTICLES; MICELLEPLEXES; INTERFERONS;
NANOVACCINE; ACTIVATION
AB Neoantigen-based cancer vaccines are promising for boosting cytotoxic T lymphocyte (CTL) responses. However, the therapeutic effect of cancer vaccines is severely blunted by functional suppression of the dendritic cells (DCs). Herein, we demonstrated an acid-responsive polymeric nanovaccine for activating the stimulator of interferon genes (STING) pathway and improving cancer immunotherapy. The nanovaccines were fabricated by integrating an acid-activatable polymeric conjugate

of the STING agonist and neoantigen into one single nanoplatform. The nanovaccines efficiently accumulated at the lymph nodes for promoting DC uptake and facilitating cytosol release of the neoantigens. Meanwhile, the STING agonist activated the STING pathway in the DCs to elicit interferon-beta secretion and to boost T-cell priming with the neoantigen. The nanovaccine dramatically inhibited tumor growth and occurrence of B16-OVA melanoma and 4T1 breast tumors in immunocompetent mouse models. Combination immunotherapy with the nanovaccines and anti-PD-L1 antibody demonstrated further improved antitumor efficacy in a 4T1 breast tumor model.
C1 [Zhou, Lei; Hou, Bo; Wang, Dangge; Sun, Fang; Song, Rundi; Yu, Haijun; Li, Yaping] Chinese Acad Sci, Shanghai Inst Mat Med, State Key Lab Drug Res, Shanghai 201203, Peoples R China.
[Zhou, Lei; Hou, Bo; Wang, Dangge; Sun, Fang; Song, Rundi; Yu, Haijun; Li, Yaping] Chinese Acad Sci, Shanghai Inst Mat Med, Ctr Pharmaceut, Shanghai 201203, Peoples R China.
[Yu, Haijun; Li, Yaping] Yantai Inst Mat Med, Yantai Key Lab Nanomedicole & Adv Preparat, Yantai 264000, Shandong, Peoples R China.
[Zhou, Lei; Wang, Hao] China State Inst Pharmaceut Ind, Shanghai 201203, Peoples R China.
[Shao, Qian] Shandong Inst Canc Prevent & Treatment, Jinan 250117, Shandong, Peoples R China.
C3 Chinese Academy of Sciences; Shanghai Institute of Materia Medica, CAS;
Chinese Academy of Sciences; Shanghai Institute of Materia Medica, CAS
RP Yu, HJ; Li, YP (通讯作者)，Chinese Acad Sci, Shanghai Inst Mat Med, State Key Lab Drug Res, Shanghai 201203, Peoples R China.; Yu, HJ; Li, YP (通讯作者)，Chinese Acad Sci, Shanghai Inst Mat Med, Ctr Pharmaceut, Shanghai 201203, Peoples R China.; Yu, HJ; Li, YP (通讯作者)，Yantai Inst Mat Med, Yantai Key Lab Nanomedicole & Adv Preparat, Yantai 264000, Shandong, Peoples R China.
EM hjyu@simm.ac.cn; ypli@simm.ac.cn
RI Yu, Haijun/C-5343-2016; Li, Jinjun/I-3956-2015; Wang, Dangge/HCH-6585-2022
OI Yu, Haijun/0000-0002-3398-0880; Li, Jinjun/0000-0003-4008-9022;
FU National Natural Science Foundation of China [51873228, 31671024, 81521005]; Natural Science Foundation of Shandong Province [ZR2019LZL020]; State Key Laboratory of Drug Research [SIMM2004KF-04]
FX Financial support from the National Natural Science Foundation of China (51873228, 31671024, and 81521005), the Natural Science Foundation of Shandong Province (ZR2019LZL020), and Open Fund of the State Key Laboratory of Drug Research (SIMM2004KF-04) is gratefully acknowledged. All animal procedures were carried out under the guidelines approved by the Institutional Animal Care and Use Committee (IACUC) of the Shanghai Institute of Materia Medica, Chinese Academy of Sciences.
CR Amsen D, 2018, NAT IMMUNOL, V19, P538, DOI 10.1038/s41590-018-0114-2
Anguille S, 2014, LANCET ONCOL, V15, pE257, DOI 10.1016/S1470-2045(13)70585-0
Binnewies M, 2018, NAT MED, V24, P541, DOI 10.1038/s41591-018-0014-x
Chen L, 2019, ADV MATER, V31, DOI 10.1002/adma.201904997
Chen Q, 2019, ADV MATER, V31, DOI 10.1002/adma.201802228
Corrales L, 2015, CLIN CANCER RES, V21, P4774, DOI 10.1158/1078-0432.CCR-15-1362
Corrales L, 2015, CELL REP, V11, P1018, DOI 10.1016/j.celrep.2015.04.031
Curran E, 2016, CELL REP, V15, P2357, DOI 10.1016/j.celrep.2016.05.023
da Silva JL, 2019, CANCER CHEMOTH PHARM, V84, P227, DOI 10.1007/s00280-019-03894-3
Dashti A, 2016, CANCER LETT, V374, P175, DOI 10.1016/j.canlet.2016.01.021
Dunn GP, 2006, NAT REV IMMUNOL, V6, P836, DOI 10.1038/nri1961
Eisenbarth SC, 2019, NAT REV IMMUNOL, V19, P89, DOI 10.1038/s41577-018-0088-1
Feng B, 2020, ADV FUNCT MATER, V30, DOI 10.1002/adfm.201906605
Feng B, 2019, ADV MATER, V31, DOI 10.1002/adma.201902960
Ferrone S, 2004, NAT BIOTECHNOL, V22, P1096, DOI 10.1038/nbt0904-1096
Galon J, 2019, NAT REV DRUG DISCOV, V18, P197, DOI 10.1038/s41573-018-0007-y
Geng F, 2019, VACCINE, V37, P4382, DOI 10.1016/j.vaccine.2019.06.012
Guo YY, 2015, ACS NANO, V9, P6918, DOI 10.1021/acsnano.5b01042
Guo YG, 2018, FRONT IMMUNOL, V9, DOI 10.3389/fimmu.2018.01499
Keskin DB, 2019, NATURE, V565, P234, DOI 10.1038/s41586-018-0792-9
Kranz LM, 2016, NATURE, V534, P396, DOI 10.1038/nature18300
Kuai R, 2017, NAT MATER, V16, P489, DOI [10.1038/nmat4822, 10.1038/NMAT4822]
Le Gall CM, 2018, NAT MATER, V17, P474, DOI 10.1038/s41563-018-0093-6
Li AW, 2018, NAT MATER, V17, P528, DOI 10.1038/s41563-018-0028-2
Luo M, 2017, NAT NANOTECHNOL, V12, P648, DOI [10.1038/nnano.2017.52, 10.1038/NNANO.2017.52]

Meng QY, 2019, POLYM CHEM-UK, V10, P306, DOI 10.1039/c8py01160e
Miao L, 2019, NAT BIOTECHNOL, V37, P1174, DOI 10.1038/s41587-019-0247-3
Ott PA, 2017, NATURE, V547, P217, DOI 10.1038/nature22991
Palucka K, 2012, NAT REV CANCER, V12, P265, DOI 10.1038/nrc3258
Parkhurst MR, 2019, CANCER DISCOV, V9, P1022, DOI 10.1158/2159-8290.CD-18-1494
Patel S, 2020, NAT COMMUN, V11, DOI 10.1038/s41467-020-14527-2
Phuengkham H, 2019, ADV MATER, V31, DOI 10.1002/adma.201803322
Qiu NS, 2016, ADV MATER, V28, P10613, DOI 10.1002/adma.201603095
Riley RS, 2019, NAT REV DRUG DISCOV, V18, P175, DOI 10.1038/s41573-018-0006-z
Saeed M, 2019, THERANOSTICS, V9, P7981, DOI 10.7150/thno.37568
Shae D, 2019, NAT NANOTECHNOL, V14, P269, DOI 10.1038/s41565-018-0342-5
Tan Y, 2015, BIOMATERIALS, V56, P219, DOI 10.1016/j.biomaterials.2015.03.049
Voorwerk L, 2019, NAT MED, V25, P920, DOI 10.1038/s41591-019-0432-4
Wang DG, 2019, SCI IMMUNOL, V4, DOI 10.1126/sciimmunol.aau6584
Wang DG, 2016, NANO LETT, V16, P5503, DOI 10.1021/acs.nanolett.6b01994
Wang RF, 2017, CELL RES, V27, P11, DOI 10.1038/cr.2016.155
Wang TT, 2018, NAT COMMUN, V9, DOI 10.1038/s41467-018-03915-4
Wang TT, 2016, ACS NANO, V10, P3496, DOI 10.1021/acsnano.5b07706
Wegmann F, 2012, NAT BIOTECHNOL, V30, P883, DOI 10.1038/nbt.2344
Wilson JT, 2013, ACS NANO, V7, P3912, DOI 10.1021/nn305466z
Woo SR, 2015, ANNU REV IMMUNOL, V33, P445, DOI 10.1146/annurev-immunol-032414-112043
Woo SR, 2014, IMMUNITY, V41, P830, DOI 10.1016/j.immuni.2014.10.017
Yang R, 2018, ACS NANO, V12, P5121, DOI 10.1021/acsnano.7b09041
Yang W, 2019, NAT MED, V25, P767, DOI 10.1038/s41591-019-0434-2
Yu HJ, 2011, ACS NANO, V5, P9246, DOI 10.1021/nn203503h
Zhang LH, 2019, NANO LETT, V19, P4237, DOI 10.1021/acs.nanolett.9b00030
Zhou FY, 2019, ADV MATER, V31, DOI 10.1002/adma.201805888
Zhou Q, 2019, NAT NANOTECHNOL, V14, P799, DOI 10.1038/s41565-019-0485-z
Zitvogel L, 2015, NAT REV IMMUNOL, V15, P405, DOI 10.1038/nri3845
NR 54
TC 76
Z9 77
U1 20
U2 230
PU AMER CHEMICAL SOC
PI WASHINGTON
PA 1155 16TH ST, NW, WASHINGTON, DC 20036 USA
SN 1530-6984
EI 1530-6992
J9 NANO LETT
JI Nano Lett.
PD JUN 10
PY 2020
VL 20
IS 6
BP 4393
EP 4402
DI 10.1021/acs.nanolett.0c01140
PG 10
WC Chemistry, Multidisciplinary; Chemistry, Physical; Nanoscience & Nanotechnology; Materials Science, Multidisciplinary; Physics, Applied; Physics, Condensed Matter
WE Science Citation Index Expanded (SCI-EXPANDED)
SC Chemistry; Science & Technology - Other Topics; Materials Science; Physics
GA MA1PZ
UT WOS:000541691200043
PM 32459969
DA 2023-09-14
ER

PT J
AU El-Sayed, A
Kamel, M
AF El-Sayed, Amr
Kamel, Mohamed
TI Advances in nanomedical applications: diagnostic, therapeutic, immunization, and vaccine production
SO ENVIRONMENTAL SCIENCE AND POLLUTION RESEARCH
LA English
DT Article
DE Cancer therapy; Imaging agents; Nanotechnology; Nanomedicine; Nanovaccines
ID GANGLIOSIDE-LIPOSOME IMMUNOASSAY; BLOOD-BRAIN-BARRIER; MAGNETIC NANOPARTICLES; DRUG-RESISTANCE; GENE DELIVERY; CANCER-THERAPY; QUANTUM DOTS; NUCLEIC-ACID; IRON-OXIDE; NANOTECHNOLOGY
AB In the last decades, nanotechnology-based tools started to draw the attention of research worldwide. They offer economic, rapid, effective, and highly specific solutions for most medical issues. As a result, the international demand of nanomaterials is expanding very rapidly. It was estimated that the market of nanomaterials was about $2.6 trillion in 2015. In medicine, various applications of nanotechnology proved their potential to revolutionize medical diagnosis, immunization, treatment, and even health care products. The loading substances can be coupled with a large set of nanoparticles (NPs) by many means: chemically (conjugation), physically (encapsulation), or via adsorption. The use of the suitable loading nanosubstance depends on the application purpose. They can be used to deliver various chemicals (drugs, chemotherapeutic agents, or imaging substances), or biological substances (antigens, antibodies, RNA, or DNA) through endocytosis. They can even be used to deliver light and heat to their target cells when needed. The present review provides a brief overview about the structure and shape of available NPs and discusses their applications in the medical sciences.
C1 [El-Sayed, Amr; Kamel, Mohamed] Cairo Univ, Dept Med & Infect Dis, Fac Vet Med, Giza, Egypt.
C3 Egyptian Knowledge Bank (EKB); Cairo University
RP Kamel, M (通讯作者)，Cairo Univ, Dept Med & Infect Dis, Fac Vet Med, Giza, Egypt.
EM m_salah@staff.cu.edu.eg
RI El-Sayed, Amr/AAT-4055-2020
CR Ahn-Yoon S, 2003, ANAL CHEM, V75, P2256, DOI 10.1021/ac026428t
Ahn-Yoon S, 2004, ANAL BIOANAL CHEM, V378, P68, DOI 10.1007/s00216-003-2365-4
[Anonymous], NANOTHERAPEUTICS CAN
[Anonymous], 2013, MADAME CURIE BIOSCIE
[Anonymous], 2010, HDB NANOPHYSICS NANO
[Anonymous], J EVOL TECHNOL
[Anonymous], 2017, NANOAND MICROSCALE D
Aparicio MG, 2013, REP PRACT ONCOL RADI, V2, P9
Awate S, 2013, FRONT IMMUNOL, V4, DOI 10.3389/fimmu.2013.00114
Banobre-Lopez M, 2013, REP PRACT ONCOL RADI, V18, P397, DOI 10.1016/j.rpor.2013.09.011
Beyth N, 2015, EVID-BASED COMPL ALT, V2015, DOI 10.1155/2015/246012
Blanco E, 2011, CANCER SCI, V102, P1247, DOI 10.1111/j.1349-7006.2011.01941.x
Brahim S, 2002, BIOSENS BIOELECTRON, V17, P973, DOI 10.1016/S0956-5663(02)00089-1
Braicu C, 2015, INT J NANOMED, V10, P791, DOI 10.2147/IJN.S72904
Brede C, 2013, ADV CHRONIC KIDNEY D, V20, P454, DOI 10.1053/j.ackd.2013.07.006
Cao Y, 2011, FUTURE ONCOL, V7, P1347, DOI [10.2217/fon.11.109, 10.2217/FON.11.109]
Casals E, 2017, CANCER NANOTECHNOL, V8, DOI 10.1186/s12645-017-0030-4
Cena V, 2018, NANOMEDICINE-UK, V13, P1513, DOI 10.2217/nnm-2018-0139
Chakravarthi PV, 2010, VET WORLD, V3
Chapman S, 2013, NANO TODAY, V8, P454, DOI 10.1016/j.nantod.2013.06.001
Cheng R, 2011, J CONTROL RELEASE, V152, P2, DOI 10.1016/j.jconrel.2011.01.030
Cho SK, 2013, BIOMATERIALS, V34, P10228, DOI 10.1016/j.biomaterials.2013.09.003
Chu M, 2017, WORLD SCI
Dastjerdi R, 2010, COLLOID SURFACE B, V79, P5, DOI 10.1016/j.colsurfb.2010.03.029
Dear JW, 2006, NEPHRON CLIN PRACT, V103, pC45, DOI 10.1159/000090608
Dobrovolskaia MA, 2016, TOXICOL APPL PHARM, V299, P78, DOI 10.1016/j.taap.2015.12.022
Duan LL, 2005, BMC INFECT DIS, V5, DOI 10.1186/1471-2334-5-53
Ebrahim S, 2015, SPECTROCHIM ACTA A, V150, P212, DOI 10.1016/j.saa.2015.05.042
El-Sayed A, 2018, INT J VET SCI MED, V6, P8, DOI 10.1016/j.ijvsm.2018.05.001

Elgqvist J, 2017, INT J MOL SCI, V18, DOI 10.3390/ijms18051102
Fang C, 2015, ACS APPL MATER INTER, V7, P6674, DOI 10.1021/am5092165
Fortin JP, 2008, EUR BIOPHYS J BIOPHY, V37, P223, DOI 10.1007/s00249-007-0197-4
Freitas RA, 2005, J COMPUT THEOR NANOS, V2, P1
Ganesan P, 2015, INT J NANOMED, V10, P6757, DOI 10.2147/IJN.S93918
Gao W, 2017, INT J NANOMED, V12, P1047, DOI 10.2147/IJN.S115215
Gbadamosi JK, 2002, FEBS LETT, V532, P338, DOI 10.1016/S0014-5793(02)03710-9
Goluch ED, 2006, LAB CHIP, V6, P1293, DOI 10.1039/b606294f
Gu F, 2001, CELL MOL LIFE SCI, V58, P1067, DOI 10.1007/PL00000922
Gupta A, 2016, J AM HEART ASSOC, V5, DOI 10.1161/JAHA.116.003816
Hajipour MJ, 2012, TRENDS BIOTECHNOL, V30, P499, DOI 10.1016/j.tibtech.2012.06.004
Hameed ASH, 2016, SCI REP-UK, V6, DOI 10.1038/srep24312
Herbst RS, 2014, NATURE, V515, P563, DOI 10.1038/nature14011
Hirsch LR, 2003, P NATL ACAD SCI USA, V100, P13549, DOI 10.1073/pnas.2232479100
Ishizaki T, 2016, NANOMATERIALS-BASEL, V6, DOI 10.3390/nano6090172
Jarockyte G, 2016, INT J MOL SCI, V17, DOI 10.3390/ijms17081193
Jeevanandam J, 2018, BEILSTEIN J NANOTECH, V9, P1050, DOI 10.3762/bjnano.9.98
Jurj A, 2017, DRUG DES DEV THER, V11, P2871, DOI 10.2147/DDDT.S142337
Kamel M., 2018, ENERGY, P1
Kamel M, 2019, VIRUS RES, V270, DOI 10.1016/j.virusres.2019.197648
Kamel M, 2019, ARCH VIROL, V164, P1501, DOI 10.1007/s00705-019-04216-x
Klostranec JM, 2007, NANO LETT, V7, P2812, DOI 10.1021/nl071415m
Kozielski KL, 2016, EXPERT OPIN DRUG DEL, V13, P1475, DOI 10.1080/17425247.2016.1190707
Kreuter J, 2007, INT J PHARMACEUT, V331, P1, DOI 10.1016/j.ijpharm.2006.10.021
Lakshmipathy U, 2004, STEM CELLS, V22, P531, DOI 10.1634/stemcells.22-4-531
Lee WC, 2017, CLIN PROTEOM, V14, DOI 10.1186/s12014-017-9164-y
Lehto T, 2016, ADV DRUG DELIVER REV, V106, P172, DOI 10.1016/j.addr.2016.06.008
Leoni L, 2004, ADV DRUG DELIVER REV, V56, P211, DOI 10.1016/j.addr.2003.08.014
Li D, 2014, INT J NANOMED, V9, P3347, DOI 10.2147/IJN.S61463
Li FY, 2017, BIOSENS BIOELECTRON, V87, P630, DOI 10.1016/j.bios.2016.09.018
Livney YD, 2013, ADV DRUG DELIVER REV, V65, P1716, DOI 10.1016/j.addr.2013.08.006
Luzio JP, 2007, NAT REV MOL CELL BIO, V8, P622, DOI 10.1038/nrm2217
Mahmoudi M, 2012, ACS NANO, V6, P2656, DOI 10.1021/nn300042m
Manolova V, 2008, EUR J IMMUNOL, V38, P1404, DOI 10.1002/eji.200737984
Manuja A., 2012, NANOTECHNOL DEV, V2, P4
Meena N., 2018, VET WORLD, V3, P477
Minchin RF, 2010, ENDOCRINOLOGY, V151, P474, DOI 10.1210/en.2009-1012
Mitchell LA, 2009, NAT NANOTECHNOL, V4, P451, DOI [10.1038/NNANO.2009.151, 10.1038/nnano.2009.151]
Mohantya N., 2014, VET RES, V2, P90
Monami G, 2009, AM J PATHOL, V174, P1037, DOI 10.2353/ajpath.2009.080735
Morilla MJ, 2005, J CONTROL RELEASE, V103, P599, DOI 10.1016/j.jconrel.2004.12.012
Nordly P, 2009, EXPERT OPIN DRUG DEL, V6, P657, DOI 10.1517/17425240903018863
Parvanian Sepideh, 2017, Sensing and Bio-Sensing Research, V13, P81, DOI 10.1016/j.sbsr.2016.08.002
Patil SS, 2009, VET WORLD, V2, P475, DOI 10.5455/vetworld.2009.475-477
Pedrosa P, 2015, NANOTECHNOLOGY DIAGN, P1
Peng LH, 2014, BIOMATERIALS, V35, P5605, DOI 10.1016/j.biomaterials.2014.03.062
Pineiro Y, 2015, EUR J INORG CHEM, P4495, DOI 10.1002/ejic.201500598
Probst CE, 2013, ADV DRUG DELIVER REV, V65, P703, DOI 10.1016/j.addr.2012.09.036
Qasim M, 2014, J NANOSCI NANOTECHNO, V14, P7374, DOI 10.1166/jnn.2014.9578
Qin DL, 2007, J BIOMED BIOTECHNOL, DOI 10.1155/2007/89364
Rapoport N, 2007, JNCI-J NATL CANCER I, V99, P1095, DOI 10.1093/jnci/djm043
Remiao MH, 2018, FRONT PHARMACOL, V8, DOI 10.3389/fphar.2017.00994
Rettig L, 2010, BLOOD, V115, P4533, DOI 10.1182/blood-2009-11-247817
Revia RA, 2016, MATER TODAY, V19, P157, DOI 10.1016/j.mattod.2015.08.022
Riley MK, 2017, NANOMATERIALS-BASEL, V7, DOI 10.3390/nano7050094
Rivas J, 2012, J MAGN MAGN MATER, V324, P3499, DOI 10.1016/j.jmmm.2012.02.075
Rizzo LY, 2013, CURR OPIN BIOTECH, V24, P1159, DOI 10.1016/j.copbio.2013.02.020
Rodriguez-Burneo N, 2017, NANOMATERIALS-BASEL, V7, DOI 10.3390/nano7070190
Ryan JJ, 2007, J IMMUNOL, V179, P665, DOI 10.4049/jimmunol.179.1.665

Seleem MN, 2009, FEMS MICROBIOL LETT, V294, P24, DOI 10.1111/j.1574-6968.2009.01530.x
Shangguan JF, 2015, ANALYST, V140, P4489, DOI 10.1039/c5an00535c
Sharma A, 2004, INT J ANTIMICROB AG, V24, P599, DOI 10.1016/j.ijantimicag.2004.07.010
Sheikh FA, 2015, J MECH BEHAV BIOMED, V41, P189, DOI 10.1016/j.jmbbm.2014.10.012
Son S, 2012, ACCOUNTS CHEM RES, V45, P1100, DOI 10.1021/ar200248u
Sridharan K, 2016, BRIT J CLIN PHARMACO, V82, P659, DOI 10.1111/bcp.12987
Swain PS, 2015, VET WORLD, V8, P888, DOI 10.14202/vetworld.2015.888-891
Talukdar D, 2014, CURR PHARM BIOTECHNO, V15, P1158, DOI 10.2174/1389201015666141122204859
Tang F, 2016, NANOSCALE, V8, P4688, DOI 10.1039/c5nr07424j
Torres-Sangiao E, 2016, MOLECULES, V21, DOI 10.3390/molecules21070867
Troncarelli MZ, 2013, FORMATEX, V13, P543
Turcheniuk K, 2016, RSC ADV, V6, P1600, DOI 10.1039/c5ra24662h
Underwood C, 2012, VET J, V193, P12, DOI 10.1016/j.tvjl.2012.01.002
Varkouhi AK, 2011, J CONTROL RELEASE, V151, P220, DOI 10.1016/j.jconrel.2010.11.004
Venkatesan J, 2014, J BIOMED NANOTECHNOL, V10, P3124, DOI 10.1166/jbn.2014.1893
Villaverde A, 2011, NANOPARTICLES TRANSL
Vinogradov S, 2012, NANOMEDICINE-UK, V7, P597, DOI [10.2217/NNM.12.22, 10.2217/nnm.12.22]
Walter EJ, 2016, CRIT CARE, V20, DOI 10.1186/s13054-016-1376-4
Wang K, 2016, SMALL, V12, P477, DOI 10.1002/smll.201501985
Wang QY, 2016, NANOSCALE RES LETT, V11, DOI 10.1186/s11671-016-1359-z
Williams DF, 2008, BIOMATERIALS, V29, P2941, DOI 10.1016/j.biomaterials.2008.04.023
Xiang SD, 2010, WIRES NANOMED NANOBI, V2, P205, DOI 10.1002/wnan.88
Xing RJ, 2014, PHARM RES-DORDR, V31, P1377, DOI 10.1007/s11095-013-1205-2
Xu CJ, 2013, ADV DRUG DELIVER REV, V65, P732, DOI 10.1016/j.addr.2012.10.008
Yi H, 2016, BONE RES, V4, DOI 10.1038/boneres.2016.50
Zhang CY, 2010, ANAL CHEM, V82, P1921, DOI 10.1021/ac9026675
Zhang LJ, 2009, NANO TODAY, V4, P66, DOI 10.1016/j.nantod.2008.10.014
Zhao L, 2014, VACCINE, V32, P327, DOI 10.1016/j.vaccine.2013.11.069
Zhou YQ, 2018, J CONTROL RELEASE, V270, P290, DOI 10.1016/j.jconrel.2017.12.015
NR 117
TC 75
Z9 77
U1 0
U2 12
PU SPRINGER HEIDELBERG
PI HEIDELBERG
PA TIERGARTENSTRASSE 17, D-69121 HEIDELBERG, GERMANY
SN 0944-1344
EI 1614-7499
J9 ENVIRON SCI POLLUT R
JI Environ. Sci. Pollut. Res.
PD JUN
PY 2020
VL 27
IS 16
SI SI
BP 19200
EP 19213
DI 10.1007/s11356-019-06459-2
PG 14
WC Environmental Sciences
WE Science Citation Index Expanded (SCI-EXPANDED)
SC Environmental Sciences & Ecology
GA MI4MP
UT WOS:000547384500022
PM 31529348
OA Bronze
DA 2023-09-14
ER

PT J
AU Yin, Y
Li, XY
Ma, HX
Zhang, J
Yu, D
Zhao, RF
Yu, SJ
Nie, GJ
Wang, H
AF Yin, Yue
Li, Xiaoyang
Ma, Haixia
Zhang, Jie
Yu, Di
Zhao, Ruifang
Yu, Shengji
Nie, Guangjun
Wang, Hai
TI In Situ Transforming RNA Nanovaccines from Polyethylenimine
Functionalized Graphene Oxide Hydrogel for Durable Cancer Immunotherapy
SO NANO LETTERS
LA English
DT Article
DE RNA vaccine; immunotherapy; long-term release; graphene oxide;
polyethylenimine
AB Messenger RNA (mRNA) vaccine is a promising candidate in cancer immunotherapy as it can encode tumor-associated antigens with an excellent safety profile. Unfortunately, the inherent instability of RNA and translational efficiency are major limitations of RNA vaccine. Here, we report an injectable hydrogel formed with graphene oxide (GO) and polyethylenimine (PEI), which can generate mRNA (ovalbumin, a model antigen) and adjuvants (R848)-laden nanovaccines for at least 30 days after subcutaneous injection. The released nanovaccines can protect the mRNA from degradation and confer targeted delivering capacity to lymph nodes. The data show that this transformable hydrogel can significantly increase the number of antigen-specific CD8(+) T cells and subsequently inhibit the tumor growth with only one treatment. Meanwhile, this hydrogel can generate an antigen specific antibody in the serum which in turn prevents the occurrence of metastasis. Collectively, these results demonstrate the potential of the PEI-functionalized GO transformable hydrogel for effective cancer immunotherapy.
C1 [Yin, Yue; Li, Xiaoyang; Ma, Haixia; Zhang, Jie; Zhao, Ruifang; Nie, Guangjun; Wang, Hai] Natl Ctr Nanosci & Technol, CAS Ctr Excellence Nanosci, CAS Key Lab Biomed Effects Nanomat & Nanosafety, Beijing 100190, Peoples R China.
[Li, Xiaoyang; Yu, Shengji] Chinese Acad Med Sci & Peking Union Med Coll, Dept Orthoped, Natl Canc Ctr, Natl Clin Res Ctr Canc,Canc Hosp, Beijing 100021, Peoples R China.
[Yu, Di] Uppsala Univ, Dept Immunol Genet & Pathol, Sci Life Lab, S-75185 Uppsala, Sweden.
[Nie, Guangjun; Wang, Hai] Univ Chinese Acad Sci, Beijing 100049, Peoples R China.
C3 Chinese Academy of Sciences; National Center for Nanoscience &
Technology - China; Chinese Academy of Medical Sciences - Peking Union
Medical College; Cancer Institute & Hospital - CAMS; Peking Union
Medical College; Uppsala University; Chinese Academy of Sciences;
University of Chinese Academy of Sciences, CAS
RP Nie, GJ; Wang, H (通讯作者)，Natl Ctr Nanosci & Technol, CAS Ctr Excellence Nanosci, CAS Key Lab Biomed Effects Nanomat & Nanosafety, Beijing 100190, Peoples R China.; Nie, GJ; Wang, H (通讯作者)，Univ Chinese Acad Sci, Beijing 100049, Peoples R China.
EM niegj@nanoctr.cn; wanghai@nanoctr.cn
RI Yu, Di/G-9784-2018; Ma, Haixia/ABC-4707-2021
OI Ma, Haixia/0000-0001-9080-2004; Zhang, Jie/0000-0002-9941-1568; nie,
guangjun/0000-0001-5040-9793
FU National Key R&D Program of China [2018YFE0205300, 2018YFA0208900]; NSFC
[31971307, 32000950]; Key Research Project of Frontier Science of the
Chinese Academy of Sciences [QYZDJ-SSW-SLH022]; Innovation Research
Group of National Natural Science Foundation [11621505]; K.C. Wong
Education Foundation [GJTD-2018-03]; Key Laboratory of Biomedical

Effects of Nanomaterials and Nanosafety, CAS [NSKF202009]
FX This work was financially supported by National Key R&D Program of China (2018YFE0205300, 2018YFA0208900), NSFC (31971307, 32000950), the Key Research Project of Frontier Science of the Chinese Academy of Sciences (QYZDJ-SSW-SLH022), the Innovation Research Group of National Natural Science Foundation (11621505), K.C. Wong Education Foundation (GJTD-2018-03), and the Key Laboratory of Biomedical Effects of Nanomaterials and Nanosafety, CAS (NSKF202009).
CR Alexis F, 2006, ADV MATER, V18, P2174, DOI 10.1002/adma.200502173
Benjaminsen RV, 2013, MOL THER, V21, P149, DOI 10.1038/mt.2012.185
Billingsley MM, 2020, NANO LETT, V20, P1578, DOI 10.1021/acs.nanolett.9b04246
Cao YH, 2014, ADV FUNCT MATER, V24, P6963, DOI 10.1002/adfm.201401358
Chen XL, 2019, NANO LETT, V19, P3527, DOI 10.1021/acs.nanolett.9b00374
Choi E, 2018, J CONTROL RELEASE, V279, P40, DOI 10.1016/j.jconrel.2018.04.021
Chung CK, 2020, J CONTROL RELEASE, V323, P1, DOI 10.1016/j.jconrel.2020.03.050
Da Silva CG, 2019, BIOMATERIALS, V220, DOI 10.1016/j.biomaterials.2019.119417
Dzhandzhugazyan KN, 2018, NAT MATER, V17, P475, DOI 10.1038/s41563-018-0094-5
Ellis RD, 2009, VACCINE, V27, P4104, DOI 10.1016/j.vaccine.2009.04.077
Faghfuri E, 2021, EXPERT OPIN BIOL TH, V21, P201, DOI 10.1080/14712598.2020.1815704
Feng YX, 2019, NAT COMMUN, V10, DOI 10.1038/s41467-019-10354-2
Han XX, 2020, MATTER-US, V3, P1948, DOI 10.1016/j.matt.2020.09.020
Han XX, 2020, J AM CHEM SOC, V142, P2490, DOI 10.1021/jacs.9b12232
Duong HTT, 2018, BIOMATERIALS, V185, P13, DOI 10.1016/j.biomaterials.2018.09.008
Irvine DJ, 2013, NAT MATER, V12, P978, DOI [10.1038/NMAT3775, 10.1038/nmat3775]
Li L, 2020, NANO LETT, V20, P4487, DOI 10.1021/acs.nanolett.0c01287
Mellman I, 2011, NATURE, V480, P480, DOI 10.1038/nature10673
Meng CC, 2016, ACS NANO, V10, P2203, DOI 10.1021/acsnano.5b06750
Miao L, 2019, NAT BIOTECHNOL, V37, P1174, DOI 10.1038/s41587-019-0247-3
Obaid G, 2019, NANO LETT, V19, P7573, DOI 10.1021/acs.nanolett.9b00859
Osna NA, 2009, HEPATOLOGY, V49, P1308, DOI 10.1002/hep.22787
Pourjavadi A, 2015, J IND ENG CHEM, V28, P45, DOI 10.1016/j.jiec.2015.01.021
Rodell CB, 2018, NAT BIOMED ENG, V2, P578, DOI 10.1038/s41551-018-0236-8
Singha N, 2019, CHEM SCI, V10, P5920, DOI 10.1039/c9sc01754b
Wang H, 2018, NAT COMMUN, V9, DOI 10.1038/s41467-018-02915-8
Wang H, 2017, ACS CENTRAL SCI, V3, P875, DOI 10.1021/acscentsci.7b00213
Wang H, 2013, ADV MATER, V25, P1616, DOI 10.1002/adma.201204750
Weiner GJ, 1997, P NATL ACAD SCI USA, V94, P10833, DOI 10.1073/pnas.94.20.10833
Yin Y, 2019, J IND ENG CHEM, V80, P870, DOI 10.1016/j.jiec.2019.08.038
Zhou P, 2019, BIOMATERIALS, V195, P86, DOI 10.1016/j.biomaterials.2019.01.007
NR 31
TC 72
Z9 74
U1 20
U2 203
PU AMER CHEMICAL SOC
PI WASHINGTON
PA 1155 16TH ST, NW, WASHINGTON, DC 20036 USA
SN 1530-6984
EI 1530-6992
J9 NANO LETT
JI Nano Lett.
PD MAR 10
PY 2021
VL 21
IS 5
BP 2224
EP 2231
DI 10.1021/acs.nanolett.0c05039
EA FEB 2021
PG 8
WC Chemistry, Multidisciplinary; Chemistry, Physical; Nanoscience &

Nanotechnology; Materials Science, Multidisciplinary; Physics, Applied;
Physics, Condensed Matter
WE Science Citation Index Expanded (SCI-EXPANDED)
SC Chemistry; Science & Technology - Other Topics; Materials Science;
Physics
GA QX1EB
UT WOS:000629091100043
PM 33594887
DA 2023-09-14
ER

PT J
AU Cheng, SH
Xu, C
Jin, Y
Li, Y
Zhong, C
Ma, J
Yang, JN
Zhang, N
Li, Y
Wang, C
Yang, ZY
Wang, Y
AF Cheng, Shanshan
Xu, Cong
Jin, Yue
Li, Yu
Zhong, Cheng
Ma, Jun
Yang, Jiani
Zhang, Nan
Li, Yuan
Wang, Chao
Yang, Zhiyou
Wang, Yu
TI Artificial Mini Dendritic Cells Boost T Cell-Based Immunotherapy for
Ovarian Cancer
SO ADVANCED SCIENCE
LA English
DT Article
DE dendritic cells; immunotherapy; nanovaccines; ovarian cancer; T cells
ID EPITHELIAL OVARIAN; ANTITUMOR IMMUNITY; TUMOR; NANOPARTICLES; ANTIGEN;
RESPONSES; DELIVERY; VACCINES; THERAPY
AB Ovarian cancer is the most lethal gynecological malignancy with high recurrence rates and low survival rates, remaining a disease of high unmet need. Cancer immunotherapy, which harnesses the potential of the immune system to attack tumors, has emerged as one of the most promising treatment options in recent years. As an important form of immunotherapy, dendritic cell (DC)-based vaccines have demonstrated the ability to induce an immune response, while clinical efficacy of DC vaccines remains unsubstantiated as long-term benefit is only reported in a restricted proportion of patients. Here, a biomimetic nanovaccine derived from DCs is developed through cell membrane coating nanotechnology. This nanovaccine, denoted "mini DC," inherits the ability of antigen presentation and T cells' stimulation from DCs and is shown to elicit enhanced activation of T cells both in vitro and in vivo. In a mouse model of ovarian cancer, mini DCs exhibit superior therapeutic and prophylactic efficacy against cancer including delayed tumor growth and reduced tumor metastasis compared with DC vaccine. These findings suggest that mini DCs may serve as a facile and potent vaccine to boost anticancer immunotherapy.
C1 [Cheng, Shanshan; Xu, Cong; Jin, Yue; Li, Yu; Zhong, Cheng; Ma, Jun; Yang, Jiani; Zhang, Nan; Li, Yuan; Wang, Chao; Yang, Zhiyou; Wang, Yu] Shanghai Jiao Tong Univ, Sch Med, Shanghai Key Lab Gynecol Oncol, Dept Obstet & Gynecol,Renji Hosp, Shanghai 200127, Peoples R China.
C3 Shanghai Jiao Tong University
RP Wang, Y (通讯作者)，Shanghai Jiao Tong Univ, Sch Med, Shanghai Key Lab Gynecol Oncol, Dept

Obstet & Gynecol,Renji Hosp, Shanghai 200127, Peoples R China.
EM wangyu@renji.com
FU National Natural Science Foundation of China [81572560]; Three-Year Action Plan of the Program of Shanghai Hospital Development Center for Promoting Clinical Skills and Innovative Ability of Municipal Hospitals [16CR2012A]
FX This work was supported by the National Natural Science Foundation of China (Grant No. 81572560) and Three-Year Action Plan of the Program of Shanghai Hospital Development Center for Promoting Clinical Skills and Innovative Ability of Municipal Hospitals (16CR2012A). The authors appreciate the use of Instrumentation Facility at Institute of Molecular Medicine and Central laboratory, Renji Hospital, School of Medicine, Shanghai Jiao Tong University. The authors also acknowledge Dr. Lu Mang and Yihan Bai for technical support.
CR [Anonymous], ANN RES HOSP
Azzi J, 2016, CELL REP, V15, P1202, DOI 10.1016/j.celrep.2016.04.007
Bol KF, 2016, CLIN CANCER RES, V22, P1897, DOI 10.1158/1078-0432.CCR-15-1399
Chang CC, 2006, J BIOL CHEM, V281, P18763, DOI 10.1074/jbc.M511525200
Chiang CLL, 2006, CANCER IMMUNOL IMMUN, V55, P1384, DOI 10.1007/s00262-006-0127-9
Chiang CLL, 2013, CLIN CANCER RES, V19, P4801, DOI 10.1158/1078-0432.CCR-13-1185
Cress RD, 2015, OBSTET GYNECOL, V126, P491, DOI 10.1097/AOG.0000000000000981
Cubillos-Ruiz JR, 2015, CELL, V161, P1527, DOI 10.1016/j.cell.2015.05.025
Fang RH, 2018, ADV MATER, V30, DOI 10.1002/adma.201706759
Gardner A, 2016, TRENDS IMMUNOL, V37, P855, DOI 10.1016/j.it.2016.09.006
Gottfried E, 2006, BLOOD, V107, P2013, DOI 10.1182/blood-2005-05-1795
Hamanishi J, 2007, P NATL ACAD SCI USA, V104, P3360, DOI 10.1073/pnas.0611533104
HOCHMUTH RM, 1983, SCIENCE, V220, P101, DOI 10.1126/science.6828875
Hu CMJ, 2015, NATURE, V526, P118, DOI 10.1038/nature15373
Hu CMJ, 2013, NAT NANOTECHNOL, V8, P336, DOI [10.1038/nnano.2013.54, 10.1038/NNANO.2013.54]
Hu CMJ, 2011, P NATL ACAD SCI USA, V108, P10980, DOI 10.1073/pnas.1106634108
Hu QY, 2015, ADV MATER, V27, P7043, DOI 10.1002/adma.201503323
Jain RA, 2000, BIOMATERIALS, V21, P2475, DOI 10.1016/S0142-9612(00)00115-0
Kandalaft LE, 2011, J CLIN ONCOL, V29, P925, DOI 10.1200/JCO.2009.27.2369
Kantoff PW, 2010, NEW ENGL J MED, V363, P411, DOI 10.1056/NEJMoa1001294
Karst AM, 2010, J ONCOL, V2010, DOI 10.1155/2010/932371
Kleindienst P, 2003, J IMMUNOL, V170, P2817, DOI 10.4049/jimmunol.170.6.2817
Kowalczyk DW, 2003, CANCER GENE THER, V10, P870, DOI 10.1038/sj.cgt.7700653
Ledermann JA, 2017, ANN ONCOL, V28, P46, DOI 10.1093/annonc/mdx452
Lim TS, 2011, J IMMUNOL, V187, P258, DOI 10.4049/jimmunol.1100267
Matsuo K, 2010, GYNECOL ONCOL, V116, P61, DOI 10.1016/j.ygyno.2009.09.018
Matsuo K, 2009, INT J CANCER, V125, P2721, DOI 10.1002/ijc.24654
Matza D, 2009, P NATL ACAD SCI USA, V106, P9785, DOI 10.1073/pnas.0902844106
McHugh MD, 2015, BIOMATERIALS, V59, P172, DOI 10.1016/j.biomaterials.2015.04.003
Moon JJ, 2012, P NATL ACAD SCI USA, V109, P1080, DOI 10.1073/pnas.1112648109
O'Neill LAJ, 2016, J EXP MED, V213, P15, DOI 10.1084/jem.20151570
Odunsi K, 2017, ANN ONCOL, V28, P1, DOI 10.1093/annonc/mdx444
Parodi A, 2013, NAT NANOTECHNOL, V8, P61, DOI 10.1038/nnano.2012.212
Paulis LE, 2013, CURR OPIN IMMUNOL, V25, P389, DOI 10.1016/j.coi.2013.03.001
Sabado RL, 2017, CELL RES, V27, P74, DOI 10.1038/cr.2016.157
Siegel RL, 2016, CA-CANCER J CLIN, V66, P7, DOI DOI 10.3322/CAAC.21442
Stuart GCE, 2003, GYNECOL ONCOL, V90, pS8, DOI 10.1016/S0090-8258(03)00472-4
Tanyi JL, 2018, SCI TRANSL MED, V10, DOI 10.1126/scitranslmed.aao5931
Torre LA, 2018, CA-CANCER J CLIN, V68, P284, DOI 10.3322/caac.21456
van der Woude LL, 2017, TRENDS CANCER, V3, P797, DOI 10.1016/j.trecan.2017.09.006
Verdijk P, 2009, CLIN CANCER RES, V15, P2531, DOI 10.1158/1078-0432.CCR-08-2729
Zhang C, 2019, ADV MATER, V31, DOI 10.1002/adma.201901179
Zhang QZ, 2018, NAT NANOTECHNOL, V13, P1182, DOI 10.1038/s41565-018-0254-4
NR 43
TC 72
Z9 76

U1 6
U2 104
PU WILEY
PI HOBOKEN
PA 111 RIVER ST, HOBOKEN 07030-5774, NJ USA
EI 2198-3844
J9 ADV SCI
JI Adv. Sci.
PD APR
PY 2020
VL 7
IS 7
AR 1903301
DI 10.1002/advs.201903301
EA FEB 2020
PG 14
WC Chemistry, Multidisciplinary; Nanoscience & Nanotechnology; Materials
Science, Multidisciplinary
WE Science Citation Index Expanded (SCI-EXPANDED)
SC Chemistry; Science & Technology - Other Topics; Materials Science
GA LE4TT
UT WOS:000511027600001
PM 32274314
OA gold, Green Published
DA 2023-09-14
ER

PT J
AU Wang, K
Wen, SM
He, LH
Li, A
Li, Y
Dong, HQ
Li, W
Ren, TB
Shi, DL
Li, YY
AF Wang, Kun
Wen, Shuman
He, Lianghua
Li, Ang
Li, Yan
Dong, Haiqing
Li, Wei
Ren, Tianbin
Shi, Donglu
Li, Yongyong
TI "Minimalist" Nanovaccine Constituted from Near Whole Antigen for Cancer
Immunotherapy
SO ACS NANO
LA English
DT Article
DE minimalist nanovaccine; ovalbumin; disulfide network; nanoassembly;
cancer immunoprotection
ID CELLULAR IMMUNE-RESPONSES; SYNTHETIC VACCINES; PEGYLATED NANOPARTICLES;
BIOMEDICAL APPLICATIONS; DENDRITIC CELLS; DRUG-DELIVERY; IN-SITU;
ADJUVANT; NANOCARRIERS; ENHANCEMENT
AB One of the major challenges in vaccine design has been the over dependence on incorporation of abundant adjuvants, which in fact is in violation of the "minimalist" principle. In the present study, a compact nanovaccine derived from a near whole antigen (up to 97 wt %) was developed. The

nanovaccine structure was stabilized by free cysteines within each antigen (ovalbumin, OVA), which were tempospatially exposed and heat-driven to form an extensive intermolecular disulfide network. This process enables the engineering of a nanovaccine upon integration of the danger signal (CpG-SH) into the network during the synthetic process. The 50 nm-sized nanovaccine was developed comprising approximately 500 antigen molecules per nanoparticle. The nano vaccine prophylactically protected 70% of mice from tumorigenesis (0% for the control group) in murine B16-OVA melanoma. Significant tumor inhibition was achieved by strongly nanovaccine-induced cytotoxic T lymphocytes. This strategy can be adapted for the future design of vaccine for a minimalist composition in clinical settings.
C1 [Wang, Kun; Wen, Shuman; He, Lianghua; Li, Yan; Dong, Haiqing; Li, Yongyong] Tongji Univ, Sch Med, Shanghai East Hosp, Inst Biomed Engn & Nano Sci, Shanghai 200092, Peoples R China.
[Wang, Kun; Wen, Shuman; Ren, Tianbin] Tongji Univ, Sch Mat Sci & Engn, 4800 Caoan Rd, Shanghai 201804, Peoples R China.
[Shi, Donglu] Univ Cincinnati, Coll Engn & Appl Sci, Dept Mech & Mat Engn, Mat Sci & Engn Program, Cincinnati, OH 45221 USA.
[Li, Ang] Tongji Univ, Sch Life Sci & Technol, 1239 Siping Rd, Shanghai 200092, Peoples R China.
[Li, Wei] Second Mil Med Univ, Int Joint Canc Inst, Shanghai 200433, Peoples R China.
C3 Tongji University; Tongji University; University System of Ohio;
University of Cincinnati; Tongji University; Naval Medical University
RP Li, YY (通讯作者)，Tongji Univ, Sch Med, Shanghai East Hosp, Inst Biomed Engn & Nano Sci, Shanghai 200092, Peoples R China.
EM yongyong_li@tongji.edu.cn
RI Shi, Donglu/G-1849-2018
FU National Natural Science Foundation of China [51773154, 31771090, 51473124, 81571801]; Shanghai Science and Technology Innovation [18JC1414500]; Shanghai Natural Science Foundation [17ZR1432100]; Young Hundred-Talent Program of Tongji University
FX This work was financially supported through grants from the National Natural Science Foundation of China (Nos. 51773154, 31771090, 51473124, and 81571801), Shanghai Science and Technology Innovation (Grant 18JC1414500), Shanghai Natural Science Foundation (Grant 17ZR1432100), and Young Hundred-Talent Program of Tongji University.
CR Ali OA, 2009, NAT MATER, V8, P151, DOI [10.1038/NMAT2357, 10.1038/nmat2357]
Apte SH, 2010, J IMMUNOL, V185, P998, DOI 10.4049/jimmunol.0903372
Banchereau J, 1998, NATURE, V392, P245, DOI 10.1038/32588
Banday AH, 2015, IMMUNOPHARM IMMUNOT, V37, P1, DOI 10.3109/08923973.2014.971963
Bertrand N, 2014, ADV DRUG DELIVER REV, V66, P2, DOI 10.1016/j.addr.2013.11.009
Bode C, 2011, EXPERT REV VACCINES, V10, P499, DOI [10.1586/ERV.10.174, 10.1586/erv.10.174]
Chen Q, 2016, NAT COMMUN, V7, DOI 10.1038/ncomms13193
Chong CSW, 2005, J CONTROL RELEASE, V102, P85, DOI 10.1016/j.jconrel.2004.09.014
Chou LYT, 2011, CHEM SOC REV, V40, P233, DOI 10.1039/c0cs00003e
Christensen D, 2016, HUM VACC IMMUNOTHER, V12, P2709, DOI 10.1080/21645515.2016.1219003
Dykman L, 2012, CHEM SOC REV, V41, P2256, DOI 10.1039/c1cs15166e
Forster R, 2008, NAT REV IMMUNOL, V8, P362, DOI 10.1038/nri2297
Gu Z, 2011, CHEM SOC REV, V40, P3638, DOI 10.1039/c0cs00227e
Guo YY, 2015, ACS NANO, V9, P6918, DOI 10.1021/acsnano.5b01042
Henry CE, 2016, ACTA BIOMATER, V43, P61, DOI 10.1016/j.actbio.2016.07.019
Hubbell JA, 2009, NATURE, V462, P449, DOI 10.1038/nature08604
Huntington JA, 2001, J CHROMATOGR B, V756, P189, DOI 10.1016/S0378-4347(01)00108-6
Irvine DJ, 2015, CHEM REV, V115, P11109, DOI 10.1021/acs.chemrev.5b00109
Irvine DJ, 2013, NAT MATER, V12, P978, DOI [10.1038/NMAT3775, 10.1038/nmat3775]
Joshi VB, 2013, HUM VACC IMMUNOTHER, V9, P2584, DOI 10.4161/hv.26136
Kasturi SP, 2011, NATURE, V470, P543, DOI 10.1038/nature09737
Kourtis IC, 2013, PLOS ONE, V8, DOI 10.1371/journal.pone.0061646
Kuai R, 2017, NAT MATER, V16, P489, DOI [10.1038/nmat4822, 10.1038/NMAT4822]
Leifer CA, 2004, J IMMUNOL, V173, P1179, DOI 10.4049/jimmunol.173.2.1179
Li A, 2011, BIOMATERIALS, V32, P469, DOI 10.1016/j.biomaterials.2010.08.107
Li XR, 2014, J CONTROL RELEASE, V173, P148, DOI 10.1016/j.jconrel.2013.10.032
Li-Chan E. C., 1995, EGG SCI TECHNOLOGY, V4, P105
Lizotte PH, 2016, NAT NANOTECHNOL, V11, P295, DOI [10.1038/NNANO.2015.292, 10.1038/nnano.2015.292]
Luo M, 2017, NAT NANOTECHNOL, V12, P648, DOI [10.1038/nnano.2017.52,

10.1038/NNANO.2017.52]
Ma Q, 2017, J IMMUNOTHER, V40, P11, DOI 10.1097/CJI.0000000000000145
Mamo T, 2012, VACCINE, V30, P6609, DOI 10.1016/j.vaccine.2012.08.023
Moon JJ, 2011, NAT MATER, V10, P243, DOI 10.1038/NMAT2960
Morsy MA, 2005, LAB INVEST, V85, P285, DOI 10.1038/labinvest.3700219
Nicolas J, 2013, CHEM SOC REV, V42, P1147, DOI 10.1039/c2cs35265f
Paulis LE, 2013, CURR OPIN IMMUNOL, V25, P389, DOI 10.1016/j.coi.2013.03.001
Reed SG, 2013, NAT MED, V19, P1597, DOI 10.1038/nm.3409
Riaz N, 2016, NAT GENET, V48, P1327, DOI 10.1038/ng.3677
Ruan QJ, 2014, FOOD CHEM, V156, P14, DOI 10.1016/j.foodchem.2014.01.083
Shindo G., 2013, J CANC THER, V4, P1382, DOI DOI 10.4236/JCT.2013.48164
Shiraishi K, 2016, J CONTROL RELEASE, V234, P59, DOI 10.1016/j.jconrel.2016.05.010
Smith JD, 2015, CURR OPIN BIOTECH, V34, P217, DOI 10.1016/j.copbio.2015.03.014
Vartak A, 2016, VACCINES-BASEL, V4, DOI 10.3390/vaccines4020012
Wang Y, 2015, ADV MATER, V27, P576, DOI 10.1002/adma.201401124
Wei H, 2013, PROG POLYM SCI, V38, P503, DOI 10.1016/j.progpolymsci.2012.07.002
Yu YRA, 2016, PLOS ONE, V11, DOI 10.1371/journal.pone.0150606
Zhao L, 2014, VACCINE, V32, P327, DOI 10.1016/j.vaccine.2013.11.069
NR 46
TC 72
Z9 73
U1 8
U2 272
PU AMER CHEMICAL SOC
PI WASHINGTON
PA 1155 16TH ST, NW, WASHINGTON, DC 20036 USA
SN 1936-0851
EI 1936-086X
J9 ACS NANO
JI ACS Nano
PD JUL
PY 2018
VL 12
IS 7
BP 6398
EP 6409
DI 10.1021/acsnano.8b00558
PG 12
WC Chemistry, Multidisciplinary; Chemistry, Physical; Nanoscience &
Nanotechnology; Materials Science, Multidisciplinary
WE Science Citation Index Expanded (SCI-EXPANDED)
SC Chemistry; Science & Technology - Other Topics; Materials Science
GA GP0MT
UT WOS:000440505000006
PM 29927574
DA 2023-09-14
ER

PT J
AU Ruiz-de-Angulo, A
Zabaleta, A
Gomez-Vallejo, V
Llop, J
Mareque-Rivas, JC
AF Ruiz-de-Angulo, Ane
Zabaleta, Aintzane
Gomez-Vallejo, Vanessa
Llop, Jordi
Mareque-Rivas, Juan C.
TI Microdosed Lipid-Coated Ga-67-Magnetite Enhances Antigen-Specific
Immunity by Image Tracked Delivery of Antigen and CpG to Lymph Nodes

SO ACS NANO
LA English
DT Article
DE multifunctional nanoparticles; multimodal imaging synthetic vaccines;
toll-like receptors; drug delivery; immunotherapy; theranostics
ID CU-64-LABELED MAGNETIC NANOPARTICLES; IN-VIVO EVALUATION; DENDRITIC
CELLS; GOLD NANOPARTICLE; RADIOLABELED NANOPARTICLES; CELLULAR-IMMUNITY;
FILLED MICELLES; VACCINES; IMMUNOTHERAPY; ACTIVATION
AB Development of vaccines to prevent and treat emerging new pathogens and re-emerging infections and cancer remains a major challenge. An attractive approach is to build the vaccine upon a biocompatible NP that simultaneously acts as accurate delivery vehicle and radiotracer for PET/SPECT imaging for ultrasensitive and quantitative in vivo imaging of NP delivery to target tissues/organs. Success in developing these nanovaccines will depend in part on having a "correct" NP size and accommodating and suitably displaying antigen and/or adjuvants (e.g., TLR agonists). Here we develop and evaluate a NP vaccine based on iron oxide-selective radio-gallium labeling suitable for SPECT(Ga-67)/PET(Ga-68) imaging 2 Targeted image-tracked nanovaccines and efficient delivery of antigen (OVA) and TLR 9 agonists (CpGs) using lipid-coated magnetite micelles. OVA, CpGs and rhodamine are easily accommodated in the hybrid micelles, and the average size of the construct can be controlled to be ca. 40 mu in diameter to target direct lymphatic delivery of the vaccine cargo to antigen presenting cells (APCs) in the lymph nodes (LNs). While the OVA/CpG-loaded construct showed effective delivery to endosomal TLR 9 in APCs, SPECT imaging demonstrated migration from the injection" site to regional and nonregional LNs. In correlation with the imaging results, a range of in vitro and in vivo studies demonstrate that by using this microdosed nanosystem the cellular and humoral immune responses are greatly enhanced and provide protection against tumor challenge. These results suggest that these nanosystems have considerable potential for image-guided development of targeted vaccines that are more effective and limit toxicity.
C1 [Ruiz-de-Angulo, Ane; Zabaleta, Aintzane; Mareque-Rivas, Juan C.] Cooperat Ctr Res Biomat CIC biomaGUNE, Theranost Nanomed Lab, Paseo Miramon 182, San Sebastian 20009, Spain.
[Gomez-Vallejo, Vanessa] Cooperat Ctr Res Biomat CIC biomaGUNE, Radiochem Platform, Paseo Miramon 182, San Sebastian 20009, Spain.
[Llop, Jordi] Cooperat Ctr Res Biomat CIC biomaGUNE, Radiochem & Nucl Imaging Lab, Paseo Miramon 182, San Sebastian 20009, Spain.
[Mareque-Rivas, Juan C.] Ikerbasque, Basque Fdn Sci, Bilbao 48011, Spain.
[Mareque-Rivas, Juan C.] Univ Aberdeen, Sch Engn, Aberdeen AB24 3UE, Scotland.
C3 CIC biomaGUNE; CIC biomaGUNE; CIC biomaGUNE; Basque Foundation for
Science; University of Aberdeen
RP Mareque-Rivas, JC (通讯作者)，Cooperat Ctr Res Biomat CIC biomaGUNE, Theranost Nanomed Lab, Paseo Miramon 182, San Sebastian 20009, Spain.; Mareque-Rivas, JC (通讯作者)，Ikerbasque, Basque Fdn Sci, Bilbao 48011, Spain.; Mareque-Rivas, JC (通讯作者)，Univ Aberdeen, Sch Engn, Aberdeen AB24 3UE, Scotland.
EM jmareque@cicbiomagune.es
RI Llop, Jordi/F-5496-2014; Zabaleta, Aintzane/K-8936-2014; Rivas, Juan
Mareque/M-6171-2014; Gómez-Vallejo, Vanessa/H-9960-2015; de Angulo, Ane
Ruiz/I-3867-2016; biomaGUNE, CIC/J-9136-2014
OI Llop, Jordi/0000-0002-0821-9838; Rivas, Juan
Mareque/0000-0002-7405-3338; Gómez-Vallejo, Vanessa/0000-0003-3995-9596;
de Angulo, Ane Ruiz/0000-0001-6581-4038; Zabaleta,
Aintzane/0000-0003-3474-3040; biomaGUNE, CIC/0000-0001-7690-0660
FU Spanish Ministry of Economy and Competitiveness [PRI-PIBIN-2011-0812,
CTQ2014-54761-R, MAT2013-48169-R]; Department of Industry of the Basque
Country (ETORTEK); Diputacion de Guipuzcoa [Exp 63/15]; Department of
Education, Language Policy and Culture of the Basque Government
FX This work was financed by Spanish Ministry of Economy and
Competitiveness (grants PRI-PIBIN-2011-0812, CTQ2014-54761-R and
MAT2013-48169-R), the Department of Industry of the Basque Country
(grant ETORTEK) and Diputacion de Guipuzcoa (Exp 63/15). We thank Dr. J.
Hernandez-Gil for carrying out TEM and DOTA conjugation to QDs, Mikel
Gonzalez Esparza for radiolabeling studies and Dr Boguslaw Szczupak and
Zurine Baz Maldonado for processing and reconstructing the SPECT/CT
images and Dr. Pablo Sarobe (CIMA, University of Navarra) for the
B16-F10-OVA cells. A. R. de A. acknowledges a PhD studentship from the

Department of Education, Language Policy and Culture of the Basque
Government.
CR Adema GJ, 2005, CURR OPIN IMMUNOL, V17, P170, DOI 10.1016/j.coi.2005.01.004
Bernstein LR, 1998, PHARMACOL REV, V50, P665
Berry DJ, 2011, CHEM COMMUN, V47, P7068, DOI 10.1039/c1cc12123e
Berzofsky JA, 2001, NAT REV IMMUNOL, V1, P209, DOI 10.1038/35105075
Blasius AL, 2010, IMMUNITY, V32, P305, DOI 10.1016/j.immuni.2010.03.012
Bonifaz LC, 2004, J EXP MED, V199, P815, DOI 10.1084/jem.20032220
Boros E, 2015, CHEM SCI, V6, P225, DOI 10.1039/c4sc02778g
Bourquin C, 2008, J IMMUNOL, V181, P2990, DOI 10.4049/jimmunol.181.5.2990
Broaders KE, 2009, P NATL ACAD SCI USA, V106, P5497, DOI 10.1073/pnas.0901592106
Carion O, 2007, NAT PROTOC, V2, P2383, DOI 10.1038/nprot.2007.351
Cavanagh LL, 2002, IMMUNOL CELL BIOL, V80, P448, DOI 10.1046/j.1440-1711.2002.01119.x
Chakravarty R, 2014, ADV MATER, V26, P5119, DOI 10.1002/adma.201401372
Chen F, 2013, ANGEW CHEM INT EDIT, V52, P13319, DOI 10.1002/anie.201306306
Chen N, 2014, SMALL, V10, P368, DOI 10.1002/smll.201300903
Cobaleda-Siles M, 2014, SMALL, V10, P5054, DOI 10.1002/smll.201401353
Colombo M, 2012, CHEM SOC REV, V41, P4306, DOI 10.1039/c2cs15337h
Cunha L, 2014, DRUG DISCOV TODAY, V19, P936, DOI 10.1016/j.drudis.2014.01.003
de Rosales RTM, 2011, ANGEW CHEM INT EDIT, V50, P5509, DOI 10.1002/anie.201007894
de Rosales RTM, 2011, BIOCONJUGATE CHEM, V22, P455, DOI 10.1021/bc100483k
de Titta A, 2013, P NATL ACAD SCI USA, V110, P19902, DOI 10.1073/pnas.1313152110
Dudziak D, 2007, SCIENCE, V315, P107, DOI 10.1126/science.1136080
Gao JH, 2009, ACCOUNTS CHEM RES, V42, P1097, DOI 10.1021/ar9000026
Glaus C, 2010, BIOCONJUGATE CHEM, V21, P715, DOI 10.1021/bc900511j
Goel S, 2014, SMALL, V10, P3825, DOI 10.1002/smll.201401048
Blanco NG, 2012, CHEM COMMUN, V48, P4211, DOI 10.1039/c2cc31045g
Hainfeld JF, 2011, BRIT J RADIOL, V84, P526, DOI 10.1259/bjr/42612922
Harrell MI, 2008, J IMMUNOL METHODS, V332, P170, DOI 10.1016/j.jim.2007.11.012
Harrell MI, 2007, AM J PATHOL, V170, P774, DOI 10.2353/ajpath.2007.060761
Hu H, 2014, BIOMATERIALS, V35, P9868, DOI 10.1016/j.biomaterials.2014.08.038
Hubbell JA, 2009, NATURE, V462, P449, DOI 10.1038/nature08604
Hwang DW, 2010, J NUCL MED, V51, P98, DOI 10.2967/jnumed.109.069880
Jarrett BR, 2008, BIOCONJUGATE CHEM, V19, P1496, DOI 10.1021/bc800108v
Jewell CM, 2011, P NATL ACAD SCI USA, V108, P15745, DOI 10.1073/pnas.1105200108
Kamaly N, 2012, CHEM SOC REV, V41, P2971, DOI 10.1039/c2cs15344k
Kaneko Y, 2007, J CLIN INVEST, V117, P877, DOI 10.1172/JCI30783
Kasturi SP, 2011, NATURE, V470, P543, DOI 10.1038/nature09737
Kim T, 2011, J AM CHEM SOC, V133, P2955, DOI 10.1021/ja1084095
Krieg AM, 2012, NUCLEIC ACID THER, V22, P77, DOI 10.1089/nat.2012.0340
Kwon YJ, 2005, P NATL ACAD SCI USA, V102, P18264, DOI 10.1073/pnas.0509541102
Lee HY, 2008, J NUCL MED, V49, P1371, DOI 10.2967/jnumed.108.051243
Lee IH, 2012, ANGEW CHEM INT EDIT, V51, P8800, DOI 10.1002/anie.201203193
Lee JH, 2007, NAT MED, V13, P95, DOI [10.1038/nm1467, 10.20659/jfp.13.1_95]
Lee JH, 2011, NAT NANOTECHNOL, V6, P418, DOI [10.1038/nnano.2011.95, 10.1038/NNANO.2011.95]
Lewin M, 2000, NAT BIOTECHNOL, V18, P410, DOI 10.1038/74464
Li HY, 2011, NAT NANOTECHNOL, V6, P645, DOI [10.1038/nnano.2011.153,
10.1038/NNANO.2011.153]
Lin AY, 2013, PLOS ONE, V8, DOI 10.1371/journal.pone.0063550
Lutz MB, 1999, J IMMUNOL METHODS, V223, P77, DOI 10.1016/S0022-1759(98)00204-X
Maldonado CR, 2013, CHEM COMMUN, V49, P3985, DOI 10.1039/c3cc39104c
Manolova V, 2008, EUR J IMMUNOL, V38, P1404, DOI 10.1002/eji.200737984
Mellman I, 2001, CELL, V106, P255, DOI 10.1016/S0092-8674(01)00449-4
Moon JJ, 2012, ADV MATER, V24, P3724, DOI 10.1002/adma.201200446
Moon JJ, 2011, NAT MATER, V10, P243, DOI 10.1038/NMAT2960
Morens DM, 2004, NATURE, V430, P242, DOI 10.1038/nature02759
Na HB, 2009, ADV MATER, V21, P2133, DOI 10.1002/adma.200802366
Nimmerjahn F, 2005, SCIENCE, V310, P1510, DOI 10.1126/science.1118948
Ogawa K, 2006, J NUCL MED, V47, P2042
Overwijk W W, 2001, Curr Protoc Immunol, VChapter 20, DOI 10.1002/0471142735.im2001s39
Pashine A, 2005, NAT MED, V11, pS63, DOI 10.1038/nm1210

Patel D, 2011, BIOMATERIALS, V32, P1167, DOI 10.1016/j.biomaterials.2010.10.013
Pohar J, 2015, J IMMUNOL, V194, P3901, DOI 10.4049/jimmunol.1402755
Randolph GJ, 2005, NAT REV IMMUNOL, V5, P617, DOI 10.1038/nri1670
Reddy ST, 2007, NAT BIOTECHNOL, V25, P1159, DOI [10.1038/nbt1332, 10.1038/nbtl332]
Rudin M, 2003, NAT REV DRUG DISCOV, V2, P123, DOI 10.1038/nrd1007
SCASNAR V, 1993, EUR J NUCL MED, V20, P273, DOI 10.1007/BF00170012
SHANNON RD, 1976, ACTA CRYSTALLOGR A, V32, P751, DOI 10.1107/S0567739476001551
Skitzki JJ, 2009, CURR OPIN INVEST DR, V10, P550
Smith DM, 2013, NAT REV IMMUNOL, V13, P592, DOI 10.1038/nri3488
Sugiyama Y, 2011, ADV DRUG DELIVER REV, V63, P494, DOI 10.1016/j.addr.2010.09.010
Sun HZ, 1999, CHEM REV, V99, P2817, DOI 10.1021/cr980430w
Sun SH, 2004, J AM CHEM SOC, V126, P273, DOI 10.1021/ja0380852
Tao Y, 2014, ADV FUNCT MATER, V24, P1004, DOI 10.1002/adfm.201302347
Toraya-Brown S, 2014, INT J HYPERTHER, V30, P531, DOI 10.3109/02656736.2014.968640
Trumpfheller C, 2006, J EXP MED, V203, P607, DOI 10.1084/jem.20052005
Velikyan I, 2014, THERANOSTICS, V4, P47, DOI 10.7150/thno.7447
Weissleder Ralph, 2000, Nature Medicine, V6, P351, DOI 10.1038/73219
Willmann JK, 2008, NAT REV DRUG DISCOV, V7, P591, DOI 10.1038/nrd2290
Wilson JT, 2013, ACS NANO, V7, P3912, DOI 10.1021/nn305466z
Xie YM, 2009, EXPERT OPIN DRUG DEL, V6, P785, DOI 10.1517/17425240903085128
Xing Y, 2014, THERANOSTICS, V4, P290, DOI 10.7150/thno.7341
Yee C, 2002, P NATL ACAD SCI USA, V99, P16168, DOI 10.1073/pnas.242600099
Zambre A, 2014, J NUCL MED, V55
NR 81
TC 71
Z9 72
U1 2
U2 128
PU AMER CHEMICAL SOC
PI WASHINGTON
PA 1155 16TH ST, NW, WASHINGTON, DC 20036 USA
SN 1936-0851
EI 1936-086X
J9 ACS NANO
JI ACS Nano
PD JAN
PY 2016
VL 10
IS 1
BP 1602
EP 1618
DI 10.1021/acsnano.5b07253
PG 17
WC Chemistry, Multidisciplinary; Chemistry, Physical; Nanoscience & Nanotechnology; Materials Science, Multidisciplinary
WE Science Citation Index Expanded (SCI-EXPANDED)
SC Chemistry; Science & Technology - Other Topics; Materials Science
GA DC3JO
UT WOS:000369115800179
PM 26678549
OA Green Submitted
DA 2023-09-14
ER

PT J
AU Fusciello, M
Fontana, F
Tahtinen, S
Capasso, C
Feola, S
Martins, B

Chiaro, J
Peltonen, K
Ylosmaki, L
Ylosmaki, E
Hamdan, F
Kari, OK
Ndika, J
Alenius, H
Urtti, A
Hirvonen, JT
Santos, HA
Cerullo, V
AF Fusciello, Manlio
Fontana, Flavia
Tahtinen, Siri
Capasso, Cristian
Feola, Sara
Martins, Beatriz
Chiaro, Jacopo
Peltonen, Karita
Ylosmaki, Leena
Ylosmaki, Erkko
Hamdan, Firas
Kari, Otto K.
Ndika, Joseph
Alenius, Harri
Urtti, Arto
Hirvonen, Jouni T.
Santos, Helder A.
Cerullo, Vincenzo
TI Artificially cloaked viral nanovaccine for cancer immunotherapy
SO NATURE COMMUNICATIONS
LA English
DT Article
ID T-CELL THERAPY; OVARIAN-CANCER; GENE-TRANSFER; SOLID TUMORS;
CO-DELIVERY; IN-VIVO; ADENOVIRUS; NANOPARTICLES; ANTIGEN; VACCINATION
AB Virus-based cancer vaccines are nowadays considered an interesting approach in the field of cancer immunotherapy, despite the observation that the majority of the immune responses they elicit are against the virus and not against the tumor. In contrast, targeting tumor associated antigens is effective, however the identification of these antigens remains challenging. Here, we describe ExtraCRAd, a multi-vaccination strategy focused on an oncolytic virus artificially wrapped with tumor cancer membranes carrying tumor antigens. We demonstrate that ExtraCRAd displays increased infectivity and oncolytic effect in vitro and in vivo. We show that this nanoparticle platform controls the growth of aggressive melanoma and lung tumors in vivo both in preventive and therapeutic setting, creating a highly specific anti-cancer immune response. In conclusion, ExtraCRAd might serve as the next generation of personalized cancer vaccines with enhanced features over standard vaccination regimens, representing an alternative way to target cancer.
C1 [Fusciello, Manlio; Tahtinen, Siri; Capasso, Cristian; Feola, Sara; Martins, Beatriz; Chiaro, Jacopo; Peltonen, Karita; Ylosmaki, Leena; Ylosmaki, Erkko; Hamdan, Firas; Kari, Otto K.; Urtti, Arto; Cerullo, Vincenzo] Univ Helsinki, Fac Pharm, Drug Res Program, Div Pharmaceut Biosci & Digital Precis Canc Med F, FI-00140 Helsinki, Finland.
[Fontana, Flavia; Hirvonen, Jouni T.; Santos, Helder A.] Univ Helsinki, Fac Pharm, Drug Res Program, Div Pharmaceut Chem & Technol, FI-00140 Helsinki, Finland.
[Ndika, Joseph; Alenius, Harri] Univ Helsinki, Dept Bacteriol & Immunol, Syst Toxicol Grp, Medicum, FI-00140 Helsinki, Finland.
[Alenius, Harri] Karolinska Inst, Inst Environm Med, SE-17177 Stockholm, Sweden.
[Urtti, Arto] Univ Eastern Finland, Fac Hlth Sci, Sch Pharm, FI-70211 Kuopio, Finland.
[Santos, Helder A.; Cerullo, Vincenzo] Univ Helsinki, Helsinki Inst Life Sci HiLIFE, FI-00014 Helsinki, Finland.
[Cerullo, Vincenzo] Naples Univ Federico II, Dept Mol Med & Med Biotechnol, I-80131 Naples, Italy.
[Cerullo, Vincenzo] Naples Univ Federico II, CEINGE, I-80131 Naples, Italy.

C3 University of Helsinki; University of Helsinki; University of Helsinki;
Karolinska Institutet; University of Helsinki; CEINGE Biotecnologie
Avanzate
RP Cerullo, V (通讯作者)，Univ Helsinki, Fac Pharm, Drug Res Program, Div Pharmaceut Biosci & Digital Precis Canc Med F, FI-00140 Helsinki, Finland.; Santos, HA (通讯作者)，Univ Helsinki, Fac Pharm, Drug Res Program, Div Pharmaceut Chem & Technol, FI-00140 Helsinki, Finland.; Santos, HA; Cerullo, V (通讯作者)，Univ Helsinki, Helsinki Inst Life Sci HiLIFE, FI-00014 Helsinki, Finland.; Cerullo, V (通讯作者)，Naples Univ Federico II, Dept Mol Med & Med Biotechnol, I-80131 Naples, Italy.; Cerullo, V (通讯作者)，Naples Univ Federico II, CEINGE, I-80131 Naples, Italy.
EM helder.santos@helsinki.fi; vincenzo.cerullo@helsinki.fi
RI Santos, Hélder A./AAC-6347-2022; Cerullo, Vincenzo/B-9075-2015; Fontana, Flavia/AAB-4898-2020; Ndika, Joseph/AAI-1074-2020
OI Santos, Hélder A./0000-0001-7850-6309; Cerullo, Vincenzo/0000-0003-4901-3796; Fontana, Flavia/0000-0001-9112-6917; Ndika, Joseph/0000-0003-0669-1300; Hirvonen, Jouni Tapio/0000-0002-5029-1657; Feola, Sara/0000-0002-4012-4310; Hamdan, Firas/0000-0003-4678-7382; Peltonen, Karita Dolores/0000-0002-3063-451X; Alenius, Harri/0000-0003-0106-8923; Kari, Otto/0000-0002-1201-5010; Fusciello, Manlio/0000-0002-7166-3018
FU Doctoral Program in Drug Research for doctoral scholarships at the Faculty of Pharmacy, Helsinki University; HiLIFE Research Funds; Sigrid Juselius Foundation; Academy of Finland [317042]; European Research Council [825020, 681219]; Jane and Aatos Erkko Foundation [4705796]; HiLIFE [797011004]; Cancer Finnish Foundation [4706116]; Magnus Ehrnrooth Foundation [4706235]; European Research Council (ERC) [825020]
Funding Source: European Research Council (ERC)
FX We thank the Extracellular Vesicle Core Facility at the University of Helsinki, Finland, for providing the NTA service. We acknowledge the provision of facilities and technical support by Aalto University at OtaNano- Nanomicroscopy Center (Aalto-NMC). We thank Smart Servier Medical Art for the contribution to Fig. 1 (https://smart.servier.com). M.F.and C.C. thank the Doctoral Program in Drug Research for doctoral scholarships at the Faculty of Pharmacy, Helsinki University. H.A.S. acknowledges financial support from the HiLIFE Research Funds, the Sigrid Juselius Foundation, the Academy of Finland (grant no. 317042), and the European Research Council Proof-of-Concept Research Grant (grant no. 825020). V.C. acknowledges the European Research Council under the Horizon 2020 framework (https://erc.europa.eu), ERC-consolidator Grant (Agreement no. 681219), Jane and Aatos Erkko Foundation (Project no. 4705796), HiLIFE Fellow (project no. 797011004) Cancer Finnish Foundation (project No. 4706116), Magnus Ehrnrooth Foundation (project No. 4706235).
CR Rojas LA, 2016, J CONTROL RELEASE, V237, P78, DOI 10.1016/j.jconrel.2016.07.004
Banchereau J, 2018, NAT REV CLIN ONCOL, V15, P9, DOI 10.1038/nrclinonc.2017.149
Bass-Stringer S, 2018, HEART LUNG CIRC, V27, P1285, DOI 10.1016/j.hlc.2018.03.005
Baudino Troy A, 2015, Curr Drug Discov Technol, V12, P3
Bencherif SA, 2015, NAT COMMUN, V6, DOI 10.1038/ncomms8556
Capasso C, 2016, ONCOIMMUNOLOGY, V5, DOI 10.1080/2162402X.2015.1105429
Cerullo V, 2012, ONCOIMMUNOLOGY, V1, P979, DOI 10.4161/onci.20172
Cerullo V, 2012, MOL THER, V20, P2076, DOI 10.1038/mt.2012.137
Champiat S, 2018, NAT REV CLIN ONCOL, V15, P748, DOI 10.1038/s41571-018-0111-2
Cox J, 2014, MOL CELL PROTEOMICS, V13, P2513, DOI 10.1074/mcp.M113.031591
dos Santos T, 2011, PLOS ONE, V6, DOI 10.1371/journal.pone.0024438
Douglas JT, 2001, CANCER RES, V61, P813
Fang RH, 2014, NANO LETT, V14, P2181, DOI 10.1021/nl500618u
Feola S, 2018, ONCOIMMUNOLOGY, V7, DOI 10.1080/2162402X.2018.1457596
Fontana F, 2017, ADV MATER, V29, DOI 10.1002/adma.201603239
Fontana F, 2017, WIRES NANOMED NANOBI, V9, DOI 10.1002/wnan.1421
Gonzalez FE, 2014, HUM VACC IMMUNOTHER, V10, P3261, DOI 10.4161/21645515.2014.982996
Hamdy S, 2008, VACCINE, V26, P5046, DOI 10.1016/j.vaccine.2008.07.035
Hayward C, 2015, HEART, V101, pA24, DOI 10.1136/heartjnl-2015-308066.41

He CB, 2010, BIOMATERIALS, V31, P3657, DOI 10.1016/j.biomaterials.2010.01.065
Hu CMJ, 2011, P NATL ACAD SCI USA, V108, P10980, DOI 10.1073/pnas.1106634108
Iversen TG, 2011, NANO TODAY, V6, P176, DOI 10.1016/j.nantod.2011.02.003
Kanerva A, 2002, CLIN CANCER RES, V8, P275
Kanerva A, 2003, MOL THER, V8, P449, DOI 10.1016/S1525-0016(03)00200-4
Kapadia CH, 2018, J CONTROL RELEASE, V269, P393, DOI 10.1016/j.jconrel.2017.11.020
Kaufman HL, 2015, NAT REV DRUG DISCOV, V14, P642, DOI 10.1038/nrd4663
Kroll AV, 2017, BIOCONJUGATE CHEM, V28, P23, DOI 10.1021/acs.bioconjchem.6b00569
Lee HM, 2017, SCI REP-UK, V7, DOI 10.1038/s41598-017-13501-1
Lesniak A, 2012, ACS NANO, V6, P5845, DOI 10.1021/nn300223w
Li AW, 2018, NAT MATER, V17, P528, DOI 10.1038/s41563-018-0028-2
Lichty BD, 2014, NAT REV CANCER, V14, P559, DOI 10.1038/nrc3770
Lizotte PH, 2016, NAT NANOTECHNOL, V11, P295, DOI [10.1038/NNANO.2015.292, 10.1038/nnano.2015.292]
Luk BT, 2015, J CONTROL RELEASE, V220, P600, DOI 10.1016/j.jconrel.2015.07.019
Melero I, 2014, CANCER DISCOV, V4, P522, DOI 10.1158/2159-8290.CD-13-0985
Mendez N, 2014, BIOMATERIALS, V35, P9554, DOI 10.1016/j.biomaterials.2014.08.010
Mi HY, 2017, NUCLEIC ACIDS RES, V45, pD183, DOI 10.1093/nar/gkw1138
Morishita M, 2016, BIOMATERIALS, V111, P55, DOI 10.1016/j.biomaterials.2016.09.031
Newick K, 2016, MOL THER-ONCOLYTICS, V3, DOI 10.1038/mto.2016.6
Oh N, 2014, INT J NANOMED, V9, P51, DOI 10.2147/IJN.S26592
Okegawa T, 2001, CANCER RES, V61, P6592
Pol J, 2016, ONCOIMMUNOLOGY, V5, DOI 10.1080/2162402X.2015.1117740
Pol JG, 2018, ONCOIMMUNOLOGY, V7, DOI 10.1080/2162402X.2018.1503032
Rainone V, 2016, PLOS ONE, V11, DOI 10.1371/journal.pone.0146622
Rekik R, 2015, CELL MOL IMMUNOL, V12, P648, DOI 10.1038/cmi.2014.104
Ribas A, 2018, SCIENCE, V359, P1350, DOI 10.1126/science.aar4060
Ribas A, 2017, CELL, V170, P1109, DOI 10.1016/j.cell.2017.08.027
Roberts DM, 2006, NATURE, V441, P239, DOI 10.1038/nature04721
Rosenberg SA, 2014, NAT REV CLIN ONCOL, V11, P630, DOI 10.1038/nrclinonc.2014.174
Russell SJ, 2012, NAT BIOTECHNOL, V30, P658, DOI 10.1038/nbt.2287
Shan YP, 2015, CHEM SOC REV, V44, P3617, DOI 10.1039/c4cs00508b
Spitzer MH, 2017, CELL, V168, P487, DOI 10.1016/j.cell.2016.12.022
Tuve S, 2009, VACCINE, V27, P4225, DOI 10.1016/j.vaccine.2009.03.074
Twumasi-Boateng K, 2018, NAT REV CANCER, V18, P419, DOI 10.1038/s41568-018-0009-4
Tyanova S, 2016, NAT METHODS, V13, P731, DOI [10.1038/NMETH.3901, 10.1038/nmeth.3901]
van der Burg SH, 2016, NAT REV CANCER, V16, P219, DOI 10.1038/nrc.2016.16
Wu A, 2007, J IMMUNOTHER, V30, P789, DOI 10.1097/CJI.0b013e318155a0f6
Xia AL, 2017, ONCOTARGET, V8, P90521, DOI 10.18632/oncotarget.19361
Yamamoto M, 2010, MOL THER, V18, P243, DOI 10.1038/mt.2009.266
Yang R, 2018, ACS NANO, V12, P5121, DOI 10.1021/acsnano.7b09041
Yang YS, 2017, ADV MATER, V29, DOI 10.1002/adma.201605604
Ylosmaki E, 2018, MOL THER, V26, P2315, DOI 10.1016/j.ymthe.2018.06.008
Yotnda P, 2002, MOL THER, V5, P233, DOI 10.1006/mthe.2002.0545
Zeimet AG, 2003, LANCET ONCOL, V4, P415, DOI 10.1016/S1470-2045(03)01139-2
Zhong ZR, 2010, MOL PHARMACEUT, V7, P105, DOI 10.1021/mp900151k
NR 64
TC 70
Z9 74
U1 7
U2 81
PU NATURE PUBLISHING GROUP
PI LONDON
PA MACMILLAN BUILDING, 4 CRINAN ST, LONDON N1 9XW, ENGLAND
SN 2041-1723
J9 NAT COMMUN
JI Nat. Commun.
PD DEC 17
PY 2019
VL 10
AR 5747

DI 10.1038/s41467-019-13744-8
PG 13
WC Multidisciplinary Sciences
WE Science Citation Index Expanded (SCI-EXPANDED)
SC Science & Technology - Other Topics
GA JW4FO
UT WOS:000503009300004
PM 31848338
OA Green Published, gold
DA 2023-09-14
ER

PT J
AU Singha, S
Shao, K
Ellestad, KK
Yang, Y
Santamaria, P
AF Singha, Santiswarup
Shao, Kun
Ellestad, Kristofor K.
Yang, Yang
Santamaria, Pere
TI Nanoparticles for Immune Stimulation Against Infection, Cancer, and Autoimmunity
SO ACS NANO
LA English
DT Review
DE nanoparticles; nanodesign; nanovaccine; infectious diseases; cancer; vaccine; immune modulation; translational potential
ID IRON-OXIDE NANOPARTICLES; ANTIGEN-PRESENTING CELLS; VIRUS-LIKE PARTICLES; LYMPH-NODE; CO-DELIVERY; PROTEIN NANOPARTICLES; SILICA NANOPARTICLES; SYNTHETIC VACCINES; NASAL DELIVERY; STING PATHWAY
AB Vaccination using nanocarrier-based delivery systems has recently emerged as a promising approach for meeting the continued challenge posed by infectious diseases and cancer. A diverse portfolio of nanocarriers of various sizes, compositions, and physical parameters have now been developed, and this diversity provides an opportunity for the rational design of vaccines that can mediate targeted delivery of various antigens and adjuvants or immune regulatory agents in ways unachievable with classical vaccination approaches. This flexibility allows control over the characteristics of vaccine-elicited immune responses such that they can be tailored to be effective in circumstances where classical vaccines have failed. Furthermore, the utility of nanocarrier-based immune modulation extends to the treatment of autoimmune disease where precisely targeted inhibition of immune responses is desirable. Clearly, the selection of appropriate nanocarriers, antigens, adjuvants, and other components underpins the efficacy of these nanoimmune interventions. Herein, we provide an overview of currently available nanocarriers of various types and their physical and pharmacological properties with the goal of providing a resource for researchers exploring nanomaterial-based approaches for immune modulation and identify some information gaps and unexplored questions to help guide future investigation.
C1 [Singha, Santiswarup; Shao, Kun; Ellestad, Kristofor K.; Yang, Yang; Santamaria, Pere] Univ Calgary, JMDRC, Cumming Sch Med, Calgary, AB T2N 4N1, Canada.
[Singha, Santiswarup; Shao, Kun; Ellestad, Kristofor K.; Yang, Yang; Santamaria, Pere] Univ Calgary, Dept Microbiol Immunol & Infect Dis, Cumming Sch Med, Snyder Inst Chron Dis, Calgary, AB T2N 4N1, Canada.
[Singha, Santiswarup; Shao, Kun; Ellestad, Kristofor K.; Yang, Yang; Santamaria, Pere] Univ Calgary, Hotchkiss Brain Inst, Cumming Sch Med, Calgary, AB T2N 4N1, Canada.
[Yang, Yang] Univ Calgary, Cumming Sch Med, Dept Biochem & Mol Biol, Calgary, AB T2N 4N1, Canada.
[Santamaria, Pere] Inst Invest Biomed August Pi i Sunyer, Barcelona 08036, Spain.
C3 University of Calgary; University of Calgary; University of Calgary; University of Calgary; University of Barcelona; Hospital Clinic de Barcelona; IDIBAPS
RP Yang, Y; Santamaria, P (通讯作者)，Univ Calgary, JMDRC, Cumming Sch Med, Calgary, AB T2N 4N1,

Canada.; Yang, Y; Santamaria, P (通讯作者)，Univ Calgary, Dept Microbiol Immunol & Infect Dis, Cumming Sch Med, Snyder Inst Chron Dis, Calgary, AB T2N 4N1, Canada.; Yang, Y; Santamaria, P (通讯作者)，Univ Calgary, Hotchkiss Brain Inst, Cumming Sch Med, Calgary, AB T2N 4N1, Canada.; Yang, Y (通讯作者)，Univ Calgary, Cumming Sch Med, Dept Biochem & Mol Biol, Calgary, AB T2N 4N1, Canada.; Santamaria, P (通讯作者)，Inst Invest Biomed August Pi i Sunyer, Barcelona 08036, Spain.
EM yyang@ucalgary.ca; psantama@ucalgary.ca
RI Santamaria, Pere/ABF-6823-2021
OI Ellestad, Kristofor/0000-0001-9350-4977; Shao, Kun/0000-0001-6876-4697
FU Canadian Institutes of Health Research (CIHR) [FRN 136866]; Diabetes Canada [NOD_OG-3-15-4963-PS]; Crohn's and Colitis Foundation of Canada; Multiple Sclerosis Society of Canada (MSSC) [EGID 2641]; ISCIII; FEDER [NEURON-FP-715-018]; MINECO [SAF2015-68187-R]; Generalitat de Catalunya [2017-SGR-338]; Diabetes Association (Foothills)
FX We thank the members of our laboratory for their contributions and insights. We apologize to those authors whose work could not be referenced here owing to space limitations. The authors' work summarized here was funded by the Canadian Institutes of Health Research (CIHR; FRN 136866), Diabetes Canada (NOD_OG-3-15-4963-PS), the Crohn's and Colitis Foundation of Canada, the Multiple Sclerosis Society of Canada (MSSC; EGID 2641), ISCIII and FEDER, NEURON-FP-715-018, MINECO (SAF2015-68187-R), and Generalitat de Catalunya (SGR and CERCA Programmes; 2017-SGR-338). The JMDRC was supported by the Diabetes Association (Foothills) and currently by Diabetes Canada.
CR An M, 2017, ACS APPL MATER INTER, V9, P23466, DOI 10.1021/acsami.7b06024
Anselmo AC, 2015, AAPS J, V17, P1041, DOI 10.1208/s12248-015-9780-2
Aroh C, 2017, J IMMUNOL, V199, P3840, DOI 10.4049/jimmunol.1700972
Beljanski V, 2015, J VIROL, V89, P10612, DOI 10.1128/JVI.01526-15
Black M, 2012, ADV MATER, V24, P3845, DOI 10.1002/adma.201200209
Bourquin C, 2008, J IMMUNOL, V181, P2990, DOI 10.4049/jimmunol.181.5.2990
Cevher E, 2015, J MICROENCAPSUL, V32, P755, DOI 10.3109/02652048.2015.1073392
Chen Q, 2016, NAT IMMUNOL, V17, P1142, DOI 10.1038/ni.3558
Chen Q, 2016, NAT COMMUN, V7, DOI 10.1038/ncomms13193
Clemente-Casares X, 2016, NATURE, V530, P434, DOI 10.1038/nature16962
Deng L, 2018, NAT COMMUN, V9, DOI 10.1038/s41467-017-02725-4
Dinauer N, 2005, BIOMATERIALS, V26, P5898, DOI 10.1016/j.biomaterials.2005.02.038
Diwan M, 2002, J CONTROL RELEASE, V85, P247, DOI 10.1016/S0168-3659(02)00275-4
El Bissati K, 2017, NPJ VACCINES, V2, DOI 10.1038/s41541-017-0024-6
Fadel TR, 2014, NAT NANOTECHNOL, V9, P639, DOI [10.1038/NNANO.2014.154, 10.1038/nnano.2014.154]
Feng GZ, 2013, PLOS ONE, V8, DOI 10.1371/journal.pone.0061135
Galarza JM, 2005, VIRAL IMMUNOL, V18, P244, DOI 10.1089/vim.2005.18.244
Gerriets VA, 2016, NAT IMMUNOL, V17, P1459, DOI 10.1038/ni.3577
Glass JJ, 2016, NANOSCALE, V8, P8255, DOI 10.1039/c6nr00506c
Gottardi R, 2013, J NANOBIOTECHNOL, V11, DOI 10.1186/1477-3155-11-30
Grgacic EVL, 2006, METHODS, V40, P60, DOI 10.1016/j.ymeth.2006.07.018
Ha NY, 2016, J NANOBIOTECHNOL, V14, DOI 10.1186/s12951-016-0229-2
Hamdy S, 2008, VACCINE, V26, P5046, DOI 10.1016/j.vaccine.2008.07.035
Hanson MC, 2015, J CLIN INVEST, V125, P2532, DOI 10.1172/JCI79915
Haynes BF, 2017, SCIENCE, V355, P1129, DOI 10.1126/science.aan0662
He Q, 2002, CLIN DIAGN LAB IMMUN, V9, P1021, DOI 10.1128/CDLI.9.5.1021-1024.2002
Heo MB, 2014, BIOMATERIALS, V35, P590, DOI 10.1016/j.biomaterials.2013.10.009
Hickey JW, 2017, NANO LETT, V17, P7045, DOI 10.1021/acs.nanolett.7b03734
Hudalla GA, 2013, ADV HEALTHC MATER, V2, P1114, DOI 10.1002/adhm.201200435
Irvine DJ, 2013, NAT MATER, V12, P978, DOI [10.1038/NMAT3775, 10.1038/nmat3775]
Iwasaki A, 2004, NAT IMMUNOL, V5, P987, DOI 10.1038/ni1112
Joyappa DH, 2009, VET MICROBIOL, V139, P58, DOI 10.1016/j.vetmic.2009.05.004
Junkins RD, 2018, J CONTROL RELEASE, V270, P1, DOI 10.1016/j.jconrel.2017.11.030
Kanekiyo M, 2013, NATURE, V499, P102, DOI 10.1038/nature12202
Kang S, 2017, J CONTROL RELEASE, V256, P56, DOI 10.1016/j.jconrel.2017.04.024
Kasturi SP, 2011, NATURE, V470, P543, DOI 10.1038/nature09737
Kavosi A, 2018, SCI REP-UK, V8, DOI 10.1038/s41598-018-26790-x

Kawai T, 2009, INT IMMUNOL, V21, P317, DOI 10.1093/intimm/dxp017
Khatri K, 2008, VACCINE, V26, P2225, DOI 10.1016/j.vaccine.2008.02.058
Kim SY, 2017, BIOMATERIALS, V130, P56, DOI 10.1016/j.biomaterials.2017.03.034
Kuai R, 2017, NAT MATER, V16, P489, DOI [10.1038/nmat4822, 10.1038/NMAT4822]
Lemos H, 2015, EXPERT REV CLIN IMMU, V11, P155, DOI 10.1586/1744666X.2015.995097
Li AV, 2013, SCI TRANSL MED, V5, DOI 10.1126/scitranslmed.3006516
Li HY, 2011, NAT NANOTECHNOL, V6, P645, DOI [10.1038/nnano.2011.153, 10.1038/NNANO.2011.153]
Li P, 2013, J CONTROL RELEASE, V168, P271, DOI 10.1016/j.jconrel.2013.03.025
Lollo G, 2015, INT J PHARMACEUT, V495, P972, DOI 10.1016/j.ijpharm.2015.09.062
Luo M, 2017, NAT NANOTECHNOL, V12, P648, DOI [10.1038/nnano.2017.52, 10.1038/NNANO.2017.52]
Maji M, 2016, SCI REP-UK, V6, DOI 10.1038/srep27206
Maldonado RA, 2015, P NATL ACAD SCI USA, V112, pE156, DOI 10.1073/pnas.1408686111
Manish M, 2013, PLOS ONE, V8, DOI 10.1371/journal.pone.0061885
Marshak-Rothstein A, 2006, NAT REV IMMUNOL, V6, P823, DOI 10.1038/nri1957
Maus MV, 2003, CLIN IMMUNOL, V106, P16, DOI 10.1016/S1521-6616(02)00017-7
McHugh MD, 2015, BIOMATERIALS, V59, P172, DOI 10.1016/j.biomaterials.2015.04.003
McKee AS, 2017, CURR OPIN IMMUNOL, V47, P44, DOI 10.1016/j.coi.2017.06.005
Meyer T, 2008, EXPERT OPIN INV DRUG, V17, P1051, DOI 10.1517/13543784.17.7.1051
Mody KT, 2013, NANOSCALE, V5, P5167, DOI 10.1039/c3nr00357d
Mohsen MO, 2017, J CONTROL RELEASE, V251, P92, DOI 10.1016/j.jconrel.2017.02.031
Molino NM, 2016, BIOMATERIALS, V86, P83, DOI 10.1016/j.biomaterials.2016.01.056
Molino NM, 2013, ACS NANO, V7, P9743, DOI 10.1021/nn403085w
Moon JJ, 2012, PLOS ONE, V7, DOI 10.1371/journal.pone.0031472
Moon JJ, 2011, NAT MATER, V10, P243, DOI 10.1038/NMAT2960
Nakamura T, 2015, J CONTROL RELEASE, V216, P149, DOI 10.1016/j.jconrel.2015.08.026
Nakanishi T, 1999, J CONTROL RELEASE, V61, P233, DOI 10.1016/S0168-3659(99)00097-8
Netea MG, 2005, ANTIMICROB AGENTS CH, V49, P3991, DOI 10.1128/AAC.49.10.3991-3996.2005
Niikura K, 2013, ACS NANO, V7, P3926, DOI 10.1021/nn3057005
Pashine A, 2005, NAT MED, V11, pS63, DOI 10.1038/nm1210
Passlick D, 2018, J CONTROL RELEASE, V289, P23, DOI 10.1016/j.jconrel.2018.09.008
Perica K, 2014, NANOMED-NANOTECHNOL, V10, P119, DOI 10.1016/j.nano.2013.06.015
Powles LM, 2017, J IMMUNOL, V198
Pulendran B, 2004, IMMUNOL REV, V199, P227, DOI 10.1111/j.0105-2896.2004.00144.x
Pusic K, 2013, FASEB J, V27, P1153, DOI 10.1096/fj.12-218362
Reddy ST, 2007, NAT BIOTECHNOL, V25, P1159, DOI [10.1038/nbt1332, 10.1038/nbtl332]
Roldao A, 2010, EXPERT REV VACCINES, V9, P1149, DOI [10.1586/erv.10.115, 10.1586/ERV.10.115]
Roy A, 2013, INT J PHARMACEUT, V445, P171, DOI 10.1016/j.ijpharm.2013.01.045
Rudra JS, 2010, P NATL ACAD SCI USA, V107, P622, DOI 10.1073/pnas.0912124107
Sanchez-Rodriguez SP, 2012, BIOCHIMIE, V94, P870, DOI 10.1016/j.biochi.2011.12.006
Schlosser E, 2008, VACCINE, V26, P1626, DOI 10.1016/j.vaccine.2008.01.030
Schottler S, 2016, NAT NANOTECHNOL, V11, P372, DOI [10.1038/NNANO.2015.330, 10.1038/nnano.2015.330]
Shao K, 2015, ACS NANO, V9, P16, DOI 10.1021/nn5062029
Singha S, 2017, NAT NANOTECHNOL, V12, P701, DOI [10.1038/NNANO.2017.56, 10.1038/nnano.2017.56]
Skrastina D, 2014, PLOS ONE, V9, DOI 10.1371/journal.pone.0114006
Stano A, 2013, BIOMATERIALS, V34, P4339, DOI 10.1016/j.biomaterials.2013.02.024
Steenblock ER, 2008, MOL THER, V16, P765, DOI 10.1038/mt.2008.11
Sungsuwan S, 2015, ACS APPL MATER INTER, V7, P17535, DOI 10.1021/acsami.5b05497
Sunshine JC, 2014, BIOMATERIALS, V35, P269, DOI 10.1016/j.biomaterials.2013.09.050
Sutmuller RPM, 2006, J CLIN INVEST, V116, P485, DOI 10.1172/JCI25439
Tao Y, 2015, NANOSCALE, V7, P12419, DOI 10.1039/c5nr02240a
Thomas C, 2011, MOL PHARMACEUT, V8, P405, DOI 10.1021/mp100255c
Thomas SN, 2014, BIOMATERIALS, V35, P814, DOI 10.1016/j.biomaterials.2013.10.003
Verbeke R, 2017, J CONTROL RELEASE, V266, P287, DOI 10.1016/j.jconrel.2017.09.041
Wang H, 2018, NAT MATER, V17, P761, DOI 10.1038/s41563-018-0147-9
Wang TY, 2012, INT J PHARMACEUT, V436, P351, DOI 10.1016/j.ijpharm.2012.06.028
Wang TY, 2011, EUR J PHARM SCI, V44, P653, DOI 10.1016/j.ejps.2011.10.012
Wilson DR, 2018, NANOMED-NANOTECHNOL, V14, P237, DOI 10.1016/j.nano.2017.10.013

Wolchok JD, 2013, NEW ENGL J MED, V369, P122, DOI 10.1056/NEJMoa1302369
Xu ZH, 2013, J CONTROL RELEASE, V172, P259, DOI 10.1016/j.jconrel.2013.08.021
Xu ZC, 2009, CHEM MATER, V21, P1778, DOI 10.1021/cm802978z
Yassine HM, 2015, NAT MED, V21, P1065, DOI 10.1038/nm.3927
Yen HJ, 2009, SMALL, V5, P1553, DOI 10.1002/smll.200900126
Yeste A, 2016, SCI SIGNAL, V9, DOI 10.1126/scisignal.aad0612
Zanganeh S, 2016, NAT NANOTECHNOL, V11, P986, DOI [10.1038/nnano.2016.168, 10.1038/NNANO.2016.168]
Zeng Q, 2017, BIOMATERIALS, V122, P105, DOI 10.1016/j.biomaterials.2017.01.010
Zhong ZH, 2010, INT J PHARMACEUT, V399, P156, DOI 10.1016/j.ijpharm.2010.07.053
Zhou SW, 2010, CANCER LETT, V287, P75, DOI 10.1016/j.canlet.2009.05.037
Zhuang XT, 2016, J CONTROL RELEASE, V228, P26, DOI 10.1016/j.jconrel.2016.02.035
NR 105
TC 70
Z9 71
U1 7
U2 127
PU AMER CHEMICAL SOC
PI WASHINGTON
PA 1155 16TH ST, NW, WASHINGTON, DC 20036 USA
SN 1936-0851
EI 1936-086X
J9 ACS NANO
JI ACS Nano
PD NOV
PY 2018
VL 12
IS 11
BP 10621
EP 10635
DI 10.1021/acsnano.8b05950
PG 15
WC Chemistry, Multidisciplinary; Chemistry, Physical; Nanoscience &
Nanotechnology; Materials Science, Multidisciplinary
WE Science Citation Index Expanded (SCI-EXPANDED)
SC Chemistry; Science & Technology - Other Topics; Materials Science
GA HC4QL
UT WOS:000451789200007
PM 30481968
DA 2023-09-14
ER

PT J
AU Zhang, Y
Lin, SB
Wang, XY
Zhu, GZ
AF Zhang, Yu
Lin, Shuibin
Wang, Xiang-Yang
Zhu, Guizhi
TI Nanovaccines for cancer immunotherapy
SO WILEY INTERDISCIPLINARY REVIEWS-NANOMEDICINE AND NANOBIOTECHNOLOGY
LA English
DT Review
DE cancer immunotherapy; co-delivery; mRNA vaccines; nanocarriers;
nanovaccine; neoantigen vaccines; STING agonists
ID ANTIGEN-PRESENTING CELLS; INNATE IMMUNE RECOGNITION; ENVELOPED POLYMERIC
NANOPARTICLES; CD8(+) T-CELLS; RECEPTOR AGONISTS; CELLULAR-IMMUNITY;
VACCINE DELIVERY; NUCLEIC-ACIDS; RNA DELIVERY; MOUSE MODEL
AB The past few decades have witnessed the booming field of cancer immunotherapy. Cancer therapeutic

vaccines, either alone or in combination with other immunotherapies such as adoptive cell therapy or immune checkpoint blockade therapy, are an attractive class of cancer immunotherapeutics. However, cancer vaccines have thus far shown suboptimal efficacy in the clinic. Nanomedicines offer unique opportunities to improve the efficacy of these vaccines. A variety of nanoplatforms have been investigated to deliver molecular or cellular or subcellular vaccines to target lymphoid tissues and cells, thereby promoting the potency and durability of anti-tumor immunity while reducing adverse side effects. In this article, we reviewed the key parameters and features of nanovaccines for cancer immunotherapy; we highlighted recent advances in the development of cancer nanovaccines based on synthetic nanocarriers, biogenic nanocarriers, as well as semi-biogenic nanocarriers; and we summarized newly emerging types of nanovaccines, such as those based on stimulator of interferon genes agonists, cancer neoantigens, mRNA vaccines, as well as artificial antigen-presenting cells. This article is categorized under: Therapeutic Approaches and Drug Discovery > Nanomedicine for Oncologic Disease
C1 [Zhang, Yu; Lin, Shuibin] Sun Yat Sen Univ, Affiliated Hosp 1, Ctr Translat Med, Precis Med Inst,Dept Rehabil Med, Guangzhou 510080, Guangdong, Peoples R China.
[Zhang, Yu; Zhu, Guizhi] Virginia Commonwealth Univ, Dept Pharmaceut, Sch Pharm, Ctr Pharmaceut Engn & Sci, Richmond, VA 23298 USA.
[Wang, Xiang-Yang] Virginia Commonwealth Univ, Dept Human & Mol Genet, Richmond, VA 23298 USA.
[Wang, Xiang-Yang] Virginia Commonwealth Univ, Inst Mol Med, Richmond, VA 23298 USA.
[Wang, Xiang-Yang] Virginia Commonwealth Univ, Massey Canc Ctr, Richmond, VA 23298 USA.
[Zhu, Guizhi] Virginia Commonwealth Univ, Massey Canc Ctr, Dev Therapeut Program, Richmond, VA 23298 USA.
C3 Sun Yat Sen University; Virginia Commonwealth University; Virginia Commonwealth University; Virginia Commonwealth University; Virginia Commonwealth University; Virginia Commonwealth University
RP Lin, SB (通讯作者)，Sun Yat Sen Univ, Affiliated Hosp 1, Ctr Translat Med, Precis Med Inst,Dept Rehabil Med, Guangzhou 510080, Guangdong, Peoples R China.; Zhu, GZ (通讯作者)，Virginia Commonwealth Univ, Ctr Pharmaceut Engn & Sci, Massey Canc Ctr, Dept Pharmaceut, 410 North 12th St, Richmond, VA 23298 USA.
EM linshb6@mail.sysu.edu.cn; gzhu2@vcu.edu
RI Zhu, Guizhi/AAN-2888-2020
OI Zhu, Guizhi/0000-0002-2945-7982
FU NCATS NIH HHS [UL1TR002649, UL1 TR002649, KL2 TR002648] Funding Source: Medline; NCI NIH HHS [R01 CA175033, R01 CA229812, R01 CA099326] Funding Source: Medline; NIH HHS [P30 CA106059] Funding Source: Medline
CR Adams S, 2009, IMMUNOTHERAPY-UK, V1, P949, DOI [10.2217/imt.09.70, 10.2217/IMT.09.70]
Ahn S, 2014, ADV HEALTHC MATER, V3, P1194, DOI 10.1002/adhm.201300597
An MG, 2018, NANOSCALE, V10, P9311, DOI 10.1039/c8nr01376d
Bachmann MF, 2010, NAT REV IMMUNOL, V10, P787, DOI 10.1038/nri2868
Bacon A, 2002, J LIPOSOME RES, V12, P173, DOI 10.1081/LPR-120004791
Basle E, 2010, CHEM BIOL, V17, P213, DOI 10.1016/j.chembiol.2010.02.008
Behzadi S, 2017, CHEM SOC REV, V46, P4218, DOI 10.1039/c6cs00636a
Bellier B, 2009, VACCINE, V27, P5772, DOI 10.1016/j.vaccine.2009.07.059
Blattman JN, 2004, SCIENCE, V305, P200, DOI 10.1126/science.1100369
Boczkowski D, 1996, J EXP MED, V184, P465, DOI 10.1084/jem.184.2.465
Burdette DL, 2013, NAT IMMUNOL, V14, P19, DOI 10.1038/ni.2491
Burdette DL, 2011, NATURE, V478, P515, DOI 10.1038/nature10429
Cai JY, 2019, ADV BIOSYST, V3, DOI 10.1002/adbi.201800287
Chen PF, 2016, HUM VACC IMMUNOTHER, V12, P612, DOI 10.1080/21645515.2015.1105415
Cheung AS, 2018, NAT BIOTECHNOL, V36, P160, DOI 10.1038/nbt.4047
Copland MJ, 2005, IMMUNOL CELL BIOL, V83, P97, DOI 10.1111/j.1440-1711.2005.01315.x
COULIE PG, 1995, P NATL ACAD SCI USA, V92, P7976, DOI 10.1073/pnas.92.17.7976
Diebold SS, 2004, SCIENCE, V303, P1529, DOI 10.1126/science.1093616
Eggermont LJ, 2014, TRENDS BIOTECHNOL, V32, P456, DOI 10.1016/j.tibtech.2014.06.007
Fadel TR, 2014, NAT NANOTECHNOL, V9, P639, DOI [10.1038/NNANO.2014.154, 10.1038/nnano.2014.154]
Fang RH, 2014, NANO LETT, V14, P2181, DOI 10.1021/nl500618u
Felnerova D, 2004, CURR OPIN BIOTECH, V15, P518, DOI 10.1016/j.copbio.2004.10.005
Fioretti D, 2010, J BIOMED BIOTECHNOL, DOI 10.1155/2010/174378
Fisher JPH, 2014, CLIN CANCER RES, V20, P5720, DOI 10.1158/1078-0432.CCR-13-3464
Gall A, 2012, IMMUNITY, V36, P120, DOI 10.1016/j.immuni.2011.11.018

Galluzzi L, 2012, ONCOIMMUNOLOGY, V1, P1111, DOI 10.4161/onci.21494
Garrone P, 2011, SCI TRANSL MED, V3, DOI 10.1126/scitranslmed.3002330
Geall AJ, 2012, P NATL ACAD SCI USA, V109, P14604, DOI 10.1073/pnas.1209367109
Gnjatic S, 2010, CANCER J, V16, P382, DOI 10.1097/PPO.0b013e3181eaca65
Goforth R, 2009, CANCER IMMUNOL IMMUN, V58, P517, DOI 10.1007/s00262-008-0574-6
Gou ML, 2014, NAT COMMUN, V5, DOI 10.1038/ncomms4774
Guo YY, 2015, ACS NANO, V9, P6918, DOI 10.1021/acsnano.5b01042
Hanson MC, 2015, J CLIN INVEST, V125, P2532, DOI 10.1172/JCI79915
Hasan AN, 2016, CLIN EXP IMMUNOL, V186, P249, DOI 10.1111/cei.12816
Heo MB, 2014, BIOMATERIALS, V35, P590, DOI 10.1016/j.biomaterials.2013.10.009
Hickey JW, 2017, NANO LETT, V17, P7045, DOI 10.1021/acs.nanolett.7b03734
Holm CK, 2012, NAT IMMUNOL, V13, P737, DOI 10.1038/ni.2350
Huang J, 2013, IMMUNITY, V39, P846, DOI 10.1016/j.immuni.2013.08.036
Ignatius R, 2000, BLOOD, V96, P3505
Irvine DJ, 2015, CHEM REV, V115, P11109, DOI 10.1021/acs.chemrev.5b00109
Ishikawa H, 2008, NATURE, V455, P674, DOI 10.1038/nature07317
Ishikawa H, 2009, NATURE, V461, P788, DOI 10.1038/nature08476
Jella KK, 2018, VACCINES-BASEL, V6, DOI 10.3390/vaccines6040069
Jones JW, 2010, P NATL ACAD SCI USA, V107, P9771, DOI 10.1073/pnas.1003738107
Junkins RD, 2018, J CONTROL RELEASE, V270, P1, DOI 10.1016/j.jconrel.2017.11.030
Kamps JAAM, 1996, BBA-BIOMEMBRANES, V1278, P183, DOI 10.1016/0005-2736(95)00218-9
Kim OY, 2017, NAT COMMUN, V8, DOI 10.1038/s41467-017-00729-8
Koshy ST, 2017, ADV BIOSYST, V1, DOI 10.1002/adbi.201600013
Kosmides AK, 2017, BIOMATERIALS, V118, P16, DOI 10.1016/j.biomaterials.2016.11.038
Kranz LM, 2016, NATURE, V534, P396, DOI 10.1038/nature18300
Krishnamachari Y, 2009, ADV DRUG DELIVER REV, V61, P205, DOI 10.1016/j.addr.2008.12.013
Kuai R, 2017, NAT MATER, V16, P489, DOI [10.1038/nmat4822, 10.1038/NMAT4822]
Lee YS, 2011, EXP MOL MED, V43, P281, DOI 10.3858/emm.2011.43.5.029
Li HY, 2011, NAT NANOTECHNOL, V6, P645, DOI [10.1038/nnano.2011.153, 10.1038/NNANO.2011.153]
Li L, 2017, ANN ONCOL, V28, P11, DOI 10.1093/annonc/mdx681
Lin LCW, 2018, ADV HEALTHC MATER, V7, DOI 10.1002/adhm.201701395
Liu HP, 2014, NATURE, V507, P519, DOI 10.1038/nature12978
Lowe DB, 2007, CELL MOL LIFE SCI, V64, P2391, DOI 10.1007/s00018-007-7165-0
Luk BT, 2015, J CONTROL RELEASE, V220, P600, DOI 10.1016/j.jconrel.2015.07.019
Luo M, 2017, NAT NANOTECHNOL, V12, P648, DOI [10.1038/nnano.2017.52, 10.1038/NNANO.2017.52]
Melief CJM, 2007, NATURE, V450, P803, DOI 10.1038/nature06363
Mellman I, 2011, NATURE, V480, P480, DOI 10.1038/nature10673
Meng J, 2008, SMALL, V4, P1364, DOI 10.1002/smll.200701059
Min YZ, 2017, NAT NANOTECHNOL, V12, P877, DOI [10.1038/nnano.2017.113, 10.1038/NNANO.2017.113]
Montomoli E, 2011, EXPERT REV VACCINES, V10, P1053, DOI [10.1586/erv.11.48, 10.1586/ERV.11.48]
Nakamura T, 2015, J CONTROL RELEASE, V216, P149, DOI 10.1016/j.jconrel.2015.08.026
Ng KW, 2018, TRENDS IMMUNOL, V39, P44, DOI 10.1016/j.it.2017.07.013
Oberli MA, 2017, NANO LETT, V17, P1326, DOI 10.1021/acs.nanolett.6b03329
Ott PA, 2017, NATURE, V547, P217, DOI 10.1038/nature22991
Parmiani G, 2007, J IMMUNOL, V178, P1975, DOI 10.4049/jimmunol.178.4.1975
Peng YB, 2017, ANGEW CHEM INT EDIT, V56, P10845, DOI 10.1002/anie.201701366
Petersen TR, 2010, CRIT REV IMMUNOL, V30, P345, DOI 10.1615/CritRevImmunol.v30.i4.30
Radovic-Moreno AF, 2015, P NATL ACAD SCI USA, V112, P3892, DOI 10.1073/pnas.1502850112
Reddy ST, 2007, NAT BIOTECHNOL, V25, P1159, DOI [10.1038/nbt1332, 10.1038/nbtl332]
Rietscher R, 2016, EUR J PHARM BIOPHARM, V102, P20, DOI 10.1016/j.ejpb.2016.02.014
Roberts ZJ, 2007, J EXP MED, V204, P1559, DOI 10.1084/jem.20061845
Rosenthal JA, 2014, PLOS ONE, V9, DOI 10.1371/journal.pone.0112802
Ross JS, 1998, STEM CELLS, V16, P413, DOI 10.1002/stem.160413
Roy P, 2008, HUM VACCINES, V4, P5, DOI 10.4161/hv.4.1.5559
Sah H, 2013, INT J NANOMED, V8, P747, DOI 10.2147/IJN.S40579
Sahin U, 2017, NATURE, V547, P222, DOI 10.1038/nature23003
Schijns VEJC, 2003, VACCINE, V21, P829, DOI 10.1016/S0264-410X(02)00527-3
Schneider IC, 2018, BIOTECHNOL J, V13, DOI 10.1002/biot.201700345

Schwaninger R, 2004, CANCER IMMUNOL IMMUN, V53, P1005, DOI 10.1007/s00262-004-0545-5
Shae D, 2019, NAT NANOTECHNOL, V14, P269, DOI 10.1038/s41565-018-0342-5
Shannahan Jonathan H, 2015, Receptors Clin Investig, V2, pe811
Sharma S, 2011, IMMUNITY, V35, P194, DOI 10.1016/j.immuni.2011.05.016
Siefert AL, 2017, METHODS MOL BIOL, V1530, P343, DOI 10.1007/978-1-4939-6646-2_21
STIENEKER F, 1991, AIDS, V5, P431, DOI 10.1097/00002030-199104000-00012
Sundararaman SK, 2018, IMMUNITY, V49, P585, DOI 10.1016/j.immuni.2018.10.001
Sykulev Y, 1996, IMMUNITY, V4, P565, DOI 10.1016/S1074-7613(00)80483-5
Syn NL, 2017, TRENDS BIOTECHNOL, V35, P665, DOI 10.1016/j.tibtech.2017.03.004
Tan A, 2010, INT J NANOMED, V5, DOI 10.2147/IJN.S13402
Tan SW, 2014, NANOMEDICINE-UK, V9, P635, DOI [10.2217/NNM.13.67, 10.2217/nnm.13.67]
Taneichi M, 2006, J IMMUNOL, V177, P2324, DOI 10.4049/jimmunol.177.4.2324
Temizoz B, 2016, INT IMMUNOL, V28, P329, DOI 10.1093/intimm/dxw015
Thery C, 2002, NAT IMMUNOL, V3, P1156, DOI 10.1038/ni854
Tian LL, 2017, THERANOSTICS, V7, P614, DOI 10.7150/thno.17381
van der Bruggen P, 2002, IMMUNOL REV, V188, P51, DOI 10.1034/j.1600-065X.2002.18806.x
Wang SJ, 2017, INT J NANOMED, V12, P6813, DOI 10.2147/IJN.S143264
Wang SH, 2019, WIRES NANOMED NANOBI, V11, DOI 10.1002/wnan.1523
Watson RO, 2012, CELL, V150, P803, DOI 10.1016/j.cell.2012.06.040
Wen ZS, 2011, MAR DRUGS, V9, P1038, DOI 10.3390/md9061038
Williams N, 1996, SCIENCE, V272, P28, DOI 10.1126/science.272.5258.28
Wilson DR, 2018, NANOMED-NANOTECHNOL, V14, P237, DOI 10.1016/j.nano.2017.10.013
Woo SR, 2014, IMMUNITY, V41, P830, DOI 10.1016/j.immuni.2014.10.017
Zang XL, 2017, EUR J PHARM BIOPHARM, V115, P243, DOI 10.1016/j.ejpb.2017.03.013
Zhang YJ, 2019, J CANCER RES CLIN, V145, P281, DOI 10.1007/s00432-019-02840-3
Zhu GZ, 2017, NAT COMMUN, V8, DOI 10.1038/s41467-017-02191-y
Zhu GZ, 2017, NAT COMMUN, V8, DOI 10.1038/s41467-017-01386-7
Zhu GZ, 2017, ACS NANO, V11, P2387, DOI 10.1021/acsnano.7b00978
NR 111
TC 69
Z9 69
U1 4
U2 132
PU WILEY
PI HOBOKEN
PA 111 RIVER ST, HOBOKEN 07030-5774, NJ USA
SN 1939-5116
EI 1939-0041
J9 WIRES NANOMED NANOBI
JI Wiley Interdiscip. Rev.-Nanomed. Nanobiotechnol.
PD SEP-OCT
PY 2019
VL 11
IS 5
AR e1559
DI 10.1002/wnan.1559
PG 20
WC Nanoscience & Nanotechnology; Medicine, Research & Experimental
WE Science Citation Index Expanded (SCI-EXPANDED)
SC Science & Technology - Other Topics; Research & Experimental Medicine
GA IR6AA
UT WOS:000481519900007
PM 31172659
OA Green Accepted
DA 2023-09-14
ER

PT J
AU Shae, D
Baljon, JJ
Wehbe, M

Christov, PP
Becker, KW
Kumar, A
Suryadevara, N
Carson, CS
Palmer, CR
Knight, FC
Joyce, S
Wilson, JT
AF Shae, Daniel
Baljon, Jessalyn J.
Wehbe, Mohamed
Christov, Plamen P.
Becker, Kyle W.
Kumar, Amrendra
Suryadevara, Naveenchandra
Carson, Carcia S.
Palmer, Christian R.
Knight, Frances C.
Joyce, Sebastian
Wilson, John T.
TI Co-delivery of Peptide Neoantigens and Stimulator of Interferon Genes Agonists Enhances Response to Cancer Vaccines
SO ACS NANO
LA English
DT Article
DE neoantigen; cancer vaccine; immune checkpoint blockade; immunotherapy; polymer nanoparticle
ID DI-GMP; STING PATHWAY; DENDRITIC CELLS; IMMUNOTHERAPY; ACTIVATION; IMMUNOGENICITY; NANOPARTICLES; TRANSPORT; IMMUNITY; INNATE
AB Cancer vaccines targeting patient-specific neo-antigens have emerged as a promising strategy for improving responses to immune checkpoint blockade. However, neo-antigenic peptides are poorly immunogenic and inept at stimulating CD8(+) T cell responses, motivating a need for new vaccine technologies that enhance their immunogenicity. The stimulator of interferon genes (STING) pathway is an endogenous mechanism by which the innate immune system generates an immunological context for priming and mobilizing neoantigen-specific T cells. Owing to this critical role in tumor immune surveillance, a synthetic cancer nanovaccine platform (nanoSTING-vax) was developed that mimics immunogenic cancer cells in its capacity to efficiently promote co-delivery of peptide antigens and the STING agonist, cGAMP. The co-loading of cGAMP and peptides into pH-responsive, endosomolytic polymersomes promoted the coordinated delivery of both cGAMP and peptide antigens to the cytosol, thereby eliciting inflammatory cytokine production, co-stimulatory marker expression, and antigen cross-presentation. Consequently, nanoSTING-vax significantly enhanced CD8(+) T cell responses to a range of peptide antigens. Therapeutic immunization with nanoSTING-vax, in combination with immune checkpoint blockade, inhibited tumor growth in multiple murine tumor models, even leading to complete tumor rejection and generation of durable antitumor immune memory. Collectively, this work establishes nanoSTING-vax as a versatile platform for enhancing immune responses to neoantigen-targeted cancer vaccines.
C1 [Shae, Daniel; Wehbe, Mohamed; Becker, Kyle W.; Palmer, Christian R.; Wilson, John T.] Vanderbilt Univ, Dept Chem & Biomol Engn, Nashville, TN 37235 USA.
[Baljon, Jessalyn J.; Carson, Carcia S.; Knight, Frances C.; Wilson, John T.] Vanderbilt Univ, Dept Biomed Engn, Nashville, TN 37235 USA.
[Christov, Plamen P.; Wilson, John T.] Vanderbilt Univ, Vanderbilt Inst Chem Biol, Nashville, TN 37232 USA.
[Kumar, Amrendra; Suryadevara, Naveenchandra; Joyce, Sebastian; Wilson, John T.] Vanderbilt Univ, Dept Pathol Microbiol & Immunol, Med Ctr, Nashville, TN 37232 USA.
[Kumar, Amrendra; Suryadevara, Naveenchandra; Joyce, Sebastian; Wilson, John T.] Tennessee Valley Healthcare Syst, Dept Vet Affairs, Nashville, TN 37212 USA.
[Joyce, Sebastian; Wilson, John T.] Vanderbilt Univ, Med Ctr, Vanderbilt Inst Infect Immunol & Inflammat, Dept Pathol Microbiol & Immunol, Nashville, TN 37232 USA.
[Joyce, Sebastian; Wilson, John T.] Vanderbilt Univ, Vanderbilt Ctr Immunobiol, Med Ctr, Nashville, TN 37232 USA.

[Wilson, John T.] Vanderbilt Univ, Vanderbilt Ingram Canc Ctr, Med Ctr, Nashville, TN 37232 USA.
C3 Vanderbilt University; Vanderbilt University; Vanderbilt University; Vanderbilt University; US Department of Veterans Affairs; Veterans Health Administration (VHA); VA Tennessee Valley Healthcare System; Vanderbilt University; Vanderbilt University; Vanderbilt University
RP Wilson, JT (通讯作者)，Vanderbilt Univ, Dept Chem & Biomol Engn, Nashville, TN 37235 USA.; Wilson, JT (通讯作者)，Vanderbilt Univ, Dept Biomed Engn, Nashville, TN 37235 USA.; Wilson, JT (通讯作者)，Vanderbilt Univ, Vanderbilt Inst Chem Biol, Nashville, TN 37232 USA.; Wilson, JT (通讯作者)，Vanderbilt Univ, Dept Pathol Microbiol & Immunol, Med Ctr, Nashville, TN 37232 USA.; Wilson, JT (通讯作者)，Tennessee Valley Healthcare Syst, Dept Vet Affairs, Nashville, TN 37212 USA.; Wilson, JT (通讯作者)，Vanderbilt Univ, Med Ctr, Vanderbilt Inst Infect Immunol & Inflammat, Dept Pathol Microbiol & Immunol, Nashville, TN 37232 USA.; Wilson, JT (通讯作者)，Vanderbilt Univ, Vanderbilt Ctr Immunobiol, Med Ctr, Nashville, TN 37232 USA.; Wilson, JT (通讯作者)，Vanderbilt Univ, Vanderbilt Ingram Canc Ctr, Med Ctr, Nashville, TN 37232 USA.
EM john.t.wilson@vanderbilt.edu
RI Suryadevara, Naveenchandra/AAQ-7626-2021
OI suryadevara, naveenchandra/0000-0003-2571-1290; Palmer, Christian/0000-0002-4184-0095; Becker, Kyle/0000-0003-1627-2724
FU Vanderbilt Ingram Cancer Center [P30 CA68485]; Vanderbilt Digestive Disease Research Center [DK058404]; National Science Foundation [CBET-1554623]; Vanderbilt Ingram Cancer Center (VICC) Ambassador Discovery Grant; VICC-Vanderbilt Center for Immunobiology Pilot Grant; National Institutes of Health (NIH) [5R21AI121626, 5R01DE027749]; Vanderbilt University; Veteran's Affairs Merit Award [BX001444]; Stand Up To Cancer Innovative Research Grant [SU2C-AACR-IRG 20-17]; Canadian Institutes of Health Research (CIHR); National Science Foundation Graduate Research Fellowship [DGE-1445197, DGE-1937963]
FX We gratefully acknowledge Kenneth Rock for providing DC2.4 cells, N. Shastri for providing B3Z T cells, A. Lund for providing B16-Ova cells, D. Beauchamp for providing MC38 cells, A. Richmond for providing B16.F10 cells, L. Glickman for consultation on peptide antigens, and C. Duvall for the use of gel permeation chromatography equipment and IVIS imaging system. We also thank Fairman Studios for illustrating the Table of Contents image and Figure 1. The following reagent was obtained through the NIH Tetramer Core Facility: PE-labeled SIINFEKL/H-2Kb tetramer. We thank the core facilities of the Vanderbilt Institute of Nanoscale Sciences and Engineering (VINSE) for use of dynamic light scattering and electron microscopy and the VUMC Flow Cytometry Shared Resource, supported by the Vanderbilt Ingram Cancer Center (P30 CA68485) and the Vanderbilt Digestive Disease Research Center (DK058404). 2'3'-cGAMP was provided by the Vanderbilt Institute of Chemical Biology Chemical Synthesis Core. This research was supported by grants from the National Science Foundation CBET-1554623 (J.T.W.), a Vanderbilt Ingram Cancer Center (VICC) Ambassador Discovery Grant (J.T.W.), a VICC-Vanderbilt Center for Immunobiology Pilot Grant (J.T.W.), the National Institutes of Health (NIH) 5R21AI121626 (J. T. W.) and 5R01DE027749 (S.J.), the Vanderbilt University Discovery Grant Program (J.T.W, S.J.), a Veteran's Affairs Merit Award BX001444 (S.J.), and also supported by a Stand Up To Cancer Innovative Research Grant, grant number SU2C-AACR-IRG 20-17 (J.T.W.). Stand Up To Cancer (SU2C) is a program of the Entertainment Industry Foundation. Research grants are administered by the American Association for Cancer Research, the scientific partner of SU2C. M.W. acknowledges a postdoctoral fellowship from the Canadian Institutes of Health Research (CIHR). C.S.C. acknowledges a National Science Foundation Graduate Research Fellowship under grant numbers DGE-1445197 and DGE-1937963.
CR Ahn J, 2018, CANCER CELL, V33, P862, DOI 10.1016/j.ccell.2018.03.027
An MG, 2018, NANOSCALE, V10, P9311, DOI 10.1039/c8nr01376d
Atukorale PU, 2019, CANCER RES, V79, P5394, DOI 10.1158/0008-5472.CAN-19-0381
Audiger C, 2017, J IMMUNOL, V198, P2223, DOI 10.4049/jimmunol.1601629
Binnewies M, 2018, NAT MED, V24, P541, DOI 10.1038/s41591-018-0014-x

Chandra D, 2014, CANCER IMMUNOL RES, V2, P901, DOI 10.1158/2326-6066.CIR-13-0123
Chen DS, 2017, NATURE, V541, P321, DOI 10.1038/nature21349
Cheng N, 2018, JCI INSIGHT, V3, DOI 10.1172/jci.insight.120638
Collier MA, 2018, MOL PHARMACEUT, V15, P4933, DOI 10.1021/acs.molpharmaceut.8b00579
Deng LF, 2014, IMMUNITY, V41, P843, DOI 10.1016/j.immuni.2014.10.019
Dubensky Thomas W Jr, 2013, Ther Adv Vaccines, V1, P131, DOI 10.1177/2051013613501988
Eppler HB, 2020, ADV MATER, V32, DOI 10.1002/adma.201903367
Feng XX, 2019, ADV SCI, V6, DOI 10.1002/advs.201802040
Fridman WH, 2017, NAT REV CLIN ONCOL, V14, P717, DOI 10.1038/nrclinonc.2017.101
Fu J, 2015, SCI TRANSL MED, V7, DOI 10.1126/scitranslmed.aaa4306
Gaffney BL, 2010, ORG LETT, V12, P3269, DOI 10.1021/ol101236b
Gutjahr A, 2019, JCI INSIGHT, V4, DOI 10.1172/jci.insight.125107
Hanson MC, 2015, J CLIN INVEST, V125, P2532, DOI 10.1172/JCI79915
Hilf N, 2019, NATURE, V565, P240, DOI 10.1038/s41586-018-0810-y
Hubbell JA, 2019, NAT NANOTECHNOL, V14, P196, DOI 10.1038/s41565-019-0380-7
Irvine DJ, 2013, NAT MATER, V12, P978, DOI [10.1038/NMAT3775, 10.1038/nmat3775]
KARTTUNEN J, 1992, P NATL ACAD SCI USA, V89, P6020, DOI 10.1073/pnas.89.13.6020
Kinkead HL, 2018, JCI INSIGHT, V3, DOI 10.1172/jci.insight.122857
Kitai Y, 2017, J IMMUNOL, V198, P1649, DOI 10.4049/jimmunol.1601694
Knight FC, 2019, ACS NANO, V13, P10939, DOI 10.1021/acsnano.9b00326
Koshy ST, 2017, ADV BIOSYST, V1, DOI 10.1002/adbi.201600013
Kourtis IC, 2013, PLOS ONE, V8, DOI 10.1371/journal.pone.0061646
Kranz LM, 2016, NATURE, V534, P396, DOI 10.1038/nature18300
Kreiter S, 2015, NATURE, V520, P692, DOI 10.1038/nature14426
Kuai R, 2017, NAT MATER, V16, P489, DOI [10.1038/nmat4822, 10.1038/NMAT4822]
Kwon J, 2020, CANCER DISCOV, V10, P26, DOI 10.1158/2159-8290.CD-19-0761
Li AW, 2018, NAT MATER, V17, P528, DOI 10.1038/s41563-018-0028-2
Lin LCW, 2019, ADV FUNCT MATER, V29, DOI 10.1002/adfm.201807616
Lirussi D, 2017, EBIOMEDICINE, V22, P100, DOI 10.1016/j.ebiom.2017.07.016
Liu HP, 2014, NATURE, V507, P519, DOI 10.1038/nature12978
Liu Y, 2019, NAT COMMUN, V10, DOI 10.1038/s41467-019-13094-5
Luo M, 2017, NAT NANOTECHNOL, V12, P648, DOI [10.1038/nnano.2017.52, 10.1038/NNANO.2017.52]
Lynn GM, 2020, NAT BIOTECHNOL, V38, P320, DOI 10.1038/s41587-019-0390-x
Manicassamy S, 2011, IMMUNOL REV, V241, P206, DOI 10.1111/j.1600-065X.2011.01015.x
Mehta NK, 2015, CANCER IMMUNOL RES, V3, P836, DOI 10.1158/2326-6066.CIR-15-0112
Melief CJM, 2008, NAT REV CANCER, V8, P351, DOI 10.1038/nrc2373
Nakamura T, 2015, J CONTROL RELEASE, V216, P149, DOI 10.1016/j.jconrel.2015.08.026
O'Donnell JS, 2019, NAT REV CLIN ONCOL, V16, P151, DOI 10.1038/s41571-018-0142-8
Ott PA, 2019, CANCER DISCOV, V9, P476, DOI 10.1158/2159-8290.CD-18-1357
Ott PA, 2017, NATURE, V547, P217, DOI 10.1038/nature22991
Planas O, 2020, SCIENCE, V367, DOI 10.1126/science.abb2416
Qiu F, 2018, BIOMATERIALS, V182, P82, DOI 10.1016/j.biomaterials.2018.07.052
Reddy ST, 2007, NAT BIOTECHNOL, V25, P1159, DOI [10.1038/nbt1332, 10.1038/nbtl332]
Ribas A, 2018, SCIENCE, V359, P1350, DOI 10.1126/science.aar4060
Romero P, 2016, SCI TRANSL MED, V8, DOI 10.1126/scitranslmed.aaf0685
Scheetz L, 2019, NAT BIOMED ENG, V3, P768, DOI 10.1038/s41551-019-0436-x
Schumacher TN, 2015, SCIENCE, V348, P69, DOI 10.1126/science.aaa4971
Shae D, 2020, J LEUKOCYTE BIOL, V108, P1435, DOI 10.1002/JLB.5BT0119-016R
Shae D, 2019, NAT NANOTECHNOL, V14, P269, DOI 10.1038/s41565-018-0342-5
Sharma P, 2015, SCIENCE, V348, P56, DOI 10.1126/science.aaa8172
Swartz MA, 2012, SCI TRANSL MED, V4, DOI 10.1126/scitranslmed.3003763
Taylor MA, 2019, J IMMUNOTHER CANCER, V7, DOI 10.1186/s40425-019-0794-7
Temizoz B, 2015, EUR J IMMUNOL, V45, P1159, DOI 10.1002/eji.201445132
Thomas SN, 2015, CURR OPIN CHEM ENG, V7, P65, DOI 10.1016/j.coche.2014.11.003
Tureci O, 2018, NAT BIOMED ENG, V2, P566, DOI 10.1038/s41551-018-0266-2
Wang H., 2017, P NATL ACAD SCI US, V114, P1637
Wang ZL, 2015, CANCER IMMUNOL IMMUN, V64, P1057, DOI 10.1007/s00262-015-1713-5
Wilson DR, 2018, NANOMED-NANOTECHNOL, V14, P237, DOI 10.1016/j.nano.2017.10.013
Woo SR, 2015, TRENDS IMMUNOL, V36, P250, DOI 10.1016/j.it.2015.02.003
Woo SR, 2014, IMMUNITY, V41, P830, DOI 10.1016/j.immuni.2014.10.017

Xu C, 2019, ACS NANO, V13, P12148, DOI 10.1021/acsnano.9b06691
Yadav M, 2014, NATURE, V515, P572, DOI 10.1038/nature14001
Yewdell JW, 2010, CURR OPIN IMMUNOL, V22, P402, DOI 10.1016/j.coi.2010.04.002
Zhang F, 2018, CANCER RES, V78, P3718, DOI 10.1158/0008-5472.CAN-18-0306
Zhou JR, 2020, ADV MATER, V32, DOI 10.1002/adma.201901255
Zhu GZ, 2017, NAT COMMUN, V8, DOI 10.1038/s41467-017-01386-7
NR 71
TC 68
Z9 68
U1 15
U2 209
PU AMER CHEMICAL SOC
PI WASHINGTON
PA 1155 16TH ST, NW, WASHINGTON, DC 20036 USA
SN 1936-0851
EI 1936-086X
J9 ACS NANO
JI ACS Nano
PD AUG 25
PY 2020
VL 14
IS 8
BP 9904
EP 9916
DI 10.1021/acsnano.0c02765
PG 13
WC Chemistry, Multidisciplinary; Chemistry, Physical; Nanoscience &
Nanotechnology; Materials Science, Multidisciplinary
WE Science Citation Index Expanded (SCI-EXPANDED)
SC Chemistry; Science & Technology - Other Topics; Materials Science
GA NJ9CF
UT WOS:000566341000055
PM 32701257
OA Green Accepted
DA 2023-09-14
ER

PT J
AU de Faria, PCB
dos Santos, LI
Coelho, JP
Ribeiro, HB
Pimenta, MA
Ladeira, LO
Gomes, DA
Furtado, CA
Gazzinelli, RT
AF Batista de Faria, Paula Cristina
dos Santos, Luara Isabela
Coelho, Joao Paulo
Ribeiro, Henrique Buecker
Pimenta, Marcos Assuncao
Ladeira, Luiz Orlando
Gomes, Dawidson Assis
Furtado, Clascidia Aparecida
Gazzinelli, Ricardo Tostes
TI Oxidized Multiwalled Carbon Nanotubes as Antigen Delivery System to
Promote Superior CD8(+) T Cell Response and Protection against Cancer
SO NANO LETTERS
LA English
DT Article

DE Nanovaccine; carbon nanotubes; NY-ESO-1; cancer; CD8(+) activation
ID DNA; PROTEINS; NY-ESO-1; TRANSPORTERS; DISPERSION; ENHANCE; QUALITY;
BINDING; BASE
AB Properties like high interfacial area with cellular membranes, unique ability to incorporate multiple functionalization, as well as compatibility and transport in biological fluids make carbon nanotubes (CNTs) useful for a variety of therapeutic and drug-delivery applications. Here we used a totally synthetic hybrid supramolecule as an anticancer vaccine formulation. This complex structure comprises CNTs as delivery system for the Cancer Testis Antigen named NY-ESO-1, allied to a synthetic Toll-Like Receptor agonist. The CNT constructs were rapidly internalized into dendritic cells, both in vitro and in vivo, and served as an intracellular antigen depot. This property favored the induction of strong CD4(+) T as well as CD8(+) T cell-mediated immune responses against the NY-ESO-1. Importantly, the vaccination significantly delayed the tumor development and prolonged the mice survival, highlighting the potential application of CNTs as a vaccine delivery system to provide superior immunogenicity and strong protection against cancer
C1 [Batista de Faria, Paula Cristina; dos Santos, Luara Isabela; Gazzinelli, Ricardo Tostes] Fundacao Oswaldo Cruz, Inst Pesquisa Rene Rachou, BR-30190002 Belo Horizonte, MG, Brazil.
[Batista de Faria, Paula Cristina] Univ Fed Uberlandia, Inst Genet & Bioquim, BR-38400902 Uberlandia, MG, Brazil.
[dos Santos, Luara Isabela; Gomes, Dawidson Assis; Gazzinelli, Ricardo Tostes] Univ Fed Minas Gerais, Dept Bioquim & Imunol, BR-31270001 Belo Horizonte, MG, Brazil.
[Coelho, Joao Paulo; Furtado, Clascidia Aparecida] CDTN CNEN, Ctr Desenvolvimento Tecnol Nucl, BR-31270001 Belo Horizonte, MG, Brazil.
[Ribeiro, Henrique Buecker] Univ Presbiteriana Mackenzie, BR-01302907 Sao Paulo, Brazil.
[Pimenta, Marcos Assuncao; Ladeira, Luiz Orlando] Univ Fed Minas Gerais, Dept Fis, BR-31270001 Belo Horizonte, MG, Brazil.
[Gazzinelli, Ricardo Tostes] Univ Massachusetts, Sch Med, Div Infect Dis & Immunol, Worcester, MA 01605 USA.
C3 Fundacao Oswaldo Cruz; Universidade Federal de Uberlandia; Universidade
Federal de Minas Gerais; Centro de Desenvolvimento da Tecnologia Nuclear
(CDTN); Comissao Nacional de Energia Nuclear (CNEN); Universidade
Presbiteriana Mackenzie; Universidade Federal de Minas Gerais;
University of Massachusetts System; University of Massachusetts
Worcester
RP Furtado, CA (通讯作者)，CDTN CNEN, Ctr Desenvolvimento Tecnol Nucl, BR-31270001 Belo Horizonte, MG, Brazil.
EM clas@cdtn.br; ritoga@cpqrr.fiocruz.br
RI Gomes, Dawidson Assis/O-1372-2013; Ladeira, Luiz Orlando/AAJ-4918-2021;
Ribeiro, Henrique/D-3779-2015; Pimenta, Marcos/S-3520-2019; Dos Santos,
Luara Isabela/D-5031-2014; Coelho, Joao Paulo/AAI-2303-2019; Furtado,
Clascidia/AAE-2801-2020; Pimenta, Marcos A/F-2122-2010
OI Gomes, Dawidson Assis/0000-0001-7714-991X; Ladeira, Luiz
Orlando/0000-0002-4935-6070; Dos Santos, Luara
Isabela/0000-0002-6212-2164; Coelho, Joao Paulo/0000-0003-0120-8834;
Pimenta, Marcos/0000-0002-3389-0682
FU Fundacao de Amparo a Pesquisa de Minas Gerais (FAPEMIG); Fundacao
Oswaldo Cruz; National Institute of Science and Technology for
Vaccines/Conselho Nacional de Desenvolvimento Cientifico e Tecnologico
(CNPq); National Institute of Science and Technology for Carbon
Nanomaterials/CNPq; Nanotoxicology Network (CNPq) [552131/2011-3]; NIH
[1R03TW008709]
FX We thank Adelina Pinheiro Santos from Centro de Desenvolvimento da
Tecnologia Nuclear and Cristiano Fantini from Federal University of
Minas Gerais for scientific discussions and suggestions during the
development of this work; Mariana Botelho Barbosa from Centro de
Desenvolvimento da Tecnologia Nuclear for help with MWCNT dispersions
preparation; Kinulpe Honorato Sampaio from Microscopy Center of Federal
University of Minas Gerais for scientific discussions and suggestions on
TEM analyzes; Bruno Galvao Filho from Federal University of Minas Gerais
for help with animal experiments; the LICR Tetramer Facility for
tetramers synthesis; the LICR Cornell University for the recombinant
NY-ESO-1 protein; Dr. Jonathan Cebon from LICR Melbourne for the

B16-NY-ESO-1 cell line; and Dr. Hiroyoshi Nishikawa from Mie University
Medical School for the CT26-NY-ESO-1cell line. This study was funded by
Fundacao de Amparo a Pesquisa de Minas Gerais (FAPEMIG), Fundacao
Oswaldo Cruz, the National Institute of Science and Technology for
Vaccines/Conselho Nacional de Desenvolvimento Cientifico e Tecnologico
(CNPq), the National Institute of Science and Technology for Carbon
Nanomaterials/CNPq, and the Nanotoxicology Network (CNPq process number
552131/2011-3). This work was also supported by NIH grant 1R03TW008709.
CR Almeida LG, 2009, NUCLEIC ACIDS RES, V37, pD816, DOI 10.1093/nar/gkn673
Bertulli C, 2013, NANOTECHNOLOGY, V24, DOI 10.1088/0957-4484/24/26/265102
Bianco A, 2005, J AM CHEM SOC, V127, P58, DOI 10.1021/ja044293y
Bianco A, 2005, CURR OPIN CHEM BIOL, V9, P674, DOI 10.1016/j.cbpa.2005.10.005
Bloomfield V. A., 2000, NUCL ACIDS STRUCTURE, P173
Bolhassani A, 2011, MOL CANCER, V10, DOI 10.1186/1476-4598-10-3
Chen YL, 2011, PLOS ONE, V6, DOI [10.1371/journal.pone.0023237, 10.1371/journal.pone.0017876]
CHOU PJ, 1993, J AM CHEM SOC, V115, P1205, DOI 10.1021/ja00057a001
Desai N, 2012, AAPS J, V14, P282, DOI 10.1208/s12248-012-9339-4
Dumortier H, 2006, NANO LETT, V6, P1522, DOI 10.1021/nl061160x
Fabbro C, 2012, CHEM COMMUN, V48, P3911, DOI 10.1039/c2cc17995d
Fadel TR, 2013, SMALL, V9, P666, DOI 10.1002/smll.201201684
Ge CC, 2011, P NATL ACAD SCI USA, V108, P16968, DOI 10.1073/pnas.1105270108
Han ZJ, 2011, NANOTECHNOLOGY, V22, DOI 10.1088/0957-4484/22/29/295712
Hemminger JA, 2013, MODERN PATHOL, V26, P282, DOI 10.1038/modpathol.2012.133
Hughes ME, 2007, NANO LETT, V7, P1191, DOI 10.1021/nl062906u
Huleatt JW, 2007, VACCINE, V25, P763, DOI 10.1016/j.vaccine.2006.08.013
Jager E, 1998, J EXP MED, V187, P265, DOI 10.1084/jem.187.2.265
Jain S., 2012, J NANOMED NANOTECHOL, V3, P2, DOI [10.4172/2157-7439.1000140, DOI
10.4172/2157-7439.1000140]
Jungbluth AA, 2001, INT J CANCER, V92, P856, DOI 10.1002/ijc.1282
Junqueira C, 2012, PLOS ONE, V7, DOI 10.1371/journal.pone.0036245
Kagan VE, 2010, NAT NANOTECHNOL, V5, P354, DOI [10.1038/NNANO.2010.44,
10.1038/nnano.2010.44]
Kakimi K, 2011, INT J CANCER, V129, P2836, DOI 10.1002/ijc.25955
Kam NWS, 2006, ANGEW CHEM INT EDIT, V45, P577, DOI 10.1002/anie.200503389
Kam NWS, 2005, J AM CHEM SOC, V127, P6021, DOI 10.1021/ja050062v
Kim UJ, 2005, J AM CHEM SOC, V127, P15437, DOI 10.1021/ja052951o
Ladeira MS, 2010, NANOTECHNOLOGY, V21, DOI 10.1088/0957-4484/21/38/385101
Liu J, 1998, SCIENCE, V280, P1253, DOI 10.1126/science.280.5367.1253
Liu Z, 2008, CANCER RES, V68, P6652, DOI 10.1158/0008-5472.CAN-08-1468
Mocan T, 2011, INT J NANOMED, V6, P1945, DOI 10.2147/IJN.S24060
Mu QX, 2008, J PHYS CHEM C, V112, P3300, DOI 10.1021/jp710541j
Nicholaou T, 2011, CANCER IMMUNOL IMMUN, V60, P1625, DOI 10.1007/s00262-011-1041-3
Pack DW, 2004, NAT MATER, V3, P133, DOI 10.1038/nmat1084
Pantarotto D, 2003, CHEM BIOL, V10, P961, DOI 10.1016/j.chembiol.2003.09.011
Pantarotto D, 2004, ANGEW CHEM INT EDIT, V43, P5242, DOI 10.1002/anie.200460437
Pantarotto D, 2004, CHEM COMMUN, P16, DOI 10.1039/b311254c
Porter AE, 2009, ACS NANO, V3, P1485, DOI 10.1021/nn900416z
Randolph GJ, 2005, NAT REV IMMUNOL, V5, P617, DOI 10.1038/nri1670
Reddy ST, 2007, NAT BIOTECHNOL, V25, P1159, DOI [10.1038/nbt1332, 10.1038/nbtl332]
Santosh M, 2012, J CHEM PHYS, V136, DOI 10.1063/1.3682780
Sun Z, 2008, J PHYS CHEM C, V112, P10692, DOI 10.1021/jp8021634
Villa CH, 2011, ACS NANO, V5, P5300, DOI 10.1021/nn200182x
Wille-Reece U, 2005, P NATL ACAD SCI USA, V102, P15190, DOI 10.1073/pnas.0507484102
Worle-Knirsch JM, 2006, NANO LETT, V6, P1261, DOI 10.1021/nl060177c
Wu W, 2005, ANGEW CHEM INT EDIT, V44, P6358, DOI 10.1002/anie.200501613
Wu YR, 2008, ACS NANO, V2, P2023, DOI 10.1021/nn800325a
Yang RH, 2008, ANAL CHEM, V80, P7408, DOI 10.1021/ac801118p
Zhao DC, 2011, CLIN CANCER RES, V17, P771, DOI 10.1158/1078-0432.CCR-10-2444
Zheng M, 2003, NAT MATER, V2, P338, DOI 10.1038/nmat877
Zhu MT, 2013, ACCOUNTS CHEM RES, V46, P622, DOI 10.1021/ar300031y
NR 50

TC 68
Z9 68
U1 1
U2 83
PU AMER CHEMICAL SOC
PI WASHINGTON
PA 1155 16TH ST, NW, WASHINGTON, DC 20036 USA
SN 1530-6984
EI 1530-6992
J9 NANO LETT
JI Nano Lett.
PD SEP
PY 2014
VL 14
IS 9
BP 5458
EP 5470
DI 10.1021/nl502911a
PG 13
WC Chemistry, Multidisciplinary; Chemistry, Physical; Nanoscience &
   Nanotechnology; Materials Science, Multidisciplinary; Physics, Applied;
   Physics, Condensed Matter
WE Science Citation Index Expanded (SCI-EXPANDED)
SC Chemistry; Science & Technology - Other Topics; Materials Science;
   Physics
GA AO7PG
UT WOS:000341544500081
PM 25115645
DA 2023-09-14
ER

PT J
AU Wen, R
   Umeano, AC
   Kou, Y
   Xu, J
   Farooqi, AA
AF Wen, Ru
   Umeano, Afoma C.
   Kou, Yi
   Xu, Jian
   Farooqi, Ammad Ahmad
TI Nanoparticle systems for cancer vaccine
SO NANOMEDICINE
LA English
DT Review
DE antigen delivery; cancer vaccine; delivery systems; nanoparticle;
   nanotechnology; nanovaccine
ID T-CELL RESPONSES; UP-CONVERSION NANOPARTICLES; IN-VITRO
   CHARACTERIZATION; LOW-DENSITY LIPOPROTEINS; SYNTHETIC LONG PEPTIDE;
   DRUG-DELIVERY SYSTEMS; DENDRITIC CELL; LYMPH-NODE; CATIONIC LIPOSOMES;
   DNA VACCINE
AB As effective tools for public health, vaccines prevent disease by priming the body's adaptive and innate immune responses against an infection. Due to advances in understanding cancers and their relationship with the immune system, there is a growing interest in priming host immune defenses for a targeted and complete antitumor response. Nanoparticle systems have shown to be promising tools for effective antigen delivery as vaccines and/or for potentiating immune response as adjuvants. Here, we highlight relevant physiological processes involved in vaccine delivery, review recent advances in the use of nanoparticle systems for vaccines and discuss pertinent challenges to viably translate nanoparticle-based vaccines and adjuvants for public use.
C1 [Wen, Ru] Univ Georgia, Dept Chem, Athens, GA 30602 USA.

[Umeano, Afoma C.] Univ Miami, Miller Sch Med, Dept Mol & Cellular Pharmacol, Miami, FL 33136 USA.
[Kou, Yi] Univ Southern Calif, Dept Mol & Computat Biol, Los Angeles, CA 90089 USA.
[Xu, Jian] NCI, Lab Canc Biol & Genet, Ctr Canc Res, Bethesda, MD 20892 USA.
[Farooqi, Ammad Ahmad] IBGE, Islamabad 54000, Pakistan.
C3 University System of Georgia; University of Georgia; University of Miami; University of Southern California; National Institutes of Health (NIH) - USA; NIH National Cancer Institute (NCI)
RP Wen, R (通讯作者)，Univ Georgia, Dept Chem, Athens, GA 30602 USA.
EM r.wen3344@gmail.com
RI Farooqi, Ammad A/H-1610-2016; Farooqi, Ammad Ahmad/AAF-5325-2021
OI Farooqi, Ammad/0000-0003-2899-5014
CR Adams JR, 2015, ACTA BIOMATER, V14, P104, DOI 10.1016/j.actbio.2014.11.050
AL-Deen FMN, 2014, GENE THER, V21, P212, DOI 10.1038/gt.2013.77
Al-Deen FMN, 2017, NANOMATERIALS-BASEL, V7, DOI 10.3390/nano7020030
Al-Deen FN, 2014, METHODS MOL BIOL, V1143, P181, DOI 10.1007/978-1-4939-0410-5_12
ALLISON AC, 1974, NATURE, V252, P252, DOI 10.1038/252252a0
Almeida JPM, 2015, SMALL, V11, P1453, DOI 10.1002/smll.201402179
Alric C, 2013, NANOSCALE, V5, P5930, DOI 10.1039/c3nr00012e
An M, 2017, ACS APPL MATER INTER, V9, P23466, DOI 10.1021/acsami.7b06024
Bachmann MF, 2010, NAT REV IMMUNOL, V10, P787, DOI 10.1038/nri2868
Banik B, 2018, NANOSCALE, V10, P366, DOI 10.1039/c7nr06295h
BAREL A, 1986, CANCER LETT, V32, P145, DOI 10.1016/0304-3835(86)90112-6
Binauld S, 2013, CHEM COMMUN, V49, P2082, DOI 10.1039/c2cc36589h
Brunsveld L, 2001, CHEM REV, V101, P4071, DOI 10.1021/cr990125q
Bulte JWM, 2014, NANOMED-NANOTECHNOL, V10, P1385, DOI 10.1016/j.nano.2014.05.001
Butts C, 2014, LANCET ONCOL, V15, P59, DOI 10.1016/S1470-2045(13)70510-2
Cao JY, 2015, BIOMATER SCI-UK, V3, P1545, DOI 10.1039/c5bm00161g
Cao Y, 2013, TRANSPORT RES REC, P22, DOI 10.3141/2325-03
Cao YZ, 2017, MRS COMMUN, V7, P672, DOI 10.1557/mrc.2017.57
Cao YZ, 2016, CEMENT CONCRETE COMP, V74, P164, DOI 10.1016/j.cemconcomp.2016.09.008
Cao YZ, 2016, CONSTR BUILD MATER, V119, P71, DOI 10.1016/j.conbuildmat.2016.03.077
Cao Y, 2015, CEMENT CONCRETE COMP, V56, P73, DOI 10.1016/j.cemconcomp.2014.11.008
Choi HS, 2007, NAT BIOTECHNOL, V25, P1165, DOI 10.1038/nbt1340
Cormode DP, 2008, NANO LETT, V8, P3715, DOI 10.1021/nl801958b
De Koker S, 2016, ANGEW CHEM INT EDIT, V55, P1334, DOI 10.1002/anie.201508626
Fan YC, 2015, VACCINES-BASEL, V3, P662, DOI 10.3390/vaccines3030662
Fan YC, 2015, J CONTROL RELEASE, V208, P121, DOI 10.1016/j.jconrel.2015.04.010
Fang RH, 2014, NANO LETT, V14, P2181, DOI 10.1021/nl500618u
Finn OJ, 2003, NAT REV IMMUNOL, V3, P630, DOI 10.1038/nri1150
FU HL, 2018, SUSTAINABILITY BASEL, V10
Fujita Y, 2011, CHEM CENT J, V5, DOI 10.1186/1752-153X-5-48
Gao J, 2017, INT J NANOMED, V12, P1251, DOI 10.2147/IJN.S125866
Garcia KP, 2014, SMALL, V10, P2516, DOI 10.1002/smll.201303540
Garinot M, 2007, J CONTROL RELEASE, V120, P195, DOI 10.1016/j.jconrel.2007.04.021
Garu A, 2016, MOL THER, V24, P385, DOI 10.1038/mt.2015.215
Gregory AE, 2013, FRONT CELL INFECT MI, V3, DOI 10.3389/fcimb.2013.00013
Guo HC, 2012, APPL MICROBIOL BIOT, V95, P1253, DOI 10.1007/s00253-012-4042-z
Harper DM, 2006, LANCET, V367, P1247, DOI 10.1016/S0140-6736(06)68439-0
Hassan HAFM, 2016, BIOMATERIALS, V104, P310, DOI 10.1016/j.biomaterials.2016.07.005
Hassan HAFM, 2016, J CONTROL RELEASE, V225, P205, DOI 10.1016/j.jconrel.2016.01.030
He QJ, 2010, BIOMATERIALS, V31, P1085, DOI 10.1016/j.biomaterials.2009.10.046
Heegaard PMH, 2010, BIOCONJUGATE CHEM, V21, P405, DOI 10.1021/bc900290d
Hirn S, 2011, EUR J PHARM BIOPHARM, V77, P407, DOI 10.1016/j.ejpb.2010.12.029
Hjalmsdottir A, 2016, MOL PHARMACEUT, V13, P320, DOI 10.1021/acs.molpharmaceut.5b00394
Hoebe K, 2004, NAT IMMUNOL, V5, P971, DOI 10.1038/ni1004-971
Hu R, 2017, J BIOMED NANOTECHNOL, V13, P1097, DOI 10.1166/jbn.2017.2407
Hu R, 2017, J BIOMED NANOTECHNOL, V13, P1058, DOI 10.1166/jbn.2017.2406
Hu XM, 2017, J CONTROL RELEASE, V256, P26, DOI 10.1016/j.jconrel.2017.04.026
Huang JL, 2016, PHARMAZIE, V71, P709, DOI 10.1691/ph.2016.6081
Iwama T, 2016, BIOCHEM BIOPH RES CO, V469, P138, DOI 10.1016/j.bbrc.2015.11.084
Jazayeri SD, 2012, J CONTROL RELEASE, V161, P116, DOI 10.1016/j.jconrel.2012.04.015

Jiang PL, 2015, ACTA BIOMATER, V11, P356, DOI 10.1016/j.actbio.2014.09.019
Jin HL, 2016, THERANOSTICS, V6, P2000, DOI 10.7150/thno.15102
Joura EA, 2015, NEW ENGL J MED, V372, P711, DOI 10.1056/NEJMoa1405044
Kanekiyo M, 2015, CELL, V162, P1090, DOI 10.1016/j.cell.2015.07.043
Kang S, 2017, J CONTROL RELEASE, V256, P56, DOI 10.1016/j.jconrel.2017.04.024
Karmakar P, 2016, BIOCONJUGATE CHEM, V27, P110, DOI 10.1021/acs.bioconjchem.5b00528
Kloxin AM, 2009, SCIENCE, V324, P59, DOI 10.1126/science.1169494
Koag MC, 2014, NUCLEIC ACIDS RES, V42, P8755, DOI 10.1093/nar/gku554
Kobayashi T, 2014, NANOMEDICINE-UK, V9, P1715, DOI [10.2217/NNM.14.106, 10.2217/nnm.14.106]
Korsholm KS, 2014, VACCINE, V32, P3927, DOI 10.1016/j.vaccine.2014.05.050
Kou Y, 2015, J AM CHEM SOC, V137, P14067, DOI 10.1021/jacs.5b10172
KRIEGER M, 1979, J SUPRAMOL STR CELL, V10, P467, DOI 10.1002/jss.400100409
Kuai R, 2017, INT J NANOMED, V12, P6581, DOI 10.2147/IJN.S140591
Kuai R, 2017, NAT MATER, V16, P489, DOI [10.1038/nmat4822, 10.1038/NMAT4822]
Kumar CSSR, 2011, ADV DRUG DELIVER REV, V63, P789, DOI 10.1016/j.addr.2011.03.008
Labhasetwar V, 1997, ADV DRUG DELIVER REV, V24, P63, DOI 10.1016/S0169-409X(96)00483-8
Li DD, 2017, ACS BIOMATER SCI ENG, V3, P42, DOI 10.1021/acsbiomaterials.6b00651
Li DD, 2015, ADV FUNCT MATER, V25, P2993, DOI 10.1002/adfm.201500894
Li HY, 2011, NAT NANOTECHNOL, V6, P645, DOI [10.1038/nnano.2011.153, 10.1038/NNANO.2011.153]
Li MH, 2014, J MATER CHEM B, V2, P1619, DOI 10.1039/c3tb21423k
Li SD, 2009, BBA-BIOMEMBRANES, V1788, P2259, DOI 10.1016/j.bbamem.2009.06.022
Li XR, 2014, J CONTROL RELEASE, V173, P148, DOI 10.1016/j.jconrel.2013.10.032
Lin AY, 2013, NANOSCALE RES LETT, V8, DOI 10.1186/1556-276X-8-72
Liu TY, 2013, BIOMACROMOLECULES, V14, P2798, DOI 10.1021/bm400626w
Liu Z, 2008, P NATL ACAD SCI USA, V105, P1410, DOI 10.1073/pnas.0707654105
Lizotte PH, 2016, NAT NANOTECHNOL, V11, P295, DOI [10.1038/NNANO.2015.292, 10.1038/nnano.2015.292]
Lu Y, 2015, P NATL ACAD SCI USA, V112, P12360, DOI 10.1073/pnas.1510533112
Luo ZC, 2015, BIOMATERIALS, V38, P50, DOI 10.1016/j.biomaterials.2014.10.050
Luthi AJ, 2010, TRENDS MOL MED, V16, P553, DOI 10.1016/j.molmed.2010.10.006
Lybaert L, 2016, BIOMACROMOLECULES, V17, P874, DOI 10.1021/acs.biomac.5b01571
Mahony D, 2013, SMALL, V9, P3138, DOI 10.1002/smll.201300012
Makadia HK, 2011, POLYMERS-BASEL, V3, P1377, DOI 10.3390/polym3031377
MEISTER A, 1983, ANNU REV BIOCHEM, V52, P711, DOI 10.1146/annurev.bi.52.070183.003431
Miyabe H, 2014, J CONTROL RELEASE, V184, P20, DOI 10.1016/j.jconrel.2014.04.004
Mo ZC, 2016, ADV DRUG DELIVER REV, V106, P132, DOI 10.1016/j.addr.2016.04.030
Moghimi SM, 1998, ADV DRUG DELIVER REV, V32, P45, DOI 10.1016/S0169-409X(97)00131-2
Molino NM, 2016, BIOMATERIALS, V86, P83, DOI 10.1016/j.biomaterials.2016.01.056
Moretta A, 2008, CELL DEATH DIFFER, V15, P226, DOI 10.1038/sj.cdd.4402170
MORGAN AG, 1975, CLIN EXP IMMUNOL, V20, P489
Nandwana V, 2017, CHEM MATER, V29, P2276, DOI 10.1021/acs.chemmater.6b05357
Neumann S, 2015, VACCINE, V33, P5838, DOI 10.1016/j.vaccine.2015.08.083
Niikura K, 2013, ACS NANO, V7, P3926, DOI 10.1021/nn3057005
Nikanjam M, 2007, INT J PHARMACEUT, V328, P86, DOI 10.1016/j.ijpharm.2006.07.046
Oskuee RK, 2016, ADV PHARM BULL, V6, P515, DOI 10.15171/apb.2016.065
Parry AL, 2013, J AM CHEM SOC, V135, P9362, DOI 10.1021/ja4046857
Pashine A, 2005, NAT MED, V11, pS63, DOI 10.1038/nm1210
Pathak RK, 2017, MOL CANCER THER, V16, P625, DOI 10.1158/1535-7163.MCT-16-0445
Peng LL, 2017, NAT COMMUN, V8, DOI 10.1038/ncomms15139
Qian Y, 2017, ACS NANO, V11, P9536, DOI 10.1021/acsnano.7b05465
Qian Y, 2016, BIOMATERIALS, V98, P171, DOI 10.1016/j.biomaterials.2016.05.008
Rahimian S, 2015, J CONTROL RELEASE, V203, P16, DOI 10.1016/j.jconrel.2015.02.006
Redhead HM, 2001, J CONTROL RELEASE, V70, P353, DOI 10.1016/S0168-3659(00)00367-9
Riabov V, 2015, VACCINE, V33, P5386, DOI 10.1016/j.vaccine.2015.08.062
Rosalia RA, 2015, BIOMATERIALS, V40, P88, DOI 10.1016/j.biomaterials.2014.10.053
Rueda F, 2017, IMMUNOBIOLOGY, V222, P989, DOI 10.1016/j.imbio.2017.06.002
Ruge CA, 2012, PLOS ONE, V7, DOI 10.1371/journal.pone.0040775
Sarmento B, 2011, CARBOHYD POLYM, V84, P919, DOI 10.1016/j.carbpol.2010.12.042
Sather S, 2007, BLOOD, V109, P1026, DOI 10.1182/blood-2006-05-021634
Schiller JT, 2012, VACCINE, V30, pF123, DOI 10.1016/j.vaccine.2012.04.108

Shariat S, 2014, CANCER LETT, V355, P54, DOI 10.1016/j.canlet.2014.09.016
Siegel RL., 2020, CA-CANCER J CLIN, V70, P7, DOI DOI 10.3322/CAAC.21654
SINGER C, 2017, AACR, V77
Smith JD, 2015, CURR OPIN BIOTECH, V34, P217, DOI 10.1016/j.copbio.2015.03.014
Sokolova V, 2017, ACTA BIOMATER, V64, P401, DOI 10.1016/j.actbio.2017.09.037
Stanley M, 2017, PHILOS T R SOC B, V372, DOI 10.1098/rstb.2016.0268
Sun YW, 2018, ONCOTARGET, V9, P5459, DOI 10.18632/oncotarget.23616
Sunasee R, 2016, EXPERT OPIN DRUG DEL, V13, P1243, DOI 10.1080/17425247.2016.1182491
SWARTZ TA, 1989, EUR J EPIDEMIOL, V5, P143, DOI 10.1007/BF00156819
Tanaka K, 2005, INT J CANCER, V116, P624, DOI 10.1002/ijc.21061
Tang SH, 2014, SMALL, V10, P3139, DOI 10.1002/smll.201303631
Taylor GS, 2014, CLIN CANCER RES, V20, P5009, DOI 10.1158/1078-0432.CCR-14-1122-T
Nguyen TX, 2016, NANOMEDICINE-UK, V11, P1169, DOI 10.2217/nnm.16.9
Tian Y, 2014, NANO LETT, V14, P1439, DOI 10.1021/nl404560v
Unger WWJ, 2012, J CONTROL RELEASE, V160, P88, DOI 10.1016/j.jconrel.2012.02.007
van der Lubben IM, 2001, EUR J PHARM SCI, V14, P201, DOI 10.1016/S0928-0987(01)00172-5
Varypataki EM, 2015, AAPS J, V17, P216, DOI 10.1208/s12248-014-9686-4
Vinluan RD, 2014, ACS APPL MATER INTER, V6, P11829, DOI 10.1021/am5031374
Virginio VG, 2017, HELIYON, V3, DOI 10.1016/j.heliyon.2016.e00225
Wang C, 2014, VACCINE, V32, P5475, DOI 10.1016/j.vaccine.2014.07.081
Wang T, 2015, ACS APPL MATER INTER, V7, P6391, DOI 10.1021/acsami.5b00348
Watarai S, 2014, VET IMMUNOL IMMUNOP, V158, P62, DOI 10.1016/j.vetimm.2013.05.005
Wei H, 2013, INTEGR BIOL-UK, V5, P108, DOI 10.1039/c2ib20142a
WEN R, 2017, TRENDS IMMUNOTHER, V1, P104
Wen R, 2018, CRIT REV THER DRUG, V35, P521, DOI
10.1615/CritRevTherDrugCarrierSyst.2018021124
Wen R, 2016, FUND BIOMED TECHNOL, V8, P229, DOI 10.1007/978-3-319-43525-1_9
Wen R, 2016, VACCINES-BASEL, V4, DOI 10.3390/vaccines4020018
Wen R, 2016, CHEM SCI, V7, P5559, DOI 10.1039/c6sc00481d
Wen R, 2016, ADV DRUG DELIVER REV, V99, P52, DOI 10.1016/j.addr.2015.12.024
Wen R, 2014, CHINESE SCI BULL, V59, P2162, DOI 10.1007/s11434-014-0262-1
Wilhelm S, 2016, NAT REV MATER, V1, DOI 10.1038/natrevmats.2016.14
Wilson JT, 2013, ACS NANO, V7, P3912, DOI 10.1021/nn305466z
Xiang J, 2015, ACS NANO, V9, P6401, DOI 10.1021/acsnano.5b02014
Xu J, 2017, PHARMACOL RES, V126, P66, DOI 10.1016/j.phrs.2017.04.001
Xu J, 2015, ONCOTARGET, V6, P26322, DOI 10.18632/oncotarget.4709
Xu ZH, 2014, ACS NANO, V8, P3636, DOI 10.1021/nn500216y
Yang PP, 2012, CHEM SOC REV, V41, P3679, DOI 10.1039/c2cs15308d
Yang SY, 2015, BIOCONJUGATE CHEM, V26, P511, DOI 10.1021/acs.bioconjchem.5b00003
Yin K, 2017, NANOSCALE, V9, P14229, DOI 10.1039/c7nr04582d
Yu SS, 2012, INT J NANOMED, V7, P799, DOI 10.2147/IJN.S28531
Yuba E, 2014, BIOMATERIALS, V35, P3091, DOI 10.1016/j.biomaterials.2013.12.024
Zeng Q, 2017, BIOMATERIALS, V122, P105, DOI 10.1016/j.biomaterials.2017.01.010
Zhang XD, 2014, INT J NANOMED, V9, P2069, DOI 10.2147/IJN.S64301
Zhang XQ, 2016, J BIOMED NANOTECHNOL, V12, P1688, DOI 10.1166/jbn.2016.2278
Zhang ZH, 2009, ANGEW CHEM INT EDIT, V48, P9171, DOI 10.1002/anie.200903112
Zhao K, 2016, SCI REP-UK, V6, DOI 10.1038/srep25720
Zheng H, 2015, J BIOMED NANOTECHNOL, V11, P2197, DOI 10.1166/jbn.2015.2156
Zhou C, 2011, ANGEW CHEM INT EDIT, V50, P3168, DOI 10.1002/anie.201007321
Zhou SS, 2017, LANGMUIR, V33, P14156, DOI 10.1021/acs.langmuir.7b03844
Zhou SS, 2017, J BIOTECHNOL, V241, P42, DOI 10.1016/j.jbiotec.2016.11.008
Zhou ZF, 2014, ORG BIOMOL CHEM, V12, P3238, DOI 10.1039/c4ob00390j
Zhu CL, 2017, ANGEW CHEM INT EDIT, V56, P10399, DOI 10.1002/anie.201704674
2018, NANOMED NANOTECH BIO, V14, P835
2014, CIRC RES, V114, P171
NR 163
TC 67
Z9 68
U1 10
U2 127
PU FUTURE MEDICINE LTD

PI LONDON
PA UNITEC HOUSE, 3RD FLOOR, 2 ALBERT PLACE, FINCHLEY CENTRAL, LONDON, N3
1QB, ENGLAND
SN 1743-5889
EI 1748-6963
J9 NANOMEDICINE-UK
JI Nanomedicine
PD MAR
PY 2019
VL 14
IS 5
BP 627
EP 648
DI 10.2217/nnm-2018-0147
PG 22
WC Biotechnology & Applied Microbiology; Nanoscience & Nanotechnology
WE Science Citation Index Expanded (SCI-EXPANDED)
SC Biotechnology & Applied Microbiology; Science & Technology - Other
Topics
GA HU9JJ
UT WOS:000465610400008
PM 30806568
OA Green Published
DA 2023-09-14
ER

PT J
AU Kroll, AV
Jiang, Y
Zhou, JR
Holay, M
Fang, RH
Zhang, LF
AF Kroll, Ashley, V
Jiang, Yao
Zhou, Jiarong
Holay, Maya
Fang, Ronnie H.
Zhang, Liangfang
TI Biomimetic Nanoparticle Vaccines for Cancer Therapy
SO ADVANCED BIOSYSTEMS
LA English
DT Article
DE anticancer vaccination; antigen presentation; biomimetic nanoparticle;
immunotherapy; nanomedicine
ID ANTIGEN-PRESENTING CELLS; MEMBRANE-COATED NANOPARTICLES; IN-SITU
VACCINATION; TARGETING DENDRITIC CELLS; POLYMERIC NANOPARTICLES;
CHECKPOINT BLOCKADE; IMMUNE-RESPONSE; LYMPH-NODE; ANTITUMOR IMMUNITY;
ANTIBODY-RESPONSES
AB It is currently understood that in order for a tumor to successfully grow, it must evolve means of evading immune surveillance. In the past several decades, researchers have leveraged increases in the knowledge of tumor immunology to develop therapies capable of augmenting endogenous immunity and eliciting strong antitumor responses. In particular, the goal of anticancer vaccination is to train the immune system to properly utilize its own resources in the fight against cancer. Although attractive in principle, there are currently only limited examples of anticancer vaccines that are successfully translated to the clinic. Recently, there has been a significant push toward the use of nanotechnology for designing vaccine candidates that exhibit enhanced potency and specificity. Here, the recent developments in the field of anticancer nanovaccines are discussed. By taking advantage of the flexibility offered by nanomedicine to purposefully program immune responses, this new generation of vaccines has the potential to address many of the hurdles facing traditional platforms. A specific emphasis is placed on the emergence of cell membrane-coated nanoparticles, a novel biomimetic platform that can be used to

generate personalized nanovaccines that elicit strong, multiantigenic antitumor responses.
C1 [Kroll, Ashley, V; Jiang, Yao; Zhou, Jiarong; Holay, Maya; Fang, Ronnie H.; Zhang, Liangfang] Univ Calif San Diego, Dept Nanoengn, La Jolla, CA 92093 USA.
[Kroll, Ashley, V; Jiang, Yao; Zhou, Jiarong; Holay, Maya; Fang, Ronnie H.; Zhang, Liangfang] Univ Calif San Diego, Moores Canc Ctr, La Jolla, CA 92093 USA.
C3 University of California System; University of California San Diego;
University of California System; University of California San Diego
RP Fang, RH; Zhang, LF (通讯作者)，Univ Calif San Diego, Dept Nanoengn, La Jolla, CA 92093 USA.;
Fang, RH; Zhang, LF (通讯作者)，Univ Calif San Diego, Moores Canc Ctr, La Jolla, CA 92093 USA.
EM rhfang@ucsd.edu; zhang@ucsd.edu
RI Holay, Maya/AAB-1553-2019; Fang, Ronnie H./HCH-5747-2022
OI Zhang, Liangfang/0000-0003-0637-0654
FU Defense Threat Reduction Agency Joint Science and Technology Office for
Chemical and Biological Defense [HDTRA1-18-1-0014]; National Institutes
of Health [5T32CA153915]; National Cancer Institute
FX This work was supported by the Defense Threat Reduction Agency Joint
Science and Technology Office for Chemical and Biological Defense under
Grant Number HDTRA1-18-1-0014. J.Z. was supported by a National
Institutes of Health 5T32CA153915 training grant from the National
Cancer Institute.
CR An MG, 2018, NANOSCALE, V10, P9311, DOI 10.1039/c8nr01376d
An M, 2017, ACS APPL MATER INTER, V9, P23466, DOI 10.1021/acsami.7b06024
Angsantikul P, 2018, BIOCONJUGATE CHEM, V29, P604, DOI 10.1021/acs.bioconjchem.7b00692
Angsantikul P, 2015, VACCINES, V3, P814, DOI 10.3390/vaccines3040814
Arias CA, 2009, NEW ENGL J MED, V360, P439, DOI 10.1056/NEJMp0804651
Aryal S, 2013, NANOMEDICINE-UK, V8, P1271, DOI [10.2217/NNM.12.153, 10.2217/nnm.12.153]
Bachmann MF, 2010, NAT REV IMMUNOL, V10, P787, DOI 10.1038/nri2868
Bale SS, 2010, ACS NANO, V4, P1493, DOI 10.1021/nn901586e
Banchereau J, 2005, NAT REV IMMUNOL, V5, P296, DOI 10.1038/nri1592
Bandyopadhyay A, 2011, BIOMATERIALS, V32, P3094, DOI 10.1016/j.biomaterials.2010.12.054
Behzadi S, 2017, CHEM SOC REV, V46, P4218, DOI 10.1039/c6cs00636a
Bishop CJ, 2015, J CONTROL RELEASE, V219, P488, DOI 10.1016/j.jconrel.2015.09.046
BOUSSIF O, 1995, P NATL ACAD SCI USA, V92, P7297, DOI 10.1073/pnas.92.16.7297
Brahmer JR, 2012, NEW ENGL J MED, V366, P2455, DOI 10.1056/NEJMoa1200694
Brudno JN, 2018, NAT REV CLIN ONCOL, V15, P31, DOI 10.1038/nrclinonc.2017.128
Castelli C, 2000, J CELL PHYSIOL, V182, P323, DOI 10.1002/(SICI)1097-4652(200003)182:3<323::AID-JCP2>3.0.CO;2-#
Chada S, 2003, CURR OPIN MOL THER, V5, P463
Chai ZL, 2017, J CONTROL RELEASE, V264, P102, DOI 10.1016/j.jconrel.2017.08.027
Chaudhary B, 2016, VACCINES-BASEL, V4, DOI 10.3390/vaccines4030028
Cheever MA, 2011, CLIN CANCER RES, V17, P3520, DOI 10.1158/1078-0432.CCR-10-3126
Chen PF, 2016, HUM VACC IMMUNOTHER, V12, P612, DOI 10.1080/21645515.2015.1105415
Chen YJ, 2018, ADV HEALTHC MATER, V7, DOI 10.1002/adhm.201701366
Chinnathambi S, 2012, SCI REP-UK, V2, DOI 10.1038/srep00534
Dagogo-Jack I, 2018, NAT REV CLIN ONCOL, V15, P81, DOI 10.1038/nrclinonc.2017.166
de Titta A, 2013, P NATL ACAD SCI USA, V110, P19902, DOI 10.1073/pnas.1313152110
Dehaini D, 2017, ADV MATER, V29, DOI 10.1002/adma.201606209
Dehaini D, 2016, BIOENG TRANSL MED, V1, P30, DOI 10.1002/btm2.10004
Deng L, 2018, NAT COMMUN, V9, DOI 10.1038/s41467-017-02725-4
Diwan Manish, 2003, J Drug Target, V11, P495
Dominguez AL, 2010, VACCINE, V28, P1383, DOI 10.1016/j.vaccine.2009.10.153
Dowling DJ, 2017, J ALLERGY CLIN IMMUN, V140, P1339, DOI 10.1016/j.jaci.2016.12.985
Dubensky TW, 2010, SEMIN IMMUNOL, V22, P155, DOI 10.1016/j.smim.2010.04.007
Eggermont LJ, 2014, TRENDS BIOTECHNOL, V32, P456, DOI 10.1016/j.tibtech.2014.06.007
Elamanchili P, 2007, J IMMUNOTHER, V30, P378, DOI 10.1097/CJI.0b013e31802cf3e3
Fan YC, 2017, NANO LETT, V17, P7387, DOI 10.1021/acs.nanolett.7b03218
Fang RH, 2018, ADV MATER, V30, DOI 10.1002/adma.201706759
Fang RH, 2017, BIOMATERIALS, V128, P69, DOI 10.1016/j.biomaterials.2017.02.041
Fang RNH, 2016, ANNU REV CHEM BIOMOL, V7, P305, DOI 10.1146/annurev-chembioeng-080615-034446
Fang RH, 2015, SMALL, V11, P5483, DOI 10.1002/smll.201501284

Fang RH, 2015, ADV DRUG DELIVER REV, V90, P69, DOI 10.1016/j.addr.2015.04.001
Fang RH, 2014, NANO LETT, V14, P2181, DOI 10.1021/nl500618u
Fang RNH, 2013, NANOSCALE, V5, P8884, DOI 10.1039/c3nr03064d
Fang RH, 2012, EXPERT OPIN BIOL TH, V12, P385, DOI 10.1517/14712598.2012.661710
Farkona S, 2016, BMC MED, V14, DOI 10.1186/s12916-016-0623-5
Fesnak AD, 2016, NAT REV CANCER, V16, P566, DOI 10.1038/nrc.2016.97
Fiering S, 2017, NAT NANOTECHNOL, V12, P615, DOI 10.1038/nnano.2017.89
Finn OJ, 2003, NAT REV IMMUNOL, V3, P630, DOI 10.1038/nri1150
Fischer NO, 2013, J AM CHEM SOC, V135, P2044, DOI 10.1021/ja3063293
Foged C, 2005, INT J PHARMACEUT, V298, P315, DOI 10.1016/j.ijpharm.2005.03.035
Fox CB, 2014, J NANOBIOTECHNOL, V12, DOI 10.1186/1477-3155-12-17
Gao WW, 2015, NANO LETT, V15, P1403, DOI 10.1021/nl504798g
Gao WW, 2013, ADV MATER, V25, P3549, DOI 10.1002/adma.201300638
Gerber HP, 2016, BIOCHEM PHARMACOL, V102, P1, DOI 10.1016/j.bcp.2015.12.008
Gilbert SC, 2001, MOL BIOTECHNOL, V19, P169, DOI 10.1385/MB:19:2:169
Goodwin TJ, 2017, VACCINE, V35, P2550, DOI 10.1016/j.vaccine.2017.03.067
Graddis TJ, 2011, INT J CLIN EXP PATHO, V4, P295
Gros A, 2016, NAT MED, V22, P433, DOI 10.1038/nm.4051
Guo CQ, 2013, ADV CANCER RES, V119, P421, DOI 10.1016/B978-0-12-407190-2.00007-1
Guo YY, 2015, ACS NANO, V9, P6918, DOI 10.1021/acsnano.5b01042
Hammerich L, 2015, MOL ONCOL, V9, P1966, DOI 10.1016/j.molonc.2015.10.016
Hanson MC, 2015, J CLIN INVEST, V125, P2532, DOI 10.1172/JCI79915
Hanson MC, 2014, BIOMACROMOLECULES, V15, P2475, DOI 10.1021/bm500337r
He Q, 2000, CLIN DIAGN LAB IMMUN, V7, P899, DOI 10.1128/CDLI.7.6.899-903.2000
Hodi FS, 2010, NEW ENGL J MED, V363, P711, DOI 10.1056/NEJMoa1003466
Hu CMJ, 2015, NATURE, V526, P118, DOI 10.1038/nature15373
Hu CMJ, 2013, NAT NANOTECHNOL, V8, P933, DOI [10.1038/nnano.2013.254, 10.1038/NNANO.2013.254]
Hu CMJ, 2013, NAT NANOTECHNOL, V8, P336, DOI [10.1038/nnano.2013.54, 10.1038/NNANO.2013.54]
Hu CMJ, 2013, NANOSCALE, V5, P2664, DOI 10.1039/c3nr00015j
Hu CMJ, 2012, ADV HEALTHC MATER, V1, P537, DOI 10.1002/adhm.201200138
Hu CMJ, 2011, P NATL ACAD SCI USA, V108, P10980, DOI 10.1073/pnas.1106634108
Ilyinskii PO, 2014, VACCINE, V32, P2882, DOI 10.1016/j.vaccine.2014.02.027
Jobsri J, 2015, PLOS ONE, V10, DOI 10.1371/journal.pone.0118096
John ALS, 2012, NAT MATER, V11, P250, DOI [10.1038/NMAT3222, 10.1038/nmat3222]
Joshi VB, 2013, AAPS J, V15, P85, DOI 10.1208/s12248-012-9418-6
Kang T, 2018, BIOMATERIALS, V164, P80, DOI 10.1016/j.biomaterials.2018.02.033
Kantoff PW, 2010, NEW ENGL J MED, V363, P411, DOI 10.1056/NEJMoa1001294
Keenan BP, 2012, SEMIN ONCOL, V39, P276, DOI 10.1053/j.seminoncol.2012.02.007
Kim R, 2007, IMMUNOLOGY, V121, P1, DOI 10.1111/j.1365-2567.2007.02587.x
Kosmides AK, 2017, ACS NANO, V11, P5417, DOI 10.1021/acsnano.6b08152
Kranz LM, 2016, NATURE, V534, P396, DOI 10.1038/nature18300
Kroll AV, 2017, ADV MATER, V29, DOI 10.1002/adma.201703969
Kroll AV, 2017, BIOCONJUGATE CHEM, V28, P23, DOI 10.1021/acs.bioconjchem.6b00569
Kuai R, 2017, NAT MATER, V16, P489, DOI [10.1038/nmat4822, 10.1038/NMAT4822]
Lai PY, 2015, RSC ADV, V5, P98222, DOI 10.1039/c5ra17447c
LAW SKA, 1991, CLIN EXP IMMUNOL, V85, P1
Lebel ME, 2016, NANO LETT, V16, P1826, DOI 10.1021/acs.nanolett.5b04877
Lee KL, 2017, NANO LETT, V17, P4019, DOI [10.1021/acs.nanolett.7b00107, 10.1021/acs.nanolett.7600107]
Leight JL, 2017, ANNU REV CANC BIOL, V1, P313, DOI 10.1146/annurev-cancerbio-050216-034431
Li DD, 2016, NANOSCALE, V8, P19592, DOI 10.1039/c6nr05583d
Li HY, 2011, NAT NANOTECHNOL, V6, P645, DOI [10.1038/nnano.2011.153, 10.1038/NNANO.2011.153]
Liang RJ, 2017, BIOMATERIALS, V149, P41, DOI 10.1016/j.biomaterials.2017.09.029
Liu Q, 2018, ACS NANO, V12, P1250, DOI [10.1021/acsnano.7b07384, 10.1021/acsnano.7607384]
Lizotte PH, 2016, NAT NANOTECHNOL, V11, P295, DOI [10.1038/NNANO.2015.292, 10.1038/nnano.2015.292]
Luo M, 2017, NAT NANOTECHNOL, V12, P648, DOI [10.1038/nnano.2017.52, 10.1038/NNANO.2017.52]

Luo ZC, 2017, ADV MATER, V29, DOI 10.1002/adma.201601776
Manolova V, 2008, EUR J IMMUNOL, V38, P1404, DOI 10.1002/eji.200737984
Margaroni M, 2016, EUR J PHARM BIOPHARM, V105, P18, DOI 10.1016/j.ejpb.2016.05.018
Mellman I, 2011, NATURE, V480, P480, DOI 10.1038/nature10673
Mendoza N, 2009, DERMATOL THER, V22, P129, DOI 10.1111/j.1529-8019.2009.01225.x
Meyer RA, 2015, SMALL, V11, P1519, DOI 10.1002/smll.201402369
Mi Y, 2018, ADV MATER, V30, DOI 10.1002/adma.201706098
Min YZ, 2017, NAT NANOTECHNOL, V12, P877, DOI [10.1038/nnano.2017.113, 10.1038/NNANO.2017.113]
Mocellin S, 2001, J IMMUNOTHER, V24, P392, DOI 10.1097/00002371-200109000-00002
Molino NM, 2017, ACS BIOMATER SCI ENG, V3, P496, DOI 10.1021/acsbiomaterials.7b00148
Moon JJ, 2011, NAT MATER, V10, P243, DOI 10.1038/NMAT2960
Morse MA, 2015, VACCINE, V33, P7377, DOI 10.1016/j.vaccine.2015.10.057
Mullard A, 2013, NAT REV DRUG DISCOV, V12, P489, DOI 10.1038/nrd4066
Nakai T, 2012, MACROMOL BIOSCI, V12, P475, DOI 10.1002/mabi.201100352
Nembrini C, 2011, P NATL ACAD SCI USA, V108, pE989, DOI 10.1073/pnas.1104264108
Nochi T, 2010, NAT MATER, V9, P572, DOI [10.1038/NMAT2784, 10.1038/nmat2784]
Oberli MA, 2017, NANO LETT, V17, P1326, DOI 10.1021/acs.nanolett.6b03329
Overwijk WW, 2017, CURR OPIN IMMUNOL, V47, P103, DOI 10.1016/j.coi.2017.07.015
Oyewumi MO, 2010, EXPERT REV VACCINES, V9, P1095, DOI [10.1586/erv.10.89, 10.1586/ERV.10.89]
Parato KA, 2005, NAT REV CANCER, V5, P965, DOI 10.1038/nrc1750
Pardoll DM, 2012, NAT REV CANCER, V12, P252, DOI 10.1038/nrc3239
Perica K, 2014, NANOMED-NANOTECHNOL, V10, P119, DOI 10.1016/j.nano.2013.06.015
Peruzzi PP, 2016, NAT NANOTECHNOL, V11, P214, DOI 10.1038/nnano.2015.306
Petersen TR, 2010, CRIT REV IMMUNOL, V30, P345, DOI 10.1615/CritRevImmunol.v30.i4.30
Petousis-Harris H, 2017, LANCET, V390, P1603, DOI 10.1016/S0140-6736(17)31449-6
Petrovsky N, 2015, DRUG SAFETY, V38, P1059, DOI 10.1007/s40264-015-0350-4
Plotkin S, 2014, P NATL ACAD SCI USA, V111, P12283, DOI 10.1073/pnas.1400472111
Pradhan P, 2014, BIOMATERIALS, V35, P5491, DOI 10.1016/j.biomaterials.2014.03.039
Purwada A, 2016, ADV HEALTHC MATER, V5, P1413, DOI 10.1002/adhm.201501062
Qian Y, 2016, BIOMATERIALS, V98, P171, DOI 10.1016/j.biomaterials.2016.05.008
Rabinovich GA, 2007, ANNU REV IMMUNOL, V25, P267, DOI 10.1146/annurev.immunol.25.022106.141609
Radovic-Moreno AF, 2015, P NATL ACAD SCI USA, V112, P3892, DOI 10.1073/pnas.1502850112
Reddy ST, 2007, NAT BIOTECHNOL, V25, P1159, DOI [10.1038/nbt1332, 10.1038/nbtl332]
Ribas A, 2018, SCIENCE, V359, P1350, DOI 10.1126/science.aar4060
Rosenberg SA, 2004, NAT MED, V10, P909, DOI 10.1038/nm1100
Rosenberg SA, 2008, NAT REV CANCER, V8, P299, DOI 10.1038/nrc2355
Rosenberg SA, 2014, J IMMUNOL, V192, P5451, DOI 10.4049/jimmunol.1490019
Rosenblum MD, 2015, J CLIN INVEST, V125, P2228, DOI 10.1172/JCI78088
Rosenthal JA, 2014, CURR OPIN BIOTECH, V28, P51, DOI 10.1016/j.copbio.2013.11.005
Rudra JS, 2012, BIOMATERIALS, V33, P6476, DOI 10.1016/j.biomaterials.2012.05.041
Sagiv-Barfi I, 2018, SCI TRANSL MED, V10, DOI 10.1126/scitranslmed.aan4488
Sakr OS, 2016, DRUG DELIV, V23, P2747, DOI 10.3109/10717544.2015.1069422
Saluja SS, 2014, INT J NANOMED, V9, P5231, DOI 10.2147/IJN.S66639
Schaaf MB, 2018, CELL DEATH DIS, V9, DOI 10.1038/s41419-017-0061-0
Schmid D, 2017, NAT COMMUN, V8, DOI 10.1038/s41467-017-01830-8
Schutz C, 2016, ONCOTARGET, V7, P68503, DOI 10.18632/oncotarget.11785
Schumacher R, 2004, VACCINE, V22, P714, DOI 10.1016/j.vaccine.2003.08.019
Schumacher TN, 2015, SCIENCE, V348, P69, DOI 10.1126/science.aaa4971
Shannahan Jonathan H, 2015, Receptors Clin Investig, V2, pe811
Shi GN, 2017, BIOMATERIALS, V113, P191, DOI 10.1016/j.biomaterials.2016.10.047
Silva AKA, 2013, NANOSCALE, V5, P11374, DOI 10.1039/c3nr01541f
Silva JM, 2015, J CONTROL RELEASE, V198, P91, DOI 10.1016/j.jconrel.2014.11.033
Singh A, 2014, ADV HEALTHC MATER, V3, P1562, DOI 10.1002/adhm.201400051
Slingluff CL, 2011, CANCER J, V17, P343, DOI 10.1097/PPO.0b013e318233e5b2
Solbrig CM, 2007, MOL PHARMACEUT, V4, P47, DOI 10.1021/mp060107e
Song JG, 2016, INT J NANOMED, V11, P6609, DOI 10.2147/IJN.S122726
Soni KS, 2016, J CONTROL RELEASE, V240, P109, DOI 10.1016/j.jconrel.2015.11.009
Steenblock ER, 2008, MOL THER, V16, P765, DOI 10.1038/mt.2008.11
Steinbach JM, 2016, ACTA BIOMATER, V30, P49, DOI 10.1016/j.actbio.2015.11.029

Suen H, 2015, LEUKEMIA, V29, P1621, DOI 10.1038/leu.2015.104
Swann JB, 2007, J CLIN INVEST, V117, P1137, DOI 10.1172/JCI31405
Tam HH, 2016, P NATL ACAD SCI USA, V113, pE6639, DOI 10.1073/pnas.1606050113
Thamphiwatana S, 2017, P NATL ACAD SCI USA, V114, P11488, DOI 10.1073/pnas.1714267114
Thomas SN, 2014, BIOMATERIALS, V35, P814, DOI 10.1016/j.biomaterials.2013.10.003
Topalian SL, 2012, NEW ENGL J MED, V366, P2443, DOI 10.1056/NEJMoa1200690
Turtle CJ, 2010, CANCER J, V16, P374, DOI 10.1097/PPO.0b013e3181eb33a6
Umeki Y, 2015, ADV FUNCT MATER, V25, P5758, DOI 10.1002/adfm.201502139
Uto Tomofumi, 2013, Results Immunol, V3, P1, DOI 10.1016/j.rinim.2012.11.002
van der Burg SH, 2016, NAT REV CANCER, V16, P219, DOI 10.1038/nrc.2016.16
Wang C, 2016, BIOMATERIALS, V79, P88, DOI 10.1016/j.biomaterials.2015.11.040
Wang C, 2017, NAT BIOMED ENG, V1, DOI 10.1038/s41551-016-0011
Wang F, 2016, ADV FUNCT MATER, V26, P1628, DOI 10.1002/adfm.201505231
Wang HM, 2016, ADV FUNCT MATER, V26, P1822, DOI 10.1002/adfm.201505188
Wang KW, 2018, MOL PHARMACEUT, V15, P975, DOI 10.1021/acs.molpharmaceut.7b00905
Wang LF, 2015, J INTERN MED, V278, P369, DOI 10.1111/joim.12395
Wang SH, 2019, WIRES NANOMED NANOBI, V11, DOI 10.1002/wnan.1523
Wang YH, 2018, MOL THER, V26, P420, DOI 10.1016/j.ymthe.2017.11.009
Wei XL, 2018, ACS NANO, V12, P109, DOI 10.1021/acsnano.7b07720
Wei XL, 2017, ADV MATER, V29, DOI 10.1002/adma.201701644
Weiner LM, 2010, NAT REV IMMUNOL, V10, P317, DOI 10.1038/nri2744
Whitney CG, 2014, MMWR-MORBID MORTAL W, V63, P352
Wilson DR, 2018, NANOMED-NANOTECHNOL, V14, P237, DOI 10.1016/j.nano.2017.10.013
Wu ZJ, 2012, NANOSCALE, V4, P3567, DOI 10.1039/c2nr30366c
Xu ZH, 2014, ACS NANO, V8, P3636, DOI 10.1021/nn500216y
Xu ZH, 2013, J CONTROL RELEASE, V172, P259, DOI 10.1016/j.jconrel.2013.08.021
Yang R, 2018, ACS NANO, V12, P5121, DOI 10.1021/acsnano.7b09041
Yarchoan M, 2017, NEW ENGL J MED, V377, P2500, DOI 10.1056/NEJMc1713444
Yoo JW, 2011, NAT REV DRUG DISCOV, V10, P521, DOI 10.1038/nrd3499
Yuan HF, 2017, NAT NANOTECHNOL, V12, P763, DOI [10.1038/nnano.2017.69, 10.1038/NNANO.2017.69]
Zhang QB, 2017, ACS NANO, V11, P10724, DOI 10.1021/acsnano.7b04955
Zhang YC, 2018, NAT COMMUN, V9, DOI 10.1038/s41467-017-02522-z
NR 185
TC 67
Z9 68
U1 7
U2 94
PU WILEY-V C H VERLAG GMBH
PI WEINHEIM
PA POSTFACH 101161, 69451 WEINHEIM, GERMANY
EI 2366-7478
J9 ADV BIOSYST
JI Adv. Biosyst.
PD JAN
PY 2019
VL 3
IS 1
AR 1800219
DI 10.1002/adbi.201800219
PG 17
WC Materials Science, Biomaterials
WE Science Citation Index Expanded (SCI-EXPANDED)
SC Materials Science
GA HH6BF
UT WOS:000455812800001
PM 31728404
OA Green Accepted, Bronze
DA 2023-09-14
ER

PT J
AU Kang, T
Huang, YK
Zhu, QQ
Cheng, H
Pei, YY
Feng, JX
Xu, MJ
Jiang, G
Song, QX
Jiang, TZ
Chen, HZ
Gao, XL
Chen, J
AF Kang, Ting
Huang, Yukun
Zhu, Qianqian
Cheng, Hao
Pei, Yuanyuan
Feng, Jingxian
Xu, Minjun
Jiang, Gan
Song, Qingxiang
Jiang, Tianze
Chen, Hongzhuan
Gao, Xiaoling
Chen, Jun
TI Necroptotic cancer cells-mimicry nanovaccine boosts anti-tumor immunity with tailored immune-stimulatory modality
SO BIOMATERIALS
LA English
DT Article
DE Biomimetic nanotechnology; Anticancer vaccines; HSP70 functional peptide; Anti-PD-1 therapy; NK cells
ID TUMOR MICROENVIRONMENT; NK CELLS; CHECKPOINT BLOCKADE; ADVANCED MELANOMA; DENDRITIC CELLS; LUNG-CANCER; IMMUNOTHERAPY; NANOPARTICLES; THERAPY; DELIVERY
AB Recent breakthroughs in cancer immunotherapy offer new paradigm-shifting therapeutic options for combating cancer. Personalized therapeutic anti-cancer vaccines training T cells to directly fight against tumor cells endogenously offer tremendous benefits in working synergistically with immune checkpoint inhibitors. Biomimetic nanotechnology offers a versatile platform to boost anticancer immunity by efficiently co-delivering optimized immunogenic antigen materials and adjuvants to antigen presenting cells (APC). Necroptotic tumor cells can release danger associated molecule patterns (DAMPs) like heat shock proteins, being more immunogenic than naive tumor cells. Here, nano-size "artificial necroptotic cancer cell" (alpha HSP70p-CM-CaP) composing of phospholipid bilayer and a phosphate calcium core was designed as a flexible vaccine platform for co-delivering cancer membrane proteins (CM), DAMPs signal augmenting element alpha-helix HSP70 functional peptide (alpha HSP70p) and CpG to both natural killer (NK) cells and APC. Mechanically, immunogenic B16OVA tumor cells membrane-associated antigens and alpha HSP70p were reconstituted in artificial outer phospholipid bilayer membrane via one-step hydration and CpG encapsulated in the phosphate calcium core. The resulted alpha HSP70p-CM-CaP exhibited 30 nm in diameter with the immunogenic membrane proteins reserved in the particles to produce synergistic effect on bone marrow derived dendritic cells maturation and antigen-presentation. Following alpha HSP70p-CM-CaP vaccination, efficient lymph node trafficking and multi-epitope-T cells response was observed in mice. Vitally, alpha HSP70p-CM-CaP was also able to induce expansion of IFN-gamma-expressing CD8(+) T cells and NKG2D(+) NK cells subsets. Most promisingly, alpha HSP70p-CM-CaP vaccination led to the killing of target cells and tumor regression in vivo when combined with anti-PD-1 antibody treatment on mice B16OVA melanoma models. Altogether, we demonstrated proof-of-concept evidence for the feasibility, capability and safety of a nanovaccine platform towards efficient personalized anticancer application. (C) 2018 Elsevier Ltd. All rights reserved.
C1 [Kang, Ting; Huang, Yukun; Zhu, Qianqian; Pei, Yuanyuan; Feng, Jingxian; Xu, Minjun; Jiang, Tianze; Chen, Jun] Fudan Univ, Sch Pharm, Minist Educ, Key Lab Smart Drug Delivery, Lane 826,Zhangheng Rd,

Shanghai 201203, Peoples R China.
[Jiang, Gan; Song, Qingxiang; Chen, Hongzhuan; Gao, Xiaoling] Shanghai Jiao Tong Univ, Sch Med, Fac Basic Med, Dept Pharmacol & Chem Biol, 280 South Chongqing Rd, Shanghai 200025, Peoples R China.
[Cheng, Hao] Shanghai Jiao Tong Univ, Bio X Inst, Shanghai 200240, Peoples R China.
C3 Fudan University; Shanghai Jiao Tong University; Shanghai Jiao Tong University
RP Chen, J (通讯作者)，Fudan Univ, Sch Pharm, Minist Educ, Key Lab Smart Drug Delivery, Lane 826,Zhangheng Rd, Shanghai 201203, Peoples R China.; Gao, XL (通讯作者)，Shanghai Jiao Tong Univ, Sch Med, Fac Basic Med, Dept Pharmacol & Chem Biol, 280 South Chongqing Rd, Shanghai 200025, Peoples R China.
EM shellygao1@sjtu.edu.cn; chenjun@fudan.edu.cn
RI Jiang, Gan/AAO-2173-2020; Gao, xiao ling/GQI-2790-2022
OI Jiang, Gan/0000-0003-2011-741X; huang, yu kun/0000-0002-3711-367X; Jiang, Tianze/0000-0002-4303-8768
FU National Natural Science Foundation of China [81673019, 8169260021, 81573382, 81722043]; Shanghai Science and Technology Committee [13NM1400500, 15540723700]; National Youth Talent Support Program; Shanghai Municipal Education Commission; Shanghai Education Development Foundation [15SG14]; "Shu Guang" project
FX This work was supported by National Natural Science Foundation of China (81673019, 8169260021, 81573382, 81722043), Grants from Shanghai Science and Technology Committee (13NM1400500, 15540723700), the National Youth Talent Support Program, and "Shu Guang" project supported by Shanghai Municipal Education Commission and Shanghai Education Development Foundation (15SG14). We appreciate Professor Qiang Zou, Professor Bin Li and Dr. Dan Li from Shanghai Institute of Immunology for their instructions in the BMDC extraction and IFN-gamma measurement. We thank Dr. Shimin An, Chunlei Zhou at Merck Chemicals (Shanghai) Co Ltd for their assistance in Imaging Flow Cytometry analysis of the BMDC.
CR Aaes TL, 2016, CELL REP, V15, P274, DOI 10.1016/j.celrep.2016.03.037
Chen Q, 2017, BIOMATERIALS, V148, P69, DOI 10.1016/j.biomaterials.2017.09.021
Chen Q, 2016, NAT COMMUN, V7, DOI 10.1038/ncomms13193
De Henau O, 2016, NATURE, V539, P443, DOI 10.1038/nature20554
Didelot C, 2007, CURR MED CHEM, V14, P2839, DOI 10.2174/092986707782360079
Fang RH, 2017, BIOMATERIALS, V128, P69, DOI 10.1016/j.biomaterials.2017.02.041
Fang RH, 2015, SMALL, V11, P5483, DOI 10.1002/smll.201501284
Fang RH, 2014, NANO LETT, V14, P2181, DOI 10.1021/nl500618u
Gao M, 2017, ADV MATER, V29, DOI 10.1002/adma.201701429
Gastpar R, 2004, J IMMUNOL, V172, P972, DOI 10.4049/jimmunol.172.2.972
Gfeller D, 2016, ONCOIMMUNOLOGY, V5, DOI 10.1080/2162402X.2016.1177691
Guzhova IV, 2016, HUM VACC IMMUNOTHER, V12, P2529, DOI 10.1080/21645515.2016.1190057
Hamid O, 2013, NEW ENGL J MED, V369, P134, DOI 10.1056/NEJMoa1305133
Hodge G, 2014, CLIN EXP IMMUNOL, V178, P79, DOI 10.1111/cei.12392
Hu XM, 2017, J CONTROL RELEASE, V256, P26, DOI 10.1016/j.jconrel.2017.04.026
Huang JL, 2017, NAT COMMUN, V8, DOI 10.1038/ncomms15144
Inoges S, 2017, J TRANSL MED, V15, DOI 10.1186/s12967-017-1202-z
Kaech SM, 2002, NAT REV IMMUNOL, V2, P251, DOI 10.1038/nri778
Kang T, 2017, ACS NANO, V11, P1397, DOI 10.1021/acsnano.6b06477
Krneta T, 2016, CELL MOL IMMUNOL, V13, P628, DOI 10.1038/cmi.2015.42
Le DT, 2015, NEW ENGL J MED, V372, P2509, DOI 10.1056/NEJMoa1500596
Lee MS, 2014, J CONTROL RELEASE, V192, P122, DOI 10.1016/j.jconrel.2014.06.049
Madaan A., 2014, ETHOLOGY, V1, P68
Mellman I, 2001, CELL, V106, P255, DOI 10.1016/S0092-8674(01)00449-4
Mellman I, 2011, NATURE, V480, P480, DOI 10.1038/nature10673
Muthumani K, 2017, CANCER IMMUNOL IMMUN, V66, P1577, DOI 10.1007/s00262-017-2042-7
Ott PA, 2017, NATURE, V547, P217, DOI 10.1038/nature22991
Pardoll DM, 2012, NAT REV CANCER, V12, P252, DOI 10.1038/nrc3239
Sahin U, 2017, NATURE, V547, P222, DOI 10.1038/nature23003
Sharma P, 2015, CELL, V161, P205, DOI 10.1016/j.cell.2015.03.030
Soares KC, 2015, J IMMUNOTHER, V38, P1, DOI 10.1097/CJI.0000000000000062

Specht HM, 2015, FRONT IMMUNOL, V6, DOI 10.3389/fimmu.2015.00162
Spranger S, 2013, SCI TRANSL MED, V5, DOI 10.1126/scitranslmed.3006504
Suski JM, 2014, NAT PROTOC, V9, P312, DOI 10.1038/nprot.2014.016
Topalian SL, 2012, NEW ENGL J MED, V366, P2443, DOI 10.1056/NEJMoa1200690
Vasileiou S, 2017, J IMMUNOTHER, V40, P253, DOI 10.1097/CJI.0000000000000182
Velpurisiva Praveena, 2017, J Biomed (Syd), V2, P64, DOI 10.7150/jbm.18877
Wang C, 2017, MATH PROBL ENG, V2017, DOI 10.1155/2017/3405619
Wang C, 2017, ADV MATER, V29, DOI 10.1002/adma.201606036
Weber JS, 2015, LANCET ONCOL, V16, P375, DOI 10.1016/S1470-2045(15)70076-8
Whitmore MM, 2001, CANCER IMMUNOL IMMUN, V50, P503, DOI 10.1007/s002620100227
Xu ZH, 2014, ACS NANO, V8, P3636, DOI 10.1021/nn500216y
Zhai YH, 2017, THERANOSTICS, V7, P2575, DOI 10.7150/thno.20118
Zhu GZ, 2017, ACS NANO, V11, P2387, DOI 10.1021/acsnano.7b00978
Zhuang XT, 2016, J CONTROL RELEASE, V228, P26, DOI 10.1016/j.jconrel.2016.02.035
NR 45
TC 67
Z9 69
U1 5
U2 212
PU ELSEVIER SCI LTD
PI OXFORD
PA THE BOULEVARD, LANGFORD LANE, KIDLINGTON, OXFORD OX5 1GB, OXON, ENGLAND
SN 0142-9612
EI 1878-5905
J9 BIOMATERIALS
JI Biomaterials
PD MAY
PY 2018
VL 164
BP 80
EP 97
DI 10.1016/j.biomaterials.2018.02.033
PG 18
WC Engineering, Biomedical; Materials Science, Biomaterials
WE Science Citation Index Expanded (SCI-EXPANDED)
SC Engineering; Materials Science
GA GA6YW
UT WOS:000428482200007
PM 29499438
DA 2023-09-14
ER

PT J
AU Zhao, H
Xu, J
Li, Y
Guan, XX
Han, X
Xu, YY
Zhou, HT
Peng, R
Wang, J
Liu, Z
AF Zhao, He
Xu, Jun
Li, Yan
Guan, Xinxian
Han, Xiao
Xu, Yunyun
Zhou, Huiting
Peng, Rui

Wang, Jian
Liu, Zhuang
TI Nanoscale Coordination Polymer Based Nanovaccine for Tumor Immunotherapy
SO ACS NANO
LA English
DT Article
DE Nanovaccine; nanoscale coordination polymer; Nod1 agonist; checkpoint blockade; cancer immunotherapy
ID CANCER; IMMUNE; NANOPARTICLES; COMBINATIONS; BLOCKADE; VACCINES; THERAPY; NOD1
AB Tumor vaccines to induce robust immunity for cancer treatment have attracted tremendous interests in cancer immunotherapy. In this work, a type of cancer vaccine is prepared by using nanoscale coordination polymer (NCP) formed between Mn2+ ions and a nucleotide oligomerization binding domain 1 (Nod1) agonist, meso-2,6-diaminopimelic acid (DAP), as the organic ligand, to encapsulate a model protein antigen, ovalbumin (OVA). The obtained OVA@Mn-DAP nanoparticles could act as an effective tumor vaccine to promote the maturation of dendritic cells (DCs) as well as their antigen cross-presentation via increasing the cellular uptake of antigen and stimulating Nod1 pathway with DAP. Such OVA@Mn-DAP vaccine could migrate into lymph nodes after local injection, as revealed by in vivo magnetic resonance (MR) and fluorescence imaging. Importantly, vaccination with OVA@Mn-DAP could not only offer prophylactic to protect mice from challenged B16-OVA tumors but also result in significant therapeutic effect to inhibit growth of already-established tumors if in combination with anti-programmed cell death protein 1 antibody (alpha-PD-1) immune checkpoint blockade therapy. Therefore, this work presents an innovative platform to construct effective nanovaccine for tumor immunotherapy.
C1 [Zhao, He; Li, Yan; Guan, Xinxian; Xu, Yunyun; Zhou, Huiting; Wang, Jian] Soochow Univ, Pediat Res Inst, Childrens Hosp, Suzhou 215123, Jiangsu, Peoples R China.
[Xu, Jun; Han, Xiao; Peng, Rui; Liu, Zhuang] Soochow Univ, Jiangsu Key Lab Carbon Based Funct Mat & Devices, Inst Funct Nano & Soft Mat FUNSOM, Suzhou 215123, Jiangsu, Peoples R China.
C3 Soochow University - China; Soochow University - China
RP Wang, J (通讯作者)，Soochow Univ, Pediat Res Inst, Childrens Hosp, Suzhou 215123, Jiangsu, Peoples R China.; Liu, Z (通讯作者)，Soochow Univ, Jiangsu Key Lab Carbon Based Funct Mat & Devices, Inst Funct Nano & Soft Mat FUNSOM, Suzhou 215123, Jiangsu, Peoples R China.
EM wj196312@vip.163.com; zliu@suda.edu.cn
RI Liu, Zhuang/H-4352-2011; li, yan/IVH-2367-2023
OI Liu, Zhuang/0000-0002-1629-1039; li, yan/0000-0002-0561-308X
FU National Basic Research Programs of China [2016YFA0201200]; National Natural Science Foundation of China [81602181, 51525203, 51761145041]; Suzhou Municipal Science and Technology Project [SYS201759]; Collaborative Innovation Center of Suzhou Nano Science and Technology, Postgraduate Research&Practice Innovation Program of Jiangsu Province [KYCX19_1995]; Priority Academic Program Development (PAPD) of Jiangsu Higher Education Institutions
FX This work was supported by the National Basic Research Programs of China (2016YFA0201200), the National Natural Science Foundation of China (81602181, 51525203, 51761145041), the Suzhou Municipal Science and Technology Project (SYS201759), the Collaborative Innovation Center of Suzhou Nano Science and Technology, Postgraduate Research&Practice Innovation Program of Jiangsu Province (KYCX19_1995), and a project funded by the Priority Academic Program Development (PAPD) of Jiangsu Higher Education Institutions.
CR Asano J, 2010, J IMMUNOL, V184, P736, DOI 10.4049/jimmunol.0900726
Banchereau J, 2001, CANCER RES, V61, P6451
Bertrand MJM, 2009, IMMUNITY, V30, P789, DOI 10.1016/j.immuni.2009.04.011
Chao Y, 2018, ACS NANO, V12, P7519, DOI 10.1021/acsnano.8b02400
Chen GY, 2008, CANCER RES, V68, P10060, DOI 10.1158/0008-5472.CAN-08-2061
Chen Q, 2019, NAT NANOTECHNOL, V14, P89, DOI 10.1038/s41565-018-0319-4
Chen Q, 2016, NAT COMMUN, V7, DOI 10.1038/ncomms13193
Correia JD, 2006, P NATL ACAD SCI USA, V103, P1840, DOI 10.1073/pnas.0509228103
Crittenden M, 2015, SEMIN RADIAT ONCOL, V25, P54, DOI 10.1016/j.semradonc.2014.07.003
Duan F, 2017, BIOMATERIALS, V122, P23, DOI 10.1016/j.biomaterials.2017.01.017
Egen JG, 2002, NAT IMMUNOL, V3, P611, DOI 10.1038/ni0702-611
Girardin SE, 2003, J BIOL CHEM, V278, P41702, DOI 10.1074/jbc.M307198200

Guermonprez P, 2003, NATURE, V425, P397, DOI 10.1038/nature01911
He CB, 2016, NAT COMMUN, V7, DOI 10.1038/ncomms12499
Hu XM, 2019, ADV HEALTHC MATER, V8, DOI 10.1002/adhm.201800837
Irvine DJ, 2015, CHEM REV, V115, P11109, DOI 10.1021/acs.chemrev.5b00109
Khalil DN, 2016, NAT REV CLIN ONCOL, V13, P273, DOI 10.1038/nrclinonc.2016.25
Kuai R, 2017, NAT MATER, V16, P489, DOI [10.1038/nmat4822, 10.1038/NMAT4822]
Lan GX, 2018, J AM CHEM SOC, V140, P16971, DOI 10.1021/jacs.8b11593
Lan GX, 2018, J AM CHEM SOC, V140, P5670, DOI 10.1021/jacs.8b01072
Lanzavecchia A, 2001, CELL, V106, P263, DOI 10.1016/S0092-8674(01)00455-X
Li PZ, 2019, COORDIN CHEM REV, V380, P484, DOI 10.1016/j.ccr.2018.11.006
Li PY, 2018, BIOCONJUGATE CHEM, V29, P624, DOI 10.1021/acs.bioconjchem.7b00669
Liu J, 2018, ACS CATAL, V8, P3865, DOI 10.1021/acscatal.8b00267
Liu JJ, 2018, ACS APPL MATER INTER, V10, P43493, DOI 10.1021/acsami.8b17684
Matsuo Y, 2012, CURR PHARM DESIGN, V18, P2416, DOI 10.2174/13816128112092416
Melero I, 2015, NAT REV CANCER, V15, P457, DOI 10.1038/nrc3973
Melero I, 2014, NAT REV CLIN ONCOL, V11, P509, DOI 10.1038/nrclinonc.2014.111
Pardoll DM, 2012, NAT REV CANCER, V12, P252, DOI 10.1038/nrc3239
Paulis LE, 2013, CURR OPIN IMMUNOL, V25, P389, DOI 10.1016/j.coi.2013.03.001
Rosenberg SA, 2004, NAT MED, V10, P909, DOI 10.1038/nm1100
Topalian SL, 2012, NEW ENGL J MED, V366, P2443, DOI 10.1056/NEJMoa1200690
Xiang J, 2015, ACS NANO, V9, P6401, DOI 10.1021/acsnano.5b02014
Xu LG, 2013, ADV MATER, V25, P5928, DOI 10.1002/adma.201300583
Yang GB, 2017, NAT COMMUN, V8, DOI 10.1038/s41467-017-01050-0
Yang R, 2018, ACS NANO, V12, P5121, DOI 10.1021/acsnano.7b09041
Zhang XD, 2018, ADV MATER, V30, DOI 10.1002/adma.201707112
Zhang Y, 2016, ADV FUNCT MATER, V26, P6454, DOI 10.1002/adfm.201600650
Zhao H, 2016, THERANOSTICS, V6, P1833, DOI 10.7150/thno.16047
Zhao L, 2014, VACCINE, V32, P327, DOI 10.1016/j.vaccine.2013.11.069
Zhao YD, 2018, J CONTROL RELEASE, V269, P322, DOI 10.1016/j.jconrel.2017.11.037
Zhu GZ, 2017, NAT COMMUN, V8, DOI 10.1038/s41467-017-02191-y
Zhu GZ, 2017, NAT COMMUN, V8, DOI 10.1038/s41467-017-01386-7
NR 43
TC 66
Z9 68
U1 13
U2 277
PU AMER CHEMICAL SOC
PI WASHINGTON
PA 1155 16TH ST, NW, WASHINGTON, DC 20036 USA
SN 1936-0851
EI 1936-086X
J9 ACS NANO
JI ACS Nano
PD NOV
PY 2019
VL 13
IS 11
BP 13127
EP 13135
DI 10.1021/acsnano.9b05974
PG 9
WC Chemistry, Multidisciplinary; Chemistry, Physical; Nanoscience &
Nanotechnology; Materials Science, Multidisciplinary
WE Science Citation Index Expanded (SCI-EXPANDED)
SC Chemistry; Science & Technology - Other Topics; Materials Science
GA JS9WN
UT WOS:000500650000085
PM 31710460
DA 2023-09-14
ER

PT J
AU Zang, XL
Zhao, XL
Hu, HY
Qiao, MC
Deng, YH
Chen, DE
AF Zang, Xinlong
Zhao, Xiuli
Hu, Haiyang
Qiao, Mingxi
Deng, Yihui
Chen, Dawei
TI Nanoparticles for tumor immunotherapy
SO EUROPEAN JOURNAL OF PHARMACEUTICS AND BIOPHARMACEUTICS
LA English
DT Review
DE Tumor immunotherapy; Antitumor immune response; Immunosuppression;
Nanoparticles; Nanovaccines; Artificial antigen presenting cells;
Targeting to immunosuppressive cells
ID ANTIGEN-PRESENTING CELLS; DENDRITIC CELLS; SUPPRESSOR-CELLS;
NITRIC-OXIDE; TARGETING NANOPARTICLES; MESENCHYMAL TRANSITION; BACCATIN
III; LYMPH-NODES; TGF-BETA; IN-VITRO
AB Although most researches and therapies have been focused on the tumor itself, it is becoming clear that immune cells can not only suppress tumor development but support and maintain their malignant type. Promising recent developments in immunology will provide opportunities for tumor-specific immunotherapy, which can orchestrate the patients immune system to target, fight and eradicate cancer cells without destroying healthy cells. However, antitumor immunity driven by self-immune system alone may be therapeutically insufficient. Developments in nanoparticle based drug delivery system can promote immunotherapy and re-educate immunosuppressive tumor microenvironment (TME), which provide promising strategies for cancer therapy. In this review, we will focus on nanoparticle-based immunotherapeutic approaches against cancer, ranging from nanovaccines, artificial antigen presenting cells (aAPCs) to nanoparticles reversing tumor immunosuppressive microenvironment. (C) 2017 Elsevier B.V. All rights reserved.
C1 [Zang, Xinlong; Zhao, Xiuli; Hu, Haiyang; Qiao, Mingxi; Deng, Yihui; Chen, Dawei] Shenyang Pharmaceut Univ, Sch Pharm, Dept Pharmaceut, 103 Wenhua Rd, Shenyang 110016, Peoples R China.
C3 Shenyang Pharmaceutical University
RP Deng, YH; Chen, DE (通讯作者)，Shenyang Pharmaceut Univ, Sch Pharm, Dept Pharmaceut, 103 Wenhua Rd, Shenyang 110016, Peoples R China.
EM dds-666@163.com; chendawei@syphu.edu.cn
CR Andersen R., 2014, J IMMUNOTHER CANCER, V2, pS3, DOI [10.1186/2051-1426-2-S3-P1, DOI 10.1186/2051-1426-2-S3-P1]
Anne J.M.N., 2014, EXP DERMATOL, V20, P430
Batrakova EV, 2015, J CONTROL RELEASE, V219, P396, DOI 10.1016/j.jconrel.2015.07.030
Bell BM, 2016, NANOMED-NANOTECHNOL, V12, P163, DOI 10.1016/j.nano.2015.09.011
Chanmee T, 2014, CANCERS, V6, P1670, DOI 10.3390/cancers6031670
Chen B, 2014, ACS APPL MATER INTER, V6, P18435, DOI 10.1021/am505510m
Cheung AS, 2015, NANO TODAY, V10, P511, DOI 10.1016/j.nantod.2015.06.007
Choi J, 2012, BIOMATERIALS, V33, P4195, DOI 10.1016/j.biomaterials.2012.02.022
Cruz LJ, 2014, MOL PHARMACEUT, V11, P4299, DOI 10.1021/mp400717r
Cruz LJ, 2014, J CONTROL RELEASE, V192, P209, DOI 10.1016/j.jconrel.2014.07.040
De la Pena H, 2009, J IMMUNOL METHODS, V344, P121, DOI 10.1016/j.jim.2009.03.011
Dermot E.R., 2015, EUR RESPIR J, V46, P1641
Dong L, 2009, J CONTROL RELEASE, V134, P214, DOI 10.1016/j.jconrel.2008.11.013
Dunn GP, 2004, ANNU REV IMMUNOL, V22, P329, DOI 10.1146/annurev.immunol.22.012703.104803
Eggermont LJ, 2014, TRENDS BIOTECHNOL, V32, P456, DOI 10.1016/j.tibtech.2014.06.007
Elkord E, 2010, EXPERT OPIN BIOL TH, V10, P1573, DOI 10.1517/14712598.2010.529126
Fadel TR, 2008, NANO LETT, V8, P2070, DOI 10.1021/nl080332i
Fadel TR, 2010, LANGMUIR, V26, P5645, DOI 10.1021/la902068z
Fan QM, 2014, CANCER LETT, V352, P160, DOI 10.1016/j.canlet.2014.05.008
Fang RNH, 2016, ANNU REV CHEM BIOMOL, V7, P305, DOI 10.1146/annurev-chembioeng-080615-

034446


Fontana F., 2017, WILEY INTERDISCIP RE, V9
Frey AB, 2015, VACCINE, V33, P7393, DOI 10.1016/j.vaccine.2015.08.096
Gao HL, 2016, ACTA PHARM SIN B, V6, P268, DOI 10.1016/j.apsb.2016.05.013
Gerald D, 2013, CANCER RES, V73, P1649, DOI 10.1158/0008-5472.CAN-12-4697
Giannoni F, 2005, J IMMUNOL, V174, P3204, DOI 10.4049/jimmunol.174.6.3204
Gilham DE, 2012, TRENDS MOL MED, V18, P377, DOI 10.1016/j.molmed.2012.04.009
Hao NB, 2012, CLIN DEV IMMUNOL, DOI 10.1155/2012/948098
Hirosue S, 2010, VACCINE, V28, P7897, DOI 10.1016/j.vaccine.2010.09.077
Huang Z, 2012, J CONTROL RELEASE, V158, P286, DOI 10.1016/j.jconrel.2011.11.013
Hubbell JA, 2009, NATURE, V462, P449, DOI 10.1038/nature08604
Irvine D.J., 2015, NAT REV MATER, V1, P1
Irvine DJ, 2015, CHEM REV, V115, P11109, DOI 10.1021/acs.chemrev.5b00109
Jeanbart L, 2015, CANCER IMMUNOL IMMUN, V64, P1033, DOI 10.1007/s00262-015-1702-8
John S.K., 2013, ACS NANO, V7, P3912
Kapadia CH, 2015, J CONTROL RELEASE, V219, P167, DOI 10.1016/j.jconrel.2015.09.062
Katz SC, 2013, ANN SURG ONCOL, V20, P946, DOI 10.1245/s10434-012-2668-9
Khallou-Laschet J, 2010, PLOS ONE, V5, DOI 10.1371/journal.pone.0008852
Knutson KL, 2015, CANCER METAST REV, V34, P53, DOI 10.1007/s10555-014-9540-2
Kojima C, 2015, BIOCONJUGATE CHEM, V26, P1804, DOI 10.1021/acs.bioconjchem.5b00325
Komohara Y, 2016, ADV DRUG DELIVER REV, V99, P180, DOI 10.1016/j.addr.2015.11.009
Koshy ST, 2016, CURR OPIN BIOTECH, V40, P1, DOI 10.1016/j.copbio.2016.02.001
Kudo S, 2015, J CONTROL RELEASE, V217, P256, DOI 10.1016/j.jconrel.2015.09.019
Kullberg M, 2015, NANOMED-NANOTECHNOL, V11, P1355, DOI 10.1016/j.nano.2015.03.010
Kumari Preeti, 2016, J Drug Target, V24, P179, DOI 10.3109/1061186X.2015.1051049
Kvistborg D.P. Pia, 2014, IMMUNOTHERAPY, V6, P128
Lee KH, 2002, SCIENCE, V295, P1539, DOI 10.1126/science.1067710
Lee YH, 2014, INT IMMUNOPHARMACOL, V21, P487, DOI 10.1016/j.intimp.2014.06.012
Lee YH, 2011, INT IMMUNOPHARMACOL, V11, P985, DOI 10.1016/j.intimp.2011.02.013
Leleux J, 2013, ADV HEALTHC MATER, V2, P72, DOI 10.1002/adhm.201200268
Lewis CE, 2006, CANCER RES, V66, P605, DOI 10.1158/0008-5472.CAN-05-4005
Li KX, 2015, INT J PHARMACEUT, V491, P105, DOI 10.1016/j.ijpharm.2015.05.068
Lin L, 2015, ONCOTARGET, V6, P34758, DOI 10.18632/oncotarget.5325
Lin WW, 2007, J CLIN INVEST, V117, P1175, DOI 10.1172/JCI31537
Liu SY, 2013, BIOMATERIALS, V34, P8291, DOI 10.1016/j.biomaterials.2013.07.020
Rowdo FPM, 2015, FRONT IMMUNOL, V6, P1, DOI 10.3389/fimmu.2015.00127
Maes W, 2009, NEURO-ONCOLOGY, V11, P529, DOI 10.1215/15228517-2009-004
Mak IWY, 2014, AM J TRANSL RES, V6, P114
Mandal S, 2013, CHEM SCI, V4, P4168, DOI 10.1039/c3sc51399h
Matheu MP, 2015, NAT COMMUN, V6, DOI 10.1038/ncomms7219
Meyer RA, 2015, SMALL, V11, P1519, DOI 10.1002/smll.201402369
Mirza N, 2006, CANCER RES, V66, P9299, DOI 10.1158/0008-5472.CAN-06-1690
Mittal SK, 2015, CURR OPIN IMMUNOL, V34, P22, DOI 10.1016/j.coi.2014.12.009
Miyabe H, 2014, J CONTROL RELEASE, V184, P20, DOI 10.1016/j.jconrel.2014.04.004
Moyer TJ, 2016, J CLIN INVEST, V126, P799, DOI 10.1172/JCI81083
Munn DH, 2016, CURR OPIN IMMUNOL, V39, P1, DOI 10.1016/j.coi.2015.10.009
Murray PJ, 2011, J LEUKOCYTE BIOL, V89, P557, DOI 10.1189/jlb.0710409
Nagy G, 2007, FREE RADICAL BIO MED, V42, P1625, DOI 10.1016/j.freeradbiomed.2007.02.026
Nair SK, 2015, J NEURO-ONCOL, V125, P65, DOI 10.1007/s11060-015-1890-2
Niikura K, 2013, ACS NANO, V7, P3926, DOI 10.1021/nn3057005
Noy R, 2014, IMMUNITY, V41, P49, DOI 10.1016/j.immuni.2014.06.010
Oh SJ, 2016, EXPERT OPIN INV DRUG, V25, P667, DOI 10.1517/13543784.2016.1163336
Parhi P, 2012, DRUG DISCOV TODAY, V17, P1044, DOI 10.1016/j.drudis.2012.05.010
Parker KH, 2015, ADV CANCER RES, V128, P95, DOI 10.1016/bs.acr.2015.04.002
Perica K, 2014, ACS NANO, V8, P2252, DOI 10.1021/nn405520d
Perica K, 2014, NANOMED-NANOTECHNOL, V10, P119, DOI 10.1016/j.nano.2013.06.015
Prakken B, 2000, NAT MED, V6, P1406, DOI 10.1038/82231
Prasad S, 2011, NANOMED-NANOTECHNOL, V7, P1, DOI 10.1016/j.nano.2010.07.002
Preston CC, 2013, PLOS ONE, V8, DOI 10.1371/journal.pone.0080063
Qian BZ, 2010, CELL, V141, P39, DOI 10.1016/j.cell.2010.03.014
Radford KJ, 2014, CURR OPIN IMMUNOL, V27, P26, DOI 10.1016/j.coi.2014.01.005

Rahimian S, 2015, BIOMATERIALS, V37, P469, DOI 10.1016/j.biomaterials.2014.10.043
Reddy ST, 2006, J CONTROL RELEASE, V112, P26, DOI 10.1016/j.jconrel.2006.01.006
Rei M, 2014, P NATL ACAD SCI USA, V111, pE3562, DOI 10.1073/pnas.1403424111
Ricciardi M, 2015, BRIT J CANCER, V112, P1067, DOI 10.1038/bjc.2015.29
Rosalia RA, 2015, BIOMATERIALS, V40, P88, DOI 10.1016/j.biomaterials.2014.10.053
Ruffell B, 2012, TRENDS IMMUNOL, V33, P119, DOI 10.1016/j.it.2011.12.001
Ryan GM, 2014, J CONTROL RELEASE, V193, P241, DOI 10.1016/j.jconrel.2014.04.051
Sacchetti C, 2013, BIOCONJUGATE CHEM, V24, P852, DOI 10.1021/bc400070q
Sakamoto T, 2004, GENE THER, V11, P317, DOI 10.1038/sj.gt.3302171
Sato Y, 2012, J CONTROL RELEASE, V163, P267, DOI 10.1016/j.jconrel.2012.09.009
Schmitt N, 2015, CURR OPIN IMMUNOL, V34, P130, DOI 10.1016/j.coi.2015.03.007
Schreibelt G, 2016, CLIN CANCER RES, V22, P2155, DOI 10.1158/1078-0432.CCR-15-2205
Sengupta N, 2010, PATHOL RES PRACT, V206, P1, DOI 10.1016/j.prp.2009.10.001
Sevko A, 2013, J IMMUNOL, V190, P2464, DOI 10.4049/jimmunol.1202781
Shao K, 2015, ACS NANO, V9, P16, DOI 10.1021/nn5062029
Sharma G, 2010, J CONTROL RELEASE, V147, P408, DOI 10.1016/j.jconrel.2010.07.116
Skobe M, 2001, NAT MED, V7, P192, DOI 10.1038/84643
Smith DM, 2013, NAT REV IMMUNOL, V13, P592, DOI 10.1038/nri3488
Stano A, 2013, BIOMATERIALS, V34, P4339, DOI 10.1016/j.biomaterials.2013.02.024
Steenblock ER, 2008, MOL THER, V16, P765, DOI 10.1038/mt.2008.11
Sunshine JC, 2014, BIOMATERIALS, V35, P269, DOI 10.1016/j.biomaterials.2013.09.050
Tanaka A, 2017, CELL RES, V27, P109, DOI 10.1038/cr.2016.151
Tham M, 2014, ONCOTARGET, V5, P12027, DOI 10.18632/oncotarget.2482
Thomas SN, 2014, BIOMATERIALS, V35, P814, DOI 10.1016/j.biomaterials.2013.10.003
Vasievich EA, 2011, MOL PHARMACEUT, V8, P635, DOI 10.1021/mp1004228
Wang MY, 2013, BIOMATERIALS, V34, P10120, DOI 10.1016/j.biomaterials.2013.09.006
Wang Y, 2016, CELL PHYSIOL BIOCHEM, V38, P306, DOI 10.1159/000438631
Wang YJ, 2014, MACROMOL BIOSCI, V14, P369, DOI 10.1002/mabi.201300295
Weigert A, 2008, NITRIC OXIDE-BIOL CH, V19, P95, DOI 10.1016/j.niox.2008.04.021
Weller P, 2014, EUR J CANCER, V50, P1291, DOI 10.1016/j.ejca.2014.02.016
Yang L, 2010, TRENDS IMMUNOL, V31, P220, DOI 10.1016/j.it.2010.04.002
Yoshizaki Y, 2016, MOLECULES, V21, DOI 10.3390/molecules21101284
Yuba E, 2015, BIOMATERIALS, V67, P214, DOI 10.1016/j.biomaterials.2015.07.031
Yuba E, 2011, J CONTROL RELEASE, V149, P72, DOI 10.1016/j.jconrel.2010.03.001
Zappasodi R, 2008, HAEMATOL-HEMATOL J, V93, P1523, DOI 10.3324/haematol.12521
Zaric M, 2013, ACS NANO, V7, P2042, DOI 10.1021/nn304235j
Zhan XD, 2014, BIOMATERIALS, V35, P10046, DOI 10.1016/j.biomaterials.2014.09.007
Zhang J, 2015, AM J TRANSL RES, V7, P1699
Zhao ZG, 2015, CANCER CELL, V28, P415, DOI 10.1016/j.ccell.2015.09.004
Zhou QQ, 2016, ACS NANO, V10, P2678, DOI 10.1021/acsnano.5b07716
NR 120
TC 66
Z9 69
U1 3
U2 138
PU ELSEVIER
PI AMSTERDAM
PA RADARWEG 29, 1043 NX AMSTERDAM, NETHERLANDS
SN 0939-6411
EI 1873-3441
J9 EUR J PHARM BIOPHARM
JI Eur. J. Pharm. Biopharm.
PD JUN
PY 2017
VL 115
BP 243
EP 256
DI 10.1016/j.ejpb.2017.03.013
PG 14
WC Pharmacology & Pharmacy
WE Science Citation Index Expanded (SCI-EXPANDED)

SC Pharmacology & Pharmacy
GA EV3VA
UT WOS:000401686000024
PM 28323111
DA 2023-09-14
ER

PT J
AU Cruz, LJ
Tacken, PJ
Bonetto, F
Buschow, SI
Croes, HJ
Wijers, M
de Vries, IJ
Figdor, CG
AF Cruz, Luis J.
Tacken, Paul J.
Bonetto, Fernando
Buschow, Sonja I.
Croes, Huib J.
Wijers, Mietske
de Vries, I. Jolanda
Figdor, Carl G.
TI Multimodal Imaging of Nanovaccine Carriers Targeted to Human Dendritic Cells
SO MOLECULAR PHARMACEUTICS
LA English
DT Article
DE imaging; nanocarriers; biocompatible materials; contrast agents; fluorescence; magnetic resonance imaging; nanoparticles; dentritic cells
ID IN-VIVO; MAGNETIC NANOPARTICLES; LYMPH-NODES; MELANOMA PATIENTS; CANCER VACCINES; DRUG-DELIVERY; TRACKING; MIGRATION; ANTIBODY; ANTIGEN
AB Dendritic cells (DCs) are key players in the initiation of adaptive immune responses and are currently exploited in immunotherapy against cancer and infectious diseases. The targeted delivery of nanovaccine particles (NPs) to DCs in vivo is a promising strategy to enhance immune responses. Here, targeted nanovaccine carriers were generated that allow multimodal imaging of nanocarrier-DC interactions from the subcellular to the organism level. These carriers were made of biodegradable poly(D,L-lactide-co-glycolide) harboring superparamagnetic iron oxide particles (SPIO) and fluorescently labeled antigen in a single particle. Targeted delivery was facilitated by coating the NPs with antibodies recognizing the DC-specific receptor DC-SIGN. The fluorescent label allowed for rapid analysis and quantification of specific versus nonspecific uptake of targeted NPs by DCs compared to other blood cells. In addition, it showed that part of the encapsulated antigen reached the lysosomal compartment of DCs within 24 h. Moreover, the presence of fluorescent label did not prevent the antigen from being presented to antigen-specific T cells. The incorporated SPIO was applied to track the NPs at subcellular cell organel level using transmission electron microscopy (TEM). NPs were found within endolysosomal compartments, where part of the SPIO was already released within 24 h. Furthermore, part of the NPs seemed to localize within the cytoplasm. Ex vivo loading of DCs with NPs resulted in efficient labeling and detection by MRI and did not abolish cell migration within collagen scaffolds. In conclusion, incorporation of two imaging agents within a single carrier allows tracking of targeted nanovaccines on a subcellular, cellular and possibly organism level, thereby facilitating rational design of in vivo targeted vaccination strategies.
C1 [Cruz, Luis J.; Tacken, Paul J.; Bonetto, Fernando; Buschow, Sonja I.; de Vries, I. Jolanda; Figdor, Carl G.] Univ Med Ctr, Nijmegen Ctr Mol Life Sci, Dept Tumor Immunol, Nijmegen, Netherlands.
[Croes, Huib J.; Wijers, Mietske] Radboud Univ Nijmegen, Med Ctr, Nijmegen Ctr Mol Life Sci, Dept Cell Biol, NL-6500 HB Nijmegen, Netherlands.
C3 Radboud University Nijmegen; Radboud University Nijmegen
RP Figdor, CG (通讯作者)，Radboud Univ Nijmegen, Med Ctr, Nijmegen Ctr Mol Life Sci, Dept Tumor Immunol, Postbox 9101, NL-6500 HB Nijmegen, Netherlands.
EM c.figdor@ncmls.ru.nl
RI Figdor, Carl G/A-4232-2010; Tacken, Paul J/A-7224-2008; Wijers-Rouw, Mietske/G-4456-2016; Croes, Huib JE/G-4012-2016; Buschow, Sonja

I/F-3777-2010; Buschow, Sonja/AAD-3567-2020; de Vries, Jolanda
I.J.M./F-5563-2010
OI Figdor, Carl G/0000-0002-2366-9212; de Vries, Jolanda
I.J.M./0000-0002-8653-4040; Buschow, Sonja/0000-0002-7907-8315
FU Marie Curie Research Training Network (RTN) Immunanomap
FX The authors wish to thank the technicians of the NCMLS Tumor Immunology
Department Clinical DC group for assistance. This work was supported by
Marie Curie Research Training Network (RTN) Immunanomap.
CR Adema GJ, 2005, CURR OPIN IMMUNOL, V17, P170, DOI 10.1016/j.coi.2005.01.004
Baumjohann D, 2006, EUR J IMMUNOL, V36, P2544, DOI 10.1002/eji.200535742
Beaune G, 2007, ANGEW CHEM INT EDIT, V46, P5421, DOI 10.1002/anie.200700581
BORRASCUESTA F, 1988, EUR J IMMUNOL, V18, P199, DOI 10.1002/eji.1830180203
BRADFORD MM, 1976, ANAL BIOCHEM, V72, P248, DOI 10.1016/0003-2697(76)90527-3
Cruz LJ, 2004, BIOCONJUGATE CHEM, V15, P112, DOI 10.1021/bc034119j
CRUZ LJ, 2004, J CONTROL RELEASE, V144, P118
de Vries IJM, 2005, NAT BIOTECHNOL, V23, P1407, DOI 10.1038/nbt1154
de Vries IJM, 2003, CANCER RES, V63, P12
Gupta AK, 2005, BIOMATERIALS, V26, P1565, DOI 10.1016/j.biomaterials.2004.05.022
Harisinghani MG, 2004, RADIOGRAPHICS, V24, P867, DOI 10.1148/rg.243035190
HOUGHTEN RA, 1986, INT J PEPT PROT RES, V27, P673
Huang SH, 2003, BIOTECHNOL PROGR, V19, P1095, DOI 10.1021/bp025587v
Jain TK, 2008, BIOMATERIALS, V29, P4012, DOI 10.1016/j.biomaterials.2008.07.004
KAISER E, 1970, ANAL BIOCHEM, V34, P595, DOI 10.1016/0003-2697(70)90146-6
Kim DH, 2006, BIOMATERIALS, V27, P3031, DOI 10.1016/j.biomaterials.2005.12.021
Kumar CSSR, 2004, J NANOSCI NANOTECHNO, V4, P245, DOI 10.1166/jnn.2004.085
LEE PW, BIOMATERIALS, V31, P1316
Lesterhuis WJ, 2008, CRIT REV ONCOL HEMAT, V66, P118, DOI 10.1016/j.critrevonc.2007.12.007
Lim YT, 2008, SMALL, V4, P1640, DOI 10.1002/smll.200800582
Lu CW, 2007, NANO LETT, V7, P149, DOI 10.1021/nl0624263
Martina MS, 2007, BIOMATERIALS, V28, P4143, DOI 10.1016/j.biomaterials.2007.05.025
McCarthy JR, 2006, SMALL, V2, P983, DOI 10.1002/smll.200600139
Mulder WJM, 2007, NANOMEDICINE-UK, V2, P307, DOI 10.2217/17435889.2.3.307
OHAGAN DT, 1991, IMMUNOLOGY, V73, P239
Okassa LN, 2007, EUR J PHARM BIOPHARM, V67, P31, DOI 10.1016/j.ejpb.2006.12.020
Okassa LN, 2005, INT J PHARMACEUT, V302, P187, DOI 10.1016/j.ijpharm.2005.06.024
Olasz EB, 2002, J IMMUNOL METHODS, V260, P137, DOI 10.1016/S0022-1759(01)00528-2
Panyam J, 2002, FASEB J, V16, DOI 10.1096/fj.02-0088com
QUARANTA V, 1980, J IMMUNOL, V125, P1421
Reichardt W, 2008, J IMMUNOL, V181, P4770, DOI 10.4049/jimmunol.181.7.4770
Schimmelpfennig CH, 2005, AM J PATHOL, V167, P1321, DOI 10.1016/S0002-9440(10)61219-9
Shen H, 2006, IMMUNOLOGY, V117, P78, DOI 10.1111/j.1365-2567.2005.02268.x
Tacken PJ, 2007, NAT REV IMMUNOL, V7, P790, DOI 10.1038/nri2173
Tacken PJ, 2005, BLOOD, V106, P1278, DOI 10.1182/blood-2005-01-0318
Thomas TC, 1996, MOL IMMUNOL, V33, P1389, DOI 10.1016/S0161-5890(96)00078-8
Uzgiris EE, 2006, TECHNOL CANCER RES T, V5, P301, DOI 10.1177/153303460600500401
Veiseh O, 2005, NANO LETT, V5, P1003, DOI 10.1021/nl0502569
Verdijk P, 2007, INT J CANCER, V120, P978, DOI 10.1002/ijc.22385
Verdijk P, 2009, CLIN CANCER RES, V15, P2531, DOI 10.1158/1078-0432.CCR-08-2729
VITIELLO A, 1995, J CLIN INVEST, V95, P341, DOI 10.1172/JCI117662
NR 41
TC 65
Z9 66
U1 0
U2 42
PU AMER CHEMICAL SOC
PI WASHINGTON
PA 1155 16TH ST, NW, WASHINGTON, DC 20036 USA
SN 1543-8384
J9 MOL PHARMACEUT
JI Mol. Pharm.
PD MAR-APR

PY 2011
VL 8
IS 2
BP 520
EP 531
DI 10.1021/mp100356k
PG 12
WC Medicine, Research & Experimental; Pharmacology & Pharmacy
WE Science Citation Index Expanded (SCI-EXPANDED)
SC Research & Experimental Medicine; Pharmacology & Pharmacy
GA 743HJ
UT WOS:000289008600021
PM 21381651
OA Green Submitted
DA 2023-09-14
ER

PT J
AU Mainini, F
Eccles, MR
AF Mainini, Francesco
Eccles, Michael R.
TI Lipid and Polymer-Based Nanoparticle siRNA Delivery Systems for Cancer Therapy
SO MOLECULES
LA English
DT Review
DE nanoparticle; intracellular delivery; siRNA; cancer therapy
ID INTERFERING RNA DELIVERY; CATIONIC LIPOSOME; IMMUNE-RESPONSE; HYALURONIC-ACID; CO-DELIVERY; IN-VITRO; CHEMOTHERAPY; TARGET; STIMULATION; PERFORMANCE
AB RNA interference (RNAi) uses small interfering RNAs (siRNAs) to mediate gene-silencing in cells and represents an emerging strategy for cancer therapy. Successful RNAi-mediated gene silencing requires overcoming multiple physiological barriers to achieve efficient delivery of siRNAs into cells in vivo, including into tumor and/or host cells in the tumor micro-environment (TME). Consequently, lipid and polymer-based nanoparticle siRNA delivery systems have been developed to surmount these physiological barriers. In this article, we review the strategies that have been developed to facilitate siRNA survival in the circulatory system, siRNA movement from the blood into tissues and the TME, targeted siRNA delivery to the tumor or specific cell types, cellular uptake, and escape from endosomal degradation. We also discuss the use of various types of lipid and polymer-based carriers for cancer therapy, including a section on anti-tumor nanovaccines enhanced by siRNAs. Finally, we review current and recent clinical trials using NPs loaded with siRNAs for cancer therapy. The siRNA cancer therapeutics field is rapidly evolving, and it is conceivable that precision cancer therapy could, in the relatively near future, benefit from the combined use of cancer therapies, for example immune checkpoint blockade together with gene-targeting siRNAs, personalized for enhancing and fine-tuning a patient's therapeutic response.
C1 [Mainini, Francesco; Eccles, Michael R.] Univ Otago, Dunedin Sch Med, Dept Pathol, Dunedin 9054, New Zealand.
C3 University of Otago
RP Eccles, MR (通讯作者)，Univ Otago, Dunedin Sch Med, Dept Pathol, Dunedin 9054, New Zealand.
EM francesco.mainini@gmail.com; michael.eccles@otago.ac.nz
RI Mainini, Francesco/AAN-1958-2020
OI Mainini, Francesco/0000-0001-7743-952X; Eccles, Michael/0000-0002-6824-8761
FU Otago Medical Research Foundation [AG303]; Cancer Society of New Zealand; New Zealand Institute for Cancer Research Trust
FX This work was supported by grants from the Otago Medical Research Foundation, grant number AG303, and from the Cancer Society of New Zealand, the MauriceWilkins Centre for Molecular Biodiscovery, and the New Zealand Institute for Cancer Research Trust.
CR Acharya R, 2019, MAT SCI ENG C-MATER, V104, DOI 10.1016/j.msec.2019.109928
Ambros V, 2004, NATURE, V431, P350, DOI 10.1038/nature02871

Ameres SL, 2007, CELL, V130, P101, DOI 10.1016/j.cell.2007.04.037
Anfray C., 2020, NANOPARTICLES IMMUNO, V16, P265
[Anonymous], 2015, J CLIN ONCOL S
Bartlett DW, 2007, BIOTECHNOL BIOENG, V97, P909, DOI 10.1002/bit.21285
Berg K., 2010, POTENTIAL PHOTOCHEMI, P311
Buyens K, 2010, J CONTROL RELEASE, V141, P38, DOI 10.1016/j.jconrel.2009.08.026
Campbell RB, 2002, CANCER RES, V62, P6831
Cerqueira NMFSA, 2005, CURR MED CHEM, V12, P1283, DOI 10.2174/0929867054020981
Chen Z, 2010, TRENDS MOL MED, V16, P594, DOI 10.1016/j.molmed.2010.08.001
Choi KY, 2011, ACS NANO, V5, P8591, DOI 10.1021/nn202070n
Cupic KI, 2019, NANOMEDICINE-UK, V14, P215, DOI 10.2217/nnm-2018-0326
Dacoba TG, 2017, SEMIN IMMUNOL, V34, P78, DOI 10.1016/j.smim.2017.09.007
Davis ME, 2010, NATURE, V464, P1067, DOI 10.1038/nature08956
Degors IMS, 2019, ACCOUNTS CHEM RES, V52, P1750, DOI 10.1021/acs.accounts.9b00177
Dong YZ, 2019, ADV DRUG DELIVER REV, V144, P133, DOI 10.1016/j.addr.2019.05.004
Du BJ, 2018, NAT REV MATER, V3, P358, DOI 10.1038/s41578-018-0038-3
Du XF, 2018, DRUG DELIV, V25, P1516, DOI 10.1080/10717544.2018.1480674
Fadeel B, 2019, FRONT IMMUNOL, V10, DOI 10.3389/fimmu.2019.00133
Fire A, 1998, NATURE, V391, P806, DOI 10.1038/35888
Galluzzi L, 2018, SCI TRANSL MED, V10, DOI 10.1126/scitranslmed.aat7807
Ganesh S, 2013, BIOMATERIALS, V34, P3489, DOI 10.1016/j.biomaterials.2013.01.077
Golan T, 2015, ONCOTARGET, V6, P24560, DOI 10.18632/oncotarget.4183
Greish K, 2010, METHODS MOL BIOL, V624, P25, DOI 10.1007/978-1-60761-609-2_3
Guan JB, 2017, NANOSCALE, V9, P9190, DOI 10.1039/c7nr02663c
Hatakeyama H, 2007, GENE THER, V14, P68, DOI 10.1038/sj.gt.3302843
Hayes ME, 2006, BBA-BIOMEMBRANES, V1758, P429, DOI 10.1016/j.bbamem.2006.03.020
Huang HQ, 2020, BIOMATER SCI-UK, V8, P1759, DOI 10.1039/c9bm01822k
Huang KW, 2020, SCI ADV, V6, DOI 10.1126/sciadv.aax5032
Huh MS, 2010, J CONTROL RELEASE, V144, P134, DOI 10.1016/j.jconrel.2010.02.023
Hussain Z, 2019, DRUG DELIV TRANSL RE, V9, P721, DOI 10.1007/s13346-019-00631-4
Judge AD, 2005, NAT BIOTECHNOL, V23, P457, DOI 10.1038/nbt1081
Kedmi R, 2018, NAT NANOTECHNOL, V13, P214, DOI 10.1038/s41565-017-0043-5
Kim TH, 2005, J CONTROL RELEASE, V105, P354, DOI 10.1016/j.jconrel.2005.03.024
Koynova R, 2006, P NATL ACAD SCI USA, V103, P14373, DOI 10.1073/pnas.0603085103
Lazarovits J, 2015, CHEM COMMUN, V51, P2756, DOI 10.1039/c4cc07644c
Lee J, 2020, J CONTROL RELEASE, V321, P184, DOI 10.1016/j.jconrel.2020.02.013
Lin SY, 2012, BIOMACROMOLECULES, V13, P664, DOI 10.1021/bm201746t
Liu DQ, 2018, J CONTROL RELEASE, V273, P1, DOI 10.1016/j.jconrel.2018.01.013
Liu JJ, 2020, ASIAN J PHARM SCI, V15, P576, DOI 10.1016/j.ajps.2019.10.006
Liu YL, 2013, ADV MATER, V25, P1353, DOI 10.1002/adma.201204683
Lu S, 2019, J PHARMACOL EXP THER, V370, P902, DOI 10.1124/jpet.119.256909
Lu ZX, 2011, INT J NANOMED, V6, P1661, DOI 10.2147/IJN.S22293
Mainini F, 2018, NUCLEIC ACID THER, V28, P225, DOI 10.1089/nat.2017.0695
Mainini F, 2015, PLOS ONE, V10, DOI 10.1371/journal.pone.0145403
Malhotra M, 2018, EUR J PHARM SCI, V114, P30, DOI 10.1016/j.ejps.2017.11.024
Markosyan N, 2019, J CLIN INVEST, V129, P3594, DOI 10.1172/JCI127755
MATSUMURA Y, 1986, CANCER RES, V46, P6387
Vicentini FTMD, 2013, PHARM RES-DORDR, V30, P915, DOI 10.1007/s11095-013-0971-1
Naseri N, 2018, ARTIF CELL NANOMED B, V46, P1111, DOI 10.1080/21691401.2017.1379014
Nel AE, 2009, NAT MATER, V8, P543, DOI [10.1038/nmat2442, 10.1038/NMAT2442]
O'Neill CP, 2020, CELLS-BASEL, V9, DOI 10.3390/cells9020521
Paddison PJ, 2002, GENE DEV, V16, P948, DOI 10.1101/gad.981002
Pandey AP, 2016, MAT SCI ENG C-MATER, V68, P904, DOI 10.1016/j.msec.2016.07.066
Parvani JG, 2015, CANCER RES, V75, P2316, DOI 10.1158/0008-5472.CAN-14-3485
Qi RG, 2012, J CONTROL RELEASE, V159, P251, DOI 10.1016/j.jconrel.2012.01.015
Rittner K, 2002, MOL THER, V5, P104, DOI 10.1006/mthe.2002.0523
Robb GB, 2007, MOL CELL, V26, P523, DOI 10.1016/j.molcel.2007.04.016
Roedig H, 2020, SEMIN CANCER BIOL, V62, P31, DOI 10.1016/j.semcancer.2019.07.026
Roncato F, 2018, NAT COMMUN, V9, DOI 10.1038/s41467-018-06602-6
Rubel F, 2018, J INVEST DERMATOL, V138, P679, DOI 10.1016/j.jid.2017.09.036
Schultheis B, 2014, J CLIN ONCOL, V32, P4141, DOI 10.1200/JCO.2013.55.0376

Siegler E. L., 2016, J CELL IMMUNOTHER, V2, P69
Song EW, 2005, NAT BIOTECHNOL, V23, P709, DOI 10.1038/nbt1101
Stylianopoulos T, 2010, BIOPHYS J, V99, P1342, DOI 10.1016/j.bpj.2010.06.016
Subramanian N, 2015, J BIOMED SCI, V22, DOI 10.1186/s12929-014-0108-9
Sun WT, 2018, BIOMACROMOLECULES, V19, P2248, DOI 10.1021/acs.biomac.8b00272
Tabernero J, 2013, CANCER DISCOV, V3, P406, DOI 10.1158/2159-8290.CD-12-0429
Tang YX, 2019, ACS NANO, V13, P13015, DOI 10.1021/acsnano.9b05679
Tengood JE, 2016, EXP BIOL MED, V241, P1033, DOI 10.1177/1535370216647130
Tynga IM, 2018, NANOMATERIALS-BASEL, V8, DOI 10.3390/nano8110923
van den Boorn JG, 2012, ADV IMMUNOL, V114, P1, DOI 10.1016/B978-0-12-396548-6.00001-9
Varkouhi AK, 2011, J CONTROL RELEASE, V151, P220, DOI 10.1016/j.jconrel.2010.11.004
Wagner MJ, 2017, MOL CANCER THER, V16, P1114, DOI 10.1158/1535-7163.MCT-16-0541
Walkey CD, 2012, J AM CHEM SOC, V134, P2139, DOI 10.1021/ja2084338
Wang DG, 2016, SMALL, V12, P488, DOI 10.1002/smll.201502913
Wang GM, 2017, INT J NANOMED, V12, P3591, DOI 10.2147/IJN.S131078
Wang YB, 2019, J BIOMED NANOTECHNOL, V15, P966, DOI 10.1166/jbn.2019.2762
Wang YH, 2018, MOL THER, V26, P420, DOI 10.1016/j.ymthe.2017.11.009
Weinstein S, 2016, P NATL ACAD SCI USA, V113, pE16, DOI 10.1073/pnas.1519273113
Whitehead KA, 2011, ANNU REV CHEM BIOMOL, V2, P77, DOI 10.1146/annurev-chembioeng-061010-114133
Xiao H, 2017, J AM CHEM SOC, V139, P3033, DOI 10.1021/jacs.6b12108
Yang H, 2014, SCI REP-UK, V4, DOI 10.1038/srep07072
Yang J, 2014, BIOTECHNOL ADV, V32, P804, DOI 10.1016/j.biotechadv.2013.11.004
Yang X, 2018, INT J NANOMED, V13, P4333, DOI 10.2147/IJN.S161908
Yu S, 2019, J BIOMED NANOTECHNOL, V15, P1135, DOI 10.1166/jbn.2019.2751
Zhang BF, 2015, BIOMATERIALS, V61, P178, DOI 10.1016/j.biomaterials.2015.05.027
Zhao CY, 2018, MOLECULES, V23, DOI 10.3390/molecules23040826
Zhao J, 2019, FRONT PHARMACOL, V10, DOI 10.3389/fphar.2019.01184
Zwicke GL, 2012, NANO REV EXP, V3, DOI 10.3402/nano.v3i0.18496
NR 91
TC 64
Z9 66
U1 19
U2 108
PU MDPI
PI BASEL
PA ST ALBAN-ANLAGE 66, CH-4052 BASEL, SWITZERLAND
EI 1420-3049
J9 MOLECULES
JI Molecules
PD JUN
PY 2020
VL 25
IS 11
AR 2692
DI 10.3390/molecules25112692
PG 18
WC Biochemistry & Molecular Biology; Chemistry, Multidisciplinary
WE Science Citation Index Expanded (SCI-EXPANDED)
SC Biochemistry & Molecular Biology; Chemistry
GA MR8RU
UT WOS:000553858800229
PM 32532030
OA Green Published, gold
DA 2023-09-14
ER

PT J
AU Bhardwaj, P
Bhatia, E
Sharma, S

Ahamad, N
Banerjee, R
AF Bhardwaj, Prateek
Bhatia, Eshant
Sharma, Shivam
Ahamad, Nadim
Banerjee, Rinti
TI Advancements in prophylactic and therapeutic nanovaccines
SO ACTA BIOMATERIALIA
LA English
DT Review
DE Nanovaccine; Prophylactic; Therapeutic; Biomimetic; Antibody-mediated immunity; Cell-mediated immunity; Immune activation; Memory responses
ID PROTEIN NANOPARTICLE VACCINE; PEPTIDE AMPHIPHILE MICELLES; PROTECTIVE IMMUNE-RESPONSES; IRON-OXIDE NANOPARTICLES; INFLUENZA-A VIRUS; T-CELL RESPONSES; GOLD NANOPARTICLES; DELIVERY-SYSTEM; DENDRITIC CELLS; IN-VITRO
AB Vaccines activate suitable immune responses to fight against diseases but can possess limitations such as compromised efficacy and immunogenic responses, poor stability, and requirement of adherence to multiple doses. 'Nanovaccines' have been explored to elicit a strong immune response with the advantages of nano-sized range, high antigen loading, enhanced immunogenicity, controlled antigen presentation, more retention in lymph nodes and promote patient compliance by a lower frequency of dosing. Various types of nanoparticles with diverse pathogenic or foreign antigens can help to overcome immunotolerance and alleviate the need of booster doses as required with conventional vaccines. Nanovaccines have the potential to induce both cell-mediated and antibody-mediated immunity and can render long-lasting immunogenic memory. With such properties, nanovaccines have shown high potential for the prevention of infectious diseases such as acquired immunodeficiency syndrome (AIDS), malaria, tuberculosis, influenza, and cancer. Their therapeutic potential has also been explored in the treatment of cancer. The various kinds of nanomaterials used for vaccine development and their effects on immune system activation have been discussed with special relevance to their implications in various pathological conditions.

Statement of Significance

Interaction of nanoparticles with the immune system has opened multiple avenues to combat a variety of infectious and non-infectious pathological conditions. Limitations of conventional vaccines have paved the path for nanomedicine associated benefits with a hope of producing effective nanovaccines. This review highlights the role of different types of nanovaccines and the role of nanoparticles in modulating the immune response of vaccines. The applications of nanovaccines in infectious and non-infectious diseases like malaria, tuberculosis, AIDS, influenza, and cancers have been discussed. It will help the readers develop an understanding of mechanisms of immune activation by nanovaccines and design appropriate strategies for novel nanovaccines. (C) 2020 Acta Materialia Inc. Published by Elsevier Ltd. All rights reserved.
C1 [Bhardwaj, Prateek; Bhatia, Eshant; Sharma, Shivam; Ahamad, Nadim; Banerjee, Rinti] Indian Inst Technol, Dept Biosci & Bioengn, Nanomed Lab, Mumbai 400076, Maharashtra, India.
C3 Indian Institute of Technology System (IIT System); Indian Institute of Technology (IIT) - Bombay
RP Banerjee, R (通讯作者)，Indian Inst Technol, Dept Biosci & Bioengn, Nanomed Lab, Mumbai 400076, Maharashtra, India.
EM rinti@iitb.ac.in
RI Bhardwaj, Prateek/AGR-5470-2022; Ahamad, Nadim/ABC-9696-2021
OI Bhardwaj, Prateek/0000-0001-5546-7829; Ahamad, Nadim/0000-0002-8418-2459; Banerjee, Rinti/0000-0002-6400-5906; Sharma, Shivam/0000-0002-7633-1557
FU IIT Bombay, Ministry of Human Resource and Development, Government of India
FX Authors would like to thank IIT Bombay, Ministry of Human Resource and Development, Government of India, for their research fellowships.
CR Aikins ME, 2017, NANOMEDICINE-UK, V12, P673, DOI 10.2217/nnm-2016-0381
Almeida JPM, 2015, SMALL, V11, P1453, DOI 10.1002/smll.201402179
Alvarez M. J., 2002, Clinical and Experimental Allergy, V32, P1574, DOI 10.1046/j.1365-2222.2002.01514.x
Amorij JP, 2012, J CONTROL RELEASE, V161, P363, DOI 10.1016/j.jconrel.2011.12.039

[Anonymous], 2014, WHO INFL SEAS, P473
[Anonymous], 2010, MULT EXT DRUG RES TB, P466
Ansari MA, 2011, PLOS ONE, V6, DOI 10.1371/journal.pone.0022889
Asgary V, 2016, INT J NANOMED, V11, P3597, DOI 10.2147/IJN.S109098
Athanasiou E, 2017, FRONT IMMUNOL, V8, DOI 10.3389/fimmu.2017.00684
Awate S, 2013, FRONT IMMUNOL, V4, DOI 10.3389/fimmu.2013.00114
Babapoor Sankhiros, 2011, Influenza Res Treat, V2011, P126794, DOI 10.1155/2011/126794
Bachmann MF, 2010, NAT REV IMMUNOL, V10, P787, DOI 10.1038/nri2868
Badiee A, 2012, EXP PARASITOL, V132, P403, DOI 10.1016/j.exppara.2012.09.001
Badiee A, 2009, COLLOID SURFACE B, V74, P37, DOI 10.1016/j.colsurfb.2009.06.025
Barhate G, 2014, J PHARM SCI-US, V103, P3448, DOI 10.1002/jps.24161
Barriga HMG, 2019, ANGEW CHEM INT EDIT, V58, P2958, DOI 10.1002/anie.201804067
Bayon E, 2018, NPJ VACCINES, V3, DOI 10.1038/s41541-018-0086-0
Beeh KM, 2013, J ALLERGY CLIN IMMUN, V131, P866, DOI 10.1016/j.jaci.2012.12.1561
Berkowska MA, 2011, BLOOD, V118, P2150, DOI 10.1182/blood-2011-04-345579
Black M, 2012, ADV MATER, V24, P3845, DOI 10.1002/adma.201200209
Blackburne BP, 2008, PLOS PATHOG, V4, DOI 10.1371/journal.ppat.1000058
Blattman JN, 2004, SCIENCE, V305, P200, DOI 10.1126/science.1100369
Bloch O, 2014, NEURO-ONCOLOGY, V16, P274, DOI 10.1093/neuonc/not203
Blok BA, 2015, J LEUKOCYTE BIOL, V98, P347, DOI 10.1189/jlb.5RI0315-096R
Blom RAM, 2016, PLOS ONE, V11, DOI 10.1371/journal.pone.0163539
Borges O, 2008, EUR J PHARM BIOPHARM, V69, P405, DOI 10.1016/j.ejpb.2008.01.019
Casanas A, 2012, CURR OPIN BIOTECH, V23, P972, DOI 10.1016/j.copbio.2012.05.004
Chaowanachan T, 2013, PLOS ONE, V8, DOI 10.1371/journal.pone.0061416
Cheever MA, 2011, CLIN CANCER RES, V17, P3520, DOI 10.1158/1078-0432.CCR-10-3126
Chen JZ, 2009, VIROL J, V6, DOI 10.1186/1743-422X-6-30
Chen QQ, 2019, DRUG DELIV TRANSL RE, V9, P1095, DOI 10.1007/s13346-019-00652-z
Chen YS, 2010, NANOTECHNOLOGY, V21, DOI 10.1088/0957-4484/21/19/195101
Chesson CB, 2018, SCI REP-UK, V8, DOI 10.1038/s41598-018-31089-y
Chiodo F, 2015, CARBOHYD RES, V405, P102, DOI 10.1016/j.carres.2014.07.012
Clemente-Casares X, 2011, J MOL MED, V89, P733, DOI 10.1007/s00109-011-0757-z
Climent N, 2018, NANOMED-NANOTECHNOL, V14, P339, DOI 10.1016/j.nano.2017.11.009
Da Silva DM, 2001, VIROLOGY, V290, P350, DOI 10.1006/viro.2001.1179
Dacoba TG, 2019, ACS NANO, V13, P4947, DOI 10.1021/acsnano.8b07662
Das I, 2017, NANOTECHNOLOGY, V28, DOI 10.1088/1361-6528/aa60fd
Date AA, 2015, J BIOMED NANOTECHNOL, V11, P416, DOI 10.1166/jbn.2015.1942
Date AA, 2012, ANTIVIR RES, V96, P430, DOI 10.1016/j.antiviral.2012.09.015
De Gregorio E, 2013, FRONT IMMUNOL, V4, DOI 10.3389/fimmu.2013.00214
de Titta A, 2013, P NATL ACAD SCI USA, V110, P19902, DOI 10.1073/pnas.1313152110
Demento SL, 2012, BIOMATERIALS, V33, P4957, DOI 10.1016/j.biomaterials.2012.03.041
Demento SL, 2011, TRENDS BIOTECHNOL, V29, P294, DOI 10.1016/j.tibtech.2011.02.004
Deng L, 2018, NAT COMMUN, V9, DOI 10.1038/s41467-017-02725-4
Dhanasooraj D, 2013, INT J NANOMED, V8, P835, DOI 10.2147/IJN.S40238
Ding PY, 2019, INT J NANOMED, V14, P7533, DOI 10.2147/IJN.S218057
Diogo GR, 2019, FRONT IMMUNOL, V10, DOI 10.3389/fimmu.2019.01349
Diwan M, 2002, J CONTROL RELEASE, V85, P247, DOI 10.1016/S0168-3659(02)00275-4
Doll TAPF, 2015, J NANOBIOTECHNOL, V13, DOI 10.1186/s12951-015-0119-z
Dong X, 2019, ACS APPL MATER INTER, V11, P4876, DOI 10.1021/acsami.8b20364
Duffy PE, 2012, EXPERT REV VACCINES, V11, P1261, DOI [10.1586/erv.12.92, 10.1586/ERV.12.92]
El Bissati K, 2017, NPJ VACCINES, V2, DOI 10.1038/s41541-017-0024-6
El Bissati K, 2016, JCI INSIGHT, V1, DOI 10.1172/jci.insight.85955
El Bissati K, 2014, VACCINE, V32, P3243, DOI 10.1016/j.vaccine.2014.03.092
Feng GZ, 2013, PLOS ONE, V8, DOI 10.1371/journal.pone.0061135
Fusciello M, 2019, NAT COMMUN, V10, DOI 10.1038/s41467-019-13744-8
Gajewski TF, 2013, NAT IMMUNOL, V14, P1014, DOI 10.1038/ni.2703
Giessen TW, 2016, CURR OPIN CHEM BIOL, V34, P1, DOI 10.1016/j.cbpa.2016.05.013
Glaffig M, 2014, CHEM-EUR J, V20, P4232, DOI 10.1002/chem.201400256
Greenwood B, 2014, PHILOS T R SOC B, V369, DOI 10.1098/rstb.2013.0433
Gregory AE, 2013, FRONT CELL INFECT MI, V3, DOI 10.3389/fcimb.2013.00013
Gross BP, 2014, AAPS J, V16, P1194, DOI 10.1208/s12248-014-9662-z
Guo HC, 2012, VIROL J, V9, DOI 10.1186/1743-422X-9-108

Guo YY, 2015, ACS NANO, V9, P6918, DOI 10.1021/acsnano.5b01042
Hamilton Erika, 2012, J Transl Med, V10, P28, DOI 10.1186/1479-5876-10-28
Hasegawa K, 2006, CLIN CANCER RES, V12, P1921, DOI 10.1158/1078-0432.CCR-05-1900
Henriksen-Lacey M, 2010, J CONTROL RELEASE, V145, P102, DOI 10.1016/j.jconrel.2010.03.027
Houser K, 2015, CELL HOST MICROBE, V17, P295, DOI 10.1016/j.chom.2015.02.012
Huang HC, 2010, P NATL ACAD SCI USA, V107, P4658, DOI 10.1073/pnas.1001074107
Huang WC, 2018, NAT NANOTECHNOL, V13, P1174, DOI 10.1038/s41565-018-0271-3
Huntimer L, 2013, J BIOMED MATER RES B, V101B, P91, DOI 10.1002/jbm.b.32820
Hussein WM, 2016, BIOORGAN MED CHEM, V24, P4372, DOI 10.1016/j.bmc.2016.07.036
Jiang J., 2017, VACCINES, V5, P141
Kaba SA, 2012, PLOS ONE, V7, DOI 10.1371/journal.pone.0048304
Kabiri M, 2018, INT J PHARMACEUT, V549, P404, DOI 10.1016/j.ijpharm.2018.07.069
Kalluru R, 2013, J CELL SCI, V126, P3043, DOI 10.1242/jcs.121814
Kanchan V, 2009, BIOMATERIALS, V30, P4763, DOI 10.1016/j.biomaterials.2009.05.075
Karch CP, 2019, FUTURE VIROL, V14, P51, DOI 10.2217/fvl-2018-0174
Karch CP, 2017, NANOMED-NANOTECHNOL, V13, P241, DOI 10.1016/j.nano.2016.08.030
Karuturi BVK, 2017, MOL PHARMACEUT, V14, P1469, DOI 10.1021/acs.molpharmaceut.6b01088
Kaur R, 2012, J CONTROL RELEASE, V164, P331, DOI 10.1016/j.jconrel.2012.07.012
Kawasaki T, 2014, FRONT IMMUNOL, V5, DOI 10.3389/fimmu.2014.00461
Keijzer C, 2013, FRONT IMMUNOL, V4, DOI 10.3389/fimmu.2013.00245
Kerkmann M, 2003, J IMMUNOL, V170, P4465, DOI 10.4049/jimmunol.170.9.4465
Kester KE, 2009, J INFECT DIS, V200, P337, DOI 10.1086/600120
Kim SY, 2017, BIOMATERIALS, V130, P56, DOI 10.1016/j.biomaterials.2017.03.034
Kuai R, 2018, BIOCONJUGATE CHEM, V29, P771, DOI 10.1021/acs.bioconjchem.7b00761
Kuai R, 2017, NAT MATER, V16, P489, DOI [10.1038/nmat4822, 10.1038/NMAT4822]
Kumar R, 2015, VACCINE, V33, P5064, DOI 10.1016/j.vaccine.2015.08.025
Lagoutte P, 2018, VACCINE, V36, P3622, DOI 10.1016/j.vaccine.2018.05.034
Leleux J, 2013, ADV HEALTHC MATER, V2, P72, DOI 10.1002/adhm.201200268
Lell B, 2009, PLOS ONE, V4, DOI 10.1371/journal.pone.0007611
LEVINE TP, 1991, CRIT REV BIOCHEM MOL, V26, P439, DOI 10.3109/10409239109086790
Li F, 2019, ACS CENTRAL SCI, V5, P796, DOI 10.1021/acscentsci.9b00060
Li P, 2013, J CONTROL RELEASE, V168, P271, DOI 10.1016/j.jconrel.2013.03.025
Li WD, 2014, VACCINES-BASEL, V2, P515, DOI 10.3390/vaccines2030515
Li WY, 2018, ACS APPL MATER INTER, V10, P28494, DOI 10.1021/acsami.8b10948
Li XH, 2001, PHARMACEUT RES, V18, P117, DOI 10.1023/A:1011043230573
Lima VMF, 2001, INFECT IMMUN, V69, P5305, DOI 10.1128/IAI.69.9.5305-5312.2001
Lin LCW, 2018, ADV HEALTHC MATER, V7, DOI 10.1002/adhm.201701395
Liu G, 2015, CLIN VACCINE IMMUNOL, V22, P516, DOI 10.1128/CVI.00770-14
Liu WL, 2019, NAT COMMUN, V10, DOI 10.1038/s41467-019-11157-1
Luo M, 2019, J CONTROL RELEASE, V300, P154, DOI 10.1016/j.jconrel.2019.02.036
Luo M, 2017, NAT NANOTECHNOL, V12, P648, DOI [10.1038/nnano.2017.52, 10.1038/NNANO.2017.52]
Maisonneuve C, 2014, P NATL ACAD SCI USA, V111, P12294, DOI 10.1073/pnas.1400478111
Makidon PE, 2010, MED MICROBIOL IMMUN, V199, P81, DOI 10.1007/s00430-009-0137-2
Malik T, 2018, ARTIF CELL NANOMED B, V46, pS79, DOI 10.1080/21691401.2017.1414054
Manish M, 2013, PLOS ONE, V8, DOI 10.1371/journal.pone.0061885
Marasini N, 2016, INT J PHARMACEUT, V513, P410, DOI 10.1016/j.ijpharm.2016.09.057
McCoy ME, 2013, MALARIA J, V12, DOI 10.1186/1475-2875-12-136
McHugh KJ, 2015, J CONTROL RELEASE, V219, P596, DOI 10.1016/j.jconrel.2015.07.029
Miura Kazutoyo, 2016, Expert Rev Vaccines, V15, P765, DOI 10.1586/14760584.2016.1141680
Mody KT, 2015, PLOS ONE, V10, DOI 10.1371/journal.pone.0143507
Mohsen MO, 2019, FRONT IMMUNOL, V10, DOI 10.3389/fimmu.2019.01015
Molino NM, 2016, BIOMATERIALS, V86, P83, DOI 10.1016/j.biomaterials.2016.01.056
Molino NM, 2013, ACS NANO, V7, P9743, DOI 10.1021/nn403085w
Montoya J, 2013, J CLIN IMMUNOL, V33, P1360, DOI 10.1007/s10875-013-9949-3
Najer A, 2014, ACS NANO, V8, P12560, DOI 10.1021/nn5054206
Nandy A, 2016, INT J MOL SCI, V17, DOI 10.3390/ijms17050666
Nevagi RJ, 2018, ACTA BIOMATER, V80, P278, DOI 10.1016/j.actbio.2018.09.037
Pham NB, 2017, J NANOBIOTECHNOL, V15, DOI 10.1186/s12951-017-0305-2
Nguyen DN, 2012, P NATL ACAD SCI USA, V109, pE797, DOI 10.1073/pnas.1121423109
Nguyen F, 2018, CLIN CANCER RES, V24, P2585, DOI 10.1158/1078-0432.CCR-17-3811

Niikura K, 2013, ACS NANO, V7, P3926, DOI 10.1021/nn3057005
Niu L, 2019, J CONTROL RELEASE, V294, P268, DOI 10.1016/j.jconrel.2018.12.026
Parry AL, 2013, J AM CHEM SOC, V135, P9362, DOI 10.1021/ja4046857
Pati R, 2018, FRONT IMMUNOL, V9, DOI 10.3389/fimmu.2018.02224
Paulis LE, 2013, CURR OPIN IMMUNOL, V25, P389, DOI 10.1016/j.coi.2013.03.001
Petersen LK, 2012, MOL PHARMACEUT, V9, P874, DOI 10.1021/mp2004059
Pimentel TAPF, 2009, CHEM BIOL DRUG DES, V73, P53, DOI 10.1111/j.1747-0285.2008.00746.x
Polhemus ME, 2009, PLOS ONE, V4, DOI 10.1371/journal.pone.0006465
Polonskaya Z, 2017, J CLIN INVEST, V127, P1491, DOI 10.1172/JCI91192
Pompano RR, 2014, ADV HEALTHC MATER, V3, P1898, DOI 10.1002/adhm.201400137
Prego C, 2010, VACCINE, V28, P2607, DOI 10.1016/j.vaccine.2010.01.011
Pulendran B, 2011, NAT IMMUNOL, V12, P509, DOI 10.1038/ni.2039
Pusic K, 2013, FASEB J, V27, P1153, DOI 10.1096/fj.12-218362
Qi M, 2018, SMALL, V14, DOI 10.1002/smll.201703207
Ramirez A, 2018, VACCINE, V36, P873, DOI 10.1016/j.vaccine.2017.12.053
Ramirez JEV, 2014, J BIOMAT SCI-POLYM E, V25, P1387, DOI 10.1080/09205063.2014.940243
Reddy ST, 2007, NAT BIOTECHNOL, V25, P1159, DOI [10.1038/nbt1332, 10.1038/nbtl332]
Restifo NP, 2012, NAT REV IMMUNOL, V12, P269, DOI 10.1038/nri3191
Rice-Ficht AC, 2010, CURR OPIN MICROBIOL, V13, P106, DOI 10.1016/j.mib.2009.12.001
Rizwan SB, 2013, J CONTROL RELEASE, V165, P16, DOI 10.1016/j.jconrel.2012.10.020
Rodrigues L, 2018, J CONTROL RELEASE, V285, P12, DOI 10.1016/j.jconrel.2018.06.028
Roman VRG, 2013, AIDS RES HUM RETROV, V29, P1504, DOI [10.1089/aid.2013.0076, 10.1089/AID.2013.0076]
Rosado MM, 2011, EUR J IMMUNOL, V41, P1800, DOI 10.1002/eji.201041187
Rosalia RA, 2015, BIOMATERIALS, V40, P88, DOI 10.1016/j.biomaterials.2014.10.053
Ross KA, 2015, INT J NANOMED, V10, P229, DOI 10.2147/IJN.S72264
Rostami H, 2017, IMMUNOL LETT, V187, P19, DOI 10.1016/j.imlet.2017.05.002
Rudra JS, 2012, BIOMATERIALS, V33, P6476, DOI 10.1016/j.biomaterials.2012.05.041
Rueda F, 2017, IMMUNOBIOLOGY, V222, P989, DOI 10.1016/j.imbio.2017.06.002
Saade F, 2013, VACCINE, V31, P1999, DOI 10.1016/j.vaccine.2012.12.077
Sanchez-Villamil JI, 2019, MBIO, V10, DOI 10.1128/mBio.01869-19
Sartorius R, 2018, FRONT IMMUNOL, V9, DOI 10.3389/fimmu.2018.01496
Schmidt ST, 2016, PHARMACEUTICS, V8, DOI 10.3390/pharmaceutics8010007
Schulze K, 2017, NANOMED-NANOTECHNOL, V13, P2463, DOI 10.1016/j.nano.2017.08.015
Schwarz B, 2016, ACS BIOMATER SCI ENG, V2, P2324, DOI 10.1021/acsbiomaterials.6b00532
Seidman JC, 2012, INFLUENZA OTHER RESP, V6, P52, DOI 10.1111/j.1750-2659.2011.00268.x
Seth L, 2017, VACCINE, V35, P5448, DOI 10.1016/j.vaccine.2017.02.040
Shen H, 2006, IMMUNOLOGY, V117, P78, DOI 10.1111/j.1365-2567.2005.02268.x
Shevtsov M, 2016, FRONT IMMUNOL, V7, DOI 10.3389/fimmu.2016.00171
Shukla GS, 2018, J IMMUNOL METHODS, V460, P51, DOI 10.1016/j.jim.2018.06.009
Si YH, 2018, J CONTROL RELEASE, V282, P120, DOI 10.1016/j.jconrel.2018.04.031
Siefert AL, 2016, BIOMATERIALS, V97, P85, DOI 10.1016/j.biomaterials.2016.03.039
Smith DM, 2013, NAT REV IMMUNOL, V13, P592, DOI 10.1038/nri3488
Song WT, 2017, BIOMATERIALS, V148, P16, DOI 10.1016/j.biomaterials.2017.09.017
Sungsuwan S, 2015, ACS APPL MATER INTER, V7, P17535, DOI 10.1021/acsami.5b05497
Swaminathan G, 2016, VACCINE, V34, P110, DOI 10.1016/j.vaccine.2015.10.132
Tacken PJ, 2007, NAT REV IMMUNOL, V7, P790, DOI 10.1038/nri2173
Tagliamonte M, 2014, HUM VACC IMMUNOTHER, V10, P3332, DOI 10.4161/21645515.2014.973317
TAN L, 1991, ASIAN PAC J ALLERGY, V9, P25
Tang J, 2012, VACCINE, V30, P1071, DOI 10.1016/j.vaccine.2011.12.029
Tao WQ, 2015, VACCINE, V33, P2307, DOI 10.1016/j.vaccine.2015.03.063
Tao WQ, 2014, NANOMEDICINE-UK, V9, P237, DOI 10.2217/nnm.13.58
Teran-Navarro H, 2019, ONCOIMMUNOLOGY, V8, DOI 10.1080/2162402X.2018.1541534
Thomas C, 2011, MOL PHARMACEUT, V8, P405, DOI 10.1021/mp100255c
Toy R, 2016, BIOENG TRANSL MED, V1, P47, DOI 10.1002/btm2.10005
Tran E, 2014, SCIENCE, V344, P641, DOI 10.1126/science.1251102
Trent A, 2015, AAPS J, V17, P380, DOI 10.1208/s12248-014-9707-3
Tsai SE, 2010, IMMUNITY, V32, P568, DOI 10.1016/j.immuni.2010.03.015
Tsoras AN, 2019, ANNU REV CHEM BIOMOL, V10, P337, DOI 10.1146/annurev-chembioeng-060718-030347
Ulery BD, 2011, PLOS ONE, V6, DOI 10.1371/journal.pone.0017642

van Dissel JT, 2014, VACCINE, V32, P7098, DOI 10.1016/j.vaccine.2014.10.036
Vasievich EA, 2011, CANCER IMMUNOL IMMUN, V60, P629, DOI 10.1007/s00262-011-0970-1
Vignali DAA, 2008, NAT REV IMMUNOL, V8, P523, DOI 10.1038/nri2343
Villa CH, 2011, ACS NANO, V5, P5300, DOI 10.1021/nn200182x
Wagner-Muniz DA, 2018, FRONT IMMUNOL, V9, DOI 10.3389/fimmu.2018.00325
Wahome N, 2012, CHEM BIOL DRUG DES, V80, P349, DOI 10.1111/j.1747-0285.2012.01423.x
Walsh KP, 2013, TRENDS IMMUNOL, V34, P521, DOI 10.1016/j.it.2013.07.006
Wang L, 2017, NANOMED-NANOTECHNOL, V13, P253, DOI 10.1016/j.nano.2016.09.001
Wang L, 2014, NANOMED-NANOTECHNOL, V10, P473, DOI 10.1016/j.nano.2013.08.005
Wang TY, 2011, EUR J PHARM SCI, V44, P653, DOI 10.1016/j.ejps.2011.10.012
Woodland DL, 2001, IMMUNOL RES, V24, P53, DOI 10.1385/IR:24:1:53
World Health Organization, 2017, WORLD MAL REP 2017, P196
Wu XJ, 2019, J MATER CHEM B, V7, P4854, DOI 10.1039/c9tb00448c
Wu YY, 2017, ACS BIOMATER SCI ENG, V3, P3128, DOI 10.1021/acsbiomaterials.7b00561
Wu YL, 2011, BMC CANCER, V11, DOI 10.1186/1471-2407-11-430
Xiang SD, 2015, T ROY SOC TROP MED H, V109, P70, DOI 10.1093/trstmh/tru182
Xu LG, 2012, NANO LETT, V12, P2003, DOI 10.1021/nl300027p
Yating L., 2019, ACS APPL BIO MAT, V2, P1241
Yin ZJ, 2018, ACS CHEM BIOL, V13, P1668, DOI 10.1021/acschembio.8b00313
Zaman M, 2013, METHODS, V60, P226, DOI 10.1016/j.ymeth.2013.04.014
Zepp F, 2010, VACCINE, V28, pC14, DOI 10.1016/j.vaccine.2010.07.020
Zhang QB, 2017, ACS NANO, V11, P10724, DOI 10.1021/acsnano.7b04955
Zhang S, 2018, INT J NANOMED, V13, P2377, DOI 10.2147/IJN.S158536
Zhang XD, 2010, INT J NANOMED, V5, P771, DOI 10.2147/IJN.S8428
Zhang ZP, 2011, BIOMATERIALS, V32, P3666, DOI 10.1016/j.biomaterials.2011.01.067
Zhao K, 2012, PLOS ONE, V7, DOI 10.1371/journal.pone.0053314
Zhou J., 2019, ADV MATER
Zhu MT, 2014, HUM VACC IMMUNOTHER, V10, P2761, DOI 10.4161/hv.29589
Zollinger WD, 2012, VACCINE, V30, P712, DOI 10.1016/j.vaccine.2011.11.084
NR 213
TC 64
Z9 66
U1 5
U2 57
PU ELSEVIER SCI LTD
PI OXFORD
PA THE BOULEVARD, LANGFORD LANE, KIDLINGTON, OXFORD OX5 1GB, OXON, ENGLAND
SN 1742-7061
EI 1878-7568
J9 ACTA BIOMATER
JI Acta Biomater.
PD MAY
PY 2020
VL 108
BP 1
EP 21
DI 10.1016/j.actbio.2020.03.020
PG 21
WC Engineering, Biomedical; Materials Science, Biomaterials
WE Science Citation Index Expanded (SCI-EXPANDED)
SC Engineering; Materials Science
GA LJ6DD
UT WOS:000530252800001
PM 32268235
OA Green Published, Bronze
DA 2023-09-14
ER

PT J
AU Zhao, Y
Huo, MR

Xu, ZH
Wang, YH
Huang, L
AF Zhao, Yan
Huo, Meirong
Xu, Zhenghong
Wang, Yuhua
Huang, Leaf
TI Nanoparticle delivery of CDDO-Me remodels the tumor microenvironment and enhances vaccine therapy for melanoma
SO BIOMATERIALS
LA English
DT Article
DE Nanoparticle; CDDO-Me; Peptide vaccine; Tumor microenvironment; Melanoma
ID REGULATORY T-CELLS; SUPPRESSOR-CELLS; IMMUNOSUPPRESSIVE ACTIVITY; CANCER-IMMUNOTHERAPY; IMMUNE-RESPONSE; DENDRITIC CELLS; DNA VACCINE; ANTITUMOR; SIRNA; CHEMOTHERAPY
AB Lipid-calcium-phosphate nanoparticle (NP) delivery of Trp2 peptide vaccine is one of the most effective vaccine strategies against melanoma. However, due to the immunosuppressive microenvironment in the tumor, the achievement of potent immune responses remains a major challenge. NP delivery systems provide an opportunity to deliver chemotherapy agent to modulate the tumor microenvironment (TME) and improve the vaccine activity. Anti-inflammatory triterpenoid methyl-2-cyano-3,12-dioxooleana-1,9(11)-dien-28-oate (CDDO-Me) is a broad spectrum inhibitor of several signaling pathways that are important in both cancer cells and cells in the TME. Intravenous delivery of CDDO-Me using poly-lactic-glycolic-acid NP combination with subcutaneous Trp2 vaccine resulted in an increase of antitumor efficacy and apoptotic tumor tissue than Trp2 vaccine alone in B16F10 melanoma. There was a significant decrease of both Treg cells and MDSCs and a concomitant increase in the cytotoxic T-lymphocyte infiltration in TEM of the vaccinated animals. Also, CDDO-Me remodeled the tumor associated fibroblasts, collagen and vessel in TME, meanwhile, enhanced the Fas signaling pathway which could sensitize the tumor cells for cytotoxic T lymphocyte mediated killing. The combination of systemic induction of antigen-specific immune response using Trp2 nanovaccine and targeted modification of the TME with the NP delivered CDDO-Me offers a powerful combination therapy for melanoma. (C) 2015 Elsevier Ltd. All rights reserved.
C1 [Zhao, Yan; Huo, Meirong; Xu, Zhenghong; Wang, Yuhua; Huang, Leaf] Univ N Carolina, Eshelman Sch Pharm, Ctr Nanotechnol Drug Delivery, Div Mol Pharmaceut, Chapel Hill, NC 27599 USA.
[Zhao, Yan] China Med Univ, Sch Pharm, Dept Pharmaceut, Shenyang 110122, Peoples R China.
[Huo, Meirong] China Pharmaceut Univ, Dept Pharmaceut, State Key Lab Nat Med, Nanjing 210009, Jiangsu, Peoples R China.
C3 University of North Carolina; University of North Carolina Chapel Hill; China Medical University; China Pharmaceutical University
RP Huang, L (通讯作者)，Univ N Carolina, Eshelman Sch Pharm, Ctr Nanotechnol Drug Delivery, Div Mol Pharmaceut, Chapel Hill, NC 27599 USA.
EM alwong1982@gmail.com; leafh@unc.edu
RI , Pathology Services Core/CEZ-2246-2022
FU NIH [CA149363, CA149387, CA151652, DK100664]
FX The authors graciously thank NIH for funding (grants CA149363, CA149387, CA151652 and DK100664). We thank Yi Zhao, Lei Miao and Shutao Guo for their technical assistance in the experiment and Andrew Mackenzie Blair for his assistance in manuscript preparation.
CR Alizadeh D, 2014, CANCER RES, V74, P2663, DOI 10.1158/0008-5472.CAN-14-0301
Buhrman JD, 2013, IMMUNOL RES, V55, P34, DOI 10.1007/s12026-012-8348-9
Chen CA, 2010, MOL THER, V18, P1233, DOI 10.1038/mt.2010.34
Deeb Dorrah, 2010, Cancers (Basel), V2, P1779, DOI 10.3390/cancers2041779
Deeb D, 2009, PROSTATE, V69, P851, DOI 10.1002/pros.20937
Denies S, 2014, J CONTROL RELEASE, V187, P175, DOI 10.1016/j.jconrel.2014.05.045
Devaud C, 2013, ONCOIMMUNOLOGY, V2, DOI 10.4161/onci.25961
Diaz-Montero CM, 2014, SEMIN ONCOL, V41, P174, DOI 10.1053/j.seminoncol.2014.02.003
Emens LA, 2009, J CLIN ONCOL, V27, P5911, DOI 10.1200/JCO.2009.23.3494
Gameiro SR, 2011, CANCER IMMUNOL IMMUN, V60, P1227, DOI 10.1007/s00262-011-1020-8
Gao XH, 2013, ANTICANCER RES, V33, P215
Guo ST, 2014, ACS NANO, V8, P4996, DOI 10.1021/nn5010815

Hu YX, 2013, ACS NANO, V7, P5376, DOI 10.1021/nn4012384
Ishida E, 2004, PHARM RES-DORDR, V21, P932, DOI 10.1023/B:PHAM.0000029280.39882.ff
Johansson Anna, 2012, Cancers (Basel), V4, P340, DOI 10.3390/cancers4020340
Kashimura S, 2012, GASTRIC CANCER, V15, P144, DOI 10.1007/s10120-011-0090-9
Kim S, 2014, J BREAST CANCER, V17, P8, DOI 10.4048/jbc.2014.17.1.8
Konkankit VV, 2011, J TRANSL MED, V9, DOI 10.1186/1479-5876-9-192
Kuriyan AE, 2012, EXP EYE RES, V94, P136, DOI 10.1016/j.exer.2011.11.018
Li J, 2012, J CONTROL RELEASE, V158, P108, DOI 10.1016/j.jconrel.2011.10.020
Liao D, 2011, CANCER RES, V71, P5688, DOI 10.1158/0008-5472.CAN-11-1264
Liby KT, 2012, PHARMACOL REV, V64, P972, DOI 10.1124/pr.111.004846
Liu YB, 2012, BIOCHEM BIOPH RES CO, V417, P570, DOI 10.1016/j.bbrc.2011.12.007
Matejuk A, 2013, ARCH IMMUNOL THER EX, V61, P285, DOI 10.1007/s00005-013-0231-4
Mattarollo SR, 2013, IMMUNOL CELL BIOL, V91, P105, DOI 10.1038/icb.2012.58
Mellman I, 2011, NATURE, V480, P480, DOI 10.1038/nature10673
Nagaraj S, 2010, CLIN CANCER RES, V16, P1812, DOI 10.1158/1078-0432.CCR-09-3272
Neninger E, 2009, J IMMUNOTHER, V32, P92, DOI 10.1097/CJI.0b013e31818fe167
Obermajer N, 2012, IMMUNOL INVEST, V41, P635, DOI 10.3109/08820139.2012.695417
Ostrand-Rosenberg S, 2012, SEMIN CANCER BIOL, V22, P275, DOI 10.1016/j.semcancer.2012.01.011
Qin Y, 2013, PIGM CELL MELANOMA R, V26, P97, DOI 10.1111/pcmr.12031
Ryu K, 2010, BMC CANCER, V10, DOI 10.1186/1471-2407-10-187
Schwartzentruber DJ, 2011, NEW ENGL J MED, V364, P2119, DOI 10.1056/NEJMoa1012863
Sharma P, 2011, NAT REV CANCER, V11, P805, DOI 10.1038/nrc3153
Shirota Y, 2012, J IMMUNOL, V188, P1592, DOI 10.4049/jimmunol.1101304
Shishodia S, 2006, CLIN CANCER RES, V12, P1828, DOI 10.1158/1078-0432.CCR-05-2044
Sinha P, 2005, CANCER RES, V65, P11743, DOI 10.1158/0008-5472.CAN-05-0045
Slingluff CL, 2011, J CLIN ONCOL, V29, P2924, DOI 10.1200/JCO.2010.33.8053
Solary E, 1998, B CANCER, V85, P685
Tan ZW, 2014, CANCER RES, V74, P6010, DOI 10.1158/0008-5472.CAN-14-0473
Venanzi F, 2013, ONCOTARGET, V4, P1829, DOI 10.18632/oncotarget.1397
Viard-Leveugle I, 2013, J INVEST DERMATOL, V133, P489, DOI 10.1038/jid.2012.330
Wallecha A, 2013, J IMMUNOTHER, V36, P468, DOI 10.1097/CJI.0000000000000000
Wang YY, 2014, MOL CANCER, V13, DOI [10.1186/1476-4598-13-30, 10.1186/s12934-014-0151-y]
Weber JS, 2013, J CLIN ONCOL, V31, P4311, DOI 10.1200/JCO.2013.51.4802
Weed DT, 2015, CLIN CANCER RES, V21, P39, DOI 10.1158/1078-0432.CCR-14-1711
Whiteside TL, 2008, ONCOGENE, V27, P5904, DOI 10.1038/onc.2008.271
Wu A, 2010, NEURO-ONCOLOGY, V12, P1113, DOI 10.1093/neuonc/noq082
Xu ZH, 2014, ACS NANO, V8, P3636, DOI 10.1021/nn500216y
Xu ZH, 2013, J CONTROL RELEASE, V172, P259, DOI 10.1016/j.jconrel.2013.08.021
Zhang J, 2014, J CONTROL RELEASE, V182, P90, DOI 10.1016/j.jconrel.2014.03.016
Zhang Y, 2013, BIOMATERIALS, V34, P8459, DOI 10.1016/j.biomaterials.2013.07.050
Zhang Y, 2013, MOL THER, V21, P1559, DOI 10.1038/mt.2013.120
NR 53
TC 64
Z9 65
U1 5
U2 183
PU ELSEVIER SCI LTD
PI OXFORD
PA THE BOULEVARD, LANGFORD LANE, KIDLINGTON, OXFORD OX5 1GB, OXON, ENGLAND
SN 0142-9612
EI 1878-5905
J9 BIOMATERIALS
JI Biomaterials
PD NOV
PY 2015
VL 68
BP 54
EP 66
DI 10.1016/j.biomaterials.2015.07.053
PG 13
WC Engineering, Biomedical; Materials Science, Biomaterials

WE Science Citation Index Expanded (SCI-EXPANDED)
SC Engineering; Materials Science
GA CR2MY
UT WOS:000361165400006
PM 26264646
OA Green Submitted, Green Accepted
DA 2023-09-14
ER

PT J
AU Liu, C
Liu, X
Xiang, XC
Pang, X
Chen, SY
Zhang, YM
Ren, E
Zhang, LL
Liu, X
Lv, P
Wang, XY
Luo, WX
Xia, NS
Chen, XY
Liu, G
AF Liu, Chao
Liu, Xue
Xiang, Xinchu
Pang, Xin
Chen, Siyuan
Zhang, Yunming
Ren, En
Zhang, Lili
Liu, Xuan
Lv, Peng
Wang, Xiaoyong
Luo, Wenxin
Xia, Ningshao
Chen, Xiaoyuan
Liu, Gang
TI A nanovaccine for antigen self-presentation and immunosuppression
reversal as a personalized cancer immunotherapy strategy
SO NATURE NANOTECHNOLOGY
LA English
DT Article
ID DENDRITIC CELLS; CROSS-PRESENTATION; T-CELLS; RESPONSES; SAFETY;
ANTIBODY; IMMUNITY; VACCINE
AB Cancer vaccines based on endogenous modified dendritic cells can activate cytotoxic T cells in an antigen-specific manner, but the short life of dendritic cells on injection in the body limits the efficacy of the strategies. Here the authors design biomimetic nanovesicles derived from antigen-presenting dendritic cell membranes for cancer vaccination and the simultaneous delivery of immune co-stimulatory molecules, showing robust antitumour activity in animal models.
The strategy of combining a vaccine with immune checkpoint inhibitors has been widely investigated in cancer management, but the complete response rate for this strategy is still unresolved. We describe a genetically engineered cell membrane nanovesicle that integrates antigen self-presentation and immunosuppression reversal (ASPIRE) for cancer immunotherapy. The ASPIRE nanovaccine is derived from recombinant adenovirus-infected dendritic cells in which specific peptide-major histocompatibility complex class I (pMHC-I), anti-PD1 antibody and B7 co-stimulatory molecules are simultaneously anchored by a programmed process. ASPIRE can markedly improve antigen delivery to lymphoid organs and generate broad-spectrum T-cell responses that eliminate established tumours. This work presents a powerful vaccine formula that can directly activate both native T cells and exhausted T cells, and

suggests a general strategy for personalized cancer immunotherapy.
C1 [Liu, Chao; Liu, Xue; Xiang, Xinchu; Pang, Xin; Chen, Siyuan; Zhang, Yunming; Ren, En; Zhang, Lili; Liu, Xuan; Lv, Peng; Wang, Xiaoyong; Luo, Wenxin; Xia, Ningshao; Liu, Gang] Xiamen Univ, State Key Lab Mol Vaccinol & Mol Diagnost, Natl Inst Diagnost & Vaccine Dev Infect Dis, Ctr Mol Imaging & Translat Med,Sch Publ Hlth, Xiamen, Peoples R China.
[Wang, Xiaoyong; Luo, Wenxin; Xia, Ningshao; Liu, Gang] Xiamen Univ, Sch Life Sci, Innovat Ctr Cell Biol, State Key Lab Cellular Stress Biol, Xiamen, Peoples R China.
[Chen, Xiaoyuan] Natl Univ Singapore, Yong Loo Lin Sch Med, Nanomed Translat Res Program, Dept Diagnost Radiol,Clin Imaging Res Ctr, Singapore, Singapore.
[Chen, Xiaoyuan] Natl Univ Singapore, Yong Loo Lin Sch Med, Nanomed Translat Res Program, Dept Surg,Clin Imaging Res Ctr, Singapore, Singapore.
[Chen, Xiaoyuan] Natl Univ Singapore, Yong Loo Lin Sch Med, Nanomed Translat Res Program, Dept Chem,Clin Imaging Res Ctr, Singapore, Singapore.
[Chen, Xiaoyuan] Natl Univ Singapore, Yong Loo Lin Sch Med, Nanomed Translat Res Program, Dept Biomol Engn,Clin Imaging Res Ctr, Singapore, Singapore.
[Chen, Xiaoyuan] Natl Univ Singapore, Yong Loo Lin Sch Med, Nanomed Translat Res Program, Dept Biomed Engn,Clin Imaging Res Ctr, Singapore, Singapore.
[Liu, Gang] Xiamen Univ, Coll Chem & Chem Engn, MOE Key Lab Spectrochem Anal & Instrumentat, Xiamen, Peoples R China.
C3 Xiamen University; Xiamen University; National University of Singapore;
National University of Singapore; National University of Singapore;
National University of Singapore; National University of Singapore;
Xiamen University
RP Liu, G (通讯作者)，Xiamen Univ, State Key Lab Mol Vaccinol & Mol Diagnost, Natl Inst Diagnost & Vaccine Dev Infect Dis, Ctr Mol Imaging & Translat Med,Sch Publ Hlth, Xiamen, Peoples R China.; Liu, G (通讯作者)，Xiamen Univ, Sch Life Sci, Innovat Ctr Cell Biol, State Key Lab Cellular Stress Biol, Xiamen, Peoples R China.; Chen, XY (通讯作者)，Natl Univ Singapore, Yong Loo Lin Sch Med, Nanomed Translat Res Program, Dept Diagnost Radiol,Clin Imaging Res Ctr, Singapore, Singapore.; Chen, XY (通讯作者)，Natl Univ Singapore, Yong Loo Lin Sch Med, Nanomed Translat Res Program, Dept Surg,Clin Imaging Res Ctr, Singapore, Singapore.; Chen, XY (通讯作者)，Natl Univ Singapore, Yong Loo Lin Sch Med, Nanomed Translat Res Program, Dept Chem,Clin Imaging Res Ctr, Singapore, Singapore.; Chen, XY (通讯作者)，Natl Univ Singapore, Yong Loo Lin Sch Med, Nanomed Translat Res Program, Dept Biomol Engn,Clin Imaging Res Ctr, Singapore, Singapore.; Chen, XY (通讯作者)，Natl Univ Singapore, Yong Loo Lin Sch Med, Nanomed Translat Res Program, Dept Biomed Engn,Clin Imaging Res Ctr, Singapore, Singapore.; Liu, G (通讯作者)，Xiamen Univ, Coll Chem & Chem Engn, MOE Key Lab Spectrochem Anal & Instrumentat, Xiamen, Peoples R China.
EM chen.shawn@nus.edu.sg; gangliu.cmitm@xmu.edu.cn
RI Chen, Xiao/GQA-8928-2022; chen, xia/GXM-5435-2022; Chen,
Siqi/IZE-8631-2023; chen, xi/GXH-3653-2022; chen, xia/GYR-3948-2022;
chen, xin/IQW-3432-2023; wang, yu/IUQ-6654-2023; CHEN, SI/GZL-4800-2022;
Luo, Wen/GRO-5989-2022
OI Chen, Xiao/0000-0002-9797-8384;
FU Major State Basic Research Development Program of China
[2017YFA0205201]; National Natural Science Foundation of China
[81925019, 81422023, U1705281]; National University of Singapore Startup
Fund [NUHSRO/2020/133/Startup/08]; Nanomedicine Translational Research
Programme [NUHSRO/2021/034/TRP/09/Nanomedicine]; Fundamental Research
Funds for the Central Universities [20720190088, 20720200019]; China
Postdoctoral Science Foundation [2021M702738, 2021M700115]; Program for
New Century Excellent Talents in University, China [NCET-13-0502]
FX This work was supported by the Major State Basic Research Development
Program of China (2017YFA0205201) (G.L.), the National Natural Science
Foundation of China (81925019, 81422023 and U1705281) (G.L.), the
National University of Singapore Startup Fund
(NUHSRO/2020/133/Startup/08) (X.C.), the Nanomedicine Translational
Research Programme (NUHSRO/2021/034/TRP/09/Nanomedicine) (X.C.), the
Fundamental Research Funds for the Central Universities (20720190088 and
20720200019) (G.L.), the China Postdoctoral Science Foundation
(2021M702738) (C.L.) and (2021M700115) (Xue Liu) and the Program for New
Century Excellent Talents in University, China (NCET-13-0502) (G.L.).
CR Albert ML, 1998, IMMUNOLOGIST, V6, P194

Banchereau J, 1998, NATURE, V392, P245, DOI 10.1038/32588
Bertrand F, 2017, NAT COMMUN, V8, DOI 10.1038/s41467-017-02358-7
Blackburn SD, 2009, NAT IMMUNOL, V10, P29, DOI 10.1038/ni.1679
Brahmer JR, 2012, NEW ENGL J MED, V366, P2455, DOI 10.1056/NEJMoa1200694
Bulik-Sullivan B, 2019, NAT BIOTECHNOL, V37, P55, DOI 10.1038/nbt.4313
Chen DS, 2013, IMMUNITY, V39, P1, DOI 10.1016/j.immuni.2013.07.012
Chen LP, 2013, NAT REV IMMUNOL, V13, P227, DOI 10.1038/nri3405
Chen LM, 2018, CANCER DISCOV, V8, P1156, DOI 10.1158/2159-8290.CD-17-1033
Chen ZW, 2019, ACCOUNTS CHEM RES, V52, P1255, DOI 10.1021/acs.accounts.9b00079
Couzin-Frankel J, 2013, SCIENCE, V342, P1432, DOI 10.1126/science.342.6165.1432
Flach TL, 2011, NAT MED, V17, P479, DOI 10.1038/nm.2306
Fuertes MB, 2011, J EXP MED, V208, P2005, DOI 10.1084/jem.20101159
Garon EB, 2015, NEW ENGL J MED, V372, P2018, DOI 10.1056/NEJMoa1501824
Hamid O, 2013, NEW ENGL J MED, V369, P134, DOI 10.1056/NEJMoa1305133
Heath WR, 2001, ANNU REV IMMUNOL, V19, P47, DOI 10.1146/annurev.immunol.19.1.47
Heath WR, 2001, NAT REV IMMUNOL, V1, P126, DOI 10.1038/35100512
Hu CMJ, 2015, NATURE, V526, P118, DOI 10.1038/nature15373
Hui EF, 2017, SCIENCE, V355, P1428, DOI 10.1126/science.aaf1292
Huppa JB, 2003, NAT REV IMMUNOL, V3, P973, DOI 10.1038/nri1245
Im SJ, 2016, NATURE, V537, P417, DOI 10.1038/nature19330
Kamphorst AO, 2017, SCIENCE, V355, P1423, DOI 10.1126/science.aaf0683
Leslie M, 2013, SCIENCE, V341, P26, DOI 10.1126/science.341.6141.26
Li HY, 2011, NAT NANOTECHNOL, V6, P645, DOI [10.1038/nnano.2011.153, 10.1038/NNANO.2011.153]
Li HY, 2017, CANCER IMMUNOL RES, V5, P767, DOI 10.1158/2326-6066.CIR-16-0365
Liu XS, 2017, CELL, V168, P600, DOI 10.1016/j.cell.2017.01.014
Liu X, 2018, ANGEW CHEM INT EDIT, V57, P12499, DOI 10.1002/anie.201807212
Liu X, 2019, ADV MATER, V31, DOI 10.1002/adma.201808294
MUNRO JM, 1993, EUR HEART J, V14, P72
Nopora A, 2002, J IMMUNOL, V169, P3006, DOI 10.4049/jimmunol.169.6.3006
Palucka AK, 2006, J IMMUNOTHER, V29, P545, DOI 10.1097/01.cji.0000211309.90621.8b
Palucka K, 2012, NAT REV CANCER, V12, P265, DOI 10.1038/nrc3258
Park D, 2006, NAT BIOTECHNOL, V24, P1581, DOI 10.1038/nbt1262
Ribas A, 2018, SCIENCE, V359, P1350, DOI 10.1126/science.aar4060
Robbins PF, 2013, NAT MED, V19, P747, DOI 10.1038/nm.3161
Sallusto F, 1999, NATURE, V401, P708, DOI 10.1038/44385
Schumacher TN, 2015, SCIENCE, V348, P69, DOI 10.1126/science.aaa4971
Shah NN, 2019, NAT REV CLIN ONCOL, V16, P372, DOI 10.1038/s41571-019-0184-6
Steinman RM, 2007, NATURE, V449, P419, DOI 10.1038/nature06175
Steinman RM, 2012, ANNU REV IMMUNOL, V30, P1, DOI 10.1146/annurev-immunol-100311-102839
Verdegaal EME, 2016, NATURE, V536, P91, DOI 10.1038/nature18945
Verma V, 2019, NAT IMMUNOL, V20, P1555, DOI 10.1038/s41590-019-0519-6
Wheeler CM, 2016, LANCET INFECT DIS, V16, P1154, DOI 10.1016/S1473-3099(16)30120-7
Wolchok JD, 2013, NEW ENGL J MED, V369, P122, DOI 10.1056/NEJMoa1302369
Worbs T, 2017, NAT REV IMMUNOL, V17, P30, DOI 10.1038/nri.2016.116
Yadav M, 2014, NATURE, V515, P572, DOI 10.1038/nature14001
Zhang PF, 2018, ADV MATER, V30, DOI 10.1002/adma.201705350
Zhang PF, 2015, P NATL ACAD SCI USA, V112, pE6129, DOI 10.1073/pnas.1505799112
Zhu FC, 2010, LANCET, V376, P895, DOI 10.1016/S0140-6736(10)61030-6
NR 49
TC 63
Z9 64
U1 64
U2 248
PU NATURE PORTFOLIO
PI BERLIN
PA HEIDELBERGER PLATZ 3, BERLIN, 14197, GERMANY
SN 1748-3387
EI 1748-3395
J9 NAT NANOTECHNOL
JI Nat. Nanotechnol.

PD MAY
PY 2022
VL 17
IS 5
BP 531
EP +
DI 10.1038/s41565-022-01098-0
EA APR 2022
PG 18
WC Nanoscience & Nanotechnology; Materials Science, Multidisciplinary
WE Science Citation Index Expanded (SCI-EXPANDED)
SC Science & Technology - Other Topics; Materials Science
GA 1J0HI
UT WOS:000780825800002
PM 35410368
OA Bronze
HC Y
HP N
DA 2023-09-14
ER

PT J
AU Serra, P
   Santamaria, P
AF Serra, Pau
   Santamaria, Pere
TI Nanoparticle-based autoimmune disease therapy
SO CLINICAL IMMUNOLOGY
LA English
DT Article
DE Nanotechnology; Nanomaterials; Nanovaccines; Autoimmunity;
   Immunotherapy; Regulatory T-cells
ID CD8(+) T-CELLS; ANTIGEN-PRESENTING CELLS; DENDRITIC CELLS; CARBON
   NANOTUBES; IMMUNE DEVIATION; PEPTIDE; TOLERANCE; ENCEPHALOMYELITIS;
   INDUCTION; INFLAMMATION
AB The goal of immunotherapy against autoimmunity is to block pathogenic inflammation without impairing immunity against infections and tumours. Regulatory T-cells (Tregs) play a central role in maintaining immune homeostasis, and autoimmune inflammation is frequently associated with decreased numbers and/or function of these T-cells. Therapies harnessing Tregs to treat autoimmune inflammation remain under-developed with caveats ranging from the lack of antigenic and disease specificity to the potential phenotypic and functional instability of in vitro-expanded Treg cells in vivo. Here, we review nanotechnology-based approaches designed to promote immune tolerance through various mechanisms, ranging from systemic or local suppression of antigen-presenting cells and deletion of antigen-specific T-cells, to the systemic expansion of antigen- and disease-specific Treg cells in vivo. (C) 2015 Elsevier Inc. All rights reserved.
C1 [Serra, Pau; Santamaria, Pere] Inst Invest Biomed August Pi & Sunyer, Barcelona 08036, Spain.
   [Santamaria, Pere] Univ Calgary, Julia McFarlane Diabet Res Ctr JMDRC, Calgary, AB T2N 4N1, Canada.
   [Santamaria, Pere] Univ Calgary, Dept Microbiol Immunol & Infect Dis, Snyder Inst Chron Dis, Calgary, AB T2N 4N1, Canada.
   [Santamaria, Pere] Univ Calgary, Hotchkiss Brain Inst, Cummings Sch Med, Calgary, AB T2N 4N1, Canada.
C3 University of Barcelona; Hospital Clinic de Barcelona; IDIBAPS;
   University of Calgary; University of Calgary; University of Calgary
RP Serra, P (通讯作者)，Inst Invest Biomed August Pi & Sunyer, Barcelona 08036, Spain.
EM pserra1@clinic.ub.es; psantama@ucalgary.ca
RI Santamaria, Pere/ABF-6823-2021
FU Canadian Institutes of Health Research (CIHR); Natural Sciences and
   Engineering Research Council of Canada (NSERC); Diabetes Research
   Foundation (DRF); Juvenile Diabetes Research Foundation (JDRF); Canadian
   Diabetes Association (CDA); Multiple Sclerosis Society of Canada (MSSC);
   Brawn Family Foundation; Ministerio de Economia y Competitividad of

Spain (MINECO); Neuron-ERANET program; European Association for the
study of diabetes (EASD); Sarda Farriol Research Programme; European
Community's Seventh Framework Programme [229673]; JDRF; Diabetes
Association (Foothills); CDA
FX We apologize to the authors of papers relevant to this review that could
not be cited due to space constrains. We thank past and present members
of our laboratories for insights discussed herein. The author's work
relevant to this review was funded by the Canadian Institutes of Health
Research (CIHR), the Natural Sciences and Engineering Research Council
of Canada (NSERC), the Diabetes Research Foundation (DRF), the Juvenile
Diabetes Research Foundation (JDRF), the Canadian Diabetes Association
(CDA), the Multiple Sclerosis Society of Canada (MSSC), the Brawn Family
Foundation, the Ministerio de Economia y Competitividad of Spain
(MINECO), the Neuron-ERANET program, the European Association for the
study of diabetes (EASD), the Sarda Farriol Research Programme, and the
European Community's Seventh Framework Programme (EC FP7/2007-2013;
#229673). P. Serra is an investigator of the Ramon y Cajal
re-integration program of MINECO and is supported by a JDRF Career
Development Award. P. Santamaria is a Scientist of the Alberta
Innovates-Health Solutions and a Scholar of the Instituto de
Investigaciones Sanitarias Carlos III. The JMDRC is supported by the
Diabetes Association (Foothills) and the CDA.
CR Ahsan FL, 2002, J CONTROL RELEASE, V79, P29, DOI 10.1016/S0168-3659(01)00549-1
Amir Y, 2014, NAT NANOTECHNOL, V9, P353, DOI [10.1038/NNANO.2014.58, 10.1038/nnano.2014.58]
Anderton SM, 2001, IMMUNOLOGY, V104, P367, DOI 10.1046/j.1365-2567.2001.01324.x
Azzi J, 2010, FASEB J, V24, P3927, DOI 10.1096/fj.10-154690
Baeten K, 2008, J NEUROIMMUNOL, V195, P1, DOI 10.1016/j.jneuroim.2007.11.008
Bonifaz L, 2002, J EXP MED, V196, P1627, DOI 10.1084/jem.20021598
Bouaziz JD, 2012, CURR MOL MED, V12, P519
Bourrinet P, 2006, INVEST RADIOL, V41, P313, DOI 10.1097/01.rli.0000197669.80475.dd
Burton BR, 2014, NAT COMMUN, V5, DOI 10.1038/ncomms5741
Champion JA, 2007, J CONTROL RELEASE, V121, P3, DOI 10.1016/j.jconrel.2007.03.022
Chen H, 2013, PLOS ONE, V8, DOI 10.1371/journal.pone.0058208
Chithrani BD, 2006, NANO LETT, V6, P662, DOI 10.1021/nl052396o
Clemente-Casares X, 2014, IMMUNOL LETT, V158, P167, DOI 10.1016/j.imlet.2013.12.018
Clemente-Casares X, 2012, CSH PERSPECT MED, V2, DOI 10.1101/cshperspect.a007773
Clemente-Casares X, 2011, J MOL MED, V89, P733, DOI 10.1007/s00109-011-0757-z
Creusot RJ, 2009, BLOOD, V113, P6638, DOI 10.1182/blood-2009-02-204321
CRITCHFIELD JM, 1994, SCIENCE, V263, P1139, DOI 10.1126/science.7509084
Das MP, 1997, J EXP MED, V186, P867, DOI 10.1084/jem.186.6.867
Denis MC, 2004, P NATL ACAD SCI USA, V101, P12634, DOI 10.1073/pnas.0404307101
Doshi N, 2011, ADV MATER, V23, pH105, DOI 10.1002/adma.201004074
Douglas SM, 2009, NATURE, V459, P414, DOI 10.1038/nature08016
Fadel TR, 2014, NAT NANOTECHNOL, V9, P639, DOI [10.1038/NNANO.2014.154,
10.1038/nnano.2014.154]
Fischer HC, 2007, CURR OPIN BIOTECH, V18, P565, DOI 10.1016/j.copbio.2007.11.008
Floris S, 2004, BRAIN, V127, P616, DOI 10.1093/brain/awh068
Gao W, 2009, CELL CYCLE, V8, P1444, DOI 10.4161/cc.8.9.8348
GAUR A, 1992, SCIENCE, V258, P1491, DOI 10.1126/science.1279812
Genain CP, 1996, SCIENCE, V274, P2054, DOI 10.1126/science.274.5295.2054
Getts DR, 2013, J IMMUNOL, V191, P5341, DOI 10.4049/jimmunol.1302070
Getts DR, 2012, NAT BIOTECHNOL, V30, P1217, DOI 10.1038/nbt.2434
Getts DR, 2011, J IMMUNOL, V187, P2405, DOI 10.4049/jimmunol.1004175
Gilbert LA, 2014, CELL, V159, P647, DOI 10.1016/j.cell.2014.09.029
Grant CR, 2015, AUTOIMMUN REV, V14, P105, DOI 10.1016/j.autrev.2014.10.012
Han BY, 2005, J CLIN INVEST, V115, P1879, DOI 10.1172/JCI24219
Han BY, 2005, NAT MED, V11, P645, DOI 10.1038/nm1250
Hawiger D, 2001, J EXP MED, V194, P769, DOI 10.1084/jem.194.6.769
Haxhinasto S, 2008, J EXP MED, V205, P565, DOI 10.1084/jem.20071477
Hayder M, 2011, SCI TRANSL MED, V3, DOI 10.1126/scitranslmed.3002212
Hester J, 2012, AM J TRANSPLANT, V12, P2008, DOI 10.1111/j.1600-6143.2012.04065.x

Hirst SM, 2009, SMALL, V5, P2848, DOI 10.1002/smll.200901048
Hong CA, 2014, THERANOSTICS, V4, P1211, DOI 10.7150/thno.8491
JENKINS MK, 1987, J EXP MED, V165, P302, DOI 10.1084/jem.165.2.302
Kaewsaneha C, 2013, ACS APPL MATER INTER, V5, P1857, DOI 10.1021/am302528g
Kontos S, 2013, P NATL ACAD SCI USA, V110, pE60, DOI 10.1073/pnas.1216353110
Kourtis IC, 2013, PLOS ONE, V8, DOI 10.1371/journal.pone.0061646
Kuklina EM, 2013, BIOCHEMISTRY-MOSCOW+, V78, P144, DOI 10.1134/S000629791302003X
Leuschner F, 2011, NAT BIOTECHNOL, V29, P1005, DOI 10.1038/nbt.1989
Lieberman SM, 2003, P NATL ACAD SCI USA, V100, P8384, DOI 10.1073/pnas.0932778100
Liu G, 2013, SMALL, V9, P1533, DOI 10.1002/smll.201201531
Look M, 2013, J CLIN INVEST, V123, P1741, DOI 10.1172/JCI65907
Macauley MS, 2013, J CLIN INVEST, V123, P3074, DOI 10.1172/JCI69187
Machen J, 2004, J IMMUNOL, V173, P4331, DOI 10.4049/jimmunol.173.7.4331
Mailander V, 2009, BIOMACROMOLECULES, V10, P2379, DOI 10.1021/bm900266r
Maile R, 2001, J IMMUNOL, V167, P3708, DOI 10.4049/jimmunol.167.7.3708
Maldonado RA, 2015, P NATL ACAD SCI USA, V112, pE156, DOI 10.1073/pnas.1408686111
Manolova V, 2008, EUR J IMMUNOL, V38, P1404, DOI 10.1002/eji.200737984
Medarova Z, 2008, MAGN RESON MED, V59, P712, DOI 10.1002/mrm.21494
Metcalfe SM, 2011, GENES IMMUN, V12, P157, DOI 10.1038/gene.2011.9
Metcalfe SM, 2005, TRANSPLANTATION, V79, P726, DOI 10.1097/01.TP.0000149324.42994.38
Miller S D, 1992, Int Rev Immunol, V9, P203, DOI 10.3109/08830189209061791
MILLER SD, 1979, J EXP MED, V149, P758, DOI 10.1084/jem.149.3.758
Mitchell LA, 2009, NAT NANOTECHNOL, V4, P451, DOI [10.1038/NNANO.2009.151, 10.1038/nnano.2009.151]
Mitchell LA, 2007, TOXICOL SCI, V100, P203, DOI 10.1093/toxsci/kfm196
Moore A, 2004, DIABETES, V53, P1459, DOI 10.2337/diabetes.53.6.1459
Muthu MS, 2014, NANOMEDICINE-UK, V9, P1277, DOI [10.2217/NNM.14.83, 10.2217/nnm.14.83]
Nicholson LB, 1997, P NATL ACAD SCI USA, V94, P9279, DOI 10.1073/pnas.94.17.9279
NICHOLSON LB, 1995, IMMUNITY, V3, P397, DOI 10.1016/1074-7613(95)90169-8
Oldenborg PA, 2000, SCIENCE, V288, P2051, DOI 10.1126/science.288.5473.2051
Mackern-Oberti JP, 2015, AUTOIMMUN REV, V14, P127, DOI 10.1016/j.autrev.2014.10.010
Pagliari F, 2012, ACS NANO, V6, P3767, DOI 10.1021/nn2048069
Park J, 2011, MOL PHARMACEUT, V8, P143, DOI 10.1021/mp100203a
Phillips B, 2008, DIABETES, V57, P1544, DOI 10.2337/db07-0507
Preda I, 2005, EUR J IMMUNOL, V35, P2762, DOI 10.1002/eji.200526158
Probst HC, 2014, EUR J IMMUNOL, V44, P927, DOI 10.1002/eji.201343862
Rausch M, 2003, MAGNET RESON MED, V50, P309, DOI 10.1002/mrm.10541
Schanen BC, 2013, PLOS ONE, V8, DOI 10.1371/journal.pone.0062816
Schutz C, 2008, BLOOD, V111, P3546, DOI 10.1182/blood-2007-09-113522
Schutz C, 2014, JOVE-J VIS EXP, DOI 10.3791/51859
Schutz C, 2010, IMMUNOTHERAPY-UK, V2, P539, DOI [10.2217/imt.10.26, 10.2217/IMT.10.26]
Schweingruber N, 2011, J IMMUNOL, V187, P4310, DOI 10.4049/jimmunol.1101604
Serres S, 2011, FASEB J, V25, P4415, DOI 10.1096/fj.11-183772
Shameli A, 2013, EUR J IMMUNOL, V43, P394, DOI 10.1002/eji.201242845
Shameli A, 2011, J IMMUNOL, V187, P2859, DOI 10.4049/jimmunol.1101117
Shen CL, 2011, IMMUNOL LETT, V138, P144, DOI 10.1016/j.imlet.2011.04.002
Singh M, 2007, EXPERT REV VACCINES, V6, P797, DOI 10.1586/14760584.6.5.797
Singh P, 2015, GENETICS, V199, P1, DOI 10.1534/genetics.114.169771
Smith CE, 2005, P NATL ACAD SCI USA, V102, P9595, DOI 10.1073/pnas.0504131102
Smith MJ, 2014, TOXICOL SCI, V138, P249, DOI 10.1093/toxsci/kfu005
Steinman RM, 2003, ANNU REV IMMUNOL, V21, P685, DOI 10.1146/annurev.immunol.21.120601.141040
Sumbayev VV, 2013, SMALL, V9, P472, DOI 10.1002/smll.201201528
Taner T, 2005, AM J TRANSPLANT, V5, P228, DOI 10.1046/j.1600-6143.2004.00673.x
Thomas TP, 2011, ARTHRITIS RHEUM-US, V63, P2671, DOI 10.1002/art.30459
Tkach AV, 2011, ACS NANO, V5, P5755, DOI 10.1021/nn2014479
Tran LTC, 2014, EXPERT OPIN DRUG DEL, V11, P1061, DOI 10.1517/17425247.2014.915806
Tsai SE, 2010, IMMUNITY, V32, P568, DOI 10.1016/j.immuni.2010.03.015
Turley DM, 2007, J IMMUNOL, V178, P2212, DOI 10.4049/jimmunol.178.4.2212
Turvey SE, 2005, J CLIN INVEST, V115, P2454, DOI 10.1172/JCI25048
Vincent BG, 2010, J IMMUNOL, V184, P4196, DOI 10.4049/jimmunol.0903931

Wahajuddin, 2012, INT J NANOMED, V7, P3445, DOI 10.2147/IJN.S30320
Wang LS, 2012, INT J NANOMED, V7, P4679, DOI 10.2147/IJN.S33065
Wang P, 2012, DIABETES, V61, P3247, DOI 10.2337/db12-0441
Wang P, 2011, DIABETES, V60, P565, DOI 10.2337/db10-1400
WETZIG R, 1979, J IMMUNOL METHODS, V28, P361, DOI 10.1016/0022-1759(79)90201-1
Yang CY, 2011, PLOS ONE, V6, DOI 10.1371/journal.pone.0025524
Yeste A, 2012, P NATL ACAD SCI USA, V109, P11270, DOI 10.1073/pnas.1120611109
Young DA, 2000, J IMMUNOL, V164, P3563, DOI 10.4049/jimmunol.164.7.3563
NR 105
TC 61
Z9 64
U1 0
U2 38
PU ACADEMIC PRESS INC ELSEVIER SCIENCE
PI SAN DIEGO
PA 525 B ST, STE 1900, SAN DIEGO, CA 92101-4495 USA
SN 1521-6616
EI 1521-7035
J9 CLIN IMMUNOL
JI Clin. Immunol.
PD SEP
PY 2015
VL 160
IS 1
BP 3
EP 13
DI 10.1016/j.clim.2015.02.003
PG 11
WC Immunology
WE Science Citation Index Expanded (SCI-EXPANDED)
SC Immunology
GA CQ7JU
UT WOS:000360780600002
PM 25704658
DA 2023-09-14
ER

PT J
AU Zhang, CN
Zhang, J
Shi, GN
Song, HJ
Shi, SB
Zhang, XY
Huang, PS
Wang, ZH
Wang, WW
Wang, C
Kong, DL
Li, C
AF Zhang, Chuangnian
Zhang, Ju
Shi, Gaona
Song, Huijuan
Shi, Shengbin
Zhang, Xiuyuan
Huang, Pingsheng
Wang, Zhihong
Wang, Weiwei
Wang, Chun
Kong, Deling

Li, Chen
TI A Light Responsive Nanoparticle-Based Delivery System Using Pheophorbide A Graft Polyethylenimine for Dendritic Cell-Based Cancer Immunotherapy
SO MOLECULAR PHARMACEUTICS
LA English
DT Article
DE photochemical internalization; endosome escape; cross-presentation; antigen delivery; immunotherapy
ID PHOTODYNAMIC THERAPY; PHOTOSENSITIZER CONJUGATE; CROSS-PRESENTATION; STIMULATION; INTERNALIZATION; NANOVACCINE; ANTIGENS; VACCINES
AB In this study, the photochemical internalization (PCI) technique was adopted in a nanoparticle-based antigen delivery system to enhance antigen-specific CD8(+) T cell immune response for cancer immunotherapy. Pheophorbide A, a hydrophobic photosensitizer, grafted with polyethylenimine (PheoA-PEI) with endosome escape activity and near-infrared imaging capability was prepared. A model antigen ovalbumin (OVA) was then complexed with PheoA-PEI to form PheoA-PEI/OVA nanoparticles (PheoA-PEI/OVA NPs) that are responsive to light. Flow cytometry analysis revealed increased endocytosis in a murine dendritic cell line (DC2.4) that was treated with PheoA-PEI/OVA NPs compared to free OVA. Generation of reactive oxygen species (ROS) in DC2.4 cells was also confirmed quantitatively and qualitatively using 2',7'-dichlorodihydrofluorescein diacetate (DCFH-DA). Confocal laser scanning microscopy (CLSM) further demonstrated that the PheoA-PEI/OVA NPs enhanced cytosolic antigen release after light stimulation. Moreover, PheoA-PEI/OVA NP treated DC2.4 cells exhibited enhanced cross-presentation to B3Z T cell hybridoma in vitro after light irradiation, substantially increased compared to those treated with free OVA. Consistently, in vivo results revealed upregulation of CD3(+)CD8(+)T lymphocytes in tumors of mice treated with dendritic cells plus PheoA-PEI/OVA NPs and light irradiation. The activated T cell response is partly responsible for the inhibitory effect on E.G7 tumor growth in mice immunized with dendritic cells plus PheoA-PEI/OVA NPs and light irradiation. Our results demonstrate the feasibility to enhance antigen-specific CD8(+) T cell immune response by light -responsive nanoparticle-based vaccine delivery for cancer immunotherapy.
C1 [Zhang, Chuangnian; Shi, Gaona; Song, Huijuan; Shi, Shengbin; Zhang, Xiuyuan; Huang, Pingsheng; Wang, Zhihong; Wang, Weiwei; Wang, Chun; Kong, Deling; Li, Chen] Chinese Acad Med Sci, Inst Biomed Engn, Tianjin Key Lab Biomat Res, Tianjin 300192, Peoples R China.
[Zhang, Chuangnian; Shi, Gaona; Song, Huijuan; Shi, Shengbin; Zhang, Xiuyuan; Huang, Pingsheng; Wang, Zhihong; Wang, Weiwei; Wang, Chun; Kong, Deling; Li, Chen] Peking Union Med Coll, Tianjin 300192, Peoples R China.
[Zhang, Ju] Qingdao Univ, Sch Nursing, Basic Nursing T&R Sect, Qingdao 26000, Shandong, Peoples R China.
[Wang, Chun] Univ Minnesota, Dept Biomed Engn, 7-105 Hasselmo Hall,312 Church St South East, Minneapolis, MN 55455 USA.
[Kong, Deling] Nankai Univ, State Key Lab Med Chem Biol, Key Lab Bioact Mat, Minist Educ, Tianjin 300071, Peoples R China.
C3 Chinese Academy of Medical Sciences - Peking Union Medical College; Institute of BioMedical Engineering - CAMS; Chinese Academy of Medical Sciences - Peking Union Medical College; Peking Union Medical College; Qingdao University; University of Minnesota System; University of Minnesota Twin Cities; Nankai University
RP Wang, C; Kong, DL (通讯作者), Chinese Acad Med Sci, Inst Biomed Engn, Tianjin Key Lab Biomat Res, Tianjin 300192, Peoples R China.; Wang, C; Kong, DL (通讯作者), Peking Union Med Coll, Tianjin 300192, Peoples R China.; Wang, C (通讯作者), Univ Minnesota, Dept Biomed Engn, 7-105 Hasselmo Hall,312 Church St South East, Minneapolis, MN 55455 USA.; Kong, DL (通讯作者), Nankai Univ, State Key Lab Med Chem Biol, Key Lab Bioact Mat, Minist Educ, Tianjin 300071, Peoples R China.
EM wangx504@umn.edu; kongdeling@nankai.edu.cn
RI Wang, Weiwei/GRF-3625-2022
FU National Natural Science Foundation of China [21604095, 31300732, 51373199, 31400833, 31670977]; China Postdoctoral Science Foundation [2015M580066]; Program for Innovative Research Team in Peking Union Medical College; CAMS Initiative for Innovative Medicine [2016-I2M-3-022]; Open Fund of Key Laboratory of Functional Polymer Materials, Ministry of Education, Nankai University [201606]
FX This work was financially supported by the National Natural Science Foundation of China (21604095, 31300732, 51373199, 31400833, 31670977), China Postdoctoral Science Foundation (2015M580066), Program for

Innovative Research Team in Peking Union Medical College, CAMS
Initiative for Innovative Medicine (2016-I2M-3-022), and the Open Fund
of Key Laboratory of Functional Polymer Materials, Ministry of
Education, Nankai University (201606).
CR Adigbli DK, 2012, CURR OPIN PHARMACOL, V12, P434, DOI 10.1016/j.coph.2012.04.005
Bae BC, 2010, BIOMATERIALS, V31, P6325, DOI 10.1016/j.biomaterials.2010.04.030
Battogtokh G, 2015, J MATER CHEM B, V3, P9349, DOI 10.1039/c5tb01719j
Bayona AMD, 2016, INT J CANCER, V138, P1049, DOI 10.1002/ijc.29510
Beack S, 2015, ACTA BIOMATER, V26, P295, DOI 10.1016/j.actbio.2015.08.027
Bruno C, 2015, J IMMUNOL, V195, P166, DOI 10.4049/jimmunol.1500431
Chen JA, 2011, INT J NANOMED, V6, P77, DOI 10.2147/IJN.S15457
Cho H, 2014, MACROMOL BIOSCI, V14, P1483, DOI 10.1002/mabi.201400145
Demana PH, 2004, IMMUNOL CELL BIOL, V82, P547, DOI 10.1111/j.0818-9641.2004.01276.x
Fang RH, 2015, SMALL, V11, P5483, DOI 10.1002/smll.201501284
Guo YY, 2015, ACS NANO, V9, P6918, DOI 10.1021/acsnano.5b01042
Hakerud M, 2015, J CONTROL RELEASE, V198, P10, DOI 10.1016/j.jconrel.2014.11.032
Hakerud M, 2014, J CONTROL RELEASE, V174, P143, DOI 10.1016/j.jconrel.2013.11.017
Hirosue S, 2010, VACCINE, V28, P7897, DOI 10.1016/j.vaccine.2010.09.077
Hjalmsdottir A, 2016, MOL PHARMACEUT, V13, P320, DOI 10.1021/acs.molpharmaceut.5b00394
Hu J, 2015, SMALL, V11, P5860, DOI 10.1002/smll.201501923
Irvine DJ, 2015, CHEM REV, V115, P11109, DOI 10.1021/acs.chemrev.5b00109
Joyce JA, 2015, SCIENCE, V348, P74, DOI 10.1126/science.aaa6204
Keller S, 2014, J CONTROL RELEASE, V191, P24, DOI 10.1016/j.jconrel.2014.03.041
Li DD, 2015, ADV FUNCT MATER, V25, P2993, DOI 10.1002/adfm.201500894
Li L, 2015, CARBOHYD POLYM, V121, P122, DOI 10.1016/j.carbpol.2014.12.035
Mellman I, 2011, NATURE, V480, P480, DOI 10.1038/nature10673
Norum OJ, 2009, J PHOTOCH PHOTOBIO B, V96, P83, DOI 10.1016/j.jphotobiol.2009.04.012
Oh IH, 2013, BIOMATERIALS, V34, P6454, DOI 10.1016/j.biomaterials.2013.05.017
Palucka K, 2012, NAT REV CANCER, V12, P265, DOI 10.1038/nrc3258
Park SJ, 2012, BIOMATERIALS, V33, P6485, DOI 10.1016/j.biomaterials.2012.05.040
Proudfoot O, 2007, MOL PHARMACEUT, V4, P58, DOI 10.1021/mp0601087
Radford KJ, 2014, CURR OPIN IMMUNOL, V27, P26, DOI 10.1016/j.coi.2014.01.005
Rosenberg SA, 2014, NAT REV CLIN ONCOL, V11, P630, DOI 10.1038/nrclinonc.2014.174
Selbo PK, 2015, PHOTOCH PHOTOBIO SCI, V14, P1433, DOI 10.1039/c5pp00027k
Selbo PK, 2010, J CONTROL RELEASE, V148, P2, DOI 10.1016/j.jconrel.2010.06.008
Shao K, 2015, ACS NANO, V9, P16, DOI 10.1021/nn5062029
Shibolet O, 2003, INT J CANCER, V106, P236, DOI 10.1002/ijc.11201
Tahara Y, 2015, ADV DRUG DELIVER REV, V95, P65, DOI 10.1016/j.addr.2015.10.004
Varkouhi AK, 2011, J CONTROL RELEASE, V151, P220, DOI 10.1016/j.jconrel.2010.11.004
Waeckerle-Men Y, 2013, EUR J PHARM BIOPHARM, V85, P34, DOI 10.1016/j.ejpb.2013.02.002
Wang C, 2016, BIOMATERIALS, V79, P88, DOI 10.1016/j.biomaterials.2015.11.040
Wang FZ, 2016, MOL PHARMACEUT, V13, P1298, DOI 10.1021/acs.molpharmaceut.5b00879
Wayteck L, 2016, BIOMATERIALS, V77, P243, DOI 10.1016/j.biomaterials.2015.11.016
Zhao L, 2015, INT J POLYM MATER PO, V64, P733, DOI 10.1080/00914037.2014.1002131
Zhou L, 2010, INT J PHARMACEUT, V386, P131, DOI 10.1016/j.ijpharm.2009.11.010
NR 41
TC 60
Z9 62
U1 3
U2 139
PU AMER CHEMICAL SOC
PI WASHINGTON
PA 1155 16TH ST, NW, WASHINGTON, DC 20036 USA
SN 1543-8384
EI 1543-8392
J9 MOL PHARMACEUT
JI Mol. Pharm.
PD MAY
PY 2017
VL 14
IS 5

BP 1760
EP 1770
DI 10.1021/acs.molpharmaceut.7b00015
PG 11
WC Medicine, Research & Experimental; Pharmacology & Pharmacy
WE Science Citation Index Expanded (SCI-EXPANDED)
SC Research & Experimental Medicine; Pharmacology & Pharmacy
GA ET9ND
UT WOS:000400633300041
PM 28296410
DA 2023-09-14
ER

PT J
AU Fontana, F
Liu, DF
Hirvonen, J
Santos, HA
AF Fontana, Flavia
Liu, Dongfei
Hirvonen, Jouni
Santos, Helder A.
TI Delivery of therapeutics with nanoparticles: what's new in cancer immunotherapy?
SO WILEY INTERDISCIPLINARY REVIEWS-NANOMEDICINE AND NANOBIOTECHNOLOGY
LA English
DT Review
ID MESOPOROUS SILICA NANOPARTICLES; T-CELL RESPONSE; POLY(PROPYLENE SULFIDE) NANOPARTICLES; ACETALATED DEXTRAN MICROPARTICLES; POTENT ANTITUMOR RESPONSES; TARGETING DENDRITIC CELLS; ENHANCED IMMUNE-RESPONSE; ANTIGEN-PRESENTING CELLS; DRUG-DELIVERY; IN-VIVO
AB The application of nanotechnology to the treatment of cancer or other diseases has been boosted during the last decades due to the possibility to precise deliver drugs where needed, enabling a decrease in the drug's side effects. Nanocarriers are particularly valuable for potentiating the simultaneous co-delivery of multiple drugs in the same particle for the treatment of heavily burdening diseases like cancer. Immunotherapy represents a new concept in the treatment of cancer and has shown outstanding results in patients treated with check-point inhibitors. Thereby, researchers are applying nanotechnology to cancer immunotherapy toward the development of nanocarriers for delivery of cancer vaccines and chemo-immunotherapies. Cancer nanovaccines can be envisioned as nanocarriers co-delivering antigens and adjuvants, molecules often presenting different physicochemical properties, in cancer therapy. A wide range of nanocarriers (e.g., polymeric, lipid-based and inorganic) allow the co-formulation of these molecules, or the delivery of chemo- and immune-therapeutics in the same system. Finally, there is a trend toward the use of biologically inspired and derived nanocarriers. In this review, we present the recent developments in the field of immunotherapy, describing the different systems proposed by categories: polymeric nanoparticles, lipid-based nanosystems, metallic and inorganic nanosystems and, finally, biologically inspired and derived nanovaccines. WIREs Nanomed Nanobiotechnol 2017, 9:e1421. doi: 10.1002/wnan.1421 For further resources related to this article, please visit the .
C1 [Fontana, Flavia; Liu, Dongfei; Hirvonen, Jouni; Santos, Helder A.] Univ Helsinki, Div Pharmaceut Chem & Technol, Fac Pharm, Helsinki, Finland.
C3 University of Helsinki
RP Santos, HA (通讯作者)，Univ Helsinki, Div Pharmaceut Chem & Technol, Fac Pharm, Helsinki, Finland.
EM helder.santos@helsinki.fi
RI Santos, Hélder A./B-5253-2009; Liu, Dongfei/B-1087-2011; Fontana, Flavia/AAB-4898-2020; Santos, Hélder A./AAC-6347-2022
OI Santos, Hélder A./0000-0001-7850-6309; Liu, Dongfei/0000-0002-2426-134X; Fontana, Flavia/0000-0001-9112-6917; Santos, Hélder A./0000-0001-7850-6309; Hirvonen, Jouni Tapio/0000-0002-5029-1657
FU Academy of Finland [252215, 281300]; University of Helsinki Research Funds; Biocentrum Helsinki; European Research Council under the European Union's Seventh Framework Programme (FP) [310892]; Sigrid Juselius Foundation; Academy of Finland (AKA) [281300, 281300] Funding Source:

Academy of Finland (AKA)
FX Dr. H. A. Santos acknowledges financial support from the Academy of Finland (decision nos. 252215 and 281300), the University of Helsinki Research Funds, the Biocentrum Helsinki, the European Research Council under the European Union's Seventh Framework Programme (FP/2007-2013, Grant No. 310892), and the Sigrid Juselius Foundation.
CR Ahmed KK, 2016, J PHARM SCI-US, V105, P1173, DOI 10.1016/j.xphs.2015.11.042
Almeida JPM, 2014, NANOMED-NANOTECHNOL, V10, P503, DOI 10.1016/j.nano.2013.09.011
Almeida JPM, 2014, SMALL, V10, P812, DOI 10.1002/smll.201301998
[Anonymous], 1893, AM J MED SCI
Bachelder EM, 2010, MOL PHARMACEUT, V7, P826, DOI 10.1021/mp900311x
Bachmann MF, 2010, NAT REV IMMUNOL, V10, P787, DOI 10.1038/nri2868
de Faria PCB, 2014, NANO LETT, V14, P5458, DOI 10.1021/nl502911a
Bershteyn A, 2012, J CONTROL RELEASE, V157, P354, DOI 10.1016/j.jconrel.2011.07.029
Broaders KE, 2009, P NATL ACAD SCI USA, V106, P5497, DOI 10.1073/pnas.0901592106
Bruno C, 2015, J IMMUNOL, V195, P166, DOI 10.4049/jimmunol.1500431
Chen G, 2013, CANCER IMMUNOL IMMUN, V62, P203, DOI 10.1007/s00262-012-1388-0
Cheung AS, 2016, SMALL, V12, P2321, DOI 10.1002/smll.201600061
Childs RW, 2015, NAT REV DRUG DISCOV, V14, P487, DOI 10.1038/nrd4506
Cohen JL, 2011, BIOCONJUGATE CHEM, V22, P1056, DOI 10.1021/bc100542r
Couzin-Frankel J, 2013, SCIENCE, V342, P1432, DOI 10.1126/science.342.6165.1432
Cruz LJ, 2014, J CONTROL RELEASE, V192, P209, DOI 10.1016/j.jconrel.2014.07.040
Cui LN, 2011, BIOCONJUGATE CHEM, V22, P949, DOI 10.1021/bc100596w
Da Silva CG, 2016, BIOMATERIALS, V83, P308, DOI 10.1016/j.biomaterials.2016.01.006
De Koker S, 2011, CHEM SOC REV, V40, P320, DOI 10.1039/b914943k
De Temmerman ML, 2011, BIOMATERIALS, V32, P9128, DOI 10.1016/j.biomaterials.2011.08.024
Demento SL, 2012, BIOMATERIALS, V33, P4957, DOI 10.1016/j.biomaterials.2012.03.041
Dewitte H, 2014, J CONTROL RELEASE, V194, P28, DOI 10.1016/j.jconrel.2014.08.011
Dolen Y, 2016, ONCOIMMUNOLOGY, V5, DOI 10.1080/2162402X.2015.1068493
Elamanchili P, 2007, J IMMUNOTHER, V30, P378, DOI 10.1097/CJI.0b013e31802cf3e3
Elsabahy M, 2012, CHEM SOC REV, V41, P2545, DOI 10.1039/c2cs15327k
Erikci E, 2011, BIOMATERIALS, V32, P1715, DOI 10.1016/j.biomaterials.2010.10.054
Fang RH, 2015, SMALL, V11, P5483, DOI 10.1002/smll.201501284
Fang RH, 2014, NANO LETT, V14, P2181, DOI 10.1021/nl500618u
Farokhzad OC, 2009, ACS NANO, V3, P16, DOI 10.1021/nn900002m
Fernandez EM, 2012, INT J PHARMACEUT, V423, P45, DOI 10.1016/j.ijpharm.2011.04.068
Ferrari M, 2005, NAT REV CANCER, V5, P161, DOI 10.1038/nrc1566
Flanary S, 2009, BIOCONJUGATE CHEM, V20, P241, DOI 10.1021/bc800317a
Foged C, 2012, EUR J PHARM SCI, V45, P482, DOI 10.1016/j.ejps.2011.08.016
Fox CB, 2014, J NANOBIOTECHNOL, V12, DOI 10.1186/1477-3155-12-17
Frohlich E, 2012, INT J NANOMED, V7, P5577, DOI 10.2147/IJN.S36111
Ghebeh H, 2010, BREAST CANCER RES, V12, DOI 10.1186/bcr2605
Ghosh P, 2008, ADV DRUG DELIVER REV, V60, P1307, DOI 10.1016/j.addr.2008.03.016
Guo LR, 2014, ACS NANO, V8, P5670, DOI 10.1021/nn5002112
Hamdy S, 2008, VACCINE, V26, P5046, DOI 10.1016/j.vaccine.2008.07.035
Hamdy S, 2007, J BIOMED MATER RES A, V81A, P652, DOI 10.1002/jbm.a.31019
Hanlon DJ, 2011, AM J REPROD IMMUNOL, V65, P597, DOI 10.1111/j.1600-0897.2010.00968.x
He CL, 2016, ADV DRUG DELIVER REV, V98, P64, DOI 10.1016/j.addr.2015.10.021
Heit A, 2007, EUR J IMMUNOL, V37, P2063, DOI 10.1002/eji.200737169
Hirosue S, 2010, VACCINE, V28, P7897, DOI 10.1016/j.vaccine.2010.09.077
Huang BN, 2015, SCI TRANSL MED, V7, DOI 10.1126/scitranslmed.aaa5447
Ilyinskii PO, 2014, VACCINE, V32, P2882, DOI 10.1016/j.vaccine.2014.02.027
Irvine DJ, 2015, CHEM REV, V115, P11109, DOI 10.1021/acs.chemrev.5b00109
Jain S, 2005, BIOMACROMOLECULES, V6, P2590, DOI 10.1021/bm0503221
Jia F, 2013, J CONTROL RELEASE, V172, P1020, DOI 10.1016/j.jconrel.2013.10.012
Joffre OP, 2012, NAT REV IMMUNOL, V12, P557, DOI 10.1038/nri3254
Kaech SM, 2002, NAT REV IMMUNOL, V2, P251, DOI 10.1038/nri778
Kaminskas LM, 2011, ADV DRUG DELIVER REV, V63, P890, DOI 10.1016/j.addr.2011.05.016
Kemp JA, 2016, ADV DRUG DELIVER REV, V98, P3, DOI 10.1016/j.addr.2015.10.019
Khan TA, 2014, ACTA BIOMATER, V10, P1720, DOI 10.1016/j.actbio.2013.12.011
Kim J, 2015, NAT BIOTECHNOL, V33, P64, DOI 10.1038/nbt.3071

KOVACSOVICSBANKOWSKI M, 1993, P NATL ACAD SCI USA, V90, P4942, DOI 10.1073/pnas.90.11.4942
Kumar A, 2013, BIOTECHNOL ADV, V31, P593, DOI 10.1016/j.biotechadv.2012.10.002
Kwong B, 2011, BIOMATERIALS, V32, P5134, DOI 10.1016/j.biomaterials.2011.03.067
Lakshminarayanan V, 2012, P NATL ACAD SCI USA, V109, P261, DOI 10.1073/pnas.1115166109
Langer R, 2003, AICHE J, V49, P2990, DOI 10.1002/aic.690491202
Langer R, 1998, NATURE, V392, P5
Lee IH, 2012, ANGEW CHEM INT EDIT, V51, P8800, DOI 10.1002/anie.201203193
Letchford K, 2007, EUR J PHARM BIOPHARM, V65, P259, DOI 10.1016/j.ejpb.2006.11.009
Li P, 2013, J CONTROL RELEASE, V168, P271, DOI 10.1016/j.jconrel.2013.03.025
Li ZX, 2012, CHEM SOC REV, V41, P2590, DOI 10.1039/c1cs15246g
Liu DF, 2015, BIOMATERIALS, V39, P249, DOI 10.1016/j.biomaterials.2014.10.079
Lollo G, 2015, INT J PHARMACEUT, V495, P972, DOI 10.1016/j.ijpharm.2015.09.062
Lu YZ, 2012, CHEM SOC REV, V41, P3594, DOI 10.1039/c2cs15325d
Luo ZC, 2015, BIOMATERIALS, V38, P50, DOI 10.1016/j.biomaterials.2014.10.050
Lynn GM, 2015, NAT BIOTECHNOL, V33, P1201, DOI 10.1038/nbt.3371
Macho-Fernandez E, 2014, J IMMUNOL, V193, P961, DOI 10.4049/jimmunol.1303029
Makadia HK, 2011, POLYMERS-BASEL, V3, P1377, DOI 10.3390/polym3031377
Makkouk A, 2015, AAPS J, V17, P184, DOI 10.1208/s12248-014-9676-6
Mandal M, 2002, BBA-BIOMEMBRANES, V1563, P7, DOI 10.1016/S0005-2736(02)00368-1
Mbow ML, 2010, CURR OPIN IMMUNOL, V22, P411, DOI 10.1016/j.coi.2010.04.004
Mellman I, 2011, NATURE, V480, P480, DOI 10.1038/nature10673
Miyabe H, 2014, J CONTROL RELEASE, V184, P20, DOI 10.1016/j.jconrel.2014.04.004
Mody KT, 2013, NANOSCALE, V5, P5167, DOI 10.1039/c3nr00357d
Molino NM, 2016, BIOMATERIALS, V86, P83, DOI 10.1016/j.biomaterials.2016.01.056
Molino NM, 2013, ACS NANO, V7, P9743, DOI 10.1021/nn403085w
Morefield GL, 2005, VACCINE, V23, P1588, DOI 10.1016/j.vaccine.2004.07.050
Morvan MG, 2016, NAT REV CANCER, V16, P7, DOI 10.1038/nrc.2015.5
Mueller M, 2012, J CONTROL RELEASE, V162, P159, DOI 10.1016/j.jconrel.2012.06.015
Muller RH, 1996, J DRUG TARGET, V4, P161, DOI 10.3109/10611869609015973
Neumann S, 2015, VACCINE, V33, P5838, DOI 10.1016/j.vaccine.2015.08.083
Nikitczuk Kevin P, 2013, J Cancer Ther, V4, P280
Noh KH, 2014, BMC IMMUNOL, V15, DOI 10.1186/s12865-014-0048-x
Park J, 2012, NAT MATER, V11, P895, DOI [10.1038/NMAT3355, 10.1038/nmat3355]
Patel JM, 2016, BIOMATERIALS, V74, P231, DOI 10.1016/j.biomaterials.2015.09.031
Peine KJ, 2013, MOL PHARMACEUT, V10, P2849, DOI 10.1021/mp300643d
Perica K, 2014, ACS NANO, V8, P2252, DOI 10.1021/nn405520d
Reddy ST, 2007, NAT BIOTECHNOL, V25, P1159, DOI [10.1038/nbt1332, 10.1038/nbtl332]
Reddy ST, 2006, TRENDS IMMUNOL, V27, P573, DOI 10.1016/j.it.2006.10.005
Reddy ST, 2006, J CONTROL RELEASE, V112, P26, DOI 10.1016/j.jconrel.2006.01.006
Rosalia RA, 2015, BIOMATERIALS, V40, P88, DOI 10.1016/j.biomaterials.2014.10.053
Roy A, 2013, INT J PHARMACEUT, V445, P171, DOI 10.1016/j.ijpharm.2013.01.045
Roy A, 2010, MOL PHARMACEUT, V7, P1778, DOI 10.1021/mp100153r
Sahdev P, 2014, PHARM RES-DORDR, V31, P2563, DOI 10.1007/s11095-014-1419-y
Santos HA, 2011, J DRUG DELIV SCI TEC, V21, P139, DOI 10.1016/S1773-2247(11)50016-4
Santos HA, 2014, NANOMEDICINE-UK, V9, P535, DOI [10.2217/NNM.13.223, 10.2217/nnm.13.223]
Santos HA, 2013, CURR PHARM BIOTECHNO, V14, P926
Santos HA, 2013, J MATER RES, V28, P152, DOI 10.1557/jmr.2012.271
Santos Helder A, 2011, Curr Drug Discov Technol, V8, P228
Sarkar S, 2013, BIOCONJUGATE CHEM, V24, P363, DOI 10.1021/bc300422a
Schwendener Reto A, 2014, Ther Adv Vaccines, V2, P159, DOI 10.1177/2051013614541440
Serda RE, 2013, INT J NANOMED, V8, P1683, DOI 10.2147/IJN.S31756
Seth A, 2014, BIOMATERIALS, V35, P7992, DOI 10.1016/j.biomaterials.2014.05.076
Shahbazi MA, 2015, NANO RES, V8, P1505, DOI 10.1007/s12274-014-0635-4
Shahbazi MA, 2014, BIOMATERIALS, V35, P9224, DOI 10.1016/j.biomaterials.2014.07.050
Shahbazi MA, 2013, BIOMATERIALS, V34, P7776, DOI 10.1016/j.biomaterials.2013.06.052
Shahbazi Mohammad-Ali, 2012, Biomatter, V2, P296, DOI 10.4161/biom.22347
Shen LM, 2013, PLOS ONE, V8, DOI 10.1371/journal.pone.0080904
Shi JJ, 2010, NANO LETT, V10, P3223, DOI 10.1021/nl102184c
Silva JM, 2013, J CONTROL RELEASE, V168, P179, DOI 10.1016/j.jconrel.2013.03.010
Slowing II, 2008, ADV DRUG DELIVER REV, V60, P1278, DOI 10.1016/j.addr.2008.03.012

Solbrig CM, 2007, MOL PHARMACEUT, V4, P47, DOI 10.1021/mp060107e
Steichen SD, 2013, EUR J PHARM SCI, V48, P416, DOI 10.1016/j.ejps.2012.12.006
Stephan MT, 2010, NAT MED, V16, P1035, DOI 10.1038/nm.2198
Storni T, 2005, ADV DRUG DELIVER REV, V57, P333, DOI 10.1016/j.addr.2004.09.008
Tao Y, 2015, NANOSCALE, V7, P12419, DOI 10.1039/c5nr02240a
Tao Y, 2014, BIOMATERIALS, V35, P6646, DOI 10.1016/j.biomaterials.2014.04.073
Tao Y, 2014, ADV FUNCT MATER, V24, P1004, DOI 10.1002/adfm.201302347
Temizoz B, 2016, INT IMMUNOL, V28, P329, DOI 10.1093/intimm/dxw015
Thomann-Harwood LJ, 2013, J CONTROL RELEASE, V166, P95, DOI 10.1016/j.jconrel.2012.11.015
Tian X, 2012, BIOMATERIALS, V33, P6147, DOI 10.1016/j.biomaterials.2012.05.034
Torchilin VP, 2005, NAT REV DRUG DISCOV, V4, P145, DOI 10.1038/nrd1632
van Broekhoven CL, 2004, CANCER RES, V64, P4357, DOI 10.1158/0008-5472.CAN-04-0138
Vanneman M, 2012, NAT REV CANCER, V12, P237, DOI 10.1038/nrc3237
Vincent J, 2010, CANCER RES, V70, P3052, DOI 10.1158/0008-5472.CAN-09-3690
Wang C, 2014, VACCINE, V32, P5475, DOI 10.1016/j.vaccine.2014.07.081
Wang C, 2012, VACCINE, V30, P4790, DOI 10.1016/j.vaccine.2012.05.027
Wilson JT, 2013, ACS NANO, V7, P3912, DOI 10.1021/nn305466z
Xiang SD, 2006, METHODS, V40, P1, DOI 10.1016/j.ymeth.2006.05.016
Xu ZH, 2014, ACS NANO, V8, P3636, DOI 10.1021/nn500216y
Yoshizaki Y, 2014, BIOMATERIALS, V35, P8186, DOI 10.1016/j.biomaterials.2014.05.077
Yu SS, 2012, INT J NANOMED, V7, P799, DOI 10.2147/IJN.S28531
Yuba E, 2015, BIOMATERIALS, V67, P214, DOI 10.1016/j.biomaterials.2015.07.031
Yuba E, 2014, BIOMATERIALS, V35, P3091, DOI 10.1016/j.biomaterials.2013.12.024
Yuba E, 2013, BIOMATERIALS, V34, P3042, DOI 10.1016/j.biomaterials.2012.12.031
Zhang PP, 2015, ACS NANO, V9, P6465, DOI 10.1021/acsnano.5b02153
Zhang ZP, 2011, BIOMATERIALS, V32, P3666, DOI 10.1016/j.biomaterials.2011.01.067
Zhong ZH, 2010, INT J PHARMACEUT, V399, P156, DOI 10.1016/j.ijpharm.2010.07.053
Zhu YF, 2011, J PHYS CHEM C, V115, P447, DOI 10.1021/jp109535d
Zitvogel L, 2013, IMMUNITY, V39, P74, DOI 10.1016/j.immuni.2013.06.014
Zitvogel L, 2011, NAT REV CLIN ONCOL, V8, P151, DOI 10.1038/nrclinonc.2010.223
NR 145
TC 60
Z9 63
U1 2
U2 85
PU WILEY
PI HOBOKEN
PA 111 RIVER ST, HOBOKEN 07030-5774, NJ USA
SN 1939-5116
EI 1939-0041
J9 WIRES NANOMED NANOBI
JI Wiley Interdiscip. Rev.-Nanomed. Nanobiotechnol.
PD JAN-FEB
PY 2017
VL 9
IS 1
AR e1421
DI 10.1002/wnan.1421
PG 26
WC Nanoscience & Nanotechnology; Medicine, Research & Experimental
WE Science Citation Index Expanded (SCI-EXPANDED)
SC Science & Technology - Other Topics; Research & Experimental Medicine
GA EI5BQ
UT WOS:000392508900009
PM 27470448
DA 2023-09-14
ER

PT J
AU Zhuang, XT
Wu, TT

Zhao, YD
Hu, XM
Bao, YL
Guo, YY
Song, QL
Li, G
Tan, SW
Zhang, ZP
AF Zhuang, Xiangting
Wu, Tingting
Zhao, Yongdan
Hu, Xiaomeng
Bao, Yuling
Guo, Yuanyuan
Song, Qingle
Li, Gao
Tan, Songwei
Zhang, Zhiping
TI Lipid-enveloped zinc phosphate hybrid nanoparticles for codelivery of H-2K(b) and H-2D(b)-restricted antigenic peptides and monophosphoryl lipid A to induce antitumor immunity against melanoma
SO JOURNAL OF CONTROLLED RELEASE
LA English
DT Article
DE Nanoparticles; Adjuvant; Dendritic cell; Tumor antigen; Cancer immunotherapy
ID ORGANIC-MOLECULES; TRP2 PEPTIDE; VACCINE; CELLS; ADJUVANT; DELIVERY; IONS; LYMPHOCYTES; PARTICLES; INNATE
AB Nanoimmunotherapy, the application of nanotechnology for sustained and targeted delivery of antigens to dendritic cells (DCs), has attracted much attention in stimulating antigen-specific immune response for antitumor therapy. In order to in situ deliver antigens to DCs for efficient antigen presentation and subsequent induction of strong cytotoxic T lymphocytes (CTL) response, here we developed a multi-peptide (TRP2180-188 and HGP10025-33) and toll-like receptor 4 agonist (monophosphoryl lipid A) codelivery system based on lipid-coated zinc phosphate hybrid nanoparticles (LZnP NPs). This delivery system equips with the chelating property of zinc to realize the high encapsulation efficiency with antigenic peptides and the influence on immune system with adjuvant-like feature. The combination of H-2K(b) and H-2D(b)-restricted peptides could providemultiple epitopes as the target of specific MHC alleles, making tumor more difficult to escape from the surveillance of immune system. The formulated LZnP nano-vaccine with the size of 30 nm and outer leaflet lipid exhibited antitumor immunity as the secretion of cytokines in vitro and increased CD8(+) T cell response from IFN-gamma ELISPOT analysis ex vivo. The antitumor effects were further evidenced from the prophylactic, therapeutic and metastatic melanoma tumor models compared with free antigens and single peptide-loaded nano-vaccines. These results validate the benefit of LZnP-based vaccine for antitumor immunity and indicate that co-delivery of tumor antigens along with adjuvant may be an optimized strategy for tumor immunotherapy. (C) 2016 Published by Elsevier B.V.
C1 [Zhuang, Xiangting; Wu, Tingting; Zhao, Yongdan; Hu, Xiaomeng; Bao, Yuling; Guo, Yuanyuan; Song, Qingle; Li, Gao; Tan, Songwei; Zhang, Zhiping] Tongji Sch Pharm, Shanghai, Peoples R China.
[Li, Gao; Zhang, Zhiping] Natl Engn Res Ctr Nanomed, Wuhan, Peoples R China.
[Li, Gao; Zhang, Zhiping] Huazhong Univ Sci & Technol, Hubei Engn Res Ctr Novel Drug Delivery Syst, Wuhan 430030, Peoples R China.
C3 Huazhong University of Science & Technology
RP Zhang, ZP (通讯作者)，Tongji Sch Pharm, Shanghai, Peoples R China.
EM zhipingzhang@hust.edu.cn
RI zhang, zhiping/H-1786-2011; Tan, Songwei/H-1789-2011; Wu, Tingting/IWM-4143-2023; Guo, Yuan/HKV-9606-2023; TAN, Songwei/K-2222-2019
OI zhang, zhiping/0000-0001-9975-4862; Tan, Songwei/0000-0002-2986-8968; Wu, Tingting/0000-0003-3437-8899; TAN, Songwei/0000-0002-2986-8968; Hu, Xiaomeng/0000-0001-9494-9003
FU National Basic Research Program of China [2012CB932501]; Nature Science Fund of China [81373360]; Ministry of Education of China

[20120142120093]; Fundamental Research Funds for the Central
Universities (HUST) [2014TS091, 2015YGYL024]
FX This research is supported by National Basic Research Program of China
(2012CB932501), Nature Science Fund of China (81373360), Doctoral Fund
of Ministry of Education of China (20120142120093), and the Fundamental
Research Funds for the Central Universities (HUST, 2014TS091,
2015YGYL024). We thank HuaZhong University of Science & Technology
Analytical and testing center for the characterization of ZnP and LZnP
NPs.
CR Ahn S, 2014, ADV HEALTHC MATER, V3, P1194, DOI 10.1002/adhm.201300597
Bao B, 2003, AM J PHYSIOL-ENDOC M, V285, pE1095, DOI 10.1152/ajpendo.00545.2002
Black M, 2012, ADV MATER, V24, P3845, DOI 10.1002/adma.201200209
Calzascia T, 2007, J CLIN INVEST, V117, P3833, DOI 10.1172/JCI32567
Celluzzi CM, 1996, J EXP MED, V183, P283, DOI 10.1084/jem.183.1.283
Chan CW, 2006, NAT MED, V12, P207, DOI 10.1038/nm1352
Choi KY, 2014, ACS NANO, V8, P4559, DOI 10.1021/nn500085k
Couzin-Frankel J, 2013, SCIENCE, V342, P1432, DOI 10.1126/science.342.6165.1432
Dash S, 2008, ADV COLLOID INTERFAC, V140, P77, DOI 10.1016/j.cis.2007.12.006
DASILVA LP, 1991, BIOCHEM BIOPH RES CO, V178, P1359, DOI 10.1016/0006-291X(91)91043-C
Del Vecchio M, 2007, CLIN CANCER RES, V13, P4677, DOI 10.1158/1078-0432.CCR-07-0776
DeLisser HM, 1997, AM J PATHOL, V151, P671
Feyerabend S, 2009, PROSTATE, V69, P917, DOI 10.1002/pros.20941
Garcia-Lora A, 2003, J CELL PHYSIOL, V195, P346, DOI 10.1002/jcp.10290
Garzon-Rodriguez W, 1999, BIOORG MED CHEM LETT, V9, P2243, DOI 10.1016/S0960-
894X(99)00357-1
Guo ZQ, 2014, NAT PROTOC, V9, P1245, DOI 10.1038/nprot.2014.086
Harmeyer S, 1998, J GEN VIROL, V79, P937, DOI 10.1099/0022-1317-79-4-937
Hawiger D, 2001, J EXP MED, V194, P769, DOI 10.1084/jem.194.6.769
Hribar G, 2011, J NANOPART RES, V13, P3019, DOI 10.1007/s11051-010-0199-5
Hwang L, 2015, J AM CHEM SOC, V137, P2432, DOI 10.1021/ja511833y
Kabelitz Dieter, 1997, Frontiers in Bioscience (online), V2, pD61
Khong HT, 2004, J IMMUNOTHER, V27, P184, DOI 10.1097/00002371-200405000-00002
Li J, 2012, J CONTROL RELEASE, V158, P108, DOI 10.1016/j.jconrel.2011.10.020
Liu DM, 2014, NAT COMMUN, V5, DOI 10.1038/ncomms5182
Liu P, 2015, J MATER CHEM B, V3, P1301, DOI 10.1039/c4tb01049c
Liu SY, 2013, BIOMATERIALS, V34, P8291, DOI 10.1016/j.biomaterials.2013.07.020
Mahony D, 2013, SMALL, V9, P3138, DOI 10.1002/smll.201300012
Mellman I, 2011, NATURE, V480, P480, DOI 10.1038/nature10673
Mendiratta SK, 2001, CANCER RES, V61, P859
Morgan TT, 2008, NANO LETT, V8, P4108, DOI 10.1021/nl8019888
Palucka K, 2013, IMMUNITY, V39, P38, DOI 10.1016/j.immuni.2013.07.004
PANTEL K, 1991, CANCER RES, V51, P4712
Pashine A, 2005, NAT MED, V11, pS63, DOI 10.1038/nm1210
Pilla L, 2009, EXPERT OPIN BIOL TH, V9, P1043, DOI 10.1517/14712590903085109
Prasad AS, 2009, CURR OPIN CLIN NUTR, V12, P646, DOI 10.1097/MCO.0b013e3283312956
Randolph GJ, 2005, NAT REV IMMUNOL, V5, P617, DOI 10.1038/nri1670
Rose K, 1995, MOL IMMUNOL, V32, P1031, DOI 10.1016/0161-5890(95)00090-9
Sabado RL, 2013, ANN NY ACAD SCI, V1284, P31, DOI 10.1111/nyas.12125
Shankaran V, 2001, NATURE, V410, P1107, DOI 10.1038/35074122
Tian X, 2012, BIOMATERIALS, V33, P6147, DOI 10.1016/j.biomaterials.2012.05.034
Tuting T, 2013, PIGM CELL MELANOMA R, V26, DOI 10.1111/pcmr.12111
Wan LJ, 2000, LANGMUIR, V16, P2164, DOI 10.1021/1a991069r
Wang C, 2014, ADV MATER, V26, P8154, DOI 10.1002/adma.201402996
Wang P, 2015, J MATER CHEM B, V3, P3617, DOI 10.1039/c5tb00142k
Willmann JK, 2008, RADIOLOGY, V246, P508, DOI 10.1148/radiol.2462070536
Xu ZH, 2013, J CONTROL RELEASE, V172, P259, DOI 10.1016/j.jconrel.2013.08.021
Yu XY, 2013, CLIN DEV IMMUNOL, DOI 10.1155/2013/238035
Zeng Q, 2015, J CONTROL RELEASE, V200, P1, DOI 10.1016/j.jconrel.2014.12.024
Zhang ZP, 2011, BIOMATERIALS, V32, P3666, DOI 10.1016/j.biomaterials.2011.01.067
Zhao L, 2014, VACCINE, V32, P327, DOI 10.1016/j.vaccine.2013.11.069
Zheng JN, 2015, J MATER CHEM B, V3, P2185, DOI 10.1039/c4tb02007c

NR 51
TC 60
Z9 65
U1 5
U2 113
PU ELSEVIER SCIENCE BV
PI AMSTERDAM
PA PO BOX 211, 1000 AE AMSTERDAM, NETHERLANDS
SN 0168-3659
EI 1873-4995
J9 J CONTROL RELEASE
JI J. Control. Release
PD APR 28
PY 2016
VL 228
BP 26
EP 37
DI 10.1016/j.jconrel.2016.02.035
PG 12
WC Chemistry, Multidisciplinary; Pharmacology & Pharmacy
WE Science Citation Index Expanded (SCI-EXPANDED)
SC Chemistry; Pharmacology & Pharmacy
GA DI3WC
UT WOS:000373429600004
PM 26921522
DA 2023-09-14
ER

PT J
AU Wagner, J
   Gossl, D
   Ustyanovska, N
   Xiong, MY
   Hauser, D
   Zhuzhgova, O
   Hocevar, S
   Taskoparan, B
   Poller, L
   Datz, S
   Engelke, H
   Daali, Y
   Bein, T
   Bourquin, C
AF Wagner, Julia
   Goessl, Dorothee
   Ustyanovska, Natasha
   Xiong, Mengyao
   Hauser, Daniel
   Zhuzhgova, Olga
   Hocevar, Sandra
   Taskoparan, Betuel
   Poller, Laura
   Datz, Stefan
   Engelke, Hanna
   Daali, Youssef
   Bein, Thomas
   Bourquin, Carole
TI Mesoporous Silica Nanoparticles as pH-Responsive Carrier for the
   Immune-Activating Drug Resiquimod Enhance the Local Immune Response in
   Mice
SO ACS NANO

LA English
DT Article
DE mesoporous silica nanoparticles; drug delivery; immunotherapy;
stimuli-responsive capping system; Toll-like receptor 7 agonist;
dendritic cell activation; nanovaccine
ID EGG-WHITE AVIDIN; POLYMERIC NANOPARTICLES; DELIVERY-SYSTEM;
DUAL-DELIVERY; TLR7 LIGAND; CO-DELIVERY; LYMPH-NODE; VACCINES; PEPTIDE;
ANTIGEN
AB Nanoparticle-based delivery systems for cancer immunotherapies aim to improve the safety and efficacy of these treatments through local delivery to specialized antigen-presenting cells (APCs). Multifunctional mesoporous silica nanoparticles (MSNs), with their large surface areas, their tunable particle and pore sizes, and their spatially controlled functionalization, represent a safe and versatile carrier system. In this study, we demonstrate the potential of MSNs as a pH-responsive drug carrier system for the anticancer immune-stimulant R848 (resiquimod), a synthetic Toll-like receptor 7 and 8 agonist. Equipped with a biotin-avidin cap, the tailor-made nanoparticles showed efficient stimuli-responsive release of their R848 cargo in an environmental pH of 5.5 or below. We showed that the MSNs loaded with R848 were rapidly taken up by APCs into the acidic environment of the lysosome and that they potently activated the immune cells. Upon subcutaneous injection into mice, the particles accumulated in migratory dendritic cells (DCs) in the draining lymph nodes, where they strongly enhanced the activation of the DCs. Furthermore, simultaneous delivery of the model antigen OVA and the adjuvant R848 by MSNs resulted in an augmented antigen-specific T-cell response. The MSNs significantly improved the pharmacokinetic profile of R848 in mice, as the half-life of the drug was increased 6-fold, and at the same time, the systemic exposure was reduced. In summary, we demonstrate that MSNs represent a promising tool for targeted delivery of the immune modulator R848 to APCs and hold considerable potential as a carrier for cancer vaccines.
C1 [Wagner, Julia; Hocevar, Sandra; Taskoparan, Betuel; Daali, Youssef; Bourquin, Carole] Univ Geneva, Inst Pharmaceut Sci Western Switzerland, CH-1211 Geneva, Switzerland.
[Wagner, Julia; Hocevar, Sandra; Taskoparan, Betuel; Daali, Youssef; Bourquin, Carole] Univ Geneva, Sch Pharmaceut Sci, CH-1211 Geneva, Switzerland.
[Goessl, Dorothee; Ustyanovska, Natasha; Xiong, Mengyao; Zhuzhgova, Olga; Poller, Laura; Datz, Stefan; Engelke, Hanna; Bein, Thomas] Ludwig Maximilians Univ LMU Munchen, Dept Chem, D-81377 Munich, Germany.
[Goessl, Dorothee; Ustyanovska, Natasha; Xiong, Mengyao; Zhuzhgova, Olga; Poller, Laura; Datz, Stefan; Engelke, Hanna; Bein, Thomas] Ludwig Maximilians Univ LMU Munchen, Ctr NanoSci CeNS, D-81377 Munich, Germany.
[Hauser, Daniel] Univ Fribourg, Adolphe Merkle Inst, CH-1700 Fribourg, Switzerland.
[Bourquin, Carole] Univ Geneva, Dept Anaesthesiol Pharmacol Intens Care & Emergen, CH-1211 Geneva, Switzerland.
C3 University of Geneva; University of Geneva; University of Munich;
University of Munich; University of Fribourg; University of Geneva
RP Bourquin, C (通讯作者)，Univ Geneva, Inst Pharmaceut Sci Western Switzerland, CH-1211 Geneva, Switzerland.; Bourquin, C (通讯作者)，Univ Geneva, Sch Pharmaceut Sci, CH-1211 Geneva, Switzerland.; Bein, T (通讯作者)，Ludwig Maximilians Univ LMU Munchen, Dept Chem, D-81377 Munich, Germany.; Bein, T (通讯作者)，Ludwig Maximilians Univ LMU Munchen, Ctr NanoSci CeNS, D-81377 Munich, Germany.; Bourquin, C (通讯作者)，Univ Geneva, Dept Anaesthesiol Pharmacol Intens Care & Emergen, CH-1211 Geneva, Switzerland.
EM bein@lmu.de; carole.bourquin@unige.ch
RI Bourquin, Carole/AAU-1418-2020
OI Bourquin, Carole/0000-0003-3862-4583; Hocevar,
Sandra/0000-0002-9770-8229; Taskoparan Yaglikci,
Betul/0000-0003-1890-1547; Ustyanovska Avtenyuk,
Natasha/0000-0003-3783-7587; Engelke, Hanna/0000-0001-9529-9436; Hauser,
Daniel/0000-0003-2148-5820; Xiong, Mengyao/0000-0001-8425-5200; Wagner,
Julia/0000-0002-5019-6932
FU German Research Foundation (DFG) [SFB 1032]; Center for NanoScience
(CeNS); Nanosystems Initiative Munich (NIM); Swiss National Science
Foundation [156372, 182317]
FX We are grateful to Ine`s Mottas for her advice. We thank Steffen Schmidt
for the TEM images. D.G., N.U., M.X., O.Z., L.P., S.D., H.E., and T.B.
thank the German Research Foundation (DFG, SFB 1032, B05), the Center
for NanoScience (CeNS) and the Nanosystems Initiative Munich (NIM) for

financial support. J.W., B.T., S.H., and C.B. thank the Swiss National
Science Foundation (projects 156372 and 182317) for financial support.
CR Ali OA, 2016, CANCER IMMUNOL RES, V4, P95, DOI 10.1158/2326-6066.CIR-14-0126
Argyo C, 2014, CHEM MATER, V26, P435, DOI 10.1021/cm402592t
Baldwin RL, 1996, BIOPHYS J, V71, P2056, DOI 10.1016/S0006-3495(96)79404-3
Bobo D, 2016, PHARM RES-DORDR, V33, P2373, DOI 10.1007/s11095-016-1958-5
Bourquin C, 2020, PHARMACOL RES, V154, DOI 10.1016/j.phrs.2019.03.001
Bulbake U, 2017, PHARMACEUTICS, V9, DOI 10.3390/pharmaceutics9020012
BUNKER BC, 1994, J NON-CRYST SOLIDS, V179, P300, DOI 10.1016/0022-3093(94)90708-0
Cacace MG, 1997, Q REV BIOPHYS, V30, P241, DOI 10.1017/S0033583597003363
Cauda V, 2010, NANO LETT, V10, P2484, DOI 10.1021/nl100991w
Cauda V, 2009, J AM CHEM SOC, V131, P11361, DOI 10.1021/ja809346n
Cha BG, 2018, ACS CENTRAL SCI, V4, P484, DOI 10.1021/acscentsci.8b00035
Chang JH, 2017, J MATER CHEM B, V5, P3012, DOI 10.1039/c7tb00351j
Chen DS, 2013, IMMUNITY, V39, P1, DOI 10.1016/j.immuni.2013.07.012
Chou CC, 2017, ACS APPL MATER INTER, V9, P22235, DOI 10.1021/acsami.7b05359
Colilla M, 2013, BIOMATER SCI-UK, V1, P114, DOI 10.1039/c2bm00085g
Couzin-Frankel J, 2013, SCIENCE, V342, P1432, DOI 10.1126/science.342.6165.1432
Croissant JG, 2017, ADV MATER, V29, DOI 10.1002/adma.201604634
Datz S, 2016, NANOSCALE, V8, P8101, DOI 10.1039/c5nr08163g
Deckwer M., 1999, ROMPP LEXIKON BIOTEC, P376
DELANGE RJ, 1971, J BIOL CHEM, V246, P698
Dockrell DH, 2001, J ANTIMICROB CHEMOTH, V48, P751, DOI 10.1093/jac/48.6.751
Dowling David J, 2018, Immunohorizons, V2, P185, DOI 10.4049/immunohorizons.1700063
Ducharme J, 1996, CLIN PHARMACOKINET, V31, P257, DOI 10.2165/00003088-199631040-00003
Duperret EK, 2018, CLIN CANCER RES, V24, P6015, DOI 10.1158/1078-0432.CCR-18-1013
Eisenbarth SC, 2019, NAT REV IMMUNOL, V19, P89, DOI 10.1038/s41577-018-0088-1
Franco S, 2020, CANCERS, V12, DOI 10.3390/cancers12102892
GEISOW MJ, 1984, EXP CELL RES, V150, P36, DOI 10.1016/0014-4827(84)90699-2
Geisse J, 2004, J AM ACAD DERMATOL, V50, P722, DOI 10.1016/j.jaad.2003.11.066
Goldstein D, 1998, J INFECT DIS, V178, P858, DOI 10.1086/515343
Havel HA, 2016, AAPS J, V18, P1351, DOI 10.1208/s12248-016-9970-6
Heidegger S, 2016, NANOSCALE, V8, P938, DOI 10.1039/c5nr06122a
Helft J, 2015, IMMUNITY, V42, P1197, DOI 10.1016/j.immuni.2015.05.018
Hocevar S, 2019, ACS NANO, V13, P6790, DOI 10.1021/acsnano.9b01492
Hotz C, 2016, ONCOIMMUNOLOGY, V5, DOI 10.1080/2162402X.2016.1232219
Hudson SP, 2008, BIOMATERIALS, V29, P4045, DOI 10.1016/j.biomaterials.2008.07.007
Huh S, 2003, CHEM MATER, V15, P4247, DOI 10.1021/cm0210041
Ilyinskii PO, 2014, VACCINE, V32, P2882, DOI 10.1016/j.vaccine.2014.02.027
Irvine DJ, 2015, CHEM REV, V115, P11109, DOI 10.1021/acs.chemrev.5b00109
Kecht J, 2008, CHEM MATER, V20, P7207, DOI 10.1021/cm801484r
KENNEDY RM, 1990, METHOD ENZYMOL, V182, P339
Kim H, 2018, BIOMATERIALS, V164, P38, DOI 10.1016/j.biomaterials.2018.02.034
Kitano M, 2016, P NATL ACAD SCI USA, V113, P1044, DOI 10.1073/pnas.1513607113
Kniep R. d., 1996, WISSENSCHAFT AKTUELL, V30, P46
Kobler J, 2008, ACS NANO, V2, P791, DOI 10.1021/nn700008s
Kourtis IC, 2013, PLOS ONE, V8, DOI 10.1371/journal.pone.0061646
Liu Linda N., 2008, V423, P139, DOI 10.1007/978-1-59745-194-9_9
Liu R, 2010, J AM CHEM SOC, V132, P1500, DOI 10.1021/ja907838s
LIVNAH O, 1993, P NATL ACAD SCI USA, V90, P5076, DOI 10.1073/pnas.90.11.5076
Lu J, 2010, SMALL, V6, P1794, DOI 10.1002/smll.201000538
Lucke M, 2018, BIOMATERIALS, V172, P105, DOI 10.1016/j.biomaterials.2018.04.008
Lynn GM, 2015, NAT BIOTECHNOL, V33, P1201, DOI 10.1038/nbt.3371
Mahjub R, 2018, J CONTROL RELEASE, V288, P239, DOI 10.1016/j.jconrel.2018.09.010
Mirakabad FST, 2014, ASIAN PAC J CANCER P, V15, P517, DOI 10.7314/APJCP.2014.15.2.517
Moller K, 2019, CHEM MATER, V31, P4364, DOI 10.1021/acs.chemmater.9b00221
Mottas I, 2019, BIOMATERIALS, V190, P111, DOI 10.1016/j.biomaterials.2018.10.031
Nie YJ, 2017, SCI REP-UK, V7, DOI 10.1038/s41598-017-14655-8
Oh JY, 2018, NAT COMMUN, V9, DOI 10.1038/s41467-018-06979-4
Oliveira CL, 2017, COLLOID SURFACE B, V150, P326, DOI 10.1016/j.colsurfb.2016.10.046
Petronzelli F, 2010, CANCER BIOTHER RADIO, V25, P563, DOI 10.1089/cbr.2010.0797

PORATH J, 1973, NATURE, V245, P465, DOI 10.1038/245465a0
PUGLIESE L, 1993, J MOL BIOL, V231, P698, DOI 10.1006/jmbi.1993.1321
RABINOVITCH M, 1995, TRENDS CELL BIOL, V5, P85, DOI 10.1016/S0962-8924(00)88955-2
Rahimian S, 2015, J CONTROL RELEASE, V203, P16, DOI 10.1016/j.jconrel.2015.02.006
Rook AH, 2015, BLOOD, V126, P1452, DOI 10.1182/blood-2015-02-630335
Rosalia RA, 2015, BIOMATERIALS, V40, P88, DOI 10.1016/j.biomaterials.2014.10.053
Rueda F, 2017, IMMUNOBIOLOGY, V222, P989, DOI 10.1016/j.imbio.2017.06.002
Sahin U, 2018, SCIENCE, V359, P1355, DOI 10.1126/science.aar7112
Schlossbauer A, 2009, ANGEW CHEM INT EDIT, V48, P3092, DOI 10.1002/anie.200805818
Schwendener Reto A, 2014, Ther Adv Vaccines, V2, P159, DOI 10.1177/2051013614541440
Slingluff CL, 2007, CLIN CANCER RES, V13, P6386, DOI 10.1158/1078-0432.CCR-07-0486
Smith M, 2018, ONCOIMMUNOLOGY, V7, DOI 10.1080/2162402X.2018.1526250
Tarn D, 2013, ACCOUNTS CHEM RES, V46, P792, DOI 10.1021/ar3000986
Thauvin C, 2019, EUR J PHARM BIOPHARM, V139, P253, DOI 10.1016/j.ejpb.2019.04.007
Thomas SN, 2014, BIOMATERIALS, V35, P814, DOI 10.1016/j.biomaterials.2013.10.003
Tomura M, 2014, SCI REP-UK, V4, DOI 10.1038/srep06030
Vallet-Regi M, 2018, MOLECULES, V23, DOI 10.3390/molecules23010047
van Rijt SH, 2016, NANOSCALE, V8, P8058, DOI 10.1039/c5nr04119h
Widmer J, 2018, INT J PHARMACEUT, V535, P444, DOI 10.1016/j.ijpharm.2017.11.031
Wilson JT, 2013, ACS NANO, V7, P3912, DOI 10.1021/nn305466z
Zhang R, 2018, ACS BIOMATER SCI ENG, V4, P2463, DOI 10.1021/acsbiomaterials.8b00511
Zhang XY, 2015, CELL BIOCHEM BIOPHYS, V72, P771, DOI 10.1007/s12013-015-0529-4
Zhao YN, 2009, J AM CHEM SOC, V131, P8398, DOI 10.1021/ja901831u
Zupancic E, 2014, PROCEDIA VACCINOL, V8, P62, DOI 10.1016/j.provac.2014.07.011
NR 83
TC 59
Z9 61
U1 22
U2 151
PU AMER CHEMICAL SOC
PI WASHINGTON
PA 1155 16TH ST, NW, WASHINGTON, DC 20036 USA
SN 1936-0851
EI 1936-086X
J9 ACS NANO
JI ACS Nano
PD MAR 23
PY 2021
VL 15
IS 3
BP 4450
EP 4466
DI 10.1021/acsnano.0c08384
EA MAR 2021
PG 17
WC Chemistry, Multidisciplinary; Chemistry, Physical; Nanoscience &
Nanotechnology; Materials Science, Multidisciplinary
WE Science Citation Index Expanded (SCI-EXPANDED)
SC Chemistry; Science & Technology - Other Topics; Materials Science
GA RF0WQ
UT WOS:000634569100063
PM 33648336
OA Green Published, hybrid
DA 2023-09-14
ER

PT J
AU Vijayan, V
Mohapatra, A
Uthaman, S
Park, IK

AF Vijayan, Veena
Mohapatra, Adityanarayan
Uthaman, Saji
Park, In-Kyu
TI Recent Advances in Nanovaccines Using Biomimetic Immunomodulatory Materials
SO PHARMACEUTICS
LA English
DT Review
DE nanovaccines; biomimetic; antigens; adjuvants; antigen-presenting cells
ID VIRUS-LIKE PARTICLES; ELICITED ANTITUMOR IMMUNITY; DRUG-DELIVERY SYSTEM; VACCINE DELIVERY; GOLD NANOPARTICLES; MEMBRANE-VESICLES; PROTEIN CORONA; LIPOSOMES; ADJUVANT; ANTIGENS
AB The development of vaccines plays a vital role in the effective control of several fatal diseases. However, effective prophylactic and therapeutic vaccines have yet to be developed for completely curing deadly diseases, such as cancer, malaria, HIV, and serious microbial infections. Thus, suitable vaccine candidates need to be designed to elicit appropriate immune responses. Nanotechnology has been found to play a unique role in the design of vaccines, providing them with enhanced specificity and potency. Nano-scaled materials, such as virus-like particles, liposomes, polymeric nanoparticles (NPs), and protein NPs, have received considerable attention over the past decade as potential carriers for the delivery of vaccine antigens and adjuvants, due to their beneficial advantages, like improved antigen stability, targeted delivery, and long-time release, for which antigens/adjuvants are either encapsulated within, or decorated on, the NP surface. Flexibility in the design of nanomedicine allows for the programming of immune responses, thereby addressing the many challenges encountered in vaccine development. Biomimetic NPs have emerged as innovative natural mimicking biosystems that can be used for a wide range of biomedical applications. In this review, we discuss the recent advances in biomimetic nanovaccines, and their use in anti-bacterial therapy, anti-HIV therapy, anti-malarial therapy, anti-melittin therapy, and anti-tumor immunity.
C1 [Vijayan, Veena; Mohapatra, Adityanarayan; Park, In-Kyu] Chonnam Natl Univ, Dept Biomed Sci, Med Sch, Gwangju 58128, South Korea.
[Uthaman, Saji] Chungnam Natl Univ, Dept Polymer Sci & Engn, Daehak Ro 99, Daejeon 34134, South Korea.
C3 Chonnam National University; Chungnam National University
RP Park, IK (通讯作者)，Chonnam Natl Univ, Dept Biomed Sci, Med Sch, Gwangju 58128, South Korea.
EM veenavj4392@gmail.com; mkaditya55@gmail.com; sajiuthaman@gmail.com; pik96@jnu.ac.kr
RI Park, In-Kyu/W-1613-2017; Uthaman, Saji/T-6068-2017
OI Park, In-Kyu/0000-0002-8522-8236; Uthaman, Saji/0000-0001-6387-7510; Park, In-Kyu/0000-0003-1616-9153
FU National Research Foundation of Korea (NRF) - Korean government, MSIP [NRF-2017M3A9F5030940, NRF-2017M3A9E2056374]; Pioneer Research Center Program through the National Research Foundation of Korea - Ministry of Science, ICT & Future Planning [2014M3C1A3053035]; National Research Foundation of Korea (NRF) - Korea government (MSIT) [2018R1A5A2024181, NRF-2019M3E5D1A02068082]; National Research Foundation of Korea [2019M3E5D1A02068082, 2017M3A9E2056374] Funding Source: Korea Institute of Science & Technology Information (KISTI), National Science & Technology Information Service (NTIS)
FX This work was financially supported by the Bio & Medical Technology Development Program (No. NRF-2017M3A9F5030940 and NRF-2017M3A9E2056374,) through the National Research Foundation of Korea (NRF) funded by the Korean government, MSIP; and the Pioneer Research Center Program through the National Research Foundation of Korea funded by the Ministry of Science, ICT & Future Planning (2014M3C1A3053035). This work was supported by the National Research Foundation of Korea (NRF) grant funded by the Korea government (MSIT) (No. 2018R1A5A2024181 and NRF-2019M3E5D1A02068082).
CR Anderson RP, 2013, CURR OPIN IMMUNOL, V25, P410, DOI 10.1016/j.coi.2013.02.004
Andersson AMC, 2016, VACCINE, V34, P5344, DOI 10.1016/j.vaccine.2016.08.089
Anderton SM, 1998, EUR J IMMUNOL, V28, P1251, DOI 10.1002/(SICI)1521-4141(199804)28:04<1251::AID-IMMU1251>3.0.CO;2-O

ANDRE FE, 1990, VACCINE, V8, pS74, DOI 10.1016/0264-410X(90)90222-8
Andrews SC, 2010, BBA-GEN SUBJECTS, V1800, P691, DOI 10.1016/j.bbagen.2010.05.010
Angsantikul P, 2018, ADV THER-GERMANY, V1, DOI 10.1002/adtp.201800016
Arias CA, 2009, NEW ENGL J MED, V360, P439, DOI 10.1056/NEJMp0804651
Arms L, 2018, FRONT PHARMACOL, V9, DOI 10.3389/fphar.2018.00802
Bachmann MF, 2010, NAT REV IMMUNOL, V10, P787, DOI 10.1038/nri2868
Bai H, 2019, BMC CANCER, V19, DOI 10.1186/s12885-019-5665-6
Bai J, 2019, ENZYME MICROB TECH, V128, P40, DOI 10.1016/j.enzmictec.2019.05.006
Bishop CJ, 2015, J CONTROL RELEASE, V219, P488, DOI 10.1016/j.jconrel.2015.09.046
Bose Rajendran Jc, 2016, Biomater Res, V20, P34, DOI 10.1186/s40824-016-0081-3
Bovier PA, 2008, EXPERT REV VACCINES, V7, P1141, DOI 10.1586/14760584.7.8.1141
Bradbury PA, 2008, J THORAC ONCOL, V3, pS164, DOI 10.1097/JTO.0b013e318174e9a7
BUI T, 1994, J ACQ IMMUN DEF SYND, V7, P799
Campbell EM, 2015, NAT REV MICROBIOL, V13, P471, DOI 10.1038/nrmicro3503
Caston Jose R, 2013, Subcell Biochem, V68, P53, DOI 10.1007/978-94-007-6552-8_2
Champion CI, 2009, PLOS ONE, V4, DOI 10.1371/journal.pone.0005409
Chasteen ND, 1999, J STRUCT BIOL, V126, P182, DOI 10.1006/jsbi.1999.4118
Chattopadhyay Saborni, 2017, Nanotheranostics, V1, P244, DOI 10.7150/ntno.19796
Chen MS, 2019, ARTIF CELL NANOMED B, V47, P1635, DOI 10.1080/21691401.2019.1608219
Chen PF, 2016, HUM VACC IMMUNOTHER, V12, P612, DOI 10.1080/21645515.2015.1105415
Chen YJ, 2018, ADV HEALTHC MATER, V7, DOI 10.1002/adhm.201701366
Collins KA, 2017, SCI REP-UK, V7, DOI 10.1038/srep46621
Corbo C, 2017, BIOMATER SCI-UK, V5, P378, DOI 10.1039/c6bm00921b
Corbo C, 2016, NANOMEDICINE-UK, V11, P81, DOI 10.2217/nnm.15.188
Cox JC, 1997, VACCINE, V15, P248, DOI 10.1016/S0264-410X(96)00183-1
Cruz LJ, 2014, NANOMEDICINE-UK, V9, P435, DOI [10.2217/NNM.13.66, 10.2217/nnm.13.66]
Cutts FT, 2007, B WORLD HEALTH ORGAN, V85, P719, DOI 10.2471/BLT.06.038414
Delany I, 2014, EMBO MOL MED, V6, P708, DOI 10.1002/emmm.201403876
Deng L, 2018, NAT COMMUN, V9, DOI 10.1038/s41467-017-02725-4
Dennis E., 2015, J INFECT DIS THER, V3, P229, DOI DOI 10.4172/2332-0877.1000229
Dhanasooraj D, 2013, INT J NANOMED, V8, P835, DOI 10.2147/IJN.S40238
Didierlaurent AM, 2017, EXPERT REV VACCINES, V16, P55, DOI 10.1080/14760584.2016.1213632
Dykman LA, 2015, GOLD BULL, V48, P93, DOI 10.1007/s13404-015-0165-1
Este JA, 2010, ANTIVIR RES, V85, P25, DOI 10.1016/j.antiviral.2009.10.007
Fang RH, 2018, ADV MATER, V30, DOI 10.1002/adma.201706759
Fantappie L, 2014, J EXTRACELL VESICLES, V3, DOI 10.3402/jev.v3.24015
Fraser CK, 2007, EXPERT REV VACCINES, V6, P559, DOI 10.1586/14760584.6.4.559
Gao M, 2017, ADV MATER, V29, DOI 10.1002/adma.201701429
Gao WW, 2015, NANO LETT, V15, P1403, DOI 10.1021/nl504798g
Gao WW, 2013, ADV MATER, V25, P3549, DOI 10.1002/adma.201300638
Gerke C, 2015, PLOS ONE, V10, DOI 10.1371/journal.pone.0134478
Glenny AT, 1926, J PATHOL BACTERIOL, V29, P31, DOI 10.1002/path.1700290106
Gluck R, 2002, VACCINE, V20, pB10, DOI 10.1016/S0264-410X(02)00513-3
Grenni P, 2018, MICROCHEM J, V136, P25, DOI 10.1016/j.microc.2017.02.006
Grgacic EVL, 2006, METHODS, V40, P60, DOI 10.1016/j.ymeth.2006.07.018
Han SW, 2018, TISSUE ENG REGEN MED, V15, P365, DOI 10.1007/s13770-018-0128-8
Hanson MC, 2015, VACCINE, V33, P861, DOI 10.1016/j.vaccine.2014.12.045
He HL, 2018, NANO LETT, V18, P6164, DOI 10.1021/acs.nanolett.8b01892
He WP, 2016, ACS APPL MATER INTER, V8, P4407, DOI 10.1021/acsami.5b10885
Henriksen-Lacey M, 2011, EXPERT OPIN DRUG DEL, V8, P505, DOI 10.1517/17425247.2011.558081
Heurtault B, 2010, EXPERT OPIN DRUG DEL, V7, P829, DOI 10.1517/17425247.2010.488687
Hoebe K, 2004, NAT IMMUNOL, V5, P971, DOI 10.1038/ni1004-971
Hokmabad V, 2018, TISSUE ENG REGEN MED, V15, P735, DOI 10.1007/s13770-018-0140-z
Hu CMJ, 2015, NATURE, V526, P118, DOI 10.1038/nature15373
Hu CMJ, 2013, NAT NANOTECHNOL, V8, P933, DOI [10.1038/nnano.2013.254, 10.1038/NNANO.2013.254]
Hu CMJ, 2012, ADV HEALTHC MATER, V1, P537, DOI 10.1002/adhm.201200138
Hu CMJ, 2011, P NATL ACAD SCI USA, V108, P10980, DOI 10.1073/pnas.1106634108
Hu YC, 2005, ACTA PHARMACOL SIN, V26, P405, DOI 10.1111/j.1745-7254.2005.00078.x
Ibrahim S, 2017, BIOL PHARM BULL, V40, P815, DOI 10.1248/bpb.b16-00914
Ichihashi T, 2013, PLOS ONE, V8, DOI 10.1371/journal.pone.0060068

Joshi VB, 2013, AAPS J, V15, P85, DOI 10.1208/s12248-012-9418-6
Jung SW, 2019, TISSUE ENG REGEN MED, V16, P479, DOI 10.1007/s13770-019-00206-x
Kaczmarczyk SJ, 2011, P NATL ACAD SCI USA, V108, P16998, DOI 10.1073/pnas.1101874108
Kanekiyo M, 2013, NATURE, V499, P102, DOI 10.1038/nature12202
Kang SM, 2009, CURR TOP MICROBIOL, V333, P269, DOI 10.1007/978-3-540-92165-3_14
Kang TY, 2018, INT J NANOMED, V13, P3251, DOI 10.2147/IJN.S156346
Khatri K, 2008, VACCINE, V26, P2225, DOI 10.1016/j.vaccine.2008.02.058
Kimiz-Gebologlu I, 2018, MOL BIOL REP, V45, P2935, DOI 10.1007/s11033-018-4427-x
KINGSMAN SM, 1988, VACCINE, V6, P304, DOI 10.1016/0264-410X(88)90174-0
Kovacs JM, 2012, P NATL ACAD SCI USA, V109, P12111, DOI 10.1073/pnas.1204533109
Krishnamachari Y, 2011, PHARM RES-DORDR, V28, P215, DOI 10.1007/s11095-010-0241-4
Kroemer G, 2013, ONCOIMMUNOLOGY, V2, DOI 10.4161/onci.23687
Kroll AV, 2019, ADV BIOSYST, V3, DOI 10.1002/adbi.201800219
Kroll AV, 2017, ADV MATER, V29, DOI 10.1002/adma.201703969
Kuehn MJ, 2005, GENE DEV, V19, P2645, DOI 10.1101/gad.1299905
Kunjachan S, 2015, CHEM REV, V115, P10907, DOI 10.1021/cr500314d
Larche M, 2005, NAT MED, V11, pS69, DOI 10.1038/nm1226
Leach A, 2011, MALARIA J, V10, DOI 10.1186/1475-2875-10-224
Lee HB, 2018, TISSUE ENG REGEN MED, V15, P1, DOI 10.1007/s13770-017-0082-x
Lee LA, 2006, NANOMED-NANOTECHNOL, V2, P137, DOI 10.1016/j.nano.2006.07.009
Li LL, 2014, ACS NANO, V8, P4975, DOI 10.1021/nn501040h
Li S, 1998, GENE THER, V5, P930, DOI 10.1038/sj.gt.3300683
Li S, 1997, GENE THER, V4, P891, DOI 10.1038/sj.gt.3300482
Lim W, 2019, TISSUE ENG REGEN MED, V16, P213, DOI 10.1007/s13770-019-00190-2
Lin LCW, 2019, ADV FUNCT MATER, V29, DOI 10.1002/adfm.201807616
Lizotte PH, 2016, NAT NANOTECHNOL, V11, P295, DOI [10.1038/NNANO.2015.292, 10.1038/nnano.2015.292]
Lou J, 2019, ADV THER-GERMANY, V2, DOI 10.1002/adtp.201800128
Mamo T, 2012, VACCINE, V30, P6609, DOI 10.1016/j.vaccine.2012.08.023
Mamo T, 2010, NANOMEDICINE-UK, V5, P269, DOI [10.2217/nnm.10.1, 10.2217/NNM.10.1]
Marques J, 2017, NANOMED-NANOTECHNOL, V13, P515, DOI 10.1016/j.nano.2016.09.010
Maurer P, 2005, EUR J IMMUNOL, V35, P2031, DOI 10.1002/eji.200526285
Moon JJ, 2012, PLOS ONE, V7, DOI 10.1371/journal.pone.0031472
Moser C, 2011, EXPERT REV VACCINES, V10, P437, DOI [10.1586/erv.11.15, 10.1586/ERV.11.15]
Nayak D, 2017, MAT SCI ENG C-MATER, V73, P406, DOI 10.1016/j.msec.2016.12.073
Noad R, 2003, TRENDS MICROBIOL, V11, P438, DOI 10.1016/S0966-842X(03)00208-7
Oberg AL, 2011, CURR OPIN IMMUNOL, V23, P436, DOI 10.1016/j.coi.2011.04.005
Opie EL, 1937, J EXP MED, V66, P761, DOI 10.1084/jem.66.6.761
Park Wooram, 2018, Biomater Res, V22, P24, DOI 10.1186/s40824-018-0133-y
Patel JM, 2015, BIOTECHNOL BIOENG, V112, P1102, DOI 10.1002/bit.25537
Peer D, 2007, NAT NANOTECHNOL, V2, P751, DOI 10.1038/nnano.2007.387
PEETERS PAM, 1989, PARASITOLOGY, V98, P381, DOI 10.1017/S003118200006145X
Pejawar-Gaddy S, 2014, BIOCONJUGATE CHEM, V25, P1470, DOI 10.1021/bc5002246
Petrovsky N, 2015, DRUG SAFETY, V38, P1059, DOI 10.1007/s40264-015-0350-4
Pillarisetti S, 2019, TISSUE ENG REGEN MED, V16, P451, DOI 10.1007/s13770-019-00218-7
Pitchaimani A, 2019, ADV FUNCT MATER, V29, DOI 10.1002/adfm.201806817
Plotkin S, 2014, P NATL ACAD SCI USA, V111, P12283, DOI 10.1073/pnas.1400472111
Poteet E, 2016, VACCINE, V34, P5886, DOI 10.1016/j.vaccine.2016.10.036
Le QV, 2019, ACS NANO, V13, P7442, DOI 10.1021/acsnano.9b02071
Rahman K, 2019, INT J NANOMED, V14, P1401, DOI 10.2147/IJN.S190692
Rajendrakumar SK, 2018, POLYMERS-BASEL, V10, DOI 10.3390/polym10101063
Rao M, 2013, TISSUE ENG REGEN MED, V10, P223, DOI 10.1007/s13770-013-1081-1
Riitho V, 2017, SCI REP-UK, V7, DOI 10.1038/s41598-017-13915-x
Roldao A, 2010, EXPERT REV VACCINES, V9, P1149, DOI [10.1586/erv.10.115, 10.1586/ERV.10.115]
Rosenblum MD, 2015, J CLIN INVEST, V125, P2228, DOI 10.1172/JCI78088
Sahu KK, 2016, INT IMMUNOPHARMACOL, V39, P343, DOI 10.1016/j.intimp.2016.08.007
Schuchat A., 2011, MMWR-MORBID MORTAL W, V63, P352
Sercombe L, 2015, FRONT PHARMACOL, V6, DOI 10.3389/fphar.2015.00286
Seth L, 2017, VACCINE, V35, P5448, DOI 10.1016/j.vaccine.2017.02.040
Shariat S, 2014, CANCER LETT, V355, P54, DOI 10.1016/j.canlet.2014.09.016
Sharma S, 2009, J PHARM SCI-US, V98, P812, DOI 10.1002/jps.21493

Shimizu T, 2018, J IMMUNOL, V201, P2969, DOI 10.4049/jimmunol.1701351
Siefert AL, 2016, BIOMATERIALS, V97, P85, DOI 10.1016/j.biomaterials.2016.03.039
Singh RK, 2013, TISSUE ENG REGEN MED, V10, P296, DOI 10.1007/s13770-013-1092-y
Smith LR, 2010, VACCINE, V28, P2565, DOI 10.1016/j.vaccine.2010.01.029
Stahl AL, 2019, PEDIATR NEPHROL, V34, P11, DOI 10.1007/s00467-017-3816-z
Sun BB, 2016, J MATER CHEM B, V4, P5496, DOI [10.1039/C6TB01131D, 10.1039/c6tb01131d]
Talesh GA, 2016, IMMUNOL LETT, V176, P57, DOI 10.1016/j.imlet.2016.05.016
Teng ZD, 2018, VACCINE, V36, P6752, DOI 10.1016/j.vaccine.2018.09.030
Thery C, 2009, NAT REV IMMUNOL, V9, P581, DOI 10.1038/nri2567
Tyagi RK, 2016, HUM VACC IMMUNOTHER, V12, P990, DOI 10.1080/21645515.2015.1116656
Urban P, 2011, J CONTROL RELEASE, V151, P202, DOI 10.1016/j.jconrel.2011.01.001
Vijayan V, 2018, POLYMERS-BASEL, V10, DOI 10.3390/polym10090983
Wahome N, 2012, CHEM BIOL DRUG DES, V80, P349, DOI 10.1111/j.1747-0285.2012.01423.x
Wang C, 2012, VACCINE, V30, P4790, DOI 10.1016/j.vaccine.2012.05.027
Wang C, 2019, ADV THER-GERMANY, V2, DOI 10.1002/adtp.201900003
Wang DD, 2018, ACS NANO, V12, P5241, DOI 10.1021/acsnano.7b08355
Wang F, 2016, ADV FUNCT MATER, V26, P1628, DOI 10.1002/adfm.201505231
Wang LF, 2015, J INTERN MED, V278, P369, DOI 10.1111/joim.12395
Wang LL, 2017, INT J NANOMED, V12, P1227, DOI 10.2147/IJN.S121956
Wei XL, 2019, NANO LETT, V19, P4760, DOI 10.1021/acs.nanolett.9b01844
Wei XL, 2018, ADV MATER, V30, DOI 10.1002/adma.201802233
Wraith DC, 2009, IMMUNOL LETT, V122, P134, DOI 10.1016/j.imlet.2008.11.013
Wu CY, 2012, PLOS ONE, V7, DOI 10.1371/journal.pone.0042363
Xia Q, 2019, ACTA PHARM SIN B, V9, P675, DOI 10.1016/j.apsb.2019.01.011
Xuan MJ, 2015, ADV HEALTHC MATER, V4, P1645, DOI 10.1002/adhm.201500129
Yahyapour R, 2018, TISSUE ENG REGEN MED, V15, P249, DOI 10.1007/s13770-017-0112-8
Yang CG, 2019, TISSUE ENG REGEN MED, V16, P433, DOI 10.1007/s13770-019-00219-6
Yang R, 2018, ACS NANO, V12, P5121, DOI 10.1021/acsnano.7b09041
YATVIN MB, 1978, SCIENCE, V202, P1290, DOI 10.1126/science.364652
Yu WQ, 2019, BIOMATERIALS, V217, DOI 10.1016/j.biomaterials.2019.119309
Zhang LF, 2000, VACCINE, V18, P1051, DOI 10.1016/S0264-410X(99)00351-5
Zhang WC, 2018, FRONT IMMUNOL, V9, DOI 10.3389/fimmu.2018.00090
Zhang XQ, 2007, J IMMUNOTHER, V30, P469, DOI 10.1097/CJI.0b013e31802fd8c6
Zhou BY, 2019, NANOSCALE, V11, P7996, DOI 10.1039/c9nr01434a
Zhu JY, 2016, NANO LETT, V16, P5895, DOI 10.1021/acs.nanolett.6b02786
NR 158
TC 57
Z9 59
U1 3
U2 81
PU MDPI
PI BASEL
PA ST ALBAN-ANLAGE 66, CH-4052 BASEL, SWITZERLAND
EI 1999-4923
J9 PHARMACEUTICS
JI Pharmaceutics
PD OCT
PY 2019
VL 11
IS 10
AR 534
DI 10.3390/pharmaceutics11100534
PG 27
WC Pharmacology & Pharmacy
WE Science Citation Index Expanded (SCI-EXPANDED)
SC Pharmacology & Pharmacy
GA JP6SE
UT WOS:000498392300047
PM 31615112
OA Green Published, gold
DA 2023-09-14

ER

PT J
AU Jin, HL
Qian, Y
Dai, YF
Qiao, S
Huang, C
Lu, LS
Luo, QM
Chen, J
Zhang, ZH
AF Jin, Honglin
Qian, Yuan
Dai, Yanfeng
Qiao, Sha
Huang, Chuan
Lu, Lisen
Luo, Qingming
Chen, Jing
Zhang, Zhihong
TI Magnetic Enrichment of Dendritic Cell Vaccine in Lymph Node with Fluorescent-Magnetic Nanoparticles Enhanced Cancer Immunotherapy
SO THERANOSTICS
LA English
DT Article
DE DC vaccine; immunotherapy; antigen delivery; DC migration tracking; magnetic targeting
ID IN-VIVO; MIGRATION; TRACKING; THERANOSTICS; NANOVACCINE; RESONANCE; ANTIGENS; TARGET; TUMORS; PROBE
AB Dendritic cell (DC) migration to the lymph node is a key component of DC-based immunotherapy. However, the DC homing rate to the lymphoid tissues is poor, thus hindering the DC-mediated activation of antigen-specific T cells. Here, we developed a system using fluorescent magnetic nanoparticles (alpha-AP-fmNPs; loaded with antigen peptide, iron oxide nanoparticles, and indocyanine green) in combination with magnetic pull force (MPF) to successfully manipulate DC migration in vitro and in vivo. alpha-AP-fmNPs endowed DCs with MPF-responsiveness, antigen presentation, and simultaneous optical and magnetic resonance imaging detectability. We showed for the first time that alpha-AP-fmNP-loaded DCs were sensitive to MPF, and their migration efficiency could be dramatically improved both in vitro and in vivo through MPF treatment. Due to the enhanced migration of DCs, MPF treatment significantly augmented antitumor efficacy of the nanoparticle-loaded DCs. Therefore, we have developed a biocompatible approach with which to improve the homing efficiency of DCs and subsequent anti-tumor efficacy, and track their migration by multi-modality imaging, with great potential applications for DC-based cancer immunotherapy.
C1 [Jin, Honglin; Qian, Yuan; Dai, Yanfeng; Qiao, Sha; Huang, Chuan; Lu, Lisen; Luo, Qingming; Zhang, Zhihong] Huazhong Univ Sci & Technol, Britton Chance Ctr Biomed Photon, Wuhan Natl Lab Optoelect, Wuhan 430074, Peoples R China.
[Jin, Honglin; Qian, Yuan; Dai, Yanfeng; Qiao, Sha; Huang, Chuan; Lu, Lisen; Luo, Qingming; Zhang, Zhihong] Huazhong Univ Sci & Technol, Dept Biomed Engn, MoE Key Lab Biomed Photon, Wuhan 430074, Peoples R China.
[Chen, Jing] Huazhong Univ Sci & Technol, Union Hosp, Tongji Med Coll, Ctr Canc, 1277 JieFang Ave, Wuhan 430022, Peoples R China.
C3 Huazhong University of Science & Technology; Huazhong University of Science & Technology; Huazhong University of Science & Technology
RP Zhang, ZH (通讯作者)，Huazhong Univ Sci & Technol, Britton Chance Ctr Biomed Photon, Wuhan Natl Lab Optoelect, Wuhan 430074, Peoples R China.; Zhang, ZH (通讯作者)，Huazhong Univ Sci & Technol, Dept Biomed Engn, MoE Key Lab Biomed Photon, Wuhan 430074, Peoples R China.
EM czyzzh@mail.hust.edu.cn
RI Qian, Yuan/I-6922-2017
OI Qian, Yuan/0000-0002-3967-7727; Qiao, Sha/0000-0002-1400-9802; Luo, Qingming/0000-0002-6725-9311; Zhang, Zhihong/0000-0001-5227-8926; Jin, Honglin/0000-0002-4398-9539

FU National Natural Science Foundation of China [91442201]; China
Postdoctoral Science Foundation [2013T60721]; State Key Laboratory of
Bioelectronics of Southeast University; Fundamental Research Funds for
the Central Universities [HUST: 2015ZDTD014]; WNLO
FX We thank Dr. Gang Zheng (University of Toronto, Toronto, ON, Canada) for
paper discussion and Dr. Yu Zhang lab (Southeast University) for
providing oleic-acid stabilized iron oxide nanocrystals. We thank the
Optical Bioimaging Core Facility of WNLO-HUST and the Center for
Nanoscale Characterization & Devices (CNCD, Tecnai G20 U-Twin) of
WNLO-HUST for the support in data acquisition, and the Analytical and
Testing Center of HUST for spectral measurements. We also thank the
Wuhan Center for Magnetic Resonance, State Key Laboratory of Magnetic
Resonance and Atomic and Molecular Physics, Wuhan Institute of Physics
and Mathematics, Chinese Academy of Sciences for the assistance of MRI
studies. This study was supported by the Major Research plan of the
National Natural Science Foundation of China (Grant No. 91442201), the
China Postdoctoral Science Foundation funded project (Grant No.
2013T60721), the Open Research Fund of State Key Laboratory of
Bioelectronics of Southeast University, the Fundamental Research Funds
for the Central Universities (HUST: 2015ZDTD014), and the Director Fund
of WNLO.
CR Aarntzen EHJG, 2013, ONCOIMMUNOLOGY, V2, DOI 10.4161/onci.24661
Akasaka K, 2005, J VASC SURG, V41, P517, DOI 10.1016/j.jvs.2004.12.043
Bai J, 2016, THERANOSTICS, V6, P342, DOI 10.7150/thno.11918
Baumjohann D, 2006, IMMUNOBIOLOGY, V211, P587, DOI 10.1016/j.imbio.2006.05.011
Chen YC, 2014, CYTOTHERAPY, V16, P699, DOI 10.1016/j.jcyt.2013.09.006
Cho NH, 2011, NAT NANOTECHNOL, V6, P675, DOI [10.1038/NNANO.2011.149,
10.1038/nnano.2011.149]
Chou LYT, 2011, CHEM SOC REV, V40, P233, DOI 10.1039/c0cs00003e
Cormode DP, 2009, ARTERIOSCL THROM VAS, V29, P992, DOI 10.1161/ATVBAHA.108.165506
Cruz LJ, 2011, MOL PHARMACEUT, V8, P520, DOI 10.1021/mp100356k
Desmettre T, 2000, SURV OPHTHALMOL, V45, P15, DOI 10.1016/S0039-6257(00)00123-5
Foy SP, 2010, ACS NANO, V4, P5217, DOI 10.1021/nn101427t
Janib SM, 2010, ADV DRUG DELIVER REV, V62, P1052, DOI 10.1016/j.addr.2010.08.004
Jewell CM, 2011, P NATL ACAD SCI USA, V108, P15745, DOI 10.1073/pnas.1105200108
Kastenmuller W, 2014, NAT REV IMMUNOL, V14, P705, DOI 10.1038/nri3727
Lim YT, 2008, SMALL, V4, P1640, DOI 10.1002/smll.200800582
Liu HP, 2014, NATURE, V507, P519, DOI 10.1038/nature12978
Luciani A, 2009, EUR RADIOL, V19, P1087, DOI 10.1007/s00330-008-1262-9
Mackay PS, 2011, NANOMED-NANOTECHNOL, V7, P489, DOI 10.1016/j.nano.2010.12.004
Martin-Fontecha A, 2003, J EXP MED, V198, P615, DOI 10.1084/jem.20030448
Muthana M, 2015, NAT COMMUN, V6, DOI 10.1038/ncomms9009
Noh YW, 2011, BIOMATERIALS, V32, P6254, DOI 10.1016/j.biomaterials.2011.05.013
Okada N, 2005, GENE THER, V12, P129, DOI 10.1038/sj.gt.3302358
Pinto AC, 2011, PROSTATE, V71, P81, DOI 10.1002/pros.21224
Qian Y, 2016, BIOMATERIALS, V98, P171, DOI 10.1016/j.biomaterials.2016.05.008
Randolph GJ, 2005, NAT REV IMMUNOL, V5, P617, DOI 10.1038/nri1670
Reddy ST, 2007, NAT BIOTECHNOL, V25, P1159, DOI [10.1038/nbt1332, 10.1038/nbtl332]
Ridolfi Ruggero, 2004, J Transl Med, V2, P27
Sabado RL, 2015, NATURE, V519, P300, DOI 10.1038/nature14211
Sarradin PM, 2005, TALANTA, V66, P1131, DOI 10.1016/j.talanta.2005.01.012
Schreiber HA, 2010, J IMMUNOL METHODS, V356, P47, DOI 10.1016/j.jim.2010.02.009
Shao S, 2015, NAT CHEM, V7, P438, DOI [10.1038/nchem.2236, 10.1038/NCHEM.2236]
Skajaa T, 2011, BIOMATERIALS, V32, P206, DOI 10.1016/j.biomaterials.2010.08.078
Suzuki H, 2013, J TRANSL MED, V11, DOI 10.1186/1479-5876-11-97
Tacken PJ, 2007, NAT REV IMMUNOL, V7, P790, DOI 10.1038/nri2173
van den Ancker W, 2010, IMMUNOTHERAPY-UK, V2, P69, DOI 10.2217/IMT.09.85
Verdijk P, 2008, EXPERT OPIN BIOL TH, V8, P865, DOI [10.1517/14712598.8.7.865,
10.1517/14712598.8.7.865 ]
Xie J, 2012, THERANOSTICS, V2, P122, DOI 10.7150/thno.4051
Xie J, 2014, BIOMATERIALS, V35, P9126, DOI 10.1016/j.biomaterials.2014.07.019

Xu Hongmei, 2011, Front Med, V5, P323, DOI 10.1007/s11684-011-0172-4
Zhang PF, 2015, P NATL ACAD SCI USA, V112, pE6129, DOI 10.1073/pnas.1505799112
Zhang ZH, 2010, SMALL, V6, P430, DOI 10.1002/smll.200901515
Zheng Y, 2013, THERANOSTICS, V3, P477, DOI 10.7150/thno.6617
Zhou FF, 2016, J INNOV OPT HEAL SCI, V9, DOI 10.1142/S1793545816300019
NR 43
TC 57
Z9 60
U1 3
U2 85
PU IVYSPRING INT PUBL
PI LAKE HAVEN
PA PO BOX 4546, LAKE HAVEN, NSW 2263, AUSTRALIA
SN 1838-7640
J9 THERANOSTICS
JI Theranostics
PY 2016
VL 6
IS 11
BP 2000
EP 2014
DI 10.7150/thno.15102
PG 15
WC Medicine, Research & Experimental
WE Science Citation Index Expanded (SCI-EXPANDED)
SC Research & Experimental Medicine
GA DX1EN
UT WOS:000384108900019
PM 27698936
OA Green Published, gold, Green Submitted
DA 2023-09-14
ER

PT J
AU Lin, AY
Lunsford, J
Bear, AS
Young, JK
Eckels, P
Luo, L
Foster, AE
Drezek, RA
AF Lin, Adam Yuh
Lunsford, Jessica
Bear, Adham Sean
Young, Joseph Keith
Eckels, Phillip
Luo, Laureen
Foster, Aaron Edward
Drezek, Rebekah Anna
TI High-density sub-100-nm peptide-gold nanoparticle complexes improve vaccine presentation by dendritic cells in vitro
SO NANOSCALE RESEARCH LETTERS
LA English
DT Article
DE Vaccines; Gold nanoparticles; ELISPOTs; Immunotherapy; Dendritic cells; Self-assembled monolayer
ID CANCER VACCINES; T-CELLS; TUMOR-REGRESSION; TANDEM REPEATS; VIVO; LYMPHOCYTES; ACTIVATION; INDUCTION; DELIVERY; PLATFORM
AB Nanocarriers have been explored to improve the delivery of tumor antigens to dendritic cells (DCs).
Gold nanoparticles are attractive nanocarriers because they are inert, non-toxic, and can be readily

endocytosed by DCs. Here, we designed novel gold-based nanovaccines (AuNVs) using a simple self-assembling bottom-up conjugation method to generate high-peptide density delivery and effective immune responses with limited toxicity. AuNVs were synthesized using a self-assembling conjugation method and optimized using DC-to-splenocyte interferon-gamma enzyme-linked immunosorbent spot assays. The AuNV design has shown successful peptide conjugation with approximately 90% yield while remaining smaller than 80 nm in diameter. DCs uptake AuNVs with minimal toxicity and are able to process the vaccine peptides on the particles to stimulate cytotoxic T lymphocytes (CTLs). These high-peptide density AuNVs can stimulate CTLs better than free peptides and have great potential as carriers for various vaccine types.
C1 [Lin, Adam Yuh; Luo, Laureen; Drezek, Rebekah Anna] Rice Univ, Dept Bioengn, Houston, TX 77005 USA.
[Lunsford, Jessica; Bear, Adham Sean; Eckels, Phillip; Foster, Aaron Edward] Baylor Coll Med, Ctr Cell & Gene Therapy, Houston, TX 77030 USA.
[Young, Joseph Keith; Drezek, Rebekah Anna] Rice Univ, Dept Elect & Comp Engn, Houston, TX 77005 USA.
C3 Rice University; Baylor College of Medicine; Rice University
RP Drezek, RA (通讯作者)，Rice Univ, Dept Bioengn, Houston, TX 77005 USA.
EM drezek@rice.edu
OI Lin, Adam/0000-0001-9359-882X
FU Cell and Gene Therapy Center; Cancer Prevention Research Institute of Texas (CPRIT); Department of Defense Congressionally Directed Medical Research Program [USAMRAA W81XWH-07-1-0428]; Ruth L. Kirschstein National Research Service Awards [F30CA165686]; Medical Scientist Training Program at Baylor College of Medicine
FX We thank A. Chen for his help with the hyperspectral data acquisition and editing. We thank J. Mattos and L. Balaoing for their help in editing the manuscript. We thank the American Journal Experts for their professional editing. We also gratefully acknowledge the Cell and Gene Therapy Center, the Cancer Prevention Research Institute of Texas (CPRIT), the Department of Defense Congressionally Directed Medical Research Program (USAMRAA W81XWH-07-1-0428), the Ruth L. Kirschstein National Research Service Awards for Individual Predoctoral MD/PhD Fellows (F30CA165686), and the Medical Scientist Training Program at Baylor College of Medicine for training support or funding.
CR Abbas AK., 2006, BASIC IMMUNOLOGY FUN
Arnaiz B, 2012, BIOCONJUGATE CHEM, V23, P814, DOI 10.1021/bc200663r
Bastus NG, 2009, MOL IMMUNOL, V46, P743, DOI 10.1016/j.molimm.2008.08.277
Bergen JM, 2006, MACROMOL BIOSCI, V6, P506, DOI 10.1002/mabi.200600075
Demers LM, 2000, ANAL CHEM, V72, P5535, DOI 10.1021/ac0006627
GOOD NE, 1966, BIOCHEMISTRY-US, V5, P467, DOI 10.1021/bi00866a011
GRABAREK Z, 1990, ANAL BIOCHEM, V185, P131, DOI 10.1016/0003-2697(90)90267-D
Hermanson GT, 2008, BIOCONJUGATE TECHNIQUES, 2ND EDITION, P1, DOI 10.1016/B978-0-12-370501-3.00001-1
KAWAKAMI Y, 1995, J IMMUNOL, V154, P3961
Kennedy LC, 2011, NANOSCALE RES LETT, V6, DOI 10.1186/1556-276X-6-283
Kim HD, 2005, BIOCHEM BIOPH RES CO, V336, P84, DOI 10.1016/j.bbrc.2005.08.044
Krishnamachari Y, 2011, PHARM RES-DORDR, V28, P215, DOI 10.1007/s11095-010-0241-4
Mansour M, 2007, J TRANSL MED, V5, DOI 10.1186/1479-5876-5-20
Moon JJ, 2011, NAT MATER, V10, P243, DOI 10.1038/NMAT2960
Ojeda R, 2007, CARBOHYD RES, V342, P448, DOI 10.1016/j.carres.2006.11.018
Overwijk WW, 2003, J EXP MED, V198, P569, DOI 10.1084/jem.20030590
Overwijk WW, 1998, J EXP MED, V188, P277, DOI 10.1084/jem.188.2.277
Palucka K, 2011, J IMMUNOL, V186, P1325, DOI 10.4049/jimmunol.0902539
Pejawar-Gaddy S, 2008, CRIT REV ONCOL HEMAT, V67, P93, DOI 10.1016/j.critrevonc.2008.02.010
Qian XM, 2008, NAT BIOTECHNOL, V26, P83, DOI 10.1038/nbt1377
Reddy ST, 2007, NAT BIOTECHNOL, V25, P1159, DOI [10.1038/nbt1332, 10.1038/nbtl332]
Reddy ST, 2006, J CONTROL RELEASE, V112, P26, DOI 10.1016/j.jconrel.2006.01.006
Rosenberg SA, 2004, NAT MED, V10, P909, DOI 10.1038/nm1100
Saleem IY, 2005, J CONTROL RELEASE, V102, P551, DOI 10.1016/j.jconrel.2004.10.034
Schlom J, 2007, CLIN CANCER RES, V13, P3776, DOI 10.1158/1078-0432.CCR-07-0588
Schmittel A, 1997, J IMMUNOL METHODS, V210, P167, DOI 10.1016/S0022-1759(97)00184-1

Schwartzentruber D, 2009, J CLIN ONCOL, V27, P209
Slingluff CL, 2001, CLIN CANCER RES, V7, P3012
Villiers CL, 2010, J NANOPART RES, V12, P55, DOI 10.1007/s11051-009-9692-0
Yankai Z, 2006, BIOCHEM BIOPH RES CO, V345, P1365, DOI 10.1016/j.bbrc.2006.05.022
Zhang GD, 2009, BIOMATERIALS, V30, P1928, DOI 10.1016/j.biomaterials.2008.12.038
NR 31
TC 57
Z9 62
U1 0
U2 66
PU SPRINGER
PI NEW YORK
PA 233 SPRING ST, NEW YORK, NY 10013 USA
SN 1556-276X
J9 NANOSCALE RES LETT
JI Nanoscale Res. Lett.
PD FEB 12
PY 2013
VL 8
AR 72
DI 10.1186/1556-276X-8-72
PG 11
WC Nanoscience & Nanotechnology; Materials Science, Multidisciplinary;
Physics, Applied
WE Science Citation Index Expanded (SCI-EXPANDED)
SC Science & Technology - Other Topics; Materials Science; Physics
GA 097QZ
UT WOS:000315489900001
PM 23402570
OA Green Published, gold
DA 2023-09-14
ER

PT J
AU Chen, FM
Wang, YJ
Gao, J
Saeed, M
Li, TL
Wang, WQ
Yu, HJ
AF Chen, Fangmin
Wang, Yingjie
Gao, Jing
Saeed, Madiha
Li, Tianliang
Wang, Weiqi
Yu, Haijun
TI Nanobiomaterial-based vaccination immunotherapy of cancer
SO BIOMATERIALS
LA English
DT Review
DE Cancer immunotherapy; Nanovaccine; Lymph node-targeting; Biomimetic
nanobiomaterials; Antitumor immune response
ID OUTER-MEMBRANE VESICLES; ANTIGEN-PRESENTING CELLS; VIRUS-LIKE
NANOPARTICLES; MESSENGER-RNA; NEOANTIGEN VACCINE; DNA VACCINES; IN-VIVO;
T-CELLS; POLYMERIC NANOPARTICLES; COATED NANOPARTICLES
AB Cancer immunotherapies including cancer vaccines, immune checkpoint blockade or chimeric antigen receptor T cells have been exploited as the attractive treatment modalities in recent years. Among these approaches, cancer vaccines that designed to deliver tumor antigens and adjuvants to activate the antigen presenting cells (APCs) and induce antitumor immune responses, have shown significant efficacy

in inhibiting tumor growth, preventing tumor relapse and metastasis. Despite the potential of cancer vaccination strategies, the therapeutic outcomes in preclinical trials are failed to promote their clinical translation, which is in part due to their inefficient vaccination cascade of five critical steps: antigen identification, antigen encapsulation, antigen delivery, antigen release and antigen presentation to T cells. In recent years, it has been demonstrated that various nanobiomaterials hold great potential to enhance cancer vaccination cascade and improve their antitumor performance and reduce the off-target effect. We summarize the cutting-edge advances of nanobiomaterials-based vaccination immunotherapy of cancer in this review. The various cancer nanovaccines including antigen peptide/adjuvant-based nanovaccines, nucleic acid-based nanovaccines as well as biomimetic nanobiomaterials-based nanovaccines are discussed in detail. We also provide some challenges and perspectives associated with the clinical translation of cancer nanovaccines.
C1 [Chen, Fangmin; Wang, Yingjie; Gao, Jing; Saeed, Madiha; Li, Tianliang; Wang, Weiqi; Yu, Haijun] Chinese Acad Sci, Shanghai Inst Mat Med, State Key Lab Drug Res, Shanghai 201203, Peoples R China.
[Chen, Fangmin; Wang, Yingjie; Gao, Jing; Saeed, Madiha; Li, Tianliang; Wang, Weiqi; Yu, Haijun] Chinese Acad Sci, Shanghai Inst Mat Med, Ctr Pharmaceut, Shanghai 201203, Peoples R China.
[Wang, Yingjie] Univ Sci & Technol China, Nano Sci & Technol Inst, Suzhou 215123, Peoples R China.
[Yu, Haijun] Univ Chinese Acad Sci, Beijing 100049, Peoples R China.
C3 Chinese Academy of Sciences; Shanghai Institute of Materia Medica, CAS;
Chinese Academy of Sciences; Shanghai Institute of Materia Medica, CAS;
Chinese Academy of Sciences; University of Science & Technology of
China, CAS; Chinese Academy of Sciences; University of Chinese Academy
of Sciences, CAS
RP Yu, HJ (通讯作者)，Chinese Acad Sci, Shanghai Inst Mat Med, State Key Lab Drug Res, Shanghai 201203, Peoples R China.; Yu, HJ (通讯作者)，Chinese Acad Sci, Shanghai Inst Mat Med, Ctr Pharmaceut, Shanghai 201203, Peoples R China.
EM hjyu@simm.ac.cn
RI li, yao/IYJ-1364-2023; jiang, lei/IWE-1124-2023; Yang,
Ying/ABD-2481-2022; Li, Ly/JCD-4746-2023; Jiang, Tao/IWM-7503-2023; wei,
xiao/ISB-6027-2023; SAEED, MADIHA/HTL-2346-2023
OI SAEED, MADIHA/0000-0003-2355-5167
FU National Natural Science Foundation of China [32050410287, 51873228];
International Cooperation Project of Science and Technology Commission
of Shanghai Municipality [20430711800]; Youth Innovation Promotion
Association of CAS [2014218]; CAS President's International Fellowship
Initiative Program [2019PB0023]; Postdoctoral Science Foundation of
China [2019M661667]; Shanghai Post-Doctoral Excellence Program [2019116]
FX F. Chen and Y. Wang contributed equally to this manuscript. This work
was supported by the National Natural Science Foundation of China
(32050410287, 51873228), International Cooperation Project of Science
and Technology Commission of Shanghai Municipality (20430711800), the
Youth Innovation Promotion Association of CAS (2014218), CAS President's
International Fellowship Initiative Program (2019PB0023), Postdoctoral
Science Foundation of China (Grant No. 2019M661667), and Shanghai
Post-Doctoral Excellence Program (Grant No. 2019116).
CR Abbaraju PL, 2018, CHEM COMMUN, V54, P2020, DOI 10.1039/c8cc00327k
Adachi K, 2018, NAT BIOTECHNOL, V36, P346, DOI 10.1038/nbt.4086
Aiga T, 2020, ANGEW CHEM INT EDIT, V59, P17705, DOI 10.1002/anie.202007999
Aldous AR, 2018, BIOORGAN MED CHEM, V26, P2842, DOI 10.1016/j.bmc.2017.10.021
Allen TM, 2013, ADV DRUG DELIVER REV, V65, P36, DOI 10.1016/j.addr.2012.09.037
An FF, 2017, THERANOSTICS, V7, P3667, DOI 10.7150/thno.19365
An M., 2017, SMALL, V13
Andre F, 2004, J IMMUNOL, V172, P2126, DOI 10.4049/jimmunol.172.4.2126
Auslander N, 2018, NAT MED, V24, P1545, DOI 10.1038/s41591-018-0157-9
Ball RL, 2018, NANO LETT, V18, P3814, DOI 10.1021/acs.nanolett.8b01101
Banchereau J, 2018, NAT REV CLIN ONCOL, V15, P9, DOI 10.1038/nrclinonc.2017.149
Batrakova EV, 2015, J CONTROL RELEASE, V219, P396, DOI 10.1016/j.jconrel.2015.07.030
Batty Cole J., 2018, Pharmaceutical Nanotechnology, V6, P232, DOI 10.2174/2211738506666180918122337
Belnoue E, 2019, JCI INSIGHT, V4, DOI 10.1172/jci.insight.127305
Benci JL, 2019, CELL, V178, P933, DOI 10.1016/j.cell.2019.07.019
Bender E, 2017, NATURE, V552, pS61

Berraondo P, 2019, BRIT J CANCER, V120, P6, DOI 10.1038/s41416-018-0328-y
Binnewies M, 2018, NAT MED, V24, P541, DOI 10.1038/s41591-018-0014-x
Bookstaver ML, 2018, TRENDS IMMUNOL, V39, P135, DOI 10.1016/j.it.2017.10.002
Buonsanti C, 2016, SCI REP-UK, V6, DOI 10.1038/srep29063
Burgdorf S, 2007, SCIENCE, V316, P612, DOI 10.1126/science.1137971
Butler MO, 2007, CLIN CANCER RES, V13, P1857, DOI 10.1158/1078-0432.CCR-06-1905
Chen Q, 2020, ADV MATER, V32, DOI 10.1002/adma.201908185
Chen Q, 2020, NANO LETT, V20, P11, DOI 10.1021/acs.nanolett.9b02182
Chen Q, 2019, CHEM SOC REV, V48, P5506, DOI 10.1039/c9cs00271e
Chen Q, 2016, NAT COMMUN, V7, DOI 10.1038/ncomms13193
Chen Z, 2016, ACS NANO, V10, P10049, DOI 10.1021/acsnano.6b04695
Cheng R, 2013, BIOMATERIALS, V34, P3647, DOI 10.1016/j.biomaterials.2013.01.084
Cheng SH, 2020, ADV SCI, V7, DOI 10.1002/advs.201903301
Cheung AS, 2015, NANO TODAY, V10, P511, DOI 10.1016/j.nantod.2015.06.007
Chin EN, 2020, SCIENCE, V369, P993, DOI 10.1126/science.abb4255
Chiu YC, 2015, ACS BIOMATER SCI ENG, V1, P1200, DOI 10.1021/acsbiomaterials.5b00375
Cong YL, 2020, NANOSCALE, V12, P7134, DOI 10.1039/c9nr08380d
Conniot J, 2019, NAT NANOTECHNOL, V14, P891, DOI 10.1038/s41565-019-0512-0
Dai ZW, 2017, SMALL, V13, DOI 10.1002/smll.201602881
Morais SMD, 2018, VACCINE, V36, P6408, DOI 10.1016/j.vaccine.2018.08.070
Deng CF, 2019, ADV SCI, V6, DOI 10.1002/advs.201801868
Ding B., 2020, NAT MATER
Ding BB, 2020, ANGEW CHEM INT EDIT, V59, P16381, DOI 10.1002/anie.202005111
Ding BB, 2018, ADV MATER, V30, DOI 10.1002/adma.201802479
Ding SY, 2016, NAT REV MATER, V1, DOI [10.1038/natrevmats.2016.71, 10.1038/natrevmats.2016.21]
Drutskaya MS, 2018, CYTOKINE, V101, P33, DOI 10.1016/j.cyto.2016.08.022
Du GS, 2020, CURR OPIN BIOTECH, V66, P113, DOI 10.1016/j.copbio.2020.06.015
Fan Q, 2020, SCI ADV, V6, DOI 10.1126/sciadv.abb4639
Fan YN, 2018, BIOMATER SCI-UK, V6, P3009, DOI 10.1039/c8bm00908b
Fang RH, 2014, NANO LETT, V14, P2181, DOI 10.1021/nl500618u
Farris E, 2017, J CONTROL RELEASE, V249, P150, DOI 10.1016/j.jconrel.2017.01.035
Feng B, 2019, ADV MATER, V31, DOI 10.1002/adma.201902960
Forner M, 2020, J ORG CHEM, V85, P1626, DOI 10.1021/acs.joc.9b02798
Frankiw L, 2019, NAT REV IMMUNOL, V19, P675, DOI 10.1038/s41577-019-0195-7
Fusciello M, 2019, NAT COMMUN, V10, DOI 10.1038/s41467-019-13744-8
Gajewski TF, 2013, NAT IMMUNOL, V14, P1014, DOI 10.1038/ni.2703
Gao A, 2020, NANO LETT, V20, P353, DOI 10.1021/acs.nanolett.9b04012
Gao A, 2019, ACTA PHARMACOL SIN, V40, P1129, DOI 10.1038/s41401-019-0281-1
Gnopo YMD, 2017, ADV DRUG DELIVER REV, V114, P132, DOI 10.1016/j.addr.2017.05.003
Gong NQ, 2020, NAT NANOTECHNOL, V15, P1053, DOI 10.1038/s41565-020-00782-3
Granot-Matok Y, 2019, J CONTROL RELEASE, V305, P165, DOI 10.1016/j.jconrel.2019.05.032
Grotzke JE, 2017, P NATL ACAD SCI USA, V114, pE5910, DOI 10.1073/pnas.1705242114
Gulla SK, 2019, BIOMATER SCI-UK, V7, P773, DOI 10.1039/c8bm01272e
Guo YG, 2018, FRONT IMMUNOL, V9, DOI 10.3389/fimmu.2018.01499
Guo ZS, 2019, J IMMUNOTHER CANCER, V7, DOI 10.1186/s40425-018-0495-7
Gurbatri CR, 2020, SCI TRANSL MED, V12, DOI 10.1126/scitranslmed.aax0876
Hailemichael Y, 2013, NAT MED, V19, P465, DOI 10.1038/nm.3105
Han X, 2019, SCI ADV, V5, DOI 10.1126/sciadv.aaw6870
He LZ, 2015, CELL MOL IMMUNOL, V12, P719, DOI 10.1038/cmi.2014.100
He LL, 2016, NAT COMMUN, V7, DOI 10.1038/ncomms12041
Hickey JW, 2020, NANO LETT, V20, P6289, DOI 10.1021/acs.nanolett.0c01511
Hickey JW, 2018, BIOMATERIALS, V187, P105, DOI 10.1016/j.biomaterials.2018.09.029
Hickey JW, 2017, NANO LETT, V17, P7045, DOI 10.1021/acs.nanolett.7b03734
Hu J, 2018, CHEM-ASIAN J, V13, P3448, DOI 10.1002/asia.201801088
Hu YY, 2020, BIOMATERIALS, V252, DOI 10.1016/j.biomaterials.2020.120114
Hu ZT, 2021, NAT MED, V27, P515, DOI 10.1038/s41591-020-01206-4
Huang H, 2020, NAT BIOTECHNOL, V38, P1194, DOI 10.1038/s41587-020-0505-4
Huang HY, 2019, NAT COMMUN, V10, DOI 10.1038/s41467-019-12794-2
Huang WW, 2020, J CONTROL RELEASE, V317, P1, DOI 10.1016/j.jconrel.2019.11.017
Huang Y, 2018, ADV EXP MED BIOL, V1056, P109, DOI 10.1007/978-3-319-74470-4_7
Hume HKC, 2017, VACCINE, V35, P4480, DOI 10.1016/j.vaccine.2017.02.069

Duong HTT, 2019, ACS APPL MATER INTER, V11, P13058, DOI 10.1021/acsami.8b21185
Duong HTT, 2018, BIOMATERIALS, V185, P13, DOI 10.1016/j.biomaterials.2018.09.008
Isapour G, 2018, ADV MATER, V30, DOI 10.1002/adma.201707069
Jiang H, 2017, J CONTROL RELEASE, V267, P47, DOI 10.1016/j.jconrel.2017.08.009
Jiang Y, 2020, ADV MATER, V32, DOI 10.1002/adma.202001808
Kaczmarek JC, 2016, ANGEW CHEM INT EDIT, V55, P13808, DOI 10.1002/anie.201608450
Kalluri R, 2020, SCIENCE, V367, P640, DOI 10.1126/science.aau6977
Kamada T, 2019, P NATL ACAD SCI USA, V116, P9999, DOI 10.1073/pnas.1822001116
Kamaly N, 2016, CHEM REV, V116, P2602, DOI 10.1021/acs.chemrev.5b00346
Kang T, 2018, BIOMATERIALS, V164, P80, DOI 10.1016/j.biomaterials.2018.02.033
Kauffman KJ, 2016, J CONTROL RELEASE, V240, P227, DOI 10.1016/j.jconrel.2015.12.032
Kightlinger W, 2019, NAT COMMUN, V10, DOI 10.1038/s41467-019-12024-9
Kim JV, 2004, NAT BIOTECHNOL, V22, P403, DOI 10.1038/nbt955
Kim OY, 2017, NAT COMMUN, V8, DOI 10.1038/s41467-017-00729-8
Knight FC, 2019, ACS NANO, V13, P10939, DOI 10.1021/acsnano.9b00326
Knutson KL, 2015, VACCINE, V33, P7376, DOI 10.1016/j.vaccine.2015.09.086
Kosmides AK, 2018, NANO LETT, V18, P1916, DOI 10.1021/acs.nanolett.7b05284
Kowalski PS, 2019, MOL THER, V27, P710, DOI 10.1016/j.ymthe.2019.02.012
Kranz LM, 2016, NATURE, V534, P396, DOI 10.1038/nature18300
Kroll AV, 2017, ADV MATER, V29, DOI 10.1002/adma.201703969
Krysko DV, 2012, NAT REV CANCER, V12, P860, DOI 10.1038/nrc3380
Kuai R, 2017, NAT MATER, V16, P489, DOI [10.1038/nmat4822, 10.1038/NMAT4822]
Kudrin A, 2012, HUM VACC IMMUNOTHER, V8, P1135, DOI 10.4161/hv.20474
Kwong GA, 2009, J AM CHEM SOC, V131, P9695, DOI 10.1021/ja9006707
Lai JP, 2015, ACS NANO, V9, P5234, DOI 10.1021/acsnano.5b00641
Landesman-Milo D, 2014, DRUG DELIV TRANSL RE, V4, P96, DOI 10.1007/s13346-013-0158-7
Lee TY, 2017, VACCINE, V35, P586, DOI 10.1016/j.vaccine.2016.12.025
Lerner MI, 2018, NANO LETT, V18, P5401, DOI 10.1021/acs.nanolett.8b01592
Li AW, 2018, NAT MATER, V17, P528, DOI 10.1038/s41563-018-0028-2
Li JC, 2021, ADV MATER, V33, DOI 10.1002/adma.202003458
Li JC, 2019, ANGEW CHEM INT EDIT, V58, P12680, DOI 10.1002/anie.201906288
Li M, 2020, J CONTROL RELEASE, V323, P253, DOI 10.1016/j.jconrel.2020.04.031
Li PY, 2018, BIOCONJUGATE CHEM, V29, P624, DOI 10.1021/acs.bioconjchem.7b00669
Li XY, 2017, ADV EXP MED BIOL, V1026, P315, DOI 10.1007/978-981-10-6020-5_15
Li X, 2020, NANO LETT, V20, P4454, DOI 10.1021/acs.nanolett.0c01230
Li Y, 2013, ADV HEALTHC MATER, V2, P388, DOI 10.1002/adhm.201200313
Liang C, 2019, ADV MATER, V31, DOI 10.1002/adma.201904197
Liang JJ, 2020, SCI ADV, V6, DOI 10.1126/sciadv.abc3646
Liao W, 2019, ACTA BIOMATER, V86, P1, DOI 10.1016/j.actbio.2018.12.045
Lin LCW, 2019, ADV FUNCT MATER, V29, DOI 10.1002/adfm.201807616
Lin LCW, 2018, ADV HEALTHC MATER, V7, DOI 10.1002/adhm.201701395
Lin MH, 2015, ANGEW CHEM INT EDIT, V54, P2151, DOI 10.1002/anie.201410720
Linares-Fernandez S, 2020, TRENDS MOL MED, V26, P311, DOI 10.1016/j.molmed.2019.10.002
Lindsay KE, 2019, NAT BIOMED ENG, V3, P371, DOI 10.1038/s41551-019-0378-3
Liu HP, 2014, NATURE, V507, P519, DOI 10.1038/nature12978
Liu MA, 2011, IMMUNOL REV, V239, P62, DOI 10.1111/j.1600-065X.2010.00980.x
Liu WL, 2019, NAT COMMUN, V10, DOI 10.1038/s41467-019-11157-1
Liu X, 2020, BIOMATERIALS, V230, DOI 10.1016/j.biomaterials.2019.119649
Lizotte PH, 2016, NAT NANOTECHNOL, V11, P295, DOI [10.1038/NNANO.2015.292, 10.1038/nnano.2015.292]
Lopes A, 2019, J EXP CLIN CANC RES, V38, DOI 10.1186/s13046-019-1154-7
Lou B, 2019, J CONTROL RELEASE, V293, P48, DOI 10.1016/j.jconrel.2018.11.006
Lu JQ, 2017, NAT COMMUN, V8, DOI 10.1038/s41467-017-01651-9
Lu Z, 2017, J HEPATOL, V67, P739, DOI 10.1016/j.jhep.2017.05.019
Luan X, 2017, ACTA PHARMACOL SIN, V38, P754, DOI 10.1038/aps.2017.12
Luk BT, 2015, J CONTROL RELEASE, V220, P600, DOI 10.1016/j.jconrel.2015.07.019
Luo M, 2017, NAT NANOTECHNOL, V12, P648, DOI [10.1038/nnano.2017.52, 10.1038/NNANO.2017.52]
Lv HT, 2006, J CONTROL RELEASE, V114, P100, DOI 10.1016/j.jconrel.2006.04.014
Lynn GM, 2020, NAT BIOTECHNOL, V38, P320, DOI 10.1038/s41587-019-0390-x
Lynn GM, 2015, NAT BIOTECHNOL, V33, P1201, DOI 10.1038/nbt.3371

Lyu Y, 2017, ADV SCI, V4, DOI 10.1002/advs.201600481
Ma L, 2019, SCIENCE, V365, P162, DOI 10.1126/science.aav8692
Majedi FS, 2019, NANO LETT, V19, P6945, DOI 10.1021/acs.nanolett.9b02252
Mascola JR, 2020, NAT REV IMMUNOL, V20, P87, DOI 10.1038/s41577-019-0243-3
Maus MV, 2002, NAT BIOTECHNOL, V20, P143, DOI 10.1038/nbt0202-143
McGranahan N, 2019, SCI TRANSL MED, V11, DOI 10.1126/scitranslmed.aax7918
McKinlay CJ, 2018, P NATL ACAD SCI USA, V115, pE5859, DOI 10.1073/pnas.1805358115
McLaughlin M, 2020, NAT REV CANCER, V20, P203, DOI 10.1038/s41568-020-0246-1
McNamara MA, 2015, J IMMUNOL RES, V2015, DOI 10.1155/2015/794528
Meyer RA, 2015, SMALL, V11, P1519, DOI 10.1002/smll.201402369
Miao L, 2019, NAT BIOTECHNOL, V37, P1174, DOI 10.1038/s41587-019-0247-3
Mikkilineni L, 2017, BLOOD, V130, P2594, DOI 10.1182/blood-2017-06-793869
Min YZ, 2017, NAT NANOTECHNOL, V12, P877, DOI [10.1038/nnano.2017.113, 10.1038/NNANO.2017.113]
Morris C, 2019, ADV FUNCT MATER, V29, DOI 10.1002/adfm.201807357
Moyer TJ, 2020, NAT MED, V26, P430, DOI 10.1038/s41591-020-0753-3
Mura S, 2013, NAT MATER, V12, P991, DOI [10.1038/NMAT3776, 10.1038/nmat3776]
Nakao R, 2016, VACCINE, V34, P4626, DOI 10.1016/j.vaccine.2016.06.016
Narain A, 2017, NANOMEDICINE-UK, V12, P2677, DOI 10.2217/nnm-2017-0225
Neelapu SS, 2018, NAT REV CLIN ONCOL, V15, P47, DOI 10.1038/nrclinonc.2017.148
Newick K, 2017, ANNU REV MED, V68, P139, DOI 10.1146/annurev-med-062315-120245
Newman JH, 2020, P NATL ACAD SCI USA, V117, P1119, DOI 10.1073/pnas.1904022116
O'Donnell JS, 2019, NAT REV CLIN ONCOL, V16, P151, DOI 10.1038/s41571-018-0142-8
Oberli MA, 2017, NANO LETT, V17, P1326, DOI 10.1021/acs.nanolett.6b03329
Ochyl LJ, 2018, BIOMATERIALS, V182, P157, DOI 10.1016/j.biomaterials.2018.08.016
Olden BR, 2019, ADV HEALTHC MATER, V8, DOI 10.1002/adhm.201801188
Oosten LEM, 2004, BLOOD, V104, P224, DOI 10.1182/blood-2003-07-2461
Ott PA, 2017, NATURE, V547, P217, DOI 10.1038/nature22991
Pan BS, 2020, SCIENCE, V369, P935, DOI 10.1126/science.aba6098
Pan C, 2020, ADV MATER, V32, DOI 10.1002/adma.202002940
Pardi N, 2018, NAT REV DRUG DISCOV, V17, P261, DOI 10.1038/nrd.2017.243
Pardi N, 2015, J CONTROL RELEASE, V217, P345, DOI 10.1016/j.jconrel.2015.08.007
Pardoll DM, 2012, NAT REV CANCER, V12, P252, DOI 10.1038/nrc3239
Parodi A, 2017, BIOMATERIALS, V147, P155, DOI 10.1016/j.biomaterials.2017.09.020
Patel RB, 2019, ADV MATER, V31, DOI 10.1002/adma.201902626
Patel S, 2019, J CONTROL RELEASE, V303, P91, DOI 10.1016/j.jconrel.2019.04.015
Patel S, 2017, NANO LETT, V17, P5711, DOI 10.1021/acs.nanolett.7b02664
Peng M, 2019, MOL CANCER, V18, DOI 10.1186/s12943-019-1055-6
Peng YB, 2017, ANGEW CHEM INT EDIT, V56, P10845, DOI 10.1002/anie.201701366
Perez CR, 2019, NAT COMMUN, V10, DOI 10.1038/s41467-019-13368-y
Phua KKL, 2014, NANOSCALE, V6, P7715, DOI 10.1039/c4nr01346h
Phuengkham H, 2019, ADV MATER, V31, DOI 10.1002/adma.201903242
Porter CJH, 2007, NAT REV DRUG DISCOV, V6, P231, DOI 10.1038/nrd2197
Qin L, 2020, SCI ADV, V6, DOI 10.1126/sciadv.abb3116
Qing S, 2020, ADV MATER, V32, DOI 10.1002/adma.202002085
Rajagopal P, 2018, J GENE MED, V20, DOI 10.1002/jgm.3011
Reichmuth AM, 2016, THER DELIV, V7, P319, DOI 10.4155/tde-2016-0006
Reinhard K, 2020, SCIENCE, V367, P446, DOI 10.1126/science.aay5967
Riley RS, 2019, NAT REV DRUG DISCOV, V18, P175, DOI 10.1038/s41573-018-0006-z
Rosenberg SA, 2014, J IMMUNOL, V192, P5451, DOI 10.4049/jimmunol.1490019
Saeed M, 2020, BIOCONJUGATE CHEM, V31, P404, DOI 10.1021/acs.bioconjchem.9b00851
Saeed M, 2019, THERANOSTICS, V9, P7981, DOI 10.7150/thno.37568
Sahin U, 2018, SCIENCE, V359, P1355, DOI 10.1126/science.aar7112
Sainsbury F, 2017, THER DELIV, V8, P1019, DOI 10.4155/tde-2017-0098
Saleh T, 2016, HUM VACC IMMUNOTHER, V12, P1863, DOI 10.1080/21645515.2016.1147635
Sayour EJ, 2018, NANO LETT, V18, P6195, DOI 10.1021/acs.nanolett.8b02179
Schlake T, 2012, RNA BIOL, V9, P1319, DOI 10.4161/rna.22269
Schudel A, 2020, NAT NANOTECHNOL, V15, P491, DOI 10.1038/s41565-020-0679-4
Schumacher TN, 2019, ANNU REV IMMUNOL, V37, P173, DOI 10.1146/annurev-immunol-042617-053402
Shae D, 2020, ACS NANO, V14, P9904, DOI 10.1021/acsnano.0c02765

Shao K, 2015, ACS NANO, V9, P16, DOI 10.1021/nn5062029
Shi Y, 2019, ACCOUNTS CHEM RES, V52, P1543, DOI 10.1021/acs.accounts.9b00148
Sun L., 2020, ANGEW CHEM INT ED EN
Sun QX, 2019, CHEM SOC REV, V48, P351, DOI 10.1039/c8cs00473k
Sun XQ, 2017, SMALL, V13, DOI 10.1002/smll.201701864
Tan YL, 2018, DRUG DISCOV TODAY, V23, P1108, DOI 10.1016/j.drudis.2018.01.051
Tang-Huau TL, 2018, NAT COMMUN, V9, DOI 10.1038/s41467-018-04985-0
Tardon MC, 2019, CURR OPIN PHARMACOL, V47, P20, DOI 10.1016/j.coph.2019.01.007
Nguyen TL, 2020, ACS NANO, V14, P11623, DOI 10.1021/acsnano.0c04188
Nguyen TL, 2020, BIOMATERIALS, V239, DOI 10.1016/j.biomaterials.2020.119859
Thomas S, 2016, METHODS MOL BIOL, V1403, P755, DOI 10.1007/978-1-4939-3387-7_43
Tian LL, 2017, THERANOSTICS, V7, P614, DOI 10.7150/thno.17381
Tohumeken S, 2017, ADV BIOSYST, V1, DOI 10.1002/adbi.201700015
Tzeng SY, 2020, P NATL ACAD SCI USA, V117, P4043, DOI 10.1073/pnas.1916039117
Van de Sande L, 2020, DRUG DELIV, V27, P40, DOI 10.1080/10717544.2019.1704945
Vauthier C, 2009, PHARM RES-DORDR, V26, P1025, DOI 10.1007/s11095-008-9800-3
Vazquez-Gonzalez M, 2020, ANGEW CHEM INT EDIT, V59, P15342, DOI 10.1002/anie.201907670
Veiga N, 2018, NAT COMMUN, V9, DOI 10.1038/s41467-018-06936-1
Wang DG, 2019, SCI IMMUNOL, V4, DOI 10.1126/sciimmunol.aau6584
Wang DD, 2020, ACS APPL MATER INTER, V12, P41138, DOI 10.1021/acsami.0c13169
Wang FH, 2020, NAT BIOMED ENG, V4, P1090, DOI 10.1038/s41551-020-0597-7
Wang HJ, 2020, BIOMATER SCI-UK, V8, P552, DOI 10.1039/c9bm01392j
Wang ML, 2019, ACS NANO, V13, P7010, DOI 10.1021/acsnano.9b02096
Wang RF, 2017, CELL RES, V27, P11, DOI 10.1038/cr.2016.155
Wang SJ, 2017, INT J NANOMED, V12, P6813, DOI 10.2147/IJN.S143264
Wang SH, 2019, WIRES NANOMED NANOBI, V11, DOI 10.1002/wnan.1523
Wang TT, 2018, NAT COMMUN, V9, DOI 10.1038/s41467-018-03915-4
Wang XH, 2019, CHEM REV, V119, P1138, DOI 10.1021/acs.chemrev.8b00209
Wang ZT, 2019, BIOMATER SCI-UK, V7, P1794, DOI 10.1039/c9bm00098d
Warrier VU, 2019, BIOSCIENCE REP, V39, DOI 10.1042/BSR20193220
Weichselbaum RR, 2017, NAT REV CLIN ONCOL, V14, P365, DOI 10.1038/nrclinonc.2016.211
Wilson DS, 2019, NAT MATER, V18, P175, DOI 10.1038/s41563-018-0256-5
Wong KK, 2016, ADV IMMUNOL, V130, P191, DOI 10.1016/bs.ai.2015.12.001
Wu P, 2018, J TRANSL MED, V16, DOI 10.1186/s12967-018-1402-1
Xi XB, 2018, J IMMUNOL RES, V2018, DOI 10.1155/2018/3714960
Xia YF, 2018, NAT MATER, V17, P187, DOI [10.1038/NMAT5057, 10.1038/nmat5057]
Xie YQ, 2018, WIRES NANOMED NANOBI, V10, DOI 10.1002/wnan.1506
Xu J, 2020, NAT NANOTECHNOL, V15, P1043, DOI 10.1038/s41565-020-00781-4
Xu J, 2019, BIOMATERIALS, V207, P1, DOI 10.1016/j.biomaterials.2019.03.037
Xu J, 2017, ACS NANO, V11, P4463, DOI 10.1021/acsnano.7b00715
Xu T, 2020, ACS NANO, V14, P3414, DOI 10.1021/acsnano.9b09426
Xu ZH, 2018, BIOCONJUGATE CHEM, V29, P572, DOI 10.1021/acs.bioconjchem.7b00478
Xu ZY, 2020, ADV SCI, V7, DOI 10.1002/advs.201902802
Xu-Monette ZY, 2018, BLOOD, V131, P68, DOI 10.1182/blood-2017-07-740993
Yamamoto TN, 2019, NAT MED, V25, P1488, DOI 10.1038/s41591-019-0596-y
Yang B, 2014, HUM VACC IMMUNOTHER, V10, P3153, DOI 10.4161/21645515.2014.980686
Yang R, 2018, ACS NANO, V12, P5121, DOI 10.1021/acsnano.7b09041
Yang YP, 2015, J CLIN INVEST, V125, P3335, DOI 10.1172/JCI83871
Zahm CD, 2017, PHARMACOL THERAPEUT, V174, P27, DOI 10.1016/j.pharmthera.2017.02.016
Zeng ZL, 2021, ADV MATER, V33, DOI 10.1002/adma.202007247
Zeng ZL, 2020, ADV FUNCT MATER, V30, DOI 10.1002/adfm.202004397
Zhai YJ, 2014, J CONTROL RELEASE, V193, P90, DOI 10.1016/j.jconrel.2014.05.054
Zhang C, 2020, CHEM SOC REV, V49, P4234, DOI 10.1039/c9cs00773c
Zhang F, 2019, ACS NANO, V13, P5662, DOI 10.1021/acsnano.9b00892
Zhang QB, 2017, ACS NANO, V11, P10724, DOI 10.1021/acsnano.7b04955
Zhang XD, 2018, ADV MATER, V30, DOI 10.1002/adma.201707112
Zhang Y, 2020, ADV MATER, V32, DOI 10.1002/adma.202002661
Zhang Y, 2021, ADV MATER, V33, DOI 10.1002/adma.202007293
Zhang Y, 2019, WIRES NANOMED NANOBI, V11, DOI 10.1002/wnan.1559
Zhang YX, 2019, NANO LETT, V19, P2774, DOI 10.1021/acs.nanolett.8b04296
Zheng DW, 2020, NAT COMMUN, V11, DOI 10.1038/s41467-020-15927-0

Zhitnyuk Y, 2018, BIOCHEM BIOPH RES CO, V505, P1097, DOI 10.1016/j.bbrc.2018.09.113
Zhong XF, 2019, J CONTROL RELEASE, V300, P81, DOI 10.1016/j.jconrel.2019.02.035
Zhou FY, 2019, ADV MATER, V31, DOI 10.1002/adma.201805888
Zhou JR, 2020, ADV MATER, V32, DOI 10.1002/adma.201901255
Zhou L, 2020, NANO LETT, V20, P4393, DOI 10.1021/acs.nanolett.0c01140
Zhu GZ, 2017, NAT COMMUN, V8, DOI 10.1038/s41467-017-02191-y
Zhu GZ, 2017, ACS NANO, V11, P2387, DOI 10.1021/acsnano.7b00978
Zitvogel L, 1998, NAT MED, V4, P594, DOI 10.1038/nm0598-594
Zitvogel L, 2008, NAT REV IMMUNOL, V8, P59, DOI 10.1038/nri2216
NR 265
TC 56
Z9 56
U1 24
U2 196
PU ELSEVIER SCI LTD
PI OXFORD
PA THE BOULEVARD, LANGFORD LANE, KIDLINGTON, OXFORD OX5 1GB, OXON, ENGLAND
SN 0142-9612
EI 1878-5905
J9 BIOMATERIALS
JI Biomaterials
PD MAR
PY 2021
VL 270
AR 120709
DI 10.1016/j.biomaterials.2021.120709
EA FEB 2021
PG 26
WC Engineering, Biomedical; Materials Science, Biomaterials
WE Science Citation Index Expanded (SCI-EXPANDED)
SC Engineering; Materials Science
GA QL6LD
UT WOS:000621194400002
PM 33581608
DA 2023-09-14
ER

PT J
AU Wang, MF
Hou, ZY
Liu, SN
Liang, S
Ding, BB
Zhao, YJ
Chang, MY
Han, G
Kheraif, AAA
Lin, J
AF Wang, Meifang
Hou, Zhiyao
Liu, Sainan
Liang, Shuang
Ding, Binbin
Zhao, Yajie
Chang, Mengyu
Han, Gang
Kheraif, Abdulaziz A. Al
Lin, Jun
TI A Multifunctional Nanovaccine based on L-Arginine-Loaded Black
Mesoporous Titania: Ultrasound-Triggered Synergistic Cancer Sonodynamic
Therapy/Gas Therapy/Immunotherapy with Remarkably Enhanced Efficacy

SO SMALL
LA English
DT Article
DE black mesoporous titania; gas therapy; immunotherapy; nanovaccines;
sonodynamic therapy
ID IMMUNOTHERAPY; CHEMOTHERAPY; RESISTANCE; GENERATION; APOPTOSIS;
BREAKING; PLATFORM; RELEASE; ESCAPE
AB In order to achieve better antitumor therapeutic efficacy and inhibit tumor metastasis, a multifunctional nanovaccine based on L-arginine (LA)-loaded black mesoporous titania (BMT) is fabricated. In this system, LA is utilized as the exogenous NO supplementation for gas therapy, and BMT is served as acoustic sensitizer for sonodynamic therapy. The ultrasound (US) as the exogenous stimulus can simultaneously trigger BMT and LA to produce singlet oxygen (O-1(2)) and NO gas at tumor sites, respectively. Interestingly, O-1(2) from US-excited BMT can promote the oxidation of LA to produce more NO. The high concentration of O-1(2) and NO in cancer cell can cause intracellular strong oxidative stress level and DNA double-strand breaks to induce cancer cell apoptosis ultimately. The US-triggered BMT@LA "nanovaccine" combining with immune checkpoint inhibitor PD-L1 antibody (alpha PD-L1) can induce strong antitumor immune response thus effectively killing primary tumors and further inhibiting metastatic tumors. Hence, BMT@LA-based "nanovaccine" combining with alpha PD-L1 checkpoint blockade treatment can realize synergetic sonodynamic/gas/immunotherapy with enhanced antitumor therapeutic effects.
C1 [Wang, Meifang; Liu, Sainan; Liang, Shuang; Ding, Binbin; Zhao, Yajie; Chang, Mengyu; Lin, Jun] Chinese Acad Sci, Changchun Inst Appl Chem, State Key Lab Rare Earth Resource Utilizat, Changchun 130022, Peoples R China.
[Hou, Zhiyao] Guangzhou Med Univ, Affiliated Canc Hosp & Inst, Dept Abdominal Surg, Guangzhou 510095, Peoples R China.
[Hou, Zhiyao] Guangzhou Med Univ, Sch Basic Med Sci, Guangzhou Municipal & Guangdong Prov Key Lab Prot, Guangzhou 511436, Peoples R China.
[Liu, Sainan; Liang, Shuang; Zhao, Yajie; Chang, Mengyu; Lin, Jun] Univ Sci & Technol China, Sch Appl Chem & Engn, Hefei 230026, Peoples R China.
[Han, Gang] Univ Massachusetts, Med Sch, Dept Biochem & Mol Pharmacol, Worcester, MA 01605 USA.
[Kheraif, Abdulaziz A. Al] King Saud Univ, Coll Appl Med Sci, Dent Hlth Dept, Riyadh 12372, Saudi Arabia.
C3 Chinese Academy of Sciences; Changchun Institute of Applied Chemistry,
CAS; Guangzhou Medical University; Guangzhou Medical University; Chinese
Academy of Sciences; University of Science & Technology of China, CAS;
University of Massachusetts System; University of Massachusetts
Worcester; King Saud University
RP Hou, ZY (通讯作者)，Guangzhou Med Univ, Affiliated Canc Hosp & Inst, Dept Abdominal Surg, Guangzhou 510095, Peoples R China.; Hou, ZY (通讯作者)，Guangzhou Med Univ, Sch Basic Med Sci, Guangzhou Municipal & Guangdong Prov Key Lab Prot, Guangzhou 511436, Peoples R China.
EM zyhou@gzhmu.edu.cn; jlin@ciac.ac.cn
RI han, gang/B-7274-2013; Chang, Mengyu/HLG-3876-2023
OI han, gang/0000-0002-2300-5862; Chang, Mengyu/0000-0001-6112-4451
FU National Natural Science Foundation of China [NSFC 52072082, 51672268,
51972138, 51720105015, 51929201, 81972918]; Science and Technology
Cooperation Project between Chinese and Australian Governments
[2017YFE0132300]; Guangdong Natural Science Foundation
[2019A1515012214]; Projects for Science and Technology Development Plan
of Jilin province [20190201232JC]; Distinguished Scientist Fellowship
Program of King Saud University
FX This project is financially supported by the National Natural Science
Foundation of China (NSFC 52072082, 51672268, 51972138, 51720105015,
51929201, 81972918), Science and Technology Cooperation Project between
Chinese and Australian Governments (2017YFE0132300), Guangdong Natural
Science Foundation (2019A1515012214), and Projects for Science and
Technology Development Plan of Jilin province (20190201232JC), the
Distinguished Scientist Fellowship Program of King Saud University. All
animal studies were conducted in accordance with the guidelines of the
National Regulation of China for Care and Use of Laboratory Animals.
CR An J, 2020, BIOMATERIALS, V234, DOI 10.1016/j.biomaterials.2020.119761
Arora H, 2018, P NATL ACAD SCI USA, V115, P11298, DOI 10.1073/pnas.1812704115

Basudhar D, 2017, P NATL ACAD SCI USA, V114, P13030, DOI 10.1073/pnas.1709119114
Cao Y, 2019, CHEM MATER, V31, P9105, DOI 10.1021/acs.chemmater.9b03430
Chen AP, 2019, ADV MATER, V31, DOI 10.1002/adma.201803241
Chen CD, 2019, ADV SCI, V6, DOI 10.1002/advs.201801189
Chen Q, 2016, NAT COMMUN, V7, DOI 10.1038/ncomms13193
Chen YL, 2017, CANCER LETT, V400, P282, DOI 10.1016/j.canlet.2017.01.040
Deepagan VG, 2016, NANO LETT, V16, P6257, DOI 10.1021/acs.nanolett.6b02547
Fan W., 2017, ANGEW CHEM, V129, P1249, DOI DOI 10.1002/ange.201610682
Fan WP, 2018, ANGEW CHEM INT EDIT, V57, P8383, DOI 10.1002/anie.201800594
Fan WP, 2015, ANGEW CHEM INT EDIT, V54, P14026, DOI 10.1002/anie.201504536
Feig C, 2013, P NATL ACAD SCI USA, V110, P20212, DOI 10.1073/pnas.1320318110
Fu LH, 2019, ADV MATER, V31, DOI 10.1002/adma.201808325
Gao FL, 2019, NANOSCALE, V11, P2374, DOI 10.1039/c8nr07188h
Guan X, 2020, ADV FUNCT MATER, V30, DOI 10.1002/adfm.202000326
Han XX, 2018, ACS NANO, V12, P4545, DOI 10.1021/acsnano.8b00899
He CB, 2016, NAT COMMUN, V7, DOI 10.1038/ncomms12499
Heidari A., 2017, INT J BIOMED DATA MI, V6, pe103
Igney FH, 2002, J LEUKOCYTE BIOL, V71, P907
Iwai Y, 2002, P NATL ACAD SCI USA, V99, P12293, DOI 10.1073/pnas.192461099
Jia XB, 2018, ADV MATER, V30, DOI 10.1002/adma.201704490
Kang J, 2016, J IMMUNOTHER CANCER, V4, DOI 10.1186/s40425-016-0156-7
Lau J, 2017, NAT COMMUN, V8, DOI 10.1038/ncomms14572
Li CH, 2020, ACS APPL MATER INTER, V12, P34505, DOI 10.1021/acsami.0c04666
Li SH, 2019, ACS NANO, V13, P2103, DOI 10.1021/acsnano.8b08700
Liang S, 2020, ADV FUNCT MATER, V30, DOI 10.1002/adfm.201908598
Liu YD, 2018, ADV MATER, V30, DOI 10.1002/adma.201704524
Maj T, 2017, NAT IMMUNOL, V18, P1332, DOI 10.1038/ni.3868
Masters GA, 2015, J CLIN ONCOL, V33, P786, DOI 10.1200/JCO.2014.59.9746
Mellman I, 2011, NATURE, V480, P480, DOI 10.1038/nature10673
Moncada S, 2002, NAT REV MOL CELL BIO, V3, P214, DOI 10.1038/nrm762
MONCADA S, 1993, NEW ENGL J MED, V329, P2002
Ng CW, 2018, ADV FUNCT MATER, V28, DOI 10.1002/adfm.201804688
Nishijima TF, 2017, ONCOLOGIST, V22, P470, DOI 10.1634/theoncologist.2016-0419
Nomikou N, 2010, CANCER LETT, V296, P133, DOI 10.1016/j.canlet.2010.06.002
Qian XQ, 2016, ADV MATER, V28, P8097, DOI 10.1002/adma.201602012
RADOMSKI MW, 1990, P NATL ACAD SCI USA, V87, P5193, DOI 10.1073/pnas.87.13.5193
Serre R, 2016, CANCER RES, V76, P4931, DOI 10.1158/0008-5472.CAN-15-3567
Sharma P, 2015, SCIENCE, V348, P56, DOI 10.1126/science.aaa8172
Son S, 2020, CHEM SOC REV, V49, P3244, DOI 10.1039/c9cs00648f
Song QL, 2017, NANO LETT, V17, P6366, DOI 10.1021/acs.nanolett.7b03186
Song XJ, 2016, NANO LETT, V16, P6145, DOI 10.1021/acs.nanolett.6b02365
Sun R, 2018, LANCET ONCOL, V19, P1180, DOI 10.1016/S1470-2045(18)30413-3
Szabo C, 2016, NAT REV DRUG DISCOV, V15, P185, DOI 10.1038/nrd.2015.1
Tang W, 2018, ACS NANO, V12, P2610, DOI 10.1021/acsnano.7b08628
Wan SS, 2018, BIOMATERIALS, V185, P51, DOI 10.1016/j.biomaterials.2018.09.004
Wang MF, 2019, ACS APPL MATER INTER, V11, P47730, DOI 10.1021/acsami.9b17375
Wang XW, 2020, J AM CHEM SOC, V142, P6527, DOI 10.1021/jacs.9b10228
Wang Z, 2019, ADV SCI, V6, DOI 10.1002/advs.201901690
Zhang D, 2019, SMALL, V15, DOI 10.1002/smll.201902636
Zhang D, 2019, ACS APPL MATER INTER, V11, P32633, DOI 10.1021/acsami.9b09568
Zhang K, 2016, ACS NANO, V10, P10816, DOI 10.1021/acsnano.6b04921
Zheng DW, 2016, NANO LETT, V16, P4341, DOI 10.1021/acs.nanolett.6b01432
Zhong XY, 2020, ADV FUNCT MATER, V30, DOI 10.1002/adfm.201907954
Zhu P, 2018, ACS NANO, V12, P3780, DOI 10.1021/acsnano.8b00999
NR 56
TC 56
Z9 56
U1 14
U2 294
PU WILEY-V C H VERLAG GMBH
PI WEINHEIM

PA POSTFACH 101161, 69451 WEINHEIM, GERMANY
SN 1613-6810
EI 1613-6829
J9 SMALL
JI Small
PD FEB
PY 2021
VL 17
IS 6
AR 2005728
DI 10.1002/smll.202005728
EA JAN 2021
PG 11
WC Chemistry, Multidisciplinary; Chemistry, Physical; Nanoscience &
Nanotechnology; Materials Science, Multidisciplinary; Physics, Applied;
Physics, Condensed Matter
WE Science Citation Index Expanded (SCI-EXPANDED)
SC Chemistry; Science & Technology - Other Topics; Materials Science;
Physics
GA QK1VL
UT WOS:000608845900001
PM 33470521
DA 2023-09-14
ER

PT J
AU Arkaban, H
Barani, M
Akbarizadeh, MR
Chauhan, NPS
Jadoun, S
Soltani, MD
Zarrintaj, P
AF Arkaban, Hassan
Barani, Mahmood
Akbarizadeh, Majid Reza
Chauhan, Narendra Pal Singh
Jadoun, Sapana
Soltani, Maryam Dehghani
Zarrintaj, Payam
TI Polyacrylic Acid Nanoplatforms: Antimicrobial, Tissue Engineering, and
Cancer Theranostic Applications
SO POLYMERS
LA English
DT Article
DE polyacrylic acid (PAA); synthesize; polymerizations; antimicrobial;
anticancer; biosensing
ID IRON-OXIDE NANOPARTICLES; DRUG-DELIVERY SYSTEM; POLY(ACRYLIC ACID);
ACRYLIC-ACID; RAFT POLYMERIZATION; MESOPOROUS SILICA; IN-VITRO; RADICAL
POLYMERIZATION; GOLD NANOPARTICLES; RESPONSIVE NANOCARRIER
AB Polyacrylic acid (PAA) is a non-toxic, biocompatible, and biodegradable polymer that gained lots of interest in recent years. PAA nano-derivatives can be obtained by chemical modification of carboxyl groups with superior chemical properties in comparison to unmodified PAA. For example, nano-particles produced from PAA derivatives can be used to deliver drugs due to their stability and biocompatibility. PAA and its nanoconjugates could also be regarded as stimuli-responsive platforms that make them ideal for drug delivery and antimicrobial applications. These properties make PAA a good candidate for conventional and novel drug carrier systems. Here, we started with synthesis approaches, structure characteristics, and other architectures of PAA nanoplatforms. Then, different conjugations of PAA/nanostructures and their potential in various fields of nanomedicine such as antimicrobial, anticancer, imaging, biosensor, and tissue engineering were discussed. Finally, biocompatibility and challenges of PAA nanoplatforms were highlighted. This review will provide fundamental knowledge and

current information connected to the PAA nanoplatforms and their applications in biological fields for a broad audience of researchers, engineers, and newcomers. In this light, PAA nanoplatforms could have great potential for the research and development of new nano vaccines and nano drugs in the future.
C1 [Arkaban, Hassan] Univ Isfahan, Dept Chem, Esfahan 8174673441, Iran.
[Barani, Mahmood] Kerman Univ Med Sci, Med Mycol & Bacteriol Res Ctr, Kerman 7616913555, Iran.
[Akbarizadeh, Majid Reza] Zabol Univ Med Sci, Amir Al Momenin Hosp, Dept Pediat, Zabol 9861663335, Iran.
[Chauhan, Narendra Pal Singh] Bhupal Nobless Univ, Fac Sci, Dept Chem, Udaipur 313002, Rajasthan, India.
[Jadoun, Sapana] Univ Concepcion, Fac Sci, Dept Analyt & Inorgan Chem, Edmundo Larenas 129, Concepcion 4070371, Chile.
[Soltani, Maryam Dehghani] Yazd Univ, Fac Sci, Dept Chem, Yazd 8915818411, Iran.
[Zarrintaj, Payam] Oklahoma State Univ, Sch Chem Engn, 420 Engn North, Stillwater, OK 74078 USA.
C3 University of Isfahan; Kerman University of Medical Sciences;
Universidad de Concepcion; University of Yazd; Oklahoma State University
System; Oklahoma State University - Stillwater
RP Barani, M (通讯作者)，Kerman Univ Med Sci, Med Mycol & Bacteriol Res Ctr, Kerman 7616913555, Iran.; Akbarizadeh, MR (通讯作者)，Zabol Univ Med Sci, Amir Al Momenin Hosp, Dept Pediat, Zabol 9861663335, Iran.
EM hassan.arkaban@gmail.com; mahmoodbarani7@gmail.com;
akbarizadeh63@gmail.com; narendrapalsingh14@gmail.com;
sjadoun022@gmail.com; maryam.dehghanisoltani@gmail.com;
payam.zarrintaj@gmail.com
RI Akbarizadeh, Majid Reza/V-2342-2017; Chauhan, Narendra P S/P-6971-2019;
Chauhan, Narendra Pal Singh/Q-7246-2018; Barani, Mahmood/N-5927-2018;
arkaban, hassan/GQZ-6322-2022; Jadoun, Sapana/K-7638-2019; Zarintaj,
Payam/W-7364-2018
OI Chauhan, Narendra P S/0000-0003-3241-7460; Chauhan, Narendra Pal
Singh/0000-0003-3241-7460; Barani, Mahmood/0000-0002-1711-2522; arkaban,
hassan/0000-0002-8859-4290; Jadoun, Sapana/0000-0002-3572-7934;
Zarintaj, Payam/0000-0002-1609-7519; Akbarizadeh,
Majidreza/0000-0003-4782-3867
CR Abolhasani M., 2021, Journal of Islamic Dental Association of Iran, V33, P17
Akkahat P, 2012, LANGMUIR, V28, P5302, DOI 10.1021/la204542e
Akkahat P, 2011, COLLOID SURFACE B, V86, P198, DOI 10.1016/j.colsurfb.2011.03.042
Alahri MB, 2021, INORG CHEM COMMUN, V134, DOI 10.1016/j.inoche.2021.108997
Amini-Fazl MS, 2019, INT J BIOL MACROMOL, V132, P506, DOI 10.1016/j.ijbiomac.2019.04.005
[Anonymous], 2015, J CHEM PHARM RES
Gonzalez-Gomez MA, 2019, NANOMATERIALS-BASEL, V9, DOI 10.3390/nano9111626
Aqil A, 2007, CHEM MATER, V19, P2150, DOI 10.1021/cm062152k
Arik N, 2019, POLYM BULL, V76, P797, DOI 10.1007/s00289-018-2409-8
Arkaban H, 2021, INORG CHEM COMMUN, V128, DOI 10.1016/j.inoche.2021.108566
Arkaban H, 2021, NANOTECHNOLOGY, V32, DOI 10.1088/1361-6528/abf878
Khan MUA, 2020, POLYMERS-BASEL, V12, DOI 10.3390/polym12061238
Babiker DMD, 2019, SURF COAT TECH, V367, P49, DOI 10.1016/j.surfcoat.2019.03.051
Bagheri N, 2020, RSC ADV, V10, P25290, DOI 10.1039/d0ra03779f
Baldim V, 2020, ACS APPL MATER INTER, V12, P42056, DOI 10.1021/acsami.0c08778
Bardajee GR, 2021, COLLOID SURFACE A, V616, DOI 10.1016/j.colsurfa.2021.126350
Bardajee GR, 2017, J POLYM RES, V24, DOI 10.1007/s10965-017-1197-4
Barner-Kowollik C, 2006, J POLYM SCI POL CHEM, V44, P5809, DOI 10.1002/pola.21589
Bazzazzadeh A, 2020, INT J PHARMACEUT, V587, DOI 10.1016/j.ijpharm.2020.119674
Belbekhouche S, 2018, J COLLOID INTERF SCI, V522, P183, DOI 10.1016/j.jcis.2018.03.061
Bian RX, 2015, BIOMATER SCI-UK, V3, P1270, DOI 10.1039/c5bm00186b
Bin-Dahman OA, 2015, STARCH-STARKE, V67, P1061, DOI 10.1002/star.201500011
Brogden KA, 2005, NAT REV MICROBIOL, V3, P238, DOI 10.1038/nrmicro1098
Cabana S, 2017, J DRUG DELIV SCI TEC, V42, P245, DOI 10.1016/j.jddst.2017.04.006
Cerqueira MA, 2014, FOOD ENG REV, V6, P1, DOI 10.1007/s12393-013-9074-3
Chandekar KV, 2021, MAT SCI ENG C-MATER, V122, DOI 10.1016/j.msec.2021.111898
Changez M, 2004, BIOMATERIALS, V25, P139, DOI 10.1016/S0142-9612(03)00466-6
Chanprapanon W., 2020, ENG APPL SCI RES, V47, P222
Chen LL, 2013, ACS APPL MATER INTER, V5, P7282, DOI 10.1021/am401627u

Chen Y, 2015, J BIOMED NANOTECHNOL, V11, P771, DOI 10.1166/jbn.2015.2001
Chen YP, 2013, J NANOMATER, V2013, DOI 10.1155/2013/345738
Cheng W, 2019, ACS NANO, V13, P8537, DOI 10.1021/acsnano.9b04436
Cheng YZ, 2020, J BIOMAT SCI-POLYM E, V31, P1836, DOI 10.1080/09205063.2020.1782023
Chiang CS, 2015, ADV HEALTHC MATER, V4, P1066, DOI 10.1002/adhm.201400794
Chinthamreddy A, 2021, INORG CHEM COMMUN, V133, DOI 10.1016/j.inoche.2021.108928
Cho MH, 2017, FRONT CHEM, V5, DOI 10.3389/fchem.2017.00109
Chrisostomo DA, 2020, ORAL HLTH PREV DENT, V18, P747, DOI 10.3290/j.ohpd.a45078
Chu YM, 2022, APPL MATH COMPUT, V419, DOI 10.1016/j.amc.2021.126883
Chu YM, 2021, FRACTAL FRACT, V5, DOI 10.3390/fractalfract5030119
Couto D, 2015, ARCH TOXICOL, V89, P1759, DOI 10.1007/s00204-014-1325-4
Couto D, 2014, TOXICOL LETT, V225, P57, DOI 10.1016/j.toxlet.2013.11.020
Couvreur L, 2003, MACROMOLECULES, V36, P8260, DOI 10.1021/ma035043p
Cui YF, 2016, INT J NANOMED, V11, P5671, DOI 10.2147/IJN.S115357
Dai YL, 2012, BIOMATERIALS, V33, P2583, DOI 10.1016/j.biomaterials.2011.12.014
Daneshvari G, 2021, BIOINTERFACE RES APP, V11, P13866, DOI 10.33263/BRIAC116.1386613877
Das D, 2015, ACS APPL MATER INTER, V7, P4791, DOI 10.1021/am508712e
Dashtizadeh A, 2012, J PLAST FILM SHEET, V28, P120, DOI 10.1177/8756087911421702
De la Garza D, 2019, J APPL POLYM SCI, V136, DOI 10.1002/app.47480
Deng ZJ, 2012, ACS NANO, V6, P8962, DOI 10.1021/nn3029953
Deng ZJ, 2011, NAT NANOTECHNOL, V6, P39, DOI 10.1038/NNANO.2010.250
Dil NN, 2018, J HAZARD MATER, V351, P38, DOI 10.1016/j.jhazmat.2018.02.017
Dilip D.S., 2021, INT J SCI RES DENT M, V3, P122
Dinita Devi N., 2021, INT J SCI RES DENT M, V3, P153
Doppalapudi S, 2014, POLYM ADVAN TECHNOL, V25, P427, DOI 10.1002/pat.3305
Duan ZJ, 2020, INT J ADV MANUF TECH, V107, P1277, DOI 10.1007/s00170-020-04969-9
Ebadian Behnaz, 2021, Int J Dent, V2021, P8461579, DOI 10.1155/2021/8461579
Ekwebene O.C., 2021, INT J SCI RES DENT M, V3, P12
Escudero A, 2016, NANOSCALE, V8, P12221, DOI 10.1039/c6nr03369e
Esfahanian M, 2019, ARTIF CELL NANOMED B, V47, P2024, DOI 10.1080/21691401.2019.1617729
Fathi A, 2022, MED BALEAR, V37, P29, DOI 10.3306/AJHS.2022.37.01.29
Foroushani MS, 2021, COLLOID SURFACE B, V202, DOI 10.1016/j.colsurfb.2021.111712
Foroushani MS, 2019, BIOELECTROCHEMISTRY, V130, DOI 10.1016/j.bioelechem.2019.107347
Gao J, 1998, J MACROMOL SCI R M C, VC38, P651
Ghaffari-Bohlouli P, 2020, INT J BIOL MACROMOL, V165, P2363, DOI 10.1016/j.ijbiomac.2020.10.078
Ghazanfari A, 2019, COLLOID SURFACE A, V576, P73, DOI 10.1016/j.colsurfa.2019.05.033
Ghimire A, 2015, SENSORS-BASEL, V15, P23868, DOI 10.3390/s150923868
Ghorbaniazar P, 2015, ADV PHARM BULL, V5, P269, DOI 10.15171/apb.2015.037
Giraldo-Villegas M, 2019, FUTUR SCI OA, V5, DOI 10.2144/fsoa-2019-0066
Gomaa OM, 2018, J FLUORESC, V28, P41, DOI 10.1007/s10895-017-2158-3
Gratzl G, 2015, COLLOID SURFACE B, V126, P98, DOI 10.1016/j.colsurfb.2014.12.016
Gratzl G, 2014, MAT SCI ENG C-MATER, V38, P94, DOI 10.1016/j.msec.2014.01.050
Greindl M, 2006, PHARM RES-DORDR, V23, P2183, DOI 10.1007/s11095-006-9087-1
Guo SM, 2017, J CLEAN PROD, V140, P1060, DOI 10.1016/j.jclepro.2016.10.073
Hacker MC, 2011, PRINCIPLES OF REGENERATIVE MEDICINE, 2ND EDITION, P587, DOI 10.1016/B978-0-12-381422-7.10033-1
Hajikhani M, 2021, J FOOD PROCESS PRES, V45, DOI 10.1111/jfpp.15055
Hamidian K, 2022, J MOL STRUCT, V1251, DOI 10.1016/j.molstruc.2021.131962
Han J.-H., 2019, KOREA IND CHEM SOC, V2019, P194
Hanafy N.A.N., 2018, J NANOMED RES, V7, P235, DOI DOI 10.15406/JNMR.2018.07.00193
Hao NJ, 2016, BIOMATER SCI-UK, V4, P575, DOI 10.1039/c5bm00589b
Hassan S, 2014, RADIAT PHYS CHEM, V97, P292, DOI 10.1016/j.radphyschem.2013.12.024
He WD, 2011, MACROMOLECULES, V44, P3358, DOI 10.1021/ma2000674
Hisamatsu N, 2014, SENSOR ACTUAT B-CHEM, V203, P289, DOI 10.1016/j.snb.2014.06.123
Hooshmand S, 2021, CURR MED CHEM, V28, P777, DOI 10.2174/0929867327666200123152006
Hu Y, 2002, BIOMATERIALS, V23, P3193, DOI 10.1016/S0142-9612(02)00071-6
Huang HY, 2013, J CONTROL RELEASE, V172, P118, DOI 10.1016/j.jconrel.2013.07.029
Huang JQ, 2020, BIOMATERIALS, V262, DOI 10.1016/j.biomaterials.2020.120346
Irfan M, 2021, J MAGN MAGN MATER, V537, DOI 10.1016/j.jmmm.2021.168150
Ishizone T., 2012, ANIONIC POLYM PROTEC
Islam M. Touhidul, 2021, INT J SCI RES DENT M, V3, P18, DOI DOI

10.30485/IJSRDMS.2021.270577.1107
Iversen NK, 2013, TOXICOL APPL PHARM, V266, P276, DOI 10.1016/j.taap.2012.10.014
Jamali S., 2020, INT J SCI RES DENT M, V2, P52, DOI [10.30485/ijsrdms.2020.233746.1062, DOI 10.30485/IJSRDMS.2020.233746.1062]
Jamali S., 2020, INT J SCI RES DENT M, V2, P6
Javakhishvili I, 2009, BIOMACROMOLECULES, V10, P74, DOI 10.1021/bm800860t
Ji J, 2010, J MACROMOL SCI A, V47, P445, DOI 10.1080/10601321003659705
Ji XJ, 2019, FRONT MATER SCI, V13, P87, DOI 10.1007/s11706-019-0448-1
Jia XK, 2013, J BIOMED NANOTECHNOL, V9, P2063, DOI 10.1166/jbn.2013.1764
Jiang LM, 2010, DRUG DEV IND PHARM, V36, P323, DOI [10.1080/03639040903170750, 10.3109/03639040903170750]
Jin X, 2005, POLYMER, V46, P5149, DOI 10.1016/j.polymer.2005.04.066
Jin XQ, 2017, MICROPOR MESOPOR MAT, V254, P77, DOI 10.1016/j.micromeso.2017.05.003
Jing X, 2021, ACS SUSTAIN CHEM ENG, V9, P9209, DOI 10.1021/acssuschemeng.1c00949
Jing ZX, 2019, POLYMERS-BASEL, V11, DOI 10.3390/polym11060952
Johnson KCC, 2012, ANAL CHIM ACTA, V739, P83, DOI 10.1016/j.aca.2012.06.025
Jose J, 2014, POLYM BULL, V71, P2787, DOI 10.1007/s00289-014-1221-3
Kadlubowski S, 2003, MACROMOLECULES, V36, P2484, DOI 10.1021/ma021628s
Kahveci M., 2012, WELL DEFINED BLOCK C
Kakroudi M. Safari, 2019, INT J SCI RES DENT M, V1, P72
Kang XJ, 2012, CHEM-EUR J, V18, P15676, DOI 10.1002/chem.201202433
Kargozar S, 2018, MATER TODAY-PROC, V5, P15492, DOI 10.1016/j.matpr.2018.04.155
Kariduraganavar MY, 2014, NATURAL AND SYNTHETIC BIOMEDICAL POLYMERS, P1
Kausar A, 2020, J PLAST FILM SHEET, V36, P409, DOI 10.1177/8756087920917177
Khajeh HG, 2022, COLLOID SURFACE A, V633, DOI 10.1016/j.colsurfa.2021.127891
Khamisi N, 2022, MED BALEAR, V37, P136, DOI 10.3306/AJHS.2022.37.01.136
Khan IM, 2020, SENSOR ACTUAT B-CHEM, V321, DOI 10.1016/j.snb.2020.128470
Khan MUA, 2021, ARAB J CHEM, V14, DOI 10.1016/j.arabjc.2020.102924
Khanlari S, 2013, MACROMOL REACT ENG, V7, P573, DOI 10.1002/mren.201300114
Kumar A, 2021, COLLOID SURFACE A, V617, DOI 10.1016/j.colsurfa.2021.126358
Kurkuri MD, 2004, J CONTROL RELEASE, V96, P9, DOI 10.1016/j.jconrel.2003.12.025
Ladaviere C, 2001, MACROMOLECULES, V34, P5370, DOI 10.1021/ma010358v
Lai WF, 2021, MOL PHARMACEUT, V18, P1833, DOI 10.1021/acs.molpharmaceut.0c00874
Laraib U, 2022, INT J BIOL MACROMOL, V195, P356, DOI 10.1016/j.ijbiomac.2021.12.052
Larsson M, 2014, J NANOMATER, V2014, DOI 10.1155/2014/371307
Lee Kyung Dong, 2013, Int J Nanomedicine, V8, P2835, DOI 10.2147/IJN.S48367
Li BK, 2017, J CLEAN PROD, V154, P1, DOI 10.1016/j.jclepro.2017.03.213
Li D, 2020, ADV THER-GERMANY, V3, DOI 10.1002/adtp.202000091
Li GL, 2018, NANO-MICRO LETT, V10, DOI 10.1007/s40820-017-0155-3
Li L, 2015, SMALL, V11, P3162, DOI 10.1002/smll.201403517
Li L, 2013, NANOSCALE, V5, P2249, DOI 10.1039/c3nr33695f
Li YJ, 2019, BIOMATER SCI-UK, V7, P1507, DOI 10.1039/c8bm01371c
Lin IC, 2011, BIOMACROMOLECULES, V12, P1339, DOI 10.1021/bm200116z
Liu AH, 2006, BIOSENS BIOELECTRON, V22, P694, DOI 10.1016/j.bios.2006.02.006
Liu D, 2014, J COAT TECHNOL RES, V11, P595, DOI 10.1007/s11998-014-9569-8
Liu JH, 2019, CHINESE J ANAL CHEM, V47, P678, DOI 10.1016/S1872-2040(19)61158-8
Liu LF, 2021, J SAUDI CHEM SOC, V25, DOI 10.1016/j.jscs.2021.101344
Liu MY, 2016, NANOSCALE, V8, P16819, DOI 10.1039/c5nr09078d
Liu XX, 2019, J COLLOID INTERF SCI, V538, P507, DOI 10.1016/j.jcis.2018.12.023
Loiseau J, 2003, MACROMOLECULES, V36, P3066, DOI 10.1021/ma0256744
Lojk J, 2015, INT J NANOMED, V10, P1449, DOI 10.2147/IJN.S76134
Lu Y, 2007, J PHYS CHEM C, V111, P7676, DOI 10.1021/jp070973m
Ma Y, 2018, ACS BIOMATER SCI ENG, V4, P547, DOI 10.1021/acsbiomaterials.7b00878
Ma YD, 2013, LANGMUIR, V29, P2946, DOI 10.1021/la305137m
Ma YF, 2016, ACTA BIOMATER, V42, P199, DOI 10.1016/j.actbio.2016.07.030
Ma YH, 2009, BIOMATERIALS, V30, P3343, DOI 10.1016/j.biomaterials.2009.02.034
Maalekipour M, 2021, INT J DENT, V2021, DOI 10.1155/2021/3178536
Mahmoodzadeh F, 2018, MAT SCI ENG C-MATER, V93, P880, DOI 10.1016/j.msec.2018.08.067
Mallawarachchi S, 2019, DRUG DELIV TRANSL RE, V9, P783, DOI 10.1007/s13346-019-00620-7
Mandavian AR, 2010, CHEM ENG J, V159, P264, DOI 10.1016/j.cej.2010.02.041
Masuda T, 2020, POLYM J, V52, P1407, DOI 10.1038/s41428-020-00401-x

Matyjaszewski K., 2012, COPPER MEDIATED ATOM
Mazloomi-Rezvani M, 2018, COLLOID SURFACE B, V166, P144, DOI 10.1016/j.colsurfb.2018.03.013
Millard PE, 2006, MACROMOL RAPID COMM, V27, P821, DOI 10.1002/marc.200600115
Moad G, 2005, POLYMER, V46, P8458, DOI 10.1016/j.polymer.2004.12.061
Mohammadi L, 2021, J MOL STRUCT, V1229, DOI 10.1016/j.molstruc.2020.129857
Molnar RM, 2009, COLLOID POLYM SCI, V287, P739, DOI 10.1007/s00396-009-2033-0
Monirifard R., 2019, Journal of Islamic Dental Association of Iran, V31, P182, DOI 10.30699/jidai.31.4.182
Mori H, 2003, J AM CHEM SOC, V125, P3712, DOI 10.1021/ja0297887
Morita K, 2018, J BIOSCI BIOENG, V126, P119, DOI 10.1016/j.jbiosc.2018.01.012
Moscoso-Londono O, 2013, EUR POLYM J, V49, P279, DOI 10.1016/j.eurpolymj.2012.11.007
Mosharraf Ramin, 2021, Int J Dent, V2021, P5977994, DOI 10.1155/2021/5977994
Mrowczynski R, 2016, RSC ADV, V6, P5936, DOI 10.1039/c5ra24222c
Muller C, 2013, J NANOPART RES, V15, DOI 10.1007/s11051-012-1353-z
Mutar M.A., 2012, AL QADISIYAH J PURE, V17, P71
Nan JY, 2014, NANO-MICRO LETT, V6, P200, DOI 10.5101/nml140022a
Nazaripour E, 2022, REND LINCEI-SCI FIS, V33, P127, DOI 10.1007/s12210-021-01042-9
Nazeer M, 2022, APPL MATH COMPUT, V420, DOI 10.1016/j.amc.2021.126868
Nie L, 2021, COLLOID SURFACE B, V197, DOI 10.1016/j.colsurfb.2020.111385
Nnaemeka O.D., 2021, IJSRDMS, V3, P66
Nunez NO, 2020, NANOMATERIALS-BASEL, V10, DOI 10.3390/nano10010149
Nunez NO, 2014, EUR J INORG CHEM, P6075, DOI 10.1002/ejic.201402690
Nurkeeva ZS, 2004, EUR J PHARM BIOPHARM, V57, P245, DOI 10.1016/s0939-6411(03)00149-8
Ortega V, 2020, ENVIRON SCI-NANO, V7, P1912, DOI 10.1039/d0en00229a
Padwal P, 2014, LANGMUIR, V30, P15266, DOI 10.1021/la503808d
Pan S, 2018, DOSE-RESPONSE, V16, DOI 10.1177/1559325818789841
Partenhauser A, 2016, DRUG DISCOV TODAY, V21, P1051, DOI 10.1016/j.drudis.2016.02.013
Patten TE, 1998, ADV MATER, V10, P901, DOI 10.1002/(SICI)1521-4095(199808)10:12<901::AID-ADMA901>3.0.CO;2-B
Pavlinec U, 2019, J PLAST FILM SHEET, V35, P239, DOI 10.1177/8756087918820904
Perkin KK, 2005, NANO LETT, V5, P1457, DOI 10.1021/nl050817w
Posha B, 2020, ANALYST, V145, P3939, DOI 10.1039/d0an00567c
Rahdar S, 2021, POLYMERS-BASEL, V13, DOI 10.3390/polym13071161
Ranjbari E, 2020, POLYM TEST, V84, DOI 10.1016/j.polymertesting.2020.106403
Rather A., 2021, INT J SCI RES DENT M, V3, P86, DOI DOI 10.30485/IJSRDMS.2021.285539.1153
Rauf A, 2021, ARAB J CHEM, V14, DOI 10.1016/j.arabjc.2021.103321
Reddy B. H. N., 2018, INT J APPL PHARM, V10, P141, DOI [10.1088/2053-1591/aaac4c, DOI 10.1088/2053-1591/AAAC4C]
Rivas BL, 2003, PROG POLYM SCI, V28, P173, DOI 10.1016/S0079-6700(02)00028-X
Rodrigues D, 2017, NANOTOXICOLOGY, V11, P256, DOI 10.1080/17435390.2017.1291865
Rong QF, 2016, BIOSENS BIOELECTRON, V75, P148, DOI 10.1016/j.bios.2015.08.041
Roostaee M., 2020, CURRENT BIOCH ENG, V6, P91, DOI DOI 10.2174/2212711906666200316163207
Roostaee M, 2022, TOP CATAL, V65, P633, DOI 10.1007/s11244-021-01504-2
Roostaee M, 2022, ENVIRON RES, V205, DOI 10.1016/j.envres.2021.112510
Roostaee M, 2022, J FOOD MEAS CHARACT, V16, P731, DOI 10.1007/s11694-021-01200-5
Roostaee M, 2022, J FOOD MEAS CHARACT, V16, P523, DOI 10.1007/s11694-021-01180-6
Runowski M, 2016, J LUMIN, V170, P484, DOI 10.1016/j.jlumin.2015.05.037
Sanaei Z, 2019, INTERJ SCI RES DENT, V1, P13
Sanchez LM, 2018, COLLOID SURFACE A, V543, P28, DOI 10.1016/j.colsurfa.2018.01.050
Santiago-Morales J, 2016, COLLOID SURFACE B, V146, P144, DOI 10.1016/j.colsurfb.2016.04.052
Sargazi G, 2019, POLYM BULL, V76, P5661, DOI 10.1007/s00289-019-02677-3
Satoh K., 2012, TRANSITION METAL COM
Yilmaz SS, 2020, MATERIALS, V13, DOI 10.3390/ma13194390
Scarioti GD, 2011, COLLOID SURFACE B, V87, P267, DOI 10.1016/j.colsurfb.2011.05.027
Schilli CM, 2004, MACROMOLECULES, V37, P7861, DOI 10.1021/ma035838w
Sennakesavan G, 2020, POLYM DEGRAD STABIL, V180, DOI 10.1016/j.polymdegradstab.2020.109308
Sethy Pramod K., 2021, Nano-Structures & Nano-Objects, V26, P370, DOI 10.1016/j.nanoso.2021.100747
Sgarlata C, 2016, COMPUT THEOR CHEM, V1096, P1, DOI 10.1016/j.comptc.2016.09.026
Shangloo P., 2021, INT J SCI RES DENT M, V3, P166
Sharma S, 2020, J MOL STRUCT, V1215, DOI 10.1016/j.molstruc.2020.128298

Shen LG, 2019, J COLLOID INTERF SCI, V543, P64, DOI 10.1016/j.jcis.2019.02.013
Shi HZ, 2015, CRYSTENGCOMM, V17, P4768, DOI 10.1039/c5ce00708a
Shibraen MHMA, 2021, COLLOID SURFACE A, V610, DOI 10.1016/j.colsurfa.2020.125921
Shimizu T, 2005, CHEM REV, V105, P1401, DOI 10.1021/cr030072j
Shukla NB, 2009, J APPL POLYM SCI, V112, P991, DOI 10.1002/app.29460
Song CY, 2018, J MATER CHEM B, V6, P3030, DOI 10.1039/c8tb00587g
Song YQ, 2015, J INEQUAL APPL, DOI 10.1186/s13660-015-0927-6
Spagnol C, 2012, CARBOHYD POLYM, V87, P2038, DOI 10.1016/j.carbpol.2011.10.017
Suarez-Garcia S, 2021, COORDIN CHEM REV, V441, DOI 10.1016/j.ccr.2021.213977
Sun SL, 2012, LANGMUIR, V28, P3542, DOI 10.1021/la204529u
Surendra MK, 2014, J NANOPART RES, V16, DOI 10.1007/s11051-014-2773-8
Sutekin SD, 2020, POLYM INT, V69, P693, DOI 10.1002/pi.6004
Swilem AE, 2020, MAT SCI ENG C-MATER, V110, DOI 10.1016/j.msec.2020.110726
Takada K, 2011, SENSOR ACTUAT B-CHEM, V160, P1586, DOI 10.1016/j.snb.2011.09.031
Talusan Timothy Jemuel E., 2017, Applied Mechanics and Materials, V863, P84, DOI 10.4028/www.scientific.net/AMM.863.84
Tan S, 2017, DRUG DES DEV THER, V11, P3519, DOI 10.2147/DDDT.S151422
Tang LZ, 2022, CHIN J MECH ENG-EN, V35, DOI 10.1186/s10033-021-00667-z
Tian BS, 2017, COLLOID SURFACE B, V154, P287, DOI 10.1016/j.colsurfb.2017.03.024
Tian CX, 2015, ACS APPL MATER INTER, V7, P17765, DOI 10.1021/acsami.5b03998
Tian F, 2017, SENSOR ACTUAT B-CHEM, V252, P831, DOI 10.1016/j.snb.2017.06.048
Tran VA, 2021, RSC ADV, V11, P9222, DOI 10.1039/d0ra10423j
Wang FZ, 2022, FRACTALS, V30, DOI 10.1142/S0218348X22400515
Wang LM, 2019, INT J NANOMED, V14, P1817, DOI 10.2147/IJN.S192520
Wang Q, 2017, MATER CHEM PHYS, V195, P213, DOI 10.1016/j.matchemphys.2017.04.026
Wang RM, 2010, POLYM ADVAN TECHNOL, V21, P685, DOI 10.1002/pat.1481
Wang YG, 2016, TRIBOL INT, V99, P198, DOI 10.1016/j.triboint.2016.03.023
Wang Y, 2017, J NANOBIOTECHNOL, V15, DOI 10.1186/s12951-017-0309-y
Wu L, 2021, COMPOS COMMUN, V24, DOI 10.1016/j.coco.2021.100669
Wu XT, 2015, J MATER CHEM B, V3, P2421, DOI 10.1039/c4tb02009j
Xia Z, 2017, MATER DESIGN, V119, P85, DOI 10.1016/j.matdes.2017.01.022
Xiong JH, 2021, ACS APPL MATER INTER, V13, P28304, DOI 10.1021/acsami.1c06683
Xu M, 2016, ACS NANO, V10, P3267, DOI 10.1021/acsnano.6b00539
Xu XY, 2020, COLLOID SURFACE B, V194, DOI 10.1016/j.colsurfb.2020.111206
Xu Y, 2013, BIOMED RES INT, V2013, DOI 10.1155/2013/462589
Xue ZL, 2018, BIOMATERIALS, V171, P153, DOI 10.1016/j.biomaterials.2018.04.037
Yan LF, 2008, POLYM ADVAN TECHNOL, V19, P221, DOI 10.1002/pat.1000
Yang F, 2019, J IND ENG CHEM, V69, P422, DOI 10.1016/j.jiec.2018.10.004
Yang YY, 2021, J MATER PROCESS TECH, V291, DOI 10.1016/j.jmatprotec.2020.117023
Yee S.Y.Y., 2021, MALAYSIAN J SUSTAIN, V5, P82
YIGIT F, 1989, RADIAT PHYS CHEM, V33, P97
Yin ZZ, 2010, BIOSENS BIOELECTRON, V25, P1319, DOI 10.1016/j.bios.2009.10.021
Yuan SM, 2016, MACROMOL BIOSCI, V16, P341, DOI 10.1002/mabi.201500302
Zaquen N, 2020, PROG POLYM SCI, V107, DOI 10.1016/j.progpolymsci.2020.101256
Zenoozi S, 2020, EUR POLYM J, V140, DOI 10.1016/j.eurpolymj.2020.109974
Zhang K, 2020, INT J NANOMED, V15, P5517, DOI 10.2147/IJN.S257906
Zhang KJ, 2021, J BIOMAT SCI-POLYM E, V32, P695, DOI 10.1080/09205063.2020.1861734
Zhang QC, 2020, CHEM ENG J, V391, DOI 10.1016/j.cej.2019.123525
Zhang WJ, 2021, J MACROMOL SCI A, V58, P860, DOI 10.1080/10601325.2021.1964368
Zhang Y, 2015, CHEM SOC REV, V44, P315, DOI 10.1039/c4cs00199k
Zhang ZC, 2022, INT J ADV MANUF TECH, V119, P5671, DOI 10.1007/s00170-022-08660-z
Zhang ZQ, 2020, AM J CANCER RES, V10, P350
Zhang ZH, 2015, BIOSENS BIOELECTRON, V74, P384, DOI 10.1016/j.bios.2015.06.062
Zhang ZL, 2022, COMPUT BIOL MED, V140, DOI 10.1016/j.compbiomed.2021.105092
Zhao S, 2020, NANOSCALE, V12, P695, DOI 10.1039/c9nr09401f
Zhao TH, 2021, APPL COMPUT MATH-BAK, V20, P160
Zhao TH, 2023, MATH METHOD APPL SCI, V46, P3012, DOI 10.1002/mma.7310
Zhao YF, 2020, J CHROMATOGR A, V1610, DOI 10.1016/j.chroma.2019.460543
Zhao Y, 2021, NANOSCALE, V13, P16251, DOI 10.1039/d1nr04067g
Zhu Q, 2021, J HAZARD MATER, V403, DOI 10.1016/j.jhazmat.2020.123941
Zong X, 2021, NUCLEIC ACIDS RES, V49, P5537, DOI 10.1093/nar/gkab343

Zuo YW, 2020, CRYSTALS, V10, DOI 10.3390/cryst10050406
NR 265
TC 55
Z9 55
U1 57
U2 161
PU MDPI
PI BASEL
PA ST ALBAN-ANLAGE 66, CH-4052 BASEL, SWITZERLAND
EI 2073-4360
J9 POLYMERS-BASEL
JI Polymers
PD MAR
PY 2022
VL 14
IS 6
AR 1259
DI 10.3390/polym14061259
PG 33
WC Polymer Science
WE Science Citation Index Expanded (SCI-EXPANDED)
SC Polymer Science
GA 0B6KI
UT WOS:000774741000001
PM 35335590
OA Green Published, gold
HC Y
HP N
DA 2023-09-14
ER

PT J
AU Qin, H
Zhao, RF
Qin, YT
Zhu, J
Chen, L
Di, CZ
Han, XX
Cheng, KM
Zhang, YL
Zhao, Y
Shi, J
Anderson, GJ
Zhao, YL
Nie, GJ
AF Qin, Hao
Zhao, Ruifang
Qin, Yuting
Zhu, Jin
Chen, Long
Di, Chunzhi
Han, Xuexiang
Cheng, Keman
Zhang, Yinlong
Zhao, Ying
Shi, Jian
Anderson, Gregory J.
Zhao, Yuliang
Nie, Guangjun
TI Development of a Cancer Vaccine Using In Vivo Click-Chemistry-Mediated

Active Lymph Node Accumulation for Improved Immunotherapy
SO ADVANCED MATERIALS
LA English
DT Article
DE cancer vaccines; click chemistry; immunotherapy; lymph node targeting
ID DENDRITIC CELLS; DELIVERY; NANOVACCINE; ANTIGEN
AB Due to their ability to elicit a potent immune reaction with low systemic toxicity, cancer vaccines represent a promising strategy for treating tumors. Considerable effort has been directed toward improving the in vivo efficacy of cancer vaccines, with direct lymph node (LN) targeting being the most promising approach. Here, a click-chemistry-based active LN accumulation system (ALAS) is developed by surface modification of lymphatic endothelial cells with an azide group, which provide targets for dibenzocyclooctyne (DBCO)-modified liposomes, to improve the delivery of encapsulated antigen and adjuvant to LNs. When loading with OVA(257-264) peptide and poly(I:C), the formulation elicits an enhanced CD8(+) T cell response in vivo, resulting in a much more efficient therapeutic effect and prolonged median survival of mice. Compared to treatment with DBCO-conjugated liposomes (DL)-Ag/Ad without the azide targeting, the percent survival of ALAS-vaccine-treated mice improves by 100% over 60 days. Altogether, the findings indicate that the novel ALAS approach is a powerful strategy to deliver vaccine components to LNs for enhanced antitumor immunity.
C1 [Qin, Hao; Zhao, Ruifang; Qin, Yuting; Zhu, Jin; Chen, Long; Di, Chunzhi; Han, Xuexiang; Cheng, Keman; Zhang, Yinlong; Zhao, Ying; Shi, Jian; Zhao, Yuliang; Nie, Guangjun] Natl Ctr Nanosci & Technol, CAS Ctr Excellence Nanosci, CAS Key Lab Biol Effects Nanomat & Nanosafety, Beijing 100190, Peoples R China.
[Qin, Hao; Zhao, Ruifang; Qin, Yuting; Zhu, Jin; Chen, Long; Di, Chunzhi; Han, Xuexiang; Zhang, Yinlong; Zhao, Ying; Shi, Jian; Zhao, Yuliang; Nie, Guangjun] Univ Chinese Acad Sci, Ctr Mat Sci & Optoelect Engn, Beijing 100049, Peoples R China.
[Anderson, Gregory J.] QIMR Berghofer Med Res Inst, Iron Metab Lab, Brisbane, Qld 4006, Australia.
[Zhao, Ruifang; Zhao, Ying; Nie, Guangjun] GBA Natl Inst Nanotechnol Innovat, Guangzhou 510700, Guangdong, Peoples R China.
C3 Chinese Academy of Sciences; National Center for Nanoscience & Technology - China; Chinese Academy of Sciences; University of Chinese Academy of Sciences, CAS; QIMR Berghofer Medical Research Institute
RP Zhao, RF; Nie, GJ (通讯作者)，Natl Ctr Nanosci & Technol, CAS Ctr Excellence Nanosci, CAS Key Lab Biol Effects Nanomat & Nanosafety, Beijing 100190, Peoples R China.; Zhao, RF; Nie, GJ (通讯作者)，Univ Chinese Acad Sci, Ctr Mat Sci & Optoelect Engn, Beijing 100049, Peoples R China.; Zhao, RF; Nie, GJ (通讯作者)，GBA Natl Inst Nanotechnol Innovat, Guangzhou 510700, Guangdong, Peoples R China.
EM zhaorf@nanoctr.cn; niegj@nanoctr.cn
FU National Basic Research Plan of China [2018YFA0208900, 2018YFE0205300]; Strategic Priority Research Program of the Chinese Academy of Sciences [XDB36000000]; National Natural Science Foundation of China [31730032, 31820103004, 11621505, 31800799, 31571021]; K.C. Wong Education Foundation [GJTD-2018-03]
FX This work was supported by grants from the National Basic Research Plan of China (2018YFA0208900, 2018YFE0205300), the Strategic Priority Research Program of the Chinese Academy of Sciences (XDB36000000), the National Natural Science Foundation of China (31730032, 31820103004, 11621505, 31800799, and 31571021), the K.C. Wong Education Foundation (GJTD-2018-03).
CR Alexopoulou L, 2001, NATURE, V413, P732, DOI 10.1038/35099560
Cho HJ, 2000, NAT BIOTECHNOL, V18, P509, DOI 10.1038/75365
Dieterich LC, 2016, ADV DRUG DELIVER REV, V99, P148, DOI 10.1016/j.addr.2015.12.011
Jewett JC, 2010, J AM CHEM SOC, V132, P3688, DOI 10.1021/ja100014q
Kastenmuller W, 2014, NAT REV IMMUNOL, V14, P705, DOI 10.1038/nri3727
Koo H, 2012, ANGEW CHEM INT EDIT, V51, P11836, DOI 10.1002/anie.201206703
Krempski J, 2011, J IMMUNOL, V186, P6905, DOI 10.4049/jimmunol.1100274
Kuai R, 2017, NAT MATER, V16, P489, DOI [10.1038/nmat4822, 10.1038/NMAT4822]
Liu HP, 2014, NATURE, V507, P519, DOI 10.1038/nature12978
Liu HP, 2011, ANGEW CHEM INT EDIT, V50, P7052, DOI 10.1002/anie.201101266
Luo M, 2017, NAT NANOTECHNOL, V12, P648, DOI [10.1038/nnano.2017.52, 10.1038/NNANO.2017.52]
Melief CJM, 2008, NAT REV CANCER, V8, P351, DOI 10.1038/nrc2373

Merad M, 2013, ANNU REV IMMUNOL, V31, P563, DOI 10.1146/annurev-immunol-020711-074950
Ni QQ, 2020, SCI ADV, V6, DOI 10.1126/sciadv.aaw6071
Nuhn L, 2018, ADV MATER, V30, DOI 10.1002/adma.201803397
Ochoa MC, 2013, CANCER RES, V73, P139, DOI 10.1158/0008-5472.CAN-12-2660
Randolph GJ, 2005, NAT REV IMMUNOL, V5, P617, DOI 10.1038/nri1670
Reddy ST, 2006, J CONTROL RELEASE, V112, P26, DOI 10.1016/j.jconrel.2006.01.006
ROTZSCHKE O, 1991, EUR J IMMUNOL, V21, P2891, DOI 10.1002/eji.1830211136
Schudel A, 2019, NAT REV MATER, V4, P415, DOI 10.1038/s41578-019-0110-7
Stacker SA, 2014, NAT REV CANCER, V14, P159, DOI 10.1038/nrc3677
Van Herck S, 2018, J AM CHEM SOC, V140, P14300, DOI 10.1021/jacs.8b08595
Wang RF, 2002, NAT BIOTECHNOL, V20, P149, DOI 10.1038/nbt0202-149
Wang Y, 2015, P NATL ACAD SCI USA, V112, P208, DOI 10.1073/pnas.1414821112
Xu J, 2019, BIOMATERIALS, V207, P1, DOI 10.1016/j.biomaterials.2019.03.037
Zhu GZ, 2017, NAT COMMUN, V8, DOI 10.1038/s41467-017-02191-y
Zhu GZ, 2017, ACS NANO, V11, P2387, DOI 10.1021/acsnano.7b00978
Zhu MT, 2018, J CONTROL RELEASE, V272, P72, DOI 10.1016/j.jconrel.2018.01.004
NR 28
TC 55
Z9 59
U1 23
U2 222
PU WILEY-V C H VERLAG GMBH
PI WEINHEIM
PA POSTFACH 101161, 69451 WEINHEIM, GERMANY
SN 0935-9648
EI 1521-4095
J9 ADV MATER
JI Adv. Mater.
PD MAY
PY 2021
VL 33
IS 20
AR 2006007
DI 10.1002/adma.202006007
EA MAR 2021
PG 15
WC Chemistry, Multidisciplinary; Chemistry, Physical; Nanoscience &
Nanotechnology; Materials Science, Multidisciplinary; Physics, Applied;
Physics, Condensed Matter
WE Science Citation Index Expanded (SCI-EXPANDED)
SC Chemistry; Science & Technology - Other Topics; Materials Science;
Physics
GA SE2PH
UT WOS:000635431600001
PM 33792097
DA 2023-09-14
ER

PT J
AU Liu, Y
Guo, JF
Huang, L
AF Liu, Yun
Guo, Jianfeng
Huang, Leaf
TI Modulation of tumor microenvironment for immunotherapy: focus on
nanomaterial-based strategies
SO THERANOSTICS
LA English
DT Review
DE tumor immunology; characterization and quantification of

immunoregulatory cells; nanoparticles; drug delivery; combination therapy
ID REGULATORY T-CELLS; SUPPRESSOR-CELLS; FLOW-CYTOMETRY; ENGINEERING NANOPARTICLES; ANTITUMOR IMMUNITY; B-CELLS; CANCER; MACROPHAGES; NANOVACCINE; ANTIGEN
AB Recent advances in the field of immunotherapy have profoundly opened up the potential for improved cancer therapy and reduced side effects. However, the tumor microenvironment (TME) is highly immunosuppressive, therefore, clinical outcomes of currently available cancer immunotherapy are still poor. Recently, nanomaterial-based strategies have been developed to modulate the TME for robust immunotherapeutic responses. In this review, the immunoregulatory cell types (cells relating to the regulation of immune responses) inside the TME in terms of stimulatory and suppressive roles are described, and the technologies used to identify and quantify these cells are provided. In addition, recent examples of nanomaterial-based cancer immunotherapy are discussed, with particular emphasis on those designed to overcome barriers caused by the complexity and diversity of TME.
C1 [Liu, Yun; Guo, Jianfeng; Huang, Leaf] Univ N Carolina, Eshelman Sch Pharm, Div Pharmacoengn & Mol Pharmaceut, Chapel Hill, NC 27515 USA.
[Guo, Jianfeng] Jilin Univ, Sch Pharmaceut Sci, Changchun 130021, Peoples R China.
C3 University of North Carolina; University of North Carolina Chapel Hill; Jilin University
RP Guo, JF; Huang, L (通讯作者)，Univ N Carolina, Eshelman Sch Pharm, Div Pharmacoengn & Mol Pharmaceut, Chapel Hill, NC 27515 USA.; Guo, JF (通讯作者)，Jilin Univ, Sch Pharmaceut Sci, Changchun 130021, Peoples R China.
EM jguo@jlu.edu.cn; leafh@email.unc.edu
OI Liu, Yun/0000-0002-6763-496X
FU Carolina Center for Cancer Nanotechnology Excellence (NIH) [CA198999]; "Talents Cultivation Program" of Jilin University
FX We acknowledge the Carolina Center for Cancer Nanotechnology Excellence (NIH grant CA198999) for the support in funding our research. This work is also supported by "Talents Cultivation Program" of Jilin University.
CR Adan A, 2017, CRIT REV BIOTECHNOL, V37, P163, DOI 10.3109/07388551.2015.1128876
Adkins I, 2014, ONCOIMMUNOLOGY, V3, DOI 10.4161/21624011.2014.968434
AlJanahi AA, 2018, MOL THER-METH CLIN D, V10, P189, DOI 10.1016/j.omtm.2018.07.003
Asadzadeh Z, 2017, CELL IMMUNOL, V322, P15, DOI 10.1016/j.cellimm.2017.10.015
Belli C, 2018, CANCER TREAT REV, V65, P22, DOI 10.1016/j.ctrv.2018.02.004
Bi JC, 2019, FRONT IMMUNOL, V10, DOI 10.3389/fimmu.2019.01999
Binnewies M, 2018, NAT MED, V24, P541, DOI 10.1038/s41591-018-0014-x
Biswas SK, 2010, NAT IMMUNOL, V11, P889, DOI 10.1038/ni.1937
Cao XF, 2007, IMMUNITY, V27, P635, DOI 10.1016/j.immuni.2007.08.014
Cariappa A, 2007, J IMMUNOL, V179, P2270, DOI 10.4049/jimmunol.179.4.2270
Chen DS, 2013, IMMUNITY, V39, P1, DOI 10.1016/j.immuni.2013.07.012
Chen Q, 2019, CHEM SOC REV, V48, P5506, DOI 10.1039/c9cs00271e
Chen Q, 2019, ADV MATER, V31, DOI 10.1002/adma.201900192
Chen Q, 2019, ADV MATER, V31, DOI 10.1002/adma.201802228
Chen Q, 2019, NAT NANOTECHNOL, V14, P89, DOI 10.1038/s41565-018-0319-4
Chen YZ, 2019, ACS NANO, V13, P1751, DOI 10.1021/acsnano.8b07830
Chen YP, 2017, THERANOSTICS, V7, P3585, DOI 10.7150/thno.21471
Cheng KM, 2018, NANO LETT, V18, P3250, DOI 10.1021/acs.nanolett.8b01071
Chinen T, 2016, NAT IMMUNOL, V17, P1322, DOI 10.1038/ni.3540
Chiswick EL, 2012, METHODS MOL BIOL, V844, P15, DOI 10.1007/978-1-61779-527-5_2
Cho NH, 2011, NAT NANOTECHNOL, V6, P675, DOI [10.1038/NNANO.2011.149, 10.1038/nnano.2011.149]
Condamine T, 2016, SCI IMMUNOL, V1, DOI 10.1126/sciimmunol.aaf8943
Condamine T, 2011, TRENDS IMMUNOL, V32, P19, DOI 10.1016/j.it.2010.10.002
Corrales L, 2015, CELL REP, V11, P1018, DOI 10.1016/j.celrep.2015.04.031
Curran MA, 2019, ANNU REV MED, V70, P409, DOI 10.1146/annurev-med-050217-121900
D'Aloia MM, 2018, CELL DEATH DIS, V9, DOI 10.1038/s41419-018-0278-6
Dai Q, 2018, ACS NANO, V12, P8423, DOI 10.1021/acsnano.8b03900
de Matos LL, 2010, BIOMARK INSIGHTS, V5, P9
de Palma M, 2017, NAT REV CANCER, V17, P457, DOI 10.1038/nrc.2017.51
Ding L, 2014, NAT REV GENET, V15, P556, DOI 10.1038/nrg3767
Drobek A, 2018, EMBO J, V37, DOI 10.15252/embj.201798518

Duan XP, 2019, NAT COMMUN, V10, DOI 10.1038/s41467-019-09221-x
Duan XP, 2019, ANGEW CHEM INT EDIT, V58, P670, DOI 10.1002/anie.201804882
Dudek AM, 2013, FRONT IMMUNOL, V4, DOI 10.3389/fimmu.2013.00438
Dudek AM, 2013, CYTOKINE GROWTH F R, V24, P319, DOI 10.1016/j.cytogfr.2013.01.005
Ernsting MJ, 2015, J CONTROL RELEASE, V206, P122, DOI 10.1016/j.jconrel.2015.03.023
Farhood B, 2019, J CELL PHYSIOL, V234, P8509, DOI 10.1002/jcp.27782
Feng B, 2018, ADV MATER, V30, DOI 10.1002/adma.201803001
Finotello F, 2019, NAT REV GENET, V20, P724, DOI 10.1038/s41576-019-0166-7
Flood BA, 2019, IMMUNOL REV, V290, P24, DOI 10.1111/imr.12765
Fontenot JD, 2017, J IMMUNOL, V198, P986, DOI 10.1038/ni904
Fridman WH, 2017, NAT REV CLIN ONCOL, V14, P717, DOI 10.1038/nrclinonc.2017.101
Gabrilovich DI, 2017, CANCER IMMUNOL RES, V5, P3, DOI 10.1158/2326-6066.CIR-16-0297
Gabrilovich DI, 2012, NAT REV IMMUNOL, V12, P253, DOI 10.1038/nri3175
Gao S, 2019, THERANOSTICS, V9, P126, DOI 10.7150/thno.29431
Gerdes MJ, 2013, P NATL ACAD SCI USA, V110, P11982, DOI 10.1073/pnas.1300136110
Goldberg MS, 2019, NAT REV CANCER, V19, P587, DOI 10.1038/s41568-019-0186-9
Goodwin TJ, 2016, SCI TRANSL MED, V8, DOI 10.1126/scitranslmed.aag2306
Granot Z, 2019, FRONT IMMUNOL, V10, DOI 10.3389/fimmu.2019.01710
Guillerey C, 2016, NAT IMMUNOL, V17, P1025, DOI 10.1038/ni.3518
Guo FF, 2019, J ONCOL, V2019, DOI 10.1155/2019/2592419
Guo YY, 2015, ACS NANO, V9, P6918, DOI 10.1021/acsnano.5b01042
Habif G, 2019, CELL MOL IMMUNOL, V16, P415, DOI 10.1038/s41423-019-0224-2
Hackl H, 2016, NAT REV GENET, V17, P441, DOI 10.1038/nrg.2016.67
Hagen JB, 2000, NAT REV GENET, V1, P231, DOI 10.1038/35042090
Han QS, 2017, NANOSCALE, V9, P5927, DOI 10.1039/c7nr01460k
Han S, 2019, FRONT ONCOL, V9, DOI 10.3389/fonc.2019.00279
Han SY, 2020, NANOSCALE, V12, P413, DOI 10.1039/c9nr08086d
Han XX, 2018, NAT COMMUN, V9, DOI 10.1038/s41467-018-05906-x
Han YY, 2016, LAB CHIP, V16, P4639, DOI 10.1039/c6lc01063f
Hanahan D, 2012, CANCER CELL, V21, P309, DOI 10.1016/j.ccr.2012.02.022
Hinshaw DC, 2019, CANCER RES, V79, P4557, DOI 10.1158/0008-5472.CAN-18-3962
Hu MY, 2019, NAT COMMUN, V10, DOI 10.1038/s41467-019-10893-8
Hu QL, 2015, NANO LETT, V15, P2732, DOI 10.1021/acs.nanolett.5b00570
Huang YH, 2012, P NATL ACAD SCI USA, V109, P17561, DOI 10.1073/pnas.1215397109
Hwang B, 2018, EXP MOL MED, V50, DOI 10.1038/s12276-018-0071-8
Ishii G, 2016, ADV DRUG DELIVER REV, V99, P186, DOI 10.1016/j.addr.2015.07.007
Ishikawa H, 2009, NATURE, V461, P788, DOI 10.1038/nature08476
Janetzki S, 2015, NAT PROTOC, V10, P1098, DOI 10.1038/nprot.2015.068
Jensen-Jarolim E, 2015, CANCER IMMUNOL IMMUN, V64, P531, DOI 10.1007/s00262-015-1684-6
Kalluri R, 2016, NAT REV CANCER, V16, P582, DOI 10.1038/nrc.2016.73
Kaminski DA, 2012, FRONT IMMUNOL, V3, DOI 10.3389/fimmu.2012.00302
Kennedy R, 2008, IMMUNOL REV, V222, P129, DOI 10.1111/j.1600-065X.2008.00616.x
Kessel A, 2012, AUTOIMMUN REV, V11, P670, DOI 10.1016/j.autrev.2011.11.018
Khan AR, 2015, NAT COMMUN, V6, DOI 10.1038/ncomms6997
Kim HJ, 2014, CANCER IMMUNOL RES, V2, P91, DOI 10.1158/2326-6066.CIR-13-0216
Kim SW, 2016, J PATHOL TRANSL MED, V50, P411, DOI 10.4132/jptm.2016.08.08
Kobayashi H, 2019, NAT REV GASTRO HEPAT, V16, P282, DOI 10.1038/s41575-019-0115-0
Kondelkova Katerina, 2010, Acta Medica (Hradec Kralove), V53, P73
Koyama S, 2016, NAT COMMUN, V7, DOI 10.1038/ncomms10501
Kroll AV, 2019, ADV BIOSYST, V3, DOI 10.1002/adbi.201800219
Kroll AV, 2017, ADV MATER, V29, DOI 10.1002/adma.201703969
Kruger P, 2015, PLOS PATHOG, V11, DOI 10.1371/journal.ppat.1004651
Krysko DV, 2012, NAT REV CANCER, V12, P860, DOI 10.1038/nrc3380
Kuai R, 2018, SCI ADV, V4, DOI 10.1126/sciadv.aao1736
Kuai R, 2017, NAT MATER, V16, P489, DOI [10.1038/nmat4822, 10.1038/NMAT4822]
Kumar V, 2016, TRENDS IMMUNOL, V37, P208, DOI 10.1016/j.it.2016.01.004
Landskron G, 2014, J IMMUNOL RES, V2014, DOI 10.1155/2014/149185
LeBien TW, 2008, BLOOD, V112, P1570, DOI 10.1182/blood-2008-02-078071
Lee C, 2019, NANOSCALE, V11, P13878, DOI 10.1039/c9nr03510a
Li XY, 2019, NAT REV CLIN ONCOL, V16, P425, DOI 10.1038/s41571-019-0203-7
Lim S, 2019, THERANOSTICS, V9, P7906, DOI 10.7150/thno.38425

Lin JR, 2018, ELIFE, V7, DOI 10.7554/eLife.31657
Liu Q, 2018, ACS NANO, V12, P7812, DOI 10.1021/acsnano.8b01890
Liu Q, 2018, CANCER IMMUNOL IMMUN, V67, P299, DOI 10.1007/s00262-017-2079-7
Liu XD, 2010, BLOOD, V115, P3520, DOI 10.1182/blood-2009-09-246124
Lu Y, 2018, CANCER CELL, V33, P1048, DOI 10.1016/j.ccell.2018.05.004
Luo M, 2017, J CONTROL RELEASE, V263, P200, DOI 10.1016/j.jconrel.2017.03.033
Luo M, 2017, NAT NANOTECHNOL, V12, P648, DOI [10.1038/nnano.2017.52, 10.1038/NNANO.2017.52]
Luo ZC, 2015, BIOMATERIALS, V38, P50, DOI 10.1016/j.biomaterials.2014.10.050
Ma L, 2019, SCIENCE, V365, P162, DOI 10.1126/science.aav8692
Maino VC, 1998, CYTOMETRY, V34, P207, DOI 10.1002/(SICI)1097-0320(19981015)34:5<207::AID-CYTO1>3.0.CO;2-J
Mandruzzato S, 2016, CANCER IMMUNOL IMMUN, V65, P161, DOI 10.1007/s00262-015-1782-5
Mantovani A, 2017, NAT REV CLIN ONCOL, V14, P399, DOI 10.1038/nrclinonc.2016.217
Mantovani A, 2015, J EXP MED, V212, P435, DOI 10.1084/jem.20150295
Marvel D, 2015, J CLIN INVEST, V125, P3356, DOI 10.1172/JCI80005
Mellman I, 2013, CANCER IMMUNOL RES, V1, P145, DOI 10.1158/2326-6066.CIR-13-0102
Mellor AL, 2008, NAT REV IMMUNOL, V8, P74, DOI 10.1038/nri2233
Meng ZQ, 2019, ADV MATER, V31, DOI 10.1002/adma.201900927
Mi Y, 2018, ADV MATER, V30, DOI 10.1002/adma.201706098
Min H, 2019, ADV MATER, V31, DOI 10.1002/adma.201808200
Min YZ, 2017, NAT NANOTECHNOL, V12, P877, DOI [10.1038/nnano.2017.113, 10.1038/NNANO.2017.113]
Mukherjee S, 2019, THERANOSTICS, V9, P7730, DOI 10.7150/thno.37306
Munn DH, 2013, TRENDS IMMUNOL, V34, P137, DOI 10.1016/j.it.2012.10.001
Murray PJ, 2014, IMMUNITY, V41, P14, DOI 10.1016/j.immuni.2014.06.008
Nelson BH, 2010, J IMMUNOL, V185, P4977, DOI 10.4049/jimmunol.1001323
Nishikawa H, 2010, INT J CANCER, V127, P759, DOI 10.1002/ijc.25429
Noy R, 2014, IMMUNITY, V41, P49, DOI 10.1016/j.immuni.2014.06.010
O'Donnell JS, 2019, NAT REV CLIN ONCOL, V16, P151, DOI 10.1038/s41571-018-0142-8
Pahl J, 2017, IMMUNOBIOLOGY, V222, P11, DOI 10.1016/j.imbio.2015.07.012
Paiva AE, 2018, CANCER RES, V78, P2779, DOI 10.1158/0008-5472.CAN-17-3883
Park J, 2019, THERANOSTICS, V9, P8073, DOI 10.7150/thno.37198
Paul S, 2017, FRONT IMMUNOL, V8, DOI 10.3389/fimmu.2017.01124
Pawelec G, 2019, FRONT IMMUNOL, V10, DOI 10.3389/fimmu.2019.01099
Peng B, 2018, CELL DEATH DIS, V9, DOI 10.1038/s41419-017-0152-y
Phuengkham H, 2019, ADV MATER, V31, DOI 10.1002/adma.201803322
Qian Y, 2017, ACS NANO, V11, P9536, DOI 10.1021/acsnano.7b05465
Quail DF, 2019, NAT REV ENDOCRINOL, V15, P139, DOI 10.1038/s41574-018-0126-x
Le QV, 2019, AAPS J, V21, DOI 10.1208/s12248-019-0333-y
Qureshi OS, 2011, SCIENCE, V332, P600, DOI 10.1126/science.1202947
Riabov V, 2014, FRONT PHYSIOL, V5, DOI 10.3389/fphys.2014.00075
Rimm DL, 2014, NAT METHODS, V11, P381, DOI 10.1038/nmeth.2896
Robert C, 2015, NEW ENGL J MED, V372, P2521, DOI 10.1056/NEJMoa1503093
Robillard N, 2014, FRONT IMMUNOL, V5, DOI 10.3389/fimmu.2014.00137
Rodell CB, 2018, NAT BIOMED ENG, V2, P578, DOI 10.1038/s41551-018-0236-8
Rodriguez R, 2014, NAT REV GENET, V15, P783, DOI 10.1038/nrg3796
Rosser EC, 2015, IMMUNITY, V42, P607, DOI 10.1016/j.immuni.2015.04.005
Ruan HT, 2019, ADV MATER, V31, DOI 10.1002/adma.201806957
Saeed M, 2019, THERANOSTICS, V9, P7981, DOI 10.7150/thno.37568
Sahin U, 2018, SCIENCE, V359, P1355, DOI 10.1126/science.aar7112
Sang W, 2019, CHEM SOC REV, V48, P3771, DOI 10.1039/c8cs00896e
Sanmamed MF, 2018, CELL, V175, P313, DOI 10.1016/j.cell.2018.09.035
Sarvaria A, 2017, CELL MOL IMMUNOL, V14, P662, DOI 10.1038/cmi.2017.35
Sautes-Fridman C, 2019, NAT REV CANCER, V19, P307, DOI 10.1038/s41568-019-0144-6
Schaaf MB, 2018, CELL DEATH DIS, V9, DOI 10.1038/s41419-017-0061-0
Schumacher TN, 2019, ANNU REV IMMUNOL, V37, P173, DOI 10.1146/annurev-immunol-042617-053402
Shae D, 2019, NAT NANOTECHNOL, V14, P269, DOI 10.1038/s41565-018-0342-5
Shalek AK, 2013, NATURE, V498, P236, DOI 10.1038/nature12172
Shen LM, 2018, ACS NANO, V12, P9830, DOI 10.1021/acsnano.8b00967

Slaney CY, 2014, CANCER RES, V74, P7168, DOI 10.1158/0008-5472.CAN-14-2458
Song WT, 2018, ADV MATER, V30, DOI 10.1002/adma.201805007
Song WT, 2018, NAT COMMUN, V9, DOI 10.1038/s41467-018-04605-x
Song WT, 2017, BIOMATERIALS, V148, P16, DOI 10.1016/j.biomaterials.2017.09.017
Spitzer MH, 2017, CELL, V168, P487, DOI 10.1016/j.cell.2016.12.022
Stephan MT, 2012, BIOMATERIALS, V33, P5776, DOI 10.1016/j.biomaterials.2012.04.029
Stephan MT, 2010, NAT MED, V16, P1035, DOI 10.1038/nm.2198
Su T, 2019, THERANOSTICS, V9, P7759, DOI 10.7150/thno.37574
Sung YC, 2019, NAT NANOTECHNOL, V14, P1160, DOI 10.1038/s41565-019-0570-3
Szabo PA, 2019, NAT COMMUN, V10, DOI 10.1038/s41467-019-12464-3
Takahashi T, 2000, J EXP MED, V192, P303, DOI 10.1084/jem.192.2.303
Tanaka A, 2017, CELL RES, V27, P109, DOI 10.1038/cr.2016.151
Tang L, 2018, NAT BIOTECHNOL, V36, P707, DOI 10.1038/nbt.4181
Tsou PL, 2016, CANCER RES, V76, P5597, DOI 10.1158/0008-5472.CAN-16-0431
Turley SJ, 2015, NAT REV IMMUNOL, V15, P669, DOI 10.1038/nri3902
Ugel S, 2015, J CLIN INVEST, V125, P3365, DOI 10.1172/JCI80006
Uyttenhove C, 2003, NAT MED, V9, P1269, DOI 10.1038/nm934
Veglia F, 2018, NAT IMMUNOL, V19, P108, DOI 10.1038/s41590-017-0022-x
Veluchamy JP, 2017, SCI REP-UK, V7, DOI 10.1038/srep43873
Verma V, 2019, NAT IMMUNOL, V20, P1231, DOI 10.1038/s41590-019-0441-y
Vesely MD, 2011, ANNU REV IMMUNOL, V29, P235, DOI 10.1146/annurev-immunol-031210-101324
Vijayan V, 2019, PHARMACEUTICS, V11, DOI 10.3390/pharmaceutics11100534
Vivier E, 2008, NAT IMMUNOL, V9, P503, DOI 10.1038/ni1582
Wang C, 2017, NAT BIOMED ENG, V1, DOI 10.1038/s41551-016-0011
Wang XP, 2016, ANGEW CHEM INT EDIT, V55, P1899, DOI 10.1002/anie.201506179
Wang YC, 2010, CANCER RES, V70, P4840, DOI 10.1158/0008-5472.CAN-10-0269
Wang YH, 2018, MOL THER, V26, P420, DOI 10.1016/j.ymthe.2017.11.009
Wculek SK, 2020, NAT REV IMMUNOL, V20, P7, DOI 10.1038/s41577-019-0210-z
Weiner LM, 2010, NAT REV IMMUNOL, V10, P317, DOI 10.1038/nri2744
Workenhe ST, 2014, MOL THER, V22, P251, DOI 10.1038/mt.2013.220
Xiong Y, 2019, NANOMED-NANOTECHNOL, V21, DOI 10.1016/j.nano.2019.102034
Xu X, 2019, THERANOSTICS, V9, P7889, DOI 10.7150/thno.38583
Yang GB, 2017, NAT COMMUN, V8, DOI 10.1038/s41467-017-01050-0
Yang R, 2018, ACS NANO, V12, P5121, DOI 10.1021/acsnano.7b09041
Yang VC, 2019, J CONTROL RELEASE, V311, P322, DOI 10.1016/j.jconrel.2019.10.021
Yata T, 2017, BIOMATERIALS, V146, P136, DOI 10.1016/j.biomaterials.2017.09.014
Zhang B, 2017, FRONT PHARMACOL, V8, DOI 10.3389/fphar.2017.00952
Zhang J, 2019, ACS NANO, V13, P12511, DOI 10.1021/acsnano.9b02875
Zhang N, 2011, IMMUNITY, V35, P161, DOI 10.1016/j.immuni.2011.07.010
Zhang PC, 2019, ADV MATER, V31, DOI 10.1002/adma.201904156
Zhang XQ, 2019, J CONTROL RELEASE, V309, P173, DOI 10.1016/j.jconrel.2019.07.039
Zhang Y, 2019, WIRES NANOMED NANOBI, V11, DOI 10.1002/wnan.1559
Zhou J, 2019, ADV MATER
Zhu GZ, 2017, ACS NANO, V11, P2387, DOI 10.1021/acsnano.7b00978
Zhu JF, 2008, BLOOD, V112, P1557, DOI 10.1182/blood-2008-05-078154
Ziani L, 2018, FRONT IMMUNOL, V9, DOI 10.3389/fimmu.2018.00414
Zou WP, 2006, NAT REV IMMUNOL, V6, P295, DOI 10.1038/nri1806
NR 196
TC 55
Z9 57
U1 1
U2 52
PU IVYSPRING INT PUBL
PI LAKE HAVEN
PA PO BOX 4546, LAKE HAVEN, NSW 2263, AUSTRALIA
SN 1838-7640
J9 THERANOSTICS
JI Theranostics
PY 2020
VL 10
IS 7

BP 3099
EP 3117
DI 10.7150/thno.42998
PG 19
WC Medicine, Research & Experimental
WE Science Citation Index Expanded (SCI-EXPANDED)
SC Research & Experimental Medicine
GA KM9HS
UT WOS:000514452800016
PM 32194857
OA Green Published, gold
DA 2023-09-14
ER

PT J
AU Zhu, DW
Hu, CY
Fan, F
Qin, Y
Huang, CL
Zhang, ZM
Lu, L
Wang, H
Sun, HF
Leng, XG
Wang, C
Kong, DL
Zhang, LH
AF Zhu, Dunwan
Hu, Chunyan
Fan, Fan
Qin, Yu
Huang, Chenlu
Zhang, Zhiming
Lu, Lu
Wang, Hai
Sun, Hongfan
Leng, Xigang
Wang, Chun
Kong, Deling
Zhang, Linhua
TI Co-delivery of antigen and dual agonists by programmed mannose-targeted cationic lipid-hybrid polymersomes for enhanced vaccination
SO BIOMATERIALS
LA English
DT Article
DE Lipid-hybrid polymersomes; Mannose-receptor targeting; Antigen delivery; Imiquimod; Monophosphoryl lipid A; Cancer vaccine
ID MATURE DENDRITIC CELLS; NANOPARTICLE VACCINES; CROSS-PRESENTATION; IMMUNE-RESPONSES; LIPOSOMES; MEMORY; SYSTEM; SIZE; DIFFERENTIATION; ACTIVATION
AB Exploiting Toll-like receptor (TLR) agonists or their certain combinations can enhance the immune potency of subunit vaccine. Nevertheless, the design of co-delivery systems which can act in a synergistic and spatio-temporal way to achieve effective and durable specific immune response is still challenging. Here we fabricated mannose-functionalized lipid-hybrid polymersomes (MAN-IMO-PS) for co-delivery of ovalbumin antigen both inside the inner core and outside the lipid layer, TLR7/8 agonist imiquimod within the hydrophobic membrane, TLR4 agonist monophosphoryl lipid A in the lipid layer as programmed nanovaccine to synergistically activate immune responses for improving vaccine efficacy. After efficiently internalized by dendritic cells via mannose targeting and TLR4 ligating, MAN-IMO-PS significantly enhanced cross-presentation and cytokine production. In addition, MAN-IMO-PS showed depot effect at the injection site and enhanced migration to draining lymph nodes. Mice immunized with MAN-IMO-PS

elicited greater lymphocyte activation, CD4(+) and CD8(+) T cell response, effector cytokines secretion, and induced Th-1 biased humoral responses. More importantly, prophylactic vaccination by MAN-IMO-PS significantly delayed tumor occurrence, suppressed tumor growth with prolonged survival, and achieved long-term immune effect. The present study demonstrates a rationally designed nanovaccine for combining antigen, different TLR agonists, and targeting moiety in a programmed manner to induce synergistic antitumor immune response.
C1 [Zhu, Dunwan; Hu, Chunyan; Fan, Fan; Qin, Yu; Huang, Chenlu; Zhang, Zhiming; Wang, Hai; Sun, Hongfan; Leng, Xigang; Zhang, Linhua] Chinese Acad Med Sci, Inst Biomed Engn, Tianjin 300192, Peoples R China.
[Zhu, Dunwan; Hu, Chunyan; Fan, Fan; Qin, Yu; Huang, Chenlu; Zhang, Zhiming; Wang, Hai; Sun, Hongfan; Leng, Xigang; Zhang, Linhua] Peking Union Med Coll, Tianjin Key Lab Biomed Mat, Tianjin 300192, Peoples R China.
[Lu, Lu] Chinese Acad Med Sci, Inst Radiat Med, Tianjin 300192, Peoples R China.
[Lu, Lu] Peking Union Med Coll, Tianjin Key Lab Radiat Med & Mol Nucl Med, Tianjin 300192, Peoples R China.
[Wang, Chun] Univ Minnesota, Dept Biomed Engn, 7-116 Hasselmo Hall,312 Church St SE, Minneapolis, MN 55455 USA.
[Kong, Deling] Nankai Univ, Coll Life Sci, Minist Educ, Key Lab Bioact Mat, Tianjin 300071, Peoples R China.
[Kong, Deling] Xuzhou Med Univ, Canc Inst, Jiangsu Ctr Collaborat & Innovat Canc Biotherapy, Xuzhou 221004, Jiangsu, Peoples R China.
C3 Chinese Academy of Medical Sciences - Peking Union Medical College; Institute of BioMedical Engineering - CAMS; Chinese Academy of Medical Sciences - Peking Union Medical College; Peking Union Medical College; Chinese Academy of Medical Sciences - Peking Union Medical College; Institute of Radiation Medicine - CAMS; Chinese Academy of Medical Sciences - Peking Union Medical College; Peking Union Medical College; University of Minnesota System; University of Minnesota Twin Cities; Nankai University; Xuzhou Medical University
RP Zhang, LH (通讯作者)，Chinese Acad Med Sci, Inst Biomed Engn, Tianjin 300192, Peoples R China.; Zhang, LH (通讯作者)，Peking Union Med Coll, Tianjin Key Lab Biomed Mat, Tianjin 300192, Peoples R China.
EM zhanglinhua@bme.pumc.edu.cn
OI Qin, Yu/0000-0003-1130-7115
FU National Natural Science Foundation of China [81571793, 81671806, 51103180, 81100100]; CAMS Initiative for Innovative Medicine [2017-I2M-3-020, 2017-I2M-4-001]; Tianjin Municipal Natural Science Foundation [15JCZDJC38300]
FX This work was financially supported by National Natural Science Foundation of China (81571793, 81671806, 51103180, 81100100), CAMS Initiative for Innovative Medicine (2017-I2M-3-020, 2017-I2M-4-001), Tianjin Municipal Natural Science Foundation (15JCZDJC38300).
CR Akagi T, 2007, BIOMATERIALS, V28, P3427, DOI 10.1016/j.biomaterials.2007.04.023
Alving CR, 2012, EXPERT REV VACCINES, V11, P733, DOI [10.1586/ERV.12.35, 10.1586/erv.12.35]
Arifin WN, 2017, MALAYS J MED SCI, V24, P101, DOI 10.21315/mjms2017.24.5.11
Bachmann MF, 2010, NAT REV IMMUNOL, V10, P787, DOI 10.1038/nri2868
Banchereau J, 1998, NATURE, V392, P245, DOI 10.1038/32588
Bayyurt B, 2017, J CONTROL RELEASE, V247, P134, DOI 10.1016/j.jconrel.2017.01.004
Blander JM, 2006, NATURE, V440, P808, DOI 10.1038/nature04596
Cai S, 2011, ADV DRUG DELIVER REV, V63, P901, DOI 10.1016/j.addr.2011.05.017
Crotty S, 2015, NAT REV IMMUNOL, V15, P185, DOI 10.1038/nri3803
Demento SL, 2012, BIOMATERIALS, V33, P4957, DOI 10.1016/j.biomaterials.2012.03.041
Discher BM, 1999, SCIENCE, V284, P1143, DOI 10.1126/science.284.5417.1143
Dunn GP, 2006, NAT REV IMMUNOL, V6, P836, DOI 10.1038/nri1961
Fan YC, 2015, J CONTROL RELEASE, V208, P121, DOI 10.1016/j.jconrel.2015.04.010
Foged C, 2005, INT J PHARMACEUT, V298, P315, DOI 10.1016/j.ijpharm.2005.03.035
Hanson MC, 2014, BIOMACROMOLECULES, V15, P2475, DOI 10.1021/bm500337r
Heo MB, 2014, BIOMATERIALS, V35, P590, DOI 10.1016/j.biomaterials.2013.10.009
Iwasaki A, 2004, NAT IMMUNOL, V5, P987, DOI 10.1038/ni1112
Joffre OP, 2012, NAT REV IMMUNOL, V12, P557, DOI 10.1038/nri3254
Jones CH, 2015, BIOMATERIALS, V37, P333, DOI 10.1016/j.biomaterials.2014.10.037

Kaech SM, 2002, NAT REV IMMUNOL, V2, P251, DOI 10.1038/nri778
Kasturi SP, 2011, NATURE, V470, P543, DOI 10.1038/nature09737
Keler T, 2004, EXPERT OPIN BIOL TH, V4, P1953, DOI 10.1517/14712598.4.12.1953
Kennedy R, 2008, IMMUNOL REV, V222, P129, DOI 10.1111/j.1600-065X.2008.00616.x
Kuai R, 2018, J CONTROL RELEASE, V282, P131, DOI 10.1016/j.jconrel.2018.04.041
Larsson M, 2000, J IMMUNOL, V165, P1182, DOI 10.4049/jimmunol.165.3.1182
Lee JS, 2012, J CONTROL RELEASE, V161, P473, DOI 10.1016/j.jconrel.2011.10.005
Liu Q, 2015, ACS NANO, V9, P4925, DOI 10.1021/nn5066793
Lutz MB, 2002, TRENDS IMMUNOL, V23, P445, DOI 10.1016/S1471-4906(02)02281-0
Mohty M, 2003, J IMMUNOL, V171, P3385, DOI 10.4049/jimmunol.171.7.3385
Moon JJ, 2012, ADV MATER, V24, P3724, DOI 10.1002/adma.201200446
Moon JJ, 2011, NAT MATER, V10, P243, DOI 10.1038/NMAT2960
MOORE MW, 1988, CELL, V54, P777, DOI 10.1016/S0092-8674(88)91043-4
Napolitani G, 2005, NAT IMMUNOL, V6, P769, DOI 10.1038/ni1223
Primard C, 2013, MOL PHARMACEUT, V10, P2996, DOI 10.1021/mp400092y
Randolph GJ, 2005, NAT REV IMMUNOL, V5, P617, DOI 10.1038/nri1670
Sallusto F, 2004, ANNU REV IMMUNOL, V22, P745, DOI 10.1146/annurev.immunol.22.012703.104702
Sau S, 2018, J CONTROL RELEASE, V274, P24, DOI 10.1016/j.jconrel.2018.01.028
Sharma R, 2017, NANOMEDICINE-UK, V12, P1945, DOI 10.2217/nnm-2017-0088
Siefert AL, 2016, BIOMATERIALS, V97, P85, DOI 10.1016/j.biomaterials.2016.03.039
Silva JM, 2013, J CONTROL RELEASE, V168, P179, DOI 10.1016/j.jconrel.2013.03.010
STAHL P, 1980, CELL, V19, P207, DOI 10.1016/0092-8674(80)90402-X
STEVENS TL, 1988, NATURE, V334, P255, DOI 10.1038/334255a0
Trinchieri G, 2007, NAT REV IMMUNOL, V7, P179, DOI 10.1038/nri2038
Vedove ED, 2018, ADV HEALTHC MATER, V7, DOI 10.1002/adhm.201701398
Walch M, 2014, CELL, V157, P1309, DOI 10.1016/j.cell.2014.03.062
Wang C, 2014, VACCINE, V32, P5475, DOI 10.1016/j.vaccine.2014.07.081
Wang YQ, 2014, ADV HEALTHC MATER, V3, P670, DOI 10.1002/adhm.201300335
Wilson JT, 2013, ACS NANO, V7, P3912, DOI 10.1021/nn305466z
Yoshizaki Y, 2014, BIOMATERIALS, V35, P8186, DOI 10.1016/j.biomaterials.2014.05.077
Zehner M, 2013, MOL IMMUNOL, V55, P146, DOI 10.1016/j.molimm.2012.10.010
Zhang LH, 2015, INT J NANOMED, V10, P2101, DOI 10.2147/IJN.S77667
Zhang WD, 2003, IMMUNOLOGY, V108, P177, DOI 10.1046/j.1365-2567.2003.01489.x
Zhang WF, 2014, BIOMATERIALS, V35, P6086, DOI 10.1016/j.biomaterials.2014.04.022
Zhu DW, 2018, ACTA BIOMATER, V75, P386, DOI 10.1016/j.actbio.2018.05.033
Zhu DW, 2017, ACTA BIOMATER, V58, P399, DOI 10.1016/j.actbio.2017.06.017
2018, BIOMATERIALS, V170, P95, DOI DOI 10.1016/J.BIOMATERIALS.2018.04.003
NR 56
TC 55
Z9 55
U1 9
U2 154
PU ELSEVIER SCI LTD
PI OXFORD
PA THE BOULEVARD, LANGFORD LANE, KIDLINGTON, OXFORD OX5 1GB, OXON, ENGLAND
SN 0142-9612
EI 1878-5905
J9 BIOMATERIALS
JI Biomaterials
PD JUN
PY 2019
VL 206
BP 25
EP 40
DI 10.1016/j.biomaterials.2019.03.012
PG 16
WC Engineering, Biomedical; Materials Science, Biomaterials
WE Science Citation Index Expanded (SCI-EXPANDED)
SC Engineering; Materials Science
GA HX8QF
UT WOS:000467669700003

PM 30925286
DA 2023-09-14
ER

PT J
AU Bros, M
Nuhn, L
Simon, J
Moll, L
Mailander, V
Landfester, K
Grabbe, S
AF Bros, Matthias
Nuhn, Lutz
Simon, Johanna
Moll, Lorna
Mailaender, Volker
Landfester, Katharina
Grabbe, Stephan
TI The Protein Corona as a Confounding variable of nanoparticle-Mediated
Targeted vaccine Delivery
SO FRONTIERS IN IMMUNOLOGY
LA English
DT Review
DE nanocarriers; cancer vaccines; immunotherapy; protein corona;
cell-specific targeting
ID DENDRITIC CELLS; GOLD NANOPARTICLES; IN-VIVO; BIOMOLECULAR CORONA;
CELLULAR UPTAKE; POLY(ETHYLENE GLYCOL); PLASMA-PROTEINS;
SURFACE-DENSITY; DRUG-DELIVERY; RECEPTOR
AB Nanocarriers (NC) are very promising tools for cancer immunotherapy. Whereas conventional vaccines are based on the administration of an antigen and an adjuvant in an independent fashion, nanovaccines can facilitate cell-specific co-delivery of antigen and adjuvant. Furthermore, nanovaccines can be decorated on their surface with molecules that facilitate target-specific antigen delivery to certain antigen-presenting cell types or tumor cells. However, the target cell-specific uptake of nanovaccines is highly dependent on the modifications of the nanocarrier itself. One of these is the formation of a protein corona around NC after in vivo administration, which may potently affect cell-specific targeting and uptake of the NC. Understanding the formation and composition of the protein corona is, therefore, of major importance for the use of nanocarriers in vaccine approaches. This Mini Review will give a short overview of potential non-specific interactions of NC with body fluids or cell surfaces that need to be considered for the design of NC vaccines for immunotherapy of cancer.
C1 [Bros, Matthias; Simon, Johanna; Moll, Lorna; Mailaender, Volker; Grabbe, Stephan] Johannes Gutenberg Univ Mainz, Univ Med Ctr, Dept Dermatol, Mainz, Germany.
[Nuhn, Lutz; Simon, Johanna; Moll, Lorna; Mailaender, Volker; Landfester, Katharina] Max Planck Inst Polymer Res, Mainz, Germany.
C3 Johannes Gutenberg University of Mainz; Max Planck Society
RP Grabbe, S (通讯作者)，Johannes Gutenberg Univ Mainz, Univ Med Ctr, Dept Dermatol, Mainz, Germany.
EM stephan.grabbe@unimedizin-mainz.de
RI Landfester, Katharina/F-9449-2011; Mailänder, Volker/H-3589-2012; Nuhn, Lutz/HPF-6598-2023; Grabe, Stephan/AAJ-4578-2021; Nuhn, Lutz/R-7324-2018; Nuhn, Lutz/P-5150-2019; Mailänder, Volker/AAD-1722-2019
OI Landfester, Katharina/0000-0001-9591-4638; Mailänder, Volker/0000-0001-6583-8136; Nuhn, Lutz/0000-0003-0761-1106; Grabe, Stephan/0000-0002-6863-8719; Nuhn, Lutz/0000-0003-0761-1106; Nuhn, Lutz/0000-0003-0761-1106; Mailänder, Volker/0000-0001-6583-8136; Moll, Lorna/0000-0003-1194-7323
FU Deutsche Forschungsgemeinschaft [SFB1066]
FX Work of MB, LN, VM, KL, and SG is funded by the Deutsche
Forschungsgemeinschaft (SFB1066).
CR Aggarwal P, 2009, ADV DRUG DELIVER REV, V61, P428, DOI 10.1016/j.addr.2009.03.009

Amoozgar Z, 2015, ADV DRUG DELIVER REV, V91, P38, DOI 10.1016/j.addr.2014.09.007
Aoyama M, 2016, BIOCHEM BIOPH RES CO, V480, P690, DOI 10.1016/j.bbrc.2016.10.121
Bamberger D, 2017, MOL PHARMACEUT, V14, P4403, DOI 10.1021/acs.molpharmaceut.7b00507
da Silva FLB, 2014, LANGMUIR, V30, P4078, DOI 10.1021/la500027f
Barz M, 2011, POLYM CHEM-UK, V2, P1900, DOI 10.1039/c0py00406e
Baumann D, 2013, NANOMEDICINE-UK, V8, P699, DOI [10.2217/NNM.12.111, 10.2217/nnm.12.111]
BAZILE D, 1995, J PHARM SCI, V84, P493, DOI 10.1002/jps.2600840420
Bonifaz L, 2002, J EXP MED, V196, P1627, DOI 10.1084/jem.20021598
Bose RJC, 2018, DRUG DISCOV TODAY, V23, P891, DOI 10.1016/j.drudis.2018.02.001
Brauweiler AM, 2003, BIOCHEM SOC T, V31, P281, DOI 10.1042/bst0310281
Brown GC, 2012, TRENDS BIOCHEM SCI, V37, P325, DOI 10.1016/j.tibs.2012.05.002
Burgdorf S, 2006, J IMMUNOL, V176, P6770, DOI 10.4049/jimmunol.176.11.6770
Canton J, 2013, NAT REV IMMUNOL, V13, P621, DOI 10.1038/nri3515
Caracciolo G, 2017, TRENDS BIOTECHNOL, V35, P257, DOI 10.1016/j.tibtech.2016.08.011
Caracciolo G, 2015, LANGMUIR, V31, P10764, DOI 10.1021/acs.langmuir.5b02158
Chakraborti S, 2012, LANGMUIR, V28, P11142, DOI 10.1021/la3007603
Chakraborty S, 2011, LANGMUIR, V27, P7722, DOI 10.1021/la200787t
Champion JA, 2008, PHARM RES-DORDR, V25, P1815, DOI 10.1007/s11095-008-9562-y
Chao Y, 2012, BIOCONJUGATE CHEM, V23, P1003, DOI 10.1021/bc200685a
Chapman RG, 2000, J AM CHEM SOC, V122, P8303, DOI 10.1021/ja000774f
Chatterjee B, 2012, BLOOD, V120, P2011, DOI 10.1182/blood-2012-01-402370
Chen FF, 2017, NAT NANOTECHNOL, V12, P387, DOI [10.1038/nnano.2016.269, 10.1038/NNANO.2016.269]
Daeron M, 2014, CURR TOP MICROBIOL, V382, P131, DOI 10.1007/978-3-319-07911-0_7
Dai Q, 2014, ANGEW CHEM INT EDIT, V53, P5093, DOI 10.1002/anie.201309464
den Haan JMM, 2000, J EXP MED, V192, P1685, DOI 10.1084/jem.192.12.1685
Duan XP, 2013, SMALL, V9, P1521, DOI 10.1002/smll.201201390
Erdei A, 2019, SEMIN CELL DEV BIOL, V85, P110, DOI 10.1016/j.semcdb.2017.11.025
Fleischer CC, 2014, ACCOUNTS CHEM RES, V47, P2651, DOI 10.1021/ar500190q
Francian A, 2017, INT J NANOMED, V12, P5149, DOI 10.2147/IJN.S138787
Freeman SA, 2014, IMMUNOL REV, V262, P193, DOI 10.1111/imr.12212
Futosi K, 2013, INT IMMUNOPHARMACOL, V17, P638, DOI 10.1016/j.intimp.2013.06.034
Gallud A, 2017, BIOMATERIALS, V121, P28, DOI 10.1016/j.biomaterials.2016.12.029
Garcia-Alvarez R, 2018, NANOSCALE, V10, P1256, DOI 10.1039/c7nr08322j
Gessner A, 2003, J BIOMED MATER RES A, V65A, P319, DOI 10.1002/jbm.a.10371
Gomes AC, 2017, VACCINES-BASEL, V5, DOI 10.3390/vaccines5010006
Gowda NM, 2013, PLOS ONE, V8, DOI 10.1371/journal.pone.0077604
Gref R, 2000, COLLOID SURFACE B, V18, P301, DOI 10.1016/S0927-7765(99)00156-3
Hadjidemetriou M, 2016, NANOSCALE, V8, P6948, DOI 10.1039/c5nr09158f
Hajipour MJ, 2014, BIOMATER SCI-UK, V2, P1210, DOI 10.1039/c4bm00131a
Hak S, 2012, ACS NANO, V6, P5648, DOI 10.1021/nn301630n
Harris JM, 2003, NAT REV DRUG DISCOV, V2, P214, DOI 10.1038/nrd1033
Hartung E, 2015, J IMMUNOL, V194, P1069, DOI 10.4049/jimmunol.1401903
Hartwell BL, 2015, J PHARM SCI-US, V104, P346, DOI 10.1002/jps.24273
Heikenwalder M, 2004, NAT MED, V10, P187, DOI 10.1038/nm987
Heinz H, 2017, SURF SCI REP, V72, P1, DOI 10.1016/j.surfrep.2017.02.001
Heo R, 2017, BIOMATERIALS, V131, P15, DOI 10.1016/j.biomaterials.2017.03.044
Irache JM, 2008, EXPERT OPIN DRUG DEL, V5, P703, DOI [10.1517/17425247.5.6.703, 10.1517/17425247.5.6.703 ]
Iurescia S, 2018, FRONT IMMUNOL, V9, DOI 10.3389/fimmu.2018.00711
Jin Y, 2002, ELECTROPHORESIS, V23, P3385, DOI 10.1002/1522-2683(200210)23:19<3385::AID-ELPS3385>3.0.CO;2-H
Joffre O, 2009, IMMUNOL REV, V227, P234, DOI 10.1111/j.1600-065X.2008.00718.x
Kanno S, 2007, TOXICOL SCI, V97, P398, DOI 10.1093/toxsci/kfm050
Kawasaki N, 2014, J IMMUNOL, V193, P1560, DOI 10.4049/jimmunol.1303278
Kelly PM, 2015, NAT NANOTECHNOL, V10, P472, DOI [10.1038/nnano.2015.47, 10.1038/NNANO.2015.47]
Kim J, 2017, BIOMATERIALS, V147, P14, DOI 10.1016/j.biomaterials.2017.08.045
Klippstein R, 2010, NANOMED-NANOTECHNOL, V6, P523, DOI 10.1016/j.nano.2010.01.001
Knop K, 2010, ANGEW CHEM INT EDIT, V49, P6288, DOI 10.1002/anie.200902672
Kokkinopoulou M, 2017, NANOSCALE, V9, P8858, DOI 10.1039/c7nr02977b


file:///D/Joyce/计量学/frontiers/修/Supplementary materials/savedrecs (94).txt[2024/6/27 16:48:08]

Kreuter J, 2002, J DRUG TARGET, V10, P317, DOI 10.1080/10611860290031877
Kuhn P, 2016, PROTEOM CLIN APPL, V10, P922, DOI 10.1002/prca.201600002
Lacerda SHD, 2010, ACS NANO, V4, P365, DOI 10.1021/nn9011187
Lahoud MH, 2012, P NATL ACAD SCI USA, V109, P16270, DOI 10.1073/pnas.1208796109
Lara S, 2017, ACS NANO, V11, P1884, DOI 10.1021/acsnano.6b07933
Laurent S, 2013, J COLLOID INTERF SCI, V392, P431, DOI 10.1016/j.jcis.2012.10.005
Lee C, 2018, CLIN EXP VACCINE RES, V7, P16, DOI 10.7774/cevr.2018.7.1.16
Li HY, 2017, INT J PHARMACEUT, V532, P647, DOI 10.1016/j.ijpharm.2017.08.074
Li Y, 2017, NANOTOXICOLOGY, V11, P1157, DOI 10.1080/17435390.2017.1401142
Li Y, 2016, NANOMEDICINE-UK, V11, P269, DOI 10.2217/nnm.15.196
Lundqvist M, 2008, P NATL ACAD SCI USA, V105, P14265, DOI 10.1073/pnas.0805135105
Macri C, 2016, CLIN TRANSL IMMUNOL, V5, DOI 10.1038/cti.2016.6
Mariam J, 2017, J BIOMOL STRUCT DYN, V35, P368, DOI 10.1080/07391102.2016.1144223
Mirshafiee V, 2016, INT J BIOCHEM CELL B, V75, P188, DOI 10.1016/j.biocel.2015.11.019
Monopoli MP, 2011, J AM CHEM SOC, V133, P2525, DOI 10.1021/ja107583h
Mortimer GM, 2014, ACS NANO, V8, P3357, DOI 10.1021/nn405830g
Moyer TJ, 2016, J CLIN INVEST, V126, P799, DOI 10.1172/JCI81083
Muller LK, 2018, BIOMACROMOLECULES, V19, P374, DOI 10.1021/acs.biomac.7b01472
Murata Y, 2014, J BIOCHEM, V155, P335, DOI 10.1093/jb/mvu017
Murshid A, 2016, FRONT IMMUNOL, V7, DOI 10.3389/fimmu.2016.00226
Nuhn L, 2018, BIOMATERIALS, V178, P643, DOI 10.1016/j.biomaterials.2018.03.026
Nuhn L, 2016, P NATL ACAD SCI USA, V113, P8098, DOI 10.1073/pnas.1600816113
Nuhn L, 2014, BIOMACROMOLECULES, V15, P1526, DOI 10.1021/bm500199h
Ostuni E, 2001, LANGMUIR, V17, P5605, DOI 10.1021/la010384m
Owens DE, 2006, INT J PHARMACEUT, V307, P93, DOI 10.1016/j.ijpharm.2005.10.010
Palchetti S, 2017, COLLOID SURFACE B, V153, P263, DOI 10.1016/j.colsurfb.2017.02.037
Patel PC, 2010, BIOCONJUGATE CHEM, V21, P2250, DOI 10.1021/bc1002423
Pieper R, 2003, PROTEOMICS, V3, P1345, DOI 10.1002/pmic.200300449
Podila R, 2012, APPL PHYS LETT, V101, DOI 10.1063/1.4772509
Prantner AM, 2014, J NANOSCI NANOTECHNO, V14, P115, DOI 10.1166/jnn.2014.8899
Rahman S.L.M., 2013, BIOPHYSICS, V15, P45
Rezaeipoor R, 2009, J INNOV OPT HEAL SCI, V2, P387, DOI 10.1142/S1793545809000693
Ritz S, 2015, BIOMACROMOLECULES, V16, P1311, DOI 10.1021/acs.biomac.5b00108
Villegas MR, 2018, MOLECULES, V23, DOI 10.3390/molecules23051008
Rodriguez PL, 2013, SCIENCE, V339, P971, DOI 10.1126/science.1229568
Roozendaal R, 2007, IMMUNOL REV, V219, P157, DOI 10.1111/j.1600-065X.2007.00556.x
Rosi NL, 2006, SCIENCE, V312, P1027, DOI 10.1126/science.1125559
Salvati A, 2013, NAT NANOTECHNOL, V8, P137, DOI [10.1038/nnano.2012.237, 10.1038/NNANO.2012.237]
Schottler S, 2016, NANOSCALE, V8, P5526, DOI 10.1039/c5nr08196c
Schottler S, 2016, NAT NANOTECHNOL, V11, P372, DOI [10.1038/NNANO.2015.330, 10.1038/nnano.2015.330]
Sharma P, 2015, CELL, V161, P205, DOI 10.1016/j.cell.2015.03.030
Shen LM, 2018, J ALLERGY CLIN IMMUN, V142, P1558, DOI 10.1016/j.jaci.2017.08.049
Shen LM, 2016, NANOMEDICINE-UK, V11, P2647, DOI 10.2217/nnm-2016-0174
Shirota H, 2014, EXPERT REV VACCINES, V13, P299, DOI 10.1586/14760584.2014.863715
Sobczynski DJ, 2017, BIOENG TRANSL MED, V2, P180, DOI 10.1002/btm2.10064
Subramaniam DS, 2016, DISCOV MED, V21, P267
Suk JS, 2016, ADV DRUG DELIVER REV, V99, P28, DOI 10.1016/j.addr.2015.09.012
Takeda KM, 2017, BIOMACROMOLECULES, V18, P36, DOI 10.1021/acs.biomac.6b01247
Takeuchi T, 2017, ANGEW CHEM INT EDIT, V56, P7088, DOI 10.1002/anie.201700647
Tenzer S, 2013, NAT NANOTECHNOL, V8, P772, DOI 10.1038/nnano.2013.181
Tockary TA, 2013, MACROMOLECULES, V46, P6585, DOI 10.1021/ma401093z
Tocut M, 2018, AUTOIMMUN REV, V17, P610, DOI 10.1016/j.autrev.2018.01.010
Tsvetkova Y, 2017, NANO LETT, V17, P4665, DOI 10.1021/acs.nanolett.7b01171
Tran TH, 2018, INT J PHARMACEUT, V542, P253, DOI 10.1016/j.ijpharm.2018.03.029
Tullett KM, 2014, FRONT IMMUNOL, V5, P1, DOI 10.3389/fimmu.2014.00239
van Liempt E, 2006, FEBS LETT, V580, P6123, DOI 10.1016/j.febslet.2006.10.009
Veronese FM, 2005, DRUG DISCOV TODAY, V10, P1451, DOI 10.1016/S1359-6446(05)03575-0
Walkey CD, 2012, CHEM SOC REV, V41, P2780, DOI 10.1039/c1cs15233e
Wang B, 2009, ANN NY ACAD SCI, V1174, P6, DOI 10.1111/j.1749-6632.2009.04933.x

Wei Q, 2014, ANGEW CHEM INT EDIT, V53, P8004, DOI 10.1002/anie.201400546
Yan Y, 2013, ACS NANO, V7, P10960, DOI 10.1021/nn404481f
Yang ST, 2013, SMALL, V9, P1635, DOI 10.1002/smll.201201492
Yue H, 2012, PHARMACOL RES, V65, P189, DOI 10.1016/j.phrs.2011.09.008
Zeng BJ, 2018, J CLIN INVEST, V128, P1971, DOI 10.1172/JCI96791
Zhang ZY, 2015, ADV FUNCT MATER, V25, P3433, DOI 10.1002/adfm.201500904
NR 123
TC 55
Z9 56
U1 3
U2 41
PU FRONTIERS MEDIA SA
PI LAUSANNE
PA AVENUE DU TRIBUNAL FEDERAL 34, LAUSANNE, CH-1015, SWITZERLAND
SN 1664-3224
J9 FRONT IMMUNOL
JI Front. Immunol.
PD AUG 2
PY 2018
VL 9
AR 1760
DI 10.3389/fimmu.2018.01760
PG 10
WC Immunology
WE Science Citation Index Expanded (SCI-EXPANDED)
SC Immunology
GA GP1PG
UT WOS:000440587700001
PM 30116246
OA gold, Green Published
DA 2023-09-14
ER

PT J
AU Saeed, M
Chen, FM
Ye, JY
Shi, Y
Lammers, T
De Geest, BG
Xu, ZP
Yu, HJ
AF Saeed, Madiha
Chen, Fangming
Ye, Jiayi
Shi, Yang
Lammers, Twan
De Geest, Bruno G.
Xu, Zhi Ping
Yu, Haijun
TI From Design to Clinic: Engineered Nanobiomaterials for Immune
Normalization Therapy of Cancer
SO ADVANCED MATERIALS
LA English
DT Review
DE clinical translation; nanobiomaterials; nanovaccines; normalization
cancer immunotherapy; primary and adaptive resistance; tumor immune
microenvironment
ID TUMOR-ASSOCIATED MACROPHAGES; IMMUNOGENIC CELL-DEATH; HEMATOPOIETIC
STEM-CELLS; SMALL INTERFERING RNA; EXTRACELLULAR-MATRIX; CHECKPOINT
BLOCKADE; ANTIANGIOGENIC THERAPY; VESSEL NORMALIZATION; IN-VIVO;

NANOPARTICLES
AB The tumor immune microenvironment (TIME) is comprised of a complex milieu that contributes to stunting antitumor immune responses by restricting T cells to accumulate in the vicinity of the tumor. Nanomedicine-based strategies are being proposed as a salvage effort to reinvigorate antitumor immunity. Various strategies, however, often fail to unleash the antitumor immune response because of the paucity of appropriate therapeutic targets in the complex TIME, invigorating a fervor of investigation into mechanisms underlying the TIME to resist nanomedicines. In this review article, effective nano/biomaterial-based delivery and TIME normalization approaches that promote T cell-mediated antitumor immune response will be discussed, with a focus on emerging preclinical and clinical strategies for immune normalization. Based on currently available evidence, it seems as if the ultimate success of cancer immunotherapy and nanomedicine hinges on the capacity to normalize the TIME. Here, how nanomedicines target immunosuppressive cells and signaling pathways to broaden the impact of cancer immunotherapy are explored. Acquisition of the urgently needed knowledge of nanomedicine-mediated immune normalization will guide researchers and scientists towards clinical applications of cancer immunotherapy.
C1 [Saeed, Madiha; Chen, Fangming; Ye, Jiayi; Yu, Haijun] Chinese Acad Sci, Shanghai Inst Mat Med, State Key Lab Drug Res, 501 Haike Rd, Shanghai 201203, Peoples R China.
[Saeed, Madiha; Chen, Fangming; Ye, Jiayi; Yu, Haijun] Chinese Acad Sci, Shanghai Inst Mat Med, Ctr Pharmaceut, 501 Haike Rd, Shanghai 201203, Peoples R China.
[Shi, Yang; Lammers, Twan] Rhein Westfal TH Aachen, Dept Nanomed & Theranost, Inst Expt Mol Imaging, Fac Med,Uniklin RWTH Aachen, D-52074 Aachen, Germany.
[Shi, Yang; Lammers, Twan] Rhein Westfal TH Aachen, Fac Med, Helmholtz Inst Biomed Engn, D-52074 Aachen, Germany.
[De Geest, Bruno G.] Univ Ghent, Dept Pharmaceut, B-9000 Ghent, Belgium.
[De Geest, Bruno G.] Univ Ghent, Canc Res Inst Ghent CRIG, B-9000 Ghent, Belgium.
[Xu, Zhi Ping] Univ Queensland, Australian Inst Bioengn & Nanotechnol, St Lucia, Qld 4072, Australia.
C3 Chinese Academy of Sciences; Shanghai Institute of Materia Medica, CAS;
Chinese Academy of Sciences; Shanghai Institute of Materia Medica, CAS;
RWTH Aachen University; RWTH Aachen University Hospital; Helmholtz
Association; RWTH Aachen University; Ghent University; Ghent University;
University of Queensland
RP Yu, HJ (通讯作者), Chinese Acad Sci, Shanghai Inst Mat Med, State Key Lab Drug Res, 501 Haike Rd, Shanghai 201203, Peoples R China.; Yu, HJ (通讯作者), Chinese Acad Sci, Shanghai Inst Mat Med, Ctr Pharmaceut, 501 Haike Rd, Shanghai 201203, Peoples R China.
EM hjyu@simm.ac.cn
RI De Geest, Bruno G./C-1868-2008; Lammers, Twan/U-2794-2018; SAEED,
MADIHA/HTL-2346-2023; Yu, Haijun/C-5343-2016
OI De Geest, Bruno G./0000-0001-9826-6170; Lammers,
Twan/0000-0002-1090-6805; SAEED, MADIHA/0000-0003-2355-5167; Yu,
Haijun/0000-0002-3398-0880
FU National Natural Science Foundation of China [32050410287, 51873228];
CAS President's International Fellowship Initiative Program
[2019PB0023]; International Cooperation Project of Science and
Technology Commission of Shanghai Municipality [20430711800]; Youth
Innovation Promotion Association of CAS [2014218]; Open Fund of the
State Key Laboratory of Drug Research [SIMM2004KF-04]
FX Financial supports from the National Natural Science Foundation of China
(32050410287 to M.S. and 51873228 to H.Y.), CAS President's
International Fellowship Initiative Program (2019PB0023 to M.S.),
International Cooperation Project of Science and Technology Commission
of Shanghai Municipality (20430711800), the Youth Innovation Promotion
Association of CAS (2014218) and Open Fund of the State Key Laboratory
of Drug Research (SIMM2004KF-04) are gratefully acknowledged.
CR Ablasser A, 2013, NATURE, V498, P380, DOI 10.1038/nature12306
Adiseshaiah PP, 2016, NAT REV CLIN ONCOL, V13, P750, DOI 10.1038/nrclinonc.2016.119
Allen BM, 2020, NAT MED, V26, P1125, DOI 10.1038/s41591-020-0892-6
Amorim S, 2021, TRENDS BIOTECHNOL, V39, P90, DOI 10.1016/j.tibtech.2020.06.003
Anchordoquy TJ, 2017, ACS NANO, V11, P12, DOI 10.1021/acsnano.6b08244
Anselmo AC, 2019, BIOENG TRANSL MED, V4, DOI 10.1002/btm2.10143
Anselmo AC, 2016, BIOENG TRANSL MED, V1, P10, DOI 10.1002/btm2.10003
Bae YH, 2011, J CONTROL RELEASE, V153, P198, DOI 10.1016/j.jconrel.2011.06.001

Baumeister SH, 2016, ANNU REV IMMUNOL, V34, P539, DOI 10.1146/annurev-immunol-032414-112049
Bencherif SA, 2015, NAT COMMUN, V6, DOI 10.1038/ncomms8556
Bentebibel SE, 2019, CANCER DISCOV, V9, P711, DOI 10.1158/2159-8290.CD-18-1495
Beri P, 2018, NAT REV MATER, V3, P418, DOI 10.1038/s41578-018-0051-6
Blackburn SD, 2009, NAT IMMUNOL, V10, P29, DOI 10.1038/ni.1679
Blanco E, 2015, NAT BIOTECHNOL, V33, P941, DOI 10.1038/nbt.3330
Bobo D, 2016, PHARM RES-DORDR, V33, P2373, DOI 10.1007/s11095-016-1958-5
Bonnans C, 2014, NAT REV MOL CELL BIO, V15, P786, DOI 10.1038/nrm3904
Bonvalot S, 2019, LANCET ONCOL, V20, P1148, DOI 10.1016/S1470-2045(19)30326-2
Boussiotis VA, 2016, NEW ENGL J MED, V375, P1767, DOI 10.1056/NEJMra1514296
Certo M, 2021, NAT REV IMMUNOL, V21, P151, DOI 10.1038/s41577-020-0406-2
Chakravarthy A, 2018, NAT COMMUN, V9, DOI 10.1038/s41467-018-06654-8
Chao MP, 2012, NAT REV CANCER, V12, P58, DOI 10.1038/nrc3171
Charych DH, 2016, CLIN CANCER RES, V22, P680, DOI 10.1158/1078-0432.CCR-15-1631
Chauhan VP, 2019, P NATL ACAD SCI USA, V116, P10674, DOI 10.1073/pnas.1819889116
Chauhan VP, 2013, NAT MATER, V12, P958, DOI 10.1038/nmat3792
Chen BF, 2020, BIOMATERIALS, V255, DOI 10.1016/j.biomaterials.2020.120187
Chen HM, 2017, NAT REV MATER, V2, DOI 10.1038/natrevmats.2017.24
Chen IX, 2019, P NATL ACAD SCI USA, V116, P4558, DOI 10.1073/pnas.1815515116
Chen Q, 2019, ADV MATER, V31, DOI 10.1002/adma.201802228
Chen Q, 2019, NAT NANOTECHNOL, V14, P89, DOI 10.1038/s41565-018-0319-4
Chen Q, 2016, ADV MATER, V28, P7129, DOI 10.1002/adma.201601902
Chen XM, 2019, NAT REV DRUG DISCOV, V18, P99, DOI 10.1038/s41573-018-0004-1
Cheng KM, 2018, NANO LETT, V18, P3250, DOI 10.1021/acs.nanolett.8b01071
Chung CH, 2020, BIOMATERIALS, V257, DOI 10.1016/j.biomaterials.2020.120227
Cifuentes-Rius A, 2021, NAT NANOTECHNOL, V16, P37, DOI 10.1038/s41565-020-00810-2
ClinicalTrials.gov, 2018, GLOB STUD ASS EFF OS
Conde J, 2015, ADV FUNCT MATER, V25, P4183, DOI 10.1002/adfm.201501283
Conniot J, 2019, NAT NANOTECHNOL, V14, P891, DOI 10.1038/s41565-019-0512-0
Cox TR, 2011, DIS MODEL MECH, V4, P165, DOI 10.1242/dmm.004077
Cummins EP, 2016, MOL ASPECTS MED, V47-48, P24, DOI 10.1016/j.mam.2015.12.004
D'Mello SR, 2017, NAT NANOTECHNOL, V12, P523, DOI [10.1038/nnano.2017.67, 10.1038/NNANO.2017.67]
Dai Q, 2018, ACS NANO, V12, P8423, DOI 10.1021/acsnano.8b03900
de Lazaro I, 2020, NAT MATER, V19, P486, DOI 10.1038/s41563-020-0669-9
Dellacherie MO, 2019, NAT REV MATER, V4, P379, DOI 10.1038/s41578-019-0106-3
DeNardo DG, 2019, NAT REV IMMUNOL, V19, P369, DOI 10.1038/s41577-019-0127-6
Denko NC, 2008, NAT REV CANCER, V8, P705, DOI 10.1038/nrc2468
Derynck R, 2021, NAT REV CLIN ONCOL, V18, P9, DOI 10.1038/s41571-020-0403-1
Duan XP, 2019, NAT COMMUN, V10, DOI 10.1038/s41467-019-09221-x
Fan WP, 2015, ADV MATER, V27, P4155, DOI 10.1002/adma.201405141
Fan YC, 2017, NANO LETT, V17, P7387, DOI 10.1021/acs.nanolett.7b03218
Feig C, 2013, P NATL ACAD SCI USA, V110, P20212, DOI 10.1073/pnas.1320318110
Feng B, 2019, ADV MATER, V31, DOI 10.1002/adma.201902960
Feng B, 2018, ADV MATER, V30, DOI 10.1002/adma.201803001
Feng MY, 2019, NAT REV CANCER, V19, P568, DOI 10.1038/s41568-019-0183-z
Fenton OS, 2018, ADV MATER, V30, DOI 10.1002/adma.201705328
Fukumura D, 2007, J CELL BIOCHEM, V101, P937, DOI 10.1002/jcb.21187
Galluzzi L, 2017, NAT REV IMMUNOL, V17, P97, DOI 10.1038/nri.2016.107
Gao A, 2020, NANO LETT, V20, P353, DOI 10.1021/acs.nanolett.9b04012
Gao Y, 2020, NAT BIOMED ENG, V4, P1
Genard G, 2017, FRONT IMMUNOL, V8, DOI 10.3389/fimmu.2017.00828
Gilkes DM, 2014, NAT REV CANCER, V14, P430, DOI 10.1038/nrc3726
Goldberg MS, 2019, NAT REV CANCER, V19, P587, DOI 10.1038/s41568-019-0186-9
Gong NQ, 2021, NAT NANOTECHNOL, V16, P25, DOI 10.1038/s41565-020-00822-y
Gosselin EA, 2018, NAT MATER, V17, P484, DOI 10.1038/s41563-018-0077-6
Han X, 2018, NAT COMMUN, V9, DOI 10.1038/s41467-018-03689-9
Han XX, 2020, J AM CHEM SOC, V142, P2490, DOI 10.1021/jacs.9b12232
Hanahan D, 2011, CELL, V144, P646, DOI 10.1016/j.cell.2011.02.013
Hanson MC, 2015, J CLIN INVEST, V125, P2532, DOI 10.1172/JCI79915

Hay E.D., 2013, CELL BIOL EXTRACELLU
He YP, 2020, SCI ADV, V6, DOI 10.1126/sciadv.aba7589
Hewitt SL, 2019, SCI TRANSL MED, V11, DOI 10.1126/scitranslmed.aat9143
Hiller JG, 2018, NAT REV CLIN ONCOL, V15, P205, DOI 10.1038/nrclinonc.2017.194
Holmgaard RB, 2015, CELL REP, V13, P412, DOI 10.1016/j.celrep.2015.08.077
Hopfner KP, 2020, NAT REV MOL CELL BIO, V21, P501, DOI 10.1038/s41580-020-0244-x
Hou B, 2020, ADV MATER, V32, DOI 10.1002/adma.201907210
Hu MJ, 2019, NAT COMMUN, V10, DOI 10.1038/s41467-019-10703-1
Hu QY, 2018, NAT BIOMED ENG, V2, P831, DOI 10.1038/s41551-018-0310-2
Hu XL, 2020, ADV SCI, V7, DOI 10.1002/advs.201903332
Huang KW, 2020, SCI ADV, V6, DOI 10.1126/sciadv.aax5032
Huang YQ, 2020, NAT COMMUN, V11, DOI 10.1038/s41467-020-19392-7
Huo MR, 2017, J CONTROL RELEASE, V245, P81, DOI 10.1016/j.jconrel.2016.11.013
Hynes RO, 2009, SCIENCE, V326, P1216, DOI 10.1126/science.1176009
Insua-Rodriguez J, 2016, ADV DRUG DELIVER REV, V97, P41, DOI 10.1016/j.addr.2015.12.017
Irvine DJ, 2020, NAT REV IMMUNOL, V20, P321, DOI 10.1038/s41577-019-0269-6
Ishihara J, 2019, SCI TRANSL MED, V11, DOI 10.1126/scitranslmed.aau3259
Ishihara J, 2017, SCI TRANSL MED, V9, DOI 10.1126/scitranslmed.aan0401
Ishikawa H, 2008, NATURE, V455, P674, DOI 10.1038/nature07317
Jaillon S, 2020, NAT REV CANCER, V20, P485, DOI 10.1038/s41568-020-0281-y
Jain RK, 2013, J CLIN ONCOL, V31, P2205, DOI 10.1200/JCO.2012.46.3653
Jain RK, 2010, NAT REV CLIN ONCOL, V7, P653, DOI 10.1038/nrclinonc.2010.139
Jain RK, 2005, SCIENCE, V307, P58, DOI 10.1126/science.1104819
Jaiswal S, 2009, CELL, V138, P271, DOI 10.1016/j.cell.2009.05.046
Jaynes JM, 2020, SCI TRANSL MED, V12, DOI 10.1126/scitranslmed.aax6337
Ji TJ, 2015, ADV MATER, V27, P1865, DOI 10.1002/adma.201404715
Jiang W, 2017, NAT BIOMED ENG, V1, DOI 10.1038/s41551-017-0029
Johansson-Percival A, 2017, NAT IMMUNOL, V18, P1207, DOI 10.1038/ni.3836
Joyce JA, 2015, SCIENCE, V348, P74, DOI 10.1126/science.aaa6204
Kadiyala P, 2019, ACS NANO, V13, P1365, DOI 10.1021/acsnano.8b06842
Kageyama S, 2013, J TRANSL MED, V11, DOI 10.1186/1479-5876-11-246
Kalluri R, 2016, NAT REV CANCER, V16, P582, DOI 10.1038/nrc.2016.73
Kammertoens T, 2005, TRENDS MOL MED, V11, P225, DOI 10.1016/j.molmed.2005.03.002
Kang T, 2017, ACS NANO, V11, P1397, DOI 10.1021/acsnano.6b06477
Karsdal MA, 2015, AM J PHYSIOL-GASTR L, V308, pG807, DOI 10.1152/ajpgi.00447.2014
Keir ME, 2006, J EXP MED, V203, P883, DOI 10.1084/jem.20051776
Kerbel RS, 2006, SCIENCE, V312, P1171, DOI 10.1126/science.1125950
Khan KA, 2018, NAT REV CLIN ONCOL, V15, P310, DOI 10.1038/nrclinonc.2018.9
Kim J, 2017, J AM CHEM SOC, V139, P10992, DOI 10.1021/jacs.7b05559
Krausgruber T, 2011, NAT IMMUNOL, V12, P231, DOI 10.1038/ni.1990
Kroemer G, 2013, ANNU REV IMMUNOL, V31, P51, DOI 10.1146/annurev-immunol-032712-100008
Krysko DV, 2012, NAT REV CANCER, V12, P860, DOI 10.1038/nrc3380
Kuai R, 2017, NAT MATER, V16, P489, DOI [10.1038/nmat4822, 10.1038/NMAT4822]
Kulkarni A, 2018, NAT BIOMED ENG, V2, P589, DOI 10.1038/s41551-018-0254-6
Kwong B, 2013, CANCER RES, V73, P1547, DOI 10.1158/0008-5472.CAN-12-3343
Ng LG, 2019, NAT REV IMMUNOL, V19, P255, DOI 10.1038/s41577-019-0141-8
Lampi MC, 2018, SCI TRANSL MED, V10, DOI 10.1126/scitranslmed.aao0475
Lan GX, 2018, J AM CHEM SOC, V140, P5670, DOI 10.1021/jacs.8b01072
Lang JY, 2019, ACS NANO, V13, P12357, DOI 10.1021/acsnano.9b04857
Lang TQ, 2018, ADV FUNCT MATER, V28, DOI 10.1002/adfm.201707289
Lemos H, 2019, NAT REV CANCER, V19, P162, DOI 10.1038/s41568-019-0106-z
Leventhal DS, 2020, NAT COMMUN, V11, DOI 10.1038/s41467-020-16602-0
Li J, 2020, ADV MATER, V32, DOI 10.1002/adma.202002380
Li JC, 2019, ANGEW CHEM INT EDIT, V58, P12680, DOI 10.1002/anie.201906288
Li S., 2020, NAT BIOMED ENG, V4, P1
Li SP, 2018, NAT BIOTECHNOL, V36, P258, DOI 10.1038/nbt.4071
Li X., 2020, ANGEW CHEM
Li XY, 2019, NAT REV CLIN ONCOL, V16, P425, DOI 10.1038/s41571-019-0203-7
Li Y, 2020, ADV MATER, V32, DOI 10.1002/adma.202001260
Li YZ, 2020, NAT CANCER, V1, P882, DOI 10.1038/s43018-020-0095-6
Li Y, 2020, NAT COMMUN, V11, DOI 10.1038/s41467-020-14802-2

Li Y, 2020, ADV SCI, V7, DOI 10.1002/advs.202000023
Li ZT, 2020, SCI ADV, V6, DOI 10.1126/sciadv.aaz9240
Libutti SK, 2010, CLIN CANCER RES, V16, P6139, DOI 10.1158/1078-0432.CCR-10-0978
Liu Q, 2018, ACS NANO, V12, P7812, DOI 10.1021/acsnano.8b01890
Liu XJ, 2015, NAT MED, V21, P1209, DOI 10.1038/nm.3931
Liu YF, 2020, SCI ADV, V6, DOI 10.1126/sciadv.abb2695
Liu ZG, 2019, TRENDS PHARMACOL SCI, V40, P613, DOI 10.1016/j.tips.2019.07.001
Lu KD, 2018, NAT BIOMED ENG, V2, P600, DOI 10.1038/s41551-018-0203-4
Lu PF, 2012, J CELL BIOL, V196, P395, DOI 10.1083/jcb.201102147
Lu XG, 2020, SCI TRANSL MED, V12, DOI 10.1126/scitranslmed.aaz6606
Luo M, 2017, NAT NANOTECHNOL, V12, P648, DOI [10.1038/nnano.2017.52, 10.1038/NNANO.2017.52]
Lynn GM, 2020, NAT BIOTECHNOL, V38, P320, DOI 10.1038/s41587-019-0390-x
Lynn GM, 2019, CANCER LETT, V459, P192, DOI 10.1016/j.canlet.2019.114427
Mahoney KM, 2015, NAT REV DRUG DISCOV, V14, P561, DOI 10.1038/nrd4591
Majeti R, 2009, CELL, V138, P286, DOI 10.1016/j.cell.2009.05.045
Mansurov A, 2020, NAT BIOMED ENG, DOI 10.1038/s41551-020-0549-2
Mantovani A, 2017, NAT REV CLIN ONCOL, V14, P399, DOI 10.1038/nrclinonc.2016.217
Mantovani A, 2011, NAT REV IMMUNOL, V11, P519, DOI 10.1038/nri3024
Mardhian DF, 2018, J CONTROL RELEASE, V290, P1, DOI 10.1016/j.jconrel.2018.09.031
Marill J, 2019, RADIOTHER ONCOL, V141, P262, DOI 10.1016/j.radonc.2019.07.029
Martin JD, 2020, NAT REV CLIN ONCOL, V17, P251, DOI 10.1038/s41571-019-0308-z
Martin JD, 2019, ANNU REV PHYSIOL, V81, P505, DOI 10.1146/annurev-physiol-020518-114700
Mellor AL, 2004, NAT REV IMMUNOL, V4, P762, DOI 10.1038/nri1457
Mellor AL, 2017, FRONT IMMUNOL, V8, DOI 10.3389/fimmu.2017.01360
Meng LT, 2018, ACS NANO, V12, P8308, DOI 10.1021/acsnano.8b03590
Meric-Bernstam F, 2019, J CLIN ONCOL, V37, DOI 10.1200/JCO.2019.37.15_suppl.2507
Miao L, 2019, NAT BIOTECHNOL, V37, P1174, DOI 10.1038/s41587-019-0247-3
Milhem MM, 2019, J CLIN ONCOL, V37, DOI 10.1200/JCO.2019.37.15_suppl.9534
Miller MA, 2017, SCI TRANSL MED, V9, DOI 10.1126/scitranslmed.aal0225
Miller MA, 2015, NAT COMMUN, V6, DOI 10.1038/ncomms9692
Milling L, 2017, ADV DRUG DELIVER REV, V114, P79, DOI 10.1016/j.addr.2017.05.011
Mitchell MJ, 2021, NAT REV DRUG DISCOV, V20, P101, DOI 10.1038/s41573-020-0090-8
Mohme M, 2017, NAT REV CLIN ONCOL, V14, P155, DOI 10.1038/nrclinonc.2016.144
Moynihan KD, 2016, NAT MED, V22, P1402, DOI 10.1038/nm.4200
Munn DH, 2016, CURR OPIN IMMUNOL, V39, P1, DOI 10.1016/j.coi.2015.10.009
Naidoo J, 2015, ANN ONCOL, V26, P2375, DOI 10.1093/annonc/mdv383
Nam J, 2019, NAT REV MATER, V4, P398, DOI 10.1038/s41578-019-0108-1
Nemeth T, 2020, NAT REV DRUG DISCOV, V19, P253, DOI 10.1038/s41573-019-0054-z
Nuhn L, 2018, ADV MATER, V30, DOI 10.1002/adma.201803397
O'Donnell JS, 2019, NAT REV CLIN ONCOL, V16, P151, DOI 10.1038/s41571-018-0142-8
Parayath NN, 2018, NANO LETT, V18, P3571, DOI 10.1021/acs.nanolett.8b00689
Pardoll DM, 2012, NAT REV CANCER, V12, P252, DOI 10.1038/nrc3239
Park J, 2012, NAT MATER, V11, P895, DOI [10.1038/NMAT3355, 10.1038/nmat3355]
Peng HG, 2017, NANO LETT, V17, P7684, DOI 10.1021/acs.nanolett.7b03756
Phung C.D., 2020, J CONTROLLED RELEASE
Poon W, 2020, NAT NANOTECHNOL, V15, P819, DOI 10.1038/s41565-020-0759-5
Postow MA, 2018, NEW ENGL J MED, V378, P158, DOI [10.1056/NEJMc1801663, 10.1056/NEJMra1703481]
Pu K., 2020, ANGEW CHEM, V132, P2034, DOI [10.1002/ange.201912524, DOI 10.1002/ANGE.201912524]
Qian Y, 2017, ACS NANO, V11, P9536, DOI 10.1021/acsnano.7b05465
Qiao Y, 2020, SCI ADV, V6, DOI 10.1126/sciadv.aba5996
Rahbari NN, 2016, SCI TRANSL MED, V8, DOI 10.1126/scitranslmed.aaf5219
Ries CH, 2014, CANCER CELL, V25, P846, DOI 10.1016/j.ccr.2014.05.016
Riley RS, 2019, NAT REV DRUG DISCOV, V18, P175, DOI 10.1038/s41573-018-0006-z
Rodell CB, 2018, NAT BIOMED ENG, V2, P578, DOI 10.1038/s41551-018-0236-8
Rosenberg JE, 2016, LANCET, V387, P1909, DOI 10.1016/S0140-6736(16)00561-4
Rosenblum D, 2018, NAT COMMUN, V9, DOI 10.1038/s41467-018-03705-y
Ruffell B, 2015, CANCER CELL, V27, P462, DOI 10.1016/j.ccell.2015.02.015
Saeed M, 2020, BIOCONJUGATE CHEM, V31, P404, DOI 10.1021/acs.bioconjchem.9b00851

Saeed M, 2019, THERANOSTICS, V9, P7981, DOI 10.7150/thno.37568
Saeed M, 2018, BIOMATER SCI-UK, V6, P708, DOI 10.1039/c7bm00999b
Sasaki K, 2019, SCI ADV, V5, DOI 10.1126/sciadv.aaw6081
Scheetz L, 2020, CLIN CANCER RES, V26, P4369, DOI 10.1158/1078-0432.CCR-20-0341
Schmid D, 2017, NAT COMMUN, V8, DOI 10.1038/s41467-017-01830-8
Schmid P, 2018, NEW ENGL J MED, V379, P2108, DOI 10.1056/NEJMoa1809615
Shae D, 2019, NAT NANOTECHNOL, V14, P269, DOI 10.1038/s41565-018-0342-5
Shah NJ, 2020, NAT BIOMED ENG, V4, P40, DOI 10.1038/s41551-019-0503-3
Shao YL, 2020, J AM CHEM SOC, V142, P3939, DOI 10.1021/jacs.9b12788
Sharma P, 2017, CELL, V168, P707, DOI 10.1016/j.cell.2017.01.017
Shen LM, 2018, ACS NANO, V12, P9830, DOI 10.1021/acsnano.8b00967
Shen N, 2019, NANO LETT, V19, P8021, DOI 10.1021/acs.nanolett.9b03214
Shi JJ, 2017, NAT REV CANCER, V17, P20, DOI 10.1038/nrc.2016.108
Shi Y, 2020, THERANOSTICS, V10, P7921, DOI 10.7150/thno.49577
Shields CW, 2020, SCI ADV, V6, DOI 10.1126/sciadv.aaz6579
Sindhwani S, 2020, NAT MATER, V19, P566, DOI 10.1038/s41563-019-0566-2
Song C, 2019, NAT COMMUN, V10, DOI 10.1038/s41467-019-11730-8
Song WT, 2018, NAT COMMUN, V9, DOI 10.1038/s41467-018-04605-x
Stetson DB, 2006, IMMUNITY, V24, P93, DOI 10.1016/j.immuni.2005.12.003
Sun LJ, 2013, SCIENCE, V339, P786, DOI 10.1126/science.1232458
Sun QX, 2019, CHEM SOC REV, V48, P351, DOI 10.1039/c8cs00473k
Sung YC, 2019, NAT NANOTECHNOL, V14, P1160, DOI 10.1038/s41565-019-0570-3
Sykes EA, 2016, P NATL ACAD SCI USA, V113, pE1142, DOI 10.1073/pnas.1521265113
Tan T, 2019, NAT COMMUN, V10, DOI 10.1038/s41467-019-11235-4
Tang J., 2020, ANGEW CHEM
Tang LG, 2020, ADV MATER, V32, DOI 10.1002/adma.202002739
Tauriello DVF, 2018, NATURE, V554, P538, DOI 10.1038/nature25492
Topalian SL, 2020, SCIENCE, V367, P525, DOI 10.1126/science.aax0182
Topalian SL, 2015, CANCER CELL, V27, P450, DOI 10.1016/j.ccell.2015.03.001
Topalian SL, 2012, CURR OPIN IMMUNOL, V24, P207, DOI 10.1016/j.coi.2011.12.009
Tumeh PC, 2014, NATURE, V515, P568, DOI 10.1038/nature13954
Turley SJ, 2015, NAT REV IMMUNOL, V15, P669, DOI 10.1038/nri3902
US National Library of Medicine, 2015, US NATL LIB MED
US National Library of Medicine ClinicalTrials.gov, 2012, US NATL LIB MED CLIN
US National Library of Medicine ClinicalTrials.gov, 2013, **NON-TRADITIONAL**
US National Library of Medicine ClinicalTrials.gov, 2021, EFF DIET EX IMMUNOTH
US National Library of Medicine ClinicalTrials.gov, US NATL LIB MED CLIN
US National Library of Medicine ClinicalTrials.gov, 2019, US NATL LIB MED CLIN
Valkenburg KC, 2018, NAT REV CLIN ONCOL, V15, P366, DOI 10.1038/s41571-018-0007-1
van der Meel R, 2019, NAT NANOTECHNOL, V14, P1007, DOI 10.1038/s41565-019-0567-y
Vanpouille-Box C, 2019, NAT REV DRUG DISCOV, V18, P845, DOI 10.1038/s41573-019-0043-2
Waldman AD, 2020, NAT REV IMMUNOL, V20, P651, DOI 10.1038/s41577-020-0306-5
Wang B, 2018, BIOMATERIALS, V175, P110, DOI 10.1016/j.biomaterials.2018.05.034
Wang C, 2017, NAT BIOMED ENG, V1, DOI 10.1038/s41551-016-0011
Wang DG, 2019, SCI IMMUNOL, V4, DOI 10.1126/sciimmunol.aau6584
Wang FH, 2020, NAT BIOMED ENG, V4, P1090, DOI 10.1038/s41551-020-0597-7
Wang HR, 2019, ADV FUNCT MATER, V29, DOI 10.1002/adfm.201902440
Wang H, 2020, NAT MATER, V19, P1244, DOI 10.1038/s41563-020-0680-1
Wang SJ, 2020, SCI ADV, V6, DOI 10.1126/sciadv.aaz8521
Wang ZY, 2018, NAT COMMUN, V9, DOI 10.1038/s41467-018-04467-3
Weber JS, 2017, J CLIN ONCOL, V35, P785, DOI 10.1200/JCO.2015.66.1389
Wherry EJ, 2011, NAT IMMUNOL, V12, P492, DOI 10.1038/ni.2035
Wilhelm S, 2016, NAT REV MATER, V1, DOI 10.1038/natrevmats.2016.14
Willingham SB, 2012, P NATL ACAD SCI USA, V109, P6662, DOI 10.1073/pnas.1121623109
Wilson DS, 2019, NAT MATER, V18, P175, DOI 10.1038/s41563-018-0256-5
Wilson WR, 2011, NAT REV CANCER, V11, P393, DOI 10.1038/nrc3064
Xu BL, 2020, ADV MATER, V32, DOI 10.1002/adma.202003563
Xu H, 2020, BIOMATERIALS, V235, DOI 10.1016/j.biomaterials.2020.119769
Yang GB, 2018, NANO LETT, V18, P2475, DOI 10.1021/acs.nanolett.8b00040
Yang GB, 2017, NAT COMMUN, V8, DOI 10.1038/s41467-017-01050-0
Zanganeh S, 2016, NAT NANOTECHNOL, V11, P986, DOI [10.1038/nnano.2016.168,

10.1038/NNANO.2016.168]
Zappasodi R, 2018, CANCER CELL, V33, P581, DOI 10.1016/j.ccell.2018.03.005
Zeisberger SM, 2006, BRIT J CANCER, V95, P272, DOI 10.1038/sj.bjc.6603240
Zhang C, 2020, ADV FUNCT MATER, V30, DOI 10.1002/adfm.202000189
Zhang C, 2020, CHEM SOC REV, V49, P4234, DOI 10.1039/c9cs00773c
Zhang F, 2019, NAT COMMUN, V10, DOI 10.1038/s41467-019-11911-5
Zhang F, 2019, ACS NANO, V13, P5662, DOI 10.1021/acsnano.9b00892
Zhang XHF, 2013, CELL, V154, P1060, DOI 10.1016/j.cell.2013.07.036
Zhang Y, 2018, NAT COMMUN, V9, DOI [10.1038/s41467-017-02251-3, 10.1038/s41467-018-06126-z]
Zhao J, 2018, ACS NANO, V12, P9881, DOI 10.1021/acsnano.8b02481
Zhao J, 2018, BIOMATERIALS, V159, P215, DOI 10.1016/j.biomaterials.2018.01.014
Zhen ZP, 2017, NANO LETT, V17, P862, DOI 10.1021/acs.nanolett.6b04150
Zheng D. W., 2020, NAT COMMUN, V11, P1
Zhou FY, 2019, ADV MATER, V31, DOI 10.1002/adma.201805888
Zhou L., 2020, NANOLETT
Zhu GZ, 2017, NAT COMMUN, V8, DOI 10.1038/s41467-017-02191-y
Zhu HD, 2020, NANOSCALE, V12, P3400, DOI 10.1039/c9nr09610h
NR 263
TC 54
Z9 54
U1 13
U2 191
PU WILEY-V C H VERLAG GMBH
PI WEINHEIM
PA POSTFACH 101161, 69451 WEINHEIM, GERMANY
SN 0935-9648
EI 1521-4095
J9 ADV MATER
JI Adv. Mater.
PD JUL
PY 2021
VL 33
IS 30
AR 2008094
DI 10.1002/adma.202008094
EA MAY 2021
PG 37
WC Chemistry, Multidisciplinary; Chemistry, Physical; Nanoscience &
Nanotechnology; Materials Science, Multidisciplinary; Physics, Applied;
Physics, Condensed Matter
WE Science Citation Index Expanded (SCI-EXPANDED)
SC Chemistry; Science & Technology - Other Topics; Materials Science;
Physics
GA TQ2LU
UT WOS:000655690600001
PM 34048101
DA 2023-09-14
ER

PT J
AU Hu, YY
Lin, L
Chen, J
Maruyama, A
Tian, HY
Chen, XS
AF Hu, Yingying
Lin, Lin
Chen, Jie
Maruyama, Atsushi
Tian, Huayu

Chen, Xuesi
TI Synergistic tumor immunological strategy by combining tumor nanovaccine
with gene-mediated extracellular matrix scavenger
SO BIOMATERIALS
LA English
DT Article
DE Immunotherapy; Nanovaccines; Gene therapy; M2-like macrophages; CTL
infiltration; ECM
ID CANCER-IMMUNOTHERAPY; IMMUNE; ADJUVANT; CELLS
AB The antitumor efficacy of tumor vaccines is often limited by weak T cell responses and poor activated T cells infiltration. Herein, we reported a novel synergistic strategy to simultaneously overcome these two obstacles to realize enhanced tumor elimination. To induce the robust T cell responses, we designed a minimalist tumor nanovaccine based on stepwise electrostatic interactions. The dual-functional delivery system PEI/CaCO3 (polyethylenimine coated CaCO3), could not only act as a vaccine carrier that absorbed antigen ovalbumin (OVA) and adjuvant unmethylated cytosine-phosphate-guanine (CpG) with high efficiency, but also work as an underlying adjuvant to activate bone marrow-derived dendritic cells (BMDCs). Therefore, the formed PEI/CaCO3/OVA/CpG vaccines (NVs) realized the significant enhancement of both the BMDCs activation and the specific responses of T cells in vivo. In addition, to enhance the infiltration of activated T cells in the tumor sites, the Spam1 gene, which could express hyaluronidase (HAase), was explored using PEI as the gene carrier shielded with aldehyde modified polyethylene glycol (CHO-PEG-CHO) through pH responsive Schiff base bonds. PEG/PEI/pSpam1 (pSpam1@NPs) could achieve a high HAase expression in the tumor sites to further degrade the tumor extracellular matrix, thus promoting the infiltration of immune cells. Besides, the degradation of extracellular matrix increased blood perfusion and relieved the tumor hypoxia to modulate the immune-suppressive microenvironment. Highly enhanced antitumor efficiency and tumor re-challenge prevention were achieved by combined NVs with pSpam1@NPs in B16-OVA bearing mice. The facile synergistic strategy we presented here is expected to be further used for personalized immunotherapy in the future.
C1 [Hu, Yingying; Lin, Lin; Chen, Jie; Tian, Huayu; Chen, Xuesi] Chinese Acad Sci, Changchun Inst Appl Chem, Key Lab Polymer Ecomat, Changchun 130022, Peoples R China.
[Hu, Yingying; Chen, Jie; Tian, Huayu; Chen, Xuesi] Univ Sci & Technol China, Hefei 230026, Peoples R China.
[Hu, Yingying; Lin, Lin; Chen, Jie; Tian, Huayu; Chen, Xuesi] Jilin Biomed Polymers Engn Lab, Changchun 130022, Peoples R China.
[Maruyama, Atsushi] Tokyo Inst Technol, Dept Life Sci & Technol, Midori Ku, Yokohama, Kanagawa 2268501, Japan.
C3 Chinese Academy of Sciences; Changchun Institute of Applied Chemistry,
CAS; Chinese Academy of Sciences; University of Science & Technology of
China, CAS; Tokyo Institute of Technology
RP Chen, J; Tian, HY (通讯作者)，Chinese Acad Sci, Changchun Inst Appl Chem, Key Lab Polymer Ecomat, Changchun 130022, Peoples R China.
EM thy@ciac.ac.cn
RI hu, ying/IVV-6516-2023; Chen, Xuesi/ABB-5039-2020; Chen,
Jie/R-6737-2016; Maruyama, Atsushi/C-9186-2015; Hu, Ying/GQZ-8100-2022
OI Maruyama, Atsushi/0000-0002-7495-2974;
FU National Natural Science Foundation of China [51925305, 51873208,
51520105004, 51833010, 51973217, 51803210]; National Science and
Technology Major Projects for Major New Drugs Innovation and Development
[2018ZX09711003-012]; Jilin province science and technology development
program [20180414027GH, 20200201075JC]
FX This work was supported by the National Natural Science Foundation of
China (Grant Nos. 51925305, 51873208, 51520105004, 51833010, 51973217
and 51803210), National Science and Technology Major Projects for Major
New Drugs Innovation and Development (2018ZX09711003-012), Jilin
province science and technology development program (Grant Nos.
20180414027GH and 20200201075JC).
CR Adachi K, 2018, NAT BIOTECHNOL, V36, P346, DOI 10.1038/nbt.4086
Banchereau J, 2005, NAT REV IMMUNOL, V5, P296, DOI 10.1038/nri1592
Chen DS, 2013, IMMUNITY, V39, P1, DOI 10.1016/j.immuni.2013.07.012
Chen HB, 2018, SCI CHINA CHEM, V61, P1503, DOI 10.1007/s11426-018-9397-5
Chen J, 2015, CHINESE J POLYM SCI, V33, P830, DOI 10.1007/s10118-015-1632-0
Gong H, 2016, NANO LETT, V16, P2512, DOI 10.1021/acs.nanolett.6b00068

Guan XW, 2019, J CONTROL RELEASE, V293, P104, DOI 10.1016/j.jconrel.2018.11.022
Guan XW, 2018, BIOMATERIALS, V171, P198, DOI 10.1016/j.biomaterials.2018.04.039
Guan XW, 2017, BIOMACROMOLECULES, V18, P1342, DOI 10.1021/acs.biomac.7b00080
Guan XW, 2016, NANO LETT, V16, P6823, DOI 10.1021/acs.nanolett.6b02536
Hariharan K, 1998, ADV DRUG DELIVER REV, V32, P187, DOI 10.1016/S0169-409X(98)00010-6
Hu CH, 2017, BIOMATERIALS, V144, P60, DOI 10.1016/j.biomaterials.2017.08.009
Itano N, 2008, SEMIN CANCER BIOL, V18, P268, DOI 10.1016/j.semcancer.2008.03.006
June CH, 2018, SCIENCE, V359, P1361, DOI 10.1126/science.aar6711
Klausen U, 2018, FRONT IMMUNOL, V9, DOI 10.3389/fimmu.2018.02264
Li AW, 2018, NAT MATER, V17, P528, DOI 10.1038/s41563-018-0028-2
Li F, 2019, ACS CENTRAL SCI, V5, P796, DOI 10.1021/acscentsci.9b00060
Li F, 2017, ADV MATER, V29, DOI 10.1002/adma.201602432
Lu Y, 2018, BIOMATERIALS, V175, P82, DOI 10.1016/j.biomaterials.2018.05.025
Mellman I, 2011, NATURE, V480, P480, DOI 10.1038/nature10673
Messenheimer DJ, 2017, CLIN CANCER RES, V23, P6165, DOI 10.1158/1078-0432.CCR-16-2677
Motz GT, 2013, IMMUNITY, V39, P61, DOI 10.1016/j.immuni.2013.07.005
Palucka K, 2012, NAT REV CANCER, V12, P265, DOI 10.1038/nrc3258
Purcell AW, 2007, NAT REV DRUG DISCOV, V6, P404, DOI 10.1038/nrd2224
Rosenberg SA, 2008, NAT REV CANCER, V8, P299, DOI 10.1038/nrc2355
Sharma P, 2017, CELL, V168, P707, DOI 10.1016/j.cell.2017.01.017
Sironen RK, 2011, EXP CELL RES, V317, P383, DOI 10.1016/j.yexcr.2010.11.017
Song WT, 2018, NAT COMMUN, V9, DOI 10.1038/s41467-018-04605-x
Wang HR, 2019, ADV FUNCT MATER, V29, DOI 10.1002/adfm.201902440
Wang K, 2018, ACS NANO, V12, P6398, DOI 10.1021/acsnano.8b00558
Xu C, 2019, ACS NANO, V13, P12148, DOI 10.1021/acsnano.9b06691
Ye YQ, 2017, SCI IMMUNOL, V2, DOI 10.1126/sciimmunol.aan5692
Yu SJ, 2018, ADV MATER, V30, DOI 10.1002/adma.201801527
Zhang Y, 2016, ADV FUNCT MATER, V26, P6454, DOI 10.1002/adfm.201600650
Zhou H, 2016, NANO LETT, V16, P3268, DOI 10.1021/acs.nanolett.6b00820
Zhou ZF, 2018, CHINESE CHEM LETT, V29, P19, DOI 10.1016/j.cclet.2017.09.047
Zucca P, 2014, MOLECULES, V19, P14139, DOI 10.3390/molecules190914139
NR 37
TC 54
Z9 54
U1 10
U2 333
PU ELSEVIER SCI LTD
PI OXFORD
PA THE BOULEVARD, LANGFORD LANE, KIDLINGTON, OXFORD OX5 1GB, OXON, ENGLAND
SN 0142-9612
EI 1878-5905
J9 BIOMATERIALS
JI Biomaterials
PD SEP
PY 2020
VL 252
AR 120114
DI 10.1016/j.biomaterials.2020.120114
PG 13
WC Engineering, Biomedical; Materials Science, Biomaterials
WE Science Citation Index Expanded (SCI-EXPANDED)
SC Engineering; Materials Science
GA LT2GJ
UT WOS:000536890500012
PM 32422491
DA 2023-09-14
ER

PT J
AU Qian, HQ
Liu, BR

Jiang, XQ
AF Qian, Hanqing
Liu, Baorui
Jiang, Xiqun
TI Application of nanomaterials in cancer immunotherapy
SO MATERIALS TODAY CHEMISTRY
LA English
DT Review
DE Nanomaterials; Cancer immunotherapy; Nanovaccines; Tumor microenvironment
ID REGULATORY T-CELLS; ANTIGEN-PRESENTING CELLS; CHECKPOINT BLOCKADE; DENDRITIC CELLS; NANOPARTICLE VACCINES; IN-VIVO; TARGETING NANOPARTICLES; PHOTOTHERMAL THERAPY; CROSS-PRESENTATION; SUPPRESSOR-CELLS
AB Cancer has become one of the major public health problems all over the word. Cancer immunotherapy is the treatment that utilizing the patient's own immune system to fight cancer. Although promising outcomes of cancer immunotherapy have been achieved nowadays, it is still a challenge to develop a therapeutic pattern with low toxicities, high specificity, and long-lasting efficacy in this field. Nano-materials can be employed as vehicles to deliver antigens or immune modulating therapeutics. Nanomaterials can enhance the efficacy of cancer immunotherapy by protecting their payload during circulation, promoting the delivery of antigen to antigen presenting cell, triggering the activation of antigen-specific T cell, and regulating the immunosuppressive tumor microenvironment. In this review, we briefly summarize the application of nanomaterials in cancer immunotherapy, including nanovaccines, tumor immune environment modulation, and other application. Without a doubt, with the advances of the interdisciplinary research focused for materials science, immunology and tumor biology, cancer immunotherapy will further enhance the therapeutic benefits and reduce side effects in the future. (C) 2018 Elsevier Ltd. All rights reserved.
C1 [Qian, Hanqing; Liu, Baorui] Nanjing Univ, Sch Med, Drum Tower Hosp, Ctr Comprehens Canc, Nanjing 210008, Peoples R China.
[Qian, Hanqing; Liu, Baorui] Nanjing Univ, Clin Canc Inst, Nanjing 210008, Peoples R China.
[Jiang, Xiqun] Nanjing Univ, Coll Chem & Chem Engn, Dept Polymer Sci & Engn, Nanjing 210093, Jiangsu, Peoples R China.
C3 Nanjing University; Nanjing University; Nanjing University
RP Jiang, XQ (通讯作者)，Nanjing Univ, Coll Chem & Chem Engn, Dept Polymer Sci & Engn, Nanjing 210093, Jiangsu, Peoples R China.
EM jiangx@nju.edu.cn
RI Qian, Hanqing/AAR-8994-2021; Jiang, Xiqun/I-8128-2014
OI Jiang, Xiqun/0000-0003-0483-0282; Qian, Hanqing/0000-0002-7743-6019
FU National Natural Science Foundation of China [81602106, 51690153, 21474045, 51273090, 51422303]; National Key R&D Program of China [2017YFA0205400]
FX This review is supported by the National Natural Science Foundation of China (nos. 81602106, 51690153, 21474045, 51273090 and 51422303), and National Key R&D Program of China (2017YFA0205400).
CR Ali OA, 2009, NAT MATER, V8, P151, DOI [10.1038/NMAT2357, 10.1038/nmat2357]
Amoozgar Z, 2015, ADV DRUG DELIVER REV, V91, P38, DOI 10.1016/j.addr.2014.09.007
Baay M, 2011, CLIN DEV IMMUNOL, DOI 10.1155/2011/565187
Bachmann MF, 2010, NAT REV IMMUNOL, V10, P787, DOI 10.1038/nri2868
Bennewitz NL, 2005, BIOMATERIALS, V26, P2991, DOI 10.1016/j.biomaterials.2004.08.023
Bohm S, 2016, CLIN CANCER RES, V22, P3025, DOI 10.1158/1078-0432.CCR-15-2657
Broaders KE, 2009, P NATL ACAD SCI USA, V106, P5497, DOI 10.1073/pnas.0901592106
Chen DS, 2013, IMMUNITY, V39, P1, DOI 10.1016/j.immuni.2013.07.012
Chen Q, 2016, NAT COMMUN, V7, DOI 10.1038/ncomms13193
Cheung AS, 2016, SMALL, V12, P2321, DOI 10.1002/smll.201600061
Cheung AS, 2015, NANO TODAY, V10, P511, DOI 10.1016/j.nantod.2015.06.007
Cieslewicz M, 2013, P NATL ACAD SCI USA, V110, P15919, DOI 10.1073/pnas.1312197110
COLEY WB, 1991, CLIN ORTHOP RELAT R, P3
Coussens LM, 2013, SCIENCE, V339, P286, DOI 10.1126/science.1232227
Cruz LJ, 2014, J CONTROL RELEASE, V192, P209, DOI 10.1016/j.jconrel.2014.07.040
de Titta A, 2013, P NATL ACAD SCI USA, V110, P19902, DOI 10.1073/pnas.1313152110
Demento SL, 2012, BIOMATERIALS, V33, P4957, DOI 10.1016/j.biomaterials.2012.03.041

Dewitte H, 2014, NANO TODAY, V9, P743, DOI 10.1016/j.nantod.2014.10.001
Draghiciu O, 2014, INT J CANCER, V134, P859, DOI 10.1002/ijc.28418
Dreaden EC, 2014, ACS NANO, V8, P8374, DOI 10.1021/nn502861t
Dudley ME, 2002, SCIENCE, V298, P850, DOI 10.1126/science.1076514
Engblom C, 2016, NAT REV CANCER, V16, P447, DOI 10.1038/nrc.2016.54
Fadel TR, 2014, NAT NANOTECHNOL, V9, P639, DOI [10.1038/NNANO.2014.154, 10.1038/nnano.2014.154]
Fan YC, 2015, VACCINES-BASEL, V3, P662, DOI 10.3390/vaccines3030662
Fan Z, 2012, ACS NANO, V6, P1065, DOI 10.1021/nn2045246
Fang RH, 2015, SMALL, V11, P5483, DOI 10.1002/smll.201501284
Fang RH, 2014, NANO LETT, V14, P2181, DOI 10.1021/nl500618u
Feig C, 2013, P NATL ACAD SCI USA, V110, P20212, DOI 10.1073/pnas.1320318110
Fridman WH, 2012, NAT REV CANCER, V12, P298, DOI 10.1038/nrc3245
Goldberg MS, 2015, CELL, V161, P201, DOI 10.1016/j.cell.2015.03.037
Guo LR, 2014, ACS NANO, V8, P5670, DOI 10.1021/nn5002112
Hanson MC, 2015, J CLIN INVEST, V125, P2532, DOI 10.1172/JCI79915
He CB, 2016, NAT COMMUN, V7, DOI 10.1038/ncomms12499
Hirosue S, 2010, VACCINE, V28, P7897, DOI 10.1016/j.vaccine.2010.09.077
Irvine DJ, 2015, CHEM REV, V115, P11109, DOI 10.1021/acs.chemrev.5b00109
Irvine DJ, 2013, NAT MATER, V12, P978, DOI [10.1038/NMAT3775, 10.1038/nmat3775]
Jeanbart L, 2015, CANCER IMMUNOL IMMUN, V64, P1033, DOI 10.1007/s00262-015-1702-8
Kapadia CH, 2015, J CONTROL RELEASE, V219, P167, DOI 10.1016/j.jconrel.2015.09.062
Kim J, 2015, NAT BIOTECHNOL, V33, P64, DOI 10.1038/nbt.3071
Kim SE, 2016, NAT NANOTECHNOL, V11, P977, DOI 10.1038/nnano.2016.164
Kitamura T, 2015, NAT REV IMMUNOL, V15, P73, DOI 10.1038/nri3789
Kuai R, 2017, NAT MATER, V16, P489, DOI [10.1038/nmat4822, 10.1038/NMAT4822]
Lei CH, 2010, J AM CHEM SOC, V132, P6906, DOI 10.1021/ja102414t
Li HY, 2011, NAT NANOTECHNOL, V6, P645, DOI [10.1038/nnano.2011.153, 10.1038/NNANO.2011.153]
Liakou CI, 2008, P NATL ACAD SCI USA, V105, P14987, DOI 10.1073/pnas.0806075105
Liao D, 2011, CANCER RES, V71, P5688, DOI 10.1158/0008-5472.CAN-11-1264
Liu GW, 2015, BIOCONJUGATE CHEM, V26, P1811, DOI 10.1021/acs.bioconjchem.5b00344
Liu HP, 2014, NATURE, V507, P519, DOI 10.1038/nature12978
Luo M, 2017, J CONTROL RELEASE, V263, P200, DOI 10.1016/j.jconrel.2017.03.033
Luo M, 2017, NAT NANOTECHNOL, V12, P648, DOI [10.1038/nnano.2017.52, 10.1038/NNANO.2017.52]
Manolova V, 2008, EUR J IMMUNOL, V38, P1404, DOI 10.1002/eji.200737984
Matijevic M, 2011, CELL IMMUNOL, V270, P62, DOI 10.1016/j.cellimm.2011.04.005
Mellman I, 2011, NATURE, V480, P480, DOI 10.1038/nature10673
Meyer RA, 2015, SMALL, V11, P1519, DOI 10.1002/smll.201402369
Min YZ, 2017, NAT NANOTECHNOL, V12, P877, DOI [10.1038/nnano.2017.113, 10.1038/NNANO.2017.113]
Minn AJ, 2016, CELL, V165, P272, DOI 10.1016/j.cell.2016.03.031
Molon B, 2011, J EXP MED, V208, P1949, DOI 10.1084/jem.20101956
Moynihan KD, 2016, NAT MED, V22, P1402, DOI 10.1038/nm.4200
Mueller M, 2012, J CONTROL RELEASE, V162, P159, DOI 10.1016/j.jconrel.2012.06.015
Nguyen DN, 2009, ADV MATER, V21, P847, DOI 10.1002/adma.200801478
Orecchioni M, 2015, THERANOSTICS, V5, P710, DOI 10.7150/thno.11387
Park J, 2012, NAT MATER, V11, P895, DOI [10.1038/NMAT3355, 10.1038/nmat3355]
Parker KH, 2015, ADV CANCER RES, V128, P95, DOI 10.1016/bs.acr.2015.04.002
Patel JM, 2016, BIOMATERIALS, V74, P231, DOI 10.1016/j.biomaterials.2015.09.031
Perica K, 2014, ACS NANO, V8, P2252, DOI 10.1021/nn405520d
Perica K, 2014, NANOMED-NANOTECHNOL, V10, P119, DOI 10.1016/j.nano.2013.06.015
Perret R, 2013, CANCER RES, V73, P6597, DOI 10.1158/0008-5472.CAN-13-0875
Qian Y, 2017, ACS NANO, V11, P9536, DOI 10.1021/acsnano.7b05465
Quail DF, 2013, NAT MED, V19, P1423, DOI 10.1038/nm.3394
Reddy ST, 2007, NAT BIOTECHNOL, V25, P1159, DOI [10.1038/nbt1332, 10.1038/nbtl332]
Reddy ST, 2006, J CONTROL RELEASE, V112, P26, DOI 10.1016/j.jconrel.2006.01.006
Robert C, 2015, NEW ENGL J MED, V372, P2521, DOI 10.1056/NEJMoa1503093
Rosalia RA, 2015, BIOMATERIALS, V40, P88, DOI 10.1016/j.biomaterials.2014.10.053
Rosenberg JE, 2016, LANCET, V387, P1909, DOI 10.1016/S0140-6736(16)00561-4

Sacchetti C, 2013, BIOCONJUGATE CHEM, V24, P852, DOI 10.1021/bc400070q
SEUNG LP, 1995, P NATL ACAD SCI USA, V92, P6254, DOI 10.1073/pnas.92.14.6254
Sharabi AB, 2015, LANCET ONCOL, V16, pE498, DOI 10.1016/S1470-2045(15)00007-8
Silva JM, 2013, J CONTROL RELEASE, V168, P179, DOI 10.1016/j.jconrel.2013.03.010
Singh N, 2011, J AM CHEM SOC, V133, P19582, DOI 10.1021/ja206998x
Smith TT, 2017, NAT NANOTECHNOL, V12, P813, DOI [10.1038/NNANO.2017.57, 10.1038/nnano.2017.57]
Speiser DE, 2016, NAT REV IMMUNOL, V16, P500, DOI 10.1038/nri.2016.80
Srinivas R, 2012, BIOMATERIALS, V33, P6220, DOI 10.1016/j.biomaterials.2012.05.006
Steenblock ER, 2008, MOL THER, V16, P765, DOI 10.1038/mt.2008.11
Tarangelo A, 2016, NAT NANOTECHNOL, V11, P921, DOI 10.1038/nnano.2016.199
Teo PY, 2015, ADV HEALTHC MATER, V4, P1180, DOI 10.1002/adhm.201500089
Tian Y, 2016, SMALL, V12, P721, DOI 10.1002/smll.201503319
Vignali DAA, 2008, NAT REV IMMUNOL, V8, P523, DOI 10.1038/nri2343
Wang C, 2017, NAT BIOMED ENG, V1, DOI 10.1038/s41551-016-0011
Wang C, 2014, ADV MATER, V26, P8154, DOI 10.1002/adma.201402996
Weichselbaum RR, 2017, NAT REV CLIN ONCOL, V14, P365, DOI 10.1038/nrclinonc.2016.211
Wilson JT, 2013, ACS NANO, V7, P3912, DOI 10.1021/nn305466z
Wrzesinski SH, 2007, CLIN CANCER RES, V13, P5262, DOI 10.1158/1078-0432.CCR-07-1157
Xu J, 2017, ACS NANO, V11, P4463, DOI 10.1021/acsnano.7b00715
Yuan HF, 2017, NAT NANOTECHNOL, V12, P763, DOI [10.1038/nnano.2017.69, 10.1038/NNANO.2017.69]
Zanganeh S, 2016, NAT NANOTECHNOL, V11, P986, DOI [10.1038/nnano.2016.168, 10.1038/NNANO.2016.168]
Zhang LR, 2017, INT J NANOMED, V12, P2129, DOI 10.2147/IJN.S126016
Zhang PP, 2015, ACS NANO, V9, P6465, DOI 10.1021/acsnano.5b02153
Zhang ZP, 2011, BIOMATERIALS, V32, P3666, DOI 10.1016/j.biomaterials.2011.01.067
Zhu GZ, 2017, ACS NANO, V11, P2387, DOI 10.1021/acsnano.7b00978
Zhu SJ, 2013, MOL PHARMACEUT, V10, P3525, DOI 10.1021/mp400216r
NR 100
TC 54
Z9 55
U1 3
U2 106
PU ELSEVIER SCI LTD
PI OXFORD
PA THE BOULEVARD, LANGFORD LANE, KIDLINGTON, OXFORD OX5 1GB, OXON, ENGLAND
SN 2468-5194
J9 MATER TODAY CHEM
JI Mater. Today Chem.
PD MAR
PY 2018
VL 7
BP 53
EP 64
DI 10.1016/j.mtchem.2018.01.001
PG 12
WC Chemistry, Multidisciplinary; Materials Science, Multidisciplinary
WE Science Citation Index Expanded (SCI-EXPANDED)
SC Chemistry; Materials Science
GA GN1TA
UT WOS:000438775800007
DA 2023-09-14
ER

PT J
AU Li, ZL
Cai, H
Li, ZQ
Ren, L
Ma, XL

Zhu, HY
Gong, QY
Zhang, H
Gu, ZW
Luo, K
AF Li, Zhilin
Cai, Hao
Li, Zhiqian
Ren, Long
Ma, Xuelei
Zhu, Hongyan
Gong, Qiyong
Zhang, Hu
Gu, Zhongwei
Luo, Kui
TI A tumor cell membrane-coated self-amplified nanosystem as a nanovaccine to boost the therapeutic effect of anti-PD-L1 antibody
SO BIOACTIVE MATERIALS
LA English
DT Article
DE Cell membrane; Biomimetic nanosystem; Immunogenic cell death; Immunotherapy; anti-PD-L1 antibody
ID BIOMIMETIC NANOPARTICLES; CALRETICULIN EXPOSURE; CANCER; DEATH
AB To improve the response rate of immune checkpoint inhibitors such as anti-PD-L1 antibody in immunosup-pressive cancers like triple-negative breast cancer (TNBC), induction of immunogenic cell death (ICD) at tumor sites can increase the antigenicity and adjuvanticity to activate the immune microenvironment so that tumors become sensitive to the intervention of immune checkpoint inhibitors. Herein, a self-amplified biomimetic nanosystem, mEHGZ, was constructed by encapsulation of epirubicin (EPI), glucose oxidase (Gox) and hemin in ZIF-8 nanoparticles and coating of the nanoparticles with calreticulin (CRT) over-expressed tumor cell mem-brane. EPI acts as an ICD inducer, Gox and hemin medicate the cascade generation of reactive oxygen species (ROS) to strengthen the ICD effect, and CRT-rich membrane as "eat me" signal promote presentation of the released antigens by dendritic cells (DCs) to invoke the tumor-immunity cycle. The biomimetic delivery system displays an amplified ICD effect via Gox oxidation, hydroxyl radical generation and glutathione (GSH) depletion. The induced potent ICD effect promotes DCs maturation and cytotoxic T lymphocytes (CTLs) infiltration, reversing an immunosuppressive tumor microenvironment to an immunoresponsive one. Treatment with the nanosystem in combination with anti-PD-L1 antibody results in distinctive inhibition of tumor growth and lung metastasis, supporting that a potent ICD effect can significantly boost the therapeutic efficacy of the anti-PD-L1 antibody. This self-amplified biomimetic nanoplatform offers a promising means of raising the response rate of immune checkpoint inhibitors.
C1 [Li, Zhilin; Cai, Hao; Li, Zhiqian; Ren, Long; Ma, Xuelei; Zhu, Hongyan; Gong, Qiyong; Gu, Zhongwei; Luo, Kui] Sichuan Univ, West China Hosp, Natl Clin Res Ctr Geriatr, Dept Radiol,Huaxi MR Res Centner HMRRC,Dept Biothe, Chengdu 610041, Peoples R China.
[Zhang, Hu] Keck Grad Inst, Amgen Bioproc Ctr, Claremont, CA 91711 USA.
[Gong, Qiyong] Chinese Acad Med Sci, Funct & Mol Imaging Key Lab Sichuan Prov, Chengdu 610041, Peoples R China.
[Gong, Qiyong] Chinese Acad Med Sci, Res Unit Psychoradiol, Chengdu 610041, Peoples R China.
C3 Sichuan University; Claremont Colleges; Keck Graduate Institute; Chinese Academy of Medical Sciences - Peking Union Medical College; Chinese Academy of Medical Sciences - Peking Union Medical College
RP Luo, K (通讯作者)，Sichuan Univ, West China Hosp, Natl Clin Res Ctr Geriatr, Dept Radiol,Huaxi MR Res Centner HMRRC,Dept Biothe, Chengdu 610041, Peoples R China.
EM hyzhu_hmrrc@126.com; luokui@scu.edu.cn
RI Gong, Qiyong/W-3052-2019; gu, zhongwei/HPC-3816-2023; Zhang, Hu/F-9815-2010
OI Gong, Qiyong/0000-0002-5912-4871; Gu, Zhongwei/0000-0003-1547-6880; Zhang, Hu/0000-0003-4178-6401
FU National Natural Science Foundation of China [52073193, 51873120, 81621003]; 1.3.5 Project for Disciplines of Excellence, West China Hospital, Sichuan University [ZYJC21013]
FX This work was supported by National Natural Science Foundation of China

(52073193, 51873120 and 81621003), and 1.3.5 Project for Disciplines of
Excellence, West China Hospital, Sichuan University (ZYJC21013). Thanks
to Sisi Wu, Hongying Chen, Zhiqian Li, Yu Ding, Xuemei Chen, Li Fu,
Huaiqiang Sun, Shenglan You and Qing Yang (Research Core Facility, West
China Hospital, Sichuan University) for their help in cell studies and
histological studies. Thanks to Wentong Meng and Xue Li (Laboratory of
Stem Cell Biology, West China Hospital, Sichuan University) for work in
flow cytometer.
CR Alzeibak R, 2021, J IMMUNOTHER CANCER, V9, DOI 10.1136/jitc-2020-001926
Bahmani B, 2021, NAT COMMUN, V12, DOI 10.1038/s41467-021-22311-z
Bansal A, 2018, J CELL BIOL, V217, P2291, DOI 10.1083/jcb.201804161
Banstola A, 2021, J CONTROL RELEASE, V337, P505, DOI 10.1016/j.jconrel.2021.07.038
Chao Y, 2020, SCI ADV, V6, DOI 10.1126/sciadv.aaz4204
Chen Z, 2016, ACS NANO, V10, P10049, DOI 10.1021/acsnano.6b04695
Deng GJ, 2018, ACS NANO, V12, P12096, DOI 10.1021/acsnano.8b05292
Deng HZ, 2020, NANO LETT, V20, P1928, DOI 10.1021/acs.nanolett.9b05210
Duan XP, 2019, NAT COMMUN, V10, DOI 10.1038/s41467-019-09221-x
Duan ZY, 2021, ADV MATER, V33, DOI 10.1002/adma.202104594
Fu LH, 2019, ADV MATER, V31, DOI 10.1002/adma.201808325
Fucikova J, 2020, CELL DEATH DIS, V11, DOI 10.1038/s41419-020-03221-2
Fuertes MB, 2011, J EXP MED, V208, P2005, DOI 10.1084/jem.20101159
Hotamisligil GS, 2010, CELL, V140, P900, DOI 10.1016/j.cell.2010.02.034
Johnson AP, 2022, J CONTROL RELEASE, V343, P724, DOI 10.1016/j.jconrel.2022.02.013
Kroemer G, 2022, NAT IMMUNOL, V23, P487, DOI 10.1038/s41590-022-01132-2
Kroemer G, 2013, ANNU REV IMMUNOL, V31, P51, DOI 10.1146/annurev-immunol-032712-100008
Kroll AV, 2017, ADV MATER, V29, DOI 10.1002/adma.201703969
Krysko DV, 2012, NAT REV CANCER, V12, P860, DOI 10.1038/nrc3380
Kunde SS, 2021, INT J PHARMACEUT, V598, DOI 10.1016/j.ijpharm.2021.120395
Lee Sylvia, 2011, Cancers (Basel), V3, P3856, DOI 10.3390/cancers3043856
Li YH, 2021, CHEM COMMUN, V57, P12087, DOI 10.1039/d1cc04604g
Liu LZ, 2021, NAT COMMUN, V12, DOI 10.1038/s41467-021-26052-x
Luo Q, 2022, ADV FUNCT MATER, V32, DOI 10.1002/adfm.202110408
Luo QH, 2019, CANCER LETT, V454, P191, DOI 10.1016/j.canlet.2019.04.017
Mei H, 2022, BIOACT MATER, V8, P220, DOI 10.1016/j.bioactmat.2021.06.035
Mellman I, 2011, NATURE, V480, P480, DOI 10.1038/nature10673
Nam J, 2019, NAT REV MATER, V4, P398, DOI 10.1038/s41578-019-0108-1
Obeid M, 2007, NAT MED, V13, P54, DOI 10.1038/nm1523
Oroojalian F, 2021, SMALL, V17, DOI 10.1002/smll.202006484
Pan YC, 2011, CHEM COMMUN, V47, P2071, DOI 10.1039/c0cc05002d
Panaretakis T, 2009, EMBO J, V28, P578, DOI 10.1038/emboj.2009.1
Sang W, 2019, CHEM SOC REV, V48, P3771, DOI 10.1039/c8cs00896e
Sharma P, 2017, CELL, V168, P707, DOI 10.1016/j.cell.2017.01.017
Smyth MJ, 2016, NAT REV CLIN ONCOL, V13, P143, DOI 10.1038/nrclinonc.2015.209
Sun F, 2021, ADV SCI, V8, DOI 10.1002/advs.202002746
Sun HP, 2016, ADV MATER, V28, P9581, DOI 10.1002/adma.201602173
Tang ZM, 2019, ANGEW CHEM INT EDIT, V58, P946, DOI 10.1002/anie.201805664
Topalian SL, 2016, NAT REV CANCER, V16, P275, DOI 10.1038/nrc.2016.36
Weinberg F, 2019, CANCERS, V11, DOI 10.3390/cancers11081191
Xiong X, 2021, NANO LETT, V21, P8418, DOI 10.1021/acs.nanolett.1c03004
Xu C, 2021, ADV MATER, V33, DOI 10.1002/adma.202008061
Yang WJ, 2019, ACS NANO, V13, P3083, DOI 10.1021/acsnano.8b08346
Yu JX, 2020, NAT REV DRUG DISCOV, V19, P163, DOI 10.1038/d41573-019-00182-w
Zeng ZL, 2020, ADV FUNCT MATER, V30, DOI 10.1002/adfm.202004397
Zhang WY, 2022, CHEM ENG J, V433, DOI 10.1016/j.cej.2021.133498
Zheng HQ, 2016, J AM CHEM SOC, V138, P962, DOI 10.1021/jacs.5b11720
Zhou FY, 2019, ADV MATER, V31, DOI 10.1002/adma.201805888
NR 48
TC 53
Z9 53
U1 69
U2 196

PU KEAI PUBLISHING LTD
PI BEIJING
PA 16 DONGHUANGCHENGGEN NORTH ST, Building 5, Room 411, BEIJING, DONGCHENG
   DISTRICT 100009, PEOPLES R CHINA
EI 2452-199X
J9 BIOACT MATER
JI Bioact. Mater.
PD MAR
PY 2023
VL 21
BP 299
EP 312
DI 10.1016/j.bioactmat.2022.08.028
EA SEP 2022
PG 14
WC Engineering, Biomedical; Materials Science, Biomaterials
WE Science Citation Index Expanded (SCI-EXPANDED)
SC Engineering; Materials Science
GA 4Z9HQ
UT WOS:000862510600001
PM 36157245
OA Green Published, gold
HC Y
HP Y
DA 2023-09-14
ER

PT J
AU Hu, SC
   Ma, JH
   Su, C
   Chen, YW
   Shu, YH
   Qi, ZB
   Zhang, B
   Shi, G
   Zhang, Y
   Zhang, YW
   Huang, AL
   Kuang, YT
   Cheng, P
AF Hu, Shichuan
   Ma, Jinhu
   Su, Chao
   Chen, Yanwei
   Shu, Yongheng
   Qi, Zhongbing
   Zhang, Bin
   Shi, Gang
   Zhang, Yan
   Zhang, Yuwei
   Huang, Anliang
   Kuang, Yueting
   Cheng, Ping
TI Engineered exosome-like nanovesicles suppress tumor growth by
   reprogramming tumor microenvironment and promoting tumor ferroptosis
SO ACTA BIOMATERIALIA
LA English
DT Article
DE Cancer-associated fibroblasts; Exosome-like vaccine; FAP; Ferroptosis;
   Tumor microenvironment

ID FIBROBLAST ACTIVATION PROTEIN; CELL-DERIVED EXOSOMES; SERINE-PROTEASE;
T-CELLS; CANCER; MODELS; IMMUNOSUPPRESSION; ENVIRONMENT; EXPRESSION;
SYNERGIZES

AB Tumor vaccines that induce effective and sustained antitumor immunity are highly promising for cancer therapy. However, the antitumor potential of these vaccines is weakened due to the immunosuppressive characteristics of the tumor microenvironment (TME). Cancer-associated fibroblasts (CAFs) are the most abundant stromal cells within the TME; they play an important role in tumor growth, metastasis, immunosuppression, and drug resistance. Fibroblast activation protein-alpha (FAP) is overexpressed in CAFs in more than 90% of human tumor tissues. Further, FAP +CAFs are an ideal interstitial target for the immunotherapy of solid tumors. Exosomes derived from tumor cells contain many tumor antigens, which can be used as the basis of tumor vaccines that elicit strong antitumor immunity. Almost all exosomebased cancer vaccines have been designed and developed for tumor parenchymal cells. Moreover, the exosome production is very low and the purification is very difficult, limiting their clinical application as tumor vaccines. In this study, we developed FAP gene-engineered tumor cell-derived exosome-like nanovesicles (eNVs-FAP) as a tumor vaccine that can be prepared easily and in large quantities. The eNVs-FAP vaccine inhibited tumor growth by inducing strong and specific cytotoxic T lymphocyte (CTL) immune responses against tumor cells and FAP +CAFs and reprogramming the immunosuppressive TME in the colon, melanoma, lung, and breast cancer models. Moreover, eNVs-FAP vaccine-activated cellular immune responses could promote tumor ferroptosis by releasing interferon-gamma (IFN-gamma) from CTLs and depleting FAP +CAFs. Thus, eNVs-FAP is a candidate tumor vaccine targeting both the tumor parenchyma and the stroma.

Statement of significance

Nanovaccines can activate immune cells and promote an antitumor immune response. In this study, we developed the fibroblast activation protein-alpha (FAP) gene-engineered tumor cell-derived exosomelike vesicle vaccines (eNVs-FAP). A large number of eNVs-FAP were obtained by continuously squeezing FAP gene-engineered tumor cells. eNVs-FAP showed excellent antitumor effects in a variety of tumor bearing mouse models. The mechanistic analysis showed that eNVs-FAP promoted the maturation of dendritic cells (DCs), increased the infiltration of effector T cells into target tumor cells and FAP-positive cancer-associated fibroblasts (FAP +CAFs), and reduced the proportion of immunosuppressive cells, including M2-like tumor-associated macrophages (M2-TAMs), myeloid-derived suppressor cells (MDSCs), and regulatory T cells (Tregs), in the tumor microenvironment (TME). Moreover, the clearance of FAP +CAFs helped enhance interferon-gamma-induced tumor cell ferroptosis. (C) 2021 Acta Materialia Inc. Published by Elsevier Ltd. All rights reserved.

C1 [Hu, Shichuan; Ma, Jinhu; Su, Chao; Chen, Yanwei; Shu, Yongheng; Qi, Zhongbing; Zhang, Bin; Shi, Gang; Zhang, Yan; Kuang, Yueting; Cheng, Ping] Sichuan Univ, West China Hosp, State Key Lab Biotherapy, 17 Peoples South Rd, Chengdu 610041, Peoples R China.

[Hu, Shichuan; Ma, Jinhu; Su, Chao; Chen, Yanwei; Shu, Yongheng; Qi, Zhongbing; Zhang, Bin; Shi, Gang; Kuang, Yueting; Cheng, Ping] Sichuan Univ, West China Hosp, Canc Ctr, Collaborat Innovat Ctr Biotherapy, Chengdu 610041, Peoples R China.

[Zhang, Yan] Sichuan Univ, West China Hosp, Dept Thorac Oncol, Canc Ctr, Chengdu 610041, Peoples R China.

[Zhang, Yuwei] Sichuan Univ, West China Hosp, State Key Lab Biotherapy, Div Endocrinol & Metab, Chengdu 610041, Peoples R China.

[Huang, Anliang] Chengdu Fifth Peoples Hosp, Dept Pathol, Chengdu 610041, Peoples R China.

C3 Sichuan University; Sichuan University; Sichuan University; Sichuan
University

RP Cheng, P (通讯作者)，Sichuan Univ, West China Hosp, State Key Lab Biotherapy, 17 Peoples South Rd, Chengdu 610041, Peoples R China.; Cheng, P (通讯作者)，Sichuan Univ, West China Hosp, Canc Ctr, 17 Peoples South Rd, Chengdu 610041, Peoples R China.; Cheng, P (通讯作者)，Collaborat Innovat Ctr Biotherapy, 17 Peoples South Rd, Chengdu 610041, Peoples R China.

EM ping.cheng@foxmail.com

OI Ma, Jinhu/0000-0001-7681-3081

FU National Science and Technology Major Projects of New Drugs
[2018ZX09201018-013]; National Science and Technology Major Project for
Infectious Diseases Control [2017ZX10203206-004]; National Natural
Science Foundation of China [81101728, 82070660]; Sichuan Regional
Innovation Cooperation Project [20QYCX0100]; Innovation Spark Project of
Sichuan University [2018SCUH0084]

FX This work was supported by the National Science and Tech-nology Major
Projects of New Drugs (2018ZX09201018-013) , the National Science and
Technology Major Project for Infectious Dis-eases Control

(2017ZX10203206-004) , the National Natural Science Foundation of China
(81101728 and 82070 6 60) , Sichuan Regional Innovation Cooperation
Project (20QYCX0100) , and the Innovation Spark Project of Sichuan
University (2018SCUH0084) .
CR Andersen MH, 2006, J INVEST DERMATOL, V126, P32, DOI 10.1038/sj.jid.5700001
Bartoschek M, 2018, NAT COMMUN, V9, DOI 10.1038/s41467-018-07582-3
Biagini G., 2019, PLOS ONE, V14
Brunker P, 2016, MOL CANCER THER, V15, P946, DOI 10.1158/1535-7163.MCT-15-0647
Chen MH, 2015, SCI REP-UK, V5, DOI 10.1038/srep14421
Cheng JD, 2002, CANCER RES, V62, P4767
Choo YW, 2018, ACS NANO, V12, P8977, DOI 10.1021/acsnano.8b02446
Costa A, 2018, CANCER CELL, V33, P463, DOI 10.1016/j.ccell.2018.01.011
Cukierman E, 2010, SEMIN CANCER BIOL, V20, P139, DOI 10.1016/j.semcancer.2010.04.004
Duperret EK, 2018, CLIN CANCER RES, V24, P1190, DOI 10.1158/1078-0432.CCR-17-2033
Fang JX, 2016, INT J CANCER, V138, P1013, DOI 10.1002/ijc.29831
Fang XX, 2020, CIRC RES, V127, P486, DOI 10.1161/CIRCRESAHA.120.316509
Feig C, 2013, P NATL ACAD SCI USA, V110, P20212, DOI 10.1073/pnas.1320318110
Flavell RA, 2010, NAT REV IMMUNOL, V10, P554, DOI 10.1038/nri2808
Gaggioli C, 2007, NAT CELL BIOL, V9, P1392, DOI 10.1038/ncb1658
Geng F, 2019, VACCINE, V37, P4382, DOI 10.1016/j.vaccine.2019.06.012
Gu XY, 2015, INT J CANCER, V136, pE74, DOI 10.1002/ijc.29100
Guo J., 2017, CANC RES TREAT OFFIC, V50
Haaglim C., 2018, CLIN CANC RES OFFICI
Huber MA, 2003, J INVEST DERMATOL, V120, P182, DOI 10.1046/j.1523-1747.2003.12035.x
Jang SC, 2014, ACS NANO, V8, P1073, DOI 10.1021/nn406580h
Joyce JA, 2015, SCIENCE, V348, P74, DOI 10.1126/science.aaa6204
Kakarla S, 2013, MOL THER, V21, P1611, DOI 10.1038/mt.2013.110
Kalluri R, 2016, NAT REV CANCER, V16, P582, DOI 10.1038/nrc.2016.73
Kato T., 2018, TRANSLAT ONCOL, V11, P890
Kobayashi H, 2019, NAT REV GASTRO HEPAT, V16, P282, DOI 10.1038/s41575-019-0115-0
Lakins MA, 2018, NAT COMMUN, V9, DOI 10.1038/s41467-018-03347-0
Laklai H, 2016, NAT MED, V22, P497, DOI 10.1038/nm.4082
Lin YL, 2019, NEOPLASIA, V21, P1133, DOI 10.1016/j.neo.2019.10.005
Loeffler M, 2006, J CLIN INVEST, V116, P1955, DOI 10.1172/JCI26532
Lu Z, 2017, J HEPATOL, V67, P739, DOI 10.1016/j.jhep.2017.05.019
Lunavat TR, 2016, BIOMATERIALS, V102, P231, DOI 10.1016/j.biomaterials.2016.06.024
Lv P, 2019, NANO LETT, V19, P2993, DOI 10.1021/acs.nanolett.9b00145
Martinez FO, 2008, FRONT BIOSCI-LANDMRK, V13, P453, DOI 10.2741/2692
Mentlein R, 2011, BIOL CHEM, V392, P199, DOI 10.1515/BC.2010.119
Morishita M, 2016, BIOMATERIALS, V111, P55, DOI 10.1016/j.biomaterials.2016.09.031
Ozdemir BC, 2014, CANCER CELL, V25, P719, DOI 10.1016/j.ccr.2014.04.005
Ostermann E, 2008, CLIN CANCER RES, V14, P4584, DOI 10.1158/1078-0432.CCR-07-5211
Ouyang LQ, 2016, SCI REP-UK, V6, DOI 10.1038/srep39541
Park JE, 1999, J BIOL CHEM, V274, P36505, DOI 10.1074/jbc.274.51.36505
Quail DF, 2013, NAT MED, V19, P1423, DOI 10.1038/nm.3394
Rao Q, 2016, HEPATOLOGY, V64, P456, DOI 10.1002/hep.28549
Seiler A, 2008, CELL METAB, V8, P237, DOI 10.1016/j.cmet.2008.07.005
Sun XF, 2016, HEPATOLOGY, V64, P488, DOI 10.1002/hep.28574
Sun Y, 2014, CLIN THER, V36, P863, DOI 10.1016/j.clinthera.2014.04.018
Wang H, 2017, HEPATOLOGY, V66, P449, DOI 10.1002/hep.29117
Wang LCS, 2014, CANCER IMMUNOL RES, V2, P154, DOI 10.1158/2326-6066.CIR-13-0027
Wang WM, 2019, NATURE, V569, P270, DOI 10.1038/s41586-019-1170-y
WELT S, 1994, J CLIN ONCOL, V12, P1193, DOI 10.1200/JCO.1994.12.6.1193
Wolfers J, 2001, NAT MED, V7, P297, DOI 10.1038/85438
Wu JY, 2018, SCI REP-UK, V8, DOI 10.1038/s41598-018-20505-y
Yang WS, 2014, CELL, V156, P317, DOI 10.1016/j.cell.2013.12.010
Yang XG, 2016, CANCER RES, V76, P4124, DOI 10.1158/0008-5472.CAN-15-2973
Zhang HY, 2020, MOL CANCER, V19, DOI 10.1186/s12943-020-01168-8
NR 54
TC 53
Z9 55

U1 18
U2 97
PU ELSEVIER SCI LTD
PI OXFORD
PA THE BOULEVARD, LANGFORD LANE, KIDLINGTON, OXFORD OX5 1GB, OXON, ENGLAND
SN 1742-7061
EI 1878-7568
J9 ACTA BIOMATER
JI Acta Biomater.
PD NOV
PY 2021
VL 135
BP 567
EP 581
DI 10.1016/j.actbio.2021.09.003
EA NOV 2021
PG 15
WC Engineering, Biomedical; Materials Science, Biomaterials
WE Science Citation Index Expanded (SCI-EXPANDED)
SC Engineering; Materials Science
GA WX9BZ
UT WOS:000718884500005
PM 34506976
DA 2023-09-14
ER

PT J
AU Xu, C
Hong, H
Lee, Y
Park, KS
Sun, MJ
Wang, TR
Aikins, ME
Xu, Y
Moon, JJ
AF Xu, Cheng
Hong, Hao
Lee, Yonghyun
Park, Kyung Soo
Sun, Mingjiao
Wang, Tianrui
Aikins, Marisa E.
Xu, Yao
Moon, James J.
TI Efficient Lymph Node-Targeted Delivery of Personalized Cancer Vaccines
with Reactive Oxygen Species-Inducing Reduced Graphene Oxide Nanosheets
SO ACS NANO
LA English
DT Article
DE reduced graphene wade; nanosheet; cancer vaccine; cancer ammunotherapy;
positron emission tomography
ID CROSS-PRESENTATION; DENDRITIC CELLS; OXIDATIVE STRESS; DRUG-DELIVERY;
T-CELLS; RESPONSES; IMMUNOGENICITY; DEGRADATION; COMBINATION; ANTIGENS
AB Therapeutic cancer vaccines require robust cellular immunity for the efficient killing of tumor cells, and recent advances in neoantigen discovery may provide safe and promising targets for cancer vaccines. However, elicitation of T cells with strong antitumor efficacy requires intricate multistep processes that have been difficult to attain with traditional vaccination approaches. Here, a multifunctional nanovaccine platform has been developed for direct delivery of neoantigens and adjuvants to lymph nodes (LNs) and highly efficient induction of neoantigen-specific T cell responses. A PEGylated reduced graphene oxide nanosheet (RGO-PEG, 20-30 nm in diameter) is a highly modular and biodegradable platform for facile

preparation of neoantigen vaccines within 2 h. RGO-PEG exhibits rapid, efficient (15-20% ID/g), and sustained (up to 72 h) accumulation in LNs, achieving >100-fold improvement in LN-targeted delivery, compared with soluble vaccines. Moreover, RGO-PEG induces intracellular reactive oxygen species in dendritic cells, guiding antigen processing and presentation to T cells. Importantly, a single injection of RGO-PEG vaccine elicits potent neoantigen-specific T cell responses lasting up to 30 days and eradicates established MC-38 colon carcinoma. Further combination with anti-PD-1 therapy achieved great therapeutic improvements against B16F10 melanoma. RGO-PEG may serve a powerful delivery platform for personalized cancer vaccination.
C1 [Xu, Cheng; Lee, Yonghyun; Sun, Mingjiao; Wang, Tianrui; Aikins, Marisa E.; Xu, Yao; Moon, James J.] Univ Michigan, Dept Pharmaceut Sci, Ann Arbor, MI 48109 USA.
[Xu, Cheng; Lee, Yonghyun; Park, Kyung Soo; Aikins, Marisa E.; Xu, Yao; Moon, James J.] Univ Michigan, Biointerfaces Inst, Ann Arbor, MI 48109 USA.
[Hong, Hao] Nanjing Univ, Sch Med, Nanjing 210093, Jiangsu, Peoples R China.
[Lee, Yonghyun] Ewha Womans Univ, Grad Sch, Coll Pharm & Pharm, Dept Pharm, Seoul 03760, South Korea.
[Park, Kyung Soo; Moon, James J.] Univ Michigan, Dept Biomed Engn, Ann Arbor, MI 48109 USA.
C3 University of Michigan System; University of Michigan; University of Michigan System; University of Michigan; Nanjing University; Ewha Womans University; University of Michigan System; University of Michigan
RP Moon, JJ (通讯作者)，Univ Michigan, Dept Pharmaceut Sci, Ann Arbor, MI 48109 USA.; Moon, JJ (通讯作者)，Univ Michigan, Biointerfaces Inst, Ann Arbor, MI 48109 USA.; Moon, JJ (通讯作者)，Univ Michigan, Dept Biomed Engn, Ann Arbor, MI 48109 USA.
EM moonjj@umich.edu
RI Moon, James J/ABE-4429-2021; Xu, Cheng/ACX-9795-2022
OI Moon, James J/0000-0003-2238-2372; Xu, Cheng/0000-0003-1000-733X
FU NIH [R01AI127070, R01EB022563, R01CA210273, R01DK125087, U01CA210152]; DoD/CDMRP Peer Reviewed Cancer Research Program [W81XWH-16-1-0369]; NSF CAREER Award [1553831]; UM TEAM Training Program from NIDCR [DE007057]; NIH Tetramer Core Facility [HHSN272201300006C]
FX This work was supported in part by the NIH (R01AI127070, R01EB022563, R01CA210273, R01DK125087, U01CA210152). JJ.M. is supported by DoD/CDMRP Peer Reviewed Cancer Research Program (W81XWH-16-1-0369) and NSF CAREER Award (1553831). K.S.P. acknowledges financial support from the UM TEAM Training Program (DE007057 from NIDCR). We acknowledge the NIH Tetramer Core Facility (contract HHSN272201300006C) for the provision of MHC-I tetramers. Opinions interpretations, conclusions, and recommendations are those of the authors and are not necessarily endorsed by the Department of Defense. Some figures were created by the authors with BioRender.com.
CR Accapezzato D, 2005, J EXP MED, V202, P817, DOI 10.1084/jem.20051106
Alspach E, 2019, NATURE, V574, P696, DOI 10.1038/s41586-019-1671-8
Arya N, 2013, NANOSCALE, V5, P2818, DOI 10.1039/c3nr33190c
Curran MA, 2010, P NATL ACAD SCI USA, V107, P4275, DOI 10.1073/pnas.0915174107
Delamarre L, 2005, SCIENCE, V307, P1630, DOI 10.1126/science.1108003
Gao J, 2017, INT J NANOMED, V12, P1251, DOI 10.2147/IJN.S125866
Gurunathan S, 2012, INT J NANOMED, V7, P5901, DOI 10.2147/IJN.S37397
Henrickson SE, 2008, NAT IMMUNOL, V9, P282, DOI 10.1038/ni1559
Hong H, 2012, ACS NANO, V6, P2361, DOI 10.1021/nn204625e
Joffre OP, 2012, NAT REV IMMUNOL, V12, P557, DOI 10.1038/nri3254
Johansen P, 2008, P NATL ACAD SCI USA, V105, P5189, DOI 10.1073/pnas.0706296105
Kaminskas LM, 2009, J CONTROL RELEASE, V140, P108, DOI 10.1016/j.jconrel.2009.08.005
Keskin DB, 2019, NATURE, V565, P234, DOI 10.1038/s41586-018-0792-9
Kim H, 2013, ACS NANO, V7, P6735, DOI 10.1021/nn403096s
Kreiter S, 2015, NATURE, V520, P692, DOI 10.1038/nature14426
Kuai R, 2017, NAT MATER, V16, P489, DOI [10.1038/nmat4822, 10.1038/NMAT4822]
Laverny G, 2013, TOXICOL LETT, V217, P91, DOI 10.1016/j.toxlet.2012.12.008
Li AW, 2018, NAT MATER, V17, P528, DOI 10.1038/s41563-018-0028-2
Li H, 2016, ACS APPL MATER INTER, V8, P1147, DOI 10.1021/acsami.5b08963
Liu HP, 2014, NATURE, V507, P519, DOI 10.1038/nature12978
Liu JQ, 2013, ACTA BIOMATER, V9, P9243, DOI 10.1016/j.actbio.2013.08.016
Luo M, 2017, NAT NANOTECHNOL, V12, P648, DOI [10.1038/nnano.2017.52,

10.1038/NNANO.2017.52]
Lutz MB, 1999, J IMMUNOL METHODS, V223, P77, DOI 10.1016/S0022-1759(98)00204-X
Lynn GM, 2020, NAT BIOTECHNOL, V38, P320, DOI 10.1038/s41587-019-0390-x
Ma J, 2015, ACS NANO, V9, P10498, DOI 10.1021/acsnano.5b04751
Miao W, 2013, BIOMATERIALS, V34, P9638, DOI 10.1016/j.biomaterials.2013.08.058
Mukherjee SP, 2018, NANOSCALE, V10, P1180, DOI 10.1039/c7nr03552g
Nathan C, 2013, NAT REV IMMUNOL, V13, P349, DOI 10.1038/nri3423
Ott PA, 2017, NATURE, V547, P217, DOI 10.1038/nature22991
Qiu F, 2018, BIOMATERIALS, V182, P82, DOI 10.1016/j.biomaterials.2018.07.052
Reddy ST, 2007, NAT BIOTECHNOL, V25, P1159, DOI [10.1038/nbt1332, 10.1038/nbtl332]
Robinson JT, 2011, J AM CHEM SOC, V133, P6825, DOI 10.1021/ja2010175
Sahin U, 2017, NATURE, V547, P222, DOI 10.1038/nature23003
Scheetz L, 2019, NAT BIOMED ENG, V3, P768, DOI 10.1038/s41551-019-0436-x
Schumacher TN, 2015, SCIENCE, V348, P69, DOI 10.1126/science.aaa4971
Shen LJ, 2006, CURR OPIN IMMUNOL, V18, P85, DOI 10.1016/j.coi.2005.11.003
Son S, 2020, NANO LETT, V20, P1499, DOI 10.1021/acs.nanolett.9b03483
Song C, 2016, INT J NANOMED, V11, P3753, DOI 10.2147/IJN.S110796
Tao Y, 2014, BIOMATERIALS, V35, P9963, DOI 10.1016/j.biomaterials.2014.08.036
Wang XL, 2019, ACTA BIOMATER, V83, P390, DOI 10.1016/j.actbio.2018.11.023
Xu C, 2013, ACS APPL MATER INTER, V5, P12911, DOI 10.1021/am404714w
Yadav M, 2014, NATURE, V515, P572, DOI 10.1038/nature14001
Yang K, 2012, ADV MATER, V24, P1868, DOI 10.1002/adma.201104964
Zhu GZ, 2017, NAT COMMUN, V8, DOI 10.1038/s41467-017-01386-7
NR 44
TC 53
Z9 53
U1 9
U2 107
PU AMER CHEMICAL SOC
PI WASHINGTON
PA 1155 16TH ST, NW, WASHINGTON, DC 20036 USA
SN 1936-0851
EI 1936-086X
J9 ACS NANO
JI ACS Nano
PD OCT 27
PY 2020
VL 14
IS 10
BP 13268
EP 13278
DI 10.1021/acsnano.0c05062
PG 11
WC Chemistry, Multidisciplinary; Chemistry, Physical; Nanoscience &
Nanotechnology; Materials Science, Multidisciplinary
WE Science Citation Index Expanded (SCI-EXPANDED)
SC Chemistry; Science & Technology - Other Topics; Materials Science
GA ON6FD
UT WOS:000586793400078
PM 32902245
OA Green Accepted
DA 2023-09-14
ER

PT J
AU Luo, M
Liu, ZD
Zhang, XY
Han, CH
Samandi, LZ
Dong, CB

Sumer, BD
Lea, J
Fu, YX
Gao, JM
AF Luo, Min
Liu, Zhida
Zhang, Xinyi
Han, Chuanhui
Samandi, Layla Z.
Dong, Chunbo
Sumer, Baran D.
Lea, Jayanthi
Fu, Yang-Xin
Gao, Jinming
TI Synergistic STING activation by PC7A nanovaccine and ionizing radiation improves cancer immunotherapy
SO JOURNAL OF CONTROLLED RELEASE
LA English
DT Article
DE STING; Polymeric nanovaccine; Radiotherapy (RT); Tumor infiltrating lymphocytes; Cancer immunotherapy
ID CD8(+) T-CELLS; PHASE-III; ANTITUMOR IMMUNITY; FOLLOW-UP; TUMOR; RADIOTHERAPY; REGRESSION; RECOGNITION; ERADICATION; IRRADIATION
AB Solid cancers are able to escape immune surveillance and are resistant to current treatment in immunotherapy. Recent evidence indicates the critical role of the stimulator of interferon genes (STING) pathway in antitumor immunity. STING-targeted activation is extensively investigated as a new strategy for cancer therapy. Previously, we reported a safe and efficacious STING-activating nanovaccine to boost systemic tumor-specific T cell responses in multiple tumor models. Local radiotherapy has been reported to not only reduce tumor burden but also enhance local antitumor immunity in a STING-dependent manner. In this study, we demonstrate that combination of these two modalities leads to a synergistic response with long-term regression of large established tumors in two mouse tumor models. The percentage of CD8(+) T cells increased significantly in primary tumors after combination therapy. Mechanistically, the augmented T cell responses of radiotherapy and nanovaccine is STING pathway dependent. Furthermore, nanovaccine synergizes with radiotherapy to achieve a better therapeutic effect in distal tumors. These findings suggest that combination of local radiotherapy with systemic PC7A nanovaccine offers a useful strategy to improve the therapeutic outcome of late stage solid cancers.
C1 [Luo, Min; Liu, Zhida; Zhang, Xinyi; Samandi, Layla Z.; Gao, Jinming] Univ Texas Southwestern Med Ctr Dallas, Dept Pharmacol, Simmons Comprehens Canc Ctr, 6001 Forest Pk Rd, Dallas, TX 75390 USA.
[Luo, Min] Fudan Univ, Childrens Hosp, Shanghai 200032, Peoples R China.
[Luo, Min] Fudan Univ, Inst Biomed Sci, Shanghai 200032, Peoples R China.
[Liu, Zhida; Han, Chuanhui; Dong, Chunbo; Fu, Yang-Xin] Univ Texas Southwestern Med Ctr Dallas, Dept Pathol, 6001 Forest Pk Rd, Dallas, TX 75390 USA.
[Sumer, Baran D.] Univ Texas Southwestern Med Ctr Dallas, Dept Otolaryngol, Dallas, TX 75390 USA.
[Lea, Jayanthi] Univ Texas Southwestern Med Ctr Dallas, Dept Obstet & Gynecol, Dallas, TX 75390 USA.
C3 University of Texas System; University of Texas Southwestern Medical Center Dallas; Fudan University; Fudan University; University of Texas System; University of Texas Southwestern Medical Center Dallas; University of Texas System; University of Texas Southwestern Medical Center Dallas; University of Texas System; University of Texas Southwestern Medical Center Dallas
RP Gao, JM (通讯作者)，Univ Texas Southwestern Med Ctr Dallas, Dept Pharmacol, Simmons Comprehens Canc Ctr, 6001 Forest Pk Rd, Dallas, TX 75390 USA.; Fu, YX (通讯作者)，Univ Texas Southwestern Med Ctr Dallas, Dept Pathol, 6001 Forest Pk Rd, Dallas, TX 75390 USA.
EM yang-xin.fu@utsouthwestern.edu; jinming.gao@utsouthwestern.edu
RI Zhang, Xinyi/AAJ-4858-2021
OI Zhang, Xinyi/0000-0002-7175-426X; Lea, Jayanthi/0000-0002-8657-4309; Samandi, Layla/0000-0001-8142-5841; Liu, Zhida/0000-0002-3980-8440
FU National Institutes of Health, United States [R01CA216839, U01CA218422]; National Nature Science Foundation of China [81873922]
FX This work was supported by grants from the National Institutes of

Health, United States (R01CA216839 and U01CA218422) and National Nature Science Foundation of China (81873922 to M.L.). We thank T.C. Wu for kindly providing the TC-1 tumor cells, P. Hwu for the B16-OVA cancer cells, and the molecular pathology core of UT Southwestern for histology analysis.
CR Begg AC, 2011, NAT REV CANCER, V11, P239, DOI 10.1038/nrc3007
Corrales L, 2015, CELL REP, V11, P1018, DOI 10.1016/j.celrep.2015.04.031
Deng LF, 2014, IMMUNITY, V41, P843, DOI 10.1016/j.immuni.2014.10.019
Deng LF, 2014, J CLIN INVEST, V124, P687, DOI 10.1172/JCI67313
Eggermont AMM, 2013, J CLIN ONCOL, V31, P3831, DOI 10.1200/JCO.2012.47.9303
Garcia-Barros M, 2003, SCIENCE, V300, P1155, DOI 10.1126/science.1082504
Giaccone G, 2005, J CLIN ONCOL, V23, P6854, DOI 10.1200/JCO.2005.17.186
Gupta A, 2012, J IMMUNOL, V189, P558, DOI 10.4049/jimmunol.1200563
HELLSTROM KE, 1978, J EXP MED, V148, P799, DOI 10.1084/jem.148.3.799
HOOVER HC, 1993, J CLIN ONCOL, V11, P390, DOI 10.1200/JCO.1993.11.3.390
Le DT, 2015, J CLIN ONCOL, V33, P1325, DOI 10.1200/JCO.2014.57.4244
Lee YJ, 2009, BLOOD, V114, P589, DOI 10.1182/blood-2009-02-206870
Li XD, 2013, SCIENCE, V341, P1390, DOI 10.1126/science.1244040
Liauw SL, 2013, SCI TRANSL MED, V5, DOI 10.1126/scitranslmed.3005148
Luo M, 2017, NAT NANOTECHNOL, V12, P648, DOI [10.1038/nnano.2017.52, 10.1038/NNANO.2017.52]
Moynihan KD, 2016, NAT MED, V22, P1402, DOI 10.1038/nm.4200
Nabhan C, 2010, NEW ENGL J MED, V363, P1966, DOI 10.1056/NEJMc1009982
Ng J, 2016, ANN TRANSL MED, V4, DOI 10.21037/atm.2016.01.32
NORTH RJ, 1986, J EXP MED, V164, P1652, DOI 10.1084/jem.164.5.1652
Park HJ, 2012, RADIAT RES, V177, P311, DOI 10.1667/RR2773.1
Rosen EM, 1999, CANCER INVEST, V17, P56
Siva S, 2013, J THORAC ONCOL, V8, pE71, DOI 10.1097/JTO.0b013e318292c55a
Takeuchi O, 2010, CELL, V140, P805, DOI 10.1016/j.cell.2010.01.022
Tsarevsky NV, 2007, CHEM REV, V107, P2270, DOI 10.1021/cr050947p
Vanpouille-Box C, 2017, NAT COMMUN, V8, DOI 10.1038/ncomms15618
Vassilaros S, 2013, IMMUNOTHERAPY-UK, V5, P1177, DOI [10.2217/IMT.13.126, 10.2217/imt.13.126]
Vatner RE, 2014, FRONT ONCOL, V4, DOI 10.3389/fonc.2014.00325
Walter S, 2012, NAT MED, V18, P1254, DOI 10.1038/nm.2883
Wattenberg MM, 2014, RADIAT RES, V182, P126, DOI 10.1667/RR13374.1
Woo SR, 2014, IMMUNITY, V41, P830, DOI 10.1016/j.immuni.2014.10.017
Zhang B, 2007, J EXP MED, V204, P49, DOI 10.1084/jem.20062056
Zhou KJ, 2012, J AM CHEM SOC, V134, P7803, DOI 10.1021/ja300176w
Zhou KJ, 2011, ANGEW CHEM INT EDIT, V50, P6109, DOI 10.1002/anie.201100884
NR 33
TC 53
Z9 56
U1 2
U2 89
PU ELSEVIER
PI AMSTERDAM
PA RADARWEG 29, 1043 NX AMSTERDAM, NETHERLANDS
SN 0168-3659
EI 1873-4995
J9 J CONTROL RELEASE
JI J. Control. Release
PD APR 28
PY 2019
VL 300
BP 154
EP 160
DI 10.1016/j.jconrel.2019.02.036
PG 7
WC Chemistry, Multidisciplinary; Pharmacology & Pharmacy
WE Science Citation Index Expanded (SCI-EXPANDED)
SC Chemistry; Pharmacology & Pharmacy

GA HS6UO
UT WOS:000464007000013
PM 30844475
DA 2023-09-14
ER

PT J
AU Pan, C
Wu, J
Qing, S
Zhang, X
Zhang, LL
Yue, H
Zeng, M
Wang, B
Yuan, Z
Qiu, YF
Ye, HH
Wang, DS
Liu, XK
Sun, P
Liu, B
Feng, EL
Gao, XY
Zhu, L
Wei, W
Ma, GH
Wang, HL
AF Pan, Chao
Wu, Jun
Qing, Shuang
Zhang, Xiao
Zhang, Lulu
Yue, Hua
Zeng, Ming
Wang, Bin
Yuan, Zheng
Qiu, Yefeng
Ye, Huahu
Wang, Dongshu
Liu, Xiankai
Sun, Peng
Liu, Bo
Feng, Erling
Gao, Xiaoyong
Zhu, Li
Wei, Wei
Ma, Guanghui
Wang, Hengliang
TI Biosynthesis of Self-Assembled Proteinaceous Nanoparticles for Vaccination
SO ADVANCED MATERIALS
LA English
DT Article
DE AB(5)toxins; biosynthesis; conjugate vaccines; nanovaccines; self-assembled proteins
ID VIRUS-LIKE PARTICLES; CHOLERA-TOXIN; O-ANTIGEN; DELIVERY; VACCINES; NANOTECHNOLOGY; ACTIVATION; RECEPTOR; DESIGN; CELLS
AB Recent years have seen enormous advances in nanovaccines for both prophylactic and therapeutic applications, but most of these technologies employ chemical or hybrid semi-biosynthetic production methods. Thus, production of nanovaccines has to date failed to exploit biology-only processes like

complex sequential post-translational biochemical modifications and scalability, limiting the realization of the initial promise for offering major performance advantages and improved therapeutic outcomes over conventional vaccines. A Nano-B5 platform for in vivo production of fully protein-based, self-assembling, stable nanovaccines bearing diverse antigens including peptides and polysaccharides is presented here. Combined with the self-assembly capacities of pentamer domains from the bacterial AB(5)toxin and unnatural trimer peptides, diverse nanovaccine structures can be produced in commonEscherichia colistrains and in attenuated pathogenic strains. Notably, the chassis of these nanovaccines functions as an immunostimulant. After showing excellent lymph node targeting and immunoresponse elicitation and safety performance in both mouse and monkey models, the strong prophylactic effects of these nanovaccines against infection, as well as their efficient therapeutic effects against tumors are further demonstrated. Thus, the Nano-B5 platform can efficiently combine diverse modular components and antigen cargos to efficiently generate a potentially very large diversity of nanovaccine structures using many bacterial species.
C1 [Pan, Chao; Wu, Jun; Zhang, Lulu; Wang, Dongshu; Liu, Xiankai; Sun, Peng; Liu, Bo; Feng, Erling; Zhu, Li; Wang, Hengliang] Beijing Inst Biotechnol, State Key Lab Pathogen & Biosecur, 20 Dongda St, Beijing 100071, Peoples R China.
[Qing, Shuang; Zhang, Xiao; Yue, Hua; Gao, Xiaoyong; Wei, Wei; Ma, Guanghui] Chinese Acad Sci, Inst Proc Engn, State Key Lab Biochem Engn, 1 Bei Er Tiao, Beijing 100190, Peoples R China.
[Qing, Shuang; Wei, Wei; Ma, Guanghui] Univ Chinese Acad Sci, Sch Chem Engn, 19A Yuquan Rd, Beijing 100049, Peoples R China.
[Zeng, Ming; Wang, Bin] Natl Inst Food & Drug Control, 31 Huatuo Rd, Beijing 102629, Peoples R China.
[Yuan, Zheng; Qiu, Yefeng; Ye, Huahu] Acad Mil Med Sci, Anim Ctr, 20 Dongda St, Beijing 100071, Peoples R China.
C3 Chinese Academy of Sciences; Institute of Process Engineering, CAS; Chinese Academy of Sciences; University of Chinese Academy of Sciences, CAS; National Institute of Food & Drug Control - China; Academy of Military Medical Sciences - China
RP Zhu, L; Wang, HL (通讯作者)，Beijing Inst Biotechnol, State Key Lab Pathogen & Biosecur, 20 Dongda St, Beijing 100071, Peoples R China.; Wei, W; Ma, GH (通讯作者)，Chinese Acad Sci, Inst Proc Engn, State Key Lab Biochem Engn, 1 Bei Er Tiao, Beijing 100190, Peoples R China.; Wei, W; Ma, GH (通讯作者)，Univ Chinese Acad Sci, Sch Chem Engn, 19A Yuquan Rd, Beijing 100049, Peoples R China.
EM jewly54@bmi.ac.cn; weiwei@ipe.ac.cn; ghma@ipe.ac.cn; wanghl@bmi.ac.cn
RI Wang, Hengliang/AAT-4586-2021; Yue, H/L-4578-2017; liu, xiankai/HZJ-3228-2023; Qing, Shuang/AFV-8842-2022; wei, wei/IQW-1347-2023; Zeng, Ming/GXF-3628-2022; Zhang, Lulu/AGG-4328-2022; wei, wei/HHR-8613-2022; Wang, Hengliang/AAL-5340-2021
OI Yue, H/0000-0003-2621-8326; Zeng, Ming/0000-0003-2836-9240; Wang, Hengliang/0000-0001-8476-4320; zhang, xiao/0000-0003-1267-2034; Chao, Pan/0000-0002-0912-5171; Wei, Wei/0000-0002-6244-3187
FU National Natural Science Foundation of China [81930122, 81871314, 31700802, 21622608, 81773619, 21821005]; National Science and Technology Major Project [2018ZX10101003-005, 2018ZX09711003-013]; National Key R&D Program of China [2017YFA0207900]; Strategic Priority Research Program of the Chinese Academy of Sciences [XDB29040303]
FX C.P., J.W., and S.Q. contributed equally to this work. This work was supported by the National Natural Science Foundation of China (Nos. 81930122, 81871314, 31700802, 21622608, 81773619, and 21821005), the National Science and Technology Major Project (2018ZX10101003-005 and 2018ZX09711003-013), the National Key R&D Program of China (2017YFA0207900), and the Strategic Priority Research Program of the Chinese Academy of Sciences (XDB29040303). All animal experiments were approved and performed in accordance with the institutional guidelines of the Academy of Military Medical Sciences Institutional Animal Care and Use Committee. The mice used in this work were obtained from Beijing Vital River Laboratory Animal Technology Co., Ltd. and were housed in Animal Center of the Academy of Military Medical Sciences (approval number: SYXK 2017-0022). Cynomolgus monkeys were obtained from the Animal Center of the Academy of Military Medical Sciences (approval number: SYXK 2017-0022) and were also housed in the Center.
CR Beddoe T, 2010, TRENDS BIOCHEM SCI, V35, P411, DOI 10.1016/j.tibs.2010.02.003

Chen Q, 2019, NAT NANOTECHNOL, V14, P89, DOI 10.1038/s41565-018-0319-4
Choi B, 2016, ACS NANO, V10, P7339, DOI 10.1021/acsnano.5b08084
de Haan L, 2002, INFECT IMMUN, V70, P3249, DOI 10.1128/IAI.70.6.3249-3258.2002
El Bissati K, 2017, NPJ VACCINES, V2, DOI 10.1038/s41541-017-0024-6
Eriksson K, 2004, EUR J IMMUNOL, V34, P1272, DOI 10.1002/eji.200324368
Fuenmayor J, 2017, NEW BIOTECHNOL, V39, P174, DOI 10.1016/j.nbt.2017.07.010
Goldberg MS, 2019, NAT REV CANCER, V19, P587, DOI 10.1038/s41568-019-0186-9
Grgacic EVL, 2006, METHODS, V40, P60, DOI 10.1016/j.ymeth.2006.07.018
Harding CM, 2019, NAT COMMUN, V10, DOI 10.1038/s41467-019-08869-9
He LL, 2016, NAT COMMUN, V7, DOI 10.1038/ncomms12041
HOLMGREN J, 1973, INFECT IMMUN, V8, P208, DOI 10.1128/IAI.8.2.208-214.1973
Hsia Y, 2016, NATURE, V535, P136, DOI 10.1038/nature18010
Huang WC, 2018, NAT NANOTECHNOL, V13, P1174, DOI 10.1038/s41565-018-0271-3
Irvine DJ, 2015, CHEM REV, V115, P11109, DOI 10.1021/acs.chemrev.5b00109
Jardine J, 2013, SCIENCE, V340, P711, DOI 10.1126/science.1234150
Kaba SA, 2012, PLOS ONE, V7, DOI 10.1371/journal.pone.0048304
Kaba SA, 2009, J IMMUNOL, V183, P7268, DOI 10.4049/jimmunol.0901957
Kanekiyo M, 2015, CELL, V162, P1090, DOI 10.1016/j.cell.2015.07.043
Kanekiyo M, 2013, NATURE, V499, P102, DOI 10.1038/nature12202
Kay E, 2019, NPJ VACCINES, V4, DOI 10.1038/s41541-019-0110-z
Kuai R, 2017, NAT MATER, V16, P489, DOI [10.1038/nmat4822, 10.1038/NMAT4822]
Li AW, 2018, NAT MATER, V17, P528, DOI 10.1038/s41563-018-0028-2
Li F, 2019, ACS CENTRAL SCI, V5, P796, DOI 10.1021/acscentsci.9b00060
Lizotte PH, 2016, NAT NANOTECHNOL, V11, P295, DOI [10.1038/NNANO.2015.292, 10.1038/nnano.2015.292]
Lynn GM, 2015, NAT BIOTECHNOL, V33, P1201, DOI 10.1038/nbt.3371
Mamo T, 2012, VACCINE, V30, P6609, DOI 10.1016/j.vaccine.2012.08.023
Manolova V, 2008, EUR J IMMUNOL, V38, P1404, DOI 10.1002/eji.200737984
MORONA R, 1995, J BACTERIOL, V177, P1059, DOI 10.1128/jb.177.4.1059-1068.1995
Pan C, 2016, MBIO, V7, DOI 10.1128/mBio.00443-16
Pati R, 2018, FRONT IMMUNOL, V9, DOI 10.3389/fimmu.2018.02224
Phalipon A, 2006, J IMMUNOL, V176, P1686, DOI 10.4049/jimmunol.176.3.1686
Reddy ST, 2007, NAT BIOTECHNOL, V25, P1159, DOI [10.1038/nbt1332, 10.1038/nbtl332]
Sharma R, 2015, BIOTECHNOL ADV, V33, P64, DOI 10.1016/j.biotechadv.2014.12.004
Sliepen K, 2015, RETROVIROLOGY, V12, DOI 10.1186/s12977-015-0210-4
Smith DM, 2013, NAT REV IMMUNOL, V13, P592, DOI 10.1038/nri3488
Stratmann T, 2015, VACCINES-BASEL, V3, P579, DOI 10.3390/vaccines3030579
Sun P, 2018, NPJ VACCINES, V3, DOI 10.1038/s41541-017-0037-1
Tang L, 2018, NAT BIOTECHNOL, V36, P707, DOI 10.1038/nbt.4181
Terra VS, 2012, J MED MICROBIOL, V61, P919, DOI 10.1099/jmm.0.039438-0
Tokatlian T, 2019, SCIENCE, V363, P649, DOI 10.1126/science.aat9120
Trimaille T, 2015, VACCINES, V3, P803, DOI 10.3390/vaccines3040803
Xia YF, 2018, NAT MATER, V17, P187, DOI [10.1038/NMAT5057, 10.1038/nmat5057]
NR 43
TC 52
Z9 53
U1 13
U2 141
PU WILEY-V C H VERLAG GMBH
PI WEINHEIM
PA POSTFACH 101161, 69451 WEINHEIM, GERMANY
SN 0935-9648
EI 1521-4095
J9 ADV MATER
JI Adv. Mater.
PD OCT 22
PY 2020
VL 32
IS 42
AR 2002940
DI 10.1002/adma.202002940

EA SEP 2020
PG 13
WC Chemistry, Multidisciplinary; Chemistry, Physical; Nanoscience &
   Nanotechnology; Materials Science, Multidisciplinary; Physics, Applied;
   Physics, Condensed Matter
WE Science Citation Index Expanded (SCI-EXPANDED)
SC Chemistry; Science & Technology - Other Topics; Materials Science;
   Physics
GA OE4OP
UT WOS:000565425700001
PM 32881121
OA hybrid
DA 2023-09-14
ER

PT J
AU Gan, JY
   Du, GS
   He, CT
   Jiang, M
   Mou, XY
   Xue, J
   Sun, X
AF Gan, Jingyao
   Du, Guangsheng
   He, Chunting
   Jiang, Min
   Mou, Xingyue
   Xue, Jiao
   Sun, Xun
TI Tumor cell membrane enveloped aluminum phosphate nanoparticles for
   enhanced cancer vaccination
SO JOURNAL OF CONTROLLED RELEASE
LA English
DT Article
DE Aluminum phosphate nanoparticles; Cancer vaccine; Lymph node targeting;
   Tumor cell membrane
ID LYMPH-NODE; IMMUNE-RESPONSES; DENDRITIC CELLS; VACCINES; MECHANISMS;
   ADSORPTION; ADJUVANT; DNA; MACROPHAGES; IMMUNOLOGY
AB An ideal cancer vaccine should contain both strongly immunogenic cancer-specific antigen and potent adjuvant for stimulating robust cellular immunity which are pivotal for clearance of cancer cells. However, most of commercially available adjuvants such as aluminum phosphate gel cannot stimulate robust cellular immune response. In the current study, we reformed microscale aluminum phosphate gel adjuvant into nanoscale and fabricated CpG loaded and B16F10 tumor cell membrane coated aluminum phosphate nanoparticles (APMC). The resultant nano-vaccines showed a size of around 60 nm and a negative surface charge of - 40 mV. Tumor cell membrane not only served as tumor antigens but also effectively improved the colloidal dispersion of aluminum phosphate nanoparticles. Subcutaneously injected APMC were efficiently drained to mouse lymph nodes, significantly increased co-uptake of tumor antigen and CpG by lymph node resident antigen presenting cells, promoted maturation of these cells and enhanced lysosomal antigen escape. After immunizing mice, they triggered robust cellular immunity, including potent IFN-gamma(+)CD4(+)T cells, IFN-gamma(+)CD8(+)T cells, cytotoxic T lymphocytes and cytokine excretion in spleen and lymph node cells. The elicited responses significantly suppressed tumor growth and prolonged survival of mice in both prophylactic and therapeutic melanoma models. This promising vaccine delivery system shows great potential to clinical transformation and can be further developed for personalized cancer vaccines.
C1 [Gan, Jingyao; Du, Guangsheng; He, Chunting; Jiang, Min; Mou, Xingyue; Xue, Jiao; Sun, Xun] Sichuan Univ, West China Sch Pharm, Key Lab Drug Targeting & Drug Delivery Syst, Minist Educ, Chengdu 610041, Peoples R China.
C3 Sichuan University
RP Sun, X (通讯作者)，Sichuan Univ, West China Sch Pharm, Key Lab Drug Targeting & Drug Delivery Syst, Minist Educ, Chengdu 610041, Peoples R China.

EM sunxun@scu.edu.cn
OI Gan, Jingyao/0000-0001-6404-4764; Du, Guangsheng/0000-0003-4401-4845
FU National Natural Science Foundation of China [81925036, 81673362];
Science & Technology Major Project of Sichuan Province [2018SZDZX0018];
Program Sichuan Veterinary Medicine and Drug Innovation Group of China
Agricultural Research System [SCCXTD-2020-18]; Fundamental Research
Funds for the Central Universities
FX We acknowledge the financial support of the National Natural Science
Foundation of China (No. 81925036 & 81673362), the Science & Technology
Major Project of Sichuan Province (2018SZDZX0018), the Program Sichuan
Veterinary Medicine and Drug Innovation Group of China Agricultural
Research System (SCCXTD-2020-18) and the Fundamental Research Funds for
the Central Universities.
CR Aimanianda V, 2009, TRENDS PHARMACOL SCI, V30, P287, DOI 10.1016/j.tips.2009.03.005
ALSHAKHSHIR RH, 1995, VACCINE, V13, P41, DOI 10.1016/0264-410X(95)80009-3
Andreu P, 2010, CANCER CELL, V17, P121, DOI 10.1016/j.ccr.2009.12.019
Becker M.D., 2010, CANC VACCINE THERAPI, P34
Bindea G, 2013, IMMUNITY, V39, P782, DOI 10.1016/j.immuni.2013.10.003
BLEAM WF, 1991, LANGMUIR, V7, P1702, DOI 10.1021/la00056a023
Bode C, 2011, EXPERT REV VACCINES, V10, P499, DOI [10.1586/ERV.10.174, 10.1586/erv.10.174]
Boedtkjer E, 2020, ANNU REV PHYSIOL, V82, P103, DOI 10.1146/annurev-physiol-021119-034627
Borst J, 2018, NAT REV IMMUNOL, V18, P635, DOI 10.1038/s41577-018-0044-0
Bot A., 2018, CANC VACCINES, P161
Brewer JM, 2006, IMMUNOL LETT, V102, P10, DOI 10.1016/j.imlet.2005.08.002
Butterfield LH, 2015, BMJ-BRIT MED J, V350, DOI 10.1136/bmj.h988
Charles River Laboratories, C57BL 6 MICE
Chen DS, 2013, IMMUNITY, V39, P1, DOI 10.1016/j.immuni.2013.07.012
Chiang CLL, 2010, SEMIN IMMUNOL, V22, P132, DOI 10.1016/j.smim.2010.02.004
Curran MA, 2019, ANNU REV MED, V70, P409, DOI 10.1146/annurev-med-050217-121900
De Temmerman ML, 2011, DRUG DISCOV TODAY, V16, P569, DOI 10.1016/j.drudis.2011.04.006
DeMaria PJ, 2019, HEMATOL ONCOL CLIN N, V33, P199, DOI 10.1016/j.hoc.2018.12.001
Farkona S, 2016, BMC MED, V14, DOI 10.1186/s12916-016-0623-5
Fifis T, 2004, J IMMUNOL, V173, P3148, DOI 10.4049/jimmunol.173.5.3148
Finn OJ, 2003, NAT REV IMMUNOL, V3, P630, DOI 10.1038/nri1150
Frietze KM, 2016, CURR OPIN VIROL, V18, P44, DOI 10.1016/j.coviro.2016.03.001
Galon J, 2020, IMMUNITY, V52, P55, DOI 10.1016/j.immuni.2019.12.018
Gatti-Mays ME, 2017, HUM VACC IMMUNOTHER, V13, P2561, DOI 10.1080/21645515.2017.1364322
Goldberg MS, 2019, NAT REV CANCER, V19, P587, DOI 10.1038/s41568-019-0186-9
Gong J, 2018, J IMMUNOTHER CANCER, V6, DOI 10.1186/s40425-018-0316-z
Grenier JM, 2018, FRONT IMMUNOL, V9, DOI 10.3389/fimmu.2018.00610
Gutierrez-Martinez E, 2015, FRONT IMMUNOL, V6, DOI 10.3389/fimmu.2015.00363
Hjorto GM, 2016, FRONT IMMUNOL, V7, DOI 10.3389/fimmu.2016.00568
Hu Z, 2018, NAT REV IMMUNOL, V18, P168, DOI 10.1038/nri.2017.131
Hubbell JA, 2009, NATURE, V462, P449, DOI 10.1038/nature08604
Jiang H, 2018, ADV SCI, V5, DOI 10.1002/advs.201700426
Jiang H, 2017, J CONTROL RELEASE, V267, P47, DOI 10.1016/j.jconrel.2017.08.009
Krieg AM, 2002, ANNU REV IMMUNOL, V20, P709, DOI 10.1146/annurev.immunol.20.100301.064842
Kroll AV, 2017, ADV MATER, V29, DOI 10.1002/adma.201703969
Kwak M, 2019, SURG ONCOL CLIN N AM, V28, P337, DOI 10.1016/j.soc.2019.02.003
Lindblad EB, 2004, VACCINE, V22, P3658, DOI 10.1016/j.vaccine.2004.03.032
Luk BT, 2014, NANOSCALE, V6, P2730, DOI 10.1039/c3nr06371b
Lutz MB, 1999, J IMMUNOL METHODS, V223, P77, DOI 10.1016/S0022-1759(98)00204-X
Manolova V, 2008, EUR J IMMUNOL, V38, P1404, DOI 10.1002/eji.200737984
Martinez-Pomares L, 2012, TRENDS IMMUNOL, V33, P66, DOI 10.1016/j.it.2011.11.001
Melief CJM, 2015, J CLIN INVEST, V125, P3401, DOI 10.1172/JCI80009
Mohsen MO, 2020, WIRES NANOMED NANOBI, V12, DOI 10.1002/wnan.1579
Nam J, 2019, NAT REV MATER, V4, P398, DOI 10.1038/s41578-019-0108-1
Neek M, 2018, BIOMATERIALS, V156, P194, DOI 10.1016/j.biomaterials.2017.11.022
O'Neill NA, 2018, CURR OPIN ORGAN TRAN, V23, P73, DOI 10.1097/MOT.0000000000000488
Othman N, 2019, FRONT IMMUNOL, V10, DOI 10.3389/fimmu.2019.02103
Palucka K, 2012, NAT REV CANCER, V12, P265, DOI 10.1038/nrc3258

Pucci F, 2016, SCIENCE, V352, P242, DOI 10.1126/science.aaf1328
Reddy ST, 2007, NAT BIOTECHNOL, V25, P1159, DOI [10.1038/nbt1332, 10.1038/nbtl332]
Reddy ST, 2006, J CONTROL RELEASE, V112, P26, DOI 10.1016/j.jconrel.2006.01.006
SEEBER SJ, 1991, VACCINE, V9, P201, DOI 10.1016/0264-410X(91)90154-X
Silva JM, 2013, J CONTROL RELEASE, V168, P179, DOI 10.1016/j.jconrel.2013.03.010
Suchorska WM, 2016, ONCOL REP, V35, P1237, DOI 10.3892/or.2015.4507
Tang J, 2015, J MATER CHEM B, V3, P6805, DOI 10.1039/c5tb00912j
Tardon MC, 2019, CURR OPIN PHARMACOL, V47, P20, DOI 10.1016/j.coph.2019.01.007
Temperton NJ, 2003, J MED VIROL, V70, P86, DOI 10.1002/jmv.10357
Vermaelen K, 2019, FRONT IMMUNOL, V10, DOI 10.3389/fimmu.2019.00008
Vollmer J, 2009, ADV DRUG DELIVER REV, V61, P195, DOI 10.1016/j.addr.2008.12.008
von Andrian UH, 2003, NAT REV IMMUNOL, V3, P867, DOI 10.1038/nri1222
Wellenstein MD, 2018, IMMUNITY, V48, P399, DOI 10.1016/j.immuni.2018.03.004
Xie FT, 2019, MOL CANCER, V18, DOI 10.1186/s12943-019-1074-3
Yan Y, 2019, FRONT CELL DEV BIOL, V7, DOI 10.3389/fcell.2019.00212
Zahavi DJ, 2019, PROG MOL BIOL TRANSL, V164, P61, DOI 10.1016/bs.pmbts.2019.03.009
Zhang HJ, 2017, MAT SCI ENG C-MATER, V70, P935, DOI 10.1016/j.msec.2016.03.045
Zhang WD, 2017, BIOCONJUGATE CHEM, V28, P1993, DOI 10.1021/acs.bioconjchem.7b00313
Zhong XF, 2019, J CONTROL RELEASE, V300, P81, DOI 10.1016/j.jconrel.2019.02.035
Zhuang XT, 2016, J CONTROL RELEASE, V228, P26, DOI 10.1016/j.jconrel.2016.02.035
NR 68
TC 51
Z9 51
U1 6
U2 142
PU ELSEVIER
PI AMSTERDAM
PA RADARWEG 29a, 1043 NX AMSTERDAM, NETHERLANDS
SN 0168-3659
EI 1873-4995
J9 J CONTROL RELEASE
JI J. Control. Release
PD OCT 10
PY 2020
VL 326
BP 297
EP 309
DI 10.1016/j.jconrel.2020.07.008
PG 13
WC Chemistry, Multidisciplinary; Pharmacology & Pharmacy
WE Science Citation Index Expanded (SCI-EXPANDED)
SC Chemistry; Pharmacology & Pharmacy
GA NS2FF
UT WOS:000572080200002
PM 32659330
DA 2023-09-14
ER

PT J
AU Verbeke, R
Lentacker, I
Breckpot, K
Janssens, J
Van Calenbergh, S
De Smedt, SC
Dewitte, H
AF Verbeke, Rein
Lentacker, Ine
Breckpot, Karine
Janssens, Jonas
Van Calenbergh, Serge

De Smedt, Stefaan C.
Dewitte, Heleen
TI Broadening the Message: A Nanovaccine Co-loaded with Messenger RNA and alpha-GalCer Induces Antitumor Immunity through Conventional and Natural Killer T Cells
SO ACS NANO
LA English
DT Article
DE iNKT cells; T cell; mRNA vaccine; nanoparticle; checkpoint inhibition; alpha-galactosylceramide; modified nucleotides
ID ACTIVATED NKT CELLS; DENDRITIC CELLS; CLINICAL-APPLICATIONS; ANERGY INDUCTION; CANCER VACCINES; CD1D EXPRESSION; BETA-CATENIN; TUMOR-CELLS; INKT CELLS; IMMUNOGENICITY
AB Messenger RNA encoding tumor antigens has the potential to evoke effective antitumor immunity. This study reports on a nanoparticle platform, named mRNA Galsomes, that successfully co-delivers nucleoside-modified antigen-encoding mRNA and the glycolipid antigen and immunopotentiator alpha-galactosylceramide (alpha-GC) to antigen-presenting cells after intravenous administration. By co-formulating low doses of alpha-GC, mRNA Galsomes induce a pluripotent innate and adaptive tumor specific immune response in mice, with invariant natural killer T cells (iNKT) as a driving force. In comparison, mRNA Galsomes exhibit advantages over the state-of-the-art cancer vaccines using unmodified ovalbumin (OVA)-encoding mRNA, as we observed up to seven times more tumor-infiltrating antigen-specific cytotoxic T cells, combined with a strong iNKT cell and NK cell activation. In addition, the presence of suppressive myeloid cells (myeloid-derived suppressor cells and tumor-associated macrophages) in the tumor microenvironment was significantly lowered. Owing to these antitumor effects, OVA mRNA Galsomes significantly reduced tumor growth in established E.G7-OVA lymphoma, with a complete tumor rejection in 40% of the animals. Moreover, therapeutic vaccination with mRNA Galsomes enhanced the responsiveness to treatment with a PD-L1 checkpoint inhibitor in B16-OVA melanoma, as evidenced by a synergistic reduction of tumor outgrowth and a significantly prolonged median survival. Taken together, these data show that intravenously administered mRNA Galsomes can provide controllable, multifaceted, and effective antitumor immunity, especially when combined with checkpoint inhibition.
C1 [Verbeke, Rein; Lentacker, Ine; De Smedt, Stefaan C.; Dewitte, Heleen] Univ Ghent, Ghent Res Grp Nanomed, Fac Pharm, B-9000 Ghent, Belgium.
[Verbeke, Rein; Lentacker, Ine; De Smedt, Stefaan C.; Dewitte, Heleen] Univ Ghent, Ghent Univ Hosp, Canc Res Inst Ghent CRIG, B-9000 Ghent, Belgium.
[Breckpot, Karine; Dewitte, Heleen] Vrije Univ Brussel VUB, Dept Biomed Sci, Lab Mol & Cellular Therapy, B-1090 Jette, Belgium.
[Janssens, Jonas; Van Calenbergh, Serge] Univ Ghent, Lab Med Chem, Fac Pharm, B-9000 Ghent, Belgium.
C3 Ghent University; Ghent University; Ghent University Hospital; Vrije Universiteit Brussel; Ghent University
RP De Smedt, SC (通讯作者)，Univ Ghent, Ghent Res Grp Nanomed, Fac Pharm, B-9000 Ghent, Belgium.; De Smedt, SC (通讯作者)，Univ Ghent, Ghent Univ Hosp, Canc Res Inst Ghent CRIG, B-9000 Ghent, Belgium.
EM Stefaan.DeSmedt@UGent.be
RI Van Calenbergh, Serge/A-3167-2008; Lentacker, Ine/AAH-3128-2019; Breckpot, Karine RP/A-4298-2008
OI Van Calenbergh, Serge/0000-0002-4201-1264; Lentacker, Ine/0000-0001-7492-6568; Breckpot, Karine RP/0000-0003-4331-3480; Verbeke, Rein/0000-0003-1849-5411; De Smedt, Stefaan/0000-0002-8653-2598; Dewitte, Heleen/0000-0002-1791-770X
FU Research Foundation-Flanders, Belgium (FWO-Vlaanderen) [12E3916N]; FWO grant [G016513N, G0B2814N]; Flemish Agency for Innovation through Science and Technology (IWT SBO NanoComit)
FX The authors would like thank E. Thuy Linh Achten for her help with the experimental work, L. Pieters and H. Declercq for the sample preparation for the histopathology study, and M. Van Bockstal for the histopathological analysis. They further wish to thank the NIH Tetramer Core Facility for providing H-2Kb/SIINFEKL tetramer and mCD1d PBS-57 tetramers. H.D. is a postdoctoral fellow of the Research Foundation-Flanders, Belgium (FWO-Vlaanderen; grant no. 12E3916N). This

research is supported by the FWO grant nos. G016513N and G0B2814N and
the Flemish Agency for Innovation through Science and Technology (IWT
SBO NanoComit).
CR Abraham Meike-Kristin, 2017, Nanotheranostics, V1, P154, DOI 10.7150/ntno.19449
Alberer M, 2017, LANCET, V390, P1511, DOI 10.1016/S0140-6736(17)31665-3
Anderson BR, 2010, NUCLEIC ACIDS RES, V38, P5884, DOI 10.1093/nar/gkq347
Anderson KG, 2017, CANCER CELL, V31, P311, DOI 10.1016/j.ccell.2017.02.008
Andries O, 2015, J CONTROL RELEASE, V217, P337, DOI 10.1016/j.jconrel.2015.08.051
Bae EA, 2018, CANCER RES, V78, P5315, DOI 10.1158/0008-5472.CAN-18-0734
Bahl K, 2017, MOL THER, V25, P1316, DOI 10.1016/j.ymthe.2017.03.035
Bedard M, 2017, FRONT IMMUNOL, V8, DOI 10.3389/fimmu.2017.01829
Berga-Bolanos R, 2015, BMC IMMUNOL, V16, DOI 10.1186/s12865-015-0121-0
Broos Katrijn, 2016, Mol Ther Nucleic Acids, V5, pe326, DOI 10.1038/mtna.2016.38
Cortesi F, 2018, CELL REP, V22, P3006, DOI 10.1016/j.celrep.2018.02.058
Crouse J, 2015, NAT REV IMMUNOL, V15, P231, DOI 10.1038/nri3806
De Beuckelaer A, 2017, TRENDS MOL MED, V23, P216, DOI 10.1016/j.molmed.2017.01.006
De Beuckelaer A, 2016, MOL THER, V24, P2012, DOI 10.1038/mt.2016.161
Devoldere J, 2016, DRUG DISCOV TODAY, V21, P11, DOI 10.1016/j.drudis.2015.07.009
Diken M, 2011, GENE THER, V18, P702, DOI 10.1038/gt.2011.17
Durgan K, 2011, CANCER IMMUNOL IMMUN, V60, P547, DOI 10.1007/s00262-010-0963-5
Favreau M, 2016, ONCOTARGET, V7, P23128, DOI 10.18632/oncotarget.7440
Fotin-Mleczek M, 2012, J GENE MED, V14, P428, DOI 10.1002/jgm.2605
Fujii S, 2003, J EXP MED, V198, P267, DOI 10.1084/jem.20030324
Garcia-Diaz A, 2017, CELL REP, V19, P1189, DOI 10.1016/j.celrep.2017.04.031
Garris CS, 2018, IMMUNITY, V49, P1148, DOI 10.1016/j.immuni.2018.09.024
Godfrey DI, 2018, IMMUNITY, V48, P453, DOI 10.1016/j.immuni.2018.03.009
Gottschalk C, 2015, FRONT IMMUNOL, V6, DOI 10.3389/fimmu.2015.00379
Grabbe S, 2016, NANOMEDICINE-UK, V11, P2723, DOI 10.2217/nnm-2016-0275
Haraguchi K, 2006, LEUKEMIA LYMPHOMA, V47, P2218, DOI 10.1080/10428190600682688
Huang JR, 2014, J IMMUNOL, V192, P1972, DOI 10.4049/jimmunol.1302623
Iavarone C, 2017, EXPERT REV VACCINES, V16, P871, DOI 10.1080/14760584.2017.1355245
Im JS, 2009, IMMUNITY, V30, P888, DOI 10.1016/j.immuni.2009.03.022
Iyoda T, 2010, INT IMMUNOL, V22, P905, DOI 10.1093/intimm/dxq444
Jonasch E, 2001, ONCOLOGIST, V6, P34, DOI 10.1634/theoncologist.6-1-34
Juneja VR, 2017, J EXP MED, V214, P895, DOI 10.1084/jem.20160801
Kariko K, 2005, IMMUNITY, V23, P165, DOI 10.1016/j.immuni.2005.06.008
Kariko K, 2008, MOL THER, V16, P1833, DOI 10.1038/mt.2008.200
Kling JC, 2018, FRONT IMMUNOL, V9, DOI 10.3389/fimmu.2018.00483
Ko HJ, 2009, J IMMUNOL, V182, P1818, DOI 10.4049/jimmunol.0802430
Kojo S, 2009, P NATL ACAD SCI USA, V106, P17847, DOI 10.1073/pnas.0904078106
Kranz LM, 2016, NATURE, V534, P396, DOI 10.1038/nature18300
Krijgsman D, 2018, FRONT IMMUNOL, V9, DOI 10.3389/fimmu.2018.00367
Lam PY, 2017, FRONT IMMUNOL, V8, DOI 10.3389/fimmu.2017.01355
Liang F, 2017, MOL THER, V25, P2635, DOI 10.1016/j.ymthe.2017.08.006
Liu LN, 2018, MOL THER, V26, P45, DOI 10.1016/j.ymthe.2017.10.020
Metelitsa LS, 2011, CLIN IMMUNOL, V140, P119, DOI 10.1016/j.clim.2010.10.005
Murugaiyan G, 2013, TRENDS MOL MED, V19, P108, DOI 10.1016/j.molmed.2012.12.002
Nowyhed HN, 2017, SCI REP-UK, V7, DOI 10.1038/srep40273
Oberli MA, 2017, NANO LETT, V17, P1326, DOI 10.1021/acs.nanolett.6b03329
Pardi N, 2018, J EXP MED, V215, P1571, DOI 10.1084/jem.20171450
Pardi N, 2018, NAT REV DRUG DISCOV, V17, P261, DOI 10.1038/nrd.2017.243
Parekh VV, 2009, J IMMUNOL, V182, P2816, DOI 10.4049/jimmunol.0803648
Park YK, 2005, BIOCHEM BIOPH RES CO, V327, P1143, DOI 10.1016/j.bbrc.2004.12.121
Pennock ND, 2014, P NATL ACAD SCI USA, V111, P16472, DOI 10.1073/pnas.1407393111
Pepini T, 2017, J IMMUNOL, V198, P4012, DOI 10.4049/jimmunol.1601877
Phua KKL, 2014, SCI REP-UK, V4, DOI 10.1038/srep05128
Plettenburg O, 2002, J ORG CHEM, V67, P4559, DOI 10.1021/jo0201530
Riese P, 2015, EUR J IMMUNOL, V45, P1794, DOI 10.1002/eji.201445209
Rosigkeit S, 2018, MOL PHARMACEUT, V15, P3909, DOI 10.1021/acs.molpharmaceut.8b00370
Sahin U, 2014, NAT REV DRUG DISCOV, V13, P759, DOI 10.1038/nrd4278
Sayour E. J., 2017, ONCOIMMUNOLOGY, V6

Sayour EJ, 2018, NANO LETT, V18, P6195, DOI 10.1021/acs.nanolett.8b02179
Semmling V, 2010, NAT IMMUNOL, V11, P313, DOI 10.1038/ni.1848
Sharma P, 2017, CELL, V168, P707, DOI 10.1016/j.cell.2017.01.017
Spitzer MH, 2017, CELL, V168, P487, DOI 10.1016/j.cell.2016.12.022
Starbeck-Miller GR, 2014, J EXP MED, V211, P105, DOI 10.1084/jem.20130901
Sun C, 2018, IMMUNITY, V48, P434, DOI 10.1016/j.immuni.2018.03.014
Tang HD, 2018, J CLIN INVEST, V128, P580, DOI 10.1172/JCI96061
Theofilopoulos AN, 2005, ANNU REV IMMUNOL, V23, P307, DOI 10.1146/annurev.immunol.23.021704.115843
Torreno-Pina JA, 2016, P NATL ACAD SCI USA, V113, pE772, DOI 10.1073/pnas.1514530113
Van Meirvenne S, 2002, CANCER GENE THER, V9, P787, DOI 10.1038/sj.cgt.7700499
Verbeke R, 2017, J CONTROL RELEASE, V266, P287, DOI 10.1016/j.jconrel.2017.09.041
Wang JX, 2009, J IMMUNOL, V182, P6644, DOI 10.4049/jimmunol.0804050
Wei J, 2013, J IMMUNOL, V191, P500, DOI 10.4049/jimmunol.1300328
Wilson MT, 2003, P NATL ACAD SCI USA, V100, P10913, DOI 10.1073/pnas.1833166100
Wingender G, 2015, J IMMUNOL, V195, P3838, DOI 10.4049/jimmunol.1500203
NR 73
TC 51
Z9 56
U1 6
U2 135
PU AMER CHEMICAL SOC
PI WASHINGTON
PA 1155 16TH ST, NW, WASHINGTON, DC 20036 USA
SN 1936-0851
EI 1936-086X
J9 ACS NANO
JI ACS Nano
PD FEB
PY 2019
VL 13
IS 2
BP 1655
EP 1669
DI 10.1021/acsnano.8b07660
PG 15
WC Chemistry, Multidisciplinary; Chemistry, Physical; Nanoscience & Nanotechnology; Materials Science, Multidisciplinary
WE Science Citation Index Expanded (SCI-EXPANDED)
SC Chemistry; Science & Technology - Other Topics; Materials Science
GA HN5CA
UT WOS:000460199400066
PM 30742405
OA Green Published
DA 2023-09-14
ER

PT J
AU Shi, Y
AF Shi, Yang
TI Clinical Translation of Nanomedicine and Biomaterials for Cancer Immunotherapy: Progress and Perspectives
SO ADVANCED THERAPEUTICS
LA English
DT Review
DE nanomedicine; biomaterials; cancer immunotherapy; cancer vaccines; checkpoint blockade therapies; clinical translation; immune biomarkers
ID PEGYLATED LIPOSOMAL DOXORUBICIN; ALBUMIN-BOUND PACLITAXEL; METASTATIC BREAST-CANCER; COMBINATION; CELLS; NANOPARTICLES; BIOMARKERS; SCAFFOLDS; DELIVERY; VACCINES
AB Advances in immuno-oncology have significantly improved cancer therapy outcomes. Unfortunately,

potent responses to immunotherapy have only been achieved in subsets of patients. Nanomedicines and biomaterials have enormous potential for making immunotherapy work better in more patients, which has been suggested by a large number of preclinical studies. However, it remains unclear whether and how these materials will really benefit cancer patients treated by immunotherapy. Therefore, reviewing current clinical studies on nanomedicines and biomaterials in immuno-oncology may provide valuable information for future translation research. In this work, the clinical landscape of cancer immunotherapy involving nanomedicines and biomaterials, mainly Abraxane, Doxil, messenger ribonucleic acid (mRNA) nanovaccines, and a biomaterial scaffold-supported autologous vaccine, is reviewed. Clinical results demonstrate the high potential of nano/biomaterials in immunotherapy, achieved by rationalized utilization of their targeted delivery and reduced toxicities/side effects. Future clinical translation in this field will likely benefit from designing rational combinations of nanomedicines and biomaterials with the right immunotherapeutics applied in the right patients/tumors, and this should be achieved with the help of immune biomarker analysis. By rationally utilizing the unique features and advantages of nanomedicines and biomaterials, they will highly impact patient care in combination with immunotherapy.
C1 [Shi, Yang] Rhein Westfal TH Aachen, Inst Expt Mol Imaging, Uniklin RWTH Aachen, Dept Nanomed & Theranost,Fac Med, D-52074 Aachen, Germany.
[Shi, Yang] Rhein Westfal TH Aachen, Fac Med, Helmholtz Inst Biomed Engn, D-52074 Aachen, Germany.
C3 RWTH Aachen University; RWTH Aachen University Hospital; RWTH Aachen University; Helmholtz Association
RP Shi, Y (通讯作者)，Rhein Westfal TH Aachen, Inst Expt Mol Imaging, Uniklin RWTH Aachen, Dept Nanomed & Theranost,Fac Med, D-52074 Aachen, Germany.; Shi, Y (通讯作者)，Rhein Westfal TH Aachen, Fac Med, Helmholtz Inst Biomed Engn, D-52074 Aachen, Germany.
EM yshi@ukaachen.de
FU Aachen Interdisciplinary Center for Clinical Research (IZKF) [O3-2];
EU-EFRE: European Fund for Regional Development: I3-STM [0800387]
FX Aachen Interdisciplinary Center for Clinical Research (IZKF; Project O3-2) and EU-EFRE: European Fund for Regional Development: I<SUP>3</SUP>-STM (0800387) are acknowledged.
CR Aigner J, 2013, ANTICANCER RES, V33, P3407
Alizadeh D, 2014, CANCER RES, V74, P104, DOI 10.1158/0008-5472.CAN-13-1545
[Anonymous], 1994, ANN ONCOL
[Anonymous], 2018, NAT REV DRUG DISCOV
[Anonymous], 2016, NAT REV MATER
Arbour KC, 2018, J CLIN ONCOL, V36, P2872, DOI 10.1200/JCO.2018.79.0006
Barenholz Y, 2012, J CONTROL RELEASE, V160, P117, DOI 10.1016/j.jconrel.2012.03.020
Bennett M. S. C., 2019, CANC NETWORK
Blank CU, 2016, SCIENCE, V352, P658, DOI 10.1126/science.aaf2834
Burris HA, 2019, J CLIN ONCOL, V37, DOI 10.1200/JCO.2019.37.15_suppl.2523
Carson WE, 2004, CLIN CANCER RES, V10, P3401, DOI 10.1158/1078-0432.CCR-1016-03
De Geest BG, 2018, MOL IMMUNOL, V98, P25, DOI 10.1016/j.molimm.2018.02.015
Demaria S, 2001, CLIN CANCER RES, V7, P3025
Dirix LY, 2018, BREAST CANCER RES TR, V167, P671, DOI 10.1007/s10549-017-4537-5
Emens LA, 2019, JAMA ONCOL, V5, P74, DOI 10.1001/jamaoncol.2018.4224
Fan Q, 2018, ADV FUNCT MATER, V28, DOI 10.1002/adfm.201802540
Feng X., 2019, ADV SCI, V6
Gabizon AA, 2001, CANCER INVEST, V19, P424, DOI 10.1081/CNV-100103136
Galon J, 2019, NAT REV DRUG DISCOV, V18, P197, DOI 10.1038/s41573-018-0007-y
Gibney GT, 2016, LANCET ONCOL, V17, pE542, DOI 10.1016/S1470-2045(16)30406-5
Gradishar WJ, 2006, EXPERT OPIN PHARMACO, V7, P1041, DOI 10.1517/14656566.7.8.1041
Gradishar WJ, 2005, J CLIN ONCOL, V23, P7794, DOI 10.1200/JCO.2005.04.937
Havel JJ, 2019, NAT REV CANCER, V19, P133, DOI 10.1038/s41568-019-0116-x
Irvine DJ, 2016, NAT REV MATER, V1, DOI 10.1038/natrevmats.2015.8
John J, 2010, BMC IMMUNOL, V11, DOI 10.1186/1471-2172-11-14
Kim J, 2015, NAT BIOTECHNOL, V33, P64, DOI 10.1038/nbt.3071
Kowalski PS, 2019, MOL THER, V27, P710, DOI 10.1016/j.ymthe.2019.02.012
Kranz LM, 2016, NATURE, V534, P396, DOI 10.1038/nature18300
Kulkarni JA, 2018, NUCLEIC ACID THER, V28, P146, DOI 10.1089/nat.2018.0721
Lammers T, 2016, NAT REV MATER, V1, DOI 10.1038/natrevmats.2016.69
Li AW, 2018, NAT MATER, V17, P528, DOI 10.1038/s41563-018-0028-2
Li F, 2017, ADV MATER, V29, DOI 10.1002/adma.201602432

Little RF, 2007, BLOOD, V110, P4165, DOI 10.1182/blood-2007-06-097568
Majzner RG, 2019, NAT MED, V25, P1341, DOI 10.1038/s41591-019-0564-6
Marina NM, 2002, CLIN CANCER RES, V8, P413
Mi Y, 2019, ADV SCI, V6, DOI 10.1002/advs.201801847
Monk BJ, 2017, ANN ONCOL, V28, P996, DOI 10.1093/annonc/mdx049
Nam J, 2019, NAT REV MATER, V4, P398, DOI 10.1038/s41578-019-0108-1
Nanda R, 2016, J CLIN ONCOL, V34, P2460, DOI 10.1200/JCO.2015.64.8931
Obeid M, 2007, NAT MED, V13, P54, DOI 10.1038/nm1523
Ovais M, 2019, ADV MATER, V31, DOI 10.1002/adma.201808303
PAGE DB, 2019, J CLIN ONCOL S, V37
Pardi N, 2018, NAT REV DRUG DISCOV, V17, P261, DOI 10.1038/nrd.2017.243
Park JH, 2018, NEW ENGL J MED, V378, P449, DOI 10.1056/NEJMoa1709919
Paz-Ares L, 2018, NEW ENGL J MED, V379, P2040, DOI 10.1056/NEJMoa1810865
Pfannenstiel LW, 2010, CELL IMMUNOL, V263, P79, DOI 10.1016/j.cellimm.2010.03.001
Ramakrishnan R, 2010, J CLIN INVEST, V120, P1111, DOI 10.1172/JCI40269
Ran S, 2015, CANCER RES, V75, P2405, DOI 10.1158/0008-5472.CAN-14-3525
Ribas A, 2018, SCIENCE, V359, P1350, DOI 10.1126/science.aar4060
Riley RS, 2019, NAT REV DRUG DISCOV, V18, P175, DOI 10.1038/s41573-018-0006-z
Rios-Doria J, 2015, NEOPLASIA, V17, P661, DOI 10.1016/j.neo.2015.08.004
Robert C, 2019, LANCET ONCOL, V20, P1239, DOI 10.1016/S1470-2045(19)30388-2
Saeed M, 2019, THERANOSTICS, V9, P7981, DOI 10.7150/thno.37568
Sahin U, 2018, SCIENCE, V359, P1355, DOI 10.1126/science.aar7112
Sang W, 2019, CHEM SOC REV, V48, P3771, DOI 10.1039/c8cs00896e
Schmid P, 2018, NEW ENGL J MED, V379, P2108, DOI 10.1056/NEJMoa1809615
Schumacher TN, 2015, CANCER CELL, V27, P12, DOI 10.1016/j.ccell.2014.12.004
Shi Y., UNPUB
Shi Y, 2019, ACCOUNTS CHEM RES, V52, P1543, DOI 10.1021/acs.accounts.9b00148
SIMPKINS F, 2012, J CLIN ONCOL S, V30
Singh A, 2014, ADV MATER, V26, P6530, DOI 10.1002/adma.201402105
Smyth MJ, 2016, NAT REV CLIN ONCOL, V13, P143, DOI 10.1038/nrclinonc.2015.209
Soliman HH, 2017, ONCOTARGETS THER, V10, P101, DOI 10.2147/OTT.S122974
Stinchcombe TE, 2007, NANOMEDICINE-UK, V2, P415, DOI 10.2217/17435889.2.4.415
Sun QX, 2019, CHEM SOC REV, V48, P351, DOI 10.1039/c8cs00473k
Torrice M, 2016, ACS CENTRAL SCI, V2, P434, DOI 10.1021/acscentsci.6b00190
Vacchelli E, 2013, ONCOIMMUNOLOGY, V2, DOI 10.4161/onci.23510
van der Meel R, 2019, NAT NANOTECHNOL, V14, P1007, DOI 10.1038/s41565-019-0567-y
Vicari AP, 2009, CANCER IMMUNOL IMMUN, V58, P615, DOI 10.1007/s00262-008-0586-2
Voorwerk L, 2019, NAT MED, V25, P920, DOI 10.1038/s41591-019-0432-4
Wang H, 2018, NAT MATER, V17, P761, DOI 10.1038/s41563-018-0147-9
West H, 2019, LANCET ONCOL, V20, P924, DOI 10.1016/S1470-2045(19)30167-6
Wilhelm S, 2016, NAT REV MATER, V1, DOI 10.1038/natrevmats.2016.14
Zhang PC, 2019, ADV MATER, V31, DOI 10.1002/adma.201904156
Zhang YL, 2020, MED RES REV, V40, P1084, DOI 10.1002/med.21644
NR 75
TC 50
Z9 52
U1 5
U2 46
PU WILEY
PI HOBOKEN
PA 111 RIVER ST, HOBOKEN 07030-5774, NJ USA
EI 2366-3987
J9 ADV THER-GERMANY
JI Adv. Therap.
PD SEP
PY 2020
VL 3
IS 9
AR UNSP 1900215
DI 10.1002/adtp.201900215
EA JUL 2020

PG 9
WC Pharmacology & Pharmacy
WE Science Citation Index Expanded (SCI-EXPANDED)
SC Pharmacology & Pharmacy
GA NL7DZ
UT WOS:000547936500001
OA hybrid
DA 2023-09-14
ER

PT J
AU Karimi, H
Soleimanjahi, H
Abdoli, A
Banijamali, RS
AF Karimi, Hesam
Soleimanjahi, Hoorieh
Abdoli, Asghar
Banijamali, Razieh Sadat
TI Combination therapy using human papillomavirus L1/E6/E7 genes and archaeosome: a nanovaccine confer immuneadjuvanting effects to fight cervical cancer
SO SCIENTIFIC REPORTS
LA English
DT Article
ID VACCINE ADJUVANTS; DNA; TRANSFECTION; INFECTIONS; INDUCTION; STABILITY; LIPOSOMES; RESPONSES; PROTEIN; CELLS
AB Cancer is a leading cause of death worldwide. Cervical cancer caused by human papillomavirus (HPV) is a major health problem in women. DNA vaccines are a perfect approach to immunization, but their potency in clinical trials has been insufficient for generating effective immunity, which may be related to the degradation of the DNA via nucleases, poor delivery to antigen-presenting cells (APCs), and insufficient uptake of DNA plasmids by cells upon injection. Archaeosome is a nano-delivery systems based on liposomes with their immunological role have been developed for gene delivery. In this study, human papillomavirus type 16 genes, containing truncated L1, E6, and E7, were simultaneously used in combination therapy with archaeosome and assessed in vivo. Findings supported that archaeosomes promotes immune responses to DNA vaccines and a long-term CTL response was generated with a low antigen dose. Combination therapy with archaeosome/L1/E6/E7 vaccines exhibited a strong cytolytic activity against tumor cells and induced prophylactic and therapeutic effect against the development of tumor in the animal model.
C1 [Karimi, Hesam; Soleimanjahi, Hoorieh; Banijamali, Razieh Sadat] Tarbiat Modares Univ, Fac Med Sci, Dept Virol, Tehran, Iran.
[Abdoli, Asghar] Pasteur Inst Iran, Dept Hepatitis & AIDS, Tehran, Iran.
C3 Tarbiat Modares University; Le Reseau International des Instituts Pasteur (RIIP); Pasteur Institute of Iran
RP Soleimanjahi, H (通讯作者), Tarbiat Modares Univ, Fac Med Sci, Dept Virol, Tehran, Iran.
EM soleim_h@modares.ac.ir
RI Soleimanjahi, Hoorieh/B-8945-2017
OI Soleimanjahi, Hoorieh/0000-0003-1931-7801
FU Tarbiat Modares University, Faculty of Medical Sciences [52/8611]
FX The results described in this paper were part of student thesis, which was supported by the grant number 52/8611 from the Research Deputy of Tarbiat Modares University, Faculty of Medical Sciences, and we gratefully acknowledge the support.
CR Allen Theresa M., 2006, Anti-Cancer Agents in Medicinal Chemistry, V6, P513, DOI 10.2174/187152006778699121
Amador-Molina A, 2013, VIRUSES-BASEL, V5, P2624, DOI 10.3390/v5112624
Araldi RP, 2018, BIOMED PHARMACOTHER, V106, P1537, DOI 10.1016/j.biopha.2018.06.149
Attar A, 2016, ARTIF CELL NANOMED B, V44, P710, DOI 10.3109/21691401.2014.982800
Bellone S, 2009, J VIROL, V83, P6779, DOI 10.1128/JVI.02443-08
Benvegnu Thierry, 2009, Recent Pat Drug Deliv Formul, V3, P206
Bharti Alok Chandra, 2018, Front Biosci (Elite Ed), V10, P15

Bolhassani A., 2018, HPV INFECT DIAGNOSIS, V3, P182
Brown DA, 2009, CHEM PHYS LIPIDS, V159, P95, DOI 10.1016/j.chemphyslip.2009.03.004
Budri T, 2006, ASMC PROC, P17
Faisal SM, 2011, VACCINE, V29, P2381, DOI 10.1016/j.vaccine.2011.01.110
Ferlay J., 2018, CANC TODAY
Garnett M. C., 1999, CRITICAL REV THERAPE, V16
Hancock G, 2018, BEST PRACT RES CL OB, V47, P59, DOI 10.1016/j.bpobgyn.2017.09.008
Haq K, 2016, EXPERT REV VACCINES, V15, P1557, DOI 10.1080/14760584.2016.1195265
Inoh Y, 2017, EUR J PHARM SCI, V102, P230, DOI 10.1016/j.ejps.2017.03.023
Jia YM, 2015, EXPERT OPIN DRUG DEL, V12, P993, DOI 10.1517/17425247.2015.1044435
Kaarthigeyan K, 2012, INDIAN J MED PAEDIAT, V33, P7, DOI 10.4103/0971-5851.96961
Karimi H., 2017, PATHOBIOLOGY RES, V19, P71
Kaur G, 2016, DRUG DELIV, V23, P2497, DOI 10.3109/10717544.2015.1019653
Krishnamachari Y, 2011, PHARM RES-DORDR, V28, P215, DOI 10.1007/s11095-010-0241-4
Krishnan L, 2000, INFECT IMMUN, V68, P54, DOI 10.1128/IAI.68.1.54-63.2000
Krishnan L., 2007, VACCINE ADJUVANTS DE, P263
Krishnan L, 2008, VACCINE, V26, P2043, DOI 10.1016/j.vaccine.2008.02.026
Levine RM, 2013, LANGMUIR, V29, P9208, DOI 10.1021/la400859e
Lin CT, 2006, J BIOMED SCI, V13, P481, DOI 10.1007/s11373-006-9086-6
LIN YL, 1992, VIROLOGY, V187, P612, DOI 10.1016/0042-6822(92)90463-Y
Liu WJ, 2002, VIROLOGY, V301, P43, DOI 10.1006/viro.2002.1584
Liu Z, 2007, ANGEW CHEM INT EDIT, V46, P2023, DOI 10.1002/anie.200604295
Lowy DR, 2016, J CLIN INVEST, V126, P5, DOI 10.1172/JCI85446
Mirshahabi H, 2012, IRAN J CANCER PREV, V5, P16
Moghimipour E, 2013, ARCHAEA, V2013, DOI 10.1155/2013/782012
Rethore G, 2007, CHEM COMMUN, P2054, DOI 10.1039/b618568a
Saadat P., 2017, INT J CANC MANAGEMEN, V10
Saade F, 2012, EXPERT REV VACCINES, V11, P189, DOI [10.1586/erv.11.188, 10.1586/ERV.11.188]
Schiller JT, 2012, VACCINE, V30, pF123, DOI 10.1016/j.vaccine.2012.04.108
Schwendener Reto A, 2014, Ther Adv Vaccines, V2, P159, DOI 10.1177/2051013614541440
SENIOR J, 1982, LIFE SCI, V30, P2123, DOI 10.1016/0024-3205(82)90455-6
Simoes S, 2000, BBA-BIOMEMBRANES, V1463, P459, DOI 10.1016/S0005-2736(99)00238-2
Singh M., 2007, VACCINE ADJUVANTS DE
Stanley MA, 2012, CLIN MICROBIOL REV, V25, P215, DOI 10.1128/CMR.05028-11
Stanley MA, 2012, J GEN VIROL, V93, P681, DOI 10.1099/vir.0.039677-0
Stern PL, 2012, VACCINE, V30, pF71, DOI 10.1016/j.vaccine.2012.05.091
Vazzana M, 2017, CARRIER-MEDIATED DERMAL DELIVERY: APPLICATIONS IN THE PREVENTION AND TREATMENT OF SKIN DISORDERS, P323
Zapatka M., 2018, 465757 BIORXIV
NR 45
TC 50
Z9 50
U1 0
U2 7
PU NATURE PORTFOLIO
PI BERLIN
PA HEIDELBERGER PLATZ 3, BERLIN, 14197, GERMANY
SN 2045-2322
J9 SCI REP-UK
JI Sci Rep
PD APR 1
PY 2020
VL 10
IS 1
AR 5787
DI 10.1038/s41598-020-62448-3
PG 15
WC Multidisciplinary Sciences
WE Science Citation Index Expanded (SCI-EXPANDED)
SC Science & Technology - Other Topics
GA NF7HJ

UT WOS:000563465100022
PM 32238821
OA gold, Green Published
DA 2023-09-14
ER

PT J
AU Dong, X
Liang, J
Yang, AF
Qian, ZY
Kong, DL
Lv, F
AF Dong, Xia
Liang, Jie
Yang, Afeng
Qian, Zhiyong
Kong, Deling
Lv, Feng
TI A Visible Codelivery Nanovaccine of Antigen and Adjuvant with Self-Carrier for Cancer Immunotherapy
SO ACS APPLIED MATERIALS & INTERFACES
LA English
DT Article
DE nanovaccine; codelivery; cancer immunotherapy; fluorescence imaging; self-carrier
ID GUIDED PHOTOTHERMAL THERAPY; IN-VIVO TRACKING; POLYMERIC NANOPARTICLES; DENDRITIC CELLS; DRUG-DELIVERY; DUAL-DELIVERY; CO-DELIVERY; CPG; VACCINES; RELEASE
AB Codelivery nanovaccines of antigens and adjuvants have achieved positive therapy for cancer immunotherapy. The insufficient immunogenicity of these vaccines leads to the difficulty of eliciting robust immune effects for immune clearance due to the inadequate loading efficiency, complex preparation processes, low safety concerns, and weak immune responses. Herein, a visible codelivery nanovaccine of an antigen and adjuvant based on self-cross-linked antigen nanoparticles (ovalbumin nanoparticles (ONPs)) combined with the adjuvant (CpG) for cancer immunotherapy was prepared using antigens themselves as carriers. ONPs not only provide sufficient antigens for continuous simulation of the immune response with high antigen loading efficiency but also serve as natural carriers of CpG. In vitro and in vivo experiments proved that ONPs-CpG can elicit a robust immune response including DC maturity, T cell activation, and IFN-y production. ONPs-CpG induced strong tumor-specific immunity and exhibited remarkable antitumor immunotherapy effects in vivo using mouse models of lymphoma. Furthermore, to perform the precise vaccine delivery, the dual fluorescent codelivery nanovaccine was monitored in real time in vivo by the visible imaging method. With regard to migration tracking, fluorescence imaging allowed for both high resolution and sensitivity of visible detection based on the fluorescence of ONPs and CpG. The multifunctional nanovaccine could function as a robust platform for cancer immunotherapy and a visible system for antigen adjuvant tracking.
C1 [Dong, Xia; Liang, Jie; Yang, Afeng; Kong, Deling] Chinese Acad Med Sci, Inst Biomed Engn, Tianjin Key Lab Biomed Mat, Tianjin 300192, Peoples R China.
[Dong, Xia; Liang, Jie; Yang, Afeng; Kong, Deling; Lv, Feng] Peking Union Med Coll, Tianjin 300192, Peoples R China.
[Qian, Zhiyong; Lv, Feng] Sichuan Univ, West China Hosp, State Key Lab Biotherapy, Chengdu 610041, Sichuan, Peoples R China.
[Qian, Zhiyong; Lv, Feng] Sichuan Univ, Collaborat Innovat Ctr Biotherapy, Chengdu 610041, Sichuan, Peoples R China.
C3 Chinese Academy of Medical Sciences - Peking Union Medical College; Institute of BioMedical Engineering - CAMS; Chinese Academy of Medical Sciences - Peking Union Medical College; Peking Union Medical College; Sichuan University; Sichuan University
EM lvfeng2002@163.com
FU National Natural Science Foundation of China [81601595, 31870951]; Natural Science Foundation of Tianjin, China [16JCYBJC27800, 18JCYBJC17400]; CAMS Innovation Fund for Medical Sciences [CIFMS

2017-I2M-3-020]
FX This work was supported by the National Natural Science Foundation of
China (Nos. 81601595, 31870951), the Natural Science Foundation of
Tianjin, China (Nos.16JCYBJC27800, 18JCYBJC17400), and the CAMS
Innovation Fund for Medical Sciences (CIFMS 2017-I2M-3-020).
CR Antonia SJ, 2014, CLIN CANCER RES, V20, P6258, DOI 10.1158/1078-0432.CCR-14-1457
Appel AA, 2013, BIOMATERIALS, V34, P6615, DOI 10.1016/j.biomaterials.2013.05.033
Bashour KT, 2014, P NATL ACAD SCI USA, V111, P2241, DOI 10.1073/pnas.1315606111
Beatty GL, 2014, CANCER IMMUNOL RES, V2, P112, DOI 10.1158/2326-6066.CIR-13-0170
Chang TZ, 2017, BIOMATER SCI-UK, V5, P223, DOI 10.1039/c6bm00500d
Chen H, 2015, NANOSCALE, V7, P15477, DOI 10.1039/c5nr03454j
Dong X, 2018, ACS APPL MATER INTER, V10, P21861, DOI 10.1021/acsami.8b04867
Dong X, 2018, NANOMED-NANOTECHNOL, V14, P1087, DOI 10.1016/j.nano.2018.02.007
Dong X, 2017, DRUG DELIV, V24, P641, DOI 10.1080/10717544.2017.1289570
Dong X, 2016, ACS BIOMATER SCI ENG, V2, P2001, DOI 10.1021/acsbiomaterials.6b00403
Du GS, 2017, J CONTROL RELEASE, V266, P109, DOI 10.1016/j.jconrel.2017.09.021
Fang Y, 2018, J BIOMED NANOTECHNOL, V14, P575, DOI 10.1166/jbn.2018.2560
Han L, 2016, BIOMATERIALS, V105, P185, DOI 10.1016/j.biomaterials.2016.08.007
Heo MB, 2014, BIOMATERIALS, V35, P590, DOI 10.1016/j.biomaterials.2013.10.009
Hoffmann S, 2012, BIOMACROMOLECULES, V13, P652, DOI 10.1021/bm2015027
Hotaling NA, 2015, ANNU REV BIOMED ENG, V17, P317, DOI 10.1146/annurev-bioeng-071813-104814
Jo DH, 2016, BIOMATERIALS, V109, P23, DOI 10.1016/j.biomaterials.2016.09.008
Kapadia CH, 2018, J CONTROL RELEASE, V269, P393, DOI 10.1016/j.jconrel.2017.11.020
Krieg AM, 2012, NUCLEIC ACID THER, V22, P77, DOI 10.1089/nat.2012.0340
Li AW, 2018, NAT MATER, V17, P528, DOI 10.1038/s41563-018-0028-2
Liu T, 2018, J BIOMED NANOTECHNOL, V14, P1107, DOI 10.1166/jbn.2018.2573
Moon JJ, 2012, ADV MATER, V24, P3724, DOI 10.1002/adma.201200446
Moon JJ, 2011, NAT MATER, V10, P243, DOI 10.1038/NMAT2960
Morishita M, 2016, BIOMATERIALS, V111, P55, DOI 10.1016/j.biomaterials.2016.09.031
Noh YW, 2011, BIOMATERIALS, V32, P6254, DOI 10.1016/j.biomaterials.2011.05.013
Pan JB, 2018, ADV MATER, V30, DOI 10.1002/adma.201704408
Peng JR, 2018, ADV SCI, V5, DOI 10.1002/advs.201700891
Peng JR, 2017, ACS APPL MATER INTER, V9, P13875, DOI 10.1021/acsami.7b01365
Peske JD, 2015, ADV CANCER RES, V128, P263, DOI 10.1016/bs.acr.2015.05.001
Rahimian S, 2015, J CONTROL RELEASE, V203, P16, DOI 10.1016/j.jconrel.2015.02.006
Rahimian S, 2015, BIOMATERIALS, V37, P469, DOI 10.1016/j.biomaterials.2014.10.043
Restifo NP, 2012, NAT REV IMMUNOL, V12, P269, DOI 10.1038/nri3191
Rosenberg SA, 2015, SCIENCE, V348, P62, DOI 10.1126/science.aaa4967
Ruiz-de-Angulo A, 2016, ACS NANO, V10, P1602, DOI 10.1021/acsnano.5b07253
Schreiber RD, 2011, SCIENCE, V331, P1565, DOI 10.1126/science.1203486
Shi ZY, 2018, J BIOMED NANOTECHNOL, V14, P1934, DOI 10.1166/jbn.2018.2636
Song HJ, 2018, BIOMATERIALS, V159, P119, DOI 10.1016/j.biomaterials.2018.01.004
Sun ZT, 2018, ACS APPL MATER INTER, V10, P20315, DOI 10.1021/acsami.8b04312
Wang ZJ, 2017, J AM CHEM SOC, V139, P15784, DOI 10.1021/jacs.7b07895
Wayteck L, 2016, BIOMATERIALS, V77, P243, DOI 10.1016/j.biomaterials.2015.11.016
Wilson JT, 2013, ACS NANO, V7, P3912, DOI 10.1021/nn305466z
Xia QM, 2018, J BIOMED NANOTECHNOL, V14, P1613, DOI 10.1166/jbn.2018.2605
Xiang J, 2015, ACS NANO, V9, P6401, DOI 10.1021/acsnano.5b02014
Yang R, 2018, ACS NANO, V12, P5121, DOI 10.1021/acsnano.7b09041
Zeng Q, 2017, BIOMATERIALS, V122, P105, DOI 10.1016/j.biomaterials.2017.01.010
Zhang Y, 2016, ADV MATER, V28, P2699, DOI 10.1002/adma.201506232
NR 46
TC 50
Z9 50
U1 2
U2 138
PU AMER CHEMICAL SOC
PI WASHINGTON
PA 1155 16TH ST, NW, WASHINGTON, DC 20036 USA
SN 1944-8244
EI 1944-8252

J9 ACS APPL MATER INTER
JI ACS Appl. Mater. Interfaces
PD FEB 6
PY 2019
VL 11
IS 5
BP 4876
EP 4888
DI 10.1021/acsami.8b20364
PG 13
WC Nanoscience & Nanotechnology; Materials Science, Multidisciplinary
WE Science Citation Index Expanded (SCI-EXPANDED)
SC Science & Technology - Other Topics; Materials Science
GA HK9YD
UT WOS:000458347900019
PM 30628437
DA 2023-09-14
ER

PT J
AU Li, CX
Zhang, XX
Chen, Q
Zhang, JL
Li, WP
Hu, HY
Zhao, XL
Qiao, MX
Chen, DW
AF Li, Chenxi
Zhang, Xiaoxu
Chen, Qing
Zhang, Jiulong
Li, Wenpan
Hu, Haiyang
Zhao, Xiuli
Qiao, Mingxi
Chen, Dawei
TI Synthetic Polymeric Mixed Micelles Targeting Lymph Nodes Trigger
Enhanced Cellular and Humoral Immune Responses
SO ACS APPLIED MATERIALS & INTERFACES
LA English
DT Article
DE polymeric mixed micelles; lymph node; surface functionalization;
dendritic cell targeting; endosomal rupture; immune responses
ID CANCER-IMMUNOTHERAPY; DENDRITIC CELLS; ANTIGEN DELIVERY; NANOPARTICLE
VACCINES; MULTIDRUG-RESISTANCE; ANTITUMOR IMMUNITY; CARBON NANOTUBES; DC
ACTIVATION; NANOVACCINE; LIPOSOMES
AB It has been widely accepted that lymph nodes (LNs) are critical targets of cancer vaccines because antigen presentation and initiation of T-cell-mediated immune responses occur primarily at these locations. In this study, amphiphilic diblock copolymer poly(2-ethyl-2-oxazoline)-poly-(D,L-lactide) (PEOz-PLA) combined with carboxylterminated-Pluronic F127 was used to construct mixed micelles [carboxylated-nanoparticles (NPs)] for codelivery of antigen ovalbumin (OVA) and Toll-like receptor-7 agonist CL264 (carboxylated-NPs/OVA/CL264) to the LN-resident dendritic cells (DCs). The results showed that the small, sub-60 nm size of the self-assembled mixed micelles enables them to rapidly penetrate into lymphatic vessels and reach draining lymph nodes after subcutaneous injection. Furthermore, the surface modification with carboxylic groups imparted the carboxylated-NPs with endocytic receptor-targeting ability, allowing for DC internalization of carboxylated-NPs/OVA/CL264 via the scavenger receptor-mediated pathway. Because stimulation of CL264 in early endosomes will lead to a more effective immune response than that in late endo/lysosomes, the mass ratio of PEOz-PLA to carboxylated-Pluronic F127 in the mixed micelles was adjusted to release the encapsulated CL264 to the

early endosome, resulting in increased expression of costimulatory molecules and secretion of stimulited cytokines by DCs. Moreover, the incorporation of PEOz outside the micellar shell effectively augmented MHC I antigen presentation through facilitating endosome escape and cytosolic release of antigens. This in turn evoked potent immune responses in vivo, including activation of antigen-specific T-cell responses, production of antigen-specific IgG antibodies, and generation of cytotoxic T-lymphocyte responses. Finally, immunization with the codelivery system in E.G7-OVA tumor-bearing mice could not only significantly inhibit tumor growth but also markedly prolong the survival of tumor-bearing mice. Taken together, carboxylated-NPs/OVA/CL264 have demonstrated great potential for clinical applications as an effective antitumor vaccine for further immunotherapy.
C1 [Li, Chenxi; Zhang, Xiaoxu; Chen, Qing; Zhang, Jiulong; Li, Wenpan; Hu, Haiyang; Zhao, Xiuli; Qiao, Mingxi; Chen, Dawei] Shenyang Pharmaceut Univ, Sch Pharm, Dept Pharmaceut, Shenyang 110016, Liaoning, Peoples R China.
C3 Shenyang Pharmaceutical University
RP Qiao, MX; Chen, DW (通讯作者)，Shenyang Pharmaceut Univ, Sch Pharm, Dept Pharmaceut, Shenyang 110016, Liaoning, Peoples R China.
EM qiaomingxi@l63.com; chendawei@syphu.edu.cn
RI Li, Wenpan/HGB-6464-2022
FU National Natural Science Foundation of China [81573370]
FX The authors are grateful to the financial support from the National
Natural Science Foundation of China (81573370).
CR Akash MSH, 2015, J CONTROL RELEASE, V209, P120, DOI 10.1016/j.jconrel.2015.04.032
An M, 2017, ACS APPL MATER INTER, V9, P23466, DOI 10.1021/acsami.7b06024
Batrakova EV, 2008, J CONTROL RELEASE, V130, P98, DOI 10.1016/j.jconrel.2008.04.013
De Coen R, 2016, BIOMACROMOLECULES, V17, P2479, DOI 10.1021/acs.biomac.6b00685
Frizzell H, 2017, CELL IMMUNOL, V311, P54, DOI 10.1016/j.cellimm.2016.10.004
Gallud A, 2017, BIOMATERIALS, V121, P28, DOI 10.1016/j.biomaterials.2016.12.029
Gao NN, 2011, ACS NANO, V5, P4581, DOI 10.1021/nn200283g
Gao YJ, 2015, ACTA BIOMATER, V23, P127, DOI 10.1016/j.actbio.2015.05.021
Garg SM, 2017, MOL PHARMACEUT, V14, P2570, DOI 10.1021/acs.molpharmaceut.6b01119
Hong W, 2013, BIOMATERIALS, V34, P9602, DOI 10.1016/j.biomaterials.2013.08.032
Huang Z, 2016, BIOMATERIALS, V90, P72, DOI 10.1016/j.biomaterials.2016.03.009
Jin JO, 2017, BIOMATERIALS, V115, P81, DOI 10.1016/j.biomaterials.2016.11.020
Kim SY, 2017, BIOMATERIALS, V130, P56, DOI 10.1016/j.biomaterials.2017.03.034
Kim WG, 2016, BIOCONJUGATE CHEM, V27, P2007, DOI 10.1021/acs.bioconjchem.6b00277
Klauber TCB, 2017, ACTA BIOMATER, V53, P367, DOI 10.1016/j.actbio.2017.01.072
Li M, 2017, ACTA BIOMATER, V64, P237, DOI 10.1016/j.actbio.2017.10.019
Lin WJ, 2017, ACTA BIOMATER, V48, P378, DOI 10.1016/j.actbio.2016.10.038
Liu LX, 2016, ACS APPL MATER INTER, V8, P11969, DOI 10.1021/acsami.6b01135
Liu Q, 2015, ACS NANO, V9, P4925, DOI 10.1021/nn5066793
Mintern JD, 2013, ADV HEALTHC MATER, V2, P940, DOI 10.1002/adhm.201200441
Mueller SN, 2015, MOL PHARMACEUT, V12, P1356, DOI 10.1021/mp500589c
Qian Y, 2016, BIOMATERIALS, V98, P171, DOI 10.1016/j.biomaterials.2016.05.008
Qiu LP, 2014, BIOMATERIALS, V35, P9877, DOI 10.1016/j.biomaterials.2014.08.008
Reddy ST, 2007, NAT BIOTECHNOL, V25, P1159, DOI [10.1038/nbt1332, 10.1038/nbtl332]
Rincon-Restrepo M, 2017, BIOMATERIALS, V132, P48, DOI 10.1016/j.biomaterials.2017.03.047
Sekiya T, 2017, BIOMATERIALS, V137, P61, DOI 10.1016/j.biomaterials.2017.05.018
Shi GN, 2017, BIOMATERIALS, V113, P191, DOI 10.1016/j.biomaterials.2016.10.047
Shima F, 2013, BIOCONJUGATE CHEM, V24, P926, DOI 10.1021/bc300611b
Sun BB, 2017, ACS APPL MATER INTER, V9, P21697, DOI 10.1021/acsami.7b05817
van der Vlies AJ, 2010, BIOCONJUGATE CHEM, V21, P653, DOI 10.1021/bc9004443
Walters AA, 2015, VACCINE, V33, P6588, DOI 10.1016/j.vaccine.2015.10.093
Wang C, 2016, BIOMATERIALS, V79, P88, DOI 10.1016/j.biomaterials.2015.11.040
Wang HY, 2012, ACS NANO, V6, P7122, DOI 10.1021/nn302186n
Wilson JT, 2013, ACS NANO, V7, P3912, DOI 10.1021/nn305466z
Xing J, 2016, ACS APPL MATER INTER, V8, P19276, DOI 10.1021/acsami.6b04591
Yamasaki Y, 2007, VACCINE, V25, P85, DOI 10.1016/j.vaccine.2006.07.017
Yang ZS, 2017, BIOMATERIALS, V134, P51, DOI 10.1016/j.biomaterials.2017.04.035
Yoshizaki Y, 2014, BIOMATERIALS, V35, P8186, DOI 10.1016/j.biomaterials.2014.05.077
Yuba E, 2017, BIOMATERIALS, V120, P32, DOI 10.1016/j.biomaterials.2016.12.021
Yuba E, 2014, BIOMATERIALS, V35, P3091, DOI 10.1016/j.biomaterials.2013.12.024
Zeng Q, 2017, BIOMATERIALS, V122, P105, DOI 10.1016/j.biomaterials.2017.01.010

   Zhang CN, 2017, J CONTROL RELEASE, V256, P170, DOI 10.1016/j.jconrel.2017.04.020
   Zhang CN, 2017, MOL PHARMACEUT, V14, P1760, DOI 10.1021/acs.molpharmaceut.7b00015
   Zhang WD, 2017, BIOCONJUGATE CHEM, V28, P1993, DOI 10.1021/acs.bioconjchem.7b00313
   Zhang XL, 2017, ACS MACRO LETT, V6, P442, DOI 10.1021/acsmacrolett.7b00049
   Zhao Y, 2015, ACTA BIOMATER, V17, P182, DOI 10.1016/j.actbio.2015.01.010
   Zhu GZ, 2017, ACS NANO, V11, P2387, DOI 10.1021/acsnano.7b00978
NR 47
TC 50
Z9 52
U1 5
U2 157
PU AMER CHEMICAL SOC
PI WASHINGTON
PA 1155 16TH ST, NW, WASHINGTON, DC 20036 USA
SN 1944-8244
J9 ACS APPL MATER INTER
JI ACS Appl. Mater. Interfaces
PD JAN 24
PY 2018
VL 10
IS 3
BP 2874
EP 2889
DI 10.1021/acsami.7b14004
PG 16
WC Nanoscience & Nanotechnology; Materials Science, Multidisciplinary
WE Science Citation Index Expanded (SCI-EXPANDED)
SC Science & Technology - Other Topics; Materials Science
GA FT9TZ
UT WOS:000423496500079
PM 29285934
DA 2023-09-14
ER

PT J
AU Mahjub, R
   Jatana, S
   Lee, SE
   Qin, Z
   Pauli, G
   Soleimani, M
   Madadi, S
   Li, SD
AF Mahjub, Reza
   Jatana, Sukhdeep
   Lee, Suen Ern
   Qin, Zhu
   Pauli, Griffin
   Soleimani, Meysam
   Madadi, Soheil
   Li, Shyh-Dar
TI Recent advances in applying nanotechnologies for cancer immunotherapy
SO JOURNAL OF CONTROLLED RELEASE
LA English
DT Review
DE Cancer Immunotherapy; Nanoparticles; Antigenic Peptides;
   Oligodeoxynucleotides; Immunomodulatory siRNA; Antigenic pDNA; Immune
   check point inhibitor
ID CELL-DERIVED EXOSOMES; TUMOR-ASSOCIATED MACROPHAGES; ANTITUMOR
   IMMUNE-RESPONSES; TARGETING DENDRITIC CELLS; ANTIGEN-DELIVERY-SYSTEM;
   MEDIATED GENE DELIVERY; IN-VIVO; GOLD NANOPARTICLES; T-CELLS; POLYMERIC

NANOPARTICLES
AB Cancer immunotherapy aimed at boosting cancer-specific immunoresponses to eradicate tumor cells has evolved as a new treatment modality. Nanoparticles incorporating antigens and immunomodulatory agents can activate immune cells and modulate the tumor microenvironment to enhance anti-tumor immunity. The nanotechnology approach has been demonstrated to be superior to standard formulations in in-vivo settings. In this article, we focus on recent advances made within the last 5 years in nanoparticle-based cancer immunotherapy, including peptide- and nucleic acid-based nanovaccines, nanomedicines containing an immunoadjuvant to activate antitumor immunity, nanoparticle delivery of immune checkpoint inhibitors and the combination of the above approaches. Encouraging results and new emerging nanotechnologies in drug delivery promise the continuous growth of this field and ultimately clinical translation of enhanced immunotherapy of cancer.
C1 [Mahjub, Reza; Jatana, Sukhdeep; Lee, Suen Ern; Qin, Zhu; Pauli, Griffin; Li, Shyh-Dar] Univ British Columbia, Fac Pharmaceut Sci, Vancouver Campus,5519-2405 Wesbrook Mall, Vancouver, BC V6T 1Z3, Canada.
[Mahjub, Reza] Hamadan Univ Med Sci, Sch Pharm, Dept Pharmaceut, Hamadan, Iran.
[Qin, Zhu] Jiangsu Acad Agr Sci, Inst Vet Immunol & Engn, Nanjing 210014, Jiangsu, Peoples R China.
[Soleimani, Meysam; Madadi, Soheil] Hamadan Univ Med Sci, Sch Pharm, Dept Pharmacognosy & Pharmaceut Biotechnol, Hamadan, Iran.
C3 University of British Columbia; Hamadan University of Medical Sciences;
Jiangsu Academy of Agricultural Sciences; Hamadan University of Medical
Sciences
RP Li, SD (通讯作者)，Univ British Columbia, Fac Pharmaceut Sci, Vancouver Campus,5519-2405 Wesbrook Mall, Vancouver, BC V6T 1Z3, Canada.
EM shyh-dar.li@ubc.ca
RI Li, Shyh-Dar/HKO-4870-2023; Madadi, Soheil/AAC-5211-2019; Mahjub, Reza/N-5034-2017; Soleimani, Meysam/M-8240-2017
OI Mahjub, Reza/0000-0002-7056-2020; Soleimani, Meysam/0000-0003-2505-8834; Jatana, Sukhdeep/0000-0002-0033-5949; Li, Shyh-Dar/0000-0002-1763-2667
FU Canadian Institutes of Health Research (CIHR); National Institutes of Health; Natural Sciences and Engineering Research Council of Canada; Canada Foundation for Innovation; CIHR New Investigator Award; Angiotech Professorship in Drug Delivery; Prostate Cancer Foundation
FX The Li lab has been supported by continuous research grants from multiple agencies, including Canadian Institutes of Health Research (CIHR), National Institutes of Health, Natural Sciences and Engineering Research Council of Canada, Canada Foundation for Innovation and Prostate Cancer Foundation. SDL is a recipient of the CIHR New Investigator Award and the Angiotech Professorship in Drug Delivery.
CR Ahmed KK, 2016, J PHARM SCI-US, V105, P1173, DOI 10.1016/j.xphs.2015.11.042
Ahn S, 2014, ADV HEALTHC MATER, V3, P1194, DOI 10.1002/adhm.201300597
Akita H, 2011, J CONTROL RELEASE, V149, P58, DOI 10.1016/j.jconrel.2010.08.023
Ali AA, 2017, NANOMED-NANOTECHNOL, V13, P921, DOI 10.1016/j.nano.2016.11.019
Almeida JPM, 2014, NANOMED-NANOTECHNOL, V10, P503, DOI 10.1016/j.nano.2013.09.011
Almeida JPM, 2014, SMALL, V10, P812, DOI 10.1002/smll.201301998
Anz D, 2011, INT J CANCER, V129, P2417, DOI 10.1002/ijc.25902
Aryani A, 2016, MOL NEUROBIOL, V53, P818, DOI 10.1007/s12035-014-9054-5
Bahrami B, 2017, IMMUNOL LETT, V190, P64, DOI 10.1016/j.imlet.2017.07.015
Bally MB, 1999, ADV DRUG DELIVER REV, V38, P291, DOI 10.1016/S0169-409X(99)00034-4
Bear AS, 2013, PLOS ONE, V8, DOI 10.1371/journal.pone.0069073
Beaudette TT, 2009, MOL PHARMACEUT, V6, P1160, DOI 10.1021/mp900038e
Begley J, 2008, CLIN CANCER RES, V14, P4385, DOI 10.1158/1078-0432.CCR-07-4804
Bhargava A, 2016, MOL IMMUNOL, V79, P98, DOI 10.1016/j.molimm.2016.10.003
Bhavsar MD, 2007, EXPERT OPIN DRUG DEL, V4, P197, DOI 10.1517/17425247.4.3.197
Boks MA, 2015, J CONTROL RELEASE, V216, P37, DOI 10.1016/j.jconrel.2015.06.033
Bonifaz L, 2002, J EXP MED, V196, P1627, DOI 10.1084/jem.20021598
Bourquin C, 2008, J IMMUNOL, V181, P2990, DOI 10.4049/jimmunol.181.5.2990
Bourquin C, 2010, J IMMUNOTHER, V33, P935, DOI 10.1097/CJI.0b013e3181f5dfa7
Boushehri MAS, 2018, DRUG DELIV, V25, P1414, DOI 10.1080/10717544.2018.1469684
Cano-Mejia J, 2017, NANOMED-NANOTECHNOL, V13, P771, DOI 10.1016/j.nano.2016.10.015
Carson WE, 2000, BLOOD, V96, P1465, DOI 10.1182/blood.V96.4.1465.h8001465_1465_1473
Carter RW, 2006, J IMMUNOL, V177, P2276, DOI 10.4049/jimmunol.177.4.2276

Cassetta L, 2018, FRONT CELL DEV BIOL, V6, DOI 10.3389/fcell.2018.00038
Chen D, 2014, J IMMUNOTHER, V37, P123, DOI 10.1097/CJI.0000000000000022
Chen Q, 2016, NAT COMMUN, V7, DOI 10.1038/ncomms13193
Chen WR, 2001, CANCER RES, V61, P459
Cheng BB, 2018, EUR J MED CHEM, V157, P582, DOI 10.1016/j.ejmech.2018.08.028
Chieppa M, 2003, J IMMUNOL, V171, P4552, DOI 10.4049/jimmunol.171.9.4552
Cruz LJ, 2014, MOL PHARMACEUT, V11, P4299, DOI 10.1021/mp400717r
Cruz LJ, 2011, BIOMATERIALS, V32, P6791, DOI 10.1016/j.biomaterials.2011.04.082
Cruz LJ, 2011, MOL PHARMACEUT, V8, P104, DOI 10.1021/mp100178k
Cubillos-Ruiz JR, 2009, J CLIN INVEST, V119, P2231, DOI 10.1172/JCI37716
Daftarian P, 2011, CANCER RES, V71, P7452, DOI 10.1158/0008-5472.CAN-11-1766
Daldrup-Link HE, 2011, CLIN CANCER RES, V17, P5695, DOI 10.1158/1078-0432.CCR-10-3420
DAVIS D, 1987, IMMUNOLOGY, V61, P229
De Koker S, 2011, CHEM SOC REV, V40, P320, DOI 10.1039/b914943k
Deaglio S, 2007, J EXP MED, V204, P1257, DOI 10.1084/jem.20062512
Delneste Y, 2002, IMMUNITY, V17, P353, DOI 10.1016/S1074-7613(02)00388-6
Desmet CJ, 2012, NAT REV IMMUNOL, V12, P479, DOI 10.1038/nri3247
Dolmans DEJGJ, 2003, NAT REV CANCER, V3, P380, DOI 10.1038/nrc1071
Dykman LA, 2011, ACTA NATURAE, V3, P34, DOI 10.32607/20758251-2011-3-2-34-56
Elgueta R, 2009, IMMUNOL REV, V229, P152, DOI 10.1111/j.1600-065X.2009.00782.x
Ercolini AM, 2005, J EXP MED, V201, P1591, DOI 10.1084/jem.20042167
Ernsting MJ, 2013, J CONTROL RELEASE, V172, P782, DOI 10.1016/j.jconrel.2013.09.013
Fayolle C, 1996, J IMMUNOL, V156, P4697
Flanary S, 2009, BIOCONJUGATE CHEM, V20, P241, DOI 10.1021/bc800317a
Gabrilovich DI, 2007, CANCER RES, V67, P425, DOI 10.1158/0008-5472.CAN-06-3037
Gabrilovich Dmitry I., 2009, P143, DOI 10.1007/978-0-387-88611-4_10
Gabrilovich DI, 2009, NAT REV IMMUNOL, V9, P162, DOI 10.1038/nri2506
Gallina G, 2006, J CLIN INVEST, V116, P2777, DOI 10.1172/JCI28828
Gao J, 2017, INT J NANOMED, V12, P1251, DOI 10.2147/IJN.S125866
Garg SM, 2017, MOL PHARMACEUT, V14, P2570, DOI 10.1021/acs.molpharmaceut.6b01119
Garu A, 2016, MOL THER, V24, P385, DOI 10.1038/mt.2015.215
Gdowski A, 2015, INT J MOL SCI, V16, P3990, DOI 10.3390/ijms16023990
Geary SM, 2015, J CONTROL RELEASE, V220, P682, DOI 10.1016/j.jconrel.2015.09.002
Ghaemi A, 2007, COMP IMMUNOL MICROB, V30, P197, DOI 10.1016/j.cimid.2007.01.002
Ghaemi A, 2011, INTERVIROLOGY, V54, P105, DOI 10.1159/000320197
Goldinger SM, 2012, EUR J IMMUNOL, V42, P3049, DOI 10.1002/eji.201142361
Grabbe S, 2016, NANOMEDICINE-UK, V11, P2723, DOI 10.2217/nnm-2016-0275
Guan X, 2010, J GENE MED, V12, P301, DOI 10.1002/jgm.1432
Guo HC, 2012, VIROL J, V9, DOI 10.1186/1743-422X-9-108
Gurnani K, 2004, J IMMUNOL, V173, P566, DOI 10.4049/jimmunol.173.1.566
Hamdy S, 2008, VACCINE, V26, P5046, DOI 10.1016/j.vaccine.2008.07.035
Hamid O, 2013, NEW ENGL J MED, V369, P134, DOI 10.1056/NEJMoa1305133
Hassan HAFM, 2016, BIOMATERIALS, V104, P310, DOI 10.1016/j.biomaterials.2016.07.005
Hassan HAFM, 2016, J CONTROL RELEASE, V225, P205, DOI 10.1016/j.jconrel.2016.01.030
Havel HA, 2016, AAPS J, V18, P1351, DOI 10.1208/s12248-016-9970-6
Heo MB, 2015, INT J NANOMED, V10, DOI 10.2147/IJN.S90104
Heo MB, 2014, ACTA BIOMATER, V10, P2169, DOI 10.1016/j.actbio.2013.12.050
Heo MB, 2014, BIOMATERIALS, V35, P590, DOI 10.1016/j.biomaterials.2013.10.009
Hirosue S, 2010, VACCINE, V28, P7897, DOI 10.1016/j.vaccine.2010.09.077
Hoare TR, 2008, POLYMER, V49, P1993, DOI 10.1016/j.polymer.2008.01.027
Hobo W, 2013, CANCER IMMUNOL IMMUN, V62, P285, DOI 10.1007/s00262-012-1334-1
Hodi FS, 2010, NEW ENGL J MED, V363, P711, DOI 10.1056/NEJMoa1003466
Hu QL, 2015, NANO LETT, V15, P2732, DOI 10.1021/acs.nanolett.5b00570
Huang F, 2017, CANCER IMMUNOL IMMUN, V66, P1321, DOI 10.1007/s00262-017-2028-5
Huang XF, 2003, CANCER RES, V63, P7321
Huang XH, 2007, NANOMEDICINE-UK, V2, P681, DOI 10.2217/17435889.2.5.681
Huang YH, 2013, CANCER RES, V73, P2943, DOI 10.1158/0008-5472.CAN-12-4354
Huo MR, 2017, J CONTROL RELEASE, V245, P81, DOI 10.1016/j.jconrel.2016.11.013
Jadidi-Niaragh F, 2017, J CONTROL RELEASE, V246, P46, DOI 10.1016/j.jconrel.2016.12.012
Jeanbart L, 2015, CANCER IMMUNOL IMMUN, V64, P1033, DOI 10.1007/s00262-015-1702-8
Jokerst JV, 2011, NANOMEDICINE-UK, V6, P715, DOI [10.2217/NNM.11.19, 10.2217/nnm.11.19]

Jones MC, 1999, EUR J PHARM BIOPHARM, V48, P101, DOI 10.1016/S0939-6411(99)00039-9
Juliano R, 2009, MOL PHARMACEUT, V6, P686, DOI 10.1021/mp900093r
Kabilova TO, 2016, PLOS ONE, V11, DOI 10.1371/journal.pone.0150751
Kallen Karl-Josef, 2014, Ther Adv Vaccines, V2, P10, DOI 10.1177/2051013613508729
Kang HZ, 2011, LANGMUIR, V27, P399, DOI 10.1021/la1037553
Kaufman HL, 2004, J CLIN INVEST, V113, P664, DOI 10.1172/JCI200421148
Kennedy LC, 2011, SMALL, V7, P169, DOI 10.1002/smll.201000134
Kim ES, 2017, DRUGS, V77, P929, DOI 10.1007/s40265-017-0749-6
Kim J, 2015, NAT BIOTECHNOL, V33, P64, DOI 10.1038/nbt.3071
Kim TH, 2006, J NANOSCI NANOTECHNO, V6, P2796, DOI 10.1166/jnn.2006.434
Kitano S, 2006, CLIN CANCER RES, V12, P7397, DOI 10.1158/1078-0432.CCR-06-1546
Knutson KL, 2015, CANCER METAST REV, V34, P53, DOI 10.1007/s10555-014-9540-2
Ko JS, 2009, CLIN CANCER RES, V15, P2148, DOI 10.1158/1078-0432.CCR-08-1332
Kokate RA, 2016, NANOMEDICINE-UK, V11, P479, DOI 10.2217/nnm.15.213
Kong M, 2017, THERANOSTICS, V7, P3276, DOI 10.7150/thno.19987
Kostarelos K, 2008, NAT BIOTECHNOL, V26, P774, DOI 10.1038/nbt0708-774
Kranz LM, 2016, NATURE, V534, P396, DOI 10.1038/nature18300
Krishnamachari Y, 2011, PHARM RES-DORDR, V28, P215, DOI 10.1007/s11095-010-0241-4
Krishnan L, 2004, J DRUG TARGET, V11, P515, DOI 10.1080/10611860410001670044
Krishnan L, 2001, J IMMUNOL, V166, P1885, DOI 10.4049/jimmunol.166.3.1885
Krishnan L, 2008, VACCINE, V26, P2043, DOI 10.1016/j.vaccine.2008.02.026
Kuai R, 2018, BIOCONJUGATE CHEM, V29, P771, DOI 10.1021/acs.bioconjchem.7b00761
Kuai R, 2017, NAT MATER, V16, P489, DOI [10.1038/nmat4822, 10.1038/NMAT4822]
Kwong B, 2013, CANCER RES, V73, P1547, DOI 10.1158/0008-5472.CAN-12-3343
Kyi C, 2014, FEBS LETT, V588, P368, DOI 10.1016/j.febslet.2013.10.015
Lai CH, 2014, J BIOMED NANOTECHNOL, V10, P3576, DOI 10.1166/jbn.2014.2051
Lee CS, 2013, BRIT J CLIN PHARMACO, V76, P233, DOI 10.1111/bcp.12164
Li P, 2013, J CONTROL RELEASE, V168, P271, DOI 10.1016/j.jconrel.2013.03.025
Li SY, 2016, J CONTROL RELEASE, V231, P17, DOI 10.1016/j.jconrel.2016.01.044
Li XX, 2016, ACS CHEM BIOL, V11, P1205, DOI 10.1021/acschembio.6b00173
Liang RJ, 2017, BIOMATERIALS, V149, P41, DOI 10.1016/j.biomaterials.2017.09.029
Liang SY, 2016, INT J NANOMED, V11, P6753, DOI 10.2147/IJN.S122047
Lin AY, 2013, NANOSCALE RES LETT, V8, DOI 10.1186/1556-276X-8-72
Liu H., 2018, ONCOTARGET, V9, P2897
Liu HY, 2017, CANCER LETT, V411, P182, DOI 10.1016/j.canlet.2017.09.022
Liu LN, 2018, MOL THER, V26, P45, DOI 10.1016/j.ymthe.2017.10.020
Liu YJ, 2017, J DRUG TARGET, V25, P275, DOI 10.1080/1061186X.2016.1258569
Liu ZD, 2017, CELL DISCOV, V3, DOI 10.1038/celldisc.2017.7
Lu JQ, 2017, NAT COMMUN, V8, DOI 10.1038/s41467-017-01651-9
Luo ZC, 2015, BIOMATERIALS, V38, P50, DOI 10.1016/j.biomaterials.2014.10.050
Luo ZC, 2013, J CONTROL RELEASE, V170, P259, DOI 10.1016/j.jconrel.2013.05.027
Rowdo FPM, 2015, FRONT IMMUNOL, V6, P1, DOI 10.3389/fimmu.2015.00127
Maurer N, 2001, BIOPHYS J, V80, P2310, DOI 10.1016/S0006-3495(01)76202-9
McNamara MA, 2015, J IMMUNOL RES, V2015, DOI 10.1155/2015/794528
Menay F, 2017, FRONT IMMUNOL, V8, DOI 10.3389/fimmu.2017.00286
Meng J, 2008, SMALL, V4, P1364, DOI 10.1002/smll.200701059
Min YZ, 2017, NAT NANOTECHNOL, V12, P877, DOI [10.1038/nnano.2017.113, 10.1038/NNANO.2017.113]
Miyabe H, 2014, J CONTROL RELEASE, V184, P20, DOI 10.1016/j.jconrel.2014.04.004
Moore C, 2017, INT J CANCER, V141, P428, DOI 10.1002/ijc.30672
Moron G, 2004, TRENDS IMMUNOL, V25, P92, DOI 10.1016/j.it.2003.11.008
Muddineti OS, 2017, EXPERT OPIN DRUG DEL, V14, P715, DOI 10.1080/17425247.2016.1229300
Mukaida N, 2018, WORLD J GASTROENTERO, V24, P1839, DOI 10.3748/wjg.v24.i17.1839
Munich S, 2012, ONCOIMMUNOLOGY, V1, P1074, DOI 10.4161/onci.20897
Musolino C, 2017, MEDIAT INFLAMM, V2017, DOI 10.1155/2017/1852517
Nabel GJ, 1996, P NATL ACAD SCI USA, V93, P15388, DOI 10.1073/pnas.93.26.15388
Nefedova Y, 2004, J IMMUNOL, V172, P464, DOI 10.4049/jimmunol.172.1.464
Olejniczak M, 2012, RNA, V18, P930, DOI 10.1261/rna.025627.110
Oliveira CL, 2017, COLLOID SURFACE B, V150, P326, DOI 10.1016/j.colsurfb.2016.10.046
Ou W, 2018, J CONTROL RELEASE, V281, P84, DOI 10.1016/j.jconrel.2018.05.018
Ozao-Choy J, 2009, CANCER RES, V69, P2514, DOI 10.1158/0008-5472.CAN-08-4709

Pardi N, 2017, NATURE, V543, P248, DOI 10.1038/nature21428
PARDOLL DM, 1995, ANNU REV IMMUNOL, V13, P399, DOI 10.1146/annurev.iy.13.040195.002151
Park J, 2012, NAT MATER, V11, P895, DOI [10.1038/NMAT3355, 10.1038/nmat3355]
Pashine A, 2005, NAT MED, V11, pS63, DOI 10.1038/nm1210
Paudel KS, 2010, THER DELIV, V1, P109, DOI 10.4155/TDE.10.16
Paulis LE, 2013, CURR OPIN IMMUNOL, V25, P389, DOI 10.1016/j.coi.2013.03.001
Persano S, 2017, BIOMATERIALS, V125, P81, DOI 10.1016/j.biomaterials.2017.02.019
Phan GQ, 2003, P NATL ACAD SCI USA, V100, P8372, DOI 10.1073/pnas.1533209100
Popova TG, 2015, NANOMATERIALS-BASEL, V5, P298, DOI 10.3390/nano5010298
Qian Y, 2017, ACS NANO, V11, P9536, DOI 10.1021/acsnano.7b05465
Rahimian S, 2015, BIOMATERIALS, V37, P469, DOI 10.1016/j.biomaterials.2014.10.043
Rai R, 2005, J SURG RES, V129, P147, DOI 10.1016/j.jss.2005.03.020
Rech AJ, 2012, SCI TRANSL MED, V4, DOI 10.1126/scitranslmed.3003330
Richner JM, 2017, CELL, V168, P1114, DOI 10.1016/j.cell.2017.02.017
Robert C, 2015, NEW ENGL J MED, V372, P2521, DOI 10.1056/NEJMoa1503093
Robert C, 2011, NEW ENGL J MED, V364, P2517, DOI 10.1056/NEJMoa1104621
Rosalia RA, 2015, BIOMATERIALS, V40, P88, DOI 10.1016/j.biomaterials.2014.10.053
Rosenberg SA, 2004, NAT MED, V10, P909, DOI 10.1038/nm1100
Sacchetti C, 2013, BIOCONJUGATE CHEM, V24, P852, DOI 10.1021/bc400070q
Saleem IY, 2005, J CONTROL RELEASE, V102, P551, DOI 10.1016/j.jconrel.2004.10.034
Sarkar S, 2013, BIOCONJUGATE CHEM, V24, P363, DOI 10.1021/bc300422a
Sarnaik AA, 2009, CANCER J, V15, P169, DOI 10.1097/PPO.0b013e3181a7450f
Sauter B, 2000, J EXP MED, V191, P423, DOI 10.1084/jem.191.3.423
Sayour EJ, 2017, ONCOIMMUNOLOGY, V6, DOI 10.1080/2162402X.2016.1256527
Schmid D, 2017, NAT COMMUN, V8, DOI 10.1038/s41467-017-01830-8
Semple SC, 2000, METHOD ENZYMOL, V313, P322
Seth A, 2017, ONCOTARGET, V8, P5371, DOI 10.18632/oncotarget.14260
Shahsavari Sh, 2014, Drug Res (Stuttg), V64, P151, DOI 10.1055/s-0033-1354372
SHAHUM E, 1995, INT J IMMUNOPHARMACO, V17, P9, DOI 10.1016/0192-0561(94)00082-Y
Sharma HS, 2007, PROG BRAIN RES, V162, P245, DOI 10.1016/S0079-6123(06)62013-X
Shi Y, 2000, P NATL ACAD SCI USA, V97, P14590, DOI 10.1073/pnas.260497597
Shimizu T, 2008, BIOCHEM BIOPH RES CO, V367, P330, DOI 10.1016/j.bbrc.2007.12.112
Shitara K, 2018, ANN NY ACAD SCI, V1417, P104, DOI 10.1111/nyas.13625
Shukla V, 2017, NANOMED-NANOTECHNOL, V13, P1833, DOI 10.1016/j.nano.2017.03.004
Silva JM, 2015, J CONTROL RELEASE, V198, P91, DOI 10.1016/j.jconrel.2014.11.033
Silva JM, 2013, J CONTROL RELEASE, V168, P179, DOI 10.1016/j.jconrel.2013.03.010
Simhadri VR, 2008, PLOS ONE, V3, DOI 10.1371/journal.pone.0003377
Sirkka T., 2016, WOUND PH DEPENDS ACT
Slingluff CL, 2006, CLIN CANCER RES, V12, p2342S, DOI 10.1158/1078-0432.CCR-05-2541
Speiser DE, 2010, J IMMUNOTHER, V33, P848, DOI 10.1097/CJI.0b013e3181f1d614
Suzuki H, 2010, EUR J IMMUNOL, V40, P197, DOI 10.1002/eji.200939887
Syed YY, 2017, DRUGS, V77, P1369, DOI 10.1007/s40265-017-0782-5
Tang WL, 2018, DRUG DISCOV TODAY, V23, P1159, DOI 10.1016/j.drudis.2018.04.007
Tang WL, 2018, BIOMATERIALS, V166, P13, DOI 10.1016/j.biomaterials.2018.03.004
Tang WL, 2017, MOL PHARMACEUT, V14, P1969, DOI 10.1021/acs.molpharmaceut.7b00077
Tang WL, 2016, PHARM RES-DORDR, V33, P1104, DOI 10.1007/s11095-015-1854-4
Teo PY, 2015, ADV HEALTHC MATER, V4, P1180, DOI 10.1002/adhm.201500089
Terme M, 2013, CANCER RES, V73, P539, DOI 10.1158/0008-5472.CAN-12-2325
Tran TH, 2015, CLIN IMMUNOL, V160, P46, DOI 10.1016/j.clim.2015.03.021
Thery C, 2001, J IMMUNOL, V166, P7309, DOI 10.4049/jimmunol.166.12.7309
Thery C, 1999, J CELL BIOL, V147, P599, DOI 10.1083/jcb.147.3.599
Thery C, 2009, NAT REV IMMUNOL, V9, P581, DOI 10.1038/nri2567
Thibaudau F, 2012, J PHYS CHEM LETT, V3, P902, DOI 10.1021/jz3001213
Thomann-Harwood LJ, 2013, J CONTROL RELEASE, V166, P95, DOI 10.1016/j.jconrel.2012.11.015
Topalian SL, 2012, NEW ENGL J MED, V366, P2443, DOI 10.1056/NEJMoa1200690
Toy R, 2016, BIOENG TRANSL MED, V1, P47, DOI 10.1002/btm2.10005
Tsai YS, 2013, SMALL, V9, P2119, DOI 10.1002/smll.201202755
Utsugi-Kobukai S, 2003, IMMUNOL LETT, V89, P125, DOI 10.1016/S0165-2478(03)00128-7
van Broekhoven CL, 2004, CANCER RES, V64, P4357, DOI 10.1158/0008-5472.CAN-04-0138
Van Woensel M, 2017, SCI REP-UK, V7, DOI 10.1038/s41598-017-01279-1
Vangasseri DP, 2006, MOL MEMBR BIOL, V23, P385, DOI 10.1080/09687860600790537

Viaud S, 2009, PLOS ONE, V4, DOI 10.1371/journal.pone.0004942
von Maltzahn G, 2009, CANCER RES, V69, P3892, DOI 10.1158/0008-5472.CAN-08-4242
Voshavar C, 2017, J MED CHEM, V60, P1605, DOI 10.1021/acs.jmedchem.6b01556
Waeckerle-Men Y, 2005, ADV DRUG DELIVER REV, V57, P475, DOI 10.1016/j.addr.2004.09.007
Wang C, 2016, NANO LETT, V16, P2334, DOI 10.1021/acs.nanolett.5b05030
Wang DG, 2016, NANO LETT, V16, P5503, DOI 10.1021/acs.nanolett.6b01994
Watson DS, 2012, VACCINE, V30, P2256, DOI 10.1016/j.vaccine.2012.01.070
Weiner LM, 2009, LANCET, V373, P1033, DOI 10.1016/S0140-6736(09)60251-8
Widmer J, 2018, INT J PHARMACEUT, V535, P444, DOI 10.1016/j.ijpharm.2017.11.031
Wilson JT, 2013, ACS NANO, V7, P3912, DOI 10.1021/nn305466z
Wolfram J, 2015, CURR DRUG TARGETS, V16, P1671, DOI 10.2174/1389450115666140804124808
Xin H, 2009, CANCER RES, V69, P2506, DOI 10.1158/0008-5472.CAN-08-4323
Xiong H, 2008, NEOPLASIA, V10, P287, DOI 10.1593/neo.07971
Xu J, 2017, ACS NANO, V11, P4463, DOI 10.1021/acsnano.7b00715
Xu ZH, 2013, J CONTROL RELEASE, V172, P259, DOI 10.1016/j.jconrel.2013.08.021
Yamaguchi T, 2007, IMMUNITY, V27, P145, DOI 10.1016/j.immuni.2007.04.017
Yamano T, 2007, CANCER GENE THER, V14, P451, DOI 10.1038/sj.cgt.7701035
Yan WL, 2008, J CONTROL RELEASE, V130, P22, DOI 10.1016/j.jconrel.2008.05.005
Yan WL, 2007, MOL IMMUNOL, V44, P3672, DOI 10.1016/j.molimm.2007.04.009
Yang Chuan He, 2010, Int J Interferon Cytokine Mediat Res, V2010, P1
Yang JP, 1998, GENE THER, V5, P380, DOI 10.1038/sj.gt.3300596
Yang M, 2012, THERANOSTICS, V2, P258, DOI 10.7150/thno.3629
Yang R, 2018, ACS NANO, V12, P5121, DOI 10.1021/acsnano.7b09041
Yao WJ, 2013, MOL PHARMACEUT, V10, P2904, DOI 10.1021/mp4000053
Yata T, 2017, BIOMATERIALS, V146, P136, DOI 10.1016/j.biomaterials.2017.09.014
Yen HJ, 2009, SMALL, V5, P1553, DOI 10.1002/smll.200900126
Yuba E, 2018, MOL IMMUNOL, V98, P8, DOI 10.1016/j.molimm.2017.11.001
Yuba E, 2017, BIOMATERIALS, V120, P32, DOI 10.1016/j.biomaterials.2016.12.021
Yuba E, 2015, BIOMATERIALS, V67, P214, DOI 10.1016/j.biomaterials.2015.07.031
Yuba E, 2014, BIOMATERIALS, V35, P3091, DOI 10.1016/j.biomaterials.2013.12.024
Yuba E, 2011, J CONTROL RELEASE, V149, P72, DOI 10.1016/j.jconrel.2010.03.001
Yuba E, 2010, BIOMATERIALS, V31, P943, DOI 10.1016/j.biomaterials.2009.10.006
Zang XL, 2017, EUR J PHARM BIOPHARM, V115, P243, DOI 10.1016/j.ejpb.2017.03.013
Zhang B, 2010, CANCER RES, V70, P6407, DOI 10.1158/0008-5472.CAN-10-1544
Zhang LX, 2003, P NATL ACAD SCI USA, V100, P15101, DOI 10.1073/pnas.2135379100
Zhang PP, 2015, ACS NANO, V9, P6465, DOI 10.1021/acsnano.5b02153
Zhang XY, 2015, CELL BIOCHEM BIOPHYS, V72, P771, DOI 10.1007/s12013-015-0529-4
Zhang ZP, 2011, BIOMATERIALS, V32, P3666, DOI 10.1016/j.biomaterials.2011.01.067
Zhou WZ, 1999, GENE THER, V6, P1768, DOI 10.1038/sj.gt.3300998
Zhu GZ, 2017, NAT COMMUN, V8, DOI 10.1038/s41467-017-01386-7
Zhu SJ, 2013, MOL PHARMACEUT, V10, P3525, DOI 10.1021/mp400216r
NR 246
TC 49
Z9 52
U1 3
U2 168
PU ELSEVIER
PI AMSTERDAM
PA RADARWEG 29, 1043 NX AMSTERDAM, NETHERLANDS
SN 0168-3659
EI 1873-4995
J9 J CONTROL RELEASE
JI J. Control. Release
PD OCT 28
PY 2018
VL 288
BP 239
EP 263
DI 10.1016/j.jconrel.2018.09.010
PG 25
WC Chemistry, Multidisciplinary; Pharmacology & Pharmacy

WE Science Citation Index Expanded (SCI-EXPANDED)
SC Chemistry; Pharmacology & Pharmacy
GA GV6QF
UT WOS:000446237700019
PM 30223043
DA 2023-09-14
ER

PT J
AU Qiao, DD
Liu, LX
Chen, Y
Xue, CB
Gao, Q
Mao, HQ
Leong, KW
Chen, YM
AF Qiao, Dongdong
Liu, Lixin
Chen, Yi
Xue, Chenbao
Gao, Qiang
Mao, Hai-Quan
Leong, Kam W.
Chen, Yongming
TI Potency of a Scalable Nanoparticulate Subunit Vaccine
SO NANO LETTERS
LA English
DT Article
DE Adjuvant; codelivery; cross-presentation; lymph node targeting; subunit nanovaccine
ID ENTEROVIRUS 71 VACCINE; IMMUNE-RESPONSES; CAPSID PROTEIN; TNF-ALPHA; DELIVERY; ADJUVANT; CHITOSAN; SIZE; IMMUNOGENICITY; MICROPARTICLES
AB Nanoparticulate vaccines can potentiate immune responses by site-specific drainage to lymph nodes (LNs). This approach may benefit from a nanoparticle engineering method with fine control over size and codelivery of antigen and adjuvant. Here, we applied the flash nanocomplexation (FNC) method to prepare nanovaccines via polyelectrolyte complexation of chitosan and heparin to coencapsulate the VP I protein antigen from enterovirus 71, which causes hand-foot-mouth disease (HFMD), with tumor necrosis factor a (TNF) or CpG as adjuvants. FNC allows for reduction of the nanovaccine size to range from 90 to 130 nm with relatively narrower size distribution and a high payload capacity. These nanovaccines reached both proximal and distal LNs via subcutaneous injection and subsequently exhibited prolonged retention in the LNs. The codelivery induced strong immune activation toward a Th1 response in addition to a potent Th2 response, and conferred effective protection against lethal virus challenge comparable to that of an approved inactivated viral vaccine in mouse models of both passive and active immunization setting. In addition, these nanovaccines also elicited strong IgA titers, which may offer unique advantages for mucosal protection. This study addresses the issues of size control, antigen bioactivity retention, and biomanufacturing to demonstrate the translational potential of a subunit nanovaccine design.
C1 [Qiao, Dongdong; Liu, Lixin; Chen, Yi; Mao, Hai-Quan; Leong, Kam W.; Chen, Yongming] Sun Yat Sen Univ, Ctr Funct Biomat, Sch Mat Sci & Engn, Key Lab Polymer Composite & Funct Mat,Minist Educ, Guangzhou 510275, Guangdong, Peoples R China.
[Xue, Chenbao; Gao, Qiang] Sinovac Biotech Co Ltd, 39 Shangdi Xi Rd, Beijing 100085, Peoples R China.
[Mao, Hai-Quan] Johns Hopkins Univ, Dept Mat Sci & Engn, Baltimore, MD 21218 USA.
[Mao, Hai-Quan] Johns Hopkins Univ, Inst NanoBioTechnol, Baltimore, MD 21218 USA.
[Leong, Kam W.] Columbia Univ, Dept Biomed Engn, New York, NY 10027 USA.
C3 Sun Yat Sen University; Johns Hopkins University; Johns Hopkins University; Columbia University
RP Liu, LX; Mao, HQ; Leong, KW (通讯作者)，Sun Yat Sen Univ, Ctr Funct Biomat, Sch Mat Sci & Engn, Key Lab Polymer Composite & Funct Mat,Minist Educ, Guangzhou 510275, Guangdong, Peoples R China.; Mao, HQ (通讯作者)，Johns Hopkins Univ, Dept Mat Sci & Engn, Baltimore, MD 21218 USA.; Mao, HQ (通

讯作者)，Johns Hopkins Univ, Inst NanoBioTechnol, Baltimore, MD 21218 USA.; Leong, KW (通讯作者)，Columbia Univ, Dept Biomed Engn, New York, NY 10027 USA.
RI Chen, Yongming/E-3656-2015; Leong, Kam W/O-9302-2019; Mao, Hai-Quan/A-3350-2010
OI Chen, Yongming/0000-0003-2843-5543; Leong, Kam W/0000-0002-8133-4955; Mao, Hai-Quan/0000-0002-4262-9988
FU Guangdong Innovative and Entrepreneurial Research Team Program [2013S086]; National Natural Science Foundation of China [51533009]; Natural Science Foundation of Guangdong Province [2014A030312018]; NIH [AI096305, AI133533, AI114609]
FX We thank Dr. Yan Su for critical reading of the manuscript and helpful discussions. This study is supported by the Guangdong Innovative and Entrepreneurial Research Team Program (2013S086), the National Natural Science Foundation of China (51533009), the Natural Science Foundation of Guangdong Province (2014A030312018). Partial support from NIH grants AI096305, AI133533, and AI114609 is also acknowledged.
CR Akira S, 2001, NAT IMMUNOL, V2, P675, DOI 10.1038/90609
Aswathyraj S, 2016, MED MICROBIOL IMMUN, V205, P397, DOI 10.1007/s00430-016-0465-y
Bachmann MF, 2010, NAT REV IMMUNOL, V10, P787, DOI 10.1038/nri2868
Banchereau J, 2000, ANNU REV IMMUNOL, V18, P767, DOI 10.1146/annurev.immunol.18.1.767
Benne N, 2016, J CONTROL RELEASE, V234, P124, DOI 10.1016/j.jconrel.2016.05.033
Bobbala S, 2016, PHARM RES-DORDR, V33, P2078, DOI 10.1007/s11095-016-1979-0
BRUNDA MJ, 1993, J EXP MED, V178, P1223, DOI 10.1084/jem.178.4.1223
Carreno BM, 2013, J CLIN INVEST, V123, P3383, DOI 10.1172/JCI68395
Carroll EC, 2016, IMMUNITY, V44, P597, DOI 10.1016/j.immuni.2016.02.004
Chen XM, 2014, MOL PHARMACEUT, V11, P1772, DOI 10.1021/mp400597z
Chua BY, 2012, MOL PHARMACEUT, V9, P81, DOI 10.1021/mp200264m
De Temmerman ML, 2011, DRUG DISCOV TODAY, V16, P569, DOI 10.1016/j.drudis.2011.04.006
Fan YC, 2017, WIRES NANOMED NANOBI, V9, DOI 10.1002/wnan.1403
FARIA PCB, 2014, NANO LETT, V14, P5458
Gause KT, 2017, ACS NANO, V11, P54, DOI 10.1021/acsnano.6b07343
Gupta R K, 1995, Pharm Biotechnol, V6, P229
Gupta RK, 1998, ADV DRUG DELIVER REV, V32, P155, DOI 10.1016/S0169-409X(98)00008-8
Gustafsson K, 2008, CANCER RES, V68, P5965, DOI 10.1158/0008-5472.CAN-07-6494
Gutjahr A, 2016, VACCINES-BASEL, V4, DOI 10.3390/vaccines4040034
He ZY, 2017, BIOMATERIALS, V130, P28, DOI 10.1016/j.biomaterials.2017.03.028
Hemmi H, 2000, NATURE, V408, P740, DOI 10.1038/35047123
John ALS, 2012, NAT MATER, V11, P250, DOI [10.1038/NMAT3222, 10.1038/nmat3222]
Jorquera PA, 2016, VACCINES-BASEL, V4, DOI 10.3390/vaccines4040045
Klinman Dennis M, 2003, Expert Rev Vaccines, V2, P305, DOI 10.1586/14760584.2.2.305
Kolpe AB, 2012, VIRUS RES, V168, P64, DOI 10.1016/j.virusres.2012.06.014
Kool M, 2012, J MED MICROBIOL, V61, P927, DOI 10.1099/jmm.0.038943-0
Lee LF, 2005, P NATL ACAD SCI USA, V102, P15995, DOI 10.1073/pnas.0508122102
Liang ZL, 2014, CLIN TRANSL IMMUNOL, V3, DOI 10.1038/cti.2014.24
Liu Q, 2016, SMALL, V12, P1744, DOI 10.1002/smll.201503662
Luo Z., 2017, ADV MATER, V29, P1601776
Manolova V, 2008, EUR J IMMUNOL, V38, P1404, DOI 10.1002/eji.200737984
Mant A, 2012, IMMUNOLOGY, V136, P163, DOI 10.1111/j.1365-2567.2012.03558.x
Moon JJ, 2012, ADV MATER, V24, P3724, DOI 10.1002/adma.201200446
Moon JJ, 2011, NAT MATER, V10, P243, DOI 10.1038/NMAT2960
Mulloy B, 2006, BIOCHEM SOC T, V34, P409, DOI 10.1042/BST0340409
NISHIMURA K, 1985, VACCINE, V3, P379, DOI 10.1016/0264-410X(85)90127-6
Oberste MS, 1999, J VIROL, V73, P1941, DOI 10.1128/JVI.73.3.1941-1948.1999
Oleszycka E, 2014, CURR OPIN IMMUNOL, V28, P1, DOI 10.1016/j.coi.2013.12.007
Rabenstein DL, 2002, NAT PROD REP, V19, P312, DOI 10.1039/b100916h
Rao DA, 2010, J PHARM SCI-US, V99, P2018, DOI 10.1002/jps.21970
Reddy ST, 2007, NAT BIOTECHNOL, V25, P1159, DOI [10.1038/nbt1332, 10.1038/nbtl332]
Reed Z, 2016, VACCINE, V34, P2967, DOI 10.1016/j.vaccine.2016.02.077
Riteau N, 2016, IMMUNITY, V44, P522, DOI 10.1016/j.immuni.2016.03.002
Scheiermann J, 2014, VACCINE, V32, P6377, DOI 10.1016/j.vaccine.2014.06.065
Schmidt ST, 2016, PHARMACEUTICS, V8, DOI 10.3390/pharmaceutics8010007

Silva AL, 2016, HUM VACC IMMUNOTHER, V12, P1056, DOI 10.1080/21645515.2015.1117714
Smith DM, 2013, NAT REV IMMUNOL, V13, P592, DOI 10.1038/nri3488
Sui ZW, 2010, ARCH VIROL, V155, P535, DOI 10.1007/s00705-010-0621-4
Swartz MA, 2012, SCI TRANSL MED, V4, DOI 10.1126/scitranslmed.3003763
Tan CS, 2007, ARCH VIROL, V152, P1069, DOI 10.1007/s00705-007-0941-1
Trevejo JM, 2001, P NATL ACAD SCI USA, V98, P12162, DOI 10.1073/pnas.211423598
Wilson JT, 2013, ACS NANO, V7, P3912, DOI 10.1021/nn305466z
Xue L, 2017, BIOCHEM ENG J, V117, P139, DOI 10.1016/j.bej.2016.10.009
Yi EJ, 2017, CLIN EXP VACCINE RES, V6, P4, DOI [10.7774/cevr.2017.6.1.4, 10.7774/cevr.2017.6.1.4.]
Yuan H, 2017, VIROL J, V14, DOI 10.1186/s12985-017-0897-z
Yue ZG, 2011, BIOMACROMOLECULES, V12, P2440, DOI 10.1021/bm101482r
Zhu FC, 2014, NEW ENGL J MED, V370, P818, DOI 10.1056/NEJMoa1304923
NR 58
TC 49
Z9 52
U1 6
U2 142
PU AMER CHEMICAL SOC
PI WASHINGTON
PA 1155 16TH ST, NW, WASHINGTON, DC 20036 USA
SN 1530-6984
EI 1530-6992
J9 NANO LETT
JI Nano Lett.
PD MAY
PY 2018
VL 18
IS 5
BP 3007
EP 3016
DI 10.1021/acs.nanolett.8b00478
PG 10
WC Chemistry, Multidisciplinary; Chemistry, Physical; Nanoscience &
Nanotechnology; Materials Science, Multidisciplinary; Physics, Applied;
Physics, Condensed Matter
WE Science Citation Index Expanded (SCI-EXPANDED)
SC Chemistry; Science & Technology - Other Topics; Materials Science;
Physics
GA GF6QT
UT WOS:000432093200038
PM 29694053
DA 2023-09-14
ER

PT J
AU Zhou, SL
Huang, YK
Chen, Y
Liu, SS
Xu, MJ
Jiang, TZ
Song, QX
Jiang, G
Gu, X
Gao, XL
Chen, J
AF Zhou, Songlei
Huang, Yukun
Chen, Yu
Liu, Shanshan
Xu, Minjun

Jiang, Tianze
Song, Qingxiang
Jiang, Gan
Gu, Xiao
Gao, Xiaoling
Chen, Jun
TI Engineering ApoE3-incorporated biomimetic nanoparticle for efficient vaccine delivery to dendritic cells via macropinocytosis to enhance cancer immunotherapy
SO BIOMATERIALS
LA English
DT Article
DE ApoE3; Macropinocytosis; Immunotherapy; Vaccine; Biomimetic
ID MHC CLASS-I; SILICA NANOPARTICLE; CROSS-PRESENTATION; APOLIPOPROTEIN-E; IMMUNE-RESPONSE; CO-DELIVERY; ANTIGEN; ADJUVANT; TARGET; VIVO
AB Efficient delivery of vaccines to dendritic cells (DCs) is critical for inducing sufficient immune response and realizing effective cancer immunotherapy. In the past decade, researchers have spent tremendous effort in delivering vaccines by using nanoparticles. However, most of the present strategies are designed based on receptor-mediated endocytosis to increase nanovaccines uptake by DCs, and underestimate the role of macropinocytosis in taking up exogenous antigen. Here, we proposed that macropinocytosis, an efficient pathway for DCs to internalize extracellular fluid-phase solutes, might be utilized as a highly-effective approach to facilitate nanovaccines uptake in DCs. Accordingly, we designed a biomimetic nanovaccine (R837-alpha OVA-ApoE3-HNP), composing of a poly-(D, L-lactide-co-glycolide) (PLGA) core to encapsulate adjuvant imiquimod (R837), a phospholipid membrane to load antigen peptide (alpha OVA), and apolipoprotein E3 (ApoE3), to boost the internalization of antigens into DCs. The nanovaccine exhibited highly efficient cellular uptake into DCs through the macropinocytosis pathway, and significantly promoted DCs maturation and antigen presentation. After subcutaneous injection, the nanovaccine was efficiently drained to lymph nodes. Strong T cell immune responses including the generation of antigen-specific CD8(+) T cells, expansion of IFN-gamma(+) CD8(+) T cells and the secretion of IFN-gamma(+) were observed after the vaccination of R837-alpha OVA-ApoE3-HNP. It also efficiently inhibited the formation of tumor metastasis in lung as a prevention vaccine, and exerted superior therapeutic efficiency on B16-OVA tumor-bearing mice when in combination with alpha PD-1 therapy. Overall, our work demonstrated that by utilizing the macropinocytosis pathway, ApoE3-incorporated biomimetic nanoparticle has great potential to function as a feasible, effective, and safe nanovaccine for cancer immunotherapy.
C1 [Zhou, Songlei; Huang, Yukun; Chen, Yu; Liu, Shanshan; Xu, Minjun; Jiang, Tianze; Chen, Jun] Fudan Univ, Shanghai Pudong Hosp, Lane 826,Zhangheng Rd, Shanghai 201203, Peoples R China.
[Zhou, Songlei; Huang, Yukun; Chen, Yu; Liu, Shanshan; Xu, Minjun; Jiang, Tianze; Chen, Jun] Fudan Univ, Dept Pharmaceut, Sch Pharm, Lane 826,Zhangheng Rd, Shanghai 201203, Peoples R China.
[Song, Qingxiang; Jiang, Gan; Gu, Xiao; Gao, Xiaoling] Shanghai Jiao Tong Univ, Dept Pharmacol & Chem Biol, Sch Med, 280 South Chongqing Rd, Shanghai 200025, Peoples R China.
[Zhou, Songlei; Huang, Yukun; Chen, Yu; Liu, Shanshan; Xu, Minjun; Jiang, Tianze; Chen, Jun] Fudan Univ, Sch Pharm, Key Lab Smart Drug Delivery, Minist Educ, Shanghai, Peoples R China.
C3 Fudan University; Fudan University; Shanghai Jiao Tong University; Fudan University
RP Chen, J (通讯作者)，Fudan Univ, Shanghai Pudong Hosp, Lane 826,Zhangheng Rd, Shanghai 201203, Peoples R China.; Chen, J (通讯作者)，Fudan Univ, Dept Pharmaceut, Sch Pharm, Lane 826,Zhangheng Rd, Shanghai 201203, Peoples R China.; Gao, XL (通讯作者)，Shanghai Jiao Tong Univ, Dept Pharmacol & Chem Biol, Sch Med, 280 South Chongqing Rd, Shanghai 200025, Peoples R China.
EM shellygao1@sjtu.edu.cn; chenjun@fudan.edu.cn
RI Jiang, Gan/AAO-2173-2020; Gao, xiao ling/GQI-2790-2022; Jiang, Gan/J-6421-2017
OI Jiang, Gan/0000-0003-2011-741X; Jiang, Tianze/0000-0002-4303-8768; Liu, Shanshan/0000-0003-0533-933X
FU National Natural Science Foundation of China [81690263, 81673019, 81872498, 81573382, 81722043, 81973272]; Shanghai Science and Technology Committee [19410710100]; National Science and Technology Major Project [2018ZX09734005, 2017ZX09304016]; National Youth Talent Support Program; Shanghai Municipal Education Commission; Shanghai Education Development Foundation [15SG14]
FX This work was supported by National Natural Science Foundation of China

(No. 81690263, 81673019, 81872498, 81573382, 81722043, 81973272), grant from Shanghai Science and Technology Committee (19410710100), National Science and Technology Major Project (2018ZX09734005, 2017ZX09304016), National Youth Talent Support Program, and "Shu Guang" project supported by Shanghai Municipal Education Commission and Shanghai Education Development Foundation (15SG14).
CR Almeida JPM, 2015, SMALL, V11, P1453, DOI 10.1002/smll.201402179
An M, 2017, ACS APPL MATER INTER, V9, P23466, DOI 10.1021/acsami.7b06024
Bandyopadhyay A, 2011, BIOMATERIALS, V32, P3094, DOI 10.1016/j.biomaterials.2010.12.054
Basu S, 2001, IMMUNITY, V14, P303, DOI 10.1016/S1074-7613(01)00111-X
Buteau C, 2002, MAYO CLIN PROC, V77, P339, DOI 10.4065/77.4.339
Carmona-Ribeiro AM, 1999, COLLOID SURFACE A, V153, P355, DOI 10.1016/S0927-7757(98)00532-9
Chen Q, 2016, NAT COMMUN, V7, DOI 10.1038/ncomms13193
Conniot J, 2019, NAT NANOTECHNOL, V14, P891, DOI 10.1038/s41565-019-0512-0
Cruz LJ, 2014, J CONTROL RELEASE, V192, P209, DOI 10.1016/j.jconrel.2014.07.040
Finn OJ, 2018, NAT REV IMMUNOL, V18, P183, DOI 10.1038/nri.2017.140
Garrett WS, 2000, CELL, V102, P325, DOI 10.1016/S0092-8674(00)00038-6
Guo JW, 2011, BIOMATERIALS, V32, P8010, DOI 10.1016/j.biomaterials.2011.07.004
INABA K, 1992, J EXP MED, V176, P1693, DOI 10.1084/jem.176.6.1693
Irvine DJ, 2015, CHEM REV, V115, P11109, DOI 10.1021/acs.chemrev.5b00109
Irvine DJ, 2013, NAT MATER, V12, P978, DOI [10.1038/NMAT3775, 10.1038/nmat3775]
Jiang H, 2017, J CONTROL RELEASE, V267, P47, DOI 10.1016/j.jconrel.2017.08.009
Kang T, 2017, ACS NANO, V11, P1397, DOI 10.1021/acsnano.6b06477
Kaufman HL, 2015, NAT REV DRUG DISCOV, V14, P642, DOI 10.1038/nrd4663
Kim SY, 2017, BIOMATERIALS, V130, P56, DOI 10.1016/j.biomaterials.2017.03.034
Kirkwood JM, 2012, CA-CANCER J CLIN, V62, P309, DOI 10.3322/caac.20132
Koivusalo M, 2010, J CELL BIOL, V188, P547, DOI 10.1083/jcb.200908086
Kuai R, 2017, NAT MATER, V16, P489, DOI [10.1038/nmat4822, 10.1038/NMAT4822]
Lepenies B, 2013, ADV DRUG DELIVER REV, V65, P1271, DOI 10.1016/j.addr.2013.05.007
Li FF, 2017, NAT COMMUN, V8, DOI 10.1038/s41467-017-01565-6
Li HY, 2011, NAT NANOTECHNOL, V6, P645, DOI [10.1038/nnano.2011.153, 10.1038/NNANO.2011.153]
Liu Y, 2014, SMALL, V10, P4505, DOI 10.1002/smll.201401707
Liu YJ, 2019, ADV HEALTHC MATER, V8, DOI 10.1002/adhm.201900660
Luo M, 2017, NAT NANOTECHNOL, V12, P648, DOI [10.1038/nnano.2017.52, 10.1038/NNANO.2017.52]
Luo ZC, 2013, J CONTROL RELEASE, V170, P259, DOI 10.1016/j.jconrel.2013.05.027
Lynn GM, 2015, NAT BIOTECHNOL, V33, P1201, DOI 10.1038/nbt.3371
Melero I, 2014, NAT REV CLIN ONCOL, V11, P509, DOI 10.1038/nrclinonc.2014.111
Meng H, 2011, ACS NANO, V5, P4434, DOI 10.1021/nn103344k
Molino NM, 2016, BIOMATERIALS, V86, P83, DOI 10.1016/j.biomaterials.2016.01.056
Nakamura T, 2008, MOL THER, V16, P1507, DOI 10.1038/mt.2008.122
Nakamura T, 2014, MOL PHARMACEUT, V11, P2787, DOI 10.1021/mp500147y
Narayanaswami V, 2004, J BIOL CHEM, V279, P14273, DOI 10.1074/jbc.M313318200
Neefjes J, 2011, NAT REV IMMUNOL, V11, P823, DOI 10.1038/nri3084
Neek M, 2018, BIOMATERIALS, V156, P194, DOI 10.1016/j.biomaterials.2017.11.022
Niikura K, 2013, ACS NANO, V7, P3926, DOI 10.1021/nn3057005
Ning N, 2012, CANCER RES, V72, P1853, DOI 10.1158/0008-5472.CAN-11-1400
Norbury CC, 2006, IMMUNOLOGY, V117, P443, DOI 10.1111/j.1365-2567.2006.02335.x
Norbury CC, 1997, EUR J IMMUNOL, V27, P280, DOI 10.1002/eji.1830270141
O'Shea JJ, 2010, SCIENCE, V327, P1098, DOI 10.1126/science.1178334
Palucka K, 2013, IMMUNITY, V39, P38, DOI 10.1016/j.immuni.2013.07.004
Palucka K, 2012, NAT REV CANCER, V12, P265, DOI 10.1038/nrc3258
Passlick D, 2018, J CONTROL RELEASE, V289, P23, DOI 10.1016/j.jconrel.2018.09.008
Purwada A, 2016, ADV HEALTHC MATER, V5, P1413, DOI 10.1002/adhm.201501062
Rahimian S, 2015, J CONTROL RELEASE, V203, P16, DOI 10.1016/j.jconrel.2015.02.006
Rajora MA, 2017, CHEM SCI, V8, P5371, DOI 10.1039/c7sc00732a
Rosalia RA, 2015, BIOMATERIALS, V40, P88, DOI 10.1016/j.biomaterials.2014.10.053
Rosenberg SA, 2015, SCIENCE, V348, P62, DOI 10.1126/science.aaa4967
SALLUSTO F, 1995, J EXP MED, V182, P389, DOI 10.1084/jem.182.2.389
Savina A, 2007, IMMUNOL REV, V219, P143, DOI 10.1111/j.1600-065X.2007.00552.x

Sharma P, 2015, SCIENCE, V348, P56, DOI 10.1126/science.aaa8172
Shima F, 2013, BIOMATERIALS, V34, P9709, DOI 10.1016/j.biomaterials.2013.08.064
Skwarczynski M, 2011, CURR DRUG DELIV, V8, P282, DOI 10.2174/156720111795256192
Steinman RM, 2008, IMMUNITY, V29, P319, DOI 10.1016/j.immuni.2008.08.001
Torchilin VP, 2005, NAT REV DRUG DISCOV, V4, P145, DOI 10.1038/nrd1632
Trombetta ES, 2005, ANNU REV IMMUNOL, V23, P975, DOI 10.1146/annurev.immunol.22.012703.104538
Tumeh PC, 2014, NATURE, V515, P568, DOI 10.1038/nature13954
van den Elzen P, 2005, NATURE, V437, P906, DOI 10.1038/nature04001
Wang C, 2016, BIOMATERIALS, V79, P88, DOI 10.1016/j.biomaterials.2015.11.040
Wang H, 2018, NAT MATER, V17, P761, DOI 10.1038/s41563-018-0147-9
WESTERLUND JA, 1993, J BIOL CHEM, V268, P15745
Xiang J, 2015, ACS NANO, V9, P6401, DOI 10.1021/acsnano.5b02014
Xu J, 2019, BIOMATERIALS, V207, P1, DOI 10.1016/j.biomaterials.2019.03.037
Yang R, 2018, ACS NANO, V12, P5121, DOI 10.1021/acsnano.7b09041
Zupancic E, 2017, J CONTROL RELEASE, V258, P182, DOI 10.1016/j.jconrel.2017.05.014
NR 68
TC 48
Z9 50
U1 3
U2 135
PU ELSEVIER SCI LTD
PI OXFORD
PA THE BOULEVARD, LANGFORD LANE, KIDLINGTON, OXFORD OX5 1GB, OXON, ENGLAND
SN 0142-9612
EI 1878-5905
J9 BIOMATERIALS
JI Biomaterials
PD MAR
PY 2020
VL 235
AR 119795
DI 10.1016/j.biomaterials.2020.119795
PG 17
WC Engineering, Biomedical; Materials Science, Biomaterials
WE Science Citation Index Expanded (SCI-EXPANDED)
SC Engineering; Materials Science
GA KR8FP
UT WOS:000517851700012
PM 32014739
DA 2023-09-14
ER

PT J
AU Shevtsov, MA
Nikolaev, BP
Yakovleva, LY
Parr, MA
Marchenko, YY
Eliseev, I
Yudenko, A
Dobrodumov, AV
Zlobina, O
Zhakhov, A
Ischenko, AM
Pitkin, E
Multhoff, G
AF Shevtsov, Maxim A.
Nikolaev, Boris P.
Yakovleva, Liudmila Y.
Parr, Marina A.

Marchenko, Yaroslav Y.
Eliseev, Igor
Yudenko, Anna
Dobrodumov, Anatolii V.
Zlobina, Olga
Zhakhov, Alexander
Ischenko, Alexander M.
Pitkin, Emil
Multhoff, Gabriele
TI 70-kDa heat shock protein coated magnetic nanocarriers as a nanovaccine for induction of anti-tumor immune response in experimental glioma
SO JOURNAL OF CONTROLLED RELEASE
LA English
DT Article
DE Magnetic nanoparticles; Hsp70; Vaccine; Brain tumor; Immunotherapy
ID IRON-OXIDE NANOPARTICLES; DENDRITIC CELL; MOLECULAR CHAPERONES; SUBSTRATE-BINDING; ADVANCED MELANOMA; HSP70 CHAPERONE; PEPTIDE; HEAT-SHOCK-PROTEIN-70; IMMUNOTHERAPY; INNATE
AB Nanovaccines based on superparamagnetic iron oxide nanoparticles (SPIONs) provide a novel approach to induce the humoral and cell-based immune system to fight cancer. Herein, we increased the immunostimulatory capacity of SPIONs by coating them with recombinant heat shock protein 70 (Hsp70) which is known to chaperone antigenic peptides. After binding, Hsp70-SPIONs deliver immunogenic peptides from tumor lysates to dendriti? cells (DCs) and thus stimulate a tumor-specific, CD8+ cytotoxic T cell response. We could show that binding activity of Hsp70-SPIONs to the substrate-binding domain (SBD) is highly dependent on the ATPase activity of its nucleotide-binding domain NBD), as shown by P-31 NMR spectroscopy. Immunization of C6 glioma-bearing rats with DCs pulsed with Hsp70-SPIONs and tumor lysates resulted in a delayed tumor progression (as measured by MRI) and an increased overall survival. In parallel an increased IFN gamma secretion were detected in the serum of these animals and immunohistological analysis of subsequent cryosections of the glioma revealed an enhanced infiltration of memory CD45RO+ and cytotoxic CD8+ T cells. Taken together the study demonstrates that magnetic nanocarriers such as SPIONs coated with Hsp70 can be applied as a platform for boosting anti-cancer immune responses. (C) 2015 Elseviei B.V. All rights reserved.
C1 [Shevtsov, Maxim A.] Russian Acad Sci, Inst Cytol, St Petersburg 194064, Russia.
[Shevtsov, Maxim A.] IP Pavlov State Med Univ St Petersburg, St Petersburg 197022, Russia.
[Shevtsov, Maxim A.] AL Polenov Russian Res Sci Inst Neurosurg, St Petersburg 191014, Russia.
[Shevtsov, Maxim A.; Multhoff, Gabriele] Tech Univ Munich, Klinikum Rechts Isar, D-81675 Munich, Germany.
[Nikolaev, Boris P.; Yakovleva, Liudmila Y.; Marchenko, Yaroslav Y.; Zlobina, Olga; Zhakhov, Alexander; Ischenko, Alexander M.] Res Inst Highly Pure Biopreparat, St Petersburg 191014, Russia.
[Parr, Marina A.] St Petersburg State Univ, VF Fock Inst Phys, St Petersburg 199034, Russia.
[Eliseev, Igor; Yudenko, Anna] Russian Acad Sci, Acad Univ, St Petersburg 194021, Russia.
[Dobrodumov, Anatolii V.] Russian Acad Sci, Inst Macromol Cpds, St Petersburg 199004, Russia.
[Pitkin, Emil] Univ Penn, Wharton Sch, Philadelphia, PA 19104 USA.
C3 Russian Academy of Sciences; St. Petersburg Scientific Centre of the Russian Academy of Sciences; Institute of Cytology RAS; Technical University of Munich; Saint Petersburg State University; Russian Academy of Sciences; Russian Academy of Sciences; Institute of Macromolecular Compounds of the Russian Academy of Sciences; University of Pennsylvania
RP Shevtsov, MA (通讯作者)，Russian Acad Sci, Inst Cytol, Tikhoretsky Ave 4, St Petersburg 194064, Russia.
EM shevtsov-max@mail.ru
RI Parr, Marina/U-1269-2019; Multhoff, Gabriele/AAW-9796-2021; Yudenko, Anna/AAN-1903-2021; Shevtsov, Maxim/U-8830-2019; Eliseev, Igor E./E-2488-2014; Dobrodumov, Anatoliy V/I-9151-2017
OI Multhoff, Gabriele/0000-0002-2616-3137; Shevtsov, Maxim/0000-0002-8539-2239; Eliseev, Igor E./0000-0002-3344-2513; Dobrodumov, Anatoliy V/0000-0002-4554-6002; Marchenko, Yaroslav/0000-0002-7923-2175; Yudenko, Anna/0000-0002-3115-0015
FU Russian Foundation for Basic Research [15-08-08148A]; Russian Science Foundation [14-50-00068]; Federal Agency of Scientific Organizations, Russia; DFG in Germany [SFB824/2]; Russian Science Foundation

[14-50-00068] Funding Source: Russian Science Foundation
FX The authors thank Nan-Jong Lee for schematic presentation of
Hsp70-SPIONs; Olga G. Genbach for assistance in animal experiments. The
study was supported by a grant from the Russian Foundation for Basic
Research 15-08-08148A, animal experiments were supported in part by a
grant of the Russian Science Foundation 14-50-00068 and by the Federal
Agency of Scientific Organizations, Russia and by the DFG SFB824/2 in
Germany.
CR AL-Deen FMN, 2014, GENE THER, V21, P212, DOI 10.1038/gt.2013.77
[Anonymous], TUMOUR BIOL
Bertelsen EB, 2009, P NATL ACAD SCI USA, V106, P8471, DOI 10.1073/pnas.0903503106
Binder RJ, 2014, J IMMUNOL, V193, P5765, DOI 10.4049/jimmunol.1401417
BUCHBERGER A, 1995, J BIOL CHEM, V270, P16903, DOI 10.1074/jbc.270.28.16903
Campbell D, 2015, BREAST CANCER RES, V17, DOI 10.1186/s13058-015-0552-9
Candeias S.M., 2015, ANTICANCER AGENTS ME
CHEETHAM ME, 1994, EUR J BIOCHEM, V226, P99, DOI 10.1111/j.1432-1033.1994.tb20030.x
Dhodapkar MV, 2014, ONCOIMMUNOLOGY, V3, DOI 10.4161/21624011.2014.954832
Dunn GP, 2002, NAT IMMUNOL, V3, P991, DOI 10.1038/ni1102-991
Guo YY, 2015, ACS NANO, V9, P6918, DOI 10.1021/acsnano.5b01042
Guzhova IV, 2013, INT J HYPERTHER, V29, P399, DOI 10.3109/02656736.2013.807439
Hernandez-Gil J, 2015, ADV HEALTHC MATER, V4, P1034, DOI 10.1002/adhm.201500080
Hoang MD, 2015, J IMMUNOL RES, V2015, DOI 10.1155/2015/706379
Ito A, 2004, CANCER IMMUNOL IMMUN, V53, P26, DOI 10.1007/s00262-003-0416-5
Kim Y, 2015, PLOS ONE, V10, DOI 10.1371/journal.pone.0122183
Lee YT, 2015, INT J NANOMED, V10, P4491, DOI 10.2147/IJN.S83493
Li A, 2010, BIOMATERIALS, V31, P748, DOI 10.1016/j.biomaterials.2009.09.095
Lu FJ, 2015, J CONTROL RELEASE, V204, P51, DOI 10.1016/j.jconrel.2015.03.002
Mattoo RUH, 2014, CELL MOL LIFE SCI, V71, P3311, DOI 10.1007/s00018-014-1627-y
McNulty S, 2013, IMMUNOLOGY, V139, P407, DOI 10.1111/imm.12104
Misselwitz B, 1998, MOL CELL, V2, P593, DOI 10.1016/S1097-2765(00)80158-6
Multhoff G, 2009, INT J HYPERTHER, V25, P169, DOI 10.1080/02656730902902001
Nakai N, 2006, J DERMATOL, V33, P462, DOI 10.1111/j.1346-8138.2006.00110.x
Nikolaev BP, 2013, IEEE T MAGN, V49, P429, DOI 10.1109/TMAG.2012.2223203
Nishikawa M, 2010, MOL THER, V18, P421, DOI 10.1038/mt.2009.203
Paulis LE, 2013, CURR OPIN IMMUNOL, V25, P389, DOI 10.1016/j.coi.2013.03.001
Priya S, 2013, FEBS LETT, V587, P1981, DOI 10.1016/j.febslet.2013.05.014
Revington M, 2004, J BIOL CHEM, V279, P33958, DOI 10.1074/jbc.M313967200
Sato K, 2001, BLOOD, V98, P1852, DOI 10.1182/blood.V98.6.1852
Sehgal K, 2014, J IMMUNOL, V193, P2297, DOI 10.4049/jimmunol.1400489
Shevtsov MA, 2014, INT J CANCER, V135, P2118, DOI 10.1002/ijc.28858
Shevtsov MA, 2014, ONCOTARGET, V5, P3101, DOI 10.18632/oncotarget.1820
Shevtsov MA, 2014, NEURO-ONCOLOGY, V16, P38, DOI 10.1093/neuonc/not141
Srivastava P, 2002, NAT REV IMMUNOL, V2, P185, DOI 10.1038/nri749
Srivastava PK, 2009, EXPERT OPIN BIOL TH, V9, P179, DOI [10.1517/14712590802633918, 10.1517/14712590802633918 ]
Tamura Y, 2012, CURR MOL MED, V12, P1198, DOI 10.2174/156652412803306710
Tassa C, 2011, ACCOUNTS CHEM RES, V44, P842, DOI 10.1021/ar200084x
Theriault JR, 2005, FEBS LETT, V579, P1951, DOI 10.1016/j.febslet.2005.02.046
Toki S, 2013, NANOMED-NANOTECHNOL, V9, P1235, DOI 10.1016/j.nano.2013.05.010
Tosti G, 2014, EXPERT OPIN BIOL TH, V14, P955, DOI 10.1517/14712598.2014.902928
UDONO H, 1993, J EXP MED, V178, P1391, DOI 10.1084/jem.178.4.1391
Udono H, 2012, ACTA MED OKAYAMA, V66, P1
Yajima R., 2015, BREAST CANC
Zhang Z, 2015, CANCER GENE THER, V22, P198, DOI 10.1038/cgt.2015.7
Zhu RR, 2014, MAT SCI ENG C-MATER, V39, P305, DOI 10.1016/j.msec.2014.03.005
NR 46
TC 48
Z9 49
U1 1
U2 70
PU ELSEVIER SCIENCE BV

PI AMSTERDAM
PA PO BOX 211, 1000 AE AMSTERDAM, NETHERLANDS
SN 0168-3659
EI 1873-4995
J9 J CONTROL RELEASE
JI J. Control. Release
PD DEC 28
PY 2015
VL 220
BP 329
EP 340
DI 10.1016/j.jconrel.2015.10.051
PN A
PG 12
WC Chemistry, Multidisciplinary; Pharmacology & Pharmacy
WE Science Citation Index Expanded (SCI-EXPANDED)
SC Chemistry; Pharmacology & Pharmacy
GA CY0TN
UT WOS:000366119100036
PM 26522072
DA 2023-09-14
ER

PT J
AU Werengowska-Ciecwierz, K
   Wisniewski, M
   Terzyk, AP
   Furmaniak, S
AF Werengowska-Ciecwierz, Karolina
   Wisniewski, Marek
   Terzyk, Artur P.
   Furmaniak, Sylwester
TI The Chemistry of Bioconjugation in Nanoparticles-Based Drug Delivery
   System
SO ADVANCES IN CONDENSED MATTER PHYSICS
LA English
DT Review
ID WALLED CARBON NANOTUBES; POLY(L-GLUTAMIC ACID)-PACLITAXEL CONJUGATE;
   MESOPOROUS SILICA NANOPARTICLES; STERICALLY STABILIZED LIPOSOMES;
   PEPTIDE-BASED NANOPARTICLES; BREAST-CANCER CELLS; IN-VIVO; TARGETED
   DELIVERY; POLYMERIC MICELLES; PHASE-I
AB Nanomedicine is, generally, the application of nanotechnology to medicine. The term nanomedicine includes monitoring, construction of novel drug delivery systems, and any possible future applications of nanotechnology and nanovaccinology. In this review, the most important ligand-nanocarrier and drug-nanocarrier bioconjugations are described. The detailed characterizations of covalently formed bonds between targeted ligand and nanocarrier, including amide, thioether, disulfide, acetyl-hydrazone and polycyclic groups, are described. Also, the coupling of small elements and heteroatoms in the form of R-X-R the "click chemistry" groups is shown. Physical adsorption and chemical bonding of drug to nanocarrier surface involving drug on the internal or external surfaces of nanocarriers are described throughout possibility of the formation of the above-mentioned functionalities. Moreover, the most popular nanostructures (liposomes, micelles, polymeric nanoparticles, dendrimers, carbon nanotubes, and nanohorns) are characterized as nanocarriers. Building of modern drug carrier is a new method which could be effectively applied in targeted anticancer therapy.
C1 [Werengowska-Ciecwierz, Karolina; Wisniewski, Marek; Terzyk, Artur P.; Furmaniak, Sylwester] Nicholas Copernicus Univ, Physicochem Carbon Mat Res Grp, Fac Chem, PL-87100 Torun, Poland.
   [Wisniewski, Marek] INVEST TECH R&D Ctr, PL-87100 Torun, Poland.
C3 Nicolaus Copernicus University
RP Furmaniak, S (通讯作者)，Nicholas Copernicus Univ, Physicochem Carbon Mat Res Grp, Fac Chem, Gagarin St 7, PL-87100 Torun, Poland.
EM sf@chem.umk.pl
RI Furmaniak, Sylwester/E-8320-2014; Werengowska, Karolina/P-3351-2015;

Wisniewski, Marek/E-5503-2014; Furmaniak, Sylwester/AFT-5493-2022;
Terzyk, Artur P./M-6445-2014
OI Furmaniak, Sylwester/0000-0001-7443-9129; Wisniewski,
Marek/0000-0003-3478-5371; Terzyk, Artur/0000-0003-0622-1771
FU NSC [DEC-2011/01/B/ST5/01192]
FX The paper was supported by NSC Grant DEC-2011/01/B/ST5/01192.
CR Ajima K, 2008, ACS NANO, V2, P2057, DOI 10.1021/nn800395t
Ajima K, 2005, MOL PHARMACEUT, V2, P475, DOI 10.1021/mp0500566
Ali-Boucetta H, 2008, CHEM COMMUN, P459, DOI 10.1039/b712350g
ALLEN TM, 1995, BBA-BIOMEMBRANES, V1237, P99, DOI 10.1016/0005-2736(95)00085-H
Altintas I, 2013, J CONTROL RELEASE, V165, P110, DOI 10.1016/j.jconrel.2012.11.007
Anhorn MG, 2008, BIOCONJUGATE CHEM, V19, P2321, DOI 10.1021/bc8002452
[Anonymous], 2010, J CANC SCI THER
Arlt M, 2010, NANOTECHNOLOGY, V21, DOI 10.1088/0957-4484/21/33/335101
Arukuusk P, 2013, BBA-BIOMEMBRANES, V1828, P1365, DOI 10.1016/j.bbamem.2013.01.011
Aryal S, 2010, SMALL, V6, P1442, DOI 10.1002/smll.201000631
Aryal S, 2010, ACS NANO, V4, P251, DOI 10.1021/nn9014032
Attia ABE, 2013, BIOMATERIALS, V34, P3132, DOI 10.1016/j.biomaterials.2013.01.042
Barroug A, 2004, J ORTHOP RES, V22, P703, DOI 10.1016/j.orthres.2003.10.016
Bertrand N, 2014, ADV DRUG DELIVER REV, V66, P2, DOI 10.1016/j.addr.2013.11.009
BLUME G, 1993, BIOCHIM BIOPHYS ACTA, V1149, P180, DOI 10.1016/0005-2736(93)90039-3
Brime B, 2003, J ANTIMICROB CHEMOTH, V52, P103, DOI 10.1093/jac/dkg266
Bronich TK, 1999, COLLOID SURFACE B, V16, P243, DOI 10.1016/S0927-7765(99)00075-2
Cambon A, 2013, INT J PHARMACEUT, V445, P47, DOI 10.1016/j.ijpharm.2013.01.056
CHAMOW SM, 1992, J BIOL CHEM, V267, P15916
Chan JM, 2011, P NATL ACAD SCI USA, V108, P19347, DOI 10.1073/pnas.1115945108
Che E., 2012, AJPS, V7, P85
Chen JY, 2008, J AM CHEM SOC, V130, P16778, DOI 10.1021/ja805570f
Chiu SH, 2004, J CONTROL RELEASE, V97, P357, DOI 10.1016/j.jconrel.2004.03.019
Chou HT, 2013, MAT SCI ENG C-MATER, V33, P989, DOI 10.1016/j.msec.2012.11.035
Chouhan R, 2010, NANOMED-NANOTECHNOL, V6, P453, DOI 10.1016/j.nano.2009.11.006
CHUA MM, 1984, BIOCHIM BIOPHYS ACTA, V800, P291
Cirillo G, 2014, BIOMED RES INT, V2014, DOI 10.1155/2014/825017
Crombez L, 2008, CURR PHARM DESIGN, V14, P3656, DOI 10.2174/138161208786898842
Danson S, 2004, BRIT J CANCER, V90, P2085, DOI 10.1038/sj.bjc.6601856
de Araujo AD, 2006, ANGEW CHEM INT EDIT, V45, P296, DOI 10.1002/anie.200502266
de Menezes DEL, 1998, CANCER RES, V58, P3320
De P, 2008, BIOMACROMOLECULES, V9, P1064, DOI 10.1021/bm701255v
Deshayes S, 2012, SMALL, V8, P2184, DOI 10.1002/smll.201102413
Di Crescenzo A, 2011, NANOSCALE, V3, P925, DOI 10.1039/c0nr00444h
Duma A, 2014, UNIV POLITEH BUCHAR, V76, P49
Dvir T, 2011, NANO LETT, V11, P4411, DOI 10.1021/nl2025882
Etrych T, 2008, J APPL POLYM SCI, V109, P3050, DOI 10.1002/app.28466
Etzerodt A, 2012, J CONTROL RELEASE, V160, P72, DOI 10.1016/j.jconrel.2012.01.034
Farokhzad OC, 2004, CANCER RES, V64, P7668, DOI 10.1158/0008-5472.CAN-04-2550
Feazell RP, 2007, J AM CHEM SOC, V129, P8438, DOI 10.1021/ja073231f
Firme CP, 2010, NANOMED-NANOTECHNOL, V6, P245, DOI 10.1016/j.nano.2009.07.003
Flynn T, 2005, NANOMED-NANOTECHNOL, V1, P47, DOI 10.1016/j.nano.2004.11.010
Foldvari M, 2008, NANOMED-NANOTECHNOL, V4, P183, DOI 10.1016/j.nano.2008.04.003
Fonseca C, 2002, J CONTROL RELEASE, V83, P273, DOI 10.1016/S0168-3659(02)00212-2
Freitas RA, 2005, NANOMED-NANOTECHNOL, V1, P2, DOI 10.1016/j.nano.2004.11.003
Gabizon AA, 2001, CANCER INVEST, V19, P424, DOI 10.1081/CNV-100103136
Gindy ME, 2008, BIOMACROMOLECULES, V9, P2705, DOI 10.1021/bm8002013
Graf N, 2012, ACS NANO, V6, P4530, DOI 10.1021/nn301148e
Greish K, 2004, J CONTROL RELEASE, V97, P219, DOI 10.1016/j.jconrel.2004.03.027
Gryparis EC, 2007, EUR J PHARM BIOPHARM, V67, P1, DOI 10.1016/j.ejpb.2006.12.017
Gu MQ, 2007, CARBOHYD POLYM, V67, P417, DOI 10.1016/j.carbpol.2006.06.019
Gupta U, 2006, NANOMED-NANOTECHNOL, V2, P66, DOI 10.1016/j.nano.2006.04.002
Hamaguchi T, 2007, BRIT J CANCER, V97, P170, DOI 10.1038/sj.bjc.6603855
Hampel S, 2008, NANOMEDICINE-UK, V3, P175, DOI 10.2217/17435889.3.2.175
Han Shuang, 2013, Sheng Wu Gong Cheng Xue Bao, V29, P10

HANSEN CB, 1995, BBA-BIOMEMBRANES, V1239, P133, DOI 10.1016/0005-2736(95)00138-S
Harding JA, 1997, BBA-BIOMEMBRANES, V1327, P181, DOI 10.1016/S0005-2736(97)00056-4
Hassane FS, 2006, BIOCONJUGATE CHEM, V17, P849, DOI 10.1021/bc0503081
He W, 2013, INT J PHARMACEUT, V445, P69, DOI 10.1016/j.ijpharm.2013.01.072
Hein CD, 2008, PHARM RES-DORDR, V25, P2216, DOI 10.1007/s11095-008-9616-1
Heister E, 2009, CARBON, V47, P2152, DOI 10.1016/j.carbon.2009.03.057
Hermanson GT, 2008, BIOCONJUGATE TECHNIQUES, 2ND EDITION, P1, DOI 10.1016/B978-0-12-370501-3.00001-1
Hosseinzadeh H, 2012, INT J NANOMED, V7, P1851, DOI 10.2147/IJN.S26365
Hou KK, 2013, BIOMATERIALS, V34, P3110, DOI 10.1016/j.biomaterials.2013.01.037
Hruby M, 2005, J CONTROL RELEASE, V103, P137, DOI 10.1016/j.jconrel.2004.11.017
Hughes GA, 2005, NANOMED-NANOTECHNOL, V1, P22, DOI 10.1016/j.nano.2004.11.009
Hwang JY, 2013, NANOSCALE, V5, P487, DOI 10.1039/c2nr31581e
Ishida O, 2001, PHARMACEUT RES, V18, P1042, DOI 10.1023/A:1010960900254
IVANOV VO, 1985, BIOCHIM BIOPHYS ACTA, V846, P76, DOI 10.1016/0167-4889(85)90112-0
Jain AK, 2009, NANOMED-NANOTECHNOL, V5, P432, DOI 10.1016/j.nano.2009.03.001
Jeong B, 1997, NATURE, V388, P860, DOI 10.1038/42218
Jeong YI, 2008, J PHARM SCI-US, V97, P1268, DOI 10.1002/jps.21103
Juillerat-Jeanneret L, 2008, DRUG DISCOV TODAY, V13, P1099, DOI 10.1016/j.drudis.2008.09.005
Kam NWS, 2006, PHYS STATUS SOLIDI B, V243, P3561, DOI 10.1002/pssb.200669226
Kam NWS, 2004, J AM CHEM SOC, V126, P6850, DOI 10.1021/ja0486059
Kam NWS, 2005, P NATL ACAD SCI USA, V102, P11600, DOI 10.1073/pnas.0502680102
Kang KW, 2010, NANOMED-NANOTECHNOL, V6, P210, DOI 10.1016/j.nano.2009.12.006
Kataoka K, 2000, J CONTROL RELEASE, V64, P143, DOI 10.1016/S0168-3659(99)00133-9
Kayser O, 2003, INT J PHARM, V254, P73, DOI 10.1016/S0378-5173(02)00686-5
Kedar U, 2010, NANOMED-NANOTECHNOL, V6, P714, DOI 10.1016/j.nano.2010.05.005
Kim KY, 2007, NANOMED-NANOTECHNOL, V3, P103, DOI 10.1016/j.nano.2006.12.002
Kim TY, 2004, CLIN CANCER RES, V10, P3708, DOI 10.1158/1078-0432.CCR-03-0655
Kirpotin D, 1997, BIOCHEMISTRY-US, V36, P66, DOI 10.1021/bi962148u
Kocbek P, 2007, J CONTROL RELEASE, V120, P18, DOI 10.1016/j.jconrel.2007.03.012
Kolb HC, 2001, ANGEW CHEM INT EDIT, V40, P2004, DOI 10.1002/1521-3773(20010601)40:11<2004::AID-ANIE2004>3.0.CO;2-5
Koo OM, 2005, NANOMED-NANOTECHNOL, V1, P193, DOI 10.1016/j.nano.2005.06.004
Koren E, 2012, J CONTROL RELEASE, V160, P264, DOI 10.1016/j.jconrel.2011.12.002
Koshkaryev A, 2012, CANCER BIOL THER, V13, P50, DOI 10.4161/cbt.13.1.18871
Kostarelos K, 2006, NANOMEDICINE-UK, V1, P1, DOI 10.2217/17435889.1.1.1
Koziara JM, 2004, J CONTROL RELEASE, V99, P259, DOI 10.1016/j.jconrel.2004.07.006
Lai PS, 2007, J CONTROL RELEASE, V122, P39, DOI 10.1016/j.jconrel.2007.06.012
Lehner R, 2013, NANOMED-NANOTECHNOL, V9, P742, DOI 10.1016/j.nano.2013.01.012
Li C, 2000, CANCER CHEMOTH PHARM, V46, P416, DOI 10.1007/s002800000168
Li J, 2012, CARBON, V50, P1625, DOI 10.1016/j.carbon.2011.11.043
Li RB, 2010, ACS NANO, V4, P1399, DOI 10.1021/nn9011225
Lince F, 2011, CHEM ENG RES DES, V89, P2410, DOI 10.1016/j.cherd.2011.03.010
Liu CX, 2013, BIOMATERIALS, V34, P2547, DOI 10.1016/j.biomaterials.2012.12.038
Liu YY, 2007, INT J CANCER, V120, P2527, DOI 10.1002/ijc.22709
Liu Z, 2008, CANCER RES, V68, P6652, DOI 10.1158/0008-5472.CAN-08-1468
Liu Z, 2007, ACS NANO, V1, P50, DOI 10.1021/nn700040t
Liu Z, 2011, MATER TODAY, V14, P316, DOI 10.1016/S1369-7021(11)70161-4
Liu Z, 2009, ANGEW CHEM INT EDIT, V48, P7668, DOI 10.1002/anie.200902612
Liu Z, 2009, NAT PROTOC, V4, P1372, DOI 10.1038/nprot.2009.146
Liu Z, 2009, NANO RES, V2, P85, DOI 10.1007/s12274-009-9009-8
Lodhi N, 2013, J DRUG TARGET, V21, P67, DOI 10.3109/1061186X.2012.729213
Lu J, 2009, BIOCONJUGATE CHEM, V20, P87, DOI 10.1021/bc8003167
Lu YJ, 2012, COLLOID SURFACE B, V89, P1, DOI 10.1016/j.colsurfb.2011.08.001
Ma XN, 2014, J NANOPART RES, V16, DOI 10.1007/s11051-014-2497-9
Mai WX, 2013, INTEGR BIOL-UK, V5, P19, DOI 10.1039/c2ib20137b
Marchan V, 2006, NUCLEIC ACIDS RES, V34, DOI 10.1093/nar/gnj020
Marega R, 2014, NEW J CHEM, V38, P22, DOI 10.1039/c3nj01008b
MARTIN FJ, 1981, BIOCHEMISTRY-US, V20, P4229, DOI 10.1021/bi00517a043
MARTIN FJ, 1982, J BIOL CHEM, V257, P286
Maruyama K, 1997, ADV DRUG DELIVER REV, V24, P235, DOI 10.1016/S0169-409X(96)00463-2

MARUYAMA K, 1995, BBA-BIOMEMBRANES, V1234, P74, DOI 10.1016/0005-2736(94)00263-O
Matsumura Y, 2004, ANN ONCOL, V15, P517, DOI 10.1093/annonc/mdh092
Mehra NK, 2014, BIOMATERIALS, V35, P1267, DOI 10.1016/j.biomaterials.2013.10.032
Mendes RG, 2013, J MATER CHEM B, V1, P401, DOI 10.1039/c2tb00085g
Milas L, 2003, INT J RADIAT ONCOL, V55, P707, DOI 10.1016/S0360-3016(02)04153-6
Minotti G, 2004, PHARMACOL REV, V56, P185, DOI 10.1124/pr.56.2.6
Mishra B, 2010, NANOMED-NANOTECHNOL, V6, P9, DOI 10.1016/j.nano.2009.04.008
Mitra S, 2001, J CONTROL RELEASE, V74, P317, DOI 10.1016/S0168-3659(01)00342-X
Mizumura Y, 2001, JPN J CANCER RES, V92, P328, DOI 10.1111/j.1349-7006.2001.tb01099.x
Mody N, 2014, AAPS PHARMSCITECH, V15, P388, DOI 10.1208/s12249-014-0073-3
Moffat Bradford A, 2003, Mol Imaging, V2, P324, DOI 10.1162/15353500322750664
Moore TL, 2013, PART PART SYST CHAR, V30, P365, DOI 10.1002/ppsc.201200145
Newman MS, 1999, CANCER CHEMOTH PHARM, V43, P1, DOI 10.1007/s002800050855
Nishiyama N, 2001, J CONTROL RELEASE, V74, P83, DOI 10.1016/S0168-3659(01)00314-5
Niu LY, 2013, MACROMOL BIOSCI, V13, P735, DOI 10.1002/mabi.201200475
Nobs L, 2004, J PHARM SCI-US, V93, P1980, DOI 10.1002/jps.20098
Oberoi HS, 2013, ADV DRUG DELIVER REV, V65, P1667, DOI 10.1016/j.addr.2013.09.014
Ou ZM, 2009, NANOTECHNOLOGY, V20, DOI 10.1088/0957-4484/20/10/105102
Paik PK, 2011, CANCER CHEMOTH PHARM, V68, P1331, DOI 10.1007/s00280-011-1621-0
Park H, 2009, ACS NANO, V3, P2919, DOI 10.1021/nn900215k
PARK JW, 1995, P NATL ACAD SCI USA, V92, P1327, DOI 10.1073/pnas.92.5.1327
Parveen S, 2012, NANOMED-NANOTECHNOL, V8, P147, DOI 10.1016/j.nano.2011.05.016
Pastorin G, 2006, CHEM COMMUN, P1182, DOI 10.1039/b516309a
Peer D, 2007, NAT NANOTECHNOL, V2, P751, DOI 10.1038/nnano.2007.387
Perche F, 2012, J CONTROL RELEASE, V164, P95, DOI 10.1016/j.jconrel.2012.09.003
Reis CP, 2006, NANOMED-NANOTECHNOL, V2, P8, DOI 10.1016/j.nano.2005.12.003
Ren JF, 2012, BIOMATERIALS, V33, P3324, DOI 10.1016/j.biomaterials.2012.01.025
Ren YP, 2008, ADV MATER, V20, P2031, DOI 10.1002/adma.200702292
Rodrigues PCA, 2003, BIOORG MED CHEM LETT, V13, P355, DOI 10.1016/S0960-894X(02)01002-8
Roy I, 2003, J AM CHEM SOC, V125, P7860, DOI 10.1021/ja0343095
Ruan G, 2003, BIOMATERIALS, V24, P5037, DOI 10.1016/S0142-9612(03)00419-8
Sahoo SK, 2007, NANOMED-NANOTECHNOL, V3, P20, DOI 10.1016/j.nano.2006.11.008
Samori C, 2010, CHEM COMMUN, V46, P1494, DOI 10.1039/b923560d
Sawant RR, 2012, AAPS J, V14, P303, DOI 10.1208/s12248-012-9330-0
Schroeder A, 2009, J CONTROL RELEASE, V137, P63, DOI 10.1016/j.jconrel.2009.03.007
Shahi A., 2014, INT J PHARM BIO SCI, V5, P298
Shaik MS, 2001, J CONTROL RELEASE, V76, P285, DOI 10.1016/S0168-3659(01)00450-3
Shi JJ, 2011, ACCOUNTS CHEM RES, V44, P1123, DOI 10.1021/ar200054n
Shi M, 2007, ANGEW CHEM INT EDIT, V46, P6126, DOI 10.1002/anie.200701032
Shirazi AN, 2013, MOL PHARMACEUT, V10, P488, DOI 10.1021/mp3004034
Sinha R, 2006, MOL CANCER THER, V5, P1909, DOI 10.1158/1535-7163.MCT-06-0141
Sui L, 2014, INT J PHARMACEUT, V471, P157, DOI 10.1016/j.ijpharm.2014.05.022
Talelli M, 2013, BIOMATERIALS, V34, P1255, DOI 10.1016/j.biomaterials.2012.09.064
Tian Z, 2011, NANO BIOMED ENG, V3, P157, DOI DOI 10.5101/NBE.V3I3.P157-162
Torchilin V, 2009, EUR J PHARM BIOPHARM, V71, P431, DOI 10.1016/j.ejpb.2008.09.026
Torchilin VP, 2006, ADV DRUG DELIVER REV, V58, P1532, DOI 10.1016/j.addr.2006.09.009
Tripisciano C, 2010, EUR PHYS J B, V75, P141, DOI 10.1140/epjb/e2010-00037-2
Tripisciano C, 2009, CHEM PHYS LETT, V478, P200, DOI 10.1016/j.cplett.2009.07.071
Tsai E., 2011, COSMOS, V8, P1
Vashist SK, 2011, BIOTECHNOL ADV, V29, P169, DOI 10.1016/j.biotechadv.2010.10.002
VERWEIJ J, 1994, ANN ONCOL, V5, P495, DOI 10.1093/oxfordjournals.annonc.a058903
Vetvicka D, 2009, BIOCONJUGATE CHEM, V20, P2090, DOI 10.1021/bc900212k
Vinogradov SV, 2004, BIOCONJUGATE CHEM, V15, P50, DOI 10.1021/bc034164r
Wang CY, 2012, J CONTROL RELEASE, V163, P82, DOI 10.1016/j.jconrel.2012.04.030
Wang FH, 2012, INT J NANOMED, V7, P325, DOI 10.2147/IJN.S27823
Wang SY, 2013, ADV EXP MED BIOL, V765, P315, DOI 10.1007/978-1-4614-4989-8_44
Wartlick H, 2004, J CONTROL RELEASE, V96, P483, DOI 10.1016/j.jconrel.2004.01.029
Wei CM, 2005, NANOMED-NANOTECHNOL, V1, P285, DOI 10.1016/j.nano.2005.10.010
Wen SH, 2013, ADV HEALTHC MATER, V2, P1267, DOI 10.1002/adhm.201200389
Werengowska-Ciecwierz K, 2014, CARBON, V70, P46, DOI 10.1016/j.carbon.2013.12.060
West Kevin R, 2005, Curr Drug Discov Technol, V2, P123, DOI 10.2174/1570163054866882

Wilson B, 2010, NANOMED-NANOTECHNOL, V6, P144, DOI 10.1016/j.nano.2009.04.001
Wong BS, 2013, ADV DRUG DELIVER REV, V65, P1964, DOI 10.1016/j.addr.2013.08.005
Wu WT, 2011, BIOMATERIALS, V32, P9876, DOI 10.1016/j.biomaterials.2011.08.082
Wu Y, 2012, INT J NANOMED, V7, P3221, DOI 10.2147/IJN.S31858
Xiao ZY, 2012, ACS NANO, V6, P696, DOI 10.1021/nn204165v
Yan Z., 2013, CHINESE GERMAN J CLI, V12, P30
Yoo HS, 1999, PHARMACEUT RES, V16, P1114, DOI 10.1023/A:1018908421434
Yoo HS, 2000, J CONTROL RELEASE, V68, P419, DOI 10.1016/S0168-3659(00)00280-7
Yoo HS, 2002, J CONTROL RELEASE, V82, P17, DOI 10.1016/S0168-3659(02)00088-3
Yuan DM, 2012, THORAC CANCER, V3, P341, DOI 10.1111/j.1759-7714.2012.00113.x
Yudasaka M, 2003, CHEM PHYS LETT, V380, P42, DOI 10.1016/j.cplett.2003.08.095
Zalipsky S., 1993, POLYM MATER SCI ENG, V67, P519
Zeng FQ, 2006, BIOCONJUGATE CHEM, V17, P399, DOI 10.1021/bc050350g
Zhang BL, 2010, COLLOID SURFACE B, V80, P18, DOI 10.1016/j.colsurfb.2010.05.023
Zhang HL, 2009, CHEM COMMUN, P3032, DOI 10.1039/b822420j
Zhang HJ, 2013, J DRUG TARGET, V21, P312, DOI 10.3109/1061186X.2012.749880
Zhang LF, 2007, CHEMMEDCHEM, V2, P1268, DOI 10.1002/cmdc.200700121
Zhang M, 2014, CARBON, V69, P626, DOI 10.1016/j.carbon.2013.12.015
Zhang Q, 2012, ADV FUNCT MATER, V22, P5144, DOI 10.1002/adfm.201201316
Zhang XK, 2009, BIOMATERIALS, V30, P6041, DOI 10.1016/j.biomaterials.2009.07.025
Zhao YZ, 2011, CANCER LETT, V311, P187, DOI 10.1016/j.canlet.2011.07.013
NR 196
TC 48
Z9 49
U1 1
U2 19
PU HINDAWI LTD
PI LONDON
PA ADAM HOUSE, 3RD FLR, 1 FITZROY SQ, LONDON, W1T 5HF, ENGLAND
SN 1687-8108
EI 1687-8124
J9 ADV COND MATTER PHYS
JI Adv. Condens. Matter Phys.
PY 2015
VL 2015
AR 198175
DI 10.1155/2015/198175
PG 27
WC Physics, Condensed Matter
WE Science Citation Index Expanded (SCI-EXPANDED)
SC Physics
GA CD4PH
UT WOS:000351065200001
OA Green Submitted, gold
DA 2023-09-14
ER

PT J
AU Zhan, GT
Xu, QB
Zhang, ZL
Wei, ZH
Yong, TY
Bie, NN
Zhang, XQ
Li, X
Li, JY
Gan, L
Yang, XL
AF Zhan, Guiting
Xu, Qingbo

Zhang, Zelong
Wei, Zhaohan
Yong, Tuying
Bie, Nana
Zhang, Xiaoqiong
Li, Xin
Li, Jianye
Gan, Lu
Yang, Xiangliang
TI Biomimetic sonodynamic therapy-nanovaccine integration platform potentiates Anti-PD-1 therapy in hypoxic tumors
SO NANO TODAY
LA English
DT Article
DE Sonodynamic therapy; Nanovaccine; Immunogenic cell death; Anti-PD-1 antibody; Hypoxic tumors
ID IMMUNOGENIC CELL-DEATH; CANCER; NANOPARTICLES; MELANOMA
AB Immune checkpoint blockade (ICB) therapy has emerged as novel therapeutic modality for hard-to-treat solid tumors. However, inadequate T cell infiltration in hypoxic solid tumors limits its anticancer efficacy. Here we develop a sonodynamic therapy (SDT)-nanovaccine integration platform constructed by binding a manganese porphyrin-based metal-organic frameworks (Mn-MOF) with an immune adjuvant CpG and then coating with cell membranes derived from ovalbumin (OVA)-overexpressing melanoma B16 cells (cMnMOF@CM) for potentiating anti-programmed death receptor 1 (PD-1) antibody in malignant melanoma therapy. cMn-MOF@CM with prolonged blood circulation and enhanced tumor targeting efficiently relieves tumor hypoxia and generates strong SDT effects and immunogenic cell death. The tumor-associated antigens both in situ derived from SDT and OVA exhibit vaccine-like functions together with the immune adjuvant CpG, eliciting a strong tumor-specific immune response by promoting DC maturation and T cell activation. Importantly, the combination of cMn-MOF@CM-triggered SDT and anti-PD-1 antibody induces stronger systemic immune response and long-term immunological memory function to prevent tumor growth and recurrence. These findings reveal that cMn-MOF@CM with US irradiation may be a potential candidate to potentiate ICB therapy for hypoxic cancer therapy. (c) 2021 Elsevier Ltd. All rights reserved.
C1 [Zhan, Guiting; Xu, Qingbo; Zhang, Zelong; Wei, Zhaohan; Yong, Tuying; Bie, Nana; Zhang, Xiaoqiong; Li, Xin; Li, Jianye; Gan, Lu; Yang, Xiangliang] Huazhong Univ Sci & Technol, Coll Life Sci & Technol, Natl Engn Res Ctr Nanomed, Wuhan 430074, Peoples R China.
[Gan, Lu; Yang, Xiangliang] Huazhong Univ Sci & Technol, Coll Life Sci & Technol, Key Lab Mol Biophys, Minist Educ, Wuhan 430074, Peoples R China.
[Gan, Lu; Yang, Xiangliang] Huazhong Univ Sci & Technol, Hubei Key Lab Bioinorgan Chem & Mat Med, Wuhan 430074, Peoples R China.
C3 Huazhong University of Science & Technology; Huazhong University of Science & Technology; Huazhong University of Science & Technology
RP Gan, L; Yang, XL (通讯作者)，Huazhong Univ Sci & Technol, Coll Life Sci & Technol, Natl Engn Res Ctr Nanomed, Wuhan 430074, Peoples R China.
EM lugan@mail.hust.edu.cn; yangxl@mail.hust.edu.cn
RI Wei, Zhaohan/GRR-8941-2022; Bie, Nana/ABG-4380-2021
OI Yang, Xiangliang/0000-0003-0683-8763
FU National Key R&D Program of China [2020YFA0710700, 2018YFA0208900]; National Natural Science Foundation of China [81974459, 81627901, 81773653, 82073796]
FX This work was supported by National Key R&D Program of China (2020YFA0710700 and 2018YFA0208900), National Natural Science Foundation of China (81974459, 81627901, 81773653 and 82073796). We thank the Research Core Facilities for Life Science (HUST), the Analytical and Testing Center of Huazhong University of Science and Technology, Optical Bioimaging Core Facility of WNLO-HUST and Wuhan institute of biotechnology for related analysis.
CR Binnewies M, 2018, NAT MED, V24, P541, DOI 10.1038/s41591-018-0014-x
Bray F, 2018, CA-CANCER J CLIN, V68, P394, DOI 10.3322/caac.21492
Chen Z, 2016, ACS NANO, V10, P10049, DOI 10.1021/acsnano.6b04695
Chen ZK, 2018, ACS NANO, V12, P8633, DOI 10.1021/acsnano.8b04371
Danilova L, 2016, P NATL ACAD SCI USA, V113, pE7769, DOI 10.1073/pnas.1607836113

Eisenbarth SC, 2019, NAT REV IMMUNOL, V19, P89, DOI 10.1038/s41577-018-0088-1
Fan WP, 2017, CHEM REV, V117, P13566, DOI 10.1021/acs.chemrev.7b00258
Fang RH, 2018, ADV MATER, V30, DOI 10.1002/adma.201706759
Finn OJ, 2018, NAT REV IMMUNOL, V18, P183, DOI 10.1038/nri.2017.140
Fusciello M, 2019, NAT COMMUN, V10, DOI 10.1038/s41467-019-13744-8
Galluzzi L, 2017, NAT REV IMMUNOL, V17, P97, DOI 10.1038/nri.2016.107
Gide TN, 2018, CLIN CANCER RES, V24, P1260, DOI 10.1158/1078-0432.CCR-17-2267
Gong NQ, 2020, NAT NANOTECHNOL, V15, P1053, DOI 10.1038/s41565-020-00782-3
Harari A, 2020, NAT REV DRUG DISCOV, V19, P635, DOI 10.1038/s41573-020-0074-8
Kang T, 2018, BIOMATERIALS, V164, P80, DOI 10.1016/j.biomaterials.2018.02.033
Kleffel S, 2015, CELL, V162, P1242, DOI 10.1016/j.cell.2015.08.052
Krysko DV, 2012, NAT REV CANCER, V12, P860, DOI 10.1038/nrc3380
Liang S, 2020, ADV MATER, V32, DOI 10.1002/adma.202003214
Liu Q, 2018, ADV DRUG DELIVER REV, V127, P208, DOI 10.1016/j.addr.2017.09.016
Luk BT, 2014, NANOSCALE, V6, P2730, DOI 10.1039/c3nr06371b
Luke JJ, 2017, NAT REV CLIN ONCOL, V14, P463, DOI 10.1038/nrclinonc.2017.43
Ma AQ, 2019, SMALL, V15, DOI 10.1002/smll.201804028
Marzagalli M, 2019, SEMIN CANCER BIOL, V59, P236, DOI 10.1016/j.semcancer.2019.08.002
Murciano-Goroff YR, 2020, CELL RES, V30, P507, DOI 10.1038/s41422-020-0337-2
Musetti S, 2018, ACS NANO, V12, P11740, DOI 10.1021/acsnano.8b05893
Nam J, 2019, NAT REV MATER, V4, P398, DOI 10.1038/s41578-019-0108-1
Ni KY, 2020, ACS CENTRAL SCI, V6, P861, DOI 10.1021/acscentsci.0c00397
Rao L, 2019, ADV FUNCT MATER, V29, DOI 10.1002/adfm.201905671
Schadendorf D, 2015, NAT REV DIS PRIMERS, V1, DOI 10.1038/nrdp.2015.3
Shain AH, 2016, NAT REV CANCER, V16, P345, DOI 10.1038/nrc.2016.37
Simon-Yarza T, 2018, ADV MATER, V30, DOI 10.1002/adma.201707365
Sun HP, 2016, ADV MATER, V28, P9581, DOI 10.1002/adma.201602173
Sun L, 2020, ADV MATER, V32, DOI 10.1002/adma.202005295
van der Burg SH, 2016, NAT REV CANCER, V16, P219, DOI 10.1038/nrc.2016.16
Wang CX, 2020, ADV FUNCT MATER, V30, DOI 10.1002/adfm.201909369
Wang DD, 2020, ACS NANO, V14, P13500, DOI 10.1021/acsnano.0c05499
Wang DD, 2018, ADV SCI, V5, DOI 10.1002/advs.201800287
Xie W, 2019, INT J NANOMED, V14, P9377, DOI 10.2147/IJN.S208404
Xie W, 2019, ACS NANO, V13, P2849, DOI 10.1021/acsnano.8b03788
Xu QB, 2021, THERANOSTICS, V11, P1937, DOI 10.7150/thno.45511
Yang R, 2018, ACS NANO, V12, P5121, DOI 10.1021/acsnano.7b09041
Yue WW, 2019, NAT COMMUN, V10, DOI 10.1038/s41467-019-09760-3
Zhang QY, 2018, CANCER SCI, V109, P1330, DOI 10.1111/cas.13578
Zhang YY, 2020, CELL MOL IMMUNOL, V17, P807, DOI 10.1038/s41423-020-0488-6
Zhong XY, 2020, ADV FUNCT MATER, V30, DOI 10.1002/adfm.201907954
Zhu P, 2018, ACS NANO, V12, P3780, DOI 10.1021/acsnano.8b00999
NR 46
TC 46
Z9 46
U1 34
U2 282
PU ELSEVIER SCI LTD
PI OXFORD
PA THE BOULEVARD, LANGFORD LANE, KIDLINGTON, OXFORD OX5 1GB, OXON, ENGLAND
SN 1748-0132
EI 1878-044X
J9 NANO TODAY
JI Nano Today
PD JUN
PY 2021
VL 38
AR 101195
DI 10.1016/j.nantod.2021.101195
EA MAY 2021
PG 16
WC Chemistry, Multidisciplinary; Nanoscience & Nanotechnology; Materials

Science, Multidisciplinary
WE Science Citation Index Expanded (SCI-EXPANDED)
SC Chemistry; Science & Technology - Other Topics; Materials Science
GA TE9IC
UT WOS:000670318800003
DA 2023-09-14
ER

PT J
AU Huang, CL
Zhang, L
Guo, Q
Zuo, YY
Wang, NN
Wang, H
Kong, DL
Zhu, DW
Zhang, LH
AF Huang, Chenlu
Zhang, Li
Guo, Qing
Zuo, Yueyue
Wang, Nannan
Wang, Hai
Kong, Deling
Zhu, Dunwan
Zhang, Linhua
TI Robust Nanovaccine Based on Polydopamine-Coated Mesoporous Silica Nanoparticles for Effective Photothermal-Immunotherapy Against Melanoma
SO ADVANCED FUNCTIONAL MATERIALS
LA English
DT Article
DE immunotherapy; melanoma; mesoporous silica nanoparticles; nanovaccine; photothermal therapy
AB Nanoparticle-based combination therapy strategy of photothermal therapy (PTT) and immunotherapy is an attractive cancer treatment for ablating tumors and eliciting host immune responses. However, this strategy is often hampered by tedious treatment process and limited immune response, and usually needs to be combined with checkpoint blockades to enhance therapeutic effect. Herein, a nanoplatform with mesoporous silica nanoparticles (MSNs) as a vector, which integrated photothermal agent polydopamine (PDA), model antigen ovalbumin (OVA), and antigen release promoter ammonium bicarbonate (ABC) in an easy way for melanoma PTT-immunotherapy is designed. The formulated MSNs-ABC@PDA-OVA nanovaccine exhibits excellent photothermal properties and effectively eliminates primary tumors. Under laser irradiation, the MSNs-ABC@PDA-OVA nanovaccine realizes rapid antigen release and endosome escape, enhances dendritic cells activation and maturation, facilitates migration to tumor-draining lymph nodes, and induces robust antitumor immune responses. Impressively, single injection of MSNs-ABC@PDA-OVA combines with single round of PTT successfully eradicates melanoma tumors with a cure rate of 75% and generates strong immunological memory to inhibit tumor recurrence and lung metastasis. Hence, the research offers a simple and promising strategy of synergistic PTT-immunotherapy to effectively treat cancer.
C1 [Huang, Chenlu; Zhang, Li; Guo, Qing; Zuo, Yueyue; Wang, Nannan; Wang, Hai; Kong, Deling; Zhu, Dunwan; Zhang, Linhua] Chinese Acad Med Sci & Peking Union Med Coll, Inst Biomed Engn, Tianjin Key Lab Biomed Mat, Key Lab Biomat & Nanotechnol Canc Immunotherapy, Tianjin 300192, Peoples R China.
[Kong, Deling] Nankai Univ, Coll Life Sci, Key Lab Bioact Mat, Minist Educ, Tianjin 300071, Peoples R China.
[Kong, Deling] Xuzhou Med Univ, Jiangsu Ctr Collaborat & Innovat Canc Biotherapy, Canc Inst, Xuzhou 221004, Jiangsu, Peoples R China.
C3 Chinese Academy of Medical Sciences - Peking Union Medical College; Peking Union Medical College; Institute of BioMedical Engineering - CAMS; Nankai University; Xuzhou Medical University
RP Kong, DL; Zhu, DW; Zhang, LH (通讯作者), Chinese Acad Med Sci & Peking Union Med Coll, Inst Biomed Engn, Tianjin Key Lab Biomed Mat, Key Lab Biomat & Nanotechnol Canc Immunotherapy, Tianjin

300192, Peoples R China.; Kong, DL (通讯作者)，Nankai Univ, Coll Life Sci, Key Lab Bioact Mat, Minist Educ, Tianjin 300071, Peoples R China.; Kong, DL (通讯作者)，Xuzhou Med Univ, Jiangsu Ctr Collaborat & Innovat Canc Biotherapy, Canc Inst, Xuzhou 221004, Jiangsu, Peoples R China.
EM kongdeling@nankai.edu.cn; zhudunwan@bme.pumc.edu.cn;
zhanglinhua@bme.pumc.edu.cn
RI zhang, yu/HNS-5948-2023
FU National Natural Science Foundation of China [82072059, 81671806];
Fundamental Research Funds for the Central Universities [2019PT320028,
2019-0831-03]; Tianjin Municipal Natural Science Foundation
[20JCYBJC00030]; Technology & Innovation Commission of Shenzhen
Municipality [JCYJ20180507181654186, JCYJ20170818162637217]
FX This work was financially supported by National Natural Science
Foundation of China (Nos. 82072059 and 81671806), the Fundamental
Research Funds for the Central Universities (Nos. 2019PT320028 and
2019-0831-03), Tianjin Municipal Natural Science Foundation (No.
20JCYBJC00030), Technology & Innovation Commission of Shenzhen
Municipality (Nos. JCYJ20180507181654186 and JCYJ20170818162637217).
CR Bardhan R, 2011, ACCOUNTS CHEM RES, V44, P936, DOI 10.1021/ar200023x
Basta S, 2007, SCAND J IMMUNOL, V65, P311, DOI 10.1111/j.1365-3083.2007.01909.x
Chen WF, 2018, THERANOSTICS, V8, P2229, DOI 10.7150/thno.24073
Chen Z, 2016, ACS NANO, V10, P10049, DOI 10.1021/acsnano.6b04695
Cheng W, 2019, J GEOPHYS RES-ATMOS, V124, P12773, DOI 10.1029/2018JD030237
Cheng W, 2017, SMALL, V13, DOI 10.1002/smll.201700623
Cheng W, 2017, ACS APPL MATER INTER, V9, P18462, DOI 10.1021/acsami.7b02457
Christensen D, 2012, J CONTROL RELEASE, V160, P468, DOI 10.1016/j.jconrel.2012.03.016
Chu KF, 2014, NAT REV CANCER, V14, P199, DOI 10.1038/nrc3672
da Silva JL, 2019, CANCER CHEMOTH PHARM, V84, P227, DOI 10.1007/s00280-019-03894-3
Demento SL, 2012, BIOMATERIALS, V33, P4957, DOI 10.1016/j.biomaterials.2012.03.041
Deng RH, 2019, ACS NANO, V13, P8618, DOI 10.1021/acsnano.9b02993
Ding F, 2020, BIOMATERIALS, V245, DOI 10.1016/j.biomaterials.2020.119976
Ding JX, 2019, BIOMACROMOLECULES, V20, P4258, DOI 10.1021/acs.biomac.9b01263
Ding L, 2017, NANO LETT, V17, P6790, DOI 10.1021/acs.nanolett.7b03021
Ding T, 2019, NANOSCALE HORIZ, V4, P652, DOI 10.1039/c8nh00351c
Foged C, 2005, INT J PHARMACEUT, V298, P315, DOI 10.1016/j.ijpharm.2005.03.035
Gao Q, 2018, CHINESE CHEM LETT, V29, P1804, DOI 10.1016/j.cclet.2018.09.006
Gao ZF, 2016, DALTON T, V45, P19519, DOI 10.1039/c6dt03897b
Gosselin EA, 2018, NAT MATER, V17, P484, DOI 10.1038/s41563-018-0077-6
Gotwals P, 2017, NAT REV CANCER, V17, P286, DOI 10.1038/nrc.2017.17
Hu YY, 2017, ADV MATER, V29, DOI 10.1002/adma.201700448
Huang LP, 2019, NAT COMMUN, V10, DOI 10.1038/s41467-019-12771-9
Islam MA, 2021, BIOMATERIALS, V266, DOI 10.1016/j.biomaterials.2020.120431
Ji CW, 2019, J BIOMED NANOTECHNOL, V15, P311, DOI 10.1166/jbn.2019.2685
Ji QJ, 2016, NAT COMMUN, V7, DOI 10.1038/ncomms11000
Kapadia CH, 2018, J CONTROL RELEASE, V269, P393, DOI 10.1016/j.jconrel.2017.11.020
Kimura MY, 2017, IMMUNOL REV, V278, P87, DOI 10.1111/imr.12559
Li AW, 2018, NAT MATER, V17, P528, DOI 10.1038/s41563-018-0028-2
Li M., 2018, ADV SCI, V5
Li W, 2019, NAT COMMUN, V10, DOI 10.1038/s41467-019-11269-8
Liang X, 2019, J CONTROL RELEASE, V296, P150, DOI 10.1016/j.jconrel.2019.01.027
Lin C., 2020, SMART MAT MED, V1, P150, DOI DOI 10.1016/J.SMAIM.2020.09.001
Liu H, 2020, ADV SCI, V7, DOI 10.1002/advs.201903129
Liu Q, 2016, SMALL, V12, P1744, DOI 10.1002/smll.201503662
Liu Q, 2015, ACS NANO, V9, P4925, DOI 10.1021/nn5066793
Lovell JF, 2011, NAT MATER, V10, P324, DOI [10.1038/NMAT2986, 10.1038/nmat2986]
Nam J, 2018, NAT COMMUN, V9, DOI 10.1038/s41467-018-03473-9
Palucka K, 2012, NAT REV CANCER, V12, P265, DOI 10.1038/nrc3258
Park J, 2014, ACS NANO, V8, P3347, DOI 10.1021/nn405809c
Paulis LE, 2013, CURR OPIN IMMUNOL, V25, P389, DOI 10.1016/j.coi.2013.03.001
Peng JR, 2018, ADV SCI, V5, DOI 10.1002/advs.201700891
Qian M, 2019, NANO LETT, V19, P8409, DOI 10.1021/acs.nanolett.9b02448
Randolph GJ, 2005, NAT REV IMMUNOL, V5, P617, DOI 10.1038/nri1670

Sheng ZH, 2014, ACS NANO, V8, P12310, DOI 10.1021/nn5062386
Stenger S, 1998, SCIENCE, V282, P121, DOI 10.1126/science.282.5386.121
Sun W, 2019, BIOMATERIALS, V217, DOI 10.1016/j.biomaterials.2019.119264
Sweeney EE, 2018, SMALL, V14, DOI 10.1002/smll.201800678
Nguyen TL, 2020, BIOMATERIALS, V239, DOI 10.1016/j.biomaterials.2020.119859
Nguyen TL, 2019, ADV MATER, V31, DOI 10.1002/adma.201803953
Thibaut R, 2020, NAT CANCER, V1, P302, DOI 10.1038/s43018-020-0038-2
Wang H, 2018, NAT MATER, V17, P761, DOI 10.1038/s41563-018-0147-9
Wang SW, 2016, ACS APPL MATER INTER, V8, P24368, DOI 10.1021/acsami.6b05907
Wang XJ, 2017, BIOMATERIALS, V143, P130, DOI 10.1016/j.biomaterials.2017.08.004
Wang Z, 2020, SMALL, V16, DOI 10.1002/smll.201907042
Wu D, 2019, ADV FUNCT MATER, V29, DOI 10.1002/adfm.201900095
Wu D, 2020, BIOMATERIALS, V238, DOI 10.1016/j.biomaterials.2020.119847
Xu J, 2017, ACS NANO, V11, P4463, DOI 10.1021/acsnano.7b00715
Xu LG, 2013, ADV MATER, V25, P5928, DOI 10.1002/adma.201300583
Yan SQ, 2019, ADV MATER, V31, DOI 10.1002/adma.201905825
Ye Q, 2011, CHEM SOC REV, V40, P4244, DOI 10.1039/c1cs15026j
Ye XY, 2019, ACS NANO, V13, P2956, DOI 10.1021/acsnano.8b07371
Yuan Z, 2019, BIOMATERIALS, V223, DOI 10.1016/j.biomaterials.2019.119479
Zhang D, 2019, SMALL, V15, DOI 10.1002/smll.201902636
Zhang D, 2019, ACS APPL MATER INTER, V11, P32633, DOI 10.1021/acsami.9b09568
Zhang F, 2020, J CONTROL RELEASE, V326, P131, DOI 10.1016/j.jconrel.2020.06.015
Zhang J, 2019, J BIOMED NANOTECHNOL, V15, P1546, DOI 10.1166/jbn.2019.2788
Zhou L, 2020, NANO LETT, V20, P4393, DOI 10.1021/acs.nanolett.0c01140
Zhou SL, 2020, BIOMATERIALS, V235, DOI 10.1016/j.biomaterials.2020.119795
NR 69
TC 46
Z9 47
U1 41
U2 318
PU WILEY-V C H VERLAG GMBH
PI WEINHEIM
PA POSTFACH 101161, 69451 WEINHEIM, GERMANY
SN 1616-301X
EI 1616-3028
J9 ADV FUNCT MATER
JI Adv. Funct. Mater.
PD MAY
PY 2021
VL 31
IS 18
AR 2010637
DI 10.1002/adfm.202010637
EA FEB 2021
PG 17
WC Chemistry, Multidisciplinary; Chemistry, Physical; Nanoscience & Nanotechnology; Materials Science, Multidisciplinary; Physics, Applied; Physics, Condensed Matter
WE Science Citation Index Expanded (SCI-EXPANDED)
SC Chemistry; Science & Technology - Other Topics; Materials Science; Physics
GA TK4HK
UT WOS:000621060200001
DA 2023-09-14
ER

PT J
AU Hassan, HAFM
Diebold, SS
Smyth, LA
Walters, AA

Lombardi, G
Al-Jamal, KT
AF Hassan, Hatem A. F. M.
Diebold, Sandra S.
Smyth, Lesley A.
Walters, Adam A.
Lombardi, Giovanna
Al-Jamal, Khuloud T.
TI Application of carbon nanotubes in cancer vaccines: Achievements, challenges and chances
SO JOURNAL OF CONTROLLED RELEASE
LA English
DT Article; Proceedings Paper
CT 15th European Symposium on Controlled Drug Delivery
CY APR 11-13, 2018
CL Egmond aan Zee, NETHERLANDS
DE Cancer immunotherapy; Antigen presenting cells; Nanomedicine; Vaccine delivery; Carbon nanotube
ID MESOPOROUS SILICA NANOPARTICLES; PATTERN-RECOGNITION RECEPTORS; DENDRITIC CELLS; GRAPHENE OXIDE; PLGA NANOPARTICLES; ANTIGEN DELIVERY; PROTEIN ENHANCE; CELLULAR UPTAKE; TUMOR-ANTIGENS; SOLVENT-FREE
AB Tumour-specific, immuno-based therapeutic interventions can be considered as safe and effective approaches for cancer therapy. Exploitation of nano-vaccinology to intensify the cancer vaccine potency may overcome the need for administration of high vaccine doses or additional adjuvants and therefore could be a more efficient approach. Carbon nanotube (CNT) can be described as carbon sheet(s) rolled up into a cylinder that is nanometers wide and nanometers to micrometers long. Stemming from the observed capacities of CNTs to enter various types of cells via diversified mechanisms utilising energy-dependent and/or passive routes of cell uptake, the use of CNTs for the delivery of therapeutic agents has drawn increasing interests over the last decade. Here we review the previous studies that demonstrated the possible benefits of these cylindrical nano-vectors as cancer vaccine delivery systems as well as the obstacles their clinical application is facing.
C1 [Hassan, Hatem A. F. M.; Walters, Adam A.; Al-Jamal, Khuloud T.] Kings Coll London, Fac Life Sci & Med, Sch Canc & Pharmaceut Sci, Inst Pharmaceut Sci, Franklin Wilkins Bldg, London SE1 9NH, England.
[Diebold, Sandra S.] NIBSC, Biotherapeut Div, Blanche Lane, Potters Bar EN6 3QG, Herts, England.
[Smyth, Lesley A.] Univ East London, Sch Hlth Sport & Biosci, Stratford Campus,Water Lane, London E15 4LZ, England.
[Lombardi, Giovanna] Kings Coll London, Guys Hosp, Sch Immunol & Microbial Sci, London SE1 9RT, England.
[Hassan, Hatem A. F. M.] Queens Univ Belfast, Sch Pharm, 97 Lisburn Rd, Belfast BT9 7BL, Antrim, North Ireland.
C3 University of London; King's College London; National Institute for Biological Standards & Control; University of East London; Guy's & St Thomas' NHS Foundation Trust; University of London; King's College London; Queens University Belfast
RP Al-Jamal, KT (通讯作者)，Kings Coll London, Sch Canc & Pharmaceut Sci, Chair Drug Delivery & Nanomed, Franklin Wilkins Bldg,150 Stamford St, London SE1 9NH, England.
EM Khuloud.al-jamal@kcl.ac.uk
RI Al-Jamal, Khuloud/ABD-5668-2021; Ahmed, Hassan Elzain Hassan/AGA-7917-2022
OI AL-JAMAL, KHULOUD/0000-0001-5165-2699; Hassan, Hatem/0009-0009-7493-8564
FU YCCF; Biotechnology and Biological Sciences Research Council [BB/J008656/1]; Worldwide Cancer Research [12- 1054]; BBSRC [BB/J008656/1] Funding Source: UKRI
FX HAFMH would like to thank YCCF for PhD funding. Partial funding from Biotechnology and Biological Sciences Research Council (BB/J008656/1) and Worldwide Cancer Research (12- 1054) are acknowledged. The authors declare that they have no competing interests.
CR Ahmed KK, 2016, J PHARM SCI-US, V105, P1173, DOI 10.1016/j.xphs.2015.11.042
Al-Jamal KT, 2012, ANGEW CHEM INT EDIT, V51, P6389, DOI 10.1002/anie.201201991
Alidori S, 2016, SCI TRANSL MED, V8, DOI 10.1126/scitranslmed.aac9647
Allen BL, 2008, NANO LETT, V8, P3899, DOI 10.1021/nl802315h

Andon FT, 2013, SMALL, V9, P2721, DOI 10.1002/smll.201202508
Bachmann MF, 2010, NAT REV IMMUNOL, V10, P787, DOI 10.1038/nri2868
de Faria PCB, 2014, NANO LETT, V14, P5458, DOI 10.1021/nl502911a
Cai XN, 2013, NANOMED-NANOTECHNOL, V9, P583, DOI 10.1016/j.nano.2012.09.004
Cartiera MS, 2009, BIOMATERIALS, V30, P2790, DOI 10.1016/j.biomaterials.2009.01.057
Cha BG, 2018, ACS CENTRAL SCI, V4, P484, DOI 10.1021/acscentsci.8b00035
Chatterjee B, 2012, BLOOD, V120, P2011, DOI 10.1182/blood-2012-01-402370
Chen WS, 2008, CANCER IMMUNOL IMMUN, V57, P517, DOI 10.1007/s00262-007-0390-4
Cherukuri P, 2004, J AM CHEM SOC, V126, P15638, DOI 10.1021/ja0466311
Coccini T, 2010, TOXICOLOGY, V269, P41, DOI 10.1016/j.tox.2010.01.005
Cohn L, 2013, J EXP MED, V210, P1049, DOI 10.1084/jem.20121251
Coulie PG, 2014, NAT REV CANCER, V14, P135, DOI 10.1038/nrc3670
Crawford J, 2004, CANCER-AM CANCER SOC, V100, P228, DOI 10.1002/cncr.11882
Cui DX, 2005, TOXICOL LETT, V155, P73, DOI 10.1016/j.toxlet.2004.08.015
De Paoli SH, 2014, BIOMATERIALS, V35, P6182, DOI 10.1016/j.biomaterials.2014.04.067
De Ruysscher D, 2007, ANN ONCOL, V18, P909, DOI 10.1093/annonc/mdm005
De Temmerman ML, 2011, DRUG DISCOV TODAY, V16, P569, DOI 10.1016/j.drudis.2011.04.006
Diebold SS, 2008, IMMUNOL CELL BIOL, V86, P389, DOI 10.1038/icb.2008.26
Dumortier H, 2006, NANO LETT, V6, P1522, DOI 10.1021/nl061160x
Dunn GP, 2002, NAT IMMUNOL, V3, P991, DOI 10.1038/ni1102-991
Fan HT, 2012, CLIN CANCER RES, V18, P5628, DOI 10.1158/1078-0432.CCR-12-1911
Finn OJ, 2003, NAT REV IMMUNOL, V3, P630, DOI 10.1038/nri1150
Geary SM, 2015, J CONTROL RELEASE, V220, P682, DOI 10.1016/j.jconrel.2015.09.002
Gordon S, 2002, CELL, V111, P927, DOI 10.1016/S0092-8674(02)01201-1
Guo JX, 2007, NUCL MED BIOL, V34, P579, DOI 10.1016/j.nucmedbio.2007.03.003
Hamdy S, 2008, VACCINE, V26, P5046, DOI 10.1016/j.vaccine.2008.07.035
Hassan HAFM, 2016, BIOMATERIALS, V104, P310, DOI 10.1016/j.biomaterials.2016.07.005
Hassan HAFM, 2016, J CONTROL RELEASE, V225, P205, DOI 10.1016/j.jconrel.2016.01.030
He Y, 2016, TRENDS BIOCHEM SCI, V41, P1012, DOI 10.1016/j.tibs.2016.09.002
Hirsch A, 2010, NAT MATER, V9, P868, DOI 10.1038/nmat2885
Hou PX, 2008, CARBON, V46, P2003, DOI 10.1016/j.carbon.2008.09.009
Huang XL, 2010, BIOMATERIALS, V31, P438, DOI 10.1016/j.biomaterials.2009.09.060
IIJIMA S, 1991, NATURE, V354, P56, DOI 10.1038/354056a0
Irvine DJ, 2013, NAT MATER, V12, P978, DOI [10.1038/NMAT3775, 10.1038/nmat3775]
Janeway CA, 2002, ANNU REV IMMUNOL, V20, P197, DOI 10.1146/annurev.immunol.20.083001.084359
Kafa H, 2016, J CONTROL RELEASE, V225, P217, DOI 10.1016/j.jconrel.2016.01.031
Kagan VE, 2006, TOXICOL LETT, V165, P88, DOI 10.1016/j.toxlet.2006.02.001
Kagan VE, 2010, NAT NANOTECHNOL, V5, P354, DOI [10.1038/NNANO.2010.44, 10.1038/nnano.2010.44]
Kam NWS, 2004, J AM CHEM SOC, V126, P6850, DOI 10.1021/ja0486059
Kam NWS, 2005, J AM CHEM SOC, V127, P12492, DOI 10.1021/ja053962k
Kastenmuller W, 2014, NAT REV IMMUNOL, V14, P705, DOI 10.1038/nri3727
Klinman DM, 2004, NAT REV IMMUNOL, V4, P248, DOI 10.1038/nri1329
Kosmides AK, 2017, ACS NANO, V11, P5417, DOI 10.1021/acsnano.6b08152
Kostarelos K, 2007, NAT NANOTECHNOL, V2, P108, DOI 10.1038/nnano.2006.209
Krieg AM, 2008, ONCOGENE, V27, P161, DOI 10.1038/sj.onc.1210911
Krishnamachari Y, 2011, PHARM RES-DORDR, V28, P215, DOI 10.1007/s11095-010-0241-4
KROTO HW, 1985, NATURE, V318, P162, DOI 10.1038/318162a0
Kuai R, 2017, NAT MATER, V16, P489, DOI [10.1038/nmat4822, 10.1038/NMAT4822]
Li RB, 2013, ACS NANO, V7, P2352, DOI 10.1021/nn305567s
Liu J, 1998, SCIENCE, V280, P1253, DOI 10.1126/science.280.5367.1253
Liu Z, 2008, P NATL ACAD SCI USA, V105, P1410, DOI 10.1073/pnas.0707654105
Lizotte PH, 2016, NAT NANOTECHNOL, V11, P295, DOI [10.1038/NNANO.2015.292, 10.1038/nnano.2015.292]
Lopez CF, 2004, P NATL ACAD SCI USA, V101, P4431, DOI 10.1073/pnas.0400352101
Ludewig B, 2000, VACCINE, V19, P23, DOI 10.1016/S0264-410X(00)00163-8
MacManus M, 1997, BLOOD, V89, P2303, DOI 10.1182/blood.V89.7.2303
Mao S, 2005, PHARM RES-DORDR, V22, P2058, DOI 10.1007/s11095-005-8175-y
Medepalli K, 2011, NANOMED-NANOTECHNOL, V7, P983, DOI 10.1016/j.nano.2011.04.002
Mei KC, 2018, ADV MATER, V30, DOI 10.1002/adma.201802732

Mei KC, 2015, CHEM COMMUN, V51, P14981, DOI 10.1039/c5cc05412e
Mei KC, 2015, ACS APPL MATER INTER, V7, P14176, DOI 10.1021/acsami.5b03577
Melero I, 2007, NAT REV CANCER, V7, P95, DOI 10.1038/nrc2051
Mellman I, 2011, NATURE, V480, P480, DOI 10.1038/nature10673
Meng J, 2008, SMALL, V4, P1364, DOI 10.1002/smll.200701059
Meng J, 2011, NANOTOXICOLOGY, V5, P583, DOI 10.3109/17435390.2010.523483
Min YZ, 2017, NAT NANOTECHNOL, V12, P877, DOI [10.1038/nnano.2017.113, 10.1038/NNANO.2017.113]
Molino NM, 2016, BIOMATERIALS, V86, P83, DOI 10.1016/j.biomaterials.2016.01.056
Mu QX, 2009, NANO LETT, V9, P4370, DOI 10.1021/nl902647x
Mueller M, 2012, J CONTROL RELEASE, V162, P159, DOI 10.1016/j.jconrel.2012.06.015
Mueller M, 2011, INT J CANCER, V129, P407, DOI 10.1002/ijc.25914
Murray AA, 2018, MOL PHARMACEUT, V15, P3700, DOI 10.1021/acs.molpharmaceut.8b00316
Novoselov KS, 2004, SCIENCE, V306, P666, DOI 10.1126/science.1102896
Nunes A, 2012, NANOMEDICINE-UK, V7, P1485, DOI [10.2217/NNM.12.33, 10.2217/nnm.12.33]
Ouyang M, 2016, PLOS ONE, V11, DOI 10.1371/journal.pone.0148139
Pages F, 2005, NEW ENGL J MED, V353, P2654, DOI 10.1056/NEJMoa051424
Palm NW, 2009, IMMUNOL REV, V227, P221, DOI 10.1111/j.1600-065X.2008.00731.x
Palomaki J, 2011, ACS NANO, V5, P6861, DOI 10.1021/nn200595c
Palucka K, 2012, NAT REV CANCER, V12, P265, DOI 10.1038/nrc3258
Pantarotto D, 2004, CHEM COMMUN, P16, DOI 10.1039/b311254c
Parish CR, 2003, IMMUNOL CELL BIOL, V81, P106, DOI 10.1046/j.0818-9641.2003.01151.x
Pescatori M, 2013, BIOMATERIALS, V34, P4395, DOI 10.1016/j.biomaterials.2013.02.052
Peters WA, 2000, J CLIN ONCOL, V18, P1606, DOI 10.1200/JCO.2000.18.8.1606
Porter AE, 2009, ACS NANO, V3, P1485, DOI 10.1021/nn900416z
Pulskamp K, 2007, TOXICOL LETT, V168, P58, DOI 10.1016/j.toxlet.2006.11.001
Ramanathan T, 2005, CHEM MATER, V17, P1290, DOI 10.1021/cm048357f
Ribas A, 2018, SCIENCE, V359, P1350, DOI 10.1126/science.aar4060
Rosalia RA, 2015, BIOMATERIALS, V40, P88, DOI 10.1016/j.biomaterials.2014.10.053
Rotoli BM, 2008, TOXICOL LETT, V178, P95, DOI 10.1016/j.toxlet.2008.02.007
Rubio N, 2015, CHEM COMMUN, V51, P9366, DOI 10.1039/c5cc02675j
Rubio N, 2015, ACS APPL MATER INTER, V7, P18920, DOI 10.1021/acsami.5b06250
Sagiv-Barfi I, 2018, SCI TRANSL MED, V10, DOI 10.1126/scitranslmed.aan4488
Schipper ML, 2008, NAT NANOTECHNOL, V3, P216, DOI 10.1038/nnano.2008.68
Schlom J, 2007, CLIN CANCER RES, V13, P3776, DOI 10.1158/1078-0432.CCR-07-0588
Shariat S, 2014, CANCER LETT, V355, P54, DOI 10.1016/j.canlet.2014.09.016
Shvedova AA, 2012, PLOS ONE, V7, DOI 10.1371/journal.pone.0030923
Singh P, 2009, CHEM SOC REV, V38, P2214, DOI 10.1039/b518111a
Sinha A, 2017, CHEM MATER, V29, P6883, DOI 10.1021/acs.chemmater.7b02197
Smith DM, 2013, NAT REV IMMUNOL, V13, P592, DOI 10.1038/nri3488
Smyth MJ, 2016, NAT REV CLIN ONCOL, V13, P143, DOI 10.1038/nrclinonc.2015.209
Steinhagen F, 2011, VACCINE, V29, P3341, DOI 10.1016/j.vaccine.2010.08.002
Stratton MR, 2009, NATURE, V458, P719, DOI 10.1038/nature07943
Summers HD, 2017, NANOSCALE, V9, P6800, DOI 10.1039/c7nr01561e
Sun Z, 2010, CELL RES, V20, P1170, DOI 10.1038/cr.2010.133
Tao Y, 2014, BIOMATERIALS, V35, P9963, DOI 10.1016/j.biomaterials.2014.08.036
Tasis D, 2006, CHEM REV, V106, P1105, DOI 10.1021/cr050569o
Thostenson ET, 2001, COMPOS SCI TECHNOL, V61, P1899, DOI 10.1016/S0266-3538(01)00094-X
Torchilin VP, 2005, NAT REV DRUG DISCOV, V4, P145, DOI 10.1038/nrd1632
Trewyn BG, 2008, CHEM ENG J, V137, P23, DOI 10.1016/j.cej.2007.09.045
Tschopp J, 2010, NAT REV IMMUNOL, V10, P210, DOI 10.1038/nri2725
Van Braeckel-Budimir N, 2014, FRONT MICROBIOL, V5, DOI 10.3389/fmicb.2014.00272
van Kooten C, 2000, J LEUKOCYTE BIOL, V67, P2, DOI 10.1002/jlb.67.1.2
Villa CH, 2011, ACS NANO, V5, P5300, DOI 10.1021/nn200182x
Villadangos JA, 2007, NAT REV IMMUNOL, V7, P543, DOI 10.1038/nri2103
Vukovic G, 2009, APPL SURF SCI, V255, P8067, DOI 10.1016/j.apsusc.2009.05.016
Wakita D, 2006, INT IMMUNOL, V18, P425, DOI 10.1093/intimm/dxh381
Wang J, 2009, CARBON, V47, P1752, DOI 10.1016/j.carbon.2009.03.003
Wang X, 2010, ACS NANO, V4, P7241, DOI 10.1021/nn102112b
Wen J, 2017, NAT COMMUN, V8, DOI 10.1038/s41467-017-01669-z
Whitmore MM, 2001, CANCER IMMUNOL IMMUN, V50, P503, DOI 10.1007/s002620100227

Xu LG, 2013, ADV MATER, V25, P5928, DOI 10.1002/adma.201300583
Yang ST, 2007, J PHYS CHEM C, V111, P17761, DOI 10.1021/jp070712c
Yue H, 2015, NANOSCALE, V7, P19949, DOI 10.1039/c5nr04986e
Zeltins A, 2013, MOL BIOTECHNOL, V53, P92, DOI 10.1007/s12033-012-9598-4
Zhang LW, 2007, INT J TOXICOL, V26, P103, DOI 10.1080/10915810701225133
Zhang XY, 2012, TOXICOL RES-UK, V1, P62, DOI 10.1039/c2tx20006f
Zhang ZP, 2011, BIOMATERIALS, V32, P3666, DOI 10.1016/j.biomaterials.2011.01.067
Zhao DC, 2011, CLIN CANCER RES, V17, P771, DOI 10.1158/1078-0432.CCR-10-2444
Zheng M, 2003, NAT MATER, V2, P338, DOI 10.1038/nmat877
Zhu GZ, 2017, NAT COMMUN, V8, DOI 10.1038/s41467-017-02191-y
Zou WP, 2005, NAT REV CANCER, V5, P263, DOI 10.1038/nrc1586
NR 133
TC 46
Z9 47
U1 7
U2 92
PU ELSEVIER SCIENCE BV
PI AMSTERDAM
PA PO BOX 211, 1000 AE AMSTERDAM, NETHERLANDS
SN 0168-3659
EI 1873-4995
J9 J CONTROL RELEASE
JI J. Control. Release
PD MAR 10
PY 2019
VL 297
BP 79
EP 90
DI 10.1016/j.jconrel.2019.01.017
PG 12
WC Chemistry, Multidisciplinary; Pharmacology & Pharmacy
WE Science Citation Index Expanded (SCI-EXPANDED); Conference Proceedings Citation Index - Science (CPCI-S)
SC Chemistry; Pharmacology & Pharmacy
GA HN0PG
UT WOS:000459890300009
PM 30659906
OA Green Accepted
DA 2023-09-14
ER

PT J
AU Athanasiou, E
Agallou, M
Tastsoglou, S
Kammona, O
Hatzigeorgiou, A
Kiparissides, C
Karagouni, E
AF Athanasiou, Evita
Agallou, Maria
Tastsoglou, Spyros
Kammona, Olga
Hatzigeorgiou, Artemis
Kiparissides, Costas
Karagouni, Evdokia
TI A Poly(Lactic-co-Glycolic) Acid Nanovaccine Based on Chimeric Peptides
from Different Leishmania infantum Proteins Induces Dendritic Cells
Maturation and Promotes Peptide-Specific IFN gamma-Producing CD8(+) T
Cells Essential for the Protection against Experimental Visceral
Leishmaniasis

SO FRONTIERS IN IMMUNOLOGY
LA English
DT Article
DE Leishmania; chimeric peptides; peptide-based vaccine;
poly(lactic-co-glycolic) acid nanoparticles; dendritic cell
transcriptome
ID MHC CLASS-I; PLGA NANOPARTICLES; CROSS-PRESENTATION; ANTIGEN DELIVERY;
IMMUNE-RESPONSES; SUBUNIT VACCINES; EPITOPES; VITRO; MODEL; TH1
AB Visceral leishmaniasis, caused by Leishmania (L.) donovani and L. infantum protozoan parasites, can provoke overwhelming and protracted epidemics, with high case-fatality rates. An effective vaccine against the disease must rely on the generation of a strong and long-lasting T cell immunity, mediated by CD4(+) T-H1 and CD8(+) T cells. Multi-epitope peptide-based vaccine development is manifesting as the new era of vaccination strategies against Leishmania infection. In this study, we designed chimeric peptides containing HLA-restricted epitopes from three immunogenic L. infantum proteins (cysteine peptidase A, histone H1, and kinetoplastid membrane protein 11), in order to be encapsulated in poly(lactic-co-glycolic) acid nanoparticles with or without the adjuvant monophosphoryl lipid A (MPLA) or surface modification with an octapeptide targeting the tumor necrosis factor receptor II. We aimed to construct differentially functionalized peptide-based nanovaccine candidates and investigate their capacity to stimulate the immunomodulatory properties of dendritic cells (DCs), which are critical regulators of adaptive immunity generated upon vaccination. According to our results, DCs stimulation with the peptide-based nanovaccine candidates with MPLA incorporation or surface modification induced an enhanced maturation profile with prominent IL-12 production, promoting allogeneic T cell proliferation and intracellular production of IFN gamma by CD4(+) and CD8(+) T cell subsets. In addition, DCs stimulated with the peptide-based nanovaccine candidate with MPLA incorporation exhibited a robust transcriptional activation, characterized by upregulated genes indicative of vaccine-driven DCs differentiation toward type 1 phenotype. Immunization of HLA A2.1 transgenic mice with this peptide-based nanovaccine candidate induced peptide-specific IFN gamma-producing CD8(+) T cells and conferred significant protection against L. infantum infection. Concluding, our findings supported that encapsulation of more than one chimeric multi-epitope peptides from different immunogenic L. infantum proteins in a proper biocompatible delivery system with the right adjuvant is considered as an improved promising approach for the development of a vaccine against VL.
C1 [Athanasiou, Evita; Agallou, Maria; Karagouni, Evdokia] Hellenic Pasteur Inst, Dept Microbiol, Lab Cellular Immunol, Athens, Greece.
[Tastsoglou, Spyros; Hatzigeorgiou, Artemis] Hellenic Pasteur Inst, DIANA Lab, Athens, Greece.
[Kammona, Olga; Kiparissides, Costas] Ctr Res & Technol Hellas, Chem Proc & Energy Resources Inst, Lab Polymer React Engn, Thessaloniki, Greece.
[Kiparissides, Costas] Aristotle Univ Thessaloniki, Dept Chem Engn, Lab Chem Engn B, Thessaloniki, Greece.
C3 Centre for Research & Technology Hellas; Aristotle University of
Thessaloniki
RP Karagouni, E (通讯作者)，Hellenic Pasteur Inst, Dept Microbiol, Lab Cellular Immunol, Athens, Greece.
EM ekaragouni@pasteur.gr
RI Hatzigeorgiou, Artemis/Z-1976-2019; Agallou, Maria/AAC-6502-2020
OI Agallou, Maria/0000-0003-0241-1340; Karagouni,
Evdokia/0000-0002-0987-4877; Kiparissides, Costas/0000-0003-4649-0001;
Tastsoglou, Spyros/0000-0002-0316-6239; Kammona,
Olga/0000-0003-0730-507X
FU European Union; National Ministry of Education and Religion Affairs;
"NGS-infect" grant - SIEMENS AG; Hellenic Pasteur Institute; State
Scholarships Foundation (IKY) [SR22954]; SYNERGASIA [09SYN-14-643];
KRIPIS [MIS 450598]
FX This work was mainly supported by the grants "SYNERGASIA" (09SYN-14-643)
and "KRIPIS" (MIS 450598) co-financed by the European Union and the
National Ministry of Education and Religion Affairs under the
Operational Strategic Reference Framework (NSFR 2007-2013), awarded to
EK. The work was also partially supported by "NGS-infect" grant financed
by SIEMENS AG. EA and MA were awarded post-doctoral fellowships by the
Hellenic Pasteur Institute and the State Scholarships Foundation (IKY)
under "IKY FELLOWSHIPS OF EXCELLENCE FOR POSTGRADUATE STUDIES IN
GREECE-SIEMENS PROGRAM" (Contract No.: SR22954), respectively.
CR Agallou M, 2017, PLOS NEGLECT TROP D, V11, DOI 10.1371/journal.pntd.0005311

Agallou M, 2014, FRONT IMMUNOL, V5, DOI 10.3389/fimmu.2014.00268
Agallou M, 2011, VACCINE, V29, P5053, DOI 10.1016/j.vaccine.2011.04.089
Akhoon BA, 2011, MICROB PATHOGENESIS, V51, P77, DOI 10.1016/j.micpath.2011.01.011
Alexander J, 1998, IMMUNOL RES, V18, P79, DOI 10.1007/BF02788751
Alvar J, 2012, PLOS ONE, V7, DOI 10.1371/journal.pone.0035671
Alves-Silva MV, 2017, FRONT IMMUNOL, V8, P1, DOI 10.3389/fimmu.2017.00100
Banchereau R, 2014, NAT COMMUN, V5, DOI 10.1038/ncomms6283
Basu R, 2007, J INFECT DIS, V195, P1373, DOI 10.1086/513439
Bergmann CC, 1996, J IMMUNOL, V157, P3242
Bijker MS, 2007, J IMMUNOL, V179, P5033, DOI 10.4049/jimmunol.179.8.5033
Black M, 2010, EXPERT REV VACCINES, V9, P157, DOI [10.1586/erv.09.160, 10.1586/ERV.09.160]
Blake JA, 2015, NUCLEIC ACIDS RES, V43, pD1049, DOI 10.1093/nar/gku1179
Bougneres L, 2009, IMMUNITY, V31, P232, DOI 10.1016/j.immuni.2009.06.022
Bousso P, 2008, NAT REV IMMUNOL, V8, P675, DOI 10.1038/nri2379
Burkett PR, 2004, J EXP MED, V200, P825, DOI 10.1084/jem.20041389
Callahan MK, 2008, P NATL ACAD SCI USA, V105, P1662, DOI 10.1073/pnas.0711365105
Carrion J, 2008, VACCINE, V26, P2537, DOI 10.1016/j.vaccine.2008.03.003
Chen KY, 2012, IMMUNOL RES, V53, P182, DOI 10.1007/s12026-012-8295-5
Chong CSW, 2005, J CONTROL RELEASE, V102, P85, DOI 10.1016/j.jconrel.2004.09.014
Lima SAC, 2012, NANOMEDICINE-UK, V7, P1839, DOI [10.2217/NNM.12.74, 10.2217/nnm.12.74]
Costa V, 2017, INT J MOL SCI, V18, DOI 10.3390/ijms18030494
Danhier F, 2012, J CONTROL RELEASE, V161, P505, DOI 10.1016/j.jconrel.2012.01.043
De Groot AS, 2002, IMMUNOL CELL BIOL, V80, P255, DOI 10.1046/j.1440-1711.2002.01092.x
de Oliveira LC, 2015, PLOS ONE, V10, DOI 10.1371/journal.pone.0118225
Diwan Manish, 2004, Current Drug Delivery, V1, P405, DOI 10.2174/1567201043334597
Dolenc I, 1998, FEBS LETT, V434, P357, DOI 10.1016/S0014-5793(98)01010-2
Doytchinova IA, 2006, BMC BIOINFORMATICS, V7, DOI 10.1186/1471-2105-7-131
El Bissati K, 2016, JCI INSIGHT, V1, DOI 10.1172/jci.insight.85955
Elamanchili P, 2007, J IMMUNOTHER, V30, P378, DOI 10.1097/CJI.0b013e31802cf3e3
Elfaki MEE, 2012, HUM VACC IMMUNOTHER, V8, P1769, DOI 10.4161/hv.21881
Etna MP, 2014, ALTEX-ALTERN ANIM EX, V31, P397, DOI 10.14573/altex.1311041
Farhadi T, 2017, NETW MODEL ANAL HLTH, V6, DOI 10.1007/s13721-016-0142-5
Ferrington DA, 2012, PROG MOL BIOL TRANSL, V109, P75, DOI 10.1016/B978-0-12-397863-9.00003-1
Firat H, 1999, EUR J IMMUNOL, V29, P3112, DOI 10.1002/(SICI)1521-4141(199910)29:10<3112::AID-IMMU3112>3.0.CO;2-Q
Ghaffari-Nazari H, 2015, PLOS ONE, V10, DOI 10.1371/journal.pone.0142563
Gouzelou E, 2013, PARASITE VECTOR, V6, DOI 10.1186/1756-3305-6-342
Hamdy S, 2007, J BIOMED MATER RES A, V81A, P652, DOI 10.1002/jbm.a.31019
Hamdy S, 2011, ADV DRUG DELIVER REV, V63, P943, DOI 10.1016/j.addr.2011.05.021
Hirosue S, 2010, VACCINE, V28, P7897, DOI 10.1016/j.vaccine.2010.09.077
Ichiyanagi T, 2010, J IMMUNOL, V185, P2693, DOI 10.4049/jimmunol.1000821
Iranpour S, 2016, J EXP CLIN CANC RES, V35, DOI 10.1186/s13046-016-0444-6
Joshi MD, 2012, J CONTROL RELEASE, V161, P25, DOI 10.1016/j.jconrel.2012.05.010
Jung ID, 2010, BIOCHEM BIOPH RES CO, V394, P272, DOI 10.1016/j.bbrc.2010.02.153
Kamhawi S, 2014, SCI TRANSL MED, V6, DOI 10.1126/scitranslmed.3009118
Kapsenberg ML, 2003, NAT REV IMMUNOL, V3, P984, DOI 10.1038/nri1246
Kaye P, 2011, NAT REV MICROBIOL, V9, P604, DOI 10.1038/nrmicro2608
Kazzaz J, 2006, J CONTROL RELEASE, V110, P566, DOI 10.1016/j.jconrel.2005.10.010
Kotturi Maya F, 2009, Immunome Res, V5, P3, DOI 10.1186/1745-7580-5-3
Lee H, 1998, BIOTECHNOL PROGR, V14, P508, DOI 10.1021/bp9701224
Li YP, 2001, J CONTROL RELEASE, V71, P203, DOI 10.1016/S0168-3659(01)00218-8
Loeuillet C, 2016, PARASITE VECTOR, V9, DOI 10.1186/s13071-016-1413-9
Longmate J, 2001, IMMUNOGENETICS, V52, P165, DOI 10.1007/s002510000271
Lopes C, 2015, VISC LEISHM CONTR ST
Lundegaard C, 2007, BIOINFORMATICS, V23, P3265, DOI 10.1093/bioinformatics/btm471
Luo WJ, 2013, BIOINFORMATICS, V29, P1830, DOI 10.1093/bioinformatics/btt285
Lutsiak MEC, 2006, J PHARM PHARMACOL, V58, P739, DOI 10.1211/jpp.58.6.0004
Maertzdorf J, 2015, VACCINE, V33, P5256, DOI 10.1016/j.vaccine.2015.03.075
Margaroni M, 2016, EUR J PHARM BIOPHARM, V105, P18, DOI 10.1016/j.ejpb.2016.05.018
Martins VT, 2017, CELL IMMUNOL, V313, P32, DOI 10.1016/j.cellimm.2016.12.008
Melief CJM, 2008, NAT REV CANCER, V8, P351, DOI 10.1038/nrc2373

Meng LJ, 2016, J IMMUNOL, V196, P1070, DOI 10.4049/jimmunol.1501310
Miah MA, 2012, EUR J IMMUNOL, V42, P58, DOI 10.1002/eji.201141846
NEISIG A, 1995, J IMMUNOL, V154, P1273
Nezafat N, 2014, J THEOR BIOL, V349, P121, DOI 10.1016/j.jtbi.2014.01.018
Nishimura H, 2005, J IMMUNOL, V175, P4627, DOI 10.4049/jimmunol.175.7.4627
Pajot A, 2004, EUR J IMMUNOL, V34, P3060, DOI 10.1002/eji.200425463
Palatnik-De-Sousa CB, 2008, VACCINE, V26, P1709, DOI 10.1016/j.vaccine.2008.01.023
Panda AK, 2011, EXPERT REV VACCINES, V10, P155, DOI [10.1586/erv.10.164, 10.1586/ERV.10.164]
Pascolo S, 2005, EXPERT OPIN BIOL TH, V5, P919, DOI 10.1517/14712598.5.7.919
Patronov A, 2013, OPEN BIOL, V3, DOI 10.1098/rsob.120139
Rammensee HG, 1999, IMMUNOGENETICS, V50, P213, DOI 10.1007/s002510050595
Ready PD, 2014, CLIN EPIDEMIOL, V6, P147, DOI 10.2147/CLEP.S44267
Resende DM, 2008, VACCINE, V26, P4585, DOI 10.1016/j.vaccine.2008.05.091
Rezvan H, 2012, IRAN J PARASITOL, V7, P27
Rodrigues V, 2016, PARASITE VECTOR, V9, DOI 10.1186/s13071-016-1412-x
Sandau MM, 2004, J IMMUNOL, V173, P6537, DOI 10.4049/jimmunol.173.11.6537
Santos Diego M, 2012, Int J Nanomedicine, V7, P2115, DOI 10.2147/IJN.S30093
Schubert B, 2016, GENOME MED, V8, DOI 10.1186/s13073-016-0263-6
Seyed N, 2016, FRONT MICROBIOL, V7, DOI 10.3389/fmicb.2016.00467
Seyed N, 2011, PLOS NEGLECT TROP D, V5, DOI 10.1371/journal.pntd.0001295
Silva AL, 2016, HUM VACC IMMUNOTHER, V12, P1056, DOI 10.1080/21645515.2015.1117714
Stager S, 2012, FRONT IMMUNOL, V3, DOI 10.3389/fimmu.2012.00005
Stoel M, 2015, PLOS ONE, V10, DOI 10.1371/journal.pone.0125228
Suthar MS, 2012, IMMUNITY, V37, P235, DOI 10.1016/j.immuni.2012.07.004
Tafaghodi M, 2011, PARASITOL RES, V108, P1265, DOI 10.1007/s00436-010-2176-4
Taki A, 2015, VACCINES, V3, P638, DOI 10.3390/vaccines3030638
TITUS RG, 1985, PARASITE IMMUNOL, V7, P545, DOI 10.1111/j.1365-3024.1985.tb00098.x
Varypataki EM, 2016, J CONTROL RELEASE, V226, P98, DOI 10.1016/j.jconrel.2016.02.018
Velders MP, 2001, J IMMUNOL, V166, P5366, DOI 10.4049/jimmunol.166.9.5366
Vigne S, 2012, BLOOD, V120, P3478, DOI 10.1182/blood-2012-06-439026
Wang QM, 2004, SCAND J IMMUNOL, V60, P219, DOI 10.1111/j.0300-9475.2004.01442.x
Weissenbock A, 2004, J CONTROL RELEASE, V99, P383, DOI 10.1016/j.jconrel.2004.07.025
Yan M, 2006, BIOCHEM ENG J, V30, P48, DOI 10.1016/j.bej.2006.02.001
Yang AX, 2014, PLOS ONE, V9, DOI 10.1371/journal.pone.0086306
Yu GC, 2012, OMICS, V16, P284, DOI 10.1089/omi.2011.0118
NR 96
TC 46
Z9 46
U1 0
U2 26
PU FRONTIERS MEDIA SA
PI LAUSANNE
PA AVENUE DU TRIBUNAL FEDERAL 34, LAUSANNE, CH-1015, SWITZERLAND
SN 1664-3224
J9 FRONT IMMUNOL
JI Front. Immunol.
PD JUN 13
PY 2017
VL 8
AR 684
DI 10.3389/fimmu.2017.00684
PG 25
WC Immunology
WE Science Citation Index Expanded (SCI-EXPANDED)
SC Immunology
GA EX3EC
UT WOS:000403113800001
PM 28659922
OA gold, Green Published
DA 2023-09-14
ER

PT J
AU Liu, JL
Liu, XX
Han, YF
Zhang, J
Liu, D
Ma, GL
Li, C
Liu, LX
Kong, DL
AF Liu, Jiale
Liu, Xiaoxuan
Han, Yanfeng
Zhang, Jing
Liu, Dan
Ma, Guilei
Li, Chen
Liu, Lanxia
Kong, Deling
TI Nanovaccine Incorporated with Hydroxychloroquine Enhances Antigen
Cross-Presentation and Promotes Antitumor Immune Responses
SO ACS APPLIED MATERIALS & INTERFACES
LA English
DT Article
DE vaccine; nanovaccine; hydroxychloroquine; immune response; PLGA
nanoparticles
ID TARGETING DENDRITIC CELLS; POLYMERIC NANOPARTICLES; T-CELLS; CANCER;
AUTOPHAGY; ADJUVANT; IMMUNOTHERAPY; TEMOZOLOMIDE; DELIVERY; AGONISTS
AB Induction of effective antigen-specific CDS8(+) T cell responses is critical for cancer immunotherapy success. Hydroxychloroquine (HCQ) is a widely used classical antimalarial and antirheumatic drug. HCQ is also an endosomal membrane disrupting agent that can lead to vesicular swelling and membrane permeabilization, which likely facilitates the release of therapeutic agents from lysosomes into the cytoplasm. Here, we develop a minimalistic nanovaccine, which is composed of poly(lactide-co-glycolide)acid (PLGA) nanoparticles (NPs) encapsulating a physical mixture of ovalbumin (OVA, a model antigen) and HCQ (HCQ OVA PLGA NPs). spatiotemporally control the cytosolic delivery of antigens, enhance antigen processing histocompatibility complex (MHC)-I pathway, and thus generate a sufficient antitumor cytotoxic T-cell response. The results of in vitro experiments showed that HCQ OVA PLGA NPs significantly enhanced OVA escape from lysosomes into the cytoplasm within bone-marrow-derived dendritic cells. We also observed that HCQ OVA PLGA NPs enhanced the expression level of MHC-I on dendritic cells and improved cross-presentation of antigen, compared to free OVA or OVA PLGA NPs. Results of in vivo experiments confirmed that HCQinitiated Thl-type responses and strong CDS8(+) T-cell responses that induced tumor cell apoptosis. Moreover, vaccination of mice with HCQ OVA PLGA NPs effectively generated memory immune responses in vivo and prevented tumor progression. We conclude that co-encapsulation of HCQ with antigens in nanovaccines can boost antigen-specific antitumor immune responses, particularly through CDS8(+) T-cells, serving as a simple and effective platform for the treatment of tumors and infectious diseases. We tested whether HCQ could and presentation via the major
C1 [Liu, Jiale; Liu, Xiaoxuan; Zhang, Jing; Liu, Dan; Ma, Guilei; Li, Chen; Liu, Lanxia; Kong, Deling] Peking Union Med Coll, Tianjin Key Lab Biomat, Inst Biomed Engn, Tianjin 300192, Peoples R China.
[Liu, Jiale; Liu, Xiaoxuan; Zhang, Jing; Liu, Dan; Ma, Guilei; Li, Chen; Liu, Lanxia; Kong, Deling] Chinese Acad Med Sci, Tianjin 300192, Peoples R China.
[Han, Yanfeng] Guangdong Univ Technol, Inst Biomed & Pharmaceut Sci, Guangzhou 510006, Guangdong, Peoples R China.
[Han, Yanfeng] Univ Queensland, Sch Biomed Sci, Brisbane, Qld 4072, Australia.
[Kong, Deling] Nankai Univ, Coll Life Sci, State Key Lab Med Chem Biol, Tianjin 300071, Peoples R China.
C3 Chinese Academy of Medical Sciences - Peking Union Medical College;
Institute of BioMedical Engineering - CAMS; Peking Union Medical
College; Chinese Academy of Medical Sciences - Peking Union Medical
College; Guangdong University of Technology; University of Queensland;

Nankai University
RP Liu, LX; Kong, DL (通讯作者)，Peking Union Med Coll, Tianjin Key Lab Biomat, Inst Biomed Engn, Tianjin 300192, Peoples R China.; Liu, LX; Kong, DL (通讯作者)，Chinese Acad Med Sci, Tianjin 300192, Peoples R China.; Kong, DL (通讯作者)，Nankai Univ, Coll Life Sci, State Key Lab Med Chem Biol, Tianjin 300071, Peoples R China.
EM liulanxiabme@163.com; kongdeling@nankai.edu.cn
RI liu, jiale/HTO-3772-2023
OI Han, Yanfeng/0000-0001-8423-0409
FU Natural Science Foundation of China [31670948, 81673027, 51373199];
Tianjin Natural Science Foundation [15JCYBJC47600, 15JCYBJC18400,
15JCQNJC46200]; CAMS Innovation Fund for Medical Sciences [CAMS
I2M-3-004]
FX This work was supported by the Natural Science Foundation of China (nos.
31670948, 81673027, and 51373199), the Tianjin Natural Science
Foundation project (nos. 15JCYBJC47600, 15JCYBJC18400, and
15JCQNJC46200), and CAMS Innovation Fund for Medical Sciences (CAMS
I2M-3-004). We thanks to Dr. Adam C. Midgley's kind help in improving
the writing.
CR Adachi K, 2018, NAT BIOTECHNOL, V36, P346, DOI 10.1038/nbt.4086
Barnaby Stacey N, 2015, Cancer Treat Res, V166, P23, DOI 10.1007/978-3-319-16555-4_2
Bolhassani A, 2014, HUM VACC IMMUNOTHER, V10, P321, DOI 10.4161/hv.26796
Cook KL, 2014, CLIN CANCER RES, V20, P3222, DOI 10.1158/1078-0432.CCR-13-3227
Duffy A, 2015, CANCER CHEMOTH PHARM, V75, P439, DOI 10.1007/s00280-014-2637-z
Esser MT, 2003, VACCINE, V21, P419, DOI 10.1016/S0264-410X(02)00407-3
Finn OJ, 2012, ANN ONCOL, V23, P6, DOI 10.1093/annonc/mds256
Gewirtz DA, 2014, CANCER RES, V74, P647, DOI 10.1158/0008-5472.CAN-13-2966
Guo YY, 2015, ACS NANO, V9, P6918, DOI 10.1021/acsnano.5b01042
Hamdy S, 2011, ADV DRUG DELIVER REV, V63, P943, DOI 10.1016/j.addr.2011.05.021
Huang L, 2014, CURR OPIN ONCOL, V26, P92, DOI 10.1097/CCO.0000000000000035
Jindal V, 2018, MED ONCOL, V35, DOI 10.1007/s12032-018-1131-6
Joffre OP, 2012, NAT REV IMMUNOL, V12, P557, DOI 10.1038/nri3254
Kim H, 2018, BIOMATERIALS, V164, P38, DOI 10.1016/j.biomaterials.2018.02.034
Krishnan L, 2007, J IMMUNOL, V178, P2396, DOI 10.4049/jimmunol.178.4.2396
Liu LX, 2016, ACS APPL MATER INTER, V8, P11969, DOI 10.1021/acsami.6b01135
Liu LX, 2016, J CONTROL RELEASE, V225, P230, DOI 10.1016/j.jconrel.2016.01.050
Liu Q, 2015, ACS NANO, V9, P4925, DOI 10.1021/nn5066793
Luo M, 2017, J CONTROL RELEASE, V263, P200, DOI 10.1016/j.jconrel.2017.03.033
Luo M, 2017, NAT NANOTECHNOL, V12, P648, DOI [10.1038/nnano.2017.52,
10.1038/NNANO.2017.52]
Mellman I, 2013, CANCER IMMUNOL RES, V1, P145, DOI 10.1158/2326-6066.CIR-13-0102
Perche F, 2016, BIOMATERIALS, V90, P62, DOI 10.1016/j.biomaterials.2016.02.027
Qian Y, 2016, BIOMATERIALS, V98, P171, DOI 10.1016/j.biomaterials.2016.05.008
Qiu LP, 2018, SMALL, V14, DOI 10.1002/smll.201703539
Radovic-Moreno AF, 2015, P NATL ACAD SCI USA, V112, P3892, DOI 10.1073/pnas.1502850112
Rangwala R, 2014, AUTOPHAGY, V10, P1369, DOI 10.4161/auto.29118
Rosenfeld MR, 2014, AUTOPHAGY, V10, P1359, DOI 10.4161/auto.28984
Cordeiro AS, 2015, BIOTECHNOL ADV, V33, P1279, DOI 10.1016/j.biotechadv.2015.05.010
Schreiber RD, 2011, SCIENCE, V331, P1565, DOI 10.1126/science.1203486
Seya T, 2015, CANCER SCI, V106, P1659, DOI 10.1111/cas.12824
Shao K, 2015, ACS NANO, V9, P16, DOI 10.1021/nn5062029
Shi GN, 2017, BIOMATERIALS, V113, P191, DOI 10.1016/j.biomaterials.2016.10.047
Tang SY, 2013, COLLOID SURFACE B, V102, P653, DOI 10.1016/j.colsurfb.2012.08.036
Topalian SL, 2017, JAMA-J AM MED ASSOC, V318, P1647, DOI 10.1001/jama.2017.14155
Torres MP, 2011, ACTA BIOMATER, V7, P2857, DOI 10.1016/j.actbio.2011.03.023
Wang C, 2016, BIOMATERIALS, V79, P88, DOI 10.1016/j.biomaterials.2015.11.040
Wang MJ, 2014, IMMUNOTHERAPY-UK, V6, P1265, DOI 10.2217/imt.14.86
Waqar SN, 2017, PHARMACOL THERAPEUT, V180, P16, DOI 10.1016/j.pharmthera.2017.06.002
Zappasodi R, 2018, CANCER CELL, V33, P581, DOI 10.1016/j.ccell.2018.03.005
Zhang WF, 2014, BIOMATERIALS, V35, P6086, DOI 10.1016/j.biomaterials.2014.04.022
Zhang Y, 2014, ADV EXP MED BIOL, V841, P45, DOI 10.1007/978-94-017-9487-9_3
Zhang Y, 2014, ADV EXP MED BIOL, V841, P15, DOI 10.1007/978-94-017-9487-9_2

Zhao L, 2014, VACCINE, V32, P327, DOI 10.1016/j.vaccine.2013.11.069
Zhu GZ, 2017, ACS NANO, V11, P2387, DOI 10.1021/acsnano.7b00978
NR 44
TC 45
Z9 46
U1 3
U2 88
PU AMER CHEMICAL SOC
PI WASHINGTON
PA 1155 16TH ST, NW, WASHINGTON, DC 20036 USA
SN 1944-8244
EI 1944-8252
J9 ACS APPL MATER INTER
JI ACS Appl. Mater. Interfaces
PD SEP 19
PY 2018
VL 10
IS 37
BP 30983
EP 30993
DI 10.1021/acsami.8b09348
PG 11
WC Nanoscience & Nanotechnology; Materials Science, Multidisciplinary
WE Science Citation Index Expanded (SCI-EXPANDED)
SC Science & Technology - Other Topics; Materials Science
GA GU6QE
UT WOS:000445439900009
PM 30136844
DA 2023-09-14
ER

PT J
AU Zhu, GZ
Liu, YJ
Yang, XY
Kim, YH
Zhang, HM
Jia, R
Liao, HS
Jin, A
Lin, J
Aronova, M
Leapman, R
Nie, ZH
Niu, G
Chen, XY
AF Zhu, Guizhi
Liu, Yijing
Yang, Xiangyu
Kim, Young-Hwa
Zhang, Huimin
Jia, Rui
Liao, Hsien-Shun
Jin, Albert
Lin, Jing
Aronova, Maria
Leapman, Richard
Nie, Zhihong
Niu, Gang
Chen, Xiaoyuan
TI DNA-inorganic hybrid nanovaccine for cancer immunotherapy

SO NANOSCALE
LA English
DT Article
ID CPG DNA; DELIVERY; OLIGONUCLEOTIDES; SIGNALS; PARTICLES; ADJUVANTS; TLR9
AB Cancer evolves to evade or compromise the surveillance of the immune system, and cancer immunotherapy aims to harness the immune system in order to inhibit cancer development. Unmethylated CpG dinucleotide-containing oligonucleotides (CpG), a class of potent adjuvants that activate the toll-like receptor 9 (TLR9) located in the endolysosome of many antigen-presenting cells (APCs), are promising for cancer immunotherapy. However, clinical application of synthetic CpG confronts many challenges such as suboptimal delivery into APCs, unfavorable pharmacokinetics caused by limited biostability and short in vivo half-life, and side effects associated with leaking of CpG into the systemic circulation. Here we present DNA-inorganic hybrid nanovaccines (hNVs) for efficient uptake into APCs, prolonged tumor retention, and potent immunostimulation and cancer immunotherapy. hNVs were self-assembled from concatemer CpG analogs and magnesium pyrophosphate (Mg(2)PPi). Mg(2)PPi renders hNVs resistant to nuclease degradation and thermal denaturation, both of which are demanding characteristics for effective vaccination and the storage and transportation of vaccines. Fluorophore-labeled hNVs were tracked to be efficiently internalized into the endolysosomes of APCs, where Mg(2)PPi was dissolved in an acidic environment and thus CpG analogs were exposed to hNVs. Internalized hNVs in APCs led to (1) elevated secretion of proinflammatory factors, and (2) elevated expression of co-stimulatory factors. Compared with molecular CpG, hNVs dramatically prolonged the tissue retention of CpG analogs and reduced splenomegaly, a common side effect of CpG. In a melanoma mouse model, two injections of hNVs significantly inhibited the tumor growth and outperformed the molecular CpG. These results suggest hNVs are promising for cancer immunotherapy.
C1 [Zhu, Guizhi; Liu, Yijing; Yang, Xiangyu; Kim, Young-Hwa; Zhang, Huimin; Niu, Gang; Chen, Xiaoyuan] NIBIB, Lab Mol Imaging & Nanomed, NIH, Bethesda, MD 20892 USA.
[Jia, Rui] NICHHD, Sect Intracellular Prot Trafficking, Cell Biol & Metab Program, NIH, Bethesda, MD 20892 USA.
[Liao, Hsien-Shun; Jin, Albert; Lin, Jing; Aronova, Maria; Leapman, Richard] NIBIB, Lab Cellular Imaging & Macromol Biophys, NIH, Bethesda, MD 20892 USA.
[Nie, Zhihong] Univ Maryland, Dept Chem & Biochem, College Pk, MD 20742 USA.
C3 National Institutes of Health (NIH) - USA; NIH National Institute of Biomedical Imaging & Bioengineering (NIBIB); National Institutes of Health (NIH) - USA; NIH Eunice Kennedy Shriver National Institute of Child Health & Human Development (NICHD); National Institutes of Health (NIH) - USA; NIH National Institute of Biomedical Imaging & Bioengineering (NIBIB); University System of Maryland; University of Maryland College Park
RP Chen, XY (通讯作者)，NIBIB, Lab Mol Imaging & Nanomed, NIH, Bethesda, MD 20892 USA.
EM shawn.chen@nih.gov
RI chen, xi/GXH-3653-2022; chen, xia/GYR-3948-2022; Zhu, Guizhi/AAN-2888-2020; Jin, Albert/HKV-3135-2023; Liu, Yijing/D-6666-2016; chen, xia/GXM-5435-2022; LIN, JING/CAF-2178-2022; Nie, Zhihong/D-7495-2011
OI Jin, Albert/0000-0003-3826-1081; Nie, Zhihong/0000-0001-9639-905X; Jia, Rui/0000-0002-1797-4069; lin, jing/0000-0001-9865-2098; Liu, Yijing/0000-0002-7503-3910; Zhu, Guizhi/0000-0002-2945-7982; Leapman, Richard/0000-0002-8575-4084
FU National Institute of Biomedical Imaging and Bioengineering (NIBIB), National Institutes of Health (NIH)
FX This work was supported by the intramural research program of the National Institute of Biomedical Imaging and Bioengineering (NIBIB), National Institutes of Health (NIH).
CR Appelqvist H, 2013, J MOL CELL BIOL, V5, P214, DOI 10.1093/jmcb/mjt022
Chockalingam A, 2009, IMMUNOL CELL BIOL, V87, P209, DOI 10.1038/icb.2008.101
Darrell J. I., 2015, CHEM REV
FDA, 2011, AG RESP LETT GRAS NO
FDA, 2011, GRAS NOT 000383
FOGHANDERSEN N, 1995, CLIN CHEM, V41, P1522
Gregory M. B., 2005, NAT IMMUNOL, P7
Hartmann G, 1999, P NATL ACAD SCI USA, V96, P9305, DOI 10.1073/pnas.96.16.9305
Irvine DJ, 2013, NAT MATER, V12, P978, DOI [10.1038/NMAT3775, 10.1038/nmat3775]

Kevin E. S., 2014, SMALL, V10, P1623
Kim JH, 2012, ANGEW CHEM INT EDIT, V51, P9670, DOI 10.1002/anie.201204989
Klinman DM, 2004, NAT REV IMMUNOL, V4, P248, DOI 10.1038/nri1329
Klinman DM, 1996, P NATL ACAD SCI USA, V93, P2879, DOI 10.1073/pnas.93.7.2879
Krieg AM, 2006, NAT REV DRUG DISCOV, V5, P471, DOI 10.1038/nrd2059
Latz E, 2004, NAT IMMUNOL, V5, P190, DOI 10.1038/ni1028
Lee JB, 2012, NAT MATER, V11, P316, DOI [10.1038/NMAT3253, 10.1038/nmat3253]
Li J, 2011, ACS NANO, V5, P8783, DOI 10.1021/nn202774x
Liu HP, 2014, NATURE, V507, P519, DOI 10.1038/nature12978
Liu HP, 2011, ANGEW CHEM INT EDIT, V50, P7052, DOI 10.1002/anie.201101266
Liu XW, 2012, NANO LETT, V12, P4254, DOI 10.1021/nl301877k
Lv YF, 2015, NAT PROTOC, V10, P1508, DOI 10.1038/nprot.2015.078
Nguyen DN, 2012, P NATL ACAD SCI USA, V109, pE797, DOI 10.1073/pnas.1121423109
Nishikawa M, 2011, BIOMATERIALS, V32, P488, DOI 10.1016/j.biomaterials.2010.09.013
Palucka K, 2012, NAT REV CANCER, V12, P265, DOI 10.1038/nrc3258
Roh YH, 2014, ACS NANO, V8, P9767, DOI 10.1021/nn502596b
Rosenberg SA, 2004, NAT MED, V10, P909, DOI 10.1038/nm1100
Scheiermann J, 2014, VACCINE, V32, P6377, DOI 10.1016/j.vaccine.2014.06.065
Sester DP, 2006, J IMMUNOL, V177, P4473, DOI 10.4049/jimmunol.177.7.4473
Sharma P, 2015, SCIENCE, V348, P56, DOI 10.1126/science.aaa8172
Sharp FA, 2009, P NATL ACAD SCI USA, V106, P870, DOI 10.1073/pnas.0804897106
Sparwasser T, 1999, J IMMUNOL, V162, P2368
Storni T, 2004, J IMMUNOL, V172, P1777, DOI 10.4049/jimmunol.172.3.1777
Thomas B., 2012, NAT REV CANCER, V12, P307
Titta D. A., 2013, P NATL ACAD SCI USA, P19902
Zhang L., 2015, ACS APPL MATER INTER, P24069
Zhang PP, 2015, ACS NANO, V9, P6465, DOI 10.1021/acsnano.5b02153
Zhu GZ, 2013, J AM CHEM SOC, V135, P16438, DOI 10.1021/ja406115e
NR 37
TC 45
Z9 46
U1 3
U2 109
PU ROYAL SOC CHEMISTRY
PI CAMBRIDGE
PA THOMAS GRAHAM HOUSE, SCIENCE PARK, MILTON RD, CAMBRIDGE CB4 0WF, CAMBS, ENGLAND
SN 2040-3364
EI 2040-3372
J9 NANOSCALE
JI Nanoscale
PY 2016
VL 8
IS 12
BP 6684
EP 6692
DI 10.1039/c5nr08821f
PG 9
WC Chemistry, Multidisciplinary; Nanoscience & Nanotechnology; Materials Science, Multidisciplinary; Physics, Applied
WE Science Citation Index Expanded (SCI-EXPANDED)
SC Chemistry; Science & Technology - Other Topics; Materials Science; Physics
GA DH5SG
UT WOS:000372851500054
PM 26947116
OA Green Accepted
DA 2023-09-14
ER

PT J

AU Xiong, X
Zhao, JY
Pan, JM
Liu, CP
Guo, X
Zhou, SB
AF Xiong, Xiang
Zhao, Jingya
Pan, Jingmei
Liu, Chunping
Guo, Xing
Zhou, Shaobing
TI Personalized Nanovaccine Coated with Calcinetin-Expressed Cancer Cell Membrane Antigen for Cancer Immunotherapy
SO NANO LETTERS
LA English
DT Article
DE Tumor cell membrane; immunogenic cell death; nanoparticles; nanovaccine; immunotherapy
ID T-CELLS; DEATH; HETEROGENEITY; NANOPARTICLES; NANOMATERIALS; POLARIZATION; VACCINES
AB A cancer vaccine has been widely applied in clinical tumor therapy as one of the main strategies of immunotherapy. However, the traditional cancer vaccine for a single antigen has a low benefit rate due to the individual differences in patients. Here, we report a R837-loaded poly(lactic-co-glycolic acid) nanovaccine coated with a calcinetin (CRT)-expressed cancer cell membrane antigen for immunotherapy. The cell membrane antigen that possessed a complete antigen array was obtained by inducing immunogenic cell death in vitro, avoiding the severe systemic toxicity of chemotherapy in vivo. The nanovaccine codelivers the adjuvant R837 and the Luc-4T1 membrane antigen, triggering a personalized immune response to the corresponding tumor. Moreover, the calcinetin exposed on the surface of the nanovaccine induces the active uptake of dendritic cells, consequently enhancing the antitumor effect. Meanwhile, the nanovaccine activates immune memory cells to provide long-term protection. Our work provides a new strategy for a clinical personalized antitumor vaccine.
C1 [Xiong, Xiang; Zhao, Jingya; Pan, Jingmei; Liu, Chunping; Guo, Xing; Zhou, Shaobing] Southwest Jiaotong Univ, Sch Mat Sci & Engn, Key Lab Adv Technol Mat, Minist Educ, Chengdu 610031, Peoples R China.
C3 Southwest Jiaotong University
RP Guo, X; Zhou, SB (通讯作者)，Southwest Jiaotong Univ, Sch Mat Sci & Engn, Key Lab Adv Technol Mat, Minist Educ, Chengdu 610031, Peoples R China.
EM xingguo@swjtu.edu.cn; shaobingzhou@swjtu.edu.cn
FU National Natural Science Foundation of China [51725303, 81701831, 52033007, 52073236]; 5th Chinese Association for Science and Technology Young Talents Support Project
FX This work was supported by the National Natural Science Foundation of China (51725303, 81701831, 52033007, and 52073236) and the 5th Chinese Association for Science and Technology Young Talents Support Project. All animal experiments met the ethical requirements for animal experiments and were approved by the Institutional Animal Care and Use Committee of Sichuan University. The authors thank the Analysis and Testing Center of Southwest Jiaotong University.
CR Burrell RA, 2013, NATURE, V501, P338, DOI 10.1038/nature12625
Byrne A, 2020, NAT REV CLIN ONCOL, V17, P341, DOI 10.1038/s41571-020-0333-y
Cabeza-Cabrerizo M, 2021, ANNU REV IMMUNOL, V39, P131, DOI 10.1146/annurev-immunol-061020-053707
Couzin-Frankel J, 2013, SCIENCE, V342, P1432, DOI 10.1126/science.342.6165.1432
Duan XP, 2019, ANGEW CHEM INT EDIT, V58, P670, DOI 10.1002/anie.201804882
Emens LA, 2017, EUR J CANCER, V81, P116, DOI 10.1016/j.ejca.2017.01.035
Fan Q, 2020, SCI ADV, V6, DOI 10.1126/sciadv.abb4639
Fang RH, 2018, ADV MATER, V30, DOI 10.1002/adma.201706759
Fang RH, 2017, BIOMATERIALS, V128, P69, DOI 10.1016/j.biomaterials.2017.02.041
Fang RH, 2014, NANO LETT, V14, P2181, DOI 10.1021/nl500618u
Farkona S, 2016, BMC MED, V14, DOI 10.1186/s12916-016-0623-5

Feng B, 2019, ADV MATER, V31, DOI 10.1002/adma.201902960
Feng B, 2018, ADV MATER, V30, DOI 10.1002/adma.201803001
Galluzzi L, 2017, NAT REV IMMUNOL, V17, P97, DOI 10.1038/nri.2016.107
Garg AD, 2010, BBA-REV CANCER, V1805, P53, DOI 10.1016/j.bbcan.2009.08.003
Han X, 2019, SCI ADV, V5, DOI 10.1126/sciadv.aaw6870
Huang XH, 2021, ADV SCI, V8, DOI 10.1002/advs.202003572
Irvine DJ, 2020, NAT REV IMMUNOL, V20, P321, DOI 10.1038/s41577-019-0269-6
Jackson HJ, 2016, NAT REV CLIN ONCOL, V13, P370, DOI 10.1038/nrclinonc.2016.36
June CH, 2018, SCIENCE, V359, P1361, DOI 10.1126/science.aar6711
Krysko DV, 2012, NAT REV CANCER, V12, P860, DOI 10.1038/nrc3380
Laidlaw BJ, 2016, NAT REV IMMUNOL, V16, P102, DOI 10.1038/nri.2015.10
Lawrence MS, 2013, NATURE, V499, P214, DOI 10.1038/nature12213
Le Moignic A, 2018, J CONTROL RELEASE, V278, P110, DOI 10.1016/j.jconrel.2018.03.035
Liu LQ, 2020, SMALL, V16, DOI 10.1002/smll.202003543
Panaretakis T, 2009, EMBO J, V28, P578, DOI 10.1038/emboj.2009.1
Ribas A, 2018, SCIENCE, V359, P1350, DOI 10.1126/science.aar4060
Rosenberg SA, 2004, NAT MED, V10, P909, DOI 10.1038/nm1100
Rosenberg SA, 2008, NAT REV CANCER, V8, P299, DOI 10.1038/nrc2355
Roth F, 2012, CANCER RES, V72, P1373, DOI 10.1158/0008-5472.CAN-11-2772
Sabado RL, 2017, CELL RES, V27, P74, DOI 10.1038/cr.2016.157
Sahin U, 2018, SCIENCE, V359, P1355, DOI 10.1126/science.aar7112
Schumacher TN, 2015, SCIENCE, V348, P69, DOI 10.1126/science.aaa4971
Song WT, 2017, BIOMATERIALS, V148, P16, DOI 10.1016/j.biomaterials.2017.09.017
Togashi Y, 2019, NAT REV CLIN ONCOL, V16, P356, DOI 10.1038/s41571-019-0175-7
Wculek SK, 2020, NAT REV IMMUNOL, V20, P7, DOI 10.1038/s41577-019-0210-z
Wei BC, 2021, NANO LETT, V21, P4231, DOI 10.1021/acs.nanolett.1c00209
Xiang SD, 2008, EXPERT REV VACCINES, V7, P1103, DOI 10.1586/14760584.7.7.1103
Xiong X, 2021, NANO TODAY, V39, DOI 10.1016/j.nantod.2021.101225
Yang R, 2018, ACS NANO, V12, P5121, DOI 10.1021/acsnano.7b09041
Zanganeh S, 2016, NAT NANOTECHNOL, V11, P986, DOI [10.1038/nnano.2016.168, 10.1038/NNANO.2016.168]
Zhang HM, 2018, J CANCER, V9, P1773, DOI 10.7150/jca.24577
NR 42
TC 44
Z9 44
U1 16
U2 130
PU AMER CHEMICAL SOC
PI WASHINGTON
PA 1155 16TH ST, NW, WASHINGTON, DC 20036 USA
SN 1530-6984
EI 1530-6992
J9 NANO LETT
JI Nano Lett.
PD OCT 13
PY 2021
VL 21
IS 19
BP 8418
EP 8425
DI 10.1021/acs.nanolett.1c03004
EA SEP 2021
PG 8
WC Chemistry, Multidisciplinary; Chemistry, Physical; Nanoscience & Nanotechnology; Materials Science, Multidisciplinary; Physics, Applied; Physics, Condensed Matter
WE Science Citation Index Expanded (SCI-EXPANDED)
SC Chemistry; Science & Technology - Other Topics; Materials Science; Physics
GA WK2GK
UT WOS:000709549100067

PM 34546061
DA 2023-09-14
ER

PT J
AU Kim, CG
Kye, YC
Yun, CH
AF Kim, Cheol Gyun
Kye, Yoon-Chul
Yun, Cheol-Heui
TI The Role of Nanovaccine in Cross-Presentation of Antigen-Presenting Cells for the Activation of CD8(+) T Cell Responses
SO PHARMACEUTICS
LA English
DT Review
DE nanovaccine; cross-presentation; CD8(+) T cell; cytotoxic T lymphocyte; cancer vaccine; dendritic cell
ID PH-SENSITIVE LIPOSOMES; DENDRITIC CELLS; DELIVERY-SYSTEM; LYMPH-NODE; PHOTOCHEMICAL INTERNALIZATION; FUNCTIONALIZED NANOPARTICLES; CPG OLIGONUCLEOTIDES; PARTICULATE VACCINES; TARGETED DELIVERY; IMMUNE-RESPONSES
AB Explosive growth in nanotechnology has merged with vaccine development in the battle against diseases caused by bacterial or viral infections and malignant tumors. Due to physicochemical characteristics including size, viscosity, density and electrostatic properties, nanomaterials have been applied to various vaccination strategies. Nanovaccines, as they are called, have been the subject of many studies, including review papers from a material science point of view, although a mode of action based on a biological and immunological understanding has yet to emerge. In this review, we discuss nanovaccines in terms of CD8(+) T cell responses, which are essential for antiviral and anticancer therapies. We focus mainly on the role and mechanism, with particular attention to the functional aspects, of nanovaccines in inducing cross-presentation, an unconventional type of antigen-presentation that activates CD8(+) T cells upon administration of exogenous antigens, in dendritic cells followed by activation of antigen-specific CD8(+) T cell responses. Two major intracellular mechanisms that nanovaccines harness for cross-presentation are described; one is endosomal swelling and rupture, and the other is membrane fusion. Both processes eventually allow exogenous vaccine antigens to be exported from phagosomes to the cytosol followed by loading on major histocompatibility complex class I, triggering clonal expansion of CD8(+) T cells. Advancement of nanotechnology with an enhanced understanding of how nanovaccines work will contribute to the design of more effective and safer nanovaccines.
C1 [Kim, Cheol Gyun; Kye, Yoon-Chul; Yun, Cheol-Heui] Seoul Natl Univ, Dept Agr Biotechnol, Seoul 08826, South Korea.
[Kim, Cheol Gyun; Kye, Yoon-Chul; Yun, Cheol-Heui] Seoul Natl Univ, Res Inst Agr & Life Sci, Seoul 08826, South Korea.
[Yun, Cheol-Heui] Seoul Natl Univ, Ctr Food & Bioconvergence, Seoul 08826, South Korea.
[Yun, Cheol-Heui] Seoul Natl Univ, Inst Green Bio Sci & Technol, Pyeongchang 25354, Gangwon Do, South Korea.
C3 Seoul National University (SNU); Seoul National University (SNU); Seoul National University (SNU); Seoul National University (SNU)
RP Yun, CH (通讯作者)，Seoul Natl Univ, Dept Agr Biotechnol, Seoul 08826, South Korea.; Yun, CH (通讯作者)，Seoul Natl Univ, Res Inst Agr & Life Sci, Seoul 08826, South Korea.; Yun, CH (通讯作者)，Seoul Natl Univ, Ctr Food & Bioconvergence, Seoul 08826, South Korea.; Yun, CH (通讯作者)，Seoul Natl Univ, Inst Green Bio Sci & Technol, Pyeongchang 25354, Gangwon Do, South Korea.
EM cjfrbs73@snu.ac.kr; highspect@snu.ac.kr; cyun@snu.ac.kr
RI YUN, Cheol-Heui/AAR-2775-2020
OI YUN, Cheol-Heui/0000-0002-0041-2887; Kye, Yoon-Chul/0000-0003-0261-5247
FU Cooperative Research Program for Agriculture Science and Technology Development, Rural Development Administration, Republic of Korea [PJ01336401]; National Research Foundation of Korea, Republic of Korea [2018R1A2B2006793]; Korea Institute of Planning and Evaluation for Technology in Food, Agriculture and Forestry (IPET) through Animal Disease Management Technology Development Program - Ministry of

Agriculture, Food and Rural Affairs (MAFRA) [319081-03]; BK21 Plus
Program of the Department of Agricultural Biotechnology, Seoul National
University, Seoul, Republic of Korea; National Research Foundation of
Korea [2018R1A2B2006793] Funding Source: Korea Institute of Science &
Technology Information (KISTI), National Science & Technology
Information Service (NTIS)
FX This work was carried out with the support of Cooperative Research
Program for Agriculture Science and Technology Development (PJ01336401),
Rural Development Administration, Republic of Korea. This work was also
supported by the Basic Science Research Program through the National
Research Foundation of Korea (2018R1A2B2006793), Republic of Korea, and
Korea Institute of Planning and Evaluation for Technology in Food,
Agriculture and Forestry (IPET) through Animal Disease Management
Technology Development Program, funded by Ministry of Agriculture, Food
and Rural Affairs (MAFRA) (319081-03). Cheol Gyun Kim was supported by
the BK21 Plus Program of the Department of Agricultural Biotechnology,
Seoul National University, Seoul, Republic of Korea.
CR Ackerman AL, 2003, P NATL ACAD SCI USA, V100, P12889, DOI 10.1073/pnas.1735556100
Adigbli DK, 2012, CURR OPIN PHARMACOL, V12, P434, DOI 10.1016/j.coph.2012.04.005
ALLISON AC, 1974, NATURE, V252, P252, DOI 10.1038/252252a0
Alloatti A, 2016, IMMUNOL REV, V272, P97, DOI 10.1111/imr.12432
Arosio D, 2014, BIOCONJUGATE CHEM, V25, P2244, DOI 10.1021/bc500467u
Asadzadeh Z, 2020, CANCERS, V12, DOI 10.3390/cancers12041047
Barker CIS, 2014, LANCET INFECT DIS, V14, P227, DOI 10.1016/S1473-3099(13)70238-X
Bayona AMD, 2016, INT J CANCER, V138, P1049, DOI 10.1002/ijc.29510
Behr JP, 1997, CHIMIA, V51, P34
Benjaminsen RV, 2013, MOL THER, V21, P149, DOI 10.1038/mt.2012.185
Gondan AIB, 2018, BIOMATERIALS, V170, P95, DOI 10.1016/j.biomaterials.2018.04.003
Bode C, 2011, EXPERT REV VACCINES, V10, P499, DOI [10.1586/ERV.10.174, 10.1586/erv.10.174]
Bourquin C, 2008, J IMMUNOL, V181, P2990, DOI 10.4049/jimmunol.181.5.2990
Bruno C, 2015, J IMMUNOL, V195, P166, DOI 10.4049/jimmunol.1500431
BULLOUGH PA, 1994, NATURE, V371, P37, DOI 10.1038/371037a0
Bungener L, 2002, VACCINE, V20, P2287, DOI 10.1016/S0264-410X(02)00103-2
Castellino F, 2006, NATURE, V440, P890, DOI 10.1038/nature04651
Chen HC, 2011, BIOMATERIALS, V32, P1731, DOI 10.1016/j.biomaterials.2010.10.059
Chen JA, 2011, INT J NANOMED, V6, P77, DOI 10.2147/IJN.S15457
Choi B, 2016, ACS NANO, V10, P7339, DOI 10.1021/acsnano.5b08084
de Gruijl TD, 2008, CANCER IMMUNOL IMMUN, V57, P1569, DOI 10.1007/s00262-008-0536-z
de Jong S, 2007, CANCER IMMUNOL IMMUN, V56, P1251, DOI 10.1007/s00262-006-0276-x
De Smedt SC, 2000, PHARMACEUT RES, V17, P113, DOI 10.1023/A:1007548826495
de Titta A, 2013, P NATL ACAD SCI USA, V110, P19902, DOI 10.1073/pnas.1313152110
Del Giudice G, 2018, SEMIN IMMUNOL, V39, P14, DOI 10.1016/j.smim.2018.05.001
Delamarre L, 2005, CELL STRUCT FUNCT, V30, P74
Di Marco M, 2010, INT J NANOMED, V5, P37
Dong H, 2018, INT J NANOMED, V13, P3353, DOI 10.2147/IJN.S164097
Duthie MS, 2012, VACCINE, V30, P134, DOI 10.1016/j.vaccine.2011.11.005
El-Sayed A, 2009, AAPS J, V11, P13, DOI 10.1208/s12248-008-9071-2
Embgenbroich M, 2018, FRONT IMMUNOL, V9, DOI 10.3389/fimmu.2018.01643
Falcone S, 2006, J CELL SCI, V119, P4758, DOI 10.1242/jcs.03238
Fehres CM, 2017, CELL MOL IMMUNOL, V14, P360, DOI 10.1038/cmi.2015.87
Fifis T, 2004, J IMMUNOL, V173, P3148, DOI 10.4049/jimmunol.173.5.3148
Firdous J, 2014, ACTA BIOMATER, V10, P4606, DOI 10.1016/j.actbio.2014.07.034
Franciszkiewicz K, 2012, CANCER RES, V72, P6325, DOI 10.1158/0008-5472.CAN-12-2027
Gao S, 2019, THERANOSTICS, V9, P126, DOI 10.7150/thno.29431
Gause KT, 2017, ACS NANO, V11, P54, DOI 10.1021/acsnano.6b07343
Gazi U, 2009, IMMUNOBIOLOGY, V214, P554, DOI 10.1016/j.imbio.2008.11.004
Giannini SL, 2006, VACCINE, V24, P5937, DOI 10.1016/j.vaccine.2006.06.005
Goldinger SM, 2012, EUR J IMMUNOL, V42, P3049, DOI 10.1002/eji.201142361
Gonzalez FE, 2014, HUM VACC IMMUNOTHER, V10, P3261, DOI 10.4161/21645515.2014.982996
Gros M, 2019, FRONT IMMUNOL, V10, DOI 10.3389/fimmu.2019.00041
Gulla SK, 2019, BIOMATER SCI-UK, V7, P773, DOI 10.1039/c8bm01272e

Gunn MD, 1998, P NATL ACAD SCI USA, V95, P258, DOI 10.1073/pnas.95.1.258
Hakerud M, 2015, J CONTROL RELEASE, V198, P10, DOI 10.1016/j.jconrel.2014.11.032
Hakerud M, 2014, J CONTROL RELEASE, V174, P143, DOI 10.1016/j.jconrel.2013.11.017
Hamdy S, 2008, VACCINE, V26, P5046, DOI 10.1016/j.vaccine.2008.07.035
Hart P, 2018, MOL THER, V26, P822, DOI 10.1016/j.ymthe.2017.12.016
He Q, 2000, CLIN DIAGN LAB IMMUN, V7, P899, DOI 10.1128/CDLI.7.6.899-903.2000
Henriksen-Lacey M, 2010, J CONTROL RELEASE, V145, P102, DOI 10.1016/j.jconrel.2010.03.027
Hirosue S, 2010, VACCINE, V28, P7897, DOI 10.1016/j.vaccine.2010.09.077
Hjalmsdottir A, 2016, MOL PHARMACEUT, V13, P320, DOI 10.1021/acs.molpharmaceut.5b00394
Homhuan A, 2008, ASIAN BIOMED, V2, P233
Immordino ML, 2006, INT J NANOMED, V1, P297
Irache JM, 2008, EXPERT OPIN DRUG DEL, V5, P703, DOI [10.1517/17425247.5.6.703, 10.1517/17425247.5.6.703 ]
Jahn R, 2003, CELL, V112, P519, DOI 10.1016/S0092-8674(03)00112-0
Jeanbart L, 2014, CANCER IMMUNOL RES, V2, P436, DOI 10.1158/2326-6066.CIR-14-0019-T
Jewell CM, 2011, P NATL ACAD SCI USA, V108, P15745, DOI 10.1073/pnas.1105200108
Ji Y, 2018, J MATER CHEM B, V6, P1930, DOI [10.1039/c7tb03233a, 10.1039/c7tb03319b]
Jia JB, 2018, FRONT ONCOL, V8, DOI 10.3389/fonc.2018.00404
Jiang DD, 2018, ACS APPL MATER INTER, V10, P37797, DOI 10.1021/acsami.8b09946
Jiang H, 2018, ADV SCI, V5, DOI 10.1002/advs.201700426
Jimenez-Sanchez G, 2015, PHARM RES-DORDR, V32, P311, DOI 10.1007/s11095-014-1465-5
Joffre OP, 2012, NAT REV IMMUNOL, V12, P557, DOI 10.1038/nri3254
Jones LH, 2015, NAT CHEM, V7, P952, DOI [10.1038/NCHEM.2396, 10.1038/nchem.2396]
Kasturi SP, 2011, NATURE, V470, P543, DOI 10.1038/nature09737
Khan I, 2019, ARAB J CHEM, V12, P908, DOI 10.1016/j.arabjc.2017.05.011
Kim MG, 2014, ASIAN J PHARM SCI, V9, P227, DOI 10.1016/j.ajps.2014.06.002
Kitahata Y, 2016, ONCOTARGET, V7, P48860, DOI 10.18632/oncotarget.10379
Kobiyama K, 2014, P NATL ACAD SCI USA, V111, P3086, DOI 10.1073/pnas.1319268111
Kolumam GA, 2005, J EXP MED, V202, P637, DOI 10.1084/jem.20050821
Kono K, 1997, BBA-BIOMEMBRANES, V1325, P143, DOI 10.1016/S0005-2736(96)00244-1
Koppolu BP, 2014, BIOMATERIALS, V35, P4382, DOI 10.1016/j.biomaterials.2014.01.078
Koppolu B, 2013, BIOMATERIALS, V34, P2359, DOI 10.1016/j.biomaterials.2012.11.066
Kourtis IC, 2013, PLOS ONE, V8, DOI 10.1371/journal.pone.0061646
Kunisawa J, 2001, J IMMUNOL, V167, P1406, DOI 10.4049/jimmunol.167.3.1406
Kye YC, 2019, ACTA BIOMATER, V90, P362, DOI 10.1016/j.actbio.2019.03.049
Lee YM, 2010, ARCH PHARM RES, V33, P231, DOI 10.1007/s12272-010-0207-4
Li P, 2013, J CONTROL RELEASE, V168, P271, DOI 10.1016/j.jconrel.2013.03.025
Lu FJ, 2017, NPJ VACCINES, V2, DOI 10.1038/s41541-017-0007-7
Lu FJ, 2015, J CONTROL RELEASE, V204, P51, DOI 10.1016/j.jconrel.2015.03.002
McHutchison JG, 2007, HEPATOLOGY, V46, P1341, DOI 10.1002/hep.21773
Meng CC, 2016, ACS NANO, V10, P2203, DOI 10.1021/acsnano.5b06750
Mody KT, 2013, NANOSCALE, V5, P5167, DOI 10.1039/c3nr00357d
Moon H, 2014, BIOMACROMOLECULES, V15, P3794, DOI 10.1021/bm501066m
Mumper RJ, 2003, J DISPER SCI TECHNOL, V24, P569, DOI 10.1081/DIS-120021814
Murthy N, 1999, J CONTROL RELEASE, V61, P137, DOI 10.1016/S0168-3659(99)00114-5
Nakamura T, 2008, MOL THER, V16, P1507, DOI 10.1038/mt.2008.122
Nakamura T, 2017, THER DELIV, V8, P987, DOI 10.4155/tde-2017-0071
Nam J, 2017, CELL MOL BIOENG, V10, P341, DOI 10.1007/s12195-017-0505-8
Nembrini C, 2011, P NATL ACAD SCI USA, V108, pE989, DOI 10.1073/pnas.1104264108
Norum OJ, 2009, J PHOTOCH PHOTOBIO B, V96, P83, DOI 10.1016/j.jphotobiol.2009.04.012
Ochyl LJ, 2018, BIOMATERIALS, V182, P157, DOI 10.1016/j.biomaterials.2018.08.016
Oishi M, 2006, BIOCONJUGATE CHEM, V17, P677, DOI 10.1021/bc050364m
Oyewumi MO, 2010, EXPERT REV VACCINES, V9, P1095, DOI [10.1586/erv.10.89, 10.1586/ERV.10.89]
Palm NW, 2009, IMMUNOL REV, V227, P221, DOI 10.1111/j.1600-065X.2008.00731.x
Pati R, 2018, FRONT IMMUNOL, V9, DOI 10.3389/fimmu.2018.02224
Puig M, 2006, NUCLEIC ACIDS RES, V34, P6488, DOI 10.1093/nar/gkl867
Pulendran B, 2011, NAT IMMUNOL, V12, P509, DOI 10.1038/ni.2039
REDDY R, 1991, J IMMUNOL METHODS, V141, P157, DOI 10.1016/0022-1759(91)90142-3
Roman BS, 2008, EUR J PHARM BIOPHARM, V70, P98, DOI 10.1016/j.ejpb.2008.03.015
Roopngam PE, 2019, NANOMED J, V6, P1, DOI 10.22038/nmj.2019.06.001
Sakaguchi N, 2008, BIOCONJUGATE CHEM, V19, P1040, DOI 10.1021/bc7004736

Sallusto F, 2000, IMMUNOL REV, V177, P134, DOI 10.1034/j.1600-065X.2000.17717.x
Samie M, 2015, NAT IMMUNOL, V16, P729, DOI 10.1038/ni.3196
SEKI K, 1984, MACROMOLECULES, V17, P1692, DOI 10.1021/ma00139a009
Selbo PK, 2015, PHOTOCH PHOTOBIO SCI, V14, P1433, DOI 10.1039/c5pp00027k
Sharma N, 2015, J IMMUNOL, V195, P5227, DOI 10.4049/jimmunol.1500993
Shen LM, 2013, PLOS ONE, V8, DOI 10.1371/journal.pone.0080904
Silva AL, 2015, VACCINE, V33, P847, DOI 10.1016/j.vaccine.2014.12.059
Silva JM, 2014, NANOMEDICINE-UK, V9, P2639, DOI [10.2217/NNM.14.135, 10.2217/nnm.14.135]
Slutter B, 2010, J CONTROL RELEASE, V143, P207, DOI 10.1016/j.jconrel.2010.01.007
Spadaro F, 2012, BLOOD, V119, P1407, DOI 10.1182/blood-2011-06-363564
Tachibana R, 1998, BIOCHEM BIOPH RES CO, V251, P538, DOI 10.1006/bbrc.1998.9460
Tambe P, 2017, ACS APPL MATER INTER, V9, P35562, DOI 10.1021/acsami.7b11024
van der Vlies AJ, 2010, BIOCONJUGATE CHEM, V21, P653, DOI 10.1021/bc9004443
Vieira OV, 2002, BIOCHEM J, V366, P689, DOI 10.1042/BJ20020691
Vollmer J, 2009, ADV DRUG DELIVER REV, V61, P195, DOI 10.1016/j.addr.2008.12.008
Waeckerle-Men Y, 2013, EUR J PHARM BIOPHARM, V85, P34, DOI 10.1016/j.ejpb.2013.02.002
Wamhoff EC, 2019, ACS CENTRAL SCI, V5, P808, DOI 10.1021/acscentsci.9b00093
Wang MY, 2013, BIOMATERIALS, V34, P10120, DOI 10.1016/j.biomaterials.2013.09.006
Wendorf J, 2006, J PHARM SCI-US, V95, P2738, DOI 10.1002/jps.20728
Wilkins AL, 2017, FRONT IMMUNOL, V8, DOI 10.3389/fimmu.2017.01760
Wilson NS, 2005, ADV IMMUNOL, V86, P241, DOI 10.1016/S0065-2776(04)86007-3
Xiang SD, 2006, METHODS, V40, P1, DOI 10.1016/j.ymeth.2006.05.016
Yoshikawa T, 2006, BIOL PHARM BULL, V29, P100, DOI 10.1248/bpb.29.100
Yoshizaki Y, 2014, BIOMATERIALS, V35, P8186, DOI 10.1016/j.biomaterials.2014.05.077
Yuba E, 2014, BIOMATERIALS, V35, P3091, DOI 10.1016/j.biomaterials.2013.12.024
Yuba E, 2013, BIOMATERIALS, V34, P3042, DOI 10.1016/j.biomaterials.2012.12.031
Yuba E, 2011, J CONTROL RELEASE, V149, P72, DOI 10.1016/j.jconrel.2010.03.001
Zaric M, 2015, J INVEST DERMATOL, V135, P425, DOI 10.1038/jid.2014.415
Zhang CN, 2017, MOL PHARMACEUT, V14, P1760, DOI 10.1021/acs.molpharmaceut.7b00015
Zhang XQ, 2012, ADV DRUG DELIVER REV, V64, P1363, DOI 10.1016/j.addr.2012.08.005
Zhang YJ, 2018, INT J CANCER, V143, P2039, DOI 10.1002/ijc.31588
Zhao L, 2014, VACCINE, V32, P327, DOI 10.1016/j.vaccine.2013.11.069
Zhuang XT, 2016, J CONTROL RELEASE, V228, P26, DOI 10.1016/j.jconrel.2016.02.035
Zupancic E, 2017, J CONTROL RELEASE, V258, P182, DOI 10.1016/j.jconrel.2017.05.014
NR 138
TC 44
Z9 45
U1 3
U2 36
PU MDPI
PI BASEL
PA ST ALBAN-ANLAGE 66, CH-4052 BASEL, SWITZERLAND
EI 1999-4923
J9 PHARMACEUTICS
JI Pharmaceutics
PD NOV
PY 2019
VL 11
IS 11
AR 612
DI 10.3390/pharmaceutics11110612
PG 21
WC Pharmacology & Pharmacy
WE Science Citation Index Expanded (SCI-EXPANDED)
SC Pharmacology & Pharmacy
GA JV3QF
UT WOS:000502280100064
PM 31731667
OA Green Published, gold
DA 2023-09-14
ER

PT J
AU Grimaldi, AM
Incoronato, M
Salvatore, M
Soricelli, A
AF Grimaldi, Anna Maria
Incoronato, Mariarosaria
Salvatore, Marco
Soricelli, Andrea
TI Nanoparticle-based strategies for cancer immunotherapy and immunodiagnostics
SO NANOMEDICINE
LA English
DT Review
DE adjuvant; antigen delivery; cancer; immune response; immune targeting; immunotherapy; NPs; tumor microenvironment; vaccines
ID HUMAN DENDRITIC CELLS; POLY(GAMMA-GLUTAMIC ACID) NANOPARTICLES; TARGETED CATIONIC LIPOSOMES; TERM IMMUNOLOGICAL MEMORY; ENHANCES VACCINE THERAPY; IN-VIVO TRACKING; IMMUNE-RESPONSES; TUMOR MICROENVIRONMENT; COMPLEMENT ACTIVATION; GOLD NANOPARTICLES
AB Although recent successes in clinical trials are strengthening research focused on cancer immunology, the poor immunogenicity and off-target side effects of immunotherapeutics remain major challenges in translating these promising approaches to clinically feasible therapies in the treatment of a large range of tumors. Nanotechnology offers target-based approaches, which have shown significant improvements in the rapidly advancing field of cancer immunotherapy. Here, we first discuss the chemical and physical features of nanoparticulate systems that can be tuned to address the anticancer immune response, and then review recent, key examples of the exploited strategies, ranging from nanovaccines to NPs revising the tumor immunosuppressive microenvironment, up to immunotherapeutic multimodal NPs. Finally, the paper concludes by identifying the promising and outstanding challenges the field of emerging nanotechnologies is facing for cancer immunotherapy.
C1 [Grimaldi, Anna Maria; Incoronato, Mariarosaria; Salvatore, Marco; Soricelli, Andrea] IRCCS SDN, Via Gianturco 113, I-80143 Naples, Italy.
[Soricelli, Andrea] Univ Naples Parthenope, Dept Motor Sci & Healthiness, Via Medina 40, I-80133 Naples, Italy.
C3 IRCCS Istituto di Ricerca Diagnostica e Nucleare (SDN); Parthenope University Naples
RP Grimaldi, AM (通讯作者)，IRCCS SDN, Via Gianturco 113, I-80143 Naples, Italy.
EM agrimaldi@sdn-napoli.it
RI Salvatore, Mirella/K-6691-2016; soricelli, andrea/C-1133-2009; Incoronato, Mariarosaria/K-8727-2016; Incoronato, Mariarosaria/AAA-7541-2021; Grimaldi, Anna Maria/K-2078-2019; Salvatore, Marco/K-8083-2016
OI Salvatore, Mirella/0000-0002-8296-0376; soricelli, andrea/0000-0001-7011-7667; Incoronato, Mariarosaria/0000-0001-7019-0581; Incoronato, Mariarosaria/0000-0001-7019-0581; Grimaldi, Anna Maria/0000-0002-3957-9780; Salvatore, Marco/0000-0001-9734-7702
CR Agarwal R, 2015, ADV HEALTHC MATER, V4, P2269, DOI 10.1002/adhm.201500441
Aggarwal P, ADV DRUG DEL REV, V61, P428
Andorko JI, 2017, J BIOMED MATER RES A, V105, P1219, DOI 10.1002/jbm.a.35970
[Anonymous], 2016, NATURAL POLYM DRUG D
Anselmo AC, 2017, ADV DRUG DELIVER REV, V108, P51, DOI 10.1016/j.addr.2016.01.007
Anselmo AC, 2015, ACS NANO, V9, P3169, DOI 10.1021/acsnano.5b00147
Barua S, 2013, P NATL ACAD SCI USA, V110, P3270, DOI 10.1073/pnas.1216893110
Brahmer JR, 2012, NEW ENGL J MED, V366, P2455, DOI 10.1056/NEJMoa1200694
Briley-Saebo KC, 2010, MAGN RESON MED, V64, P1510, DOI 10.1002/mrm.22519
Car BD, 1999, TOXICOL PATHOL, V27, P58, DOI 10.1177/019262339902700112
Carboni E, 2014, AAPS PHARMSCITECH, V15, P762, DOI 10.1208/s12249-014-0099-6
Champion JA, 2006, P NATL ACAD SCI USA, V103, P4930, DOI 10.1073/pnas.0600997103
Chen YC, 2014, CYTOTHERAPY, V16, P699, DOI 10.1016/j.jcyt.2013.09.006

Cho NH, 2011, NAT NANOTECHNOL, V6, P675, DOI [10.1038/NNANO.2011.149, 10.1038/nnano.2011.149]
Christian DA, 2012, IMMUNOTHERAPY-UK, V4, P425, DOI [10.2217/imt.12.26, 10.2217/IMT.12.26]
Cohen K, 2014, ACS NANO, V8, P2183, DOI 10.1021/nn500205b
Conniot J, 2014, FRONT CHEM, V2, DOI 10.3389/fchem.2014.00105
Cruz LJ, 2011, MOL PHARMACEUT, V8, P520, DOI 10.1021/mp100356k
Da Silva CA, 2009, J IMMUNOL, V182, P3573, DOI 10.4049/jimmunol.0802113
de Chickera SN, 2011, CONTRAST MEDIA MOL I, V6, P314, DOI 10.1002/cmmi.433
Demento SL, 2012, BIOMATERIALS, V33, P4957, DOI 10.1016/j.biomaterials.2012.03.041
Demento SL, 2009, VACCINE, V27, P3013, DOI 10.1016/j.vaccine.2009.03.034
Dewitte H, 2014, NANO TODAY, V9, P743, DOI 10.1016/j.nantod.2014.10.001
Dobrovolskaia MA, 2008, MOL PHARMACEUT, V5, P487, DOI 10.1021/mp800032f
Duan F, 2017, BIOMATERIALS, V122, P23, DOI 10.1016/j.biomaterials.2017.01.017
Fahmy TM, 2008, NANOMEDICINE-UK, V3, P343, DOI 10.2217/17435889.3.3.343
Fan YC, 2015, VACCINES-BASEL, V3, P662, DOI 10.3390/vaccines3030662
Fang RH, 2014, NANO LETT, V14, P2181, DOI 10.1021/nl500618u
Fifis T, 2004, VACCINE, V23, P258, DOI 10.1016/j.vaccine.2004.05.022
Fifis T, 2004, J IMMUNOL, V173, P3148, DOI 10.4049/jimmunol.173.5.3148
Fischer S, 2007, BIOMATERIALS, V28, P994, DOI 10.1016/j.biomaterials.2006.10.034
Florez L, 2012, SMALL, V8, P2222, DOI 10.1002/smll.201102002
Foged C, 2005, INT J PHARMACEUT, V298, P315, DOI 10.1016/j.ijpharm.2005.03.035
Gao Y, 2015, LANGMUIR, V31, P2833, DOI 10.1021/la504668c
Ge W, 2009, ONCOL REP, V22, P915, DOI 10.3892/or_00000517
Ge W, 2009, CANCER IMMUNOL IMMUN, V58, P201, DOI 10.1007/s00262-008-0539-9
Gentile F, 2008, J BIOMECH, V41, P2312, DOI 10.1016/j.jbiomech.2008.03.021
Getts DR, 2016, TRENDS IMMUNOL, V37, P715, DOI 10.1016/j.it.2016.08.003
Getts DR, 2014, SCI TRANSL MED, V6, DOI 10.1126/scitranslmed.3007563
Goodman CM, 2004, BIOCONJUGATE CHEM, V15, P897, DOI 10.1021/bc049951i
Gratton SEA, 2008, P NATL ACAD SCI USA, V105, P11613, DOI 10.1073/pnas.0801763105
Hamid O, 2013, NEW ENGL J MED, V369, P134, DOI 10.1056/NEJMoa1305133
Huang Z, 2012, J CONTROL RELEASE, V158, P286, DOI 10.1016/j.jconrel.2011.11.013
Hunter Z, 2014, ACS NANO, V8, P2148, DOI 10.1021/nn405033r
Huo MR, 2017, J CONTROL RELEASE, V245, P81, DOI 10.1016/j.jconrel.2016.11.013
Jeanbart L, 2015, CANCER IMMUNOL IMMUN, V64, P1033, DOI 10.1007/s00262-015-1702-8
Jilek S, 2004, PHARM RES-DORDR, V21, P1240, DOI 10.1023/B:PHAM.0000033012.16152.5d
Johansen P, 2008, P NATL ACAD SCI USA, V105, P5189, DOI 10.1073/pnas.0706296105
Johnston D, 2006, CANCER IMMUNOL IMMUN, V55, P412, DOI 10.1007/s00262-005-0013-x
Kaminskas LM, 2011, ADV DRUG DELIVER REV, V63, P890, DOI 10.1016/j.addr.2011.05.016
Kantoff PW, 2010, NEW ENGL J MED, V363, P411, DOI 10.1056/NEJMoa1001294
Kapadia CH, 2015, J CONTROL RELEASE, V219, P167, DOI 10.1016/j.jconrel.2015.09.062
Kim J, 2015, NAT BIOTECHNOL, V33, P64, DOI 10.1038/nbt.3071
Kortylewski M, 2009, CANCER CELL, V15, P114, DOI 10.1016/j.ccr.2008.12.018
Koshy ST, 2016, CURR OPIN BIOTECH, V40, P1, DOI 10.1016/j.copbio.2016.02.001
Kourtis IC, 2013, PLOS ONE, V8, DOI 10.1371/journal.pone.0061646
Kulkarni A, 2016, P NATL ACAD SCI USA, V113, pE2104, DOI 10.1073/pnas.1603455113
Kumar S, 2015, J CONTROL RELEASE, V220, P141, DOI 10.1016/j.jconrel.2015.09.069
Lee IH, 2012, ANGEW CHEM INT EDIT, V51, P8800, DOI 10.1002/anie.201203193
Lee Sylvia, 2011, Cancers (Basel), V3, P3856, DOI 10.3390/cancers3043856
Li B, 2016, INT J NANOMED, V11, P6103, DOI 10.2147/IJN.S112432
Liao D, 2011, CANCER RES, V71, P5688, DOI 10.1158/0008-5472.CAN-11-1264
Lim YT, 2008, SMALL, V4, P1640, DOI 10.1002/smll.200800582
Lin AY, 2013, PLOS ONE, V8, DOI 10.1371/journal.pone.0063550
Ma YF, 2011, NANOSCALE, V3, P2307, DOI 10.1039/c1nr10166h
Mackay PS, 2011, NANOMED-NANOTECHNOL, V7, P489, DOI 10.1016/j.nano.2010.12.004
Manolova V, 2008, EUR J IMMUNOL, V38, P1404, DOI 10.1002/eji.200737984
Neto LMM, 2017, FRONT IMMUNOL, V8, DOI 10.3389/fimmu.2017.00239
Meir R, 2015, ACS NANO, V9, P6363, DOI 10.1021/acsnano.5b01939
Mellman I, 2011, NATURE, V480, P480, DOI 10.1038/nature10673
Mizrahy S, 2014, NANOSCALE, V6, P3742, DOI 10.1039/c3nr06102g
Mizrahy S, 2012, CHEM SOC REV, V41, P2623, DOI 10.1039/c1cs15239d
Moghimi SM, 2006, FASEB J, V20, P2591, DOI 10.1096/fj.06-6186fje

Mohanan D, 2010, J CONTROL RELEASE, V147, P342, DOI 10.1016/j.jconrel.2010.08.012
Mueller SN, 2015, MOL PHARMACEUT, V12, P1356, DOI 10.1021/mp500589c
Noh YW, 2011, BIOMATERIALS, V32, P6254, DOI 10.1016/j.biomaterials.2011.05.013
Oussoren C, 1997, BBA-BIOMEMBRANES, V1328, P261, DOI 10.1016/S0005-2736(97)00122-3
Oyewumi MO, 2010, EXPERT REV VACCINES, V9, P1095, DOI [10.1586/erv.10.89, 10.1586/ERV.10.89]
Park J, 2012, ACTA BIOMATER, V8, P3606, DOI 10.1016/j.actbio.2012.06.006
Park J, 2012, NAT MATER, V11, P895, DOI [10.1038/NMAT3355, 10.1038/nmat3355]
Parvaniana S, 2017, SENS BIOSENSING RES, V13, P81
Popescu MC, 2007, BLOOD, V109, P5407, DOI 10.1182/blood-2006-08-039446
Reddy ST, 2007, NAT BIOTECHNOL, V25, P1159, DOI [10.1038/nbt1332, 10.1038/nbtl332]
Reddy ST, 2006, TRENDS IMMUNOL, V27, P573, DOI 10.1016/j.it.2006.10.005
Reddy ST, 2006, J CONTROL RELEASE, V112, P26, DOI 10.1016/j.jconrel.2006.01.006
Ruiz-de-Angulo A, 2016, ACS NANO, V10, P1602, DOI 10.1021/acsnano.5b07253
Schlosser E, 2008, VACCINE, V26, P1626, DOI 10.1016/j.vaccine.2008.01.030
Sharp FA, 2009, P NATL ACAD SCI USA, V106, P870, DOI 10.1073/pnas.0804897106
Shen H, 2006, IMMUNOLOGY, V117, P78, DOI 10.1111/j.1365-2567.2005.02268.x
Shima F, 2013, BIOMATERIALS, V34, P9709, DOI 10.1016/j.biomaterials.2013.08.064
Stano A, 2013, BIOMATERIALS, V34, P4339, DOI 10.1016/j.biomaterials.2013.02.024
Stephan MT, 2010, NAT MED, V16, P1035, DOI 10.1038/nm.2198
Sun BB, 2013, ACS NANO, V7, P10834, DOI 10.1021/nn404211j
Szebeni J, 2002, J LIPOSOME RES, V12, P165, DOI 10.1081/LPR-120004790
Tan SW, 2014, NANOMEDICINE-UK, V9, P635, DOI [10.2217/NNM.13.67, 10.2217/nnm.13.67]
Thiele L, 2003, PHARM RES-DORDR, V20, P221, DOI 10.1023/A:1022271020390
Thiele L, 2001, J CONTROL RELEASE, V76, P59, DOI 10.1016/S0168-3659(01)00412-6
Toy R, 2011, NANOTECHNOLOGY, V22, DOI 10.1088/0957-4484/22/11/115101
Tsai KK, 2015, J HEMATOL ONCOL, V8, DOI 10.1186/s13045-015-0219-0
Uto T, 2007, J IMMUNOL, V178, P2979, DOI 10.4049/jimmunol.178.5.2979
van der Veen AH, 1998, INT J CANCER, V77, P901
Villanueva A, 2009, NANOTECHNOLOGY, V20, DOI 10.1088/0957-4484/20/11/115103
Wang C, 2015, J CONTROL RELEASE, V213, pE16, DOI 10.1016/j.jconrel.2015.05.022
Wang C, 2014, VACCINE, V32, P5475, DOI 10.1016/j.vaccine.2014.07.081
Wei M, 2012, ANGEW CHEM INT EDIT, V51, P1202, DOI 10.1002/anie.201105187
Wischke C, 2006, J CONTROL RELEASE, V114, P359, DOI 10.1016/j.jconrel.2006.06.020
Xiang SD, 2006, METHODS, V40, P1, DOI 10.1016/j.ymeth.2006.05.016
Xu Y, 2015, BIOMATERIALS, V58, P63, DOI 10.1016/j.biomaterials.2015.04.016
Xu ZH, 2014, ACS NANO, V8, P3636, DOI 10.1021/nn500216y
Xu ZH, 2013, J CONTROL RELEASE, V172, P259, DOI 10.1016/j.jconrel.2013.08.021
Yanasarn N, 2011, MOL PHARMACEUT, V8, P1174, DOI 10.1021/mp200016d
Yoshizaki Y, 2014, BIOMATERIALS, V35, P8186, DOI 10.1016/j.biomaterials.2014.05.077
Yue ZG, 2011, BIOMACROMOLECULES, V12, P2440, DOI 10.1021/bm101482r
Zaks K, 2006, J IMMUNOL, V176, P7335, DOI 10.4049/jimmunol.176.12.7335
Zhang WF, 2014, PHARM RES-DORDR, V31, P1015, DOI 10.1007/s11095-013-1224-z
Zhang ZP, 2011, BIOMATERIALS, V32, P3666, DOI 10.1016/j.biomaterials.2011.01.067
Zhao Y, 2015, BIOMATERIALS, V68, P54, DOI 10.1016/j.biomaterials.2015.07.053
Zhuang Y, 2012, J CONTROL RELEASE, V159, P135, DOI 10.1016/j.jconrel.2011.12.017
NR 118
TC 44
Z9 44
U1 1
U2 87
PU FUTURE MEDICINE LTD
PI LONDON
PA UNITEC HOUSE, 3RD FLOOR, 2 ALBERT PLACE, FINCHLEY CENTRAL, LONDON, N3
1QB, ENGLAND
SN 1743-5889
EI 1748-6963
J9 NANOMEDICINE-UK
JI Nanomedicine
PD OCT
PY 2017
VL 12

IS 19
BP 2349
EP 2365
DI 10.2217/nnm-2017-0208
PG 17
WC Biotechnology & Applied Microbiology; Nanoscience & Nanotechnology
WE Science Citation Index Expanded (SCI-EXPANDED)
SC Biotechnology & Applied Microbiology; Science & Technology - Other
   Topics
GA FI3FM
UT WOS:000411843400006
PM 28868980
DA 2023-09-14
ER

PT J
AU Qin, L
   Zhang, HL
   Zhou, Y
   Umeshappa, CS
   Gao, HL
AF Qin, Lin
   Zhang, Huilin
   Zhou, Yang
   Umeshappa, Channakeshava Sokke
   Gao, Huile
TI Nanovaccine-Based Strategies to Overcome Challenges in the Whole
   Vaccination Cascade for Tumor Immunotherapy
SO SMALL
LA English
DT Review
DE antigen‐ specific approach; CD8; (+) cytotoxic T lymphocytes;
   cross‐ presentation; DC maturation; lymph target; nanovaccine;
   tumor immunotherapy
ID HUMAN DENDRITIC CELLS; CROSS-PRESENTATION; CANCER VACCINE;
   PARTICLE-SIZE; IN-VITRO; T-CELLS; ANTIGEN PRESENTATION; NANOPARTICLES;
   DELIVERY; RESPONSES
AB Nanovaccine-based immunotherapy (NBI) has received greater attention recently for its potential to prime tumor-specific immunity and establish a long-term immune memory that prevents tumor recurrence. Despite encouraging results in the recent studies, there are still numerous challenges to be tackled for eliciting potent antitumor immunity using NBI strategies. Based on the principles that govern immune response, here it is proposed that these challenges need to be addressed at the five critical cascading events: Loading tumor-specific antigens by nanoscale drug delivery systems (L); Draining tumor antigens to lymph nodes (D); Internalization by dendritic cells (DCs) (I); Maturation of DCs by costimulatory signaling (M); and Presenting tumor-peptide-major histocompatibility complexes to T cells (P) (LDIMP cascade in short). This review provides a detailed and objective overview of emerging NBI strategies to improve the efficacy of nanovaccines in each step of the LDIMP cascade. It is concluded that the balance between each step must be optimized by delicate designing and modification of nanovaccines and by combining with complementary approaches to provide a synergistic immunity in the fight against cancer.
C1 [Qin, Lin; Zhang, Huilin; Zhou, Yang; Gao, Huile] Sichuan Univ, West China Sch Pharm, Key Lab Drug Targeting & Drug Delivery Syst, Educ Minist, Chengdu 610041, Peoples R China.
   [Qin, Lin; Zhang, Huilin; Zhou, Yang; Gao, Huile] Sichuan Univ, West China Sch Pharm, Sichuan Prov Sichuan Engn Lab Plant Sourced Drug, Chengdu 610041, Peoples R China.
   [Qin, Lin; Zhang, Huilin; Zhou, Yang; Gao, Huile] Sichuan Univ, West China Sch Pharm, Sichuan Res Ctr Drug Precis Ind Technol, Chengdu 610041, Peoples R China.
   [Qin, Lin] Chongqing Vocat Coll Transportat, Chongqing 400715, Peoples R China.
   [Umeshappa, Channakeshava Sokke] Univ Calgary, Cumming Sch Med, Snyder Inst Chron Dis, Dept Microbiol Immunol & Infect Dis, Calgary, AB T2N 4N1, Canada.
C3 Sichuan University; Sichuan University; Sichuan University; University
   of Calgary

file:///D/Joyce/计量学/frontiers/修/Supplementary materials/savedrecs (94).txt[2024/6/27 16:48:08]

RP Gao, HL (通讯作者)，Sichuan Univ, West China Sch Pharm, Key Lab Drug Targeting & Drug Delivery Syst, Educ Minist, Chengdu 610041, Peoples R China.; Gao, HL (通讯作者)，Sichuan Univ, West China Sch Pharm, Sichuan Prov Sichuan Engn Lab Plant Sourced Drug, Chengdu 610041, Peoples R China.; Gao, HL (通讯作者)，Sichuan Univ, West China Sch Pharm, Sichuan Res Ctr Drug Precis Ind Technol, Chengdu 610041, Peoples R China.; Umeshappa, CS (通讯作者)，Univ Calgary, Cumming Sch Med, Snyder Inst Chron Dis, Dept Microbiol Immunol & Infect Dis, Calgary, AB T2N 4N1, Canada.
EM csumesha@ucalgary.ca; gaohuile@scu.edu.cn
RI Gao, Huile/H-9206-2019; gao, huile/G-8290-2012
OI Gao, Huile/0000-0002-5355-7238; gao, huile/0000-0002-5355-7238
FU National Natural Science Foundation of China [81872806, 81961138009];
Programme of Introducing Talents of Discipline to Universities [B18035];
"Fundamentals of Research Funds" for the Central Universities; Key
Research and Development Program of Science and Technology Department of
Sichuan Province [2020YFS0570]
FX L.Q. and H.Z. contributed equally to this work. The work was supported
by National Natural Science Foundation of China (81872806, 81961138009),
the Programme of Introducing Talents of Discipline to Universities (No.
B18035), the "Fundamentals of Research Funds" for the Central
Universities and the Key Research and Development Program of Science and
Technology Department of Sichuan Province (No. 2020YFS0570).
CR Accapezzato D, 2005, J EXP MED, V202, P817, DOI 10.1084/jem.20051106
Aguilar JC, 2007, VACCINE, V25, P3752, DOI 10.1016/j.vaccine.2007.01.111
Alexis F, 2008, MOL PHARMACEUT, V5, P505, DOI 10.1021/mp800051m
Alloatti A, 2016, IMMUNOL REV, V272, P97, DOI 10.1111/imr.12432
Ammi R, 2015, PHARMACOL THERAPEUT, V146, P120, DOI 10.1016/j.pharmthera.2014.09.010
Andorko JI, 2015, AAPS J, V17, P323, DOI 10.1208/s12248-014-9708-2
Apostolico JD, 2016, J IMMUNOL RES, V2016, DOI 10.1155/2016/1459394
Barthel FP, 2019, NATURE, V576, P112, DOI 10.1038/s41586-019-1775-1
Bencherif SA, 2015, NAT COMMUN, V6, DOI 10.1038/ncomms8556
Blander JM, 2006, NATURE, V440, P808, DOI 10.1038/nature04596
Bonifaz LC, 2004, J EXP MED, V199, P815, DOI 10.1084/jem.20032220
Burgdorf S, 2007, SCIENCE, V316, P612, DOI 10.1126/science.1137971
Cao FQ, 2018, ADV HEALTHC MATER, V7, DOI 10.1002/adhm.201701439
Carbone K., 2006, P NATL ACAD SCI USA, V103, P10729
Carreno V., 2015, SCIENCE, V348, P803
Cawlfield A, 2019, VACCINE, V37, P3793, DOI 10.1016/j.vaccine.2019.05.059
Champion JA, 2008, PHARM RES-DORDR, V25, P1815, DOI 10.1007/s11095-008-9562-y
Chang K, 1996, P NATL ACAD SCI USA, V93, P136, DOI 10.1073/pnas.93.1.136
Chaurasiya S, 2018, CURR OPIN IMMUNOL, V51, P83, DOI 10.1016/j.coi.2018.03.008
Chen Q, 2016, NAT COMMUN, V7, DOI 10.1038/ncomms13193
Chen WS, 2008, CANCER IMMUNOL IMMUN, V57, P517, DOI 10.1007/s00262-007-0390-4
Cho HI, 2009, CANCER RES, V69, P9012, DOI 10.1158/0008-5472.CAN-09-2019
Coulie PG, 2014, NAT REV CANCER, V14, P135, DOI 10.1038/nrc3670
Cruz LJ, 2014, J CONTROL RELEASE, V192, P209, DOI 10.1016/j.jconrel.2014.07.040
D.M, 2012, NAT REV CANCER, V12, P252
Dingjan I, 2016, SCI REP-UK, V6, DOI 10.1038/srep22064
Disis ML, 2009, J CLIN ONCOL, V27, P4685, DOI 10.1200/JCO.2008.20.6789
Dong X, 2018, NANOMED-NANOTECHNOL, V14, P1087, DOI 10.1016/j.nano.2018.02.007
Dunn GP, 2004, IMMUNITY, V21, P137, DOI 10.1016/j.immuni.2004.07.017
Engering AJ, 1997, EUR J IMMUNOL, V27, P2417, DOI 10.1002/eji.1830270941
Feng LL, 2010, J DRUG TARGET, V18, P168, DOI 10.3109/10611860903318126
Finn OJ, 2011, IMMUNOL RES, V50, P261, DOI 10.1007/s12026-011-8214-1
Foged C, 2005, INT J PHARMACEUT, V298, P315, DOI 10.1016/j.ijpharm.2005.03.035
Fromen CA, 2015, P NATL ACAD SCI USA, V112, P488, DOI 10.1073/pnas.1422923112
Gao S, 2019, THERANOSTICS, V9, P126, DOI 10.7150/thno.29431
Garrett WS, 2000, CELL, V102, P325, DOI 10.1016/S0092-8674(00)00038-6
Gause KT, 2017, ACS NANO, V11, P54, DOI 10.1021/acsnano.6b07343
Germeau C, 2005, J EXP MED, V201, P241, DOI 10.1084/jem.20041379
Gil-Torregrosa BC, 2004, EUR J IMMUNOL, V34, P398, DOI 10.1002/eji.200324508
Gill S, 2015, IMMUNOL REV, V263, P68, DOI 10.1111/imr.12243
Golos Aleksandra, 2015, Przegl Epidemiol, V69, P731

Haniffa M, 2015, SEMIN CELL DEV BIOL, V41, P59, DOI 10.1016/j.semcdb.2015.05.004
Harvey AJ, 2011, PHARM RES-DORDR, V28, P107, DOI 10.1007/s11095-010-0123-9
He LZ, 2007, J IMMUNOL, V178, P6259, DOI 10.4049/jimmunol.178.10.6259
He P, 2015, HUM VACC IMMUNOTHER, V11, P477, DOI 10.1080/21645515.2014.1004026
Heidel JD, 2011, PHARM RES-DORDR, V28, P187, DOI 10.1007/s11095-010-0178-7
Heo MB, 2014, BIOMATERIALS, V35, P590, DOI 10.1016/j.biomaterials.2013.10.009
Hjalmsdottir A, 2016, MOL PHARMACEUT, V13, P320, DOI 10.1021/acs.molpharmaceut.5b00394
Hollingsworth RE, 2019, NPJ VACCINES, V4, DOI 10.1038/s41541-019-0103-y
Hotaling NA, 2015, ANNU REV BIOMED ENG, V17, P317, DOI 10.1146/annurev-bioeng-071813-104814
Hu C, 2018, ACS APPL MATER INTER, V10, P22571, DOI 10.1021/acsami.8b04847
Irvine DJ, 2015, CHEM REV, V115, P11109, DOI 10.1021/acs.chemrev.5b00109
Jiang H, 2017, J CONTROL RELEASE, V267, P47, DOI 10.1016/j.jconrel.2017.08.009
Joffre OP, 2012, NAT REV IMMUNOL, V12, P557, DOI 10.1038/nri3254
Jokerst JV, 2011, NANOMEDICINE-UK, V6, P715, DOI [10.2217/NNM.11.19, 10.2217/nnm.11.19]
Joshi VB, 2014, AAPS J, V16, P975, DOI 10.1208/s12248-014-9624-5
June CH, 2007, J CLIN INVEST, V117, P1204, DOI 10.1172/JCI31446
Kapadia CH, 2015, J CONTROL RELEASE, V219, P167, DOI 10.1016/j.jconrel.2015.09.062
Karasaki T, 2017, CANCER SCI, V108, P170, DOI 10.1111/cas.13131
Kim CG, 2019, PHARMACEUTICS, V11, DOI 10.3390/pharmaceutics11110612
Kim H, 2010, ADV FUNCT MATER, V20, P3925, DOI 10.1002/adfm.201000021
Kratz F, 2008, J CONTROL RELEASE, V132, P171, DOI 10.1016/j.jconrel.2008.05.010
Kreiter S, 2012, ONCOIMMUNOLOGY, V1, P768, DOI 10.4161/onci.19727
Kuballa P, 2012, ANNU REV IMMUNOL, V30, P611, DOI 10.1146/annurev-immunol-020711-074948
Kugler A, 2000, NAT MED, V6, P332, DOI 10.1038/73193
Lee JH, 2013, CELL MOL IMMUNOL, V10, P275, DOI 10.1038/cmi.2012.74
Li AW, 2018, NAT MATER, V17, P528, DOI 10.1038/s41563-018-0028-2
Li C, 2019, ACTA PHARM SIN B, V9, P1145, DOI 10.1016/j.apsb.2019.08.003
Li F, 2019, ACS CENTRAL SCI, V5, P796, DOI 10.1021/acscentsci.9b00060
Li M, 2017, ACTA BIOMATER, V64, P237, DOI 10.1016/j.actbio.2017.10.019
Li P, 2013, J CONTROL RELEASE, V168, P271, DOI 10.1016/j.jconrel.2013.03.025
Li YH, 2008, CANCER RES, V68, P6889, DOI 10.1158/0008-5472.CAN-08-0161
Lin CC, 2020, ACTA PHARM SIN B, V10, P2348, DOI 10.1016/j.apsb.2020.10.009
Lindquist RL, 2004, NAT IMMUNOL, V5, P1243, DOI 10.1038/ni1139
Liu HP, 2014, NATURE, V507, P519, DOI 10.1038/nature12978
Liu J, 2019, ACS NANO, V13, P8648, DOI 10.1021/acsnano.9b03472
Liu WL, 2019, NAT COMMUN, V10, DOI 10.1038/s41467-019-11157-1
Liu YT, 2019, ACS APPL BIO MATER, V2, P1241, DOI 10.1021/acsabm.8b00811
Liu Y, 2013, J MATER CHEM B, V1, P3888, DOI 10.1039/c3tb20383b
Lurquin C, 2005, J EXP MED, V201, P249, DOI 10.1084/jem.20041378
Mant A, 2012, IMMUNOLOGY, V136, P163, DOI 10.1111/j.1365-2567.2012.03558.x
Merad M, 2013, ANNU REV IMMUNOL, V31, P563, DOI 10.1146/annurev-immunol-020711-074950
Min YZ, 2017, NAT NANOTECHNOL, V12, P877, DOI [10.1038/nnano.2017.113, 10.1038/NNANO.2017.113]
Mockey M, 2007, CANCER GENE THER, V14, P802, DOI 10.1038/sj.cgt.7701072
Moyer TJ, 2016, J CLIN INVEST, V126, P799, DOI 10.1172/JCI81083
Niikura K, 2013, ACS NANO, V7, P3926, DOI 10.1021/nn3057005
Norum OJ, 2009, J PHOTOCH PHOTOBIO B, V96, P83, DOI 10.1016/j.jphotobiol.2009.04.012
Ott PA, 2017, NATURE, V547, P217, DOI 10.1038/nature22991
Palucka K, 2010, IMMUNITY, V33, P464, DOI 10.1016/j.immuni.2010.10.007
PARKER JC, 1985, J APPL PHYSIOL, V59, P1128, DOI 10.1152/jappl.1985.59.4.1128
Parkhurst MR, 2011, MOL THER, V19, P620, DOI 10.1038/mt.2010.272
Parkhurst MR, 1998, CANCER RES, V58, P4895
Patel SG, 2019, SCI REP-UK, V9, DOI 10.1038/s41598-019-42456-8
Pedersen GK, 2018, SEMIN IMMUNOL, V39, P4, DOI 10.1016/j.smim.2018.10.003
Perrie Y, 2008, INT J PHARMACEUT, V364, P272, DOI 10.1016/j.ijpharm.2008.04.036
Platt CD, 2010, P NATL ACAD SCI USA, V107, P4287, DOI 10.1073/pnas.0910609107
Pratt A., 2011, NAT REV CANCER, V12, P39
Rafiq K, 2002, J CLIN INVEST, V110, P71, DOI 10.1172/JCI200215640
Reddy ST, 2007, NAT BIOTECHNOL, V25, P1159, DOI [10.1038/nbt1332, 10.1038/nbtl332]
Reed SG, 2018, SEMIN IMMUNOL, V39, P22, DOI 10.1016/j.smim.2018.10.004
Rettig L, 2010, BLOOD, V115, P4533, DOI 10.1182/blood-2009-11-247817

Rohner NA, 2017, ACS BIOMATER SCI ENG, V3, P153, DOI 10.1021/acsbiomaterials.6b00438
Safar HA, 2020, PLOS ONE, V15, DOI 10.1371/journal.pone.0228381
Saharinen P, 2004, TRENDS IMMUNOL, V25, P387, DOI 10.1016/j.it.2004.05.003
Sahin U, 2017, NATURE, V547, P222, DOI 10.1038/nature23003
Salcedo M., 2006, IMMUNOTHER, V55, P819
Schudel A, 2019, NAT REV MATER, V4, P415, DOI 10.1038/s41578-019-0110-7
Schwartzentruber D.H., 2011, ENGL J MED, V364, P2119
Seong SY, 2004, NAT REV IMMUNOL, V4, P469, DOI 10.1038/nri1372
Sharma P, 2015, SCIENCE, V348, P56, DOI 10.1126/science.aaa8172
Singh M, 2003, INT J PARASITOL, V33, P469, DOI 10.1016/S0020-7519(03)00053-5
Singh M, 1999, NAT BIOTECHNOL, V17, P1075, DOI 10.1038/15058
Singha S, 2018, ACS NANO, V12, P10621, DOI 10.1021/acsnano.8b05950
Stary G, 2015, SCIENCE, V348, DOI 10.1126/science.aaa8205
SUPERSAXO A, 1990, PHARMACEUT RES, V7, P167, DOI 10.1023/A:1015880819328
Tacken PJ, 2007, NAT REV IMMUNOL, V7, P790, DOI 10.1038/nri2173
Tacken PJ, 2005, BLOOD, V106, P1278, DOI 10.1182/blood-2005-01-0318
Tao CL, 2015, MICROPOR MESOPOR MAT, V204, P91, DOI 10.1016/j.micromeso.2014.11.007
Thomas SN, 2015, CURR OPIN CHEM ENG, V7, P65, DOI 10.1016/j.coche.2014.11.003
Tian XY, 2018, FRONT IMMUNOL, V9, DOI [10.3389/fimmu.2018.00473, 10.3389/fimmu.2018.01683]
Toke ER, 2014, GENE THER, V21, P566, DOI 10.1038/gt.2014.29
Trevaskis NL, 2015, NAT REV DRUG DISCOV, V14, P781, DOI 10.1038/nrd4608
Trombetta ES, 2005, ANNU REV IMMUNOL, V23, P975, DOI 10.1146/annurev.immunol.22.012703.104538
Tureci O, 2018, NAT BIOMED ENG, V2, P566, DOI 10.1038/s41551-018-0266-2
Twumasi-Boateng K, 2018, NAT REV CANCER, V18, P419, DOI 10.1038/s41568-018-0009-4
Umeshappa CS, 2013, IMMUNOLOGY, V138, P298, DOI 10.1111/imm.12033
Umeshappa CS, 2018, ACS APPL MATER INTER, V10, P38659, DOI 10.1021/acsami.8b11346
Umeshappa CS, 2013, PLOS ONE, V8, DOI 10.1371/journal.pone.0064787
Umeshappa CS, 2011, IMMUNOL CELL BIOL, V89, P578, DOI 10.1038/icb.2011.28
van Broekhoven CL, 2004, CANCER RES, V64, P4357, DOI 10.1158/0008-5472.CAN-04-0138
van der Burg SH, 2016, NAT REV CANCER, V16, P219, DOI 10.1038/nrc.2016.16
Vigneron N, 2015, BIOMED RES INT, V2015, DOI 10.1155/2015/948501
Wang K, 2018, ACS NANO, V12, P6398, DOI 10.1021/acsnano.8b00558
Wang S, 2018, SMALL, V14, DOI 10.1002/smll.201704272
Wang XP, 2019, ACS NANO, V13, P7705, DOI 10.1021/acsnano.9b01271
Warrier VU, 2019, BIOSCIENCE REP, V39, DOI 10.1042/BSR20193220
Wegmann F, 2012, NAT BIOTECHNOL, V30, P883, DOI 10.1038/nbt.2344
Wiig H, 2012, PHYSIOL REV, V92, P1005, DOI 10.1152/physrev.00037.2011
Xiang J, 2015, ACS NANO, V9, P6401, DOI 10.1021/acsnano.5b02014
Yamano T, 2015, IMMUNITY, V42, P1048, DOI 10.1016/j.immuni.2015.05.013
Yang JN, 2019, NANOSCALE, V11, P21782, DOI 10.1039/c9nr05475h
Yang PX, 2018, NANO LETT, V18, P4377, DOI 10.1021/acs.nanolett.8b01406
Yang R, 2018, ACS NANO, V12, P5121, DOI 10.1021/acsnano.7b09041
Yang WJ, 2019, ACS NANO, V13, P3083, DOI 10.1021/acsnano.8b08346
Yang XQ, 2018, ACS APPL MATER INTER, V10, P33532, DOI 10.1021/acsami.8b10081
Yi SJ, 2016, ACS NANO, V10, P11290, DOI 10.1021/acsnano.6b06451
You YW, 2013, AIP ADV, V3, DOI 10.1063/1.4789547
Zariri A, 2015, EXPERT REV VACCINES, V14, P861, DOI 10.1586/14760584.2015.1026808
Zhang CN, 2017, MOL PHARMACEUT, V14, P1760, DOI 10.1021/acs.molpharmaceut.7b00015
Zhang LX, 2003, P NATL ACAD SCI USA, V100, P15101, DOI 10.1073/pnas.2135379100
Zhang N, 2011, IMMUNITY, V35, P161, DOI 10.1016/j.immuni.2011.07.010
Zhang QB, 2017, ACS NANO, V11, P10724, DOI 10.1021/acsnano.7b04955
Zhao XY, 2019, ACTA PHARM SIN B, V9, P1050, DOI 10.1016/j.apsb.2019.06.001
Zhu GZ, 2017, NAT COMMUN, V8, DOI 10.1038/s41467-017-02191-y
Zhu GZ, 2017, NAT COMMUN, V8, DOI 10.1038/s41467-017-01386-7
Zhu GZ, 2017, ACS NANO, V11, P2387, DOI 10.1021/acsnano.7b00978
Zhuang Y, 2012, J CONTROL RELEASE, V159, P135, DOI 10.1016/j.jconrel.2011.12.017
NR 157
TC 43
Z9 44
U1 9

U2 125
PU WILEY-V C H VERLAG GMBH
PI WEINHEIM
PA POSTFACH 101161, 69451 WEINHEIM, GERMANY
SN 1613-6810
EI 1613-6829
J9 SMALL
JI Small
PD JUL
PY 2021
VL 17
IS 28
AR 2006000
DI 10.1002/smll.202006000
EA MAR 2021
PG 21
WC Chemistry, Multidisciplinary; Chemistry, Physical; Nanoscience &
Nanotechnology; Materials Science, Multidisciplinary; Physics, Applied;
Physics, Condensed Matter
WE Science Citation Index Expanded (SCI-EXPANDED)
SC Chemistry; Science & Technology - Other Topics; Materials Science;
Physics
GA TJ4YO
UT WOS:000632824500001
PM 33768693
DA 2023-09-14
ER

PT J
AU Li, X
Cai, XY
Zhang, ZZ
Ding, YX
Ma, RJ
Huang, F
Liu, Y
Liu, JF
Shi, LQ
AF Li, Xue
Cai, Xiaoyao
Zhang, Zhanzhan
Ding, Yuxun
Ma, Rujiang
Huang, Fan
Liu, Yang
Liu, Jianfeng
Shi, Linqi
TI Mimetic Heat Shock Protein Mediated Immune Process to Enhance Cancer
Immunotherapy
SO NANO LETTERS
LA English
DT Article
DE cancer nanovaccines; heat shock proteins; immunotherapy;
cross-presentation; polymeric micelles
ID CHECKPOINT BLOCKADE; CROSS-PRESENTATION; VACCINE DELIVERY; CELLS;
CHAPERONES; ADJUVANT; SCAFFOLDS; HYDROGELS; SYSTEM
AB Inspired by heat shock proteins (HSPs), a self-assembly nanochaperone (nChap) is developed as a novel nanovaccine for boosting antitumor immune responses. Taking advantage of HSP-like microdomains and surface-decorated mannose, this nChap can efficiently capture antigens and ferry them into the dendritic cells (DCs). Subsequently, the nChap can blast lysosomes by transforming the structure and property of surface microdomains, thereby promoting antigen escape and enhancing their

cross-presentation in cytoplasm. As a result, the nChap-based nanovaccine can elicit both CD4+ and CD8+ T cell-based immune responses and shows an excellent preventive effect on melanoma. Further combination of the nanovaccine with antiprogrammed death-1 (anti-PD-1) checkpoint blockade offers effective inhibition on the growth of already-established melanoma. Therefore, this nC ap-based nanovaccine provides a simple and robust strategy in mimicking HSPs to realize structure-assisted antigen capture, surface-receptor-mediated DC internalization, and both activation of humoral immunity and cellular immunity, promising for efficient cancer immunotherapy.
C1 [Li, Xue; Zhang, Zhanzhan; Ding, Yuxun; Ma, Rujiang; Liu, Yang; Shi, Linqi] Nankai Univ, Coll Chem, Minist Educ, State Key Lab Med Chem Biol,Key Lab Funct Polymer, Tianjin 300071, Peoples R China.
[Cai, Xiaoyao] First Mobile Armed Police Crops, Tianjin 300192, Peoples R China.
[Huang, Fan; Liu, Jianfeng] Chinese Acad Med Sci & Peking Union Med Coll, Inst Radiat Med, Tianjin Key Lab Radiat Med & Mol Nucl Med, Tianjin 300192, Peoples R China.
C3 Nankai University; Chinese Academy of Medical Sciences - Peking Union
Medical College; Peking Union Medical College; Institute of Radiation
Medicine - CAMS
RP Liu, Y; Shi, LQ (通讯作者)，Nankai Univ, Coll Chem, Minist Educ, State Key Lab Med Chem Biol,Key Lab Funct Polymer, Tianjin 300071, Peoples R China.; Huang, F; Liu, JF (通讯作者)，Chinese Acad Med Sci & Peking Union Med Coll, Inst Radiat Med, Tianjin Key Lab Radiat Med & Mol Nucl Med, Tianjin 300192, Peoples R China.
EM huangfan@irm-cams.ac.cn; yliu@nankai.edu.cn; liujianfeng@irm-cams.ac.cn;
shilinqi@nankai.edu.cn
RI Ma, Rujiang/H-8997-2013; Liu, Yang/AAY-7115-2020
OI Ma, Rujiang/0000-0002-2021-3850; Liu, Yang/0000-0001-5752-5180
FU National Natural Science Foundation of China [51933006, 21620102005,
81722026, 51603231, 51773099]; National Key Research and Development
Programs of China [2018YFA0209700]; CAMS Innovation Fund for Medical
Sciences [2016-I2M-3-022]; Nonprofit Central Research Institute Fund of
Chinese Academy of Medical Sciences [2019-RC-HL-014]
FX This work was financially supported by the National Natural Science
Foundation of China (Nos. 51933006, 21620102005, 81722026, 51603231, and
51773099), National Key Research and Development Programs of China
(2018YFA0209700), the CAMS Innovation Fund for Medical Sciences
(2016-I2M-3-022) and the Nonprofit Central Research Institute Fund of
Chinese Academy of Medical Sciences (2019-RC-HL-014).
CR Alter G, 2004, J IMMUNOL METHODS, V294, P15, DOI 10.1016/j.jim.2004.08.008
Ampie L, 2015, J NEURO-ONCOL, V123, P441, DOI 10.1007/s11060-015-1837-7
Bachmann MF, 2010, NAT REV IMMUNOL, V10, P787, DOI 10.1038/nri2868
Banday AH, 2015, IMMUNOPHARM IMMUNOT, V37, P1, DOI 10.3109/08923973.2014.971963
Bhowmick S, 2010, J CONTROL RELEASE, V141, P199, DOI 10.1016/j.jconrel.2009.09.018
Bolhassani A, 2013, HUM VACC IMMUNOTHER, V9, P153, DOI 10.4161/hv.22248
Boudier A, 2011, J CONTROL RELEASE, V154, P156, DOI 10.1016/j.jconrel.2011.05.016
Buchbinder EI, 2016, NAT REV CLIN ONCOL, V13, P77, DOI 10.1038/nrclinonc.2015.237
Burgdorf S, 2008, NAT IMMUNOL, V9, P558, DOI 10.1038/ni.1601
Couzin-Frankel J, 2013, SCIENCE, V342, P1432, DOI 10.1126/science.342.6165.1432
Farkona S, 2016, BMC MED, V14, DOI 10.1186/s12916-016-0623-5
Fesnak AD, 2016, NAT REV CANCER, V16, P566, DOI 10.1038/nrc.2016.97
Finn OJ, 2003, NAT REV IMMUNOL, V3, P630, DOI 10.1038/nri1150
Guzhova IV, 2016, HUM VACC IMMUNOTHER, V12, P2529, DOI 10.1080/21645515.2016.1190057
Hartl FU, 2011, NATURE, V475, P324, DOI 10.1038/nature10317
Hu Z, 2018, NAT REV IMMUNOL, V18, P168, DOI 10.1038/nri.2017.131
Huang F, 2014, ANGEW CHEM INT EDIT, V53, P8985, DOI 10.1002/anie.201400735
June CH, 2018, SCIENCE, V359, P1361, DOI 10.1126/science.aar6711
Kim J, 2015, NAT BIOTECHNOL, V33, P64, DOI 10.1038/nbt.3071
Larmonier N, 2008, J LEUKOCYTE BIOL, V83, P1049, DOI 10.1189/jlb.0907635
Li C, 2020, NANO LETT, V20, P1755, DOI 10.1021/acs.nanolett.9b04966
Liu TL, 2013, J COLLOID INTERF SCI, V400, P168, DOI 10.1016/j.jcis.2013.03.005
Luo ZC, 2013, J CONTROL RELEASE, V170, P259, DOI 10.1016/j.jconrel.2013.05.027
Mahoney KM, 2015, NAT REV DRUG DISCOV, V14, P561, DOI 10.1038/nrd4591
Mellman I, 2011, NATURE, V480, P480, DOI 10.1038/nature10673
Mercuri LP, 2006, SMALL, V2, P254, DOI 10.1002/smll.200500274
Moon JJ, 2011, NAT MATER, V10, P243, DOI 10.1038/NMAT2960

   Nicchitta CV, 2003, NAT REV IMMUNOL, V3, P427, DOI 10.1038/nri1089
   Pavot V, 2013, J CONTROL RELEASE, V167, P60, DOI 10.1016/j.jconrel.2013.01.015
   Rock KL, 2005, IMMUNOL REV, V207, P166, DOI 10.1111/j.0105-2896.2005.00301.x
   Rosenberg SA, 2015, SCIENCE, V348, P62, DOI 10.1126/science.aaa4967
   Rosenzweig R, 2019, NAT REV MOL CELL BIO, V20, P665, DOI 10.1038/s41580-019-0133-3
   Sharma P, 2015, CELL, V161, P205, DOI 10.1016/j.cell.2015.03.030
   Singh A, 2014, ADV MATER, V26, P6530, DOI 10.1002/adma.201402105
   Smits ELJM, 2010, CANCER IMMUNOL IMMUN, V59, P35, DOI 10.1007/s00262-009-0721-8
   Srivastava P, 2002, NAT REV IMMUNOL, V2, P185, DOI 10.1038/nri749
   Topalian SL, 2016, NAT REV CANCER, V16, P275, DOI 10.1038/nrc.2016.36
   Trimaille T, 2015, VACCINES, V3, P803, DOI 10.3390/vaccines3040803
   Wang HM, 2016, ADV FUNCT MATER, V26, P1822, DOI 10.1002/adfm.201505188
   Wang K, 2018, ACS NANO, V12, P6398, DOI 10.1021/acsnano.8b00558
   Wu YB, 2012, BIOMATERIALS, V33, P2351, DOI 10.1016/j.biomaterials.2011.11.068
   Xu J, 2019, BIOMATERIALS, V207, P1, DOI 10.1016/j.biomaterials.2019.03.037
   Yang HR, 2019, ADV SCI, V6, DOI 10.1002/advs.201901844
   Yang R, 2018, ACS NANO, V12, P5121, DOI 10.1021/acsnano.7b09041
NR 44
TC 42
Z9 43
U1 7
U2 100
PU AMER CHEMICAL SOC
PI WASHINGTON
PA 1155 16TH ST, NW, WASHINGTON, DC 20036 USA
SN 1530-6984
EI 1530-6992
J9 NANO LETT
JI Nano Lett.
PD JUN 10
PY 2020
VL 20
IS 6
BP 4454
EP 4463
DI 10.1021/acs.nanolett.0c01230
PG 10
WC Chemistry, Multidisciplinary; Chemistry, Physical; Nanoscience &
   Nanotechnology; Materials Science, Multidisciplinary; Physics, Applied;
   Physics, Condensed Matter
WE Science Citation Index Expanded (SCI-EXPANDED)
SC Chemistry; Science & Technology - Other Topics; Materials Science;
   Physics
GA MA1PZ
UT WOS:000541691200051
PM 32401534
DA 2023-09-14
ER

PT J
AU Li, SX
   Feng, XR
   Wang, JX
   Xu, WG
   Islam, MA
   Sun, TM
   Xie, ZG
   Wang, CX
   Ding, JX
   Chen, XS
AF Li, Shengxian

Feng, Xiangru
Wang, Jixue
Xu, Weiguo
Islam, Mohammad Ariful
Sun, Tianmeng
Xie, Zhigang
Wang, Chunxi
Ding, Jianxun
Chen, Xuesi
TI Multiantigenic Nanoformulations Activate Anticancer Immunity Depending on Size
SO ADVANCED FUNCTIONAL MATERIALS
LA English
DT Article
DE anticancer immunity; cancer cell membrane; cancer prevention and therapy; immunoadjuvant; multiantigenic nanovaccine
ID NANOPARTICLES; MICROPARTICLES; METASTASIS; CELLS
AB Nanoparticle-adjuvanted cancer vaccines are attracting increasing attention because they can induce an effective anticancer immune response. Single-antigen vaccines are inefficient to inhibit cancer progression due to the heterogeneity of tumors and the antigenicity alteration of tumor-associated antigens. Therefore, the efficient delivery of multiple antigens to antigen-presenting cells is an excellent opportunity for strong anticancer immunity. In this study, three immunoadjuvant-loaded multiantigenic nanoparticles MANPs/R837 with different diameters, i.e., 83, 103, and 122 nm, are prepared through coating of the cancer cell membrane as a source of multiple antigens onto the imiquimod R837-loaded poly(lactic-co-glycolic acid) nanoparticles. The MANP/R837 with a diameter of 83 nm (MANP83/R837) shows the most efficient delivery of the payload to the draining lymph nodes and achieves the best antigen presentation to T lymphocytes. Compared with the other two nanovaccines, MANP83/R837 has a stronger inhibitory effect on tumor growth and metastasis. In the combination therapy with checkpoint blockade therapy using programmed cell death-1 antibody, MANPs/R837 show effective inhibition against tumor progression, and MANP83/R837 achieves the most exciting effect. Therefore, MANPs/R837, as a promising therapeutic cancer vaccine, demonstrates great prospects in cancer immunotherapy.
C1 [Li, Shengxian; Feng, Xiangru; Wang, Jixue; Xu, Weiguo; Ding, Jianxun; Chen, Xuesi] Chinese Acad Sci, Changchun Inst Appl Chem, Key Lab Polymer Ecomat, 5625 Renmin St, Changchun 130022, Jilin, Peoples R China.
[Li, Shengxian; Wang, Jixue; Wang, Chunxi] Jilin Univ, Hosp 1, Dept Urol, 71 Xinmin St, Changchun 130021, Jilin, Peoples R China.
[Feng, Xiangru; Xu, Weiguo; Ding, Jianxun; Chen, Xuesi] Jilin Biomed Polymers Engn Lab, 5625 Renmin St, Changchun 130022, Jilin, Peoples R China.
[Islam, Mohammad Ariful] Harvard Med Sch, Brigham & Womens Hosp, Ctr Nanomed, 60 Fenwood Rd, Boston, MA 02115 USA.
[Islam, Mohammad Ariful] Harvard Med Sch, Brigham & Womens Hosp, Dept Anesthesiol, 60 Fenwood Rd, Boston, MA 02115 USA.
[Sun, Tianmeng] Jilin Univ, Hosp 1, 519 Dongminzhu St, Changchun 130061, Jilin, Peoples R China.
[Sun, Tianmeng] Jilin Univ, Inst Immunol, 519 Dongminzhu St, Changchun 130061, Jilin, Peoples R China.
[Xie, Zhigang] Chinese Acad Sci, Changchun Inst Appl Chem, State Key Lab Polymer Phys & Chem, 5625 Renmin St, Changchun 130022, Jilin, Peoples R China.
C3 Chinese Academy of Sciences; Changchun Institute of Applied Chemistry, CAS; Jilin University; Harvard University; Harvard Medical School; Brigham & Women's Hospital; Harvard University; Brigham & Women's Hospital; Harvard Medical School; Jilin University; Jilin University; Chinese Academy of Sciences; Changchun Institute of Applied Chemistry, CAS
RP Ding, JX (通讯作者)，Chinese Acad Sci, Changchun Inst Appl Chem, Key Lab Polymer Ecomat, 5625 Renmin St, Changchun 130022, Jilin, Peoples R China.; Ding, JX (通讯作者)，Jilin Biomed Polymers Engn Lab, 5625 Renmin St, Changchun 130022, Jilin, Peoples R China.
EM jxding@ciac.ac.cn
RI Ding, Jianxun/A-6155-2013; Sun, Tianmeng/C-7853-2014; Xie, Zhigang/AAF-6102-2019; Chen, Xuesi/ABB-5039-2020
OI Ding, Jianxun/0000-0002-5232-8863; Xie, Zhigang/0000-0003-2974-1825; feng, xiangru/0000-0001-7671-3144

FU National Natural Science Foundation of China [51873207, 51803006, 51773083, 51673190, 51673187, 51603204, 51503076, 51473165, 51520105004]; Science and Technology Development Program of Jilin Province [20190201068JC, 20180101200JC]
FX S.L. and X.F. contributed equally to this work. This study was financially supported by the National Natural Science Foundation of China (Grant Nos. 51873207, 51803006, 51773083, 51673190, 51673187, 51603204, 51503076, 51473165, and 51520105004) and the Science and Technology Development Program of Jilin Province (Grant Nos. 20190201068JC and 20180101200JC). All animal experiments in this work were handled under protocols approved by the Institutional Animal Care and Use Committee of Jilin University. All in vivo studies were performed in a blinded manner.
CR Akagi T, 2007, BIOMATERIALS, V28, P3427, DOI 10.1016/j.biomaterials.2007.04.023
Barve A, 2014, J CONTROL RELEASE, V187, P118, DOI 10.1016/j.jconrel.2014.05.035
Dane KY, 2011, J CONTROL RELEASE, V156, P154, DOI 10.1016/j.jconrel.2011.08.009
Eng HL, 2018, BIOCHEM BIOPH RES CO, V497, P319, DOI 10.1016/j.bbrc.2018.02.079
Fang RH, 2014, NANO LETT, V14, P2181, DOI 10.1021/nl500618u
Farkona S, 2016, BMC MED, V14, DOI 10.1186/s12916-016-0623-5
Fontana F, 2017, ADV MATER, V29, DOI 10.1002/adma.201603239
Heo MB, 2014, BIOMATERIALS, V35, P590, DOI 10.1016/j.biomaterials.2013.10.009
Joshi VB, 2014, EXPERT REV VACCINES, V13, P9, DOI 10.1586/14760584.2014.851606
Kroll AV, 2017, ADV MATER, V29, DOI 10.1002/adma.201703969
Li SX, 2018, NANO RES, V11, P5769, DOI 10.1007/s12274-018-2124-7
Liu HP, 2014, NATURE, V507, P519, DOI 10.1038/nature12978
Manolova V, 2008, EUR J IMMUNOL, V38, P1404, DOI 10.1002/eji.200737984
Shi GL, 2018, CANCERS, V10, DOI 10.3390/cancers10120498
Yang R, 2018, ACS NANO, V12, P5121, DOI 10.1021/acsnano.7b09041
Yu XH, 2017, ACS NANO, V11, P10147, DOI 10.1021/acsnano.7b04736
Zhang WF, 2015, PHARM RES-DORDR, V32, P2837, DOI 10.1007/s11095-015-1666-6
Zhao KD, 2017, BIOMATERIALS, V116, P82, DOI 10.1016/j.biomaterials.2016.11.030
NR 18
TC 42
Z9 42
U1 11
U2 224
PU WILEY-V C H VERLAG GMBH
PI WEINHEIM
PA POSTFACH 101161, 69451 WEINHEIM, GERMANY
SN 1616-301X
EI 1616-3028
J9 ADV FUNCT MATER
JI Adv. Funct. Mater.
PD DEC
PY 2019
VL 29
IS 49
AR 1903391
DI 10.1002/adfm.201903391
EA SEP 2019
PG 11
WC Chemistry, Multidisciplinary; Chemistry, Physical; Nanoscience & Nanotechnology; Materials Science, Multidisciplinary; Physics, Applied; Physics, Condensed Matter
WE Science Citation Index Expanded (SCI-EXPANDED)
SC Chemistry; Science & Technology - Other Topics; Materials Science; Physics
GA JT7BU
UT WOS:000487621900001
DA 2023-09-14
ER

PT J
AU Liu, LL
He, HM
Liang, RJ
Yi, HQ
Meng, XQ
Chen, ZK
Pan, H
Ma, YF
Cai, LT
AF Liu, Lanlan
He, Huamei
Liang, Ruijing
Yi, Huqiang
Meng, Xiaoqing
Chen, Zhikuan
Pan, Hong
Ma, Yifan
Cai, Lintao
TI ROS-Inducing Micelles Sensitize Tumor-Associated Macrophages to TLR3 Stimulation for Potent Immunotherapy
SO BIOMACROMOLECULES
LA English
DT Article
ID OXIDATIVE STRESS; DENDRITIC CELLS; PROGRESSION; NANOVACCINE; STAT3; INHIBITION; DELIVERY; TARGET; CANCER
AB One approach to cancer immunotherapy is the repolarization of immunosuppressive tumor-associated macrophages (TAMs) to antitumor M1 macrophages. The present study developed galactose-functionalized zinc protoporphyrin IX (ZnPP) grafted poly(L-lysine)-b-poly(ethyleneglycol) polypeptide micelles (ZnPP PM) for TAM-targeted immunopotentiator delivery, which aimed at in vivo repolarization of TAMs to antitumor M1 macrophages. The outcomes revealed that ROS-inducing ZnPP PM demonstrated specificity for the in vitro and in vivo targeting of macrophages, elevated the level of ROS, and lowered STAT3 expression in BM-TAMs. Poly I:C (PIC, a TLR3 agonist)-loaded ZnPP PM (ZnPP PM/PIC) efficiently repolarized TAMs to M1 macrophages, which were reliant on ROS generation. Further, ZnPP PM/PIC substantially elevated the activated NK cells and T lymphocytes in B16-F10 melanoma tumors, which caused vigorous tumor regression. Therefore, the TAM-targeted transport of an immunologic adjuvant with ZnPP-grafted nanovectors may be a potential strategy to repolarize TAMs to Ml macrophages in situ for effective cancer immunotherapy.
C1 [Liu, Lanlan; He, Huamei; Liang, Ruijing; Yi, Huqiang; Meng, Xiaoqing; Chen, Zhikuan; Pan, Hong; Ma, Yifan; Cai, Lintao] Chinese Acad Sci, Shenzhen Inst Adv Technol, Guangdong Key Lab Nanomed, CAS Key Lab Hlth Informat,Inst Biomed & Biotechno, Shenzhen 518055, Peoples R China.
[Liu, Lanlan; Meng, Xiaoqing; Chen, Zhikuan; Pan, Hong] Univ Chinese Acad Sci, Beijing 100049, Peoples R China.
[Yi, Huqiang] Southern Univ Sci & Technol, Dept Mat Sci & Engn, Shenzhen 518055, Peoples R China.
[Ma, Yifan] HRYZ Biotech Co, Shanghai 200122, Peoples R China.
C3 Chinese Academy of Sciences; Shenzhen Institute of Advanced Technology, CAS; Chinese Academy of Sciences; University of Chinese Academy of Sciences, CAS; Southern University of Science & Technology
RP Ma, YF; Cai, LT (通讯作者)，Chinese Acad Sci, Shenzhen Inst Adv Technol, Guangdong Key Lab Nanomed, CAS Key Lab Hlth Informat,Inst Biomed & Biotechno, Shenzhen 518055, Peoples R China.; Ma, YF (通讯作者)，HRYZ Biotech Co, Shanghai 200122, Peoples R China.
EM yf.ma@siat.ac.cn; lt.cai@siat.ac.cn
RI Pan, Hong/AAC-2326-2019; meng, xiaoqing/L-2435-2019; Liang, Ruijing/AED-9686-2022
OI meng, xiaoqing/0000-0002-4335-9238; Cai, Lintao/0000-0002-2461-6390; Liang, Ruijing/0000-0003-3248-2780
FU National Natural Science Foundation of China [81371679, 81701816, 81671758, 31571013, 51502333, 81501580]; Key International S&T Cooperation Project [2015DFH50230]; Natural Science Foundation of Guangdong Province [2017A030313079, 2017A030313726]; Guangdong Natural

Science Foundation of Research Team [2016A030312006]; Shanghai Science and technology Program [16DZ1910900]; Dongguan Project on Social Science and Technology Development [2015108101019]; Shenzhen Science and Technology Program [JSGG20160331185422390, JCYJ20160429191503002]; China Postdoctoral Science Foundation [2017M612776]; SIAT Innovation Program for Excellent Young Researchers [Y7G005]
FX This work was supported by the National Natural Science Foundation of China (81371679, 81701816, 81671758, 31571013, 51502333, and 81501580), Key International S&T Cooperation Project (2015DFH50230), Natural Science Foundation of Guangdong Province (2017A030313079 and 2017A030313726), Guangdong Natural Science Foundation of Research Team (2016A030312006), Shanghai Science and technology Program (16DZ1910900), Dongguan Project on Social Science and Technology Development (2015108101019), Shenzhen Science and Technology Program (JSGG20160331185422390 and JCYJ20160429191503002), China Postdoctoral Science Foundation (2017M612776), and SIAT Innovation Program for Excellent Young Researchers (Y7G005).
CR Allavena P, 2008, CRIT REV ONCOL HEMAT, V66, P1, DOI 10.1016/j.critrevonc.2007.07.004
Apel K, 2004, ANNU REV PLANT BIOL, V55, P373, DOI 10.1146/annurev.arplant.55.031903.141701
Chanmee T, 2014, CANCERS, V6, P1670, DOI 10.3390/cancers6031670
Cheng FD, 2003, IMMUNITY, V19, P425, DOI 10.1016/S1074-7613(03)00232-2
Chevre R, 2011, NUCLEIC ACIDS RES, V39, P1610, DOI 10.1093/nar/gkq922
Choi SH, 2012, J NEUROCHEM, V120, P292, DOI 10.1111/j.1471-4159.2011.07572.x
Condeelis J, 2006, CELL, V124, P263, DOI 10.1016/j.cell.2006.01.007
Deng JZ, 2012, BIOMACROMOLECULES, V13, P3795, DOI 10.1021/bm3012538
El Kasmi KC, 2015, SEMIN IMMUNOL, V27, P267, DOI 10.1016/j.smim.2015.09.001
Fang J, 2004, APOPTOSIS, V9, P27, DOI 10.1023/B:APPT.0000012119.83734.4e
Frohner IE, 2009, MOL MICROBIOL, V71, P240, DOI 10.1111/j.1365-2958.2008.06528.x
Gordon S, 2005, NAT REV IMMUNOL, V5, P953, DOI 10.1038/nri1733
He CB, 2010, BIOMATERIALS, V31, P3657, DOI 10.1016/j.biomaterials.2010.01.065
Huang Z, 2012, BIOMATERIALS, V33, P7605, DOI 10.1016/j.biomaterials.2012.06.074
Lee AS, 2014, BIOCHEM BIOPH RES CO, V450, P1363, DOI 10.1016/j.bbrc.2014.06.135
Lewis CE, 2006, CANCER RES, V66, P605, DOI 10.1158/0008-5472.CAN-05-4005
Liu B, 2016, ASIAN PAC J TROP MED, V9, P470, DOI 10.1016/j.apjtm.2016.03.019
Liu LL, 2017, BIOMATERIALS, V134, P166, DOI 10.1016/j.biomaterials.2017.04.043
Liu LL, 2016, J IMMUNOL, V197, P1231, DOI 10.4049/jimmunol.1600182
Luo ZC, 2015, BIOMATERIALS, V38, P50, DOI 10.1016/j.biomaterials.2014.10.050
Molina-Cruz A, 2008, J BIOL CHEM, V283, P3217, DOI 10.1074/jbc.M705873200
Nathan C, 2013, NAT REV IMMUNOL, V13, P349, DOI 10.1038/nri3423
Padgett LE, 2015, DIABETES, V64, P937, DOI 10.2337/db14-0929
Qian BZ, 2010, CELL, V141, P39, DOI 10.1016/j.cell.2010.03.014
Shime H, 2012, P NATL ACAD SCI USA, V109, P2066, DOI 10.1073/pnas.1113099109
Solinas G, 2009, J LEUKOCYTE BIOL, V86, P1065, DOI 10.1189/jlb.0609385
Tan HY, 2016, OXID MED CELL LONGEV, V2016, DOI 10.1155/2016/2795090
Tanaka S, 2003, BRIT J CANCER, V88, P902, DOI 10.1038/sj.bjc.6600830
Tang XQ, 2013, IMMUNOLOGY, V138, P93, DOI 10.1111/imm.12023
Wang C, 2016, BIOMATERIALS, V79, P88, DOI 10.1016/j.biomaterials.2015.11.040
Yi HQ, 2016, BIOMACROMOLECULES, V17, P1737, DOI 10.1021/acs.biomac.6b00141
Yi L, 2012, AM J PATHOL, V180, P1049, DOI 10.1016/j.ajpath.2011.11.019
Yu H, 2007, NAT REV IMMUNOL, V7, P41, DOI 10.1038/nri1995
NR 33
TC 42
Z9 44
U1 4
U2 82
PU AMER CHEMICAL SOC
PI WASHINGTON
PA 1155 16TH ST, NW, WASHINGTON, DC 20036 USA
SN 1525-7797
EI 1526-4602
J9 BIOMACROMOLECULES

JI Biomacromolecules
PD JUN
PY 2018
VL 19
IS 6
SI SI
BP 2146
EP 2155
DI 10.1021/acs.biomac.8b00239
PG 10
WC Biochemistry & Molecular Biology; Chemistry, Organic; Polymer Science
WE Science Citation Index Expanded (SCI-EXPANDED)
SC Biochemistry & Molecular Biology; Chemistry; Polymer Science
GA GJ3VF
UT WOS:000435226200038
PM 29669207
DA 2023-09-14
ER

PT J
AU Cao, FQ
Yan, MM
Liu, YJ
Liu, LX
Ma, GL
AF Cao, Fengqiang
Yan, Mengmeng
Liu, Yijia
Liu, Lanxia
Ma, Guilei
TI Photothermally Controlled MHC Class I Restricted CD8(+) T-Cell Responses Elicited by Hyaluronic Acid Decorated Gold Nanoparticles as a Vaccine for Cancer Immunotherapy
SO ADVANCED HEALTHCARE MATERIALS
LA English
DT Article
DE cancer immunotherapy; cytosolic delivery; gold nanoparticles; near-infrared light; vaccine
ID ANTIGEN PRESENTATION; CROSS-PRESENTATION; DENDRITIC CELLS; DELIVERY; THERAPY; RELEASE; ENDOCYTOSIS; NANOSHELLS; PROTEASOME; IMMUNITY
AB Cancer vaccines aim to induce a strong major histocompatibility complex class I (MHC-I)-restricted CD8(+) cytotoxic T-cell response, which is an important prerequisite for successful cancer immunotherapy. Herein, a hyaluronic acid (HA) and antigen (ovalbumin, OVA)-decorated gold nanoparticle (AuNPs)-based (HA-OVA-AuNPs) vaccine is developed for photothermally controlled cytosolic antigen delivery using near-infrared (NIR) irradiation and is found to induce antigen-specific CD8(+) T-cell responses. Chemical binding of thiolated HA and OVA to AuNPs facilitates antigen uptake of dendritic cells via receptor-mediated endocytosis. HA-OVA-AuNPs exhibit enhanced NIR absorption and thermal energy translation. Cytosolic antigen delivery is then permitted through the photothermally controlled process of local heat-mediated endo/lysosome disruption by laser irradiation along with reactive oxygen species generation, which helps to augment proteasome activity and downstream MHC I antigen presentation. Consequently, the HA-OVA-AuNPs nanovaccine can effectively evoke a potent anticancer immune response in mice under laser irradiation. This NIR-responsive nanovaccine is promising as a potent vaccination method for improving cancer vaccine efficacy.
C1 [Cao, Fengqiang; Yan, Mengmeng; Liu, Yijia; Liu, Lanxia; Ma, Guilei] Chinese Acad Med Sci, Peking Union Med Coll, Inst Biomed Engn, Tianjin Key Lab Biomat, 236 Baidi Rd, Tianjin 300192, Peoples R China.
C3 Chinese Academy of Medical Sciences - Peking Union Medical College; Institute of BioMedical Engineering - CAMS; Peking Union Medical College
RP Ma, GL (通讯作者)，Chinese Acad Med Sci, Peking Union Med Coll, Inst Biomed Engn, Tianjin Key Lab Biomat, 236 Baidi Rd, Tianjin 300192, Peoples R China.
EM bmemgl@126.com

OI Cao, Fengqiang/0000-0002-0458-2306
FU Natural Science Foundation of China [81673027, 31670948]; CAMS
Innovation Fund for Medical Sciences [CAMS-I2M-3-026, CAMS-I2M-3-004];
Tianjin Natural Science Fund for Distinguished Young Scholars
[17JCJQJC46400]; Science and Technology Support Program of Tianjin
[14RCGFSY00146]
FX The authors acknowledge the financial support from the Natural Science
Foundation of China (Grant Nos. 81673027 and 31670948), CAMS Innovation
Fund for Medical Sciences (Grant Nos. CAMS-I2M-3-026 and
CAMS-I2M-3-004), Tianjin Natural Science Fund for Distinguished Young
Scholars (Grant No. 17JCJQJC46400), and the Science and Technology
Support Program of Tianjin (Grant No. 14RCGFSY00146).
CR Abadeer NS, 2016, J PHYS CHEM C, V120, P4691, DOI 10.1021/acs.jpcc.5b11232
Aiken CT, 2011, MOL CELL PROTEOMICS, V10, DOI 10.1074/mcp.M110.006924
AL-Deen FMN, 2014, GENE THER, V21, P212, DOI 10.1038/gt.2013.77
Banchereau J, 1998, NATURE, V392, P245, DOI 10.1038/32588
Burgdorf S, 2008, CURR OPIN IMMUNOL, V20, P89, DOI 10.1016/j.coi.2007.12.002
Chen S, 2017, BIOMATERIALS, V117, P92, DOI 10.1016/j.biomaterials.2016.11.056
Crownover EF, 2011, POLYM CHEM-UK, V2, P1499, DOI 10.1039/c1py00060h
De Gregorio E, 2012, MICROB BIOTECHNOL, V5, P149, DOI 10.1111/j.1751-7915.2011.00276.x
Delany I, 2014, EMBO MOL MED, V6, P708, DOI 10.1002/emmm.201403876
Dosio F, 2016, ADV DRUG DELIVER REV, V97, P204, DOI 10.1016/j.addr.2015.11.011
Dreaden EC, 2012, CHEM SOC REV, V41, P2740, DOI 10.1039/c1cs15237h
Dubbini A, 2015, EUR POLYM J, V72, P423, DOI 10.1016/j.eurpolymj.2015.07.036
Fan YC, 2015, J CONTROL RELEASE, V208, P121, DOI 10.1016/j.jconrel.2015.04.010
Foged C, 2012, EUR J PHARM SCI, V45, P482, DOI 10.1016/j.ejps.2011.08.016
Ghosh P, 2008, ADV DRUG DELIVER REV, V60, P1307, DOI 10.1016/j.addr.2008.03.016
Hosseinzadeh H, 2017, INT J PHARMACEUT, V526, P339, DOI 10.1016/j.ijpharm.2017.04.060
HUA Q, 1993, J CELL SCI, V106, P365
Huang XH, 2008, LASER MED SCI, V23, P217, DOI 10.1007/s10103-007-0470-x
Huschka R, 2011, J AM CHEM SOC, V133, P12247, DOI 10.1021/ja204578e
Jeong EH, 2014, CHEM COMMUN, V50, P13388, DOI 10.1039/c4cc07155g
Jiang DH, 2011, PHYSIOL REV, V91, P221, DOI 10.1152/physrev.00052.2009
Joffre OP, 2012, NAT REV IMMUNOL, V12, P557, DOI 10.1038/nri3254
Ju EG, 2015, ADV FUNCT MATER, V25, P1574, DOI 10.1002/adfm.201403885
Keller S, 2014, J CONTROL RELEASE, V191, P24, DOI 10.1016/j.jconrel.2014.03.041
Kim J, 2016, ADV DRUG DELIVER REV, V98, P99, DOI 10.1016/j.addr.2015.12.018
Kloetzel PM, 2004, CURR OPIN IMMUNOL, V16, P76, DOI 10.1016/j.coi.2003.11.004
Li J, 2012, BIOMATERIALS, V33, P2310, DOI 10.1016/j.biomaterials.2011.11.022
Lin ML, 2008, IMMUNOL CELL BIOL, V86, P353, DOI 10.1038/icb.2008.3
Ling LU, 2011, CELL DEATH DIS, V2, DOI 10.1038/cddis.2011.12
Liu LX, 2016, ACS APPL MATER INTER, V8, P11969, DOI 10.1021/acsami.6b01135
Liu Q, 2015, ACS NANO, V9, P4925, DOI 10.1021/nn5066793
Lucey DR, 1996, CLIN MICROBIOL REV, V9, P532, DOI 10.1128/CMR.9.4.532
Ma Y, 2013, ADV FUNCT MATER, V23, P815, DOI 10.1002/adfm.201201663
Melief CJM, 2015, J CLIN INVEST, V125, P3401, DOI 10.1172/JCI80009
Seifert U, 2010, CELL, V142, P613, DOI 10.1016/j.cell.2010.07.036
Shang L, 2007, LANGMUIR, V23, P2714, DOI 10.1021/la062064e
Trinchieri G, 2003, NAT REV IMMUNOL, V3, P133, DOI 10.1038/nri1001
Ulmer JB, 2006, NAT BIOTECHNOL, V24, P1377, DOI 10.1038/nbt1261
Vigneron N, 2014, BIOMOLECULES, V4, P994, DOI 10.3390/biom4040994
Wang C, 2016, BIOMATERIALS, V79, P88, DOI 10.1016/j.biomaterials.2015.11.040
Wang YQ, 2014, ADV HEALTHC MATER, V3, P670, DOI 10.1002/adhm.201300335
NR 41
TC 42
Z9 44
U1 4
U2 106
PU WILEY
PI HOBOKEN
PA 111 RIVER ST, HOBOKEN 07030-5774, NJ USA

SN 2192-2640
EI 2192-2659
J9 ADV HEALTHC MATER
JI Adv. Healthc. Mater.
PD MAY 23
PY 2018
VL 7
IS 10
AR 1701439
DI 10.1002/adhm.201701439
PG 12
WC Engineering, Biomedical; Nanoscience & Nanotechnology; Materials
Science, Biomaterials
WE Science Citation Index Expanded (SCI-EXPANDED)
SC Engineering; Science & Technology - Other Topics; Materials Science
GA GI0AM
UT WOS:000434031600009
PM 29508543
DA 2023-09-14
ER

PT J
AU Zhang, T
Xiong, HG
Ma, XB
Gao, Y
Xue, P
Kang, YJ
Sun, ZJ
Xu, ZG
AF Zhang, Tian
Xiong, Honggang
Ma, Xianbin
Gao, Yuan
Xue, Peng
Kang, Yuejun
Sun, Zhi-Jun
Xu, Zhigang
TI Supramolecular Tadalafil Nanovaccine for Cancer Immunotherapy by
Alleviating Myeloid-Derived Suppressor Cells and Heightening
Immunogenicity
SO SMALL METHODS
LA English
DT Article
DE myeloid-derived suppressor cells; nano-immunotherapy; phototherapy;
stimuli activation; tadalafil
ID CHECKPOINT BLOCKADE; IMMUNITY; DEATH; CHEMOTHERAPY; RESISTANCE;
DELIVERY; HEAD
AB Tumor-induced immune suppression mediated by myeloid-derived suppressor cells (MDSCs) and insufficient immunogenicity are two major factors for the poor overall response rate to the immune checkpoint blockade (ICB). Here, a tumor microenvironment responsive nanoprodrug (FIT nanoparticles) is presented for co-delivering tadalafil (TAD) and indocyanine green (ICG) photosensitizer to simultaneously targeting intratumor MDSCs and amplifying tumor immunogenicity. The resulting nanoprodrug shows high drug loading (nearly 100%), tumor-specific release, and robust therapeutic efficacy by virtue of promoting immunogenic cell death (ICD) induction and alleviation of MDSCs for augmenting the photothermal immunotherapy. In an in vivo colon tumor model, the released TAD in the tumor can effectively ameliorate MDSCs immunosuppressive activity, while the photosensitizer ICG is capable of inducing ICD to promote sufficient dendritic cells maturation and T cell infiltration. The results reported here may provide a superior candidate of adjuvants for strengthening immune response and ICB efficacy.
C1 [Zhang, Tian; Ma, Xianbin; Gao, Yuan; Xue, Peng; Kang, Yuejun; Xu, Zhigang] Southwest Univ, Key

Lab Luminescence Anal & Mol Sensing, Sch Mat & Energy, Minist Educ, Chongqing 400715, Peoples R China.
[Zhang, Tian; Ma, Xianbin; Gao, Yuan; Xue, Peng; Kang, Yuejun; Xu, Zhigang] Southwest Univ, Chongqing Engn Res Ctr Micro Nano Biomed Mat & De, Chongqing 400715, Peoples R China.
[Xiong, Honggang; Sun, Zhi-Jun] Wuhan Univ, State Key Lab Breeding Base Basic Sci Stomatol Hu, Minist Educ, Sch & Hosp Stomatol, Wuhan 430079, Peoples R China.
[Xiong, Honggang; Sun, Zhi-Jun] Wuhan Univ, Key Lab Oral Biomed, Minist Educ, Sch & Hosp Stomatol, Wuhan 430079, Peoples R China.
C3 Southwest University - China; Southwest University - China; Wuhan University; Wuhan University
RP Xu, ZG (通讯作者)，Southwest Univ, Key Lab Luminescence Anal & Mol Sensing, Sch Mat & Energy, Minist Educ, Chongqing 400715, Peoples R China.; Xu, ZG (通讯作者)，Southwest Univ, Chongqing Engn Res Ctr Micro Nano Biomed Mat & De, Chongqing 400715, Peoples R China.; Sun, ZJ (通讯作者)，Wuhan Univ, State Key Lab Breeding Base Basic Sci Stomatol Hu, Minist Educ, Sch & Hosp Stomatol, Wuhan 430079, Peoples R China.; Sun, ZJ (通讯作者)，Wuhan Univ, Key Lab Oral Biomed, Minist Educ, Sch & Hosp Stomatol, Wuhan 430079, Peoples R China.
EM sunzj@whu.edu.cn; zgxu@swu.edu.cn
RI Xu, Zhigang/AAU-2388-2021; Sun, Zhi-Jun/AAD-8791-2022; kang, yue/GYA-2003-2022
OI Xu, Zhigang/0000-0003-1805-5061; Sun, Zhi-Jun/0000-0003-0932-8013; Gao, Yuan/0000-0003-1859-2158
FU National Natural Science Foundation of China [51703187, 81874131, 82072996]; National Key Research and Development Program [2017YFSF090107]; Chongqing Talent Plan for Young TopNotch Talents [CQYC2020057686]; Hubei Province Natural Science Funds for Distinguished Young Scholar [2017CFA062]
FX T.Z. and H.X. contributed equally to this work. This work was financially supported by National Natural Science Foundation of China (51703187 (Z.-G.X), 81874131 (Z.-J.S.), 82072996 (Z.-J.S.), National Key Research and Development Program (2017YFSF090107 (Z.-J.S.)), the Chongqing Talent Plan for Young TopNotch Talents (CQYC2020057686) (Z.-G.X) and the Hubei Province Natural Science Funds for Distinguished Young Scholar (2017CFA062 (Z.-J.S.)).
CR Bai J, 2018, J AM CHEM SOC, V140, P106, DOI 10.1021/jacs.7b11114
Bertollotto GM, 2018, J PHARMACOL EXP THER, V367, P138, DOI 10.1124/jpet.118.250076
Blanco E, 2015, NAT BIOTECHNOL, V33, P941, DOI 10.1038/nbt.3330
Borst J, 2018, NAT REV IMMUNOL, V18, P635, DOI 10.1038/s41577-018-0044-0
Califano JA, 2015, CLIN CANCER RES, V21, P30, DOI 10.1158/1078-0432.CCR-14-1716
Chao Y, 2020, ADV FUNCT MATER, V30, DOI 10.1002/adfm.201902785
Chen JJ, 2012, CANCER SCI, V103, P1531, DOI 10.1111/j.1349-7006.2012.02328.x
Chen Q, 2019, NANO LETT, V19, P4879, DOI 10.1021/acs.nanolett.9b00584
Chen Q, 2019, NAT NANOTECHNOL, V14, P89, DOI 10.1038/s41565-018-0319-4
Chen WH, 2021, ANGEW CHEM INT EDIT, V60, P5626, DOI 10.1002/anie.201914511
Chen YZ, 2019, ACS NANO, V13, P1751, DOI 10.1021/acsnano.8b07830
Chihara N, 2018, NATURE, V558, P454, DOI 10.1038/s41586-018-0206-z
Deng KR, 2015, ADV FUNCT MATER, V25, P7280, DOI 10.1002/adfm.201503046
Draghiciu O, 2015, ONCOIMMUNOLOGY, V4, DOI 10.4161/2162402X.2014.989764
Elliott MR, 2009, NATURE, V461, P282, DOI 10.1038/nature08296
Fan Q, 2018, ADV FUNCT MATER, V28, DOI 10.1002/adfm.201802540
Feng B, 2020, ADV FUNCT MATER, V30, DOI 10.1002/adfm.201906605
Fontana F, 2018, SMALL METHODS, V2, DOI 10.1002/smtd.201700347
Gabrilovich DI, 2012, NAT REV IMMUNOL, V12, P253, DOI 10.1038/nri3175
Galluzzi L, 2017, NAT REV IMMUNOL, V17, P402, DOI 10.1038/nri.2017.48
Galluzzi L, 2017, NAT REV IMMUNOL, V17, P97, DOI 10.1038/nri.2016.107
Galluzzi L, 2015, CANCER CELL, V28, P690, DOI 10.1016/j.ccell.2015.10.012
Gan SJ, 2019, ADV FUNCT MATER, V29, DOI 10.1002/adfm.201902757
Garg AD, 2014, PHOTOCH PHOTOBIO SCI, V13, P474, DOI 10.1039/c3pp50333j
Garg AD, 2012, CANCER IMMUNOL IMMUN, V61, P215, DOI 10.1007/s00262-011-1184-2
Granier C., 2018, JOVE-J VIS EXP, V2, P132
Han XX, 2020, J AM CHEM SOC, V142, P2490, DOI 10.1021/jacs.9b12232
Hassel JC, 2017, ONCOIMMUNOLOGY, V6, DOI 10.1080/2162402X.2017.1326440

Hou B, 2020, ADV MATER, V32, DOI 10.1002/adma.201907210
Hu DR, 2020, VIEW-CHINA, V1, DOI 10.1002/viw2.6
Im S, 2019, ACS NANO, V13, P476, DOI 10.1021/acsnano.8b07045
Jia D, 2021, CHINESE CHEM LETT, V32, P162, DOI 10.1016/j.cclet.2020.11.052
Jiang J, 2019, ADV MATER, V31, DOI 10.1002/adma.201904278
Jiang YY, 2019, ANGEW CHEM INT EDIT, V58, P8161, DOI 10.1002/anie.201903968
Jing LH, 2020, VIEW-CHINA, V1, DOI 10.1002/viw2.19
Kalbasi Anusha, 2020, Nat Rev Immunol, V20, P25, DOI 10.1038/s41577-019-0218-4
Kim J, 2008, ANGEW CHEM INT EDIT, V47, P8438, DOI 10.1002/anie.200802469
Li SX, 2019, ADV FUNCT MATER, V29, DOI 10.1002/adfm.201903391
Liu FH, 2018, NANO LETT, V18, P6577, DOI 10.1021/acs.nanolett.8b03174
Liu LL, 2020, ADV FUNCT MATER, V30, DOI 10.1002/adfm.201910176
Ma S, 2020, NANO LETT, V20, P2514, DOI 10.1021/acs.nanolett.9b05265
Melero I, 2014, CANCER DISCOV, V4, P522, DOI 10.1158/2159-8290.CD-13-0985
Mellman I, 2011, NATURE, V480, P480, DOI 10.1038/nature10673
Meng ZQ, 2019, ADV MATER, V31, DOI 10.1002/adma.201900927
Munn LL, 2019, SCIENCE, V365, P544, DOI 10.1126/science.aaw7875
Ng CW, 2018, ADV FUNCT MATER, V28, DOI 10.1002/adfm.201804688
Nishino M, 2017, NAT REV CLIN ONCOL, V14, P655, DOI 10.1038/nrclinonc.2017.88
Ou CJ, 2021, ANGEW CHEM INT EDIT, V60, P8157, DOI 10.1002/anie.202014852
Pang X, 2019, ADV MATER, V31, DOI 10.1002/adma.201902530
Ribas A, 2018, SCIENCE, V359, P1350, DOI 10.1126/science.aar4060
Riley RS, 2019, NAT REV DRUG DISCOV, V18, P175, DOI 10.1038/s41573-018-0006-z
Sanmamed MF, 2018, CELL, V175, P313, DOI 10.1016/j.cell.2018.09.035
Scott AC, 2019, NATURE, V571, P270, DOI 10.1038/s41586-019-1324-y
Shao D, 2020, ADV MATER, V32, DOI 10.1002/adma.202004385
Sharma P, 2017, CELL, V168, P707, DOI 10.1016/j.cell.2017.01.017
Shi XJ, 2020, SMALL METHODS, V4, DOI 10.1002/smtd.202000046
Shi Y, 2019, ACCOUNTS CHEM RES, V52, P1543, DOI 10.1021/acs.accounts.9b00148
Song QL, 2017, NANO LETT, V17, P6366, DOI 10.1021/acs.nanolett.7b03186
Srivastava MK, 2012, ONCOIMMUNOLOGY, V1, P1650, DOI 10.4161/onci.21970
Stewart MP, 2016, NATURE, V538, P183, DOI 10.1038/nature19764
Tai LH, 2018, ONCOIMMUNOLOGY, V7, DOI 10.1080/2162402X.2018.1431082
Timko BP, 2010, ADV MATER, V22, P4925, DOI 10.1002/adma.201002072
van der Meel R, 2019, NAT NANOTECHNOL, V14, P1007, DOI 10.1038/s41565-019-0567-y
Veglia F, 2018, NAT IMMUNOL, V19, P108, DOI 10.1038/s41590-017-0022-x
Wang HR, 2019, ADV FUNCT MATER, V29, DOI 10.1002/adfm.201902440
Wang H, 2018, NAT MATER, V17, P761, DOI 10.1038/s41563-018-0147-9
Wang M, 2019, ADV SCI, V6, DOI 10.1002/advs.201802157
Wang Q, 2019, ADV FUNCT MATER, V29, DOI 10.1002/adfm.201901480
WCULEK SK, 2020, NAT REV IMMUNOL, V20, P7, DOI DOI 10.1038/S41577-019-0210-Z
Weed DT, 2015, CLIN CANCER RES, V21, P39, DOI 10.1158/1078-0432.CCR-14-1711
Wilhelm S, 2016, NAT REV MATER, V1, DOI 10.1038/natrevmats.2016.14
Wu C, 2017, ADV FUNCT MATER, V27, DOI 10.1002/adfm.201700338
Yang WJ, 2020, ACS NANO, V14, P620, DOI 10.1021/acsnano.9b07212
Yu GT, 2019, ACS NANO, V13, P12830, DOI 10.1021/acsnano.9b05038
Zhang DF, 2017, NAT MED, V23, P1036, DOI 10.1038/nm.4375
NR 75
TC 41
Z9 41
U1 6
U2 87
PU WILEY-V C H VERLAG GMBH
PI WEINHEIM
PA POSTFACH 101161, 69451 WEINHEIM, GERMANY
SN 2366-9608
J9 SMALL METHODS
JI Small Methods
PD JUN
PY 2021
VL 5

IS 6
AR 2100115
DI 10.1002/smtd.202100115
EA MAY 2021
PG 14
WC Chemistry, Physical; Nanoscience & Nanotechnology; Materials Science, Multidisciplinary
WE Science Citation Index Expanded (SCI-EXPANDED)
SC Chemistry; Science & Technology - Other Topics; Materials Science
GA SP7BS
UT WOS:000649799400001
PM 34927922
DA 2023-09-14
ER

PT J
AU Meng, ZQ
Zhang, YJ
She, JL
Zhou, XF
Xu, J
Han, X
Wang, C
Zhu, MF
Liu, Z
AF Meng, Zhouqi
Zhang, Yaojia
She, Jialin
Zhou, Xuanfang
Xu, Jun
Han, Xiao
Wang, Chao
Zhu, Meifang
Liu, Zhuang
TI Ultrasound-Mediated Remotely Controlled Nanovaccine Delivery for Tumor Vaccination and Individualized Cancer Immunotherapy
SO NANO LETTERS
LA English
DT Article
DE Ultrasound responsive; Self-healing nanocomposite hydrogels; Remotely controllable delivery; Nanovaccines; Cancer immunotherapy
AB Vaccines are one of utmost important weapons in modern medicine to fight a wide range of diseases. To achieve optimal vaccination effects, repeated injections of vaccines are often required, which would largely decrease patient comfort. Herein, an ultrasound-responsive self-healing hydrogel system loaded with nanovaccines is designed for remotely controlled tumor vaccine release and individualized cancer immunotherapy. The gel could be transformed into sol status in response to ultrasound treatment, allowing a burst release of nanovaccines, and self-healed to gel afterward. For mice with a single subcutaneous injection of nanovaccine-loaded gel and multiple ultrasound treatments, repeatedly released nanovaccines could elicit antitumor immune responses, which in combination with immune checkpoint blockade could effectively inhibit established tumors, and prevent postoperative tumor metastases and recurrence based on our personalized nanovaccine system. This work presents an easy-to-operate strategy to realize controllable and durable delivery of vaccines against cancer and potentially other types of diseases.
C1 [Meng, Zhouqi; Zhang, Yaojia; She, Jialin; Zhou, Xuanfang; Xu, Jun; Han, Xiao; Wang, Chao; Liu, Zhuang] Soochow Univ, Inst Funct Nano & Soft Mat FUNSOM, Jiangsu Key Lab Carbon Based Funct Mat & Devices, Suzhou 215123, Jiangsu, Peoples R China.
[Zhu, Meifang] Donghua Univ, Coll Mat Sci & Engn, State Key Lab Modificat Chem Fibers & Polymer Mat, Shanghai 201620, Peoples R China.
C3 Soochow University - China; Donghua University
RP Liu, Z (通讯作者)，Soochow Univ, Inst Funct Nano & Soft Mat FUNSOM, Jiangsu Key Lab Carbon Based Funct Mat & Devices, Suzhou 215123, Jiangsu, Peoples R China.

EM zliu@suda.edu.cn
RI Wang, Chao/G-5951-2012; zhou, xuan/GZA-8157-2022; Liu, Zhuang/H-4352-2011
OI Wang, Chao/0000-0002-8054-3472; Liu, Zhuang/0000-0002-1629-1039
FU National Basic Research Programs of China [2016YFA0201200]; National Natural Science Foundation of China [21907073, 52032008, 51803146]; Jiangsu Social Development Project [BE2019658]; Collaborative Innovation Center of Suzhou Nano Science and Technology; "111" program from the Ministry of Education of China; China Postdoctoral Science Foundation [7131703518]
FX This work was partially supported by the National Basic Research Programs of China (2016YFA0201200), the National Natural Science Foundation of China (21907073, 52032008, 51803146), the Jiangsu Social Development Project (BE2019658), a Collaborative Innovation Center of Suzhou Nano Science and Technology, the "111" program from the Ministry of Education of China, and the Project funded by China Postdoctoral Science Foundation (7131703518).
CR Alarcon CDH, 2005, CHEM SOC REV, V34, P276, DOI 10.1039/b406727d
Alter G, 2004, J IMMUNOL METHODS, V294, P15, DOI 10.1016/j.jim.2004.08.008
Chao Y, 2018, NAT BIOMED ENG, V2, P611, DOI 10.1038/s41551-018-0262-6
Chen Q, 2019, CHEM SOC REV, V48, P5506, DOI 10.1039/c9cs00271e
Chen Q, 2016, NAT COMMUN, V7, DOI 10.1038/ncomms13193
Cleland JL, 1999, TRENDS BIOTECHNOL, V17, P25, DOI 10.1016/S0167-7799(98)01272-4
Conniot J, 2019, NAT NANOTECHNOL, V14, P891, DOI 10.1038/s41565-019-0512-0
Fang RH, 2014, NANO LETT, V14, P2181, DOI 10.1021/nl500618u
Ghiringhelli F, 2007, CANCER IMMUNOL IMMUN, V56, P641, DOI 10.1007/s00262-006-0225-8
Goldberg MS, 2015, CELL, V161, P201, DOI 10.1016/j.cell.2015.03.037
Guermonprez P, 2003, NATURE, V425, P397, DOI 10.1038/nature01911
Haraguchi K, 2011, MACROMOL RAPID COMM, V32, P1253, DOI 10.1002/marc.201100248
Hu CMJ, 2013, NAT NANOTECHNOL, V8, P336, DOI [10.1038/nnano.2013.54, 10.1038/NNANO.2013.54]
Huebsch N, 2014, P NATL ACAD SCI USA, V111, P9762, DOI 10.1073/pnas.1405469111
JANEWAY CA, 1994, CELL, V76, P275, DOI 10.1016/0092-8674(94)90335-2
Klebanoff CA, 2006, IMMUNOL REV, V211, P214, DOI 10.1111/j.0105-2896.2006.00391.x
Kuai R, 2017, NAT MATER, V16, P489, DOI [10.1038/nmat4822, 10.1038/NMAT4822]
Lee ES, 2019, ADV HEALTHC MATER, V8, DOI 10.1002/adhm.201801320
Lin CY, 2015, HUM VACC IMMUNOTHER, V11, P650, DOI 10.1080/21645515.2015.1009345
McLean HQ, 2014, CLIN INFECT DIS, V59, P1375, DOI 10.1093/cid/ciu680
Melero I, 2015, NAT REV CANCER, V15, P457, DOI 10.1038/nrc3973
Meng ZQ, 2019, ADV MATER, V31, DOI 10.1002/adma.201900927
Miyata T, 1999, NATURE, V399, P766, DOI 10.1038/21619
Murdan S, 2003, J CONTROL RELEASE, V92, P1, DOI 10.1016/S0168-3659(03)00303-1
Nam J, 2019, NAT REV MATER, V4, P398, DOI 10.1038/s41578-019-0108-1
Palucka K, 2011, J IMMUNOL, V186, P1325, DOI 10.4049/jimmunol.0902539
Pardoll DM, 2012, NAT REV CANCER, V12, P252, DOI 10.1038/nrc3239
Park CG, 2018, SCI TRANSL MED, V10, DOI 10.1126/scitranslmed.aar1916
Paulis LE, 2013, CURR OPIN IMMUNOL, V25, P389, DOI 10.1016/j.coi.2013.03.001
Qiu Y, 2001, ADV DRUG DELIVER REV, V53, P321, DOI 10.1016/S0169-409X(01)00203-4
Randolph GJ, 2005, NAT REV IMMUNOL, V5, P617, DOI 10.1038/nri1670
Scheetz L, 2019, NAT BIOMED ENG, V3, P768, DOI 10.1038/s41551-019-0436-x
Siegrist CA, 2009, NAT REV IMMUNOL, V9, P185, DOI 10.1038/nri2508
Singh A, 2014, ADV MATER, V26, P6530, DOI 10.1002/adma.201402105
SINGH M, 1991, PHARMACEUT RES, V8, P958, DOI 10.1023/A:1015832302605
Steinman RM, 2007, NAT MED, V13, P1155, DOI 10.1038/nm1643
Topalian SL, 2016, NAT REV CANCER, V16, P275, DOI 10.1038/nrc.2016.36
Trepanier P, 2014, IMMUNOLOGY, V141, P233, DOI 10.1111/imm.12189
Ukidve A, 2020, P NATL ACAD SCI USA, V117, P17727, DOI 10.1073/pnas.2002880117
van der Burg SH, 2016, NAT REV CANCER, V16, P219, DOI 10.1038/nrc.2016.16
Wang H, 2020, NAT MATER, V19, P1244, DOI 10.1038/s41563-020-0680-1
Wattrang E, 2015, J IMMUNOL METHODS, V419, P35, DOI 10.1016/j.jim.2015.02.011
WRAITH DC, 1989, CELL, V59, P247, DOI 10.1016/0092-8674(89)90287-0

   Xia MG, 2015, MACROMOL RAPID COMM, V36, P477, DOI 10.1002/marc.201400665
   Xia YF, 2018, NAT MATER, V17, P187, DOI [10.1038/NMAT5057, 10.1038/nmat5057]
   Xu J, 2019, BIOMATERIALS, V207, P1, DOI 10.1016/j.biomaterials.2019.03.037
   Xu LG, 2013, ADV MATER, V25, P5928, DOI 10.1002/adma.201300583
   Yang R, 2018, ACS NANO, V12, P5121, DOI 10.1021/acsnano.7b09041
   Yang YY, 2000, J CONTROL RELEASE, V69, P81, DOI 10.1016/S0168-3659(00)00291-1
   Zhang XL, 2012, ANGEW CHEM INT EDIT, V51, P4388, DOI 10.1002/anie.201108612
   Zhu GZ, 2017, ACS NANO, V11, P2387, DOI 10.1021/acsnano.7b00978
   Zou WP, 2006, NAT REV IMMUNOL, V6, P295, DOI 10.1038/nri1806
NR 52
TC 41
Z9 41
U1 21
U2 217
PU AMER CHEMICAL SOC
PI WASHINGTON
PA 1155 16TH ST, NW, WASHINGTON, DC 20036 USA
SN 1530-6984
EI 1530-6992
J9 NANO LETT
JI Nano Lett.
PD FEB 10
PY 2021
VL 21
IS 3
BP 1228
EP 1237
DI 10.1021/acs.nanolett.0c03646
EA FEB 2021
PG 10
WC Chemistry, Multidisciplinary; Chemistry, Physical; Nanoscience &
   Nanotechnology; Materials Science, Multidisciplinary; Physics, Applied;
   Physics, Condensed Matter
WE Science Citation Index Expanded (SCI-EXPANDED)
SC Chemistry; Science & Technology - Other Topics; Materials Science;
   Physics
GA QJ4CG
UT WOS:000619638600005
PM 33522825
DA 2023-09-14
ER

PT J
AU Affandi, AJ
   Grabowska, J
   Olesek, K
   Venegas, ML
   Barbaria, A
   Rodriguez, E
   Mulder, PPG
   Pijffers, HJ
   Ambrosini, M
   Kalay, H
   O'Toole, T
   Zwart, ES
   Kazemier, G
   Nazmi, K
   Bikker, FJ
   Stockl, J
   van den Eertwegh, AJM
   de Gruijl, TD

Storm, G
van Kooyk, Y
den Haan, JMM
AF Affandi, Alsya J.
Grabowska, Joanna
Olesek, Katarzyna
Venegas, Miguel Lopez
Barbaria, Arnaud
Rodriguez, Ernesto
Mulder, Patrick P. G.
Pijffers, Helen J.
Ambrosini, Martino
Kalay, Hakan
O'Toole, Tom
Zwart, Eline S.
Kazemier, Geert
Nazmi, Kamran
Bikker, Floris J.
Stoeckl, Johannes
van den Eertwegh, Alfons J. M.
de Gruijl, Tanja D.
Storm, Gert
van Kooyk, Yvette
den Haan, Joke M. M.
TI Selective tumor antigen vaccine delivery to human CD169(+) antigen-presenting cells using ganglioside-liposomes
SO PROCEEDINGS OF THE NATIONAL ACADEMY OF SCIENCES OF THE UNITED STATES OF AMERICA
LA English
DT Article
DE Siglec-1; CD169; dendritic cells; CD8(+) T cells; vaccination
ID ACCELERATED BLOOD CLEARANCE; TARGETING DC-SIGN; DENDRITIC CELLS; TRANS-INFECTION; HUMAN SPLEEN; MACROPHAGES; SIALOADHESIN; BINDING; SPECIFICITIES; EXPRESSION
AB Priming of CD8(+) T cells by dendritic cells (DCs) is crucial for the generation of effective antitumor immune responses. Here, we describe a liposomal vaccine carrier that delivers tumor antigens to human CD169/Siglec-1(+) antigen-presenting cells using gangliosides as targeting ligands. Ganglioside-liposomes specifically bound to CD169 and were internalized by in vitro-generated monocyte-derived DCs (moDCs) and macrophages and by ex vivo-isolated splenic macrophages in a CD169-dependent manner. In blood, high-dimensional reduction analysis revealed that ganglioside-liposomes specifically targeted CD14(+) CD169(+) monocytes and Axl(+) CD169(+) DCs. Liposomal codelivery of tumor antigen and Toll-like receptor ligand to CD169(+) moDCs and Axl(+) CD169(+) DCs led to cytokine production and robust cross-presentation and activation of tumor antigen-specific CD8(+) T cells. Finally, Axl(+) CD169(+) DCs were present in cancer patients and efficiently captured ganglioside-liposomes. Our findings demonstrate a nanovaccine platform targeting CD169(+) DCs to drive antitumor T cell responses.
C1 [Affandi, Alsya J.; Grabowska, Joanna; Olesek, Katarzyna; Venegas, Miguel Lopez; Barbaria, Arnaud; Rodriguez, Ernesto; Mulder, Patrick P. G.; Pijffers, Helen J.; Ambrosini, Martino; Kalay, Hakan; O'Toole, Tom; Zwart, Eline S.; van Kooyk, Yvette; den Haan, Joke M. M.] Vrije Univ Amsterdam, Canc Ctr Amsterdam, Amsterdam Infect & Immun Inst, Dept Mol Cell Biol & Immunol,Amsterdam UMC, NL-1081 HZ Amsterdam, Netherlands.
[Venegas, Miguel Lopez; van Kooyk, Yvette] DC4U, NL-3621 ZA Breukelen, Netherlands.
[Zwart, Eline S.; Kazemier, Geert] Vrije Univ Amsterdam, Canc Ctr Amsterdam, Dept Surg, Amsterdam UMC, NL-1081 HV Amsterdam, Netherlands.
[Nazmi, Kamran; Bikker, Floris J.] Vrije Univ Amsterdam, Acad Ctr Dent Amsterdam ACTA, Dept Oral Biochem, NL-1081 LA Amsterdam, Netherlands.
[Nazmi, Kamran; Bikker, Floris J.] Univ Amsterdam, NL-1081 LA Amsterdam, Netherlands.
[Stoeckl, Johannes] Med Univ Vienna, Ctr Pathophysiol Infectiol & Immunol, Inst Immunol, A-1090 Vienna, Austria.
[van den Eertwegh, Alfons J. M.; de Gruijl, Tanja D.] Vrije Univ Amsterdam, Canc Ctr Amsterdam, Dept Med Oncol, Amsterdam UMC, NL-1081 HV Amsterdam, Netherlands.
[Storm, Gert] Univ Utrecht, Utrecht Inst Pharmaceut Sci, Dept Pharmaceut, NL-3508 TB Utrecht,

Netherlands.
[Storm, Gert] Univ Twente, Fac Sci & Technol, Dept Biomat Sci & Technol, NL-7522 NB Enschede, Netherlands.
C3 Vrije Universiteit Amsterdam; University of Amsterdam; University of Amsterdam; Vrije Universiteit Amsterdam; Vrije Universiteit Amsterdam; University of Amsterdam; Medical University of Vienna; University of Amsterdam; Vrije Universiteit Amsterdam; Utrecht University; University of Twente
RP den Haan, JMM (通讯作者)，Vrije Univ Amsterdam, Canc Ctr Amsterdam, Amsterdam Infect & Immun Inst, Dept Mol Cell Biol & Immunol,Amsterdam UMC, NL-1081 HZ Amsterdam, Netherlands.
EM j.denhaan@amsterdamumc.nl
RI Bikker, Floris/R-1941-2019; Mulder, Patrick P.G./D-1699-2019; den Haan, Joke MM/A-4281-2010; giovannetti, elisa/F-4261-2011
OI Bikker, Floris/0000-0002-9453-4630; Mulder, Patrick P.G./0000-0002-0811-8658; den Haan, Joke MM/0000-0002-4421-6917; giovannetti, elisa/0000-0002-7565-7504; Barbaria, Arnaud/0000-0003-2784-5129; Affandi, Alsya/0000-0003-0859-2322; Zwart, Eline/0000-0002-2121-8039
FU Dutch Cancer Society [VU2016-10449]; Phospholipid Research Center; NWO ZonMW [TOP 91218024]; EU Research Framework Program [H2020-MSCA-ITN-2014-ETN-642870]
FX This work was supported by grants from the Dutch Cancer Society (VU2016-10449) to J.M.M.d.H., Y.v.K., and T.D.d.G.; from the Phospholipid Research Center to J.M.M.d.H. and Y.v.K.; from NWO ZonMW (TOP 91218024) to J.M.M.d.H. and G.S.; and by an EU Research Framework Program Grant (H2020-MSCA-ITN-2014-ETN-642870) to DC4U/M.L.V. We thank Sven Bruijns, Larissa Klaase (Amsterdam UMC), and Louis van Bloois (Utrecht University) for their technical advice and expertise. We acknowledge Jan Verhoeff, Dr. Juan Garcia Vallejo, and the Microscopy and Cytometry Core Facility at the Amsterdam UMC-Location VUmc for providing assistance in microscopy and cytometry. We thank Daan J. Brinkman (Amsterdam UMC, Catharina Hospital) and Wouter J. de Jonge (Amsterdam UMC) for additional patient material.
CR Akiyama H, 2014, J VIROL, V88, P8813, DOI 10.1128/JVI.00992-14
Alcantara-Hernandez M, 2017, IMMUNITY, V47, P1037, DOI 10.1016/j.immuni.2017.11.001
Alculumbre SG, 2018, NAT IMMUNOL, V19, P63, DOI 10.1038/s41590-017-0012-z
Anguille S, 2014, LANCET ONCOL, V15, pE257, DOI 10.1016/S1470-2045(13)70585-0
Asano T, 2018, CANCER SCI, V109, P1723, DOI 10.1111/cas.13565
Baars A, 2000, ANN ONCOL, V11, P965, DOI 10.1023/A:1008363601515
Backer R, 2010, P NATL ACAD SCI USA, V107, P216, DOI 10.1073/pnas.0909541107
Balachandran VP, 2017, NATURE, V551, P512, DOI 10.1038/nature24462
Bernhard CA, 2015, P NATL ACAD SCI USA, V112, P5461, DOI 10.1073/pnas.1423356112
Black LVC, 2016, IMMUNOL CELL BIOL, V94, P430, DOI 10.1038/icb.2015.111
Boks MA, 2015, J CONTROL RELEASE, V216, P37, DOI 10.1016/j.jconrel.2015.06.033
Bol KF, 2016, CLIN CANCER RES, V22, P1897, DOI 10.1158/1078-0432.CCR-15-1399
Chen WHC, 2012, PLOS ONE, V7, DOI 10.1371/journal.pone.0039039
Collin M, 2018, IMMUNOLOGY, V154, P3, DOI 10.1111/imm.12888
Collins BE, 1997, J BIOL CHEM, V272, P16889, DOI 10.1074/jbc.272.27.16889
CROCKER PR, 1991, EMBO J, V10, P1661, DOI 10.1002/j.1460-2075.1991.tb07689.x
Dams ETM, 2000, J PHARMACOL EXP THER, V292, P1071
Danilova L, 2019, CANCER IMMUNOL RES, V7, P886, DOI 10.1158/2326-6066.CIR-18-0822
Darrah PA, 2020, NATURE, V577, P95, DOI 10.1038/s41586-019-1817-8
Dhodapkar MV, 2014, SCI TRANSL MED, V6, DOI 10.1126/scitranslmed.3008068
Duinkerken S, 2019, THERANOSTICS, V9, P5797, DOI 10.7150/thno.35059
Erikson E, 2015, J BIOL CHEM, V290, P27345, DOI 10.1074/jbc.M115.681338
Fehres CM, 2015, J CONTROL RELEASE, V203, P67, DOI 10.1016/j.jconrel.2015.01.040
Geijtenbeek TBH, 2000, CELL, V100, P587, DOI 10.1016/S0092-8674(00)80694-7
Grabowska J, 2018, FRONT IMMUNOL, V9, DOI 10.3389/fimmu.2018.02472
Guilliams M, 2016, IMMUNITY, V45, P669, DOI 10.1016/j.immuni.2016.08.015
Hashimoto Y, 1998, J BIOCHEM-TOKYO, V123, P468
Haslam A, 2019, JAMA NETW OPEN, V2, DOI 10.1001/jamanetworkopen.2019.2535

Horrevorts SK, 2018, FRONT IMMUNOL, V9, DOI 10.3389/fimmu.2018.01231
Huber A, 2018, FRONT IMMUNOL, V9, DOI 10.3389/fimmu.2018.02804
Ino Y, 2013, BRIT J CANCER, V108, P914, DOI 10.1038/bjc.2013.32
Izquierdo-Useros N, 2012, PLOS BIOL, V10, DOI 10.1371/journal.pbio.1001448
Kawasaki N, 2013, P NATL ACAD SCI USA, V110, P7826, DOI 10.1073/pnas.1219888110
Klaas M, 2012, SEMIN IMMUNOPATHOL, V34, P353, DOI 10.1007/s00281-012-0310-3
Kranz LM, 2016, NATURE, V534, P396, DOI 10.1038/nature18300
Kuball J, 2007, BLOOD, V109, P2331, DOI 10.1182/blood-2006-05-023069
Laverman P, 2001, J PHARMACOL EXP THER, V298, P607
Leylek R, 2019, CELL REP, V29, P3736, DOI 10.1016/j.celrep.2019.11.042
Louie DAP, 2019, FRONT IMMUNOL, V10, DOI 10.3389/fimmu.2019.00347
Lubbers J, 2018, FRONT IMMUNOL, V9, DOI 10.3389/fimmu.2018.02807
Macauley MS, 2014, NAT REV IMMUNOL, V14, P653, DOI 10.1038/nri3737
Mitragotri S, 2014, NAT REV DRUG DISCOV, V13, P655, DOI 10.1038/nrd4363
Mohamed M, 2019, SCI TECHNOL ADV MAT, V20, P710, DOI 10.1080/14686996.2019.1627174
Mordmuller B, 2017, NATURE, V542, P445, DOI 10.1038/nature21060
Nagelkerke SQ, 2018, BLOOD ADV, V2, P941, DOI 10.1182/bloodadvances.2017015008
Nevala-Plagemann C., 2019, NAT REV CLIN ONCOL
Nycholat CM, 2012, J AM CHEM SOC, V134, P15696, DOI 10.1021/ja307501e
Ohnishi K, 2013, CANCER SCI, V104, P1236, DOI 10.1111/cas.12212
Pack M, 2008, IMMUNOLOGY, V123, P438, DOI 10.1111/j.1365-2567.2007.02710.x
Parmar R, 2016, VACCINE, V34, P3229, DOI 10.1016/j.vaccine.2016.04.095
Pattni BS, 2015, CHEM REV, V115, P10938, DOI 10.1021/acs.chemrev.5b00046
Perez-Zsolt D., 2019, VIRUSES, V12, pE8
Perez-Zsolt D, 2019, NAT MICROBIOL, V4, P1558, DOI 10.1038/s41564-019-0453-2
Pucci F, 2016, SCIENCE, V352, P242, DOI 10.1126/science.aaf1328
Puryear WB, 2013, PLOS PATHOG, V9, DOI 10.1371/journal.ppat.1003291
Puryear WB, 2012, P NATL ACAD SCI USA, V109, P7475, DOI 10.1073/pnas.1201104109
Rempel H, 2008, PLOS ONE, V3, DOI 10.1371/journal.pone.0001967
Ruffin N, 2019, P NATL ACAD SCI USA, V116, P21685, DOI 10.1073/pnas.1911007116
Saito Y, 2015, CANCER IMMUNOL RES, V3, P1356, DOI 10.1158/2326-6066.CIR-14-0180
Schaft N, 2003, J IMMUNOL, V170, P2186, DOI 10.4049/jimmunol.170.4.2186
Schetters STT, 2018, FRONT IMMUNOL, V9, DOI 10.3389/fimmu.2018.00990
See P, 2017, SCIENCE, V356, DOI 10.1126/science.aag3009
Sewald X, 2015, SCIENCE, V350, P563, DOI 10.1126/science.aab2749
Silvin A, 2017, SCI IMMUNOL, V2, DOI 10.1126/sciimmunol.aai8071
Steiniger B, 1997, IMMUNOLOGY, V92, P307, DOI 10.1046/j.1365-2567.1997.00328.x
Tacken PJ, 2011, BLOOD, V118, P4111, DOI 10.1182/blood-2011-04-346957
Tullett KM, 2016, JCI INSIGHT, V1, DOI 10.1172/jci.insight.87102
U'Ren L, 2006, CANCER GENE THER, V13, P1033, DOI 10.1038/sj.cgt.7700982
Uchil PD, 2019, CELL HOST MICROBE, V25, P87, DOI 10.1016/j.chom.2018.11.011
Unger WWJ, 2012, J CONTROL RELEASE, V160, P88, DOI 10.1016/j.jconrel.2012.02.007
Van der Jeught K, 2018, ACS NANO, V12, P9815, DOI 10.1021/acsnano.8b00966
van Dinther D, 2019, CANCERS, V11, DOI 10.3390/cancers11020183
van Dinther D, 2018, FRONT IMMUNOL, V9, DOI 10.3389/fimmu.2018.01997
van Dinther D, 2018, CELL REP, V22, P1484, DOI 10.1016/j.celrep.2018.01.021
van Dinther D, 2017, J LEUKOCYTE BIOL, V102, P1017, DOI [10.1189/jlb.5MR0217-059RR, 10.1189/jlb.5mr0217-059rr]
Villani AC, 2017, SCIENCE, V356, DOI 10.1126/science.aah4573
Vonderheide RH, 2018, CANCER CELL, V33, P563, DOI 10.1016/j.ccell.2018.03.008
Wu AA, 2019, CURR ONCOL REP, V21, DOI 10.1007/s11912-019-0811-5
Xu F, 2018, P NATL ACAD SCI USA, V115, pE9041, DOI 10.1073/pnas.1804292115
Yarchoan M, 2017, NEW ENGL J MED, V377, P2500, DOI 10.1056/NEJMc1713444
York MR, 2007, ARTHRITIS RHEUM-US, V56, P1010, DOI 10.1002/art.22382
Yu XW, 2014, NAT COMMUN, V5, DOI 10.1038/ncomms5136
Zhang Y, 2016, ELIFE, V5, DOI 10.7554/eLife.18156
NR 83
TC 40
Z9 42
U1 1
U2 29

PU NATL ACAD SCIENCES
PI WASHINGTON
PA 2101 CONSTITUTION AVE NW, WASHINGTON, DC 20418 USA
SN 0027-8424
J9 P NATL ACAD SCI USA
JI Proc. Natl. Acad. Sci. U. S. A.
PD NOV 3
PY 2020
VL 117
IS 44
BP 27528
EP 27539
DI 10.1073/pnas.2006186117
PG 12
WC Multidisciplinary Sciences
WE Science Citation Index Expanded (SCI-EXPANDED)
SC Science & Technology - Other Topics
GA OO6QW
UT WOS:000587503000063
PM 33067394
OA Green Published, hybrid
DA 2023-09-14
ER

PT J
AU Liu, Q
Fan, TJ
Zheng, YY
Yang, SL
Yu, ZQ
Duo, YH
Zhang, YH
Adah, D
Shi, LL
Sun, Z
Wang, D
Xie, JL
Wu, H
Wu, ZZ
Ge, CC
Qiao, LJ
Wei, CY
Huang, LD
Yan, QT
Yang, QH
Bao, SY
Liu, LP
Zhang, H
AF Liu, Quan
Fan, Taojian
Zheng, Yuanyuan
Yang, Sheng-li
Yu, Zhiqiang
Duo, Yanhong
Zhang, Yuhua
Adah, Dickson
Shi, Lulin
Sun, Zhe
Wang, Dou
Xie, Jianlei
Wu, Hong

Wu, Zongze
Ge, Chenchen
Qiao, Lijun
Wei, Chaoying
Huang, Luodan
Yan, Qiaoting
Yang, Qinhe
Bao, Shiyun
Liu, Li-Ping
Zhang, Han
TI Immunogenic exosome-encapsulated black phosphorus nanoparticles as an effective anticancer photo-nanovaccine
SO NANOSCALE
LA English
DT Article
ID TUMOR-DERIVED EXOSOMES; PHOTOTHERMAL THERAPY; QUANTUM DOTS; CANCER; IMMUNOTHERAPY; CELLS; NANOCARRIERS; DELIVERY; VACCINES; IMMUNITY
AB Tumor vaccines are a promising form of cancer immunotherapy, but difficulties such as neo-antigen identification, activation of immune cells, and tumor infiltration prevent their clinical breakthrough. Interestingly, nanotechnology-based photothermal therapy (PTT) has great potential to overcome these barriers. Previous studies have shown that serum exosomes (hEX) from hyperthermia-treated tumor-bearing mice displayed an array of patient-specific tumor-associated antigens (TAAs), and strong immunoregulatory abilities in promoting dendritic cell (DC) differentiation and maturation. Here, we developed a tumor vaccine (hEX@BP) by encapsulating black phosphorus quantum dots (BPQDs) with exosomes (hEX) against a murine subcutaneous lung cancer model. In comparison with BPQDs alone (BP), hEX@BP demonstrated better long-term PTT performance, greater elevation of tumor temperature and tumor targeting efficacyin vivo. Vaccination with hEX@BP in combination with PTT further demonstrated an outstanding therapeutic efficacy against established lung cancer, and promoted the infiltration of T lymphocytes into the tumor tissue. Our findings demonstrated that hEX@BP might be an innovative cancer photo-nanovaccine that offers effective immuno-PTT against cancers.
C1 [Liu, Quan; Zhang, Yuhua; Shi, Lulin; Sun, Zhe; Wang, Dou; Wu, Hong; Wu, Zongze; Ge, Chenchen; Qiao, Lijun; Wei, Chaoying; Huang, Luodan; Yan, Qiaoting; Bao, Shiyun; Liu, Li-Ping] Jinan Univ, Clin Med Coll 2, Shenzhen Peoples Hosp, Dept Hepatohiliary & Pancrease Surg, Shenzhen 518020, Guangdong, Peoples R China.
[Liu, Quan; Zhang, Yuhua; Shi, Lulin; Sun, Zhe; Wang, Dou; Wu, Hong; Wu, Zongze; Ge, Chenchen; Qiao, Lijun; Wei, Chaoying; Huang, Luodan; Yan, Qiaoting; Bao, Shiyun; Liu, Li-Ping] Southern Univ Sci & Technol, Affiliated Hosp 1, Shenzhen 518020, Guangdong, Peoples R China.
[Liu, Quan; Sun, Zhe; Ge, Chenchen; Yang, Qinhe] Jinan Univ, Sch Tradit Chinese Med, Guangzhou 510632, Guangdong, Peoples R China.
[Liu, Quan; Sun, Zhe; Ge, Chenchen; Qiao, Lijun] Jinan Univ, Integrated Chinese & Western Med Postdoctoral Res, Guangzhou 510632, Guangdong, Peoples R China.
[Fan, Taojian; Duo, Yanhong; Xie, Jianlei; Zhang, Han] Shenzhen Univ, Affiliated Hosp 1, Shenzhen Peoples Hosp 2, Shenzhen 518060, Peoples R China.
[Fan, Taojian; Duo, Yanhong; Xie, Jianlei; Zhang, Han] Shenzhen Univ, Collaborat Innovat Centerfor Optoelect Sci & Tech, Shenzhen 518060, Peoples R China.
[Zheng, Yuanyuan] Jinan Univ, Shenzhen Peoples Hosp, Clin Med Coll 2, Translat Med Collaborat Innovat Ctr, Shenzhen 518020, Peoples R China.
[Yang, Sheng-li] Huazhong Univ Sci & Technol, Union Hosp, Tongji Med Coll, Canc Ctr, Wuhan 430022, Peoples R China.
[Yu, Zhiqiang; Zhang, Yuhua; Wu, Hong; Huang, Luodan] Southern Med Univ, Sch Pharmaceut Sci, Guangdong Prov Key Lab New Drug Screening, Guangzhou 510515, Guangdong, Peoples R China.
[Adah, Dickson] Chinese Acad Sci, Guangzhou Inst Biomed & Hlth, Dept Infect & Immun, Stale Key Lab Resp Dis, Guangzhou 510530, Guangdong, Peoples R China.
[Liu, Li-Ping] Shenzhen Publ Serv Platform Tumor Precis Med & Mo, Shenzhen 518020, Guangdong, Peoples R China.
C3 Jinan University; Southern University of Science & Technology; Jinan University; Jinan University; Shenzhen University; Shenzhen University; Jinan University; Huazhong University of Science & Technology; Southern Medical University - China; Chinese Academy of Sciences; Guangzhou Institute of Biomedicine & Health, CAS
RP Bao, SY; Liu, LP (通讯作者)，Jinan Univ, Clin Med Coll 2, Shenzhen Peoples Hosp, Dept Hepatohiliary

& Pancrease Surg, Shenzhen 518020, Guangdong, Peoples R China.; Bao, SY; Liu, LP (通讯作者), Southern Univ Sci & Technol, Affiliated Hosp 1, Shenzhen 518020, Guangdong, Peoples R China.; Yang, QH (通讯作者), Jinan Univ, Sch Tradit Chinese Med, Guangzhou 510632, Guangdong, Peoples R China.; Zhang, H (通讯作者), Shenzhen Univ, Affiliated Hosp 1, Shenzhen Peoples Hosp 2, Shenzhen 518060, Peoples R China.; Zhang, H (通讯作者), Shenzhen Univ, Collaborat Innovat Centerfor Optoelect Sci & Tech, Shenzhen 518060, Peoples R China.; Liu, LP (通讯作者), Shenzhen Publ Serv Platform Tumor Precis Med & Mo, Shenzhen 518020, Guangdong, Peoples R China.
EM tyangqh@jnu.edu.cn; baomi94@163.com; liu.liping@szhospital.com;
hzhang@szu.edu.cn
RI wu, tong/ITV-6896-2023; Jiang, Tao/IWM-7503-2023; zhang, han/I-8297-2015
OI zhang, han/0000-0002-0166-1973; Xie, Jianlei/0000-0002-7357-4676; Quan, Liu/0000-0001-9994-3743
FU National Natural Science Foundation of China [61575089, 61435010, 81903013]; Science and Technology Innovation Commission of Shenzhen [KQJSCX20180321164801762, KQTD2015032416270385, JCYJ20150625103619275, JCYJ20180507181817604]; China Postdoctoral Science Foundation [2018M643364, 2019M663377, 2019M663372]; PhD Start-up Fund of the Natural Science Foundation of Guangdong Province, China [2018A030310040]
FX This study was supported by the National Natural Science Foundation of China (61575089 and 61435010, H. Z., 81903013, L. J. Q.), the Science and Technology Innovation Commission of Shenzhen (KQJSCX20180321164801762, L. P. L.; KQTD2015032416270385 and JCYJ20150625103619275, H. Z.; and JCYJ20180507181817604, S. Y. B.), the China Postdoctoral Science Foundation (2018M643364, Q. L., 2019M663377, Z. S., 2019M663372, L. J. Q.), the and PhD Start-up Fund of the Natural Science Foundation of Guangdong Province, China (2018A030310040, Q. L.).
CR Ainsworth C, 2018, NATURE, V563, pS52, DOI 10.1038/d41586-018-07366-1
Alvarez-Erviti L, 2011, NAT BIOTECHNOL, V29, P341, DOI 10.1038/nbt.1807
Chen DS, 2017, NATURE, V541, P321, DOI 10.1038/nature21349
Chen Q, 2019, ADV MATER, V31, DOI 10.1002/adma.201900192
Chen Q, 2016, NAT COMMUN, V7, DOI 10.1038/ncomms13193
Chen WS, 2018, ADV MATER, V30, DOI 10.1002/adma.201703458
den Haan JMM, 2000, J EXP MED, V192, P1685, DOI 10.1084/jem.192.12.1685
Evans SS, 2015, NAT REV IMMUNOL, V15, P335, DOI 10.1038/nri3843
Galluzzi L, 2018, SCI TRANSL MED, V10, DOI 10.1126/scitranslmed.aat7807
Ge XX, 2019, ADV FUNCT MATER, V29, DOI 10.1002/adfm.201900318
Geng SY, 2018, CHEM COMMUN, V54, P6060, DOI 10.1039/c8cc03423k
Jiang XC, 2017, INT J PHARMACEUT, V521, P167, DOI 10.1016/j.ijpharm.2017.02.038
Johnsen KB, 2014, BBA-REV CANCER, V1846, P75, DOI 10.1016/j.bbcan.2014.04.005
Jyoti K, 2020, J MICROENCAPSUL, V37, P14, DOI 10.1080/02652048.2019.1692943
Kalluri R, 2020, SCIENCE, V367, P640, DOI 10.1126/science.aau6977
Li Y, 2017, ACS APPL MATER INTER, V9, P25098, DOI 10.1021/acsami.7b05824
Liang X, 2019, J CONTROL RELEASE, V296, P150, DOI 10.1016/j.jconrel.2019.01.027
Liu Q, 2017, ONCOTARGET, V8, P24785, DOI 10.18632/oncotarget.15806
Lizotte PH, 2016, NAT NANOTECHNOL, V11, P295, DOI [10.1038/NNANO.2015.292, 10.1038/nnano.2015.292]
Melief CJM, 2008, NAT REV CANCER, V8, P351, DOI 10.1038/nrc2373
Melo SA, 2015, NATURE, V523, P177, DOI 10.1038/nature14581
Mo JB, 2018, NAT COMMUN, V9, DOI 10.1038/s41467-018-04873-7
Moy AJ, 2017, ADV DRUG DELIVER REV, V114, P175, DOI 10.1016/j.addr.2017.06.008
Mu XY, 2017, ACS APPL MATER INTER, V9, P20399, DOI 10.1021/acsami.7b02900
Nabhan C, 2010, NEW ENGL J MED, V363, P1966, DOI 10.1056/NEJMc1009982
Nam J, 2018, NAT COMMUN, V9, DOI 10.1038/s41467-018-03473-9
Ning YL, 2018, IMMUNOL LETT, V199, P36, DOI 10.1016/j.imlet.2018.05.002
Niu LM, 2019, CANCER SCI, V110, P433, DOI 10.1111/cas.13862
Pan D, 2019, INT J MOL SCI, V20, DOI 10.3390/ijms20020323
Qiu LP, 2018, SMALL, V14, DOI 10.1002/smll.201703539
Qiu M, 2018, P NATL ACAD SCI USA, V115, P501, DOI 10.1073/pnas.1714421115
Rao Q, 2016, HEPATOLOGY, V64, P456, DOI 10.1002/hep.28549
Ribas A, 2018, SCIENCE, V359, P1350, DOI 10.1126/science.aar4060
Sahin U, 2018, SCIENCE, V359, P1355, DOI 10.1126/science.aar7112

Shang YH, 2019, ACS APPL MATER INTER, V11, P28254, DOI 10.1021/acsami.9b04735
Sun ZB, 2015, ANGEW CHEM INT EDIT, V54, P11526, DOI 10.1002/anie.201506154
TAO W, 2017, ANGEW CHEM INT EDIT, V56, DOI DOI 10.1002/ANIE.201703657
Tao W, 2017, ADV MATER, V29, DOI 10.1002/adma.201603276
Thery C, 2002, NAT REV IMMUNOL, V2, P569, DOI 10.1038/nri855
Thery C, 2009, NAT REV IMMUNOL, V9, P581, DOI 10.1038/nri2567
Valadi H, 2007, NAT CELL BIOL, V9, P654, DOI 10.1038/ncb1596
van der Burg SH, 2016, NAT REV CANCER, V16, P219, DOI 10.1038/nrc.2016.16
Wang C, 2014, ADV MATER, V26, P8154, DOI 10.1002/adma.201402996
Wang H, 2019, ACS NANO, V13, P9206, DOI 10.1021/acsnano.9b03531
Wang QS, 2019, ADV MATER, V31, DOI 10.1002/adma.201904836
Wang ZM, 2019, SMALL, V15, DOI 10.1002/smll.201901560
Wolfers J, 2001, NAT MED, V7, P297, DOI 10.1038/85438
Yang R, 2018, ACS NANO, V12, P5121, DOI 10.1021/acsnano.7b09041
Yang YS, 2017, ADV MATER, V29, DOI 10.1002/adma.201605604
Ye XY, 2019, ACS NANO, V13, P2956, DOI 10.1021/acsnano.8b07371
Zhang XY, 2016, SMALL, V12, P3578, DOI 10.1002/smll.201600618
Zhou L, 2017, CANCER LETT, V407, P84, DOI 10.1016/j.canlet.2017.08.003
Zhou WH, 2018, ANGEW CHEM INT EDIT, V57, P10268, DOI 10.1002/anie.201806941
NR 53
TC 40
Z9 42
U1 12
U2 139
PU ROYAL SOC CHEMISTRY
PI CAMBRIDGE
PA THOMAS GRAHAM HOUSE, SCIENCE PARK, MILTON RD, CAMBRIDGE CB4 0WF, CAMBS,
ENGLAND
SN 2040-3364
EI 2040-3372
J9 NANOSCALE
JI Nanoscale
PD OCT 14
PY 2020
VL 12
IS 38
BP 19939
EP 19952
DI 10.1039/d0nr05953f
PG 14
WC Chemistry, Multidisciplinary; Nanoscience & Nanotechnology; Materials
Science, Multidisciplinary; Physics, Applied
WE Science Citation Index Expanded (SCI-EXPANDED)
SC Chemistry; Science & Technology - Other Topics; Materials Science;
Physics
GA OA9LD
UT WOS:000578100000038
PM 32991664
DA 2023-09-14
ER

PT J
AU Najafabadi, AH
Zhang, J
Aikins, ME
Abadi, ZIN
Liao, F
Qin, Y
Okeke, EB
Scheetz, LM
Nam, J

Xu, Y
Adams, D
Lester, P
Hetrick, T
Schwendeman, A
Wicha, MS
Chang, AE
Li, Q
Moon, JJ
AF Najafabadi, Alireza Hassani
Zhang, Jing
Aikins, Marisa E.
Abadi, Zeynab Izadi Najaf
Liao, Fei
Qin, You
Okeke, Emeka B.
Scheetz, Lindsay M.
Nam, Jutaek
Xu, Yao
Adams, David
Lester, Patrick
Hetrick, Taryn
Schwendeman, Anna
Wicha, Max S.
Chang, Alfred E.
Li, Qiao
Moon, James J.
TI Cancer Immunotherapy via Targeting Cancer Stem Cells Using Vaccine Nanodiscs
SO NANO LETTERS
LA English
DT Article
DE Cancer stem cells; cancer vaccine; breast cancer; melanoma; aldehyde dehydrogenase
ID ALDEHYDE DEHYDROGENASE-ACTIVITY; IMMUNE CHECKPOINT BLOCKADE; COMBINATION; THERAPY; METASTASIS; DISEASE; MARKER
AB Cancer stem cells (CSCs) proliferate extensively and drive tumor metastasis and recurrence. CSCs have been identified in over 20 cancer types to date, but it remains unknown how to target and eliminate CSCs in vivo. Aldehyde dehydrogenase (ALDH) is a marker that has been used extensively for isolating CSCs. Here we present a novel approach to target and reduce the frequency of ALDH(hig)(h) CSCs by vaccination against ALDH. We have identified ALDH1-A1 and ALDH1-A3 epitopes from CSCs and developed synthetic high-density lipoprotein nanodiscs for vaccination against ALDH(high) CSCs. Nanodiscs increased antigen trafficking to lymph nodes and generated robust ALDH-specific T cell responses. Nanodisc vaccination against ALDH(high) CSCs combined with anti-PD-L1 therapy exerted potent antitumor efficacy and prolonged animal survival in multiple murine models. Overall, this is the first demonstration of a simple nanovaccine strategy against CSCs and may lead to new avenues for cancer immunotherapy against CSCs.
C1 [Najafabadi, Alireza Hassani; Aikins, Marisa E.; Abadi, Zeynab Izadi Najaf; Okeke, Emeka B.; Scheetz, Lindsay M.; Nam, Jutaek; Xu, Yao; Schwendeman, Anna; Moon, James J.] Univ Michigan, Dept Pharmaceut Sci, Ann Arbor, MI 48109 USA.
[Najafabadi, Alireza Hassani; Aikins, Marisa E.; Abadi, Zeynab Izadi Najaf; Okeke, Emeka B.; Scheetz, Lindsay M.; Nam, Jutaek; Xu, Yao; Schwendeman, Anna; Moon, James J.] Univ Michigan, Biointerfaces Inst, Ann Arbor, MI 48109 USA.
[Zhang, Jing; Liao, Fei; Qin, You; Wicha, Max S.; Chang, Alfred E.; Li, Qiao] Univ Michigan, Dept Surg, Ann Arbor, MI 48109 USA.
[Zhang, Jing] Huazhong Univ Sci & Technol, Hubei Canc Hosp, Tongji Med Coll, Dept Med Oncol, Wuhan 430079, Hubei, Peoples R China.
[Liao, Fei] Wuhan Univ, Gastroenterol Dept, Renmin Hosp, Wuhan 430060, Hubei, Peoples R China.
[Qin, You] Huazhong Univ Sci & Technol, Union Hosp, Tongji Med Coll, Canc Ctr, Wuhan 430022, Hubei, Peoples R China.
[Adams, David] Univ Michigan, Biomed Res Core Facil, Ann Arbor, MI 48109 USA.

[Lester, Patrick; Hetrick, Taryn] Univ Michigan, Unit Lab Anim Med, Ann Arbor, MI 48109 USA.
[Moon, James J.] Univ Michigan, Dept Biomed Engn, Ann Arbor, MI 48109 USA.
C3 University of Michigan System; University of Michigan; University of Michigan System; University of Michigan; University of Michigan System; University of Michigan; Huazhong University of Science & Technology; Wuhan University; Huazhong University of Science & Technology; University of Michigan System; University of Michigan; University of Michigan System; University of Michigan; University of Michigan System; University of Michigan
RP Moon, JJ (通讯作者)，Univ Michigan, Dept Pharmaceut Sci, Ann Arbor, MI 48109 USA.; Moon, JJ (通讯作者)，Univ Michigan, Biointerfaces Inst, Ann Arbor, MI 48109 USA.; Li, Q (通讯作者)，Univ Michigan, Dept Surg, Ann Arbor, MI 48109 USA.; Moon, JJ (通讯作者)，Univ Michigan, Dept Biomed Engn, Ann Arbor, MI 48109 USA.
EM qiaoli@med.umich.edu; moonjj@med.umich.edu
RI Moon, James J/ABE-4429-2021
OI Moon, James J/0000-0003-2238-2372; Nam, Jutaek/0000-0002-0397-1449
FU NIH [R01EB022563, R01CA210273, R01AI127070, R01DK125087, R21NS091555, R01HL134569, R35CA197585]; MTRAC for Life Sciences Hub; Emerald Foundation; Gillson Longenbaugh Foundation; Breast Cancer Research Foundation; NSF CAREER Award [1553831]; Hubei Cancer Hospital; China Scholarship Council; Wuhan Union Hospital; NSERC Postdoctoral Fellowship; CIHR Postdoctoral Fellowship; Cancer Center Support Grant [P30 CA046592]; UM Forbes Institute for Cancer Discovery Pilot Grant
FX This work was supported in part by NIH (R01EB022563, R01CA210273, R01AI127070, and R01DK125087 to J.J.M. R21NS091555 and R01HL134569 to A.S. R35CA197585 to M.W.), the MTRAC for Life Sciences Hub, a UM Forbes Institute for Cancer Discovery Pilot Grant, the Emerald Foundation, the Gillson Longenbaugh Foundation (Q.L. and A.E.C.), and Breast Cancer Research Foundation (M.W.). J.J.M. was supported by an NSF CAREER Award (1553831). J.Z., F.L., and Y.Q. were supported in part by Hubei Cancer Hospital, the China Scholarship Council, and Wuhan Union Hospital, respectively. E.B.O. was supported by an NSERC Postdoctoral Fellowship and a CIHR Postdoctoral Fellowship. D.A. was supported by a Cancer Center Support Grant (P30 CA046592). Some figures were created by the authors using BioRender.com.
CR Batlle E, 2017, NAT MED, V23, P1124, DOI 10.1038/nm.4409
Charafe-Jauffret E, 2010, CLIN CANCER RES, V16, P45, DOI 10.1158/1078-0432.CCR-09-1630
De Henau O, 2016, NATURE, V539, P443, DOI 10.1038/nature20554
Deng ZL, 2015, BMC IMMUNOL, V16, DOI 10.1186/s12865-014-0064-x
Duarte S, 2013, STEM CELLS, V31, P423, DOI 10.1002/stem.1292
Ginestier C, 2007, CELL STEM CELL, V1, P555, DOI 10.1016/j.stem.2007.08.014
Hu YY, 2016, CANCER RES, V76, P4661, DOI 10.1158/0008-5472.CAN-15-2664
Jordan CT, 2006, NEW ENGL J MED, V355, P1253, DOI 10.1056/NEJMra061808
Kim RJ, 2013, CANCER LETT, V333, P18, DOI 10.1016/j.canlet.2012.11.026
Klein O, 2011, EXPERT REV VACCINES, V10, P853, DOI [10.1586/ERV.11.74, 10.1586/erv.11.74]
Kuai R, 2018, J CONTROL RELEASE, V282, P131, DOI 10.1016/j.jconrel.2018.04.041
Kuai R, 2018, BIOCONJUGATE CHEM, V29, P771, DOI 10.1021/acs.bioconjchem.7b00761
Kuai R, 2017, NAT MATER, V16, P489, DOI [10.1038/nmat4822, 10.1038/NMAT4822]
Kuai R, 2016, ACS NANO, V10, P3015, DOI 10.1021/acsnano.5b07522
Liu SL, 2014, STEM CELL REP, V2, P78, DOI 10.1016/j.stemcr.2013.11.009
Lu L, 2015, ONCOIMMUNOLOGY, V4, DOI 10.4161/2162402X.2014.990767
Luo YC, 2012, STEM CELLS, V30, P2100, DOI 10.1002/stem.1193
Marcato P, 2011, CELL CYCLE, V10, P1378, DOI 10.4161/cc.10.9.15486
Marcato P, 2011, STEM CELLS, V29, P32, DOI 10.1002/stem.563
Mei Y, 2020, NAT COMMUN, V11, DOI 10.1038/s41467-020-16911-4
Melief CJM, 2008, NAT REV CANCER, V8, P351, DOI 10.1038/nrc2373
Miao YX, 2019, CELL, V177, P1172, DOI 10.1016/j.cell.2019.03.025
Nam J, 2019, NAT REV MATER, V4, P398, DOI 10.1038/s41578-019-0108-1
Ning N, 2012, CANCER RES, V72, P1853, DOI 10.1158/0008-5472.CAN-11-1400
Ochyl LJ, 2018, BIOMATERIALS, V182, P157, DOI 10.1016/j.biomaterials.2018.08.016
Pattabiraman DR, 2014, NAT REV DRUG DISCOV, V13, P497, DOI 10.1038/nrd4253

Pearce DJ, 2005, STEM CELLS, V23, P752, DOI 10.1634/stemcells.2004-0292
Reya T, 2001, NATURE, V414, P105, DOI 10.1038/35102167
Robert C, 2015, NEW ENGL J MED, V372, P2521, DOI 10.1056/NEJMoa1503093
Sales KM, 2007, STEM CELL REV, V3, P249, DOI 10.1007/s12015-007-9002-0
Schatton T, 2009, ANN NY ACAD SCI, V1176, P154, DOI 10.1111/j.1749-6632.2009.04568.x
Scheetz L, 2019, NAT BIOMED ENG, V3, P768, DOI 10.1038/s41551-019-0436-x
Schumacher TN, 2019, ANNU REV IMMUNOL, V37, P173, DOI 10.1146/annurev-immunol-042617-053402
Sharma P, 2015, CELL, V161, P205, DOI 10.1016/j.cell.2015.03.030
Shen S, 2016, BIOMATERIALS, V74, P1, DOI 10.1016/j.biomaterials.2015.09.037
Shi SJ, 2013, ANGEW CHEM INT EDIT, V52, P3901, DOI 10.1002/anie.201208077
Silva IA, 2011, CANCER RES, V71, P3991, DOI 10.1158/0008-5472.CAN-10-3175
Su WJ, 2019, CANCER CELL, V36, P139, DOI 10.1016/j.ccell.2019.06.009
Sun TM, 2014, BIOMATERIALS, V35, P836, DOI 10.1016/j.biomaterials.2013.10.011
Topalian SL, 2016, NAT REV CANCER, V16, P275, DOI 10.1038/nrc.2016.36
Topalian SL, 2015, CANCER CELL, V27, P450, DOI 10.1016/j.ccell.2015.03.001
Visus C, 2011, CLIN CANCER RES, V17, P6174, DOI 10.1158/1078-0432.CCR-11-1111
Wu D, 2015, J OVARIAN RES, V8, DOI 10.1186/s13048-015-0196-5
Zhuang XF, 2012, BMC CANCER, V12, DOI 10.1186/1471-2407-12-549
NR 44
TC 40
Z9 40
U1 0
U2 62
PU AMER CHEMICAL SOC
PI WASHINGTON
PA 1155 16TH ST, NW, WASHINGTON, DC 20036 USA
SN 1530-6984
EI 1530-6992
J9 NANO LETT
JI Nano Lett.
PD OCT 14
PY 2020
VL 20
IS 10
BP 7783
EP 7792
DI 10.1021/acs.nanolett.0c03414
PG 10
WC Chemistry, Multidisciplinary; Chemistry, Physical; Nanoscience & Nanotechnology; Materials Science, Multidisciplinary; Physics, Applied; Physics, Condensed Matter
WE Science Citation Index Expanded (SCI-EXPANDED)
SC Chemistry; Science & Technology - Other Topics; Materials Science; Physics
GA PZ9NM
UT WOS:000613073900035
PM 32926633
OA Green Accepted
DA 2023-09-14
ER

PT J
AU Ratput, MKS
Kesharwani, SS
Kumar, S
Muley, P
Narisetty, S
Tummala, H
AF Ratput, Mrigendra K. S.
Kesharwani, Siddharth S.

Kumar, Sunny
Muley, Pratik
Narisetty, Susmitha
Tummala, Hemachand
TI Dendritic Cell-Targeted Nanovaccine Delivery System Prepared with an Immune-Active Polymer
SO ACS APPLIED MATERIALS & INTERFACES
LA English
DT Article
DE dendritic cell targeting; cancer immunotherapy; nanoparticulate vaccine; humoral and cellular immunity; inulin acetate; toll-like receptor-4 agonists
ID VACCINE ADJUVANTS; RATIONAL DESIGN; ADAPTIVE IMMUNITY; ANTITUMOR IMMUNITY; INULIN ACETATE; IN-VIVO; ANTIGEN; NANOPARTICLES; RESPONSES; IMMUNOTHERAPY
AB Targeting dendritic cells (DCs), either ex vivo (Ex. Sipuleucel-T) or in vivo, for stimulating cellular immunity has been a leading approach for cancer vaccines. We have rationally engineered a nanoparticle (NP)-based delivery system for vaccines (InAc-NPs) using inulin acetate (InAc) as the polymer to target DCs. The material and the antigen-encapsulated InAc-NPs (similar to 190 nm in diameter) were characterized for their physicochemical properties. As a potent vaccine adjuvant, InAc-NPs activated TLR4 on multiple immune cells, including DCs and primary swine and human cells, to secrete various cytokines as detected by enzyme-linked immunosorbent assay and quantitative polymerase chain reaction. In addition, InAc-NPs promoted the maturation of DCs as observed by a decreased phagocytic ability and enhanced capability to activate various maturation markers (MHC-I, MHC-II, CD40, and CD80) quantified using flow cytometry. In mice, the InAc-NPs produced strong serum antibody titers (total IgG, IgG1, and IgG2a) against the encapsulated antigen (ovalbumin) similar to complete Freund's adjuvant. Additionally, as a dose-sparing delivery system, antigen delivered through InAc-NPs generated higher antibody titers (IgG1, 1.57 times; IgG-total, 1.66 times; and IgG2a, 29.8 times) even at 100 times less antigen dose. High amounts of cytokines representing both humoral (IL4 and IL10) and cell-mediated (IL2 and IFN-gamma) immunities were secreted from splenocytes of mice immunized with InAc-NPs. Importantly, InAc-NPs provided complete protection in 100% of the vaccinated mice from metastasis of intravenously injected melanoma cells (B-16-F-10) to lungs. In addition, the InAc-NPs were cleared from the injection site within 30 h of injection (in vivo imaging) and displayed no toxicity at the injection site (histology). The current study demonstrates that the multifunctional InAc-based nanovaccine delivery system has potential applications in cancer immunotherapy and delivering vaccines against various infectious diseases.
C1 [Ratput, Mrigendra K. S.; Kesharwani, Siddharth S.; Kumar, Sunny; Muley, Pratik; Narisetty, Susmitha; Tummala, Hemachand] South Dakota State Univ, Dept Pharmaceut Sci, Box 2202C, Brookings, SD 57007 USA.
[Ratput, Mrigendra K. S.] Arkansas Tech Univ, Dept Biol Sci, Russellville, AR 72801 USA.
C3 South Dakota State University; Arkansas Tech University
RP Tummala, H (通讯作者)，South Dakota State Univ, Dept Pharmaceut Sci, Box 2202C, Brookings, SD 57007 USA.
EM hemachand.tummala@sdstate.edu
RI Kesharwani, Siddharth/AAE-6148-2020
OI TUMMALA, HEMACHAND/0000-0001-9850-276X; Rajput, mrigendra/0000-0002-0762-4188
FU USDA National Institute of Food and Agriculture [SD00G647-17]; college of pharmacy, SDSU
FX The work was supported by the Animal Health and Production and Animal Products: Animal Health and Disease [SD00G647-17] from the USDA National Institute of Food and Agriculture and the college of pharmacy, SDSU.
CR Awate S, 2013, FRONT IMMUNOL, V4, DOI 10.3389/fimmu.2013.00114
Azzawi M, 2016, NANOMEDICINE-UK, V11, P2025, DOI 10.2217/nnm-2016-8000
Baras B, 2008, INFLUENZA OTHER RESP, V2, P251, DOI 10.1111/j.1750-2659.2008.00054.x
Blair DA, 2011, J IMMUNOL, V187, P2310, DOI 10.4049/jimmunol.1100363
Cooper P D, 1995, Pharm Biotechnol, V6, P559
Dowling JK, 2016, CLIN TRANSL IMMUNOL, V5, DOI 10.1038/cti.2016.22
Fujii SI, 2004, J EXP MED, V199, P1607, DOI 10.1084/jem.20040317
Gregory AE, 2013, FRONT CELL INFECT MI, V3, DOI 10.3389/fcimb.2013.00013
Griffiths KL, 2016, NAT COMMUN, V7, DOI 10.1038/ncomms13894

Hailemichael Y, 2013, NAT MED, V19, P465, DOI 10.1038/nm.3105
Irvine DJ, 2013, NAT MATER, V12, P978, DOI [10.1038/NMAT3775, 10.1038/nmat3775]
Iwasaki A, 2004, NAT IMMUNOL, V5, P987, DOI 10.1038/ni1112
Iwasaki A, 2015, NAT IMMUNOL, V16, P343, DOI 10.1038/ni.3123
Jiang H, 2017, J CONTROL RELEASE, V267, P47, DOI 10.1016/j.jconrel.2017.08.009
Klinman DM, 2016, WIRES NANOMED NANOBI, V8, P631, DOI 10.1002/wnan.1382
Klippstein R, 2010, NANOMED-NANOTECHNOL, V6, P523, DOI 10.1016/j.nano.2010.01.001
Kumar S, 2017, J CONTROL RELEASE, V261, P263, DOI 10.1016/j.jconrel.2017.06.026
Kumar S, 2016, J MATER CHEM B, V4, P7950, DOI 10.1039/c6tb02181f
Kumar S, 2013, MOL PHARMACEUT, V10, P1845, DOI 10.1021/mp3006374
Leleux J, 2013, ADV HEALTHC MATER, V2, P72, DOI 10.1002/adhm.201200268
Lewis KL, 2012, CSH PERSPECT BIOL, V4, DOI 10.1101/cshperspect.a007401
Li PL, 2015, DRUG DISCOV THER, V9, P88, DOI 10.5582/ddt.2015.01025
Lu YC, 2008, CYTOKINE, V42, P145, DOI 10.1016/j.cyto.2008.01.006
Mallapragada SK, 2008, INT J PHARMACEUT, V364, P265, DOI 10.1016/j.ijpharm.2008.06.030
Morefield GL, 2007, CLIN VACCINE IMMUNOL, V14, P1499, DOI 10.1128/CVI.00153-07
Moreno-Mendieta S, 2017, CARBOHYD POLYM, V165, P103, DOI 10.1016/j.carbpol.2017.02.030
Muley P, 2016, INT J PHARMACEUT, V500, P32, DOI 10.1016/j.ijpharm.2016.01.005
Nakamura T, 2013, INT J PHARMACEUT, V441, P476, DOI 10.1016/j.ijpharm.2012.11.006
Narasimhan B, 2016, ANNU REV BIOMED ENG, V18, P25, DOI 10.1146/annurev-bioeng-082615-030519
O'Hagan DT, 2015, VACCINE, V33, pB14, DOI 10.1016/j.vaccine.2015.01.088
Palese P, 2006, EMERG INFECT DIS, V12, P61, DOI 10.3201/eid1201.051043
Park BS, 2009, NATURE, V458, P1191, DOI 10.1038/nature07830
Reed SG, 2016, CURR OPIN IMMUNOL, V41, P85, DOI 10.1016/j.coi.2016.06.007
Rizwan SB, 2013, J CONTROL RELEASE, V165, P16, DOI 10.1016/j.jconrel.2012.10.020
Rodriguez-Cerdeira C, 2017, MEDIAT INFLAMM, V2017
Sahdev P, 2014, PHARM RES-DORDR, V31, P2563, DOI 10.1007/s11095-014-1419-y
Cordeiro AS, 2015, BIOTECHNOL ADV, V33, P1279, DOI 10.1016/j.biotechadv.2015.05.010
Schoenberger SP, 1998, NATURE, V393, P480, DOI 10.1038/31002
Shen ZH, 1997, J IMMUNOL, V158, P2723
Shi GN, 2017, BIOMATERIALS, V113, P191, DOI 10.1016/j.biomaterials.2016.10.047
Steinman RM, 2006, CURR TOP MICROBIOL, V311, P17
Steinman RM, 2008, IMMUNITY, V29, P319, DOI 10.1016/j.immuni.2008.08.001
Thompson AG, 2002, IMMUNOL CELL BIOL, V80, P509, DOI 10.1046/j.1440-1711.2002.01114.x
Ulery BD, 2011, SCI REP-UK, V1, DOI 10.1038/srep00198
van Stipdonk MJB, 2003, NAT IMMUNOL, V4, P361, DOI 10.1038/ni912
Villiers C, 2009, MOL CELL PROTEOMICS, V8, P1252, DOI 10.1074/mcp.M800589-MCP200
Wang C, 2012, VACCINE, V30, P4790, DOI 10.1016/j.vaccine.2012.05.027
Zhang CN, 2017, J CONTROL RELEASE, V256, P170, DOI 10.1016/j.jconrel.2017.04.020
Zhao L, 2014, VACCINE, V32, P327, DOI 10.1016/j.vaccine.2013.11.069
Zupancic E, 2017, J CONTROL RELEASE, V258, P182, DOI 10.1016/j.jconrel.2017.05.014
NR 50
TC 40
Z9 40
U1 5
U2 65
PU AMER CHEMICAL SOC
PI WASHINGTON
PA 1155 16TH ST, NW, WASHINGTON, DC 20036 USA
SN 1944-8244
J9 ACS APPL MATER INTER
JI ACS Appl. Mater. Interfaces
PD AUG 22
PY 2018
VL 10
IS 33
BP 27589
EP 27602
DI 10.1021/acsami.8b02019
PG 14
WC Nanoscience & Nanotechnology; Materials Science, Multidisciplinary

WE Science Citation Index Expanded (SCI-EXPANDED)
SC Science & Technology - Other Topics; Materials Science
GA GR5UE
UT WOS:000442706600007
PM 30048112
DA 2023-09-14
ER

PT J
AU Ma, L
Diao, L
Peng, ZF
Jia, Y
Xie, HM
Li, BS
Ma, JT
Zhang, M
Cheng, LF
Ding, DW
Zhang, XO
Chen, HB
Mo, FF
Jiang, H
Xu, GQ
Meng, FH
Zhong, ZY
Liu, M
AF Ma, Lin
Diao, Lu
Peng, Zuofu
Jia, Yun
Xie, Huimin
Li, Baisong
Ma, Jianting
Zhang, Meng
Cheng, Lifang
Ding, Dawei
Zhang, Xuenong
Chen, Huabing
Mo, Fengfeng
Jiang, Honglv
Xu, Guoqiang
Meng, Fenghua
Zhong, Zhiyuan
Liu, Mi
TI Immunotherapy and Prevention of Cancer by Nanovaccines Loaded with
Whole-Cell Components of Tumor Tissues or Cells
SO ADVANCED MATERIALS
LA English
DT Article
DE cancer immunotherapy; cancer prevention; cell lysate; drug delivery;
nanovaccines; tumor tissues; whole cell components
ID HUMAN DENDRITIC CELLS; B-CELLS; VACCINES; NEOANTIGENS; CHALLENGES;
MUTANOME; SURVIVAL; IMMUNITY; IMPROVE
AB Tumor tissues/cells are the best sources of antigens to prepare cancer vaccines. However, due to the difficulty of solubilization and delivery of water-insoluble antigens in tumor tissues/cells, including water-insoluble antigens into cancer vaccines and delivering such vaccines efficiently to antigen-presenting cells (APCs) remain challenging. To solve these problems, herein, water-insoluble components of tumor tissues/cells are solubilized by 8 m urea and thus whole components of macrometer-sized tumor cells are reasssembled into nanosized nanovaccines. To induce maximized immunization efficacy, various antigens are loaded both inside and on the surface of nanovaccines. By encapsulating both water-insoluble and

water-soluble components of tumor tissues/cells into nanovaccines, the nanovaccines are efficiently phagocytosed by APCs and showed better therapeutic efficacy than the nanovaccine loaded with only water-soluble components in melanoma and breast cancer. Anti-PD-1 antibody and metformin can improve the efficacy of nanovaccines. In addition, the nanovaccines can prevent lung cancer (100%) and melanoma (70%) efficiently in mice. T cell analysis and tumor microenvironment analysis indicate that tumor-specific T cells are induced by nanovaccines and both adaptive and innate immune responses against cancer cells are activated by nanovaccines. Overall, this study demonstrates a universal method to make tumor-cell-based nanovaccines for cancer immunotherapy and prevention.
C1 [Ma, Lin; Diao, Lu; Xie, Huimin; Li, Baisong; Ma, Jianting; Zhang, Meng; Cheng, Lifang; Ding, Dawei; Zhang, Xuenong; Chen, Huabing; Jiang, Honglv; Xu, Guoqiang; Liu, Mi] Soochow Univ, Coll Pharmaceut Sci, Dept Pharmaceut, Suzhou 215123, Peoples R China.
[Peng, Zuofu; Jia, Yun] Alpha X Beijing Biotech Co Ltd, Beijing 102600, Peoples R China.
[Mo, Fengfeng] Naval Med Univ, Fac Naval Med, Dept Naval Nutr & Food Hyg, Shanghai 200433, Peoples R China.
[Meng, Fenghua; Zhong, Zhiyuan] Soochow Univ, Coll Chem Chem Engn & Mat Sci, Biomed Polymers Lab, Suzhou 215123, Peoples R China.
C3 Soochow University - China; Naval Medical University; Soochow University - China
RP Liu, M (通讯作者)，Soochow Univ, Coll Pharmaceut Sci, Dept Pharmaceut, Suzhou 215123, Peoples R China.
EM mi.liu@suda.edu.cn
RI liu, Mi/GNW-4569-2022; Chen, Huabing/B-3687-2011; Zhong, Zhiyuan/D-8508-2011; Li, Baisong/AAO-1571-2021
OI Zhong, Zhiyuan/0000-0003-4175-4741; Li, Baisong/0000-0002-1293-3938; Liu, Mi/0000-0002-5185-5872
FU startup package of Soochow University, Priority Academic Program Development (PAPD) of Jiangsu Higher Education Institutions; Jiangsu Key Laboratory of Advanced Functional Polymer Design and Application
FX The research was funded by the startup package of Soochow University, Priority Academic Program Development (PAPD) of Jiangsu Higher Education Institutions and Jiangsu Key Laboratory of Advanced Functional Polymer Design and Application. The authors thank the Pediatric Tumor Research Center of College of Pharmaceutical Sciences (Soochow University) for the assistance in performing the flow cytometry analyses.
CR Alspach E, 2019, NATURE, V574, P696, DOI 10.1038/s41586-019-1671-8
Bianchini G, 2016, NAT REV CLIN ONCOL, V13, P674, DOI 10.1038/nrclinonc.2016.66
Cabrita R, 2020, NATURE, V577, P561, DOI 10.1038/s41586-019-1914-8
Castle JC, 2012, CANCER RES, V72, P1081, DOI 10.1158/0008-5472.CAN-11-3722
Cedervall T, 2007, P NATL ACAD SCI USA, V104, P2050, DOI 10.1073/pnas.0608582104
Cha JH, 2018, MOL CELL, V71, P606, DOI 10.1016/j.molcel.2018.07.030
Chen FM, 2021, BIOMATERIALS, V270, DOI 10.1016/j.biomaterials.2021.120709
Conniot J, 2019, NAT NANOTECHNOL, V14, P891, DOI 10.1038/s41565-019-0512-0
Eikawa S, 2015, P NATL ACAD SCI USA, V112, P1809, DOI 10.1073/pnas.1417636112
Finn OJ, 2018, NAT REV IMMUNOL, V18, P183, DOI 10.1038/nri.2017.140
Foged C, 2005, INT J PHARMACEUT, V298, P315, DOI 10.1016/j.ijpharm.2005.03.035
Ge CC, 2015, ARCH TOXICOL, V89, P519, DOI 10.1007/s00204-015-1458-0
Gide TN, 2020, ONCOIMMUNOLOGY, V9, DOI 10.1080/2162402X.2019.1659093
Gubin MM, 2015, J CLIN INVEST, V125, P3413, DOI 10.1172/JCI80008
Helmink BA, 2020, NATURE, V577, P549, DOI 10.1038/s41586-019-1922-8
Ho NI, 2018, FRONT IMMUNOL, V9, DOI 10.3389/fimmu.2018.02874
Hu Z, 2018, NAT REV IMMUNOL, V18, P168, DOI 10.1038/nri.2017.131
Joffre OP, 2012, NAT REV IMMUNOL, V12, P557, DOI 10.1038/nri3254
Kalra J, 2017, METHODS MOL BIOL, V1554, P237, DOI 10.1007/978-1-4939-6759-9_17
Kardos J, 2016, JCI INSIGHT, V1, DOI 10.1172/jci.insight.85902
Kreiter S, 2015, NATURE, V520, P692, DOI 10.1038/nature14426
Li Q, 2000, CLIN IMMUNOL, V94, P64, DOI 10.1006/clim.1999.4820
Lutsiak MEC, 2002, PHARM RES-DORDR, V19, P1480, DOI 10.1023/A:1020452531828
Ma W, 2018, FRONT MICROBIOL, V9, DOI 10.3389/fmicb.2018.01336
Melief CJM, 2015, J CLIN INVEST, V125, P3401, DOI 10.1172/JCI80009
Min YZ, 2017, NAT NANOTECHNOL, V12, P877, DOI [10.1038/nnano.2017.113, 10.1038/NNANO.2017.113]

Nel AE, 2009, NAT MATER, V8, P543, DOI [10.1038/nmat2442, 10.1038/NMAT2442]
Nolan E, 2017, SCI TRANSL MED, V9, DOI 10.1126/scitranslmed.aal4922
Ott PA, 2017, NATURE, V547, P217, DOI 10.1038/nature22991
Pareja F, 2018, NAT REV CLIN ONCOL, V15, P347, DOI 10.1038/s41571-018-0001-7
Petitprez F, 2020, NATURE, V577, P556, DOI 10.1038/s41586-019-1906-8
Sahin U, 2017, NATURE, V547, P222, DOI 10.1038/nature23003
Schumacher TN, 2015, SCIENCE, V348, P69, DOI 10.1126/science.aaa4971
Tenzer S, 2013, NAT NANOTECHNOL, V8, P772, DOI 10.1038/nnano.2013.181
Tran E, 2014, SCIENCE, V344, P641, DOI 10.1126/science.1251102
van der Burg SH, 2016, NAT REV CANCER, V16, P219, DOI 10.1038/nrc.2016.16
Wang TT, 2018, NAT COMMUN, V9, DOI 10.1038/s41467-018-03915-4
Wu TD, 2020, NATURE, V579, P274, DOI 10.1038/s41586-020-2056-8
Zhou L, 2020, NANO LETT, V20, P4393, DOI 10.1021/acs.nanolett.0c01140
Zhu Y, 2018, J PROTEOME RES, V17, P1509, DOI 10.1021/acs.jproteome.7b00843
NR 40
TC 39
Z9 39
U1 23
U2 205
PU WILEY-V C H VERLAG GMBH
PI WEINHEIM
PA POSTFACH 101161, 69451 WEINHEIM, GERMANY
SN 0935-9648
EI 1521-4095
J9 ADV MATER
JI Adv. Mater.
PD OCT
PY 2021
VL 33
IS 43
AR 2104849
DI 10.1002/adma.202104849
EA SEP 2021
PG 14
WC Chemistry, Multidisciplinary; Chemistry, Physical; Nanoscience & Nanotechnology; Materials Science, Multidisciplinary; Physics, Applied; Physics, Condensed Matter
WE Science Citation Index Expanded (SCI-EXPANDED)
SC Chemistry; Science & Technology - Other Topics; Materials Science; Physics
GA WL8BW
UT WOS:000696902100001
PM 34536044
DA 2023-09-14
ER

PT J
AU Nam, J
Son, S
Park, KS
Moon, JJ
AF Nam, Jutaek
Son, Sejin
Park, Kyung Soo
Moon, James J.
TI Modularly Programmable Nanoparticle Vaccine Based on Polyethyleneimine for Personalized Cancer Immunotherapy
SO ADVANCED SCIENCE
LA English
DT Article
DE cancer vaccines; immunotherapy; nanoparticles; neoantigens

ID TOLL-LIKE RECEPTORS; DENDRITIC CELLS; IN-VIVO; GENE DELIVERY;
IMMUNE-RESPONSES; TUMOR; POTENT; CONJUGATION; ADJUVANT; MACROPHAGES

AB Nanoparticles (NPs) can serve as a promising vaccine delivery platform for improving pharmacological property and codelivery of antigens and adjuvants. However, NP-based vaccines are generally associated with complex synthesis and postmodification procedures, which pose technical and manufacturing challenges for tailor-made vaccine production. Here, modularly programmed, polyethyleneimine (PEI)-based NP vaccines are reported for simple production of personalized cancer vaccines. Briefly, PEI is conjugated with neoantigens by facile coupling chemistry, followed by electrostatic assembly with CpG adjuvants, leading to the self-assembly of nontoxic, sub-50 nm PEI NPs. Importantly, PEI NPs promote activation and antigen cross-presentation of antigen-presenting cells and cross-priming of neoantigen-specific CD8(+) T cells. Surprisingly, after only a single intratumoral injection, PEI NPs with optimal PEGylation elicit as high as approximate to 30% neoantigen-specific CD8(+) T cell response in the systemic circulation and sustain elevated CD8(+) T cell response over 3 weeks. PEI-based nanovaccines exert potent antitumor efficacy against pre-established local tumors as well as highly aggressive metastatic tumors. PEI engineering for modular incorporation of neoantigens and adjuvants offers a promising strategy for rapid and facile production of personalized cancer vaccines.

C1 [Nam, Jutaek; Son, Sejin] Univ Michigan, Dept Pharmaceut Sci, Biointerfaces Inst, Ann Arbor, MI 48109 USA.
[Park, Kyung Soo] Univ Michigan, Dept Biomed Engn, Biointerfaces Inst, Ann Arbor, MI 48109 USA.
[Moon, James J.] Univ Michigan, Dept Pharmaceut Sci, Biointerfaces Inst, Dept Biomed Engn, Ann Arbor, MI 48109 USA.
C3 University of Michigan System; University of Michigan; University of Michigan System; University of Michigan; University of Michigan System; University of Michigan
RP Moon, JJ (通讯作者)，Univ Michigan, Dept Pharmaceut Sci, Biointerfaces Inst, Dept Biomed Engn, Ann Arbor, MI 48109 USA.
EM moonjj@umich.edu
RI Moon, James J/ABE-4429-2021; Son, Sejin/ABC-5818-2021
OI Moon, James J/0000-0003-2238-2372; Son, Sejin/0000-0003-1523-2990

FU NIH [R01EB022563, R01AI127070, R01CA210273, R01DK125087, U01CA210152]; MTRAC for Life Sciences Hub; Emerald Foundation; DoD/CDMRP Peer Reviewed Cancer Research Program [W81XWH-16-1-0369]; NSF CAREER Award [1553831]; UM TEAM Training Program (from NIDCR) [DE007057]; NIH Tetramer Core Facility [HHSN272201300006C]
FX J.N. and S.S. contributed equally to this work. This work was supported in part by NIH (Grant Nos. R01EB022563, R01AI127070, R01CA210273, R01DK125087, and U01CA210152), MTRAC for Life Sciences Hub, and Emerald Foundation. J.J.M. was supported by DoD/CDMRP Peer Reviewed Cancer Research Program (Grant No. W81XWH-16-1-0369) and NSF CAREER Award (Grant No. 1553831). K.S.P. acknowledges financial support from the UM TEAM Training Program (DE007057 from NIDCR). The authors acknowledge the NIH Tetramer Core Facility (contract HHSN272201300006C) for provision of MHC-I tetramers. Opinions interpretations, conclusions, and recommendations are those of the author and are not necessarily endorsed by the Department of Defense. The authors thank Marisa Aikins for critical review of the manuscript.

CR Akinc A, 2005, J GENE MED, V7, P657, DOI 10.1002/jgm.696
Aznar MA, 2017, J IMMUNOL, V198, P31, DOI 10.4049/jimmunol.1601145
Aras S, 2017, BRIT J CANCER, V117, P1583, DOI 10.1038/bjc.2017.356
Bivas-Benita M, 2010, J VIROL, V84, P5764, DOI 10.1128/JVI.02202-09
Blander JM, 2006, NAT IMMUNOL, V7, P1029, DOI 10.1038/ni1006-1029
BOUSSIF O, 1995, P NATL ACAD SCI USA, V92, P7297, DOI 10.1073/pnas.92.16.7297
Chen DS, 2013, IMMUNITY, V39, P1, DOI 10.1016/j.immuni.2013.07.012
de Titta A, 2013, P NATL ACAD SCI USA, V110, P19902, DOI 10.1073/pnas.1313152110
Gabrielson NP, 2006, BIOMACROMOLECULES, V7, P2427, DOI 10.1021/bm060300u
Garzon MR, 2005, VACCINE, V23, P1384, DOI 10.1016/j.vaccine.2004.09.009
Grant EV, 2012, EUR J IMMUNOL, V42, P2937, DOI 10.1002/eji.201242410
Hilf N, 2019, NATURE, V565, P240, DOI 10.1038/s41586-018-0810-y
Jager M, 2012, CHEM SOC REV, V41, P4755, DOI 10.1039/c2cs35146c
Jaynes JM, 2020, SCI TRANSL MED, V12, DOI 10.1126/scitranslmed.aax6337
Jeanbart L, 2014, CANCER IMMUNOL RES, V2, P436, DOI 10.1158/2326-6066.CIR-14-0019-T

Joffre OP, 2012, NAT REV IMMUNOL, V12, P557, DOI 10.1038/nri3254
Kandimalla ER, 2002, BIOCONJUGATE CHEM, V13, P966, DOI 10.1021/bc0200374
Kawai T, 2011, IMMUNITY, V34, P637, DOI 10.1016/j.immuni.2011.05.006
Keskin DB, 2019, NATURE, V565, P234, DOI 10.1038/s41586-018-0792-9
Kranz LM, 2016, NATURE, V534, P396, DOI 10.1038/nature18300
Kreiter S, 2015, NATURE, V520, P692, DOI 10.1038/nature14426
Kuai R, 2017, NAT MATER, V16, P489, DOI [10.1038/nmat4822, 10.1038/NMAT4822]
Kursa M, 2003, BIOCONJUGATE CHEM, V14, P222, DOI 10.1021/bc0256087
Li QF, 2020, RSC ADV, V10, P16537, DOI 10.1039/c9ra10563h
Liu HP, 2014, NATURE, V507, P519, DOI 10.1038/nature12978
Lungwitz U, 2005, EUR J PHARM BIOPHARM, V60, P247, DOI 10.1016/j.ejpb.2004.11.011
Lutz MB, 1999, J IMMUNOL METHODS, V223, P77, DOI 10.1016/S0022-1759(98)00204-X
Lynn GM, 2020, NAT BIOTECHNOL, V38, P320, DOI 10.1038/s41587-019-0390-x
Lynn GM, 2015, NAT BIOTECHNOL, V33, P1201, DOI 10.1038/nbt.3371
Ma YF, 2010, EUR J PHARM SCI, V40, P75, DOI 10.1016/j.ejps.2010.02.009
Marabelle A, 2017, ANN ONCOL, V28, P33, DOI 10.1093/annonc/mdx683
Moghimi SM, 2005, MOL THER, V11, P990, DOI 10.1016/j.ymthe.2005.02.010
Moon JJ, 2011, NAT MATER, V10, P243, DOI 10.1038/NMAT2960
Nair-Gupta P, 2014, CELL, V158, P506, DOI 10.1016/j.cell.2014.04.054
Nam J, 2019, NAT REV MATER, V4, P398, DOI 10.1038/s41578-019-0108-1
Nam J, 2017, CELL MOL BIOENG, V10, P341, DOI 10.1007/s12195-017-0505-8
Nam J, 2013, ADV DRUG DELIVER REV, V65, P622, DOI 10.1016/j.addr.2012.08.015
Nierkens S, 2008, CANCER RES, V68, P5390, DOI 10.1158/0008-5472.CAN-07-6023
Nierkens S, 2009, PLOS ONE, V4, DOI 10.1371/journal.pone.0008368
Norbury CC, 2006, IMMUNOLOGY, V117, P443, DOI 10.1111/j.1365-2567.2006.02335.x
O'Neill LAJ, 2013, NAT REV IMMUNOL, V13, P453, DOI 10.1038/nri3446
Ogris M, 2003, J CONTROL RELEASE, V91, P173, DOI 10.1016/S0168-3659(03)00230-X
Ogris M, 1999, GENE THER, V6, P595, DOI 10.1038/sj.gt.3300900
Ott PA, 2017, NATURE, V547, P217, DOI 10.1038/nature22991
Palumbo RN, 2012, J CONTROL RELEASE, V157, P86, DOI 10.1016/j.jconrel.2011.08.037
Petersen H, 2002, MACROMOLECULES, V35, P6867, DOI 10.1021/ma012060a
Petersen H, 2002, BIOCONJUGATE CHEM, V13, P845, DOI 10.1021/bc025529v
Putta MR, 2010, BIOCONJUGATE CHEM, V21, P39, DOI 10.1021/bc900425s
Raimondo TM, 2018, P NATL ACAD SCI USA, V115, P10648, DOI 10.1073/pnas.1806908115
Regnstrom K, 2003, GENE THER, V10, P1575, DOI 10.1038/sj.gt.3302054
Sahin U, 2018, SCIENCE, V359, P1355, DOI 10.1126/science.aar7112
Sahin U, 2017, NATURE, V547, P222, DOI 10.1038/nature23003
Scheetz L, 2019, NAT BIOMED ENG, V3, P768, DOI 10.1038/s41551-019-0436-x
Schumacher TN, 2015, SCIENCE, V348, P69, DOI 10.1126/science.aaa4971
Shen C, 2017, INT J NANOMED, V12, P5443, DOI 10.2147/IJN.S137980
Sheppard NC, 2014, INT IMMUNOL, V26, P531, DOI 10.1093/intimm/dxu055
Shim BS, 2010, BMC IMMUNOL, V11, DOI 10.1186/1471-2172-11-65
SNYDER SL, 1975, ANAL BIOCHEM, V64, P284, DOI 10.1016/0003-2697(75)90431-5
Steinman RM, 2002, P NATL ACAD SCI USA, V99, P351, DOI 10.1073/pnas.231606698
Suk JS, 2016, ADV DRUG DELIVER REV, V99, P28, DOI 10.1016/j.addr.2015.09.012
Sun ZQ, 2014, MOL MED REP, V10, P2657, DOI 10.3892/mmr.2014.2537
van der Burg SH, 2016, NAT REV CANCER, V16, P219, DOI 10.1038/nrc.2016.16
Veglia F, 2017, NAT COMMUN, V8, DOI 10.1038/s41467-017-02186-9
Wegmann F, 2012, NAT BIOTECHNOL, V30, P883, DOI 10.1038/nbt.2344
Xu C, 2019, ACS NANO, V13, P12148, DOI 10.1021/acsnano.9b06691
Yadav M, 2014, NATURE, V515, P572, DOI 10.1038/nature14001
Yang AC, 2008, J CHEM PHYS, V129, DOI 10.1063/1.2996179
Zhu GZ, 2017, NAT COMMUN, V8, DOI 10.1038/s41467-017-02191-y
NR 68
TC 39
Z9 38
U1 11
U2 92
PU WILEY
PI HOBOKEN
PA 111 RIVER ST, HOBOKEN 07030-5774, NJ USA

EI 2198-3844
J9 ADV SCI
JI Adv. Sci.
PD MAR
PY 2021
VL 8
IS 5
AR 2002577
DI 10.1002/advs.202002577
EA JAN 2021
PG 15
WC Chemistry, Multidisciplinary; Nanoscience & Nanotechnology; Materials
Science, Multidisciplinary
WE Science Citation Index Expanded (SCI-EXPANDED)
SC Chemistry; Science & Technology - Other Topics; Materials Science
GA QS7SK
UT WOS:000605169900001
PM 33717838
OA Green Published
DA 2023-09-14
ER

PT J
AU Wang, D
Wang, SY
Zhou, ZD
Bai, D
Zhang, QZ
Ai, XZ
Gao, WW
Zhang, LF
AF Wang, Dan
Wang, Shuyan
Zhou, Zhidong
Bai, Dean
Zhang, Qiangzhe
Ai, Xiangzhao
Gao, Weiwei
Zhang, Liangfang
TI White Blood Cell Membrane-Coated Nanoparticles: Recent Development and
Medical Applications
SO ADVANCED HEALTHCARE MATERIALS
LA English
DT Review
DE cell membrane-coating; detoxification; drug delivery; nanovaccines;
white blood cells
ID MACROPHAGE-MEMBRANE; BIOMIMETIC NANOPARTICLES; POLYMERIC NANOPARTICLES;
RHEUMATOID-ARTHRITIS; CANCER; THERAPY; NEUTROPHILS; INFLAMMATION;
PROGRESSION; NANOSPONGES
AB White blood cells (WBCs) are immune cells that play essential roles in critical diseases including cancers, infections, and inflammatory disorders. Their dynamic and diverse functions have inspired the development of WBC membrane-coated nanoparticles (denoted "WBC-NPs"), which are formed by fusing the plasma membranes of WBCs, such as macrophages, neutrophils, T cells, and natural killer cells, onto synthetic nanoparticle cores. Inheriting the entire source cell antigens, WBC-NPs act as source cell decoys and simulate their broad biointerfacing properties with intriguing therapeutic potentials. Herein, the recent development and medical applications of WBC-NPs focusing on four areas, including WBC-NPs as carriers for drug delivery, as countermeasures for biological neutralization, as nanovaccines for immune modulation, and as tools for the isolation of circulating tumor cells and fundamental research is reviewed. Overall, the recent development and studies of WBC-NPs have established the platform as versatile nanotherapeutics and tools with broad medical application potentials.
C1 [Wang, Dan; Wang, Shuyan; Zhou, Zhidong; Bai, Dean; Zhang, Qiangzhe; Ai, Xiangzhao; Gao,

Weiwei; Zhang, Liangfang] Univ Calif San Diego, Dept NanoEngn, Chem Engn Program, Moores Canc Ctr, San Diego, CA 92093 USA.
C3 University of California System; University of California San Diego
RP Gao, WW; Zhang, LF (通讯作者), Univ Calif San Diego, Dept NanoEngn, Chem Engn Program, Moores Canc Ctr, San Diego, CA 92093 USA.
EM w5gao@ucsd.edu; zhang@ucsd.edu
RI Wang, Shuyan/GRN-8467-2022
OI Wang, Shuyan/0009-0007-6329-3153; Zhang, Liangfang/0000-0003-0637-0654
FU Defense Threat Reduction Agency Joint Science and Technology Office for
Chemical and Biological Defense [HDTRA1-18-1-0014, HDTRA1-21-1-0010];
National Science Foundation [DMR-1904702]
FX This work was supported by the Defense Threat Reduction Agency Joint
Science and Technology Office for Chemical and Biological Defense under
Grant Nos. HDTRA1-18-1-0014 and HDTRA1-21-1-0010, the National Science
Foundation, Grant No. DMR-1904702,
CR Ai XZ, 2021, BIOCHEMISTRY-US, V60, P941, DOI 10.1021/acs.biochem.0c00343
Akira S, 2001, NAT IMMUNOL, V2, P675, DOI 10.1038/90609
Angsantikul P, 2015, VACCINES, V3, P814, DOI 10.3390/vaccines3040814
Aryal S, 2013, NANOMEDICINE-UK, V8, P1271, DOI [10.2217/NNM.12.153, 10.2217/nnm.12.153]
Cao HQ, 2016, ACS NANO, V10, P7738, DOI 10.1021/acsnano.6b03148
Cao J, 2020, ADV DRUG DELIVER REV, V167, P170, DOI 10.1016/j.addr.2020.06.030
Cao X, 2019, ACTA PHARM SIN B, V9, P575, DOI 10.1016/j.apsb.2018.12.009
Capucetti A, 2020, FRONT IMMUNOL, V11, DOI 10.3389/fimmu.2020.01259
Cedergren J, 2007, J RHEUMATOL, V34, P2162
Chakravarti A, 2009, BLOOD, V114, P1633, DOI 10.1182/blood-2008-09-178301
Chen YJ, 2019, ACS NANO, V13, P7209, DOI 10.1021/acsnano.9b02773
Colombo S, 2018, ADV DRUG DELIVER REV, V128, P115, DOI 10.1016/j.addr.2018.04.004
Copp JA, 2014, P NATL ACAD SCI USA, V111, P13481, DOI 10.1073/pnas.1412420111
Dehaini D, 2017, ADV MATER, V29, DOI 10.1002/adma.201606209
Delano MJ, 2016, IMMUNOL REV, V274, P330, DOI 10.1111/imr.12499
Deng GJ, 2018, ACS NANO, V12, P12096, DOI 10.1021/acsnano.8b05292
Fang RH, 2018, ADV MATER, V30, DOI 10.1002/adma.201706759
Fang RNH, 2013, NANOSCALE, V5, P8884, DOI 10.1039/c3nr03064d
Feng J, 2019, J TRANSL MED, V17, DOI 10.1186/s12967-019-1945-9
Feng LS, 2021, ACS NANO, V15, P2263, DOI 10.1021/acsnano.0c07973
Fontana F, 2018, ADV FUNCT MATER, V28, DOI 10.1002/adfm.201801355
Freed EO, 2015, NAT REV MICROBIOL, V13, P484, DOI 10.1038/nrmicro3490
Gao C, 2020, NAT COMMUN, V11, DOI 10.1038/s41467-020-16439-7
Glenn A, 2019, ANAEST INTENS CARE M, V20, P170, DOI 10.1016/j.mpaic.2019.01.001
Gong CA, 2020, J NANOBIOTECHNOL, V18, DOI 10.1186/s12951-020-00649-8
Gong H, 2021, NANO LETT, V21, P2603, DOI 10.1021/acs.nanolett.1c00238
Gong H, 2019, ACS NANO, V13, P3714, DOI 10.1021/acsnano.9b00911
Grant RA, 2021, NATURE, V590, P635, DOI 10.1038/s41586-020-03148-w
Guan WJ, 2020, EUR RESPIR J, V55, DOI 10.1183/13993003.00597-2020
Han Y, 2021, BIOACT MATER, V6, P529, DOI 10.1016/j.bioactmat.2020.08.017
Han YT, 2019, ADV SCI, V6, DOI 10.1002/advs.201900251
Harjunpaa H, 2019, FRONT IMMUNOL, V10, DOI 10.3389/fimmu.2019.01078
Harouaka R, 2014, PHARMACOL THERAPEUT, V141, P209, DOI 10.1016/j.pharmthera.2013.10.004
Hassanzadeh P, 2021, LIFE SCI, V270, DOI 10.1016/j.lfs.2021.119128
Hu CMJ, 2013, NAT NANOTECHNOL, V8, P933, DOI [10.1038/nnano.2013.254, 10.1038/NNANO.2013.254]
Hu CMJ, 2013, NAT NANOTECHNOL, V8, P336, DOI [10.1038/nnano.2013.54, 10.1038/NNANO.2013.54]
Hu CMJ, 2011, P NATL ACAD SCI USA, V108, P10980, DOI 10.1073/pnas.1106634108
Hu C, 2020, BIOMATERIALS, V255, DOI 10.1016/j.biomaterials.2020.120159
Jiang Y, 2020, ADV MATER, V32, DOI 10.1002/adma.202001808
Jin T, 2009, IMMUNOL RES, V43, P118, DOI 10.1007/s12026-008-8056-7
Kang T, 2017, ACS NANO, V11, P1397, DOI 10.1021/acsnano.6b06477
Krishnamurthy S, 2016, NANOSCALE, V8, P6981, DOI 10.1039/c5nr07588b
Kroll AV, 2019, ADV BIOSYST, V3, DOI 10.1002/adbi.201800219
Li RX, 2019, NANO LETT, V19, P124, DOI 10.1021/acs.nanolett.8b03439

Li SY, 2020, NANO LETT, V20, P242, DOI 10.1021/acs.nanolett.9b03753
Ma WJ, 2020, THERANOSTICS, V10, P1281, DOI 10.7150/thno.40291
Ma YD, 2021, BIOMATERIALS, V274, DOI 10.1016/j.biomaterials.2021.120865
Madsen CD, 2010, DEV CELL, V19, P13, DOI 10.1016/j.devcel.2010.06.013
McEver RP, 2015, CARDIOVASC RES, V107, P331, DOI 10.1093/cvr/cvv154
Mitchell MJ, 2021, NAT REV DRUG DISCOV, V20, P101, DOI 10.1038/s41573-020-0090-8
Mohtar MA, 2020, BIOMOLECULES, V10, DOI 10.3390/biom10020255
Pang ZQ, 2015, ACS NANO, V9, P6450, DOI 10.1021/acsnano.5b02132
Parodi A, 2013, NAT NANOTECHNOL, V8, P61, DOI 10.1038/nnano.2012.212
Peppas N.A., 2020, BIOMATER SCI-UK, V4th ed., P153, DOI DOI 10.1016/B978-0-12-816137-1.00014-3
Pitchaimani A, 2019, ADV FUNCT MATER, V29, DOI 10.1002/adfm.201806817
Rao L, 2020, P NATL ACAD SCI USA, V117, P27141, DOI 10.1073/pnas.2014352117
Rao L, 2018, ADV FUNCT MATER, V28, DOI 10.1002/adfm.201803531
Rieckmann JC, 2017, NAT IMMUNOL, V18, P583, DOI 10.1038/ni.3693
Ruegg C, 2006, J LEUKOCYTE BIOL, V80, P682, DOI 10.1189/jlb.0606394
Rushton AJ, 2021, CANCERS, V13, DOI 10.3390/cancers13050970
Shen S, 2019, BIOMATERIALS, V189, P60, DOI 10.1016/j.biomaterials.2018.10.029
Shi MS, 2021, THERANOSTICS, V11, P2349, DOI 10.7150/thno.48407
Thamphiwatana S, 2017, P NATL ACAD SCI USA, V114, P11488, DOI 10.1073/pnas.1714267114
Tu YS, 2013, NAT NANOTECHNOL, V8, P594, DOI [10.1038/NNANO.2013.125, 10.1038/nnano.2013.125]
Wang C, 2018, ADV MATER, V30, DOI 10.1002/adma.201804023
Wang DL, 2020, RESEARCH-CHINA, V2020, DOI 10.34133/2020/3676954
Wang F, 2015, ADV MATER, V27, P3437, DOI 10.1002/adma.201501071
Wang JS, 2021, INT J NANOMED, V16, P1175, DOI 10.2147/IJN.S288636
Wang LX, 2013, NANO TODAY, V8, P374, DOI 10.1016/j.nantod.2013.07.001
Wang Y, 2021, THERANOSTICS, V11, P164, DOI 10.7150/thno.47841
Wei XL, 2019, NANO LETT, V19, P4760, DOI 10.1021/acs.nanolett.9b01844
Wei XL, 2018, ADV MATER, V30, DOI 10.1002/adma.201802233
Winkler J, 2020, NAT COMMUN, V11, DOI 10.1038/s41467-020-18794-x
Wipke BT, 2001, J IMMUNOL, V167, P1601, DOI 10.4049/jimmunol.167.3.1601
Wu GH, 2020, BIOMATERIALS, V250, DOI 10.1016/j.biomaterials.2020.119963
Wu TT, 2017, ADV FUNCT MATER, V27, DOI 10.1002/adfm.201703191
Xiao P, 2021, NANO LETT, V21, P2094, DOI 10.1021/acs.nanolett.0c04783
Xiong F, 2019, NANO LETT, V19, P3256, DOI 10.1021/acs.nanolett.9b00824
Xiong K, 2016, ADV MATER, V28, P7929, DOI 10.1002/adma.201601643
Xuan MJ, 2016, ACS APPL MATER INTER, V8, P9610, DOI 10.1021/acsami.6b00853
Xuan MJ, 2015, ADV HEALTHC MATER, V4, P1645, DOI 10.1002/adhm.201500129
Yin CC, 2020, ACTA BIOMATER, V102, P416, DOI 10.1016/j.actbio.2019.11.025
Yu GT, 2018, ADV FUNCT MATER, V28, DOI 10.1002/adfm.201801389
Yu YQ, 2018, ACS NANO, V12, P11871, DOI 10.1021/acsnano.8b04880
Zhang C, 2019, ADV MATER, V31, DOI 10.1002/adma.201901179
Zhang F, 2019, ACS NANO, V13, P5662, DOI 10.1021/acsnano.9b00892
Zhang F, 2018, ADV FUNCT MATER, V28, DOI 10.1002/adfm.201703326
Zhang G, 2020, MBIO, V11, DOI 10.1128/mBio.00903-20
Zhang J, 2021, NAT NANOTECHNOL, V16, P538, DOI 10.1038/s41565-020-00843-7
Zhang JH, 2021, CELL MOL IMMUNOL, V18, P1305, DOI 10.1038/s41423-021-00665-0
Zhang LR, 2017, INT J NANOMED, V12, P2129, DOI 10.2147/IJN.S126016
Zhang QZ, 2020, NANO LETT, V20, P5570, DOI 10.1021/acs.nanolett.0c02278
Zhang QZ, 2018, NAT NANOTECHNOL, V13, P1182, DOI 10.1038/s41565-018-0254-4
Zhang Y, 2018, NANO LETT, V18, P1908, DOI 10.1021/acs.nanolett.7b05263
Zhang Y, 2017, ACS NANO, V11, P11923, DOI 10.1021/acsnano.7b06968
Zhao HJ, 2018, ACS APPL MATER INTER, V10, P31124, DOI 10.1021/acsami.8b11645
Zhou JR, 2020, ADV MATER, V32, DOI 10.1002/adma.201901255
Zhou XX, 2019, SMALL, V15, DOI 10.1002/smll.201900558
Zhou X, 2019, J BIOMED NANOTECHNOL, V15, P993, DOI 10.1166/jbn.2019.2739
Zhou X, 2019, MOL PHARMACEUT, V16, P1397, DOI 10.1021/acs.molpharmaceut.8b01342
NR 100
TC 38
Z9 39

U1 23
U2 165
PU WILEY
PI HOBOKEN
PA 111 RIVER ST, HOBOKEN 07030-5774, NJ USA
SN 2192-2640
EI 2192-2659
J9 ADV HEALTHC MATER
JI Adv. Healthc. Mater.
PD APR
PY 2022
VL 11
IS 7
SI SI
AR 2101349
DI 10.1002/adhm.202101349
EA AUG 2021
PG 16
WC Engineering, Biomedical; Nanoscience & Nanotechnology; Materials
Science, Biomaterials
WE Science Citation Index Expanded (SCI-EXPANDED)
SC Engineering; Science & Technology - Other Topics; Materials Science
GA 0I8OV
UT WOS:000693917600001
PM 34468090
DA 2023-09-14
ER

PT J
AU Ge, W
Li, Y
Li, ZS
Zhang, SH
Sun, YJ
Hu, PZ
Wang, XM
Huang, Y
Si, SY
Zhang, XM
Sui, YF
AF Ge, Wei
Li, Yuan
Li, Zeng-Shan
Zhang, Shan-Hong
Sun, Yu-Jing
Hu, Pei-Zhen
Wang, Xiao-Ming
Huang, Yang
Si, Shao-Yan
Zhang, Xiu-Min
Sui, Yan-Fang
TI The antitumor immune responses induced by nanoemulsion-encapsulated
MAGE1-HSP70/SEA complex protein vaccine following peroral administration
route
SO CANCER IMMUNOLOGY IMMUNOTHERAPY
LA English
DT Article
DE Tumor vaccine; Nanoemulsion; Peroral (po; )
ID INTESTINAL PERMEABILITY ENHANCEMENT; IN-OIL MICROEMULSIONS;
DRUG-DELIVERY-SYSTEM; ORAL DELIVERY; POLYALKYLCYANOACRYLATE
NANOCAPSULES; NANOPARTICLES; IMMUNIZATION; ABSORPTION; VIVO; PEPTIDE

AB Previous studies have shown that there are profuse lymphatic tissues under the intestinal mucous membrane. Moreover, vaccine administered orally can elicit both mucous membrane and system immune response simultaneously, accordingly induce tumor-specific cytotoxic T lymphocyte. As a result, the oral route is constituted the preferred immune route for vaccine delivery theoretically. However, numerous vaccines especially protein/peptide vaccines remain poorly available when administered by this route. Nanoemulsion has been shown as a useful vehicle can be developed to enhance the antitumor immune response against antigens encapsulated in it and it is good for the different administration routes. Of particular interest is whether the protein vaccine following peroral route using nanoemulsion as delivery carrier can induce the same, so much as stronger antitumor immune response to following conventional ways such as subcutaneous (sc.) or not. Hence, in the present study, we encapsulated the MAGE1-HSP70 and SEA complex protein in nanoemulsion as nanovaccine NE (MHS) using magnetic ultrasound method. We then immuned C57BL/6 mice with NE (MHS), MHS alone or NE (-) via po. or sc. route and detected the cellular immunocompetence by using ELISpot assay and LDH release assay. The therapeutic and tumor challenge assay were examined then. The results showed that compared with vaccination with MHS or NE (-), the cellular immune responses against MAGE-1 could be elicited fiercely by vaccination with NE (MHS) nanoemulsion. Furthermore, encapsulating MHS in nanoemulsion could delay tumor growth and defer tumor occurrence of mice challenged with B16-MAGE-1 tumor cells. Especially, the peroral administration of NE (MHS) could induce approximately similar antitumor immune responses to the sc. administration, but the MHS unencapsulated with nanoemulsion via po. could induce significantly weaker antitumor immune responses than that via sc., suggesting nanoemulsion as a promising carrier can exert potent antitumor immunity against antigen encapsulated in it and make the tumor protein vaccine immunizing via po. route feasible and effective. It may have a broad application in tumor protein vaccine.
C1 [Ge, Wei; Li, Zeng-Shan; Sun, Yu-Jing; Hu, Pei-Zhen; Huang, Yang; Si, Shao-Yan; Zhang, Xiu-Min; Sui, Yan-Fang] Fourth Mil Med Univ, Xijing Hosp, Dept Pathol, State Key Lab Canc Biol, Xian 710032, Shaanxi Prov, Peoples R China.
[Ge, Wei; Li, Yuan; Zhang, Shan-Hong; Wang, Xiao-Ming] Fourth Mil Med Univ, Xijing Hosp, Dept Geriatr, Xian 710032, Peoples R China.
C3 Air Force Military Medical University; Air Force Military Medical University
RP Sui, YF (通讯作者)，Fourth Mil Med Univ, Xijing Hosp, Dept Pathol, State Key Lab Canc Biol, Xian 710032, Shaanxi Prov, Peoples R China.
EM geweidr@fmmu.edu.cn; suiyanf@fmmu.edu.cn
CR ALPAR HO, 1989, J PHARM PHARMACOL, V41, P50
AMMOURY N, 1991, PHARMACEUT RES, V8, P101, DOI 10.1023/A:1015846810474
BECK PH, 1994, EUR J PHARM BIOPHARM, V40, P134
Bonduelle S, 1996, EUR J PHARM BIOPHARM, V42, P313
Challacombe SJ, 1997, VACCINE, V15, P169, DOI 10.1016/S0264-410X(96)00159-4
CONSTANTINIDES PP, 1995, J CONTROL RELEASE, V34, P109, DOI 10.1016/0168-3659(94)00129-I
CONSTANTINIDES PP, 1994, PHARMACEUT RES, V11, P1385, DOI 10.1023/A:1018927402875
CONSTANTINIDES PP, 1995, PHARMACEUT RES, V12, P1561, DOI 10.1023/A:1016268311867
Damge C, 1997, J PHARM PHARMACOL, V49, P949, DOI 10.1111/j.2042-7158.1997.tb06022.x
DAMGE C, 1990, J CONTROL RELEASE, V13, P233, DOI 10.1016/0168-3659(90)90013-J
DAMGE C, 1988, DIABETES, V37, P246, DOI 10.2337/diabetes.37.2.246
DAMGE C, 1987, INT J PHARM, V36, P121, DOI 10.1016/0378-5173(87)90146-3
des Rieux A, 2006, J CONTROL RELEASE, V116, P1, DOI 10.1016/j.jconrel.2006.08.013
DEVISSAGUET JP, 1992, E HORWOOD SER PHARM, P71
ESPARZA I, 1992, VACCINE, V10, P714, DOI 10.1016/0264-410X(92)90094-Z
FLEISCHER B, 1988, J EXP MED, V167, P1697, DOI 10.1084/jem.167.5.1697
Galindo-Rodriguez SA, 2005, CRIT REV THER DRUG, V22, P419, DOI 10.1615/CritRevTherDrugCarrierSyst.v22.i5.10
Ge W, 2006, CANCER IMMUNOL IMMUN, V55, P841, DOI 10.1007/s00262-005-0073-y
Hamman JH, 2005, BIODRUGS, V19, P165, DOI 10.2165/00063030-200519030-00003
HUBERT B, 1991, PHARMACEUT RES, V8, P734, DOI 10.1023/A:1015897900363
Jores K, 2004, J CONTROL RELEASE, V95, P217, DOI 10.1016/j.jconrel.2003.11.012
Jung T, 2001, PHARM RES-DORDR, V18, P352, DOI 10.1023/A:1011063232257
Kim SY, 1999, HELICOBACTER, V4, P33, DOI 10.1046/j.1523-5378.1999.09046.x
Ma JH, 2005, CANCER IMMUNOL IMMUN, V54, P907, DOI 10.1007/s00262-004-0660-3
MAINCENT P, 1986, J PHARM SCI, V75, P955, DOI 10.1002/jps.2600751009
MALOY KJ, 1994, IMMUNOLOGY, V81, P661
Mathiowitz E, 1997, NATURE, V386, P410, DOI 10.1038/386410a0

MELIEF CJM, 1995, IMMUNOL REV, V145, P167, DOI 10.1111/j.1600-065X.1995.tb00081.x
Mutwiri George, 2005, Expert Opin Drug Deliv, V2, P791, DOI 10.1517/17425247.2.5.791
Niidome T, 2002, GENE THER, V9, P1647, DOI 10.1038/sj.gt.3301923
O'HAGAN D T, 1990, Advanced Drug Delivery Reviews, V5, P265, DOI 10.1016/0169-409X(90)90020-S
Ottenbrite RM, 1996, MACROMOL SYMP, V101, P379, DOI 10.1002/masy.19961010143
PAPPO J, 1989, CLIN EXP IMMUNOL, V76, P144
Ponchel G, 1998, ADV DRUG DELIVER REV, V34, P191, DOI 10.1016/S0169-409X(98)00040-4
Pouton CW, 1997, ADV DRUG DELIVER REV, V25, P47, DOI 10.1016/S0169-409X(96)00490-5
SARCIAUX JM, 1995, INT J PHARMACEUT, V120, P127, DOI 10.1016/0378-5173(94)00386-J
Storni T, 2005, ADV DRUG DELIVER REV, V57, P333, DOI 10.1016/j.addr.2004.09.008
Streatfield SJ, 2006, METHODS, V38, P150, DOI 10.1016/j.ymeth.2005.09.013
Sudo T, 1997, J ORTHOPAED RES, V15, P128, DOI 10.1002/jor.1100150119
SWENSON ES, 1994, PHARMACEUT RES, V11, P1132, DOI 10.1023/A:1018984731584
SWENSON ES, 1992, ADV DRUG DELIVER REV, V8, P39
Torres BA, 2001, EXP BIOL MED, V226, P164, DOI 10.1177/153537020122600303
Vinogradov SV, 2002, ADV DRUG DELIVER REV, V54, P135, DOI 10.1016/S0169-409X(01)00245-9
Vyas SP, 2007, EXPERT REV VACCINES, V6, P401, DOI 10.1586/14760584.6.3.401
Ye J, 2004, CANCER IMMUNOL IMMUN, V53, P825, DOI 10.1007/s00262-004-0536-6
NR 45
TC 38
Z9 40
U1 0
U2 16
PU SPRINGER
PI NEW YORK
PA 233 SPRING ST, NEW YORK, NY 10013 USA
SN 0340-7004
J9 CANCER IMMUNOL IMMUN
JI Cancer Immunol. Immunother.
PD FEB
PY 2009
VL 58
IS 2
BP 201
EP 208
DI 10.1007/s00262-008-0539-9
PG 8
WC Oncology; Immunology
WE Science Citation Index Expanded (SCI-EXPANDED)
SC Oncology; Immunology
GA 374QX
UT WOS:000261059900004
PM 18523770
DA 2023-09-14
ER

PT J
AU Yang, WJ
Deng, HZ
Zhu, SJ
Lau, J
Tian, R
Wang, S
Zhou, ZJ
Yu, GC
Rao, L
He, LC
Ma, Y
Chen, XY
AF Yang, Weijing
Deng, Hongzhang

Zhu, Shoujun
Lau, Joseph
Tian, Rui
Wang, Sheng
Zhou, Zijian
Yu, Guocan
Rao, Lang
He, Liangcan
Ma, Ying
Chen, Xiaoyuan
TI Size-transformable antigen-presenting cell-mimicking nanovesicles potentiate effective cancer immunotherapy
SO SCIENCE ADVANCES
LA English
DT Article
ID DEATH; NANOPARTICLES; NANOVACCINE; LYMPHOCYTES; ACTIVATION; EXPANSION; INDUCTION; EFFICIENT; DELIVERY; VACCINES
AB Artificial antigen-presenting cells (aAPCs) can stimulate CD8(+) T cell activation. While nanosized aAPCs (naAPCs) have a better safety profile than microsized (maAPCs), they generally induce a weaker T cell response. Treatment with aAPCs alone is insufficient due to the lack of autologous antigen-specific CD8(+) T cells. Here, we devised a nanovaccine for antigen-specific CD8(+) T cell preactivation in vivo, followed by reactivation of CD8(+) T cells via size-transformable naAPCs. naAPCs can be converted to maAPCs in tumor tissue when encountering preactivated CD8(+) T cells with high surface redox potential. In vivo study revealed that naAPC's combination with nanovaccine had an impressive antitumor efficacy. The methodology can also be applied to chemotherapy and photodynamic therapy. Our findings provide a generalizable approach for using size-transformable naAPCs in vivo for immunotherapy in combination with nanotechnologies that can activate CD8(+) T cells.
C1 [Yang, Weijing; Deng, Hongzhang; Zhu, Shoujun; Lau, Joseph; Tian, Rui; Wang, Sheng; Zhou, Zijian; Yu, Guocan; Rao, Lang; He, Liangcan; Ma, Ying; Chen, Xiaoyuan] Natl Inst Biomed Imaging & Bioengn NIBIB, Lab Mol Imaging & Nanomed LOMIN, NIH, Bethesda, MD 20892 USA.
[Chen, Xiaoyuan] Natl Univ Singapore, Yong Loo Lin Sch Med, Singapore 117597, Singapore.
[Chen, Xiaoyuan] Natl Univ Singapore, Fac Engn, Singapore 117597, Singapore.
C3 National Institutes of Health (NIH) - USA; NIH National Institute of Biomedical Imaging & Bioengineering (NIBIB); National University of Singapore; National University of Singapore
RP Chen, XY (通讯作者)，Natl Inst Biomed Imaging & Bioengn NIBIB, Lab Mol Imaging & Nanomed LOMIN, NIH, Bethesda, MD 20892 USA.; Chen, XY (通讯作者)，Natl Univ Singapore, Yong Loo Lin Sch Med, Singapore 117597, Singapore.; Chen, XY (通讯作者)，Natl Univ Singapore, Fac Engn, Singapore 117597, Singapore.
EM chen9647@gmail.com
RI chen, xia/GXM-5435-2022; Yu, Guocan/D-2963-2014; he, liangcan/AFC-8442-2022; chen, xin/IQW-3432-2023; chen, xia/GYR-3948-2022; Wang, Sheng/ABE-4580-2020; Ma, Ying/HKN-2215-2023; zhu, shoujun/S-8058-2017; chen, xi/GXH-3653-2022; Rao, Lang/ABD-4926-2021
OI Yu, Guocan/0000-0003-1157-4184; he, liangcan/0000-0002-9415-9535; Wang, Sheng/0000-0002-4797-5310; zhu, shoujun/0000-0002-2412-6121; Rao, Lang/0000-0001-5010-0729; Tian, Rui/0000-0003-4965-9861
FU Intramural Research Program (IRP) of NIBIB, NIH
FX This work was supported by the Intramural Research Program (IRP) of the NIBIB, NIH.
CR Chen HM, 2017, NAT REV MATER, V2, DOI 10.1038/natrevmats.2017.24
Chen ZK, 2018, ACS NANO, V12, P8633, DOI 10.1021/acsnano.8b04371
Cheng R, 2015, NANO TODAY, V10, P656, DOI 10.1016/j.nantod.2015.09.005
Cheng WY, 2017, CHEM MATER, V29, P4218, DOI 10.1021/acs.chemmater.7b00047
Cheung AS, 2018, NAT BIOTECHNOL, V36, P160, DOI 10.1038/nbt.4047
Conniot J, 2019, NAT NANOTECHNOL, V14, P891, DOI 10.1038/s41565-019-0512-0
Deng HZ, 2020, NANO LETT, V20, P1928, DOI 10.1021/acs.nanolett.9b05210
Duncan R, 2010, ADV DRUG DELIVER REV, V62, P272, DOI 10.1016/j.addr.2009.12.005
Eggermont LJ, 2014, TRENDS BIOTECHNOL, V32, P456, DOI 10.1016/j.tibtech.2014.06.007
Friedl P, 2005, NAT REV IMMUNOL, V5, P532, DOI 10.1038/nri1647

Galluzzi L, 2017, NAT REV IMMUNOL, V17, P97, DOI 10.1038/nri.2016.107
Goldberg MS, 2019, NAT REV CANCER, V19, P587, DOI 10.1038/s41568-019-0186-9
Han X, 2019, SCI ADV, V5, DOI 10.1126/sciadv.aaw6870
He CB, 2016, NAT COMMUN, V7, DOI 10.1038/ncomms12499
Hickey JW, 2017, NANO LETT, V17, P7045, DOI 10.1021/acs.nanolett.7b03734
Hong EP, 2019, ADV DRUG DELIVER REV, V141, P3, DOI 10.1016/j.addr.2018.01.005
Krysko DV, 2012, NAT REV CANCER, V12, P860, DOI 10.1038/nrc3380
Latouche JB, 2000, NAT BIOTECHNOL, V18, P405, DOI 10.1038/74455
Lawrence DA, 1996, J LEUKOCYTE BIOL, V60, P611, DOI 10.1002/jlb.60.5.611
Li AW, 2018, NAT MATER, V17, P528, DOI 10.1038/s41563-018-0028-2
Li CY, 2017, ADV MATER, V29, DOI 10.1002/adma.201605754
Li SX, 2018, NANO RES, V11, P5769, DOI 10.1007/s12274-018-2124-7
Liu WL, 2019, NAT COMMUN, V10, DOI 10.1038/s41467-019-11157-1
Lovell JF, 2010, CHEM REV, V110, P2839, DOI 10.1021/cr900236h
Luo M, 2017, NAT NANOTECHNOL, V12, P648, DOI [10.1038/nnano.2017.52, 10.1038/NNANO.2017.52]
Martin JD, 2020, NAT REV CLIN ONCOL, V17, P251, DOI 10.1038/s41571-019-0308-z
Master A, 2013, J CONTROL RELEASE, V168, P88, DOI 10.1016/j.jconrel.2013.02.020
Melero I, 2014, NAT REV CLIN ONCOL, V11, P509, DOI 10.1038/nrclinonc.2014.111
Meng ZQ, 2019, ADV MATER, V31, DOI 10.1002/adma.201900927
Mi P, 2020, THERANOSTICS, V10, P4557, DOI 10.7150/thno.38069
Ni QQ, 2020, SCI ADV, V6, DOI 10.1126/sciadv.aaw6071
Oelke M, 2003, NAT MED, V9, P619, DOI 10.1038/nm869
Pellom ST, 2013, PLOS ONE, V8, DOI 10.1371/journal.pone.0081134
Perica K, 2014, ACS NANO, V8, P2252, DOI 10.1021/nn405520d
Riley RS, 2019, NAT REV DRUG DISCOV, V18, P175, DOI 10.1038/s41573-018-0006-z
Sahin U, 2018, SCIENCE, V359, P1355, DOI 10.1126/science.aar7112
Shi JJ, 2017, NAT REV CANCER, V17, P20, DOI 10.1038/nrc.2016.108
Song WT, 2018, NAT COMMUN, V9, DOI 10.1038/s41467-018-04605-x
Tang L, 2018, NAT BIOTECHNOL, V36, P707, DOI 10.1038/nbt.4181
Wang C, 2017, THERANOSTICS, V7, P3504, DOI 10.7150/thno.19017
Wang H, 2018, NAT MATER, V17, P761, DOI 10.1038/s41563-018-0147-9
Wang S, 2019, ANGEW CHEM INT EDIT, V58, P14758, DOI 10.1002/anie.201908997
Yang BW, 2019, CHEM REV, V119, P4881, DOI 10.1021/acs.chemrev.8b00626
Yang GB, 2018, NANO LETT, V18, P2475, DOI 10.1021/acs.nanolett.8b00040
Yang WJ, 2019, NANO TODAY, V27, P28, DOI 10.1016/j.nantod.2019.05.004
Yang WJ, 2019, ACS NANO, V13, P3083, DOI 10.1021/acsnano.8b08346
Yang WJ, 2016, ADV MATER, V28, P8234, DOI 10.1002/adma.201600065
Ye CX, 2018, SIGNAL TRANSDUCT TAR, V3, DOI 10.1038/s41392-017-0002-5
Ye YQ, 2017, SCI IMMUNOL, V2, DOI 10.1126/sciimmunol.aan5692
Zhang YN, 2016, J CONTROL RELEASE, V240, P332, DOI 10.1016/j.jconrel.2016.01.020
Zhao MY, 2018, ADV MATER, V30, DOI 10.1002/adma.201804982
Zhou FY, 2019, ADV MATER, V31, DOI 10.1002/adma.201805888
Zhu GZ, 2017, ACS NANO, V11, P2387, DOI 10.1021/acsnano.7b00978
NR 53
TC 37
Z9 38
U1 9
U2 100
PU AMER ASSOC ADVANCEMENT SCIENCE
PI WASHINGTON
PA 1200 NEW YORK AVE, NW, WASHINGTON, DC 20005 USA
SN 2375-2548
J9 SCI ADV
JI Sci. Adv.
PD DEC
PY 2020
VL 6
IS 50
AR eabd1631
DI 10.1126/sciadv.abd1631

PG 14
WC Multidisciplinary Sciences
WE Science Citation Index Expanded (SCI-EXPANDED)
SC Science & Technology - Other Topics
GA PG7HZ
UT WOS:000599903600015
PM 33310853
OA Green Published, gold
DA 2023-09-14
ER

PT J
AU Chang, R
Yan, XH
AF Chang, Rui
Yan, Xuehai
TI Supramolecular Immunotherapy of Cancer Based on the Self-Assembling Peptide Design
SO SMALL STRUCTURES
LA English
DT Review
DE immunogenicity; peptides; regulation; self-assembly; supramolecular immunotherapy
ID IMMUNE CHECKPOINT INHIBITORS; DELIVERY; VACCINES; HYDROGELS; IMMUNOGENICITY; NANOVACCINES; STABILITY; RESPONSES; THERAPY
AB Supramolecular peptide assemblies with specific motifs have the advantages of high immunogenicity, regulated immune response, and increased multiple functions in improving the performance of cancer immunotherapy, compared with molecular peptide immunotherapy. These peptide-based assemblies with structural and functional versatility can serve as immunotherapeutic agents such as antigens, adjuvants, drug delivery carriers, or immunomodulators. This article reviews the effects of supramolecular peptide assemblies on cancer immunotherapy and highlights the unique role of supramolecular assembly strategies in regulating the immunotherapeutic performance. The mechanism of how the peptide supramolecular self-assembly affects the performance of immunotherapy is critically emphasized. The summary of peptide supramolecular immunotherapy provides a reference for the precise design of adjustable and multifunctional immunotherapeutic agents, and shows that peptide self-assembly has great application prospects in cancer immunotherapy.
C1 [Chang, Rui; Yan, Xuehai] Chinese Acad Sci, Inst Proc Engn, State Key Lab Biochem Engn, Beijing 100190, Peoples R China.
[Chang, Rui; Yan, Xuehai] Univ Chinese Acad Sci, Sch Chem Engn, Beijing 100049, Peoples R China.
[Yan, Xuehai] Chinese Acad Sci, Inst Proc Engn, Ctr Mesosci, Beijing 100190, Peoples R China.
C3 Institute of Process Engineering, CAS; Chinese Academy of Sciences; Chinese Academy of Sciences; University of Chinese Academy of Sciences, CAS; Chinese Academy of Sciences; Institute of Process Engineering, CAS
RP Yan, XH (通讯作者)，Chinese Acad Sci, Inst Proc Engn, State Key Lab Biochem Engn, Beijing 100190, Peoples R China.; Yan, XH (通讯作者)，Univ Chinese Acad Sci, Sch Chem Engn, Beijing 100049, Peoples R China.
EM yanxh@ipe.ac.cn
RI Yan, Xuehai/E-8722-2012
OI Yan, Xuehai/0000-0002-0890-0340
FU Innovation Research Community Science Fund [21821005]; National Natural Science Fund BRICS STI Framework Programme [51861145304]; Key Research Program of Frontier Sciences of Chinese Academy of Sciences (CAS) [QYZDB-SSW-JSC034]; CAS President's International Fellowship Initiative [2018VEA0005]
FX This work was financially supported by the Innovation Research Community Science Fund (grant no. 21821005), the National Natural Science Fund BRICS STI Framework Programme (grant no. 51861145304), the Key Research Program of Frontier Sciences of Chinese Academy of Sciences (CAS; grant no. QYZDB-SSW-JSC034), and the CAS President's International Fellowship Initiative (grant no. 2018VEA0005).
CR Abbas M, 2017, ADV MATER, V29, DOI 10.1002/adma.201605021

Adler-Abramovich L, 2014, CHEM SOC REV, V43, P6881, DOI 10.1039/c4cs00164h
Afridi S, 2016, IMMUNOL REV, V272, P151, DOI 10.1111/imr.12435
BenMohamed L, 2002, LANCET INFECT DIS, V2, P425, DOI 10.1016/S1473-3099(02)00318-3
Black M, 2012, ADV MATER, V24, P3845, DOI 10.1002/adma.201200209
Black M, 2010, EXPERT REV VACCINES, V9, P157, DOI [10.1586/erv.09.160, 10.1586/ERV.09.160]
Branco MC, 2011, CURR OPIN CHEM BIOL, V15, P427, DOI 10.1016/j.cbpa.2011.03.021
Cai Y, 2020, BIOMATER SCI-UK, V8, P1045, DOI 10.1039/c9bm01380f
Chang HN, 2015, ANGEW CHEM INT EDIT, V54, P11760, DOI 10.1002/anie.201506225
Chang R, 2019, ADV THER-GERMANY, V2, DOI 10.1002/adtp.201900048
Chen JJ, 2013, BIOMATERIALS, V34, P8776, DOI 10.1016/j.biomaterials.2013.07.063
Chen WF, 2020, ADV FUNCT MATER, V30, DOI 10.1002/adfm.201906623
Cheng H, 2019, MACROMOL BIOSCI, V19, DOI 10.1002/mabi.201800410
Cheng KM, 2018, NANO LETT, V18, P3250, DOI 10.1021/acs.nanolett.8b01071
Chesson CB, 2014, VACCINE, V32, P1174, DOI 10.1016/j.vaccine.2013.11.047
Dellacherie MO, 2018, BIOCONJUGATE CHEM, V29, P733, DOI 10.1021/acs.bioconjchem.7b00656
Di Lorenzo G, 2011, NAT REV CLIN ONCOL, V8, P551, DOI 10.1038/nrclinonc.2011.72
Goldberg MS, 2019, NAT REV CANCER, V19, P587, DOI 10.1038/s41568-019-0186-9
Grohmann U, 1999, CANCER IMMUNOL IMMUN, V48, P195, DOI 10.1007/s002620050565
Habibi N, 2016, NANO TODAY, V11, P41, DOI 10.1016/j.nantod.2016.02.004
He WX, 2019, ADV FUNCT MATER, V29, DOI 10.1002/adfm.201807736
Hos BJ, 2018, FRONT IMMUNOL, V9, DOI 10.3389/fimmu.2018.00884
Hudalla GA, 2013, ADV HEALTHC MATER, V2, P1114, DOI 10.1002/adhm.201200435
Im S, 2019, ACS NANO, V13, P476, DOI 10.1021/acsnano.8b07045
Jeong WJ, 2020, J AM CHEM SOC, V142, P1832, DOI 10.1021/jacs.9b10160
Ji TJ, 2019, ADV MATER, V31, DOI 10.1002/adma.201804395
Jin HL, 2018, ACS NANO, V12, P3295, DOI 10.1021/acsnano.7b08148
Kakwere H, 2017, BIOCONJUGATE CHEM, V28, P2756, DOI 10.1021/acs.bioconjchem.7b00502
Kuang J, 2018, ADV FUNCT MATER, V28, DOI 10.1002/adfm.201800025
Lao XZ, 2014, BIOCHIMIE, V107, P277, DOI 10.1016/j.biochi.2014.09.013
Li JY, 2016, NAT REV MATER, V1, DOI 10.1038/natrevmats.2016.71
Li M, 2018, NANOMED-NANOTECHNOL, V14, P2541, DOI 10.1016/j.nano.2018.08.008
Li MM, 2019, SCI CHINA MATER, V62, P1759, DOI 10.1007/s40843-019-9451-7
Li SK, 2018, CURR OPIN COLLOID IN, V35, P17, DOI 10.1016/j.cocis.2017.12.004
Li YX, 2018, ANGEW CHEM INT EDIT, V57, P17084, DOI 10.1002/anie.201810087
Liu HJ, 2019, ACS NANO, V13, P12638, DOI 10.1021/acsnano.9b04181
Liu JX, 2019, NANO LETT, V19, P8708, DOI 10.1021/acs.nanolett.9b03414
Liu K, 2016, ANGEW CHEM INT EDIT, V55, P3036, DOI 10.1002/anie.201509810
Liu YT, 2019, ACS APPL BIO MATER, V2, P1241, DOI 10.1021/acsabm.8b00811
Liu YJ, 2020, BIOMATERIALS, V242, DOI 10.1016/j.biomaterials.2020.119933
Luo LH, 2018, ACS NANO, V12, P7647, DOI 10.1021/acsnano.8b00204
Luo LH, 2018, J CONTROL RELEASE, V278, P87, DOI 10.1016/j.jconrel.2018.04.002
Luo ZC, 2017, ADV MATER, V29, DOI 10.1002/adma.201601776
Lynn GM, 2020, NAT BIOTECHNOL, V38, P320, DOI 10.1038/s41587-019-0390-x
Mei EC, 2019, COLLOID SURFACE A, V577, P570, DOI 10.1016/j.colsurfa.2019.06.023
Mora-Solano C, 2017, BIOMATERIALS, V149, P1, DOI 10.1016/j.biomaterials.2017.09.031
Ng CW, 2018, ADV FUNCT MATER, V28, DOI 10.1002/adfm.201804688
Patel SP, 2015, MOL CANCER THER, V14, P847, DOI 10.1158/1535-7163.MCT-14-0983
Qian Y, 2016, BIOMATERIALS, V98, P171, DOI 10.1016/j.biomaterials.2016.05.008
Rai XK, 2019, ANGEW CHEM INT EDIT, V58, P5872, DOI 10.1002/anie.201814575
Rudra JS, 2012, ACS NANO, V6, P1557, DOI 10.1021/nn204530r
Rudra JS, 2010, P NATL ACAD SCI USA, V107, P622, DOI 10.1073/pnas.0912124107
Sang W, 2019, CHEM SOC REV, V48, P3771, DOI 10.1039/c8cs00896e
Schumacher TN, 2015, SCIENCE, V348, P69, DOI 10.1126/science.aaa4971
Skwarczynski M, 2016, CHEM SCI, V7, P842, DOI 10.1039/c5sc03892h
Song HJ, 2019, THERANOSTICS, V9, P2299, DOI 10.7150/thno.30577
Song W, 2018, ACS NANO, V12, P1978, DOI 10.1021/acsnano.7b09112
Sun T, 2016, ACTA BIOMATER, V30, P62, DOI 10.1016/j.actbio.2015.11.019
Tahara Y, 2015, ADV DRUG DELIVER REV, V95, P65, DOI 10.1016/j.addr.2015.10.004
Tardon MC, 2019, CURR OPIN PHARMACOL, V47, P20, DOI 10.1016/j.coph.2019.01.007
Tesauro D, 2019, MOLECULES, V24, DOI 10.3390/molecules24020351
TISCHIO JP, 1979, INT J PEPT PROT RES, V14, P479

Torchilin VP, 2003, DRUG DISCOV TODAY, V8, P259, DOI 10.1016/S1359-6446(03)02623-0
Tsoras AN, 2018, BIOCONJUGATE CHEM, V29, P776, DOI 10.1021/acs.bioconjchem.8b00079
Villadolid J, 2015, TRANSL LUNG CANCER R, V4, P560, DOI 10.3978/j.issn.2218-6751.2015.06.06
Wang HM, 2016, ADV FUNCT MATER, V26, P1822, DOI 10.1002/adfm.201505188
Wang J, 2016, CHEM SOC REV, V45, P5589, DOI 10.1039/c6cs00176a
Wang TT, 2018, NAT COMMUN, V9, DOI 10.1038/s41467-018-03915-4
Wang Y, 2019, ACS NANO, V13, P7568, DOI 10.1021/acsnano.9b00143
Wang ZY, 2017, NANOSCALE, V9, P14058, DOI 10.1039/c7nr04990k
Wen Y, 2016, ACS NANO, V10, P9274, DOI 10.1021/acsnano.6b03409
Wen Y, 2015, CURR OPIN IMMUNOL, V35, P73, DOI 10.1016/j.coi.2015.06.007
Wu DD, 2014, CANCER LETT, V351, P13, DOI 10.1016/j.canlet.2014.05.002
Wu YY, 2017, ACS BIOMATER SCI ENG, V3, P3128, DOI 10.1021/acsbiomaterials.7b00561
Xing RR, 2019, ADV MATER, V31, DOI 10.1002/adma.201900822
Xing RR, 2017, BIOMACROMOLECULES, V18, P3514, DOI 10.1021/acs.biomac.7b00787
Xu CF, 2019, ADV FUNCT MATER, V29, DOI 10.1002/adfm.201905213
Xu C, 2019, ACS NANO, V13, P12148, DOI 10.1021/acsnano.9b06691
Xu Y, 2019, THERANOSTICS, V9, P3388, DOI 10.7150/thno.34031
Yang PX, 2018, NANO LETT, V18, P4377, DOI 10.1021/acs.nanolett.8b01406
Yang Q, 2019, J CONTROL RELEASE, V308, P29, DOI 10.1016/j.jconrel.2019.06.031
Yang WJ, 2019, NANO TODAY, V27, P28, DOI 10.1016/j.nantod.2019.05.004
Yanover C, 2011, P NATL ACAD SCI USA, V108, P6981, DOI 10.1073/pnas.1018165108
Yu SJ, 2018, MACROMOL BIOSCI, V18, DOI 10.1002/mabi.201800240
Yu ZY, 2004, J CLIN INVEST, V114, P551, DOI 10.1172/JCI200421695
Yuan CQ, 2019, NAT REV CHEM, V3, P567, DOI 10.1038/s41570-019-0129-8
Yuan CQ, 2019, ANGEW CHEM INT EDIT, V58, P18116, DOI 10.1002/anie.201911782
Zhang C, 2019, ACS NANO, V13, P11249, DOI 10.1021/acsnano.9b04315
Zhang C, 2018, ADV FUNCT MATER, V28, DOI 10.1002/adfm.201804492
Zhang L, 2020, NAT NANOTECHNOL, V15, P145, DOI 10.1038/s41565-019-0626-4
Zhang L, 2019, THERANOSTICS, V9, P7807, DOI 10.7150/thno.37194
Zhang T, 2019, ACTA PHARMACOL SIN, V40, P514, DOI 10.1038/s41401-018-0085-8
Zhang YK, 2018, J MATER CHEM B, V6, P7335, DOI 10.1039/c8tb01487f
Zhu GZ, 2017, ACS NANO, V11, P2387, DOI 10.1021/acsnano.7b00978
Zou QL, 2017, J AM CHEM SOC, V139, P1921, DOI 10.1021/jacs.6b11382
NR 95
TC 37
Z9 37
U1 11
U2 66
PU WILEY
PI HOBOKEN
PA 111 RIVER ST, HOBOKEN 07030-5774, NJ USA
EI 2688-4062
J9 SMALL STRUCT
JI Small Struct.
PD NOV
PY 2020
VL 1
IS 2
AR 2000068
DI 10.1002/sstr.202000068
PG 14
WC Chemistry, Physical; Nanoscience & Nanotechnology; Materials Science, Multidisciplinary
WE Science Citation Index Expanded (SCI-EXPANDED)
SC Chemistry; Science & Technology - Other Topics; Materials Science
GA XX9BN
UT WOS:000736581700012
OA Bronze
DA 2023-09-14
ER

PT J
AU El-Sayed, A
Kamel, M
AF El-Sayed, Amr
Kamel, Mohamed
TI Advanced applications of nanotechnology in veterinary medicine
SO ENVIRONMENTAL SCIENCE AND POLLUTION RESEARCH
LA English
DT Article
DE Nanotechnology; Nanomedicine; Nanovaccines; Nanoadjuvants;
Nanoparticles; Nanomaterials; Nanominerals; Veterinary; Pet care
ID VIRUS-LIKE PARTICLES; ORAL IMMUNIZATION; VIRAL NANOPARTICLES;
CONTROLLED-RELEASE; CARBON NANOTUBES; IMMUNE-RESPONSE; DELIVERY SYSTEM;
NANO; NANOMEDICINE; ANTIGENS
AB The invention of new techniques to manipulate materials at their nanoscale had an evolutionary effect on various medical sciences. At the time, there are thousands of nanomaterials which can be divided according to their shape, origin, or their application. The nanotechnology provided new solutions for old problems. In medical sciences, they are used for diagnostic or therapeutic purposes. They can also be applied in the preparation of nanovaccines and nanoadjuvants. Their use in the treatment of cancer and in gene therapy opened the door for a new era in medicine. Recently, various applications of nanotechnology started to find their way in the veterinary sector. They increasingly invade animal therapeutics, diagnostics, production of veterinary vaccines, farm disinfectants, for animal breeding and reproduction, and even the field of animal nutrition. Their replacement of commonly used antibiotics directly reflects on the public health. By so doing, they minimize the problem of drug resistance in both human and veterinary medicine, and the problem of drug residues in milk and meat. In addition, they have a great economic impact, by minimizing the amounts of discarded milk and the number of culled calves in dairy herds. Nanotechnology was also applied to develop pet care products and hygienic articles. The present review discusses the advantage of using nanomaterials compared to their counterparts, the various classes of nanoparticles, and illustrates the applications and the role of nanotechnology in the field of veterinary medicine.
C1 [El-Sayed, Amr; Kamel, Mohamed] Cairo Univ, Fac Vet Med, Dept Med & Infect Dis, Giza, Egypt.
C3 Egyptian Knowledge Bank (EKB); Cairo University
RP Kamel, M (通讯作者)，Cairo Univ, Fac Vet Med, Dept Med & Infect Dis, Giza, Egypt.
EM drm_salah201011@yahoo.com
RI El-Sayed, Amr/AAT-4055-2020
CR Alshatwi AA, 2015, J MATER SCI-MATER M, V26, DOI 10.1007/s10856-015-5468-5
Amiji MM., 2007, NANOTECHNOLOGIES LIF
Anand M, 2015, INT J ADV RES COMPUT, V5, P1271
[Anonymous], J EVOL TECHNOL
Anton N, 2011, PHARM RES-DORDR, V28, P978, DOI 10.1007/s11095-010-0309-1
Awate S, 2013, FRONT IMMUNOL, V4, DOI 10.3389/fimmu.2013.00114
Bakker-Woudenberg IAJM, 2005, METHOD ENZYMOL, V391, P228, DOI 10.1016/S0076-6879(05)91014-8
Bellocq NC, 2003, BIOCONJUGATE CHEM, V14, P1122, DOI 10.1021/bc034125f
Bielinska AU, 2007, INFECT IMMUN, V75, P4020, DOI 10.1128/IAI.00070-07
Casals E, 2017, CANCER NANOTECHNOL, V8, DOI 10.1186/s12645-017-0030-4
Chakravarthi PV, 2010, VET WORLD, V3, P477
Chapman S, 2013, NANO TODAY, V8, P454, DOI 10.1016/j.nantod.2013.06.001
Chen SC, 1998, J VIROL, V72, P5757, DOI 10.1128/JVI.72.7.5757-5761.1998
Chen YS, 2010, NANOTECHNOLOGY, V21, DOI 10.1088/0957-4484/21/19/195101
Chowdhury A, 2017, INT NANO LETT, V7, P91, DOI 10.1007/s40089-017-0208-0
Conway MA, 2001, VACCINE, V19, P1940, DOI 10.1016/S0264-410X(00)00433-3
Cormode DP, 2013, NMR BIOMED, V26, P766, DOI 10.1002/nbm.2909
Danesh-Bahreini MA, 2011, INT J NANOMED, V6, P835, DOI 10.2147/IJN.S16805
Dobrovolskaia MA, 2016, TOXICOL APPL PHARM, V299, P78, DOI 10.1016/j.taap.2015.12.022
Elgqvist J, 2017, INT J MOL SCI, V18, DOI 10.3390/ijms18051102
Erathodiyil N, 2011, ACCOUNTS CHEM RES, V44, P925, DOI 10.1021/ar2000327
Fakhri A, 2017, J PHOTOCH PHOTOBIO B, V169, P21, DOI 10.1016/j.jphotobiol.2017.02.014
Fernando GJP, 2010, PLOS ONE, V5, DOI 10.1371/journal.pone.0010266
Florindo HF, 2009, BIOMATERIALS, V30, P879, DOI 10.1016/j.biomaterials.2008.10.035
Freitas RA, 2005, J COMPUT THEOR NANOS, V2, P1

Greenwood DLV, 2008, VACCINE, V26, P2706, DOI 10.1016/j.vaccine.2008.03.025
Grgacic EVL, 2006, METHODS, V40, P60, DOI 10.1016/j.ymeth.2006.07.018
Hamouda T, 2011, CLIN VACCINE IMMUNOL, V18, P1167, DOI 10.1128/CVI.00035-11
Harvey J, 2010, NANOTECHNOLOGY NEW R
He Q, 2002, CLIN DIAGN LAB IMMUN, V9, P1021, DOI 10.1128/CDLI.9.5.1021-1024.2002
Hirsch LR, 2003, P NATL ACAD SCI USA, V100, P13549, DOI 10.1073/pnas.2232479100
Illum L, 2001, ADV DRUG DELIVER REV, V51, P81, DOI 10.1016/S0169-409X(01)00171-5
Jahanshahi M, 2008, AFR J BIOTECHNOL, V7, P4926
Jain SK, 2011, VET WORLD, V4, P35, DOI 10.5455/vetworld.2011.35-40
Joanitti GA, 2014, CURR DRUG TARGETS, V15, P478, DOI 10.2174/13894501113159990171
Johansson HKL, 2017, PART FIBRE TOXICOL, V14, DOI 10.1186/s12989-017-0197-1
Jung T, 2001, PHARM RES-DORDR, V18, P352, DOI 10.1023/A:1011063232257
Jurj A, 2017, DRUG DES DEV THER, V11, P2871, DOI 10.2147/DDDT.S142337
Kanekiyo M, 2013, NATURE, V499, P102, DOI 10.1038/nature12202
Kang BK, 2004, INT J PHARMACEUT, V286, P147, DOI 10.1016/j.ijpharm.2004.08.008
Karimi M, 2013, J NANOPART RES, V15, DOI 10.1007/s11051-013-1651-0
Kim BYS, 2010, NEW ENGL J MED, V363, P2434, DOI 10.1056/NEJMra0912273
Kim SY, 1999, HELICOBACTER, V4, P33, DOI 10.1046/j.1523-5378.1999.09046.x
Knapp CM, 2016, EXP BIOL MED, V241, P1007, DOI 10.1177/1535370216640944
Krishnan Samaya R., 2014, PHARM THERAPEUTICS
Kroubi M, 2010, NANOTECHNOLOGY, V21, DOI 10.1088/0957-4484/21/50/505102
Kudela D, 2015, ANGEW CHEM INT EDIT, V54, P4018, DOI 10.1002/anie.201409639
Landers JJ, 2002, J INFECT DIS, V186, P1222, DOI 10.1086/344316
Latour R.A., 2003, ADHESION SPECIFIC NA
Lee SJ, 2013, J CONTROL RELEASE, V172, P358, DOI 10.1016/j.jconrel.2013.09.002
Liao CD, 2011, EUR J RADIOL, V80, P699, DOI 10.1016/j.ejrad.2010.08.005
Manuja A., 2012, NANOTECHNOL DEV, V2, P4
Maree S, 2016, VIRUS RES, V213, P184, DOI 10.1016/j.virusres.2015.12.006
Meena NS., 2018, J ENTOMOL ZOOL STUD, V6, P167
Mieszawska AJ, 2013, MOL PHARMACEUT, V10, P831, DOI 10.1021/mp3005885
Mishra Akash, 2014, Indian Journal of Animal Nutrition, V31, P384
Mishra B, 2010, NANOMED-NANOTECHNOL, V6, P9, DOI 10.1016/j.nano.2009.04.008
Mittal AK, 2014, J COLLOID INTERF SCI, V431, P194, DOI 10.1016/j.jcis.2014.06.030
Moghaddam S.H., 2010, NANOCON, V10, P12
Mohantya N., 2014, VET RES, V2, P90
Moon JS, 2016, NANOMED-NANOTECHNOL, V12, P1489, DOI 10.1016/j.nano.2016.02.015
Moyer TJ, 2016, J CLIN INVEST, V126, P799, DOI 10.1172/JCI81083
Noad R, 2003, TRENDS MICROBIOL, V11, P438, DOI 10.1016/S0966-842X(03)00208-7
Ochoa-Reparaz J, 2004, VET RES, V35, P291, DOI 10.1051/vetres:2004011
Okamoto S, 2009, VACCINE, V27, P5896, DOI 10.1016/j.vaccine.2009.07.037
Patil SS, 2009, VET WORLD, V2, P475, DOI 10.5455/vetworld.2009.475-477
Prabhu RH, 2015, INT J NANOMED, V10, P1001, DOI 10.2147/IJN.S56932
Pushko P, 2013, INTERVIROLOGY, V56, P141, DOI 10.1159/000346773
Rajendran D, 2013, RES J BIOTECHNOL, V8, P1
Rapoport N, 2007, JNCI-J NATL CANCER I, V99, P1095, DOI 10.1093/jnci/djm043
Read RC, 2005, VACCINE, V23, P4367, DOI 10.1016/j.vaccine.2005.04.021
Reilly RM, 2007, J NUCL MED, V48, P1039, DOI 10.2967/jnumed.107.041723
Revia RA, 2016, MATER TODAY, V19, P157, DOI 10.1016/j.mattod.2015.08.022
Riley MK, 2017, NANOMATERIALS-BASEL, V7, DOI 10.3390/nano7050094
Rizzo LY, 2013, CURR OPIN BIOTECH, V24, P1159, DOI 10.1016/j.copbio.2013.02.020
Rodriguez-Burneo N, 2017, NANOMATERIALS-BASEL, V7, DOI 10.3390/nano7070190
Sailor MJ, 2012, ADV MATER, V24, P3779, DOI 10.1002/adma.201200653
Saragusty J, 2011, REPRODUCTION, V141, P1, DOI 10.1530/REP-10-0236
Schneider M, 2009, DERM-ENDOCRINOL, V1, P197, DOI 10.4161/derm.1.4.9501
Scott NR, 2007, VET RES COMMUN, V31, P139, DOI 10.1007/s11259-007-0083-7
Shchepelina O, 2011, ADV MATER, V23, P4655, DOI 10.1002/adma.201102234
Shukla R, 2001, IND CROP PROD, V13, P171, DOI 10.1016/S0926-6690(00)00064-9
Soppimath KS, 2002, DRUG DEV IND PHARM, V28, P957, DOI 10.1081/DDC-120006428
Sridharan K, 2016, BRIT J CLIN PHARMACO, V82, P659, DOI 10.1111/bcp.12987
Steinmetz NF, 2011, SMALL, V7, P1664, DOI 10.1002/smll.201000435
Steinmetz NF, 2010, NANOMED-NANOTECHNOL, V6, P634, DOI 10.1016/j.nano.2010.04.005

Suzuki Y, 1998, J BIOMED MATER RES, V42, P112, DOI 10.1002/(SICI)1097-4636(199810)42:1<112::AID-JBM14>3.3.CO;2-8
Swain PS, 2015, VET WORLD, V8, P888, DOI 10.14202/vetworld.2015.888-891
Talukdar D, 2014, CURR PHARM BIOTECHNO, V15, P1158, DOI 10.2174/1389201015666141122204859
Thulasi A, 2013, NANOBIOTECHNOLOGY AN
Torres-Sangiao E, 2016, MOLECULES, V21, DOI 10.3390/molecules21070867
Troncarelli MZ, 2013, FORMATEX, V13, P543
de Ilarduya CT, 2010, EUR J PHARM SCI, V40, P159, DOI 10.1016/j.ejps.2010.03.019
Underwood C, 2012, VET J, V193, P12, DOI 10.1016/j.tvjl.2012.01.002
Uskokovic D. P, 2018, NANOTECHNOLOGIES PRE, P1, DOI [10.1016/B978-0-323-48063-5.00001-0, DOI 10.1016/B978-0-323-48063-5.00001-0]
Venkatesan J, 2014, J BIOMED NANOTECHNOL, V10, P3124, DOI 10.1166/jbn.2014.1893
Vila A, 2004, J AEROSOL MED, V17, P174, DOI 10.1089/0894268041457183
Williams DF, 2008, BIOMATERIALS, V29, P2941, DOI 10.1016/j.biomaterials.2008.04.023
Xu JH, 2011, CLIN VACCINE IMMUNOL, V18, P75, DOI 10.1128/CVI.00263-10
Yang XQ, 2010, BIOMATERIALS, V31, P9065, DOI 10.1016/j.biomaterials.2010.08.039
Yang Z. P., 2006, J SHANXI AGR SCI, V3, P577
Yi H, 2016, BONE RES, V4, DOI 10.1038/boneres.2016.50
Zhao G, 2017, BIOTECHNOL ADV, V35, P323, DOI 10.1016/j.biotechadv.2017.01.006
Zhao L, 2014, VACCINE, V32, P327, DOI 10.1016/j.vaccine.2013.11.069
NR 104
TC 37
Z9 40
U1 6
U2 29
PU SPRINGER HEIDELBERG
PI HEIDELBERG
PA TIERGARTENSTRASSE 17, D-69121 HEIDELBERG, GERMANY
SN 0944-1344
EI 1614-7499
J9 ENVIRON SCI POLLUT R
JI Environ. Sci. Pollut. Res.
PD JUN
PY 2020
VL 27
IS 16
SI SI
BP 19073
EP 19086
DI 10.1007/s11356-018-3913-y
PG 14
WC Environmental Sciences
WE Science Citation Index Expanded (SCI-EXPANDED)
SC Environmental Sciences & Ecology
GA MI4MP
UT WOS:000547384500011
PM 30547342
DA 2023-09-14
ER

PT J
AU Gala, RP
Zaman, RU
D'Souza, MJ
Zughaier, SM
AF Gala, Rikhav P.
Zaman, Rokon Uz
D'Souza, Martin J.
Zughaier, Susu M.
TI Novel Whole-Cell Inactivated Neisseria Gonorrhoeae Microparticles as

file:///D/Joyce/计量学/frontiers/修/Supplementary materials/savedrecs (94).txt[2024/6/27 16:48:08]

Vaccine Formulation in Microneedle-Based Transdermal Immunization
SO VACCINES
LA English
DT Article
DE Neisseria gonorrhoeae; gonorrhea; vaccine; microneedle; skin patch; nanotechnology; antigen-specific antibody; antigen-specific CD4 T lymphocytes
ID POLYMERIC NANOCARRIERS; CANCER VACCINE; ORAL DELIVERY; SHAPE; SUSCEPTIBILITY; PREVALENCE; RESPONSES; MEASLES; ROUTE
AB Neisseria gonorrhoeae is a strict human pathogen responsible for more than 100 million new sexually transmitted infections worldwide each year. Due to the global emergence of antibiotic resistance, the Center for Disease control (CDC) recently listed N. gonorrhoeae as an urgent threat to public health. No vaccine is available in spite of the huge disease burden and the possibility of untreatable gonorrhea. The aim of this study is to investigate the immunogenicity of a novel whole-cell-based inactivated gonococcal microparticle vaccine formulation loaded in dissolvable microneedles for transdermal administration. The nanotechnology-based vaccine formulation consists of inactivated whole-cell gonococci strain CDC-F62, spray dried and encapsulated into biodegradable cross-linked albumin matrix with sustained slow antigen release. The dry vaccine nanoparticles were then loaded in a dissolvable microneedle skin patch for transdermal delivery. The efficacy of the whole-cell microparticles vaccine formulation loaded in microneedles was assessed in vitro using dendritic cells and macrophages as well as in vivo mouse model. Antibody titers were measured using an enzyme immunosorbent assay (ELISA) and antigen-specific T lymphocytes were assessed in spleens and lymph nodes. Here we report that whole-cell-based gonococcal microparticle vaccine loaded in dissolvable microneedles for transdermal administration induced significant increase in antigen-specific IgG antibody titers and antigen-specific CD4 and CD8 T lymphocytes in mice compared to gonococcal antigens in solution or empty microneedles. Significant increase in antigen-specific IgG antibody levels was observed at the end of week 2 in groups that received the vaccine compared to the group receiving empty nanoparticles. The advantages of using formalin-fixed whole-cell gonococci that all immunogenic epitopes are covered and preserved from degradation. The spherical shaped micro and nanoparticles are biological mimics of gonococci, therefore present to the immune system as invaders but without the ability to suppress adaptive immunity. In conclusion, the transdermal delivery of microparticles vaccine via a microneedle patch was shown to be an effective system for vaccine delivery. The novel gonorrhea nanovaccine is cheap to produce in a stable dry powder and can be delivered in microneedle skin patch obviating the need for needle use or the cold chain.
C1 [Gala, Rikhav P.; Zaman, Rokon Uz; D'Souza, Martin J.] Mercer Univ, Coll Pharm, Dept Pharmaceut Sci, Vaccine Nanotechnol Lab, Atlanta, GA 30341 USA.
[Zughaier, Susu M.] Qatar Univ, Coll Med, Dept Basic Med Sci, Doha 2713, Qatar.
C3 Mercer University; Qatar University
RP Zughaier, SM (通讯作者)，Qatar Univ, Coll Med, Dept Basic Med Sci, Doha 2713, Qatar.
EM rikhav.praful.gala@gmail.com; rokonuz.zaman@live.mercer.edu; DSOUZA_MJ@mercer.edu; szughaier@qu.edu.qa
RI Gala, Rikhav/U-7138-2019; Zughaier, Susu/GLQ-9288-2022; Zughaier, Susu/N-3185-2019
OI Zughaier, Susu/0000-0001-7487-8770
FU Development Research Program grant from the Henry M. Jackson Foundation through NIH grant [1U19AI113170-01]; Mercer University
FX This research received no external funding. S.M.Z. was funded by Development Research Program grant from the Henry M. Jackson Foundation through NIH grant 1U19AI113170-01 (Ann E. Jerse, PI, Uniformed Services University, Bethesda, MD). M.J.D. was funded by Mercer University internal grants.
CR Agarwal R, 2013, THER DELIV, V4, P705, DOI [10.4155/TDE.13.37, 10.4155/tde.13.37]
Agarwal R, 2012, ACS NANO, V6, P2524, DOI 10.1021/nn2049152
Akalkotkar A, 2015, J MICROENCAPSUL, V32, P281, DOI 10.3109/02652048.2015.1017615
[Anonymous], 2013, ANTIBIOTIC RESISTANC
Bejugam NK, 2013, J MICROENCAPSUL, V30, P274, DOI 10.3109/02652048.2012.720724
Bejugam NK, 2008, J MICROENCAPSUL, V25, P577, DOI 10.1080/02652040802476187
Carbone A, 2013, GIORN ITAL DERMAT V, V148, P465
Chablani L, 2012, J PHARM SCI-US, V101, P3661, DOI 10.1002/jps.23275
Chablani L, 2012, J MICROENCAPSUL, V29, P388, DOI 10.3109/02652048.2011.651503
Craig AP, 2015, VACCINE, V33, P4520, DOI 10.1016/j.vaccine.2015.07.015

Duncan JA, 2009, J IMMUNOL, V182, P6460, DOI 10.4049/jimmunol.0802696
Edwards J. L., 2016, CRIT REV MICROBIOL, V3, P1
Feinen B, 2010, MUCOSAL IMMUNOL, V3, P312, DOI 10.1038/mi.2009.139
Gala R. P., 2015, NANOPARTICULATE VACC, P195
Gala RP, 2017, AAPS PHARMSCITECH, V18, P283, DOI 10.1208/s12249-016-0566-3
Gala RP, 2015, NANOPARTICULATE VACCINE DELIVERY SYSTEMS, P127
Gala RP, 2015, INT J PHARMACEUT, V486, P153, DOI 10.1016/j.ijpharm.2015.03.038
Gao Hui, 2008, Zhonghua Yufang Yixue Zazhi, V42, P663
Glangchai LC, 2008, J CONTROL RELEASE, V125, P263, DOI 10.1016/j.jconrel.2007.10.021
Gottlieb SL, 2016, VACCINE, V34, P2939, DOI 10.1016/j.vaccine.2016.03.111
GREENBERG L, 1974, CAN J PUBLIC HEALTH, V65, P29
GREENBERG L, 1975, J REPROD MED, V14, P34
Ison C. A., 2013, SEX TRANSM INFECT
Jerse AE, 2014, VACCINE, V32, P1579, DOI 10.1016/j.vaccine.2013.08.067
Kaveh DA, 2012, PLOS ONE, V7, DOI 10.1371/journal.pone.0038926
Kim YC, 2012, ADV DRUG DELIVER REV, V64, P1547, DOI 10.1016/j.addr.2012.04.005
Kirkcaldy RD, 2016, MMWR SURVEILL SUMM, V65, P1, DOI 10.15585/mmwr.ss6507a1
Liu Y, 2012, MUCOSAL IMMUNOL, V5, P320, DOI 10.1038/mi.2012.12
Petousis-Harris H, 2017, LANCET, V390, P1603, DOI 10.1016/S0140-6736(17)31449-6
Plans P, 2015, EUR J CLIN MICROBIOL, V34, P1161, DOI 10.1007/s10096-015-2339-4
Russell MW, 2018, FUTURE MICROBIOL, V13, P495, DOI 10.2217/fmb-2017-0262
SHAFER WM, 1990, MOL MICROBIOL, V4, P1269, DOI 10.1111/j.1365-2958.1990.tb00706.x
Shastri PN, 2012, J PHARM SCI-US, V101, P3623, DOI 10.1002/jps.23220
Singh M., 2014, NOVEL APPROACHES STR
Tawde SA, 2016, J CONTROL RELEASE, V235, P147, DOI 10.1016/j.jconrel.2016.05.058
Tawde SA, 2012, VACCINE, V30, P5675, DOI 10.1016/j.vaccine.2012.05.073
Ubale RV, 2014, AAPS J, V16, P986, DOI 10.1208/s12248-014-9635-2
Ubale RV, 2013, J MICROENCAPSUL, V30, P28, DOI 10.3109/02652048.2012.692402
Uddin AN, 2009, J DRUG TARGET, V17, P553, DOI 10.1080/10611860903067301
Unemo M, 2016, MICROBIOL SPECTR, V4, DOI 10.1128/microbiolspec.EI10-0009-2015
Wetzler LM, 2016, CLIN VACCINE IMMUNOL, V23, P656, DOI 10.1128/CVI.00230-16
Zhu WY, 2012, PLOS ONE, V7, DOI 10.1371/journal.pone.0041260
Zhu WY, 2011, FRONT MICROBIOL, V2, DOI 10.3389/fmicb.2011.00124
Zhu ZZ, 2014, PHARM RES-DORDR, V31, P3348, DOI 10.1007/s11095-014-1424-1
Zughaier SM, 2004, INFECT IMMUN, V72, P371, DOI 10.1128/IAI.72.1.371-380.2004
Zughaier SM, 2015, PLOS ONE, V10, DOI 10.1371/journal.pone.0144347
Zughaier SM, 2014, PLOS ONE, V9, DOI 10.1371/journal.pone.0087688
NR 47
TC 37
Z9 39
U1 0
U2 25
PU MDPI
PI BASEL
PA ST ALBAN-ANLAGE 66, CH-4052 BASEL, SWITZERLAND
SN 2076-393X
J9 VACCINES-BASEL
JI Vaccines
PD SEP
PY 2018
VL 6
IS 3
AR 60
DI 10.3390/vaccines6030060
PG 17
WC Immunology; Medicine, Research & Experimental
WE Science Citation Index Expanded (SCI-EXPANDED)
SC Immunology; Research & Experimental Medicine
GA GS8CG
UT WOS:000443934000026
PM 30181504

OA Green Submitted, Green Published, gold
DA 2023-09-14
ER

PT J
AU Kim, Y
Kang, S
Shin, H
Kim, T
Yu, B
Kim, J
Yoo, D
Jon, S
AF Kim, Yujin
Kang, Sukmo
Shin, Hocheol
Kim, Taewoo
Yu, Byeongjun
Kim, Jinjoo
Yoo, Dohyun
Jon, Sangyong
TI Sequential and Timely Combination of a Cancer Nanovaccine with Immune Checkpoint Blockade Effectively Inhibits Tumor Growth and Relapse
SO ANGEWANDTE CHEMIE-INTERNATIONAL EDITION
LA English
DT Article
DE cancer immunotherapy; immune checkpoint blockades; lipid nanoparticles; nanoparticle vaccines; peptide antigens
ID CROSS-PRESENTATION; PD-1 BLOCKADE; NANOPARTICLES; ANTIGEN; DELIVERY; VACCINES; CELLS
AB We describe a small lipid nanoparticle (SLNP)-based nanovaccine platform and a new combination treatment regimen. Tumor antigen-displaying, CpG adjuvant-embedded SLNPs (OVA(PEP)-SLNP@CpG) were prepared from biocompatible phospholipids and a cationic cholesterol derivative. The resulting nanovaccine showed highly potent antitumor efficacy in both prophylactic and therapeutic E.G7 tumor models. However, this vaccine induced T cell exhaustion by elevating PD-L1 expression, leading to tumor recurrence. Thus, the nanovaccine was combined with simultaneous anti-PD-1 antibody treatment, but the therapeutic efficacy of this regimen was comparable to that of the nanovaccine alone. Finally, mice that showed a good therapeutic response after the first cycle of immunization with the nanovaccine underwent a second cycle together with anti-PD-1 therapy, resulting in suppression of tumor relapse. This suggests that the antitumor efficacy of combinations of nanovaccines with immune checkpoint blockade therapy is dependent on treatment sequence and the timing of each modality.
C1 [Kim, Yujin; Kang, Sukmo; Shin, Hocheol; Kim, Taewoo; Yu, Byeongjun; Kim, Jinjoo; Yoo, Dohyun; Jon, Sangyong] Korea Adv Inst Sci & Technol, Dept Biol Sci, 291 Daehak Ro, Daejeon 34141, South Korea.
[Kim, Yujin; Shin, Hocheol; Kim, Taewoo; Yu, Byeongjun; Kim, Jinjoo; Yoo, Dohyun; Jon, Sangyong] Korea Adv Inst Sci & Technol, Ctr Precis Bionanomed, Daejeon 34141, South Korea.
[Kim, Yujin; Kang, Sukmo; Shin, Hocheol; Kim, Taewoo; Yu, Byeongjun; Kim, Jinjoo; Yoo, Dohyun; Jon, Sangyong] Korea Adv Inst Sci & Technol, Inst BioCentury, 291 Daehak Ro, Daejeon 34141, South Korea.
C3 Korea Advanced Institute of Science & Technology (KAIST); Korea Advanced Institute of Science & Technology (KAIST); Korea Advanced Institute of Science & Technology (KAIST)
RP Jon, S (通讯作者)，Korea Adv Inst Sci & Technol, Dept Biol Sci, 291 Daehak Ro, Daejeon 34141, South Korea.; Jon, S (通讯作者)，Korea Adv Inst Sci & Technol, Ctr Precis Bionanomed, Daejeon 34141, South Korea.; Jon, S (通讯作者)，Korea Adv Inst Sci & Technol, Inst BioCentury, 291 Daehak Ro, Daejeon 34141, South Korea.
EM syjon@kaist.ac.kr
OI Kim, Yujin/0000-0002-7186-221X; Yu, Byeongjun/0000-0002-3539-9416; Yoo, Dohyun/0000-0002-6911-7181; Jon, Sangyong/0000-0002-6971-586X; Kim, Tae Woo/0000-0002-9255-4355; Kim, Jinjoo/0000-0002-2702-6871; Shin, Hocheol/0000-0002-9774-0704
FU Basic Science Research Program through the National Research Foundation

of Korea (NRF) - Ministry of Science and ICT [NRF-2018R1A3B1052661]; Bio & Medical Technology Development Program of the National Research Foundation (NRF) - Korean government (MSIT) [NRF-2018M3A9B5023527]; National Research Foundation of Korea [2018M3A9B5023527] Funding Source: Korea Institute of Science & Technology Information (KISTI), National Science & Technology Information Service (NTIS)
FX This research was supported by Basic Science Research Program through the National Research Foundation of Korea (NRF) funded by the Ministry of Science and ICT (NRF-2018R1A3B1052661) and the Bio & Medical Technology Development Program of the National Research Foundation (NRF) funded by the Korean government (MSIT) (NRF-2018M3A9B5023527).
CR Adamus Tomasz, 2018, Contemp Oncol (Pozn), V22, P56, DOI 10.5114/wo.2018.73887
Belizaire R, 2009, P NATL ACAD SCI USA, V106, P17463, DOI 10.1073/pnas.0908583106
De Koker S., 2016, ANGEW CHEM-GER EDIT, V128, P1356, DOI [10.1002/ange.201508626, DOI 10.1002/ANGE.201508626]
De Koker S, 2016, ANGEW CHEM INT EDIT, V55, P1334, DOI 10.1002/anie.201508626
Fourcade J, 2014, CANCER RES, V74, P1045, DOI 10.1158/0008-5472.CAN-13-2908
Galluzzi L, 2018, SCI TRANSL MED, V10, DOI 10.1126/scitranslmed.aat7807
Hirosue S, 2010, VACCINE, V28, P7897, DOI 10.1016/j.vaccine.2010.09.077
Ilyinskii PO, 2014, VACCINE, V32, P2882, DOI 10.1016/j.vaccine.2014.02.027
Irvine DJ, 2013, NAT MATER, V12, P978, DOI [10.1038/NMAT3775, 10.1038/nmat3775]
Joffre OP, 2012, NAT REV IMMUNOL, V12, P557, DOI 10.1038/nri3254
Kapadia CH, 2016, MOL PHARMACEUT, V13, P3381, DOI 10.1021/acs.molpharmaceut.6b00288
Kim C, 2017, CLIN EXP IMMUNOL, V187, P71, DOI 10.1111/cei.12829
Knutson KL, 2002, CLIN CANCER RES, V8, P1014
Krieg AM, 2006, NAT REV DRUG DISCOV, V5, P471, DOI 10.1038/nrd2059
Kroll AV, 2017, ADV MATER, V29, DOI 10.1002/adma.201703969
Kuai R, 2017, NAT MATER, V16, P489, DOI [10.1038/nmat4822, 10.1038/NMAT4822]
Lee J, 2016, THERANOSTICS, V6, P192, DOI 10.7150/thno.13657
Li AW, 2018, NAT MATER, V17, P528, DOI 10.1038/s41563-018-0028-2
Liu HP, 2014, NATURE, V507, P519, DOI 10.1038/nature12978
Mandai M, 2016, CLIN CANCER RES, V22, P2329, DOI 10.1158/1078-0432.CCR-16-0224
Manolova V, 2008, EUR J IMMUNOL, V38, P1404, DOI 10.1002/eji.200737984
Melero I, 2014, NAT REV CLIN ONCOL, V11, P509, DOI 10.1038/nrclinonc.2014.111
Messenheimer DJ, 2017, CLIN CANCER RES, V23, P6165, DOI 10.1158/1078-0432.CCR-16-2677
Min YZ, 2017, NAT NANOTECHNOL, V12, P877, DOI [10.1038/nnano.2017.113, 10.1038/NNANO.2017.113]
Nam J, 2019, NAT REV MATER, V4, P398, DOI 10.1038/s41578-019-0108-1
Pardoll DM, 2012, NAT REV CANCER, V12, P252, DOI 10.1038/nrc3239
Rothschilds AM, 2019, TRENDS IMMUNOL, V40, P12, DOI 10.1016/j.it.2018.11.003
Sharma P, 2017, CELL, V168, P707, DOI 10.1016/j.cell.2017.01.017
Spranger S, 2013, SCI TRANSL MED, V5, DOI 10.1126/scitranslmed.3006504
Taube JM, 2012, SCI TRANSL MED, V4, DOI 10.1126/scitranslmed.3003689
Topalian SL, 2015, CANCER CELL, V27, P450, DOI 10.1016/j.ccell.2015.03.001
Tumeh PC, 2014, NATURE, V515, P568, DOI 10.1038/nature13954
Verma V, 2019, NAT IMMUNOL, V20, P1231, DOI 10.1038/s41590-019-0441-y
Wilson Kaley D, 2009, Expert Rev Clin Pharmacol, V2, P181, DOI 10.1586/17512433.2.2.181
Zhan X, 2012, MOL PHARMACEUT, V9, P3442, DOI 10.1021/mp300190g
NR 35
TC 36
Z9 36
U1 4
U2 107
PU WILEY-V C H VERLAG GMBH
PI WEINHEIM
PA POSTFACH 101161, 69451 WEINHEIM, GERMANY
SN 1433-7851
EI 1521-3773
J9 ANGEW CHEM INT EDIT
JI Angew. Chem.-Int. Edit.
PD AUG 17

PY 2020
VL 59
IS 34
BP 14628
EP 14638
DI 10.1002/anie.202006117
EA JUL 2020
PG 11
WC Chemistry, Multidisciplinary
WE Science Citation Index Expanded (SCI-EXPANDED)
SC Chemistry
GA NG4RP
UT WOS:000545713000001
PM 32430981
DA 2023-09-14
ER

PT J
AU Traini, G
Ruiz-de-Angulo, A
Blanco-Canosa, JB
Bascaran, KZ
Molinaro, A
Silipo, A
Escors, D
Mareque-Rivas, JC
AF Traini, Giordano
Ruiz-de-Angulo, Ane
Bautista Blanco-Canosa, Juan
Zamacola Bascaran, Kepa
Molinaro, Antonio
Silipo, Alba
Escors, David
Mareque-Rivas, Juan C.
TI Cancer Immunotherapy of TLR4 Agonist-Antigen Constructs Enhanced with Pathogen-Mimicking Magnetite Nanoparticles and Checkpoint Blockade of PD-L1
SO SMALL
LA English
DT Article
DE checkpoint inhibition; drug delivery; immunotherapy; magnetic nanoparticles; Toll-like receptor 4 agonists; vaccines
ID IRON-OXIDE NANOPARTICLES; TOLL-LIKE RECEPTORS; DENDRITIC CELLS; CELLULAR-IMMUNITY; T-CELLS; VACCINES; DELIVERY; INNATE; LIPOOLIGOSACCHARIDE; LIPOPOLYSACCHARIDES
AB Despite the tremendous potential of Toll-like receptor 4 (TLR4) agonists in vaccines, their efficacy as monotherapy to treat cancer has been limited. Only some lipopolysaccharides (LPS) isolated from particular bacterial strains or structures like monophosphoryl lipid A (MPLA) derived from lipooligosaccharide (LOS), avoid toxic overactivation of innate immune responses while retaining adequate immunogenicity to act as adjuvants. Here, different LOS structures are incorporated into nanoparticle-filled phospholipid micelles for efficient vaccine delivery and more potent cancer immunotherapy. The structurally unique LOS of the plant pathogen Xcc is incorporated into phospholipid micelles encapsulating iron oxide nanoparticles, producing stable pathogen-mimicking nanostructures suitable for targeting antigen presenting cells in the lymph nodes. The antigen is conjugated via a hydrazone bond, enabling rapid, easy-to-monitor and high-yield antigen ligation at low concentrations. The protective effect of these constructs is investigated against a highly aggressive model for tumor immunotherapy. The results show that the nanovaccines lead to a higher-level antigen-specific cytotoxic T lymphocyte (CTL) effector and memory responses, which when combined with abrogation of the immunosuppressive programmed death-ligand 1 (PD-L1), provide 100% long-term protection against repeated tumor challenge. This nanovaccine platform in combination with checkpoint inhibition of PD-L1 represents a promising approach to improve the cancer immunotherapy of TLR4 agonists.

C1 [Traini, Giordano; Ruiz-de-Angulo, Ane; Bautista Blanco-Canosa, Juan; Zamacola Bascaran, Kepa] CIC biomaGUNE, Paseo Miramon 182, San Sebastian 20014, Spain.
[Molinaro, Antonio; Silipo, Alba] Univ Napoli Federico II, Dipartimento Sci Chim, Complesso Univ Monte St Angelo,Via Cintia 4, I-80126 Naples, Italy.
[Escors, David] Complejo Hosp Navarra, Navarrabiomed Biomed Res Ctr, Fdn Miguel Servet IdISNA, Pamplona 31008, Spain.
[Mareque-Rivas, Juan C.] Swansea Univ, Dept Chem, Singleton Pk, Swansea SA2 8PP, W Glam, Wales.
[Mareque-Rivas, Juan C.] Swansea Univ, Ctr NanoHlth, Singleton Pk, Swansea SA2 8PP, W Glam, Wales.
C3 CIC biomaGUNE; University of Naples Federico II; Navarrabiomed; Servicio Navarro de Salud - Osasunbidea; Swansea University; Swansea University
RP Mareque-Rivas, JC (通讯作者)，Swansea Univ, Dept Chem, Singleton Pk, Swansea SA2 8PP, W Glam, Wales.; Mareque-Rivas, JC (通讯作者)，Swansea Univ, Ctr NanoHlth, Singleton Pk, Swansea SA2 8PP, W Glam, Wales.
EM juan.mareque-rivas@swansea.ac.uk
RI de Angulo, Ane Ruiz/I-3867-2016; Silipo, Alba/L-5929-2014; Escors, David/C-1549-2008; Rivas, Juan Mareque/M-6171-2014; Blanco-Canosa, Juan B./H-4544-2015
OI de Angulo, Ane Ruiz/0000-0001-6581-4038; Escors, David/0000-0003-2828-4458; Rivas, Juan Mareque/0000-0002-7405-3338; Blanco-Canosa, Juan B./0000-0001-5738-6993
FU H2020-MSCA-ITN-2014-ETN TOLLerant [642157]; M-ERA.NET-2015 MediSURF [3193]; Spanish Ministry of Economy and Competitiveness [CTQ2014-54761-R, PCIN-2016-085]
FX This work was supported by H2020-MSCA-ITN-2014-ETN TOLLerant (grant agreement 642157), M-ERA.NET-2015 MediSURF (Reference Number: project 3193), and by the Spanish Ministry of Economy and Competitiveness Grant CTQ2014-54761-R and PCIN-2016-085. The authors are grateful to Dr. Pablo Sarobe (Centre for Applied Medical Research, CIMA, Universidad de Navarra, Pamplona, Spain) for providing the B16-F10(OVA) cells.
CR Adams PG, 2014, BIOPHYS J, V106, P2395, DOI 10.1016/j.bpj.2014.04.016
Ahsan FL, 2002, J CONTROL RELEASE, V79, P29, DOI 10.1016/S0168-3659(01)00549-1
Akira S, 2001, NAT IMMUNOL, V2, P675, DOI 10.1038/90609
Akira S, 2004, NAT REV IMMUNOL, V4, P499, DOI 10.1038/nri1391
Algar WR, 2011, BIOCONJUGATE CHEM, V22, P825, DOI 10.1021/bc200065z
Almeida JPM, 2015, SMALL, V11, P1453, DOI 10.1002/smll.201402179
Anselmo AC, 2015, AAPS J, V17, P1041, DOI 10.1208/s12248-015-9780-2
Baxevanis CN, 2013, IMMUNOTHERAPY-UK, V5, P497, DOI [10.2217/IMT.13.24, 10.2217/imt.13.24]
Blanco-Canosa JB, 2010, J AM CHEM SOC, V132, P10027, DOI 10.1021/ja910988d
Gondan AIB, 2018, BIOMATERIALS, V170, P95, DOI 10.1016/j.biomaterials.2018.04.003
Butler NS, 2011, CELL MICROBIOL, V13, P925, DOI 10.1111/j.1462-5822.2011.01594.x
Champiat S, 2017, CLIN CANCER RES, V23, P1920, DOI 10.1158/1078-0432.CCR-16-1741
Chang CC, 2004, CURR OPIN IMMUNOL, V16, P644, DOI 10.1016/j.coi.2004.07.015
Cho NH, 2011, NAT NANOTECHNOL, V6, P675, DOI [10.1038/NNANO.2011.149, 10.1038/nnano.2011.149]
Cho YJ, 2006, VACCINE, V24, P5862, DOI 10.1016/j.vaccine.2006.04.048
Cluff CW, 2009, ADV EXP MED BIOL, V667, P111, DOI 10.1007/978-1-4419-1603-7_10
Cobaleda-Siles M, 2014, SMALL, V10, P5054, DOI 10.1002/smll.201401353
de Titta A, 2013, P NATL ACAD SCI USA, V110, P19902, DOI 10.1073/pnas.1313152110
Di Lorenzo F, 2016, CARBOHYD RES, V427, P38, DOI 10.1016/j.carres.2016.03.026
Didierlaurent AM, 2009, J IMMUNOL, V183, P6186, DOI 10.4049/jimmunol.0901474
Dowling JK, 2016, CLIN TRANSL IMMUNOL, V5, DOI 10.1038/cti.2016.22
Francisco LM, 2009, J EXP MED, V206, P3015, DOI 10.1084/jem.20090847
Friedman CF, 2016, JAMA ONCOL, V2, P1346, DOI 10.1001/jamaoncol.2016.1051
Fukaya T, 2010, BLOOD, V116, P2266, DOI 10.1182/blood-2009-10-250472
Gato-Canas M, 2017, CELL REP, V20, P1818, DOI 10.1016/j.celrep.2017.07.075
Giannini SL, 2006, VACCINE, V24, P5937, DOI 10.1016/j.vaccine.2006.06.005
Gregory AE, 2012, VACCINE, V30, P6777, DOI 10.1016/j.vaccine.2012.09.021
Husebye H, 2006, EMBO J, V25, P683, DOI 10.1038/sj.emboj.7600991
Jenkins RW, 2018, BRIT J CANCER, V118, P9, DOI 10.1038/bjc.2017.434
Kaczanowska S, 2013, J LEUKOCYTE BIOL, V93, P847, DOI 10.1189/jlb.1012501

Karaki S, 2016, VACCINES-BASEL, V4, DOI 10.3390/vaccines4040037
Kato S, 2017, CLIN CANCER RES, V23, P4242, DOI 10.1158/1078-0432.CCR-16-3133
Lau J, 2017, NAT COMMUN, V8, DOI 10.1038/ncomms14572
Lee IH, 2012, ANGEW CHEM INT EDIT, V51, P8800, DOI 10.1002/anie.201203193
Lee N, 2012, NANO LETT, V12, P3127, DOI 10.1021/nl3010308
Li HY, 2011, NAT NANOTECHNOL, V6, P645, DOI [10.1038/nnano.2011.153, 10.1038/NNANO.2011.153]
Liu HP, 2015, BIOCONJUGATE CHEM, V26, P791, DOI 10.1021/acs.bioconjchem.5b00103
Liu HP, 2014, NATURE, V507, P519, DOI 10.1038/nature12978
Lu HL, 2014, FRONT IMMUNOL, V5, DOI 10.3389/fimmu.2014.00083
Lutz MB, 1999, J IMMUNOL METHODS, V223, P77, DOI 10.1016/S0022-1759(98)00204-X
MacLeod MKL, 2011, P NATL ACAD SCI USA, V108, P7914, DOI 10.1073/pnas.1104588108
Maisonneuve C, 2014, P NATL ACAD SCI USA, V111, P12294, DOI 10.1073/pnas.1400478111
Mastelic Beatris, 2010, Biologicals, V38, P594, DOI 10.1016/j.biologicals.2010.06.002
Moon JJ, 2011, NAT MATER, V10, P243, DOI 10.1038/NMAT2960
Mueller M, 2004, J BIOL CHEM, V279, P26307, DOI 10.1074/jbc.M401231200
Nembrini C, 2011, P NATL ACAD SCI USA, V108, pE989, DOI 10.1073/pnas.1104264108
Pardoll DM, 2012, NAT REV CANCER, V12, P252, DOI 10.1038/nrc3239
Paulos CM, 2007, CLIN CANCER RES, V13, P5280, DOI 10.1158/1078-0432.CCR-07-1378
Postow MA, 2015, J CLIN ONCOL, V33, P1974, DOI 10.1200/JCO.2014.59.4358
Pulko V, 2009, J IMMUNOL, V183, P3634, DOI 10.4049/jimmunol.0900974
Rao DA, 2010, J PHARM SCI-US, V99, P2018, DOI 10.1002/jps.21970
Reddy ST, 2007, NAT BIOTECHNOL, V25, P1159, DOI [10.1038/nbt1332, 10.1038/nbtl332]
Revia RA, 2016, MATER TODAY, V19, P157, DOI 10.1016/j.mattod.2015.08.022
Rice A, 2018, BIOPHYS J, V114, P1389, DOI 10.1016/j.bpj.2018.02.006
Rosen JE, 2012, NANOMED-NANOTECHNOL, V8, P275, DOI 10.1016/j.nano.2011.08.017
Rumenapp C, 2012, PHARM RES-DORDR, V29, P1165, DOI 10.1007/s11095-012-0711-y
Ruiz-de-Angulo A, 2016, ACS NANO, V10, P1602, DOI 10.1021/acsnano.5b07253
Sallusto F, 1999, NATURE, V401, P708, DOI 10.1038/44385
Shields BD, 2017, SCI REP-UK, V7, DOI 10.1038/s41598-017-01000-2
Shouheng S, 2004, J AM CHEM SOC, V126, P273
Silipo A, 2005, J BIOL CHEM, V280, P33660, DOI 10.1074/jbc.M506254200
Silipo A, 2004, EUR J ORG CHEM, V2004, P1336, DOI 10.1002/ejoc.200300721
Smulders S, 2012, PART FIBRE TOXICOL, V9, DOI 10.1186/1743-8977-9-41
Stephen ZR, 2011, MATER TODAY, V14, P330, DOI 10.1016/S1369-7021(11)70163-8
TABATA Y, 1988, BIOMATERIALS, V9, P356, DOI 10.1016/0142-9612(88)90033-6
Varthaman A, 2016, PLOS ONE, V11, DOI 10.1371/journal.pone.0167057
Wilson JT, 2013, ACS NANO, V7, P3912, DOI 10.1021/nn305466z
Xiang J, 2015, ACS NANO, V9, P6401, DOI 10.1021/acsnano.5b02014
Yan SY, 2014, BIOMATERIALS, V35, P9508, DOI 10.1016/j.biomaterials.2014.07.055
Yu WW, 2003, CHEM MATER, V15, P2854, DOI 10.1021/cm034081k
Zanganeh S, 2016, NAT NANOTECHNOL, V11, P986, DOI [10.1038/nnano.2016.168, 10.1038/NNANO.2016.168]
NR 71
TC 36
Z9 38
U1 4
U2 79
PU WILEY-V C H VERLAG GMBH
PI WEINHEIM
PA POSTFACH 101161, 69451 WEINHEIM, GERMANY
SN 1613-6810
EI 1613-6829
J9 SMALL
JI Small
PD JAN
PY 2019
VL 15
IS 4
AR 1803993
DI 10.1002/smll.201803993

PG 16
WC Chemistry, Multidisciplinary; Chemistry, Physical; Nanoscience &
   Nanotechnology; Materials Science, Multidisciplinary; Physics, Applied;
   Physics, Condensed Matter
WE Science Citation Index Expanded (SCI-EXPANDED)
SC Chemistry; Science & Technology - Other Topics; Materials Science;
   Physics
GA HJ0KR
UT WOS:000456849600006
PM 30569516
OA hybrid, Green Published
DA 2023-09-14
ER

PT J
AU Hu, HZ
   Yang, C
   Zhang, F
   Li, MQ
   Tu, ZX
   Mu, LZ
   Dawulieti, J
   Lao, YH
   Xiao, ZX
   Yan, HZ
   Sun, W
   Shao, D
   Leong, KW
AF Hu, Hanze
   Yang, Chao
   Zhang, Fan
   Li, Mingqiang
   Tu, Zhaoxu
   Mu, Lizhong
   Dawulieti, Jianati
   Lao, Yeh-Hsing
   Xiao, Zixuan
   Yan, Huize
   Sun, Wen
   Shao, Dan
   Leong, Kam W.
TI A Versatile and Robust Platform for the Scalable Manufacture of
   Biomimetic Nanovaccines
SO ADVANCED SCIENCE
LA English
DT Article
DE biomimetics; cancer vaccine; cell membrane; flash nanocomplexation;
   mesoporous silica nanoparticles
AB Biomimetic strategies are useful for designing potent vaccines. Decorating a nanoparticulate adjuvant with cell membrane fragments as the antigen-presenting source exemplifies, such as a promising strategy. For translation, a standardizable, consistent, and scalable approach for coating nanoadjuvant with the cell membrane is important. Here a turbulent mixing and self-assembly method called flash nanocomplexation (FNC) for producing cell membrane-coated nanovaccines in a scalable manner is demonstrated. The broad applicability of this FNC technique compared with bulk-sonication by using ten different core materials and multiple cell membrane types is shown. FNC-produced biomimetic nanoparticles have promising colloidal stability and narrow particle polydispersity, indicating an equal or more homogeneous coating compared to the bulk-sonication method. The potency of a nanovaccine comprised of B16-F10 cancer cell membrane decorating mesoporous silica nanoparticles loaded with the adjuvant CpG is then demonstrated. The FNC-fabricated nanovaccines when combined with anti-CTLA-4 show potency in lymph node targeting, DC antigen presentation, and T cell immune activation, leading to prophylactic and therapeutic efficacy in a melanoma mouse model. This study advances the design of a

biomimetic nanovaccine enabled by a robust and versatile nanomanufacturing technique.
C1 [Hu, Hanze; Yang, Chao; Tu, Zhaoxu; Lao, Yeh-Hsing; Xiao, Zixuan; Yan, Huize; Leong, Kam W.] Columbia Univ, Dept Biomed Engn, New York, NY 10027 USA.
[Yang, Chao; Zhang, Fan; Tu, Zhaoxu; Dawulieti, Jianati; Shao, Dan] South China Univ Technol, Sch Biomed Sci & Engn, Inst Life Sci, Guangzhou Int Campus, Guangzhou 510630, Guangdong, Peoples R China.
[Yang, Chao; Dawulieti, Jianati; Shao, Dan] South China Univ Technol, Key Lab Biomed Engn Guangdong Prov, Natl Engn Res Ctr Tissue Restorat & Reconstruct, Guangzhou 510006, Peoples R China.
[Li, Mingqiang] Sun Yat Sen Univ, Affiliated Hosp 3, Lab Biomat & Translat Med, Guangzhou 510006, Guangdong, Peoples R China.
[Mu, Lizhong] Dalian Univ Technol, Sch Energy & Power Engn, Dalian 116024, Liaoning, Peoples R China.
[Sun, Wen] Dalian Univ Technol, State Key Lab Fine Chem, Dalian 116024, Liaoning, Peoples R China.
[Leong, Kam W.] Columbia Univ, Dept Syst Biol, New York, NY 10032 USA.
C3 Columbia University; South China University of Technology; South China University of Technology; Sun Yat Sen University; Dalian University of Technology; Dalian University of Technology; Columbia University
RP Leong, KW (通讯作者)，Columbia Univ, Dept Biomed Engn, New York, NY 10027 USA.; Shao, D (通讯作者)，South China Univ Technol, Sch Biomed Sci & Engn, Inst Life Sci, Guangzhou Int Campus, Guangzhou 510630, Guangdong, Peoples R China.; Shao, D (通讯作者)，South China Univ Technol, Key Lab Biomed Engn Guangdong Prov, Natl Engn Res Ctr Tissue Restorat & Reconstruct, Guangzhou 510006, Peoples R China.; Leong, KW (通讯作者)，Columbia Univ, Dept Syst Biol, New York, NY 10032 USA.
EM stanauagate@outlook.com; kam.leong@columbia.edu
RI Leong, Kam W/O-9302-2019; Li, Mingqiang/L-1182-2019; Hu, Hanze/AED-0011-2022
OI Leong, Kam W/0000-0002-8133-4955; Li, Mingqiang/0000-0002-5178-4138; Xiao, Zixuan/0009-0005-2107-2067
FU NIH [UG3NS115598, AR073935]; DARPA [HR001118S0037-PA-008]
FX H.H. and C.Y. contributed equally to this work. The authors thank Xing Meng, Kunbao Zhang, Naoto Yoshinaga, Yumei Chen, and Sydney Shannon for scientific discussion and preparation of the manuscript. Support by NIH (Nos. UG3NS115598 and AR073935) and DARPA HR001118S0037-PA-008 is acknowledged.
CR Akbulut M, 2009, ADV FUNCT MATER, V19, P718, DOI 10.1002/adfm.200801583
Bang KH, 2019, CANCERS, V11, DOI 10.3390/cancers11060807
Chai ZL, 2019, ACS NANO, V13, P5591, DOI 10.1021/acsnano.9b00661
Chen Z, 2016, ACS NANO, V10, P10049, DOI 10.1021/acsnano.6b04695
Copp JA, 2014, P NATL ACAD SCI USA, V111, P13481, DOI 10.1073/pnas.1412420111
Dawulieti J, 2020, SCI ADV, V6, DOI 10.1126/sciadv.aay7148
Fang RH, 2018, ADV MATER, V30, DOI 10.1002/adma.201706759
Fang RH, 2017, BIOMATERIALS, V128, P69, DOI 10.1016/j.biomaterials.2017.02.041
Feng J, 2019, J TRANSL MED, V17, DOI 10.1186/s12967-019-1945-9
Finn OJ, 2003, NAT REV IMMUNOL, V3, P630, DOI 10.1038/nri1150
Gao WW, 2015, NANO LETT, V15, P1403, DOI 10.1021/nl504798g
Guo YY, 2015, ACS NANO, V9, P6918, DOI 10.1021/acsnano.5b01042
He W., 2019, ADV DRUG DELIVER REV, V165, P1540
He ZY, 2018, ACTA BIOMATER, V81, P195, DOI 10.1016/j.actbio.2018.09.047
He ZY, 2018, NANOSCALE, V10, P3307, DOI 10.1039/c7nr08047f
He ZY, 2017, BIOMATERIALS, V130, P28, DOI 10.1016/j.biomaterials.2017.03.028
Howard GP, 2019, NANO RES, V12, P837, DOI 10.1007/s12274-019-2301-3
Hu CMJ, 2011, P NATL ACAD SCI USA, V108, P10980, DOI 10.1073/pnas.1106634108
Hu HZ, 2021, MATER TODAY, V42, P99, DOI 10.1016/j.mattod.2020.08.019
Hu YZ, 2019, ACS NANO, V13, P10161, DOI 10.1021/acsnano.9b03334
Duong HTT, 2018, BIOMATERIALS, V185, P13, DOI 10.1016/j.biomaterials.2018.09.008
Kim B, 2019, ADV MATER, V31, DOI 10.1002/adma.201902952
Kroll AV, 2017, ADV MATER, V29, DOI 10.1002/adma.201703969
Kumar V, 2010, SMALL, V6, P2907, DOI 10.1002/smll.201001199
Li RX, 2018, ACTA PHARM SIN B, V8, P14, DOI 10.1016/j.apsb.2017.11.009
Li SY, 2017, BIOMATERIALS, V142, P149, DOI 10.1016/j.biomaterials.2017.07.026
Liu C, 2019, NANO LETT, V19, P7836, DOI 10.1021/acs.nanolett.9b02841
Liu Y, 2008, CHEM ENG SCI, V63, P2829, DOI 10.1016/j.ces.2007.10.020

Liu ZP, 2015, IND ENG CHEM RES, V54, P4512, DOI 10.1021/ie5041836
Luk BT, 2014, NANOSCALE, V6, P2730, DOI 10.1039/c3nr06371b
Ma PX, 2008, ADV DRUG DELIVER REV, V60, P184, DOI 10.1016/j.addr.2007.08.041
Markwalter CE, 2018, J PHARM SCI-US, V107, P2465, DOI 10.1016/j.xphs.2018.05.003
Mellman I, 2011, NATURE, V480, P480, DOI 10.1038/nature10673
Nie TQ, 2019, ACS APPL MATER INTER, V11, P29593, DOI 10.1021/acsami.9b10294
Parodi A, 2013, NAT NANOTECHNOL, V8, P61, DOI 10.1038/nnano.2012.212
Rao L, 2017, ACS NANO, V11, P3496, DOI 10.1021/acsnano.7b00133
Santos JL, 2016, SMALL, V12, P6214, DOI 10.1002/smll.201601425
Sevencan C, 2020, ADV THER-GERMANY, V3, DOI 10.1002/adtp.201900201
Shao D, 2020, ADV MATER, V32, DOI 10.1002/adma.202004385
Shao D, 2018, ADV MATER, V30, DOI 10.1002/adma.201801198
Shen H, 2011, J NANOPART RES, V13, P4109, DOI 10.1007/s11051-011-0354-7
Silva AKA, 2013, NANOSCALE, V5, P11374, DOI 10.1039/c3nr01541f
Tian HK, 2018, ADV HEALTHC MATER, V7, DOI 10.1002/adhm.201800285
Tseng Y.-H., 2019, CHEM ENG SCI X, V1, P100002
Wang Z, 2019, ADV SCI, V6, DOI 10.1002/advs.201901690
Xu CH, 2020, ACTA BIOMATER, V105, P1, DOI 10.1016/j.actbio.2020.01.036
Yan HZ, 2019, ADV SCI, V6, DOI 10.1002/advs.201900605
Zeng ZP, 2018, ACS APPL MATER INTER, V10, P24969, DOI 10.1021/acsami.8b07024
Zhang SL, 2009, ADV MATER, V21, P419, DOI 10.1002/adma.200801393
Zhou X, 2017, NANO LETT, V17, P1056, DOI 10.1021/acs.nanolett.6b04523
Zhu JY, 2016, NANO LETT, V16, P5895, DOI 10.1021/acs.nanolett.6b02786
NR 51
TC 35
Z9 35
U1 13
U2 88
PU WILEY
PI HOBOKEN
PA 111 RIVER ST, HOBOKEN 07030-5774, NJ USA
EI 2198-3844
J9 ADV SCI
JI Adv. Sci.
PD AUG
PY 2021
VL 8
IS 15
AR 2002020
DI 10.1002/advs.202002020
EA MAY 2021
PG 8
WC Chemistry, Multidisciplinary; Nanoscience & Nanotechnology; Materials
Science, Multidisciplinary
WE Science Citation Index Expanded (SCI-EXPANDED)
SC Chemistry; Science & Technology - Other Topics; Materials Science
GA TU3VQ
UT WOS:000645932300001
PM 34386315
OA Green Published, gold
DA 2023-09-14
ER

PT J
AU Du, GS
Sun, X
AF Du, Guangsheng
Sun, Xun
TI Engineering nanoparticulate vaccines for enhancing antigen
cross-presentation
SO CURRENT OPINION IN BIOTECHNOLOGY

LA English
DT Review
ID POLYMERIC HYBRID MICELLES; HUMAN DENDRITIC CELLS; EXOGENOUS ANTIGENS;
ANTITUMOR IMMUNITY; PARTICLE-SIZE; DELIVERY; INDUCTION; PATHWAY; SYSTEM;
POTENCY
AB Efficient cross-presentation is pivotal for vaccination against cancer and infection by intracellular virus and bacteria. Recently, various types of nanoparticle vaccines have been developed and investigated for efficiently and specifically improving cross-presentation and CD8(+) T cell priming. In this review, we will summarize the known intracellular pathways involved in cross-presentation, and focus on several nanoparticle strategies that have been reported for enhancing cross-presentation, including designing multifunctional nano-vaccines for increasing endosomal escape, designing nano-vaccines that can target lymph nodes to improve antigen uptake by lymph node resident CD8 alpha(+) dendritic cells, and co-delivering immune modulators for upregulating cross-presentation related intracellular components. We will also briefly discuss the future prospects of cross-presentation based nano-vaccine strategy for curing diseases.
C1 [Du, Guangsheng; Sun, Xun] Sichuan Univ, West China Sch Pharm, Sichuan Engn Lab Plant Sourced Drug,Educ Minist, Key Lab Drug Targeting & Drug Delivery Syst, Chengdu 610064, Peoples R China.
[Du, Guangsheng; Sun, Xun] Sichuan Univ, West China Sch Pharm, Sichuan Res Ctr Drug Precis Ind Technol, Chengdu 610064, Peoples R China.
C3 Sichuan University; Sichuan University
RP Sun, X (通讯作者)，Sichuan Univ, West China Sch Pharm, Sichuan Engn Lab Plant Sourced Drug,Educ Minist, Key Lab Drug Targeting & Drug Delivery Syst, Chengdu 610064, Peoples R China.
EM sunxun@scu.edu.cn
OI Du, Guangsheng/0000-0003-4401-4845
FU National Natural Science Foundation of China [81925036, 81961130395];
Science and Technology Major Project of Sichuan Province
[2018SZDZX0018]; Fundamental Research Funds for the Central Universities
FX We acknowledge the financial support of the National Natural Science
Foundation of China (No. 81925036 & 81961130395), Science and Technology
Major Project of Sichuan Province (No. 2018SZDZX0018) and the
Fundamental Research Funds for the Central Universities.
CR Alloatti A, 2015, IMMUNITY, V43, P1087, DOI 10.1016/j.immuni.2015.11.006
Asadzadeh Z, 2020, CANCERS, V12, DOI 10.3390/cancers12041047
Asano J, 2010, J IMMUNOL, V184, P736, DOI 10.4049/jimmunol.0900726
Benjaminsen RV, 2013, MOL THER, V21, P149, DOI 10.1038/mt.2012.185
BEVAN MJ, 1976, J EXP MED, V143, P1283, DOI 10.1084/jem.143.5.1283
Blander JM, 2016, IMMUNOL REV, V272, P65, DOI 10.1111/imr.12428
Chen JA, 2011, INT J NANOMED, V6, P77, DOI 10.2147/IJN.S15457
Cruz FM, 2017, ANNU REV IMMUNOL, V35, P149, DOI 10.1146/annurev-immunol-041015-055254
Dong H, 2018, INT J NANOMED, V13, P3353, DOI 10.2147/IJN.S164097
Du GS, 2020, WOODH PUBL SER BIOM, P527, DOI 10.1016/B978-0-08-102983-1.00019-3
FALO LD, 1995, NAT MED, V1, P649, DOI 10.1038/nm0795-649
Ferrari R, 2018, NANOSCALE, V10, P22701, DOI 10.1039/c8nr05933k
Foged C, 2005, INT J PHARMACEUT, V298, P315, DOI 10.1016/j.ijpharm.2005.03.035
Germain RN, 2004, CURR OPIN IMMUNOL, V16, P120, DOI 10.1016/j.coi.2003.11.001
Graham BS, 2018, NAT IMMUNOL, V19, P20, DOI 10.1038/s41590-017-0007-9
Gros M, 2019, FRONT IMMUNOL, V10, DOI 10.3389/fimmu.2019.00041
Guo YY, 2015, ACS NANO, V9, P6918, DOI 10.1021/acsnano.5b01042
Haniffa M, 2012, IMMUNITY, V37, P60, DOI 10.1016/j.immuni.2012.04.012
He CB, 2010, BIOMATERIALS, V31, P3657, DOI 10.1016/j.biomaterials.2010.01.065
Hoffmann E, 2012, P NATL ACAD SCI USA, V109, P14556, DOI 10.1073/pnas.1203912109
Howard GP, 2019, NANO RES, V12, P837, DOI 10.1007/s12274-019-2301-3
HUANG AYC, 1994, SCIENCE, V264, P961, DOI 10.1126/science.7513904
Irvine DJ, 2013, NAT MATER, V12, P978, DOI [10.1038/NMAT3775, 10.1038/nmat3775]
Ji Y, 2018, J MATER CHEM B, V6, P1930, DOI [10.1039/c7tb03233a, 10.1039/c7tb03319b]
Jiang H, 2018, ADV SCI, V5, DOI 10.1002/advs.201700426
Kim CG, 2019, PHARMACEUTICS, V11, DOI 10.3390/pharmaceutics11110612
KOVACSOVICSBANKOWSKI M, 1995, SCIENCE, V267, P243, DOI 10.1126/science.7809629
Li CX, 2018, ACS APPL MATER INTER, V10, P2874, DOI 10.1021/acsami.7b14004
Li HM, 2017, THERANOSTICS, V7, P4383, DOI 10.7150/thno.20745
Ma WB, 2016, J IMMUNOL, V196, P1711, DOI 10.4049/jimmunol.1501574

Maisel K, 2017, ADV DRUG DELIVER REV, V114, P43, DOI 10.1016/j.addr.2017.07.005
Mant A, 2012, IMMUNOLOGY, V136, P163, DOI 10.1111/j.1365-2567.2012.03558.x
Miyazaki M, 2019, ACS BIOMATER SCI ENG, V5, P5790, DOI 10.1021/acsbiomaterials.9b01278
Montealegre S, 2019, FRONT IMMUNOL, V9, DOI 10.3389/fimmu.2018.03098
Mukai Y, 2011, J IMMUNOL, V187, P6249, DOI 10.4049/jimmunol.1001093
Nair-Gupta P, 2014, CELL, V158, P506, DOI 10.1016/j.cell.2014.04.054
Pauwels AM, 2017, TRENDS IMMUNOL, V38, P407, DOI 10.1016/j.it.2017.03.006
ROCK KL, 1990, SCIENCE, V249, P918, DOI 10.1126/science.2392683
Selbo PK, 2010, J CONTROL RELEASE, V148, P2, DOI 10.1016/j.jconrel.2010.06.008
Shen H, 2006, IMMUNOLOGY, V117, P78, DOI 10.1111/j.1365-2567.2005.02268.x
Shreffler JW, 2019, INT J MOL SCI, V20, DOI 10.3390/ijms20236056
Sigal LJ, 1999, NATURE, V398, P77, DOI 10.1038/18038
Swartz MA, 2001, ADV DRUG DELIVER REV, V50, P3, DOI 10.1016/S0169-409X(01)00150-8
Tiwari N, 2007, J IMMUNOL, V178, P7932, DOI 10.4049/jimmunol.178.12.7932
Trevaskis NL, 2015, NAT REV DRUG DISCOV, V14, P781, DOI 10.1038/nrd4608
Tumeh PC, 2014, NATURE, V515, P568, DOI 10.1038/nature13954
Wang HJ, 2020, BIOMATERIALS, V244, DOI 10.1016/j.biomaterials.2020.119964
Wang T, 2015, ACS APPL MATER INTER, V7, P6391, DOI 10.1021/acsami.5b00348
Warrier VU, 2019, BIOSCIENCE REP, V39, DOI 10.1042/BSR20193220
Xia Y, 2018, CELL, V175, P1059, DOI 10.1016/j.cell.2018.08.070
Xu J, 2019, BIOMATERIALS, V207, P1, DOI 10.1016/j.biomaterials.2019.03.037
Xu ZH, 2013, J CONTROL RELEASE, V172, P259, DOI 10.1016/j.jconrel.2013.08.021
Yoshizaki Y, 2017, BIOMATERIALS, V141, P272, DOI 10.1016/j.biomaterials.2017.07.001
Yuba E, 2017, BIOMATERIALS, V120, P32, DOI 10.1016/j.biomaterials.2016.12.021
Yuba E, 2013, BIOMATERIALS, V34, P3042, DOI 10.1016/j.biomaterials.2012.12.031
Zeng Q, 2017, BIOMATERIALS, V122, P105, DOI 10.1016/j.biomaterials.2017.01.010
Zhang CN, 2017, J CONTROL RELEASE, V256, P170, DOI 10.1016/j.jconrel.2017.04.020
Zhang LH, 2019, NANO LETT, V19, P4237, DOI 10.1021/acs.nanolett.9b00030
Zhang R, 2018, J CONTROL RELEASE, V292, P256, DOI 10.1016/j.jconrel.2018.10.008
Zhang S, 2019, CARBOHYD POLYM, V213, P100, DOI 10.1016/j.carbpol.2019.02.093
Zhao H, 2019, ACS NANO, V13, P13127, DOI 10.1021/acsnano.9b05974
Zhong XF, 2019, J CONTROL RELEASE, V300, P81, DOI 10.1016/j.jconrel.2019.02.035
Zhuang XT, 2016, J CONTROL RELEASE, V228, P26, DOI 10.1016/j.jconrel.2016.02.035
NR 63
TC 35
Z9 35
U1 7
U2 72
PU ELSEVIER SCI LTD
PI OXFORD
PA THE BOULEVARD, LANGFORD LANE, KIDLINGTON, OXFORD OX5 1GB, OXON, ENGLAND
SN 0958-1669
EI 1879-0429
J9 CURR OPIN BIOTECH
JI Curr. Opin. Biotechnol.
PD DEC
PY 2020
VL 66
BP 113
EP 122
DI 10.1016/j.copbio.2020.06.015
PG 10
WC Biochemical Research Methods; Biotechnology & Applied Microbiology
WE Science Citation Index Expanded (SCI-EXPANDED)
SC Biochemistry & Molecular Biology; Biotechnology & Applied Microbiology
GA PI3NG
UT WOS:000601001000015
PM 32745889
DA 2023-09-14
ER

PT J
AU Huang, WW
Shu, CY
Hua, LQ
Zhao, YL
Xie, HH
Qi, JL
Gao, FL
Gao, RY
Chen, YJ
Zhang, QS
Li, WR
Yuan, MC
Ye, C
Ma, YB
AF Huang, Weiwei
Shu, Congyan
Hua, Liangqun
Zhao, Yilin
Xie, Hanghang
Qi, Jialong
Gao, Fulan
Gao, Ruiyu
Chen, Yongjun
Zhang, Qishu
Li, Weiran
Yuan, Mingcui
Ye, Chao
Ma, Yanbing
TI Modified bacterial outer membrane vesicles induce autoantibodies for
tumor therapy
SO ACTA BIOMATERIALIA
LA English
DT Article
DE Outer membrane vesicle; Biomembrane; Antiangiogenesis; Basic fibroblast
growth factor; Autoantibody; Tumor immunotherapy
ID FIBROBLAST-GROWTH-FACTOR; MOUSE MODEL; MYELOID-LEUKEMIA; ANGIOGENESIS;
ACTIVATION; CELLS; PROLIFERATION; RESISTANCE; INHIBITOR; MIGRATION
AB Using monoclonal antibodies to block tumor angiogenesis has yielded effective antitumor effects. How-ever, this treatment method has long cycles and is very expensive; therefore, its long-term and extensive application is limited. In this study, we developed a nanovaccine using bacterial biomembranes as carriers for antitumor therapy. The whole basic fibroblast growth factor (BFGF) molecule (154 amino acids (aa)) was loaded onto bacterial outer membrane vesicles (OMVs) using gene recombination technology. The strong adjuvant effect of OMVs was used to induce the host to produce anti-BFGF autoantibodies. We proved that persistent anti-BFGF autoantibodies can be induced in mice after only 3 immunizations to antagonize BFGF functions. The effects included multiple tumor suppression functions, including inhibition of tumor angiogenesis, induction of tumor cell apoptosis, reversal of tumor immune barriers, and promotion of tumor-specific cytotoxic T lymphocytes (CTLs), eventually causing tumor regression. We confirmed that bacterial biomembranes can be used as a vaccine delivery system to induce the production of antibodies against autoantigens, which may be used for tumor therapy. This study expands the application fields of bacterial biomembrane systems and provides insight for tumor immunotherapy other than monoclonal antibody technology.
Statement of Significance
In this study, we proved that bacteria-released outer membrane vesicles (OMVs) modified via genetic engineering can be used as a vaccine carrier to break autoimmune tolerance and induce the body to produce autoantibodies to antagonize pathological molecules and block pathological signaling pathways for tumor therapy. OMVs naturally released by bacteria were used to successfully load the full-length BFGF protein (154 aa). We proved that persistent anti-BFGF autoantibodies can be induced in tumor-bearing mice after only 3 immunizations to effectively inhibit tumors. Furthermore, the production of these antibodies successfully inhibited tumor angiogenesis, promoted tumor cell apoptosis, reversed the tumor immunosuppressive microenvironment, increased the cytotoxic T lymphocyte (CTL) reaction, and

eventually inhibited tumor growth. (C) 2020 Acta Materialia Inc. Published by Elsevier Ltd. All rights reserved.
C1 [Huang, Weiwei; Hua, Liangqun; Xie, Hanghang; Qi, Jialong; Gao, Fulan; Gao, Ruiyu; Chen, Yongjun; Zhang, Qishu; Li, Weiran; Yuan, Mingcui; Ye, Chao; Ma, Yanbing] Chinese Acad Med Sci & Peking Union Med Coll, Inst Med Biol, Lab Mol Immunol, Kunming, Yunnan, Peoples R China.
[Shu, Congyan] Sichuan Inst Food & Drug Control, Chengdu, Peoples R China.
[Hua, Liangqun] Yunnan Univ, Kunming, Yunnan, Peoples R China.
[Zhao, Yilin] Sichuan Univ, West China Univ Hosp 2, State Key Lab Biotherapy, Chengdu, Peoples R China.
[Zhao, Yilin] Collaborat Innovat Ctr Biotherapy, Chengdu, Peoples R China.
C3 Chinese Academy of Medical Sciences - Peking Union Medical College;
Peking Union Medical College; Institute of Medical Biology - CAMS;
Yunnan University; Sichuan University
RP Ma, YB (通讯作者)，Chinese Acad Med Sci & Peking Union Med Coll, Inst Med Biol, Lab Mol Immunol, Kunming, Yunnan, Peoples R China.
EM yanbingma1969@126.com
RI Li, Weiran/HZJ-3346-2023
OI jialong, qi/0000-0001-6361-7479
FU CAMS Initiative for Innovative Medicine [2016-I2M-1-019]; Science and Technology Project of Yunnan Province [2019FB045, 2016FA049]; Fundamental Research Funds for the Central Universities; PUMC Youth Fund [3332020063]; National Natural Science Foundation of China [81773270]; Yunnan High-level Health and Technical Personnel Project [H-2018069]
FX This research was financially supported by the CAMS Initiative for Innovative Medicine (2016-I2M-1-019), the Science and Technology Project of Yunnan Province (2019FB045 and 2016FA049), the Fundamental Research Funds for the Central Universities and PUMC Youth Fund (3332020063), the National Natural Science Foundation of China (81773270), and Yunnan High-level Health and Technical Personnel Project (H-2018069).
CR Ahrends T, 2017, IMMUNITY, V47, P848, DOI 10.1016/j.immuni.2017.10.009
Akl MR, 2016, ONCOTARGET, V7, P44735, DOI 10.18632/oncotarget.8203
Amano A, 2010, MICROBES INFECT, V12, P791, DOI 10.1016/j.micinf.2010.05.008
[Anonymous], J CONTROL RELEASE
Arunkumar P, 2019, NANOMATERIALS-BASEL, V9, DOI 10.3390/nano9071037
Bachmann MF, 2010, NAT REV IMMUNOL, V10, P787, DOI 10.1038/nri2868
BAIRD A, 1988, P NATL ACAD SCI USA, V85, P2324, DOI 10.1073/pnas.85.7.2324
Beveridge TJ, 1999, J BACTERIOL, V181, P4725, DOI 10.1128/JB.181.16.4725-4733.1999
Casanovas O, 2005, CANCER CELL, V8, P299, DOI 10.1016/j.ccr.2005.09.005
Datta SK, 2004, IMMUNOL REV, V199, P217, DOI 10.1111/j.0105-2896.2004.00149.x
de Palma M, 2017, NAT REV CANCER, V17, P457, DOI 10.1038/nrc.2017.51
De Silva NS, 2015, NAT REV IMMUNOL, V15, P137, DOI 10.1038/nri3804
DENIS M, 1994, IMMUNOLOGY, V82, P584
Di Pilato M, 2019, NATURE, V570, P112, DOI 10.1038/s41586-019-1215-2
Ellis TN, 2010, MICROBIOL MOL BIOL R, V74, P81, DOI 10.1128/MMBR.00031-09
Fabian KL, 2017, ADV EXP MED BIOL, V1036, P191, DOI 10.1007/978-3-319-67577-0_13
Gacche RN, 2018, DRUG RESIST UPDATE, V36, P47, DOI 10.1016/j.drup.2018.01.002
Gavalas NG, 2013, INT J MOL SCI, V14, P15885, DOI 10.3390/ijms140815885
Giavazzi R, 2001, CANCER RES, V61, P309
Gordon-Weeks AN, 2017, HEPATOLOGY, V65, P1920, DOI 10.1002/hep.29088
Gujrati V, 2014, ACS NANO, V8, P1525, DOI 10.1021/nn405724x
Harding TC, 2013, SCI TRANSL MED, V5, DOI 10.1126/scitranslmed.3005414
Heras B, 2009, NAT REV MICROBIOL, V7, P215, DOI 10.1038/nrmicro2087
Horstman AL, 2000, J BIOL CHEM, V275, P12489, DOI 10.1074/jbc.275.17.12489
Houssiau FA, 2011, ARTHRITIS RHEUM-US, V63, pS963
Hu MM, 2016, PLOS ONE, V11, DOI 10.1371/journal.pone.0147374
Huang WW, 2020, J CONTROL RELEASE, V317, P1, DOI 10.1016/j.jconrel.2019.11.017
Huang WW, 2016, SCI REP-UK, V6, DOI 10.1038/srep37242
Huang WW, 2016, SCI REP-UK, V6, DOI 10.1038/srep20724
Jain RK, 2014, CANCER CELL, V26, P605, DOI 10.1016/j.ccell.2014.10.006
Jayson GC, 2016, LANCET, V388, P518, DOI 10.1016/S0140-6736(15)01088-0
Joos S, 2000, CANCER RES, V60, P549

Karajannis MA, 2006, LEUKEMIA, V20, P979, DOI 10.1038/sj.leu.2404203
Kesty NC, 2004, J BIOL CHEM, V279, P2069, DOI 10.1074/jbc.M307628200
Kuhn H, 2004, LUNG CANCER-J IASLC, V44, P167, DOI 10.1016/j.lungcan.2003.11.005
Kuipers ME, 2018, FRONT MICROBIOL, V9, DOI 10.3389/fmicb.2018.02182
Kumar RK, 2004, AM J RESP CRIT CARE, V170, P1043, DOI 10.1164/rccm.200405-681OC
Li D, 2014, BRIT J CANCER, V111, P68, DOI 10.1038/bjc.2014.282
Li XR, 2018, SCIENCE, V359, P1335, DOI 10.1126/science.aar5557
Manfe V, 2010, J NEUROCHEM, V114, P74, DOI 10.1111/j.1471-4159.2010.06718.x
Mathian A, 2011, ANN RHEUM DIS, V70, P1138, DOI 10.1136/ard.2010.141101
Matsui J, 2008, CLIN CANCER RES, V14, P5459, DOI 10.1158/1078-0432.CCR-07-5270
O'Hare T, 2009, CANCER CELL, V16, P401, DOI 10.1016/j.ccr.2009.09.028
Palm AKE, 2019, FRONT IMMUNOL, V10, DOI 10.3389/fimmu.2019.01787
PIGIET VP, 1986, P NATL ACAD SCI USA, V83, P7643, DOI 10.1073/pnas.83.20.7643
Pradhan P, 2014, BIOMATERIALS, V35, P5491, DOI 10.1016/j.biomaterials.2014.03.039
PRESTA M, 1991, CELL REGUL, V2, P719, DOI 10.1091/mbc.2.9.719
Presta M, 2005, CYTOKINE GROWTH F R, V16, P159, DOI 10.1016/j.cytogfr.2005.01.004
Presta M, 2017, PHARMACOL THERAPEUT, V179, P171, DOI 10.1016/j.pharmthera.2017.05.013
Robbins PD, 2014, NAT REV IMMUNOL, V14, P195, DOI 10.1038/nri3622
Schetters STT, 2019, ACTA BIOMATER, V91, P248, DOI 10.1016/j.actbio.2019.04.033
Schmittnaegel M, 2017, TRENDS CANCER, V3, P809, DOI 10.1016/j.trecan.2017.10.002
Schwechheimer C, 2015, NAT REV MICROBIOL, V13, P605, DOI 10.1038/nrmicro3525
Simon T, 2017, TRENDS MOL MED, V23, P282, DOI 10.1016/j.molmed.2017.01.002
Sun DD, 2013, BIOMATERIALS, V34, P171, DOI 10.1016/j.biomaterials.2012.09.031
Takase N, 2016, CANCER LETT, V380, P47, DOI 10.1016/j.canlet.2016.06.009
Turner N, 2010, NAT REV CANCER, V10, P116, DOI 10.1038/nrc2780
Vandepapeliere P, 2011, GASTROENTEROLOGY, V140, pS123
Viallard C, 2017, ANGIOGENESIS, V20, P409, DOI 10.1007/s10456-017-9562-9
Wang SJ, 2017, INT J NANOMED, V12, P6813, DOI 10.2147/IJN.S143264
Wroblewski M, 2017, NAT COMMUN, V8, DOI 10.1038/s41467-017-00327-8
Yamakawa M, 2018, MED RES REV, V38, P1769, DOI 10.1002/med.21496
Yanez-Mo M, 2015, J EXTRACELL VESICLES, V4, DOI 10.3402/jev.v4.27066
NR 63
TC 35
Z9 36
U1 14
U2 53
PU ELSEVIER SCI LTD
PI OXFORD
PA THE BOULEVARD, LANGFORD LANE, KIDLINGTON, OXFORD OX5 1GB, OXON, ENGLAND
SN 1742-7061
EI 1878-7568
J9 ACTA BIOMATER
JI Acta Biomater.
PD MAY
PY 2020
VL 108
BP 300
EP 312
DI 10.1016/j.actbio.2020.03.030
PG 13
WC Engineering, Biomedical; Materials Science, Biomaterials
WE Science Citation Index Expanded (SCI-EXPANDED)
SC Engineering; Materials Science
GA LJ6DD
UT WOS:000530252800023
PM 32251780
DA 2023-09-14
ER

PT J
AU Shan, WJ

Zheng, HP
Fu, GF
Liu, CF
Li, ZZ
Ye, YH
Zhao, J
Xu, D
Sun, LP
Wang, X
Chen, XL
Bi, SL
Ren, L
Fu, G
AF Shan, Wenjun
Zheng, Haiping
Fu, Guofeng
Liu, Chenfeng
Li, Zizhen
Ye, Yuhan
Zhao, Jie
Xu, Dan
Sun, Liping
Wang, Xin
Chen, Xiao Lei
Bi, Shengli
Ren, Lei
Fu, Guo
TI Bioengineered Nanocage from HBc Protein for Combination Cancer Immunotherapy
SO NANO LETTERS
LA English
DT Article
DE Protein nanocage; Hepatitis B core protein; antigen delivery; vaccination; cancer immuotherapy
ID VIRUS-LIKE PARTICLES; T-CELLS; DENDRITIC CELLS; VACCINE; IMMUNITY; RECOGNITION; CD8(+); MODEL; SIZE
AB Protein nanocages are promising multifunctional platforms for nanomedicine owing to the ability to decorate their surfaces with multiple functionalities through genetic and/or chemical modification to achieve desired properties for therapeutic and diagnostic purposes. Here, we describe a model antigen (OVA peptide) that was conjugated to the surface of a naturally occurring hepatitis B core protein nanocage (HBc NC) by genetic modification. The engineered OVA-HBc nanocages (OVA-HBc NCs), displaying high density repetitive array of epitopes in a limited space by self-assembling into symmetrical structure, not only can induce bone marrow derived dendritic cells (BMDC) maturation effectively but also can be enriched in the draining lymph nodes. Naive C57BL/6 mice immunized with OVA-HBc NCs are able to generate significant and specific cytotoxic T lymphocyte (CTL) responses. Moreover, OVA-HBc NCs as a robust nanovaccine can trigger preventive antitumor immunity and significantly delay tumor growth. When combined with a low-dose chemotherapy drug (paclitaxel), OVA-HBc NCs could specifically inhibit progression of an established tumor. Our findings support HBc-based nanocages with modularity and scalability as an attractive nanoplatform for combination cancer immunotherapy.
C1 [Shan, Wenjun; Zhao, Jie; Xu, Dan; Sun, Liping; Ren, Lei] Xiamen Univ, Coll Mat, Dept Biomat, Key Lab Biomed Engn Fujian Prov,State Key Lab Phy, Xiamen 361005, Fujian, Peoples R China.
[Zheng, Haiping; Fu, Guofeng; Liu, Chenfeng; Chen, Xiao Lei; Fu, Guo] Xiamen Univ, State Key Lab Cellular Stress Biol, Innovat Ctr Cell Signaling Network, Sch Life Sci, Xiamen 361102, Fujian, Peoples R China.
[Li, Zizhen; Wang, Xin] Xiamen Univ, Sch Publ Hlth, Natl Inst Diagnost & Vaccine Dev Infect Dis, Xiamen 361102, Fujian, Peoples R China.
[Ye, Yuhan] Xiamen Univ, Zhongshan Hosp, Xiamen 361005, Fujian, Peoples R China.
[Bi, Shengli] Chinese Ctr Dis Control & Prevent, Inst Viral Dis Control & Prevent, Beijing 102206, Peoples R China.
[Fu, Guo] Xiamen Univ, Canc Res Ctr, Xiamen 361102, Fujian, Peoples R China.
C3 Xiamen University; Xiamen University; Xiamen University; Xiamen

University; Chinese Center for Disease Control & Prevention; Xiamen
University
RP Ren, L (通讯作者)，Xiamen Univ, Coll Mat, Dept Biomat, Key Lab Biomed Engn Fujian Prov,State Key Lab Phy, Xiamen 361005, Fujian, Peoples R China.; Fu, G (通讯作者)，Xiamen Univ, State Key Lab Cellular Stress Biol, Innovat Ctr Cell Signaling Network, Sch Life Sci, Xiamen 361102, Fujian, Peoples R China.; Fu, G (通讯作者)，Xiamen Univ, Canc Res Ctr, Xiamen 361102, Fujian, Peoples R China.
EM renlei@xmu.edu.cn; guofu@xmu.edu.cn
RI ; Lei, Ren/G-3459-2010
OI Bi, Shengli/0000-0002-0380-5790; Lei, Ren/0000-0003-2131-1601; shan,
wenjun/0000-0001-5983-8455
FU National Natural Science Foundation of China [U1505228, 31870994,
81571764, 31770952, 31570911, 2017ZX10202203-003-001]
FX This work was supported by National Natural Science Foundation of China
(grants U1505228 and 31870994 to L.R., 81571764 to L.S., 31770952,
31570911 and 2017ZX10202203-003-001 to Guo Fu). We thank Wenzhu Fan,
Guangxi Wu, and Yazhen Hong for technical help.
CR Baumeister SH, 2016, ANNU REV IMMUNOL, V34, P539, DOI 10.1146/annurev-immunol-032414-112049
Bhaskar S, 2017, NPG ASIA MATER, V9, DOI 10.1038/am.2016.128
Cohen JA, 2009, BIOCONJUGATE CHEM, V20, P111, DOI 10.1021/bc800338n
Condotta SA, 2017, PLOS PATHOG, V13, DOI 10.1371/journal.ppat.1006618
den Haan JMM, 2000, J EXP MED, V192, P1685, DOI 10.1084/jem.192.12.1685
Deng W, 2018, NAT COMMUN, V9, DOI 10.1038/s41467-018-05118-3
Emens LA, 2015, CANCER IMMUNOL RES, V3, P436, DOI 10.1158/2326-6066.CIR-15-0064
Fukurnura D, 2018, NAT REV CLIN ONCOL, V15, P325, DOI 10.1038/nrclinonc.2018.29
Gardner A, 2016, TRENDS IMMUNOL, V37, P855, DOI 10.1016/j.it.2016.09.006
Hua Z, 2007, CLIN CANCER RES, V13, P5455, DOI 10.1158/1078-0432.CCR-07-0517
Kaiser J, 2017, SCIENCE, V356, P122, DOI 10.1126/science.356.6334.122
Kershaw MH, 2013, ONCOIMMUNOLOGY, V2, DOI 10.4161/onci.25962
Klebanoff CA, 2005, P NATL ACAD SCI USA, V102, P9571, DOI 10.1073/pnas.0503726102
Kreiter S, 2015, NATURE, V520, P692, DOI 10.1038/nature14426
Kushnir N, 2012, VACCINE, V31, P58, DOI 10.1016/j.vaccine.2012.10.083
Lee EJ, 2016, ADV DRUG DELIVER REV, V106, P157, DOI 10.1016/j.addr.2016.03.002
Li Q, 2016, FEBS J, V283, P2836, DOI 10.1111/febs.13767
Ludwig C, 2007, CURR OPIN BIOTECH, V18, P537, DOI 10.1016/j.copbio.2007.10.013
Mellman I, 2011, NATURE, V480, P480, DOI 10.1038/nature10673
Mottram PL, 2007, MOL PHARMACEUT, V4, P73, DOI 10.1021/mp060096p
Nencioni A, 2018, NAT REV CANCER, V18, P707, DOI 10.1038/s41568-018-0061-0
Pardoll DM, 2012, NAT REV CANCER, V12, P252, DOI 10.1038/nrc3239
Pumpens P, 1999, FEBS LETT, V442, P1, DOI 10.1016/S0014-5793(98)01599-3
Rajasagi M, 2014, BLOOD, V124, P453, DOI 10.1182/blood-2014-04-567933
Ramakrishnan R, 2010, J CLIN INVEST, V120, P1111, DOI 10.1172/JCI40269
Roldao A, 2010, EXPERT REV VACCINES, V9, P1149, DOI [10.1586/erv.10.115, 10.1586/ERV.10.115]
SHIMONKEVITZ R, 1983, J EXP MED, V158, P303, DOI 10.1084/jem.158.2.303
Slifka MK, 2014, VACCINE, V32, P2948, DOI 10.1016/j.vaccine.2014.03.078
Sun WH, 2018, CELL MOL IMMUNOL, V15, P768, DOI 10.1038/cmi.2017.155
Tang-Huau TL, 2018, NAT COMMUN, V9, DOI 10.1038/s41467-018-04985-0
Wherry EJ, 2003, NAT IMMUNOL, V4, P225, DOI 10.1038/ni889
Xiang SD, 2006, METHODS, V40, P1, DOI 10.1016/j.ymeth.2006.05.016
Zhu GZ, 2017, ACS NANO, V11, P2387, DOI 10.1021/acsnano.7b00978
NR 33
TC 35
Z9 38
U1 2
U2 131
PU AMER CHEMICAL SOC
PI WASHINGTON
PA 1155 16TH ST, NW, WASHINGTON, DC 20036 USA
SN 1530-6984
EI 1530-6992
J9 NANO LETT

JI Nano Lett.
PD MAR
PY 2019
VL 19
IS 3
BP 1719
EP 1727
DI 10.1021/acs.nanolett.8b04722
PG 9
WC Chemistry, Multidisciplinary; Chemistry, Physical; Nanoscience &
Nanotechnology; Materials Science, Multidisciplinary; Physics, Applied;
Physics, Condensed Matter
WE Science Citation Index Expanded (SCI-EXPANDED)
SC Chemistry; Science & Technology - Other Topics; Materials Science;
Physics
GA HP2XB
UT WOS:000461537600040
PM 30724087
DA 2023-09-14
ER

PT J
AU Liu, ZD
Zhou, C
Qin, Y
Wang, ZH
Wang, LY
Wei, XL
Zhou, YJ
Li, QC
Zhou, H
Wang, WJ
Fu, YX
Zhu, MZ
Liang, W
AF Liu, Zhida
Zhou, Chang
Qin, Yan
Wang, Zihao
Wang, Luyao
Wei, Xiuli
Zhou, Yinjian
Li, Qicheng
Zhou, Hang
Wang, Wenjun
Fu, Yang-Xin
Zhu, Mingzhao
Liang, Wei
TI Coordinating antigen cytosolic delivery and danger signaling to program
potent cross-priming by micelle-based nanovaccine
SO CELL DISCOVERY
LA English
DT Article
DE micelle; cancer therapeutic vaccine; antigen cytosolic delivery; cross
priming; lymph node targeting; dendritic cell; CTL
ID IMMUNE CHECKPOINT BLOCKADE; DENDRITIC CELLS; RECEPTOR 4-MD-2; IN-VIVO;
PEPTIDES; VACCINES; AGGREGATION; GENERATION; MECHANISM; DYNAMICS
AB Although re-activating cytotoxic T-cell (CTLs) response inside tumor tissues by checkpoint blockade has demonstrated great success in tumor immunotherapy, active induction of efficient endogenous CTL response by therapeutic vaccines has been largely hampered by inefficient cytosolic delivery of antigens and coordinated activation of dendritic cells (DCs) in lymph nodes. Here we show that polyethylene

glycol-phosphatidylethanolamine (PEG-PE) micelles transform soluble peptides into a-helix to enable their efficient cytosolic delivery. The same PEG-PE micelles also serve as chaperon of TLR4 signaling to coordinate its adjuvant effect on the same DCs. Furthermore, these nanovaccines effectively target lymph node DCs. Thus, PEG-PE micelle vaccines program at multiple key aspects for inducing strong CTL responses and build up a foundation for combinational tumor therapy.
C1 [Liu, Zhida; Zhou, Hang; Wang, Wenjun; Fu, Yang-Xin; Zhu, Mingzhao] Chinese Acad Sci, Inst Biophys, Key Lab Infect & Immun, Beijing, Peoples R China.
[Liu, Zhida; Zhou, Chang; Wang, Zihao; Wang, Luyao; Zhou, Hang; Wang, Wenjun; Zhu, Mingzhao; Liang, Wei] Univ Chinese Acad Sci, Beijing, Peoples R China.
[Liu, Zhida; Fu, Yang-Xin] UT Southwestern Med Ctr, Dept Pathol & Immunol, Dallas, TX 75390 USA.
[Zhou, Chang; Qin, Yan; Wang, Zihao; Wang, Luyao; Wei, Xiuli; Zhou, Yinjian; Li, Qicheng; Liang, Wei] Chinese Acad Sci, Inst Biophys, Prot & Peptide Pharmaceut Lab, Beijing, Peoples R China.
C3 Chinese Academy of Sciences; Institute of Biophysics, CAS; Chinese Academy of Sciences; University of Chinese Academy of Sciences, CAS; University of Texas System; University of Texas Southwestern Medical Center Dallas; Chinese Academy of Sciences; Institute of Biophysics, CAS
RP Fu, YX; Zhu, MZ (通讯作者)，Chinese Acad Sci, Inst Biophys, Key Lab Infect & Immun, Beijing, Peoples R China.; Zhu, MZ; Liang, W (通讯作者)，Univ Chinese Acad Sci, Beijing, Peoples R China.; Fu, YX (通讯作者)，UT Southwestern Med Ctr, Dept Pathol & Immunol, Dallas, TX 75390 USA.; Liang, W (通讯作者)，Chinese Acad Sci, Inst Biophys, Prot & Peptide Pharmaceut Lab, Beijing, Peoples R China.
EM Yang-Xin.Fu@UTSouthwestern.edu; zhumz@ibp.ac.cn; weixx@sun5.ibp.ac.cn
RI Zhu, Mingzhao/L-8662-2019
OI Liu, Zhida/0000-0002-3980-8440; liang, wei/0000-0002-0739-6648
FU Chinese Academy of Sciences [XDA09030303]; Ministry of Science and Technology [2013ZX10004606]; National Natural Science Foundation of China [81261130022, 31570888, 81173012]
FX We thank Dr Xuemei Xu (Institute of Basic Medical Sciences, Chinese Academy of Medical Sciences and School of Basic Medicine, Peking Union Medical College) for TC-1 tumor cell line, Dr Li Tang (Key Laboratory of Protein Engineering, Academy of Military Medical Sciences) for DC2.4 cell line. We are grateful for technical support from the Core Facilities of Institute of Biophysics, Chinese Academy of Sciences. We thank Yongsheng Chen for Transmission Electron Microscopy, Yihui Xu for IVIS Spectrum Imaging System and Confocal Microscopy work, Yan Teng and Chunli Jiang for Confocal Microscopy work, Junying Jia and Junjing Yu for flow cytometry analysis and cell sorting. We thank Animal Research Center of Institute of Biophysics for technical assistance. This work was supported by grants from the Strategic Priority Research Programs of the Chinese Academy of Sciences (XDA09030303 to WL), Ministry of Science and Technology (2013ZX10004606 to MZ), National Natural Science Foundation of China (81261130022 and 31570888 to MZ, 81173012 to XW).
CR Akashi S, 2003, J EXP MED, V198, P1035, DOI 10.1084/jem.20031076
Akashi S, 2000, J IMMUNOL, V164, P3471, DOI 10.4049/jimmunol.164.7.3471
[Anonymous], 2012, FRONT ONCOL
Apellaniz B, 2014, CHEM PHYS LIPIDS, V181, P40, DOI 10.1016/j.chemphyslip.2014.03.003
Bachmann MF, 2010, NAT REV IMMUNOL, V10, P787, DOI 10.1038/nri2868
Beaudette TT, 2009, MOL PHARMACEUT, V6, P1160, DOI 10.1021/mp900038e
Blum JS, 2013, ANNU REV IMMUNOL, V31, P443, DOI 10.1146/annurev-immunol-032712-095910
Chen DS, 2013, IMMUNITY, V39, P1, DOI 10.1016/j.immuni.2013.07.012
de Titta A, 2013, P NATL ACAD SCI USA, V110, P19902, DOI 10.1073/pnas.1313152110
Demaria S, 2015, JAMA ONCOL, V1, P1325, DOI 10.1001/jamaoncol.2015.2756
Dirndorfer D, 2013, J BIOL CHEM, V288, P13961, DOI 10.1074/jbc.M112.430264
Fang XC, 2016, BIOMATERIALS, V77, P139, DOI 10.1016/j.biomaterials.2015.11.007
Fonseca SB, 2009, ADV DRUG DELIVER REV, V61, P953, DOI 10.1016/j.addr.2009.06.001
Galluzzi L, 2015, CANCER CELL, V28, P690, DOI 10.1016/j.ccell.2015.10.012
Garnacho C, 2016, CURR PHARM DESIGN, V22, P1210, DOI 10.2174/1381612822666151216151021
HAILMAN E, 1994, J EXP MED, V179, P269, DOI 10.1084/jem.179.1.269
Hilinski GJ, 2014, J AM CHEM SOC, V136, P12314, DOI 10.1021/ja505141j
Hubbell JA, 2009, NATURE, V462, P449, DOI 10.1038/nature08604
Irvine DJ, 2013, NAT MATER, V12, P978, DOI [10.1038/NMAT3775, 10.1038/nmat3775]
Janeway CA, 2002, ANNU REV IMMUNOL, V20, P197, DOI

10.1146/annurev.immunol.20.083001.084359
Kauffman WB, 2015, TRENDS BIOCHEM SCI, V40, P749, DOI 10.1016/j.tibs.2015.10.004
Kim J, 2015, NAT BIOTECHNOL, V33, P64, DOI 10.1038/nbt.3071
Kobayashi M, 2006, J IMMUNOL, V176, P6211, DOI 10.4049/jimmunol.176.10.6211
Li AV, 2013, SCI TRANSL MED, V5, DOI 10.1126/scitranslmed.3006516
Liu HP, 2014, NATURE, V507, P519, DOI 10.1038/nature12978
Lundberg M, 2001, NAT BIOTECHNOL, V19, P713, DOI 10.1038/90741
Mitchell DA, 2015, NATURE, V519, P366, DOI 10.1038/nature14320
Niidome T, 1999, BIOCONJUGATE CHEM, V10, P773, DOI 10.1021/bc990012d
Park BS, 2009, NATURE, V458, P1191, DOI 10.1038/nature07830
Raghuraman H, 2006, BIOPHYS CHEM, V124, P115, DOI 10.1016/j.bpc.2006.06.011
Raghuraman H, 2004, EUR BIOPHYS J BIOPHY, V33, P611, DOI 10.1007/s00249-004-0402-7
Ramsay AG, 2013, BRIT J HAEMATOL, V162, P313, DOI 10.1111/bjh.12380
Reddy ST, 2007, NAT BIOTECHNOL, V25, P1159, DOI [10.1038/nbt1332, 10.1038/nbtl332]
Reddy ST, 2006, TRENDS IMMUNOL, V27, P573, DOI 10.1016/j.it.2006.10.005
Reddy ST, 2006, J CONTROL RELEASE, V112, P26, DOI 10.1016/j.jconrel.2006.01.006
Santos NC, 2003, CHEMBIOCHEM, V4, P96, DOI 10.1002/cbic.200390020
Schlosser E, 2008, VACCINE, V26, P1626, DOI 10.1016/j.vaccine.2008.01.030
SCHOLTZ JM, 1992, ANNU REV BIOPH BIOM, V21, P95, DOI 10.1146/annurev.bb.21.060192.000523
Schulke S, 2015, ALLERGY, V70, P1259, DOI 10.1111/all.12675
Swartz MA, 2012, SCI TRANSL MED, V4, DOI 10.1126/scitranslmed.3003763
Tang N, 2007, JNCI-J NATL CANCER I, V99, P1004, DOI 10.1093/jnci/djm027
Topalian SL, 2015, CANCER CELL, V27, P450, DOI 10.1016/j.ccell.2015.03.001
Tsukamoto H, 2010, INT IMMUNOL, V22, P271, DOI 10.1093/intimm/dxq005
Tumeh PC, 2014, NATURE, V515, P568, DOI 10.1038/nature13954
Verdine GL, 2012, METHOD ENZYMOL, V503, P3, DOI 10.1016/B978-0-12-396962-0.00001-X
Wang J, 2013, PHILOS T R SOC A, V371, DOI 10.1098/rsta.2012.0309
Wang J, 2012, ACS NANO, V6, P5018, DOI 10.1021/nn300571c
Wang J, 2012, J CONTROL RELEASE, V160, P637, DOI 10.1016/j.jconrel.2012.02.021
Wang WM, 2016, CELL, V165, P1092, DOI 10.1016/j.cell.2016.04.009
Wilson JT, 2013, ACS NANO, V7, P3912, DOI 10.1021/nn305466z
Yamashita H, 2016, CHEMBIOCHEM, V17, P137, DOI 10.1002/cbic.201500468
Yamashita H, 2014, BIOORGAN MED CHEM, V22, P2403, DOI 10.1016/j.bmc.2014.03.005
Yu LL, 2006, ANAL CHIM ACTA, V556, P216, DOI 10.1016/j.aca.2005.09.008
NR 53
TC 34
Z9 35
U1 2
U2 36
PU NATURE PUBLISHING GROUP
PI LONDON
PA MACMILLAN BUILDING, 4 CRINAN ST, LONDON N1 9XW, ENGLAND
EI 2056-5968
J9 CELL DISCOV
JI Cell Discov.
PD APR 4
PY 2017
VL 3
AR 17007
DI 10.1038/celldisc.2017.7
PG 14
WC Cell Biology
WE Science Citation Index Expanded (SCI-EXPANDED)
SC Cell Biology
GA FM3KO
UT WOS:000414904200001
PM 28417012
OA Green Published, gold
DA 2023-09-14
ER

PT J
AU Liu, LL
Yi, HQ
Wang, C
He, HM
Li, P
Pan, H
Sheng, N
Ji, MY
Cai, LT
Ma, YF
AF Liu, Lanlan
Yi, Huqiang
Wang, Ce
He, Huamei
Li, Ping
Pan, Hong
Sheng, Nan
Ji, Manyi
Cai, Lintao
Ma, Yifan
TI Integrated Nanovaccine with MicroRNA-148a Inhibition Reprograms
Tumor-Associated Dendritic Cells by Modulating miR-148a/DNMT1/SOCS1 Axis
SO JOURNAL OF IMMUNOLOGY
LA English
DT Article
ID DNA METHYLATION; SIGNALING PATHWAYS; ANTITUMOR IMMUNITY; CANCER-CELLS;
MATURATION; DIFFERENTIATION; RESPONSES; STAT3; 5-AZACYTIDINE;
MACROPHAGES
AB Immunosuppressive tumor-associated dendritic cells (TADCs) are potential targets for cancer therapy. However, their poor responsiveness to TLR stimulation is a major obstacle for achieving successful cancer immunotherapy. In the current study, we reported a dysregulated miR-148a/DNA methyltransferase (DNMT) 1/suppressor of cytokine signaling (SOCS) 1 axis as a unique mechanism for dampened TLR stimulation in TADCs. The results showed that aberrantly elevated miR-148a in bone marrow-derived TADC (BM-TADC) abolished polyinosinic-polycytidylic acid (poly I:C) or LPS-induced dendritic cell maturation through directly suppressing DNMT1 gene, which consequently led to the hypomethylation and upregulation of SOCS1, the suppressor of TLR signaling. In contrast, miR-148a inhibitor (miR-148ai) effectively rescued the expression of DNMT1 and decreased SOCS1 in BM-TADCs, thereby recovering their sensitivity to TLR3 or TLR4 stimulation. To further reprogram TADCs in vivo, miR-148ai was coencapsulated with poly I:C and OVA by cationic polypeptide micelles to generate integrated polypeptide micelle/poly I:C (PMP)/OVA/148ai nanovaccine, which was designed to simultaneously inhibit miR-148a and activate TLR3 signaling in TADCs. The immunization of PMP/OVA/148ai nanovaccine not only effectively modulated the miR-148a/DNMT1/SOCS1 axis in the spleen, but also significantly increased mature dendritic cells both in the spleen and in tumor microenvironment. Moreover, PMP/OVA/148ai ameliorated tumor immunosuppression through reducing regulatory T cells and myeloid-derived suppressor cells, thereby leading to potent anticancer immune responses and robust tumor regression with prolonged survival. This study proposes a nanovaccine-based immunogene therapy with the integration of miR-148a inhibition and TLR3 stimulation as a novel therapeutic approach to boost anticancer immunity by reprogramming TADCs in vivo.
C1 [Liu, Lanlan; Yi, Huqiang; Wang, Ce; He, Huamei; Li, Ping; Pan, Hong; Sheng, Nan; Ji, Manyi; Cai, Lintao; Ma, Yifan] Chinese Acad Sci, Key Lab Hlth Informat, Guangdong Key Lab Nanomed, Shenzhen Inst Adv Technol, Shenzhen 518055, Peoples R China.
C3 Chinese Academy of Sciences; Shenzhen Institute of Advanced Technology,
CAS
RP Ma, YF (通讯作者)，Chinese Acad Sci, Shenzhen Inst Adv Technol, 1068 Xueyuan Ave, Shenzhen 518055, Peoples R China.
EM yf.ma@siat.ac.cn
RI Pan, Hong/AAC-2326-2019
OI Cai, Lintao/0000-0002-2461-6390
CR Alizadeh D, 2014, CANCER RES, V74, P2663, DOI 10.1158/0008-5472.CAN-14-0301
Aranda F, 2013, ONCOIMMUNOLOGY, V2, DOI 10.4161/onci.26621

Bartel DP, 2009, CELL, V136, P215, DOI 10.1016/j.cell.2009.01.002
Baxevanis CN, 2013, IMMUNOTHERAPY-UK, V5, P497, DOI [10.2217/IMT.13.24, 10.2217/imt.13.24]
Pampena MB, 2015, FRONT IMMUNOL, V6, DOI 10.3389/fimmu.2015.00013
Cheng C, 2014, TOXICOL LETT, V225, P488, DOI 10.1016/j.toxlet.2013.12.023
Chiba S, 2012, NAT IMMUNOL, V13, P832, DOI 10.1038/ni.2376
Cui TX, 2013, IMMUNITY, V39, P611, DOI 10.1016/j.immuni.2013.08.025
Dai XJ, 2006, J INVEST DERMATOL, V126, P1574, DOI 10.1038/sj.jid.5700294
Deng JZ, 2012, BIOMACROMOLECULES, V13, P3795, DOI 10.1021/bm3012538
Djuranovic S, 2011, SCIENCE, V331, P550, DOI 10.1126/science.1191138
Fraszczak J, 2010, J IMMUNOL, V184, P1876, DOI 10.4049/jimmunol.0900831
Frikeche J, 2011, EXP HEMATOL, V39, P1056, DOI 10.1016/j.exphem.2011.08.004
Gal-Yam EN, 2008, ANNU REV MED, V59, P267, DOI 10.1146/annurev.med.59.061606.095816
Hargadon KM, 2013, FRONT IMMUNOL, V4, DOI 10.3389/fimmu.2013.00192
Harimoto H, 2013, IMMUNOL CELL BIOL, V91, P545, DOI 10.1038/icb.2013.38
Herman JG, 1996, P NATL ACAD SCI USA, V93, P9821, DOI 10.1073/pnas.93.18.9821
Hurwitz AA, 2012, CANCER IMMUNOL IMMUN, V61, P289, DOI 10.1007/s00262-011-1181-5
Idoyaga J, 2007, CANCER IMMUNOL IMMUN, V56, P1237, DOI 10.1007/s00262-006-0275-y
Janco JMT, 2015, J IMMUNOL, V194, P2985, DOI 10.4049/jimmunol.1403134
Kalinski P, 2005, MOL IMMUNOL, V42, P535, DOI 10.1016/j.molimm.2004.07.038
Karrich JJ, 2013, BLOOD, V122, P3001, DOI 10.1182/blood-2012-12-475087
Kinjyo I, 2002, IMMUNITY, V17, P583, DOI 10.1016/S1074-7613(02)00446-6
Lay FD, 2015, GENOME RES, V25, P467, DOI 10.1101/gr.183368.114
Li CR, 2013, J BIOL CHEM, V288, P19127, DOI 10.1074/jbc.M113.453357
Liu XG, 2010, J IMMUNOL, V185, P7244, DOI 10.4049/jimmunol.1001573
Lombard AP, 2016, MOL CARCINOGEN, V55, P757, DOI 10.1002/mc.22319
Lujambio A, 2008, P NATL ACAD SCI USA, V105, P13556, DOI 10.1073/pnas.0803055105
Luo ZC, 2015, BIOMATERIALS, V38, P50, DOI 10.1016/j.biomaterials.2014.10.050
Luo ZC, 2013, J CONTROL RELEASE, V170, P259, DOI 10.1016/j.jconrel.2013.05.027
Maurya N, 2014, J IMMUNOL, V193, P3417, DOI 10.4049/jimmunol.1400395
Min SP, 2013, J IMMUNOL, V190, P2437, DOI 10.4049/jimmunol.1202282
Nefedova Y, 2004, J IMMUNOL, V172, P464, DOI 10.4049/jimmunol.172.1.464
Norian LA, 2009, CANCER RES, V69, P3086, DOI 10.1158/0008-5472.CAN-08-2826
O'Connell RM, 2010, NAT REV IMMUNOL, V10, P111, DOI 10.1038/nri2708
Pyfferoen L, 2014, INT J CANCER, V135, P2868, DOI 10.1002/ijc.28945
Restifo NP, 2012, NAT REV IMMUNOL, V12, P269, DOI 10.1038/nri3191
Rothschild Sacha I, 2014, Mol Cell Ther, V2, P7, DOI 10.1186/2052-8426-2-7
Shurin GV, 2013, CANCER MICROENVIRON, V6, P159, DOI 10.1007/s12307-013-0133-3
Smyth LA, 2015, IMMUNOLOGY, V144, P197, DOI 10.1111/imm.12390
Stepanek I, 2011, J BIOL REG HOMEOS AG, V25, P517
Sun YP, 2013, J IMMUNOL, V190, P4005, DOI 10.4049/jimmunol.1202911
Topalian SL, 2015, CELL, V161, P185
Wan SS, 2014, GASTROENTEROLOGY, V147, P1393, DOI 10.1053/j.gastro.2014.08.039
Wang C, 2012, VACCINE, V30, P4790, DOI 10.1016/j.vaccine.2012.05.027
Watkins SK, 2011, J CLIN INVEST, V121, P1361, DOI 10.1172/JCI44325
Wolffe AP, 1999, SCIENCE, V286, P481, DOI 10.1126/science.286.5439.481
Xia JZ, 2014, J CANCER RES CLIN, V140, P1451, DOI 10.1007/s00432-014-1649-8
Xu Q, 2013, J MOL CELL BIOL, V5, P3, DOI 10.1093/jmcb/mjs049
Xue XC, 2011, J IMMUNOL, V187, P5879, DOI 10.4049/jimmunol.1100535
Yin H, 2014, NAT REV GENET, V15, P541, DOI 10.1038/nrg3763
Zhang X, 2014, EPIGENET CHROMATIN, V7, DOI 10.1186/1756-8935-7-21
Zheng J, 2012, ALLERGY, V67, P362, DOI 10.1111/j.1398-9995.2011.02776.x
NR 53
TC 34
Z9 35
U1 0
U2 36
PU AMER ASSOC IMMUNOLOGISTS
PI BETHESDA
PA 9650 ROCKVILLE PIKE, BETHESDA, MD 20814 USA
SN 0022-1767
EI 1550-6606

J9 J IMMUNOL
JI J. Immunol.
PD AUG 15
PY 2016
VL 197
IS 4
BP 1231
EP 1241
DI 10.4049/jimmunol.1600182
PG 11
WC Immunology
WE Science Citation Index Expanded (SCI-EXPANDED)
SC Immunology
GA DY3NB
UT WOS:000384999100023
PM 27421476
OA Bronze
DA 2023-09-14
ER

PT J
AU Lv, J
   Wang, H
   Rong, GY
   Cheng, YY
AF Lv, Jia
   Wang, Hui
   Rong, Guangyu
   Cheng, Yiyun
TI Fluorination Promotes the Cytosolic Delivery of Genes, Proteins, and
   Peptides
SO ACCOUNTS OF CHEMICAL RESEARCH
LA English
DT Review
ID SURFACE-ENGINEERED DENDRIMERS; TRANSFECTION EFFICACY; HIGHLY EFFICIENT;
   CELL-PENETRATION; IN-VITRO; SIRNA; DESIGN; THERAPEUTICS; DRUG; DNA
AB The cytosolic delivery of biomolecules such as genes, proteins, and peptides is of great importance for biotherapy but usually limited by multiple barriers during the process. Cell membrane with high hydrophobic character is one of the representative biological barriers for cytosolic delivery. The introduction of hydrophobic ligands such as aliphatic lipids onto materials or biomolecules could improve their membrane permeability. However, these ligands are lipophilic and tend to interact with the phospholipids in the membrane as well as serum proteins, which may hinder efficient intracellular delivery. To solve this issue, our research group proposed the use of fluorous ligands with both hydrophobicity and lipophobicity as ideal alternatives to aliphatic lipids to promote cytosolic delivery.
   In our first attempt, fluorous ligands were conjugated onto cationic polymers to increase their gene delivery efficacy. The fluorination dramatically increased the gene delivery performance at low polymer doses. In addition, the strategy greatly improved the serum tolerance of cationic polymers, which is critical for efficient gene delivery in vivo. Besides serum tolerance, mechanism studies revealed that fluorination increases multiple steps such as cellular uptake and endosomal escape. Fluorination also allowed the assembly of low-molecular-weight polymers and achieved highly efficient gene delivery with minimal material toxicity. The method showed robust efficiency for polymers, induding linear polymers, branched polymers, dendrimers, bola amphiphilies, and dendronized polymers.
   Besides gene delivery, fluorinated polymers were also used for intracellular protein delivery via a coassembly strategy. For this purpose, two lead fluoropolymers were screened from a library of amphiphilic materials. The fluoropolymers are greatly superior to their nonfluorinated analogues conjugated with aliphatic lipids. The fluorous lipids are beneficial for polymer assembly and protein encapsulation, reduced protein denaturation, facilitated endocytosis, and decreased polymer toxicity compared to nonfluorinated lipids. The materials exhibited potent efficacy in therapeutic protein and peptide delivery to achieve cancer therapy and were able to fabricate a personalized nanovaccine for cancer immunotherapy. Finally, the fluorous lipids were directly conjugated to peptides via a disulfide bond for cytosolic peptide delivery. Fluorous lipids drive the assembly of cargo peptides into uniform

nanoparticles with much improved proteolytic stability and promote their delivery into various types of cells. The delivery efficacy of this strategy is greatly superior to traditional techniques such as cell-penetrating peptides both in vitro and in vivo. Overall, the fluorination techniques provide efficient and promising strategies for the cytosolic delivery of biomolecules.
C1 [Lv, Jia; Cheng, Yiyun] East China Normal Univ, Shanghai Frontiers Sci Ctr Genome Editing & Cell, Sch Life Sci, Shanghai Key Lab Regulatory Biol, Shanghai 200241, Peoples R China.
[Wang, Hui; Rong, Guangyu] South China Univ Technol, Sch Mol Sci & Engn, South China Adv Inst Soft Matter Sci & Technol, Guangdong Prov Key Lab Funct & Intelligent Hybrid, Guangzhou 510640, Peoples R China.
C3 East China Normal University; South China University of Technology
RP Cheng, YY (通讯作者)，East China Normal Univ, Shanghai Frontiers Sci Ctr Genome Editing & Cell, Sch Life Sci, Shanghai Key Lab Regulatory Biol, Shanghai 200241, Peoples R China.
EM yycheng@mail.ustc.edu.cn
FU National Key R&D Program of China, Synthetic Biology Research [2019YFA0904500]; National Natural Science Foundation of China [22135002, 21725402, 22005101]; Basic Research Program of Science and Technology Commission of Shanghai Municipality [21JC1401800]; Shanghai Frontiers Science Center of Genome Editing and Cell Therapy
FX The authors are grateful for support from the National Key R&D Program of China, Synthetic Biology Research (no. 2019YFA0904500), the National Natural Science Foundation of China (22135002, 21725402, and 22005101), the Basic Research Program of Science and Technology Commission of Shanghai Municipality (21JC1401800), and the Shanghai Frontiers Science Center of Genome Editing and Cell Therapy.
CR Ai Q, 2019, ADV MATER INTERFACES, V6, DOI 10.1002/admi.201901187
Akishiba M, 2017, NAT CHEM, V9, P751, DOI [10.1038/NCHEM.2779, 10.1038/nchem.2779]
Bilgicer B, 2004, P NATL ACAD SCI USA, V101, P15324, DOI 10.1073/pnas.0403314101
Cai XJ, 2017, ACS APPL MATER INTER, V9, P9402, DOI 10.1021/acsami.6b16689
Chang H, 2017, NANO LETT, V17, P1678, DOI 10.1021/acs.nanolett.6b04955
Chang H, 2014, BIOCONJUGATE CHEM, V25, P342, DOI 10.1021/bc400496u
Chen G, 2018, ACS NANO, V12, P6620, DOI 10.1021/acsnano.8b01482
Chen G, 2018, ADV HEALTHC MATER, V7, DOI 10.1002/adhm.201700978
Cheng YY, 2017, ACTA POLYM SIN, P1234, DOI 10.11777/j.issn1000-3304.2017.17095
Cheng YY, 2021, CHINESE J CHEM, V39, P1443, DOI 10.1002/cjoc.202000655
Eldredge AC, 2018, BIOMATERIALS, V178, P458, DOI 10.1016/j.biomaterials.2018.04.024
Fan QQ, 2021, ADV FUNCT MATER, V31, DOI 10.1002/adfm.202101646
Fu CK, 2020, ANGEW CHEM INT EDIT, V59, P4729, DOI 10.1002/anie.201914119
Gaucheron J, 2001, BIOCONJUGATE CHEM, V12, P949, DOI 10.1021/bc010033j
Ge CL, 2020, NANO LETT, V20, P1738, DOI 10.1021/acs.nanolett.9b04957
Guo JJ, 2021, ACS CENTRAL SCI, V7, P990, DOI 10.1021/acscentsci.0c01648
He BW, 2015, ACTA BIOMATER, V22, P111, DOI 10.1016/j.actbio.2015.04.037
Jean SR, 2016, ACCOUNTS CHEM RES, V49, P1893, DOI 10.1021/acs.accounts.6b00277
Ji TJ, 2015, ADV MATER, V27, P1865, DOI 10.1002/adma.201404715
Kasuya MCZ, 2011, J FLUORINE CHEM, V132, P202, DOI 10.1016/j.jfluchem.2011.01.004
Klein E, 2010, BIOMATERIALS, V31, P4781, DOI 10.1016/j.biomaterials.2010.02.047
Klein E, 2010, BIOCONJUGATE CHEM, V21, P360, DOI 10.1021/bc900469z
Krafft MP, 2001, ADV DRUG DELIVER REV, V47, P209, DOI 10.1016/S0169-409X(01)00107-7
Kretzmann JA, 2019, CHEM SCI, V10, P7718, DOI 10.1039/c9sc01432b
Kretzmann JA, 2017, CHEM SCI, V8, P2923, DOI 10.1039/c7sc00097a
Lachelt U, 2015, CHEM REV, V115, P11043, DOI 10.1021/cr5006793
Lee YW, 2019, THERANOSTICS, V9, P3280, DOI 10.7150/thno.34412
Li G., 2020, ACS NANO
Li GZ, 2019, ADV FUNCT MATER, V29, DOI 10.1002/adfm.201901932
Li GZ, 2019, SMALL, V15, DOI 10.1002/smll.201900936
Li L, 2017, ACS NANO, V11, P95, DOI 10.1021/acsnano.6b04261
Li S, 2017, CHEM SCI, V8, P7552, DOI 10.1039/c7sc02616a
Li ZT, 2020, SCI ADV, V6, DOI 10.1126/sciadv.aaz9240
Liu CY, 2019, SCI ADV, V5, DOI 10.1126/sciadv.aaw8922
Liu HM, 2016, SCI REP-UK, V6, DOI 10.1038/srep25069
Liu HM, 2014, BIOMATERIALS, V35, P5407, DOI 10.1016/j.biomaterials.2014.03.040
Lostale-Seijo I, 2018, NAT REV CHEM, V2, P258, DOI 10.1038/s41570-018-0039-1

Lv J, 2021, CHEM SOC REV, V50, P5435, DOI 10.1039/d0cs00258e
Lv J, 2020, NANO LETT, V20, P8600, DOI 10.1021/acs.nanolett.0c03287
Lv J, 2020, SCI CHINA MATER, V63, P620, DOI 10.1007/s40843-019-1213-2
Lv J, 2019, BIOMATERIALS, V218, DOI 10.1016/j.biomaterials.2019.119358
Lv J, 2018, BIOMATERIALS, V182, P167, DOI 10.1016/j.biomaterials.2018.08.023
Lv J, 2015, J MATER CHEM B, V3, P642, DOI 10.1039/c4tb01447b
Naarmann N, 2006, ANGEW CHEM INT EDIT, V45, P2588, DOI 10.1002/anie.200503567
Newcomb CJ, 2014, NAT COMMUN, V5, DOI 10.1038/ncomms4321
Ojima I, 2013, J ORG CHEM, V78, P6358, DOI 10.1021/jo400301u
Peraro L, 2018, ANGEW CHEM INT EDIT, V57, P11868, DOI 10.1002/anie.201801361
Qi Y, 2018, SMALL, V14, DOI 10.1002/smll.201803061
Ren LF, 2022, BIOACT MATER, V9, P44, DOI 10.1016/j.bioactmat.2021.08.006
Ren LF, 2020, ANGEW CHEM INT EDIT, V59, P4711, DOI 10.1002/anie.201914970
Rong GY, 2020, SCI ADV, V6, DOI 10.1126/sciadv.aaz1774
Sanchez-Navarro M, 2017, ACCOUNTS CHEM RES, V50, P1847, DOI 10.1021/acs.accounts.7b00204
Schrenk D, 2020, EFSA J, V18, DOI 10.2903/j.efsa.2020.6223
Shen WW, 2020, CCS CHEM, V2, P146, DOI 10.31635/ccschem.020.201900084
Shen WW, 2016, J MATER CHEM B, V4, P6468, DOI 10.1039/c6tb01929c
Stewart MP, 2018, CHEM REV, V118, P7409, DOI 10.1021/acs.chemrev.7b00678
Tan EC, 2018, J MATER CHEM B, V6, P7230, DOI 10.1039/c8tb01470a
Tirotta I, 2015, CHEM REV, V115, P1106, DOI 10.1021/cr500286d
Vierling P, 2001, J FLUORINE CHEM, V107, P337, DOI 10.1016/S0022-1139(00)00378-X
Wang H, 2015, ANGEW CHEM INT EDIT, V54, P11647, DOI 10.1002/anie.201501461
Wang H, 2016, POLYM CHEM-UK, V7, P2319, DOI 10.1039/c6py00153j
Wang LH, 2016, ANGEW CHEM INT EDIT, V55, P755, DOI 10.1002/anie.201508695
Wang MM, 2016, ACTA BIOMATER, V46, P204, DOI 10.1016/j.actbio.2016.09.023
Wang MM, 2014, BIOMATERIALS, V35, P6603, DOI 10.1016/j.biomaterials.2014.04.065
Wang MM, 2014, NAT COMMUN, V5, DOI 10.1038/ncomms4053
Wang XY, 2020, ACS APPL MATER INTER, V12, P45763, DOI 10.1021/acsami.0c11967
Wang Y, 2021, ADV MATER, V33, DOI 10.1002/adma.202103307
Wang YT, 2016, J MATER CHEM B, V4, P1354, DOI 10.1039/c5tb02712h
Wei H, 2013, ANGEW CHEM INT EDIT, V52, P5377, DOI 10.1002/anie.201301896
Wu PK, 2020, BIOMATERIALS, V261, DOI 10.1016/j.biomaterials.2020.120311
Xu JK, 2021, ADV MATER, V33, DOI 10.1002/adma.202104355
Xu J, 2020, NAT NANOTECHNOL, V15, P1043, DOI 10.1038/s41565-020-00781-4
Yan Y, 2022, BIOACT MATER, V7, P333, DOI 10.1016/j.bioactmat.2021.05.041
Yang JP, 2015, CHEM REV, V115, P5274, DOI 10.1021/cr500542t
Zeng HX, 2015, ACS CENTRAL SCI, V1, P303, DOI 10.1021/acscentsci.5b00233
Zhang C, 2022, CHEM REV, V122, P167, DOI 10.1021/acs.chemrev.1c00632
Zhang S, 2021, NANO LETT, V21, P7855, DOI 10.1021/acs.nanolett.1c03031
Zhang S, 2021, THERANOSTICS, V11, P614, DOI 10.7150/thno.47007
Zhang TB, 2018, NANO LETT, V18, P6301, DOI 10.1021/acs.nanolett.8b02553
Zhang ZJ, 2018, NAT COMMUN, V9, DOI 10.1038/s41467-018-03779-8
NR 80
TC 33
Z9 34
U1 50
U2 165
PU AMER CHEMICAL SOC
PI WASHINGTON
PA 1155 16TH ST, NW, WASHINGTON, DC 20036 USA
SN 0001-4842
EI 1520-4898
J9 ACCOUNTS CHEM RES
JI Accounts Chem. Res.
PD MAR 1
PY 2022
VL 55
IS 5
BP 722
EP 733

DI 10.1021/acs.accounts.1c00766
PG 12
WC Chemistry, Multidisciplinary
WE Science Citation Index Expanded (SCI-EXPANDED)
SC Chemistry
GA 0A1YA
UT WOS:000773756100004
PM 35175741
HC Y
HP N
DA 2023-09-14
ER

PT J
AU Liu, X
Su, Q
Song, HJ
Shi, XG
Zhang, YM
Zhang, CN
Huang, PS
Dong, AJ
Kong, DL
Wang, WW
AF Liu, Xiang
Su, Qi
Song, Huijuan
Shi, Xiaoguang
Zhang, Yuming
Zhang, Chuangnian
Huang, Pingsheng
Dong, Anjie
Kong, Deling
Wang, Weiwei
TI PolyTLR7/8a-conjugated, antigen-trapping gold nanorods elicit anticancer immunity against abscopal tumors by photothermal therapy-induced in situ vaccination
SO BIOMATERIALS
LA English
DT Article
DE Vaccination; Photothermal therapy; Gold nanorods; Antitumor immunity; Cancer immunotherapy
ID VACCINES; CELLS; NANOPARTICLES; MECHANISMS; ABLATION; DELIVERY; AGONISTS; ROLES; SIZE
AB Nanovaccine can elicit antigen-specific immune responses against tumor cells expressing homologous antigens and has attracted enormous attention in cancer immunotherapy. However, tumor heterogeneity remarkably hinders the development of nanovaccines. Here we demonstrate that PTT-induced in situ vaccination cancer therapy can elicit potent antitumor immunity against disseminated and metastatic tumors. Gold nanorods (AuNRs) covalently coupled with amphiphilic polyTLR7/8a and MMP-2-sensitive R9-PEG conjugate (AuNRsIMQD-R9-PEG) were developed as a new biocompatible PTT agent with favorable photothermal efficiency and stability. Importantly, AuNRs-IMQD-R9-PEG can effectively absorb tumor-derived protein antigens, forming nanovaccines directly in vivo and enhance the activation of host dendritic cells (DCs), thereby amplifying adaptive antitumor T-cell responses, triggering effector memory immune responses, and activating innate antitumor immunity. Remarkably, peri-tumoral administration of low-dose multifunctional AuNRs followed by noninvasive near-infrared (NIR) laser irradiation enables efficient tandem PTT-vaccination treatment modality that can inhibit local as well as untreated distant and metastatic tumors in mice inoculated with poorly immunogenic, highly metastatic 4T1 tumors. Our findings indicate that AuNRs-IMQD-R9-PEG-mediated in situ cancer vaccination provides a powerful immunotherapy characterized by markedly increased infiltration of effector CD8+ T, natural killer T (NKT) cells in tumors and long-term animal survival, thus, offering a promising therapeutic strategy for advanced, disseminated cancers.

C1 [Liu, Xiang; Su, Qi; Dong, Anjie] Tianjin Univ, Sch Chem Engn & Technol, Dept Polymer Sci & Engn, Minist Educ,Key Lab Syst Bioengn, Tianjin 300072, Peoples R China.
[Song, Huijuan; Zhang, Chuangnian; Huang, Pingsheng; Wang, Weiwei] Chinese Acad Med Sci & Peking Union Med Coll, Tianjin Key Lab Biomat Res, Inst Biomed Engn, Tianjin 300192, Peoples R China.
[Shi, Xiaoguang] Nankai Univ, Coll Chem, State Key Lab Med Chem Biol & Inst Polymer Chem, Key Lab Funct Polymer Mat Minist Educ, Tianjin 300071, Peoples R China.
[Dong, Anjie] Collaborat Innovat Ctr Chem Sci & Engn Tianjin, Tianjin 300072, Peoples R China.
[Zhang, Yuming] Chinese Acad Med Sci & Peking Union Med Coll, Tianjin Key Lab Radiat Med & Mol Nucl Med, Inst Radiat Med, Tianjin 300192, Peoples R China.
[Kong, Deling] Nankai Univ, Coll Life Sci, State Key Lab Med Chem Biol, Tianjin 300071, Peoples R China.
C3 Tianjin University; Chinese Academy of Medical Sciences - Peking Union Medical College; Peking Union Medical College; Institute of BioMedical Engineering - CAMS; Nankai University; Chinese Academy of Medical Sciences - Peking Union Medical College; Institute of Radiation Medicine - CAMS; Peking Union Medical College; Nankai University
RP Dong, AJ (通讯作者)，Tianjin Univ, Sch Chem Engn & Technol, Dept Polymer Sci & Engn, Minist Educ,Key Lab Syst Bioengn, Tianjin 300072, Peoples R China.; Wang, WW (通讯作者)，Chinese Acad Med Sci & Peking Union Med Coll, Tianjin Key Lab Biomat Res, Inst Biomed Engn, Tianjin 300192, Peoples R China.; Zhang, YM (通讯作者)，Chinese Acad Med Sci & Peking Union Med Coll, Tianjin Key Lab Radiat Med & Mol Nucl Med, Inst Radiat Med, Tianjin 300192, Peoples R China.
EM zhangyumin21@163.com; ajdong@tju.edu.cn; wwwangtj@163.com
RI zhang, yu/HNS-5948-2023; Wang, Weiwei/GRF-3625-2022; Li, Li/IAQ-0885-2023; ZHANG, YUTING/HOH-4131-2023
FU National Natural Science Foundation of China [31971306, 31870950, 82001954]; Young Elite Scientists Sponsorship Program by Tianjin [TJSQNTJ-2018-01]; Tianjin Innovation and Promotion Plan Key Innovation Team of Immunoreactive Biomaterials; Specific Program for High-Tech Leader & Team of Tianjin Government
FX We acknowledge the financial support from National Natural Science Foundation of China (No. 31971306, 31870950, 82001954) , Young Elite Scientists Sponsorship Program by Tianjin (TJSQNTJ-2018-01) , Tianjin Innovation and Promotion Plan Key Innovation Team of Immunoreactive Biomaterials, and Specific Program for High-Tech Leader & Team of Tianjin Government.
CR Bachmann MF, 2010, NAT REV IMMUNOL, V10, P787, DOI 10.1038/nri2868
Banchereau J, 1998, NATURE, V392, P245, DOI 10.1038/32588
Bear AS, 2013, PLOS ONE, V8, DOI 10.1371/journal.pone.0069073
Bekeredjian-Ding I, 2006, J IMMUNOL, V176, P7438, DOI 10.4049/jimmunol.176.12.7438
Bray F, 2018, CA-CANCER J CLIN, V68, P394, DOI 10.3322/caac.21492
Chao Y, 2020, SCI ADV, V6, DOI 10.1126/sciadv.aaz4204
Chen Q, 2019, ADV MATER, V31, DOI 10.1002/adma.201900192
Chen Q, 2019, ADV MATER, V31, DOI 10.1002/adma.201802228
Chen Q, 2016, NAT COMMUN, V7, DOI 10.1038/ncomms13193
Chu KF, 2014, NAT REV CANCER, V14, P199, DOI 10.1038/nrc3672
Fang L, 2020, SCI ADV, V6, DOI 10.1126/sciadv.aba4024
Finn OJ, 2018, NAT REV IMMUNOL, V18, P183, DOI 10.1038/nri.2017.140
Frucht DM, 2001, TRENDS IMMUNOL, V22, P556, DOI 10.1016/S1471-4906(01)02005-1
Glaffig M, 2015, ORG BIOMOL CHEM, V13, P10150, DOI 10.1039/c5ob01255d
Guo LR, 2014, ACS NANO, V8, P5670, DOI 10.1021/nn5002112
Hammerich L, 2019, NAT MED, V25, P814, DOI 10.1038/s41591-019-0410-x
Hilf N, 2019, NATURE, V565, P240, DOI 10.1038/s41586-018-0810-y
Hu Z, 2018, NAT REV IMMUNOL, V18, P168, DOI 10.1038/nri.2017.131
Lee IH, 2012, ANGEW CHEM INT EDIT, V51, P8800, DOI 10.1002/anie.201203193
Li W, 2019, NAT COMMUN, V10, DOI 10.1038/s41467-019-11269-8
Li WS, 2017, CHEM COMMUN, V53, P5565, DOI 10.1039/c7cc01128h
Liu X, 2020, BIOMATERIALS, V230, DOI 10.1016/j.biomaterials.2019.119649
Lynn GM, 2020, NAT BIOTECHNOL, V38, P320, DOI 10.1038/s41587-019-0390-x
Mackey MA, 2014, J PHYS CHEM B, V118, P1319, DOI 10.1021/jp409298f
Maisonneuve C, 2014, P NATL ACAD SCI USA, V111, P12294, DOI 10.1073/pnas.1400478111
Meacham CE, 2013, NATURE, V501, P328, DOI 10.1038/nature12624

Melief CJM, 2015, J CLIN INVEST, V125, P3401, DOI 10.1172/JCI80009
Min YZ, 2017, NAT NANOTECHNOL, V12, P877, DOI [10.1038/nnano.2017.113, 10.1038/NNANO.2017.113]
Nam J, 2019, NAT REV MATER, V4, P398, DOI 10.1038/s41578-019-0108-1
Nam J, 2018, NAT COMMUN, V9, DOI 10.1038/s41467-018-03473-9
Nam J, 2013, ACS NANO, V7, P3388, DOI 10.1021/nn400223a
Ni KY, 2020, ANGEW CHEM INT EDIT, V59, P1108, DOI 10.1002/anie.201911429
Ni QQ, 2020, SCI ADV, V6, DOI 10.1126/sciadv.aaw6071
Ott PA, 2017, NATURE, V547, P217, DOI 10.1038/nature22991
Peng JR, 2018, ADV SCI, V5, DOI 10.1002/advs.201700891
Rastinehad AR, 2019, P NATL ACAD SCI USA, V116, P18590, DOI 10.1073/pnas.1906929116
Reed SG, 2013, NAT MED, V19, P1597, DOI 10.1038/nm.3409
Sallusto F, 2004, ANNU REV IMMUNOL, V22, P745, DOI 10.1146/annurev.immunol.22.012703.104702
Smith TT, 2017, J CLIN INVEST, V127, P2176, DOI 10.1172/JCI87624
Song C, 2019, NAT COMMUN, V10, DOI 10.1038/s41467-019-11730-8
Srivastava P, 2002, NAT REV IMMUNOL, V2, P185, DOI 10.1038/nri749
Sweeney EE, 2018, SMALL, V14, DOI 10.1002/smll.201800678
Tao Y, 2014, ADV FUNCT MATER, V24, P1004, DOI 10.1002/adfm.201302347
Toraya-Brown S, 2014, NANOMED-NANOTECHNOL, V10, P1273, DOI 10.1016/j.nano.2014.01.011
Uvyn A, 2019, ANGEW CHEM INT EDIT, V58, P12988, DOI 10.1002/anie.201905093
Wang C, 2016, ADV MATER, V28, P8912, DOI 10.1002/adma.201506312
Wilson DS, 2019, NAT MATER, V18, P175, DOI 10.1038/s41563-018-0256-5
Yamaoka A, 2010, J CONTROL RELEASE, V142, P411, DOI 10.1016/j.jconrel.2009.11.005
Ye XY, 2019, ACS NANO, V13, P2956, DOI 10.1021/acsnano.8b07371
Zeng JY, 2018, ADV FUNCT MATER, V28, DOI 10.1002/adfm.201705451
Zhang ST, 2020, NANO-MICRO LETT, V12, DOI 10.1007/s40820-020-00516-z
Zheng LY, 2020, J AM CHEM SOC, V142, P382, DOI 10.1021/jacs.9b11046
Zhou FY, 2019, ADV MATER, V31, DOI 10.1002/adma.201805888
NR 53
TC 33
Z9 34
U1 11
U2 85
PU ELSEVIER SCI LTD
PI OXFORD
PA THE BOULEVARD, LANGFORD LANE, KIDLINGTON, OXFORD OX5 1GB, OXON, ENGLAND
SN 0142-9612
EI 1878-5905
J9 BIOMATERIALS
JI Biomaterials
PD AUG
PY 2021
VL 275
AR 120921
DI 10.1016/j.biomaterials.2021.120921
EA JUN 2021
PG 12
WC Engineering, Biomedical; Materials Science, Biomaterials
WE Science Citation Index Expanded (SCI-EXPANDED)
SC Engineering; Materials Science
GA TU8TR
UT WOS:000681304000003
PM 34139508
DA 2023-09-14
ER

PT J
AU Li, WH
Wu, JJ
Wu, L
Zhang, BD

Hu, HG
Zhao, L
Li, ZB
Yu, XF
Li, YM
AF Li, Wen-Hao
Wu, Jun-Jun
Wu, Lie
Zhang, Bo-Dou
Hu, Hong-Guo
Zhao, Lang
Li, Zhi-Bin
Yu, Xue-Feng
Li, Yan-Mei
TI Black phosphorous nanosheet: A novel immune-potentiating nanoadjuvant for near-infrared-improved immunotherapy
SO BIOMATERIALS
LA English
DT Article
DE Antitumor vaccines; Adjuvant materials; Black phosphorus; Cancer immunotherapy; Two-dimensional materials
ID SILICA NANOPARTICLES; STIMULATION; VACCINES; ADJUVANT; FUTURE
AB Intrinsic immune behaviors of nanomaterials and immune systems promote research on their adjuvanticity and the design of next generation nanovaccine-based immunotherapies. Herein, we report a promising multifunctional nanoadjuvant by exploring the immune-potentiating effects of black phosphorus nanosheets (BPs) in vitro and in vivo. The facile coating of BPs with phenylalanine-lysine-phenylalanine (FKF) tripeptide-modified antigen epitopes (FKF-OVAp@BP) enables the generation of a minimalized nanovaccine by integrating high loading capacity, efficient drug delivery, comprehensive dendritic cell (DC) activation, and biocompatibility for cancer immunotherapy. Systemic immunization elicits potent antitumor cellular immunity and significantly augments checkpoint blockade (CPB) against melanoma in a mouse model. Furthermore, near-infrared (NIR) photothermal effects of BPs create an immune-favorable microenvironment for improved local immunization. This study offers new insight into the integration of immunoactivity and photothermal effects for enhanced cancer immunotherapy by using a nanoadjuvant and thus potentially advances the design and application of multifunctional adjuvant materials for cancer nanotreatment.
C1 [Li, Wen-Hao; Wu, Jun-Jun; Zhang, Bo-Dou; Hu, Hong-Guo; Zhao, Lang; Li, Yan-Mei] Tsinghua Univ, Dept Chem, Minist Educ, Key Lab Bioorgan Phosphorus Chem & Chem Biol, Beijing 100084, Peoples R China.
[Wu, Lie; Li, Zhi-Bin; Yu, Xue-Feng] Chinese Acad Sci, Shenzhen Inst Adv Technol, Mat & Interfaces Ctr, Shenzhen 518055, Peoples R China.
[Li, Yan-Mei] Beijing Inst Brain Disorders, Beijing 100069, Peoples R China.
[Li, Yan-Mei] Tsinghua Univ, Ctr Synthet & Syst Biol, Beijing 100084, Peoples R China.
C3 Tsinghua University; Chinese Academy of Sciences; Shenzhen Institute of Advanced Technology, CAS; Tsinghua University
RP Li, YM (通讯作者)，Tsinghua Univ, Dept Chem, Minist Educ, Key Lab Bioorgan Phosphorus Chem & Chem Biol, Beijing 100084, Peoples R China.; Yu, XF (通讯作者)，Chinese Acad Sci, Shenzhen Inst Adv Technol, Mat & Interfaces Ctr, Shenzhen 518055, Peoples R China.
EM xf.yu@siat.ac.cn; liym@mail.tsinghua.edu.cn
RI yu, xuefeng/AGT-5190-2022
OI Yu (yu), Xuefeng (xue feng)/0000-0003-2566-6194; Wu, Jun-Jun/0000-0001-6113-8976; Li, Zhibin/0000-0003-4752-5528; Wu, Lie/0000-0003-0184-2106; Lang, Zhao/0000-0002-2266-8728
FU National Key R&D Program of China [2019YFA0904200, 2018YFA0507600]; National Natural Science Foundation of China [21672126, 21975280]; Guangdong Special Support Program [2017TX04C096]
FX This work was supported by the National Key R&D Program of China (2019YFA0904200, 2018YFA0507600), and the National Natural Science Foundation of China (21672126, 21975280), and Guangdong Special Support Program (2017TX04C096).
CR Abbaraju PL, 2018, CHEM COMMUN, V54, P2020, DOI 10.1039/c8cc00327k
Abbaraju PL, 2017, J AM CHEM SOC, V139, P6321, DOI 10.1021/jacs.6b12622

Blander JM, 2018, ANNU REV IMMUNOL, V36, P717, DOI 10.1146/annurev-immunol-041015-055523
Cao FQ, 2018, ADV HEALTHC MATER, V7, DOI 10.1002/adhm.201701439
Cao YH, 2014, ADV FUNCT MATER, V24, P6963, DOI 10.1002/adfm.201401358
Chen Q, 2019, ADV MATER, V31, DOI 10.1002/adma.201900192
Chen WS, 2017, ADV MATER, V29, DOI 10.1002/adma.201603864
Clatworthy MR, 2014, NAT MED, V20, P1458, DOI 10.1038/nm.3709
Deng ZJ, 2011, NAT NANOTECHNOL, V6, P39, DOI 10.1038/NNANO.2010.250
EUHUS DM, 1986, J SURG ONCOL, V31, P229, DOI 10.1002/jso.2930310402
Gu ZY, 2019, J AM CHEM SOC, V141, P6122, DOI 10.1021/jacs.8b10904
Guo ZN, 2015, ADV FUNCT MATER, V25, P6996, DOI 10.1002/adfm.201502902
Nguyen HT, 2019, ACS APPL MATER INTER, V11, P24959, DOI 10.1021/acsami.9b04632
Houde M, 2003, NATURE, V425, P402, DOI 10.1038/nature01912
Hu Z, 2018, NAT REV IMMUNOL, V18, P168, DOI 10.1038/nri.2017.131
Huang LP, 2019, NAT COMMUN, V10, DOI 10.1038/s41467-019-12771-9
Irvine DJ, 2016, NAT REV MATER, V1, DOI 10.1038/natrevmats.2015.8
Irvine DJ, 2015, CHEM REV, V115, P11109, DOI 10.1021/acs.chemrev.5b00109
Kitano M, 2016, P NATL ACAD SCI USA, V113, P1044, DOI 10.1073/pnas.1513607113
Li WH, 2020, CHEM REV, V120, P11420, DOI 10.1021/acs.chemrev.9b00833
Li XS, 2020, NAT REV CLIN ONCOL, V17, P657, DOI 10.1038/s41571-020-0410-2
Li Y, 2018, ADV SCI, V5, DOI 10.1002/advs.201700805
Li Z, 2020, ADV FUNCT MATER, V30, DOI 10.1002/adfm.201905758
Liang F, 2017, SCI TRANSL MED, V9, DOI 10.1126/scitranslmed.aal2094
Liang X, 2019, J CONTROL RELEASE, V296, P150, DOI 10.1016/j.jconrel.2019.01.027
Luo M, 2017, NAT NANOTECHNOL, V12, P648, DOI [10.1038/nnano.2017.52, 10.1038/NNANO.2017.52]
Lutz MB, 1999, J IMMUNOL METHODS, V223, P77, DOI 10.1016/S0022-1759(98)00204-X
Manolova V, 2008, EUR J IMMUNOL, V38, P1404, DOI 10.1002/eji.200737984
Melief CJM, 2015, J CLIN INVEST, V125, P3401, DOI 10.1172/JCI80009
Meng CC, 2016, ACS NANO, V10, P2203, DOI 10.1021/acsnano.5b06750
Miao L, 2019, NAT BIOTECHNOL, V37, P1174, DOI 10.1038/s41587-019-0247-3
Mo JB, 2020, NANOSCALE, V12, P1742, DOI 10.1039/c9nr08570j
Mo JB, 2018, NAT COMMUN, V9, DOI 10.1038/s41467-018-04873-7
Moyer TJ, 2016, J CLIN INVEST, V126, P799, DOI 10.1172/JCI81083
Ou W, 2019, THERANOSTICS, V9, P6780, DOI 10.7150/thno.37123
Plutnar J, 2018, ACS NANO, V12, P8390, DOI 10.1021/acsnano.8b03740
Qu GB, 2020, CHEM REV, V120, P2288, DOI 10.1021/acs.chemrev.9b00445
Qu GB, 2017, ANGEW CHEM INT EDIT, V56, P14488, DOI 10.1002/anie.201706228
Skwarczynski M, 2020, SCI ADV, V6, DOI 10.1126/sciadv.aax2285
Su YJ, 2020, MATER HORIZ, V7, P574, DOI 10.1039/c9mh01068h
Swartz MA, 2012, SCI TRANSL MED, V4, DOI 10.1126/scitranslmed.3003763
Tao W, 2017, ADV MATER, V29, DOI 10.1002/adma.201603276
TOMAYKO MM, 1989, CANCER CHEMOTH PHARM, V24, P148, DOI 10.1007/BF00300234
Trevaskis NL, 2015, NAT REV DRUG DISCOV, V14, P781, DOI 10.1038/nrd4608
Wang HY, 2018, SMALL, V14, DOI 10.1002/smll.201801701
Wang J, 2018, NAT NANOTECHNOL, V13, P1078, DOI 10.1038/s41565-018-0274-0
Wang XP, 2017, SMALL, V13, DOI 10.1002/smll.201701816
Wang XP, 2016, ANGEW CHEM INT EDIT, V55, P1899, DOI 10.1002/anie.201506179
Xiang J, 2015, ACS NANO, V9, P6401, DOI 10.1021/acsnano.5b02014
Xu LG, 2016, NANOSCALE, V8, P3785, DOI 10.1039/c5nr09208f
Yata T, 2017, BIOMATERIALS, V146, P136, DOI 10.1016/j.biomaterials.2017.09.014
Ye XY, 2019, ACS NANO, V13, P2956, DOI 10.1021/acsnano.8b07371
Zanganeh S, 2016, NAT NANOTECHNOL, V11, P986, DOI [10.1038/nnano.2016.168, 10.1038/NNANO.2016.168]
Zhao HN, 2020, CHEM ENG J, V394, DOI 10.1016/j.cej.2020.124314
Zhou WH, 2018, ANGEW CHEM INT EDIT, V57, P10268, DOI 10.1002/anie.201806941
NR 55
TC 33
Z9 33
U1 20
U2 127
PU ELSEVIER SCI LTD

PI OXFORD
PA THE BOULEVARD, LANGFORD LANE, KIDLINGTON, OXFORD OX5 1GB, OXON, ENGLAND
SN 0142-9612
EI 1878-5905
J9 BIOMATERIALS
JI Biomaterials
PD JUN
PY 2021
VL 273
AR 120788
DI 10.1016/j.biomaterials.2021.120788
EA APR 2021
PG 15
WC Engineering, Biomedical; Materials Science, Biomaterials
WE Science Citation Index Expanded (SCI-EXPANDED)
SC Engineering; Materials Science
GA SB0CH
UT WOS:000649669800004
PM 33933912
DA 2023-09-14
ER

PT J
AU Mohsen, MO
   Heath, MD
   Cabral-Miranda, G
   Lipp, C
   Zeltins, A
   Sande, M
   Stein, JV
   Riether, C
   Roesti, E
   Zha, LS
   Engroff, P
   El-Turabi, A
   Kundig, TM
   Vogel, M
   Skinner, MA
   Speiser, DE
   Knuth, A
   Kramer, MF
   Bachmann, MF
AF Mohsen, Mona O.
   Heath, Matthew D.
   Cabral-Miranda, Gustavo
   Lipp, Cyrill
   Zeltins, Andris
   Sande, Marcos
   Stein, Jens, V
   Riether, Carsten
   Roesti, Elisa
   Zha, Lisha
   Engroff, Paul
   El-Turabi, Aadil
   Kundig, Thomas M.
   Vogel, Monique
   Skinner, Murray A.
   Speiser, Daniel E.
   Knuth, Alexander
   Kramer, Matthias F.
   Bachmann, Martin F.

TI Vaccination with nanoparticles combined with micro-adjuvants protects against cancer
SO JOURNAL FOR IMMUNOTHERAPY OF CANCER
LA English
DT Article
DE Cucumber-mosaic virus CuMV; Virus-like particle VLP; Microcrystalline tyrosine MCT; Nano-vaccine
ID T-CELL RESPONSES; TUMOR-INFILTRATING LYMPHOCYTES; FREE CLICK CHEMISTRY; VIRUS-LIKE PARTICLES; MELANOMA; TYROSINE; EFFICACY; PEPTIDE; ANTIGEN; OFFERS
AB Background: Induction of strong T cell responses, in particular cytotoxic T cells, is a key for the generation of efficacious therapeutic cancer vaccines which yet, remains a major challenge for the vaccine developing world. Here we demonstrate that it is possible to harness the physiological properties of the lymphatic system to optimize the induction of a protective T cell response. Indeed, the lymphatic system sharply distinguishes between nanoscale and microscale particles. The former reaches the fenestrated lymphatic system via diffusion, while the latter either need to be transported by dendritic cells or form a local depot.

Methods: Our previously developed cucumber-mosaic virus-derived nanoparticles termed (CuMVTT-VLPs) incorporating a universal Tetanus toxoid epitope TT830-843 were assessed for their draining kinetics using stereomicroscopic imaging. A nano-vaccine has been generated by coupling p33 epitope as a model antigen to CuMVTT-VLPs using bio-orthogonal Cu-free click chemistry. The CuMVTT-p33 nano-sized vaccine has been next formulated with the micron-sized microcrystalline tyrosine (MCT) adjuvant and the formed depot effect was studied using confocal microscopy and trafficking experiments. The immunogenicity of the nanoparticles combined with the micron-sized adjuvant was next assessed in an aggressive transplanted murine melanoma model. The obtained results were compared to other commonly used adjuvants such as B type CpGs and Alum.

Results: Our results showed that CuMVTT-VLPs can efficiently and rapidly drain into the lymphatic system due to their nano-size of similar to 30 nm. However, formulating the nanoparticles with the micron-sized MCT adjuvant of similar to 5 mu M resulted in a local depot for the nanoparticles and a longer exposure time for the immune system. The preclinical nano-vaccine CuMVTT-p33 formulated with the micron-sized MCT adjuvant has enhanced the specific T cell response in the stringent B16F10p33 murine melanoma model. Furthermore, the micron-sized MCT adjuvant was as potent as B type CpGs and clearly superior to the commonly used Alum adjuvant when total CD8(+), specific p33 T cell response or tumour protection were assessed.

Conclusion: The combination of nano- and micro-particles may optimally harness the physiological properties of the lymphatic system. Since the nanoparticles are well defined virus-like particles and the micron-sized adjuvant MCT has been used for decades in allergen-specific desensitization, this approach may readily be translated to the clinic.
C1 [Mohsen, Mona O.; El-Turabi, Aadil; Bachmann, Martin F.] Univ Oxford, Jenner Inst, Nuffield Dept Med, Oxford, England.

[Mohsen, Mona O.; Cabral-Miranda, Gustavo; Lipp, Cyrill; Roesti, Elisa; Zha, Lisha; Engroff, Paul; Vogel, Monique; Bachmann, Martin F.] Univ Bern, Dept BioMed Res Immunol RIA, Inselspital, Bern, Switzerland.

[Heath, Matthew D.; Skinner, Murray A.; Kramer, Matthias F.] Bencard Adjuvant Syst, Dominion Way, Worthing, England.

[Zeltins, Andris] Latvian Biomed Res & Study Ctr, Riga, Latvia.

[Sande, Marcos] Univ Bern, Inst Anat, Bern, Switzerland.

[Stein, Jens, V] Univ Bern, Theodor Kocher Inst, Bern, Switzerland.

[Riether, Carsten] Univ Bern, Bern Univ Hosp, Dept Med Oncol, Bern, Switzerland.

[Zha, Lisha] Anhui Agr Univ, Int Immunol Ctr, Hefei, Anhui, Peoples R China.

[Kundig, Thomas M.] Univ Zurich, Dept Dermatol, Bern, Switzerland.

[Speiser, Daniel E.] Univ Lausanne, Dept Oncol, Bern, Switzerland.

[Mohsen, Mona O.; Knuth, Alexander] NCCCR, Doha, State Of Qatar, Qatar.
C3 University of Oxford; Jenner Institute; University of Bern; Latvian Biomedical Research & Study Centre; University of Bern; University of Bern; University of Bern; University Hospital of Bern; Anhui Agricultural University; University of Zurich; University of Lausanne; Hamad Medical Corporation
RP Mohsen, MO (通讯作者)，Univ Oxford, Jenner Inst, Nuffield Dept Med, Oxford, England.; Mohsen, MO (通讯作者)，Univ Bern, Dept BioMed Res Immunol RIA, Inselspital, Bern, Switzerland.; Mohsen, MO (通讯作者)，NCCCR, Doha, State Of Qatar, Qatar.
EM mona.mohsen@dbmr.unibe.ch

RI knuth, alexander/H-2608-2014; Speiser, Daniel E./F-2173-2013; Engeroff, Paul/AGS-3183-2022; Kramer, Matthias F./AAK-9279-2021; Riether, Carsten/K-8603-2013; Cabral-Miranda, Gustavo/AAH-5494-2019; Zeltins, Andris/AAI-1017-2021; Heath, Matthew/A-8886-2010; Bachmann, Martin Fabian/IAP-4113-2023; Riether, Carsten/ABC-7699-2021; Bachmann, Martin/AFO-8025-2022
OI knuth, alexander/0000-0002-9318-2784; Engeroff, Paul/0000-0002-3409-512X; Kramer, Matthias F./0000-0002-3740-4733; Riether, Carsten/0000-0001-7512-513X; Cabral-Miranda, Gustavo/0000-0001-8351-4880; Riether, Carsten/0000-0001-7512-513X; Sande-Melon, Marcos/0000-0003-3844-0143; Stein, Jens V/0000-0002-8199-9586; Heath, Matthew/0000-0002-6095-4098
FU Bencard Adjuvant Systems, Dominion Way, UK; Swiss Cancer League [KFS-4132-02-2017]; Qatar Foundation
FX This work was supported by Bencard Adjuvant Systems, Dominion Way, UK, the Swiss Cancer League (KFS-4132-02-2017) and Qatar Foundation.
CR Ambuhl PM, 2007, J HYPERTENS, V25, P63, DOI 10.1097/HJH.0b013e32800ff5d6
Ammi R, 2015, PHARMACOL THERAPEUT, V146, P120, DOI 10.1016/j.pharmthera.2014.09.010
Bachmann MF, 2010, NAT REV IMMUNOL, V10, P787, DOI 10.1038/nri2868
Bachmann MF, 2018, J ALLERGY CLIN IMMUN
Baskin JM, 2007, P NATL ACAD SCI USA, V104, P16793, DOI 10.1073/pnas.0707090104
Bastola R, 2017, ARCH PHARM RES, V40, P1238, DOI 10.1007/s12272-017-0969-z
Baumgaertner P, 2016, ONCOIMMUNOLOGY, V5, DOI 10.1080/2162402X.2016.1216290
Brewer KD, 2018, J BIOMED SCI, V25, DOI 10.1186/s12929-018-0413-9
Cabral-Miranda G, 2017, VACCINES-BASEL, V5, DOI 10.3390/vaccines5040032
Cabral-Miranda G, 2017, VACCINES-BASEL, V5, DOI 10.3390/vaccines5020010
Cavelti-Weder C, 2016, MOL THER, V24, P1003, DOI 10.1038/mt.2015.227
Chang PV, 2010, P NATL ACAD SCI USA, V107, P1821, DOI 10.1073/pnas.0911116107
Cox JC, 1997, VACCINE, V15, P248, DOI 10.1016/S0264-410X(96)00183-1
Fettelschoss-Gabriel A, 2018, J ALLERGY CLIN IMMUN
Goldinger SM, 2012, EUR J IMMUNOL, V42, P3049, DOI 10.1002/eji.201142361
Hailemichael Y, 2013, NAT MED, V19, P465, DOI 10.1038/nm.3105
Heath MD, 2017, BMC INFECT DIS, V17, DOI 10.1186/s12879-017-2329-5
Hu Z, 2018, NAT REV IMMUNOL, V18, P168, DOI 10.1038/nri.2017.131
Kakwere H, 2017, BIOCONJUGATE CHEM, V28, P2756, DOI 10.1021/acs.bioconjchem.7b00502
Khong H, 2016, J IMMUNOTHER CANCER, V4, DOI 10.1186/s40425-016-0160-y
Klimek L, 2017, EXPERT REV CLIN IMMU, V13, P599, DOI 10.1080/1744666X.2017.1292133
Koster BD, 2017, CLIN CANCER RES, V23, P5679, DOI 10.1158/1078-0432.CCR-17-0944
Krieg AM, 2006, NAT REV DRUG DISCOV, V5, P471, DOI 10.1038/nrd2059
Lai CH, 2018, THERANOSTICS, V8, P1723, DOI 10.7150/thno.22056
Lebel ME, 2015, VACCINES-BASEL, V3, P620, DOI 10.3390/vaccines3030620
Lee N, 2016, PATHOLOGY, V48, P177, DOI 10.1016/j.pathol.2015.12.006
Leuthard DS, 2018, J IMMUNOL, V200, P3151, DOI 10.4049/jimmunol.1800035
Mellor AL, 2017, FRONT IMMUNOL, V8, DOI 10.3389/fimmu.2017.01360
Mempel TR, 2004, NATURE, V427, P154, DOI 10.1038/nature02238
Mihm MC, 2015, CANCER IMMUNOL RES, V3, P827, DOI 10.1158/2326-6066.CIR-15-0143
Mohsen MO, 2017, SEMIN IMMUNOL, V34, P123, DOI 10.1016/j.smim.2017.08.014
Mohsen MO, 2017, J CONTROL RELEASE, V251, P92, DOI 10.1016/j.jconrel.2017.02.031
Nuzzaci M, 2007, ARCH VIROL, V152, P915, DOI 10.1007/s00705-006-0916-7
O'Hagan DT, 2017, CURR OPIN IMMUNOL, V47, P93, DOI 10.1016/j.coi.2017.07.010
Piazzolla G, 2005, J CLIN IMMUNOL, V25, P142, DOI 10.1007/s10875-005-2820-4
Rahbar M, 2015, INDIAN J DERMATOL, V60, DOI 10.4103/0019-5154.152571
Rath M, 2014, FRONT IMMUNOL, V5, DOI 10.3389/fimmu.2014.00532
Sparwasser T, 1997, NATURE, V386, P336, DOI 10.1038/386336a0
Sparwasser T, 1999, J IMMUNOL, V162, P2368
Speiser DE, 2005, J CLIN INVEST, V115, P739, DOI 10.1172/JCI200523373
Stills HF, 2005, ILAR J, V46, P280, DOI 10.1093/ilar.46.3.280
Storni T, 2004, J IMMUNOL, V172, P1777, DOI 10.4049/jimmunol.172.3.1777
Tainaka K, 2014, CELL, V159, P911, DOI 10.1016/j.cell.2014.10.034
Temizoz B, 2016, INT IMMUNOL, V28, P329, DOI 10.1093/intimm/dxw015
Tissot AC, 2008, LANCET, V371, P821, DOI 10.1016/S0140-6736(08)60381-5

Wheeler AW, 2001, INT ARCH ALLERGY IMM, V126, P135, DOI 10.1159/000049504
WHEELER AW, 1982, INT ARCH ALLER A IMM, V69, P113, DOI 10.1159/000233157
Worm M, 2018, ALLERGY, V73, P1812, DOI 10.1111/all.13478
Zeltins A, 2017, NPJ VACCINES, V2, DOI 10.1038/s41541-017-0030-8
NR 49
TC 33
Z9 34
U1 0
U2 21
PU BMC
PI LONDON
PA CAMPUS, 4 CRINAN ST, LONDON N1 9XW, ENGLAND
SN 2051-1426
J9 J IMMUNOTHER CANCER
JI J. Immunother. Cancer
PD APR 26
PY 2019
VL 7
AR 114
DI 10.1186/s40425-019-0587-z
PG 12
WC Oncology; Immunology
WE Science Citation Index Expanded (SCI-EXPANDED)
SC Oncology; Immunology
GA HW7RT
UT WOS:000466887900001
PM 31027511
OA Green Published, gold
DA 2023-09-14
ER

PT J
AU Yang, YN
Jambhrunkar, M
Abbaraju, PL
Yu, MH
Zhang, M
Yu, CZ
AF Yang, Yannan
Jambhrunkar, Manasi
Abbaraju, Prasanna Lakshmi
Yu, Meihua
Zhang, Min
Yu, Chengzhong
TI Understanding the Effect of Surface Chemistry of Mesoporous Silica Nanorods on Their Vaccine Adjuvant Potency
SO ADVANCED HEALTHCARE MATERIALS
LA English
DT Article
DE hydrophobic; mesoporous silica; surface chemistry; vaccine delivery
ID IN-VIVO; IMMUNE-RESPONSE; CANCER-IMMUNOTHERAPY; PARTICULATE VACCINES; TUMOR-IMMUNOTHERAPY; ACID) NANOPARTICLES; GOLD NANOPARTICLES; ANTIGEN RELEASE; PARTICLE-SIZE; DELIVERY
AB Mesoporous silica nanoparticles are reported as adjuvants in nanovaccines in generating robust antigen-specific immunity. However, the effect of surface chemistry in initiating and modulating the immune response remains largely unexplored. In this study, mesoporous silica nanorods (MSNRs) are modified with -NH2 and -C-18 groups to investigate the influence of surface functional groups (-OH, -NH2, and -C-18) on their adjuvant efficacy. It is found that compared to -OH and -NH2 groups, the hydrophobic -C-18 modification significantly enhances antigen uptake by antigen presenting cells and endosomal-lysosomal escape in vitro, dendritic cells, and macrophages maturation ex vivo, and elicits secretion of interferon-gamma level and antibody response in immunized mice. Moreover, bare MSNR and

MSNR -NH2 exhibit T-helper 2 biased immune response, while MSNR -C-18 shows a T-helper 1 biased immune response. These findings suggest that the surface chemistry of nanostructured adjuvants has profound impact on the immune response, which provides useful guidance for the design of effective nanomaterial based vaccines.
C1 [Yang, Yannan; Jambhrunkar, Manasi; Abbaraju, Prasanna Lakshmi; Yu, Meihua; Zhang, Min; Yu, Chengzhong] Univ Queensland, Australian Inst Bioengn & Nanotechnol, St Lucia, Qld 4072, Australia.
C3 University of Queensland
RP Yu, CZ (通讯作者)，Univ Queensland, Australian Inst Bioengn & Nanotechnol, St Lucia, Qld 4072, Australia.
EM c.yu@uq.edu.au
RI Yu, Meihua/E-2355-2012; Yu, Chengzhong/I-8663-2012
OI Yu, Meihua/0000-0003-3696-7267; Yu, Chengzhong/0000-0003-3707-0785;
Yang, Yannan/0000-0001-6696-3879; Mantri Jambhrunkar,
Manasi/0000-0001-8344-3824
FU Australian Research Council; Queensland Government
FX Y.Y. and M.J. contributed equally to this work. The authors acknowledge the financial support from the Australian Research Council and the Queensland Government. The authors also appreciate facilities and the technical assistance of the Australian National Fabrication Facility and the Australian Microscopy and Microanalysis Research Facility at the Centre for Microscopy and Microanalysis and The University of Queensland.
CR Ahsan FL, 2002, J CONTROL RELEASE, V79, P29, DOI 10.1016/S0168-3659(01)00549-1
Bachmann MF, 2010, NAT REV IMMUNOL, V10, P787, DOI 10.1038/nri2868
Bale SS, 2010, ACS NANO, V4, P1493, DOI 10.1021/nn901586e
Broaders KE, 2009, P NATL ACAD SCI USA, V106, P5497, DOI 10.1073/pnas.0901592106
De Temmerman ML, 2011, DRUG DISCOV TODAY, V16, P569, DOI 10.1016/j.drudis.2011.04.006
Demento SL, 2012, BIOMATERIALS, V33, P4957, DOI 10.1016/j.biomaterials.2012.03.041
Dougan M, 2009, ANNU REV IMMUNOL, V27, P83, DOI 10.1146/annurev.immunol.021908.132544
Foged C, 2005, INT J PHARMACEUT, V298, P315, DOI 10.1016/j.ijpharm.2005.03.035
Hassan HAFM, 2016, J CONTROL RELEASE, V225, P205, DOI 10.1016/j.jconrel.2016.01.030
Huang HY, 2008, MICROPOR MESOPOR MAT, V111, P254, DOI 10.1016/j.micromeso.2007.07.038
Irvine DJ, 2013, NAT MATER, V12, P978, DOI [10.1038/NMAT3775, 10.1038/nmat3775]
Jennette JC, 2006, J AM SOC NEPHROL, V17, P1235, DOI 10.1681/ASN.2005101048
Kailasam K, 2008, J CHROMATOGR A, V1191, P125, DOI 10.1016/j.chroma.2008.02.026
Kupferschmidt N, 2014, NANOMEDICINE-UK, V9, P1835, DOI [10.2217/NNM.13.170, 10.2217/nnm.13.170]
Li JS, 2008, J COLLOID INTERF SCI, V318, P309, DOI 10.1016/j.jcis.2007.09.093
Li WA, 2016, BIOMATERIALS, V83, P249, DOI 10.1016/j.biomaterials.2016.01.026
Li WA, 2013, CURR OPIN IMMUNOL, V25, P238, DOI 10.1016/j.coi.2012.12.008
Li X, 2013, ADV HEALTHC MATER, V2, P863, DOI 10.1002/adhm.201200149
Liu Q, 2015, ACS NANO, V9, P4925, DOI 10.1021/nn5066793
Liu Y, 2013, J MATER CHEM B, V1, P3888, DOI 10.1039/c3tb20383b
Mahadik DB, 2011, J COLLOID INTERF SCI, V356, P298, DOI 10.1016/j.jcis.2010.12.088
Mahony D, 2013, SMALL, V9, P3138, DOI 10.1002/smll.201300012
Mamaeva V, 2013, ADV DRUG DELIVER REV, V65, P689, DOI 10.1016/j.addr.2012.07.018
Mercuri LP, 2006, SMALL, V2, P254, DOI 10.1002/smll.200500274
Mody KT, 2014, BIOMATERIALS, V35, P9972, DOI 10.1016/j.biomaterials.2014.08.044
Mody KT, 2013, NANOSCALE, V5, P5167, DOI 10.1039/c3nr00357d
Morishige T, 2010, BIOMATERIALS, V31, P6833, DOI 10.1016/j.biomaterials.2010.05.036
Moyano DF, 2012, J AM CHEM SOC, V134, P3965, DOI 10.1021/ja2108905
Niikura K, 2013, ACS NANO, V7, P3926, DOI 10.1021/nn3057005
Niu YT, 2016, J MATER CHEM B, V4, P212, DOI 10.1039/c5tb01911g
Niu YT, 2015, J MATER CHEM B, V3, P8477, DOI 10.1039/c5tb01405k
Olafsdottir TA, 2009, SCAND J IMMUNOL, V69, P194, DOI 10.1111/j.1365-3083.2008.02225.x
Oyewumi MO, 2010, EXPERT REV VACCINES, V9, P1095, DOI [10.1586/erv.10.89, 10.1586/ERV.10.89]
Pulendran B, 2011, NAT IMMUNOL, V12, P509, DOI 10.1038/ni.2039
Schijns VEJC, 2007, CLIN PHARMACOL THER, V82, P750, DOI 10.1038/sj.clpt.6100394
Shao K, 2015, ACS NANO, V9, P16, DOI 10.1021/nn5062029
Shima F, 2013, BIOMATERIALS, V34, P9709, DOI 10.1016/j.biomaterials.2013.08.064
Shukla R, 2005, LANGMUIR, V21, P10644, DOI 10.1021/la0513712

Sun BB, 2013, ACS NANO, V7, P10834, DOI 10.1021/nn404211j
Tang FQ, 2012, ADV MATER, V24, P1504, DOI 10.1002/adma.201104763
Tritto E, 2009, VACCINE, V27, P3331, DOI 10.1016/j.vaccine.2009.01.084
Ulmer JB, 2006, NAT BIOTECHNOL, V24, P1377, DOI 10.1038/nbt1261
Umeki Y, 2015, ADV FUNCT MATER, V25, P5758, DOI 10.1002/adfm.201502139
Wang XP, 2016, SMALL, V12, P3510, DOI 10.1002/smll.201600677
Wang XP, 2016, ADV HEALTHC MATER, V5, P1169, DOI 10.1002/adhm.201501013
Wang XP, 2016, ANGEW CHEM INT EDIT, V55, P1899, DOI 10.1002/anie.201506179
Wang XP, 2013, ACTA BIOMATER, V9, P7480, DOI 10.1016/j.actbio.2013.03.031
Wolfs TGAM, 2002, J IMMUNOL, V168, P1286, DOI 10.4049/jimmunol.168.3.1286
Wu SH, 2013, CHEM SOC REV, V42, P3862, DOI 10.1039/c3cs35405a
Xiang SD, 2006, METHODS, V40, P1, DOI 10.1016/j.ymeth.2006.05.016
Xu LG, 2012, NANO LETT, V12, P2003, DOI 10.1021/nl300027p
Yang YN, 2014, J MATER CHEM B, V2, P4929, DOI 10.1039/c4tb00595c
Yao YT, 2013, RSC ADV, V3, P12026, DOI 10.1039/c3ra42356e
Yu T, 2012, ACS NANO, V6, P2289, DOI 10.1021/nn2043803
Zhang J, 2014, SMALL, V10, P5068, DOI 10.1002/smll.201401538
Zhang J, 2010, J MATER RES, V25, P648, DOI 10.1557/JMR.2010.0085
Zhao L, 2016, PLOS ONE, V11, DOI 10.1371/journal.pone.0154295
NR 57
TC 33
Z9 34
U1 3
U2 101
PU WILEY
PI HOBOKEN
PA 111 RIVER ST, HOBOKEN 07030-5774, NJ USA
SN 2192-2640
EI 2192-2659
J9 ADV HEALTHC MATER
JI Adv. Healthc. Mater.
PD SEP 6
PY 2017
VL 6
IS 17
AR 1700466
DI 10.1002/adhm.201700466
PG 12
WC Engineering, Biomedical; Nanoscience & Nanotechnology; Materials
Science, Biomaterials
WE Science Citation Index Expanded (SCI-EXPANDED)
SC Engineering; Science & Technology - Other Topics; Materials Science
GA FG0IZ
UT WOS:000409410500015
PM 28557331
DA 2023-09-14
ER

PT J
AU Zhao, JY
Xu, YD
Ma, S
Wang, YB
Huang, ZC
Qu, HY
Yao, HC
Zhang, Y
Wu, GL
Huang, LA
Song, WT
Tang, ZH

Chen, XS
AF Zhao, Jiayu
Xu, Yudi
Ma, Sheng
Wang, Yibo
Huang, Zichao
Qu, Haoyuan
Yao, Haochen
Zhang, Yu
Wu, Guanglu
Huang, Leaf
Song, Wantong
Tang, Zhaohui
Chen, Xuesi
TI A Minimalist Binary Vaccine Carrier for Personalized Postoperative Cancer Vaccine Therapy
SO ADVANCED MATERIALS
LA English
DT Article
DE cancer immunotherapy; cancer vaccine; nanovaccine; polymeric carriers; postoperative therapy
ID IMMUNOTHERAPY
AB In recent years, significant evolutions have been made in applying nanotechnologies for prophylactic and therapeutic cancer vaccine design. However, the clinical translation of nanovaccines is still limited owing to their complicated compositions and difficulties in the spatiotemporal coordination of antigen-presenting cell activation and antigen cross-presentation. Herein, a minimalist binary nanovaccine (BiVax) is designed that integrates innate stimulating activity into the carrier to elicit robust antitumor immunity. The authors started by making a series of azole molecules end-capped polyethylenimine (PEI-M), and were surprised to find that over 60% of the PEI-M polymers have innate stimulating activity via activation of the stimulator of interferon genes pathway. PEI-4BImi, a PEI-M obtained from a series of polymers, elicits robust antitumor immune responses when used as a subcutaneously injected nanovaccine by simply mixing with ovalbumin antigens, and this BiVax system performs much better than the traditional ternary vaccine system, as well as, commercialized aluminum-containing adjuvants. This system also enables the fast preparation of personalized BiVax by compositing PEI-4BImi with autologous tumor cell membrane protein antigens, and a 60% postoperative cure rate is observed when combined with immune checkpoint inhibitors.
C1 [Zhao, Jiayu; Xu, Yudi; Ma, Sheng; Huang, Zichao; Yao, Haochen; Zhang, Yu; Song, Wantong; Tang, Zhaohui; Chen, Xuesi] Chinese Acad Sci, Changchun Inst Appl Chem, Key Lab Polymer Ecomat, 5625 Renmin St, Changchun 130022, Peoples R China.
[Zhao, Jiayu; Huang, Zichao; Tang, Zhaohui; Chen, Xuesi] Univ Sci & Technol China, 96 Jinzhai Rd, Hefei 230026, Peoples R China.
[Ma, Sheng; Zhang, Yu; Song, Wantong; Tang, Zhaohui; Chen, Xuesi] Jilin Biomed Polymers Engn Lab, 5625 Renmin St, Changchun 130022, Peoples R China.
[Wang, Yibo] Chinese Acad Sci, Changchun Inst Appl Chem, Lab Chem Biol, 5625 Renmin St, Changchun 130022, Peoples R China.
[Qu, Haoyuan; Wu, Guanglu] Jilin Univ, Coll Chem, State Key Lab Supramol Struct & Mat, 2699 Qianjin St, Changchun 130012, Peoples R China.
[Yao, Haochen] Jilin Univ, Coll Basic Med Sci, Chinese Minist Educ, Key Lab Zoonosis, 126 Xinmin Streey, Changchun 130021, Peoples R China.
[Huang, Leaf] Univ N Carolina, Eshelman Sch Pharm, Div Pharmacoengn & Mol Pharmaceut, 301 Pharm Lane, Chapel Hill, NC 27599 USA.
C3 Chinese Academy of Sciences; Changchun Institute of Applied Chemistry, CAS; Chinese Academy of Sciences; University of Science & Technology of China, CAS; Chinese Academy of Sciences; Changchun Institute of Applied Chemistry, CAS; Jilin University; Jilin University; University of North Carolina; University of North Carolina Chapel Hill
RP Song, WT (通讯作者)，Chinese Acad Sci, Changchun Inst Appl Chem, Key Lab Polymer Ecomat, 5625 Renmin St, Changchun 130022, Peoples R China.; Song, WT (通讯作者)，Jilin Biomed Polymers Engn Lab, 5625 Renmin St, Changchun 130022, Peoples R China.
EM wtsong@ciac.ac.cn
OI Wang, Yibo/0000-0003-1262-4497

FU Jilin Province Science and Technology Development Plan
[YDZJ202101ZYTS131]; National Natural Science Foundation of China
[51973215, 52025035, 52003268, 52103194, 22105199, 51829302, 51833010];
Bureau of International Cooperation Chinese Academy of Sciences
[121522KYSB20200029]; Youth Innovation Promotion Association of Chinese
Academy of Sciences [2020232]; Fundamental Research Funds for the
Central Universities; National Institutes of Health [CA189999]
FX This work was financially supported by the Jilin Province Science and
Technology Development Plan (YDZJ202101ZYTS131), the National Natural
Science Foundation of China (51973215, 52025035, 52003268, 52103194,
22105199, 51829302, and 51833010), Bureau of International Cooperation
Chinese Academy of Sciences (121522KYSB20200029), the Youth Innovation
Promotion Association of Chinese Academy of Sciences (2020232), the
Fundamental Research Funds for the Central Universities, as well as, the
support from the National Institutes of Health (CA189999). All animal
studies were carried out according to the guidelines approved by the
Animal Welfare and Ethics Committee of Changchun Institute of Applied
Chemistry, Chinese Academy of Sciences.
CR Anassi Enock, 2011, P T, V36, P197
Bachmann MF, 2010, NAT REV IMMUNOL, V10, P787, DOI 10.1038/nri2868
Chin EN, 2020, SCIENCE, V369, P993, DOI 10.1126/science.abb4255
Dong X, 2020, BIOMATERIALS, V230, DOI 10.1016/j.biomaterials.2019.119659
Fan ZY, 2021, BIOMACROMOLECULES, V22, P5074, DOI 10.1021/acs.biomac.1c01052
Gros A, 2016, NAT MED, V22, P433, DOI 10.1038/nm.4051
Hopfner KP, 2020, NAT REV MOL CELL BIO, V21, P501, DOI 10.1038/s41580-020-0244-x
Irvine DJ, 2020, ADV DRUG DELIVER REV, V158, P91, DOI 10.1016/j.addr.2020.06.019
Ji GF, 2021, ADV MATER, V33, DOI 10.1002/adma.202004559
Kim CG, 2019, PHARMACEUTICS, V11, DOI 10.3390/pharmaceutics11110612
Koshy ST, 2017, ADV BIOSYST, V1, DOI 10.1002/adbi.201600013
Kuai R, 2018, SCI ADV, V4, DOI 10.1126/sciadv.aao1736
Kuai R, 2017, NAT MATER, V16, P489, DOI [10.1038/nmat4822, 10.1038/NMAT4822]
Li SX, 2021, NAT BIOMED ENG, V5, P455, DOI 10.1038/s41551-020-00675-9
Liu Q, 2018, CANCER IMMUNOL IMMUN, V67, P299, DOI 10.1007/s00262-017-2079-7
Luo M, 2017, NAT NANOTECHNOL, V12, P648, DOI [10.1038/nnano.2017.52,
10.1038/NNANO.2017.52]
Manna S, 2018, ACS CENTRAL SCI, V4, P982, DOI 10.1021/acscentsci.8b00218
Mellman I, 2011, NATURE, V480, P480, DOI 10.1038/nature10673
Miao L, 2019, NAT BIOTECHNOL, V37, P1174, DOI 10.1038/s41587-019-0247-3
Mukai K, 2016, NAT COMMUN, V7, DOI 10.1038/ncomms11932
Ott PA, 2017, NATURE, V547, P217, DOI 10.1038/nature22991
Pardoll DM, 2012, NAT REV CANCER, V12, P252, DOI 10.1038/nrc3239
Ramanjulu JM, 2018, NATURE, V564, P439, DOI 10.1038/s41586-018-0705-y
Reddy ST, 2007, NAT BIOTECHNOL, V25, P1159, DOI [10.1038/nbt1332, 10.1038/nbtl332]
Rosenberg SA, 2004, NAT MED, V10, P909, DOI 10.1038/nm1100
Rosenberg SA, 2015, SCIENCE, V348, P62, DOI 10.1126/science.aaa4967
Sahin U, 2017, NATURE, V547, P222, DOI 10.1038/nature23003
Shae D, 2019, NAT NANOTECHNOL, V14, P269, DOI 10.1038/s41565-018-0342-5
Song W, 2019, MATER TODAY NANO, V5, DOI 10.1016/j.mtnano.2019.100029
Song WT, 2020, TRENDS CANCER, V6, P288, DOI 10.1016/j.trecan.2020.01.011
Song WT, 2017, BIOMATERIALS, V148, P16, DOI 10.1016/j.biomaterials.2017.09.017
Tanaka Y, 2012, SCI SIGNAL, V5, DOI 10.1126/scisignal.2002521
Tian XY, 2018, FRONT IMMUNOL, V9, DOI [10.3389/fimmu.2018.00473, 10.3389/fimmu.2018.01683]
van der Burg SH, 2016, NAT REV CANCER, V16, P219, DOI 10.1038/nrc.2016.16
Wen R, 2019, NANOMEDICINE-UK, V14, P627, DOI 10.2217/nnm-2018-0147
Williams MA, 2007, ANNU REV IMMUNOL, V25, P171, DOI
10.1146/annurev.immunol.25.022106.141548
Xu J, 2020, NAT NANOTECHNOL, V15, P1043, DOI 10.1038/s41565-020-00781-4
Yang Y, 2021, VIEW-CHINA, V2, DOI 10.1002/VIW.20200042
Yu ZT, 2021, BIOMATER SCI-UK, V9, P6879, DOI 10.1039/d1bm01000j
Zhang CG, 2019, NATURE, V567, P394, DOI 10.1038/s41586-019-1000-2
Zhang X, 2013, MOL CELL, V51, P226, DOI 10.1016/j.molcel.2013.05.022

Zhang Y, 2021, ADV MATER, V33, DOI 10.1002/adma.202007293
Zhou KC, 2019, J PHARMACOL SCI, V139, P15, DOI 10.1016/j.jphs.2018.10.012
NR 43
TC 32
Z9 32
U1 55
U2 275
PU WILEY-V C H VERLAG GMBH
PI WEINHEIM
PA POSTFACH 101161, 69451 WEINHEIM, GERMANY
SN 0935-9648
EI 1521-4095
J9 ADV MATER
JI Adv. Mater.
PD MAR
PY 2022
VL 34
IS 10
AR 2109254
DI 10.1002/adma.202109254
EA JAN 2022
PG 11
WC Chemistry, Multidisciplinary; Chemistry, Physical; Nanoscience & Nanotechnology; Materials Science, Multidisciplinary; Physics, Applied; Physics, Condensed Matter
WE Science Citation Index Expanded (SCI-EXPANDED)
SC Chemistry; Science & Technology - Other Topics; Materials Science; Physics
GA ZP8II
UT WOS:000748798600001
PM 34984753
DA 2023-09-14
ER

PT J
AU Chen, J
Fang, HP
Hu, YY
Wu, JY
Zhang, SJ
Feng, YJ
Lin, L
Tian, HY
Chen, XS
AF Chen, Jie
Fang, Huapan
Hu, Yingying
Wu, Jiayan
Zhang, Sijia
Feng, Yuanji
Lin, Lin
Tian, Huayu
Chen, Xuesi
TI Combining mannose receptor mediated nanovaccines and gene regulated PD-L1 blockade for boosting cancer immunotherapy
SO BIOACTIVE MATERIALS
LA English
DT Article
DE Gene delivery; PD-L1 blockade; Targeted vaccine; Nanovaccines; Tumor immunotherapy
ID TARGETING DENDRITIC CELLS; DELIVERY; ANTIGEN; NANOPARTICLES; VACCINES;

THERAPY; POLYMER; PROGRESS
AB Tumor nanovaccines have potential applications in the prevention and treatment of malignant tumors. However, it remains a longstanding challenge in exploiting efficient nanocarriers for inducing potent specifically cellular immune responses. Toward this objective, we herein explore an intensive tumor immunotherapeutic strategy by combining mannosylated nanovaccines and gene regulated PD-L1 blockade for immune stimulation and killing activity. Here, we fabricate a mannose modified PLL-RT (Man-PLL-RT) mediated nanovaccines with dendritic cells (DCs) targeting capacity. Man-PLL-RT is capable of co-encapsulating with antigen (ovalbumin, OVA) and adjuvant (unmethylated cytosine-phosphate-guanine, CpG) by electrostatic interaction. This positively charged Man-PLL-RT/OVA/CpG nanovaccines can facilitate the endocytosis, maturation and cross presentation in DCs. However, the nanovaccines arouse limited inhibition of tumor growth, which is mainly due to the immunosuppressed microenvironment of tumors. Combining tumor nanovaccines with gene regulated PD-L1 blockade leads to an obvious tumor remission in B16F10 melanoma bearing mice. The collaborative strategy provides essential insights to boost the benefits of tumor vaccines by regulating the checkpoint blockade with gene therapy.
C1 [Chen, Jie; Fang, Huapan; Hu, Yingying; Wu, Jiayan; Zhang, Sijia; Feng, Yuanji; Lin, Lin; Tian, Huayu; Chen, Xuesi] Chinese Acad Sci, Changchun Inst Appl Chem, Key Lab Polymer Ecomat, Changchun 130022, Peoples R China.
[Chen, Jie; Fang, Huapan; Hu, Yingying; Wu, Jiayan; Zhang, Sijia; Feng, Yuanji; Lin, Lin; Tian, Huayu; Chen, Xuesi] Univ Sci & Technol China, Hefei 230026, Peoples R China.
[Chen, Jie; Lin, Lin; Tian, Huayu; Chen, Xuesi] Jilin Biomed Polymers Engn Lab, Changchun 130022, Peoples R China.
[Fang, Huapan] Soochow Univ, Inst Funct Nano & Soft Mat FUNSOM, Jiangsu Key Lab CarbonBased Funct Mat & Devices, Suzhou 215123, Jiangsu, Peoples R China.
C3 Chinese Academy of Sciences; Changchun Institute of Applied Chemistry, CAS; Chinese Academy of Sciences; University of Science & Technology of China, CAS; Soochow University - China
RP Tian, HY (通讯作者)，Chinese Acad Sci, Changchun Inst Appl Chem, Key Lab Polymer Ecomat, Changchun 130022, Peoples R China.
EM chenjie@ciac.ac.cn; hpfang@suda.edu.cn; yyhu@ciac.ac.cn; wujiayan@ciac.ac.cn; sjzhang@ciac.ac.cn; yjfeng@ciac.ac.cn; linlin@ciac.ac.cn; thy@ciac.ac.cn; xschen@ciac.ac.cn
RI Wu, Jiayan/HZJ-2894-2023; Zhang, Sijia/ISS-6609-2023
FU National Natural Science Foundation of China [51925305, 51873208, 51973217, 51520105004, 51803210]; National Science and Technology Major Projects for Major New Drugs Innovation and Development [2018ZX09711003-012]; Jilin Province Science and Technology Development Program [20180414027GH, 20200201075JC]
FX This work was supported by the National Natural Science Foundation of China (51925305, 51873208, 51973217, 51520105004 and 51803210), National Science and Technology Major Projects for Major New Drugs Innovation and Development (2018ZX09711003-012), and Jilin Province Science and Technology Development Program (20180414027GH and 20200201075JC).
CR Chen J, 2020, ACS APPL MATER INTER, V12, P19295, DOI 10.1021/acsami.0c02769
Chen J, 2019, BIOCONJUGATE CHEM, V30, P338, DOI 10.1021/acs.bioconjchem.8b00688
Chen J, 2016, MOL THER-METH CLIN D, V3, DOI 10.1038/mtm.2016.23
Chen Q, 2019, NANO LETT, V19, P4879, DOI 10.1021/acs.nanolett.9b00584
Conniot J, 2019, NAT NANOTECHNOL, V14, P891, DOI 10.1038/s41565-019-0512-0
de Miguel M, 2020, CANCER CELL, V38, P326, DOI 10.1016/j.ccell.2020.07.004
El-Sayed N, 2021, INT J PHARMACEUT, V593, DOI 10.1016/j.ijpharm.2020.120123
Fang HP, 2018, J AM CHEM SOC, V140, P11992, DOI 10.1021/jacs.8b05341
Foster S, 2010, BIOCONJUGATE CHEM, V21, P2205, DOI 10.1021/bc100204m
Fox CB, 2013, EXPERT REV VACCINES, V12, P747, DOI 10.1586/14760584.2013.811188
Guan XW, 2018, BIOMATERIALS, V171, P198, DOI 10.1016/j.biomaterials.2018.04.039
Hu YY, 2021, CHINESE CHEM LETT, V32, P1770, DOI 10.1016/j.cclet.2020.12.055
Hu YY, 2020, ACS APPL MATER INTER, V12, P41127, DOI 10.1021/acsami.0c12734
Hu YY, 2020, BIOMATERIALS, V252, DOI 10.1016/j.biomaterials.2020.120114
Huang H, 2019, NANO LETT, V19, P5356, DOI 10.1021/acs.nanolett.9b01807
Li JW, 2020, SCI CHINA CHEM, V63, P546, DOI 10.1007/s11426-019-9673-4
Li P, 2013, NANOTECHNOLOGY, V24, DOI 10.1088/0957-4484/24/29/295101
Liang Y, 2021, BIOACT MATER, V6, P433, DOI 10.1016/j.bioactmat.2020.08.019

Liu JF, 2019, J BIOMED NANOTECHNOL, V15, P1454, DOI 10.1166/jbn.2019.2790
Liu Q, 2015, ACS NANO, V9, P4925, DOI 10.1021/nn5066793
Lynn GM, 2020, NAT BIOTECHNOL, V38, P320, DOI 10.1038/s41587-019-0390-x
Ma L, 2019, SCIENCE, V365, P162, DOI 10.1126/science.aav8692
Malonis RJ, 2020, CHEM REV, V120, P3210, DOI 10.1021/acs.chemrev.9b00472
Qian Y, 2016, BIOMATERIALS, V98, P171, DOI 10.1016/j.biomaterials.2016.05.008
Schaffert D, 2008, GENE THER, V15, P1131, DOI 10.1038/gt.2008.105
Scott EA, 2012, BIOMATERIALS, V33, P6211, DOI 10.1016/j.biomaterials.2012.04.060
Shi GN, 2017, BIOMATERIALS, V113, P191, DOI 10.1016/j.biomaterials.2016.10.047
Singh SK, 2011, EUR J IMMUNOL, V41, P916, DOI 10.1002/eji.201040762
Wang DG, 2019, SCI IMMUNOL, V4, DOI 10.1126/sciimmunol.aau6584
Wang Y, 2020, CHINESE J POLYM SCI, V38, P830, DOI 10.1007/s10118-020-2393-y
Wu JY, 2020, SCI ADV, V6, DOI 10.1126/sciadv.abc7828
Wu PK, 2021, SCI CHINA MATER, V64, P241, DOI 10.1007/s40843-020-1366-2
Xi XB, 2020, SCI ADV, V6, DOI 10.1126/sciadv.aay7735
Xu J, 2019, BIOMATERIALS, V207, P1, DOI 10.1016/j.biomaterials.2019.03.037
Yang PP, 2017, ADV MATER, V29, DOI 10.1002/adma.201605869
Yang R, 2020, BIOACT MATER, V5, P990, DOI 10.1016/j.bioactmat.2020.06.004
Yu X, 2020, NAT COMMUN, V11, DOI 10.1038/s41467-020-14906-9
Zhang LH, 2019, NANO LETT, V19, P4237, DOI 10.1021/acs.nanolett.9b00030
Zhang WF, 2014, BIOMATERIALS, V35, P6086, DOI 10.1016/j.biomaterials.2014.04.022
Zhang XD, 2018, ADV MATER, V30, DOI 10.1002/adma.201707112
Zhou ZX, 2017, ADV DRUG DELIVER REV, V115, P115, DOI 10.1016/j.addr.2017.07.021
Zhu JH, 2019, AAPS PHARMSCITECH, V21, DOI 10.1208/s12249-019-1526-5
NR 42
TC 32
Z9 33
U1 32
U2 192
PU KEAI PUBLISHING LTD
PI BEIJING
PA 16 DONGHUANGCHENGGEN NORTH ST, BEIJING, DONGHENG DISTRICT 100717,
PEOPLES R CHINA
EI 2452-199X
J9 BIOACT MATER
JI Bioact. Mater.
PD JAN
PY 2022
VL 7
BP 167
EP 180
DI 10.1016/j.bioactmat.2021.05.036
PG 14
WC Engineering, Biomedical; Materials Science, Biomaterials
WE Science Citation Index Expanded (SCI-EXPANDED)
SC Engineering; Materials Science
GA WJ9QC
UT WOS:000709370300016
PM 34466725
OA gold, Green Published
HC Y
HP N
DA 2023-09-14
ER

PT J
AU Warrier, VU
Makandar, AI
Garg, M
Sethi, G
Kant, R

Pal, JK
Yuba, E
Gupta, RK
AF Warrier, Vaishnavi U.
Makandar, Amina I.
Garg, Manoj
Sethi, Gautam
Kant, Ravi
Pal, Jayanta K.
Yuba, Eiji
Gupta, Rajesh Kumar
TI Engineering anti-cancer nanovaccine based on antigen cross-presentation
SO BIOSCIENCE REPORTS
LA English
DT Review
ID MHC CLASS-I; VIRUS-LIKE PARTICLES; T-CELL RESPONSES; IRON-OXIDE NANOPARTICLES; MUTANT TERT PROMOTER; DENDRITIC CELLS; INVARIANT CHAIN; DC-SIGN; ENDOPLASMIC-RETICULUM; PROTECTIVE IMMUNITY
AB Dendritic cells (DCs) present exogenous antigens on major histocompatibility complex (MHC) class I molecules, thereby activating CD8(+) T cells, contributing to tumor elimination through a mechanism known as antigen cross-presentation. A variety of factors such as maturation state of DCs, co-stimulatory signals, T-cell microenvironment, antigen internalization routes and adjuvants regulate the process of DC-mediated antigen cross-presentation. Recently, the development of successful cancer immunotherapies may be attributed to the ability of DCs to cross-present tumor antigens. In this review article, we focus on the underlying mechanism of antigen cross-presentation and ways to improve antigen cross-presentation in different DC subsets. We have critically summarized the recent developments in the generation of novel nanovaccines for robust CD8(+) T-cell response in cancer. In this context, we have reviewed nanocarriers that have been used for cancer immunotherapeutics based on antigen cross-presentation mechanism. Additionally, we have also expressed our views on the future applications of this mechanism in curing cancer.
C1 [Warrier, Vaishnavi U.; Makandar, Amina I.; Pal, Jayanta K.; Gupta, Rajesh Kumar] Dr DY Patil Vidyapeeth Deemed Univ, Dr DY Patil Biotechnol & Bioinformat Inst, Prot Biochem Res Lab, Pune 411033, Maharashtra, India.
[Garg, Manoj] Amity Univ, AIMMSCR, Noida 201313, Uttar Pradesh, India.
[Sethi, Gautam] Natl Univ Singapore, Yong Loo Lin Sch Med, Dept Pharmacol, Singapore 117600, Singapore.
[Kant, Ravi] Tech Univ Munich, Klinikum Rechts Isar, Expt Neuroimmunol, Munich, Germany.
[Yuba, Eiji] Osaka Prefecture Univ, Grad Sch Engn, Dept Appl Chem, Naka Ku, 1-1 Gakuen Cho, Sakai, Osaka 5998531, Japan.
C3 Dr DY Patil Vidyapeeth Pune; Dr D Y Patil Biotechnology & Bioinformatics Institute; Amity University Noida; National University of Singapore; Technical University of Munich; Osaka Metropolitan University
RP Gupta, RK (通讯作者), Dr DY Patil Vidyapeeth Deemed Univ, Dr DY Patil Biotechnol & Bioinformat Inst, Prot Biochem Res Lab, Pune 411033, Maharashtra, India.; Garg, M (通讯作者), Amity Univ, AIMMSCR, Noida 201313, Uttar Pradesh, India.; Sethi, G (通讯作者), Natl Univ Singapore, Yong Loo Lin Sch Med, Dept Pharmacol, Singapore 117600, Singapore.
EM mgarg@amity.edu; phcgs@nus.edu.sg; guptarajesh76@gmail.com
RI Warrier, Vaishnavi/ISS-5306-2023; Kant, Ravi/F-7025-2013; Sethi, Gautam/F-2372-2011
OI Warrier, Vaishnavi/0000-0001-5166-6409; Kant, Ravi/0000-0003-3878-9775; Sethi, Gautam/0000-0002-8677-8475; Eiji, Yuba/0000-0003-4984-2113; Garg, Manoj/0000-0002-3492-2957; Gupta, Rajesh Kumar/0000-0001-8820-0661; Pal, Jayanta/0000-0003-1840-6458
FU Department of Science and Technology-Science and Engineering Research Board (DST-SERB), Government of India under ECRA scheme [ECR/2016/001187]; Department of Biotechnology (DBT), Science and Engineering Research Board-Department of Science and Technology, Government of India [BT/RLF/Re-entry/24/2014]; ECRA scheme (SERB) [ECR/2016/001519]; Dr. D.Y. Patil Vidyapeeth (Deemed to be University) [DPU/106/18/2015, DPU/17/2016]
FX This work was supported by the Department of Science and

Technology-Science and Engineering Research Board (DST-SERB), Government of India under ECRA scheme [grant number ECR/2016/001187]; Dr. D.Y. Patil Vidyapeeth (Deemed to be University) [grant numbers DPU/106/18/2015, DPU/17/2016 (to Rajesh Kumar Gupta)]; the Department of Biotechnology (DBT), Science and Engineering Research Board-Department of Science and Technology, Government of India under its Ramalingaswami Fellowship [grant number BT/RLF/Re-entry/24/2014]; and the ECRA scheme (SERB) [grant number ECR/2016/001519 (to Manoj Garg)].
CR Abele R, 2011, ESSAYS BIOCHEM, V50, P249, DOI [10.1042/BSE0500249, 10.1042/bse0500249]
Accapezzato D, 2005, J EXP MED, V202, P817, DOI 10.1084/jem.20051106
Ackerman AL, 2005, NAT IMMUNOL, V6, P107, DOI 10.1038/ni1147
Adamina M, 2004, BRIT J CANCER, V90, P263, DOI 10.1038/sj.bjc.6601473
Ahn S, 2014, ADV HEALTHC MATER, V3, P1194, DOI 10.1002/adhm.201300597
Akincilar SC, 2016, CANCER DISCOV, V6, P1276, DOI 10.1158/2159-8290.CD-16-0177
Akincilar SC, 2015, FEBS LETT, V589, P974, DOI 10.1016/j.febslet.2015.02.035
Albert ML, 1998, NATURE, V392, P86, DOI 10.1038/32183
Andersen CS, 2007, INFECT IMMUN, V75, P408, DOI 10.1128/IAI.01290-06
Arasaki K, 2010, TRAFFIC, V11, P587, DOI 10.1111/j.1600-0854.2010.01050.x
Bachmann MF, 1996, EUR J IMMUNOL, V26, P2595, DOI 10.1002/eji.1830261109
Baek SH, 2017, J CELL PHYSIOL, V232, P346, DOI 10.1002/jcp.25426
Banchereau J, 1998, NATURE, V392, P245, DOI 10.1038/32588
Belizaire R, 2009, P NATL ACAD SCI USA, V106, P17463, DOI 10.1073/pnas.0908583106
Bellone M, 1997, J IMMUNOL, V159, P5391
BEVAN MJ, 1976, J EXP MED, V143, P1283, DOI 10.1084/jem.143.5.1283
Bindhya S, 2019, INT J BIOCHEM CELL B, V107, P62, DOI 10.1016/j.biocel.2018.12.008
Boks MA, 2015, J CONTROL RELEASE, V216, P37, DOI 10.1016/j.jconrel.2015.06.033
Bonifaz L, 2002, J EXP MED, V196, P1627, DOI 10.1084/jem.20021598
Caminschi I, 2012, MOL IMMUNOL, V50, P9, DOI 10.1016/j.molimm.2011.11.008
Campana S, 2015, IMMUNOL LETT, V168, P349, DOI 10.1016/j.imlet.2015.11.002
Campbell-Valois FX, 2012, MOL CELL PROTEOMICS, V11, DOI 10.1074/mcp.M111.016378
Cebon J, 2018, MAMM GENOME, V29, P703, DOI 10.1007/s00335-018-9786-z
Cebrian I, 2011, CELL, V147, P1355, DOI 10.1016/j.cell.2011.11.021
Chakraborty S, 2017, CELL REP, V18, P2464, DOI 10.1016/j.celrep.2017.02.041
CHEN LP, 1993, IMMUNOL TODAY, V14, P483, DOI 10.1016/0167-5699(93)90262-J
Chew CL, 2018, TRENDS MOL MED, V24, P66, DOI 10.1016/j.molmed.2017.11.003
Chien WW, 2014, ONCOTARGET, V5, P4881, DOI 10.18632/oncotarget.2051
Chithrani BD, 2006, NANO LETT, V6, P662, DOI 10.1021/nl052396o
Chua AWL, 2010, BIOCHEM PHARMACOL, V80, P1553, DOI 10.1016/j.bcp.2010.07.045
Cildir G, 2017, J EXP MED, V214, P2491, DOI 10.1084/jem.20170910
Cleland JL, 1999, TRENDS BIOTECHNOL, V17, P25, DOI 10.1016/S0167-7799(98)01272-4
Cruz FM, 2017, ANNU REV IMMUNOL, V35, P149, DOI 10.1146/annurev-immunol-041015-055254
Datta SK, 2003, J IMMUNOL, V170, P4102, DOI 10.4049/jimmunol.170.8.4102
De la Fuente JM, 2006, BBA-GEN SUBJECTS, V1760, P636, DOI 10.1016/j.bbagen.2005.12.001
DEBRICK JE, 1991, J IMMUNOL, V147, P2846
Delamarre L, 2005, SCIENCE, V307, P1630, DOI 10.1126/science.1108003
Dingjan I, 2016, SCI REP-UK, V6, DOI 10.1038/srep22064
Dong H, 2013, MUCOSAL IMMUNOL, V6, P522, DOI 10.1038/mi.2012.92
Eggermont LJ, 2014, TRENDS BIOTECHNOL, V32, P456, DOI 10.1016/j.tibtech.2014.06.007
Fasting C, 2012, ANGEW CHEM INT EDIT, V51, P10472, DOI 10.1002/anie.201201114
Faure F, 2009, EUR J IMMUNOL, V39, P380, DOI 10.1002/eji.200838669
Fehres CM, 2014, EUR J IMMUNOL, V44, P2415, DOI 10.1002/eji.201344094
Garcia-Vallejo JJ, 2013, MOL IMMUNOL, V53, P387, DOI 10.1016/j.molimm.2012.09.012
Garg M, 2017, SCI REP-UK, V7, DOI 10.1038/s41598-017-10325-x
Garg M, 2015, BLOOD, V126, P2491, DOI 10.1182/blood-2015-05-646240
Garg M, 2015, J CLIN ENDOCR METAB, V100, P725, DOI 10.1210/jc.2014-2359
Garg M, 2010, CANCER-AM CANCER SOC, V116, P3785, DOI 10.1002/cncr.25218
Garulli B, 2008, CLIN VACCINE IMMUNOL, V15, P1497, DOI 10.1128/CVI.00166-08
Girotti AW, 1998, J LIPID RES, V39, P1529
Golnik R, 2016, J BIOL CHEM, V291, P8805, DOI 10.1074/jbc.M115.705178
Gottardi R, 2013, J NANOBIOTECHNOL, V11, DOI 10.1186/1477-3155-11-30
Greenfield EA, 1998, CRIT REV IMMUNOL, V18, P389, DOI 10.1615/CritRevImmunol.v18.i5.10

Gupta RK, 2012, EUR J PHARM SCI, V45, P282, DOI 10.1016/j.ejps.2011.11.022
Ha NY, 2016, J NANOBIOTECHNOL, V14, DOI 10.1186/s12951-016-0229-2
Hattori A, 2013, J BIOCHEM, V154, P219, DOI 10.1093/jb/mvt066
He HN, 2013, PHARM RES-DORDR, V30, P2445, DOI 10.1007/s11095-013-0982-y
He J, 2016, THERANOSTICS, V6, P752, DOI 10.7150/thno.14527
He Q, 2002, CLIN DIAGN LAB IMMUN, V9, P1021, DOI 10.1128/CDLI.9.5.1021-1024.2002
Hodi FS, 2010, NEW ENGL J MED, V363, P711, DOI 10.1056/NEJMoa1003466
Hoffmann TK, 2001, J IMMUNOTHER, V24, P162, DOI 10.1097/00002371-200103000-00011
Hwu P, 2000, J IMMUNOL, V164, P3596, DOI 10.4049/jimmunol.164.7.3596
INABA K, 1992, J EXP MED, V176, P1693, DOI 10.1084/jem.176.6.1693
Janikashvili N, 2010, IMMUNOTHERAPY-UK, V2, P57, DOI 10.2217/IMT.09.78
Jiang WL, 2005, ADV DRUG DELIVER REV, V57, P391, DOI 10.1016/j.addr.2004.09.003
Joffre OP, 2012, NAT REV IMMUNOL, V12, P557, DOI 10.1038/nri3254
Joshi MD, 2012, J CONTROL RELEASE, V161, P25, DOI 10.1016/j.jconrel.2012.05.010
Kanchan V, 2007, BIOMATERIALS, V28, P5344, DOI 10.1016/j.biomaterials.2007.08.015
Kanojia D, 2017, J HEMATOL ONCOL, V10, DOI 10.1186/s13045-017-0540-x
Katare YK, 2006, VACCINE, V24, P3599, DOI 10.1016/j.vaccine.2006.01.066
Kelly A, 2019, IMMUNOGENETICS, V71, P161, DOI 10.1007/s00251-018-1082-2
Khattar E, 2016, J CLIN INVEST, V126, P4045, DOI 10.1172/JCI86042
Kim H, 2019, J PHARMACOL EXP THER, V370, P715, DOI 10.1124/jpet.118.254953
Kitano M, 2016, P NATL ACAD SCI USA, V113, P1044, DOI 10.1073/pnas.1513607113
Krasnova L, 2016, ANNU REV BIOCHEM, V85, P599, DOI 10.1146/annurev-biochem-060614-034420
Larmonier N, 2008, J IMMUNOL, V181, P6955, DOI 10.4049/jimmunol.181.10.6955
Laurent S, 2008, CHEM REV, V108, P2064, DOI 10.1021/cr068445e
Lawand M, 2016, J IMMUNOL, V197, P3454, DOI 10.4049/jimmunol.1501925
Lehmann CHK, 2016, VACCINES-BASEL, V4, DOI 10.3390/vaccines4020008
Li YH, 2016, P NATL ACAD SCI USA, V113, P14402, DOI 10.1073/pnas.1611106113
Lyalina T., 2016, NANOBIOMEDICINE, V3, P1, DOI [10.1177/1849543516667355, DOI 10.1177/1849543516667355]
Maji M, 2016, SCI REP-UK, V6, DOI 10.1038/srep27206
McKee AS, 2017, CURR OPIN IMMUNOL, V47, P44, DOI 10.1016/j.coi.2017.06.005
Menager J, 2014, PLOS ONE, V9, DOI 10.1371/journal.pone.0089897
Milould T, 2010, METHODS MOL BIOL, V595, P21, DOI 10.1007/978-1-60761-421-0_2
Miyazaki M, 2018, BIOCONJUGATE CHEM, V29, P44, DOI 10.1021/acs.bioconjchem.7b00551
Mody KT, 2013, NANOSCALE, V5, P5167, DOI 10.1039/c3nr00357d
Moon JJ, 2011, NAT MATER, V10, P243, DOI 10.1038/NMAT2960
Mou YB, 2011, INT J NANOMED, V6, P1779, DOI 10.2147/IJN.S23240
MUELLER DL, 1989, ANNU REV IMMUNOL, V7, P445, DOI 10.1146/annurev.iy.07.040189.002305
Nair AS, 2006, J IMMUNOL, V177, P5612, DOI 10.4049/jimmunol.177.8.5612
Nair P, 2011, TRAFFIC, V12, P1669, DOI 10.1111/j.1600-0854.2011.01251.x
Nair-Gupta P, 2014, CELL, V158, P506, DOI 10.1016/j.cell.2014.04.054
Naskalska A, 2015, POL J MICROBIOL, V64, P3
Neal Lillian R, 2017, J Immunol Res Ther, V2, P68
Neefjes J, 2011, NAT REV IMMUNOL, V11, P823, DOI 10.1038/nri3084
Neijssen J, 2005, NATURE, V434, P83, DOI 10.1038/nature03290
Nierkens S, 2013, TRENDS IMMUNOL, V34, P361, DOI 10.1016/j.it.2013.02.007
Nowak AK, 2006, ADV DRUG DELIVER REV, V58, P975, DOI 10.1016/j.addr.2006.04.002
ODORIZZI CG, 1994, J CELL BIOL, V126, P317, DOI 10.1083/jcb.126.2.317
Okubo M, 2019, BIOCONJUGATE CHEM, V30, P1518, DOI 10.1021/acs.bioconjchem.9b00221
OLD LJ, 1988, SCI AM, V258, P59, DOI 10.1038/scientificamerican0588-59
Palucka K, 2012, NAT REV CANCER, V12, P265, DOI 10.1038/nrc3258
Panda AK, 2011, EXPERT REV VACCINES, V10, P155, DOI [10.1586/erv.10.164, 10.1586/ERV.10.164]
Peaper DR, 2008, P NATL ACAD SCI USA, V105, P10477, DOI 10.1073/pnas.0805044105
PFEIFER JD, 1993, NATURE, V361, P359, DOI 10.1038/361359a0
Platt CD, 2010, P NATL ACAD SCI USA, V107, P4287, DOI 10.1073/pnas.0910609107
Prasad V, 2017, SEMIN ONCOL, V44, P132, DOI 10.1053/j.seminoncol.2017.06.007
Proudfoot O, 2007, MOL PHARMACEUT, V4, P58, DOI 10.1021/mp0601087
Quakkelaar ED, 2012, ADV IMMUNOL, V114, P77, DOI 10.1016/B978-0-12-396548-6.00004-4
Ramanathan HN, 2012, CELL RES, V22, P346, DOI 10.1038/cr.2011.80
Reeves E, 2018, BIOSCIENCE REP, V38, DOI 10.1042/BSR20171503
Rohovie MJ, 2017, BIOENG TRANSL MED, V2, P43, DOI 10.1002/btm2.10049

Saccheri F, 2010, SCI TRANSL MED, V2, DOI 10.1126/scitranslmed.3000739
Sahin A, 2018, PLOS ONE, V13, DOI 10.1371/journal.pone.0199414
Salem ML, 2006, VACCINE, V24, P5119, DOI 10.1016/j.vaccine.2006.04.010
Savina A, 2007, IMMUNOL REV, V219, P143, DOI 10.1111/j.1600-065X.2007.00552.x
Savina A, 2006, CELL, V126, P205, DOI 10.1016/j.cell.2006.05.035
Schutz CA, 2013, NANOMEDICINE-UK, V8, P449, DOI [10.2217/nnm.13.8, 10.2217/NNM.13.8]
Selby MJ, 2013, CANCER IMMUNOL RES, V1, P32, DOI 10.1158/2326-6066.CIR-13-0013
Sethi G, 2014, MOL CANCER, V13, DOI 10.1186/1476-4598-13-66
Sharma R, 2017, NANOMEDICINE-UK, V12, P1945, DOI 10.2217/nnm-2017-0088
Shen LJ, 2004, IMMUNITY, V21, P155, DOI 10.1016/j.immuni.2004.07.004
Sheng KC, 2008, EUR J IMMUNOL, V38, P424, DOI 10.1002/eji.200737578
Singh SS, 2018, ONCOGENE, V37, P1142, DOI 10.1038/s41388-017-0046-6
Singh SS, 2015, WORLD J GASTROENTERO, V21, P12261, DOI 10.3748/wjg.v21.i43.12261
Snapper CM, 2018, FRONT IMMUNOL, V9, DOI 10.3389/fimmu.2018.00598
Subramaniam A, 2013, BRIT J PHARMACOL, V170, P807, DOI 10.1111/bph.12302
SUGITA M, 1995, J BIOL CHEM, V270, P1443, DOI 10.1074/jbc.270.3.1443
Sungsuwan S, 2015, ACS APPL MATER INTER, V7, P17535, DOI 10.1021/acsami.5b05497
Tel J, 2013, J IMMUNOL, V191, P5005, DOI 10.4049/jimmunol.1300787
Temizoz B, 2016, INT IMMUNOL, V28, P329, DOI 10.1093/intimm/dxw015
Torres-Sangiao E, 2016, MOLECULES, V21, DOI 10.3390/molecules21070867
Toubaji A, 2007, VACCINE, V25, P5882, DOI 10.1016/j.vaccine.2007.05.040
Tuyaerts S, 2007, CANCER IMMUNOL IMMUN, V56, P1513, DOI 10.1007/s00262-007-0334-z
Unger WWJ, 2012, J CONTROL RELEASE, V160, P88, DOI 10.1016/j.jconrel.2012.02.007
Van Braeckel-Budimir N, 2013, FRONT IMMUNOL, V4, DOI 10.3389/fimmu.2013.00282
van Kooyk Y, 2013, MOL IMMUNOL, V55, P143, DOI 10.1016/j.molimm.2012.10.031
van Liempt E, 2006, FEBS LETT, V580, P6123, DOI 10.1016/j.febslet.2006.10.009
Vigna JL, 1996, J IMMUNOL, V157, P4503
Wang CQ, 2014, CELL REP, V8, P767, DOI 10.1016/j.celrep.2014.06.046
Wang FL, 2016, INT J ENV RES PUB HE, V13, DOI 10.3390/ijerph13121182
Wattendorf U, 2008, LANGMUIR, V24, P11790, DOI 10.1021/la801085d
Woo CC, 2013, PLOS ONE, V8, DOI 10.1371/journal.pone.0075356
Xu WW, 2016, BIOSCIENCE REP, V36, DOI 10.1042/BSR20160204
York IA, 2019, BIOSCIENCE REP, V39, DOI 10.1042/BSR20171505
Young PA, 2014, SEMIN ONCOL, V41, P623, DOI 10.1053/j.seminoncol.2014.08.002
Yuba E, 2018, MOL IMMUNOL, V98, P8, DOI 10.1016/j.molimm.2017.11.001
Yuba E, 2017, MEMBRANES-BASEL, V7, DOI 10.3390/membranes7030041
Yuba E, 2015, BIOMATERIALS, V67, P214, DOI 10.1016/j.biomaterials.2015.07.031
NR 150
TC 32
Z9 33
U1 1
U2 23
PU PORTLAND PRESS LTD
PI LONDON
PA 1ST FLR, 10 QUEEN STREET PLACE, LONDON, ENGLAND
SN 0144-8463
EI 1573-4935
J9 BIOSCIENCE REP
JI Biosci. Rep.
PD OCT 18
PY 2019
VL 39
AR BSR20193220
DI 10.1042/BSR20193220
PN 10
PG 19
WC Biochemistry & Molecular Biology; Cell Biology
WE Science Citation Index Expanded (SCI-EXPANDED)
SC Biochemistry & Molecular Biology; Cell Biology
GA JF0XF
UT WOS:000491109800001

PM 31652460
OA Green Published, gold
DA 2023-09-14
ER

PT J
AU Fontana, F
Fusciello, M
Groeneveldt, C
Capasso, C
Chiaro, J
Feola, S
Liu, ZH
Makila, EM
Salonen, JJ
Hirvonen, JT
Cerullo, V
Santos, HA
AF Fontana, Flavia
Fusciello, Manlio
Groeneveldt, Christianne
Capasso, Cristian
Chiaro, Jacopo
Feola, Sara
Liu, Zehua
Makila, Ermei M.
Salonen, Jarno J.
Hirvonen, Jouni T.
Cerullo, Vincenzo
Santos, Helder A.
TI Biohybrid Vaccines for Improved Treatment of Aggressive Melanoma with Checkpoint Inhibitor
SO ACS NANO
LA English
DT Article
DE biohybrid; cancer vaccine; cell membrane; microfluidics; melanoma
ID TUMOR-CELL VACCINE; ACETALATED DEXTRAN; CANCER-IMMUNOTHERAPY; SURFACE-CHEMISTRY; IMMUNE CELLS; BLOCKADE; NANOPARTICLES; EFFICACY; DELIVERY; ANTIGEN
AB Recent approaches in the treatment of cancer focus on involving the immune system to control the tumor growth. The administration of immunotherapies, like checkpoint inhibitors, has shown impressive results in the long term survival of patients. Cancer vaccines are being investigated as further tools to prime tumor-specific immunity. Biomaterials show potential as adjuvants in the formulation of vaccines, and biomimetic elements derived from the membrane of tumor cells may widen the range of antigens contained in the vaccine. Here, we show how mice presenting an aggressive melanoma tumor model treated twice with the complete nanovaccine formulation showed control on the tumor progression, while in a less aggressive model, the animals showed remission and control on the tumor progression, with a modification in the immunological profile of the tumor microenvironment. We also prove that co-administration of the nanovaccine together with a checkpoint inhibitor increases the efficacy of the treatment (87.5% of the animals responding, with 2 remissions) compared to the checkpoint inhibitor alone in the B16.0VA model. Our platform thereby shows potential applications as a cancer nanovaccine in combination with the standard clinical care treatment for melanoma cancers.
C1 [Fontana, Flavia; Liu, Zehua; Hirvonen, Jouni T.; Santos, Helder A.] Univ Helsinki, Fac Pharm, Div Pharmaceut Chem & Technol, Drug Res Program, FI-00014 Helsinki, Finland.
[Fusciello, Manlio; Capasso, Cristian; Chiaro, Jacopo; Feola, Sara; Cerullo, Vincenzo] Univ Helsinki, Fac Pharm, Div Pharmaceut Biosci, Drug Res Program, FI-00014 Helsinki, Finland.
[Groeneveldt, Christianne] Leiden Univ, LACDR, Div Biotherapeut, NL-2300 RA Leiden, Netherlands.
[Makila, Ermei M.; Salonen, Jarno J.] Univ Turku, Dept Phys & Astron, Lab Ind Phys, FI-20014 Turku, Finland.
[Cerullo, Vincenzo; Santos, Helder A.] Univ Helsinki, Helsinki Inst Life Sci HiLIFE, FI-00014 Helsinki, Finland.

C3 University of Helsinki; University of Helsinki; Leiden University - Excl
LUMC; Leiden University; University of Turku; University of Helsinki
RP Santos, HA (通讯作者)，Univ Helsinki, Fac Pharm, Div Pharmaceut Chem & Technol, Drug Res Program, FI-00014 Helsinki, Finland.; Cerullo, V (通讯作者)，Univ Helsinki, Fac Pharm, Div Pharmaceut Biosci, Drug Res Program, FI-00014 Helsinki, Finland.; Cerullo, V; Santos, HA (通讯作者)，Univ Helsinki, Helsinki Inst Life Sci HiLIFE, FI-00014 Helsinki, Finland.
EM vincenzo.cerullo@helsinki.fi; helder.santos@helsinki.fi
RI Santos, Hélder A./AAC-6347-2022; Cerullo, Vincenzo/B-9075-2015; Fontana,
Flavia/AAB-4898-2020
OI Santos, Hélder A./0000-0001-7850-6309; Cerullo,
Vincenzo/0000-0003-4901-3796; Fontana, Flavia/0000-0001-9112-6917;
Feola, Sara/0000-0002-4012-4310; Salonen, Jarno/0000-0002-5245-742X;
Fusciello, Manlio/0000-0002-7166-3018; Groeneveldt,
Christianne/0000-0003-1742-1517; Makila, Ermei/0000-0002-8300-6533;
Hirvonen, Jouni Tapio/0000-0002-5029-1657
FU Faculty of Pharmacy of the University of Helsinki; Drug Research
Doctoral Program of the University of Helsinki; ULLA Consortium; Jo Kolk
Studiefonds; LUF International Study Fund; HiLIFE Research Funds; Jane
and Aatos Erkko Foundation; European Research Council under the European
Union's Horizon 2020 Program [681219]; University of Helsinki Research
Funds; Sigrid Juselius Foundation [4704580]; European Research Council
[825020]; European Research Council (ERC) [825020] Funding Source:
European Research Council (ERC)
FX We thank Ms. Nazanin Zanjanizadeh Ezazi for assistance in the
preparation of Scheme 1. F.F. acknowledges the Faculty of Pharmacy of
the University of Helsinki for an assistant research grant. M.F. and
C.C. acknowledge the Drug Research Doctoral Program of the University of
Helsinki for a Ph.D. grant. C.G. would like to acknowledge financial
support from the ULLA Consortium, the Jo Kolk Studiefonds, and the LUF
International Study Fund. V.C. acknowledges financial support from the
HiLIFE Research Funds, Jane and Aatos Erkko Foundation, and the European
Research Council under the European Union's Horizon 2020 Program (grant
no. 681219). H.A.S. acknowledges financial support from the University
of Helsinki Research Funds, the Sigrid Juselius Foundation (decision no.
4704580), the HiLIFE Research Funds, and the European Research Council
proof-of-concept grant (decision no. 825020).
CR Ali OA, 2016, CANCER IMMUNOL RES, V4, P95, DOI 10.1158/2326-6066.CIR-14-0126
Ascierto PA, 2013, J TRANSL MED, V11, DOI 10.1186/1479-5876-11-54
Bachelder EM, 2017, CHEM REV, V117, P1915, DOI 10.1021/acs.chemrev.6b00532
Bauleth-Ramos T, 2017, ADV FUNCT MATER, V27, DOI 10.1002/adfm.201703303
Bimbo LM, 2011, BIOMATERIALS, V32, P2625, DOI 10.1016/j.biomaterials.2010.12.011
Branca MA, 2016, NAT BIOTECHNOL, V34, P1019, DOI 10.1038/nbt.3690
Broaders KE, 2009, P NATL ACAD SCI USA, V106, P5497, DOI 10.1073/pnas.0901592106
Campana S, 2015, IMMUNOL LETT, V168, P349, DOI 10.1016/j.imlet.2015.11.002
Chen Q, 2018, ADV HEALTHC MATER, V7, DOI 10.1002/adhm.201800424
Cheung AS, 2016, SMALL, V12, P2321, DOI 10.1002/smll.201600061
Chiang CLL, 2011, INT REV IMMUNOL, V30, P150, DOI 10.3109/08830185.2011.572210
Crespo J, 2013, CURR OPIN IMMUNOL, V25, P214, DOI 10.1016/j.coi.2012.12.003
Engeland CE, 2014, MOL THER, V22, P1949, DOI 10.1038/mt.2014.160
EUHUS DM, 1989, CANCER IMMUNOL IMMUN, V29, P247
Fan Q, 2018, ADV FUNCT MATER, V28, DOI 10.1002/adfm.201802540
Fang RH, 2014, NANO LETT, V14, P2181, DOI 10.1021/nl500618u
Fishman M, 2008, J IMMUNOTHER, V31, P72, DOI 10.1097/CJI.0b013e31815ba792
Fontana F, 2017, ADV MATER, V29, DOI 10.1002/adma.201603239
Fontana F, 2017, WIRES NANOMED NANOBI, V9, DOI 10.1002/wnan.1421
Francis DM, 2017, ADV DRUG DELIVER REV, V114, P33, DOI 10.1016/j.addr.2017.04.011
Fridman WH, 2017, NAT REV CLIN ONCOL, V14, P717, DOI 10.1038/nrclinonc.2017.101
Gajewski TF, 2013, NAT IMMUNOL, V14, P1014, DOI 10.1038/ni.2703
Gros A, 2014, J CLIN INVEST, V124, P2246, DOI [10.1172/JC173639, 10.1172/JCI73639]
Herranz-Blanco B, 2017, INT J PHARMACEUT, V516, P100, DOI 10.1016/j.ijpharm.2016.11.024
Inozume T, 2010, J IMMUNOTHER, V33, P956, DOI 10.1097/CJI.0b013e3181fad2b0

Irvine DJ, 2015, CHEM REV, V115, P11109, DOI 10.1021/acs.chemrev.5b00109
Jain RK, 2014, ANNU REV BIOMED ENG, V16, P321, DOI 10.1146/annurev-bioeng-071813-105259
Joyce JA, 2015, SCIENCE, V348, P74, DOI 10.1126/science.aaa6204
Kapadia CH, 2015, J CONTROL RELEASE, V219, P167, DOI 10.1016/j.jconrel.2015.09.062
Koshy ST, 2016, CURR OPIN BIOTECH, V40, P1, DOI 10.1016/j.copbio.2016.02.001
Koyama S, 2016, NAT COMMUN, V7, DOI 10.1038/ncomms10501
Liang Y, 2015, INT J CLIN EXP MED, V8, P6914
Liu DF, 2017, NANO LETT, V17, P606, DOI 10.1021/acs.nanolett.6b03251
Liu DF, 2015, ADV MATER, V27, P2298, DOI 10.1002/adma.201405408
Liu DF, 2015, BIOMATERIALS, V39, P249, DOI 10.1016/j.biomaterials.2014.10.079
Liu JW, 2016, J IMMUNOTHER, V39, P171, DOI 10.1097/CJI.0000000000000122
Ott PA, 2017, NATURE, V547, P217, DOI 10.1038/nature22991
Pardoll DM, 2012, NAT REV CANCER, V12, P252, DOI 10.1038/nrc3239
Rashidian M, 2017, J EXP MED, V214, P2243, DOI 10.1084/jem.20161950
Roh W, 2017, SCI TRANSL MED, V9, DOI 10.1126/scitranslmed.aah3560
Sahin U, 2017, NATURE, V547, P222, DOI 10.1038/nature23003
Santos HA, 2010, ACTA BIOMATER, V6, P2721, DOI 10.1016/j.actbio.2009.12.043
Schreiber RD, 2011, SCIENCE, V331, P1565, DOI 10.1126/science.1203486
Shahbazi MA, 2014, BIOMATERIALS, V35, P9224, DOI 10.1016/j.biomaterials.2014.07.050
Simon S, 2018, ONCOIMMUNOLOGY, V7, DOI 10.1080/2162402X.2017.1364828
Sloan AE, 2000, NEUROSURG FOCUS, V9, P1
Smyth MJ, 2016, NAT REV CLIN ONCOL, V13, P143, DOI 10.1038/nrclinonc.2015.209
Tahtinen S, 2015, CANCER IMMUNOL RES, V3, P915, DOI 10.1158/2326-6066.CIR-14-0220-T
van der Burg SH, 2016, NAT REV CANCER, V16, P219, DOI 10.1038/nrc.2016.16
Verdeil G, 2016, BBA-REV CANCER, V1865, P49, DOI 10.1016/j.bbcan.2015.06.007
Wakim LM, 2011, NATURE, V471, P629, DOI 10.1038/nature09863
Wang C, 2016, BIOMATERIALS, V79, P88, DOI 10.1016/j.biomaterials.2015.11.040
Wang C, 2017, ADV MATER, V29, DOI 10.1002/adma.201606036
Wang C, 2017, NAT BIOMED ENG, V1, DOI 10.1038/s41551-016-0011
Wang C, 2016, NANO LETT, V16, P2334, DOI 10.1021/acs.nanolett.5b05030
Xia XJ, 2015, CELL REP, V11, P957, DOI 10.1016/j.celrep.2015.04.009
Yang W, 2009, BIOMED PHARMACOTHER, V63, P404, DOI 10.1016/j.biopha.2008.08.020
Yarchoan M, 2017, NAT REV CANCER, V17, P209, DOI 10.1038/nrc.2016.154
Zheng WX, 2016, ONCOTARGET, V7, P43039, DOI 10.18632/oncotarget.9915
Zhu GZ, 2017, ACS NANO, V11, P2387, DOI 10.1021/acsnano.7b00978
Zitvogel L, 2012, ONCOIMMUNOLOGY, V1, P1223, DOI 10.4161/onci.21335
Zou WP, 2005, NAT REV CANCER, V5, P263, DOI 10.1038/nrc1586
NR 62
TC 32
Z9 34
U1 4
U2 75
PU AMER CHEMICAL SOC
PI WASHINGTON
PA 1155 16TH ST, NW, WASHINGTON, DC 20036 USA
SN 1936-0851
EI 1936-086X
J9 ACS NANO
JI ACS Nano
PD JUN
PY 2019
VL 13
IS 6
BP 6477
EP 6490
DI 10.1021/acsnano.8b09613
PG 14
WC Chemistry, Multidisciplinary; Chemistry, Physical; Nanoscience & Nanotechnology; Materials Science, Multidisciplinary
WE Science Citation Index Expanded (SCI-EXPANDED)
SC Chemistry; Science & Technology - Other Topics; Materials Science

GA IF7GA
UT WOS:000473248300034
PM 31100004
OA Green Published, hybrid
DA 2023-09-14
ER

PT J
AU Chen, CY
Tong, YH
Zheng, YS
Shi, YJ
Chen, ZW
Li, J
Liu, XL
Zhang, D
Yang, HH
AF Chen, Chengyun
Tong, Yuhong
Zheng, Youshi
Shi, Yingjun
Chen, Zhaowei
Li, Juan
Liu, Xiaolong
Zhang, Da
Yang, Huanghao
TI Cytosolic Delivery of Thiolated Mn-cGAMP Nanovaccine to Enhance the Antitumor Immune Responses
SO SMALL
LA English
DT Article
DE cancer immunotherapy; cGAMP; cytosolic delivery; manganese; STING pathway
ID STING PATHWAY
AB As a stimulator of interferon gene (STING), cyclic dinucleotide activates a broad cellular immune response for anti-cancer immunotherapy (CIT). However, the inherent of instability of 2 ' 3 '-cyclic-GMP-AMP (cGAMP) with poor cellular targeting, rapid clearance, and inefficient transport to the cytoplasm seriously hinders cGAMP potency. Here, a thiolated and Mn2+ coordinated cyclic dinucleotide nanovaccine (termed as Mn-cGAMP NVs) to enable direct cytosolic co-delivery of cGAMP and Mn2+ to potentiate the antitumor immune response is presented. In the NVs, the fixation cGAMP with Mn2+ ions not only improve its stability, but also potentiate the activation of STING. Meanwhile, the presence of polysulfides on the NVs surface allowed direct cytosolic delivery while avoiding degradation. In this way, the production of cytokines for activating T cells immunity is greatly elevated, which in turn suppressed the primary and distal tumors growth through long-term immune memory and led to long-term survival of poorly immunogenic B16F10 melanoma mice. Moreover, by further combining with anti-PD-L1 monoclonal antibody, synergistic T cells antitumor immune response is elicited. This work offers a promising strategy to enhance the potency of cGAMP, holding a considerable potential for CIT applications.
C1 [Chen, Chengyun; Tong, Yuhong; Chen, Zhaowei; Li, Juan; Zhang, Da; Yang, Huanghao] Fuzhou Univ, MOE Key Lab Analyt Sci Food Safety & Biol, State Key Lab Photocatalysis Energy & Environm, Coll Chem, Fuzhou 350116, Peoples R China.
[Zheng, Youshi; Shi, Yingjun; Liu, Xiaolong; Zhang, Da] Fujian Med Univ, United Innovat Mengchao Hepatobiliary Technol, Key Lab Fujian Prov, Mengchao Hepatobiliary Hosp, Fuzhou 350025, Peoples R China.
C3 Fuzhou University; Fujian Medical University
RP Zhang, D; Yang, HH (通讯作者)，Fuzhou Univ, MOE Key Lab Analyt Sci Food Safety & Biol, State Key Lab Photocatalysis Energy & Environm, Coll Chem, Fuzhou 350116, Peoples R China.; Zhang, D (通讯作者)，Fujian Med Univ, United Innovat Mengchao Hepatobiliary Technol, Key Lab Fujian Prov, Mengchao Hepatobiliary Hosp, Fuzhou 350025, Peoples R China.
EM zdluoman1987@163.com; hhyang@fzu.edu.cn
RI wu, jun/ISB-8607-2023; Chen, Zhaowei/U-3297-2019
OI Chen, Zhaowei/0000-0001-9007-5513; Zhang, Da/0000-0002-2613-1354

FU Natural Science Foundation of China [61805041, 91959102]; Natural
Science Foundation of Fujian Province of China [2020J011154]
FX This work was supported by the Natural Science Foundation of China
(Grant Nos. 61805041, 91959102) and Natural Science Foundation of Fujian
Province of China (Grant No. 2020J011154). All procedures of animal
studies were approved by the Ethics Committee for Animal Research of
Mengchao Hepatobiliary Hospital of Fujian Medical University, Fuzhou,
Fujian, China (SYXK 2019-0007).
CR Adler J, 2010, CYTOM PART A, V77A, P733, DOI 10.1002/cyto.a.20896
Alspach E, 2019, NATURE, V574, P696, DOI 10.1038/s41586-019-1671-8
Bai JL, 2019, DIABETES, V68, P1099, DOI 10.2337/dbi18-0052
Binnewies M, 2018, NAT MED, V24, P541, DOI 10.1038/s41591-018-0014-x
Chamian S.L., J TRANSL MED, V5, P27
Chen CY, 2021, SMALL, V17, DOI 10.1002/smll.202006970
Chen Q, 2016, NAT IMMUNOL, V17, P1142, DOI 10.1038/ni.3558
He MY, 2019, ACTA BIOMATER, V84, P114, DOI 10.1016/j.actbio.2018.11.053
Koshy ST, 2017, ADV BIOSYST, V1, DOI 10.1002/adbi.201600013
Leong M.L., THER ADV VACCINES, V1, P131
Lu XG, 2020, SCI TRANSL MED, V12, DOI 10.1126/scitranslmed.aaz6606
Lv MZ, 2020, CELL RES, V30, P966, DOI 10.1038/s41422-020-00395-4
Moral JA, 2020, NATURE, V579, P130, DOI 10.1038/s41586-020-2015-4
Moynihan KD, 2016, NAT MED, V22, P1402, DOI 10.1038/nm.4200
Reddy A.J., NAT BIOTECHNOL, V25, P1159
Wang CG, 2018, IMMUNITY, V48, P675, DOI 10.1016/j.immuni.2018.03.017
Watkins-Schulz P., BIOMATERIALS, V205, P94
Wu JX, 2013, SCIENCE, V339, P826, DOI 10.1126/science.1229963
NR 18
TC 31
Z9 33
U1 26
U2 163
PU WILEY-V C H VERLAG GMBH
PI WEINHEIM
PA POSTFACH 101161, 69451 WEINHEIM, GERMANY
SN 1613-6810
EI 1613-6829
J9 SMALL
JI Small
PD APR
PY 2021
VL 17
IS 17
AR 2006970
DI 10.1002/smll.202006970
EA MAR 2021
PG 9
WC Chemistry, Multidisciplinary; Chemistry, Physical; Nanoscience &
Nanotechnology; Materials Science, Multidisciplinary; Physics, Applied;
Physics, Condensed Matter
WE Science Citation Index Expanded (SCI-EXPANDED)
SC Chemistry; Science & Technology - Other Topics; Materials Science;
Physics
GA RT5KM
UT WOS:000628814300001
PM 33719177
DA 2023-09-14
ER

PT J
AU Liu, F
Sun, JL

Yu, WQ
Jiang, QY
Pan, M
Xu, Z
Mo, FY
Liu, XQ
AF Liu, Feng
Sun, Junlin
Yu, Wenqian
Jiang, Qunying
Pan, Min
Xu, Zhen
Mo, Fengye
Liu, Xiaoqing
TI Quantum dot-pulsed dendritic cell vaccines plus macrophage polarization for amplified cancer immunotherapy
SO BIOMATERIALS
LA English
DT Article
DE Quantum dots; Cancer therapy; Macrophage polarization; Therapeutic cancer vaccines; Immunotherapy
ID TUMOR-ASSOCIATED MACROPHAGES; ANTITUMOR IMMUNITY; NALP3 INFLAMMASOME; DELIVERY; MICROENVIRONMENTS; METHACRYLATE); NANOVACCINE; MECHANISMS; SYSTEM
AB Dendritic cell (DC) vaccines hold great potential in cancer immunotherapy, but the suboptimal design of DC vaccines and the immunosuppressive tumor microenvironment largely impair their anti-tumor efficacy. Here, quantum dot (QD) pulsed-DC vaccines integrating with tumor-associated macrophage polarization are developed for amplified anti-tumor immunity. Semiconductor QDs are engineered with diverse functions to act as fluorescence nanoprobes, immunomodulatory adjuvants, and nanocarriers to load tumor antigens and Toll-like receptor 9 agonists. The QD-pulsed DC vaccines enable spatiotemporal tracking of lymphatic drainage and efficacy evaluation of DC immunotherapy, and trigger potent immunoactivation. Specifically, designer DC vaccine plus macrophage polarization elicits potent immune response to stimulate innate and adaptive antitumor immunity and ameliorate the immunosuppressive tumor microenvironment. As a new combination therapy, this strategy greatly boosts antigen-specific T-cell immunity and thus strongly inhibits local tumor growth and tumor metastasis in vivo. This study may provide an applicable treatment for cancer immunotherapy.
C1 [Liu, Feng; Sun, Junlin; Yu, Wenqian; Jiang, Qunying; Pan, Min; Xu, Zhen; Mo, Fengye; Liu, Xiaoqing] Wuhan Univ, Coll Chem & Mol Sci, Key Lab Analyt Chem Biol & Med, Minist Educ, Wuhan 430072, Peoples R China.
C3 Wuhan University
RP Liu, XQ (通讯作者)，Wuhan Univ, Coll Chem & Mol Sci, Key Lab Analyt Chem Biol & Med, Minist Educ, Wuhan 430072, Peoples R China.
EM xiaoqingliu@whu.edu.cn
RI Xu, Zhen/AAU-2599-2021; Liu, Xiaoqing/A-3080-2015; Liu, Xiaoqing/C-5752-2009
OI Xu, Zhen/0000-0003-1408-5976; Liu, Xiaoqing/0000-0002-1309-5454
FU National Natural Science Foundation of China [81602610]; Fundamental Research Funds for the Central Universities [2042018kf1006]
FX The research was supported by the National Natural Science Foundation of China (No. 81602610), and Fundamental Research Funds for the Central Universities (No. 2042018kf1006).
CR Anguille S, 2014, LANCET ONCOL, V15, pE257, DOI 10.1016/S1470-2045(13)70585-0
Bai J, 2018, J AM CHEM SOC, V140, P106, DOI 10.1021/jacs.7b11114
Belderbos RA, 2019, MOL THER-ONCOLYTICS, V13, P67, DOI 10.1016/j.omto.2019.03.007
Chen DG, 2018, NAT COMMUN, V9, DOI 10.1038/s41467-018-03225-9
Dammeijer F, 2017, CANCER IMMUNOL RES, V5, P535, DOI 10.1158/2326-6066.CIR-16-0309
de Brito CA, 2009, IMMUNOTHERAPY-UK, V1, P883, DOI 10.2217/IMT.09.38
Eisenbarth SC, 2008, NATURE, V453, P1122, DOI 10.1038/nature06939
Feng B, 2018, ADV MATER, V30, DOI 10.1002/adma.201803001
Flinsenberg TWH, 2011, CLIN EXP IMMUNOL, V165, P8, DOI 10.1111/j.1365-2249.2011.04411.x
Garg AD, 2017, TRENDS IMMUNOL, V38, P577, DOI 10.1016/j.it.2017.05.006

Georgoudaki AM, 2016, CELL REP, V15, P2000, DOI 10.1016/j.celrep.2016.04.084
Harrell MI, 2008, J IMMUNOL METHODS, V332, P170, DOI 10.1016/j.jim.2007.11.012
Hess KL, 2017, ADV FUNCT MATER, V27, DOI 10.1002/adfm.201700290
Hornung V, 2008, NAT IMMUNOL, V9, P847, DOI 10.1038/ni.1631
Kaiko GE, 2008, IMMUNOLOGY, V123, P326, DOI 10.1111/j.1365-2567.2007.02719.x
Kersten K, 2015, FRONT IMMUNOL, V6, DOI 10.3389/fimmu.2015.00516
Kitamura T, 2015, J EXP MED, V212, P1043, DOI 10.1084/jem.20141836
Kitamura T, 2015, NAT REV IMMUNOL, V15, P73, DOI 10.1038/nri3789
Li AW, 2018, NAT MATER, V17, P528, DOI 10.1038/s41563-018-0028-2
Li YH, 2019, NANO LETT, V19, P5806, DOI 10.1021/acs.nanolett.9b02769
Linde N, 2018, NAT COMMUN, V9, DOI 10.1038/s41467-017-02481-5
Liu F, 2016, NANOSCALE, V8, P4387, DOI 10.1039/c5nr07436c
Liu Q, 2018, ACS NANO, V12, P1250, DOI [10.1021/acsnano.7b07384, 10.1021/acsnano.7607384]
Liu SY, 2001, J AM CHEM SOC, V123, P9910, DOI 10.1021/ja011206i
Luo M, 2017, J CONTROL RELEASE, V263, P200, DOI 10.1016/j.jconrel.2017.03.033
Luo M, 2017, NAT NANOTECHNOL, V12, P648, DOI [10.1038/nnano.2017.52, 10.1038/NNANO.2017.52]
Mantovani A, 2017, NAT REV CLIN ONCOL, V14, P399, DOI 10.1038/nrclinonc.2016.217
Mondanelli G, 2017, IMMUNITY, V46, P233, DOI 10.1016/j.immuni.2017.01.005
Munn DH, 2016, CURR OPIN IMMUNOL, V39, P1, DOI 10.1016/j.coi.2015.10.009
Noy R, 2014, IMMUNITY, V41, P49, DOI 10.1016/j.immuni.2014.06.010
Phuengkham H, 2019, ADV MATER, V31, DOI 10.1002/adma.201803322
Qian BZ, 2011, NATURE, V475, P222, DOI 10.1038/nature10138
Qiu LP, 2018, SMALL, V14, DOI 10.1002/smll.201703539
Rodell CB, 2018, NAT BIOMED ENG, V2, P578, DOI 10.1038/s41551-018-0236-8
Ruiz-de-Angulo A, 2016, ACS NANO, V10, P1602, DOI 10.1021/acsnano.5b07253
Santos PM, 2018, J IMMUNOL, V200, P443, DOI 10.4049/jimmunol.1701024
Saxena M, 2018, NAT BIOMED ENG, V2, P341, DOI 10.1038/s41551-018-0250-x
Saxena M, 2018, TRENDS CANCER, V4, P119, DOI 10.1016/j.trecan.2017.12.007
Sharma P, 2015, SCIENCE, V348, P56, DOI 10.1126/science.aaa8172
Shi GN, 2017, BIOMATERIALS, V113, P191, DOI 10.1016/j.biomaterials.2016.10.047
Sun L, 2018, ANGEW CHEM INT EDIT, V57, P10666, DOI 10.1002/anie.201806611
van der Burg SH, 2016, NAT REV CANCER, V16, P219, DOI 10.1038/nrc.2016.16
van Gulijk M, 2018, FRONT IMMUNOL, V9, DOI 10.3389/fimmu.2018.02759
Veglia F, 2017, CURR OPIN IMMUNOL, V45, P43, DOI 10.1016/j.coi.2017.01.002
Versteven M, 2018, FRONT IMMUNOL, V9, DOI 10.3389/fimmu.2018.00394
Wang C, 2017, ADV MATER, V29, DOI 10.1002/adma.201606036
Wang J, 2018, NAT NANOTECHNOL, V13, P1078, DOI 10.1038/s41565-018-0274-0
Wang MF, 2009, LANGMUIR, V25, P11732, DOI 10.1021/la900614e
Wang XS, 2004, J AM CHEM SOC, V126, P7784, DOI 10.1021/ja0489339
Xia FF, 2018, BIOMATERIALS, V170, P1, DOI 10.1016/j.biomaterials.2018.03.048
Xu FJ, 2008, BIOMATERIALS, V29, P3023, DOI 10.1016/j.biomaterials.2008.03.041
Yang L, 2017, J HEMATOL ONCOL, V10, DOI 10.1186/s13045-017-0430-2
Yue H, 2015, VACCINE, V33, P5927, DOI 10.1016/j.vaccine.2015.07.100
Zhu GZ, 2017, ACS NANO, V11, P2387, DOI 10.1021/acsnano.7b00978
Zong JB, 2016, CANCER IMMUNOL IMMUN, V65, P821, DOI 10.1007/s00262-016-1820-y
NR 55
TC 31
Z9 32
U1 4
U2 121
PU ELSEVIER SCI LTD
PI OXFORD
PA THE BOULEVARD, LANGFORD LANE, KIDLINGTON, OXFORD OX5 1GB, OXON, ENGLAND
SN 0142-9612
EI 1878-5905
J9 BIOMATERIALS
JI Biomaterials
PD JUN
PY 2020
VL 242

AR 119928
DI 10.1016/j.biomaterials.2020.119928
PG 12
WC Engineering, Biomedical; Materials Science, Biomaterials
WE Science Citation Index Expanded (SCI-EXPANDED)
SC Engineering; Materials Science
GA KZ9FP
UT WOS:000523564800008
PM 32145508
DA 2023-09-14
ER

PT J
AU Mohsen, MO
Vogel, M
Riether, C
Muller, J
Salatinos, S
Ternette, N
Gomes, AC
Cabral-Miranda, G
El-Turabi, A
Ruedl, C
Kundig, TM
Dermime, S
Knuth, A
Speiser, DE
Bachmann, MF
AF Mohsen, Mona O.
Vogel, Monique
Riether, Carsten
Muller, Julius
Salatinos, Silvia
Ternette, Nicola
Gomes, Ariane C.
Cabral-Miranda, Gustavo
El-Turabi, Aadil
Ruedl, Christiane
Kundig, Thomas M.
Dermime, Said
Knuth, Alexander
Speiser, Daniel E.
Bachmann, Martin F.
TI Targeting Mutated Plus Germline Epitopes Confers Pre-clinical Efficacy
of an Instantly Formulated Cancer Nano-Vaccine
SO FRONTIERS IN IMMUNOLOGY
LA English
DT Article
DE virus-like particles; vaccine; personalized; melanoma; neoantigen;
mutated; germline
ID TUMOR-INFILTRATING LYMPHOCYTES; FREE CLICK CHEMISTRY; T-CELL RESPONSES;
MELANOMA; IMMUNITY; IMMUNOTHERAPY; ERADICATION; MUTANOME; ANTIGENS;
PROTEIN
AB Personalized cancer vaccines hold promises for future cancer therapy. Targeting neoantigens is perceived as more beneficial compared to germline, non-mutated antigens. However, it is a practical challenge to identify and vaccinate patients with neoantigens. Here we asked whether two neoantigens are sufficient, and whether the addition of germline antigens would enhance the therapeutic efficacy. We developed and used a personalized cancer nano-vaccine platform based on virus-like particles loaded with toll-like receptor ligands. We generated three sets of multi-target vaccines (MTV) to immunize against the aggressive B16F10 murine melanoma: one set based on germline epitopes (GL-MTV) identified by immunopeptidomics, another set based on mutated epitopes (Mutated-MTV) predicted by whole exome

sequencing and a last set combines both germline and mutated epitopes (Mix-MTV). Our results demonstrate that both germline and mutated epitopes induced protection but the best therapeutic effect was achieved with the combination of both. Our platform is based on Cu-free click chemistry used for peptide-VLP coupling, thus enabling bedside production of a personalized cancer vaccine, ready for clinical translation.
C1 [Mohsen, Mona O.; Muller, Julius; Ternette, Nicola; Gomes, Ariane C.; El-Turabi, Aadil; Bachmann, Martin F.] Univ Oxford, Jenner Inst, Nuffield Dept Med, Oxford, England.
[Mohsen, Mona O.; Vogel, Monique; Cabral-Miranda, Gustavo; Bachmann, Martin F.] Univ Hosp Bern, Dept BioMed Res, Immunol RIA, Bern, Switzerland.
[Mohsen, Mona O.; Dermime, Said; Knuth, Alexander] Natl Ctr Canc Care & Res, Doha, Qatar.
[Riether, Carsten] Univ Hosp Bern, Dept Med Oncol, Bern, Switzerland.
[Salatinos, Silvia] Univ Oxford, Wellcome Ctr Human Genet, Oxford, England.
[Ruedl, Christiane] Nanyang Technol Univ, Div Mol Genet & Cell Biol, Singapore, Singapore.
[Kundig, Thomas M.] Univ Zurich, Dept Dermatol, Zurich, Switzerland.
[Speiser, Daniel E.] Univ Lausanne, Dept Oncol, Lausanne, Switzerland.
C3 University of Oxford; Jenner Institute; University of Bern; University Hospital of Bern; Hamad Medical Corporation; University of Bern; University Hospital of Bern; University of Oxford; Wellcome Centre for Human Genetics; Nanyang Technological University & National Institute of Education (NIE) Singapore; Nanyang Technological University; University of Zurich; University of Lausanne
RP Mohsen, MO (通讯作者)，Univ Oxford, Jenner Inst, Nuffield Dept Med, Oxford, England.; Mohsen, MO (通讯作者)，Univ Hosp Bern, Dept BioMed Res, Immunol RIA, Bern, Switzerland.; Mohsen, MO (通讯作者)，Natl Ctr Canc Care & Res, Doha, Qatar.
EM mona.mohsen@dbmr.unibe.ch
RI Bachmann, Martin/AFO-8025-2022; Riether, Carsten/K-8603-2013; El-Turabi, Aadil/AAH-4786-2020; Riether, Carsten/ABC-7699-2021; Speiser, Daniel E./F-2173-2013; Salatino, Silvia/HZK-7289-2023; Bachmann, Martin Fabian/IAP-4113-2023; Cabral-Miranda, Gustavo/AAH-5494-2019; knuth, alexander/H-2608-2014; Dermime, Said/C-6235-2009
OI Riether, Carsten/0000-0001-7512-513X; El-Turabi, Aadil/0000-0003-1092-8155; Riether, Carsten/0000-0001-7512-513X; Salatino, Silvia/0000-0003-1286-3076; Cabral-Miranda, Gustavo/0000-0001-8351-4880; knuth, alexander/0000-0002-9318-2784; Dermime, Said/0000-0002-5526-7496; Muller, Julius/0000-0002-1046-2968; Ternette, Nicola/0000-0002-9283-0743; Cruz Gomes, Ariane Karla/0000-0002-3974-4088
FU Swiss Cancer League [KFS-4132-02-2017]; Qatar National Research Fund (PDRA grant) [PDRA4-0118-18002]
FX This work was supported by the Swiss Cancer League (KFS-4132-02-2017) and Qatar National Research Fund (PDRA grant PDRA4-0118-18002).
CR Ahi YS, 2011, CURR GENE THER, V11, P307
Akira S, 2006, CELL, V124, P783, DOI 10.1016/j.cell.2006.02.015
Alexandrov LB, 2013, NATURE, V500, P415, DOI 10.1038/nature12477
[Anonymous], ALIGNING SEQUENCE RE, DOI DOI 10.48550/ARXIV.1303.3997
Bachmann MF, 2010, NAT REV IMMUNOL, V10, P787, DOI 10.1038/nri2868
Baskin JM, 2007, P NATL ACAD SCI USA, V104, P16793, DOI 10.1073/pnas.0707090104
Carreno BM, 2015, SCIENCE, V348, P803, DOI 10.1126/science.aaa3828
Castle JC, 2012, CANCER RES, V72, P1081, DOI 10.1158/0008-5472.CAN-11-3722
Chang PV, 2010, P NATL ACAD SCI USA, V107, P1821, DOI 10.1073/pnas.0911116107
Chen DS, 2013, IMMUNITY, V39, P1, DOI 10.1016/j.immuni.2013.07.012
Cielens I, 2000, FEBS LETT, V482, P261, DOI 10.1016/S0014-5793(00)02061-5
Donaldson B, 2017, J IMMUNOTHER CANCER, V5, DOI 10.1186/s40425-017-0270-1
Goldinger SM, 2012, EUR J IMMUNOL, V42, P3049, DOI 10.1002/eji.201142361
Gomes AC, 2017, FRONT IMMUNOL, V8, DOI 10.3389/fimmu.2017.00226
Gooden MJM, 2011, BRIT J CANCER, V105, P93, DOI 10.1038/bjc.2011.189
Haanen JBAG, 2006, CANCER IMMUNOL IMMUN, V55, P451, DOI 10.1007/s00262-005-0018-5
Hacohen N, 2013, CANCER IMMUNOL RES, V1, P11, DOI 10.1158/2326-6066.CIR-13-0022
Hu Z, 2018, NAT REV IMMUNOL, V18, P168, DOI 10.1038/nri.2017.131
Jewett JC, 2010, CHEM SOC REV, V39, P1272, DOI 10.1039/b901970g
Kim K, 2014, P NATL ACAD SCI USA, V111, P11774, DOI 10.1073/pnas.1410626111

Kozlovska TM, 1996, INTERVIROLOGY, V39, P9, DOI 10.1159/000150469
Kroemer G, 2012, ONCOIMMUNOLOGY, V1, P579, DOI 10.4161/onci.20730
Kruit WHJ, 2013, J CLIN ONCOL, V31, P2413, DOI 10.1200/JCO.2012.43.7111
Lee N, 2016, PATHOLOGY, V48, P177, DOI 10.1016/j.pathol.2015.12.006
Li H, 2009, BIOINFORMATICS, V25, P1754, DOI 10.1093/bioinformatics/btp324
Luo C, 2017, CANCER LETT, V397, P120, DOI 10.1016/j.canlet.2017.03.037
Makkouk A, 2015, CANCER RES, V75, P5, DOI 10.1158/0008-5472.CAN-14-2538
Martinez-Bosch N, 2018, CANCERS, V10, DOI 10.3390/cancers10010006
McLaren W, 2016, GENOME BIOL, V17, DOI 10.1186/s13059-016-0974-4
Mohsen MO, 2017, SEMIN IMMUNOL, V34, P123, DOI 10.1016/j.smim.2017.08.014
Mohsen MO, 2017, J CONTROL RELEASE, V251, P92, DOI 10.1016/j.jconrel.2017.02.031
Moynihan KD, 2016, NAT MED, V22, P1402, DOI 10.1038/nm.4200
Murray AA, 2018, MOL PHARMACEUT, V15, P3700, DOI 10.1021/acs.molpharmaceut.8b00316
Okuyama R, 2013, ONCOIMMUNOLOGY, V2, DOI 10.4161/onci.27010
Ott PA, 2017, NATURE, V547, P217, DOI 10.1038/nature22991
Rimmer A, 2014, NAT GENET, V46, P912, DOI 10.1038/ng.3036
Sahin U, 2017, NATURE, V547, P222, DOI 10.1038/nature23003
Schwarz K, 2005, EUR J IMMUNOL, V35, P816, DOI 10.1002/eji.200425755
Speiser DE, 2010, J IMMUNOTHER, V33, P848, DOI 10.1097/CJI.0b013e3181f1d614
Stefanetti G, 2015, BIOCONJUGATE CHEM, V26, P2507, DOI 10.1021/acs.bioconjchem.5b00521
Storni T, 2004, J IMMUNOL, V172, P6129, DOI 10.4049/jimmunol.172.10.6129
Storni T, 2002, J IMMUNOL, V168, P2880, DOI 10.4049/jimmunol.168.6.2880
Strasner A, 2015, FRONT ONCOL, V5, DOI 10.3389/fonc.2015.00128
Vasquez M, 2017, ANN TRANSL MED, V5, DOI 10.21037/atm.2017.09.09
Vogelstein B, 2013, SCIENCE, V339, P1546, DOI 10.1126/science.1235122
Yoon HI, 2016, BIOCONJUGATE CHEM, V27, P927, DOI 10.1021/acs.bioconjchem.6b00010
NR 46
TC 31
Z9 34
U1 1
U2 15
PU FRONTIERS MEDIA SA
PI LAUSANNE
PA AVENUE DU TRIBUNAL FEDERAL 34, LAUSANNE, CH-1015, SWITZERLAND
SN 1664-3224
J9 FRONT IMMUNOL
JI Front. Immunol.
PD MAY 15
PY 2019
VL 10
AR 1015
DI 10.3389/fimmu.2019.01015
PG 14
WC Immunology
WE Science Citation Index Expanded (SCI-EXPANDED)
SC Immunology
GA HY2SC
UT WOS:000467972200001
PM 31156619
OA Green Published, gold, Green Accepted
DA 2023-09-14
ER

PT J
AU Gou, SS
Liu, WW
Wang, S
Chen, GY
Chen, ZZ
Qiu, L
Zhou, XM

Wu, YH
Qi, YM
Gao, YF
AF Gou, Shanshan
Liu, Wenwen
Wang, Shuai
Chen, Guanyu
Chen, Zhenzhen
Qiu, Lu
Zhou, Xiuman
Wu, Yahong
Qi, Yuanming
Gao, Yanfeng
TI Engineered Nanovaccine Targeting Clec9a(+) Dendritic Cells Remarkably Enhances the Cancer Immunotherapy Effects of STING Agonist
SO NANO LETTERS
LA English
DT Article
DE STING agonist; engineered biomimetic membrane; nanovaccine; Clec9a; cancer immunotherapy
ID TUMOR MICROENVIRONMENT; PATHWAY; NANOPARTICLES; ACTIVATION; RESISTANCE; RADIATION; THERAPY
AB Agonists of the stimulator of interferon gene (STING) are considered as promising therapeutics for cancer immunotherapy. However, drug-delivery barriers and adverse effects limit the clinical application of STING agonists. Therefore, it is an urgent need to develop an ideal delivery system to deliver STING agonists and avoid side effects. Here, we discovered that STING agonists significantly stimulated type I interferon (IFN) secretion in Clec9a(+) dendritic cells (DCs). Then, we designed an engineered peptide-expressed biomimetic cancer cell membrane (EPBM)-coated nanovaccine drug-delivery system (PLGA/STING@EPBM) to deliver STING agonists and tumor antigens to Clec9a(+) DCs. The PLGA/STING@EPBM nanovaccine significantly enhanced IFN-stimulated expression of genes and antigen cross-presentation of Clec9a(+) DCs, thus eliciting strong antitumor effects in both anti-PD-1-responsive and -resistant tumor models without obvious cytotoxicity. Moreover, the PLGA/STING@EPBM nanovaccine combined with radiotherapy exhibited remarkable synergistic antitumor effects. Our work highlights the great potential of a EPBM-coated nanovaccine for systemic STING agonist delivery as an attractive tool for cancer immunotherapy.
C1 [Gou, Shanshan; Liu, Wenwen; Wang, Shuai; Chen, Zhenzhen; Qiu, Lu; Wu, Yahong; Qi, Yuanming] Zhengzhou Univ, Sch Life Sci, Zhengzhou 450001, Peoples R China.
[Chen, Guanyu; Zhou, Xiuman; Gao, Yanfeng] Sun Yat Sen Univ, Sch Pharmaceut Sci Shenzhen, Shenzhen 518107, Peoples R China.
[Chen, Zhenzhen; Qiu, Lu; Wu, Yahong; Qi, Yuanming] Zhengzhou Univ, Henan Key Lab Bioact Macromol & Int Joint Lab, Zhengzhou 450001, Peoples R China.
C3 Zhengzhou University; Sun Yat Sen University; Zhengzhou University
RP Gao, YF (通讯作者)，Sun Yat Sen Univ, Sch Pharmaceut Sci Shenzhen, Shenzhen 518107, Peoples R China.
EM gaoyf29@mail.sysu.edu.cn
RI GAO, YANFENG/E-1461-2014; Dunn, Shawn/HPF-6836-2023
OI GAO, YANFENG/0000-0001-5533-7100; yahong, Wu/0000-0001-6381-611X
FU National Natural Science Foundation of China [U20A20369, 81822043]; Pearl River Talent Plan" Innovation and Entrepreneurship Team Project of Guangdong Province [2019ZT08Y464]; Shenzhen Science and Technology Program [KQTD20190929173853397]
FX We would like to thank Prof. Shengdian Wang and Hua Peng for providing cell lines and Prof. Xuanming Yang for providing the mice. This work was supported by grants from the National Natural Science Foundation of China (U20A20369, 81822043), "Pearl River Talent Plan" Innovation and Entrepreneurship Team Project of Guangdong Province (2019ZT08Y464), and Shenzhen Science and Technology Program (KQTD20190929173853397).
CR [Anonymous], 2018, J IMMUNOTHER CANCER, DOI DOI 10.1186/S40425-018-0423-X
Barker HE, 2015, NAT REV CANCER, V15, P409, DOI 10.1038/nrc3958
Chang HN, 2015, ANGEW CHEM INT EDIT, V54, P11760, DOI 10.1002/anie.201506225
Chen WF, 2020, ADV FUNCT MATER, V30, DOI 10.1002/adfm.201906623

Chin EN, 2020, SCIENCE, V369, P993, DOI 10.1126/science.abb4255
Corrales L, 2016, J CLIN INVEST, V126, P2404, DOI 10.1172/JCI86892
Feng X, 2020, DRUG DISCOV TODAY, V25, P230, DOI 10.1016/j.drudis.2019.11.007
Flood BA, 2019, IMMUNOL REV, V290, P24, DOI 10.1111/imr.12765
Gajewski TF, 2020, SCIENCE, V369, P921, DOI 10.1126/science.abc6622
Goldberg MS, 2019, NAT REV CANCER, V19, P587, DOI 10.1038/s41568-019-0186-9
Gou SS, 2021, THERANOSTICS, V11, P7308, DOI 10.7150/thno.56406
Guo YY, 2015, ACS NANO, V9, P6918, DOI 10.1021/acsnano.5b01042
Haniffa M, 2012, IMMUNITY, V37, P60, DOI 10.1016/j.immuni.2012.04.012
Hartl CA, 2019, J IMMUNOTHER CANCER, V7, DOI 10.1186/s40425-019-0654-5
Irvine DJ, 2013, NAT MATER, V12, P978, DOI [10.1038/NMAT3775, 10.1038/nmat3775]
Karaolis DKR, 2005, BIOCHEM BIOPH RES CO, V329, P40, DOI 10.1016/j.bbrc.2005.01.093
Kawazoe A, 2020, LANCET ONCOL, V21, P1057, DOI 10.1016/S1470-2045(20)30271-0
Koshy ST, 2017, ADV BIOSYST, V1, DOI 10.1002/adbi.201600013
Kwon J, 2020, CANCER DISCOV, V10, P26, DOI 10.1158/2159-8290.CD-19-0761
Lee YJ, 2009, BLOOD, V114, P589, DOI 10.1182/blood-2009-02-206870
Li AP, 2019, J HEMATOL ONCOL, V12, DOI 10.1186/s13045-019-0721-x
Li RX, 2018, ACTA PHARM SIN B, V8, P14, DOI 10.1016/j.apsb.2017.11.009
Li SX, 2021, NAT BIOMED ENG, V5, P455, DOI 10.1038/s41551-020-00675-9
Li TJ, 2016, SCI REP-UK, V6, DOI 10.1038/srep19049
Li Y, 2020, ACS NANO, V14, P16698, DOI 10.1021/acsnano.0c03776
Lugade AA, 2008, J IMMUNOL, V180, P3132, DOI 10.4049/jimmunol.180.5.3132
Lv MZ, 2020, CELL RES, V30, P966, DOI 10.1038/s41422-020-00395-4
Merad M, 2013, ANNU REV IMMUNOL, V31, P563, DOI 10.1146/annurev-immunol-020711-074950
Pan BS, 2020, SCIENCE, V369, P935, DOI 10.1126/science.aba6098
Pan CX, 2020, J HEMATOL ONCOL, V13, DOI 10.1186/s13045-020-00862-w
Ramanjulu JM, 2018, NATURE, V564, P439, DOI 10.1038/s41586-018-0705-y
Sancho D, 2008, J CLIN INVEST, V118, P2098, DOI 10.1172/JCI34584
Sang W, 2019, CHEM SOC REV, V48, P3771, DOI 10.1039/c8cs00896e
Shae D, 2019, NAT NANOTECHNOL, V14, P269, DOI 10.1038/s41565-018-0342-5
Su T, 2019, THERANOSTICS, V9, P7759, DOI 10.7150/thno.37574
Swartz MA, 2012, SCI TRANSL MED, V4, DOI 10.1126/scitranslmed.3003763
Tang CHA, 2016, CANCER RES, V76, P2137, DOI 10.1158/0008-5472.CAN-15-1885
Tang HD, 2016, CANCER CELL, V29, P285, DOI 10.1016/j.ccell.2016.02.004
Trujillo JA, 2018, CANCER IMMUNOL RES, V6, P990, DOI 10.1158/2326-6066.CIR-18-0277
Wang HF, 2020, J IMMUNOTHER CANCER, V8, DOI 10.1136/jitc-2020-000905
Wang J, 2020, SCIENCE, V367, P869, DOI 10.1126/science.aau0810
Wang ZQ, 2019, ADV MATER, V31, DOI 10.1002/adma.201807175
Wang-Bishop L, 2020, J IMMUNOTHER CANCER, V8, DOI 10.1136/jitc-2019-000282
Wiklander OPB, 2019, SCI TRANSL MED, V11, DOI 10.1126/scitranslmed.aav8521
Yaman S, 2020, FRONT BIOENG BIOTECH, V8, DOI 10.3389/fbioe.2020.00943
Yan ZY, 2016, ONCOTARGET, V7, P40437, DOI 10.18632/oncotarget.9624
Yang R, 2018, ACS NANO, V12, P5121, DOI 10.1021/acsnano.7b09041
Yang Y, 2019, ADV DRUG DELIVER REV, V151, P2, DOI 10.1016/j.addr.2019.05.007
Yu L, 2020, NANO LETT, V20, P8903, DOI 10.1021/acs.nanolett.0c03982
Zander R, 2019, IMMUNITY, V51, P1028, DOI 10.1016/j.immuni.2019.10.009
Zhai WJ, 2020, ACTA PHARMACOL SIN B, V10, P1047, DOI 10.1016/j.apsb.2020.01.005
Zhao JY, 2021, BIOMATERIALS, V268, DOI 10.1016/j.biomaterials.2020.120542
Zhou XM, 2018, FRONT IMMUNOL, V9, DOI 10.3389/fimmu.2018.02821
Zhou XM, 2020, ANGEW CHEM INT EDIT, V59, P15114, DOI 10.1002/anie.202002783
Zhu YY, 2019, MOL CANCER, V18, DOI 10.1186/s12943-019-1087-y
NR 55
TC 30
Z9 31
U1 28
U2 147
PU AMER CHEMICAL SOC
PI WASHINGTON
PA 1155 16TH ST, NW, WASHINGTON, DC 20036 USA
SN 1530-6984
EI 1530-6992

J9 NANO LETT
JI Nano Lett.
PD DEC 8
PY 2021
VL 21
IS 23
BP 9939
EP 9950
DI 10.1021/acs.nanolett.1c03243
PG 12
WC Chemistry, Multidisciplinary; Chemistry, Physical; Nanoscience &
   Nanotechnology; Materials Science, Multidisciplinary; Physics, Applied;
   Physics, Condensed Matter
WE Science Citation Index Expanded (SCI-EXPANDED)
SC Chemistry; Science & Technology - Other Topics; Materials Science;
   Physics
GA ZA9KU
UT WOS:000756475000016
PM 34779631
DA 2023-09-14
ER

PT J
AU Wei, ZW
   Huang, LF
   Cui, L
   Zhu, X
AF Wei, Zewei
   Huang, Lianfang
   Cui, Liao
   Zhu, Xiao
TI Mannose: Good player and assister in pharmacotherapy
SO BIOMEDICINE & PHARMACOTHERAPY
LA English
DT Review
DE Mannose; Cancer; Nanovaccine; Photodynamic therapy; Medicinal function
ID PANCREATIC FISTULA; ESCHERICHIA-COLI; DRUG-THERAPY; GD-DTPA;
   METHOTREXATE; GUT; PANCREATICODUODENECTOMY; PANCREATICOJEJUNOSTOMY;
   NANOPARTICLES; HYALURONAN
AB Mannose is a monosaccharide widely distributed in body fluids and tissues, especially in the nerve, skin, testicles, retina, liver and intestines. It is used to synthesize glycoproteins and participate in immune regulation. In recent years, mannose has been applied more and more widely in the biomedical context as people have a deeper understanding of its biological effects. This review introduces the use of mannose in treating various diseases (including cancer, urinary tract infections, type 1 diabetes, and diabetic wounds), preventing pancreatic fistula, and improving magnetic resonance imaging for acute pancreatitis. We also demonstrate that mannose has the potential for clinical applications.
C1 [Wei, Zewei; Huang, Lianfang; Cui, Liao; Zhu, Xiao] Guangdong Med Univ, Marine Med Res Inst Guangdong Zhanjiang GDZJMMRI, Southern Marine Sci & Engn Guangdong Lab Zhanjian, Zhanjiang, Peoples R China.
   [Wei, Zewei; Huang, Lianfang; Cui, Liao; Zhu, Xiao] Guangdong Med Univ, Sch Pharmaceut Sci, Guangdong Key Lab Res & Dev Nat Drugs, Zhanjiang, Peoples R China.
C3 Southern Marine Science & Engineering Guangdong Laboratory; Southern
   Marine Science & Engineering Guangdong Laboratory (Zhanjiang); Guangdong
   Medical University; Guangdong Medical University
RP Cui, L; Zhu, X (通讯作者)，Guangdong Med Univ, Marine Med Res Inst Guangdong Zhanjiang GDZJMMRI, Southern Marine Sci & Engn Guangdong Lab Zhanjian, Zhanjiang, Peoples R China.
EM cuiliao@163.com; xzhu@gdmu.edu.cn
OI Zhu, Xiao/0000-0002-1737-3386
FU National Natural Science Foundation of China [81541153]; Guangdong
   Science and Technology Department [2016A050503046, 2015A050502048,
   2016B030309002]; Southern Science and Engineering Guangdong Laboratory

Zhanjiang [ZJW-2019-07]; Zhanjiang Science and Technology Plan [2017A06012]; "Grouptype" Special Supporting Project for Educational Talents in Universities [4SG19057G]; Public Service Platform of South China for R&D Marine Biomedicine Resources [GDMUK201808]
FX This work was supported partly by National Natural Science Foundation of China (81541153); Guangdong Science and Technology Department (2016A050503046, 2015A050502048 and 2016B030309002); Southern Science and Engineering Guangdong Laboratory Zhanjiang (ZJW-2019-07); The Public Service Platform of South China for R&D Marine Biomedicine Resources (GDMUK201808); Zhanjiang Science and Technology Plan (2017A06012); and "Grouptype" Special Supporting Project for Educational Talents in Universities (4SG19057G). The funders had no role in the design of the study; the collection, analysis, and interpretation of the data; the writing of the manuscript; and the decision to submit the manuscript for publication.
CR Akbarshahi H, 2012, PLOS ONE, V7, DOI 10.1371/journal.pone.0042654
Andrianello S, 2016, PANCREATOLOGY, V16, P138, DOI 10.1016/j.pan.2015.11.004
Bassi C, 2005, SURGERY, V138, P8, DOI 10.1016/j.surg.2005.05.001
Bouffard E, 2019, INT J MOL SCI, V20, DOI 10.3390/ijms20112809
Chan KC, 2008, EXP EYE RES, V87, P334, DOI 10.1016/j.exer.2008.06.015
Crafa Francesco, 2018, Ann Hepatobiliary Pancreat Surg, V22, P248, DOI 10.14701/ahbps.2018.22.3.248
de la Motte CA, 1999, J BIOL CHEM, V274, P30747, DOI 10.1074/jbc.274.43.30747
Dervenis C, 1999, INT J PANCREATOL, V25, P195
Ding CZ, 2012, CURR THER RES CLIN E, V73, P123, DOI 10.1016/j.curtheres.2012.06.001
DJERASSI I, 1967, CANCER, V20, P233, DOI 10.1002/1097-0142(1967)20:2<233::AID-CNCR2820200209>3.0.CO;2-8
Dobson J, 2018, VET J, V233, P8, DOI 10.1016/j.tvjl.2017.11.012
Dong Z., 2019, CHEMISTRYOPEN, V8
Engering AJ, 1997, ADV EXP MED BIOL, V417, P183
Fan ZX, 2020, J MATER CHEM B, V8, P1922, DOI 10.1039/d0tb00049c
Gan JJ, 2019, BIOMATERIALS, V219, DOI 10.1016/j.biomaterials.2019.119340
Gan JJ, 2018, BIOMATERIALS, V178, P95, DOI 10.1016/j.biomaterials.2018.06.015
Genovese C, 2018, J CHEMOTHERAPY, V30, P107, DOI 10.1080/1120009X.2017.1393587
Gonzalez PS, 2018, NATURE, V563, P719, DOI 10.1038/s41586-018-0729-3
Gupta K, 2007, J UROLOGY, V177, P2357, DOI 10.1016/j.juro.2007.01.114
Hou YP, 2017, BIOMED RES INT, V2017, DOI 10.1155/2017/7585989
Jokela TA, 2013, WOUND REPAIR REGEN, V21, P247, DOI 10.1111/wrr.12022
Kaneko H, 2017, SURGERY, V161, P1561, DOI 10.1016/j.surg.2016.12.022
Katayama K, 2015, MOD RHEUMATOL, V25, P50, DOI 10.3109/14397595.2014.924385
Kikuchi S, 2005, J BIOL CHEM, V280, P15398, DOI 10.1074/jbc.M414048200
Kim S, 2009, NMR BIOMED, V22, P303, DOI 10.1002/nbm.1324
Kossi J, 1999, EUR SURG RES, V31, P74, DOI 10.1159/000008623
KROGFELT KA, 1990, INFECT IMMUN, V58, P1995, DOI 10.1128/IAI.58.6.1995-1998.1990
Kuroshima N, 2016, PANCREATOLOGY, V16, P917, DOI 10.1016/j.pan.2016.06.011
Ley RE, 2006, NATURE, V444, P1022, DOI 10.1038/4441022a
Liu M, 2018, LANGMUIR, V34, P1574, DOI 10.1021/acs.langmuir.7b03556
Milandri R, 2019, UROLOGIA J, V86, P122, DOI 10.1177/0391560318798291
Min YZ, 2017, NAT NANOTECHNOL, V12, P877, DOI [10.1038/nnano.2017.113, 10.1038/NNANO.2017.113]
Miwa Y, 2016, INTERNAL MED, V55, P2581, DOI 10.2169/internalmedicine.55.6872
Movahedi K, 2012, CANCER RES, V72, P4165, DOI 10.1158/0008-5472.CAN-11-2994
Naz F, 2019, INT J PHARMACEUT, V569, DOI 10.1016/j.ijpharm.2019.118561
Niemela E, 2020, EUR J PHARM BIOPHARM, V148, P1, DOI 10.1016/j.ejpb.2019.12.015
Russo E, 2020, CLIMACTERIC, V23, P201, DOI 10.1080/13697137.2019.1679110
Sharma V, 2018, CELL REP, V24, P3087, DOI 10.1016/j.celrep.2018.08.064
Spaulding CN, 2017, NATURE, V546, P528, DOI 10.1038/nature22972
Standop J, 2005, WORLD J SURG, V29, P505, DOI 10.1007/s00268-004-7741-5
Strassburger D, 2018, CHEMBIOCHEM, V19, P912, DOI 10.1002/cbic.201800114
Tian B, 2017, INT J NANOMED, V12, P1127, DOI 10.2147/IJN.S123290
Tran K, 2002, ANN SURG, V236, P422, DOI 10.1097/00000658-200210000-00004

Wei CW, 2019, ONCOL REP, V41, P2060, DOI 10.3892/or.2019.6958
Yamamoto S, 1997, J UROLOGY, V157, P1127, DOI 10.1016/S0022-5347(01)65154-1
Yang R, 2018, ACS NANO, V12, P5121, DOI 10.1021/acsnano.7b09041
Zhang DY, 2017, CARBON LETT, V23, P9, DOI 10.5714/CL.2017.23.009
Zhang Q, 2017, CHEM-EUR J, V23, P14307, DOI 10.1002/chem.201702935
Zhao L, 2017, FOOD FUNCT, V8, P4644, DOI [10.1039/c7fo01383c, 10.1039/C7FO01383C]
Zhu X, 2019, CELL BIOSCI, V9, DOI 10.1186/s13578-019-0356-1
NR 50
TC 30
Z9 31
U1 2
U2 25
PU ELSEVIER FRANCE-EDITIONS SCIENTIFIQUES MEDICALES ELSEVIER
PI ISSY-LES-MOULINEAUX
PA 65 RUE CAMILLE DESMOULINS, CS50083, 92442 ISSY-LES-MOULINEAUX, FRANCE
SN 0753-3322
EI 1950-6007
J9 BIOMED PHARMACOTHER
JI Biomed. Pharmacother.
PD SEP
PY 2020
VL 129
AR 110420
DI 10.1016/j.biopha.2020.110420
PG 6
WC Medicine, Research & Experimental; Pharmacology & Pharmacy
WE Science Citation Index Expanded (SCI-EXPANDED)
SC Research & Experimental Medicine; Pharmacology & Pharmacy
GA NJ9SF
UT WOS:000566382800011
PM 32563989
OA gold
DA 2023-09-14
ER

PT J
AU Yan, YF
Ding, HW
AF Yan, Yunfeng
Ding, Hangwei
TI pH-Responsive Nanoparticles for Cancer Immunotherapy: A Brief Review
SO NANOMATERIALS
LA English
DT Review
DE nanoparticle; cancer; immunotherapy; pH-responsive; drug delivery
ID TUMOR-ASSOCIATED MACROPHAGES; DENDRITIC CELLS; BREAST-CANCER; DELIVERY; CHEMOTHERAPY; NANOVACCINE
AB Immunotherapy has recently become a promising strategy for the treatment of a wide range of cancers. However, the broad implementation of cancer immunotherapy suffers from inadequate efficacy and toxic side effects. Integrating pH-responsive nanoparticles into immunotherapy is a powerful approach to tackle these challenges because they are able to target the tumor tissues and organelles of antigen-presenting cells (APCs) which have a characteristic acidic microenvironment. The spatiotemporal control of immunotherapeutic drugs using pH-responsive nanoparticles endows cancer immunotherapy with enhanced antitumor immunity and reduced off-tumor immunity. In this review, we first discuss the cancer-immunity circle and how nanoparticles can modulate the key steps in this circle. Then, we highlight the recent advances in cancer immunotherapy with pH-responsive nanoparticles and discuss the perspective for this emerging area.
C1 [Yan, Yunfeng; Ding, Hangwei] Zhejiang Univ Technol, Coll Biotechnol & Bioengn, Hangzhou 310032, Peoples R China.
C3 Zhejiang University of Technology
RP Yan, YF (通讯作者)，Zhejiang Univ Technol, Coll Biotechnol & Bioengn, Hangzhou 310032, Peoples R

China.
EM yfyan@zjut.edu.cn; dhwpao@163.com
RI Yan, Yunfeng/J-4627-2013; Yan, Yunfeng/JBJ-8628-2023
OI Yan, Yunfeng/0000-0001-8983-640X
FU National Natural Science Foundation of China [U1932164]; Zhejiang
Provincial Natural Science Foundation [LY19B040004]
FX We gratefully acknowledge the financial support from the National
Natural Science Foundation of China (U1932164) and the Zhejiang
Provincial Natural Science Foundation (LY19B040004).
CR Blanco E, 2015, NAT BIOTECHNOL, V33, P941, DOI 10.1038/nbt.3330
Chen DS, 2013, IMMUNITY, V39, P1, DOI 10.1016/j.immuni.2013.07.012
Cheng Q, 2020, NAT NANOTECHNOL, V15, P313, DOI 10.1038/s41565-020-0669-6
Chi HJ, 2017, FRONT PHARMACOL, V8, DOI 10.3389/fphar.2017.00304
Dai LL, 2018, ADV FUNCT MATER, V28, DOI 10.1002/adfm.201707249
Davis ME, 2008, NAT REV DRUG DISCOV, V7, P771, DOI 10.1038/nrd2614
Du JZ, 2015, J CONTROL RELEASE, V219, P205, DOI 10.1016/j.jconrel.2015.08.050
Duan F, 2017, BIOMATERIALS, V122, P23, DOI 10.1016/j.biomaterials.2017.01.017
Feng B, 2018, ADV MATER, V30, DOI 10.1002/adma.201803001
Goldberg MS, 2019, NAT REV CANCER, V19, P587, DOI 10.1038/s41568-019-0186-9
Hajj KA, 2019, SMALL, V15, DOI 10.1002/smll.201805097
Hu Y, 2007, NANO LETT, V7, P3056, DOI 10.1021/nl071542i
Huang Z, 2012, J CONTROL RELEASE, V158, P286, DOI 10.1016/j.jconrel.2011.11.013
Irvine DJ, 2020, NAT REV IMMUNOL, V20, P321, DOI 10.1038/s41577-019-0269-6
Jacobson ME, 2019, BIOMATER SCI-UK, V7, P547, DOI 10.1039/c8bm01064a
Kanamala M, 2016, BIOMATERIALS, V85, P152, DOI 10.1016/j.biomaterials.2016.01.061
Kim H, 2018, NANOSCALE, V10, P20851, DOI 10.1039/c8nr07201a
Kwon YJ, 2005, P NATL ACAD SCI USA, V102, P18264, DOI 10.1073/pnas.0509541102
Liu Q, 2015, ACS NANO, V9, P4925, DOI 10.1021/nn5066793
Liu YY, 2018, ACTA BIOMATER, V66, P310, DOI 10.1016/j.actbio.2017.11.010
Luo M, 2017, NAT NANOTECHNOL, V12, P648, DOI [10.1038/nnano.2017.52,
10.1038/NNANO.2017.52]
Ma XP, 2014, J AM CHEM SOC, V136, P11085, DOI 10.1021/ja5053158
Martin JD, 2020, NAT REV CLIN ONCOL, V17, P251, DOI 10.1038/s41571-019-0308-z
Martins F, 2019, NAT REV CLIN ONCOL, V16, P563, DOI 10.1038/s41571-019-0218-0
Maude SL, 2018, NEW ENGL J MED, V378, P439, DOI 10.1056/NEJMoa1709866
McKinlay CJ, 2018, P NATL ACAD SCI USA, V115, pE5859, DOI 10.1073/pnas.1805358115
Mellman I, 2011, NATURE, V480, P480, DOI 10.1038/nature10673
Molavi L, 2010, MOL PHARMACEUT, V7, P364, DOI 10.1021/mp900145g
Nam J, 2019, NAT REV MATER, V4, P398, DOI 10.1038/s41578-019-0108-1
Neelapu SS, 2017, NEW ENGL J MED, V377, P2531, DOI 10.1056/NEJMoa1707447
Neelapu SS, 2018, NAT REV CLIN ONCOL, V15, P47, DOI 10.1038/nrclinonc.2017.148
Palucka K, 2013, IMMUNITY, V39, P38, DOI 10.1016/j.immuni.2013.07.004
Paquette RL, 1998, J LEUKOCYTE BIOL, V64, P358, DOI 10.1002/jlb.64.3.358
Ramanjulu JM, 2018, NATURE, V564, P439, DOI 10.1038/s41586-018-0705-y
Riley RS, 2019, NAT REV DRUG DISCOV, V18, P175, DOI 10.1038/s41573-018-0006-z
Rosenberg SA, 2015, SCIENCE, V348, P62, DOI 10.1126/science.aaa4967
Rosenberg SA, 2014, J IMMUNOL, V192, P5451, DOI 10.4049/jimmunol.1490019
Shen S, 2017, NANO LETT, V17, P3822, DOI 10.1021/acs.nanolett.7b01193
Shen Youqing, 2008, V437, P183, DOI 10.1007/978-1-59745-210-6_10
Solbrig CM, 2007, MOL PHARMACEUT, V4, P47, DOI 10.1021/mp060107e
Song QL, 2017, NANO LETT, V17, P6366, DOI 10.1021/acs.nanolett.7b03186
Song YD, 2018, BIOMATERIALS, V185, P117, DOI 10.1016/j.biomaterials.2018.09.017
Varkouhi AK, 2011, J CONTROL RELEASE, V151, P220, DOI 10.1016/j.jconrel.2010.11.004
Wang C, 2016, BIOMATERIALS, V79, P88, DOI 10.1016/j.biomaterials.2015.11.040
Wang DG, 2016, NANO LETT, V16, P5503, DOI 10.1021/acs.nanolett.6b01994
Wang Y, 2017, BIOMATERIALS, V112, P153, DOI 10.1016/j.biomaterials.2016.09.034
Xiong QQ, 2018, NANO RES, V11, P5281, DOI 10.1007/s12274-018-2146-1
Yan YF, 2017, BIOMACROMOLECULES, V18, P4307, DOI 10.1021/acs.biomac.7b01356
Yang GB, 2018, NANO LETT, V18, P2475, DOI 10.1021/acs.nanolett.8b00040
Yuba E, 2014, BIOMATERIALS, V35, P3091, DOI 10.1016/j.biomaterials.2013.12.024
Zhang CN, 2017, J CONTROL RELEASE, V256, P170, DOI 10.1016/j.jconrel.2017.04.020

Zhao T, 2017, NAT BIOMED ENG, V1, DOI 10.1038/s41551-016-0006
Zheng DW, 2016, NANO LETT, V16, P4341, DOI 10.1021/acs.nanolett.6b01432
Zhu SJ, 2013, MOL PHARMACEUT, V10, P3525, DOI 10.1021/mp400216r
NR 54
TC 30
Z9 29
U1 6
U2 86
PU MDPI
PI BASEL
PA ST ALBAN-ANLAGE 66, CH-4052 BASEL, SWITZERLAND
EI 2079-4991
J9 NANOMATERIALS-BASEL
JI Nanomaterials
PD AUG
PY 2020
VL 10
IS 8
AR 1613
DI 10.3390/nano10081613
PG 15
WC Chemistry, Multidisciplinary; Nanoscience & Nanotechnology; Materials
Science, Multidisciplinary; Physics, Applied
WE Science Citation Index Expanded (SCI-EXPANDED)
SC Chemistry; Science & Technology - Other Topics; Materials Science;
Physics
GA NH7PB
UT WOS:000564856800001
PM 32824578
OA Green Published, gold
DA 2023-09-14
ER

PT J
AU Zhou, ZZ
Pang, JH
Wu, XJ
Wu, W
Chen, XG
Kong, M
AF Zhou, Zhongzheng
Pang, Jianhui
Wu, Xuanjin
Wu, Wei
Chen, Xiguang
Kong, Ming
TI Reverse immune suppressive microenvironment in tumor draining lymph
nodes to enhance anti-PD1 immunotherapy via nanovaccine complexed
microneedle
SO NANO RESEARCH
LA English
DT Article
DE transfersomes; microneedles; immunosuppressive microenvironment; tumor
draining lymph node; alpha PD1
ID DENDRITIC-CELL; CANCER-IMMUNOTHERAPY; TARGETED DELIVERY; RESPONSES;
CHITOSAN; NANOCARRIERS; IPILIMUMAB; VACCINES; THERAPY; ANTIGEN
AB The maturation of dendritic cells (DCs) and infiltration effector T cells in tumor-draining lymph node (tdLN) and tumor tissue are crucial for immunotherapy. Despite constructive progresses have been made with anti-programmed death-1 (anti-PD1) checkpoint blockade for immunotherapy, the efficacy of PD1/PD-L1 therapy deserves to be improved. Here, we constructed a novel transfersomes based nanovaccine complexed microneedles to enhance anti-PD1 immunotherapy via transdermal immunization

for skin tumor therapy. Transfersomes were functionalized with DCs targeting moiety alpha CD40, co-encapsulated with antigens and adjuvant poly I:C. Moreover, transdermal administration promoted accumulation in tumor-draining lymph nodes (tdLN), which could facilitate cellular uptake, activate DCs maturation and enhance Th1 immune responses. Using a mouse melanoma model, combined therapy of such nanovaccine complexed microneedles with pembrolizumab (alpha PD1) was able to enhance cytotoxic T lymphocytes activation, promote infiltration and reduce regulatory T cells frequency in tdLN and tumor tissues, which achieved reversion of the immunosuppressive microenvironment into immune activation. This study highlighted the potential of transfersomes based nanovaccines complexed microneedles as an attractive platform for tumor immunotherapy.
C1 [Zhou, Zhongzheng; Pang, Jianhui; Wu, Xuanjin; Wu, Wei; Chen, Xiguang; Kong, Ming] Ocean Univ China, Coll Marine Life Sci, 5 Yushan Rd, Qingdao 266003, Peoples R China.
[Chen, Xiguang] Qingdao Natl Lab Marine Sci & Technol, Wenhai Rd, Qingdao 266237, Peoples R China.
C3 Ocean University of China; Laoshan Laboratory
RP Kong, M (通讯作者)，Ocean Univ China, Coll Marine Life Sci, 5 Yushan Rd, Qingdao 266003, Peoples R China.
EM kongming@ouc.edu.cn
RI chen, xi/GXH-3653-2022; chen, xin/IQW-3432-2023
FU National Natural Science Foundation of China [31670972]; Taishan Scholar Program, China
FX work was supported by the National Natural Science Foundation of China (No. 31670972), and the Taishan Scholar Program, China.
CR AL-Deen FMN, 2014, GENE THER, V21, P212, DOI 10.1038/gt.2013.77
Chandrasekaran S, 2014, INT J MOL SCI, V15, P20209, DOI 10.3390/ijms151120209
Chen GJ, 2020, P NATL ACAD SCI USA, V117, P3687, DOI 10.1073/pnas.1917891117
Danhier F, 2010, J CONTROL RELEASE, V148, P135, DOI 10.1016/j.jconrel.2010.08.027
de Wilt JHW, 2008, SURG ONCOL, V17, P175, DOI 10.1016/j.suronc.2008.06.004
Frank-Bertoncelj M, 2018, FRONT IMMUNOL, V9, DOI 10.3389/fimmu.2018.00028
Fransen MF, 2013, INT J CANCER, V132, P1971, DOI 10.1002/ijc.27755
Habijanic J, 2015, NEW BIOTECHNOL, V32, P85, DOI 10.1016/j.nbt.2014.07.007
Hao Y, 2017, J BIOMED NANOTECHNOL, V13, P1581, DOI 10.1166/jbn.2017.2474
Higa K, 2005, CORNEA, V24, P206, DOI 10.1097/01.ico.0000133999.45262.83
Hong Y, 2015, CANCER RES, V75, P656, DOI 10.1158/0008-5472.CAN-14-2377
Jeanbart L, 2014, CANCER IMMUNOL RES, V2, P436, DOI 10.1158/2326-6066.CIR-14-0019-T
Joffre OP, 2012, NAT REV IMMUNOL, V12, P557, DOI 10.1038/nri3254
Kane LP, 2001, NAT IMMUNOL, V2, P37, DOI 10.1038/83144
Kong M, 2017, CARBOHYD POLYM, V169, P341, DOI 10.1016/j.carbpol.2017.03.090
Kong M, 2015, CHEM COMMUN, V51, P1453, DOI 10.1039/c4cc08746a
Kong M, 2011, CARBOHYD POLYM, V83, P1303, DOI 10.1016/j.carbpol.2010.09.041
Kong M, 2011, CARBOHYD POLYM, V83, P462, DOI 10.1016/j.carbpol.2010.08.001
Lau WH, 2017, ONCOTARGET, V8, P32884, DOI 10.18632/oncotarget.15862
Liu LX, 2016, ACS APPL MATER INTER, V8, P11969, DOI 10.1021/acsami.6b01135
Ma GJ, 2017, J CONTROL RELEASE, V251, P11, DOI 10.1016/j.jconrel.2017.02.011
Malissen B, 2014, NAT REV IMMUNOL, V14, P417, DOI 10.1038/nri3683
Rahimian S, 2015, BIOMATERIALS, V61, P33, DOI 10.1016/j.biomaterials.2015.04.043
Rahimian S, 2015, BIOMATERIALS, V37, P469, DOI 10.1016/j.biomaterials.2014.10.043
Randolph GJ, 2005, NAT REV IMMUNOL, V5, P617, DOI 10.1038/nri1670
Rao SB, 1997, J BIOMED MATER RES, V34, P21, DOI 10.1002/(SICI)1097-4636(199701)34:1<21::AID-JBM4>3.0.CO;2-P
Robert C, 2015, NEW ENGL J MED, V372, P2521, DOI 10.1056/NEJMoa1503093
Rosalia RA, 2015, BIOMATERIALS, V40, P88, DOI 10.1016/j.biomaterials.2014.10.053
Schjetne KW, 2007, J IMMUNOL, V178, P4169, DOI 10.4049/jimmunol.178.7.4169
Sharma P, 2015, CELL, V161, P205, DOI 10.1016/j.cell.2015.03.030
Sun GH, 2017, INT J BIOL MACROMOL, V105, P566, DOI 10.1016/j.ijbiomac.2017.07.082
Tacken PJ, 2011, BLOOD, V118, P6836, DOI 10.1182/blood-2011-07-367615
Takemura R, 2015, CANCER IMMUNOL RES, V3, P902, DOI 10.1158/2326-6066.CIR-14-0219
Tang HD, 2016, CANCER LETT, V370, P85, DOI 10.1016/j.canlet.2015.10.009
Thomas SN, 2014, BIOMATERIALS, V35, P814, DOI 10.1016/j.biomaterials.2013.10.003
Valsecchi ME, 2015, NEW ENGL J MED, V373, P1270, DOI [10.1056/NEJMoa1504030, 10.1056/NEJMc1509660]
Wang C, 2016, NANO LETT, V16, P2334, DOI 10.1021/acs.nanolett.5b05030

Wu XJ, 2019, J MATER CHEM B, V7, P4854, DOI 10.1039/c9tb00448c
Yang HJ, 2019, INT J BIOL MACROMOL, V125, P9, DOI 10.1016/j.ijbiomac.2018.11.230
Yang YP, 2015, J CLIN INVEST, V125, P3335, DOI 10.1172/JCI83871
Ye YQ, 2018, ADV DRUG DELIVER REV, V127, P106, DOI 10.1016/j.addr.2018.01.015
Ye YQ, 2017, SCI IMMUNOL, V2, DOI 10.1126/sciimmunol.aan5692
Ye YQ, 2016, ACS NANO, V10, P8956, DOI 10.1021/acsnano.6b04989
NR 43
TC 30
Z9 30
U1 10
U2 138
PU TSINGHUA UNIV PRESS
PI BEIJING
PA B605D, XUE YAN BUILDING, BEIJING, 100084, PEOPLES R CHINA
SN 1998-0124
EI 1998-0000
J9 NANO RES
JI Nano Res.
PD JUN
PY 2020
VL 13
IS 6
BP 1509
EP 1518
DI 10.1007/s12274-020-2737-5
EA APR 2020
PG 10
WC Chemistry, Physical; Nanoscience & Nanotechnology; Materials Science,
Multidisciplinary; Physics, Applied
WE Science Citation Index Expanded (SCI-EXPANDED)
SC Chemistry; Science & Technology - Other Topics; Materials Science;
Physics
GA MA4MO
UT WOS:000525164600007
DA 2023-09-14
ER

PT J
AU Yang, JN
Arya, S
Lung, PS
Lin, QB
Huang, JD
Li, Q
AF Yang, Jingnan
Arya, Smriti
Lung, Pingsai
Lin, Qiubin
Huang, Jiandong
Li, Quan
TI Hybrid nanovaccine for the co-delivery of the mRNA antigen and adjuvant
SO NANOSCALE
LA English
DT Article
ID IN-VIVO; NONVIRAL TRANSFECTION; CANCER-IMMUNOTHERAPY; DENDRITIC CELLS;
NANOPARTICLES; AGONISTS
AB For efficient cancer vaccines, the antitumor function largely relies on cytotoxic T cells, whose activation can be effectively induced via antigen-encoding mRNA, making mRNA-based cancer vaccines an attractive approach for personalized cancer therapy. While the liposome-based delivery system enables the systemic delivery and transfection of mRNA, incorporating an adjuvant that is non-lipid like remains challenging, although the co-delivery of mRNA (antigen) and effective adjuvant is key to the

activation of the cytotoxic T cells. This is because the presence of an adjuvant is important for dendritic cell maturation-another necessity for cytotoxic T cell activation. In the present work, we designed a poly (lactic-co-glycolic acid) (PLGA)-core/lipid-shell hybrid nanoparticle carrier for the co-delivery of mRNA and gardiquimod (adjuvant that cannot be incorporated into the lipid shell). We demonstrated in the present work that the co-delivery of mRNA and gardiquimod led to the effective antigen expression and DC maturation in vitro. The intravenous administration of the hybrid nanovaccine resulted in the enrichment of mRNA expression in the spleen and a strong immune response in vivo. The simultaneous delivery of the antigen and adjuvant both spatially and temporally via the core/shell nanoparticle carrier is found to be beneficial for tumor growth inhibition.
C1 [Yang, Jingnan; Lung, Pingsai; Li, Quan] Chinese Univ Hong Kong, Dept Phys, Shatin, Hong Kong, Peoples R China.
[Arya, Smriti; Lin, Qiubin; Huang, Jiandong] Univ Hong Kong, Sch Biomed Sci, Hong Kong, Peoples R China.
C3 Chinese University of Hong Kong; University of Hong Kong
RP Li, Q (通讯作者)，Chinese Univ Hong Kong, Dept Phys, Shatin, Hong Kong, Peoples R China.; Huang, JD (通讯作者)，Univ Hong Kong, Sch Biomed Sci, Hong Kong, Peoples R China.
EM jdhuang@hku.hk; liquan@phy.cuhk.edu.hk
RI Li, Quan/G-5082-2013
OI Li, Quan/0000-0001-8563-1802; Lin, Qiubin/0000-0002-4621-304X
FU HMRF [18170262]; Shenzhen Peacock project [KQTD2015033117210153]; Shenzhen Science and Technology Innovation Committee Basic Science Research Grant [JCYJ20170413154523577]
FX The authors are grateful for financial support from the HMRF under project no. 18170262. This study was also supported by the Shenzhen Peacock project (KQTD2015033117210153) and the Shenzhen Science and Technology Innovation Committee Basic Science Research Grant (JCYJ20170413154523577). We also thank Wenlong Zuo and Yilin Wu for the assistance with fluorescence microscopy imaging and Jun Xu and Zhuang Liu for kindly providing B16-OVA cells.
CR Akinc A, 2005, J GENE MED, V7, P657, DOI 10.1002/jgm.696
Awasthi S, 2003, BIOTECHNIQUES, V35, P600, DOI 10.2144/03353dd03
BOUSSIF O, 1995, P NATL ACAD SCI USA, V92, P7297, DOI 10.1073/pnas.92.16.7297
Coffman RL, 2010, IMMUNITY, V33, P492, DOI 10.1016/j.immuni.2010.10.002
Goldberg MS, 2015, CELL, V161, P201, DOI 10.1016/j.cell.2015.03.037
Hajj KA, 2017, NAT REV MATER, V2, DOI 10.1038/natrevmats.2017.56
Heil F, 2004, SCIENCE, V303, P1526, DOI 10.1126/science.1093620
Irvine AS, 2000, NAT BIOTECHNOL, V18, P1273, DOI 10.1038/82383
Kim H, 2018, NANOSCALE, V10, P20851, DOI 10.1039/c8nr07201a
Kim H, 2018, BIOMATERIALS, V164, P38, DOI 10.1016/j.biomaterials.2018.02.034
Kranz LM, 2016, NATURE, V534, P396, DOI 10.1038/nature18300
Kuai R, 2017, NAT MATER, V16, P489, DOI [10.1038/nmat4822, 10.1038/NMAT4822]
MALONE RW, 1989, P NATL ACAD SCI USA, V86, P6077, DOI 10.1073/pnas.86.16.6077
Melief CJM, 2015, J CLIN INVEST, V125, P3401, DOI 10.1172/JCI80009
Meyer O, 2000, EUR J PHARM BIOPHARM, V50, P353, DOI 10.1016/S0939-6411(00)00115-6
Nuhn L, 2016, P NATL ACAD SCI USA, V113, P8098, DOI 10.1073/pnas.1600816113
Oberli MA, 2017, NANO LETT, V17, P1326, DOI 10.1021/acs.nanolett.6b03329
Palucka K, 2012, NAT REV CANCER, V12, P265, DOI 10.1038/nrc3258
Reed SG, 2013, NAT MED, V19, P1597, DOI 10.1038/nm.3409
Rietwyk S, 2017, ACS NANO, V11, P7572, DOI 10.1021/acsnano.7b04734
Sahin U, 2014, NAT REV DRUG DISCOV, V13, P759, DOI 10.1038/nrd4278
Su XF, 2011, MOL PHARMACEUT, V8, P774, DOI 10.1021/mp100390w
Tan SW, 2013, NANOSCALE, V5, P860, DOI 10.1039/c2nr32880a
Troutier AL, 2005, LANGMUIR, V21, P9901, DOI 10.1021/la050796l
Uchida S, 2016, BIOMATERIALS, V82, P221, DOI 10.1016/j.biomaterials.2015.12.031
Verbeke R, 2019, ACS NANO, V13, P1655, DOI 10.1021/acsnano.8b07660
Verbeke R, 2017, J CONTROL RELEASE, V266, P287, DOI 10.1016/j.jconrel.2017.09.041
Xing RJ, 2011, CHEM COMMUN, V47, P12152, DOI 10.1039/c1cc15408g
Zhang LF, 2008, ACS NANO, V2, P1696, DOI 10.1021/nn800275r
Zhu GZ, 2017, ACS NANO, V11, P2387, DOI 10.1021/acsnano.7b00978
NR 30
TC 30

Z9 31
U1 11
U2 165
PU ROYAL SOC CHEMISTRY
PI CAMBRIDGE
PA THOMAS GRAHAM HOUSE, SCIENCE PARK, MILTON RD, CAMBRIDGE CB4 0WF, CAMBS, ENGLAND
SN 2040-3364
EI 2040-3372
J9 NANOSCALE
JI Nanoscale
PD DEC 7
PY 2019
VL 11
IS 45
BP 21782
EP 21789
DI 10.1039/c9nr05475h
PG 8
WC Chemistry, Multidisciplinary; Nanoscience & Nanotechnology; Materials Science, Multidisciplinary; Physics, Applied
WE Science Citation Index Expanded (SCI-EXPANDED)
SC Chemistry; Science & Technology - Other Topics; Materials Science; Physics
GA JT1SU
UT WOS:000500778500009
PM 31709434
DA 2023-09-14
ER

PT J
AU Yang, WJ
Zhou, LJ
Lau, J
Hu, S
Chen, XY
AF Yang, Weijing
Zhou, Lijian
Lau, Joseph
Hu, Shuo
Chen, Xiaoyuan
TI Functional T cell activation by smart nanosystems for effective cancer immunotherapy
SO NANO TODAY
LA English
DT Review
DE Cancer immunotherapy; Smart nanosystems; Artificial antigen presenting cells; Cancer nanovaccine; Tumor microenvironment modulation; Combination therapy
ID ANTIGEN-PRESENTING CELLS; PHOTODYNAMIC THERAPY; TUMOR MICROENVIRONMENT; DENDRITIC CELLS; CHECKPOINT BLOCKADE; IMMUNE CELLS; NANOPARTICLES; VACCINES; DELIVERY; METASTASIS
AB Immunotherapy (e.g., checkpoint blockade, adoptive T cell therapy, cancer vaccine) has emerged as one of the most important treatment modalities for cancer in the past two decades. While these approaches are promising, the response rates observed in clinical trials are less than 30% due to tumor heterogeneity and complexity of the tumor microenvironment. Smart nanosystems have the potential to address some of the limitations associated with immunotherapy. Herein, we review applications of nanosystem-mediated immunotherapy for functional T cell activation, including formation of artificial antigen presenting cells, cancer nanovaccination, tumor microenvironment modulation, and combination therapy. Finally, we propose areas of research for the future development of nanosystem-mediated cancer immunotherapy. (C) 2019 Published by Elsevier Ltd.

C1 [Yang, Weijing; Hu, Shuo] Cent S Univ, Xiangya Hosp, Dept PET Ctr, Changsha 410008, Hunan, Peoples R China.
[Hu, Shuo] Cent S Univ, Xiangya Hosp, Natl Clin Res Ctr Geriatr Disorders XIANGYA, Changsha 410008, Hunan, Peoples R China.
[Yang, Weijing; Zhou, Lijian; Lau, Joseph; Chen, Xiaoyuan] NIBIB, Lab Mol Imaging & Nanomed LOMIN, NIH, Bethesda, MD 20892 USA.
C3 Central South University; Central South University; National Institutes of Health (NIH) - USA; NIH National Institute of Biomedical Imaging & Bioengineering (NIBIB)
RP Hu, S (通讯作者)，Cent S Univ, Xiangya Hosp, Dept PET Ctr, Changsha 410008, Hunan, Peoples R China.; Chen, XY (通讯作者)，NIBIB, Lab Mol Imaging & Nanomed LOMIN, NIH, Bethesda, MD 20892 USA.
EM hushuo_xy@163.com; shawn.chen@nih.gov
RI chen, xia/GXM-5435-2022; chen, xi/GXH-3653-2022; chen, xin/IQW-3432-2023; chen, xia/GYR-3948-2022
FU Intramural Research Program (IRP) of the National Institute of Biomedical Imaging and Bioengineering (NIBIB), National Institutes of Health (NIH); National Natural Science Foundation of China (NSFC) [81471689, 81771873, 91859207]
FX This work was supported by the Intramural Research Program (IRP) of the National Institute of Biomedical Imaging and Bioengineering (NIBIB), National Institutes of Health (NIH) and National Natural Science Foundation of China (NSFC 81471689, 81771873 and 91859207).
CR [Anonymous], 2008, NANO LETT, V8, P2070
Baeuerle PA, 2009, CANCER RES, V69, P4941, DOI 10.1158/0008-5472.CAN-09-0547
Banchereau J, 2005, NAT REV IMMUNOL, V5, P296, DOI 10.1038/nri1592
Banchereau J, 2018, NAT REV CLIN ONCOL, V15, P9, DOI 10.1038/nrclinonc.2017.149
Bernstein MB, 2016, NAT REV CLIN ONCOL, V13, P516, DOI 10.1038/nrclinonc.2016.30
Castano AP, 2008, P NATL ACAD SCI USA, V105, P5495, DOI 10.1073/pnas.0709256105
Castano AP, 2006, NAT REV CANCER, V6, P535, DOI 10.1038/nrc1894
Chao Y, 2018, NAT BIOMED ENG, V2, P611, DOI 10.1038/s41551-018-0262-6
Chatterjee DK, 2008, ADV DRUG DELIVER REV, V60, P1627, DOI 10.1016/j.addr.2008.08.003
Cheever MA, 2011, CLIN CANCER RES, V17, P3520, DOI 10.1158/1078-0432.CCR-10-3126
Chen B, 2014, ACS APPL MATER INTER, V6, P18435, DOI 10.1021/am505510m
Chen HM, 2017, NAT REV MATER, V2, DOI 10.1038/natrevmats.2017.24
Chen Q, 2019, NAT NANOTECHNOL, V14, P89, DOI 10.1038/s41565-018-0319-4
Chen Q, 2016, NAT COMMUN, V7, DOI 10.1038/ncomms13193
Cheung AS, 2018, NAT BIOTECHNOL, V36, P160, DOI 10.1038/nbt.4047
Chiang CS, 2018, NAT NANOTECHNOL, V13, P746, DOI 10.1038/s41565-018-0146-7
Chow MT, 2014, CANCER IMMUNOL RES, V2, P1125, DOI 10.1158/2326-6066.CIR-14-0160
Chu YH, 2018, THERANOSTICS, V8, P4238, DOI 10.7150/thno.24387
Couzin-Frankel J, 2013, SCIENCE, V342, P1432, DOI 10.1126/science.342.6165.1432
Dai LL, 2018, ADV FUNCT MATER, V28, DOI 10.1002/adfm.201707249
Dai YL, 2017, CHEM SOC REV, V46, P3830, DOI 10.1039/c6cs00592f
Ding BB, 2018, ADV MATER, V30, DOI 10.1002/adma.201802479
Domling A, 2014, ANGEW CHEM INT EDIT, V53, P2286, DOI 10.1002/anie.201307906
Dolmans DEJGJ, 2003, NAT REV CANCER, V3, P380, DOI 10.1038/nrc1071
Drake CG, 2014, NAT REV CLIN ONCOL, V11, P24, DOI 10.1038/nrclinonc.2013.208
Durymanov MO, 2015, THERANOSTICS, V5, P1007, DOI 10.7150/thno.11742
Eggermont LJ, 2014, TRENDS BIOTECHNOL, V32, P456, DOI 10.1016/j.tibtech.2014.06.007
Fan WP, 2017, CHEM REV, V117, P13566, DOI 10.1021/acs.chemrev.7b00258
Fan YC, 2017, NANO LETT, V17, P7387, DOI 10.1021/acs.nanolett.7b03218
Fang RH, 2014, NANO LETT, V14, P2181, DOI 10.1021/nl500618u
Fesnak AD, 2016, NAT REV CANCER, V16, P566, DOI 10.1038/nrc.2016.97
Figdor CG, 2004, NAT MED, V10, P475, DOI 10.1038/nm1039
Gajewski TF, 2013, NAT IMMUNOL, V14, P1014, DOI 10.1038/ni.2703
Garon EB, 2015, NEW ENGL J MED, V372, P2018, DOI 10.1056/NEJMoa1501824
Goldberg MS, 2015, CELL, V161, P201, DOI 10.1016/j.cell.2015.03.037
Grupp SA, 2013, NEW ENGL J MED, V368, P1509, DOI 10.1056/NEJMoa1215134
Guo CQ, 2013, ADV CANCER RES, V119, P421, DOI 10.1016/B978-0-12-407190-2.00007-1
Hamid O, 2013, NEW ENGL J MED, V369, P134, DOI 10.1056/NEJMoa1305133

He CB, 2016, NAT COMMUN, V7, DOI 10.1038/ncomms12499
He QJ, 2015, CHEM SOC REV, V44, P6258, DOI 10.1039/c4cs00511b
Hem SL, 2007, EXPERT REV VACCINES, V6, P685, DOI 10.1586/14760584.6.5.685
Hinrichs CS, 2013, NAT BIOTECHNOL, V31, P999, DOI 10.1038/nbt.2725
Hu Z, 2018, NAT REV IMMUNOL, V18, P168, DOI 10.1038/nri.2017.131
Irvine DJ, 2015, CHEM REV, V115, P11109, DOI 10.1021/acs.chemrev.5b00109
Ji TJ, 2013, ADV MATER, V25, P3508, DOI 10.1002/adma.201300299
Jo SD, 2017, NANO TODAY, V17, P23, DOI 10.1016/j.nantod.2017.10.008
Junttila MR, 2013, NATURE, V501, P346, DOI 10.1038/nature12626
Kapadia CH, 2015, J CONTROL RELEASE, V219, P167, DOI 10.1016/j.jconrel.2015.09.062
Kim J, 2015, NAT BIOTECHNOL, V33, P64, DOI 10.1038/nbt.3071
Kim KS, 2016, ADV FUNCT MATER, V26, P2512, DOI 10.1002/adfm.201504879
Kosmides AK, 2017, BIOMATERIALS, V118, P16, DOI 10.1016/j.biomaterials.2016.11.038
Kroll AV, 2017, ADV MATER, V29, DOI 10.1002/adma.201703969
Kuai R, 2018, J CONTROL RELEASE, V282, P131, DOI 10.1016/j.jconrel.2018.04.041
Kuai R, 2017, NAT MATER, V16, P489, DOI [10.1038/nmat4822, 10.1038/NMAT4822]
La Cava A, 2006, TRENDS IMMUNOL, V27, P322, DOI 10.1016/j.it.2006.05.003
Li AW, 2018, NAT MATER, V17, P528, DOI 10.1038/s41563-018-0028-2
Li XR, 2014, J CONTROL RELEASE, V173, P148, DOI 10.1016/j.jconrel.2013.10.032
Liang C, 2016, CHEM SOC REV, V45, P6250, DOI 10.1039/c6cs00458j
Liangran G., 2014, ACS NANO, V8, P5670
Liu JJ, 2018, NANO TODAY, V21, P55, DOI 10.1016/j.nantod.2018.06.008
Liu Q, 2018, ACS NANO, V12, P7812, DOI 10.1021/acsnano.8b01890
Liu Q, 2018, ACS NANO, V12, P1250, DOI [10.1021/acsnano.7b07384, 10.1021/acsnano.7607384]
Liu Y., 2018, CHEM SOC REV, DOI [10.1039/c1038cs00618k, DOI 10.1039/C1038CS00618K]
Lovell JF, 2010, CHEM REV, V110, P2839, DOI 10.1021/cr900236h
Lu JQ, 2017, NAT COMMUN, V8, DOI 10.1038/s41467-017-01651-9
Lu KD, 2018, NAT BIOMED ENG, V2, P600, DOI 10.1038/s41551-018-0203-4
Luo LH, 2018, ACS NANO, V12, P7647, DOI 10.1021/acsnano.8b00204
Luo M, 2017, J CONTROL RELEASE, V263, P200, DOI 10.1016/j.jconrel.2017.03.033
Luo M, 2017, NAT NANOTECHNOL, V12, P648, DOI [10.1038/nnano.2017.52, 10.1038/NNANO.2017.52]
Mandal S, 2013, CHEM SCI, V4, P4168, DOI 10.1039/c3sc51399h
Maude SL, 2014, NEW ENGL J MED, V371, P1507, DOI 10.1056/NEJMoa1407222
Melero I, 2014, NAT REV CLIN ONCOL, V11, P509, DOI 10.1038/nrclinonc.2014.111
Mellman I, 2011, NATURE, V480, P480, DOI 10.1038/nature10673
Meyer RA, 2015, SMALL, V11, P1519, DOI 10.1002/smll.201402369
Miao L, 2017, ACS NANO, V11, P8690, DOI 10.1021/acsnano.7b01786
Min YZ, 2017, NAT NANOTECHNOL, V12, P877, DOI [10.1038/nnano.2017.113, 10.1038/NNANO.2017.113]
Mody KT, 2013, NANOSCALE, V5, P5167, DOI 10.1039/c3nr00357d
Moy AJ, 2017, ADV DRUG DELIVER REV, V114, P175, DOI 10.1016/j.addr.2017.06.008
Nam J, 2018, NAT COMMUN, V9, DOI 10.1038/s41467-018-03473-9
Nikolai P., 2004, IMMUNOL CELL BIOL, V82, P488
Noy R, 2014, IMMUNITY, V41, P49, DOI 10.1016/j.immuni.2014.06.010
Oelke M, 2005, TRENDS MOL MED, V11, P412, DOI 10.1016/j.molmed.2005.07.005
Ohaegbulam KC, 2015, TRENDS MOL MED, V21, P24, DOI 10.1016/j.molmed.2014.10.009
Ou W, 2018, THERANOSTICS, V8, P4574, DOI 10.7150/thno.26758
Palucka K, 2012, NAT REV CANCER, V12, P265, DOI 10.1038/nrc3258
Pardoll DM, 2012, NAT REV CANCER, V12, P252, DOI 10.1038/nrc3239
Park J, 2012, NAT MATER, V11, P895, DOI [10.1038/NMAT3355, 10.1038/nmat3355]
Pashine A, 2005, NAT MED, V11, pS63, DOI 10.1038/nm1210
Peng JR, 2018, ADV SCI, V5, DOI 10.1002/advs.201700891
Perica K, 2014, ACS NANO, V8, P2252, DOI 10.1021/nn405520d
Qian Y, 2017, ACS NANO, V11, P9536, DOI 10.1021/acsnano.7b05465
Qiu LP, 2018, SMALL, V14, DOI 10.1002/smll.201703539
Reddy ST, 2007, NAT BIOTECHNOL, V25, P1159, DOI [10.1038/nbt1332, 10.1038/nbtl332]
Restifo NP, 2012, NAT REV IMMUNOL, V12, P269, DOI 10.1038/nri3191
Rosenberg SA, 2008, NAT REV CANCER, V8, P299, DOI 10.1038/nrc2355
Scott EA, 2012, BIOMATERIALS, V33, P6211, DOI 10.1016/j.biomaterials.2012.04.060
Sharp FA, 2009, P NATL ACAD SCI USA, V106, P870, DOI 10.1073/pnas.0804897106

Shaw CA, 2009, J INORG BIOCHEM, V103, P1555, DOI 10.1016/j.jinorgbio.2009.05.019
Shi CR, 2018, NANO LETT, V18, P7330, DOI 10.1021/acs.nanolett.8b03568
Shi JJ, 2017, NAT REV CANCER, V17, P20, DOI 10.1038/nrc.2016.108
Sinha A, 2017, CHEM MATER, V29, P6883, DOI 10.1021/acs.chemmater.7b02197
Smyth MJ, 2004, IMMUNOL REV, V202, P275, DOI 10.1111/j.0105-2896.2004.00199.x
Snyder A, 2014, NEW ENGL J MED, V371, P2189, DOI 10.1056/NEJMoa1406498
Song QL, 2017, NANO LETT, V17, P6366, DOI 10.1021/acs.nanolett.7b03186
Song W, 2018, ACS NANO, V12, P1978, DOI 10.1021/acsnano.7b09112
Steenblock ER, 2008, MOL THER, V16, P765, DOI 10.1038/mt.2008.11
Steenblock ER, 2009, EXPERT OPIN BIOL TH, V9, P451, DOI [10.1517/14712590902849216, 10.1517/14712590902849216 ]
Steinhagen F, 2011, VACCINE, V29, P3341, DOI 10.1016/j.vaccine.2010.08.002
Sunshine JC, 2014, BIOMATERIALS, V35, P269, DOI 10.1016/j.biomaterials.2013.09.050
Tanaka A, 2017, CELL RES, V27, P109, DOI 10.1038/cr.2016.151
Tang L, 2014, P NATL ACAD SCI USA, V111, P15344, DOI 10.1073/pnas.1411499111
von Roemeling C, 2017, TRENDS BIOTECHNOL, V35, P159, DOI 10.1016/j.tibtech.2016.07.006
Wang C, 2016, BIOMATERIALS, V79, P88, DOI 10.1016/j.biomaterials.2015.11.040
Wang C, 2018, SCI TRANSL MED, V10, DOI 10.1126/scitranslmed.aan3682
Wang C, 2017, THERANOSTICS, V7, P3504, DOI 10.7150/thno.19017
Wang C, 2017, ADV MATER, V29, DOI 10.1002/adma.201606036
Wang DG, 2016, NANO LETT, V16, P5503, DOI 10.1021/acs.nanolett.6b01994
Wang H, 2018, NAT MATER, V17, P761, DOI 10.1038/s41563-018-0147-9
Wang TT, 2018, NAT COMMUN, V9, DOI 10.1038/s41467-018-03915-4
Weijden J. V. D., 2014, CHEM SCI, V5, P3355
Willmann JK, 2008, NAT REV DRUG DISCOV, V7, P591, DOI 10.1038/nrd2290
Xia YF, 2018, NAT MATER, V17, P187, DOI [10.1038/NMAT5057, 10.1038/nmat5057]
Yang GB, 2017, NAT COMMUN, V8, DOI 10.1038/s41467-017-01050-0
Yang R, 2018, ACS NANO, V12, P5121, DOI 10.1021/acsnano.7b09041
Yang YN, 2018, ANGEW CHEM INT EDIT, V57, P11764, DOI 10.1002/anie.201807595
Yu XH, 2017, ACS NANO, V11, P10147, DOI 10.1021/acsnano.7b04736
Zhang L, 2018, NANO LETT, V18, P7092, DOI 10.1021/acs.nanolett.8b03096
Zhang PP, 2015, ACS NANO, V9, P6465, DOI 10.1021/acsnano.5b02153
Zhang XX, 2012, J CONTROL RELEASE, V159, P2, DOI 10.1016/j.jconrel.2011.10.023
Zhang XD, 2018, NANO LETT, V18, P5716, DOI 10.1021/acs.nanolett.8b02321
Zhang XD, 2018, ADV MATER, V30, DOI 10.1002/adma.201707112
Zhao PF, 2018, CHEM SCI, V9, P2674, DOI 10.1039/c7sc04853j
Zhu GZ, 2017, NAT COMMUN, V8, DOI 10.1038/s41467-017-02191-y
Zhu GZ, 2017, NAT COMMUN, V8, DOI 10.1038/s41467-017-01386-7
Zhu GZ, 2017, ACS NANO, V11, P2387, DOI 10.1021/acsnano.7b00978
Zou WP, 2005, NAT REV CANCER, V5, P263, DOI 10.1038/nrc1586
NR 136
TC 30
Z9 30
U1 7
U2 198
PU ELSEVIER SCI LTD
PI OXFORD
PA THE BOULEVARD, LANGFORD LANE, KIDLINGTON, OXFORD OX5 1GB, OXON, ENGLAND
SN 1748-0132
EI 1878-044X
J9 NANO TODAY
JI Nano Today
PD AUG
PY 2019
VL 27
BP 28
EP 47
DI 10.1016/j.nantod.2019.05.004
PG 20
WC Chemistry, Multidisciplinary; Nanoscience & Nanotechnology; Materials Science, Multidisciplinary

WE Science Citation Index Expanded (SCI-EXPANDED)
SC Chemistry; Science & Technology - Other Topics; Materials Science
GA IT0HC
UT WOS:000482526300007
DA 2023-09-14
ER

PT J
AU Kakwere, H
Ingham, ES
Allen, R
Mahakian, LM
Tam, SM
Zhang, H
Silvestrini, MT
Lewis, JS
Ferrara, KW
AF Kakwere, Hamilton
Ingham, Elizabeth S.
Allen, Riley
Mahakian, Lisa M.
Tam, Sarah M.
Zhang, Hua
Silvestrini, Matthew T.
Lewis, Jamal S.
Ferrara, Katherine W.
TI Toward Personalized Peptide-Based Cancer Nanovaccines: A Facile and Versatile Synthetic Approach
SO BIOCONJUGATE CHEMISTRY
LA English
DT Article
ID DENDRITIC CELLS; ADVANCED MELANOMA; IMMUNE-RESPONSES; EPITOPE PEPTIDES; VACCINE; DELIVERY; NANOPARTICLES; IMMUNOTHERAPY; IMMUNOGENICITY; POLYMERIZATION
AB Personalized cancer vaccines (PCVs) are receiving attention as an avenue for cancer immunotherapy. PCVs employ immunogenic peptide epitopes capable of stimulating the immune system to destroy cancer cells with great specificity. Challenges associated with effective delivery of these peptides include poor solubility of hydrophobic sequences, rapid clearance, and poor immunogenicity, among others. The incorporation of peptides into nanoparticles has the potential to overcome these challenges, but the broad range of functionalities found in amino acids presents a challenge to conjugation due to possible interferences and lack of reaction specificity. Herein, a facile and versatile approach to generating nanosized PCVs under mild nonstringent conditions is reported. Following a simple two-step semibatch synthetic approach, amphiphilic hyperbranched polymer-peptide conjugates were prepared by the conjugation of melanoma antigen peptides, either TRP2 (hydrophobic) or MUT30 (hydrophilic), to an alkyne functionalized core via strain-promoted azide-alkyne click chemistry. Self-assembly of the amphiphiles gave spherical nanovaccines (by transmission electron microscopy) with sizes in the range of 10-30 nm (by dynamic light scattering). Fluorescently labeled nanovaccines were prepared to investigate the cellular uptake by antigen presenting cells (dendritic cells), and uptake was confirmed by flow cytometry and microscopy. The TRP2 nanovaccine was taken up the most followed by MUT30 nanoparticles and, finally, nanoparticles without peptide. The nanovaccines showed good biocompatibility against B16-F10 cells, yet the TRP2 peptide showed signs of toxicity, possibly due to its hydrophobicity. A test for immunogenicity revealed that the nanovaccines were poorly immunogenic, implying the need for an adjuvant when administered in vivo. Treatment of mice with melanoma tumors showed that in combination with adjuvant, CpG, groups with the peptide nanovaccines slowed tumor growth and improved survival (up to 24 days, TRP2) compared to the untreated group (14 days).
C1 [Kakwere, Hamilton; Ingham, Elizabeth S.; Allen, Riley; Mahakian, Lisa M.; Tam, Sarah M.; Zhang, Hua; Silvestrini, Matthew T.; Lewis, Jamal S.; Ferrara, Katherine W.] Univ Calif Davis, Dept Biomed Engn, Davis, CA 95616 USA.
C3 University of California System; University of California Davis
RP Ferrara, KW (通讯作者)，Univ Calif Davis, Dept Biomed Engn, Davis, CA 95616 USA.
EM kwferrara@ucdavis.edu

RI kakwere, hamilton/A-4902-2010; Ferrara, Katherine W/H-5503-2011; Lewis, Jamal S/B-1734-2013; Ferrara, Kathy/ISR-8452-2023
OI kakwere, hamilton/0000-0002-1934-4992; Ferrara, Kathy/0000-0002-4976-9107
FU National Institutes of Health [NIHR01CA112356, NIHR01CA199658, NIHR01CA211602, NIHR01CA210553, NIHR01-CA134659]
FX We gratefully acknowledge the support of the National Institutes of Health (NIHR01CA112356, NIHR01CA199658, NIHR01CA211602, NIHR01CA210553, and NIHR01-CA134659).
CR Agard NJ, 2006, ACS CHEM BIOL, V1, P644, DOI 10.1021/cb6003228
Agard NJ, 2004, J AM CHEM SOC, V126, P15046, DOI 10.1021/ja044996f
Alexander J, 2000, J IMMUNOL, V164, P1625, DOI 10.4049/jimmunol.164.3.1625
Banchereau J, 2000, ANNU REV IMMUNOL, V18, P767, DOI 10.1146/annurev.immunol.18.1.767
Bhattacharjee S, 2012, PART FIBRE TOXICOL, V9, DOI 10.1186/1743-8977-9-11
Braunecker WA, 2007, PROG POLYM SCI, V32, P93, DOI 10.1016/j.progpolymsci.2006.11.002
Carreno BM, 2015, SCIENCE, V348, P803, DOI 10.1126/science.aaa3828
Castle JC, 2012, CANCER RES, V72, P1081, DOI 10.1158/0008-5472.CAN-11-3722
Caucheteux SM, 2016, J INVEST DERMATOL, V136, P1172, DOI 10.1016/j.jid.2016.01.033
Chen DS, 2013, IMMUNITY, V39, P1, DOI 10.1016/j.immuni.2013.07.012
Couzin-Frankel J, 2013, SCIENCE, V342, P1432, DOI 10.1126/science.342.6165.1432
Dagvadorj N, 2017, CANCER IMMUNOL IMMUN, V66, P319, DOI 10.1007/s00262-016-1938-y
Dechy-Cabaret O, 2004, CHEM REV, V104, P6147, DOI 10.1021/cr040002s
Diebold SS, 1999, J BIOL CHEM, V274, P19087, DOI 10.1074/jbc.274.27.19087
Fan YC, 2015, VACCINES-BASEL, V3, P662, DOI 10.3390/vaccines3030662
Fraser CC, 2014, VACCINE, V32, P2896, DOI 10.1016/j.vaccine.2014.02.024
Glaffig M, 2015, ORG BIOMOL CHEM, V13, P10150, DOI 10.1039/c5ob01255d
Glaffig M, 2014, CHEM-EUR J, V20, P4232, DOI 10.1002/chem.201400256
Griewank KG, 2017, AM J CLIN DERMATOL, V18, P303, DOI 10.1007/s40257-017-0260-6
Heegaard PMH, 2010, BIOCONJUGATE CHEM, V21, P405, DOI 10.1021/bc900290d
Herrmann A, 2010, CANCER RES, V70, P7455, DOI 10.1158/0008-5472.CAN-10-0736
HILBERT A, 1994, SCAND J IMMUNOL, V40, P609, DOI 10.1111/j.1365-3083.1994.tb03512.x
Huang ZH, 2014, VACCINES, V2, P549, DOI 10.3390/vaccines2030549
Irvine DJ, 2015, CHEM REV, V115, P11109, DOI 10.1021/acs.chemrev.5b00109
Jewett JC, 2010, J AM CHEM SOC, V132, P3688, DOI 10.1021/ja100014q
Kakwere H, 2011, SOFT MATTER, V7, P3754, DOI 10.1039/c0sm01237h
Kapadia CH, 2015, J CONTROL RELEASE, V219, P167, DOI 10.1016/j.jconrel.2015.09.062
KARIN M, 1981, J BIOL CHEM, V256, P3245
Kimura T, 2017, NAT REV UROL, V14, P501, DOI 10.1038/nrurol.2017.77
Kreiter S, 2015, NATURE, V520, P692, DOI 10.1038/nature14426
Kuai R, 2017, NAT MATER, V16, P489, DOI [10.1038/nmat4822, 10.1038/NMAT4822]
Kurniasih IN, 2015, CHEM SOC REV, V44, P4145, DOI 10.1039/c4cs00333k
Lewis JS, 2014, J MATER CHEM B, V2, P2562, DOI 10.1039/c3tb21460e
Lewis JS, 2013, BIOMATERIALS, V34, P9063, DOI 10.1016/j.biomaterials.2013.08.021
Lin YL, 2010, BIOMATERIALS, V31, P7150, DOI 10.1016/j.biomaterials.2010.05.048
Liu HP, 2015, BIOCONJUGATE CHEM, V26, P791, DOI 10.1021/acs.bioconjchem.5b00103
Liu TY, 2015, J MED CHEM, V58, P888, DOI 10.1021/jm501514h
Liu TY, 2013, BIOMACROMOLECULES, V14, P2798, DOI 10.1021/bm400626w
Matyjaszewski K., 2009, CONTROLLED LIVING PO
Mezo G, 2002, J PEPT SCI, V8, P107, DOI 10.1002/psc.373
Mezo G, 2000, BIOCONJUGATE CHEM, V11, P484, DOI 10.1021/bc990116o
Miksa M, 2009, J IMMUNOL METHODS, V342, P71, DOI 10.1016/j.jim.2008.11.019
Morris AS, 2017, MICRO NANO TECHNOL, P171
Moynihan KD, 2016, NAT MED, V22, P1402, DOI 10.1038/nm.4200
Ning XH, 2008, ANGEW CHEM INT EDIT, V47, P2253, DOI 10.1002/anie.200705456
Ott PA, 2017, NATURE, V547, P217, DOI 10.1038/nature22991
Palucka K, 2013, IMMUNITY, V39, P38, DOI 10.1016/j.immuni.2013.07.004
Parry AL, 2013, J AM CHEM SOC, V135, P9362, DOI 10.1021/ja4046857
Postow MA, 2015, J CLIN ONCOL, V33, P1974, DOI 10.1200/JCO.2014.59.4358
Rad-Malekshahi M, 2017, MOL PHARMACEUT, V14, P1482, DOI 10.1021/acs.molpharmaceut.6b01003
Reddy ST, 2007, NAT BIOTECHNOL, V25, P1159, DOI [10.1038/nbt1332, 10.1038/nbtl332]
Restifo NP, 2012, NAT REV IMMUNOL, V12, P269, DOI 10.1038/nri3191

Rostovtsev VV, 2002, ANGEW CHEM INT EDIT, V41, P2596, DOI 10.1002/1521-3773(20020715)41:14<2596::AID-ANIE2596>3.0.CO;2-4
Rubino JT, 2011, METALLOMICS, V3, P61, DOI 10.1039/c0mt00044b
Sahin U, 2017, NATURE, V547, P222, DOI 10.1038/nature23003
Saleh T, 2016, HUM VACC IMMUNOTHER, V12, P1863, DOI 10.1080/21645515.2016.1147635
Schumacher TN, 2015, SCIENCE, V348, P69, DOI 10.1126/science.aaa4971
Shao K, 2015, ACS NANO, V9, P16, DOI 10.1021/nn5062029
Skwarczynski M, 2010, ANGEW CHEM INT EDIT, V49, P5742, DOI 10.1002/anie.201002221
Slingluff CL, 2011, CANCER J, V17, P343, DOI 10.1097/PPO.0b013e318233e5b2
Stockwin LH, 2000, IMMUNOL CELL BIOL, V78, P91, DOI 10.1046/j.1440-1711.2000.00888.x
Tillman BW, 1999, J IMMUNOL, V162, P6378
Tornoe CW, 2002, J ORG CHEM, V67, P3057, DOI 10.1021/jo011148j
Vasievich EA, 2012, MOL PHARMACEUT, V9, P261, DOI 10.1021/mp200350n
Wilkinson KA, 1999, EUR J IMMUNOL, V29, P2788, DOI 10.1002/(SICI)1521-4141(199909)29:09<2788::AID-IMMU2788>3.0.CO;2-4
Wilkinson KA, 1998, BIOCONJUGATE CHEM, V9, P539, DOI 10.1021/bc970159+
Xu ZH, 2014, ACS NANO, V8, P3636, DOI 10.1021/nn500216y
Ye ZB, 2013, CHEM COMMUN, V49, P6235, DOI 10.1039/c3cc42517g
Yoshizaki Y, 2014, BIOMATERIALS, V35, P8186, DOI 10.1016/j.biomaterials.2014.05.077
Zhao GZ, 2017, EUR POLYM J, V93, P670, DOI 10.1016/j.eurpolymj.2017.02.014
Zhao L, 2014, VACCINE, V32, P327, DOI 10.1016/j.vaccine.2013.11.069
Zhu GZ, 2017, ACS NANO, V11, P2387, DOI 10.1021/acsnano.7b00978
NR 72
TC 30
Z9 30
U1 0
U2 62
PU AMER CHEMICAL SOC
PI WASHINGTON
PA 1155 16TH ST, NW, WASHINGTON, DC 20036 USA
SN 1043-1802
EI 1520-4812
J9 BIOCONJUGATE CHEM
JI Bioconjugate Chem.
PD NOV
PY 2017
VL 28
IS 11
BP 2756
EP 2771
DI 10.1021/acs.bioconjchem.7b00502
PG 16
WC Biochemical Research Methods; Biochemistry & Molecular Biology;
Chemistry, Multidisciplinary; Chemistry, Organic
WE Science Citation Index Expanded (SCI-EXPANDED)
SC Biochemistry & Molecular Biology; Chemistry
GA FN1XX
UT WOS:000415785600011
PM 28956907
OA Green Accepted, Green Submitted
DA 2023-09-14
ER

PT J
AU Das, A
Ali, N
AF Das, Amrita
Ali, Nahid
TI Nanovaccine: an emerging strategy
SO EXPERT REVIEW OF VACCINES
LA English

DT Article
DE Nanoparticle; nanovaccine; adjuvant; infection; cancer; COVID-19
ID SOLID LIPID NANOPARTICLES; CONFER PROTECTIVE IMMUNITY; MESSENGER-RNA VACCINES; IN-VIVO; CHITOSAN NANOPARTICLES; DELIVERY-SYSTEM; DENDRITIC CELLS; GOLD NANOPARTICLES; CELLULAR-IMMUNITY; A VACCINE
AB Introduction Vaccination is so far the most effective way of eradicating infections. Rapidly emerging drug resistance against infectious diseases and chemotherapy-related toxicities in cancer warrant immediate vaccine development to save mankind. Subunit vaccines alone, however, fail to elicit sufficiently strong and long-lasting protective immunity against deadly pathogens. Nanoparticle (NP)-based delivery vehicles like microemulsions, liposomes, virosomes, nanogels, micelles and dendrimers offer promising strategies to overcome limitations of traditional vaccine adjuvants. Nanovaccines can improve targeted delivery, antigen presentation, stimulation of body's innate immunity, strong T cell response combined with safety to combat infectious diseases and cancers. Further, nanovaccines can be highly beneficial to generate effective immutherapeutic formulations against cancer. Areas covered This review summarizes the emerging nanoparticle strategies highlighting their success and challenges in preclinical and clinical trials in infectious diseases and cancer. It provides a concise overview of current nanoparticle-based vaccines, their adjuvant potential and their cellular delivery mechanisms. Expert opinion The nanovaccines (50-250 nm in size) are most efficient in terms of tissue targeting, prolonged circulation and preferential uptake by the professional APCs chiefly due to their small size. More rational designing, improved antigen loading, extensive functionalization and targeted delivery are some of the future goals of ideal nanovaccines.
C1 [Das, Amrita; Ali, Nahid] CSIR Indian Inst Chem Biol, Infect Dis & Immunol Div, 4,Raja SC Mullick Rd, Kolkata 700032, W Bengal, India.
C3 Council of Scientific & Industrial Research (CSIR) - India; CSIR - Indian Institute of Chemical Biology (IICB)
RP Ali, N (通讯作者)，CSIR Indian Inst Chem Biol, Infect Dis & Immunol Div, 4,Raja SC Mullick Rd, Kolkata 700032, W Bengal, India.
EM nali@iicb.res.in
FU Council of Scientific and Industrial Research (CSIR) grant [BSC0114]; UK Research and Innovation [MR/P027989/1]
FX This work was supported by the Council of Scientific and Industrial Research (CSIR) grant [No. BSC0114]. The work has been partly supported by UK Research and Innovation under the grant 'A global Network for Neglected Tropical Diseases' [No. MR/P027989/1].
CR AbdelAllah NH, 2020, INT J BIOL MACROMOL, V152, P904, DOI 10.1016/j.ijbiomac.2020.02.287
Abouelmagd SA, 2015, J DRUG TARGET, V23, P725, DOI 10.3109/1061186X.2015.1054829
Adams JR, 2015, ACTA BIOMATER, V14, P104, DOI 10.1016/j.actbio.2014.11.050
Admyre C, 2006, EUR J IMMUNOL, V36, P1772, DOI 10.1002/eji.200535615
Aikins ME, 2020, ACCOUNTS CHEM RES, V53, P2094, DOI 10.1021/acs.accounts.0c00456
Akkaya M, 2020, NAT REV IMMUNOL, V20, P229, DOI 10.1038/s41577-019-0244-2
Alkie TN, 2018, PLOS ONE, V13, DOI 10.1371/journal.pone.0206324
Almeida JPM, 2015, SMALL, V11, P1453, DOI 10.1002/smll.201402179
Amacker M, 2020, NPJ VACCINES, V5, DOI 10.1038/s41541-020-0190-9
Amin MK, 2019, THER DELIV, V10, P493, DOI 10.4155/tde-2019-0038
Ardolino L, 2019, AUST PRESCR, V42, P62, DOI 10.18773/austprescr.2019.012
Aroh C, 2017, J IMMUNOL, V199, P3840, DOI 10.4049/jimmunol.1700972
Baden LR, 2021, NEW ENGL J MED, V384, P403, DOI 10.1056/NEJMoa2035389
Ballou B, 2007, BIOCONJUGATE CHEM, V18, P389, DOI 10.1021/bc060261j
Barry MA, 2016, HUM VACC IMMUNOTHER, V12, P976, DOI 10.1080/21645515.2015.1119346
Bayyurt B, 2017, J CONTROL RELEASE, V247, P134, DOI 10.1016/j.jconrel.2017.01.004
Bazzill JD, 2019, NANOMED-NANOTECHNOL, V18, P414, DOI 10.1016/j.nano.2018.11.005
Beauvillain C, 2009, VACCINE, V27, P1750, DOI 10.1016/j.vaccine.2009.01.022
Bento D, 2015, EUR J PHARM BIOPHARM, V93, P149, DOI 10.1016/j.ejpb.2015.03.024
Bernasconi V, 2016, J IMMUNOL RES, V2016, DOI 10.1155/2016/5482087
Bhowmick S, 2009, INFECT IMMUN, V77, P1514, DOI 10.1128/IAI.01739-07
Bhowmick S, 2010, J CONTROL RELEASE, V141, P199, DOI 10.1016/j.jconrel.2009.09.018
Blom RAM, 2016, PLOS ONE, V11, DOI 10.1371/journal.pone.0163539
Blumenthal KG, 2021, JAMA-J AM MED ASSOC, V325, P1562, DOI 10.1001/jama.2021.3976
Boks MA, 2015, J INVEST DERMATOL, V135, P2697, DOI 10.1038/jid.2015.226
Branco LM, 2010, VIROL J, V7, DOI 10.1186/1743-422X-7-279
Brignole C, 2004, JNCI-J NATL CANCER I, V96, P1171, DOI 10.1093/jnci/djh221

Brouwer PJM, 2019, NAT COMMUN, V10, DOI 10.1038/s41467-019-12080-1
Bulbake U, 2017, PHARMACEUTICS, V9, DOI 10.3390/pharmaceutics9020012
Bungener L, 2005, VACCINE, V23, P1232, DOI 10.1016/j.vaccine.2004.09.002
Butts C, 2011, J CANCER RES CLIN, V137, P1337, DOI 10.1007/s00432-011-1003-3
Carrasco YR, 2007, IMMUNITY, V27, P160, DOI 10.1016/j.immuni.2007.06.007
Cech PG, 2011, PLOS ONE, V6, DOI 10.1371/journal.pone.0022273
Cecilio P, 2017, PLOS NEGLECT TROP D, V11, DOI 10.1371/journal.pntd.0005951
Chen G, 2018, NATURE, V560, P382, DOI 10.1038/s41586-018-0392-8
Cheng RY, 2020, ACS APPL MATER INTER, V12, P44554, DOI 10.1021/acsami.0c15057
Cheung AS, 2016, SMALL, V12, P2321, DOI 10.1002/smll.201600061
Chis AA, 2020, MOLECULES, V25, DOI 10.3390/molecules25173982
Climent N, 2018, NANOMED-NANOTECHNOL, V14, P339, DOI 10.1016/j.nano.2017.11.009
Coleman CM, 2017, VACCINE, V35, P1586, DOI 10.1016/j.vaccine.2017.02.012
Dan JM, 2021, SCIENCE, V371, P587, DOI 10.1126/science.abf4063
Danhier F, 2016, J CONTROL RELEASE, V244, P108, DOI 10.1016/j.jconrel.2016.11.015
Das A, 2018, ACS APPL BIO MATER, V1, P999, DOI 10.1021/acsabm.8b00184
Das A, 2012, FRONT IMMUNOL, V3, DOI 10.3389/fimmu.2012.00099
Das Amrita, 2015, PLoS Negl Trop Dis, V9, pe0004185, DOI 10.1371/journal.pntd.0004185
De Serrano LO, 2017, J NANOBIOTECHNOL, V15, DOI 10.1186/s12951-017-0319-9
Debache K, 2011, PARASITE IMMUNOL, V33, P81, DOI 10.1111/j.1365-3024.2010.01255.x
Deshpande PP, 2013, NANOMEDICINE-UK, V8, P1509, DOI [10.2217/NNM.13.118, 10.2217/nnm.13.118]
Dianzani C, 2014, BIOMED RES INT, V2014, DOI 10.1155/2014/895986
Donaldson B, 2018, EXPERT REV VACCINES, V17, P833, DOI 10.1080/14760584.2018.1516552
Doroud D, 2010, J CONTROL RELEASE, V148, pE105, DOI 10.1016/j.jconrel.2010.07.079
Duan XP, 2019, NAT COMMUN, V10, DOI 10.1038/s41467-019-09221-x
Elashiry M, 2020, J EXTRACELL VESICLES, V9, DOI 10.1080/20013078.2020.1795362
Fan YC, 2019, ACS NANO, V13, P11087, DOI 10.1021/acsnano.9b03660
Fontana F, 2019, ACS NANO, V13, P6477, DOI 10.1021/acsnano.8b09613
Fries Chelsea N., 2021, Nature Nanotechnology, V16, DOI 10.1038/s41565-020-0739-9
Fries L, 2020, J INFECT DIS, V222, P572, DOI 10.1093/infdis/jiz518
Fu S, 2019, J DRUG TARGET, V27, P1, DOI 10.1080/1061186X.2018.1455841
Gao Y, 2020, MOLECULES, V25, DOI 10.3390/molecules25112505
Garinot M, 2020, INT J PHARM-X, V2, DOI 10.1016/j.ijpx.2020.100054
Genito CJ, 2021, ADV NANOBIOMED RES, V1, DOI 10.1002/anbr.202000041
Genton B, 2007, PLOS ONE, V2, DOI 10.1371/journal.pone.0001018
Ghiringhelli F, 2009, NAT MED, V15, P1170, DOI 10.1038/nm.2028
Goel RR, 2021, SCI IMMUNOL, V6, DOI 10.1126/sciimmunol.abi6950
Greenwood B, 2014, PHILOS T R SOC B, V369, DOI 10.1098/rstb.2013.0433
Gu ZL, 2020, PHARMACEUTICS, V12, DOI 10.3390/pharmaceutics12111054
Guevara ML, 2020, FRONT CHEM, V8, DOI 10.3389/fchem.2020.589959
Gulla SK, 2019, BIOMATER SCI-UK, V7, P773, DOI 10.1039/c8bm01272e
Guo YG, 2019, ACS CENTRAL SCI, V5, P747, DOI 10.1021/acscentsci.9b00325
Hiltbrunner S, 2016, ONCOTARGET, V7, P38707, DOI 10.18632/oncotarget.9585
HogenEsch H, 2018, NPJ VACCINES, V3, DOI 10.1038/s41541-018-0089-x
Hojatizade Mansure, 2018, Iran J Immunol, V15, P281, DOI 10.22034/IJI.2018.39397
Hood JL, 2012, WIRES NANOMED NANOBI, V4, P458, DOI 10.1002/wnan.1174
Jackson LA, 2020, NEW ENGL J MED, V383, P1920, DOI 10.1056/NEJMoa2022483
Jeyanathan M, 2020, NAT REV IMMUNOL, V20, P615, DOI 10.1038/s41577-020-00434-6
Jia CC, 2018, ACS APPL MATER INTER, V10, P35745, DOI 10.1021/acsami.8b11556
Jones RB, 2017, BIOMATERIALS, V117, P44, DOI 10.1016/j.biomaterials.2016.11.048
Kaczanowska S, 2013, J LEUKOCYTE BIOL, V93, P847, DOI 10.1189/jlb.1012501
Kanekiyo M, 2013, NATURE, V499, P102, DOI 10.1038/nature12202
Kang MH, 2015, MOL PHARMACEUT, V12, P4200, DOI 10.1021/acs.molpharmaceut.5b00399
Kasturi SP, 2017, J VIROL, V91, DOI 10.1128/JVI.01844-16
Kasturi SP, 2011, NATURE, V470, P543, DOI 10.1038/nature09737
Kim H, 2019, J PHARMACOL EXP THER, V370, P715, DOI 10.1124/jpet.118.254953
Kounis NG, 2021, VACCINES-BASEL, V9, DOI 10.3390/vaccines9030221
Kulkarni JA, 2020, NANOSCALE, V12, P23959, DOI 10.1039/d0nr06816k
Kumar R, 2015, VACCINE, V33, P5064, DOI 10.1016/j.vaccine.2015.08.025
Kwon YJ, 2005, P NATL ACAD SCI USA, V102, P18264, DOI 10.1073/pnas.0509541102

Lai SK, 2007, BIOMATERIALS, V28, P2876, DOI 10.1016/j.biomaterials.2007.02.021
Lei YY, 2021, CIRC RES, V128, P1323, DOI 10.1161/CIRCRESAHA.121.318902
Li B, 2019, WIRES NANOMED NANOBI, V11, DOI 10.1002/wnan.1530
Li S, 2020, VACCINE, V38, P2478, DOI 10.1016/j.vaccine.2020.02.004
Liu CY, 2020, MOL THER-METH CLIN D, V18, P751, DOI 10.1016/j.omtm.2020.07.015
Liu LN, 2018, MOL THER, V26, P45, DOI 10.1016/j.ymthe.2017.10.020
Lo MK, 2020, J INFECT DIS, V221, pS493, DOI 10.1093/infdis/jiz553
Lonez C, 2012, ADV DRUG DELIVER REV, V64, P1749, DOI 10.1016/j.addr.2012.05.009
Luo ZC, 2015, BIOMATERIALS, V38, P50, DOI 10.1016/j.biomaterials.2014.10.050
MacLaughlin FC, 1998, J CONTROL RELEASE, V56, P259, DOI 10.1016/S0168-3659(98)00097-2
Maji M, 2016, SCI REP-UK, V6, DOI 10.1038/srep27206
Malik A, 2019, INT J NANOMED, V14, P3129, DOI 10.2147/IJN.S172391
Mandal S, 2017, POLYMERS-BASEL, V9, DOI 10.3390/polym9090423
Manolova V, 2008, EUR J IMMUNOL, V38, P1404, DOI 10.1002/eji.200737984
Mantovani A, 2020, NEW ENGL J MED, V383, P1078, DOI 10.1056/NEJMcibr2011679
Mateu Ferrando Ruth, 2020, Drug Discov Today Technol, V38, P57, DOI 10.1016/j.ddtec.2021.02.001
Mazumdar T, 2005, J PARASITOL, V91, P269, DOI 10.1645/GE-356R1
Mazumder S, 2011, PLOS NEGLECT TROP D, V5, DOI 10.1371/journal.pntd.0001429
Mecke A, 2005, LANGMUIR, V21, P10348, DOI 10.1021/la050629l
Medhi R, 2020, ACS APPL NANO MATER, V3, P8557, DOI 10.1021/acsanm.0c01978
Milane L, 2021, DRUG DELIV TRANSL RE, V11, P1309, DOI 10.1007/s13346-021-00911-y
Moerk SK., 2019, ANN ONCOL, V30, DOI [10.1093/annonc/mdz451.019, DOI 10.1093/ANNONC/MDZ451.019]
Moon JJ, 2012, PLOS ONE, V7, DOI 10.1371/journal.pone.0031472
Moon JJ, 2012, P NATL ACAD SCI USA, V109, P1080, DOI 10.1073/pnas.1112648109
Morelli AB, 2012, J MED MICROBIOL, V61, P935, DOI 10.1099/jmm.0.040857-0
Morse MA, 2005, J TRANSL MED, V3, DOI 10.1186/1479-5876-3-9
Moser C, 2013, EXPERT REV VACCINES, V12, P779, DOI 10.1586/14760584.2013.811195
MULLER RH, 1995, EUR J PHARM BIOPHARM, V41, P62
Mumper Russell J, 2014, J Control Release, V190, P46
Naseri M, 2020, ONCOIMMUNOLOGY, V9, DOI 10.1080/2162402X.2020.1779991
Ni L, 2020, IMMUNITY, V52, P971, DOI 10.1016/j.immuni.2020.04.023
Nisini R, 2018, FRONT IMMUNOL, V9, DOI 10.3389/fimmu.2018.00155
Novio F, 2020, MOLECULES, V25, DOI 10.3390/molecules25153449
O'Hagan DT, 2017, CURR OPIN IMMUNOL, V47, P93, DOI 10.1016/j.coi.2017.07.010
Okitsu SL, 2007, PLOS ONE, V2, DOI 10.1371/journal.pone.0001278
Orr MT, 2019, NPJ VACCINES, V4, DOI 10.1038/s41541-018-0094-0
Painter MM, 2021, IMMUNITY, V54, P2133, DOI 10.1016/j.immuni.2021.08.001
Pardi N, 2019, MOL THER-NUCL ACIDS, V15, P36, DOI 10.1016/j.omtn.2019.03.003
Pardi N, 2018, NAT REV DRUG DISCOV, V17, P261, DOI 10.1038/nrd.2017.243
Patel JM, 2016, BIOMATERIALS, V74, P231, DOI 10.1016/j.biomaterials.2015.09.031
Pavot V, 2021, HUM VACC IMMUNOTHER, V17, P2336, DOI 10.1080/21645515.2020.1855956
Pawar D, 2013, EUR J PHARM BIOPHARM, V85, P550, DOI 10.1016/j.ejpb.2013.06.017
Petkar KC, 2021, PHARMACEUTICS, V13, DOI 10.3390/pharmaceutics13040455
Polack FP, 2020, NEW ENGL J MED, V383, P2603, DOI 10.1056/NEJMoa2034577
Pollard AJ, 2021, NAT REV IMMUNOL, V21, P83, DOI 10.1038/s41577-020-00479-7
Poon C, 2020, NANO EXPRESS, V1, DOI 10.1088/2632-959X/ab8075
Pulendran B, 2021, NAT REV DRUG DISCOV, V20, P454, DOI 10.1038/s41573-021-00163-y
Raghuwanshi D, 2012, MOL PHARMACEUT, V9, P946, DOI 10.1021/mp200553x
Romanowski B, 2009, LANCET, V374, P1975, DOI 10.1016/S0140-6736(09)61567-1
Sabur A, 2018, FRONT IMMUNOL, V9, DOI 10.3389/fimmu.2018.00018
Montoto SS, 2020, FRONT MOL BIOSCI, V7, DOI 10.3389/fmolb.2020.587997
Senel S, 2020, DRUG DELIV TRANSL RE, V10, P1644, DOI 10.1007/s13346-020-00806-4
Setaro F, 2015, NANO LETT, V15, P1245, DOI 10.1021/nl5044055
Shah S, 2020, ADV DRUG DELIVER REV, V154, P102, DOI 10.1016/j.addr.2020.07.002
Shahbazi R, 2019, NAT MATER, V18, P1124, DOI 10.1038/s41563-019-0385-5
Sharma J, 2020, ACS APPL MATER INTER, V12, P18211, DOI 10.1021/acsami.9b21776
Shenoda BB, 2016, CLIN MED INSIGHTS-PA, V9, P1, DOI 10.4137/CPath.S39925
Sherje AP, 2018, INT J PHARMACEUT, V548, P707, DOI 10.1016/j.ijpharm.2018.07.030
Shin MD, 2020, NAT NANOTECHNOL, V15, P646, DOI 10.1038/s41565-020-0737-y
Silva AL, 2016, HUM VACC IMMUNOTHER, V12, P1056, DOI 10.1080/21645515.2015.1117714

Singh K, 2020, J VIROL, V94, DOI 10.1128/JVI.01884-19
Slutter B, 2010, VACCINE, V28, P6282, DOI 10.1016/j.vaccine.2010.06.121
Sun BB, 2013, ACS NANO, V7, P10834, DOI 10.1021/nn404211j
Talamini L, 2017, ACS NANO, V11, P5519, DOI 10.1021/acsnano.7b00497
Tao WQ, 2015, VACCINE, V33, P2307, DOI 10.1016/j.vaccine.2015.03.063
Thi TTH, 2021, VACCINES-BASEL, V9, DOI 10.3390/vaccines9040359
Tie Y, 2020, SIGNAL TRANSDUCT TAR, V5, DOI 10.1038/s41392-020-0115-0
Tosyali OA, 2021, MAT SCI ENG C-MATER, V120, DOI 10.1016/j.msec.2020.111684
Tukhvatulin A, 2020, VACCINES-BASEL, V8, DOI 10.3390/vaccines8030519
Turner JS, 2021, NATURE, V596, P109, DOI 10.1038/s41586-021-03738-2
Villa LL, 2005, LANCET ONCOL, V6, P271, DOI 10.1016/S1470-2045(05)70101-7
VOSIKA GJ, 1984, CANCER IMMUNOL IMMUN, V18, P107
Wang N, 2020, J INORG ORGANOMET P, V30, P4203, DOI 10.1007/s10904-020-01572-z
Wang N, 2019, J CONTROL RELEASE, V303, P130, DOI 10.1016/j.jconrel.2019.04.025
Wegmann F, 2015, CLIN VACCINE IMMUNOL, V22, P1004, DOI 10.1128/CVI.00736-14
Whiteside TL, 2011, B CANCER, V98, pE25, DOI 10.1684/bdc.2010.1294
Wiedermann U, 2010, BREAST CANCER RES TR, V119, P673, DOI 10.1007/s10549-009-0666-9
Wu ZM, 2021, PHARM RES-DORDR, V38, P473, DOI 10.1007/s11095-021-03015-x
Wui SR, 2021, PHARMACEUTICS, V13, DOI 10.3390/pharmaceutics13030390
Xu J, 2019, BIOMATERIALS, V207, P1, DOI 10.1016/j.biomaterials.2019.03.037
Xu Yingying, 2014, Pharmaceutics, V6, P378, DOI 10.3390/pharmaceutics6030378
Xu ZH, 2013, J CONTROL RELEASE, V172, P259, DOI 10.1016/j.jconrel.2013.08.021
Xu ZJ, 2020, MOL CANCER, V19, DOI 10.1186/s12943-020-01278-3
Yan W, 2020, TRENDS CANCER, V6, P506, DOI 10.1016/j.trecan.2020.02.013
Yousefi M, 2020, ADV COLLOID INTERFAC, V278, DOI 10.1016/j.cis.2020.102125
Yuan YM, 2016, BIOMED RES INT, V2016, DOI 10.1155/2016/1287128
Zaman M, 2011, NANOMED-NANOTECHNOL, V7, P168, DOI 10.1016/j.nano.2010.10.002
Zhao L, 2014, VACCINE, V32, P327, DOI 10.1016/j.vaccine.2013.11.069
Zhao Y, 2020, INT J NANOMED, V15, P9571, DOI 10.2147/IJN.S275670
Zhong ZH, 2010, INT J PHARMACEUT, V399, P156, DOI 10.1016/j.ijpharm.2010.07.053
Zhu MT, 2014, HUM VACC IMMUNOTHER, V10, P2761, DOI 10.4161/hv.29589
Zupancic E, 2018, NANOMED-NANOTECHNOL, V14, P835, DOI 10.1016/j.nano.2017.12.011
NR 179
TC 29
Z9 29
U1 5
U2 43
PU TAYLOR & FRANCIS LTD
PI ABINGDON
PA 2-4 PARK SQUARE, MILTON PARK, ABINGDON OR14 4RN, OXON, ENGLAND
SN 1476-0584
EI 1744-8395
J9 EXPERT REV VACCINES
JI Expert Rev. Vaccines
PD OCT 3
PY 2021
VL 20
IS 10
BP 1273
EP 1290
DI 10.1080/14760584.2021.1984890
EA OCT 2021
PG 18
WC Immunology
WE Science Citation Index Expanded (SCI-EXPANDED)
SC Immunology
GA WP9MJ
UT WOS:000702262600001
PM 34550859
DA 2023-09-14
ER

PT J
AU Zeng, LM
Liao, ZL
Li, WW
Yuan, QJ
Wu, P
Gu, ZP
Liu, ZQ
Liao, GC
AF Zeng, Liming
Liao, Zonglang
Li, Wenwei
Yuan, Qijuan
Wu, Peng
Gu, Zhipeng
Liu, Zhongqiu
Liao, Guochao
TI Non-covalent glycosylated gold nanoparticles/peptides nanovaccine as
potential cancer vaccines
SO CHINESE CHEMICAL LETTERS
LA English
DT Article
DE Non-covalent; Glycosylation; Nanoparticles/peptides nanovaccine;
beta-Cyclodextrin; Host-guest recognitions; Cancer vaccines
ID IN-VITRO; IMMUNOGENICITY; SIZE
AB Herein, we firstly developed a non-covalent glycosylated gold nanoparticles/peptides nanovaccine which is assembled by beta-cyclodextrin (beta-CD) based host-guest recognitions. This nanovaccine can generate significant titers of antibodies and improve the therapeutic effect against melanoma, suggesting the immunogenicity of peptide antigens can be improved by loading with this carrier. The novel vaccine carrier provides a platform for the transport of various antigens especially T cell-independent antigens.
(C) 2019 Chinese Chemical Society and Institute of Materia Medica, Chinese Academy of Medical Sciences. Published by Elsevier B.V. All rights reserved.
C1 [Zeng, Liming; Liao, Zonglang; Li, Wenwei; Wu, Peng; Liu, Zhongqiu; Liao, Guochao] Guangzhou Univ Chinese Med, Int Inst Translat Chinese Med, Joint Lab Translat Canc Res Chinese Med, Minist Educ Peoples Republ China, Guangzhou 510006, Peoples R China.
[Yuan, Qijuan; Gu, Zhipeng] Sun Yat Sen Univ, Sch Biomed Engn, Guangzhou 510006, Peoples R China.
C3 Ministry of Education, China; Guangzhou University of Chinese Medicine;
Sun Yat Sen University
RP Liu, ZQ; Liao, GC (通讯作者)，Guangzhou Univ Chinese Med, Int Inst Translat Chinese Med, Joint Lab Translat Canc Res Chinese Med, Minist Educ Peoples Republ China, Guangzhou 510006, Peoples R China.
EM liuzq@gzucm.edu.cn; liao@gzucm.edu.cn
RI Liu, Zhongqiu/AHC-9661-2022; Liu, Zhongqiu/ABB-8204-2021; Wu,
Peng/AAO-9756-2021; Gu, Zhi peng/E-5108-2013
OI Liu, Zhongqiu/0000-0001-6986-9677; Gu, Zhi peng/0000-0003-4533-6539
FU Guangdong Key Laboratory for Translational Cancer Research of Chinese
Medicine [2018B030322011]; National Natural Science Foundation of China
[81773580]
FX It is thankful that the Guangdong Key Laboratory for Translational
Cancer Research of Chinese Medicine (No. 2018B030322011) and the
National Natural Science Foundation of China (No. 81773580) offered
financial support for this work.
CR Anderson RJ, 2014, NAT CHEM BIOL, V10, P943, DOI [10.1038/NCHEMBIO.1640, 10.1038/nchembio.1640]
Bai Y, 2018, CHEM-ASIAN J, V13, P3903, DOI 10.1002/asia.201801366
Benne N, 2016, J CONTROL RELEASE, V234, P124, DOI 10.1016/j.jconrel.2016.05.033
Botelho NK, 2019, FRONT IMMUNOL, V10, DOI 10.3389/fimmu.2019.00294
Chen J, 2018, ANAL CHIM ACTA, V1023, P89, DOI 10.1016/j.aca.2018.02.082
Du ZK, 2018, LANGMUIR, V34, P5606, DOI 10.1021/acs.langmuir.8b00470
Ganda IS, 2017, INT J PHARMACEUT, V527, P79, DOI 10.1016/j.ijpharm.2017.05.045
Gasser O, 2018, CANCER IMMUNOL IMMUN, V67, P285, DOI 10.1007/s00262-017-2085-9

Jia YP, 2017, CHINESE CHEM LETT, V28, P691, DOI 10.1016/j.cclet.2017.01.021
Kang S, 2017, J CONTROL RELEASE, V256, P56, DOI 10.1016/j.jconrel.2017.04.024
Khamehchian S, 2016, J BIOMED MATER RES A, V104, P2693, DOI 10.1002/jbm.a.35806
Kuai R, 2017, NAT MATER, V16, P489, DOI [10.1038/nmat4822, 10.1038/NMAT4822]
Li AW, 2018, NAT MATER, V17, P528, DOI 10.1038/s41563-018-0028-2
Liao GC, 2016, ACS CENTRAL SCI, V2, P210, DOI 10.1021/acscentsci.5b00364
Lin WJ, 2018, J COLLOID INTERF SCI, V528, P135, DOI 10.1016/j.jcis.2018.05.082
Liu YH, 2017, CHEM COMMUN, V53, P9486, DOI 10.1039/c7cc04386d
Liu YH, 2016, CHEM COMMUN, V52, P10886, DOI 10.1039/c6cc04623a
Luo M, 2017, NAT NANOTECHNOL, V12, P648, DOI [10.1038/nnano.2017.52, 10.1038/NNANO.2017.52]
Mi Y, 2018, ADV MATER, V30, DOI 10.1002/adma.201706098
Narita Y, 2019, NEURO-ONCOLOGY, V21, P348, DOI 10.1093/neuonc/noy200
Ott PA, 2017, NATURE, V547, P217, DOI 10.1038/nature22991
Sahin U, 2017, NATURE, V547, P222, DOI 10.1038/nature23003
Shelley H, 2018, J PHARM SCI-US, V107, P1741, DOI 10.1016/j.xphs.2018.03.021
Tsuboi A, 2019, CANCER IMMUNOL IMMUN, V68, P331, DOI 10.1007/s00262-018-2274-1
Vetro M, 2017, NANOMEDICINE-UK, V12, P13, DOI 10.2217/nnm-2016-0306
Wang HX, 2019, CHEM COMMUN, V55, P253, DOI 10.1039/c8cc07691j
Wang ZJ, 2018, MATER HORIZ, V5, P344, DOI 10.1039/c7mh00991g
Yu MH, 2019, ACS APPL MATER INTER, V11, P15103, DOI 10.1021/acsami.8b20817
Zhang FB, 2019, SCI REP-UK, V9, DOI 10.1038/s41598-019-40084-w
NR 29
TC 29
Z9 29
U1 2
U2 50
PU ELSEVIER SCIENCE INC
PI NEW YORK
PA STE 800, 230 PARK AVE, NEW YORK, NY 10169 USA
SN 1001-8417
EI 1878-5964
J9 CHINESE CHEM LETT
JI Chin. Chem. Lett.
PD MAY
PY 2020
VL 31
IS 5
BP 1162
EP 1164
DI 10.1016/j.cclet.2019.10.015
PG 3
WC Chemistry, Multidisciplinary
WE Science Citation Index Expanded (SCI-EXPANDED)
SC Chemistry
GA LK7QO
UT WOS:000531056900023
DA 2023-09-14
ER

PT J
AU Zottig, X
Cote-Cyr, M
Arpin, D
Archambault, D
Bourgault, S
AF Zottig, Ximena
Cote-Cyr, Melanie
Arpin, Dominic
Archambault, Denis
Bourgault, Steve

TI Protein Supramolecular Structures: From Self-Assembly to Nanovaccine Design
SO NANOMATERIALS
LA English
DT Review
DE self-assembly; vaccine; supramolecular structure; nanovaccine; nanoassemblies; immune response; adjuvant; protein
ID SURFACTANT-LIKE PEPTIDES; NUCLEOPROTEIN-RNA COMPLEX; HUMORAL IMMUNE-RESPONSE; TOLL-LIKE RECEPTORS; DENDRITIC CELLS; T-CELL; NANOPARTICLE INTERACTION; ACID) NANOPARTICLES; VACCINE DEVELOPMENT; RATIONAL DESIGN
AB Life-inspired protein supramolecular assemblies have recently attracted considerable attention for the development of next-generation vaccines to fight against infectious diseases, as well as autoimmune diseases and cancer. Protein self-assembly enables atomic scale precision over the final architecture, with a remarkable diversity of structures and functionalities. Self-assembling protein nanovaccines are associated with numerous advantages, including biocompatibility, stability, molecular specificity and multivalency. Owing to their nanoscale size, proteinaceous nature, symmetrical organization and repetitive antigen display, protein assemblies closely mimic most invading pathogens, serving as danger signals for the immune system. Elucidating how the structural and physicochemical properties of the assemblies modulate the potency and the polarization of the immune responses is critical for bottom-up design of vaccines. In this context, this review briefly covers the fundamentals of supramolecular interactions involved in protein self-assembly and presents the strategies to design and functionalize these assemblies. Examples of advanced nanovaccines are presented, and properties of protein supramolecular structures enabling modulation of the immune responses are discussed. Combining the understanding of the self-assembly process at the molecular level with knowledge regarding the activation of the innate and adaptive immune responses will support the design of safe and effective nanovaccines.
C1 [Zottig, Ximena; Cote-Cyr, Melanie; Arpin, Dominic; Bourgault, Steve] Univ Quebec Montreal, Dept Chem, Montreal, PQ H2L 2C4, Canada.
[Zottig, Ximena; Cote-Cyr, Melanie; Arpin, Dominic; Bourgault, Steve] PROTEO, Quebec Network Res Prot Funct Engn & Applicat, Quebec City, PQ G1V 0A6, Canada.
[Zottig, Ximena; Cote-Cyr, Melanie; Arpin, Dominic; Archambault, Denis; Bourgault, Steve] CRIPA, Swine & Poultry Infect Dis Res Ctr, St Hyacinthe, PQ J2S 2M2, Canada.
[Archambault, Denis] Univ Quebec Montreal, Dept Biol Sci, Montreal, PQ H2L 2C4, Canada.
C3 University of Quebec; University of Quebec Montreal; University of Quebec; University of Quebec Montreal
RP Bourgault, S (通讯作者)，Univ Quebec Montreal, Dept Chem, Montreal, PQ H2L 2C4, Canada.; Bourgault, S (通讯作者)，PROTEO, Quebec Network Res Prot Funct Engn & Applicat, Quebec City, PQ G1V 0A6, Canada.; Archambault, D; Bourgault, S (通讯作者)，CRIPA, Swine & Poultry Infect Dis Res Ctr, St Hyacinthe, PQ J2S 2M2, Canada.; Archambault, D (通讯作者)，Univ Quebec Montreal, Dept Biol Sci, Montreal, PQ H2L 2C4, Canada.
EM zottig.ximena_alumine@courrier.uqam.ca; cote-cyr.melanie@courrier.uqam.ca; arpin.dominic@courrier.uqam.ca; archambault.denis@uqam.ca; bourgault.steve@uqam.ca
OI Cote-Cyr, Melanie/0000-0002-9807-2680; Zottig, Ximena/0000-0002-2344-5163
FU Natural Sciences and Engineering Research Council of Canada (NSERC) [RGPIN-2018-06209]; International Development Research Centre (IDRC) [108517]
FX This research was funded by the Natural Sciences and Engineering Research Council of Canada (NSERC), grant number RGPIN-2018-06209, and by the International Development Research Centre (IDRC), grant number 108517.
CR ABRAM D, 1964, J MOL BIOL, V9, P168, DOI 10.1016/S0022-2836(64)80098-X
Aggarwal P, 2009, ADV DRUG DELIVER REV, V61, P428, DOI 10.1016/j.addr.2009.03.009
Aguilar JC, 2007, VACCINE, V25, P3752, DOI 10.1016/j.vaccine.2007.01.111
Aida T, 2012, SCIENCE, V335, P813, DOI 10.1126/science.1205962
Al-Halifa S, 2019, FRONT IMMUNOL, V10, DOI 10.3389/fimmu.2019.00022
Al-Halifa S, 2019, PEPTIDE SCI, V111, DOI 10.1002/pep2.24095
Amit M, 2018, ADV MATER, V30, DOI 10.1002/adma.201707083
Andersson AMC, 2019, SCI REP-UK, V9, DOI 10.1038/s41598-019-40985-w

Audette GF, 2019, BIOMEDICINES, V7, DOI 10.3390/biomedicines7020046
Awate S, 2013, FRONT IMMUNOL, V4, DOI 10.3389/fimmu.2013.00114
Azmi F, 2014, BIOORGAN MED CHEM, V22, P6401, DOI 10.1016/j.bmc.2014.09.042
Babych M, 2018, NANOSCALE, V10, P19547, DOI 10.1039/c8nr05948a
Bachmann MF, 2010, NAT REV IMMUNOL, V10, P787, DOI 10.1038/nri2868
Bachmann MF, 1997, ANNU REV IMMUNOL, V15, P235, DOI 10.1146/annurev.immunol.15.1.235
Baker EG, 2015, NAT CHEM BIOL, V11, P221, DOI 10.1038/nchembio.1739
Bakker SE, 2013, J GEN VIROL, V94, P1734, DOI 10.1099/vir.0.053025-0
Banchereau J, 1998, NATURE, V392, P245, DOI 10.1038/32588
Bechara C, 2013, FEBS LETT, V587, P1693, DOI 10.1016/j.febslet.2013.04.031
Beniac DR, 2012, PLOS ONE, V7, DOI 10.1371/journal.pone.0029608
Bennett KM, 2015, BMC BIOTECHNOL, V15, DOI 10.1186/s12896-015-0194-0
Biedermann F, 2014, ANGEW CHEM INT EDIT, V53, P11158, DOI 10.1002/anie.201310958
Blander JM, 2006, NATURE, V440, P808, DOI 10.1038/nature04596
Blom AM, 2004, MOL IMMUNOL, V40, P1333, DOI 10.1016/j.molimm.2003.12.002
Bonilla FA, 2010, J ALLERGY CLIN IMMUN, V125, pS33, DOI 10.1016/j.jaci.2009.09.017
Bozic S, 2013, CURR OPIN CHEM BIOL, V17, P940, DOI 10.1016/j.cbpa.2013.10.014
Brown JC, 2011, CURR OPIN VIROL, V1, P142, DOI 10.1016/j.coviro.2011.06.003
Brune KD, 2018, FRONT IMMUNOL, V9, DOI 10.3389/fimmu.2018.01432
Brune KD, 2017, BIOCONJUGATE CHEM, V28, P1544, DOI 10.1021/acs.bioconjchem.7b00174
Brune KD, 2016, SCI REP-UK, V6, DOI 10.1038/srep19234
Cella M, 1997, NATURE, V388, P782, DOI 10.1038/42030
Chackerian B, 2001, J CLIN INVEST, V108, P415, DOI 10.1172/JCI11849
Chandler D, 2005, NATURE, V437, P640, DOI 10.1038/nature04162
Chaplin DD, 2010, J ALLERGY CLIN IMMUN, V125, pS3, DOI 10.1016/j.jaci.2009.12.980
Chen Q, 2015, CHEM COMMUN, V51, P12107, DOI 10.1039/c5cc03769g
Chesson CB, 2014, VACCINE, V32, P1174, DOI 10.1016/j.vaccine.2013.11.047
Chiti F, 2017, ANNU REV BIOCHEM, V86, P27, DOI 10.1146/annurev-biochem-061516-045115
Colino J, 2014, EUR J IMMUNOL, V44, P1433, DOI 10.1002/eji.201344266
Cormier AR, 2013, ACS NANO, V7, P7562, DOI 10.1021/nn401562f
Davis BK, 2011, ANNU REV IMMUNOL, V29, P707, DOI 10.1146/annurev-immunol-031210-101405
Delamarre L, 2003, J EXP MED, V198, P111, DOI 10.1084/jem.20021542
Denroche HC, 2018, J MOL ENDOCRINOL, V60, pR57, DOI 10.1530/JME-17-0138
Dieterich DC, 2007, NAT PROTOC, V2, P532, DOI 10.1038/nprot.2007.52
Dill KA, 2005, ANNU REV BIOPH BIOM, V34, P173, DOI 10.1146/annurev.biophys.34.040204.144517
Dobrovolskaia MA, 2008, MOL PHARMACEUT, V5, P487, DOI 10.1021/mp800032f
Dobrovolskaia MA, 2007, NAT NANOTECHNOL, V2, P469, DOI 10.1038/nnano.2007.223
Dong H, 2008, J AM CHEM SOC, V130, P13691, DOI 10.1021/ja8037323
El Bissati K, 2014, VACCINE, V32, P3243, DOI 10.1016/j.vaccine.2014.03.092
El Omari K, 2011, ACTA CRYSTALLOGR F, V67, P1179, DOI [10.1107/S1744309111029228, 10.1107/S17443091110292281179]
Elrad OM, 2008, NANO LETT, V8, P3850, DOI 10.1021/nl802269a
Eskandari S, 2017, ADV DRUG DELIVER REV, V110, P169, DOI 10.1016/j.addr.2016.06.013
Fang YF, 1998, J IMMUNOL, V160, P5273
FELDMANN M, 1971, J EXP MED, V134, P103, DOI 10.1084/jem.134.1.103
Fifis T, 2004, J IMMUNOL, V173, P3148, DOI 10.4049/jimmunol.173.5.3148
Fischer NO, 2013, J AM CHEM SOC, V135, P2044, DOI 10.1021/ja3063293
Fitzpatrick AWP, 2013, P NATL ACAD SCI USA, V110, P5468, DOI 10.1073/pnas.1219476110
Fletcher NL, 2011, SOFT MATTER, V7, P10210, DOI 10.1039/c1sm06261a
Foged C, 2011, THER DELIV, V2, P1057, DOI 10.4155/TDE.11.68
Gamvrellis A, 2004, IMMUNOL CELL BIOL, V82, P506, DOI 10.1111/j.0818-9641.2004.01271.x
Gatto D, 2007, J IMMUNOL, V178, P67, DOI 10.4049/jimmunol.178.1.67
Gebbie MA, 2017, NAT CHEM, V9, P473, DOI [10.1038/NCHEM.2720, 10.1038/nchem.2720]
Goodsell DS, 2000, ANNU REV BIOPH BIOM, V29, P105, DOI 10.1146/annurev.biophys.29.1.105
Gordon DJ, 2003, BIOCHEMISTRY-US, V42, P475, DOI 10.1021/bi0259857
Gowder SM, 2014, SCI WORLD J, DOI 10.1155/2014/971258
Hainfeld JF, 1999, J STRUCT BIOL, V127, P185, DOI 10.1006/jsbi.1999.4149
Han JY, 2018, POLYMERS-BASEL, V10, DOI 10.3390/polym10010031
Herve PL, 2014, J VIROL, V88, P325, DOI 10.1128/JVI.01141-13
Hills RD, 2007, J MOL BIOL, V368, P894, DOI 10.1016/j.jmb.2007.02.043
Huang ZH, 2012, J AM CHEM SOC, V134, P8730, DOI 10.1021/ja211725s

Hubbard R. E., 2010, ELS, DOI [10.1002/9780470015902.a0003011.pub2, DOI 10.1002/9780470015902.A0003011.PUB2]
Huleatt JW, 2008, VACCINE, V26, P201, DOI 10.1016/j.vaccine.2007.10.062
Indelicato G, 2017, ROY SOC OPEN SCI, V4, DOI 10.1098/rsos.161092
Irvine DJ, 2013, NAT MATER, V12, P978, DOI [10.1038/NMAT3775, 10.1038/nmat3775]
Jahn TR, 2010, J MOL BIOL, V395, P717, DOI 10.1016/j.jmb.2009.09.039
Jain S, 2005, BIOMACROMOLECULES, V6, P2590, DOI 10.1021/bm0503221
Janabi M, 2000, ARTERIOSCL THROM VAS, V20, P1953, DOI 10.1161/01.ATV.20.8.1953
Jardine J, 2013, SCIENCE, V340, P711, DOI 10.1126/science.1234150
Jardine JG, 2015, SCIENCE, V349, P156, DOI 10.1126/science.aac5894
Joffre OP, 2012, NAT REV IMMUNOL, V12, P557, DOI 10.1038/nri3254
Kaba SA, 2012, PLOS ONE, V7, DOI 10.1371/journal.pone.0048304
Kaba SA, 2009, J IMMUNOL, V183, P7268, DOI 10.4049/jimmunol.0901957
Kanchan V, 2007, BIOMATERIALS, V28, P5344, DOI 10.1016/j.biomaterials.2007.08.015
Kanekiyo M, 2015, CELL, V162, P1090, DOI 10.1016/j.cell.2015.07.043
Kanekiyo M, 2013, NATURE, V499, P102, DOI 10.1038/nature12202
Kang HJ, 2007, SCIENCE, V318, P1625, DOI 10.1126/science.1145806
Kar K, 2008, PROTEIN SCI, V17, P1086, DOI 10.1110/ps.083441308
Kar K, 2009, BIOCHEMISTRY-US, V48, P7959, DOI 10.1021/bi900496m
Karch CP, 2016, BIOCHEM PHARMACOL, V120, P1, DOI 10.1016/j.bcp.2016.05.001
Kask L, 2002, BIOCHEMISTRY-US, V41, P9349, DOI 10.1021/bi025980+
Kawai T, 2011, IMMUNITY, V34, P637, DOI 10.1016/j.immuni.2011.05.006
Kim H, 2010, ADV FUNCT MATER, V20, P3925, DOI 10.1002/adfm.201000021
King NP, 2013, CURR OPIN STRUC BIOL, V23, P632, DOI 10.1016/j.sbi.2013.06.002
King NP, 2012, SCIENCE, V336, P1171, DOI 10.1126/science.1219364
Klug A, 1999, PHILOS T ROY SOC B, V354, P531, DOI 10.1098/rstb.1999.0404
Knowles TPJ, 2016, ADV MATER, V28, P6546, DOI 10.1002/adma.201505961
Koho T, 2015, EUR J PHARM BIOPHARM, V96, P22, DOI 10.1016/j.ejpb.2015.07.002
Kolappan S, 2016, NAT COMMUN, V7, DOI 10.1038/ncomms13015
Kumar S, 2019, FRONT IMMUNOL, V10, DOI 10.3389/fimmu.2019.01144
Le DHT, 2019, ACS NANO, V13, P2501, DOI 10.1021/acsnano.8b09462
Leblanc P, 2014, HUM VACC IMMUNOTHER, V10, P3022, DOI 10.4161/hv.34413
Lee EB, 2019, NANOMED-NANOTECHNOL, V17, P223, DOI 10.1016/j.nano.2019.01.012
Lehn JM, 2009, EUR REV, V17, P263, DOI 10.1017/S1062798709000805
LEHN JM, 1988, ANGEW CHEM INT EDIT, V27, P89, DOI 10.1002/anie.198800891
Leon MA, 2019, ACS CHEM BIOL, V14, P1436, DOI 10.1021/acschembio.9b00090
Li L, 2014, J MOL BIOL, V426, P309, DOI 10.1016/j.jmb.2013.10.021
Liu SR, 2012, J IMMUNOL, V188, P1098, DOI 10.4049/jimmunol.1101121
Liu WL, 2005, EUR J IMMUNOL, V35, P505, DOI 10.1002/eji.200425749
Lockner JW, 2015, MOL PHARMACEUT, V12, P653, DOI 10.1021/mp500520r
Lopez-Sagaseta J, 2016, COMPUT STRUCT BIOTEC, V14, P58, DOI 10.1016/j.csbj.2015.11.001
Ma BY, 2012, J MOL BIOL, V421, P172, DOI 10.1016/j.jmb.2011.11.023
Maki-Yonekura S, 2010, NAT STRUCT MOL BIOL, V17, P417, DOI 10.1038/nsmb.1774
Mallapragada SK, 2008, INT J PHARMACEUT, V364, P265, DOI 10.1016/j.ijpharm.2008.06.030
Marsh JA, 2015, ANNU REV BIOCHEM, V84, P551, DOI 10.1146/annurev-biochem-060614-034142
Martinez CR, 2012, CHEM SCI, V3, P2191, DOI 10.1039/c2sc20045g
Matsuura K, 2005, J AM CHEM SOC, V127, P10148, DOI 10.1021/ja052644i
Matsuura K, 2018, CHEM COMMUN, V54, P8944, DOI 10.1039/c8cc03844a
Matsuura K, 2010, ANGEW CHEM INT EDIT, V49, P9662, DOI 10.1002/anie.201004606
McCullers Jonathan A, 2008, P T, V33, P35
Miller KD, 2014, PLOS ONE, V9, DOI 10.1371/journal.pone.0114366
Miller RA, 2007, J AM CHEM SOC, V129, P3104, DOI 10.1021/ja063887t
Miranda FF, 2009, SMALL, V5, P2077, DOI 10.1002/smll.200900667
Molino NM, 2016, BIOMATERIALS, V86, P83, DOI 10.1016/j.biomaterials.2016.01.056
Molino NM, 2013, ACS NANO, V7, P9743, DOI 10.1021/nn403085w
MOND JJ, 1995, CURR OPIN IMMUNOL, V7, P349, DOI 10.1016/0952-7915(95)80109-X
Moon JJ, 2012, P NATL ACAD SCI USA, V109, P1080, DOI 10.1073/pnas.1112648109
Morales R, 2013, PLOS PATHOG, V9, DOI 10.1371/journal.ppat.1003537
Morales R, 2010, J NEUROSCI, V30, P4528, DOI 10.1523/JNEUROSCI.5924-09.2010
Mottram PL, 2007, MOL PHARMACEUT, V4, P73, DOI 10.1021/mp060096p
Moyano DF, 2012, J AM CHEM SOC, V134, P3965, DOI 10.1021/ja2108905

Muller-Dethlefs K, 2000, CHEM REV, V100, P143, DOI 10.1021/cr9900331
Nemerow GR, 2012, CURR OPIN VIROL, V2, P115, DOI 10.1016/j.coviro.2011.12.008
Nguyen HD, 2009, J AM CHEM SOC, V131, P2606, DOI 10.1021/ja807730x
Nunes-Correia I, 2004, CELL MOL BIOL LETT, V9, P47
Ogun SA, 2008, INFECT IMMUN, V76, P3817, DOI 10.1128/IAI.01369-07
Otzen D, 2019, CSH PERSPECT BIOL, V11, DOI 10.1101/cshperspect.a033860
Oyewumi MO, 2010, EXPERT REV VACCINES, V9, P1095, DOI [10.1586/erv.10.89, 10.1586/ERV.10.89]
Pacheco P, 2013, PLOS ONE, V8, DOI 10.1371/journal.pone.0060989
Padilla JE, 2001, P NATL ACAD SCI USA, V98, P2217, DOI 10.1073/pnas.041614998
Pandya MJ, 2000, BIOCHEMISTRY-US, V39, P8728, DOI 10.1021/bi000246g
Patel KG, 2011, BIOCONJUGATE CHEM, V22, P376, DOI 10.1021/bc100367u
PAULING L, 1951, P NATL ACAD SCI USA, V37, P729, DOI 10.1073/pnas.37.11.729
PAULING L, 1951, P NATL ACAD SCI USA, V37, P205, DOI 10.1073/pnas.37.4.205
Pearce EJ, 2015, NAT REV IMMUNOL, V15, P18, DOI 10.1038/nri3771
Nguyen PT, 2015, J DIABETES RES, V2015, DOI 10.1155/2015/515307
Platt N, 2001, J CLIN INVEST, V108, P649, DOI 10.1172/JCI13903
Plotkin SA, 1999, CR ACAD SCI III-VIE, V322, P943, DOI 10.1016/S0764-4469(00)87191-7
Pohl G, 2010, J PHYS CHEM B, V114, P9338, DOI 10.1021/jp100955u
Pompano RR, 2014, ADV HEALTHC MATER, V3, P1898, DOI 10.1002/adhm.201400137
Pozzi LAM, 2005, J IMMUNOL, V175, P2071, DOI 10.4049/jimmunol.175.4.2071
Ra JS, 2014, CLIN EXP VACCINE RES, V3, P227, DOI 10.7774/cevr.2014.3.2.227
Raman S, 2006, NANOMED-NANOTECHNOL, V2, P95, DOI 10.1016/j.nano.2006.04.007
Reddington SC, 2015, CURR OPIN CHEM BIOL, V29, P94, DOI 10.1016/j.cbpa.2015.10.002
Reddy ST, 2007, NAT BIOTECHNOL, V25, P1159, DOI [10.1038/nbt1332, 10.1038/nbtl332]
Restuccia A, 2019, COMMUN CHEM, V2, DOI 10.1038/s42004-019-0154-z
Rudra JS, 2012, BIOMATERIALS, V33, P6476, DOI 10.1016/j.biomaterials.2012.05.041
Rudra JS, 2010, BIOMATERIALS, V31, P8475, DOI 10.1016/j.biomaterials.2010.07.068
Rudra JS, 2010, P NATL ACAD SCI USA, V107, P622, DOI 10.1073/pnas.0912124107
SALLUSTO F, 1995, J EXP MED, V182, P389, DOI 10.1084/jem.182.2.389
Sallusto F, 2009, EUR J IMMUNOL, V39, P2076, DOI 10.1002/eji.200939722
Sanner M. F., 2005, NANOBIOTECHNOL, V1, P7, DOI DOI 10.1385/NBT:1:1:007
Santoso S, 2002, NANO LETT, V2, P687, DOI 10.1021/n1025563i
Seong SY, 2004, NAT REV IMMUNOL, V4, P469, DOI 10.1038/nri1372
Shen YB, 2018, MEDCHEMCOMM, V9, P226, DOI 10.1039/c7md00158d
Shima F, 2013, ACTA BIOMATER, V9, P8894, DOI 10.1016/j.actbio.2013.06.010
Slutter B, 2016, EXPERT OPIN DRUG DEL, V13, P167, DOI 10.1517/17425247.2016.1121989
Southall NT, 2002, J PHYS CHEM B, V106, P521, DOI 10.1021/jp015514e
Stepanova LA, 2018, J BIOMED SCI, V25, DOI 10.1186/s12929-018-0433-5
Sun Q, 2015, ACS NANO, V9, P8554, DOI 10.1021/acsnano.5b03688
Tacken PJ, 2011, BLOOD, V118, P6836, DOI 10.1182/blood-2011-07-367615
TANFORD C, 1978, SCIENCE, V200, P1012, DOI 10.1126/science.653353
Tang SB, 2016, SCI REP-UK, V6, DOI 10.1038/srep25741
Tawar RG, 2009, SCIENCE, V326, P1279, DOI 10.1126/science.1177634
Thirumurugan P, 2013, CHEM REV, V113, P4905, DOI 10.1021/cr200409f
Thomas F, 2016, ANGEW CHEM INT EDIT, V55, P987, DOI 10.1002/anie.201509304
Thrane S, 2015, PLOS ONE, V10, DOI 10.1371/journal.pone.0143071
Toy R, 2016, BIOENG TRANSL MED, V1, P47, DOI 10.1002/btm2.10005
Trabulo S, 2010, PHARMACEUTICALS, V3, P961, DOI 10.3390/ph3040961
Tukel C, 2010, CELL MICROBIOL, V12, P1495, DOI 10.1111/j.1462-5822.2010.01485.x
Turley CB, 2011, VACCINE, V29, P5145, DOI 10.1016/j.vaccine.2011.05.041
Vauthey S, 2002, P NATL ACAD SCI USA, V99, P5355, DOI 10.1073/pnas.072089599
von Maltzahn G, 2003, LANGMUIR, V19, P4332, DOI 10.1021/la026526+
Vos Q, 2000, IMMUNOL REV, V176, P154
Walker A, 2011, SCI REP-UK, V1, DOI 10.1038/srep00005
Walshaw J, 2001, J MOL BIOL, V307, P1427, DOI 10.1006/jmbi.2001.4545
Wang D, 2015, HUM IMMUNOL, V76, P442, DOI 10.1016/j.humimm.2015.03.012
Wang F, 2017, NAT COMMUN, V8, DOI 10.1038/s41467-017-01075-5
Wang L, 2019, NANOMATERIALS-BASEL, V9, DOI 10.3390/nano9020285
Wang L, 2017, NANOMED-NANOTECHNOL, V13, P253, DOI 10.1016/j.nano.2016.09.001
Wang L, 2014, NANOMED-NANOTECHNOL, V10, P473, DOI 10.1016/j.nano.2013.08.005
Wang ST, 2017, ACS NANO, V11, P8579, DOI 10.1021/acsnano.7b02325

Webber MJ, 2016, NAT MATER, V15, P13, DOI [10.1038/nmat4474, 10.1038/NMAT4474]
Wen Y, 2016, ACS NANO, V10, P9274, DOI 10.1021/acsnano.6b03409
Wen Y, 2015, CURR OPIN IMMUNOL, V35, P73, DOI 10.1016/j.coi.2015.06.007
Werling D, 1999, J LEUKOCYTE BIOL, V66, P50, DOI 10.1002/jlb.66.1.50
West MA, 2004, SCIENCE, V305, P1153, DOI 10.1126/science.1099153
Westergard L, 2014, MOL MICROBIOL, V92, P698, DOI 10.1111/mmi.12579
Westwell-Roper C, 2016, J BIOL CHEM, V291, P8908, DOI 10.1074/jbc.M115.712455
WHITESIDES GM, 1991, SCIENCE, V254, P1312, DOI 10.1126/science.1962191
Woolfson DN, 2010, BIOPOLYMERS, V94, P118, DOI 10.1002/bip.21345
Woolfson DN, 2005, ADV PROTEIN CHEM, V70, P79, DOI 10.1016/S0065-3233(04)70004-2
Wu YY, 2017, ACS BIOMATER SCI ENG, V3, P3128, DOI 10.1021/acsbiomaterials.7b00561
Yeates TO, 2017, ANNU REV BIOPHYS, V46, P23, DOI 10.1146/annurev-biophys-070816-033928
Yeates TO, 2016, CURR OPIN STRUC BIOL, V39, P134, DOI 10.1016/j.sbi.2016.07.003
Yu YB, 2002, ADV DRUG DELIVER REV, V54, P1113, DOI 10.1016/S0169-409X(02)00058-3
Zakeri B, 2012, P NATL ACAD SCI USA, V109, pE690, DOI 10.1073/pnas.1115485109
Zelzer M, 2010, CHEM SOC REV, V39, P3351, DOI 10.1039/c0cs00035c
Zhang HY, 2017, ACTA BIOMATER, V55, P183, DOI 10.1016/j.actbio.2017.03.041
Zhang Y, 2011, INT J MOL SCI, V12, P5406, DOI 10.3390/ijms12085406
Zinkernagel RM, 2003, ANNU REV IMMUNOL, V21, P515, DOI
10.1146/annurev.immunol.21.120601.141045
Zottig X, 2019, SMALL, V15, DOI 10.1002/smll.201901806
NR 209
TC 29
Z9 29
U1 7
U2 65
PU MDPI
PI BASEL
PA ST ALBAN-ANLAGE 66, CH-4052 BASEL, SWITZERLAND
EI 2079-4991
J9 NANOMATERIALS-BASEL
JI Nanomaterials
PD MAY
PY 2020
VL 10
IS 5
AR 1008
DI 10.3390/nano10051008
PG 28
WC Chemistry, Multidisciplinary; Nanoscience & Nanotechnology; Materials
Science, Multidisciplinary; Physics, Applied
WE Science Citation Index Expanded (SCI-EXPANDED)
SC Chemistry; Science & Technology - Other Topics; Materials Science;
Physics
GA LY8MG
UT WOS:000540781800191
PM 32466176
OA Green Published, gold, Green Accepted
DA 2023-09-14
ER

PT J
AU Liang, XY
Li, XL
Duan, JW
Chen, YL
Wang, XL
Pang, LY
Kong, DL
Song, B
Li, C

Yang, J
AF Liang, Xiaoyu
Li, Xuanling
Duan, Jianwei
Chen, Youlu
Wang, Xiaoli
Pang, Liyun
Kong, Deling
Song, Bing
Li, Chen
Yang, Jing
TI Nanoparticles with CD44 Targeting and ROS Triggering Properties as Effective in Vivo Antigen Delivery System
SO MOLECULAR PHARMACEUTICS
LA English
DT Article
DE ROS triggered; hyaluronic acid modified; nanoparticles; immune response
ID HYALURONIC-ACID; DENDRITIC CELL; CANCER-IMMUNOTHERAPY; CROSS-PRESENTATION; DRUG-DELIVERY; IMMUNITY; PROTEIN; RESPONSES; GENE; SIZE
AB Currently, development of subunit vaccine based on recombinant antigens or peptides has gradually become an important alternative option for traditional vaccine. However, induction of potent immune response with desired efficacy remains a major challenge. The nanoparticle-based antigen delivery system has been considered a potential carrier system to improve the efficacy of subunit vaccine. In the present study, we have designed an immune-stimulatory delivery system by conjugating three-armed PLGA to PEG via the peroxalate ester bond which is sensitive to hydrogen peroxide (H2O2), a major reactive oxygen species (ROS). Hyaluronic acid (HA), a ligand for CD44 receptors was also modified onto the outer shell of the 3s-PLGA-PEG nanoparticles to promote immune cell uptake. For in vitro and in vivo immune response assessment, a model antigen ovalbumin (OVA) was enclosed within the core of the 3s-PLGA-PEG nanoparticles to form 3s-PLGA-PO-PEG/HA nanoparticles (PHO NPs). Our results showed that the PHO NPs enhanced dendritic cell maturation, antigen uptake, and antigen presentation in vitro, likely due to enhanced lysosomal escape. In vivo experiments further revealed that the PHO nanovaccine robustly promoted OVA-specific antibody production and T cell response accompanied by modest stimulation of memory T cells. In summary, the ROS-responsive PHO NPs with modified HA may be an effective vehicle antigen delivery system to promote antigen-induced immune response.
C1 [Liang, Xiaoyu; Li, Xuanling; Duan, Jianwei; Chen, Youlu; Wang, Xiaoli; Pang, Liyun; Kong, Deling; Li, Chen; Yang, Jing] Chinese Acad Med Sci, Inst Biomed Engn, Tianjin Key Lab Biomat Res, Tianjin 300192, Peoples R China.
[Liang, Xiaoyu; Li, Xuanling; Duan, Jianwei; Chen, Youlu; Wang, Xiaoli; Pang, Liyun; Kong, Deling; Li, Chen; Yang, Jing] Peking Union Med Coll, Tianjin 300192, Peoples R China.
[Kong, Deling] Nankai Univ, Minist Educ, Key Lab Bioact Mat, Tianjin 300071, Peoples R China.
[Song, Bing] Cardiff Univ, Coll Biomed & Life Sci, Sch Dent, Heath Pk, Cardiff CF14 4XY, S Glam, Wales.
C3 Chinese Academy of Medical Sciences - Peking Union Medical College; Institute of BioMedical Engineering - CAMS; Chinese Academy of Medical Sciences - Peking Union Medical College; Peking Union Medical College; Nankai University; Cardiff University
RP Li, C; Yang, J (通讯作者)，Chinese Acad Med Sci, Inst Biomed Engn, Tianjin Key Lab Biomat Res, Tianjin 300192, Peoples R China.; Li, C; Yang, J (通讯作者)，Peking Union Med Coll, Tianjin 300192, Peoples R China.
EM cli0616826@126.com; yangjing37@hotmail.com
RI wang, xiao/HZI-9156-2023; wang, xiao/HGB-7081-2022
OI wang, xiao/0000-0002-4088-3341
FU National Natural Science Funds of China [31771097, 31770968, 31600743]; Tianjin Research Program of Application Foundation and Advanced Technology [17JCZDJC37400, 17JCZDJC33400, 16JCQNJC13900]; Science and Technology Support Program of Tianjin [14RCGFSY00146]; CAMS Innovation Fund for Medical Sciences [CIFMS 2017-I2M-1-016]; European Research Council [243261]; British Council Global Innovation Initiative grant
FX The authors thank for the National Natural Science Funds of China (31771097, 31770968, 31600743), Tianjin Research Program of Application Foundation and Advanced Technology (17JCZDJC37400, 17JCZDJC33400,

16JCQNJC13900), Science and Technology Support Program of Tianjin (14RCGFSY00146), CAMS Innovation Fund for Medical Sciences (CIFMS 2017-I2M-1-016), European Research Council FP7 StG grant243261, and British Council Global Innovation Initiative grant.
CR Bae S, 2016, J AM HEART ASSOC, V5, DOI 10.1161/JAHA.116.003697
Chen J, 2017, ACS APPL MATER INTER, V9, P24140, DOI 10.1021/acsami.7b06879
Choi KY, 2011, ACS NANO, V5, P8591, DOI 10.1021/nn202070n
Dobrovolskaia MA, 2007, NAT NANOTECHNOL, V2, P469, DOI 10.1038/nnano.2007.223
Dzieciuch-Rojek M, 2017, MOL PHARMACEUT, V14, P1057, DOI 10.1021/acs.molpharmaceut.6b00969
Fonseca SB, 2009, ADV DRUG DELIVER REV, V61, P953, DOI 10.1016/j.addr.2009.06.001
Harisinghani MG, 2003, NEW ENGL J MED, V348, P2491, DOI 10.1056/NEJMoa022749
Heath WR, 2001, NAT REV IMMUNOL, V1, P126, DOI 10.1038/35100512
Jensen PV, 2004, HISTOCHEM CELL BIOL, V121, P361, DOI 10.1007/s00418-004-0648-2
Jiang DH, 2011, PHYSIOL REV, V91, P221, DOI 10.1152/physrev.00052.2009
Jiang H, 2017, J CONTROL RELEASE, V267, P47, DOI 10.1016/j.jconrel.2017.08.009
Kikuchi S, 2005, J BIOL CHEM, V280, P15398, DOI 10.1074/jbc.M414048200
Kim S, 2011, BIOMATERIALS, V32, P3021, DOI 10.1016/j.biomaterials.2010.11.033
Kipnis A, 2005, INFECT IMMUN, V73, P7759, DOI 10.1128/IAI.73.11.7759-7764.2005
Lallana E, 2017, MOL PHARMACEUT, V14, P2422, DOI 10.1021/acs.molpharmaceut.7b00320
Liu LX, 2016, J CONTROL RELEASE, V225, P230, DOI 10.1016/j.jconrel.2016.01.050
Ma YF, 2011, NANOSCALE, V3, P2307, DOI 10.1039/c1nr10166h
Manolova V, 2008, EUR J IMMUNOL, V38, P1404, DOI 10.1002/eji.200737984
Mesa C, 2006, VACCINE, V24, P2692, DOI 10.1016/j.vaccine.2005.08.111
Mueller SN, 2015, MOL PHARMACEUT, V12, P1356, DOI 10.1021/mp500589c
Panagioti E, 2016, PLOS PATHOG, V12, DOI 10.1371/journal.ppat.1005895
PURKERSON J, 1992, FASEB J, V6, P3245, DOI 10.1096/fasebj.6.14.1385241
Singh D, 2015, MOL IMMUNOL, V68, P272, DOI 10.1016/j.molimm.2015.09.011
Wang C, 2012, VACCINE, V30, P4790, DOI 10.1016/j.vaccine.2012.05.027
Yang YW, 2008, BIOMATERIALS, V29, P2516, DOI 10.1016/j.biomaterials.2008.02.015
Yoo JW, 2011, NAT REV DRUG DISCOV, V10, P521, DOI 10.1038/nrd3499
Yun YH, 2004, BIOMATERIALS, V25, P147, DOI 10.1016/S0142-9612(03)00467-8
Zamboni WC, 2012, CLIN CANCER RES, V18, P3229, DOI 10.1158/1078-0432.CCR-11-2938
Zhang CN, 2017, J CONTROL RELEASE, V256, P170, DOI 10.1016/j.jconrel.2017.04.020
Zhang CN, 2017, MOL PHARMACEUT, V14, P1760, DOI 10.1021/acs.molpharmaceut.7b00015
Zhu XW, 2017, ACS APPL MATER INTER, V9, P27522, DOI 10.1021/acsami.7b08312
Zhu XW, 2016, INT J NANOMED, V11, P6065, DOI 10.2147/IJN.S119446
NR 32
TC 29
Z9 32
U1 2
U2 97
PU AMER CHEMICAL SOC
PI WASHINGTON
PA 1155 16TH ST, NW, WASHINGTON, DC 20036 USA
SN 1543-8384
J9 MOL PHARMACEUT
JI Mol. Pharm.
PD FEB
PY 2018
VL 15
IS 2
BP 508
EP 518
DI 10.1021/acs.molpharmaceut.7b00890
PG 11
WC Medicine, Research & Experimental; Pharmacology & Pharmacy
WE Science Citation Index Expanded (SCI-EXPANDED)
SC Research & Experimental Medicine; Pharmacology & Pharmacy
GA FV6ZM
UT WOS:000424730900017
PM 29323913

OA Green Accepted
DA 2023-09-14
ER

PT J
AU Wang, YH
Zhao, QF
Zhao, BY
Zheng, YS
Zhuang, QY
Liao, NS
Wang, PY
Cai, ZX
Zhang, D
Zeng, YY
Liu, XL
AF Wang, Yunhao
Zhao, Qingfu
Zhao, Binyu
Zheng, Youshi
Zhuang, Qiuyu
Liao, Naishun
Wang, Peiyuan
Cai, Zhixiong
Zhang, Da
Zeng, Yongyi
Liu, Xiaolong
TI Remodeling Tumor-Associated Neutrophils to Enhance Dendritic Cell-Based HCC Neoantigen Nano-Vaccine Efficiency
SO ADVANCED SCIENCE
LA English
DT Article
DE dendritic cell-based vaccine; immunotherapy; neoantigen; neutrophils; photodynamic therapy
ID HEPATOCELLULAR-CARCINOMA; CANCER; DIAGNOSIS
AB Hepatocellular carcinoma (HCC) commonly emerges in an immunologically "cold" state, thereafter protects it away from cytolytic attack by tumor-infiltrating lymphocytes, resulting in poor response to immunotherapy. Herein, an acidic/photo-sensitive dendritic cell (DCs)-based neoantigen nano-vaccine has been explored to convert tumor immune "cold" state into "hot", and remodel tumor-associated neutrophils to potentiate anticancer immune response for enhancing immunotherapy efficiency. The nano-vaccine is constructed by SiPCCl2-hybridized mesoporous silica with coordination of Fe(III)-captopril, and coating with exfoliated membrane of matured DCs by H22-specific neoantigen stimulation. The nano-vaccines actively target H22 tumors and induce immunological cell death to boost tumor-associated antigen release by the generation of excess O-1(2) through photodynamic therapy, which act as in situ tumor vaccination to strengthen antitumor T-cell response against primary H22 tumor growth. Interestingly, the nano-vaccines are also home to lymph nodes to directly induce the activation and proliferation of neoantigen-specific T cells to suppress the primary/distal tumor growth. Moreover, the acidic-triggered captopril release in tumor microenvironment can polarize the protumoral N2 phenotype neutrophils to antitumor N1 phenotype for improving the immune effects to achieve complete tumor regression (83%) in H22-bearing mice and prolong the survival time. This work provides an alternative approach for developing novel HCC immunotherapy strategies.
C1 [Wang, Yunhao; Zhao, Qingfu; Zhao, Binyu; Zheng, Youshi; Zhuang, Qiuyu; Liao, Naishun; Cai, Zhixiong; Zhang, Da; Zeng, Yongyi; Liu, Xiaolong] Fujian Med Univ, United Innovat Mengchao Hepatobiliary Technol Key, Mengchao Hepatobiliary Hosp, Fuzhou 350025, Peoples R China.
[Zheng, Youshi; Zhuang, Qiuyu; Liao, Naishun; Cai, Zhixiong; Zhang, Da; Liu, Xiaolong] Fuzhou Univ, Mengchao Med X Ctr, Fuzhou 350116, Peoples R China.
[Wang, Peiyuan; Liu, Xiaolong] Chinese Acad Sci, CAS Key Lab Design & Assembly Funct Nanostruct, Fujian Inst Res Struct Matter, Fuzhou 350002, Peoples R China.
[Zeng, Yongyi] Fujian Med Univ, Liver Dis Ctr, Affiliated Hosp 1, Fuzhou 350005, Peoples R China.
C3 Fujian Medical University; Fuzhou University; Chinese Academy of Sciences; Fujian Institute of Research on the Structure of Matter, CAS;

Fujian Medical University
RP Zhang, D; Zeng, YY; Liu, XL (通讯作者)，Fujian Med Univ, United Innovat Mengchao Hepatobiliary Technol Key, Mengchao Hepatobiliary Hosp, Fuzhou 350025, Peoples R China.; Zhang, D; Liu, XL (通讯作者)，Fuzhou Univ, Mengchao Med X Ctr, Fuzhou 350116, Peoples R China.; Liu, XL (通讯作者)，Chinese Acad Sci, CAS Key Lab Design & Assembly Funct Nanostruct, Fujian Inst Res Struct Matter, Fuzhou 350002, Peoples R China.; Zeng, YY (通讯作者)，Fujian Med Univ, Liver Dis Ctr, Affiliated Hosp 1, Fuzhou 350005, Peoples R China.
EM zdluoman1987@163.com; lamp197311@126.com; xiaoloong.liu@gmail.com
RI Wang, Peiyuan/IWD-6781-2023; zhao, qingfu/ABE-7714-2022
OI Wang, Peiyuan/0000-0003-1700-1061; Liao, Naishun/0000-0002-2781-4229
FU National Natural Science Foundation of China [62175031, 61875141];
Natural Science Foundation of Fujian Province of China [2020J011154];
Scientific Foundation of Fuzhou Municipal Health commission
[2021-S-wp1]; Young and Middle-aged Talent Training Project of Fujian
Provincial Health Commission [2021GGB020]
FX Y.W. and Q.Z. contributed equally to this work. The authors thank the
Key Laboratory of Optoelectronic Science and Technology for Medicine of
Ministry of Education, Fujian Provincial Key Laboratory of Photonics
Technology, Fujian Normal University, Fuzhou 350007, P. R. China in 1O2
detection. This work was supported by the National Natural Science
Foundation of China (grant no. 62175031, and 61875141); Natural Science
Foundation of Fujian Province of China (grant no. 2020J011154);
Scientific Foundation of Fuzhou Municipal Health commission (grant no.
2021-S-wp1); Young and Middle-aged Talent Training Project of Fujian
Provincial Health Commission (grant no. 2021GGB020).
CR Agostinis P, 2011, CA-CANCER J CLIN, V61, P250, DOI 10.3322/caac.20114
Alzeibak, 2021, INT J CANCER, V9
Bol KF, 2016, CANCER IMMUNOL IMMUN, V65, P327, DOI 10.1007/s00262-016-1796-7
Cai ZX, 2021, MOL CANCER, V20, DOI 10.1186/s12943-021-01467-8
Chen CL, 2021, NANO LETT, V21, P5522, DOI 10.1021/acs.nanolett.1c00818
Ding L, 2020, ACS APPL MATER INTER, V12, P36906, DOI 10.1021/acsami.0c09657
Ding ZY, 2021, SIGNAL TRANSDUCT TAR, V6, DOI 10.1038/s41392-020-00448-5
Fomer A, 2014, NAT REV CLIN ONCOL, V11, P525, DOI 10.1038/nrclinonc.2014.122
Fridlender ZG, 2009, CANCER CELL, V16, P183, DOI 10.1016/j.ccr.2009.06.017
Gao R, 2018, NAT COMMUN, V9, DOI 10.1038/s41467-018-05223-3
Giermasz AS, 2009, CANCER IMMUNOL IMMUN, V58, P1329, DOI 10.1007/s00262-008-0648-5
Gravitz, 2013, NATURE, P504
Jiang Y, 2020, ADV MATER, V32, DOI 10.1002/adma.202001808
Kelley RK, 2021, J CLIN ONCOL, V39, P2991, DOI 10.1200/JCO.20.03555
Keskin DB, 2019, NATURE, V565, P234, DOI 10.1038/s41586-018-0792-9
Kroll AV, 2017, ADV MATER, V29, DOI 10.1002/adma.201703969
Kuang DM, 2011, J HEPATOL, V54, P948, DOI 10.1016/j.jhep.2010.08.041
Lan SY, 2019, ACS APPL MATER INTER, V11, P9804, DOI 10.1021/acsami.8b21820
Llovet JM, 2021, NAT REV GASTRO HEPAT, V18, P293, DOI 10.1038/s41575-020-00395-0
Llovet JM, 2021, NAT REV DIS PRIMERS, V7, DOI 10.1038/s41572-020-00240-3
Llovet JM, 2018, NAT REV CLIN ONCOL, V15, P599, DOI 10.1038/s41571-018-0073-4
Mellman I, 2011, NATURE, V480, P480, DOI 10.1038/nature10673
Obermajer N, 2018, NAT PROTOC, V13, P335, DOI 10.1038/nprot.2017.130
Ohms M, 2020, FRONT IMMUNOL, V11, DOI 10.3389/fimmu.2020.00532
Omeroglu I, 2017, BIOORGAN MED CHEM, V25, P5415, DOI 10.1016/j.bmc.2017.07.065
Oroojalian F, 2021, SMALL, V17, DOI 10.1002/smll.202006484
Ott PA, 2017, NATURE, V547, P217, DOI 10.1038/nature22991
Pan H, 2021, ADV MATER, V33, DOI 10.1002/adma.202100241
Pylaeva E, 2019, INT J CANCER, V144, P136, DOI 10.1002/ijc.31808
Sangro B, 2020, J HEPATOL, V72, P320, DOI 10.1016/j.jhep.2019.10.021
Saxena M, 2021, NAT REV CANCER, V21, P360, DOI 10.1038/s41568-021-00346-0
Shrestha S, 2016, ONCOIMMUNOLOGY, V5, DOI 10.1080/2162402X.2015.1067744
Vogel A, 2019, ANN ONCOL, V30, P871, DOI 10.1093/annonc/mdy510
Waghray A, 2015, WORLD J HEPATOL, V7, P1020, DOI 10.4254/wjh.v7.i8.1020
Wu M, 2020, ISCIENCE, V23, DOI 10.1016/j.isci.2020.101341
Xie W, 2019, ACS NANO, V13, P2849, DOI 10.1021/acsnano.8b03788

Zeng YY, 2020, J IMMUNOTHER CANCER, V8, DOI 10.1136/jitc-2019-000217
Zhan GT, 2021, NANO TODAY, V38, DOI 10.1016/j.nantod.2021.101195
Zhang D, 2021, ADV SCI, V8, DOI 10.1002/advs.202003504
Zhang D, 2020, ACS NANO, V14, P8985, DOI 10.1021/acsnano.0c03833
Zhang D, 2018, CHEM SCI, V9, P7390, DOI 10.1039/c8sc02408a
Zhang D, 2018, ADV SCI, V5, DOI 10.1002/advs.201700648
Zhang H, 2007, J IMMUNOL, V179, P4910, DOI 10.4049/jimmunol.179.7.4910
Zheng DY, 2020, NANO-MICRO LETT, V12, DOI 10.1007/s40820-020-00429-x
Zhu AX, 2019, LANCET ONCOL, V20, P282, DOI 10.1016/S1470-2045(18)30937-9
NR 45
TC 28
Z9 28
U1 45
U2 153
PU WILEY
PI HOBOKEN
PA 111 RIVER ST, HOBOKEN 07030-5774, NJ USA
EI 2198-3844
J9 ADV SCI
JI Adv. Sci.
PD APR
PY 2022
VL 9
IS 11
AR 2105631
DI 10.1002/advs.202105631
EA FEB 2022
PG 14
WC Chemistry, Multidisciplinary; Nanoscience & Nanotechnology; Materials
Science, Multidisciplinary
WE Science Citation Index Expanded (SCI-EXPANDED)
SC Chemistry; Science & Technology - Other Topics; Materials Science
GA 0M9AF
UT WOS:000753477600001
PM 35142445
OA gold, Green Published
HC Y
HP N
DA 2023-09-14
ER

PT J
AU Meng, ZQ
Zhang, YJ
Zhou, XF
Ji, JS
Liu, Z
AF Meng, Zhouqi
Zhang, Yaojia
Zhou, Xuanfang
Ji, Jiansong
Liu, Zhuang
TI Nanovaccines with cell-derived components for cancer immunotherapy
SO ADVANCED DRUG DELIVERY REVIEWS
LA English
DT Review
DE Nanovaccine; Cell lysates; Whole cell vaccine; Cell-derived
nanovesicles; Cell membranes-based nanovaccine; Cancer immunotherapy
ID UP-CONVERSION NANOPARTICLES; ANTITUMOR IMMUNE-RESPONSES; OUTER-MEMBRANE
VESICLES; MONOPHOSPHORYL-LIPID-A; DENDRITIC CELLS; PHOTOTHERMAL THERAPY;
CHECKPOINT BLOCKADE; DRUG-DELIVERY; PHOTODYNAMIC THERAPY; CPG

OLIGONUCLEOTIDES
AB Cancer nanovaccines as one of immunotherapeutic approaches are able to attack tumors by stimulating tumor-specific immunological responses. However, there still exist multiple challenges to be tackled for cancer nanovaccines to evoke potent antitumor immunity. Particularly, the administration of exogenous materials may cause the off-target immunotherapy responses. In recent years, biomimetic nanovaccines by using cell lysates, cell-derived nanovesicles, or extracted cell membranes as the functional compo-nents have received extensive attention. Such nanovaccines based on cell-derived components would show many unique advantages including inherent biocompatibility and the ability to trigger immune responses against a range of tumor-associated antigens. In this review article, we will introduce the recent research progresses of those cell-derived biomimetic nanovaccines for cancer immunotherapy, and discuss the perspectives and challenges associated with the future clinical translation of these emerging vaccine platforms. (c) 2022 Elsevier B.V. All rights reserved.
C1 [Meng, Zhouqi; Zhang, Yaojia; Zhou, Xuanfang; Liu, Zhuang] Soochow Univ, Inst Funct Nano & Soft Mat FUNSOM, Jiangsu Key Lab Carbon Based Funct Mat & Devices, 199 Renai Rd, Suzhou, Jiangsu, Peoples R China.
[Ji, Jiansong] Zhejiang Univ, Key Lab Imaging Diag & Minimally Invas Intervent, Lishui Hosp, Lishui 323000, Peoples R China.
C3 Soochow University - China; Zhejiang University
RP Liu, Z (通讯作者)，Soochow Univ, Inst Funct Nano & Soft Mat FUNSOM, Jiangsu Key Lab Carbon Based Funct Mat & Devices, 199 Renai Rd, Suzhou, Jiangsu, Peoples R China.; Ji, JS (通讯作者)，Zhejiang Univ, Key Lab Imaging Diag & Minimally Invas Intervent, Lishui Hosp, Lishui 323000, Peoples R China.
EM jijiansong@zju.edu.cn; zliu@suda.edu.cn
RI Liu, Zhuang/H-4352-2011; zhou, xuan/GZA-8157-2022
OI Liu, Zhuang/0000-0002-1629-1039;
FU National Natural Science Foundation of China [21907073, 52032008, 51803146]; 111 Project; Jiangsu Social Development Project [BE2019658]; Collaborative Innovation Center of Suzhou Nano Science and Technology (Nano-CIC)
FX This work was partially supported by the National Natural Science Foundation of China (21907073, 52032008, 51803146), the 111 Project, the Jiangsu Social Development Project (BE2019658), and the Collaborative Innovation Center of Suzhou Nano Science and Technology (Nano-CIC).
CR Acevedo R, 2014, FRONT IMMUNOL, V5, DOI 10.3389/fimmu.2014.00121
Ai XZ, 2018, BIOCONJUGATE CHEM, V29, P838, DOI 10.1021/acs.bioconjchem.8b00103
Aikins ME, 2020, ACCOUNTS CHEM RES, V53, P2094, DOI 10.1021/acs.accounts.0c00456
Andre F, 2004, J IMMUNOL, V172, P2126, DOI 10.4049/jimmunol.172.4.2126
Angsantikul P, 2018, ADV THER-GERMANY, V1, DOI 10.1002/adtp.201800016
Bahmani B, 2021, NAT COMMUN, V12, DOI 10.1038/s41467-021-22311-z
Banchereau J, 2005, NAT REV IMMUNOL, V5, P296, DOI 10.1038/nri1592
Barkal AA, 2018, NAT IMMUNOL, V19, P76, DOI 10.1038/s41590-017-0004-z
Bencherif SA, 2015, NAT COMMUN, V6, DOI 10.1038/ncomms8556
Blass E., NATURE REV CLIN ONCO, V18, P215
Bol KF, 2016, CLIN CANCER RES, V22, P1897, DOI 10.1158/1078-0432.CCR-15-1399
Califice S, 2004, INT J ONCOL, V25, P983
Camussi G, 2010, KIDNEY INT, V78, P838, DOI 10.1038/ki.2010.278
Cao HQ, 2016, ACS NANO, V10, P7738, DOI 10.1021/acsnano.6b03148
Carlring J, 2005, CURR OPIN MOL THER, V7, P73
Casella CR, 2008, CELL MOL LIFE SCI, V65, P3231, DOI 10.1007/s00018-008-8228-6
Chang Sam S, 2004, Rev Urol, V6 Suppl 10, pS13
Chao Y, 2020, SCI ADV, V6, DOI 10.1126/sciadv.aaz4204
Chao Y, 2019, NANO LETT, V19, P4287, DOI 10.1021/acs.nanolett.9b00579
Chao Y, 2018, NAT BIOMED ENG, V2, P611, DOI 10.1038/s41551-018-0262-6
Chen FM, 2021, BIOMATERIALS, V270, DOI 10.1016/j.biomaterials.2021.120709
Chen Q, 2020, ADV MATER, V32, DOI 10.1002/adma.201908185
Chen Q, 2019, ADV MATER, V31, DOI 10.1002/adma.201802228
Chen Q, 2016, NAT COMMUN, V7, DOI 10.1038/ncomms13193
Cheng LF, 2017, MOL THER, V25, P1665, DOI 10.1016/j.ymthe.2017.02.007
Cheng SH, 2020, ADV SCI, V7, DOI 10.1002/advs.201903301
Chester J, 2002, NAT BIOTECHNOL, V20, P256, DOI 10.1038/nbt0302-256
Cheung AS, 2016, SMALL, V12, P2321, DOI 10.1002/smll.201600061

Chiang CLL, 2011, INT REV IMMUNOL, V30, P150, DOI 10.3109/08830185.2011.572210
Chiang CLL, 2010, SEMIN IMMUNOL, V22, P132, DOI 10.1016/j.smim.2010.02.004
Choo YW, 2018, ACS NANO, V12, P8977, DOI 10.1021/acsnano.8b02446
Chu DF, 2016, ADV HEALTHC MATER, V5, P1088, DOI 10.1002/adhm.201500998
Cools-Lartigue J, 2013, J CLIN INVEST, V123, P3446, DOI 10.1172/JCI67484
Copier J, 2006, INT REV IMMUNOL, V25, P297, DOI 10.1080/08830180600992472
Corrigan RM, 2013, NAT REV MICROBIOL, V11, P513, DOI 10.1038/nrmicro3069
Couzin-Frankel J, 2013, SCIENCE, V342, P1432, DOI 10.1126/science.342.6165.1432
Crooke ST, 2017, NAT BIOTECHNOL, V35, P230, DOI 10.1038/nbt.3779
Dash P, 2020, J CONTROL RELEASE, V327, P546, DOI 10.1016/j.jconrel.2020.09.012
DeNardo DG, 2019, NAT REV IMMUNOL, V19, P369, DOI 10.1038/s41577-019-0127-6
Diehl L, 1999, NAT MED, V5, P774, DOI 10.1038/10495
Esposito M, 2021, NAT CANCER, V2, P258, DOI 10.1038/s43018-021-00181-0
Fan Q, 2020, SCI ADV, V6, DOI 10.1126/sciadv.abb4639
Fang L, 2020, SCI ADV, V6, DOI 10.1126/sciadv.aba4024
Fang R.H., 2018, CELL MEMBR COAT NANO, V30
Fang RH, 2014, NANO LETT, V14, P2181, DOI 10.1021/nl500618u
Farkona S, 2016, BMC MED, V14, DOI 10.1186/s12916-016-0623-5
Fields RC, 1998, P NATL ACAD SCI USA, V95, P9482, DOI 10.1073/pnas.95.16.9482
Finn OJ, 2003, NAT REV IMMUNOL, V3, P630, DOI 10.1038/nri1150
Fu Q, 2015, NANOSCALE, V7, P4020, DOI 10.1039/c4nr07027e
Fusciello M, 2019, NAT COMMUN, V10, DOI 10.1038/s41467-019-13744-8
Gan JY, 2020, J CONTROL RELEASE, V326, P297, DOI 10.1016/j.jconrel.2020.07.008
Gao CY, 2016, NANOSCALE, V8, P3548, DOI 10.1039/c5nr08407e
Garbers C, 2018, NAT REV DRUG DISCOV, V17, P395, DOI 10.1038/nrd.2018.45
Gause KT, 2017, ACS NANO, V11, P54, DOI 10.1021/acsnano.6b07343
Gilboa E, 2007, J CLIN INVEST, V117, P1195, DOI 10.1172/JCI31205
Goldberg MS, 2019, NAT REV CANCER, V19, P587, DOI 10.1038/s41568-019-0186-9
Gong NQ, 2020, NAT NANOTECHNOL, V15, P1053, DOI 10.1038/s41565-020-00782-3
Grotzke JE, 2017, P NATL ACAD SCI USA, V114, pE5910, DOI 10.1073/pnas.1705242114
Grouard G, 1996, NATURE, V384, P364, DOI 10.1038/384364a0
Guo YY, 2015, ACS NANO, V9, P6918, DOI 10.1021/acsnano.5b01042
GUTTERMAN JU, 1994, P NATL ACAD SCI USA, V91, P1198, DOI 10.1073/pnas.91.4.1198
Han X., 2019, SCI ADV
Han X, 2019, SCI ADV, V5, DOI 10.1126/sciadv.aaw6870
Han X, 2019, BIOMATERIALS, V224, DOI 10.1016/j.biomaterials.2019.119490
Han X, 2018, BIOCONJUGATE CHEM, V29, P852, DOI 10.1021/acs.bioconjchem.7b00758
Ho-Tin-Noe B, 2011, J THROMB HAEMOST, V9, P56, DOI 10.1111/j.1538-7836.2011.04317.x
Hu CMJ, 2015, NATURE, V526, P118, DOI 10.1038/nature15373
Hu QY, 2015, ADV MATER, V27, P7043, DOI 10.1002/adma.201503323
ISRAELI RS, 1994, CANCER RES, V54, P1807
Jin JF, 2019, ACS APPL MATER INTER, V11, P7850, DOI 10.1021/acsami.8b22309
Jing LJ, 2018, THERANOSTICS, V8, P2683, DOI 10.7150/thno.23654
Jo W, 2014, NANOSCALE, V6, P12056, DOI 10.1039/c4nr02391a
Kang T, 2017, ACS NANO, V11, P1397, DOI 10.1021/acsnano.6b06477
Keenan BP, 2012, SEMIN ONCOL, V39, P276, DOI 10.1053/j.seminoncol.2012.02.007
Kim HY, 2019, NANO LETT, V19, P5185, DOI 10.1021/acs.nanolett.9b01571
Kim OY, 2017, NAT COMMUN, V8, DOI 10.1038/s41467-017-00729-8
Klichinsky M, 2020, NAT BIOTECHNOL, V38, P947, DOI 10.1038/s41587-020-0462-y
Klinman Dennis M, 2004, Nat Rev Immunol, V4, P249, DOI 10.1038/nri1329
Klinman DM, 2009, ADV DRUG DELIVER REV, V61, P248, DOI 10.1016/j.addr.2008.12.012
Koido S, 2013, ONCOIMMUNOLOGY, V2, DOI 10.4161/onci.24437
Kosmides AK, 2017, ACS NANO, V11, P5417, DOI 10.1021/acsnano.6b08152
Kroll AV, 2019, ADV BIOSYST, V3, DOI 10.1002/adbi.201800219
Kroll AV, 2017, ADV MATER, V29, DOI 10.1002/adma.201703969
Kuai R, 2018, SCI ADV, V4, DOI 10.1126/sciadv.aao1736
Kwak G, 2017, ACS NANO, V11, P10135, DOI 10.1021/acsnano.7b04717
Lang TQ, 2018, ADV THER-GERMANY, V1, DOI 10.1002/adtp.201800053
Leavy O, 2011, NAT REV IMMUNOL, V11, DOI 10.1038/nri2966
Li F, 2019, ACS CENTRAL SCI, V5, P796, DOI 10.1021/acscentsci.9b00060
Li SX, 2019, ADV FUNCT MATER, V29, DOI 10.1002/adfm.201903391

Li SY, 2017, ACS NANO, V11, P7006, DOI 10.1021/acsnano.7b02533
Li YY, 2019, BIOMATER SCI-UK, V7, P3652, DOI 10.1039/c9bm00630c
Liao YY, 2020, NANOSCALE HORIZ, V5, P1293, DOI 10.1039/d0nh00267d
Liu CM, 2019, COLLOID SURFACE B, V175, P477, DOI 10.1016/j.colsurfb.2018.12.038
Liu CY, 2019, THERANOSTICS, V9, P1015, DOI 10.7150/thno.30853
Liu JP, 2019, SMALL, V15, DOI 10.1002/smll.201900262
Liu Q, 2020, NANOSCALE, V12, P19939, DOI 10.1039/d0nr05953f
Liu WL, 2019, NAT COMMUN, V10, DOI 10.1038/s41467-019-11157-1
Liu WL, 2019, ADV MATER, V31, DOI 10.1002/adma.201900499
Luk BT, 2015, J CONTROL RELEASE, V220, P600, DOI 10.1016/j.jconrel.2015.07.019
Luo M, 2017, J CONTROL RELEASE, V263, P200, DOI 10.1016/j.jconrel.2017.03.033
Luo M, 2017, NAT NANOTECHNOL, V12, P648, DOI [10.1038/nnano.2017.52, 10.1038/NNANO.2017.52]
Ma JN, 2020, NANO LETT, V20, P4084, DOI 10.1021/acs.nanolett.9b05171
Martin JD, 2020, NAT REV CLIN ONCOL, V17, P251, DOI 10.1038/s41571-019-0308-z
Martinez F.O., 2009, PERSPECTIVE, V27, P451
Mata-Haro V, 2007, SCIENCE, V316, P1628, DOI 10.1126/science.1138963
Matsuo K, 2021, CANCERS, V13, DOI 10.3390/cancers13102495
Melero I, 2014, NAT REV CLIN ONCOL, V11, P509, DOI 10.1038/nrclinonc.2014.111
Melief CJM, 2015, J CLIN INVEST, V125, P3401, DOI 10.1172/JCI80009
Mellman I, 2011, NATURE, V480, P480, DOI 10.1038/nature10673
Meng Z., NANO LETT, V21, P1228
Meng ZQ, 2019, ADV MATER, V31, DOI 10.1002/adma.201900927
Miller KD, 2019, CA-CANCER J CLIN, V69, P363, DOI 10.3322/caac.21565
Mitsunaga M, 2011, NAT MED, V17, P1685, DOI 10.1038/nm.2554
Montecalvo A, 2012, BLOOD, V119, P756, DOI 10.1182/blood-2011-02-338004
Moon GD, 2011, J AM CHEM SOC, V133, P4762, DOI 10.1021/ja200894u
Morse MA, 2005, NAT CLIN PRACT ONCOL, V2, P108, DOI 10.1038/ncponc0098
Muzykantov VR, 2010, EXPERT OPIN DRUG DEL, V7, P403, DOI 10.1517/17425241003610633
Naseri M, 2020, ONCOIMMUNOLOGY, V9, DOI 10.1080/2162402X.2020.1779991
Natasha G, 2014, CLIN THER, V36, P820, DOI 10.1016/j.clinthera.2014.04.019
Ni QQ, 2020, SCI ADV, V6, DOI 10.1126/sciadv.aaw6071
Noh YW, 2017, ADV FUNCT MATER, V27, DOI 10.1002/adfm.201605398
Ochyl LJ, 2018, BIOMATERIALS, V182, P157, DOI 10.1016/j.biomaterials.2018.08.016
OHANNESIAN DW, 1995, CANCER RES, V55, P2191
Oroojalian F, 2021, SMALL, V17, DOI 10.1002/smll.202006484
Ott PA, 2017, NATURE, V547, P217, DOI 10.1038/nature22991
Palucka K, 2012, NAT REV CANCER, V12, P265, DOI 10.1038/nrc3258
Pardoll DM, 1998, NAT MED, V4, P525, DOI 10.1038/nm0598supp-525
Pitt JM, 2016, J CLIN INVEST, V126, P1224, DOI 10.1172/JCI81137
Postow MA, 2015, J CLIN ONCOL, V33, P1974, DOI 10.1200/JCO.2014.59.4358
Powell, 1995, VACCINE DESIGN SUBUN, P495
Qi SH, 2020, THERANOSTICS, V10, P1814, DOI 10.7150/thno.38515
Qin L, 2021, SMALL, V17, DOI 10.1002/smll.202006000
Rao L, 2020, NAT COMMUN, V11, DOI 10.1038/s41467-020-18626-y
Rao L, 2018, ANGEW CHEM INT EDIT, V57, P986, DOI 10.1002/anie.201709457
Rao L, 2018, ADV FUNCT MATER, V28, DOI 10.1002/adfm.201803531
Rao L, 2017, ACS APPL MATER INTER, V9, P2159, DOI 10.1021/acsami.6b14450
Raza F, 2021, ADV HEALTHC MATER, V10, DOI 10.1002/adhm.202002081
Reuven EM, 2019, ACS NANO, V13, P2936, DOI 10.1021/acsnano.8b07241
*REV, 2007, CANCER BIOTHER RADI, V22, P692
Ruella M, 2018, NAT MED, V24, P1499, DOI 10.1038/s41591-018-0201-9
Sabado RL, 2017, CELL RES, V27, P74, DOI 10.1038/cr.2016.157
Sahin U, 2018, SCIENCE, V359, P1355, DOI 10.1126/science.aar7112
Saxena M, 2018, NAT BIOMED ENG, V2, P341, DOI 10.1038/s41551-018-0250-x
Scheetz L, 2019, NAT BIOMED ENG, V3, P768, DOI 10.1038/s41551-019-0436-x
Seo N, 2018, NAT COMMUN, V9, DOI 10.1038/s41467-018-02865-1
Shirota H, 2015, VACCINES-BASEL, V3, P390, DOI 10.3390/vaccines3020390
Song C, 2020, ADV THER-GERMANY, V3, DOI 10.1002/adtp.201900115
Srivatsan S, 2014, HUM VACC IMMUNOTHER, V10, P52, DOI 10.4161/hv.26568
Stanley MA, 2002, CLIN EXP DERMATOL, V27, P571, DOI 10.1046/j.1365-2230.2002.01151.x

Stoorvogel W, 2012, BLOOD, V119, P646, DOI 10.1182/blood-2011-11-389478
Sun DM, 2013, ADV DRUG DELIVER REV, V65, P342, DOI 10.1016/j.addr.2012.07.002
Sun HP, 2017, ADV FUNCT MATER, V27, DOI 10.1002/adfm.201604300
Tan A, 2010, INT J NANOMED, V5, DOI 10.2147/IJN.S13402
Tan SW, 2015, THERANOSTICS, V5, P863, DOI 10.7150/thno.11852
Tang-Huau TL, 2018, NAT COMMUN, V9, DOI 10.1038/s41467-018-04985-0
Thamphiwatana S, 2017, P NATL ACAD SCI USA, V114, P11488, DOI 10.1073/pnas.1714267114
Tran TH, 2015, CLIN IMMUNOL, V160, P46, DOI 10.1016/j.clim.2015.03.021
Thim HL, 2014, VACCINES-BASEL, V2, P228, DOI 10.3390/vaccines2020228
Tian H, 2017, ADV FUNCT MATER, V27, DOI 10.1002/adfm.201703197
Tian XY, 2018, FRONT IMMUNOL, V9, DOI [10.3389/fimmu.2018.00473, 10.3389/fimmu.2018.01683]
Topalian SL, 2015, CANCER CELL, V27, P450, DOI 10.1016/j.ccell.2015.03.001
Troyanskaya O, 2020, NAT CANCER, V1, P149, DOI 10.1038/s43018-020-0034-6
van der Pol L, 2015, BIOTECHNOL J, V10, P1689, DOI 10.1002/biot.201400395
Vanneman M, 2012, NAT REV CANCER, V12, P237, DOI 10.1038/nrc3237
Vijayan V, 2019, PHARMACEUTICS, V11, DOI 10.3390/pharmaceutics11100534
Vollmer J, 2009, ADV DRUG DELIVER REV, V61, P195, DOI 10.1016/j.addr.2008.12.008
Wang C, 2020, INT J BIOL SCI, V16, P633, DOI 10.7150/ijbs.38414
Wang DD, 2020, ACS APPL MATER INTER, V12, P41138, DOI 10.1021/acsami.0c13169
Wang DD, 2018, ACS NANO, V12, P5241, DOI 10.1021/acsnano.7b08355
Wang H, 2018, NAT MATER, V17, P761, DOI 10.1038/s41563-018-0147-9
Wang N, 2018, ANGEW CHEM INT EDIT, V57, P3426, DOI 10.1002/anie.201800422
Wang RF, 1999, IMMUNOL REV, V170, P85, DOI 10.1111/j.1600-065X.1999.tb01331.x
Warrier VU, 2019, BIOSCIENCE REP, V39, DOI 10.1042/BSR20193220
Wei XL, 2018, ACS NANO, V12, P109, DOI 10.1021/acsnano.7b07720
Wei XL, 2016, BIOMATERIALS, V111, P116, DOI 10.1016/j.biomaterials.2016.10.003
Wiklander OPB, 2019, SCI TRANSL MED, V11, DOI 10.1126/scitranslmed.aav8521
Woodward JJ, 2010, SCIENCE, V328, P1703, DOI 10.1126/science.1189801
Wu J, 2016, ACS APPL MATER INTER, V8, P17927, DOI 10.1021/acsami.6b05677
Wu M, 2019, ACS APPL MATER INTER, V11, P9850, DOI 10.1021/acsami.9b00294
Xia YQ, 2020, ADV MATER, V32, DOI 10.1002/adma.202002054
Xiao L, 2021, NANOSCALE, V13, P3594, DOI 10.1039/d0nr08978h
Xie J, 2019, ACS NANO, V13, P5268, DOI 10.1021/acsnano.8b09681
Xie W, 2019, ACS NANO, V13, P2849, DOI 10.1021/acsnano.8b03788
Xu J, 2020, NAT NANOTECHNOL, V15, P1043, DOI 10.1038/s41565-020-00781-4
Xu J, 2017, ACS NANO, V11, P4463, DOI 10.1021/acsnano.7b00715
Xu R, 2018, NAT REV CLIN ONCOL, V15, P617, DOI 10.1038/s41571-018-0036-9
Xue X, 2021, SCI CHINA MATER, V64, P2045, DOI 10.1007/s40843-020-1591-1
Yang F, 2020, ACTA PHARMACOL SIN, V41, P911, DOI 10.1038/s41401-020-0372-z
Yang R, 2018, ACS NANO, V12, P5121, DOI 10.1021/acsnano.7b09041
Yao S, 2013, NAT REV DRUG DISCOV, V12, P130, DOI 10.1038/nrd3877
Ye YQ, 2017, SCI IMMUNOL, V2, DOI 10.1126/sciimmunol.aan5692
Ying M, 2018, ADV FUNCT MATER, V28, DOI 10.1002/adfm.201801032
Yoo JW, 2011, NAT REV DRUG DISCOV, V10, P521, DOI 10.1038/nrd3499
Yu JS, 2004, CANCER RES, V64, P4973, DOI 10.1158/0008-5472.CAN-03-3505
Yu XH, 2017, ACS NANO, V11, P10147, DOI 10.1021/acsnano.7b04736
Zeng ZL, 2020, ADV FUNCT MATER, V30, DOI 10.1002/adfm.202004397
Zhang D, 2021, ADV SCI, V8, DOI 10.1002/advs.202003504
Zhang JW, 2019, ARTIF CELL NANOMED B, V47, P2298, DOI 10.1080/21691401.2019.1622554
Zhang K, 2019, ADV MATER, V31, DOI 10.1002/adma.201807888
Zhang PF, 2017, NANO TODAY, V13, P7, DOI 10.1016/j.nantod.2016.10.008
Zhang QB, 2017, ACS NANO, V11, P10724, DOI 10.1021/acsnano.7b04955
Zhang X, 2008, J PATHOL, V214, P161, DOI 10.1002/path.2284
Zhang Y, 2020, CHINESE J CHEM, V38, P595, DOI 10.1002/cjoc.201900557
Zhang Y, 2019, WIRES NANOMED NANOBI, V11, DOI 10.1002/wnan.1559
Zhang ZQ, 2020, INT J BIOL MACROMOL, V165, P239, DOI 10.1016/j.ijbiomac.2020.09.187
Zhou HM, 2021, BIOMATERIALS, V269, DOI 10.1016/j.biomaterials.2021.120670
Zhou JR, 2020, ADV MATER, V32, DOI 10.1002/adma.201901255
Zhu GZ, 2017, ACS NANO, V11, P2387, DOI 10.1021/acsnano.7b00978
Zhu JY, 2018, BIOMACROMOLECULES, V19, P2043, DOI 10.1021/acs.biomac.8b00242
Zhu L, 2017, THERANOSTICS, V7, P2732, DOI 10.7150/thno.18752

Zitvogel L, 1998, NAT MED, V4, P594, DOI 10.1038/nm0598-594
NR 211
TC 28
Z9 28
U1 39
U2 164
PU ELSEVIER
PI AMSTERDAM
PA RADARWEG 29, 1043 NX AMSTERDAM, NETHERLANDS
SN 0169-409X
EI 1872-8294
J9 ADV DRUG DELIVER REV
JI Adv. Drug Deliv. Rev.
PD MAR
PY 2022
VL 182
AR 114107
DI 10.1016/j.addr.2021.114107
EA JAN 2022
PG 22
WC Pharmacology & Pharmacy
WE Science Citation Index Expanded (SCI-EXPANDED)
SC Pharmacology & Pharmacy
GA 1D4QR
UT WOS:000793787100009
PM 34995678
HC Y
HP N
DA 2023-09-14
ER

PT J
AU Asadi, K
Gholami, A
AF Asadi, Khatereh
Gholami, Ahmad
TI Virosome-based nanovaccines; a promising bioinspiration and biomimetic approach for preventing viral diseases: A review
SO INTERNATIONAL JOURNAL OF BIOLOGICAL MACROMOLECULES
LA English
DT Review
DE Nanovaccine; Virosome; Viral infections; SARS-CoV-2; Bioinspiration
ID SUBUNIT INFLUENZA VACCINE; ANTIGEN-DELIVERY-SYSTEM; PROTEIN NANOPARTICLES; MEDIATED DELIVERY; VIRUS VIROSOMES; DENDRITIC CELLS; DRUG-DELIVERY; IN-VIVO; CANCER; EFFICIENT
AB Vaccination is the most effective means of controlling infectious disease-related morbidity and mortality. However, due to low immunogenicity of viral antigens, nanomedicine as a new opportunity in new generation of vaccine advancement attracted researcher encouragement. Virosome is a lipidic nanomaterial emerging as FDA approved nanocarriers with promising bioinspiration and biomimetic potency against viral infections. Virosome surface modification with critical viral fusion proteins is the cornerstone of vaccine development. Surface antigens at virosomes innovatively interact with targeted receptors on host cells that evoke humoral or cellular immune responses through antibody-producing B cell and internalization by endocytosis-mediated pathways. To date, several nanovaccine based on virosome formulations have been commercialized against widespread and life-threatening infections. Recently, Great efforts were made to fabricate a virosome-based vaccine platform against a new severe acute respiratory syndrome coronavirus 2 (SARS-CoV-2) virus. Thus, this review provides a novel overview of the virosome based nanovaccine production, properties, and application on the viral disease, especially its importance in SARS-CoV-2 vaccine discovery. (c) 2021 Elsevier B.V. All rights reserved.
C1 [Asadi, Khatereh] Shiraz Univ Med Sci, Biotchnol Res Ctr, Shiraz, Iran.
[Asadi, Khatereh] Guilan Univ Med Sci, Guilan Rd Trauma Res Ctr, Rasht, Iran.
[Gholami, Ahmad] Shiraz Univ Med Sci, Pharmaceut Sci Res Ctr, Shiraz, Iran.

[Gholami, Ahmad] Shiraz Univ Med Sci, Sch Adv Med Sci & Technol, Dept Med Nanotedmol, Shiraz, Iran.
C3 Shiraz University of Medical Science; Tehran University of Medical Sciences; Shiraz University of Medical Science; Shiraz University of Medical Science
RP Gholami, A (通讯作者)，Shiraz Univ Med Sci, Pharmaceut Sci Res Ctr, Shiraz, Iran.
EM Gholami@sums.ac.ir
RI Gholami, Ahmad/I-1094-2016
OI Gholami, Ahmad/0000-0003-1851-159X
CR Abdoli A, 2014, PATHOG DIS, V72, P197, DOI 10.1111/2049-632X.12206
Adamina M, 2008, CONTEMP CLIN TRIALS, V29, P165, DOI 10.1016/j.cct.2007.07.002
Amacker M, 2005, INT IMMUNOL, V17, P695, DOI 10.1093/intimm/dxh249
Amacker M, 2020, NPJ VACCINES, V5, DOI 10.1038/s41541-020-0190-9
Angel J, 2007, VACCINE, V25, P3913, DOI 10.1016/j.vaccine.2007.01.101
Bernasconi V, 2016, J IMMUNOL RES, V2016, DOI 10.1155/2016/5482087
Bhardwaj P, 2020, ACTA BIOMATER, V108, P1, DOI 10.1016/j.actbio.2020.03.020
Bomsel M, 2011, IMMUNITY, V34, P269, DOI 10.1016/j.immuni.2011.01.015
Bovier PA, 2008, EXPERT REV VACCINES, V7, P1141, DOI 10.1586/14760584.7.8.1141
Brouwer PJM, 2019, CURR OPIN HIV AIDS, V14, P302, DOI 10.1097/COH.0000000000000549
Bungener L, 2005, VACCINE, V23, P1232, DOI 10.1016/j.vaccine.2004.09.002
Bungener L, 2006, ANTIVIR THER, V11, P717
Calzas C, 2019, FRONT IMMUNOL, V10, DOI 10.3389/fimmu.2019.01605
Cech PG, 2011, PLOS ONE, V6, DOI 10.1371/journal.pone.0022273
Chan CK, 2019, J ONCOL, V2019, DOI 10.1155/2019/3257939
Clementi N, 2019, J IMMUNOL RES, V8, P303
Climent N, 2018, NANOMED-NANOTECHNOL, V14, P339, DOI 10.1016/j.nano.2017.11.009
Coleman CM, 2014, VACCINE, V32, P3169, DOI 10.1016/j.vaccine.2014.04.016
Dacoba TG, 2020, DRUG DELIV TRANSL RE, V10, P621, DOI 10.1007/s13346-020-00721-8
Dastjerdi MB, 2020, INT J BIOL MACROMOL, V158, P894, DOI 10.1016/j.ijbiomac.2020.05.024
Delany I, 2014, EMBO MOL MED, V6, P708, DOI 10.1002/emmm.201403876
Duchemin M, 2020, FRONT IMMUNOL, V11, DOI 10.3389/fimmu.2020.01141
Duinkerken S, 2019, THERANOSTICS, V9, P5797, DOI 10.7150/thno.35059
Ebensen T, 2017, FRONT IMMUNOL, V8, DOI 10.3389/fimmu.2017.01223
Emadi F, 2020, CURR PHARM BIOTECHNO, V21, P1016, DOI 10.2174/1389201021666200318131422
Facciola A, 2019, EUR REV MED PHARMACO, V23, P7163, DOI 10.26355/eurrev_201908_18763
Gao Y, 2018, VIRUSES-BASEL, V10, DOI 10.3390/v10040167
Garg H, 2020, SCI REP-UK, V10, DOI 10.1038/s41598-020-61103-1
Gholami A, 2020, DRUG METAB REV, V52, P205, DOI 10.1080/03602532.2020.1726943
GLUCK R, 1994, LANCET, V344, P160, DOI 10.1016/S0140-6736(94)92758-8
Gomes AC, 2017, VACCINES-BASEL, V5, DOI 10.3390/vaccines5010006
Gupta T, 2020, INT IMMUNOPHARMACOL, V86, DOI 10.1016/j.intimp.2020.106717
Hajizade A., 2014, J APPL BIOTECHNOL RE, V1, P125
Hashemi SA, 2021, BIOSENS BIOELECTRON, V171, DOI 10.1016/j.bios.2020.112731
Hatz C, 2011, CLIN VACCINE IMMUNOL, V18, P885, DOI 10.1128/CVI.00358-10
He H, 2019, PHARMACEUTICS, V11, DOI 10.3390/pharmaceutics11030110
He Q, 2002, CLIN DIAGN LAB IMMUN, V9, P1021, DOI 10.1128/CDLI.9.5.1021-1024.2002
Huckriede A, 2003, VACCINE, V21, P925, DOI 10.1016/S0264-410X(02)00542-X
Huckriede A, 2005, VACCINE, V23, pS26, DOI 10.1016/j.vaccine.2005.04.026
Jazayeri SD, 2019, VACCINES-BASEL, V7, DOI 10.3390/vaccines7040169
Kalra N., 2013, AM J ADV DRUG DELIV, V1, P29
Kammer AR, 2007, VACCINE, V25, P7065, DOI 10.1016/j.vaccine.2007.07.052
Kaneda Y, 2000, ADV DRUG DELIVER REV, V43, P197, DOI 10.1016/S0169-409X(00)00069-7
Kaneda Y, 2002, MOL THER, V6, P219, DOI 10.1006/mthe.2002.0647
Kennedy RB, 2020, FRONT IMMUNOL, V11, DOI 10.3389/fimmu.2020.01181
Khalaj-Hedayati A, 2020, INFLUENZA OTHER RESP, V14, P92, DOI 10.1111/irv.12697
Kole S, 2019, VACCINE, V37, P973, DOI 10.1016/j.vaccine.2018.12.063
Kozlowski Pamela A., 2019, Current Immunology Reviews, V15, P102, DOI 10.2174/1573395514666180605092054
Kuate S, 2007, VIROLOGY, V362, P26, DOI 10.1016/j.virol.2006.12.011
Lederhofer J, 2018, PHARM RES-DORDR, V35, DOI 10.1007/s11095-018-2453-y
Lee NH, 2012, CLIN EXP VACCINE RES, V1, P18, DOI 10.7774/cevr.2012.1.1.18

Li P, 2013, J CONTROL RELEASE, V168, P271, DOI 10.1016/j.jconrel.2013.03.025
Liu HQ, 2015, ACTA PHARMACEUT, V65, P105, DOI 10.1515/acph-2015-0019
Lopez-Sagaseta J, 2016, COMPUT STRUCT BIOTEC, V14, P58, DOI 10.1016/j.csbj.2015.11.001
Mastrobattista E, 2001, FEBS LETT, V509, P71, DOI 10.1016/S0014-5793(01)03112-X
Mayorga O, 2016, J INFECT DIS, V214, P1498, DOI 10.1093/infdis/jiw411
Medjitna TDE, 2006, DEV BIOLOGICALS, V126, P261
Mischler R, 2002, VACCINE, V20, pB17, DOI 10.1016/S0264-410X(02)00512-1
Mousavi SM, 2021, DRUG METAB REV, V53, P141, DOI 10.1080/03602532.2020.1845201
Mousavi SM, 2020, DRUG METAB REV, V52, P299, DOI 10.1080/03602532.2020.1734021
Olczak P, 2020, VACCINES-BASEL, V8, DOI 10.3390/vaccines8040568
Olive C, 2006, J INFECT DIS, V194, P316, DOI 10.1086/505580
Otten G, 2020, VACCINE, V38, P578, DOI 10.1016/j.vaccine.2019.10.058
Pastorino B, 2015, PLOS ONE, V10, DOI 10.1371/journal.pone.0118963
Pati R, 2018, FRONT IMMUNOL, V9, DOI 10.3389/fimmu.2018.02224
Pluschke G, 2012, FUTURE VIROL, V7, P779, DOI 10.2217/FVL.12.74
Poltl-Frank F, 1999, CLIN EXP IMMUNOL, V117, P496
Qi M, 2018, SMALL, V14, DOI 10.1002/smll.201703207
Qian CY, 2020, VACCINES-BASEL, V8, DOI 10.3390/vaccines8010139
Rauch S, 2018, FRONT IMMUNOL, V9, DOI 10.3389/fimmu.2018.01963
Ruprecht RM, 2017, CURR OPIN HIV AIDS, V12, P222, DOI 10.1097/COH.0000000000000369
Saga K, 2013, BIOMED RES INT, V2013, DOI 10.1155/2013/764706
SCHEULE RK, 1986, BIOCHEMISTRY-US, V25, P4223, DOI 10.1021/bi00363a009
Soema PC, 2015, EUR J PHARM BIOPHARM, V94, P251, DOI 10.1016/j.ejpb.2015.05.023
Stegmann, US Patent App, Patent No. [16 (/696) (2020) 752, 166962020752]
Stegmann T, 2010, VACCINE, V28, P5543, DOI 10.1016/j.vaccine.2010.06.041
Sulczewski FB, 2018, ARCH VIROL, V163, P2313, DOI 10.1007/s00705-018-3856-0
Summerfield A, 2009, VIRUSES-BASEL, V1, P1022, DOI 10.3390/v1031022
Tao WQ, 2014, NANOMEDICINE-UK, V9, P237, DOI 10.2217/nnm.13.58
Thomas C, 2011, MOL PHARMACEUT, V8, P405, DOI 10.1021/mp100255c
Thompson FM, 2008, PLOS ONE, V3, DOI 10.1371/journal.pone.0001493
Tian Y, 2014, NANO LETT, V14, P1439, DOI 10.1021/nl404560v
Touze A, 2010, J CLIN MICROBIOL, V48, P1767, DOI 10.1128/JCM.01691-09
Tu YF, 2020, INT J MOL SCI, V21, DOI 10.3390/ijms21072657
Urakami A, 2017, CLIN VACCINE IMMUNOL, V24, DOI 10.1128/CVI.00090-17
Vijayan V, 2019, PHARMACEUTICS, V11, DOI 10.3390/pharmaceutics11100534
W.H. Organization, 2004, GUID CLIN EV VACC RE, P035
W.H. Organization, 2005, GUID INT PACK SHIPP
Waelti E, 2002, CANCER RES, V62, P437
Waelti ER, 1998, INT J CANCER, V77, P728, DOI 10.1002/(SICI)1097-0215(19980831)77:5<728::AID-IJC11>3.0.CO;2-Y
Wang N, 2019, J CONTROL RELEASE, V303, P130, DOI 10.1016/j.jconrel.2019.04.025
Wang ZB, 2020, VACCINES-BASEL, V8, DOI 10.3390/vaccines8010128
White JM, 2016, TRAFFIC, V17, P593, DOI 10.1111/tra.12389
Wilschut J, 2008, J LIPOSOME RES, V3, P767
Yang XB, 2020, LANCET RESP MED, V8, P475, DOI 10.1016/S2213-2600(20)30079-5
Zhao LH, 2020, INT J NANOMED, V15, P2071, DOI [10.2147/JN.S232677, 10.2147/IJN.S232677]
NR 96
TC 28
Z9 31
U1 2
U2 19
PU ELSEVIER
PI AMSTERDAM
PA RADARWEG 29, 1043 NX AMSTERDAM, NETHERLANDS
SN 0141-8130
EI 1879-0003
J9 INT J BIOL MACROMOL
JI Int. J. Biol. Macromol.
PD JUL 1
PY 2021
VL 182

BP 648
EP 658
DI 10.1016/j.ijbiomac.2021.04.005
EA APR 2021
PG 11
WC Biochemistry & Molecular Biology; Chemistry, Applied; Polymer Science
WE Science Citation Index Expanded (SCI-EXPANDED)
SC Biochemistry & Molecular Biology; Chemistry; Polymer Science
GA TD7RN
UT WOS:000669519300004
PM 33862071
OA Green Published, Bronze
DA 2023-09-14
ER

PT J
AU Chen, WQ
Jiang, MX
Yu, WJ
Xu, ZW
Liu, XY
Jia, QM
Guan, XW
Zhang, WF
AF Chen, Wenqiang
Jiang, Mingxia
Yu, Wenjing
Xu, Zhiwei
Liu, Xinyue
Jia, Qingmiao
Guan, Xiuwen
Zhang, Weifen
TI CpG-Based Nanovaccines for Cancer Immunotherapy
SO INTERNATIONAL JOURNAL OF NANOMEDICINE
LA English
DT Review
DE cancer immunotherapy; nanovaccine; nanoparticle; adjuvant; CpG
ID T-CELL RESPONSES; DENDRITIC CELLS; INFECTIOUS-DISEASES;
ANTITUMOR-ACTIVITY; SUBUNIT VACCINES; RATIONAL DESIGN; ANTIGEN PEPTIDE;
B-CELL; DNA; OLIGODEOXYNUCLEOTIDES
AB Cancer has been a serious health hazard to the people all over the world with its high incidence and horrible mortality. In recent years, tumor vaccines in immunotherapy have become a hotspot in cancer therapy due to their many practical advantages and good therapeutic potentials. Among the various vaccines, nanovaccine utilized nanoparticles (NPs) as the carrier and/or adjuvant has presented significant therapeutic effect in cancer treatment. For tumor nanovaccines, unmethylated cytosine-phosphate-guanine oligodeoxynucleotide (CpG ODN) is a commonly used adjuvant. It has been reported that CpG ODN was the most effective immune stimulant among the currently known adjuvants. It could be recognized by toll-like receptor 9 (TLR9) to activate humoral and cellular immunity for preventing or treating cancer. In this review, the topic of CpG-based nanovaccines for cancer immunotherapy will be focused. The types and properties of different CpG will be introduced in detail first, and then some representative tumor nanovaccines will be reviewed according to the diverse loading modes of CpG, such as electrostatic adsorption, covalent bonding, hydrophilic and hydrophobic interaction, and DNA self-assembly, for summarizing the current progress of CpG-based tumor nanovaccines. Finally, the challenges and future perspectives will be discussed. It is hoped that this review will provide valuable references for the development of nanovaccines in cancer immunotherapy.
C1 [Chen, Wenqiang; Jiang, Mingxia; Yu, Wenjing; Xu, Zhiwei; Liu, Xinyue; Jia, Qingmiao; Guan, Xiuwen; Zhang, Weifen] Weifang Med Univ, Coll Pharm, Weifang 261053, Peoples R China.
[Guan, Xiuwen; Zhang, Weifen] Weifang Med Univ, Collaborat Innovat Ctr Target Drug Delivery Syst, Weifang 261053, Peoples R China.
[Guan, Xiuwen; Zhang, Weifen] Weifang Med Univ, Shandong Engn Res Ctr Smart Mat & Regenerat Med, Weifang 261053, Peoples R China.

C3 Weifang Medical University; Weifang Medical University; Weifang Medical
University
RP Guan, XW; Zhang, WF (通讯作者)，Weifang Med Univ, Coll Pharm, Weifang 261053, Peoples R China.; Guan, XW; Zhang, WF (通讯作者)，Weifang Med Univ, Collaborat Innovat Ctr Target Drug Delivery Syst, Weifang 261053, Peoples R China.; Guan, XW; Zhang, WF (通讯作者)，Weifang Med Univ, Shandong Engn Res Ctr Smart Mat & Regenerat Med, Weifang 261053, Peoples R China.
EM gxw2603@wfmc.edu.cn; zhangwf@wfmc.edu.cn
OI Guan, Xiuwen/0000-0003-1796-8993
FU National Natural Science Foundation of China [81973671]; Natural Science
Foundation of Shandong Province [ZR2019BEM003]
FX The authors are thankful to the National Natural Science Foundation of
China (81973671) and the Natural Science Foundation of Shandong Province
(ZR2019BEM003) for financial support to this work.
CR Adamus Tomasz, 2018, Contemp Oncol (Pozn), V22, P56, DOI 10.5114/wo.2018.73887
Aghebati-Maleki A, 2020, J CELL PHYSIOL, V235, P1962, DOI 10.1002/jcp.29126
Ansari MT, 2020, CURR PHARM DESIGN, V26, P4272, DOI 10.2174/1381612826666200720235752
Arruebo Manuel, 2011, Cancers (Basel), V3, P3279, DOI 10.3390/cancers3033279
Ballas ZK, 2007, IMMUNOL RES, V39, P15, DOI 10.1007/s12026-007-0066-3
Banchereau J, 1998, NATURE, V392, P245, DOI 10.1038/32588
Bavananthasivam J, 2018, VACCINE, V36, P4070, DOI 10.1016/j.vaccine.2018.05.091
Cai ZX, 2020, ADV HEALTHC MATER, V9, DOI 10.1002/adhm.201900996
Campbell JD, 2017, METHODS MOL BIOL, V1494, P15, DOI 10.1007/978-1-4939-6445-1_2
Crooks J, 2017, MEDIAT INFLAMM, V2017, DOI 10.1155/2017/1380615
Dalpke AH, 2002, IMMUNOLOGY, V106, P102, DOI 10.1046/j.1365-2567.2002.01410.x
Dong X, 2019, ACS APPL MATER INTER, V11, P4876, DOI 10.1021/acsami.8b20364
Dubois B, 2001, CELL IMMUNOL, V214, P173, DOI 10.1006/cimm.2001.1898
Farkona S, 2016, BMC MED, V14, DOI 10.1186/s12916-016-0623-5
Foged C, 2011, THER DELIV, V2, P1057, DOI 10.4155/TDE.11.68
Fourcade J, 2008, J IMMUNOTHER, V31, P781, DOI 10.1097/CJI.0b013e318183af0b
Gause KT, 2017, ACS NANO, V11, P54, DOI 10.1021/acsnano.6b07343
Geary SM, 2015, J CONTROL RELEASE, V220, P682, DOI 10.1016/j.jconrel.2015.09.002
Gonzalez H, 2018, GENE DEV, V32, P1267, DOI 10.1101/gad.314617.118
Gray RC, 2007, J LEUKOCYTE BIOL, V81, P1075, DOI 10.1189/jlb.1006606
Guan XW, 2018, BIOMATERIALS, V171, P198, DOI 10.1016/j.biomaterials.2018.04.039
Gursel M, 2002, J LEUKOCYTE BIOL, V71, P813
Han SL, 2019, NANOSCALE, V11, P20206, DOI 10.1039/c9nr03353j
Hanagata N, 2017, INT J NANOMED, V12, P515, DOI 10.2147/IJN.S114477
Hartmann G, 1999, P NATL ACAD SCI USA, V96, P9305, DOI 10.1073/pnas.96.16.9305
Hartmann G, 2003, EUR J IMMUNOL, V33, P1633, DOI 10.1002/eji.200323813
Hayat SMG, 2019, J CELL PHYSIOL, V234, P12530, DOI 10.1002/jcp.28120
Heeg K, 2008, INT J MED MICROBIOL, V298, P33, DOI 10.1016/j.ijmm.2007.07.007
Ingelsson B, 2018, P NATL ACAD SCI USA, V115, pE478, DOI 10.1073/pnas.1711950115
Jurk M, 2004, IMMUNOBIOLOGY, V209, P141, DOI 10.1016/j.imbio.2004.02.006
Karbach J, 2010, INT J CANCER, V126, P909, DOI 10.1002/ijc.24850
Katsuda M, 2011, INT J ONCOL, V39, P1295, DOI 10.3892/ijo.2011.1146
Kim R, 2007, IMMUNOLOGY, V121, P1, DOI 10.1111/j.1365-2567.2007.02587.x
Kim Y, 2020, ANGEW CHEM INT EDIT, V59, P14628, DOI 10.1002/anie.202006117
Klinman DM, 2004, NAT REV IMMUNOL, V4, P248, DOI 10.1038/nri1329
Kobayashi N, 2013, J IMMUNOL, V190, P1882, DOI 10.4049/jimmunol.1201063
Kokate RA, 2016, NANOMEDICINE-UK, V11, P479, DOI 10.2217/nnm.15.213
Kokate RA, 2015, NANOMEDICINE-UK, V10, P915, DOI [10.2217/NNM.14.144, 10.2217/nnm.14.144]
Krieg AM, 2002, ANNU REV IMMUNOL, V20, P709, DOI 10.1146/annurev.immunol.20.100301.064842
Krieg AM, 1998, P NATL ACAD SCI USA, V95, P12631, DOI 10.1073/pnas.95.21.12631
Krieg AM, 2006, NAT REV DRUG DISCOV, V5, P471, DOI 10.1038/nrd2059
Krishnamachari Y, 2011, PHARM RES-DORDR, V28, P215, DOI 10.1007/s11095-010-0241-4
Krug A, 2001, EUR J IMMUNOL, V31, P2154, DOI 10.1002/1521-4141(200107)31:7<2154::AID-IMMU2154>3.0.CO;2-U
Krug A, 2001, EUR J IMMUNOL, V31, P3026, DOI 10.1002/1521-4141(2001010)31:10<3026::AID-IMMU3026>3.0.CO;2-H
Kuai R, 2017, NAT MATER, V16, P489, DOI [10.1038/nmat4822, 10.1038/NMAT4822]
Lemke-Miltner CD, 2020, J IMMUNOL, V204, P1386, DOI 10.4049/jimmunol.1900742

file:///D/Joyce/计量学/frontiers/修/Supplementary materials/savedrecs (94).txt[2024/6/27 16:48:08]

Li LN, 2019, J CELL BIOCHEM, V120, P7868, DOI 10.1002/jcb.28061
Li TT, 2020, PHARMACOL RES, V161, DOI 10.1016/j.phrs.2020.105293
Li TT, 2020, FRONT PHARMACOL, V11, DOI 10.3389/fphar.2020.00008
Liang XQ, 2010, BLOOD, V115, P5041, DOI 10.1182/blood-2009-03-213363
Liu D, 2021, BIOMATER SCI-UK, V9, P84, DOI 10.1039/d0bm01333a
Liu H, 2020, BIOMATERIALS, V255, DOI 10.1016/j.biomaterials.2020.120158
Liu X, 2020, BIOMATERIALS, V230, DOI 10.1016/j.biomaterials.2019.119649
Liu YT, 2019, ACS APPL BIO MATER, V2, P1241, DOI 10.1021/acsabm.8b00811
Lv YF, 2015, NAT PROTOC, V10, P1508, DOI 10.1038/nprot.2015.078
Ma Xiaomei, 2006, Yale J Biol Med, V79, P85
Mailliard Robbie B., 2000, Immunological Investigations, V29, P177, DOI 10.3109/08820130009062302
Maina TW, 2020, FRONT BIOENG BIOTECH, V8, DOI 10.3389/fbioe.2020.608050
Marshall JD, 2003, J LEUKOCYTE BIOL, V73, P781, DOI 10.1189/jlb.1202630
Mathur S, 2011, SHOCK, V36, P478, DOI 10.1097/SHK.0b013e31822d6442
Mellman I, 2011, NATURE, V480, P480, DOI 10.1038/nature10673
Ming J, 2020, NANOSCALE, V12, P3916, DOI 10.1039/c9nr09402d
Moore JA, 2021, NANO EXPRESS, V2, DOI 10.1088/2632-959X/abddd3
Moyle PM, 2013, CHEMMEDCHEM, V8, P360, DOI 10.1002/cmdc.201200487
Mueller M, 2012, J CONTROL RELEASE, V162, P159, DOI 10.1016/j.jconrel.2012.06.015
Mutwiri GK, 2004, J CONTROL RELEASE, V97, P1, DOI 10.1016/j.jcornel.2004.02.022
Ni QQ, 2020, SCI ADV, V6, DOI 10.1126/sciadv.aaw6071
Purcell AW, 2007, NAT REV DRUG DISCOV, V6, P404, DOI 10.1038/nrd2224
Qian Y, 2016, BIOMATERIALS, V98, P171, DOI 10.1016/j.biomaterials.2016.05.008
Riley RS, 2019, NAT REV DRUG DISCOV, V18, P175, DOI 10.1038/s41573-018-0006-z
Rosenberg SA, 2004, NAT MED, V10, P909, DOI 10.1038/nm1100
Ruiz-de-Angulo A, 2016, ACS NANO, V10, P1602, DOI 10.1021/acsnano.5b07253
Sainz V, 2018, ACTA BIOMATER, V76, P193, DOI 10.1016/j.actbio.2018.06.029
Scheiermann J, 2014, VACCINE, V32, P6377, DOI 10.1016/j.vaccine.2014.06.065
Sharma P, 2011, NAT REV CANCER, V11, P805, DOI 10.1038/nrc3153
Shi R, 2005, CANCER BIOL THER, V4, P218
Shi XG, 2020, CHEM ENG J, V399, DOI 10.1016/j.cej.2020.125854
Shirota H, 2005, J IMMUNOL, V174, P4579, DOI 10.4049/jimmunol.174.8.4579
Shirota H, 2014, EXPERT REV VACCINES, V13, P299, DOI 10.1586/14760584.2014.863715
Siddique S, 2020, NANOMATERIALS-BASEL, V10, DOI 10.3390/nano10091700
Siddique S, 2020, APPL SCI-BASEL, V10, DOI 10.3390/app10113824
Sorski L, 2020, CANCER IMMUNOL IMMUN, V69, P2021, DOI 10.1007/s00262-020-02596-7
Speiser DE, 2005, J CLIN INVEST, V115, P739, DOI 10.1172/JCI200523373
Valmori D, 2007, P NATL ACAD SCI USA, V104, P8947, DOI 10.1073/pnas.0703395104
van Ojik HH, 2003, CANCER RES, V63, P5595
van Pul KM, 2020, J IMMUNOTHER CANCER, V8, DOI 10.1136/jitc-2020-000761
Verthelyi D, 2001, J IMMUNOL, V166, P2372, DOI 10.4049/jimmunol.166.4.2372
Vijayan V, 2019, PHARMACEUTICS, V11, DOI 10.3390/pharmaceutics11100534
Vollmer J, 2004, EUR J IMMUNOL, V34, P251, DOI 10.1002/eji.200324032
Vollmer J, 2009, ADV DRUG DELIVER REV, V61, P195, DOI 10.1016/j.addr.2008.12.008
Wang JH, 2020, ADV SCI, V7, DOI 10.1002/advs.202001108
Wang K, 2018, ACS NANO, V12, P6398, DOI 10.1021/acsnano.8b00558
Wang YR, 2016, NANOSCALE RES LETT, V11, DOI 10.1186/s11671-016-1554-y
Wilson HL, 2006, INT REV IMMUNOL, V25, P183, DOI 10.1080/08830180600785868
Wooldridge James E., 2003, Current Opinion in Oncology, V15, P440, DOI 10.1097/00001622-200311000-00006
Yamamoto Y, 2017, FRONT IMMUNOL, V8, DOI 10.3389/fimmu.2017.01049
Zang XL, 2017, EUR J PHARM BIOPHARM, V115, P243, DOI 10.1016/j.ejpb.2017.03.013
Zhang LQ, 2015, ACS APPL MATER INTER, V7, P24069, DOI 10.1021/acsami.5b06987
Zhang Y, 2019, WIRES NANOMED NANOBI, V11, DOI 10.1002/wnan.1559
Zhang Y, 2016, ADV MATER, V28, P2699, DOI 10.1002/adma.201506232
Zhao Y, 2020, INT J NANOMED, V15, P9571, DOI 10.2147/IJN.S275670
Zhu GZ, 2017, NAT COMMUN, V8, DOI 10.1038/s41467-017-02191-y
Zhu GZ, 2017, NAT COMMUN, V8, DOI 10.1038/s41467-017-01386-7
Zhu GZ, 2017, ACS NANO, V11, P2387, DOI 10.1021/acsnano.7b00978
Zhu GZ, 2016, NANOSCALE, V8, P6684, DOI 10.1039/c5nr08821f
Zhu GZ, 2013, J AM CHEM SOC, V135, P16438, DOI 10.1021/ja406115e

Zupancic E, 2018, NANOMED-NANOTECHNOL, V14, P835, DOI 10.1016/j.nano.2017.12.011
NR 107
TC 28
Z9 28
U1 23
U2 79
PU DOVE MEDICAL PRESS LTD
PI ALBANY
PA PO BOX 300-008, ALBANY, AUCKLAND 0752, NEW ZEALAND
SN 1178-2013
J9 INT J NANOMED
JI Int. J. Nanomed.
PY 2021
VL 16
BP 5281
EP 5299
DI 10.2147/IJN.S317626
PG 19
WC Nanoscience & Nanotechnology; Pharmacology & Pharmacy
WE Science Citation Index Expanded (SCI-EXPANDED)
SC Science & Technology - Other Topics; Pharmacology & Pharmacy
GA TU9DN
UT WOS:000681329800001
PM 34385817
OA Green Published, gold
DA 2023-09-14
ER

PT J
AU Wang, JH
Wang, S
Ye, T
Li, F
Gao, XY
Wang, Y
Ye, P
Qing, S
Wang, CL
Yue, H
Wu, J
Wei, W
Ma, GH
AF Wang, Jianghua
Wang, Shuang
Ye, Tong
Li, Feng
Gao, Xiaoyong
Wang, Yan
Ye, Peng
Qing, Shuang
Wang, Changlong
Yue, Hua
Wu, Jie
Wei, Wei
Ma, Guanghui
TI Choice of Nanovaccine Delivery Mode Has Profound Impacts on the Intralymph Node Spatiotemporal Distribution and Immunotherapy Efficacy
SO ADVANCED SCIENCE
LA English
DT Article
DE delivery modes; hydrogels; lymph nodes; nanovaccines; tumor

immunotherapy
ID CELLS IN-VIVO; DENDRITIC CELLS; LYMPH-NODE; QUATERNIZED CHITOSAN; VACCINE DELIVERY; HYDROGEL; NANOPARTICLES; ENCAPSULATION; BIOMATERIALS; ANTIGENS
AB Nanovaccines have attracted booming interests in vaccinology studies, but the profound impacts of their delivery mode on immune response remain unrealized. Herein, immunostimulatory CpG-modified tumor-derived nanovesicles (CNVs) are used as a nanovaccine testbed to initially evaluate the impacts of three distinct delivery modes, including mono-pulse CNVs, staggered-pulse CNVs, and gel-confined CNVs. Fundamentally, delivery mode has enormous impacts on the immunomodulatory effects, altering the spatiotemporal distribution of nanovaccine residence and dendritic cell-T cell interaction in lymph nodes, and finally affecting subsequent T cell-mediated immune performance. As a result, the gel-confined delivery mode offers the best therapeutic performance in multiple tumor models. When extending evaluation to examine how the various delivery modes impact the performance of liposome-based nanovaccines, similar trends in intralymph node distribution and antitumor effect are observed. This work provides a strong empirical foundation that nanovaccine researchers should position delivery mode near the top of their considerations for the experimental design, which should speed up nanovaccine development and facilitate efficient selection of appropriate delivery modes in the clinic.
C1 [Wang, Jianghua; Wang, Shuang; Ye, Tong; Li, Feng; Gao, Xiaoyong; Wang, Yan; Ye, Peng; Qing, Shuang; Wang, Changlong; Yue, Hua; Wu, Jie; Wei, Wei; Ma, Guanghui] Chinese Acad Sci, Inst Proc Engn, State Key Lab Biochem Engn, 1 North 2nd St, Beijing 100190, Peoples R China.
[Wang, Jianghua; Ye, Tong; Li, Feng; Wang, Yan; Qing, Shuang; Wang, Changlong; Wei, Wei; Ma, Guanghui] Univ Chinese Acad Sci, 19A Yuquan Rd, Beijing 100049, Peoples R China.
C3 Chinese Academy of Sciences; Institute of Process Engineering, CAS; Chinese Academy of Sciences; University of Chinese Academy of Sciences, CAS
RP Wei, W; Ma, GH (通讯作者)，Chinese Acad Sci, Inst Proc Engn, State Key Lab Biochem Engn, 1 North 2nd St, Beijing 100190, Peoples R China.; Ma, GH (通讯作者)，Univ Chinese Acad Sci, 19A Yuquan Rd, Beijing 100049, Peoples R China.
EM weiwei@ipe.ac.cn; ghma@ipe.ac.cn
RI wei, wei/HHR-8613-2022; wei, wei/IQW-1347-2023; Yue, H/L-4578-2017
OI Yue, H/0000-0003-2621-8326; Wei, Wei/0000-0002-6244-3187; Li, Feng/0000-0002-0045-1501
FU Innovative Research Group Project of the National Natural Science Foundation of China [21821005]; Strategic Priority Research Program of the Chinese Academy of Sciences [XDB29040303]; National Key Research and Development Program of China [2017YFA0207900]; Beijing Municipal Natural Science Foundation [2202056]
FX This work was supported by Innovative Research Group Project of the National Natural Science Foundation of China (21821005), the Strategic Priority Research Program of the Chinese Academy of Sciences (XDB29040303), the National Key Research and Development Program of China (2017YFA0207900), and Beijing Municipal Natural Science Foundation (2202056).
CR Ali OA, 2009, SCI TRANSL MED, V1, DOI 10.1126/scitranslmed.3000359
Ali OA, 2009, NAT MATER, V8, P151, DOI [10.1038/NMAT2357, 10.1038/nmat2357]
Bachmann MF, 2010, NAT REV IMMUNOL, V10, P787, DOI 10.1038/nri2868
Bayyurt B, 2017, J CONTROL RELEASE, V247, P134, DOI 10.1016/j.jconrel.2017.01.004
Bencherif SA, 2015, NAT COMMUN, V6, DOI 10.1038/ncomms8556
Chen Q, 2020, NANO LETT, V20, P11, DOI 10.1021/acs.nanolett.9b02182
Cheng Q, 2018, J AM CHEM SOC, V140, P16413, DOI 10.1021/jacs.8b10047
Cheung AS, 2016, SMALL, V12, P2321, DOI 10.1002/smll.201600061
Couzin-Frankel J, 2013, SCIENCE, V342, P1432, DOI 10.1126/science.342.6165.1432
De Koker S, 2016, ANGEW CHEM INT EDIT, V55, P1334, DOI 10.1002/anie.201508626
de Titta A, 2013, P NATL ACAD SCI USA, V110, P19902, DOI 10.1073/pnas.1313152110
Dellacherie MO, 2019, NAT REV MATER, V4, P379, DOI 10.1038/s41578-019-0106-3
Han Z. W., 2020, ACS APPL BIOMATER, V3, P2666
Hor Y, 2009, BIOMATERIALS, V30, P6757, DOI 10.1016/j.biomaterials.2009.08.037
Huang AC, 2017, NATURE, V545, P60, DOI 10.1038/nature22079
Irvine DJ, 2015, CHEM REV, V115, P11109, DOI 10.1021/acs.chemrev.5b00109
Irvine DJ, 2013, NAT MATER, V12, P978, DOI [10.1038/NMAT3775, 10.1038/nmat3775]
Iwasaki A, 2004, NAT IMMUNOL, V5, P987, DOI 10.1038/ni1112

Kim J, 2015, NAT BIOTECHNOL, V33, P64, DOI 10.1038/nbt.3071
Kim YC, 2012, ADV DRUG DELIVER REV, V64, P1547, DOI 10.1016/j.addr.2012.04.005
Kuai R, 2017, NAT MATER, V16, P489, DOI [10.1038/nmat4822, 10.1038/NMAT4822]
Li F, 2019, ACS CENTRAL SCI, V5, P796, DOI 10.1021/acscentsci.9b00060
Liang RJ, 2017, BIOMATERIALS, V149, P41, DOI 10.1016/j.biomaterials.2017.09.029
Liu C, 2019, NANO LETT, V19, P7836, DOI 10.1021/acs.nanolett.9b02841
Liu HP, 2014, NATURE, V507, P519, DOI 10.1038/nature12978
Lu S, 2009, CURR OPIN IMMUNOL, V21, P346, DOI 10.1016/j.coi.2009.05.016
Luo M, 2017, J CONTROL RELEASE, V263, P200, DOI 10.1016/j.jconrel.2017.03.033
Luo M, 2017, NAT NANOTECHNOL, V12, P648, DOI [10.1038/nnano.2017.52, 10.1038/NNANO.2017.52]
Manolova V, 2008, EUR J IMMUNOL, V38, P1404, DOI 10.1002/eji.200737984
Martin-Fontecha A, 2003, J EXP MED, V198, P615, DOI 10.1084/jem.20030448
McHugh KJ, 2015, J CONTROL RELEASE, V219, P596, DOI 10.1016/j.jconrel.2015.07.029
McKee AS, 2017, CURR OPIN IMMUNOL, V47, P44, DOI 10.1016/j.coi.2017.06.005
Moreira AF, 2019, PHARMACOL RES, V148, DOI 10.1016/j.phrs.2019.104438
Morishita M, 2016, BIOMATERIALS, V111, P55, DOI 10.1016/j.biomaterials.2016.09.031
Muraoka D, 2014, ACS NANO, V8, P9209, DOI 10.1021/nn502975r
Noh YW, 2017, ADV FUNCT MATER, V27, DOI 10.1002/adfm.201605398
Oleszycka E, 2014, CURR OPIN IMMUNOL, V28, P1, DOI 10.1016/j.coi.2013.12.007
Parry AL, 2013, J AM CHEM SOC, V135, P9362, DOI 10.1021/ja4046857
Peng LH, 2015, ACS APPL MATER INTER, V7, P18628, DOI 10.1021/acsami.5b05065
Radovic-Moreno AF, 2015, P NATL ACAD SCI USA, V112, P3892, DOI 10.1073/pnas.1502850112
Reddy ST, 2007, NAT BIOTECHNOL, V25, P1159, DOI [10.1038/nbt1332, 10.1038/nbtl332]
Reddy ST, 2006, TRENDS IMMUNOL, V27, P573, DOI 10.1016/j.it.2006.10.005
Reddy ST, 2006, J CONTROL RELEASE, V112, P26, DOI 10.1016/j.jconrel.2006.01.006
Sallusto F, 2010, IMMUNITY, V33, P451, DOI 10.1016/j.immuni.2010.10.008
Schudel A, 2019, NAT REV MATER, V4, P415, DOI 10.1038/s41578-019-0110-7
Smith DM, 2013, NAT REV IMMUNOL, V13, P592, DOI 10.1038/nri3488
Song HJ, 2018, BIOMATERIALS, V159, P119, DOI 10.1016/j.biomaterials.2018.01.004
Stoll S, 2002, SCIENCE, V296, P1873, DOI 10.1126/science.1071065
Nguyen TL, 2019, ADV MATER, V31, DOI 10.1002/adma.201803953
Wang K, 2018, ACS NANO, V12, P6398, DOI 10.1021/acsnano.8b00558
Wu J, 2007, BIOMATERIALS, V28, P2220, DOI 10.1016/j.biomaterials.2006.12.024
Wu J, 2015, J CONTROL RELEASE, V213, pE51, DOI 10.1016/j.jconrel.2015.05.084
Wu YB, 2012, BIOMATERIALS, V33, P2351, DOI 10.1016/j.biomaterials.2011.11.068
Xi XB, 2020, SCI ADV, V6, DOI 10.1126/sciadv.aay7735
Xia YF, 2018, NAT MATER, V17, P187, DOI [10.1038/NMAT5057, 10.1038/nmat5057]
Yang F, 2020, APPL MATER TODAY, V19, DOI 10.1016/j.apmt.2020.100608
Yang R, 2018, ACS NANO, V12, P5121, DOI 10.1021/acsnano.7b09041
Yue WW, 2019, NAT COMMUN, V10, DOI 10.1038/s41467-019-09760-3
Zhang XD, 2018, ADV MATER, V30, DOI 10.1002/adma.201707112
Zhu GZ, 2017, NAT COMMUN, V8, DOI 10.1038/s41467-017-01386-7
Zhu GZ, 2017, ACS NANO, V11, P2387, DOI 10.1021/acsnano.7b00978
NR 61
TC 28
Z9 29
U1 13
U2 97
PU WILEY
PI HOBOKEN
PA 111 RIVER ST, HOBOKEN 07030-5774, NJ USA
EI 2198-3844
J9 ADV SCI
JI Adv. Sci.
PD OCT
PY 2020
VL 7
IS 19
AR 2001108
DI 10.1002/advs.202001108

EA AUG 2020
PG 17
WC Chemistry, Multidisciplinary; Nanoscience & Nanotechnology; Materials
Science, Multidisciplinary
WE Science Citation Index Expanded (SCI-EXPANDED)
SC Chemistry; Science & Technology - Other Topics; Materials Science
GA OB6WU
UT WOS:000565309100001
PM 33042743
OA Green Published, gold
DA 2023-09-14
ER

PT J
AU Wang, C
Huang, X
Wu, YJ
Wang, JB
Li, FR
Guo, GQ
AF Wang, Ce
Huang, Xue
Wu, Yingjuan
Wang, Jingbo
Li, Furong
Guo, Guoqing
TI Tumor Cell-associated Exosomes Robustly Elicit Anti-tumor Immune
Responses through Modulating Dendritic Cell Vaccines in Lung Tumor
SO INTERNATIONAL JOURNAL OF BIOLOGICAL SCIENCES
LA English
DT Article
DE tumor cell-associated exosome; immune response; DC vaccine;
immunosuppression; lung tumor
ID T-CELLS; EFFICIENT INDUCTION; CANCER; BLOCKADE; DELIVERY; LIGANDS; GENE;
PERSPECTIVES; SUPPRESSION; NANOVACCINE
AB DC vaccine-based immunotherapy is emerging as a novel therapeutic strategy for cancer treatment, however, antitumor effect of DC vaccines based on tumor cell lysates (TCLs) remains unsatisfactory due to poor immunogenicity of tumor antigens. Although tumor-associated exosomes (TAEs) have been reported as a promising antigen for DC vaccines, it remains unclear how TAE-based DC vaccine induced antitumor immunity in lung cancer.
Methods: In the present study, we extracted TAEs from the supernatant of tumor cell culture medium, and compared the effect of TAEs with TCLs on DCs. To further evaluate the therapeutic effect of DCTAE, we used immunofluorescence and flow cytometry to evaluate the apoptosis of tumor tissue, tumor-infiltrating CD8(+) T cells and Tregs in TDLNs and spleen. Then the levels of cytokines of IL-12, IFN-gamma, L-10 and TGF-beta were quantified by ELISA assays.
Results: Our data showed that TAEs were more potent than TCLs to promote DC maturation and enhance MHC cross presentation, which directly contributed to more robust tumor-specific cytotoxic T lymphocyte (CTL) response. More importantly, TAEs reduced the expression of PD-L1 of DCs, thereby led to down-regulated population of Tregs in vitro. Moreover, DCTAE remarkably suppressed the tumor growth and prolonged survival rate in vivo, due to participance of CD8(+) T cells and decreased Tregs in TDLNs and spleen.
Conclusion: TAEs could serve to improve vaccine-elicited immunotherapy by triggering stronger DC-mediated immune responses and decreasing Tregs in the tumor microenvironment.
C1 [Wang, Ce; Guo, Guoqing] Jinan Univ, Sch Med, Dept Anat, 601 West Whampoa Ave, Guangzhou 510632, Peoples R China.
[Wang, Ce; Huang, Xue; Wu, Yingjuan; Wang, Jingbo; Li, Furong] Jinan Univ, Shenzhen Peoples Hosp,Guangdong Engn Technol Res, Translat Med Collaborat Innovat Ct,Clin Med Coll, Shenzhen Key Lab Stem Cell Res & Clin Transformat, Shenzhen 518020, Guangdong, Peoples R China.
C3 Jinan University; Jinan University
RP Guo, GQ (通讯作者)，Jinan Univ, Sch Med, Dept Anat, 601 West Whampoa Ave, Guangzhou 510632, Peoples R China.; Li, FR (通讯作者)，Jinan Univ, Shenzhen Peoples Hosp, Clin Med Coll 2, Translat Med

Collaborat Innovat Ctr, 1017 Dongmen North Rd, Shenzhen 518020, Peoples R China.
FU National Natural Science Foundation of China [81602695]; Science and
Technology Project of Shenzhen [JCYJ20170307095822325, GJHZ201703
10090257380, JCYJ20170413092711058, JCYJ20180228 164407689]; Shenzhen
Municipal Health and Family Planning Commission Research Project
[SZXJ2018016]
FX This project was supported by the National Natural Science Foundation of
China (Grant No. 81602695), the Science and Technology Project of
Shenzhen (No. JCYJ20170307095822325, GJHZ201703 10090257380,
JCYJ20170413092711058, JCYJ20180228 164407689), Shenzhen Municipal
Health and Family Planning Commission Research Project (No.
SZXJ2018016).
CR Apte SH, 2010, J IMMUNOL, V185, P998, DOI 10.4049/jimmunol.0903372
Brown JA, 2003, J IMMUNOL, V170, P1257, DOI 10.4049/jimmunol.170.3.1257
Chen ZZ, 2018, J EXP CLIN CANC RES, V37, DOI 10.1186/s13046-018-0863-7
Chew V, 2012, J ONCOL, V2012, DOI 10.1155/2012/608406
Chiang CLL, 2015, EXPERT OPIN BIOL TH, V15, P569, DOI 10.1517/14712598.2015.1000298
Chistiakov DA, 2016, CURR PHARM DESIGN, V22, P2431, DOI 10.2174/1381612822666160203141740
Colombo M, 2014, ANNU REV CELL DEV BI, V30, P255, DOI 10.1146/annurev-cellbio-101512-122326
Curiel TJ, 2003, NAT MED, V9, P562, DOI 10.1038/nm863
Dance A., 2017, SCIENCE, V355, P1220, DOI [10.1126/science.355.6330.1220., DOI
10.1126/science.355.6330.1220]
den Haan JMM, 2014, IMMUNOL LETT, V162, P103, DOI 10.1016/j.imlet.2014.10.011
Deng LF, 2010, CLIN CANCER RES, V16, P4105, DOI 10.1158/1078-0432.CCR-10-1073
EL Andaloussi S, 2013, NAT REV DRUG DISCOV, V12, P348, DOI 10.1038/nrd3978
El-Bohy M, 2018, ANN ALLERG ASTHMA IM, V121, pS97, DOI 10.1016/j.anai.2018.09.318
Gao XJ, 2018, SCI TRANSL MED, V10, DOI 10.1126/scitranslmed.aat0195
Gehrmann U, 2014, SEMIN CANCER BIOL, V28, P58, DOI 10.1016/j.semcancer.2014.05.003
Ha D, 2016, ACTA PHARM SIN B, V6, P287, DOI 10.1016/j.apsb.2016.02.001
Hamanishi J, 2016, INT J CLIN ONCOL, V21, P462, DOI 10.1007/s10147-016-0959-z
Hargadon KM, 2013, FRONT IMMUNOL, V4, DOI 10.3389/fimmu.2013.00192
Huang SC, 2017, EMBO MOL MED, V9, P1660, DOI 10.15252/emmm.201606914
Keir ME, 2008, ANNU REV IMMUNOL, V26, P677, DOI 10.1146/annurev.immunol.26.021607.090331
Layek B, 2015, J CONTROL RELEASE, V207, P143, DOI 10.1016/j.jconrel.2015.04.014
Lee YS, 2011, EXP MOL MED, V43, P281, DOI 10.3858/emm.2011.43.5.029
Levy Michelle, 2017, Med Sci (Basel), V5, DOI 10.3390/medsci5040034
Li P, 2013, J CONTROL RELEASE, V168, P271, DOI 10.1016/j.jconrel.2013.03.025
Li WH, 2013, MOL MED REP, V8, P1876, DOI 10.3892/mmr.2013.1738
Luo ZC, 2015, BIOMATERIALS, V38, P50, DOI 10.1016/j.biomaterials.2014.10.050
Medema JP, 2013, NAT CELL BIOL, V15, P338, DOI 10.1038/ncb2717
Pitt JM, 2014, J IMMUNOL, V193, P1006, DOI 10.4049/jimmunol.1400703
Rao Q, 2016, HEPATOLOGY, V64, P456, DOI 10.1002/hep.28549
Rebelatto MC, 2016, DIAGN PATHOL, V11, DOI 10.1186/s13000-016-0545-8
Romagnoli GG, 2015, FRONT IMMUNOL, V5, DOI 10.3389/fimmu.2014.00692
Rotman J, 2019, CANCER IMMUNOL IMMUN, V68, P1681, DOI 10.1007/s00262-019-02330-y
Saha B, 2010, CYTOKINE, V50, P1, DOI 10.1016/j.cyto.2009.11.021
Salido J, 2018, INT J INFECT DIS, V73, P85, DOI 10.1016/j.ijid.2018.04.3619
Selenko-Gebauer N, 2003, J IMMUNOL, V170, P3637, DOI 10.4049/jimmunol.170.7.3637
Shi XJ, 2018, INT J CANCER, V142, P2106, DOI 10.1002/ijc.31219
Shurin GV, 2013, CANCER MICROENVIRON, V6, P159, DOI 10.1007/s12307-013-0133-3
Tamura Y, 2012, IMMUNOTHERAPY-UK, V4, P841, DOI [10.2217/IMT.12.75, 10.2217/imt.12.75]
Umar A, 2012, NAT REV CANCER, V12, P835, DOI 10.1038/nrc3397
Vandenberk L, 2016, FRONT IMMUNOL, V6, DOI 10.3389/fimmu.2015.00663
Wahlund CJE, 2017, SCI REP-UK, V7, DOI 10.1038/s41598-017-16609-6
Wang C, 2016, BIOMATERIALS, V79, P88, DOI 10.1016/j.biomaterials.2015.11.040
Wang C, 2012, VACCINE, V30, P4790, DOI 10.1016/j.vaccine.2012.05.027
Wang HJ, 2010, J HUAZHONG U SCI-MED, V30, P415, DOI 10.1007/s11596-010-0441-5
Wang JL, 2014, MOL MED REP, V9, P125, DOI 10.3892/mmr.2013.1759
Weis SM, 2011, NAT MED, V17, P1359, DOI 10.1038/nm.2537
NR 46
TC 28

Z9 35
U1 2
U2 37
PU IVYSPRING INT PUBL
PI LAKE HAVEN
PA PO BOX 4546, LAKE HAVEN, NSW 2263, AUSTRALIA
SN 1449-2288
J9 INT J BIOL SCI
JI Int. J. Biol. Sci.
PY 2020
VL 16
IS 4
BP 633
EP 643
DI 10.7150/ijbs.38414
PG 11
WC Biochemistry & Molecular Biology; Biology
WE Science Citation Index Expanded (SCI-EXPANDED)
SC Biochemistry & Molecular Biology; Life Sciences & Biomedicine - Other
   Topics
GA KD8NL
UT WOS:000508119300008
PM 32025211
OA Green Published, gold
DA 2023-09-14
ER

PT J
AU Wu, XJ
   Li, Y
   Chen, XG
   Zhou, ZZ
   Pang, JH
   Luo, X
   Kong, M
AF Wu, Xuanjin
   Li, Yang
   Chen, Xiguang
   Zhou, Zhongzheng
   Pang, Jianhui
   Luo, Xin
   Kong, Ming
TI A surface charge dependent enhanced Th1 antigen-specific immune response
   in lymph nodes by transfersome-based nanovaccine-loaded dissolving
   microneedle-assisted transdermal immunization
SO JOURNAL OF MATERIALS CHEMISTRY B
LA English
DT Article
ID DENDRITIC CELLS; TRANSCUTANEOUS IMMUNIZATION; CANCER-IMMUNOTHERAPY;
   NANOPARTICLE SIZE; DRUG-DELIVERY; IFN-GAMMA; IN-VIVO; VACCINE;
   LIPOSOMES; MUCOSAL
AB The efficient delivery of vaccines to draining lymph nodes and the induction of robust local immune responses are crucial for immunotherapy. Transdermal administration has been evidenced to facilitate the delivery of ingredients to lymph nodes. In this study, transfersomes with opposite surface charges were applied for antigen encapsulation and these were integrated with dissolving microneedles to investigate their effects on immune responses via transdermal immunization. The microneedles were easily inserted into mouse skin and achieved the local release of nanovaccines into the dermis through dissolution. Although anionic nanovaccines promoted cellular uptake via DC2.4, cationic nanovaccines exhibited stronger escape capacities from endocytic compartments, facilitating antigen processing via an MHC-I presentation pathway, and formed larger accumulations in lymph nodes. Compared with their anionic counterparts, the cationic nanovaccines more efficiently activated DC maturation and induced Th1

immunity; this was suggested by the significantly increased IgG2a/IgG1 ratio and elevated cytokine secretion from Th1 cells, without an enhancement in the Th2 response. Such an enhanced Th1 antigen-specific immune response in lymph nodes via a transdermal vaccine delivery platform is beneficial for potential immunotherapy approaches.
C1 [Wu, Xuanjin; Li, Yang; Chen, Xiguang; Zhou, Zhongzheng; Pang, Jianhui; Luo, Xin; Kong, Ming] Ocean Univ China, Coll Marine Life Sci, 5 Yushan Rd, Qingdao 266003, Shandong, Peoples R China.
[Chen, Xiguang] Qingdao Natl Lab Marine Sci & Technol, Qingdao 266000, Shandong, Peoples R China.
C3 Ocean University of China; Laoshan Laboratory
RP Kong, M (通讯作者)，Ocean Univ China, Coll Marine Life Sci, 5 Yushan Rd, Qingdao 266003, Shandong, Peoples R China.
EM kongming@ouc.edu.cn
RI chen, xi/GXH-3653-2022; chen, xin/IQW-3432-2023
FU National Natural Science Foundation of China [31670972]; Taishan Scholar Program, China
FX This work was supported by the National Natural Science Foundation of China (31670972), and the Taishan Scholar Program, China.
CR AL-Deen FMN, 2014, GENE THER, V21, P212, DOI 10.1038/gt.2013.77
Bai XY, 2018, J MATER CHEM B, V6, P5910, DOI 10.1039/c8tb02038h
Belyakov IM, 2004, J CLIN INVEST, V113, P998, DOI 10.1172/JCI200420261
Borges O, 2006, J CONTROL RELEASE, V114, P348, DOI 10.1016/j.jconrel.2006.06.011
Brewer JM, 1998, J IMMUNOL, V161, P4000
Brgles M, 2009, VACCINE, V27, P5435, DOI 10.1016/j.vaccine.2009.07.012
Butterfield LH, 2015, BMJ-BRIT MED J, V350, DOI 10.1136/bmj.h988
Cai S, 2011, ADV DRUG DELIVER REV, V63, P901, DOI 10.1016/j.addr.2011.05.017
Christensen D, 2007, EXPERT REV VACCINES, V6, P785, DOI 10.1586/14760584.6.5.785
Cortesi R, 1996, INT J PHARM, V139, P69, DOI 10.1016/0378-5173(96)04574-7
Dobrovolskaia MA, 2008, MOL PHARMACEUT, V5, P487, DOI 10.1021/mp800032f
Du GS, 2017, J CONTROL RELEASE, V266, P109, DOI 10.1016/j.jconrel.2017.09.021
FINKELMAN FD, 1988, J IMMUNOL, V140, P1022
Foged C, 2004, VACCINE, V22, P1903, DOI 10.1016/j.vaccine.2003.11.008
Fromen CA, 2015, P NATL ACAD SCI USA, V112, P488, DOI 10.1073/pnas.1422923112
Garg AD, 2017, TRENDS IMMUNOL, V38, P577, DOI 10.1016/j.it.2017.05.006
Guo L, 2013, INT J PHARMACEUT, V447, P22, DOI 10.1016/j.ijpharm.2013.02.006
HENNEY CS, 1981, NATURE, V291, P335, DOI 10.1038/291335a0
Hirobe S, 2015, BIOMATERIALS, V57, P50, DOI 10.1016/j.biomaterials.2015.04.007
Hokey DA, 2005, CANCER RES, V65, P10059, DOI 10.1158/0008-5472.CAN-05-1692
Jiao XM, 2003, HEPATOLOGY, V37, P452, DOI 10.1053/jhep.2003.50051
Jusforgues-Saklani H, 2008, J IMMUNOL, V181, P3067, DOI 10.4049/jimmunol.181.5.3067
Kennedy J, 2017, J CONTROL RELEASE, V265, P57, DOI 10.1016/j.jconrel.2017.04.022
Kong M, 2015, CHEM COMMUN, V51, P1453, DOI 10.1039/c4cc08746a
Kong M, 2011, CARBOHYD POLYM, V83, P462, DOI 10.1016/j.carbpol.2010.08.001
Kumar A, 2012, J CONTROL RELEASE, V163, P230, DOI 10.1016/j.jconrel.2012.08.011
Kuwana M, 2005, BLOOD, V105, P1552, DOI 10.1182/blood-2004-08-3145
Liu LX, 2016, ACS APPL MATER INTER, V8, P11969, DOI 10.1021/acsami.6b01135
Liu S, 2016, MOL PHARMACEUT, V13, P272, DOI 10.1021/acs.molpharmaceut.5b00765
Liu S, 2014, EUR J PHARM BIOPHARM, V86, P267, DOI 10.1016/j.ejpb.2013.10.001
Lowenfeld L, 2017, CLIN CANCER RES, V23, P2961, DOI 10.1158/1078-0432.CCR-16-1924
Lundqvist M, 2008, P NATL ACAD SCI USA, V105, P14265, DOI 10.1073/pnas.0805135105
Lutz MB, 1999, J IMMUNOL METHODS, V223, P77, DOI 10.1016/S0022-1759(98)00204-X
Lv HT, 2006, J CONTROL RELEASE, V114, P100, DOI 10.1016/j.jconrel.2006.04.014
Ma XJ, 2001, MICROBES INFECT, V3, P121, DOI 10.1016/S1286-4579(00)01359-9
MACATONIA SE, 1995, J IMMUNOL, V154, P5071
Mosmann TR, 1996, IMMUNOL TODAY, V17, P138, DOI 10.1016/0167-5699(96)80606-2
Mottram PL, 2007, MOL PHARMACEUT, V4, P73, DOI 10.1021/mp060096p
Nakanishi T, 1999, J CONTROL RELEASE, V61, P233, DOI 10.1016/S0168-3659(99)00097-8
Ott PA, 2017, NATURE, V547, P217, DOI 10.1038/nature22991
Qiao DD, 2018, NANO LETT, V18, P3007, DOI 10.1021/acs.nanolett.8b00478
Rode A, 2018, ASIAN J PHARM, V12, pS13
Rosenberg SA, 2004, NAT MED, V10, P909, DOI 10.1038/nm1100
Sahin U, 2017, NATURE, V547, P222, DOI 10.1038/nature23003
SNAPPER CM, 1988, IMMUNOL REV, V102, P51, DOI 10.1111/j.1600-065X.1988.tb00741.x

Soema PC, 2015, EUR J PHARM BIOPHARM, V94, P427, DOI 10.1016/j.ejpb.2015.06.026
Viallard JF, 1999, CLIN EXP IMMUNOL, V115, P189
Vinod KR, 2012, ACTA SCI POLON-TECHN, V11, P67
Wang C, 2016, NANO LETT, V16, P2334, DOI 10.1021/acs.nanolett.5b05030
Wang T, 2015, COLLOID SURFACE B, V126, P520, DOI 10.1016/j.colsurfb.2015.01.005
Widera G, 2006, VACCINE, V24, P1653, DOI 10.1016/j.vaccine.2005.09.049
Woof JM, 2005, SCIENCE, V310, P1442, DOI 10.1126/science.1122009
Yang HJ, 2019, INT J BIOL MACROMOL, V125, P9, DOI 10.1016/j.ijbiomac.2018.11.230
YOSHIHARA E, 1986, BIOCHIM BIOPHYS ACTA, V854, P93, DOI 10.1016/0005-2736(86)90068-4
Zaric M, 2013, ACS NANO, V7, P2042, DOI 10.1021/nn304235j
NR 55
TC 28
Z9 29
U1 2
U2 79
PU ROYAL SOC CHEMISTRY
PI CAMBRIDGE
PA THOMAS GRAHAM HOUSE, SCIENCE PARK, MILTON RD, CAMBRIDGE CB4 0WF, CAMBS, ENGLAND
SN 2050-750X
EI 2050-7518
J9 J MATER CHEM B
JI J. Mat. Chem. B
PD AUG 21
PY 2019
VL 7
IS 31
BP 4854
EP 4866
DI 10.1039/c9tb00448c
PG 13
WC Materials Science, Biomaterials
WE Science Citation Index Expanded (SCI-EXPANDED)
SC Materials Science
GA IO2XE
UT WOS:000479244300012
PM 31389952
DA 2023-09-14
ER

PT J
AU Passlick, D
Piradashvili, K
Bamberger, D
Li, MY
Jiang, S
Strand, D
Wich, PR
Landfester, K
Bros, M
Grabbe, S
Mailander, V
AF Passlick, David
Piradashvili, Keti
Bamberger, Denise
Li, Mengyi
Jiang, Shuai
Strand, Dennis
Wich, Peter R.
Landfester, Katharina
Bros, Matthias

Grabbe, Stephan
Mailaender, Volker
TI Delivering all in one: Antigen-nanocapsule loaded with dual adjuvant yields superadditive effects by DC-directed T cell stimulation
SO JOURNAL OF CONTROLLED RELEASE
LA English
DT Article
DE Nanocarrier; Vaccine; Adjuvants; Antigen; R848; MDP
ID DENDRITIC CELLS; IMMUNE-RESPONSES; ACETALATED DEXTRAN; RECEPTOR AGONISTS; CANCER VACCINES; VIRUS; NANOPARTICLES; VACCINATION; INDUCTION; LIGANDS
AB Therapeutic vaccination is and remains a major challenge, particularly in cancer treatment. In this process, the effective activation of dendritic cells by a combination of distinctly acting adjuvants and an antigen is crucial for success. While most common vaccine formulations lack the efficiency to trigger sufficient T cell responses in a therapeutic tumor treatment, nanovaccines offer unique properties to tackle that challenge.

Here, we report the stepwise development of a nanocapsule for vaccination approaches, comprising a shell consisting of antigen and loaded with a superadditive adjuvant combination. In a first initial step, we identified the combination of resiquimod (R848) and muramyl dipeptide (MDP) to have a superadditive stimulatory potential. Particulated in Spermine-modified dextran-nanoparticles, the dual-adjuvant maintains its superadditive character and stimulates murine dendritic cells (DC) stronger than the soluble equivalents. The second step was to evaluate a protein-based nanocapsule as suitable antigen source for the induction of antigen-specific T cell responses. Therefore, the DC-mediated antigen-specific T cell proliferation upon treatment with nanocapsules, whose shell consists of ovalbumin (OVA), was assessed. At least, the superadditive adjuvant combination was encapsulated into OVA-nanocapsules to create the final nanovaccine. Its immunostimulatory potential for DC was extensively tested by measuring the expression of co-stimulatory surface markers, the secretion of pro-inflammatory cytokines and the capability to mediate OVA-specific T cell responses.

The developed nanovaccine triggers strong superadditive dendritic cell stimulation and potent antigen-specific CD4(+) and CD8(+) T cell proliferation. Combined with a high modifiability, an excellent biocompatibility, low cytotoxicity and an enormous loading capacity, the introduced antigen-nanocapsule provides an enormous potential for the effective delivery of superadditive adjuvant combinations, particularly when they target intracellular receptors.
C1 [Passlick, David; Bros, Matthias; Grabbe, Stephan; Mailaender, Volker] Univ Med Ctr Mainz, Dermatol Clin, Langenbeckstr 1, D-55131 Mainz, Germany.
[Passlick, David; Piradashvili, Keti; Li, Mengyi; Jiang, Shuai; Landfester, Katharina; Mailaender, Volker] Max Planck Inst Polymer Res, Ackermannweg 10, D-55128 Mainz, Germany.
[Bamberger, Denise; Wich, Peter R.] Johannes Gutenberg Univ Mainz, Inst Pharm & Biochem, Staudingerweg 5, D-55128 Mainz, Germany.
[Strand, Dennis] Univ Med Ctr Mainz, Med Clin 1, Langenbeckstr 1, D-55131 Mainz, Germany.
C3 Johannes Gutenberg University of Mainz; Max Planck Society; Johannes Gutenberg University of Mainz; Johannes Gutenberg University of Mainz
RP Mailander, V (通讯作者)，Univ Med Ctr Mainz, Dermatol Clin, Langenbeckstr 1, D-55131 Mainz, Germany.
EM volker.mailaender@mpip-mainz.mpg.de
RI Grabe, Stephan/AAJ-4578-2021; Landfester, Katharina/F-9449-2011; Wich, Peter/D-4005-2014; Mailänder, Volker/H-3589-2012; Mailänder, Volker/AAD-1722-2019; Jiang, Shuai/N-9387-2017
OI Grabe, Stephan/0000-0002-6863-8719; Landfester, Katharina/0000-0001-9591-4638; Wich, Peter/0000-0003-1522-0340; Mailänder, Volker/0000-0001-6583-8136; Mailänder, Volker/0000-0001-6583-8136; Jiang, Shuai/0000-0002-8639-3495
FU Max Planck Graduate School (MPGC); [DFG/SFB1066]
FX This work was funded by the Max Planck Graduate School (MPGC) and the DFG/SFB1066 ('Nanodimensional polymer therapeutics for tumor therapy').
CR Almeida JPM, 2015, SMALL, V11, P1453, DOI 10.1002/smll.201402179
Bachelder EM, 2010, MOL PHARMACEUT, V7, P826, DOI 10.1021/mp900311x
Bergmann-Leitner ES, 2014, VACCINES-BASEL, V2, P252, DOI 10.3390/vaccines2020252
Broaders KE, 2009, P NATL ACAD SCI USA, V106, P5497, DOI 10.1073/pnas.0901592106
Bros M, 2007, BLOOD, V109, P3820, DOI 10.1182/blood-2006-07-035576
Bros M, 2016, INT IMMUNOPHARMACOL, V35, P174, DOI 10.1016/j.intimp.2016.03.025

Cardona PJ, 2016, FRONT MICROBIOL, V7, DOI 10.3389/fmicb.2016.01536
Cohen JL, 2011, BIOCONJUGATE CHEM, V22, P1056, DOI 10.1021/bc100542r
Elster JD, 2016, HUM VACC IMMUNOTHER, V12, P2232, DOI 10.1080/21645515.2016.1179844
Escamilla-Tilch M, 2013, IMMUNOL CELL BIOL, V91, P601, DOI 10.1038/icb.2013.58
Fichter M, 2016, BIOMATERIALS, V108, P1, DOI 10.1016/j.biomaterials.2016.08.046
Foerster F, 2016, NANOMEDICINE-UK, V11, P2663, DOI 10.2217/nnm-2016-0156
Fritz JH, 2005, EUR J IMMUNOL, V35, P2459, DOI 10.1002/eji.200526286
Gardner A, 2016, TRENDS IMMUNOL, V37, P855, DOI 10.1016/j.it.2016.09.006
Garrett WS, 2000, CELL, V102, P325, DOI 10.1016/S0092-8674(00)00038-6
Goldinger SM, 2012, EUR J IMMUNOL, V42, P3049, DOI 10.1002/eji.201142361
GREENBERG PD, 1991, ADV IMMUNOL, V49, P281
Hickman-Miller HD, 2006, NAT IMMUNOL, V7, P125, DOI 10.1038/ni0206-125
Khan H, 2015, J ONCOL, V2015, DOI 10.1155/2015/847383
Krummen M, 2010, J LEUKOCYTE BIOL, V88, P189, DOI 10.1189/jlb.0408228
Lim TS, 2012, PLOS ONE, V7, DOI 10.1371/journal.pone.0045185
Marina-Garcia N, 2009, J IMMUNOL, V182, P4321, DOI 10.4049/jimmunol.0802197
McArthur HL, 2016, CLIN ADV HEMATOL ONC, V14, P922
Nejad EB, 2017, EXPERT OPIN BIOL TH, V17, P87, DOI 10.1080/14712598.2017.1256388
Ornelas-Megiatto C, 2012, MOL PHARMACEUT, V9, P3012, DOI 10.1021/mp3004379
Pavot V, 2014, J IMMUNOL, V193, P5781, DOI 10.4049/jimmunol.1402184
Pavot V, 2013, J CONTROL RELEASE, V167, P60, DOI 10.1016/j.jconrel.2013.01.015
Pietrzak-Nguyen A, 2016, NANOMED-NANOTECHNOL, V12, P2383, DOI 10.1016/j.nano.2016.07.010
Piradashvili K, 2015, BIOMACROMOLECULES, V16, P815, DOI 10.1021/bm5016915
ROBINS RA, 1986, BIOCHIM BIOPHYS ACTA, V865, P289, DOI 10.1016/0304-419X(86)90019-3
Santana-Davila R, 2017, JAMA ONCOL, V3, P106, DOI 10.1001/jamaoncol.2016.4574
Satoh T., 2016, MICROBIOL SPECTR, P4
Schlaad H, 2001, MACROMOLECULES, V34, P4302, DOI 10.1021/ma010311+
Schwarz H, 2013, IMMUNOBIOLOGY, V218, P533, DOI 10.1016/j.imbio.2012.06.007
Shafique M, 2012, VACCINE, V30, P597, DOI 10.1016/j.vaccine.2011.11.054
Silva JM, 2015, J CONTROL RELEASE, V198, P91, DOI 10.1016/j.jconrel.2014.11.033
Singh SM, 2016, VACCINE, V34, P4807, DOI 10.1016/j.vaccine.2016.08.009
Sprent J, 2002, ANNU REV IMMUNOL, V20, P551, DOI 10.1146/annurev.immunol.20.100101.151926
Sultan H, 2017, CANCER IMMUNOL IMMUN, V66, P203, DOI 10.1007/s00262-016-1834-5
Tada H, 2005, INFECT IMMUN, V73, P7967, DOI 10.1128/IAI.73.12.7967-7976.2005
Thiel M, 2010, IMMUNOLOGY, V129, P28, DOI 10.1111/j.1365-2567.2009.03155.x
Toussirot Eric, 2015, Inflammation & Allergy Drug Targets, V14, P94, DOI 10.2174/1871528114666160105113046
Trombetta ES, 2005, ANNU REV IMMUNOL, V23, P975, DOI 10.1146/annurev.immunol.22.012703.104538
Tsukahara T, 2016, PATHOL INT, V66, P547, DOI 10.1111/pin.12438
Uehara A, 2005, CELL MICROBIOL, V7, P53, DOI 10.1111/j.1462-5822.2004.00433.x
Vajjhala PR, 2017, MOL IMMUNOL, V86, P23, DOI 10.1016/j.molimm.2017.02.012
van Heel DA, 2005, LANCET, V365, P1794, DOI 10.1016/S0140-6736(05)66582-8
Vesely MD, 2011, ANNU REV IMMUNOL, V29, P235, DOI 10.1146/annurev-immunol-031210-101324
ZAL T, 1994, J EXP MED, V180, P2089, DOI 10.1084/jem.180.6.2089
Zou Y, 2016, FEMS MICROBIOL LETT, V363, DOI 10.1093/femsle/fnw170
NR 50
TC 28
Z9 31
U1 0
U2 63
PU ELSEVIER SCIENCE BV
PI AMSTERDAM
PA PO BOX 211, 1000 AE AMSTERDAM, NETHERLANDS
SN 0168-3659
EI 1873-4995
J9 J CONTROL RELEASE
JI J. Control. Release
PD NOV 10
PY 2018
VL 289

BP 23
EP 34
DI 10.1016/j.jconrel.2018.09.008
PG 12
WC Chemistry, Multidisciplinary; Pharmacology & Pharmacy
WE Science Citation Index Expanded (SCI-EXPANDED)
SC Chemistry; Pharmacology & Pharmacy
GA GY2TI
UT WOS:000448396000004
PM 30219277
DA 2023-09-14
ER

PT J
AU Zhao, Z
Powers, K
Hu, Y
Raleigh, M
Pentel, P
Zhang, CM
AF Zhao, Zongmin
Powers, Kristen
Hu, Yun
Raleigh, Michael
Pentel, Paul
Zhang, Chenming
TI Engineering of a hybrid nanoparticle-based nicotine nanovaccine as a
next-generation immunotherapeutic strategy against nicotine addiction: A
focus on hapten density
SO BIOMATERIALS
LA English
DT Article
DE Nicotine addiction; Nicotine vaccine; Hybrid nanoparticle; Antibody;
Hapten density
ID SMOKING-CESSATION; DENDRITIC CELLS; CANCER-IMMUNOTHERAPY; REPLACEMENT
THERAPY; VACCINE; IMMUNOGENICITY; DELIVERY; SIZE; ANTIBODIES; EFFICACY
AB Although vaccination is a promising way to combat nicotine addiction, most traditional hapten-protein conjugate nicotine vaccines only show limited efficacy due to their poor recognition and uptake by immune cells. This study aimed to develop a hybrid nanoparticle-based nicotine vaccine with improved efficacy. The focus was to study the impact of hapten density on the immunological efficacy of the proposed hybrid nanovaccine. It was shown that the nanovaccine nanoparticles were taken up by the dendritic cells more efficiently than the conjugate vaccine, regardless of the hapten density on the nanoparticles. At a similar hapten density, the nanovaccine induced a significantly stronger immune response against nicotine than the conjugate vaccine in mice. Moreover, the high- and medium-density nanovaccines resulted in significantly higher anti-nicotine antibody titers than their low-density counterpart. Specifically, the high-density nanovaccine exhibited better immunogenic efficacy, resulting in higher anti-nicotine antibody titers and lower anti-carrier protein antibody titers than the medium- and low-density versions. The high-density nanovaccine also had the best ability to retain nicotine in serum and to block nicotine from entering the brain. These results suggest that the hybrid nanoparticle-based nicotine vaccine can elicit strong immunogenicity by modulating the hapten density, thereby providing a promising next-generation immunotherapeutic strategy against nicotine addiction. (C) 2017 Elsevier Ltd. All rights reserved.
C1 [Zhao, Zongmin; Zhang, Chenming] Virginia Tech, Dept Biol Syst Engn, Blacksburg, VA 24061 USA.
[Powers, Kristen] Virginia Tech, Dept Biol Sci, Blacksburg, VA 24061 USA.
[Raleigh, Michael; Pentel, Paul] Minneapolis Med Res Fdn Inc, 2211 Pk Ave S, Minneapolis, MN 55404 USA.
C3 Virginia Polytechnic Institute & State University; Virginia Polytechnic
Institute & State University; Minneapolis Medical Research Foundation
RP Zhang, CM (通讯作者)，Virginia Tech, Dept Biol Syst Engn, Blacksburg, VA 24061 USA.
EM chzhang2@vt.edu
RI Zhao, Zongmin/I-9864-2019

OI Zhao, Zongmin/0000-0001-8979-844X
FU National Institute on Drug Abuse [U01DA036850]
FX This work was financially supported by National Institute on Drug Abuse
(U01DA036850). We thank Ms. Kathy Lowe for her assistance on TEM imaging
of NPs. We thank Dr. Tanya LeRoith for providing help on
histopathological analysis.
CR Bachmann MF, 2010, NAT REV IMMUNOL, V10, P787, DOI 10.1038/nri2868
Banchereau J, 1998, NATURE, V392, P245, DOI 10.1038/32588
Benne N, 2016, J CONTROL RELEASE, V234, P124, DOI 10.1016/j.jconrel.2016.05.033
Benowitz NL, 2010, NEW ENGL J MED, V362, P2295, DOI 10.1056/NEJMra0809890
Carpenter MJ, 2013, DRUGS, V73, P407, DOI 10.1007/s40265-013-0038-y
Chen XY, 2012, VACCINE, V31, P159, DOI 10.1016/j.vaccine.2012.10.069
Collins KC, 2014, BIOCONJUGATE CHEM, V25, P593, DOI 10.1021/bc500016k
Cornish KE, 2013, PLOS ONE, V8, DOI 10.1371/journal.pone.0082557
Cornuz J, 2008, PLOS ONE, V3, DOI 10.1371/journal.pone.0002547
De Biasi M, 2014, DRUG ALCOHOL DEPEN, V141, P107, DOI 10.1016/j.drugalcdep.2014.05.013
de Villiers SHL, 2013, VACCINE, V31, P6185, DOI 10.1016/j.vaccine.2013.10.051
de Villiers SHL, 2010, VACCINE, V28, P2161, DOI 10.1016/j.vaccine.2009.12.051
Foged C, 2005, INT J PHARMACEUT, V298, P315, DOI 10.1016/j.ijpharm.2005.03.035
Harris JR, 1999, MICRON, V30, P597, DOI 10.1016/S0968-4328(99)00036-0
Hatsukami DK, 2011, CLIN PHARMACOL THER, V89, P392, DOI 10.1038/clpt.2010.317
Hieda Y, 1997, J PHARMACOL EXP THER, V283, P1076
Hu Y, 2014, NANOSCALE RES LETT, V9, DOI 10.1186/1556-276X-9-434
Keyler DE, 2008, INT IMMUNOPHARMACOL, V8, P1589, DOI 10.1016/j.intimp.2008.07.001
Liu J, 2016, BIOMATERIALS, V91, P44, DOI 10.1016/j.biomaterials.2016.03.013
Liu XW, 2012, NANO LETT, V12, P4254, DOI 10.1021/nl301877k
Lockner JW, 2015, J MED CHEM, V58, P1005, DOI 10.1021/jm501625j
Lockner JW, 2013, BIOORG MED CHEM LETT, V23, P975, DOI 10.1016/j.bmcl.2012.12.048
Maurer P, 2005, EUR J IMMUNOL, V35, P2031, DOI 10.1002/eji.200526285
McCarthy DE, 2008, NICOTINE TOB RES, V10, P717, DOI 10.1080/14622200801968343
McCluskie MJ, 2015, INT IMMUNOPHARMACOL, V25, P518, DOI 10.1016/j.intimp.2015.02.030
Meijler MM, 2003, J AM CHEM SOC, V125, P7164, DOI 10.1021/ja034805t
Miller K. D., 2014, PLOS ONE, V9
Oussoren C, 1997, BBA-BIOMEMBRANES, V1328, P261, DOI 10.1016/S0005-2736(97)00122-3
Pentel PR, 2014, ADV PHARMACOL, V69, P553, DOI 10.1016/B978-0-12-420118-7.00014-7
Pravetoni M, 2012, BIOCHEM PHARMACOL, V83, P543, DOI 10.1016/j.bcp.2011.11.004
Pravetoni M, 2011, BIOCHEM PHARMACOL, V81, P1164, DOI 10.1016/j.bcp.2011.02.004
Pryde DC, 2013, PLOS ONE, V8, DOI 10.1371/journal.pone.0076557
Qian Y, 2016, BIOMATERIALS, V98, P171, DOI 10.1016/j.biomaterials.2016.05.008
Rahimian S, 2015, BIOMATERIALS, V37, P469, DOI 10.1016/j.biomaterials.2014.10.043
Raupach Tobias, 2012, Drugs, V72, pe1, DOI 10.2165/11599900-000000000-00000
Reddy ST, 2007, NAT BIOTECHNOL, V25, P1159, DOI [10.1038/nbt1332, 10.1038/nbtl332]
Rosalia RA, 2015, BIOMATERIALS, V40, P88, DOI 10.1016/j.biomaterials.2014.10.053
Saylor K, 2016, TOXICOL APPL PHARM, V307, P150, DOI 10.1016/j.taap.2016.07.017
Shen XY, 2012, CLIN PHARMACOL THER, V91, P60, DOI 10.1038/clpt.2011.281
Stapleton JA, 2008, ADDICTION, V103, P146, DOI 10.1111/j.1360-0443.2007.02083.x
Storni T, 2005, ADV DRUG DELIVER REV, V57, P333, DOI 10.1016/j.addr.2004.09.008
Thangavel S, 2016, BIOMATERIALS, V99, P109, DOI 10.1016/j.biomaterials.2016.05.001
Wang C, 2016, BIOMATERIALS, V79, P88, DOI 10.1016/j.biomaterials.2015.11.040
Yearn J. H., 2016, BIOMATERIALS, V104, P43
Zheng H, 2015, J BIOMED NANOTECHNOL, V11, P2197, DOI 10.1166/jbn.2015.2156
NR 45
TC 28
Z9 28
U1 1
U2 48
PU ELSEVIER SCI LTD
PI OXFORD
PA THE BOULEVARD, LANGFORD LANE, KIDLINGTON, OXFORD OX5 1GB, OXON, ENGLAND
SN 0142-9612
EI 1878-5905

J9 BIOMATERIALS
JI Biomaterials
PD APR
PY 2017
VL 123
BP 107
EP 117
DI 10.1016/j.biomaterials.2017.01.038
PG 11
WC Engineering, Biomedical; Materials Science, Biomaterials
WE Science Citation Index Expanded (SCI-EXPANDED)
SC Engineering; Materials Science
GA EM8YO
UT WOS:000395597800009
PM 28167389
OA Green Accepted
DA 2023-09-14
ER

PT J
AU Zhang, D
Lin, ZG
Wu, M
Cai, ZX
Zheng, YS
He, L
Li, ZL
Zhou, J
Sun, LQ
Chen, G
Zeng, YY
Li, J
Liu, JF
Yang, HH
Liu, XL
AF Zhang, Da
Lin, Ziguo
Wu, Ming
Cai, Zhixiong
Zheng, Youshi
He, Lei
Li, Zhenli
Zhou, Jie
Sun, Liqin
Chen, Geng
Zeng, Yongyi
Li, Juan
Liu, Jingfeng
Yang, Huanghao
Liu, Xiaolong
TI Cytosolic Delivery of Thiolated Neoantigen Nano-Vaccine Combined with
Immune Checkpoint Blockade to Boost Anti-Cancer T Cell Immunity
SO ADVANCED SCIENCE
LA English
DT Article
DE cytosolic delivery; immune checkpoint; neoantigen; T-cells immunity;
thiolated nano-vaccine
ID CROSS-PRESENTATION; CANCER; PD-1; IMMUNOTHERAPY
AB Although tumor-specific neoantigen-based cancer vaccines hold tremendous potential, it still faces low cross-presentation associated with severe degradation via endocytosis pathway. Herein, a thiolated nano-vaccine allowing direct cytosolic delivery of neoantigen and Toll like receptor 9 agonist CpG-ODN is

developed. This approach is capable of bypassing the endo-/lysosome degradation, increasing uptake and local concentration of neoantigen and CpG-ODN to activate antigen-presenting cells, significantly strengthening the anti-cancer T-cell immunity. In vivo immunization with thiolated nano-vaccine enhanced the lymph organ homing and promoted the antigen presentation on dendritic cells, effectively inhibited tumor growth, and significantly prolonged the survival of H22-bearing mice. Strikingly, further combination of the thiolated nano-vaccine with anti-programmed cell death protein-1 antibody (alpha PD-1) could efficiently reverse immunosuppression and enhance response rate of tumors, which led to enhanced tumor elimination, complete prevention of tumor re-challenge, and long-term survival above 150 d. Collectively, a versatile methodology to design cancer vaccines for strengthening anti-cancer T-cell immunity in solid tumors is presented, which could be further remarkably enhanced by combining with immune checkpoint inhibitors.
C1 [Zhang, Da; Lin, Ziguo; Wu, Ming; Cai, Zhixiong; Zheng, Youshi; He, Lei; Li, Zhenli; Chen, Geng; Zeng, Yongyi; Liu, Jingfeng; Liu, Xiaolong] Fujian Med Univ, United Innovat Mengchao Hepatobiliary Technol, Key Lab Fujian Prov, Mengchao Hepatobiliary Hosp, Fuzhou 350025, Peoples R China.
[Zhang, Da; Zhou, Jie; Sun, Liqin; Li, Juan; Yang, Huanghao] Fuzhou Univ, Key Lab Anal & Detect Technol Food Safety MOE, Fujian Prov Key Lab Anal & Detect Technol Food Sa, Coll Chem, Fuzhou 350002, Peoples R China.
[Wu, Ming; Cai, Zhixiong; Zheng, Youshi; He, Lei; Li, Zhenli; Chen, Geng; Zeng, Yongyi; Liu, Jingfeng; Liu, Xiaolong] Fuzhou Univ, Mengchao Med X Ctr, Fuzhou 350116, Peoples R China.
[Liu, Jingfeng; Liu, Xiaolong] Chinese Acad Sci, CAS Key Lab Design & Assembly Funct Nanostruct, Fujian Inst Res Struct Matter, Fuzhou 350002, Peoples R China.
C3 Fujian Medical University; Fuzhou University; Fuzhou University; Chinese Academy of Sciences; Fujian Institute of Research on the Structure of Matter, CAS
RP Liu, JF; Liu, XL (通讯作者)，Fujian Med Univ, United Innovat Mengchao Hepatobiliary Technol, Key Lab Fujian Prov, Mengchao Hepatobiliary Hosp, Fuzhou 350025, Peoples R China.; Yang, HH (通讯作者)，Fuzhou Univ, Key Lab Anal & Detect Technol Food Safety MOE, Fujian Prov Key Lab Anal & Detect Technol Food Sa, Coll Chem, Fuzhou 350002, Peoples R China.; Liu, JF; Liu, XL (通讯作者)，Fuzhou Univ, Mengchao Med X Ctr, Fuzhou 350116, Peoples R China.; Liu, JF; Liu, XL (通讯作者)，Chinese Acad Sci, CAS Key Lab Design & Assembly Funct Nanostruct, Fujian Inst Res Struct Matter, Fuzhou 350002, Peoples R China.
EM drjingfeng@126.com; hhyang@fzu.edu.cn; xiaoloong.liu@gmail.com
RI Cai, Zhixiong/ABA-9580-2021; lin, ziguo/IZQ-2952-2023
OI Zhang, Da/0000-0002-2613-1354
FU National Natural Science Foundation of China [61727823, 81871483, 61805041, 91959102]; Program for Changjiang Scholars and Innovative Research Team in University [IRT15R11]; Joint Research Projects of Health and Education Commission of Fujian Province [2019-WJ-12, 2019-WJ-20]; Major State Basic Research Development Program of China [2017YFA0205201]; medical innovation project of Fujian province [2018-CX-49]; Guiding Project of Fujian Provincial Department of Science Technology [2019Y0013]; Natural Science Foundation of Fujian Province of China [2020J011154]
FX D.Z., Zi.L., and M.W. contributed equally to this work. The authors thank Prof. Zhaowei Chen of Fuzhou University to improve the English. This work was supported by the National Natural Science Foundation of China (grant no. 61727823, 81871483, 61805041, 91959102); the Program for Changjiang Scholars and Innovative Research Team in University (No. IRT15R11); the Joint Research Projects of Health and Education Commission of Fujian Province (2019-WJ-12, 2019-WJ-20); the Major State Basic Research Development Program of China (2017YFA0205201); the medical innovation project of Fujian province (grant no. 2018-CX-49); the Guiding Project of Fujian Provincial Department of Science & Technology (grant no. 2019Y0013). Natural Science Foundation of Fujian Province of China (Grant No. 2020J011154).
CR Ackerman AL, 2004, NAT IMMUNOL, V5, P678, DOI 10.1038/ni1082
Allahyari M, 2016, HUM VACC IMMUNOTHER, V12, P806, DOI 10.1080/21645515.2015.1102804
Bavandpour AK, 2020, INT J PHARMACEUT, V573, DOI 10.1016/j.ijpharm.2019.118868
Chuard N, 2015, ORG BIOMOL CHEM, V13, P64, DOI 10.1039/c4ob02060j
Conniot J, 2019, NAT NANOTECHNOL, V14, P891, DOI 10.1038/s41565-019-0512-0
Fan ZJ, 2021, BIOACT MATER, V6, P312, DOI 10.1016/j.bioactmat.2020.08.005

Gajewski TF, 2013, NAT IMMUNOL, V14, P1014, DOI 10.1038/ni.2703
Hellmann MD, 2019, NAT MED, V25, P203, DOI 10.1038/s41591-019-0350-5
Hilton HG, 2019, PLOS BIOL, V17, DOI 10.1371/journal.pbio.3000528
Hung S.-I., 2018, J IMMUNOL RES, V2018, P2314
Keskin DB, 2019, NATURE, V565, P234, DOI 10.1038/s41586-018-0792-9
Kuai R, 2018, SCI ADV, V4, DOI 10.1126/sciadv.aao1736
Kuai R, 2017, NAT MATER, V16, P489, DOI [10.1038/nmat4822, 10.1038/NMAT4822]
Labanieh L, 2018, NAT BIOMED ENG, V2, P377, DOI 10.1038/s41551-018-0235-9
Lee CH, 2018, TRENDS IMMUNOL, V39, P536, DOI 10.1016/j.it.2018.04.005
Li F, 2017, ADV MATER, V29, DOI 10.1002/adma.201602432
Liang LL, 2018, CELL MOL BIOL LETT, V23, DOI 10.1186/s11658-018-0122-0
Llovet JM, 2003, LANCET, V362, P1907, DOI 10.1016/S0140-6736(03)14964-1
Lynn GM, 2020, NAT BIOTECHNOL, V38, P320, DOI 10.1038/s41587-019-0390-x
Matsiko A, 2018, NAT MATER, V17, P472, DOI 10.1038/s41563-018-0091-8
Melief CJM, 2008, NAT REV CANCER, V8, P351, DOI 10.1038/nrc2373
Min YZ, 2017, NAT NANOTECHNOL, V12, P877, DOI [10.1038/nnano.2017.113, 10.1038/NNANO.2017.113]
Ni QQ, 2020, SCI ADV, V6, DOI 10.1126/sciadv.aaw6071
Rizvi NA, 2015, SCIENCE, V348, P124, DOI 10.1126/science.aaa1348
Schumacher TN, 2019, ANNU REV IMMUNOL, V37, P173, DOI 10.1146/annurev-immunol-042617-053402
Schumacher TN, 2015, SCIENCE, V348, P69, DOI 10.1126/science.aaa4971
Shae D, 2019, NAT NANOTECHNOL, V14, P269, DOI 10.1038/s41565-018-0342-5
Shah NJ, 2020, NAT BIOMED ENG, V4, P40, DOI 10.1038/s41551-019-0503-3
Shen H, 2006, IMMUNOLOGY, V117, P78, DOI 10.1111/j.1365-2567.2005.02268.x
Snyder A, 2015, CURR OPIN GENET DEV, V30, P7, DOI 10.1016/j.gde.2014.12.003
van Kooyk Y, 2019, NAT MATER, V18, P94, DOI 10.1038/s41563-018-0267-2
Vartak A, 2016, VACCINES-BASEL, V4, DOI 10.3390/vaccines4020012
Villanueva A, 2019, NEW ENGL J MED, V380, P1450, DOI 10.1056/NEJMra1713263
Vivier E, 2012, NAT REV IMMUNOL, V12, P239, DOI 10.1038/nri3174
Wilson DS, 2019, NAT MATER, V18, P175, DOI 10.1038/s41563-018-0256-5
Wu M, 2020, ISCIENCE, V23, DOI 10.1016/j.isci.2020.101341
Yang JD, 2019, NAT REV GASTRO HEPAT, V16, P589, DOI 10.1038/s41575-019-0186-y
Yarchoan M, 2019, JCI INSIGHT, V4, DOI 10.1172/jci.insight.126908
Zak KM, 2017, STRUCTURE, V25, P1163, DOI 10.1016/j.str.2017.06.011
Zeng YY, 2020, J IMMUNOTHER CANCER, V8, DOI 10.1136/jitc-2019-000217
Zhang D, 2021, ADV SCI, V8, DOI 10.1002/advs.202003504
Zhang D, 2020, ACS NANO, V14, P8985, DOI 10.1021/acsnano.0c03833
Zhang YY, 2020, CELL MOL IMMUNOL, V17, P807, DOI 10.1038/s41423-020-0488-6
Zhou J, 2019, ANGEW CHEM INT EDIT, V58, P5236, DOI 10.1002/anie.201813665
Zhu GZ, 2017, ACS NANO, V11, P2387, DOI 10.1021/acsnano.7b00978
Zou WP, 2016, SCI TRANSL MED, V8, DOI 10.1126/scitranslmed.aad7118
NR 46
TC 27
Z9 28
U1 6
U2 107
PU WILEY
PI HOBOKEN
PA 111 RIVER ST, HOBOKEN 07030-5774, NJ USA
EI 2198-3844
J9 ADV SCI
JI Adv. Sci.
PD MAR
PY 2021
VL 8
IS 6
AR 2003504
DI 10.1002/advs.202003504
EA JAN 2021
PG 12

WC Chemistry, Multidisciplinary; Nanoscience & Nanotechnology; Materials
Science, Multidisciplinary
WE Science Citation Index Expanded (SCI-EXPANDED)
SC Chemistry; Science & Technology - Other Topics; Materials Science
GA QY5DL
UT WOS:000612809500001
PM 33747739
OA gold, Green Published
DA 2023-09-14
ER

PT J
AU Ruiz-de-Angulo, A
Bilbao-Asensio, M
Cronin, J
Evans, SJ
Clift, MJD
Llop, J
Feiner, IVJ
Beadman, R
Bascaran, KZ
Mareque-Rivas, JC
AF Ruiz-de-Angulo, Ane
Bilbao-Asensio, Marc
Cronin, James
Evans, Stephen J.
Clift, Martin J. D.
Llop, Jordi
Feiner, Irene V. J.
Beadman, Rhiannon
Bascaran, Kepa Zamacola
Mareque-Rivas, Juan C.
TI Chemically Programmed Vaccines: Iron Catalysis in Nanoparticles Enhances
Combination Immunotherapy and Immunotherapy-Promoted Tumor Ferroptosis
SO ISCIENCE
LA English
DT Article
ID IFN-GAMMA; CANCER-IMMUNOTHERAPY; PD-1 BLOCKADE; IMMUNITY; ANTIGEN;
CELLS; MICROENVIRONMENT; DELIVERY; NANOMEDICINE; CONJUGATION
AB Immunotherapy has yielded impressive results, but only for a minority of patients with cancer. Therefore, new approaches that potentiate immunotherapy are a pressing medical need. Ferroptosis is a newly described type of programmed cell death driven by iron-dependent phospholipid peroxidation via Fenton chemistry. Here, we developed iron oxide-loaded nanovaccines (IONVs), which, chemically programmed to integrate iron catalysis, drug delivery, and tracking exploiting the characteristics of the tumor microenvironment (TME), improves immunotherapy and activation of ferroptosis. The IONVs trigger danger signals and use molecular disassembly and reversible covalent bonds for targeted antigen delivery and improved immunostimulatory capacity and catalytic iron for targeting tumor cell ferroptosis. IONV- and antibody-mediated TME modulation interfaced with imaging was important toward achieving complete eradication of aggressive and established tumors, eliciting long-lived protective antitumor immunity with no toxicities. This work establishes the feasibility of using nanoparticle iron catalytic activity as a versatile and effective feature for enhancing immunotherapy.
C1 [Bilbao-Asensio, Marc; Mareque-Rivas, Juan C.] Swansea Univ, Dept Chem, Singleton Pk, Swansea SA2 8PP, W Glam, Wales.
[Bilbao-Asensio, Marc; Mareque-Rivas, Juan C.] Swansea Univ, Ctr NanoHlth, Singleton Pk, Swansea SA2 8PP, W Glam, Wales.
[Llop, Jordi; Feiner, Irene V. J.; Bascaran, Kepa Zamacola] CIC BiomaGUNE, Radiochem & Nucl Imaging Lab, Paseo Miramon 182, San Sebastian 20014, Spain.
[Ruiz-de-Angulo, Ane] CIC BioGUNE, Chem Immunol Lab, Bldg 801A, Derio 48160, Spain.
[Cronin, James; Evans, Stephen J.; Clift, Martin J. D.; Beadman, Rhiannon] Swansea Univ, Sch Med, Inst Life Sci, Singleton Pk, Swansea SA2 8PP, W Glam, Wales.
C3 Swansea University; Swansea University; CIC biomaGUNE; CIC bioGUNE;

Swansea University
RP Mareque-Rivas, JC (通讯作者)，Swansea Univ, Dept Chem, Singleton Pk, Swansea SA2 8PP, W Glam, Wales.; Mareque-Rivas, JC (通讯作者)，Swansea Univ, Ctr NanoHlth, Singleton Pk, Swansea SA2 8PP, W Glam, Wales.; Ruiz-de-Angulo, A (通讯作者)，CIC BioGUNE, Chem Immunol Lab, Bldg 801A, Derio 48160, Spain.
EM aruizdeangulo@cicbiogune.es; juan.mareque-rivas@swansea.ac.uk
RI Llop, Jordi/F-5496-2014; Rivas, Juan Mareque/M-6171-2014; Ruiz de Angulo, Ane/I-3867-2016
OI Llop, Jordi/0000-0002-0821-9838; Rivas, Juan Mareque/0000-0002-7405-3338; Ruiz de Angulo, Ane/0000-0001-6581-4038; Bilbao-Asensio, Marc/0000-0002-2862-403X; Cronin, James/0000-0002-0590-9462; CLIFT, MARTIN/0000-0001-6133-3368; Feiner, Irene V. J./0000-0001-6938-9745
FU EPSRC PhD Studentship [EP/N509553/1, EP/R51312X/1]; Spanish Ministry of Economy and Competitiveness (MINECO) [CTQ2017-87637-R, PCIN2016-085]
FX J.C.M.-R. and J.L. thank MediSURF project (PCIN2016-085) from the Spanish Ministry of Economy and Competitiveness (MINECO). This work was supported by an EPSRC PhD Studentship to M.B.-A. and R.B. (EP/N509553/1 and EP/R51312X/1). J.L. acknowledges financial support from the Spanish Ministry of Economy and Competitiveness (MINECO), grant number CTQ2017-87637-R. The authors would like to thank Dr. Vanessa Go ' mez-Vallejo and Vi ' ctor Salinas for <SUP>89</SUP>Zr production, Zurine Baz for assistance in image quantification, April Rees for flow cytometry studies, and Dr. Asi ' s Palazo ' n (bioGUNE) and Dr. Nick Jones (Swansea Medical School) for critically reading the manuscript.
CR Almeida JPM, 2014, NANOMED-NANOTECHNOL, V10, P503, DOI 10.1016/j.nano.2013.09.011
Anderson KG, 2017, CANCER CELL, V31, P311, DOI 10.1016/j.ccell.2017.02.008
Aspeslagh S, 2016, EUR J CANCER, V52, P50, DOI 10.1016/j.ejca.2015.08.021
Gondan AIB, 2018, BIOMATERIALS, V170, P95, DOI 10.1016/j.biomaterials.2018.04.003
Bourzac K, 2016, P NATL ACAD SCI USA, V113, P12600, DOI 10.1073/pnas.1616895113
Bronte V, 2015, NAT MED, V21, P117, DOI 10.1038/nm.3794
Carion O, 2007, NAT PROTOC, V2, P2383, DOI 10.1038/nprot.2007.351
Chao Y, 2019, NANO LETT, V19, P4287, DOI 10.1021/acs.nanolett.9b00579
Chen H, 2009, P NATL ACAD SCI USA, V106, P2729, DOI 10.1073/pnas.0813175106
Chiang CS, 2018, NAT NANOTECHNOL, V13, P746, DOI 10.1038/s41565-018-0146-7
Cluff C.W., 2009, MONOPHOSPHORYL LIPID, P111
Cobaleda-Siles M, 2014, SMALL, V10, P5054, DOI 10.1002/smll.201401353
Dingjan I, 2016, SCI REP-UK, V6, DOI 10.1038/srep22064
Embgenbroich M, 2018, FRONT IMMUNOL, V9, DOI 10.3389/fimmu.2018.01643
Eranki A, 2020, CLIN CANCER RES, V26, P1152, DOI 10.1158/1078-0432.CCR-19-1604
Friedman CF, 2016, JAMA ONCOL, V2, P1346, DOI 10.1001/jamaoncol.2016.1051
Gabrilovich DI, 2012, NAT REV IMMUNOL, V12, P253, DOI 10.1038/nri3175
Gajewski TF, 2013, NAT IMMUNOL, V14, P1014, DOI 10.1038/ni.2703
Galon J, 2019, NAT REV DRUG DISCOV, V18, P197, DOI 10.1038/s41573-018-0007-y
Arguinzoniz AG, 2015, DALTON T, V44, P7135, DOI 10.1039/c5dt00175g
Blanco NG, 2012, CHEM COMMUN, V48, P4211, DOI 10.1039/c2cc31045g
Hare JI, 2017, ADV DRUG DELIVER REV, V108, P25, DOI 10.1016/j.addr.2016.04.025
Hebb JPO, 2016, BLOOD, V128, DOI 10.1182/blood.V128.22.4172.4172
Huo MF, 2017, NAT COMMUN, V8, DOI 10.1038/s41467-017-00424-8
Irvine DJ, 2013, NAT MATER, V12, P978, DOI [10.1038/NMAT3775, 10.1038/nmat3775]
Jenkins RW, 2018, BRIT J CANCER, V118, P9, DOI 10.1038/bjc.2017.434
Kang TH, 2013, CANCER RES, V73, P2493, DOI 10.1158/0008-5472.CAN-12-4241
Kapralov AA, 2020, NAT CHEM BIOL, V16, P278, DOI 10.1038/s41589-019-0462-8
Kim SE, 2016, NAT NANOTECHNOL, V11, P977, DOI 10.1038/nnano.2016.164
Kuai R, 2017, NAT MATER, V16, P489, DOI [10.1038/nmat4822, 10.1038/NMAT4822]
Lee IH, 2012, ANGEW CHEM INT EDIT, V51, P8800, DOI 10.1002/anie.201203193
Li CX, 2019, ADV MATER, V31, DOI 10.1002/adma.201807211
Liang X, 2018, NANOSCALE, V10, P4500, DOI 10.1039/c7nr09585f
Lin H, 2018, CHEM SOC REV, V47, P1938, DOI 10.1039/c7cs00471k
Ling X, 2019, ACS NANO, V13, P357, DOI 10.1021/acsnano.8b06400
Liu Y, 2018, NAT COMMUN, V9, DOI 10.1038/s41467-018-05342-x

Lucky SS, 2015, CHEM REV, V115, P1990, DOI 10.1021/cr5004198
Lutz MB, 1999, J IMMUNOL METHODS, V223, P77, DOI 10.1016/S0022-1759(98)00204-X
Mahoney KM, 2015, NAT REV DRUG DISCOV, V14, P561, DOI 10.1038/nrd4591
Maldonado RA, 2015, P NATL ACAD SCI USA, V112, pE156, DOI 10.1073/pnas.1408686111
Mandai M, 2016, CLIN CANCER RES, V22, P2329, DOI 10.1158/1078-0432.CCR-16-0224
Marabelle A, 2013, J CLIN INVEST, V123, P2447, DOI 10.1172/JCI64859
Matsushita M, 2015, J EXP MED, V212, P555, DOI 10.1084/jem.20140857
Messenheimer DJ, 2017, CLIN CANCER RES, V23, P6165, DOI 10.1158/1078-0432.CCR-16-2677
Min YZ, 2017, NAT NANOTECHNOL, V12, P877, DOI [10.1038/nnano.2017.113, 10.1038/NNANO.2017.113]
Moros M, 2010, NANOSCALE, V2, P1746, DOI 10.1039/c0nr00104j
Moslehi JJ, 2018, LANCET, V391, P933, DOI 10.1016/S0140-6736(18)30533-6
Ni KY, 2018, NAT COMMUN, V9, DOI 10.1038/s41467-018-04703-w
Peng WY, 2012, CANCER RES, V72, P5209, DOI 10.1158/0008-5472.CAN-12-1187
Pitt JM, 2016, ANN ONCOL, V27, P1482, DOI 10.1093/annonc/mdw168
Revia RA, 2016, MATER TODAY, V19, P157, DOI 10.1016/j.mattod.2015.08.022
Riley RS, 2019, NAT REV DRUG DISCOV, V18, P175, DOI 10.1038/s41573-018-0006-z
Rosalia RA, 2015, BIOMATERIALS, V40, P88, DOI 10.1016/j.biomaterials.2014.10.053
Ruiz-de-Angulo A, 2016, ACS NANO, V10, P1602, DOI 10.1021/acsnano.5b07253
SCASNAR V, 1993, EUR J NUCL MED, V20, P273, DOI 10.1007/BF00170012
Schmidt C, 2006, J NATL CANCER I, V98, P574, DOI 10.1093/jnci/djj198
Schudel A, 2019, NAT REV MATER, V4, P415, DOI 10.1038/s41578-019-0110-7
Seong SY, 2004, NAT REV IMMUNOL, V4, P469, DOI 10.1038/nri1372
Shapiro B, 2015, WIRES NANOMED NANOBI, V7, P446, DOI 10.1002/wnan.1311
Shen C, 2017, INT J NANOMED, V12, P5443, DOI 10.2147/IJN.S137980
Shiao SL, 2011, GENE DEV, V25, P2559, DOI 10.1101/gad.169029.111
Shields BD, 2017, SCI REP-UK, V7, DOI 10.1038/s41598-017-01000-2
Song WJ, 2010, SMALL, V6, P239, DOI 10.1002/smll.200901513
Storni T, 2005, ADV DRUG DELIVER REV, V57, P333, DOI 10.1016/j.addr.2004.09.008
Sun SH, 2004, J AM CHEM SOC, V126, P273, DOI 10.1021/ja0380852
SZATROWSKI TP, 1991, CANCER RES, V51, P794
Thomas M, 2003, P NATL ACAD SCI USA, V100, P9138, DOI 10.1073/pnas.1233634100
Traini G, 2019, SMALL, V15, DOI 10.1002/smll.201803993
Trujillo-Alonso V, 2019, NAT NANOTECHNOL, V14, P616, DOI 10.1038/s41565-019-0406-1
Nguyen VH, 2017, INT J NANOMED, V12, P3137, DOI 10.2147/IJN.S129300
Varypataki EM, 2016, J CONTROL RELEASE, V226, P98, DOI 10.1016/j.jconrel.2016.02.018
Vosjan MJWD, 2010, NAT PROTOC, V5, P739, DOI 10.1038/nprot.2010.13
Wang WM, 2019, NATURE, V569, P270, DOI 10.1038/s41586-019-1170-y
Wang XP, 2016, ANGEW CHEM INT EDIT, V55, P1899, DOI 10.1002/anie.201506179
Ward PS, 2012, CANCER CELL, V21, P297, DOI 10.1016/j.ccr.2012.02.014
Wei SC, 2018, CANCER DISCOV, V8, P1069, DOI 10.1158/2159-8290.CD-18-0367
Xu XD, 2017, NANO LETT, V17, P4427, DOI 10.1021/acs.nanolett.7b01571
Yamanaka K, 2012, RSC ADV, V2, P7894, DOI 10.1039/c2ra20816d
Yang WS, 2016, TRENDS CELL BIOL, V26, P165, DOI 10.1016/j.tcb.2015.10.014
Yu WW, 2006, NANOTECHNOLOGY, V17, P4483, DOI 10.1088/0957-4484/17/17/033
Zanganeh S, 2016, NAT NANOTECHNOL, V11, P986, DOI [10.1038/nnano.2016.168, 10.1038/NNANO.2016.168]
Zhang YJ, 2020, NANOSCALE, V12, P4988, DOI 10.1039/c9nr10620k
NR 82
TC 27
Z9 27
U1 2
U2 51
PU CELL PRESS
PI CAMBRIDGE
PA 50 HAMPSHIRE ST, FLOOR 5, CAMBRIDGE, MA 02139 USA
EI 2589-0042
J9 ISCIENCE
JI iScience
PD SEP 25
PY 2020

VL 23
IS 9
AR 101499
DI 10.1016/j.isci.2020.101499
PG 42
WC Multidisciplinary Sciences
WE Science Citation Index Expanded (SCI-EXPANDED)
SC Science & Technology - Other Topics
GA NZ4VX
UT WOS:000577095400006
PM 32919370
OA gold, Green Published
DA 2023-09-14
ER

PT J
AU Dolen, Y
Valente, M
Tagit, O
Jager, E
Van Dinther, EAW
van Riessen, NK
Hruby, M
Gileadi, U
Cerundolo, V
Figdor, CG
AF Dolen, Yusuf
Valente, Michael
Tagit, Oya
Jager, Eliezer
Van Dinther, Eric A. W.
van Riessen, N. Koen
Hruby, Martin
Gileadi, Uzi
Cerundolo, Vincenzo
Figdor, Carl G.
TI Nanovaccine administration route is critical to obtain pertinent iNKt cell help for robust anti-tumor T and B cell responses
SO ONCOIMMUNOLOGY
LA English
DT Article
DE Cancer vaccines; nanoparticle biodistribution; iNKT cells; PD-1; 4-1BB; checkpoint blockade
ID INVARIANT NKT CELLS; ALPHA-GALACTOSYLCERAMIDE; PROTECTIVE IMMUNITY; PHASE-I; ACTIVATION; SUBSETS; VACCINATION; INDUCTION; DELIVERY; ANTIBODY
AB Nanovaccines, co-delivering antigen and invariant natural killer T (iNKT) cell agonists, proved to be very effective in inducing anti-tumor T cell responses due to their exceptional helper function. However, it is known that iNKT cells are not equally present in all lymphoid organs and nanoparticles do not get evenly distributed to all immune compartments. In this study, we evaluated the effect of the vaccination route on iNKT cell help to T and B cell responses for the first time in an antigen and agonist co-delivery setting. Intravenous administration of PLGA nanoparticles was mainly targeting liver and spleen where iNKT1 cells are abundant and induced the highest serum IFN-γ levels, T cell cytotoxicity, and Th-1 type antibody responses. In comparison, after subcutaneous or intranodal injections, nanoparticles mostly drained or remained in regional lymph nodes where iNKT17 cells were abundant. After subcutaneous and intranodal injections, antigen-specific IgG2 c production was hampered and IFN-γ production, as well as cytotoxic T cell responses, depended on sporadic systemic drainage. Therapeutic anti-tumor experiments also demonstrated a clear advantage of intravenous injection over intranodal or subcutaneous vaccinations. Moreover, tumor control could be further improved by PD-1 immune checkpoint blockade after intravenous vaccination, but not by intranodal vaccination. Anti PD-1 antibody combination mainly exerts its effect by prolonging the cytotoxicity of T cells. Nanovaccines also demonstrated synergism with anti-4-1BB agonistic antibody treatment in controlling tumor growth. We conclude that nanovaccines

containing iNKT cell agonists shall be preferentially administered intravenously, to optimally reach cellular partners for inducing effective anti-tumor immune responses.
C1 [Dolen, Yusuf; Valente, Michael; Tagit, Oya; Van Dinther, Eric A. W.; van Riessen, N. Koen; Figdor, Carl G.] Radboud Univ Nijmegen, Med Ctr, Radboud Inst Mol Life Sci, Dept Tumor Immunol, Geert Grootepl 26-28 Route 278, NL-6525 GA Nijmegen, Netherlands.
[Dolen, Yusuf; Valente, Michael; Tagit, Oya; Van Dinther, Eric A. W.; van Riessen, N. Koen; Figdor, Carl G.] Oncode Inst, Geert Grootepl 26-28 Route 278, NL-6525 GA Nijmegen, Netherlands.
[Jager, Eliezer; Hruby, Martin] Acad Sci Czech Republ, Vvi, Inst Macromol Chem, Prague 6, Czech Republic.
[Gileadi, Uzi; Cerundolo, Vincenzo] Univ Oxford, Weatherall Inst Mol Med, MRC Human Immunol Unit, Oxford, England.
C3 Radboud University Nijmegen; Czech Academy of Sciences; Institute of
Macromolecular Chemistry of the Czech Academy of Sciences; University of
Oxford
RP Figdor, CG (通讯作者)，Radboud Univ Nijmegen, Med Ctr, Radboud Inst Mol Life Sci, Dept Tumor Immunol, Geert Grootepl 26-28 Route 278, NL-6525 GA Nijmegen, Netherlands.; Figdor, CG (通讯作者)，Oncode Inst, Geert Grootepl 26-28 Route 278, NL-6525 GA Nijmegen, Netherlands.
EM carl.figdor@radboudumc.nl
RI Dolen, Yusuf/H-9945-2018; van Dinther, Eric A.W./D-9638-2016; Hruby,
Martin/H-6479-2014; Jäger, Eliézer/D-8924-2016; Tagit, Oya/A-2194-2016
OI Dolen, Yusuf/0000-0001-8211-9811; Hruby, Martin/0000-0002-5075-261X;
Jäger, Eliézer/0000-0001-9939-2355; Tagit, Oya/0000-0002-5773-6647;
valente, michael/0000-0002-0844-9799
FU Horizon 2020 project PRECIOUS [686089]; Medical Research Council (UK);
NIHR Oxford Biomedical Research Centre; Cancer Research UK [C399/A2291];
European Research Council (ERC) [834618]; Nederlandse Organisatie voor
Wetenschappelijk Onderzoek (NWO) Spinoza award; MRC [MC_U137884181]
Funding Source: UKRI
FX This work was supported by Horizon 2020 project PRECIOUS
(H2020-NMP-2015two-stage, grant #686089) and Medical Research Council
(UK), NIHR Oxford Biomedical Research Centre, and Cancer Research UK
(C399/A2291). CF is a recipient of European Research Council (ERC)
Advanced grant ARTimmune (#834618) and the Nederlandse Organisatie voor
Wetenschappelijk Onderzoek (NWO) Spinoza award.
CR Artiaga BL, 2016, SCI REP-UK, V6, DOI 10.1038/srep23593
Ayers M, 2017, J CLIN INVEST, V127, P2930, DOI 10.1172/JCI91190
Barral P, 2008, P NATL ACAD SCI USA, V105, P8345, DOI 10.1073/pnas.0802968105
Bartkowiak T, 2015, P NATL ACAD SCI USA, V112, pE5290, DOI 10.1073/pnas.1514418112
Bontkes HJ, 2010, CLIN IMMUNOL, V136, P364, DOI 10.1016/j.clim.2010.04.019
Brennan PJ, 2013, NAT REV IMMUNOL, V13, P101, DOI 10.1038/nri3369
Carnaud C, 1999, J IMMUNOL, V163, P4647
Cerundolo V, 2009, NAT REV IMMUNOL, V9, P28, DOI 10.1038/nri2451
Coquet JM, 2008, P NATL ACAD SCI USA, V105, P11287, DOI 10.1073/pnas.0801631105
Courtney AN, 2011, EUR J IMMUNOL, V41, P3312, DOI 10.1002/eji.201041359
Crosby CM, 2018, NAT REV IMMUNOL, V18, P559, DOI 10.1038/s41577-018-0034-2
Crowe NY, 2005, J EXP MED, V202, P1279, DOI 10.1084/jem.20050953
Doisne JM, 2009, J IMMUNOL, V183, P2142, DOI 10.4049/jimmunol.0901059
Dolen Y, 2016, ONCOIMMUNOLOGY, V5, DOI 10.1080/2162402X.2015.1068493
Fotouhi F, 2017, ARCH VIROL, V162, P1251, DOI 10.1007/s00705-017-3230-7
Gasser O, 2018, CANCER IMMUNOL IMMUN, V67, P285, DOI 10.1007/s00262-017-2085-9
Giaccone G, 2002, CLIN CANCER RES, V8, P3702
Hasegawa H, 2016, ANTICANCER RES, V36, P3667
Hermans IF, 2003, J IMMUNOL, V171, P5140, DOI 10.4049/jimmunol.171.10.5140
Jukes JP, 2016, EUR J IMMUNOL, V46, P1224, DOI 10.1002/eji.201546114
King LA, 2018, FRONT IMMUNOL, V9, DOI 10.3389/fimmu.2018.01519
Kinjo Y, 2005, NATURE, V434, P520, DOI 10.1038/nature03407
Kitamura H, 2000, CELL IMMUNOL, V199, P37, DOI 10.1006/cimm.1999.1602
Lee YJ, 2015, IMMUNITY, V43, P566, DOI 10.1016/j.immuni.2015.06.025
Lee YJ, 2013, NAT IMMUNOL, V14, P1146, DOI 10.1038/ni.2731
Lindqvist M, 2009, J IMMUNOL, V182, P6435, DOI 10.4049/jimmunol.0900136
Macho-Fernandez E, 2014, J IMMUNOL, V193, P961, DOI 10.4049/jimmunol.1303029

Nagaraj S, 2006, INT IMMUNOL, V18, P1279, DOI 10.1093/intimm/dxl059
Neparidze N, 2009, ANN NY ACAD SCI, V1174, P61, DOI 10.1111/j.1749-6632.2009.04931.x
Nicol AJ, 2011, CLIN CANCER RES, V17, P5140, DOI 10.1158/1078-0432.CCR-10-3105
Parekh VV, 2005, J CLIN INVEST, V115, P2572, DOI 10.1172/JCI24762
Slauenwhite D, 2015, FRONT IMMUNOL, V6, DOI 10.3389/fimmu.2015.00255
Smyth MJ, 2005, J EXP MED, V201, P1973, DOI 10.1084/jem.20042280
Sprengers D, 2008, GASTROENTEROLOGY, V134, P2132, DOI 10.1053/j.gastro.2008.02.037
Stetson DB, 2003, J EXP MED, V198, P1069, DOI 10.1084/jem.20030630
Tsao CC, 2016, PLOS ONE, V11, DOI 10.1371/journal.pone.0147710
Veldt BJ, 2007, J HEPATOL, V47, P356, DOI 10.1016/j.jhep.2007.04.018
WESTERMANN J, 1992, CLIN INVESTIGATOR, V70, P539
Wilson MT, 2003, P NATL ACAD SCI USA, V100, P10913, DOI 10.1073/pnas.1833166100
Yoshiga Y, 2011, CLIN EXP IMMUNOL, V164, P236, DOI 10.1111/j.1365-2249.2011.04369.x
NR 40
TC 27
Z9 29
U1 4
U2 15
PU TAYLOR & FRANCIS INC
PI PHILADELPHIA
PA 530 WALNUT STREET, STE 850, PHILADELPHIA, PA 19106 USA
SN 2162-402X
J9 ONCOIMMUNOLOGY
JI OncoImmunology
PY 2020
VL 9
IS 1
AR 1738813
DI 10.1080/2162402X.2020.1738813
PG 14
WC Oncology; Immunology
WE Science Citation Index Expanded (SCI-EXPANDED)
SC Oncology; Immunology
GA KU8SX
UT WOS:000519983200001
PM 33457086
OA Green Published, gold, Green Submitted
DA 2023-09-14
ER

PT J
AU Kim, H
Khanna, V
Kucaba, TA
Zhang, WQ
Ferguson, DM
Griffith, TS
Panyam, J
AF Kim, Hyunjoon
Khanna, Vidhi
Kucaba, Tamara A.
Zhang, Wenqiu
Ferguson, David M.
Griffith, Thomas S.
Panyam, Jayanth
TI Combination of Sunitinib and PD-L1 Blockade Enhances Anticancer Efficacy
of TLR7/8 Agonist-Based Nanovaccine
SO MOLECULAR PHARMACEUTICS
LA English
DT Article
DE TLR7/8 agonist; sunitinib; checkpoint blockade; nanoparticle; cancer

vaccine
ID RENAL-CELL CARCINOMA; SUPPRESSOR-CELLS; RECEPTORS 7; IFN-GAMMA; CANCER;
RESIQUIMOD; RESISTANCE; INDUCTION; DELIVERY; MICROENVIRONMENT
AB Cancer vaccines composed of tumor-associated antigens (TAAs) and toll-like receptor (TLR) agonists have shown promising antitumor efficacy in preclinical studies by generating antigen-specific CD8 T cells, but translation of cancer vaccines to the clinic has been limited due to variables responses and development of resistance. The tumor microenvironment deploys various immune escape mechanisms that neutralize CD8 T cell-mediated tumor rejection. Therefore, we hypothesized that modulation of the tumor microenvironment can augment CD8 T cell activation and enhance therapeutic efficacy of cancer vaccines. To accomplish this, we aimed to eliminate immune suppressive cells and block their inhibitory signaling. Combination of the tyrosine kinase inhibitor (TKI) sunitinib with a nanoparticle-based cancer vaccine (nanovaccine) resulted in the reduction of immune-suppressive myeloid-derived suppressive cells (MDSCs) and regulatory T cells (Tregs). Blockade of programmed death-ligand 1 (PD-L1) using anti-PD-L1 antibody was used to reduce CD8 T cell exhaustion. Combination of nanovaccine+sunitinib+PD-L1 antibody treatment reduced PD-L1(high) M2 macrophages and MDSCs and upregulated activation of CD8 T cells in the tumor. Nanovaccine+sunitinib+PD-L1 antibody treatment also stimulated antigen-specific CD8 T cell response, which led to improved therapeutic efficacy in MB49 and B16F10 murine tumor models. These results suggest that modulation of tumor microenvironment using sunitinib and PD-L1 blockade can significantly enhance the antitumor efficacy of cancer nanovaccine.s
C1 [Kim, Hyunjoon; Khanna, Vidhi; Zhang, Wenqiu; Panyam, Jayanth] Univ Minnesota, Dept Pharmaceut, Minneapolis, MN 55455 USA.
[Kucaba, Tamara A.; Griffith, Thomas S.] Univ Minnesota, Dept Urol, Minneapolis, MN 55455 USA.
[Ferguson, David M.] Univ Minnesota, Dept Med Chem, Minneapolis, MN 55455 USA.
[Griffith, Thomas S.; Panyam, Jayanth] Univ Minnesota, Masonic Canc Ctr, Minneapolis, MN 55455 USA.
[Griffith, Thomas S.] Univ Minnesota, Ctr Immunol, Minneapolis, MN 55455 USA.
[Griffith, Thomas S.] Univ Minnesota, Microbiol Immunol & Canc Biol Grad Program, Minneapolis, MN 55455 USA.
C3 University of Minnesota System; University of Minnesota Twin Cities;
University of Minnesota System; University of Minnesota Twin Cities;
University of Minnesota System; University of Minnesota Twin Cities;
University of Minnesota System; University of Minnesota Twin Cities;
University of Minnesota System; University of Minnesota Twin Cities;
University of Minnesota System; University of Minnesota Twin Cities
RP Panyam, J (通讯作者)，Univ Minnesota, Dept Pharmaceut, Minneapolis, MN 55455 USA.; Panyam, J (通讯作者)，Univ Minnesota, Masonic Canc Ctr, Minneapolis, MN 55455 USA.
EM jpanyam@umn.edu
RI Griffith, Thomas/ABF-8225-2020
OI Ferguson, David/0000-0001-6294-8057; Kim, Hyunjoon/0000-0002-9481-3218;
Panyam, Jayanth/0000-0002-8656-2244
FU University of Minnesota; GAP award; Masonic Cancer Center, University of
Minnesota; Prostate and Urological Cancer Translational Working Group;
Randy Shaver Cancer Research Community Fund
FX This work was supported by the Grant in aid program, University of
Minnesota (J.P.), GAP award (J.P.), Masonic Cancer Center, University of
Minnesota (T.S.G.), the Prostate and Urological Cancer Translational
Working Group (T.S.G.), and the Randy Shaver Cancer Research & Community
Fund (T.S.G., D.M.F.)
CR Abiko K, 2015, BRIT J CANCER, V112, P1501, DOI 10.1038/bjc.2015.101
Adelaiye R, 2015, MOL CANCER THER, V14, P513, DOI 10.1158/1535-7163.MCT-14-0208
Baaten BJG, 2010, IMMUNITY, V32, P104, DOI 10.1016/j.immuni.2009.10.011
Bachmann MF, 2010, NAT REV IMMUNOL, V10, P787, DOI 10.1038/nri2868
Blank C, 2005, CANCER IMMUNOL IMMUN, V54, P307, DOI 10.1007/s00262-004-0593-x
Bose A, 2011, INT J CANCER, V129, P2158, DOI 10.1002/ijc.25863
Brahmer JR, 2012, NEW ENGL J MED, V366, P2455, DOI 10.1056/NEJMoa1200694
Chan LJ, 2015, MOL PHARMACEUT, V12, P794, DOI 10.1021/mp5006189
Chen LP, 2015, J CLIN INVEST, V125, P3384, DOI 10.1172/JCI80011
Chen YC, 2018, MOL PHARMACEUT, V15, P5162, DOI 10.1021/acs.molpharmaceut.8b00717
Collier MA, 2018, MOL PHARMACEUT, V15, P4933, DOI 10.1021/acs.molpharmaceut.8b00579
Davis RJ, 2017, CANCER RES, V77, P2607, DOI 10.1158/0008-5472.CAN-16-2534
Dewan MZ, 2012, CLIN CANCER RES, V18, P6668, DOI 10.1158/1078-0432.CCR-12-0984
Draghiciu O, 2015, ONCOIMMUNOLOGY, V4, DOI 10.4161/21624011.2014.954829

Duong AD, 2013, MOL PHARMACEUT, V10, P1045, DOI 10.1021/mp3005098
Gabrilovich DI, 2009, NAT REV IMMUNOL, V9, P162, DOI 10.1038/nri2506
Gao NN, 2016, SCI REP-UK, V6, DOI 10.1038/srep39598
Gorgun GT, 2013, BLOOD, V121, P2975, DOI 10.1182/blood-2012-08-448548
Granot Z, 2015, CANCER RES, V75, P4441, DOI 10.1158/0008-5472.CAN-15-1502
Hipp MM, 2008, BLOOD, V111, P5610, DOI 10.1182/blood-2007-02-075945
Huo MR, 2017, J CONTROL RELEASE, V245, P81, DOI 10.1016/j.jconrel.2016.11.013
Iwata T, 2016, SCI REP, V6, P1
Jaini R, 2014, INT J CANCER, V134, P1695, DOI 10.1002/ijc.28488
James BR, 2014, CANCER IMMUNOL IMMUN, V63, P685, DOI 10.1007/s00262-014-1548-5
Jenkins RW, 2018, BRIT J CANCER, V118, P9, DOI 10.1038/bjc.2017.434
Jha BK, 2013, MOL THER, V21, P1749, DOI 10.1038/mt.2013.112
Kim H, 2018, BIOMATERIALS, V164, P38, DOI 10.1016/j.biomaterials.2018.02.034
Kleffel S, 2015, CELL, V162, P1242, DOI 10.1016/j.cell.2015.08.052
Ko JS, 2009, CLIN CANCER RES, V15, P2148, DOI 10.1158/1078-0432.CCR-08-1332
Larson P, 2017, ACS MED CHEM LETT, V8, P1148, DOI 10.1021/acsmedchemlett.7b00256
Loke P, 2003, P NATL ACAD SCI USA, V100, P5336, DOI 10.1073/pnas.0931259100
Lu HL, 2010, J IMMUNOL, V184, P5360, DOI 10.4049/jimmunol.0902997
Lynn GM, 2015, NAT BIOTECHNOL, V33, P1201, DOI 10.1038/nbt.3371
Makadia HK, 2011, POLYMERS-BASEL, V3, P1377, DOI 10.3390/polym3031377
Mandai M, 2016, CLIN CANCER RES, V22, P2329, DOI 10.1158/1078-0432.CCR-16-0224
Mangsbo SM, 2010, J IMMUNOTHER, V33, P225, DOI 10.1097/CJI.0b013e3181c01fcb
Naidoo J, 2015, ANN ONCOL, V26, P2375, DOI 10.1093/annonc/mdv383
Nawaz A, 2017, NAT COMMUN, V8, DOI 10.1038/s41467-017-00231-1
Nishii Naoto, 2018, Oncotarget, V9, P13301, DOI 10.18632/oncotarget.24327
Panyam J, 2004, J PHARM SCI-US, V93, P1804, DOI 10.1002/jps.20094
PARKER JC, 1985, J APPL PHYSIOL, V59, P1128, DOI 10.1152/jappl.1985.59.4.1128
Patel SP, 2015, MOL CANCER THER, V14, P847, DOI 10.1158/1535-7163.MCT-14-0983
Rai D, 2009, J IMMUNOL, V183, P7672, DOI 10.4049/jimmunol.0902874
Rook AH, 2015, BLOOD, V126, P1452, DOI 10.1182/blood-2015-02-630335
Sabado RL, 2015, CANCER IMMUNOL RES, V3, P278, DOI 10.1158/2326-6066.CIR-14-0202
Sabbatini P, 2012, CLIN CANCER RES, V18, P6497, DOI 10.1158/1078-0432.CCR-12-2189
Sato-Kaneko F, 2017, JCI INSIGHT, V2, DOI 10.1172/jci.insight.93397
Schiaffo CE, 2014, J MED CHEM, V57, P339, DOI 10.1021/jm4004957
Spinetti T, 2016, ONCOIMMUNOLOGY, V5, P1
SUPERSAXO A, 1990, PHARMACEUT RES, V7, P167, DOI 10.1023/A:1015880819328
Swartz MA, 2001, ADV DRUG DELIVER REV, V50, P3, DOI 10.1016/S0169-409X(01)00150-8
Vandeveer AJ, 2016, CANCER IMMUNOL RES, V4, P452, DOI 10.1158/2326-6066.CIR-15-0176
Wang WR, 2012, DRUG METAB DISPOS, V40, P952, DOI 10.1124/dmd.111.043604
Whiteside TL, 2008, ONCOGENE, V27, P5904, DOI 10.1038/onc.2008.271
Wongkajornsilp A, 2013, PLOS ONE, V8
Xin H, 2009, CANCER RES, V69, P2506, DOI 10.1158/0008-5472.CAN-08-4323
Zhao XD, 2017, CANCER RES, V77, P817, DOI 10.1158/0008-5472.CAN-16-2379
Zou WP, 2008, NAT REV IMMUNOL, V8, P467, DOI 10.1038/nri2326
NR 58
TC 27
Z9 27
U1 3
U2 53
PU AMER CHEMICAL SOC
PI WASHINGTON
PA 1155 16TH ST, NW, WASHINGTON, DC 20036 USA
SN 1543-8384
J9 MOL PHARMACEUT
JI Mol. Pharm.
PD MAR
PY 2019
VL 16
IS 3
BP 1200
EP 1210

DI 10.1021/acs.molpharmaceut.8b01165
PG 11
WC Medicine, Research & Experimental; Pharmacology & Pharmacy
WE Science Citation Index Expanded (SCI-EXPANDED)
SC Research & Experimental Medicine; Pharmacology & Pharmacy
GA HO0NM
UT WOS:000460600400024
PM 30620878
DA 2023-09-14
ER

PT J
AU Qiu, LP
Valente, M
Dolen, Y
Jager, E
ter Beest, M
Zheng, LY
Figdor, CG
Verdoes, M
AF Qiu, Liping
Valente, Michael
Dolen, Yusuf
Jager, Eliezer
ter Beest, Martin
Zheng, Liyan
Figdor, Carl G.
Verdoes, Martijn
TI Endolysosomal-Escape Nanovaccines through Adjuvant-Induced Tumor Antigen Assembly for Enhanced Effector CD8(+) T Cell Activation
SO SMALL
LA English
DT Article
DE antigen; adjuvant codelivery; cancer nanovaccines; cross-presentation; endolysosomal escape; T cell activation
ID DENDRITIC CELLS; CANCER-IMMUNOTHERAPY; ANTITUMOR IMMUNITY; CROSS-PRESENTATION; MELANOMA PATIENTS; DRUG-DELIVERY; NANOPARTICLES; RESPONSES; VACCINES; GENERATION
AB The activation of tumor-specific effector immune cells is key for successful immunotherapy and vaccination is a powerful strategy to induce such adaptive immune responses. However, the generation of effective anticancer vaccines is challenging. To overcome these challenges, a novel straight-forward strategy of adjuvant-induced tumor antigen assembly to generate nanovaccines with superior antigen/adjuvant loading efficiency is developed. To protect nanovaccines in circulation and to introduce additional functionalities, a biocompatible polyphenol coating is installed. The resulting functionalizable nanovaccines are equipped with a pH (low) insertion peptide (pHLIP) to facilitate endolysosomal escape and to promote cytoplasmic localization, with the aim to enhance cross-presentation of the antigen by dendritic cells to effectively activate CD8(+) T cell. The results demonstrate that pHLIP-functionalized model nanovaccine can induce endolysosomal escape and enhance CD8(+) T cell activation both in vitro and in vivo. Furthermore, based on the adjuvant-induced antigen assembly, nanovaccines of the clinically relevant tumor-associated antigen NY-ESO-1 are generated and show excellent capacity to elicit NY-ESO-1-specific CD8(+) T cell activation, demonstrating a high potential of this functionalizable nanovaccine formulation strategy for clinical applications.
C1 [Qiu, Liping; Zheng, Liyan] Hunan Univ, Coll Chem & Chem Engn, State Key Lab Chemo Biosensing & Chemometr, Mol Sci & Biomed Lab, Changsha 410082, Hunan, Peoples R China.
[Qiu, Liping; Valente, Michael; Dolen, Yusuf; Jager, Eliezer; ter Beest, Martin; Figdor, Carl G.; Verdoes, Martijn] Radboud Univ Nijmegen, Med Ctr, Inst Mol Life Sci, Dept Tumor Immunol, NL-6500 HB Nijmegen, Netherlands.
[Jager, Eliezer] Acad Sci Czech Republ, Vvi, Inst Macromol Chem, Heyrovsky Sq 2, Prague 16206 6, Czech Republic.
C3 Hunan University; Radboud University Nijmegen; Czech Academy of Sciences; Institute of Macromolecular Chemistry of the Czech Academy of

Sciences
RP Figdor, CG; Verdoes, M (通讯作者)，Radboud Univ Nijmegen, Med Ctr, Inst Mol Life Sci, Dept Tumor Immunol, NL-6500 HB Nijmegen, Netherlands.
EM Carl.Figdor@radboudumc.nl; Martijn.Verdoes@radboudumc.nl
RI Figdor, Carl G/A-4232-2010; Dolen, Yusuf/H-9945-2018; Verdoes, Martijn/A-4911-2015; Verdoes, Martijn/AAD-5239-2022; Jäger, Eliézer/D-8924-2016
OI Figdor, Carl G/0000-0002-2366-9212; Dolen, Yusuf/0000-0001-8211-9811; Verdoes, Martijn/0000-0001-8753-3528; Verdoes, Martijn/0000-0001-8753-3528; Jäger, Eliézer/0000-0001-9939-2355; valente, michael/0000-0002-0844-9799
FU National Key Scientific Program of China [21505039]; ERC Advanced grant PATHFINDER [269019]; Spinoza grant from the Netherlands Organisation for Scientific Research (NWO); Dutch Cancer Society KWO grant [2009-4402]; ERC Starting grant CHEMCHECK [679921]; NWO; Czech Academy of Sciences grant [MSM200501602]
FX The authors thank the Ludwig Institute for Cancer Research for providing the recombinant NY-ESO-1 protein used in the study. mRNA encoding for a TCR specific for HLA-A2-NY-ESO-I peptide p157-165 was kindly provided by Prof. U. Sahin. This work was supported by the National Key Scientific Program of China (Grant 21505039), ERC Advanced grant PATHFINDER (269019), and a Spinoza grant from the Netherlands Organisation for Scientific Research (NWO). C.F. is recipient of Dutch Cancer Society KWO grant 2009-4402. M.V. is recipient of ERC Starting grant CHEMCHECK (679921) and a Gravity Program Institute for Chemical Immunology tenure track grant by NWO. E.J. received a Czech Academy of Sciences grant (MSM200501602).
CR An M, 2010, P NATL ACAD SCI USA, V107, P20246, DOI 10.1073/pnas.1014403107
Andreev OA, 2010, P NATL ACAD SCI USA, V107, P4081, DOI 10.1073/pnas.0914330107
Baghirova S, 2015, METHODSX, V2, P440, DOI 10.1016/j.mex.2015.11.001
Chen Q, 2016, BIOMATERIALS, V98, P23, DOI 10.1016/j.biomaterials.2016.04.041
Chiu YH, 1999, J EXP MED, V189, P103, DOI 10.1084/jem.189.1.103
de Vries IJM, 2003, CANCER RES, V63, P12
Dolen Y, 2016, ONCOIMMUNOLOGY, V5, DOI 10.1080/2162402X.2015.1068493
Fu Q, 2009, RECENT PAT ANTI-CANC, V4, P262, DOI 10.2174/157489209789206869
Gradishar WJ, 2006, EXPERT OPIN PHARMACO, V7, P1041, DOI 10.1517/14656566.7.8.1041
Gutscher M, 2008, NAT METHODS, V5, P553, DOI 10.1038/NMETH.1212
Hamdy S, 2008, VACCINE, V26, P5046, DOI 10.1016/j.vaccine.2008.07.035
Hamdy S, 2011, ADV DRUG DELIVER REV, V63, P943, DOI 10.1016/j.addr.2011.05.021
Heath WR, 2004, IMMUNOL REV, V199, P9, DOI 10.1111/j.0105-2896.2004.00142.x
Hemmi H, 2000, NATURE, V408, P740, DOI 10.1038/35047123
Joffre OP, 2012, NAT REV IMMUNOL, V12, P557, DOI 10.1038/nri3254
Kazzaz J, 2006, J CONTROL RELEASE, V110, P566, DOI 10.1016/j.jconrel.2005.10.010
Klebanoff CA, 2005, P NATL ACAD SCI USA, V102, P9571, DOI 10.1073/pnas.0503726102
Li J, 2016, NANOSCALE, V8, P8600, DOI 10.1039/c6nr00600k
Li YS, 1999, NAT MED, V5, P1298, DOI 10.1038/15256
Lutz MB, 1999, J IMMUNOL METHODS, V223, P77, DOI 10.1016/S0022-1759(98)00204-X
Palucka K., 2014, INTERACTION IMMUNE C, P75
Perrie Y, 2008, INT J PHARMACEUT, V364, P272, DOI 10.1016/j.ijpharm.2008.04.036
Powell, 1995, VACCINE DESIGN SUBUN, P495
Pritchard GH, 2016, NAT IMMUNOL, V17, P471, DOI 10.1038/ni.3411
Quideau S, 2011, ANGEW CHEM INT EDIT, V50, P586, DOI 10.1002/anie.201000044
Rahimian S, 2015, BIOMATERIALS, V37, P469, DOI 10.1016/j.biomaterials.2014.10.043
Rapoport AP, 2015, NAT MED, V21, P914, DOI 10.1038/nm.3910
Restifo NP, 2012, NAT REV IMMUNOL, V12, P269, DOI 10.1038/nri3191
Rosenberg SA, 2004, NAT MED, V10, P909, DOI 10.1038/nm1100
Sang SM, 2005, CHEM RES TOXICOL, V18, P1762, DOI 10.1021/tx050151l
Saupe A, 2006, EXPERT OPIN DRUG DEL, V3, P345, DOI 10.1517/17425247.3.3.345
Schmid DA, 2010, J IMMUNOL, V184, P4936, DOI 10.4049/jimmunol.1000173
Sileika TS, 2013, ANGEW CHEM INT EDIT, V52, P10766, DOI 10.1002/anie.201304922
Tacken PJ, 2007, NAT REV IMMUNOL, V7, P790, DOI 10.1038/nri2173

Takayanagi H, 2000, NATURE, V408, P600, DOI 10.1038/35046102
Valente M, 2014, EUR J IMMUNOL, V44, P2274, DOI 10.1002/eji.201344191
vansde Laar L., 2014, BLOOD, V124, P3036
Wadle A, 2010, YEAST, V27, P919, DOI 10.1002/yea.1796
Weerakkody D, 2013, P NATL ACAD SCI USA, V110, P5834, DOI 10.1073/pnas.1303708110
Xiang SD, 2008, EXPERT REV VACCINES, V7, P1103, DOI 10.1586/14760584.7.7.1103
Xu YK, 2007, J IMMUNOL, V179, P7577, DOI 10.4049/jimmunol.179.11.7577
Yang CS, 2009, NAT REV CANCER, V9, P429, DOI 10.1038/nrc2641
Yuan JD, 2011, P NATL ACAD SCI USA, V108, P16723, DOI 10.1073/pnas.1110814108
Zhu GZ, 2017, ACS NANO, V11, P2387, DOI 10.1021/acsnano.7b00978
NR 44
TC 27
Z9 28
U1 2
U2 63
PU WILEY-V C H VERLAG GMBH
PI WEINHEIM
PA POSTFACH 101161, 69451 WEINHEIM, GERMANY
SN 1613-6810
EI 1613-6829
J9 SMALL
JI Small
PD APR 12
PY 2018
VL 14
IS 15
AR 1703539
DI 10.1002/smll.201703539
PG 11
WC Chemistry, Multidisciplinary; Chemistry, Physical; Nanoscience &
Nanotechnology; Materials Science, Multidisciplinary; Physics, Applied;
Physics, Condensed Matter
WE Science Citation Index Expanded (SCI-EXPANDED)
SC Chemistry; Science & Technology - Other Topics; Materials Science;
Physics
GA GD0JH
UT WOS:000430186600007
PM 29493121
OA Green Submitted
DA 2023-09-14
ER

PT J
AU Clemente-Casares, X
Tsai, S
Yang, Y
Santamaria, P
AF Clemente-Casares, Xavier
Tsai, Sue
Yang, Yang
Santamaria, Pere
TI Peptide-MHC-based nanovaccines for the treatment of autoimmunity: a "one
size fits all" approach?
SO JOURNAL OF MOLECULAR MEDICINE-JMM
LA English
DT Review
DE Autoimmunity; Beta cells; Diabetes; Immune tolerance; Lymphocyte
ID T-CELL-RECEPTOR; PROMISING THERAPEUTIC APPROACH; ANTIGEN-SPECIFIC
APOPTOSIS; RECOMBINANT TCR LIGANDS; CLASS-II MOLECULES;
MYASTHENIA-GRAVIS; IN-VITRO; MULTIPLE-SCLEROSIS; CYTOKINE SWITCH;
TRANSGENIC MICE

AB Nanotechnology offers enormous potential in drug delivery and in vivo imaging. Nanoparticles (NPs), for example, are being extensively tested as scaffolds to deliver anti-cancer therapeutics or imaging tags. Our recent work, discussed herein, indicates that an opportunity exists to use NPs to deliver ligands for, and trigger, cognate receptors on T lymphocytes as a way to induce therapeutic immune responses in vivo. Specifically, systemic delivery of NPs coated with Type 1 diabetes (T1D)-relevant peptide-major histocompatibility complex molecules triggered the expansion of cognate memory autoregulatory (disease-suppressing) T cells, suppressed the progression of autoimmune attack against insulin-producing beta cells, and restored glucose homeostasis. This therapeutic avenue exploits a new paradigm in the progression of chronic autoimmune responses that enables the rational design of disease-specific "nanovaccines" capable of blunting autoimmunity without impairing systemic immunity, a long sought-after goal in the therapy of these disorders. Here, we discuss the research paths that led to the discovery of this therapeutic avenue and highlight the features that make it an attractive approach for the treatment, in an antigen-specific manner, of a whole host of autoimmune diseases.
C1 [Clemente-Casares, Xavier; Tsai, Sue; Yang, Yang; Santamaria, Pere] Univ Calgary, Fac Med, Inst Inflammat Infect & Immun, Julia McFarlane Diabet Res Ctr, Calgary, AB T2N 4N1, Canada.
[Clemente-Casares, Xavier; Tsai, Sue; Yang, Yang; Santamaria, Pere] Univ Calgary, Fac Med, Inst Inflammat Infect & Immun, Dept Microbiol & Infect Dis, Calgary, AB T2N 4N1, Canada.
C3 University of Calgary; University of Calgary
RP Santamaria, P (通讯作者)，Univ Calgary, Fac Med, Inst Inflammat Infect & Immun, Julia McFarlane Diabet Res Ctr, 3330 Hosp Dr NW, Calgary, AB T2N 4N1, Canada.
EM psantama@ucalgary.ca
RI Santamaria, Pere/ABF-6823-2021
OI Clemente-Casares, Xavier/0000-0002-8627-4971; Tsai, Sue/0000-0002-6261-5233
FU Canadian Institutes of Health Research (CIHR); National and Engineering Research Council of Canada (NSERC); Juvenile Diabetes Research Foundation (JDRF); Diabetes Association (Foothills); Canadian Diabetes Association; AXA Research Fund Foundation; Alberta Innovates-Health Solutions (AIHS, formerly AHFMR)
FX The work described here was funded by grants from the Canadian Institutes of Health Research (CIHR), the National and Engineering Research Council of Canada (NSERC), the Juvenile Diabetes Research Foundation (JDRF), the Diabetes Association (Foothills), and the Canadian Diabetes Association. X.C.C. and S.T. are supported by studentships from the AXA Research Fund Foundation and Alberta Innovates-Health Solutions (AIHS, formerly AHFMR), respectively. P.S. is a Scientist of AIHS and a JDRF Scholar. The JMDRC is supported by the Diabetes Association (Foothills).
CR ABASTADO JP, 1995, J EXP MED, V182, P439, DOI 10.1084/jem.182.2.439
Adamus G, 2006, INVEST OPHTH VIS SCI, V47, P2555, DOI 10.1167/iovs.05-1242
Adamus G, 2010, OPHTHAL RES, V44, P24, DOI 10.1159/000281815
AICHELE P, 1994, P NATL ACAD SCI USA, V91, P444, DOI 10.1073/pnas.91.2.444
Amrani A, 2000, NATURE, V406, P739, DOI 10.1038/35021081
Appel H, 2001, J IMMUNOL, V166, P5279, DOI 10.4049/jimmunol.166.8.5279
Arimilli S, 1996, IMMUNOL CELL BIOL, V74, P96, DOI 10.1038/icb.1996.13
Bond AP, 1998, J NEUROIMMUNOL, V82, P182, DOI 10.1016/S0165-5728(97)00202-6
Burrows GG, 2000, J IMMUNOL, V164, P6366, DOI 10.4049/jimmunol.164.12.6366
Burrows GG, 1998, J IMMUNOL, V161, P5987
Burrows GG, 1999, PROTEIN ENG, V12, P771, DOI 10.1093/protein/12.9.771
Casares S, 2002, NAT IMMUNOL, V3, P383, DOI 10.1038/ni770
Casares S, 2008, TRANSPLANTATION, V85, P1717, DOI 10.1097/TP.0b013e31817752cc
Fan Y, 2009, EMBO J, V28, P2812, DOI 10.1038/emboj.2009.212
Fontenot AP, 2006, J IMMUNOL, V177, P3874, DOI 10.4049/jimmunol.177.6.3874
Goodkin DE, 2000, NEUROLOGY, V54, P1414, DOI 10.1212/WNL.54.7.1414
Groux H, 1997, NATURE, V389, P737, DOI 10.1038/39614
Han BY, 2005, NAT MED, V11, P645, DOI 10.1038/nm1250
HERRMANN SH, 1986, J IMMUNOL, V136, P2816
Huan J, 2011, MUCOSAL IMMUNOL, V4, P112, DOI 10.1038/mi.2010.44
Huan J, 2008, J IMMUNOL, V180, P1249, DOI 10.4049/jimmunol.180.2.1249
Huan JY, 2004, J IMMUNOL, V172, P4556, DOI 10.4049/jimmunol.172.7.4556
JENKINS MK, 1987, IMMUNOL REV, V95, P113, DOI 10.1111/j.1600-065X.1987.tb00502.x

Jiang H, 2009, ADV IMMUNOL, V102, P95, DOI 10.1016/S0065-2776(09)01202-4
Kavanaugh A, 2003, J RHEUMATOL, V30, P449
Li L, 2009, J IMMUNOL, V183, P4809, DOI 10.4049/jimmunol.0901616
Lieberman SM, 2004, J IMMUNOL, V173, P6727, DOI 10.4049/jimmunol.173.11.6727
Lieberman SM, 2003, TISSUE ANTIGENS, V62, P359, DOI 10.1034/j.1399-0039.2003.00152.x
Lin M, 2010, EUR J IMMUNOL, V40, P2277, DOI 10.1002/eji.200940094
Link JM, 2007, CLIN IMMUNOL, V123, P95, DOI 10.1016/j.clim.2006.12.002
Masteller EL, 2003, J IMMUNOL, V171, P5587, DOI 10.4049/jimmunol.171.10.5587
MCCLUSKEY J, 1989, TRANSPLANT P, V21, P591
Nag B, 1996, CELL IMMUNOL, V170, P25, DOI 10.1006/cimm.1996.0130
Nag B, 1996, J BIOL CHEM, V271, P10413, DOI 10.1074/jbc.271.17.10413
Nakayama M, 2005, NATURE, V435, P220, DOI 10.1038/nature03523
NICOLLE MW, 1994, J CLIN INVEST, V93, P1361, DOI 10.1172/JCI117112
NICOLLE MW, 1993, ANN NY ACAD SCI, V681, P577, DOI 10.1111/j.1749-6632.1993.tb22946.x
O'Herrin SM, 2001, J IMMUNOL, V167, P2555, DOI 10.4049/jimmunol.167.5.2555
Offner H, 2005, J IMMUNOL, V175, P4103, DOI 10.4049/jimmunol.175.6.4103
Offner H, 2011, J NEUROIMMUNOL, V231, P7, DOI 10.1016/j.jneuroim.2010.09.013
Preda I, 2005, EUR J IMMUNOL, V35, P2762, DOI 10.1002/eji.200526158
SANGKON O, 2004, P NATL ACAD SCI USA, V101, P15154
Santamaria P, 2010, IMMUNITY, V32, P437, DOI 10.1016/j.immuni.2010.04.003
SHARMA SD, 1991, P NATL ACAD SCI USA, V88, P11465, DOI 10.1073/pnas.88.24.11465
Sinha S, 2007, J NEUROSCI, V27, P12531, DOI 10.1523/JNEUROSCI.3599-07.2007
Sinha S, 2010, J NEUROIMMUNE PHARM, V5, P231, DOI 10.1007/s11481-009-9175-1
Sinha S, 2009, J NEUROSCI, V29, P3816, DOI 10.1523/JNEUROSCI.5812-08.2009
Spack EG, 1995, J AUTOIMMUN, V8, P787, DOI 10.1016/S0896-8411(95)80018-2
Subramanian S, 2009, STROKE, V40, P2539, DOI 10.1161/STROKEAHA.108.543991
Thornton AM, 1998, J EXP MED, V188, P287, DOI 10.1084/jem.188.2.287
Toes REM, 1996, P NATL ACAD SCI USA, V93, P7855, DOI 10.1073/pnas.93.15.7855
Tsai SE, 2010, IMMUNITY, V32, P568, DOI 10.1016/j.immuni.2010.03.015
Tsai S, 2008, ADV IMMUNOL, V100, P79, DOI 10.1016/S0065-2776(08)00804-3
Vandenbark AA, 2003, J IMMUNOL, V171, P127, DOI 10.4049/jimmunol.171.1.127
Wang CH, 2006, J NEUROCHEM, V98, P1817, DOI 10.1111/j.1471-4159.2006.04081.x
Wang JG, 2010, P NATL ACAD SCI USA, V107, P9317, DOI 10.1073/pnas.0913835107
Yadav V, 2010, NEUROLOGY, V74, pA293
Yamanouchi J, 2003, J IMMUNOL, V171, P6900, DOI 10.4049/jimmunol.171.12.6900
Zuo L, 2002, J IMMUNOL, V168, P2554, DOI 10.4049/jimmunol.168.5.2554
NR 59
TC 27
Z9 36
U1 0
U2 17
PU SPRINGER
PI NEW YORK
PA 233 SPRING ST, NEW YORK, NY 10013 USA
SN 0946-2716
J9 J MOL MED
JI J. Mol. Med.
PD AUG
PY 2011
VL 89
IS 8
BP 733
EP 742
DI 10.1007/s00109-011-0757-z
PG 10
WC Genetics & Heredity; Medicine, Research & Experimental
WE Science Citation Index Expanded (SCI-EXPANDED)
SC Genetics & Heredity; Research & Experimental Medicine
GA 804FH
UT WOS:000293639200002
PM 21499734

DA 2023-09-14
ER

PT J
AU Feng, C
   Li, YJ
   Ferdows, BE
   Patel, DN
   Ouyang, J
   Tang, ZM
   Kong, N
   Chen, EG
   Tao, W
AF Feng, Chan
   Li, Yongjiang
   Ferdows, Bijan Emiliano
   Patel, Dylan Neal
   Ouyang, Jiang
   Tang, Zhongmin
   Kong, Na
   Chen, Enguo
   Tao, Wei
TI Emerging vaccine nanotechnology: From defense against infection to
   sniping cancer
SO ACTA PHARMACEUTICA SINICA B
LA English
DT Review
DE Vaccine; Nanotechnology; Infection; Cancer; Nanovaccines; Vaccination;
   Diseases prevention; Immunotherapy
ID ANTIGEN-PRESENTING CELLS; IN-SITU VACCINATION; VIRUS-LIKE PARTICLES;
   DENDRITIC CELLS; IMMUNE-RESPONSES; MESSENGER-RNA; POLYMERIC
   NANOPARTICLES; ANTITUMOR IMMUNITY; MEMBRANE-VESICLES; DELIVERY-SYSTEMS
AB Looking retrospectively at the development of humanity, vaccination is an unprecedented medical landmark that saves lives by harnessing the human immune system. During the ongoing coronavirus disease 2019 (COVID-19) pandemic, vaccination is still the most effective defense modality. The successful clinical application of the lipid nanoparticle-based Pfizer/BioNTech and Moderna mRNA COVID-19 vaccines highlights promising future of nanotechnology in vaccine development. Compared with conventional vaccines, nanovaccines are supposed to have advantages in lymph node accumulation, antigen assembly, and antigen presentation; they also have, unique pathogen biomimicry properties because of well-organized combination of multiple immune factors. Beyond infectious diseases, vaccine nanotechnology also exhibits considerable potential for cancer treatment. The ultimate goal of cancer vaccines is to fully mobilize the potency of the immune system as a living therapeutic to recognize tumor antigens and eliminate tumor cells, and nanotechnologies have the requisite properties to realize this goal. In this review, we summarize the recent advances in vaccine nanotechnology from infectious disease prevention to cancer immunotherapy and highlight the different types of materials, mechanisms, administration methods, as well as future perspectives. (C) 2022 Chinese Pharmaceutical Association and Institute of Materia Medica, Chinese Academy of Medical Sciences. Production and hosting by Elsevier B.V. This is an open access article under the CC BY-NC-ND license (http://creativecommons.org/licenses/by-nc-nd/4.0/).
C1 [Feng, Chan; Chen, Enguo] Zhejiang Univ, Sir Run Run Shaw Hosp, Dept Resp Med, Sch Med, Hangzhou 310016, Peoples R China.
   [Feng, Chan; Li, Yongjiang; Ferdows, Bijan Emiliano; Patel, Dylan Neal; Ouyang, Jiang; Tang, Zhongmin; Kong, Na; Tao, Wei] Harvard Med Sch, Ctr Nanomed & Dept Anesthesiol, Brigham & Womens Hosp, Boston, MA 02115 USA.
   [Feng, Chan; Chen, Enguo] Zhejiang Univ, Canc Ctr, Hangzhou 310058, Peoples R China.
   [Li, Yongjiang] Cent South Univ, Dept Pharm, Xiangya Hosp 2, Changsha 410011, Peoples R China.
C3 Zhejiang University; Harvard University; Brigham & Women's Hospital;
   Harvard Medical School; Zhejiang University; Central South University
RP Chen, EG (通讯作者)，Zhejiang Univ, Sir Run Run Shaw Hosp, Dept Resp Med, Sch Med, Hangzhou 310016, Peoples R China.; Tao, W (通讯作者)，Harvard Med Sch, Ctr Nanomed & Dept Anesthesiol, Brigham & Womens Hosp, Boston, MA 02115 USA.; Chen, EG (通讯作者)，Zhejiang Univ, Canc Ctr,

Hangzhou 310058, Peoples R China.
EM 3195024@zju.edu.cn; wtao@bwh.harvard.edu
RI Li, Yong-Jiang/AAF-9313-2020; Tang, Zhong-min/AAQ-2526-2021; Chen, Enguo/ABA-2991-2021; tao, wei/GXN-4445-2022; Tao, Wei/AAE-7955-2020
OI Li, Yong-Jiang/0000-0001-6882-7862; Tao, Wei/0000-0002-4277-3728; Patel, Dylan/0000-0002-7479-5534; Ferdows, Bijan/0000-0002-0247-5224
FU US METAvivor Early Career Investigator Award [2018A020560]; Harvard Medical School/Brigham and Women's Hospital Department of Anesthesiology Basic Scientist Grant [2420 BPA075]; Center for Neuroscience Research Fund [2019A014810]; Khoury Innovation Award [2020A003219]; American Heart Association (AHA) Collaborative Science Award [2018A004190]; Gillian Reny Stepping Strong Center for Trauma Innovation Break-through Innovator Award [113548]
FX The authors acknowledged the support from the US METAvivor Early Career Investigator Award (No. 2018A020560; to W.T., USA) , Harvard Medical School/Brigham and Women's Hospital Department of Anesthesiology Basic Scientist Grant (No. 2420 BPA075; to W.T., USA) and Center for Neuroscience Research Fund (No. 2019A014810; to W.T., USA) . W.T. is a recipient of the Khoury Innovation Award (No. 2020A003219, USA) , Gillian Reny Stepping Strong Center for Trauma Innovation Break-through Innovator Award (No. 113548, USA) , and American Heart Association (AHA) Collaborative Science Award (No. 2018A004190, USA) . W.T. also received a start-up package (for 3 years) from the Department of Anesthesiology, Perioperative and Pain Medicine to establish his independent research laboratory at Harvard Medical School and Brigham and Women?s Hospital.
CR Abdelwahab WM, 2021, INT J PHARMACEUT, V593, DOI 10.1016/j.ijpharm.2020.120119
Adepoju P, 2019, LANCET, V393, P1685, DOI 10.1016/S0140-6736(19)30937-7
Al-Deen FN, 2011, LANGMUIR, V27, P3703, DOI 10.1021/la104479c
An M, 2017, ACS APPL MATER INTER, V9, P23466, DOI 10.1021/acsami.7b06024
Anderson BR, 2010, NUCLEIC ACIDS RES, V38, P5884, DOI 10.1093/nar/gkq347
Anderson EJ, 2020, NEW ENGL J MED, V383, P2427, DOI 10.1056/NEJMoa2028436
Andries O, 2015, J CONTROL RELEASE, V217, P337, DOI 10.1016/j.jconrel.2015.08.051
Asadi K, 2021, INT J BIOL MACROMOL, V182, P648, DOI 10.1016/j.ijbiomac.2021.04.005
Babapoor Sankhiros, 2011, Influenza Res Treat, V2011, P126794, DOI 10.1155/2011/126794
Bachmann MF, 2010, NAT REV IMMUNOL, V10, P787, DOI 10.1038/nri2868
Bailey BA, 2017, MOL PHARMACEUT, V14, P3228, DOI 10.1021/acs.molpharmaceut.7b00586
Bailey BA, 2017, ADV HEALTHC MATER, V6, DOI 10.1002/adhm.201601418
Bakkari MA, 2021, MOL PHARMACEUT, V18, P2233, DOI 10.1021/acs.molpharmaceut.0c01256
Barrett JC, 2017, ACS BIOMATER SCI ENG, V3, P144, DOI 10.1021/acsbiomaterials.6b00422
Batista FD, 2000, EMBO J, V19, P513, DOI 10.1093/emboj/19.4.513
Batty Cole J., 2018, Pharmaceutical Nanotechnology, V6, P232, DOI
10.2174/2211738506666180918122337
Bazzill JD, 2018, NANO LETT, V18, P7832, DOI 10.1021/acs.nanolett.8b03601
Bernocchi B, 2017, INT J PHARMACEUT, V530, P128, DOI 10.1016/j.ijpharm.2017.07.012
Blass E, 2021, NAT REV CLIN ONCOL, V18, P215, DOI 10.1038/s41571-020-00460-2
Bomsel M, 2011, IMMUNITY, V34, P269, DOI 10.1016/j.immuni.2011.01.015
Boone CE, 2020, ACS APPL NANO MATER, V3, P8037, DOI 10.1021/acsanm.0c01506
Brouwer PJM, 2019, NAT COMMUN, V10, DOI 10.1038/s41467-019-12080-1
Burris HA, 2019, J CLIN ONCOL, V37, DOI 10.1200/JCO.2019.37.15_suppl.2523
Butkovich N, 2021, WIRES NANOMED NANOBI, V13, DOI 10.1002/wnan.1681
Casella CR, 2008, CELL MOL LIFE SCI, V65, P3231, DOI 10.1007/s00018-008-8228-6
Champion JA, 2006, P NATL ACAD SCI USA, V103, P4930, DOI 10.1073/pnas.0600997103
Chen DS, 2013, IMMUNITY, V39, P1, DOI 10.1016/j.immuni.2013.07.012
Chen FM, 2021, BIOMATERIALS, V270, DOI 10.1016/j.biomaterials.2021.120709
Chen JJ, 2013, BIOMATERIALS, V34, P8776, DOI 10.1016/j.biomaterials.2013.07.063
Chen L, 2021, SCI TRANSL MED, V13, DOI 10.1126/scitranslmed.abc2816
Chen Q, 2016, NAT COMMUN, V7, DOI 10.1038/ncomms13193
Chuan L, 2020, MATTER-US, V3, P12, DOI 10.1016/j.matt.2020.06.004
Climent N, 2018, NANOMED-NANOTECHNOL, V14, P339, DOI 10.1016/j.nano.2017.11.009
Coffman RL, 2010, IMMUNITY, V33, P492, DOI 10.1016/j.immuni.2010.10.002
Dagogo-Jack I, 2018, NAT REV CLIN ONCOL, V15, P81, DOI 10.1038/nrclinonc.2017.166

Das A, 2021, EXPERT REV VACCINES, V20, P1273, DOI 10.1080/14760584.2021.1984890
Morais SMD, 2018, VACCINE, V36, P6408, DOI 10.1016/j.vaccine.2018.08.070
Dehaini D, 2016, BIOENG TRANSL MED, V1, P30, DOI 10.1002/btm2.10004
Demento SL, 2012, BIOMATERIALS, V33, P4957, DOI 10.1016/j.biomaterials.2012.03.041
Deng GJ, 2018, ACS NANO, V12, P12096, DOI 10.1021/acsnano.8b05292
Deng L, 2018, NAT COMMUN, V9, DOI 10.1038/s41467-017-02725-4
Diego-Gonzalez L, 2020, PHARMACEUTICS, V12, DOI 10.3390/pharmaceutics12060489
Dong X, 2020, SCI ADV, V6, DOI 10.1126/sciadv.aba1590
Duan XP, 2019, ANGEW CHEM INT EDIT, V58, P670, DOI 10.1002/anie.201804882
Dubrovskaya V, 2019, IMMUNITY, V51, P915, DOI 10.1016/j.immuni.2019.10.008
Dykman LA, 2020, EXPERT REV VACCINES, V19, P465, DOI 10.1080/14760584.2020.1758070
Eggermont LJ, 2014, TRENDS BIOTECHNOL, V32, P456, DOI 10.1016/j.tibtech.2014.06.007
Eppler HB, 2020, ADV MATER, V32, DOI 10.1002/adma.201903367
Fan YC, 2017, NANO LETT, V17, P7387, DOI 10.1021/acs.nanolett.7b03218
Fang L, 2020, SCI ADV, V6, DOI 10.1126/sciadv.aba4024
Fang RH, 2018, ADV MATER, V30, DOI 10.1002/adma.201706759
Felnerova D, 2004, CURR OPIN BIOTECH, V15, P518, DOI 10.1016/j.copbio.2004.10.005
Figdor CG, 2002, NAT REV IMMUNOL, V2, P77, DOI 10.1038/nri723
Fontana F, 2017, ADV MATER, V29, DOI 10.1002/adma.201603239
Fries CN, 2021, NAT NANOTECHNOL, V16, P385, DOI 10.1038/s41565-020-0739-9
Frietze KM, 2016, CURR OPIN VIROL, V18, P44, DOI 10.1016/j.coviro.2016.03.001
Galluzzi L, 2017, NAT REV IMMUNOL, V17, P97, DOI 10.1038/nri.2016.107
Ge W, 2009, CANCER IMMUNOL IMMUN, V58, P201, DOI 10.1007/s00262-008-0539-9
Gnopo YMD, 2017, ADV DRUG DELIVER REV, V114, P132, DOI 10.1016/j.addr.2017.05.003
Gong H, 2021, NANO LETT, V21, P2603, DOI 10.1021/acs.nanolett.1c00238
Gonzalez-Ballesteros N, 2019, J MATER CHEM B, V7, P4677, DOI 10.1039/c9tb00215d
Guo YY, 2015, ACS NANO, V9, P6918, DOI 10.1021/acsnano.5b01042
Hajj KA, 2017, NAT REV MATER, V2, DOI 10.1038/natrevmats.2017.56
Hess KL, 2019, NANO TODAY, V27, P73, DOI 10.1016/j.nantod.2019.04.005
Ho W, 2021, ADV HEALTHC MATER, V10, DOI 10.1002/adhm.202001812
Hong XY, 2020, SCI ADV, V6, DOI 10.1126/sciadv.aaz4462
Hou XC, 2021, NAT REV MATER, V6, P1078, DOI 10.1038/s41578-021-00358-0
Hu HZ, 2021, ADV SCI, V8, DOI 10.1002/advs.202002020
Huang W, 2020, MATTER-US, V3, P1725, DOI 10.1016/j.matt.2020.08.027
Huang WC, 2018, NAT NANOTECHNOL, V13, P1174, DOI 10.1038/s41565-018-0271-3
Ichikawa J, 2020, CLIN CANCER RES, V26, P3384, DOI 10.1158/1078-0432.CCR-19-3487
Irene C, 2019, P NATL ACAD SCI USA, V116, P21780, DOI 10.1073/pnas.1905112116
Islam MA, 2021, BIOMATERIALS, V266, DOI 10.1016/j.biomaterials.2020.120431
Islam MA, 2018, NAT BIOMED ENG, V2, P850, DOI 10.1038/s41551-018-0284-0
Jardine J, 2013, SCIENCE, V340, P711, DOI 10.1126/science.1234150
Ji XY, 2019, ADV SCI, V6, DOI 10.1002/advs.201901211
Kamp HD, 2020, NPJ VACCINES, V5, DOI 10.1038/s41541-020-0183-8
Kang H, 2020, ADV HEALTHC MATER, V9, DOI 10.1002/adhm.201901223
Karch CP, 2019, NANOMED-NANOTECHNOL, V16, P206, DOI 10.1016/j.nano.2018.12.001
Karlsson J, 2018, ANNU REV CHEM BIOMOL, V9, P105, DOI 10.1146/annurev-chembioeng-060817-084055
Kasturi SP, 2020, SCI IMMUNOL, V5, DOI 10.1126/sciimmunol.abb1025
Khurana A, 2021, NANO TODAY, V38, DOI 10.1016/j.nantod.2021.101142
Kim OY, 2017, NAT COMMUN, V8, DOI 10.1038/s41467-017-00729-8
Kim YM, 2006, P NATL ACAD SCI USA, V103, P3327, DOI 10.1073/pnas.0511315103
Kingwell Katie, 2019, Nat Rev Drug Discov, V18, P171, DOI 10.1038/d41573-019-00025-8
Kirtane AR, 2021, NAT NANOTECHNOL, V16, P369, DOI 10.1038/s41565-021-00866-8
Kong N, 2021, NAT COMMUN, V12, DOI 10.1038/s41467-021-24961-5
Kong N, 2020, NANO LETT, V20, P3943, DOI 10.1021/acs.nanolett.0c01098
Kong N, 2019, SCI TRANSL MED, V11, DOI 10.1126/scitranslmed.aaw1565
Kosmides AK, 2018, NANO LETT, V18, P1916, DOI 10.1021/acs.nanolett.7b05284
Kranz LM, 2016, NATURE, V534, P396, DOI 10.1038/nature18300
Kroll AV, 2017, ADV MATER, V29, DOI 10.1002/adma.201703969
Kuai R, 2017, NAT MATER, V16, P489, DOI [10.1038/nmat4822, 10.1038/NMAT4822]
Kulp DW, 2017, NAT COMMUN, V8, DOI 10.1038/s41467-017-01549-6
Lai SK, 2009, ADV DRUG DELIVER REV, V61, P158, DOI 10.1016/j.addr.2008.11.002

Laurens MB, 2020, HUM VACC IMMUNOTHER, V16, P480, DOI 10.1080/21645515.2019.1669415
Lebel ME, 2016, NANO LETT, V16, P1826, DOI 10.1021/acs.nanolett.5b04877
Lee JY, 2020, ACS APPL MATER INTER, V12, P34658, DOI 10.1021/acsami.0c09484
Lee KL, 2017, NANO LETT, V17, P4019, DOI [10.1021/acs.nanolett.7b00107, 10.1021/acs.nanolett.7600107]
Li W, 2019, NAT COMMUN, V10, DOI 10.1038/s41467-019-11269-8
Li XM, 2018, COLLOID SURFACE B, V167, P260, DOI 10.1016/j.colsurfb.2018.04.005
Linares-Fernandez S, 2020, TRENDS MOL MED, V26, P311, DOI 10.1016/j.molmed.2019.10.002
Liu C, 2021, ADV MATER, V33, DOI 10.1002/adma.202102054
Liu C, 2021, CHEM SOC REV, V50, P2260, DOI 10.1039/d0cs01175d
Liu HP, 2014, NATURE, V507, P519, DOI 10.1038/nature12978
Liu QF, 2014, VACCINE, V32, P2582, DOI 10.1016/j.vaccine.2014.03.041
Liu SL, 2021, NAT MATER, V20, P421, DOI 10.1038/s41563-020-0793-6
Liu X, 2020, BIOMATERIALS, V230, DOI 10.1016/j.biomaterials.2019.119649
Lizotte PH, 2016, NAT NANOTECHNOL, V11, P295, DOI [10.1038/NNANO.2015.292, 10.1038/nnano.2015.292]
Lopes A, 2019, J EXP CLIN CANC RES, V38, DOI 10.1186/s13046-019-1154-7
Luo LH, 2021, BIOMATERIALS, V270, DOI 10.1016/j.biomaterials.2021.120678
Maier MA, 2013, MOL THER, V21, P1570, DOI 10.1038/mt.2013.124
Marshall JD, 2004, CELL IMMUNOL, V229, P93, DOI 10.1016/j.cellimm.2004.04.009
Mateu Ferrando Ruth, 2020, Drug Discov Today Technol, V38, P57, DOI 10.1016/j.ddtec.2021.02.001
McLeod HL, 2020, J CLIN ONCOL, V38, P103, DOI 10.1200/JCO.19.01712
McNamara MA, 2015, J IMMUNOL RES, V2015, DOI 10.1155/2015/794528
Meyer RA, 2015, SMALL, V11, P1519, DOI 10.1002/smll.201402369
Mody KT, 2013, NANOSCALE, V5, P5167, DOI 10.1039/c3nr00357d
Mohsen MO, 2017, SEMIN IMMUNOL, V34, P123, DOI 10.1016/j.smim.2017.08.014
Molino NM, 2016, BIOMATERIALS, V86, P83, DOI 10.1016/j.biomaterials.2016.01.056
Moon JJ, 2012, P NATL ACAD SCI USA, V109, P1080, DOI 10.1073/pnas.1112648109
Mora-Solano C, 2017, BIOMATERIALS, V149, P1, DOI 10.1016/j.biomaterials.2017.09.031
Neek M, 2019, NANOMED-NANOTECHNOL, V15, P164, DOI 10.1016/j.nano.2018.09.004
O'Connor G, 2019, EUR J PHARM BIOPHARM, V134, P153, DOI 10.1016/j.ejpb.2018.10.020
O'Neill CL, 2021, ACTA BIOMATER, V133, P153, DOI 10.1016/j.actbio.2021.05.003
Ochyl LJ, 2018, BIOMATERIALS, V182, P157, DOI 10.1016/j.biomaterials.2018.08.016
Oroojalian F, 2021, SMALL, V17, DOI 10.1002/smll.202006484
Ouyang J, 2020, NANO TODAY, V35, DOI 10.1016/j.nantod.2020.100949
Ouyang J, 2020, P NATL ACAD SCI USA, V117, P28667, DOI 10.1073/pnas.2016268117
Ouyang XM, 2020, BIOMATER SCI-UK, V8, P1160, DOI 10.1039/c9bm01401b
Pardi N, 2018, NAT REV DRUG DISCOV, V17, P261, DOI 10.1038/nrd.2017.243
Patel MR, 2020, J CLIN ONCOL, V38
Patel R, 2018, ACS OMEGA, V3, P3702, DOI 10.1021/acsomega.8b00227
Pejawar-Gaddy S, 2014, BIOCONJUGATE CHEM, V25, P1470, DOI 10.1021/bc5002246
Perica K, 2014, NANOMED-NANOTECHNOL, V10, P119, DOI 10.1016/j.nano.2013.06.015
Phuengkham H, 2018, ADV MATER, V30, DOI 10.1002/adma.201706719
Polack FP, 2020, NEW ENGL J MED, V383, P2603, DOI 10.1056/NEJMoa2034577
Puffer EB, 2007, ACS CHEM BIOL, V2, P252, DOI 10.1021/cb600489g
Qing S, 2020, ADV MATER, V32, DOI 10.1002/adma.202002085
Rao L, 2019, ADV FUNCT MATER, V29, DOI 10.1002/adfm.201905671
Rao L, 2015, SMALL, V11, P6225, DOI 10.1002/smll.201502388
Reddy ST, 2007, NAT BIOTECHNOL, V25, P1159, DOI [10.1038/nbt1332, 10.1038/nbtl332]
Rietscher R, 2016, EUR J PHARM BIOPHARM, V102, P20, DOI 10.1016/j.ejpb.2016.02.014
Rohner NA, 2017, ACS BIOMATER SCI ENG, V3, P153, DOI 10.1021/acsbiomaterials.6b00438
Samaridou E, 2020, ADV DRUG DELIVER REV, V154, P37, DOI 10.1016/j.addr.2020.06.002
Schudel A, 2020, NAT NANOTECHNOL, V15, P491, DOI 10.1038/s41565-020-0679-4
Schuster BS, 2013, BIOMATERIALS, V34, P3439, DOI 10.1016/j.biomaterials.2013.01.064
Sedic M, 2018, VET PATHOL, V55, P341, DOI 10.1177/0300985817738095
Shao S, 2020, ANN BIOMED ENG, V48, P1991, DOI 10.1007/s10439-019-02398-8
Sharma M, 2013, NANOSCALE, V5, P3747, DOI 10.1039/c3nr30071d
Shi GN, 2017, BIOMATERIALS, V113, P191, DOI 10.1016/j.biomaterials.2016.10.047
Shi SR, 2020, CHEM ENG J, V388, DOI 10.1016/j.cej.2020.124268
Shields CW, 2020, ADV MATER, V32, DOI 10.1002/adma.201901633
Shukla S, 2017, BIOMATERIALS, V121, P15, DOI 10.1016/j.biomaterials.2016.12.030

Simon-Vazquez R, 2020, EXPERT OPIN DRUG DEL, V17, P1007, DOI 10.1080/17425247.2020.1776259
Singh A, 2021, NAT NANOTECHNOL, V16, P16, DOI 10.1038/s41565-020-00790-3
Singh B, 2018, INT J BIOL MACROMOL, V110, P54, DOI 10.1016/j.ijbiomac.2017.10.101
Sixt M, 2005, IMMUNITY, V22, P19, DOI 10.1016/j.immuni.2004.11.013
Sliepen K, 2015, RETROVIROLOGY, V12, DOI 10.1186/s12977-015-0210-4
Tait DR, 2019, NEW ENGL J MED, V381, P2429, DOI 10.1056/NEJMoa1909953
Takamura S, 2004, GENE THER, V11, P628, DOI 10.1038/sj.gt.3302193
Tang ZM, 2021, ACTA PHARM SIN B, V11, P3447, DOI 10.1016/j.apsb.2021.05.004
Tang ZM, 2021, NANO TODAY, V36, DOI 10.1016/j.nantod.2020.101019
Tang ZM, 2020, NAT REV MATER, V5, P847, DOI 10.1038/s41578-020-00247-y
Tao W, 2020, SCI TRANSL MED, V12, DOI 10.1126/scitranslmed.aay1063
Tesarova B, 2020, J CONTROL RELEASE, V325, P176, DOI 10.1016/j.jconrel.2020.06.026
Tokatlian T, 2019, SCIENCE, V363, P649, DOI 10.1126/science.aat9120
Tran KTM, 2021, NAT BIOMED ENG, V5, P998, DOI 10.1038/s41551-020-00650-4
Turajlic S, 2019, NAT REV GENET, V20, P404, DOI 10.1038/s41576-019-0114-6
Wang DA, 2010, VACCINE, V28, P3134, DOI 10.1016/j.vaccine.2010.02.058
Wang J, 2021, ADV DRUG DELIVER REV, V178, DOI 10.1016/j.addr.2021.113974
Wei XL, 2018, ADV MATER, V30, DOI 10.1002/adma.201802233
Wu DJ, 2020, CARBOHYD POLYM, V238, DOI 10.1016/j.carbpol.2020.116126
Xiang SD, 2006, METHODS, V40, P1, DOI 10.1016/j.ymeth.2006.05.016
Xiao YF, 2020, ANGEW CHEM INT EDIT, V59, P19787, DOI 10.1002/anie.202008879
Xu SX, 2021, ANGEW CHEM INT EDIT, V60, P3858, DOI 10.1002/anie.202005309
Xu ZY, 2020, ADV SCI, V7, DOI 10.1002/advs.201902802
Yang JL, 2021, PROG MATER SCI, V118, DOI 10.1016/j.pmatsci.2020.100768
Yang R, 2018, ACS NANO, V12, P5121, DOI 10.1021/acsnano.7b09041
Yang Y, 2021, INT J BIOL MACROMOL, V183, P1891, DOI 10.1016/j.ijbiomac.2021.05.158
Yenkoidiok-Douti L, 2021, ACS BIOMATER SCI ENG, V7, P1854, DOI 10.1021/acsbiomaterials.0c01363
Yu FL, 2012, NANOMED-NANOTECHNOL, V8, P1337, DOI 10.1016/j.nano.2012.02.015
Yu GT, 2018, ADV FUNCT MATER, V28, DOI 10.1002/adfm.201801389
Yu SS, 2012, INT J NANOMED, V7, P799, DOI 10.2147/IJN.S28531
Zhang DKY, 2020, NAT PROTOC, V15, P773, DOI 10.1038/s41596-019-0249-0
Zhang QB, 2017, ACS NANO, V11, P10724, DOI 10.1021/acsnano.7b04955
Zhang YW, 2021, J CONTROL RELEASE, V330, P554, DOI 10.1016/j.jconrel.2020.12.038
Zhang Y, 2021, ADV MATER, V33, DOI 10.1002/adma.202007293
Zhao Y, 2018, MOL PHARMACEUT, V15, P1791, DOI 10.1021/acs.molpharmaceut.7b01103
Zheng B, 2021, CHEM ENG J, V418, DOI 10.1016/j.cej.2021.129392
Zheng DW, 2020, NAT COMMUN, V11, DOI 10.1038/s41467-020-15927-0
Zhong XF, 2019, J CONTROL RELEASE, V300, P81, DOI 10.1016/j.jconrel.2019.02.035
Zhou JR, 2020, ADV MATER, V32, DOI 10.1002/adma.201901255
Zhou L, 2020, ACCOUNTS CHEM RES, V53, P1761, DOI 10.1021/acs.accounts.0c00254
NR 194
TC 26
Z9 27
U1 9
U2 52
PU INST MATERIA MEDICA, CHINESE ACAD MEDICAL SCIENCES
PI BEIJING
PA C/O EDITORIAL BOARD OF ACTA PHARMACEUTICA SINICA, 1 XIANNONGTAN ST,
BEIJING, 100050, PEOPLES R CHINA
SN 2211-3835
EI 2211-3843
J9 ACTA PHARM SIN B
JI Acta Pharm. Sin. B
PD MAY
PY 2022
VL 12
IS 5
BP 2206
EP 2223
DI 10.1016/j.apsb.2021.12.0212211-3835
PG 18

WC Pharmacology & Pharmacy
WE Science Citation Index Expanded (SCI-EXPANDED)
SC Pharmacology & Pharmacy
GA 2C5DP
UT WOS:000810889400005
PM 35013704
DA 2023-09-14
ER

PT J
AU Liu, H
Chen, HL
Liu, ZJ
Le, ZC
Nie, TQ
Qiao, DD
Su, Y
Mai, HQ
Chen, YM
Liu, LX
AF Liu, Hong
Chen, Haolin
Liu, Zhijia
Le, Zhicheng
Nie, Tianqi
Qiao, Dongdong
Su, Yan
Mai, Haiqiang
Chen, Yongming
Liu, Lixin
TI Therapeutic nanovaccines sensitize EBV-associated tumors to checkpoint blockade therapy
SO BIOMATERIALS
LA English
DT Article
DE Checkpoint inhibitor; Immunotherapy; Nanovaccine; Subunit antigen; Tannic acid; Tumors
ID EPSTEIN-BARR-VIRUS; NASOPHARYNGEAL CARCINOMA; IMMUNE-RESPONSE; VACCINE; NIVOLUMAB; EXPRESSION; RESISTANCE; ADJUVANT; DELIVERY; PROTEIN
AB For successful treatment of EBV-associated tumors immune tolerance must be broken. While most studies of EBV-associated tumor vaccines have focused on augmenting tumor-specific effector T cells, the effects of these vaccines on the immune-suppressive tumor microenvironment have not been investigated. Here, we describe the manufacture of a nanovaccine using tannic acid (TA) and a newly constructed protein antigen for EBV-associated tumors with interferon-alpha (IFN-alpha) or CpG as adjuvants. TA as a biocompatible material from plant self-assembles with antigens and adjuvants via hydrogen bonding to form well-defined nanoparticulate vaccines by flash nanocomplexation, a scalable yet controllable technique. By targeting lymph nodes, the nanovaccine co-loaded with CpG adjuvant induces strong immune activation and exhibits efficient inhibition tumorigenesis. Moreover, the nanovaccine combining with anti-PD-L1 results a marked decrease in tumor size and prolonged survival of tumor-bearing mice by decreasing infiltration of regulatory T cells to the tumor lesion. This suggests that the nanovaccine can reverse immune checkpoint inhibitor resistance by remodeling the tumor microenvironment. Thus, this study shows a promising strategy for treatment of EBV-positive tumors in patients.
C1 [Liu, Hong; Chen, Haolin; Liu, Zhijia; Le, Zhicheng; Nie, Tianqi; Qiao, Dongdong; Chen, Yongming; Liu, Lixin] Sun Yat Sen Univ, Key Lab Polymer Composite & Funct Mat, Sch Mat Sci & Engn, Ctr Funct Biomat,Minist Educ, Guangzhou, Peoples R China.
[Su, Yan] Biotao Therapeut Inc, Beijing, Peoples R China.
[Mai, Haiqiang] Sun Yat Sen Univ, Ctr Canc, Guangzhou, Peoples R China.
C3 Sun Yat Sen University; Sun Yat Sen University
RP Liu, LX (通讯作者), Sun Yat Sen Univ, Key Lab Polymer Composite & Funct Mat, Sch Mat Sci & Engn, Ctr Funct Biomat,Minist Educ, Guangzhou, Peoples R China.

EM liulixin@mail.sysu.edu.cn
RI LE, Zhicheng/IUN-8004-2023
OI LE, Zhicheng/0000-0002-4931-5500; Liu, Hong/0000-0003-0350-7475
FU National Natural Science Foundation of China [51820105004]; Guangdong Innovative and Entrepreneurial Research Team Program [2013S086]
FX We appreciate discussion and suggestion from Kam W. Leong of Columbia University and Hai-quan Mao of Johns Hopkins University. Financial support from the National Natural Science Foundation of China (51820105004) and the Guangdong Innovative and Entrepreneurial Research Team Program (2013S086) is gratefully acknowledged.
CR Al Moustafa AE, 2018, FRONT ONCOL, V8, DOI 10.3389/fonc.2018.00528
Balfour HH, 2013, J INFECT DIS, V207, P80, DOI 10.1093/infdis/jis646
Bellone M, 2017, CYTOKINE GROWTH F R, V36, P17, DOI 10.1016/j.cytogfr.2017.06.002
Bonaventura P, 2019, FRONT IMMUNOL, V10, DOI 10.3389/fimmu.2019.00168
CHENG HM, 1991, J CLIN MICROBIOL, V29, P2180, DOI 10.1128/JCM.29.10.2180-2186.1991
Conniot J, 2019, NAT NANOTECHNOL, V14, P891, DOI 10.1038/s41565-019-0512-0
de-The G, 1975, IARC Sci Publ, P3
Eroglu Z, 2018, NATURE, V553, P347, DOI 10.1038/nature25187
Flanagan J, 2003, J GEN VIROL, V84, P1871, DOI 10.1099/vir.0.18944-0
Fu J, 2015, SCI TRANSL MED, V7, DOI 10.1126/scitranslmed.aaa4306
Gajewski TF, 2017, ADV EXP MED BIOL, V1036, P19, DOI 10.1007/978-3-319-67577-0_2
Gibney GT, 2015, CLIN CANCER RES, V21, P712, DOI 10.1158/1078-0432.CCR-14-2468
He ZY, 2017, BIOMATERIALS, V130, P28, DOI 10.1016/j.biomaterials.2017.03.028
Holmgaard RB, 2015, CELL REP, V13, P412, DOI 10.1016/j.celrep.2015.08.077
Huang C, 2016, SCI REP-UK, V6, DOI 10.1038/srep28497
Leskowitz R, 2014, J VIROL, V88, P4721, DOI 10.1128/JVI.03744-13
Li J, 2015, ONCOIMMUNOLOGY, V4, DOI 10.4161/23723556.2014.976507
Lin PH, 2018, MUCOSAL IMMUNOL, V11, P1239, DOI 10.1038/s41385-018-0004-9
Ma BBY, 2018, J CLIN ONCOL, V36, P1412, DOI 10.1200/JCO.2017.77.0388
Motsch N, 2007, RNA BIOL, V4, P131, DOI 10.4161/rna.4.3.5206
Munz C, 2000, J EXP MED, V191, P1649, DOI 10.1084/jem.191.10.1649
O'Donnell JS, 2017, CANCER TREAT REV, V52, P71, DOI 10.1016/j.ctrv.2016.11.007
Oweida A, 2017, ONCOIMMUNOLOGY, V6, DOI 10.1080/2162402X.2017.1356153
Prochetto E, 2017, ONCOTARGET, V8, P58003, DOI 10.18632/oncotarget.18217
Proietti E, 2002, J IMMUNOL, V169, P375, DOI 10.4049/jimmunol.169.1.375
Qiao DD, 2018, NANO LETT, V18, P3007, DOI 10.1021/acs.nanolett.8b00478
Quah BJC, 2007, NAT PROTOC, V2, P2049, DOI 10.1038/nprot.2007.296
Ribas A, 2018, SCIENCE, V359, P1350, DOI 10.1126/science.aar4060
Taylor GS, 2016, CHIN CLIN ONCOL, V5, DOI 10.21037/cco.2016.03.20
Taylor GS, 2004, J VIROL, V78, P768, DOI 10.1128/JVI.78.2.768-778.2004
Van Allen EM, 2015, SCIENCE, V350, P207, DOI 10.1126/science.aad0095
Verma V, 2019, NAT IMMUNOL, V20, P1231, DOI 10.1038/s41590-019-0441-y
Voo KS, 2004, J EXP MED, V199, P459, DOI 10.1084/jem.20031219
Wang M, 2013, APPL MICROBIOL BIOT, V97, P6251, DOI 10.1007/s00253-013-4967-x
Wang Y, 2018, P NATL ACAD SCI USA, V115, pE8698, DOI 10.1073/pnas.1809232115
Weber JS, 2013, J CLIN ONCOL, V31, P4311, DOI 10.1200/JCO.2013.51.4802
Yin Y, 2003, SCIENCE, V301, P1371, DOI 10.1126/science.1088902
Young LS, 2004, NAT REV CANCER, V4, P757, DOI 10.1038/nrc1452
Zhao BC, 2018, FRONT IMMUNOL, V9, DOI 10.3389/fimmu.2018.00932
Zhou QQ, 2016, ACS NANO, V10, P2678, DOI 10.1021/acsnano.5b07716
Zhu GZ, 2017, ACS NANO, V11, P2387, DOI 10.1021/acsnano.7b00978
NR 41
TC 26
Z9 26
U1 8
U2 82
PU ELSEVIER SCI LTD
PI OXFORD
PA THE BOULEVARD, LANGFORD LANE, KIDLINGTON, OXFORD OX5 1GB, OXON, ENGLAND
SN 0142-9612
EI 1878-5905

J9 BIOMATERIALS
JI Biomaterials
PD OCT
PY 2020
VL 255
AR 120158
DI 10.1016/j.biomaterials.2020.120158
PG 12
WC Engineering, Biomedical; Materials Science, Biomaterials
WE Science Citation Index Expanded (SCI-EXPANDED)
SC Engineering; Materials Science
GA MU5DQ
UT WOS:000555693800013
PM 32544717
DA 2023-09-14
ER

PT S
AU Hagan, CT
   Medik, YB
   Wang, AZ
AF Hagan, C. Tilden
   Medik, Yusra B.
   Wang, Andrew Z.
BE Broome, AM
TI Nanotechnology Approaches to Improving Cancer Immunotherapy
SO CANCER NANOTECHNOLOGY-BOOK
SE Advances in Cancer Research
LA English
DT Review; Book Chapter
ID DENDRITIC CELLS; TARGETED DELIVERY; IMMUNE-RESPONSE; ANTITUMOR IMMUNITY;
   RNA DELIVERY; NANOPARTICLES; VACCINES; SYSTEM; VACCINATION; NANOVACCINE
AB Cancer immunotherapy is a powerful, growing treatment approach to cancer that can be combined with chemotherapy, radiotherapy, and oncosurgery. Modulating the immune system to enhance anticancer response by several strategies has yielded improved cancer survival. Despite this progress, the success rate for immunotherapy has been below expectations due to unpredictable efficacy and off-target side effects from systemic dosing. Nanotechnology offers numerous different materials and targeting properties to overcome many of these challenges in immunotherapy. In this chapter, we review current immunotherapy and its challenges as well as the latest nanotechnology applications in cancer immunotherapy.
C1 [Hagan, C. Tilden; Medik, Yusra B.; Wang, Andrew Z.] Univ North Carolina Chapel Hill, Carolina Ctr Canc Nanotechnol Excellence, Lab Nano & Translat Med, Lineberger Comprehens Canc Ctr,Carolina Inst Nano, Chapel Hill, NC 27599 USA.
   [Hagan, C. Tilden; Medik, Yusra B.; Wang, Andrew Z.] Univ North Carolina Chapel Hill, Lineberger Comprehens Canc Ctr, Dept Radiat Oncol, Chapel Hill, NC 27599 USA.
   [Hagan, C. Tilden] Univ North Carolina Chapel Hill, UNC NCSU Joint Dept Biomed Engn, Chapel Hill, NC USA.
C3 University of North Carolina School of Medicine; University of North
   Carolina; University of North Carolina Chapel Hill; University of North
   Carolina; University of North Carolina Chapel Hill; University of North
   Carolina School of Medicine; University of North Carolina School of
   Medicine; North Carolina State University; University of North Carolina;
   University of North Carolina Chapel Hill
RP Wang, AZ (通讯作者)，Univ North Carolina Chapel Hill, Carolina Ctr Canc Nanotechnol Excellence, Lab Nano & Translat Med, Lineberger Comprehens Canc Ctr,Carolina Inst Nano, Chapel Hill, NC 27599 USA.; Wang, AZ (通讯作者)，Univ North Carolina Chapel Hill, Lineberger Comprehens Canc Ctr, Dept Radiat Oncol, Chapel Hill, NC 27599 USA.
EM zawang@med.unc.edu
RI Wang, Andrew zhuang/AAU-7549-2020
OI Wang, Andrew zhuang/0000-0002-9781-4494
FU NCI NIH HHS [U54 CA198999] Funding Source: Medline

CR Bauleth-Ramos T, 2017, ADV FUNCT MATER, V27, DOI 10.1002/adfm.201703303
Gondan AIB, 2018, BIOMATERIALS, V170, P95, DOI 10.1016/j.biomaterials.2018.04.003
Bolli E, 2018, ONCOIMMUNOLOGY, V7, DOI 10.1080/2162402X.2017.1408746
Boutros C, 2016, NAT REV CLIN ONCOL, V13, P473, DOI 10.1038/nrclinonc.2016.58
Buonaguro L, 2011, EXPERT REV VACCINES, V10, P1569, DOI [10.1586/erv.11.135, 10.1586/ERV.11.135]
Chakraborty A, 2018, BIOCONJUGATE CHEM, V29, P657, DOI 10.1021/acs.bioconjchem.7b00455
Chen DS, 2013, IMMUNITY, V39, P1, DOI 10.1016/j.immuni.2013.07.012
Chiavenna SM, 2017, J BIOMED SCI, V24, DOI 10.1186/s12929-016-0311-y
Christian DA, 2012, IMMUNOTHERAPY-UK, V4, P425, DOI [10.2217/imt.12.26, 10.2217/IMT.12.26]
COLEY W.B., 1898, J AMER MED ASSOC, VXXXI, P389
Colzani B, 2018, INT J NANOMED, V13, P957, DOI 10.2147/IJN.S152742
Cruz LJ, 2014, J CONTROL RELEASE, V192, P209, DOI 10.1016/j.jconrel.2014.07.040
Czapar AE, 2018, ADV SCI, V5, DOI 10.1002/advs.201700991
Demento SL, 2012, BIOMATERIALS, V33, P4957, DOI 10.1016/j.biomaterials.2012.03.041
Duan F, 2017, BIOMATERIALS, V122, P23, DOI 10.1016/j.biomaterials.2017.01.017
Dunn GP, 2002, NAT IMMUNOL, V3, P991, DOI 10.1038/ni1102-991
Dwivedi PD, 2011, J BIOMED NANOTECHNOL, V7, P193, DOI 10.1166/jbn.2011.1264
Fogli S, 2017, NANOMEDICINE-UK, V12, P1647, DOI 10.2217/nnm-2017-0061
Fontana F, 2017, WIRES NANOMED NANOBI, V9, DOI 10.1002/wnan.1421
Garcia-Vallejo JJ, 2013, MOL IMMUNOL, V53, P387, DOI 10.1016/j.molimm.2012.09.012
Grimaldi AM, 2017, NANOMEDICINE-UK, V12, P2349, DOI 10.2217/nnm-2017-0208
Guimaraes PPG, 2018, ACS NANO, V12, P912, DOI 10.1021/acsnano.7b05876
Hansel TT, 2010, NAT REV DRUG DISCOV, V9, P325, DOI 10.1038/nrd3003
Hassan HAFM, 2016, BIOMATERIALS, V104, P310, DOI 10.1016/j.biomaterials.2016.07.005
Hercus TR, 2009, BLOOD, V114, P1289, DOI 10.1182/blood-2008-12-164004
Hobo W, 2013, CANCER IMMUNOL IMMUN, V62, P285, DOI 10.1007/s00262-012-1334-1
Irvine DJ, 2015, CHEM REV, V115, P11109, DOI 10.1021/acs.chemrev.5b00109
Jahan ST, 2018, INT J NANOMED, V13, P367, DOI 10.2147/IJN.S144266
Kang T, 2018, BIOMATERIALS, V164, P80, DOI 10.1016/j.biomaterials.2018.02.033
Kapadia CH, 2015, J CONTROL RELEASE, V219, P167, DOI 10.1016/j.jconrel.2015.09.062
Khalil DN, 2016, NAT REV CLIN ONCOL, V13, P273, DOI 10.1038/nrclinonc.2016.25
Kim H, 2018, BIOMATERIALS, V164, P38, DOI 10.1016/j.biomaterials.2018.02.034
Kranz LM, 2016, NATURE, V534, P396, DOI 10.1038/nature18300
Kuai R, 2017, NAT MATER, V16, P489, DOI [10.1038/nmat4822, 10.1038/NMAT4822]
Lai CH, 2018, THERANOSTICS, V8, P1723, DOI 10.7150/thno.22056
Li GF, 2018, GASTROENTEROLOGY, V154, P1024, DOI 10.1053/j.gastro.2017.10.050
Liang RJ, 2017, BIOMATERIALS, V149, P41, DOI 10.1016/j.biomaterials.2017.09.029
Liu LL, 2016, J IMMUNOL, V197, P1231, DOI 10.4049/jimmunol.1600182
Lizotte PH, 2016, NAT NANOTECHNOL, V11, P295, DOI [10.1038/NNANO.2015.292, 10.1038/nnano.2015.292]
Mi Y, 2018, ADV MATER, V30, DOI 10.1002/adma.201706098
Min YZ, 2017, NAT NANOTECHNOL, V12, P877, DOI [10.1038/nnano.2017.113, 10.1038/NNANO.2017.113]
Moura LIF, 2017, J DRUG TARGET, V25, P873, DOI 10.1080/1061186X.2017.1363213
Oberli MA, 2017, NANO LETT, V17, P1326, DOI 10.1021/acs.nanolett.6b03329
Pardoll DM, 2012, NAT REV CANCER, V12, P252, DOI 10.1038/nrc3239
Patel SA, 2018, IMMUNITY, V48, P417, DOI 10.1016/j.immuni.2018.03.007
Qiu H, 2017, WIRES NANOMED NANOBI, V9, DOI 10.1002/wnan.1456
Ray M, 2018, BIOCONJUGATE CHEM, V29, P445, DOI 10.1021/acs.bioconjchem.7b00768
Rosenberg SA, 2014, J IMMUNOL, V192, P5451, DOI 10.4049/jimmunol.1490019
Sahin U, 2014, NAT REV DRUG DISCOV, V13, P759, DOI 10.1038/nrd4278
Schreiber RD, 2011, SCIENCE, V331, P1565, DOI 10.1126/science.1203486
Silva JM, 2013, J CONTROL RELEASE, V168, P179, DOI 10.1016/j.jconrel.2013.03.010
Smith DM, 2013, NAT REV IMMUNOL, V13, P592, DOI 10.1038/nri3488
Tang JL, 2016, NANOMED-NANOTECHNOL, V12, P945, DOI 10.1016/j.nano.2015.11.021
van der Burg SH, 2016, NAT REV CANCER, V16, P219, DOI 10.1038/nrc.2016.16
Vasievich EA, 2012, MOL PHARMACEUT, V9, P261, DOI 10.1021/mp200350n
Wang X, 2018, INT J MOL MED, V41, P3671, DOI 10.3892/ijmm.2018.3570
Xie YQ, 2018, WIRES NANOMED NANOBI, V10, DOI 10.1002/wnan.1506
Yan WL, 2017, IMMUNOTHERAPY-UK, V9, P347, DOI 10.2217/imt-2016-0141

Yin H, 2014, NAT REV GENET, V15, P541, DOI 10.1038/nrg3763
Zhang PF, 2015, P NATL ACAD SCI USA, V112, pE6129, DOI 10.1073/pnas.1505799112
Zhuang XT, 2016, J CONTROL RELEASE, V228, P26, DOI 10.1016/j.jconrel.2016.02.035
Zolnik BS, 2010, ENDOCRINOLOGY, V151, P458, DOI 10.1210/en.2009-1082
NR 62
TC 26
Z9 28
U1 0
U2 33
PU ELSEVIER ACADEMIC PRESS INC
PI SAN DIEGO
PA 525 B STREET, SUITE 1900, SAN DIEGO, CA 92101-4495 USA
SN 0065-230X
BN 978-0-12-814169-4
J9 ADV CANCER RES
JI Adv.Cancer Res.
PY 2018
VL 139
BP 35
EP 56
DI 10.1016/bs.acr.2018.05.003
PG 22
WC Oncology; Nanoscience & Nanotechnology
WE Book Citation Index– Science (BKCI-S); Science Citation Index Expanded (SCI-EXPANDED)
SC Oncology; Science & Technology - Other Topics
GA BL6EB
UT WOS:000453659500003
PM 29941106
DA 2023-09-14
ER

PT J
AU Wen, M
Liu, MJ
Xue, WT
Yang, K
Chen, GJ
Zhang, WD
AF Wen, Ming
Liu, Mengjie
Xue, Wentao
Yang, Kai
Chen, Gaojian
Zhang, Weidong
TI Simple and Green Strategy for the Synthesis of "Pathogen-Mimetic" Glycoadjuvant@AuNPs by Combination of Photoinduced RAFT and Bioinspired Dopamine Chemistry
SO ACS MACRO LETTERS
LA English
DT Article
ID CANCER-IMMUNOTHERAPY; LYMPH-NODE; NANOPARTICLES; POLYMERIZATION; BINDING; FUNCTIONALIZATION; GLYCOPOLYMERS; NANOVACCINE; PARTICLES; SURFACES
AB Innate immune responses recognizing pathogen associated molecular patterns (PAMPs) play a crucial role in adaptive immunity. Toll-like receptors (TLRs) and C-type lectin receptors (CLRs) contribute to antigen capture, uptake, presentation and activation of immune responses. In this contribution, metal-free reversible addition-fragmentation chain transfer (RAFT) polymerization of N-3,4-dihydroxybenzenethyl methacrylamide (DMA) and 2-(methacrylamido) glucopyranose (MAG) under sunlight irradiation using 2-cyanoprop-2-yl-alpha-dithionaphthalate (CPDN) as iniferter agent, can be employed to fabricate the multivalent glycopolymer containing bioresponsive sugar group and multifunctional catechol functionalities. The polymerization behavior is investigated and it presents

controlled features. Moreover, bioinspired dopamine chemistry can be successfully utilized to form in situ glycopolymer-coated gold nanoparticles (AuNPs) without the need of additional reducing reagent, design "pathogen-mimetic" glycoadjuvant recognized by both CLRs and TLRs. The synthetic glycoadjuvant is found to enhance the adjuvant activity as "infected signals" in vitro.
C1 [Chen, Gaojian; Zhang, Weidong] Soochow Univ, Ctr Soft Condensed Matter Phys & Interdisciplinar, Suzhou 215006, Peoples R China.
Soochow Univ, State & Local Joint Engn Lab Novel Funct Polymer, Suzhou 215123, Peoples R China.
C3 Soochow University - China; Soochow University - China
RP Chen, GJ; Zhang, WD (通讯作者)，Soochow Univ, Ctr Soft Condensed Matter Phys & Interdisciplinar, Suzhou 215006, Peoples R China.
EM gchen@suda.edu.cn; zhangweidong@suda.edu.cn
RI Chen, Gaojian/C-2802-2008; zhang, weidong/B-3866-2012
OI Chen, Gaojian/0000-0002-5877-3159; zhang, weidong/0000-0002-6837-3060
FU Natural Science Foundation of Jiangsu Province [BK20171208]; National
Natural Science Foundation of China [21774084]
FX The authors thank the Natural Science Foundation of Jiangsu Province
(No. BK20171208) and the National Natural Science Foundation of China
(No. 21774084) for financial support.
CR Becer CR, 2010, J AM CHEM SOC, V132, P15130, DOI 10.1021/ja1056714
Chen K, 2016, POLYM CHEM-UK, V7, P2565, DOI 10.1039/c6py00129g
Chen M, 2015, ACS MACRO LETT, V4, P566, DOI 10.1021/acsmacrolett.5b00241
Chen Y, 2015, MACROMOLECULES, V48, P346, DOI 10.1021/ma501742v
Faure E, 2013, PROG POLYM SCI, V38, P236, DOI 10.1016/j.progpolymsci.2012.06.004
Goldberg MS, 2015, CELL, V161, P201, DOI 10.1016/j.cell.2015.03.037
Holten-Andersen N, 2011, P NATL ACAD SCI USA, V108, P2651, DOI 10.1073/pnas.1015862108
Isakova A, 2014, MACROMOLECULES, V47, P2561, DOI 10.1021/ma500336u
Julier Z, 2016, VACCINE, V34, P2453, DOI 10.1016/j.vaccine.2016.03.057
Vang KB, 2017, SCI REP-UK, V7, DOI 10.1038/s41598-017-04459-1
Lee H, 2009, ADV MATER, V21, P431, DOI 10.1002/adma.200801222
Lepenies B, 2013, ADV DRUG DELIVER REV, V65, P1271, DOI 10.1016/j.addr.2013.05.007
Li X, 2015, POLYM CHEM-UK, V6, P1417, DOI 10.1039/c4py01740d
Li X, 2014, J MATER CHEM B, V2, P5569, DOI 10.1039/c4tb00852a
Liu HP, 2015, BIOCONJUGATE CHEM, V26, P791, DOI 10.1021/acs.bioconjchem.5b00103
Liu HP, 2014, NATURE, V507, P519, DOI 10.1038/nature12978
Lu JW, 2014, POLYM CHEM-UK, V5, P2326, DOI 10.1039/c3py01526b
McKenzie TG, 2016, POLYM CHEM-UK, V7, P4246, DOI 10.1039/c6py00808a
Miura Y, 2016, CHEM REV, V116, P1673, DOI 10.1021/acs.chemrev.5b00247
Mrowczynski R, 2018, ACS APPL MATER INTER, V10, P7541, DOI 10.1021/acsami.7b08392
Neves A, 2002, INORG CHEM, V41, P1788, DOI 10.1021/ic010708u
Sedo J, 2013, ADV MATER, V25, P653, DOI 10.1002/adma.201202343
Son S, 2002, J AGR FOOD CHEM, V50, P468, DOI 10.1021/jf010830b
Sunasee R, 2013, MACROMOL BIOSCI, V13, P9, DOI 10.1002/mabi.201200222
Thomas SN, 2014, BIOMATERIALS, V35, P814, DOI 10.1016/j.biomaterials.2013.10.003
Ting SRS, 2010, MACROMOLECULES, V43, P5211, DOI 10.1021/ma1004937
Wang C, 2016, BIOMATERIALS, V79, P88, DOI 10.1016/j.biomaterials.2015.11.040
Wang J, 2016, ACS MACRO LETT, V5, P1278, DOI 10.1021/acsmacrolett.6b00818
Wang JL, 2014, MACROMOL RAPID COMM, V35, P1061, DOI 10.1002/marc.201400029
Wu LB, 2017, J AM CHEM SOC, V139, P14684, DOI 10.1021/jacs.7b07768
Xu LQ, 2012, POLYM CHEM-UK, V3, P920, DOI 10.1039/c2py00552b
Yan HM, 2015, FRONT IMMUNOL, V6, DOI 10.3389/fimmu.2015.00408
Yeow J, 2016, ACS MACRO LETT, V5, P558, DOI 10.1021/acsmacrolett.6b00235
Zhang Q, 2016, POLYM CHEM-UK, V7, P7002, DOI 10.1039/c6py01709f
Zhang WD, 2017, BIOCONJUGATE CHEM, V28, P1993, DOI 10.1021/acs.bioconjchem.7b00313
Zhu GZ, 2017, ACS NANO, V11, P2387, DOI 10.1021/acsnano.7b00978
NR 36
TC 26
Z9 26
U1 1
U2 68
PU AMER CHEMICAL SOC
PI WASHINGTON

PA 1155 16TH ST, NW, WASHINGTON, DC 20036 USA
SN 2161-1653
J9 ACS MACRO LETT
JI ACS Macro Lett.
PD JAN
PY 2018
VL 7
IS 1
BP 70
EP 74
DI 10.1021/acsmacrolett.7b00837
PG 5
WC Polymer Science
WE Science Citation Index Expanded (SCI-EXPANDED)
SC Polymer Science
GA FT3AC
UT WOS:000423016200013
PM 35610919
DA 2023-09-14
ER

PT J
AU Shi, R
Hong, L
Wu, DC
Ning, XX
Chen, Y
Lin, T
Fan, DM
Wu, KC
AF Shi, R
Hong, L
Wu, DC
Ning, XX
Chen, Y
Lin, T
Fan, DM
Wu, KC
TI Enhanced immune response to gastric cancer specific antigen peptide by
coencapsulation with CpG oligodeoxynucleotides in nanoemulsion
SO CANCER BIOLOGY & THERAPY
LA English
DT Article
DE gastric cancer; coencapsulate; CPG; nanovaccine; peptide
ID IMMUNOSTIMULATORY DNA-SEQUENCES; B SURFACE-ANTIGEN; BACTERIAL-DNA;
DENDRITIC CELLS; MG7 ANTIGEN; VACCINES; MICE; PROTEIN; TRANSFECTION;
IMMUNIZATION
AB CpG oligodeoxynucleotides (CpG ODN) have been shown to have potent adjuvant activity for a wide range of antigens. Of particular interest is their improved activity when closely associated with the antigen. The purpose of this study is to construct a nanovaccine coencapsulated with a gastric cancer specific antigen MG7 mimotope peptide and adjuvant CpG ODN 1645 using new nanotechnology as nanoemulsion and evaluate its immunocompetence. Nanoemulsion vaccine was prepared using magnetic ultrasound methods. BALB/c mice were immunized and the in vivo effectiveness was evaluated using tumor challenge assay. It was shown that the tumor masses formed in the mice immunized with coencapsulated nanovaccine (0.0825 g) markedly smaller ($P < 0.01$) than those formed in the mice immunized with nanovaccine encapsulated with antigen peptide alone (0.4465 g). A tumor inhibiting rate as high as 82.5% of the coencapsulated nanovaccine was obtained, while nanovaccine encapsulated with peptide only could not achieve the same effect (28.5%) ($P < 0.01$). Enzyme-linked immunospot assay (ELISPOT) showed that immunization using MG7 mimotope peptide coencapsulated with CpG ODN within the same nonoemulsion enhanced the frequency of splenocytes secreting IFN-gamma significantly ($P <$ 0.01) when compared with immunization using MG7 peptide encapsulated in nanoemulsion alone (197

spots/1 x 10(6) vs. 73 spots/l x 10(6)). Cellular ELISA indicated that serum titer of antibody against MG7-Ag was significantly higher (P < 0.01) in mice immunized with coencapsulation form nanovaccine (0.7884) than that in the group immunized with nanovaccine encapsulated with MG7 peptide alone (0.3616). Using intracellular flow cytometric analysis, it was found that the IFN-gamma response was contributed by CD4(+) T-cells. Our experiments suggest that a vaccinal approach using nano-delivery system carrying in tumoral epitope and CpG ODN as adjuvant may have important implications for cancer therapy.
C1 Fourth Mil Med Univ, Xiling Hosp, Inst Digest Dis, Natl Key Lab Gi Canc Biol, Xian 710032, Shaanxi Prov, Peoples R China.
Fourth Mil Med Univ, Dept Chem, Xian, Shaanxi Prov, Peoples R China.
C3 Air Force Military Medical University; Air Force Military Medical University
RP Wu, KC (通讯作者)，Fourth Mil Med Univ, Xiling Hosp, Inst Digest Dis, Natl Key Lab Gi Canc Biol, Xian 710032, Shaanxi Prov, Peoples R China.
EM kaicwu@fmmu.edu.cn
RI Wu, Daocheng/H-5661-2017
OI Wu, Kaichun/0000-0002-0699-9666; Wu, Daocheng/0000-0002-6183-539X
CR Baines J, 2003, CLIN CANCER RES, V9, P2693
Bauer S, 2001, P NATL ACAD SCI USA, V98, P9237, DOI 10.1073/pnas.161293498
Chaix C, 2003, COLLOID SURFACE B, V29, P39, DOI 10.1016/S0927-7765(02)00177-7
Cho HJ, 2002, J IMMUNOL, V168, P4907, DOI 10.4049/jimmunol.168.10.4907
Cho HJ, 2000, NAT BIOTECHNOL, V18, P509, DOI 10.1038/75365
Fengtian He, 2002, Chin Med Sci J, V17, P215
Gramzinski RA, 2002, INFECT IMMUN, V70, P5338, DOI 10.1128/IAI.70.9.5338.2002
Guo CC, 2003, WORLD J GASTROENTERO, V9, P1191, DOI 10.3748/wjg.v9.i6.1191
Guo DL, 2002, WORLD J GASTROENTERO, V8, P1009, DOI 10.3748/wjg.v8.i6.1009
Guo Dong-li, 2003, Zhonghua Liu Xing Bing Xue Za Zhi, V24, P494
Hancock GE, 2001, VACCINE, V19, P4874, DOI 10.1016/S0264-410X(01)00228-6
Hang Quan-Li, 2003, Xi Bao Yu Fen Zi Mian Yi Xue Za Zhi, V19, P308
Heckelsmiller K, 2002, J IMMUNOL, V169, P3892, DOI 10.4049/jimmunol.169.7.3892
Hirunpetcharat C, 2003, VACCINE, V21, P2923, DOI 10.1016/S0264-410X(03)00132-4
Horner AA, 2001, IMMUNOL REV, V179, P102, DOI 10.1034/j.1600-065X.2001.790111.x
Jores K, 2004, J CONTROL RELEASE, V95, P217, DOI 10.1016/j.jconrel.2003.11.012
Joseph A, 2002, VACCINE, V20, P3342, DOI 10.1016/S0264-410X(02)00295-5
Krieg AM, 2000, SPRINGER SEMIN IMMUN, V22, P97, DOI 10.1007/s002810050019
Lawrence MJ, 2000, ADV DRUG DELIVER REV, V45, P89, DOI 10.1016/S0169-409X(00)00103-4
Li Song, 2001, Current Gene Therapy, V1, P201, DOI 10.2174/1566523013348814
Li WM, 2003, VACCINE, V21, P3319, DOI 10.1016/S0264-410X(03)00172-5
Liu J, 2002, INT J CLIN PRACT, V56, P169
Liu NS, 2003, NAT IMMUNOL, V4, P687, DOI 10.1038/ni941
Marquez L., 2003, COLLOIDI TPOINT, V2, P12
McCluskie MJ, 2002, FEMS IMMUNOL MED MIC, V32, P179, DOI 10.1016/S0928-8244(01)00305-4
McCluskie MJ, 2000, SPRINGER SEMIN IMMUN, V22, P125, DOI 10.1007/s002810000014
Millan CLB, 1998, P NATL ACAD SCI USA, V95, P15553, DOI 10.1073/pnas.95.26.15553
Morisaki Takashi, 2003, Hum Cell, V16, P175, DOI 10.1111/j.1749-0774.2003.tb00151.x
Myc A, 2003, VACCINE, V21, P3801, DOI 10.1016/S0264-410X(03)00381-5
Niidome T, 2002, GENE THER, V9, P1647, DOI 10.1038/sj.gt.3301923
NING XX, 2003, J TUMOR MARKER ONCOL, V18, P53
Pan GL, 2003, PHARM RES-DORDR, V20, P738, DOI 10.1023/A:1023477317668
Pichyangkul S, 2001, J IMMUNOL METHODS, V247, P83, DOI 10.1016/S0022-1759(00)00320-3
Seki J, 2004, INT J PHARM, V273, P75, DOI 10.1016/j.ijpharm.2003.12.022
SERGUEI V, 2002, ADV DRUG DELIVERY RE, V54, P135
Shirota H, 2001, J IMMUNOL, V167, P66, DOI 10.4049/jimmunol.167.1.66
Singh M, 1999, NAT BIOTECHNOL, V17, P1075, DOI 10.1038/15058
Sparwasser T, 1998, EUR J IMMUNOL, V28, P2045, DOI 10.1002/(SICI)1521-4141(199806)28:06<2045::AID-IMMU2045>3.0.CO;2-8
Tighe H, 2000, J ALLERGY CLIN IMMUN, V106, P124, DOI 10.1067/mai.2000.107927
Tighe H, 2000, EUR J IMMUNOL, V30, P1939, DOI 10.1002/1521-4141(200007)30:7<1939::AID-IMMU1939>3.0.CO;2-#
Wagner H, 2000, SPRINGER SEMIN IMMUN, V22, P147, DOI 10.1007/s002810000025
Weijzen S, 2001, J IMMUNOL, V166, P7151, DOI 10.4049/jimmunol.166.12.7151

Weiner GJ, 2000, SPRINGER SEMIN IMMUN, V22, P107, DOI 10.1007/s002810000022
Wu HL, 2001, INT J PHARM, V221, P23, DOI 10.1016/S0378-5173(01)00672-X
Xu L, 2003, MOL CANCER THER, V2, P301
Xu L, 2000, Zhonghua Yi Xue Za Zhi, V80, P304
Yamamoto S, 2000, SPRINGER SEMIN IMMUN, V22, P35, DOI 10.1007/s002810050013
Yi AK, 1998, J IMMUNOL, V160, P4755
Zhang Y, 2003, VACCINE, V21, P3307, DOI 10.1016/S0264-410X(03)00176-2
NR 49
TC 26
Z9 31
U1 0
U2 9
PU TAYLOR & FRANCIS INC
PI PHILADELPHIA
PA 530 WALNUT STREET, STE 850, PHILADELPHIA, PA 19106 USA
SN 1538-4047
EI 1555-8576
J9 CANCER BIOL THER
JI Cancer Biol. Ther.
PD FEB
PY 2005
VL 4
IS 2
BP 218
EP 224
PG 7
WC Oncology
WE Science Citation Index Expanded (SCI-EXPANDED)
SC Oncology
GA 957GX
UT WOS:000231359400014
PM 15753659
DA 2023-09-14
ER

PT J
AU Zhang, LM
Xu, LF
Wang, Y
Liu, JY
Tan, GH
Huang, FY
He, NY
Lu, ZX
AF Zhang, Liming
Xu, Lingfeng
Wang, Yi
Liu, Jieyu
Tan, Guanghong
Huang, Fengying
He, Nongyue
Lu, Zhuoxuan
TI A novel therapeutic vaccine based on graphene oxide nanocomposite for tumor immunotherapy
SO CHINESE CHEMICAL LETTERS
LA English
DT Article
DE Nanovaccine; Graphene Oxide; Adjuvant; Tumor-specific antigen; NLG919
ID DENDRITIC CELLS; DELIVERY-SYSTEM; CANCER; NANOPARTICLES; NANOMATERIALS; PROGRESS; BIOLOGY
AB With an intensive understanding of the mechanism of immune system, developing a therapeutic tumor

vaccine is one of the most perspective strategy of cancer immunotherapy. In this study, we report a facile approach to prepare graphene oxide (GO)-based therapeutic cancer-nanovaccine. The model antigen (ovalbumin, OVA) and adjuvant (CpG ODN), are conjugated with GO-PEI nanosheet through electrostatic interaction. The addition of PEG can improve biocompatibility and prevent nanoparticle aggregation. The prepared GO-based nanovaccine, GO-PEI-OVA-PEG-CpG, exhibits good biocompatibility and low toxicity both in vivo and in vitro. More importantly, it can efficiently induce the maturation of dendritic cells (DCs), the enhancement of antigen cross-presentation ability, and the amplification of cytokine production of immune cells. Impressively, this nanovaccine shows a remarkable therapeutic effect against preestablished B16-OVA-melanoma tumors, which can significantly inhibit tumor growth and prolong the survival time of the OVA-expressed tumor-bearing mice. Moreover, combining GO-PEI-OVA-PEG-CpG with NLG919, an IDO-1 (indoleamine-2,3-dioxygenase) inhibitor which can regulate the tumor microenvironment, displays a synergistic therapeutic effect. These findings indicate the GO-PEI-OVA-PEG-CpG nanovaccine actively induces an antigen-specific antitumor immune response and it combined with NLG919 could achieve better therapeutic outcomes. (C) 2022 Published by Elsevier B.V. on behalf of Chinese Chemical Society and Institute of Materia Medica, Chinese Academy of Medical Sciences.
C1 [Zhang, Liming; Xu, Lingfeng; Wang, Yi; Liu, Jieyu; Tan, Guanghong; Huang, Fengying; Lu, Zhuoxuan] Hainan Med Univ, Key Lab Trop Translat Med, Minist Educ, Haikou 571199, Hainan, Peoples R China.
[Zhang, Liming; Xu, Lingfeng; Wang, Yi; Liu, Jieyu; Tan, Guanghong; Huang, Fengying; Lu, Zhuoxuan] Hainan Med Univ, Hainan Prov Key Lab Trop Med, Haikou 571199, Hainan, Peoples R China.
[He, Nongyue] Southeast Univ, Sch Biol Sci & Med Engn, State Key Lab Bioelect, Nanjing 210096, Peoples R China.
[Xu, Lingfeng] Univ Chinese Acad Sci, HwaMei Hosp, Dept Clin Lab, Ningbo 315000, Peoples R China.
C3 Hainan Medical University; Hainan Medical University; Southeast University - China; Chinese Academy of Sciences; University of Chinese Academy of Sciences, CAS
RP Lu, ZX (通讯作者)，Hainan Med Univ, Key Lab Trop Translat Med, Minist Educ, Haikou 571199, Hainan, Peoples R China.; Lu, ZX (通讯作者)，Hainan Med Univ, Hainan Prov Key Lab Trop Med, Haikou 571199, Hainan, Peoples R China.; He, NY (通讯作者)，Southeast Univ, Sch Biol Sci & Med Engn, State Key Lab Bioelect, Nanjing 210096, Peoples R China.
EM nyhe19584@163.com; Luzhuoxuan1981@163.com
FU Basic and Applied Basic Research Program of Hainan Province [2019RC209, 820RC646]; National Natural Science Foundation of China [81860037]; China Postdoctoral Science Special Foundations [2015T80488, 2014T70459]
FX We acknowledge the financial support from Basic and Applied Basic Research Program of Hainan Province (Nos. 2019RC209 and 820RC646), the National Natural Science Foundation of China (No. 81860037) and China Postdoctoral Science Special Foundations (Nos. 2015T80488 and 2014T70459).
CR Ahmad H, 2019, MATER EXPRESS, V9, P265, DOI 10.1166/mex.2019.1494
Andersen MH, 2019, SEMIN IMMUNOPATHOL, V41, P1, DOI 10.1007/s00281-018-0711-z
Bethune MT, 2017, CURR OPIN BIOTECH, V48, P142, DOI 10.1016/j.copbio.2017.03.024
Chen FM, 2021, BIOMATERIALS, V270, DOI 10.1016/j.biomaterials.2021.120709
Couper KN, 2008, J IMMUNOL, V180, P5771, DOI 10.4049/jimmunol.180.9.5771
Deshmane SL, 2009, J INTERF CYTOK RES, V29, P313, DOI 10.1089/jir.2008.0027
Frohlich E, 2012, INT J NANOMED, V7, P5577, DOI 10.2147/IJN.S36111
Hou KX, 2021, MATER ADV, V2, P3441, DOI 10.1039/d1ma00174d
Jalili Ahmad, 2007, Iran J Immunol, V4, P127
Kayraklioglu N, 2021, METHODS MOL BIOL, V2197, P51, DOI 10.1007/978-1-0716-0872-2_4
Le Gall CM, 2018, NAT MATER, V17, P474, DOI 10.1038/s41563-018-0093-6
Liang F, 2017, MOL THER, V25, P2635, DOI 10.1016/j.ymthe.2017.08.006
Lou J, 2019, ADV THER-GERMANY, V2, DOI 10.1002/adtp.201800128
Luo KP, 2021, CHEM ENG J, V412, DOI 10.1016/j.cej.2021.128659
Ma DY, 2009, SEMIN IMMUNOL, V21, P265, DOI 10.1016/j.smim.2009.05.010
Mautino M.R., 2013, CANCER RES, V73, P491, DOI DOI 10.1158/1538-7445.AM2013-491
Mu WW, 2020, NANO-MICRO LETT, V12, DOI 10.1007/s40820-020-00482-6
Ni K, 1997, IMMUNOL CELL BIOL, V75, P223, DOI 10.1038/icb.1997.35
OKADA M, 1988, J IMMUNOL, V141, P1543
Otsuka H, 2003, ADV DRUG DELIVER REV, V55, P403, DOI 10.1016/S0169-409X(02)00226-0
Polak ME, 2005, J PATHOL, V207, P83, DOI 10.1002/path.1809
Priyadarsini S, 2018, J NANOSTRUCTURE CHEM, V8, P123, DOI 10.1007/s40097-018-0265-6

Sau S, 2018, J CONTROL RELEASE, V274, P24, DOI 10.1016/j.jconrel.2018.01.028
Scheetz L, 2019, NAT BIOMED ENG, V3, P768, DOI 10.1038/s41551-019-0436-x
Sharma P, 2011, NAT REV CANCER, V11, P805, DOI 10.1038/nrc3153
Shen LJ, 2020, CHINESE CHEM LETT, V31, P1709, DOI 10.1016/j.cclet.2020.02.007
Shirota H, 2015, VACCINES-BASEL, V3, P390, DOI 10.3390/vaccines3020390
SIDHU RS, 1993, PHARMACOL THERAPEUT, V57, P79, DOI 10.1016/0163-7258(93)90037-E
Su H, 2018, J APPL TOXICOL, V38, P3, DOI 10.1002/jat.3476
Sun CR, 2010, ACS NANO, V4, P2402, DOI 10.1021/nn100190v
Sun K, 2021, SMALL, V17, DOI 10.1002/smll.202101897
Wang YY, 2020, J HEMATOL ONCOL, V13, DOI 10.1186/s13045-020-00939-6
Wang ZY, 2021, CHINESE CHEM LETT, V32, P1888, DOI 10.1016/j.cclet.2021.01.036
Wei C, 2019, J BIOMED NANOTECHNOL, V15, P100, DOI 10.1166/jbn.2019.2669
Xiong ZY, 2016, FOOD HYDROCOLLOID, V60, P29, DOI 10.1016/j.foodhyd.2016.03.007
Xu LG, 2016, NANOSCALE, V8, P3785, DOI 10.1039/c5nr09208f
Yang F, 2021, BIOACT MATER, V6, P3036, DOI 10.1016/j.bioactmat.2021.03.003
Yang F, 2020, APPL MATER TODAY, V19, DOI 10.1016/j.apmt.2020.100608
Zeng LM, 2020, CHINESE CHEM LETT, V31, P1162, DOI 10.1016/j.cclet.2019.10.015
Zhang HR, 2019, J BIOMED NANOTECHNOL, V15, P1839, DOI 10.1166/jbn.2019.2827
Zhang LM, 2011, SMALL, V7, P460, DOI 10.1002/smll.201001522
Zhang LM, 2010, SMALL, V6, P537, DOI 10.1002/smll.200901680
Zhang Y, 2019, WIRES NANOMED NANOBI, V11, DOI 10.1002/wnan.1559
Zhao H, 2019, ACS NANO, V13, P13127, DOI 10.1021/acsnano.9b05974
Zhao L, 2014, VACCINE, V32, P327, DOI 10.1016/j.vaccine.2013.11.069
Zhou TL, 2021, BIOACT MATER, V6, P697, DOI 10.1016/j.bioactmat.2020.09.002
NR 46
TC 25
Z9 26
U1 13
U2 40
PU ELSEVIER SCIENCE INC
PI NEW YORK
PA STE 800, 230 PARK AVE, NEW YORK, NY 10169 USA
SN 1001-8417
EI 1878-5964
J9 CHINESE CHEM LETT
JI Chin. Chem. Lett.
PD AUG
PY 2022
VL 33
IS 8
BP 4089
EP 4095
DI 10.1016/j.cclet.2022.01.071
EA JUN 2022
PG 7
WC Chemistry, Multidisciplinary
WE Science Citation Index Expanded (SCI-EXPANDED)
SC Chemistry
GA 2I7DT
UT WOS:000815135400087
DA 2023-09-14
ER

PT J
AU Cai, T
Liu, HN
Zhang, S
Hu, J
Zhang, LX
AF Cai, Ting
Liu, Huina

Zhang, Shun
Hu, Jing
Zhang, Lingxiao
TI Delivery of nanovaccine towards lymphoid organs: recent strategies in enhancing cancer immunotherapy
SO JOURNAL OF NANOBIOTECHNOLOGY
LA English
DT Review
DE Nanovaccine; Cancer immunotherapy; Lymphoid organ; Vaccine delivery; Immune barrier
ID SUBCAPSULAR SINUS MACROPHAGES; IN-SITU VACCINATION; THERAPEUTIC VACCINES; DENDRITIC CELLS; RNA DELIVERY; T-CELLS; NANOPARTICLES; ANTIGEN; SIZE; TRAFFICKING
AB With the in-depth exploration on cancer therapeutic nanovaccines, increasing evidence shows that the poor delivery of nanovaccines to lymphoid organs has become the culprit limiting the rapid induction of anti-tumor immune response. Unlike the conventional prophylactic vaccines that mainly form a depot at the injection site to gradually trigger durable immune response, the rapid proliferation of tumors requires an efficient delivery of nanovaccines to lymphoid organs for rapid induction of anti-tumor immunity. Optimization of the physicochemical properties of nanovaccine (e.g., size, shape, charge, colloidal stability and surface ligands) is an effective strategy to enhance their accumulation in lymphoid organs, and nanovaccines with dynamic structures are also designed for precise targeted delivery of lymphoid organs or their subregions. The recent progress of these nanovaccine delivery strategies is highlighted in this review, and the challenges and future direction are also discussed.
C1 [Cai, Ting; Liu, Huina; Zhang, Shun; Zhang, Lingxiao] Univ Chinese Acad Sci, Ningbo Hwa Mei Hosp, Ningbo Clin Res Ctr Digest Syst Tumors, Ningbo 315010, Peoples R China.
[Cai, Ting; Liu, Huina; Zhang, Shun; Zhang, Lingxiao] Univ Chinese Acad Sci, Ningbo Hwa Mei Hosp, Key Lab Diag & Treatment Digest Syst Tumors Zheji, Ningbo 315010, Peoples R China.
[Cai, Ting; Liu, Huina; Zhang, Shun; Zhang, Lingxiao] Univ Chinese Acad Sci, Ningbo Inst Life & Hlth Ind, Ningbo 315010, Peoples R China.
[Hu, Jing] Zhejiang Univ, Coll Chem & Biol Engn, Key Lab Biomass Chem Engn, Minist Educ, Hangzhou 310027, Peoples R China.
[Hu, Jing] Zhejiang Univ, Hangzhou Global Sci & Technol Innovat Ctr, Hangzhou 211200, Peoples R China.
[Zhang, Lingxiao] Zhejiang Univ, Coll Pharmaceut Sci, Hangzhou 310058, Peoples R China.
C3 Chinese Academy of Sciences; University of Chinese Academy of Sciences, CAS; Chinese Academy of Sciences; University of Chinese Academy of Sciences, CAS; Chinese Academy of Sciences; University of Chinese Academy of Sciences, CAS; Zhejiang University; Zhejiang University; Zhejiang University
RP Zhang, LX (通讯作者)，Univ Chinese Acad Sci, Ningbo Hwa Mei Hosp, Ningbo Clin Res Ctr Digest Syst Tumors, Ningbo 315010, Peoples R China.; Zhang, LX (通讯作者)，Univ Chinese Acad Sci, Ningbo Hwa Mei Hosp, Key Lab Diag & Treatment Digest Syst Tumors Zheji, Ningbo 315010, Peoples R China.; Zhang, LX (通讯作者)，Univ Chinese Acad Sci, Ningbo Inst Life & Hlth Ind, Ningbo 315010, Peoples R China.; Hu, J (通讯作者)，Zhejiang Univ, Coll Chem & Biol Engn, Key Lab Biomass Chem Engn, Minist Educ, Hangzhou 310027, Peoples R China.; Hu, J (通讯作者)，Zhejiang Univ, Hangzhou Global Sci & Technol Innovat Ctr, Hangzhou 211200, Peoples R China.; Zhang, LX (通讯作者)，Zhejiang Univ, Coll Pharmaceut Sci, Hangzhou 310058, Peoples R China.
EM hujing10@zju.edu.cn; zhanglx913@zju.edu.cn
OI Zhang, Lingxiao/0000-0002-7066-6065
FU National Natural Science Foundation of China [32101123]; Ningbo Clinical Research Center for Digestive System Tumors [2019A21003]; Natural Science Foundation of Ningbo [2021J320]; China Postdoctoral Science Foundation [2021M702816]
FX This work was financially supported by National Natural Science Foundation of China (Grant Number 32101123), Ningbo Clinical Research Center for Digestive System Tumors (Grant Number 2019A21003), Natural Science Foundation of Ningbo (Grant Number 2021J320) and China Postdoctoral Science Foundation (Grant Number 2021M702816).
CR An JY, 2020, ACS NANO, V14, P7639, DOI 10.1021/acsnano.0c03881
Bachmann MF, 2010, NAT REV IMMUNOL, V10, P787, DOI 10.1038/nri2868
Bronte V, 2013, IMMUNITY, V39, P806, DOI 10.1016/j.immuni.2013.10.010

Carrasco YR, 2007, IMMUNITY, V27, P160, DOI 10.1016/j.immuni.2007.06.007
Cataldi M, 2017, INT J MOL SCI, V18, DOI 10.3390/ijms18061249
Chen CY, 2020, SMALL, V16, DOI 10.1002/smll.202004934
Chen CY, 2020, SMALL, V16, DOI 10.1002/smll.202002503
Chen FM, 2021, BIOMATERIALS, V270, DOI 10.1016/j.biomaterials.2021.120709
Chen Q, 2019, NAT NANOTECHNOL, V14, P89, DOI 10.1038/s41565-018-0319-4
Chen WY, 2018, SMALL, V14, DOI 10.1002/smll.201704465
Chen WY, 2016, SMALL, V12, P1627, DOI 10.1002/smll.201503359
Chen WF, 2018, THERANOSTICS, V8, P2229, DOI 10.7150/thno.24073
Cho NH, 2011, NAT NANOTECHNOL, V6, P675, DOI [10.1038/NNANO.2011.149, 10.1038/nnano.2011.149]
Chu YH, 2018, THERANOSTICS, V8, P4238, DOI 10.7150/thno.24387
Cooks T, 2018, NAT COMMUN, V9, DOI 10.1038/s41467-018-03224-w
Corbet C, 2017, NAT REV CANCER, V17, P577, DOI 10.1038/nrc.2017.77
Cyster JG, 2010, NAT IMMUNOL, V11, P989, DOI 10.1038/ni.1946
Demoy M, 1999, LIFE SCI, V64, P1329, DOI 10.1016/S0024-3205(99)00068-5
den Haan JMM, 2013, SEMIN IMMUNOPATHOL, V35, P541, DOI 10.1007/s00281-013-0378-4
Ding YY, 2021, ADV DRUG DELIVER REV, V179, DOI 10.1016/j.addr.2021.113914
Ernsting MJ, 2013, J CONTROL RELEASE, V172, P782, DOI 10.1016/j.jconrel.2013.09.013
Fan YC, 2015, VACCINES-BASEL, V3, P662, DOI 10.3390/vaccines3030662
Girard JP, 2012, NAT REV IMMUNOL, V12, P762, DOI 10.1038/nri3298
Gong NQ, 2019, ADV FUNCT MATER, V29, DOI 10.1002/adfm.201806087
Gonzalez SF, 2011, ANNU REV IMMUNOL, V29, P215, DOI 10.1146/annurev-immunol-031210-101255
Gu WY, 2019, BIOMACROMOLECULES, V20, P625, DOI 10.1021/acs.biomac.8b01196
GUPTA RK, 1993, VACCINE, V11, P293, DOI 10.1016/0264-410X(93)90190-9
Guy B, 2007, NAT REV MICROBIOL, V5, P505, DOI 10.1038/nrmicro1681
Hammerich L, 2015, MOL ONCOL, V9, P1966, DOI 10.1016/j.molonc.2015.10.016
Han X, 2019, SCI ADV, V5, DOI 10.1126/sciadv.aaw6870
Howard GP, 2019, NANO RES, V12, P837, DOI 10.1007/s12274-019-2301-3
Hu Z, 2018, NAT REV IMMUNOL, V18, P168, DOI 10.1038/nri.2017.131
Irvine DJ, 2015, CHEM REV, V115, P11109, DOI 10.1021/acs.chemrev.5b00109
Itano AA, 2003, NAT IMMUNOL, V4, P733, DOI 10.1038/ni957
Jennings GT, 2007, CURR MOL MED, V7, P143, DOI 10.2174/156652407780059140
Jiang H, 2017, J CONTROL RELEASE, V267, P47, DOI 10.1016/j.jconrel.2017.08.009
Jiang LW, 2021, NANO TODAY, V36, DOI 10.1016/j.nantod.2020.101045
Junt T, 2007, NATURE, V450, P110, DOI 10.1038/nature06287
Karabin NB, 2018, NAT COMMUN, V9, DOI 10.1038/s41467-018-03001-9
Kim J, 2015, NAT BIOTECHNOL, V33, P64, DOI 10.1038/nbt.3071
Kool M, 2012, J MED MICROBIOL, V61, P927, DOI 10.1099/jmm.0.038943-0
Kranz LM, 2016, NATURE, V534, P396, DOI 10.1038/nature18300
Kuai R, 2017, NAT MATER, V16, P489, DOI [10.1038/nmat4822, 10.1038/NMAT4822]
Kuka M, 2018, NAT REV IMMUNOL, V18, P255, DOI 10.1038/nri.2017.133
LEAK LV, 1971, J CELL BIOL, V50, P300, DOI 10.1083/jcb.50.2.300
Li F, 2019, INT J NANOMED, V14, P9917, DOI 10.2147/IJN.S226466
Li SX, 2019, ADV FUNCT MATER, V29, DOI 10.1002/adfm.201903391
Liu GN, 2021, ADV DRUG DELIVER REV, V176, DOI 10.1016/j.addr.2021.113889
Liu JP, 2019, SMALL, V15, DOI 10.1002/smll.201900262
Liu J, 2019, ACS NANO, V13, P8648, DOI 10.1021/acsnano.9b03472
Lu XF, 2019, NAT NANOTECHNOL, V14, P719, DOI 10.1038/s41565-019-0472-4
Manolova V, 2008, EUR J IMMUNOL, V38, P1404, DOI 10.1002/eji.200737984
Mao CK, 2020, INT J HYPERTHER, V37, P4, DOI 10.1080/02656736.2020.1810333
Marcandalli J, 2019, CELL, V176, P1420, DOI 10.1016/j.cell.2019.01.046
MARCHESI VT, 1964, PROC R SOC SER B-BIO, V159, P283, DOI 10.1098/rspb.1964.0002
Margaris KN, 2012, J R SOC INTERFACE, V9, P601, DOI 10.1098/rsif.2011.0751
Melero I, 2014, NAT REV CLIN ONCOL, V11, P509, DOI 10.1038/nrclinonc.2014.111
Melief CJM, 2015, J CLIN INVEST, V125, P3401, DOI 10.1172/JCI80009
Milling L, 2017, ADV DRUG DELIVER REV, V114, P79, DOI 10.1016/j.addr.2017.05.011
Min YZ, 2017, NAT NANOTECHNOL, V12, P877, DOI [10.1038/nnano.2017.113, 10.1038/NNANO.2017.113]
Moghimi SM, 2012, ANNU REV PHARMACOL, V52, P481, DOI 10.1146/annurev-pharmtox-010611-134623

MOGHIMI SM, 1993, BIOCHIM BIOPHYS ACTA, V1157, P233, DOI 10.1016/0304-4165(93)90105-H
Mondino A, 1996, P NATL ACAD SCI USA, V93, P2245, DOI 10.1073/pnas.93.6.2245
Moore JE, 2018, ANNU REV FLUID MECH, V50, P459, DOI 10.1146/annurev-fluid-122316-045259
Moyer TJ, 2016, J CLIN INVEST, V126, P799, DOI 10.1172/JCI81083
Nikitin MP, 2020, NAT BIOMED ENG, V4, P717, DOI 10.1038/s41551-020-0581-2
Noy R, 2014, IMMUNITY, V41, P49, DOI 10.1016/j.immuni.2014.06.010
Oberli MA, 2017, NANO LETT, V17, P1326, DOI 10.1021/acs.nanolett.6b03329
Oussoren C, 1997, BBA-BIOMEMBRANES, V1328, P261, DOI 10.1016/S0005-2736(97)00122-3
Ouyang B, 2020, NAT MATER, V19, P1362, DOI 10.1038/s41563-020-0755-z
Palucka K, 2012, NAT REV CANCER, V12, P265, DOI 10.1038/nrc3258
Peng F, 2019, NAT NANOTECHNOL, V14, P279, DOI 10.1038/s41565-018-0356-z
Pflicke H, 2009, J EXP MED, V206, P2925, DOI 10.1084/jem.20091739
Phan TG, 2007, NAT IMMUNOL, V8, P992, DOI 10.1038/ni1494
Phan TG, 2009, NAT IMMUNOL, V10, P786, DOI 10.1038/ni.1745
Qin H, 2021, ADV MATER, V33, DOI 10.1002/adma.202006007
Reddy ST, 2006, J CONTROL RELEASE, V112, P26, DOI 10.1016/j.jconrel.2006.01.006
Reed SG, 2013, NAT MED, V19, P1597, DOI 10.1038/nm.3409
Schudel A, 2020, NAT NANOTECHNOL, V15, P491, DOI 10.1038/s41565-020-0679-4
Schudel A, 2019, NAT REV MATER, V4, P415, DOI 10.1038/s41578-019-0110-7
Schumacher TN, 2015, SCIENCE, V348, P69, DOI 10.1126/science.aaa4971
Singh M, 2007, EXPERT REV VACCINES, V6, P797, DOI 10.1586/14760584.6.5.797
Song TT, 2021, ADV MATER, V33, DOI 10.1002/adma.202100106
Sultan H, 2019, CANCER IMMUNOL IMMUN, V68, P455, DOI 10.1007/s00262-018-02294-5
Swartz MA, 2001, ADV DRUG DELIVER REV, V50, P3, DOI 10.1016/S0169-409X(01)00150-8
Tavares AJ, 2017, P NATL ACAD SCI USA, V114, pE10871, DOI 10.1073/pnas.1713390114
Theurl I, 2016, NAT MED, V22, P945, DOI 10.1038/nm.4146
Ukidve A, 2020, P NATL ACAD SCI USA, V117, P17727, DOI 10.1073/pnas.2002880117
Verbeke CS, 2015, ADV HEALTHC MATER, V4, P2677, DOI 10.1002/adhm.201500618
Verdegaal EME, 2016, NATURE, V536, P91, DOI 10.1038/nature18945
von Andrian UH, 2003, NAT REV IMMUNOL, V3, P867, DOI 10.1038/nri1222
Wang H, 2018, NAT MATER, V17, P761, DOI 10.1038/s41563-018-0147-9
Wang L, 2020, BIOMATERIALS, V255, DOI 10.1016/j.biomaterials.2020.120208
Wang S, 2021, SCI TRANSL MED, V13, DOI 10.1126/scitranslmed.abb6981
Wang YC, 2021, NAT COMMUN, V12, DOI 10.1038/s41467-021-25197-z
Willard-Mack CL, 2006, TOXICOL PATHOL, V34, P409, DOI 10.1080/01926230600867727
Xia YF, 2018, NAT MATER, V17, P187, DOI [10.1038/NMAT5057, 10.1038/nmat5057]
Xu C, 2020, ACS NANO, V14, P13268, DOI 10.1021/acsnano.0c05062
Yang WJ, 2020, SCI ADV, V6, DOI 10.1126/sciadv.abd1631
Yang WJ, 2019, ACS NANO, V13, P3083, DOI 10.1021/acsnano.8b08346
Yin Y, 2021, NANO LETT, V21, P2224, DOI 10.1021/acs.nanolett.0c05039
Zhang LX, 2020, NANO TODAY, V35, DOI 10.1016/j.nantod.2020.100923
Zhang LX, 2021, NANO RES, V14, P1326, DOI 10.1007/s12274-020-3175-0
Zhang LX, 2019, ACS APPL MATER INTER, V11, P35566, DOI 10.1021/acsami.9b11746
Zhang LX, 2018, BIOMATERIALS, V174, P54, DOI 10.1016/j.biomaterials.2018.05.015
Zhang YN, 2020, ACS NANO, V14, P9478, DOI 10.1021/acsnano.0c02240
Zhang YW, 2020, J AM CHEM SOC, V142, P8827, DOI 10.1021/jacs.0c01853
Zhu GZ, 2017, ACS NANO, V11, P2387, DOI 10.1021/acsnano.7b00978
NR 108
TC 25
Z9 25
U1 9
U2 73
PU BMC
PI LONDON
PA CAMPUS, 4 CRINAN ST, LONDON N1 9XW, ENGLAND
EI 1477-3155
J9 J NANOBIOTECHNOL
JI J. Nanobiotechnol.
PD NOV 25
PY 2021
VL 19

IS 1
AR 389
DI 10.1186/s12951-021-01146-2
PG 23
WC Biotechnology & Applied Microbiology; Nanoscience & Nanotechnology
WE Science Citation Index Expanded (SCI-EXPANDED)
SC Biotechnology & Applied Microbiology; Science & Technology - Other
Topics
GA XD3QK
UT WOS:000722626400001
PM 34823541
OA gold, Green Published
DA 2023-09-14
ER

PT S
AU Tejeda-Mansir, A
Garcia-Rendon, A
Guerrero-German, P
AF Tejeda-Mansir, Armando
Garcia-Rendon, Aurora
Guerrero-German, Patricia
BE Mayes, S
TI Plasmid-DNA lipid and polymeric nanovaccines: a new strategic in
vaccines development
SO BIOTECHNOLOGY AND GENETIC ENGINEERING REVIEWS, VOL 35
SE Biotechnology & Genetic Engineering Reviews
LA English
DT Review; Book Chapter
DE Plasmid DNA; bioprocesses; liposomes; nanoparticles; vaccines;
nanovaccines
ID CATIONIC LIPOSOME; HYALURONIC-ACID; NANOPARTICLES; DELIVERY; GENE;
CHITOSAN; IMPACT; EFFICACY; PROTEIN; DRUG
AB Vaccination is the most effective and least expensive technique used for human diseases prevention and eradication. The need for more vaccine doses and the rapid establishment of facilities for the development of new vaccines are stimulating significate changes in the vaccine industry, which is gradually moving towards cell culture production. One approach is the third generation of vaccines, which are based on the use of plasmid DNA (pDNA) containing transgenes that encode an antigen capable of mimicking intracellular pathogenic infection and triggering both humoral and cellular immune responses. Plasmid DNA vaccination has distinct advantages over other vaccine technologies in terms of safety, ease of fabrication and stability. The effectiveness of pDNA vaccines against viruses, bacteria, parasites and cancer cells has been demonstrated in preclinical and clinical assays. Furthermore, currently there are a few veterinary pDNA vaccines in the market. The application of a simple formulation of naked pDNA as a vaccine is attractive, but a low transfection efficiency is often obtained. The use of nanoparticles to increase transfection efficiency is an approach that has been tested clinically. This review provides a summary of vaccine production, advances and major challenges associated with pDNA lipid and polymeric nanovaccines applications.
C1 [Tejeda-Mansir, Armando] Univ Sonora, Dept Sci & Technol Res, Hermosillo 83000, Sonora, Mexico.
[Garcia-Rendon, Aurora; Guerrero-German, Patricia] Univ Sonora, Sci & Engn Grad Program, Hermosillo, Sonora, Mexico.
C3 Universidad de Sonora; Universidad de Sonora
RP Tejeda-Mansir, A (通讯作者)，Univ Sonora, Dept Sci & Technol Res, Hermosillo 83000, Sonora, Mexico.
EM atejeda@guayacan.uson.mx
RI Guerrero, Patricia/AAA-4496-2020; Tejeda-Mansir, Armando/HJG-7460-2022;
Tejeda-Mansir, Armando/B-2468-2008
OI Guerrero, Patricia/0000-0002-3835-7342; Tejeda-Mansir,
Armando/0000-0003-2225-467X; Garcia Rendon, Aurora/0000-0002-2367-3182
FU CONACYT [257411CB 2016]
FX This work was supported by the CONACYT [257411CB 2016];
CR Abbink P, 2016, SCIENCE, V353, P1129, DOI 10.1126/science.aah6157

Ai WQ, 2013, MICROBIOL IMMUNOL, V57, P224, DOI 10.1111/1348-0421.12027
Akbarzadeh A, 2013, NANOSCALE RES LETT, V8, DOI 10.1186/1556-276X-8-102
[Anonymous], 1986, ANIMALS SCI PROCEDUR
Balbino TA, 2015, COLLOID SURFACE A, V484, P262, DOI 10.1016/j.colsurfa.2015.08.005
Barran-Berdon AL, 2014, LANGMUIR, V30, P11704, DOI 10.1021/la502823z
Benjaminsen RV, 2013, MOL THER, V21, P149, DOI 10.1038/mt.2012.185
Bhise NS, 2012, SMALL, V8, P367, DOI 10.1002/smll.201101718
Bivas-Benita M, 2003, INT J PHARM, V266, P17, DOI 10.1016/S0378-5173(03)00377-6
Bohle K, 2011, BIOTECHNOL BIOENG, V108, P2099, DOI 10.1002/bit.23138
Bolhassani A, 2014, HUM VACC IMMUNOTHER, V10, P321, DOI 10.4161/hv.26796
Bordelon H., 2011, J NANOMATER, V211, P1
Bozo T, 2016, COLLOID SURFACE B, V147, P467, DOI 10.1016/j.colsurfb.2016.06.056
Brown D. M., 2017, J IMMUNOL, V198
Chen L, 2013, PLOS ONE, V8, DOI 10.1371/journal.pone.0053949
Cherif MS, 2016, J BIOACT COMPAT POL, V31, P334, DOI 10.1177/0883911515614011
Choi YS, 2014, MOL CELL TOXICOL, V10, P1, DOI 10.1007/s13273-014-0001-3
Correia-Pinto JF, 2013, INT J PHARMACEUT, V440, P27, DOI 10.1016/j.ijpharm.2012.04.047
Dadwal M., 2014, J ADV PHARM ED RES, V4, P20
De La Vega J, 2013, J PHARM SCI-US, V102, P3932, DOI 10.1002/jps.23709
Di Cola E, 2016, PHARMACEUTICS, V8, DOI 10.3390/pharmaceutics8020010
EMEA, 2012, CONC PAP GUID PDNA V
Fan YC, 2015, J CONTROL RELEASE, V208, P121, DOI 10.1016/j.jconrel.2015.04.010
FDA, 2015, LIP DRUG PROD
Fda, 2004, PHARM CGMP 21 CENT R
FDA, 2016, IN INGR SEARCH APPR
Feng G, 2013, PLOS ONE, V8, P1
Ferraro B, 2011, CLIN INFECT DIS, V53, P296, DOI 10.1093/cid/cir334
Garcia-Diez R, 2016, LANGMUIR, V32, P772, DOI 10.1021/acs.langmuir.5b02261
Ghanem A, 2013, ANAL CHIM ACTA, V760, P1, DOI 10.1016/j.aca.2012.11.006
Goncalves GAL, 2014, J BIOTECHNOL, V186, P119, DOI 10.1016/j.jbiotec.2014.06.008
Grunwald T, 2015, CLIN EXP VACCINE RES, V4, P1, DOI 10.7774/cevr.2015.4.1.1
Hasson Sidgi Syed Anwer Abdo, 2015, Asian Pacific Journal of Tropical Biomedicine, V5, P344, DOI 10.1016/S2221-1691(15)30366-X
Hung CF, 2012, GENE VACCINES, P37, DOI 10.1007/978-3-7091-0439-2_2
Ingolotti M, 2010, EXPERT REV VACCINES, V9, P747, DOI 10.1586/ERV.10.57
Ismail R, 2012, VACCINE, V30, P5914, DOI 10.1016/j.vaccine.2012.02.061
Kalra J., 2013, FUNDAMENTALS PHARM N, P27, DOI [10.1007/978-1-4614-9164-4_3, DOI 10.1007/978-1-4614-9164-4_3]
Klug B, 2012, GENE VACCINES, P285, DOI 10.1007/978-3-7091-0439-2_14
Knuschke T, 2014, HUM VACC IMMUNOTHER, V10, P164, DOI 10.4161/hv.26203
Kolate A, 2014, J CONTROL RELEASE, V192, P67, DOI 10.1016/j.jconrel.2014.06.046
Kutzler MA, 2008, NAT REV GENET, V9, P776, DOI 10.1038/nrg2432
Lai P, 2014, COLLOID SURFACE B, V118, P154, DOI 10.1016/j.colsurfb.2014.03.017
Le TMH, 2012, J EXP NANOSCI, V7, P189, DOI 10.1080/17458080.2010.515247
Li GP, 2009, CELL MOL IMMUNOL, V6, P45, DOI 10.1038/cmi.2009.6
Li M, 2013, VACCINE, V31, P1264, DOI 10.1016/j.vaccine.2012.12.056
Lin PC, 2014, BIOTECHNOL ADV, V32, P711, DOI 10.1016/j.biotechadv.2013.11.006
Liu MA, 2011, IMMUNOL REV, V239, P62, DOI 10.1111/j.1600-065X.2010.00980.x
Mahor S, 2007, INT J PHARMACEUT, V340, P13, DOI 10.1016/j.ijpharm.2007.03.006
Mahut M, 2013, ANAL CHEM, V85, P2913, DOI 10.1021/ac3034823
Mbanefo EC, 2015, PARASITOL INT, V64, P24, DOI 10.1016/j.parint.2015.01.005
McCullers Jonathan A, 2008, P T, V33, P35
McDonnell M., 1999, MEDSCAPE GEN MED, V1, P1
Meerak J, 2013, VACCINE, V31, P784, DOI 10.1016/j.vaccine.2012.11.075
Menon JU, 2014, ACTA BIOMATER, V10, P2643, DOI 10.1016/j.actbio.2014.01.033
MIURA N, 2015, NUCLEIC ACIDS RES, V43, P1317, DOI DOI 10.1093/NAR/GKV008
Mourdikoudis S, 2018, NANOSCALE, V10, P12871, DOI 10.1039/c8nr02278j
Munguia-Soto R, 2015, BIOTECHNOL APPL BIOC, V62, P795, DOI 10.1002/bab.1339
Munye M.M., 2015, SCI REP-UK, V5, P1
Nakayama Y, 2015, VACCINES-BASEL, V3, P186, DOI 10.3390/vaccines3010186
Newman MJ, 2011, BIOPHARM INT, V24, P42

Passarinha LA, 2006, J MICROBIOL BIOTECHN, V16, P20
Peetla C, 2009, MOL PHARMACEUT, V6, P1264, DOI 10.1021/mp9000662
Pereira VB, 2014, WORLD J VACCIN, V4, P50, DOI [10.4236/wjv.2014.42008, DOI 10.4236/WJV.2014.42008]
Peterson M, 2008, BIOPHARM INT, P16
Poecheim J, 2016, VACCINES-BASEL, V4, DOI 10.3390/vaccines4030032
Prego C, 2010, VACCINE, V28, P2607, DOI 10.1016/j.vaccine.2010.01.011
Qiao Y, 2010, BIOMATERIALS, V31, P115, DOI 10.1016/j.biomaterials.2009.09.032
Raghuwanshi D, 2012, MOL PHARMACEUT, V9, P946, DOI 10.1021/mp200553x
Sahdev P, 2014, PHARM RES-DORDR, V31, P2563, DOI 10.1007/s11095-014-1419-y
Saleh T, 2015, VACCINE, V33, P3164, DOI 10.1016/j.vaccine.2015.05.015
Cordeiro AS, 2015, BIOTECHNOL ADV, V33, P1279, DOI 10.1016/j.biotechadv.2015.05.010
Shah MAA, 2015, J NANOSCI NANOTECHNO, V15, P41, DOI 10.1166/jnn.2015.9603
Shim BS, 2010, BMC IMMUNOL, V11, DOI 10.1186/1471-2172-11-65
Song YB, 2014, BIOMATER SCI-UK, V2, P1440, DOI 10.1039/c4bm00202d
Tregoning JS, 2014, MICROBIOL SPECTR, V2, DOI 10.1128/microbiolspec.PLAS-0028-2014
Urthaler J, 2012, GENE VACCINES, P311, DOI 10.1007/978-3-7091-0439-2_16
US Food and Drug Administration, 2007, GUID IND CONS PLASM
Wang CQ, 2014, MOL BIOSYST, V10, P672, DOI 10.1039/c3mb70502a
Welser K, 2013, MOL PHARMACEUT, V10, P127, DOI 10.1021/mp300187t
WHO, 2012, GUID ASS QUAL NONCL
Williams JA, 2013, VACCINES, V1, P225, DOI 10.3390/vaccines1030225
Yang Jun, 2015, Biomater Res, V19, P5, DOI 10.1186/s40824-014-0025-8
Yu FL, 2017, INT J CLIN EXP MED, V10, P12123
Zaman M, 2013, METHODS, V60, P226, DOI 10.1016/j.ymeth.2013.04.014
Zhai YZ, 2015, MOL MED REP, V12, P199, DOI 10.3892/mmr.2015.3419
Zhang B, 2010, ANAL BIOANAL CHEM, V396, P973, DOI 10.1007/s00216-009-2996-1
Zhang QL, 2008, BIOTECHNOL APPL BIOC, V49, P11, DOI 10.1042/BA20060251
Zhao L, 2014, VACCINE, V32, P327, DOI 10.1016/j.vaccine.2013.11.069
NR 88
TC 25
Z9 27
U1 1
U2 44
PU TAYLOR & FRANCIS LTD
PI LONDON
PA 11 NEW FETTER LANE, LONDON EC4P 4EE, ENGLAND
SN 0264-8725
EI 2046-5556
J9 BIOTECHNOL GENET ENG
JI Biotechnol. Genet. Eng. Rev.
PY 2019
VL 35
BP 46
EP 68
DI 10.1080/02648725.2018.1560552
PG 23
WC Biotechnology & Applied Microbiology; Genetics & Heredity
WE Book Citation Index– Science (BKCI-S); Science Citation Index Expanded (SCI-EXPANDED)
SC Biotechnology & Applied Microbiology; Genetics & Heredity
GA BO1NW
UT WOS:000501594600003
PM 30587085
OA Bronze
DA 2023-09-14
ER

PT J
AU Gupta, B
Sadaria, D
Warrier, VU

Kirtonia, A
Kant, R
Awasthi, A
Baligar, P
Pal, JK
Yuba, E
Sethi, G
Garg, M
Gupta, RK
AF Gupta, Bhavika
Sadaria, Daizy
Warrier, Vaishnavi U.
Kirtonia, Anuradha
Kant, Ravi
Awasthi, Amit
Baligar, Prakash
Pal, Jayanta K.
Yuba, Eiji
Sethi, Gautam
Garg, Manoj
Gupta, Rajesh Kumar
TI Plant lectins and their usage in preparing targeted nanovaccines for cancer immunotherapy
SO SEMINARS IN CANCER BIOLOGY
LA English
DT Review
DE Lectin; Plant glycoprotein lectin; Glycans; DC-SIGN; C-type lectin receptor; Cross- presentation; Cancer immunotherapy
ID C-TYPE LECTIN; T-CELL RESPONSES; CARBOHYDRATE-RECOGNITION DOMAIN; REGULATED GENE-EXPRESSION; N-LINKED OLIGOSACCHARIDES; DC-SIGN ANTAGONISTS; TOLL-LIKE RECEPTORS; WINGED-BEAN LECTIN; SOYBEAN AGGLUTININ; CROSS-PRESENTATION
AB Plant lectins, a natural source of glycans with a therapeutic potential may lead to the discovery of new targeted therapies. Glycans extracted from plant lectins are known to act as ligands for C-type lectin receptors (CLRs) that are primarily present on immune cells. Plant-derived glycosylated lectins offer diversity in their N-linked oligosaccharide structures that can serve as a unique source of homogenous and heterogenous glycans. Among the plant lectins-derived glycan motifs, Man9GlcNAc2Asn exhibits high-affinity interactions with CLRs that may resemble glycan motifs of pathogens. Thus, such glycan domains when presented along with antigens complexed with a nanocarrier of choice may bewilder the immune cells and direct antigen cross-presentation - a cytotoxic T lymphocyte immune response mediated by CD8(+) T cells. Glycan structure analysis has attracted considerable interest as glycans are looked upon as better therapeutic alternatives than monoclonal antibodies due to their cost-effectiveness, reduced toxicity and side effects, and high specificity. Furthermore, this approach will be useful to understand whether the multivalent glycan presentation on the surface of nanocarriers can overcome the low-affinity lectin-ligand interaction and thereby modulation of CLR-dependent immune response. Besides this, understanding how the heterogeneity of glycan structure impacts the antigen cross-presentation is pivotal to develop alternative targeted therapies. In the present review, we discuss the findings on structural analysis of glycans from natural lectins performed using GlycanBuilder2 - a software tool based on a thorough literature review of natural lectins. Additionally, we discuss how multiple parameters like the orientation of glycan ligands, ligand density, simultaneous targeting of multiple CLRs and design of antigen delivery nanocarriers may influence the CLR targeting efficacy. Integrating this information will eventually set the ground for new generation immunotherapeutic vaccine design for the treatment of various human malignancies.
C1 [Gupta, Bhavika; Sadaria, Daizy; Warrier, Vaishnavi U.; Pal, Jayanta K.; Gupta, Rajesh Kumar] Dr DY Patil Vidyapeeth Deemed Univ, Dr DY Patil Biotechnol & Bioinformat Inst, Prot Biochem Res Lab, Pune 411033, Maharashtra, India.
[Kirtonia, Anuradha; Baligar, Prakash; Garg, Manoj] Amity Univ Uttar Pradesh, Amity Inst Mol Med & Stem Cell Res AIMMSCR, Noida 201313, India.
[Kant, Ravi] Tech Univ Munich, Dept Expt Neuroimmunol, Klinikum Rechts Isar, Ismaninger Str 22, D-81675 Munich, Germany.
[Awasthi, Amit] Translat Hlth Sci & Technol Inst, Immunobiol Lab, NCR Biotech Sci Cluster, 3rd

Milestone Gurgaon Faridabad Expressway, Faridabad 121001, Haryana, India.
[Yuba, Eiji] Osaka Prefecture Univ, Grad Sch Engn, Dept Appl Chem, Naka Ku, 1-1 Gakuen Cho, Sakai, Osaka 5998531, Japan.
[Sethi, Gautam] Natl Univ Singapore, Yong Loo Lin Sch Med, Dept Pharmacol, Singapore 117600, Singapore.
C3 Dr DY Patil Vidyapeeth Pune; Dr D Y Patil Biotechnology & Bioinformatics Institute; Amity University Noida; Technical University of Munich; Department of Biotechnology (DBT) India; Translational Health Science & Technology Institute (THSTI); Osaka Metropolitan University; National University of Singapore
RP Sethi, G (通讯作者)，Natl Univ Singapore, Yong Loo Lin Sch Med, Dept Pharmacol, Singapore 117600, Singapore.; Gupta, RK (通讯作者)，Dr DY Patil Vidyapeeth, Dr DY Patil Biotechnol & Bioinformat Inst, Pune 411033, Maharashtra, India.; Garg, M (通讯作者)，Amity Univ, Amity Inst Mol Med & Stem Cell Res AIMMSCR, Noida 201313, Uttar Pradesh, India.
EM phecgs@nus.edu.sg; mgarg@amity.edu; rajeshkumar.gupta@dpu.edu.in
RI Kant, Ravi/F-7025-2013; Warrier, Vaishnavi/ISS-5306-2023; Sethi, Gautam/F-2372-2011; BALIGAR, PRAKASH/AAB-7404-2021; Kirtonia, Anuradha/IQS-7868-2023
OI Kant, Ravi/0000-0003-3878-9775; Warrier, Vaishnavi/0000-0001-5166-6409; Sethi, Gautam/0000-0002-8677-8475; BALIGAR, PRAKASH/0000-0002-0553-4090; Eiji, Yuba/0000-0003-4984-2113; Gupta, Rajesh Kumar/0000-0001-8820-0661
FU Department of Science and Technology-Science and Engineering Research Board (DST-SERB), Government of India under ECRA scheme [ECR/2016/001187]; Department of Biotechnology (DBT), under its Ramalingaswami Fellowship [BT/RLF/Re-entry/24/2014]; Science and Engineering Research Board under its ECRA scheme (SERB) [ECR/2016/001519]; Dr. D.Y. Patil Vidyapeeth (Deemed to be University) [DPU/106/18/2015, DPU/17/2016]
FX This work was supported by the Department of Science and Technology-Science and Engineering Research Board (DST-SERB) ,Government of India under ECRA scheme [grant number ECR/2016/001187] ; Dr. D.Y. Patil Vidyapeeth (Deemed to be University) [grant numbers DPU/106/18/2015, DPU/17/2016 (to Dr. Rajesh Kumar Gupta) ] . This work acknowledges the Department of Biotechnology (DBT) , under its Ramalingaswami Fellowship number BT/RLF/Re-entry/24/2014 and Science and Engineering Research Board under its ECRA scheme (SERB File No. ECR/2016/001519) award to Dr. Manoj Garg.
CR Agrawal SB, 2019, ARCH BIOCHEM BIOPHYS, V662, P134, DOI 10.1016/j.abb.2018.12.003
Ahn KS, 2008, BIOCHEM PHARMACOL, V75, P907, DOI 10.1016/j.bcp.2007.10.010
ALLEN AK, 1976, BIOCHEM J, V155, P127, DOI 10.1042/bj1550127
ALLEN AK, 1978, BIOCHEM J, V171, P665, DOI 10.1042/bj1710665
Arosio D, 2014, BIOCONJUGATE CHEM, V25, P2244, DOI 10.1021/bc500467u
ASHFORD D, 1982, BIOCHEM J, V201, P199, DOI 10.1042/bj2010199
ASHFORD D, 1987, EUR J BIOCHEM, V166, P311, DOI 10.1111/j.1432-1033.1987.tb13516.x
ASHFORD DA, 1991, CARBOHYD RES, V213, P215, DOI 10.1016/S0008-6215(00)90610-4
Audette GF, 2000, J MOL BIOL, V304, P423, DOI 10.1006/jmbi.2000.4214
Babino A, 2003, FEBS LETT, V536, P106, DOI 10.1016/S0014-5793(03)00037-1
Baccala R, 2009, NAT REV RHEUMATOL, V5, P448, DOI 10.1038/nrrheum.2009.136
Becer CR, 2012, MACROMOL RAPID COMM, V33, P742, DOI 10.1002/marc.201200055
Bertolotti B, 2016, CARBOHYD RES, V435, P7, DOI 10.1016/j.carres.2016.09.005
Bhutia SK, 2019, PHARMACOL RES, V144, P8, DOI 10.1016/j.phrs.2019.04.001
Bindhya S, 2019, INT J BIOCHEM CELL B, V107, P62, DOI 10.1016/j.biocel.2018.12.008
Blattes E, 2013, P NATL ACAD SCI USA, V110, P8795, DOI 10.1073/pnas.1221708110
Bloem K, 2014, IMMUNOL LETT, V158, P33, DOI 10.1016/j.imlet.2013.11.007
Boks MA, 2015, J INVEST DERMATOL, V135, P2697, DOI 10.1038/jid.2015.226
Boks MA, 2015, J CONTROL RELEASE, V216, P37, DOI 10.1016/j.jconrel.2015.06.033
Bosch D, 2010, EXPERT REV VACCINES, V9, P835, DOI 10.1586/erv.10.83
BOYD WC, 1954, SCIENCE, V119, P419, DOI 10.1126/science.119.3091.419
Brabletz T, 2018, NAT REV CANCER, V18, P128, DOI 10.1038/nrc.2017.118
Bray F, 2018, CA-CANCER J CLIN, V68, P394, DOI 10.3322/caac.21492
BROEKAERT WF, 1984, BIOCHEM J, V221, P163, DOI 10.1042/bj2210163

Brown GD, 2001, NATURE, V413, P36, DOI 10.1038/35092620
Bryant CE, 2015, MOL IMMUNOL, V63, P162, DOI 10.1016/j.molimm.2014.06.033
CARTER WG, 1975, BIOCHEMISTRY-US, V14, P5118, DOI 10.1021/bi00694a015
Ceroni A, 2007, SOURCE CODE BIOL MED, V2, DOI 10.1186/1751-0473-2-3
Chen KW, 2013, J PROTEOME RES, V12, P1408, DOI 10.1021/pr3010474
Chiodo F, 2014, BEILSTEIN J ORG CHEM, V10, P1339, DOI 10.3762/bjoc.10.136
CROWLEY JF, 1984, ARCH BIOCHEM BIOPHYS, V231, P524, DOI 10.1016/0003-9861(84)90417-X
Cummings R.D., 2015, ESSENTIALS GLYCOBIOL, P373
da Silva LCN, 2014, FRONT MICROBIOL, V5, DOI 10.3389/fmicb.2014.00020
Datta SK, 2003, J IMMUNOL, V170, P4102, DOI 10.4049/jimmunol.170.8.4102
Demento SL, 2011, TRENDS BIOTECHNOL, V29, P294, DOI 10.1016/j.tibtech.2011.02.004
DENNIS JW, 1987, SCIENCE, V236, P582, DOI 10.1126/science.2953071
DESAI NN, 1981, BIOCHEM J, V197, P345, DOI 10.1042/bj1970345
Donadei A, 2015, MOL PHARMACEUT, V12, P1662, DOI 10.1021/acs.molpharmaceut.5b00072
DORLAND L, 1981, J BIOL CHEM, V256, P7708
Du CL, 2013, CHEM-ASIAN J, V8, P736, DOI 10.1002/asia.201201088
Duinkerken S, 2019, CURR OPIN CHEM BIOL, V53, P167, DOI 10.1016/j.cbpa.2019.10.001
Engering A, 2002, J IMMUNOL, V168, P2118, DOI 10.4049/jimmunol.168.5.2118
Engering AJ, 1997, ADV EXP MED BIOL, V417, P183
Eriksson M, 2014, CHEMBIOCHEM, V15, P844, DOI 10.1002/cbic.201300764
Espuelas S, 2008, BIOCONJUGATE CHEM, V19, P2385, DOI 10.1021/bc8002524
Fasting C, 2012, ANGEW CHEM INT EDIT, V51, P10472, DOI 10.1002/anie.201201114
Fehres CM, 2015, J CONTROL RELEASE, V203, P67, DOI 10.1016/j.jconrel.2015.01.040
Fehres CM, 2014, EUR J IMMUNOL, V44, P2415, DOI 10.1002/eji.201344094
FERNANDES B, 1991, CANCER RES, V51, P718
Figdor CG, 2002, NAT REV IMMUNOL, V2, P77, DOI 10.1038/nri723
Fouquaert E, 2008, PLANT PHYSIOL, V147, P1316, DOI 10.1104/pp.108.116764
FOURNET B, 1987, EUR J BIOCHEM, V166, P321, DOI 10.1111/j.1432-1033.1987.tb13517.x
Frenz T, 2014, J IMMUNOL, V193, P2496, DOI 10.4049/jimmunol.1400215
Fritz JH, 2005, EUR J IMMUNOL, V35, P2459, DOI 10.1002/eji.200526286
Fuster MM, 2005, NAT REV CANCER, V5, P526, DOI 10.1038/nrc1649
Galustian C, 2004, INT IMMUNOL, V16, P853, DOI 10.1093/intimm/dxh089
Gao JM, 2013, BIOCONJUGATE CHEM, V24, P1332, DOI 10.1021/bc400011v
Garcia-Vallejo JJ, 2015, FRONT IMMUNOL, V6, DOI 10.3389/fimmu.2015.00087
Garcia-Vallejo JJ, 2013, ONCOIMMUNOLOGY, V2, DOI 10.4161/onci.23040
Garcia-Vallejo JJ, 2013, INT IMMUNOL, V25, P221, DOI 10.1093/intimm/dxs115
Garcia-Vallejo JJ, 2013, MOL IMMUNOL, V53, P387, DOI 10.1016/j.molimm.2012.09.012
Garg M, 2017, ONCOTARGET, V8, P7521, DOI 10.18632/oncotarget.13485
Garg M, 2015, J CLIN ENDOCR METAB, V100, P725, DOI 10.1210/jc.2014-2359
Garg M, 2014, EXPERT OPIN THER TAR, V18, P979, DOI 10.1517/14728222.2014.934814
Geijtenbeek TBH, 2004, ANNU REV IMMUNOL, V22, P33, DOI
10.1146/annurev.immunol.22.012703.104558
Goldstein I J, 1978, Adv Carbohydr Chem Biochem, V35, P127
Gringhuis SI, 2007, IMMUNITY, V26, P605, DOI 10.1016/j.immuni.2007.03.012
Guo Y, 2004, NAT STRUCT MOL BIOL, V11, P591, DOI 10.1038/nsmb784
Gupta RK, 2012, EUR J PHARM SCI, V45, P282, DOI 10.1016/j.ejps.2011.11.022
Gupta RK, 2006, FEBS LETT, V580, P1691, DOI 10.1016/j.febslet.2006.02.006
Halder S, 2015, GLYCOCONJUGATE J, V32, P371, DOI 10.1007/s10719-015-9601-y
HAMMARSTROM S, 1982, P NATL ACAD SCI-BIOL, V79, P1611, DOI 10.1073/pnas.79.5.1611
Hanahan D, 2011, CELL, V144, P646, DOI 10.1016/j.cell.2011.02.013
Hashim OH, 2017, PEERJ, V5, DOI 10.7717/peerj.3784
HAYES CE, 1974, J BIOL CHEM, V249, P1904
Holla A, 2011, PROTEIN ENG DES SEL, V24, P659, DOI 10.1093/protein/gzr016
Hou H, 2017, MED CHEM, V13, P536, DOI 10.2174/1573406413666170510103030
IGLESIAS JL, 1982, EUR J BIOCHEM, V123, P247, DOI 10.1111/j.1432-1033.1982.tb19760.x
Ignacio BJ, 2018, BIOCONJUGATE CHEM, V29, P587, DOI 10.1021/acs.bioconjchem.7b00808
Imberty A, 2000, J BIOL CHEM, V275, P17541, DOI 10.1074/jbc.M000560200
Imperiali B, 1999, CURR OPIN CHEM BIOL, V3, P643, DOI 10.1016/S1367-5931(99)00021-6
IRIMURA T, 1972, ARCH BIOCHEM BIOPHYS, V151, P475, DOI 10.1016/0003-9861(72)90524-3
Janeway CA, 2002, ANNU REV IMMUNOL, V20, P197, DOI
10.1146/annurev.immunol.20.083001.084359

Jegouzo SAF, 2015, J BIOL CHEM, V290, P16759, DOI 10.1074/jbc.M115.660613
Jiang QL, 2015, CELL PROLIFERAT, V48, P17, DOI 10.1111/cpr.12155
Jiang SY, 2010, BMC EVOL BIOL, V10, DOI 10.1186/1471-2148-10-79
Johannssen T, 2017, TRENDS BIOTECHNOL, V35, P334, DOI 10.1016/j.tibtech.2016.10.002
Joshi MD, 2012, J CONTROL RELEASE, V161, P25, DOI 10.1016/j.jconrel.2012.05.010
Ju TZ, 2014, CANCER BIOMARK, V14, P63, DOI 10.3233/CBM-130375
KABIR S, 1994, IMMUNOL INVEST, V23, P167, DOI 10.3109/08820139409087798
KAKU H, 1990, ARCH BIOCHEM BIOPHYS, V277, P255, DOI 10.1016/0003-9861(90)90576-K
Kang B, 2015, ANGEW CHEM INT EDIT, V54, P7436, DOI 10.1002/anie.201502398
KATZIN BJ, 1991, PROTEINS, V10, P251, DOI 10.1002/prot.340100309
Khan S, 2007, J BIOL CHEM, V282, P21145, DOI 10.1074/jbc.M701705200
Kim BS, 2015, BBA-GEN SUBJECTS, V1850, P1815, DOI 10.1016/j.bbagen.2015.05.011
Kim H, 2019, J PHARMACOL EXP THER, V370, P715, DOI 10.1124/jpet.118.254953
Kim YS, 2009, PROTEIN PEPTIDE LETT, V16, P499, DOI 10.2174/092986609788167798
Kirwan A, 2015, BIOMED RES INT, V2015, DOI 10.1155/2015/490531
KONAMI Y, 1990, FEBS LETT, V268, P281, DOI 10.1016/0014-5793(90)81028-M
KUROKAWA T, 1976, J BIOL CHEM, V251, P5686
Lang R, 2011, IMMUNOBIOLOGY, V216, P1184, DOI 10.1016/j.imbio.2011.06.005
Lehmann CHK, 2016, VACCINES-BASEL, V4, DOI 10.3390/vaccines4020008
Lepenies B, 2013, ADV DRUG DELIVER REV, V65, P1271, DOI 10.1016/j.addr.2013.05.007
Lescar J, 2002, J BIOL CHEM, V277, P6608, DOI 10.1074/jbc.M109867200
Li RJE, 2018, CURR OPIN BIOTECH, V51, P24, DOI 10.1016/j.copbio.2017.11.003
Li RJE, 2019, FRONT CHEM, V7, DOI 10.3389/fchem.2019.00650
LIS H, 1978, J BIOL CHEM, V253, P3468
LORD JM, 1994, FASEB J, V8, P201, DOI 10.1096/fasebj.8.2.8119491
LOTAN R, 1974, J BIOL CHEM, V249, P1219
Luczkowiak J, 2013, BIOMACROMOLECULES, V14, P431, DOI 10.1021/bm3016658
Maglinao M, 2014, J CONTROL RELEASE, V175, P36, DOI 10.1016/j.jconrel.2013.12.011
Mahnke K, 2000, J CELL BIOL, V151, P673, DOI 10.1083/jcb.151.3.673
Manna SK, 2007, CLIN CANCER RES, V13, P2290, DOI 10.1158/1078-0432.CCR-06-2394
Manoj N, 1999, ACTA CRYSTALLOGR D, V55, P794, DOI 10.1107/S090744499900044X
Martinez-Pomares L, 2012, J LEUKOCYTE BIOL, V92, P1177, DOI 10.1189/jlb.0512231
Matsui T, 2011, GLYCOBIOLOGY, V21, P994, DOI 10.1093/glycob/cwq198
Maveyraud L, 2009, PROTEINS, V75, P89, DOI 10.1002/prot.22222
McGreal EP, 2005, CURR OPIN IMMUNOL, V17, P18, DOI 10.1016/j.coi.2004.12.001
Meany Danni L, 2011, Clin Proteomics, V8, P7, DOI 10.1186/1559-0275-8-7
Medzhitov R, 2001, NAT REV IMMUNOL, V1, P135, DOI 10.1038/35100529
Moreno FBMB, 2008, J STRUCT BIOL, V161, P133, DOI 10.1016/j.jsb.2007.10.002
Mitra N, 2006, TRENDS BIOCHEM SCI, V31, P156, DOI 10.1016/j.tibs.2006.01.003
Miyazaki M, 2018, BIOCONJUGATE CHEM, V29, P44, DOI 10.1021/acs.bioconjchem.7b00551
Miyoshi E, 2012, BIOMOLECULES, V2, P34, DOI 10.3390/biom2010034
MONTFORT W, 1987, J BIOL CHEM, V262, P5398
Munkley J, 2016, ONCOTARGET, V7, P35478, DOI 10.18632/oncotarget.8155
Munoz-Wolf N, 2016, METHODS MOL BIOL, V1417, P1, DOI 10.1007/978-1-4939-3566-6_1
Muraille E, 1999, CELL IMMUNOL, V191, P1, DOI 10.1006/cimm.1998.1401
NACHBAR MS, 1980, J BIOL CHEM, V255, P2056
Nair AS, 2006, J IMMUNOL, V177, P5612, DOI 10.4049/jimmunol.177.8.5612
Nalivaeva NN, 2001, PROTEOMICS, V1, P735, DOI 10.1002/1615-9861(200106)1:6<735::AID-PROT735>3.3.CO;2-#
Nascimento KS, 2012, J MOL RECOGNIT, V25, P527, DOI 10.1002/jmr.2200
Noda K, 1998, HEPATOLOGY, V28, P944, DOI 10.1002/hep.510280408
Oguri S, 2008, BIOSCI BIOTECH BIOCH, V72, P2640, DOI 10.1271/bbb.80310
Okubo M, 2019, BIOCONJUGATE CHEM, V30, P1518, DOI 10.1021/acs.bioconjchem.9b00221
OLSNES S, 1975, EUR J BIOCHEM, V60, P281, DOI 10.1111/j.1432-1033.1975.tb21001.x
Osinaga E, 1997, FEBS LETT, V412, P190, DOI 10.1016/S0014-5793(97)00677-7
Panda AK, 2011, EXPERT REV VACCINES, V10, P155, DOI [10.1586/erv.10.164, 10.1586/ERV.10.164]
Park HJ, 2010, BIOCHEM BIOPH RES CO, V396, P721, DOI 10.1016/j.bbrc.2010.04.169
Pearce OMT, 2018, GLYCOBIOLOGY, V28, P670, DOI 10.1093/glycob/cwy023
Pearson A.J., 1928, METHODS MOL BIOL, V2019, P389
Peracaula R, 2003, GLYCOBIOLOGY, V13, P457, DOI 10.1093/glycob/cwg041
Peracaula R, 2008, DIS MARKERS, V25, P207, DOI 10.1155/2008/797629

PETRYNIAK J, 1977, ARCH BIOCHEM BIOPHYS, V178, P118, DOI 10.1016/0003-9861(77)90176-X
PEUMANS WJ, 1995, PLANT PHYSIOL, V109, P347, DOI 10.1104/pp.109.2.347
Peumans WJ, 2001, ADV EXP MED BIOL, V491, P27
Pinho SS, 2015, NAT REV CANCER, V15, P540, DOI 10.1038/nrc3982
Prabu MM, 1998, J MOL BIOL, V276, P787, DOI 10.1006/jmbi.1997.1568
Prost LR, 2012, ACS CHEM BIOL, V7, P1603, DOI 10.1021/cb300260p
Puar YR, 2018, BIOMEDICINES, V6, DOI 10.3390/biomedicines6030082
Ramachandran L, 2012, J BIOL CHEM, V287, DOI 10.1074/jbc.M112.388702
Rana R, 2006, HUM REPROD, V21, P2894, DOI 10.1093/humrep/del068
Rao RSP, 2010, BIOINFORMATION, V5, P208, DOI 10.6026/97320630005208
Ricardo S, 2015, MOL ONCOL, V9, P503, DOI 10.1016/j.molonc.2014.10.005
Ricci-Azevedo R, 2017, FRONT IMMUNOL, V8, DOI 10.3389/fimmu.2017.01820
Rodriguez E, 2018, NAT REV IMMUNOL, V18, P204, DOI 10.1038/nri.2018.3
Rudiger H, 2001, GLYCOCONJUGATE J, V18, P589, DOI 10.1023/A:1020687518999
RUTENBER E, 1991, PROTEINS, V10, P260, DOI 10.1002/prot.340100310
SATO M, 1992, J BIOCHEM-TOKYO, V111, P331, DOI 10.1093/oxfordjournals.jbchem.a123758
SATO Y, 1993, NEW ENGL J MED, V328, P1802, DOI 10.1056/NEJM199306243282502
SCHNELL DJ, 1987, J BIOL CHEM, V262, P7220
Scott E, 2020, CLIN CHIM ACTA, V502, P167, DOI 10.1016/j.cca.2019.12.015
Sethi G, 2014, MOL CANCER, V13, DOI 10.1186/1476-4598-13-66
Sharon N, 2004, GLYCOBIOLOGY, V14, p53R, DOI 10.1093/glycob/cwh122
Sharon N, 2008, BIOCHEM SOC T, V36, P1457, DOI 10.1042/BST0361457
Shibue T, 2017, NAT REV CLIN ONCOL, V14, P611, DOI 10.1038/nrclinonc.2017.44
Shin EM, 2014, J CLIN INVEST, V124, P3807, DOI 10.1172/JCI73451
Sidhanth C, 2018, BIOCHEM CELL BIOL, V96, P295, DOI 10.1139/bcb-2017-0020
Silva MLS, 2019, ADV CLIN CHEM, V93, P1, DOI 10.1016/bs.acc.2019.07.001
Singh SK, 2009, MOL IMMUNOL, V47, P164, DOI 10.1016/j.molimm.2009.09.026
Singh SK, 2009, MOL IMMUNOL, V46, P1240, DOI 10.1016/j.molimm.2008.11.021
Sodhi A, 2007, INT IMMUNOPHARMACOL, V7, P454, DOI 10.1016/j.intimp.2006.11.014
Souza MA, 2013, GLYCOCONJUGATE J, V30, P641, DOI 10.1007/s10719-012-9464-4
Spiro RG, 2002, GLYCOBIOLOGY, V12, p43R, DOI 10.1093/glycob/12.4.43R
Stambach NS, 2003, GLYCOBIOLOGY, V13, P401, DOI 10.1093/glycob/cwg045
Steinhagen F, 2011, VACCINE, V29, P3341, DOI 10.1016/j.vaccine.2010.08.002
Stowell SR, 2015, ANNU REV PATHOL-MECH, V10, P473, DOI 10.1146/annurev-pathol-012414-040438
Strasser R, 2016, GLYCOBIOLOGY, V26, P926, DOI 10.1093/glycob/cww023
Streng-Ouwehand I, 2016, ELIFE, V5, DOI 10.7554/eLife.11765
STURM A, 1986, PLANT PHYSIOL, V81, P320, DOI 10.1104/pp.81.1.320
STURM A, 1992, EUR J BIOCHEM, V204, P313, DOI 10.1111/j.1432-1033.1992.tb16639.x
Sumner JB, 1936, J BACTERIOL, V32, P227, DOI 10.1128/JB.32.2.227-237.1936
Sutkeviciute I, 2014, ACS CHEM BIOL, V9, P1377, DOI 10.1021/cb500054h
Suzuki N, 1996, J IMMUNOL, V156, P128
Syed P, 2016, PROTEOMICS, V16, P1257, DOI 10.1002/pmic.201500404
Sze KL, 2011, APPL MICROBIOL BIOT, V89, P45, DOI 10.1007/s00253-010-2892-9
Tabarani G, 2006, FEBS LETT, V580, P2402, DOI 10.1016/j.febslet.2006.03.061
TAKANO R, 1990, AM J PATHOL, V137, P1007
Tateno H, 2010, J BIOL CHEM, V285, P6390, DOI 10.1074/jbc.M109.041863
Tempel W, 2002, J BIOL CHEM, V277, P6615, DOI 10.1074/jbc.M109919200
Thepaut M, 2013, J AM CHEM SOC, V135, P2518, DOI 10.1021/ja3053305
TOLLEFSEN SE, 1983, J BIOL CHEM, V258, P5165
Tomasic T, 2014, EUR J MED CHEM, V75, P308, DOI 10.1016/j.ejmech.2014.01.047
Tsuiji M, 2002, J BIOL CHEM, V277, P28892, DOI 10.1074/jbc.M203774200
Ujita M, 2009, BIOSCI BIOTECH BIOCH, V73, P237, DOI 10.1271/bbb.80503
Unger WWJ, 2012, J CONTROL RELEASE, V160, P88, DOI 10.1016/j.jconrel.2012.02.007
Unitt J, 2011, BIOCHEM PHARMACOL, V81, P1324, DOI 10.1016/j.bcp.2011.03.010
Vajaria B, 2017, GLYCOCONJUGATE J, V34, P147, DOI 10.1007/s10719-016-9755-2
Van den Steen P, 1998, CRIT REV BIOCHEM MOL, V33, P151
van Dinther D, 2017, J LEUKOCYTE BIOL, V102, P1017, DOI [10.1189/jlb.5MR0217-059RR, 10.1189/jlb.5mr0217-059rr]
van Kooyk Y, 2013, MOL IMMUNOL, V55, P143, DOI 10.1016/j.molimm.2012.10.031
van Liempt E, 2006, FEBS LETT, V580, P6123, DOI 10.1016/j.febslet.2006.10.009
van Vliet SJ, 2008, TRENDS IMMUNOL, V29, P83, DOI 10.1016/j.it.2007.10.010

van Vliet SJ, 2005, INT IMMUNOL, V17, P661, DOI 10.1093/intimm/dxh246
VANDONSELAAR M, 1994, J MOL BIOL, V243, P345, DOI 10.1006/jmbi.1994.1658
Vogelstein B, 2004, NAT MED, V10, P789, DOI 10.1038/nm1087
Wang C, 2018, CANCER TREAT REV, V62, P29, DOI 10.1016/j.ctrv.2017.10.014
Warrier VU, 2019, BIOSCIENCE REP, V39, DOI 10.1042/BSR20193220
Weck MM, 2008, BLOOD, V111, P4264, DOI 10.1182/blood-2006-10-051375
Weis WI, 1998, IMMUNOL REV, V163, P19, DOI 10.1111/j.1600-065X.1998.tb01185.x
Wells CA, 2008, J IMMUNOL, V180, P7404, DOI 10.4049/jimmunol.180.11.7404
Yadav P, 2016, INT J BIOL MACROMOL, V91, P75, DOI 10.1016/j.ijbiomac.2016.05.047
Yau T, 2015, MOLECULES, V20, P3791, DOI 10.3390/molecules20033791
Yu H., 2020, EXPERT REV PROTEOMIC
Yuba E, 2018, MOL IMMUNOL, V98, P8, DOI 10.1016/j.molimm.2017.11.001
Yuba E, 2017, VACCINES-BASEL, V5, DOI 10.3390/vaccines5040041
Yuba E, 2017, BIOMATERIALS, V120, P32, DOI 10.1016/j.biomaterials.2016.12.021
Yuba E, 2015, BIOMATERIALS, V67, P214, DOI 10.1016/j.biomaterials.2015.07.031
Yuba E, 2014, BIOMATERIALS, V35, P3091, DOI 10.1016/j.biomaterials.2013.12.024
Yuba E, 2013, BIOMATERIALS, V34, P5711, DOI 10.1016/j.biomaterials.2013.04.007
Yuba E, 2013, BIOMATERIALS, V34, P3042, DOI 10.1016/j.biomaterials.2012.12.031
Zhang Q, 2013, ANGEW CHEM INT EDIT, V52, P4435, DOI 10.1002/anie.201300068
Zimmermann Stephanie, 2015, Methods Mol Biol, V1331, P11, DOI 10.1007/978-1-4939-2874-3_2
NR 223
TC 24
Z9 26
U1 15
U2 56
PU ACADEMIC PRESS LTD- ELSEVIER SCIENCE LTD
PI LONDON
PA 24-28 OVAL RD, LONDON NW1 7DX, ENGLAND
SN 1044-579X
EI 1096-3650
J9 SEMIN CANCER BIOL
JI Semin. Cancer Biol.
PD MAY
PY 2022
VL 80
BP 87
EP 106
DI 10.1016/j.semcancer.2020.02.005
EA MAR 2022
PG 20
WC Oncology
WE Science Citation Index Expanded (SCI-EXPANDED)
SC Oncology
GA ZQ6AN
UT WOS:000767185600006
PM 32068087
HC Y
HP N
DA 2023-09-14
ER

PT J
AU Zhai, YW
He, XR
Li, Y
Han, R
Ma, YY
Gao, P
Qian, ZY
Gu, YQ
Li, SW

AF Zhai, Yuewen
He, Xiaorong
Li, Ying
Han, Ran
Ma, Yuying
Gao, Peng
Qian, Zhiyu
Gu, Yueqing
Li, Siwen
TI A splenic-targeted versatile antigen courier: iPSC wrapped in coalescent erythrocyte-liposome as tumor nanovaccine
SO SCIENCE ADVANCES
LA English
DT Article
ID DENDRITIC CELLS; VACCINES; NANOPARTICLES; VACCINATION; THERAPY
AB The major obstacles for tumor vaccine to be surmounted are the lack of versatile property and immunity-inducing effectiveness. Induced pluripotent stem cells (iPSCs) expressed various antigens the same as multiple types of tumors, providing a promising source of wide-spectrum cancer vaccines. The damaged erythrocyte membrane entrapped by spleen could be developed as antigen deliverer for enhancing acquired immunity. Here, the modified lipid materials were used to dilate erythrocyte membrane to fabricate coalescent nanovector, which not only preserved the biological characteristics of erythrocyte membrane but also remedied the defect of insufficient drug loading capacity. After wrapping iPSC protein, the nanovaccine iPSC@RBC-Mlipo exhibited obvious splenic accumulation, systemic specific antitumor immunity evocation, and effective tumor expansion and metastasis inhibition in mice. Hence, our research may provide a prospective strategy of efficient tumor vaccine for clinical practice.
C1 [Zhai, Yuewen; He, Xiaorong; Li, Ying; Han, Ran; Ma, Yuying; Gu, Yueqing; Li, Siwen] China Pharmaceut Univ, Dept Biomed Engn, Sch Engn, State Key Lab Nat Med,Jiangsu Key Lab Drug Screen, 639 Longmian Ave, Nanjing 211198, Jiangsu, Peoples R China.
[Gao, Peng] Chia Tai Tianqing Pharmaceut Grp Co Ltd, 1099 Fuying Rd, Nanjing 211106, Jiangsu, Peoples R China.
[Qian, Zhiyu] Nanjing Univ Aeronaut & Astronaut, Sch Automat, Dept Biomed Engn, 29th JiangJun St, Nanjing 211106, Jiangsu, Peoples R China.
C3 China Pharmaceutical University; Nanjing University of Aeronautics & Astronautics
RP Gu, YQ; Li, SW (通讯作者)，China Pharmaceut Univ, Dept Biomed Engn, Sch Engn, State Key Lab Nat Med,Jiangsu Key Lab Drug Screen, 639 Longmian Ave, Nanjing 211198, Jiangsu, Peoples R China.
EM lsw@cpu.edu.cn
RI wang, yi/HOF-6668-2023
FU National Natural Science Foundation of China [NSFC 81971743, 81801835, 81827803, 8172900016, 81727804, 91859204]; Natural Science Foundation of Jiangsu Province, China [BK20200083]
FX We are grateful to National Natural Science Foundation of China (NSFC 81971743, 81801835, 81827803, 8172900016, 81727804, and 91859204) and project supported by the Natural Science Foundation of Jiangsu Province, China (BK20200083) for their financial support.
CR Alipour E, 2017, ANNU REV PHYS CHEM, V68, P261, DOI 10.1146/annurev-physchem-040215-112634
Bowen WS, 2018, EXPERT REV VACCINES, V17, P207, DOI 10.1080/14760584.2018.1434000
Christensen K, 2018, ADV BIOCHEM ENG BIOT, V163, P207, DOI 10.1007/10_2017_23
Chu YQ, 2020, DRUG DELIV TRANSL RE, V10, P1278, DOI 10.1007/s13346-020-00780-x
Chung CK, 2018, J CONTROL RELEASE, V285, P56, DOI 10.1016/j.jconrel.2018.07.011
Cruz LJ, 2014, J CONTROL RELEASE, V192, P209, DOI 10.1016/j.jconrel.2014.07.040
de Jesus BB, 2020, CANCERS, V12, DOI 10.3390/cancers12123581
de Titta A, 2013, P NATL ACAD SCI USA, V110, P19902, DOI 10.1073/pnas.1313152110
Dogra P, 2020, CELL, V180, P749, DOI 10.1016/j.cell.2020.01.022
Dovydenko IS, 2019, J PHYS CHEM B, V123, P8829, DOI 10.1021/acs.jpcb.9b06006
Fox CB, 2014, J NANOBIOTECHNOL, V12, DOI 10.1186/1477-3155-12-17
Gibbins JD, 2015, J IMMUNOTHER, V38, P321, DOI 10.1097/CJI.0000000000000096
Hailemichael Y, 2018, TRENDS MOL MED, V24, P524, DOI 10.1016/j.molmed.2018.04.006
Han X, 2019, SCI ADV, V5, DOI 10.1126/sciadv.aaw6870
Irvine DJ, 2015, CHEM REV, V115, P11109, DOI 10.1021/acs.chemrev.5b00109
Irvine DJ, 2013, NAT MATER, V12, P978, DOI [10.1038/NMAT3775, 10.1038/nmat3775]

Kondo Y, 2018, J DIABETES INVEST, V9, P234, DOI 10.1111/jdi.12702
Kooreman NG, 2018, CELL STEM CELL, V22, P501, DOI 10.1016/j.stem.2018.01.016
Li H, 2018, P NATL ACAD SCI USA, V115, P9574, DOI 10.1073/pnas.1806501115
Li J, 2020, FRONT BIOENG BIOTECH, V8, DOI 10.3389/fbioe.2020.612950
Li MY, 2019, EUR J MED CHEM, V164, P640, DOI 10.1016/j.ejmech.2019.01.007
Li WJ, 2017, J AGR FOOD CHEM, V65, P348, DOI 10.1021/acs.jafc.6b04888
Liu B, 2019, BIOMATERIALS, V217, DOI 10.1016/j.biomaterials.2019.119301
Lloyd DM., 2010, SURG OXFORD, V28, P229
Malik Nasir, 2013, Methods Mol Biol, V997, P23, DOI 10.1007/978-1-62703-348-0_3
Mitchell A, 2020, AM J TRANSL RES, V12, P292
Patel A, 2017, CHIN CLIN ONCOL, V6, DOI 10.21037/cco.2017.02.04
Peng JR, 2017, ACS APPL MATER INTER, V9, P44410, DOI 10.1021/acsami.7b17022
Peng M, 2019, MOL CANCER, V18, DOI 10.1186/s12943-019-1055-6
Riley RS, 2019, NAT REV DRUG DISCOV, V18, P175, DOI 10.1038/s41573-018-0006-z
Sahin U, 2018, SCIENCE, V359, P1355, DOI 10.1126/science.aar7112
Nguyen TX, 2016, NANOMEDICINE-UK, V11, P1169, DOI 10.2217/nnm.16.9
Thomas S, 2016, METHODS MOL BIOL, V1403, P755, DOI 10.1007/978-1-4939-3387-7_43
Uchi SH, 2019, PLOS ONE, V14, DOI 10.1371/journal.pone.0219405
Wang DG, 2019, SCI IMMUNOL, V4, DOI 10.1126/sciimmunol.aau6584
Xia AL, 2019, J MED GENET, V56, P1, DOI 10.1136/jmedgenet-2018-105852
Xu ZH, 2014, ACS NANO, V8, P3636, DOI 10.1021/nn500216y
Yaddanapudi Kavitha, 2018, Stem Cell Investig, V5, P23, DOI 10.21037/sci.2018.07.03
Yang YP, 2015, J CLIN INVEST, V125, P3335, DOI 10.1172/JCI83871
Yuba E, 2020, PHARMACEUTICS, V12, DOI 10.3390/pharmaceutics12080754
Zhao ZW, 2019, J HEMATOL ONCOL, V12, DOI 10.1186/s13045-019-0817-3
Zhou YF, 2018, J ALLERGY CLIN IMMUN, V141, P350, DOI 10.1016/j.jaci.2017.04.049
NR 42
TC 24
Z9 24
U1 20
U2 114
PU AMER ASSOC ADVANCEMENT SCIENCE
PI WASHINGTON
PA 1200 NEW YORK AVE, NW, WASHINGTON, DC 20005 USA
SN 2375-2548
J9 SCI ADV
JI Sci. Adv.
PD AUG
PY 2021
VL 7
IS 35
AR eabi6326
DI 10.1126/sciadv.abi6326
PG 12
WC Multidisciplinary Sciences
WE Science Citation Index Expanded (SCI-EXPANDED)
SC Science & Technology - Other Topics
GA UH1YL
UT WOS:000689735500018
PM 34433569
OA Green Published
DA 2023-09-14
ER

PT J
AU Li, YB
Jiang, MY
Deng, ZM
Zeng, SJ
Hao, JH
AF Li, Youbin

Jiang, Mingyang
Deng, Zhiming
Zeng, Songjun
Hao, Jianhua
TI Low Dose Soft X-Ray Remotely Triggered Lanthanide Nanovaccine for Deep Tissue CO Gas Release and Activation of Systemic Anti-Tumor Immunoresponse
SO ADVANCED SCIENCE
LA English
DT Article
DE activated systemic anti‐ tumor immunotherapy; elicited tumor oxidative stress; gas therapy; lanthanide scintillator‐ based nanovaccine; reversed immunosuppressive tumor microenvironment; soft X‐ ray‐ activated CO release
AB Gas-based therapy has emerged as a new green therapy strategy for anti-tumor treatment. However, the therapeutic efficacy is still restricted by the deep tissue controlled release, poor lymphocytic infiltration, and inherent immunosuppressive tumor microenvironment (TME). Herein, a new type of nanovaccine is designed by integrating low dose soft X-ray-triggered CO releasing lanthanide scintillator nanoparticles (ScNPs: NaLuF4:Gd,Tb@NaLuF4) with photo-responsive CO releasing moiety (PhotoCORM) for synergistic CO gas/immuno-therapy of tumors. The designed nanovaccine presents significantly boosted radioluminescence and enables deep tissue CO generation at unprecedented tissue depths of 5 cm under soft X-ray irradiation. Intriguingly, CO as a superior immunogenic cell death (ICD) inducer further reverses the deep tissue immunosuppressive TME and concurrently activates adaptive anti-tumor immunity through efficient reactive oxygen species (ROS) generation. More importantly, the designed nanovaccine presents efficient growth inhibition of both local and distant tumors via a soft X-ray activated systemic anti-tumor immunoresponse. This work provides a new strategy of designing anti-tumor nanovaccines for synergistic deep tissue gas-therapy and remote soft X-ray photoactivation of the immune response.
C1 [Li, Youbin; Jiang, Mingyang; Deng, Zhiming; Zeng, Songjun] Hunan Normal Univ, Key Lab Low Dimens Quantum Struct & Quantum Contr, Synerget Innovat Ctr Quantum Effects & Applicat,M, Key Lab Matter Microstruct & Funct Hunan Prov,Sch, Changsha 410081, Peoples R China.
[Hao, Jianhua] Hong Kong Polytech Univ, Dept Appl Phys, Hong Kong 999077, Peoples R China.
C3 Hunan Normal University; Hong Kong Polytechnic University
RP Zeng, SJ (通讯作者)，Hunan Normal Univ, Key Lab Low Dimens Quantum Struct & Quantum Contr, Synerget Innovat Ctr Quantum Effects & Applicat,M, Key Lab Matter Microstruct & Funct Hunan Prov,Sch, Changsha 410081, Peoples R China.; Hao, JH (通讯作者)，Hong Kong Polytech Univ, Dept Appl Phys, Hong Kong 999077, Peoples R China.
EM songjunz@hunnu.edu.cn; jh.hao@polyu.edu.hk
RI Hao, Jianhua/J-2806-2012; zeng, songjun/G-6955-2011
OI Hao, Jianhua/0000-0002-6186-5169; zeng, songjun/0000-0001-8663-6090
FU National Natural Science Foundation of China [21671064]; Science and Technology Planning Project of Hunan Province [2017RS3031]; Scientific Research Fund of Hunan Provincial Education Department [19A329]; Natural Science Foundation of Hunan Province, China [2019JJ10002]; Hunan Provincial Innovation Foundation for Postgraduate [CX20190338]; Furong Scholars Programme of Hunan Province
FX This work was supported by the National Natural Science Foundation of China (No.21671064), the Science and Technology Planning Project of Hunan Province (No. 2017RS3031), the Scientific Research Fund of Hunan Provincial Education Department (19A329), Natural Science Foundation of Hunan Province, China (No. 2019JJ10002), the Hunan Provincial Innovation Foundation for Postgraduate (CX20190338) and the Furong Scholars Programme of Hunan Province.
CR Attia AMM, 2020, RADIAT ENVIRON BIOPH, V59, P131, DOI 10.1007/s00411-019-00821-1
Chakraborty I, 2015, CHEM MATER, V27, P8387, DOI 10.1021/acs.chemmater.5b03859
Chen XF, 2019, CHEM SOC REV, V48, P3073, DOI 10.1039/c8cs00921j
Diring S, 2017, CHEM SCI, V8, P2381, DOI 10.1039/c6sc04824b
Fan WP, 2019, NAT COMMUN, V10, DOI 10.1038/s41467-019-09158-1
Feng B, 2018, ADV MATER, V30, DOI 10.1002/adma.201803001
Fukurnura D, 2018, NAT REV CLIN ONCOL, V15, P325, DOI 10.1038/nrclinonc.2018.29
Galluzzi L, 2017, NAT REV IMMUNOL, V17, P97, DOI 10.1038/nri.2016.107

Galluzzi L, 2012, EMBO J, V31, P1055, DOI 10.1038/emboj.2012.2
He QJ, 2015, ADV MATER, V27, P6741, DOI 10.1002/adma.201502762
Karnkaew A, 2016, ACS NANO, V10, P3918, DOI 10.1021/acsnano.6b01401
Krysko DV, 2012, NAT REV CANCER, V12, P860, DOI 10.1038/nrc3380
Li JC, 2019, ANGEW CHEM INT EDIT, V58, P12680, DOI 10.1002/anie.201906288
Li WP, 2016, ACS NANO, V10, P11027, DOI 10.1021/acsnano.6b05858
Li XL, 2018, NANOSCALE, V10, P342, DOI 10.1039/c7nr02926h
Li YB, 2019, ACS NANO, V13, P248, DOI 10.1021/acsnano.8b05431
Liu Q, 2011, J AM CHEM SOC, V133, P17122, DOI 10.1021/ja207078s
Lu KD, 2016, J AM CHEM SOC, V138, P12502, DOI 10.1021/jacs.6b06663
Ma S, 2020, NANO LETT, V20, P2514, DOI 10.1021/acs.nanolett.9b05265
Ma YC, 2019, ACS NANO, V13, P11967, DOI 10.1021/acsnano.9b06040
Mao D, 2020, SCI ADV, V6, DOI 10.1126/sciadv.abb2712
Meng J, 2020, SCI ADV, V6, DOI 10.1126/sciadv.aba1362
Oh SB, 2013, BRIT J PHARMACOL, V168, P421, DOI 10.1111/j.1476-5381.2012.02142.x
Ouyang J, 2019, CHEM COMMUN, V55, P4877, DOI 10.1039/c9cc01129c
Palao E, 2016, J AM CHEM SOC, V138, P126, DOI 10.1021/jacs.5b10800
Panzarini E, 2013, BIOMED RES INT, V2013, DOI 10.1155/2013/482160
Pardoll DM, 2012, NAT REV CANCER, V12, P252, DOI 10.1038/nrc3239
Pierri AE, 2015, CHEM COMMUN, V51, P2072, DOI 10.1039/c4cc06766e
Pittet MJ, 2009, CURR OPIN ONCOL, V21, P53, DOI 10.1097/CCO.0b013e32831bc38a
Popova M, 2018, J AM CHEM SOC, V140, P9721, DOI 10.1021/jacs.8b06011
SHANNON RD, 1976, ACTA CRYSTALLOGR A, V32, P751, DOI 10.1107/S0567739476001551
Shi CR, 2018, NANO LETT, V18, P7330, DOI 10.1021/acs.nanolett.8b03568
Shi Y, 2019, ACCOUNTS CHEM RES, V52, P1543, DOI 10.1021/acs.accounts.9b00148
Soboleva T, 2017, J AM CHEM SOC, V139, P9435, DOI 10.1021/jacs.7b04077
Song GS, 2017, ADV MATER, V29, DOI 10.1002/adma.201700996
Song W, 2018, ACS NANO, V12, P1978, DOI 10.1021/acsnano.7b09112
Taha MS, 2019, NANO LETT, V19, P8333, DOI 10.1021/acs.nanolett.9b04147
Tak PP, 2001, J CLIN INVEST, V107, P7, DOI 10.1172/JCI11830
Terry S, 2017, FRONT IMMUNOL, V8, DOI 10.3389/fimmu.2017.01625
Tian LL, 2019, MATTER-US, V1, P1061, DOI 10.1016/j.matt.2019.07.007
Wang K, 2019, CHEM MATER, V31, P3313, DOI 10.1021/acs.chemmater.9b00265
Wang R, 2014, ANGEW CHEM INT EDIT, V53, P12086, DOI 10.1002/anie.201407420
Wang SB, 2019, ACS NANO, V13, P5523, DOI 10.1021/acsnano.9b00345
Wen M, 2019, ANGEW CHEM INT EDIT, V58, P17425, DOI 10.1002/anie.201909729
Yang JX, 2020, ADV SCI, V7, DOI 10.1002/advs.202001088
Yang WJ, 2020, ACS NANO, V14, P620, DOI 10.1021/acsnano.9b07212
Zanganeh S, 2016, NAT NANOTECHNOL, V11, P986, DOI [10.1038/nnano.2016.168, 10.1038/NNANO.2016.168]
Zheng DW, 2017, ADV MATER, V29, DOI 10.1002/adma.201703822
NR 48
TC 24
Z9 24
U1 10
U2 98
PU WILEY
PI HOBOKEN
PA 111 RIVER ST, HOBOKEN 07030-5774, NJ USA
EI 2198-3844
J9 ADV SCI
JI Adv. Sci.
PD JUN
PY 2021
VL 8
IS 12
AR 2004391
DI 10.1002/advs.202004391
EA APR 2021
PG 13
WC Chemistry, Multidisciplinary; Nanoscience & Nanotechnology; Materials

Science, Multidisciplinary
WE Science Citation Index Expanded (SCI-EXPANDED)
SC Chemistry; Science & Technology - Other Topics; Materials Science
GA SX4UB
UT WOS:000637688400001
PM 34165903
OA Green Published, gold
DA 2023-09-14
ER

PT J
AU Sun, B
Zhao, XH
Wu, YH
Cao, P
Movahedi, F
Liu, J
Wang, JJ
Xu, ZP
Gu, WY
AF Sun, Bing
Zhao, Xiaohui
Wu, Yanheng
Cao, Pei
Movahedi, Fatemeh
Liu, Jie
Wang, Jingjing
Xu, Zhi Ping
Gu, Wenyi
TI Mannose-Functionalized Biodegradable Nanoparticles Efficiently Deliver
DNA Vaccine and Promote Anti-tumor Immunity
SO ACS APPLIED MATERIALS & INTERFACES
LA English
DT Article
DE DNA vaccines; calcium phosphate nanoparticles; cancer immunotherapy;
bisphosphonate; mannose
ID CALCIUM-PHOSPHATE NANOPARTICLES; LAYERED DOUBLE HYDROXIDES; BOVINE
SERUM-ALBUMIN; LECTIN RECEPTORS; DENDRITIC CELLS; COATED NANOPARTICLES;
GENE DELIVERY; PLASMID DNA; DRUG; TRANSFECTION
AB Cancer vaccines have attracted increasing attention for their application in tumor immunotherapy. DNA vaccines are one of them that have been proven very promising with the advantages of safety, rapid design, and low cost. However, the low stability, ineffective cell internalization, and low immunostimulation hinder their wide application. Thus, developing targeted and safe systems to effectively deliver DNA vaccines becomes a vital step. In this study, we report the development of mannose- and bisphosphonate (BP)modified calcium phosphate (CP) nanoparticles (NPs) as efficient vaccine delivery vehicles by targeting C-type lectin receptors (CLRs) on antigen-presenting cells (APCs). Using a model antigen ovalbumin (OVA)-encoded plasmid DNA (pOVA) as a model vaccine, we demonstrate that mannose-modified and BP-stabilized CP (MBCP) nanoparticles are mono-dispersed for enhanced uptake by APCs and subsequently induce OVA antigen presentation and immunostimulation. Mice immunized with MBCP-pOVA nanovaccines show a significantly stronger anti-OVA antibody response with a quicker IgG1 and IgG2a antibody production than unmodified NPs. Moreover, MBCP-pOVA immunization significantly inhibits the growth of OVA-expressing E.G7 tumor cells in C57BL/6J mice. Our data collectively suggest that the modifications to enhance the stability and targeting ability of MBCP NPs are essential for effective delivery of DNA vaccines and promote robust anti-tumor immunity.
C1 [Sun, Bing; Zhao, Xiaohui; Wu, Yanheng; Cao, Pei; Movahedi, Fatemeh; Liu, Jie; Wang, Jingjing; Xu, Zhi Ping; Gu, Wenyi] Univ Queensland, Australian Inst Bioengn & Nanotechnol, St Lucia, Qld 4072, Australia.
[Zhao, Xiaohui] Guangzhou Med Univ, GMU GIBH Joint Sch Life Sci, Guangzhou 511436, Peoples R China.
[Wu, Yanheng] Gill ITM Res Inst, Guangzhou 510530, Peoples R China.
C3 University of Queensland; Guangzhou Medical University

RP Xu, ZP; Gu, WY (通讯作者)，Univ Queensland, Australian Inst Bioengn & Nanotechnol, St Lucia, Qld 4072, Australia.
EM gordonxu@uq.edu.au; w.gu@uq.edu.au
RI Movahedi, Fatemeh/GOK-1797-2022; wang, jiahui/IXD-1197-2023; wang, jing/GRS-7509-2022; wang, jie/HTQ-4920-2023; Gu, Wenyi/O-5018-2019; wang, jian/HRB-9588-2023; Wang, Jin/GYA-2019-2022; wang, jing/HJA-5384-2022; wang, juan/IUO-6218-2023; wang, xu/IAN-4886-2023; wang, jing/GVT-8700-2022; WANG, JINGYI/GSJ-1241-2022
OI Movahedi, Fatemeh/0000-0002-5554-2849; Gu, Wenyi/0000-0003-3360-1390; Sun, Bing/0000-0002-4407-3135
FU Australian Research Council (ARC) [DP190103486]
FX The authors acknowledge the financial support from the Australian Research Council (ARC) Discovery Project (DP190103486). The authors also acknowledge the facilities and the scientific and technical assistance of the Australian Microscopy and Microanalysis Research Facility at the Centre for Microscopy and Microanalysis (CMM) and Australian National Fabrication Facility (ANFF, Qld Node), University of Queensland.
CR Abkar M, 2019, IMMUNOL LETT, V207, P28, DOI 10.1016/j.imlet.2019.01.010
Adams EW, 2008, CHEMBIOCHEM, V9, P294, DOI 10.1002/cbic.200700310
Ahmadpour E, 2017, IMMUNOL LETT, V185, P40, DOI 10.1016/j.imlet.2017.03.006
Akinc A, 2005, J GENE MED, V7, P657, DOI 10.1002/jgm.696
Bolhassani Azam, 2009, Avicenna Journal of Medical Biotechnology, V1, P71
Carrillo-Conde B, 2011, MOL PHARMACEUT, V8, P1877, DOI 10.1021/mp200213r
Chen WY, 2016, SMALL, V12, P1627, DOI 10.1002/smll.201503359
Cheng HN, 2012, POLYM REV, V52, P81, DOI 10.1080/15583724.2012.668154
Coban C, 2013, HUM VACC IMMUNOTHER, V9, P2216, DOI 10.4161/hv.25893
Costa A, 2018, EUR J PHARM SCI, V114, P103, DOI 10.1016/j.ejps.2017.12.006
COURSAGET P, 1986, INFECT IMMUN, V51, P784, DOI 10.1128/IAI.51.3.784-787.1986
Diebold SS, 2001, GENE THER, V8, P487, DOI 10.1038/sj.gt.3301433
Dong X, 2019, ACS APPL MATER INTER, V11, P4876, DOI 10.1021/acsami.8b20364
Elias DR, 2013, NANOMED-NANOTECHNOL, V9, P194, DOI 10.1016/j.nano.2012.05.015
Fakhari A, 2011, J PHARM SCI-US, V100, P1045, DOI 10.1002/jps.22342
Fang RH, 2014, NANO LETT, V14, P2181, DOI 10.1021/nl500618u
Figdor CG, 2002, NAT REV IMMUNOL, V2, P77, DOI 10.1038/nri723
Finn OJ, 2003, NAT REV IMMUNOL, V3, P630, DOI 10.1038/nri1150
Ganbold T, 2018, ACS APPL MATER INTER, V10, P14463, DOI 10.1021/acsami.8b02073
Geijtenbeek TBH, 2009, NAT REV IMMUNOL, V9, P465, DOI 10.1038/nri2569
Hobernik D, 2018, INT J MOL SCI, V19, DOI 10.3390/ijms19113605
Hong XY, 2020, SCI ADV, V6, DOI 10.1126/sciadv.aaz4462
Hu CMJ, 2011, P NATL ACAD SCI USA, V108, P10980, DOI 10.1073/pnas.1106634108
Joffre O, 2009, IMMUNOL REV, V227, P234, DOI 10.1111/j.1600-065X.2008.00718.x
Keler T, 2004, EXPERT OPIN BIOL TH, V4, P1953, DOI 10.1517/14712598.4.12.1953
Khalifehzadeh R, 2020, NANOSCALE, V12, P9603, DOI 10.1039/c9nr09782a
Li A, 2011, BIOMATERIALS, V32, P469, DOI 10.1016/j.biomaterials.2010.08.107
Li XR, 2014, J CONTROL RELEASE, V173, P148, DOI 10.1016/j.jconrel.2013.10.032
Liang RJ, 2017, BIOMATERIALS, V149, P41, DOI 10.1016/j.biomaterials.2017.09.029
Lin H, 2020, CHEM COMMUN, V56, P13959, DOI 10.1039/d0cc05263a
Lindblad EB, 2017, IMMUNOPOTENTIATORS IN MODERN VACCINES, 2ND EDITION, P347, DOI 10.1016/B978-0-12-804019-5.00018-9
Liu MA, 2003, J INTERN MED, V253, P402, DOI 10.1046/j.1365-2796.2003.01140.x
Lorenz JN, 1999, AM J PHYSIOL-RENAL, V276, pF172, DOI 10.1152/ajprenal.1999.276.1.F172
MCREYNOLDS L, 1978, NATURE, V273, P723, DOI 10.1038/273723a0
Melief CJM, 2015, J CLIN INVEST, V125, P3401, DOI 10.1172/JCI80009
Minigo G, 2007, VACCINE, V25, P1316, DOI 10.1016/j.vaccine.2006.09.086
Nakamura M, 2018, COLLOID SURFACE B, V162, P135, DOI 10.1016/j.colsurfb.2017.11.034
Palucka K, 2012, NAT REV CANCER, V12, P265, DOI 10.1038/nrc3258
Pashine A, 2005, NAT MED, V11, pS63, DOI 10.1038/nm1210
Pietrella D, 2005, INFECT IMMUN, V73, P820, DOI 10.1128/IAI.73.2.820-827.2005
Relyveld E H, 1986, Dev Biol Stand, V65, P131
Rice J, 2008, NAT REV CANCER, V8, P108, DOI 10.1038/nrc2326
Scheffel F, 2020, VACCINES-BASEL, V8, DOI 10.3390/vaccines8010110

Sharma P, 2015, SCIENCE, V348, P56, DOI 10.1126/science.aaa8172
Song H, 2020, ADV THER-GERMANY, V3, DOI 10.1002/adtp.201900154
Stahl PD, 1998, CURR OPIN IMMUNOL, V10, P50, DOI 10.1016/S0952-7915(98)80031-9
Sun B, 2020, ACS APPL BIO MATER, V3, P986, DOI 10.1021/acsabm.9b00994
Takeda Y, 2018, CANCER SCI, V109, P2119, DOI 10.1111/cas.13649
Thamake SI, 2012, BIOMATERIALS, V33, P7164, DOI 10.1016/j.biomaterials.2012.06.026
Torralba D, 2018, NAT COMMUN, V9, DOI 10.1038/s41467-018-05077-9
Ulmer JB, 2006, TRENDS MOL MED, V12, P216, DOI 10.1016/j.molmed.2006.03.007
Un K, 2010, BIOMATERIALS, V31, P7813, DOI 10.1016/j.biomaterials.2010.06.058
Uskokovic V, 2011, J BIOMED MATER RES B, V96B, P152, DOI 10.1002/jbm.b.31746
Van der Jeught K, 2018, ACS NANO, V12, P9815, DOI 10.1021/acsnano.8b00966
van Kooyk Y, 2008, NAT IMMUNOL, V9, P593, DOI 10.1038/ni.f.203
Wang YH, 2018, MOL THER, V26, P420, DOI 10.1016/j.ymthe.2017.11.009
Weide B, 2008, IMMUNOL LETT, V115, P33, DOI 10.1016/j.imlet.2007.09.012
WOLFF JA, 1990, SCIENCE, V247, P1465, DOI 10.1126/science.1690918
Yan SY, 2014, BIOMATERIALS, V35, P9508, DOI 10.1016/j.biomaterials.2014.07.055
Yang R, 2018, ACS NANO, V12, P5121, DOI 10.1021/acsnano.7b09041
Yingchoncharoen P, 2016, PHARMACOL REV, V68, P701, DOI 10.1124/pr.115.012070
Zhang MZ, 2009, NANO TODAY, V4, P508, DOI 10.1016/j.nantod.2009.10.009
Zhou SL, 2020, BIOMATERIALS, V235, DOI 10.1016/j.biomaterials.2020.119795
NR 63
TC 24
Z9 24
U1 10
U2 85
PU AMER CHEMICAL SOC
PI WASHINGTON
PA 1155 16TH ST, NW, WASHINGTON, DC 20036 USA
SN 1944-8244
EI 1944-8252
J9 ACS APPL MATER INTER
JI ACS Appl. Mater. Interfaces
PD MAR 31
PY 2021
VL 13
IS 12
BP 14015
EP 14027
DI 10.1021/acsami.1c01401
EA MAR 2021
PG 13
WC Nanoscience & Nanotechnology; Materials Science, Multidisciplinary
WE Science Citation Index Expanded (SCI-EXPANDED)
SC Science & Technology - Other Topics; Materials Science
GA RI1RB
UT WOS:000636686200010
PM 33751882
DA 2023-09-14
ER

PT J
AU Zaheer, T
Pal, K
Zaheer, I
AF Zaheer, Tean
Pal, Kaushik
Zaheer, Iqra
TI Topical review on nano-vaccinology: Biochemical promises and key
challenges
SO PROCESS BIOCHEMISTRY
LA English

DT Review
DE Immunity; Nanoparticles; Vaccine; Pathogens; Prevention
ID VIRUS-LIKE PARTICLES; MOLECULAR-PATTERNS; NANOPARTICLES; VACCINES;
NANOVACCINE; ANTIBODY; ANTIBACTERIAL; EXPRESSION; PROTECTION; RESPONSES
AB Nanomaterials have wide-ranging biomedical applications in prevention, treatment and control of diseases. Nanoparticle based vaccines have proven prodigious prophylaxis of various infectious and non-infectious diseases of human and animal concern. Nano-vaccines outnumber the conventional vaccines by virtue of plasticity in physio-chemical properties and ease of administration. The efficacy of nano-based vaccines may be attributed to the improved antigen stability, minimum immuno-toxicity, sustained release, enhanced immunogenicity and the flexibility of physical features of nanoparticles. Based on these, the nano-based vaccines have potential to evoke both cellular and humoral immune responses. Targeted and highly specific immunological pathways required for solid and long lasting immunity may be achieved with specially engineered nano-vaccines. This review presents an insight into the prevention of infectious diseases (of bacterial, viral and parasitic origin) and non-infectious diseases (cancer, auto-immune diseases) using nano-vaccinology. Additionally, key challenges to the effective utilization of nano-vaccines from bench to clinical settings have been highlighted as research domains for future.
C1 [Zaheer, Tean] Univ Agr Faisalabad, Dept Parasitol, Faisalabad 38040, Pakistan.
[Pal, Kaushik] Univ Fed Rio de Janeiro, Cidade Univ, BR-21941901 Rio De Janeiro, RJ, Brazil.
[Pal, Kaushik] Wuhan Univ, 8 East Lake South Rd, Wuchang 430072, Hubei, Peoples R China.
[Zaheer, Iqra] Univ Agr Faisalabad, Dept Pathol, Faisalabad 38040, Pakistan.
C3 University of Agriculture Faisalabad; Universidade Federal do Rio de
Janeiro; Wuhan University; University of Agriculture Faisalabad
RP Pal, K (通讯作者)，Univ Fed Rio de Janeiro, Lab Tecnol, Cidade Univ, BR-21941901 Rio De Janeiro, RJ, Brazil.
EM kaushikphysics@gmail.com
RI Zaheer, Tean/ABI-6123-2020
OI Zaheer, Tean/0000-0001-8248-6451; Zaheer, Iqra/0000-0003-3707-6685
CR Abdelkader DH, 2018, DRUG DELIV TRANSL RE, V8, P1053, DOI 10.1007/s13346-018-0554-0
Aljabali AAA, 2020, CANCERS, V12, DOI 10.3390/cancers12010113
Alkie TN, 2018, PLOS ONE, V13, DOI 10.1371/journal.pone.0206324
Amanat F, 2020, IMMUNITY, V52, P583, DOI 10.1016/j.immuni.2020.03.007
Amawi H, 2020, THER DELIV, V11, P245, DOI 10.4155/tde-2020-0035
Asiya SI, 2020, MATER TODAY CHEM, V17, DOI 10.1016/j.mtchem.2020.100327
Bento D, 2015, EUR J PHARM BIOPHARM, V93, P149, DOI 10.1016/j.ejpb.2015.03.024
Bhardwaj P, 2020, ACTA BIOMATER, V108, P1, DOI 10.1016/j.actbio.2020.03.020
Brady JM, 2017, IMMUNOL REV, V275, P324, DOI 10.1111/imr.12478
Chauhan G, 2020, ACS NANO, V14, P7760, DOI 10.1021/acsnano.0c04006
Coleman CM, 2014, VACCINE, V32, P3169, DOI 10.1016/j.vaccine.2014.04.016
Collins KA, 2017, SCI REP-UK, V7, DOI 10.1038/srep46621
Cunningham AL, 2016, NEW ENGL J MED, V375, P1019, DOI 10.1056/NEJMoa1603800
Demento SL, 2012, BIOMATERIALS, V33, P4957, DOI 10.1016/j.biomaterials.2012.03.041
Demento SL, 2011, TRENDS BIOTECHNOL, V29, P294, DOI 10.1016/j.tibtech.2011.02.004
Ding PY, 2019, INT J NANOMED, V14, P7533, DOI 10.2147/IJN.S218057
El Bissati K, 2017, NPJ VACCINES, V2, DOI 10.1038/s41541-017-0024-6
El-Sissi AF, 2020, 3 BIOTECH, V10, DOI 10.1007/s13205-020-2076-y
Firouzmand H, 2013, ACTA TROP, V128, P528, DOI 10.1016/j.actatropica.2013.07.021
Gao WW, 2015, NANO LETT, V15, P1403, DOI 10.1021/nl504798g
Gardner MR, 2020, FRONT CELL INFECT MI, V10, DOI 10.3389/fcimb.2020.00176
Hanson MC, 2015, VACCINE, V33, P861, DOI 10.1016/j.vaccine.2014.12.045
Huertas-Diaz MC, 2019, VACCINE, V37, P2925, DOI 10.1016/j.vaccine.2019.04.042
Janson O, 2019, MAT SCI ENG C-MATER, V96, P272, DOI 10.1016/j.msec.2018.11.021
Jeevanandam J, 2020, MICRO NANOTECHNOLOGI, P187
Jeevanandam J, 2019, BIOCHIMIE, V157, P38, DOI 10.1016/j.biochi.2018.11.001
Khan MN, 2018, CURR DRUG DELIV, V15, P286, DOI 10.2174/1567201814666171019104002
Kim MY, 2017, PLANT BIOTECHNOL J, V15, P1590, DOI 10.1111/pbi.12741
Liu L., 2020, PREPRINT, DOI [10.26434/chemrxiv.12301157.v1, DOI 10.26434/CHEMRXIV.12301157.V1]
Lokugamage N, 2020, VACCINES-BASEL, V8, DOI 10.3390/vaccines8010096
Lurie N, 2020, NEW ENGL J MED, V382, P1969, DOI 10.1056/NEJMp2005630
Maleki M, 2019, ARTIF CELL NANOMED B, V47, P4248, DOI 10.1080/21691401.2019.1687490
Marsian J, 2017, NAT COMMUN, V8, DOI 10.1038/s41467-017-00090-w

Moura APV, 2017, ACS CENTRAL SCI, V3, P1026, DOI 10.1021/acscentsci.7b00311
Nevagi RJ, 2018, ACTA BIOMATER, V80, P278, DOI 10.1016/j.actbio.2018.09.037
Ni QQ, 2020, SCI ADV, V6, DOI 10.1126/sciadv.aaw6071
Oun AA, 2020, CRIT REV FOOD SCI, V60, P435, DOI 10.1080/10408398.2018.1536966
Page A, 2020, CANCERS, V12, DOI 10.3390/cancers12040962
Park HJ, 2017, J IMMUNOL, V198
Qi M, 2018, SMALL, V14, DOI 10.1002/smll.201703207
Renu S, 2020, INT J NANOMED, V15, P761, DOI 10.2147/IJN.S238445
Rodrigues-Jesus MJ, 2019, BRAZ J MICROBIOL, V50, P67, DOI 10.1007/s42770-018-0035-0
Sadeghi Z, 2020, INT J NANOMED, V15, P3877, DOI 10.2147/IJN.S249942
Sanchez-Villamil JI, 2019, MBIO, V10, DOI 10.1128/mBio.01869-19
Sekimukai H, 2020, MICROBIOL IMMUNOL, V64, P33, DOI 10.1111/1348-0421.12754
Serradell MC, 2019, NAT COMMUN, V10, DOI 10.1038/s41467-018-08265-9
Sharma J, 2020, ACS APPL MATER INTER, V12, P18211, DOI 10.1021/acsami.9b21776
Shin MD, 2020, NAT NANOTECHNOL, V15, P646, DOI 10.1038/s41565-020-0737-y
Shukla S, 2019, ADV THER-GERMANY, V2, DOI 10.1002/adtp.201800139
Siefert AL, 2016, BIOMATERIALS, V97, P85, DOI 10.1016/j.biomaterials.2016.03.039
Syomin B V, 2019, Mol Biol (Mosk), V53, P367, DOI 10.1134/S0026898419030157
Tan ZL, 2017, EUR J PHARM BIOPHARM, V111, P33, DOI 10.1016/j.ejpb.2016.11.007
Tejeda-Mansir A, 2019, BIOTECHNOL GENET ENG, V35, P46, DOI 10.1080/02648725.2018.1560552
Le TT, 2020, NAT REV DRUG DISCOV, V19, P305, DOI 10.1038/d41573-020-00073-5
Thukral Akanksha, 2020, NPJ Vaccines, V5, P15, DOI 10.1038/s41541-020-0164-y
Vandoolaeghe P, 2016, EXPERT REV VACCINES, V15, P1481, DOI 10.1080/14760584.2016.1236689
Wang F, 2016, ADV FUNCT MATER, V26, P1628, DOI 10.1002/adfm.201505231
Wang WJ, 2020, NAT NANOTECHNOL, V15, P406, DOI 10.1038/s41565-020-0648-y
Wang XT, 2014, PLOS ONE, V9, DOI 10.1371/journal.pone.0086578
Wang YR, 2016, NANOSCALE RES LETT, V11, DOI 10.1186/s11671-016-1554-y
Watson DS, 2012, VACCINE, V30, P2256, DOI 10.1016/j.vaccine.2012.01.070
Wei HL, 2017, NPJ VACCINES, V2, DOI 10.1038/s41541-017-0031-7
Wu ML, 2017, FRONT CELL INFECT MI, V7, DOI 10.3389/fcimb.2017.00445
Xiang SD, 2018, FRONT IMMUNOL, V9, DOI 10.3389/fimmu.2018.02968
Yaqoob AA, 2020, FRONT CHEM, V8, DOI 10.3389/fchem.2020.00341
Youssef FS, 2019, INT J VET SCI MED, V7, P78, DOI 10.1080/23144599.2019.1691379
Zeng LM, 2020, CHINESE CHEM LETT, V31, P1162, DOI 10.1016/j.cclet.2019.10.015
Zhang B, 2016, NANOMATERIALS-BASEL, V6, DOI 10.3390/nano6110201
Zhang C, 2020, J NANOBIOTECHNOL, V18, DOI 10.1186/s12951-020-0584-x
Zhao QJ, 2013, TRENDS BIOTECHNOL, V31, P654, DOI 10.1016/j.tibtech.2013.09.002
Zhu GZ, 2017, NAT COMMUN, V8, DOI 10.1038/s41467-017-02191-y
NR 71
TC 24
Z9 25
U1 10
U2 77
PU ELSEVIER SCI LTD
PI OXFORD
PA THE BOULEVARD, LANGFORD LANE, KIDLINGTON, OXFORD OX5 1GB, OXON, ENGLAND
SN 1359-5113
EI 1873-3298
J9 PROCESS BIOCHEM
JI Process Biochem.
PD JAN
PY 2021
VL 100
BP 237
EP 244
DI 10.1016/j.procbio.2020.09.028
PG 8
WC Biochemistry & Molecular Biology; Biotechnology & Applied Microbiology;
Engineering, Chemical
WE Science Citation Index Expanded (SCI-EXPANDED)
SC Biochemistry & Molecular Biology; Biotechnology & Applied Microbiology;

Engineering
GA RC3WO
UT WOS:000632735000003
PM 33013180
OA Bronze, Green Published
DA 2023-09-14
ER

PT J
AU Zhang, LX
Sun, XM
Jia, YB
Liu, XG
Dong, MD
Xu, ZP
Liu, RT
AF Zhang, Ling-xiao
Sun, Xia-mei
Jia, Ying-bo
Liu, Xiao-ge
Dong, Mingdong
Xu, Zhi Ping
Liu, Rui-tian
TI Nanovaccine's rapid induction of anti-tumor immunity significantly improves malignant cancer immunotherapy
SO NANO TODAY
LA English
DT Article
DE Malignant cancer immunotherapy; Layered double hydroxide based nanovaccine; Vaccination routes; Quick induction of anti-tumor immunity
ID DOUBLE HYDROXIDE NANOPARTICLES; DENDRITIC CELLS; THERAPEUTIC VACCINES; ADMINISTRATION ROUTE; MELANOMA TUMOR; CO-DELIVERY; EXPRESSION; EFFICIENT; RESPONSES; ADJUVANT
AB The advantage of rapid tumor growth over slow immune induction often limits the efficiency of cancer therapeutic vaccines. Many functionalized micro-/nano-vaccines are shown to enhance cancer immunotherapy, but few studies correlate the therapeutic efficacy of these micro-/nano-vaccines to the vaccination route such as intravenous (IV), subcutaneous (SC) injection or their combination. Herein, we employed classical "priming + boosting" vaccination strategy to investigate the influence of four vaccination combinations (IV + IV, IV + SC, SC + IV and SC + SC) of nanovaccines on the anti-tumor therapeutic efficacy in mice bearing E.G7-OVA-lymphoma and B16F10-melanoma. The nanovaccines were constructed by loading antigen and CpG onto layered double hydroxide (LDH) nanoparticles. Our experimental data indicate that "IV-priming + SC-boosting" vaccination combination most efficiently inhibits the growth of early stage tumors, with the tumor volume reduced by >75-90 % in comparison with the control group. Based on these findings, a novel vaccination strategy, i.e. simultaneous IV and SC injection was proposed, which significantly delayed the progression of both early stage and advanced Bl6F10 tumors. Our current research for the first time correlates the vaccination route of nanovaccines to the anti-tumor therapeutic efficacy, and the highest efficacy of optimal vaccination combination (IV + SC) is benefited from that fact that IV priming quickly induces strong anti-tumor responses that are well sustained by subsequent SC boosting. Moreover, the simultaneous IV/SC vaccination potentially provides a novel vaccination strategy for enhanced immunotherapy of early stage and advanced tumors. (C) 2020 Elsevier Ltd. All rights reserved.
C1 [Zhang, Ling-xiao; Sun, Xia-mei; Jia, Ying-bo; Liu, Xiao-ge; Liu, Rui-tian] Chinese Acad Sci, Inst Proc Engn, State Key Lab Biochem Engn, Beijing 100190, Peoples R China.
[Zhang, Ling-xiao; Xu, Zhi Ping] Univ Queensland, Australian Inst Bioengn & Nanotechnol, St Lucia, Qld 4072, Australia.
[Dong, Mingdong] Aarhus Univ, Interdisciplinary Nanosci Ctr iNANO, Sino Danish Ctr Educ & Res SDC, DK-8000 Aarhus C, Denmark.
[Zhang, Ling-xiao; Jia, Ying-bo; Liu, Xiao-ge] Univ Chinese Acad Sci, Sch Chem Engn, Beijing 100049, Peoples R China.
C3 Institute of Process Engineering, CAS; Chinese Academy of Sciences; University of Queensland; Aarhus University; Chinese Academy of

Sciences; University of Chinese Academy of Sciences, CAS
RP Liu, RT (通讯作者)，Chinese Acad Sci, Inst Proc Engn, State Key Lab Biochem Engn, Beijing 100190, Peoples R China.; Xu, ZP (通讯作者)，Univ Queensland, Australian Inst Bioengn & Nanotechnol, St Lucia, Qld 4072, Australia.
EM gordonxu@uq.edu.au; rtliu@ipe.ac.cn
RI Zhang, Lingxiao/AAR-8876-2021; Dong, Mingdong/B-3341-2010
OI Dong, Mingdong/0000-0002-2025-2171; Zhang, Lingxiao/0000-0002-7066-6065
FU International Partnership Program of Chinese Academy of Sciences [122111KYSB20180005]; Australian Research Council (ARC) [DP170104643, DP190103486]; Danish National Research Foundation [904000219B]; European Union [734174]; UCAS Joint PhD Training Program [2018-23]; Marie Curie Actions (MSCA) [734174] Funding Source: Marie Curie Actions (MSCA)
FX This work was financially supported by the International Partnership Program of Chinese Academy of Sciences [grant number 122111KYSB20180005], the Australian Research Council (ARC) Discovery Project [grant number DP170104643 and DP190103486], the Danish National Research Foundation [grant number 904000219B], European Union's Horizon 2020 research and innovation program [grant number 734174 MNR4S Cell project]. L.X. Zhang acknowledges the financial support from The UCAS Joint PhD Training Program [2018-23].
CR Banchereau J, 2005, NAT REV IMMUNOL, V5, P296, DOI 10.1038/nri1592
Bronte V, 2013, IMMUNITY, V39, P806, DOI 10.1016/j.immuni.2013.10.010
Cao ZB, 2018, ADV SCI, V5, DOI 10.1002/advs.201801155
Chen Q, 2019, NAT NANOTECHNOL, V14, P89, DOI 10.1038/s41565-018-0319-4
Chen WY, 2018, J CONTROL RELEASE, V292, P196, DOI 10.1016/j.jconrel.2018.11.008
Chen WY, 2018, SMALL, V14, DOI 10.1002/smll.201704465
Chen WY, 2016, SMALL, V12, P1627, DOI 10.1002/smll.201503359
Choi SJ, 2010, J NANOSCI NANOTECHNO, V10, P2913, DOI 10.1166/jnn.2010.1415
Conniot J, 2019, NAT NANOTECHNOL, V14, P891, DOI 10.1038/s41565-019-0512-0
Courant T, 2017, BIOMATERIALS, V136, P29, DOI 10.1016/j.biomaterials.2017.05.001
Cyster JG, 1999, J EXP MED, V189, P447, DOI 10.1084/jem.189.3.447
Epstein JE, 2011, SCIENCE, V334, P475, DOI 10.1126/science.1211548
Gu Z, 2015, J MATER CHEM B, V3, P3331, DOI 10.1039/c5tb00248f
Gunn MD, 1999, J EXP MED, V189, P451, DOI 10.1084/jem.189.3.451
Guy B, 2007, NAT REV MICROBIOL, V5, P505, DOI 10.1038/nrmicro1681
Han X, 2019, SCI ADV, V5, DOI 10.1126/sciadv.aaw6870
Itano AA, 2003, NAT IMMUNOL, V4, P733, DOI 10.1038/ni957
Kranz LM, 2016, NATURE, V534, P396, DOI 10.1038/nature18300
Kuai R, 2017, NAT MATER, V16, P489, DOI [10.1038/nmat4822, 10.1038/NMAT4822]
Kwak SY, 2004, BIOMATERIALS, V25, P5995, DOI 10.1016/j.biomaterials.2004.01.056
Li AW, 2018, NAT MATER, V17, P528, DOI 10.1038/s41563-018-0028-2
Li A, 2010, BIOMATERIALS, V31, P748, DOI 10.1016/j.biomaterials.2009.09.095
Li B, 2018, BIOMATERIALS, V177, P40, DOI 10.1016/j.biomaterials.2018.05.055
Li F, 2019, INT J NANOMED, V14, P9917, DOI 10.2147/IJN.S226466
Liu DQ, 2018, J CONTROL RELEASE, V273, P1, DOI 10.1016/j.jconrel.2018.01.013
Liu J, 2011, J MATER CHEM, V21, P10641, DOI 10.1039/c1jm12054a
Lu Y, 2018, BIOMATERIALS, V175, P82, DOI 10.1016/j.biomaterials.2018.05.025
Mebius RE, 2005, NAT REV IMMUNOL, V5, P606, DOI 10.1038/nri1669
Melero I, 2014, NAT REV CLIN ONCOL, V11, P509, DOI 10.1038/nrclinonc.2014.111
Okada N, 2001, BRIT J CANCER, V84, P1564, DOI 10.1054/bjoc.2001.1801
Pardoll DM, 2012, NAT REV CANCER, V12, P252, DOI 10.1038/nrc3239
Pavlovic M, 2019, J COLLOID INTERF SCI, V543, P174, DOI 10.1016/j.jcis.2019.02.050
Pavlovic M, 2018, J COLLOID INTERF SCI, V524, P114, DOI 10.1016/j.jcis.2018.04.007
Pearson RM, 2017, MOL THER, V25, P1655, DOI 10.1016/j.ymthe.2017.04.015
Peng F, 2019, NAT NANOTECHNOL, V14, P279, DOI 10.1038/s41565-018-0356-z
Raeesi V, 2016, ADV MATER, V28, P8511, DOI 10.1002/adma.201600773
Reed SG, 2013, NAT MED, V19, P1597, DOI 10.1038/nm.3409
Seder RA, 2013, SCIENCE, V341, P1359, DOI 10.1126/science.1241800
Shafik HM, 2014, J RADIOANAL NUCL CH, V301, P81, DOI 10.1007/s10967-014-3155-5
Sharma P, 2015, CELL, V161, P205, DOI 10.1016/j.cell.2015.03.030
Sultan H, 2019, CANCER IMMUNOL IMMUN, V68, P455, DOI 10.1007/s00262-018-02294-5

von Andrian UH, 2003, NAT REV IMMUNOL, V3, P867, DOI 10.1038/nri1222
Wang H, 2018, NAT MATER, V17, P761, DOI 10.1038/s41563-018-0147-9
Wang S, 2018, SMALL, V14, DOI 10.1002/smll.201704272
Wei PR, 2012, J MATER CHEM, V22, P5503, DOI 10.1039/c2jm16447g
Xia YF, 2018, NAT MATER, V17, P187, DOI [10.1038/NMAT5057, 10.1038/nmat5057]
Xu ZP, 2008, J CONTROL RELEASE, V130, P86, DOI 10.1016/j.jconrel.2008.05.021
Yan SY, 2014, BIOMATERIALS, V35, P9508, DOI 10.1016/j.biomaterials.2014.07.055
Zhang LX, 2019, ACS APPL MATER INTER, V11, P35566, DOI 10.1021/acsami.9b11746
Zhang LX, 2018, BIOMATERIALS, V174, P54, DOI 10.1016/j.biomaterials.2018.05.015
Zhang LX, 2017, J MATER CHEM B, V5, P6266, DOI 10.1039/c7tb00819h
NR 51
TC 24
Z9 25
U1 9
U2 65
PU ELSEVIER SCI LTD
PI OXFORD
PA THE BOULEVARD, LANGFORD LANE, KIDLINGTON, OXFORD OX5 1GB, OXON, ENGLAND
SN 1748-0132
EI 1878-044X
J9 NANO TODAY
JI Nano Today
PD DEC
PY 2020
VL 35
AR 100923
DI 10.1016/j.nantod.2020.100923
PG 12
WC Chemistry, Multidisciplinary; Nanoscience & Nanotechnology; Materials
Science, Multidisciplinary
WE Science Citation Index Expanded (SCI-EXPANDED)
SC Chemistry; Science & Technology - Other Topics; Materials Science
GA PI1HL
UT WOS:000600849700004
DA 2023-09-14
ER

PT J
AU Li, T
Chen, GJ
Xiao, ZC
Li, B
Zhong, HH
Lin, MZ
Cai, YJ
Huang, J
Xie, X
Shuai, XT
AF Li, Tan
Chen, Gengjia
Xiao, Zecong
Li, Bo
Zhong, Huihai
Lin, Minzhao
Cai, Yujun
Huang, Jing
Xie, Xi
Shuai, Xintao
TI Surgical Tumor-Derived Photothermal Nanovaccine for Personalized Cancer
Therapy and Prevention
SO NANO LETTERS

LA English
DT Article
DE surgical tumor-derived cell membranes; mesoporous polydopamine;
photothermal immunotherapy; cancer nanovaccine; prevention
ID BIOMIMETIC NANOPARTICLES; CHECKPOINT BLOCKADE; IMMUNOTHERAPY
AB Recent breakthroughs in cell membrane-fabricated nanovaccine offer innovateive therapeutic options for preventing tumor metastasies and recurrence, yet the treatment of patient-specific solid tumor remained challenging owing to the immunosuppressive tumor microenvironment. Herein, we developed a personalized photothermal nanovaccine based on the surgical loaded mesoporous polydopamine (MPDA) nanoparticles for targeting tumor photothermal immunotherapy and prevention. The demonstrates outstanding imaging-guided photothermal immunotherapy efficacy to eradicate solid tumors under near-IR laser irradiation and further inhibiting metastasis tumors by the resulted antitumor immunities, especially in combination with programmed death-ligand 1 antibody therapy (aPD-L1). Furthermore, from in vivo prophylactic testing results, it is confirmed that the 4T1 cells rechallenge can be prevented 100% in postsurgical tumor model after vaccination of the photothermal nanovaccine. Our work fabricates a personalized photothermal nanovaccine that possesses great potential for tumor-specific treatment and for preventing postoperative tumor recurrence.
C1 [Li, Tan; Chen, Gengjia; Zhong, Huihai; Lin, Minzhao; Cai, Yujun; Huang, Jing; Shuai, Xintao] Sun Yat Sen Univ, PCFM Lab Minist Educ, Sch Mat Sci & Engn, Guangzhou 510275, Peoples R China.
[Xiao, Zecong] Guangzhou Med Univ, Dept Minimally Invas Intervent Radiol, Affiliated Hosp 2, Guangzhou 510275, Peoples R China.
[Xiao, Zecong] Guangzhou Med Univ, Lab Intervent Radiol, Affiliated Hosp 2, Guangzhou 510275, Peoples R China.
[Li, Bo; Shuai, Xintao] Sun Yat Sen Univ, Nanomed Res Ctr, Affiliated Hosp 3, Guangzhou 510630, Peoples R China.
[Xie, Xi] Yat Sen Univ, Affiliated Hosp 1, Sun Yat Sen Univ, State Key Lab Optoelect Mat & Technol,Sch Elect &, Guangzhou 510275, Peoples R China.
C3 Sun Yat Sen University; Guangzhou Medical University; Guangzhou Medical
University; Sun Yat Sen University; Sun Yat Sen University
RP Shuai, XT (通讯作者)，Sun Yat Sen Univ, PCFM Lab Minist Educ, Sch Mat Sci & Engn, Guangzhou 510275, Peoples R China.; Shuai, XT (通讯作者)，Sun Yat Sen Univ, Nanomed Res Ctr, Affiliated Hosp 3, Guangzhou 510630, Peoples R China.; Xie, X (通讯作者)，Yat Sen Univ, Affiliated Hosp 1, Sun Yat Sen Univ, State Key Lab Optoelect Mat & Technol,Sch Elect &, Guangzhou 510275, Peoples R China.
EM xiexi27@mail.sysu.edu.cn; shuaixt@mail.sysu.edu.cn
RI Shuai, Xintao/C-5819-2008; lin, minzhao/IAP-7017-2023
OI Shuai, Xintao/0000-0003-4271-0310; Li, Bo/0000-0002-5575-3582; Xiao,
Zecong/0000-0002-7923-5309
FU National Natural Science Foundation of China [51933011, 31971296];
Science and Technology Program of Guangzhou [202007020006]
FX We greatly appreciate the financial support from the National Natural
Science Foundation of China (Grant Nos. 51933011 and 31971296) and the
Science and Technology Program of Guangzhou (Grant No. 202007020006).
CR Barkal AA, 2019, NATURE, V572, P392, DOI 10.1038/s41586-019-1456-0
Chen XT, 2020, THERANOSTICS, V10, P6011, DOI 10.7150/thno.38742
Chen Z, 2016, ACS NANO, V10, P10049, DOI 10.1021/acsnano.6b04695
Cheng YY, 2020, ACS NANO, V14, P15161, DOI 10.1021/acsnano.0c05392
Duan XP, 2019, ANGEW CHEM INT EDIT, V58, P670, DOI 10.1002/anie.201804882
Guan BY, 2016, J AM CHEM SOC, V138, P11306, DOI 10.1021/jacs.6b06558
Hong JY, 2019, INT J NANOMED, V14, P7053, DOI 10.2147/IJN.S215415
Huang CL, 2021, ADV FUNCT MATER, V31, DOI 10.1002/adfm.202010637
Huang LP, 2019, NAT COMMUN, V10, DOI 10.1038/s41467-019-12771-9
Huang XY, 2021, THERANOSTICS, V11, P7546, DOI 10.7150/thno.56482
Kreiter S, 2015, NATURE, V520, P692, DOI 10.1038/nature14426
Liu WL, 2019, ADV MATER, V31, DOI 10.1002/adma.201900499
Lu QL, 2019, J MATER CHEM B, V7, P2499, DOI 10.1039/c9tb00089e
Ma L, 2021, ADV MATER, V33, DOI 10.1002/adma.202104849
Nam J, 2021, ADV THER-GERMANY, V4, DOI 10.1002/adtp.202100093
Nam J, 2021, ADV SCI, V8, DOI 10.1002/advs.202002577
Ng CW, 2018, ADV FUNCT MATER, V28, DOI 10.1002/adfm.201804688
Pan JB, 2018, ADV MATER, V30, DOI 10.1002/adma.201704408
Rao L., 2019, ADV FUNCT MATER, V29

Rao L, 2016, ADV MATER, V28, P3460, DOI 10.1002/adma.201506086
Sun HP, 2016, ADV MATER, V28, P9581, DOI 10.1002/adma.201602173
Wu D, 2019, ADV FUNCT MATER, V29, DOI 10.1002/adfm.201900095
Xiao P, 2021, ADV FUNCT MATER, V31, DOI 10.1002/adfm.202104068
Xiao ZC, 2020, SCI ADV, V6, DOI 10.1126/sciadv.aay7785
Xie W, 2019, ACS NANO, V13, P2849, DOI 10.1021/acsnano.8b03788
Xu J, 2020, NAT NANOTECHNOL, V15, P1043, DOI 10.1038/s41565-020-00781-4
Xu J, 2017, ACS NANO, V11, P4463, DOI 10.1021/acsnano.7b00715
Yang R, 2018, ACS NANO, V12, P5121, DOI 10.1021/acsnano.7b09041
Ye XY, 2019, ACS NANO, V13, P2956, DOI 10.1021/acsnano.8b07371
Zhang C, 2019, BIOMATERIALS, V223, DOI 10.1016/j.biomaterials.2019.119472
Zhu ML, 2021, J NANOBIOTECHNOL, V19, DOI 10.1186/s12951-021-01131-9
NR 31
TC 23
Z9 23
U1 25
U2 132
PU AMER CHEMICAL SOC
PI WASHINGTON
PA 1155 16TH ST, NW, WASHINGTON, DC 20036 USA
SN 1530-6984
EI 1530-6992
J9 NANO LETT
JI Nano Lett.
PD MAR 31
PY 2022
VL 22
IS 7
BP 3095
EP 3103
DI 10.1021/acs.nanolett.2c00500
PG 9
WC Chemistry, Multidisciplinary; Chemistry, Physical; Nanoscience &
Nanotechnology; Materials Science, Multidisciplinary; Physics, Applied;
Physics, Condensed Matter
WE Science Citation Index Expanded (SCI-EXPANDED)
SC Chemistry; Science & Technology - Other Topics; Materials Science;
Physics
GA 2F2KT
UT WOS:000812743200001
PM 35357839
DA 2023-09-14
ER

PT J
AU Li, M
Zhou, H
Jiang, W
Yang, C
Miao, H
Wang, YC
AF Li, Min
Zhou, Han
Jiang, Wei
Yang, Chen
Miao, Hui
Wang, Yucai
TI Nanovaccines integrating endogenous antigens and pathogenic adjuvants
elicit potent antitumor immunity
SO NANO TODAY
LA English

DT Article
DE Cancer nanovaccine; Outer membrane vesicles; Tumor cell membrane;
Checkpoint blockade therapy; Cancer immunotherapy
ID NANOPARTICLES; DELIVERY; VACCINE; IMMUNOTHERAPY; NANOMEDICINE
AB Cancer nanovaccines have the ability to train the immune system to specifically recognize and eliminate tumor cells. However, the vaccine's efficacy is hampered by limited access to draining lymph nodes, poor recognition and internalization by dendritic cells (DCs), as well as insufficient immunogenicity to elicit the DC maturation. To overcome these challenges, we developed potent vesicular cancer nanovaccines by fusing bacterial outer membrane vesicles (OMVs) and tumor cell membranes (TCMs), namely BTs. The bacteria-derived pathogenic adjuvants in BTs enabled DC targeting, promoted full maturation of DCs and antigen presentation by DCs. The TCMs in BTs assembled a multitude of diverse tumor endogenous antigens as training cues to generate multiantigenic antitumor immunity. Integrating the two components via a lipid bilayer assembly avoids the premature separation in the biological environment and enables the high spatial and temporal delivery of the antigens and adjuvants into the same DCs. Immunization with BTs containing as low as 1 mu g of OMVs confers an excellent tumor prophylactic effect with similar to 30 % of treated mice maintaining a tumor-free status for over 100 days. Moreover, the nanovaccines exhibit a remarkable therapeutic effect in treating established solid tumors and potentiate immune checkpoint blockade therapy. Overall, the BTs are promising as personalized cancer nanovaccines and carry no systemic side effects. (C) 2020 Elsevier Ltd. All rights reserved.
C1 [Li, Min; Wang, Yucai] Univ Sci & Technol China, Affiliated Hosp USTC 1, Intelligent Nanomed Inst, Div Life Sci & Med, Hefei 230001, Anhui, Peoples R China.
[Li, Min; Zhou, Han; Jiang, Wei; Yang, Chen; Miao, Hui; Wang, Yucai] Univ Sci & Technol China, Sch Life Sci, Hefei Natl Lab Phys Sci Microscale, Div Mol Med,CAS Key Lab Innate Immun & Chron Dis, Hefei 230027, Anhui, Peoples R China.
C3 Chinese Academy of Sciences; University of Science & Technology of
China, CAS; Chinese Academy of Sciences; University of Science &
Technology of China, CAS
RP Wang, YC (通讯作者)，Univ Sci & Technol China, Sch Life Sci, CAS Key Lab Innate Immun & Chron Dis, Hefei 230027, Anhui, Peoples R China.
EM yucaiwang@ustc.edu.cn
RI Zhou, Han/ISU-2803-2023
FU National Key R&D Program of China [2017YFA0205600]; National Natural
Science Foundation of China [5202500650, 51961145109, 91942310,
51773191, 81901683]; Fundamental Research Funds for the Central
Universities [WK3520000009]; Open Project of Key Laboratory of
Biomedical Engineering of Guangdong Province [KLBEMGD201701]
FX This work was supported by the National Key R&D Program of China
(2017YFA0205600), the National Natural Science Foundation of China
(5202500650, 51961145109, 91942310, 51773191 and 81901683), the
Fundamental Research Funds for the Central Universities (WK3520000009),
and the Open Project of Key Laboratory of Biomedical Engineering of
Guangdong Province (KLBEMGD201701). This work was partially carried out
at the USTC Center for Micro and Nanoscale Research and Fabrication.
CR Agrawal S, 2004, CELL IMMUNOL, V228, P130, DOI 10.1016/j.cellimm.2004.04.006
Bookstaver ML, 2018, TRENDS IMMUNOL, V39, P135, DOI 10.1016/j.it.2017.10.002
Chen Q, 2020, ADV MATER, V32, DOI 10.1002/adma.201908185
Chen Q, 2020, NANO LETT, V20, P11, DOI 10.1021/acs.nanolett.9b02182
Cubas R, 2009, J IMMUNOTHER, V32, P118, DOI 10.1097/CJI.0b013e31818f13c4
De Koker S, 2016, ANGEW CHEM INT EDIT, V55, P1334, DOI 10.1002/anie.201508626
Fan YC, 2015, VACCINES-BASEL, V3, P662, DOI 10.3390/vaccines3030662
Fang RH, 2014, NANO LETT, V14, P2181, DOI 10.1021/nl500618u
Gao WW, 2015, NANO LETT, V15, P1403, DOI 10.1021/nl504798g
Gardner A, 2016, TRENDS IMMUNOL, V37, P855, DOI 10.1016/j.it.2016.09.006
Hadjidemetriou M, 2017, NAT NANOTECHNOL, V12, P288, DOI 10.1038/nnano.2017.61
Han X, 2019, SCI ADV, V5, DOI 10.1126/sciadv.aaw6870
Harayama T, 2018, NAT REV MOL CELL BIO, V19, P281, DOI 10.1038/nrm.2017.138
Hu CMJ, 2011, P NATL ACAD SCI USA, V108, P10980, DOI 10.1073/pnas.1106634108
Hui Y, 2018, ACS NANO, V12, P2846, DOI 10.1021/acsnano.8b00242
Irvine DJ, 2015, CHEM REV, V115, P11109, DOI 10.1021/acs.chemrev.5b00109
Kalbasi A, 2018, CLIN CANCER RES, V24, P3239, DOI 10.1158/1078-0432.CCR-18-0698

Kaparakis-Liaskos M, 2015, NAT REV IMMUNOL, V15, P375, DOI 10.1038/nri3837
Kapsenberg ML, 2003, NAT REV IMMUNOL, V3, P984, DOI 10.1038/nri1246
Ke XY, 2019, ADV DRUG DELIVER REV, V151, P72, DOI 10.1016/j.addr.2019.09.005
Kieser KJ, 2017, NAT REV IMMUNOL, V17, P376, DOI 10.1038/nri.2017.25
Kim JH, 2015, DEV CELL, V32, P561, DOI 10.1016/j.devcel.2015.01.005
Kim OY, 2017, NAT COMMUN, V8, DOI 10.1038/s41467-017-00729-8
Kroll AV, 2017, ADV MATER, V29, DOI 10.1002/adma.201703969
Li M, 2020, J CONTROL RELEASE, V323, P253, DOI 10.1016/j.jconrel.2020.04.031
Li M, 2020, NAT COMMUN, V11, DOI 10.1038/s41467-020-14963-0
Liang F, 2017, SCI TRANSL MED, V9, DOI 10.1126/scitranslmed.aal2094
Liu HP, 2014, NATURE, V507, P519, DOI 10.1038/nature12978
Liu WL, 2019, NAT COMMUN, V10, DOI 10.1038/s41467-019-11157-1
Mildner A, 2014, IMMUNITY, V40, P642, DOI 10.1016/j.immuni.2014.04.016
Moyer TJ, 2016, J CLIN INVEST, V126, P799, DOI 10.1172/JCI81083
Mukhopadhyay A, 2019, GENE THER, V26, P1, DOI 10.1038/s41434-018-0044-5
Nair-Gupta P, 2014, CELL, V158, P506, DOI 10.1016/j.cell.2014.04.054
Nair-Gupta P, 2013, FRONT IMMUNOL, V4, DOI 10.3389/fimmu.2013.00401
Palomba R, 2018, ACS NANO, V12, P1433, DOI 10.1021/acsnano.7b07797
Pulendran B, 2013, IMMUNOL REV, V255, P243, DOI 10.1111/imr.12099
Pulendran B, 2009, NAT REV IMMUNOL, V9, P741, DOI 10.1038/nri2629
Querec T, 2006, J EXP MED, V203, P413, DOI 10.1084/jem.20051720
Schuijs MJ, 2019, TRENDS IMMUNOL, V40, P22, DOI 10.1016/j.it.2018.11.001
Sioniorou C.G., 2017, MEMBRANES, V7, p3 8
Siontorou CG, 2017, MEMBRANES-BASEL, V7, DOI 10.3390/membranes7030038
Stead SO, 2018, ACS NANO, V12, P6637, DOI 10.1021/acsnano.8b01625
Sudhof TC, 2009, SCIENCE, V323, P474, DOI 10.1126/science.1161748
Sun JS, 2015, ADV MATER, V27, P1402, DOI 10.1002/adma.201404788
Tom JK, 2019, TRENDS BIOTECHNOL, V37, P373, DOI 10.1016/j.tibtech.2018.10.004
Yang R, 2018, ACS NANO, V12, P5121, DOI 10.1021/acsnano.7b09041
Yu MR, 2018, NAT COMMUN, V9, DOI 10.1038/s41467-018-05061-3
Zhang LH, 2019, NANO LETT, V19, P4237, DOI 10.1021/acs.nanolett.9b00030
Zhang PC, 2019, ADV MATER, V31, DOI 10.1002/adma.201904156
Zheng BB, 2019, ADV FUNCT MATER, V29, DOI 10.1002/adfm.201901437
NR 50
TC 23
Z9 24
U1 7
U2 105
PU ELSEVIER SCI LTD
PI OXFORD
PA THE BOULEVARD, LANGFORD LANE, KIDLINGTON, OXFORD OX5 1GB, OXON, ENGLAND
SN 1748-0132
EI 1878-044X
J9 NANO TODAY
JI Nano Today
PD DEC
PY 2020
VL 35
AR 101007
DI 10.1016/j.nantod.2020.101007
PG 12
WC Chemistry, Multidisciplinary; Nanoscience & Nanotechnology; Materials
Science, Multidisciplinary
WE Science Citation Index Expanded (SCI-EXPANDED)
SC Chemistry; Science & Technology - Other Topics; Materials Science
GA PI1HL
UT WOS:000600849700063
DA 2023-09-14
ER

PT J

AU Liu, L
Kshirsagar, P
Christiansen, J
Gautam, SK
Aithal, A
Gulati, M
Kumar, S
Solheim, JC
Batra, SK
Jain, M
Wannemuehler, MJ
Narasimhan, B
AF Liu, Luman
Kshirsagar, Prakash
Christiansen, John
Gautam, Shailendra K.
Aithal, Abhijit
Gulati, Mansi
Kumar, Sushil
Solheim, Joyce C.
Batra, Surinder K.
Jain, Maneesh
Wannemuehler, Michael J.
Narasimhan, Balaji
TI Polyanhydride nanoparticles stabilize pancreatic cancer antigenMUC4 beta
SO JOURNAL OF BIOMEDICAL MATERIALS RESEARCH PART A
LA English
DT Article
DE antigenicity; immunogenicity; MUC4; nanoparticle; pancreatic cancer;
polyanhydride; protein stability
ID MUC4 EXPRESSION; CLINICAL-TRIALS; DRUG; ANTIGEN; VACCINES;
IMMUNOTHERAPY; PROGRESSION; ACTIVATION; MECHANISMS; GENERATION
AB Pancreatic cancer (PC) is one of the most lethal malignancies and represents an increasing and challenging threat, especially with an aging population. The identification of immunogenic PC-specific upregulated antigens and an enhanced understanding of the immunosuppressive tumor microenvironment have provided opportunities to enable the immune system to recognize cancer cells. Due to its differential upregulation and functional role in PC, the transmembrane mucin MUC4 is an attractive target for immunotherapy. In the current study we characterized the antigen stability, antigenicity and release kinetics of a MUC4 beta-nanovaccine to guide further optimization and, in vivo evaluation. Amphiphilic polyanhydride copolymers based on 20 mol % 1,8-bis(p-carboxyphenoxy)-3,6-dioxaoctane and 80 mol % 1,6-bis(p-carboxyphenoxy)hexane were used to synthesize nanoparticles. Structurally stable MUC4 beta protein was released from the particles in a sustained manner and characterized by gel electrophoresis and fluorescence spectroscopy. Modest levels of protein degradation were observed upon release. The released protein was also analyzed by MUC4 beta-specific monoclonal antibodies using ELISA and showed no significant loss of epitope availability. Further, mice immunized with multiple formulations of combination vaccines containing MUC4 beta-loaded nanoparticles generated MUC4 beta-specific antibody responses. These results indicate that polyanhydride nanoparticles are viable MUC4 beta vaccine carriers, laying the foundation for evaluation of this platform for PC immunotherapy.
C1 [Liu, Luman; Narasimhan, Balaji] Iowa State Univ, Dept Chem & Biol Engn, 2035 Sweeney Hall,618 Bissell Rd, Ames, IA 50011 USA.
[Kshirsagar, Prakash; Gautam, Shailendra K.; Aithal, Abhijit; Gulati, Mansi; Kumar, Sushil; Batra, Surinder K.; Jain, Maneesh] Univ Nebraska Med Ctr, Dept Biochem & Mol Biol, Omaha, NE USA.
[Christiansen, John; Wannemuehler, Michael J.] Iowa State Univ, Dept Vet Microbiol & Preventat Med, Ames, IA USA.
[Solheim, Joyce C.; Batra, Surinder K.; Jain, Maneesh; Wannemuehler, Michael J.; Narasimhan, Balaji] Iowa State Univ, Nanovaccine Inst, Ames, IA USA.
[Solheim, Joyce C.] Univ Nebraska Med Ctr, Eppley Inst Res Canc & Allied Dis, Omaha, NE USA.
[Solheim, Joyce C.; Batra, Surinder K.; Jain, Maneesh] Univ Nebraska Med Ctr, Fred & Pamela Buffett Canc Ctr, Omaha, NE USA.
C3 Iowa State University; University of Nebraska System; University of
Nebraska Medical Center; Iowa State University; Iowa State University;

University of Nebraska System; University of Nebraska Medical Center;
University of Nebraska System; University of Nebraska Medical Center
RP Narasimhan, B (通讯作者)，Iowa State Univ, Dept Chem & Biol Engn, 2035 Sweeney Hall,618 Bissell Rd, Ames, IA 50011 USA.
EM nbalaji@iastate.edu
RI Aithal, Abhijit/AAR-9856-2021; Narasimhan, Balaji/V-9018-2018; Gautam, Shailendra/ABD-2991-2021; Kshirsagar, Prakash/X-8420-2019; Gautam, Shailendra/GLQ-9528-2022
OI Narasimhan, Balaji/0000-0003-2609-9320; Gautam, Shailendra/0000-0002-8959-1331; Kshirsagar, Prakash/0000-0002-0110-0797; Aithal, Abhijit/0000-0001-9035-7529
FU National Cancer Institute [U01 CA213862]
FX National Cancer Institute, Grant/Award Number: U01 CA213862
CR Aithal A, 2018, PLOS ONE, V13, DOI 10.1371/journal.pone.0193907
Banerjee K, 2018, CANCER LETT, V417, P35, DOI 10.1016/j.canlet.2017.12.012
Banerjee S, 2019, BIOSCOPE, V10, P52, DOI 10.1177/0974927619855452
Bhatia R, 2019, CANCER METAST REV, V38, P223, DOI 10.1007/s10555-018-09775-0
Bobo D, 2016, PHARM RES-DORDR, V33, P2373, DOI 10.1007/s11095-016-1958-5
Carrillo-Conde B, 2010, ACTA BIOMATER, V6, P3110, DOI 10.1016/j.actbio.2010.01.040
Chaturvedi P, 2008, FASEB J, V22, P966, DOI 10.1096/fj.07-9673rev
D'Addio SM, 2011, ADV DRUG DELIVER REV, V63, P417, DOI 10.1016/j.addr.2011.04.005
Danhier F, 2012, J CONTROL RELEASE, V161, P505, DOI 10.1016/j.jconrel.2012.01.043
Determan AS, 2006, J MICROENCAPSUL, V23, P832, DOI 10.1080/02652040601033841
Duque L, 2018, INT J PHARMACEUT, V538, P139, DOI 10.1016/j.ijpharm.2018.01.026
Erkan M, 2012, NAT REV GASTRO HEPAT, V9, P454, DOI 10.1038/nrgastro.2012.115
Fan YC, 2015, VACCINES-BASEL, V3, P662, DOI 10.3390/vaccines3030662
Gautam SK, 2017, EXPERT OPIN THER TAR, V21, P657, DOI 10.1080/14728222.2017.1323880
Gopferich A, 2002, ADV DRUG DELIVER REV, V54, P911, DOI 10.1016/s0169-409x(02)00051-0
Gresham GK, 2014, BMC CANCER, V14, DOI 10.1186/1471-2407-14-471
Hailemichael Y, 2013, NAT MED, V19, P465, DOI 10.1038/nm.3105
Han RL, 2011, J BIOMED MATER RES A, V96A, P142, DOI 10.1002/jbm.a.32860
Haughney SL, 2013, ACTA BIOMATER, V9, P8262, DOI 10.1016/j.actbio.2013.06.006
Hinoda Y, 2003, J GASTROENTEROL, V38, P1162, DOI 10.1007/s00535-003-1224-6
Horn A, 2013, ARCH PATHOL LAB MED, V137, P546, DOI 10.5858/arpa.2011-0229-OA
Huntimer LM, 2014, TECHNOLOGY, V2, P171, DOI 10.1142/S2339547814500162
Jain M, 2011, PLOS ONE, V6, P2
Joshi VB, 2013, ACTA BIOMATER, V9, P5583, DOI 10.1016/j.actbio.2012.11.001
Kamaly N, 2016, CHEM REV, V116, P2602, DOI 10.1021/acs.chemrev.5b00346
Kaur S, 2013, NAT REV GASTRO HEPAT, V10, P607, DOI 10.1038/nrgastro.2013.120
Kipper MJ, 2002, BIOMATERIALS, V23, P4405, DOI 10.1016/S0142-9612(02)00181-3
Klibanov AM, 2001, NATURE, V409, P241, DOI 10.1038/35051719
Koido S, 2011, CLIN DEV IMMUNOL, DOI 10.1155/2011/267539
Moniaux N, 2004, J HISTOCHEM CYTOCHEM, V52, P253, DOI 10.1177/002215540405200213
Narasimhan B, 2016, ANNU REV BIOMED ENG, V18, P25, DOI 10.1146/annurev-bioeng-082615-030519
Oberstein PE, 2013, THER ADV GASTROENTER, V6, P321, DOI 10.1177/1756283X13478680
Petersen LK, 2012, MOL PHARMACEUT, V9, P874, DOI 10.1021/mp2004059
Petersen LK, 2011, BIOMATERIALS, V32, P6815, DOI 10.1016/j.biomaterials.2011.05.063
Petersen LK, 2009, BIOMATERIALS, V30, P5131, DOI 10.1016/j.biomaterials.2009.05.069
Plate J, 2011, EXPERT REV VACCINES, V10, P825, DOI [10.1586/ERV.11.77, 10.1586/erv.11.77]
Rachagani S, 2012, CARCINOGENESIS, V33, P1953, DOI 10.1093/carcin/bgs225
Ramirez JEV, 2014, J BIOMAT SCI-POLYM E, V25, P1387, DOI 10.1080/09205063.2014.940243
Remmers N, 2013, CLIN CANCER RES, V19, P1981, DOI 10.1158/1078-0432.CCR-12-2662
Ross K, 2019, BIOMATER SCI-UK, V7, P809, DOI 10.1039/c8bm01443d
Ross KA, 2014, J BIOMED MATER RES A, V102, P4161, DOI 10.1002/jbm.a.35086
Saitou M, 2005, J CLIN PATHOL, V58, P845, DOI 10.1136/jcp.2004.023572
Schaut RG, 2018, VACCINE, V36, P1016, DOI 10.1016/j.vaccine.2017.12.050
Sideras K, 2014, CANCER TREAT REV, V40, P513, DOI 10.1016/j.ctrv.2013.11.005
Siegel RL, 2016, CA-CANCER J CLIN, V66, P7, DOI DOI 10.3322/CAAC.21442
Swartz MJ, 2002, AM J CLIN PATHOL, V117, P791
Torres MP, 2007, BIOMATERIALS, V28, P108, DOI 10.1016/j.biomaterials.2006.08.047
Torres MP, 2012, CURR PHARM DESIGN, V18, P2472

Torres MP, 2006, J BIOMED MATER RES A, V76A, P102, DOI 10.1002/jbm.a.30510
Ulery BD, 2011, SCI REP-UK, V1, DOI 10.1038/srep00198
Ulery BD, 2011, PLOS ONE, V6, DOI 10.1371/journal.pone.0017642
Vivian JT, 2001, BIOPHYS J, V80, P2093, DOI 10.1016/S0006-3495(01)76183-8
Wafa EI, 2019, NANOMED-NANOTECHNOL, V21, DOI 10.1016/j.nano.2019.102055
Wafa EI, 2017, ACTA BIOMATER, V50, P417, DOI 10.1016/j.actbio.2017.01.005
Westgaard A, 2009, HISTOPATHOLOGY, V54, P337, DOI 10.1111/j.1365-2559.2009.03227.x
Wilson DR, 2018, NANOMED-NANOTECHNOL, V14, P237, DOI 10.1016/j.nano.2017.10.013
Zacharias ZR, 2018, FRONT IMMUNOL, V9, DOI 10.3389/fimmu.2018.01953
Zhao H, 2016, J MATER CHEM B, V4, P4060, DOI 10.1039/c6tb00308g
NR 58
TC 23
Z9 24
U1 3
U2 10
PU WILEY
PI HOBOKEN
PA 111 RIVER ST, HOBOKEN 07030-5774, NJ USA
SN 1549-3296
EI 1552-4965
J9 J BIOMED MATER RES A
JI J. Biomed. Mater. Res. Part A
PD JUN
PY 2021
VL 109
IS 6
BP 893
EP 902
DI 10.1002/jbm.a.37080
EA AUG 2020
PG 10
WC Engineering, Biomedical; Materials Science, Biomaterials
WE Science Citation Index Expanded (SCI-EXPANDED)
SC Engineering; Materials Science
GA RH1KM
UT WOS:000562323700001
PM 32776461
OA Green Published, Green Accepted
DA 2023-09-14
ER

PT J
AU Meng, JL
Zhang, PS
Chen, QZ
Wang, ZH
Gu, Y
Ma, J
Li, W
Yang, C
Qiao, YY
Hou, Y
Jing, LH
Wang, Y
Gu, Z
Zhu, LC
Xu, HZ
Lu, XG
Gao, MY
AF Meng, Junli
Zhang, Peisen

Chen, Qizhe
Wang, Zihua
Gu, Yuan
Ma, Jie
Li, Wang
Yang, Chen
Qiao, Yuanyuan
Hou, Yi
Jing, Lihong
Wang, Yong
Gu, Zi
Zhu, Lichong
Xu, Haozhen
Lu, Xueguang
Gao, Mingyuan
TI Two-Pronged Intracellular Co-Delivery of Antigen and Adjuvant for Synergistic Cancer Immunotherapy
SO ADVANCED MATERIALS
LA English
DT Article
DE cancer immunotherapy; CpG; iron oxide nanoparticles; membrane fusion; nanovaccines
ID DENDRITIC CELLS; CPG DNA; NANOPARTICLES; VACCINATION; CD11C/CD18; MECHANISMS; PEPTIDES; INDUCE
AB Nanovaccines have emerged as promising alternatives or complements to conventional cancer treatments. Despite the progresses, specific co-delivery of antigen and adjuvant to their corresponding intracellular destinations for maximizing the activation of antitumor immune responses remains a challenge. Herein, a lipid-coated iron oxide nanoparticle is delivered as nanovaccine (IONP-C/O@LP) that can co-deliver peptide antigen and adjuvant (CpG DNA) into cytosol and lysosomes of dendritic cells (DCs) through both membrane fusion and endosome-mediated endocytosis. Such two-pronged cellular uptake pattern enables IONP-C/O@LP to synergistically activate immature DCs. Iron oxide nanoparticle also exhibits adjuvant effects by generating intracellular reactive oxygen species, which further promotes DC maturation. IONP-C/O@LP accumulated in the DCs of draining lymph nodes effectively increases the antigen-specific T cells in both tumor and spleen, inhibits tumor growth, and improves animal survival. Moreover, it is demonstrated that this nanovaccine is a general platform of delivering clinically relevant peptide antigens derived from human papilloma virus 16 to trigger antigen-specific immune responses in vivo.
C1 [Meng, Junli; Zhang, Peisen; Chen, Qizhe; Wang, Zihua; Li, Wang; Yang, Chen; Qiao, Yuanyuan; Hou, Yi; Jing, Lihong; Zhu, Lichong; Lu, Xueguang; Gao, Mingyuan] Chinese Acad Sci, Inst Chem, Key Lab Colloid Interface & Chem Thermodynam, Beijing 100190, Peoples R China.
[Meng, Junli; Zhang, Peisen; Li, Wang; Yang, Chen] Univ Chinese Acad Sci, Sch Chem & Chem Engn, Beijing 100049, Peoples R China.
[Gu, Yuan; Ma, Jie; Wang, Yong; Gao, Mingyuan] Soochow Univ, Ctr Mol Imaging, Suzhou 215123, Peoples R China.
[Gu, Yuan; Ma, Jie; Wang, Yong; Gao, Mingyuan] Soochow Univ, Nucl Med Sch Radiol & Interdisciplinary Sci RAD X, Suzhou 215123, Peoples R China.
[Gu, Zi] Univ New South Wales, Sch Chem Engn, Sydney, NSW 2052, Australia.
[Gu, Zi] Univ New South Wales, Australian Ctr NanoMed ACN, Sydney, NSW 2052, Australia.
[Xu, Haozhen] Arizona State Univ, Sch Math & Stat Sci, Tempe, AZ 85287 USA.
C3 Chinese Academy of Sciences; Institute of Chemistry, CAS; Chinese Academy of Sciences; University of Chinese Academy of Sciences, CAS; Soochow University - China; Soochow University - China; University of New South Wales Sydney; University of New South Wales Sydney; Arizona State University; Arizona State University-Tempe
RP Lu, XG; Gao, MY (通讯作者)，Chinese Acad Sci, Inst Chem, Key Lab Colloid Interface & Chem Thermodynam, Beijing 100190, Peoples R China.; Gao, MY (通讯作者)，Soochow Univ, Ctr Mol Imaging, Suzhou 215123, Peoples R China.; Gao, MY (通讯作者)，Soochow Univ, Nucl Med Sch Radiol & Interdisciplinary Sci RAD X, Suzhou 215123, Peoples R China.
EM xueguang@iccas.ac.cn; gaomy@iccas.ac.cn
RI wang, ziqi/IQS-5011-2023; Wang, Zhiquan/HHS-6768-2022; Jing, Lihong/N-8341-2013; Qiao, Yuanyuan/AAB-9230-2020; Wang,

Zilong/IQV-2260-2023; wang, ziwei/HQY-9921-2023; , Gao MY/ABC-2193-2021;
Zhang, Peisen/IZQ-6173-2023
OI Qiao, Yuanyuan/0000-0002-3573-9847; Zhang, Peisen/0000-0003-0569-0576;
gao, ming yuan/0000-0002-7360-3684; /0000-0001-6115-2743
FU National Key Research and Development Program of China [2018YFA0208800];
National Natural Science Foundation of China (NSFC) [81720108024,
81530057, 81801766, 81671754, 22177115, GJHZ201963]; Youth Innovation
Promotion Association CAS [2018042]; CAS-VPST Silk Road Science Fund
2019 [GJHZ201963]; UNSW-CAS collaborative research grant
FX J.M. and P.Z. contributed equally to this work. The authors acknowledge
the financial support from the National Key Research and Development
Program of China (2018YFA0208800), National Natural Science Foundation
of China (NSFC) (81720108024, 81530057, 81801766, 81671754, 22177115),
CAS-VPST Silk Road Science Fund 2019 (GJHZ201963), the Youth Innovation
Promotion Association CAS (2018042), and UNSW-CAS collaborative research
grant.
CR Akira S, 2004, NAT REV IMMUNOL, V4, P499, DOI 10.1038/nri1391
Bode C, 2011, EXPERT REV VACCINES, V10, P499, DOI [10.1586/ERV.10.174, 10.1586/erv.10.174]
Cao ZB, 2020, SMALL METHODS, V4, DOI 10.1002/smtd.201900343
Cresswell P, 2005, IMMUNOL REV, V207, P145, DOI 10.1111/j.0105-2896.2005.00316.x
Du Y, 2020, SMALL, V16, DOI 10.1002/smll.202002537
Faham A, 2011, INT J CANCER, V129, P1391, DOI 10.1002/ijc.25810
Frick C, 2005, EUR J IMMUNOL, V35, P3610, DOI 10.1002/eji.200425914
Gao Z, 2017, ADV MATER, V29, DOI 10.1002/adma.201700018
Giljohann DA, 2010, ANGEW CHEM INT EDIT, V49, P3280, DOI 10.1002/anie.200904359
Goldberg MS, 2015, CELL, V161, P201, DOI 10.1016/j.cell.2015.03.037
Gu ZY, 2019, J AM CHEM SOC, V141, P6122, DOI 10.1021/jacs.8b10904
He YP, 2018, ACS NANO, V12, P10272, DOI 10.1021/acsnano.8b05373
Hossen MN, 2020, SCI ADV, V6, DOI 10.1126/sciadv.aba5379
Irvine DJ, 2015, CHEM REV, V115, P11109, DOI 10.1021/acs.chemrev.5b00109
Ji HX, 1998, INT J CANCER, V78, P41, DOI 10.1002/(SICI)1097-0215(19980925)78:1<41::AID-IJC8>3.0.CO;2-X
Jokerst JV, 2011, NANOMEDICINE-UK, V6, P715, DOI [10.2217/NNM.11.19, 10.2217/nnm.11.19]
Kantengwa S, 2003, AM J RESP CRIT CARE, V167, P431, DOI 10.1164/rccm.200205-425OC
Kim B, 2019, ADV MATER, V31, DOI 10.1002/adma.201902952
Kroll AV, 2017, ADV MATER, V29, DOI 10.1002/adma.201703969
Kuai R, 2017, NAT MATER, V16, P489, DOI [10.1038/nmat4822, 10.1038/NMAT4822]
Lai CH, 2018, THERANOSTICS, V8, P1723, DOI 10.7150/thno.22056
Latz E, 2004, NAT IMMUNOL, V5, P190, DOI 10.1038/ni1028
Li F, 2019, ACS CENTRAL SCI, V5, P796, DOI 10.1021/acscentsci.9b00060
Li WQ, 2021, NAT COMMUN, V12, DOI 10.1038/s41467-021-27434-x
Liao JB, 2008, CLIN VACCINE IMMUNOL, V15, P817, DOI 10.1128/CVI.00343-07
Liu B, 2017, ADV SCI, V4, DOI 10.1002/advs.201600540
Luo LJ, 2019, BIOMATERIALS, V223, DOI 10.1016/j.biomaterials.2019.119464
Met O, 2003, CELL IMMUNOL, V222, P126, DOI 10.1016/S0008-8749(03)00128-X
Meyer RA, 2015, TRENDS BIOTECHNOL, V33, P514, DOI 10.1016/j.tibtech.2015.07.001
Mout R, 2017, ACS NANO, V11, P2452, DOI 10.1021/acsnano.6b07600
Neshat SY, 2020, CURR OPIN BIOTECH, V66, P1, DOI 10.1016/j.copbio.2020.05.008
Pardo A, 2020, CHEM MATER, V32, P2220, DOI 10.1021/acs.chemmater.9b04848
Reddy ST, 2006, TRENDS IMMUNOL, V27, P573, DOI 10.1016/j.it.2006.10.005
Rock KL, 2005, IMMUNOL REV, V207, P166, DOI 10.1111/j.0105-2896.2005.00301.x
Rutault K, 1999, FREE RADICAL BIO MED, V26, P232, DOI 10.1016/S0891-5849(98)00194-4
Santhosh PB, 2014, COLLOID SURFACE A, V460, P248, DOI 10.1016/j.colsurfa.2014.02.035
Schudel A, 2019, NAT REV MATER, V4, P415, DOI 10.1038/s41578-019-0110-7
Shen LJ, 2004, IMMUNITY, V21, P155, DOI 10.1016/j.immuni.2004.07.004
Shi C, 2020, BIOMATERIALS, V233, DOI 10.1016/j.biomaterials.2020.119755
Speir M, 2017, SCI REP-UK, V7, DOI 10.1038/s41598-017-14690-5
Stewart MP, 2016, NATURE, V538, P183, DOI 10.1038/nature19764
Verhasselt V, 1998, EUR J IMMUNOL, V28, P3886
Wang H, 2020, NAT COMMUN, V11, DOI 10.1038/s41467-020-19540-z
Wang H, 2018, NAT MATER, V17, P761, DOI 10.1038/s41563-018-0147-9

WCULEK SK, 2020, NAT REV IMMUNOL, V20, P7, DOI DOI 10.1038/S41577-019-0210-Z
Xiang J, 2015, ACS NANO, V9, P6401, DOI 10.1021/acsnano.5b02014
Xie J, 2017, SMALL, V13, DOI 10.1002/smll.201701741
Xu HL, 2018, ADV HEALTHC MATER, V7, DOI 10.1002/adhm.201701130
Xu JL, 2022, NAT COMMUN, V13, DOI 10.1038/s41467-021-27750-2
Yang BW, 2019, ADV MATER, V31, DOI 10.1002/adma.201901778
Yang R, 2018, ACS NANO, V12, P5121, DOI 10.1021/acsnano.7b09041
Yang XQ, 2011, BIOMATERIALS, V32, P4151, DOI 10.1016/j.biomaterials.2011.02.006
Yu SJ, 2018, ADV MATER, V30, DOI 10.1002/adma.201801527
Zanganeh S, 2016, NAT NANOTECHNOL, V11, P986, DOI [10.1038/nnano.2016.168, 10.1038/NNANO.2016.168]
Zhang C, 2020, CHEM SOC REV, V49, P4234, DOI 10.1039/c9cs00773c
Zhang Y, 2019, WIRES NANOMED NANOBI, V11, DOI 10.1002/wnan.1559
Zhou J, 2020, ACS APPL BIO MATER, V3, P2686, DOI 10.1021/acsabm.9b01131
Zhou QQ, 2016, ACS NANO, V10, P2678, DOI 10.1021/acsnano.5b07716
NR 58
TC 22
Z9 22
U1 25
U2 142
PU WILEY-V C H VERLAG GMBH
PI WEINHEIM
PA POSTFACH 101161, 69451 WEINHEIM, GERMANY
SN 0935-9648
EI 1521-4095
J9 ADV MATER
JI Adv. Mater.
PD MAY
PY 2022
VL 34
IS 21
AR 2202168
DI 10.1002/adma.202202168
EA APR 2022
PG 14
WC Chemistry, Multidisciplinary; Chemistry, Physical; Nanoscience & Nanotechnology; Materials Science, Multidisciplinary; Physics, Applied; Physics, Condensed Matter
WE Science Citation Index Expanded (SCI-EXPANDED)
SC Chemistry; Science & Technology - Other Topics; Materials Science; Physics
GA 1M8UD
UT WOS:000786413700001
PM 35362203
DA 2023-09-14
ER

PT J
AU Li, JC
Huang, D
Cheng, RY
Figueiredo, P
Fontana, F
Correia, A
Wang, SQ
Liu, ZH
Kemell, M
Torrieri, G
Makila, EM
Salonen, JJ
Hirvonen, J

Gao, Y
Li, JL
Luo, ZY
Santos, HA
Xia, B
AF Li, Jiachen
Huang, Di
Cheng, Ruoyu
Figueiredo, Patricia
Fontana, Flavia
Correia, Alexandra
Wang, Shiqi
Liu, Zehua
Kemell, Marianna
Torrieri, Giulia
Makila, Ermei M.
Salonen, Jarno J.
Hirvonen, Jouni
Gao, Yan
Li, Jialiang
Luo, Zhenyang
Santos, Helder A.
Xia, Bing
TI Multifunctional Biomimetic Nanovaccines Based on Photothermal and Weak-Immunostimulatory Nanoparticulate Cores for the Immunotherapy of Solid Tumors
SO ADVANCED MATERIALS
LA English
DT Article
DE antitumor immune response; biomimetic nanovaccines; cancer cell membranes; photothermal synergized immunotherapy; porous silicon@Au nanocomposites
ID CANCER VACCINES; CELL; THERAPY
AB An alternative strategy of choosing photothermal and weak-immunostimulatory porous silicon@Au nanocomposites as particulate cores to prepare a biomimetic nanovaccine is reported to improve its biosafety and immunotherapeutic efficacy for solid tumors. A quantitative analysis method is used to calculate the loading amount of cancer cell membranes onto porous silicon@Au nanocomposites. Assisted with foreign-body responses, these exogenous nanoparticulate cores with weak immunostimulatory effect can still efficiently deliver cancer cell membranes into dendritic cells to activate them and the downstream antitumor immunity, resulting in no occurrence of solid tumors and the survival of all immunized mice during 55 day observation. In addition, this nanovaccine, as a photothermal therapeutic agent, synergized with additional immunotherapies can significantly inhibit the growth and metastasis of established solid tumors, via the initiation of the antitumor immune responses in the body and the reversion of their immunosuppressive microenvironments. Considering the versatile surface engineering of porous silicon nanoparticles, the strategy developed here is beneficial to construct multifunctional nanovaccines with better biosafety and more diagnosis or therapeutic modalities against the occurrence, recurrence, or metastasis of solid tumors in future clinical practice.
C1 [Li, Jiachen; Huang, Di; Gao, Yan; Li, Jialiang; Luo, Zhenyang; Xia, Bing] Nanjing Forestry Univ, Coll Sci, Nanjing 210037, Peoples R China.
[Li, Jiachen; Cheng, Ruoyu; Figueiredo, Patricia; Fontana, Flavia; Correia, Alexandra; Wang, Shiqi; Liu, Zehua; Torrieri, Giulia; Hirvonen, Jouni; Santos, Helder A.] Univ Helsinki, Div Pharmaceut Chem & Technol, Drug Res Program, FL-00014 Helsinki, Finland.
[Li, Jiachen; Santos, Helder A.] Univ Groningen, Dept Biomed Engn, Univ Med Ctr Groningen, Ant Deusinglaan 1, NL-9713 AV Groningen, Netherlands.
[Li, Jiachen; Santos, Helder A.] Univ Groningen, WJ Kolff Inst Biomed Engn & Mat Sci, Univ Med Ctr Groningen, Ant Deusinglaan 1, NL-9713 AV Groningen, Netherlands.
[Kemell, Marianna] Univ Helsinki, Dept Chem, FI-00014 Helsinki, Finland.
[Makila, Ermei M.; Salonen, Jarno J.] Univ Turku, Dept Phys & Astron, Lab Ind Phys, FL-20014 Turku, Finland.
C3 Nanjing Forestry University; University of Helsinki; University of Groningen; University of Groningen; University of Helsinki; University

of Turku
RP Luo, ZY; Xia, B (通讯作者)，Nanjing Forestry Univ, Coll Sci, Nanjing 210037, Peoples R China.; Santos, HA (通讯作者)，Univ Helsinki, Div Pharmaceut Chem & Technol, Drug Res Program, FL-00014 Helsinki, Finland.; Santos, HA (通讯作者)，Univ Groningen, Dept Biomed Engn, Univ Med Ctr Groningen, Ant Deusinglaan 1, NL-9713 AV Groningen, Netherlands.; Santos, HA (通讯作者)，Univ Groningen, WJ Kolff Inst Biomed Engn & Mat Sci, Univ Med Ctr Groningen, Ant Deusinglaan 1, NL-9713 AV Groningen, Netherlands.
EM luozhenyang@njfu.edu.cn; h.a.santos@umcg.nl; xiabing@njfu.edu.cn
RI Kemell, Marianna L/C-1512-2014; Fontana, Flavia/AAB-4898-2020; Wang, Shiqi/N-8753-2018; Santos, Hélder A./AAC-6347-2022; Huang, Di/JBJ-3541-2023
OI Kemell, Marianna L/0000-0002-3583-2064; Fontana, Flavia/0000-0001-9112-6917; Wang, Shiqi/0000-0003-2010-0132; Santos, Hélder A./0000-0001-7850-6309; Huang, Di/0000-0001-7877-7301; Torrieri, Giulia/0000-0001-8300-4661; Figueiredo, Patricia/0000-0002-3689-3280; Hirvonen, Jouni Tapio/0000-0002-5029-1657
FU HiLIFE Research Funds; Sigrid Juselius Foundation; Academy of Finland [317042, 331151, 331106]; Priority Academic Program Development (PAPD) of Jiangsu Higher Education Institutions; National Natural Science Foundation of China [31000164]; Natural Science Foundation of Jiangsu Province [BK20130964]; Bilateral Chinese-Croatian Scientific Project [6-5]
FX H.A.S. acknowledges financial support from the HiLIFE Research Funds, the Sigrid Juselius Foundation, and the Academy of Finland (Grant Nos. 317042 and 331151). This work utilized the ALD Center Finland Research Infrastructure at University of Helsinki. Z.Y.L. acknowledges financial support from Priority Academic Program Development (PAPD) of Jiangsu Higher Education Institutions. B.X. acknowledges financial support from National Natural Science Foundation of China (Grant No. 31000164), Natural Science Foundation of Jiangsu Province (Grant No. BK20130964), and Bilateral Chinese-Croatian Scientific Project (Project No. 6-5). S.W. acknowledges the financial support the Academy of Finland (Grant No. 331106). The authors thank Dr. Christos Tapeinos from University of Helsinki for the kind help on the TEM observation with the negative staining. They also thank Prof. Pingping Shen and Dr. Jiqiang Fan from Nanjing University very much for their valuable discussions about the mechanism of immune responses. All animal experiments were reviewed and approved by the Experimental Animal Committee of the KeyGEN Biotechnology Co. Ltd., China (Animal Ethics Review Approval Numbers: IACUC-007-1and IACUC-007-2).
CR Alhmoud H, 2017, ACS APPL MATER INTER, V9, P33707, DOI 10.1021/acsami.7b13278
Asadzadeh Z, 2020, CANCERS, V12, DOI 10.3390/cancers12041047
Belardelli F, 2002, TRENDS IMMUNOL, V23, P201, DOI 10.1016/S1471-4906(02)02195-6
Bodey B, 2000, ANTICANCER RES, V20, P2665
Chang MY, 2021, ADV MATER, V33, DOI 10.1002/adma.202004788
Chen DS, 2013, IMMUNITY, V39, P1, DOI 10.1016/j.immuni.2013.07.012
Chen Q, 2016, NAT COMMUN, V7, DOI 10.1038/ncomms13193
Colby AH, 2021, J CONTROL RELEASE, V337, P144, DOI 10.1016/j.jconrel.2021.07.012
Fan YC, 2015, VACCINES-BASEL, V3, P662, DOI 10.3390/vaccines3030662
Fang RH, 2018, ADV MATER, V30, DOI 10.1002/adma.201706759
Fernandez TD, 2015, BIOMATERIALS, V43, P1, DOI 10.1016/j.biomaterials.2014.11.045
Fontana F, 2020, ADV HEALTHC MATER, V9, DOI 10.1002/adhm.202000529
Fontana F, 2019, ACS NANO, V13, P6477, DOI 10.1021/acsnano.8b09613
Fontana F, 2017, ADV MATER, V29, DOI 10.1002/adma.201603239
Gong NQ, 2021, NAT NANOTECHNOL, V16, P25, DOI 10.1038/s41565-020-00822-y
Gong NQ, 2020, NAT NANOTECHNOL, V15, P1053, DOI 10.1038/s41565-020-00782-3
Han X, 2019, SCI ADV, V5, DOI 10.1126/sciadv.aaw6870
Hu Z, 2018, NAT REV IMMUNOL, V18, P168, DOI 10.1038/nri.2017.131
Irvine DJ, 2015, CHEM REV, V115, P11109, DOI 10.1021/acs.chemrev.5b00109
Kolaczkowska E, 2013, NAT REV IMMUNOL, V13, P159, DOI 10.1038/nri3399
Kroll AV, 2019, ADV BIOSYST, V3, DOI 10.1002/adbi.201800219

Li AW, 2018, NAT MATER, V17, P528, DOI 10.1038/s41563-018-0028-2
Li J, 2021, ADV MATER, V33, DOI 10.1002/adma.202008481
Li XS, 2020, NAT REV CLIN ONCOL, V17, P657, DOI 10.1038/s41571-020-0410-2
Liu HP, 2014, NATURE, V507, P519, DOI 10.1038/nature12978
Liu SL, 2021, NAT MATER, V20, P421, DOI 10.1038/s41563-020-0793-6
Lokhov PG, 2010, J CANCER, V1, P230
Najibi AJ, 2020, ADV DRUG DELIVER REV, V161, P42, DOI 10.1016/j.addr.2020.07.023
Ng CW, 2018, ADV FUNCT MATER, V28, DOI 10.1002/adfm.201804688
Orchyl L.J., 2018, BIOMATERIALS, V182, P157
Palucka K, 2011, J IMMUNOL, V186, P1325, DOI 10.4049/jimmunol.0902539
Riley RS, 2019, NAT REV DRUG DISCOV, V18, P175, DOI 10.1038/s41573-018-0006-z
Sahin U, 2018, SCIENCE, V359, P1355, DOI 10.1126/science.aar7112
Sallusto F, 2004, ANNU REV IMMUNOL, V22, P745, DOI 10.1146/annurev.immunol.22.012703.104702
Shahbazi MA, 2014, BIOMATERIALS, V35, P9224, DOI 10.1016/j.biomaterials.2014.07.050
Shen HF, 2012, ADV HEALTHC MATER, V1, P84, DOI 10.1002/adhm.201100005
Su YY, 2012, NANO LETT, V12, P1845, DOI 10.1021/nl204203t
Tonigold M, 2016, NANOMEDICINE-UK, V11, P2625, DOI 10.2217/nnm-2016-0195
Ukidve A, 2021, DRUG DELIV TRANSL RE, V11, P2276, DOI 10.1007/s13346-021-00923-8
van der Burg SH, 2016, NAT REV CANCER, V16, P219, DOI 10.1038/nrc.2016.16
Van Gool SW, 1999, EUR J IMMUNOL, V29, P2367, DOI 10.1002/(SICI)1521-4141(199908)29:08<2367::AID-IMMU2367>3.3.CO;2-V
Veiseh O, 2019, ADV DRUG DELIVER REV, V144, P148, DOI 10.1016/j.addr.2019.08.010
Wherry EJ, 2015, NAT REV IMMUNOL, V15, P486, DOI 10.1038/nri3862
Xia B, 2019, ACS BIOMATER SCI ENG, V5, P1857, DOI 10.1021/acsbiomaterials.8b01533
Xu J, 2020, NAT NANOTECHNOL, V15, P1043, DOI 10.1038/s41565-020-00781-4
Xuan MJ, 2019, NATL SCI REV, V6, P551, DOI 10.1093/nsr/nwz037
Yue YL, 2021, ACS NANO, V15, P15166, DOI 10.1021/acsnano.1c05618
Zhang L, 2019, ADV MATER, V31, DOI 10.1002/adma.201805936
Zhou JR, 2020, ADV MATER, V32, DOI 10.1002/adma.201901255
Zhu GX, 2016, ACS APPL MATER INTER, V8, P11881, DOI 10.1021/acsami.6b03008
NR 50
TC 22
Z9 22
U1 10
U2 171
PU WILEY-V C H VERLAG GMBH
PI WEINHEIM
PA POSTFACH 101161, 69451 WEINHEIM, GERMANY
SN 0935-9648
EI 1521-4095
J9 ADV MATER
JI Adv. Mater.
PD MAR
PY 2022
VL 34
IS 9
AR 2108012
DI 10.1002/adma.202108012
EA JAN 2022
PG 12
WC Chemistry, Multidisciplinary; Chemistry, Physical; Nanoscience & Nanotechnology; Materials Science, Multidisciplinary; Physics, Applied; Physics, Condensed Matter
WE Science Citation Index Expanded (SCI-EXPANDED)
SC Chemistry; Science & Technology - Other Topics; Materials Science; Physics
GA ZL1XK
UT WOS:000744494300001
PM 34877724
OA Green Published
DA 2023-09-14

ER

PT J
AU Liu, B
Yang, Y
Chao, Y
Xiao, ZS
Xu, JL
Wang, CJ
Dong, ZL
Hou, LQ
Li, QF
Liu, Z
AF Liu, Bo
Yang, Yu
Chao, Yu
Xiao, Zhisheng
Xu, Jialu
Wang, Chunjie
Dong, Ziliang
Hou, Linqian
Li, Qiaofeng
Liu, Zhuang
TI Equipping Cancer Cell Membrane Vesicles with Functional DNA as a Targeted Vaccine for Cancer Immunotherapy
SO NANO LETTERS
LA English
DT Article
DE cell membrane vesicles; cancer vaccine; cancer immunotherapy; aptamer targeting; immune checkpoint blockade
ID NANOPARTICLES; NEOANTIGENS
AB By inducing tumor-specific immune responses, tumor vaccines have recently aroused great research interest. Herein, we design a targeted nanovaccine by equipping cell membrane vesicles (CMVs) harvested from tumor cells with functional DNA including CpG oligonucleotide, an agonist for toll-like receptor 9, as well as an aptamer targeting the dendritic cell (DC)specific intercellular adhesion molecule (ICAM)-3 grabbing nonintegrin (DC-SIGN) receptor overexpressed on DCs. Such DNA-modified CMVs could target DCs and further stimulate their maturation. Notably, our nanovaccines could trigger robust antitumor immune responses to effective delay the tumor growth. Moreover, the combination of CMV-based nanovaccines with an immune checkpoint blockade could result in improved therapeutic responses by eliminating the majority of the tumors as well as long-term immune memory to prevent tumor recurrence. Therefore, by simply assembling functional DNA on CMVs harvested from tumor cells, we propose a general platform of DC-targeted personalized cancer vaccines for effective and specific cancer immunotherapy.
C1 [Liu, Bo; Chao, Yu; Xiao, Zhisheng; Xu, Jialu; Wang, Chunjie; Dong, Ziliang; Hou, Linqian; Li, Qiaofeng; Liu, Zhuang] Soochow Univ, Inst Funct Nano & Soft Mat FUNSOM, Jiangsu Key Lab Carbon Based Funct Mat & Devices, Suzhou 215123, Jiangsu, Peoples R China.
[Liu, Zhuang] Macau Univ Sci & Technol, Macao Inst Mat Sci & Engn, Taipa 999078, Macau Special A, Peoples R China.
[Yang, Yu] Shanghai Jiao Tong Univ, Renji Hosp, Sch Med, Inst Mol Med IMM, Shanghai 200240, Peoples R China.
C3 Soochow University - China; Macau University of Science & Technology; Shanghai Jiao Tong University
RP Liu, Z (通讯作者)，Soochow Univ, Inst Funct Nano & Soft Mat FUNSOM, Jiangsu Key Lab Carbon Based Funct Mat & Devices, Suzhou 215123, Jiangsu, Peoples R China.; Liu, Z (通讯作者)，Macau Univ Sci & Technol, Macao Inst Mat Sci & Engn, Taipa 999078, Macau Special A, Peoples R China.; Yang, Y (通讯作者)，Shanghai Jiao Tong Univ, Renji Hosp, Sch Med, Inst Mol Med IMM, Shanghai 200240, Peoples R China.
EM yuyang@shsmu.edu.cn; zliu@suda.edu.cn
RI Dong, Ziliang/AEH-7615-2022; Liu, Zhuang/H-4352-2011; Yang, Yu/GYJ-1931-2022
OI Liu, Zhuang/0000-0002-1629-1039; Yang, Yu/0000-0002-4518-3299

FU National Basic Research Programs of China (973 Program)
[2016YFA0201200]; National Natural Science Foundation of China
[51525203, 51761145041]; Collaborative Innovation Center of Suzhou Nano
Science and Technology; "111" program from the Ministry of Education of
China
FX This work was partially supported by the National Basic Research
Programs of China (973 Program) (2016YFA0201200), the National Natural
Science Foundation of China (51525203 and 51761145041), the
Collaborative Innovation Center of Suzhou Nano Science and Technology,
and the "111" program from the Ministry of Education of China.
CR Bassani-Sternberg M, 2018, METHODS MOL BIOL, V1719, P209, DOI 10.1007/978-1-4939-7537-2_14
Blass E, 2021, NAT REV CLIN ONCOL, V18, P215, DOI 10.1038/s41571-020-00460-2
Bobisse S, 2016, ANN TRANSL MED, V4, DOI 10.21037/atm.2016.06.17
Couzin-Frankel J, 2013, SCIENCE, V342, P1432, DOI 10.1126/science.342.6165.1432
Desrichard A, 2016, CLIN CANCER RES, V22, P807, DOI 10.1158/1078-0432.CCR-14-3175
Fan YQ, 2019, ONCOL REP, V41, P3, DOI 10.3892/or.2018.6819
Fang RH, 2014, NANO LETT, V14, P2181, DOI 10.1021/nl500618u
Farkona S, 2016, BMC MED, V14, DOI 10.1186/s12916-016-0623-5
Gao WW, 2015, J DRUG TARGET, V23, P619, DOI 10.3109/1061186X.2015.1052074
Gonzalez FE, 2014, HUM VACC IMMUNOTHER, V10, P3261, DOI 10.4161/21645515.2014.982996
Gubin MM, 2015, J CLIN INVEST, V125, P3413, DOI 10.1172/JCI80008
Guo YG, 2018, FRONT IMMUNOL, V9, DOI 10.3389/fimmu.2018.01499
June CH, 2018, SCIENCE, V359, P1361, DOI 10.1126/science.aar6711
Klichinsky M, 2020, NAT BIOTECHNOL, V38, P947, DOI 10.1038/s41587-020-0462-y
Klinman DM, 2004, NAT REV IMMUNOL, V4, P248, DOI 10.1038/nri1329
Kuai R, 2017, NAT MATER, V16, P489, DOI [10.1038/nmat4822, 10.1038/NMAT4822]
Li L, 2017, ANN ONCOL, V28, P11, DOI 10.1093/annonc/mdx681
Liu YJ, 2001, CELL, V106, P259, DOI 10.1016/S0092-8674(01)00456-1
Lu YC, 2016, SEMIN IMMUNOL, V28, P22, DOI 10.1016/j.smim.2015.11.002
Manolova V, 2008, EUR J IMMUNOL, V38, P1404, DOI 10.1002/eji.200737984
Ochyl LJ, 2018, BIOMATERIALS, V182, P157, DOI 10.1016/j.biomaterials.2018.08.016
Palucka K, 2012, NAT REV CANCER, V12, P265, DOI 10.1038/nrc3258
Peng M, 2019, MOL CANCER, V18, DOI 10.1186/s12943-019-1055-6
Postow MA, 2015, J CLIN ONCOL, V33, P1974, DOI 10.1200/JCO.2014.59.4358
Rao L, 2019, ADV FUNCT MATER, V29, DOI 10.1002/adfm.201905671
Rosenberg SA, 2004, NAT MED, V10, P909, DOI 10.1038/nm1100
Schumacher TN, 2015, SCIENCE, V348, P69, DOI 10.1126/science.aaa4971
Shi GN, 2017, BIOMATERIALS, V113, P191, DOI 10.1016/j.biomaterials.2016.10.047
Vergati M, 2010, J BIOMED BIOTECHNOL, DOI 10.1155/2010/596432
Wang WX, 2020, CANCER LETT, V472, P175, DOI 10.1016/j.canlet.2019.11.033
Wang ZJ, 2018, MATER HORIZ, V5, P344, DOI 10.1039/c7mh00991g
Xu J, 2019, BIOMATERIALS, V207, P1, DOI 10.1016/j.biomaterials.2019.03.037
Yan H, 2007, MOL CELL BIOCHEM, V306, P71, DOI 10.1007/s11010-007-9555-x
Yang R, 2018, ACS NANO, V12, P5121, DOI 10.1021/acsnano.7b09041
Yarchoan M, 2017, NAT REV CANCER, V17, DOI 10.1038/nrc.2017.74
Zeng ZL, 2020, ADV FUNCT MATER, V30, DOI 10.1002/adfm.202004397
Zhang D, 2020, ANGEW CHEM INT EDIT, V59, P12022, DOI 10.1002/anie.202002145
Zhang HM, 2018, J CANCER, V9, P1773, DOI 10.7150/jca.24577
Zhao H, 2019, ACS NANO, V13, P13127, DOI 10.1021/acsnano.9b05974
NR 39
TC 22
Z9 22
U1 18
U2 105
PU AMER CHEMICAL SOC
PI WASHINGTON
PA 1155 16TH ST, NW, WASHINGTON, DC 20036 USA
SN 1530-6984
EI 1530-6992
J9 NANO LETT
JI Nano Lett.

PD NOV 24
PY 2021
VL 21
IS 22
BP 9410
EP 9418
DI 10.1021/acs.nanolett.1c02582
PG 9
WC Chemistry, Multidisciplinary; Chemistry, Physical; Nanoscience &
Nanotechnology; Materials Science, Multidisciplinary; Physics, Applied;
Physics, Condensed Matter
WE Science Citation Index Expanded (SCI-EXPANDED)
SC Chemistry; Science & Technology - Other Topics; Materials Science;
Physics
GA ZA8QL
UT WOS:000756421600007
PM 34730968
DA 2023-09-14
ER

PT J
AU Xiao, B
Li, DD
Xu, HX
Zhou, XX
Xu, XD
Qian, Y
Yu, FD
Hu, HJ
Zhou, ZX
Liu, XR
Gao, JQ
Slater, NKH
Shen, YQ
Tang, JB
AF Xiao, Bing
Li, Dongdong
Xu, Hongxia
Zhou, Xiaoxuan
Xu, Xiaodan
Qian, Yue
Yu, Feidan
Hu, Hongjie
Zhou, Zhuxian
Liu, Xiangrui
Gao, Jianqing
Slater, Nigel K. H.
Shen, Youqing
Tang, Jianbin
TI An MRI-trackable therapeutic nanovaccine preventing cancer liver
metastasis
SO BIOMATERIALS
LA English
DT Article
DE Nanovaccine; Magnetic resonance imaging; Photothermal therapy;
Immunotherapy; Tumor prevention
ID NANOPARTICLES; POLYDOPAMINE
AB Cancer vaccines consisting of tumor-associated antigens (TAAs) can initiate a powerful antitumor immune response through antigen-presenting cells, such as dendritic cells (DCs) and macrophages, and have shown great potential in cancer prevention and therapy. However, poor anticancer efficacy and an uncertain immunization process have hitherto limited the application of cancer vaccines. Herein, a

multifunctional nanovaccine comprising ovalbumin (OVA), MnO2, and polydopamine (OMPN) was prepared by a facile one-pot method. OMPN displayed excellent anticancer efficacy against an orthotopic melanoma and could also prevent liver metastasis in a tumor re-challenge mice model. Additionally, the migration behavior of DCs in the inguinal lymph node after vaccination was tracked by MRI contrasted with OMPN, indicating successful DC activation and immune response. The superior anticancer efficacy, especially the high efficiency against tumor metastasis, and the capability of tracking the immunization process make OMPN a very promising multifunctional nanovaccine for cancer therapy.
C1 [Xiao, Bing; Li, Dongdong; Xu, Hongxia; Xu, Xiaodan; Zhou, Zhuxian; Liu, Xiangrui; Shen, Youqing; Tang, Jianbin] Zhejiang Univ, Ctr Bionanoengn, Minist Educ, Key Lab Biomass Chem Engn, Hangzhou 310027, Zhejiang, Peoples R China.
[Xiao, Bing; Li, Dongdong; Xu, Hongxia; Xu, Xiaodan; Zhou, Zhuxian; Liu, Xiangrui; Shen, Youqing; Tang, Jianbin] Zhejiang Univ, Coll Chem & Biol Engn, Hangzhou 310027, Zhejiang, Peoples R China.
[Zhou, Xiaoxuan; Qian, Yue; Yu, Feidan; Hu, Hongjie] Zhejiang Univ, Sch Med, Sir Run Run Shaw Hosp SRRSH, Dept Radiol, Hangzhou 310027, Zhejiang, Peoples R China.
[Xiao, Bing; Gao, Jianqing] Zhejiang Univ, Coll Pharmaceut Sci, Zhejiang Prov Key Lab Anticanc Drug Res, Hangzhou 310058, Peoples R China.
[Slater, Nigel K. H.] Univ Cambridge, Dept Chem Engn & Biotechnol, Cambridge, England.
C3 Zhejiang University; Zhejiang University; Zhejiang University; Zhejiang University; University of Cambridge
RP Tang, JB (通讯作者)，Zhejiang Univ, Ctr Bionanoengn, Minist Educ, Key Lab Biomass Chem Engn, Hangzhou 310027, Zhejiang, Peoples R China.; Tang, JB (通讯作者)，Zhejiang Univ, Coll Chem & Biol Engn, Hangzhou 310027, Zhejiang, Peoples R China.
EM jianbin@zju.edu.cn
RI Zhou, Xiaoxuan/ABD-8708-2021; Zhou, Zhuxian/F-5412-2015
OI Zhou, Zhuxian/0000-0002-7104-9915
FU National Natural Science Foundation of China [21774109, 51973188, 51522304]; Zhejiang Provincial Natural Science Foundation of China [LR18E030002]; Zhejiang University Education Foundation Global Partnership Fund; China Postdoctoral Science Foundation [2019M662088]; National Postdoctoral Program for Innovative Talent [BX20190297]
FX This work was financially supported by the National Natural Science Foundation of China (21774109, 51973188, 51522304) , the Zhejiang Provincial Natural Science Foundation of China (LR18E030002) , the Zhejiang University Education Foundation Global Partnership Fund. The China Postdoctoral Science Foundation (2019M662088) , the National Postdoctoral Program for Innovative Talent (BX20190297) , the Zhejiang Provincial Natural Science Foundation of China (LR18E030002) and the Zhejiang University Education Foundation Global Partnership Fund. All animal studies were approved by the Institutional Ethical Committee of Animal Experimentation of Zhejiang University in China, and performed according to the guidelines established by the Institute for Experimental Animals of Zhejiang University.
CR Ao LJ, 2018, ACS APPL MATER INTER, V10, P12544, DOI 10.1021/acsami.8b02973
Chen Q, 2019, ADV MATER, V31, DOI 10.1002/adma.201802228
Chen Q, 2016, ADV MATER, V28, P7129, DOI 10.1002/adma.201601902
Deng GJ, 2018, ACS NANO, V12, P12096, DOI 10.1021/acsnano.8b05292
Dong X, 2018, ACS APPL MATER INTER, V10, P21861, DOI 10.1021/acsami.8b04867
Duan XP, 2019, ANGEW CHEM INT EDIT, V58, P670, DOI 10.1002/anie.201804882
Evans SS, 2015, NAT REV IMMUNOL, V15, P335, DOI 10.1038/nri3843
Feng B, 2018, ADV MATER, V30, DOI 10.1002/adma.201803001
Kantoff PW, 2010, NEW ENGL J MED, V363, P411, DOI 10.1056/NEJMoa1001294
Katagiri W, 2019, ADV HEALTHC MATER, V8, DOI 10.1002/adhm.201900035
Khalil DN, 2016, NAT REV CLIN ONCOL, V13, P273, DOI 10.1038/nrclinonc.2016.25
Krysko DV, 2012, NAT REV CANCER, V12, P860, DOI 10.1038/nrc3380
Li AW, 2018, NAT MATER, V17, P528, DOI 10.1038/s41563-018-0028-2
Li DW, 2014, ACS APPL MATER INTER, V6, P5144, DOI 10.1021/am500375n
Li X, 2018, CHEM SOC REV, V47, P4954, DOI 10.1039/c8cs00028j
Li Y, 2018, ADV SCI, V5, DOI 10.1002/advs.201700805
Lindsay KE, 2019, NAT BIOMED ENG, V3, P371, DOI 10.1038/s41551-019-0378-3
Lv MZ, 2020, CELL RES, V30, P966, DOI 10.1038/s41422-020-00395-4
Ma HS, 2019, ACS NANO, V13, P4302, DOI 10.1021/acsnano.8b09496

Melief CJM, 2015, J CLIN INVEST, V125, P3401, DOI 10.1172/JCI80009
Ng CW, 2018, ADV FUNCT MATER, V28, DOI 10.1002/adfm.201804688
Ott PA, 2017, NATURE, V547, P217, DOI 10.1038/nature22991
Pan JB, 2018, ADV MATER, V30, DOI 10.1002/adma.201704408
Pan JB, 2017, ADV FUNCT MATER, V27, DOI 10.1002/adfm.201603440
Reuven EM, 2019, ACS NANO, V13, P2936, DOI 10.1021/acsnano.8b07241
Sato K, 2016, SCI TRANSL MED, V8, DOI 10.1126/scitranslmed.aaf6843
Sun QH, 2017, ADV MATER, V29, DOI 10.1002/adma.201606628
Verbeke R, 2019, ACS NANO, V13, P1655, DOI 10.1021/acsnano.8b07660
Wang CY, 2020, SMALL, V16, DOI 10.1002/smll.202000589
Wang H, 2018, NAT MATER, V17, P761, DOI 10.1038/s41563-018-0147-9
Wang K, 2018, ACS NANO, V12, P6398, DOI 10.1021/acsnano.8b00558
Wang N, 2019, ACS BIOMATER SCI ENG, V5, P2330, DOI 10.1021/acsbiomaterials.9b00359
Wang TT, 2018, NAT COMMUN, V9, DOI 10.1038/s41467-018-03915-4
Xiao B, 2018, ACS NANO, V12, P12682, DOI 10.1021/acsnano.8b07584
Xie W, 2019, ACS NANO, V13, P2849, DOI 10.1021/acsnano.8b03788
Yang GB, 2017, NAT COMMUN, V8, DOI 10.1038/s41467-017-01050-0
Yang R, 2018, ACS NANO, V12, P5121, DOI 10.1021/acsnano.7b09041
Yang XQ, 2020, BIOMACROMOLECULES, V21, P2818, DOI 10.1021/acs.biomac.0c00518
Ye MZ, 2012, J MATER CHEM, V22, P14369, DOI 10.1039/c2jm32211k
Ye XY, 2019, ACS NANO, V13, P2956, DOI 10.1021/acsnano.8b07371
Ye YQ, 2017, SCI IMMUNOL, V2, DOI 10.1126/sciimmunol.aan5692
Yu GT, 2018, ADV FUNCT MATER, V28, DOI 10.1002/adfm.201801389
Zhang D, 2019, NANO LETT, V19, P6635, DOI 10.1021/acs.nanolett.9b02903
Zhang L, 2016, ACS NANO, V10, P10404, DOI 10.1021/acsnano.6b06267
Zhao H, 2019, ACS NANO, V13, P13127, DOI 10.1021/acsnano.9b05974
NR 45
TC 22
Z9 22
U1 9
U2 140
PU ELSEVIER SCI LTD
PI OXFORD
PA THE BOULEVARD, LANGFORD LANE, KIDLINGTON, OXFORD OX5 1GB, OXON, ENGLAND
SN 0142-9612
EI 1878-5905
J9 BIOMATERIALS
JI Biomaterials
PD JUL
PY 2021
VL 274
AR 120893
DI 10.1016/j.biomaterials.2021.120893
EA MAY 2021
PG 9
WC Engineering, Biomedical; Materials Science, Biomaterials
WE Science Citation Index Expanded (SCI-EXPANDED)
SC Engineering; Materials Science
GA SV1LG
UT WOS:000663586100001
PM 34029913
DA 2023-09-14
ER

PT J
AU Sang, MM
Liu, FL
Wang, Y
Luo, RJ
Huan, XX
Han, LF

Zhang, ZT
Feng, F
Qu, W
Liu, WY
Zheng, F
AF Sang, Mang Mang
Liu, Fu Lei
Wang, Yang
Luo, Ren Jie
Huan, Xiao Xian
Han, Ling Fei
Zhang, Zhong Tao
Feng, Feng
Qu, Wei
Liu, Wenyuan
Zheng, Feng
TI A novel redox/pH dual-responsive and hyaluronic acid-decorated multifunctional magnetic complex micelle for targeted gambogic acid delivery for the treatment of triple negative breast cancer
SO DRUG DELIVERY
LA English
DT Article
DE Magnetic; redox/pH dual-responsive; hyaluronic acid; gambogic acid; TNBC
ID MULTIDRUG-RESISTANCE; DENDRITIC CELLS; TUMOR; NANOPARTICLES; NANOCARRIERS; NANOVACCINE; PARTICLES; RELEASE; BIOLOGY; CARRIER
AB Gambogic acid (GA) is a naturally derived potent anticancer agent with extremely poor biocompatibility. In the present study, a novel of redox/pH dual-responsive multifunctional magnetic complex micelle (sPEG/HA/CSO-SS-Hex/Fe3O4/GA), which consisted of a reducible hexadecanol-modified chitosan oligosaccharide polymer micelle (CSO-SS-Hex) coated with hyaluronic acid (HA) and DCA grafted sheddable PEG-PLL (sPEG) copolymers and loaded with gambogic acid (GA) and Fe3O4 nanoparticles were developed for parenteral delivery for the treatment of triple negative breast cancer (TNBC). The ex vivo study showed that the sPEG shielded cationic HA/CSO-SS-Hex/Fe3O4/GA core at physiological pH but quickly shed off to re-expose the core due to its charge reversible property. The sPEG/HA/CSO-SS-Hex/Fe3O4/GA micelles effectively facilitated tumor-targeted GA delivery by HA, which is a targeting ligand for CD44 receptor of TNBC cells, meanwhile increase GA uptake at the acidic condition but diminished the drug uptake at neutral pH. The in vitro cellular uptake study and in vivo biodistribution and antitumor activity of the formulations were determined, all results showed that the complex micelle enhanced TNBC tumor cellular uptake and fast drug release due to the combined effect of magnet targeting, CD44 receptor-mediated internalization and redox/pH dual-responsive drug release. Hence, tumor-targeted delivery of GA with redox/pH dual-responsive multifunctional magnetic complex micelle sPEG/HA/CSO-SS-Hex/Fe3O4/GA might have potential implications for the chemotherapy of TNBC.
C1 [Sang, Mang Mang; Wang, Yang; Luo, Ren Jie; Han, Ling Fei; Liu, Wenyuan; Zheng, Feng] China Pharmaceut Univ, Key Lab Drug Qual Control & Pharmacovigilance, Minist Educ, Nanjing 210009, Jiangsu, Peoples R China.
[Sang, Mang Mang; Wang, Yang; Luo, Ren Jie; Han, Ling Fei; Liu, Wenyuan; Zheng, Feng] China Pharmaceut Univ, Dept Pharmaceut Anal, Nanjing, Jiangsu, Peoples R China.
[Liu, Fu Lei; Huan, Xiao Xian; Zhang, Zhong Tao; Feng, Feng; Qu, Wei] China Pharmaceut Univ, Dept Nat Med Chem, Nanjing, Jiangsu, Peoples R China.
C3 China Pharmaceutical University; China Pharmaceutical University; China Pharmaceutical University
RP Liu, WY; Zheng, F (通讯作者)，China Pharmaceut Univ, Key Lab Drug Qual Control & Pharmacovigilance, Minist Educ, Nanjing 210009, Jiangsu, Peoples R China.
EM liuwenyuan8506@163.com; cpu_analyst@126.com
RI Zhang, Zhongtao/AAE-2185-2021; liu, wen/HGE-3071-2022
OI Zhang, Zhongtao/0000-0003-2375-764X;
FU National Natural Science Foundation of China [81573557]; Fund of 2018 Graduate Student Research Innovation Program Project [3152080037]
FX This is study was supported by the National Natural Science Foundation of China (No. 81573557) and the Fund of 2018 Graduate Student Research Innovation Program Project (NO. 3152080037).
CR AUTERHOFF H, 1962, ARCHIV PHARM BERICHT, V295, P833, DOI 10.1002/ardp.19622951106

Bauhuber S, 2009, ADV MATER, V21, P3286, DOI 10.1002/adma.200802453
Brewster AM, 2014, LANCET ONCOL, V15, pE625, DOI 10.1016/S1470-2045(14)70364-X
Carey LA, 2007, CLIN CANCER RES, V13, P2329, DOI 10.1158/1078-0432.CCR-06-1109
Chen HP, 2015, J MED CHEM, V58, P3704, DOI 10.1021/jm501996y
Cheng D, 2011, J MATER CHEM, V21, P4796, DOI 10.1039/c0jm03783d
Coley HM, 2008, CANCER TREAT REV, V34, P378, DOI 10.1016/j.ctrv.2008.01.007
DALY WH, 1988, TETRAHEDRON LETT, V29, P5859, DOI 10.1016/S0040-4039(00)82209-1
Dey A, 2016, POLYMER, V93, P44, DOI 10.1016/j.polymer.2016.04.027
Doddapaneni R, 2016, DRUG DELIV, V23, P1232, DOI 10.3109/10717544.2015.1124472
Gelmon K, 2012, ANN ONCOL, V23, P2223, DOI 10.1093/annonc/mds067
Gu HY, 2009, J CANCER RES CLIN, V135, P1777, DOI 10.1007/s00432-009-0624-2
Guo X, 2015, ADV MATER, V27, P6450, DOI 10.1002/adma.201502865
Guo X, 2014, CHEM MATER, V26, P4405, DOI 10.1021/cm5012718
Hauser AK, 2016, BIOMATERIALS, V105, P127, DOI 10.1016/j.biomaterials.2016.07.032
Hu YW, 2015, J CONTROL RELEASE, V206, P91, DOI 10.1016/j.jconrel.2015.03.018
Huang R, 2018, DRUG DELIV, V25, P757, DOI 10.1080/10717544.2018.1446474
Kang YA, 2018, ACTA BIOMATER, V68, P137, DOI 10.1016/j.actbio.2017.12.028
Kline DG., 2008, KLINE HUDSONS NERVE
Lee J, 1996, J COLLOID INTERF SCI, V177, P490, DOI 10.1006/jcis.1996.0062
Liu LL, 2017, BIOMATERIALS, V134, P166, DOI 10.1016/j.biomaterials.2017.04.043
Liu LL, 2016, J IMMUNOL, V197, P1231, DOI 10.4049/jimmunol.1600182
Luo ZC, 2015, BIOMATERIALS, V38, P50, DOI 10.1016/j.biomaterials.2014.10.050
Palmer JR, 2014, JNCI-J NATL CANCER I, V106, DOI 10.1093/jnci/dju237
Price C, 2014, GENES DIS, V1, P53, DOI 10.1016/j.gendis.2014.06.004
Qi Q, 2008, BIOCHEM CELL BIOL, V86, P386, DOI 10.1139/o08-104
Rekha MR, 2015, J BIOMED NANOTECHNOL, V11, P165, DOI 10.1166/jbn.2015.1904
Sang MM, 2018, J BIOMED NANOTECHNOL, V14, P477, DOI 10.1166/jbn.2018.2541
Sata F, 2007, EPIDEMIOLOGY, V18, pS56
Sharma KK, 2006, INT C GROUNDN AFL MA
Song SS, 2014, PHARM RES-DORDR, V31, P1032, DOI 10.1007/s11095-013-1225-y
Turner NC, 2013, CLIN CANCER RES, V19, P6380, DOI 10.1158/1078-0432.CCR-13-0915
Wang JN, 2016, J MATER CHEM B, V4, P2954, DOI 10.1039/c5tb02450a
Wang SP, 2017, DRUG DELIV, V24, P1791, DOI 10.1080/10717544.2017.1406558
Wang X, 2013, BASIC CLIN PHARMACOL, V112, P25, DOI 10.1111/j.1742-7843.2012.00921.x
Wang Y, 2017, SMALL, V14
Wang Y, 2017, ADV MATER, V29, DOI 10.1002/adma.201605357
Yang Y, 2007, CANCER LETT, V256, P259, DOI 10.1016/j.canlet.2007.06.014
Yi HQ, 2016, NANOSCALE, V8, P5985, DOI 10.1039/c5nr07348k
Zheng CF, 2013, BIOMATERIALS, V34, P3431, DOI 10.1016/j.biomaterials.2013.01.053
NR 40
TC 22
Z9 24
U1 5
U2 53
PU TAYLOR & FRANCIS LTD
PI ABINGDON
PA 2-4 PARK SQUARE, MILTON PARK, ABINGDON OR14 4RN, OXON, ENGLAND
SN 1071-7544
EI 1521-0464
J9 DRUG DELIV
JI Drug Deliv.
PD OCT 18
PY 2018
VL 25
IS 1
BP 1846
EP 1857
DI 10.1080/10717544.2018.1486472
PG 12
WC Pharmacology & Pharmacy
WE Science Citation Index Expanded (SCI-EXPANDED)

SC Pharmacology & Pharmacy
GA GX4MF
UT WOS:000447706700001
PM 30334478
OA gold, Green Published, Green Submitted
DA 2023-09-14
ER

PT J
AU Shahbazi, S
Bolhassani, A
AF Shahbazi, Sepideh
Bolhassani, Azam
TI Comparison of six cell penetrating peptides with different properties for in vitro and in vivo delivery of HPV16 E7 antigen in therapeutic vaccines
SO INTERNATIONAL IMMUNOPHARMACOLOGY
LA English
DT Article
DE Human papillomavirus; E7 protein; Cervical cancer; Cell penetrating peptide; Therapeutic vaccine
ID MAMMALIAN-CELLS; SUPERCHARGED PROTEINS; EFFICIENT DELIVERY; GENE DELIVERY; NUCLEIC-ACID; STRATEGY; VECTOR; IMMUNOTHERAPY; NANOPARTICLE; VACCINATION
AB The high risk human papillomavirus (HPV) types 16 and 18 are globally linked to > 50% and 20% of all cervical cancers, respectively. The HPV E7 oncoprotein was determined as a therapeutic vaccine target due to its constitutive expression by HPV-infected cells. The findings demonstrated the efficiency of therapeutic HPV DNA- and protein-based vaccines in preclinical and clinical trials. However, there are limitations for penetration of DNA and protein constructs into the cells without a suitable delivery system. Recently, several cell penetrating peptides (CPPs) have been suggested for delivery of nucleic acids and proteins into cells through covalent or noncovalent fashion. In this study, we determined highly efficient CPPs for the controlled delivery of HPV16 E7 antigen, in vitro and in vivo. Our data indicated the effective delivery of E7 protein by Pep-1, Cady-2, P28 and hPP10, and E7 DNA by MPG and + 36 GFP CPPs in HEK-293T cell line at certain ratios. Moreover, immunization with the heterologous MPG + E7 DNA prime/P28 + rE7 protein boost elicited a higher Th1 cellular immune response with a predominant IFN-gamma profile and strong Granzyme B secretion than those induced by other groups in a murine tumor model. Indeed, the groups vaccinated with rE7 + P28/rE7 + P28, MPG + E7 DNA/P28 + rE7, and E7 DNA + MPG/E7 DNA + MPG nanovaccines displayed complete protection and remained tumor-free > 60 days after treatment. These data suggest P28 and MPG as promising protein and gene delivery systems for development of HPV therapeutic vaccines.
C1 [Shahbazi, Sepideh; Bolhassani, Azam] Pasteur Inst Iran, Dept Hepatitis & AIDS, Tehran, Iran.
C3 Le Reseau International des Instituts Pasteur (RIIP); Pasteur Institute of Iran
RP Bolhassani, A (通讯作者)，Pasteur Inst Iran, Dept Hepatitis & AIDS, Tehran, Iran.
EM A_bolhasani@pasteur.ac.ir
RI Bolhassani, Azam/HDN-7200-2022
OI Bolhassani, Azam/0000-0001-7363-7406
CR Bolhassani A, 2008, VACCINE, V26, P3362, DOI 10.1016/j.vaccine.2008.03.082
Chang M.D.-T, 2015, BIOMED RES INT, V2015, P1
Deshayes S, 2008, ADV DRUG DELIVER REV, V60, P537, DOI 10.1016/j.addr.2007.09.005
Durzynska J, 2015, J PHARMACOL EXP THER, V354, P32, DOI 10.1124/jpet.115.223305
Gros E, 2006, BBA-BIOMEMBRANES, V1758, P384, DOI 10.1016/j.bbamem.2006.02.006
Heitz F, 2009, BRIT J PHARMACOL, V157, P195, DOI 10.1111/j.1476-5381.2009.00057.x
Jafarzade BS, 2017, INT J PEPT RES THER, V23, P145, DOI 10.1007/s10989-016-9547-3
Ji HX, 1998, INT J CANCER, V78, P41, DOI 10.1002/(SICI)1097-0215(19980925)78:1<41::AID-IJC8>3.0.CO;2-X
Kadkhodayan S, 2016, FOLIA BIOL-PRAGUE, V62, P103
Kadkhodayan S, 2017, CURR DRUG DELIV, V14, P536, DOI 10.2174/1567201813666161006114448
Kadkhodayan S, 2017, IMMUNOL LETT, V188, P38, DOI 10.1016/j.imlet.2017.06.003
Keller AA, 2013, PHARMACEUTICALS, V6, P184, DOI 10.3390/ph6020184
Kristensen M, 2016, INT J MOL SCI, V17, DOI 10.3390/ijms17020185

   Kurzawa L, 2010, BBA-BIOMEMBRANES, V1798, P2274, DOI 10.1016/j.bbamem.2010.02.027
   Layek B, 2015, INT J MOL SCI, V16, P28912, DOI 10.3390/ijms161226142
   Lee SJ, 2016, J GYNECOL ONCOL, V27, DOI 10.3802/jgo.2016.27.e51
   Li Y, 2006, VACCINE, V24, P5360, DOI 10.1016/j.vaccine.2006.04.028
   Lin L, 2014, BMC IMMUNOL, V15, DOI 10.1186/s12865-014-0036-1
   Lulla R.R., 2016, NEURO-ONCOLOGY, P1
   Mardani G, 2016, IUBMB LIFE, V68, P459, DOI 10.1002/iub.1503
   McNaughton BR, 2009, P NATL ACAD SCI USA, V106, P6111, DOI 10.1073/pnas.0807883106
   Mehta RR, 2011, ANGIOGENESIS, V14, P355, DOI 10.1007/s10456-011-9220-6
   Morris MC, 1999, NUCLEIC ACIDS RES, V27, P3510, DOI 10.1093/nar/27.17.3510
   Morris MC, 1997, NUCLEIC ACIDS RES, V25, P2730, DOI 10.1093/nar/25.14.2730
   Motevalli F, 2018, IMMUNOL LETT, V194, P29, DOI 10.1016/j.imlet.2017.12.005
   Munoz-Morris MA, 2007, BIOCHEM BIOPH RES CO, V355, P877, DOI 10.1016/j.bbrc.2007.02.046
   Rathnayake P V G M, 2017, J Biophys, V2017, P1059216, DOI 10.1155/2017/1059216
   Sadeghian F, 2012, INT J PHARMACEUT, V427, P393, DOI 10.1016/j.ijpharm.2012.01.062
   Saleh T, 2015, VACCINE, V33, P3164, DOI 10.1016/j.vaccine.2015.05.015
   Shafer-Weaver K., 2003, J TRANSL MED, P1
   Thompson DB, 2012, METHOD ENZYMOL, V503, P293, DOI 10.1016/B978-0-12-396962-0.00012-4
   Yang A, 2016, J BIOMED SCI, V23, DOI 10.1186/s12929-016-0293-9
   Yu F, 2016, ONCOTARGET, V7, P79800, DOI 10.18632/oncotarget.12973
   Zahid M, 2015, MOLECULES, V20, P13055, DOI 10.3390/molecules200713055
NR 34
TC 22
Z9 23
U1 1
U2 21
PU ELSEVIER SCIENCE BV
PI AMSTERDAM
PA PO BOX 211, 1000 AE AMSTERDAM, NETHERLANDS
SN 1567-5769
EI 1878-1705
J9 INT IMMUNOPHARMACOL
JI Int. Immunopharmacol.
PD SEP
PY 2018
VL 62
BP 170
EP 180
DI 10.1016/j.intimp.2018.07.006
PG 11
WC Immunology; Pharmacology & Pharmacy
WE Science Citation Index Expanded (SCI-EXPANDED)
SC Immunology; Pharmacology & Pharmacy
GA GU5IA
UT WOS:000445318000021
PM 30015237
DA 2023-09-14
ER

PT J
AU Zhou, WX
   Chen, XL
   Zhou, Y
   Shi, S
   Liang, C
   Yu, XJ
   Chen, HY
   Guo, Q
   Zhang, YW
   Liu, PX
   Li, C

Chu, YC
Luo, YF
Wang, Y
Zhou, Z
Zhao, ZH
Chen, QJ
Sun, T
Jiang, C
AF Zhou, Wenxi
Chen, Xinli
Zhou, Yu
Shi, Si
Liang, Chen
Yu, Xianjun
Chen, Hongyi
Guo, Qin
Zhang, Yiwen
Liu, Peixin
Li, Chao
Chu, Yongchao
Luo, Yifan
Wang, Yu
Zhou, Zheng
Zhao, Zhenhao
Chen, Qinjun
Sun, Tao
Jiang, Chen
TI Exosomes derived from immunogenically dying tumor cells as a versatile tool for vaccination against pancreatic cancer
SO BIOMATERIALS
LA English
DT Article
DE Pancreatic cancer immunotherapy; Exosomes; Nanovaccines; Lymphatics; CCR4; CCL22 axis
ID DENDRITIC CELLS; IMMUNE; CHEMOTHERAPY; RESPONSES; RECRUITMENT; BLOCKADE; LEADS
AB Despite tremendous progress achieved in immunotherapy, many critical challenges in treating pancreatic ductal adenocarcinoma (PDAC) persist. Considering the poor vascularization of PDAC, after intramuscular adminis-tration exosomes can targeted deliver "cargos" to pancreatic tumors and bypass obstructions of the intrinsic overexpressed stroma through lymphatics. Herein, we propose a strategy to derive exosomes from immuno-genically dying tumor cells and exploit their properties for several purposes, including antigen presentation, adjuvant supply, and "cargo" delivery of vaccines against pancreatic cancer via intramuscular injection. To enhance the immunostimulatory effects, the MART-1 peptide is modified to the exosomes to expand T-cell-related responses. Furthermore, CCL22 siRNA is electroporated into the exosomes (referred to as spMEXO) to hinder the CCR4/CCL22 axis between DCs and Tregs, thereby suppressing Treg expansion. Both in vitro and in vivo studies demonstrate that spMEXO can serve as an effective prophylactic vaccine to delay tumor growth, whereas combining spMEXO with PDAC first-line chemotherapeutics (co-administration of gemcitabine with albumin-paclitaxel) demonstrated significantly enhanced therapeutic effects in established PANC-02 tumors. Therefore, the present work provides an effective strategy to employ cancer vaccines through intramuscular injection in PDAC and highlights the potential of exosomes derived from immunogenically dying tumor cells as a versatile tool to develop nanovaccines for immunotherapy.
C1 [Zhou, Wenxi; Chen, Xinli; Chen, Hongyi; Guo, Qin; Zhang, Yiwen; Liu, Peixin; Li, Chao; Chu, Yongchao; Luo, Yifan; Wang, Yu; Zhou, Zheng; Zhao, Zhenhao; Chen, Qinjun; Sun, Tao; Jiang, Chen] Fudan Univ, MOE Frontiers Ctr Brain Sci, Sch Pharm, Dept Pharmaceut, Shanghai 201203, Peoples R China.
[Zhou, Yu] Shanghai Jiao Tong Univ, Ruijin Hosp, Sch Med, Dept Intervent Radiol, Shanghai 200025, Peoples R China.
[Shi, Si; Liang, Chen; Yu, Xianjun] Fudan Univ, Shanghai Canc Ctr, Dept Pancreat & Hepatobiliary Surg, 270 Dongan Rd, Shanghai 200032, Peoples R China.
[Shi, Si; Liang, Chen; Yu, Xianjun] Fudan Univ, Shanghai Med Coll, Dept Oncol, Shanghai 200032,

Peoples R China.
[Zhou, Wenxi; Chen, Xinli; Chen, Hongyi; Guo, Qin; Zhang, Yiwen; Liu, Peixin; Li, Chao; Chu, Yongchao; Luo, Yifan; Wang, Yu; Zhou, Zheng; Zhao, Zhenhao; Chen, Qinjun; Sun, Tao; Jiang, Chen] Fudan Univ, Key Lab Smart Drug Delivery, State Key Lab Med Neurobiol, Minist Educ, Shanghai 201203, Peoples R China.
C3 Fudan University; Shanghai Jiao Tong University; Fudan University; Fudan University; Fudan University
RP Jiang, C (通讯作者)，Fudan Univ, MOE Frontiers Ctr Brain Sci, Sch Pharm, Dept Pharmaceut, Shanghai 201203, Peoples R China.; Jiang, C (通讯作者)，Fudan Univ, Key Lab Smart Drug Delivery, State Key Lab Med Neurobiol, Minist Educ, Shanghai 201203, Peoples R China.
EM jiangchen@shmu.edu.cn
RI Chen, Qinjun/J-1223-2019; jiang, chen/HZL-9276-2023; Liang, Chen/HNP-5916-2023; chen, hongyi/HTS-1467-2023
FU National Natural Science Foundation of China [32030059]; Program of Shanghai Academic Research Leader [18XD1400500]; Key projects of Shanghai Science Foundation [19JC1410800]; Shanghai Municipal Science and Technology Major Project and ZJLab [2018SHZDZX01]
FX We acknowledge Prof. Zhigang Zhang at Shanghai Cancer Institute for providing the PANC02 cell lines. We also acknowledge the support from the National Natural Science Foundation of China (grant No. 32030059) , Program of Shanghai Academic Research Leader (18XD1400500) , Key projects of Shanghai Science Foundation (19JC1410800) and Shanghai Municipal Science and Technology Major Project (No. 2018SHZDZX01) and ZJLab.
CR Apetoh L, 2007, NAT MED, V13, P1050, DOI 10.1038/nm1622
Banchereau J, 2005, NAT REV IMMUNOL, V5, P296, DOI 10.1038/nri1592
Bazhin AV, 2014, CANCER IMMUNOL IMMUN, V63, P59, DOI 10.1007/s00262-013-1485-8
Brunet LR, 2016, ONCOIMMUNOLOGY, V5, DOI 10.1080/2162402X.2015.1112942
Cheng LF, 2017, MOL THER, V25, P1665, DOI 10.1016/j.ymthe.2017.02.007
Christensen O, 2009, J IMMUNOTHER, V32, P613, DOI 10.1097/CJI.0b013e3181a95198
Curiel TJ, 2004, NAT MED, V10, P942, DOI 10.1038/nm1093
Ekeruche-Makinde J, 2012, J BIOL CHEM, V287, P37269, DOI 10.1074/jbc.M112.386409
Faget J, 2011, CANCER RES, V71, P6143, DOI 10.1158/0008-5472.CAN-11-0573
Fan YC, 2017, NANO LETT, V17, P7387, DOI 10.1021/acs.nanolett.7b03218
Fridlender ZG, 2010, MOL THER, V18, P1947, DOI 10.1038/mt.2010.159
Gao XJ, 2018, SCI TRANSL MED, V10, DOI 10.1126/scitranslmed.aat0195
Joffre OP, 2012, NAT REV IMMUNOL, V12, P557, DOI 10.1038/nri3254
Kamerkar S, 2017, NATURE, V546, P498, DOI 10.1038/nature22341
Kim MS, 2016, NANOMED-NANOTECHNOL, V12, P655, DOI 10.1016/j.nano.2015.10.012
Lai CP, 2014, ACS NANO, V8, P483, DOI 10.1021/nn404945r
Liu HP, 2014, NATURE, V507, P519, DOI 10.1038/nature12978
Luo ZC, 2015, BIOMATERIALS, V38, P50, DOI 10.1016/j.biomaterials.2014.10.050
Madura F, 2015, EUR J IMMUNOL, V45, P584, DOI 10.1002/eji.201445114
Maisel K, 2017, ADV DRUG DELIVER REV, V114, P43, DOI 10.1016/j.addr.2017.07.005
Montecalvo A, 2012, BLOOD, V119, P756, DOI 10.1182/blood-2011-02-338004
Nowak AK, 2002, CANCER RES, V62, P2353
Obeid M, 2007, NAT MED, V13, P54, DOI 10.1038/nm1523
Pennock GK, 2015, ONCOLOGIST, V20, P812, DOI 10.1634/theoncologist.2014-0422
Pitt JM, 2014, J IMMUNOL, V193, P1006, DOI 10.4049/jimmunol.1400703
Rakoff-Nahoum S, 2007, SCIENCE, V317, P124, DOI 10.1126/science.1140488
Rucki AA, 2014, WORLD J GASTROENTERO, V20, P2237, DOI 10.3748/wjg.v20.i9.2237
Sodium, 2014, NEW ENGL J MED, V371, P2134
Srinivasan S, 2016, SCI REP-UK, V6, DOI 10.1038/srep24436
Steinman RM, 2007, NATURE, V449, P419, DOI 10.1038/nature06175
Sugiyama D., P NATL ACAD SCI
Tran TH, 2015, CLIN IMMUNOL, V160, P46, DOI 10.1016/j.clim.2015.03.021
Topalian SL, 2015, CANCER CELL, V27, P450, DOI 10.1016/j.ccell.2015.03.001
Tumeh PC, 2014, NATURE, V515, P568, DOI 10.1038/nature13954
Vonderheide RH, 2013, CURR OPIN IMMUNOL, V25, P200, DOI 10.1016/j.coi.2013.01.006
WCULEK SK, 2020, NAT REV IMMUNOL, V20, P7, DOI DOI 10.1038/S41577-019-0210-Z
Wolfers J, 2001, NAT MED, V7, P297, DOI 10.1038/85438

Wu AA, 2020, CLIN CANCER RES, V26, P5129, DOI 10.1158/1078-0432.CCR-20-1025
Yadav D, 2013, GASTROENTEROLOGY, V144, P1252, DOI 10.1053/j.gastro.2013.01.068
Zhou WX, 2021, BIOMATERIALS, V268, DOI 10.1016/j.biomaterials.2020.120546
Zhou Y, 2020, ACTA PHARM SIN B, V10, P1563, DOI 10.1016/j.apsb.2019.11.013
NR 41
TC 21
Z9 23
U1 12
U2 57
PU ELSEVIER SCI LTD
PI OXFORD
PA THE BOULEVARD, LANGFORD LANE, KIDLINGTON, OXFORD OX5 1GB, OXON, ENGLAND
SN 0142-9612
EI 1878-5905
J9 BIOMATERIALS
JI Biomaterials
PD JAN
PY 2022
VL 280
AR 121306
DI 10.1016/j.biomaterials.2021.121306
PG 12
WC Engineering, Biomedical; Materials Science, Biomaterials
WE Science Citation Index Expanded (SCI-EXPANDED)
SC Engineering; Materials Science
GA 1A8QI
UT WOS:000792013700001
PM 34952381
DA 2023-09-14
ER

PT J
AU Ding, BB
Yue, J
Zheng, P
Ma, PA
Lin, J
AF Ding, Binbin
Yue, Jun
Zheng, Pan
Ma, Ping'an
Lin, Jun
TI Manganese oxide nanomaterials boost cancer immunotherapy
SO JOURNAL OF MATERIALS CHEMISTRY B
LA English
DT Review
ID TUMOR-ASSOCIATED MACROPHAGES; ENHANCED PHOTODYNAMIC THERAPY; IMMUNOGENIC CELL-DEATH; MNO2 NANOSHEETS; CHECKPOINT BLOCKADE; STING PATHWAY; NANOPARTICLES; MICROENVIRONMENT; HYPOXIA; DELIVERY
AB Immunotherapy, a strategy that leverages the host immune function to fight against cancer, plays an increasingly important role in clinical tumor therapy. In spite of the great success achieved in not only clinical treatment but also basic research, cancer immunotherapy still faces many huge challenges. Manganese oxide nanomaterials (MONs), as ideal tumor microenvironment (TME)-responsive biomaterials, are able to dramatically elicit anti-tumor immune responses in multiple ways, indicating great prospects for immunotherapy. In this review, on the basis of different mechanisms to boost immunotherapy, major highlighted topics are presented, covering adjusting an immunosuppressive TME by generating O-2 (like O-2-sensitized photodynamic therapy (PDT), programmed cell death ligand-1 (PD-L1) expression downregulation, reprogramming tumor-associated macrophages (TAMs), and restraining tumor angiogenesis and lactic acid exhaustion), inducing immunogenic cell death (ICD), photothermal therapy (PTT) induction, activating the stimulator of interferon gene (STING) pathway and immunoadjuvants for nanovaccines. We hope that this review will provide holistic understanding about

MONs and their application in cancer immunotherapy, and thus pave the way to the translation from bench to bedside in the future.
C1 [Ding, Binbin; Zheng, Pan; Ma, Ping'an; Lin, Jun] Chinese Acad Sci, Changchun Inst Appl Chem, State Key Lab Rare Earth Resource Utilizat, Changchun 130022, Peoples R China.
[Yue, Jun] Sun Yat Sen Univ, Sch Biomed Engn, Guangzhou 510006, Peoples R China.
[Zheng, Pan] Shandong Univ, Inst Frontier & Interdisciplinar Sci, Qingdao 266237, Peoples R China.
[Zheng, Pan] Shandong Univ, Inst Mol Sci & Engn, Qingdao 266237, Peoples R China.
[Ma, Ping'an; Lin, Jun] Univ Sci & Technol China, Hefei 230026, Peoples R China.
C3 Chinese Academy of Sciences; Changchun Institute of Applied Chemistry, CAS; Sun Yat Sen University; Shandong University; Shandong University; Chinese Academy of Sciences; University of Science & Technology of China, CAS
RP Ma, PA; Lin, J (通讯作者)，Chinese Acad Sci, Changchun Inst Appl Chem, State Key Lab Rare Earth Resource Utilizat, Changchun 130022, Peoples R China.; Ma, PA; Lin, J (通讯作者)，Univ Sci & Technol China, Hefei 230026, Peoples R China.
EM mapa675@ciac.ac.cn; jlin@ciac.ac.cn
RI , Jun/M-1049-2018
OI , Jun/0000-0001-8038-3555
FU Chinese Government [2017YFE0132300]; National Natural Science Foundation of China [NSFC 51720105015, 51929201, 51922097, 51772124, 51872282]; Key Research Program of Frontier Sciences, CAS [YZDY-SSW-JSC018]; Postdoctoral Innovative Talents Support Program [BX2021360]; Youth Innovation Promotion Association of CAS [2017273]; Australian Government [2017YFE0132300]
FX This work is financially supported by the Science and Technology Cooperation Project between Chinese and Australian Governments (2017YFE0132300), the National Natural Science Foundation of China (Grant No. NSFC 51720105015, 51929201, 51922097, 51772124 and 51872282), the Key Research Program of Frontier Sciences, CAS (YZDY-SSW-JSC018), the Postdoctoral Innovative Talents Support Program (BX2021360) and the Youth Innovation Promotion Association of CAS (Grant No. 2017273).
CR Ai XZ, 2018, BIOCONJUGATE CHEM, V29, P928, DOI 10.1021/acs.bioconjchem.8b00068
Aikins ME, 2020, ACCOUNTS CHEM RES, V53, P2094, DOI 10.1021/acs.accounts.0c00456
Barsoum IB, 2014, CANCER RES, V74, P665, DOI 10.1158/0008-5472.CAN-13-0992
Bhattarai P, 2018, NANOSCALE, V10, P5393, DOI 10.1039/c7nr09612g
Bohn T, 2018, NAT IMMUNOL, V19, P1319, DOI 10.1038/s41590-018-0226-8
Cabral H, 2020, ACCOUNTS CHEM RES, V53, P2765, DOI 10.1021/acs.accounts.0c00518
Castano AP, 2006, NAT REV CANCER, V6, P535, DOI 10.1038/nrc1894
Chang CC, 2020, ACS APPL MATER INTER, V12, P44407, DOI 10.1021/acsami.0c08473
Chang MY, 2021, ADV MATER, V33, DOI 10.1002/adma.202004788
Chen HC, 2015, J AM CHEM SOC, V137, P1539, DOI 10.1021/ja511420n
Chen Q, 2019, CHEM SOC REV, V48, P5506, DOI 10.1039/c9cs00271e
Chen Q, 2019, ADV MATER, V31, DOI 10.1002/adma.201900192
Chen Q, 2016, NAT COMMUN, V7, DOI 10.1038/ncomms13193
Chen Y, 2014, ADV MATER, V26, P7019, DOI 10.1002/adma.201402572
Cheung AS, 2015, NANO TODAY, V10, P511, DOI 10.1016/j.nantod.2015.06.007
Chung CH, 2020, BIOMATERIALS, V257, DOI 10.1016/j.biomaterials.2020.120227
Colegio OR, 2014, NATURE, V513, P559, DOI 10.1038/nature13490
Deng RR, 2011, J AM CHEM SOC, V133, P20168, DOI 10.1021/ja2100774
Ding BB, 2020, ANGEW CHEM INT EDIT, V59, P16381, DOI 10.1002/anie.202005111
Ding BB, 2020, ADV MATER, V32, DOI 10.1002/adma.201905823
Ding BB, 2019, CHEM MATER, V31, P2651, DOI 10.1021/acs.chemmater.9b00893
Ding BB, 2018, ADV MATER, V30, DOI 10.1002/adma.201802479
Duan XP, 2019, ANGEW CHEM INT EDIT, V58, P670, DOI 10.1002/anie.201804882
Eisenbarth SC, 2008, NATURE, V453, P1122, DOI 10.1038/nature06939
Fan HH, 2016, ANGEW CHEM INT EDIT, V55, P5477, DOI 10.1002/anie.201510748
Fan M, 2021, ADV FUNCT MATER, V31, DOI 10.1002/adfm.202010587
Fan WP, 2015, ADV MATER, V27, P4155, DOI 10.1002/adma.201405141
Fang RH, 2015, SMALL, V11, P5483, DOI 10.1002/smll.201501284
Flach TL, 2011, NAT MED, V17, P479, DOI 10.1038/nm.2306
Gao F, 2019, ADV MATER, V31, DOI 10.1002/adma.201904639

Gu GZ, 2014, BIOMATERIALS, V35, P8215, DOI 10.1016/j.biomaterials.2014.06.022
Han X, 2020, ACCOUNTS CHEM RES, V53, P2521, DOI 10.1021/acs.accounts.0c00339
He CB, 2016, NAT COMMUN, V7, DOI 10.1038/ncomms12499
He LZ, 2019, ADV FUNCT MATER, V29, DOI 10.1002/adfm.201901240
He QY, 2020, NANOSCALE, V12, P14788, DOI 10.1039/d0nr02750b
Hou L, 2020, ACS NANO, V14, P3927, DOI 10.1021/acsnano.9b06111
Keskin DB, 2019, NATURE, V565, P234, DOI 10.1038/s41586-018-0792-9
Krysko DV, 2012, NAT REV CANCER, V12, P860, DOI 10.1038/nrc3380
Kuai R, 2017, NAT MATER, V16, P489, DOI [10.1038/nmat4822, 10.1038/NMAT4822]
Lan GX, 2018, J AM CHEM SOC, V140, P5670, DOI 10.1021/jacs.8b01072
Li W, 2018, ADV MATER, V30, DOI 10.1002/adma.201703740
Liang RJ, 2018, BIOMATERIALS, V177, P149, DOI 10.1016/j.biomaterials.2018.05.051
Lin LS, 2018, ANGEW CHEM INT EDIT, V57, P4902, DOI 10.1002/anie.201712027
Liu J, 2017, BIOSENS BIOELECTRON, V90, P69, DOI 10.1016/j.bios.2016.11.046
Liu SY, 2013, BIOMATERIALS, V34, P8291, DOI 10.1016/j.biomaterials.2013.07.020
Liu XM, 2020, SMALL METHODS, V4, DOI 10.1002/smtd.202000566
Liu YL, 2020, NANO-MICRO LETT, V12, DOI 10.1007/s40820-020-00452-y
Liu YL, 2019, THERANOSTICS, V9, P6867, DOI 10.7150/thno.37586
Liu Z, 2018, BIOMATERIALS, V155, P54, DOI 10.1016/j.biomaterials.2017.11.015
Lu JQ, 2018, ACS NANO, V12, P11041, DOI 10.1021/acsnano.8b05189
Lu KD, 2016, J AM CHEM SOC, V138, P12502, DOI 10.1021/jacs.6b06663
Luo M, 2017, NAT NANOTECHNOL, V12, P648, DOI [10.1038/nnano.2017.52, 10.1038/NNANO.2017.52]
Lv MZ, 2020, CELL RES, V30, P966, DOI 10.1038/s41422-020-00395-4
Mellman I, 2011, NATURE, V480, P480, DOI 10.1038/nature10673
Meng LT, 2018, ACS NANO, V12, P8308, DOI 10.1021/acsnano.8b03590
Min YZ, 2017, NAT NANOTECHNOL, V12, P877, DOI [10.1038/nnano.2017.113, 10.1038/NNANO.2017.113]
Ng CW, 2018, ADV FUNCT MATER, V28, DOI 10.1002/adfm.201804688
Noman MZ, 2014, J EXP MED, V211, P781, DOI 10.1084/jem.20131916
Obeid M, 2007, NAT MED, V13, P54, DOI 10.1038/nm1523
Pardoll DM, 2012, NAT REV CANCER, V12, P252, DOI 10.1038/nrc3239
Phuengkham H, 2019, ADV MATER, V31, DOI 10.1002/adma.201803322
Qian XQ, 2020, ADV FUNCT MATER, V30, DOI 10.1002/adfm.201907066
Rosenberg SA, 2004, NAT MED, V10, P909, DOI 10.1038/nm1100
Ruan SB, 2015, BIOMATERIALS, V37, P425, DOI 10.1016/j.biomaterials.2014.10.007
Saeed AFUH, 2020, ADV SCI, V7, DOI 10.1002/advs.201902599
Sahin U, 2020, NATURE, V585, P107, DOI 10.1038/s41586-020-2537-9
Sang W, 2019, CHEM SOC REV, V48, P3771, DOI 10.1039/c8cs00896e
Schumacher TN, 2015, SCIENCE, V348, P69, DOI 10.1126/science.aaa4971
Shae D, 2019, NAT NANOTECHNOL, V14, P269, DOI 10.1038/s41565-018-0342-5
Shen ZJ, 2020, THERANOSTICS, V10, P9132, DOI 10.7150/thno.46076
Shi Y, 2019, ACCOUNTS CHEM RES, V52, P1543, DOI 10.1021/acs.accounts.9b00148
Song ML, 2016, ACS NANO, V10, P633, DOI 10.1021/acsnano.5b06779
Song WT, 2017, BIOMATERIALS, V148, P16, DOI 10.1016/j.biomaterials.2017.09.017
Sun QX, 2019, CHEM SOC REV, V48, P351, DOI 10.1039/c8cs00473k
Tang ZM, 2017, ADV MATER, V29, DOI 10.1002/adma.201701683
Wang CG, 2018, IMMUNITY, V48, P675, DOI 10.1016/j.immuni.2018.03.017
Wang CY, 2020, SMALL, V16, DOI 10.1002/smll.202000589
Wang DG, 2016, NANO LETT, V16, P5503, DOI 10.1021/acs.nanolett.6b01994
Wang JX, 2020, INT J MOL SCI, V21, DOI 10.3390/ijms21218363
Wang L, 2019, ACS APPL MATER INTER, V11, P6267, DOI 10.1021/acsami.8b20639
Wang SF, 2018, ACS NANO, V12, P12380, DOI 10.1021/acsnano.8b06399
Wang YL, 2021, NANO TODAY, V38, DOI 10.1016/j.nantod.2021.101139
Wang Y, 2017, BIOMATERIALS, V112, P153, DOI 10.1016/j.biomaterials.2016.09.034
Wei Z, 2019, CHEM SCI, V10, P2778, DOI 10.1039/c8sc04123g
Xu J, 2017, ACS NANO, V11, P4463, DOI 10.1021/acsnano.7b00715
Yan SQ, 2020, ANGEW CHEM INT EDIT, V59, P17332, DOI 10.1002/anie.202002780
Yang GB, 2017, NAT COMMUN, V8, DOI 10.1038/s41467-017-01050-0
Yang X, 2021, NANO TODAY, V38, DOI 10.1016/j.nantod.2021.101109
Yang YN, 2021, ADV SCI, V8, DOI 10.1002/advs.202002667

Yi X, 2016, NANO RES, V9, P3267, DOI 10.1007/s12274-016-1205-8
Yu GT, 2018, ADV FUNCT MATER, V28, DOI 10.1002/adfm.201801389
Yu M, 2019, ADV SCI, V6, DOI 10.1002/advs.201900037
Zanganeh S, 2016, NAT NANOTECHNOL, V11, P986, DOI [10.1038/nnano.2016.168, 10.1038/NNANO.2016.168]
Zhang C, 2020, CHEM SOC REV, V49, P4234, DOI 10.1039/c9cs00773c
Zhang Y, 2020, CHEM ENG J, V390, DOI 10.1016/j.cej.2020.124521
Zhao JY, 2021, BIOMATERIALS, V268, DOI 10.1016/j.biomaterials.2020.120542
Zhao ZL, 2014, J AM CHEM SOC, V136, P11220, DOI 10.1021/ja5029364
Zheng P, 2021, NANO LETT, V21, P2088, DOI 10.1021/acs.nanolett.0c04778
Zhou FY, 2019, ADV MATER, V31, DOI 10.1002/adma.201805888
Zhou L, 2020, NANO LETT, V20, P4393, DOI 10.1021/acs.nanolett.0c01140
Zhou LL, 2020, COMMUN BIOL, V3, DOI 10.1038/s42003-020-1070-6
Zhu DM, 2021, J NEUROSURG, V134, P499, DOI 10.3171/2019.10.JNS191959
Zhu WW, 2016, ADV FUNCT MATER, V26, P5490, DOI 10.1002/adfm.201600676
Zhu YJ, 2017, ACTA PHARMACOL SIN, V38, P614, DOI 10.1038/aps.2017.5
NR 104
TC 21
Z9 21
U1 15
U2 183
PU ROYAL SOC CHEMISTRY
PI CAMBRIDGE
PA THOMAS GRAHAM HOUSE, SCIENCE PARK, MILTON RD, CAMBRIDGE CB4 0WF, CAMBS, ENGLAND
SN 2050-750X
EI 2050-7518
J9 J MATER CHEM B
JI J. Mat. Chem. B
PD SEP 21
PY 2021
VL 9
IS 35
BP 7117
EP 7131
DI 10.1039/d1tb01001h
EA JUN 2021
PG 15
WC Materials Science, Biomaterials
WE Science Citation Index Expanded (SCI-EXPANDED)
SC Materials Science
GA UQ5MU
UT WOS:000674372200001
PM 34279012
OA Bronze
DA 2023-09-14
ER

PT J
AU Acebes-Fernandez, V
Landeira-Vinuela, A
Juanes-Velasco, P
Hernandez, AP
Otazo-Perez, A
Manzano-Roman, R
Gongora, R
Fuentes, M
AF Acebes-Fernandez, Vanessa
Landeira-Vinuela, Alicia
Juanes-Velasco, Pablo
Hernandez, Angela-Patricia

Otazo-Perez, Andrea
Manzano-Roman, Raul
Gongora, Rafael
Fuentes, Manuel
TI Nanomedicine and Onco-Immunotherapy: From the Bench to Bedside to Biomarkers
SO NANOMATERIALS
LA English
DT Review
DE nanomaterials; nanomedicine; immunotherapy; oncotherapy; immune-checkpoint inhibitors; immunogenic cell death; nano-vaccines; nano-conjugates; immune response
ID IMMUNOGENIC CELL-DEATH; ANTIGEN-PRESENTING CELLS; IMMUNE-CHECKPOINT BLOCKADE; TUMOR-ASSOCIATED ANTIGENS; ACTIVE SPECIFIC IMMUNOTHERAPY; IRON-OXIDE NANOPARTICLES; CYTOTOXIC T-LYMPHOCYTES; NATURAL-KILLER-CELLS; CANCER-IMMUNOTHERAPY; DENDRITIC CELLS
AB The broad relationship between the immune system and cancer is opening a new hallmark to explore for nanomedicine. Here, all the common and synergy points between both areas are reviewed and described, and the recent approaches which show the progress from the bench to the beside to biomarkers developed in nanomedicine and onco-immunotherapy.
C1 [Acebes-Fernandez, Vanessa; Landeira-Vinuela, Alicia; Juanes-Velasco, Pablo; Hernandez, Angela-Patricia; Otazo-Perez, Andrea; Gongora, Rafael; Fuentes, Manuel] USAL, Dept Med & Cytometry Gen Serv Nucleus, CIBERONC, CB16 12 00400,Canc Res Ctr,IBMCC,CSIC,IBSAL, Salamanca 37007, Spain.
[Manzano-Roman, Raul; Fuentes, Manuel] USAL, Prote Unit, Canc Res Ctr, IBMCC,CSIC,IBSAL, Salamanca 37007, Spain.
C3 CIBER - Centro de Investigacion Biomedica en Red; CIBERONC; University of Salamanca; Consejo Superior de Investigaciones Cientificas (CSIC); CSIC-USAL - Instituto de Biologia Molecular y Celular del Cancer de Salamanca (IBMCC); University of Salamanca; Consejo Superior de Investigaciones Cientificas (CSIC); CSIC-USAL - Instituto de Biologia Molecular y Celular del Cancer de Salamanca (IBMCC)
RP Fuentes, M (通讯作者)，USAL, Dept Med & Cytometry Gen Serv Nucleus, CIBERONC, CB16 12 00400,Canc Res Ctr,IBMCC,CSIC,IBSAL, Salamanca 37007, Spain.; Fuentes, M (通讯作者)，USAL, Prote Unit, Canc Res Ctr, IBMCC,CSIC,IBSAL, Salamanca 37007, Spain.
EM vanessaacebes@usal.es; alavi29@usal.es; pablojuanesvelasco@usal.es; angytahg@usal.es; andreaotazopz@gmail.com; rmanzano@usal.es; rgongora@usal.es; mfuentes@usal.es
RI Hernandez, Ángela-Patricia/D-6342-2018; Gongora, Rafael/M-4891-2018
OI Hernandez, Ángela-Patricia/0000-0002-5585-9918; Gongora, Rafael/0000-0002-8046-4301; Juanes-Velasco, Pablo/0000-0001-9435-2952; Fuentes, Manuel/0000-0002-7305-3766; Otazo Perez, Andrea/0000-0002-4437-3832; ACEBES FERNANDEZ, VANESSA/0000-0001-6166-8002
FU Spanish Health Institute Carlos III (ISCIII) [FIS PI17/01930, CB16/12/00400]; Fundacion Solorzano [FS/38-2017]; PE I + D + I 2017-2020 - ISCIII [PT17/0019/0023]; FEDER; VII Centenario-USAL PhD Program; IBSAL PhD Program; "Retencion Talentos" Programm; Ayuntamiento Salamanca (Salamanca, Spain)
FX We gratefully acknowledge financial support from the Spanish Health Institute Carlos III (ISCIII) for the grants: FIS PI17/01930 and CB16/12/00400. Fundacion Solorzano FS/38-2017. The Proteomics Unit belongs to ProteoRed, PRB3-ISCIII, supported by grant PT17/0019/0023, of the PE I + D + I 2017-2020, funded by ISCIII and FEDER. A. Landeira-Vinuela is supported by VII Centenario-USAL PhD Program, P. Juanes-Velasco is supported by IBSAL PhD Program, V-Acebes-Fernandez is supported by "Retencion Talentos" Programm sponsorized by Ayuntamiento Salamanca (Salamanca, Spain).
CR Aaes TL, 2016, CELL REP, V15, P274, DOI 10.1016/j.celrep.2016.03.037
Ahmadzadeh M, 2009, BLOOD, V114, P1537, DOI 10.1182/blood-2008-12-195792
AKIYOSHI K, 1993, MACROMOLECULES, V26, P3062, DOI 10.1021/ma00064a011
Ambrosi A, 2010, ANAL CHEM, V82, P1151, DOI 10.1021/ac902492c

Anderson JM, 2008, SEMIN IMMUNOL, V20, P86, DOI 10.1016/j.smim.2007.11.004
Andersson U, 2011, ANNU REV IMMUNOL, V29, P139, DOI 10.1146/annurev-immunol-030409-101323
Andreatta M, 2015, IMMUNOGENETICS, V67, P641, DOI 10.1007/s00251-015-0873-y
Antonia SJ, 2002, J UROLOGY, V167, P1995, DOI 10.1016/S0022-5347(05)65071-9
Apetoh L, 2007, NAT MED, V13, P1050, DOI 10.1038/nm1622
Apetoh L, 2015, ONCOIMMUNOLOGY, V4, DOI 10.1080/2162402X.2014.998538
Arab D, 2013, COLLOID SURFACE A, V436, P803, DOI 10.1016/j.colsurfa.2013.08.022
Auria-Soro C, 2019, NANOMATERIALS-BASEL, V9, DOI 10.3390/nano9101365
Axelrod ML, 2019, CLIN CANCER RES, V25, P2392, DOI 10.1158/1078-0432.CCR-18-3200
Bald T, 2014, CANCER DISCOV, V4, P674, DOI 10.1158/2159-8290.CD-13-0458
Banchereau J, 2005, NAT REV IMMUNOL, V5, P296, DOI 10.1038/nri1592
Bao CC, 2014, NANOMEDICINE-UK, V9, P2353, DOI [10.2217/NNM.14.155, 10.2217/nnm.14.155]
Bassani-Sternberg M, 2010, P NATL ACAD SCI USA, V107, P18769, DOI 10.1073/pnas.1008501107
Baughman RH, 2002, SCIENCE, V297, P787, DOI 10.1126/science.1060928
Bear AS, 2013, PLOS ONE, V8, DOI 10.1371/journal.pone.0069073
Benmebarek MR, 2019, INT J MOL SCI, V20, DOI 10.3390/ijms20061283
BERD D, 1990, J CLIN ONCOL, V8, P1858, DOI 10.1200/JCO.1990.8.11.1858
Berger M, 2007, J PHARM PHARM SCI, V10, P144
Berraondo P, 2019, BRIT J CANCER, V120, P6, DOI 10.1038/s41416-018-0328-y
Binnewies M, 2018, NAT MED, V24, P541, DOI 10.1038/s41591-018-0014-x
BLAY JY, 1992, CANCER RES, V52, P3317
Bobisse S, 2016, ANN TRANSL MED, V4, DOI 10.21037/atm.2016.06.17
Bonnemain B, 1998, J DRUG TARGET, V6, P167, DOI 10.3109/10611869808997890
Brennick CA, 2017, IMMUNOTHERAPY-UK, V9, P361, DOI 10.2217/imt-2016-0146
Brentjens RJ, 2013, SCI TRANSL MED, V5, DOI 10.1126/scitranslmed.3005930
Britten CM, 2013, NAT BIOTECHNOL, V31, P880, DOI 10.1038/nbt.2708
Broz P, 2013, NAT REV IMMUNOL, V13, P551, DOI 10.1038/nri3479
Bunggulawa EJ, 2018, J NANOBIOTECHNOL, V16, DOI 10.1186/s12951-018-0403-9
Butler MO, 2014, IMMUNOL REV, V257, P191, DOI 10.1111/imr.12129
Butterfield LH, 2015, BMJ-BRIT MED J, V350, DOI 10.1136/bmj.h988
Byun DJ, 2017, NAT REV ENDOCRINOL, V13, P195, DOI 10.1038/nrendo.2016.205
Califf RM, 2018, EXP BIOL MED, V243, P213, DOI 10.1177/1535370217750088
Cao XT, 2016, NAT REV IMMUNOL, V16, P35, DOI 10.1038/nri.2015.8
Carlino MS, 2016, CLIN CANCER RES, V22, P3992, DOI 10.1158/1078-0432.CCR-15-2944
Castle JC, 2012, CANCER RES, V72, P1081, DOI 10.1158/0008-5472.CAN-11-3722
Centi S, 2014, J NANOBIOTECHNOL, V12, DOI 10.1186/s12951-014-0055-3
Chalmers ZR, 2017, GENOME MED, V9, DOI 10.1186/s13073-017-0424-2
Chan TA, 2015, NEW ENGL J MED, V373, P1984, DOI 10.1056/NEJMc1508163
Cheever MA, 2011, CLIN CANCER RES, V17, P3520, DOI 10.1158/1078-0432.CCR-10-3126
Chen DS, 2013, IMMUNITY, V39, P1, DOI 10.1016/j.immuni.2013.07.012
Chen WL, 2014, BIOMATERIALS, V35, P1686, DOI 10.1016/j.biomaterials.2013.11.004
Chen YP, 2017, THERANOSTICS, V7, P3585, DOI 10.7150/thno.21471
Chiang CLL, 2015, VACCINES-BASEL, V3, P344, DOI 10.3390/vaccines3020344
Chiang CS, 2018, NAT NANOTECHNOL, V13, P746, DOI 10.1038/s41565-018-0146-7
Chowell D, 2018, SCIENCE, V359, P582, DOI 10.1126/science.aao4572
Christian DA, 2012, IMMUNOTHERAPY-UK, V4, P425, DOI [10.2217/imt.12.26, 10.2217/IMT.12.26]
Cirenajwis H, 2015, ONCOTARGET, V6, P12297, DOI 10.18632/oncotarget.3655
Clynes R, 1998, P NATL ACAD SCI USA, V95, P652, DOI 10.1073/pnas.95.2.652
Cobbold M, 2013, SCI TRANSL MED, V5, DOI 10.1126/scitranslmed.3006061
Colombo M, 2012, CHEM SOC REV, V41, P4306, DOI 10.1039/c2cs15337h
Conlan RS, 2017, TRENDS MOL MED, V23, P636, DOI 10.1016/j.molmed.2017.05.003
Conlon KC, 2019, J INTERF CYTOK RES, V39, P6, DOI 10.1089/jir.2018.0019
Crane CA, 2013, CLIN CANCER RES, V19, P205, DOI 10.1158/1078-0432.CCR-11-3358
CRAWFORD LV, 1982, INT J CANCER, V30, P403, DOI 10.1002/ijc.2910300404
Curnis F, 2016, NANO RES, V9, P1393, DOI 10.1007/s12274-016-1035-8
Dai WB, 2012, NANOMED-NANOTECHNOL, V8, P1152, DOI 10.1016/j.nano.2012.01.003
Dall'Ozzo S, 2004, CANCER RES, V64, P4664, DOI 10.1158/0008-5472.CAN-03-2862
Das DK, 2015, P NATL ACAD SCI USA, V112, P1517, DOI 10.1073/pnas.1424829112
De Vries IJM, 2007, CANCER IMMUNOL IMMUN, V56, P1667, DOI 10.1007/s00262-007-0304-5
Decuzzi P, 2010, J CONTROL RELEASE, V141, P320, DOI 10.1016/j.jconrel.2009.10.014
di Pietro A, 2008, EXPERT OPIN BIOL TH, V8, P1973, DOI [10.1517/14712590802517970,

10.1517/14712590802517970 ]
Diez P, 2017, NANOSCALE, V9, P9960, DOI 10.1039/c7nr02196h
Ding BB, 2018, ADV MATER, V30, DOI 10.1002/adma.201802479
Dizaj SM, 2015, EXPERT OPIN DRUG DEL, V12, P1649, DOI 10.1517/17425247.2015.1049530
Doan A., 2018, PEDIAT BLOOD CANC, V65, DOI 10.1002/pbc.26914
Doyle D.J., 2013, ADV ANESTH, V31, P181, DOI DOI 10.1016/J.AAN.2013.08.010
Duan XP, 2016, J AM CHEM SOC, V138, P16686, DOI 10.1021/jacs.6b09538
Dudek AM, 2013, CYTOKINE GROWTH F R, V24, P319, DOI 10.1016/j.cytogfr.2013.01.005
Emens LA, 2017, EUR J CANCER, V81, P116, DOI 10.1016/j.ejca.2017.01.035
Fan YC, 2017, NANO LETT, V17, P7387, DOI 10.1021/acs.nanolett.7b03218
FDA, 2017, BEST BIOM ENDPOINTS, V55, DOI [10.1164/rccm.201301-0153OC, DOI 10.1164/RCCM.201301-0153OC]
Feldman SA, 2015, SEMIN ONCOL, V42, P626, DOI 10.1053/j.seminoncol.2015.05.005
Fernandes C, 2018, FRONT PHARMACOL, V9, DOI 10.3389/fphar.2018.01230
Ferrara N, 2004, NAT REV DRUG DISCOV, V3, P391, DOI 10.1038/nrd1381
Fleming TP, 2000, ANN NY ACAD SCI, V923, P78, DOI 10.1111/j.1749-6632.2000.tb05521.x
Fortier MH, 2008, J EXP MED, V205, P595, DOI 10.1084/jem.20071985
Franklin MC, 2004, CANCER CELL, V5, P317, DOI 10.1016/S1535-6108(04)00083-2
Fridman WH, 2012, NAT REV CANCER, V12, P298, DOI 10.1038/nrc3245
Fritsche J, 2018, PROTEOMICS, V18, DOI 10.1002/pmic.201700284
Fuchs Y, 2015, NAT REV MOL CELL BIO, V16, P329, DOI 10.1038/nrm3999
Fucikova J, 2014, INT J CANCER, V135, P1165, DOI 10.1002/ijc.28766
Gajewski TF, 2013, NAT IMMUNOL, V14, P1014, DOI 10.1038/ni.2703
Galluzzi L, 2017, NAT REV IMMUNOL, V17, P97, DOI 10.1038/nri.2016.107
Galluzzi L, 2014, ONCOTARGET, V5, P12472, DOI 10.18632/oncotarget.2998
Galon J, 2013, IMMUNITY, V39, P11, DOI 10.1016/j.immuni.2013.07.008
Gao S, 2019, THERANOSTICS, V9, P126, DOI 10.7150/thno.29431
Garg AD, 2017, TRENDS IMMUNOL, V38, P577, DOI 10.1016/j.it.2017.05.006
Garg AD, 2012, CANCER IMMUNOL IMMUN, V61, P215, DOI 10.1007/s00262-011-1184-2
Garg AD, 2010, BBA-REV CANCER, V1805, P53, DOI 10.1016/j.bbcan.2009.08.003
Garon EB, 2015, NEW ENGL J MED, V372, P2018, DOI 10.1056/NEJMoa1501824
Geary SM, 2013, ONCOIMMUNOLOGY, V2, DOI 10.4161/onci.24523
Germain C, 2015, FRONT IMMUNOL, V6, DOI 10.3389/fimmu.2015.00067
Germain C, 2014, AM J RESP CRIT CARE, V189, P832, DOI 10.1164/rccm.201309-1611OC
Geyer PE, 2017, MOL SYST BIOL, V13, DOI 10.15252/msb.20156297
Ghanem G, 2011, MOL ONCOL, V5, P150, DOI 10.1016/j.molonc.2011.01.006
Gillison ML, 2000, JNCI-J NATL CANCER I, V92, P709, DOI 10.1093/jnci/92.9.709
Gnjatic S, 2017, J IMMUNOTHER CANCER, V5, DOI 10.1186/s40425-017-0243-4
Grupp SA, 2013, NEW ENGL J MED, V368, P1509, DOI 10.1056/NEJMoa1215134
Guerrero S, 2012, BIOCONJUGATE CHEM, V23, P399, DOI 10.1021/bc200362a
Gulley JL, 2017, JNCI-J NATL CANCER I, V109, DOI 10.1093/jnci/djw261
Guo CQ, 2013, ADV CANCER RES, V119, P421, DOI 10.1016/B978-0-12-407190-2.00007-1
Guo PX, 2010, NAT NANOTECHNOL, V5, P833, DOI [10.1038/nnano.2010.231, 10.1038/NNANO.2010.231]
Hanahan D, 2000, CELL, V100, P57, DOI 10.1016/S0092-8674(00)81683-9
Hanahan D, 2011, CELL, V144, P646, DOI 10.1016/j.cell.2011.02.013
Hanna MG, 2001, VACCINE, V19, P2576, DOI 10.1016/S0264-410X(00)00485-0
Hanna MG, 2012, HUM VACC IMMUNOTHER, V8, P1156, DOI 10.4161/hv.20740
Hannani D, 2015, CELL RES, V25, P399, DOI [10.1038/cr.2015.3, 10.1038/cr.2015.28]
Hashemi R, 2014, APPL ENERG, V133, P374, DOI 10.1016/j.apenergy.2014.07.069
He CB, 2016, NAT COMMUN, V7, DOI 10.1038/ncomms12499
He CB, 2010, BIOMATERIALS, V31, P3657, DOI 10.1016/j.biomaterials.2010.01.065
Health N, 2013, NATURE, V500, P1, DOI [10.1038/nature, DOI 10.1038/NATURE]
Heo MB, 2014, BIOMATERIALS, V35, P590, DOI 10.1016/j.biomaterials.2013.10.009
Herbst RS, 2014, NATURE, V515, P563, DOI 10.1038/nature14011
Hickey JW, 2017, NANO LETT, V17, P7045, DOI 10.1021/acs.nanolett.7b03734
Hirsh SL, 2013, COLLOID SURFACE B, V103, P395, DOI 10.1016/j.colsurfb.2012.10.039
Hodi FS, 2010, NEW ENGL J MED, V363, P711, DOI 10.1056/NEJMoa1003466
Holtl L, 1999, J UROLOGY, V161, P777, DOI 10.1016/S0022-5347(01)61767-1
Hoo LS, 2002, ONCOGENE, V21, P5006, DOI 10.1038/sj.onc.1205625
Hood JL, 2016, NANOMEDICINE-UK, V11, P1745, DOI 10.2217/nnm-2016-0102

Hood JL, 2012, WIRES NANOMED NANOBI, V4, P458, DOI 10.1002/wnan.1174
IMAI H, 1993, CANCER-AM CANCER SOC, V71, P26, DOI 10.1002/1097-0142(19930101)71:1<26::AID-CNCR2820710106>3.0.CO;2-N
INABA K, 1992, J EXP MED, V176, P1693, DOI 10.1084/jem.176.6.1693
Irache JM, 2008, EXPERT OPIN DRUG DEL, V5, P703, DOI [10.1517/17425247.5.6.703, 10.1517/17425247.5.6.703 ]
JANEWAY CA, 1992, IMMUNOL TODAY, V13, P11, DOI 10.1016/0167-5699(92)90198-G
JANEWAY CA, 1989, COLD SH Q B, V54, P1
Ji QJ, 2016, NAT COMMUN, V7, DOI 10.1038/ncomms11000
Ji RR, 2012, CANCER IMMUNOL IMMUN, V61, P1019, DOI 10.1007/s00262-011-1172-6
Jiang WL, 2005, ADV DRUG DELIVER REV, V57, P391, DOI 10.1016/j.addr.2004.09.003
Johnson DB, 2016, NAT COMMUN, V7, DOI 10.1038/ncomms10582
Johnson LE, 2018, ONCOIMMUNOLOGY, V7, DOI 10.1080/2162402X.2018.1456603
Johnstone TC, 2016, CHEM REV, V116, P3436, DOI 10.1021/acs.chemrev.5b00597
June CH, 2017, NAT MED, V23, P540, DOI 10.1038/nm.4321
Kalbasi A, 2013, J CLIN INVEST, V123, P2756, DOI 10.1172/JCI69219
Kantoff PW, 2010, NEW ENGL J MED, V363, P411, DOI 10.1056/NEJMoa1001294
Kao H, 2001, J EXP MED, V194, P1313, DOI 10.1084/jem.194.9.1313
Kauffman KJ, 2016, J CONTROL RELEASE, V240, P227, DOI 10.1016/j.jconrel.2015.12.032
Keenan BP, 2012, SEMIN ONCOL, V39, P276, DOI 10.1053/j.seminoncol.2012.02.007
Khong H, 2016, J IMMUNOTHER CANCER, V4, DOI 10.1186/s40425-016-0160-y
Kim MS, 2018, NANOMED-NANOTECHNOL, V14, P195, DOI 10.1016/j.nano.2017.09.011
Kim Y, 2013, NATURE, V500, P59, DOI 10.1038/nature12401
Kloor M, 2010, INT J CANCER, V127, P1001, DOI 10.1002/ijc.25283
Knochelmann HM, 2018, FRONT IMMUNOL, V9, DOI 10.3389/fimmu.2018.01740
Kochenderfer JN, 2015, J CLIN ONCOL, V33, P540, DOI 10.1200/JCO.2014.56.2025
Kochenderfer JN, 2012, BLOOD, V119, P2709, DOI 10.1182/blood-2011-10-384388
Koo YEL, 2004, ANAL CHEM, V76, P2498, DOI 10.1021/ac035493f
KOVACSOVICSBANKOWSKI M, 1995, SCIENCE, V267, P243, DOI 10.1126/science.7809629
Kranz LM, 2016, NATURE, V534, P396, DOI 10.1038/nature18300
Kreiter S, 2015, NATURE, V520, P692, DOI 10.1038/nature14426
Kreiter S, 2012, ONCOIMMUNOLOGY, V1, P768, DOI 10.4161/onci.19727
Kroemer G, 2013, ANNU REV IMMUNOL, V31, P51, DOI 10.1146/annurev-immunol-032712-100008
Kuai R, 2018, SCI ADV, V4, DOI 10.1126/sciadv.aao1736
Kuai R, 2017, NAT MATER, V16, P489, DOI [10.1038/nmat4822, 10.1038/NMAT4822]
Kurokawa T, 2001, INT J CANCER, V91, P749, DOI 10.1002/1097-0215(200002)9999:9999<::AID-IJC1141>3.0.CO;2-X
Lambert LH, 2017, NANO LETT, V17, P821, DOI 10.1021/acs.nanolett.6b04071
Lawrence MS, 2013, NATURE, V499, P214, DOI 10.1038/nature12213
Lechuga L., 2011, NANOMEDICINA APLICAC
Lee HT, 2019, MOLECULES, V24, DOI 10.3390/molecules24061190
Lee JK, 2013, PLOS ONE, V8, DOI [10.1371/journal.pone.0066555, 10.1371/journal.pone.0084256]
Lee J, 2009, SMALL, V5, P1008, DOI 10.1002/smll.200801242
Lee SH, 2015, HUM VACC IMMUNOTHER, V11, P1889, DOI 10.1080/21645515.2015.1035502
Lee Sylvia, 2011, Cancers (Basel), V3, P3856, DOI 10.3390/cancers3043856
Lee Y, 2007, J AM CHEM SOC, V129, P5362, DOI 10.1021/ja071090b
Lefebvre ML, 2006, J IMMUNOTHER, V29, P388, DOI 10.1097/01.cji.0000203081.43235.d7
LEUVERING JHW, 1981, J IMMUNOL METHODS, V45, P183, DOI 10.1016/0022-1759(81)90212-X
Li GM, 2008, CELL RES, V18, P1, DOI 10.1038/cr.2008.9
Li L, 2017, ANN ONCOL, V28, P11, DOI 10.1093/annonc/mdx681
Li P, 2013, J CONTROL RELEASE, V168, P271, DOI 10.1016/j.jconrel.2013.03.025
Li W, 2016, SCI REP-UK, V6, DOI 10.1038/srep30619
Li XY, 2018, TUMORI J, V104, P1, DOI 10.5301/tj.5000675
Linette GP, 2013, BLOOD, V122, P863, DOI 10.1182/blood-2013-03-490565
Liu W, 2013, NANOSCALE, V5, P1658, DOI 10.1039/c2nr33611a
Liu XS, 2013, ACS NANO, V7, P6244, DOI 10.1021/nn402201w
Liu XX, 2017, NANOMED-NANOTECHNOL, V13, P1993, DOI 10.1016/j.nano.2017.04.006
Liu YL, 2018, CURR ONCOL REP, V20, DOI 10.1007/s11912-018-0740-8
Liu ZG, 2018, NANO LETT, V18, P6655, DOI 10.1021/acs.nanolett.8b02340
Lopez-Bertoni H, 2018, NANO LETT, V18, P4086, DOI 10.1021/acs.nanolett.8b00390
Lovat V, 2005, NANO LETT, V5, P1107, DOI 10.1021/nl050637m


file:///D/Joyce/计量学/frontiers/修/Supplementary materials/savedrecs (94).txt[2024/6/27 16:48:08]

Lu JQ, 2017, NAT COMMUN, V8, DOI 10.1038/s41467-017-01651-9
Lu ML, 2001, AM J PATHOL, V159, P945, DOI 10.1016/S0002-9440(10)61770-1
LUBIN R, 1995, NAT MED, V1, P701, DOI 10.1038/nm0795-701
Lundegaard C, 2011, J IMMUNOL METHODS, V374, P26, DOI 10.1016/j.jim.2010.10.011
Ma YT, 2011, J EXP MED, V208, DOI 10.1084/jem.201002692084c
MacFarlane AW, 2014, CANCER IMMUNOL RES, V2, P320, DOI 10.1158/2326-6066.CIR-13-0133
Madsen CB, 2012, PLOS ONE, V7, DOI 10.1371/journal.pone.0050139
Magherini Francesca, 2018, Oncotarget, V9, P28042, DOI 10.18632/oncotarget.25556
MaHam A, 2009, SMALL, V5, P1706, DOI 10.1002/smll.200801602
Mahmoudi M, 2011, ADV DRUG DELIVER REV, V63, P24, DOI 10.1016/j.addr.2010.05.006
Markham A, 2018, DRUGS, V78, P1841, DOI 10.1007/s40265-018-1012-5
Martins I, 2011, ONCOGENE, V30, P1147, DOI 10.1038/onc.2010.500
Marzo T, 2019, ACS MED CHEM LETT, V10, P656, DOI 10.1021/acsmedchemlett.9b00007
Masucci GV, 2016, J IMMUNOTHER CANCER, V4, DOI 10.1186/s40425-016-0178-1
Mattson MP, 2000, J MOL NEUROSCI, V14, P175, DOI 10.1385/JMN:14:3:175
McNamara MA, 2015, J IMMUNOL RES, V2015, DOI 10.1155/2015/794528
MEEKER TC, 1985, BLOOD, V65, P1349, DOI 10.1182/blood.V65.6.1349.bloodjournal6561349
Meir R, 2017, ACS NANO, V11, P11127, DOI 10.1021/acsnano.7b05299
Melendez B, 2018, TRANSL LUNG CANCER R, V7, P661, DOI 10.21037/tlcr.2018.08.02
Meng XJ, 2015, CANCER TREAT REV, V41, P868, DOI 10.1016/j.ctrv.2015.11.001
Meng ZJ, 2013, NANOSCALE RES LETT, V8, DOI 10.1186/1556-276X-8-118
Menger L, 2012, SCI TRANSL MED, V4, DOI 10.1126/scitranslmed.3003807
Michaud M, 2011, SCIENCE, V334, P1573, DOI 10.1126/science.1208347
Milling L, 2017, ADV DRUG DELIVER REV, V114, P79, DOI 10.1016/j.addr.2017.05.011
Min YZ, 2017, NAT NANOTECHNOL, V12, P877, DOI [10.1038/nnano.2017.113, 10.1038/NNANO.2017.113]
Mocellin S, 2009, CURR MED CHEM, V16, P4779, DOI 10.2174/092986709789909648
Mohammadizadeh M, 2014, MAT SCI SEMICON PROC, V20, P74, DOI 10.1016/j.mssp.2013.12.032
Mokarizadeh Aram, 2012, Vet Res Forum, V3, P257
Mokarizadeh A, 2012, IMMUNOL LETT, V147, P47, DOI 10.1016/j.imlet.2012.06.001
Momen-Heravi F, 2014, NANOMED-NANOTECHNOL, V10, P1517, DOI 10.1016/j.nano.2014.03.014
Montico B, 2017, ONCOIMMUNOLOGY, V6, DOI 10.1080/2162402X.2017.1356964
Morgan RA, 2013, J IMMUNOTHER, V36, P133, DOI 10.1097/CJI.0b013e3182829903
MORTON DL, 1992, ANN SURG, V216, P463, DOI 10.1097/00000658-199210000-00010
Mullard A, 2017, NAT REV DRUG DISCOV, V16, P669, DOI 10.1038/nrd.2017.196
Munich S, 2012, ONCOIMMUNOLOGY, V1, P1074, DOI 10.4161/onci.20897
Muraoka D, 2014, ACS NANO, V8, P9209, DOI 10.1021/nn502975r
Murphy G, 1996, PROSTATE, V29, P371
Naahidi S, 2013, J CONTROL RELEASE, V166, P182, DOI 10.1016/j.jconrel.2012.12.013
Nel AE, 2009, NAT MATER, V8, P543, DOI [10.1038/nmat2442, 10.1038/NMAT2442]
Nelson D, 2014, J IMMUNOL RES, V2014, DOI 10.1155/2014/789069
Nestle FO, 1998, NAT MED, V4, P328, DOI 10.1038/nm0398-328
Nielsen M, 2009, BMC BIOINFORMATICS, V10, DOI 10.1186/1471-2105-10-296
Nishino M, 2017, NAT REV CLIN ONCOL, V14, P655, DOI 10.1038/nrclinonc.2017.88
Noguchi T, 2012, CANCER RES, V72, P1672, DOI 10.1158/0008-5472.CAN-11-3072
O'Rourke DM, 2017, SCI TRANSL MED, V9, DOI 10.1126/scitranslmed.aaa0984
Obeid M, 2007, NAT MED, V13, P54, DOI 10.1038/nm1523
Oberli MA, 2017, NANO LETT, V17, P1326, DOI 10.1021/acs.nanolett.6b03329
Ochs M, 2013, ANGEW CHEM INT EDIT, V52, P695, DOI 10.1002/anie.201206696
Ochsenbein AF, 2004, J EXP MED, V200, P1407, DOI 10.1084/jem.20040717
Ock CY, 2016, CLIN CANCER RES, V22, P2261, DOI 10.1158/1078-0432.CCR-15-2834
Osada T, 2012, SEMIN ONCOL, V39, P305, DOI 10.1053/j.seminoncol.2012.02.013
Ott PA, 2017, NATURE, V547, P217, DOI 10.1038/nature22991
Pan RY, 2018, J IMMUNOL RES, V2018, DOI 10.1155/2018/4325874
Pawluczkowycz AW, 2009, J IMMUNOL, V183, P749, DOI 10.4049/jimmunol.0900632
Pedersen AW, 2018, CHIN CLIN ONCOL, V7, DOI 10.21037/cco.2018.01.03
Pelaz B, 2017, ACS NANO, V11, P2313, DOI 10.1021/acsnano.6b06040
Peng Q, 2013, BIOMATERIALS, V34, P8521, DOI 10.1016/j.biomaterials.2013.07.102
Peng RQ, 2010, WORLD J GASTROENTERO, V16, P2428, DOI 10.3748/wjg.v16.i19.2428
Perica K, 2015, ACS NANO, V9, P6861, DOI 10.1021/acsnano.5b02829
Perica K, 2014, NANOMED-NANOTECHNOL, V10, P119, DOI 10.1016/j.nano.2013.06.015

Petros RA, 2010, NAT REV DRUG DISCOV, V9, P615, DOI 10.1038/nrd2591
Porter DL, 2015, SCI TRANSL MED, V7, DOI 10.1126/scitranslmed.aac5415
Porter DL, 2011, NEW ENGL J MED, V365, P725, DOI 10.1056/NEJMoa1103849
Powell DJ, 2005, BLOOD, V105, P241, DOI 10.1182/blood-2004-06-2482
Rabinovich GA, 2007, ANNU REV IMMUNOL, V25, P267, DOI 10.1146/annurev.immunol.25.022106.141609
Rahimian S, 2015, BIOMATERIALS, V61, P33, DOI 10.1016/j.biomaterials.2015.04.043
Raimondo F, 2011, PROTEOMICS, V11, P709, DOI 10.1002/pmic.201000422
Rajasagi M, 2014, BLOOD, V124, P453, DOI 10.1182/blood-2014-04-567933
Raposo G, 1996, J EXP MED, V183, P1161, DOI 10.1084/jem.183.3.1161
RATAIN MJ, 1985, BLOOD, V65, P644
Reinherz EL, 2015, CANCER IMMUNOL RES, V3, P305, DOI 10.1158/2326-6066.CIR-15-0042
Restifo NP, 2016, NAT REV CANCER, V16, P121, DOI 10.1038/nrc.2016.2
Rhodes KR, 2018, MOL IMMUNOL, V98, P13, DOI 10.1016/j.molimm.2018.02.016
Riley RS, 2019, NAT REV DRUG DISCOV, V18, P175, DOI 10.1038/s41573-018-0006-z
Rizvi NA, 2015, SCIENCE, V348, P124, DOI 10.1126/science.aaa1348
Robert L, 2014, ONCOIMMUNOLOGY, V3, DOI 10.4161/onci.29244
Robert L, 2014, CLIN CANCER RES, V20, P2424, DOI 10.1158/1078-0432.CCR-13-2648
Robins HS, 2009, BLOOD, V114, P4099, DOI 10.1182/blood-2009-04-217604
Rodig SJ, 2018, SCI TRANSL MED, V10, DOI 10.1126/scitranslmed.aar3342
Romano E, 2011, CLIN CANCER RES, V17, P1984, DOI 10.1158/1078-0432.CCR-10-3421
Rosenberg SA, 2004, NAT MED, V10, P909, DOI 10.1038/nm1100
ROSENBERG SA, 1988, NEW ENGL J MED, V319, P1676, DOI 10.1056/NEJM198812223192527
ROSENBERG SA, 1986, SCIENCE, V233, P1318, DOI 10.1126/science.3489291
Ruttinger D, 2007, J TRANSL MED, V5, DOI 10.1186/1479-5876-5-43
Sabado RL, 2015, NATURE, V519, P300, DOI 10.1038/nature14211
Sabatino M, 2009, J CLIN ONCOL, V27, P2645, DOI 10.1200/JCO.2008.19.1106
Sahin U, 2017, NATURE, V547, P222, DOI 10.1038/nature23003
Sanmamed MF, 2018, CELL, V175, P313, DOI 10.1016/j.cell.2018.09.035
Sapsford KE, 2011, ANAL CHEM, V83, P4453, DOI 10.1021/ac200853a
Saunderson SC, 2008, J IMMUNOL, V180, P8146, DOI 10.4049/jimmunol.180.12.8146
Schmidt H, 2005, BRIT J CANCER, V93, P273, DOI 10.1038/sj.bjc.6602702
Schumacher TN, 2015, SCIENCE, V348, P69, DOI 10.1126/science.aaa4971
Schwartzentruber DJ, 2011, NEW ENGL J MED, V364, P2119, DOI 10.1056/NEJMoa1012863
Sennino B, 2012, NAT REV CANCER, V12, P699, DOI 10.1038/nrc3366
Serrano-del Valle A, 2019, FRONT CELL DEV BIOL, V7, DOI 10.3389/fcell.2019.00050
SETTE A, 1994, J IMMUNOL, V153, P5586
Shao WG, 2018, NUCLEIC ACIDS RES, V46, pD1237, DOI 10.1093/nar/gkx664
Sharifi M, 2019, BIOSENS BIOELECTRON, V126, P773, DOI 10.1016/j.bios.2018.11.026
Sharma P, 2015, SCIENCE, V348, P56, DOI 10.1126/science.aaa8172
Shimizu T, 2008, BIOCHEM BIOPH RES CO, V367, P330, DOI 10.1016/j.bbrc.2007.12.112
Shin SR, 2013, ACS NANO, V7, P2369, DOI 10.1021/nn305559j
Shukla S, 2019, ADV THER-GERMANY, V2, DOI 10.1002/adtp.201800139
Simeone E, 2014, CANCER IMMUNOL IMMUN, V63, P675, DOI 10.1007/s00262-014-1545-8
Singh R, 2009, EXP MOL PATHOL, V86, P215, DOI 10.1016/j.yexmp.2008.12.004
Skirtach AG, 2006, ANGEW CHEM INT EDIT, V45, P4612, DOI 10.1002/anie.200504599
Small EJ, 2006, J CLIN ONCOL, V24, P3089, DOI 10.1200/JCO.2005.04.5252
Smith TT, 2017, NAT NANOTECHNOL, V12, P813, DOI [10.1038/NNANO.2017.57, 10.1038/nnano.2017.57]
Smith-Garvin JE, 2009, ANNU REV IMMUNOL, V27, P591, DOI 10.1146/annurev.immunol.021908.132706
Snyder A, 2014, NEW ENGL J MED, V371, P2189, DOI 10.1056/NEJMoa1406498
Soares MM, 2001, J IMMUNOL, V166, P6555, DOI 10.4049/jimmunol.166.11.6555
Song MJ, 2018, CHINESE J CANCER RES, V30, P157, DOI 10.21147/j.issn.1000-9604.2018.02.01
Song WT, 2018, NAT COMMUN, V9, DOI 10.1038/s41467-018-04605-x
Spencer Kristen R, 2016, Am Soc Clin Oncol Educ Book, V35, pe493, DOI 10.14694/EDBK_160766
SRIVASTAVA PK, 1993, ADV CANCER RES, V62, P153, DOI 10.1016/S0065-230X(08)60318-8
Stenzinger A, 2019, GENE CHROMOSOME CANC, V58, P578, DOI 10.1002/gcc.22733
Stirland DL, 2013, J CONTROL RELEASE, V172, P1045, DOI 10.1016/j.jconrel.2013.09.026
Stoll G, 2014, ONCOIMMUNOLOGY, V3, DOI 10.4161/onci.27884
Stone JD, 2015, CURR OPIN IMMUNOL, V33, P16, DOI 10.1016/j.coi.2015.01.003

Stronen E, 2016, SCIENCE, V352, P1337, DOI 10.1126/science.aaf2288
Sugahara KN, 2010, SCIENCE, V328, P1031, DOI 10.1126/science.1183057
Sun JS, 2015, ADV MATER, V27, P1402, DOI 10.1002/adma.201404788
Sunshine J, 2015, CURR OPIN PHARMACOL, V23, P32, DOI 10.1016/j.coph.2015.05.011
Suzuki H, 2005, CLIN CANCER RES, V11, P1521, DOI 10.1158/1078-0432.CCR-04-0538
TAN EM, 1989, ADV IMMUNOL, V44, P93, DOI 10.1016/S0065-2776(08)60641-0
Tan EM, 2003, CLIN EXP IMMUNOL, V134, P169, DOI 10.1046/j.1365-2249.2003.02259.x
Tan EM, 2008, IMMUNOL REV, V222, P328, DOI 10.1111/j.1600-065X.2008.00611.x
Tartour E, 1996, J CLIN ONCOL, V14, P1697, DOI 10.1200/JCO.1996.14.5.1697
Taube JM, 2014, CLIN CANCER RES, V20, P5064, DOI 10.1158/1078-0432.CCR-13-3271
Tay CY, 2014, NANO LETT, V14, P83, DOI 10.1021/nl4032549
Teng MWL, 2015, CANCER RES, V75, P2139, DOI 10.1158/0008-5472.CAN-15-0255
Thery C, 2009, NAT REV IMMUNOL, V9, P581, DOI 10.1038/nri2567
Thurner B, 1999, J EXP MED, V190, P1669, DOI 10.1084/jem.190.11.1669
Tian YH, 2014, BIOMATERIALS, V35, P2383, DOI 10.1016/j.biomaterials.2013.11.083
Torchilin VP, 2007, PHARM RES-DORDR, V24, P1, DOI 10.1007/s11095-006-9132-0
Tray N, 2018, CANCER IMMUNOL RES, V6, P1122, DOI 10.1158/2326-6066.CIR-18-0214
TRIVERS GE, 1995, J NATL CANCER I, V87, P1400, DOI 10.1093/jnci/87.18.1400
Trivers GE, 1996, CLIN CANCER RES, V2, P1767
Tutt AL, 1998, J IMMUNOL, V161, P3176
Valadi H, 2007, NAT CELL BIOL, V9, P654, DOI 10.1038/ncb1596
Van Allen EM, 2015, SCIENCE, V350, P207, DOI 10.1126/science.aad0095
van Kempen TS, 2015, NAT REV RHEUMATOL, V11, P483, DOI 10.1038/nrrheum.2015.60
van Rooij N, 2013, J CLIN ONCOL, V31, pE439, DOI 10.1200/JCO.2012.47.7521
van Willigen WW, 2018, FRONT IMMUNOL, V9, DOI 10.3389/fimmu.2018.02265
Vermaelen K, 2019, FRONT IMMUNOL, V10, DOI 10.3389/fimmu.2019.00008
Vigneron N, 2004, SCIENCE, V304, P587, DOI 10.1126/science.1095522
Vita R, 2015, NUCLEIC ACIDS RES, V43, pD405, DOI 10.1093/nar/gku938
Vogelstein B, 2013, SCIENCE, V339, P1546, DOI 10.1126/science.1235122
Vogeser M, 2016, TRAC-TREND ANAL CHEM, V84, P1, DOI 10.1016/j.trac.2016.04.018
Wahlgren J, 2012, NUCLEIC ACIDS RES, V40, DOI 10.1093/nar/gks463
Waldmann TA, 2018, CSH PERSPECT BIOL, V10, DOI 10.1101/cshperspect.a028472
Wang C, 2016, BIOMATERIALS, V79, P88, DOI 10.1016/j.biomaterials.2015.11.040
Wang C, 2017, NAT BIOMED ENG, V1, DOI 10.1038/s41551-016-0011
Wang C, 2014, ADV MATER, V26, P4794, DOI 10.1002/adma.201400158
Wang D, 2017, NUCLEIC ACIDS RES, V45, pD769, DOI 10.1093/nar/gkw946
Wang HY, 2014, MOL CELL, V54, P133, DOI 10.1016/j.molcel.2014.03.003
Wang XG, 2007, PLOS ONE, V2, DOI 10.1371/journal.pone.0001114
Wei SC, 2018, CANCER DISCOV, V8, P1069, DOI 10.1158/2159-8290.CD-18-0367
Weiner GJ, 2015, NAT REV CANCER, V15, P361, DOI 10.1038/nrc3930
Weinzierl AO, 2007, MOL CELL PROTEOMICS, V6, P102, DOI 10.1074/mcp.M600310-MCP200
Wemeau M, 2010, CELL DEATH DIS, V1, DOI 10.1038/cddis.2010.82
Wick DA, 2014, CLIN CANCER RES, V20, P1125, DOI 10.1158/1078-0432.CCR-13-2147
Wild D., 2013, IMMUNOASSAY HDB THEO
Williams DF, 2008, BIOMATERIALS, V29, P2941, DOI 10.1016/j.biomaterials.2008.04.023
WINTER SF, 1992, CANCER RES, V52, P4168
Wirth TC, 2017, FRONT IMMUNOL, V8, DOI 10.3389/fimmu.2017.01848
Witwer KW, 2013, J EXTRACELL VESICLES, V2, DOI 10.3402/jev.v2i0.20360
WOESSNER RD, 1991, CELL GROWTH DIFFER, V2, P209
Wolfers J, 2001, NAT MED, V7, P297, DOI 10.1038/85438
Wong C, 2011, P NATL ACAD SCI USA, V108, P2426, DOI 10.1073/pnas.1018382108
Wrangle JM, 2018, J INTERF CYTOK RES, V38, P45, DOI 10.1089/jir.2017.0101
Xia WJ, 2014, J THORAC DIS, V6, P1513, DOI 10.3978/j.issn.2072-1439.2014.08.17
Xie YJ, 2019, P NATL ACAD SCI USA, V116, P7624, DOI 10.1073/pnas.1817147116
Xu XY, 2010, SMALL, V6, P623, DOI [10.1002/Smll.200901691, 10.1002/smll.200901691]
Xue JW, 2017, NAT NANOTECHNOL, V12, P692, DOI [10.1038/nnano.2017.54, 10.1038/NNANO.2017.54]
Yadav M, 2014, NATURE, V515, P572, DOI 10.1038/nature14001
Yang B, 2014, HUM VACC IMMUNOTHER, V10, P3153, DOI 10.4161/21645515.2014.980686
Yang H, 2016, ONCOIMMUNOLOGY, V5, DOI 10.1080/2162402X.2015.1078061
Yang R, 2018, ACS NANO, V12, P5121, DOI 10.1021/acsnano.7b09041

Yarchoan M, 2017, NEW ENGL J MED, V377, P2500, DOI 10.1056/NEJMc1713444
Yarden Y, 2012, NAT REV CANCER, V12, P553, DOI 10.1038/nrc3309
Yeo RWY, 2013, ADV DRUG DELIVER REV, V65, P336, DOI 10.1016/j.addr.2012.07.001
Yu B, 2014, INT J MOL SCI, V15, P4142, DOI 10.3390/ijms15034142
Yu JS, 2004, CANCER RES, V64, P4973, DOI 10.1158/0008-5472.CAN-03-3505
Yu MK, 2012, THERANOSTICS, V2, P3, DOI 10.7150/thno.3463
Yu SJ, 2005, J AM CHEM SOC, V127, P17604, DOI 10.1021/ja0567081
Yuan HF, 2017, NAT NANOTECHNOL, V12, P763, DOI [10.1038/nnano.2017.69, 10.1038/NNANO.2017.69]
Yuan JD, 2011, P NATL ACAD SCI USA, V108, P16723, DOI 10.1073/pnas.1110814108
Zappa C, 2016, TRANSL LUNG CANCER R, V5, P288, DOI 10.21037/tlcr.2016.06.07
Zappasodi R, 2010, CANCER RES, V70, P9062, DOI 10.1158/0008-5472.CAN-10-1825
Zavazava N, 1996, NAT MED, V2, P1267
Zhang B, 2014, STEM CELLS DEV, V23, P1233, DOI 10.1089/scd.2013.0479
Zhang Jian-Ying, 2009, V520, P1, DOI 10.1007/978-1-60327-811-9_1
Zhang N, 2011, IMMUNITY, V35, P161, DOI 10.1016/j.immuni.2011.07.010
Zhang QW, 2012, BIOMATERIALS, V33, P5067, DOI 10.1016/j.biomaterials.2012.03.063
Zhang YC, 2018, NAT COMMUN, V9, DOI 10.1038/s41467-017-02522-z
Zhou SA, 2008, PHYS MEDICA, V24, P129, DOI 10.1016/j.ejmp.2008.05.006
Zhu GZ, 2017, ACS NANO, V11, P2387, DOI 10.1021/acsnano.7b00978
Zhu L, 2017, NANOMEDICINE-UK, V12, P73, DOI 10.2217/nnm-2016-0316
Zhu W, 2012, CANCER LETT, V315, P28, DOI 10.1016/j.canlet.2011.10.002
Zhuang J, 2019, THERANOSTICS, V9, P7826, DOI 10.7150/thno.37216
Zitvogel L, 1998, NAT MED, V4, P594, DOI 10.1038/nm0598-594
Zoller M, 2009, NAT REV CANCER, V9, P40, DOI 10.1038/nrc2543
Zou WP, 2016, SCI TRANSL MED, V8, DOI 10.1126/scitranslmed.aad7118
Zyuzin MV, 2017, BIOCONJUGATE CHEM, V28, P556, DOI 10.1021/acs.bioconjchem.6b00657
NR 385
TC 21
Z9 21
U1 6
U2 41
PU MDPI
PI BASEL
PA ST ALBAN-ANLAGE 66, CH-4052 BASEL, SWITZERLAND
EI 2079-4991
J9 NANOMATERIALS-BASEL
JI Nanomaterials
PD JUL
PY 2020
VL 10
IS 7
AR 1274
DI 10.3390/nano10071274
PG 72
WC Chemistry, Multidisciplinary; Nanoscience & Nanotechnology; Materials
Science, Multidisciplinary; Physics, Applied
WE Science Citation Index Expanded (SCI-EXPANDED)
SC Chemistry; Science & Technology - Other Topics; Materials Science;
Physics
GA MY4TI
UT WOS:000558411500001
PM 32610601
OA gold, Green Submitted, Green Published
DA 2023-09-14
ER

PT J
AU Zhang, Y
Liu, MD
Li, CX

Li, B
Zhang, XZ
AF Zhang, Yu
Liu, Miao-Deng
Li, Chu-Xin
Li, Bin
Zhang, Xian-Zheng
TI Tumor Cell Membrane-Coated Liquid Metal Nanovaccine for Tumor Prevention
SO CHINESE JOURNAL OF CHEMISTRY
LA English
DT Article
DE Nanovaccine; Liquid metal; Tumor cell membrane; Photothermal;
Immunotherapy
ID ANTIGENS; VACCINES
AB The of main observation and conclusion
Herein, we report on a tumor nanovaccine LMNP@CM that enhances the process of antigen-presenting by stimulating the immune system to uptake tumor antigens actively. The nanovaccine is comprised of polyethylene glycol modified liquid metal nanoparticles (LMNP) and tumor cell membranes (CM) as antigens. Under 808 nm irradiation, the photothermal conversion effect of injected LMNP can cause mild local inflammation, and subsequently induces antigen-presenting cells active recruitment and facilitates cellular uptake of tumor antigens. Meanwhile, because of the immune adjuvant effect of metal materials, the nanovaccine LMNP@CM promotes the maturation and activation of antigen-presenting cells and induces anti-tumor immune response effectively. By priming the host immune recognition of tumor antigens, this nanovaccine displays prophylactic effects and significantly suppresses tumor growth in a mouse breast tumor model. The nanovaccine LMNP@CM represents a novel strategy of utilizing light-controlled means to actively induce anti-tumor immune processes, showing advanced therapeutic potentials and robust adaptability for treating multiple tumors by changing the loaded antigens.
C1 [Zhang, Yu; Liu, Miao-Deng; Li, Chu-Xin; Li, Bin; Zhang, Xian-Zheng] Wuhan Univ, Minist Educ, Key Lab Biomed Polymers, Wuhan 430072, Hubei, Peoples R China.
[Zhang, Yu; Liu, Miao-Deng; Li, Chu-Xin; Li, Bin; Zhang, Xian-Zheng] Wuhan Univ, Dept Chem, Wuhan 430072, Hubei, Peoples R China.
C3 Wuhan University; Wuhan University
RP Zhang, XZ (通讯作者)，Wuhan Univ, Minist Educ, Key Lab Biomed Polymers, Wuhan 430072, Hubei, Peoples R China.
EM xz-zhang@whu.edu.cn
RI zhang, xian/GYA-0290-2022; Zhang, XZ/HJA-4189-2022
FU National Natural Science Foundation of China [51833007, 21721005, 51690152]
FX This work was financially supported by the National Natural Science Foundation of China (Nos. 51833007, 21721005 and 51690152).
CR Antonios D, 2009, TOXICOL IN VITRO, V23, P227, DOI 10.1016/j.tiv.2008.11.009
Aucouturier Jerome, 2002, Expert Rev Vaccines, V1, P111, DOI 10.1586/14760584.1.1.111
Bachmann MF, 2010, NAT REV IMMUNOL, V10, P787, DOI 10.1038/nri2868
Black M, 2012, ADV MATER, V24, P3845, DOI 10.1002/adma.201200209
Buonaguro L, 2011, CLIN VACCINE IMMUNOL, V18, P23, DOI 10.1128/CVI.00286-10
Chen Q, 2016, NAT COMMUN, V7, DOI 10.1038/ncomms13193
Conniot J, 2019, NAT NANOTECHNOL, V14, P891, DOI 10.1038/s41565-019-0512-0
Fritsch EF, 2014, CANCER IMMUNOL RES, V2, P522, DOI 10.1158/2326-6066.CIR-13-0227
Ghassemi S., 2017, HEATING CANC VACCINE, V2, P1
HogenEsch H, 2013, FRONT IMMUNOL, V3, DOI 10.3389/fimmu.2012.00406
Hu Z, 2018, NAT REV IMMUNOL, V18, P168, DOI 10.1038/nri.2017.131
Irvine DJ, 2013, NAT MATER, V12, P978, DOI [10.1038/NMAT3775, 10.1038/nmat3775]
Joffre OP, 2012, NAT REV IMMUNOL, V12, P557, DOI 10.1038/nri3254
Kourtis IC, 2013, PLOS ONE, V8, DOI 10.1371/journal.pone.0061646
Kroll AV, 2017, ADV MATER, V29, DOI 10.1002/adma.201703969
Luo M, 2017, NAT NANOTECHNOL, V12, P648, DOI [10.1038/nnano.2017.52, 10.1038/NNANO.2017.52]
Maisonneuve C, 2014, P NATL ACAD SCI USA, V111, P12294, DOI 10.1073/pnas.1400478111
Mellman I, 2001, CELL, V106, P255, DOI 10.1016/S0092-8674(01)00449-4
Novellino L, 2005, CANCER IMMUNOL IMMUN, V54, P187, DOI 10.1007/s00262-004-0560-6
Swartz MA, 2012, SCI TRANSL MED, V4, DOI 10.1126/scitranslmed.3003763

Xu ZH, 2014, ACS NANO, V8, P3636, DOI 10.1021/nn500216y
Yan JJ, 2019, NANO RES, V12, P1313, DOI 10.1007/s12274-018-2262-y
Yang R, 2018, ACS NANO, V12, P5121, DOI 10.1021/acsnano.7b09041
Zhang LP, 2019, ADV MATER, V31, DOI 10.1002/adma.201805252
NR 24
TC 21
Z9 21
U1 8
U2 86
PU WILEY-V C H VERLAG GMBH
PI WEINHEIM
PA POSTFACH 101161, 69451 WEINHEIM, GERMANY
SN 1001-604X
EI 1614-7065
J9 CHINESE J CHEM
JI Chin. J. Chem.
PD JUN
PY 2020
VL 38
IS 6
BP 595
EP 600
DI 10.1002/cjoc.201900557
EA APR 2020
PG 6
WC Chemistry, Multidisciplinary
WE Science Citation Index Expanded (SCI-EXPANDED)
SC Chemistry
GA LL5DP
UT WOS:000526160700001
DA 2023-09-14
ER

PT J
AU Zhu, B
Zhang, C
Zhao, Z
Wang, GX
AF Zhu, Bin
Zhang, Chen
Zhao, Zhao
Wang, Gao-Xue
TI Targeted Delivery of Mannosylated Nanoparticles Improve Prophylactic Efficacy of Immersion Vaccine against Fish Viral Disease
SO VACCINES
LA English
DT Article
DE targeted nanovaccine; mannose; carbon nanotubes; viral disease; immune response
ID WALLED CARBON NANOTUBES; GRASS CARP REOVIRUS; IMMUNE-RESPONSES; CANCER-CELLS; DNA VACCINE; IN-VITRO; RECEPTOR; ANTIGEN; VIRUS; OPTIMIZATION
AB Immersion vaccination is considered as the most effective method for juvenile fish in preventing viral disease, due to its convenience for mass vaccination and stress-free administration. However, immune responses following immersion vaccination are generally less robust and of shorter duration than those induced through intraperitoneal injection. Herein, to improve the efficacy of the immersion vaccine, we constructed a targeted single-walled carbon nanotubes-based immersion vaccine delivery system (CNTs-M-VP7), the surface of which are modified with mannose to allow antigen-presenting cells' (APCs) targeting. The targeting ability of CNTs-M-VP7 was confirmed in vivo and in vitro. Critically, this immersion CNTs-M-VP7 vaccine could cross into the fish body through mucosal tissues (skin, gill, and intestine), and then present to immune-related tissues. Moreover, CNTs-M-VP7 could significantly induce

the maturation and presenting process of APCs, which would then trigger robust immune responses. Altogether, this study demonstrates that the single-walled carbon nanotubes (SWCNTs)-based targeted nanovaccine delivery system shows the potential to be an effective prophylactic against fish viral disease.
C1 [Zhu, Bin; Zhang, Chen; Zhao, Zhao; Wang, Gao-Xue] Northwest A&F Univ, Coll Anim Sci & Technol, Dept Aquaculture, Yangling 712100, Shaanxi, Peoples R China.
C3 Northwest A&F University - China
RP Zhu, B (通讯作者)，Northwest A&F Univ, Coll Anim Sci & Technol, Dept Aquaculture, Yangling 712100, Shaanxi, Peoples R China.
EM zhubin@nwafu.edu.cn; bestzc@nwafu.edu.cn; zhaoz96122@nwafu.edu.cn;
wanggaoxue@126.com
RI zhao, zhao/JAC-1686-2023; ZHU, BIN/IYJ-3847-2023; Zhu, Bin/ABC-2439-2020
OI Wang, Gaoxue/0000-0001-9465-108X
FU National Natural Science Foundation of China [31602204]; Fundamental Research Funds for the Central Universities [2452017078]; Special Funds for Talents in Northwest A F University [Z111021510]
FX This work was supported by the National Natural Science Foundation of China (Program No. 31602204), the Fundamental Research Funds for the Central Universities (2452017078) and the Special Funds for Talents in Northwest A & F University to B. Zhu (Program No. Z111021510).
CR [Anonymous], 1981, FISH BIOL SERODIAGNO
Ashley CE, 2011, NAT MATER, V10, P389, DOI [10.1038/NMAT2992, 10.1038/nmat2992]
de Faria PCB, 2014, NANO LETT, V14, P5458, DOI 10.1021/nl502911a
Bedendo G, 2018, J FISH DIS, V41, P1063, DOI 10.1111/jfd.12797
BLACK JG, 1980, CAN J COMP MED, V44, P169
Carrillo-Conde B, 2011, MOL PHARMACEUT, V8, P1877, DOI 10.1021/mp200213r
Caruffo M, 2016, FISH SHELLFISH IMMUN, V54, P54, DOI 10.1016/j.fsi.2016.03.009
Christensen D, 2012, J CONTROL RELEASE, V160, P468, DOI 10.1016/j.jconrel.2012.03.016
Colic M, 2014, CARBON, V67, P273, DOI 10.1016/j.carbon.2013.09.090
Dineshkumar B, 2015, INDIAN J CANCER, V52, P262, DOI 10.4103/0019-509X.176720
Duffield JS, 2010, SEMIN NEPHROL, V30, P234, DOI 10.1016/j.semnephrol.2010.03.003
Dumortier H, 2006, NANO LETT, V6, P1522, DOI 10.1021/nl061160x
Embregts CWE, 2019, FISH SHELLFISH IMMUN, V85, P66, DOI 10.1016/j.fsi.2018.03.028
Fang Q, 2008, ARCH VIROL, V153, P675, DOI 10.1007/s00705-008-0048-3
Gottardi R, 2013, J NANOBIOTECHNOL, V11, DOI 10.1186/1477-3155-11-30
Guo Q, 2017, NANOSCALE, V9, P10832, DOI 10.1039/c7nr02682j
HARDONK MJ, 1992, J LEUKOCYTE BIOL, V52, P296, DOI 10.1002/jlb.52.3.296
Hassan HAFM, 2019, J CONTROL RELEASE, V297, P79, DOI 10.1016/j.jconrel.2019.01.017
HERNANDEZPANDO R, 1994, IMMUNOLOGY, V82, P591
Hoare R, 2017, VET RES, V48, DOI 10.1186/s13567-017-0448-z
Jechlinger W, 2006, EXPERT REV VACCINES, V5, P803, DOI 10.1586/14760584.5.6.803
Kafa H, 2015, BIOMATERIALS, V53, P437, DOI 10.1016/j.biomaterials.2015.02.083
Kobayashi N, 2011, INHAL TOXICOL, V23, P814, DOI 10.3109/08958378.2011.614968
Kreutz FT, 1997, CLIN CHEM, V43, P649
Liu WS, 2017, ARCH VIROL, V162, P2375, DOI 10.1007/s00705-017-3354-9
Montes-Fonseca SL, 2012, NEW BIOTECHNOL, V29, pS203, DOI 10.1016/j.nbt.2012.08.571
Mishra PK, 2009, DRUG DELIV, V16, P437, DOI 10.3109/10717540903271391
Mulvey JJ, 2013, NAT NANOTECHNOL, V8, P763, DOI 10.1038/nnano.2013.190
Pikarsky E, 2004, J VIROL, V78, P9544, DOI 10.1128/JVI.78.17.9544-9551.2004
Plant KP, 2011, DEV COMP IMMUNOL, V35, P1256, DOI 10.1016/j.dci.2011.03.007
Rastogi V, 2014, J DRUG DELIV, V2014, DOI 10.1155/2014/670815
Ratput MKS, 2018, ACS APPL MATER INTER, V10, P27589, DOI 10.1021/acsami.8b02019
Reczek D, 2007, CELL, V131, P770, DOI 10.1016/j.cell.2007.10.018
Rombout JHWM, 2014, FISH SHELLFISH IMMUN, V40, P634, DOI 10.1016/j.fsi.2014.08.020
Sallusto F, 2010, IMMUNITY, V33, P451, DOI 10.1016/j.immuni.2010.10.008
Sansonetti PJ, 2000, IMMUNITY, V12, P581, DOI 10.1016/S1074-7613(00)80209-5
Shi X., 2010, J BIOMED MATER RES A, V86, P813
Skinner LA, 2010, FISH SHELLFISH IMMUN, V28, P579, DOI 10.1016/j.fsi.2009.12.017
Smart SK, 2006, CARBON, V44, P1034, DOI 10.1016/j.carbon.2005.10.011
Su H, 2017, ONCOTARGET, V8, P86576, DOI 10.18632/oncotarget.21245
Sudheesh PS, 2016, FISH SHELLFISH IMMUN, V56, P169, DOI 10.1016/j.fsi.2016.07.004
Tunuguntla RH, 2017, SCIENCE, V357, P792, DOI 10.1126/science.aan2438

Valladao GMR, 2017, AQUAC RES, V48, P5640, DOI 10.1111/are.13386
van Hateren Andy, 2017, F1000Res, V6, P158, DOI 10.12688/f1000research.10474.1
Vono M, 2017, BLOOD, V129, P1991, DOI 10.1182/blood-2016-10-744441
Wang L, 2014, DEV COMP IMMUNOL, V43, P54, DOI 10.1016/j.dci.2013.10.006
Wang Y, 2011, POLYM CHEM-UK, V2, P1754, DOI 10.1039/c1py00179e
Warheit DB, 2004, TOXICOL SCI, V77, P117, DOI 10.1093/toxsci/kfg228
Wu HX, 2014, BIOMATERIALS, V35, P5369, DOI 10.1016/j.biomaterials.2014.03.038
Xu TJ, 2018, J VIROL, V92, DOI 10.1128/JVI.01594-17
Xu Z, 2016, NAT COMMUN, V7, DOI 10.1038/ncomms10728
Xu Z, 2013, P NATL ACAD SCI USA, V110, P13097, DOI 10.1073/pnas.1304319110
Yang R, 2018, ACS NANO, V12, P5121, DOI 10.1021/acsnano.7b09041
Ye YQ, 2017, SCI IMMUNOL, V2, DOI 10.1126/sciimmunol.aan5692
Yin F, 2015, FISH SHELLFISH IMMUN, V47, P344, DOI 10.1016/j.fsi.2015.09.013
Zarghami N, 1997, CLIN CHEM, V43, P1862
Zhang C, 2018, VACCINE, V36, P6334, DOI 10.1016/j.vaccine.2018.08.003
Zhang C, 2017, FISH SHELLFISH IMMUN, V71, P191, DOI 10.1016/j.fsi.2017.10.012
Zhao YC, 2012, FISH SHELLFISH IMMUN, V32, P750, DOI 10.1016/j.fsi.2012.01.027
Zhu B, 2015, FISH SHELLFISH IMMUN, V42, P325, DOI 10.1016/j.fsi.2014.11.026
Zhu B, 2014, FISH SHELLFISH IMMUN, V41, P279, DOI 10.1016/j.fsi.2014.09.014
NR 61
TC 21
Z9 21
U1 4
U2 21
PU MDPI
PI BASEL
PA ST ALBAN-ANLAGE 66, CH-4052 BASEL, SWITZERLAND
EI 2076-393X
J9 VACCINES-BASEL
JI Vaccines
PD MAR
PY 2020
VL 8
IS 1
AR 87
DI 10.3390/vaccines8010087
PG 17
WC Immunology; Medicine, Research & Experimental
WE Science Citation Index Expanded (SCI-EXPANDED)
SC Immunology; Research & Experimental Medicine
GA LI4EV
UT WOS:000529439800086
PM 32075291
OA Green Published, gold
DA 2023-09-14
ER

PT J
AU Liu, XX
Liu, JL
Liu, D
Han, YF
Xu, HY
Liu, LX
Leng, XG
Kong, DL
AF Liu, Xiaoxuan
Liu, Jiale
Liu, Dan
Han, Yanfeng
Xu, Haiyan

Liu, Lanxia
Leng, Xigang
Kong, Deling
TI A cell-penetrating peptide-assisted nanovaccine promotes antigen cross-presentation and anti-tumor immune response
SO BIOMATERIALS SCIENCE
LA English
DT Article
ID DELIVERY; NANOPARTICLES; VACCINES; MECHANISMS; CHALLENGES; KINETICS; RELEASE; BIOLOGY
AB Exogenous antigens processed in the cytosol and subsequently cross-presented on major histocompatibility complex class I (MHC-I) molecules activate cytotoxic CD8(+) lymphocytes (CTL), which are crucial in cancer immunotherapy. Here, we reported a nanovaccine, which was produced by encapsulating OVA (ovalbumin, a model antigen) chemically modified with MPG(Delta NLS) (MPG(Delta NLS)-OVA conjugate) into poly(lactide-co-glycolide) acid (PLGA) nanoparticles. We hypothesized that after the uptake of the nanovaccine into immune cells, MPG(Delta NLS), a cell-penetrating peptide (CPP), would assist the escape of the antigens from lysosomes into the cytosol, increase the amount of antigens processed in the cytosol and subsequently enhance antigen cross-presentation via MHC-I molecules to elicit cytotoxic CD8(+) T cell responses. The results of the in vitro experiments demonstrated that the MPG(Delta NLS)-OVA-loaded PLGA NPs not only elevated the release of OVA into the cytosol of dendritic cells (BMDCs), but also promoted the maturation and activation of BMDCs. It was also observed in mice vaccinated with MPG(Delta NLS)-OVA-loaded PLGA NPs that the MPG(Delta NLS) modification could stimulate the expansion of OVA-specific T-cells, generation of OVA-specific IgG antibodies and proliferation of OVA-specific memory T cells. Moreover, the treatment of E.G7-OVA tumor-bearing mice with MPG(Delta NLS)-OVA-loaded PLGA NPs resulted in significantly suppressed tumor growth and prolonged survival periods of the mice compared to the treatment with unmodified OVA-PLGA NPs or free OVA. In summary, cell-penetrating peptides linked with antigens encapsulated in nanovaccines can spatiotemporally affect the intracellular localization of antigens, promote antigen cross-presentation and stimulate antigen-specific immune responses, especially CTL responses. Therefore, the CPP modification on antigens is an innovative approach to enhance the efficacy of nanovaccines for cancer immunotherapy.
C1 [Liu, Xiaoxuan; Liu, Jiale; Liu, Dan; Liu, Lanxia; Leng, Xigang] Peking Union Med Coll, Inst Biomed Engn, Tianjin Key Lab Biomat, Tianjin 300192, Peoples R China.
[Liu, Xiaoxuan; Liu, Jiale; Liu, Dan; Liu, Lanxia; Leng, Xigang] Chinese Acad Med Sci, Tianjin 300192, Peoples R China.
[Han, Yanfeng] Univ Queensland, Sch Biomed Sci, St Lucia, Qld 4072, Australia.
[Xu, Haiyan] Chinese Acad Med Sci, Inst Basic Med Sci, Sch Basic Med, Peking Union Med Coll, Beijing 100730, Peoples R China.
[Kong, Deling] Nankai Univ, Key Lab Bioact Mat, State Key Lab Med Chem Biol, Minist Educ,Coll Life Sci, Tianjin 300071, Peoples R China.
[Kong, Deling] Xuzhou Med Univ, Jiangsu Ctr Collaborat & Innovat Canc Biotherapy, Canc Inst, Xuzhou 221004, Jiangsu, Peoples R China.
C3 Chinese Academy of Medical Sciences - Peking Union Medical College; Peking Union Medical College; Institute of BioMedical Engineering - CAMS; Chinese Academy of Medical Sciences - Peking Union Medical College; University of Queensland; Institute of Basic Medical Sciences - CAMS; Chinese Academy of Medical Sciences - Peking Union Medical College; Peking Union Medical College; Nankai University; Xuzhou Medical University
RP Liu, LX; Leng, XG (通讯作者)，Peking Union Med Coll, Inst Biomed Engn, Tianjin Key Lab Biomat, Tianjin 300192, Peoples R China.
EM liulanxiabme@163.com
RI liu, jiale/HTO-3772-2023
OI Han, Yanfeng/0000-0001-8423-0409
FU Natural Science Foundation of China [31670948, 31870920]; Tianjin Natural Science Foundation [15JCYBJC47600]; CAMS Innovation Fund for Medical Sciences [CAMS-I2M-3-004]
FX This work was supported by the Natural Science Foundation of China (No. 31670948 and 31870920), the Tianjin Natural Science Foundation project (No. 15JCYBJC47600), and the CAMS Innovation Fund for Medical Sciences (CAMS-I2M-3-004).

CR Banchereau J, 1998, NATURE, V392, P245, DOI 10.1038/32588
Burgdorf S, 2008, CURR OPIN IMMUNOL, V20, P89, DOI 10.1016/j.coi.2007.12.002
Constant SL, 1997, ANNU REV IMMUNOL, V15, P297, DOI 10.1146/annurev.immunol.15.1.297
De Gregorio E, 2012, MICROB BIOTECHNOL, V5, P149, DOI 10.1111/j.1751-7915.2011.00276.x
Delany I, 2014, EMBO MOL MED, V6, P708, DOI 10.1002/emmm.201403876
Demento SL, 2012, BIOMATERIALS, V33, P4957, DOI 10.1016/j.biomaterials.2012.03.041
Endoh T, 2009, ADV DRUG DELIVER REV, V61, P704, DOI 10.1016/j.addr.2009.04.005
Erazo-Oliveras A, 2012, PHARMACEUTICALS-BASE, V5, P1177, DOI 10.3390/ph5111177
Esser MT, 2003, VACCINE, V21, P419, DOI 10.1016/S0264-410X(02)00407-3
Fahmy TM, 2008, NANOMEDICINE-UK, V3, P343, DOI 10.2217/17435889.3.3.343
Fan YC, 2015, J CONTROL RELEASE, V208, P121, DOI 10.1016/j.jconrel.2015.04.010
Foged C, 2012, EUR J PHARM SCI, V45, P482, DOI 10.1016/j.ejps.2011.08.016
Gao YH, 2012, MACROMOL BIOSCI, V12, P251, DOI 10.1002/mabi.201100208
Heit A, 2007, EUR J IMMUNOL, V37, P2063, DOI 10.1002/eji.200737169
Joffre OP, 2012, NAT REV IMMUNOL, V12, P557, DOI 10.1038/nri3254
Jusforgues-Saklani H, 2008, J IMMUNOL, V181, P3067, DOI 10.4049/jimmunol.181.5.3067
Keller S, 2014, J CONTROL RELEASE, V191, P24, DOI 10.1016/j.jconrel.2014.03.041
Kristensen M, 2016, INT J MOL SCI, V17, DOI 10.3390/ijms17020185
Kurrikoff K, 2016, EXPERT OPIN DRUG DEL, V13, P373, DOI 10.1517/17425247.2016.1125879
Lin ML, 2008, IMMUNOL CELL BIOL, V86, P353, DOI 10.1038/icb.2008.3
Liu JL, 2018, ACS APPL MATER INTER, V10, P30983, DOI 10.1021/acsami.8b09348
Liu LX, 2016, ACS APPL MATER INTER, V8, P11969, DOI 10.1021/acsami.6b01135
Liu LX, 2016, J CONTROL RELEASE, V225, P230, DOI 10.1016/j.jconrel.2016.01.050
Liu Q, 2015, ACS NANO, V9, P4925, DOI 10.1021/nn5066793
Lucey DR, 1996, CLIN MICROBIOL REV, V9, P532, DOI 10.1128/CMR.9.4.532
Lundberg P, 2007, FASEB J, V21, P2664, DOI 10.1096/fj.06-6502com
Mager I, 2010, BBA-BIOMEMBRANES, V1798, P338, DOI 10.1016/j.bbamem.2009.11.001
Melief CJM, 2015, J CLIN INVEST, V125, P3401, DOI 10.1172/JCI80009
Morris MC, 1997, NUCLEIC ACIDS RES, V25, P2730, DOI 10.1093/nar/25.14.2730
Radovic-Moreno AF, 2015, P NATL ACAD SCI USA, V112, P3892, DOI 10.1073/pnas.1502850112
Ruiz-de-Angulo A, 2016, ACS NANO, V10, P1602, DOI 10.1021/acsnano.5b07253
Shah MAA, 2014, J BIOMED NANOTECHNOL, V10, P2332, DOI 10.1166/jbn.2014.1981
Shedlock DJ, 2003, SCIENCE, V300, P337, DOI 10.1126/science.1082305
Skotland T, 2015, MOLECULES, V20, P13313, DOI 10.3390/molecules200713313
Smith JD, 2015, CURR OPIN BIOTECH, V34, P217, DOI 10.1016/j.copbio.2015.03.014
Trinchieri G, 2003, NAT REV IMMUNOL, V3, P133, DOI 10.1038/nri1001
Ulmer JB, 2006, NAT BIOTECHNOL, V24, P1377, DOI 10.1038/nbt1261
Wang Z, 2015, EUR J PHARM BIOPHARM, V90, P53, DOI 10.1016/j.ejpb.2014.11.002
Woof JM, 2005, SCIENCE, V310, P1442, DOI 10.1126/science.1122009
Zhang WF, 2014, BIOMATERIALS, V35, P6086, DOI 10.1016/j.biomaterials.2014.04.022
Zhang ZP, 2011, BIOMATERIALS, V32, P3666, DOI 10.1016/j.biomaterials.2011.01.067
Zorko M, 2005, ADV DRUG DELIVER REV, V57, P529, DOI 10.1016/j.addr.2004.10.010
NR 42
TC 21
Z9 21
U1 4
U2 73
PU ROYAL SOC CHEMISTRY
PI CAMBRIDGE
PA THOMAS GRAHAM HOUSE, SCIENCE PARK, MILTON RD, CAMBRIDGE CB4 0WF, CAMBS, ENGLAND
SN 2047-4830
EI 2047-4849
J9 BIOMATER SCI-UK
JI Biomater. Sci.
PD DEC 1
PY 2019
VL 7
IS 12
BP 5516
EP 5527

DI 10.1039/c9bm01183h
PG 12
WC Materials Science, Biomaterials
WE Science Citation Index Expanded (SCI-EXPANDED)
SC Materials Science
GA JY2KQ
UT WOS:000504250100050
PM 31670734
DA 2023-09-14
ER

PT J
AU Wang, N
Yang, Y
Wang, XL
Tian, XX
Qin, WJ
Wang, XX
Liang, JY
Zhang, HL
Leng, XG
AF Wang, Ning
Yang, Ying
Wang, Xiaoli
Tian, Xinxin
Qin, Wenjuan
Wang, Xiaoxiao
Liang, Jiayi
Zhang, Hailing
Leng, Xigang
TI Polydopamine as the Antigen Delivery Nanocarrier for Enhanced Immune Response in Tumor Immunotherapy
SO ACS BIOMATERIALS SCIENCE & ENGINEERING
LA English
DT Article
DE cancer immunotherapy; polydopamine; nanoparticles; colon cancer
ID TARGETING DENDRITIC CELLS; T-CELLS; INTERFERON-GAMMA; LYMPH-NODE; NANOPARTICLES; DOPAMINE; SYSTEM; NANOVACCINE; VACCINES; TRAFFICKING
AB This study aimed to investigate the efficacy of polydopamine nanoparticles (Pdop-NPs) as a subcutaneous antigen delivery vehicle in antitumor therapy. The nanoparticles were prepared by self-polymerization of dopamine in an aerobic and weak alkaline solution, and the tumor model antigen-ovalbumin (OVA) was grafted onto the nanoparticles to form OVA@Pdop nanoparticles (OVA@Pdop-NPs). The particle size of OVA@Pdop-NPs was 232.8 nm with a zeta potential of -23.4 mV, and the loading capacity of OVA protein was 754 mu g mg(-1). OVA@Pdop-NPs were essentially noncytotoxic and even demonstrated a slightly viability effect on bone-marrow-derived dendritic cells (BMDCs). As compared to free OVA, OVA@Pdop-NPs exhibited higher cellular uptake and were easier to migrate to lymph nodes in vivo. Both in vitro and in vivo experiments showed that OVA@ Pdop-NPs promoted the maturation of DCs with up-regulated expression of major histocompatibility complex (MHC), costimulatory molecules, and cytokines. When used to treat the mice bearing OVA-MC38 colon tumor, OVA@Pdop-NPs could effectively activate OVA-specific cytotoxic CD8(+) T cells and induce the production of memory CD4(+) and CD8(+) T cells and thus led to significantly suppressed tumor growth. All the preliminary data demonstrated the application potential of OVA@PdopNPs as a vaccine vector in cancer immunotherapy.
C1 [Zhang, Hailing; Leng, Xigang] Chinese Acad Med Sci, Inst Biomed Engn, Tianjin Key Lab Biomat, Tianjin 300192, Peoples R China.
Peking Union Med Coll, Tianjin 300192, Peoples R China.
C3 Chinese Academy of Medical Sciences - Peking Union Medical College; Institute of BioMedical Engineering - CAMS; Chinese Academy of Medical Sciences - Peking Union Medical College; Peking Union Medical College
RP Zhang, HL; Leng, XG (通讯作者)，Chinese Acad Med Sci, Inst Biomed Engn, Tianjin Key Lab Biomat, Tianjin 300192, Peoples R China.
EM zhlyky@163.com; lengxgyky@163.com

RI wang, xiao/HGB-7081-2022; wang, jiahui/IXD-1197-2023
OI wang, xiao/0000-0002-4088-3341; Zhang, Hailing/0000-0001-6050-0697
FU National Natural Science Foundation of China [31300794, 31600743]; Natural Science Foundation of Tianjin City [16JCZDJC35800, 16JCQNJC13900]; Tianjin Innovation and Promotion Plan Key Innovation Team of Immunoreactive Biomaterials
FX The authors are particularly grateful to the National Natural Science Foundation of China (31300794, 31600743), Natural Science Foundation of Tianjin City (16JCZDJC35800, 16JCQNJC13900) and Tianjin Innovation and Promotion Plan Key Innovation Team of Immunoreactive Biomaterials for the financial support.
CR Azuma M, 2016, ONCOIMMUNOLOGY, V5, DOI 10.1080/2162402X.2016.1188244
Beaulieu JM, 2011, PHARMACOL REV, V63, P182, DOI 10.1124/pr.110.002642
Blanc C, 2018, FRONT IMMUNOL, V9, DOI 10.3389/fimmu.2018.01722
Cao FQ, 2018, ADV HEALTHC MATER, V7, DOI 10.1002/adhm.201701439
Cao XO, 2016, CURR BIOL, V26, P2329, DOI 10.1016/j.cub.2016.06.036
Chakroborty D, 2009, CANCER RES, V69, P3727, DOI 10.1158/0008-5472.CAN-08-4289
Chang TZ, 2017, BIOMATER SCI-UK, V5, P223, DOI 10.1039/c6bm00500d
Cosentino M, 2007, BLOOD, V109, P632, DOI 10.1182/blood-2006-01-028423
Cruz LJ, 2010, J CONTROL RELEASE, V144, P118, DOI 10.1016/j.jconrel.2010.02.013
Deng JZ, 2012, BIOMACROMOLECULES, V13, P3795, DOI 10.1021/bm3012538
Georgoudaki AM, 2016, CELL REP, V15, P2000, DOI 10.1016/j.celrep.2016.04.084
Ham B, 2015, CANCER RES, V75, P5235, DOI 10.1158/0008-5472.CAN-14-3173
Hong S, 2011, NANOMEDICINE-UK, V6, P793, DOI [10.2217/nnm.11.76, 10.2217/NNM.11.76]
Hornung V, 2008, NAT IMMUNOL, V9, P847, DOI 10.1038/ni.1631
Hughes R, 2015, CANCER RES, V75, P3479, DOI 10.1158/0008-5472.CAN-14-3587
Jia JL, 2017, J MATER CHEM B, V5, P1611, DOI 10.1039/c6tb02845d
Kammertoens T, 2017, NATURE, V545, P98, DOI 10.1038/nature22311
Kaplanski G, 2003, TRENDS IMMUNOL, V24, P25, DOI 10.1016/S1471-4906(02)00013-3
Keller S, 2014, J CONTROL RELEASE, V191, P24, DOI 10.1016/j.jconrel.2014.03.041
Klippstein R, 2010, NANOMED-NANOTECHNOL, V6, P523, DOI 10.1016/j.nano.2010.01.001
Knee DA, 2016, EUR J CANCER, V67, P1, DOI 10.1016/j.ejca.2016.06.028
Knocke S, 2016, CELL REP, V17, P2234, DOI 10.1016/j.celrep.2016.10.086
Lamalle-Bernard D, 2006, J CONTROL RELEASE, V115, P57, DOI 10.1016/j.jconrel.2006.07.006
Lemke CD, 2013, BIOMATERIALS, V34, P2524, DOI 10.1016/j.biomaterials.2012.12.030
Levite M, 2016, ACTA PHYSIOL, V216, P42, DOI 10.1111/apha.12476
Levite M, 2017, J NEURAL TRANSM, V124, P525, DOI 10.1007/s00702-016-1640-4
Liu LX, 2016, ACS APPL MATER INTER, V8, P11969, DOI 10.1021/acsami.6b01135
Liu LX, 2016, J CONTROL RELEASE, V225, P230, DOI 10.1016/j.jconrel.2016.01.050
Liu Q, 2016, SMALL, V12, P1744, DOI 10.1002/smll.201503662
Liu XS, 2013, ACS NANO, V7, P9384, DOI 10.1021/nn404117j
Luo M, 2017, NAT NANOTECHNOL, V12, P648, DOI [10.1038/nnano.2017.52, 10.1038/NNANO.2017.52]
Lv HT, 2006, J CONTROL RELEASE, V114, P100, DOI 10.1016/j.jconrel.2006.04.014
Manolova V, 2008, EUR J IMMUNOL, V38, P1404, DOI 10.1002/eji.200737984
Mardiana S, 2017, CANCER RES, V77, P1296, DOI 10.1158/0008-5472.CAN-16-1831
Miao ZH, 2015, ACS APPL MATER INTER, V7, P16946, DOI 10.1021/acsami.5b06265
Moon JJ, 2011, NAT MATER, V10, P243, DOI 10.1038/NMAT2960
Oda Y, 2012, J CONTROL RELEASE, V160, P362, DOI 10.1016/j.jconrel.2011.12.003
Qian Y, 2016, BIOMATERIALS, V98, P171, DOI 10.1016/j.biomaterials.2016.05.008
Roberts EW, 2016, CANCER CELL, V30, P324, DOI 10.1016/j.ccell.2016.06.003
Rock KL, 2005, IMMUNOL REV, V207, P166, DOI 10.1111/j.0105-2896.2005.00301.x
Saei A, 2014, IRAN J ALLERGY ASTHM, V13, P1
Cordeiro AS, 2015, BIOTECHNOL ADV, V33, P1279, DOI 10.1016/j.biotechadv.2015.05.010
Sarkar C, 2008, CLIN CANCER RES, V14, P2502, DOI 10.1158/1078-0432.CCR-07-1778
Sayour E.J., 2017, ONCOIMMUNOL, V6
Sekiya T, 2017, BIOMATERIALS, V137, P61, DOI 10.1016/j.biomaterials.2017.05.018
Shi GN, 2017, BIOMATERIALS, V113, P191, DOI 10.1016/j.biomaterials.2016.10.047
Silva JM, 2013, J CONTROL RELEASE, V168, P179, DOI 10.1016/j.jconrel.2013.03.010
Sojka DK, 2011, P NATL ACAD SCI USA, V108, P18336, DOI 10.1073/pnas.1110566108
Tanaka T, 2014, CSH PERSPECT BIOL, V6, DOI 10.1101/cshperspect.a016295

Thomas SN, 2014, BIOMATERIALS, V35, P814, DOI 10.1016/j.biomaterials.2013.10.003
Wang S, 2018, SMALL, V14, DOI 10.1002/smll.201704272
Wang YQ, 2014, ADV HEALTHC MATER, V3, P670, DOI 10.1002/adhm.201300335
Wen M, 2018, ACS MACRO LETT, V7, P70, DOI 10.1021/acsmacrolett.7b00837
Wu J, 2015, J LEUKOCYTE BIOL, V97, P191, DOI 10.1189/jlb.5A1113-626RR
Yin ZN, 2019, INT J CANCER, V144, P933, DOI 10.1002/ijc.31744
Zaidi MR, 2011, CLIN CANCER RES, V17, P6118, DOI 10.1158/1078-0432.CCR-11-0482
Zhang CN, 2017, MOL PHARMACEUT, V14, P1760, DOI 10.1021/acs.molpharmaceut.7b00015
Zhang XY, 2012, NANOSCALE, V4, P5581, DOI 10.1039/c2nr31281f
Zhang ZP, 2011, BIOMATERIALS, V32, P3666, DOI 10.1016/j.biomaterials.2011.01.067
Zhao Z, 2017, BIOMATERIALS, V123, P107, DOI 10.1016/j.biomaterials.2017.01.038
Zhu GZ, 2017, ACS NANO, V11, P2387, DOI 10.1021/acsnano.7b00978
Zou AH, 2018, J AGR FOOD CHEM, V66, P6262, DOI 10.1021/acs.jafc.8b00877
2004, EXPERT OPIN BIOL TH, V4, P1735, DOI DOI 10.1517/14712598.4.11.1735
2017, J CONTROL RELEASE, V256, P170, DOI DOI 10.1016/J.JCONREL.2017.04.020
NR 64
TC 21
Z9 21
U1 4
U2 89
PU AMER CHEMICAL SOC
PI WASHINGTON
PA 1155 16TH ST, NW, WASHINGTON, DC 20036 USA
SN 2373-9878
J9 ACS BIOMATER SCI ENG
JI ACS Biomater. Sci. Eng.
PD MAY
PY 2019
VL 5
IS 5
BP 2330
EP 2342
DI 10.1021/acsbiomaterials.9b00359
PG 25
WC Materials Science, Biomaterials
WE Science Citation Index Expanded (SCI-EXPANDED)
SC Materials Science
GA HY4TE
UT WOS:000468120000025
PM 33405783
DA 2023-09-14
ER

PT J
AU Mohr, N
Kappel, C
Kramer, S
Bros, M
Grabbe, S
Zentel, R
AF Mohr, Nicole
Kappel, Cinja
Kramer, Stefan
Bros, Matthias
Grabbe, Stephan
Zentel, Rudolf
TI Targeting cells of the immune system: mannosylated HPMA-LMA
block-copolymer micelles for targeting of dendritic cells
SO NANOMEDICINE
LA English
DT Article

DE dendritic cell; HPMA block copolymer; immune system; mannose as targeting ligand
ID LECTIN RECEPTORS; DRUG-DELIVERY; IN-VIVO; POLYMERS; NANOPARTICLES; ANTIGEN; HEMAGGLUTINATION; POLYMERIZATION; GLYCOPOLYMERS; ENDOCYTOSIS
AB Background: Successful tumor immunotherapy depends on the induction of strong and sustained tumor antigen-specific immune responses by activated antigen-presenting cells (APCs) such as dendritic cells (DCs). Since nanoparticles have the potential to codeliver tumor-specific antigen and DC-stimulating adjuvant in a DC-targeting manner, we wanted to assess the suitability of mannosylated HPMA-LMA block polymers for immunotherapy. Materials & methods: Fluorescence-labeled block copolymer micelles derived from P(HPMA)-block-P(LMA) copolymers and according statistical copolymers were synthesized via RAFT polymerization, and loaded with the APC activator L18-MDP. Both types of copolymers were conjugated with D-mannose to target the mannose receptor as expressed by DCs and macrophages. The extent and specificity of micelle binding and activation of APCs was monitored using mouse spleen cells and bone marrow-derived DC (BMDC). Results: Nontargeting HPMA-LMA statistical copolymers showed strong unspecific cell binding. HPMA-LMA block copolymers bound DC only when conjugated with mannose, and in a mannose receptor-specific manner. Mannosylated HPMA-LMA block copolymers were internalized by DC. DC-targeting HPMA-LMA block copolymers mediated DC activation when loaded with L18-MDP. Conclusion: Mannosylated HPMA-LMA block copolymers are a promising candidate for the delvopment of DC-targeting nanovaccines.
C1 [Mohr, Nicole; Kramer, Stefan; Zentel, Rudolf] Johannes Gutenberg Univ Mainz, Inst Organ Chem, Duesbergweg 10-14, D-55099 Mainz, Germany.
[Kappel, Cinja; Bros, Matthias; Grabbe, Stephan] Johannes Gutenberg Univ Mainz, Univ Med Ctr, Dept Dermatol, Obere Zahlbacher Str 63, D-55131 Mainz, Germany.
C3 Johannes Gutenberg University of Mainz; Johannes Gutenberg University of Mainz
RP Zentel, R (通讯作者)，Johannes Gutenberg Univ Mainz, Inst Organ Chem, Duesbergweg 10-14, D-55099 Mainz, Germany.; Bros, M; Grabbe, S (通讯作者)，Johannes Gutenberg Univ Mainz, Univ Med Ctr, Dept Dermatol, Obere Zahlbacher Str 63, D-55131 Mainz, Germany.
EM mbros@uni-mainz.de; stephan.grabbe@unimedizin-mainz.de; zentel@uni-mainz.de
RI Zentel, Rudolf/D-4542-2011; Grabe, Stephan/AAJ-4578-2021
OI Grabe, Stephan/0000-0002-6863-8719
FU DFG [SFB 1066]
FX The authors acknowledge funding by the SFB 1066 (DFG), especially projects B4 and B5. The authors have no other relevant affiliations or financial involvement with any organization or entity with a financial interest in or financial conflict with the subject matter or materials discussed in the manuscript apart from those disclosed.
CR Allmeroth M, 2013, J CONTROL RELEASE, V172, P77, DOI 10.1016/j.jconrel.2013.07.027
Allmeroth M, 2011, BIOMACROMOLECULES, V12, P2841, DOI 10.1021/bm2005774
[Anonymous], 2014, J APPL MATH
Balkow S, 2007, BLOOD, V110, P3949, DOI 10.1182/blood-2007-05-092189
Barz M, 2008, BIOMACROMOLECULES, V9, P3114, DOI 10.1021/bm800684b
Barz M, 2009, BIOMATERIALS, V30, P5682, DOI 10.1016/j.biomaterials.2009.06.058
Brown J, 2011, J DENT RES, V90, P417, DOI 10.1177/0022034510381264
Cai H, ANGEW CHEM INT ED EN, V52, P6106
Carrillo-Conde B, 2011, MOL PHARMACEUT, V8, P1877, DOI 10.1021/mp200213r
Chavez-Santoscoy AV, 2012, BIOMATERIALS, V33, P4762, DOI 10.1016/j.biomaterials.2012.03.027
Cui J, 2014, HUM VACC IMMUNOTHER, V10, P3270, DOI 10.4161/21645515.2014.979640
Cureton DK, 2009, PLOS PATHOG, V5, DOI 10.1371/journal.ppat.1000394
Duncan R, 2003, NAT REV DRUG DISCOV, V2, P347, DOI 10.1038/nrd1088
Duncan R, 1988, J BIOACT COMPAT POL, V3, P4, DOI 10.1177/088391158800300102
Duncan R, 2010, ADV DRUG DELIVER REV, V62, P272, DOI 10.1016/j.addr.2009.12.005
Eberhardt M, 2005, EUR POLYM J, V41, P1569, DOI 10.1016/j.eurpolymj.2005.01.025
Ercolani G, 2011, ANGEW CHEM INT EDIT, V50, P1762, DOI 10.1002/anie.201004201
Frenz T, 2015, EUR J PHARM BIOPHARM, V95, P13, DOI 10.1016/j.ejpb.2015.02.008
Fukuda I, 2015, BIOMED RES INT, V2015, DOI 10.1155/2015/350580
Gestwicki JE, 2002, J AM CHEM SOC, V124, P14922, DOI 10.1021/ja027184x
Gi M, 2009, SENSORS-BASEL, V9, P6730, DOI 10.3390/s90906730
Gibson MI, 2009, J POLYM SCI POL CHEM, V47, P4332, DOI 10.1002/pola.23486
Gorden KB, 2005, J IMMUNOL, V174, P1259, DOI 10.4049/jimmunol.174.3.1259

GUNTHER W, 1992, CARBOHYD RES, V228, P217, DOI 10.1016/S0008-6215(00)90561-5
Hartmann S, 2015, ADV HEALTHC MATER, V4, P522, DOI 10.1002/adhm.201400460
Hemmelmann M, 2013, MOL PHARMACEUT, V10, P3769, DOI 10.1021/mp400254b
Hemmelmann M, 2011, MACROMOL RAPID COMM, V32, P712, DOI 10.1002/marc.201000810
Irache JM, 2008, EXPERT OPIN DRUG DEL, V5, P703, DOI [10.1517/17425247.5.6.703, 10.1517/17425247.5.6.703 ]
Karlsen K, 2014, NANOMEDICINE-UK, V9, P2625, DOI [10.2217/NNM.14.197, 10.2217/nnm.14.197]
Kiessling LL, 2006, ANGEW CHEM INT EDIT, V45, P2348, DOI 10.1002/anie.200502794
Kopecek J, 2010, ADV DRUG DELIVER REV, V62, P122, DOI 10.1016/j.addr.2009.10.004
Kramer JR, 2014, POLYM CHEM-UK, V5, P671, DOI 10.1039/c3py01081c
Krummen M, 2010, J LEUKOCYTE BIOL, V88, P189, DOI 10.1189/jlb.0408228
Lepeltier EA, 2015, NANOMEDICINE-UK, V10, P3147, DOI 10.2217/nnm.15.132
Li HY, 2002, MED RES REV, V22, P225, DOI 10.1002/med.10008
Lundquist JJ, 2002, CHEM REV, V102, P555, DOI 10.1021/cr000418f
Mai YY, 2012, CHEM SOC REV, V41, P5969, DOI 10.1039/c2cs35115c
MAMMEN M, 1995, J MED CHEM, V38, P4179, DOI 10.1021/jm00021a007
Martinez-Pomares L, 2012, J LEUKOCYTE BIOL, V92, P1177, DOI 10.1189/jlb.0512231
Moad G, 2008, POLYMER, V49, P1079, DOI 10.1016/j.polymer.2007.11.020
Mohr N, 2014, MACROMOL RAPID COMM, V35, P1522, DOI 10.1002/marc.201400249
Nobrega C, 2003, TETRAHEDRON-ASYMMETR, V14, P2793, DOI 10.1016/S0957-4166(03)00623-2
Nuhn L, 2014, MACROMOL BIOSCI, V14, P607, DOI 10.1002/mabi.201400028
Nuhn L, 2013, ANGEW CHEM INT EDIT, V52, P10652, DOI 10.1002/anie.201304212
Papp I, 2011, CHEMBIOCHEM, V12, P1075, DOI 10.1002/cbic.201000718
Pavot V, 2014, J IMMUNOL, V193, P5781, DOI 10.4049/jimmunol.1402184
Pavot V, 2013, J CONTROL RELEASE, V167, P60, DOI 10.1016/j.jconrel.2013.01.015
Pearce EJ, 2006, CHEM IMMUNOL ALLERGY, V90, P82, DOI 10.1159/000088882
Pelkmans L, 2001, NAT CELL BIOL, V3, P473, DOI 10.1038/35074539
Ponader D, 2012, BIOMACROMOLECULES, V13, P1845, DOI 10.1021/bm300331z
Qian XP, 2002, ANAL CHEM, V74, P1805, DOI 10.1021/ac011042o
Qian ZM, 2002, PHARMACOL REV, V54, P561, DOI 10.1124/pr.54.4.561
Rahimian S, 2015, J CONTROL RELEASE, V203, P16, DOI 10.1016/j.jconrel.2015.02.006
Rauen J, 2014, PLOS ONE, V9, DOI 10.1371/journal.pone.0103755
Reddy ST, 2007, NAT BIOTECHNOL, V25, P1159, DOI [10.1038/nbt1332, 10.1038/nbtl332]
Reddy ST, 2006, J CONTROL RELEASE, V112, P26, DOI 10.1016/j.jconrel.2006.01.006
SALLUSTO F, 1995, J EXP MED, V182, P389, DOI 10.1084/jem.182.2.389
Sato T, 2011, INNATE IMMUN-LONDON, V17, P54, DOI 10.1177/1753425909351904
Schraml BU, 2015, CURR OPIN IMMUNOL, V32, P13, DOI 10.1016/j.coi.2014.11.001
Shannahan Jonathan H, 2015, Receptors Clin Investig, V2, pe811
Shao K, 2015, ACS NANO, V9, P16, DOI 10.1021/nn5062029
Spain SG, 2011, POLYM CHEM-UK, V2, P60, DOI 10.1039/c0py00149j
SPARKS MA, 1993, J MED CHEM, V36, P778, DOI 10.1021/jm00058a016
Tada H, 2005, INFECT IMMUN, V73, P7967, DOI 10.1128/IAI.73.12.7967-7976.2005
Talelli M, 2015, NANO TODAY, V10, P93, DOI 10.1016/j.nantod.2015.01.005
Tappertzhofen K, 2014, MACROMOL BIOSCI, V14, P1444, DOI 10.1002/mabi.201400190
Ting SRS, 2010, POLYM CHEM-UK, V1, P1392, DOI 10.1039/c0py00141d
Tong C, 2016, SCAND J IMMUNOL, V83, P10, DOI 10.1111/sji.12369
Toth I, 2014, NANOMEDICINE-UK, V9, P2607, DOI [10.2217/NNM.14.199, 10.2217/nnm.14.199]
Van Parijs L, 1998, SCIENCE, V280, P243
Weilbacher M, 2013, J BIOMED NANOTECHNOL, V9, P1
WILLNER D, 1993, BIOCONJUGATE CHEM, V4, P521, DOI 10.1021/bc00024a015
Xie HZ, 2005, CHEM BIOL, V12, P109, DOI 10.1016/j.chembiol.2004.11.013
Zaric M, 2015, J INVEST DERMATOL, V135, P425, DOI 10.1038/jid.2014.415
Zhang J, 2000, POLYM J, V32, P694, DOI 10.1295/polymj.32.694
NR 75
TC 21
Z9 22
U1 4
U2 60
PU FUTURE MEDICINE LTD
PI LONDON
PA UNITEC HOUSE, 3RD FLOOR, 2 ALBERT PLACE, FINCHLEY CENTRAL, LONDON, N3

1QB, ENGLAND
SN 1743-5889
EI 1748-6963
J9 NANOMEDICINE-UK
JI Nanomedicine
PD OCT
PY 2016
VL 11
IS 20
BP 2679
EP 2697
DI 10.2217/nnm-2016-0167
PG 19
WC Biotechnology & Applied Microbiology; Nanoscience & Nanotechnology
WE Science Citation Index Expanded (SCI-EXPANDED)
SC Biotechnology & Applied Microbiology; Science & Technology - Other
Topics
GA DZ2EM
UT WOS:000385655100007
PM 27628185
DA 2023-09-14
ER

PT J
AU Sun, ZY
Chen, PG
Liu, YF
Zhang, BD
Wu, JJ
Chen, YX
Zhao, YF
Li, YM
AF Sun, Z. Y.
Chen, P. G.
Liu, Y. F.
Zhang, B. D.
Wu, J. J.
Chen, Y. X.
Zhao, Y. F.
Li, Y. M.
TI Multi-component self-assembled anti-tumor nano-vaccines based on MUC1
glycopeptides
SO CHEMICAL COMMUNICATIONS
LA English
DT Article
ID SYNTHETIC VACCINES; CELL EPITOPES; TUMOR-CELLS; IMMUNE; ANTIGEN; CANCER;
THERAPY; MUCINS
AB Novel multi-component self-assembled nano-vaccines containing both Pam(3)CSK(4) and CpG were developed for the first time. These multi-component vaccines could effectively activate the macrophages in vitro and elicit strong antibody immune responses and anti-tumor immune responses in vivo.
C1 [Sun, Z. Y.; Chen, P. G.; Liu, Y. F.; Zhang, B. D.; Wu, J. J.; Chen, Y. X.; Zhao, Y. F.; Li, Y. M.] Tsinghua Univ, Dept Chem, Minist Educ, Key Lab Bioorgan Phosphorus Chem & Chem Biol, Beijing 100084, Peoples R China.
C3 Tsinghua University
RP Li, YM (通讯作者)，Tsinghua Univ, Dept Chem, Minist Educ, Key Lab Bioorgan Phosphorus Chem & Chem Biol, Beijing 100084, Peoples R China.
EM liym@mail.tsinghua.edu.cn
RI li, yong/HDN-3885-2022; CHEN, YONG-XIANG/D-4792-2017
OI CHEN, YONG-XIANG/0000-0003-3518-0139; Wu, Jun-Jun/0000-0001-6113-8976;
Li, Yan-Mei/0000-0003-1215-5010
FU Major State Basic Research Development Program of China [2013CB910700,

2012CB821600]; National Natural Science Foundation of China [21332006]
FX This research was supported by the Major State Basic Research Development Program of China (2013CB910700 and 2012CB821600) and the National Natural Science Foundation of China (21332006).
CR Abdel-Aal AM, 2014, CHEMBIOCHEM, V15, P1508, DOI 10.1002/cbic.201402077
Buskas T, 2009, CHEM COMMUN, P5335, DOI 10.1039/b908664c
Cai H, 2014, ANGEW CHEM INT EDIT, V53, P1699, DOI 10.1002/anie.201308875
Cai H, 2013, ANGEW CHEM INT EDIT, V52, P6106, DOI 10.1002/anie.201300390
Cai H, 2013, CHEM-EUR J, V19, P1962, DOI 10.1002/chem.201203709
Cai H, 2012, ANGEW CHEM INT EDIT, V51, P1719, DOI 10.1002/anie.201106396
Cai H, 2011, CHEM-EUR J, V17, P6396, DOI 10.1002/chem.201100217
Diehl SA, 2012, IMMUNOL CELL BIOL, V90, P802, DOI 10.1038/icb.2012.17
Dziadek S, 2005, ANGEW CHEM INT EDIT, V44, P7624, DOI 10.1002/anie.200501593
Gao Y, 2014, CHEM-EUR J, V20, P13541, DOI 10.1002/chem.201404013
Hartmann S, 2015, ADV HEALTHC MATER, V4, P522, DOI 10.1002/adhm.201400460
Hoffmann-Roder A, 2010, ANGEW CHEM INT EDIT, V49, P8498, DOI 10.1002/anie.201003810
Hoffmann-Roder A, 2011, CHEM COMMUN, V47, P9903, DOI 10.1039/c1cc13184b
Hoffmann-Roder A, 2011, CHEM COMMUN, V47, P382, DOI 10.1039/c0cc02250k
Hollingsworth MA, 2004, NAT REV CANCER, V4, P45, DOI 10.1038/nrc1251
Huang ZH, 2012, J AM CHEM SOC, V134, P8730, DOI 10.1021/ja211725s
Ingale S, 2007, NAT CHEM BIOL, V3, P663, DOI 10.1038/nchembio.2007.25
Jones LH, 2015, NAT CHEM, V7, P952, DOI [10.1038/NCHEM.2396, 10.1038/nchem.2396]
Kornbluth RS, 2006, J LEUKOCYTE BIOL, V80, P1084, DOI 10.1189/jlb.0306147
Kufe DW, 2009, NAT REV CANCER, V9, P874, DOI 10.1038/nrc2761
Lakshminarayanan V, 2012, P NATL ACAD SCI USA, V109, P261, DOI 10.1073/pnas.1115166109
Liu YF, 2015, BIOCONJUGATE CHEM, V26, P1439, DOI 10.1021/acs.bioconjchem.5b00150
Mancini RJ, 2014, ANGEW CHEM INT EDIT, V53, P189, DOI 10.1002/anie.201306551
Palitzsch B, 2014, ANGEW CHEM INT EDIT, V53, P14245, DOI 10.1002/anie.201406843
Pavot V, 2014, J IMMUNOL, V193, P5781, DOI 10.4049/jimmunol.1402184
Schulz EG, 2009, IMMUNITY, V30, P673, DOI 10.1016/j.immuni.2009.03.013
Skwarczynski M, 2016, CHEM SCI, V7, P842, DOI 10.1039/c5sc03892h
Thompson P, 2015, CHEM COMMUN, V51, P10214, DOI 10.1039/c5cc02199e
Wilkinson BL, 2011, ANGEW CHEM INT EDIT, V50, P1635, DOI 10.1002/anie.201006115
Wilkinson BL, 2010, CHEM COMMUN, V46, P6249, DOI 10.1039/c0cc01360a
Wilson R. M., 2012, J AM CHEM SOC, V134, P8730
NR 31
TC 21
Z9 21
U1 2
U2 49
PU ROYAL SOC CHEMISTRY
PI CAMBRIDGE
PA THOMAS GRAHAM HOUSE, SCIENCE PARK, MILTON RD, CAMBRIDGE CB4 0WF, CAMBS, ENGLAND
SN 1359-7345
EI 1364-548X
J9 CHEM COMMUN
JI Chem. Commun.
PY 2016
VL 52
IS 48
BP 7572
EP 7575
DI 10.1039/c6cc02000c
PG 4
WC Chemistry, Multidisciplinary
WE Science Citation Index Expanded (SCI-EXPANDED)
SC Chemistry
GA DP1IP
UT WOS:000378244000010
PM 27216415

DA 2023-09-14
ER

PT J
AU Zuo, BF
Zhang, Y
Zhao, KJ
Wu, L
Qi, H
Yang, R
Gao, XJ
Geng, MY
Wu, YJ
Jing, RW
Zhou, QB
Seow, YQ
Yin, HF
AF Zuo, Bingfeng
Zhang, Yang
Zhao, Kangjie
Wu, Li
Qi, Han
Yang, Rong
Gao, Xianjun
Geng, Mengyuan
Wu, Yingjie
Jing, Renwei
Zhou, Qibing
Seow, Yiqi
Yin, HaiFang
TI Universal immunotherapeutic strategy for hepatocellular carcinoma with exosome vaccines that engage adaptive and innate immune responses
SO JOURNAL OF HEMATOLOGY & ONCOLOGY
LA English
DT Article
DE Exosome; Hepatocellular carcinoma; Personalized immunotherapy; Adaptive and innate immunity
ID DENDRITIC CELLS; CROSS-PRESENTATION; ANTIGEN; CANCER; MELANOMA; SUBSETS; THERAPY; MEMORY; FLT3L
AB Background Personalized immunotherapy utilizing cancer vaccines tailored to the tumors of individual patients holds promise for tumors with high genetic heterogeneity, potentially enabling eradication of the tumor in its entirety. Methods Here, we demonstrate a general strategy for biological nanovaccines that trigger tailored tumor-specific immune responses for hepatocellular carcinoma (HCC). Dendritic cell (DC)-derived exosomes (DEX) are painted with a HCC-targeting peptide (P47-P), an alpha-fetoprotein epitope (AFP212-A2) and a functional domain of high mobility group nucleosome-binding protein 1 (N1ND-N), an immunoadjuvant for DC recruitment and activation, via an exosomal anchor peptide to form a "trigger" DEX vaccine (DEXP&A2&N). Results DEXP&A2&N specifically promoted recruitment, accumulation and activation of DCs in mice with orthotopic HCC tumor, resulting in enhanced cross-presentation of tumor neoantigens and de novo T cell response. DEXP&A2&N elicited significant tumor retardation and tumor-specific immune responses in HCC mice with large tumor burdens. Importantly, tumor eradication was achieved in orthotopic HCC mice when antigenic AFP peptide was replaced with the full-length AFP (A) to form DEXP&A&N. Supplementation of Fms-related tyrosine kinase 3 ligand greatly augmented the antitumor immunity of DEXP&A&N by increasing immunological memory against tumor re-challenge in orthotopic HCC mice. Depletion of T cells, cross-presenting DCs and other innate immune cells abrogated the functionality of DEXP&A&N. Conclusions These findings demonstrate the capacity of universal DEX vaccines to induce tumor-specific immune responses by triggering an immune response tailored to the tumors of each individual, thus presenting a generalizable approach for personalized immunotherapy of HCC, by extension of other tumors, without the need to identify tumor antigens.
C1 [Zuo, Bingfeng; Zhang, Yang; Zhao, Kangjie; Wu, Li; Qi, Han; Gao, Xianjun; Geng, Mengyuan; Wu, Yingjie; Jing, Renwei; Yin, HaiFang] Tianjin Med Univ, Prov & Minist Cosponsored Collaborat Innovat Ctr, Qixiangtai Rd, Tianjin 300070, Peoples R China.

[Zuo, Bingfeng; Zhang, Yang; Zhao, Kangjie; Wu, Li; Qi, Han; Gao, Xianjun; Geng, Mengyuan; Wu, Yingjie; Jing, Renwei; Yin, HaiFang] Tianjin Med Univ, Key Lab Immune Microenvironm & Dis, Minist Educ, Qixiangtai Rd, Tianjin 300070, Peoples R China.
[Zuo, Bingfeng; Zhang, Yang; Zhao, Kangjie; Wu, Li; Qi, Han; Gao, Xianjun; Geng, Mengyuan; Wu, Yingjie; Jing, Renwei; Yin, HaiFang] Tianjin Med Univ, Sch Med Technol, Qixiangtai Rd, Tianjin 300070, Peoples R China.
[Zuo, Bingfeng; Zhang, Yang; Zhao, Kangjie; Wu, Li; Qi, Han; Gao, Xianjun; Geng, Mengyuan; Wu, Yingjie; Jing, Renwei; Yin, HaiFang] Tianjin Med Univ, Sch Basic Med Sci, Qixiangtai Rd, Tianjin 300070, Peoples R China.
[Yang, Rong; Zhou, Qibing] Huazhong Univ Sci & Technol, Natl Engn Res Ctr Nanomed, Dept Nanomed & Biopharmaceut, Wuhan 430074, Hubei, Peoples R China.
[Seow, Yiqi] Inst Bioengn & Bioimaging, 31 Biopolis Way, Singapore 138669, Singapore.
[Seow, Yiqi] Inst Mol & Cell Biol, 61 Biopolis Way, Singapore 138668, Singapore.
C3 Tianjin Medical University; Tianjin Medical University; Tianjin Medical University; Tianjin Medical University; Huazhong University of Science & Technology; Agency for Science Technology & Research (A*STAR); A*STAR - Institute of Bioengineering & Bioimaging (IBB); Agency for Science Technology & Research (A*STAR); A*STAR - Institute of Molecular & Cell Biology (IMCB)
RP Yin, HF (通讯作者), Tianjin Med Univ, Prov & Minist Cosponsored Collaborat Innovat Ctr, Qixiangtai Rd, Tianjin 300070, Peoples R China.; Yin, HF (通讯作者), Tianjin Med Univ, Key Lab Immune Microenvironm & Dis, Minist Educ, Qixiangtai Rd, Tianjin 300070, Peoples R China.; Yin, HF (通讯作者), Tianjin Med Univ, Sch Med Technol, Qixiangtai Rd, Tianjin 300070, Peoples R China.; Yin, HF (通讯作者), Tianjin Med Univ, Sch Basic Med Sci, Qixiangtai Rd, Tianjin 300070, Peoples R China.
EM haifangyin@tmu.edu.cn
OI Seow, Yiqi/0000-0001-7040-3006
FU National Natural Science Foundation of China [82030054, 81803416]; Tianjin Municipal 13th five-year plan (Tianjin Medical University Talent Project)
FX This study was supported by the National Natural Science Foundation of China (Nos. 82030054 and 81803416) and Tianjin Municipal 13th five-year plan (Tianjin Medical University Talent Project).
CR Anandasabapathy N, 2015, BONE MARROW TRANSPL, V50, P924, DOI 10.1038/bmt.2015.74
Aslan C, 2021, BMC BIOTECHNOL, V21, DOI 10.1186/s12896-021-00683-w
Badovinac VP, 2005, NAT MED, V11, P748, DOI 10.1038/nm1257
Balan S, 2020, CANCER CELL, V37, P751, DOI 10.1016/j.ccell.2020.05.013
Bhardwaj N, 2020, NAT CANCER, V1, P1204, DOI 10.1038/s43018-020-00143-y
Blass E, 2021, NAT REV CLIN ONCOL, V18, P215, DOI 10.1038/s41571-020-00460-2
Cheng AL, 2020, J HEPATOL, V72, P307, DOI 10.1016/j.jhep.2019.09.025
Chiang MC, 2016, EUR J IMMUNOL, V46, P329, DOI 10.1002/eji.201546023
de Vries IJM, 2003, CANCER RES, V63, P12
Dunn GP, 2006, NAT REV IMMUNOL, V6, P836, DOI 10.1038/nri1961
Edelson BT, 2010, J EXP MED, V207, P823, DOI 10.1084/jem.20091627
Gao XJ, 2018, SCI TRANSL MED, V10, DOI 10.1126/scitranslmed.aat0195
Gardner A, 2016, TRENDS IMMUNOL, V37, P855, DOI 10.1016/j.it.2016.09.006
Habif G, 2019, CELL MOL IMMUNOL, V16, P415, DOI 10.1038/s41423-019-0224-2
Hammerich L, 2019, NAT MED, V25, P814, DOI 10.1038/s41591-019-0410-x
Hildner K, 2008, SCIENCE, V322, P1097, DOI 10.1126/science.1164206
Hong Y, 2014, HEPATOLOGY, V59, P1448, DOI 10.1002/hep.26893
Iwama T, 2013, INT J ONCOL, V42, P831, DOI 10.3892/ijo.2013.1793
Jing RW, 2018, HEPATOLOGY, V68, P1391, DOI 10.1002/hep.29829
Joffre OP, 2012, NAT REV IMMUNOL, V12, P557, DOI 10.1038/nri3254
Johnston MP, 2019, WORLD J GASTROENTERO, V25, P2977, DOI 10.3748/wjg.v25.i24.2977
Kreiter S, 2015, NATURE, V520, P692, DOI 10.1038/nature14426
Lai JY, 2020, NAT IMMUNOL, V21, P914, DOI 10.1038/s41590-020-0676-7
Li AW, 2018, NAT MATER, V17, P528, DOI 10.1038/s41563-018-0028-2
Liu K, 2010, IMMUNOL REV, V234, P45, DOI 10.1111/j.0105-2896.2009.00879.x
Loffler MW, 2019, GENOME MED, V11, DOI 10.1186/s13073-019-0636-8
Lotvall J, 2014, J EXTRACELL VESICLES, V3, DOI 10.3402/jev.v3.26913
Lu LG, 2021, HEPATOLOGY, V73, P414, DOI 10.1002/hep.31279
Lu Z, 2017, J HEPATOL, V67, P739, DOI 10.1016/j.jhep.2017.05.019

Marzagalli M, 2019, SEMIN CANCER BIOL, V59, P236, DOI 10.1016/j.semcancer.2019.08.002
Merad M, 2013, ANNU REV IMMUNOL, V31, P563, DOI 10.1146/annurev-immunol-020711-074950
Moynihan KD, 2016, NAT MED, V22, P1402, DOI 10.1038/nm.4200
Nikfarjam S, 2020, J EXP CLIN CANC RES, V39, DOI 10.1186/s13046-020-01781-x
Ott PA, 2020, CELL, V183, P347, DOI 10.1016/j.cell.2020.08.053
Pinter M, 2021, JAMA ONCOL, V7, P113, DOI 10.1001/jamaoncol.2020.3381
Rao Q, 2016, HEPATOLOGY, V64, P456, DOI 10.1002/hep.28549
Roberts EW, 2016, CANCER CELL, V30, P324, DOI 10.1016/j.ccell.2016.06.003
Sallusto F, 2004, ANNU REV IMMUNOL, V22, P745, DOI 10.1146/annurev.immunol.22.012703.104702
Salmon H, 2016, IMMUNITY, V44, P924, DOI 10.1016/j.immuni.2016.03.012
Schmid MA, 2010, IMMUNOL REV, V234, P32, DOI 10.1111/j.0105-2896.2009.00877.x
Shen ZH, 1997, J IMMUNOL, V158, P2723
Viaud S, 2011, J IMMUNOTHER, V34, P65, DOI 10.1097/CJI.0b013e3181fe535b
Vitale M, 2014, EUR J IMMUNOL, V44, P1582, DOI 10.1002/eji.201344272
Vivier E, 2018, CELL, V174, P1054, DOI 10.1016/j.cell.2018.07.017
Weigel BJ, 2002, BLOOD, V100, P4169, DOI 10.1182/blood-2002-04-1063
Xu WQ, 2019, THER ADV MED ONCOL, V11, DOI 10.1177/1758835919862692
Xu ZJ, 2020, MOL CANCER, V19, DOI 10.1186/s12943-020-01278-3
Yang D, 2012, J EXP MED, V209, P157, DOI 10.1084/jem.20101354
Zhu GZ, 2017, NAT COMMUN, V8, DOI 10.1038/s41467-017-02191-y
Zhu RX, 2016, GUT LIVER, V10, P332, DOI 10.5009/gnl15257
Zuo BF, 2020, NAT COMMUN, V11, DOI 10.1038/s41467-020-15569-2
NR 51
TC 20
Z9 23
U1 10
U2 39
PU BMC
PI LONDON
PA CAMPUS, 4 CRINAN ST, LONDON N1 9XW, ENGLAND
EI 1756-8722
J9 J HEMATOL ONCOL
JI J. Hematol. Oncol.
PD APR 29
PY 2022
VL 15
IS 1
AR 46
DI 10.1186/s13045-022-01266-8
PG 21
WC Oncology; Hematology
WE Science Citation Index Expanded (SCI-EXPANDED)
SC Oncology; Hematology
GA 0W2TV
UT WOS:000788886800002
PM 35488312
OA gold, Green Published
DA 2023-09-14
ER

PT J
AU Li, JX
Ren, H
Zhang, YM
AF Li, Jiexin
Ren, He
Zhang, Yumiao
TI Metal-based nano-vaccines for cancer immunotherapy
SO COORDINATION CHEMISTRY REVIEWS
LA English
DT Review

DE Metal-based biomaterials; Vaccines; Metalloimmunotherapy; Cancer
treatments
ID NLRP3 INFLAMMASOME ACTIVATION; TUMOR-ASSOCIATED MACROPHAGES; ENHANCED
PHOTODYNAMIC THERAPY; IRON-OXIDE NANOPARTICLES; MONOPHOSPHORYL-LIPID-A;
CGAS-STING PATHWAY; CELL LUNG-CANCER; T-CELL; GOLD NANOPARTICLES;
DENDRITIC CELLS
AB Cancer vaccination is an effective way to prevent or cure cancers by inducing strong cellular and humoral immunity with the aid of delivery carriers or adjuvants. Increasing new delivery vehicles and vaccine adjuvants have been developed for immunotherapy as they can enhance immune responses with reduced doses and side effects. In spite of many advantages of cancer immunotherapy, the clinical outcomes of cancer vaccines are still not satisfactory due to insufficient induction of antigen specific T cells. Metalloimmunology may revitalize cancer vaccines and improve their clinical applications considering that nutritional metal ions such as Ca2+, K+, Fe2+/3+, Zn2+, Mn2+ play important roles in many key immune processes. As good adjuvants, a wide variety of metals can be incorporated in the formulations of nano-vaccines for the enhanced immunity. Metal-based nanomaterials can also efficiently deliver antigens to antigen-presenting cells for the generation of immunity. In this review, we comprehensively summarized different types of commonly-used metal nanomaterials, as adjuvants or delivery carriers, for the development of effective cancer vaccines. Other applications such as use as contrast agents for bioimaging were also briefly reviewed. Then, their applications for cancer immunotherapy, along with their action mechanisms were systematically discussed. (C)& nbsp;2021 Elsevier B.V. All rights reserved.
C1 [Li, Jiexin; Ren, He; Zhang, Yumiao] Tianjin Univ, Sch Chem Engn & Technol, Key Lab Syst Bioengn, Minist Educ, Tianjin 300350, Peoples R China.
C3 Tianjin University
RP Zhang, YM (通讯作者)，Tianjin Univ, Sch Chem Engn & Technol, Key Lab Syst Bioengn, Minist Educ, Tianjin 300350, Peoples R China.
EM ymzhang88@tju.edu.cn
OI Zhang, Yumiao/0000-0002-6166-0470
FU National Key Research and Development Program [2021YFC2102300]; National
Natural Science Foundation of China [32071384]; Tianjin University;
One-thousand Young Talent Program of China
FX This work was supported by the National Key Research and Development
Program (2021YFC2102300) , National Natural Science Foundation of China
(32071384) , Start-up Grant at Tianjin University and One-thousand Young
Talent Program of China.
CR Abkar M, 2019, IMMUNOL LETT, V207, P28, DOI 10.1016/j.imlet.2019.01.010
Adler LN, 2017, FRONT IMMUNOL, V8, DOI 10.3389/fimmu.2017.00319
Afroz S, 2017, NANOSCALE, V9, P14641, DOI 10.1039/c7nr03697c
Aguilar JC, 2007, VACCINE, V25, P3752, DOI 10.1016/j.vaccine.2007.01.111
Ahn S, 2014, ADV HEALTHC MATER, V3, P1194, DOI 10.1002/adhm.201300597
Ai XZ, 2018, BIOCONJUGATE CHEM, V29, P928, DOI 10.1021/acs.bioconjchem.8b00068
Akagi T, 2007, BIOMATERIALS, V28, P3427, DOI 10.1016/j.biomaterials.2007.04.023
Akram MW, 2018, J NANOMATER, V2018, DOI 10.1155/2018/4210920
Aldous AR, 2018, BIOORGAN MED CHEM, V26, P2842, DOI 10.1016/j.bmc.2017.10.021
Allavena P, 2004, CRIT REV IMMUNOL, V24, P179, DOI 10.1615/CritRevImmunol.v24.i3.20
Almeida JPM, 2015, SMALL, V11, P1453, DOI 10.1002/smll.201402179
Angelova N, 2017, COLLOID SURFACE A, V535, P184, DOI 10.1016/j.colsurfa.2017.09.043
Antoine TE, 2016, J IMMUNOL, V196, P4566, DOI 10.4049/jimmunol.1502373
Arnau J, 2006, PROTEIN EXPRES PURIF, V48, P1, DOI 10.1016/j.pep.2005.12.002
Bachmann MF, 2010, NAT REV IMMUNOL, V10, P787, DOI 10.1038/nri2868
Bansal V, 2015, VACCINE, V33, P5623, DOI 10.1016/j.vaccine.2015.08.076
Batista-Duharte A, 2014, INT IMMUNOPHARMACOL, V20, P170, DOI 10.1016/j.intimp.2014.02.033
Baylor NW, 2002, VACCINE, V20, pS18, DOI 10.1016/S0264-410X(02)00166-4
BECK L, 1989, CELL IMMUNOL, V123, P1, DOI 10.1016/0008-8749(89)90263-3
Behera T, 2011, CELL IMMUNOL, V271, P350, DOI 10.1016/j.cellimm.2011.07.015
Beran J, 2008, EXPERT OPIN BIOL TH, V8, P235, DOI [10.1517/14712598.8.2.235, 10.1517/14712598.8.2.235 ]
Bessman NJ, 2020, SCIENCE, V368, P186, DOI 10.1126/science.aau6481
Bhakta NR, 2005, NAT IMMUNOL, V6, P143, DOI 10.1038/ni1161
Bilyy R, 2019, MATER TODAY, V22, P58, DOI 10.1016/j.mattod.2018.10.034
Blum JS, 2013, ANNU REV IMMUNOL, V31, P443, DOI 10.1146/annurev-immunol-032712-095910
Bonaventura P, 2015, AUTOIMMUN REV, V14, P277, DOI 10.1016/j.autrev.2014.11.008

Bondy SC, 2014, TOXICOLOGY, V315, P1, DOI 10.1016/j.tox.2013.10.008
Bose T, 2015, CELL DEATH DIS, V6, DOI 10.1038/cddis.2015.23
Bray F, 2018, CA-CANCER J CLIN, V68, P394, DOI 10.3322/caac.21492
Brieger A, 2013, J IMMUNOL, V191, P1808, DOI 10.4049/jimmunol.1301261
Burin AM, 2019, POULTRY SCI, V98, P2160, DOI 10.3382/ps/pey570
Butts C, 2011, J CANCER RES CLIN, V137, P1337, DOI 10.1007/s00432-011-1003-3
Cai H, 2016, BIOORGAN MED CHEM, V24, P1132, DOI 10.1016/j.bmc.2016.01.044
Calderon-Gonzalez R, 2017, NANOSCALE, V9, P10721, DOI 10.1039/c7nr02494k
Cao FQ, 2018, ADV HEALTHC MATER, V7, DOI 10.1002/adhm.201701439
Chaigne-Delalande B, 2013, SCIENCE, V341, P186, DOI 10.1126/science.1240094
Chandy KG, 2016, NATURE, V537, P497, DOI 10.1038/nature19467
Chang CC, 2020, ACS APPL MATER INTER, V12, P44407, DOI 10.1021/acsami.0c08473
Chang H, 2013, EXP TOXICOL PATHOL, V65, P887, DOI 10.1016/j.etp.2013.01.001
Chang MY, 2020, ADV MATER, V32, DOI 10.1002/adma.202004647
Chen SM, 2013, PLOS ONE, V8, DOI 10.1371/journal.pone.0082561
Chen WY, 2016, SMALL, V12, P1627, DOI 10.1002/smll.201503359
Chen YL, 2018, ACS APPL MATER INTER, V10, P43533, DOI 10.1021/acsami.8b18164
Chen Y, 2014, ADV MATER, V26, P7019, DOI 10.1002/adma.201402572
Chen Y, 2012, BIOMATERIALS, V33, P7126, DOI 10.1016/j.biomaterials.2012.06.059
Chen YC, 2014, CYTOTHERAPY, V16, P699, DOI 10.1016/j.jcyt.2013.09.006
Cho NH, 2011, NAT NANOTECHNOL, V6, P675, DOI [10.1038/NNANO.2011.149, 10.1038/nnano.2011.149]
Choi JY, 2008, CARCINOGENESIS, V29, P964, DOI 10.1093/carcin/bgn056
Coffman RL, 2010, IMMUNITY, V33, P492, DOI 10.1016/j.immuni.2010.10.002
Coley W B, 1891, Ann Surg, V14, P199, DOI 10.1097/00000658-189112000-00015
Corrales L, 2015, CELL REP, V11, P1018, DOI 10.1016/j.celrep.2015.04.031
CROMME FV, 1994, J EXP MED, V179, P335, DOI 10.1084/jem.179.1.335
Crowe WE, 2004, AM J PHYSIOL-CELL PH, V287, pC1023, DOI 10.1152/ajpcell.00511.2003
Cruz LJ, 2011, MOL PHARMACEUT, V8, P520, DOI 10.1021/mp100356k
Didierlaurent AM, 2009, J IMMUNOL, V183, P6186, DOI 10.4049/jimmunol.0901474
Ding BB, 2021, J MATER CHEM B, V9, P7117, DOI 10.1039/d1tb01001h
Ding BB, 2020, ANGEW CHEM INT EDIT, V59, P16381, DOI 10.1002/anie.202005111
Dobrovolskaia MA, 2007, NAT NANOTECHNOL, V2, P469, DOI 10.1038/nnano.2007.223
Dong H, 2018, INT J NANOMED, V13, P3353, DOI 10.2147/IJN.S164097
Du MJ, 2018, SCIENCE, V361, P704, DOI 10.1126/science.aat1022
Duan F, 2017, BIOMATERIALS, V122, P23, DOI 10.1016/j.biomaterials.2017.01.017
Duan QQ, 2020, TRENDS CANCER, V6, P605, DOI 10.1016/j.trecan.2020.02.022
Dunn LL, 2007, TRENDS CELL BIOL, V17, P93, DOI 10.1016/j.tcb.2006.12.003
Eisenbarth SC, 2019, NAT REV IMMUNOL, V19, P89, DOI 10.1038/s41577-018-0088-1
Elliott EI, 2015, IMMUNOL REV, V265, P35, DOI 10.1111/imr.12286
Embgenbroich M, 2018, FRONT IMMUNOL, V9, DOI 10.3389/fimmu.2018.01643
Exley C, 2010, TRENDS IMMUNOL, V31, P103, DOI 10.1016/j.it.2009.12.009
Fan HH, 2016, ANGEW CHEM INT EDIT, V55, P5477, DOI 10.1002/anie.201510748
Fan M, 2021, ADV FUNCT MATER, V31, DOI 10.1002/adfm.202010587
Feske S, 2001, NAT IMMUNOL, V2, P316, DOI 10.1038/86318
Flach TL, 2011, NAT MED, V17, P479, DOI 10.1038/nm.2306
Flood BA, 2019, IMMUNOL REV, V290, P24, DOI 10.1111/imr.12765
Gao M, 2021, ADV THER-GERMANY, V4, DOI 10.1002/adtp.202100065
Gao P, 2013, CELL, V153, P1094, DOI 10.1016/j.cell.2013.04.046
Gardai SJ, 2005, CELL, V123, P321, DOI 10.1016/j.cell.2005.08.032
Gerner MY, 2017, J EXP MED, V214, P3105, DOI 10.1084/jem.20170335
Glenny AT, 1926, J PATHOL BACTERIOL, V29, P31, DOI 10.1002/path.1700290106
Goodwin TJ, 2017, VACCINE, V35, P2550, DOI 10.1016/j.vaccine.2017.03.067
Green DR, 2009, NAT REV IMMUNOL, V9, P353, DOI 10.1038/nri2545
Gregory AE, 2012, VACCINE, V30, P6777, DOI 10.1016/j.vaccine.2012.09.021
Guilliams M, 2014, NAT REV IMMUNOL, V14, P94, DOI 10.1038/nri3582
Hamid O, 2007, CLIN CANCER RES, V13, P215, DOI 10.1158/1078-0432.CCR-06-1450
Hanson MC, 2015, J CLIN INVEST, V125, P2532, DOI 10.1172/JCI79915
Hayashi M, 2016, VACCINE, V34, P306, DOI 10.1016/j.vaccine.2015.11.059
He HM, 2020, ACTA BIOMATER, V104, P188, DOI 10.1016/j.actbio.2020.01.012
He Q, 2002, CLIN DIAGN LAB IMMUN, V9, P1021, DOI 10.1128/CDLI.9.5.1021-1024.2002

He Q, 2000, CLIN DIAGN LAB IMMUN, V7, P899, DOI 10.1128/CDLI.7.6.899-903.2000
He XD, 2021, SMALL, V17, DOI 10.1002/smll.202007165
He XD, 2021, ACS NANO, V15, P4357, DOI 10.1021/acsnano.0c07680
He XD, 2018, ADV THER-GERMANY, V1, DOI 10.1002/adtp.201800060
HogenEsch H, 2013, FRONT IMMUNOL, V3, DOI 10.3389/fimmu.2012.00406
Hood MI, 2012, NAT REV MICROBIOL, V10, P525, DOI 10.1038/nrmicro2836
Horcajada P, 2012, CHEM REV, V112, P1232, DOI 10.1021/cr200256v
Horcajada P, 2010, NAT MATER, V9, P172, DOI [10.1038/NMAT260B, 10.1038/NMAT2608, 10.1038/nmat2608]
Horning KJ, 2015, ANNU REV NUTR, V35, P71, DOI 10.1146/annurev-nutr-071714-034419
Hornung V, 2008, NAT IMMUNOL, V9, P847, DOI 10.1038/ni.1631
Hu CL, 2020, BIOCONJUGATE CHEM, V31, P1661, DOI 10.1021/acs.bioconjchem.0c00209
Huang WC, 2020, ADV MATER, V32, DOI 10.1002/adma.202005637
Huang WC, 2018, NAT NANOTECHNOL, V13, P1174, DOI 10.1038/s41565-018-0271-3
Huang XF, 2017, HUM VACC IMMUNOTHER, V13, P2612, DOI 10.1080/21645515.2017.1369639
Huo MF, 2017, NAT COMMUN, V8, DOI 10.1038/s41467-017-00424-8
Irvine DJ, 2020, CURR OPIN IMMUNOL, V65, P1, DOI 10.1016/j.coi.2020.01.007
Ishikawa H, 2008, NATURE, V455, P674, DOI 10.1038/nature07317
Iyer S, 2003, VACCINE, V21, P1219, DOI 10.1016/S0264-410X(02)00556-X
Jemal A, 2009, CA-CANCER J CLIN, V59, P225, DOI [10.3322/caac.20006, 10.3322/caac.21601, 10.3322/caac.21254, 10.3322/caac.21387, 10.3322/caac.20073]
Jiang H, 2018, ADV SCI, V5, DOI 10.1002/advs.201700426
Jin HL, 2016, THERANOSTICS, V6, P2000, DOI 10.7150/thno.15102
Joyappa DH, 2009, VET MICROBIOL, V139, P58, DOI 10.1016/j.vetmic.2009.05.004
Ju EG, 2016, ANGEW CHEM INT EDIT, V55, P11467, DOI 10.1002/anie.201605509
Kalos M, 2013, IMMUNITY, V39, P49, DOI 10.1016/j.immuni.2013.07.002
Kang S, 2017, J CONTROL RELEASE, V256, P56, DOI 10.1016/j.jconrel.2017.04.024
Kershaw MH, 2013, NAT REV CANCER, V13, P525, DOI 10.1038/nrc3565
Knuschke T, 2016, RETROVIROLOGY, V13, DOI 10.1186/s12977-016-0258-9
Koping-Hoggard M, 2004, GENE THER, V11, P1441, DOI 10.1038/sj.gt.3302312
Koppad S, 2011, RES VET SCI, V91, P384, DOI 10.1016/j.rvsc.2010.09.008
Koshy ST, 2016, CURR OPIN BIOTECH, V40, P1, DOI 10.1016/j.copbio.2016.02.001
Koury MJ, 2015, NAT REV NEPHROL, V11, P394, DOI 10.1038/nrneph.2015.82
Kozlova Diana, 2014, Virologica Sinica, V29, P33, DOI 10.1007/s12250-014-3379-0
Kranzusch PJ, 2019, CURR OPIN STRUC BIOL, V59, P178, DOI 10.1016/j.sbi.2019.08.003
Kuai R, 2017, NAT MATER, V16, P489, DOI [10.1038/nmat4822, 10.1038/NMAT4822]
Kwissa M, 2003, J MOL MED-JMM, V81, P502, DOI 10.1007/s00109-003-0452-9
Kwon J, 2020, CANCER DISCOV, V10, P26, DOI 10.1158/2159-8290.CD-19-0761
Ladewig K, 2010, BIOMATERIALS, V31, P1821, DOI 10.1016/j.biomaterials.2009.10.058
Lan GX, 2018, J AM CHEM SOC, V140, P5670, DOI 10.1021/jacs.8b01072
LANZAVECCHIA A, 1985, NATURE, V314, P537, DOI 10.1038/314537a0
Lebre F, 2018, INT J PHARMACEUT, V552, P7, DOI 10.1016/j.ijpharm.2018.09.044
Lebre F, 2017, INT J PHARMACEUT, V527, P103, DOI 10.1016/j.ijpharm.2017.05.028
Lee BY, 2012, VACCINE, V30, P5637, DOI 10.1016/j.vaccine.2012.06.087
Lee IH, 2012, ANGEW CHEM INT EDIT, V51, P8800, DOI 10.1002/anie.201203193
Lei J, 2020, J CELL PHYSIOL, V235, P4814, DOI 10.1002/jcp.29358
Leibl H, 1999, VACCINE, V17, P1017, DOI 10.1016/S0264-410X(98)00201-1
Leopold LF, 2019, MOLECULES, V24, DOI 10.3390/molecules24224145
Lewis R., 1980, REGISTRY TOXIC EFFEC, V1, P252
Li A, 2010, BIOMATERIALS, V31, P748, DOI 10.1016/j.biomaterials.2009.09.095
Li FY, 2011, NATURE, V475, P471, DOI 10.1038/nature10246
Li HY, 2011, NAT NANOTECHNOL, V6, P645, DOI [10.1038/nnano.2011.153, 10.1038/NNANO.2011.153]
Li JX, 2021, CHEM MATER, V33, P9780, DOI 10.1021/acs.chemmater.1c03681
Li L, 2014, BIOMATERIALS, V35, P3331, DOI 10.1016/j.biomaterials.2013.12.095
Li N., 2018, NANOSCALE RES LETT, V13, P1
Li WP, 2016, ACS NANO, V10, P2017, DOI 10.1021/acsnano.5b06175
Li X, 2017, SCI REP-UK, V7, DOI [10.1038/s41598-017-00596-9, 10.1038/s41598-017-08465-1]
Li XR, 2014, J CONTROL RELEASE, V173, P148, DOI 10.1016/j.jconrel.2013.10.032
Li X, 2017, ACS APPL MATER INTER, V9, P22893, DOI 10.1021/acsami.7b03965
Li YH, 2020, CHEM SCI, V11, P7429, DOI 10.1039/d0sc00293c

Liang K, 2015, NAT COMMUN, V6, DOI 10.1038/ncomms8240
Liang RJ, 2017, BIOMATERIALS, V149, P41, DOI 10.1016/j.biomaterials.2017.09.029
Lin AY, 2013, PLOS ONE, V8, DOI 10.1371/journal.pone.0063550
Lin TS, 2018, THERANOSTICS, V8, P990, DOI 10.7150/thno.22465
Lindblad EB, 2004, VACCINE, V22, P3658, DOI 10.1016/j.vaccine.2004.03.032
Liu CC, 2017, EUR J CANCER CARE, V26, DOI 10.1111/ecc.12446
Liu F, 2019, ADV MATER, V31, DOI 10.1002/adma.201902885
Liu H, 2018, BIOMATERIALS, V167, P32, DOI 10.1016/j.biomaterials.2018.03.014
Liu TL, 2013, J COLLOID INTERF SCI, V400, P168, DOI 10.1016/j.jcis.2013.03.005
Liu Y., 2019, ADV HEALTHC MATER, V8
Liu ZG, 2008, J NANOPART RES, V10, P1271, DOI 10.1007/s11051-008-9379-y
Ljutic B, 2012, VACCINE, V30, P2981, DOI 10.1016/j.vaccine.2012.02.038
Luckheeram RV, 2012, CLIN DEV IMMUNOL, DOI 10.1155/2012/925135
Luo LJ, 2019, BIOMATERIALS, V223, DOI 10.1016/j.biomaterials.2019.119464
Luo M, 2017, CELL RES, V27, P586, DOI 10.1038/cr.2017.14
Lv MZ, 2020, CELL RES, V30, P966, DOI 10.1038/s41422-020-00395-4
Ma XY, 2019, BIOMATERIALS, V214, DOI 10.1016/j.biomaterials.2019.119223
Macian F, 2005, NAT REV IMMUNOL, V5, P472, DOI 10.1038/nri1632
Malhotra S, 2009, J BIOL CHEM, V284, P24088, DOI 10.1074/jbc.M109.014209
Neto LMM, 2018, HUM VACC IMMUNOTHER, V14, P2786, DOI 10.1080/21645515.2018.1489192
Martinez-Boubeta C, 2010, NANOMED-NANOTECHNOL, V6, P362, DOI 10.1016/j.nano.2009.09.003
Mata-Haro V, 2007, SCIENCE, V316, P1628, DOI 10.1126/science.1138963
Mathew R, 2009, CELL, V137, P1062, DOI 10.1016/j.cell.2009.03.048
Matsumura M, 2010, IMMUNOPHARM IMMUNOT, V32, P56, DOI 10.3109/08923970903124627
MCMILLAN RM, 1980, J CELL SCI, V44, P299
MILLER AB, 1981, CANCER, V47, P207, DOI 10.1002/1097-0142(19810101)47:1<207::AID-CNCR2820470134>3.0.CO;2-6
Mocan T, 2015, J CANCER, V6, P583, DOI 10.7150/jca.11567
Morefield GL, 2005, VACCINE, V23, P1502, DOI 10.1016/j.vaccine.2004.08.048
Munoz-Planillo R, 2013, IMMUNITY, V38, P1142, DOI 10.1016/j.immuni.2013.05.016
Nairz M, 2018, J TRACE ELEM MED BIO, V48, P118, DOI 10.1016/j.jtemb.2018.03.007
Nemunaitis J, 2006, J CLIN ONCOL, V24, P4721, DOI 10.1200/JCO.2005.05.5335
Ni KY, 2019, CHEM-US, V5, P1892, DOI 10.1016/j.chempr.2019.05.013
Nobs L, 2004, J PHARM SCI-US, V93, P1980, DOI 10.1002/jps.20098
Noh YW, 2011, BIOMATERIALS, V32, P6254, DOI 10.1016/j.biomaterials.2011.05.013
O'Neal Stefanie L, 2015, Curr Environ Health Rep, V2, P315, DOI 10.1007/s40572-015-0056-x
Okada N, 2005, GENE THER, V12, P129, DOI 10.1038/sj.gt.3302358
Oleszycka E, 2014, CURR OPIN IMMUNOL, V28, P1, DOI 10.1016/j.coi.2013.12.007
Palomaki J, 2010, TOXICOLOGY, V267, P125, DOI 10.1016/j.tox.2009.10.034
Pan DPJ, 2011, WIRES NANOMED NANOBI, V3, P162, DOI 10.1002/wnan.116
Pan JB, 2018, ADV MATER, V30, DOI 10.1002/adma.201704408
Pandey NK, 2019, J MATER CHEM B, V7, P6630, DOI 10.1039/c9tb01566c
Parry AL, 2013, J AM CHEM SOC, V135, P9362, DOI 10.1021/ja4046857
Pazar B, 2011, J IMMUNOL, V186, P2495, DOI 10.4049/jimmunol.1001284
Petersen DR, 2005, ALCOHOL, V35, P243, DOI 10.1016/j.alcohol.2005.03.013
Platt V, 2010, BIOCONJUGATE CHEM, V21, P892, DOI 10.1021/bc900448f
Poranen MM, 2008, NUCLEIC ACIDS RES, V36, P6633, DOI 10.1093/nar/gkn632
Powell TJ, 2011, VACCINE, V29, P558, DOI 10.1016/j.vaccine.2010.10.001
Pradeu T, 2016, SCI IMMUNOL, V1, DOI 10.1126/sciimmunol.aag0479
Prashant CK, 2014, J BIOMED NANOTECHNOL, V10, P2317, DOI 10.1166/jbn.2014.1991
Rahimi MT, 2017, PARASITOL RES, V116, P609, DOI 10.1007/s00436-016-5325-6
Ramlogan-Steel CA, 2014, TRANSL LUNG CANCER R, V3, P46, DOI 10.3978/j.issn.2218-6751.2013.12.01
Reeves E, 2017, IMMUNOLOGY, V150, P16, DOI 10.1111/imm.12675
Rhie GE, 2005, FEMS IMMUNOL MED MIC, V45, P341, DOI 10.1016/j.femsim.2005.05.009
Ridolfi Ruggero, 2004, J Transl Med, V2, P27
Rink L, 2000, P NUTR SOC, V59, P541, DOI 10.1017/S0029665100000781
Roche PA, 2015, NAT REV IMMUNOL, V15, P203, DOI 10.1038/nri3818
Rojas-Sepulveda D, 2018, CANCER IMMUNOL IMMUN, V67, P1897, DOI 10.1007/s00262-018-2157-5
Rossol M, 2012, NAT COMMUN, V3, DOI 10.1038/ncomms2339
Roy R, 2014, TOXICOL LETT, V230, P421, DOI 10.1016/j.toxlet.2014.08.008

Ruiz-de-Angulo A, 2016, ACS NANO, V10, P1602, DOI 10.1021/acsnano.5b07253
Saeed MI, 2015, CLIN EXP VACCINE RES, V4, P88, DOI 10.7774/cevr.2015.4.1.88
Safari D, 2012, NANOMEDICINE-UK, V7, P651, DOI [10.2217/nnm.11.151, 10.2217/NNM.11.151]
Sahdev P, 2014, PHARM RES-DORDR, V31, P2563, DOI 10.1007/s11095-014-1419-y
Savina A, 2007, IMMUNOL REV, V219, P143, DOI 10.1111/j.1600-065X.2007.00552.x
Schumacher TN, 2015, SCIENCE, V348, P69, DOI 10.1126/science.aaa4971
Scott A, 2018, NANOTECHNOL REV, V7, P69, DOI 10.1515/ntrev-2017-0159
Shao S, 2015, NAT CHEM, V7, P438, DOI [10.1038/nchem.2236, 10.1038/NCHEM.2236]
Sharma P, 2015, SCIENCE, V348, P56, DOI 10.1126/science.aaa8172
Sharma P, 2019, PHARMACEUTICS, V11, DOI 10.3390/pharmaceutics11100493
Sharma P, 2019, NANOSCALE, V11, P4591, DOI 10.1039/c8nr08704k
Shepherd F, 2013, EUR J CANCER, V49, pS802
Shi GP, 1999, IMMUNITY, V10, P197, DOI 10.1016/S1074-7613(00)80020-5
Shi XS, 2013, NATURE, V493, P111, DOI 10.1038/nature11699
Shukla R, 2005, LANGMUIR, V21, P10644, DOI 10.1021/la0513712
Small EJ, 2006, J CLIN ONCOL, V24, P3089, DOI 10.1200/JCO.2005.04.5252
Sokolovska A, 2007, VACCINE, V25, P4575, DOI 10.1016/j.vaccine.2007.03.045
Solomons NW, 1998, NUTR REV, V56, P27, DOI 10.1111/j.1753-4887.1998.tb01656.x
Song ML, 2016, ACS NANO, V10, P633, DOI 10.1021/acsnano.5b06779
Song RX, 2018, BIOMATERIALS, V175, P123, DOI 10.1016/j.biomaterials.2018.05.018
Sonnweber T, 2011, NEPHROL DIAL TRANSPL, V26, P977, DOI 10.1093/ndt/gfq483
Srikanth S, 2019, NAT IMMUNOL, V20, P152, DOI 10.1038/s41590-018-0287-8
Stumptner P, 1997, EMBO J, V16, P5807, DOI 10.1038/sj.emboj.7590555
Sun BB, 2013, ACS NANO, V7, P10834, DOI 10.1021/nn404211j
Sun LJ, 2013, SCIENCE, V339, P786, DOI 10.1126/science.1232458
Sun S, 2020, ANGEW CHEM INT EDIT, V59, P21041, DOI 10.1002/anie.202007786
Sun S, 2016, CHEM MATER, V28, P8659, DOI 10.1021/acs.chemmater.6b03695
Sun XQ, 2021, NAT NANOTECHNOL, V16, P1260, DOI 10.1038/s41565-021-00962-9
Sun YZ, 2021, NANO RES, V14, P1260, DOI 10.1007/s12274-020-3243-5
Sungsuwan S, 2015, ACS APPL MATER INTER, V7, P17535, DOI 10.1021/acsami.5b05497
Tao WQ, 2015, VACCINE, V33, P2307, DOI 10.1016/j.vaccine.2015.03.063
Tao Y, 2014, ADV FUNCT MATER, V24, P1004, DOI 10.1002/adfm.201302347
Tassa C, 2011, ACCOUNTS CHEM RES, V44, P842, DOI 10.1021/ar200084x
Terpe K, 2003, APPL MICROBIOL BIOT, V60, P523, DOI 10.1007/s00253-002-1158-6
Tschopp J, 2010, NAT REV IMMUNOL, V10, P210, DOI 10.1038/nri2725
Vaeth M, 2017, IMMUNITY, V47, P664, DOI 10.1016/j.immuni.2017.09.003
van der Lubben IM, 2001, ADV DRUG DELIVER REV, V52, P139, DOI 10.1016/S0169-409X(01)00197-1
van Hateren A, 2010, TISSUE ANTIGENS, V76, P259, DOI 10.1111/j.1399-0039.2010.01550.x
Vries I., 2016, NAT BIOTECHNOL, V23, P1407
Wang BH, 2018, SMALL, V14, DOI 10.1002/smll.201801372
Wang CG, 2020, ADV IMMUNOL, V145, P187, DOI 10.1016/bs.ai.2019.11.007
Wang CG, 2018, IMMUNITY, V48, P675, DOI 10.1016/j.immuni.2018.03.017
Wang J, 2013, INT J PHARMACEUT, V446, P205, DOI 10.1016/j.ijpharm.2013.02.028
Wang QT, 2020, CANCER IMMUNOL IMMUN, V69, P1375, DOI 10.1007/s00262-020-02496-w
Wang S, 2000, VACCINE, V18, P1227, DOI 10.1016/S0264-410X(99)00391-6
Wang T, 2015, ACS APPL MATER INTER, V7, P6391, DOI 10.1021/acsami.5b00348
Wang XP, 2017, ACS APPL MATER INTER, V9, P43538, DOI 10.1021/acsami.7b16118
Wang XP, 2016, COLLOID SURFACE B, V139, P10, DOI 10.1016/j.colsurfb.2015.12.004
Wang XP, 2013, SCI REP-UK, V3, DOI 10.1038/srep02203
Wang YL, 2021, NANO TODAY, V38, DOI 10.1016/j.nantod.2021.101139
Wang YT, 2011, BIOMATERIALS, V32, P7988, DOI 10.1016/j.biomaterials.2011.07.009
Weber KS, 2010, J IMMUNOL, V185, P2836, DOI 10.4049/jimmunol.1000880
Wei M, 2012, ANGEW CHEM INT EDIT, V51, P1202, DOI 10.1002/anie.201105187
Weintraub K, 2013, NATURE, V495, pS14, DOI 10.1038/495S14a
West MA, 2000, CURR BIOL, V10, P839, DOI 10.1016/S0960-9822(00)00595-9
Xiang J, 2015, ACS NANO, V9, P6401, DOI 10.1021/acsnano.5b02014
Xu J, 2019, BIOMATERIALS, V207, P1, DOI 10.1016/j.biomaterials.2019.03.037
Yan SY, 2014, BIOMATERIALS, V35, P9508, DOI 10.1016/j.biomaterials.2014.07.055
Yang GB, 2017, NAT COMMUN, V8, DOI 10.1038/s41467-017-01050-0
Yang X, 2021, NANO TODAY, V38, DOI 10.1016/j.nantod.2021.101109
Yang Y, 2020, ANGEW CHEM INT EDIT, V59, P19610, DOI 10.1002/anie.202006861

Yang YN, 2021, ADV SCI, V8, DOI 10.1002/advs.202002667
Yang Y, 2015, ADV FUNCT MATER, V25, P812, DOI 10.1002/adfm.201402764
Yu MC, 2011, J EXP MED, V208, P775, DOI 10.1084/jem.20100031
Yu Y, 2011, BLOOD, V118, P5011, DOI 10.1182/blood-2011-03-340315
Yuan HF, 2017, NAT NANOTECHNOL, V12, P763, DOI [10.1038/nnano.2017.69, 10.1038/NNANO.2017.69]
Yue J, 2018, ACS APPL MATER INTER, V10, P21920, DOI 10.1021/acsami.8b06633
Yun JW, 2015, J APPL TOXICOL, V35, P593, DOI 10.1002/jat.3102
Zanganeh S, 2017, IMMUNOTHERAPY-UK, V9, P819, DOI 10.2217/imt-2017-0041
Zeng Q, 2017, BIOMATERIALS, V122, P105, DOI 10.1016/j.biomaterials.2017.01.010
Zhan GT, 2021, NANO TODAY, V38, DOI 10.1016/j.nantod.2021.101195
Zhang J, 2017, CELL DEATH DIS, V8, DOI 10.1038/cddis.2017.337
Zhang LX, 2018, BIOMATERIALS, V174, P54, DOI 10.1016/j.biomaterials.2018.05.015
Zhang MX, 2015, ACS APPL MATER INTER, V7, P4650, DOI 10.1021/am5080453
Zhang M, 2018, CHEM ENG J, V338, P526, DOI 10.1016/j.cej.2018.01.081
Zhang PP, 2015, ACS NANO, V9, P6465, DOI 10.1021/acsnano.5b02153
Zhang Y, 2018, ACS NANO, V12, P651, DOI 10.1021/acsnano.7b07746
Zhang Y, 2017, CHEM COMMUN, V53, P1840, DOI 10.1039/c6cc09280b
Zhang Y, 2016, ADV FUNCT MATER, V26, P6454, DOI 10.1002/adfm.201600650
Zhao H, 2019, ACS NANO, V13, P13127, DOI 10.1021/acsnano.9b05974
Zhao L, 2014, VACCINE, V32, P327, DOI 10.1016/j.vaccine.2013.11.069
[truncated: 667,858 more chars]
